# The Novel Regulatory Role of lncRNA-miRNA-mRNA Axis in Amyotrophic Lateral Sclerosis: An Integrated Bioinformatics Analysis

**DOI:** 10.1155/2021/5526179

**Published:** 2021-04-15

**Authors:** Dingsheng Liu, Xiaojia Zuo, Peng Zhang, Rui Zhao, Donglin Lai, Kaijie Chen, Yuru Han, Guoqing Wan, Yanjun Zheng, Changlian Lu, Xuefeng Gu

**Affiliations:** ^1^Department of Oncology and Hematology, Shanghai University of Medicine and Health Sciences Affiliated Zhoupu Hospital, Shanghai, China; ^2^Shanghai Key Laboratory of Molecular Imaging, Shanghai University of Medicine and Health Sciences, Shanghai, China; ^3^Research Department, Shanghai University of Medicine and Health Sciences Affiliated Zhoupu Hospital, Shanghai, China

## Abstract

Amyotrophic lateral sclerosis (ALS) is an incurable neurodegenerative disease that primarily affects motor neurons, causing muscle atrophy, bulbar palsy, and pyramidal tract signs. However, the aetiology and pathogenesis of ALS have not been elucidated to date. In this study, a competitive endogenous RNA (ceRNA) network was constructed by analyzing the expression profiles of messenger RNAs (mRNAs) and long noncoding RNAs (lncRNAs) that were matched by 7 ALS samples and 4 control samples, and then a protein-protein interaction (PPI) network was constructed to identify the genes related to ALS. Gene Ontology (GO) was used to study the potential functions of differentially expressed mRNAs (DEmRNAs) in the ceRNA network. For the ALS and control groups, 247177 potential lncRNA-mRNA ceRNA relationship pairs were screened. Analysis of significant relationship pairs demonstrated that the PPI modules formed by the *MALAT1*-regulated *SYNRG*, *ITSN2*, *PICALM*, *AP3B1*, and *AAK1* genes may play important roles in the pathogenesis of ALS, and these results may help to characterize the pathogenesis of ALS.

## 1. Introduction

Amyotrophic lateral sclerosis (ALS) is an incurable chronic neurological disease that can lead to the continuous death of upper and lower motor neurons, resulting in muscle atrophy and fatigue that affect the patient's limb movement until the death of the patient [1–4]. This disease is most likely to occur in middle-aged people, with an incidence rate of 1.5-2.7 per 100,000 people observed; in addition, most patients die within 5 years after the onset of the disease, which has a severe impact on the physical and mental health of patients [1, 5, 6]. Although the aetiology and pathogenesis of ALS have not been fully elucidated to date, they may be related to genetics, the immune/inflammatory response [7, 8], sphingolipid metabolism [9], oxidative stress [10], and glutamate excitotoxicity [5].

ALS includes two types: familial ALS and sporadic ALS. Familial ALS accounts for only 5%-10% of ALS cases, while sporadic ALS accounts for 90%-95% of cases [11]. Regardless of whether there is a family history, the disease pathogenesis is related to mutations in genes including *SOD1* [7, 12], *OPTN* [6, 13], *UBQLN2* [11], *C9orf72* [8], *SQSTM1* [2], *SETX* [2], *GARP* [9], *PFN1* [14], and *SPG7* [15] and genes that encode RNA-binding proteins [16], such as *TARDBP*, *hnRNPA2B1*, *hnRNPA1*, and *FUS*.

With the development of microarray and next-generation sequencing technologies, noncoding RNAs (ncRNAs) and other indirectly pathogenic genes have received extensive attention from researchers. Numerous studies [1, 17–23] have analysed long ncRNAs (lncRNAs), messenger RNAs (mRNAs), and microRNAs (miRNAs) in different specimens, such as serum and cerebrospinal fluid samples and muscle biopsies from ALS patients, and found that large numbers of miRNAs are differentially regulated. mRNAs, lncRNAs, and other RNA transcripts can act as endogenous miRNA sponges to inhibit miRNA functions. These interactions can be explained by the well-known competing endogenous RNA (ceRNA) hypothesis proposed by Salmena et al., which has been applied to many fields [24, 25]. Continued analysis of ceRNA networks may help to elucidate how different subtypes of ncRNAs interact.

In this study, we performed a comprehensive analysis of the mRNA and lncRNA expression profiles in ALS. Then, we constructed an ALS-specific ceRNA network using a large number of research objects from online databases. As far as we know, this study is the earliest to create an lncRNA-miRNA-mRNA ceRNA network in ALS. This study helps to characterize the molecular pathogenesis of ALS and thus provides promising clues for clinical treatment. Interestingly, the *SYNRG*, *ITSN2*, *AAK1*, *PICALM*, and *AP3B1* modules in the protein-protein interaction (PPI) network, which are regulated by the lncRNA *MALAT1*, may play important roles in the pathogenesis of ALS.

## 2. Materials and Methods

### 2.1. Data Collection and Analysis

Data on miRNA-lncRNA regulatory relationships were downloaded from the experimental module of the lncBase database (http://carolina.imis.athena-innovation.gr/diana_tools/web/index.php?r=lncbasev2%2Findex), and miRNA-mRNA regulatory relationships were confirmed by experimental data downloaded from miRTarBase (http://mirtarbase.cuhk.edu.cn/php/index.php) to ensure the accuracy of miRNA target gene prediction. With regard to the potential ceRNA regulatory relationships between lncRNAs and mRNAs, if the number of common target miRNAs between lncRNAs and mRNAs was more than 3 and significance was indicated by a hypergeometric test, a potential ceRNA relationship between the lncRNAs and mRNAs was accepted. The equation for the hypergeometric test is shown in

(1) $$\begin{array}{c} P=1-F\left(x\;|\;N,L,M\right)=1-\overset{x-1}{\underset{k=0}{\sum }}\frac{\left(\begin{array}{c} L\\ k \end{array}\right)\left(\begin{array}{c} N-L\\ M-t \end{array}\right)}{\left(\begin{array}{c} N\\ M \end{array}\right)}, \end{array}$$

where *N* represents the total number of miRNAs, *L* represents the number of miRNAs targeting lncRNAs, *M* represents the number of miRNAs targeting mRNAs, and *x* represents the number of miRNAs targeting both lncRNAs and mRNAs. During screening, a *P* < 0.05 was considered to indicate a potential ceRNA relationship between lncRNAs and mRNAs.

Sample RNA-seq data (GSE115259) were downloaded from the Gene Expression Omnibus (GEO, http://www.ncbi.nlm.nih.gov/geo/) database; the data were analysed by Illumina RNA-seq on peripheral blood mononuclear cells from 7 ALS patients (including sporadic and mutated ALS patients) and 4 controls. The lncRNAs in the extracted expression profile were considered to be the expressed lncRNAs, and the expression profile was further filtered to move the mRNAs and lncRNAs expressed in more than half of the samples. Subsequent analysis was performed on the remaining lncRNAs and mRNAs. The correlation between lncRNAs and mRNAs was calculated by the R language cor.test function, and the correlation was calculated by the Spearman method. The lncRNA-mRNA regulatory relationships with *P* < 0.01 and *R* > 0.5 were screened.

### 2.2. Gene Ontology (GO) and Pathway Enrichment Analysis

The GO database is aimed at establishing a language vocabulary standard that strictly defines and comprehensively describes the gene and protein functions of any organization and can be dynamically updated with the continuous deepening of research. The GO system is an internationally standardized gene function classification system with three categories: molecular functions, cellular components, and biological processes.

In this study, the R language circlize package was used for visualization. The functions of network genes were analysed with Metascape.

### 2.3. PPI Network

All differentially expressed genes (DEGs) were imported into a search tool for identifying gene interactions, known as STRING 10.5 (https://string-db.org/) to construct the network, and Cytoscape software 3.6 (https://www.cytoscape.org) was employed for visualization. The colours of the edges in the network represent the types of protein-protein relationships: light blue and purple show known interactions determined from the planning database and experiments, respectively; dark green/red/dark blue represents predicted interactions through gene neighbourhood/gene fusion/gene sharing, respectively; and light green/black/blue represents text mining/coexpression/protein homology.

### 2.4. Statistical Analysis

We used SPSS 11.0 (SPSS, Chicago, IL) to analyse the data sets from RNA-seq experiments. *P* values < 0.05 were considered to indicate significance.

## 3. Results

### 3.1. Construction of a ceRNA Network with ALS and Control Sample

**Figure 1:**
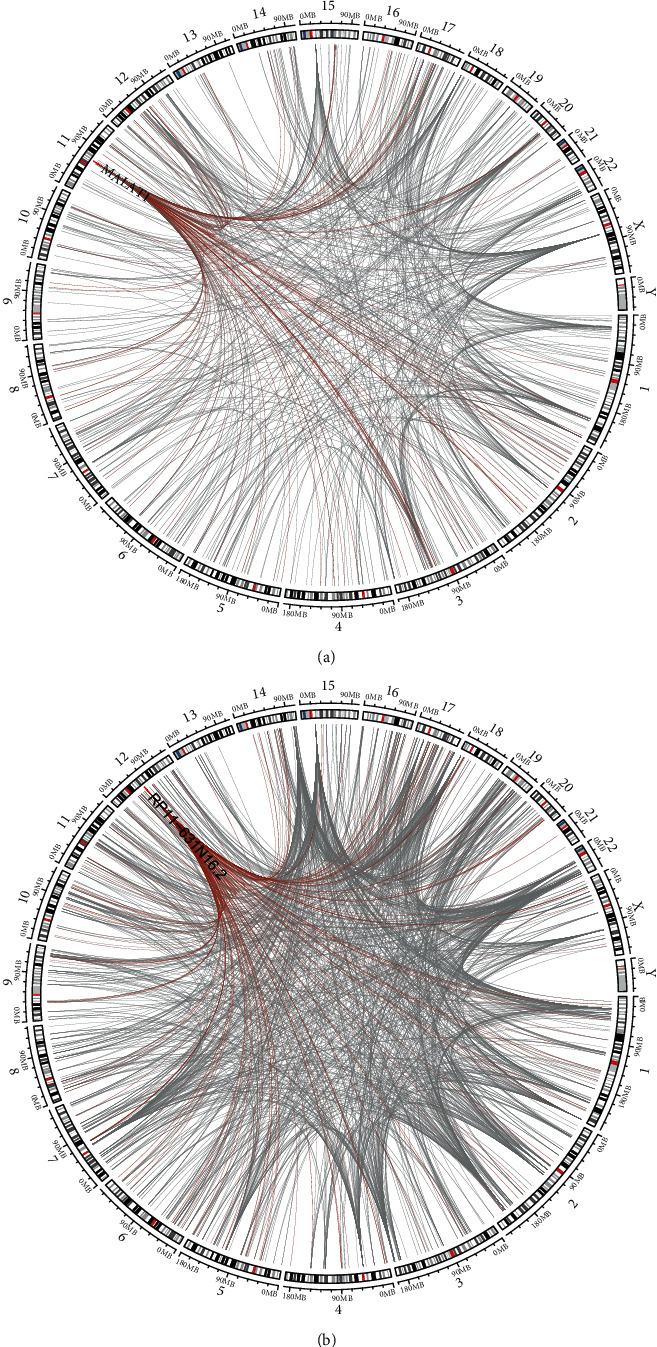
Differential ceRNA networks in ALS and control samples.

We found that ALS samples and control samples were separated using *t*-Distributed Stochastic Neighbour Embedding (*t*-SNE) for dimensionality reduction visualization (Figure S1). Next, the target miRNAs of lncRNAs were obtained from the lncBase database, which consists of 100727 miRNA-lncRNA regulatory pairs composed of 1420 miRNAs and 8217 lncRNAs. Furthermore, the miRNA-mRNA target relationships were obtained from miRTarBase, which consists of 243613 pairs of miRNA-mRNA target relationships consisting of 2585 miRNAs and 13618 mRNAs. Screening for a lncRNA-mRNA shared miRNA number greater than 3 and a significant result of the hypergeometric test (*P* < 0.05) left 247177 potential lncRNA-mRNA ceRNA relationship pairs (Table S1). The correlations between the lncRNA and mRNA expression in potential ceRNA pairs in ALS and the control samples were calculated, which revealed a significant lncRNA-mRNA ceRNA pair (Table S2), as shown in Figure 1.  Figure 1(a) represents the ceRNA network in ALS patient samples, and Figure 1(b) represents the ceRNA network in control samples. We found that most lncRNAs and mRNAs were located on different chromosomes; in other words, the regulatory relationships among lncRNAs, mRNAs, and miRNAs involve trans mechanisms and miRNA sponging functions.

**Figure 2:**
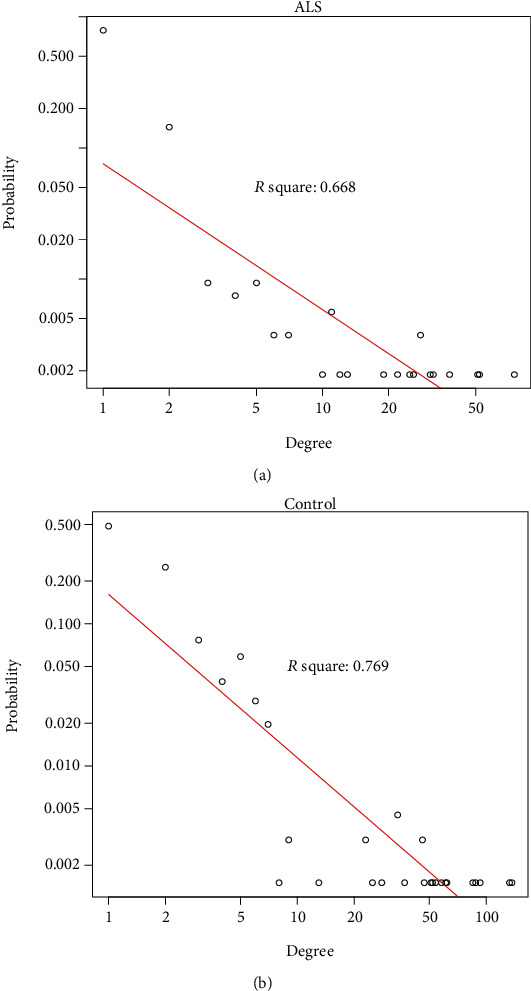
The degree distribution of the ceRNA network formed between lncRNA-mRNA in the ALS group and in the control group.

Further analysis was performed to characterize the degree distribution of the ceRNA network-formed lncRNA-mRNA interactions. It was found that the degree distribution of the network approximately obeyed the power-law distribution: namely, the degree of most of the nodes was relatively small, and the degree of a small proportion of the nodes was relatively large (Figures 2(a) and 2(b)), which is in keeping with the nature of conventional biological networks. There were some nodes with large degrees in the network. The nodes with relatively large degrees may play important roles in the network. For example, the lncRNAs *MALAT1* and *RP11-631N16.2* in the two networks of ALS and control samples may play important regulatory roles.

### 3.2. ceRNA Network Function Annotation

**Figure 3:**
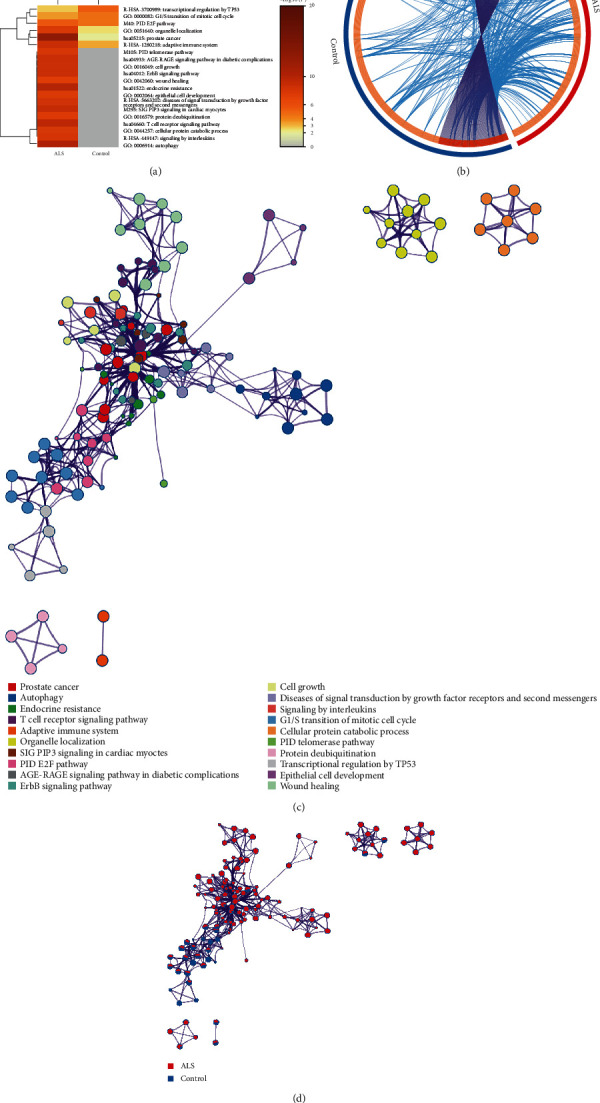
ceRNA network function annotation. (a) Biological processes associated with genes in the ceRNA network in ALS cases and control cases. (b) Comparison of 493 genes between ALS cases and control cases in the ceRNA network. The red/blue/light orange/dark orange colour represents ALS group genes/controls genes/differentially expressed genes/common genes, respectively. (c) Further analysis of the relationship among top 20 GO terms. (d) Most terms were enriched in different categories in the ALS group.

To study the functions associated with the ceRNA networks of ALS and control samples, the genes in these two networks were analysed with Metascape, and it was found that the genes in the ALS network were associated with GO biological processes such as autophagy (Figure 3(a)). Furthermore, some genes were the same between the regulatory networks of the ALS and control samples, but most of the genes were different (Figure 3(b)). Some of the different genes were associated with common GO terms (Figure 3(b), blue line). Further analysis was performed to determine the relationships in the top 20 terms.  Figure 3(c) shows the connections among the terms, where different colours indicate different categories. Compared with the terms for the control samples, most terms for the ALS samples were significantly enriched in different categories (Figure 3(d)).

### 3.3. Hub lncRNA Function in the Network

**Figure 4:**
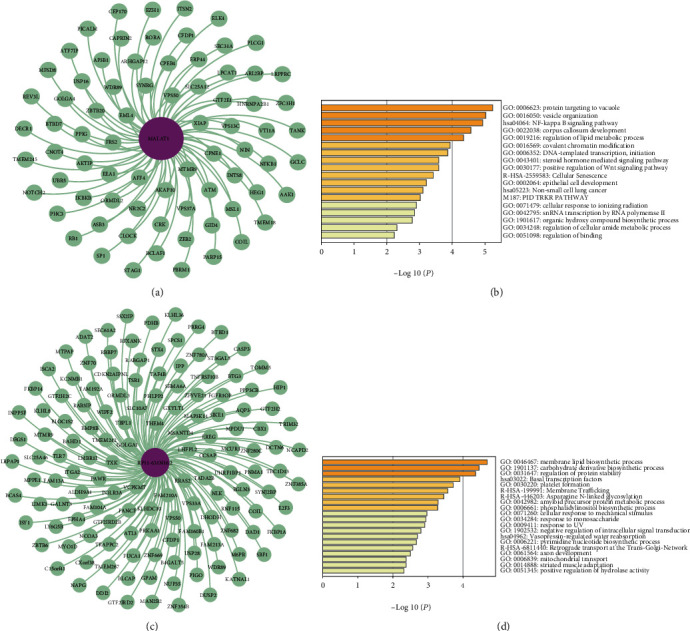
Hub lncRNA functions in the network: (a) seventy-five genes regulated by *MALAT1* in ALS; (b) enriched GO terms for ALS patients in different categories; (c) one hundred thirty-seven genes regulated by *RP11-631N16.2* in control samples; (d) enriched GO terms for control samples in different categories.

In both ALS and control samples, there were important hub nodes that played important roles in the networks. To study the functions of lncRNAs in ALS and control samples, the lncRNAs were functionally annotated with the genes they regulated. The lncRNAs were first analysed in the two ceRNA networks. *MALAT1* was determined to serve as a miRNA sponge to regulate 75 genes in ALS samples (Figure 4(a)). Through functional enrichment analysis of these regulated genes, we found enrichment for the following terms: GO:0006623: protein targeting to vacuole, GO: 0016050: vesicle organization, hsa04064: *NF-κB* signalling pathway, and GO: 0006352: DNA-templated transcription and initiation (Figure 4(b)). Notably, it has been reported that some genes regulated by *MALAT1* are involved in the pathogenesis of ALS, including *DECR1* [26], *CPEB4* [27], *VPS37A* [28], *SP1* [10, 29], *EEA1* [30], *RB1* [31], and *GCLC* [32] (Figure 4(a)).

In control samples, *RP11-631N16.2* was observed to exhibit an important function by regulating 137 genes (Figure 4(c)), and it was associated with GO terms, such as GO:0046467: membrane lipid biosynthetic process, and GO:0031647: regulation of protein stability (Figure 4(d)). *STX4* (Figure 4(c)), one of the genes regulated by the lncRNA *RP11-631N16.2*, encodes a protein that plays a vital role in the control regulation of glucose metabolism uptake in skeletal muscle. Reductions in the *STX4* protein expression levels lead to decreases in systemic hormone-stimulated glucose metabolism. Another gene regulated by the lncRNA *RP11-631N16.2* is *CASP3* (Figure 4(c)), which encodes cysteine-aspartic acid protease; this protein plays a central role in the execution phase of apoptosis, which is associated with neuronal death in Alzheimer's disease (AD).

### 3.4. PPIs Regulated by MALAT1

**Figure 5:**
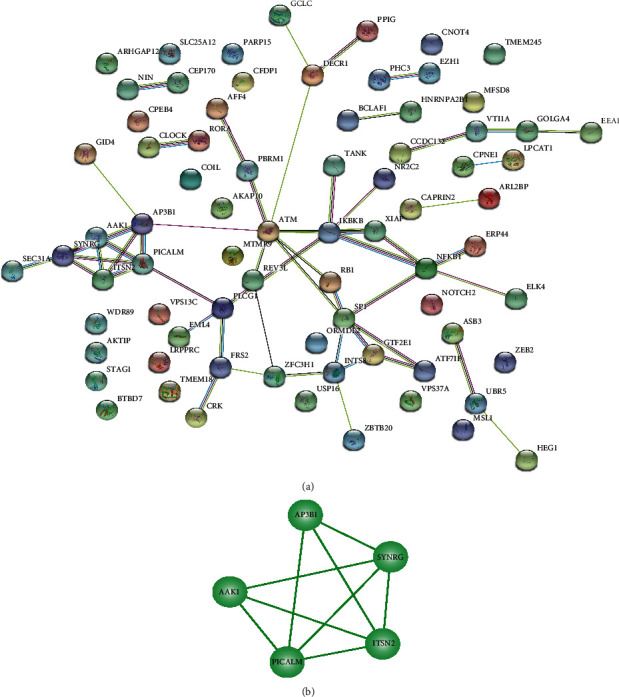
PPI network: (a) the red and green nodes represent the upregulated and downregulated genes, respectively; (b) modules formed by *SYNRG*, *ITSN2*, *AAK1*, *PICALM*, and *AP3B1* in the PPI network.

Since *MALAT1* plays an important role in the network, it was hypothesized that the genes regulated by *MALAT1* interact at the protein level. We depicted the PPI network of the genes regulated by *MALAT1* using the STRING database (Figure 5(a)). The MCODE plug-in of Cytoscape was further used to mine the PPI modules, and it was determined that the modules formed by *SYNRG*, *ITSN2*, *AAK1*, *PICALM*, and *AP3B1* may play important roles in ALS (Figure 5(b)).

## 4. Discussion

The typical manifestations of ALS are muscle weakness and atrophy, which severely affect the physical and mental health of patients. In this study, a number of meaningful findings were obtained regarding the pathogenesis of ALS. The lncRNAs in the ceRNA network have diverse functions and can regulate a variety of genes. GO analysis of genes regulated by *MALAT1* revealed enrichment for several GO terms related to ALS.

In Figure 4(b), the terms GO:0006623: protein targeting to vacuole, and GO:0016050: vesicle organization, are depicted. A previous study has shown that miRNA signals in the plasma of ALS patients (PALS) can cross the blood-brain barrier and enter the circulatory system [1]. Analysis of the differentially expressed miRNAs in extracellular vesicles (EVs) revealed elevated levels of 5 miRNAs and decreased levels of 22 miRNAs in EVs collected from PALS samples compared with control samples [1]. Four unregulated miRNAs associated with ALS involving miR-9-5p, miR-183-5p, miR-338-3p, and miR-1246 [1]. These results emphasize the diagnostic relevance of miR-15a-5p for distinguishing samples from healthy individuals from PALS samples and of miR-193a-5p for distinguishing among patients with low vs. high Revised ALS Functional Rating Scale (ALSFRS-R) scores [1]. The data in Table S2 suggest that miR-9-5p is related to the *AP3B1* and *NF-κB* genes, that miR-15a-5p is related to the *SYNRG* and *AP3B1* genes, that miR-193a-5p is related to the TMEM245 gene, and that *SYNRG*, *AP3B1*, *NF-κB*, and *TMEM245* are regulated by *MALAT1* (Figure 4(a)).

Another term depicted in Figure 4(b) is hsa04064: *NF-κB* signalling pathway. *NF-κB* is a pleiotropic transcription factor that exists in almost all cell types and is the terminus of a series of signal transduction events. Growth, tumorigenesis, and apoptosis are triggered by numerous stimuli related to many biological processes, such as inflammation and immunity. *OPTN* is known as an *NF-κB* basic regulation-related protein and is involved in maintaining the morphology of the Golgi apparatus and in regulating exocytosis, endoplasmic reticulum stress, membrane receptor levels, type-I interferon response, cell death, and autophagy; nonsense and missense mutations of the *OPTN* gene abolish the inhibitory effect of *NF-κB* activation, and *NF-κB* inhibitors can be used to treat ALS [6, 13].

A third term depicted in Figure 4(b) is GO:0006352: DNA-templated transcription and initiation. *TDP-43* can bind to *MALAT1*. *TDP-43* is a DNA/RNA binding protein encoded by the *TARDBP* gene that has been determined to be an ALS ubiquitination aggregate. Importantly, the given neuroinflammation is a pathological feature of ALS, and mutations to genes such as TARDBP enhance this neuroinflammation [7, 33–36]. In addition, the *MALAT-1*-regulated gene *hnRNPA2/B1* encodes an RNA-binding protein that is associated with neurodegeneration [16, 37–40]. Martinez et al. found that *hnRNPA2/B1 D290V* mutant fibroblasts and motor neurons differentiated from induced pluripotent stem cells obtained from ALS patients showed abnormal splicing changes and that the survival rates of induced pluripotent stem cell with mutations were decreased in long-term culture, which aggravated changes in gene expression and splicing, when placing stress on the cells [38].

Another gene regulated by *MALAT1* is *XIAP* (shown in Figure 4(a)). Many studies [41–44] have shown that different pathways (including the mitochondrial apoptosis pathway, the glutamate excitotoxicity pathway, and the HGF overexpression pathway) increase the levels of apoptotic proteases and decrease the expression levels of *XIAP* protein in the spinal cord, which is closely related to motor neuron degeneration in the process of ALS-related apoptosis due to mutant *SOD-1*. Importantly, changes in the expression levels of the *XIAP* protein may play important roles in the late stage of ALS.

The *ATM* gene is also regulated by *MALAT1*, as depicted in Figure 4(a). Studies [45, 46] have revealed that *ATM* plays an important role in the response to DNA damage observed in the pathogenesis of ALS. Moreover, neuronal dysfunction and neuronal death in ALS patients may be related to continuous DNA damage and the activation of the *ATM* and *p53* proapoptotic signalling pathways.

Interestingly, in this study, the modules formed by *SYNRG*, *ITSN2*, *PICALM*, *AP3B1*, and *AAK1* in the PPI network regulated by *MALAT1* were closely related to one another (Figure 5(b)).

*ITSN2*, shown in Figure 5(b), is an adapter protein and a member of the conserved family of clathrin-mediated endocytosis proteins. *ITSN2* may be involved in regulating the formation of clathrin-coated vesicles and may also participate in the maturation of clathrin-coated vesicles [47, 48].

*AAK1* is an adapter-related regulatory protein in the endocytic pathway of clathrin-coated vesicles. This protein selectively interacts with *SOD1* mutants, rather than wild-type *SOD1*. Adapter receptor-associated protein complex 2 plays a role in receptor-mediated endocytosis, triggering clathrin assembly, interacting with membrane-bound receptors, and recruiting coding cofactors. The kinase activity of this complex is stimulated by clathrin or transcriptional splice variants, but its biological effectiveness has not been determined. The *AP2M1/Mu2* subunit of adaptor protein complex 2 is phosphorylated to regulate clathrin-mediated endocytosis, which ensures the high-affinity binding of *AP2* to cargo membrane proteins in the initial stage of endocytosis. Research on this gene in animal models has confirmed that abnormal functioning of components in the endoplasmic and synaptic vesicle recycling pathway is related to the pathology of ALS [49].

It has been reported in the literature that *SYNRG* gene mutations appear in the *17q12* microdeletion syndrome, which is related to cognitive impairment and abnormal brain structure [50, 51]. AD and ALS are both neurodegenerative diseases, and *PICALM* has been proven to be the causative gene of AD [52–54]. In addition, there are a large number of published studies confirming that *AP3B1* is related to type 2 Hermansky-Pudlak syndrome [55–64]. Although these four genes (*SYNRG*, *ITSN2*, *PICALM*, and *AP3B1*) have not been reported in the literature on ALS, the *SYNRG*, *ITSN2*, *PICALM*, *AP3B1*, and *AAK1* genes are regulated by *MALAT1*, and the proteins encoded by these genes are all related to clathrin. As far as we know, the clathrin may participate in the formation of early autophagosomes, and autophagy played a role in the pathogenesis of neurodegenerative diseases. Thus, we speculated that the PPI module formed by these five genes (*SYNRG*, *ITSN2*, *PICALM*, *AP3B1*, and *AAK1*) is related to the autophagy and may play an important role in the pathogenesis of ALS.

## 5. Conclusion

**Figure 6:**
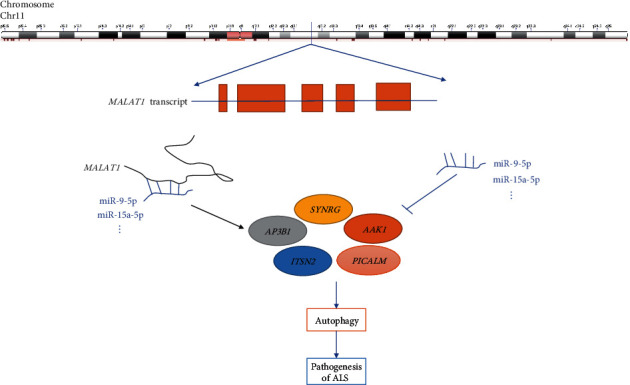
Potential mechanism of differentially expressed lncRNA sponges.

In this study, we constructed a ceRNA network and found that isogenic regulation by *MALAT1* may play an important role in the pathogenesis of ALS (Figure 6). We further observed that the modules formed by the *MALAT1*-regulated *SYNRG*, *ITSN2*, *AAK1*, *PICALM*, and *AP3B1* ALS genes are potentially important roles of participants in the pathogenesis of ALS. Our findings provide a new perspective for understanding the mechanism underlying ALS. The *AAK1*-encoding gene has been proven to be related to ALS, and further research is warranted to determine whether the other four genes are related to ALS.

## Data Availability

Sample RNA-seq data (GSE115259) were downloaded from the Gene Expression Omnibus (GEO, http://www.ncbi.nlm.nih.gov/geo/) database.

## Supplementary Materials

Supplementary 1 Figure S1: samples are separated by dimensionality reduction using the *t*-Distributed Stochastic Neighbour Embedding (*t*-SNE) algorithm.

Supplementary 2 Table S1: potential lncRNA-mRNA ceRNA relationship pairs (Excel file).

Supplementary 3 Table S2: significant lncRNA-mRNA ceRNA relationship pairs.
