# Supplementary figures and images for: The Novel Regulatory Role of lncRNA-miRNA-mRNA Axis in Amyotrophic Lateral Sclerosis: An Integrated Bioinformatics Analysis
Source: Comput Math Methods Med. 2021 Apr 15;2021:5526179. doi: 10.1155/2021/5526179 (PMC8067776; doi:10.1155/2021/5526179)

Figure S1

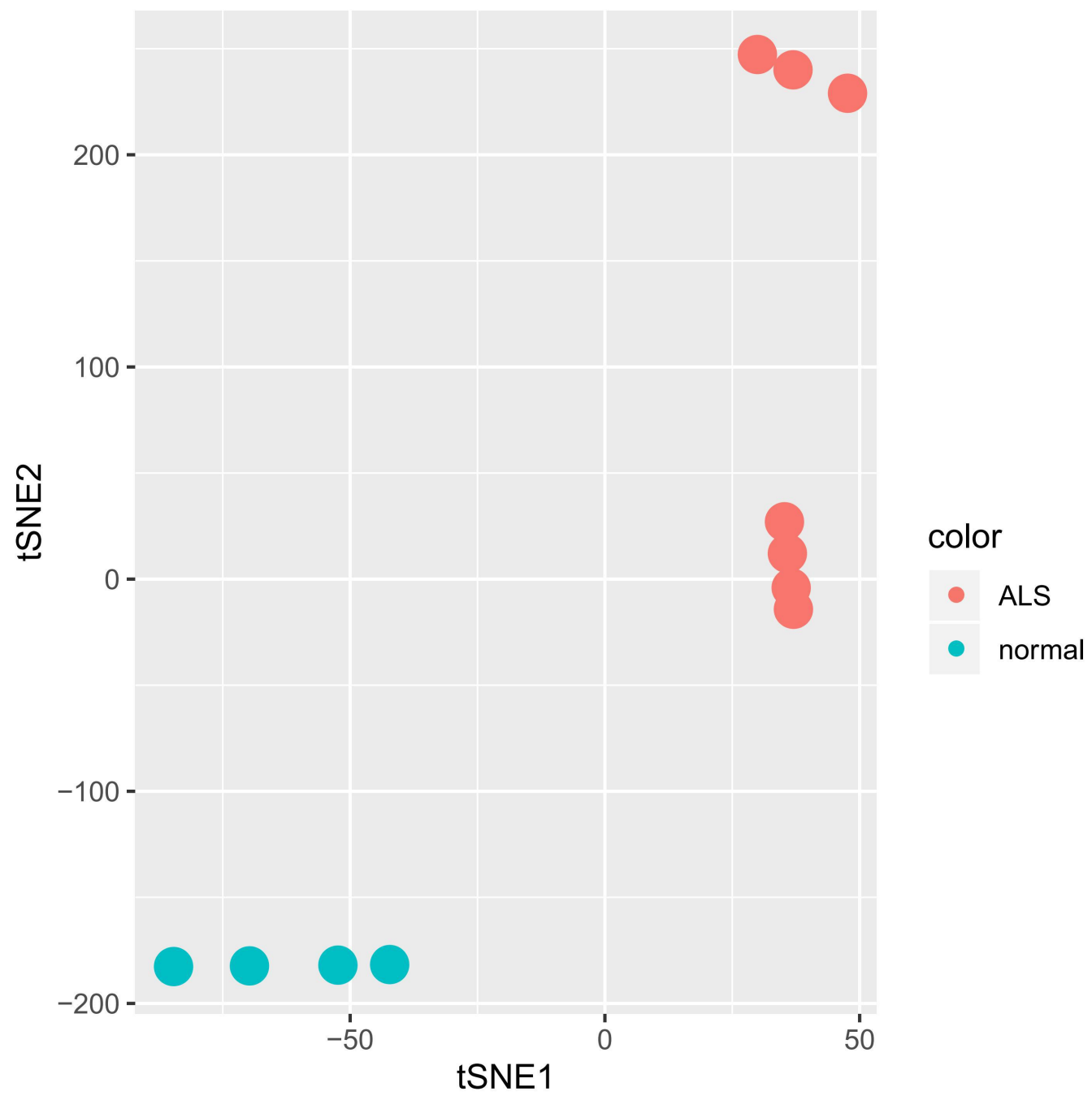

Supplement: Supplementary 1 — Figure S1: samples are separated by dimensionality reduction using the t-Distributed Stochastic Neighbour Embedding (t-SNE) algorithm. [file 5526179.f1.pdf]
