# Supplementary material for: The Novel Regulatory Role of lncRNA-miRNA-mRNA Axis in Amyotrophic Lateral Sclerosis: An Integrated Bioinformatics Analysis
Source: Comput Math Methods Med. 2021 Apr 15;2021:5526179. doi: 10.1155/2021/5526179 (PMC8067776; doi:10.1155/2021/5526179)
Supplement: Supplementary 3 — Table S2: significant lncRNA-mRNA ceRNA relationship pairs. [file 5526179.f3.pdf]

| lncRNA | miRNAs | mRNA |
| --- | --- | --- |
| RP11-332H14.2 | hsa-miR-500a-5p;hsa- | SCYL3 |
| MALAT1 | hsa-miR-101-3p;hsa-m | GCLC |
| RP11-458F8.4 | hsa-let-7a-5p;hsa-let- | PLXND1 |
| RP11-78O7.2 | hsa-let-7a-5p;hsa-let- | PLXND1 |
| MALAT1 | hsa-miR-106a-5p;hsa- | VPS50 |
| LINC00910 | hsa-miR-15a-5p;hsa-m | LAMP2 |
| CTC-459F4.1 | hsa-miR-148a-3p;hsa- | MAP3K9 |
| LINC00662 | hsa-miR-101-3p;hsa-m | COX10 |
| LINC01355 | hsa-miR-30a-5p;hsa-m | UBE3C |
| NEAT1 | hsa-miR-136-3p;hsa-m | UBE3C |
| MALAT1 | hsa-miR-124-5p;hsa-m | REV3L |
| NEAT1 | hsa-miR-124-5p;hsa-m | REV3L |
| FTX | hsa-let-7a-5p;hsa-let- | ZNF200 |
| LINC01355 | hsa-miR-30a-5p;hsa-m | ZNF200 |
| RP11-488L18.10 | hsa-let-7a-5p;hsa-let- | ZNF200 |
| NEAT1 | hsa-miR-10b-5p;hsa-m | HIVEP2 |
| MALAT1 | hsa-miR-106b-5p;hsa- | BTBD7 |
| XIST | hsa-miR-106b-5p;hsa- | BTBD7 |
| NEAT1 | hsa-miR-1224-3p;hsa- | PIK3C2A |
| LINC00106 | hsa-miR-124-3p;hsa-m | MAN2B2 |
| LINC01355 | hsa-miR-30a-5p;hsa-m | PGM3 |
| NEAT1 | hsa-miR-126-5p;hsa-m | PGM3 |
| LINC01355 | hsa-miR-15a-5p;hsa-m | CCDC88C |
| MALAT1 | hsa-miR-100-3p;hsa-m | ERP44 |
| LINC00467 | hsa-miR-130a-3p;hsa- | RRAGD |
| MALAT1 | hsa-miR-142-3p;hsa-m | BCLAF1 |
| PWAR6 | hsa-miR-17-5p;hsa-mi | SLC4A7 |
| RP11-332H14.2 | hsa-miR-30b-5p;hsa-m | SLC4A7 |
| PWAR6 | hsa-miR-135a-5p;hsa- | PHLPP2 |
| RP11-332H14.2 | hsa-miR-30a-5p;hsa-m | JADE2 |
| NEAT1 | hsa-let-7a-3p;hsa-let- | ARAP2 |
| FTX | hsa-let-7a-5p;hsa-let- | C12orf4 |
| XIST | hsa-let-7g-3p;hsa-miR | USP28 |
| NEAT1 | hsa-let-7a-3p;hsa-let- | ZNF800 |
| RP11-701H24.4 | hsa-miR-17-5p;hsa-mi | ZNF800 |
| C1orf132 | hsa-let-7a-5p;hsa-let- | HERPUD1 |
| LINC00667 | hsa-miR-106b-5p;hsa- | SIKE1 |
| MEG3 | hsa-miR-106a-5p;hsa- | TRAF3IP2 |
| RP11-631N16.2 | hsa-miR-106b-5p;hsa- | DCBLD2 |
| XIST | hsa-miR-15a-5p;hsa-m | SEC61A1 |
| LINC00662 | hsa-miR-15a-5p;hsa-m | CDK17 |
| LINC00662 | hsa-miR-378a-3p;hsa- | SPA17 |
| RP11-352M15.2 | hsa-miR-126-5p;hsa-m | BTBD1 |
| LINC01355 | hsa-miR-30a-5p;hsa-m | SEC61A2 |
| LINC00667 | hsa-miR-181a-5p;hsa- | ASB1 |
| XIST | hsa-miR-106b-5p;hsa- | ATG2B |
| MIRLET7BHG | hsa-miR-188-3p;hsa-m | SDK2 |
| MALAT1 | hsa-miR-101-3p;hsa-m | RORA |
| LINC00667 | hsa-miR-181a-5p;hsa- | AP3M2 |
| NORAD | hsa-miR-224-5p;hsa-m | CDC42 |
| XIST | hsa-let-7a-5p;hsa-let- | RAD18 |
| NEAT1 | hsa-miR-106b-5p;hsa- | ATP2B1 |
| NORAD | hsa-miR-129-5p;hsa-m | RDH11 |

| | | |
|---|---|---|
| MALAT1 | hsa-miR-142-3p;hsa-m | AFF4 |
| MALAT1 | hsa-miR-1-3p;hsa-miR | PICALM |
| RP11-631N16.2 | hsa-miR-106a-5p;hsa- | TSG101 |
| PKI55 | hsa-miR-15a-5p;hsa-m | FRYL |
| LINC01355 | hsa-miR-15a-5p;hsa-m | FRYL |
| C17orf100 | hsa-let-7a-5p;hsa-let- | MAP2K7 |
| LINC00910 | hsa-let-7a-5p;hsa-let- | RABL2B |
| C1orf132 | hsa-miR-106a-5p;hsa- | SESN1 |
| LINC00667 | hsa-miR-106b-5p;hsa- | SESN1 |
| MALAT1 | hsa-miR-106a-5p;hsa- | CNOT4 |
| LINC00667 | hsa-miR-148b-3p;hsa- | HSP90AA1 |
| LINC00910 | hsa-miR-15a-5p;hsa-m | GSK3B |
| RNU12 | hsa-miR-548a-5p;hsa- | LYRM2 |
| PWAR6 | hsa-miR-126-5p;hsa-m | CYLD |
| PWAR6 | hsa-miR-15a-5p;hsa-m | YTHDC1 |
| RP11-78O7.2 | hsa-let-7a-5p;hsa-let- | NOA1 |
| NEAT1 | hsa-miR-106a-5p;hsa- | RRN3 |
| CTD-2587M2.1 | hsa-miR-25-3p;hsa-mi | RRN3 |
| XIST | hsa-miR-124-3p;hsa-m | B4GALT1 |
| C1orf132 | hsa-let-7a-5p;hsa-let- | TXLNG |
| RP11-332H14.2 | hsa-miR-30a-5p;hsa-m | ALG9 |
| RP11-498C9.15 | hsa-miR-27a-3p;hsa-m | ALG9 |
| PKI55 | hsa-miR-101-3p;hsa-m | L2HGDH |
| NEAT1 | hsa-miR-1-3p;hsa-miR | KLHL42 |
| NORAD | hsa-miR-106b-5p;hsa- | GNAS |
| SNHG8 | hsa-miR-19a-3p;hsa-m | SNX5 |
| PWAR6 | hsa-miR-15a-5p;hsa-m | GANAB |
| C1orf132 | hsa-miR-136-5p;hsa-m | GANAB |
| PWAR6 | hsa-miR-124-3p;hsa-m | USP48 |
| NEAT1 | hsa-miR-125a-3p;hsa- | RBM27 |
| C1orf132 | hsa-let-7a-5p;hsa-let- | CDV3 |
| C1orf132 | hsa-miR-129-5p;hsa-m | ESR1 |
| RP11-332H14.2 | hsa-miR-30a-5p;hsa-m | SEMA6A |
| RNU12 | hsa-miR-15a-5p;hsa-m | CLSPN |
| RP11-488L18.10 | hsa-let-7a-5p;hsa-let- | CBX5 |
| RP11-138A9.1 | hsa-miR-200c-3p;hsa- | SIRT1 |
| NEAT1 | hsa-miR-142-3p;hsa-m | DDX17 |
| LINC00106 | hsa-miR-185-3p;hsa-m | SBF1 |
| PWAR6 | hsa-miR-19a-3p;hsa-m | CBX7 |
| C1orf132 | hsa-let-7a-5p;hsa-let- | PDGFB |
| XIST | hsa-miR-148a-3p;hsa- | SOS2 |
| XIST | hsa-miR-106a-5p;hsa- | NIN |
| MALAT1 | hsa-miR-106a-5p;hsa- | NIN |
| LINC00969 | hsa-miR-145-5p;hsa-m | HIF1A |
| RP11-332H14.2 | hsa-miR-30a-5p;hsa-m | ZMYND8 |
| PWAR6 | hsa-miR-143-3p;hsa-m | JAG1 |
| NORAD | hsa-let-7a-5p;hsa-miR | E2F1 |
| LINC00910 | hsa-miR-15a-5p;hsa-m | RPRD1B |
| GS1-124K5.11 | hsa-miR-15a-5p;hsa-m | RPRD1B |
| PWAR6 | hsa-miR-15a-5p;hsa-m | RNMT |
| PWAR6 | hsa-miR-15a-5p;hsa-m | MIB1 |
| RP11-332H14.2 | hsa-miR-30a-5p;hsa-m | MIB1 |
| RNU12 | hsa-miR-130a-5p;hsa- | STS |
| NORAD | hsa-let-7a-5p;hsa-let- | PGRMC1 |

| | | |
|---|---|---|
| LINC00467 | hsa-miR-130a-3p;hsa- | NKAP |
| MALAT1 | hsa-miR-103a-2-5p;hs | XIAP |
| MALAT1 | hsa-miR-101-3p;hsa-m | EEA1 |
| MIRLET7BHG | hsa-miR-34a-5p;hsa-m | CD40LG |
| NORAD | hsa-miR-1825;hsa-miR | TSC22D1 |
| MALAT1 | hsa-miR-30a-3p;hsa-m | ARL2BP |
| RP11-488L18.10 | hsa-let-7a-5p;hsa-let-7 | SLC38A7 |
| NEAT1 | hsa-miR-145-5p;hsa-m | ABCC1 |
| C17orf100 | hsa-let-7a-5p;hsa-let-7 | GABPB1 |
| MALAT1 | hsa-miR-103a-3p;hsa- | DECR1 |
| MALAT1 | hsa-miR-148a-3p;hsa- | IKBKB |
| MALAT1 | hsa-miR-106b-5p;hsa- | UBR5 |
| NEAT1 | hsa-miR-106b-5p;hsa- | UBR5 |
| MALAT1 | hsa-miR-106a-5p;hsa- | MTMR9 |
| NEAT1 | hsa-miR-106a-5p;hsa- | MTMR9 |
| NORAD | hsa-miR-106a-5p;hsa- | TGFB1 |
| LINC00467 | hsa-miR-130a-3p;hsa- | KDELR1 |
| LINC01355 | hsa-miR-15a-5p;hsa-m | AVL9 |
| C1orf132 | hsa-miR-125a-3p;hsa- | LFNG |
| RP11-67L2.2 | hsa-let-7d-5p;hsa-miR | WASL |
| PKI55 | hsa-let-7a-5p;hsa-let-7 | AP1S1 |
| C17orf100 | hsa-let-7a-5p;hsa-let-7 | AP1S1 |
| FTX | hsa-let-7a-5p;hsa-let-7 | AP1S1 |
| LINC00969 | hsa-miR-129-5p;hsa-m | GLCCI1 |
| MALAT1 | hsa-miR-129-5p;hsa-m | TMEM245 |
| RP11-498C9.15 | hsa-miR-125a-3p;hsa- | TBC1D13 |
| PKI55 | hsa-miR-1-3p;hsa-miR | RAB11FIP2 |
| RP11-631N16.2 | hsa-miR-30a-5p;hsa-m | PPP3CB |
| LINC00667 | hsa-miR-103a-3p;hsa- | CPEB3 |
| RP11-67L2.2 | hsa-miR-25-3p;hsa-mi | CPEB3 |
| PWAR6 | hsa-miR-15a-5p;hsa-m | CUL2 |
| C1orf132 | hsa-miR-15a-5p;hsa-m | CUL2 |
| RP11-488L18.10 | hsa-let-7a-5p;hsa-let-7 | FBXL20 |
| LINC01355 | hsa-miR-15a-5p;hsa-m | MTMR4 |
| MALAT1 | hsa-miR-10a-3p;hsa-m | AKAP10 |
| PWAR6 | hsa-miR-15a-5p;hsa-m | PEX12 |
| MALAT1 | hsa-miR-106b-5p;hsa- | EZH1 |
| NORAD | hsa-miR-106a-5p;hsa- | PRKAR1A |
| MALAT1 | hsa-miR-138-5p;hsa-m | NFKB1 |
| NEAT1 | hsa-miR-138-5p;hsa-m | NFKB1 |
| PWAR6 | hsa-miR-15a-5p;hsa-m | PPP6R3 |
| NORAD | hsa-let-7a-5p;hsa-let-7 | EIF4G2 |
| LINC00969 | hsa-miR-129-5p;hsa-m | EXPH5 |
| XIST | hsa-miR-1260a;hsa-mi | PRDM4 |
| DUBR | hsa-miR-1-3p;hsa-miR | CORO1C |
| MALAT1 | hsa-miR-106a-5p;hsa- | CAPRIN2 |
| NEAT1 | hsa-miR-106a-5p;hsa- | CAPRIN2 |
| XIST | hsa-miR-106b-5p;hsa- | PTGES3 |
| C1orf132 | hsa-miR-15a-5p;hsa-m | CREBL2 |
| LINC00667 | hsa-miR-148b-3p;hsa- | CDKN1B |
| RP11-67L2.2 | hsa-miR-196b-5p;hsa- | CDKN1B |
| AC004951.6 | hsa-miR-30a-3p;hsa-m | CDKN1B |
| GS1-124K5.11 | hsa-miR-15a-5p;hsa-m | NAA25 |
| NEAT1 | hsa-miR-1277-5p;hsa- | BTN3A3 |

| | | |
|---|---|---|
| LINC01355 | hsa-miR-15a-5p;hsa-m | BTN3A3 |
| RP11-352M15.2 | hsa-miR-21-5p;hsa-mi | E2F3 |
| NORAD | hsa-let-7g-3p;hsa-miR | PTP4A1 |
| LINC00667 | hsa-miR-181a-5p;hsa- | TMEM30A |
| RP11-121C2.2 | hsa-miR-106a-5p;hsa- | ENPP5 |
| PVT1 | hsa-miR-148a-3p;hsa- | MRPS27 |
| LINC00910 | hsa-miR-15a-5p;hsa-m | SEC24A |
| GS1-124K5.11 | hsa-miR-15a-5p;hsa-m | SEC24A |
| NEAT1 | hsa-miR-1267;hsa-miR | CPEB4 |
| MALAT1 | hsa-miR-1267;hsa-miR | CPEB4 |
| RP11-488L18.10 | hsa-miR-181a-5p;hsa- | CPEB4 |
| NORAD | hsa-miR-106a-5p;hsa- | MORC1 |
| NEAT1 | hsa-miR-122-5p;hsa-m | FOXP1 |
| XIST | hsa-miR-124-3p;hsa-m | INO80D |
| LINC00667 | hsa-miR-181a-5p;hsa- | INO80D |
| PKI55 | hsa-let-7a-5p;hsa-let- | SNX17 |
| MALAT1 | hsa-miR-193a-3p;hsa- | ASB3 |
| C1orf132 | hsa-miR-15a-5p;hsa-m | LANCL1 |
| PWAR6 | hsa-miR-15a-5p;hsa-m | LANCL1 |
| RP11-631N16.2 | hsa-miR-30a-5p;hsa-m | KDM3A |
| RP11-332H14.2 | hsa-miR-30b-5p;hsa-m | STRN |
| MALAT1 | hsa-miR-100-3p;hsa-m | SLC25A12 |
| NEAT1 | hsa-miR-100-3p;hsa-m | SLC25A12 |
| MALAT1 | hsa-miR-106b-5p;hsa- | AAK1 |
| RP11-78O7.2 | hsa-let-7a-5p;hsa-let- | PLEKHA3 |
| NEAT1 | hsa-let-7a-5p;hsa-let- | EPHA4 |
| MIRLET7BHG | hsa-miR-140-3p;hsa-m | RPL22 |
| RP11-67L2.2 | hsa-let-7d-5p;hsa-miR | MEF2D |
| PWAR6 | hsa-miR-145-5p;hsa-m | IVNS1ABP |
| NEAT1 | hsa-miR-107;hsa-miR- | RIMS3 |
| XIST | hsa-miR-106b-5p;hsa- | GBP3 |
| C1orf132 | hsa-miR-1224-3p;hsa- | RCAN3 |
| C1orf132 | hsa-miR-125b-5p;hsa- | RPS6KA1 |
| XIST | hsa-miR-132-3p;hsa-m | STAG1 |
| MALAT1 | hsa-miR-132-3p;hsa-m | STAG1 |
| LINC00106 | hsa-miR-1914-3p;hsa- | CREB1 |
| RP11-631N16.2 | hsa-miR-106b-5p;hsa- | ANKRD13C |
| RP11-631N16.2 | hsa-miR-106a-5p;hsa- | DNAL1 |
| XIST | hsa-miR-103a-3p;hsa- | TTLL5 |
| LINC00667 | hsa-miR-181a-5p;hsa- | RBM25 |
| RP11-67L2.2 | hsa-miR-137;hsa-miR- | SUPT7L |
| MEG3 | hsa-miR-106b-5p;hsa- | GPAM |
| RNU12 | hsa-miR-17-5p;hsa-mi | MXI1 |
| RP11-701H24.4 | hsa-miR-3065-5p;hsa- | HOXB3 |
| NEAT1 | hsa-miR-103a-2-5p;hs | CD274 |
| NEAT1 | hsa-miR-1271-5p;hsa- | NUP43 |
| MIRLET7BHG | hsa-miR-24-3p;hsa-mi | WDR55 |
| MALAT1 | hsa-let-7a-5p;hsa-let- | COIL |
| RP11-488L18.10 | hsa-let-7a-5p;hsa-let- | COIL |
| C1orf132 | hsa-miR-146a-3p;hsa- | CXCR4 |
| RP11-488L18.10 | hsa-let-7b-5p;hsa-let- | ZNF644 |
| NEAT1 | hsa-let-7b-5p;hsa-let- | ZNF644 |
| MALAT1 | hsa-miR-107;hsa-miR- | HNRNPA2B1 |
| NORAD | hsa-miR-101-3p;hsa-m | NT5C3A |

| | | |
|---|---|---|
| C17orf100 | hsa-let-7a-5p;hsa-let-7 | ACOT9 |
| MALAT1 | hsa-miR-1260a;hsa-mi | ORMDL2 |
| PWAR6 | hsa-miR-101-3p;hsa-m | RAP2C |
| MAPKAPK5-AS1 | hsa-miR-101-3p;hsa-m | RAP2C |
| MALAT1 | hsa-miR-103a-2-5p;hs | PLCG1 |
| RP11-332H14.2 | hsa-miR-30b-5p;hsa-m | STX16 |
| PWAR6 | hsa-miR-101-3p;hsa-m | STX16 |
| MAPKAPK5-AS1 | hsa-miR-101-3p;hsa-m | STX16 |
| C1orf132 | hsa-miR-146a-3p;hsa- | ZNF391 |
| MIRLET7BHG | hsa-miR-29a-3p;hsa-m | KLHDC3 |
| XIST | hsa-let-7a-5p;hsa-let-7 | GLO1 |
| LINC00662 | hsa-miR-15a-5p;hsa-m | SLC35B3 |
| NEAT1 | hsa-miR-103a-3p;hsa- | RUNX2 |
| PWAR6 | hsa-miR-15a-5p;hsa-m | TM9SF2 |
| RP11-78O7.2 | hsa-let-7a-5p;hsa-let-7 | OPA3 |
| C1orf132 | hsa-miR-15a-5p;hsa-m | TBC1D20 |
| PWAR6 | hsa-miR-15a-5p;hsa-m | TBC1D20 |
| RP11-458F8.4 | hsa-let-7a-5p;hsa-let-7 | PLAGL2 |
| LINC00667 | hsa-miR-30a-3p;hsa-m | BECN1 |
| XIST | hsa-miR-129-5p;hsa-m | ZNF384 |
| LINC00667 | hsa-miR-106b-5p;hsa- | ATG14 |
| XIST | hsa-miR-106b-5p;hsa- | ATG14 |
| LINC01355 | hsa-miR-15a-5p;hsa-m | IPPK |
| RP11-67L2.2 | hsa-miR-25-3p;hsa-mi | BCL11B |
| MIRLET7BHG | hsa-miR-24-3p;hsa-mi | RBM48 |
| MIR222HG | hsa-miR-34a-5p;hsa-m | MKLN1 |
| MALAT1 | hsa-miR-106b-5p;hsa- | VPS13C |
| NEAT1 | hsa-miR-106b-5p;hsa- | VPS13C |
| C1orf132 | hsa-miR-1226-3p;hsa- | MBD4 |
| RP11-67L2.2 | hsa-miR-137;hsa-miR- | SPCS3 |
| NORAD | hsa-miR-106a-5p;hsa- | EGLN3 |
| RP11-78O7.2 | hsa-let-7a-5p;hsa-let-7 | THEM6 |
| RP11-458F8.4 | hsa-let-7a-5p;hsa-let-7 | THEM6 |
| C1orf132 | hsa-miR-145-5p;hsa-m | SESN2 |
| MIR222HG | hsa-miR-1224-5p;hsa- | ZNF317 |
| NEAT1 | hsa-miR-106a-5p;hsa- | ZNF426 |
| XIST | hsa-miR-106a-5p;hsa- | RLIM |
| LINC00667 | hsa-miR-106b-5p;hsa- | RLIM |
| XIST | hsa-miR-1293;hsa-miR | DIAPH1 |
| FTX | hsa-miR-125a-5p;hsa- | RAF1 |
| XIST | hsa-miR-106a-5p;hsa- | RAN |
| LINC00910 | hsa-miR-15a-5p;hsa-m | PRKAA1 |
| RP11-498C9.15 | hsa-miR-548am-5p;hsa | ANKEF1 |
| MALAT1 | hsa-miR-1305;hsa-miR | AP3B1 |
| RP11-488L18.10 | hsa-miR-26a-5p;hsa-m | DCTN4 |
| PWAR6 | hsa-miR-17-5p;hsa-mi | RFXAP |
| NORAD | hsa-miR-106b-5p;hsa- | MKRN1 |
| MALAT1 | hsa-miR-25-3p;hsa-mi | ZFC3H1 |
| XIST | hsa-let-7a-5p;hsa-let-7 | MBD2 |
| LINC00969 | hsa-miR-15a-5p;hsa-m | CD180 |
| GS1-124K5.11 | hsa-miR-103a-3p;hsa- | CD180 |
| C17orf100 | hsa-let-7a-5p;hsa-let-7 | ARL8B |
| MALAT1 | hsa-miR-1271-5p;hsa- | NOTCH2 |
| XIST | hsa-miR-16-5p;hsa-mi | NAPG |

| | | |
|---|---|---|
| XIST | hsa-miR-1269b;hsa-mi | SLC38A2 |
| MALAT1 | hsa-miR-107;hsa-miR- | CLOCK |
| XIST | hsa-miR-107;hsa-miR- | CLOCK |
| LINC00910 | hsa-miR-15a-5p;hsa-m | ARHGAP32 |
| XIST | hsa-miR-139-5p;hsa-m | TMED7 |
| LINC00662 | hsa-miR-107;hsa-miR- | MAP3K7 |
| PKI55 | hsa-miR-15a-5p;hsa-m | MAP3K7 |
| RNU12 | hsa-miR-195-5p;hsa-m | CDK4 |
| MIRLET7BHG | hsa-miR-21-3p;hsa-mi | CPM |
| NEAT1 | hsa-miR-101-3p;hsa-m | DYNC1LI2 |
| LINC00667 | hsa-miR-103a-3p;hsa- | STX6 |
| XIST | hsa-miR-103a-3p;hsa- | STX6 |
| LINC01355 | hsa-miR-30a-5p;hsa-m | USP37 |
| PWAR6 | hsa-miR-15a-5p;hsa-m | SRSF1 |
| MALAT1 | hsa-miR-25-3p;hsa-mi | TANK |
| RP11-78O7.2 | hsa-let-7a-5p;hsa-let- | TRMO |
| C1orf132 | hsa-let-7a-5p;hsa-let- | MYC |
| RP11-631N16.2 | hsa-miR-519a-3p;hsa- | HADHB |
| MALAT1 | hsa-miR-1270;hsa-miR | LRPPRC |
| LINC01355 | hsa-miR-30a-5p;hsa-m | ENTPD1 |
| PWAR6 | hsa-miR-15a-5p;hsa-m | ASCC1 |
| MALAT1 | hsa-miR-103a-3p;hsa- | PPIG |
| LINC00667 | hsa-miR-1275;hsa-miR | SECISBP2L |
| MALAT1 | hsa-miR-127-3p;hsa-m | SEC31A |
| RP11-631N16.2 | hsa-miR-1277-5p;hsa- | G3BP2 |
| LINC00667 | hsa-miR-181a-5p;hsa- | SLC7A1 |
| LINC01355 | hsa-miR-30a-5p;hsa-m | LMBR1L |
| MIR222HG | hsa-miR-146a-5p;hsa- | ESD |
| MALAT1 | hsa-miR-106a-5p;hsa- | RB1 |
| XIST | hsa-miR-106b-5p;hsa- | WDR89 |
| MALAT1 | hsa-miR-106b-5p;hsa- | WDR89 |
| NORAD | hsa-miR-106a-5p;hsa- | BNIP2 |
| MALAT1 | hsa-miR-1271-5p;hsa- | GID4 |
| XIST | hsa-miR-197-3p;hsa-m | TAF4B |
| LINC00662 | hsa-miR-15a-5p;hsa-m | GNAL |
| PKI55 | hsa-miR-15a-5p;hsa-m | GNAL |
| XIST | hsa-miR-101-3p;hsa-m | SLC39A6 |
| PKI55 | hsa-miR-15a-5p;hsa-m | RAB40B |
| PWAR6 | hsa-miR-125a-5p;hsa- | SMAD4 |
| LINC00969 | hsa-miR-3613-3p;hsa- | BRD4 |
| NORAD | hsa-let-7a-5p;hsa-let- | APP |
| PWAR6 | hsa-miR-124-3p;hsa-m | ALDH9A1 |
| PWAR6 | hsa-miR-15a-5p;hsa-m | GOLPH3L |
| MALAT1 | hsa-miR-106a-5p;hsa- | CEP170 |
| LINC00667 | hsa-miR-19a-3p;hsa-m | SDE2 |
| RP11-67L2.2 | hsa-miR-25-3p;hsa-mi | C1orf35 |
| NEAT1 | hsa-miR-103a-3p;hsa- | EML4 |
| MALAT1 | hsa-miR-103a-3p;hsa- | EML4 |
| RP11-78O7.2 | hsa-let-7a-5p;hsa-let- | RABL2A |
| RP11-332H14.2 | hsa-miR-30a-5p;hsa-m | UBXN4 |
| LINC00910 | hsa-let-7a-5p;hsa-let- | POLR2D |
| MALAT1 | hsa-let-7a-5p;hsa-let- | GOLGA4 |
| RP11-78O7.2 | hsa-let-7a-5p;hsa-let- | GOLGA4 |
| NORAD | hsa-miR-100-5p;hsa-m | CTDSPL |

| | | |
|---|---|---|
| XIST | hsa-miR-181a-5p;hsa- | TMF1 |
| LINC00667 | hsa-miR-181a-5p;hsa- | TMF1 |
| LINC01355 | hsa-miR-15a-5p;hsa-m | SRPRB |
| C1orf132 | hsa-miR-1247-5p;hsa- | EIF2B5 |
| RP11-488L18.10 | hsa-let-7a-5p;hsa-let- | CDKAL1 |
| RP11-67L2.2 | hsa-let-7d-5p;hsa-miR | RNF44 |
| RP11-631N16.2 | hsa-miR-106a-5p;hsa- | PURB |
| NEAT1 | hsa-miR-106b-5p;hsa- | C7orf43 |
| LINC01355 | hsa-miR-30a-5p;hsa-m | C7orf43 |
| RP11-78O7.2 | hsa-let-7a-5p;hsa-let- | NOM1 |
| NORAD | hsa-miR-106a-5p;hsa- | NFIB |
| PWAR6 | hsa-miR-17-5p;hsa-mi | FBXO10 |
| LINC00667 | hsa-miR-143-3p;hsa-m | SLC25A25 |
| MEG3 | hsa-miR-149-3p;hsa-m | MIGA2 |
| MAPKAPK5-AS1 | hsa-miR-200b-3p;hsa- | ZEB1 |
| RP11-67L2.2 | hsa-miR-25-3p;hsa-mi | EIF4EBP2 |
| LINC00910 | hsa-miR-103a-3p;hsa- | LIN7C |
| RP11-488L18.10 | hsa-let-7a-5p;hsa-let- | CELF1 |
| NEAT1 | hsa-let-7f-2-3p;hsa-m | SESN3 |
| RP11-488L18.10 | hsa-miR-181a-5p;hsa- | ATM |
| MALAT1 | hsa-miR-101-3p;hsa-m | ATM |
| NORAD | hsa-miR-106a-5p;hsa- | LAMTOR1 |
| C1orf132 | hsa-miR-103a-3p;hsa- | EI24 |
| PWAR6 | hsa-miR-126-5p;hsa-m | ARID5B |
| RP11-67L2.2 | hsa-miR-25-3p;hsa-mi | TIRAP |
| RP11-488L18.10 | hsa-let-7a-5p;hsa-let- | KIAA1328 |
| XIST | hsa-miR-129-2-3p;hsa | PDCD4 |
| XIST | hsa-miR-1271-5p;hsa- | FOXO1 |
| MALAT1 | hsa-miR-199a-3p;hsa- | TMEM18 |
| LINC00667 | hsa-miR-181a-5p;hsa- | EPS8 |
| MALAT1 | hsa-miR-106b-5p;hsa- | VTI1A |
| MAPKAPK5-AS1 | hsa-miR-129-5p;hsa-m | ADAM17 |
| RP11-631N16.2 | hsa-miR-106a-5p;hsa- | RABGAP1L |
| RP11-332H14.2 | hsa-miR-30a-5p;hsa-m | EPG5 |
| RP11-488L18.10 | hsa-let-7a-5p;hsa-let- | TGOLN2 |
| FTX | hsa-let-7a-5p;hsa-let- | TGOLN2 |
| NEAT1 | hsa-let-7a-3p;hsa-let- | ZNF256 |
| C1orf132 | hsa-miR-126-5p;hsa-m | HNRNPDL |
| MALAT1 | hsa-miR-181a-5p;hsa- | LPCAT1 |
| NEAT1 | hsa-miR-124-3p;hsa-m | GTF2E1 |
| MALAT1 | hsa-miR-124-3p;hsa-m | GTF2E1 |
| MALAT1 | hsa-miR-27a-3p;hsa-m | CFDP1 |
| NORAD | hsa-miR-140-3p;hsa-m | DDAH1 |
| XIST | hsa-let-7a-5p;hsa-let- | MSI2 |
| PWAR6 | hsa-miR-143-3p;hsa-m | VOPP1 |
| MALAT1 | hsa-miR-298;hsa-miR- | VPS37A |
| XIST | hsa-miR-106b-5p;hsa- | USP16 |
| MALAT1 | hsa-miR-106b-5p;hsa- | USP16 |
| NORAD | hsa-miR-1277-5p;hsa- | NPTN |
| PWAR6 | hsa-miR-17-5p;hsa-mi | EIF4A2 |
| RP11-488L18.10 | hsa-let-7a-5p;hsa-let- | PAFAH2 |
| RP11-488L18.10 | hsa-let-7a-5p;hsa-let- | AHCYL2 |
| C1orf132 | hsa-let-7a-5p;hsa-let- | TMED4 |
| MALAT1 | hsa-miR-103a-3p;hsa- | ELK4 |

| | | |
|---|---|---|
| RP11-332H14.2 | hsa-miR-30a-5p;hsa-m | EPB41 |
| LINC00667 | hsa-miR-181a-5p;hsa- | EFCAB14 |
| MIRLET7BHG | hsa-miR-221-5p;hsa-m | PDXK |
| FTX | hsa-let-7a-5p;hsa-let- | TRAPPC10 |
| LINC01355 | hsa-miR-15a-5p;hsa-m | VAV2 |
| RP11-458F8.4 | hsa-let-7a-5p;hsa-let- | C19orf47 |
| RP11-488L18.10 | hsa-let-7a-5p;hsa-let- | C19orf47 |
| C1orf132 | hsa-let-7a-5p;hsa-let- | IL6R |
| RP11-488L18.10 | hsa-let-7a-5p;hsa-let- | IKZF3 |
| RP11-488L18.10 | hsa-let-7a-5p;hsa-let- | FMNL3 |
| PWAR6 | hsa-miR-15a-5p;hsa-m | RACGAP1 |
| SNHG8 | hsa-miR-124-3p;hsa-m | RAVER2 |
| RP11-631N16.2 | hsa-miR-106a-5p;hsa- | LZIC |
| LINC00667 | hsa-miR-103a-3p;hsa- | REL |
| LINC00910 | hsa-let-7a-5p;hsa-let- | PDLIM5 |
| LINC00969 | hsa-miR-129-5p;hsa-m | CDC42EP3 |
| MEG3 | hsa-miR-106a-5p;hsa- | ICA1L |
| LINC00667 | hsa-miR-181a-5p;hsa- | TOPBP1 |
| LINC00667 | hsa-miR-106b-5p;hsa- | FYCO1 |
| RP11-488L18.10 | hsa-miR-181a-5p;hsa- | PBRM1 |
| MALAT1 | hsa-miR-181a-5p;hsa- | PBRM1 |
| XIST | hsa-miR-103a-3p;hsa- | RNF168 |
| XIST | hsa-miR-186-5p;hsa-m | AIMP1 |
| MALAT1 | hsa-let-7a-5p;hsa-let- | MFSD8 |
| RP11-78O7.2 | hsa-let-7a-5p;hsa-let- | MFSD8 |
| RP11-284N8.3 | hsa-miR-19a-3p;hsa-m | OTUD4 |
| XIST | hsa-miR-101-5p;hsa-m | TMEM161B |
| NORAD | hsa-miR-106a-5p;hsa- | ANKRD33B |
| RP11-332H14.2 | hsa-miR-30a-5p;hsa-m | CASP3 |
| NEAT1 | hsa-miR-106b-5p;hsa- | ERAP1 |
| RP11-332H14.2 | hsa-miR-30a-5p;hsa-m | ANKRA2 |
| RP11-631N16.2 | hsa-miR-106b-5p;hsa- | BMT2 |
| NORAD | hsa-miR-204-5p;hsa-m | KIAA1324L |
| LINC00106 | hsa-miR-17-3p;hsa-mi | FOXK1 |
| NORAD | hsa-let-7a-5p;hsa-let- | YWHAZ |
| MALAT1 | hsa-miR-183-3p;hsa-m | INTS8 |
| LINC00667 | hsa-miR-181a-5p;hsa- | KIAA0196 |
| MALAT1 | hsa-miR-106a-5p;hsa- | ARHGAP12 |
| XIST | hsa-miR-106a-5p;hsa- | ARHGAP12 |
| LINC00467 | hsa-miR-130a-3p;hsa- | KBTBD6 |
| RP11-631N16.2 | hsa-miR-30a-5p;hsa-m | KBTBD6 |
| RP11-488L18.10 | hsa-let-7a-5p;hsa-let- | NSD1 |
| PVT1 | hsa-miR-27a-3p;hsa-m | NSD1 |
| RP11-488L18.10 | hsa-let-7a-5p;hsa-let- | PMPCA |
| FTX | hsa-let-7a-5p;hsa-let- | PMPCA |
| RP11-67L2.2 | hsa-miR-25-3p;hsa-mi | MOAP1 |
| C17orf100 | hsa-let-7a-5p;hsa-let- | POLL |
| PWAR6 | hsa-miR-101-3p;hsa-m | SGPL1 |
| NEAT1 | hsa-miR-106a-5p;hsa- | FRS2 |
| MALAT1 | hsa-miR-106a-5p;hsa- | FRS2 |
| RP11-631N16.2 | hsa-miR-106a-5p;hsa- | PRKCB |
| RP11-332H14.2 | hsa-miR-30a-5p;hsa-m | SLFN5 |
| RP11-284N8.3 | hsa-miR-23a-3p;hsa-m | TMEM170A |
| NEAT1 | hsa-miR-145-3p;hsa-m | SMAD3 |

| | | |
|---|---|---|
| MALAT1 | hsa-miR-106b-5p;hsa- | AKTIP |
| LINC00667 | hsa-miR-106b-5p;hsa- | FAM102A |
| SNHG8 | hsa-miR-19a-3p;hsa-m | CERCAM |
| PWAR6 | hsa-miR-17-5p;hsa-mi | CERCAM |
| MALAT1 | hsa-miR-106a-5p;hsa- | CRK |
| LINC01355 | hsa-miR-101-5p;hsa-m | TMEM68 |
| LINC00667 | hsa-miR-106b-5p;hsa- | ZNF597 |
| RP11-67L2.2 | hsa-miR-25-3p;hsa-mi | RBPJ |
| RP11-631N16.2 | hsa-miR-20a-5p;hsa-m | IRF2 |
| C1orf132 | hsa-let-7a-5p;hsa-let- | SEMA4C |
| NORAD | hsa-let-7a-5p;hsa-let- | ZBTB5 |
| LINC00667 | hsa-miR-548ak;hsa-mi | TNIP2 |
| RP11-488L18.10 | hsa-let-7a-5p;hsa-let- | CEP120 |
| SNHG8 | hsa-miR-19a-3p;hsa-m | MFF |
| RP11-332H14.2 | hsa-miR-30b-5p;hsa-m | CSNK1G1 |
| NORAD | hsa-miR-135a-5p;hsa- | PTK2 |
| NORAD | hsa-miR-101-3p;hsa-m | CLIC4 |
| MALAT1 | hsa-let-7d-5p;hsa-miR | ZEB2 |
| XIST | hsa-miR-148a-3p;hsa- | ALCAM |
| LINC01355 | hsa-miR-15a-5p;hsa-m | RALGAPB |
| RP11-488L18.10 | hsa-let-7a-5p;hsa-let- | STAT2 |
| NEAT1 | hsa-let-7a-5p;hsa-let- | STAT2 |
| XIST | hsa-miR-106a-5p;hsa- | USP32 |
| LINC01355 | hsa-miR-15a-5p;hsa-m | MSANTD4 |
| RP11-498C9.15 | hsa-miR-30b-5p;hsa-m | MSANTD4 |
| FTX | hsa-miR-17-5p;hsa-mi | MCC |
| RP11-498C9.15 | hsa-miR-106a-5p;hsa- | MCC |
| LINC01355 | hsa-miR-15a-5p;hsa-m | ASXL1 |
| PWAR6 | hsa-miR-15a-5p;hsa-m | PLRG1 |
| MALAT1 | hsa-miR-1304-3p;hsa- | ATF7IP |
| NEAT1 | hsa-miR-1304-3p;hsa- | ATF7IP |
| PWAR6 | hsa-miR-125b-5p;hsa- | BCL2 |
| XIST | hsa-miR-103a-3p;hsa- | PTEN |
| C1orf132 | hsa-miR-1299;hsa-miR | BSG |
| RP11-488L18.10 | hsa-let-7a-5p;hsa-let- | FNDC9 |
| C1orf132 | hsa-let-7a-5p;hsa-let- | FUT10 |
| RP11-67L2.2 | hsa-let-7d-5p;hsa-miR | FUT10 |
| LINC00667 | hsa-miR-181a-5p;hsa- | FAM192A |
| C1orf132 | hsa-miR-124-3p;hsa-m | TADA2B |
| MALAT1 | hsa-miR-129-5p;hsa-m | PARP15 |
| RP11-332H14.2 | hsa-miR-30a-5p;hsa-m | IQCB1 |
| XIST | hsa-let-7a-5p;hsa-let- | NAA20 |
| RP11-67L2.2 | hsa-let-7d-5p;hsa-miR | ZNF417 |
| GS1-124K5.11 | hsa-miR-15a-5p;hsa-m | ZNF622 |
| LINC00969 | hsa-miR-15a-5p;hsa-m | ZNF622 |
| MALAT1 | hsa-miR-129-5p;hsa-m | HEG1 |
| MALAT1 | hsa-miR-15a-5p;hsa-m | PHC3 |
| RP11-332H14.2 | hsa-miR-30b-5p;hsa-m | ATP2A2 |
| LINC00467 | hsa-miR-27a-3p;hsa-m | GCSAM |
| LINC01355 | hsa-miR-27a-3p;hsa-m | GCSAM |
| XIST | hsa-let-7a-5p;hsa-let- | CEP135 |
| LINC01355 | hsa-miR-15a-5p;hsa-m | KLC2 |
| LINC00910 | hsa-let-7a-5p;hsa-let- | MARCKSL1 |
| RP11-67L2.2 | hsa-miR-25-3p;hsa-mi | GOLGA8A |

| | | |
|---|---|---|
| RP11-67L2.2 | hsa-miR-137;hsa-miR- | LONRF3 |
| XIST | hsa-let-7a-5p;hsa-let-7 | ZBTB8OS |
| PWAR6 | hsa-miR-145-5p;hsa-m | RPS6KA3 |
| MALAT1 | hsa-miR-106a-5p;hsa- | NR2C2 |
| MIRLET7BHG | hsa-miR-221-5p;hsa-m | AGTRAP |
| RP11-332H14.2 | hsa-miR-30a-5p;hsa-m | FUCA1 |
| NEAT1 | hsa-miR-30a-5p;hsa-m | FUCA1 |
| NORAD | hsa-miR-29a-3p;hsa-m | ZFPM1 |
| RP11-631N16.2 | hsa-miR-519a-3p;hsa- | PAK2 |
| NEAT1 | hsa-miR-1271-5p;hsa- | MIGA1 |
| LINC00667 | hsa-miR-106b-5p;hsa- | YOD1 |
| XIST | hsa-miR-106b-5p;hsa- | CMTR2 |
| RP11-67L2.2 | hsa-miR-25-3p;hsa-mi | AEN |
| MALAT1 | hsa-miR-1306-5p;hsa- | ZBTB20 |
| NEAT1 | hsa-miR-19a-3p;hsa-m | ZNF721 |
| LINC01355 | hsa-miR-30a-5p;hsa-m | FANCF |
| LINC00969 | hsa-miR-15a-5p;hsa-m | SMDT1 |
| NEAT1 | hsa-let-7a-3p;hsa-let-7 | RBM12B |
| FTX | hsa-let-7a-5p;hsa-let-7 | RBM12B |
| NEAT1 | hsa-miR-15a-5p;hsa-m | VPS33B |
| RP11-78O7.2 | hsa-let-7a-5p;hsa-let-7 | BRI3BP |
| RP11-488L18.10 | hsa-let-7a-5p;hsa-let-7 | ZBTB37 |
| GS1-124K5.11 | hsa-miR-212-5p;hsa-m | SP140L |
| MALAT1 | hsa-miR-124-3p;hsa-m | SP1 |
| LINC00106 | hsa-miR-124-3p;hsa-m | SP1 |
| PWAR6 | hsa-miR-15a-5p;hsa-m | ZNF267 |
| LINC01355 | hsa-miR-15a-5p;hsa-m | GPATCH8 |
| NEAT1 | hsa-miR-124-3p;hsa-m | GPATCH8 |
| MIR222HG | hsa-miR-26a-5p;hsa-m | UBE2H |
| C17orf100 | hsa-let-7a-5p;hsa-let-7 | RHD |
| PKI55 | hsa-let-7a-5p;hsa-let-7 | RHD |
| NORAD | hsa-let-7a-5p;hsa-let-7 | NAP1L1 |
| RP11-332H14.2 | hsa-miR-30a-5p;hsa-m | PTAR1 |
| PWAR6 | hsa-miR-15a-5p;hsa-m | RPL14 |
| MALAT1 | hsa-miR-15a-5p;hsa-m | MSL1 |
| LL22NC03-2H8.5 | hsa-let-7a-5p;hsa-let-7 | RNFT1 |
| RP11-67L2.2 | hsa-miR-25-3p;hsa-mi | NUGGC |
| LINC01355 | hsa-miR-15a-5p;hsa-m | SLC35E2B |
| LINC00667 | hsa-miR-181a-5p;hsa- | BLOC1S2 |
| RP11-332H14.2 | hsa-miR-1224-3p;hsa- | ZNF699 |
| RP11-67L2.2 | hsa-miR-101-5p;hsa-m | LCOR |
| FTX | hsa-miR-17-5p;hsa-mi | ZNF107 |
| PWAR6 | hsa-miR-101-3p;hsa-m | TSC22D2 |
| PKI55 | hsa-let-7a-5p;hsa-let-7 | ZNF799 |
| XIST | hsa-miR-107;hsa-miR- | NF1 |
| MIRLET7BHG | hsa-miR-24-3p;hsa-mi | PPTC7 |
| RP11-488L18.10 | hsa-let-7a-5p;hsa-let-7 | NHLRC2 |
| RP11-498C9.15 | hsa-miR-548am-5p;hsa | FAM3C |
| RP11-67L2.2 | hsa-miR-137;hsa-miR- | FUT11 |
| CTD-2587M2.1 | hsa-miR-25-3p;hsa-mi | IPP |
| RP11-631N16.2 | hsa-miR-106a-5p;hsa- | RPF2 |
| XIST | hsa-miR-106b-5p;hsa- | TOPORS |
| RP11-488L18.10 | hsa-let-7a-5p;hsa-let-7 | ZNF460 |
| MIRLET7BHG | hsa-miR-24-3p;hsa-mi | SGTB |

| | | |
|---|---|---|
| RP11-67L2.2 | hsa-miR-25-3p;hsa-mi | FAM49A |
| MEG3 | hsa-miR-106a-5p;hsa- | NKIRAS1 |
| NORAD | hsa-let-7a-5p;hsa-let- | HIST1H2BK |
| RP11-78O7.2 | hsa-let-7a-5p;hsa-let- | PLCG2 |
| MALAT1 | hsa-miR-126-5p;hsa-m | ITSN2 |
| NEAT1 | hsa-let-7a-3p;hsa-let- | ZNF652 |
| RP11-67L2.2 | hsa-miR-25-3p;hsa-mi | TMEM184B |
| NEAT1 | hsa-miR-125a-5p;hsa- | MTOR |
| XIST | hsa-miR-1224-5p;hsa- | ZNF277 |
| RP11-701H24.4 | hsa-miR-106a-5p;hsa- | FICD |
| NEAT1 | hsa-miR-106a-5p;hsa- | FICD |
| NORAD | hsa-miR-106a-5p;hsa- | CAPZA2 |
| RP11-67L2.2 | hsa-miR-25-3p;hsa-mi | TATDN3 |
| PKI55 | hsa-let-7a-5p;hsa-let- | PHACTR4 |
| FTX | hsa-let-7a-5p;hsa-let- | PHACTR4 |
| RP11-78O7.2 | hsa-let-7a-5p;hsa-let- | PBX2 |
| RP11-67L2.2 | hsa-miR-25-3p;hsa-mi | TBC1D8 |
| RP11-488L18.10 | hsa-let-7a-5p;hsa-let- | ATXN2 |
| RP11-631N16.2 | hsa-miR-106b-5p;hsa- | FBXO48 |
| LINC00969 | hsa-miR-1283;hsa-miR | C5orf51 |
| XIST | hsa-let-7a-5p;hsa-let- | NRAS |
| XIST | hsa-miR-15a-5p;hsa-m | CHUK |
| MALAT1 | hsa-miR-15a-5p;hsa-m | CPNE1 |
| RP11-332H14.2 | hsa-miR-378a-3p;hsa- | HAUS3 |
| LINC00910 | hsa-let-7a-5p;hsa-let- | UBXN2B |
| RP11-458F8.4 | hsa-let-7a-5p;hsa-let- | TIAF1 |
| RP11-631N16.2 | hsa-miR-106a-5p;hsa- | SMIM13 |
| RP11-701H24.4 | hsa-miR-106a-5p;hsa- | CDKN2AIPNL |
| NEAT1 | hsa-miR-106a-5p;hsa- | CDKN2AIPNL |
| XIST | hsa-miR-21-5p;hsa-mi | RNF103 |
| GS1-124K5.11 | hsa-miR-15a-5p;hsa-m | PISD |
| RP11-67L2.2 | hsa-miR-25-3p;hsa-mi | C15orf38-AP3S2 |
| NORAD | hsa-miR-106a-5p;hsa- | CCDC71L |
| NEAT1 | hsa-miR-130a-3p;hsa- | ZNF224 |
| LINC00969 | hsa-miR-129-5p;hsa-m | MLLT6 |
| MALAT1 | hsa-miR-103a-3p;hsa- | SYNRG |
| XIST | hsa-let-7b-5p;hsa-miR | UHRF1 |

| lncRNA | miRNAs | mRNA |
|---|---|---|
| RP11-631N16.2 | hsa-miR-106a-5p; | M6PR |
| RNU12 | hsa-miR-17-5p;hs | VPS50 |
| RP11-631N16.2 | hsa-miR-106a-5p; | VPS50 |
| LINC00667 | hsa-miR-103a-3p; | DHX33 |
| RP11-488L18.10 | hsa-let-7a-5p;hsa | ITGA3 |
| RP11-78O7.2 | hsa-let-7a-5p;hsa | ITGA3 |
| PKI55 | hsa-let-7a-5p;hsa | ITGA3 |
| RP11-1055B8.4 | hsa-miR-199a-3p; | ITGA3 |
| AC009404.2 | hsa-let-7a-5p;hsa | ITGA3 |
| RP11-121C2.2 | hsa-let-7a-5p;hsa | ITGA3 |
| RNU12 | hsa-miR-17-5p;hs | MAP3K14 |
| LINC00667 | hsa-miR-106b-5p | MAP3K14 |
| RP11-631N16.2 | hsa-miR-106b-5p | MAP3K14 |
| MIRLET7BHG | hsa-miR-30c-1-3p | TBXA2R |
| MEG3 | hsa-miR-106b-5p | PNPLA4 |
| RP11-701H24.4 | hsa-miR-125b-5p | E2F2 |
| RP11-121C2.2 | hsa-let-7a-5p;hsa | E2F2 |
| PWAR6 | hsa-miR-125b-5p | E2F2 |
| MEG3 | hsa-let-7a-5p;hsa | E2F2 |
| PKI55 | hsa-let-7a-5p;hsa | E2F2 |
| LINC00969 | hsa-miR-15a-5p;h | SYPL1 |
| RP11-332H14.2 | hsa-miR-30a-5p;h | CELSR3 |
| RP11-121C2.2 | hsa-miR-548ak;hsa | CELSR3 |
| RNU12 | hsa-miR-548a-5p; | PKD1 |
| RP11-498C9.15 | hsa-miR-377-3p;h | PKD1 |
| RP11-631N16.2 | hsa-miR-106b-5p | NCAPD2 |
| RP11-631N16.2 | hsa-miR-1277-5p; | RABGAP1 |
| RNU12 | hsa-miR-15a-5p;h | BRCA1 |
| RNU12 | hsa-miR-150-5p;h | MAN2B2 |
| RP11-631N16.2 | hsa-miR-106a-5p; | MAN2B2 |
| LINC00467 | hsa-miR-130a-3p; | RRAGD |
| RP11-701H24.4 | hsa-miR-106a-5p; | RRAGD |
| SNHG8 | hsa-miR-19a-3p;h | RRAGD |
| MEG3 | hsa-miR-106a-5p; | RRAGD |
| PWAR6 | hsa-miR-17-5p;hs | RRAGD |
| LINC00630 | hsa-miR-25-3p;hs | B4GALT7 |
| CTD-2587M2.1 | hsa-miR-25-3p;hs | B4GALT7 |
| RP11-631N16.2 | hsa-miR-1277-5p; | TBPL1 |
| LINC00667 | hsa-miR-103a-3p; | TBPL1 |
| PWAR6 | hsa-miR-15a-5p;h | TFB1M |
| LINC01355 | hsa-miR-15a-5p;h | TFB1M |
| GS1-124K5.11 | hsa-miR-15a-5p;h | TFB1M |
| PKI55 | hsa-miR-15a-5p;h | TFB1M |
| MEG3 | hsa-miR-15a-5p;h | TFB1M |
| PKI55 | hsa-miR-21-5p;hs | FAM136A |
| LINC00969 | hsa-miR-15a-5p;h | PHLPP2 |
| RNU12 | hsa-miR-15a-5p;h | PHLPP2 |
| RP11-631N16.2 | hsa-miR-106b-5p | PHLPP2 |
| RP11-701H24.4 | hsa-miR-106a-5p; | GEMIN8 |
| MEG3 | hsa-miR-106a-5p; | GEMIN8 |
| RP11-121C2.2 | hsa-let-7a-5p;hsa | C12orf4 |
| RP11-488L18.10 | hsa-let-7a-5p;hsa | C12orf4 |
| AC009404.2 | hsa-let-7a-5p;hsa | C12orf4 |
| PKI55 | hsa-let-7a-5p;hsa | C12orf4 |
| RP11-78O7.2 | hsa-let-7a-5p;hsa | C12orf4 |
| RP11-631N16.2 | hsa-miR-106b-5p | USP28 |
| LINC00467 | hsa-miR-130a-3p; | RFC2 |

| | | |
|---|---|---|
| RP11-78O7.2 | hsa-let-7a-5p;hsa | RFC2 |
| RP11-121C2.2 | hsa-let-7a-5p;hsa | RFC2 |
| AC009404.2 | hsa-let-7a-5p;hsa | RFC2 |
| RP11-488L18.10 | hsa-let-7a-5p;hsa | RFC2 |
| RP11-631N16.2 | hsa-miR-519a-3p; | MCUR1 |
| RP11-701H24.4 | hsa-miR-106a-5p; | TNIP3 |
| MEG3 | hsa-miR-106a-5p; | TNIP3 |
| RP11-631N16.2 | hsa-miR-106b-5p | SIKE1 |
| LINC00667 | hsa-miR-106b-5p | SIKE1 |
| GS1-124K5.11 | hsa-miR-30c-1-3p | MCF2L2 |
| AC009404.2 | hsa-let-7a-5p;hsa | MCF2L2 |
| RP11-488L18.10 | hsa-let-7a-5p;hsa | MCF2L2 |
| RP11-121C2.2 | hsa-let-7a-5p;hsa | MCF2L2 |
| LINC00630 | hsa-miR-25-3p;hs | MCF2L2 |
| MEG3 | hsa-let-7a-5p;hsa | MCF2L2 |
| CTD-2587M2.1 | hsa-miR-25-3p;hs | MCF2L2 |
| RP11-78O7.2 | hsa-let-7a-5p;hsa | MCF2L2 |
| MIRLET7BHG | hsa-miR-30c-1-3p | MCF2L2 |
| MIRLET7BHG | hsa-miR-24-3p;hs | PLEKHH1 |
| RP11-631N16.2 | hsa-miR-106b-5p | ZNF280C |
| RP11-701H24.4 | hsa-miR-106a-5p; | TRAF3IP2 |
| MEG3 | hsa-miR-106a-5p; | TRAF3IP2 |
| SNHG8 | hsa-miR-197-3p;h | DCBLD2 |
| LINC00467 | hsa-miR-130a-3p; | DCBLD2 |
| RP11-79H23.3 | hsa-miR-181a-5p; | DCBLD2 |
| RP11-701H24.4 | hsa-miR-101-3p;h | DCBLD2 |
| MEG3 | hsa-miR-106b-5p | DCBLD2 |
| PWAR6 | hsa-miR-101-3p;h | DCBLD2 |
| PKI55 | hsa-miR-101-3p;h | DCBLD2 |
| LINC00667 | hsa-miR-19a-3p;h | ATG5 |
| LINC00969 | hsa-miR-15a-5p;h | TM7SF3 |
| RP11-332H14.2 | hsa-miR-378a-3p; | SPA17 |
| RP11-631N16.2 | hsa-miR-106b-5p | RFXANK |
| RP11-631N16.2 | hsa-miR-30a-5p;h | BTBD1 |
| RP11-352M15.2 | hsa-miR-126-5p;h | BTBD1 |
| RP11-631N16.2 | hsa-miR-30a-5p;h | UHRF1BP1 |
| RP11-498C9.15 | hsa-miR-548am-5 | MAP2K4 |
| RNU12 | hsa-miR-10a-3p;h | MAP2K4 |
| RP11-631N16.2 | hsa-miR-30a-5p;h | SEC61A2 |
| RP11-701H24.4 | hsa-miR-106a-5p; | ISOC1 |
| MEG3 | hsa-miR-106a-5p; | ISOC1 |
| MIRLET7BHG | hsa-miR-188-3p;h | SDK2 |
| LL22NC03-2H8.5 | hsa-let-7a-5p;hsa | NUCB2 |
| PWAR6 | hsa-miR-19a-3p;h | PFN2 |
| LINC01355 | hsa-miR-30a-5p;h | VASH1 |
| RP11-332H14.2 | hsa-miR-30a-5p;h | VASH1 |
| RP11-631N16.2 | hsa-miR-106a-5p; | TXK |
| RNU12 | hsa-miR-150-5p;h | TXK |
| CTD-2587M2.1 | hsa-miR-137;hsa- | GRAMD4 |
| LINC00630 | hsa-miR-25-3p;hs | GRAMD4 |
| LINC00667 | hsa-miR-19a-3p;h | UBE2A |
| RP11-332H14.2 | hsa-miR-30a-5p;h | OPHN1 |
| LINC01355 | hsa-miR-30a-5p;h | OPHN1 |
| LL22NC03-2H8.5 | hsa-let-7a-5p;hsa | RABL2B |
| LINC00667 | hsa-miR-181a-5p; | ATP8B1 |
| LL22NC03-2H8.5 | hsa-let-7a-5p;hsa | NOA1 |
| CTD-2587M2.1 | hsa-miR-25-3p;hs | AGBL5 |
| LINC00630 | hsa-miR-25-3p;hs | AGBL5 |

| | | |
|---|---|---|
| RP11-78O7.2 | hsa-let-7a-5p;hsa | CD59 |
| PKI55 | hsa-let-7a-5p;hsa | CD59 |
| RP11-121C2.2 | hsa-let-7a-5p;hsa | CD59 |
| AC009404.2 | hsa-let-7a-5p;hsa | CD59 |
| RP11-488L18.10 | hsa-let-7a-5p;hsa | CD59 |
| LINC00467 | hsa-miR-130a-3p; | CHERP |
| RP11-631N16.2 | hsa-miR-1277-5p; | NLK |
| LINC01355 | hsa-miR-101-3p;h | L2HGDH |
| GS1-124K5.11 | hsa-miR-103a-3p; | L2HGDH |
| PKI55 | hsa-miR-101-3p;h | L2HGDH |
| RP11-121C2.2 | hsa-miR-548ak;hsa | KHSRP |
| RP11-701H24.4 | hsa-miR-106a-5p; | PDRG1 |
| SNHG8 | hsa-miR-18a-3p;h | PDRG1 |
| LINC00969 | hsa-miR-137;hsa- | FKBP1A |
| RP11-631N16.2 | hsa-miR-1277-5p; | FKBP1A |
| LINC00667 | hsa-miR-181a-5p; | FKBP1A |
| GS1-124K5.11 | hsa-miR-15a-5p;h | TASP1 |
| LINC01355 | hsa-miR-15a-5p;h | TASP1 |
| PKI55 | hsa-miR-15a-5p;h | TASP1 |
| PWAR6 | hsa-miR-15a-5p;h | TASP1 |
| MEG3 | hsa-miR-15a-5p;h | TASP1 |
| RP11-701H24.4 | hsa-miR-16-1-3p; | TFAP4 |
| RNU12 | hsa-miR-130a-5p; | ZNF268 |
| RP11-631N16.2 | hsa-miR-30a-5p;h | SEMA6A |
| LINC00667 | hsa-miR-103a-3p; | SEMA6A |
| MEG3 | hsa-miR-149-3p;h | CLSPN |
| PKI55 | hsa-miR-15a-5p;h | CLSPN |
| LINC01355 | hsa-miR-15a-5p;h | CLSPN |
| GS1-124K5.11 | hsa-miR-15a-5p;h | CLSPN |
| LL22NC03-2H8.5 | hsa-let-7a-5p;hsa | ECHDC1 |
| LINC00969 | hsa-miR-200b-3p | ERMP1 |
| RP11-1055B8.4 | hsa-miR-199a-5p; | POLR2F |
| RP11-631N16.2 | hsa-miR-30a-5p;h | SBF1 |
| RP11-78O7.2 | hsa-let-7a-5p;hsa | PDGFB |
| RP11-701H24.4 | hsa-miR-106a-5p; | PDGFB |
| RP11-488L18.10 | hsa-let-7a-5p;hsa | PDGFB |
| RP11-121C2.2 | hsa-let-7a-5p;hsa | PDGFB |
| AC009404.2 | hsa-let-7a-5p;hsa | PDGFB |
| PKI55 | hsa-let-7a-5p;hsa | PDGFB |
| MEG3 | hsa-let-7a-5p;hsa | PDGFB |
| RP11-701H24.4 | hsa-miR-106a-5p; | FOXRED2 |
| LL22NC03-2H8.5 | hsa-let-7a-5p;hsa | KIAA0930 |
| RP11-631N16.2 | hsa-miR-106b-5p | VCPKMT |
| LINC00969 | hsa-miR-10a-3p;h | GNPNAT1 |
| RP11-631N16.2 | hsa-miR-106a-5p; | ZFYVE21 |
| RP11-498C9.15 | hsa-miR-548am-5 | MTHFD1 |
| RNU12 | hsa-miR-548a-5p; | MTHFD1 |
| RP11-79H23.3 | hsa-miR-181a-5p; | GSKIP |
| PWAR6 | hsa-miR-132-3p;h | MMP9 |
| PKI55 | hsa-miR-132-3p;h | MMP9 |
| MEG3 | hsa-miR-132-3p;h | MMP9 |
| RP11-701H24.4 | hsa-miR-519a-3p; | ABHD12 |
| MEG3 | hsa-let-7g-3p;hsa | ABHD12 |
| LINC01355 | hsa-miR-30a-5p;h | MYBL2 |
| RP11-332H14.2 | hsa-miR-30a-5p;h | MYBL2 |
| RP11-701H24.4 | hsa-miR-519a-3p; | SEC23B |
| RP11-498C9.15 | hsa-miR-548am-5 | CRNKL1 |
| RNU12 | hsa-miR-3613-3p; | CRNKL1 |

| | | |
|---|---|---|
| LINC00969 | hsa-miR-15a-5p;h | RPRD1B |
| RNU12 | hsa-miR-130a-5p; | STS |
| LL22NC03-2H8.5 | hsa-let-7a-5p;hsa | PGRMC1 |
| LINC00467 | hsa-miR-130a-3p; | NKAP |
| CTD-2587M2.1 | hsa-miR-25-3p;hs | NKAP |
| LINC00630 | hsa-miR-25-3p;hs | NKAP |
| RP11-631N16.2 | hsa-miR-106b-5p | RBBP7 |
| LINC00969 | hsa-miR-15a-5p;h | KPNA3 |
| RNU12 | hsa-miR-15a-5p;h | KPNA3 |
| RP11-631N16.2 | hsa-miR-106a-5p; | KATNAL1 |
| LINC00667 | hsa-miR-19a-3p;h | KATNAL1 |
| RP11-498C9.15 | hsa-miR-106a-5p; | KATNAL1 |
| RNU12 | hsa-miR-15a-5p;h | KATNAL1 |
| RP11-1055B8.4 | hsa-miR-30b-3p;h | PLLP |
| RP11-631N16.2 | hsa-miR-106a-5p; | DHODH |
| RP11-78O7.2 | hsa-let-7a-5p;hsa | SLC38A7 |
| RP11-332H14.2 | hsa-miR-30a-5p;h | SLC38A7 |
| RP11-121C2.2 | hsa-let-7a-5p;hsa | SLC38A7 |
| MEG3 | hsa-let-7a-5p;hsa | SLC38A7 |
| RP11-488L18.10 | hsa-let-7a-5p;hsa | SLC38A7 |
| LINC01355 | hsa-miR-193b-5p | SLC38A7 |
| AC009404.2 | hsa-let-7a-5p;hsa | SLC38A7 |
| RP11-701H24.4 | hsa-miR-519a-3p; | HAS3 |
| MEG3 | hsa-miR-106a-5p; | ZNF174 |
| RP11-701H24.4 | hsa-miR-106a-5p; | ZNF174 |
| RNU12 | hsa-miR-15a-5p;h | USP31 |
| LINC00969 | hsa-miR-15a-5p;h | USP31 |
| RP11-631N16.2 | hsa-miR-106a-5p; | STX4 |
| MEG3 | hsa-miR-214-3p;h | HOMER2 |
| LINC00969 | hsa-miR-194-3p;h | EMC2 |
| GS1-124K5.11 | hsa-miR-15a-5p;h | SNX16 |
| RP11-631N16.2 | hsa-miR-106a-5p; | MTMR9 |
| RNU12 | hsa-miR-150-5p;h | MTMR9 |
| RP11-631N16.2 | hsa-miR-106a-5p; | DCTN6 |
| MEG3 | hsa-let-7b-5p;hsa | GYS1 |
| LINC00969 | hsa-miR-15a-5p;h | DMPK |
| RNU12 | hsa-miR-15a-5p;h | DMPK |
| RP11-488L18.10 | hsa-let-7a-5p;hsa | C19orf53 |
| AC009404.2 | hsa-let-7a-5p;hsa | C19orf53 |
| RP11-121C2.2 | hsa-let-7a-5p;hsa | C19orf53 |
| PKI55 | hsa-let-7a-5p;hsa | C19orf53 |
| RP11-78O7.2 | hsa-let-7a-5p;hsa | C19orf53 |
| GS1-124K5.11 | hsa-miR-15a-5p;h | CCNE1 |
| PKI55 | hsa-miR-15a-5p;h | CCNE1 |
| MEG3 | hsa-miR-15a-5p;h | CCNE1 |
| PWAR6 | hsa-miR-15a-5p;h | CCNE1 |
| LINC01355 | hsa-miR-15a-5p;h | CCNE1 |
| RP11-1055B8.4 | hsa-miR-125a-3p; | TIMM50 |
| LINC00467 | hsa-miR-130a-3p; | TIMM50 |
| RP11-78O7.2 | hsa-let-7a-5p;hsa | PLD3 |
| RP11-488L18.10 | hsa-let-7a-5p;hsa | PLD3 |
| PKI55 | hsa-let-7a-5p;hsa | PLD3 |
| RP11-1055B8.4 | hsa-miR-185-5p;h | PLD3 |
| RP11-121C2.2 | hsa-let-7a-5p;hsa | PLD3 |
| AC009404.2 | hsa-let-7a-5p;hsa | PLD3 |
| MEG3 | hsa-miR-106a-5p; | SLC1A5 |
| SNHG8 | hsa-miR-186-3p;h | DNASE2 |
| MEG3 | hsa-miR-106a-5p; | SUGP1 |

| | | |
|---|---|---|
| PWAR6 | hsa-miR-17-5p;hs | SUGP1 |
| RP11-701H24.4 | hsa-miR-106a-5p; | SUGP1 |
| LINC00630 | hsa-miR-25-3p;hs | ITGB8 |
| CTD-2587M2.1 | hsa-miR-25-3p;hs | ITGB8 |
| RNU12 | hsa-miR-130a-5p; | MPP6 |
| RP11-631N16.2 | hsa-miR-106a-5p; | FKBP14 |
| LL22NC03-2H8.5 | hsa-let-7a-5p;hsa | AP1S1 |
| LINC00969 | hsa-miR-200b-3p | PHF14 |
| RP11-631N16.2 | hsa-miR-519a-3p; | LIMK1 |
| MEG3 | hsa-miR-136-5p;h | FSD1L |
| RP11-79H23.3 | hsa-miR-181a-5p; | FSD1L |
| LINC00667 | hsa-miR-181a-5p; | TBC1D13 |
| RNU12 | hsa-miR-15b-3p;h | TBC1D13 |
| RP11-498C9.15 | hsa-miR-125a-3p; | TBC1D13 |
| RP11-631N16.2 | hsa-miR-181a-5p; | TBC1D13 |
| RNU12 | hsa-miR-15a-5p;h | KANK1 |
| LINC00969 | hsa-miR-15a-5p;h | KANK1 |
| RNU12 | hsa-miR-15a-5p;h | RAB11FIP2 |
| LINC00969 | hsa-miR-1-3p;hsa | RAB11FIP2 |
| RP11-631N16.2 | hsa-miR-30a-5p;h | PPP3CB |
| RNU12 | hsa-miR-520h;hsa | LIPA |
| LINC00667 | hsa-miR-548ak;hs | LIPA |
| RP11-498C9.15 | hsa-miR-548am-5 | LIPA |
| GS1-124K5.11 | hsa-miR-103a-3p; | CPEB3 |
| MEG3 | hsa-miR-103a-3p; | CPEB3 |
| PKI55 | hsa-miR-15a-5p;h | CPEB3 |
| PWAR6 | hsa-miR-15a-5p;h | CPEB3 |
| LINC01355 | hsa-miR-15a-5p;h | CPEB3 |
| CTD-2587M2.1 | hsa-miR-25-3p;hs | CPEB3 |
| LINC00630 | hsa-miR-25-3p;hs | CPEB3 |
| LINC00667 | hsa-miR-106b-5p | MTPAP |
| RP11-631N16.2 | hsa-miR-106a-5p; | MTPAP |
| LINC01355 | hsa-miR-30a-5p;h | SH3PXD2A |
| AC004951.6 | hsa-miR-30a-3p;h | SH3PXD2A |
| RP11-332H14.2 | hsa-miR-30a-5p;h | SH3PXD2A |
| LINC00969 | hsa-miR-15a-5p;h | MTMR4 |
| RNU12 | hsa-miR-15a-5p;h | MTMR4 |
| RP11-631N16.2 | hsa-miR-106a-5p; | CBX1 |
| LINC01006 | hsa-miR-23b-5p;h | C17orf75 |
| RP11-701H24.4 | hsa-miR-106a-5p; | C17orf75 |
| MEG3 | hsa-miR-106a-5p; | C17orf75 |
| CTD-2587M2.1 | hsa-miR-25-3p;hs | C17orf75 |
| LINC00630 | hsa-miR-25-3p;hs | C17orf75 |
| RP11-78O7.2 | hsa-let-7a-5p;hsa | TBC1D19 |
| RP11-121C2.2 | hsa-let-7a-5p;hsa | TBC1D19 |
| RP11-488L18.10 | hsa-let-7a-5p;hsa | TBC1D19 |
| AC009404.2 | hsa-let-7a-5p;hsa | TBC1D19 |
| LINC00667 | hsa-miR-181a-5p; | WHSC1 |
| RNU12 | hsa-miR-15a-5p;h | ZBTB16 |
| LINC00969 | hsa-miR-15a-5p;h | ZBTB16 |
| RP11-121C2.2 | hsa-miR-548ak;hs | PANX1 |
| MIRLET7BHG | hsa-miR-29a-3p;h | NAA40 |
| RP11-37B2.1 | hsa-let-7a-5p;hsa | SLC11A2 |
| RNU12 | hsa-miR-15a-5p;h | CREBL2 |
| RP11-701H24.4 | hsa-miR-106a-5p; | GTF2H3 |
| LINC00667 | hsa-miR-181a-5p; | STX2 |
| RNU12 | hsa-miR-130a-5p; | USP5 |
| GS1-124K5.11 | hsa-miR-103a-3p; | HCFC2 |

| | | |
|---|---|---|
| MEG3 | hsa-miR-103a-3p; | HCFC2 |
| PWAR6 | hsa-miR-15a-5p;h | HCFC2 |
| LINC01355 | hsa-miR-15a-5p;h | HCFC2 |
| MEG3 | hsa-miR-214-5p;h | BAG2 |
| RP11-631N16.2 | hsa-miR-106b-5p | E2F3 |
| LINC00667 | hsa-miR-106b-5p | E2F3 |
| RP11-352M15.2 | hsa-miR-21-5p;hs | E2F3 |
| GS1-124K5.11 | hsa-miR-15a-5p;h | SLC29A1 |
| RP11-701H24.4 | hsa-miR-106a-5p; | ENPP5 |
| RP11-121C2.2 | hsa-miR-106a-5p; | ENPP5 |
| MEG3 | hsa-miR-106a-5p; | CEP72 |
| RP11-701H24.4 | hsa-miR-106a-5p; | CEP72 |
| RNU12 | hsa-miR-1224-5p; | LMNB1 |
| RP11-138A9.1 | hsa-miR-1224-5p; | LMNB1 |
| LL22NC03-2H8.5 | hsa-let-7a-5p;hsa | NUP155 |
| CTD-2587M2.1 | hsa-miR-25-3p;hs | TXNDC15 |
| LINC00630 | hsa-miR-25-3p;hs | TXNDC15 |
| MEG3 | hsa-miR-106a-5p; | WWC1 |
| RP11-701H24.4 | hsa-miR-106a-5p; | WWC1 |
| PKI55 | hsa-miR-15a-5p;h | TBCCD1 |
| LINC01355 | hsa-miR-15a-5p;h | TBCCD1 |
| GS1-124K5.11 | hsa-miR-15a-5p;h | TBCCD1 |
| MEG3 | hsa-miR-106a-5p; | MORC1 |
| RP11-701H24.4 | hsa-miR-106a-5p; | MORC1 |
| RP11-631N16.2 | hsa-miR-106a-5p; | SPCS1 |
| MEG3 | hsa-miR-106b-5p | DNAJC27 |
| LINC00969 | hsa-miR-148a-3p; | ACVR1 |
| MEG3 | hsa-let-7a-5p;hsa | SNX17 |
| RP11-121C2.2 | hsa-let-7a-5p;hsa | SNX17 |
| AC009404.2 | hsa-let-7a-5p;hsa | SNX17 |
| RP11-488L18.10 | hsa-let-7a-5p;hsa | SNX17 |
| RP11-78O7.2 | hsa-let-7a-5p;hsa | SNX17 |
| PKI55 | hsa-let-7a-5p;hsa | SNX17 |
| LINC00969 | hsa-miR-15a-5p;h | CLIP4 |
| RNU12 | hsa-miR-15a-5p;h | CLIP4 |
| RP11-631N16.2 | hsa-miR-26a-5p;h | GALNT3 |
| RNU12 | hsa-miR-15a-5p;h | LANCL1 |
| LINC00969 | hsa-miR-15a-5p;h | LANCL1 |
| MEG3 | hsa-miR-30a-5p;h | FANCL |
| LINC01355 | hsa-miR-30a-5p;h | FANCL |
| RP11-332H14.2 | hsa-miR-30a-5p;h | FANCL |
| RP11-631N16.2 | hsa-miR-30a-5p;h | ST3GAL5 |
| RNU12 | hsa-miR-15a-5p;h | SLC25A12 |
| LINC00969 | hsa-miR-15a-5p;h | SLC25A12 |
| LINC00969 | hsa-miR-1225-5p; | ORC4 |
| PWAR6 | hsa-miR-15a-5p;h | C2orf42 |
| MEG3 | hsa-miR-15a-5p;h | C2orf42 |
| PKI55 | hsa-miR-15a-5p;h | C2orf42 |
| RP11-488L18.10 | hsa-let-7a-5p;hsa | ARID3A |
| PKI55 | hsa-let-7a-5p;hsa | ARID3A |
| RP11-121C2.2 | hsa-let-7a-5p;hsa | ARID3A |
| RP11-78O7.2 | hsa-let-7a-5p;hsa | ARID3A |
| AC009404.2 | hsa-let-7a-5p;hsa | ARID3A |
| RP11-631N16.2 | hsa-miR-106a-5p; | EPHA4 |
| LL22NC03-2H8.5 | hsa-let-7a-5p;hsa | EPHA4 |
| RNU12 | hsa-miR-150-5p;h | BMP8B |
| RP11-631N16.2 | hsa-miR-106a-5p; | BMP8B |
| LINC00667 | hsa-miR-106b-5p | SSX2IP |

| | | |
|---|---|---|
| RP11-631N16.2 | hsa-miR-106b-5p | SSX2IP |
| RNU12 | hsa-miR-548a-5p; | EBNA1BP2 |
| RP11-498C9.15 | hsa-miR-339-5p;h | EBNA1BP2 |
| RP11-121C2.2 | hsa-miR-548ak;hsa | RAB32 |
| RNU12 | hsa-miR-150-5p;h | MYB |
| RNU12 | hsa-miR-324-5p;h | CYP20A1 |
| RP11-498C9.15 | hsa-miR-125a-3p; | CYP20A1 |
| RP11-631N16.2 | hsa-miR-106b-5p | TRIM32 |
| LINC00667 | hsa-miR-181a-5p; | DCAF4 |
| RP11-121C2.2 | hsa-let-7a-5p;hsa | DNAL1 |
| RP11-488L18.10 | hsa-let-7a-5p;hsa | DNAL1 |
| RP11-78O7.2 | hsa-let-7a-5p;hsa | DNAL1 |
| AC009404.2 | hsa-let-7a-5p;hsa | DNAL1 |
| MEG3 | hsa-let-7a-5p;hsa | DNAL1 |
| RP11-1055B8.4 | hsa-miR-212-5p;h | IRF2BPL |
| MEG3 | hsa-miR-150-5p;h | EIF2B2 |
| GS1-124K5.11 | hsa-miR-15a-5p;h | EIF2B2 |
| LINC00467 | hsa-miR-130a-3p; | GPR75 |
| RP11-631N16.2 | hsa-miR-106b-5p | GPAM |
| LINC00667 | hsa-miR-106b-5p | GPAM |
| RNU12 | hsa-miR-16-5p;hs | GPAM |
| RP11-701H24.4 | hsa-miR-181a-5p; | GOT1 |
| LINC00467 | hsa-miR-130a-3p; | HOXB3 |
| RP11-701H24.4 | hsa-miR-3065-5p; | HOXB3 |
| MEG3 | hsa-miR-124-3p;h | HOXB3 |
| LINC00969 | hsa-miR-15a-5p;h | CD274 |
| RNU12 | hsa-miR-15a-5p;h | CD274 |
| LINC00667 | hsa-miR-181b-5p | MRPS14 |
| LL22NC03-2H8.5 | hsa-let-7a-5p;hsa | SLC10A7 |
| RP11-631N16.2 | hsa-miR-181a-5p; | SLC10A7 |
| LINC00667 | hsa-miR-1275;hsa | SLC10A7 |
| MEG3 | hsa-miR-106a-5p; | PLS1 |
| RP11-631N16.2 | hsa-miR-106a-5p; | TNFRSF10B |
| LINC00667 | hsa-miR-106b-5p | TNFRSF10B |
| RP11-631N16.2 | hsa-miR-106a-5p; | COIL |
| LL22NC03-2H8.5 | hsa-let-7a-5p;hsa | COIL |
| RP11-701H24.4 | hsa-miR-106a-5p; | LRIF1 |
| MEG3 | hsa-miR-103a-3p; | LRIF1 |
| GS1-124K5.11 | hsa-miR-103a-3p; | LRIF1 |
| PWAR6 | hsa-miR-15a-5p;h | LRIF1 |
| RP11-701H24.4 | hsa-miR-106a-5p; | KIAA1191 |
| LINC00467 | hsa-miR-130a-3p; | KIAA1191 |
| RP11-631N16.2 | hsa-miR-106a-5p; | FAM213A |
| RNU12 | hsa-miR-150-5p;h | FAM213A |
| LINC00969 | hsa-miR-15a-5p;h | TRMT13 |
| MEG3 | hsa-miR-106a-5p; | POLM |
| PWAR6 | hsa-miR-17-5p;hs | POLM |
| RP11-701H24.4 | hsa-miR-106a-5p; | POLM |
| MEG3 | hsa-miR-100-5p;h | EGR2 |
| PWAR6 | hsa-miR-17-5p;hs | CIT |
| MEG3 | hsa-miR-106b-5p | CIT |
| AC009404.2 | hsa-let-7a-5p;hsa | ACOT9 |
| RP11-78O7.2 | hsa-let-7a-5p;hsa | ACOT9 |
| MEG3 | hsa-let-7a-5p;hsa | ACOT9 |
| PKI55 | hsa-let-7a-5p;hsa | ACOT9 |
| RP11-121C2.2 | hsa-let-7a-5p;hsa | ACOT9 |
| RP11-488L18.10 | hsa-let-7a-5p;hsa | ACOT9 |
| LINC01355 | hsa-miR-144-3p;h | NLN |

| | | |
|---|---|---|
| PKI55 | hsa-miR-19a-3p;h | ATPAF1 |
| PVT1 | hsa-miR-27a-3p;h | METTL8 |
| PWAR6 | hsa-miR-17-5p;hs | PFKFB2 |
| MEG3 | hsa-miR-106a-5p; | PFKFB2 |
| RP11-701H24.4 | hsa-miR-106a-5p; | PFKFB2 |
| RP11-631N16.2 | hsa-miR-224-5p;h | NCOA3 |
| LINC00630 | hsa-miR-25-3p;hs | PARD6B |
| CTD-2587M2.1 | hsa-miR-25-3p;hs | PARD6B |
| RP11-631N16.2 | hsa-miR-106a-5p; | BCAS4 |
| RNU12 | hsa-miR-150-5p;h | BCAS4 |
| RP11-701H24.4 | hsa-miR-106a-5p; | HIST1H2BJ |
| MEG3 | hsa-miR-106a-5p; | HIST1H2BJ |
| RP11-138A9.1 | hsa-miR-129-2-3p | SOX4 |
| RP11-631N16.2 | hsa-miR-106a-5p; | EREG |
| RP11-37B2.1 | hsa-let-7a-5p;hsa | GGA3 |
| GS1-124K5.11 | hsa-miR-30c-1-3p | C3 |
| RNU12 | hsa-miR-15a-5p;h | TBC1D20 |
| LINC00969 | hsa-miR-15a-5p;h | TBC1D20 |
| MEG3 | hsa-miR-103a-3p; | AMOT |
| GS1-124K5.11 | hsa-miR-103a-3p; | AMOT |
| PKI55 | hsa-let-7a-5p;hsa | COX6B1 |
| RP11-121C2.2 | hsa-let-7a-5p;hsa | COX6B1 |
| RP11-78O7.2 | hsa-let-7a-5p;hsa | COX6B1 |
| MEG3 | hsa-let-7a-5p;hsa | COX6B1 |
| RP11-488L18.10 | hsa-let-7a-5p;hsa | COX6B1 |
| RP11-701H24.4 | hsa-miR-106a-5p; | COX6B1 |
| AC009404.2 | hsa-let-7a-5p;hsa | COX6B1 |
| LINC00467 | hsa-miR-130a-3p; | THRA |
| LINC01355 | hsa-miR-15a-5p;h | IPPK |
| PWAR6 | hsa-miR-125a-5p; | IPPK |
| GS1-124K5.11 | hsa-miR-15a-5p;h | IPPK |
| PKI55 | hsa-miR-125a-5p; | IPPK |
| RP11-631N16.2 | hsa-miR-106a-5p; | RAB3IP |
| RP11-701H24.4 | hsa-miR-106a-5p; | F2RL3 |
| RP11-631N16.2 | hsa-miR-106a-5p; | HIP1 |
| MIRLET7BHG | hsa-miR-24-3p;hs | RBM48 |
| LINC00667 | hsa-miR-181a-5p; | ZNF780B |
| RP11-631N16.2 | hsa-miR-1305;hsa | KLHDC10 |
| RP11-498C9.15 | hsa-miR-186-5p;h | HAT1 |
| LINC00969 | hsa-miR-15a-5p;h | ANAPC13 |
| RP11-631N16.2 | hsa-miR-224-5p;h | MPDU1 |
| RP11-488L18.10 | hsa-let-7d-5p;hsa | MTUS1 |
| MEG3 | hsa-let-7d-5p;hsa | MTUS1 |
| RP11-78O7.2 | hsa-let-7d-5p;hsa | MTUS1 |
| AC009404.2 | hsa-let-7d-5p;hsa | MTUS1 |
| RP11-121C2.2 | hsa-let-7e-5p;hsa | MTUS1 |
| PKI55 | hsa-let-7f-5p;hsa- | MTUS1 |
| RP11-701H24.4 | hsa-miR-125a-5p; | MTUS1 |
| LINC00467 | hsa-miR-130a-3p; | SIGLEC9 |
| LINC00106 | hsa-miR-129-2-3p | BCL2L2 |
| RP11-1055B8.4 | hsa-miR-30b-3p;h | DTD2 |
| RP11-631N16.2 | hsa-miR-106a-5p; | EGLN3 |
| RP11-631N16.2 | hsa-miR-382-5p;h | DAD1 |
| RP11-458F8.4 | hsa-let-7a-5p;hsa | THEM6 |
| MIRLET7BHG | hsa-miR-221-5p;h | TOMM40 |
| SNHG8 | hsa-miR-125a-5p; | LSM4 |
| LINC00467 | hsa-miR-130a-3p; | MED18 |
| RP11-701H24.4 | hsa-miR-106a-5p; | MED18 |

| | | |
|---|---|---|
| RP11-701H24.4 | hsa-miR-106a-5p; | C12orf65 |
| MEG3 | hsa-miR-106a-5p; | C12orf65 |
| LINC01355 | hsa-miR-30a-5p;h | HABP4 |
| RP11-332H14.2 | hsa-miR-30a-5p;h | HABP4 |
| MEG3 | hsa-miR-30a-5p;h | HABP4 |
| LINC00969 | hsa-miR-200b-3p | ATP6V1E1 |
| RP11-701H24.4 | hsa-miR-106a-5p; | HAUS8 |
| MEG3 | hsa-miR-106a-5p; | HAUS8 |
| RP11-498C9.15 | hsa-miR-26a-5p;h | KIF3A |
| RNU12 | hsa-miR-150-5p;h | KIF3A |
| LINC00667 | hsa-miR-19a-3p;h | KIF3A |
| LINC00969 | hsa-miR-130a-3p; | PRKAA1 |
| RNU12 | hsa-miR-15a-5p;h | PRKAA1 |
| RP11-631N16.2 | hsa-miR-1305;hsa | PRKAA1 |
| LINC00467 | hsa-miR-130a-3p; | NIP7 |
| RP11-121C2.2 | hsa-miR-548ak;hsa | ANKEF1 |
| PWAR6 | hsa-miR-17-5p;hs | RFXAP |
| RP11-701H24.4 | hsa-miR-106a-5p; | RFXAP |
| MEG3 | hsa-miR-106a-5p; | RFXAP |
| LINC00467 | hsa-miR-130a-3p; | RFXAP |
| LL22NC03-2H8.5 | hsa-let-7a-5p;hsa | FAM104A |
| LINC00667 | hsa-miR-148b-3p | FAM104A |
| RP11-631N16.2 | hsa-miR-30a-5p;h | FAM104A |
| MIRLET7BHG | hsa-miR-24-3p;hs | C1QTNF6 |
| LINC01355 | hsa-miR-15a-5p;h | ACTR3B |
| PKI55 | hsa-miR-15a-5p;h | ACTR3B |
| GS1-124K5.11 | hsa-miR-15a-5p;h | ACTR3B |
| PWAR6 | hsa-miR-15a-5p;h | ACTR3B |
| MEG3 | hsa-miR-127-3p;h | ACTR3B |
| LINC00467 | hsa-miR-130a-3p; | TMTC1 |
| RP11-631N16.2 | hsa-miR-106a-5p; | RRAS2 |
| MEG3 | hsa-miR-204-5p;h | RNF122 |
| RP11-332H14.2 | hsa-miR-30a-5p;h | RNF122 |
| LINC01355 | hsa-miR-30a-5p;h | RNF122 |
| LINC00969 | hsa-miR-15a-5p;h | CD180 |
| RP11-631N16.2 | hsa-miR-30b-5p;h | NAPG |
| GS1-124K5.11 | hsa-miR-15a-5p;h | RFK |
| PWAR6 | hsa-miR-15a-5p;h | RFK |
| RP11-701H24.4 | hsa-miR-106a-5p; | RFK |
| MEG3 | hsa-miR-106a-5p; | RFK |
| LINC00969 | hsa-miR-27a-3p;h | CEP162 |
| RP11-631N16.2 | hsa-miR-106a-5p; | PRRG4 |
| LINC00630 | hsa-miR-25-3p;hs | TSPAN31 |
| CTD-2587M2.1 | hsa-miR-25-3p;hs | TSPAN31 |
| RP11-631N16.2 | hsa-miR-106a-5p; | KLHL36 |
| RNU12 | hsa-miR-1185-5p; | KLHL36 |
| PWAR6 | hsa-miR-124-3p;h | LAMC1 |
| MEG3 | hsa-miR-106b-5p | LAMC1 |
| LINC01355 | hsa-miR-15a-5p;h | LAMC1 |
| PKI55 | hsa-miR-124-3p;h | LAMC1 |
| GS1-124K5.11 | hsa-miR-15a-5p;h | LAMC1 |
| RP11-488L18.10 | hsa-let-7a-5p;hsa | IGF2BP3 |
| RP11-121C2.2 | hsa-let-7a-5p;hsa | IGF2BP3 |
| AC009404.2 | hsa-let-7a-5p;hsa | IGF2BP3 |
| PKI55 | hsa-let-7a-5p;hsa | IGF2BP3 |
| RP11-78O7.2 | hsa-let-7a-5p;hsa | IGF2BP3 |
| LINC00667 | hsa-miR-107;hsa- | SRSF1 |
| LINC00969 | hsa-miR-15a-5p;h | SRSF1 |

| | | |
|---|---|---|
| LL22NC03-2H8.5 | hsa-let-7a-5p;hsa | LIMD2 |
| MEG3 | hsa-miR-30a-3p;h | RTP4 |
| LINC00969 | hsa-miR-15a-5p;h | VPS45 |
| LINC00467 | hsa-miR-130a-3p; | PRPF4 |
| LINC00667 | hsa-miR-106b-5p | GOLGA1 |
| RP11-631N16.2 | hsa-miR-106b-5p | GOLGA1 |
| RP11-498C9.15 | hsa-miR-125a-5p; | GOLGA1 |
| RP11-352M15.2 | hsa-miR-145-5p;h | MYC |
| MEG3 | hsa-miR-129-1-3p | DNAJB5 |
| LINC00667 | hsa-miR-200c-3p; | TJAP1 |
| GS1-124K5.11 | hsa-miR-15a-5p;h | TUBB2A |
| PKI55 | hsa-let-7a-5p;hsa | TUBB2A |
| MEG3 | hsa-let-7a-5p;hsa | TUBB2A |
| AC009404.2 | hsa-let-7a-5p;hsa | TUBB2A |
| RP11-78O7.2 | hsa-let-7a-5p;hsa | TUBB2A |
| RP11-79H23.3 | hsa-miR-181a-5p; | TUBB2A |
| RP11-488L18.10 | hsa-let-7a-5p;hsa | TUBB2A |
| MEG3 | hsa-miR-106a-5p; | ATAT1 |
| RP11-701H24.4 | hsa-miR-106a-5p; | ATAT1 |
| LL22NC03-2H8.5 | hsa-let-7a-5p;hsa | RNF144B |
| RP11-498C9.15 | hsa-miR-199a-3p; | CREBZF |
| RP11-37B2.1 | hsa-let-7a-5p;hsa | RDX |
| PKI55 | hsa-let-7a-5p;hsa | SLC5A6 |
| RP11-121C2.2 | hsa-let-7a-5p;hsa | SLC5A6 |
| RP11-488L18.10 | hsa-let-7a-5p;hsa | SLC5A6 |
| AC009404.2 | hsa-let-7a-5p;hsa | SLC5A6 |
| RP11-78O7.2 | hsa-let-7a-5p;hsa | SLC5A6 |
| RP11-79H23.3 | hsa-miR-181a-5p; | CENPO |
| RNU12 | hsa-miR-130a-5p; | STAMBPL1 |
| RP11-488L18.10 | hsa-let-7a-5p;hsa | DNA2 |
| RP11-78O7.2 | hsa-let-7a-5p;hsa | DNA2 |
| RP11-121C2.2 | hsa-let-7a-5p;hsa | DNA2 |
| MEG3 | hsa-let-7a-5p;hsa | DNA2 |
| PKI55 | hsa-let-7a-5p;hsa | DNA2 |
| AC009404.2 | hsa-let-7a-5p;hsa | DNA2 |
| LL22NC03-2H8.5 | hsa-let-7a-5p;hsa | PARP16 |
| MEG3 | hsa-miR-106a-5p; | SEMA7A |
| PWAR6 | hsa-miR-126-5p;h | SEMA7A |
| RP11-701H24.4 | hsa-miR-106a-5p; | SEMA7A |
| LINC00667 | hsa-miR-181a-5p; | FAM13A |
| RP11-631N16.2 | hsa-miR-181a-5p; | FAM13A |
| RP11-121C2.2 | hsa-miR-548ak;hsa | PRKG2 |
| MEG3 | hsa-miR-136-5p;h | ZCRB1 |
| LINC01355 | hsa-miR-30a-5p;h | ZCRB1 |
| RP11-332H14.2 | hsa-miR-30a-5p;h | ZCRB1 |
| RP11-37B2.1 | hsa-let-7a-5p;hsa | C1RL |
| RP11-631N16.2 | hsa-miR-30a-5p;h | LMBR1L |
| LINC00969 | hsa-miR-129-5p;h | HNRNPA1L2 |
| RP11-631N16.2 | hsa-miR-30a-5p;h | VPS33A |
| RP11-498C9.15 | hsa-miR-199a-3p; | VPS33A |
| GS1-124K5.11 | hsa-miR-15a-5p;h | RAB15 |
| RP11-631N16.2 | hsa-miR-106b-5p | WDR89 |
| RP11-631N16.2 | hsa-miR-30a-5p;h | BAHD1 |
| RNU12 | hsa-miR-1185-5p; | TPM1 |
| RP11-1055B8.4 | hsa-miR-103a-3p; | C16orf58 |
| MEG3 | hsa-miR-103a-3p; | C16orf58 |
| PWAR6 | hsa-miR-15a-5p;h | C16orf58 |
| RP11-332H14.2 | hsa-miR-1224-3p; | C16orf58 |

| | | |
|---|---|---|
| GS1-124K5.11 | hsa-miR-103a-3p; | C16orf58 |
| RP11-631N16.2 | hsa-miR-30a-5p;h | TAF4B |
| GS1-124K5.11 | hsa-miR-15a-5p;h | GNAL |
| MEG3 | hsa-miR-15a-5p;h | GNAL |
| PKI55 | hsa-miR-15a-5p;h | GNAL |
| GS1-124K5.11 | hsa-miR-15a-5p;h | RAB40B |
| PWAR6 | hsa-miR-15a-5p;h | RAB40B |
| PKI55 | hsa-miR-15a-5p;h | RAB40B |
| LINC01355 | hsa-miR-15a-5p;h | RAB40B |
| LL22NC03-2H8.5 | hsa-let-7a-5p;hsa | EIF4A3 |
| CTD-2587M2.1 | hsa-miR-25-3p;hs | NARF |
| LINC00630 | hsa-miR-25-3p;hs | NARF |
| RP11-701H24.4 | hsa-miR-106a-5p; | TRIM65 |
| RP11-332H14.2 | hsa-miR-30b-5p;h | SH3GL1 |
| MEG3 | hsa-miR-122-5p;h | SH3GL1 |
| LINC01355 | hsa-miR-30b-5p;h | SH3GL1 |
| RP11-701H24.4 | hsa-miR-106a-5p; | DPP9 |
| LINC00667 | hsa-miR-125a-5p; | AKT1 |
| RP11-498C9.15 | hsa-miR-125a-5p; | AKT1 |
| LINC00969 | hsa-miR-15b-5p;h | CARM1 |
| RNU12 | hsa-miR-15b-5p;h | CARM1 |
| LINC00467 | hsa-miR-130a-3p; | GPR161 |
| LINC00667 | hsa-miR-106b-5p | ALDH9A1 |
| RP11-631N16.2 | hsa-miR-106b-5p | ALDH9A1 |
| SNHG8 | hsa-miR-186-3p;h | PIGM |
| RP11-458F8.4 | hsa-let-7a-5p;hsa | INTS7 |
| RP11-701H24.4 | hsa-miR-106a-5p; | SNAP47 |
| RP11-631N16.2 | hsa-miR-106a-5p; | DEGS1 |
| RNU12 | hsa-miR-150-5p;h | DEGS1 |
| AC004951.6 | hsa-miR-30a-3p;h | SYT2 |
| LINC00667 | hsa-miR-548ak;hsa | MRPS5 |
| RP11-498C9.15 | hsa-miR-548am-5 | MRPS5 |
| RNU12 | hsa-miR-548a-5p; | MRPS5 |
| RP11-488L18.10 | hsa-let-7a-5p;hsa | RABL2A |
| PKI55 | hsa-let-7a-5p;hsa | RABL2A |
| AC009404.2 | hsa-let-7a-5p;hsa | RABL2A |
| RP11-121C2.2 | hsa-let-7a-5p;hsa | RABL2A |
| MEG3 | hsa-let-7a-5p;hsa | RABL2A |
| RP11-78O7.2 | hsa-let-7a-5p;hsa | RABL2A |
| LINC00969 | hsa-miR-145-5p;h | VGLL4 |
| LINC00969 | hsa-miR-15a-5p;h | SRPRB |
| RNU12 | hsa-miR-15a-5p;h | SRPRB |
| MIRLET7BHG | hsa-miR-24-3p;hs | SCD5 |
| RP11-631N16.2 | hsa-miR-519a-3p; | KLHL8 |
| LINC00467 | hsa-miR-130a-3p; | CCNA2 |
| MIRLET7BHG | hsa-miR-24-3p;hs | CCNA2 |
| SNHG8 | hsa-miR-19a-3p;h | CCNA2 |
| PKI55 | hsa-let-7b-5p;hsa | CCNA2 |
| RP11-631N16.2 | hsa-miR-224-5p;h | LHFPL2 |
| RP11-498C9.15 | hsa-miR-137;hsa- | LHFPL2 |
| RNU12 | hsa-miR-150-5p;h | GTF2H2 |
| RP11-631N16.2 | hsa-miR-106a-5p; | GTF2H2 |
| RP11-631N16.2 | hsa-miR-106a-5p; | KCNMB1 |
| RP11-701H24.4 | hsa-miR-106a-5p; | TNFRSF21 |
| MEG3 | hsa-miR-106a-5p; | TNFRSF21 |
| PWAR6 | hsa-miR-124-3p;h | TNFRSF21 |
| LL22NC03-2H8.5 | hsa-let-7a-5p;hsa | PRIM2 |
| RP11-498C9.15 | hsa-miR-144-3p;h | RPL7L1 |

| | | |
|---|---|---|
| RNU12 | hsa-miR-130a-5p; | RPL7L1 |
| LL22NC03-2H8.5 | hsa-let-7a-5p;hsa | PM20D2 |
| LL22NC03-2H8.5 | hsa-let-7a-5p;hsa | NOM1 |
| LINC00969 | hsa-miR-15a-5p;h | CASK |
| RP11-79H23.3 | hsa-miR-181a-5p; | KRBOX4 |
| LINC00969 | hsa-miR-15a-5p;h | PROSC |
| RNU12 | hsa-miR-150-5p;h | PROSC |
| RP11-701H24.4 | hsa-miR-106a-5p; | LRP12 |
| PWAR6 | hsa-miR-15a-5p;h | NR6A1 |
| MEG3 | hsa-let-7a-5p;hsa | NR6A1 |
| AC009404.2 | hsa-let-7a-5p;hsa | NR6A1 |
| RP11-121C2.2 | hsa-let-7a-5p;hsa | NR6A1 |
| GS1-124K5.11 | hsa-miR-15a-5p;h | NR6A1 |
| RP11-78O7.2 | hsa-let-7a-5p;hsa | NR6A1 |
| RP11-488L18.10 | hsa-let-7a-5p;hsa | NR6A1 |
| PKI55 | hsa-let-7a-5p;hsa | NR6A1 |
| MIRLET7BHG | hsa-miR-1226-3p; | SURF2 |
| RP11-79H23.3 | hsa-miR-181a-5p; | MIGA2 |
| RP11-701H24.4 | hsa-miR-181a-5p; | MIGA2 |
| MEG3 | hsa-miR-149-3p;h | MIGA2 |
| RP11-121C2.2 | hsa-miR-106a-5p; | NACC2 |
| LINC00467 | hsa-miR-130a-3p; | NACC2 |
| RP11-701H24.4 | hsa-miR-106a-5p; | PARD3 |
| RP11-631N16.2 | hsa-miR-106a-5p; | POLR3A |
| RP11-121C2.2 | hsa-miR-218-5p;h | MKI67 |
| PWAR6 | hsa-miR-218-5p;h | ADAM12 |
| RP11-1055B8.4 | hsa-miR-103a-3p; | LIN7C |
| PWAR6 | hsa-miR-1-3p;hsa | LIN7C |
| RP11-332H14.2 | hsa-miR-30a-5p;h | LIN7C |
| MEG3 | hsa-miR-103a-3p; | LIN7C |
| LINC01355 | hsa-miR-30a-5p;h | LIN7C |
| LINC00969 | hsa-miR-15a-5p;h | SSRP1 |
| RNU12 | hsa-miR-15a-5p;h | SSRP1 |
| RP11-1055B8.4 | hsa-miR-149-5p;h | SERPINH1 |
| CTD-2587M2.1 | hsa-miR-25-3p;hs | INCENP |
| LINC00630 | hsa-miR-25-3p;hs | INCENP |
| RP11-1055B8.4 | hsa-miR-34b-5p;h | HIRIP3 |
| CTD-2587M2.1 | hsa-miR-25-3p;hs | TIRAP |
| LINC00630 | hsa-miR-25-3p;hs | TIRAP |
| LL22NC03-2H8.5 | hsa-let-7a-5p;hsa | KIAA1328 |
| LINC00667 | hsa-miR-19a-3p;h | MTMR12 |
| LINC00667 | hsa-miR-143-3p;h | GXYLT1 |
| RP11-631N16.2 | hsa-miR-30a-5p;h | GXYLT1 |
| RP11-352M15.2 | hsa-miR-30b-5p;h | GXYLT1 |
| LL22NC03-2H8.5 | hsa-let-7a-5p;hsa | THYN1 |
| RP11-631N16.2 | hsa-miR-106a-5p; | FAM160B1 |
| RP11-631N16.2 | hsa-miR-106b-5p | TMEM267 |
| LINC00969 | hsa-miR-141-5p;h | RNF219 |
| LINC00667 | hsa-miR-181a-5p; | SUV39H2 |
| LINC00630 | hsa-miR-25-3p;hs | GTF2E1 |
| CTD-2587M2.1 | hsa-miR-137;hsa- | GTF2E1 |
| RP11-631N16.2 | hsa-miR-30a-5p;h | CFDP1 |
| RP11-498C9.15 | hsa-miR-27a-3p;h | CFDP1 |
| LINC00630 | hsa-miR-25-3p;hs | MCOLN2 |
| LINC00667 | hsa-miR-143-3p;h | PPP2R5E |
| RP11-631N16.2 | hsa-miR-4317;hsa | CCSAP |
| PWAR6 | hsa-miR-132-3p;h | TCEB1 |
| RP11-701H24.4 | hsa-miR-106a-5p; | TCEB1 |

| | | |
|---|---|---|
| RP11-631N16.2 | hsa-miR-106a-5p; | BTG3 |
| RP11-631N16.2 | hsa-miR-106a-5p; | MPPE1 |
| GS1-124K5.11 | hsa-miR-15a-5p;h | FBXL18 |
| PKI55 | hsa-miR-125a-5p; | FBXL18 |
| PWAR6 | hsa-miR-125a-5p; | FBXL18 |
| RP11-701H24.4 | hsa-miR-106a-5p; | RHOC |
| MIRLET7BHG | hsa-miR-29a-3p;h | TMEM237 |
| LINC01355 | hsa-miR-193b-5p | SLC26A2 |
| RP11-631N16.2 | hsa-miR-106a-5p; | DUSP2 |
| LINC00630 | hsa-miR-25-3p;hs | CNNM4 |
| CTD-2587M2.1 | hsa-miR-25-3p;hs | CNNM4 |
| LL22NC03-2H8.5 | hsa-let-7a-5p;hsa | AHCYL2 |
| RP11-631N16.2 | hsa-miR-185-5p;h | B4GALT5 |
| LL22NC03-2H8.5 | hsa-let-7a-5p;hsa | TMED4 |
| LL22NC03-2H8.5 | hsa-let-7a-5p;hsa | GPAT4 |
| LINC00630 | hsa-miR-25-3p;hs | ZSCAN12 |
| LL22NC03-2H8.5 | hsa-let-7a-5p;hsa | TOMM40L |
| RP11-631N16.2 | hsa-miR-106a-5p; | THEM4 |
| RNU12 | hsa-miR-15a-5p;h | TMEM69 |
| LINC00969 | hsa-miR-15a-5p;h | TMEM69 |
| RP11-37B2.1 | hsa-let-7a-5p;hsa | ICOSLG |
| RP11-488L18.10 | hsa-let-7a-5p;hsa | C19orf47 |
| AC009404.2 | hsa-let-7a-5p;hsa | C19orf47 |
| MIRLET7BHG | hsa-miR-30c-1-3p | C19orf47 |
| PKI55 | hsa-let-7a-5p;hsa | C19orf47 |
| RP11-121C2.2 | hsa-let-7a-5p;hsa | C19orf47 |
| RP11-78O7.2 | hsa-let-7a-5p;hsa | C19orf47 |
| PKI55 | hsa-miR-125a-5p; | TOR2A |
| LINC01355 | hsa-miR-193b-5p | TOR2A |
| RNU12 | hsa-miR-1226-5p; | SLC25A44 |
| LINC00630 | hsa-miR-25-3p;hs | ORAI2 |
| CTD-2587M2.1 | hsa-miR-25-3p;hs | ORAI2 |
| RNU12 | hsa-miR-150-5p;h | PCYT1A |
| RP11-631N16.2 | hsa-miR-106a-5p; | ZNF385A |
| RP11-701H24.4 | hsa-miR-106a-5p; | ASB16 |
| GS1-124K5.11 | hsa-miR-103a-3p; | RACGAP1 |
| LINC00467 | hsa-miR-130a-3p; | RACGAP1 |
| RP11-701H24.4 | hsa-miR-106a-5p; | RACGAP1 |
| MEG3 | hsa-miR-103a-3p; | RACGAP1 |
| PWAR6 | hsa-miR-15a-5p;h | RACGAP1 |
| GS1-124K5.11 | hsa-miR-15a-5p;h | ASGR2 |
| LINC01355 | hsa-miR-15a-5p;h | ASGR2 |
| CTD-2587M2.1 | hsa-miR-137;hsa- | ASGR2 |
| PKI55 | hsa-miR-137;hsa- | ASGR2 |
| PWAR6 | hsa-miR-15a-5p;h | ASGR2 |
| LINC00630 | hsa-miR-25-3p;hs | ASGR2 |
| MEG3 | hsa-miR-137;hsa- | ASGR2 |
| MIRLET7BHG | hsa-miR-2355-5p; | CCNF |
| MIRLET7BHG | hsa-miR-3065-3p; | CLPB |
| LINC00910 | hsa-let-7a-5p;hsa | AK4 |
| LINC00969 | hsa-miR-130a-3p; | IL23R |
| RNU12 | hsa-miR-1226-5p; | OMA1 |
| LINC00969 | hsa-miR-130a-3p; | ACP6 |
| MIRLET7BHG | hsa-miR-24-3p;hs | RFTN2 |
| RP11-631N16.2 | hsa-miR-106b-5p | NUP35 |
| LINC00969 | hsa-miR-130a-3p; | EOGT |
| LINC00667 | hsa-miR-148b-3p | EOGT |
| RP11-1055B8.4 | hsa-miR-1254;hsa | CHCHD4 |

| | | |
|---|---|---|
| PVT1 | hsa-miR-27a-3p;h | EIF5A2 |
| LINC00467 | hsa-miR-130a-3p; | C4orf36 |
| RP11-121C2.2 | hsa-miR-548ak;hs | TPRA1 |
| LINC00630 | hsa-miR-25-3p;hs | TMEM41A |
| CTD-2587M2.1 | hsa-miR-137;hsa- | TMEM41A |
| LINC00467 | hsa-miR-130a-3p; | RFT1 |
| LINC00969 | hsa-miR-129-5p;h | SLBP |
| RP11-631N16.2 | hsa-miR-106a-5p; | LRPAP1 |
| RNU12 | hsa-miR-17-5p;hs | LRPAP1 |
| RP11-701H24.4 | hsa-miR-106a-5p; | EXO5 |
| RP11-701H24.4 | hsa-miR-106a-5p; | C1orf50 |
| RP11-631N16.2 | hsa-miR-106a-5p; | ITGA2 |
| LINC00467 | hsa-miR-130a-3p; | MOCS2 |
| RP11-701H24.4 | hsa-miR-519a-3p; | MOCS2 |
| RP11-631N16.2 | hsa-miR-106a-5p; | SLC25A46 |
| RP11-631N16.2 | hsa-miR-224-5p;h | CASP3 |
| SNHG8 | hsa-miR-197-3p;h | MB21D1 |
| LINC00969 | hsa-miR-1-3p;hsa | SFXN1 |
| MEG3 | hsa-miR-15a-5p;h | KIAA0895 |
| GS1-124K5.11 | hsa-miR-15a-5p;h | KIAA0895 |
| PKI55 | hsa-miR-15a-5p;h | KIAA0895 |
| PWAR6 | hsa-miR-15a-5p;h | KIAA0895 |
| LINC01355 | hsa-miR-15a-5p;h | KIAA0895 |
| LINC01355 | hsa-miR-30a-5p;h | BMT2 |
| PWAR6 | hsa-miR-17-5p;hs | BMT2 |
| MEG3 | hsa-miR-106b-5p | BMT2 |
| RP11-332H14.2 | hsa-miR-30a-5p;h | BMT2 |
| RP11-701H24.4 | hsa-miR-17-5p;hs | BMT2 |
| MIRLET7BHG | hsa-miR-34a-5p;h | SLC4A2 |
| MEG3 | hsa-miR-146b-3p | COX6C |
| RP11-701H24.4 | hsa-miR-519a-3p; | COX6C |
| MEG3 | hsa-miR-125b-5p | FZD6 |
| PKI55 | hsa-miR-125b-5p | FZD6 |
| PWAR6 | hsa-miR-125b-5p | FZD6 |
| RP11-1055B8.4 | hsa-miR-125b-5p | FZD6 |
| RP11-701H24.4 | hsa-miR-16-1-3p; | TMEM67 |
| LINC00630 | hsa-miR-25-3p;hs | CCDC171 |
| CTD-2587M2.1 | hsa-miR-25-3p;hs | CCDC171 |
| RP11-121C2.2 | hsa-let-7a-5p;hsa | FXN |
| PKI55 | hsa-let-7a-5p;hsa | FXN |
| MEG3 | hsa-let-7a-5p;hsa | FXN |
| AC009404.2 | hsa-let-7a-5p;hsa | FXN |
| RP11-488L18.10 | hsa-let-7a-5p;hsa | FXN |
| RP11-78O7.2 | hsa-let-7a-5p;hsa | FXN |
| RP11-37B2.1 | hsa-let-7a-5p;hsa | KIF27 |
| PWAR6 | hsa-miR-139-5p;h | ZNF367 |
| LINC01355 | hsa-miR-15a-5p;h | ZNF367 |
| GS1-124K5.11 | hsa-miR-15a-5p;h | ZNF367 |
| MEG3 | hsa-miR-15a-5p;h | ZNF367 |
| PKI55 | hsa-miR-15a-5p;h | ZNF367 |
| RP11-631N16.2 | hsa-miR-1277-5p; | AQP3 |
| RNU12 | hsa-miR-548a-5p; | AQP3 |
| RP11-498C9.15 | hsa-miR-548am-5 | AQP3 |
| RP11-631N16.2 | hsa-miR-106b-5p | PIGO |
| AC009404.2 | hsa-let-7a-5p;hsa | PGM2L1 |
| MEG3 | hsa-let-7a-5p;hsa | PGM2L1 |
| RP11-488L18.10 | hsa-let-7a-5p;hsa | PGM2L1 |
| RP11-78O7.2 | hsa-let-7a-5p;hsa | PGM2L1 |

| | | |
|---|---|---|
| RP11-121C2.2 | hsa-let-7a-5p;hsa | PGM2L1 |
| PKI55 | hsa-let-7a-5p;hsa | PGM2L1 |
| MEG3 | hsa-miR-106a-5p; | REEP3 |
| MIRLET7BHG | hsa-miR-139-3p;h | REEP3 |
| RP11-701H24.4 | hsa-miR-106a-5p; | REEP3 |
| RP11-332H14.2 | hsa-miR-30a-5p;h | KBTBD6 |
| RP11-701H24.4 | hsa-miR-519a-3p; | KBTBD6 |
| LINC01355 | hsa-miR-30a-5p;h | KBTBD6 |
| LINC00467 | hsa-miR-130a-3p; | KBTBD6 |
| RP11-121C2.2 | hsa-miR-548ak;hsa | VDAC2 |
| LL22NC03-2H8.5 | hsa-let-7a-5p;hsa | PMPCA |
| RP11-121C2.2 | hsa-miR-548ak;hsa | HPRT1 |
| PKI55 | hsa-miR-15a-5p;h | E2F7 |
| MEG3 | hsa-miR-15a-5p;h | E2F7 |
| LINC01355 | hsa-miR-15a-5p;h | E2F7 |
| GS1-124K5.11 | hsa-miR-15a-5p;h | E2F7 |
| RNU12 | hsa-miR-1304-3p; | ISCA2 |
| RP11-631N16.2 | hsa-miR-106a-5p; | ISCA2 |
| PKI55 | hsa-let-7a-5p;hsa | POLL |
| RP11-121C2.2 | hsa-let-7a-5p;hsa | POLL |
| RP11-488L18.10 | hsa-let-7a-5p;hsa | POLL |
| AC009404.2 | hsa-let-7a-5p;hsa | POLL |
| RP11-78O7.2 | hsa-let-7a-5p;hsa | POLL |
| MEG3 | hsa-miR-106a-5p; | ZNF202 |
| RP11-701H24.4 | hsa-miR-106a-5p; | ZNF202 |
| RP11-701H24.4 | hsa-miR-106a-5p; | CYB5A |
| MEG3 | hsa-miR-106a-5p; | CYB5A |
| RNU12 | hsa-miR-548a-5p; | TMEM41B |
| RP11-498C9.15 | hsa-miR-548am-5 | TMEM41B |
| RP11-1055B8.4 | hsa-miR-103a-3p; | MCM7 |
| GS1-124K5.11 | hsa-miR-103a-3p; | MCM7 |
| MEG3 | hsa-miR-103a-3p; | MCM7 |
| RP11-701H24.4 | hsa-miR-519a-3p; | MCM7 |
| PWAR6 | hsa-miR-15a-5p;h | TMEM135 |
| LINC01355 | hsa-miR-15a-5p;h | TMEM135 |
| MEG3 | hsa-miR-15a-5p;h | TMEM135 |
| PKI55 | hsa-miR-15a-5p;h | TMEM135 |
| GS1-124K5.11 | hsa-miR-15a-5p;h | TMEM135 |
| RP11-631N16.2 | hsa-miR-10a-3p;h | BLCAP |
| MEG3 | hsa-miR-106b-5p; | AKTIP |
| LINC00467 | hsa-miR-130a-3p; | DUSP18 |
| LINC00630 | hsa-miR-25-3p;hs | SNRPD1 |
| CTD-2587M2.1 | hsa-miR-25-3p;hs | SNRPD1 |
| CTD-2587M2.1 | hsa-miR-137;hsa- | DUS2 |
| LINC00630 | hsa-miR-25-3p;hs | DUS2 |
| LL22NC03-2H8.5 | hsa-let-7a-5p;hsa | RRM1 |
| LINC00667 | hsa-miR-181a-5p; | ZNF23 |
| RP11-701H24.4 | hsa-miR-106a-5p; | ZNF180 |
| MEG3 | hsa-miR-106a-5p; | ZNF180 |
| LINC00630 | hsa-miR-27a-3p;h | SERTAD3 |
| RNU12 | hsa-miR-17-5p;hs | TSR1 |
| RP11-631N16.2 | hsa-miR-106a-5p; | TSR1 |
| RNU12 | hsa-miR-15a-5p;h | OTUB1 |
| LINC00467 | hsa-miR-130a-3p; | CDK2AP2 |
| LINC00969 | hsa-miR-16-2-3p; | TMEM68 |
| MEG3 | hsa-miR-106b-5p; | ZNF597 |
| RP11-701H24.4 | hsa-miR-17-5p;hs | ZNF597 |
| LINC00969 | hsa-miR-15a-5p;h | MAP4K2 |

| | | |
|---|---|---|
| RP11-631N16.2 | hsa-miR-106a-5p; | PDHB |
| RNU12 | hsa-miR-150-5p;h | PDHB |
| MEG3 | hsa-miR-106a-5p; | MFSD2A |
| SNHG8 | hsa-miR-125a-3p; | MFSD2A |
| RP11-701H24.4 | hsa-miR-106a-5p; | MFSD2A |
| RP11-1055B8.4 | hsa-miR-30b-3p;h | NDUFS5 |
| RP11-1055B8.4 | hsa-miR-188-5p;h | SLC16A4 |
| GS1-124K5.11 | hsa-miR-30c-1-3p | SLC16A4 |
| AC009404.2 | hsa-let-7a-5p;hsa | SEMA4C |
| RP11-488L18.10 | hsa-let-7a-5p;hsa | SEMA4C |
| MEG3 | hsa-let-7a-5p;hsa | SEMA4C |
| RP11-78O7.2 | hsa-let-7a-5p;hsa | SEMA4C |
| PKI55 | hsa-let-7a-5p;hsa | SEMA4C |
| RP11-121C2.2 | hsa-let-7a-5p;hsa | SEMA4C |
| RP11-1055B8.4 | hsa-miR-2278;hsa | TCTN2 |
| RP11-701H24.4 | hsa-miR-106a-5p; | ABHD15 |
| MEG3 | hsa-miR-106a-5p; | ABHD15 |
| RP11-121C2.2 | hsa-miR-548ak;hsa | TNIP2 |
| RP11-458F8.4 | hsa-let-7a-5p;hsa | E2F6 |
| RP11-79H23.3 | hsa-miR-30c-1-3p | AR |
| MIRLET7BHG | hsa-miR-2110;hsa | AR |
| LINC00667 | hsa-miR-548ak;hsa | PRELID1 |
| RP11-498C9.15 | hsa-miR-548am-5 | PRELID1 |
| RNU12 | hsa-miR-548a-5p; | PRELID1 |
| LINC01355 | hsa-miR-26a-5p;h | SMAD1 |
| RP11-332H14.2 | hsa-miR-26a-5p;h | SMAD1 |
| MEG3 | hsa-miR-186-5p;h | SMAD1 |
| RP11-488L18.10 | hsa-miR-26a-5p;h | SMAD1 |
| MEG3 | hsa-miR-106a-5p; | SLC30A1 |
| PWAR6 | hsa-miR-17-5p;hs | SLC30A1 |
| RP11-701H24.4 | hsa-miR-106a-5p; | SLC30A1 |
| PKI55 | hsa-miR-21-5p;hs | SOCS6 |
| MEG3 | hsa-miR-23a-3p;h | SOCS6 |
| LINC00467 | hsa-miR-130a-3p; | CDCA4 |
| LINC01355 | hsa-miR-15a-5p;h | CDCA4 |
| GS1-124K5.11 | hsa-miR-15a-5p;h | CDCA4 |
| RP11-631N16.2 | hsa-miR-1277-5p; | MSANTD4 |
| LINC00969 | hsa-miR-130a-3p; | MSANTD4 |
| RP11-498C9.15 | hsa-miR-30b-5p;h | MSANTD4 |
| RNU12 | hsa-miR-1268a;hs | MSANTD4 |
| LINC01355 | hsa-miR-15a-5p;h | OSCAR |
| PWAR6 | hsa-miR-15a-5p;h | OSCAR |
| GS1-124K5.11 | hsa-miR-15a-5p;h | OSCAR |
| PKI55 | hsa-miR-15a-5p;h | OSCAR |
| MEG3 | hsa-miR-136-5p;h | PAQR8 |
| LINC00630 | hsa-miR-25-3p;hs | C11orf24 |
| PKI55 | hsa-miR-15a-5p;h | C11orf24 |
| GS1-124K5.11 | hsa-miR-15a-5p;h | C11orf24 |
| MEG3 | hsa-miR-15a-5p;h | C11orf24 |
| CTD-2587M2.1 | hsa-miR-25-3p;hs | C11orf24 |
| LINC01355 | hsa-miR-15a-5p;h | C11orf24 |
| PWAR6 | hsa-miR-15a-5p;h | C11orf24 |
| RP11-701H24.4 | hsa-miR-106a-5p; | NETO2 |
| PWAR6 | hsa-miR-1-3p;hsa | NETO2 |
| MEG3 | hsa-miR-106a-5p; | NETO2 |
| RP11-631N16.2 | hsa-miR-106a-5p; | WIPF2 |
| RP11-78O7.2 | hsa-let-7a-5p;hsa | GATM |
| RP11-121C2.2 | hsa-let-7a-5p;hsa | GATM |

| | | |
|---|---|---|
| AC009404.2 | hsa-let-7a-5p;hsa | GATM |
| RP11-488L18.10 | hsa-let-7a-5p;hsa | GATM |
| PKI55 | hsa-let-7a-5p;hsa | GATM |
| RP11-701H24.4 | hsa-miR-106a-5p; | RNASEH1 |
| RP11-631N16.2 | hsa-miR-106b-5p | ORMDL3 |
| PWAR6 | hsa-miR-17-5p;hs | NME6 |
| RP11-701H24.4 | hsa-miR-106a-5p; | NME6 |
| MEG3 | hsa-miR-19a-5p;h | CXCR6 |
| AC009404.2 | hsa-let-7a-5p;hsa | FNDC9 |
| MEG3 | hsa-let-7a-5p;hsa | FNDC9 |
| RP11-78O7.2 | hsa-let-7a-5p;hsa | FNDC9 |
| PKI55 | hsa-let-7a-5p;hsa | FNDC9 |
| RP11-121C2.2 | hsa-let-7a-5p;hsa | FNDC9 |
| RP11-488L18.10 | hsa-let-7a-5p;hsa | FNDC9 |
| RP11-701H24.4 | hsa-miR-106a-5p; | TMEM134 |
| MEG3 | hsa-miR-106a-5p; | TMEM134 |
| RP11-701H24.4 | hsa-miR-106a-5p; | FUT10 |
| PKI55 | hsa-let-7a-5p;hsa | FUT10 |
| MEG3 | hsa-let-7a-5p;hsa | FUT10 |
| RP11-121C2.2 | hsa-let-7a-5p;hsa | FUT10 |
| RP11-488L18.10 | hsa-let-7a-5p;hsa | FUT10 |
| AC009404.2 | hsa-let-7a-5p;hsa | FUT10 |
| RP11-78O7.2 | hsa-let-7a-5p;hsa | FUT10 |
| LINC00667 | hsa-miR-181a-5p; | FAM192A |
| RP11-631N16.2 | hsa-miR-181a-5p; | FAM192A |
| LINC00467 | hsa-miR-130a-3p; | PDP2 |
| RP11-631N16.2 | hsa-miR-106b-5p | TADA2B |
| LINC00667 | hsa-miR-103a-3p; | ZNF680 |
| RNU12 | hsa-miR-130a-5p; | FAM222B |
| LL22NC03-2H8.5 | hsa-let-7a-5p;hsa | FAM222B |
| PWAR6 | hsa-miR-15a-5p;h | RAPH1 |
| LINC01355 | hsa-miR-15a-5p;h | RAPH1 |
| MEG3 | hsa-let-7g-3p;hsa | RAPH1 |
| GS1-124K5.11 | hsa-miR-15a-5p;h | RAPH1 |
| PKI55 | hsa-miR-15a-5p;h | RAPH1 |
| LINC00667 | hsa-miR-103a-3p; | ZNF449 |
| LINC00969 | hsa-miR-15a-5p;h | ZNF622 |
| RNU12 | hsa-miR-15a-5p;h | ZNF622 |
| RNU12 | hsa-miR-1304-3p; | RBM4B |
| RP11-498C9.15 | hsa-miR-548am-5 | RBM4B |
| RP11-1055B8.4 | hsa-miR-132-3p;h | UBXN2A |
| MIRLET7BHG | hsa-miR-30c-1-3p | FBXO45 |
| CTD-2587M2.1 | hsa-miR-25-3p;hs | ZDHHC24 |
| LINC00630 | hsa-miR-25-3p;hs | ZDHHC24 |
| RNU12 | hsa-miR-17-5p;hs | GTF2IRD2B |
| RP11-631N16.2 | hsa-miR-106a-5p; | GTF2IRD2B |
| RP11-1055B8.4 | hsa-miR-30b-3p;h | MFSD4A |
| MIRLET7BHG | hsa-miR-29a-3p;h | SLC29A2 |
| LINC00969 | hsa-miR-15a-5p;h | DENND6A |
| LINC00667 | hsa-miR-19a-3p;h | DENND6A |
| RNU12 | hsa-miR-15a-5p;h | DENND6A |
| RP11-121C2.2 | hsa-let-7a-5p;hsa | MARCKSL1 |
| AC009404.2 | hsa-let-7a-5p;hsa | MARCKSL1 |
| LINC01355 | hsa-miR-30a-5p;h | MARCKSL1 |
| RP11-488L18.10 | hsa-let-7a-5p;hsa | MARCKSL1 |
| RP11-78O7.2 | hsa-let-7a-5p;hsa | MARCKSL1 |
| RP11-332H14.2 | hsa-miR-30a-5p;h | MARCKSL1 |
| PKI55 | hsa-let-7a-5p;hsa | MARCKSL1 |

| | | |
|---|---|---|
| LINC01355 | hsa-miR-199a-3p; | CSRP2 |
| RP11-631N16.2 | hsa-miR-30b-5p;h | TOMM5 |
| CTD-2587M2.1 | hsa-miR-25-3p;hs | DENND2C |
| LINC00630 | hsa-miR-25-3p;hs | DENND2C |
| RP11-78O7.2 | hsa-let-7a-5p;hsa | ZBTB8OS |
| AC009404.2 | hsa-let-7a-5p;hsa | ZBTB8OS |
| PKI55 | hsa-let-7a-5p;hsa | ZBTB8OS |
| MEG3 | hsa-let-7a-5p;hsa | ZBTB8OS |
| RP11-488L18.10 | hsa-let-7a-5p;hsa | ZBTB8OS |
| RP11-121C2.2 | hsa-let-7a-5p;hsa | ZBTB8OS |
| RP11-631N16.2 | hsa-miR-106a-5p; | MYO1D |
| CTD-2587M2.1 | hsa-miR-25-3p;hs | CDK5R1 |
| LINC00630 | hsa-miR-25-3p;hs | CDK5R1 |
| RP11-631N16.2 | hsa-miR-30a-5p;h | PNMA1 |
| PKI55 | hsa-let-7a-5p;hsa | MTX3 |
| RP11-78O7.2 | hsa-let-7a-5p;hsa | MTX3 |
| AC009404.2 | hsa-let-7a-5p;hsa | MTX3 |
| RP11-488L18.10 | hsa-let-7a-5p;hsa | MTX3 |
| RP11-121C2.2 | hsa-let-7a-5p;hsa | MTX3 |
| LINC00630 | hsa-miR-25-3p;hs | ZBTB34 |
| CTD-2587M2.1 | hsa-miR-25-3p;hs | ZBTB34 |
| RP11-701H24.4 | hsa-miR-425-5p;h | ZBTB34 |
| RP11-631N16.2 | hsa-miR-106a-5p; | FAM210A |
| RP11-631N16.2 | hsa-miR-30a-5p;h | PAWR |
| LINC00667 | hsa-miR-103a-3p; | PAWR |
| PKI55 | hsa-miR-1-3p;hsa | SLC25A22 |
| GS1-124K5.11 | hsa-miR-15a-5p;h | SLC25A22 |
| LINC01355 | hsa-miR-15a-5p;h | SLC25A22 |
| PWAR6 | hsa-miR-1-3p;hsa | SLC25A22 |
| LINC01355 | hsa-miR-30a-5p;h | SOX12 |
| MEG3 | hsa-miR-18a-5p;h | SOX12 |
| RP11-332H14.2 | hsa-miR-30a-5p;h | SOX12 |
| LINC01355 | hsa-miR-15a-5p;h | ZNF620 |
| LINC00467 | hsa-miR-130a-3p; | ZNF620 |
| GS1-124K5.11 | hsa-miR-15a-5p;h | ZNF620 |
| RP11-631N16.2 | hsa-miR-106a-5p; | ZNF354B |
| PWAR6 | hsa-miR-101-3p;h | ZNF223 |
| PWAR6 | hsa-miR-15a-5p;h | EDC3 |
| RP11-332H14.2 | hsa-miR-30a-5p;h | EDC3 |
| LINC01355 | hsa-miR-15a-5p;h | EDC3 |
| MEG3 | hsa-miR-15a-5p;h | EDC3 |
| RP11-631N16.2 | hsa-miR-30a-5p;h | FUCA1 |
| MEG3 | hsa-miR-106b-5p | C14orf28 |
| RP11-121C2.2 | hsa-miR-548ak;hsa | BBS10 |
| RP11-1055B8.4 | hsa-miR-103a-3p; | RCC1 |
| RP11-121C2.2 | hsa-miR-548ak;hsa | ZNRF2 |
| LL22NC03-2H8.5 | hsa-let-7b-5p;hsa | ZNF609 |
| RP11-701H24.4 | hsa-miR-106a-5p; | GPR157 |
| MEG3 | hsa-miR-106a-5p; | GPR157 |
| GS1-124K5.11 | hsa-miR-15a-5p;h | HIGD1A |
| MEG3 | hsa-miR-15a-5p;h | HIGD1A |
| PKI55 | hsa-miR-15a-5p;h | HIGD1A |
| RNU12 | hsa-miR-150-5p;h | FRAT2 |
| LINC00969 | hsa-miR-1225-5p; | ZNF678 |
| RP11-352M15.2 | hsa-miR-126-5p;h | PLAG1 |
| LINC00969 | hsa-miR-15a-5p;h | PLAG1 |
| LINC00667 | hsa-miR-107;hsa- | PLAG1 |
| LINC00969 | hsa-miR-15a-5p;h | YIPF6 |

| | | |
|---|---|---|
| RP11-701H24.4 | hsa-miR-519a-3p; | C5orf30 |
| PWAR6 | hsa-miR-15a-5p;h | EXT1 |
| LINC01355 | hsa-miR-15a-5p;h | EXT1 |
| PKI55 | hsa-miR-15a-5p;h | EXT1 |
| GS1-124K5.11 | hsa-miR-15a-5p;h | EXT1 |
| RP11-701H24.4 | hsa-miR-106a-5p; | CEP97 |
| LINC00106 | hsa-miR-17-3p;hs | MTA1 |
| RP11-631N16.2 | hsa-miR-30a-5p;h | FANCF |
| MEG3 | hsa-miR-125a-3p; | SMDT1 |
| PKI55 | hsa-miR-15a-5p;h | SMDT1 |
| GS1-124K5.11 | hsa-miR-15a-5p;h | SMDT1 |
| PWAR6 | hsa-miR-15a-5p;h | SMDT1 |
| LINC01355 | hsa-miR-15a-5p;h | SMDT1 |
| RP11-701H24.4 | hsa-miR-106a-5p; | CCDC125 |
| LINC00969 | hsa-miR-129-5p;h | SF3A3 |
| RNU12 | hsa-miR-150-5p;h | GTF2H2C |
| RP11-631N16.2 | hsa-miR-106a-5p; | GTF2H2C |
| GS1-124K5.11 | hsa-miR-15a-5p;h | CMTM4 |
| MEG3 | hsa-miR-1283;hsa | CMTM4 |
| LINC01355 | hsa-miR-15a-5p;h | CMTM4 |
| PWAR6 | hsa-miR-15a-5p;h | CMTM4 |
| SNHG8 | hsa-miR-124-3p;h | CHEK2 |
| RP11-121C2.2 | hsa-miR-26a-5p;h | CHEK2 |
| LINC00467 | hsa-miR-130a-3p; | CHEK2 |
| PKI55 | hsa-miR-124-3p;h | CHEK2 |
| PWAR6 | hsa-miR-15a-5p;h | VPS33B |
| PKI55 | hsa-miR-15a-5p;h | VPS33B |
| LINC01355 | hsa-miR-15a-5p;h | VPS33B |
| GS1-124K5.11 | hsa-miR-15a-5p;h | VPS33B |
| MEG3 | hsa-miR-15a-5p;h | VPS33B |
| LINC00969 | hsa-miR-199a-5p; | ZFP1 |
| RP11-631N16.2 | hsa-miR-106a-5p; | ATL3 |
| RP11-332H14.2 | hsa-miR-30a-5p;h | NDUFA12 |
| LINC01355 | hsa-miR-30a-5p;h | NDUFA12 |
| RP11-78O7.2 | hsa-let-7a-5p;hsa | SDR42E1 |
| RP11-121C2.2 | hsa-let-7a-5p;hsa | SDR42E1 |
| AC009404.2 | hsa-let-7a-5p;hsa | SDR42E1 |
| MEG3 | hsa-let-7a-5p;hsa | SDR42E1 |
| RP11-488L18.10 | hsa-let-7a-5p;hsa | SDR42E1 |
| PKI55 | hsa-let-7a-5p;hsa | SDR42E1 |
| RP11-121C2.2 | hsa-let-7a-5p;hsa | BRI3BP |
| RP11-488L18.10 | hsa-let-7a-5p;hsa | BRI3BP |
| RP11-78O7.2 | hsa-let-7a-5p;hsa | BRI3BP |
| AC009404.2 | hsa-let-7a-5p;hsa | BRI3BP |
| RP11-701H24.4 | hsa-miR-106a-5p; | BRI3BP |
| PKI55 | hsa-let-7a-5p;hsa | BRI3BP |
| MEG3 | hsa-let-7a-5p;hsa | BRI3BP |
| MEG3 | hsa-miR-106a-5p; | UBOX5 |
| RP11-701H24.4 | hsa-miR-106a-5p; | UBOX5 |
| RP11-488L18.10 | hsa-let-7a-5p;hsa | FAM43A |
| RP11-78O7.2 | hsa-let-7a-5p;hsa | FAM43A |
| RP11-121C2.2 | hsa-let-7a-5p;hsa | FAM43A |
| MEG3 | hsa-let-7a-5p;hsa | FAM43A |
| AC009404.2 | hsa-let-7a-5p;hsa | FAM43A |
| PKI55 | hsa-let-7a-5p;hsa | FAM43A |
| RNU12 | hsa-miR-15a-5p;h | PURA |
| RP11-701H24.4 | hsa-miR-106a-5p; | TNFAIP8L1 |
| RP11-332H14.2 | hsa-miR-150-5p;h | TNFAIP8L1 |

| | | |
|---|---|---|
| PWAR6 | hsa-miR-15a-5p;h | MRPL40 |
| LINC01355 | hsa-miR-15a-5p;h | MRPL40 |
| PKI55 | hsa-miR-15a-5p;h | MRPL40 |
| GS1-124K5.11 | hsa-miR-15a-5p;h | MRPL40 |
| MEG3 | hsa-miR-15a-5p;h | MRPL40 |
| RP11-631N16.2 | hsa-miR-106a-5p; | CXorf38 |
| RNU12 | hsa-miR-150-5p;h | CXorf38 |
| RP11-701H24.4 | hsa-miR-106a-5p; | WDR53 |
| MEG3 | hsa-miR-106a-5p; | WDR53 |
| RP11-78O7.2 | hsa-let-7a-5p;hsa | ZNF566 |
| RP11-121C2.2 | hsa-let-7a-5p;hsa | ZNF566 |
| PKI55 | hsa-let-7a-5p;hsa | ZNF566 |
| AC009404.2 | hsa-let-7a-5p;hsa | ZNF566 |
| RP11-488L18.10 | hsa-let-7a-5p;hsa | ZNF566 |
| RP11-631N16.2 | hsa-miR-106a-5p; | C15orf41 |
| RP11-631N16.2 | hsa-miR-106b-5p | ZBTB6 |
| RNU12 | hsa-miR-17-5p;hs | ZBTB6 |
| MEG3 | hsa-miR-15a-5p;h | ZNRF1 |
| GS1-124K5.11 | hsa-miR-15a-5p;h | ZNRF1 |
| MIRLET7BHG | hsa-miR-24-3p;hs | ZNF17 |
| PKI55 | hsa-miR-124-3p;h | BACE1 |
| MEG3 | hsa-miR-107;hsa- | BACE1 |
| PWAR6 | hsa-miR-124-3p;h | BACE1 |
| MEG3 | hsa-miR-106a-5p; | CCDC30 |
| RP11-701H24.4 | hsa-miR-106a-5p; | CCDC30 |
| RP11-498C9.15 | hsa-miR-548am-5 | ZNF395 |
| RNU12 | hsa-miR-548a-5p; | ZNF395 |
| RP11-78O7.2 | hsa-let-7a-5p;hsa | RHD |
| AC009404.2 | hsa-let-7a-5p;hsa | RHD |
| PKI55 | hsa-let-7a-5p;hsa | RHD |
| RP11-121C2.2 | hsa-let-7a-5p;hsa | RHD |
| RP11-488L18.10 | hsa-let-7a-5p;hsa | RHD |
| MEG3 | hsa-let-7a-5p;hsa | RHD |
| AC004951.6 | hsa-miR-30a-3p;h | ENTPD5 |
| MIRLET7BHG | hsa-miR-221-5p;h | DNAH17 |
| RP11-1055B8.4 | hsa-miR-221-5p;h | DNAH17 |
| RP11-631N16.2 | hsa-miR-106a-5p; | ZNF70 |
| RP11-631N16.2 | hsa-miR-181a-5p; | ZNF669 |
| LINC00969 | hsa-miR-15a-5p;h | PDCD1 |
| RNU12 | hsa-miR-15a-5p;h | PDCD1 |
| RP11-458F8.4 | hsa-let-7a-5p;hsa | BEND4 |
| LINC00630 | hsa-miR-25-3p;hs | TRMT2B |
| CTD-2587M2.1 | hsa-miR-25-3p;hs | TRMT2B |
| RP11-631N16.2 | hsa-miR-106a-5p; | ADAT2 |
| LL22NC03-2H8.5 | hsa-let-7a-5p;hsa | RNFT1 |
| SNHG8 | hsa-miR-18a-3p;h | FAM179A |
| LINC00667 | hsa-miR-181a-5p; | BLOC1S2 |
| RP11-631N16.2 | hsa-miR-181a-5p; | BLOC1S2 |
| LINC00630 | hsa-miR-25-3p;hs | SPATS2L |
| CTD-2587M2.1 | hsa-miR-25-3p;hs | SPATS2L |
| RP11-631N16.2 | hsa-miR-106a-5p; | GTF2IRD2 |
| RP11-631N16.2 | hsa-miR-106a-5p; | TRAPPC2 |
| AC009404.2 | hsa-let-7a-5p;hsa | ZNF799 |
| RP11-488L18.10 | hsa-let-7a-5p;hsa | ZNF799 |
| PKI55 | hsa-let-7a-5p;hsa | ZNF799 |
| RP11-78O7.2 | hsa-let-7a-5p;hsa | ZNF799 |
| RP11-121C2.2 | hsa-let-7a-5p;hsa | ZNF799 |
| LINC00667 | hsa-miR-135a-5p; | ZNF846 |

| | | |
|---|---|---|
| GS1-124K5.11 | hsa-miR-15a-5p;h | TECPR2 |
| PKI55 | hsa-miR-15a-5p;h | TECPR2 |
| RNU12 | hsa-miR-150-5p;h | TLR7 |
| RP11-631N16.2 | hsa-miR-106a-5p; | TLR7 |
| LL22NC03-2H8.5 | hsa-let-7a-5p;hsa | NHLRC2 |
| PKI55 | hsa-let-7a-5p;hsa | KPNA5 |
| RP11-121C2.2 | hsa-let-7a-5p;hsa | KPNA5 |
| RP11-78O7.2 | hsa-let-7a-5p;hsa | KPNA5 |
| RP11-488L18.10 | hsa-let-7a-5p;hsa | KPNA5 |
| AC009404.2 | hsa-let-7a-5p;hsa | KPNA5 |
| RP11-1055B8.4 | hsa-miR-30b-3p;h | SERTAD1 |
| LINC00969 | hsa-miR-15a-5p;h | SLC25A29 |
| RNU12 | hsa-miR-15a-5p;h | SLC25A29 |
| RNU12 | hsa-miR-1304-3p; | ZNF682 |
| RP11-631N16.2 | hsa-miR-106a-5p; | ZNF682 |
| LINC00630 | hsa-miR-25-3p;hs | ZNF772 |
| CTD-2587M2.1 | hsa-miR-137;hsa- | ZNF772 |
| RP11-79H23.3 | hsa-miR-23a-3p;h | ZNF257 |
| RP11-701H24.4 | hsa-miR-106a-5p; | ZNF785 |
| RP11-631N16.2 | hsa-miR-106a-5p; | DDI2 |
| RNU12 | hsa-miR-150-5p;h | DDI2 |
| RP11-701H24.4 | hsa-miR-106a-5p; | ZNF786 |
| RP11-631N16.2 | hsa-miR-106a-5p; | IPP |
| LINC00910 | hsa-let-7b-5p;hsa | RAB40C |
| PWAR6 | hsa-miR-101-3p;h | TAF13 |
| LINC01355 | hsa-miR-101-3p;h | TAF13 |
| GS1-124K5.11 | hsa-miR-103a-3p; | TAF13 |
| PKI55 | hsa-miR-101-3p;h | TAF13 |
| MEG3 | hsa-miR-103a-3p; | TAF13 |
| RP11-631N16.2 | hsa-miR-106a-5p; | ZNF780A |
| RP11-78O7.2 | hsa-let-7a-5p;hsa | HIST1H2BK |
| RP11-121C2.2 | hsa-let-7a-5p;hsa | HIST1H2BK |
| PKI55 | hsa-let-7a-5p;hsa | HIST1H2BK |
| RP11-488L18.10 | hsa-let-7a-5p;hsa | HIST1H2BK |
| MEG3 | hsa-let-7a-5p;hsa | HIST1H2BK |
| AC009404.2 | hsa-let-7a-5p;hsa | HIST1H2BK |
| RNU12 | hsa-miR-130a-5p; | ZNF273 |
| RP11-701H24.4 | hsa-miR-106a-5p; | PRIM1 |
| LINC00630 | hsa-miR-25-3p;hs | TOR4A |
| CTD-2587M2.1 | hsa-miR-25-3p;hs | TOR4A |
| PKI55 | hsa-miR-19a-3p;h | ASNA1 |
| SNHG8 | hsa-miR-149-3p;h | ASNA1 |
| LINC00969 | hsa-miR-1283;hsa | ZNF480 |
| GS1-124K5.11 | hsa-miR-15a-5p;h | TPM2 |
| LINC01355 | hsa-miR-15a-5p;h | TPM2 |
| PWAR6 | hsa-miR-1-3p;hsa | TPM2 |
| MEG3 | hsa-miR-125a-3p; | TPM2 |
| PKI55 | hsa-miR-1-3p;hsa | TPM2 |
| LL22NC03-2H8.5 | hsa-let-7a-5p;hsa | IPO9 |
| RP11-631N16.2 | hsa-miR-106b-5p | INPP5F |
| RP11-1055B8.4 | hsa-miR-199a-3p; | FICD |
| RP11-701H24.4 | hsa-miR-106a-5p; | FICD |
| PWAR6 | hsa-miR-17-5p;hs | FICD |
| MEG3 | hsa-miR-106a-5p; | FICD |
| LINC00467 | hsa-miR-130a-3p; | RUNDC1 |
| PKI55 | hsa-let-7a-5p;hsa | ATG9A |
| RP11-78O7.2 | hsa-let-7a-5p;hsa | ATG9A |
| RP11-121C2.2 | hsa-let-7a-5p;hsa | ATG9A |

| | | |
|---|---|---|
| MEG3 | hsa-let-7a-5p;hsa | ATG9A |
| RP11-488L18.10 | hsa-let-7a-5p;hsa | ATG9A |
| GS1-124K5.11 | hsa-miR-15a-5p;h | ATG9A |
| AC009404.2 | hsa-let-7a-5p;hsa | ATG9A |
| MEG3 | hsa-miR-186-3p;h | DMD |
| CTD-2587M2.1 | hsa-miR-25-3p;hs | TATDN3 |
| LINC00630 | hsa-miR-25-3p;hs | TATDN3 |
| RP11-498C9.15 | hsa-miR-23a-3p;h | SDHD |
| RNU12 | hsa-miR-130a-5p; | SDHD |
| LINC00969 | hsa-miR-1283;hsa | C5orf51 |
| RNU12 | hsa-miR-548a-5p; | KLRC3 |
| LINC00667 | hsa-miR-548ak;hs | KLRC3 |
| RP11-498C9.15 | hsa-miR-369-3p;h | KLRC3 |
| LINC01355 | hsa-miR-15a-5p;h | HN1L |
| MEG3 | hsa-miR-15a-5p;h | HN1L |
| PKI55 | hsa-miR-15a-5p;h | HN1L |
| PWAR6 | hsa-miR-15a-5p;h | HN1L |
| GS1-124K5.11 | hsa-miR-15a-5p;h | HN1L |
| RP11-631N16.2 | hsa-miR-106a-5p; | FGFR1OP |
| RNU12 | hsa-miR-15a-5p;h | CHUK |
| RNU12 | hsa-miR-150-5p;h | SYNJ2BP |
| RP11-631N16.2 | hsa-miR-106a-5p; | SYNJ2BP |
| LL22NC03-2H8.5 | hsa-let-7a-5p;hsa | SYNJ2BP |
| RP11-488L18.10 | hsa-let-7a-5p;hsa | NCKIPSD |
| AC009404.2 | hsa-let-7a-5p;hsa | NCKIPSD |
| RP11-78O7.2 | hsa-let-7a-5p;hsa | NCKIPSD |
| RP11-121C2.2 | hsa-let-7a-5p;hsa | NCKIPSD |
| SNHG8 | hsa-miR-125a-3p; | S1PR3 |
| PWAR6 | hsa-miR-124-3p;h | IFRD2 |
| PKI55 | hsa-miR-124-3p;h | IFRD2 |
| RP11-631N16.2 | hsa-miR-106a-5p; | TMEM242 |
| LINC00667 | hsa-miR-106b-5p | TMEM242 |
| RNU12 | hsa-miR-548a-5p; | TIAF1 |
| LL22NC03-2H8.5 | hsa-let-7a-5p;hsa | TIAF1 |
| RP11-498C9.15 | hsa-miR-548am-5 | TIAF1 |
| MEG3 | hsa-miR-106a-5p; | SMIM13 |
| RP11-701H24.4 | hsa-miR-106a-5p; | SMIM13 |
| RP11-631N16.2 | hsa-miR-106a-5p; | CDKN2AIPNL |
| RNU12 | hsa-miR-150-5p;h | CDKN2AIPNL |
| RP11-631N16.2 | hsa-miR-106a-5p; | LY6G5B |
| RNU12 | hsa-miR-150-5p;h | LY6G5B |
| RP11-631N16.2 | hsa-miR-106a-5p; | ISY1 |
| PKI55 | hsa-let-7a-5p;hsa | YAE1D1 |
| RP11-78O7.2 | hsa-let-7a-5p;hsa | YAE1D1 |
| MIRLET7BHG | hsa-miR-139-3p;h | YAE1D1 |
| RP11-488L18.10 | hsa-let-7a-5p;hsa | YAE1D1 |
| RP11-121C2.2 | hsa-let-7a-5p;hsa | YAE1D1 |
| AC009404.2 | hsa-let-7a-5p;hsa | YAE1D1 |
| RP11-121C2.2 | hsa-let-7a-5p;hsa | PEG10 |
| AC009404.2 | hsa-let-7a-5p;hsa | PEG10 |
| RP11-78O7.2 | hsa-let-7a-5p;hsa | PEG10 |
| RP11-488L18.10 | hsa-let-7a-5p;hsa | PEG10 |
| PKI55 | hsa-let-7a-5p;hsa | PEG10 |
| RNU12 | hsa-miR-15a-5p;h | AP5Z1 |
| LINC00969 | hsa-miR-137;hsa- | AP5Z1 |
| RNU12 | hsa-miR-150-5p;h | DDOST |
| RP11-121C2.2 | hsa-let-7a-5p;hsa | MARS2 |
| MEG3 | hsa-let-7a-5p;hsa | MARS2 |

| | | |
|---|---|---|
| RP11-488L18.10 | hsa-let-7a-5p;hsa | MARS2 |
| RP11-78O7.2 | hsa-let-7a-5p;hsa | MARS2 |
| PKI55 | hsa-let-7a-5p;hsa | MARS2 |
| AC009404.2 | hsa-let-7a-5p;hsa | MARS2 |
| LINC01006 | hsa-miR-199a-5p; | ZNF286B |
| MEG3 | hsa-miR-106a-5p; | CCDC71L |
| LINC01355 | hsa-miR-221-5p;h | CCDC71L |
| RP11-701H24.4 | hsa-miR-106a-5p; | CCDC71L |
| PWAR6 | hsa-miR-17-5p;hs | CCDC71L |
| RP11-332H14.2 | hsa-miR-30a-5p;h | CCDC71L |
| LINC00667 | hsa-miR-181a-5p; | ZNF253 |
| RNU12 | hsa-miR-130a-5p; | ZNF253 |
| MEG3 | hsa-miR-124-3p;h | GALNT4 |
| LINC00467 | hsa-miR-130a-3p; | GALNT4 |
| LINC00467 | hsa-miR-130a-3p; | POC1B-GALNT4 |
| LINC00969 | hsa-miR-17-3p;hs | ITGB3 |
| LINC00630 | hsa-miR-25-3p;hs | MYZAP |
| CTD-2587M2.1 | hsa-miR-25-3p;hs | MYZAP |
| LINC00667 | hsa-miR-125a-5p; | NBPF11 |
| RP11-631N16.2 | hsa-miR-106a-5p; | RNF115 |
| RNU12 | hsa-miR-150-5p;h | RNF115 |
| LINC00630 | hsa-miR-25-3p;hs | ZNF850 |
| CTD-2587M2.1 | hsa-miR-25-3p;hs | ZNF850 |
| LINC00467 | hsa-miR-130a-3p; | S1PR2 |
| MIRLET7BHG | hsa-miR-188-3p;h | NDUFA7 |
| RP11-701H24.4 | hsa-miR-106a-5p; | SPIB |
