# Supplementary material for: The Novel Regulatory Role of lncRNA-miRNA-mRNA Axis in Amyotrophic Lateral Sclerosis: An Integrated Bioinformatics Analysis
Source: Comput Math Methods Med. 2021 Apr 15;2021:5526179. doi: 10.1155/2021/5526179 (PMC8067776; doi:10.1155/2021/5526179)
Supplement: Supplementary 2 — Table S1: potential lncRNA-mRNA ceRNA relationship pairs (Excel file). [file 5526179.f2.pdf]

lncRNA	mRNA	p value	shared miRNAs
ENSG0000	ENSG0000	3.57E-07	hsa-miR-130a-3p;hsa-miR-148a-3p;hsa-miR-151a-3p;hsa-miR-
ENSG0000	ENSG0000	0.001221	hsa-miR-137;hsa-miR-196a-5p;hsa-miR-196b-5p;hsa-miR-204-
ENSG0000	ENSG0000	0.001768	hsa-miR-1224-3p;hsa-miR-124-3p;hsa-miR-17-3p;hsa-miR-49
ENSG0000	ENSG0000	0.000571	hsa-miR-1224-3p;hsa-miR-204-5p;hsa-miR-2114-5p;hsa-miR-
ENSG0000	ENSG0000	0.005446	hsa-miR-204-5p;hsa-miR-5682;hsa-miR-766-3p;hsa-miR-877-
ENSG0000	ENSG0000	0.000161	hsa-miR-124-3p;hsa-miR-2114-5p;hsa-miR-520a-5p;hsa-miR-
ENSG0000	ENSG0000	0.006907	hsa-miR-1224-3p;hsa-miR-520a-5p;hsa-miR-766-3p;hsa-miR-
ENSG0000	ENSG0000	0.003339	hsa-miR-204-5p;hsa-miR-4691-3p;hsa-miR-766-3p;hsa-miR-8
ENSG0000	ENSG0000	0.002738	hsa-miR-338-3p;hsa-miR-520a-5p;hsa-miR-5682;hsa-miR-766-
ENSG0000	ENSG0000	0.001221	hsa-miR-137;hsa-miR-196a-5p;hsa-miR-196b-5p;hsa-miR-319
ENSG0000	ENSG0000	6.57E-08	hsa-miR-181a-5p;hsa-miR-181b-5p;hsa-miR-181c-5p;hsa-miR-
ENSG0000	ENSG0000	1.44E-07	hsa-miR-181a-5p;hsa-miR-181b-5p;hsa-miR-181c-5p;hsa-miR-
ENSG0000	ENSG0000	3.07E-06	hsa-miR-181a-5p;hsa-miR-181b-5p;hsa-miR-181c-5p;hsa-miR-
ENSG0000	ENSG0000	1.30E-06	hsa-miR-181a-5p;hsa-miR-181b-5p;hsa-miR-181c-5p;hsa-miR-
ENSG0000	ENSG0000	1.30E-06	hsa-miR-181a-5p;hsa-miR-181b-5p;hsa-miR-181c-5p;hsa-miR-
ENSG0000	ENSG0000	3.18E-09	hsa-miR-181a-5p;hsa-miR-181b-5p;hsa-miR-181c-5p;hsa-miR-
ENSG0000	ENSG0000	0.001036	hsa-miR-378a-3p;hsa-miR-378c;hsa-miR-378d;hsa-miR-378i;hs
ENSG0000	ENSG0000	0.001036	hsa-miR-378a-3p;hsa-miR-378c;hsa-miR-378d;hsa-miR-378i;hs
ENSG0000	ENSG0000	0.000137	hsa-miR-185-3p;hsa-miR-378a-3p;hsa-miR-378c;hsa-miR-378
ENSG0000	ENSG0000	2.68E-06	hsa-miR-17-3p;hsa-miR-378a-3p;hsa-miR-378c;hsa-miR-378d;
ENSG0000	ENSG0000	0.001132	hsa-miR-19a-5p;hsa-miR-19b-1-5p;hsa-miR-377-5p;hsa-miR-
ENSG0000	ENSG0000	0.004969	hsa-miR-19a-5p;hsa-miR-19b-1-5p;hsa-miR-324-5p;hsa-miR-
ENSG0000	ENSG0000	0.000943	hsa-miR-19a-5p;hsa-miR-19b-1-5p;hsa-miR-3065-3p;hsa-miR-
ENSG0000	ENSG0000	6.17E-06	hsa-miR-378a-3p;hsa-miR-378c;hsa-miR-378d;hsa-miR-378i;hs
ENSG0000	ENSG0000	0.008812	hsa-miR-19a-5p;hsa-miR-19b-1-5p;hsa-miR-377-5p;hsa-miR-
ENSG0000	ENSG0000	0.008812	hsa-miR-151a-5p;hsa-miR-151b;hsa-miR-17-3p;hsa-miR-204-
ENSG0000	ENSG0000	0.00086	hsa-let-7d-5p;hsa-miR-185-5p;hsa-miR-377-5p;hsa-miR-378a-
ENSG0000	ENSG0000	0.000582	hsa-miR-378a-3p;hsa-miR-378c;hsa-miR-378d;hsa-miR-378i;hs
ENSG0000	ENSG0000	0.000569	hsa-miR-19a-5p;hsa-miR-19b-1-5p;hsa-miR-200a-5p;hsa-miR-
ENSG0000	ENSG0000	0.000569	hsa-miR-19a-5p;hsa-miR-19b-1-5p;hsa-miR-200a-5p;hsa-miR-
ENSG0000	ENSG0000	0.000569	hsa-miR-19a-5p;hsa-miR-19b-1-5p;hsa-miR-200a-5p;hsa-miR-
ENSG0000	ENSG0000	0.000569	hsa-miR-19a-5p;hsa-miR-19b-1-5p;hsa-miR-200a-5p;hsa-miR-
ENSG0000	ENSG0000	0.000569	hsa-miR-19a-5p;hsa-miR-19b-1-5p;hsa-miR-200a-5p;hsa-miR-
ENSG0000	ENSG0000	8.56E-05	hsa-miR-378a-3p;hsa-miR-378c;hsa-miR-378d;hsa-miR-378i;hs
ENSG0000	ENSG0000	2.05E-05	hsa-miR-378a-3p;hsa-miR-378c;hsa-miR-378d;hsa-miR-378i;hs
ENSG0000	ENSG0000	8.56E-05	hsa-miR-378a-3p;hsa-miR-378c;hsa-miR-378d;hsa-miR-378i;hs
ENSG0000	ENSG0000	0.006984	hsa-miR-204-3p;hsa-miR-30c-2-3p;hsa-miR-378a-3p;hsa-miR-
ENSG0000	ENSG0000	0.007199	hsa-let-7d-5p;hsa-miR-19a-5p;hsa-miR-19b-1-5p;hsa-miR-49
ENSG0000	ENSG0000	0.002069	hsa-let-7d-5p;hsa-miR-17-3p;hsa-miR-19a-5p;hsa-miR-19b-1-
ENSG0000	ENSG0000	0.006245	hsa-miR-19a-5p;hsa-miR-19b-1-5p;hsa-miR-30c-2-3p;hsa-mi
ENSG0000	ENSG0000	0.009874	hsa-miR-30c-2-3p;hsa-miR-340-3p;hsa-miR-377-5p;hsa-miR-
ENSG0000	ENSG0000	0.006245	hsa-miR-378a-3p;hsa-miR-378c;hsa-miR-378d;hsa-miR-378i;hs
ENSG0000	ENSG0000	7.46E-06	hsa-miR-324-5p;hsa-miR-378a-3p;hsa-miR-378c;hsa-miR-378
ENSG0000	ENSG0000	2.32E-05	hsa-miR-378a-3p;hsa-miR-378c;hsa-miR-378d;hsa-miR-378i;hs
ENSG0000	ENSG0000	0.000783	hsa-miR-378a-3p;hsa-miR-378c;hsa-miR-378d;hsa-miR-378i;hs
ENSG0000	ENSG0000	0.008812	hsa-miR-200a-5p;hsa-miR-200b-5p;hsa-miR-5010-3p;hsa-miR-
ENSG0000	ENSG0000	0.000903	hsa-let-7d-5p;hsa-miR-378a-3p;hsa-miR-378c;hsa-miR-378d;h
ENSG0000	ENSG0000	0.004775	hsa-miR-19a-5p;hsa-miR-19b-1-5p;hsa-miR-4691-3p;hsa-miR-
ENSG0000	ENSG0000	5.12E-05	hsa-miR-378a-3p;hsa-miR-378c;hsa-miR-378d;hsa-miR-378i;hs
ENSG0000	ENSG0000	0.004969	hsa-miR-200a-5p;hsa-miR-200b-5p;hsa-miR-30c-2-3p;hsa-mi
ENSG0000	ENSG0000	0.00624	hsa-miR-200a-5p;hsa-miR-200b-5p;hsa-miR-377-5p;hsa-miR-
ENSG0000	ENSG0000	0.00232	hsa-miR-377-5p;hsa-miR-378a-3p;hsa-miR-378c;hsa-miR-378
ENSG0000	ENSG0000	0.000117	hsa-miR-378a-3p;hsa-miR-378c;hsa-miR-378d;hsa-miR-378i;hs

ENSG0000	ENSG0000	0.000337	hsa-miR-378a-3p;hsa-miR-378c;hsa-miR-378d;hsa-miR-378i;hs
ENSG0000	ENSG0000	0.00825	hsa-miR-151a-5p;hsa-miR-151b;hsa-miR-30c-2-3p;hsa-miR-48
ENSG0000	ENSG0000	0.00825	hsa-miR-151a-5p;hsa-miR-151b;hsa-miR-30c-2-3p;hsa-miR-48
ENSG0000	ENSG0000	1.30E-06	hsa-let-7d-5p;hsa-miR-30c-2-3p;hsa-miR-378a-3p;hsa-miR-37
ENSG0000	ENSG0000	2.39E-05	hsa-miR-151a-5p;hsa-miR-151b;hsa-miR-19a-5p;hsa-miR-19b-
ENSG0000	ENSG0000	8.56E-05	hsa-miR-378a-3p;hsa-miR-378c;hsa-miR-378d;hsa-miR-378i;hs
ENSG0000	ENSG0000	1.22E-06	hsa-miR-378a-3p;hsa-miR-378c;hsa-miR-378d;hsa-miR-378i;hs
ENSG0000	ENSG0000	0.002294	hsa-miR-378a-3p;hsa-miR-378c;hsa-miR-378d;hsa-miR-378i;hs
ENSG0000	ENSG0000	0.009552	hsa-miR-30c-2-3p;hsa-miR-378a-3p;hsa-miR-378c;hsa-miR-37
ENSG0000	ENSG0000	0.000241	hsa-miR-151a-5p;hsa-miR-200a-5p;hsa-miR-200b-5p;hsa-miR-
ENSG0000	ENSG0000	3.51E-05	hsa-miR-378a-3p;hsa-miR-378c;hsa-miR-378d;hsa-miR-378i;hs
ENSG0000	ENSG0000	2.56E-06	hsa-miR-20a-3p;hsa-miR-217;hsa-miR-30c-2-3p;hsa-miR-377-
ENSG0000	ENSG0000	0.000137	hsa-miR-296-5p;hsa-miR-378c;hsa-miR-378d;hsa-miR-378i;hsa
ENSG0000	ENSG0000	0.005371	hsa-miR-200a-5p;hsa-miR-200b-5p;hsa-miR-30c-2-3p;hsa-miR
ENSG0000	ENSG0000	0.004169	hsa-miR-185-5p;hsa-miR-204-3p;hsa-miR-340-3p;hsa-miR-473
ENSG0000	ENSG0000	0.008812	hsa-miR-3065-3p;hsa-miR-30c-2-3p;hsa-miR-340-3p;hsa-miR-
ENSG0000	ENSG0000	0.001981	hsa-miR-185-5p;hsa-miR-19a-5p;hsa-miR-19b-1-5p;hsa-miR-2
ENSG0000	ENSG0000	2.86E-05	hsa-miR-378a-3p;hsa-miR-378c;hsa-miR-378d;hsa-miR-378i;hs
ENSG0000	ENSG0000	0.000299	hsa-miR-378a-3p;hsa-miR-378c;hsa-miR-378d;hsa-miR-378i;hs
ENSG0000	ENSG0000	0.006708	hsa-let-7d-5p;hsa-miR-377-5p;hsa-miR-485-5p;hsa-miR-488-3
ENSG0000	ENSG0000	0.000536	hsa-miR-377-5p;hsa-miR-378a-3p;hsa-miR-378c;hsa-miR-378c
ENSG0000	ENSG0000	1.64E-06	hsa-miR-132-3p;hsa-miR-212-3p;hsa-miR-362-3p;hsa-miR-603
ENSG0000	ENSG0000	1.08E-08	hsa-miR-132-3p;hsa-miR-212-3p;hsa-miR-362-3p;hsa-miR-603
ENSG0000	ENSG0000	2.64E-06	hsa-miR-132-3p;hsa-miR-212-3p;hsa-miR-362-3p;hsa-miR-603
ENSG0000	ENSG0000	2.64E-06	hsa-miR-132-3p;hsa-miR-212-3p;hsa-miR-362-3p;hsa-miR-603
ENSG0000	ENSG0000	0.00011	hsa-miR-17-5p;hsa-miR-20b-5p;hsa-miR-423-3p;hsa-miR-93-5
ENSG0000	ENSG0000	1.14E-05	hsa-miR-17-5p;hsa-miR-20b-5p;hsa-miR-423-3p;hsa-miR-93-5
ENSG0000	ENSG0000	1.81E-07	hsa-miR-17-5p;hsa-miR-20b-5p;hsa-miR-423-3p;hsa-miR-93-5
ENSG0000	ENSG0000	8.42E-07	hsa-miR-17-5p;hsa-miR-20b-5p;hsa-miR-423-3p;hsa-miR-93-5
ENSG0000	ENSG0000	8.55E-05	hsa-miR-19a-3p;hsa-miR-19b-3p;hsa-miR-766-3p;hsa-miR-785
ENSG0000	ENSG0000	6.20E-06	hsa-miR-19a-3p;hsa-miR-19b-3p;hsa-miR-365a-3p;hsa-miR-36
ENSG0000	ENSG0000	3.22E-05	hsa-miR-19a-3p;hsa-miR-19b-3p;hsa-miR-365a-3p;hsa-miR-36
ENSG0000	ENSG0000	9.06E-05	hsa-miR-19a-3p;hsa-miR-19b-3p;hsa-miR-365a-3p;hsa-miR-36
ENSG0000	ENSG0000	3.33E-07	hsa-miR-19a-3p;hsa-miR-19b-3p;hsa-miR-365a-3p;hsa-miR-36
ENSG0000	ENSG0000	0.000288	hsa-miR-365a-3p;hsa-miR-365b-3p;hsa-miR-766-3p;hsa-miR-7
ENSG0000	ENSG0000	0.002185	hsa-miR-126-5p;hsa-miR-142-3p;hsa-miR-212-5p;hsa-miR-502
ENSG0000	ENSG0000	1.10E-07	hsa-miR-27a-3p;hsa-miR-27b-3p;hsa-miR-30b-5p;hsa-miR-30c
ENSG0000	ENSG0000	0.000139	hsa-miR-27a-3p;hsa-miR-27b-3p;hsa-miR-30b-5p;hsa-miR-30c
ENSG0000	ENSG0000	6.35E-05	hsa-miR-27a-3p;hsa-miR-27b-3p;hsa-miR-361-5p;hsa-miR-425
ENSG0000	ENSG0000	3.46E-05	hsa-miR-218-5p;hsa-miR-27a-3p;hsa-miR-27b-3p;hsa-miR-425
ENSG0000	ENSG0000	8.07E-05	hsa-miR-218-5p;hsa-miR-27a-3p;hsa-miR-27b-3p;hsa-miR-369
ENSG0000	ENSG0000	0.002862	hsa-miR-27a-3p;hsa-miR-27b-3p;hsa-miR-30b-5p;hsa-miR-30c
ENSG0000	ENSG0000	4.91E-05	hsa-miR-218-5p;hsa-miR-27a-3p;hsa-miR-27b-3p;hsa-miR-497
ENSG0000	ENSG0000	0.000312	hsa-miR-27a-3p;hsa-miR-27b-3p;hsa-miR-30b-5p;hsa-miR-30c
ENSG0000	ENSG0000	7.76E-09	hsa-miR-27a-3p;hsa-miR-27b-3p;hsa-miR-30b-5p;hsa-miR-30c
ENSG0000	ENSG0000	0.003972	hsa-miR-27a-3p;hsa-miR-27b-3p;hsa-miR-30b-5p;hsa-miR-30c
ENSG0000	ENSG0000	2.00E-05	hsa-miR-218-5p;hsa-miR-27a-3p;hsa-miR-27b-3p;hsa-miR-497
ENSG0000	ENSG0000	0.000475	hsa-miR-218-5p;hsa-miR-30b-5p;hsa-miR-30c-5p;hsa-miR-497
ENSG0000	ENSG0000	0.000312	hsa-miR-218-5p;hsa-miR-27a-3p;hsa-miR-27b-3p;hsa-miR-497
ENSG0000	ENSG0000	0.000458	hsa-miR-27a-3p;hsa-miR-27b-3p;hsa-miR-30b-5p;hsa-miR-30c
ENSG0000	ENSG0000	0.000442	hsa-miR-126-5p;hsa-miR-218-5p;hsa-miR-27a-3p;hsa-miR-27b
ENSG0000	ENSG0000	9.55E-07	hsa-miR-27a-3p;hsa-miR-27b-3p;hsa-miR-30b-5p;hsa-miR-30c
ENSG0000	ENSG0000	9.55E-07	hsa-miR-27a-3p;hsa-miR-27b-3p;hsa-miR-30b-5p;hsa-miR-30c
ENSG0000	ENSG0000	3.46E-05	hsa-miR-27a-3p;hsa-miR-27b-3p;hsa-miR-30b-5p;hsa-miR-30c

ENSG0000	ENSG0000	3.33E-07	hsa-miR-27a-3p;hsa-miR-27b-3p;hsa-miR-30b-5p;hsa-miR-30
ENSG0000	ENSG0000	0.001155	hsa-miR-30b-5p;hsa-miR-30c-5p;hsa-miR-361-5p;hsa-miR-49
ENSG0000	ENSG0000	7.76E-09	hsa-miR-27a-3p;hsa-miR-27b-3p;hsa-miR-30b-5p;hsa-miR-30
ENSG0000	ENSG0000	7.76E-09	hsa-miR-27a-3p;hsa-miR-27b-3p;hsa-miR-30b-5p;hsa-miR-30
ENSG0000	ENSG0000	0.000115	hsa-miR-34b-5p;hsa-miR-3929;hsa-miR-449c-5p;hsa-miR-940
ENSG0000	ENSG0000	8.18E-05	hsa-miR-34b-5p;hsa-miR-3929;hsa-miR-449c-5p;hsa-miR-940
ENSG0000	ENSG0000	0.000495	hsa-miR-34b-5p;hsa-miR-449c-5p;hsa-miR-9-3p;hsa-miR-940
ENSG0000	ENSG0000	1.81E-05	hsa-miR-34b-5p;hsa-miR-3929;hsa-miR-449c-5p;hsa-miR-940
ENSG0000	ENSG0000	0.000256	hsa-miR-34b-5p;hsa-miR-3929;hsa-miR-449c-5p;hsa-miR-940
ENSG0000	ENSG0000	0.000612	hsa-miR-34b-5p;hsa-miR-3929;hsa-miR-449c-5p;hsa-miR-940
ENSG0000	ENSG0000	0.000273	hsa-miR-34b-5p;hsa-miR-3929;hsa-miR-449c-5p;hsa-miR-940
ENSG0000	ENSG0000	1.29E-08	hsa-miR-106b-5p;hsa-miR-148a-5p;hsa-miR-17-5p;hsa-miR-2
ENSG0000	ENSG0000	1.09E-07	hsa-miR-106a-5p;hsa-miR-106b-5p;hsa-miR-150-5p;hsa-miR-
ENSG0000	ENSG0000	1.54E-07	hsa-miR-106a-5p;hsa-miR-106b-5p;hsa-miR-20a-5p;hsa-miR-
ENSG0000	ENSG0000	0.001415	hsa-miR-519a-3p;hsa-miR-519b-3p;hsa-miR-519c-3p;hsa-miR
ENSG0000	ENSG0000	0.008274	hsa-miR-421;hsa-miR-519a-3p;hsa-miR-519b-3p;hsa-miR-519
ENSG0000	ENSG0000	0.000122	hsa-miR-17-5p;hsa-miR-20a-5p;hsa-miR-20b-5p;hsa-miR-421;
ENSG0000	ENSG0000	0.001521	hsa-miR-106b-5p;hsa-miR-17-5p;hsa-miR-20a-5p;hsa-miR-20
ENSG0000	ENSG0000	4.09E-14	hsa-miR-106a-5p;hsa-miR-106b-5p;hsa-miR-150-5p;hsa-miR-
ENSG0000	ENSG0000	0.0026	hsa-miR-106a-5p;hsa-miR-106b-5p;hsa-miR-17-5p;hsa-miR-2
ENSG0000	ENSG0000	6.91E-08	hsa-miR-106a-5p;hsa-miR-106b-5p;hsa-miR-17-5p;hsa-miR-2
ENSG0000	ENSG0000	3.10E-08	hsa-miR-106a-5p;hsa-miR-106b-5p;hsa-miR-17-5p;hsa-miR-2
ENSG0000	ENSG0000	4.61E-07	hsa-miR-106b-5p;hsa-miR-17-5p;hsa-miR-20a-5p;hsa-miR-20
ENSG0000	ENSG0000	4.44E-09	hsa-miR-106a-5p;hsa-miR-106b-5p;hsa-miR-150-5p;hsa-miR-
ENSG0000	ENSG0000	6.94E-05	hsa-miR-106b-5p;hsa-miR-17-5p;hsa-miR-20a-5p;hsa-miR-20
ENSG0000	ENSG0000	6.94E-05	hsa-miR-106b-5p;hsa-miR-20a-5p;hsa-miR-20b-5p;hsa-miR-5
ENSG0000	ENSG0000	1.09E-13	hsa-miR-106a-5p;hsa-miR-106b-5p;hsa-miR-150-5p;hsa-miR-
ENSG0000	ENSG0000	2.67E-09	hsa-miR-106a-5p;hsa-miR-106b-5p;hsa-miR-17-5p;hsa-miR-2
ENSG0000	ENSG0000	0.00393	hsa-miR-519a-3p;hsa-miR-519b-3p;hsa-miR-519c-3p;hsa-miR
ENSG0000	ENSG0000	0.001415	hsa-miR-186-3p;hsa-miR-30c-1-3p;hsa-miR-30c-2-3p;hsa-mi
ENSG0000	ENSG0000	1.08E-10	hsa-miR-106a-5p;hsa-miR-106b-5p;hsa-miR-1307-3p;hsa-miR
ENSG0000	ENSG0000	1.10E-05	hsa-miR-17-5p;hsa-miR-20a-5p;hsa-miR-20b-5p;hsa-miR-519
ENSG0000	ENSG0000	1.24E-10	hsa-miR-106a-5p;hsa-miR-106b-5p;hsa-miR-17-5p;hsa-miR-1
ENSG0000	ENSG0000	1.47E-05	hsa-miR-106b-5p;hsa-miR-17-5p;hsa-miR-192-5p;hsa-miR-20
ENSG0000	ENSG0000	0.000144	hsa-miR-106a-5p;hsa-miR-106b-5p;hsa-miR-17-5p;hsa-miR-2
ENSG0000	ENSG0000	6.27E-09	hsa-miR-106a-5p;hsa-miR-106b-5p;hsa-miR-17-5p;hsa-miR-2
ENSG0000	ENSG0000	0.000202	hsa-miR-519a-3p;hsa-miR-519b-3p;hsa-miR-519c-3p;hsa-miR
ENSG0000	ENSG0000	0.001521	hsa-miR-148a-5p;hsa-miR-150-5p;hsa-miR-186-3p;hsa-miR-2
ENSG0000	ENSG0000	5.33E-05	hsa-miR-106a-5p;hsa-miR-106b-5p;hsa-miR-17-5p;hsa-miR-2
ENSG0000	ENSG0000	1.30E-07	hsa-miR-106a-5p;hsa-miR-106b-5p;hsa-miR-135a-5p;hsa-miR
ENSG0000	ENSG0000	5.07E-05	hsa-miR-519a-3p;hsa-miR-519b-3p;hsa-miR-519c-3p;hsa-miR
ENSG0000	ENSG0000	3.91E-06	hsa-miR-106b-5p;hsa-miR-17-5p;hsa-miR-20a-5p;hsa-miR-20
ENSG0000	ENSG0000	6.65E-05	hsa-miR-106a-5p;hsa-miR-106b-5p;hsa-miR-20a-5p;hsa-miR-
ENSG0000	ENSG0000	2.22E-10	hsa-miR-106a-5p;hsa-miR-106b-5p;hsa-miR-150-5p;hsa-miR-
ENSG0000	ENSG0000	3.30E-06	hsa-miR-106a-5p;hsa-miR-106b-5p;hsa-miR-17-5p;hsa-miR-2
ENSG0000	ENSG0000	8.12E-13	hsa-miR-106a-5p;hsa-miR-106b-5p;hsa-miR-17-5p;hsa-miR-2
ENSG0000	ENSG0000	0.001634	hsa-miR-148a-5p;hsa-miR-186-3p;hsa-miR-30c-1-3p;hsa-miR
ENSG0000	ENSG0000	8.60E-06	hsa-miR-106b-5p;hsa-miR-17-5p;hsa-miR-20a-5p;hsa-miR-20
ENSG0000	ENSG0000	1.85E-11	hsa-miR-106a-5p;hsa-miR-106b-5p;hsa-miR-150-5p;hsa-miR-
ENSG0000	ENSG0000	0.000255	hsa-miR-519a-3p;hsa-miR-519b-3p;hsa-miR-519c-3p;hsa-miR
ENSG0000	ENSG0000	1.67E-08	hsa-miR-106a-5p;hsa-miR-106b-5p;hsa-miR-1271-3p;hsa-miR
ENSG0000	ENSG0000	6.61E-09	hsa-miR-106a-5p;hsa-miR-106b-5p;hsa-miR-17-5p;hsa-miR-2
ENSG0000	ENSG0000	3.67E-09	hsa-miR-106a-5p;hsa-miR-106b-5p;hsa-miR-17-5p;hsa-miR-2
ENSG0000	ENSG0000	2.49E-06	hsa-miR-106a-5p;hsa-miR-106b-5p;hsa-miR-17-5p;hsa-miR-2

ENSG0000	ENSG0000	1.03E-06	hsa-miR-106a-5p;hsa-miR-106b-5p;hsa-miR-135a-5p;hsa-miR-
ENSG0000	ENSG0000	6.46E-05	hsa-miR-106b-5p;hsa-miR-17-5p;hsa-miR-204-3p;hsa-miR-20
ENSG0000	ENSG0000	6.46E-05	hsa-miR-106b-5p;hsa-miR-17-5p;hsa-miR-204-3p;hsa-miR-20
ENSG0000	ENSG0000	0.00294	hsa-miR-106b-5p;hsa-miR-17-5p;hsa-miR-20a-5p;hsa-miR-20
ENSG0000	ENSG0000	1.13E-08	hsa-miR-106a-5p;hsa-miR-106b-5p;hsa-miR-17-5p;hsa-miR-20
ENSG0000	ENSG0000	5.45E-09	hsa-miR-106a-5p;hsa-miR-106b-5p;hsa-miR-17-5p;hsa-miR-20
ENSG0000	ENSG0000	9.31E-12	hsa-miR-106a-5p;hsa-miR-106b-5p;hsa-miR-17-5p;hsa-miR-20
ENSG0000	ENSG0000	1.59E-06	hsa-miR-106a-5p;hsa-miR-106b-5p;hsa-miR-17-5p;hsa-miR-20
ENSG0000	ENSG0000	0.001218	hsa-miR-519a-3p;hsa-miR-519b-3p;hsa-miR-519c-3p;hsa-miR-
ENSG0000	ENSG0000	1.07E-07	hsa-miR-106a-5p;hsa-miR-106b-5p;hsa-miR-17-5p;hsa-miR-20
ENSG0000	ENSG0000	4.65E-07	hsa-miR-106a-5p;hsa-miR-106b-5p;hsa-miR-17-5p;hsa-miR-20
ENSG0000	ENSG0000	4.44E-09	hsa-miR-106a-5p;hsa-miR-106b-5p;hsa-miR-17-5p;hsa-miR-18
ENSG0000	ENSG0000	3.14E-05	hsa-miR-192-5p;hsa-miR-20b-5p;hsa-miR-519a-3p;hsa-miR-51
ENSG0000	ENSG0000	1.25E-10	hsa-miR-106a-5p;hsa-miR-106b-5p;hsa-miR-150-5p;hsa-miR-1
ENSG0000	ENSG0000	0.000107	hsa-miR-106b-5p;hsa-miR-17-5p;hsa-miR-20a-5p;hsa-miR-20
ENSG0000	ENSG0000	4.61E-07	hsa-miR-106b-5p;hsa-miR-17-5p;hsa-miR-20a-5p;hsa-miR-20
ENSG0000	ENSG0000	2.93E-09	hsa-miR-106a-5p;hsa-miR-106b-5p;hsa-miR-150-5p;hsa-miR-1
ENSG0000	ENSG0000	7.67E-11	hsa-miR-106a-5p;hsa-miR-106b-5p;hsa-miR-17-5p;hsa-miR-20
ENSG0000	ENSG0000	1.23E-05	hsa-miR-106a-5p;hsa-miR-106b-5p;hsa-miR-17-5p;hsa-miR-20
ENSG0000	ENSG0000	2.30E-09	hsa-miR-106b-5p;hsa-miR-17-5p;hsa-miR-20a-5p;hsa-miR-20
ENSG0000	ENSG0000	2.30E-09	hsa-miR-106b-5p;hsa-miR-17-5p;hsa-miR-20a-5p;hsa-miR-20
ENSG0000	ENSG0000	2.30E-09	hsa-miR-106b-5p;hsa-miR-17-5p;hsa-miR-20a-5p;hsa-miR-20
ENSG0000	ENSG0000	2.30E-09	hsa-miR-106b-5p;hsa-miR-17-5p;hsa-miR-20a-5p;hsa-miR-20
ENSG0000	ENSG0000	2.30E-09	hsa-miR-106b-5p;hsa-miR-17-5p;hsa-miR-20a-5p;hsa-miR-20
ENSG0000	ENSG0000	2.30E-09	hsa-miR-106b-5p;hsa-miR-17-5p;hsa-miR-20a-5p;hsa-miR-20
ENSG0000	ENSG0000	2.30E-09	hsa-miR-106b-5p;hsa-miR-17-5p;hsa-miR-20a-5p;hsa-miR-20
ENSG0000	ENSG0000	7.68E-09	hsa-miR-106a-5p;hsa-miR-106b-5p;hsa-miR-1271-3p;hsa-miR
ENSG0000	ENSG0000	4.69E-08	hsa-miR-106b-5p;hsa-miR-17-5p;hsa-miR-20a-5p;hsa-miR-20
ENSG0000	ENSG0000	0.000255	hsa-miR-519a-3p;hsa-miR-519b-3p;hsa-miR-519c-3p;hsa-miR-
ENSG0000	ENSG0000	1.24E-07	hsa-miR-106a-5p;hsa-miR-106b-5p;hsa-miR-1307-3p;hsa-miR
ENSG0000	ENSG0000	7.97E-09	hsa-miR-106b-5p;hsa-miR-17-5p;hsa-miR-20a-5p;hsa-miR-20
ENSG0000	ENSG0000	1.24E-05	hsa-miR-106a-5p;hsa-miR-106b-5p;hsa-miR-186-3p;hsa-miR-2
ENSG0000	ENSG0000	0.001651	hsa-miR-106b-5p;hsa-miR-20a-5p;hsa-miR-20b-5p;hsa-miR-9
ENSG0000	ENSG0000	2.51E-05	hsa-miR-106b-5p;hsa-miR-17-5p;hsa-miR-20a-5p;hsa-miR-20
ENSG0000	ENSG0000	6.84E-11	hsa-miR-106a-5p;hsa-miR-106b-5p;hsa-miR-150-5p;hsa-miR-1
ENSG0000	ENSG0000	0.003373	hsa-miR-192-5p;hsa-miR-519a-3p;hsa-miR-519b-3p;hsa-miR-5
ENSG0000	ENSG0000	8.69E-05	hsa-miR-106a-5p;hsa-miR-106b-5p;hsa-miR-17-5p;hsa-miR-20
ENSG0000	ENSG0000	0.000317	hsa-miR-519a-3p;hsa-miR-519b-3p;hsa-miR-519c-3p;hsa-miR-
ENSG0000	ENSG0000	7.64E-05	hsa-miR-106a-5p;hsa-miR-106b-5p;hsa-miR-17-5p;hsa-miR-20
ENSG0000	ENSG0000	2.29E-11	hsa-miR-106a-5p;hsa-miR-106b-5p;hsa-miR-150-5p;hsa-miR-1
ENSG0000	ENSG0000	2.29E-11	hsa-miR-106a-5p;hsa-miR-106b-5p;hsa-miR-150-5p;hsa-miR-1
ENSG0000	ENSG0000	2.29E-11	hsa-miR-106a-5p;hsa-miR-106b-5p;hsa-miR-150-5p;hsa-miR-1
ENSG0000	ENSG0000	1.12E-11	hsa-miR-106a-5p;hsa-miR-106b-5p;hsa-miR-150-5p;hsa-miR-1
ENSG0000	ENSG0000	0.000436	hsa-miR-106a-5p;hsa-miR-106b-5p;hsa-miR-17-5p;hsa-miR-20
ENSG0000	ENSG0000	5.95E-05	hsa-miR-519a-3p;hsa-miR-519b-3p;hsa-miR-519c-3p;hsa-miR-
ENSG0000	ENSG0000	1.82E-12	hsa-miR-106a-5p;hsa-miR-106b-5p;hsa-miR-150-5p;hsa-miR-1
ENSG0000	ENSG0000	2.51E-05	hsa-miR-519a-3p;hsa-miR-519b-3p;hsa-miR-519c-3p;hsa-miR-
ENSG0000	ENSG0000	0.004916	hsa-miR-17-5p;hsa-miR-20a-5p;hsa-miR-20b-5p;hsa-miR-519
ENSG0000	ENSG0000	7.20E-11	hsa-miR-106a-5p;hsa-miR-106b-5p;hsa-miR-150-5p;hsa-miR-1
ENSG0000	ENSG0000	8.60E-06	hsa-miR-519a-3p;hsa-miR-519b-3p;hsa-miR-519c-3p;hsa-miR-
ENSG0000	ENSG0000	4.88E-08	hsa-miR-106a-5p;hsa-miR-106b-5p;hsa-miR-1307-3p;hsa-miR
ENSG0000	ENSG0000	6.48E-06	hsa-miR-106a-5p;hsa-miR-106b-5p;hsa-miR-135a-5p;hsa-miR-
ENSG0000	ENSG0000	2.88E-12	hsa-miR-106a-5p;hsa-miR-17-5p;hsa-miR-20a-5p;hsa-miR-20
ENSG0000	ENSG0000	0.001752	hsa-miR-519a-3p;hsa-miR-519b-3p;hsa-miR-519c-3p;hsa-miR-

ENSG0000	ENSG0000	2.22E-07	hsa-miR-106a-5p;hsa-miR-106b-5p;hsa-miR-17-5p;hsa-miR-2
ENSG0000	ENSG0000	2.06E-08	hsa-miR-106a-5p;hsa-miR-106b-5p;hsa-miR-17-5p;hsa-miR-2
ENSG0000	ENSG0000	5.97E-08	hsa-miR-106a-5p;hsa-miR-106b-5p;hsa-miR-150-5p;hsa-miR-
ENSG0000	ENSG0000	1.75E-06	hsa-miR-20a-5p;hsa-miR-519a-3p;hsa-miR-519b-3p;hsa-miR-
ENSG0000	ENSG0000	6.18E-07	hsa-miR-106a-5p;hsa-miR-106b-5p;hsa-miR-17-5p;hsa-miR-2
ENSG0000	ENSG0000	1.21E-06	hsa-miR-106a-5p;hsa-miR-106b-5p;hsa-miR-17-5p;hsa-miR-2
ENSG0000	ENSG0000	0.000179	hsa-miR-106b-5p;hsa-miR-17-5p;hsa-miR-20a-5p;hsa-miR-20
ENSG0000	ENSG0000	1.91E-08	hsa-miR-106a-5p;hsa-miR-106b-5p;hsa-miR-17-5p;hsa-miR-2
ENSG0000	ENSG0000	1.63E-09	hsa-miR-106a-5p;hsa-miR-106b-5p;hsa-miR-17-5p;hsa-miR-2
ENSG0000	ENSG0000	2.51E-10	hsa-miR-106a-5p;hsa-miR-106b-5p;hsa-miR-17-5p;hsa-miR-1
ENSG0000	ENSG0000	4.68E-12	hsa-miR-106a-5p;hsa-miR-106b-5p;hsa-miR-17-5p;hsa-miR-2
ENSG0000	ENSG0000	1.86E-07	hsa-miR-106a-5p;hsa-miR-106b-5p;hsa-miR-17-5p;hsa-miR-2
ENSG0000	ENSG0000	3.24E-09	hsa-miR-106a-5p;hsa-miR-106b-5p;hsa-miR-17-5p;hsa-miR-2
ENSG0000	ENSG0000	0.000887	hsa-miR-106b-5p;hsa-miR-17-5p;hsa-miR-20a-5p;hsa-miR-20
ENSG0000	ENSG0000	1.68E-06	hsa-miR-30c-1-3p;hsa-miR-30c-2-3p;hsa-miR-519a-3p;hsa-mi
ENSG0000	ENSG0000	8.75E-10	hsa-miR-106a-5p;hsa-miR-106b-5p;hsa-miR-17-5p;hsa-miR-2
ENSG0000	ENSG0000	1.76E-08	hsa-miR-106a-5p;hsa-miR-106b-5p;hsa-miR-17-5p;hsa-miR-2
ENSG0000	ENSG0000	0.000227	hsa-miR-519a-3p;hsa-miR-519b-3p;hsa-miR-519c-3p;hsa-miR
ENSG0000	ENSG0000	3.79E-10	hsa-miR-106a-5p;hsa-miR-106b-5p;hsa-miR-17-5p;hsa-miR-2
ENSG0000	ENSG0000	0.003509	hsa-miR-106b-5p;hsa-miR-17-5p;hsa-miR-20b-5p;hsa-miR-58
ENSG0000	ENSG0000	5.00E-07	hsa-miR-30c-1-3p;hsa-miR-30c-2-3p;hsa-miR-519a-3p;hsa-mi
ENSG0000	ENSG0000	2.37E-08	hsa-miR-106a-5p;hsa-miR-106b-5p;hsa-miR-17-5p;hsa-miR-2
ENSG0000	ENSG0000	1.36E-05	hsa-miR-519a-3p;hsa-miR-519b-3p;hsa-miR-519c-3p;hsa-miR
ENSG0000	ENSG0000	1.04E-06	hsa-miR-106a-5p;hsa-miR-106b-5p;hsa-miR-17-5p;hsa-miR-2
ENSG0000	ENSG0000	0.000317	hsa-miR-106b-5p;hsa-miR-17-5p;hsa-miR-20a-5p;hsa-miR-20
ENSG0000	ENSG0000	6.84E-11	hsa-miR-106a-5p;hsa-miR-106b-5p;hsa-miR-150-5p;hsa-miR-
ENSG0000	ENSG0000	0.002004	hsa-miR-106b-5p;hsa-miR-20a-5p;hsa-miR-20b-5p;hsa-miR-9
ENSG0000	ENSG0000	8.02E-09	hsa-miR-106a-5p;hsa-miR-106b-5p;hsa-miR-1307-3p;hsa-miR
ENSG0000	ENSG0000	9.99E-09	hsa-miR-106a-5p;hsa-miR-106b-5p;hsa-miR-17-5p;hsa-miR-2
ENSG0000	ENSG0000	0.000962	hsa-miR-17-5p;hsa-miR-20a-5p;hsa-miR-20b-5p;hsa-miR-93-
ENSG0000	ENSG0000	4.49E-10	hsa-miR-106a-5p;hsa-miR-106b-5p;hsa-miR-150-5p;hsa-miR-
ENSG0000	ENSG0000	1.47E-10	hsa-miR-106a-5p;hsa-miR-106b-5p;hsa-miR-17-5p;hsa-miR-2
ENSG0000	ENSG0000	3.14E-05	hsa-miR-106a-5p;hsa-miR-106b-5p;hsa-miR-17-5p;hsa-miR-2
ENSG0000	ENSG0000	4.02E-08	hsa-miR-106a-5p;hsa-miR-106b-5p;hsa-miR-17-5p;hsa-miR-2
ENSG0000	ENSG0000	1.10E-08	hsa-miR-106a-5p;hsa-miR-106b-5p;hsa-miR-17-5p;hsa-miR-2
ENSG0000	ENSG0000	1.60E-08	hsa-miR-106a-5p;hsa-miR-106b-5p;hsa-miR-17-5p;hsa-miR-2
ENSG0000	ENSG0000	2.17E-08	hsa-miR-106a-5p;hsa-miR-106b-5p;hsa-miR-17-5p;hsa-miR-2
ENSG0000	ENSG0000	0.002008	hsa-miR-519a-3p;hsa-miR-519b-3p;hsa-miR-519c-3p;hsa-miR
ENSG0000	ENSG0000	9.76E-06	hsa-miR-106b-5p;hsa-miR-17-5p;hsa-miR-188-3p;hsa-miR-20
ENSG0000	ENSG0000	0.002145	hsa-miR-519a-3p;hsa-miR-519b-3p;hsa-miR-519c-3p;hsa-miR
ENSG0000	ENSG0000	9.18E-06	hsa-miR-106a-5p;hsa-miR-106b-5p;hsa-miR-17-5p;hsa-miR-2
ENSG0000	ENSG0000	0.001079	hsa-miR-150-5p;hsa-miR-186-3p;hsa-miR-214-5p;hsa-miR-37
ENSG0000	ENSG0000	8.58E-09	hsa-miR-106a-5p;hsa-miR-106b-5p;hsa-miR-17-5p;hsa-miR-2
ENSG0000	ENSG0000	2.97E-12	hsa-miR-106a-5p;hsa-miR-106b-5p;hsa-miR-150-5p;hsa-miR-
ENSG0000	ENSG0000	9.99E-09	hsa-miR-106a-5p;hsa-miR-106b-5p;hsa-miR-148a-5p;hsa-miR
ENSG0000	ENSG0000	0.008703	hsa-miR-519a-3p;hsa-miR-519b-3p;hsa-miR-519c-3p;hsa-miR
ENSG0000	ENSG0000	5.18E-08	hsa-miR-106a-5p;hsa-miR-106b-5p;hsa-miR-17-5p;hsa-miR-2
ENSG0000	ENSG0000	2.85E-09	hsa-miR-106a-5p;hsa-miR-106b-5p;hsa-miR-17-5p;hsa-miR-2
ENSG0000	ENSG0000	1.27E-05	hsa-miR-2355-5p;hsa-miR-519a-3p;hsa-miR-519b-3p;hsa-miR
ENSG0000	ENSG0000	5.07E-05	hsa-miR-106b-5p;hsa-miR-17-5p;hsa-miR-20a-5p;hsa-miR-20
ENSG0000	ENSG0000	1.43E-06	hsa-miR-106a-5p;hsa-miR-106b-5p;hsa-miR-17-5p;hsa-miR-2
ENSG0000	ENSG0000	6.27E-09	hsa-miR-106a-5p;hsa-miR-106b-5p;hsa-miR-17-5p;hsa-miR-2
ENSG0000	ENSG0000	7.24E-07	hsa-miR-106a-5p;hsa-miR-106b-5p;hsa-miR-17-5p;hsa-miR-2
ENSG0000	ENSG0000	2.86E-07	hsa-miR-106a-5p;hsa-miR-20a-5p;hsa-miR-20b-5p;hsa-miR-5

ENSG0000	ENSG0000	1.71E-05	hsa-miR-106a-5p;hsa-miR-106b-5p;hsa-miR-17-5p;hsa-miR-2
ENSG0000	ENSG0000	0.000159	hsa-miR-106b-5p;hsa-miR-17-5p;hsa-miR-20a-5p;hsa-miR-20
ENSG0000	ENSG0000	4.30E-05	hsa-miR-106b-5p;hsa-miR-17-5p;hsa-miR-20a-5p;hsa-miR-20
ENSG0000	ENSG0000	6.62E-08	hsa-miR-106a-5p;hsa-miR-106b-5p;hsa-miR-150-5p;hsa-miR-
ENSG0000	ENSG0000	0.003943	hsa-miR-519a-3p;hsa-miR-519b-3p;hsa-miR-519c-3p;hsa-miR-
ENSG0000	ENSG0000	5.18E-08	hsa-miR-106a-5p;hsa-miR-106b-5p;hsa-miR-17-5p;hsa-miR-2
ENSG0000	ENSG0000	1.21E-06	hsa-miR-106a-5p;hsa-miR-106b-5p;hsa-miR-17-5p;hsa-miR-2
ENSG0000	ENSG0000	6.99E-06	hsa-miR-106a-5p;hsa-miR-106b-5p;hsa-miR-1307-3p;hsa-miR
ENSG0000	ENSG0000	6.20E-06	hsa-miR-106b-5p;hsa-miR-17-5p;hsa-miR-20a-5p;hsa-miR-93
ENSG0000	ENSG0000	0.001839	hsa-miR-106b-5p;hsa-miR-17-5p;hsa-miR-20a-5p;hsa-miR-51
ENSG0000	ENSG0000	0.001839	hsa-miR-106b-5p;hsa-miR-17-5p;hsa-miR-20a-5p;hsa-miR-51
ENSG0000	ENSG0000	2.30E-09	hsa-miR-106b-5p;hsa-miR-17-5p;hsa-miR-20a-5p;hsa-miR-20
ENSG0000	ENSG0000	1.96E-06	hsa-miR-106a-5p;hsa-miR-106b-5p;hsa-miR-17-5p;hsa-miR-2
ENSG0000	ENSG0000	6.61E-09	hsa-miR-106a-5p;hsa-miR-106b-5p;hsa-miR-150-5p;hsa-miR-
ENSG0000	ENSG0000	0.001415	hsa-miR-106b-5p;hsa-miR-17-5p;hsa-miR-20a-5p;hsa-miR-20
ENSG0000	ENSG0000	8.94E-08	hsa-miR-106a-5p;hsa-miR-106b-5p;hsa-miR-150-5p;hsa-miR-
ENSG0000	ENSG0000	1.25E-10	hsa-miR-106a-5p;hsa-miR-106b-5p;hsa-miR-150-5p;hsa-miR-
ENSG0000	ENSG0000	0.002766	hsa-miR-106b-5p;hsa-miR-188-3p;hsa-miR-20a-5p;hsa-miR-2
ENSG0000	ENSG0000	0.000425	hsa-miR-106a-5p;hsa-miR-106b-5p;hsa-miR-17-5p;hsa-miR-2
ENSG0000	ENSG0000	0.009302	hsa-miR-1271-3p;hsa-miR-150-5p;hsa-miR-214-5p;hsa-miR-5
ENSG0000	ENSG0000	7.35E-09	hsa-miR-106a-5p;hsa-miR-106b-5p;hsa-miR-17-5p;hsa-miR-2
ENSG0000	ENSG0000	6.62E-08	hsa-miR-106a-5p;hsa-miR-106b-5p;hsa-miR-150-5p;hsa-miR-
ENSG0000	ENSG0000	2.95E-07	hsa-miR-106a-5p;hsa-miR-106b-5p;hsa-miR-17-5p;hsa-miR-1
ENSG0000	ENSG0000	3.76E-06	hsa-miR-106b-5p;hsa-miR-17-5p;hsa-miR-20a-5p;hsa-miR-20
ENSG0000	ENSG0000	0.000816	hsa-miR-106b-5p;hsa-miR-150-5p;hsa-miR-17-5p;hsa-miR-18
ENSG0000	ENSG0000	3.13E-08	hsa-miR-106b-5p;hsa-miR-17-5p;hsa-miR-192-5p;hsa-miR-20
ENSG0000	ENSG0000	3.95E-14	hsa-miR-106a-5p;hsa-miR-106b-5p;hsa-miR-17-5p;hsa-miR-2
ENSG0000	ENSG0000	0.000809	hsa-miR-106a-5p;hsa-miR-17-5p;hsa-miR-20a-5p;hsa-miR-20
ENSG0000	ENSG0000	2.30E-09	hsa-miR-106b-5p;hsa-miR-17-5p;hsa-miR-20a-5p;hsa-miR-20
ENSG0000	ENSG0000	1.69E-09	hsa-miR-106a-5p;hsa-miR-106b-5p;hsa-miR-20a-5p;hsa-miR-
ENSG0000	ENSG0000	2.21E-10	hsa-miR-106a-5p;hsa-miR-106b-5p;hsa-miR-150-5p;hsa-miR-
ENSG0000	ENSG0000	0.002266	hsa-miR-106a-5p;hsa-miR-106b-5p;hsa-miR-17-5p;hsa-miR-2
ENSG0000	ENSG0000	8.49E-06	hsa-miR-106a-5p;hsa-miR-106b-5p;hsa-miR-17-5p;hsa-miR-2
ENSG0000	ENSG0000	7.97E-09	hsa-miR-106b-5p;hsa-miR-17-5p;hsa-miR-20a-5p;hsa-miR-20
ENSG0000	ENSG0000	2.51E-10	hsa-miR-106a-5p;hsa-miR-106b-5p;hsa-miR-17-5p;hsa-miR-1
ENSG0000	ENSG0000	2.51E-10	hsa-miR-106a-5p;hsa-miR-106b-5p;hsa-miR-17-5p;hsa-miR-1
ENSG0000	ENSG0000	0.000104	hsa-miR-106a-5p;hsa-miR-106b-5p;hsa-miR-17-5p;hsa-miR-2
ENSG0000	ENSG0000	2.82E-08	hsa-miR-106a-5p;hsa-miR-106b-5p;hsa-miR-148a-5p;hsa-miR
ENSG0000	ENSG0000	3.67E-12	hsa-miR-106a-5p;hsa-miR-106b-5p;hsa-miR-1307-3p;hsa-miR
ENSG0000	ENSG0000	1.63E-09	hsa-miR-106a-5p;hsa-miR-106b-5p;hsa-miR-150-5p;hsa-miR-
ENSG0000	ENSG0000	0.000388	hsa-miR-106a-5p;hsa-miR-106b-5p;hsa-miR-17-5p;hsa-miR-1
ENSG0000	ENSG0000	0.000388	hsa-miR-106a-5p;hsa-miR-106b-5p;hsa-miR-17-5p;hsa-miR-1
ENSG0000	ENSG0000	6.08E-05	hsa-miR-106a-5p;hsa-miR-106b-5p;hsa-miR-20a-5p;hsa-miR-
ENSG0000	ENSG0000	2.93E-09	hsa-miR-106a-5p;hsa-miR-106b-5p;hsa-miR-150-5p;hsa-miR-
ENSG0000	ENSG0000	2.00E-08	hsa-miR-106a-5p;hsa-miR-106b-5p;hsa-miR-150-5p;hsa-miR-
ENSG0000	ENSG0000	0.000816	hsa-miR-519a-3p;hsa-miR-519b-3p;hsa-miR-519c-3p;hsa-miR-
ENSG0000	ENSG0000	0.000352	hsa-miR-30c-1-3p;hsa-miR-30c-2-3p;hsa-miR-519a-3p;hsa-mi
ENSG0000	ENSG0000	7.05E-10	hsa-miR-106a-5p;hsa-miR-106b-5p;hsa-miR-150-5p;hsa-miR-
ENSG0000	ENSG0000	8.95E-10	hsa-miR-106a-5p;hsa-miR-106b-5p;hsa-miR-17-5p;hsa-miR-2
ENSG0000	ENSG0000	4.69E-08	hsa-miR-106b-5p;hsa-miR-17-5p;hsa-miR-20a-5p;hsa-miR-20
ENSG0000	ENSG0000	1.63E-09	hsa-miR-106a-5p;hsa-miR-106b-5p;hsa-miR-17-5p;hsa-miR-2
ENSG0000	ENSG0000	1.24E-12	hsa-miR-106a-5p;hsa-miR-106b-5p;hsa-miR-17-5p;hsa-miR-2
ENSG0000	ENSG0000	3.87E-06	hsa-miR-30c-1-3p;hsa-miR-30c-2-3p;hsa-miR-519a-3p;hsa-mi
ENSG0000	ENSG0000	1.03E-06	hsa-miR-150-5p;hsa-miR-519a-3p;hsa-miR-519b-3p;hsa-miR-

ENSG0000	ENSG0000	0.001128	hsa-miR-106a-5p;hsa-miR-17-5p;hsa-miR-20a-5p;hsa-miR-52
ENSG0000	ENSG0000	9.49E-08	hsa-miR-106a-5p;hsa-miR-106b-5p;hsa-miR-17-5p;hsa-miR-2
ENSG0000	ENSG0000	3.10E-08	hsa-miR-106a-5p;hsa-miR-106b-5p;hsa-miR-1307-3p;hsa-miR
ENSG0000	ENSG0000	0.00014	hsa-miR-150-5p;hsa-miR-17-5p;hsa-miR-186-3p;hsa-miR-520
ENSG0000	ENSG0000	0.000163	hsa-miR-106b-5p;hsa-miR-150-5p;hsa-miR-17-5p;hsa-miR-20
ENSG0000	ENSG0000	1.39E-11	hsa-miR-106a-5p;hsa-miR-106b-5p;hsa-miR-17-5p;hsa-miR-2
ENSG0000	ENSG0000	2.41E-09	hsa-miR-106a-5p;hsa-miR-106b-5p;hsa-miR-20b-5p;hsa-miR-
ENSG0000	ENSG0000	6.07E-11	hsa-miR-106a-5p;hsa-miR-106b-5p;hsa-miR-150-5p;hsa-miR-
ENSG0000	ENSG0000	5.75E-11	hsa-miR-106a-5p;hsa-miR-106b-5p;hsa-miR-17-5p;hsa-miR-2
ENSG0000	ENSG0000	0.008778	hsa-miR-150-5p;hsa-miR-186-3p;hsa-miR-520a-3p;hsa-miR-5
ENSG0000	ENSG0000	4.56E-05	hsa-miR-106a-5p;hsa-miR-106b-5p;hsa-miR-17-5p;hsa-miR-2
ENSG0000	ENSG0000	7.97E-09	hsa-miR-106b-5p;hsa-miR-17-5p;hsa-miR-20a-5p;hsa-miR-20
ENSG0000	ENSG0000	0.000153	hsa-miR-106a-5p;hsa-miR-106b-5p;hsa-miR-150-5p;hsa-miR-
ENSG0000	ENSG0000	6.50E-13	hsa-miR-106a-5p;hsa-miR-106b-5p;hsa-miR-150-5p;hsa-miR-
ENSG0000	ENSG0000	3.45E-07	hsa-miR-106a-5p;hsa-miR-106b-5p;hsa-miR-17-5p;hsa-miR-2
ENSG0000	ENSG0000	4.85E-10	hsa-miR-106a-5p;hsa-miR-106b-5p;hsa-miR-17-5p;hsa-miR-2
ENSG0000	ENSG0000	4.85E-10	hsa-miR-106a-5p;hsa-miR-106b-5p;hsa-miR-17-5p;hsa-miR-2
ENSG0000	ENSG0000	1.59E-09	hsa-miR-106a-5p;hsa-miR-106b-5p;hsa-miR-1307-3p;hsa-miR
ENSG0000	ENSG0000	3.00E-14	hsa-miR-106a-5p;hsa-miR-106b-5p;hsa-miR-1271-3p;hsa-miR
ENSG0000	ENSG0000	2.01E-10	hsa-miR-106a-5p;hsa-miR-106b-5p;hsa-miR-17-5p;hsa-miR-2
ENSG0000	ENSG0000	3.95E-07	hsa-miR-106a-5p;hsa-miR-106b-5p;hsa-miR-17-5p;hsa-miR-2
ENSG0000	ENSG0000	5.49E-13	hsa-miR-106a-5p;hsa-miR-106b-5p;hsa-miR-17-5p;hsa-miR-2
ENSG0000	ENSG0000	7.48E-11	hsa-miR-106a-5p;hsa-miR-106b-5p;hsa-miR-17-5p;hsa-miR-2
ENSG0000	ENSG0000	4.17E-07	hsa-miR-106a-5p;hsa-miR-106b-5p;hsa-miR-17-5p;hsa-miR-2
ENSG0000	ENSG0000	2.51E-10	hsa-miR-106a-5p;hsa-miR-106b-5p;hsa-miR-17-5p;hsa-miR-2
ENSG0000	ENSG0000	4.79E-06	hsa-miR-106a-5p;hsa-miR-106b-5p;hsa-miR-17-5p;hsa-miR-1
ENSG0000	ENSG0000	0.004916	hsa-miR-106b-5p;hsa-miR-20a-5p;hsa-miR-20b-5p;hsa-miR-9
ENSG0000	ENSG0000	1.18E-06	hsa-miR-106a-5p;hsa-miR-106b-5p;hsa-miR-17-5p;hsa-miR-2
ENSG0000	ENSG0000	1.96E-06	hsa-miR-106a-5p;hsa-miR-106b-5p;hsa-miR-17-5p;hsa-miR-2
ENSG0000	ENSG0000	9.29E-08	hsa-miR-106b-5p;hsa-miR-17-5p;hsa-miR-20a-5p;hsa-miR-20
ENSG0000	ENSG0000	2.09E-07	hsa-miR-106a-5p;hsa-miR-106b-5p;hsa-miR-17-5p;hsa-miR-2
ENSG0000	ENSG0000	1.52E-10	hsa-miR-106a-5p;hsa-miR-106b-5p;hsa-miR-150-5p;hsa-miR-
ENSG0000	ENSG0000	1.05E-06	hsa-miR-106b-5p;hsa-miR-17-5p;hsa-miR-20a-5p;hsa-miR-20
ENSG0000	ENSG0000	0.000135	hsa-miR-192-5p;hsa-miR-519a-3p;hsa-miR-519b-3p;hsa-miR-
ENSG0000	ENSG0000	2.29E-09	hsa-miR-106a-5p;hsa-miR-106b-5p;hsa-miR-17-5p;hsa-miR-2
ENSG0000	ENSG0000	2.29E-09	hsa-miR-106a-5p;hsa-miR-106b-5p;hsa-miR-17-5p;hsa-miR-2
ENSG0000	ENSG0000	2.22E-06	hsa-miR-106a-5p;hsa-miR-106b-5p;hsa-miR-17-5p;hsa-miR-1
ENSG0000	ENSG0000	2.54E-12	hsa-miR-106a-5p;hsa-miR-106b-5p;hsa-miR-150-5p;hsa-miR-
ENSG0000	ENSG0000	0.001263	hsa-miR-106a-5p;hsa-miR-106b-5p;hsa-miR-17-5p;hsa-miR-2
ENSG0000	ENSG0000	0.00393	hsa-miR-186-3p;hsa-miR-188-3p;hsa-miR-30c-1-3p;hsa-miR-
ENSG0000	ENSG0000	2.30E-09	hsa-miR-106b-5p;hsa-miR-17-5p;hsa-miR-20a-5p;hsa-miR-20
ENSG0000	ENSG0000	1.90E-05	hsa-miR-106a-5p;hsa-miR-106b-5p;hsa-miR-17-5p;hsa-miR-2
ENSG0000	ENSG0000	5.03E-12	hsa-miR-106a-5p;hsa-miR-106b-5p;hsa-miR-20a-5p;hsa-miR-
ENSG0000	ENSG0000	6.94E-05	hsa-miR-106b-5p;hsa-miR-17-5p;hsa-miR-20a-5p;hsa-miR-20
ENSG0000	ENSG0000	6.94E-05	hsa-miR-106b-5p;hsa-miR-17-5p;hsa-miR-20a-5p;hsa-miR-20
ENSG0000	ENSG0000	6.94E-05	hsa-miR-106b-5p;hsa-miR-17-5p;hsa-miR-20a-5p;hsa-miR-20
ENSG0000	ENSG0000	6.94E-05	hsa-miR-106b-5p;hsa-miR-17-5p;hsa-miR-20a-5p;hsa-miR-20
ENSG0000	ENSG0000	6.94E-05	hsa-miR-106b-5p;hsa-miR-17-5p;hsa-miR-20a-5p;hsa-miR-20
ENSG0000	ENSG0000	6.94E-05	hsa-miR-106b-5p;hsa-miR-17-5p;hsa-miR-20a-5p;hsa-miR-20
ENSG0000	ENSG0000	8.98E-05	hsa-miR-106a-5p;hsa-miR-106b-5p;hsa-miR-17-5p;hsa-miR-2
ENSG0000	ENSG0000	0.000573	hsa-miR-150-5p;hsa-miR-186-3p;hsa-miR-378a-5p;hsa-miR-5
ENSG0000	ENSG0000	2.21E-10	hsa-miR-106a-5p;hsa-miR-106b-5p;hsa-miR-150-5p;hsa-miR-
ENSG0000	ENSG0000	1.34E-05	hsa-miR-106a-5p;hsa-miR-106b-5p;hsa-miR-20a-5p;hsa-miR-
ENSG0000	ENSG0000	0.005411	hsa-miR-106a-5p;hsa-miR-519a-3p;hsa-miR-519b-3p;hsa-miR

ENSG0000	ENSG0000	2.38E-07	hsa-miR-106a-5p;hsa-miR-106b-5p;hsa-miR-17-5p;hsa-miR-2
ENSG0000	ENSG0000	4.69E-08	hsa-miR-106b-5p;hsa-miR-17-5p;hsa-miR-20a-5p;hsa-miR-20
ENSG0000	ENSG0000	4.69E-08	hsa-miR-106b-5p;hsa-miR-17-5p;hsa-miR-20a-5p;hsa-miR-20
ENSG0000	ENSG0000	4.26E-13	hsa-miR-106a-5p;hsa-miR-106b-5p;hsa-miR-150-5p;hsa-miR-
ENSG0000	ENSG0000	7.53E-09	hsa-miR-106a-5p;hsa-miR-106b-5p;hsa-miR-150-5p;hsa-miR-
ENSG0000	ENSG0000	4.85E-10	hsa-miR-106a-5p;hsa-miR-106b-5p;hsa-miR-1307-3p;hsa-miR
ENSG0000	ENSG0000	1.37E-11	hsa-miR-106a-5p;hsa-miR-106b-5p;hsa-miR-17-5p;hsa-miR-2
ENSG0000	ENSG0000	4.60E-07	hsa-miR-106b-5p;hsa-miR-17-5p;hsa-miR-20a-5p;hsa-miR-20
ENSG0000	ENSG0000	8.56E-09	hsa-miR-106a-5p;hsa-miR-106b-5p;hsa-miR-17-5p;hsa-miR-2
ENSG0000	ENSG0000	2.68E-08	hsa-miR-106a-5p;hsa-miR-106b-5p;hsa-miR-17-5p;hsa-miR-2
ENSG0000	ENSG0000	6.70E-12	hsa-miR-106a-5p;hsa-miR-106b-5p;hsa-miR-150-5p;hsa-miR-
ENSG0000	ENSG0000	1.67E-07	hsa-miR-106a-5p;hsa-miR-106b-5p;hsa-miR-17-5p;hsa-miR-2
ENSG0000	ENSG0000	0.000352	hsa-miR-106b-5p;hsa-miR-17-5p;hsa-miR-20a-5p;hsa-miR-20
ENSG0000	ENSG0000	8.06E-05	hsa-miR-148a-5p;hsa-miR-150-5p;hsa-miR-186-3p;hsa-miR-2
ENSG0000	ENSG0000	6.24E-10	hsa-miR-106a-5p;hsa-miR-106b-5p;hsa-miR-17-5p;hsa-miR-2
ENSG0000	ENSG0000	0.000352	hsa-miR-519a-3p;hsa-miR-519b-3p;hsa-miR-519c-3p;hsa-miR
ENSG0000	ENSG0000	2.03E-08	hsa-miR-106a-5p;hsa-miR-106b-5p;hsa-miR-1271-3p;hsa-miR
ENSG0000	ENSG0000	2.29E-11	hsa-miR-106a-5p;hsa-miR-106b-5p;hsa-miR-150-5p;hsa-miR-
ENSG0000	ENSG0000	2.27E-08	hsa-miR-106a-5p;hsa-miR-17-5p;hsa-miR-20a-5p;hsa-miR-20
ENSG0000	ENSG0000	9.29E-08	hsa-miR-106b-5p;hsa-miR-17-5p;hsa-miR-20a-5p;hsa-miR-20
ENSG0000	ENSG0000	3.13E-07	hsa-miR-106a-5p;hsa-miR-106b-5p;hsa-miR-17-5p;hsa-miR-2
ENSG0000	ENSG0000	0.000123	hsa-miR-17-5p;hsa-miR-20a-5p;hsa-miR-20b-5p;hsa-miR-450
ENSG0000	ENSG0000	1.52E-10	hsa-miR-106a-5p;hsa-miR-106b-5p;hsa-miR-150-5p;hsa-miR-
ENSG0000	ENSG0000	3.37E-09	hsa-miR-106a-5p;hsa-miR-106b-5p;hsa-miR-150-5p;hsa-miR-
ENSG0000	ENSG0000	0.007789	hsa-miR-106b-5p;hsa-miR-17-5p;hsa-miR-20a-5p;hsa-miR-20
ENSG0000	ENSG0000	3.39E-05	hsa-miR-106a-5p;hsa-miR-106b-5p;hsa-miR-17-5p;hsa-miR-2
ENSG0000	ENSG0000	3.62E-05	hsa-miR-106b-5p;hsa-miR-17-5p;hsa-miR-20a-5p;hsa-miR-20
ENSG0000	ENSG0000	9.12E-10	hsa-miR-106a-5p;hsa-miR-106b-5p;hsa-miR-150-5p;hsa-miR-
ENSG0000	ENSG0000	2.58E-11	hsa-miR-106a-5p;hsa-miR-106b-5p;hsa-miR-20a-5p;hsa-miR-
ENSG0000	ENSG0000	2.30E-09	hsa-miR-106b-5p;hsa-miR-17-5p;hsa-miR-20a-5p;hsa-miR-20
ENSG0000	ENSG0000	3.14E-10	hsa-miR-106a-5p;hsa-miR-106b-5p;hsa-miR-17-5p;hsa-miR-2
ENSG0000	ENSG0000	1.59E-09	hsa-miR-106a-5p;hsa-miR-106b-5p;hsa-miR-17-5p;hsa-miR-2
ENSG0000	ENSG0000	6.19E-07	hsa-miR-106a-5p;hsa-miR-17-5p;hsa-miR-20a-5p;hsa-miR-20
ENSG0000	ENSG0000	1.40E-09	hsa-miR-106a-5p;hsa-miR-106b-5p;hsa-miR-1307-3p;hsa-miR
ENSG0000	ENSG0000	0.000321	hsa-miR-106a-5p;hsa-miR-20a-5p;hsa-miR-20b-5p;hsa-miR-5
ENSG0000	ENSG0000	0.000321	hsa-miR-106a-5p;hsa-miR-20a-5p;hsa-miR-20b-5p;hsa-miR-5
ENSG0000	ENSG0000	0.000321	hsa-miR-106a-5p;hsa-miR-20a-5p;hsa-miR-20b-5p;hsa-miR-5
ENSG0000	ENSG0000	2.74E-07	hsa-miR-106a-5p;hsa-miR-106b-5p;hsa-miR-17-5p;hsa-miR-2
ENSG0000	ENSG0000	2.74E-07	hsa-miR-106a-5p;hsa-miR-106b-5p;hsa-miR-17-5p;hsa-miR-2
ENSG0000	ENSG0000	0.002862	hsa-miR-135a-5p;hsa-miR-214-5p;hsa-miR-30c-1-3p;hsa-miR
ENSG0000	ENSG0000	2.73E-07	hsa-miR-1271-3p;hsa-miR-150-5p;hsa-miR-214-5p;hsa-miR-3
ENSG0000	ENSG0000	6.14E-10	hsa-miR-106a-5p;hsa-miR-106b-5p;hsa-miR-150-5p;hsa-miR-
ENSG0000	ENSG0000	4.30E-05	hsa-miR-106b-5p;hsa-miR-17-5p;hsa-miR-20a-5p;hsa-miR-20
ENSG0000	ENSG0000	7.68E-06	hsa-miR-106b-5p;hsa-miR-17-5p;hsa-miR-20a-5p;hsa-miR-20
ENSG0000	ENSG0000	2.06E-05	hsa-miR-106b-5p;hsa-miR-17-5p;hsa-miR-20a-5p;hsa-miR-20
ENSG0000	ENSG0000	7.68E-06	hsa-miR-106a-5p;hsa-miR-106b-5p;hsa-miR-20a-5p;hsa-miR-
ENSG0000	ENSG0000	6.71E-06	hsa-miR-106b-5p;hsa-miR-17-5p;hsa-miR-20a-5p;hsa-miR-20
ENSG0000	ENSG0000	2.05E-05	hsa-miR-106b-5p;hsa-miR-17-5p;hsa-miR-20a-5p;hsa-miR-20
ENSG0000	ENSG0000	3.00E-08	hsa-miR-106a-5p;hsa-miR-106b-5p;hsa-miR-1307-3p;hsa-miR
ENSG0000	ENSG0000	0.001207	hsa-miR-150-5p;hsa-miR-186-3p;hsa-miR-520a-3p;hsa-miR-5
ENSG0000	ENSG0000	2.06E-05	hsa-miR-519a-3p;hsa-miR-519b-3p;hsa-miR-519c-3p;hsa-miR
ENSG0000	ENSG0000	2.49E-11	hsa-miR-106a-5p;hsa-miR-106b-5p;hsa-miR-17-5p;hsa-miR-1
ENSG0000	ENSG0000	7.70E-07	hsa-miR-106a-5p;hsa-miR-106b-5p;hsa-miR-17-5p;hsa-miR-2
ENSG0000	ENSG0000	1.61E-11	hsa-miR-106a-5p;hsa-miR-106b-5p;hsa-miR-150-5p;hsa-miR-

ENSG0000	ENSG0000	1.61E-11	hsa-miR-106a-5p;hsa-miR-106b-5p;hsa-miR-150-5p;hsa-miR-
ENSG0000	ENSG0000	3.61E-10	hsa-miR-106a-5p;hsa-miR-17-5p;hsa-miR-20a-5p;hsa-miR-20
ENSG0000	ENSG0000	4.50E-10	hsa-miR-106a-5p;hsa-miR-106b-5p;hsa-miR-17-5p;hsa-miR-2
ENSG0000	ENSG0000	3.14E-05	hsa-miR-106a-5p;hsa-miR-106b-5p;hsa-miR-17-5p;hsa-miR-2
ENSG0000	ENSG0000	4.85E-08	hsa-miR-106a-5p;hsa-miR-106b-5p;hsa-miR-17-5p;hsa-miR-2
ENSG0000	ENSG0000	0.000116	hsa-miR-106a-5p;hsa-miR-106b-5p;hsa-miR-20a-5p;hsa-miR-
ENSG0000	ENSG0000	2.97E-12	hsa-miR-106a-5p;hsa-miR-106b-5p;hsa-miR-150-5p;hsa-miR-
ENSG0000	ENSG0000	4.47E-11	hsa-miR-106a-5p;hsa-miR-106b-5p;hsa-miR-150-5p;hsa-miR-
ENSG0000	ENSG0000	2.30E-09	hsa-miR-106b-5p;hsa-miR-17-5p;hsa-miR-20a-5p;hsa-miR-20
ENSG0000	ENSG0000	3.94E-05	hsa-miR-106b-5p;hsa-miR-17-5p;hsa-miR-20a-5p;hsa-miR-20
ENSG0000	ENSG0000	2.13E-09	hsa-miR-106a-5p;hsa-miR-106b-5p;hsa-miR-17-5p;hsa-miR-2
ENSG0000	ENSG0000	2.13E-09	hsa-miR-106a-5p;hsa-miR-106b-5p;hsa-miR-17-5p;hsa-miR-2
ENSG0000	ENSG0000	2.82E-06	hsa-miR-106a-5p;hsa-miR-106b-5p;hsa-miR-17-5p;hsa-miR-2
ENSG0000	ENSG0000	0.001651	hsa-miR-17-5p;hsa-miR-20a-5p;hsa-miR-20b-5p;hsa-miR-93-
ENSG0000	ENSG0000	6.50E-13	hsa-miR-106a-5p;hsa-miR-106b-5p;hsa-miR-150-5p;hsa-miR-
ENSG0000	ENSG0000	1.66E-12	hsa-miR-106a-5p;hsa-miR-106b-5p;hsa-miR-150-5p;hsa-miR-
ENSG0000	ENSG0000	0.000122	hsa-miR-519a-3p;hsa-miR-519b-3p;hsa-miR-519c-3p;hsa-miR
ENSG0000	ENSG0000	0.000105	hsa-miR-106b-5p;hsa-miR-17-5p;hsa-miR-20a-5p;hsa-miR-20
ENSG0000	ENSG0000	2.30E-09	hsa-miR-106b-5p;hsa-miR-17-5p;hsa-miR-20a-5p;hsa-miR-20
ENSG0000	ENSG0000	0.007253	hsa-miR-106b-5p;hsa-miR-17-5p;hsa-miR-20a-5p;hsa-miR-20
ENSG0000	ENSG0000	1.60E-08	hsa-miR-106a-5p;hsa-miR-106b-5p;hsa-miR-17-5p;hsa-miR-2
ENSG0000	ENSG0000	1.37E-05	hsa-miR-378a-5p;hsa-miR-519a-3p;hsa-miR-519b-3p;hsa-miR
ENSG0000	ENSG0000	0.000104	hsa-miR-106a-5p;hsa-miR-106b-5p;hsa-miR-17-5p;hsa-miR-2
ENSG0000	ENSG0000	3.92E-12	hsa-miR-106a-5p;hsa-miR-106b-5p;hsa-miR-150-5p;hsa-miR-
ENSG0000	ENSG0000	7.03E-05	hsa-miR-106b-5p;hsa-miR-1307-3p;hsa-miR-150-5p;hsa-miR-
ENSG0000	ENSG0000	4.69E-08	hsa-miR-106b-5p;hsa-miR-17-5p;hsa-miR-20a-5p;hsa-miR-20
ENSG0000	ENSG0000	5.80E-09	hsa-miR-106a-5p;hsa-miR-106b-5p;hsa-miR-17-5p;hsa-miR-2
ENSG0000	ENSG0000	0.002632	hsa-miR-1271-3p;hsa-miR-214-5p;hsa-miR-30c-1-3p;hsa-miR
ENSG0000	ENSG0000	4.32E-12	hsa-miR-106a-5p;hsa-miR-106b-5p;hsa-miR-150-5p;hsa-miR-
ENSG0000	ENSG0000	2.91E-06	hsa-miR-106b-5p;hsa-miR-17-5p;hsa-miR-20a-5p;hsa-miR-20
ENSG0000	ENSG0000	3.45E-07	hsa-miR-106a-5p;hsa-miR-106b-5p;hsa-miR-17-5p;hsa-miR-2
ENSG0000	ENSG0000	3.00E-08	hsa-miR-106a-5p;hsa-miR-106b-5p;hsa-miR-150-5p;hsa-miR-
ENSG0000	ENSG0000	2.30E-09	hsa-miR-106b-5p;hsa-miR-17-5p;hsa-miR-20a-5p;hsa-miR-20
ENSG0000	ENSG0000	2.30E-09	hsa-miR-106b-5p;hsa-miR-17-5p;hsa-miR-20a-5p;hsa-miR-20
ENSG0000	ENSG0000	2.30E-09	hsa-miR-106b-5p;hsa-miR-17-5p;hsa-miR-20a-5p;hsa-miR-20
ENSG0000	ENSG0000	2.30E-09	hsa-miR-106b-5p;hsa-miR-17-5p;hsa-miR-20a-5p;hsa-miR-20
ENSG0000	ENSG0000	0.001634	hsa-miR-519a-3p;hsa-miR-519b-3p;hsa-miR-519c-3p;hsa-miR
ENSG0000	ENSG0000	6.03E-07	hsa-miR-106a-5p;hsa-miR-106b-5p;hsa-miR-17-5p;hsa-miR-2
ENSG0000	ENSG0000	3.40E-08	hsa-miR-106a-5p;hsa-miR-17-5p;hsa-miR-20a-5p;hsa-miR-20
ENSG0000	ENSG0000	3.40E-08	hsa-miR-106a-5p;hsa-miR-17-5p;hsa-miR-20a-5p;hsa-miR-20
ENSG0000	ENSG0000	8.73E-08	hsa-miR-106a-5p;hsa-miR-106b-5p;hsa-miR-17-5p;hsa-miR-2
ENSG0000	ENSG0000	3.87E-09	hsa-miR-106a-5p;hsa-miR-106b-5p;hsa-miR-150-5p;hsa-miR-
ENSG0000	ENSG0000	1.09E-09	hsa-miR-106a-5p;hsa-miR-106b-5p;hsa-miR-17-5p;hsa-miR-2
ENSG0000	ENSG0000	7.44E-10	hsa-miR-106a-5p;hsa-miR-106b-5p;hsa-miR-150-5p;hsa-miR-
ENSG0000	ENSG0000	1.10E-08	hsa-miR-106a-5p;hsa-miR-106b-5p;hsa-miR-150-5p;hsa-miR-
ENSG0000	ENSG0000	0.000816	hsa-miR-519a-3p;hsa-miR-519b-3p;hsa-miR-519c-3p;hsa-miR
ENSG0000	ENSG0000	3.91E-06	hsa-miR-106b-5p;hsa-miR-17-5p;hsa-miR-20a-5p;hsa-miR-20
ENSG0000	ENSG0000	3.97E-08	hsa-miR-106a-5p;hsa-miR-106b-5p;hsa-miR-17-5p;hsa-miR-2
ENSG0000	ENSG0000	1.05E-09	hsa-miR-106a-5p;hsa-miR-106b-5p;hsa-miR-150-5p;hsa-miR-
ENSG0000	ENSG0000	3.24E-09	hsa-miR-106a-5p;hsa-miR-106b-5p;hsa-miR-17-5p;hsa-miR-2
ENSG0000	ENSG0000	2.50E-08	hsa-miR-106a-5p;hsa-miR-106b-5p;hsa-miR-150-5p;hsa-miR-
ENSG0000	ENSG0000	0.008778	hsa-miR-1271-3p;hsa-miR-30c-1-3p;hsa-miR-30c-2-3p;hsa-mi
ENSG0000	ENSG0000	4.61E-07	hsa-miR-106b-5p;hsa-miR-17-5p;hsa-miR-20a-5p;hsa-miR-93
ENSG0000	ENSG0000	0.002008	hsa-miR-106a-5p;hsa-miR-17-5p;hsa-miR-20a-5p;hsa-miR-20

ENSG0000	ENSG0000	2.66E-06	hsa-miR-106a-5p;hsa-miR-106b-5p;hsa-miR-17-5p;hsa-miR-2
ENSG0000	ENSG0000	5.71E-08	hsa-miR-106a-5p;hsa-miR-106b-5p;hsa-miR-150-5p;hsa-miR-
ENSG0000	ENSG0000	2.50E-07	hsa-miR-106a-5p;hsa-miR-106b-5p;hsa-miR-1307-3p;hsa-miR
ENSG0000	ENSG0000	3.09E-06	hsa-miR-106b-5p;hsa-miR-148a-5p;hsa-miR-17-5p;hsa-miR-2
ENSG0000	ENSG0000	1.43E-06	hsa-miR-106a-5p;hsa-miR-106b-5p;hsa-miR-17-5p;hsa-miR-2
ENSG0000	ENSG0000	5.64E-06	hsa-miR-106b-5p;hsa-miR-1307-3p;hsa-miR-17-5p;hsa-miR-2
ENSG0000	ENSG0000	2.11E-08	hsa-miR-106b-5p;hsa-miR-17-5p;hsa-miR-20a-5p;hsa-miR-20
ENSG0000	ENSG0000	4.66E-13	hsa-miR-106a-5p;hsa-miR-106b-5p;hsa-miR-150-5p;hsa-miR-
ENSG0000	ENSG0000	0.000749	hsa-miR-150-5p;hsa-miR-30c-1-3p;hsa-miR-30c-2-3p;hsa-mi
ENSG0000	ENSG0000	7.97E-09	hsa-miR-106b-5p;hsa-miR-17-5p;hsa-miR-20a-5p;hsa-miR-20
ENSG0000	ENSG0000	0.009498	hsa-miR-519a-3p;hsa-miR-519b-3p;hsa-miR-519c-3p;hsa-miR
ENSG0000	ENSG0000	5.95E-05	hsa-miR-106b-5p;hsa-miR-17-5p;hsa-miR-20a-5p;hsa-miR-20
ENSG0000	ENSG0000	0.008778	hsa-miR-106b-5p;hsa-miR-135a-5p;hsa-miR-17-5p;hsa-miR-2
ENSG0000	ENSG0000	1.23E-07	hsa-miR-106a-5p;hsa-miR-106b-5p;hsa-miR-17-5p;hsa-miR-2
ENSG0000	ENSG0000	0.002628	hsa-miR-186-3p;hsa-miR-519a-3p;hsa-miR-519b-3p;hsa-miR-
ENSG0000	ENSG0000	0.000148	hsa-miR-106b-5p;hsa-miR-17-5p;hsa-miR-192-5p;hsa-miR-20
ENSG0000	ENSG0000	1.07E-10	hsa-miR-106a-5p;hsa-miR-106b-5p;hsa-miR-17-5p;hsa-miR-2
ENSG0000	ENSG0000	3.67E-09	hsa-miR-106a-5p;hsa-miR-106b-5p;hsa-miR-17-5p;hsa-miR-2
ENSG0000	ENSG0000	1.19E-07	hsa-miR-106a-5p;hsa-miR-106b-5p;hsa-miR-17-5p;hsa-miR-2
ENSG0000	ENSG0000	1.83E-10	hsa-miR-106a-5p;hsa-miR-106b-5p;hsa-miR-150-5p;hsa-miR-
ENSG0000	ENSG0000	4.56E-05	hsa-miR-106a-5p;hsa-miR-106b-5p;hsa-miR-17-5p;hsa-miR-2
ENSG0000	ENSG0000	0.000296	hsa-miR-186-3p;hsa-miR-519a-3p;hsa-miR-519b-3p;hsa-miR-
ENSG0000	ENSG0000	1.42E-11	hsa-miR-106a-5p;hsa-miR-106b-5p;hsa-miR-150-5p;hsa-miR-
ENSG0000	ENSG0000	0.000431	hsa-miR-519a-3p;hsa-miR-519b-3p;hsa-miR-519c-3p;hsa-miR
ENSG0000	ENSG0000	0.004576	hsa-miR-519a-3p;hsa-miR-519b-3p;hsa-miR-519c-3p;hsa-miR
ENSG0000	ENSG0000	1.84E-08	hsa-miR-106a-5p;hsa-miR-106b-5p;hsa-miR-17-5p;hsa-miR-1
ENSG0000	ENSG0000	6.84E-11	hsa-miR-106a-5p;hsa-miR-106b-5p;hsa-miR-17-5p;hsa-miR-2
ENSG0000	ENSG0000	5.79E-07	hsa-miR-106a-5p;hsa-miR-106b-5p;hsa-miR-150-5p;hsa-miR-
ENSG0000	ENSG0000	3.45E-07	hsa-miR-106a-5p;hsa-miR-106b-5p;hsa-miR-20a-5p;hsa-miR-
ENSG0000	ENSG0000	1.74E-11	hsa-miR-106a-5p;hsa-miR-106b-5p;hsa-miR-17-5p;hsa-miR-2
ENSG0000	ENSG0000	0.000403	hsa-miR-106b-5p;hsa-miR-17-5p;hsa-miR-204-3p;hsa-miR-20
ENSG0000	ENSG0000	3.24E-07	hsa-miR-106a-5p;hsa-miR-106b-5p;hsa-miR-150-5p;hsa-miR-
ENSG0000	ENSG0000	7.97E-09	hsa-miR-106b-5p;hsa-miR-17-5p;hsa-miR-20a-5p;hsa-miR-20
ENSG0000	ENSG0000	4.44E-09	hsa-miR-106a-5p;hsa-miR-106b-5p;hsa-miR-150-5p;hsa-miR-
ENSG0000	ENSG0000	4.02E-08	hsa-miR-106a-5p;hsa-miR-106b-5p;hsa-miR-150-5p;hsa-miR-
ENSG0000	ENSG0000	0.002763	hsa-miR-17-5p;hsa-miR-20a-5p;hsa-miR-20b-5p;hsa-miR-450
ENSG0000	ENSG0000	5.34E-10	hsa-miR-106a-5p;hsa-miR-106b-5p;hsa-miR-150-5p;hsa-miR-
ENSG0000	ENSG0000	8.75E-10	hsa-miR-106a-5p;hsa-miR-106b-5p;hsa-miR-150-5p;hsa-miR-
ENSG0000	ENSG0000	3.84E-06	hsa-miR-106a-5p;hsa-miR-106b-5p;hsa-miR-150-5p;hsa-miR-
ENSG0000	ENSG0000	0.000317	hsa-miR-519a-3p;hsa-miR-519b-3p;hsa-miR-519c-3p;hsa-miR
ENSG0000	ENSG0000	7.44E-10	hsa-miR-106a-5p;hsa-miR-106b-5p;hsa-miR-17-5p;hsa-miR-2
ENSG0000	ENSG0000	6.79E-06	hsa-miR-106a-5p;hsa-miR-106b-5p;hsa-miR-17-5p;hsa-miR-2
ENSG0000	ENSG0000	4.45E-06	hsa-miR-106a-5p;hsa-miR-106b-5p;hsa-miR-1307-3p;hsa-miR
ENSG0000	ENSG0000	1.09E-05	hsa-miR-519a-3p;hsa-miR-519b-3p;hsa-miR-519c-3p;hsa-miR
ENSG0000	ENSG0000	6.18E-07	hsa-miR-106a-5p;hsa-miR-106b-5p;hsa-miR-17-5p;hsa-miR-2
ENSG0000	ENSG0000	0.002862	hsa-miR-519a-3p;hsa-miR-519b-3p;hsa-miR-519c-3p;hsa-miR
ENSG0000	ENSG0000	3.38E-06	hsa-miR-106a-5p;hsa-miR-106b-5p;hsa-miR-17-5p;hsa-miR-2
ENSG0000	ENSG0000	1.74E-11	hsa-miR-106a-5p;hsa-miR-106b-5p;hsa-miR-17-5p;hsa-miR-2
ENSG0000	ENSG0000	2.11E-08	hsa-miR-106b-5p;hsa-miR-17-5p;hsa-miR-20a-5p;hsa-miR-20
ENSG0000	ENSG0000	2.11E-08	hsa-miR-106b-5p;hsa-miR-17-5p;hsa-miR-20a-5p;hsa-miR-20
ENSG0000	ENSG0000	6.71E-06	hsa-miR-106b-5p;hsa-miR-17-5p;hsa-miR-20a-5p;hsa-miR-20
ENSG0000	ENSG0000	0.007955	hsa-miR-150-5p;hsa-miR-186-3p;hsa-miR-378a-5p;hsa-miR-5
ENSG0000	ENSG0000	6.21E-09	hsa-miR-106a-5p;hsa-miR-106b-5p;hsa-miR-17-5p;hsa-miR-2
ENSG0000	ENSG0000	7.43E-09	hsa-miR-106b-5p;hsa-miR-17-5p;hsa-miR-20a-5p;hsa-miR-20

ENSG0000	ENSG0000	1.72E-10	hsa-miR-106a-5p;hsa-miR-106b-5p;hsa-miR-17-5p;hsa-miR-2
ENSG0000	ENSG0000	1.72E-10	hsa-miR-106a-5p;hsa-miR-106b-5p;hsa-miR-17-5p;hsa-miR-2
ENSG0000	ENSG0000	4.22E-05	hsa-miR-132-5p;hsa-miR-519a-3p;hsa-miR-519b-3p;hsa-miR-
ENSG0000	ENSG0000	2.58E-11	hsa-miR-106a-5p;hsa-miR-106b-5p;hsa-miR-17-5p;hsa-miR-2
ENSG0000	ENSG0000	3.92E-12	hsa-miR-106a-5p;hsa-miR-106b-5p;hsa-miR-17-5p;hsa-miR-2
ENSG0000	ENSG0000	3.92E-12	hsa-miR-106a-5p;hsa-miR-106b-5p;hsa-miR-17-5p;hsa-miR-2
ENSG0000	ENSG0000	3.92E-12	hsa-miR-106a-5p;hsa-miR-106b-5p;hsa-miR-17-5p;hsa-miR-2
ENSG0000	ENSG0000	3.32E-09	hsa-miR-106a-5p;hsa-miR-106b-5p;hsa-miR-17-5p;hsa-miR-2
ENSG0000	ENSG0000	1.37E-05	hsa-miR-106a-5p;hsa-miR-106b-5p;hsa-miR-17-5p;hsa-miR-2
ENSG0000	ENSG0000	1.91E-07	hsa-miR-106a-5p;hsa-miR-106b-5p;hsa-miR-150-5p;hsa-miR-
ENSG0000	ENSG0000	9.19E-09	hsa-miR-106a-5p;hsa-miR-106b-5p;hsa-miR-17-5p;hsa-miR-2
ENSG0000	ENSG0000	8.90E-08	hsa-miR-106a-5p;hsa-miR-106b-5p;hsa-miR-150-5p;hsa-miR-
ENSG0000	ENSG0000	0.000506	hsa-miR-150-5p;hsa-miR-186-3p;hsa-miR-520a-3p;hsa-miR-5
ENSG0000	ENSG0000	5.96E-05	hsa-miR-106b-5p;hsa-miR-17-5p;hsa-miR-186-3p;hsa-miR-20
ENSG0000	ENSG0000	3.18E-10	hsa-miR-106a-5p;hsa-miR-106b-5p;hsa-miR-17-5p;hsa-miR-1
ENSG0000	ENSG0000	2.78E-15	hsa-miR-106a-5p;hsa-miR-106b-5p;hsa-miR-17-5p;hsa-miR-2
ENSG0000	ENSG0000	1.15E-05	hsa-miR-106a-5p;hsa-miR-17-5p;hsa-miR-20a-5p;hsa-miR-20
ENSG0000	ENSG0000	2.70E-05	hsa-miR-106a-5p;hsa-miR-106b-5p;hsa-miR-17-5p;hsa-miR-2
ENSG0000	ENSG0000	1.04E-09	hsa-miR-106a-5p;hsa-miR-106b-5p;hsa-miR-17-5p;hsa-miR-2
ENSG0000	ENSG0000	1.55E-09	hsa-miR-106a-5p;hsa-miR-17-5p;hsa-miR-20a-5p;hsa-miR-20
ENSG0000	ENSG0000	0.000296	hsa-miR-106a-5p;hsa-miR-106b-5p;hsa-miR-17-5p;hsa-miR-2
ENSG0000	ENSG0000	3.15E-08	hsa-miR-106a-5p;hsa-miR-106b-5p;hsa-miR-17-5p;hsa-miR-2
ENSG0000	ENSG0000	1.20E-09	hsa-miR-106a-5p;hsa-miR-106b-5p;hsa-miR-150-5p;hsa-miR-
ENSG0000	ENSG0000	0.000202	hsa-miR-106b-5p;hsa-miR-17-5p;hsa-miR-20a-5p;hsa-miR-20
ENSG0000	ENSG0000	1.55E-07	hsa-miR-17-5p;hsa-miR-20a-5p;hsa-miR-20b-5p;hsa-miR-93-
ENSG0000	ENSG0000	1.37E-05	hsa-miR-106a-5p;hsa-miR-106b-5p;hsa-miR-17-5p;hsa-miR-2
ENSG0000	ENSG0000	5.45E-09	hsa-miR-106a-5p;hsa-miR-106b-5p;hsa-miR-17-5p;hsa-miR-2
ENSG0000	ENSG0000	2.09E-07	hsa-miR-106a-5p;hsa-miR-106b-5p;hsa-miR-150-5p;hsa-miR-
ENSG0000	ENSG0000	1.07E-05	hsa-miR-106a-5p;hsa-miR-106b-5p;hsa-miR-17-5p;hsa-miR-2
ENSG0000	ENSG0000	4.75E-05	hsa-miR-106a-5p;hsa-miR-106b-5p;hsa-miR-17-5p;hsa-miR-2
ENSG0000	ENSG0000	0.007598	hsa-miR-519a-3p;hsa-miR-519b-3p;hsa-miR-519c-3p;hsa-miR
ENSG0000	ENSG0000	1.72E-10	hsa-miR-106a-5p;hsa-miR-106b-5p;hsa-miR-17-5p;hsa-miR-2
ENSG0000	ENSG0000	2.12E-08	hsa-miR-106a-5p;hsa-miR-106b-5p;hsa-miR-17-5p;hsa-miR-2
ENSG0000	ENSG0000	1.24E-08	hsa-miR-106a-5p;hsa-miR-106b-5p;hsa-miR-17-5p;hsa-miR-2
ENSG0000	ENSG0000	1.63E-09	hsa-miR-106a-5p;hsa-miR-106b-5p;hsa-miR-150-5p;hsa-miR-
ENSG0000	ENSG0000	4.39E-11	hsa-miR-106a-5p;hsa-miR-106b-5p;hsa-miR-17-5p;hsa-miR-2
ENSG0000	ENSG0000	7.67E-11	hsa-miR-106a-5p;hsa-miR-106b-5p;hsa-miR-17-5p;hsa-miR-2
ENSG0000	ENSG0000	0.00013	hsa-miR-106b-5p;hsa-miR-17-5p;hsa-miR-20a-5p;hsa-miR-20
ENSG0000	ENSG0000	0.000962	hsa-miR-1307-3p;hsa-miR-150-5p;hsa-miR-186-3p;hsa-miR-1
ENSG0000	ENSG0000	7.09E-07	hsa-miR-106b-5p;hsa-miR-17-5p;hsa-miR-20a-5p;hsa-miR-20
ENSG0000	ENSG0000	6.50E-13	hsa-miR-106a-5p;hsa-miR-106b-5p;hsa-miR-150-5p;hsa-miR-
ENSG0000	ENSG0000	2.30E-07	hsa-miR-106a-5p;hsa-miR-106b-5p;hsa-miR-17-5p;hsa-miR-2
ENSG0000	ENSG0000	0.000988	hsa-miR-106a-5p;hsa-miR-106b-5p;hsa-miR-17-5p;hsa-miR-2
ENSG0000	ENSG0000	2.11E-06	hsa-miR-106b-5p;hsa-miR-17-5p;hsa-miR-20a-5p;hsa-miR-20
ENSG0000	ENSG0000	2.09E-07	hsa-miR-106a-5p;hsa-miR-106b-5p;hsa-miR-135a-5p;hsa-miR
ENSG0000	ENSG0000	6.19E-07	hsa-miR-106a-5p;hsa-miR-106b-5p;hsa-miR-17-5p;hsa-miR-2
ENSG0000	ENSG0000	1.52E-10	hsa-miR-106a-5p;hsa-miR-106b-5p;hsa-miR-17-5p;hsa-miR-2
ENSG0000	ENSG0000	0.000122	hsa-miR-150-5p;hsa-miR-17-5p;hsa-miR-186-3p;hsa-miR-20a
ENSG0000	ENSG0000	1.58E-08	hsa-miR-106a-5p;hsa-miR-106b-5p;hsa-miR-150-5p;hsa-miR-
ENSG0000	ENSG0000	1.55E-09	hsa-miR-106a-5p;hsa-miR-106b-5p;hsa-miR-17-5p;hsa-miR-2
ENSG0000	ENSG0000	2.30E-09	hsa-miR-106b-5p;hsa-miR-17-5p;hsa-miR-20a-5p;hsa-miR-20
ENSG0000	ENSG0000	8.97E-13	hsa-miR-106a-5p;hsa-miR-106b-5p;hsa-miR-150-5p;hsa-miR-
ENSG0000	ENSG0000	5.46E-09	hsa-miR-106a-5p;hsa-miR-106b-5p;hsa-miR-150-5p;hsa-miR-
ENSG0000	ENSG0000	5.87E-06	hsa-miR-106a-5p;hsa-miR-106b-5p;hsa-miR-17-5p;hsa-miR-2

ENSG0000	ENSG0000	0.005411	hsa-miR-106b-5p;hsa-miR-17-5p;hsa-miR-20a-5p;hsa-miR-20
ENSG0000	ENSG0000	0.000475	hsa-miR-106b-5p;hsa-miR-17-5p;hsa-miR-20a-5p;hsa-miR-20
ENSG0000	ENSG0000	3.84E-05	hsa-miR-106b-5p;hsa-miR-17-5p;hsa-miR-20a-5p;hsa-miR-20
ENSG0000	ENSG0000	8.13E-10	hsa-miR-106a-5p;hsa-miR-106b-5p;hsa-miR-150-5p;hsa-miR-
ENSG0000	ENSG0000	1.75E-06	hsa-miR-106a-5p;hsa-miR-106b-5p;hsa-miR-17-5p;hsa-miR-2
ENSG0000	ENSG0000	1.75E-06	hsa-miR-106a-5p;hsa-miR-106b-5p;hsa-miR-17-5p;hsa-miR-2
ENSG0000	ENSG0000	0.00071	hsa-miR-106a-5p;hsa-miR-106b-5p;hsa-miR-17-5p;hsa-miR-2
ENSG0000	ENSG0000	6.76E-08	hsa-miR-106a-5p;hsa-miR-106b-5p;hsa-miR-17-5p;hsa-miR-2
ENSG0000	ENSG0000	5.34E-09	hsa-miR-106a-5p;hsa-miR-106b-5p;hsa-miR-17-5p;hsa-miR-1
ENSG0000	ENSG0000	5.34E-09	hsa-miR-106a-5p;hsa-miR-106b-5p;hsa-miR-17-5p;hsa-miR-1
ENSG0000	ENSG0000	1.44E-05	hsa-miR-106a-5p;hsa-miR-106b-5p;hsa-miR-17-5p;hsa-miR-2
ENSG0000	ENSG0000	2.30E-09	hsa-miR-106b-5p;hsa-miR-17-5p;hsa-miR-20a-5p;hsa-miR-20
ENSG0000	ENSG0000	0.001877	hsa-miR-30c-1-3p;hsa-miR-30c-2-3p;hsa-miR-519a-3p;hsa-mi
ENSG0000	ENSG0000	0.004251	hsa-miR-2355-5p;hsa-miR-519a-3p;hsa-miR-519b-3p;hsa-miR
ENSG0000	ENSG0000	1.09E-05	hsa-miR-135a-5p;hsa-miR-519a-3p;hsa-miR-519b-3p;hsa-miR
ENSG0000	ENSG0000	4.03E-06	hsa-miR-186-3p;hsa-miR-519a-3p;hsa-miR-519b-3p;hsa-miR-
ENSG0000	ENSG0000	4.03E-06	hsa-miR-186-3p;hsa-miR-519a-3p;hsa-miR-519b-3p;hsa-miR-
ENSG0000	ENSG0000	4.39E-11	hsa-miR-106a-5p;hsa-miR-106b-5p;hsa-miR-17-5p;hsa-miR-2
ENSG0000	ENSG0000	6.84E-11	hsa-miR-106a-5p;hsa-miR-106b-5p;hsa-miR-150-5p;hsa-miR-
ENSG0000	ENSG0000	1.51E-08	hsa-miR-106a-5p;hsa-miR-106b-5p;hsa-miR-1307-3p;hsa-miR
ENSG0000	ENSG0000	1.51E-08	hsa-miR-106a-5p;hsa-miR-106b-5p;hsa-miR-1307-3p;hsa-miR
ENSG0000	ENSG0000	1.15E-12	hsa-miR-106a-5p;hsa-miR-106b-5p;hsa-miR-17-5p;hsa-miR-1
ENSG0000	ENSG0000	2.76E-08	hsa-miR-106a-5p;hsa-miR-106b-5p;hsa-miR-150-5p;hsa-miR-
ENSG0000	ENSG0000	1.21E-06	hsa-miR-106b-5p;hsa-miR-17-5p;hsa-miR-20a-5p;hsa-miR-20
ENSG0000	ENSG0000	2.01E-10	hsa-miR-106a-5p;hsa-miR-106b-5p;hsa-miR-17-5p;hsa-miR-2
ENSG0000	ENSG0000	1.47E-10	hsa-miR-106a-5p;hsa-miR-106b-5p;hsa-miR-17-5p;hsa-miR-2
ENSG0000	ENSG0000	2.23E-12	hsa-miR-106a-5p;hsa-miR-106b-5p;hsa-miR-150-5p;hsa-miR-
ENSG0000	ENSG0000	7.84E-06	hsa-miR-106a-5p;hsa-miR-106b-5p;hsa-miR-17-5p;hsa-miR-2
ENSG0000	ENSG0000	0.008323	hsa-miR-148a-5p;hsa-miR-2355-5p;hsa-miR-519a-3p;hsa-miR
ENSG0000	ENSG0000	8.51E-09	hsa-miR-106a-5p;hsa-miR-17-5p;hsa-miR-20a-5p;hsa-miR-20
ENSG0000	ENSG0000	6.71E-06	hsa-miR-106b-5p;hsa-miR-17-5p;hsa-miR-20a-5p;hsa-miR-20
ENSG0000	ENSG0000	1.41E-07	hsa-miR-106a-5p;hsa-miR-106b-5p;hsa-miR-17-5p;hsa-miR-2
ENSG0000	ENSG0000	0.000809	hsa-miR-106a-5p;hsa-miR-106b-5p;hsa-miR-17-5p;hsa-miR-2
ENSG0000	ENSG0000	1.65E-08	hsa-miR-106a-5p;hsa-miR-106b-5p;hsa-miR-150-5p;hsa-miR-
ENSG0000	ENSG0000	8.67E-12	hsa-miR-106a-5p;hsa-miR-106b-5p;hsa-miR-150-5p;hsa-miR-
ENSG0000	ENSG0000	5.83E-06	hsa-miR-106a-5p;hsa-miR-106b-5p;hsa-miR-150-5p;hsa-miR-
ENSG0000	ENSG0000	1.69E-07	hsa-miR-106b-5p;hsa-miR-17-5p;hsa-miR-20a-5p;hsa-miR-20
ENSG0000	ENSG0000	2.13E-09	hsa-miR-106a-5p;hsa-miR-106b-5p;hsa-miR-17-5p;hsa-miR-2
ENSG0000	ENSG0000	5.38E-08	hsa-miR-106a-5p;hsa-miR-106b-5p;hsa-miR-150-5p;hsa-miR-
ENSG0000	ENSG0000	5.38E-08	hsa-miR-106a-5p;hsa-miR-106b-5p;hsa-miR-150-5p;hsa-miR-
ENSG0000	ENSG0000	8.56E-09	hsa-miR-106a-5p;hsa-miR-106b-5p;hsa-miR-150-5p;hsa-miR-
ENSG0000	ENSG0000	0.002862	hsa-miR-378a-5p;hsa-miR-519a-3p;hsa-miR-519b-3p;hsa-miR
ENSG0000	ENSG0000	8.97E-13	hsa-miR-106a-5p;hsa-miR-106b-5p;hsa-miR-150-5p;hsa-miR-
ENSG0000	ENSG0000	9.31E-12	hsa-miR-106a-5p;hsa-miR-106b-5p;hsa-miR-150-5p;hsa-miR-
ENSG0000	ENSG0000	3.60E-05	hsa-miR-106a-5p;hsa-miR-106b-5p;hsa-miR-150-5p;hsa-miR-
ENSG0000	ENSG0000	7.13E-05	hsa-miR-106a-5p;hsa-miR-106b-5p;hsa-miR-1271-3p;hsa-miR
ENSG0000	ENSG0000	0.001836	hsa-miR-106a-5p;hsa-miR-106b-5p;hsa-miR-17-5p;hsa-miR-2
ENSG0000	ENSG0000	1.21E-06	hsa-miR-106b-5p;hsa-miR-17-5p;hsa-miR-20a-5p;hsa-miR-20
ENSG0000	ENSG0000	5.18E-08	hsa-miR-106a-5p;hsa-miR-106b-5p;hsa-miR-17-5p;hsa-miR-2
ENSG0000	ENSG0000	1.58E-08	hsa-miR-106a-5p;hsa-miR-106b-5p;hsa-miR-150-5p;hsa-miR-
ENSG0000	ENSG0000	4.49E-10	hsa-miR-106a-5p;hsa-miR-106b-5p;hsa-miR-150-5p;hsa-miR-
ENSG0000	ENSG0000	3.14E-05	hsa-miR-106b-5p;hsa-miR-150-5p;hsa-miR-17-5p;hsa-miR-20
ENSG0000	ENSG0000	5.95E-08	hsa-miR-106a-5p;hsa-miR-106b-5p;hsa-miR-17-5p;hsa-miR-2
ENSG0000	ENSG0000	2.71E-10	hsa-miR-106a-5p;hsa-miR-106b-5p;hsa-miR-17-5p;hsa-miR-2

ENSG0000	ENSG0000	4.22E-05	hsa-miR-106a-5p;hsa-miR-106b-5p;hsa-miR-17-5p;hsa-miR-2
ENSG0000	ENSG0000	2.70E-07	hsa-miR-106a-5p;hsa-miR-106b-5p;hsa-miR-17-5p;hsa-miR-2
ENSG0000	ENSG0000	1.23E-12	hsa-miR-106a-5p;hsa-miR-106b-5p;hsa-miR-150-5p;hsa-miR-
ENSG0000	ENSG0000	1.23E-12	hsa-miR-106a-5p;hsa-miR-106b-5p;hsa-miR-150-5p;hsa-miR-
ENSG0000	ENSG0000	8.60E-06	hsa-miR-106b-5p;hsa-miR-17-5p;hsa-miR-20a-5p;hsa-miR-20
ENSG0000	ENSG0000	8.60E-06	hsa-miR-106b-5p;hsa-miR-17-5p;hsa-miR-20a-5p;hsa-miR-20
ENSG0000	ENSG0000	2.30E-09	hsa-miR-106b-5p;hsa-miR-17-5p;hsa-miR-20a-5p;hsa-miR-20
ENSG0000	ENSG0000	2.30E-09	hsa-miR-106b-5p;hsa-miR-17-5p;hsa-miR-20a-5p;hsa-miR-20
ENSG0000	ENSG0000	5.80E-09	hsa-miR-106a-5p;hsa-miR-106b-5p;hsa-miR-150-5p;hsa-miR-
ENSG0000	ENSG0000	0.001521	hsa-miR-519a-3p;hsa-miR-519b-3p;hsa-miR-519c-3p;hsa-miR
ENSG0000	ENSG0000	0.005648	hsa-miR-1271-3p;hsa-miR-30c-1-3p;hsa-miR-30c-2-3p;hsa-mi
ENSG0000	ENSG0000	2.05E-11	hsa-miR-106a-5p;hsa-miR-106b-5p;hsa-miR-150-5p;hsa-miR-
ENSG0000	ENSG0000	5.45E-09	hsa-miR-106a-5p;hsa-miR-106b-5p;hsa-miR-17-5p;hsa-miR-2
ENSG0000	ENSG0000	2.70E-07	hsa-miR-106a-5p;hsa-miR-106b-5p;hsa-miR-17-5p;hsa-miR-2
ENSG0000	ENSG0000	6.76E-08	hsa-miR-106a-5p;hsa-miR-106b-5p;hsa-miR-17-5p;hsa-miR-2
ENSG0000	ENSG0000	0.000273	hsa-miR-106a-5p;hsa-miR-106b-5p;hsa-miR-17-5p;hsa-miR-2
ENSG0000	ENSG0000	1.15E-14	hsa-miR-106a-5p;hsa-miR-106b-5p;hsa-miR-17-5p;hsa-miR-2
ENSG0000	ENSG0000	2.73E-09	hsa-miR-106a-5p;hsa-miR-106b-5p;hsa-miR-17-5p;hsa-miR-2
ENSG0000	ENSG0000	1.55E-07	hsa-miR-106b-5p;hsa-miR-17-5p;hsa-miR-20a-5p;hsa-miR-20
ENSG0000	ENSG0000	0.000628	hsa-miR-106b-5p;hsa-miR-17-5p;hsa-miR-20a-5p;hsa-miR-20
ENSG0000	ENSG0000	1.59E-09	hsa-miR-106a-5p;hsa-miR-106b-5p;hsa-miR-17-5p;hsa-miR-2
ENSG0000	ENSG0000	1.55E-06	hsa-miR-106a-5p;hsa-miR-106b-5p;hsa-miR-17-5p;hsa-miR-2
ENSG0000	ENSG0000	1.52E-10	hsa-miR-106a-5p;hsa-miR-106b-5p;hsa-miR-17-5p;hsa-miR-2
ENSG0000	ENSG0000	5.63E-10	hsa-miR-106a-5p;hsa-miR-106b-5p;hsa-miR-17-5p;hsa-miR-2
ENSG0000	ENSG0000	5.63E-10	hsa-miR-106a-5p;hsa-miR-106b-5p;hsa-miR-17-5p;hsa-miR-2
ENSG0000	ENSG0000	7.35E-09	hsa-miR-106a-5p;hsa-miR-106b-5p;hsa-miR-17-5p;hsa-miR-2
ENSG0000	ENSG0000	1.01E-10	hsa-miR-106a-5p;hsa-miR-106b-5p;hsa-miR-150-5p;hsa-miR-
ENSG0000	ENSG0000	0.0026	hsa-miR-1271-3p;hsa-miR-214-5p;hsa-miR-30c-1-3p;hsa-miR
ENSG0000	ENSG0000	1.52E-10	hsa-miR-106a-5p;hsa-miR-106b-5p;hsa-miR-150-5p;hsa-miR-
ENSG0000	ENSG0000	6.54E-06	hsa-miR-106a-5p;hsa-miR-106b-5p;hsa-miR-135a-5p;hsa-miR
ENSG0000	ENSG0000	0.002766	hsa-miR-1271-3p;hsa-miR-214-5p;hsa-miR-30c-1-3p;hsa-miR
ENSG0000	ENSG0000	1.44E-06	hsa-miR-106a-5p;hsa-miR-106b-5p;hsa-miR-17-5p;hsa-miR-2
ENSG0000	ENSG0000	2.06E-05	hsa-miR-519a-3p;hsa-miR-519b-3p;hsa-miR-519c-3p;hsa-miR
ENSG0000	ENSG0000	2.91E-06	hsa-miR-106b-5p;hsa-miR-17-5p;hsa-miR-20a-5p;hsa-miR-20
ENSG0000	ENSG0000	8.64E-07	hsa-miR-106a-5p;hsa-miR-20a-5p;hsa-miR-20b-5p;hsa-miR-5
ENSG0000	ENSG0000	8.64E-07	hsa-miR-106a-5p;hsa-miR-20a-5p;hsa-miR-20b-5p;hsa-miR-5
ENSG0000	ENSG0000	1.68E-05	hsa-miR-106b-5p;hsa-miR-17-5p;hsa-miR-20a-5p;hsa-miR-20
ENSG0000	ENSG0000	1.60E-08	hsa-miR-106a-5p;hsa-miR-106b-5p;hsa-miR-17-5p;hsa-miR-2
ENSG0000	ENSG0000	1.69E-09	hsa-miR-106a-5p;hsa-miR-106b-5p;hsa-miR-17-5p;hsa-miR-2
ENSG0000	ENSG0000	1.58E-08	hsa-miR-106a-5p;hsa-miR-106b-5p;hsa-miR-17-5p;hsa-miR-1
ENSG0000	ENSG0000	3.79E-10	hsa-miR-106a-5p;hsa-miR-106b-5p;hsa-miR-17-5p;hsa-miR-2
ENSG0000	ENSG0000	2.68E-12	hsa-miR-106a-5p;hsa-miR-106b-5p;hsa-miR-150-5p;hsa-miR-
ENSG0000	ENSG0000	2.68E-12	hsa-miR-106a-5p;hsa-miR-106b-5p;hsa-miR-150-5p;hsa-miR-
ENSG0000	ENSG0000	2.68E-12	hsa-miR-106a-5p;hsa-miR-106b-5p;hsa-miR-150-5p;hsa-miR-
ENSG0000	ENSG0000	2.68E-12	hsa-miR-106a-5p;hsa-miR-106b-5p;hsa-miR-150-5p;hsa-miR-
ENSG0000	ENSG0000	2.68E-12	hsa-miR-106a-5p;hsa-miR-106b-5p;hsa-miR-150-5p;hsa-miR-
ENSG0000	ENSG0000	2.68E-12	hsa-miR-106a-5p;hsa-miR-106b-5p;hsa-miR-150-5p;hsa-miR-
ENSG0000	ENSG0000	2.68E-12	hsa-miR-106a-5p;hsa-miR-106b-5p;hsa-miR-150-5p;hsa-miR-
ENSG0000	ENSG0000	2.07E-14	hsa-miR-106a-5p;hsa-miR-106b-5p;hsa-miR-17-5p;hsa-miR-2
ENSG0000	ENSG0000	1.09E-09	hsa-miR-106a-5p;hsa-miR-106b-5p;hsa-miR-17-5p;hsa-miR-2
ENSG0000	ENSG0000	1.37E-06	hsa-miR-106a-5p;hsa-miR-106b-5p;hsa-miR-188-3p;hsa-miR-
ENSG0000	ENSG0000	2.13E-09	hsa-miR-106a-5p;hsa-miR-106b-5p;hsa-miR-17-5p;hsa-miR-2
ENSG0000	ENSG0000	0.000522	hsa-miR-519a-3p;hsa-miR-519b-3p;hsa-miR-519c-3p;hsa-miR
ENSG0000	ENSG0000	0.000749	hsa-miR-519a-3p;hsa-miR-519b-3p;hsa-miR-519c-3p;hsa-miR

ENSG0000	ENSG0000	5.97E-08	hsa-miR-106a-5p;hsa-miR-106b-5p;hsa-miR-1307-3p;hsa-miR-
ENSG0000	ENSG0000	9.25E-07	hsa-miR-106a-5p;hsa-miR-106b-5p;hsa-miR-150-5p;hsa-miR-
ENSG0000	ENSG0000	9.26E-08	hsa-miR-106a-5p;hsa-miR-106b-5p;hsa-miR-17-5p;hsa-miR-2
ENSG0000	ENSG0000	7.43E-08	hsa-miR-106a-5p;hsa-miR-106b-5p;hsa-miR-150-5p;hsa-miR-
ENSG0000	ENSG0000	7.97E-09	hsa-miR-106b-5p;hsa-miR-17-5p;hsa-miR-20a-5p;hsa-miR-20
ENSG0000	ENSG0000	7.97E-09	hsa-miR-106b-5p;hsa-miR-17-5p;hsa-miR-20a-5p;hsa-miR-20
ENSG0000	ENSG0000	0.00011	hsa-miR-106a-5p;hsa-miR-106b-5p;hsa-miR-17-5p;hsa-miR-2
ENSG0000	ENSG0000	0.001207	hsa-miR-150-5p;hsa-miR-186-3p;hsa-miR-520a-3p;hsa-miR-5
ENSG0000	ENSG0000	1.68E-05	hsa-miR-106b-5p;hsa-miR-17-5p;hsa-miR-20a-5p;hsa-miR-20
ENSG0000	ENSG0000	1.59E-09	hsa-miR-106a-5p;hsa-miR-106b-5p;hsa-miR-150-5p;hsa-miR-
ENSG0000	ENSG0000	5.08E-09	hsa-miR-106a-5p;hsa-miR-106b-5p;hsa-miR-150-5p;hsa-miR-
ENSG0000	ENSG0000	1.55E-08	hsa-miR-106a-5p;hsa-miR-106b-5p;hsa-miR-17-5p;hsa-miR-2
ENSG0000	ENSG0000	3.98E-12	hsa-miR-106a-5p;hsa-miR-106b-5p;hsa-miR-17-5p;hsa-miR-2
ENSG0000	ENSG0000	2.01E-12	hsa-miR-106a-5p;hsa-miR-106b-5p;hsa-miR-150-5p;hsa-miR-
ENSG0000	ENSG0000	2.01E-12	hsa-miR-106a-5p;hsa-miR-106b-5p;hsa-miR-150-5p;hsa-miR-
ENSG0000	ENSG0000	5.63E-10	hsa-miR-106a-5p;hsa-miR-106b-5p;hsa-miR-17-5p;hsa-miR-2
ENSG0000	ENSG0000	4.68E-12	hsa-miR-106a-5p;hsa-miR-106b-5p;hsa-miR-17-5p;hsa-miR-2
ENSG0000	ENSG0000	9.60E-08	hsa-miR-106a-5p;hsa-miR-106b-5p;hsa-miR-17-5p;hsa-miR-2
ENSG0000	ENSG0000	0.009845	hsa-miR-2355-5p;hsa-miR-519a-3p;hsa-miR-519b-3p;hsa-miR-
ENSG0000	ENSG0000	3.46E-07	hsa-miR-106a-5p;hsa-miR-106b-5p;hsa-miR-17-5p;hsa-miR-1
ENSG0000	ENSG0000	3.46E-07	hsa-miR-106a-5p;hsa-miR-106b-5p;hsa-miR-17-5p;hsa-miR-1
ENSG0000	ENSG0000	6.27E-07	hsa-miR-106a-5p;hsa-miR-106b-5p;hsa-miR-150-5p;hsa-miR-
ENSG0000	ENSG0000	2.37E-08	hsa-miR-106a-5p;hsa-miR-106b-5p;hsa-miR-17-5p;hsa-miR-2
ENSG0000	ENSG0000	0.001079	hsa-miR-106b-5p;hsa-miR-17-5p;hsa-miR-20a-5p;hsa-miR-20
ENSG0000	ENSG0000	2.24E-08	hsa-miR-106a-5p;hsa-miR-106b-5p;hsa-miR-17-5p;hsa-miR-2
ENSG0000	ENSG0000	2.30E-09	hsa-miR-106b-5p;hsa-miR-17-5p;hsa-miR-20a-5p;hsa-miR-20
ENSG0000	ENSG0000	4.47E-11	hsa-miR-106a-5p;hsa-miR-106b-5p;hsa-miR-150-5p;hsa-miR-
ENSG0000	ENSG0000	5.64E-06	hsa-miR-106a-5p;hsa-miR-106b-5p;hsa-miR-17-5p;hsa-miR-2
ENSG0000	ENSG0000	2.56E-05	hsa-miR-106b-5p;hsa-miR-17-5p;hsa-miR-20a-5p;hsa-miR-20
ENSG0000	ENSG0000	2.56E-05	hsa-miR-106b-5p;hsa-miR-17-5p;hsa-miR-20a-5p;hsa-miR-20
ENSG0000	ENSG0000	0.008703	hsa-miR-150-5p;hsa-miR-186-3p;hsa-miR-192-5p;hsa-miR-52
ENSG0000	ENSG0000	3.69E-06	hsa-miR-106a-5p;hsa-miR-106b-5p;hsa-miR-17-5p;hsa-miR-1
ENSG0000	ENSG0000	6.64E-08	hsa-miR-106a-5p;hsa-miR-106b-5p;hsa-miR-17-5p;hsa-miR-2
ENSG0000	ENSG0000	1.42E-10	hsa-miR-106a-5p;hsa-miR-106b-5p;hsa-miR-150-5p;hsa-miR-
ENSG0000	ENSG0000	0.001651	hsa-miR-106b-5p;hsa-miR-17-5p;hsa-miR-20b-5p;hsa-miR-93
ENSG0000	ENSG0000	1.03E-09	hsa-miR-106a-5p;hsa-miR-106b-5p;hsa-miR-150-5p;hsa-miR-
ENSG0000	ENSG0000	7.32E-08	hsa-miR-106a-5p;hsa-miR-106b-5p;hsa-miR-150-5p;hsa-miR-
ENSG0000	ENSG0000	3.46E-07	hsa-miR-106a-5p;hsa-miR-106b-5p;hsa-miR-17-5p;hsa-miR-2
ENSG0000	ENSG0000	8.23E-05	hsa-miR-106b-5p;hsa-miR-17-5p;hsa-miR-20a-5p;hsa-miR-20
ENSG0000	ENSG0000	3.84E-05	hsa-miR-106b-5p;hsa-miR-17-5p;hsa-miR-20a-5p;hsa-miR-20
ENSG0000	ENSG0000	6.84E-11	hsa-miR-106a-5p;hsa-miR-106b-5p;hsa-miR-150-5p;hsa-miR-
ENSG0000	ENSG0000	3.37E-09	hsa-miR-106a-5p;hsa-miR-106b-5p;hsa-miR-150-5p;hsa-miR-
ENSG0000	ENSG0000	1.85E-11	hsa-miR-106a-5p;hsa-miR-106b-5p;hsa-miR-17-5p;hsa-miR-2
ENSG0000	ENSG0000	1.85E-11	hsa-miR-106a-5p;hsa-miR-106b-5p;hsa-miR-17-5p;hsa-miR-2
ENSG0000	ENSG0000	1.85E-11	hsa-miR-106a-5p;hsa-miR-106b-5p;hsa-miR-17-5p;hsa-miR-2
ENSG0000	ENSG0000	1.85E-11	hsa-miR-106a-5p;hsa-miR-106b-5p;hsa-miR-17-5p;hsa-miR-2
ENSG0000	ENSG0000	1.85E-11	hsa-miR-106a-5p;hsa-miR-106b-5p;hsa-miR-17-5p;hsa-miR-2
ENSG0000	ENSG0000	1.85E-11	hsa-miR-106a-5p;hsa-miR-106b-5p;hsa-miR-17-5p;hsa-miR-2
ENSG0000	ENSG0000	1.85E-11	hsa-miR-106a-5p;hsa-miR-106b-5p;hsa-miR-17-5p;hsa-miR-2
ENSG0000	ENSG0000	0.000352	hsa-miR-519a-3p;hsa-miR-519b-3p;hsa-miR-519c-3p;hsa-miR-
ENSG0000	ENSG0000	2.38E-07	hsa-miR-106a-5p;hsa-miR-106b-5p;hsa-miR-17-5p;hsa-miR-2
ENSG0000	ENSG0000	1.34E-08	hsa-miR-106a-5p;hsa-miR-106b-5p;hsa-miR-150-5p;hsa-miR-
ENSG0000	ENSG0000	2.22E-16	hsa-miR-106a-5p;hsa-miR-106b-5p;hsa-miR-17-5p;hsa-miR-1
ENSG0000	ENSG0000	0.000163	hsa-miR-106b-5p;hsa-miR-148a-5p;hsa-miR-17-5p;hsa-miR-2

ENSG0000	ENSG0000	2.30E-09	hsa-miR-106b-5p;hsa-miR-17-5p;hsa-miR-20a-5p;hsa-miR-20
ENSG0000	ENSG0000	4.64E-10	hsa-miR-106a-5p;hsa-miR-106b-5p;hsa-miR-150-5p;hsa-miR-
ENSG0000	ENSG0000	5.15E-12	hsa-miR-106a-5p;hsa-miR-106b-5p;hsa-miR-150-5p;hsa-miR-
ENSG0000	ENSG0000	1.15E-12	hsa-miR-106a-5p;hsa-miR-106b-5p;hsa-miR-150-5p;hsa-miR-
ENSG0000	ENSG0000	9.52E-06	hsa-miR-106a-5p;hsa-miR-106b-5p;hsa-miR-17-5p;hsa-miR-2
ENSG0000	ENSG0000	1.60E-07	hsa-miR-106a-5p;hsa-miR-106b-5p;hsa-miR-1307-3p;hsa-miR
ENSG0000	ENSG0000	1.90E-10	hsa-miR-106a-5p;hsa-miR-106b-5p;hsa-miR-150-5p;hsa-miR-
ENSG0000	ENSG0000	1.75E-06	hsa-miR-106a-5p;hsa-miR-106b-5p;hsa-miR-17-5p;hsa-miR-2
ENSG0000	ENSG0000	3.62E-05	hsa-miR-106b-5p;hsa-miR-17-5p;hsa-miR-20a-5p;hsa-miR-20
ENSG0000	ENSG0000	2.27E-08	hsa-miR-106a-5p;hsa-miR-106b-5p;hsa-miR-17-5p;hsa-miR-2
ENSG0000	ENSG0000	2.11E-08	hsa-miR-106b-5p;hsa-miR-17-5p;hsa-miR-20a-5p;hsa-miR-20
ENSG0000	ENSG0000	9.31E-05	hsa-miR-106b-5p;hsa-miR-17-5p;hsa-miR-20a-5p;hsa-miR-20
ENSG0000	ENSG0000	1.37E-11	hsa-miR-106a-5p;hsa-miR-106b-5p;hsa-miR-17-5p;hsa-miR-2
ENSG0000	ENSG0000	1.33E-09	hsa-miR-106a-5p;hsa-miR-106b-5p;hsa-miR-17-5p;hsa-miR-2
ENSG0000	ENSG0000	1.45E-07	hsa-miR-106a-5p;hsa-miR-106b-5p;hsa-miR-150-5p;hsa-miR-
ENSG0000	ENSG0000	7.91E-09	hsa-miR-106a-5p;hsa-miR-106b-5p;hsa-miR-17-5p;hsa-miR-2
ENSG0000	ENSG0000	1.25E-08	hsa-miR-106a-5p;hsa-miR-106b-5p;hsa-miR-150-5p;hsa-miR-
ENSG0000	ENSG0000	0.001634	hsa-miR-106b-5p;hsa-miR-17-5p;hsa-miR-20a-5p;hsa-miR-20
ENSG0000	ENSG0000	0.001634	hsa-miR-106b-5p;hsa-miR-17-5p;hsa-miR-20a-5p;hsa-miR-20
ENSG0000	ENSG0000	1.13E-07	hsa-miR-106a-5p;hsa-miR-106b-5p;hsa-miR-1307-3p;hsa-miR
ENSG0000	ENSG0000	2.95E-07	hsa-miR-106b-5p;hsa-miR-17-5p;hsa-miR-20a-5p;hsa-miR-20
ENSG0000	ENSG0000	0.005692	hsa-miR-30c-1-3p;hsa-miR-30c-2-3p;hsa-miR-519a-3p;hsa-mi
ENSG0000	ENSG0000	6.70E-12	hsa-miR-106a-5p;hsa-miR-106b-5p;hsa-miR-150-5p;hsa-miR-
ENSG0000	ENSG0000	0.003497	hsa-miR-188-3p;hsa-miR-519a-3p;hsa-miR-519b-3p;hsa-miR-
ENSG0000	ENSG0000	1.78E-05	hsa-miR-106a-5p;hsa-miR-17-5p;hsa-miR-20a-5p;hsa-miR-20
ENSG0000	ENSG0000	5.85E-12	hsa-miR-106a-5p;hsa-miR-106b-5p;hsa-miR-1271-3p;hsa-miR
ENSG0000	ENSG0000	8.44E-08	hsa-miR-106a-5p;hsa-miR-106b-5p;hsa-miR-17-5p;hsa-miR-2
ENSG0000	ENSG0000	1.24E-10	hsa-miR-106a-5p;hsa-miR-106b-5p;hsa-miR-17-5p;hsa-miR-1
ENSG0000	ENSG0000	3.53E-08	hsa-miR-106a-5p;hsa-miR-106b-5p;hsa-miR-17-5p;hsa-miR-2
ENSG0000	ENSG0000	1.83E-07	hsa-miR-106a-5p;hsa-miR-106b-5p;hsa-miR-17-5p;hsa-miR-2
ENSG0000	ENSG0000	0.008778	hsa-miR-192-5p;hsa-miR-378a-5p;hsa-miR-520a-3p;hsa-miR-
ENSG0000	ENSG0000	1.18E-05	hsa-miR-106a-5p;hsa-miR-106b-5p;hsa-miR-17-5p;hsa-miR-1
ENSG0000	ENSG0000	5.97E-08	hsa-miR-106a-5p;hsa-miR-106b-5p;hsa-miR-17-5p;hsa-miR-1
ENSG0000	ENSG0000	1.52E-10	hsa-miR-106a-5p;hsa-miR-106b-5p;hsa-miR-150-5p;hsa-miR-
ENSG0000	ENSG0000	4.61E-06	hsa-miR-106a-5p;hsa-miR-106b-5p;hsa-miR-17-5p;hsa-miR-2
ENSG0000	ENSG0000	3.15E-08	hsa-miR-106a-5p;hsa-miR-106b-5p;hsa-miR-150-5p;hsa-miR-
ENSG0000	ENSG0000	7.43E-08	hsa-miR-106a-5p;hsa-miR-106b-5p;hsa-miR-17-5p;hsa-miR-2
ENSG0000	ENSG0000	3.36E-08	hsa-miR-106a-5p;hsa-miR-106b-5p;hsa-miR-1307-3p;hsa-miR
ENSG0000	ENSG0000	3.65E-09	hsa-miR-106a-5p;hsa-miR-106b-5p;hsa-miR-150-5p;hsa-miR-
ENSG0000	ENSG0000	2.95E-07	hsa-miR-106a-5p;hsa-miR-106b-5p;hsa-miR-20a-5p;hsa-miR-
ENSG0000	ENSG0000	2.88E-11	hsa-miR-106a-5p;hsa-miR-106b-5p;hsa-miR-150-5p;hsa-miR-
ENSG0000	ENSG0000	7.47E-05	hsa-miR-106a-5p;hsa-miR-106b-5p;hsa-miR-150-5p;hsa-miR-
ENSG0000	ENSG0000	0.000102	hsa-miR-106a-5p;hsa-miR-106b-5p;hsa-miR-17-5p;hsa-miR-2
ENSG0000	ENSG0000	5.11E-12	hsa-miR-106a-5p;hsa-miR-106b-5p;hsa-miR-17-5p;hsa-miR-2
ENSG0000	ENSG0000	6.27E-07	hsa-miR-106a-5p;hsa-miR-106b-5p;hsa-miR-150-5p;hsa-miR-
ENSG0000	ENSG0000	0.000301	hsa-miR-106b-5p;hsa-miR-17-5p;hsa-miR-20a-5p;hsa-miR-20
ENSG0000	ENSG0000	1.23E-07	hsa-miR-106a-5p;hsa-miR-106b-5p;hsa-miR-148a-5p;hsa-miR
ENSG0000	ENSG0000	0.000628	hsa-miR-519a-3p;hsa-miR-519b-3p;hsa-miR-519c-3p;hsa-miR
ENSG0000	ENSG0000	1.03E-10	hsa-miR-106a-5p;hsa-miR-106b-5p;hsa-miR-150-5p;hsa-miR-
ENSG0000	ENSG0000	1.12E-11	hsa-miR-106a-5p;hsa-miR-106b-5p;hsa-miR-150-5p;hsa-miR-
ENSG0000	ENSG0000	0.000816	hsa-miR-106b-5p;hsa-miR-17-5p;hsa-miR-20a-5p;hsa-miR-37
ENSG0000	ENSG0000	0.007789	hsa-miR-2355-5p;hsa-miR-378a-5p;hsa-miR-518b;hsa-miR-51
ENSG0000	ENSG0000	0.003943	hsa-miR-150-5p;hsa-miR-186-3p;hsa-miR-520a-3p;hsa-miR-5
ENSG0000	ENSG0000	9.26E-08	hsa-miR-106a-5p;hsa-miR-106b-5p;hsa-miR-17-5p;hsa-miR-2

ENSG0000	ENSG0000	0.002407	hsa-miR-150-5p;hsa-miR-186-3p;hsa-miR-204-3p;hsa-miR-37
ENSG0000	ENSG0000	2.23E-12	hsa-miR-106a-5p;hsa-miR-106b-5p;hsa-miR-150-5p;hsa-miR-
ENSG0000	ENSG0000	5.33E-05	hsa-miR-106a-5p;hsa-miR-106b-5p;hsa-miR-17-5p;hsa-miR-2
ENSG0000	ENSG0000	0.000179	hsa-miR-106b-5p;hsa-miR-17-5p;hsa-miR-20a-5p;hsa-miR-20
ENSG0000	ENSG0000	0.001043	hsa-miR-135a-5p;hsa-miR-204-3p;hsa-miR-30c-1-3p;hsa-miR
ENSG0000	ENSG0000	0.002008	hsa-miR-519a-3p;hsa-miR-519b-3p;hsa-miR-519c-3p;hsa-miR
ENSG0000	ENSG0000	1.40E-09	hsa-miR-106a-5p;hsa-miR-106b-5p;hsa-miR-150-5p;hsa-miR-
ENSG0000	ENSG0000	6.71E-06	hsa-miR-519a-3p;hsa-miR-519b-3p;hsa-miR-519c-3p;hsa-miR
ENSG0000	ENSG0000	1.39E-10	hsa-miR-106a-5p;hsa-miR-106b-5p;hsa-miR-150-5p;hsa-miR-
ENSG0000	ENSG0000	0.00294	hsa-miR-106b-5p;hsa-miR-17-5p;hsa-miR-20a-5p;hsa-miR-20
ENSG0000	ENSG0000	3.91E-10	hsa-miR-106a-5p;hsa-miR-150-5p;hsa-miR-17-5p;hsa-miR-18
ENSG0000	ENSG0000	2.22E-10	hsa-miR-106a-5p;hsa-miR-106b-5p;hsa-miR-1307-3p;hsa-miR
ENSG0000	ENSG0000	3.40E-08	hsa-miR-106a-5p;hsa-miR-106b-5p;hsa-miR-17-5p;hsa-miR-2
ENSG0000	ENSG0000	0.00039	hsa-miR-106b-5p;hsa-miR-17-5p;hsa-miR-20a-5p;hsa-miR-20
ENSG0000	ENSG0000	1.63E-10	hsa-miR-106a-5p;hsa-miR-106b-5p;hsa-miR-148a-5p;hsa-miR
ENSG0000	ENSG0000	6.79E-06	hsa-miR-106a-5p;hsa-miR-106b-5p;hsa-miR-17-5p;hsa-miR-2
ENSG0000	ENSG0000	6.84E-11	hsa-miR-106a-5p;hsa-miR-106b-5p;hsa-miR-150-5p;hsa-miR-
ENSG0000	ENSG0000	0.001314	hsa-miR-106b-5p;hsa-miR-17-5p;hsa-miR-20a-5p;hsa-miR-20
ENSG0000	ENSG0000	1.09E-13	hsa-miR-106a-5p;hsa-miR-106b-5p;hsa-miR-150-5p;hsa-miR-
ENSG0000	ENSG0000	2.30E-09	hsa-miR-106b-5p;hsa-miR-17-5p;hsa-miR-20a-5p;hsa-miR-20
ENSG0000	ENSG0000	7.70E-07	hsa-miR-106a-5p;hsa-miR-106b-5p;hsa-miR-1271-3p;hsa-miR
ENSG0000	ENSG0000	7.70E-07	hsa-miR-106a-5p;hsa-miR-106b-5p;hsa-miR-1271-3p;hsa-miR
ENSG0000	ENSG0000	0.003122	hsa-miR-106b-5p;hsa-miR-17-5p;hsa-miR-20a-5p;hsa-miR-20
ENSG0000	ENSG0000	0.000573	hsa-miR-519a-3p;hsa-miR-519b-3p;hsa-miR-519c-3p;hsa-miR
ENSG0000	ENSG0000	6.62E-08	hsa-miR-106a-5p;hsa-miR-106b-5p;hsa-miR-150-5p;hsa-miR-
ENSG0000	ENSG0000	3.19E-07	hsa-miR-106a-5p;hsa-miR-106b-5p;hsa-miR-150-5p;hsa-miR-
ENSG0000	ENSG0000	0.006596	hsa-miR-150-5p;hsa-miR-186-3p;hsa-miR-30c-1-3p;hsa-miR-
ENSG0000	ENSG0000	4.30E-05	hsa-miR-519a-3p;hsa-miR-519b-3p;hsa-miR-519c-3p;hsa-miR
ENSG0000	ENSG0000	3.81E-09	hsa-miR-106a-5p;hsa-miR-106b-5p;hsa-miR-17-5p;hsa-miR-2
ENSG0000	ENSG0000	2.86E-07	hsa-miR-106b-5p;hsa-miR-17-5p;hsa-miR-20a-5p;hsa-miR-20
ENSG0000	ENSG0000	1.69E-07	hsa-miR-106b-5p;hsa-miR-17-5p;hsa-miR-20a-5p;hsa-miR-20
ENSG0000	ENSG0000	0.001651	hsa-miR-106b-5p;hsa-miR-17-5p;hsa-miR-20b-5p;hsa-miR-93
ENSG0000	ENSG0000	1.06E-06	hsa-miR-106a-5p;hsa-miR-106b-5p;hsa-miR-17-5p;hsa-miR-2
ENSG0000	ENSG0000	3.32E-11	hsa-miR-106a-5p;hsa-miR-106b-5p;hsa-miR-17-5p;hsa-miR-2
ENSG0000	ENSG0000	0.00393	hsa-miR-106a-5p;hsa-miR-106b-5p;hsa-miR-20b-5p;hsa-miR-
ENSG0000	ENSG0000	7.67E-11	hsa-miR-106a-5p;hsa-miR-106b-5p;hsa-miR-17-5p;hsa-miR-2
ENSG0000	ENSG0000	2.30E-09	hsa-miR-106b-5p;hsa-miR-17-5p;hsa-miR-20a-5p;hsa-miR-20
ENSG0000	ENSG0000	1.75E-06	hsa-miR-106a-5p;hsa-miR-106b-5p;hsa-miR-17-5p;hsa-miR-2
ENSG0000	ENSG0000	2.11E-08	hsa-miR-106b-5p;hsa-miR-17-5p;hsa-miR-20a-5p;hsa-miR-20
ENSG0000	ENSG0000	1.11E-08	hsa-miR-106a-5p;hsa-miR-106b-5p;hsa-miR-17-5p;hsa-miR-2
ENSG0000	ENSG0000	0.00229	hsa-miR-106b-5p;hsa-miR-17-5p;hsa-miR-20a-5p;hsa-miR-20
ENSG0000	ENSG0000	1.59E-12	hsa-miR-106a-5p;hsa-miR-106b-5p;hsa-miR-1307-3p;hsa-miR
ENSG0000	ENSG0000	6.44E-05	hsa-miR-106a-5p;hsa-miR-106b-5p;hsa-miR-150-5p;hsa-miR-
ENSG0000	ENSG0000	3.40E-11	hsa-miR-106a-5p;hsa-miR-106b-5p;hsa-miR-1307-3p;hsa-miR
ENSG0000	ENSG0000	5.15E-09	hsa-miR-106a-5p;hsa-miR-106b-5p;hsa-miR-150-5p;hsa-miR-
ENSG0000	ENSG0000	1.69E-09	hsa-miR-106a-5p;hsa-miR-106b-5p;hsa-miR-17-5p;hsa-miR-2
ENSG0000	ENSG0000	1.41E-07	hsa-miR-106a-5p;hsa-miR-106b-5p;hsa-miR-17-5p;hsa-miR-2
ENSG0000	ENSG0000	2.24E-05	hsa-miR-106b-5p;hsa-miR-17-5p;hsa-miR-20a-5p;hsa-miR-20
ENSG0000	ENSG0000	6.21E-09	hsa-miR-106a-5p;hsa-miR-106b-5p;hsa-miR-17-5p;hsa-miR-2
ENSG0000	ENSG0000	7.19E-09	hsa-miR-106a-5p;hsa-miR-106b-5p;hsa-miR-150-5p;hsa-miR-
ENSG0000	ENSG0000	7.09E-07	hsa-miR-106b-5p;hsa-miR-17-5p;hsa-miR-20a-5p;hsa-miR-20
ENSG0000	ENSG0000	2.47E-05	hsa-miR-106a-5p;hsa-miR-106b-5p;hsa-miR-17-5p;hsa-miR-2
ENSG0000	ENSG0000	2.30E-09	hsa-miR-106b-5p;hsa-miR-17-5p;hsa-miR-20a-5p;hsa-miR-20
ENSG0000	ENSG0000	4.44E-16	hsa-miR-106a-5p;hsa-miR-106b-5p;hsa-miR-1271-3p;hsa-miR

ENSG0000	ENSG0000	1.76E-09	hsa-miR-106a-5p;hsa-miR-106b-5p;hsa-miR-150-5p;hsa-miR-1
ENSG0000	ENSG0000	1.32E-09	hsa-miR-106a-5p;hsa-miR-106b-5p;hsa-miR-17-5p;hsa-miR-20
ENSG0000	ENSG0000	6.14E-12	hsa-miR-106a-5p;hsa-miR-106b-5p;hsa-miR-17-5p;hsa-miR-20
ENSG0000	ENSG0000	3.24E-09	hsa-miR-106a-5p;hsa-miR-106b-5p;hsa-miR-1307-3p;hsa-miR-
ENSG0000	ENSG0000	0.007324	hsa-miR-150-5p;hsa-miR-186-3p;hsa-miR-378a-5p;hsa-miR-7
ENSG0000	ENSG0000	0.000202	hsa-miR-519a-3p;hsa-miR-519b-3p;hsa-miR-519c-3p;hsa-miR-
ENSG0000	ENSG0000	2.55E-09	hsa-miR-106a-5p;hsa-miR-106b-5p;hsa-miR-150-5p;hsa-miR-1
ENSG0000	ENSG0000	4.30E-05	hsa-miR-106b-5p;hsa-miR-17-5p;hsa-miR-20a-5p;hsa-miR-20
ENSG0000	ENSG0000	1.72E-10	hsa-miR-106a-5p;hsa-miR-106b-5p;hsa-miR-17-5p;hsa-miR-20
ENSG0000	ENSG0000	4.02E-10	hsa-miR-106a-5p;hsa-miR-106b-5p;hsa-miR-150-5p;hsa-miR-1
ENSG0000	ENSG0000	3.73E-08	hsa-miR-106b-5p;hsa-miR-204-3p;hsa-miR-20a-5p;hsa-miR-20
ENSG0000	ENSG0000	3.77E-11	hsa-miR-106a-5p;hsa-miR-106b-5p;hsa-miR-17-5p;hsa-miR-20
ENSG0000	ENSG0000	1.19E-06	hsa-miR-106a-5p;hsa-miR-106b-5p;hsa-miR-150-5p;hsa-miR-1
ENSG0000	ENSG0000	7.57E-09	hsa-miR-106a-5p;hsa-miR-106b-5p;hsa-miR-17-5p;hsa-miR-20
ENSG0000	ENSG0000	3.37E-09	hsa-miR-106a-5p;hsa-miR-106b-5p;hsa-miR-150-5p;hsa-miR-1
ENSG0000	ENSG0000	0.000291	hsa-miR-106a-5p;hsa-miR-106b-5p;hsa-miR-17-5p;hsa-miR-20
ENSG0000	ENSG0000	1.11E-11	hsa-miR-106a-5p;hsa-miR-106b-5p;hsa-miR-17-5p;hsa-miR-20
ENSG0000	ENSG0000	0.001218	hsa-miR-106b-5p;hsa-miR-17-5p;hsa-miR-20a-5p;hsa-miR-20
ENSG0000	ENSG0000	0.001218	hsa-miR-106b-5p;hsa-miR-17-5p;hsa-miR-20a-5p;hsa-miR-20
ENSG0000	ENSG0000	2.13E-06	hsa-miR-106b-5p;hsa-miR-17-5p;hsa-miR-20a-5p;hsa-miR-20
ENSG0000	ENSG0000	4.17E-07	hsa-miR-106a-5p;hsa-miR-106b-5p;hsa-miR-17-5p;hsa-miR-20
ENSG0000	ENSG0000	4.60E-07	hsa-miR-106b-5p;hsa-miR-17-5p;hsa-miR-20a-5p;hsa-miR-20
ENSG0000	ENSG0000	5.11E-08	hsa-miR-106a-5p;hsa-miR-106b-5p;hsa-miR-17-5p;hsa-miR-20
ENSG0000	ENSG0000	1.37E-06	hsa-miR-106a-5p;hsa-miR-106b-5p;hsa-miR-17-5p;hsa-miR-20
ENSG0000	ENSG0000	3.87E-09	hsa-miR-106a-5p;hsa-miR-106b-5p;hsa-miR-150-5p;hsa-miR-1
ENSG0000	ENSG0000	5.26E-07	hsa-miR-106a-5p;hsa-miR-106b-5p;hsa-miR-1307-3p;hsa-miR-
ENSG0000	ENSG0000	6.31E-10	hsa-miR-106a-5p;hsa-miR-106b-5p;hsa-miR-17-5p;hsa-miR-20
ENSG0000	ENSG0000	5.34E-10	hsa-miR-106a-5p;hsa-miR-106b-5p;hsa-miR-150-5p;hsa-miR-1
ENSG0000	ENSG0000	0.00062	hsa-miR-106b-5p;hsa-miR-17-5p;hsa-miR-192-5p;hsa-miR-20
ENSG0000	ENSG0000	2.83E-07	hsa-miR-106a-5p;hsa-miR-106b-5p;hsa-miR-17-5p;hsa-miR-1
ENSG0000	ENSG0000	1.27E-08	hsa-miR-106a-5p;hsa-miR-106b-5p;hsa-miR-1307-3p;hsa-miR-
ENSG0000	ENSG0000	1.11E-09	hsa-miR-106b-5p;hsa-miR-17-5p;hsa-miR-20a-5p;hsa-miR-20
ENSG0000	ENSG0000	7.58E-12	hsa-miR-106a-5p;hsa-miR-106b-5p;hsa-miR-150-5p;hsa-miR-1
ENSG0000	ENSG0000	0.000153	hsa-miR-106b-5p;hsa-miR-1307-3p;hsa-miR-17-5p;hsa-miR-20
ENSG0000	ENSG0000	5.19E-08	hsa-miR-106a-5p;hsa-miR-106b-5p;hsa-miR-17-5p;hsa-miR-20
ENSG0000	ENSG0000	0.000352	hsa-miR-106b-5p;hsa-miR-17-5p;hsa-miR-20a-5p;hsa-miR-20
ENSG0000	ENSG0000	1.53E-05	hsa-miR-106a-5p;hsa-miR-106b-5p;hsa-miR-17-5p;hsa-miR-20
ENSG0000	ENSG0000	0.000195	hsa-miR-450b-5p;hsa-miR-519a-3p;hsa-miR-519b-3p;hsa-miR
ENSG0000	ENSG0000	3.19E-07	hsa-miR-106a-5p;hsa-miR-106b-5p;hsa-miR-150-5p;hsa-miR-1
ENSG0000	ENSG0000	1.58E-08	hsa-miR-106a-5p;hsa-miR-106b-5p;hsa-miR-150-5p;hsa-miR-1
ENSG0000	ENSG0000	9.87E-08	hsa-miR-106a-5p;hsa-miR-106b-5p;hsa-miR-150-5p;hsa-miR-1
ENSG0000	ENSG0000	1.32E-09	hsa-miR-106a-5p;hsa-miR-106b-5p;hsa-miR-17-5p;hsa-miR-1
ENSG0000	ENSG0000	1.32E-09	hsa-miR-106a-5p;hsa-miR-106b-5p;hsa-miR-17-5p;hsa-miR-1
ENSG0000	ENSG0000	8.06E-05	hsa-miR-106b-5p;hsa-miR-17-5p;hsa-miR-20a-5p;hsa-miR-20
ENSG0000	ENSG0000	0.001634	hsa-miR-106b-5p;hsa-miR-17-5p;hsa-miR-20a-5p;hsa-miR-20
ENSG0000	ENSG0000	1.09E-05	hsa-miR-519a-3p;hsa-miR-519b-3p;hsa-miR-519c-3p;hsa-miR-
ENSG0000	ENSG0000	1.20E-09	hsa-miR-106a-5p;hsa-miR-106b-5p;hsa-miR-17-5p;hsa-miR-20
ENSG0000	ENSG0000	7.49E-08	hsa-miR-106a-5p;hsa-miR-106b-5p;hsa-miR-135a-5p;hsa-miR-
ENSG0000	ENSG0000	2.17E-08	hsa-miR-106a-5p;hsa-miR-106b-5p;hsa-miR-17-5p;hsa-miR-20
ENSG0000	ENSG0000	0.000245	hsa-miR-106a-5p;hsa-miR-106b-5p;hsa-miR-1307-3p;hsa-miR-
ENSG0000	ENSG0000	6.18E-07	hsa-miR-106a-5p;hsa-miR-17-5p;hsa-miR-20a-5p;hsa-miR-20
ENSG0000	ENSG0000	4.76E-10	hsa-miR-106a-5p;hsa-miR-106b-5p;hsa-miR-17-5p;hsa-miR-20
ENSG0000	ENSG0000	0.007253	hsa-miR-106b-5p;hsa-miR-17-5p;hsa-miR-20a-5p;hsa-miR-20
ENSG0000	ENSG0000	2.34E-05	hsa-miR-188-3p;hsa-miR-378a-5p;hsa-miR-519a-3p;hsa-miR-5

ENSG0000	ENSG0000	1.23E-07	hsa-miR-106a-5p;hsa-miR-106b-5p;hsa-miR-17-5p;hsa-miR-2
ENSG0000	ENSG0000	1.23E-07	hsa-miR-106a-5p;hsa-miR-106b-5p;hsa-miR-17-5p;hsa-miR-2
ENSG0000	ENSG0000	3.10E-08	hsa-miR-106a-5p;hsa-miR-106b-5p;hsa-miR-17-5p;hsa-miR-2
ENSG0000	ENSG0000	9.48E-10	hsa-miR-106a-5p;hsa-miR-106b-5p;hsa-miR-17-5p;hsa-miR-2
ENSG0000	ENSG0000	5.11E-12	hsa-miR-106a-5p;hsa-miR-106b-5p;hsa-miR-17-5p;hsa-miR-2
ENSG0000	ENSG0000	4.61E-06	hsa-miR-106a-5p;hsa-miR-106b-5p;hsa-miR-17-5p;hsa-miR-2
ENSG0000	ENSG0000	1.40E-09	hsa-miR-106a-5p;hsa-miR-106b-5p;hsa-miR-150-5p;hsa-miR-
ENSG0000	ENSG0000	1.40E-09	hsa-miR-106a-5p;hsa-miR-106b-5p;hsa-miR-150-5p;hsa-miR-
ENSG0000	ENSG0000	1.58E-10	hsa-miR-106a-5p;hsa-miR-106b-5p;hsa-miR-17-5p;hsa-miR-1
ENSG0000	ENSG0000	2.65E-10	hsa-miR-106a-5p;hsa-miR-106b-5p;hsa-miR-17-5p;hsa-miR-2
ENSG0000	ENSG0000	0.000125	hsa-miR-106b-5p;hsa-miR-150-5p;hsa-miR-17-5p;hsa-miR-18
ENSG0000	ENSG0000	0.003339	hsa-miR-106a-5p;hsa-miR-106b-5p;hsa-miR-17-5p;hsa-miR-2
ENSG0000	ENSG0000	2.21E-10	hsa-miR-106a-5p;hsa-miR-106b-5p;hsa-miR-150-5p;hsa-miR-
ENSG0000	ENSG0000	2.54E-09	hsa-miR-106a-5p;hsa-miR-106b-5p;hsa-miR-150-5p;hsa-miR-
ENSG0000	ENSG0000	6.03E-07	hsa-miR-106a-5p;hsa-miR-106b-5p;hsa-miR-17-5p;hsa-miR-2
ENSG0000	ENSG0000	6.03E-07	hsa-miR-106a-5p;hsa-miR-106b-5p;hsa-miR-17-5p;hsa-miR-2
ENSG0000	ENSG0000	2.36E-05	hsa-miR-106a-5p;hsa-miR-106b-5p;hsa-miR-17-5p;hsa-miR-2
ENSG0000	ENSG0000	2.93E-09	hsa-miR-106a-5p;hsa-miR-106b-5p;hsa-miR-150-5p;hsa-miR-
ENSG0000	ENSG0000	0.001524	hsa-miR-30c-1-3p;hsa-miR-30c-2-3p;hsa-miR-519a-3p;hsa-mi
ENSG0000	ENSG0000	1.43E-06	hsa-miR-106a-5p;hsa-miR-106b-5p;hsa-miR-17-5p;hsa-miR-2
ENSG0000	ENSG0000	6.50E-13	hsa-miR-106a-5p;hsa-miR-106b-5p;hsa-miR-150-5p;hsa-miR-
ENSG0000	ENSG0000	5.00E-07	hsa-miR-106a-5p;hsa-miR-106b-5p;hsa-miR-17-5p;hsa-miR-2
ENSG0000	ENSG0000	5.00E-07	hsa-miR-106a-5p;hsa-miR-106b-5p;hsa-miR-17-5p;hsa-miR-2
ENSG0000	ENSG0000	0.00014	hsa-miR-519a-3p;hsa-miR-519b-3p;hsa-miR-519c-3p;hsa-miR-
ENSG0000	ENSG0000	1.44E-13	hsa-miR-106a-5p;hsa-miR-106b-5p;hsa-miR-150-5p;hsa-miR-
ENSG0000	ENSG0000	1.66E-12	hsa-miR-106a-5p;hsa-miR-106b-5p;hsa-miR-150-5p;hsa-miR-
ENSG0000	ENSG0000	1.83E-10	hsa-miR-106a-5p;hsa-miR-106b-5p;hsa-miR-150-5p;hsa-miR-
ENSG0000	ENSG0000	2.54E-09	hsa-miR-106a-5p;hsa-miR-106b-5p;hsa-miR-150-5p;hsa-miR-
ENSG0000	ENSG0000	7.54E-13	hsa-miR-106a-5p;hsa-miR-106b-5p;hsa-miR-150-5p;hsa-miR-
ENSG0000	ENSG0000	7.59E-13	hsa-miR-106a-5p;hsa-miR-106b-5p;hsa-miR-17-5p;hsa-miR-2
ENSG0000	ENSG0000	7.18E-12	hsa-miR-106a-5p;hsa-miR-106b-5p;hsa-miR-17-5p;hsa-miR-1
ENSG0000	ENSG0000	1.25E-10	hsa-miR-106a-5p;hsa-miR-106b-5p;hsa-miR-150-5p;hsa-miR-
ENSG0000	ENSG0000	0.000364	hsa-miR-106a-5p;hsa-miR-20b-5p;hsa-miR-520a-3p;hsa-miR-
ENSG0000	ENSG0000	0.00039	hsa-miR-1271-3p;hsa-miR-214-5p;hsa-miR-30c-1-3p;hsa-miR-
ENSG0000	ENSG0000	2.06E-05	hsa-miR-106b-5p;hsa-miR-17-5p;hsa-miR-20a-5p;hsa-miR-20
ENSG0000	ENSG0000	1.39E-11	hsa-miR-106a-5p;hsa-miR-106b-5p;hsa-miR-150-5p;hsa-miR-
ENSG0000	ENSG0000	2.79E-06	hsa-miR-106a-5p;hsa-miR-106b-5p;hsa-miR-17-5p;hsa-miR-2
ENSG0000	ENSG0000	0.003497	hsa-miR-378a-5p;hsa-miR-519a-3p;hsa-miR-519b-3p;hsa-miR-
ENSG0000	ENSG0000	4.59E-09	hsa-miR-106a-5p;hsa-miR-106b-5p;hsa-miR-150-5p;hsa-miR-
ENSG0000	ENSG0000	1.86E-07	hsa-miR-106a-5p;hsa-miR-106b-5p;hsa-miR-150-5p;hsa-miR-
ENSG0000	ENSG0000	1.83E-10	hsa-miR-106a-5p;hsa-miR-106b-5p;hsa-miR-150-5p;hsa-miR-
ENSG0000	ENSG0000	0.001218	hsa-miR-106b-5p;hsa-miR-17-5p;hsa-miR-20a-5p;hsa-miR-20
ENSG0000	ENSG0000	1.95E-13	hsa-miR-106a-5p;hsa-miR-106b-5p;hsa-miR-17-5p;hsa-miR-2
ENSG0000	ENSG0000	2.93E-08	hsa-miR-106a-5p;hsa-miR-106b-5p;hsa-miR-1307-3p;hsa-miR-
ENSG0000	ENSG0000	3.84E-09	hsa-miR-106a-5p;hsa-miR-106b-5p;hsa-miR-17-5p;hsa-miR-2
ENSG0000	ENSG0000	0.000816	hsa-miR-1271-3p;hsa-miR-135a-5p;hsa-miR-150-5p;hsa-miR-
ENSG0000	ENSG0000	2.29E-11	hsa-miR-106a-5p;hsa-miR-106b-5p;hsa-miR-150-5p;hsa-miR-
ENSG0000	ENSG0000	3.40E-13	hsa-miR-106a-5p;hsa-miR-106b-5p;hsa-miR-150-5p;hsa-miR-
ENSG0000	ENSG0000	7.59E-13	hsa-miR-106a-5p;hsa-miR-106b-5p;hsa-miR-17-5p;hsa-miR-2
ENSG0000	ENSG0000	2.58E-11	hsa-miR-106a-5p;hsa-miR-106b-5p;hsa-miR-17-5p;hsa-miR-2
ENSG0000	ENSG0000	0.000561	hsa-miR-144-3p;hsa-miR-16-2-3p;hsa-miR-195-3p;hsa-miR-3
ENSG0000	ENSG0000	0.004379	hsa-miR-148b-3p;hsa-miR-16-2-3p;hsa-miR-195-3p;hsa-miR-
ENSG0000	ENSG0000	5.00E-06	hsa-miR-16-2-3p;hsa-miR-195-3p;hsa-miR-338-5p;hsa-miR-3
ENSG0000	ENSG0000	1.82E-05	hsa-miR-16-2-3p;hsa-miR-195-3p;hsa-miR-22-5p;hsa-miR-37

ENSG0000	ENSG0000	0.005201	hsa-miR-16-2-3p;hsa-miR-195-3p;hsa-miR-3611;hsa-miR-382-
ENSG0000	ENSG0000	0.000112	hsa-miR-144-3p;hsa-miR-16-2-3p;hsa-miR-195-3p;hsa-miR-3
ENSG0000	ENSG0000	0.00157	hsa-miR-30c-1-3p;hsa-miR-497-3p;hsa-miR-548ap-3p;hsa-mi
ENSG0000	ENSG0000	0.000105	hsa-miR-103a-3p;hsa-miR-107;hsa-miR-548ap-3p;hsa-miR-54
ENSG0000	ENSG0000	0.00861	hsa-miR-103a-2-5p;hsa-miR-103a-3p;hsa-miR-107;hsa-miR-1
ENSG0000	ENSG0000	0.000109	hsa-miR-103a-3p;hsa-miR-107;hsa-miR-1301-3p;hsa-miR-500
ENSG0000	ENSG0000	0.000641	hsa-miR-101-5p;hsa-miR-103a-3p;hsa-miR-107;hsa-miR-1301-
ENSG0000	ENSG0000	0.004861	hsa-miR-103a-3p;hsa-miR-107;hsa-miR-1301-3p;hsa-miR-191-
ENSG0000	ENSG0000	0.000546	hsa-miR-30c-1-3p;hsa-miR-497-3p;hsa-miR-500a-5p;hsa-miR-
ENSG0000	ENSG0000	0.001768	hsa-miR-103a-3p;hsa-miR-107;hsa-miR-548ap-3p;hsa-miR-54
ENSG0000	ENSG0000	0.004024	hsa-miR-1301-3p;hsa-miR-30c-1-3p;hsa-miR-548ap-3p;hsa-m
ENSG0000	ENSG0000	5.44E-05	hsa-miR-103a-3p;hsa-miR-107;hsa-miR-1301-3p;hsa-miR-319
ENSG0000	ENSG0000	6.53E-05	hsa-miR-103a-3p;hsa-miR-107;hsa-miR-1301-3p;hsa-miR-548
ENSG0000	ENSG0000	0.004801	hsa-miR-103a-3p;hsa-miR-107;hsa-miR-30c-1-3p;hsa-miR-451
ENSG0000	ENSG0000	0.004801	hsa-miR-103a-3p;hsa-miR-107;hsa-miR-30c-1-3p;hsa-miR-451
ENSG0000	ENSG0000	0.001388	hsa-miR-101-5p;hsa-miR-107;hsa-miR-30c-1-3p;hsa-miR-548
ENSG0000	ENSG0000	0.00157	hsa-miR-103a-3p;hsa-miR-107;hsa-miR-500a-5p;hsa-miR-501-
ENSG0000	ENSG0000	0.001388	hsa-miR-103a-3p;hsa-miR-107;hsa-miR-548ap-3p;hsa-miR-54
ENSG0000	ENSG0000	0.001768	hsa-miR-30c-1-3p;hsa-miR-3194-5p;hsa-miR-548ap-3p;hsa-m
ENSG0000	ENSG0000	0.008014	hsa-miR-30c-1-3p;hsa-miR-3194-5p;hsa-miR-548ap-3p;hsa-m
ENSG0000	ENSG0000	6.97E-05	hsa-miR-103a-3p;hsa-miR-107;hsa-miR-500a-5p;hsa-miR-501-
ENSG0000	ENSG0000	0.00054	hsa-miR-103a-2-5p;hsa-miR-103a-3p;hsa-miR-107;hsa-miR-5
ENSG0000	ENSG0000	0.000298	hsa-miR-3194-5p;hsa-miR-500a-5p;hsa-miR-501-5p;hsa-miR-
ENSG0000	ENSG0000	0.000149	hsa-miR-103a-3p;hsa-miR-107;hsa-miR-1301-3p;hsa-miR-500
ENSG0000	ENSG0000	0.002468	hsa-miR-27a-3p;hsa-miR-30c-1-3p;hsa-miR-548ap-3p;hsa-mi
ENSG0000	ENSG0000	9.23E-05	hsa-miR-103a-3p;hsa-miR-107;hsa-miR-1301-3p;hsa-miR-314
ENSG0000	ENSG0000	0.00288	hsa-miR-103a-3p;hsa-miR-107;hsa-miR-500a-5p;hsa-miR-501-
ENSG0000	ENSG0000	0.001568	hsa-miR-146a-3p;hsa-miR-181b-5p;hsa-miR-181c-5p;hsa-miR-
ENSG0000	ENSG0000	0.00157	hsa-miR-181a-5p;hsa-miR-181b-5p;hsa-miR-181c-5p;hsa-miR-
ENSG0000	ENSG0000	1.39E-05	hsa-miR-181a-5p;hsa-miR-181b-5p;hsa-miR-181c-5p;hsa-miR-
ENSG0000	ENSG0000	2.96E-05	hsa-miR-181a-5p;hsa-miR-181b-5p;hsa-miR-181c-5p;hsa-miR-
ENSG0000	ENSG0000	0.001768	hsa-miR-181a-5p;hsa-miR-181b-5p;hsa-miR-181c-5p;hsa-miR-
ENSG0000	ENSG0000	0.004861	hsa-miR-1307-3p;hsa-miR-135a-5p;hsa-miR-135b-5p;hsa-miR-
ENSG0000	ENSG0000	1.29E-05	hsa-miR-146a-3p;hsa-miR-27a-3p;hsa-miR-27b-3p;hsa-miR-3
ENSG0000	ENSG0000	1.66E-05	hsa-miR-181a-5p;hsa-miR-181b-5p;hsa-miR-181c-5p;hsa-miR-
ENSG0000	ENSG0000	0.001049	hsa-miR-181a-5p;hsa-miR-181c-5p;hsa-miR-301a-5p;hsa-miR-
ENSG0000	ENSG0000	0.001049	hsa-miR-181a-5p;hsa-miR-181c-5p;hsa-miR-301a-5p;hsa-miR-
ENSG0000	ENSG0000	0.003339	hsa-miR-1307-3p;hsa-miR-181a-5p;hsa-miR-181b-5p;hsa-miR-
ENSG0000	ENSG0000	0.00012	hsa-miR-181a-5p;hsa-miR-181b-5p;hsa-miR-181c-5p;hsa-miR-
ENSG0000	ENSG0000	0.001706	hsa-miR-146a-3p;hsa-miR-27a-3p;hsa-miR-27b-3p;hsa-miR-3
ENSG0000	ENSG0000	0.005446	hsa-miR-135a-5p;hsa-miR-135b-5p;hsa-miR-146a-3p;hsa-miR-
ENSG0000	ENSG0000	0.001768	hsa-miR-181a-5p;hsa-miR-181b-5p;hsa-miR-181c-5p;hsa-miR-
ENSG0000	ENSG0000	0.000262	hsa-miR-148b-3p;hsa-miR-181a-5p;hsa-miR-181b-5p;hsa-miR
ENSG0000	ENSG0000	0.00501	hsa-miR-181a-5p;hsa-miR-181b-5p;hsa-miR-181c-5p;hsa-miR-
ENSG0000	ENSG0000	0.000423	hsa-miR-146a-3p;hsa-miR-27a-3p;hsa-miR-27b-3p;hsa-miR-3
ENSG0000	ENSG0000	0.005907	hsa-miR-181a-5p;hsa-miR-181b-5p;hsa-miR-181c-5p;hsa-miR-
ENSG0000	ENSG0000	1.72E-05	hsa-miR-146a-3p;hsa-miR-181a-5p;hsa-miR-181b-5p;hsa-miR-
ENSG0000	ENSG0000	7.91E-07	hsa-miR-181a-5p;hsa-miR-181b-5p;hsa-miR-181c-5p;hsa-miR-
ENSG0000	ENSG0000	0.001477	hsa-miR-181a-5p;hsa-miR-181b-5p;hsa-miR-181c-5p;hsa-miR-
ENSG0000	ENSG0000	2.12E-05	hsa-miR-181a-5p;hsa-miR-181b-5p;hsa-miR-181c-5p;hsa-miR-
ENSG0000	ENSG0000	0.001477	hsa-miR-181a-5p;hsa-miR-181b-5p;hsa-miR-181c-5p;hsa-miR-
ENSG0000	ENSG0000	3.86E-07	hsa-miR-181a-5p;hsa-miR-181b-5p;hsa-miR-181c-5p;hsa-miR-
ENSG0000	ENSG0000	0.000706	hsa-miR-146a-3p;hsa-miR-181a-5p;hsa-miR-181b-5p;hsa-miR-
ENSG0000	ENSG0000	2.65E-06	hsa-miR-181a-5p;hsa-miR-181b-5p;hsa-miR-181c-5p;hsa-miR-

ENSG0000	ENSG0000	0.001388	hsa-miR-135a-5p;hsa-miR-181a-5p;hsa-miR-27a-3p;hsa-miR-2
ENSG0000	ENSG0000	0.007727	hsa-miR-27a-3p;hsa-miR-27b-3p;hsa-miR-3179;hsa-miR-4328
ENSG0000	ENSG0000	0.000238	hsa-miR-181a-5p;hsa-miR-181b-5p;hsa-miR-181c-5p;hsa-miR-
ENSG0000	ENSG0000	0.002347	hsa-miR-135a-5p;hsa-miR-135b-5p;hsa-miR-181a-5p;hsa-miR-
ENSG0000	ENSG0000	1.66E-05	hsa-miR-181a-5p;hsa-miR-181b-5p;hsa-miR-181c-5p;hsa-miR-
ENSG0000	ENSG0000	0.001388	hsa-miR-148b-3p;hsa-miR-181a-5p;hsa-miR-181b-5p;hsa-miR
ENSG0000	ENSG0000	0.009629	hsa-miR-181a-5p;hsa-miR-181b-5p;hsa-miR-181c-5p;hsa-miR-
ENSG0000	ENSG0000	5.69E-07	hsa-miR-146a-3p;hsa-miR-181a-5p;hsa-miR-181b-5p;hsa-miR-
ENSG0000	ENSG0000	4.47E-05	hsa-miR-181b-5p;hsa-miR-181c-5p;hsa-miR-27a-3p;hsa-miR-2
ENSG0000	ENSG0000	0.000602	hsa-miR-146a-3p;hsa-miR-181a-5p;hsa-miR-181b-5p;hsa-miR-
ENSG0000	ENSG0000	0.000192	hsa-miR-181a-5p;hsa-miR-181b-5p;hsa-miR-181c-5p;hsa-miR-
ENSG0000	ENSG0000	0.000171	hsa-miR-181a-5p;hsa-miR-181b-5p;hsa-miR-181c-5p;hsa-miR-
ENSG0000	ENSG0000	0.007727	hsa-miR-181a-5p;hsa-miR-181b-5p;hsa-miR-181c-5p;hsa-miR-
ENSG0000	ENSG0000	8.25E-07	hsa-miR-181a-5p;hsa-miR-181b-5p;hsa-miR-181c-5p;hsa-miR-
ENSG0000	ENSG0000	8.25E-07	hsa-miR-181a-5p;hsa-miR-181b-5p;hsa-miR-181c-5p;hsa-miR-
ENSG0000	ENSG0000	6.63E-06	hsa-miR-181a-5p;hsa-miR-181b-5p;hsa-miR-181c-5p;hsa-miR-
ENSG0000	ENSG0000	0.000385	hsa-miR-181a-5p;hsa-miR-181b-5p;hsa-miR-181c-5p;hsa-miR-
ENSG0000	ENSG0000	7.15E-05	hsa-miR-181a-5p;hsa-miR-181b-5p;hsa-miR-181c-5p;hsa-miR-
ENSG0000	ENSG0000	2.65E-05	hsa-miR-181a-5p;hsa-miR-181b-5p;hsa-miR-181c-5p;hsa-miR-
ENSG0000	ENSG0000	4.14E-07	hsa-miR-146a-3p;hsa-miR-181a-5p;hsa-miR-181b-5p;hsa-miR-
ENSG0000	ENSG0000	0.00157	hsa-miR-1307-3p;hsa-miR-181a-5p;hsa-miR-181b-5p;hsa-miR-
ENSG0000	ENSG0000	7.45E-06	hsa-miR-181a-5p;hsa-miR-181b-5p;hsa-miR-181c-5p;hsa-miR-
ENSG0000	ENSG0000	9.23E-05	hsa-miR-181a-5p;hsa-miR-181b-5p;hsa-miR-181c-5p;hsa-miR-
ENSG0000	ENSG0000	0.000321	hsa-miR-181a-5p;hsa-miR-181b-5p;hsa-miR-181c-5p;hsa-miR-
ENSG0000	ENSG0000	0.008919	hsa-miR-181a-5p;hsa-miR-181b-5p;hsa-miR-181c-5p;hsa-miR-
ENSG0000	ENSG0000	0.008919	hsa-miR-181a-5p;hsa-miR-181b-5p;hsa-miR-181c-5p;hsa-miR-
ENSG0000	ENSG0000	0.000461	hsa-miR-146a-3p;hsa-miR-27a-3p;hsa-miR-27b-3p;hsa-miR-3
ENSG0000	ENSG0000	0.000461	hsa-miR-146a-3p;hsa-miR-27a-3p;hsa-miR-27b-3p;hsa-miR-3
ENSG0000	ENSG0000	1.59E-06	hsa-miR-135a-5p;hsa-miR-135b-5p;hsa-miR-181a-5p;hsa-miR-
ENSG0000	ENSG0000	0.00234	hsa-miR-181a-5p;hsa-miR-181b-5p;hsa-miR-181c-5p;hsa-miR-
ENSG0000	ENSG0000	0.00234	hsa-miR-181a-5p;hsa-miR-181b-5p;hsa-miR-181c-5p;hsa-miR-
ENSG0000	ENSG0000	0.00234	hsa-miR-181a-5p;hsa-miR-181b-5p;hsa-miR-181c-5p;hsa-miR-
ENSG0000	ENSG0000	0.00234	hsa-miR-181a-5p;hsa-miR-181b-5p;hsa-miR-181c-5p;hsa-miR-
ENSG0000	ENSG0000	0.00234	hsa-miR-181a-5p;hsa-miR-181b-5p;hsa-miR-181c-5p;hsa-miR-
ENSG0000	ENSG0000	0.00234	hsa-miR-181a-5p;hsa-miR-181b-5p;hsa-miR-181c-5p;hsa-miR-
ENSG0000	ENSG0000	0.00234	hsa-miR-181a-5p;hsa-miR-181b-5p;hsa-miR-181c-5p;hsa-miR-
ENSG0000	ENSG0000	0.00234	hsa-miR-181a-5p;hsa-miR-181b-5p;hsa-miR-181c-5p;hsa-miR-
ENSG0000	ENSG0000	0.003181	hsa-miR-181a-5p;hsa-miR-181b-5p;hsa-miR-181c-5p;hsa-miR-
ENSG0000	ENSG0000	0.00288	hsa-miR-1307-3p;hsa-miR-181a-5p;hsa-miR-181b-5p;hsa-miR-
ENSG0000	ENSG0000	0.005173	hsa-miR-1307-3p;hsa-miR-181a-5p;hsa-miR-181b-5p;hsa-miR-
ENSG0000	ENSG0000	0.000461	hsa-miR-181c-5p;hsa-miR-27a-3p;hsa-miR-27b-3p;hsa-miR-4
ENSG0000	ENSG0000	0.001388	hsa-miR-146a-3p;hsa-miR-181a-5p;hsa-miR-181b-5p;hsa-miR-
ENSG0000	ENSG0000	1.76E-06	hsa-miR-130b-3p;hsa-miR-301a-3p;hsa-miR-301b-3p;hsa-miR
ENSG0000	ENSG0000	3.42E-05	hsa-miR-130b-3p;hsa-miR-301a-3p;hsa-miR-301b-3p;hsa-miR
ENSG0000	ENSG0000	2.97E-06	hsa-miR-130b-3p;hsa-miR-301a-3p;hsa-miR-301b-3p;hsa-miR
ENSG0000	ENSG0000	0.00181	hsa-miR-130b-3p;hsa-miR-301a-3p;hsa-miR-301b-3p;hsa-miR
ENSG0000	ENSG0000	3.76E-08	hsa-miR-106a-5p;hsa-miR-106b-5p;hsa-miR-20a-5p;hsa-miR-
ENSG0000	ENSG0000	5.22E-06	hsa-miR-130b-3p;hsa-miR-301a-3p;hsa-miR-301b-3p;hsa-miR
ENSG0000	ENSG0000	5.24E-05	hsa-miR-130b-3p;hsa-miR-301a-3p;hsa-miR-301b-3p;hsa-miR
ENSG0000	ENSG0000	8.52E-05	hsa-miR-130b-3p;hsa-miR-301a-3p;hsa-miR-301b-3p;hsa-miR
ENSG0000	ENSG0000	2.11E-05	hsa-miR-130b-3p;hsa-miR-301a-3p;hsa-miR-301b-3p;hsa-miR
ENSG0000	ENSG0000	0.000125	hsa-miR-130b-3p;hsa-miR-301a-3p;hsa-miR-301b-3p;hsa-miR
ENSG0000	ENSG0000	1.39E-10	hsa-miR-106a-5p;hsa-miR-106b-5p;hsa-miR-130b-3p;hsa-miR
ENSG0000	ENSG0000	2.02E-06	hsa-miR-130b-3p;hsa-miR-301a-3p;hsa-miR-301b-3p;hsa-miR

ENSG0000	ENSG0000	1.32E-05	hsa-miR-130b-3p;hsa-miR-301a-3p;hsa-miR-301b-3p;hsa-miR
ENSG0000	ENSG0000	2.61E-11	hsa-miR-106a-5p;hsa-miR-106b-5p;hsa-miR-130b-3p;hsa-miR
ENSG0000	ENSG0000	1.12E-05	hsa-miR-130b-3p;hsa-miR-301a-3p;hsa-miR-301b-3p;hsa-miR
ENSG0000	ENSG0000	0.000218	hsa-miR-130b-3p;hsa-miR-301a-3p;hsa-miR-301b-3p;hsa-miR
ENSG0000	ENSG0000	7.79E-06	hsa-miR-130b-3p;hsa-miR-301a-3p;hsa-miR-301b-3p;hsa-miR
ENSG0000	ENSG0000	2.01E-07	hsa-miR-130b-3p;hsa-miR-301a-3p;hsa-miR-301b-3p;hsa-miR
ENSG0000	ENSG0000	0.000389	hsa-miR-106a-5p;hsa-miR-106b-5p;hsa-miR-20a-5p;hsa-miR-3
ENSG0000	ENSG0000	9.97E-10	hsa-miR-106a-5p;hsa-miR-106b-5p;hsa-miR-130b-3p;hsa-miR
ENSG0000	ENSG0000	1.96E-05	hsa-miR-130b-3p;hsa-miR-301a-3p;hsa-miR-301b-3p;hsa-miR
ENSG0000	ENSG0000	7.67E-10	hsa-miR-106a-5p;hsa-miR-106b-5p;hsa-miR-130b-3p;hsa-miR
ENSG0000	ENSG0000	5.24E-05	hsa-miR-130b-3p;hsa-miR-301a-3p;hsa-miR-301b-3p;hsa-miR
ENSG0000	ENSG0000	5.84E-10	hsa-miR-106a-5p;hsa-miR-106b-5p;hsa-miR-130b-3p;hsa-miR
ENSG0000	ENSG0000	4.21E-06	hsa-miR-130b-3p;hsa-miR-301a-3p;hsa-miR-301b-3p;hsa-miR
ENSG0000	ENSG0000	1.44E-05	hsa-miR-130b-3p;hsa-miR-301a-3p;hsa-miR-301b-3p;hsa-miR
ENSG0000	ENSG0000	1.68E-05	hsa-miR-130b-3p;hsa-miR-301a-3p;hsa-miR-301b-3p;hsa-miR
ENSG0000	ENSG0000	2.62E-06	hsa-miR-130b-3p;hsa-miR-301a-3p;hsa-miR-301b-3p;hsa-miR
ENSG0000	ENSG0000	1.68E-05	hsa-miR-130b-3p;hsa-miR-301a-3p;hsa-miR-301b-3p;hsa-miR
ENSG0000	ENSG0000	8.10E-05	hsa-miR-130b-3p;hsa-miR-301a-3p;hsa-miR-301b-3p;hsa-miR
ENSG0000	ENSG0000	2.31E-06	hsa-miR-130b-3p;hsa-miR-301a-3p;hsa-miR-301b-3p;hsa-miR
ENSG0000	ENSG0000	4.13E-05	hsa-miR-130b-3p;hsa-miR-301a-3p;hsa-miR-301b-3p;hsa-miR
ENSG0000	ENSG0000	1.23E-12	hsa-miR-106a-5p;hsa-miR-106b-5p;hsa-miR-130b-3p;hsa-miR
ENSG0000	ENSG0000	3.27E-10	hsa-miR-106a-5p;hsa-miR-106b-5p;hsa-miR-130b-3p;hsa-miR
ENSG0000	ENSG0000	3.20E-05	hsa-miR-130b-3p;hsa-miR-301a-3p;hsa-miR-301b-3p;hsa-miR
ENSG0000	ENSG0000	1.53E-06	hsa-miR-130b-3p;hsa-miR-301a-3p;hsa-miR-301b-3p;hsa-miR
ENSG0000	ENSG0000	3.99E-10	hsa-miR-106a-5p;hsa-miR-106b-5p;hsa-miR-130b-3p;hsa-miR
ENSG0000	ENSG0000	8.64E-11	hsa-miR-106a-5p;hsa-miR-106b-5p;hsa-miR-130b-3p;hsa-miR
ENSG0000	ENSG0000	4.13E-05	hsa-miR-130b-3p;hsa-miR-301a-3p;hsa-miR-301b-3p;hsa-miR
ENSG0000	ENSG0000	1.55E-10	hsa-miR-106a-5p;hsa-miR-106b-5p;hsa-miR-130b-3p;hsa-miR
ENSG0000	ENSG0000	0.000444	hsa-miR-130b-3p;hsa-miR-301a-3p;hsa-miR-301b-3p;hsa-miR
ENSG0000	ENSG0000	8.21E-07	hsa-miR-130b-3p;hsa-miR-301a-3p;hsa-miR-301b-3p;hsa-miR
ENSG0000	ENSG0000	1.94E-10	hsa-miR-106a-5p;hsa-miR-106b-5p;hsa-miR-130b-3p;hsa-miR
ENSG0000	ENSG0000	1.02E-05	hsa-miR-130b-3p;hsa-miR-301a-3p;hsa-miR-301b-3p;hsa-miR
ENSG0000	ENSG0000	1.02E-05	hsa-miR-130b-3p;hsa-miR-301a-3p;hsa-miR-301b-3p;hsa-miR
ENSG0000	ENSG0000	2.40E-10	hsa-miR-106a-5p;hsa-miR-106b-5p;hsa-miR-130b-3p;hsa-miR
ENSG0000	ENSG0000	0.000185	hsa-miR-130b-3p;hsa-miR-301a-3p;hsa-miR-301b-3p;hsa-miR
ENSG0000	ENSG0000	0.000193	hsa-miR-130b-3p;hsa-miR-301a-3p;hsa-miR-301b-3p;hsa-miR
ENSG0000	ENSG0000	9.99E-08	hsa-miR-106a-5p;hsa-miR-106b-5p;hsa-miR-20a-5p;hsa-miR-3
ENSG0000	ENSG0000	3.89E-09	hsa-miR-106a-5p;hsa-miR-106b-5p;hsa-miR-20a-5p;hsa-miR-3
ENSG0000	ENSG0000	2.02E-06	hsa-miR-130b-3p;hsa-miR-301a-3p;hsa-miR-301b-3p;hsa-miR
ENSG0000	ENSG0000	1.22E-05	hsa-miR-130b-3p;hsa-miR-301a-3p;hsa-miR-301b-3p;hsa-miR
ENSG0000	ENSG0000	2.97E-06	hsa-miR-130b-3p;hsa-miR-301a-3p;hsa-miR-301b-3p;hsa-miR
ENSG0000	ENSG0000	2.62E-06	hsa-miR-130b-3p;hsa-miR-301a-3p;hsa-miR-301b-3p;hsa-miR
ENSG0000	ENSG0000	5.79E-06	hsa-miR-130b-3p;hsa-miR-301a-3p;hsa-miR-301b-3p;hsa-miR
ENSG0000	ENSG0000	7.69E-05	hsa-miR-130b-3p;hsa-miR-301a-3p;hsa-miR-301b-3p;hsa-miR
ENSG0000	ENSG0000	1.32E-06	hsa-miR-106a-5p;hsa-miR-106b-5p;hsa-miR-20a-5p;hsa-miR-3
ENSG0000	ENSG0000	1.16E-07	hsa-miR-106a-5p;hsa-miR-106b-5p;hsa-miR-130b-3p;hsa-miR
ENSG0000	ENSG0000	5.55E-05	hsa-miR-130b-3p;hsa-miR-301a-3p;hsa-miR-301b-3p;hsa-miR
ENSG0000	ENSG0000	5.65E-09	hsa-miR-106a-5p;hsa-miR-106b-5p;hsa-miR-130b-3p;hsa-miR
ENSG0000	ENSG0000	8.55E-09	hsa-miR-106a-5p;hsa-miR-106b-5p;hsa-miR-20a-5p;hsa-miR-3
ENSG0000	ENSG0000	4.77E-07	hsa-miR-130b-3p;hsa-miR-301a-3p;hsa-miR-301b-3p;hsa-miR
ENSG0000	ENSG0000	1.55E-10	hsa-miR-106a-5p;hsa-miR-106b-5p;hsa-miR-130b-3p;hsa-miR
ENSG0000	ENSG0000	0.000104	hsa-miR-130b-3p;hsa-miR-301a-3p;hsa-miR-301b-3p;hsa-miR
ENSG0000	ENSG0000	3.92E-14	hsa-miR-106a-5p;hsa-miR-106b-5p;hsa-miR-130b-3p;hsa-miR
ENSG0000	ENSG0000	4.39E-05	hsa-miR-130b-3p;hsa-miR-301a-3p;hsa-miR-301b-3p;hsa-miR

ENSG0000	ENSG0000	5.10E-12	hsa-miR-106a-5p;hsa-miR-106b-5p;hsa-miR-130b-3p;hsa-miR
ENSG0000	ENSG0000	0.000284	hsa-miR-106a-5p;hsa-miR-106b-5p;hsa-miR-130b-3p;hsa-miR
ENSG0000	ENSG0000	1.69E-07	hsa-miR-106a-5p;hsa-miR-106b-5p;hsa-miR-20a-5p;hsa-miR-3
ENSG0000	ENSG0000	1.56E-07	hsa-miR-130b-3p;hsa-miR-301a-3p;hsa-miR-301b-3p;hsa-miR
ENSG0000	ENSG0000	9.37E-06	hsa-miR-130b-3p;hsa-miR-301a-3p;hsa-miR-301b-3p;hsa-miR
ENSG0000	ENSG0000	9.37E-06	hsa-miR-130b-3p;hsa-miR-301a-3p;hsa-miR-301b-3p;hsa-miR
ENSG0000	ENSG0000	2.44E-05	hsa-miR-130b-3p;hsa-miR-301a-3p;hsa-miR-301b-3p;hsa-miR
ENSG0000	ENSG0000	3.99E-10	hsa-miR-106a-5p;hsa-miR-106b-5p;hsa-miR-130b-3p;hsa-miR
ENSG0000	ENSG0000	0.000104	hsa-miR-130b-3p;hsa-miR-301a-3p;hsa-miR-301b-3p;hsa-miR
ENSG0000	ENSG0000	4.39E-05	hsa-miR-130b-3p;hsa-miR-301a-3p;hsa-miR-301b-3p;hsa-miR
ENSG0000	ENSG0000	2.31E-06	hsa-miR-130b-3p;hsa-miR-301a-3p;hsa-miR-301b-3p;hsa-miR
ENSG0000	ENSG0000	5.10E-12	hsa-miR-106a-5p;hsa-miR-106b-5p;hsa-miR-130b-3p;hsa-miR
ENSG0000	ENSG0000	0.000244	hsa-miR-130b-3p;hsa-miR-301a-3p;hsa-miR-301b-3p;hsa-miR
ENSG0000	ENSG0000	1.96E-05	hsa-miR-130b-3p;hsa-miR-301a-3p;hsa-miR-301b-3p;hsa-miR
ENSG0000	ENSG0000	0.001079	hsa-miR-130b-3p;hsa-miR-301a-3p;hsa-miR-301b-3p;hsa-miR
ENSG0000	ENSG0000	3.61E-10	hsa-miR-106a-5p;hsa-miR-106b-5p;hsa-miR-130b-3p;hsa-miR
ENSG0000	ENSG0000	3.42E-05	hsa-miR-130b-3p;hsa-miR-301a-3p;hsa-miR-301b-3p;hsa-miR
ENSG0000	ENSG0000	0.000316	hsa-miR-130b-3p;hsa-miR-301a-3p;hsa-miR-301b-3p;hsa-miR
ENSG0000	ENSG0000	1.32E-06	hsa-miR-130b-3p;hsa-miR-301a-3p;hsa-miR-301b-3p;hsa-miR
ENSG0000	ENSG0000	0.000114	hsa-miR-106a-5p;hsa-miR-106b-5p;hsa-miR-130b-3p;hsa-miR
ENSG0000	ENSG0000	8.55E-06	hsa-miR-130b-3p;hsa-miR-301a-3p;hsa-miR-301b-3p;hsa-miR
ENSG0000	ENSG0000	3.00E-05	hsa-miR-130b-3p;hsa-miR-301a-3p;hsa-miR-301b-3p;hsa-miR
ENSG0000	ENSG0000	3.76E-06	hsa-miR-130b-3p;hsa-miR-301a-3p;hsa-miR-301b-3p;hsa-miR
ENSG0000	ENSG0000	2.05E-07	hsa-miR-106a-5p;hsa-miR-106b-5p;hsa-miR-130b-3p;hsa-miR
ENSG0000	ENSG0000	1.32E-06	hsa-miR-130b-3p;hsa-miR-301a-3p;hsa-miR-301b-3p;hsa-miR
ENSG0000	ENSG0000	2.24E-09	hsa-miR-106a-5p;hsa-miR-106b-5p;hsa-miR-130b-3p;hsa-miR
ENSG0000	ENSG0000	7.82E-09	hsa-miR-106a-5p;hsa-miR-106b-5p;hsa-miR-130b-3p;hsa-miR
ENSG0000	ENSG0000	3.61E-06	hsa-miR-106a-5p;hsa-miR-130b-3p;hsa-miR-301a-3p;hsa-miR-
ENSG0000	ENSG0000	3.04E-09	hsa-miR-106a-5p;hsa-miR-106b-5p;hsa-miR-20a-5p;hsa-miR-3
ENSG0000	ENSG0000	8.38E-09	hsa-miR-130b-3p;hsa-miR-301a-3p;hsa-miR-301b-3p;hsa-miR
ENSG0000	ENSG0000	1.56E-05	hsa-miR-130b-3p;hsa-miR-301a-3p;hsa-miR-301b-3p;hsa-miR
ENSG0000	ENSG0000	3.88E-05	hsa-miR-130b-3p;hsa-miR-301a-3p;hsa-miR-301b-3p;hsa-miR
ENSG0000	ENSG0000	3.88E-05	hsa-miR-130b-3p;hsa-miR-301a-3p;hsa-miR-301b-3p;hsa-miR
ENSG0000	ENSG0000	1.51E-09	hsa-miR-106a-5p;hsa-miR-106b-5p;hsa-miR-130b-3p;hsa-miR
ENSG0000	ENSG0000	0.000178	hsa-miR-130b-3p;hsa-miR-301a-3p;hsa-miR-301b-3p;hsa-miR
ENSG0000	ENSG0000	3.76E-06	hsa-miR-130b-3p;hsa-miR-301a-3p;hsa-miR-301b-3p;hsa-miR
ENSG0000	ENSG0000	1.53E-12	hsa-miR-106a-5p;hsa-miR-106b-5p;hsa-miR-130b-3p;hsa-miR
ENSG0000	ENSG0000	1.53E-12	hsa-miR-106a-5p;hsa-miR-106b-5p;hsa-miR-130b-3p;hsa-miR
ENSG0000	ENSG0000	1.96E-05	hsa-miR-130b-3p;hsa-miR-301a-3p;hsa-miR-301b-3p;hsa-miR
ENSG0000	ENSG0000	1.16E-10	hsa-miR-106b-5p;hsa-miR-130b-3p;hsa-miR-20a-5p;hsa-miR-3
ENSG0000	ENSG0000	6.18E-09	hsa-miR-106b-5p;hsa-miR-130b-3p;hsa-miR-20a-5p;hsa-miR-3
ENSG0000	ENSG0000	8.55E-06	hsa-miR-130b-3p;hsa-miR-301a-3p;hsa-miR-301b-3p;hsa-miR
ENSG0000	ENSG0000	1.92E-07	hsa-miR-106b-5p;hsa-miR-130b-3p;hsa-miR-20a-5p;hsa-miR-3
ENSG0000	ENSG0000	1.20E-07	hsa-miR-130b-3p;hsa-miR-301a-3p;hsa-miR-301b-3p;hsa-miR
ENSG0000	ENSG0000	5.55E-05	hsa-miR-130b-3p;hsa-miR-301a-3p;hsa-miR-301b-3p;hsa-miR
ENSG0000	ENSG0000	4.66E-05	hsa-miR-130b-3p;hsa-miR-301a-3p;hsa-miR-301b-3p;hsa-miR
ENSG0000	ENSG0000	4.66E-05	hsa-miR-130b-3p;hsa-miR-301a-3p;hsa-miR-301b-3p;hsa-miR
ENSG0000	ENSG0000	9.89E-05	hsa-miR-130b-3p;hsa-miR-301a-3p;hsa-miR-301b-3p;hsa-miR
ENSG0000	ENSG0000	3.20E-05	hsa-miR-130b-3p;hsa-miR-301a-3p;hsa-miR-301b-3p;hsa-miR
ENSG0000	ENSG0000	9.05E-06	hsa-miR-130b-3p;hsa-miR-301a-3p;hsa-miR-301b-3p;hsa-miR
ENSG0000	ENSG0000	9.05E-06	hsa-miR-130b-3p;hsa-miR-301a-3p;hsa-miR-301b-3p;hsa-miR
ENSG0000	ENSG0000	0.000137	hsa-miR-130b-3p;hsa-miR-301a-3p;hsa-miR-301b-3p;hsa-miR
ENSG0000	ENSG0000	0.000352	hsa-miR-130b-3p;hsa-miR-301a-3p;hsa-miR-301b-3p;hsa-miR
ENSG0000	ENSG0000	0.000109	hsa-miR-130b-3p;hsa-miR-301a-3p;hsa-miR-301b-3p;hsa-miR

ENSG0000	ENSG0000	0.000109	hsa-miR-130b-3p;hsa-miR-301a-3p;hsa-miR-301b-3p;hsa-miR
ENSG0000	ENSG0000	0.000109	hsa-miR-130b-3p;hsa-miR-301a-3p;hsa-miR-301b-3p;hsa-miR
ENSG0000	ENSG0000	0.000109	hsa-miR-130b-3p;hsa-miR-301a-3p;hsa-miR-301b-3p;hsa-miR
ENSG0000	ENSG0000	1.61E-08	hsa-miR-106a-5p;hsa-miR-106b-5p;hsa-miR-130b-3p;hsa-miR
ENSG0000	ENSG0000	7.69E-05	hsa-miR-130b-3p;hsa-miR-301a-3p;hsa-miR-301b-3p;hsa-miR
ENSG0000	ENSG0000	0.00017	hsa-miR-130b-3p;hsa-miR-301a-3p;hsa-miR-301b-3p;hsa-miR
ENSG0000	ENSG0000	1.56E-05	hsa-miR-130b-3p;hsa-miR-301a-3p;hsa-miR-301b-3p;hsa-miR
ENSG0000	ENSG0000	3.91E-07	hsa-miR-130b-3p;hsa-miR-301a-3p;hsa-miR-301b-3p;hsa-miR
ENSG0000	ENSG0000	3.20E-05	hsa-miR-130b-3p;hsa-miR-301a-3p;hsa-miR-301b-3p;hsa-miR
ENSG0000	ENSG0000	5.24E-05	hsa-miR-130b-3p;hsa-miR-301a-3p;hsa-miR-301b-3p;hsa-miR
ENSG0000	ENSG0000	3.91E-07	hsa-miR-130b-3p;hsa-miR-301a-3p;hsa-miR-301b-3p;hsa-miR
ENSG0000	ENSG0000	3.47E-11	hsa-miR-106a-5p;hsa-miR-106b-5p;hsa-miR-130b-3p;hsa-miR
ENSG0000	ENSG0000	5.22E-06	hsa-miR-130b-3p;hsa-miR-301a-3p;hsa-miR-301b-3p;hsa-miR
ENSG0000	ENSG0000	0.000163	hsa-miR-130b-3p;hsa-miR-301a-3p;hsa-miR-301b-3p;hsa-miR
ENSG0000	ENSG0000	0.000316	hsa-miR-130b-3p;hsa-miR-301a-3p;hsa-miR-301b-3p;hsa-miR
ENSG0000	ENSG0000	1.32E-05	hsa-miR-130b-3p;hsa-miR-301a-3p;hsa-miR-301b-3p;hsa-miR
ENSG0000	ENSG0000	8.67E-12	hsa-miR-106a-5p;hsa-miR-106b-5p;hsa-miR-130b-3p;hsa-miR
ENSG0000	ENSG0000	8.67E-12	hsa-miR-106a-5p;hsa-miR-106b-5p;hsa-miR-130b-3p;hsa-miR
ENSG0000	ENSG0000	8.67E-12	hsa-miR-106a-5p;hsa-miR-106b-5p;hsa-miR-130b-3p;hsa-miR
ENSG0000	ENSG0000	0.00015	hsa-miR-130b-3p;hsa-miR-301a-3p;hsa-miR-301b-3p;hsa-miR
ENSG0000	ENSG0000	2.62E-06	hsa-miR-130b-3p;hsa-miR-301a-3p;hsa-miR-301b-3p;hsa-miR
ENSG0000	ENSG0000	2.40E-10	hsa-miR-106a-5p;hsa-miR-106b-5p;hsa-miR-130b-3p;hsa-miR
ENSG0000	ENSG0000	2.80E-09	hsa-miR-106a-5p;hsa-miR-106b-5p;hsa-miR-130b-3p;hsa-miR
ENSG0000	ENSG0000	4.21E-06	hsa-miR-130b-3p;hsa-miR-301a-3p;hsa-miR-301b-3p;hsa-miR
ENSG0000	ENSG0000	4.66E-05	hsa-miR-130b-3p;hsa-miR-301a-3p;hsa-miR-301b-3p;hsa-miR
ENSG0000	ENSG0000	6.55E-05	hsa-miR-130b-3p;hsa-miR-301a-3p;hsa-miR-301b-3p;hsa-miR
ENSG0000	ENSG0000	1.51E-09	hsa-miR-106a-5p;hsa-miR-106b-5p;hsa-miR-130b-3p;hsa-miR
ENSG0000	ENSG0000	4.23E-12	hsa-miR-106a-5p;hsa-miR-106b-5p;hsa-miR-130b-3p;hsa-miR
ENSG0000	ENSG0000	1.56E-07	hsa-miR-130b-3p;hsa-miR-301a-3p;hsa-miR-301b-3p;hsa-miR
ENSG0000	ENSG0000	4.77E-07	hsa-miR-130b-3p;hsa-miR-301a-3p;hsa-miR-301b-3p;hsa-miR
ENSG0000	ENSG0000	1.97E-09	hsa-miR-106b-5p;hsa-miR-20a-5p;hsa-miR-301a-3p;hsa-miR-3
ENSG0000	ENSG0000	1.20E-08	hsa-miR-106a-5p;hsa-miR-106b-5p;hsa-miR-130b-3p;hsa-miR
ENSG0000	ENSG0000	7.07E-06	hsa-miR-130b-3p;hsa-miR-301a-3p;hsa-miR-301b-3p;hsa-miR
ENSG0000	ENSG0000	1.00E-08	hsa-miR-106a-5p;hsa-miR-106b-5p;hsa-miR-130b-3p;hsa-miR
ENSG0000	ENSG0000	9.00E-08	hsa-miR-130b-3p;hsa-miR-301a-3p;hsa-miR-301b-3p;hsa-miR
ENSG0000	ENSG0000	8.55E-06	hsa-miR-130b-3p;hsa-miR-301a-3p;hsa-miR-301b-3p;hsa-miR
ENSG0000	ENSG0000	3.00E-05	hsa-miR-130b-3p;hsa-miR-301a-3p;hsa-miR-301b-3p;hsa-miR
ENSG0000	ENSG0000	5.79E-06	hsa-miR-130b-3p;hsa-miR-301a-3p;hsa-miR-301b-3p;hsa-miR
ENSG0000	ENSG0000	5.79E-06	hsa-miR-130b-3p;hsa-miR-301a-3p;hsa-miR-301b-3p;hsa-miR
ENSG0000	ENSG0000	0.000254	hsa-miR-130b-3p;hsa-miR-301a-3p;hsa-miR-301b-3p;hsa-miR
ENSG0000	ENSG0000	1.24E-10	hsa-miR-106a-5p;hsa-miR-106b-5p;hsa-miR-130b-3p;hsa-miR
ENSG0000	ENSG0000	1.24E-10	hsa-miR-106a-5p;hsa-miR-106b-5p;hsa-miR-130b-3p;hsa-miR
ENSG0000	ENSG0000	1.32E-05	hsa-miR-130b-3p;hsa-miR-301a-3p;hsa-miR-301b-3p;hsa-miR
ENSG0000	ENSG0000	4.23E-12	hsa-miR-106a-5p;hsa-miR-106b-5p;hsa-miR-130b-3p;hsa-miR
ENSG0000	ENSG0000	0.000143	hsa-miR-130b-3p;hsa-miR-301a-3p;hsa-miR-301b-3p;hsa-miR
ENSG0000	ENSG0000	0.00017	hsa-miR-130b-3p;hsa-miR-301a-3p;hsa-miR-301b-3p;hsa-miR
ENSG0000	ENSG0000	6.61E-08	hsa-miR-130b-3p;hsa-miR-301a-3p;hsa-miR-301b-3p;hsa-miR
ENSG0000	ENSG0000	2.31E-06	hsa-miR-106a-5p;hsa-miR-106b-5p;hsa-miR-20a-5p;hsa-miR-3
ENSG0000	ENSG0000	0.001052	hsa-miR-130b-3p;hsa-miR-301a-3p;hsa-miR-301b-3p;hsa-miR
ENSG0000	ENSG0000	6.92E-05	hsa-miR-130b-3p;hsa-miR-301a-3p;hsa-miR-301b-3p;hsa-miR
ENSG0000	ENSG0000	3.35E-06	hsa-miR-130b-3p;hsa-miR-301a-3p;hsa-miR-301b-3p;hsa-miR
ENSG0000	ENSG0000	0.000235	hsa-miR-130b-3p;hsa-miR-301a-3p;hsa-miR-301b-3p;hsa-miR
ENSG0000	ENSG0000	2.11E-05	hsa-miR-130b-3p;hsa-miR-301a-3p;hsa-miR-301b-3p;hsa-miR
ENSG0000	ENSG0000	3.64E-05	hsa-miR-130b-3p;hsa-miR-301a-3p;hsa-miR-301b-3p;hsa-miR

ENSG0000	ENSG0000	6.41E-06	hsa-miR-130b-3p;hsa-miR-301a-3p;hsa-miR-301b-3p;hsa-miR
ENSG0000	ENSG0000	1.42E-08	hsa-miR-106a-5p;hsa-miR-106b-5p;hsa-miR-130b-3p;hsa-miR
ENSG0000	ENSG0000	8.64E-11	hsa-miR-106a-5p;hsa-miR-106b-5p;hsa-miR-130b-3p;hsa-miR
ENSG0000	ENSG0000	0.002059	hsa-miR-130b-3p;hsa-miR-301a-3p;hsa-miR-301b-3p;hsa-miR
ENSG0000	ENSG0000	5.87E-05	hsa-miR-130b-3p;hsa-miR-301a-3p;hsa-miR-301b-3p;hsa-miR
ENSG0000	ENSG0000	0.000163	hsa-miR-130b-3p;hsa-miR-301a-3p;hsa-miR-301b-3p;hsa-miR
ENSG0000	ENSG0000	6.41E-06	hsa-miR-106a-5p;hsa-miR-20a-5p;hsa-miR-301a-3p;hsa-miR-3
ENSG0000	ENSG0000	6.41E-06	hsa-miR-106a-5p;hsa-miR-20a-5p;hsa-miR-301a-3p;hsa-miR-3
ENSG0000	ENSG0000	1.02E-05	hsa-miR-130b-3p;hsa-miR-301a-3p;hsa-miR-301b-3p;hsa-miR
ENSG0000	ENSG0000	3.98E-11	hsa-miR-106a-5p;hsa-miR-106b-5p;hsa-miR-130b-3p;hsa-miR
ENSG0000	ENSG0000	4.07E-07	hsa-miR-106a-5p;hsa-miR-106b-5p;hsa-miR-20a-5p;hsa-miR-3
ENSG0000	ENSG0000	6.92E-05	hsa-miR-130b-3p;hsa-miR-301a-3p;hsa-miR-301b-3p;hsa-miR
ENSG0000	ENSG0000	1.96E-05	hsa-miR-130b-3p;hsa-miR-301a-3p;hsa-miR-301b-3p;hsa-miR
ENSG0000	ENSG0000	2.79E-08	hsa-miR-106a-5p;hsa-miR-106b-5p;hsa-miR-130b-3p;hsa-miR
ENSG0000	ENSG0000	2.38E-08	hsa-miR-106a-5p;hsa-miR-106b-5p;hsa-miR-130b-3p;hsa-miR
ENSG0000	ENSG0000	0.000185	hsa-miR-130b-3p;hsa-miR-301a-3p;hsa-miR-301b-3p;hsa-miR
ENSG0000	ENSG0000	0.004442	hsa-miR-106a-5p;hsa-miR-106b-5p;hsa-miR-20a-5p;hsa-miR-3
ENSG0000	ENSG0000	6.61E-08	hsa-miR-130b-3p;hsa-miR-301a-3p;hsa-miR-301b-3p;hsa-miR
ENSG0000	ENSG0000	2.66E-10	hsa-miR-106a-5p;hsa-miR-106b-5p;hsa-miR-130b-3p;hsa-miR
ENSG0000	ENSG0000	6.74E-11	hsa-miR-106a-5p;hsa-miR-106b-5p;hsa-miR-130b-3p;hsa-miR
ENSG0000	ENSG0000	2.79E-08	hsa-miR-106a-5p;hsa-miR-106b-5p;hsa-miR-130b-3p;hsa-miR
ENSG0000	ENSG0000	3.61E-10	hsa-miR-106a-5p;hsa-miR-106b-5p;hsa-miR-130b-3p;hsa-miR
ENSG0000	ENSG0000	0.000264	hsa-miR-130b-3p;hsa-miR-301a-3p;hsa-miR-301b-3p;hsa-miR
ENSG0000	ENSG0000	1.96E-05	hsa-miR-130b-3p;hsa-miR-301a-3p;hsa-miR-301b-3p;hsa-miR
ENSG0000	ENSG0000	7.67E-10	hsa-miR-106a-5p;hsa-miR-106b-5p;hsa-miR-130b-3p;hsa-miR
ENSG0000	ENSG0000	1.32E-05	hsa-miR-130b-3p;hsa-miR-301a-3p;hsa-miR-301b-3p;hsa-miR
ENSG0000	ENSG0000	2.97E-06	hsa-miR-130b-3p;hsa-miR-301a-3p;hsa-miR-301b-3p;hsa-miR
ENSG0000	ENSG0000	7.19E-07	hsa-miR-106b-5p;hsa-miR-130b-3p;hsa-miR-20a-5p;hsa-miR-3
ENSG0000	ENSG0000	1.14E-06	hsa-miR-130b-3p;hsa-miR-301a-3p;hsa-miR-301b-3p;hsa-miR
ENSG0000	ENSG0000	4.93E-09	hsa-miR-106a-5p;hsa-miR-106b-5p;hsa-miR-20a-5p;hsa-miR-3
ENSG0000	ENSG0000	3.20E-05	hsa-miR-130b-3p;hsa-miR-301a-3p;hsa-miR-301b-3p;hsa-miR
ENSG0000	ENSG0000	0.00012	hsa-miR-130b-3p;hsa-miR-301a-3p;hsa-miR-301b-3p;hsa-miR
ENSG0000	ENSG0000	0.000328	hsa-miR-130b-3p;hsa-miR-301a-3p;hsa-miR-301b-3p;hsa-miR
ENSG0000	ENSG0000	9.15E-10	hsa-miR-106a-5p;hsa-miR-106b-5p;hsa-miR-130b-3p;hsa-miR
ENSG0000	ENSG0000	4.84E-10	hsa-miR-106a-5p;hsa-miR-106b-5p;hsa-miR-130b-3p;hsa-miR
ENSG0000	ENSG0000	2.38E-08	hsa-miR-106a-5p;hsa-miR-106b-5p;hsa-miR-130b-3p;hsa-miR
ENSG0000	ENSG0000	9.74E-13	hsa-miR-106a-5p;hsa-miR-106b-5p;hsa-miR-130b-3p;hsa-miR
ENSG0000	ENSG0000	2.27E-05	hsa-miR-130b-3p;hsa-miR-301a-3p;hsa-miR-301b-3p;hsa-miR
ENSG0000	ENSG0000	3.88E-05	hsa-miR-130b-3p;hsa-miR-301a-3p;hsa-miR-301b-3p;hsa-miR
ENSG0000	ENSG0000	2.62E-05	hsa-miR-130b-3p;hsa-miR-301a-3p;hsa-miR-301b-3p;hsa-miR
ENSG0000	ENSG0000	4.66E-05	hsa-miR-130b-3p;hsa-miR-301a-3p;hsa-miR-301b-3p;hsa-miR
ENSG0000	ENSG0000	9.69E-07	hsa-miR-130b-3p;hsa-miR-301a-3p;hsa-miR-301b-3p;hsa-miR
ENSG0000	ENSG0000	9.69E-07	hsa-miR-130b-3p;hsa-miR-301a-3p;hsa-miR-301b-3p;hsa-miR
ENSG0000	ENSG0000	4.77E-07	hsa-miR-130b-3p;hsa-miR-301a-3p;hsa-miR-301b-3p;hsa-miR
ENSG0000	ENSG0000	1.05E-08	hsa-miR-106b-5p;hsa-miR-130b-3p;hsa-miR-20a-5p;hsa-miR-3
ENSG0000	ENSG0000	9.42E-05	hsa-miR-106a-5p;hsa-miR-106b-5p;hsa-miR-20a-5p;hsa-miR-3
ENSG0000	ENSG0000	9.42E-05	hsa-miR-106a-5p;hsa-miR-106b-5p;hsa-miR-20a-5p;hsa-miR-3
ENSG0000	ENSG0000	4.94E-05	hsa-miR-130b-3p;hsa-miR-301a-3p;hsa-miR-301b-3p;hsa-miR
ENSG0000	ENSG0000	2.22E-07	hsa-miR-106a-5p;hsa-miR-106b-5p;hsa-miR-130b-3p;hsa-miR
ENSG0000	ENSG0000	0.000137	hsa-miR-130b-3p;hsa-miR-301a-3p;hsa-miR-301b-3p;hsa-miR
ENSG0000	ENSG0000	1.76E-06	hsa-miR-130b-3p;hsa-miR-301a-3p;hsa-miR-301b-3p;hsa-miR
ENSG0000	ENSG0000	1.76E-06	hsa-miR-130b-3p;hsa-miR-301a-3p;hsa-miR-301b-3p;hsa-miR
ENSG0000	ENSG0000	7.16E-07	hsa-miR-106a-5p;hsa-miR-106b-5p;hsa-miR-301a-3p;hsa-miR-
ENSG0000	ENSG0000	3.49E-12	hsa-miR-106a-5p;hsa-miR-106b-5p;hsa-miR-130b-3p;hsa-miR

ENSG0000	ENSG0000	1.16E-10	hsa-miR-106a-5p;hsa-miR-106b-5p;hsa-miR-20a-5p;hsa-miR-[illegible]
ENSG0000	ENSG0000	1.53E-06	hsa-miR-130b-3p;hsa-miR-301a-3p;hsa-miR-301b-3p;hsa-miR
ENSG0000	ENSG0000	1.76E-06	hsa-miR-130b-3p;hsa-miR-301a-3p;hsa-miR-301b-3p;hsa-miR
ENSG0000	ENSG0000	2.27E-05	hsa-miR-130b-3p;hsa-miR-301a-3p;hsa-miR-301b-3p;hsa-miR
ENSG0000	ENSG0000	6.41E-06	hsa-miR-130b-3p;hsa-miR-301a-3p;hsa-miR-301b-3p;hsa-miR
ENSG0000	ENSG0000	8.21E-07	hsa-miR-130b-3p;hsa-miR-301a-3p;hsa-miR-301b-3p;hsa-miR
ENSG0000	ENSG0000	1.92E-09	hsa-miR-106a-5p;hsa-miR-106b-5p;hsa-miR-130b-3p;hsa-miR
ENSG0000	ENSG0000	5.79E-06	hsa-miR-130b-3p;hsa-miR-301a-3p;hsa-miR-301b-3p;hsa-miR
ENSG0000	ENSG0000	1.14E-06	hsa-miR-130b-3p;hsa-miR-301a-3p;hsa-miR-301b-3p;hsa-miR
ENSG0000	ENSG0000	4.56E-11	hsa-miR-106a-5p;hsa-miR-106b-5p;hsa-miR-130b-3p;hsa-miR
ENSG0000	ENSG0000	2.62E-05	hsa-miR-130b-3p;hsa-miR-301a-3p;hsa-miR-301b-3p;hsa-miR
ENSG0000	ENSG0000	3.76E-06	hsa-miR-130b-3p;hsa-miR-301a-3p;hsa-miR-301b-3p;hsa-miR
ENSG0000	ENSG0000	0.000235	hsa-miR-130b-3p;hsa-miR-301a-3p;hsa-miR-301b-3p;hsa-miR
ENSG0000	ENSG0000	6.90E-09	hsa-miR-106b-5p;hsa-miR-130b-3p;hsa-miR-20a-5p;hsa-miR-[illegible]
ENSG0000	ENSG0000	1.53E-06	hsa-miR-130b-3p;hsa-miR-301a-3p;hsa-miR-301b-3p;hsa-miR
ENSG0000	ENSG0000	1.53E-06	hsa-miR-130b-3p;hsa-miR-301a-3p;hsa-miR-301b-3p;hsa-miR
ENSG0000	ENSG0000	4.35E-06	hsa-miR-106a-5p;hsa-miR-106b-5p;hsa-miR-20a-5p;hsa-miR-[illegible]
ENSG0000	ENSG0000	2.54E-07	hsa-miR-130b-3p;hsa-miR-301a-3p;hsa-miR-301b-3p;hsa-miR
ENSG0000	ENSG0000	4.13E-05	hsa-miR-130b-3p;hsa-miR-301a-3p;hsa-miR-301b-3p;hsa-miR
ENSG0000	ENSG0000	1.76E-06	hsa-miR-130b-3p;hsa-miR-301a-3p;hsa-miR-301b-3p;hsa-miR
ENSG0000	ENSG0000	1.32E-05	hsa-miR-130b-3p;hsa-miR-301a-3p;hsa-miR-301b-3p;hsa-miR
ENSG0000	ENSG0000	7.34E-09	hsa-miR-106a-5p;hsa-miR-106b-5p;hsa-miR-130b-3p;hsa-miR
ENSG0000	ENSG0000	3.12E-06	hsa-miR-106a-5p;hsa-miR-106b-5p;hsa-miR-20a-5p;hsa-miR-[illegible]
ENSG0000	ENSG0000	5.76E-07	hsa-miR-130b-3p;hsa-miR-301a-3p;hsa-miR-301b-3p;hsa-miR
ENSG0000	ENSG0000	3.64E-05	hsa-miR-130b-3p;hsa-miR-301a-3p;hsa-miR-301b-3p;hsa-miR
ENSG0000	ENSG0000	5.17E-08	hsa-miR-106b-5p;hsa-miR-130b-3p;hsa-miR-20a-5p;hsa-miR-[illegible]
ENSG0000	ENSG0000	2.44E-05	hsa-miR-130b-3p;hsa-miR-301a-3p;hsa-miR-301b-3p;hsa-miR
ENSG0000	ENSG0000	6.92E-05	hsa-miR-130b-3p;hsa-miR-301a-3p;hsa-miR-301b-3p;hsa-miR
ENSG0000	ENSG0000	1.02E-05	hsa-miR-130b-3p;hsa-miR-301a-3p;hsa-miR-301b-3p;hsa-miR
ENSG0000	ENSG0000	0.001052	hsa-miR-130b-3p;hsa-miR-301a-3p;hsa-miR-301b-3p;hsa-miR
ENSG0000	ENSG0000	1.80E-09	hsa-miR-106b-5p;hsa-miR-130b-3p;hsa-miR-20a-5p;hsa-miR-[illegible]
ENSG0000	ENSG0000	0.000226	hsa-miR-130b-3p;hsa-miR-301a-3p;hsa-miR-301b-3p;hsa-miR
ENSG0000	ENSG0000	0.000722	hsa-miR-130b-3p;hsa-miR-301a-3p;hsa-miR-301b-3p;hsa-miR
ENSG0000	ENSG0000	2.22E-07	hsa-miR-106a-5p;hsa-miR-106b-5p;hsa-miR-130b-3p;hsa-miR
ENSG0000	ENSG0000	1.96E-05	hsa-miR-130b-3p;hsa-miR-301a-3p;hsa-miR-301b-3p;hsa-miR
ENSG0000	ENSG0000	4.39E-05	hsa-miR-130b-3p;hsa-miR-301a-3p;hsa-miR-301b-3p;hsa-miR
ENSG0000	ENSG0000	2.27E-05	hsa-miR-130b-3p;hsa-miR-301a-3p;hsa-miR-301b-3p;hsa-miR
ENSG0000	ENSG0000	1.24E-10	hsa-miR-106a-5p;hsa-miR-106b-5p;hsa-miR-130b-3p;hsa-miR
ENSG0000	ENSG0000	7.69E-05	hsa-miR-130b-3p;hsa-miR-301a-3p;hsa-miR-301b-3p;hsa-miR
ENSG0000	ENSG0000	2.80E-05	hsa-miR-130b-3p;hsa-miR-301a-3p;hsa-miR-301b-3p;hsa-miR
ENSG0000	ENSG0000	2.80E-05	hsa-miR-130b-3p;hsa-miR-301a-3p;hsa-miR-301b-3p;hsa-miR
ENSG0000	ENSG0000	4.66E-05	hsa-miR-130b-3p;hsa-miR-301a-3p;hsa-miR-301b-3p;hsa-miR
ENSG0000	ENSG0000	0.00015	hsa-miR-130b-3p;hsa-miR-301a-3p;hsa-miR-301b-3p;hsa-miR
ENSG0000	ENSG0000	2.71E-13	hsa-miR-106a-5p;hsa-miR-106b-5p;hsa-miR-130b-3p;hsa-miR
ENSG0000	ENSG0000	2.54E-07	hsa-miR-130b-3p;hsa-miR-301a-3p;hsa-miR-301b-3p;hsa-miR
ENSG0000	ENSG0000	2.62E-05	hsa-miR-130b-3p;hsa-miR-301a-3p;hsa-miR-301b-3p;hsa-miR
ENSG0000	ENSG0000	1.22E-05	hsa-miR-130b-3p;hsa-miR-301a-3p;hsa-miR-301b-3p;hsa-miR
ENSG0000	ENSG0000	1.32E-06	hsa-miR-130b-3p;hsa-miR-301a-3p;hsa-miR-301b-3p;hsa-miR
ENSG0000	ENSG0000	1.32E-06	hsa-miR-130b-3p;hsa-miR-301a-3p;hsa-miR-301b-3p;hsa-miR
ENSG0000	ENSG0000	1.32E-06	hsa-miR-130b-3p;hsa-miR-301a-3p;hsa-miR-301b-3p;hsa-miR
ENSG0000	ENSG0000	1.32E-06	hsa-miR-130b-3p;hsa-miR-301a-3p;hsa-miR-301b-3p;hsa-miR
ENSG0000	ENSG0000	1.14E-06	hsa-miR-130b-3p;hsa-miR-301a-3p;hsa-miR-301b-3p;hsa-miR
ENSG0000	ENSG0000	3.31E-11	hsa-let-7a-5p;hsa-let-7b-5p;hsa-let-7d-5p;hsa-let-7e-5p;hsa-l
ENSG0000	ENSG0000	1.42E-07	hsa-let-7a-5p;hsa-let-7b-5p;hsa-let-7d-5p;hsa-let-7e-5p;hsa-l

ENSG0000	ENSG0000	1.03E-07	hsa-let-7a-5p;hsa-let-7b-5p;hsa-let-7d-5p;hsa-let-7e-5p;hsa-l
ENSG0000	ENSG0000	4.85E-09	hsa-let-7a-5p;hsa-let-7b-5p;hsa-let-7d-5p;hsa-let-7e-5p;hsa-l
ENSG0000	ENSG0000	8.19E-07	hsa-let-7a-5p;hsa-let-7b-5p;hsa-let-7d-5p;hsa-let-7e-5p;hsa-l
ENSG0000	ENSG0000	1.60E-08	hsa-let-7a-5p;hsa-let-7b-5p;hsa-let-7d-5p;hsa-let-7e-5p;hsa-l
ENSG0000	ENSG0000	0.00014	hsa-let-7b-5p;hsa-miR-34b-5p;hsa-miR-449c-5p;hsa-miR-940
ENSG0000	ENSG0000	0.000946	hsa-let-7a-5p;hsa-let-7b-5p;hsa-let-7d-5p;hsa-let-7e-5p;hsa-l
ENSG0000	ENSG0000	2.80E-09	hsa-let-7a-5p;hsa-let-7b-5p;hsa-let-7d-5p;hsa-let-7e-5p;hsa-l
ENSG0000	ENSG0000	2.66E-08	hsa-let-7a-5p;hsa-let-7b-5p;hsa-let-7d-5p;hsa-let-7e-5p;hsa-l
ENSG0000	ENSG0000	0.001816	hsa-let-7a-5p;hsa-let-7b-5p;hsa-miR-34b-5p;hsa-miR-449c-5p
ENSG0000	ENSG0000	2.32E-08	hsa-let-7a-5p;hsa-let-7b-5p;hsa-let-7d-5p;hsa-let-7e-5p;hsa-l
ENSG0000	ENSG0000	4.82E-11	hsa-let-7a-5p;hsa-let-7b-5p;hsa-let-7d-5p;hsa-let-7e-5p;hsa-l
ENSG0000	ENSG0000	6.30E-06	hsa-let-7a-5p;hsa-let-7b-5p;hsa-let-7d-5p;hsa-let-7e-5p;hsa-l
ENSG0000	ENSG0000	1.28E-12	hsa-let-7a-5p;hsa-let-7b-5p;hsa-let-7d-5p;hsa-let-7e-5p;hsa-l
ENSG0000	ENSG0000	4.17E-09	hsa-let-7a-5p;hsa-let-7d-5p;hsa-let-7e-5p;hsa-let-7f-5p;hsa-le
ENSG0000	ENSG0000	2.40E-10	hsa-let-7a-5p;hsa-let-7b-5p;hsa-let-7d-5p;hsa-let-7e-5p;hsa-l
ENSG0000	ENSG0000	8.21E-08	hsa-let-7a-5p;hsa-let-7b-5p;hsa-let-7d-5p;hsa-let-7e-5p;hsa-l
ENSG0000	ENSG0000	2.17E-06	hsa-let-7a-5p;hsa-let-7e-5p;hsa-let-7f-5p;hsa-let-7g-5p;hsa-le
ENSG0000	ENSG0000	9.22E-11	hsa-let-7a-5p;hsa-let-7b-5p;hsa-let-7d-5p;hsa-let-7e-5p;hsa-l
ENSG0000	ENSG0000	5.64E-10	hsa-let-7a-5p;hsa-let-7b-5p;hsa-let-7d-5p;hsa-let-7e-5p;hsa-l
ENSG0000	ENSG0000	7.88E-06	hsa-let-7a-5p;hsa-let-7d-5p;hsa-let-7e-5p;hsa-let-7f-5p;hsa-le
ENSG0000	ENSG0000	3.05E-08	hsa-let-7a-5p;hsa-let-7b-5p;hsa-let-7d-5p;hsa-let-7e-5p;hsa-l
ENSG0000	ENSG0000	0.00014	hsa-let-7a-5p;hsa-let-7e-5p;hsa-let-7f-5p;hsa-miR-582-5p
ENSG0000	ENSG0000	1.03E-07	hsa-let-7a-5p;hsa-let-7b-5p;hsa-let-7d-5p;hsa-let-7e-5p;hsa-l
ENSG0000	ENSG0000	2.25E-10	hsa-let-7a-5p;hsa-let-7b-5p;hsa-let-7d-5p;hsa-let-7e-5p;hsa-l
ENSG0000	ENSG0000	6.61E-08	hsa-let-7a-5p;hsa-let-7b-5p;hsa-let-7d-5p;hsa-let-7e-5p;hsa-l
ENSG0000	ENSG0000	6.06E-08	hsa-let-7a-5p;hsa-let-7b-5p;hsa-let-7d-5p;hsa-let-7e-5p;hsa-l
ENSG0000	ENSG0000	4.55E-06	hsa-let-7a-5p;hsa-let-7b-5p;hsa-let-7d-5p;hsa-let-7e-5p;hsa-l
ENSG0000	ENSG0000	2.00E-10	hsa-let-7a-5p;hsa-let-7b-5p;hsa-let-7d-5p;hsa-let-7e-5p;hsa-l
ENSG0000	ENSG0000	1.94E-07	hsa-let-7a-5p;hsa-let-7b-5p;hsa-let-7d-5p;hsa-let-7e-5p;hsa-l
ENSG0000	ENSG0000	6.55E-05	hsa-let-7a-5p;hsa-let-7d-5p;hsa-let-7e-5p;hsa-let-7f-5p;hsa-le
ENSG0000	ENSG0000	9.08E-08	hsa-let-7a-5p;hsa-let-7b-5p;hsa-let-7d-5p;hsa-let-7f-5p;hsa-le
ENSG0000	ENSG0000	3.44E-13	hsa-let-7a-5p;hsa-let-7b-5p;hsa-let-7d-5p;hsa-let-7e-5p;hsa-l
ENSG0000	ENSG0000	3.02E-06	hsa-let-7a-5p;hsa-let-7b-5p;hsa-let-7d-5p;hsa-let-7e-5p;hsa-l
ENSG0000	ENSG0000	1.04E-06	hsa-let-7a-5p;hsa-let-7b-5p;hsa-let-7d-5p;hsa-let-7e-5p;hsa-l
ENSG0000	ENSG0000	3.83E-10	hsa-let-7a-5p;hsa-let-7b-5p;hsa-let-7d-5p;hsa-let-7e-5p;hsa-l
ENSG0000	ENSG0000	8.11E-09	hsa-let-7d-5p;hsa-let-7f-5p;hsa-let-7g-5p;hsa-let-7i-5p
ENSG0000	ENSG0000	2.01E-06	hsa-let-7a-5p;hsa-let-7b-5p;hsa-let-7d-5p;hsa-let-7e-5p;hsa-l
ENSG0000	ENSG0000	2.68E-07	hsa-let-7a-5p;hsa-let-7b-5p;hsa-let-7e-5p;hsa-let-7f-5p;hsa-le
ENSG0000	ENSG0000	1.11E-08	hsa-let-7a-5p;hsa-let-7b-5p;hsa-let-7d-5p;hsa-let-7e-5p;hsa-l
ENSG0000	ENSG0000	1.51E-11	hsa-let-7a-5p;hsa-let-7b-5p;hsa-let-7d-5p;hsa-let-7e-5p;hsa-l
ENSG0000	ENSG0000	9.88E-12	hsa-let-7a-5p;hsa-let-7b-5p;hsa-let-7d-5p;hsa-let-7e-5p;hsa-l
ENSG0000	ENSG0000	9.22E-11	hsa-let-7a-5p;hsa-let-7b-5p;hsa-let-7d-5p;hsa-let-7e-5p;hsa-l
ENSG0000	ENSG0000	0.000146	hsa-let-7a-5p;hsa-let-7b-5p;hsa-let-7d-5p;hsa-let-7e-5p;hsa-l
ENSG0000	ENSG0000	2.21E-05	hsa-let-7a-5p;hsa-let-7d-5p;hsa-let-7e-5p;hsa-let-7f-5p
ENSG0000	ENSG0000	5.89E-07	hsa-let-7a-5p;hsa-let-7d-5p;hsa-let-7e-5p;hsa-let-7g-5p;hsa-r
ENSG0000	ENSG0000	4.82E-11	hsa-let-7a-5p;hsa-let-7b-5p;hsa-let-7d-5p;hsa-let-7e-5p;hsa-l
ENSG0000	ENSG0000	2.74E-09	hsa-let-7a-5p;hsa-let-7b-5p;hsa-let-7d-5p;hsa-let-7e-5p;hsa-l
ENSG0000	ENSG0000	1.26E-10	hsa-let-7a-5p;hsa-let-7b-5p;hsa-let-7d-5p;hsa-let-7e-5p;hsa-l
ENSG0000	ENSG0000	1.26E-10	hsa-let-7a-5p;hsa-let-7b-5p;hsa-let-7d-5p;hsa-let-7e-5p;hsa-l
ENSG0000	ENSG0000	1.26E-10	hsa-let-7a-5p;hsa-let-7b-5p;hsa-let-7d-5p;hsa-let-7e-5p;hsa-l
ENSG0000	ENSG0000	4.93E-10	hsa-let-7a-5p;hsa-let-7b-5p;hsa-let-7d-5p;hsa-let-7e-5p;hsa-l
ENSG0000	ENSG0000	3.31E-11	hsa-let-7a-5p;hsa-let-7b-5p;hsa-let-7d-5p;hsa-let-7e-5p;hsa-l
ENSG0000	ENSG0000	3.31E-11	hsa-let-7a-5p;hsa-let-7b-5p;hsa-let-7d-5p;hsa-let-7e-5p;hsa-l
ENSG0000	ENSG0000	3.31E-11	hsa-let-7a-5p;hsa-let-7b-5p;hsa-let-7d-5p;hsa-let-7e-5p;hsa-l

ENSG0000	ENSG0000	3.31E-11	hsa-let-7a-5p;hsa-let-7b-5p;hsa-let-7d-5p;hsa-let-7e-5p;hsa-l
ENSG0000	ENSG0000	3.31E-11	hsa-let-7a-5p;hsa-let-7b-5p;hsa-let-7d-5p;hsa-let-7e-5p;hsa-l
ENSG0000	ENSG0000	3.31E-11	hsa-let-7a-5p;hsa-let-7b-5p;hsa-let-7d-5p;hsa-let-7e-5p;hsa-l
ENSG0000	ENSG0000	3.31E-11	hsa-let-7a-5p;hsa-let-7b-5p;hsa-let-7d-5p;hsa-let-7e-5p;hsa-l
ENSG0000	ENSG0000	3.16E-06	hsa-let-7a-5p;hsa-let-7b-5p;hsa-let-7d-5p;hsa-let-7e-5p;hsa-l
ENSG0000	ENSG0000	0.000741	hsa-let-7a-5p;hsa-let-7d-5p;hsa-let-7e-5p;hsa-let-7f-5p;hsa-le
ENSG0000	ENSG0000	1.15E-07	hsa-let-7a-5p;hsa-let-7b-5p;hsa-let-7d-5p;hsa-let-7e-5p;hsa-l
ENSG0000	ENSG0000	9.20E-08	hsa-let-7a-5p;hsa-let-7b-5p;hsa-let-7d-5p;hsa-let-7e-5p;hsa-l
ENSG0000	ENSG0000	7.30E-06	hsa-let-7a-5p;hsa-let-7b-5p;hsa-let-7e-5p;hsa-let-7f-5p;hsa-le
ENSG0000	ENSG0000	1.11E-08	hsa-let-7a-5p;hsa-let-7b-5p;hsa-let-7d-5p;hsa-let-7e-5p;hsa-l
ENSG0000	ENSG0000	1.50E-09	hsa-let-7a-5p;hsa-let-7b-5p;hsa-let-7d-5p;hsa-let-7e-5p;hsa-l
ENSG0000	ENSG0000	5.79E-11	hsa-let-7a-5p;hsa-let-7b-5p;hsa-let-7d-5p;hsa-let-7e-5p;hsa-l
ENSG0000	ENSG0000	0.000445	hsa-miR-24-3p;hsa-miR-34b-5p;hsa-miR-449c-5p;hsa-miR-94(
ENSG0000	ENSG0000	3.85E-06	hsa-let-7a-5p;hsa-let-7d-5p;hsa-let-7f-5p;hsa-let-7g-5p;hsa-le
ENSG0000	ENSG0000	5.88E-07	hsa-let-7a-5p;hsa-let-7b-5p;hsa-let-7d-5p;hsa-let-7e-5p;hsa-l
ENSG0000	ENSG0000	1.89E-09	hsa-let-7a-5p;hsa-let-7b-5p;hsa-let-7d-5p;hsa-let-7e-5p;hsa-l
ENSG0000	ENSG0000	5.84E-14	hsa-let-7a-5p;hsa-let-7b-5p;hsa-let-7d-5p;hsa-let-7e-5p;hsa-l
ENSG0000	ENSG0000	2.14E-09	hsa-let-7a-5p;hsa-let-7b-5p;hsa-let-7d-5p;hsa-let-7e-5p;hsa-l
ENSG0000	ENSG0000	5.54E-09	hsa-let-7a-5p;hsa-let-7b-5p;hsa-let-7d-5p;hsa-let-7e-5p;hsa-l
ENSG0000	ENSG0000	0.001312	hsa-miR-185-3p;hsa-miR-2110;hsa-miR-491-5p;hsa-miR-940
ENSG0000	ENSG0000	3.48E-08	hsa-let-7a-5p;hsa-let-7d-5p;hsa-let-7e-5p;hsa-let-7f-5p;hsa-le
ENSG0000	ENSG0000	0.000938	hsa-let-7a-5p;hsa-let-7b-5p;hsa-let-7d-5p;hsa-let-7e-5p;hsa-l
ENSG0000	ENSG0000	0.000938	hsa-let-7a-5p;hsa-let-7b-5p;hsa-let-7d-5p;hsa-let-7e-5p;hsa-l
ENSG0000	ENSG0000	7.31E-08	hsa-let-7a-5p;hsa-let-7b-5p;hsa-let-7d-5p;hsa-let-7e-5p;hsa-l
ENSG0000	ENSG0000	1.15E-07	hsa-let-7a-5p;hsa-let-7b-5p;hsa-let-7d-5p;hsa-let-7e-5p;hsa-l
ENSG0000	ENSG0000	9.49E-09	hsa-let-7a-5p;hsa-let-7b-5p;hsa-let-7d-5p;hsa-let-7e-5p;hsa-l
ENSG0000	ENSG0000	1.30E-08	hsa-let-7a-5p;hsa-let-7b-5p;hsa-let-7d-5p;hsa-let-7e-5p;hsa-l
ENSG0000	ENSG0000	1.30E-08	hsa-let-7a-5p;hsa-let-7b-5p;hsa-let-7d-5p;hsa-let-7e-5p;hsa-l
ENSG0000	ENSG0000	1.51E-11	hsa-let-7a-5p;hsa-let-7b-5p;hsa-let-7d-5p;hsa-let-7e-5p;hsa-l
ENSG0000	ENSG0000	1.86E-09	hsa-let-7a-5p;hsa-let-7b-5p;hsa-let-7d-5p;hsa-let-7e-5p;hsa-l
ENSG0000	ENSG0000	0.000206	hsa-let-7b-5p;hsa-let-7e-5p;hsa-let-7f-5p;hsa-let-7i-5p;hsa-m
ENSG0000	ENSG0000	8.21E-08	hsa-let-7a-5p;hsa-let-7b-5p;hsa-let-7d-5p;hsa-let-7e-5p;hsa-l
ENSG0000	ENSG0000	0.000156	hsa-let-7a-5p;hsa-let-7b-5p;hsa-let-7d-5p;hsa-let-7e-5p;hsa-l
ENSG0000	ENSG0000	2.67E-08	hsa-let-7a-5p;hsa-let-7b-5p;hsa-let-7d-5p;hsa-let-7e-5p;hsa-l
ENSG0000	ENSG0000	1.24E-09	hsa-let-7a-5p;hsa-let-7b-5p;hsa-let-7d-5p;hsa-let-7e-5p;hsa-l
ENSG0000	ENSG0000	1.20E-06	hsa-let-7a-5p;hsa-let-7d-5p;hsa-let-7e-5p;hsa-let-7f-5p;hsa-le
ENSG0000	ENSG0000	2.35E-10	hsa-let-7a-5p;hsa-let-7b-5p;hsa-let-7d-5p;hsa-let-7e-5p;hsa-l
ENSG0000	ENSG0000	0.00016	hsa-miR-2110;hsa-miR-24-3p;hsa-miR-34b-5p;hsa-miR-449c-5
ENSG0000	ENSG0000	2.01E-08	hsa-let-7a-5p;hsa-let-7b-5p;hsa-let-7d-5p;hsa-let-7e-5p;hsa-l
ENSG0000	ENSG0000	3.61E-08	hsa-let-7a-5p;hsa-let-7b-5p;hsa-let-7e-5p;hsa-let-7f-5p;hsa-le
ENSG0000	ENSG0000	5.78E-09	hsa-let-7a-5p;hsa-let-7b-5p;hsa-let-7d-5p;hsa-let-7e-5p;hsa-l
ENSG0000	ENSG0000	2.15E-06	hsa-let-7a-5p;hsa-let-7b-5p;hsa-let-7d-5p;hsa-let-7e-5p;hsa-l
ENSG0000	ENSG0000	0.000256	hsa-let-7a-5p;hsa-let-7b-5p;hsa-let-7d-5p;hsa-let-7e-5p;hsa-l
ENSG0000	ENSG0000	9.49E-09	hsa-let-7a-5p;hsa-let-7b-5p;hsa-let-7d-5p;hsa-let-7e-5p;hsa-l
ENSG0000	ENSG0000	4.42E-06	hsa-let-7a-5p;hsa-let-7d-5p;hsa-let-7e-5p;hsa-let-7f-5p;hsa-le
ENSG0000	ENSG0000	1.75E-07	hsa-let-7a-5p;hsa-let-7b-5p;hsa-let-7d-5p;hsa-let-7e-5p;hsa-l
ENSG0000	ENSG0000	4.95E-10	hsa-let-7a-5p;hsa-let-7b-5p;hsa-let-7d-5p;hsa-let-7e-5p;hsa-l
ENSG0000	ENSG0000	2.31E-09	hsa-let-7a-5p;hsa-let-7b-5p;hsa-let-7d-5p;hsa-let-7e-5p;hsa-l
ENSG0000	ENSG0000	3.38E-09	hsa-let-7a-5p;hsa-let-7b-5p;hsa-let-7d-5p;hsa-let-7e-5p;hsa-l
ENSG0000	ENSG0000	6.66E-11	hsa-let-7a-5p;hsa-let-7b-5p;hsa-let-7d-5p;hsa-let-7e-5p;hsa-l
ENSG0000	ENSG0000	6.90E-05	hsa-let-7a-5p;hsa-let-7d-5p;hsa-let-7e-5p;hsa-let-7f-5p;hsa-le
ENSG0000	ENSG0000	6.49E-08	hsa-let-7a-5p;hsa-let-7b-5p;hsa-let-7d-5p;hsa-let-7e-5p;hsa-l
ENSG0000	ENSG0000	1.05E-09	hsa-let-7a-5p;hsa-let-7b-5p;hsa-let-7d-5p;hsa-let-7e-5p;hsa-l
ENSG0000	ENSG0000	2.14E-07	hsa-let-7a-5p;hsa-let-7b-5p;hsa-let-7d-5p;hsa-let-7e-5p;hsa-l

ENSG0000	ENSG0000	2.25E-10	hsa-let-7a-5p;hsa-let-7e-5p;hsa-let-7f-5p;hsa-let-7g-5p;hsa-le
ENSG0000	ENSG0000	2.27E-12	hsa-let-7a-5p;hsa-let-7b-5p;hsa-let-7d-5p;hsa-let-7e-5p;hsa-l
ENSG0000	ENSG0000	0.00023	hsa-let-7a-5p;hsa-let-7d-5p;hsa-let-7f-5p;hsa-let-7g-5p;hsa-le
ENSG0000	ENSG0000	8.21E-08	hsa-let-7a-5p;hsa-let-7b-5p;hsa-let-7d-5p;hsa-let-7e-5p;hsa-l
ENSG0000	ENSG0000	5.76E-08	hsa-let-7a-5p;hsa-let-7b-5p;hsa-let-7d-5p;hsa-let-7e-5p;hsa-l
ENSG0000	ENSG0000	5.76E-08	hsa-let-7a-5p;hsa-let-7b-5p;hsa-let-7d-5p;hsa-let-7e-5p;hsa-l
ENSG0000	ENSG0000	9.49E-09	hsa-let-7a-5p;hsa-let-7b-5p;hsa-let-7d-5p;hsa-let-7e-5p;hsa-l
ENSG0000	ENSG0000	3.05E-08	hsa-let-7a-5p;hsa-let-7b-5p;hsa-let-7d-5p;hsa-let-7e-5p;hsa-l
ENSG0000	ENSG0000	0	hsa-let-7a-5p;hsa-let-7b-5p;hsa-let-7d-5p;hsa-let-7e-5p;hsa-l
ENSG0000	ENSG0000	1.24E-07	hsa-let-7a-5p;hsa-let-7b-5p;hsa-let-7d-5p;hsa-let-7e-5p;hsa-l
ENSG0000	ENSG0000	8.35E-05	hsa-miR-185-3p;hsa-miR-34b-5p;hsa-miR-449c-5p;hsa-miR-94
ENSG0000	ENSG0000	2.01E-08	hsa-let-7a-5p;hsa-let-7b-5p;hsa-let-7d-5p;hsa-let-7e-5p;hsa-l
ENSG0000	ENSG0000	3.96E-08	hsa-let-7a-5p;hsa-let-7b-5p;hsa-let-7d-5p;hsa-let-7e-5p;hsa-l
ENSG0000	ENSG0000	4.74E-11	hsa-let-7a-5p;hsa-let-7b-5p;hsa-let-7d-5p;hsa-let-7e-5p;hsa-l
ENSG0000	ENSG0000	2.39E-08	hsa-let-7a-5p;hsa-let-7b-5p;hsa-let-7e-5p;hsa-let-7f-5p;hsa-le
ENSG0000	ENSG0000	2.80E-09	hsa-let-7a-5p;hsa-let-7b-5p;hsa-let-7d-5p;hsa-let-7e-5p;hsa-l
ENSG0000	ENSG0000	1.22E-09	hsa-let-7a-5p;hsa-let-7b-5p;hsa-let-7d-5p;hsa-let-7e-5p;hsa-l
ENSG0000	ENSG0000	1.12E-06	hsa-let-7a-5p;hsa-let-7b-5p;hsa-let-7d-5p;hsa-let-7e-5p;hsa-l
ENSG0000	ENSG0000	2.14E-09	hsa-let-7a-5p;hsa-let-7b-5p;hsa-let-7d-5p;hsa-let-7e-5p;hsa-l
ENSG0000	ENSG0000	2.26E-05	hsa-let-7a-5p;hsa-let-7b-5p;hsa-let-7g-5p;hsa-miR-140-5p;hsa
ENSG0000	ENSG0000	0.000186	hsa-let-7a-5p;hsa-let-7f-5p;hsa-let-7g-5p;hsa-let-7i-5p
ENSG0000	ENSG0000	1.54E-05	hsa-let-7a-5p;hsa-let-7b-5p;hsa-let-7d-5p;hsa-let-7e-5p;hsa-l
ENSG0000	ENSG0000	2.47E-06	hsa-let-7a-5p;hsa-let-7b-5p;hsa-let-7d-5p;hsa-let-7e-5p;hsa-l
ENSG0000	ENSG0000	2.47E-06	hsa-let-7a-5p;hsa-let-7b-5p;hsa-let-7d-5p;hsa-let-7e-5p;hsa-l
ENSG0000	ENSG0000	0.002265	hsa-let-7a-5p;hsa-let-7b-5p;hsa-let-7d-5p;hsa-let-7e-5p;hsa-l
ENSG0000	ENSG0000	1.26E-10	hsa-let-7a-5p;hsa-let-7b-5p;hsa-let-7d-5p;hsa-let-7e-5p;hsa-l
ENSG0000	ENSG0000	2.25E-10	hsa-let-7a-5p;hsa-let-7b-5p;hsa-let-7d-5p;hsa-let-7e-5p;hsa-l
ENSG0000	ENSG0000	2.25E-10	hsa-let-7a-5p;hsa-let-7b-5p;hsa-let-7d-5p;hsa-let-7e-5p;hsa-l
ENSG0000	ENSG0000	1.10E-06	hsa-let-7a-5p;hsa-let-7b-5p;hsa-let-7d-5p;hsa-let-7e-5p;hsa-l
ENSG0000	ENSG0000	1.74E-08	hsa-let-7a-5p;hsa-let-7b-5p;hsa-let-7d-5p;hsa-let-7e-5p;hsa-l
ENSG0000	ENSG0000	7.30E-06	hsa-let-7b-5p;hsa-let-7d-5p;hsa-let-7e-5p;hsa-let-7f-5p;hsa-le
ENSG0000	ENSG0000	6.49E-08	hsa-let-7a-5p;hsa-let-7b-5p;hsa-let-7d-5p;hsa-let-7e-5p;hsa-l
ENSG0000	ENSG0000	5.88E-07	hsa-let-7a-5p;hsa-let-7b-5p;hsa-let-7d-5p;hsa-let-7e-5p;hsa-l
ENSG0000	ENSG0000	1.51E-06	hsa-let-7a-5p;hsa-let-7b-5p;hsa-let-7d-5p;hsa-let-7e-5p;hsa-l
ENSG0000	ENSG0000	4.93E-10	hsa-let-7a-5p;hsa-let-7b-5p;hsa-let-7d-5p;hsa-let-7e-5p;hsa-l
ENSG0000	ENSG0000	2.61E-07	hsa-let-7a-5p;hsa-let-7b-5p;hsa-let-7d-5p;hsa-let-7f-5p;hsa-le
ENSG0000	ENSG0000	4.34E-07	hsa-let-7a-5p;hsa-let-7b-5p;hsa-let-7d-5p;hsa-let-7e-5p;hsa-l
ENSG0000	ENSG0000	7.31E-08	hsa-let-7a-5p;hsa-let-7b-5p;hsa-let-7d-5p;hsa-let-7e-5p;hsa-l
ENSG0000	ENSG0000	0.000358	hsa-miR-34b-5p;hsa-miR-449c-5p;hsa-miR-92a-1-5p;hsa-miR-
ENSG0000	ENSG0000	2.40E-08	hsa-let-7a-5p;hsa-let-7b-5p;hsa-let-7d-5p;hsa-let-7e-5p;hsa-l
ENSG0000	ENSG0000	0.001246	hsa-miR-24-3p;hsa-miR-34b-5p;hsa-miR-449c-5p;hsa-miR-94(
ENSG0000	ENSG0000	1.23E-05	hsa-let-7a-5p;hsa-let-7d-5p;hsa-let-7f-5p;hsa-let-7g-5p;hsa-le
ENSG0000	ENSG0000	5.23E-05	hsa-let-7d-5p;hsa-let-7e-5p;hsa-let-7f-5p;hsa-let-7g-5p;hsa-le
ENSG0000	ENSG0000	5.23E-05	hsa-let-7d-5p;hsa-let-7e-5p;hsa-let-7f-5p;hsa-let-7g-5p;hsa-le
ENSG0000	ENSG0000	8.41E-06	hsa-let-7a-5p;hsa-let-7b-5p;hsa-let-7d-5p;hsa-let-7e-5p;hsa-l
ENSG0000	ENSG0000	6.49E-08	hsa-let-7a-5p;hsa-let-7b-5p;hsa-let-7d-5p;hsa-let-7e-5p;hsa-l
ENSG0000	ENSG0000	1.51E-08	hsa-let-7a-5p;hsa-let-7b-5p;hsa-let-7d-5p;hsa-let-7e-5p;hsa-l
ENSG0000	ENSG0000	4.48E-06	hsa-let-7a-5p;hsa-let-7d-5p;hsa-let-7e-5p;hsa-let-7f-5p;hsa-le
ENSG0000	ENSG0000	6.23E-06	hsa-let-7d-5p;hsa-let-7e-5p;hsa-let-7f-5p;hsa-let-7g-5p;hsa-le
ENSG0000	ENSG0000	6.84E-09	hsa-let-7a-5p;hsa-let-7b-5p;hsa-let-7d-5p;hsa-let-7e-5p;hsa-l
ENSG0000	ENSG0000	6.84E-09	hsa-let-7a-5p;hsa-let-7b-5p;hsa-let-7d-5p;hsa-let-7e-5p;hsa-l
ENSG0000	ENSG0000	6.84E-09	hsa-let-7a-5p;hsa-let-7b-5p;hsa-let-7d-5p;hsa-let-7e-5p;hsa-l
ENSG0000	ENSG0000	9.49E-09	hsa-let-7a-5p;hsa-let-7b-5p;hsa-let-7d-5p;hsa-let-7e-5p;hsa-l
ENSG0000	ENSG0000	2.40E-08	hsa-let-7a-5p;hsa-let-7b-5p;hsa-let-7d-5p;hsa-let-7e-5p;hsa-l

ENSG0000	ENSG0000	9.98E-10	hsa-let-7a-5p;hsa-let-7b-5p;hsa-let-7d-5p;hsa-let-7e-5p;hsa-l
ENSG0000	ENSG0000	7.95E-10	hsa-let-7a-5p;hsa-let-7b-5p;hsa-let-7d-5p;hsa-let-7e-5p;hsa-l
ENSG0000	ENSG0000	1.65E-10	hsa-let-7a-5p;hsa-let-7d-5p;hsa-let-7e-5p;hsa-let-7f-5p;hsa-le
ENSG0000	ENSG0000	1.36E-05	hsa-let-7a-5p;hsa-let-7b-5p;hsa-let-7d-5p;hsa-let-7e-5p;hsa-l
ENSG0000	ENSG0000	1.36E-05	hsa-let-7a-5p;hsa-let-7b-5p;hsa-let-7d-5p;hsa-let-7e-5p;hsa-l
ENSG0000	ENSG0000	4.59E-07	hsa-let-7a-5p;hsa-let-7d-5p;hsa-let-7e-5p;hsa-let-7f-5p;hsa-le
ENSG0000	ENSG0000	1.92E-08	hsa-let-7a-5p;hsa-let-7e-5p;hsa-let-7f-5p;hsa-let-7g-5p;hsa-le
ENSG0000	ENSG0000	1.51E-11	hsa-let-7a-5p;hsa-let-7b-5p;hsa-let-7d-5p;hsa-let-7e-5p;hsa-l
ENSG0000	ENSG0000	6.29E-10	hsa-let-7a-5p;hsa-let-7b-5p;hsa-let-7d-5p;hsa-let-7e-5p;hsa-l
ENSG0000	ENSG0000	1.30E-08	hsa-let-7a-5p;hsa-let-7b-5p;hsa-let-7d-5p;hsa-let-7e-5p;hsa-l
ENSG0000	ENSG0000	4.50E-08	hsa-let-7a-5p;hsa-let-7b-5p;hsa-let-7d-5p;hsa-let-7e-5p;hsa-l
ENSG0000	ENSG0000	4.50E-08	hsa-let-7a-5p;hsa-let-7b-5p;hsa-let-7d-5p;hsa-let-7e-5p;hsa-l
ENSG0000	ENSG0000	9.98E-10	hsa-let-7a-5p;hsa-let-7b-5p;hsa-let-7d-5p;hsa-let-7e-5p;hsa-l
ENSG0000	ENSG0000	1.54E-09	hsa-let-7a-5p;hsa-let-7b-5p;hsa-let-7d-5p;hsa-let-7e-5p;hsa-l
ENSG0000	ENSG0000	9.17E-06	hsa-let-7a-5p;hsa-let-7b-5p;hsa-let-7d-5p;hsa-let-7f-5p;hsa-le
ENSG0000	ENSG0000	4.53E-07	hsa-let-7a-5p;hsa-let-7b-5p;hsa-let-7d-5p;hsa-let-7e-5p;hsa-l
ENSG0000	ENSG0000	5.89E-07	hsa-let-7a-5p;hsa-let-7b-5p;hsa-let-7e-5p;hsa-let-7f-5p;hsa-le
ENSG0000	ENSG0000	1.11E-08	hsa-let-7a-5p;hsa-let-7b-5p;hsa-let-7d-5p;hsa-let-7e-5p;hsa-l
ENSG0000	ENSG0000	8.36E-09	hsa-let-7a-5p;hsa-let-7b-5p;hsa-let-7d-5p;hsa-let-7f-5p;hsa-le
ENSG0000	ENSG0000	1.74E-08	hsa-let-7a-5p;hsa-let-7b-5p;hsa-let-7d-5p;hsa-let-7e-5p;hsa-l
ENSG0000	ENSG0000	5.63E-06	hsa-let-7a-5p;hsa-let-7b-5p;hsa-let-7d-5p;hsa-let-7e-5p;hsa-l
ENSG0000	ENSG0000	5.63E-06	hsa-let-7a-5p;hsa-let-7b-5p;hsa-let-7d-5p;hsa-let-7e-5p;hsa-l
ENSG0000	ENSG0000	2.26E-11	hsa-let-7a-5p;hsa-let-7b-5p;hsa-let-7d-5p;hsa-let-7e-5p;hsa-l
ENSG0000	ENSG0000	2.27E-12	hsa-let-7a-5p;hsa-let-7b-5p;hsa-let-7d-5p;hsa-let-7e-5p;hsa-l
ENSG0000	ENSG0000	0.000622	hsa-miR-34b-5p;hsa-miR-449c-5p;hsa-miR-491-5p;hsa-miR-94
ENSG0000	ENSG0000	8.21E-08	hsa-let-7a-5p;hsa-let-7b-5p;hsa-let-7d-5p;hsa-let-7e-5p;hsa-l
ENSG0000	ENSG0000	3.46E-07	hsa-let-7a-5p;hsa-let-7b-5p;hsa-let-7d-5p;hsa-let-7e-5p;hsa-l
ENSG0000	ENSG0000	5.09E-08	hsa-let-7a-5p;hsa-let-7b-5p;hsa-let-7d-5p;hsa-let-7e-5p;hsa-l
ENSG0000	ENSG0000	5.00E-06	hsa-let-7a-5p;hsa-let-7b-5p;hsa-let-7d-5p;hsa-let-7e-5p;hsa-l
ENSG0000	ENSG0000	6.06E-08	hsa-let-7a-5p;hsa-let-7b-5p;hsa-let-7d-5p;hsa-let-7e-5p;hsa-l
ENSG0000	ENSG0000	4.34E-06	hsa-let-7a-5p;hsa-let-7b-5p;hsa-let-7d-5p;hsa-let-7e-5p;hsa-l
ENSG0000	ENSG0000	7.87E-06	hsa-let-7a-5p;hsa-let-7b-5p;hsa-let-7d-5p;hsa-let-7e-5p;hsa-l
ENSG0000	ENSG0000	9.98E-10	hsa-let-7a-5p;hsa-let-7b-5p;hsa-let-7d-5p;hsa-let-7e-5p;hsa-l
ENSG0000	ENSG0000	3.84E-12	hsa-let-7a-5p;hsa-let-7b-5p;hsa-let-7d-5p;hsa-let-7e-5p;hsa-l
ENSG0000	ENSG0000	1.94E-07	hsa-let-7a-5p;hsa-let-7b-5p;hsa-let-7d-5p;hsa-let-7e-5p;hsa-l
ENSG0000	ENSG0000	1.11E-11	hsa-let-7a-5p;hsa-let-7b-5p;hsa-let-7d-5p;hsa-let-7e-5p;hsa-l
ENSG0000	ENSG0000	8.07E-09	hsa-let-7a-5p;hsa-let-7b-5p;hsa-let-7d-5p;hsa-let-7e-5p;hsa-l
ENSG0000	ENSG0000	6.66E-11	hsa-let-7a-5p;hsa-let-7b-5p;hsa-let-7d-5p;hsa-let-7e-5p;hsa-l
ENSG0000	ENSG0000	2.25E-10	hsa-let-7a-5p;hsa-let-7b-5p;hsa-let-7d-5p;hsa-let-7e-5p;hsa-l
ENSG0000	ENSG0000	2.25E-10	hsa-let-7a-5p;hsa-let-7b-5p;hsa-let-7d-5p;hsa-let-7e-5p;hsa-l
ENSG0000	ENSG0000	1.36E-05	hsa-let-7a-5p;hsa-let-7b-5p;hsa-let-7d-5p;hsa-let-7e-5p;hsa-l
ENSG0000	ENSG0000	1.24E-09	hsa-let-7a-5p;hsa-let-7b-5p;hsa-let-7d-5p;hsa-let-7e-5p;hsa-l
ENSG0000	ENSG0000	1.17E-08	hsa-let-7a-5p;hsa-let-7b-5p;hsa-let-7d-5p;hsa-let-7e-5p;hsa-l
ENSG0000	ENSG0000	1.04E-06	hsa-let-7a-5p;hsa-let-7b-5p;hsa-let-7d-5p;hsa-let-7e-5p;hsa-l
ENSG0000	ENSG0000	5.54E-09	hsa-let-7a-5p;hsa-let-7b-5p;hsa-let-7e-5p;hsa-let-7f-5p;hsa-le
ENSG0000	ENSG0000	2.14E-07	hsa-let-7a-5p;hsa-let-7b-5p;hsa-let-7d-5p;hsa-let-7e-5p;hsa-l
ENSG0000	ENSG0000	2.14E-07	hsa-let-7a-5p;hsa-let-7b-5p;hsa-let-7d-5p;hsa-let-7e-5p;hsa-l
ENSG0000	ENSG0000	3.35E-06	hsa-let-7d-5p;hsa-let-7e-5p;hsa-let-7f-5p;hsa-let-7g-5p;hsa-le
ENSG0000	ENSG0000	8.36E-09	hsa-let-7a-5p;hsa-let-7b-5p;hsa-let-7d-5p;hsa-let-7e-5p;hsa-l
ENSG0000	ENSG0000	7.64E-08	hsa-let-7a-5p;hsa-let-7b-5p;hsa-let-7e-5p;hsa-let-7f-5p;hsa-le
ENSG0000	ENSG0000	5.32E-08	hsa-let-7b-5p;hsa-let-7d-5p;hsa-let-7e-5p;hsa-let-7f-5p;hsa-le
ENSG0000	ENSG0000	2.27E-12	hsa-let-7a-5p;hsa-let-7b-5p;hsa-let-7d-5p;hsa-let-7e-5p;hsa-l
ENSG0000	ENSG0000	3.31E-11	hsa-let-7a-5p;hsa-let-7b-5p;hsa-let-7d-5p;hsa-let-7e-5p;hsa-l
ENSG0000	ENSG0000	3.31E-11	hsa-let-7a-5p;hsa-let-7b-5p;hsa-let-7d-5p;hsa-let-7e-5p;hsa-l

ENSG0000	ENSG0000	6.49E-08	hsa-let-7a-5p;hsa-let-7b-5p;hsa-let-7d-5p;hsa-let-7e-5p;hsa-l
ENSG0000	ENSG0000	1.63E-06	hsa-let-7a-5p;hsa-let-7b-5p;hsa-let-7d-5p;hsa-let-7e-5p;hsa-l
ENSG0000	ENSG0000	3.08E-11	hsa-let-7a-5p;hsa-let-7b-5p;hsa-let-7d-5p;hsa-let-7e-5p;hsa-l
ENSG0000	ENSG0000	1.30E-06	hsa-let-7a-5p;hsa-let-7b-5p;hsa-let-7d-5p;hsa-let-7e-5p;hsa-l
ENSG0000	ENSG0000	1.75E-07	hsa-let-7a-5p;hsa-let-7b-5p;hsa-let-7d-5p;hsa-let-7e-5p;hsa-l
ENSG0000	ENSG0000	1.84E-05	hsa-let-7a-5p;hsa-let-7d-5p;hsa-let-7e-5p;hsa-let-7f-5p;hsa-le
ENSG0000	ENSG0000	3.61E-09	hsa-let-7a-5p;hsa-let-7b-5p;hsa-let-7d-5p;hsa-let-7e-5p;hsa-l
ENSG0000	ENSG0000	1.54E-09	hsa-let-7a-5p;hsa-let-7b-5p;hsa-let-7d-5p;hsa-let-7e-5p;hsa-l
ENSG0000	ENSG0000	2.88E-07	hsa-let-7a-5p;hsa-let-7b-5p;hsa-let-7d-5p;hsa-let-7e-5p;hsa-l
ENSG0000	ENSG0000	1.21E-06	hsa-let-7a-5p;hsa-let-7b-5p;hsa-let-7d-5p;hsa-let-7e-5p;hsa-l
ENSG0000	ENSG0000	5.30E-06	hsa-let-7a-5p;hsa-let-7d-5p;hsa-let-7e-5p;hsa-let-7f-5p;hsa-le
ENSG0000	ENSG0000	9.79E-10	hsa-let-7a-5p;hsa-let-7b-5p;hsa-let-7d-5p;hsa-let-7e-5p;hsa-l
ENSG0000	ENSG0000	7.95E-10	hsa-let-7a-5p;hsa-let-7b-5p;hsa-let-7d-5p;hsa-let-7e-5p;hsa-l
ENSG0000	ENSG0000	1.24E-09	hsa-let-7a-5p;hsa-let-7b-5p;hsa-let-7d-5p;hsa-let-7e-5p;hsa-l
ENSG0000	ENSG0000	9.49E-09	hsa-let-7a-5p;hsa-let-7b-5p;hsa-let-7d-5p;hsa-let-7e-5p;hsa-l
ENSG0000	ENSG0000	6.78E-05	hsa-let-7a-5p;hsa-let-7b-5p;hsa-let-7d-5p;hsa-let-7e-5p;hsa-l
ENSG0000	ENSG0000	1.86E-09	hsa-let-7a-5p;hsa-let-7b-5p;hsa-let-7d-5p;hsa-let-7e-5p;hsa-l
ENSG0000	ENSG0000	4.93E-10	hsa-let-7a-5p;hsa-let-7b-5p;hsa-let-7d-5p;hsa-let-7e-5p;hsa-l
ENSG0000	ENSG0000	1.97E-06	hsa-let-7a-5p;hsa-let-7b-5p;hsa-let-7e-5p;hsa-let-7f-5p;hsa-le
ENSG0000	ENSG0000	4.06E-09	hsa-let-7a-5p;hsa-let-7b-5p;hsa-let-7d-5p;hsa-let-7e-5p;hsa-l
ENSG0000	ENSG0000	2.48E-05	hsa-let-7b-5p;hsa-let-7e-5p;hsa-let-7f-5p;hsa-let-7g-5p;hsa-le
ENSG0000	ENSG0000	6.61E-08	hsa-let-7a-5p;hsa-let-7b-5p;hsa-let-7d-5p;hsa-let-7e-5p;hsa-l
ENSG0000	ENSG0000	4.81E-09	hsa-let-7a-5p;hsa-let-7b-5p;hsa-let-7d-5p;hsa-let-7e-5p;hsa-l
ENSG0000	ENSG0000	2.80E-05	hsa-let-7a-5p;hsa-let-7b-5p;hsa-let-7d-5p;hsa-let-7e-5p;hsa-l
ENSG0000	ENSG0000	2.26E-11	hsa-let-7a-5p;hsa-let-7b-5p;hsa-let-7d-5p;hsa-let-7e-5p;hsa-l
ENSG0000	ENSG0000	1.15E-07	hsa-let-7a-5p;hsa-let-7b-5p;hsa-let-7d-5p;hsa-let-7e-5p;hsa-l
ENSG0000	ENSG0000	5.40E-07	hsa-let-7a-5p;hsa-let-7b-5p;hsa-let-7d-5p;hsa-let-7e-5p;hsa-l
ENSG0000	ENSG0000	4.10E-08	hsa-let-7a-5p;hsa-let-7b-5p;hsa-let-7d-5p;hsa-let-7e-5p;hsa-l
ENSG0000	ENSG0000	5.89E-07	hsa-let-7a-5p;hsa-let-7b-5p;hsa-let-7e-5p;hsa-let-7f-5p;hsa-le
ENSG0000	ENSG0000	2.48E-08	hsa-let-7a-5p;hsa-let-7b-5p;hsa-let-7d-5p;hsa-let-7e-5p;hsa-l
ENSG0000	ENSG0000	1.03E-07	hsa-let-7a-5p;hsa-let-7b-5p;hsa-let-7d-5p;hsa-let-7e-5p;hsa-l
ENSG0000	ENSG0000	3.38E-09	hsa-let-7a-5p;hsa-let-7b-5p;hsa-let-7d-5p;hsa-let-7e-5p;hsa-l
ENSG0000	ENSG0000	7.52E-06	hsa-let-7a-5p;hsa-let-7d-5p;hsa-let-7e-5p;hsa-let-7f-5p;hsa-le
ENSG0000	ENSG0000	1.75E-07	hsa-let-7a-5p;hsa-let-7b-5p;hsa-let-7d-5p;hsa-let-7e-5p;hsa-l
ENSG0000	ENSG0000	2.61E-07	hsa-let-7a-5p;hsa-let-7b-5p;hsa-let-7d-5p;hsa-let-7e-5p;hsa-l
ENSG0000	ENSG0000	4.70E-06	hsa-let-7a-5p;hsa-let-7b-5p;hsa-let-7d-5p;hsa-let-7e-5p;hsa-l
ENSG0000	ENSG0000	9.22E-11	hsa-let-7a-5p;hsa-let-7b-5p;hsa-let-7d-5p;hsa-let-7e-5p;hsa-l
ENSG0000	ENSG0000	9.88E-12	hsa-let-7a-5p;hsa-let-7b-5p;hsa-let-7d-5p;hsa-let-7e-5p;hsa-l
ENSG0000	ENSG0000	1.12E-06	hsa-let-7a-5p;hsa-let-7b-5p;hsa-let-7d-5p;hsa-let-7e-5p;hsa-l
ENSG0000	ENSG0000	0.007589	hsa-miR-185-3p;hsa-miR-2110;hsa-miR-491-5p;hsa-miR-582-5
ENSG0000	ENSG0000	3.67E-06	hsa-let-7a-5p;hsa-let-7b-5p;hsa-let-7d-5p;hsa-let-7e-5p;hsa-l
ENSG0000	ENSG0000	5.32E-08	hsa-let-7a-5p;hsa-let-7b-5p;hsa-let-7d-5p;hsa-let-7e-5p;hsa-l
ENSG0000	ENSG0000	5.76E-08	hsa-let-7a-5p;hsa-let-7b-5p;hsa-let-7d-5p;hsa-let-7e-5p;hsa-l
ENSG0000	ENSG0000	7.83E-08	hsa-let-7a-5p;hsa-let-7b-5p;hsa-let-7d-5p;hsa-let-7e-5p;hsa-l
ENSG0000	ENSG0000	1.27E-07	hsa-let-7a-5p;hsa-let-7b-5p;hsa-let-7d-5p;hsa-let-7e-5p;hsa-l
ENSG0000	ENSG0000	4.74E-11	hsa-let-7a-5p;hsa-let-7b-5p;hsa-let-7d-5p;hsa-let-7e-5p;hsa-l
ENSG0000	ENSG0000	4.06E-09	hsa-let-7a-5p;hsa-let-7b-5p;hsa-let-7d-5p;hsa-let-7e-5p;hsa-l
ENSG0000	ENSG0000	8.51E-08	hsa-let-7a-5p;hsa-let-7b-5p;hsa-let-7d-5p;hsa-let-7e-5p;hsa-l
ENSG0000	ENSG0000	6.61E-08	hsa-let-7a-5p;hsa-let-7b-5p;hsa-let-7d-5p;hsa-let-7e-5p;hsa-l
ENSG0000	ENSG0000	8.41E-06	hsa-let-7a-5p;hsa-let-7b-5p;hsa-let-7d-5p;hsa-let-7e-5p;hsa-l
ENSG0000	ENSG0000	0.000797	hsa-miR-24-3p;hsa-miR-34b-5p;hsa-miR-449c-5p;hsa-miR-94(
ENSG0000	ENSG0000	4.95E-07	hsa-let-7a-5p;hsa-let-7b-5p;hsa-let-7d-5p;hsa-let-7e-5p;hsa-l
ENSG0000	ENSG0000	4.53E-05	hsa-let-7a-5p;hsa-let-7b-5p;hsa-let-7d-5p;hsa-let-7e-5p;hsa-l
ENSG0000	ENSG0000	7.95E-10	hsa-let-7a-5p;hsa-let-7b-5p;hsa-let-7d-5p;hsa-let-7e-5p;hsa-l

ENSG0000	ENSG0000	5.76E-08	hsa-let-7a-5p;hsa-let-7b-5p;hsa-let-7d-5p;hsa-let-7e-5p;hsa-l
ENSG0000	ENSG0000	1.74E-08	hsa-let-7a-5p;hsa-let-7b-5p;hsa-let-7d-5p;hsa-let-7e-5p;hsa-l
ENSG0000	ENSG0000	2.17E-06	hsa-let-7a-5p;hsa-let-7b-5p;hsa-let-7e-5p;hsa-let-7f-5p;hsa-le
ENSG0000	ENSG0000	4.93E-10	hsa-let-7a-5p;hsa-let-7b-5p;hsa-let-7d-5p;hsa-let-7e-5p;hsa-l
ENSG0000	ENSG0000	8.59E-09	hsa-let-7a-5p;hsa-let-7b-5p;hsa-let-7d-5p;hsa-let-7e-5p;hsa-l
ENSG0000	ENSG0000	1.69E-10	hsa-let-7a-5p;hsa-let-7b-5p;hsa-let-7d-5p;hsa-let-7e-5p;hsa-l
ENSG0000	ENSG0000	5.30E-06	hsa-let-7a-5p;hsa-let-7b-5p;hsa-let-7d-5p;hsa-let-7e-5p;hsa-l
ENSG0000	ENSG0000	1.03E-07	hsa-let-7a-5p;hsa-let-7b-5p;hsa-let-7d-5p;hsa-let-7e-5p;hsa-l
ENSG0000	ENSG0000	2.13E-08	hsa-let-7a-5p;hsa-let-7b-5p;hsa-let-7e-5p;hsa-let-7f-5p;hsa-le
ENSG0000	ENSG0000	2.31E-09	hsa-let-7a-5p;hsa-let-7b-5p;hsa-let-7d-5p;hsa-let-7e-5p;hsa-l
ENSG0000	ENSG0000	1.65E-13	hsa-let-7a-5p;hsa-let-7b-5p;hsa-let-7d-5p;hsa-let-7e-5p;hsa-l
ENSG0000	ENSG0000	5.97E-06	hsa-let-7a-5p;hsa-let-7b-5p;hsa-let-7d-5p;hsa-let-7e-5p;hsa-l
ENSG0000	ENSG0000	3.05E-08	hsa-let-7a-5p;hsa-let-7b-5p;hsa-let-7d-5p;hsa-let-7e-5p;hsa-l
ENSG0000	ENSG0000	2.25E-10	hsa-let-7a-5p;hsa-let-7b-5p;hsa-let-7d-5p;hsa-let-7e-5p;hsa-l
ENSG0000	ENSG0000	1.11E-08	hsa-let-7a-5p;hsa-let-7b-5p;hsa-let-7d-5p;hsa-let-7e-5p;hsa-l
ENSG0000	ENSG0000	0.000154	hsa-miR-2110;hsa-miR-34b-5p;hsa-miR-449c-5p;hsa-miR-491-
ENSG0000	ENSG0000	1.51E-06	hsa-let-7a-5p;hsa-let-7b-5p;hsa-let-7d-5p;hsa-let-7e-5p;hsa-l
ENSG0000	ENSG0000	1.78E-08	hsa-let-7a-5p;hsa-let-7b-5p;hsa-let-7d-5p;hsa-let-7e-5p;hsa-l
ENSG0000	ENSG0000	9.49E-09	hsa-let-7a-5p;hsa-let-7b-5p;hsa-let-7d-5p;hsa-let-7e-5p;hsa-l
ENSG0000	ENSG0000	2.47E-06	hsa-let-7a-5p;hsa-let-7d-5p;hsa-let-7e-5p;hsa-let-7f-5p;hsa-le
ENSG0000	ENSG0000	2.80E-09	hsa-let-7a-5p;hsa-let-7b-5p;hsa-let-7d-5p;hsa-let-7e-5p;hsa-l
ENSG0000	ENSG0000	0.000102	hsa-let-7a-5p;hsa-let-7d-5p;hsa-let-7e-5p;hsa-let-7f-5p;hsa-le
ENSG0000	ENSG0000	3.84E-12	hsa-let-7a-5p;hsa-let-7b-5p;hsa-let-7d-5p;hsa-let-7e-5p;hsa-l
ENSG0000	ENSG0000	2.45E-09	hsa-let-7a-5p;hsa-let-7b-5p;hsa-let-7d-5p;hsa-let-7e-5p;hsa-l
ENSG0000	ENSG0000	2.80E-09	hsa-let-7a-5p;hsa-let-7b-5p;hsa-let-7d-5p;hsa-let-7e-5p;hsa-l
ENSG0000	ENSG0000	9.91E-06	hsa-let-7a-5p;hsa-let-7b-5p;hsa-let-7d-5p;hsa-let-7e-5p;hsa-l
ENSG0000	ENSG0000	3.96E-08	hsa-let-7a-5p;hsa-let-7b-5p;hsa-let-7d-5p;hsa-let-7e-5p;hsa-l
ENSG0000	ENSG0000	5.49E-06	hsa-let-7a-5p;hsa-let-7b-5p;hsa-let-7d-5p;hsa-let-7e-5p;hsa-l
ENSG0000	ENSG0000	1.76E-05	hsa-miR-17-5p;hsa-miR-20b-5p;hsa-miR-26a-5p;hsa-miR-93-5
ENSG0000	ENSG0000	0.00102	hsa-miR-107;hsa-miR-15a-5p;hsa-miR-15b-5p;hsa-miR-195-5p
ENSG0000	ENSG0000	3.42E-05	hsa-miR-15a-5p;hsa-miR-15b-5p;hsa-miR-16-5p;hsa-miR-17-5
ENSG0000	ENSG0000	0.000684	hsa-miR-15a-5p;hsa-miR-15b-5p;hsa-miR-16-5p;hsa-miR-195-
ENSG0000	ENSG0000	0.002541	hsa-miR-1-3p;hsa-miR-20b-5p;hsa-miR-27a-3p;hsa-miR-27b-3
ENSG0000	ENSG0000	1.59E-08	hsa-miR-103a-3p;hsa-miR-107;hsa-miR-15a-5p;hsa-miR-15b-5
ENSG0000	ENSG0000	0.000765	hsa-miR-15a-5p;hsa-miR-15b-5p;hsa-miR-16-5p;hsa-miR-195-
ENSG0000	ENSG0000	0.000949	hsa-miR-17-5p;hsa-miR-20b-5p;hsa-miR-27a-3p;hsa-miR-27b-
ENSG0000	ENSG0000	7.41E-08	hsa-miR-103a-3p;hsa-miR-107;hsa-miR-15a-5p;hsa-miR-15b-5
ENSG0000	ENSG0000	0.006833	hsa-miR-17-5p;hsa-miR-185-5p;hsa-miR-20b-5p;hsa-miR-93-5
ENSG0000	ENSG0000	0.003416	hsa-miR-15a-5p;hsa-miR-15b-5p;hsa-miR-16-5p;hsa-miR-195-
ENSG0000	ENSG0000	0.003416	hsa-miR-15a-5p;hsa-miR-15b-5p;hsa-miR-16-5p;hsa-miR-195-
ENSG0000	ENSG0000	0.00609	hsa-miR-138-5p;hsa-miR-17-5p;hsa-miR-20b-5p;hsa-miR-93-5
ENSG0000	ENSG0000	0.002541	hsa-miR-15a-5p;hsa-miR-15b-5p;hsa-miR-16-5p;hsa-miR-195-
ENSG0000	ENSG0000	0.000609	hsa-miR-17-5p;hsa-miR-20b-5p;hsa-miR-26a-5p;hsa-miR-93-5
ENSG0000	ENSG0000	0.005257	hsa-miR-15a-5p;hsa-miR-15b-5p;hsa-miR-16-5p;hsa-miR-195-
ENSG0000	ENSG0000	5.14E-07	hsa-miR-15a-5p;hsa-miR-15b-5p;hsa-miR-16-5p;hsa-miR-17-5
ENSG0000	ENSG0000	2.37E-05	hsa-miR-15a-5p;hsa-miR-16-5p;hsa-miR-26a-5p;hsa-miR-34a-
ENSG0000	ENSG0000	8.07E-05	hsa-miR-15a-5p;hsa-miR-15b-5p;hsa-miR-16-5p;hsa-miR-195-
ENSG0000	ENSG0000	2.95E-05	hsa-miR-15a-5p;hsa-miR-15b-5p;hsa-miR-16-5p;hsa-miR-195-
ENSG0000	ENSG0000	0.000876	hsa-miR-17-5p;hsa-miR-27a-3p;hsa-miR-27b-3p;hsa-miR-377-
ENSG0000	ENSG0000	0.00318	hsa-miR-107;hsa-miR-15a-5p;hsa-miR-15b-5p;hsa-miR-195-5p
ENSG0000	ENSG0000	1.27E-05	hsa-miR-15a-5p;hsa-miR-15b-5p;hsa-miR-16-5p;hsa-miR-195-
ENSG0000	ENSG0000	0.00235	hsa-miR-15a-5p;hsa-miR-16-5p;hsa-miR-17-5p;hsa-miR-26a-5
ENSG0000	ENSG0000	2.81E-06	hsa-miR-138-5p;hsa-miR-15b-5p;hsa-miR-16-5p;hsa-miR-195-
ENSG0000	ENSG0000	4.07E-05	hsa-miR-15a-5p;hsa-miR-15b-5p;hsa-miR-16-5p;hsa-miR-26a-

ENSG0000	ENSG0000	5.08E-05	hsa-miR-103a-3p;hsa-miR-107;hsa-miR-15a-5p;hsa-miR-15b-5
ENSG0000	ENSG0000	1.28E-06	hsa-miR-103a-3p;hsa-miR-107;hsa-miR-15a-5p;hsa-miR-15b-5
ENSG0000	ENSG0000	0.002956	hsa-miR-15a-5p;hsa-miR-15b-5p;hsa-miR-195-5p;hsa-miR-27a
ENSG0000	ENSG0000	0.000168	hsa-miR-107;hsa-miR-15a-5p;hsa-miR-15b-5p;hsa-miR-16-5p;
ENSG0000	ENSG0000	9.78E-05	hsa-miR-16-5p;hsa-miR-17-5p;hsa-miR-19a-3p;hsa-miR-19b-3
ENSG0000	ENSG0000	2.82E-07	hsa-miR-15a-5p;hsa-miR-16-5p;hsa-miR-23a-3p;hsa-miR-23b-
ENSG0000	ENSG0000	0.007633	hsa-miR-15a-5p;hsa-miR-15b-5p;hsa-miR-16-5p;hsa-miR-195-
ENSG0000	ENSG0000	6.51E-05	hsa-miR-1-3p;hsa-miR-17-5p;hsa-miR-20b-5p;hsa-miR-93-5p
ENSG0000	ENSG0000	0.000646	hsa-miR-15a-5p;hsa-miR-15b-5p;hsa-miR-16-5p;hsa-miR-195-
ENSG0000	ENSG0000	0.000365	hsa-miR-126-5p;hsa-miR-1-3p;hsa-miR-15a-5p;hsa-miR-16-5p
ENSG0000	ENSG0000	0.008055	hsa-miR-17-5p;hsa-miR-20b-5p;hsa-miR-26a-5p;hsa-miR-93-5
ENSG0000	ENSG0000	0.000699	hsa-miR-15a-5p;hsa-miR-15b-5p;hsa-miR-16-5p;hsa-miR-185-
ENSG0000	ENSG0000	0.006499	hsa-miR-103a-3p;hsa-miR-107;hsa-miR-19a-3p;hsa-miR-19b-3
ENSG0000	ENSG0000	0.000699	hsa-miR-126-5p;hsa-miR-15a-5p;hsa-miR-15b-5p;hsa-miR-16-
ENSG0000	ENSG0000	0.001541	hsa-miR-15a-5p;hsa-miR-15b-5p;hsa-miR-195-5p;hsa-miR-19a
ENSG0000	ENSG0000	1.40E-07	hsa-miR-103a-3p;hsa-miR-107;hsa-miR-15a-5p;hsa-miR-15b-5
ENSG0000	ENSG0000	0.003663	hsa-miR-15a-5p;hsa-miR-15b-5p;hsa-miR-16-5p;hsa-miR-195-
ENSG0000	ENSG0000	5.23E-08	hsa-miR-103a-3p;hsa-miR-107;hsa-miR-15a-5p;hsa-miR-15b-5
ENSG0000	ENSG0000	0.0032	hsa-miR-17-5p;hsa-miR-19a-3p;hsa-miR-19b-3p;hsa-miR-20b-
ENSG0000	ENSG0000	8.07E-05	hsa-miR-126-5p;hsa-miR-17-5p;hsa-miR-20b-5p;hsa-miR-93-5
ENSG0000	ENSG0000	0.000127	hsa-miR-135a-5p;hsa-miR-17-5p;hsa-miR-20b-5p;hsa-miR-23b
ENSG0000	ENSG0000	0.002177	hsa-miR-17-5p;hsa-miR-20b-5p;hsa-miR-24-3p;hsa-miR-26a-5
ENSG0000	ENSG0000	0.002177	hsa-miR-17-5p;hsa-miR-20b-5p;hsa-miR-24-3p;hsa-miR-26a-5
ENSG0000	ENSG0000	0.000205	hsa-miR-17-5p;hsa-miR-195-5p;hsa-miR-20b-5p;hsa-miR-34a-
ENSG0000	ENSG0000	0.000275	hsa-miR-15a-5p;hsa-miR-15b-5p;hsa-miR-195-5p;hsa-miR-20b
ENSG0000	ENSG0000	6.72E-07	hsa-miR-132-3p;hsa-miR-135a-5p;hsa-miR-1-3p;hsa-miR-16-5
ENSG0000	ENSG0000	0.000129	hsa-miR-103a-3p;hsa-miR-107;hsa-miR-1-3p;hsa-miR-15a-5p;
ENSG0000	ENSG0000	0.00054	hsa-miR-149-5p;hsa-miR-24-3p;hsa-miR-27a-3p;hsa-miR-27b-
ENSG0000	ENSG0000	0.006454	hsa-miR-17-5p;hsa-miR-20b-5p;hsa-miR-26a-5p;hsa-miR-93-5
ENSG0000	ENSG0000	5.30E-07	hsa-miR-103a-3p;hsa-miR-107;hsa-miR-15a-5p;hsa-miR-15b-5
ENSG0000	ENSG0000	0.006454	hsa-miR-107;hsa-miR-23a-3p;hsa-miR-23b-3p;hsa-miR-9-5p
ENSG0000	ENSG0000	0.002726	hsa-miR-17-5p;hsa-miR-19a-3p;hsa-miR-19b-3p;hsa-miR-20b-
ENSG0000	ENSG0000	0.001328	hsa-miR-19a-3p;hsa-miR-19b-3p;hsa-miR-20b-5p;hsa-miR-26a
ENSG0000	ENSG0000	0.000854	hsa-miR-17-5p;hsa-miR-20b-5p;hsa-miR-26a-5p;hsa-miR-93-5
ENSG0000	ENSG0000	0.001279	hsa-miR-15a-5p;hsa-miR-15b-5p;hsa-miR-16-5p;hsa-miR-195-
ENSG0000	ENSG0000	0.000949	hsa-miR-132-3p;hsa-miR-19a-3p;hsa-miR-19b-3p;hsa-miR-369
ENSG0000	ENSG0000	0.000318	hsa-miR-15a-5p;hsa-miR-15b-5p;hsa-miR-16-5p;hsa-miR-195-
ENSG0000	ENSG0000	0.000942	hsa-miR-15a-5p;hsa-miR-15b-5p;hsa-miR-16-5p;hsa-miR-195-
ENSG0000	ENSG0000	6.76E-05	hsa-miR-103a-3p;hsa-miR-107;hsa-miR-138-5p;hsa-miR-17-5p
ENSG0000	ENSG0000	5.95E-05	hsa-miR-15a-5p;hsa-miR-15b-5p;hsa-miR-16-5p;hsa-miR-195-
ENSG0000	ENSG0000	1.77E-10	hsa-miR-1-3p;hsa-miR-17-5p;hsa-miR-19a-3p;hsa-miR-19b-3p
ENSG0000	ENSG0000	0.002129	hsa-miR-135a-5p;hsa-miR-1-3p;hsa-miR-17-5p;hsa-miR-185-5
ENSG0000	ENSG0000	0.009899	hsa-miR-15a-5p;hsa-miR-15b-5p;hsa-miR-16-5p;hsa-miR-195-
ENSG0000	ENSG0000	0.003416	hsa-miR-15a-5p;hsa-miR-195-5p;hsa-miR-19a-3p;hsa-miR-19b
ENSG0000	ENSG0000	0.003416	hsa-miR-103a-3p;hsa-miR-107;hsa-miR-23a-3p;hsa-miR-23b-3
ENSG0000	ENSG0000	1.28E-06	hsa-miR-103a-3p;hsa-miR-107;hsa-miR-15a-5p;hsa-miR-15b-5
ENSG0000	ENSG0000	0.001161	hsa-miR-132-3p;hsa-miR-15a-5p;hsa-miR-15b-5p;hsa-miR-195
ENSG0000	ENSG0000	0.00235	hsa-miR-15a-5p;hsa-miR-15b-5p;hsa-miR-16-5p;hsa-miR-195-
ENSG0000	ENSG0000	0.000318	hsa-miR-138-5p;hsa-miR-17-5p;hsa-miR-20b-5p;hsa-miR-377-
ENSG0000	ENSG0000	5.67E-08	hsa-miR-15a-5p;hsa-miR-15b-5p;hsa-miR-16-5p;hsa-miR-195-
ENSG0000	ENSG0000	0.007072	hsa-miR-1-3p;hsa-miR-15a-5p;hsa-miR-16-5p;hsa-miR-27a-3p
ENSG0000	ENSG0000	0.000325	hsa-miR-107;hsa-miR-138-5p;hsa-miR-17-5p;hsa-miR-20b-5p;
ENSG0000	ENSG0000	0.00012	hsa-miR-17-5p;hsa-miR-20b-5p;hsa-miR-27b-3p;hsa-miR-93-5
ENSG0000	ENSG0000	0.003173	hsa-miR-149-5p;hsa-miR-15a-5p;hsa-miR-15b-5p;hsa-miR-16-

ENSG0000	ENSG0000	0.001405	hsa-miR-107;hsa-miR-15a-5p;hsa-miR-15b-5p;hsa-miR-195-5p
ENSG0000	ENSG0000	0.000176	hsa-miR-135a-5p;hsa-miR-15a-5p;hsa-miR-15b-5p;hsa-miR-16
ENSG0000	ENSG0000	4.47E-05	hsa-miR-17-5p;hsa-miR-20b-5p;hsa-miR-27a-3p;hsa-miR-27b-
ENSG0000	ENSG0000	1.99E-05	hsa-miR-103a-3p;hsa-miR-107;hsa-miR-15a-5p;hsa-miR-15b-5
ENSG0000	ENSG0000	0.001611	hsa-miR-15a-5p;hsa-miR-17-5p;hsa-miR-195-5p;hsa-miR-20b-
ENSG0000	ENSG0000	0.002306	hsa-miR-16-5p;hsa-miR-17-5p;hsa-miR-20b-5p;hsa-miR-26a-5
ENSG0000	ENSG0000	0.001683	hsa-miR-143-3p;hsa-miR-17-5p;hsa-miR-20b-5p;hsa-miR-93-5
ENSG0000	ENSG0000	0.001243	hsa-miR-15a-5p;hsa-miR-15b-5p;hsa-miR-195-5p;hsa-miR-19a
ENSG0000	ENSG0000	4.07E-05	hsa-miR-1-3p;hsa-miR-19a-3p;hsa-miR-19b-3p;hsa-miR-9-5p
ENSG0000	ENSG0000	0.004772	hsa-miR-132-3p;hsa-miR-15a-5p;hsa-miR-15b-5p;hsa-miR-195
ENSG0000	ENSG0000	0.000366	hsa-miR-15a-5p;hsa-miR-15b-5p;hsa-miR-16-5p;hsa-miR-195-
ENSG0000	ENSG0000	1.27E-05	hsa-miR-15a-5p;hsa-miR-15b-5p;hsa-miR-16-5p;hsa-miR-195-
ENSG0000	ENSG0000	1.27E-05	hsa-miR-15a-5p;hsa-miR-15b-5p;hsa-miR-16-5p;hsa-miR-195-
ENSG0000	ENSG0000	3.31E-06	hsa-miR-15a-5p;hsa-miR-15b-5p;hsa-miR-16-5p;hsa-miR-17-5
ENSG0000	ENSG0000	3.29E-05	hsa-miR-15a-5p;hsa-miR-15b-5p;hsa-miR-195-5p;hsa-miR-27a
ENSG0000	ENSG0000	0.008493	hsa-miR-103a-3p;hsa-miR-107;hsa-miR-1-3p;hsa-miR-185-5p
ENSG0000	ENSG0000	2.87E-05	hsa-miR-103a-3p;hsa-miR-107;hsa-miR-15a-5p;hsa-miR-15b-5
ENSG0000	ENSG0000	9.40E-07	hsa-miR-103a-3p;hsa-miR-107;hsa-miR-1-3p;hsa-miR-15a-5p;
ENSG0000	ENSG0000	0.007225	hsa-miR-132-3p;hsa-miR-15b-5p;hsa-miR-27a-3p;hsa-miR-27b
ENSG0000	ENSG0000	0.00016	hsa-miR-17-5p;hsa-miR-19a-3p;hsa-miR-26a-5p;hsa-miR-27a-
ENSG0000	ENSG0000	0.000127	hsa-miR-23a-3p;hsa-miR-23b-3p;hsa-miR-26a-5p;hsa-miR-27a
ENSG0000	ENSG0000	0.001011	hsa-miR-17-5p;hsa-miR-19a-3p;hsa-miR-19b-3p;hsa-miR-20b-
ENSG0000	ENSG0000	0.000257	hsa-miR-15a-5p;hsa-miR-16-5p;hsa-miR-17-5p;hsa-miR-20b-5
ENSG0000	ENSG0000	0.000205	hsa-miR-17-5p;hsa-miR-195-5p;hsa-miR-19a-3p;hsa-miR-19b-
ENSG0000	ENSG0000	0.000236	hsa-miR-1-3p;hsa-miR-23b-3p;hsa-miR-27b-3p;hsa-miR-34a-5
ENSG0000	ENSG0000	0.004476	hsa-miR-17-5p;hsa-miR-20b-5p;hsa-miR-7-5p;hsa-miR-93-5p
ENSG0000	ENSG0000	0.000854	hsa-miR-149-5p;hsa-miR-15a-5p;hsa-miR-15b-5p;hsa-miR-195
ENSG0000	ENSG0000	0.00936	hsa-miR-17-5p;hsa-miR-20b-5p;hsa-miR-27a-3p;hsa-miR-27b-
ENSG0000	ENSG0000	0.001162	hsa-miR-17-5p;hsa-miR-19a-3p;hsa-miR-19b-3p;hsa-miR-20b-
ENSG0000	ENSG0000	5.94E-06	hsa-miR-107;hsa-miR-15a-5p;hsa-miR-16-5p;hsa-miR-34a-5p
ENSG0000	ENSG0000	0.002956	hsa-miR-185-5p;hsa-miR-24-3p;hsa-miR-26a-5p;hsa-miR-34a-
ENSG0000	ENSG0000	0.001328	hsa-miR-17-5p;hsa-miR-20b-5p;hsa-miR-24-3p;hsa-miR-377-3
ENSG0000	ENSG0000	2.31E-06	hsa-miR-138-5p;hsa-miR-16-5p;hsa-miR-34a-5p;hsa-miR-9-5p
ENSG0000	ENSG0000	0.003416	hsa-miR-17-5p;hsa-miR-19a-3p;hsa-miR-20b-5p;hsa-miR-34a-
ENSG0000	ENSG0000	5.17E-05	hsa-miR-23b-3p;hsa-miR-24-3p;hsa-miR-27b-3p;hsa-miR-34a-
ENSG0000	ENSG0000	1.89E-06	hsa-miR-1-3p;hsa-miR-15a-5p;hsa-miR-15b-5p;hsa-miR-16-5p
ENSG0000	ENSG0000	0.004193	hsa-miR-26a-5p;hsa-miR-27a-3p;hsa-miR-27b-3p;hsa-miR-7-5
ENSG0000	ENSG0000	0.000573	hsa-miR-103a-3p;hsa-miR-107;hsa-miR-15a-5p;hsa-miR-15b-5
ENSG0000	ENSG0000	0.001085	hsa-miR-107;hsa-miR-15a-5p;hsa-miR-15b-5p;hsa-miR-195-5p
ENSG0000	ENSG0000	1.27E-05	hsa-miR-15a-5p;hsa-miR-15b-5p;hsa-miR-16-5p;hsa-miR-195-
ENSG0000	ENSG0000	1.27E-05	hsa-miR-15a-5p;hsa-miR-15b-5p;hsa-miR-16-5p;hsa-miR-195-
ENSG0000	ENSG0000	0.008055	hsa-miR-143-3p;hsa-miR-17-5p;hsa-miR-20b-5p;hsa-miR-93-5
ENSG0000	ENSG0000	1.91E-08	hsa-miR-15a-5p;hsa-miR-15b-5p;hsa-miR-16-5p;hsa-miR-195-
ENSG0000	ENSG0000	0.000318	hsa-miR-17-5p;hsa-miR-20b-5p;hsa-miR-377-3p;hsa-miR-93-5
ENSG0000	ENSG0000	3.14E-05	hsa-miR-138-5p;hsa-miR-19a-3p;hsa-miR-19b-3p;hsa-miR-7-5
ENSG0000	ENSG0000	0.000684	hsa-miR-107;hsa-miR-15a-5p;hsa-miR-15b-5p;hsa-miR-195-5p
ENSG0000	ENSG0000	3.81E-06	hsa-miR-103a-3p;hsa-miR-107;hsa-miR-1-3p;hsa-miR-7-5p
ENSG0000	ENSG0000	5.82E-07	hsa-miR-17-5p;hsa-miR-19a-3p;hsa-miR-19b-3p;hsa-miR-20b-
ENSG0000	ENSG0000	7.43E-05	hsa-miR-107;hsa-miR-126-5p;hsa-miR-15a-5p;hsa-miR-15b-5p
ENSG0000	ENSG0000	2.29E-05	hsa-miR-132-3p;hsa-miR-138-5p;hsa-miR-1-3p;hsa-miR-17-5p
ENSG0000	ENSG0000	6.17E-06	hsa-miR-15a-5p;hsa-miR-15b-5p;hsa-miR-17-5p;hsa-miR-195-
ENSG0000	ENSG0000	0.000257	hsa-miR-138-5p;hsa-miR-15a-5p;hsa-miR-15b-5p;hsa-miR-16-
ENSG0000	ENSG0000	9.48E-08	hsa-miR-15a-5p;hsa-miR-15b-5p;hsa-miR-16-5p;hsa-miR-195-
ENSG0000	ENSG0000	0.001161	hsa-miR-17-5p;hsa-miR-20b-5p;hsa-miR-369-3p;hsa-miR-93-5

ENSG0000	ENSG0000	3.63E-08	hsa-miR-107;hsa-miR-15a-5p;hsa-miR-15b-5p;hsa-miR-17-5p;
ENSG0000	ENSG0000	0.007998	hsa-miR-15a-5p;hsa-miR-16-5p;hsa-miR-17-5p;hsa-miR-20b-5
ENSG0000	ENSG0000	1.13E-06	hsa-miR-15a-5p;hsa-miR-15b-5p;hsa-miR-16-5p;hsa-miR-195-
ENSG0000	ENSG0000	0.001836	hsa-miR-15a-5p;hsa-miR-15b-5p;hsa-miR-16-5p;hsa-miR-195-
ENSG0000	ENSG0000	0.009899	hsa-miR-15a-5p;hsa-miR-15b-5p;hsa-miR-16-5p;hsa-miR-195-
ENSG0000	ENSG0000	1.36E-06	hsa-miR-103a-3p;hsa-miR-107;hsa-miR-17-5p;hsa-miR-19a-3p
ENSG0000	ENSG0000	1.36E-06	hsa-miR-103a-3p;hsa-miR-107;hsa-miR-17-5p;hsa-miR-19a-3p
ENSG0000	ENSG0000	1.43E-05	hsa-miR-17-5p;hsa-miR-20b-5p;hsa-miR-23a-3p;hsa-miR-23b-
ENSG0000	ENSG0000	8.16E-05	hsa-miR-103a-3p;hsa-miR-107;hsa-miR-17-5p;hsa-miR-20b-5p
ENSG0000	ENSG0000	4.70E-06	hsa-miR-135a-5p;hsa-miR-15a-5p;hsa-miR-15b-5p;hsa-miR-16
ENSG0000	ENSG0000	4.70E-06	hsa-miR-135a-5p;hsa-miR-15a-5p;hsa-miR-15b-5p;hsa-miR-16
ENSG0000	ENSG0000	0.001611	hsa-miR-107;hsa-miR-15a-5p;hsa-miR-15b-5p;hsa-miR-16-5p;
ENSG0000	ENSG0000	0.005655	hsa-miR-103a-3p;hsa-miR-107;hsa-miR-17-5p;hsa-miR-20b-5p
ENSG0000	ENSG0000	0.001997	hsa-miR-15a-5p;hsa-miR-15b-5p;hsa-miR-16-5p;hsa-miR-195-
ENSG0000	ENSG0000	3.85E-05	hsa-miR-132-3p;hsa-miR-195-5p;hsa-miR-19a-3p;hsa-miR-19b
ENSG0000	ENSG0000	0.001011	hsa-miR-17-5p;hsa-miR-19a-3p;hsa-miR-19b-3p;hsa-miR-20b-
ENSG0000	ENSG0000	4.70E-06	hsa-miR-132-3p;hsa-miR-17-5p;hsa-miR-23b-3p;hsa-miR-26a-
ENSG0000	ENSG0000	0.002011	hsa-miR-103a-3p;hsa-miR-107;hsa-miR-15a-5p;hsa-miR-15b-5
ENSG0000	ENSG0000	1.76E-05	hsa-miR-15a-5p;hsa-miR-15b-5p;hsa-miR-16-5p;hsa-miR-195-
ENSG0000	ENSG0000	1.76E-05	hsa-miR-15a-5p;hsa-miR-15b-5p;hsa-miR-16-5p;hsa-miR-195-
ENSG0000	ENSG0000	6.52E-07	hsa-miR-15a-5p;hsa-miR-15b-5p;hsa-miR-16-5p;hsa-miR-195-
ENSG0000	ENSG0000	3.24E-06	hsa-miR-103a-3p;hsa-miR-107;hsa-miR-15a-5p;hsa-miR-15b-5
ENSG0000	ENSG0000	0.003921	hsa-miR-107;hsa-miR-15a-5p;hsa-miR-15b-5p;hsa-miR-195-5p
ENSG0000	ENSG0000	0.001196	hsa-miR-17-5p;hsa-miR-19a-3p;hsa-miR-19b-3p;hsa-miR-20b-
ENSG0000	ENSG0000	4.32E-07	hsa-miR-107;hsa-miR-15a-5p;hsa-miR-15b-5p;hsa-miR-195-5p
ENSG0000	ENSG0000	2.73E-06	hsa-miR-103a-3p;hsa-miR-107;hsa-miR-15a-5p;hsa-miR-15b-5
ENSG0000	ENSG0000	1.13E-05	hsa-miR-15a-5p;hsa-miR-15b-5p;hsa-miR-16-5p;hsa-miR-185-
ENSG0000	ENSG0000	1.00E-08	hsa-miR-15a-5p;hsa-miR-15b-5p;hsa-miR-16-5p;hsa-miR-195-
ENSG0000	ENSG0000	0.002531	hsa-miR-17-5p;hsa-miR-185-5p;hsa-miR-20b-5p;hsa-miR-23a-
ENSG0000	ENSG0000	0.002531	hsa-miR-17-5p;hsa-miR-185-5p;hsa-miR-20b-5p;hsa-miR-23a-
ENSG0000	ENSG0000	0.001085	hsa-miR-149-5p;hsa-miR-24-3p;hsa-miR-27a-3p;hsa-miR-27b-
ENSG0000	ENSG0000	0.001085	hsa-miR-149-5p;hsa-miR-24-3p;hsa-miR-27a-3p;hsa-miR-27b-
ENSG0000	ENSG0000	0.004978	hsa-miR-17-5p;hsa-miR-20b-5p;hsa-miR-27a-3p;hsa-miR-27b-
ENSG0000	ENSG0000	0.000366	hsa-miR-15a-5p;hsa-miR-15b-5p;hsa-miR-195-5p;hsa-miR-7-5
ENSG0000	ENSG0000	0.001683	hsa-miR-15a-5p;hsa-miR-15b-5p;hsa-miR-16-5p;hsa-miR-195-
ENSG0000	ENSG0000	8.07E-05	hsa-miR-15a-5p;hsa-miR-15b-5p;hsa-miR-16-5p;hsa-miR-195-
ENSG0000	ENSG0000	0.006454	hsa-miR-149-5p;hsa-miR-23a-3p;hsa-miR-23b-3p;hsa-miR-24-
ENSG0000	ENSG0000	8.28E-06	hsa-miR-103a-3p;hsa-miR-107;hsa-miR-15a-5p;hsa-miR-15b-5
ENSG0000	ENSG0000	0.001161	hsa-miR-107;hsa-miR-15a-5p;hsa-miR-15b-5p;hsa-miR-195-5p
ENSG0000	ENSG0000	0.00039	hsa-miR-132-3p;hsa-miR-17-5p;hsa-miR-20b-5p;hsa-miR-3909
ENSG0000	ENSG0000	0.001279	hsa-miR-15a-5p;hsa-miR-15b-5p;hsa-miR-16-5p;hsa-miR-195-
ENSG0000	ENSG0000	0.003663	hsa-miR-1-3p;hsa-miR-17-5p;hsa-miR-20b-5p;hsa-miR-93-5p
ENSG0000	ENSG0000	3.48E-07	hsa-miR-103a-3p;hsa-miR-107;hsa-miR-15a-5p;hsa-miR-15b-5
ENSG0000	ENSG0000	0.0032	hsa-miR-17-5p;hsa-miR-20b-5p;hsa-miR-23a-3p;hsa-miR-23b-
ENSG0000	ENSG0000	8.07E-05	hsa-miR-15a-5p;hsa-miR-15b-5p;hsa-miR-16-5p;hsa-miR-195-
ENSG0000	ENSG0000	2.82E-07	hsa-miR-17-5p;hsa-miR-19a-3p;hsa-miR-24-3p;hsa-miR-34a-5
ENSG0000	ENSG0000	2.82E-07	hsa-miR-17-5p;hsa-miR-19a-3p;hsa-miR-24-3p;hsa-miR-34a-5
ENSG0000	ENSG0000	2.82E-07	hsa-miR-17-5p;hsa-miR-19a-3p;hsa-miR-24-3p;hsa-miR-34a-5
ENSG0000	ENSG0000	2.82E-07	hsa-miR-17-5p;hsa-miR-19a-3p;hsa-miR-24-3p;hsa-miR-34a-5
ENSG0000	ENSG0000	2.82E-07	hsa-miR-17-5p;hsa-miR-19a-3p;hsa-miR-24-3p;hsa-miR-34a-5
ENSG0000	ENSG0000	2.82E-07	hsa-miR-17-5p;hsa-miR-19a-3p;hsa-miR-24-3p;hsa-miR-34a-5
ENSG0000	ENSG0000	2.82E-07	hsa-miR-17-5p;hsa-miR-19a-3p;hsa-miR-24-3p;hsa-miR-34a-5
ENSG0000	ENSG0000	2.82E-07	hsa-miR-17-5p;hsa-miR-19a-3p;hsa-miR-24-3p;hsa-miR-34a-5
ENSG0000	ENSG0000	0.008946	hsa-miR-19a-3p;hsa-miR-19b-3p;hsa-miR-23a-3p;hsa-miR-23b

ENSG0000	ENSG0000	0.001395	hsa-miR-15a-5p;hsa-miR-16-5p;hsa-miR-185-5p;hsa-miR-19a-
ENSG0000	ENSG0000	2.37E-05	hsa-miR-1-3p;hsa-miR-15a-5p;hsa-miR-15b-5p;hsa-miR-16-5
ENSG0000	ENSG0000	0.002905	hsa-miR-1-3p;hsa-miR-15a-5p;hsa-miR-15b-5p;hsa-miR-16-5
ENSG0000	ENSG0000	5.82E-07	hsa-miR-126-5p;hsa-miR-15a-5p;hsa-miR-15b-5p;hsa-miR-16-
ENSG0000	ENSG0000	0.006875	hsa-miR-143-3p;hsa-miR-15a-5p;hsa-miR-15b-5p;hsa-miR-16-
ENSG0000	ENSG0000	0.000341	hsa-miR-15a-5p;hsa-miR-15b-5p;hsa-miR-16-5p;hsa-miR-195-
ENSG0000	ENSG0000	0.00697	hsa-miR-138-5p;hsa-miR-15a-5p;hsa-miR-15b-5p;hsa-miR-16-
ENSG0000	ENSG0000	0.000205	hsa-miR-17-5p;hsa-miR-20b-5p;hsa-miR-24-3p;hsa-miR-27a-
ENSG0000	ENSG0000	0.00609	hsa-miR-15a-5p;hsa-miR-15b-5p;hsa-miR-16-5p;hsa-miR-195-
ENSG0000	ENSG0000	0.000366	hsa-miR-15a-5p;hsa-miR-15b-5p;hsa-miR-16-5p;hsa-miR-195-
ENSG0000	ENSG0000	6.52E-07	hsa-miR-17-5p;hsa-miR-20b-5p;hsa-miR-7-5p;hsa-miR-93-5p
ENSG0000	ENSG0000	1.42E-10	hsa-miR-107;hsa-miR-126-5p;hsa-miR-1-3p;hsa-miR-15a-5p;h
ENSG0000	ENSG0000	9.67E-06	hsa-miR-15a-5p;hsa-miR-15b-5p;hsa-miR-17-5p;hsa-miR-195-
ENSG0000	ENSG0000	0.000318	hsa-miR-15a-5p;hsa-miR-15b-5p;hsa-miR-16-5p;hsa-miR-195-
ENSG0000	ENSG0000	0.007225	hsa-miR-19a-3p;hsa-miR-19b-3p;hsa-miR-26a-5p;hsa-miR-37
ENSG0000	ENSG0000	0.009899	hsa-miR-19a-3p;hsa-miR-19b-3p;hsa-miR-23a-3p;hsa-miR-23
ENSG0000	ENSG0000	0.000143	hsa-miR-15a-5p;hsa-miR-15b-5p;hsa-miR-16-5p;hsa-miR-195-
ENSG0000	ENSG0000	9.70E-07	hsa-miR-15a-5p;hsa-miR-15b-5p;hsa-miR-17-5p;hsa-miR-195-
ENSG0000	ENSG0000	0.006499	hsa-miR-17-5p;hsa-miR-20b-5p;hsa-miR-27a-3p;hsa-miR-27b-
ENSG0000	ENSG0000	0.004117	hsa-miR-107;hsa-miR-15a-5p;hsa-miR-16-5p;hsa-miR-26a-5p;
ENSG0000	ENSG0000	0.002169	hsa-miR-1-3p;hsa-miR-17-5p;hsa-miR-20b-5p;hsa-miR-93-5p
ENSG0000	ENSG0000	1.91E-08	hsa-miR-15a-5p;hsa-miR-15b-5p;hsa-miR-16-5p;hsa-miR-195-
ENSG0000	ENSG0000	1.40E-07	hsa-miR-107;hsa-miR-15a-5p;hsa-miR-15b-5p;hsa-miR-16-5p;
ENSG0000	ENSG0000	6.79E-08	hsa-miR-15a-5p;hsa-miR-15b-5p;hsa-miR-16-5p;hsa-miR-17-
ENSG0000	ENSG0000	0.00574	hsa-miR-143-3p;hsa-miR-17-5p;hsa-miR-20b-5p;hsa-miR-93-
ENSG0000	ENSG0000	3.12E-05	hsa-miR-15a-5p;hsa-miR-15b-5p;hsa-miR-16-5p;hsa-miR-195-
ENSG0000	ENSG0000	0.00574	hsa-miR-15a-5p;hsa-miR-15b-5p;hsa-miR-16-5p;hsa-miR-195-
ENSG0000	ENSG0000	8.84E-06	hsa-miR-15a-5p;hsa-miR-15b-5p;hsa-miR-16-5p;hsa-miR-195-
ENSG0000	ENSG0000	0.005082	hsa-miR-126-5p;hsa-miR-17-5p;hsa-miR-20b-5p;hsa-miR-93-
ENSG0000	ENSG0000	0.005082	hsa-miR-126-5p;hsa-miR-17-5p;hsa-miR-20b-5p;hsa-miR-93-
ENSG0000	ENSG0000	0.005082	hsa-miR-15a-5p;hsa-miR-15b-5p;hsa-miR-16-5p;hsa-miR-195-
ENSG0000	ENSG0000	1.10E-05	hsa-miR-15a-5p;hsa-miR-15b-5p;hsa-miR-16-5p;hsa-miR-195-
ENSG0000	ENSG0000	0.000876	hsa-miR-15a-5p;hsa-miR-15b-5p;hsa-miR-16-5p;hsa-miR-185-
ENSG0000	ENSG0000	9.78E-05	hsa-miR-103a-3p;hsa-miR-107;hsa-miR-17-5p;hsa-miR-20b-5
ENSG0000	ENSG0000	1.56E-06	hsa-miR-15a-5p;hsa-miR-15b-5p;hsa-miR-195-5p;hsa-miR-23
ENSG0000	ENSG0000	0.008055	hsa-miR-27a-3p;hsa-miR-27b-3p;hsa-miR-34a-5p;hsa-miR-37
ENSG0000	ENSG0000	0.005211	hsa-miR-19a-3p;hsa-miR-19b-3p;hsa-miR-20b-5p;hsa-miR-34
ENSG0000	ENSG0000	2.31E-06	hsa-miR-15a-5p;hsa-miR-15b-5p;hsa-miR-16-5p;hsa-miR-195-
ENSG0000	ENSG0000	3.85E-05	hsa-miR-17-5p;hsa-miR-20b-5p;hsa-miR-27a-3p;hsa-miR-27b-
ENSG0000	ENSG0000	3.85E-05	hsa-miR-17-5p;hsa-miR-20b-5p;hsa-miR-27a-3p;hsa-miR-27b-
ENSG0000	ENSG0000	3.85E-05	hsa-miR-17-5p;hsa-miR-20b-5p;hsa-miR-27a-3p;hsa-miR-27b-
ENSG0000	ENSG0000	3.85E-05	hsa-miR-17-5p;hsa-miR-20b-5p;hsa-miR-27a-3p;hsa-miR-27b-
ENSG0000	ENSG0000	3.85E-05	hsa-miR-17-5p;hsa-miR-20b-5p;hsa-miR-27a-3p;hsa-miR-27b-
ENSG0000	ENSG0000	3.85E-05	hsa-miR-17-5p;hsa-miR-20b-5p;hsa-miR-27a-3p;hsa-miR-27b-
ENSG0000	ENSG0000	4.01E-05	hsa-miR-143-3p;hsa-miR-17-5p;hsa-miR-20b-5p;hsa-miR-24-
ENSG0000	ENSG0000	1.91E-08	hsa-miR-15a-5p;hsa-miR-15b-5p;hsa-miR-16-5p;hsa-miR-195-
ENSG0000	ENSG0000	6.76E-05	hsa-miR-15a-5p;hsa-miR-15b-5p;hsa-miR-16-5p;hsa-miR-195-
ENSG0000	ENSG0000	0.006454	hsa-miR-17-5p;hsa-miR-20b-5p;hsa-miR-24-3p;hsa-miR-93-5
ENSG0000	ENSG0000	0.00205	hsa-miR-19a-3p;hsa-miR-19b-3p;hsa-miR-20b-5p;hsa-miR-27
ENSG0000	ENSG0000	1.77E-10	hsa-miR-17-5p;hsa-miR-19a-3p;hsa-miR-19b-3p;hsa-miR-20b-
ENSG0000	ENSG0000	1.77E-10	hsa-miR-17-5p;hsa-miR-19a-3p;hsa-miR-19b-3p;hsa-miR-20b-
ENSG0000	ENSG0000	0.000236	hsa-miR-126-5p;hsa-miR-15b-5p;hsa-miR-16-5p;hsa-miR-19b
ENSG0000	ENSG0000	0.000475	hsa-miR-15a-5p;hsa-miR-15b-5p;hsa-miR-17-5p;hsa-miR-195-
ENSG0000	ENSG0000	0.001326	hsa-miR-103a-3p;hsa-miR-107;hsa-miR-15a-5p;hsa-miR-15b-

ENSG0000	ENSG0000	5.95E-05	hsa-miR-17-5p;hsa-miR-20b-5p;hsa-miR-24-3p;hsa-miR-3909;
ENSG0000	ENSG0000	0.00012	hsa-miR-15a-5p;hsa-miR-15b-5p;hsa-miR-16-5p;hsa-miR-195-
ENSG0000	ENSG0000	6.82E-05	hsa-miR-15a-5p;hsa-miR-15b-5p;hsa-miR-195-5p;hsa-miR-26a
ENSG0000	ENSG0000	0.003547	hsa-miR-103a-3p;hsa-miR-107;hsa-miR-17-5p;hsa-miR-20b-5p
ENSG0000	ENSG0000	1.27E-05	hsa-miR-15a-5p;hsa-miR-15b-5p;hsa-miR-16-5p;hsa-miR-195-
ENSG0000	ENSG0000	0.007225	hsa-miR-185-5p;hsa-miR-19a-3p;hsa-miR-19b-3p;hsa-miR-43
ENSG0000	ENSG0000	0.007225	hsa-miR-185-5p;hsa-miR-19a-3p;hsa-miR-19b-3p;hsa-miR-43
ENSG0000	ENSG0000	0.00318	hsa-miR-1-3p;hsa-miR-19a-3p;hsa-miR-19b-3p;hsa-miR-26a-5
ENSG0000	ENSG0000	0.000539	hsa-miR-103a-3p;hsa-miR-107;hsa-miR-15a-5p;hsa-miR-15b-5
ENSG0000	ENSG0000	5.29E-06	hsa-miR-103a-3p;hsa-miR-107;hsa-miR-15a-5p;hsa-miR-15b-5
ENSG0000	ENSG0000	3.24E-06	hsa-miR-103a-3p;hsa-miR-107;hsa-miR-15a-5p;hsa-miR-15b-5
ENSG0000	ENSG0000	1.29E-06	hsa-miR-15a-5p;hsa-miR-15b-5p;hsa-miR-16-5p;hsa-miR-195-
ENSG0000	ENSG0000	6.52E-07	hsa-miR-19a-3p;hsa-miR-19b-3p;hsa-miR-34a-5p;hsa-miR-9-5
ENSG0000	ENSG0000	1.49E-07	hsa-miR-103a-3p;hsa-miR-107;hsa-miR-15a-5p;hsa-miR-15b-5
ENSG0000	ENSG0000	0.000236	hsa-miR-15a-5p;hsa-miR-15b-5p;hsa-miR-16-5p;hsa-miR-195-
ENSG0000	ENSG0000	6.14E-05	hsa-miR-107;hsa-miR-17-5p;hsa-miR-19a-3p;hsa-miR-19b-3p;
ENSG0000	ENSG0000	0.004193	hsa-miR-15a-5p;hsa-miR-15b-5p;hsa-miR-16-5p;hsa-miR-195-
ENSG0000	ENSG0000	5.56E-05	hsa-miR-107;hsa-miR-15a-5p;hsa-miR-15b-5p;hsa-miR-17-5p;
ENSG0000	ENSG0000	0.000171	hsa-miR-15a-5p;hsa-miR-15b-5p;hsa-miR-16-5p;hsa-miR-195-
ENSG0000	ENSG0000	3.83E-07	hsa-miR-132-3p;hsa-miR-15a-5p;hsa-miR-15b-5p;hsa-miR-16-
ENSG0000	ENSG0000	0.000506	hsa-miR-149-5p;hsa-miR-17-5p;hsa-miR-20b-5p;hsa-miR-24-
ENSG0000	ENSG0000	0.000418	hsa-miR-107;hsa-miR-15a-5p;hsa-miR-15b-5p;hsa-miR-195-5p
ENSG0000	ENSG0000	0.001279	hsa-miR-15a-5p;hsa-miR-15b-5p;hsa-miR-16-5p;hsa-miR-195-
ENSG0000	ENSG0000	3.67E-05	hsa-miR-103a-3p;hsa-miR-107;hsa-miR-135a-5p;hsa-miR-15a-
ENSG0000	ENSG0000	0.004476	hsa-miR-15a-5p;hsa-miR-15b-5p;hsa-miR-16-5p;hsa-miR-195-
ENSG0000	ENSG0000	5.17E-05	hsa-miR-17-5p;hsa-miR-19a-3p;hsa-miR-19b-3p;hsa-miR-20b-
ENSG0000	ENSG0000	5.17E-05	hsa-miR-17-5p;hsa-miR-19a-3p;hsa-miR-19b-3p;hsa-miR-20b-
ENSG0000	ENSG0000	0.002146	hsa-miR-15a-5p;hsa-miR-15b-5p;hsa-miR-16-5p;hsa-miR-195-
ENSG0000	ENSG0000	0.008055	hsa-miR-15a-5p;hsa-miR-15b-5p;hsa-miR-16-5p;hsa-miR-195-
ENSG0000	ENSG0000	0.001051	hsa-miR-15a-5p;hsa-miR-15b-5p;hsa-miR-16-5p;hsa-miR-195-
ENSG0000	ENSG0000	0.00373	hsa-miR-1-3p;hsa-miR-17-5p;hsa-miR-20b-5p;hsa-miR-26a-5p
ENSG0000	ENSG0000	4.54E-05	hsa-miR-15a-5p;hsa-miR-15b-5p;hsa-miR-16-5p;hsa-miR-195-
ENSG0000	ENSG0000	1.27E-05	hsa-miR-17-5p;hsa-miR-19a-3p;hsa-miR-19b-3p;hsa-miR-20b-
ENSG0000	ENSG0000	3.81E-06	hsa-miR-15a-5p;hsa-miR-15b-5p;hsa-miR-16-5p;hsa-miR-195-
ENSG0000	ENSG0000	8.84E-06	hsa-miR-15a-5p;hsa-miR-15b-5p;hsa-miR-16-5p;hsa-miR-195-
ENSG0000	ENSG0000	0.000765	hsa-miR-126-5p;hsa-miR-15a-5p;hsa-miR-15b-5p;hsa-miR-19
ENSG0000	ENSG0000	1.62E-05	hsa-miR-15a-5p;hsa-miR-15b-5p;hsa-miR-16-5p;hsa-miR-195-
ENSG0000	ENSG0000	1.62E-05	hsa-miR-15a-5p;hsa-miR-15b-5p;hsa-miR-16-5p;hsa-miR-195-
ENSG0000	ENSG0000	3.69E-05	hsa-miR-17-5p;hsa-miR-195-5p;hsa-miR-19a-3p;hsa-miR-19b-
ENSG0000	ENSG0000	0.008055	hsa-miR-17-5p;hsa-miR-20b-5p;hsa-miR-24-3p;hsa-miR-93-5p
ENSG0000	ENSG0000	0.001836	hsa-miR-1-3p;hsa-miR-149-5p;hsa-miR-34a-5p;hsa-miR-369-
ENSG0000	ENSG0000	0.001196	hsa-miR-138-5p;hsa-miR-17-5p;hsa-miR-20b-5p;hsa-miR-27a-
ENSG0000	ENSG0000	2.37E-05	hsa-miR-15a-5p;hsa-miR-15b-5p;hsa-miR-195-5p;hsa-miR-43
ENSG0000	ENSG0000	0.000854	hsa-miR-15a-5p;hsa-miR-15b-5p;hsa-miR-23a-3p;hsa-miR-23
ENSG0000	ENSG0000	1.21E-07	hsa-miR-103a-3p;hsa-miR-107;hsa-miR-1-3p;hsa-miR-17-5p;h
ENSG0000	ENSG0000	2.34E-05	hsa-miR-15a-5p;hsa-miR-15b-5p;hsa-miR-16-5p;hsa-miR-195-
ENSG0000	ENSG0000	1.89E-06	hsa-miR-15a-5p;hsa-miR-15b-5p;hsa-miR-16-5p;hsa-miR-195-
ENSG0000	ENSG0000	1.76E-05	hsa-miR-15a-5p;hsa-miR-15b-5p;hsa-miR-16-5p;hsa-miR-195-
ENSG0000	ENSG0000	2.10E-07	hsa-miR-15a-5p;hsa-miR-15b-5p;hsa-miR-16-5p;hsa-miR-17-5
ENSG0000	ENSG0000	9.88E-05	hsa-miR-15a-5p;hsa-miR-15b-5p;hsa-miR-16-5p;hsa-miR-195-
ENSG0000	ENSG0000	9.88E-05	hsa-miR-15a-5p;hsa-miR-15b-5p;hsa-miR-16-5p;hsa-miR-195-
ENSG0000	ENSG0000	1.76E-05	hsa-miR-15a-5p;hsa-miR-15b-5p;hsa-miR-16-5p;hsa-miR-195-
ENSG0000	ENSG0000	7.96E-05	hsa-miR-17-5p;hsa-miR-19a-3p;hsa-miR-19b-3p;hsa-miR-20b-
ENSG0000	ENSG0000	0.00205	hsa-miR-15a-5p;hsa-miR-15b-5p;hsa-miR-16-5p;hsa-miR-195-

ENSG0000	ENSG0000	0.00235	hsa-miR-15a-5p;hsa-miR-15b-5p;hsa-miR-16-5p;hsa-miR-195-
ENSG0000	ENSG0000	0.004978	hsa-miR-143-3p;hsa-miR-15a-5p;hsa-miR-15b-5p;hsa-miR-16-
ENSG0000	ENSG0000	8.07E-05	hsa-miR-103a-3p;hsa-miR-107;hsa-miR-23a-3p;hsa-miR-23b-
ENSG0000	ENSG0000	1.84E-05	hsa-miR-15a-5p;hsa-miR-15b-5p;hsa-miR-16-5p;hsa-miR-195-
ENSG0000	ENSG0000	0.007225	hsa-miR-138-5p;hsa-miR-1-3p;hsa-miR-19a-3p;hsa-miR-432-
ENSG0000	ENSG0000	0.004193	hsa-miR-15a-5p;hsa-miR-15b-5p;hsa-miR-16-5p;hsa-miR-195-
ENSG0000	ENSG0000	5.17E-05	hsa-miR-1-3p;hsa-miR-15a-5p;hsa-miR-15b-5p;hsa-miR-16-5
ENSG0000	ENSG0000	5.17E-05	hsa-miR-1-3p;hsa-miR-15a-5p;hsa-miR-15b-5p;hsa-miR-16-5
ENSG0000	ENSG0000	0.000202	hsa-miR-15a-5p;hsa-miR-15b-5p;hsa-miR-16-5p;hsa-miR-195-
ENSG0000	ENSG0000	0.000143	hsa-miR-1-3p;hsa-miR-15a-5p;hsa-miR-15b-5p;hsa-miR-16-5
ENSG0000	ENSG0000	0.003921	hsa-miR-17-5p;hsa-miR-20b-5p;hsa-miR-24-3p;hsa-miR-93-5
ENSG0000	ENSG0000	8.07E-05	hsa-miR-23a-3p;hsa-miR-23b-3p;hsa-miR-27a-3p;hsa-miR-27
ENSG0000	ENSG0000	9.88E-05	hsa-miR-15a-5p;hsa-miR-15b-5p;hsa-miR-195-5p;hsa-miR-39
ENSG0000	ENSG0000	2.13E-06	hsa-miR-17-5p;hsa-miR-19a-3p;hsa-miR-19b-3p;hsa-miR-20b-
ENSG0000	ENSG0000	0.000418	hsa-miR-15a-5p;hsa-miR-15b-5p;hsa-miR-16-5p;hsa-miR-195-
ENSG0000	ENSG0000	0.000699	hsa-miR-17-5p;hsa-miR-20b-5p;hsa-miR-34a-5p;hsa-miR-7-5
ENSG0000	ENSG0000	7.41E-05	hsa-miR-103a-3p;hsa-miR-107;hsa-miR-15a-5p;hsa-miR-15b-
ENSG0000	ENSG0000	2.82E-07	hsa-miR-17-5p;hsa-miR-19b-3p;hsa-miR-24-3p;hsa-miR-93-5
ENSG0000	ENSG0000	0.002054	hsa-miR-19a-3p;hsa-miR-19b-3p;hsa-miR-26a-5p;hsa-miR-27
ENSG0000	ENSG0000	0.000476	hsa-miR-15a-5p;hsa-miR-15b-5p;hsa-miR-16-5p;hsa-miR-195-
ENSG0000	ENSG0000	9.70E-07	hsa-miR-103a-3p;hsa-miR-107;hsa-miR-15a-5p;hsa-miR-15b-
ENSG0000	ENSG0000	9.70E-07	hsa-miR-103a-3p;hsa-miR-107;hsa-miR-15a-5p;hsa-miR-15b-
ENSG0000	ENSG0000	6.76E-05	hsa-miR-107;hsa-miR-15a-5p;hsa-miR-15b-5p;hsa-miR-185-5
ENSG0000	ENSG0000	0.001051	hsa-miR-15a-5p;hsa-miR-15b-5p;hsa-miR-16-5p;hsa-miR-195-
ENSG0000	ENSG0000	2.52E-08	hsa-miR-1-3p;hsa-miR-15a-5p;hsa-miR-15b-5p;hsa-miR-16-5
ENSG0000	ENSG0000	0.005211	hsa-miR-17-5p;hsa-miR-20b-5p;hsa-miR-24-3p;hsa-miR-377-
ENSG0000	ENSG0000	8.34E-10	hsa-miR-103a-3p;hsa-miR-107;hsa-miR-15a-5p;hsa-miR-15b-
ENSG0000	ENSG0000	0.001836	hsa-miR-1-3p;hsa-miR-17-5p;hsa-miR-20b-5p;hsa-miR-93-5p
ENSG0000	ENSG0000	3.94E-09	hsa-miR-107;hsa-miR-15a-5p;hsa-miR-15b-5p;hsa-miR-17-5p;
ENSG0000	ENSG0000	1.29E-06	hsa-miR-1-3p;hsa-miR-17-5p;hsa-miR-20b-5p;hsa-miR-93-5p
ENSG0000	ENSG0000	2.98E-05	hsa-miR-15a-5p;hsa-miR-15b-5p;hsa-miR-16-5p;hsa-miR-17-
ENSG0000	ENSG0000	2.98E-05	hsa-miR-15a-5p;hsa-miR-15b-5p;hsa-miR-16-5p;hsa-miR-17-
ENSG0000	ENSG0000	1.27E-05	hsa-miR-15a-5p;hsa-miR-15b-5p;hsa-miR-16-5p;hsa-miR-195-
ENSG0000	ENSG0000	0.00318	hsa-miR-19a-3p;hsa-miR-19b-3p;hsa-miR-27a-3p;hsa-miR-27
ENSG0000	ENSG0000	1.91E-08	hsa-miR-135a-5p;hsa-miR-15a-5p;hsa-miR-15b-5p;hsa-miR-1
ENSG0000	ENSG0000	0.007633	hsa-miR-17-5p;hsa-miR-20b-5p;hsa-miR-93-5p;hsa-miR-9-5p
ENSG0000	ENSG0000	4.22E-06	hsa-miR-103a-3p;hsa-miR-107;hsa-miR-15a-5p;hsa-miR-15b-
ENSG0000	ENSG0000	1.40E-07	hsa-miR-103a-3p;hsa-miR-107;hsa-miR-15a-5p;hsa-miR-15b-
ENSG0000	ENSG0000	0.001405	hsa-miR-15a-5p;hsa-miR-15b-5p;hsa-miR-16-5p;hsa-miR-195-
ENSG0000	ENSG0000	0.000476	hsa-miR-15a-5p;hsa-miR-15b-5p;hsa-miR-16-5p;hsa-miR-195-
ENSG0000	ENSG0000	0.002743	hsa-miR-19a-3p;hsa-miR-19b-3p;hsa-miR-27a-3p;hsa-miR-27
ENSG0000	ENSG0000	7.78E-05	hsa-miR-103a-3p;hsa-miR-107;hsa-miR-377-3p;hsa-miR-432-
ENSG0000	ENSG0000	3.14E-05	hsa-miR-15a-5p;hsa-miR-15b-5p;hsa-miR-16-5p;hsa-miR-195-
ENSG0000	ENSG0000	0.00012	hsa-miR-15a-5p;hsa-miR-15b-5p;hsa-miR-16-5p;hsa-miR-195-
ENSG0000	ENSG0000	0.000942	hsa-miR-15a-5p;hsa-miR-15b-5p;hsa-miR-16-5p;hsa-miR-195-
ENSG0000	ENSG0000	0.000765	hsa-miR-15a-5p;hsa-miR-15b-5p;hsa-miR-16-5p;hsa-miR-195-
ENSG0000	ENSG0000	0.002956	hsa-miR-15a-5p;hsa-miR-15b-5p;hsa-miR-19a-3p;hsa-miR-19
ENSG0000	ENSG0000	3.66E-06	hsa-miR-132-3p;hsa-miR-138-5p;hsa-miR-23b-3p;hsa-miR-34
ENSG0000	ENSG0000	0.004039	hsa-miR-126-5p;hsa-miR-17-5p;hsa-miR-19a-3p;hsa-miR-19b-
ENSG0000	ENSG0000	4.70E-06	hsa-miR-138-5p;hsa-miR-15a-5p;hsa-miR-15b-5p;hsa-miR-16-
ENSG0000	ENSG0000	6.51E-05	hsa-miR-19a-3p;hsa-miR-19b-3p;hsa-miR-7-5p;hsa-miR-9-5p
ENSG0000	ENSG0000	0.003663	hsa-miR-19a-3p;hsa-miR-19b-3p;hsa-miR-20b-5p;hsa-miR-93-
ENSG0000	ENSG0000	8.16E-05	hsa-miR-103a-3p;hsa-miR-107;hsa-miR-15a-5p;hsa-miR-15b-
ENSG0000	ENSG0000	5.69E-07	hsa-miR-107;hsa-miR-15a-5p;hsa-miR-16-5p;hsa-miR-19b-3p;

ENSG0000	ENSG0000	0.00012	hsa-miR-15a-5p;hsa-miR-15b-5p;hsa-miR-16-5p;hsa-miR-195-
ENSG0000	ENSG0000	0.000213	hsa-miR-15a-5p;hsa-miR-15b-5p;hsa-miR-17-5p;hsa-miR-195-
ENSG0000	ENSG0000	0.000205	hsa-miR-15a-5p;hsa-miR-15b-5p;hsa-miR-16-5p;hsa-miR-195-
ENSG0000	ENSG0000	0.009899	hsa-miR-143-3p;hsa-miR-17-5p;hsa-miR-20b-5p;hsa-miR-93-5
ENSG0000	ENSG0000	1.27E-05	hsa-miR-15a-5p;hsa-miR-15b-5p;hsa-miR-16-5p;hsa-miR-195-
ENSG0000	ENSG0000	0.009415	hsa-miR-17-5p;hsa-miR-20b-5p;hsa-miR-24-3p;hsa-miR-93-5p
ENSG0000	ENSG0000	5.33E-06	hsa-miR-103a-3p;hsa-miR-107;hsa-miR-15a-5p;hsa-miR-15b-5
ENSG0000	ENSG0000	0.005702	hsa-miR-15a-5p;hsa-miR-15b-5p;hsa-miR-195-5p;hsa-miR-27a
ENSG0000	ENSG0000	9.23E-06	hsa-miR-15a-5p;hsa-miR-15b-5p;hsa-miR-16-5p;hsa-miR-195-
ENSG0000	ENSG0000	0.005702	hsa-miR-15a-5p;hsa-miR-195-5p;hsa-miR-19a-3p;hsa-miR-19b
ENSG0000	ENSG0000	1.13E-08	hsa-miR-17-5p;hsa-miR-19a-3p;hsa-miR-19b-3p;hsa-miR-20b-
ENSG0000	ENSG0000	1.13E-08	hsa-miR-17-5p;hsa-miR-19a-3p;hsa-miR-19b-3p;hsa-miR-20b-
ENSG0000	ENSG0000	1.91E-08	hsa-miR-15a-5p;hsa-miR-15b-5p;hsa-miR-16-5p;hsa-miR-195-
ENSG0000	ENSG0000	9.88E-05	hsa-miR-17-5p;hsa-miR-20b-5p;hsa-miR-26a-5p;hsa-miR-93-5
ENSG0000	ENSG0000	0.00039	hsa-miR-135a-5p;hsa-miR-15a-5p;hsa-miR-15b-5p;hsa-miR-16
ENSG0000	ENSG0000	0.00235	hsa-miR-1-3p;hsa-miR-17-5p;hsa-miR-20b-5p;hsa-miR-93-5p
ENSG0000	ENSG0000	0.000171	hsa-miR-15a-5p;hsa-miR-15b-5p;hsa-miR-16-5p;hsa-miR-195-
ENSG0000	ENSG0000	0.002956	hsa-miR-1-3p;hsa-miR-19a-3p;hsa-miR-19b-3p;hsa-miR-34a-5
ENSG0000	ENSG0000	7.78E-05	hsa-miR-15a-5p;hsa-miR-15b-5p;hsa-miR-16-5p;hsa-miR-195-
ENSG0000	ENSG0000	0.000765	hsa-miR-1-3p;hsa-miR-26a-5p;hsa-miR-27a-3p;hsa-miR-27b-3
ENSG0000	ENSG0000	0.00609	hsa-miR-17-5p;hsa-miR-20b-5p;hsa-miR-377-3p;hsa-miR-93-5
ENSG0000	ENSG0000	0.001997	hsa-miR-1-3p;hsa-miR-15a-5p;hsa-miR-16-5p;hsa-miR-93-5p
ENSG0000	ENSG0000	0.002743	hsa-miR-17-5p;hsa-miR-20b-5p;hsa-miR-7-5p;hsa-miR-93-5p
ENSG0000	ENSG0000	0.003416	hsa-miR-16-5p;hsa-miR-17-5p;hsa-miR-20b-5p;hsa-miR-93-5p
ENSG0000	ENSG0000	5.69E-07	hsa-miR-103a-3p;hsa-miR-107;hsa-miR-132-3p;hsa-miR-1-3p;
ENSG0000	ENSG0000	0.003036	hsa-miR-149-5p;hsa-miR-17-5p;hsa-miR-20b-5p;hsa-miR-369-
ENSG0000	ENSG0000	0.00258	hsa-miR-103a-3p;hsa-miR-107;hsa-miR-15a-5p;hsa-miR-15b-5
ENSG0000	ENSG0000	0.009415	hsa-miR-17-5p;hsa-miR-19b-3p;hsa-miR-20b-5p;hsa-miR-369
ENSG0000	ENSG0000	0.002541	hsa-miR-15a-5p;hsa-miR-15b-5p;hsa-miR-16-5p;hsa-miR-195-
ENSG0000	ENSG0000	0.000366	hsa-miR-138-5p;hsa-miR-15a-5p;hsa-miR-15b-5p;hsa-miR-19
ENSG0000	ENSG0000	1.10E-07	hsa-miR-103a-3p;hsa-miR-107;hsa-miR-15a-5p;hsa-miR-15b-5
ENSG0000	ENSG0000	2.34E-05	hsa-miR-103a-3p;hsa-miR-107;hsa-miR-15a-5p;hsa-miR-15b-5
ENSG0000	ENSG0000	0.001405	hsa-miR-15a-5p;hsa-miR-15b-5p;hsa-miR-195-5p;hsa-miR-34a
ENSG0000	ENSG0000	5.18E-05	hsa-miR-103a-3p;hsa-miR-107;hsa-miR-27a-3p;hsa-miR-27b-3
ENSG0000	ENSG0000	0.001782	hsa-miR-143-3p;hsa-miR-17-5p;hsa-miR-20b-5p;hsa-miR-432-
ENSG0000	ENSG0000	0.001051	hsa-miR-15a-5p;hsa-miR-16-5p;hsa-miR-26a-5p;hsa-miR-9-5p
ENSG0000	ENSG0000	0.000366	hsa-miR-15a-5p;hsa-miR-15b-5p;hsa-miR-16-5p;hsa-miR-195-
ENSG0000	ENSG0000	6.51E-05	hsa-miR-15a-5p;hsa-miR-15b-5p;hsa-miR-16-5p;hsa-miR-195-
ENSG0000	ENSG0000	1.27E-05	hsa-miR-15a-5p;hsa-miR-15b-5p;hsa-miR-16-5p;hsa-miR-195-
ENSG0000	ENSG0000	2.16E-12	hsa-miR-15a-5p;hsa-miR-15b-5p;hsa-miR-16-5p;hsa-miR-195-
ENSG0000	ENSG0000	0.004117	hsa-miR-17-5p;hsa-miR-19a-3p;hsa-miR-19b-3p;hsa-miR-20b-
ENSG0000	ENSG0000	0.0001	hsa-miR-17-5p;hsa-miR-19a-3p;hsa-miR-19b-3p;hsa-miR-20b-
ENSG0000	ENSG0000	3.83E-07	hsa-miR-15a-5p;hsa-miR-15b-5p;hsa-miR-16-5p;hsa-miR-195-
ENSG0000	ENSG0000	0.00609	hsa-miR-15a-5p;hsa-miR-15b-5p;hsa-miR-16-5p;hsa-miR-195-
ENSG0000	ENSG0000	0.001051	hsa-miR-17-5p;hsa-miR-19a-3p;hsa-miR-20b-5p;hsa-miR-93-5
ENSG0000	ENSG0000	1.29E-06	hsa-miR-17-5p;hsa-miR-20b-5p;hsa-miR-27a-3p;hsa-miR-27b-
ENSG0000	ENSG0000	0.000397	hsa-miR-135a-5p;hsa-miR-17-5p;hsa-miR-19a-3p;hsa-miR-19b
ENSG0000	ENSG0000	0.004772	hsa-miR-17-5p;hsa-miR-20b-5p;hsa-miR-27a-3p;hsa-miR-27b-
ENSG0000	ENSG0000	7.17E-06	hsa-miR-15a-5p;hsa-miR-15b-5p;hsa-miR-16-5p;hsa-miR-195-
ENSG0000	ENSG0000	9.88E-05	hsa-miR-15a-5p;hsa-miR-15b-5p;hsa-miR-16-5p;hsa-miR-195-
ENSG0000	ENSG0000	3.84E-06	hsa-miR-103a-3p;hsa-miR-107;hsa-miR-15a-5p;hsa-miR-15b-5
ENSG0000	ENSG0000	0.00574	hsa-miR-15a-5p;hsa-miR-15b-5p;hsa-miR-16-5p;hsa-miR-195-
ENSG0000	ENSG0000	0.000765	hsa-miR-15a-5p;hsa-miR-15b-5p;hsa-miR-16-5p;hsa-miR-195-
ENSG0000	ENSG0000	0.00574	hsa-miR-132-3p;hsa-miR-19a-3p;hsa-miR-19b-3p;hsa-miR-26a

ENSG0000	ENSG0000	1.39E-06	hsa-miR-103a-3p;hsa-miR-107;hsa-miR-15a-5p;hsa-miR-15b-5
ENSG0000	ENSG0000	0.0032	hsa-miR-17-5p;hsa-miR-19a-3p;hsa-miR-19b-3p;hsa-miR-20b-
ENSG0000	ENSG0000	2.08E-05	hsa-miR-17-5p;hsa-miR-19a-3p;hsa-miR-19b-3p;hsa-miR-20b-
ENSG0000	ENSG0000	0.000854	hsa-miR-1-3p;hsa-miR-19a-3p;hsa-miR-19b-3p;hsa-miR-26a-5
ENSG0000	ENSG0000	0.000365	hsa-miR-17-5p;hsa-miR-19a-3p;hsa-miR-20b-5p;hsa-miR-26a-
ENSG0000	ENSG0000	4.07E-05	hsa-miR-15a-5p;hsa-miR-15b-5p;hsa-miR-16-5p;hsa-miR-195-
ENSG0000	ENSG0000	8.85E-05	hsa-miR-135a-5p;hsa-miR-15a-5p;hsa-miR-15b-5p;hsa-miR-16
ENSG0000	ENSG0000	2.07E-10	hsa-miR-15a-5p;hsa-miR-15b-5p;hsa-miR-195-5p;hsa-miR-19
ENSG0000	ENSG0000	1.29E-06	hsa-miR-15a-5p;hsa-miR-15b-5p;hsa-miR-16-5p;hsa-miR-195-
ENSG0000	ENSG0000	0.00012	hsa-miR-15a-5p;hsa-miR-15b-5p;hsa-miR-16-5p;hsa-miR-195-
ENSG0000	ENSG0000	0.000596	hsa-miR-1-3p;hsa-miR-15a-5p;hsa-miR-15b-5p;hsa-miR-16-5p
ENSG0000	ENSG0000	3.66E-06	hsa-miR-17-5p;hsa-miR-19a-3p;hsa-miR-19b-3p;hsa-miR-20b-
ENSG0000	ENSG0000	0.000876	hsa-miR-15a-5p;hsa-miR-15b-5p;hsa-miR-16-5p;hsa-miR-195-
ENSG0000	ENSG0000	8.29E-06	hsa-miR-126-5p;hsa-miR-15a-5p;hsa-miR-15b-5p;hsa-miR-17-
ENSG0000	ENSG0000	2.37E-05	hsa-miR-107;hsa-miR-15a-5p;hsa-miR-15b-5p;hsa-miR-16-5p;
ENSG0000	ENSG0000	8.16E-05	hsa-miR-15a-5p;hsa-miR-15b-5p;hsa-miR-17-5p;hsa-miR-195-
ENSG0000	ENSG0000	0.00054	hsa-miR-132-3p;hsa-miR-1-3p;hsa-miR-19a-3p;hsa-miR-19b-3
ENSG0000	ENSG0000	0.003663	hsa-miR-107;hsa-miR-15a-5p;hsa-miR-15b-5p;hsa-miR-195-5p
ENSG0000	ENSG0000	0.00154	hsa-miR-15a-5p;hsa-miR-15b-5p;hsa-miR-16-5p;hsa-miR-195-
ENSG0000	ENSG0000	2.82E-07	hsa-miR-15a-5p;hsa-miR-15b-5p;hsa-miR-16-5p;hsa-miR-195-
ENSG0000	ENSG0000	4.70E-06	hsa-miR-17-5p;hsa-miR-19a-3p;hsa-miR-19b-3p;hsa-miR-20b-
ENSG0000	ENSG0000	4.70E-06	hsa-miR-17-5p;hsa-miR-19a-3p;hsa-miR-19b-3p;hsa-miR-20b-
ENSG0000	ENSG0000	3.51E-07	hsa-miR-103a-3p;hsa-miR-107;hsa-miR-15a-5p;hsa-miR-15b-5
ENSG0000	ENSG0000	0.005404	hsa-miR-107;hsa-miR-15a-5p;hsa-miR-15b-5p;hsa-miR-195-5p
ENSG0000	ENSG0000	0.000699	hsa-miR-107;hsa-miR-15a-5p;hsa-miR-15b-5p;hsa-miR-195-5p
ENSG0000	ENSG0000	1.28E-07	hsa-miR-103a-3p;hsa-miR-107;hsa-miR-15a-5p;hsa-miR-15b-5
ENSG0000	ENSG0000	9.88E-05	hsa-miR-15a-5p;hsa-miR-15b-5p;hsa-miR-16-5p;hsa-miR-195-
ENSG0000	ENSG0000	9.88E-05	hsa-miR-15a-5p;hsa-miR-15b-5p;hsa-miR-16-5p;hsa-miR-195-
ENSG0000	ENSG0000	0.00015	hsa-miR-17-5p;hsa-miR-20b-5p;hsa-miR-27a-3p;hsa-miR-27b-
ENSG0000	ENSG0000	9.48E-08	hsa-miR-15a-5p;hsa-miR-15b-5p;hsa-miR-16-5p;hsa-miR-195-
ENSG0000	ENSG0000	9.88E-05	hsa-miR-17-5p;hsa-miR-19a-3p;hsa-miR-20b-5p;hsa-miR-93-5
ENSG0000	ENSG0000	1.91E-08	hsa-miR-15a-5p;hsa-miR-15b-5p;hsa-miR-16-5p;hsa-miR-195-
ENSG0000	ENSG0000	0.00866	hsa-miR-103a-3p;hsa-miR-107;hsa-miR-19a-3p;hsa-miR-19b-3
ENSG0000	ENSG0000	5.23E-08	hsa-miR-103a-3p;hsa-miR-107;hsa-miR-15a-5p;hsa-miR-15b-5
ENSG0000	ENSG0000	0.000476	hsa-miR-15a-5p;hsa-miR-15b-5p;hsa-miR-16-5p;hsa-miR-195-
ENSG0000	ENSG0000	1.09E-07	hsa-miR-15a-5p;hsa-miR-15b-5p;hsa-miR-16-5p;hsa-miR-17-5
ENSG0000	ENSG0000	0.000144	hsa-miR-15a-5p;hsa-miR-15b-5p;hsa-miR-16-5p;hsa-miR-195-
ENSG0000	ENSG0000	0.00012	hsa-miR-15a-5p;hsa-miR-15b-5p;hsa-miR-16-5p;hsa-miR-195-
ENSG0000	ENSG0000	9.48E-08	hsa-miR-15a-5p;hsa-miR-16-5p;hsa-miR-17-5p;hsa-miR-195-5
ENSG0000	ENSG0000	9.48E-08	hsa-miR-15a-5p;hsa-miR-16-5p;hsa-miR-17-5p;hsa-miR-195-5
ENSG0000	ENSG0000	0.000609	hsa-miR-15a-5p;hsa-miR-15b-5p;hsa-miR-16-5p;hsa-miR-195-
ENSG0000	ENSG0000	0.000699	hsa-miR-17-5p;hsa-miR-19a-3p;hsa-miR-19b-3p;hsa-miR-20b-
ENSG0000	ENSG0000	0.00596	hsa-miR-15a-5p;hsa-miR-15b-5p;hsa-miR-16-5p;hsa-miR-195-
ENSG0000	ENSG0000	0.001161	hsa-miR-1-3p;hsa-miR-19a-3p;hsa-miR-23a-3p;hsa-miR-23b-3
ENSG0000	ENSG0000	9.48E-08	hsa-miR-15a-5p;hsa-miR-15b-5p;hsa-miR-16-5p;hsa-miR-195-
ENSG0000	ENSG0000	3.84E-06	hsa-miR-103a-3p;hsa-miR-107;hsa-miR-15a-5p;hsa-miR-15b-5
ENSG0000	ENSG0000	5.56E-05	hsa-miR-15a-5p;hsa-miR-15b-5p;hsa-miR-16-5p;hsa-miR-185-
ENSG0000	ENSG0000	0.000423	hsa-miR-135a-5p;hsa-miR-15a-5p;hsa-miR-15b-5p;hsa-miR-19
ENSG0000	ENSG0000	1.89E-09	hsa-miR-149-5p;hsa-miR-15a-5p;hsa-miR-15b-5p;hsa-miR-16-
ENSG0000	ENSG0000	8.95E-09	hsa-miR-16-5p;hsa-miR-17-5p;hsa-miR-19a-3p;hsa-miR-19b-3
ENSG0000	ENSG0000	6.58E-07	hsa-miR-15a-5p;hsa-miR-15b-5p;hsa-miR-16-5p;hsa-miR-17-5
ENSG0000	ENSG0000	9.88E-05	hsa-miR-138-5p;hsa-miR-27a-3p;hsa-miR-27b-3p;hsa-miR-39
ENSG0000	ENSG0000	0.00012	hsa-miR-15a-5p;hsa-miR-15b-5p;hsa-miR-16-5p;hsa-miR-195-
ENSG0000	ENSG0000	0.003416	hsa-miR-15a-5p;hsa-miR-15b-5p;hsa-miR-16-5p;hsa-miR-195-

ENSG0000	ENSG0000	0.001161	hsa-miR-17-5p;hsa-miR-185-5p;hsa-miR-20b-5p;hsa-miR-93-
ENSG0000	ENSG0000	3.68E-05	hsa-miR-17-5p;hsa-miR-19a-3p;hsa-miR-19b-3p;hsa-miR-20b-
ENSG0000	ENSG0000	0.006833	hsa-miR-15a-5p;hsa-miR-15b-5p;hsa-miR-16-5p;hsa-miR-195-
ENSG0000	ENSG0000	0.000684	hsa-miR-15a-5p;hsa-miR-15b-5p;hsa-miR-16-5p;hsa-miR-195-
ENSG0000	ENSG0000	1.91E-08	hsa-miR-15a-5p;hsa-miR-15b-5p;hsa-miR-16-5p;hsa-miR-195-
ENSG0000	ENSG0000	1.88E-07	hsa-miR-15a-5p;hsa-miR-15b-5p;hsa-miR-16-5p;hsa-miR-17-
ENSG0000	ENSG0000	0.004032	hsa-miR-17-5p;hsa-miR-19a-3p;hsa-miR-19b-3p;hsa-miR-20b-
ENSG0000	ENSG0000	0.002878	hsa-miR-15a-5p;hsa-miR-15b-5p;hsa-miR-16-5p;hsa-miR-195-
ENSG0000	ENSG0000	0.002455	hsa-miR-103a-3p;hsa-miR-107;hsa-miR-15a-5p;hsa-miR-15b-
ENSG0000	ENSG0000	0.000275	hsa-miR-19a-3p;hsa-miR-19b-3p;hsa-miR-27a-3p;hsa-miR-27
ENSG0000	ENSG0000	0.001051	hsa-miR-15a-5p;hsa-miR-15b-5p;hsa-miR-195-5p;hsa-miR-9-
ENSG0000	ENSG0000	1.25E-05	hsa-miR-132-3p;hsa-miR-15a-5p;hsa-miR-15b-5p;hsa-miR-16-
ENSG0000	ENSG0000	0.000684	hsa-miR-15a-5p;hsa-miR-15b-5p;hsa-miR-16-5p;hsa-miR-195-
ENSG0000	ENSG0000	1.91E-08	hsa-miR-15a-5p;hsa-miR-15b-5p;hsa-miR-16-5p;hsa-miR-195-
ENSG0000	ENSG0000	0.000178	hsa-miR-15a-5p;hsa-miR-15b-5p;hsa-miR-16-5p;hsa-miR-195-
ENSG0000	ENSG0000	0.000202	hsa-miR-15a-5p;hsa-miR-15b-5p;hsa-miR-16-5p;hsa-miR-195-
ENSG0000	ENSG0000	7.72E-08	hsa-miR-15a-5p;hsa-miR-15b-5p;hsa-miR-16-5p;hsa-miR-17-
ENSG0000	ENSG0000	0.005082	hsa-miR-17-5p;hsa-miR-19a-3p;hsa-miR-19b-3p;hsa-miR-20b-
ENSG0000	ENSG0000	5.33E-06	hsa-miR-103a-3p;hsa-miR-107;hsa-miR-15a-5p;hsa-miR-15b-
ENSG0000	ENSG0000	0.005071	hsa-miR-17-5p;hsa-miR-19a-3p;hsa-miR-19b-3p;hsa-miR-20b-
ENSG0000	ENSG0000	0.004117	hsa-miR-15a-5p;hsa-miR-15b-5p;hsa-miR-16-5p;hsa-miR-195-
ENSG0000	ENSG0000	0.005702	hsa-miR-143-3p;hsa-miR-17-5p;hsa-miR-20b-5p;hsa-miR-26a-
ENSG0000	ENSG0000	0.005702	hsa-miR-143-3p;hsa-miR-17-5p;hsa-miR-20b-5p;hsa-miR-26a-
ENSG0000	ENSG0000	0.000116	hsa-miR-138-5p;hsa-miR-143-3p;hsa-miR-17-5p;hsa-miR-20b-
ENSG0000	ENSG0000	0.001714	hsa-miR-17-5p;hsa-miR-19a-3p;hsa-miR-19b-3p;hsa-miR-20b-
ENSG0000	ENSG0000	0.000476	hsa-miR-107;hsa-miR-15a-5p;hsa-miR-15b-5p;hsa-miR-195-5
ENSG0000	ENSG0000	2.92E-06	hsa-miR-103a-3p;hsa-miR-107;hsa-miR-15a-5p;hsa-miR-15b-
ENSG0000	ENSG0000	0.000121	hsa-miR-15a-5p;hsa-miR-15b-5p;hsa-miR-16-5p;hsa-miR-17-
ENSG0000	ENSG0000	0.000275	hsa-miR-15a-5p;hsa-miR-15b-5p;hsa-miR-16-5p;hsa-miR-195-
ENSG0000	ENSG0000	0.000178	hsa-miR-1-3p;hsa-miR-15a-5p;hsa-miR-15b-5p;hsa-miR-16-5
ENSG0000	ENSG0000	0.001467	hsa-miR-126-5p;hsa-miR-17-5p;hsa-miR-20b-5p;hsa-miR-27a-
ENSG0000	ENSG0000	2.37E-05	hsa-miR-1-3p;hsa-miR-17-5p;hsa-miR-20b-5p;hsa-miR-377-3
ENSG0000	ENSG0000	0.000573	hsa-miR-103a-3p;hsa-miR-107;hsa-miR-15a-5p;hsa-miR-15b-
ENSG0000	ENSG0000	0.000949	hsa-miR-17-5p;hsa-miR-20b-5p;hsa-miR-23a-3p;hsa-miR-93-
ENSG0000	ENSG0000	0.000949	hsa-miR-17-5p;hsa-miR-20b-5p;hsa-miR-23a-3p;hsa-miR-93-
ENSG0000	ENSG0000	0.000949	hsa-miR-17-5p;hsa-miR-20b-5p;hsa-miR-23a-3p;hsa-miR-93-
ENSG0000	ENSG0000	0.000949	hsa-miR-17-5p;hsa-miR-20b-5p;hsa-miR-23a-3p;hsa-miR-93-
ENSG0000	ENSG0000	0.000949	hsa-miR-17-5p;hsa-miR-20b-5p;hsa-miR-23a-3p;hsa-miR-93-
ENSG0000	ENSG0000	0.000949	hsa-miR-17-5p;hsa-miR-20b-5p;hsa-miR-23a-3p;hsa-miR-93-
ENSG0000	ENSG0000	0.000949	hsa-miR-17-5p;hsa-miR-20b-5p;hsa-miR-23a-3p;hsa-miR-93-
ENSG0000	ENSG0000	8.29E-06	hsa-miR-126-5p;hsa-miR-132-3p;hsa-miR-15a-5p;hsa-miR-15
ENSG0000	ENSG0000	0.007633	hsa-miR-15a-5p;hsa-miR-15b-5p;hsa-miR-16-5p;hsa-miR-195-
ENSG0000	ENSG0000	0.003173	hsa-miR-126-5p;hsa-miR-17-5p;hsa-miR-20b-5p;hsa-miR-27a-
ENSG0000	ENSG0000	0.00015	hsa-miR-15a-5p;hsa-miR-15b-5p;hsa-miR-16-5p;hsa-miR-17-
ENSG0000	ENSG0000	0.004193	hsa-miR-26a-5p;hsa-miR-27a-3p;hsa-miR-27b-3p;hsa-miR-37
ENSG0000	ENSG0000	0.000171	hsa-miR-143-3p;hsa-miR-15a-5p;hsa-miR-16-5p;hsa-miR-195-
ENSG0000	ENSG0000	0.001279	hsa-miR-1-3p;hsa-miR-17-5p;hsa-miR-20b-5p;hsa-miR-93-5p
ENSG0000	ENSG0000	6.72E-07	hsa-miR-103a-3p;hsa-miR-107;hsa-miR-15a-5p;hsa-miR-15b-
ENSG0000	ENSG0000	0.00022	hsa-miR-15a-5p;hsa-miR-15b-5p;hsa-miR-16-5p;hsa-miR-195-
ENSG0000	ENSG0000	0.007225	hsa-miR-17-5p;hsa-miR-20b-5p;hsa-miR-34a-5p;hsa-miR-93-
ENSG0000	ENSG0000	0.007225	hsa-miR-17-5p;hsa-miR-20b-5p;hsa-miR-34a-5p;hsa-miR-93-
ENSG0000	ENSG0000	4.70E-06	hsa-miR-17-5p;hsa-miR-20b-5p;hsa-miR-27a-3p;hsa-miR-27b-
ENSG0000	ENSG0000	0.000573	hsa-miR-15a-5p;hsa-miR-15b-5p;hsa-miR-17-5p;hsa-miR-195-
ENSG0000	ENSG0000	0.003036	hsa-miR-17-5p;hsa-miR-19a-3p;hsa-miR-19b-3p;hsa-miR-20b-

ENSG0000	ENSG0000	0.00016	hsa-miR-17-5p;hsa-miR-20b-5p;hsa-miR-27a-3p;hsa-miR-27b-
ENSG0000	ENSG0000	1.30E-06	hsa-miR-15a-5p;hsa-miR-15b-5p;hsa-miR-16-5p;hsa-miR-17-5
ENSG0000	ENSG0000	8.07E-05	hsa-miR-15a-5p;hsa-miR-15b-5p;hsa-miR-16-5p;hsa-miR-195-
ENSG0000	ENSG0000	0.003416	hsa-miR-17-5p;hsa-miR-20b-5p;hsa-miR-7-5p;hsa-miR-93-5p
ENSG0000	ENSG0000	0.00596	hsa-miR-17-5p;hsa-miR-20b-5p;hsa-miR-27a-3p;hsa-miR-27b-
ENSG0000	ENSG0000	0.00526	hsa-miR-15a-5p;hsa-miR-15b-5p;hsa-miR-16-5p;hsa-miR-195-
ENSG0000	ENSG0000	0.009006	hsa-miR-143-3p;hsa-miR-17-5p;hsa-miR-20b-5p;hsa-miR-26a-
ENSG0000	ENSG0000	0.007209	hsa-miR-143-3p;hsa-miR-149-5p;hsa-miR-17-5p;hsa-miR-20b-
ENSG0000	ENSG0000	0.006225	hsa-miR-17-5p;hsa-miR-20b-5p;hsa-miR-27a-3p;hsa-miR-27b-
ENSG0000	ENSG0000	8.84E-06	hsa-miR-15a-5p;hsa-miR-15b-5p;hsa-miR-16-5p;hsa-miR-195-
ENSG0000	ENSG0000	0.007225	hsa-miR-15a-5p;hsa-miR-15b-5p;hsa-miR-16-5p;hsa-miR-195-
ENSG0000	ENSG0000	0.001279	hsa-miR-17-5p;hsa-miR-20b-5p;hsa-miR-26a-5p;hsa-miR-93-5
ENSG0000	ENSG0000	0.000573	hsa-miR-15a-5p;hsa-miR-15b-5p;hsa-miR-16-5p;hsa-miR-195-
ENSG0000	ENSG0000	0.000318	hsa-miR-15a-5p;hsa-miR-15b-5p;hsa-miR-16-5p;hsa-miR-195-
ENSG0000	ENSG0000	3.14E-05	hsa-miR-15a-5p;hsa-miR-15b-5p;hsa-miR-16-5p;hsa-miR-195-
ENSG0000	ENSG0000	0.00258	hsa-miR-17-5p;hsa-miR-20b-5p;hsa-miR-23a-3p;hsa-miR-23b-
ENSG0000	ENSG0000	2.74E-06	hsa-miR-126-5p;hsa-miR-15a-5p;hsa-miR-15b-5p;hsa-miR-16-
ENSG0000	ENSG0000	0.00318	hsa-miR-15a-5p;hsa-miR-15b-5p;hsa-miR-16-5p;hsa-miR-195-
ENSG0000	ENSG0000	7.43E-05	hsa-miR-17-5p;hsa-miR-19a-3p;hsa-miR-19b-3p;hsa-miR-20b-
ENSG0000	ENSG0000	6.51E-05	hsa-miR-15a-5p;hsa-miR-15b-5p;hsa-miR-16-5p;hsa-miR-195-
ENSG0000	ENSG0000	6.51E-05	hsa-miR-15a-5p;hsa-miR-15b-5p;hsa-miR-16-5p;hsa-miR-195-
ENSG0000	ENSG0000	6.51E-05	hsa-miR-15a-5p;hsa-miR-15b-5p;hsa-miR-16-5p;hsa-miR-195-
ENSG0000	ENSG0000	6.51E-05	hsa-miR-15a-5p;hsa-miR-15b-5p;hsa-miR-16-5p;hsa-miR-195-
ENSG0000	ENSG0000	3.69E-05	hsa-miR-107;hsa-miR-15a-5p;hsa-miR-15b-5p;hsa-miR-17-5p;
ENSG0000	ENSG0000	3.69E-05	hsa-miR-107;hsa-miR-15a-5p;hsa-miR-15b-5p;hsa-miR-17-5p;
ENSG0000	ENSG0000	0.000445	hsa-miR-103a-3p;hsa-miR-107;hsa-miR-15a-5p;hsa-miR-15b-5
ENSG0000	ENSG0000	0.000418	hsa-miR-19a-3p;hsa-miR-19b-3p;hsa-miR-23a-3p;hsa-miR-23b
ENSG0000	ENSG0000	3.97E-07	hsa-miR-15a-5p;hsa-miR-15b-5p;hsa-miR-16-5p;hsa-miR-17-5
ENSG0000	ENSG0000	8.85E-05	hsa-miR-17-5p;hsa-miR-19a-3p;hsa-miR-19b-3p;hsa-miR-20b-
ENSG0000	ENSG0000	2.16E-12	hsa-miR-15a-5p;hsa-miR-15b-5p;hsa-miR-16-5p;hsa-miR-195-
ENSG0000	ENSG0000	5.18E-05	hsa-miR-19a-3p;hsa-miR-19b-3p;hsa-miR-27a-3p;hsa-miR-27b
ENSG0000	ENSG0000	0.00016	hsa-miR-17-5p;hsa-miR-19a-3p;hsa-miR-19b-3p;hsa-miR-20b-
ENSG0000	ENSG0000	0.00235	hsa-miR-17-5p;hsa-miR-20b-5p;hsa-miR-24-3p;hsa-miR-93-5p
ENSG0000	ENSG0000	1.91E-08	hsa-miR-15a-5p;hsa-miR-15b-5p;hsa-miR-16-5p;hsa-miR-195-
ENSG0000	ENSG0000	0.000465	hsa-miR-149-5p;hsa-miR-17-5p;hsa-miR-20b-5p;hsa-miR-24-3
ENSG0000	ENSG0000	0.000949	hsa-miR-15a-5p;hsa-miR-15b-5p;hsa-miR-16-5p;hsa-miR-195-
ENSG0000	ENSG0000	0.000949	hsa-miR-15a-5p;hsa-miR-15b-5p;hsa-miR-16-5p;hsa-miR-195-
ENSG0000	ENSG0000	0.000949	hsa-miR-15a-5p;hsa-miR-15b-5p;hsa-miR-16-5p;hsa-miR-195-
ENSG0000	ENSG0000	0.000949	hsa-miR-15a-5p;hsa-miR-15b-5p;hsa-miR-16-5p;hsa-miR-195-
ENSG0000	ENSG0000	0.000949	hsa-miR-15a-5p;hsa-miR-15b-5p;hsa-miR-16-5p;hsa-miR-195-
ENSG0000	ENSG0000	0.000949	hsa-miR-15a-5p;hsa-miR-15b-5p;hsa-miR-16-5p;hsa-miR-195-
ENSG0000	ENSG0000	0.000949	hsa-miR-15a-5p;hsa-miR-15b-5p;hsa-miR-16-5p;hsa-miR-195-
ENSG0000	ENSG0000	0.000949	hsa-miR-15a-5p;hsa-miR-15b-5p;hsa-miR-16-5p;hsa-miR-195-
ENSG0000	ENSG0000	0.000949	hsa-miR-15a-5p;hsa-miR-15b-5p;hsa-miR-16-5p;hsa-miR-195-
ENSG0000	ENSG0000	0.000949	hsa-miR-15a-5p;hsa-miR-15b-5p;hsa-miR-16-5p;hsa-miR-195-
ENSG0000	ENSG0000	1.29E-06	hsa-miR-15a-5p;hsa-miR-15b-5p;hsa-miR-16-5p;hsa-miR-195-
ENSG0000	ENSG0000	0.00012	hsa-miR-15a-5p;hsa-miR-15b-5p;hsa-miR-16-5p;hsa-miR-195-
ENSG0000	ENSG0000	0.004772	hsa-miR-19a-3p;hsa-miR-19b-3p;hsa-miR-20b-5p;hsa-miR-93-
ENSG0000	ENSG0000	0.000949	hsa-miR-15a-5p;hsa-miR-15b-5p;hsa-miR-16-5p;hsa-miR-195-
ENSG0000	ENSG0000	7.17E-06	hsa-miR-103a-3p;hsa-miR-107;hsa-miR-15a-5p;hsa-miR-15b-5
ENSG0000	ENSG0000	1.52E-05	hsa-miR-15a-5p;hsa-miR-15b-5p;hsa-miR-16-5p;hsa-miR-195-
ENSG0000	ENSG0000	0.000699	hsa-miR-132-3p;hsa-miR-17-5p;hsa-miR-20b-5p;hsa-miR-377-
ENSG0000	ENSG0000	0.000699	hsa-miR-132-3p;hsa-miR-17-5p;hsa-miR-20b-5p;hsa-miR-377-
ENSG0000	ENSG0000	4.52E-06	hsa-miR-103a-3p;hsa-miR-107;hsa-miR-15a-5p;hsa-miR-15b-5

ENSG0000	ENSG0000	0.003663	hsa-miR-15a-5p;hsa-miR-15b-5p;hsa-miR-16-5p;hsa-miR-195-
ENSG0000	ENSG0000	1.99E-05	hsa-miR-15a-5p;hsa-miR-15b-5p;hsa-miR-195-5p;hsa-miR-27a
ENSG0000	ENSG0000	0.00258	hsa-miR-149-5p;hsa-miR-15a-5p;hsa-miR-15b-5p;hsa-miR-16-
ENSG0000	ENSG0000	0.002956	hsa-miR-15a-5p;hsa-miR-15b-5p;hsa-miR-16-5p;hsa-miR-195-
ENSG0000	ENSG0000	0.00258	hsa-miR-17-5p;hsa-miR-20b-5p;hsa-miR-23a-3p;hsa-miR-23b-
ENSG0000	ENSG0000	0.008946	hsa-miR-17-5p;hsa-miR-20b-5p;hsa-miR-369-3p;hsa-miR-93-5
ENSG0000	ENSG0000	0.000684	hsa-miR-15a-5p;hsa-miR-15b-5p;hsa-miR-16-5p;hsa-miR-195-
ENSG0000	ENSG0000	0.006833	hsa-miR-15a-5p;hsa-miR-15b-5p;hsa-miR-16-5p;hsa-miR-195-
ENSG0000	ENSG0000	0.000999	hsa-miR-15a-5p;hsa-miR-15b-5p;hsa-miR-16-5p;hsa-miR-195-
ENSG0000	ENSG0000	0.001418	hsa-miR-103a-3p;hsa-miR-107;hsa-miR-23a-3p;hsa-miR-23b-3
ENSG0000	ENSG0000	0.000765	hsa-miR-23a-3p;hsa-miR-23b-3p;hsa-miR-27a-3p;hsa-miR-27b
ENSG0000	ENSG0000	0.001467	hsa-miR-17-5p;hsa-miR-20b-5p;hsa-miR-27a-3p;hsa-miR-27b-
ENSG0000	ENSG0000	6.51E-05	hsa-miR-15a-5p;hsa-miR-15b-5p;hsa-miR-16-5p;hsa-miR-195-
ENSG0000	ENSG0000	0.000186	hsa-miR-15a-5p;hsa-miR-15b-5p;hsa-miR-16-5p;hsa-miR-17-5
ENSG0000	ENSG0000	2.81E-06	hsa-miR-103a-3p;hsa-miR-107;hsa-miR-15a-5p;hsa-miR-15b-5
ENSG0000	ENSG0000	0.000476	hsa-miR-15a-5p;hsa-miR-15b-5p;hsa-miR-195-5p;hsa-miR-19a
ENSG0000	ENSG0000	2.37E-05	hsa-miR-15a-5p;hsa-miR-15b-5p;hsa-miR-16-5p;hsa-miR-195-
ENSG0000	ENSG0000	5.33E-07	hsa-miR-103a-3p;hsa-miR-107;hsa-miR-15a-5p;hsa-miR-15b-5
ENSG0000	ENSG0000	2.73E-06	hsa-miR-15a-5p;hsa-miR-15b-5p;hsa-miR-16-5p;hsa-miR-195-
ENSG0000	ENSG0000	1.43E-05	hsa-miR-17-5p;hsa-miR-19a-3p;hsa-miR-19b-3p;hsa-miR-20b-
ENSG0000	ENSG0000	6.67E-06	hsa-miR-17-5p;hsa-miR-19a-3p;hsa-miR-19b-3p;hsa-miR-20b-
ENSG0000	ENSG0000	1.13E-05	hsa-miR-15a-5p;hsa-miR-15b-5p;hsa-miR-16-5p;hsa-miR-195-
ENSG0000	ENSG0000	9.48E-08	hsa-miR-15a-5p;hsa-miR-15b-5p;hsa-miR-16-5p;hsa-miR-195-
ENSG0000	ENSG0000	0.001144	hsa-miR-103a-3p;hsa-miR-126-5p;hsa-miR-17-5p;hsa-miR-19a
ENSG0000	ENSG0000	3.24E-06	hsa-miR-15a-5p;hsa-miR-15b-5p;hsa-miR-16-5p;hsa-miR-195-
ENSG0000	ENSG0000	2.54E-07	hsa-miR-15a-5p;hsa-miR-15b-5p;hsa-miR-16-5p;hsa-miR-195-
ENSG0000	ENSG0000	0.001328	hsa-miR-15a-5p;hsa-miR-15b-5p;hsa-miR-195-5p;hsa-miR-369
ENSG0000	ENSG0000	0.001328	hsa-miR-15a-5p;hsa-miR-15b-5p;hsa-miR-195-5p;hsa-miR-369
ENSG0000	ENSG0000	0.001328	hsa-miR-15a-5p;hsa-miR-15b-5p;hsa-miR-195-5p;hsa-miR-369
ENSG0000	ENSG0000	0.001328	hsa-miR-15a-5p;hsa-miR-15b-5p;hsa-miR-195-5p;hsa-miR-369
ENSG0000	ENSG0000	0.001328	hsa-miR-15a-5p;hsa-miR-15b-5p;hsa-miR-195-5p;hsa-miR-369
ENSG0000	ENSG0000	0.001328	hsa-miR-15a-5p;hsa-miR-15b-5p;hsa-miR-195-5p;hsa-miR-369
ENSG0000	ENSG0000	0.001328	hsa-miR-15a-5p;hsa-miR-15b-5p;hsa-miR-195-5p;hsa-miR-369
ENSG0000	ENSG0000	0.001328	hsa-miR-15a-5p;hsa-miR-15b-5p;hsa-miR-195-5p;hsa-miR-369
ENSG0000	ENSG0000	2.34E-05	hsa-miR-103a-3p;hsa-miR-107;hsa-miR-15a-5p;hsa-miR-15b-5
ENSG0000	ENSG0000	1.40E-07	hsa-miR-103a-3p;hsa-miR-107;hsa-miR-15a-5p;hsa-miR-15b-5
ENSG0000	ENSG0000	0.002878	hsa-miR-135a-5p;hsa-miR-17-5p;hsa-miR-185-5p;hsa-miR-20b
ENSG0000	ENSG0000	2.37E-05	hsa-miR-15a-5p;hsa-miR-15b-5p;hsa-miR-16-5p;hsa-miR-195-
ENSG0000	ENSG0000	8.19E-06	hsa-miR-103a-3p;hsa-miR-107;hsa-miR-1-3p;hsa-miR-17-5p;h
ENSG0000	ENSG0000	0.004476	hsa-miR-15a-5p;hsa-miR-15b-5p;hsa-miR-16-5p;hsa-miR-195-
ENSG0000	ENSG0000	0.000366	hsa-miR-126-5p;hsa-miR-19a-3p;hsa-miR-19b-3p;hsa-miR-37
ENSG0000	ENSG0000	8.07E-05	hsa-miR-15a-5p;hsa-miR-15b-5p;hsa-miR-16-5p;hsa-miR-195-
ENSG0000	ENSG0000	8.07E-05	hsa-miR-15a-5p;hsa-miR-15b-5p;hsa-miR-16-5p;hsa-miR-195-
ENSG0000	ENSG0000	0.003416	hsa-miR-17-5p;hsa-miR-20b-5p;hsa-miR-34a-5p;hsa-miR-93-5
ENSG0000	ENSG0000	0.007372	hsa-miR-15a-5p;hsa-miR-15b-5p;hsa-miR-16-5p;hsa-miR-195-
ENSG0000	ENSG0000	0.001997	hsa-miR-17-5p;hsa-miR-20b-5p;hsa-miR-27a-3p;hsa-miR-27b-
ENSG0000	ENSG0000	0.000876	hsa-miR-17-5p;hsa-miR-19a-3p;hsa-miR-19b-3p;hsa-miR-20b-
ENSG0000	ENSG0000	1.99E-05	hsa-miR-15a-5p;hsa-miR-15b-5p;hsa-miR-16-5p;hsa-miR-195-
ENSG0000	ENSG0000	0.002349	hsa-miR-17-5p;hsa-miR-19a-3p;hsa-miR-19b-3p;hsa-miR-20b-
ENSG0000	ENSG0000	1.59E-08	hsa-miR-15a-5p;hsa-miR-15b-5p;hsa-miR-16-5p;hsa-miR-195-
ENSG0000	ENSG0000	8.84E-06	hsa-miR-15a-5p;hsa-miR-15b-5p;hsa-miR-16-5p;hsa-miR-195-
ENSG0000	ENSG0000	0.000127	hsa-miR-103a-3p;hsa-miR-107;hsa-miR-15a-5p;hsa-miR-15b-5
ENSG0000	ENSG0000	0.001051	hsa-miR-17-5p;hsa-miR-20b-5p;hsa-miR-369-3p;hsa-miR-93-5
ENSG0000	ENSG0000	2.37E-05	hsa-miR-15a-5p;hsa-miR-15b-5p;hsa-miR-16-5p;hsa-miR-195-

ENSG0000	ENSG0000	2.37E-05	hsa-miR-103a-3p;hsa-miR-107;hsa-miR-19a-3p;hsa-miR-19b-3
ENSG0000	ENSG0000	7.78E-05	hsa-miR-17-5p;hsa-miR-20b-5p;hsa-miR-27a-3p;hsa-miR-27b-
ENSG0000	ENSG0000	3.69E-05	hsa-miR-103a-3p;hsa-miR-107;hsa-miR-15a-5p;hsa-miR-15b-5
ENSG0000	ENSG0000	0.002956	hsa-miR-15a-5p;hsa-miR-15b-5p;hsa-miR-16-5p;hsa-miR-195-
ENSG0000	ENSG0000	0.000476	hsa-miR-15a-5p;hsa-miR-15b-5p;hsa-miR-16-5p;hsa-miR-195-
ENSG0000	ENSG0000	0.00609	hsa-miR-17-5p;hsa-miR-20b-5p;hsa-miR-24-3p;hsa-miR-93-5
ENSG0000	ENSG0000	1.73E-06	hsa-miR-15a-5p;hsa-miR-15b-5p;hsa-miR-16-5p;hsa-miR-195-
ENSG0000	ENSG0000	0.000813	hsa-miR-15a-5p;hsa-miR-15b-5p;hsa-miR-16-5p;hsa-miR-195-
ENSG0000	ENSG0000	0.00697	hsa-miR-138-5p;hsa-miR-15a-5p;hsa-miR-15b-5p;hsa-miR-16-
ENSG0000	ENSG0000	4.18E-07	hsa-miR-143-3p;hsa-miR-17-5p;hsa-miR-19a-3p;hsa-miR-19b-
ENSG0000	ENSG0000	1.91E-08	hsa-miR-15a-5p;hsa-miR-15b-5p;hsa-miR-16-5p;hsa-miR-195-
ENSG0000	ENSG0000	0.000236	hsa-miR-15a-5p;hsa-miR-15b-5p;hsa-miR-16-5p;hsa-miR-195-
ENSG0000	ENSG0000	0.006454	hsa-miR-15a-5p;hsa-miR-15b-5p;hsa-miR-16-5p;hsa-miR-195-
ENSG0000	ENSG0000	0.000754	hsa-miR-17-5p;hsa-miR-185-5p;hsa-miR-20b-5p;hsa-miR-432-
ENSG0000	ENSG0000	0.000699	hsa-miR-17-5p;hsa-miR-19a-3p;hsa-miR-19b-3p;hsa-miR-20b-
ENSG0000	ENSG0000	0.000205	hsa-miR-103a-3p;hsa-miR-107;hsa-miR-15a-5p;hsa-miR-15b-5
ENSG0000	ENSG0000	5.08E-05	hsa-miR-15a-5p;hsa-miR-15b-5p;hsa-miR-16-5p;hsa-miR-195-
ENSG0000	ENSG0000	0.000596	hsa-miR-15a-5p;hsa-miR-15b-5p;hsa-miR-16-5p;hsa-miR-195-
ENSG0000	ENSG0000	5.03E-05	hsa-miR-15a-5p;hsa-miR-15b-5p;hsa-miR-16-5p;hsa-miR-195-
ENSG0000	ENSG0000	1.82E-05	hsa-miR-130a-3p;hsa-miR-130b-3p;hsa-miR-301a-3p;hsa-miR-
ENSG0000	ENSG0000	0.000332	hsa-miR-130a-3p;hsa-miR-130b-3p;hsa-miR-301a-3p;hsa-miR-
ENSG0000	ENSG0000	3.04E-05	hsa-miR-130a-3p;hsa-miR-130b-3p;hsa-miR-301a-3p;hsa-miR-
ENSG0000	ENSG0000	2.37E-05	hsa-miR-148a-3p;hsa-miR-148b-3p;hsa-miR-30b-5p;hsa-miR-3
ENSG0000	ENSG0000	7.03E-07	hsa-miR-27a-3p;hsa-miR-27b-3p;hsa-miR-30b-5p;hsa-miR-30
ENSG0000	ENSG0000	1.59E-06	hsa-miR-130a-3p;hsa-miR-130b-3p;hsa-miR-301a-3p;hsa-miR-
ENSG0000	ENSG0000	1.18E-06	hsa-miR-130a-3p;hsa-miR-130b-3p;hsa-miR-148a-3p;hsa-miR-
ENSG0000	ENSG0000	0.000804	hsa-miR-27a-3p;hsa-miR-27b-3p;hsa-miR-30b-5p;hsa-miR-30
ENSG0000	ENSG0000	0.000804	hsa-miR-130a-3p;hsa-miR-130b-3p;hsa-miR-301a-3p;hsa-miR-
ENSG0000	ENSG0000	0.000208	hsa-miR-130a-3p;hsa-miR-130b-3p;hsa-miR-301a-3p;hsa-miR-
ENSG0000	ENSG0000	3.39E-07	hsa-miR-130a-3p;hsa-miR-148a-3p;hsa-miR-148b-3p;hsa-miR-
ENSG0000	ENSG0000	0.001164	hsa-miR-130a-3p;hsa-miR-130b-3p;hsa-miR-301a-3p;hsa-miR-
ENSG0000	ENSG0000	0.000376	hsa-miR-130a-3p;hsa-miR-130b-3p;hsa-miR-301a-3p;hsa-miR-
ENSG0000	ENSG0000	2.08E-05	hsa-miR-130a-3p;hsa-miR-130b-3p;hsa-miR-301a-3p;hsa-miR-
ENSG0000	ENSG0000	0.000132	hsa-miR-130a-3p;hsa-miR-130b-3p;hsa-miR-301a-3p;hsa-miR-
ENSG0000	ENSG0000	6.24E-06	hsa-miR-130a-3p;hsa-miR-130b-3p;hsa-miR-148a-3p;hsa-miR-
ENSG0000	ENSG0000	0.000112	hsa-miR-130a-3p;hsa-miR-130b-3p;hsa-miR-301a-3p;hsa-miR-
ENSG0000	ENSG0000	4.80E-07	hsa-miR-130a-3p;hsa-miR-130b-3p;hsa-miR-148a-3p;hsa-miR-
ENSG0000	ENSG0000	7.85E-05	hsa-miR-130a-3p;hsa-miR-130b-3p;hsa-miR-301a-3p;hsa-miR-
ENSG0000	ENSG0000	2.12E-06	hsa-miR-130a-3p;hsa-miR-130b-3p;hsa-miR-301a-3p;hsa-miR-
ENSG0000	ENSG0000	0.000196	hsa-miR-130a-3p;hsa-miR-148a-3p;hsa-miR-148b-3p;hsa-miR-
ENSG0000	ENSG0000	7.37E-05	hsa-miR-130a-3p;hsa-miR-130b-3p;hsa-miR-301a-3p;hsa-miR-
ENSG0000	ENSG0000	0.000194	hsa-miR-130a-3p;hsa-miR-130b-3p;hsa-miR-301a-3p;hsa-miR-
ENSG0000	ENSG0000	0.000928	hsa-miR-130a-3p;hsa-miR-130b-3p;hsa-miR-301a-3p;hsa-miR-
ENSG0000	ENSG0000	0.000503	hsa-miR-130a-3p;hsa-miR-130b-3p;hsa-miR-301a-3p;hsa-miR-
ENSG0000	ENSG0000	0.000804	hsa-miR-130a-3p;hsa-miR-130b-3p;hsa-miR-301a-3p;hsa-miR-
ENSG0000	ENSG0000	4.29E-05	hsa-miR-130a-3p;hsa-miR-130b-3p;hsa-miR-301a-3p;hsa-miR-
ENSG0000	ENSG0000	0.000143	hsa-miR-130a-3p;hsa-miR-130b-3p;hsa-miR-301a-3p;hsa-miR-
ENSG0000	ENSG0000	0.000167	hsa-miR-130a-3p;hsa-miR-130b-3p;hsa-miR-301a-3p;hsa-miR-
ENSG0000	ENSG0000	2.69E-05	hsa-miR-130a-3p;hsa-miR-130b-3p;hsa-miR-301a-3p;hsa-miR-
ENSG0000	ENSG0000	0.000167	hsa-miR-130a-3p;hsa-miR-130b-3p;hsa-miR-301a-3p;hsa-miR-
ENSG0000	ENSG0000	4.82E-05	hsa-miR-130a-3p;hsa-miR-130b-3p;hsa-miR-301a-3p;hsa-miR-
ENSG0000	ENSG0000	2.37E-05	hsa-miR-130a-3p;hsa-miR-130b-3p;hsa-miR-301a-3p;hsa-miR-
ENSG0000	ENSG0000	2.46E-08	hsa-miR-130a-3p;hsa-miR-130b-3p;hsa-miR-27a-3p;hsa-miR-2
ENSG0000	ENSG0000	1.47E-08	hsa-miR-130a-3p;hsa-miR-130b-3p;hsa-miR-27a-3p;hsa-miR-2

ENSG0000	ENSG0000	0.000592	hsa-miR-130a-3p;hsa-miR-130b-3p;hsa-miR-301a-3p;hsa-miR-
ENSG0000	ENSG0000	0.000312	hsa-miR-130a-3p;hsa-miR-130b-3p;hsa-miR-301a-3p;hsa-miR-
ENSG0000	ENSG0000	1.58E-05	hsa-miR-130a-3p;hsa-miR-130b-3p;hsa-miR-301a-3p;hsa-miR-
ENSG0000	ENSG0000	2.62E-05	hsa-miR-130a-3p;hsa-miR-148a-3p;hsa-miR-148b-3p;hsa-miR-
ENSG0000	ENSG0000	0.000658	hsa-miR-130a-3p;hsa-miR-130b-3p;hsa-miR-301a-3p;hsa-miR-
ENSG0000	ENSG0000	0.000292	hsa-miR-130a-3p;hsa-miR-130b-3p;hsa-miR-301a-3p;hsa-miR-
ENSG0000	ENSG0000	0.000399	hsa-miR-130a-3p;hsa-miR-130b-3p;hsa-miR-301a-3p;hsa-miR-
ENSG0000	ENSG0000	0.000399	hsa-miR-130a-3p;hsa-miR-130b-3p;hsa-miR-301a-3p;hsa-miR-
ENSG0000	ENSG0000	1.71E-06	hsa-miR-130a-3p;hsa-miR-130b-3p;hsa-miR-148a-3p;hsa-miR-
ENSG0000	ENSG0000	8.55E-06	hsa-miR-130a-3p;hsa-miR-130b-3p;hsa-miR-301a-3p;hsa-miR-
ENSG0000	ENSG0000	0.000449	hsa-miR-130a-3p;hsa-miR-130b-3p;hsa-miR-301a-3p;hsa-miR-
ENSG0000	ENSG0000	0.000103	hsa-miR-130a-3p;hsa-miR-130b-3p;hsa-miR-301a-3p;hsa-miR-
ENSG0000	ENSG0000	0.000103	hsa-miR-130a-3p;hsa-miR-130b-3p;hsa-miR-301a-3p;hsa-miR-
ENSG0000	ENSG0000	0.000503	hsa-miR-130a-3p;hsa-miR-130b-3p;hsa-miR-301a-3p;hsa-miR-
ENSG0000	ENSG0000	0.001692	hsa-miR-130a-3p;hsa-miR-130b-3p;hsa-miR-301a-3p;hsa-miR-
ENSG0000	ENSG0000	0.00176	hsa-miR-130a-3p;hsa-miR-130b-3p;hsa-miR-301a-3p;hsa-miR-
ENSG0000	ENSG0000	6.89E-06	hsa-miR-130a-3p;hsa-miR-148a-3p;hsa-miR-148b-3p;hsa-miR-
ENSG0000	ENSG0000	2.08E-05	hsa-miR-130a-3p;hsa-miR-130b-3p;hsa-miR-301a-3p;hsa-miR-
ENSG0000	ENSG0000	0.000122	hsa-miR-130a-3p;hsa-miR-130b-3p;hsa-miR-301a-3p;hsa-miR-
ENSG0000	ENSG0000	3.04E-05	hsa-miR-130a-3p;hsa-miR-130b-3p;hsa-miR-301a-3p;hsa-miR-
ENSG0000	ENSG0000	2.69E-05	hsa-miR-130a-3p;hsa-miR-130b-3p;hsa-miR-301a-3p;hsa-miR-
ENSG0000	ENSG0000	5.87E-05	hsa-miR-130a-3p;hsa-miR-130b-3p;hsa-miR-301a-3p;hsa-miR-
ENSG0000	ENSG0000	0.000728	hsa-miR-130a-3p;hsa-miR-130b-3p;hsa-miR-301a-3p;hsa-miR-
ENSG0000	ENSG0000	0.000531	hsa-miR-130a-3p;hsa-miR-130b-3p;hsa-miR-301a-3p;hsa-miR-
ENSG0000	ENSG0000	0.000235	hsa-miR-130a-3p;hsa-miR-130b-3p;hsa-miR-301a-3p;hsa-miR-
ENSG0000	ENSG0000	5.00E-06	hsa-miR-130a-3p;hsa-miR-130b-3p;hsa-miR-301a-3p;hsa-miR-
ENSG0000	ENSG0000	0.000399	hsa-miR-130a-3p;hsa-miR-130b-3p;hsa-miR-301a-3p;hsa-miR-
ENSG0000	ENSG0000	6.55E-05	hsa-miR-130a-3p;hsa-miR-130b-3p;hsa-miR-301a-3p;hsa-miR-
ENSG0000	ENSG0000	5.00E-06	hsa-miR-130a-3p;hsa-miR-130b-3p;hsa-miR-301a-3p;hsa-miR-
ENSG0000	ENSG0000	0.000423	hsa-miR-130a-3p;hsa-miR-130b-3p;hsa-miR-301a-3p;hsa-miR-
ENSG0000	ENSG0000	6.48E-05	hsa-miR-130a-3p;hsa-miR-130b-3p;hsa-miR-301a-3p;hsa-miR-
ENSG0000	ENSG0000	0.00176	hsa-miR-130a-3p;hsa-miR-29b-2-5p;hsa-miR-301a-3p;hsa-miI
ENSG0000	ENSG0000	1.65E-06	hsa-miR-130a-3p;hsa-miR-130b-3p;hsa-miR-301a-3p;hsa-miR-
ENSG0000	ENSG0000	9.41E-05	hsa-miR-130a-3p;hsa-miR-130b-3p;hsa-miR-301a-3p;hsa-miR-
ENSG0000	ENSG0000	9.41E-05	hsa-miR-130a-3p;hsa-miR-130b-3p;hsa-miR-301a-3p;hsa-miR-
ENSG0000	ENSG0000	0.000239	hsa-miR-130a-3p;hsa-miR-130b-3p;hsa-miR-301a-3p;hsa-miR-
ENSG0000	ENSG0000	0.000658	hsa-miR-130a-3p;hsa-miR-130b-3p;hsa-miR-301a-3p;hsa-miR-
ENSG0000	ENSG0000	6.55E-05	hsa-miR-130a-3p;hsa-miR-130b-3p;hsa-miR-301a-3p;hsa-miR-
ENSG0000	ENSG0000	0.000423	hsa-miR-130a-3p;hsa-miR-130b-3p;hsa-miR-301a-3p;hsa-miR-
ENSG0000	ENSG0000	1.37E-05	hsa-miR-130a-3p;hsa-miR-301a-3p;hsa-miR-301b-3p;hsa-miR-
ENSG0000	ENSG0000	0.00176	hsa-miR-27a-3p;hsa-miR-27b-3p;hsa-miR-30b-5p;hsa-miR-30
ENSG0000	ENSG0000	2.37E-05	hsa-miR-130a-3p;hsa-miR-130b-3p;hsa-miR-301a-3p;hsa-miR-
ENSG0000	ENSG0000	6.48E-05	hsa-miR-130a-3p;hsa-miR-130b-3p;hsa-miR-301a-3p;hsa-miR-
ENSG0000	ENSG0000	2.24E-08	hsa-miR-130a-3p;hsa-miR-130b-3p;hsa-miR-148a-3p;hsa-miR-
ENSG0000	ENSG0000	0.000194	hsa-miR-130a-3p;hsa-miR-130b-3p;hsa-miR-301a-3p;hsa-miR-
ENSG0000	ENSG0000	0.008896	hsa-miR-130a-3p;hsa-miR-130b-3p;hsa-miR-301a-3p;hsa-miR-
ENSG0000	ENSG0000	0.000624	hsa-miR-130a-3p;hsa-miR-130b-3p;hsa-miR-301a-3p;hsa-miR-
ENSG0000	ENSG0000	1.66E-05	hsa-miR-130a-3p;hsa-miR-130b-3p;hsa-miR-301a-3p;hsa-miR-
ENSG0000	ENSG0000	0.000257	hsa-miR-130a-3p;hsa-miR-130b-3p;hsa-miR-301a-3p;hsa-miR-
ENSG0000	ENSG0000	1.37E-05	hsa-miR-130a-3p;hsa-miR-130b-3p;hsa-miR-301a-3p;hsa-miR-
ENSG0000	ENSG0000	7.51E-08	hsa-miR-130a-3p;hsa-miR-130b-3p;hsa-miR-148a-3p;hsa-miR-
ENSG0000	ENSG0000	0.000292	hsa-miR-130a-3p;hsa-miR-130b-3p;hsa-miR-301a-3p;hsa-miR-
ENSG0000	ENSG0000	3.84E-05	hsa-miR-130a-3p;hsa-miR-130b-3p;hsa-miR-301a-3p;hsa-miR-
ENSG0000	ENSG0000	0.002457	hsa-miR-130a-3p;hsa-miR-130b-3p;hsa-miR-301a-3p;hsa-miR-

ENSG0000	ENSG0000	1.37E-05	hsa-miR-130a-3p;hsa-miR-130b-3p;hsa-miR-301a-3p;hsa-miR
ENSG0000	ENSG0000	7.44E-06	hsa-miR-130a-3p;hsa-miR-130b-3p;hsa-miR-301a-3p;hsa-miR
ENSG0000	ENSG0000	0.000844	hsa-miR-29b-2-5p;hsa-miR-30b-5p;hsa-miR-30c-5p;hsa-miR-
ENSG0000	ENSG0000	4.73E-08	hsa-miR-130a-3p;hsa-miR-130b-3p;hsa-miR-148a-3p;hsa-miR
ENSG0000	ENSG0000	0.001065	hsa-miR-130a-3p;hsa-miR-130b-3p;hsa-miR-301a-3p;hsa-miR
ENSG0000	ENSG0000	9.00E-08	hsa-miR-130a-3p;hsa-miR-130b-3p;hsa-miR-301a-3p;hsa-miR
ENSG0000	ENSG0000	0.000155	hsa-miR-130a-3p;hsa-miR-130b-3p;hsa-miR-301a-3p;hsa-miR
ENSG0000	ENSG0000	1.94E-05	hsa-miR-130a-3p;hsa-miR-130b-3p;hsa-miR-301a-3p;hsa-miR
ENSG0000	ENSG0000	1.94E-05	hsa-miR-130a-3p;hsa-miR-130b-3p;hsa-miR-301a-3p;hsa-miR
ENSG0000	ENSG0000	9.74E-05	hsa-miR-130a-3p;hsa-miR-130b-3p;hsa-miR-148a-3p;hsa-miR
ENSG0000	ENSG0000	7.44E-06	hsa-miR-130a-3p;hsa-miR-130b-3p;hsa-miR-148a-3p;hsa-miR
ENSG0000	ENSG0000	3.84E-05	hsa-miR-130a-3p;hsa-miR-130b-3p;hsa-miR-301a-3p;hsa-miR
ENSG0000	ENSG0000	0.000928	hsa-miR-130a-3p;hsa-miR-148b-3p;hsa-miR-301a-3p;hsa-miR
ENSG0000	ENSG0000	3.42E-05	hsa-miR-130a-3p;hsa-miR-130b-3p;hsa-miR-301a-3p;hsa-miR
ENSG0000	ENSG0000	3.42E-05	hsa-miR-130a-3p;hsa-miR-130b-3p;hsa-miR-301a-3p;hsa-miR
ENSG0000	ENSG0000	0.000194	hsa-miR-130a-3p;hsa-miR-130b-3p;hsa-miR-301a-3p;hsa-miR
ENSG0000	ENSG0000	0.000388	hsa-miR-130a-3p;hsa-miR-2277-3p;hsa-miR-301a-3p;hsa-miR
ENSG0000	ENSG0000	1.82E-05	hsa-miR-130a-3p;hsa-miR-130b-3p;hsa-miR-301a-3p;hsa-miR
ENSG0000	ENSG0000	0.000223	hsa-miR-130a-3p;hsa-miR-130b-3p;hsa-miR-301a-3p;hsa-miR
ENSG0000	ENSG0000	8.60E-05	hsa-miR-130a-3p;hsa-miR-130b-3p;hsa-miR-301a-3p;hsa-miR
ENSG0000	ENSG0000	9.50E-06	hsa-miR-130a-3p;hsa-miR-130b-3p;hsa-miR-148a-3p;hsa-miR
ENSG0000	ENSG0000	1.27E-06	hsa-miR-130a-3p;hsa-miR-130b-3p;hsa-miR-301a-3p;hsa-miR
ENSG0000	ENSG0000	0.000531	hsa-miR-130a-3p;hsa-miR-130b-3p;hsa-miR-301a-3p;hsa-miR
ENSG0000	ENSG0000	9.90E-07	hsa-miR-130a-3p;hsa-miR-130b-3p;hsa-miR-29b-2-5p;hsa-mi
ENSG0000	ENSG0000	9.90E-07	hsa-miR-130a-3p;hsa-miR-130b-3p;hsa-miR-29b-2-5p;hsa-mi
ENSG0000	ENSG0000	5.02E-08	hsa-miR-27a-3p;hsa-miR-27b-3p;hsa-miR-30b-5p;hsa-miR-30
ENSG0000	ENSG0000	0.000475	hsa-miR-27a-3p;hsa-miR-27b-3p;hsa-miR-361-5p;hsa-miR-57
ENSG0000	ENSG0000	0.000928	hsa-miR-130a-3p;hsa-miR-130b-3p;hsa-miR-301a-3p;hsa-miR
ENSG0000	ENSG0000	0.000312	hsa-miR-130a-3p;hsa-miR-130b-3p;hsa-miR-301a-3p;hsa-miR
ENSG0000	ENSG0000	0.003132	hsa-miR-2277-3p;hsa-miR-27a-3p;hsa-miR-27b-3p;hsa-miR-3
ENSG0000	ENSG0000	0.000179	hsa-miR-130a-3p;hsa-miR-130b-3p;hsa-miR-148a-3p;hsa-miR
ENSG0000	ENSG0000	0.000179	hsa-miR-130a-3p;hsa-miR-130b-3p;hsa-miR-148a-3p;hsa-miR
ENSG0000	ENSG0000	9.23E-05	hsa-miR-130a-3p;hsa-miR-130b-3p;hsa-miR-301a-3p;hsa-miR
ENSG0000	ENSG0000	0.003112	hsa-miR-130a-3p;hsa-miR-130b-3p;hsa-miR-301a-3p;hsa-miR
ENSG0000	ENSG0000	0.001018	hsa-miR-130a-3p;hsa-miR-130b-3p;hsa-miR-301a-3p;hsa-miR
ENSG0000	ENSG0000	0.001018	hsa-miR-130a-3p;hsa-miR-130b-3p;hsa-miR-301a-3p;hsa-miR
ENSG0000	ENSG0000	0.001018	hsa-miR-130a-3p;hsa-miR-130b-3p;hsa-miR-301a-3p;hsa-miR
ENSG0000	ENSG0000	0.001018	hsa-miR-130a-3p;hsa-miR-130b-3p;hsa-miR-301a-3p;hsa-miR
ENSG0000	ENSG0000	0.000471	hsa-miR-130a-3p;hsa-miR-130b-3p;hsa-miR-301a-3p;hsa-miR
ENSG0000	ENSG0000	2.11E-06	hsa-miR-130a-3p;hsa-miR-130b-3p;hsa-miR-27a-3p;hsa-miR-
ENSG0000	ENSG0000	6.99E-06	hsa-miR-130a-3p;hsa-miR-130b-3p;hsa-miR-148a-3p;hsa-miR
ENSG0000	ENSG0000	1.88E-07	hsa-miR-130a-3p;hsa-miR-130b-3p;hsa-miR-27a-3p;hsa-miR-
ENSG0000	ENSG0000	6.24E-06	hsa-miR-130a-3p;hsa-miR-148b-3p;hsa-miR-301a-3p;hsa-miR
ENSG0000	ENSG0000	1.66E-05	hsa-miR-148a-3p;hsa-miR-148b-3p;hsa-miR-27a-3p;hsa-miR-
ENSG0000	ENSG0000	1.13E-05	hsa-miR-130a-3p;hsa-miR-148a-3p;hsa-miR-148b-3p;hsa-miR
ENSG0000	ENSG0000	6.19E-10	hsa-miR-130a-3p;hsa-miR-130b-3p;hsa-miR-148a-3p;hsa-miR
ENSG0000	ENSG0000	0.000312	hsa-miR-130a-3p;hsa-miR-130b-3p;hsa-miR-301a-3p;hsa-miR
ENSG0000	ENSG0000	0.000503	hsa-miR-130a-3p;hsa-miR-130b-3p;hsa-miR-301a-3p;hsa-miR
ENSG0000	ENSG0000	5.65E-06	hsa-miR-130a-3p;hsa-miR-148a-3p;hsa-miR-148b-3p;hsa-miR
ENSG0000	ENSG0000	4.10E-06	hsa-miR-130a-3p;hsa-miR-130b-3p;hsa-miR-301a-3p;hsa-miR
ENSG0000	ENSG0000	2.38E-07	hsa-miR-130a-3p;hsa-miR-130b-3p;hsa-miR-148a-3p;hsa-miR
ENSG0000	ENSG0000	1.18E-05	hsa-miR-130a-3p;hsa-miR-148b-3p;hsa-miR-301a-3p;hsa-miR
ENSG0000	ENSG0000	5.30E-05	hsa-miR-130a-3p;hsa-miR-130b-3p;hsa-miR-301a-3p;hsa-miR
ENSG0000	ENSG0000	0.001501	hsa-miR-130a-3p;hsa-miR-130b-3p;hsa-miR-301a-3p;hsa-miR

ENSG0000	ENSG0000	0.002817	hsa-miR-130a-3p;hsa-miR-130b-3p;hsa-miR-301a-3p;hsa-miR-
ENSG0000	ENSG0000	0.000132	hsa-miR-130a-3p;hsa-miR-130b-3p;hsa-miR-301a-3p;hsa-miR-
ENSG0000	ENSG0000	8.60E-05	hsa-miR-130a-3p;hsa-miR-130b-3p;hsa-miR-301a-3p;hsa-miR-
ENSG0000	ENSG0000	8.60E-05	hsa-miR-130a-3p;hsa-miR-130b-3p;hsa-miR-301a-3p;hsa-miR-
ENSG0000	ENSG0000	8.60E-05	hsa-miR-130a-3p;hsa-miR-130b-3p;hsa-miR-301a-3p;hsa-miR-
ENSG0000	ENSG0000	0.000103	hsa-miR-130a-3p;hsa-miR-130b-3p;hsa-miR-301a-3p;hsa-miR-
ENSG0000	ENSG0000	2.69E-05	hsa-miR-130a-3p;hsa-miR-130b-3p;hsa-miR-301a-3p;hsa-miR-
ENSG0000	ENSG0000	1.18E-06	hsa-miR-130a-3p;hsa-miR-130b-3p;hsa-miR-301a-3p;hsa-miR-
ENSG0000	ENSG0000	0.001829	hsa-miR-130a-3p;hsa-miR-130b-3p;hsa-miR-301a-3p;hsa-miR-
ENSG0000	ENSG0000	4.29E-05	hsa-miR-130a-3p;hsa-miR-130b-3p;hsa-miR-301a-3p;hsa-miR-
ENSG0000	ENSG0000	0.000449	hsa-miR-130a-3p;hsa-miR-130b-3p;hsa-miR-301a-3p;hsa-miR-
ENSG0000	ENSG0000	0.000624	hsa-miR-130a-3p;hsa-miR-130b-3p;hsa-miR-301a-3p;hsa-miR-
ENSG0000	ENSG0000	0.002369	hsa-miR-130a-3p;hsa-miR-301a-3p;hsa-miR-301b-3p;hsa-miR-
ENSG0000	ENSG0000	0.001325	hsa-miR-130a-3p;hsa-miR-130b-3p;hsa-miR-301a-3p;hsa-miR-
ENSG0000	ENSG0000	5.87E-05	hsa-miR-130a-3p;hsa-miR-130b-3p;hsa-miR-301a-3p;hsa-miR-
ENSG0000	ENSG0000	1.65E-06	hsa-miR-130a-3p;hsa-miR-130b-3p;hsa-miR-301a-3p;hsa-miR-
ENSG0000	ENSG0000	5.00E-06	hsa-miR-130a-3p;hsa-miR-130b-3p;hsa-miR-301a-3p;hsa-miR-
ENSG0000	ENSG0000	0.000388	hsa-miR-130a-3p;hsa-miR-130b-3p;hsa-miR-301a-3p;hsa-miR-
ENSG0000	ENSG0000	7.14E-05	hsa-miR-130a-3p;hsa-miR-130b-3p;hsa-miR-301a-3p;hsa-miR-
ENSG0000	ENSG0000	0.003539	hsa-miR-130a-3p;hsa-miR-130b-3p;hsa-miR-301a-3p;hsa-miR-
ENSG0000	ENSG0000	9.55E-07	hsa-miR-130a-3p;hsa-miR-130b-3p;hsa-miR-301a-3p;hsa-miR-
ENSG0000	ENSG0000	2.08E-11	hsa-miR-130a-3p;hsa-miR-130b-3p;hsa-miR-148a-3p;hsa-miR-
ENSG0000	ENSG0000	5.07E-07	hsa-miR-130a-3p;hsa-miR-130b-3p;hsa-miR-148a-3p;hsa-miR-
ENSG0000	ENSG0000	5.87E-05	hsa-miR-130a-3p;hsa-miR-130b-3p;hsa-miR-301a-3p;hsa-miR-
ENSG0000	ENSG0000	5.87E-05	hsa-miR-130a-3p;hsa-miR-130b-3p;hsa-miR-301a-3p;hsa-miR-
ENSG0000	ENSG0000	0.000196	hsa-miR-130a-3p;hsa-miR-130b-3p;hsa-miR-301a-3p;hsa-miR-
ENSG0000	ENSG0000	6.17E-05	hsa-miR-148a-3p;hsa-miR-148b-3p;hsa-miR-27a-3p;hsa-miR-2
ENSG0000	ENSG0000	0.000354	hsa-miR-130a-3p;hsa-miR-130b-3p;hsa-miR-301a-3p;hsa-miR-
ENSG0000	ENSG0000	0.000354	hsa-miR-130a-3p;hsa-miR-130b-3p;hsa-miR-301a-3p;hsa-miR-
ENSG0000	ENSG0000	0.000132	hsa-miR-130a-3p;hsa-miR-130b-3p;hsa-miR-301a-3p;hsa-miR-
ENSG0000	ENSG0000	5.87E-05	hsa-miR-130a-3p;hsa-miR-130b-3p;hsa-miR-301a-3p;hsa-miR-
ENSG0000	ENSG0000	9.74E-05	hsa-miR-130a-3p;hsa-miR-130b-3p;hsa-miR-301a-3p;hsa-miR-
ENSG0000	ENSG0000	0.001563	hsa-miR-130a-3p;hsa-miR-130b-3p;hsa-miR-301a-3p;hsa-miR-
ENSG0000	ENSG0000	7.03E-07	hsa-miR-130a-3p;hsa-miR-130b-3p;hsa-miR-301a-3p;hsa-miR-
ENSG0000	ENSG0000	7.85E-06	hsa-miR-130a-3p;hsa-miR-130b-3p;hsa-miR-148a-3p;hsa-miR-
ENSG0000	ENSG0000	3.97E-05	hsa-miR-130a-3p;hsa-miR-130b-3p;hsa-miR-301a-3p;hsa-miR-
ENSG0000	ENSG0000	3.42E-05	hsa-miR-130a-3p;hsa-miR-130b-3p;hsa-miR-301a-3p;hsa-miR-
ENSG0000	ENSG0000	0.002126	hsa-miR-130a-3p;hsa-miR-130b-3p;hsa-miR-301a-3p;hsa-miR-
ENSG0000	ENSG0000	0.000208	hsa-miR-130a-3p;hsa-miR-130b-3p;hsa-miR-301a-3p;hsa-miR-
ENSG0000	ENSG0000	1.80E-05	hsa-miR-130a-3p;hsa-miR-130b-3p;hsa-miR-301a-3p;hsa-miR-
ENSG0000	ENSG0000	6.48E-05	hsa-miR-130a-3p;hsa-miR-130b-3p;hsa-miR-301a-3p;hsa-miR-
ENSG0000	ENSG0000	1.94E-05	hsa-miR-130a-3p;hsa-miR-130b-3p;hsa-miR-301a-3p;hsa-miR-
ENSG0000	ENSG0000	0.000292	hsa-miR-130a-3p;hsa-miR-130b-3p;hsa-miR-301a-3p;hsa-miR-
ENSG0000	ENSG0000	0.000561	hsa-miR-130a-3p;hsa-miR-130b-3p;hsa-miR-301a-3p;hsa-miR-
ENSG0000	ENSG0000	0.000114	hsa-miR-130a-3p;hsa-miR-130b-3p;hsa-miR-301a-3p;hsa-miR-
ENSG0000	ENSG0000	0.000304	hsa-miR-130a-3p;hsa-miR-130b-3p;hsa-miR-301a-3p;hsa-miR-
ENSG0000	ENSG0000	0.000103	hsa-miR-130a-3p;hsa-miR-130b-3p;hsa-miR-301a-3p;hsa-miR-
ENSG0000	ENSG0000	0.000194	hsa-miR-130a-3p;hsa-miR-130b-3p;hsa-miR-301a-3p;hsa-miR-
ENSG0000	ENSG0000	0.000658	hsa-miR-130a-3p;hsa-miR-130b-3p;hsa-miR-301a-3p;hsa-miR-
ENSG0000	ENSG0000	0.000194	hsa-miR-130a-3p;hsa-miR-130b-3p;hsa-miR-301a-3p;hsa-miR-
ENSG0000	ENSG0000	0.005961	hsa-miR-130a-3p;hsa-miR-130b-3p;hsa-miR-301a-3p;hsa-miR-
ENSG0000	ENSG0000	0.000608	hsa-miR-130a-3p;hsa-miR-130b-3p;hsa-miR-301a-3p;hsa-miR-
ENSG0000	ENSG0000	0.001692	hsa-miR-130a-3p;hsa-miR-130b-3p;hsa-miR-301a-3p;hsa-miR-
ENSG0000	ENSG0000	7.03E-07	hsa-miR-130a-3p;hsa-miR-130b-3p;hsa-miR-301a-3p;hsa-miR-

ENSG0000	ENSG0000	0.000531	hsa-miR-130a-3p;hsa-miR-130b-3p;hsa-miR-301a-3p;hsa-miR-
ENSG0000	ENSG0000	0.000256	hsa-miR-130a-3p;hsa-miR-130b-3p;hsa-miR-301a-3p;hsa-miR-
ENSG0000	ENSG0000	0.005961	hsa-miR-130a-3p;hsa-miR-130b-3p;hsa-miR-301a-3p;hsa-miR-
ENSG0000	ENSG0000	0.000624	hsa-miR-130a-3p;hsa-miR-130b-3p;hsa-miR-301a-3p;hsa-miR-
ENSG0000	ENSG0000	0.002369	hsa-miR-130a-3p;hsa-miR-130b-3p;hsa-miR-301a-3p;hsa-miR-
ENSG0000	ENSG0000	2.67E-07	hsa-miR-130a-3p;hsa-miR-130b-3p;hsa-miR-148a-3p;hsa-miR-
ENSG0000	ENSG0000	0.000928	hsa-miR-130a-3p;hsa-miR-130b-3p;hsa-miR-301a-3p;hsa-miR-
ENSG0000	ENSG0000	0.002542	hsa-miR-27a-3p;hsa-miR-27b-3p;hsa-miR-30b-5p;hsa-miR-30
ENSG0000	ENSG0000	9.80E-08	hsa-miR-27a-3p;hsa-miR-27b-3p;hsa-miR-30b-5p;hsa-miR-30
ENSG0000	ENSG0000	9.80E-08	hsa-miR-27a-3p;hsa-miR-27b-3p;hsa-miR-30b-5p;hsa-miR-30
ENSG0000	ENSG0000	0.000132	hsa-miR-130a-3p;hsa-miR-130b-3p;hsa-miR-301a-3p;hsa-miR-
ENSG0000	ENSG0000	3.04E-05	hsa-miR-130a-3p;hsa-miR-130b-3p;hsa-miR-301a-3p;hsa-miR-
ENSG0000	ENSG0000	3.39E-05	hsa-miR-130a-3p;hsa-miR-130b-3p;hsa-miR-27a-3p;hsa-miR-2
ENSG0000	ENSG0000	1.18E-05	hsa-miR-130a-3p;hsa-miR-130b-3p;hsa-miR-301a-3p;hsa-miR-
ENSG0000	ENSG0000	0.000208	hsa-miR-27a-3p;hsa-miR-27b-3p;hsa-miR-30b-5p;hsa-miR-30
ENSG0000	ENSG0000	2.12E-06	hsa-miR-27a-3p;hsa-miR-27b-3p;hsa-miR-30b-5p;hsa-miR-30
ENSG0000	ENSG0000	5.61E-07	hsa-miR-130a-3p;hsa-miR-130b-3p;hsa-miR-148a-3p;hsa-miR-
ENSG0000	ENSG0000	7.80E-05	hsa-miR-130a-3p;hsa-miR-130b-3p;hsa-miR-301a-3p;hsa-miR-
ENSG0000	ENSG0000	1.01E-05	hsa-miR-130a-3p;hsa-miR-148b-3p;hsa-miR-301a-3p;hsa-miR-
ENSG0000	ENSG0000	0.000112	hsa-miR-130a-3p;hsa-miR-29b-2-5p;hsa-miR-301a-3p;hsa-miR
ENSG0000	ENSG0000	0.000268	hsa-miR-130a-3p;hsa-miR-130b-3p;hsa-miR-301a-3p;hsa-miR-
ENSG0000	ENSG0000	6.95E-05	hsa-miR-130a-3p;hsa-miR-130b-3p;hsa-miR-301a-3p;hsa-miR-
ENSG0000	ENSG0000	0.000728	hsa-miR-130a-3p;hsa-miR-130b-3p;hsa-miR-301a-3p;hsa-miR-
ENSG0000	ENSG0000	0.005497	hsa-miR-130a-3p;hsa-miR-130b-3p;hsa-miR-301a-3p;hsa-miR-
ENSG0000	ENSG0000	6.05E-05	hsa-miR-130a-3p;hsa-miR-301a-3p;hsa-miR-301b-3p;hsa-miR-
ENSG0000	ENSG0000	2.69E-05	hsa-miR-130a-3p;hsa-miR-130b-3p;hsa-miR-301a-3p;hsa-miR-
ENSG0000	ENSG0000	0.000143	hsa-miR-148a-3p;hsa-miR-148b-3p;hsa-miR-30b-5p;hsa-miR-3
ENSG0000	ENSG0000	0.000223	hsa-miR-130a-3p;hsa-miR-130b-3p;hsa-miR-301a-3p;hsa-miR-
ENSG0000	ENSG0000	7.51E-07	hsa-miR-130a-3p;hsa-miR-130b-3p;hsa-miR-148a-3p;hsa-miR-
ENSG0000	ENSG0000	0.000256	hsa-miR-130a-3p;hsa-miR-130b-3p;hsa-miR-301a-3p;hsa-miR-
ENSG0000	ENSG0000	2.44E-05	hsa-miR-130a-3p;hsa-miR-130b-3p;hsa-miR-301a-3p;hsa-miR-
ENSG0000	ENSG0000	1.01E-05	hsa-miR-130a-3p;hsa-miR-130b-3p;hsa-miR-301a-3p;hsa-miR-
ENSG0000	ENSG0000	1.01E-05	hsa-miR-130a-3p;hsa-miR-130b-3p;hsa-miR-301a-3p;hsa-miR-
ENSG0000	ENSG0000	8.48E-10	hsa-miR-130a-3p;hsa-miR-130b-3p;hsa-miR-148a-3p;hsa-miR-
ENSG0000	ENSG0000	0.000312	hsa-miR-130a-3p;hsa-miR-130b-3p;hsa-miR-301a-3p;hsa-miR-
ENSG0000	ENSG0000	3.40E-08	hsa-miR-130a-3p;hsa-miR-130b-3p;hsa-miR-148a-3p;hsa-miR-
ENSG0000	ENSG0000	0.00127	hsa-miR-130a-3p;hsa-miR-130b-3p;hsa-miR-301a-3p;hsa-miR-
ENSG0000	ENSG0000	1.82E-05	hsa-miR-130a-3p;hsa-miR-130b-3p;hsa-miR-301a-3p;hsa-miR-
ENSG0000	ENSG0000	1.82E-05	hsa-miR-130a-3p;hsa-miR-130b-3p;hsa-miR-301a-3p;hsa-miR-
ENSG0000	ENSG0000	5.30E-05	hsa-miR-130a-3p;hsa-miR-130b-3p;hsa-miR-301a-3p;hsa-miR-
ENSG0000	ENSG0000	1.58E-05	hsa-miR-130a-3p;hsa-miR-130b-3p;hsa-miR-301a-3p;hsa-miR-
ENSG0000	ENSG0000	1.82E-05	hsa-miR-130a-3p;hsa-miR-130b-3p;hsa-miR-301a-3p;hsa-miR-
ENSG0000	ENSG0000	0.000223	hsa-miR-130a-3p;hsa-miR-130b-3p;hsa-miR-301a-3p;hsa-miR-
ENSG0000	ENSG0000	6.48E-05	hsa-miR-130a-3p;hsa-miR-130b-3p;hsa-miR-301a-3p;hsa-miR-
ENSG0000	ENSG0000	8.55E-06	hsa-miR-130a-3p;hsa-miR-130b-3p;hsa-miR-301a-3p;hsa-miR-
ENSG0000	ENSG0000	0.001501	hsa-miR-130a-3p;hsa-miR-130b-3p;hsa-miR-301a-3p;hsa-miR-
ENSG0000	ENSG0000	5.87E-05	hsa-miR-130a-3p;hsa-miR-130b-3p;hsa-miR-301a-3p;hsa-miR-
ENSG0000	ENSG0000	1.18E-05	hsa-miR-130a-3p;hsa-miR-130b-3p;hsa-miR-301a-3p;hsa-miR-
ENSG0000	ENSG0000	0.000208	hsa-miR-130a-3p;hsa-miR-130b-3p;hsa-miR-301a-3p;hsa-miR-
ENSG0000	ENSG0000	4.13E-07	hsa-miR-130a-3p;hsa-miR-130b-3p;hsa-miR-148a-3p;hsa-miR-
ENSG0000	ENSG0000	3.84E-05	hsa-miR-130a-3p;hsa-miR-130b-3p;hsa-miR-301a-3p;hsa-miR-
ENSG0000	ENSG0000	0.002126	hsa-miR-130a-3p;hsa-miR-130b-3p;hsa-miR-301a-3p;hsa-miR-
ENSG0000	ENSG0000	0.000239	hsa-miR-130a-3p;hsa-miR-130b-3p;hsa-miR-301a-3p;hsa-miR-
ENSG0000	ENSG0000	1.58E-05	hsa-miR-130a-3p;hsa-miR-130b-3p;hsa-miR-301a-3p;hsa-miR-

ENSG0000	ENSG0000	1.58E-05	hsa-miR-130a-3p;hsa-miR-130b-3p;hsa-miR-301a-3p;hsa-miR-
ENSG0000	ENSG0000	0.001749	hsa-miR-130a-3p;hsa-miR-29b-2-5p;hsa-miR-301a-3p;hsa-miR
ENSG0000	ENSG0000	2.67E-06	hsa-miR-130a-3p;hsa-miR-130b-3p;hsa-miR-301a-3p;hsa-miR-
ENSG0000	ENSG0000	2.10E-05	hsa-miR-130a-3p;hsa-miR-130b-3p;hsa-miR-301a-3p;hsa-miR-
ENSG0000	ENSG0000	1.82E-05	hsa-miR-130a-3p;hsa-miR-130b-3p;hsa-miR-301a-3p;hsa-miR-
ENSG0000	ENSG0000	0.000132	hsa-miR-130a-3p;hsa-miR-130b-3p;hsa-miR-301a-3p;hsa-miR-
ENSG0000	ENSG0000	0.00028	hsa-miR-130a-3p;hsa-miR-130b-3p;hsa-miR-301a-3p;hsa-miR-
ENSG0000	ENSG0000	1.14E-09	hsa-miR-130a-3p;hsa-miR-130b-3p;hsa-miR-148a-3p;hsa-miR-
ENSG0000	ENSG0000	0.000354	hsa-miR-130a-3p;hsa-miR-130b-3p;hsa-miR-301a-3p;hsa-miR-
ENSG0000	ENSG0000	0.000844	hsa-miR-130a-3p;hsa-miR-130b-3p;hsa-miR-301a-3p;hsa-miR-
ENSG0000	ENSG0000	0.000239	hsa-miR-130a-3p;hsa-miR-130b-3p;hsa-miR-301a-3p;hsa-miR-
ENSG0000	ENSG0000	0.000658	hsa-miR-130a-3p;hsa-miR-130b-3p;hsa-miR-301a-3p;hsa-miR-
ENSG0000	ENSG0000	0.000103	hsa-miR-130a-3p;hsa-miR-130b-3p;hsa-miR-301a-3p;hsa-miR-
ENSG0000	ENSG0000	0.008689	hsa-miR-130a-3p;hsa-miR-130b-3p;hsa-miR-301a-3p;hsa-miR-
ENSG0000	ENSG0000	3.70E-06	hsa-miR-130a-3p;hsa-miR-130b-3p;hsa-miR-27b-3p;hsa-miR-3
ENSG0000	ENSG0000	0.002048	hsa-miR-130a-3p;hsa-miR-130b-3p;hsa-miR-301a-3p;hsa-miR-
ENSG0000	ENSG0000	0.006122	hsa-miR-130a-3p;hsa-miR-130b-3p;hsa-miR-301a-3p;hsa-miR-
ENSG0000	ENSG0000	0.000304	hsa-miR-130a-3p;hsa-miR-130b-3p;hsa-miR-148a-3p;hsa-miR-
ENSG0000	ENSG0000	0.000194	hsa-miR-130a-3p;hsa-miR-130b-3p;hsa-miR-301a-3p;hsa-miR-
ENSG0000	ENSG0000	0.000423	hsa-miR-130a-3p;hsa-miR-130b-3p;hsa-miR-301a-3p;hsa-miR-
ENSG0000	ENSG0000	0.000223	hsa-miR-130a-3p;hsa-miR-130b-3p;hsa-miR-301a-3p;hsa-miR-
ENSG0000	ENSG0000	5.02E-08	hsa-miR-27a-3p;hsa-miR-27b-3p;hsa-miR-30b-5p;hsa-miR-30
ENSG0000	ENSG0000	5.02E-08	hsa-miR-27a-3p;hsa-miR-27b-3p;hsa-miR-30b-5p;hsa-miR-30
ENSG0000	ENSG0000	0.000354	hsa-miR-130a-3p;hsa-miR-130b-3p;hsa-miR-301a-3p;hsa-miR-
ENSG0000	ENSG0000	0.000728	hsa-miR-130a-3p;hsa-miR-130b-3p;hsa-miR-301a-3p;hsa-miR-
ENSG0000	ENSG0000	4.58E-07	hsa-miR-130a-3p;hsa-miR-130b-3p;hsa-miR-148a-3p;hsa-miR-
ENSG0000	ENSG0000	0.000274	hsa-miR-130a-3p;hsa-miR-130b-3p;hsa-miR-301a-3p;hsa-miR-
ENSG0000	ENSG0000	0.000449	hsa-miR-130a-3p;hsa-miR-130b-3p;hsa-miR-301a-3p;hsa-miR-
ENSG0000	ENSG0000	0.001382	hsa-miR-130a-3p;hsa-miR-130b-3p;hsa-miR-301a-3p;hsa-miR-
ENSG0000	ENSG0000	1.37E-05	hsa-miR-130a-3p;hsa-miR-130b-3p;hsa-miR-301a-3p;hsa-miR-
ENSG0000	ENSG0000	0.000728	hsa-miR-130a-3p;hsa-miR-301a-3p;hsa-miR-301b-3p;hsa-miR-
ENSG0000	ENSG0000	2.67E-06	hsa-miR-130a-3p;hsa-miR-130b-3p;hsa-miR-301a-3p;hsa-miR-
ENSG0000	ENSG0000	0.000256	hsa-miR-130a-3p;hsa-miR-130b-3p;hsa-miR-301a-3p;hsa-miR-
ENSG0000	ENSG0000	0.000122	hsa-miR-130a-3p;hsa-miR-130b-3p;hsa-miR-301a-3p;hsa-miR-
ENSG0000	ENSG0000	1.37E-05	hsa-miR-130a-3p;hsa-miR-130b-3p;hsa-miR-301a-3p;hsa-miR-
ENSG0000	ENSG0000	1.37E-05	hsa-miR-130a-3p;hsa-miR-130b-3p;hsa-miR-301a-3p;hsa-miR-
ENSG0000	ENSG0000	1.37E-05	hsa-miR-130a-3p;hsa-miR-130b-3p;hsa-miR-301a-3p;hsa-miR-
ENSG0000	ENSG0000	1.37E-05	hsa-miR-130a-3p;hsa-miR-130b-3p;hsa-miR-301a-3p;hsa-miR-
ENSG0000	ENSG0000	1.18E-05	hsa-miR-130a-3p;hsa-miR-130b-3p;hsa-miR-301a-3p;hsa-miR-
ENSG0000	ENSG0000	0.000682	hsa-miR-125b-2-3p;hsa-miR-301a-5p;hsa-miR-362-3p;hsa-miR
ENSG0000	ENSG0000	0.001411	hsa-miR-125b-2-3p;hsa-miR-18a-3p;hsa-miR-1913;hsa-miR-32
ENSG0000	ENSG0000	0.000474	hsa-miR-18a-3p;hsa-miR-1913;hsa-miR-324-3p;hsa-miR-362-3
ENSG0000	ENSG0000	0.000807	hsa-miR-125b-2-3p;hsa-miR-18a-3p;hsa-miR-1913;hsa-miR-32
ENSG0000	ENSG0000	0.000178	hsa-miR-18a-3p;hsa-miR-1913;hsa-miR-324-3p;hsa-miR-362-3
ENSG0000	ENSG0000	0.000137	hsa-miR-18a-3p;hsa-miR-1913;hsa-miR-324-3p;hsa-miR-362-3
ENSG0000	ENSG0000	0.000193	hsa-miR-18a-3p;hsa-miR-1913;hsa-miR-324-3p;hsa-miR-577
ENSG0000	ENSG0000	0.00034	hsa-miR-18a-3p;hsa-miR-1913;hsa-miR-324-3p;hsa-miR-624-3
ENSG0000	ENSG0000	0.000294	hsa-miR-1913;hsa-miR-21-3p;hsa-miR-324-3p;hsa-miR-362-3p
ENSG0000	ENSG0000	9.37E-06	hsa-miR-125b-2-3p;hsa-miR-18a-3p;hsa-miR-1913;hsa-miR-32
ENSG0000	ENSG0000	1.24E-12	hsa-let-7a-5p;hsa-let-7b-5p;hsa-let-7e-5p;hsa-let-7f-5p;hsa-le
ENSG0000	ENSG0000	1.63E-09	hsa-let-7a-5p;hsa-let-7b-5p;hsa-let-7e-5p;hsa-let-7f-5p;hsa-le
ENSG0000	ENSG0000	1.23E-09	hsa-let-7a-5p;hsa-let-7b-5p;hsa-let-7e-5p;hsa-let-7f-5p;hsa-le
ENSG0000	ENSG0000	8.64E-11	hsa-let-7a-5p;hsa-let-7b-5p;hsa-let-7e-5p;hsa-let-7f-5p;hsa-le
ENSG0000	ENSG0000	8.68E-08	hsa-let-7a-5p;hsa-let-7b-5p;hsa-let-7e-5p;hsa-let-7f-5p;hsa-le

ENSG0000	ENSG0000	4.18E-09	hsa-let-7a-5p;hsa-let-7b-5p;hsa-let-7e-5p;hsa-let-7f-5p;hsa-le
ENSG0000	ENSG0000	2.86E-05	hsa-let-7a-5p;hsa-let-7b-5p;hsa-let-7e-5p;hsa-let-7g-5p;hsa-l
ENSG0000	ENSG0000	5.38E-11	hsa-let-7a-5p;hsa-let-7b-5p;hsa-let-7e-5p;hsa-let-7f-5p;hsa-le
ENSG0000	ENSG0000	3.77E-10	hsa-let-7a-5p;hsa-let-7b-5p;hsa-let-7e-5p;hsa-let-7f-5p;hsa-le
ENSG0000	ENSG0000	3.34E-10	hsa-let-7a-5p;hsa-let-7b-5p;hsa-let-7e-5p;hsa-let-7f-5p;hsa-le
ENSG0000	ENSG0000	5.38E-11	hsa-let-7a-5p;hsa-let-7b-5p;hsa-let-7e-5p;hsa-let-7f-5p;hsa-le
ENSG0000	ENSG0000	4.34E-07	hsa-let-7a-5p;hsa-let-7b-5p;hsa-let-7e-5p;hsa-let-7f-5p;hsa-le
ENSG0000	ENSG0000	3.81E-12	hsa-let-7a-5p;hsa-let-7b-5p;hsa-let-7e-5p;hsa-let-7f-5p;hsa-le
ENSG0000	ENSG0000	7.18E-10	hsa-let-7a-5p;hsa-let-7e-5p;hsa-let-7f-5p;hsa-let-7g-5p;hsa-le
ENSG0000	ENSG0000	1.77E-10	hsa-let-7a-5p;hsa-let-7b-5p;hsa-let-7e-5p;hsa-let-7f-5p;hsa-le
ENSG0000	ENSG0000	1.01E-09	hsa-let-7a-5p;hsa-let-7b-5p;hsa-let-7e-5p;hsa-let-7f-5p;hsa-le
ENSG0000	ENSG0000	5.89E-09	hsa-let-7a-5p;hsa-let-7e-5p;hsa-let-7f-5p;hsa-let-7g-5p;hsa-le
ENSG0000	ENSG0000	2.93E-12	hsa-let-7a-5p;hsa-let-7b-5p;hsa-let-7e-5p;hsa-let-7f-5p;hsa-le
ENSG0000	ENSG0000	8.62E-13	hsa-let-7a-5p;hsa-let-7b-5p;hsa-let-7e-5p;hsa-let-7f-5p;hsa-le
ENSG0000	ENSG0000	4.07E-07	hsa-let-7a-5p;hsa-let-7e-5p;hsa-let-7f-5p;hsa-let-7g-5p;hsa-le
ENSG0000	ENSG0000	4.24E-10	hsa-let-7a-5p;hsa-let-7b-5p;hsa-let-7e-5p;hsa-let-7f-5p;hsa-le
ENSG0000	ENSG0000	1.23E-09	hsa-let-7a-5p;hsa-let-7b-5p;hsa-let-7e-5p;hsa-let-7f-5p;hsa-le
ENSG0000	ENSG0000	6.24E-12	hsa-let-7a-5p;hsa-let-7b-5p;hsa-let-7e-5p;hsa-let-7f-5p;hsa-le
ENSG0000	ENSG0000	1.23E-08	hsa-let-7a-5p;hsa-let-7b-5p;hsa-let-7e-5p;hsa-let-7f-5p;hsa-le
ENSG0000	ENSG0000	1.16E-08	hsa-let-7a-5p;hsa-let-7b-5p;hsa-let-7e-5p;hsa-let-7f-5p;hsa-le
ENSG0000	ENSG0000	3.35E-07	hsa-let-7a-5p;hsa-let-7b-5p;hsa-let-7e-5p;hsa-let-7f-5p;hsa-le
ENSG0000	ENSG0000	1.54E-10	hsa-let-7a-5p;hsa-let-7b-5p;hsa-let-7e-5p;hsa-let-7f-5p;hsa-le
ENSG0000	ENSG0000	2.14E-09	hsa-let-7a-5p;hsa-let-7b-5p;hsa-let-7e-5p;hsa-let-7f-5p;hsa-le
ENSG0000	ENSG0000	2.58E-06	hsa-let-7a-5p;hsa-let-7e-5p;hsa-let-7f-5p;hsa-let-7g-5p;hsa-le
ENSG0000	ENSG0000	9.21E-09	hsa-let-7a-5p;hsa-let-7b-5p;hsa-let-7f-5p;hsa-let-7g-5p;hsa-le
ENSG0000	ENSG0000	2.85E-14	hsa-let-7a-5p;hsa-let-7b-5p;hsa-let-7e-5p;hsa-let-7f-5p;hsa-le
ENSG0000	ENSG0000	2.45E-08	hsa-let-7a-5p;hsa-let-7b-5p;hsa-let-7e-5p;hsa-let-7f-5p;hsa-le
ENSG0000	ENSG0000	9.42E-09	hsa-let-7a-5p;hsa-let-7b-5p;hsa-let-7e-5p;hsa-let-7f-5p;hsa-le
ENSG0000	ENSG0000	9.82E-12	hsa-let-7a-5p;hsa-let-7b-5p;hsa-let-7e-5p;hsa-let-7f-5p;hsa-le
ENSG0000	ENSG0000	8.92E-11	hsa-let-7a-5p;hsa-let-7b-5p;hsa-let-7e-5p;hsa-let-7f-5p;hsa-le
ENSG0000	ENSG0000	1.16E-10	hsa-let-7a-5p;hsa-let-7b-5p;hsa-let-7e-5p;hsa-let-7f-5p;hsa-le
ENSG0000	ENSG0000	1.77E-10	hsa-let-7a-5p;hsa-let-7b-5p;hsa-let-7e-5p;hsa-let-7f-5p;hsa-le
ENSG0000	ENSG0000	6.43E-13	hsa-let-7a-5p;hsa-let-7b-5p;hsa-let-7e-5p;hsa-let-7f-5p;hsa-le
ENSG0000	ENSG0000	4.51E-13	hsa-let-7a-5p;hsa-let-7b-5p;hsa-let-7e-5p;hsa-let-7f-5p;hsa-le
ENSG0000	ENSG0000	2.93E-12	hsa-let-7a-5p;hsa-let-7b-5p;hsa-let-7e-5p;hsa-let-7f-5p;hsa-le
ENSG0000	ENSG0000	5.27E-06	hsa-let-7a-5p;hsa-let-7b-5p;hsa-let-7e-5p;hsa-let-7g-5p;hsa-l
ENSG0000	ENSG0000	5.38E-11	hsa-let-7a-5p;hsa-let-7b-5p;hsa-let-7e-5p;hsa-let-7f-5p;hsa-le
ENSG0000	ENSG0000	1.32E-08	hsa-let-7a-5p;hsa-let-7b-5p;hsa-let-7e-5p;hsa-let-7f-5p;hsa-le
ENSG0000	ENSG0000	3.81E-12	hsa-let-7a-5p;hsa-let-7b-5p;hsa-let-7e-5p;hsa-let-7f-5p;hsa-le
ENSG0000	ENSG0000	3.81E-12	hsa-let-7a-5p;hsa-let-7b-5p;hsa-let-7e-5p;hsa-let-7f-5p;hsa-le
ENSG0000	ENSG0000	3.81E-12	hsa-let-7a-5p;hsa-let-7b-5p;hsa-let-7e-5p;hsa-let-7f-5p;hsa-le
ENSG0000	ENSG0000	1.22E-11	hsa-let-7a-5p;hsa-let-7b-5p;hsa-let-7e-5p;hsa-let-7f-5p;hsa-le
ENSG0000	ENSG0000	1.24E-12	hsa-let-7a-5p;hsa-let-7b-5p;hsa-let-7e-5p;hsa-let-7f-5p;hsa-le
ENSG0000	ENSG0000	1.24E-12	hsa-let-7a-5p;hsa-let-7b-5p;hsa-let-7e-5p;hsa-let-7f-5p;hsa-le
ENSG0000	ENSG0000	1.24E-12	hsa-let-7a-5p;hsa-let-7b-5p;hsa-let-7e-5p;hsa-let-7f-5p;hsa-le
ENSG0000	ENSG0000	1.24E-12	hsa-let-7a-5p;hsa-let-7b-5p;hsa-let-7e-5p;hsa-let-7f-5p;hsa-le
ENSG0000	ENSG0000	1.24E-12	hsa-let-7a-5p;hsa-let-7b-5p;hsa-let-7e-5p;hsa-let-7f-5p;hsa-le
ENSG0000	ENSG0000	1.24E-12	hsa-let-7a-5p;hsa-let-7b-5p;hsa-let-7e-5p;hsa-let-7f-5p;hsa-le
ENSG0000	ENSG0000	1.24E-12	hsa-let-7a-5p;hsa-let-7b-5p;hsa-let-7e-5p;hsa-let-7f-5p;hsa-le
ENSG0000	ENSG0000	1.85E-07	hsa-let-7a-5p;hsa-let-7b-5p;hsa-let-7e-5p;hsa-let-7g-5p;hsa-l
ENSG0000	ENSG0000	2.29E-05	hsa-let-7a-5p;hsa-let-7e-5p;hsa-let-7f-5p;hsa-let-7g-5p;hsa-le
ENSG0000	ENSG0000	1.35E-09	hsa-let-7a-5p;hsa-let-7b-5p;hsa-let-7e-5p;hsa-let-7f-5p;hsa-le
ENSG0000	ENSG0000	1.11E-09	hsa-let-7a-5p;hsa-let-7b-5p;hsa-let-7e-5p;hsa-let-7f-5p;hsa-le
ENSG0000	ENSG0000	3.56E-09	hsa-let-7a-5p;hsa-let-7b-5p;hsa-let-7e-5p;hsa-let-7f-5p;hsa-le

ENSG0000	ENSG0000	1.77E-10	hsa-let-7a-5p;hsa-let-7b-5p;hsa-let-7e-5p;hsa-let-7f-5p;hsa-le
ENSG0000	ENSG0000	8.79E-09	hsa-let-7a-5p;hsa-let-7b-5p;hsa-let-7e-5p;hsa-let-7f-5p;hsa-le
ENSG0000	ENSG0000	1.01E-09	hsa-let-7a-5p;hsa-let-7b-5p;hsa-let-7e-5p;hsa-let-7f-5p;hsa-le
ENSG0000	ENSG0000	1.33E-06	hsa-let-7a-5p;hsa-let-7f-5p;hsa-let-7g-5p;hsa-let-7i-5p
ENSG0000	ENSG0000	5.69E-09	hsa-let-7a-5p;hsa-let-7b-5p;hsa-let-7e-5p;hsa-let-7f-5p;hsa-le
ENSG0000	ENSG0000	3.84E-11	hsa-let-7a-5p;hsa-let-7b-5p;hsa-let-7e-5p;hsa-let-7f-5p;hsa-le
ENSG0000	ENSG0000	1.60E-08	hsa-let-7a-5p;hsa-let-7b-5p;hsa-let-7e-5p;hsa-let-7f-5p;hsa-le
ENSG0000	ENSG0000	9.09E-10	hsa-let-7a-5p;hsa-let-7b-5p;hsa-let-7e-5p;hsa-let-7f-5p;hsa-le
ENSG0000	ENSG0000	9.06E-10	hsa-let-7a-5p;hsa-let-7b-5p;hsa-let-7e-5p;hsa-let-7f-5p;hsa-le
ENSG0000	ENSG0000	7.27E-08	hsa-let-7a-5p;hsa-let-7e-5p;hsa-let-7f-5p;hsa-let-7g-5p;hsa-le
ENSG0000	ENSG0000	5.13E-06	hsa-let-7a-5p;hsa-let-7b-5p;hsa-let-7e-5p;hsa-let-7f-5p;hsa-le
ENSG0000	ENSG0000	5.13E-06	hsa-let-7a-5p;hsa-let-7b-5p;hsa-let-7e-5p;hsa-let-7f-5p;hsa-le
ENSG0000	ENSG0000	9.09E-10	hsa-let-7a-5p;hsa-let-7b-5p;hsa-let-7e-5p;hsa-let-7f-5p;hsa-le
ENSG0000	ENSG0000	1.35E-09	hsa-let-7a-5p;hsa-let-7b-5p;hsa-let-7e-5p;hsa-let-7f-5p;hsa-le
ENSG0000	ENSG0000	1.54E-10	hsa-let-7a-5p;hsa-let-7b-5p;hsa-let-7e-5p;hsa-let-7f-5p;hsa-le
ENSG0000	ENSG0000	3.56E-09	hsa-let-7a-5p;hsa-let-7b-5p;hsa-let-7e-5p;hsa-let-7f-5p;hsa-le
ENSG0000	ENSG0000	3.56E-09	hsa-let-7a-5p;hsa-let-7b-5p;hsa-let-7e-5p;hsa-let-7f-5p;hsa-le
ENSG0000	ENSG0000	6.43E-13	hsa-let-7a-5p;hsa-let-7b-5p;hsa-let-7e-5p;hsa-let-7f-5p;hsa-le
ENSG0000	ENSG0000	2.49E-12	hsa-let-7a-5p;hsa-let-7b-5p;hsa-let-7e-5p;hsa-let-7f-5p;hsa-le
ENSG0000	ENSG0000	3.50E-05	hsa-let-7b-5p;hsa-let-7e-5p;hsa-let-7f-5p;hsa-let-7i-5p
ENSG0000	ENSG0000	1.01E-09	hsa-let-7a-5p;hsa-let-7b-5p;hsa-let-7e-5p;hsa-let-7f-5p;hsa-le
ENSG0000	ENSG0000	9.15E-07	hsa-let-7a-5p;hsa-let-7b-5p;hsa-let-7e-5p;hsa-let-7f-5p;hsa-le
ENSG0000	ENSG0000	6.17E-08	hsa-let-7a-5p;hsa-let-7b-5p;hsa-let-7e-5p;hsa-let-7f-5p;hsa-le
ENSG0000	ENSG0000	2.68E-11	hsa-let-7a-5p;hsa-let-7b-5p;hsa-let-7e-5p;hsa-let-7f-5p;hsa-le
ENSG0000	ENSG0000	3.45E-05	hsa-let-7a-5p;hsa-let-7e-5p;hsa-let-7f-5p;hsa-let-7g-5p;hsa-le
ENSG0000	ENSG0000	2.55E-09	hsa-let-7a-5p;hsa-let-7b-5p;hsa-let-7e-5p;hsa-let-7f-5p;hsa-le
ENSG0000	ENSG0000	2.96E-10	hsa-let-7a-5p;hsa-let-7b-5p;hsa-let-7e-5p;hsa-let-7f-5p;hsa-le
ENSG0000	ENSG0000	1.50E-11	hsa-let-7a-5p;hsa-let-7b-5p;hsa-let-7e-5p;hsa-let-7f-5p;hsa-le
ENSG0000	ENSG0000	1.00E-10	hsa-let-7a-5p;hsa-let-7b-5p;hsa-let-7e-5p;hsa-let-7f-5p;hsa-le
ENSG0000	ENSG0000	1.85E-07	hsa-let-7a-5p;hsa-let-7b-5p;hsa-let-7e-5p;hsa-let-7f-5p;hsa-le
ENSG0000	ENSG0000	4.20E-05	hsa-let-7a-5p;hsa-let-7b-5p;hsa-let-7e-5p;hsa-let-7g-5p
ENSG0000	ENSG0000	1.54E-10	hsa-let-7a-5p;hsa-let-7b-5p;hsa-let-7e-5p;hsa-let-7f-5p;hsa-le
ENSG0000	ENSG0000	2.47E-06	hsa-let-7a-5p;hsa-let-7e-5p;hsa-let-7f-5p;hsa-let-7g-5p;hsa-le
ENSG0000	ENSG0000	1.96E-09	hsa-let-7a-5p;hsa-let-7b-5p;hsa-let-7e-5p;hsa-let-7f-5p;hsa-le
ENSG0000	ENSG0000	4.18E-09	hsa-let-7a-5p;hsa-let-7b-5p;hsa-let-7e-5p;hsa-let-7f-5p;hsa-le
ENSG0000	ENSG0000	4.56E-11	hsa-let-7a-5p;hsa-let-7b-5p;hsa-let-7e-5p;hsa-let-7f-5p;hsa-le
ENSG0000	ENSG0000	6.33E-11	hsa-let-7a-5p;hsa-let-7b-5p;hsa-let-7e-5p;hsa-let-7f-5p;hsa-le
ENSG0000	ENSG0000	2.23E-12	hsa-let-7a-5p;hsa-let-7b-5p;hsa-let-7e-5p;hsa-let-7f-5p;hsa-le
ENSG0000	ENSG0000	2.70E-06	hsa-let-7a-5p;hsa-let-7e-5p;hsa-let-7f-5p;hsa-let-7g-5p;hsa-le
ENSG0000	ENSG0000	8.20E-10	hsa-let-7a-5p;hsa-let-7b-5p;hsa-let-7e-5p;hsa-let-7f-5p;hsa-le
ENSG0000	ENSG0000	5.33E-10	hsa-let-7a-5p;hsa-let-7b-5p;hsa-let-7e-5p;hsa-let-7f-5p;hsa-le
ENSG0000	ENSG0000	2.34E-09	hsa-let-7a-5p;hsa-let-7b-5p;hsa-let-7e-5p;hsa-let-7f-5p;hsa-le
ENSG0000	ENSG0000	2.09E-09	hsa-let-7a-5p;hsa-let-7e-5p;hsa-let-7f-5p;hsa-let-7g-5p;hsa-le
ENSG0000	ENSG0000	1.33E-13	hsa-let-7a-5p;hsa-let-7b-5p;hsa-let-7e-5p;hsa-let-7f-5p;hsa-le
ENSG0000	ENSG0000	3.83E-05	hsa-let-7a-5p;hsa-let-7f-5p;hsa-let-7g-5p;hsa-let-7i-5p
ENSG0000	ENSG0000	1.01E-09	hsa-let-7a-5p;hsa-let-7b-5p;hsa-let-7e-5p;hsa-let-7f-5p;hsa-le
ENSG0000	ENSG0000	7.38E-10	hsa-let-7a-5p;hsa-let-7b-5p;hsa-let-7e-5p;hsa-let-7f-5p;hsa-le
ENSG0000	ENSG0000	7.38E-10	hsa-let-7a-5p;hsa-let-7b-5p;hsa-let-7e-5p;hsa-let-7f-5p;hsa-le
ENSG0000	ENSG0000	1.54E-10	hsa-let-7a-5p;hsa-let-7b-5p;hsa-let-7e-5p;hsa-let-7f-5p;hsa-le
ENSG0000	ENSG0000	4.24E-10	hsa-let-7a-5p;hsa-let-7b-5p;hsa-let-7e-5p;hsa-let-7f-5p;hsa-le
ENSG0000	ENSG0000	3.81E-12	hsa-let-7a-5p;hsa-let-7b-5p;hsa-let-7e-5p;hsa-let-7f-5p;hsa-le
ENSG0000	ENSG0000	1.77E-07	hsa-let-7a-5p;hsa-let-7b-5p;hsa-let-7e-5p;hsa-let-7f-5p;hsa-le
ENSG0000	ENSG0000	2.96E-10	hsa-let-7a-5p;hsa-let-7b-5p;hsa-let-7e-5p;hsa-let-7f-5p;hsa-le
ENSG0000	ENSG0000	5.33E-10	hsa-let-7a-5p;hsa-let-7b-5p;hsa-let-7e-5p;hsa-let-7f-5p;hsa-le

ENSG0000	ENSG0000	1.67E-12	hsa-let-7a-5p;hsa-let-7b-5p;hsa-let-7e-5p;hsa-let-7f-5p;hsa-le
ENSG0000	ENSG0000	9.82E-12	hsa-let-7a-5p;hsa-let-7b-5p;hsa-let-7e-5p;hsa-let-7f-5p;hsa-le
ENSG0000	ENSG0000	5.38E-11	hsa-let-7a-5p;hsa-let-7b-5p;hsa-let-7e-5p;hsa-let-7f-5p;hsa-le
ENSG0000	ENSG0000	5.95E-10	hsa-let-7a-5p;hsa-let-7b-5p;hsa-let-7e-5p;hsa-let-7f-5p;hsa-le
ENSG0000	ENSG0000	1.01E-08	hsa-let-7a-5p;hsa-let-7b-5p;hsa-let-7e-5p;hsa-let-7f-5p;hsa-le
ENSG0000	ENSG0000	9.09E-10	hsa-let-7a-5p;hsa-let-7b-5p;hsa-let-7e-5p;hsa-let-7f-5p;hsa-le
ENSG0000	ENSG0000	2.44E-06	hsa-let-7a-5p;hsa-let-7f-5p;hsa-let-7g-5p;hsa-let-7i-5p
ENSG0000	ENSG0000	8.87E-07	hsa-let-7a-5p;hsa-let-7b-5p;hsa-let-7e-5p;hsa-let-7f-5p;hsa-le
ENSG0000	ENSG0000	2.05E-08	hsa-let-7a-5p;hsa-let-7b-5p;hsa-let-7e-5p;hsa-let-7f-5p;hsa-le
ENSG0000	ENSG0000	2.05E-08	hsa-let-7a-5p;hsa-let-7b-5p;hsa-let-7e-5p;hsa-let-7f-5p;hsa-le
ENSG0000	ENSG0000	1.23E-05	hsa-let-7a-5p;hsa-let-7b-5p;hsa-let-7e-5p;hsa-let-7f-5p;hsa-le
ENSG0000	ENSG0000	3.81E-12	hsa-let-7a-5p;hsa-let-7b-5p;hsa-let-7e-5p;hsa-let-7f-5p;hsa-le
ENSG0000	ENSG0000	6.24E-12	hsa-let-7a-5p;hsa-let-7b-5p;hsa-let-7e-5p;hsa-let-7f-5p;hsa-le
ENSG0000	ENSG0000	6.24E-12	hsa-let-7a-5p;hsa-let-7b-5p;hsa-let-7e-5p;hsa-let-7f-5p;hsa-le
ENSG0000	ENSG0000	1.10E-07	hsa-let-7a-5p;hsa-let-7b-5p;hsa-let-7e-5p;hsa-let-7f-5p;hsa-le
ENSG0000	ENSG0000	2.61E-10	hsa-let-7a-5p;hsa-let-7b-5p;hsa-let-7e-5p;hsa-let-7f-5p;hsa-le
ENSG0000	ENSG0000	3.81E-07	hsa-let-7b-5p;hsa-let-7e-5p;hsa-let-7f-5p;hsa-let-7g-5p;hsa-le
ENSG0000	ENSG0000	8.20E-10	hsa-let-7a-5p;hsa-let-7b-5p;hsa-let-7e-5p;hsa-let-7f-5p;hsa-le
ENSG0000	ENSG0000	5.69E-09	hsa-let-7a-5p;hsa-let-7b-5p;hsa-let-7e-5p;hsa-let-7f-5p;hsa-le
ENSG0000	ENSG0000	1.32E-08	hsa-let-7a-5p;hsa-let-7b-5p;hsa-let-7e-5p;hsa-let-7f-5p;hsa-le
ENSG0000	ENSG0000	1.22E-11	hsa-let-7a-5p;hsa-let-7b-5p;hsa-let-7e-5p;hsa-let-7f-5p;hsa-le
ENSG0000	ENSG0000	3.10E-07	hsa-let-7a-5p;hsa-let-7b-5p;hsa-let-7f-5p;hsa-let-7g-5p;hsa-le
ENSG0000	ENSG0000	5.29E-08	hsa-let-7a-5p;hsa-let-7b-5p;hsa-let-7e-5p;hsa-let-7f-5p;hsa-le
ENSG0000	ENSG0000	9.09E-10	hsa-let-7a-5p;hsa-let-7b-5p;hsa-let-7e-5p;hsa-let-7f-5p;hsa-le
ENSG0000	ENSG0000	5.69E-09	hsa-let-7a-5p;hsa-let-7b-5p;hsa-let-7e-5p;hsa-let-7f-5p;hsa-le
ENSG0000	ENSG0000	0.000177	hsa-let-7a-5p;hsa-let-7f-5p;hsa-let-7g-5p;hsa-let-7i-5p
ENSG0000	ENSG0000	1.12E-05	hsa-let-7e-5p;hsa-let-7f-5p;hsa-let-7g-5p;hsa-let-7i-5p
ENSG0000	ENSG0000	1.12E-05	hsa-let-7e-5p;hsa-let-7f-5p;hsa-let-7g-5p;hsa-let-7i-5p
ENSG0000	ENSG0000	6.17E-08	hsa-let-7a-5p;hsa-let-7b-5p;hsa-let-7e-5p;hsa-let-7f-5p;hsa-le
ENSG0000	ENSG0000	8.20E-10	hsa-let-7a-5p;hsa-let-7b-5p;hsa-let-7e-5p;hsa-let-7f-5p;hsa-le
ENSG0000	ENSG0000	2.30E-10	hsa-let-7a-5p;hsa-let-7b-5p;hsa-let-7e-5p;hsa-let-7f-5p;hsa-le
ENSG0000	ENSG0000	2.50E-07	hsa-let-7a-5p;hsa-let-7e-5p;hsa-let-7f-5p;hsa-let-7g-5p;hsa-le
ENSG0000	ENSG0000	2.01E-05	hsa-let-7e-5p;hsa-let-7f-5p;hsa-let-7g-5p;hsa-let-7i-5p
ENSG0000	ENSG0000	1.16E-10	hsa-let-7a-5p;hsa-let-7b-5p;hsa-let-7e-5p;hsa-let-7f-5p;hsa-le
ENSG0000	ENSG0000	1.16E-10	hsa-let-7a-5p;hsa-let-7b-5p;hsa-let-7e-5p;hsa-let-7f-5p;hsa-le
ENSG0000	ENSG0000	1.16E-10	hsa-let-7a-5p;hsa-let-7b-5p;hsa-let-7e-5p;hsa-let-7f-5p;hsa-le
ENSG0000	ENSG0000	1.54E-10	hsa-let-7a-5p;hsa-let-7b-5p;hsa-let-7e-5p;hsa-let-7f-5p;hsa-le
ENSG0000	ENSG0000	5.69E-09	hsa-let-7a-5p;hsa-let-7b-5p;hsa-let-7e-5p;hsa-let-7f-5p;hsa-le
ENSG0000	ENSG0000	2.22E-11	hsa-let-7a-5p;hsa-let-7b-5p;hsa-let-7e-5p;hsa-let-7f-5p;hsa-le
ENSG0000	ENSG0000	1.83E-11	hsa-let-7a-5p;hsa-let-7b-5p;hsa-let-7e-5p;hsa-let-7f-5p;hsa-le
ENSG0000	ENSG0000	2.57E-08	hsa-let-7a-5p;hsa-let-7e-5p;hsa-let-7f-5p;hsa-let-7g-5p;hsa-le
ENSG0000	ENSG0000	9.54E-08	hsa-let-7a-5p;hsa-let-7b-5p;hsa-let-7e-5p;hsa-let-7f-5p;hsa-le
ENSG0000	ENSG0000	9.54E-08	hsa-let-7a-5p;hsa-let-7b-5p;hsa-let-7e-5p;hsa-let-7f-5p;hsa-le
ENSG0000	ENSG0000	3.59E-08	hsa-let-7a-5p;hsa-let-7e-5p;hsa-let-7f-5p;hsa-let-7g-5p;hsa-le
ENSG0000	ENSG0000	2.52E-09	hsa-let-7a-5p;hsa-let-7e-5p;hsa-let-7f-5p;hsa-let-7g-5p;hsa-le
ENSG0000	ENSG0000	6.43E-13	hsa-let-7a-5p;hsa-let-7b-5p;hsa-let-7e-5p;hsa-let-7f-5p;hsa-le
ENSG0000	ENSG0000	1.50E-11	hsa-let-7a-5p;hsa-let-7b-5p;hsa-let-7e-5p;hsa-let-7f-5p;hsa-le
ENSG0000	ENSG0000	2.02E-10	hsa-let-7a-5p;hsa-let-7b-5p;hsa-let-7e-5p;hsa-let-7f-5p;hsa-le
ENSG0000	ENSG0000	5.95E-10	hsa-let-7a-5p;hsa-let-7b-5p;hsa-let-7e-5p;hsa-let-7f-5p;hsa-le
ENSG0000	ENSG0000	5.95E-10	hsa-let-7a-5p;hsa-let-7b-5p;hsa-let-7e-5p;hsa-let-7f-5p;hsa-le
ENSG0000	ENSG0000	2.22E-11	hsa-let-7a-5p;hsa-let-7b-5p;hsa-let-7e-5p;hsa-let-7f-5p;hsa-le
ENSG0000	ENSG0000	3.22E-11	hsa-let-7a-5p;hsa-let-7b-5p;hsa-let-7e-5p;hsa-let-7f-5p;hsa-le
ENSG0000	ENSG0000	4.63E-07	hsa-let-7a-5p;hsa-let-7b-5p;hsa-let-7f-5p;hsa-let-7g-5p;hsa-le
ENSG0000	ENSG0000	4.52E-09	hsa-let-7a-5p;hsa-let-7b-5p;hsa-let-7e-5p;hsa-let-7f-5p;hsa-le

ENSG0000	ENSG0000	2.61E-10	hsa-let-7a-5p;hsa-let-7b-5p;hsa-let-7e-5p;hsa-let-7f-5p;hsa-le
ENSG0000	ENSG0000	1.77E-10	hsa-let-7a-5p;hsa-let-7b-5p;hsa-let-7e-5p;hsa-let-7f-5p;hsa-le
ENSG0000	ENSG0000	2.89E-07	hsa-let-7a-5p;hsa-let-7b-5p;hsa-let-7f-5p;hsa-let-7g-5p;hsa-le
ENSG0000	ENSG0000	2.61E-10	hsa-let-7a-5p;hsa-let-7b-5p;hsa-let-7e-5p;hsa-let-7f-5p;hsa-le
ENSG0000	ENSG0000	4.29E-08	hsa-let-7a-5p;hsa-let-7b-5p;hsa-let-7e-5p;hsa-let-7f-5p;hsa-le
ENSG0000	ENSG0000	4.29E-08	hsa-let-7a-5p;hsa-let-7b-5p;hsa-let-7e-5p;hsa-let-7f-5p;hsa-le
ENSG0000	ENSG0000	9.01E-13	hsa-let-7a-5p;hsa-let-7b-5p;hsa-let-7e-5p;hsa-let-7f-5p;hsa-le
ENSG0000	ENSG0000	1.33E-13	hsa-let-7a-5p;hsa-let-7b-5p;hsa-let-7e-5p;hsa-let-7f-5p;hsa-le
ENSG0000	ENSG0000	1.01E-09	hsa-let-7a-5p;hsa-let-7b-5p;hsa-let-7e-5p;hsa-let-7f-5p;hsa-le
ENSG0000	ENSG0000	3.56E-09	hsa-let-7a-5p;hsa-let-7b-5p;hsa-let-7e-5p;hsa-let-7f-5p;hsa-le
ENSG0000	ENSG0000	6.63E-10	hsa-let-7a-5p;hsa-let-7b-5p;hsa-let-7e-5p;hsa-let-7f-5p;hsa-le
ENSG0000	ENSG0000	3.85E-08	hsa-let-7a-5p;hsa-let-7b-5p;hsa-let-7e-5p;hsa-let-7f-5p;hsa-le
ENSG0000	ENSG0000	1.16E-08	hsa-let-7a-5p;hsa-let-7b-5p;hsa-let-7e-5p;hsa-let-7f-5p;hsa-le
ENSG0000	ENSG0000	3.23E-07	hsa-let-7a-5p;hsa-let-7b-5p;hsa-let-7e-5p;hsa-let-7f-5p;hsa-le
ENSG0000	ENSG0000	5.19E-07	hsa-let-7a-5p;hsa-let-7b-5p;hsa-let-7e-5p;hsa-let-7f-5p;hsa-le
ENSG0000	ENSG0000	2.22E-11	hsa-let-7a-5p;hsa-let-7b-5p;hsa-let-7e-5p;hsa-let-7f-5p;hsa-le
ENSG0000	ENSG0000	2.06E-13	hsa-let-7a-5p;hsa-let-7b-5p;hsa-let-7e-5p;hsa-let-7f-5p;hsa-le
ENSG0000	ENSG0000	2.14E-09	hsa-let-7a-5p;hsa-let-7b-5p;hsa-let-7e-5p;hsa-let-7f-5p;hsa-le
ENSG0000	ENSG0000	1.83E-11	hsa-let-7a-5p;hsa-let-7b-5p;hsa-let-7e-5p;hsa-let-7f-5p;hsa-le
ENSG0000	ENSG0000	1.34E-10	hsa-let-7a-5p;hsa-let-7b-5p;hsa-let-7e-5p;hsa-let-7f-5p;hsa-le
ENSG0000	ENSG0000	2.23E-12	hsa-let-7a-5p;hsa-let-7b-5p;hsa-let-7e-5p;hsa-let-7f-5p;hsa-le
ENSG0000	ENSG0000	6.24E-12	hsa-let-7a-5p;hsa-let-7b-5p;hsa-let-7e-5p;hsa-let-7f-5p;hsa-le
ENSG0000	ENSG0000	6.24E-12	hsa-let-7a-5p;hsa-let-7b-5p;hsa-let-7e-5p;hsa-let-7f-5p;hsa-le
ENSG0000	ENSG0000	9.54E-08	hsa-let-7a-5p;hsa-let-7b-5p;hsa-let-7e-5p;hsa-let-7f-5p;hsa-le
ENSG0000	ENSG0000	2.68E-11	hsa-let-7a-5p;hsa-let-7b-5p;hsa-let-7e-5p;hsa-let-7f-5p;hsa-le
ENSG0000	ENSG0000	3.28E-09	hsa-let-7a-5p;hsa-let-7b-5p;hsa-let-7e-5p;hsa-let-7f-5p;hsa-le
ENSG0000	ENSG0000	9.42E-09	hsa-let-7a-5p;hsa-let-7b-5p;hsa-let-7e-5p;hsa-let-7f-5p;hsa-le
ENSG0000	ENSG0000	2.23E-12	hsa-let-7a-5p;hsa-let-7b-5p;hsa-let-7e-5p;hsa-let-7f-5p;hsa-le
ENSG0000	ENSG0000	2.34E-09	hsa-let-7a-5p;hsa-let-7b-5p;hsa-let-7e-5p;hsa-let-7f-5p;hsa-le
ENSG0000	ENSG0000	2.34E-09	hsa-let-7a-5p;hsa-let-7b-5p;hsa-let-7e-5p;hsa-let-7f-5p;hsa-le
ENSG0000	ENSG0000	1.19E-06	hsa-let-7e-5p;hsa-let-7f-5p;hsa-let-7g-5p;hsa-let-7i-5p
ENSG0000	ENSG0000	2.55E-09	hsa-let-7a-5p;hsa-let-7b-5p;hsa-let-7e-5p;hsa-let-7f-5p;hsa-le
ENSG0000	ENSG0000	3.22E-11	hsa-let-7a-5p;hsa-let-7b-5p;hsa-let-7e-5p;hsa-let-7f-5p;hsa-le
ENSG0000	ENSG0000	5.89E-09	hsa-let-7b-5p;hsa-let-7e-5p;hsa-let-7f-5p;hsa-let-7g-5p;hsa-le
ENSG0000	ENSG0000	1.33E-13	hsa-let-7a-5p;hsa-let-7b-5p;hsa-let-7e-5p;hsa-let-7f-5p;hsa-le
ENSG0000	ENSG0000	1.24E-12	hsa-let-7a-5p;hsa-let-7b-5p;hsa-let-7e-5p;hsa-let-7f-5p;hsa-le
ENSG0000	ENSG0000	1.24E-12	hsa-let-7a-5p;hsa-let-7b-5p;hsa-let-7e-5p;hsa-let-7f-5p;hsa-le
ENSG0000	ENSG0000	8.20E-10	hsa-let-7a-5p;hsa-let-7b-5p;hsa-let-7e-5p;hsa-let-7f-5p;hsa-le
ENSG0000	ENSG0000	1.41E-08	hsa-let-7a-5p;hsa-let-7b-5p;hsa-let-7e-5p;hsa-let-7f-5p;hsa-le
ENSG0000	ENSG0000	6.63E-10	hsa-let-7a-5p;hsa-let-7b-5p;hsa-let-7e-5p;hsa-let-7f-5p;hsa-le
ENSG0000	ENSG0000	1.16E-08	hsa-let-7a-5p;hsa-let-7b-5p;hsa-let-7e-5p;hsa-let-7f-5p;hsa-le
ENSG0000	ENSG0000	1.96E-09	hsa-let-7a-5p;hsa-let-7b-5p;hsa-let-7e-5p;hsa-let-7f-5p;hsa-le
ENSG0000	ENSG0000	8.48E-07	hsa-let-7a-5p;hsa-let-7e-5p;hsa-let-7f-5p;hsa-let-7g-5p;hsa-le
ENSG0000	ENSG0000	1.35E-09	hsa-let-7a-5p;hsa-let-7b-5p;hsa-let-7e-5p;hsa-let-7f-5p;hsa-le
ENSG0000	ENSG0000	3.22E-11	hsa-let-7a-5p;hsa-let-7b-5p;hsa-let-7e-5p;hsa-let-7f-5p;hsa-le
ENSG0000	ENSG0000	3.85E-08	hsa-let-7a-5p;hsa-let-7b-5p;hsa-let-7e-5p;hsa-let-7f-5p;hsa-le
ENSG0000	ENSG0000	1.08E-08	hsa-let-7a-5p;hsa-let-7b-5p;hsa-let-7e-5p;hsa-let-7f-5p;hsa-le
ENSG0000	ENSG0000	2.89E-07	hsa-let-7a-5p;hsa-let-7e-5p;hsa-let-7f-5p;hsa-let-7g-5p;hsa-le
ENSG0000	ENSG0000	6.60E-09	hsa-let-7a-5p;hsa-let-7b-5p;hsa-let-7e-5p;hsa-let-7f-5p;hsa-le
ENSG0000	ENSG0000	1.83E-11	hsa-let-7a-5p;hsa-let-7b-5p;hsa-let-7e-5p;hsa-let-7f-5p;hsa-le
ENSG0000	ENSG0000	2.68E-11	hsa-let-7a-5p;hsa-let-7b-5p;hsa-let-7e-5p;hsa-let-7f-5p;hsa-le
ENSG0000	ENSG0000	1.54E-10	hsa-let-7a-5p;hsa-let-7b-5p;hsa-let-7e-5p;hsa-let-7f-5p;hsa-le
ENSG0000	ENSG0000	4.19E-07	hsa-let-7a-5p;hsa-let-7b-5p;hsa-let-7e-5p;hsa-let-7f-5p;hsa-le
ENSG0000	ENSG0000	8.20E-10	hsa-let-7a-5p;hsa-let-7b-5p;hsa-let-7e-5p;hsa-let-7f-5p;hsa-le

ENSG0000	ENSG0000	1.22E-11	hsa-let-7a-5p;hsa-let-7b-5p;hsa-let-7e-5p;hsa-let-7f-5p;hsa-le
ENSG0000	ENSG0000	9.09E-10	hsa-let-7a-5p;hsa-let-7b-5p;hsa-let-7e-5p;hsa-let-7f-5p;hsa-le
ENSG0000	ENSG0000	7.41E-11	hsa-let-7a-5p;hsa-let-7b-5p;hsa-let-7e-5p;hsa-let-7f-5p;hsa-le
ENSG0000	ENSG0000	7.27E-08	hsa-let-7b-5p;hsa-let-7e-5p;hsa-let-7f-5p;hsa-let-7g-5p;hsa-le
ENSG0000	ENSG0000	1.23E-08	hsa-let-7a-5p;hsa-let-7b-5p;hsa-let-7e-5p;hsa-let-7f-5p;hsa-le
ENSG0000	ENSG0000	1.93E-08	hsa-let-7a-5p;hsa-let-7b-5p;hsa-let-7e-5p;hsa-let-7f-5p;hsa-le
ENSG0000	ENSG0000	1.85E-07	hsa-let-7a-5p;hsa-let-7b-5p;hsa-let-7e-5p;hsa-let-7f-5p;hsa-le
ENSG0000	ENSG0000	9.01E-13	hsa-let-7a-5p;hsa-let-7b-5p;hsa-let-7e-5p;hsa-let-7f-5p;hsa-le
ENSG0000	ENSG0000	1.35E-09	hsa-let-7a-5p;hsa-let-7b-5p;hsa-let-7e-5p;hsa-let-7f-5p;hsa-le
ENSG0000	ENSG0000	5.28E-09	hsa-let-7a-5p;hsa-let-7b-5p;hsa-let-7e-5p;hsa-let-7f-5p;hsa-le
ENSG0000	ENSG0000	8.28E-08	hsa-let-7a-5p;hsa-let-7b-5p;hsa-let-7e-5p;hsa-let-7f-5p;hsa-le
ENSG0000	ENSG0000	2.61E-10	hsa-let-7a-5p;hsa-let-7b-5p;hsa-let-7e-5p;hsa-let-7f-5p;hsa-le
ENSG0000	ENSG0000	5.86E-08	hsa-let-7a-5p;hsa-let-7b-5p;hsa-let-7e-5p;hsa-let-7f-5p;hsa-le
ENSG0000	ENSG0000	1.23E-09	hsa-let-7a-5p;hsa-let-7b-5p;hsa-let-7e-5p;hsa-let-7f-5p;hsa-le
ENSG0000	ENSG0000	6.33E-11	hsa-let-7a-5p;hsa-let-7b-5p;hsa-let-7e-5p;hsa-let-7f-5p;hsa-le
ENSG0000	ENSG0000	3.66E-06	hsa-let-7a-5p;hsa-let-7e-5p;hsa-let-7f-5p;hsa-let-7g-5p;hsa-le
ENSG0000	ENSG0000	1.96E-09	hsa-let-7a-5p;hsa-let-7b-5p;hsa-let-7e-5p;hsa-let-7f-5p;hsa-le
ENSG0000	ENSG0000	2.78E-09	hsa-let-7a-5p;hsa-let-7b-5p;hsa-let-7e-5p;hsa-let-7f-5p;hsa-le
ENSG0000	ENSG0000	3.64E-08	hsa-let-7a-5p;hsa-let-7b-5p;hsa-let-7e-5p;hsa-let-7f-5p;hsa-le
ENSG0000	ENSG0000	2.93E-12	hsa-let-7a-5p;hsa-let-7b-5p;hsa-let-7e-5p;hsa-let-7f-5p;hsa-le
ENSG0000	ENSG0000	4.51E-13	hsa-let-7a-5p;hsa-let-7b-5p;hsa-let-7e-5p;hsa-let-7f-5p;hsa-le
ENSG0000	ENSG0000	1.01E-08	hsa-let-7a-5p;hsa-let-7b-5p;hsa-let-7e-5p;hsa-let-7f-5p;hsa-le
ENSG0000	ENSG0000	2.91E-08	hsa-let-7a-5p;hsa-let-7b-5p;hsa-let-7e-5p;hsa-let-7f-5p;hsa-le
ENSG0000	ENSG0000	5.89E-09	hsa-let-7a-5p;hsa-let-7b-5p;hsa-let-7e-5p;hsa-let-7g-5p;hsa-l
ENSG0000	ENSG0000	7.38E-10	hsa-let-7a-5p;hsa-let-7b-5p;hsa-let-7e-5p;hsa-let-7f-5p;hsa-le
ENSG0000	ENSG0000	1.41E-08	hsa-let-7a-5p;hsa-let-7b-5p;hsa-let-7e-5p;hsa-let-7f-5p;hsa-le
ENSG0000	ENSG0000	2.05E-08	hsa-let-7a-5p;hsa-let-7b-5p;hsa-let-7e-5p;hsa-let-7f-5p;hsa-le
ENSG0000	ENSG0000	1.67E-12	hsa-let-7a-5p;hsa-let-7b-5p;hsa-let-7e-5p;hsa-let-7f-5p;hsa-le
ENSG0000	ENSG0000	7.41E-11	hsa-let-7a-5p;hsa-let-7b-5p;hsa-let-7e-5p;hsa-let-7f-5p;hsa-le
ENSG0000	ENSG0000	1.50E-08	hsa-let-7a-5p;hsa-let-7b-5p;hsa-let-7e-5p;hsa-let-7f-5p;hsa-le
ENSG0000	ENSG0000	1.23E-08	hsa-let-7a-5p;hsa-let-7b-5p;hsa-let-7e-5p;hsa-let-7f-5p;hsa-le
ENSG0000	ENSG0000	6.17E-08	hsa-let-7a-5p;hsa-let-7b-5p;hsa-let-7e-5p;hsa-let-7f-5p;hsa-le
ENSG0000	ENSG0000	4.89E-09	hsa-let-7a-5p;hsa-let-7b-5p;hsa-let-7e-5p;hsa-let-7f-5p;hsa-le
ENSG0000	ENSG0000	2.88E-07	hsa-let-7a-5p;hsa-let-7b-5p;hsa-let-7e-5p;hsa-let-7f-5p;hsa-le
ENSG0000	ENSG0000	1.83E-11	hsa-let-7a-5p;hsa-let-7b-5p;hsa-let-7e-5p;hsa-let-7f-5p;hsa-le
ENSG0000	ENSG0000	7.38E-10	hsa-let-7a-5p;hsa-let-7b-5p;hsa-let-7e-5p;hsa-let-7f-5p;hsa-le
ENSG0000	ENSG0000	2.61E-10	hsa-let-7a-5p;hsa-let-7b-5p;hsa-let-7e-5p;hsa-let-7f-5p;hsa-le
ENSG0000	ENSG0000	1.01E-09	hsa-let-7a-5p;hsa-let-7b-5p;hsa-let-7e-5p;hsa-let-7f-5p;hsa-le
ENSG0000	ENSG0000	1.22E-11	hsa-let-7a-5p;hsa-let-7b-5p;hsa-let-7e-5p;hsa-let-7f-5p;hsa-le
ENSG0000	ENSG0000	2.18E-07	hsa-let-7a-5p;hsa-let-7b-5p;hsa-let-7e-5p;hsa-let-7f-5p;hsa-le
ENSG0000	ENSG0000	4.90E-12	hsa-let-7a-5p;hsa-let-7b-5p;hsa-let-7e-5p;hsa-let-7f-5p;hsa-le
ENSG0000	ENSG0000	4.06E-08	hsa-let-7a-5p;hsa-let-7b-5p;hsa-let-7e-5p;hsa-let-7f-5p;hsa-le
ENSG0000	ENSG0000	1.23E-09	hsa-let-7a-5p;hsa-let-7b-5p;hsa-let-7e-5p;hsa-let-7f-5p;hsa-le
ENSG0000	ENSG0000	5.29E-08	hsa-let-7a-5p;hsa-let-7b-5p;hsa-let-7e-5p;hsa-let-7f-5p;hsa-le
ENSG0000	ENSG0000	4.56E-11	hsa-let-7a-5p;hsa-let-7b-5p;hsa-let-7e-5p;hsa-let-7f-5p;hsa-le
ENSG0000	ENSG0000	3.34E-10	hsa-let-7a-5p;hsa-let-7b-5p;hsa-let-7e-5p;hsa-let-7f-5p;hsa-le
ENSG0000	ENSG0000	4.52E-08	hsa-let-7a-5p;hsa-let-7b-5p;hsa-let-7e-5p;hsa-let-7f-5p;hsa-le
ENSG0000	ENSG0000	4.24E-10	hsa-let-7a-5p;hsa-let-7b-5p;hsa-let-7e-5p;hsa-let-7f-5p;hsa-le
ENSG0000	ENSG0000	6.24E-12	hsa-let-7a-5p;hsa-let-7b-5p;hsa-let-7e-5p;hsa-let-7f-5p;hsa-le
ENSG0000	ENSG0000	1.77E-10	hsa-let-7a-5p;hsa-let-7b-5p;hsa-let-7e-5p;hsa-let-7f-5p;hsa-le
ENSG0000	ENSG0000	1.32E-08	hsa-let-7a-5p;hsa-let-7b-5p;hsa-let-7e-5p;hsa-let-7f-5p;hsa-le
ENSG0000	ENSG0000	4.52E-09	hsa-let-7a-5p;hsa-let-7b-5p;hsa-let-7e-5p;hsa-let-7f-5p;hsa-le
ENSG0000	ENSG0000	1.54E-10	hsa-let-7a-5p;hsa-let-7b-5p;hsa-let-7e-5p;hsa-let-7f-5p;hsa-le
ENSG0000	ENSG0000	1.61E-06	hsa-let-7a-5p;hsa-let-7e-5p;hsa-let-7f-5p;hsa-let-7g-5p;hsa-le

ENSG0000	ENSG0000	5.38E-11	hsa-let-7a-5p;hsa-let-7b-5p;hsa-let-7e-5p;hsa-let-7f-5p;hsa-le
ENSG0000	ENSG0000	3.81E-06	hsa-let-7a-5p;hsa-let-7e-5p;hsa-let-7f-5p;hsa-let-7g-5p;hsa-le
ENSG0000	ENSG0000	2.06E-13	hsa-let-7a-5p;hsa-let-7b-5p;hsa-let-7e-5p;hsa-let-7f-5p;hsa-le
ENSG0000	ENSG0000	1.01E-09	hsa-let-7a-5p;hsa-let-7b-5p;hsa-let-7e-5p;hsa-let-7f-5p;hsa-le
ENSG0000	ENSG0000	5.38E-11	hsa-let-7a-5p;hsa-let-7b-5p;hsa-let-7e-5p;hsa-let-7f-5p;hsa-le
ENSG0000	ENSG0000	7.16E-08	hsa-let-7a-5p;hsa-let-7b-5p;hsa-let-7e-5p;hsa-let-7f-5p;hsa-le
ENSG0000	ENSG0000	5.33E-10	hsa-let-7a-5p;hsa-let-7b-5p;hsa-let-7e-5p;hsa-let-7f-5p;hsa-le
ENSG0000	ENSG0000	3.89E-07	hsa-let-7a-5p;hsa-let-7b-5p;hsa-let-7e-5p;hsa-let-7f-5p;hsa-le
ENSG0000	ENSG0000	8.48E-10	hsa-let-7a-5p;hsa-let-7b-5p;hsa-let-7e-5p;hsa-let-7f-5p;hsa-le
ENSG0000	ENSG0000	9.90E-07	hsa-let-7a-5p;hsa-let-7b-5p;hsa-let-7e-5p;hsa-let-7f-5p;hsa-le
ENSG0000	ENSG0000	7.51E-07	hsa-let-7a-5p;hsa-let-7b-5p;hsa-let-7e-5p;hsa-let-7f-5p;hsa-le
ENSG0000	ENSG0000	5.61E-08	hsa-let-7a-5p;hsa-let-7b-5p;hsa-let-7e-5p;hsa-let-7f-5p;hsa-le
ENSG0000	ENSG0000	1.37E-07	hsa-let-7a-5p;hsa-let-7b-5p;hsa-let-7e-5p;hsa-let-7f-5p;hsa-le
ENSG0000	ENSG0000	2.46E-06	hsa-let-7a-5p;hsa-let-7b-5p;hsa-let-7e-5p;hsa-let-7f-5p;hsa-le
ENSG0000	ENSG0000	0.000304	hsa-let-7a-5p;hsa-let-7b-5p;hsa-let-7e-5p;hsa-let-7g-5p;hsa-l
ENSG0000	ENSG0000	5.04E-07	hsa-let-7a-5p;hsa-let-7b-5p;hsa-let-7f-5p;hsa-miR-9-5p
ENSG0000	ENSG0000	3.52E-08	hsa-let-7a-5p;hsa-let-7b-5p;hsa-let-7e-5p;hsa-let-7f-5p;hsa-le
ENSG0000	ENSG0000	2.38E-07	hsa-let-7a-5p;hsa-let-7b-5p;hsa-let-7e-5p;hsa-let-7f-5p;hsa-le
ENSG0000	ENSG0000	2.11E-07	hsa-let-7a-5p;hsa-let-7b-5p;hsa-let-7e-5p;hsa-let-7f-5p;hsa-le
ENSG0000	ENSG0000	3.52E-08	hsa-let-7a-5p;hsa-let-7b-5p;hsa-let-7e-5p;hsa-let-7f-5p;hsa-le
ENSG0000	ENSG0000	0.000201	hsa-let-7a-5p;hsa-let-7b-5p;hsa-let-7e-5p;hsa-let-7f-5p;hsa-le
ENSG0000	ENSG0000	2.59E-09	hsa-let-7a-5p;hsa-let-7b-5p;hsa-let-7e-5p;hsa-let-7f-5p;hsa-le
ENSG0000	ENSG0000	9.80E-08	hsa-let-7a-5p;hsa-let-7e-5p;hsa-let-7f-5p;hsa-let-7g-5p;hsa-le
ENSG0000	ENSG0000	2.34E-09	hsa-let-7a-5p;hsa-let-7b-5p;hsa-let-7e-5p;hsa-let-7f-5p;hsa-le
ENSG0000	ENSG0000	6.19E-07	hsa-let-7a-5p;hsa-let-7b-5p;hsa-let-7e-5p;hsa-let-7f-5p;hsa-le
ENSG0000	ENSG0000	7.82E-07	hsa-let-7a-5p;hsa-let-7e-5p;hsa-let-7f-5p;hsa-let-7g-5p;hsa-le
ENSG0000	ENSG0000	2.00E-09	hsa-let-7a-5p;hsa-let-7b-5p;hsa-let-7e-5p;hsa-let-7f-5p;hsa-le
ENSG0000	ENSG0000	4.91E-09	hsa-let-7a-5p;hsa-let-7b-5p;hsa-let-7e-5p;hsa-let-7f-5p;hsa-le
ENSG0000	ENSG0000	4.82E-05	hsa-let-7a-5p;hsa-let-7e-5p;hsa-let-7f-5p;hsa-let-7g-5p;hsa-le
ENSG0000	ENSG0000	2.67E-07	hsa-let-7a-5p;hsa-let-7b-5p;hsa-let-7e-5p;hsa-let-7f-5p;hsa-le
ENSG0000	ENSG0000	7.51E-07	hsa-let-7a-5p;hsa-let-7b-5p;hsa-let-7e-5p;hsa-let-7f-5p;hsa-le
ENSG0000	ENSG0000	4.21E-09	hsa-let-7a-5p;hsa-let-7b-5p;hsa-let-7e-5p;hsa-let-7f-5p;hsa-le
ENSG0000	ENSG0000	6.99E-06	hsa-let-7a-5p;hsa-let-7b-5p;hsa-let-7e-5p;hsa-let-7f-5p;hsa-le
ENSG0000	ENSG0000	6.56E-06	hsa-let-7a-5p;hsa-let-7b-5p;hsa-let-7e-5p;hsa-let-7f-5p;hsa-le
ENSG0000	ENSG0000	0.000158	hsa-let-7a-5p;hsa-let-7b-5p;hsa-let-7e-5p;hsa-let-7f-5p;hsa-le
ENSG0000	ENSG0000	2.00E-09	hsa-let-7a-5p;hsa-let-7b-5p;hsa-let-7e-5p;hsa-let-7f-5p;hsa-le
ENSG0000	ENSG0000	1.29E-06	hsa-let-7a-5p;hsa-let-7b-5p;hsa-let-7e-5p;hsa-let-7f-5p;hsa-le
ENSG0000	ENSG0000	1.96E-05	hsa-let-7a-5p;hsa-let-7e-5p;hsa-let-7f-5p;hsa-let-7g-5p;hsa-le
ENSG0000	ENSG0000	1.21E-06	hsa-let-7a-5p;hsa-let-7b-5p;hsa-let-7f-5p;hsa-let-7g-5p;hsa-le
ENSG0000	ENSG0000	2.00E-11	hsa-let-7a-5p;hsa-let-7b-5p;hsa-let-7e-5p;hsa-let-7f-5p;hsa-le
ENSG0000	ENSG0000	1.34E-05	hsa-let-7a-5p;hsa-let-7b-5p;hsa-let-7e-5p;hsa-let-7f-5p;hsa-le
ENSG0000	ENSG0000	2.28E-07	hsa-let-7a-5p;hsa-let-7b-5p;hsa-let-7e-5p;hsa-let-7f-5p;hsa-le
ENSG0000	ENSG0000	6.59E-09	hsa-let-7a-5p;hsa-let-7b-5p;hsa-let-7e-5p;hsa-let-7f-5p;hsa-le
ENSG0000	ENSG0000	9.50E-06	hsa-let-7a-5p;hsa-let-7b-5p;hsa-let-7e-5p;hsa-let-7f-5p;hsa-le
ENSG0000	ENSG0000	7.51E-08	hsa-let-7a-5p;hsa-let-7b-5p;hsa-let-7e-5p;hsa-let-7f-5p;hsa-le
ENSG0000	ENSG0000	1.13E-07	hsa-let-7a-5p;hsa-let-7b-5p;hsa-let-7e-5p;hsa-let-7f-5p;hsa-le
ENSG0000	ENSG0000	4.43E-10	hsa-let-7a-5p;hsa-let-7b-5p;hsa-let-7e-5p;hsa-let-7f-5p;hsa-le
ENSG0000	ENSG0000	3.11E-10	hsa-let-7a-5p;hsa-let-7b-5p;hsa-let-7e-5p;hsa-let-7f-5p;hsa-le
ENSG0000	ENSG0000	2.00E-09	hsa-let-7a-5p;hsa-let-7b-5p;hsa-let-7e-5p;hsa-let-7f-5p;hsa-le
ENSG0000	ENSG0000	4.46E-05	hsa-let-7a-5p;hsa-let-7b-5p;hsa-let-7e-5p;hsa-let-7g-5p;hsa-l
ENSG0000	ENSG0000	3.52E-08	hsa-let-7a-5p;hsa-let-7b-5p;hsa-let-7e-5p;hsa-let-7f-5p;hsa-le
ENSG0000	ENSG0000	7.44E-06	hsa-let-7a-5p;hsa-let-7b-5p;hsa-let-7e-5p;hsa-let-7f-5p;hsa-le
ENSG0000	ENSG0000	2.59E-09	hsa-let-7a-5p;hsa-let-7b-5p;hsa-let-7e-5p;hsa-let-7f-5p;hsa-le
ENSG0000	ENSG0000	2.59E-11	hsa-let-7a-5p;hsa-let-7b-5p;hsa-let-7e-5p;hsa-let-7f-5p;hsa-le

ENSG0000	ENSG0000	2.59E-11	hsa-let-7a-5p;hsa-let-7b-5p;hsa-let-7e-5p;hsa-let-7f-5p;hsa-le
ENSG0000	ENSG0000	8.15E-09	hsa-let-7a-5p;hsa-let-7b-5p;hsa-let-7e-5p;hsa-let-7f-5p;hsa-le
ENSG0000	ENSG0000	8.48E-10	hsa-let-7a-5p;hsa-let-7b-5p;hsa-let-7e-5p;hsa-let-7f-5p;hsa-le
ENSG0000	ENSG0000	8.48E-10	hsa-let-7a-5p;hsa-let-7b-5p;hsa-let-7e-5p;hsa-let-7f-5p;hsa-le
ENSG0000	ENSG0000	8.48E-10	hsa-let-7a-5p;hsa-let-7b-5p;hsa-let-7e-5p;hsa-let-7f-5p;hsa-le
ENSG0000	ENSG0000	8.48E-10	hsa-let-7a-5p;hsa-let-7b-5p;hsa-let-7e-5p;hsa-let-7f-5p;hsa-le
ENSG0000	ENSG0000	8.48E-10	hsa-let-7a-5p;hsa-let-7b-5p;hsa-let-7e-5p;hsa-let-7f-5p;hsa-le
ENSG0000	ENSG0000	8.48E-10	hsa-let-7a-5p;hsa-let-7b-5p;hsa-let-7e-5p;hsa-let-7f-5p;hsa-le
ENSG0000	ENSG0000	8.48E-10	hsa-let-7a-5p;hsa-let-7b-5p;hsa-let-7e-5p;hsa-let-7f-5p;hsa-le
ENSG0000	ENSG0000	2.26E-05	hsa-let-7a-5p;hsa-let-7b-5p;hsa-let-7e-5p;hsa-let-7g-5p;hsa-l
ENSG0000	ENSG0000	0.000236	hsa-let-7a-5p;hsa-let-7e-5p;hsa-let-7f-5p;hsa-let-7g-5p;hsa-le
ENSG0000	ENSG0000	8.25E-07	hsa-let-7a-5p;hsa-let-7b-5p;hsa-let-7e-5p;hsa-let-7f-5p;hsa-le
ENSG0000	ENSG0000	6.82E-07	hsa-let-7a-5p;hsa-let-7b-5p;hsa-let-7e-5p;hsa-let-7f-5p;hsa-le
ENSG0000	ENSG0000	2.11E-06	hsa-let-7a-5p;hsa-let-7b-5p;hsa-let-7e-5p;hsa-let-7f-5p;hsa-le
ENSG0000	ENSG0000	1.13E-07	hsa-let-7a-5p;hsa-let-7b-5p;hsa-let-7e-5p;hsa-let-7f-5p;hsa-le
ENSG0000	ENSG0000	5.04E-06	hsa-let-7a-5p;hsa-let-7b-5p;hsa-let-7e-5p;hsa-let-7f-5p;hsa-le
ENSG0000	ENSG0000	6.19E-07	hsa-let-7a-5p;hsa-let-7b-5p;hsa-let-7e-5p;hsa-let-7f-5p;hsa-le
ENSG0000	ENSG0000	4.77E-05	hsa-let-7a-5p;hsa-let-7f-5p;hsa-let-7g-5p;hsa-let-7i-5p
ENSG0000	ENSG0000	3.32E-06	hsa-let-7a-5p;hsa-let-7b-5p;hsa-let-7e-5p;hsa-let-7f-5p;hsa-le
ENSG0000	ENSG0000	2.53E-08	hsa-let-7a-5p;hsa-let-7b-5p;hsa-let-7e-5p;hsa-let-7f-5p;hsa-le
ENSG0000	ENSG0000	8.95E-06	hsa-let-7a-5p;hsa-let-7b-5p;hsa-let-7e-5p;hsa-let-7f-5p;hsa-le
ENSG0000	ENSG0000	5.61E-07	hsa-let-7a-5p;hsa-let-7b-5p;hsa-let-7e-5p;hsa-let-7f-5p;hsa-le
ENSG0000	ENSG0000	1.23E-07	hsa-let-7a-5p;hsa-let-7b-5p;hsa-let-7e-5p;hsa-let-7f-5p;hsa-le
ENSG0000	ENSG0000	9.13E-06	hsa-let-7a-5p;hsa-let-7e-5p;hsa-let-7f-5p;hsa-let-7g-5p;hsa-le
ENSG0000	ENSG0000	0.000245	hsa-let-7a-5p;hsa-let-7b-5p;hsa-let-7e-5p;hsa-let-7f-5p;hsa-le
ENSG0000	ENSG0000	0.000245	hsa-let-7a-5p;hsa-let-7b-5p;hsa-let-7e-5p;hsa-let-7f-5p;hsa-le
ENSG0000	ENSG0000	5.61E-07	hsa-let-7a-5p;hsa-let-7b-5p;hsa-let-7e-5p;hsa-let-7f-5p;hsa-le
ENSG0000	ENSG0000	8.25E-07	hsa-let-7a-5p;hsa-let-7b-5p;hsa-let-7e-5p;hsa-let-7f-5p;hsa-le
ENSG0000	ENSG0000	9.91E-08	hsa-let-7a-5p;hsa-let-7b-5p;hsa-let-7e-5p;hsa-let-7f-5p;hsa-le
ENSG0000	ENSG0000	2.11E-06	hsa-let-7a-5p;hsa-let-7b-5p;hsa-let-7e-5p;hsa-let-7f-5p;hsa-le
ENSG0000	ENSG0000	2.11E-06	hsa-let-7a-5p;hsa-let-7b-5p;hsa-let-7e-5p;hsa-let-7f-5p;hsa-le
ENSG0000	ENSG0000	4.43E-10	hsa-let-7a-5p;hsa-let-7b-5p;hsa-let-7e-5p;hsa-let-7f-5p;hsa-le
ENSG0000	ENSG0000	5.07E-07	hsa-let-7a-5p;hsa-let-7b-5p;hsa-let-7e-5p;hsa-let-7f-5p;hsa-le
ENSG0000	ENSG0000	0.001114	hsa-let-7b-5p;hsa-let-7e-5p;hsa-let-7f-5p;hsa-let-7i-5p
ENSG0000	ENSG0000	6.19E-07	hsa-let-7a-5p;hsa-let-7b-5p;hsa-let-7e-5p;hsa-let-7f-5p;hsa-le
ENSG0000	ENSG0000	0.000398	hsa-let-7a-5p;hsa-let-7b-5p;hsa-let-7e-5p;hsa-let-7f-5p;hsa-le
ENSG0000	ENSG0000	1.91E-06	hsa-let-7a-5p;hsa-let-7b-5p;hsa-let-7e-5p;hsa-let-7f-5p;hsa-le
ENSG0000	ENSG0000	1.77E-08	hsa-let-7a-5p;hsa-let-7b-5p;hsa-let-7e-5p;hsa-let-7f-5p;hsa-le
ENSG0000	ENSG0000	0.000375	hsa-let-7a-5p;hsa-let-7e-5p;hsa-let-7f-5p;hsa-let-7g-5p;hsa-le
ENSG0000	ENSG0000	1.53E-06	hsa-let-7a-5p;hsa-let-7b-5p;hsa-let-7e-5p;hsa-let-7f-5p;hsa-le
ENSG0000	ENSG0000	1.88E-07	hsa-let-7a-5p;hsa-let-7b-5p;hsa-let-7e-5p;hsa-let-7f-5p;hsa-le
ENSG0000	ENSG0000	1.00E-08	hsa-let-7a-5p;hsa-let-7b-5p;hsa-let-7e-5p;hsa-let-7f-5p;hsa-le
ENSG0000	ENSG0000	6.51E-08	hsa-let-7a-5p;hsa-let-7b-5p;hsa-let-7e-5p;hsa-let-7f-5p;hsa-le
ENSG0000	ENSG0000	6.54E-06	hsa-let-7a-5p;hsa-let-7b-5p;hsa-let-7e-5p;hsa-let-7f-5p;hsa-le
ENSG0000	ENSG0000	0.001325	hsa-let-7a-5p;hsa-let-7b-5p;hsa-let-7e-5p;hsa-let-7g-5p
ENSG0000	ENSG0000	9.91E-08	hsa-let-7a-5p;hsa-let-7b-5p;hsa-let-7e-5p;hsa-let-7f-5p;hsa-le
ENSG0000	ENSG0000	0.000268	hsa-let-7a-5p;hsa-let-7e-5p;hsa-let-7f-5p;hsa-let-7g-5p;hsa-le
ENSG0000	ENSG0000	1.18E-06	hsa-let-7a-5p;hsa-let-7b-5p;hsa-let-7e-5p;hsa-let-7f-5p;hsa-le
ENSG0000	ENSG0000	2.46E-06	hsa-let-7a-5p;hsa-let-7b-5p;hsa-let-7e-5p;hsa-let-7f-5p;hsa-le
ENSG0000	ENSG0000	2.99E-08	hsa-let-7a-5p;hsa-let-7b-5p;hsa-let-7e-5p;hsa-let-7f-5p;hsa-le
ENSG0000	ENSG0000	4.13E-08	hsa-let-7a-5p;hsa-let-7b-5p;hsa-let-7e-5p;hsa-let-7f-5p;hsa-le
ENSG0000	ENSG0000	1.37E-11	hsa-let-7a-5p;hsa-let-7b-5p;hsa-let-7e-5p;hsa-let-7f-5p;hsa-le
ENSG0000	ENSG0000	0.000292	hsa-let-7a-5p;hsa-let-7e-5p;hsa-let-7f-5p;hsa-let-7g-5p;hsa-le
ENSG0000	ENSG0000	5.07E-07	hsa-let-7a-5p;hsa-let-7b-5p;hsa-let-7e-5p;hsa-let-7f-5p;hsa-le

ENSG0000	ENSG0000	3.33E-07	hsa-let-7a-5p;hsa-let-7b-5p;hsa-let-7e-5p;hsa-let-7f-5p;hsa-le
ENSG0000	ENSG0000	1.40E-06	hsa-let-7a-5p;hsa-let-7b-5p;hsa-let-7e-5p;hsa-let-7f-5p;hsa-le
ENSG0000	ENSG0000	2.82E-07	hsa-let-7a-5p;hsa-let-7e-5p;hsa-let-7f-5p;hsa-let-7g-5p;hsa-le
ENSG0000	ENSG0000	9.27E-11	hsa-let-7a-5p;hsa-let-7b-5p;hsa-let-7e-5p;hsa-let-7f-5p;hsa-le
ENSG0000	ENSG0000	8.73E-05	hsa-let-7a-5p;hsa-let-7f-5p;hsa-let-7g-5p;hsa-let-7i-5p;hsa-m
ENSG0000	ENSG0000	6.19E-07	hsa-let-7a-5p;hsa-let-7b-5p;hsa-let-7e-5p;hsa-let-7f-5p;hsa-le
ENSG0000	ENSG0000	4.58E-07	hsa-let-7a-5p;hsa-let-7b-5p;hsa-let-7e-5p;hsa-let-7f-5p;hsa-le
ENSG0000	ENSG0000	4.58E-07	hsa-let-7a-5p;hsa-let-7b-5p;hsa-let-7e-5p;hsa-let-7f-5p;hsa-le
ENSG0000	ENSG0000	9.91E-08	hsa-let-7a-5p;hsa-let-7b-5p;hsa-let-7e-5p;hsa-let-7f-5p;hsa-le
ENSG0000	ENSG0000	2.67E-07	hsa-let-7a-5p;hsa-let-7b-5p;hsa-let-7e-5p;hsa-let-7f-5p;hsa-le
ENSG0000	ENSG0000	2.59E-09	hsa-let-7a-5p;hsa-let-7b-5p;hsa-let-7e-5p;hsa-let-7f-5p;hsa-le
ENSG0000	ENSG0000	6.24E-06	hsa-let-7a-5p;hsa-let-7b-5p;hsa-let-7e-5p;hsa-let-7f-5p;hsa-le
ENSG0000	ENSG0000	1.88E-07	hsa-let-7a-5p;hsa-let-7b-5p;hsa-let-7e-5p;hsa-let-7f-5p;hsa-le
ENSG0000	ENSG0000	3.33E-07	hsa-let-7a-5p;hsa-let-7b-5p;hsa-let-7e-5p;hsa-let-7f-5p;hsa-le
ENSG0000	ENSG0000	1.14E-09	hsa-let-7a-5p;hsa-let-7b-5p;hsa-let-7e-5p;hsa-let-7f-5p;hsa-le
ENSG0000	ENSG0000	6.59E-09	hsa-let-7a-5p;hsa-let-7b-5p;hsa-let-7e-5p;hsa-let-7f-5p;hsa-le
ENSG0000	ENSG0000	3.52E-08	hsa-let-7a-5p;hsa-let-7b-5p;hsa-let-7e-5p;hsa-let-7f-5p;hsa-le
ENSG0000	ENSG0000	3.71E-07	hsa-let-7a-5p;hsa-let-7b-5p;hsa-let-7e-5p;hsa-let-7f-5p;hsa-le
ENSG0000	ENSG0000	5.76E-06	hsa-let-7a-5p;hsa-let-7b-5p;hsa-let-7e-5p;hsa-let-7f-5p;hsa-le
ENSG0000	ENSG0000	5.61E-07	hsa-let-7a-5p;hsa-let-7b-5p;hsa-let-7e-5p;hsa-let-7f-5p;hsa-le
ENSG0000	ENSG0000	0.000194	hsa-let-7a-5p;hsa-let-7b-5p;hsa-let-7g-5p;hsa-miR-9-5p
ENSG0000	ENSG0000	2.95E-06	hsa-let-7a-5p;hsa-let-7f-5p;hsa-let-7g-5p;hsa-let-7i-5p;hsa-m
ENSG0000	ENSG0000	0.000386	hsa-let-7a-5p;hsa-let-7b-5p;hsa-let-7e-5p;hsa-let-7f-5p;hsa-le
ENSG0000	ENSG0000	1.13E-05	hsa-let-7a-5p;hsa-let-7b-5p;hsa-let-7e-5p;hsa-let-7f-5p;hsa-le
ENSG0000	ENSG0000	1.13E-05	hsa-let-7a-5p;hsa-let-7b-5p;hsa-let-7e-5p;hsa-let-7f-5p;hsa-le
ENSG0000	ENSG0000	7.46E-05	hsa-let-7a-5p;hsa-let-7b-5p;hsa-let-7e-5p;hsa-let-7f-5p;hsa-le
ENSG0000	ENSG0000	2.59E-09	hsa-let-7a-5p;hsa-let-7b-5p;hsa-let-7e-5p;hsa-let-7f-5p;hsa-le
ENSG0000	ENSG0000	4.21E-09	hsa-let-7a-5p;hsa-let-7b-5p;hsa-let-7e-5p;hsa-let-7f-5p;hsa-le
ENSG0000	ENSG0000	4.21E-09	hsa-let-7a-5p;hsa-let-7b-5p;hsa-let-7e-5p;hsa-let-7f-5p;hsa-le
ENSG0000	ENSG0000	5.56E-05	hsa-let-7a-5p;hsa-let-7b-5p;hsa-let-7e-5p;hsa-let-7f-5p;hsa-le
ENSG0000	ENSG0000	0.005057	hsa-miR-18a-3p;hsa-miR-324-3p;hsa-miR-361-5p;hsa-miR-9-3
ENSG0000	ENSG0000	1.66E-07	hsa-let-7a-5p;hsa-let-7b-5p;hsa-let-7e-5p;hsa-let-7f-5p;hsa-le
ENSG0000	ENSG0000	4.53E-05	hsa-let-7b-5p;hsa-let-7e-5p;hsa-let-7f-5p;hsa-let-7g-5p;hsa-le
ENSG0000	ENSG0000	5.07E-07	hsa-let-7a-5p;hsa-let-7b-5p;hsa-let-7e-5p;hsa-let-7f-5p;hsa-le
ENSG0000	ENSG0000	3.32E-06	hsa-let-7a-5p;hsa-let-7b-5p;hsa-let-7e-5p;hsa-let-7f-5p;hsa-le
ENSG0000	ENSG0000	7.44E-06	hsa-let-7a-5p;hsa-let-7b-5p;hsa-let-7e-5p;hsa-let-7f-5p;hsa-le
ENSG0000	ENSG0000	8.15E-09	hsa-let-7a-5p;hsa-let-7b-5p;hsa-let-7e-5p;hsa-let-7f-5p;hsa-le
ENSG0000	ENSG0000	3.72E-05	hsa-let-7a-5p;hsa-let-7b-5p;hsa-let-7f-5p;hsa-let-7g-5p;hsa-le
ENSG0000	ENSG0000	2.80E-05	hsa-let-7a-5p;hsa-let-7b-5p;hsa-let-7e-5p;hsa-let-7f-5p;hsa-le
ENSG0000	ENSG0000	3.32E-10	hsa-let-7a-5p;hsa-let-7b-5p;hsa-let-7e-5p;hsa-let-7f-5p;hsa-le
ENSG0000	ENSG0000	3.32E-06	hsa-let-7a-5p;hsa-let-7b-5p;hsa-let-7e-5p;hsa-let-7f-5p;hsa-le
ENSG0000	ENSG0000	0.000546	hsa-let-7a-5p;hsa-let-7f-5p;hsa-let-7g-5p;hsa-let-7i-5p;hsa-m
ENSG0000	ENSG0000	0.000376	hsa-let-7e-5p;hsa-let-7f-5p;hsa-let-7g-5p;hsa-let-7i-5p
ENSG0000	ENSG0000	0.000376	hsa-let-7e-5p;hsa-let-7f-5p;hsa-let-7g-5p;hsa-let-7i-5p
ENSG0000	ENSG0000	3.23E-05	hsa-let-7a-5p;hsa-let-7b-5p;hsa-let-7e-5p;hsa-let-7f-5p;hsa-le
ENSG0000	ENSG0000	5.07E-07	hsa-let-7a-5p;hsa-let-7b-5p;hsa-let-7e-5p;hsa-let-7f-5p;hsa-le
ENSG0000	ENSG0000	1.47E-07	hsa-let-7a-5p;hsa-let-7b-5p;hsa-let-7e-5p;hsa-let-7f-5p;hsa-le
ENSG0000	ENSG0000	3.02E-05	hsa-let-7a-5p;hsa-let-7e-5p;hsa-let-7f-5p;hsa-let-7g-5p;hsa-le
ENSG0000	ENSG0000	0.000658	hsa-let-7e-5p;hsa-let-7f-5p;hsa-let-7g-5p;hsa-let-7i-5p
ENSG0000	ENSG0000	7.51E-08	hsa-let-7a-5p;hsa-let-7b-5p;hsa-let-7e-5p;hsa-let-7f-5p;hsa-le
ENSG0000	ENSG0000	7.51E-08	hsa-let-7a-5p;hsa-let-7b-5p;hsa-let-7e-5p;hsa-let-7f-5p;hsa-le
ENSG0000	ENSG0000	7.51E-08	hsa-let-7a-5p;hsa-let-7b-5p;hsa-let-7e-5p;hsa-let-7f-5p;hsa-le
ENSG0000	ENSG0000	9.91E-08	hsa-let-7a-5p;hsa-let-7b-5p;hsa-let-7e-5p;hsa-let-7f-5p;hsa-le
ENSG0000	ENSG0000	3.32E-06	hsa-let-7a-5p;hsa-let-7b-5p;hsa-let-7e-5p;hsa-let-7f-5p;hsa-le

ENSG0000	ENSG0000	1.47E-08	hsa-let-7a-5p;hsa-let-7b-5p;hsa-let-7e-5p;hsa-let-7f-5p;hsa-le
ENSG0000	ENSG0000	1.22E-08	hsa-let-7a-5p;hsa-let-7b-5p;hsa-let-7e-5p;hsa-let-7f-5p;hsa-le
ENSG0000	ENSG0000	3.31E-06	hsa-let-7a-5p;hsa-let-7e-5p;hsa-let-7f-5p;hsa-let-7g-5p;hsa-le
ENSG0000	ENSG0000	4.88E-05	hsa-let-7a-5p;hsa-let-7b-5p;hsa-let-7e-5p;hsa-let-7f-5p;hsa-le
ENSG0000	ENSG0000	4.88E-05	hsa-let-7a-5p;hsa-let-7b-5p;hsa-let-7e-5p;hsa-let-7f-5p;hsa-le
ENSG0000	ENSG0000	4.60E-06	hsa-let-7a-5p;hsa-let-7e-5p;hsa-let-7f-5p;hsa-let-7g-5p;hsa-le
ENSG0000	ENSG0000	3.39E-07	hsa-let-7a-5p;hsa-let-7e-5p;hsa-let-7f-5p;hsa-let-7g-5p;hsa-le
ENSG0000	ENSG0000	4.43E-10	hsa-let-7a-5p;hsa-let-7b-5p;hsa-let-7e-5p;hsa-let-7f-5p;hsa-le
ENSG0000	ENSG0000	1.00E-08	hsa-let-7a-5p;hsa-let-7b-5p;hsa-let-7e-5p;hsa-let-7f-5p;hsa-le
ENSG0000	ENSG0000	2.73E-09	hsa-let-7a-5p;hsa-let-7b-5p;hsa-let-7e-5p;hsa-let-7f-5p;hsa-le
ENSG0000	ENSG0000	3.71E-07	hsa-let-7a-5p;hsa-let-7b-5p;hsa-let-7e-5p;hsa-let-7f-5p;hsa-le
ENSG0000	ENSG0000	3.71E-07	hsa-let-7a-5p;hsa-let-7b-5p;hsa-let-7e-5p;hsa-let-7f-5p;hsa-le
ENSG0000	ENSG0000	1.47E-08	hsa-let-7a-5p;hsa-let-7b-5p;hsa-let-7e-5p;hsa-let-7f-5p;hsa-le
ENSG0000	ENSG0000	2.12E-08	hsa-let-7a-5p;hsa-let-7b-5p;hsa-let-7e-5p;hsa-let-7f-5p;hsa-le
ENSG0000	ENSG0000	5.47E-05	hsa-let-7a-5p;hsa-let-7b-5p;hsa-let-7f-5p;hsa-let-7g-5p;hsa-le
ENSG0000	ENSG0000	2.66E-06	hsa-let-7a-5p;hsa-let-7b-5p;hsa-let-7e-5p;hsa-let-7f-5p;hsa-le
ENSG0000	ENSG0000	1.66E-07	hsa-let-7a-5p;hsa-let-7b-5p;hsa-let-7e-5p;hsa-let-7f-5p;hsa-le
ENSG0000	ENSG0000	1.13E-07	hsa-let-7a-5p;hsa-let-7b-5p;hsa-let-7e-5p;hsa-let-7f-5p;hsa-le
ENSG0000	ENSG0000	3.47E-05	hsa-let-7a-5p;hsa-let-7b-5p;hsa-let-7f-5p;hsa-let-7g-5p;hsa-le
ENSG0000	ENSG0000	1.66E-07	hsa-let-7a-5p;hsa-let-7b-5p;hsa-let-7e-5p;hsa-let-7f-5p;hsa-le
ENSG0000	ENSG0000	2.29E-05	hsa-let-7a-5p;hsa-let-7b-5p;hsa-let-7e-5p;hsa-let-7f-5p;hsa-le
ENSG0000	ENSG0000	2.29E-05	hsa-let-7a-5p;hsa-let-7b-5p;hsa-let-7e-5p;hsa-let-7f-5p;hsa-le
ENSG0000	ENSG0000	6.19E-10	hsa-let-7a-5p;hsa-let-7b-5p;hsa-let-7e-5p;hsa-let-7f-5p;hsa-le
ENSG0000	ENSG0000	9.27E-11	hsa-let-7a-5p;hsa-let-7b-5p;hsa-let-7e-5p;hsa-let-7f-5p;hsa-le
ENSG0000	ENSG0000	6.19E-07	hsa-let-7a-5p;hsa-let-7b-5p;hsa-let-7e-5p;hsa-let-7f-5p;hsa-le
ENSG0000	ENSG0000	7.51E-08	hsa-let-7a-5p;hsa-let-7b-5p;hsa-let-7e-5p;hsa-let-7f-5p;hsa-le
ENSG0000	ENSG0000	1.09E-08	hsa-let-7a-5p;hsa-let-7b-5p;hsa-let-7e-5p;hsa-let-7f-5p;hsa-le
ENSG0000	ENSG0000	2.07E-05	hsa-let-7a-5p;hsa-let-7b-5p;hsa-let-7e-5p;hsa-let-7f-5p;hsa-le
ENSG0000	ENSG0000	6.56E-06	hsa-let-7a-5p;hsa-let-7b-5p;hsa-let-7e-5p;hsa-let-7f-5p;hsa-le
ENSG0000	ENSG0000	0.000153	hsa-let-7a-5p;hsa-let-7b-5p;hsa-let-7e-5p;hsa-let-7f-5p;hsa-le
ENSG0000	ENSG0000	1.38E-06	hsa-let-7a-5p;hsa-let-7b-5p;hsa-let-7e-5p;hsa-let-7f-5p;hsa-le
ENSG0000	ENSG0000	1.47E-08	hsa-let-7a-5p;hsa-let-7b-5p;hsa-let-7e-5p;hsa-let-7f-5p;hsa-le
ENSG0000	ENSG0000	1.43E-10	hsa-let-7a-5p;hsa-let-7b-5p;hsa-let-7e-5p;hsa-let-7f-5p;hsa-le
ENSG0000	ENSG0000	1.29E-06	hsa-let-7a-5p;hsa-let-7b-5p;hsa-let-7e-5p;hsa-let-7f-5p;hsa-le
ENSG0000	ENSG0000	1.22E-08	hsa-let-7a-5p;hsa-let-7b-5p;hsa-let-7e-5p;hsa-let-7f-5p;hsa-le
ENSG0000	ENSG0000	8.64E-08	hsa-let-7a-5p;hsa-let-7b-5p;hsa-let-7e-5p;hsa-let-7f-5p;hsa-le
ENSG0000	ENSG0000	1.52E-09	hsa-let-7a-5p;hsa-let-7b-5p;hsa-let-7e-5p;hsa-let-7f-5p;hsa-le
ENSG0000	ENSG0000	4.21E-09	hsa-let-7a-5p;hsa-let-7b-5p;hsa-let-7e-5p;hsa-let-7f-5p;hsa-le
ENSG0000	ENSG0000	4.21E-09	hsa-let-7a-5p;hsa-let-7b-5p;hsa-let-7e-5p;hsa-let-7f-5p;hsa-le
ENSG0000	ENSG0000	4.88E-05	hsa-let-7a-5p;hsa-let-7b-5p;hsa-let-7e-5p;hsa-let-7f-5p;hsa-le
ENSG0000	ENSG0000	1.77E-08	hsa-let-7a-5p;hsa-let-7b-5p;hsa-let-7e-5p;hsa-let-7f-5p;hsa-le
ENSG0000	ENSG0000	1.95E-06	hsa-let-7a-5p;hsa-let-7b-5p;hsa-let-7e-5p;hsa-let-7f-5p;hsa-le
ENSG0000	ENSG0000	5.39E-06	hsa-let-7a-5p;hsa-let-7b-5p;hsa-let-7e-5p;hsa-let-7f-5p;hsa-le
ENSG0000	ENSG0000	1.52E-09	hsa-let-7a-5p;hsa-let-7b-5p;hsa-let-7e-5p;hsa-let-7f-5p;hsa-le
ENSG0000	ENSG0000	1.40E-06	hsa-let-7a-5p;hsa-let-7b-5p;hsa-let-7e-5p;hsa-let-7f-5p;hsa-le
ENSG0000	ENSG0000	1.40E-06	hsa-let-7a-5p;hsa-let-7b-5p;hsa-let-7e-5p;hsa-let-7f-5p;hsa-le
ENSG0000	ENSG0000	4.29E-05	hsa-let-7e-5p;hsa-let-7f-5p;hsa-let-7g-5p;hsa-let-7i-5p
ENSG0000	ENSG0000	1.53E-06	hsa-let-7a-5p;hsa-let-7b-5p;hsa-let-7e-5p;hsa-let-7f-5p;hsa-le
ENSG0000	ENSG0000	2.12E-08	hsa-let-7a-5p;hsa-let-7b-5p;hsa-let-7e-5p;hsa-let-7f-5p;hsa-le
ENSG0000	ENSG0000	7.82E-07	hsa-let-7b-5p;hsa-let-7e-5p;hsa-let-7f-5p;hsa-let-7g-5p;hsa-le
ENSG0000	ENSG0000	9.27E-11	hsa-let-7a-5p;hsa-let-7b-5p;hsa-let-7e-5p;hsa-let-7f-5p;hsa-le
ENSG0000	ENSG0000	8.48E-10	hsa-let-7a-5p;hsa-let-7b-5p;hsa-let-7e-5p;hsa-let-7f-5p;hsa-le
ENSG0000	ENSG0000	8.48E-10	hsa-let-7a-5p;hsa-let-7b-5p;hsa-let-7e-5p;hsa-let-7f-5p;hsa-le
ENSG0000	ENSG0000	5.07E-07	hsa-let-7a-5p;hsa-let-7b-5p;hsa-let-7e-5p;hsa-let-7f-5p;hsa-le

ENSG0000	ENSG0000	7.92E-06	hsa-let-7a-5p;hsa-let-7b-5p;hsa-let-7e-5p;hsa-let-7f-5p;hsa-le
ENSG0000	ENSG0000	4.13E-07	hsa-let-7a-5p;hsa-let-7b-5p;hsa-let-7e-5p;hsa-let-7f-5p;hsa-le
ENSG0000	ENSG0000	6.56E-06	hsa-let-7a-5p;hsa-let-7b-5p;hsa-let-7e-5p;hsa-let-7f-5p;hsa-le
ENSG0000	ENSG0000	1.18E-06	hsa-let-7a-5p;hsa-let-7b-5p;hsa-let-7e-5p;hsa-let-7f-5p;hsa-le
ENSG0000	ENSG0000	9.74E-05	hsa-let-7a-5p;hsa-let-7e-5p;hsa-let-7f-5p;hsa-let-7g-5p;hsa-le
ENSG0000	ENSG0000	8.25E-07	hsa-let-7a-5p;hsa-let-7b-5p;hsa-let-7e-5p;hsa-let-7f-5p;hsa-le
ENSG0000	ENSG0000	2.12E-08	hsa-let-7a-5p;hsa-let-7b-5p;hsa-let-7e-5p;hsa-let-7f-5p;hsa-le
ENSG0000	ENSG0000	2.07E-05	hsa-let-7a-5p;hsa-let-7b-5p;hsa-let-7e-5p;hsa-let-7f-5p;hsa-le
ENSG0000	ENSG0000	6.15E-06	hsa-let-7a-5p;hsa-let-7b-5p;hsa-let-7e-5p;hsa-let-7f-5p;hsa-le
ENSG0000	ENSG0000	1.53E-06	hsa-let-7a-5p;hsa-let-7e-5p;hsa-let-7f-5p;hsa-let-7g-5p;hsa-le
ENSG0000	ENSG0000	1.52E-07	hsa-let-7a-5p;hsa-let-7b-5p;hsa-let-7e-5p;hsa-let-7f-5p;hsa-le
ENSG0000	ENSG0000	1.22E-08	hsa-let-7a-5p;hsa-let-7b-5p;hsa-let-7e-5p;hsa-let-7f-5p;hsa-le
ENSG0000	ENSG0000	1.77E-08	hsa-let-7a-5p;hsa-let-7b-5p;hsa-let-7e-5p;hsa-let-7f-5p;hsa-le
ENSG0000	ENSG0000	9.91E-08	hsa-let-7a-5p;hsa-let-7b-5p;hsa-let-7e-5p;hsa-let-7f-5p;hsa-le
ENSG0000	ENSG0000	0.000194	hsa-let-7a-5p;hsa-let-7b-5p;hsa-let-7e-5p;hsa-let-7f-5p;hsa-le
ENSG0000	ENSG0000	5.07E-07	hsa-let-7a-5p;hsa-let-7b-5p;hsa-let-7e-5p;hsa-let-7f-5p;hsa-le
ENSG0000	ENSG0000	8.15E-09	hsa-let-7a-5p;hsa-let-7b-5p;hsa-let-7e-5p;hsa-let-7f-5p;hsa-le
ENSG0000	ENSG0000	1.56E-08	hsa-let-7a-5p;hsa-let-7b-5p;hsa-let-7e-5p;hsa-let-7f-5p;hsa-le
ENSG0000	ENSG0000	4.83E-08	hsa-let-7a-5p;hsa-let-7b-5p;hsa-let-7e-5p;hsa-let-7f-5p;hsa-le
ENSG0000	ENSG0000	9.13E-06	hsa-let-7b-5p;hsa-let-7e-5p;hsa-let-7f-5p;hsa-let-7g-5p;hsa-le
ENSG0000	ENSG0000	6.99E-06	hsa-let-7a-5p;hsa-let-7b-5p;hsa-let-7e-5p;hsa-let-7f-5p;hsa-le
ENSG0000	ENSG0000	1.07E-05	hsa-let-7a-5p;hsa-let-7b-5p;hsa-let-7e-5p;hsa-let-7f-5p;hsa-le
ENSG0000	ENSG0000	9.08E-05	hsa-let-7a-5p;hsa-let-7b-5p;hsa-let-7e-5p;hsa-let-7f-5p;hsa-le
ENSG0000	ENSG0000	6.19E-10	hsa-let-7a-5p;hsa-let-7b-5p;hsa-let-7e-5p;hsa-let-7f-5p;hsa-le
ENSG0000	ENSG0000	8.25E-07	hsa-let-7a-5p;hsa-let-7b-5p;hsa-let-7e-5p;hsa-let-7f-5p;hsa-le
ENSG0000	ENSG0000	3.09E-06	hsa-let-7a-5p;hsa-let-7b-5p;hsa-let-7e-5p;hsa-let-7f-5p;hsa-le
ENSG0000	ENSG0000	4.27E-05	hsa-let-7a-5p;hsa-let-7b-5p;hsa-let-7e-5p;hsa-let-7f-5p;hsa-le
ENSG0000	ENSG0000	1.66E-07	hsa-let-7a-5p;hsa-let-7b-5p;hsa-let-7e-5p;hsa-let-7f-5p;hsa-le
ENSG0000	ENSG0000	1.81E-06	hsa-let-7a-5p;hsa-let-7b-5p;hsa-let-7e-5p;hsa-let-7f-5p;hsa-le
ENSG0000	ENSG0000	7.51E-07	hsa-let-7a-5p;hsa-let-7b-5p;hsa-let-7e-5p;hsa-let-7f-5p;hsa-le
ENSG0000	ENSG0000	4.13E-08	hsa-let-7a-5p;hsa-let-7b-5p;hsa-let-7e-5p;hsa-let-7f-5p;hsa-le
ENSG0000	ENSG0000	0.000388	hsa-let-7a-5p;hsa-let-7e-5p;hsa-let-7f-5p;hsa-let-7g-5p;hsa-le
ENSG0000	ENSG0000	1.18E-06	hsa-let-7a-5p;hsa-let-7b-5p;hsa-let-7e-5p;hsa-let-7f-5p;hsa-le
ENSG0000	ENSG0000	1.66E-06	hsa-let-7a-5p;hsa-let-7b-5p;hsa-let-7e-5p;hsa-let-7f-5p;hsa-le
ENSG0000	ENSG0000	1.96E-05	hsa-let-7a-5p;hsa-let-7b-5p;hsa-let-7e-5p;hsa-let-7f-5p;hsa-le
ENSG0000	ENSG0000	2.00E-09	hsa-let-7a-5p;hsa-let-7b-5p;hsa-let-7e-5p;hsa-let-7f-5p;hsa-le
ENSG0000	ENSG0000	3.11E-10	hsa-let-7a-5p;hsa-let-7b-5p;hsa-let-7e-5p;hsa-let-7f-5p;hsa-le
ENSG0000	ENSG0000	5.76E-06	hsa-let-7a-5p;hsa-let-7b-5p;hsa-let-7e-5p;hsa-let-7f-5p;hsa-le
ENSG0000	ENSG0000	1.59E-05	hsa-let-7a-5p;hsa-let-7b-5p;hsa-let-7e-5p;hsa-let-7f-5p;hsa-le
ENSG0000	ENSG0000	7.82E-07	hsa-let-7a-5p;hsa-let-7b-5p;hsa-let-7e-5p;hsa-let-7g-5p;hsa-l
ENSG0000	ENSG0000	4.58E-07	hsa-let-7a-5p;hsa-let-7b-5p;hsa-let-7e-5p;hsa-let-7f-5p;hsa-le
ENSG0000	ENSG0000	7.92E-06	hsa-let-7a-5p;hsa-let-7b-5p;hsa-let-7e-5p;hsa-let-7f-5p;hsa-le
ENSG0000	ENSG0000	1.13E-05	hsa-let-7a-5p;hsa-let-7b-5p;hsa-let-7e-5p;hsa-let-7f-5p;hsa-le
ENSG0000	ENSG0000	1.14E-09	hsa-let-7a-5p;hsa-let-7b-5p;hsa-let-7e-5p;hsa-let-7f-5p;hsa-le
ENSG0000	ENSG0000	4.83E-08	hsa-let-7a-5p;hsa-let-7b-5p;hsa-let-7e-5p;hsa-let-7f-5p;hsa-le
ENSG0000	ENSG0000	8.42E-06	hsa-let-7a-5p;hsa-let-7b-5p;hsa-let-7e-5p;hsa-let-7f-5p;hsa-le
ENSG0000	ENSG0000	6.99E-06	hsa-let-7a-5p;hsa-let-7b-5p;hsa-let-7e-5p;hsa-let-7f-5p;hsa-le
ENSG0000	ENSG0000	1.91E-06	hsa-let-7a-5p;hsa-let-7b-5p;hsa-let-7e-5p;hsa-let-7f-5p;hsa-le
ENSG0000	ENSG0000	2.87E-06	hsa-let-7a-5p;hsa-let-7b-5p;hsa-let-7e-5p;hsa-let-7f-5p;hsa-le
ENSG0000	ENSG0000	0.000137	hsa-let-7a-5p;hsa-let-7b-5p;hsa-let-7e-5p;hsa-let-7f-5p;hsa-le
ENSG0000	ENSG0000	1.65E-10	hsa-let-7a-5p;hsa-let-7b-5p;hsa-let-7e-5p;hsa-let-7f-5p;hsa-le
ENSG0000	ENSG0000	4.58E-07	hsa-let-7a-5p;hsa-let-7b-5p;hsa-let-7e-5p;hsa-let-7f-5p;hsa-le
ENSG0000	ENSG0000	1.66E-07	hsa-let-7a-5p;hsa-let-7b-5p;hsa-let-7e-5p;hsa-let-7f-5p;hsa-le
ENSG0000	ENSG0000	6.19E-07	hsa-let-7a-5p;hsa-let-7b-5p;hsa-let-7e-5p;hsa-let-7f-5p;hsa-le

ENSG0000	ENSG0000	8.15E-09	hsa-let-7a-5p;hsa-let-7b-5p;hsa-let-7e-5p;hsa-let-7f-5p;hsa-le
ENSG0000	ENSG0000	0.000106	hsa-let-7a-5p;hsa-let-7b-5p;hsa-let-7e-5p;hsa-let-7f-5p;hsa-le
ENSG0000	ENSG0000	3.31E-09	hsa-let-7a-5p;hsa-let-7b-5p;hsa-let-7e-5p;hsa-let-7f-5p;hsa-le
ENSG0000	ENSG0000	2.18E-05	hsa-let-7a-5p;hsa-let-7b-5p;hsa-let-7e-5p;hsa-let-7f-5p;hsa-le
ENSG0000	ENSG0000	4.96E-10	hsa-let-7a-5p;hsa-let-7b-5p;hsa-let-7e-5p;hsa-let-7f-5p;hsa-le
ENSG0000	ENSG0000	2.80E-05	hsa-let-7a-5p;hsa-let-7b-5p;hsa-let-7e-5p;hsa-let-7f-5p;hsa-le
ENSG0000	ENSG0000	2.99E-08	hsa-let-7a-5p;hsa-let-7b-5p;hsa-let-7e-5p;hsa-let-7f-5p;hsa-le
ENSG0000	ENSG0000	2.11E-07	hsa-let-7a-5p;hsa-let-7b-5p;hsa-let-7e-5p;hsa-let-7f-5p;hsa-le
ENSG0000	ENSG0000	2.41E-05	hsa-let-7a-5p;hsa-let-7b-5p;hsa-let-7e-5p;hsa-let-7f-5p;hsa-le
ENSG0000	ENSG0000	2.67E-07	hsa-let-7a-5p;hsa-let-7b-5p;hsa-let-7e-5p;hsa-let-7f-5p;hsa-le
ENSG0000	ENSG0000	4.21E-09	hsa-let-7a-5p;hsa-let-7b-5p;hsa-let-7e-5p;hsa-let-7f-5p;hsa-le
ENSG0000	ENSG0000	2.34E-09	hsa-let-7a-5p;hsa-let-7b-5p;hsa-let-7e-5p;hsa-let-7f-5p;hsa-le
ENSG0000	ENSG0000	7.44E-06	hsa-let-7a-5p;hsa-let-7b-5p;hsa-let-7e-5p;hsa-let-7f-5p;hsa-le
ENSG0000	ENSG0000	2.66E-06	hsa-let-7a-5p;hsa-let-7b-5p;hsa-let-7e-5p;hsa-let-7f-5p;hsa-le
ENSG0000	ENSG0000	9.91E-08	hsa-let-7a-5p;hsa-let-7b-5p;hsa-let-7e-5p;hsa-let-7f-5p;hsa-le
ENSG0000	ENSG0000	0.000179	hsa-let-7a-5p;hsa-let-7e-5p;hsa-let-7f-5p;hsa-let-7g-5p;hsa-le
ENSG0000	ENSG0000	3.52E-08	hsa-let-7a-5p;hsa-let-7b-5p;hsa-let-7e-5p;hsa-let-7f-5p;hsa-le
ENSG0000	ENSG0000	0.000404	hsa-let-7a-5p;hsa-let-7e-5p;hsa-let-7f-5p;hsa-let-7g-5p;hsa-le
ENSG0000	ENSG0000	1.43E-10	hsa-let-7a-5p;hsa-let-7b-5p;hsa-let-7e-5p;hsa-let-7f-5p;hsa-le
ENSG0000	ENSG0000	6.19E-07	hsa-let-7a-5p;hsa-let-7b-5p;hsa-let-7e-5p;hsa-let-7f-5p;hsa-le
ENSG0000	ENSG0000	3.52E-08	hsa-let-7a-5p;hsa-let-7b-5p;hsa-let-7e-5p;hsa-let-7f-5p;hsa-le
ENSG0000	ENSG0000	2.26E-06	hsa-let-7a-5p;hsa-let-7b-5p;hsa-let-7e-5p;hsa-let-7f-5p;hsa-le
ENSG0000	ENSG0000	3.33E-07	hsa-let-7a-5p;hsa-let-7b-5p;hsa-let-7e-5p;hsa-let-7f-5p;hsa-le
ENSG0000	ENSG0000	0.000181	hsa-let-7a-5p;hsa-let-7b-5p;hsa-let-7e-5p;hsa-let-7f-5p;hsa-le
ENSG0000	ENSG0000	3.34E-09	hsa-let-7a-5p;hsa-let-7b-5p;hsa-let-7f-5p;hsa-let-7g-5p;hsa-le
ENSG0000	ENSG0000	1.15E-06	hsa-let-7a-5p;hsa-let-7b-5p;hsa-let-7f-5p;hsa-let-7g-5p;hsa-le
ENSG0000	ENSG0000	9.14E-07	hsa-let-7a-5p;hsa-let-7b-5p;hsa-let-7f-5p;hsa-let-7g-5p;hsa-le
ENSG0000	ENSG0000	1.05E-07	hsa-let-7a-5p;hsa-let-7b-5p;hsa-let-7f-5p;hsa-let-7g-5p;hsa-le
ENSG0000	ENSG0000	2.92E-05	hsa-let-7a-5p;hsa-let-7b-5p;hsa-let-7f-5p;hsa-let-7g-5p;hsa-le
ENSG0000	ENSG0000	2.49E-06	hsa-let-7a-5p;hsa-let-7b-5p;hsa-let-7f-5p;hsa-let-7g-5p;hsa-le
ENSG0000	ENSG0000	0.002147	hsa-let-7a-5p;hsa-let-7b-5p;hsa-let-7g-5p;hsa-let-7i-5p
ENSG0000	ENSG0000	6.32E-10	hsa-let-7a-5p;hsa-let-7b-5p;hsa-let-7f-5p;hsa-let-7g-5p;hsa-le
ENSG0000	ENSG0000	4.38E-09	hsa-let-7a-5p;hsa-let-7b-5p;hsa-let-7f-5p;hsa-let-7g-5p;hsa-le
ENSG0000	ENSG0000	3.17E-07	hsa-let-7a-5p;hsa-let-7b-5p;hsa-let-7f-5p;hsa-let-7g-5p;hsa-le
ENSG0000	ENSG0000	7.13E-08	hsa-let-7a-5p;hsa-let-7b-5p;hsa-let-7f-5p;hsa-let-7g-5p;hsa-le
ENSG0000	ENSG0000	0.000107	hsa-let-7a-5p;hsa-let-7b-5p;hsa-let-7f-5p;hsa-let-7g-5p;hsa-le
ENSG0000	ENSG0000	8.29E-09	hsa-let-7a-5p;hsa-let-7b-5p;hsa-let-7f-5p;hsa-let-7g-5p;hsa-le
ENSG0000	ENSG0000	5.76E-07	hsa-let-7a-5p;hsa-let-7f-5p;hsa-let-7g-5p;hsa-let-7i-5p
ENSG0000	ENSG0000	1.88E-07	hsa-let-7a-5p;hsa-let-7b-5p;hsa-let-7f-5p;hsa-let-7g-5p;hsa-le
ENSG0000	ENSG0000	7.78E-07	hsa-let-7a-5p;hsa-let-7b-5p;hsa-let-7f-5p;hsa-let-7g-5p;hsa-le
ENSG0000	ENSG0000	2.97E-06	hsa-let-7a-5p;hsa-let-7f-5p;hsa-let-7g-5p;hsa-let-7i-5p
ENSG0000	ENSG0000	6.70E-09	hsa-let-7a-5p;hsa-let-7b-5p;hsa-let-7f-5p;hsa-let-7g-5p;hsa-le
ENSG0000	ENSG0000	3.17E-07	hsa-let-7a-5p;hsa-let-7b-5p;hsa-let-7f-5p;hsa-let-7g-5p;hsa-le
ENSG0000	ENSG0000	8.10E-05	hsa-let-7a-5p;hsa-let-7f-5p;hsa-let-7g-5p;hsa-let-7i-5p
ENSG0000	ENSG0000	3.84E-07	hsa-let-7a-5p;hsa-let-7b-5p;hsa-let-7f-5p;hsa-let-7g-5p;hsa-le
ENSG0000	ENSG0000	9.14E-07	hsa-let-7a-5p;hsa-let-7b-5p;hsa-let-7f-5p;hsa-let-7g-5p;hsa-le
ENSG0000	ENSG0000	1.24E-08	hsa-let-7a-5p;hsa-let-7b-5p;hsa-let-7f-5p;hsa-let-7g-5p;hsa-le
ENSG0000	ENSG0000	6.00E-06	hsa-let-7a-5p;hsa-let-7b-5p;hsa-let-7f-5p;hsa-let-7g-5p;hsa-le
ENSG0000	ENSG0000	5.68E-06	hsa-let-7a-5p;hsa-let-7b-5p;hsa-let-7f-5p;hsa-let-7g-5p;hsa-le
ENSG0000	ENSG0000	8.66E-05	hsa-let-7a-5p;hsa-let-7b-5p;hsa-let-7f-5p;hsa-let-7g-5p;hsa-le
ENSG0000	ENSG0000	1.68E-07	hsa-let-7a-5p;hsa-let-7b-5p;hsa-let-7f-5p;hsa-let-7g-5p;hsa-le
ENSG0000	ENSG0000	1.44E-06	hsa-let-7a-5p;hsa-let-7b-5p;hsa-let-7f-5p;hsa-let-7g-5p;hsa-le
ENSG0000	ENSG0000	1.44E-05	hsa-let-7a-5p;hsa-let-7f-5p;hsa-let-7g-5p;hsa-let-7i-5p;hsa-m
ENSG0000	ENSG0000	5.42E-08	hsa-let-7a-5p;hsa-let-7b-5p;hsa-let-7f-5p;hsa-let-7g-5p;hsa-le

ENSG0000	ENSG0000	1.65E-10	hsa-let-7a-5p;hsa-let-7b-5p;hsa-let-7f-5p;hsa-let-7g-5p;hsa-le
ENSG0000	ENSG0000	1.05E-05	hsa-let-7a-5p;hsa-let-7b-5p;hsa-let-7f-5p;hsa-let-7g-5p;hsa-le
ENSG0000	ENSG0000	4.82E-06	hsa-let-7a-5p;hsa-let-7b-5p;hsa-let-7f-5p;hsa-let-7g-5p;hsa-le
ENSG0000	ENSG0000	1.79E-08	hsa-let-7a-5p;hsa-let-7b-5p;hsa-let-7f-5p;hsa-let-7g-5p;hsa-le
ENSG0000	ENSG0000	7.79E-06	hsa-let-7a-5p;hsa-let-7b-5p;hsa-let-7f-5p;hsa-let-7g-5p;hsa-le
ENSG0000	ENSG0000	1.34E-07	hsa-let-7a-5p;hsa-let-7b-5p;hsa-let-7f-5p;hsa-let-7g-5p;hsa-le
ENSG0000	ENSG0000	1.88E-07	hsa-let-7a-5p;hsa-let-7b-5p;hsa-let-7f-5p;hsa-let-7g-5p;hsa-le
ENSG0000	ENSG0000	1.97E-09	hsa-let-7a-5p;hsa-let-7b-5p;hsa-let-7f-5p;hsa-let-7g-5p;hsa-le
ENSG0000	ENSG0000	1.48E-09	hsa-let-7a-5p;hsa-let-7b-5p;hsa-let-7f-5p;hsa-let-7g-5p;hsa-le
ENSG0000	ENSG0000	6.70E-09	hsa-let-7a-5p;hsa-let-7b-5p;hsa-let-7f-5p;hsa-let-7g-5p;hsa-le
ENSG0000	ENSG0000	0.000589	hsa-let-7a-5p;hsa-let-7b-5p;hsa-let-7g-5p;hsa-let-7i-5p
ENSG0000	ENSG0000	7.13E-08	hsa-let-7a-5p;hsa-let-7b-5p;hsa-let-7f-5p;hsa-let-7g-5p;hsa-le
ENSG0000	ENSG0000	6.33E-06	hsa-let-7a-5p;hsa-let-7b-5p;hsa-let-7f-5p;hsa-let-7g-5p;hsa-le
ENSG0000	ENSG0000	8.29E-09	hsa-let-7a-5p;hsa-let-7b-5p;hsa-let-7f-5p;hsa-let-7g-5p;hsa-le
ENSG0000	ENSG0000	8.29E-09	hsa-let-7a-5p;hsa-let-7b-5p;hsa-let-7f-5p;hsa-let-7g-5p;hsa-le
ENSG0000	ENSG0000	8.29E-09	hsa-let-7a-5p;hsa-let-7b-5p;hsa-let-7f-5p;hsa-let-7g-5p;hsa-le
ENSG0000	ENSG0000	2.13E-08	hsa-let-7a-5p;hsa-let-7b-5p;hsa-let-7f-5p;hsa-let-7g-5p;hsa-le
ENSG0000	ENSG0000	3.34E-09	hsa-let-7a-5p;hsa-let-7b-5p;hsa-let-7f-5p;hsa-let-7g-5p;hsa-le
ENSG0000	ENSG0000	3.34E-09	hsa-let-7a-5p;hsa-let-7b-5p;hsa-let-7f-5p;hsa-let-7g-5p;hsa-le
ENSG0000	ENSG0000	3.34E-09	hsa-let-7a-5p;hsa-let-7b-5p;hsa-let-7f-5p;hsa-let-7g-5p;hsa-le
ENSG0000	ENSG0000	3.34E-09	hsa-let-7a-5p;hsa-let-7b-5p;hsa-let-7f-5p;hsa-let-7g-5p;hsa-le
ENSG0000	ENSG0000	3.34E-09	hsa-let-7a-5p;hsa-let-7b-5p;hsa-let-7f-5p;hsa-let-7g-5p;hsa-le
ENSG0000	ENSG0000	3.34E-09	hsa-let-7a-5p;hsa-let-7b-5p;hsa-let-7f-5p;hsa-let-7g-5p;hsa-le
ENSG0000	ENSG0000	3.34E-09	hsa-let-7a-5p;hsa-let-7b-5p;hsa-let-7f-5p;hsa-let-7g-5p;hsa-le
ENSG0000	ENSG0000	4.39E-05	hsa-let-7a-5p;hsa-let-7b-5p;hsa-let-7g-5p;hsa-let-7i-5p
ENSG0000	ENSG0000	0.00181	hsa-let-7a-5p;hsa-let-7f-5p;hsa-let-7g-5p;hsa-let-7i-5p
ENSG0000	ENSG0000	9.89E-07	hsa-let-7a-5p;hsa-let-7b-5p;hsa-let-7f-5p;hsa-let-7g-5p;hsa-le
ENSG0000	ENSG0000	8.44E-07	hsa-let-7a-5p;hsa-let-7b-5p;hsa-let-7f-5p;hsa-let-7g-5p;hsa-le
ENSG0000	ENSG0000	2.18E-06	hsa-let-7a-5p;hsa-let-7b-5p;hsa-let-7f-5p;hsa-let-7g-5p;hsa-le
ENSG0000	ENSG0000	1.88E-07	hsa-let-7a-5p;hsa-let-7b-5p;hsa-let-7f-5p;hsa-let-7g-5p;hsa-le
ENSG0000	ENSG0000	4.55E-06	hsa-let-7a-5p;hsa-let-7b-5p;hsa-let-7f-5p;hsa-let-7g-5p;hsa-le
ENSG0000	ENSG0000	7.78E-07	hsa-let-7a-5p;hsa-let-7b-5p;hsa-let-7f-5p;hsa-let-7g-5p;hsa-le
ENSG0000	ENSG0000	4.69E-06	hsa-let-7a-5p;hsa-let-7f-5p;hsa-let-7g-5p;hsa-let-7i-5p
ENSG0000	ENSG0000	6.49E-08	hsa-let-7a-5p;hsa-let-7b-5p;hsa-let-7f-5p;hsa-let-7g-5p;hsa-le
ENSG0000	ENSG0000	5.42E-08	hsa-let-7a-5p;hsa-let-7b-5p;hsa-let-7f-5p;hsa-let-7g-5p;hsa-le
ENSG0000	ENSG0000	7.40E-06	hsa-let-7a-5p;hsa-let-7b-5p;hsa-let-7f-5p;hsa-let-7g-5p;hsa-le
ENSG0000	ENSG0000	7.16E-07	hsa-let-7a-5p;hsa-let-7b-5p;hsa-let-7f-5p;hsa-let-7g-5p;hsa-le
ENSG0000	ENSG0000	6.91E-07	hsa-let-7a-5p;hsa-let-7b-5p;hsa-let-7f-5p;hsa-let-7g-5p
ENSG0000	ENSG0000	2.11E-05	hsa-let-7a-5p;hsa-let-7f-5p;hsa-let-7g-5p;hsa-let-7i-5p
ENSG0000	ENSG0000	0.000757	hsa-let-7a-5p;hsa-let-7b-5p;hsa-let-7f-5p;hsa-let-7g-5p;hsa-le
ENSG0000	ENSG0000	0.000757	hsa-let-7a-5p;hsa-let-7b-5p;hsa-let-7f-5p;hsa-let-7g-5p;hsa-le
ENSG0000	ENSG0000	7.16E-07	hsa-let-7a-5p;hsa-let-7b-5p;hsa-let-7f-5p;hsa-let-7g-5p;hsa-le
ENSG0000	ENSG0000	9.89E-07	hsa-let-7a-5p;hsa-let-7b-5p;hsa-let-7f-5p;hsa-let-7g-5p;hsa-le
ENSG0000	ENSG0000	1.68E-07	hsa-let-7a-5p;hsa-let-7b-5p;hsa-let-7f-5p;hsa-let-7g-5p;hsa-le
ENSG0000	ENSG0000	2.18E-06	hsa-let-7a-5p;hsa-let-7b-5p;hsa-let-7f-5p;hsa-let-7g-5p;hsa-le
ENSG0000	ENSG0000	2.18E-06	hsa-let-7a-5p;hsa-let-7b-5p;hsa-let-7f-5p;hsa-let-7g-5p;hsa-le
ENSG0000	ENSG0000	1.97E-09	hsa-let-7a-5p;hsa-let-7b-5p;hsa-let-7f-5p;hsa-let-7g-5p;hsa-le
ENSG0000	ENSG0000	6.58E-07	hsa-let-7a-5p;hsa-let-7b-5p;hsa-let-7f-5p;hsa-let-7g-5p;hsa-le
ENSG0000	ENSG0000	7.78E-07	hsa-let-7a-5p;hsa-let-7b-5p;hsa-let-7f-5p;hsa-let-7g-5p;hsa-le
ENSG0000	ENSG0000	0.000193	hsa-let-7a-5p;hsa-let-7b-5p;hsa-let-7f-5p;hsa-let-7g-5p;hsa-le
ENSG0000	ENSG0000	2.21E-05	hsa-let-7a-5p;hsa-let-7b-5p;hsa-let-7f-5p;hsa-let-7g-5p;hsa-le
ENSG0000	ENSG0000	4.04E-08	hsa-let-7a-5p;hsa-let-7b-5p;hsa-let-7f-5p;hsa-let-7g-5p;hsa-le
ENSG0000	ENSG0000	0.002476	hsa-let-7a-5p;hsa-let-7f-5p;hsa-let-7g-5p;hsa-let-7i-5p
ENSG0000	ENSG0000	1.66E-06	hsa-let-7a-5p;hsa-let-7b-5p;hsa-let-7f-5p;hsa-let-7g-5p;hsa-le

ENSG0000	ENSG0000	2.87E-07	hsa-let-7a-5p;hsa-let-7b-5p;hsa-let-7f-5p;hsa-let-7g-5p;hsa-le
ENSG0000	ENSG0000	2.52E-08	hsa-let-7a-5p;hsa-let-7b-5p;hsa-let-7f-5p;hsa-let-7g-5p;hsa-le
ENSG0000	ENSG0000	1.19E-07	hsa-let-7a-5p;hsa-let-7b-5p;hsa-let-7f-5p;hsa-let-7g-5p;hsa-le
ENSG0000	ENSG0000	5.37E-05	hsa-let-7a-5p;hsa-let-7b-5p;hsa-let-7f-5p;hsa-let-7g-5p;hsa-le
ENSG0000	ENSG0000	0.000143	hsa-let-7a-5p;hsa-let-7b-5p;hsa-let-7g-5p;hsa-miR-1304-5p
ENSG0000	ENSG0000	1.68E-07	hsa-let-7a-5p;hsa-let-7b-5p;hsa-let-7f-5p;hsa-let-7g-5p;hsa-le
ENSG0000	ENSG0000	0.000328	hsa-let-7a-5p;hsa-let-7f-5p;hsa-let-7g-5p;hsa-let-7i-5p
ENSG0000	ENSG0000	2.25E-08	hsa-let-7a-5p;hsa-let-7b-5p;hsa-let-7f-5p;hsa-let-7g-5p;hsa-le
ENSG0000	ENSG0000	2.49E-06	hsa-let-7a-5p;hsa-let-7b-5p;hsa-let-7f-5p;hsa-let-7g-5p;hsa-le
ENSG0000	ENSG0000	6.23E-08	hsa-let-7a-5p;hsa-let-7b-5p;hsa-let-7f-5p;hsa-let-7g-5p;hsa-le
ENSG0000	ENSG0000	8.14E-08	hsa-let-7a-5p;hsa-let-7b-5p;hsa-let-7f-5p;hsa-let-7g-5p;hsa-le
ENSG0000	ENSG0000	5.37E-09	hsa-let-7a-5p;hsa-let-7b-5p;hsa-let-7f-5p;hsa-let-7g-5p;hsa-le
ENSG0000	ENSG0000	0.000352	hsa-let-7a-5p;hsa-let-7f-5p;hsa-let-7g-5p;hsa-let-7i-5p
ENSG0000	ENSG0000	6.58E-07	hsa-let-7a-5p;hsa-let-7b-5p;hsa-let-7f-5p;hsa-let-7g-5p;hsa-le
ENSG0000	ENSG0000	4.63E-07	hsa-let-7a-5p;hsa-let-7b-5p;hsa-let-7f-5p;hsa-let-7g-5p;hsa-le
ENSG0000	ENSG0000	1.55E-06	hsa-let-7a-5p;hsa-let-7b-5p;hsa-let-7f-5p;hsa-let-7g-5p;hsa-le
ENSG0000	ENSG0000	1.32E-06	hsa-let-7a-5p;hsa-let-7f-5p;hsa-let-7g-5p;hsa-let-7i-5p
ENSG0000	ENSG0000	5.58E-10	hsa-let-7a-5p;hsa-let-7b-5p;hsa-let-7f-5p;hsa-let-7g-5p;hsa-le
ENSG0000	ENSG0000	0.000131	hsa-let-7a-5p;hsa-let-7f-5p;hsa-let-7g-5p;hsa-let-7i-5p
ENSG0000	ENSG0000	7.78E-07	hsa-let-7a-5p;hsa-let-7b-5p;hsa-let-7f-5p;hsa-let-7g-5p;hsa-le
ENSG0000	ENSG0000	6.04E-07	hsa-let-7a-5p;hsa-let-7b-5p;hsa-let-7f-5p;hsa-let-7g-5p;hsa-le
ENSG0000	ENSG0000	6.04E-07	hsa-let-7a-5p;hsa-let-7b-5p;hsa-let-7f-5p;hsa-let-7g-5p;hsa-le
ENSG0000	ENSG0000	1.68E-07	hsa-let-7a-5p;hsa-let-7b-5p;hsa-let-7f-5p;hsa-let-7g-5p;hsa-le
ENSG0000	ENSG0000	3.84E-07	hsa-let-7a-5p;hsa-let-7b-5p;hsa-let-7f-5p;hsa-let-7g-5p;hsa-le
ENSG0000	ENSG0000	8.29E-09	hsa-let-7a-5p;hsa-let-7b-5p;hsa-let-7f-5p;hsa-let-7g-5p;hsa-le
ENSG0000	ENSG0000	5.19E-05	hsa-let-7a-5p;hsa-let-7b-5p;hsa-let-7f-5p;hsa-let-7g-5p;hsa-le
ENSG0000	ENSG0000	2.87E-07	hsa-let-7a-5p;hsa-let-7b-5p;hsa-let-7f-5p;hsa-let-7g-5p;hsa-le
ENSG0000	ENSG0000	4.63E-07	hsa-let-7a-5p;hsa-let-7b-5p;hsa-let-7f-5p;hsa-let-7g-5p;hsa-le
ENSG0000	ENSG0000	4.26E-09	hsa-let-7a-5p;hsa-let-7b-5p;hsa-let-7f-5p;hsa-let-7g-5p;hsa-le
ENSG0000	ENSG0000	1.79E-08	hsa-let-7a-5p;hsa-let-7b-5p;hsa-let-7f-5p;hsa-let-7g-5p;hsa-le
ENSG0000	ENSG0000	7.13E-08	hsa-let-7a-5p;hsa-let-7b-5p;hsa-let-7f-5p;hsa-let-7g-5p;hsa-le
ENSG0000	ENSG0000	5.07E-07	hsa-let-7a-5p;hsa-let-7b-5p;hsa-let-7f-5p;hsa-let-7g-5p;hsa-le
ENSG0000	ENSG0000	5.09E-06	hsa-let-7a-5p;hsa-let-7b-5p;hsa-let-7f-5p;hsa-let-7g-5p;hsa-le
ENSG0000	ENSG0000	7.16E-07	hsa-let-7a-5p;hsa-let-7b-5p;hsa-let-7f-5p;hsa-let-7g-5p;hsa-le
ENSG0000	ENSG0000	8.55E-06	hsa-let-7a-5p;hsa-let-7f-5p;hsa-let-7g-5p;hsa-let-7i-5p
ENSG0000	ENSG0000	0.000188	hsa-let-7a-5p;hsa-let-7b-5p;hsa-let-7f-5p;hsa-let-7g-5p;hsa-le
ENSG0000	ENSG0000	9.05E-06	hsa-let-7a-5p;hsa-let-7b-5p;hsa-let-7f-5p;hsa-let-7g-5p;hsa-le
ENSG0000	ENSG0000	9.05E-06	hsa-let-7a-5p;hsa-let-7b-5p;hsa-let-7f-5p;hsa-let-7g-5p;hsa-le
ENSG0000	ENSG0000	0.000121	hsa-let-7a-5p;hsa-let-7b-5p;hsa-let-7f-5p;hsa-let-7g-5p;hsa-le
ENSG0000	ENSG0000	8.29E-09	hsa-let-7a-5p;hsa-let-7b-5p;hsa-let-7f-5p;hsa-let-7g-5p;hsa-le
ENSG0000	ENSG0000	1.24E-08	hsa-let-7a-5p;hsa-let-7b-5p;hsa-let-7f-5p;hsa-let-7g-5p;hsa-le
ENSG0000	ENSG0000	1.24E-08	hsa-let-7a-5p;hsa-let-7b-5p;hsa-let-7f-5p;hsa-let-7g-5p;hsa-le
ENSG0000	ENSG0000	3.52E-05	hsa-let-7a-5p;hsa-let-7b-5p;hsa-let-7f-5p;hsa-let-7g-5p;hsa-le
ENSG0000	ENSG0000	2.59E-07	hsa-let-7a-5p;hsa-let-7b-5p;hsa-let-7f-5p;hsa-let-7g-5p;hsa-le
ENSG0000	ENSG0000	7.69E-05	hsa-let-7b-5p;hsa-let-7f-5p;hsa-let-7g-5p;hsa-let-7i-5p
ENSG0000	ENSG0000	6.58E-07	hsa-let-7a-5p;hsa-let-7b-5p;hsa-let-7f-5p;hsa-let-7g-5p;hsa-le
ENSG0000	ENSG0000	3.20E-06	hsa-let-7a-5p;hsa-let-7b-5p;hsa-let-7f-5p;hsa-let-7g-5p;hsa-le
ENSG0000	ENSG0000	6.33E-06	hsa-let-7a-5p;hsa-let-7b-5p;hsa-let-7f-5p;hsa-let-7g-5p;hsa-le
ENSG0000	ENSG0000	2.13E-08	hsa-let-7a-5p;hsa-let-7b-5p;hsa-let-7f-5p;hsa-let-7g-5p;hsa-le
ENSG0000	ENSG0000	1.78E-06	hsa-let-7a-5p;hsa-let-7b-5p;hsa-let-7f-5p;hsa-let-7g-5p;hsa-le
ENSG0000	ENSG0000	1.95E-05	hsa-let-7a-5p;hsa-let-7b-5p;hsa-let-7f-5p;hsa-let-7g-5p;hsa-le
ENSG0000	ENSG0000	7.16E-07	hsa-let-7a-5p;hsa-let-7b-5p;hsa-let-7f-5p;hsa-let-7g-5p;hsa-le
ENSG0000	ENSG0000	3.20E-06	hsa-let-7a-5p;hsa-let-7b-5p;hsa-let-7f-5p;hsa-let-7g-5p;hsa-le
ENSG0000	ENSG0000	2.92E-05	hsa-let-7a-5p;hsa-let-7f-5p;hsa-let-7g-5p;hsa-let-7i-5p;hsa-m

ENSG0000	ENSG0000	2.21E-05	hsa-let-7a-5p;hsa-let-7b-5p;hsa-let-7f-5p;hsa-let-7g-5p;hsa-le
ENSG0000	ENSG0000	6.58E-07	hsa-let-7a-5p;hsa-let-7b-5p;hsa-let-7f-5p;hsa-let-7g-5p;hsa-le
ENSG0000	ENSG0000	2.33E-07	hsa-let-7a-5p;hsa-let-7b-5p;hsa-let-7f-5p;hsa-let-7g-5p;hsa-le
ENSG0000	ENSG0000	5.55E-05	hsa-let-7a-5p;hsa-let-7f-5p;hsa-let-7g-5p;hsa-let-7i-5p
ENSG0000	ENSG0000	1.34E-07	hsa-let-7a-5p;hsa-let-7b-5p;hsa-let-7f-5p;hsa-let-7g-5p;hsa-le
ENSG0000	ENSG0000	1.34E-07	hsa-let-7a-5p;hsa-let-7b-5p;hsa-let-7f-5p;hsa-let-7g-5p;hsa-le
ENSG0000	ENSG0000	1.34E-07	hsa-let-7a-5p;hsa-let-7b-5p;hsa-let-7f-5p;hsa-let-7g-5p;hsa-le
ENSG0000	ENSG0000	1.68E-07	hsa-let-7a-5p;hsa-let-7b-5p;hsa-let-7f-5p;hsa-let-7g-5p;hsa-le
ENSG0000	ENSG0000	3.20E-06	hsa-let-7a-5p;hsa-let-7b-5p;hsa-let-7f-5p;hsa-let-7g-5p;hsa-le
ENSG0000	ENSG0000	3.47E-08	hsa-let-7a-5p;hsa-let-7b-5p;hsa-let-7f-5p;hsa-let-7g-5p;hsa-le
ENSG0000	ENSG0000	2.96E-08	hsa-let-7a-5p;hsa-let-7b-5p;hsa-let-7f-5p;hsa-let-7g-5p;hsa-le
ENSG0000	ENSG0000	9.37E-06	hsa-let-7a-5p;hsa-let-7f-5p;hsa-let-7g-5p;hsa-let-7i-5p
ENSG0000	ENSG0000	3.15E-05	hsa-let-7a-5p;hsa-let-7b-5p;hsa-let-7f-5p;hsa-let-7g-5p;hsa-le
ENSG0000	ENSG0000	3.15E-05	hsa-let-7a-5p;hsa-let-7b-5p;hsa-let-7f-5p;hsa-let-7g-5p;hsa-le
ENSG0000	ENSG0000	1.22E-05	hsa-let-7a-5p;hsa-let-7f-5p;hsa-let-7g-5p;hsa-let-7i-5p
ENSG0000	ENSG0000	1.49E-08	hsa-let-7a-5p;hsa-let-7f-5p;hsa-let-7g-5p;hsa-let-7i-5p;hsa-m
ENSG0000	ENSG0000	1.97E-09	hsa-let-7a-5p;hsa-let-7b-5p;hsa-let-7f-5p;hsa-let-7g-5p;hsa-le
ENSG0000	ENSG0000	2.52E-08	hsa-let-7a-5p;hsa-let-7b-5p;hsa-let-7f-5p;hsa-let-7g-5p;hsa-le
ENSG0000	ENSG0000	2.10E-07	hsa-let-7a-5p;hsa-let-7b-5p;hsa-let-7f-5p;hsa-let-7g-5p;hsa-le
ENSG0000	ENSG0000	5.07E-07	hsa-let-7a-5p;hsa-let-7b-5p;hsa-let-7f-5p;hsa-let-7g-5p;hsa-le
ENSG0000	ENSG0000	5.07E-07	hsa-let-7a-5p;hsa-let-7b-5p;hsa-let-7f-5p;hsa-let-7g-5p;hsa-le
ENSG0000	ENSG0000	3.47E-08	hsa-let-7a-5p;hsa-let-7b-5p;hsa-let-7f-5p;hsa-let-7g-5p;hsa-le
ENSG0000	ENSG0000	4.69E-08	hsa-let-7a-5p;hsa-let-7b-5p;hsa-let-7f-5p;hsa-let-7g-5p;hsa-le
ENSG0000	ENSG0000	2.65E-06	hsa-let-7a-5p;hsa-let-7b-5p;hsa-let-7f-5p;hsa-let-7g-5p;hsa-le
ENSG0000	ENSG0000	2.65E-06	hsa-let-7a-5p;hsa-let-7b-5p;hsa-let-7f-5p;hsa-let-7g-5p;hsa-le
ENSG0000	ENSG0000	2.59E-07	hsa-let-7a-5p;hsa-let-7b-5p;hsa-let-7f-5p;hsa-let-7g-5p;hsa-le
ENSG0000	ENSG0000	2.06E-09	hsa-let-7a-5p;hsa-let-7b-5p;hsa-let-7f-5p;hsa-let-7g-5p;hsa-le
ENSG0000	ENSG0000	1.66E-06	hsa-let-7a-5p;hsa-let-7b-5p;hsa-let-7f-5p;hsa-let-7g-5p;hsa-le
ENSG0000	ENSG0000	2.59E-07	hsa-let-7a-5p;hsa-let-7b-5p;hsa-let-7f-5p;hsa-let-7g-5p;hsa-le
ENSG0000	ENSG0000	1.65E-05	hsa-let-7a-5p;hsa-let-7b-5p;hsa-let-7f-5p;hsa-let-7g-5p;hsa-le
ENSG0000	ENSG0000	1.65E-05	hsa-let-7a-5p;hsa-let-7b-5p;hsa-let-7f-5p;hsa-let-7g-5p;hsa-le
ENSG0000	ENSG0000	2.58E-09	hsa-let-7a-5p;hsa-let-7b-5p;hsa-let-7f-5p;hsa-let-7g-5p;hsa-le
ENSG0000	ENSG0000	5.58E-10	hsa-let-7a-5p;hsa-let-7b-5p;hsa-let-7f-5p;hsa-let-7g-5p;hsa-le
ENSG0000	ENSG0000	7.78E-07	hsa-let-7a-5p;hsa-let-7b-5p;hsa-let-7f-5p;hsa-let-7g-5p;hsa-le
ENSG0000	ENSG0000	2.18E-06	hsa-let-7a-5p;hsa-let-7b-5p;hsa-let-7f-5p;hsa-let-7g-5p;hsa-le
ENSG0000	ENSG0000	5.54E-07	hsa-let-7a-5p;hsa-let-7b-5p;hsa-let-7f-5p;hsa-let-7g-5p;hsa-le
ENSG0000	ENSG0000	1.51E-05	hsa-let-7a-5p;hsa-let-7b-5p;hsa-let-7f-5p;hsa-let-7g-5p;hsa-le
ENSG0000	ENSG0000	5.68E-06	hsa-let-7a-5p;hsa-let-7b-5p;hsa-let-7f-5p;hsa-let-7g-5p;hsa-le
ENSG0000	ENSG0000	8.40E-05	hsa-let-7a-5p;hsa-let-7b-5p;hsa-let-7f-5p;hsa-let-7g-5p;hsa-le
ENSG0000	ENSG0000	5.56E-06	hsa-let-7a-5p;hsa-let-7b-5p;hsa-let-7f-5p;hsa-let-7g-5p;hsa-le
ENSG0000	ENSG0000	3.47E-08	hsa-let-7a-5p;hsa-let-7b-5p;hsa-let-7f-5p;hsa-let-7g-5p;hsa-le
ENSG0000	ENSG0000	7.89E-10	hsa-let-7a-5p;hsa-let-7b-5p;hsa-let-7f-5p;hsa-let-7g-5p;hsa-le
ENSG0000	ENSG0000	1.44E-06	hsa-let-7a-5p;hsa-let-7b-5p;hsa-let-7f-5p;hsa-let-7g-5p;hsa-le
ENSG0000	ENSG0000	2.96E-08	hsa-let-7a-5p;hsa-let-7b-5p;hsa-let-7f-5p;hsa-let-7g-5p;hsa-le
ENSG0000	ENSG0000	1.50E-07	hsa-let-7a-5p;hsa-let-7b-5p;hsa-let-7f-5p;hsa-let-7g-5p;hsa-le
ENSG0000	ENSG0000	5.37E-09	hsa-let-7a-5p;hsa-let-7b-5p;hsa-let-7f-5p;hsa-let-7g-5p;hsa-le
ENSG0000	ENSG0000	1.24E-08	hsa-let-7a-5p;hsa-let-7b-5p;hsa-let-7f-5p;hsa-let-7g-5p;hsa-le
ENSG0000	ENSG0000	1.24E-08	hsa-let-7a-5p;hsa-let-7b-5p;hsa-let-7f-5p;hsa-let-7g-5p;hsa-le
ENSG0000	ENSG0000	3.15E-05	hsa-let-7a-5p;hsa-let-7b-5p;hsa-let-7f-5p;hsa-let-7g-5p;hsa-le
ENSG0000	ENSG0000	3.16E-10	hsa-let-7a-5p;hsa-let-7b-5p;hsa-let-7f-5p;hsa-let-7g-5p;hsa-le
ENSG0000	ENSG0000	2.04E-06	hsa-let-7a-5p;hsa-let-7b-5p;hsa-let-7f-5p;hsa-let-7g-5p;hsa-le
ENSG0000	ENSG0000	4.82E-06	hsa-let-7a-5p;hsa-let-7b-5p;hsa-let-7f-5p;hsa-let-7g-5p;hsa-le
ENSG0000	ENSG0000	5.37E-09	hsa-let-7a-5p;hsa-let-7b-5p;hsa-let-7f-5p;hsa-let-7g-5p;hsa-le
ENSG0000	ENSG0000	1.55E-06	hsa-let-7a-5p;hsa-let-7b-5p;hsa-let-7f-5p;hsa-let-7g-5p;hsa-le

ENSG0000	ENSG0000	1.55E-06	hsa-let-7a-5p;hsa-let-7b-5p;hsa-let-7f-5p;hsa-let-7g-5p;hsa-le
ENSG0000	ENSG0000	1.66E-06	hsa-let-7a-5p;hsa-let-7b-5p;hsa-let-7f-5p;hsa-let-7g-5p;hsa-le
ENSG0000	ENSG0000	4.69E-08	hsa-let-7a-5p;hsa-let-7b-5p;hsa-let-7f-5p;hsa-let-7g-5p;hsa-le
ENSG0000	ENSG0000	2.97E-06	hsa-let-7b-5p;hsa-let-7f-5p;hsa-let-7g-5p;hsa-let-7i-5p
ENSG0000	ENSG0000	5.58E-10	hsa-let-7a-5p;hsa-let-7b-5p;hsa-let-7f-5p;hsa-let-7g-5p;hsa-le
ENSG0000	ENSG0000	3.34E-09	hsa-let-7a-5p;hsa-let-7b-5p;hsa-let-7f-5p;hsa-let-7g-5p;hsa-le
ENSG0000	ENSG0000	3.34E-09	hsa-let-7a-5p;hsa-let-7b-5p;hsa-let-7f-5p;hsa-let-7g-5p;hsa-le
ENSG0000	ENSG0000	6.58E-07	hsa-let-7a-5p;hsa-let-7b-5p;hsa-let-7f-5p;hsa-let-7g-5p;hsa-le
ENSG0000	ENSG0000	6.67E-06	hsa-let-7a-5p;hsa-let-7b-5p;hsa-let-7f-5p;hsa-let-7g-5p;hsa-le
ENSG0000	ENSG0000	5.54E-07	hsa-let-7a-5p;hsa-let-7b-5p;hsa-let-7f-5p;hsa-let-7g-5p;hsa-le
ENSG0000	ENSG0000	5.68E-06	hsa-let-7a-5p;hsa-let-7b-5p;hsa-let-7f-5p;hsa-let-7g-5p;hsa-le
ENSG0000	ENSG0000	1.34E-06	hsa-let-7a-5p;hsa-let-7b-5p;hsa-let-7f-5p;hsa-let-7g-5p;hsa-le
ENSG0000	ENSG0000	0.000143	hsa-let-7a-5p;hsa-let-7f-5p;hsa-let-7g-5p;hsa-let-7i-5p
ENSG0000	ENSG0000	9.89E-07	hsa-let-7a-5p;hsa-let-7b-5p;hsa-let-7f-5p;hsa-let-7g-5p;hsa-le
ENSG0000	ENSG0000	4.69E-08	hsa-let-7a-5p;hsa-let-7b-5p;hsa-let-7f-5p;hsa-let-7g-5p;hsa-le
ENSG0000	ENSG0000	1.51E-05	hsa-let-7a-5p;hsa-let-7b-5p;hsa-let-7f-5p;hsa-let-7g-5p;hsa-le
ENSG0000	ENSG0000	5.38E-06	hsa-let-7a-5p;hsa-let-7b-5p;hsa-let-7f-5p;hsa-let-7g-5p;hsa-le
ENSG0000	ENSG0000	6.20E-05	hsa-let-7a-5p;hsa-let-7f-5p;hsa-let-7g-5p;hsa-let-7i-5p
ENSG0000	ENSG0000	3.61E-06	hsa-let-7a-5p;hsa-let-7b-5p;hsa-let-7f-5p;hsa-let-7g-5p;hsa-le
ENSG0000	ENSG0000	2.96E-08	hsa-let-7a-5p;hsa-let-7b-5p;hsa-let-7f-5p;hsa-let-7g-5p;hsa-le
ENSG0000	ENSG0000	4.04E-08	hsa-let-7a-5p;hsa-let-7b-5p;hsa-let-7f-5p;hsa-let-7g-5p;hsa-le
ENSG0000	ENSG0000	1.68E-07	hsa-let-7a-5p;hsa-let-7b-5p;hsa-let-7f-5p;hsa-let-7g-5p;hsa-le
ENSG0000	ENSG0000	4.51E-06	hsa-let-7a-5p;hsa-let-7b-5p;hsa-let-7f-5p;hsa-let-7g-5p;hsa-le
ENSG0000	ENSG0000	6.58E-07	hsa-let-7a-5p;hsa-let-7b-5p;hsa-let-7f-5p;hsa-let-7g-5p;hsa-le
ENSG0000	ENSG0000	2.13E-08	hsa-let-7a-5p;hsa-let-7b-5p;hsa-let-7f-5p;hsa-let-7g-5p;hsa-le
ENSG0000	ENSG0000	1.05E-08	hsa-let-7a-5p;hsa-let-7b-5p;hsa-let-7f-5p;hsa-let-7g-5p;hsa-le
ENSG0000	ENSG0000	9.26E-08	hsa-let-7a-5p;hsa-let-7b-5p;hsa-let-7f-5p;hsa-let-7g-5p;hsa-le
ENSG0000	ENSG0000	2.11E-05	hsa-let-7b-5p;hsa-let-7f-5p;hsa-let-7g-5p;hsa-let-7i-5p
ENSG0000	ENSG0000	6.00E-06	hsa-let-7a-5p;hsa-let-7b-5p;hsa-let-7f-5p;hsa-let-7g-5p;hsa-le
ENSG0000	ENSG0000	8.61E-06	hsa-let-7a-5p;hsa-let-7b-5p;hsa-let-7f-5p;hsa-let-7g-5p;hsa-le
ENSG0000	ENSG0000	5.37E-05	hsa-let-7a-5p;hsa-let-7b-5p;hsa-let-7f-5p;hsa-let-7g-5p;hsa-le
ENSG0000	ENSG0000	2.58E-09	hsa-let-7a-5p;hsa-let-7b-5p;hsa-let-7f-5p;hsa-let-7g-5p;hsa-le
ENSG0000	ENSG0000	9.89E-07	hsa-let-7a-5p;hsa-let-7b-5p;hsa-let-7f-5p;hsa-let-7g-5p;hsa-le
ENSG0000	ENSG0000	3.00E-06	hsa-let-7a-5p;hsa-let-7b-5p;hsa-let-7f-5p;hsa-let-7g-5p;hsa-le
ENSG0000	ENSG0000	2.80E-05	hsa-let-7a-5p;hsa-let-7b-5p;hsa-let-7f-5p;hsa-let-7g-5p;hsa-le
ENSG0000	ENSG0000	2.59E-07	hsa-let-7a-5p;hsa-let-7b-5p;hsa-let-7f-5p;hsa-let-7g-5p;hsa-le
ENSG0000	ENSG0000	2.12E-05	hsa-let-7a-5p;hsa-let-7b-5p;hsa-let-7f-5p;hsa-let-7g-5p;hsa-le
ENSG0000	ENSG0000	9.14E-07	hsa-let-7a-5p;hsa-let-7b-5p;hsa-let-7f-5p;hsa-let-7g-5p;hsa-le
ENSG0000	ENSG0000	8.14E-08	hsa-let-7a-5p;hsa-let-7b-5p;hsa-let-7f-5p;hsa-let-7g-5p;hsa-le
ENSG0000	ENSG0000	0.000444	hsa-let-7a-5p;hsa-let-7f-5p;hsa-let-7g-5p;hsa-let-7i-5p
ENSG0000	ENSG0000	1.34E-06	hsa-let-7a-5p;hsa-let-7b-5p;hsa-let-7f-5p;hsa-let-7g-5p;hsa-le
ENSG0000	ENSG0000	1.78E-06	hsa-let-7a-5p;hsa-let-7b-5p;hsa-let-7f-5p;hsa-let-7g-5p;hsa-le
ENSG0000	ENSG0000	4.07E-07	hsa-let-7a-5p;hsa-let-7b-5p;hsa-let-7f-5p;hsa-let-7g-5p;hsa-le
ENSG0000	ENSG0000	6.70E-09	hsa-let-7a-5p;hsa-let-7b-5p;hsa-let-7f-5p;hsa-let-7g-5p;hsa-le
ENSG0000	ENSG0000	1.48E-09	hsa-let-7a-5p;hsa-let-7b-5p;hsa-let-7f-5p;hsa-let-7g-5p;hsa-le
ENSG0000	ENSG0000	5.09E-06	hsa-let-7a-5p;hsa-let-7b-5p;hsa-let-7f-5p;hsa-let-7g-5p;hsa-le
ENSG0000	ENSG0000	1.20E-05	hsa-let-7a-5p;hsa-let-7b-5p;hsa-let-7f-5p;hsa-let-7g-5p;hsa-le
ENSG0000	ENSG0000	2.97E-06	hsa-let-7a-5p;hsa-let-7b-5p;hsa-let-7g-5p;hsa-let-7i-5p
ENSG0000	ENSG0000	6.04E-07	hsa-let-7a-5p;hsa-let-7b-5p;hsa-let-7f-5p;hsa-let-7g-5p;hsa-le
ENSG0000	ENSG0000	6.67E-06	hsa-let-7a-5p;hsa-let-7b-5p;hsa-let-7f-5p;hsa-let-7g-5p;hsa-le
ENSG0000	ENSG0000	9.05E-06	hsa-let-7a-5p;hsa-let-7b-5p;hsa-let-7f-5p;hsa-let-7g-5p;hsa-le
ENSG0000	ENSG0000	4.26E-09	hsa-let-7a-5p;hsa-let-7b-5p;hsa-let-7f-5p;hsa-let-7g-5p;hsa-le
ENSG0000	ENSG0000	9.26E-08	hsa-let-7a-5p;hsa-let-7b-5p;hsa-let-7f-5p;hsa-let-7g-5p;hsa-le
ENSG0000	ENSG0000	7.03E-06	hsa-let-7a-5p;hsa-let-7b-5p;hsa-let-7f-5p;hsa-let-7g-5p;hsa-le

ENSG0000	ENSG0000	6.00E-06	hsa-let-7a-5p;hsa-let-7b-5p;hsa-let-7f-5p;hsa-let-7g-5p;hsa-le
ENSG0000	ENSG0000	2.21E-05	hsa-let-7a-5p;hsa-let-7b-5p;hsa-let-7f-5p;hsa-let-7g-5p;hsa-le
ENSG0000	ENSG0000	2.82E-06	hsa-let-7a-5p;hsa-let-7b-5p;hsa-let-7f-5p;hsa-let-7g-5p;hsa-le
ENSG0000	ENSG0000	7.66E-05	hsa-let-7a-5p;hsa-let-7b-5p;hsa-let-7f-5p;hsa-let-7g-5p;hsa-le
ENSG0000	ENSG0000	2.96E-08	hsa-let-7a-5p;hsa-let-7b-5p;hsa-let-7f-5p;hsa-let-7g-5p;hsa-le
ENSG0000	ENSG0000	6.04E-07	hsa-let-7a-5p;hsa-let-7b-5p;hsa-let-7f-5p;hsa-let-7g-5p;hsa-le
ENSG0000	ENSG0000	2.59E-07	hsa-let-7a-5p;hsa-let-7b-5p;hsa-let-7f-5p;hsa-let-7g-5p;hsa-le
ENSG0000	ENSG0000	7.78E-07	hsa-let-7a-5p;hsa-let-7b-5p;hsa-let-7f-5p;hsa-let-7g-5p;hsa-le
ENSG0000	ENSG0000	2.13E-08	hsa-let-7a-5p;hsa-let-7b-5p;hsa-let-7f-5p;hsa-let-7g-5p;hsa-le
ENSG0000	ENSG0000	6.13E-05	hsa-let-7a-5p;hsa-let-7b-5p;hsa-let-7f-5p;hsa-let-7g-5p;hsa-le
ENSG0000	ENSG0000	1.02E-08	hsa-let-7a-5p;hsa-let-7b-5p;hsa-let-7f-5p;hsa-let-7g-5p;hsa-le
ENSG0000	ENSG0000	1.58E-05	hsa-let-7a-5p;hsa-let-7b-5p;hsa-let-7f-5p;hsa-let-7g-5p;hsa-le
ENSG0000	ENSG0000	9.14E-07	hsa-let-7a-5p;hsa-let-7b-5p;hsa-let-7f-5p;hsa-let-7g-5p;hsa-le
ENSG0000	ENSG0000	1.95E-05	hsa-let-7a-5p;hsa-let-7b-5p;hsa-let-7f-5p;hsa-let-7g-5p;hsa-le
ENSG0000	ENSG0000	6.23E-08	hsa-let-7a-5p;hsa-let-7b-5p;hsa-let-7f-5p;hsa-let-7g-5p;hsa-le
ENSG0000	ENSG0000	3.17E-07	hsa-let-7a-5p;hsa-let-7b-5p;hsa-let-7f-5p;hsa-let-7g-5p;hsa-le
ENSG0000	ENSG0000	1.72E-05	hsa-let-7a-5p;hsa-let-7b-5p;hsa-let-7f-5p;hsa-let-7g-5p;hsa-le
ENSG0000	ENSG0000	3.84E-07	hsa-let-7a-5p;hsa-let-7b-5p;hsa-let-7f-5p;hsa-let-7g-5p;hsa-le
ENSG0000	ENSG0000	1.24E-08	hsa-let-7a-5p;hsa-let-7b-5p;hsa-let-7f-5p;hsa-let-7g-5p;hsa-le
ENSG0000	ENSG0000	2.06E-09	hsa-let-7a-5p;hsa-let-7b-5p;hsa-let-7f-5p;hsa-let-7g-5p;hsa-le
ENSG0000	ENSG0000	6.33E-06	hsa-let-7a-5p;hsa-let-7b-5p;hsa-let-7f-5p;hsa-let-7g-5p;hsa-le
ENSG0000	ENSG0000	2.65E-06	hsa-let-7a-5p;hsa-let-7b-5p;hsa-let-7f-5p;hsa-let-7g-5p;hsa-le
ENSG0000	ENSG0000	1.68E-07	hsa-let-7a-5p;hsa-let-7b-5p;hsa-let-7f-5p;hsa-let-7g-5p;hsa-le
ENSG0000	ENSG0000	0.000235	hsa-let-7a-5p;hsa-let-7f-5p;hsa-let-7g-5p;hsa-let-7i-5p
ENSG0000	ENSG0000	7.13E-08	hsa-let-7a-5p;hsa-let-7b-5p;hsa-let-7f-5p;hsa-let-7g-5p;hsa-le
ENSG0000	ENSG0000	0.000459	hsa-let-7a-5p;hsa-let-7f-5p;hsa-let-7g-5p;hsa-let-7i-5p
ENSG0000	ENSG0000	7.89E-10	hsa-let-7a-5p;hsa-let-7b-5p;hsa-let-7f-5p;hsa-let-7g-5p;hsa-le
ENSG0000	ENSG0000	7.78E-07	hsa-let-7a-5p;hsa-let-7b-5p;hsa-let-7f-5p;hsa-let-7g-5p;hsa-le
ENSG0000	ENSG0000	7.13E-08	hsa-let-7a-5p;hsa-let-7b-5p;hsa-let-7f-5p;hsa-let-7g-5p;hsa-le
ENSG0000	ENSG0000	2.49E-05	hsa-let-7a-5p;hsa-let-7b-5p;hsa-let-7f-5p;hsa-let-7g-5p;hsa-le
ENSG0000	ENSG0000	4.63E-07	hsa-let-7a-5p;hsa-let-7b-5p;hsa-let-7f-5p;hsa-let-7g-5p;hsa-le
ENSG0000	ENSG0000	9.76E-05	hsa-let-7a-5p;hsa-let-7b-5p;hsa-let-7f-5p;hsa-let-7g-5p;hsa-le
ENSG0000	ENSG0000	0.002511	hsa-miR-15a-5p;hsa-miR-15b-5p;hsa-miR-16-5p;hsa-miR-195-
ENSG0000	ENSG0000	4.76E-06	hsa-miR-15a-5p;hsa-miR-15b-5p;hsa-miR-16-5p;hsa-miR-188-
ENSG0000	ENSG0000	2.16E-05	hsa-miR-15a-5p;hsa-miR-15b-5p;hsa-miR-16-5p;hsa-miR-195-
ENSG0000	ENSG0000	0.000551	hsa-miR-15a-5p;hsa-miR-15b-5p;hsa-miR-195-5p;hsa-miR-36
ENSG0000	ENSG0000	0.000313	hsa-miR-15a-5p;hsa-miR-15b-5p;hsa-miR-16-5p;hsa-miR-195-
ENSG0000	ENSG0000	0.000217	hsa-miR-15a-5p;hsa-miR-15b-5p;hsa-miR-16-5p;hsa-miR-195-
ENSG0000	ENSG0000	0.000217	hsa-miR-15a-5p;hsa-miR-15b-5p;hsa-miR-16-5p;hsa-miR-195-
ENSG0000	ENSG0000	0.008766	hsa-miR-34b-5p;hsa-miR-34c-5p;hsa-miR-449a;hsa-miR-449b-
ENSG0000	ENSG0000	0.008829	hsa-miR-15a-5p;hsa-miR-15b-5p;hsa-miR-16-5p;hsa-miR-195-
ENSG0000	ENSG0000	0.001196	hsa-miR-34a-5p;hsa-miR-34c-5p;hsa-miR-449a;hsa-miR-449b-
ENSG0000	ENSG0000	2.26E-08	hsa-miR-125b-5p;hsa-miR-15a-3p;hsa-miR-15a-5p;hsa-miR-1
ENSG0000	ENSG0000	0.006167	hsa-miR-34a-5p;hsa-miR-34c-5p;hsa-miR-449a;hsa-miR-449b-
ENSG0000	ENSG0000	9.41E-05	hsa-miR-15a-5p;hsa-miR-16-5p;hsa-miR-212-3p;hsa-miR-34a-
ENSG0000	ENSG0000	0.000313	hsa-miR-15a-5p;hsa-miR-15b-5p;hsa-miR-16-5p;hsa-miR-195-
ENSG0000	ENSG0000	0.001776	hsa-miR-15a-5p;hsa-miR-15b-5p;hsa-miR-16-5p;hsa-miR-195-
ENSG0000	ENSG0000	5.05E-05	hsa-miR-15a-5p;hsa-miR-15b-5p;hsa-miR-16-5p;hsa-miR-195-
ENSG0000	ENSG0000	0.000313	hsa-miR-15b-5p;hsa-miR-16-5p;hsa-miR-195-5p;hsa-miR-34a-
ENSG0000	ENSG0000	0.000152	hsa-miR-15a-5p;hsa-miR-15b-5p;hsa-miR-16-5p;hsa-miR-195-
ENSG0000	ENSG0000	0.002511	hsa-miR-15a-5p;hsa-miR-15b-5p;hsa-miR-34a-5p;hsa-miR-44
ENSG0000	ENSG0000	1.21E-05	hsa-miR-15a-5p;hsa-miR-15b-5p;hsa-miR-16-5p;hsa-miR-195-
ENSG0000	ENSG0000	0.008829	hsa-miR-34a-5p;hsa-miR-34c-5p;hsa-miR-449a;hsa-miR-449b-
ENSG0000	ENSG0000	0.004772	hsa-miR-15a-5p;hsa-miR-15b-5p;hsa-miR-16-5p;hsa-miR-195-

ENSG0000	ENSG0000	2.39E-05	hsa-miR-34a-5p;hsa-miR-34c-5p;hsa-miR-449a;hsa-miR-449b-
ENSG0000	ENSG0000	0.001316	hsa-miR-125a-5p;hsa-miR-1260a;hsa-miR-1260b;hsa-miR-188-
ENSG0000	ENSG0000	5.68E-05	hsa-miR-15a-5p;hsa-miR-15b-5p;hsa-miR-16-5p;hsa-miR-188-
ENSG0000	ENSG0000	0.001966	hsa-miR-15a-5p;hsa-miR-15b-5p;hsa-miR-195-5p;hsa-miR-19
ENSG0000	ENSG0000	0.000461	hsa-miR-15a-5p;hsa-miR-15b-5p;hsa-miR-16-5p;hsa-miR-195-
ENSG0000	ENSG0000	2.48E-05	hsa-miR-1306-5p;hsa-miR-15a-5p;hsa-miR-15b-5p;hsa-miR-1
ENSG0000	ENSG0000	1.22E-05	hsa-miR-15a-5p;hsa-miR-15b-5p;hsa-miR-16-5p;hsa-miR-195-
ENSG0000	ENSG0000	2.42E-07	hsa-miR-125a-5p;hsa-miR-125b-5p;hsa-miR-1260a;hsa-miR-1
ENSG0000	ENSG0000	0.008288	hsa-miR-34b-5p;hsa-miR-34c-5p;hsa-miR-449a;hsa-miR-449b-
ENSG0000	ENSG0000	0.001196	hsa-miR-125b-5p;hsa-miR-132-3p;hsa-miR-16-5p;hsa-miR-7-
ENSG0000	ENSG0000	0.004587	hsa-miR-15a-5p;hsa-miR-15b-5p;hsa-miR-16-5p;hsa-miR-195-
ENSG0000	ENSG0000	0.003619	hsa-miR-1260a;hsa-miR-1260b;hsa-miR-1306-5p;hsa-miR-16-
ENSG0000	ENSG0000	9.16E-05	hsa-miR-15a-5p;hsa-miR-15b-5p;hsa-miR-16-5p;hsa-miR-195-
ENSG0000	ENSG0000	1.68E-06	hsa-miR-15a-5p;hsa-miR-15b-5p;hsa-miR-16-5p;hsa-miR-195-
ENSG0000	ENSG0000	0.006962	hsa-miR-1260a;hsa-miR-1260b;hsa-miR-188-3p;hsa-miR-34b-
ENSG0000	ENSG0000	0.001196	hsa-miR-34c-5p;hsa-miR-421;hsa-miR-449a;hsa-miR-449b-5p
ENSG0000	ENSG0000	0.001106	hsa-miR-15a-3p;hsa-miR-15a-5p;hsa-miR-15b-5p;hsa-miR-16-
ENSG0000	ENSG0000	0.008196	hsa-miR-1306-5p;hsa-miR-199a-5p;hsa-miR-199b-5p;hsa-miR
ENSG0000	ENSG0000	0.002244	hsa-miR-34a-5p;hsa-miR-34c-5p;hsa-miR-449a;hsa-miR-449b-
ENSG0000	ENSG0000	0.000653	hsa-miR-15a-5p;hsa-miR-15b-5p;hsa-miR-195-5p;hsa-miR-36
ENSG0000	ENSG0000	0.00177	hsa-miR-15a-5p;hsa-miR-15b-5p;hsa-miR-16-5p;hsa-miR-195-
ENSG0000	ENSG0000	0.000465	hsa-miR-132-3p;hsa-miR-15a-5p;hsa-miR-15b-5p;hsa-miR-19
ENSG0000	ENSG0000	0.008196	hsa-miR-15a-5p;hsa-miR-15b-5p;hsa-miR-16-5p;hsa-miR-195-
ENSG0000	ENSG0000	0.002617	hsa-miR-125a-5p;hsa-miR-125b-5p;hsa-miR-1260a;hsa-miR-1
ENSG0000	ENSG0000	0.001232	hsa-miR-15a-5p;hsa-miR-15b-5p;hsa-miR-16-5p;hsa-miR-195-
ENSG0000	ENSG0000	1.22E-05	hsa-miR-34a-5p;hsa-miR-34b-5p;hsa-miR-34c-5p;hsa-miR-44
ENSG0000	ENSG0000	0.005478	hsa-miR-15a-5p;hsa-miR-15b-5p;hsa-miR-195-5p;hsa-miR-36
ENSG0000	ENSG0000	0.000411	hsa-miR-17-3p;hsa-miR-34a-5p;hsa-miR-34c-5p;hsa-miR-449
ENSG0000	ENSG0000	1.47E-05	hsa-miR-15a-5p;hsa-miR-15b-5p;hsa-miR-195-5p;hsa-miR-34
ENSG0000	ENSG0000	0.000362	hsa-miR-132-3p;hsa-miR-15a-5p;hsa-miR-15b-5p;hsa-miR-19
ENSG0000	ENSG0000	0.00137	hsa-miR-15a-5p;hsa-miR-15b-5p;hsa-miR-16-5p;hsa-miR-195-
ENSG0000	ENSG0000	5.05E-05	hsa-miR-15a-5p;hsa-miR-15b-5p;hsa-miR-16-5p;hsa-miR-195-
ENSG0000	ENSG0000	5.05E-05	hsa-miR-15a-5p;hsa-miR-15b-5p;hsa-miR-16-5p;hsa-miR-195-
ENSG0000	ENSG0000	0.006479	hsa-miR-125a-5p;hsa-miR-125b-5p;hsa-miR-199a-5p;hsa-miR
ENSG0000	ENSG0000	0.006479	hsa-miR-125b-5p;hsa-miR-132-3p;hsa-miR-212-3p;hsa-miR-7
ENSG0000	ENSG0000	0.00137	hsa-miR-1260a;hsa-miR-1260b;hsa-miR-188-3p;hsa-miR-7-1-
ENSG0000	ENSG0000	0.001825	hsa-miR-15a-5p;hsa-miR-15b-5p;hsa-miR-16-5p;hsa-miR-195-
ENSG0000	ENSG0000	0.005794	hsa-miR-15a-5p;hsa-miR-15b-5p;hsa-miR-16-5p;hsa-miR-195-
ENSG0000	ENSG0000	0.004462	hsa-miR-125a-5p;hsa-miR-132-3p;hsa-miR-15b-5p;hsa-miR-2
ENSG0000	ENSG0000	0.006479	hsa-miR-125a-5p;hsa-miR-125b-5p;hsa-miR-199a-5p;hsa-miR
ENSG0000	ENSG0000	0.000362	hsa-miR-34b-5p;hsa-miR-34c-5p;hsa-miR-449a;hsa-miR-449b-
ENSG0000	ENSG0000	0.001705	hsa-miR-125a-5p;hsa-miR-125b-5p;hsa-miR-15a-5p;hsa-miR-
ENSG0000	ENSG0000	6.29E-05	hsa-miR-34a-5p;hsa-miR-34b-5p;hsa-miR-34c-5p;hsa-miR-44
ENSG0000	ENSG0000	9.41E-05	hsa-miR-1260a;hsa-miR-1260b;hsa-miR-15a-5p;hsa-miR-188-
ENSG0000	ENSG0000	5.37E-07	hsa-miR-15a-5p;hsa-miR-16-5p;hsa-miR-34a-5p;hsa-miR-34b-
ENSG0000	ENSG0000	0.000362	hsa-miR-34a-5p;hsa-miR-34b-5p;hsa-miR-34c-5p;hsa-miR-44
ENSG0000	ENSG0000	0.001936	hsa-miR-3180-3p;hsa-miR-34a-5p;hsa-miR-34c-5p;hsa-miR-4
ENSG0000	ENSG0000	0.000183	hsa-miR-34a-5p;hsa-miR-34b-5p;hsa-miR-34c-5p;hsa-miR-42
ENSG0000	ENSG0000	0.000217	hsa-miR-34a-5p;hsa-miR-34b-5p;hsa-miR-34c-5p;hsa-miR-44
ENSG0000	ENSG0000	0.002244	hsa-miR-15a-5p;hsa-miR-15b-5p;hsa-miR-16-5p;hsa-miR-195-
ENSG0000	ENSG0000	0.003614	hsa-miR-15a-5p;hsa-miR-15b-5p;hsa-miR-16-5p;hsa-miR-195-
ENSG0000	ENSG0000	5.05E-05	hsa-miR-15a-5p;hsa-miR-15b-5p;hsa-miR-16-5p;hsa-miR-195-
ENSG0000	ENSG0000	5.05E-05	hsa-miR-15a-5p;hsa-miR-15b-5p;hsa-miR-16-5p;hsa-miR-195-
ENSG0000	ENSG0000	0.007594	hsa-miR-15a-5p;hsa-miR-15b-5p;hsa-miR-195-5p;hsa-miR-19

ENSG0000	ENSG0000	7.93E-08	hsa-miR-15a-5p;hsa-miR-15b-5p;hsa-miR-16-5p;hsa-miR-195-
ENSG0000	ENSG0000	0.002511	hsa-miR-15a-5p;hsa-miR-15b-5p;hsa-miR-195-5p;hsa-miR-36
ENSG0000	ENSG0000	0.000526	hsa-miR-15a-5p;hsa-miR-15b-5p;hsa-miR-188-5p;hsa-miR-19
ENSG0000	ENSG0000	0.004587	hsa-miR-15a-5p;hsa-miR-15b-5p;hsa-miR-195-5p;hsa-miR-36
ENSG0000	ENSG0000	1.04E-05	hsa-miR-10a-3p;hsa-miR-125b-5p;hsa-miR-199a-5p;hsa-miR-
ENSG0000	ENSG0000	0.009263	hsa-miR-15a-5p;hsa-miR-15b-5p;hsa-miR-16-5p;hsa-miR-195-
ENSG0000	ENSG0000	3.91E-07	hsa-miR-15a-5p;hsa-miR-15b-5p;hsa-miR-16-5p;hsa-miR-195-
ENSG0000	ENSG0000	0.001561	hsa-miR-15a-5p;hsa-miR-15b-5p;hsa-miR-195-5p;hsa-miR-64
ENSG0000	ENSG0000	0.008829	hsa-miR-15a-5p;hsa-miR-15b-5p;hsa-miR-16-5p;hsa-miR-195-
ENSG0000	ENSG0000	0.000823	hsa-miR-15a-5p;hsa-miR-15b-5p;hsa-miR-16-5p;hsa-miR-195-
ENSG0000	ENSG0000	0.006556	hsa-miR-15a-5p;hsa-miR-15b-5p;hsa-miR-16-5p;hsa-miR-195-
ENSG0000	ENSG0000	0.000461	hsa-miR-15a-5p;hsa-miR-15b-5p;hsa-miR-16-5p;hsa-miR-195-
ENSG0000	ENSG0000	0.000461	hsa-miR-15a-5p;hsa-miR-15b-5p;hsa-miR-16-5p;hsa-miR-195-
ENSG0000	ENSG0000	2.26E-05	hsa-miR-1260a;hsa-miR-1260b;hsa-miR-15a-5p;hsa-miR-15b-
ENSG0000	ENSG0000	0.000465	hsa-miR-15b-5p;hsa-miR-17-3p;hsa-miR-3065-5p;hsa-miR-36
ENSG0000	ENSG0000	0.007022	hsa-miR-15a-5p;hsa-miR-15b-5p;hsa-miR-16-5p;hsa-miR-195-
ENSG0000	ENSG0000	0.000207	hsa-miR-125a-5p;hsa-miR-125b-5p;hsa-miR-132-3p;hsa-miR-
ENSG0000	ENSG0000	0.003899	hsa-miR-1260a;hsa-miR-1260b;hsa-miR-15a-5p;hsa-miR-15b-
ENSG0000	ENSG0000	6.99E-05	hsa-miR-15a-5p;hsa-miR-15b-5p;hsa-miR-16-5p;hsa-miR-195-
ENSG0000	ENSG0000	6.99E-05	hsa-miR-15a-5p;hsa-miR-15b-5p;hsa-miR-16-5p;hsa-miR-195-
ENSG0000	ENSG0000	2.67E-06	hsa-miR-15a-5p;hsa-miR-15b-5p;hsa-miR-16-5p;hsa-miR-195-
ENSG0000	ENSG0000	0.00311	hsa-miR-15a-5p;hsa-miR-15b-5p;hsa-miR-16-5p;hsa-miR-195-
ENSG0000	ENSG0000	0.0028	hsa-miR-15a-5p;hsa-miR-15b-5p;hsa-miR-16-5p;hsa-miR-195-
ENSG0000	ENSG0000	0.007022	hsa-miR-34a-5p;hsa-miR-34c-5p;hsa-miR-449a;hsa-miR-449b-
ENSG0000	ENSG0000	0.000896	hsa-miR-15a-5p;hsa-miR-15b-5p;hsa-miR-16-5p;hsa-miR-195-
ENSG0000	ENSG0000	9.41E-05	hsa-miR-15a-5p;hsa-miR-15b-5p;hsa-miR-16-5p;hsa-miR-195-
ENSG0000	ENSG0000	1.18E-06	hsa-miR-34a-5p;hsa-miR-34b-5p;hsa-miR-34c-5p;hsa-miR-44
ENSG0000	ENSG0000	0.005964	hsa-miR-15a-5p;hsa-miR-15b-5p;hsa-miR-16-5p;hsa-miR-195-
ENSG0000	ENSG0000	0.000313	hsa-miR-15a-5p;hsa-miR-15b-5p;hsa-miR-16-5p;hsa-miR-195-
ENSG0000	ENSG0000	0.000382	hsa-miR-34a-5p;hsa-miR-34c-5p;hsa-miR-449a;hsa-miR-449b-
ENSG0000	ENSG0000	0.000362	hsa-miR-15a-5p;hsa-miR-15b-5p;hsa-miR-195-5p;hsa-miR-30
ENSG0000	ENSG0000	0.004462	hsa-miR-15a-5p;hsa-miR-15b-5p;hsa-miR-16-5p;hsa-miR-195-
ENSG0000	ENSG0000	4.12E-05	hsa-miR-15a-5p;hsa-miR-15b-5p;hsa-miR-195-5p;hsa-miR-34
ENSG0000	ENSG0000	0.004587	hsa-miR-15a-5p;hsa-miR-15b-5p;hsa-miR-16-5p;hsa-miR-195-
ENSG0000	ENSG0000	0.002525	hsa-miR-34a-5p;hsa-miR-34b-5p;hsa-miR-34c-5p;hsa-miR-44
ENSG0000	ENSG0000	0.002525	hsa-miR-34a-5p;hsa-miR-34b-5p;hsa-miR-34c-5p;hsa-miR-44
ENSG0000	ENSG0000	0.002525	hsa-miR-34a-5p;hsa-miR-34b-5p;hsa-miR-34c-5p;hsa-miR-44
ENSG0000	ENSG0000	0.002525	hsa-miR-34a-5p;hsa-miR-34b-5p;hsa-miR-34c-5p;hsa-miR-44
ENSG0000	ENSG0000	0.002525	hsa-miR-34a-5p;hsa-miR-34b-5p;hsa-miR-34c-5p;hsa-miR-44
ENSG0000	ENSG0000	0.002525	hsa-miR-34a-5p;hsa-miR-34b-5p;hsa-miR-34c-5p;hsa-miR-44
ENSG0000	ENSG0000	0.002525	hsa-miR-34a-5p;hsa-miR-34b-5p;hsa-miR-34c-5p;hsa-miR-44
ENSG0000	ENSG0000	0.002525	hsa-miR-34a-5p;hsa-miR-34b-5p;hsa-miR-34c-5p;hsa-miR-44
ENSG0000	ENSG0000	0.002479	hsa-miR-10a-3p;hsa-miR-15a-5p;hsa-miR-15b-5p;hsa-miR-19
ENSG0000	ENSG0000	0.000313	hsa-miR-15a-5p;hsa-miR-15b-5p;hsa-miR-16-5p;hsa-miR-195-
ENSG0000	ENSG0000	0.001561	hsa-miR-15a-5p;hsa-miR-15b-5p;hsa-miR-16-5p;hsa-miR-195-
ENSG0000	ENSG0000	0.001462	hsa-miR-15a-5p;hsa-miR-15b-5p;hsa-miR-16-5p;hsa-miR-195-
ENSG0000	ENSG0000	0.000684	hsa-miR-10a-3p;hsa-miR-15a-5p;hsa-miR-15b-5p;hsa-miR-16-
ENSG0000	ENSG0000	0.00502	hsa-miR-15a-3p;hsa-miR-3065-5p;hsa-miR-362-3p;hsa-miR-7-
ENSG0000	ENSG0000	0.00036	hsa-miR-10a-3p;hsa-miR-15a-5p;hsa-miR-15b-5p;hsa-miR-16-
ENSG0000	ENSG0000	0.00266	hsa-miR-15a-5p;hsa-miR-15b-5p;hsa-miR-16-5p;hsa-miR-188-
ENSG0000	ENSG0000	0.003619	hsa-miR-15a-5p;hsa-miR-15b-5p;hsa-miR-16-5p;hsa-miR-195-
ENSG0000	ENSG0000	0.004181	hsa-miR-199a-5p;hsa-miR-199b-5p;hsa-miR-361-3p;hsa-miR-
ENSG0000	ENSG0000	6.83E-06	hsa-miR-15a-5p;hsa-miR-15b-5p;hsa-miR-16-5p;hsa-miR-195-
ENSG0000	ENSG0000	1.29E-05	hsa-miR-125a-5p;hsa-miR-140-5p;hsa-miR-15a-5p;hsa-miR-1

ENSG0000	ENSG0000	0.001483	hsa-miR-15a-5p;hsa-miR-16-5p;hsa-miR-212-3p;hsa-miR-368
ENSG0000	ENSG0000	0.001196	hsa-miR-15a-5p;hsa-miR-15b-5p;hsa-miR-16-5p;hsa-miR-195-
ENSG0000	ENSG0000	7.31E-05	hsa-miR-1306-5p;hsa-miR-15a-5p;hsa-miR-15b-5p;hsa-miR-1
ENSG0000	ENSG0000	0.000472	hsa-miR-16-5p;hsa-miR-34a-5p;hsa-miR-34c-5p;hsa-miR-361
ENSG0000	ENSG0000	0.000694	hsa-miR-10a-3p;hsa-miR-125b-5p;hsa-miR-15a-5p;hsa-miR-1
ENSG0000	ENSG0000	0.00059	hsa-miR-34a-5p;hsa-miR-34c-5p;hsa-miR-449a;hsa-miR-449b-
ENSG0000	ENSG0000	7.93E-08	hsa-miR-15a-5p;hsa-miR-15b-5p;hsa-miR-16-5p;hsa-miR-195-
ENSG0000	ENSG0000	1.18E-06	hsa-miR-15a-5p;hsa-miR-15b-5p;hsa-miR-16-5p;hsa-miR-195-
ENSG0000	ENSG0000	0.000129	hsa-miR-125b-5p;hsa-miR-132-3p;hsa-miR-199a-5p;hsa-miR-
ENSG0000	ENSG0000	4.61E-07	hsa-miR-15a-5p;hsa-miR-15b-5p;hsa-miR-16-5p;hsa-miR-195-
ENSG0000	ENSG0000	3.54E-05	hsa-miR-15a-5p;hsa-miR-15b-5p;hsa-miR-16-5p;hsa-miR-195-
ENSG0000	ENSG0000	1.57E-06	hsa-miR-34a-5p;hsa-miR-34b-5p;hsa-miR-34c-5p;hsa-miR-44
ENSG0000	ENSG0000	0.002898	hsa-miR-15a-5p;hsa-miR-15b-5p;hsa-miR-16-5p;hsa-miR-195-
ENSG0000	ENSG0000	0.000525	hsa-miR-34a-5p;hsa-miR-34c-5p;hsa-miR-3619-5p;hsa-miR-4
ENSG0000	ENSG0000	0.000823	hsa-miR-15a-5p;hsa-miR-15b-5p;hsa-miR-16-5p;hsa-miR-195-
ENSG0000	ENSG0000	0.004167	hsa-miR-15a-5p;hsa-miR-15b-5p;hsa-miR-16-5p;hsa-miR-195-
ENSG0000	ENSG0000	0.004462	hsa-miR-1306-5p;hsa-miR-17-3p;hsa-miR-212-3p;hsa-miR-34
ENSG0000	ENSG0000	0.001997	hsa-miR-15a-5p;hsa-miR-15b-5p;hsa-miR-195-5p;hsa-miR-36
ENSG0000	ENSG0000	0.007594	hsa-miR-1260a;hsa-miR-1260b;hsa-miR-188-3p;hsa-miR-361-
ENSG0000	ENSG0000	9.37E-06	hsa-miR-15a-5p;hsa-miR-15b-5p;hsa-miR-16-5p;hsa-miR-195-
ENSG0000	ENSG0000	7.93E-08	hsa-miR-15a-5p;hsa-miR-15b-5p;hsa-miR-16-5p;hsa-miR-195-
ENSG0000	ENSG0000	0.000465	hsa-miR-10a-3p;hsa-miR-15b-5p;hsa-miR-34b-5p;hsa-miR-42
ENSG0000	ENSG0000	0.000461	hsa-miR-15a-5p;hsa-miR-15b-5p;hsa-miR-16-5p;hsa-miR-195-
ENSG0000	ENSG0000	1.14E-07	hsa-miR-15a-5p;hsa-miR-15b-5p;hsa-miR-195-5p;hsa-miR-34
ENSG0000	ENSG0000	0.000823	hsa-miR-34a-5p;hsa-miR-34c-5p;hsa-miR-361-3p;hsa-miR-44
ENSG0000	ENSG0000	2.08E-05	hsa-miR-3199;hsa-miR-34a-5p;hsa-miR-34c-5p;hsa-miR-449a;
ENSG0000	ENSG0000	5.05E-05	hsa-miR-15a-5p;hsa-miR-15b-5p;hsa-miR-16-5p;hsa-miR-195-
ENSG0000	ENSG0000	9.37E-06	hsa-miR-34a-5p;hsa-miR-34c-5p;hsa-miR-449a;hsa-miR-449b-
ENSG0000	ENSG0000	0.000465	hsa-miR-15a-5p;hsa-miR-15b-5p;hsa-miR-16-5p;hsa-miR-195-
ENSG0000	ENSG0000	0.00311	hsa-miR-15a-5p;hsa-miR-15b-5p;hsa-miR-16-5p;hsa-miR-195-
ENSG0000	ENSG0000	5.27E-06	hsa-miR-15a-5p;hsa-miR-15b-5p;hsa-miR-16-5p;hsa-miR-195-
ENSG0000	ENSG0000	3.44E-06	hsa-miR-15a-5p;hsa-miR-15b-5p;hsa-miR-16-5p;hsa-miR-195-
ENSG0000	ENSG0000	4.17E-05	hsa-miR-15a-5p;hsa-miR-15b-5p;hsa-miR-16-5p;hsa-miR-195-
ENSG0000	ENSG0000	2.24E-06	hsa-miR-132-3p;hsa-miR-15a-5p;hsa-miR-15b-5p;hsa-miR-16-
ENSG0000	ENSG0000	0.001561	hsa-miR-15a-5p;hsa-miR-15b-5p;hsa-miR-195-5p;hsa-miR-36
ENSG0000	ENSG0000	0.004587	hsa-miR-15a-5p;hsa-miR-15b-5p;hsa-miR-16-5p;hsa-miR-195-
ENSG0000	ENSG0000	0.000896	hsa-miR-34a-5p;hsa-miR-34c-5p;hsa-miR-449a;hsa-miR-449b-
ENSG0000	ENSG0000	0.000896	hsa-miR-34a-5p;hsa-miR-34c-5p;hsa-miR-449a;hsa-miR-449b-
ENSG0000	ENSG0000	3.21E-06	hsa-miR-10a-3p;hsa-miR-1260a;hsa-miR-1260b;hsa-miR-140-
ENSG0000	ENSG0000	0.002084	hsa-miR-34a-5p;hsa-miR-34c-5p;hsa-miR-449a;hsa-miR-449b-
ENSG0000	ENSG0000	0.002856	hsa-miR-188-5p;hsa-miR-34a-5p;hsa-miR-34c-5p;hsa-miR-44
ENSG0000	ENSG0000	0.002856	hsa-miR-188-5p;hsa-miR-34a-5p;hsa-miR-34c-5p;hsa-miR-44
ENSG0000	ENSG0000	0.000329	hsa-miR-15a-5p;hsa-miR-15b-5p;hsa-miR-16-5p;hsa-miR-195-
ENSG0000	ENSG0000	0.00137	hsa-miR-15a-5p;hsa-miR-15b-5p;hsa-miR-195-5p;hsa-miR-42
ENSG0000	ENSG0000	0.0038	hsa-miR-15a-5p;hsa-miR-15b-5p;hsa-miR-16-5p;hsa-miR-195-
ENSG0000	ENSG0000	0.002479	hsa-miR-15a-5p;hsa-miR-15b-5p;hsa-miR-16-5p;hsa-miR-195-
ENSG0000	ENSG0000	1.54E-05	hsa-miR-15a-5p;hsa-miR-15b-5p;hsa-miR-16-5p;hsa-miR-195-
ENSG0000	ENSG0000	3.54E-05	hsa-miR-15a-5p;hsa-miR-15b-5p;hsa-miR-16-5p;hsa-miR-195-
ENSG0000	ENSG0000	0.0028	hsa-miR-15a-5p;hsa-miR-15b-5p;hsa-miR-195-5p;hsa-miR-54
ENSG0000	ENSG0000	0.001118	hsa-miR-15a-5p;hsa-miR-15b-5p;hsa-miR-16-5p;hsa-miR-195-
ENSG0000	ENSG0000	0.001118	hsa-miR-15a-5p;hsa-miR-15b-5p;hsa-miR-16-5p;hsa-miR-195-
ENSG0000	ENSG0000	0.000823	hsa-miR-34a-5p;hsa-miR-34c-5p;hsa-miR-449a;hsa-miR-449b-
ENSG0000	ENSG0000	0.009494	hsa-miR-15a-5p;hsa-miR-16-5p;hsa-miR-7-1-3p;hsa-miR-766-
ENSG0000	ENSG0000	0.000207	hsa-miR-15a-5p;hsa-miR-15b-5p;hsa-miR-16-5p;hsa-miR-195-

ENSG0000	ENSG0000	6.99E-05	hsa-miR-15a-5p;hsa-miR-15b-5p;hsa-miR-16-5p;hsa-miR-195-
ENSG0000	ENSG0000	0.0038	hsa-miR-15a-5p;hsa-miR-15b-5p;hsa-miR-16-5p;hsa-miR-195-
ENSG0000	ENSG0000	0.000382	hsa-miR-125a-5p;hsa-miR-125b-5p;hsa-miR-34b-5p;hsa-miR-4
ENSG0000	ENSG0000	0.001399	hsa-miR-15a-3p;hsa-miR-199a-5p;hsa-miR-199b-5p;hsa-miR-3
ENSG0000	ENSG0000	0.000382	hsa-miR-15a-5p;hsa-miR-15b-5p;hsa-miR-16-5p;hsa-miR-195-
ENSG0000	ENSG0000	0.000382	hsa-miR-15a-5p;hsa-miR-15b-5p;hsa-miR-16-5p;hsa-miR-195-
ENSG0000	ENSG0000	6.99E-05	hsa-miR-15a-5p;hsa-miR-15b-5p;hsa-miR-16-5p;hsa-miR-195-
ENSG0000	ENSG0000	0.001118	hsa-miR-15a-5p;hsa-miR-15b-5p;hsa-miR-16-5p;hsa-miR-195-
ENSG0000	ENSG0000	0.008829	hsa-miR-15a-5p;hsa-miR-15b-5p;hsa-miR-16-5p;hsa-miR-195-
ENSG0000	ENSG0000	0.0028	hsa-miR-15a-5p;hsa-miR-15b-5p;hsa-miR-16-5p;hsa-miR-195-
ENSG0000	ENSG0000	0.0028	hsa-miR-15a-5p;hsa-miR-15b-5p;hsa-miR-16-5p;hsa-miR-195-
ENSG0000	ENSG0000	0.000768	hsa-miR-15a-5p;hsa-miR-15b-5p;hsa-miR-16-5p;hsa-miR-195-
ENSG0000	ENSG0000	0.000739	hsa-miR-15a-5p;hsa-miR-15b-5p;hsa-miR-16-5p;hsa-miR-195-
ENSG0000	ENSG0000	2.84E-08	hsa-miR-1260a;hsa-miR-1260b;hsa-miR-15a-5p;hsa-miR-15b-5
ENSG0000	ENSG0000	8.43E-06	hsa-miR-1260a;hsa-miR-1260b;hsa-miR-15a-5p;hsa-miR-15b-5
ENSG0000	ENSG0000	1.62E-06	hsa-miR-15a-5p;hsa-miR-15b-5p;hsa-miR-16-5p;hsa-miR-195-
ENSG0000	ENSG0000	0.004587	hsa-miR-15a-5p;hsa-miR-15b-5p;hsa-miR-195-5p;hsa-miR-31
ENSG0000	ENSG0000	0.00177	hsa-miR-15a-5p;hsa-miR-15b-5p;hsa-miR-16-5p;hsa-miR-195-
ENSG0000	ENSG0000	0.001759	hsa-miR-199a-5p;hsa-miR-199b-5p;hsa-miR-34a-5p;hsa-miR-3
ENSG0000	ENSG0000	0.001118	hsa-miR-15a-5p;hsa-miR-15b-5p;hsa-miR-16-5p;hsa-miR-195-
ENSG0000	ENSG0000	0.001118	hsa-miR-15a-5p;hsa-miR-15b-5p;hsa-miR-16-5p;hsa-miR-195-
ENSG0000	ENSG0000	0.000481	hsa-miR-1260a;hsa-miR-1260b;hsa-miR-15a-5p;hsa-miR-15b-5
ENSG0000	ENSG0000	0.000411	hsa-miR-15a-5p;hsa-miR-15b-5p;hsa-miR-16-5p;hsa-miR-195-
ENSG0000	ENSG0000	1.03E-05	hsa-miR-34a-5p;hsa-miR-34b-5p;hsa-miR-34c-5p;hsa-miR-449
ENSG0000	ENSG0000	1.51E-05	hsa-miR-125a-5p;hsa-miR-125b-5p;hsa-miR-34a-5p;hsa-miR-3
ENSG0000	ENSG0000	9.37E-06	hsa-miR-15a-5p;hsa-miR-15b-5p;hsa-miR-16-5p;hsa-miR-195-
ENSG0000	ENSG0000	3.98E-07	hsa-miR-15a-5p;hsa-miR-15b-5p;hsa-miR-16-5p;hsa-miR-195-
ENSG0000	ENSG0000	0.00941	hsa-miR-15a-5p;hsa-miR-15b-5p;hsa-miR-16-5p;hsa-miR-195-
ENSG0000	ENSG0000	0.00941	hsa-miR-15a-5p;hsa-miR-15b-5p;hsa-miR-16-5p;hsa-miR-195-
ENSG0000	ENSG0000	5.05E-05	hsa-miR-15a-5p;hsa-miR-15b-5p;hsa-miR-16-5p;hsa-miR-195-
ENSG0000	ENSG0000	0.008766	hsa-miR-15a-3p;hsa-miR-34a-5p;hsa-miR-34c-5p;hsa-miR-449
ENSG0000	ENSG0000	0.000329	hsa-miR-34a-5p;hsa-miR-34b-5p;hsa-miR-34c-5p;hsa-miR-449
ENSG0000	ENSG0000	0.000481	hsa-miR-15a-5p;hsa-miR-15b-5p;hsa-miR-16-5p;hsa-miR-195-
ENSG0000	ENSG0000	0.002898	hsa-miR-15a-5p;hsa-miR-15b-5p;hsa-miR-16-5p;hsa-miR-188-
ENSG0000	ENSG0000	0.000461	hsa-miR-15a-5p;hsa-miR-15b-5p;hsa-miR-16-5p;hsa-miR-195-
ENSG0000	ENSG0000	0.00059	hsa-miR-15a-5p;hsa-miR-15b-5p;hsa-miR-16-5p;hsa-miR-195-
ENSG0000	ENSG0000	0.00177	hsa-miR-15a-5p;hsa-miR-15b-5p;hsa-miR-16-5p;hsa-miR-195-
ENSG0000	ENSG0000	4.76E-06	hsa-miR-15a-5p;hsa-miR-15b-5p;hsa-miR-16-5p;hsa-miR-195-
ENSG0000	ENSG0000	0.000461	hsa-miR-15a-5p;hsa-miR-15b-5p;hsa-miR-16-5p;hsa-miR-195-
ENSG0000	ENSG0000	0.004462	hsa-miR-15a-5p;hsa-miR-15b-5p;hsa-miR-16-5p;hsa-miR-195-
ENSG0000	ENSG0000	0.001561	hsa-miR-34a-5p;hsa-miR-34c-5p;hsa-miR-449a;hsa-miR-449b-
ENSG0000	ENSG0000	0.0028	hsa-miR-15a-5p;hsa-miR-15b-5p;hsa-miR-16-5p;hsa-miR-195-
ENSG0000	ENSG0000	2.08E-05	hsa-miR-132-3p;hsa-miR-199b-5p;hsa-miR-34a-5p;hsa-miR-4
ENSG0000	ENSG0000	0.000461	hsa-miR-15a-5p;hsa-miR-15b-5p;hsa-miR-16-5p;hsa-miR-195-
ENSG0000	ENSG0000	7.26E-06	hsa-miR-1260a;hsa-miR-1260b;hsa-miR-15a-5p;hsa-miR-15b-5
ENSG0000	ENSG0000	2.65E-05	hsa-miR-15a-5p;hsa-miR-15b-5p;hsa-miR-16-5p;hsa-miR-195-
ENSG0000	ENSG0000	0.0038	hsa-miR-15a-5p;hsa-miR-16-5p;hsa-miR-361-3p;hsa-miR-652-
ENSG0000	ENSG0000	0.0028	hsa-miR-15a-5p;hsa-miR-15b-5p;hsa-miR-195-5p;hsa-miR-54
ENSG0000	ENSG0000	1.46E-06	hsa-miR-15a-5p;hsa-miR-15b-5p;hsa-miR-16-5p;hsa-miR-195-
ENSG0000	ENSG0000	0.003366	hsa-miR-15a-5p;hsa-miR-15b-5p;hsa-miR-16-5p;hsa-miR-195-
ENSG0000	ENSG0000	9.09E-06	hsa-miR-15a-5p;hsa-miR-15b-5p;hsa-miR-195-5p;hsa-miR-36
ENSG0000	ENSG0000	0.000768	hsa-miR-15a-5p;hsa-miR-15b-5p;hsa-miR-16-5p;hsa-miR-195-
ENSG0000	ENSG0000	0.004045	hsa-miR-15a-5p;hsa-miR-15b-5p;hsa-miR-195-5p;hsa-miR-34
ENSG0000	ENSG0000	7.93E-08	hsa-miR-15a-5p;hsa-miR-15b-5p;hsa-miR-16-5p;hsa-miR-195-

ENSG0000	ENSG0000	1.22E-05	hsa-miR-34a-5p;hsa-miR-34c-5p;hsa-miR-449a;hsa-miR-449b-
ENSG0000	ENSG0000	0.002525	hsa-miR-15a-5p;hsa-miR-15b-5p;hsa-miR-16-5p;hsa-miR-195-
ENSG0000	ENSG0000	4.17E-05	hsa-miR-15a-5p;hsa-miR-15b-5p;hsa-miR-16-5p;hsa-miR-195-
ENSG0000	ENSG0000	0.0038	hsa-miR-15a-5p;hsa-miR-15b-5p;hsa-miR-16-5p;hsa-miR-195-
ENSG0000	ENSG0000	0.001232	hsa-miR-15a-5p;hsa-miR-15b-5p;hsa-miR-16-5p;hsa-miR-195-
ENSG0000	ENSG0000	0.007594	hsa-miR-1306-5p;hsa-miR-199a-5p;hsa-miR-199b-5p;hsa-miR-
ENSG0000	ENSG0000	4.23E-06	hsa-miR-15a-5p;hsa-miR-15b-5p;hsa-miR-195-5p;hsa-miR-34
ENSG0000	ENSG0000	0.000109	hsa-miR-15a-5p;hsa-miR-15b-5p;hsa-miR-16-5p;hsa-miR-195-
ENSG0000	ENSG0000	9.09E-06	hsa-miR-132-3p;hsa-miR-212-3p;hsa-miR-421;hsa-miR-449a;h
ENSG0000	ENSG0000	1.22E-05	hsa-miR-15a-5p;hsa-miR-15b-5p;hsa-miR-16-5p;hsa-miR-195-
ENSG0000	ENSG0000	5.05E-05	hsa-miR-15a-5p;hsa-miR-15b-5p;hsa-miR-16-5p;hsa-miR-195-
ENSG0000	ENSG0000	1.16E-06	hsa-miR-15a-5p;hsa-miR-15b-5p;hsa-miR-16-5p;hsa-miR-195-
ENSG0000	ENSG0000	6.99E-05	hsa-miR-15a-5p;hsa-miR-15b-5p;hsa-miR-16-5p;hsa-miR-195-
ENSG0000	ENSG0000	0.002244	hsa-miR-1260a;hsa-miR-1260b;hsa-miR-188-3p;hsa-miR-361-
ENSG0000	ENSG0000	0.003619	hsa-miR-15a-5p;hsa-miR-15b-5p;hsa-miR-16-5p;hsa-miR-195-
ENSG0000	ENSG0000	0.000438	hsa-miR-34a-5p;hsa-miR-34c-5p;hsa-miR-3619-5p;hsa-miR-4
ENSG0000	ENSG0000	0.00059	hsa-miR-15a-5p;hsa-miR-15b-5p;hsa-miR-16-5p;hsa-miR-195-
ENSG0000	ENSG0000	0.001997	hsa-miR-15a-5p;hsa-miR-15b-5p;hsa-miR-195-5p;hsa-miR-54
ENSG0000	ENSG0000	0.000382	hsa-miR-15a-5p;hsa-miR-15b-5p;hsa-miR-16-5p;hsa-miR-195-
ENSG0000	ENSG0000	0.000362	hsa-miR-140-5p;hsa-miR-15a-5p;hsa-miR-15b-5p;hsa-miR-16-
ENSG0000	ENSG0000	0.000481	hsa-miR-125a-5p;hsa-miR-125b-5p;hsa-miR-15a-5p;hsa-miR-
ENSG0000	ENSG0000	0.0028	hsa-miR-15a-5p;hsa-miR-15b-5p;hsa-miR-16-5p;hsa-miR-195-
ENSG0000	ENSG0000	0.00016	hsa-miR-15a-5p;hsa-miR-15b-5p;hsa-miR-16-5p;hsa-miR-195-
ENSG0000	ENSG0000	0.004181	hsa-miR-15a-5p;hsa-miR-15b-5p;hsa-miR-16-5p;hsa-miR-195-
ENSG0000	ENSG0000	5.27E-06	hsa-miR-15a-5p;hsa-miR-15b-5p;hsa-miR-16-5p;hsa-miR-195-
ENSG0000	ENSG0000	0.000461	hsa-miR-15a-5p;hsa-miR-15b-5p;hsa-miR-16-5p;hsa-miR-195-
ENSG0000	ENSG0000	0.000399	hsa-miR-15a-5p;hsa-miR-15b-5p;hsa-miR-16-5p;hsa-miR-195-
ENSG0000	ENSG0000	0.000628	hsa-miR-15a-5p;hsa-miR-15b-5p;hsa-miR-16-5p;hsa-miR-195-
ENSG0000	ENSG0000	0.006556	hsa-miR-15a-5p;hsa-miR-15b-5p;hsa-miR-195-5p;hsa-miR-48
ENSG0000	ENSG0000	8.43E-06	hsa-miR-15a-5p;hsa-miR-15b-5p;hsa-miR-16-5p;hsa-miR-188-
ENSG0000	ENSG0000	0.000661	hsa-miR-15a-5p;hsa-miR-15b-5p;hsa-miR-16-5p;hsa-miR-195-
ENSG0000	ENSG0000	1.16E-06	hsa-miR-15a-5p;hsa-miR-15b-5p;hsa-miR-16-5p;hsa-miR-195-
ENSG0000	ENSG0000	0.001936	hsa-miR-15a-5p;hsa-miR-15b-5p;hsa-miR-16-5p;hsa-miR-195-
ENSG0000	ENSG0000	8.66E-07	hsa-miR-1306-5p;hsa-miR-15a-5p;hsa-miR-15b-5p;hsa-miR-1
ENSG0000	ENSG0000	8.66E-07	hsa-miR-1306-5p;hsa-miR-15a-5p;hsa-miR-15b-5p;hsa-miR-1
ENSG0000	ENSG0000	3.91E-07	hsa-miR-15a-5p;hsa-miR-15b-5p;hsa-miR-16-5p;hsa-miR-195-
ENSG0000	ENSG0000	7.93E-08	hsa-miR-15a-5p;hsa-miR-15b-5p;hsa-miR-16-5p;hsa-miR-195-
ENSG0000	ENSG0000	0.004181	hsa-miR-125a-5p;hsa-miR-125b-5p;hsa-miR-331-3p;hsa-miR-
ENSG0000	ENSG0000	1.22E-05	hsa-miR-15a-5p;hsa-miR-15b-5p;hsa-miR-16-5p;hsa-miR-195-
ENSG0000	ENSG0000	0.00177	hsa-miR-15a-5p;hsa-miR-15b-5p;hsa-miR-16-5p;hsa-miR-195-
ENSG0000	ENSG0000	0.000551	hsa-miR-15a-5p;hsa-miR-15b-5p;hsa-miR-16-5p;hsa-miR-195-
ENSG0000	ENSG0000	0.000461	hsa-miR-15a-5p;hsa-miR-15b-5p;hsa-miR-16-5p;hsa-miR-195-
ENSG0000	ENSG0000	3.91E-07	hsa-miR-140-5p;hsa-miR-15a-5p;hsa-miR-16-5p;hsa-miR-195-
ENSG0000	ENSG0000	3.91E-07	hsa-miR-140-5p;hsa-miR-15a-5p;hsa-miR-16-5p;hsa-miR-195-
ENSG0000	ENSG0000	0.002244	hsa-miR-15a-5p;hsa-miR-15b-5p;hsa-miR-16-5p;hsa-miR-195-
ENSG0000	ENSG0000	3.91E-07	hsa-miR-15a-5p;hsa-miR-15b-5p;hsa-miR-16-5p;hsa-miR-195-
ENSG0000	ENSG0000	0.003443	hsa-miR-15a-5p;hsa-miR-15b-5p;hsa-miR-16-5p;hsa-miR-195-
ENSG0000	ENSG0000	0.002898	hsa-miR-15a-5p;hsa-miR-15b-5p;hsa-miR-16-5p;hsa-miR-195-
ENSG0000	ENSG0000	0.005612	hsa-miR-1260a;hsa-miR-1260b;hsa-miR-15a-5p;hsa-miR-15b-
ENSG0000	ENSG0000	3.54E-05	hsa-miR-15a-5p;hsa-miR-15b-5p;hsa-miR-16-5p;hsa-miR-195-
ENSG0000	ENSG0000	0.000461	hsa-miR-15a-5p;hsa-miR-15b-5p;hsa-miR-16-5p;hsa-miR-195-
ENSG0000	ENSG0000	9.37E-06	hsa-miR-15a-5p;hsa-miR-15b-5p;hsa-miR-195-5p;hsa-miR-54
ENSG0000	ENSG0000	0.004587	hsa-miR-34a-5p;hsa-miR-34c-5p;hsa-miR-449a;hsa-miR-449b-
ENSG0000	ENSG0000	0.004167	hsa-miR-15a-5p;hsa-miR-15b-5p;hsa-miR-16-5p;hsa-miR-195-

ENSG0000	ENSG0000	0.001561	hsa-miR-34a-5p;hsa-miR-34c-5p;hsa-miR-449a;hsa-miR-449b-
ENSG0000	ENSG0000	0.001561	hsa-miR-34a-5p;hsa-miR-34c-5p;hsa-miR-449a;hsa-miR-449b-
ENSG0000	ENSG0000	0.002511	hsa-miR-15a-5p;hsa-miR-15b-5p;hsa-miR-16-5p;hsa-miR-195-
ENSG0000	ENSG0000	7.93E-08	hsa-miR-15a-5p;hsa-miR-15b-5p;hsa-miR-16-5p;hsa-miR-195-
ENSG0000	ENSG0000	0.00138	hsa-miR-15a-5p;hsa-miR-15b-5p;hsa-miR-16-5p;hsa-miR-195-
ENSG0000	ENSG0000	1.85E-05	hsa-miR-1260a;hsa-miR-1260b;hsa-miR-15a-5p;hsa-miR-15b-5
ENSG0000	ENSG0000	2.47E-06	hsa-miR-125a-5p;hsa-miR-125b-5p;hsa-miR-1306-5p;hsa-miR-
ENSG0000	ENSG0000	8.11E-06	hsa-miR-132-3p;hsa-miR-15a-5p;hsa-miR-15b-5p;hsa-miR-16-
ENSG0000	ENSG0000	0.00024	hsa-miR-125b-5p;hsa-miR-15a-5p;hsa-miR-15b-5p;hsa-miR-1
ENSG0000	ENSG0000	7.93E-08	hsa-miR-15a-5p;hsa-miR-15b-5p;hsa-miR-16-5p;hsa-miR-195-
ENSG0000	ENSG0000	9.51E-05	hsa-miR-15a-5p;hsa-miR-15b-5p;hsa-miR-16-5p;hsa-miR-195-
ENSG0000	ENSG0000	0.000768	hsa-miR-15a-5p;hsa-miR-15b-5p;hsa-miR-16-5p;hsa-miR-195-
ENSG0000	ENSG0000	0.006556	hsa-miR-15a-5p;hsa-miR-15b-5p;hsa-miR-16-5p;hsa-miR-195-
ENSG0000	ENSG0000	0.003366	hsa-miR-15a-5p;hsa-miR-15b-5p;hsa-miR-16-5p;hsa-miR-195-
ENSG0000	ENSG0000	0.000743	hsa-miR-188-3p;hsa-miR-34a-5p;hsa-miR-34b-5p;hsa-miR-34
ENSG0000	ENSG0000	0.009494	hsa-miR-132-3p;hsa-miR-212-3p;hsa-miR-362-3p;hsa-miR-36
ENSG0000	ENSG0000	0.004181	hsa-miR-125a-5p;hsa-miR-125b-5p;hsa-miR-362-3p;hsa-miR-
ENSG0000	ENSG0000	0.000313	hsa-miR-1260a;hsa-miR-1260b;hsa-miR-188-3p;hsa-miR-362-
ENSG0000	ENSG0000	2.08E-05	hsa-miR-1260a;hsa-miR-1260b;hsa-miR-17-3p;hsa-miR-188-3
ENSG0000	ENSG0000	0.00177	hsa-miR-15a-5p;hsa-miR-15b-5p;hsa-miR-195-5p;hsa-miR-54
ENSG0000	ENSG0000	0.002287	hsa-miR-15a-5p;hsa-miR-15b-5p;hsa-miR-16-5p;hsa-miR-195-
ENSG0000	ENSG0000	0.001039	hsa-miR-15a-5p;hsa-miR-15b-5p;hsa-miR-16-5p;hsa-miR-195-
ENSG0000	ENSG0000	0.007022	hsa-miR-15a-5p;hsa-miR-15b-5p;hsa-miR-16-5p;hsa-miR-195-
ENSG0000	ENSG0000	0.000648	hsa-miR-1260a;hsa-miR-1260b;hsa-miR-1306-5p;hsa-miR-15a-
ENSG0000	ENSG0000	0.005017	hsa-miR-125a-5p;hsa-miR-125b-5p;hsa-miR-15a-5p;hsa-miR-
ENSG0000	ENSG0000	5.01E-06	hsa-miR-132-3p;hsa-miR-15a-5p;hsa-miR-15b-5p;hsa-miR-16-
ENSG0000	ENSG0000	0.000203	hsa-miR-34a-5p;hsa-miR-34c-5p;hsa-miR-449a;hsa-miR-449b-
ENSG0000	ENSG0000	1.04E-05	hsa-miR-15a-5p;hsa-miR-15b-5p;hsa-miR-16-5p;hsa-miR-195-
ENSG0000	ENSG0000	0.007386	hsa-miR-10a-3p;hsa-miR-34a-5p;hsa-miR-34c-5p;hsa-miR-44
ENSG0000	ENSG0000	1.18E-06	hsa-miR-34a-5p;hsa-miR-34b-5p;hsa-miR-34c-5p;hsa-miR-44
ENSG0000	ENSG0000	9.37E-06	hsa-miR-34a-5p;hsa-miR-34c-5p;hsa-miR-449a;hsa-miR-449b-
ENSG0000	ENSG0000	0.007594	hsa-miR-132-3p;hsa-miR-212-3p;hsa-miR-421;hsa-miR-766-3
ENSG0000	ENSG0000	0.001196	hsa-miR-15a-5p;hsa-miR-15b-5p;hsa-miR-16-5p;hsa-miR-195-
ENSG0000	ENSG0000	0.001118	hsa-miR-125a-5p;hsa-miR-15a-5p;hsa-miR-15b-5p;hsa-miR-1
ENSG0000	ENSG0000	8.67E-05	hsa-miR-17-3p;hsa-miR-199a-5p;hsa-miR-199b-5p;hsa-miR-3
ENSG0000	ENSG0000	8.67E-05	hsa-miR-17-3p;hsa-miR-199a-5p;hsa-miR-199b-5p;hsa-miR-3
ENSG0000	ENSG0000	0.001354	hsa-miR-15a-5p;hsa-miR-15b-5p;hsa-miR-16-5p;hsa-miR-195-
ENSG0000	ENSG0000	1.60E-05	hsa-miR-15a-5p;hsa-miR-15b-5p;hsa-miR-16-5p;hsa-miR-195-
ENSG0000	ENSG0000	0.000526	hsa-miR-34a-5p;hsa-miR-34b-5p;hsa-miR-34c-5p;hsa-miR-44
ENSG0000	ENSG0000	0.002511	hsa-miR-34a-5p;hsa-miR-34c-5p;hsa-miR-449a;hsa-miR-449b-
ENSG0000	ENSG0000	0.001396	hsa-miR-3199;hsa-miR-34a-5p;hsa-miR-34b-5p;hsa-miR-34c-
ENSG0000	ENSG0000	0.000203	hsa-miR-15a-5p;hsa-miR-16-5p;hsa-miR-195-5p;hsa-miR-361
ENSG0000	ENSG0000	9.08E-07	hsa-miR-15a-5p;hsa-miR-15b-5p;hsa-miR-16-5p;hsa-miR-195-
ENSG0000	ENSG0000	0.004462	hsa-miR-15a-5p;hsa-miR-15b-5p;hsa-miR-16-5p;hsa-miR-17-
ENSG0000	ENSG0000	0.002511	hsa-miR-34a-5p;hsa-miR-34c-5p;hsa-miR-449a;hsa-miR-449b-
ENSG0000	ENSG0000	0.002511	hsa-miR-34a-5p;hsa-miR-34c-5p;hsa-miR-449a;hsa-miR-449b-
ENSG0000	ENSG0000	0.001354	hsa-miR-1260a;hsa-miR-1260b;hsa-miR-188-3p;hsa-miR-3065-
ENSG0000	ENSG0000	0.00311	hsa-miR-199a-5p;hsa-miR-199b-5p;hsa-miR-362-3p;hsa-miR-
ENSG0000	ENSG0000	0.00311	hsa-miR-199a-5p;hsa-miR-199b-5p;hsa-miR-362-3p;hsa-miR-
ENSG0000	ENSG0000	0.000362	hsa-miR-34a-5p;hsa-miR-34c-5p;hsa-miR-449a;hsa-miR-449b-
ENSG0000	ENSG0000	1.26E-08	hsa-miR-1260a;hsa-miR-1260b;hsa-miR-15a-5p;hsa-miR-15b-5
ENSG0000	ENSG0000	9.16E-05	hsa-miR-15a-5p;hsa-miR-15b-5p;hsa-miR-16-5p;hsa-miR-195-
ENSG0000	ENSG0000	0.002244	hsa-miR-1260a;hsa-miR-1260b;hsa-miR-188-3p;hsa-miR-362-
ENSG0000	ENSG0000	0.000124	hsa-miR-15a-5p;hsa-miR-15b-5p;hsa-miR-188-3p;hsa-miR-19

ENSG0000	ENSG0000	4.76E-06	hsa-miR-15a-5p;hsa-miR-15b-5p;hsa-miR-16-5p;hsa-miR-188-
ENSG0000	ENSG0000	0.001626	hsa-miR-15a-5p;hsa-miR-15b-5p;hsa-miR-16-5p;hsa-miR-195-
ENSG0000	ENSG0000	1.22E-05	hsa-miR-15a-5p;hsa-miR-15b-5p;hsa-miR-16-5p;hsa-miR-195-
ENSG0000	ENSG0000	1.22E-05	hsa-miR-15a-5p;hsa-miR-15b-5p;hsa-miR-16-5p;hsa-miR-195-
ENSG0000	ENSG0000	1.22E-05	hsa-miR-15a-5p;hsa-miR-15b-5p;hsa-miR-16-5p;hsa-miR-195-
ENSG0000	ENSG0000	1.22E-05	hsa-miR-15a-5p;hsa-miR-15b-5p;hsa-miR-16-5p;hsa-miR-195-
ENSG0000	ENSG0000	0.002856	hsa-miR-15a-5p;hsa-miR-15b-5p;hsa-miR-16-5p;hsa-miR-195-
ENSG0000	ENSG0000	0.000526	hsa-miR-15a-5p;hsa-miR-15b-5p;hsa-miR-16-5p;hsa-miR-195-
ENSG0000	ENSG0000	0.000768	hsa-miR-34a-5p;hsa-miR-34c-5p;hsa-miR-449a;hsa-miR-449b-
ENSG0000	ENSG0000	5.05E-05	hsa-miR-15a-5p;hsa-miR-15b-5p;hsa-miR-195-5p;hsa-miR-36
ENSG0000	ENSG0000	1.16E-06	hsa-miR-15a-5p;hsa-miR-15b-5p;hsa-miR-16-5p;hsa-miR-195-
ENSG0000	ENSG0000	4.45E-07	hsa-miR-34a-5p;hsa-miR-34b-5p;hsa-miR-34c-5p;hsa-miR-44
ENSG0000	ENSG0000	4.45E-07	hsa-miR-34a-5p;hsa-miR-34b-5p;hsa-miR-34c-5p;hsa-miR-44
ENSG0000	ENSG0000	0.005964	hsa-miR-34a-5p;hsa-miR-34c-5p;hsa-miR-449a;hsa-miR-449b-
ENSG0000	ENSG0000	7.93E-08	hsa-miR-15a-5p;hsa-miR-15b-5p;hsa-miR-16-5p;hsa-miR-195-
ENSG0000	ENSG0000	0.003443	hsa-miR-15a-5p;hsa-miR-15b-5p;hsa-miR-16-5p;hsa-miR-195-
ENSG0000	ENSG0000	0.003443	hsa-miR-15a-5p;hsa-miR-15b-5p;hsa-miR-16-5p;hsa-miR-195-
ENSG0000	ENSG0000	0.003443	hsa-miR-15a-5p;hsa-miR-15b-5p;hsa-miR-16-5p;hsa-miR-195-
ENSG0000	ENSG0000	0.003443	hsa-miR-15a-5p;hsa-miR-15b-5p;hsa-miR-16-5p;hsa-miR-195-
ENSG0000	ENSG0000	0.003443	hsa-miR-15a-5p;hsa-miR-15b-5p;hsa-miR-16-5p;hsa-miR-195-
ENSG0000	ENSG0000	0.003443	hsa-miR-15a-5p;hsa-miR-15b-5p;hsa-miR-16-5p;hsa-miR-195-
ENSG0000	ENSG0000	0.003443	hsa-miR-15a-5p;hsa-miR-15b-5p;hsa-miR-16-5p;hsa-miR-195-
ENSG0000	ENSG0000	0.003443	hsa-miR-15a-5p;hsa-miR-15b-5p;hsa-miR-16-5p;hsa-miR-195-
ENSG0000	ENSG0000	0.003443	hsa-miR-15a-5p;hsa-miR-15b-5p;hsa-miR-16-5p;hsa-miR-195-
ENSG0000	ENSG0000	0.003443	hsa-miR-15a-5p;hsa-miR-15b-5p;hsa-miR-16-5p;hsa-miR-195-
ENSG0000	ENSG0000	5.27E-06	hsa-miR-15a-5p;hsa-miR-15b-5p;hsa-miR-16-5p;hsa-miR-195-
ENSG0000	ENSG0000	2.65E-05	hsa-miR-15a-5p;hsa-miR-15b-5p;hsa-miR-16-5p;hsa-miR-195-
ENSG0000	ENSG0000	0.000768	hsa-miR-15a-5p;hsa-miR-15b-5p;hsa-miR-16-5p;hsa-miR-545-
ENSG0000	ENSG0000	0.000362	hsa-miR-15a-5p;hsa-miR-15b-5p;hsa-miR-16-5p;hsa-miR-195-
ENSG0000	ENSG0000	0.00502	hsa-miR-15a-5p;hsa-miR-15b-5p;hsa-miR-16-5p;hsa-miR-195-
ENSG0000	ENSG0000	0.000411	hsa-miR-15a-5p;hsa-miR-15b-5p;hsa-miR-16-5p;hsa-miR-195-
ENSG0000	ENSG0000	0.001936	hsa-miR-15a-5p;hsa-miR-15b-5p;hsa-miR-16-5p;hsa-miR-195-
ENSG0000	ENSG0000	0.002511	hsa-miR-15a-5p;hsa-miR-15b-5p;hsa-miR-16-5p;hsa-miR-195-
ENSG0000	ENSG0000	0.004167	hsa-miR-15a-5p;hsa-miR-15b-5p;hsa-miR-16-5p;hsa-miR-195-
ENSG0000	ENSG0000	0.000896	hsa-miR-1260a;hsa-miR-1260b;hsa-miR-188-3p;hsa-miR-362-3
ENSG0000	ENSG0000	0.000343	hsa-miR-1306-5p;hsa-miR-34a-5p;hsa-miR-34b-5p;hsa-miR-3
ENSG0000	ENSG0000	0.000254	hsa-miR-15a-5p;hsa-miR-15b-5p;hsa-miR-16-5p;hsa-miR-195-
ENSG0000	ENSG0000	0.000461	hsa-miR-34a-5p;hsa-miR-34c-5p;hsa-miR-449a;hsa-miR-449b-
ENSG0000	ENSG0000	9.41E-05	hsa-miR-15a-5p;hsa-miR-15b-5p;hsa-miR-16-5p;hsa-miR-195-
ENSG0000	ENSG0000	0.008829	hsa-miR-188-3p;hsa-miR-199a-5p;hsa-miR-199b-5p;hsa-miR-
ENSG0000	ENSG0000	0.004181	hsa-miR-34a-5p;hsa-miR-34c-5p;hsa-miR-449a;hsa-miR-449b-
ENSG0000	ENSG0000	0.001039	hsa-miR-15a-5p;hsa-miR-15b-5p;hsa-miR-16-5p;hsa-miR-195-
ENSG0000	ENSG0000	0.000276	hsa-miR-15a-5p;hsa-miR-15b-5p;hsa-miR-16-5p;hsa-miR-195-
ENSG0000	ENSG0000	0.000896	hsa-miR-15a-5p;hsa-miR-15b-5p;hsa-miR-16-5p;hsa-miR-195-
ENSG0000	ENSG0000	3.91E-07	hsa-miR-15a-5p;hsa-miR-15b-5p;hsa-miR-16-5p;hsa-miR-195-
ENSG0000	ENSG0000	9.08E-08	hsa-miR-1260a;hsa-miR-1260b;hsa-miR-15a-5p;hsa-miR-15b-
ENSG0000	ENSG0000	0.000465	hsa-miR-15a-5p;hsa-miR-15b-5p;hsa-miR-16-5p;hsa-miR-195-
ENSG0000	ENSG0000	0.002898	hsa-miR-34a-5p;hsa-miR-34c-5p;hsa-miR-449a;hsa-miR-449b-
ENSG0000	ENSG0000	0.002898	hsa-miR-34a-5p;hsa-miR-34c-5p;hsa-miR-449a;hsa-miR-449b-
ENSG0000	ENSG0000	0.000461	hsa-miR-15a-5p;hsa-miR-15b-5p;hsa-miR-16-5p;hsa-miR-195-
ENSG0000	ENSG0000	9.41E-05	hsa-miR-15a-5p;hsa-miR-15b-5p;hsa-miR-16-5p;hsa-miR-195-
ENSG0000	ENSG0000	5.27E-06	hsa-miR-34a-5p;hsa-miR-34c-5p;hsa-miR-449a;hsa-miR-449b-
ENSG0000	ENSG0000	0.000313	hsa-miR-15a-5p;hsa-miR-15b-5p;hsa-miR-16-5p;hsa-miR-195-
ENSG0000	ENSG0000	0.000313	hsa-miR-15a-5p;hsa-miR-15b-5p;hsa-miR-16-5p;hsa-miR-195-

ENSG0000	ENSG0000	0.000217	hsa-miR-34a-5p;hsa-miR-34b-5p;hsa-miR-34c-5p;hsa-miR-449
ENSG0000	ENSG0000	0.00028	hsa-miR-125a-5p;hsa-miR-125b-5p;hsa-miR-15a-5p;hsa-miR-1
ENSG0000	ENSG0000	0.000109	hsa-miR-15a-5p;hsa-miR-15b-5p;hsa-miR-16-5p;hsa-miR-195-
ENSG0000	ENSG0000	0.001997	hsa-miR-188-3p;hsa-miR-361-3p;hsa-miR-362-3p;hsa-miR-545
ENSG0000	ENSG0000	0.000124	hsa-miR-15a-5p;hsa-miR-15b-5p;hsa-miR-16-5p;hsa-miR-195-
ENSG0000	ENSG0000	3.54E-05	hsa-miR-15a-5p;hsa-miR-15b-5p;hsa-miR-16-5p;hsa-miR-195-
ENSG0000	ENSG0000	0.000875	hsa-miR-15a-5p;hsa-miR-15b-5p;hsa-miR-16-5p;hsa-miR-195-
ENSG0000	ENSG0000	9.41E-05	hsa-miR-15a-5p;hsa-miR-15b-5p;hsa-miR-16-5p;hsa-miR-195-
ENSG0000	ENSG0000	0.002224	hsa-miR-34a-5p;hsa-miR-34b-5p;hsa-miR-34c-5p;hsa-miR-449
ENSG0000	ENSG0000	0.00177	hsa-miR-15a-5p;hsa-miR-15b-5p;hsa-miR-16-5p;hsa-miR-195-
ENSG0000	ENSG0000	5.05E-05	hsa-miR-34a-5p;hsa-miR-34c-5p;hsa-miR-449a;hsa-miR-449b-
ENSG0000	ENSG0000	0.005478	hsa-miR-34a-5p;hsa-miR-34c-5p;hsa-miR-449a;hsa-miR-449b-
ENSG0000	ENSG0000	1.10E-07	hsa-miR-15a-5p;hsa-miR-15b-5p;hsa-miR-16-5p;hsa-miR-195-
ENSG0000	ENSG0000	0.003886	hsa-miR-15a-5p;hsa-miR-15b-5p;hsa-miR-16-5p;hsa-miR-195-
ENSG0000	ENSG0000	0.00266	hsa-miR-15a-5p;hsa-miR-15b-5p;hsa-miR-16-5p;hsa-miR-188-
ENSG0000	ENSG0000	7.93E-08	hsa-miR-15a-5p;hsa-miR-15b-5p;hsa-miR-16-5p;hsa-miR-195-
ENSG0000	ENSG0000	0.000896	hsa-miR-15a-5p;hsa-miR-15b-5p;hsa-miR-16-5p;hsa-miR-195-
ENSG0000	ENSG0000	0.000116	hsa-miR-34a-5p;hsa-miR-34b-5p;hsa-miR-34c-5p;hsa-miR-362
ENSG0000	ENSG0000	0.003886	hsa-miR-15a-5p;hsa-miR-15b-5p;hsa-miR-16-5p;hsa-miR-195-
ENSG0000	ENSG0000	0.004186	hsa-miR-34a-5p;hsa-miR-34b-5p;hsa-miR-34c-5p;hsa-miR-449
ENSG0000	ENSG0000	1.43E-05	hsa-miR-15a-5p;hsa-miR-15b-5p;hsa-miR-16-5p;hsa-miR-195-
ENSG0000	ENSG0000	1.22E-07	hsa-miR-15a-5p;hsa-miR-15b-5p;hsa-miR-16-5p;hsa-miR-195-
ENSG0000	ENSG0000	4.82E-09	hsa-miR-15a-5p;hsa-miR-15b-5p;hsa-miR-16-5p;hsa-miR-195-
ENSG0000	ENSG0000	1.38E-07	hsa-miR-15a-5p;hsa-miR-15b-5p;hsa-miR-16-5p;hsa-miR-195-
ENSG0000	ENSG0000	1.28E-08	hsa-miR-15a-5p;hsa-miR-15b-5p;hsa-miR-16-5p;hsa-miR-195-
ENSG0000	ENSG0000	7.10E-07	hsa-miR-15a-5p;hsa-miR-15b-5p;hsa-miR-16-5p;hsa-miR-195-
ENSG0000	ENSG0000	7.10E-07	hsa-miR-15a-5p;hsa-miR-15b-5p;hsa-miR-16-5p;hsa-miR-195-
ENSG0000	ENSG0000	3.48E-05	hsa-miR-15a-5p;hsa-miR-15b-5p;hsa-miR-16-5p;hsa-miR-195-
ENSG0000	ENSG0000	5.11E-07	hsa-miR-15a-5p;hsa-miR-15b-5p;hsa-miR-16-5p;hsa-miR-195-
ENSG0000	ENSG0000	0.000106	hsa-miR-15a-5p;hsa-miR-15b-5p;hsa-miR-16-5p;hsa-miR-195-
ENSG0000	ENSG0000	1.28E-08	hsa-miR-15a-5p;hsa-miR-15b-5p;hsa-miR-16-5p;hsa-miR-195-
ENSG0000	ENSG0000	7.10E-07	hsa-miR-15a-5p;hsa-miR-15b-5p;hsa-miR-16-5p;hsa-miR-195-
ENSG0000	ENSG0000	1.90E-09	hsa-miR-15a-5p;hsa-miR-15b-5p;hsa-miR-16-5p;hsa-miR-195-
ENSG0000	ENSG0000	5.01E-06	hsa-miR-15a-5p;hsa-miR-15b-5p;hsa-miR-16-5p;hsa-miR-195-
ENSG0000	ENSG0000	8.30E-08	hsa-miR-15a-5p;hsa-miR-15b-5p;hsa-miR-16-5p;hsa-miR-195-
ENSG0000	ENSG0000	3.48E-05	hsa-miR-15a-5p;hsa-miR-15b-5p;hsa-miR-16-5p;hsa-miR-195-
ENSG0000	ENSG0000	1.77E-06	hsa-miR-15a-5p;hsa-miR-15b-5p;hsa-miR-16-5p;hsa-miR-195-
ENSG0000	ENSG0000	1.19E-06	hsa-miR-15a-5p;hsa-miR-15b-5p;hsa-miR-16-5p;hsa-miR-195-
ENSG0000	ENSG0000	1.27E-06	hsa-miR-15a-5p;hsa-miR-15b-5p;hsa-miR-16-5p;hsa-miR-195-
ENSG0000	ENSG0000	1.27E-06	hsa-miR-15a-5p;hsa-miR-15b-5p;hsa-miR-16-5p;hsa-miR-195-
ENSG0000	ENSG0000	1.94E-08	hsa-miR-15a-5p;hsa-miR-15b-5p;hsa-miR-16-5p;hsa-miR-195-
ENSG0000	ENSG0000	7.67E-07	hsa-miR-15a-5p;hsa-miR-15b-5p;hsa-miR-16-5p;hsa-miR-195-
ENSG0000	ENSG0000	1.03E-08	hsa-miR-15a-5p;hsa-miR-15b-5p;hsa-miR-16-5p;hsa-miR-195-
ENSG0000	ENSG0000	8.95E-06	hsa-miR-15a-5p;hsa-miR-15b-5p;hsa-miR-16-5p;hsa-miR-195-
ENSG0000	ENSG0000	5.77E-06	hsa-miR-15a-5p;hsa-miR-15b-5p;hsa-miR-16-5p;hsa-miR-195-
ENSG0000	ENSG0000	2.40E-07	hsa-miR-15a-5p;hsa-miR-15b-5p;hsa-miR-16-5p;hsa-miR-195-
ENSG0000	ENSG0000	5.41E-08	hsa-miR-15a-5p;hsa-miR-15b-5p;hsa-miR-16-5p;hsa-miR-195-
ENSG0000	ENSG0000	1.66E-06	hsa-miR-15a-5p;hsa-miR-15b-5p;hsa-miR-16-5p;hsa-miR-195-
ENSG0000	ENSG0000	1.55E-07	hsa-miR-15a-5p;hsa-miR-15b-5p;hsa-miR-16-5p;hsa-miR-195-
ENSG0000	ENSG0000	2.39E-06	hsa-miR-15a-5p;hsa-miR-15b-5p;hsa-miR-16-5p;hsa-miR-195-
ENSG0000	ENSG0000	8.30E-08	hsa-miR-15a-5p;hsa-miR-15b-5p;hsa-miR-16-5p;hsa-miR-195-
ENSG0000	ENSG0000	4.68E-07	hsa-miR-15a-5p;hsa-miR-15b-5p;hsa-miR-16-5p;hsa-miR-195-
ENSG0000	ENSG0000	5.25E-06	hsa-miR-15a-5p;hsa-miR-15b-5p;hsa-miR-195-5p;hsa-miR-22-
ENSG0000	ENSG0000	5.11E-07	hsa-miR-15a-5p;hsa-miR-15b-5p;hsa-miR-16-5p;hsa-miR-195-

ENSG0000	ENSG0000	2.25E-05	hsa-miR-15a-5p;hsa-miR-15b-5p;hsa-miR-16-5p;hsa-miR-195
ENSG0000	ENSG0000	2.53E-06	hsa-miR-15a-5p;hsa-miR-15b-5p;hsa-miR-16-5p;hsa-miR-195
ENSG0000	ENSG0000	6.27E-08	hsa-miR-15a-5p;hsa-miR-15b-5p;hsa-miR-16-5p;hsa-miR-195
ENSG0000	ENSG0000	1.90E-09	hsa-miR-15a-5p;hsa-miR-15b-5p;hsa-miR-16-5p;hsa-miR-195
ENSG0000	ENSG0000	1.90E-09	hsa-miR-15a-5p;hsa-miR-15b-5p;hsa-miR-16-5p;hsa-miR-195
ENSG0000	ENSG0000	9.62E-07	hsa-miR-15a-5p;hsa-miR-15b-5p;hsa-miR-16-5p;hsa-miR-195
ENSG0000	ENSG0000	3.52E-06	hsa-miR-15a-5p;hsa-miR-15b-5p;hsa-miR-16-5p;hsa-miR-195
ENSG0000	ENSG0000	2.12E-06	hsa-miR-15a-5p;hsa-miR-15b-5p;hsa-miR-16-5p;hsa-miR-195
ENSG0000	ENSG0000	1.55E-07	hsa-miR-15a-5p;hsa-miR-15b-5p;hsa-miR-195-5p;hsa-miR-22
ENSG0000	ENSG0000	0.000477	hsa-miR-15a-5p;hsa-miR-15b-5p;hsa-miR-16-5p;hsa-miR-195
ENSG0000	ENSG0000	1.08E-07	hsa-miR-15a-5p;hsa-miR-15b-5p;hsa-miR-16-5p;hsa-miR-195
ENSG0000	ENSG0000	6.04E-06	hsa-miR-15a-5p;hsa-miR-15b-5p;hsa-miR-16-5p;hsa-miR-195
ENSG0000	ENSG0000	1.90E-09	hsa-miR-15a-5p;hsa-miR-15b-5p;hsa-miR-16-5p;hsa-miR-195
ENSG0000	ENSG0000	1.90E-09	hsa-miR-15a-5p;hsa-miR-15b-5p;hsa-miR-16-5p;hsa-miR-195
ENSG0000	ENSG0000	2.66E-12	hsa-miR-15a-5p;hsa-miR-15b-5p;hsa-miR-16-5p;hsa-miR-195
ENSG0000	ENSG0000	3.34E-06	hsa-miR-15a-5p;hsa-miR-15b-5p;hsa-miR-16-5p;hsa-miR-195
ENSG0000	ENSG0000	1.33E-11	hsa-miR-15a-5p;hsa-miR-15b-5p;hsa-miR-16-5p;hsa-miR-195
ENSG0000	ENSG0000	5.11E-07	hsa-miR-15a-5p;hsa-miR-15b-5p;hsa-miR-16-5p;hsa-miR-195
ENSG0000	ENSG0000	3.57E-07	hsa-miR-15a-5p;hsa-miR-15b-5p;hsa-miR-16-5p;hsa-miR-195
ENSG0000	ENSG0000	2.39E-06	hsa-miR-15a-5p;hsa-miR-15b-5p;hsa-miR-16-5p;hsa-miR-195
ENSG0000	ENSG0000	1.94E-08	hsa-miR-15a-5p;hsa-miR-15b-5p;hsa-miR-16-5p;hsa-miR-195
ENSG0000	ENSG0000	1.94E-08	hsa-miR-15a-5p;hsa-miR-15b-5p;hsa-miR-16-5p;hsa-miR-195
ENSG0000	ENSG0000	3.91E-07	hsa-miR-15a-5p;hsa-miR-15b-5p;hsa-miR-16-5p;hsa-miR-195
ENSG0000	ENSG0000	5.43E-05	hsa-miR-15a-5p;hsa-miR-15b-5p;hsa-miR-16-5p;hsa-miR-195
ENSG0000	ENSG0000	2.66E-09	hsa-miR-15a-5p;hsa-miR-15b-5p;hsa-miR-16-5p;hsa-miR-195
ENSG0000	ENSG0000	2.66E-09	hsa-miR-15a-5p;hsa-miR-15b-5p;hsa-miR-16-5p;hsa-miR-195
ENSG0000	ENSG0000	9.30E-11	hsa-miR-15a-5p;hsa-miR-15b-5p;hsa-miR-16-5p;hsa-miR-195
ENSG0000	ENSG0000	1.55E-07	hsa-miR-15a-5p;hsa-miR-15b-5p;hsa-miR-16-5p;hsa-miR-195
ENSG0000	ENSG0000	1.38E-07	hsa-miR-15a-5p;hsa-miR-15b-5p;hsa-miR-16-5p;hsa-miR-195
ENSG0000	ENSG0000	3.95E-08	hsa-miR-15a-5p;hsa-miR-15b-5p;hsa-miR-16-5p;hsa-miR-195
ENSG0000	ENSG0000	3.62E-09	hsa-miR-15a-5p;hsa-miR-15b-5p;hsa-miR-16-5p;hsa-miR-195
ENSG0000	ENSG0000	6.27E-08	hsa-miR-15a-5p;hsa-miR-15b-5p;hsa-miR-195-5p;hsa-miR-22
ENSG0000	ENSG0000	3.24E-07	hsa-miR-15a-5p;hsa-miR-15b-5p;hsa-miR-16-5p;hsa-miR-195
ENSG0000	ENSG0000	1.28E-08	hsa-miR-15a-5p;hsa-miR-15b-5p;hsa-miR-16-5p;hsa-miR-195
ENSG0000	ENSG0000	1.66E-06	hsa-miR-15a-5p;hsa-miR-15b-5p;hsa-miR-16-5p;hsa-miR-195
ENSG0000	ENSG0000	2.40E-07	hsa-miR-15a-5p;hsa-miR-15b-5p;hsa-miR-16-5p;hsa-miR-195
ENSG0000	ENSG0000	1.27E-06	hsa-miR-15a-5p;hsa-miR-15b-5p;hsa-miR-16-5p;hsa-miR-195
ENSG0000	ENSG0000	1.28E-08	hsa-miR-15a-5p;hsa-miR-15b-5p;hsa-miR-16-5p;hsa-miR-195
ENSG0000	ENSG0000	7.23E-08	hsa-miR-15a-5p;hsa-miR-15b-5p;hsa-miR-16-5p;hsa-miR-195
ENSG0000	ENSG0000	7.00E-05	hsa-miR-15a-5p;hsa-miR-15b-5p;hsa-miR-16-5p;hsa-miR-195
ENSG0000	ENSG0000	1.66E-06	hsa-miR-15a-5p;hsa-miR-15b-5p;hsa-miR-16-5p;hsa-miR-195
ENSG0000	ENSG0000	0.000129	hsa-miR-15a-5p;hsa-miR-15b-5p;hsa-miR-16-5p;hsa-miR-195
ENSG0000	ENSG0000	4.11E-06	hsa-miR-15a-5p;hsa-miR-15b-5p;hsa-miR-16-5p;hsa-miR-195
ENSG0000	ENSG0000	4.25E-05	hsa-miR-15a-5p;hsa-miR-15b-5p;hsa-miR-16-5p;hsa-miR-195
ENSG0000	ENSG0000	1.36E-06	hsa-miR-15a-5p;hsa-miR-15b-5p;hsa-miR-16-5p;hsa-miR-195
ENSG0000	ENSG0000	6.27E-08	hsa-miR-15a-5p;hsa-miR-15b-5p;hsa-miR-16-5p;hsa-miR-195
ENSG0000	ENSG0000	6.04E-06	hsa-miR-15a-5p;hsa-miR-15b-5p;hsa-miR-16-5p;hsa-miR-195
ENSG0000	ENSG0000	5.41E-08	hsa-miR-15a-5p;hsa-miR-15b-5p;hsa-miR-16-5p;hsa-miR-195
ENSG0000	ENSG0000	3.24E-07	hsa-miR-15a-5p;hsa-miR-15b-5p;hsa-miR-16-5p;hsa-miR-195
ENSG0000	ENSG0000	0.000456	hsa-miR-15a-5p;hsa-miR-15b-5p;hsa-miR-195-5p;hsa-miR-22
ENSG0000	ENSG0000	2.66E-12	hsa-miR-15a-5p;hsa-miR-15b-5p;hsa-miR-16-5p;hsa-miR-195
ENSG0000	ENSG0000	1.94E-08	hsa-miR-15a-5p;hsa-miR-15b-5p;hsa-miR-16-5p;hsa-miR-195
ENSG0000	ENSG0000	2.25E-06	hsa-miR-15a-5p;hsa-miR-15b-5p;hsa-miR-16-5p;hsa-miR-195
ENSG0000	ENSG0000	3.71E-06	hsa-miR-15a-5p;hsa-miR-15b-5p;hsa-miR-16-5p;hsa-miR-195

ENSG0000	ENSG0000	1.27E-06	hsa-miR-15a-5p;hsa-miR-15b-5p;hsa-miR-16-5p;hsa-miR-195-
ENSG0000	ENSG0000	1.31E-09	hsa-miR-15a-5p;hsa-miR-15b-5p;hsa-miR-16-5p;hsa-miR-195-
ENSG0000	ENSG0000	1.11E-06	hsa-miR-15a-5p;hsa-miR-15b-5p;hsa-miR-16-5p;hsa-miR-195-
ENSG0000	ENSG0000	3.57E-07	hsa-miR-15a-5p;hsa-miR-15b-5p;hsa-miR-16-5p;hsa-miR-195-
ENSG0000	ENSG0000	1.56E-06	hsa-miR-15a-5p;hsa-miR-15b-5p;hsa-miR-16-5p;hsa-miR-195-
ENSG0000	ENSG0000	3.35E-10	hsa-miR-15a-5p;hsa-miR-15b-5p;hsa-miR-16-5p;hsa-miR-195-
ENSG0000	ENSG0000	2.66E-12	hsa-miR-15a-5p;hsa-miR-15b-5p;hsa-miR-16-5p;hsa-miR-195-
ENSG0000	ENSG0000	1.27E-06	hsa-miR-15a-5p;hsa-miR-15b-5p;hsa-miR-16-5p;hsa-miR-195-
ENSG0000	ENSG0000	1.14E-05	hsa-miR-15a-5p;hsa-miR-15b-5p;hsa-miR-16-5p;hsa-miR-195-
ENSG0000	ENSG0000	1.94E-08	hsa-miR-15a-5p;hsa-miR-15b-5p;hsa-miR-16-5p;hsa-miR-195-
ENSG0000	ENSG0000	1.90E-09	hsa-miR-15a-5p;hsa-miR-15b-5p;hsa-miR-16-5p;hsa-miR-195-
ENSG0000	ENSG0000	5.77E-06	hsa-miR-15a-5p;hsa-miR-15b-5p;hsa-miR-16-5p;hsa-miR-195-
ENSG0000	ENSG0000	2.16E-07	hsa-miR-15a-5p;hsa-miR-15b-5p;hsa-miR-16-5p;hsa-miR-195-
ENSG0000	ENSG0000	1.55E-07	hsa-miR-15a-5p;hsa-miR-15b-5p;hsa-miR-16-5p;hsa-miR-195-
ENSG0000	ENSG0000	1.86E-10	hsa-miR-15a-5p;hsa-miR-15b-5p;hsa-miR-16-5p;hsa-miR-195-
ENSG0000	ENSG0000	3.95E-08	hsa-miR-15a-5p;hsa-miR-15b-5p;hsa-miR-16-5p;hsa-miR-195-
ENSG0000	ENSG0000	8.93E-07	hsa-miR-15a-5p;hsa-miR-15b-5p;hsa-miR-16-5p;hsa-miR-195-
ENSG0000	ENSG0000	2.81E-08	hsa-miR-15a-5p;hsa-miR-15b-5p;hsa-miR-16-5p;hsa-miR-195-
ENSG0000	ENSG0000	2.66E-09	hsa-miR-15a-5p;hsa-miR-15b-5p;hsa-miR-16-5p;hsa-miR-195-
ENSG0000	ENSG0000	2.40E-07	hsa-miR-15a-5p;hsa-miR-15b-5p;hsa-miR-16-5p;hsa-miR-195-
ENSG0000	ENSG0000	4.37E-05	hsa-miR-15a-5p;hsa-miR-15b-5p;hsa-miR-16-5p;hsa-miR-195-
ENSG0000	ENSG0000	9.62E-07	hsa-miR-15a-5p;hsa-miR-15b-5p;hsa-miR-16-5p;hsa-miR-195-
ENSG0000	ENSG0000	1.66E-05	hsa-miR-15a-5p;hsa-miR-15b-5p;hsa-miR-16-5p;hsa-miR-195-
ENSG0000	ENSG0000	1.88E-06	hsa-miR-15a-5p;hsa-miR-15b-5p;hsa-miR-16-5p;hsa-miR-195-
ENSG0000	ENSG0000	1.94E-07	hsa-miR-15a-5p;hsa-miR-15b-5p;hsa-miR-16-5p;hsa-miR-195-
ENSG0000	ENSG0000	9.62E-07	hsa-miR-15a-5p;hsa-miR-15b-5p;hsa-miR-16-5p;hsa-miR-195-
ENSG0000	ENSG0000	5.58E-10	hsa-miR-15a-5p;hsa-miR-15b-5p;hsa-miR-16-5p;hsa-miR-195-
ENSG0000	ENSG0000	1.31E-09	hsa-miR-15a-5p;hsa-miR-15b-5p;hsa-miR-16-5p;hsa-miR-195-
ENSG0000	ENSG0000	4.68E-07	hsa-miR-15a-5p;hsa-miR-15b-5p;hsa-miR-16-5p;hsa-miR-195-
ENSG0000	ENSG0000	4.68E-07	hsa-miR-15a-5p;hsa-miR-15b-5p;hsa-miR-16-5p;hsa-miR-195-
ENSG0000	ENSG0000	6.04E-07	hsa-miR-15a-5p;hsa-miR-15b-5p;hsa-miR-16-5p;hsa-miR-195-
ENSG0000	ENSG0000	1.08E-07	hsa-miR-15a-5p;hsa-miR-15b-5p;hsa-miR-16-5p;hsa-miR-195-
ENSG0000	ENSG0000	2.66E-09	hsa-miR-15a-5p;hsa-miR-15b-5p;hsa-miR-16-5p;hsa-miR-195-
ENSG0000	ENSG0000	5.16E-10	hsa-miR-15a-5p;hsa-miR-15b-5p;hsa-miR-16-5p;hsa-miR-195-
ENSG0000	ENSG0000	1.58E-08	hsa-miR-15a-5p;hsa-miR-15b-5p;hsa-miR-16-5p;hsa-miR-195-
ENSG0000	ENSG0000	1.58E-08	hsa-miR-15a-5p;hsa-miR-15b-5p;hsa-miR-16-5p;hsa-miR-195-
ENSG0000	ENSG0000	2.66E-09	hsa-miR-15a-5p;hsa-miR-15b-5p;hsa-miR-16-5p;hsa-miR-195-
ENSG0000	ENSG0000	1.60E-05	hsa-miR-15a-5p;hsa-miR-15b-5p;hsa-miR-16-5p;hsa-miR-195-
ENSG0000	ENSG0000	4.68E-07	hsa-miR-15a-5p;hsa-miR-15b-5p;hsa-miR-16-5p;hsa-miR-195-
ENSG0000	ENSG0000	6.33E-06	hsa-miR-15a-5p;hsa-miR-15b-5p;hsa-miR-16-5p;hsa-miR-195-
ENSG0000	ENSG0000	7.55E-06	hsa-miR-15a-5p;hsa-miR-15b-5p;hsa-miR-16-5p;hsa-miR-195-
ENSG0000	ENSG0000	1.76E-05	hsa-miR-15a-5p;hsa-miR-15b-5p;hsa-miR-16-5p;hsa-miR-195-
ENSG0000	ENSG0000	5.11E-07	hsa-miR-15a-5p;hsa-miR-15b-5p;hsa-miR-16-5p;hsa-miR-195-
ENSG0000	ENSG0000	8.93E-07	hsa-miR-15a-5p;hsa-miR-15b-5p;hsa-miR-16-5p;hsa-miR-195-
ENSG0000	ENSG0000	1.38E-07	hsa-miR-15a-5p;hsa-miR-15b-5p;hsa-miR-16-5p;hsa-miR-195-
ENSG0000	ENSG0000	1.38E-07	hsa-miR-15a-5p;hsa-miR-15b-5p;hsa-miR-16-5p;hsa-miR-195-
ENSG0000	ENSG0000	3.34E-08	hsa-miR-15a-5p;hsa-miR-15b-5p;hsa-miR-16-5p;hsa-miR-195-
ENSG0000	ENSG0000	3.24E-07	hsa-miR-15a-5p;hsa-miR-15b-5p;hsa-miR-16-5p;hsa-miR-195-
ENSG0000	ENSG0000	7.23E-08	hsa-miR-15a-5p;hsa-miR-15b-5p;hsa-miR-16-5p;hsa-miR-195-
ENSG0000	ENSG0000	6.33E-06	hsa-miR-15a-5p;hsa-miR-15b-5p;hsa-miR-16-5p;hsa-miR-195-
ENSG0000	ENSG0000	8.30E-08	hsa-miR-15a-5p;hsa-miR-15b-5p;hsa-miR-16-5p;hsa-miR-195-
ENSG0000	ENSG0000	4.68E-07	hsa-miR-15a-5p;hsa-miR-15b-5p;hsa-miR-16-5p;hsa-miR-195-
ENSG0000	ENSG0000	4.68E-07	hsa-miR-15a-5p;hsa-miR-15b-5p;hsa-miR-16-5p;hsa-miR-195-
ENSG0000	ENSG0000	1.94E-07	hsa-miR-15a-5p;hsa-miR-15b-5p;hsa-miR-16-5p;hsa-miR-195-

ENSG0000	ENSG0000	3.35E-10	hsa-miR-15a-5p;hsa-miR-15b-5p;hsa-miR-16-5p;hsa-miR-195·
ENSG0000	ENSG0000	3.24E-07	hsa-miR-15a-5p;hsa-miR-15b-5p;hsa-miR-16-5p;hsa-miR-195·
ENSG0000	ENSG0000	1.33E-05	hsa-miR-15a-5p;hsa-miR-15b-5p;hsa-miR-16-5p;hsa-miR-195·
ENSG0000	ENSG0000	1.33E-05	hsa-miR-15a-5p;hsa-miR-15b-5p;hsa-miR-16-5p;hsa-miR-195·
ENSG0000	ENSG0000	1.90E-09	hsa-miR-15a-5p;hsa-miR-15b-5p;hsa-miR-16-5p;hsa-miR-195·
ENSG0000	ENSG0000	1.27E-06	hsa-miR-15a-5p;hsa-miR-15b-5p;hsa-miR-16-5p;hsa-miR-195·
ENSG0000	ENSG0000	1.11E-06	hsa-miR-15a-5p;hsa-miR-15b-5p;hsa-miR-16-5p;hsa-miR-195·
ENSG0000	ENSG0000	1.94E-08	hsa-miR-15a-5p;hsa-miR-15b-5p;hsa-miR-16-5p;hsa-miR-195·
ENSG0000	ENSG0000	2.66E-07	hsa-miR-15a-5p;hsa-miR-15b-5p;hsa-miR-16-5p;hsa-miR-195·
ENSG0000	ENSG0000	8.30E-08	hsa-miR-15a-5p;hsa-miR-15b-5p;hsa-miR-16-5p;hsa-miR-195·
ENSG0000	ENSG0000	4.82E-09	hsa-miR-15a-5p;hsa-miR-15b-5p;hsa-miR-16-5p;hsa-miR-195·
ENSG0000	ENSG0000	1.94E-08	hsa-miR-15a-5p;hsa-miR-15b-5p;hsa-miR-16-5p;hsa-miR-195·
ENSG0000	ENSG0000	1.66E-06	hsa-miR-15a-5p;hsa-miR-15b-5p;hsa-miR-16-5p;hsa-miR-195·
ENSG0000	ENSG0000	1.38E-07	hsa-miR-15a-5p;hsa-miR-15b-5p;hsa-miR-16-5p;hsa-miR-195·
ENSG0000	ENSG0000	5.43E-05	hsa-miR-15a-5p;hsa-miR-15b-5p;hsa-miR-16-5p;hsa-miR-195·
ENSG0000	ENSG0000	1.94E-08	hsa-miR-15a-5p;hsa-miR-15b-5p;hsa-miR-16-5p;hsa-miR-195·
ENSG0000	ENSG0000	1.46E-06	hsa-miR-15a-5p;hsa-miR-15b-5p;hsa-miR-16-5p;hsa-miR-195·
ENSG0000	ENSG0000	1.94E-08	hsa-miR-15a-5p;hsa-miR-15b-5p;hsa-miR-16-5p;hsa-miR-195·
ENSG0000	ENSG0000	2.83E-06	hsa-miR-15a-5p;hsa-miR-15b-5p;hsa-miR-16-5p;hsa-miR-195·
ENSG0000	ENSG0000	1.90E-09	hsa-miR-15a-5p;hsa-miR-15b-5p;hsa-miR-16-5p;hsa-miR-195·
ENSG0000	ENSG0000	1.27E-06	hsa-miR-15a-5p;hsa-miR-15b-5p;hsa-miR-16-5p;hsa-miR-195·
ENSG0000	ENSG0000	3.34E-08	hsa-miR-15a-5p;hsa-miR-15b-5p;hsa-miR-16-5p;hsa-miR-195·
ENSG0000	ENSG0000	2.66E-12	hsa-miR-15a-5p;hsa-miR-15b-5p;hsa-miR-16-5p;hsa-miR-195·
ENSG0000	ENSG0000	4.54E-06	hsa-miR-15a-5p;hsa-miR-15b-5p;hsa-miR-16-5p;hsa-miR-195·
ENSG0000	ENSG0000	2.81E-08	hsa-miR-15a-5p;hsa-miR-15b-5p;hsa-miR-16-5p;hsa-miR-195·
ENSG0000	ENSG0000	1.94E-07	hsa-miR-15a-5p;hsa-miR-15b-5p;hsa-miR-16-5p;hsa-miR-195·
ENSG0000	ENSG0000	5.11E-07	hsa-miR-15a-5p;hsa-miR-15b-5p;hsa-miR-16-5p;hsa-miR-195·
ENSG0000	ENSG0000	6.04E-07	hsa-miR-15a-5p;hsa-miR-15b-5p;hsa-miR-16-5p;hsa-miR-195·
ENSG0000	ENSG0000	6.27E-08	hsa-miR-15a-5p;hsa-miR-15b-5p;hsa-miR-16-5p;hsa-miR-195·
ENSG0000	ENSG0000	1.03E-08	hsa-miR-15a-5p;hsa-miR-15b-5p;hsa-miR-16-5p;hsa-miR-195·
ENSG0000	ENSG0000	1.90E-09	hsa-miR-15a-5p;hsa-miR-15b-5p;hsa-miR-16-5p;hsa-miR-195·
ENSG0000	ENSG0000	3.99E-11	hsa-miR-15a-5p;hsa-miR-15b-5p;hsa-miR-16-5p;hsa-miR-195·
ENSG0000	ENSG0000	2.66E-09	hsa-miR-15a-5p;hsa-miR-15b-5p;hsa-miR-16-5p;hsa-miR-195·
ENSG0000	ENSG0000	1.36E-06	hsa-miR-15a-5p;hsa-miR-15b-5p;hsa-miR-16-5p;hsa-miR-195·
ENSG0000	ENSG0000	2.66E-07	hsa-miR-15a-5p;hsa-miR-15b-5p;hsa-miR-16-5p;hsa-miR-195·
ENSG0000	ENSG0000	1.58E-08	hsa-miR-15a-5p;hsa-miR-15b-5p;hsa-miR-16-5p;hsa-miR-195·
ENSG0000	ENSG0000	1.74E-07	hsa-miR-15a-5p;hsa-miR-15b-5p;hsa-miR-16-5p;hsa-miR-195·
ENSG0000	ENSG0000	1.27E-06	hsa-miR-15a-5p;hsa-miR-15b-5p;hsa-miR-16-5p;hsa-miR-195·
ENSG0000	ENSG0000	1.38E-07	hsa-miR-15a-5p;hsa-miR-15b-5p;hsa-miR-16-5p;hsa-miR-195·
ENSG0000	ENSG0000	0.000146	hsa-miR-15a-5p;hsa-miR-15b-5p;hsa-miR-16-5p;hsa-miR-195·
ENSG0000	ENSG0000	6.31E-09	hsa-miR-15a-5p;hsa-miR-15b-5p;hsa-miR-16-5p;hsa-miR-195·
ENSG0000	ENSG0000	2.16E-07	hsa-miR-15a-5p;hsa-miR-15b-5p;hsa-miR-16-5p;hsa-miR-195·
ENSG0000	ENSG0000	1.86E-10	hsa-miR-15a-5p;hsa-miR-15b-5p;hsa-miR-16-5p;hsa-miR-195·
ENSG0000	ENSG0000	1.94E-08	hsa-miR-15a-5p;hsa-miR-15b-5p;hsa-miR-16-5p;hsa-miR-195·
ENSG0000	ENSG0000	1.11E-06	hsa-miR-15a-5p;hsa-miR-15b-5p;hsa-miR-16-5p;hsa-miR-195·
ENSG0000	ENSG0000	1.56E-06	hsa-miR-15a-5p;hsa-miR-15b-5p;hsa-miR-16-5p;hsa-miR-195·
ENSG0000	ENSG0000	3.69E-05	hsa-miR-15a-5p;hsa-miR-15b-5p;hsa-miR-16-5p;hsa-miR-195·
ENSG0000	ENSG0000	7.23E-08	hsa-miR-15a-5p;hsa-miR-15b-5p;hsa-miR-16-5p;hsa-miR-195·
ENSG0000	ENSG0000	2.94E-07	hsa-miR-15a-5p;hsa-miR-15b-5p;hsa-miR-16-5p;hsa-miR-195·
ENSG0000	ENSG0000	3.99E-11	hsa-miR-15a-5p;hsa-miR-15b-5p;hsa-miR-16-5p;hsa-miR-195·
ENSG0000	ENSG0000	4.77E-06	hsa-miR-15a-5p;hsa-miR-15b-5p;hsa-miR-16-5p;hsa-miR-195·
ENSG0000	ENSG0000	7.67E-07	hsa-miR-15a-5p;hsa-miR-15b-5p;hsa-miR-16-5p;hsa-miR-195·
ENSG0000	ENSG0000	1.58E-08	hsa-miR-15a-5p;hsa-miR-15b-5p;hsa-miR-16-5p;hsa-miR-195·
ENSG0000	ENSG0000	1.58E-08	hsa-miR-15a-5p;hsa-miR-15b-5p;hsa-miR-16-5p;hsa-miR-195·

ENSG0000	ENSG0000	1.33E-11	hsa-miR-15a-5p;hsa-miR-15b-5p;hsa-miR-16-5p;hsa-miR-195-
ENSG0000	ENSG0000	2.66E-12	hsa-miR-15a-5p;hsa-miR-15b-5p;hsa-miR-16-5p;hsa-miR-195-
ENSG0000	ENSG0000	1.03E-08	hsa-miR-15a-5p;hsa-miR-15b-5p;hsa-miR-16-5p;hsa-miR-195-
ENSG0000	ENSG0000	8.30E-08	hsa-miR-15a-5p;hsa-miR-15b-5p;hsa-miR-16-5p;hsa-miR-195-
ENSG0000	ENSG0000	7.10E-07	hsa-miR-15a-5p;hsa-miR-15b-5p;hsa-miR-16-5p;hsa-miR-195-
ENSG0000	ENSG0000	2.34E-08	hsa-miR-15a-5p;hsa-miR-15b-5p;hsa-miR-16-5p;hsa-miR-195-
ENSG0000	ENSG0000	1.94E-08	hsa-miR-15a-5p;hsa-miR-15b-5p;hsa-miR-16-5p;hsa-miR-195-
ENSG0000	ENSG0000	1.08E-07	hsa-miR-15a-5p;hsa-miR-15b-5p;hsa-miR-16-5p;hsa-miR-195-
ENSG0000	ENSG0000	8.95E-06	hsa-miR-15a-5p;hsa-miR-15b-5p;hsa-miR-16-5p;hsa-miR-195-
ENSG0000	ENSG0000	1.33E-11	hsa-miR-15a-5p;hsa-miR-15b-5p;hsa-miR-16-5p;hsa-miR-195-
ENSG0000	ENSG0000	1.74E-07	hsa-miR-15a-5p;hsa-miR-15b-5p;hsa-miR-16-5p;hsa-miR-195-
ENSG0000	ENSG0000	1.11E-06	hsa-miR-15a-5p;hsa-miR-15b-5p;hsa-miR-16-5p;hsa-miR-195-
ENSG0000	ENSG0000	1.31E-09	hsa-miR-15a-5p;hsa-miR-15b-5p;hsa-miR-16-5p;hsa-miR-195-
ENSG0000	ENSG0000	3.38E-05	hsa-miR-15a-5p;hsa-miR-15b-5p;hsa-miR-16-5p;hsa-miR-195-
ENSG0000	ENSG0000	3.38E-05	hsa-miR-15a-5p;hsa-miR-15b-5p;hsa-miR-16-5p;hsa-miR-195-
ENSG0000	ENSG0000	1.77E-06	hsa-miR-15a-5p;hsa-miR-15b-5p;hsa-miR-16-5p;hsa-miR-195-
ENSG0000	ENSG0000	1.94E-08	hsa-miR-15a-5p;hsa-miR-15b-5p;hsa-miR-16-5p;hsa-miR-195-
ENSG0000	ENSG0000	7.10E-07	hsa-miR-15a-5p;hsa-miR-15b-5p;hsa-miR-16-5p;hsa-miR-195-
ENSG0000	ENSG0000	1.56E-06	hsa-miR-15a-5p;hsa-miR-15b-5p;hsa-miR-16-5p;hsa-miR-195-
ENSG0000	ENSG0000	1.22E-07	hsa-miR-15a-5p;hsa-miR-15b-5p;hsa-miR-16-5p;hsa-miR-195-
ENSG0000	ENSG0000	2.66E-12	hsa-miR-15a-5p;hsa-miR-15b-5p;hsa-miR-16-5p;hsa-miR-195-
ENSG0000	ENSG0000	2.99E-05	hsa-miR-15a-5p;hsa-miR-15b-5p;hsa-miR-16-5p;hsa-miR-195-
ENSG0000	ENSG0000	4.54E-06	hsa-miR-15a-5p;hsa-miR-15b-5p;hsa-miR-16-5p;hsa-miR-195-
ENSG0000	ENSG0000	2.83E-06	hsa-miR-15a-5p;hsa-miR-15b-5p;hsa-miR-16-5p;hsa-miR-195-
ENSG0000	ENSG0000	1.84E-05	hsa-miR-15a-5p;hsa-miR-15b-5p;hsa-miR-16-5p;hsa-miR-195-
ENSG0000	ENSG0000	3.91E-07	hsa-miR-15a-5p;hsa-miR-15b-5p;hsa-miR-16-5p;hsa-miR-195-
ENSG0000	ENSG0000	1.22E-07	hsa-miR-15a-5p;hsa-miR-15b-5p;hsa-miR-16-5p;hsa-miR-195-
ENSG0000	ENSG0000	2.66E-12	hsa-miR-15a-5p;hsa-miR-15b-5p;hsa-miR-16-5p;hsa-miR-195-
ENSG0000	ENSG0000	3.91E-07	hsa-miR-15a-5p;hsa-miR-15b-5p;hsa-miR-16-5p;hsa-miR-195-
ENSG0000	ENSG0000	3.34E-08	hsa-miR-15a-5p;hsa-miR-15b-5p;hsa-miR-16-5p;hsa-miR-195-
ENSG0000	ENSG0000	2.39E-06	hsa-miR-15a-5p;hsa-miR-15b-5p;hsa-miR-16-5p;hsa-miR-195-
ENSG0000	ENSG0000	1.27E-06	hsa-miR-15a-5p;hsa-miR-15b-5p;hsa-miR-16-5p;hsa-miR-195-
ENSG0000	ENSG0000	6.33E-06	hsa-miR-15a-5p;hsa-miR-15b-5p;hsa-miR-16-5p;hsa-miR-195-
ENSG0000	ENSG0000	8.93E-07	hsa-miR-15a-5p;hsa-miR-15b-5p;hsa-miR-16-5p;hsa-miR-195-
ENSG0000	ENSG0000	8.59E-06	hsa-miR-15a-5p;hsa-miR-15b-5p;hsa-miR-16-5p;hsa-miR-195-
ENSG0000	ENSG0000	4.64E-08	hsa-miR-15a-5p;hsa-miR-15b-5p;hsa-miR-16-5p;hsa-miR-195-
ENSG0000	ENSG0000	3.91E-07	hsa-miR-15a-5p;hsa-miR-15b-5p;hsa-miR-16-5p;hsa-miR-195-
ENSG0000	ENSG0000	6.04E-06	hsa-miR-15a-5p;hsa-miR-15b-5p;hsa-miR-16-5p;hsa-miR-195-
ENSG0000	ENSG0000	2.94E-07	hsa-miR-15a-5p;hsa-miR-15b-5p;hsa-miR-16-5p;hsa-miR-195-
ENSG0000	ENSG0000	1.77E-06	hsa-miR-15a-5p;hsa-miR-15b-5p;hsa-miR-16-5p;hsa-miR-195-
ENSG0000	ENSG0000	8.88E-05	hsa-miR-15a-5p;hsa-miR-15b-5p;hsa-miR-16-5p;hsa-miR-195-
ENSG0000	ENSG0000	5.41E-08	hsa-miR-15a-5p;hsa-miR-15b-5p;hsa-miR-16-5p;hsa-miR-195-
ENSG0000	ENSG0000	4.68E-07	hsa-miR-15a-5p;hsa-miR-15b-5p;hsa-miR-16-5p;hsa-miR-195-
ENSG0000	ENSG0000	5.56E-07	hsa-miR-15a-5p;hsa-miR-15b-5p;hsa-miR-16-5p;hsa-miR-195-
ENSG0000	ENSG0000	1.28E-08	hsa-miR-15a-5p;hsa-miR-15b-5p;hsa-miR-16-5p;hsa-miR-195-
ENSG0000	ENSG0000	3.38E-05	hsa-miR-15a-5p;hsa-miR-15b-5p;hsa-miR-16-5p;hsa-miR-195-
ENSG0000	ENSG0000	1.31E-09	hsa-miR-15a-5p;hsa-miR-15b-5p;hsa-miR-16-5p;hsa-miR-195-
ENSG0000	ENSG0000	1.66E-06	hsa-miR-15a-5p;hsa-miR-15b-5p;hsa-miR-16-5p;hsa-miR-195-
ENSG0000	ENSG0000	6.04E-06	hsa-miR-15a-5p;hsa-miR-15b-5p;hsa-miR-16-5p;hsa-miR-195-
ENSG0000	ENSG0000	5.41E-08	hsa-miR-15a-5p;hsa-miR-15b-5p;hsa-miR-16-5p;hsa-miR-195-
ENSG0000	ENSG0000	4.82E-09	hsa-miR-15a-5p;hsa-miR-15b-5p;hsa-miR-16-5p;hsa-miR-195-
ENSG0000	ENSG0000	3.71E-06	hsa-miR-15a-5p;hsa-miR-15b-5p;hsa-miR-16-5p;hsa-miR-195-
ENSG0000	ENSG0000	6.55E-07	hsa-miR-15a-5p;hsa-miR-15b-5p;hsa-miR-16-5p;hsa-miR-195-
ENSG0000	ENSG0000	1.03E-08	hsa-miR-15a-5p;hsa-miR-15b-5p;hsa-miR-16-5p;hsa-miR-195-

ENSG0000	ENSG0000	1.03E-08	hsa-miR-15a-5p;hsa-miR-15b-5p;hsa-miR-16-5p;hsa-miR-195-
ENSG0000	ENSG0000	1.03E-08	hsa-miR-15a-5p;hsa-miR-15b-5p;hsa-miR-16-5p;hsa-miR-195-
ENSG0000	ENSG0000	1.03E-08	hsa-miR-15a-5p;hsa-miR-15b-5p;hsa-miR-16-5p;hsa-miR-195-
ENSG0000	ENSG0000	5.01E-06	hsa-miR-15a-5p;hsa-miR-15b-5p;hsa-miR-16-5p;hsa-miR-195-
ENSG0000	ENSG0000	1.36E-06	hsa-miR-15a-5p;hsa-miR-15b-5p;hsa-miR-16-5p;hsa-miR-195-
ENSG0000	ENSG0000	3.99E-11	hsa-miR-15a-5p;hsa-miR-15b-5p;hsa-miR-16-5p;hsa-miR-195-
ENSG0000	ENSG0000	2.66E-12	hsa-miR-15a-5p;hsa-miR-15b-5p;hsa-miR-16-5p;hsa-miR-195-
ENSG0000	ENSG0000	1.74E-07	hsa-miR-15a-5p;hsa-miR-15b-5p;hsa-miR-16-5p;hsa-miR-195-
ENSG0000	ENSG0000	1.74E-07	hsa-miR-15a-5p;hsa-miR-15b-5p;hsa-miR-16-5p;hsa-miR-195-
ENSG0000	ENSG0000	1.74E-07	hsa-miR-15a-5p;hsa-miR-15b-5p;hsa-miR-16-5p;hsa-miR-195-
ENSG0000	ENSG0000	1.74E-07	hsa-miR-15a-5p;hsa-miR-15b-5p;hsa-miR-16-5p;hsa-miR-195-
ENSG0000	ENSG0000	1.74E-07	hsa-miR-15a-5p;hsa-miR-15b-5p;hsa-miR-16-5p;hsa-miR-195-
ENSG0000	ENSG0000	1.74E-07	hsa-miR-15a-5p;hsa-miR-15b-5p;hsa-miR-16-5p;hsa-miR-195-
ENSG0000	ENSG0000	1.74E-07	hsa-miR-15a-5p;hsa-miR-15b-5p;hsa-miR-16-5p;hsa-miR-195-
ENSG0000	ENSG0000	1.74E-07	hsa-miR-15a-5p;hsa-miR-15b-5p;hsa-miR-16-5p;hsa-miR-195-
ENSG0000	ENSG0000	1.74E-07	hsa-miR-15a-5p;hsa-miR-15b-5p;hsa-miR-16-5p;hsa-miR-195-
ENSG0000	ENSG0000	1.74E-07	hsa-miR-15a-5p;hsa-miR-15b-5p;hsa-miR-16-5p;hsa-miR-195-
ENSG0000	ENSG0000	1.86E-10	hsa-miR-15a-5p;hsa-miR-15b-5p;hsa-miR-16-5p;hsa-miR-195-
ENSG0000	ENSG0000	1.94E-08	hsa-miR-15a-5p;hsa-miR-15b-5p;hsa-miR-16-5p;hsa-miR-195-
ENSG0000	ENSG0000	1.74E-07	hsa-miR-15a-5p;hsa-miR-15b-5p;hsa-miR-16-5p;hsa-miR-195-
ENSG0000	ENSG0000	2.66E-07	hsa-miR-15a-5p;hsa-miR-15b-5p;hsa-miR-16-5p;hsa-miR-195-
ENSG0000	ENSG0000	2.39E-06	hsa-miR-15a-5p;hsa-miR-15b-5p;hsa-miR-16-5p;hsa-miR-195-
ENSG0000	ENSG0000	1.94E-07	hsa-miR-15a-5p;hsa-miR-15b-5p;hsa-miR-16-5p;hsa-miR-195-
ENSG0000	ENSG0000	7.67E-07	hsa-miR-15a-5p;hsa-miR-15b-5p;hsa-miR-16-5p;hsa-miR-195-
ENSG0000	ENSG0000	4.11E-06	hsa-miR-15a-5p;hsa-miR-15b-5p;hsa-miR-16-5p;hsa-miR-195-
ENSG0000	ENSG0000	6.04E-07	hsa-miR-15a-5p;hsa-miR-15b-5p;hsa-miR-16-5p;hsa-miR-195-
ENSG0000	ENSG0000	1.22E-07	hsa-miR-15a-5p;hsa-miR-15b-5p;hsa-miR-16-5p;hsa-miR-195-
ENSG0000	ENSG0000	1.56E-06	hsa-miR-15a-5p;hsa-miR-15b-5p;hsa-miR-16-5p;hsa-miR-195-
ENSG0000	ENSG0000	3.38E-05	hsa-miR-15a-5p;hsa-miR-15b-5p;hsa-miR-16-5p;hsa-miR-195-
ENSG0000	ENSG0000	1.03E-08	hsa-miR-15a-5p;hsa-miR-15b-5p;hsa-miR-16-5p;hsa-miR-195-
ENSG0000	ENSG0000	3.69E-05	hsa-miR-15a-5p;hsa-miR-15b-5p;hsa-miR-16-5p;hsa-miR-195-
ENSG0000	ENSG0000	3.62E-09	hsa-miR-15a-5p;hsa-miR-15b-5p;hsa-miR-16-5p;hsa-miR-195-
ENSG0000	ENSG0000	4.64E-08	hsa-miR-15a-5p;hsa-miR-15b-5p;hsa-miR-16-5p;hsa-miR-195-
ENSG0000	ENSG0000	1.38E-07	hsa-miR-15a-5p;hsa-miR-15b-5p;hsa-miR-16-5p;hsa-miR-195-
ENSG0000	ENSG0000	3.95E-08	hsa-miR-15a-5p;hsa-miR-15b-5p;hsa-miR-16-5p;hsa-miR-195-
ENSG0000	ENSG0000	1.33E-11	hsa-miR-15a-5p;hsa-miR-15b-5p;hsa-miR-16-5p;hsa-miR-195-
ENSG0000	ENSG0000	1.55E-07	hsa-miR-15a-5p;hsa-miR-15b-5p;hsa-miR-16-5p;hsa-miR-195-
ENSG0000	ENSG0000	2.16E-07	hsa-miR-15a-5p;hsa-miR-15b-5p;hsa-miR-16-5p;hsa-miR-195-
ENSG0000	ENSG0000	6.04E-07	hsa-miR-15a-5p;hsa-miR-15b-5p;hsa-miR-16-5p;hsa-miR-195-
ENSG0000	ENSG0000	1.94E-08	hsa-miR-15a-5p;hsa-miR-15b-5p;hsa-miR-16-5p;hsa-miR-195-
ENSG0000	ENSG0000	3.62E-09	hsa-miR-15a-5p;hsa-miR-15b-5p;hsa-miR-16-5p;hsa-miR-195-
ENSG0000	ENSG0000	9.62E-07	hsa-miR-15a-5p;hsa-miR-15b-5p;hsa-miR-16-5p;hsa-miR-195-
ENSG0000	ENSG0000	1.28E-08	hsa-miR-15a-5p;hsa-miR-15b-5p;hsa-miR-16-5p;hsa-miR-195-
ENSG0000	ENSG0000	1.28E-08	hsa-miR-15a-5p;hsa-miR-15b-5p;hsa-miR-16-5p;hsa-miR-195-
ENSG0000	ENSG0000	1.10E-05	hsa-miR-15a-5p;hsa-miR-15b-5p;hsa-miR-16-5p;hsa-miR-195-
ENSG0000	ENSG0000	6.27E-08	hsa-miR-15a-5p;hsa-miR-15b-5p;hsa-miR-16-5p;hsa-miR-195-
ENSG0000	ENSG0000	4.82E-09	hsa-miR-15a-5p;hsa-miR-15b-5p;hsa-miR-16-5p;hsa-miR-195-
ENSG0000	ENSG0000	1.31E-09	hsa-miR-15a-5p;hsa-miR-15b-5p;hsa-miR-16-5p;hsa-miR-195-
ENSG0000	ENSG0000	2.00E-06	hsa-miR-15a-5p;hsa-miR-15b-5p;hsa-miR-16-5p;hsa-miR-195-
ENSG0000	ENSG0000	3.62E-09	hsa-miR-15a-5p;hsa-miR-15b-5p;hsa-miR-16-5p;hsa-miR-195-
ENSG0000	ENSG0000	4.11E-06	hsa-miR-15a-5p;hsa-miR-15b-5p;hsa-miR-16-5p;hsa-miR-195-
ENSG0000	ENSG0000	6.04E-07	hsa-miR-15a-5p;hsa-miR-15b-5p;hsa-miR-16-5p;hsa-miR-195-
ENSG0000	ENSG0000	8.30E-08	hsa-miR-15a-5p;hsa-miR-15b-5p;hsa-miR-16-5p;hsa-miR-195-
ENSG0000	ENSG0000	6.55E-07	hsa-miR-15a-5p;hsa-miR-15b-5p;hsa-miR-16-5p;hsa-miR-195-

ENSG0000	ENSG0000	1.46E-06	hsa-miR-15a-5p;hsa-miR-15b-5p;hsa-miR-16-5p;hsa-miR-195-
ENSG0000	ENSG0000	7.23E-06	hsa-miR-15a-5p;hsa-miR-15b-5p;hsa-miR-16-5p;hsa-miR-195-
ENSG0000	ENSG0000	4.25E-05	hsa-miR-15a-5p;hsa-miR-15b-5p;hsa-miR-16-5p;hsa-miR-195-
ENSG0000	ENSG0000	2.66E-12	hsa-miR-15a-5p;hsa-miR-15b-5p;hsa-miR-16-5p;hsa-miR-195-
ENSG0000	ENSG0000	3.95E-08	hsa-miR-15a-5p;hsa-miR-15b-5p;hsa-miR-16-5p;hsa-miR-195-
ENSG0000	ENSG0000	1.46E-06	hsa-miR-15a-5p;hsa-miR-15b-5p;hsa-miR-16-5p;hsa-miR-195-
ENSG0000	ENSG0000	2.83E-06	hsa-miR-15a-5p;hsa-miR-15b-5p;hsa-miR-16-5p;hsa-miR-195-
ENSG0000	ENSG0000	5.01E-06	hsa-miR-15a-5p;hsa-miR-15b-5p;hsa-miR-16-5p;hsa-miR-195-
ENSG0000	ENSG0000	1.11E-06	hsa-miR-15a-5p;hsa-miR-15b-5p;hsa-miR-16-5p;hsa-miR-195-
ENSG0000	ENSG0000	1.03E-06	hsa-miR-15a-5p;hsa-miR-15b-5p;hsa-miR-16-5p;hsa-miR-195-
ENSG0000	ENSG0000	0.000155	hsa-miR-17-5p;hsa-miR-20b-5p;hsa-miR-497-5p;hsa-miR-93-
ENSG0000	ENSG0000	1.20E-09	hsa-miR-103a-3p;hsa-miR-107;hsa-miR-17-5p;hsa-miR-20b-5
ENSG0000	ENSG0000	3.31E-08	hsa-miR-103a-3p;hsa-miR-107;hsa-miR-17-5p;hsa-miR-20b-5
ENSG0000	ENSG0000	7.87E-06	hsa-miR-17-5p;hsa-miR-20b-5p;hsa-miR-497-5p;hsa-miR-93-
ENSG0000	ENSG0000	2.84E-06	hsa-miR-17-5p;hsa-miR-20b-5p;hsa-miR-497-5p;hsa-miR-93-
ENSG0000	ENSG0000	7.13E-07	hsa-miR-17-5p;hsa-miR-20b-5p;hsa-miR-497-5p;hsa-miR-93-
ENSG0000	ENSG0000	2.15E-07	hsa-miR-107;hsa-miR-17-5p;hsa-miR-20b-5p;hsa-miR-93-5p
ENSG0000	ENSG0000	2.61E-05	hsa-miR-103a-3p;hsa-miR-107;hsa-miR-17-5p;hsa-miR-93-5p
ENSG0000	ENSG0000	2.61E-05	hsa-miR-103a-3p;hsa-miR-107;hsa-miR-17-5p;hsa-miR-93-5p
ENSG0000	ENSG0000	3.92E-08	hsa-miR-103a-3p;hsa-miR-107;hsa-miR-17-5p;hsa-miR-20b-5
ENSG0000	ENSG0000	2.21E-06	hsa-miR-103a-3p;hsa-miR-107;hsa-miR-17-5p;hsa-miR-20b-5
ENSG0000	ENSG0000	1.77E-05	hsa-miR-107;hsa-miR-17-5p;hsa-miR-20b-5p;hsa-miR-93-5p
ENSG0000	ENSG0000	6.63E-06	hsa-miR-17-5p;hsa-miR-20b-5p;hsa-miR-497-5p;hsa-miR-93-
ENSG0000	ENSG0000	4.61E-08	hsa-miR-103a-3p;hsa-miR-107;hsa-miR-17-5p;hsa-miR-20b-5
ENSG0000	ENSG0000	1.54E-05	hsa-miR-17-5p;hsa-miR-20b-5p;hsa-miR-497-5p;hsa-miR-93-
ENSG0000	ENSG0000	2.09E-07	hsa-miR-103a-3p;hsa-miR-107;hsa-miR-17-5p;hsa-miR-20b-5
ENSG0000	ENSG0000	6.15E-11	hsa-miR-103a-3p;hsa-miR-107;hsa-miR-17-5p;hsa-miR-20b-5
ENSG0000	ENSG0000	3.51E-06	hsa-miR-107;hsa-miR-17-5p;hsa-miR-20b-5p;hsa-miR-93-5p
ENSG0000	ENSG0000	3.27E-06	hsa-miR-107;hsa-miR-17-5p;hsa-miR-20b-5p;hsa-miR-497-5p
ENSG0000	ENSG0000	4.06E-07	hsa-miR-103a-3p;hsa-miR-107;hsa-miR-17-5p;hsa-miR-20b-5
ENSG0000	ENSG0000	5.77E-07	hsa-miR-17-5p;hsa-miR-20b-5p;hsa-miR-497-5p;hsa-miR-93-
ENSG0000	ENSG0000	1.98E-09	hsa-miR-107;hsa-miR-17-5p;hsa-miR-20b-5p;hsa-miR-497-5p;
ENSG0000	ENSG0000	3.58E-05	hsa-miR-17-5p;hsa-miR-20b-5p;hsa-miR-497-5p;hsa-miR-93-
ENSG0000	ENSG0000	2.35E-09	hsa-miR-103a-3p;hsa-miR-107;hsa-miR-17-5p;hsa-miR-20b-5
ENSG0000	ENSG0000	1.68E-07	hsa-miR-103a-3p;hsa-miR-107;hsa-miR-17-5p;hsa-miR-20b-5
ENSG0000	ENSG0000	4.29E-06	hsa-miR-17-5p;hsa-miR-20b-5p;hsa-miR-497-5p;hsa-miR-93-
ENSG0000	ENSG0000	1.75E-07	hsa-miR-103a-3p;hsa-miR-107;hsa-miR-17-5p;hsa-miR-20b-5
ENSG0000	ENSG0000	8.63E-05	hsa-miR-17-5p;hsa-miR-20b-5p;hsa-miR-497-5p;hsa-miR-93-
ENSG0000	ENSG0000	7.02E-06	hsa-miR-17-5p;hsa-miR-20b-5p;hsa-miR-497-5p;hsa-miR-93-
ENSG0000	ENSG0000	0.000253	hsa-miR-17-5p;hsa-miR-20b-5p;hsa-miR-497-5p;hsa-miR-93-
ENSG0000	ENSG0000	1.64E-06	hsa-miR-17-5p;hsa-miR-20b-5p;hsa-miR-497-5p;hsa-miR-93-
ENSG0000	ENSG0000	1.20E-05	hsa-miR-107;hsa-miR-17-5p;hsa-miR-20b-5p;hsa-miR-497-5p
ENSG0000	ENSG0000	1.20E-05	hsa-miR-107;hsa-miR-17-5p;hsa-miR-20b-5p;hsa-miR-497-5p
ENSG0000	ENSG0000	0.000215	hsa-miR-103a-3p;hsa-miR-107;hsa-miR-17-5p;hsa-miR-20b-5
ENSG0000	ENSG0000	0.000106	hsa-miR-17-5p;hsa-miR-20b-5p;hsa-miR-497-5p;hsa-miR-93-
ENSG0000	ENSG0000	0.000302	hsa-miR-103a-3p;hsa-miR-17-5p;hsa-miR-20b-5p;hsa-miR-93-
ENSG0000	ENSG0000	2.64E-07	hsa-miR-103a-3p;hsa-miR-107;hsa-miR-17-5p;hsa-miR-20b-5
ENSG0000	ENSG0000	9.89E-05	hsa-miR-199a-3p;hsa-miR-218-5p;hsa-miR-27a-3p;hsa-miR-2
ENSG0000	ENSG0000	2.44E-05	hsa-miR-218-5p;hsa-miR-27a-3p;hsa-miR-27b-3p;hsa-miR-62
ENSG0000	ENSG0000	4.69E-06	hsa-miR-218-5p;hsa-miR-3158-3p;hsa-miR-382-5p;hsa-miR-4
ENSG0000	ENSG0000	0.000936	hsa-miR-107;hsa-miR-143-5p;hsa-miR-15a-5p;hsa-miR-15b-5
ENSG0000	ENSG0000	0.009464	hsa-miR-33a-5p;hsa-miR-33b-5p;hsa-miR-450b-5p;hsa-miR-5
ENSG0000	ENSG0000	8.02E-07	hsa-miR-15a-5p;hsa-miR-15b-5p;hsa-miR-16-5p;hsa-miR-195-
ENSG0000	ENSG0000	3.38E-11	hsa-miR-103a-3p;hsa-miR-107;hsa-miR-15a-5p;hsa-miR-15b-

ENSG0000	ENSG0000	2.03E-09	hsa-miR-15a-5p;hsa-miR-15b-5p;hsa-miR-16-5p;hsa-miR-195-
ENSG0000	ENSG0000	4.19E-08	hsa-miR-15a-5p;hsa-miR-15b-5p;hsa-miR-195-5p;hsa-miR-21
ENSG0000	ENSG0000	3.15E-10	hsa-miR-103a-3p;hsa-miR-107;hsa-miR-15a-5p;hsa-miR-15b-
ENSG0000	ENSG0000	0.000932	hsa-miR-15a-5p;hsa-miR-15b-5p;hsa-miR-195-5p;hsa-miR-29
ENSG0000	ENSG0000	8.61E-08	hsa-miR-15a-5p;hsa-miR-15b-5p;hsa-miR-16-5p;hsa-miR-195-
ENSG0000	ENSG0000	8.61E-08	hsa-miR-15a-5p;hsa-miR-15b-5p;hsa-miR-16-5p;hsa-miR-195-
ENSG0000	ENSG0000	0.001419	hsa-miR-15a-5p;hsa-miR-15b-5p;hsa-miR-16-5p;hsa-miR-195-
ENSG0000	ENSG0000	9.24E-06	hsa-miR-15a-5p;hsa-miR-15b-5p;hsa-miR-16-5p;hsa-miR-195-
ENSG0000	ENSG0000	1.99E-05	hsa-miR-1271-5p;hsa-miR-15a-5p;hsa-miR-15b-5p;hsa-miR-1
ENSG0000	ENSG0000	5.61E-05	hsa-miR-15a-5p;hsa-miR-15b-5p;hsa-miR-16-5p;hsa-miR-182-
ENSG0000	ENSG0000	0.000262	hsa-miR-15a-5p;hsa-miR-15b-5p;hsa-miR-16-5p;hsa-miR-195-
ENSG0000	ENSG0000	5.07E-06	hsa-miR-186-3p;hsa-miR-193a-3p;hsa-miR-193b-3p;hsa-miR-
ENSG0000	ENSG0000	1.60E-05	hsa-miR-15a-5p;hsa-miR-15b-5p;hsa-miR-16-5p;hsa-miR-195-
ENSG0000	ENSG0000	1.09E-06	hsa-miR-107;hsa-miR-15a-5p;hsa-miR-15b-5p;hsa-miR-195-5
ENSG0000	ENSG0000	4.21E-05	hsa-miR-15a-5p;hsa-miR-15b-5p;hsa-miR-16-5p;hsa-miR-195-
ENSG0000	ENSG0000	0.000262	hsa-miR-15b-5p;hsa-miR-16-5p;hsa-miR-195-5p;hsa-miR-424-
ENSG0000	ENSG0000	0.000134	hsa-miR-15a-5p;hsa-miR-15b-5p;hsa-miR-16-5p;hsa-miR-424-
ENSG0000	ENSG0000	4.99E-05	hsa-miR-103a-3p;hsa-miR-107;hsa-miR-15a-5p;hsa-miR-15b-
ENSG0000	ENSG0000	0.00132	hsa-miR-103a-3p;hsa-miR-107;hsa-miR-214-3p;hsa-miR-3619-
ENSG0000	ENSG0000	6.07E-10	hsa-miR-103a-3p;hsa-miR-107;hsa-miR-15a-5p;hsa-miR-15b-
ENSG0000	ENSG0000	0.008084	hsa-miR-125a-3p;hsa-miR-223-5p;hsa-miR-33a-5p;hsa-miR-3
ENSG0000	ENSG0000	0.006162	hsa-miR-107;hsa-miR-15a-5p;hsa-miR-15b-5p;hsa-miR-16-5p;
ENSG0000	ENSG0000	6.40E-07	hsa-miR-15a-5p;hsa-miR-15b-5p;hsa-miR-16-5p;hsa-miR-195-
ENSG0000	ENSG0000	0.00034	hsa-miR-15a-5p;hsa-miR-15b-5p;hsa-miR-16-5p;hsa-miR-195-
ENSG0000	ENSG0000	0.002899	hsa-miR-15a-5p;hsa-miR-16-5p;hsa-miR-195-5p;hsa-miR-298;
ENSG0000	ENSG0000	0.000373	hsa-miR-15a-5p;hsa-miR-15b-5p;hsa-miR-16-5p;hsa-miR-195-
ENSG0000	ENSG0000	0.000373	hsa-miR-15a-5p;hsa-miR-15b-5p;hsa-miR-16-5p;hsa-miR-182-
ENSG0000	ENSG0000	0.007196	hsa-miR-15a-5p;hsa-miR-15b-5p;hsa-miR-195-5p;hsa-miR-25-
ENSG0000	ENSG0000	7.80E-10	hsa-miR-103a-3p;hsa-miR-107;hsa-miR-15a-5p;hsa-miR-15b-
ENSG0000	ENSG0000	1.48E-06	hsa-miR-130b-5p;hsa-miR-15a-5p;hsa-miR-15b-5p;hsa-miR-1
ENSG0000	ENSG0000	1.91E-10	hsa-miR-103a-3p;hsa-miR-107;hsa-miR-15a-5p;hsa-miR-15b-
ENSG0000	ENSG0000	0.001712	hsa-miR-33a-5p;hsa-miR-33b-5p;hsa-miR-450b-5p;hsa-miR-5
ENSG0000	ENSG0000	6.09E-05	hsa-miR-15a-5p;hsa-miR-15b-5p;hsa-miR-195-5p;hsa-miR-42
ENSG0000	ENSG0000	0.000141	hsa-miR-103a-3p;hsa-miR-107;hsa-miR-15a-5p;hsa-miR-15b-
ENSG0000	ENSG0000	0.005723	hsa-miR-1271-5p;hsa-miR-182-5p;hsa-miR-25-3p;hsa-miR-29
ENSG0000	ENSG0000	7.87E-06	hsa-miR-103a-3p;hsa-miR-107;hsa-miR-15a-5p;hsa-miR-15b-
ENSG0000	ENSG0000	0.000629	hsa-miR-15a-5p;hsa-miR-15b-5p;hsa-miR-195-5p;hsa-miR-42
ENSG0000	ENSG0000	0.000819	hsa-miR-193b-3p;hsa-miR-450b-5p;hsa-miR-507;hsa-miR-557;
ENSG0000	ENSG0000	3.66E-05	hsa-miR-15a-5p;hsa-miR-15b-5p;hsa-miR-16-5p;hsa-miR-195-
ENSG0000	ENSG0000	0.000134	hsa-miR-15b-5p;hsa-miR-16-5p;hsa-miR-486-3p;hsa-miR-497-
ENSG0000	ENSG0000	6.09E-05	hsa-miR-33a-5p;hsa-miR-33b-5p;hsa-miR-450b-5p;hsa-miR-5
ENSG0000	ENSG0000	7.05E-09	hsa-miR-15a-5p;hsa-miR-15b-5p;hsa-miR-16-5p;hsa-miR-195-
ENSG0000	ENSG0000	6.40E-05	hsa-miR-15a-5p;hsa-miR-15b-5p;hsa-miR-16-5p;hsa-miR-195-
ENSG0000	ENSG0000	0.000254	hsa-miR-1271-5p;hsa-miR-130b-5p;hsa-miR-182-5p;hsa-miR-
ENSG0000	ENSG0000	6.23E-08	hsa-miR-15a-5p;hsa-miR-15b-5p;hsa-miR-16-5p;hsa-miR-195-
ENSG0000	ENSG0000	0.005723	hsa-miR-103a-3p;hsa-miR-107;hsa-miR-1271-5p;hsa-miR-182-
ENSG0000	ENSG0000	0.000115	hsa-miR-15a-5p;hsa-miR-15b-5p;hsa-miR-16-5p;hsa-miR-195-
ENSG0000	ENSG0000	0.001205	hsa-miR-450b-5p;hsa-miR-507;hsa-miR-557;hsa-miR-9-5p;hsa
ENSG0000	ENSG0000	9.40E-05	hsa-miR-25-3p;hsa-miR-33a-5p;hsa-miR-33b-5p;hsa-miR-450
ENSG0000	ENSG0000	5.84E-08	hsa-miR-15a-5p;hsa-miR-15b-5p;hsa-miR-195-5p;hsa-miR-21
ENSG0000	ENSG0000	1.73E-08	hsa-miR-103a-3p;hsa-miR-107;hsa-miR-15a-5p;hsa-miR-15b-
ENSG0000	ENSG0000	0.000375	hsa-miR-15a-5p;hsa-miR-15b-5p;hsa-miR-195-5p;hsa-miR-42
ENSG0000	ENSG0000	5.66E-07	hsa-miR-15a-5p;hsa-miR-15b-5p;hsa-miR-16-5p;hsa-miR-195-
ENSG0000	ENSG0000	0.009964	hsa-miR-125a-3p;hsa-miR-130b-5p;hsa-miR-657;hsa-miR-873-

ENSG0000	ENSG0000	0.000106	hsa-miR-129-1-3p;hsa-miR-15a-5p;hsa-miR-15b-5p;hsa-miR-
ENSG0000	ENSG0000	8.90E-07	hsa-miR-15a-5p;hsa-miR-16-5p;hsa-miR-223-5p;hsa-miR-450b
ENSG0000	ENSG0000	8.90E-07	hsa-miR-15a-5p;hsa-miR-16-5p;hsa-miR-223-5p;hsa-miR-450b
ENSG0000	ENSG0000	0.005687	hsa-miR-129-1-3p;hsa-miR-129-2-3p;hsa-miR-15a-5p;hsa-miR
ENSG0000	ENSG0000	0.003365	hsa-miR-15a-5p;hsa-miR-15b-5p;hsa-miR-16-5p;hsa-miR-195-
ENSG0000	ENSG0000	4.24E-05	hsa-miR-107;hsa-miR-15a-5p;hsa-miR-15b-5p;hsa-miR-195-5p
ENSG0000	ENSG0000	4.88E-05	hsa-miR-15a-5p;hsa-miR-15b-5p;hsa-miR-195-5p;hsa-miR-424
ENSG0000	ENSG0000	0.000932	hsa-miR-15a-5p;hsa-miR-15b-5p;hsa-miR-16-5p;hsa-miR-195-
ENSG0000	ENSG0000	2.48E-07	hsa-miR-103a-3p;hsa-miR-107;hsa-miR-15a-5p;hsa-miR-15b-5
ENSG0000	ENSG0000	0.006075	hsa-miR-15a-5p;hsa-miR-195-5p;hsa-miR-33a-5p;hsa-miR-424
ENSG0000	ENSG0000	0.008682	hsa-miR-25-3p;hsa-miR-450b-5p;hsa-miR-507;hsa-miR-557
ENSG0000	ENSG0000	0.006259	hsa-miR-182-5p;hsa-miR-186-3p;hsa-miR-214-3p;hsa-miR-25-
ENSG0000	ENSG0000	0.001153	hsa-miR-125a-3p;hsa-miR-143-5p;hsa-miR-193a-3p;hsa-miR-
ENSG0000	ENSG0000	0.004754	hsa-miR-15a-5p;hsa-miR-15b-5p;hsa-miR-195-5p;hsa-miR-424
ENSG0000	ENSG0000	0.005964	hsa-miR-28-5p;hsa-miR-637;hsa-miR-708-5p;hsa-miR-873-5p
ENSG0000	ENSG0000	0.000248	hsa-miR-15a-5p;hsa-miR-15b-5p;hsa-miR-195-5p;hsa-miR-25-
ENSG0000	ENSG0000	0.002185	hsa-miR-15a-5p;hsa-miR-15b-5p;hsa-miR-195-5p;hsa-miR-424
ENSG0000	ENSG0000	5.19E-06	hsa-miR-15a-5p;hsa-miR-15b-5p;hsa-miR-16-5p;hsa-miR-195-
ENSG0000	ENSG0000	0.002956	hsa-miR-143-5p;hsa-miR-28-5p;hsa-miR-33a-5p;hsa-miR-33b-
ENSG0000	ENSG0000	3.31E-10	hsa-miR-15a-5p;hsa-miR-15b-5p;hsa-miR-16-5p;hsa-miR-195-
ENSG0000	ENSG0000	3.31E-10	hsa-miR-15a-5p;hsa-miR-15b-5p;hsa-miR-16-5p;hsa-miR-195-
ENSG0000	ENSG0000	1.99E-05	hsa-miR-214-3p;hsa-miR-3619-5p;hsa-miR-657;hsa-miR-922
ENSG0000	ENSG0000	1.69E-07	hsa-miR-15a-5p;hsa-miR-15b-5p;hsa-miR-16-5p;hsa-miR-193a
ENSG0000	ENSG0000	9.55E-06	hsa-miR-15a-5p;hsa-miR-15b-5p;hsa-miR-195-5p;hsa-miR-424
ENSG0000	ENSG0000	0.00017	hsa-miR-1271-5p;hsa-miR-182-5p;hsa-miR-9-5p;hsa-miR-96-5
ENSG0000	ENSG0000	1.44E-09	hsa-miR-103a-3p;hsa-miR-107;hsa-miR-15a-5p;hsa-miR-15b-5
ENSG0000	ENSG0000	9.51E-07	hsa-miR-103a-3p;hsa-miR-107;hsa-miR-15a-5p;hsa-miR-15b-5
ENSG0000	ENSG0000	0.002364	hsa-miR-15a-5p;hsa-miR-195-5p;hsa-miR-424-5p;hsa-miR-497
ENSG0000	ENSG0000	0.000103	hsa-miR-103a-3p;hsa-miR-107;hsa-miR-143-5p;hsa-miR-657
ENSG0000	ENSG0000	1.95E-05	hsa-miR-125a-3p;hsa-miR-15a-5p;hsa-miR-15b-5p;hsa-miR-1
ENSG0000	ENSG0000	1.99E-05	hsa-miR-107;hsa-miR-15a-5p;hsa-miR-16-5p;hsa-miR-424-5p
ENSG0000	ENSG0000	0.000579	hsa-miR-15a-5p;hsa-miR-15b-5p;hsa-miR-195-5p;hsa-miR-22
ENSG0000	ENSG0000	7.46E-10	hsa-miR-125a-3p;hsa-miR-1271-5p;hsa-miR-129-1-3p;hsa-miR
ENSG0000	ENSG0000	3.64E-11	hsa-miR-1271-5p;hsa-miR-15a-5p;hsa-miR-15b-5p;hsa-miR-1
ENSG0000	ENSG0000	9.27E-07	hsa-miR-103a-3p;hsa-miR-107;hsa-miR-143-5p;hsa-miR-15a-5
ENSG0000	ENSG0000	0.000629	hsa-miR-107;hsa-miR-15a-5p;hsa-miR-15b-5p;hsa-miR-195-5p
ENSG0000	ENSG0000	0.008084	hsa-miR-103a-3p;hsa-miR-107;hsa-miR-186-3p;hsa-miR-25-3p
ENSG0000	ENSG0000	1.28E-05	hsa-miR-15a-5p;hsa-miR-15b-5p;hsa-miR-195-5p;hsa-miR-424
ENSG0000	ENSG0000	3.31E-10	hsa-miR-15a-5p;hsa-miR-15b-5p;hsa-miR-16-5p;hsa-miR-195-
ENSG0000	ENSG0000	3.31E-10	hsa-miR-15a-5p;hsa-miR-15b-5p;hsa-miR-16-5p;hsa-miR-195-
ENSG0000	ENSG0000	0.000819	hsa-miR-15a-5p;hsa-miR-15b-5p;hsa-miR-195-5p;hsa-miR-424
ENSG0000	ENSG0000	6.57E-08	hsa-miR-15a-5p;hsa-miR-15b-5p;hsa-miR-16-5p;hsa-miR-195-
ENSG0000	ENSG0000	0.000134	hsa-miR-193a-3p;hsa-miR-193b-3p;hsa-miR-33a-5p;hsa-miR-
ENSG0000	ENSG0000	1.38E-05	hsa-miR-107;hsa-miR-15a-5p;hsa-miR-15b-5p;hsa-miR-195-5p
ENSG0000	ENSG0000	4.66E-05	hsa-miR-107;hsa-miR-15a-5p;hsa-miR-15b-5p;hsa-miR-195-5p
ENSG0000	ENSG0000	0.000423	hsa-miR-15a-5p;hsa-miR-15b-5p;hsa-miR-195-5p;hsa-miR-424
ENSG0000	ENSG0000	0.001337	hsa-miR-15a-5p;hsa-miR-15b-5p;hsa-miR-16-5p;hsa-miR-195-
ENSG0000	ENSG0000	3.24E-07	hsa-miR-15a-5p;hsa-miR-15b-5p;hsa-miR-16-5p;hsa-miR-195-
ENSG0000	ENSG0000	0.001314	hsa-miR-107;hsa-miR-15a-5p;hsa-miR-15b-5p;hsa-miR-195-5p
ENSG0000	ENSG0000	0.002362	hsa-miR-103a-3p;hsa-miR-107;hsa-miR-25-3p;hsa-miR-298;hsa
ENSG0000	ENSG0000	6.71E-07	hsa-miR-15a-5p;hsa-miR-15b-5p;hsa-miR-16-5p;hsa-miR-195-
ENSG0000	ENSG0000	0.000175	hsa-miR-129-1-3p;hsa-miR-129-2-3p;hsa-miR-15a-5p;hsa-miR
ENSG0000	ENSG0000	0.001098	hsa-miR-193a-3p;hsa-miR-33b-5p;hsa-miR-450b-5p;hsa-miR-
ENSG0000	ENSG0000	1.83E-08	hsa-miR-15a-5p;hsa-miR-15b-5p;hsa-miR-16-5p;hsa-miR-195-

ENSG0000	ENSG0000	0.005385	hsa-miR-130b-5p;hsa-miR-15a-5p;hsa-miR-15b-5p;hsa-miR-1
ENSG0000	ENSG0000	0.002628	hsa-miR-193a-3p;hsa-miR-193b-3p;hsa-miR-4690-5p;hsa-miR-
ENSG0000	ENSG0000	8.90E-07	hsa-miR-15a-5p;hsa-miR-15b-5p;hsa-miR-16-5p;hsa-miR-195-
ENSG0000	ENSG0000	8.90E-07	hsa-miR-15a-5p;hsa-miR-15b-5p;hsa-miR-16-5p;hsa-miR-195-
ENSG0000	ENSG0000	4.21E-05	hsa-miR-130b-5p;hsa-miR-450b-5p;hsa-miR-507;hsa-miR-557
ENSG0000	ENSG0000	0.006075	hsa-miR-107;hsa-miR-15a-5p;hsa-miR-15b-5p;hsa-miR-16-5p;
ENSG0000	ENSG0000	7.31E-05	hsa-miR-15a-5p;hsa-miR-15b-5p;hsa-miR-16-5p;hsa-miR-195-
ENSG0000	ENSG0000	4.59E-07	hsa-miR-103a-3p;hsa-miR-107;hsa-miR-146b-3p;hsa-miR-15a-
ENSG0000	ENSG0000	5.82E-05	hsa-miR-15a-5p;hsa-miR-15b-5p;hsa-miR-16-5p;hsa-miR-195-
ENSG0000	ENSG0000	5.82E-05	hsa-miR-15a-5p;hsa-miR-15b-5p;hsa-miR-16-5p;hsa-miR-195-
ENSG0000	ENSG0000	3.77E-06	hsa-miR-125a-3p;hsa-miR-450b-5p;hsa-miR-507;hsa-miR-557;
ENSG0000	ENSG0000	2.22E-06	hsa-miR-15a-5p;hsa-miR-15b-5p;hsa-miR-16-5p;hsa-miR-195-
ENSG0000	ENSG0000	6.23E-08	hsa-miR-103a-3p;hsa-miR-107;hsa-miR-15a-5p;hsa-miR-15b-
ENSG0000	ENSG0000	2.06E-05	hsa-miR-107;hsa-miR-15a-5p;hsa-miR-15b-5p;hsa-miR-195-5
ENSG0000	ENSG0000	0.002628	hsa-miR-223-5p;hsa-miR-450b-5p;hsa-miR-507;hsa-miR-557
ENSG0000	ENSG0000	0.000392	hsa-miR-107;hsa-miR-15a-5p;hsa-miR-15b-5p;hsa-miR-195-5
ENSG0000	ENSG0000	4.90E-08	hsa-miR-103a-3p;hsa-miR-107;hsa-miR-15a-5p;hsa-miR-15b-
ENSG0000	ENSG0000	0.000752	hsa-miR-15a-5p;hsa-miR-15b-5p;hsa-miR-16-5p;hsa-miR-195-
ENSG0000	ENSG0000	6.51E-08	hsa-miR-15a-5p;hsa-miR-15b-5p;hsa-miR-16-5p;hsa-miR-195-
ENSG0000	ENSG0000	5.61E-05	hsa-miR-15a-5p;hsa-miR-15b-5p;hsa-miR-16-5p;hsa-miR-195-
ENSG0000	ENSG0000	1.37E-08	hsa-miR-15a-5p;hsa-miR-15b-5p;hsa-miR-16-5p;hsa-miR-195-
ENSG0000	ENSG0000	0.006455	hsa-miR-130b-5p;hsa-miR-143-5p;hsa-miR-33a-5p;hsa-miR-3
ENSG0000	ENSG0000	0.000291	hsa-miR-15a-5p;hsa-miR-15b-5p;hsa-miR-195-5p;hsa-miR-42
ENSG0000	ENSG0000	6.47E-06	hsa-miR-103a-3p;hsa-miR-107;hsa-miR-15a-5p;hsa-miR-15b-
ENSG0000	ENSG0000	3.15E-05	hsa-miR-107;hsa-miR-15a-5p;hsa-miR-15b-5p;hsa-miR-195-5
ENSG0000	ENSG0000	0.009464	hsa-miR-130b-5p;hsa-miR-450b-5p;hsa-miR-507;hsa-miR-557;
ENSG0000	ENSG0000	0.003885	hsa-miR-15a-5p;hsa-miR-15b-5p;hsa-miR-16-5p;hsa-miR-195-
ENSG0000	ENSG0000	2.29E-06	hsa-miR-15a-5p;hsa-miR-15b-5p;hsa-miR-195-5p;hsa-miR-21
ENSG0000	ENSG0000	5.82E-05	hsa-miR-214-3p;hsa-miR-3619-5p;hsa-miR-4690-5p;hsa-miR-
ENSG0000	ENSG0000	3.14E-07	hsa-miR-103a-3p;hsa-miR-107;hsa-miR-130b-5p;hsa-miR-15a-
ENSG0000	ENSG0000	4.74E-07	hsa-miR-15a-5p;hsa-miR-15b-5p;hsa-miR-16-5p;hsa-miR-195-
ENSG0000	ENSG0000	3.19E-07	hsa-miR-125a-3p;hsa-miR-15a-5p;hsa-miR-15b-5p;hsa-miR-1
ENSG0000	ENSG0000	0.001037	hsa-miR-146b-3p;hsa-miR-15a-5p;hsa-miR-15b-5p;hsa-miR-1
ENSG0000	ENSG0000	0.003076	hsa-miR-15a-5p;hsa-miR-15b-5p;hsa-miR-16-5p;hsa-miR-195-
ENSG0000	ENSG0000	0.000269	hsa-miR-15a-5p;hsa-miR-15b-5p;hsa-miR-16-5p;hsa-miR-195-
ENSG0000	ENSG0000	0.000413	hsa-miR-15a-5p;hsa-miR-15b-5p;hsa-miR-16-5p;hsa-miR-195-
ENSG0000	ENSG0000	4.66E-05	hsa-miR-15a-5p;hsa-miR-15b-5p;hsa-miR-16-5p;hsa-miR-195-
ENSG0000	ENSG0000	3.09E-10	hsa-miR-15a-5p;hsa-miR-15b-5p;hsa-miR-16-5p;hsa-miR-195-
ENSG0000	ENSG0000	0.002856	hsa-miR-107;hsa-miR-125a-3p;hsa-miR-15a-5p;hsa-miR-15b-
ENSG0000	ENSG0000	0.001006	hsa-miR-15a-5p;hsa-miR-15b-5p;hsa-miR-16-5p;hsa-miR-195-
ENSG0000	ENSG0000	4.30E-06	hsa-miR-15a-5p;hsa-miR-15b-5p;hsa-miR-16-5p;hsa-miR-186-
ENSG0000	ENSG0000	0.001759	hsa-miR-143-5p;hsa-miR-326;hsa-miR-378g;hsa-miR-450b-5p;
ENSG0000	ENSG0000	0.001491	hsa-miR-1271-5p;hsa-miR-182-5p;hsa-miR-873-5p;hsa-miR-9
ENSG0000	ENSG0000	0.002628	hsa-miR-143-5p;hsa-miR-450b-5p;hsa-miR-507;hsa-miR-557
ENSG0000	ENSG0000	0.004902	hsa-miR-143-5p;hsa-miR-16-5p;hsa-miR-214-3p;hsa-miR-361
ENSG0000	ENSG0000	0.000212	hsa-miR-1271-5p;hsa-miR-182-5p;hsa-miR-223-5p;hsa-miR-9
ENSG0000	ENSG0000	0.00258	hsa-miR-15a-5p;hsa-miR-15b-5p;hsa-miR-195-5p;hsa-miR-21
ENSG0000	ENSG0000	6.57E-08	hsa-miR-15a-5p;hsa-miR-15b-5p;hsa-miR-16-5p;hsa-miR-195-
ENSG0000	ENSG0000	2.95E-08	hsa-miR-107;hsa-miR-15a-5p;hsa-miR-15b-5p;hsa-miR-16-5p;
ENSG0000	ENSG0000	0.000104	hsa-miR-15a-5p;hsa-miR-15b-5p;hsa-miR-16-5p;hsa-miR-195-
ENSG0000	ENSG0000	0.000104	hsa-miR-15a-5p;hsa-miR-15b-5p;hsa-miR-195-5p;hsa-miR-42
ENSG0000	ENSG0000	0.001532	hsa-miR-15a-5p;hsa-miR-15b-5p;hsa-miR-16-5p;hsa-miR-195-
ENSG0000	ENSG0000	0.000373	hsa-miR-15a-5p;hsa-miR-15b-5p;hsa-miR-16-5p;hsa-miR-195-
ENSG0000	ENSG0000	7.19E-07	hsa-miR-15a-5p;hsa-miR-15b-5p;hsa-miR-16-5p;hsa-miR-195-

ENSG0000	ENSG0000	2.32E-07	hsa-miR-15a-5p;hsa-miR-15b-5p;hsa-miR-16-5p;hsa-miR-195-
ENSG0000	ENSG0000	0.003885	hsa-miR-214-3p;hsa-miR-3619-5p;hsa-miR-4690-5p;hsa-miR-
ENSG0000	ENSG0000	1.77E-06	hsa-miR-15a-5p;hsa-miR-15b-5p;hsa-miR-195-5p;hsa-miR-42
ENSG0000	ENSG0000	3.33E-05	hsa-miR-129-1-3p;hsa-miR-129-2-3p;hsa-miR-182-5p;hsa-mi
ENSG0000	ENSG0000	3.31E-07	hsa-miR-129-1-3p;hsa-miR-129-2-3p;hsa-miR-15a-5p;hsa-mi
ENSG0000	ENSG0000	0.001532	hsa-miR-186-3p;hsa-miR-28-5p;hsa-miR-450b-5p;hsa-miR-50
ENSG0000	ENSG0000	0.000488	hsa-miR-15a-5p;hsa-miR-15b-5p;hsa-miR-16-5p;hsa-miR-195-
ENSG0000	ENSG0000	7.79E-06	hsa-miR-15a-5p;hsa-miR-15b-5p;hsa-miR-16-5p;hsa-miR-195-
ENSG0000	ENSG0000	0.00015	hsa-miR-15a-5p;hsa-miR-15b-5p;hsa-miR-186-3p;hsa-miR-19
ENSG0000	ENSG0000	6.57E-08	hsa-miR-15a-5p;hsa-miR-15b-5p;hsa-miR-16-5p;hsa-miR-195-
ENSG0000	ENSG0000	6.66E-11	hsa-miR-1271-5p;hsa-miR-15a-5p;hsa-miR-15b-5p;hsa-miR-1
ENSG0000	ENSG0000	0.002392	hsa-miR-15a-5p;hsa-miR-15b-5p;hsa-miR-186-3p;hsa-miR-19
ENSG0000	ENSG0000	0.000215	hsa-miR-103a-3p;hsa-miR-107;hsa-miR-15a-5p;hsa-miR-15b-
ENSG0000	ENSG0000	0.000386	hsa-miR-15a-5p;hsa-miR-15b-5p;hsa-miR-16-5p;hsa-miR-195-
ENSG0000	ENSG0000	0.000291	hsa-miR-15a-5p;hsa-miR-15b-5p;hsa-miR-195-5p;hsa-miR-42
ENSG0000	ENSG0000	3.31E-10	hsa-miR-15a-5p;hsa-miR-15b-5p;hsa-miR-16-5p;hsa-miR-195-
ENSG0000	ENSG0000	7.87E-06	hsa-miR-103a-3p;hsa-miR-107;hsa-miR-15a-5p;hsa-miR-15b-
ENSG0000	ENSG0000	1.22E-07	hsa-miR-103a-3p;hsa-miR-107;hsa-miR-15a-5p;hsa-miR-15b-
ENSG0000	ENSG0000	6.23E-08	hsa-miR-103a-3p;hsa-miR-107;hsa-miR-15a-5p;hsa-miR-15b-
ENSG0000	ENSG0000	4.38E-06	hsa-miR-15a-5p;hsa-miR-15b-5p;hsa-miR-16-5p;hsa-miR-195-
ENSG0000	ENSG0000	2.06E-05	hsa-miR-103a-3p;hsa-miR-107;hsa-miR-15a-5p;hsa-miR-15b-
ENSG0000	ENSG0000	0.000752	hsa-miR-15a-5p;hsa-miR-15b-5p;hsa-miR-16-5p;hsa-miR-195-
ENSG0000	ENSG0000	0.000291	hsa-miR-103a-3p;hsa-miR-107;hsa-miR-214-3p;hsa-miR-3619-
ENSG0000	ENSG0000	6.09E-05	hsa-miR-103a-3p;hsa-miR-107;hsa-miR-450b-5p;hsa-miR-507;
ENSG0000	ENSG0000	3.34E-05	hsa-miR-107;hsa-miR-15a-5p;hsa-miR-15b-5p;hsa-miR-195-5
ENSG0000	ENSG0000	4.28E-11	hsa-miR-15a-5p;hsa-miR-15b-5p;hsa-miR-16-5p;hsa-miR-195-
ENSG0000	ENSG0000	3.95E-08	hsa-miR-15a-5p;hsa-miR-15b-5p;hsa-miR-16-5p;hsa-miR-195-
ENSG0000	ENSG0000	3.19E-07	hsa-miR-107;hsa-miR-130b-5p;hsa-miR-15a-5p;hsa-miR-15b-
ENSG0000	ENSG0000	3.66E-05	hsa-miR-15a-5p;hsa-miR-15b-5p;hsa-miR-16-5p;hsa-miR-195-
ENSG0000	ENSG0000	8.05E-05	hsa-miR-103a-3p;hsa-miR-107;hsa-miR-146b-3p;hsa-miR-15a-
ENSG0000	ENSG0000	0.000254	hsa-miR-15a-5p;hsa-miR-15b-5p;hsa-miR-16-5p;hsa-miR-195-
ENSG0000	ENSG0000	0.00217	hsa-miR-143-5p;hsa-miR-15a-5p;hsa-miR-15b-5p;hsa-miR-16-
ENSG0000	ENSG0000	8.74E-05	hsa-miR-15a-5p;hsa-miR-15b-5p;hsa-miR-195-5p;hsa-miR-42
ENSG0000	ENSG0000	0.004177	hsa-miR-143-5p;hsa-miR-15a-5p;hsa-miR-15b-5p;hsa-miR-16-
ENSG0000	ENSG0000	1.79E-06	hsa-miR-15a-5p;hsa-miR-15b-5p;hsa-miR-16-5p;hsa-miR-195-
ENSG0000	ENSG0000	2.29E-06	hsa-miR-143-5p;hsa-miR-15a-5p;hsa-miR-15b-5p;hsa-miR-16-
ENSG0000	ENSG0000	1.28E-05	hsa-miR-15a-5p;hsa-miR-15b-5p;hsa-miR-16-5p;hsa-miR-195-
ENSG0000	ENSG0000	1.25E-08	hsa-miR-15a-5p;hsa-miR-15b-5p;hsa-miR-16-5p;hsa-miR-195-
ENSG0000	ENSG0000	0.000222	hsa-miR-15a-5p;hsa-miR-15b-5p;hsa-miR-195-5p;hsa-miR-42
ENSG0000	ENSG0000	5.66E-07	hsa-miR-15a-5p;hsa-miR-15b-5p;hsa-miR-16-5p;hsa-miR-195-
ENSG0000	ENSG0000	5.66E-07	hsa-miR-15a-5p;hsa-miR-15b-5p;hsa-miR-16-5p;hsa-miR-195-
ENSG0000	ENSG0000	0.007514	hsa-miR-107;hsa-miR-16-5p;hsa-miR-25-3p;hsa-miR-873-5p
ENSG0000	ENSG0000	0.007514	hsa-miR-107;hsa-miR-16-5p;hsa-miR-25-3p;hsa-miR-873-5p
ENSG0000	ENSG0000	0.002628	hsa-miR-15a-5p;hsa-miR-15b-5p;hsa-miR-424-5p;hsa-miR-49
ENSG0000	ENSG0000	0.000936	hsa-miR-103a-3p;hsa-miR-107;hsa-miR-130b-5p;hsa-miR-214-
ENSG0000	ENSG0000	0.004254	hsa-miR-450b-5p;hsa-miR-505-5p;hsa-miR-507;hsa-miR-557
ENSG0000	ENSG0000	1.22E-05	hsa-miR-15a-5p;hsa-miR-15b-5p;hsa-miR-16-5p;hsa-miR-195-
ENSG0000	ENSG0000	0.001892	hsa-miR-15a-5p;hsa-miR-15b-5p;hsa-miR-16-5p;hsa-miR-195-
ENSG0000	ENSG0000	3.95E-08	hsa-miR-15a-5p;hsa-miR-15b-5p;hsa-miR-16-5p;hsa-miR-195-
ENSG0000	ENSG0000	1.79E-06	hsa-miR-15a-5p;hsa-miR-15b-5p;hsa-miR-16-5p;hsa-miR-195-
ENSG0000	ENSG0000	0.000143	hsa-miR-15b-5p;hsa-miR-16-5p;hsa-miR-195-5p;hsa-miR-424-
ENSG0000	ENSG0000	1.65E-05	hsa-miR-15a-5p;hsa-miR-15b-5p;hsa-miR-16-5p;hsa-miR-193
ENSG0000	ENSG0000	1.65E-05	hsa-miR-15a-5p;hsa-miR-15b-5p;hsa-miR-16-5p;hsa-miR-193
ENSG0000	ENSG0000	1.77E-06	hsa-miR-15a-5p;hsa-miR-15b-5p;hsa-miR-16-5p;hsa-miR-195-

ENSG0000	ENSG0000	8.74E-05	hsa-miR-15a-5p;hsa-miR-15b-5p;hsa-miR-195-5p;hsa-miR-42
ENSG0000	ENSG0000	0.002061	hsa-miR-15a-5p;hsa-miR-15b-5p;hsa-miR-16-5p;hsa-miR-195
ENSG0000	ENSG0000	7.99E-06	hsa-miR-15a-5p;hsa-miR-15b-5p;hsa-miR-16-5p;hsa-miR-195
ENSG0000	ENSG0000	0.000521	hsa-miR-125a-3p;hsa-miR-15a-5p;hsa-miR-15b-5p;hsa-miR-1
ENSG0000	ENSG0000	0.000682	hsa-miR-15a-5p;hsa-miR-15b-5p;hsa-miR-16-5p;hsa-miR-195
ENSG0000	ENSG0000	0.000106	hsa-miR-15a-5p;hsa-miR-15b-5p;hsa-miR-16-5p;hsa-miR-195
ENSG0000	ENSG0000	0.00023	hsa-miR-15a-5p;hsa-miR-15b-5p;hsa-miR-16-5p;hsa-miR-195
ENSG0000	ENSG0000	0.000375	hsa-miR-1271-5p;hsa-miR-182-5p;hsa-miR-28-5p;hsa-miR-70
ENSG0000	ENSG0000	9.90E-07	hsa-miR-130b-5p;hsa-miR-15a-5p;hsa-miR-15b-5p;hsa-miR-1
ENSG0000	ENSG0000	9.90E-07	hsa-miR-130b-5p;hsa-miR-15a-5p;hsa-miR-15b-5p;hsa-miR-1
ENSG0000	ENSG0000	0.000644	hsa-miR-15a-5p;hsa-miR-15b-5p;hsa-miR-16-5p;hsa-miR-195
ENSG0000	ENSG0000	0.000597	hsa-miR-15a-5p;hsa-miR-15b-5p;hsa-miR-16-5p;hsa-miR-195
ENSG0000	ENSG0000	0.003577	hsa-miR-15a-5p;hsa-miR-15b-5p;hsa-miR-195-5p;hsa-miR-42
ENSG0000	ENSG0000	6.41E-06	hsa-miR-103a-3p;hsa-miR-107;hsa-miR-450b-5p;hsa-miR-507;
ENSG0000	ENSG0000	6.41E-06	hsa-miR-103a-3p;hsa-miR-107;hsa-miR-450b-5p;hsa-miR-507;
ENSG0000	ENSG0000	0.00017	hsa-miR-129-1-3p;hsa-miR-129-2-3p;hsa-miR-193a-3p;hsa-m
ENSG0000	ENSG0000	0.00017	hsa-miR-129-1-3p;hsa-miR-129-2-3p;hsa-miR-193a-3p;hsa-m
ENSG0000	ENSG0000	1.65E-05	hsa-miR-15a-5p;hsa-miR-15b-5p;hsa-miR-195-5p;hsa-miR-42
ENSG0000	ENSG0000	6.41E-06	hsa-miR-15a-5p;hsa-miR-15b-5p;hsa-miR-16-5p;hsa-miR-195
ENSG0000	ENSG0000	9.77E-08	hsa-miR-103a-3p;hsa-miR-107;hsa-miR-15a-5p;hsa-miR-15b-
ENSG0000	ENSG0000	4.07E-07	hsa-miR-15a-5p;hsa-miR-15b-5p;hsa-miR-16-5p;hsa-miR-186
ENSG0000	ENSG0000	0.000462	hsa-miR-25-3p;hsa-miR-450b-5p;hsa-miR-507;hsa-miR-557
ENSG0000	ENSG0000	7.58E-11	hsa-miR-103a-3p;hsa-miR-107;hsa-miR-15a-5p;hsa-miR-15b-
ENSG0000	ENSG0000	7.58E-11	hsa-miR-103a-3p;hsa-miR-107;hsa-miR-15a-5p;hsa-miR-15b-
ENSG0000	ENSG0000	0.000373	hsa-miR-107;hsa-miR-15a-5p;hsa-miR-15b-5p;hsa-miR-195-5p
ENSG0000	ENSG0000	2.70E-05	hsa-miR-15a-5p;hsa-miR-15b-5p;hsa-miR-16-5p;hsa-miR-195
ENSG0000	ENSG0000	7.79E-06	hsa-miR-15a-5p;hsa-miR-15b-5p;hsa-miR-16-5p;hsa-miR-195
ENSG0000	ENSG0000	6.71E-10	hsa-miR-103a-3p;hsa-miR-107;hsa-miR-15a-5p;hsa-miR-15b-
ENSG0000	ENSG0000	0.000222	hsa-miR-107;hsa-miR-15a-5p;hsa-miR-15b-5p;hsa-miR-424-5p
ENSG0000	ENSG0000	0.007529	hsa-miR-143-5p;hsa-miR-15a-5p;hsa-miR-15b-5p;hsa-miR-16
ENSG0000	ENSG0000	0.007529	hsa-miR-143-5p;hsa-miR-15a-5p;hsa-miR-15b-5p;hsa-miR-16
ENSG0000	ENSG0000	2.29E-08	hsa-miR-15a-5p;hsa-miR-15b-5p;hsa-miR-16-5p;hsa-miR-195
ENSG0000	ENSG0000	1.28E-09	hsa-miR-146b-3p;hsa-miR-15a-5p;hsa-miR-15b-5p;hsa-miR-1
ENSG0000	ENSG0000	0.007529	hsa-miR-15a-5p;hsa-miR-15b-5p;hsa-miR-195-5p;hsa-miR-42
ENSG0000	ENSG0000	3.02E-06	hsa-miR-103a-3p;hsa-miR-107;hsa-miR-15a-5p;hsa-miR-15b-
ENSG0000	ENSG0000	1.67E-11	hsa-miR-103a-3p;hsa-miR-107;hsa-miR-143-5p;hsa-miR-15a-
ENSG0000	ENSG0000	9.36E-09	hsa-miR-15a-5p;hsa-miR-15b-5p;hsa-miR-16-5p;hsa-miR-195
ENSG0000	ENSG0000	0.001491	hsa-miR-15a-5p;hsa-miR-15b-5p;hsa-miR-16-5p;hsa-miR-195
ENSG0000	ENSG0000	0.003214	hsa-miR-103a-3p;hsa-miR-107;hsa-miR-33a-5p;hsa-miR-33b-
ENSG0000	ENSG0000	2.13E-09	hsa-miR-15a-5p;hsa-miR-15b-5p;hsa-miR-16-5p;hsa-miR-195
ENSG0000	ENSG0000	2.11E-05	hsa-miR-146b-3p;hsa-miR-15a-5p;hsa-miR-15b-5p;hsa-miR-1
ENSG0000	ENSG0000	6.40E-05	hsa-miR-15a-5p;hsa-miR-15b-5p;hsa-miR-16-5p;hsa-miR-186
ENSG0000	ENSG0000	0.000331	hsa-miR-15a-5p;hsa-miR-15b-5p;hsa-miR-195-5p;hsa-miR-42
ENSG0000	ENSG0000	9.90E-07	hsa-miR-15a-5p;hsa-miR-15b-5p;hsa-miR-16-5p;hsa-miR-195
ENSG0000	ENSG0000	0.002899	hsa-miR-15a-5p;hsa-miR-15b-5p;hsa-miR-16-5p;hsa-miR-195
ENSG0000	ENSG0000	0.000386	hsa-miR-15a-5p;hsa-miR-15b-5p;hsa-miR-16-5p;hsa-miR-195
ENSG0000	ENSG0000	5.07E-06	hsa-miR-103a-3p;hsa-miR-107;hsa-miR-15a-5p;hsa-miR-15b-
ENSG0000	ENSG0000	0.000998	hsa-miR-1271-5p;hsa-miR-182-5p;hsa-miR-193a-3p;hsa-miR-
ENSG0000	ENSG0000	2.63E-06	hsa-miR-107;hsa-miR-15a-5p;hsa-miR-16-5p;hsa-miR-298;hsa
ENSG0000	ENSG0000	7.80E-10	hsa-miR-15a-5p;hsa-miR-15b-5p;hsa-miR-16-5p;hsa-miR-195
ENSG0000	ENSG0000	0.000222	hsa-miR-15a-5p;hsa-miR-15b-5p;hsa-miR-195-5p;hsa-miR-42
ENSG0000	ENSG0000	4.83E-06	hsa-miR-15a-5p;hsa-miR-15b-5p;hsa-miR-195-5p;hsa-miR-42
ENSG0000	ENSG0000	1.76E-05	hsa-miR-125a-3p;hsa-miR-15a-5p;hsa-miR-15b-5p;hsa-miR-1
ENSG0000	ENSG0000	2.63E-06	hsa-miR-15a-5p;hsa-miR-15b-5p;hsa-miR-195-5p;hsa-miR-42

ENSG0000	ENSG0000	4.21E-05	hsa-miR-15a-5p;hsa-miR-15b-5p;hsa-miR-16-5p;hsa-miR-195-
ENSG0000	ENSG0000	0.005964	hsa-miR-143-5p;hsa-miR-214-3p;hsa-miR-3619-5p;hsa-miR-92
ENSG0000	ENSG0000	3.93E-06	hsa-miR-103a-3p;hsa-miR-107;hsa-miR-15a-5p;hsa-miR-15b-5
ENSG0000	ENSG0000	0.005968	hsa-miR-1271-5p;hsa-miR-193a-3p;hsa-miR-193b-3p;hsa-miR-
ENSG0000	ENSG0000	1.71E-12	hsa-miR-15a-5p;hsa-miR-15b-5p;hsa-miR-195-5p;hsa-miR-214
ENSG0000	ENSG0000	7.33E-05	hsa-miR-15a-5p;hsa-miR-15b-5p;hsa-miR-195-5p;hsa-miR-424
ENSG0000	ENSG0000	0.000131	hsa-miR-15a-5p;hsa-miR-15b-5p;hsa-miR-195-5p;hsa-miR-25-
ENSG0000	ENSG0000	2.03E-06	hsa-miR-15a-5p;hsa-miR-15b-5p;hsa-miR-16-5p;hsa-miR-195-
ENSG0000	ENSG0000	0.00034	hsa-miR-130b-5p;hsa-miR-15a-5p;hsa-miR-16-5p;hsa-miR-450
ENSG0000	ENSG0000	0.00034	hsa-miR-130b-5p;hsa-miR-15a-5p;hsa-miR-16-5p;hsa-miR-450
ENSG0000	ENSG0000	0.002364	hsa-miR-450b-5p;hsa-miR-507;hsa-miR-557;hsa-miR-657
ENSG0000	ENSG0000	6.57E-08	hsa-miR-15a-5p;hsa-miR-15b-5p;hsa-miR-16-5p;hsa-miR-195-
ENSG0000	ENSG0000	4.72E-07	hsa-miR-143-5p;hsa-miR-15a-5p;hsa-miR-15b-5p;hsa-miR-16-
ENSG0000	ENSG0000	5.84E-08	hsa-miR-15a-5p;hsa-miR-15b-5p;hsa-miR-16-5p;hsa-miR-195-
ENSG0000	ENSG0000	6.09E-05	hsa-miR-129-1-3p;hsa-miR-129-2-3p;hsa-miR-214-3p;hsa-miR
ENSG0000	ENSG0000	0.003214	hsa-miR-15a-5p;hsa-miR-15b-5p;hsa-miR-16-5p;hsa-miR-195-
ENSG0000	ENSG0000	0.000103	hsa-miR-103a-3p;hsa-miR-125a-3p;hsa-miR-15a-5p;hsa-miR-1
ENSG0000	ENSG0000	0.005964	hsa-miR-129-1-3p;hsa-miR-129-2-3p;hsa-miR-15a-5p;hsa-miR
ENSG0000	ENSG0000	0.002956	hsa-miR-1271-5p;hsa-miR-143-5p;hsa-miR-182-5p;hsa-miR-92
ENSG0000	ENSG0000	0.002956	hsa-miR-1271-5p;hsa-miR-143-5p;hsa-miR-182-5p;hsa-miR-92
ENSG0000	ENSG0000	3.49E-05	hsa-miR-103a-3p;hsa-miR-107;hsa-miR-15a-5p;hsa-miR-15b-5
ENSG0000	ENSG0000	0.000548	hsa-miR-450b-5p;hsa-miR-507;hsa-miR-557;hsa-miR-657
ENSG0000	ENSG0000	4.12E-08	hsa-miR-15a-5p;hsa-miR-15b-5p;hsa-miR-16-5p;hsa-miR-195-
ENSG0000	ENSG0000	8.74E-05	hsa-miR-15a-5p;hsa-miR-15b-5p;hsa-miR-195-5p;hsa-miR-424
ENSG0000	ENSG0000	0.000141	hsa-miR-103a-3p;hsa-miR-107;hsa-miR-143-5p;hsa-miR-15a-5
ENSG0000	ENSG0000	7.33E-05	hsa-miR-15a-5p;hsa-miR-15b-5p;hsa-miR-195-5p;hsa-miR-424
ENSG0000	ENSG0000	6.01E-08	hsa-miR-103a-3p;hsa-miR-107;hsa-miR-130b-5p;hsa-miR-15a-
ENSG0000	ENSG0000	4.24E-05	hsa-miR-15a-5p;hsa-miR-15b-5p;hsa-miR-195-5p;hsa-miR-424
ENSG0000	ENSG0000	0.001205	hsa-miR-214-3p;hsa-miR-3619-5p;hsa-miR-424-5p;hsa-miR-49
ENSG0000	ENSG0000	0.003214	hsa-miR-130b-5p;hsa-miR-15a-5p;hsa-miR-16-5p;hsa-miR-9-5
ENSG0000	ENSG0000	5.19E-06	hsa-miR-15a-5p;hsa-miR-15b-5p;hsa-miR-16-5p;hsa-miR-195-
ENSG0000	ENSG0000	3.36E-07	hsa-miR-15a-5p;hsa-miR-15b-5p;hsa-miR-16-5p;hsa-miR-195-
ENSG0000	ENSG0000	2.29E-08	hsa-miR-15a-5p;hsa-miR-15b-5p;hsa-miR-16-5p;hsa-miR-195-
ENSG0000	ENSG0000	9.61E-07	hsa-miR-15a-5p;hsa-miR-15b-5p;hsa-miR-16-5p;hsa-miR-195-
ENSG0000	ENSG0000	5.82E-05	hsa-miR-15a-5p;hsa-miR-15b-5p;hsa-miR-16-5p;hsa-miR-195-
ENSG0000	ENSG0000	0.000409	hsa-miR-15a-5p;hsa-miR-15b-5p;hsa-miR-16-5p;hsa-miR-195-
ENSG0000	ENSG0000	0.004458	hsa-miR-125a-3p;hsa-miR-450b-5p;hsa-miR-486-3p;hsa-miR-5
ENSG0000	ENSG0000	0.000476	hsa-miR-15a-5p;hsa-miR-15b-5p;hsa-miR-16-5p;hsa-miR-195-
ENSG0000	ENSG0000	0.000143	hsa-miR-15a-5p;hsa-miR-15b-5p;hsa-miR-195-5p;hsa-miR-424
ENSG0000	ENSG0000	6.55E-07	hsa-miR-15a-5p;hsa-miR-15b-5p;hsa-miR-16-5p;hsa-miR-195-
ENSG0000	ENSG0000	7.85E-08	hsa-miR-103a-3p;hsa-miR-107;hsa-miR-15a-5p;hsa-miR-15b-5
ENSG0000	ENSG0000	9.90E-07	hsa-miR-15a-5p;hsa-miR-15b-5p;hsa-miR-16-5p;hsa-miR-195-
ENSG0000	ENSG0000	0.003807	hsa-miR-103a-3p;hsa-miR-107;hsa-miR-15a-5p;hsa-miR-15b-5
ENSG0000	ENSG0000	0.000143	hsa-miR-298;hsa-miR-326;hsa-miR-450b-5p;hsa-miR-507;hsa-r
ENSG0000	ENSG0000	1.77E-06	hsa-miR-15a-5p;hsa-miR-15b-5p;hsa-miR-195-5p;hsa-miR-424
ENSG0000	ENSG0000	7.85E-05	hsa-miR-223-5p;hsa-miR-450b-5p;hsa-miR-507;hsa-miR-557
ENSG0000	ENSG0000	1.57E-07	hsa-miR-15a-5p;hsa-miR-15b-5p;hsa-miR-16-5p;hsa-miR-195-
ENSG0000	ENSG0000	0.003539	hsa-miR-15a-5p;hsa-miR-15b-5p;hsa-miR-16-5p;hsa-miR-195-
ENSG0000	ENSG0000	3.97E-10	hsa-miR-15a-5p;hsa-miR-15b-5p;hsa-miR-16-5p;hsa-miR-195-
ENSG0000	ENSG0000	0.000386	hsa-miR-15a-5p;hsa-miR-15b-5p;hsa-miR-16-5p;hsa-miR-195-
ENSG0000	ENSG0000	3.02E-06	hsa-miR-143-5p;hsa-miR-15a-5p;hsa-miR-15b-5p;hsa-miR-16-
ENSG0000	ENSG0000	0.000752	hsa-miR-214-3p;hsa-miR-3619-5p;hsa-miR-4690-5p;hsa-miR-9
ENSG0000	ENSG0000	0.005385	hsa-miR-15a-5p;hsa-miR-15b-5p;hsa-miR-195-5p;hsa-miR-424
ENSG0000	ENSG0000	8.65E-06	hsa-miR-143-5p;hsa-miR-15a-5p;hsa-miR-15b-5p;hsa-miR-16-

ENSG0000	ENSG0000	0.000534	hsa-miR-15a-5p;hsa-miR-15b-5p;hsa-miR-195-5p;hsa-miR-42
ENSG0000	ENSG0000	1.22E-11	hsa-miR-107;hsa-miR-15a-5p;hsa-miR-15b-5p;hsa-miR-16-5p;
ENSG0000	ENSG0000	0.000447	hsa-miR-15a-5p;hsa-miR-15b-5p;hsa-miR-186-3p;hsa-miR-19
ENSG0000	ENSG0000	0.000534	hsa-miR-103a-3p;hsa-miR-107;hsa-miR-214-3p;hsa-miR-3619
ENSG0000	ENSG0000	0.002364	hsa-miR-130b-5p;hsa-miR-450b-5p;hsa-miR-507;hsa-miR-557
ENSG0000	ENSG0000	0.000187	hsa-miR-107;hsa-miR-15a-5p;hsa-miR-15b-5p;hsa-miR-195-5
ENSG0000	ENSG0000	4.88E-05	hsa-miR-143-5p;hsa-miR-15a-5p;hsa-miR-15b-5p;hsa-miR-16
ENSG0000	ENSG0000	9.61E-07	hsa-miR-15a-5p;hsa-miR-15b-5p;hsa-miR-16-5p;hsa-miR-195
ENSG0000	ENSG0000	0.000534	hsa-miR-33a-5p;hsa-miR-33b-5p;hsa-miR-450b-5p;hsa-miR-5
ENSG0000	ENSG0000	2.67E-05	hsa-miR-15a-5p;hsa-miR-15b-5p;hsa-miR-195-5p;hsa-miR-42
ENSG0000	ENSG0000	0.000338	hsa-miR-103a-3p;hsa-miR-107;hsa-miR-15a-5p;hsa-miR-15b-
ENSG0000	ENSG0000	3.74E-05	hsa-miR-107;hsa-miR-125a-3p;hsa-miR-15a-5p;hsa-miR-15b-
ENSG0000	ENSG0000	3.14E-07	hsa-miR-107;hsa-miR-1271-5p;hsa-miR-15a-5p;hsa-miR-15b-
ENSG0000	ENSG0000	1.48E-06	hsa-miR-103a-3p;hsa-miR-107;hsa-miR-15a-5p;hsa-miR-15b-
ENSG0000	ENSG0000	6.55E-07	hsa-miR-15a-5p;hsa-miR-15b-5p;hsa-miR-16-5p;hsa-miR-195
ENSG0000	ENSG0000	6.55E-07	hsa-miR-15a-5p;hsa-miR-15b-5p;hsa-miR-16-5p;hsa-miR-195
ENSG0000	ENSG0000	9.68E-06	hsa-miR-15a-5p;hsa-miR-15b-5p;hsa-miR-195-5p;hsa-miR-42
ENSG0000	ENSG0000	3.24E-07	hsa-miR-15a-5p;hsa-miR-15b-5p;hsa-miR-16-5p;hsa-miR-195
ENSG0000	ENSG0000	6.57E-08	hsa-miR-15a-5p;hsa-miR-15b-5p;hsa-miR-16-5p;hsa-miR-195
ENSG0000	ENSG0000	1.91E-10	hsa-miR-103a-3p;hsa-miR-107;hsa-miR-15a-5p;hsa-miR-15b-
ENSG0000	ENSG0000	4.07E-07	hsa-miR-15a-5p;hsa-miR-15b-5p;hsa-miR-16-5p;hsa-miR-195
ENSG0000	ENSG0000	0.001442	hsa-miR-15a-5p;hsa-miR-15b-5p;hsa-miR-16-5p;hsa-miR-195
ENSG0000	ENSG0000	1.19E-06	hsa-miR-15a-5p;hsa-miR-15b-5p;hsa-miR-16-5p;hsa-miR-195
ENSG0000	ENSG0000	2.95E-08	hsa-miR-15a-5p;hsa-miR-15b-5p;hsa-miR-16-5p;hsa-miR-195
ENSG0000	ENSG0000	1.15E-05	hsa-miR-15a-5p;hsa-miR-15b-5p;hsa-miR-16-5p;hsa-miR-195
ENSG0000	ENSG0000	0.004659	hsa-miR-15a-5p;hsa-miR-15b-5p;hsa-miR-16-5p;hsa-miR-195
ENSG0000	ENSG0000	3.24E-07	hsa-miR-15a-5p;hsa-miR-15b-5p;hsa-miR-16-5p;hsa-miR-195
ENSG0000	ENSG0000	7.85E-08	hsa-miR-103a-3p;hsa-miR-107;hsa-miR-15a-5p;hsa-miR-15b-
ENSG0000	ENSG0000	2.03E-08	hsa-miR-33a-5p;hsa-miR-33b-5p;hsa-miR-450b-5p;hsa-miR-5
ENSG0000	ENSG0000	1.53E-08	hsa-miR-15a-5p;hsa-miR-15b-5p;hsa-miR-16-5p;hsa-miR-195
ENSG0000	ENSG0000	5.28E-06	hsa-miR-33a-5p;hsa-miR-33b-5p;hsa-miR-450b-5p;hsa-miR-5
ENSG0000	ENSG0000	4.97E-05	hsa-miR-125a-3p;hsa-miR-146b-3p;hsa-miR-15a-5p;hsa-miR-
ENSG0000	ENSG0000	2.95E-05	hsa-miR-15a-5p;hsa-miR-15b-5p;hsa-miR-16-5p;hsa-miR-195
ENSG0000	ENSG0000	1.28E-05	hsa-miR-103a-3p;hsa-miR-107;hsa-miR-450b-5p;hsa-miR-507;
ENSG0000	ENSG0000	0.005918	hsa-miR-15a-5p;hsa-miR-15b-5p;hsa-miR-16-5p;hsa-miR-195
ENSG0000	ENSG0000	0.005918	hsa-miR-15a-5p;hsa-miR-15b-5p;hsa-miR-16-5p;hsa-miR-195
ENSG0000	ENSG0000	0.001153	hsa-miR-450b-5p;hsa-miR-507;hsa-miR-557;hsa-miR-873-5p
ENSG0000	ENSG0000	8.90E-07	hsa-miR-15a-5p;hsa-miR-15b-5p;hsa-miR-16-5p;hsa-miR-195
ENSG0000	ENSG0000	1.19E-07	hsa-miR-15a-5p;hsa-miR-15b-5p;hsa-miR-195-5p;hsa-miR-42
ENSG0000	ENSG0000	0.000168	hsa-miR-15a-5p;hsa-miR-15b-5p;hsa-miR-16-5p;hsa-miR-195
ENSG0000	ENSG0000	0.000488	hsa-miR-15a-5p;hsa-miR-15b-5p;hsa-miR-16-5p;hsa-miR-195
ENSG0000	ENSG0000	5.82E-05	hsa-miR-15a-5p;hsa-miR-15b-5p;hsa-miR-424-5p;hsa-miR-49
ENSG0000	ENSG0000	8.02E-07	hsa-miR-15a-5p;hsa-miR-15b-5p;hsa-miR-16-5p;hsa-miR-195
ENSG0000	ENSG0000	6.57E-08	hsa-miR-15a-5p;hsa-miR-15b-5p;hsa-miR-16-5p;hsa-miR-195
ENSG0000	ENSG0000	0.000212	hsa-miR-15a-5p;hsa-miR-15b-5p;hsa-miR-16-5p;hsa-miR-186
ENSG0000	ENSG0000	0.000314	hsa-miR-15a-5p;hsa-miR-15b-5p;hsa-miR-16-5p;hsa-miR-195
ENSG0000	ENSG0000	0.00015	hsa-miR-15a-5p;hsa-miR-15b-5p;hsa-miR-16-5p;hsa-miR-195
ENSG0000	ENSG0000	1.53E-06	hsa-miR-103a-3p;hsa-miR-107;hsa-miR-15a-5p;hsa-miR-15b-
ENSG0000	ENSG0000	2.70E-05	hsa-miR-15a-5p;hsa-miR-15b-5p;hsa-miR-195-5p;hsa-miR-42
ENSG0000	ENSG0000	7.31E-05	hsa-miR-15a-5p;hsa-miR-15b-5p;hsa-miR-16-5p;hsa-miR-193
ENSG0000	ENSG0000	0.001683	hsa-miR-450b-5p;hsa-miR-505-5p;hsa-miR-507;hsa-miR-557
ENSG0000	ENSG0000	0.000644	hsa-miR-129-1-3p;hsa-miR-129-2-3p;hsa-miR-28-5p;hsa-miR
ENSG0000	ENSG0000	0.002119	hsa-miR-15a-5p;hsa-miR-15b-5p;hsa-miR-16-5p;hsa-miR-195
ENSG0000	ENSG0000	6.57E-08	hsa-miR-15a-5p;hsa-miR-15b-5p;hsa-miR-16-5p;hsa-miR-195

ENSG0000	ENSG0000	3.98E-07	hsa-miR-15a-5p;hsa-miR-15b-5p;hsa-miR-16-5p;hsa-miR-195-
ENSG0000	ENSG0000	0.000644	hsa-miR-15a-5p;hsa-miR-15b-5p;hsa-miR-16-5p;hsa-miR-195-
ENSG0000	ENSG0000	0.000863	hsa-miR-15a-5p;hsa-miR-15b-5p;hsa-miR-16-5p;hsa-miR-195-
ENSG0000	ENSG0000	3.03E-12	hsa-miR-103a-3p;hsa-miR-107;hsa-miR-15a-5p;hsa-miR-15b-5
ENSG0000	ENSG0000	0.001098	hsa-miR-1271-5p;hsa-miR-143-5p;hsa-miR-182-5p;hsa-miR-87
ENSG0000	ENSG0000	7.60E-05	hsa-miR-15a-5p;hsa-miR-15b-5p;hsa-miR-16-5p;hsa-miR-195-
ENSG0000	ENSG0000	0.001491	hsa-miR-107;hsa-miR-15a-5p;hsa-miR-15b-5p;hsa-miR-195-5p
ENSG0000	ENSG0000	1.99E-06	hsa-miR-103a-3p;hsa-miR-107;hsa-miR-15a-5p;hsa-miR-15b-5
ENSG0000	ENSG0000	0.000827	hsa-miR-15a-5p;hsa-miR-15b-5p;hsa-miR-16-5p;hsa-miR-186-
ENSG0000	ENSG0000	5.10E-09	hsa-miR-15a-5p;hsa-miR-15b-5p;hsa-miR-16-5p;hsa-miR-195-
ENSG0000	ENSG0000	0.000739	hsa-miR-15a-5p;hsa-miR-15b-5p;hsa-miR-16-5p;hsa-miR-195-
ENSG0000	ENSG0000	0.000103	hsa-miR-1271-5p;hsa-miR-182-5p;hsa-miR-9-5p;hsa-miR-96-5
ENSG0000	ENSG0000	0.00506	hsa-miR-298;hsa-miR-450b-5p;hsa-miR-507;hsa-miR-557
ENSG0000	ENSG0000	7.31E-05	hsa-miR-15a-5p;hsa-miR-15b-5p;hsa-miR-195-5p;hsa-miR-424
ENSG0000	ENSG0000	0.000905	hsa-miR-15a-5p;hsa-miR-15b-5p;hsa-miR-195-5p;hsa-miR-424
ENSG0000	ENSG0000	7.01E-05	hsa-miR-103a-3p;hsa-miR-107;hsa-miR-15a-5p;hsa-miR-15b-5
ENSG0000	ENSG0000	6.41E-06	hsa-miR-15a-5p;hsa-miR-15b-5p;hsa-miR-195-5p;hsa-miR-424
ENSG0000	ENSG0000	6.41E-06	hsa-miR-15a-5p;hsa-miR-15b-5p;hsa-miR-195-5p;hsa-miR-223
ENSG0000	ENSG0000	4.88E-05	hsa-miR-15a-5p;hsa-miR-15b-5p;hsa-miR-16-5p;hsa-miR-195-
ENSG0000	ENSG0000	0.000579	hsa-miR-15a-5p;hsa-miR-15b-5p;hsa-miR-16-5p;hsa-miR-195-
ENSG0000	ENSG0000	0.000357	hsa-miR-15a-5p;hsa-miR-15b-5p;hsa-miR-16-5p;hsa-miR-195-
ENSG0000	ENSG0000	8.74E-05	hsa-miR-146b-3p;hsa-miR-214-3p;hsa-miR-3619-5p;hsa-miR-8
ENSG0000	ENSG0000	3.33E-05	hsa-miR-15a-5p;hsa-miR-16-5p;hsa-miR-195-5p;hsa-miR-424-
ENSG0000	ENSG0000	7.05E-09	hsa-miR-103a-3p;hsa-miR-107;hsa-miR-15a-5p;hsa-miR-15b-5
ENSG0000	ENSG0000	9.40E-05	hsa-miR-15a-5p;hsa-miR-15b-5p;hsa-miR-16-5p;hsa-miR-195-
ENSG0000	ENSG0000	0.003176	hsa-miR-214-3p;hsa-miR-25-3p;hsa-miR-3619-5p;hsa-miR-873
ENSG0000	ENSG0000	0.000119	hsa-miR-15a-5p;hsa-miR-15b-5p;hsa-miR-16-5p;hsa-miR-195-
ENSG0000	ENSG0000	4.74E-07	hsa-miR-15a-5p;hsa-miR-15b-5p;hsa-miR-16-5p;hsa-miR-195-
ENSG0000	ENSG0000	0.001353	hsa-miR-143-5p;hsa-miR-15a-5p;hsa-miR-15b-5p;hsa-miR-16-
ENSG0000	ENSG0000	1.12E-10	hsa-miR-15a-5p;hsa-miR-16-5p;hsa-miR-195-5p;hsa-miR-214-
ENSG0000	ENSG0000	2.95E-05	hsa-miR-15a-5p;hsa-miR-15b-5p;hsa-miR-16-5p;hsa-miR-195-
ENSG0000	ENSG0000	6.40E-05	hsa-miR-15a-5p;hsa-miR-15b-5p;hsa-miR-16-5p;hsa-miR-195-
ENSG0000	ENSG0000	0.002856	hsa-miR-15a-5p;hsa-miR-15b-5p;hsa-miR-16-5p;hsa-miR-195-
ENSG0000	ENSG0000	4.17E-06	hsa-miR-15a-5p;hsa-miR-15b-5p;hsa-miR-16-5p;hsa-miR-195-
ENSG0000	ENSG0000	1.03E-07	hsa-miR-15a-5p;hsa-miR-15b-5p;hsa-miR-16-5p;hsa-miR-195-
ENSG0000	ENSG0000	0.008517	hsa-miR-15a-5p;hsa-miR-15b-5p;hsa-miR-16-5p;hsa-miR-186-
ENSG0000	ENSG0000	0.00015	hsa-miR-15a-5p;hsa-miR-15b-5p;hsa-miR-16-5p;hsa-miR-195-
ENSG0000	ENSG0000	3.21E-12	hsa-miR-125a-3p;hsa-miR-15a-5p;hsa-miR-15b-5p;hsa-miR-16
ENSG0000	ENSG0000	3.21E-12	hsa-miR-125a-3p;hsa-miR-15a-5p;hsa-miR-15b-5p;hsa-miR-16
ENSG0000	ENSG0000	3.21E-12	hsa-miR-125a-3p;hsa-miR-15a-5p;hsa-miR-15b-5p;hsa-miR-16
ENSG0000	ENSG0000	3.21E-12	hsa-miR-125a-3p;hsa-miR-15a-5p;hsa-miR-15b-5p;hsa-miR-16
ENSG0000	ENSG0000	0.000269	hsa-miR-107;hsa-miR-143-5p;hsa-miR-15a-5p;hsa-miR-15b-5p
ENSG0000	ENSG0000	0.000269	hsa-miR-107;hsa-miR-143-5p;hsa-miR-15a-5p;hsa-miR-15b-5p
ENSG0000	ENSG0000	4.99E-05	hsa-miR-103a-3p;hsa-miR-107;hsa-miR-15a-5p;hsa-miR-15b-5
ENSG0000	ENSG0000	0.000409	hsa-miR-15a-5p;hsa-miR-15b-5p;hsa-miR-16-5p;hsa-miR-195-
ENSG0000	ENSG0000	2.97E-05	hsa-miR-214-3p;hsa-miR-3619-5p;hsa-miR-450b-5p;hsa-miR-5
ENSG0000	ENSG0000	3.50E-12	hsa-miR-15a-5p;hsa-miR-15b-5p;hsa-miR-195-5p;hsa-miR-214
ENSG0000	ENSG0000	6.09E-05	hsa-miR-33a-5p;hsa-miR-33b-5p;hsa-miR-450b-5p;hsa-miR-50
ENSG0000	ENSG0000	9.61E-07	hsa-miR-15a-5p;hsa-miR-15b-5p;hsa-miR-16-5p;hsa-miR-195-
ENSG0000	ENSG0000	0.001006	hsa-miR-143-5p;hsa-miR-450b-5p;hsa-miR-507;hsa-miR-557
ENSG0000	ENSG0000	6.57E-08	hsa-miR-15a-5p;hsa-miR-15b-5p;hsa-miR-16-5p;hsa-miR-195-
ENSG0000	ENSG0000	7.85E-08	hsa-miR-15a-5p;hsa-miR-15b-5p;hsa-miR-16-5p;hsa-miR-186-
ENSG0000	ENSG0000	7.85E-08	hsa-miR-15a-5p;hsa-miR-15b-5p;hsa-miR-16-5p;hsa-miR-186-
ENSG0000	ENSG0000	7.85E-08	hsa-miR-15a-5p;hsa-miR-15b-5p;hsa-miR-16-5p;hsa-miR-186-

ENSG0000	ENSG0000	7.85E-08	hsa-miR-15a-5p;hsa-miR-15b-5p;hsa-miR-16-5p;hsa-miR-186-
ENSG0000	ENSG0000	7.85E-08	hsa-miR-15a-5p;hsa-miR-15b-5p;hsa-miR-16-5p;hsa-miR-186-
ENSG0000	ENSG0000	7.85E-08	hsa-miR-15a-5p;hsa-miR-15b-5p;hsa-miR-16-5p;hsa-miR-186-
ENSG0000	ENSG0000	7.85E-08	hsa-miR-15a-5p;hsa-miR-15b-5p;hsa-miR-16-5p;hsa-miR-186-
ENSG0000	ENSG0000	7.85E-08	hsa-miR-15a-5p;hsa-miR-15b-5p;hsa-miR-16-5p;hsa-miR-186-
ENSG0000	ENSG0000	7.85E-08	hsa-miR-15a-5p;hsa-miR-15b-5p;hsa-miR-16-5p;hsa-miR-186-
ENSG0000	ENSG0000	7.85E-08	hsa-miR-15a-5p;hsa-miR-15b-5p;hsa-miR-16-5p;hsa-miR-186-
ENSG0000	ENSG0000	4.38E-06	hsa-miR-15a-5p;hsa-miR-15b-5p;hsa-miR-16-5p;hsa-miR-195-
ENSG0000	ENSG0000	8.90E-07	hsa-miR-15a-5p;hsa-miR-15b-5p;hsa-miR-16-5p;hsa-miR-195-
ENSG0000	ENSG0000	4.21E-05	hsa-miR-1271-5p;hsa-miR-182-5p;hsa-miR-486-3p;hsa-miR-9
ENSG0000	ENSG0000	4.11E-05	hsa-miR-15a-5p;hsa-miR-15b-5p;hsa-miR-16-5p;hsa-miR-424-
ENSG0000	ENSG0000	0.002911	hsa-miR-15a-5p;hsa-miR-15b-5p;hsa-miR-16-5p;hsa-miR-195-
ENSG0000	ENSG0000	3.33E-05	hsa-miR-33a-5p;hsa-miR-33b-5p;hsa-miR-450b-5p;hsa-miR-5
ENSG0000	ENSG0000	3.33E-05	hsa-miR-33a-5p;hsa-miR-33b-5p;hsa-miR-450b-5p;hsa-miR-5
ENSG0000	ENSG0000	0.000364	hsa-miR-15a-5p;hsa-miR-15b-5p;hsa-miR-195-5p;hsa-miR-21
ENSG0000	ENSG0000	9.36E-09	hsa-miR-103a-3p;hsa-miR-107;hsa-miR-143-5p;hsa-miR-15a-
ENSG0000	ENSG0000	9.40E-05	hsa-miR-1271-5p;hsa-miR-33a-5p;hsa-miR-33b-5p;hsa-miR-4
ENSG0000	ENSG0000	0.000863	hsa-miR-15a-5p;hsa-miR-15b-5p;hsa-miR-16-5p;hsa-miR-195-
ENSG0000	ENSG0000	4.51E-09	hsa-miR-103a-3p;hsa-miR-107;hsa-miR-130b-5p;hsa-miR-15a-
ENSG0000	ENSG0000	0.000187	hsa-miR-15a-5p;hsa-miR-15b-5p;hsa-miR-16-5p;hsa-miR-195-
ENSG0000	ENSG0000	8.74E-05	hsa-miR-15a-5p;hsa-miR-15b-5p;hsa-miR-195-5p;hsa-miR-42
ENSG0000	ENSG0000	0.000269	hsa-miR-15a-5p;hsa-miR-15b-5p;hsa-miR-16-5p;hsa-miR-195-
ENSG0000	ENSG0000	9.32E-07	hsa-miR-15a-5p;hsa-miR-15b-5p;hsa-miR-16-5p;hsa-miR-195-
ENSG0000	ENSG0000	0.002119	hsa-miR-15a-5p;hsa-miR-15b-5p;hsa-miR-16-5p;hsa-miR-195-
ENSG0000	ENSG0000	0.000187	hsa-miR-143-5p;hsa-miR-214-3p;hsa-miR-223-5p;hsa-miR-36
ENSG0000	ENSG0000	3.53E-08	hsa-miR-15a-5p;hsa-miR-15b-5p;hsa-miR-16-5p;hsa-miR-195-
ENSG0000	ENSG0000	0.000873	hsa-miR-186-3p;hsa-miR-450b-5p;hsa-miR-507;hsa-miR-557
ENSG0000	ENSG0000	3.36E-07	hsa-miR-15a-5p;hsa-miR-15b-5p;hsa-miR-16-5p;hsa-miR-195-
ENSG0000	ENSG0000	0.006673	hsa-miR-15a-5p;hsa-miR-15b-5p;hsa-miR-16-5p;hsa-miR-186-
ENSG0000	ENSG0000	1.37E-08	hsa-miR-103a-3p;hsa-miR-107;hsa-miR-15a-5p;hsa-miR-15b-
ENSG0000	ENSG0000	0.000122	hsa-miR-15a-5p;hsa-miR-15b-5p;hsa-miR-195-5p;hsa-miR-42
ENSG0000	ENSG0000	6.51E-08	hsa-miR-15a-5p;hsa-miR-15b-5p;hsa-miR-16-5p;hsa-miR-195-
ENSG0000	ENSG0000	2.63E-06	hsa-miR-15a-5p;hsa-miR-15b-5p;hsa-miR-195-5p;hsa-miR-42
ENSG0000	ENSG0000	5.10E-09	hsa-miR-103a-3p;hsa-miR-107;hsa-miR-15a-5p;hsa-miR-15b-
ENSG0000	ENSG0000	1.65E-05	hsa-miR-15a-5p;hsa-miR-15b-5p;hsa-miR-16-5p;hsa-miR-195-
ENSG0000	ENSG0000	0.000752	hsa-miR-15a-5p;hsa-miR-15b-5p;hsa-miR-16-5p;hsa-miR-195-
ENSG0000	ENSG0000	3.24E-07	hsa-miR-15a-5p;hsa-miR-15b-5p;hsa-miR-16-5p;hsa-miR-195-
ENSG0000	ENSG0000	0.000532	hsa-miR-103a-3p;hsa-miR-107;hsa-miR-130b-5p;hsa-miR-450
ENSG0000	ENSG0000	1.95E-05	hsa-miR-15a-5p;hsa-miR-15b-5p;hsa-miR-16-5p;hsa-miR-195-
ENSG0000	ENSG0000	0.001161	hsa-miR-15a-5p;hsa-miR-15b-5p;hsa-miR-195-5p;hsa-miR-25-
ENSG0000	ENSG0000	0.008084	hsa-miR-214-3p;hsa-miR-3619-5p;hsa-miR-378g;hsa-miR-922
ENSG0000	ENSG0000	0.008084	hsa-miR-214-3p;hsa-miR-3619-5p;hsa-miR-378g;hsa-miR-922
ENSG0000	ENSG0000	0.008084	hsa-miR-214-3p;hsa-miR-3619-5p;hsa-miR-378g;hsa-miR-922
ENSG0000	ENSG0000	3.15E-05	hsa-miR-15a-5p;hsa-miR-15b-5p;hsa-miR-16-5p;hsa-miR-195-
ENSG0000	ENSG0000	0.000752	hsa-miR-130b-5p;hsa-miR-25-3p;hsa-miR-33a-5p;hsa-miR-33
ENSG0000	ENSG0000	6.01E-08	hsa-miR-103a-3p;hsa-miR-107;hsa-miR-15a-5p;hsa-miR-15b-
ENSG0000	ENSG0000	7.80E-10	hsa-miR-103a-3p;hsa-miR-107;hsa-miR-15a-5p;hsa-miR-15b-
ENSG0000	ENSG0000	6.51E-08	hsa-miR-15a-5p;hsa-miR-15b-5p;hsa-miR-16-5p;hsa-miR-195-
ENSG0000	ENSG0000	0.003201	hsa-miR-103a-3p;hsa-miR-107;hsa-miR-130b-5p;hsa-miR-214-
ENSG0000	ENSG0000	2.64E-05	hsa-miR-143-5p;hsa-miR-15a-5p;hsa-miR-15b-5p;hsa-miR-16-
ENSG0000	ENSG0000	0.000262	hsa-miR-15a-5p;hsa-miR-15b-5p;hsa-miR-16-5p;hsa-miR-195-
ENSG0000	ENSG0000	0.000262	hsa-miR-15a-5p;hsa-miR-15b-5p;hsa-miR-16-5p;hsa-miR-195-
ENSG0000	ENSG0000	0.005968	hsa-miR-15a-5p;hsa-miR-15b-5p;hsa-miR-16-5p;hsa-miR-195-
ENSG0000	ENSG0000	2.48E-07	hsa-miR-15a-5p;hsa-miR-15b-5p;hsa-miR-16-5p;hsa-miR-195-

ENSG0000	ENSG0000	0.000103	hsa-miR-15a-5p;hsa-miR-15b-5p;hsa-miR-16-5p;hsa-miR-195-
ENSG0000	ENSG0000	1.25E-08	hsa-miR-15a-5p;hsa-miR-15b-5p;hsa-miR-16-5p;hsa-miR-195-
ENSG0000	ENSG0000	6.34E-13	hsa-miR-103a-3p;hsa-miR-107;hsa-miR-15a-5p;hsa-miR-15b-
ENSG0000	ENSG0000	6.51E-08	hsa-miR-15a-5p;hsa-miR-15b-5p;hsa-miR-16-5p;hsa-miR-195-
ENSG0000	ENSG0000	3.87E-06	hsa-miR-103a-3p;hsa-miR-107;hsa-miR-15a-5p;hsa-miR-15b-
ENSG0000	ENSG0000	3.31E-09	hsa-miR-146b-3p;hsa-miR-15a-5p;hsa-miR-15b-5p;hsa-miR-1
ENSG0000	ENSG0000	7.85E-06	hsa-miR-15a-5p;hsa-miR-15b-5p;hsa-miR-16-5p;hsa-miR-195-
ENSG0000	ENSG0000	0.009309	hsa-miR-1271-5p;hsa-miR-182-5p;hsa-miR-25-3p;hsa-miR-96-
ENSG0000	ENSG0000	1.40E-05	hsa-miR-1271-5p;hsa-miR-15a-5p;hsa-miR-15b-5p;hsa-miR-1
ENSG0000	ENSG0000	3.01E-08	hsa-miR-1271-5p;hsa-miR-143-5p;hsa-miR-15a-5p;hsa-miR-1
ENSG0000	ENSG0000	0.003577	hsa-miR-15a-5p;hsa-miR-15b-5p;hsa-miR-16-5p;hsa-miR-195-
ENSG0000	ENSG0000	0.000413	hsa-miR-15a-5p;hsa-miR-15b-5p;hsa-miR-16-5p;hsa-miR-195-
ENSG0000	ENSG0000	0.001878	hsa-miR-15a-5p;hsa-miR-15b-5p;hsa-miR-195-5p;hsa-miR-21
ENSG0000	ENSG0000	6.57E-08	hsa-miR-15a-5p;hsa-miR-15b-5p;hsa-miR-16-5p;hsa-miR-195-
ENSG0000	ENSG0000	1.08E-07	hsa-miR-15a-5p;hsa-miR-15b-5p;hsa-miR-16-5p;hsa-miR-195-
ENSG0000	ENSG0000	0.009309	hsa-miR-103a-3p;hsa-miR-107;hsa-miR-25-3p;hsa-miR-450b-
ENSG0000	ENSG0000	0.00254	hsa-miR-143-5p;hsa-miR-214-3p;hsa-miR-25-3p;hsa-miR-361
ENSG0000	ENSG0000	0.000447	hsa-miR-15a-5p;hsa-miR-15b-5p;hsa-miR-16-5p;hsa-miR-195-
ENSG0000	ENSG0000	1.52E-07	hsa-miR-103a-3p;hsa-miR-107;hsa-miR-143-5p;hsa-miR-15a-
ENSG0000	ENSG0000	4.99E-05	hsa-miR-130b-5p;hsa-miR-15a-5p;hsa-miR-15b-5p;hsa-miR-1
ENSG0000	ENSG0000	0.000309	hsa-miR-15a-5p;hsa-miR-15b-5p;hsa-miR-16-5p;hsa-miR-195-
ENSG0000	ENSG0000	0.000281	hsa-miR-15a-5p;hsa-miR-15b-5p;hsa-miR-16-5p;hsa-miR-195-
ENSG0000	ENSG0000	0.000905	hsa-miR-1271-5p;hsa-miR-182-5p;hsa-miR-33a-5p;hsa-miR-3
ENSG0000	ENSG0000	4.30E-11	hsa-miR-320a;hsa-miR-320b;hsa-miR-320c;hsa-miR-320d;hsa-
ENSG0000	ENSG0000	1.40E-09	hsa-miR-320a;hsa-miR-320b;hsa-miR-320c;hsa-miR-320d;hsa-
ENSG0000	ENSG0000	4.00E-08	hsa-miR-320a;hsa-miR-320b;hsa-miR-320c;hsa-miR-320d;hsa-
ENSG0000	ENSG0000	1.83E-10	hsa-miR-320a;hsa-miR-320b;hsa-miR-320c;hsa-miR-320d;hsa-
ENSG0000	ENSG0000	5.03E-09	hsa-miR-320a;hsa-miR-320b;hsa-miR-320c;hsa-miR-320d;hsa-
ENSG0000	ENSG0000	1.24E-05	hsa-miR-320a;hsa-miR-320b;hsa-miR-320c;hsa-miR-320d;hsa-
ENSG0000	ENSG0000	2.03E-08	hsa-miR-320a;hsa-miR-320b;hsa-miR-320c;hsa-miR-320d;hsa-
ENSG0000	ENSG0000	4.44E-08	hsa-miR-320a;hsa-miR-320b;hsa-miR-320c;hsa-miR-320d;hsa-
ENSG0000	ENSG0000	1.22E-08	hsa-miR-320a;hsa-miR-320b;hsa-miR-320c;hsa-miR-320d;hsa-
ENSG0000	ENSG0000	3.81E-07	hsa-miR-320a;hsa-miR-320b;hsa-miR-320c;hsa-miR-320d;hsa-
ENSG0000	ENSG0000	1.04E-07	hsa-miR-320a;hsa-miR-320b;hsa-miR-320c;hsa-miR-320d;hsa-
ENSG0000	ENSG0000	9.84E-06	hsa-miR-320b;hsa-miR-320c;hsa-miR-320d;hsa-miR-4429
ENSG0000	ENSG0000	1.46E-07	hsa-miR-320a;hsa-miR-320b;hsa-miR-320c;hsa-miR-320d;hsa-
ENSG0000	ENSG0000	4.35E-10	hsa-miR-320a;hsa-miR-320b;hsa-miR-320c;hsa-miR-320d;hsa-
ENSG0000	ENSG0000	4.35E-10	hsa-miR-320a;hsa-miR-320b;hsa-miR-320c;hsa-miR-320d;hsa-
ENSG0000	ENSG0000	1.45E-06	hsa-miR-320a;hsa-miR-320b;hsa-miR-320c;hsa-miR-320d;hsa-
ENSG0000	ENSG0000	4.35E-10	hsa-miR-320a;hsa-miR-320b;hsa-miR-320c;hsa-miR-320d;hsa-
ENSG0000	ENSG0000	3.22E-08	hsa-miR-320a;hsa-miR-320b;hsa-miR-320c;hsa-miR-320d;hsa-
ENSG0000	ENSG0000	4.30E-11	hsa-miR-320a;hsa-miR-320b;hsa-miR-320c;hsa-miR-320d;hsa-
ENSG0000	ENSG0000	1.06E-08	hsa-miR-320a;hsa-miR-320b;hsa-miR-320c;hsa-miR-320d;hsa-
ENSG0000	ENSG0000	9.21E-09	hsa-miR-320a;hsa-miR-320b;hsa-miR-320c;hsa-miR-320d;hsa-
ENSG0000	ENSG0000	9.55E-08	hsa-miR-320a;hsa-miR-320b;hsa-miR-320c;hsa-miR-320d;hsa-
ENSG0000	ENSG0000	9.55E-08	hsa-miR-320a;hsa-miR-320b;hsa-miR-320c;hsa-miR-320d;hsa-
ENSG0000	ENSG0000	2.29E-08	hsa-miR-320a;hsa-miR-320b;hsa-miR-320c;hsa-miR-320d;hsa-
ENSG0000	ENSG0000	2.49E-10	hsa-miR-320a;hsa-miR-320b;hsa-miR-320c;hsa-miR-320d;hsa-
ENSG0000	ENSG0000	5.42E-12	hsa-miR-320a;hsa-miR-320b;hsa-miR-320c;hsa-miR-320d;hsa-
ENSG0000	ENSG0000	4.92E-08	hsa-miR-320a;hsa-miR-320b;hsa-miR-320c;hsa-miR-320d;hsa-
ENSG0000	ENSG0000	4.44E-08	hsa-miR-320a;hsa-miR-320b;hsa-miR-320c;hsa-miR-320d;hsa-
ENSG0000	ENSG0000	1.85E-07	hsa-miR-320a;hsa-miR-320b;hsa-miR-320c;hsa-miR-320d;hsa-
ENSG0000	ENSG0000	2.16E-07	hsa-miR-320a;hsa-miR-320b;hsa-miR-320c;hsa-miR-320d;hsa-
ENSG0000	ENSG0000	3.48E-06	hsa-miR-320b;hsa-miR-320c;hsa-miR-320d;hsa-miR-4429

ENSG0000	ENSG0000	0.000142	hsa-miR-320b;hsa-miR-320c;hsa-miR-320d;hsa-miR-4429
ENSG0000	ENSG0000	2.76E-11	hsa-miR-320a;hsa-miR-320b;hsa-miR-320c;hsa-miR-320d;hsa-r
ENSG0000	ENSG0000	2.76E-11	hsa-miR-320a;hsa-miR-320b;hsa-miR-320c;hsa-miR-320d;hsa-r
ENSG0000	ENSG0000	5.42E-12	hsa-miR-320a;hsa-miR-320b;hsa-miR-320c;hsa-miR-320d;hsa-r
ENSG0000	ENSG0000	5.27E-06	hsa-miR-320a;hsa-miR-320b;hsa-miR-320c;hsa-miR-320d;hsa-r
ENSG0000	ENSG0000	4.30E-11	hsa-miR-320a;hsa-miR-320b;hsa-miR-320c;hsa-miR-320d;hsa-r
ENSG0000	ENSG0000	1.22E-08	hsa-miR-320a;hsa-miR-320b;hsa-miR-320c;hsa-miR-320d;hsa-r
ENSG0000	ENSG0000	1.33E-10	hsa-miR-320a;hsa-miR-320b;hsa-miR-320c;hsa-miR-320d;hsa-r
ENSG0000	ENSG0000	8.53E-06	hsa-miR-320a;hsa-miR-320b;hsa-miR-320c;hsa-miR-320d;hsa-r
ENSG0000	ENSG0000	2.29E-08	hsa-miR-320a;hsa-miR-320b;hsa-miR-320c;hsa-miR-320d;hsa-r
ENSG0000	ENSG0000	3.03E-09	hsa-miR-320a;hsa-miR-320b;hsa-miR-320c;hsa-miR-320d;hsa-r
ENSG0000	ENSG0000	1.14E-07	hsa-miR-320a;hsa-miR-320b;hsa-miR-320c;hsa-miR-320d;hsa-r
ENSG0000	ENSG0000	4.63E-07	hsa-miR-320a;hsa-miR-320b;hsa-miR-320c;hsa-miR-320d;hsa-r
ENSG0000	ENSG0000	7.18E-10	hsa-miR-320a;hsa-miR-320b;hsa-miR-320c;hsa-miR-320d;hsa-r
ENSG0000	ENSG0000	2.29E-05	hsa-miR-320a;hsa-miR-320b;hsa-miR-320c;hsa-miR-320d;hsa-r
ENSG0000	ENSG0000	3.59E-08	hsa-miR-320a;hsa-miR-320b;hsa-miR-320c;hsa-miR-320d;hsa-r
ENSG0000	ENSG0000	3.03E-09	hsa-miR-320a;hsa-miR-320b;hsa-miR-320c;hsa-miR-320d;hsa-r
ENSG0000	ENSG0000	6.48E-08	hsa-miR-1276;hsa-miR-320a;hsa-miR-320b;hsa-miR-320c;hsa-r
ENSG0000	ENSG0000	7.97E-08	hsa-miR-320a;hsa-miR-320b;hsa-miR-320c;hsa-miR-320d;hsa-r
ENSG0000	ENSG0000	1.40E-09	hsa-miR-320a;hsa-miR-320b;hsa-miR-320c;hsa-miR-320d;hsa-r
ENSG0000	ENSG0000	2.33E-07	hsa-miR-320a;hsa-miR-320b;hsa-miR-320c;hsa-miR-320d;hsa-r
ENSG0000	ENSG0000	9.06E-10	hsa-miR-320a;hsa-miR-320b;hsa-miR-320c;hsa-miR-320d;hsa-r
ENSG0000	ENSG0000	1.12E-06	hsa-miR-320a;hsa-miR-320b;hsa-miR-320c;hsa-miR-320d;hsa-r
ENSG0000	ENSG0000	1.21E-12	hsa-miR-320a;hsa-miR-320b;hsa-miR-320c;hsa-miR-320d;hsa-r
ENSG0000	ENSG0000	4.00E-08	hsa-miR-320a;hsa-miR-320b;hsa-miR-320c;hsa-miR-320d;hsa-r
ENSG0000	ENSG0000	2.69E-07	hsa-miR-320a;hsa-miR-320b;hsa-miR-320c;hsa-miR-320d;hsa-r
ENSG0000	ENSG0000	4.00E-08	hsa-miR-320a;hsa-miR-320b;hsa-miR-320c;hsa-miR-320d;hsa-r
ENSG0000	ENSG0000	1.35E-07	hsa-miR-320a;hsa-miR-320b;hsa-miR-320c;hsa-miR-320d;hsa-r
ENSG0000	ENSG0000	5.27E-06	hsa-miR-320a;hsa-miR-320b;hsa-miR-320c;hsa-miR-320d;hsa-r
ENSG0000	ENSG0000	6.61E-08	hsa-miR-320a;hsa-miR-320b;hsa-miR-320c;hsa-miR-320d;hsa-r
ENSG0000	ENSG0000	2.00E-07	hsa-miR-320a;hsa-miR-320b;hsa-miR-320c;hsa-miR-320d;hsa-r
ENSG0000	ENSG0000	1.70E-11	hsa-miR-320a;hsa-miR-320b;hsa-miR-320c;hsa-miR-320d;hsa-r
ENSG0000	ENSG0000	3.61E-09	hsa-miR-320a;hsa-miR-320b;hsa-miR-320c;hsa-miR-320d;hsa-r
ENSG0000	ENSG0000	3.61E-09	hsa-miR-320a;hsa-miR-320b;hsa-miR-320c;hsa-miR-320d;hsa-r
ENSG0000	ENSG0000	5.89E-09	hsa-miR-320a;hsa-miR-320b;hsa-miR-320c;hsa-miR-320d;hsa-r
ENSG0000	ENSG0000	0.004931	hsa-miR-148a-5p;hsa-miR-17-5p;hsa-miR-20b-5p;hsa-miR-93-
ENSG0000	ENSG0000	0.004127	hsa-miR-1293;hsa-miR-15a-5p;hsa-miR-15b-5p;hsa-miR-16-5p
ENSG0000	ENSG0000	0.001595	hsa-miR-15a-5p;hsa-miR-15b-5p;hsa-miR-16-5p;hsa-miR-195-
ENSG0000	ENSG0000	2.07E-08	hsa-miR-103a-3p;hsa-miR-107;hsa-miR-15a-5p;hsa-miR-15b-5
ENSG0000	ENSG0000	5.38E-05	hsa-miR-15a-5p;hsa-miR-15b-5p;hsa-miR-16-5p;hsa-miR-195-
ENSG0000	ENSG0000	1.24E-05	hsa-miR-15a-5p;hsa-miR-15b-5p;hsa-miR-195-5p;hsa-miR-21
ENSG0000	ENSG0000	0.003117	hsa-miR-137;hsa-miR-143-3p;hsa-miR-34a-5p;hsa-miR-34b-3
ENSG0000	ENSG0000	3.65E-06	hsa-miR-103a-3p;hsa-miR-107;hsa-miR-15a-5p;hsa-miR-15b-5
ENSG0000	ENSG0000	0.003528	hsa-miR-101-3p;hsa-miR-15a-5p;hsa-miR-15b-5p;hsa-miR-17-
ENSG0000	ENSG0000	0.000287	hsa-miR-15a-5p;hsa-miR-15b-5p;hsa-miR-16-5p;hsa-miR-195-
ENSG0000	ENSG0000	0.000287	hsa-miR-15a-5p;hsa-miR-15b-5p;hsa-miR-16-5p;hsa-miR-195-
ENSG0000	ENSG0000	0.00448	hsa-miR-1293;hsa-miR-151a-5p;hsa-miR-15a-5p;hsa-miR-15b-
ENSG0000	ENSG0000	0.006617	hsa-miR-142-3p;hsa-miR-17-5p;hsa-miR-204-5p;hsa-miR-20b-
ENSG0000	ENSG0000	1.10E-06	hsa-miR-15a-5p;hsa-miR-15b-5p;hsa-miR-16-5p;hsa-miR-17-5
ENSG0000	ENSG0000	0.006452	hsa-miR-15a-5p;hsa-miR-16-5p;hsa-miR-212-3p;hsa-miR-34a-
ENSG0000	ENSG0000	0.00889	hsa-miR-101-3p;hsa-miR-124-3p;hsa-miR-142-3p;hsa-miR-31
ENSG0000	ENSG0000	0.002799	hsa-miR-17-3p;hsa-miR-204-5p;hsa-miR-378a-3p;hsa-miR-37
ENSG0000	ENSG0000	0.006946	hsa-miR-30b-3p;hsa-miR-30c-1-3p;hsa-miR-30c-2-3p;hsa-mi
ENSG0000	ENSG0000	2.34E-07	hsa-miR-103a-3p;hsa-miR-135a-5p;hsa-miR-195-5p;hsa-miR-

ENSG0000	ENSG0000	0.005083	hsa-miR-101-3p;hsa-miR-144-5p;hsa-miR-17-5p;hsa-miR-27a-
ENSG0000	ENSG0000	0.003671	hsa-miR-15a-5p;hsa-miR-15b-5p;hsa-miR-16-5p;hsa-miR-195-
ENSG0000	ENSG0000	0.000409	hsa-miR-138-5p;hsa-miR-15b-5p;hsa-miR-16-5p;hsa-miR-195-
ENSG0000	ENSG0000	0.000149	hsa-miR-144-5p;hsa-miR-15a-5p;hsa-miR-15b-5p;hsa-miR-16-
ENSG0000	ENSG0000	0.003306	hsa-miR-101-3p;hsa-miR-30b-3p;hsa-miR-30c-1-3p;hsa-miR-
ENSG0000	ENSG0000	0.001237	hsa-miR-103a-3p;hsa-miR-107;hsa-miR-15a-5p;hsa-miR-15b-
ENSG0000	ENSG0000	1.84E-05	hsa-miR-103a-3p;hsa-miR-107;hsa-miR-15a-5p;hsa-miR-15b-
ENSG0000	ENSG0000	0.007693	hsa-miR-140-3p;hsa-miR-15a-5p;hsa-miR-15b-5p;hsa-miR-34
ENSG0000	ENSG0000	0.000149	hsa-miR-107;hsa-miR-137;hsa-miR-195-5p;hsa-miR-21-3p;hsa-
ENSG0000	ENSG0000	0.000197	hsa-miR-107;hsa-miR-124-3p;hsa-miR-137;hsa-miR-15a-5p;hs
ENSG0000	ENSG0000	0.000103	hsa-miR-15a-5p;hsa-miR-16-5p;hsa-miR-23a-3p;hsa-miR-23b-
ENSG0000	ENSG0000	0.003671	hsa-miR-137;hsa-miR-140-3p;hsa-miR-23a-3p;hsa-miR-23b-3
ENSG0000	ENSG0000	8.16E-06	hsa-miR-126-5p;hsa-miR-1267;hsa-miR-135b-3p;hsa-miR-15a-
ENSG0000	ENSG0000	0.003306	hsa-miR-103a-3p;hsa-miR-15a-5p;hsa-miR-17-3p;hsa-miR-30
ENSG0000	ENSG0000	0.000949	hsa-miR-1293;hsa-miR-15a-5p;hsa-miR-15b-5p;hsa-miR-16-5
ENSG0000	ENSG0000	0.007204	hsa-miR-103a-3p;hsa-miR-107;hsa-miR-126-5p;hsa-miR-142-
ENSG0000	ENSG0000	0.00889	hsa-miR-124-3p;hsa-miR-148a-5p;hsa-miR-148b-5p;hsa-miR-
ENSG0000	ENSG0000	6.84E-07	hsa-miR-103a-3p;hsa-miR-107;hsa-miR-15a-5p;hsa-miR-15b-
ENSG0000	ENSG0000	0.002406	hsa-miR-17-5p;hsa-miR-1976;hsa-miR-204-5p;hsa-miR-20b-5
ENSG0000	ENSG0000	0.000337	hsa-miR-1293;hsa-miR-15a-5p;hsa-miR-15b-5p;hsa-miR-16-5
ENSG0000	ENSG0000	0.00863	hsa-miR-186-3p;hsa-miR-31-5p;hsa-miR-561-3p;hsa-miR-567;
ENSG0000	ENSG0000	1.46E-07	hsa-miR-103a-3p;hsa-miR-107;hsa-miR-15a-5p;hsa-miR-15b-
ENSG0000	ENSG0000	4.20E-05	hsa-miR-135a-5p;hsa-miR-17-5p;hsa-miR-193a-3p;hsa-miR-2
ENSG0000	ENSG0000	0.006452	hsa-miR-124-3p;hsa-miR-129-1-3p;hsa-miR-129-2-3p;hsa-mi
ENSG0000	ENSG0000	0.002297	hsa-miR-15a-5p;hsa-miR-15b-5p;hsa-miR-195-5p;hsa-miR-20
ENSG0000	ENSG0000	0.002799	hsa-miR-132-3p;hsa-miR-135a-5p;hsa-miR-137;hsa-miR-16-5
ENSG0000	ENSG0000	0.004309	hsa-miR-103a-3p;hsa-miR-107;hsa-miR-1243;hsa-miR-15a-5p;
ENSG0000	ENSG0000	0.005267	hsa-miR-103a-3p;hsa-miR-107;hsa-miR-15a-5p;hsa-miR-15b-
ENSG0000	ENSG0000	0.008255	hsa-miR-124-3p;hsa-miR-142-3p;hsa-miR-4487;hsa-miR-455-
ENSG0000	ENSG0000	0.000578	hsa-miR-101-3p;hsa-miR-124-3p;hsa-miR-1260a;hsa-miR-126
ENSG0000	ENSG0000	0.000764	hsa-miR-101-3p;hsa-miR-23a-3p;hsa-miR-23b-3p;hsa-miR-36
ENSG0000	ENSG0000	6.27E-05	hsa-miR-15a-5p;hsa-miR-15b-5p;hsa-miR-16-5p;hsa-miR-195-
ENSG0000	ENSG0000	0.00159	hsa-miR-137;hsa-miR-151a-5p;hsa-miR-15a-5p;hsa-miR-15b-
ENSG0000	ENSG0000	0.008157	hsa-miR-135a-5p;hsa-miR-182-5p;hsa-miR-183-5p;hsa-miR-1
ENSG0000	ENSG0000	9.55E-06	hsa-miR-15a-5p;hsa-miR-15b-5p;hsa-miR-16-5p;hsa-miR-195-
ENSG0000	ENSG0000	0.00827	hsa-miR-21-3p;hsa-miR-30b-3p;hsa-miR-30c-1-3p;hsa-miR-3
ENSG0000	ENSG0000	0.000103	hsa-miR-222-5p;hsa-miR-30a-3p;hsa-miR-30d-3p;hsa-miR-30
ENSG0000	ENSG0000	0.003172	hsa-miR-15a-5p;hsa-miR-15b-5p;hsa-miR-16-5p;hsa-miR-17-
ENSG0000	ENSG0000	5.61E-05	hsa-miR-103a-3p;hsa-miR-107;hsa-miR-124-3p;hsa-miR-23a-
ENSG0000	ENSG0000	1.27E-07	hsa-miR-15a-5p;hsa-miR-15b-5p;hsa-miR-195-5p;hsa-miR-21
ENSG0000	ENSG0000	0.000139	hsa-miR-103a-3p;hsa-miR-107;hsa-miR-15a-5p;hsa-miR-15b-
ENSG0000	ENSG0000	0.000756	hsa-miR-132-3p;hsa-miR-15a-5p;hsa-miR-15b-5p;hsa-miR-19
ENSG0000	ENSG0000	0.007665	hsa-miR-124-3p;hsa-miR-137;hsa-miR-138-5p;hsa-miR-17-5p;
ENSG0000	ENSG0000	3.96E-06	hsa-miR-129-1-3p;hsa-miR-15a-5p;hsa-miR-15b-5p;hsa-miR-
ENSG0000	ENSG0000	0.000242	hsa-miR-107;hsa-miR-138-5p;hsa-miR-17-5p;hsa-miR-20b-5p;
ENSG0000	ENSG0000	0.004861	hsa-miR-17-5p;hsa-miR-20b-5p;hsa-miR-27b-3p;hsa-miR-545
ENSG0000	ENSG0000	0.001073	hsa-miR-107;hsa-miR-140-3p;hsa-miR-15a-5p;hsa-miR-15b-5
ENSG0000	ENSG0000	0.005612	hsa-miR-15a-5p;hsa-miR-15b-5p;hsa-miR-195-5p;hsa-miR-42
ENSG0000	ENSG0000	0.003528	hsa-miR-135a-5p;hsa-miR-15a-5p;hsa-miR-15b-5p;hsa-miR-1
ENSG0000	ENSG0000	0.001796	hsa-miR-124-3p;hsa-miR-224-5p;hsa-miR-27a-3p;hsa-miR-27
ENSG0000	ENSG0000	0.007693	hsa-miR-17-5p;hsa-miR-20b-5p;hsa-miR-27a-3p;hsa-miR-27b-
ENSG0000	ENSG0000	1.00E-05	hsa-miR-101-3p;hsa-miR-103a-3p;hsa-miR-107;hsa-miR-15a-
ENSG0000	ENSG0000	0.00452	hsa-miR-1226-3p;hsa-miR-143-3p;hsa-miR-193a-3p;hsa-miR-
ENSG0000	ENSG0000	0.000214	hsa-miR-17-3p;hsa-miR-222-5p;hsa-miR-3065-3p;hsa-miR-30

ENSG0000	ENSG0000	0.001489	hsa-miR-124-3p;hsa-miR-143-3p;hsa-miR-17-5p;hsa-miR-183-
ENSG0000	ENSG0000	0.000628	hsa-miR-17-3p;hsa-miR-204-5p;hsa-miR-30b-3p;hsa-miR-30c-
ENSG0000	ENSG0000	0.000685	hsa-miR-15a-5p;hsa-miR-15b-5p;hsa-miR-195-5p;hsa-miR-19
ENSG0000	ENSG0000	0.001415	hsa-miR-196a-5p;hsa-miR-196b-5p;hsa-miR-212-3p;hsa-miR-
ENSG0000	ENSG0000	0.003634	hsa-miR-1243;hsa-miR-129-1-3p;hsa-miR-129-2-3p;hsa-miR-
ENSG0000	ENSG0000	0.000623	hsa-miR-132-3p;hsa-miR-15a-5p;hsa-miR-15b-5p;hsa-miR-19
ENSG0000	ENSG0000	0.003378	hsa-miR-15a-5p;hsa-miR-15b-5p;hsa-miR-16-5p;hsa-miR-195-
ENSG0000	ENSG0000	2.46E-05	hsa-miR-15a-5p;hsa-miR-15b-5p;hsa-miR-16-5p;hsa-miR-195-
ENSG0000	ENSG0000	2.46E-05	hsa-miR-15a-5p;hsa-miR-15b-5p;hsa-miR-16-5p;hsa-miR-195-
ENSG0000	ENSG0000	3.78E-06	hsa-miR-124-3p;hsa-miR-142-3p;hsa-miR-15a-5p;hsa-miR-15
ENSG0000	ENSG0000	0.000177	hsa-miR-140-3p;hsa-miR-15a-5p;hsa-miR-15b-5p;hsa-miR-19
ENSG0000	ENSG0000	3.24E-05	hsa-miR-101-3p;hsa-miR-103a-3p;hsa-miR-107;hsa-miR-15a-
ENSG0000	ENSG0000	0.000166	hsa-miR-103a-3p;hsa-miR-107;hsa-miR-1293;hsa-miR-15a-5p;
ENSG0000	ENSG0000	0.005654	hsa-miR-1267;hsa-miR-132-3p;hsa-miR-15b-5p;hsa-miR-212-
ENSG0000	ENSG0000	0.007298	hsa-miR-144-5p;hsa-miR-17-5p;hsa-miR-224-5p;hsa-miR-27a-
ENSG0000	ENSG0000	0.001907	hsa-miR-15a-5p;hsa-miR-195-5p;hsa-miR-374b-3p;hsa-miR-4
ENSG0000	ENSG0000	0.001777	hsa-miR-101-3p;hsa-miR-1207-5p;hsa-miR-15a-5p;hsa-miR-1
ENSG0000	ENSG0000	0.008255	hsa-miR-140-3p;hsa-miR-182-5p;hsa-miR-183-5p;hsa-miR-19
ENSG0000	ENSG0000	0.000349	hsa-miR-132-3p;hsa-miR-143-3p;hsa-miR-15b-5p;hsa-miR-9-
ENSG0000	ENSG0000	0.000425	hsa-miR-15a-5p;hsa-miR-15b-5p;hsa-miR-195-5p;hsa-miR-42
ENSG0000	ENSG0000	0.006617	hsa-miR-17-5p;hsa-miR-20b-5p;hsa-miR-342-3p;hsa-miR-455-
ENSG0000	ENSG0000	0.000137	hsa-miR-107;hsa-miR-15a-5p;hsa-miR-16-5p;hsa-miR-34a-5p;
ENSG0000	ENSG0000	0.002672	hsa-miR-101-3p;hsa-miR-103a-3p;hsa-miR-124-3p;hsa-miR-1
ENSG0000	ENSG0000	4.07E-05	hsa-miR-138-5p;hsa-miR-16-5p;hsa-miR-34a-5p;hsa-miR-9-3
ENSG0000	ENSG0000	7.22E-06	hsa-miR-101-3p;hsa-miR-204-5p;hsa-miR-23b-3p;hsa-miR-27
ENSG0000	ENSG0000	0.000223	hsa-miR-15a-5p;hsa-miR-15b-5p;hsa-miR-16-5p;hsa-miR-195-
ENSG0000	ENSG0000	0.000719	hsa-miR-1226-3p;hsa-miR-1260a;hsa-miR-1260b;hsa-miR-129-
ENSG0000	ENSG0000	0.007311	hsa-miR-137;hsa-miR-16-5p;hsa-miR-214-3p;hsa-miR-330-3p;
ENSG0000	ENSG0000	0.0015	hsa-miR-144-5p;hsa-miR-30a-3p;hsa-miR-30d-3p;hsa-miR-30
ENSG0000	ENSG0000	0.003117	hsa-miR-15a-5p;hsa-miR-15b-5p;hsa-miR-195-5p;hsa-miR-42
ENSG0000	ENSG0000	2.46E-05	hsa-miR-15a-5p;hsa-miR-15b-5p;hsa-miR-16-5p;hsa-miR-195-
ENSG0000	ENSG0000	2.46E-05	hsa-miR-15a-5p;hsa-miR-15b-5p;hsa-miR-16-5p;hsa-miR-195-
ENSG0000	ENSG0000	0.002649	hsa-miR-103a-3p;hsa-miR-107;hsa-miR-17-3p;hsa-miR-3065-
ENSG0000	ENSG0000	7.47E-06	hsa-miR-15a-5p;hsa-miR-15b-5p;hsa-miR-16-5p;hsa-miR-195-
ENSG0000	ENSG0000	0.007693	hsa-miR-107;hsa-miR-15a-5p;hsa-miR-15b-5p;hsa-miR-195-5
ENSG0000	ENSG0000	0.000891	hsa-miR-1207-5p;hsa-miR-1267;hsa-miR-17-5p;hsa-miR-20b-
ENSG0000	ENSG0000	0.00159	hsa-miR-17-5p;hsa-miR-20b-5p;hsa-miR-27a-3p;hsa-miR-27b-
ENSG0000	ENSG0000	0.000257	hsa-miR-107;hsa-miR-126-5p;hsa-miR-15a-5p;hsa-miR-15b-5
ENSG0000	ENSG0000	0.003334	hsa-miR-132-3p;hsa-miR-138-5p;hsa-miR-17-5p;hsa-miR-182-
ENSG0000	ENSG0000	0.000903	hsa-miR-15a-5p;hsa-miR-15b-5p;hsa-miR-17-5p;hsa-miR-195-
ENSG0000	ENSG0000	0.005816	hsa-miR-138-5p;hsa-miR-15a-5p;hsa-miR-15b-5p;hsa-miR-16-
ENSG0000	ENSG0000	3.58E-05	hsa-miR-15a-5p;hsa-miR-15b-5p;hsa-miR-16-5p;hsa-miR-195-
ENSG0000	ENSG0000	0.000721	hsa-miR-107;hsa-miR-15a-5p;hsa-miR-15b-5p;hsa-miR-17-5p;
ENSG0000	ENSG0000	2.52E-05	hsa-miR-101-3p;hsa-miR-137;hsa-miR-15a-5p;hsa-miR-15b-5
ENSG0000	ENSG0000	0.000171	hsa-miR-1267;hsa-miR-129-1-3p;hsa-miR-129-2-3p;hsa-miR-
ENSG0000	ENSG0000	0.007298	hsa-miR-15a-5p;hsa-miR-15b-5p;hsa-miR-16-5p;hsa-miR-195-
ENSG0000	ENSG0000	0.004309	hsa-miR-103a-3p;hsa-miR-107;hsa-miR-17-3p;hsa-miR-17-5p;
ENSG0000	ENSG0000	0.004309	hsa-miR-103a-3p;hsa-miR-107;hsa-miR-17-3p;hsa-miR-17-5p;
ENSG0000	ENSG0000	7.79E-05	hsa-miR-101-3p;hsa-miR-137;hsa-miR-143-3p;hsa-miR-16-5p;
ENSG0000	ENSG0000	3.58E-05	hsa-miR-135a-5p;hsa-miR-138-5p;hsa-miR-193a-3p;hsa-miR-
ENSG0000	ENSG0000	0.003916	hsa-miR-30b-3p;hsa-miR-30c-1-3p;hsa-miR-30c-2-3p;hsa-mi
ENSG0000	ENSG0000	0.001236	hsa-miR-103a-3p;hsa-miR-107;hsa-miR-17-5p;hsa-miR-20b-5
ENSG0000	ENSG0000	0.003805	hsa-miR-1207-5p;hsa-miR-124-3p;hsa-miR-1267;hsa-miR-197-
ENSG0000	ENSG0000	8.45E-06	hsa-miR-135a-5p;hsa-miR-15a-5p;hsa-miR-15b-5p;hsa-miR-1

ENSG0000	ENSG0000	8.45E-06	hsa-miR-135a-5p;hsa-miR-15a-5p;hsa-miR-15b-5p;hsa-miR-16
ENSG0000	ENSG0000	0.00827	hsa-miR-15a-5p;hsa-miR-15b-5p;hsa-miR-16-5p;hsa-miR-195-
ENSG0000	ENSG0000	0.004799	hsa-miR-27a-3p;hsa-miR-27b-3p;hsa-miR-378a-3p;hsa-miR-3
ENSG0000	ENSG0000	0.000721	hsa-miR-132-3p;hsa-miR-17-5p;hsa-miR-212-3p;hsa-miR-23b-
ENSG0000	ENSG0000	0.002131	hsa-miR-101-3p;hsa-miR-103a-3p;hsa-miR-107;hsa-miR-1260a
ENSG0000	ENSG0000	0.003653	hsa-miR-378a-3p;hsa-miR-378c;hsa-miR-378d;hsa-miR-378i;hs
ENSG0000	ENSG0000	0.001877	hsa-miR-17-3p;hsa-miR-182-5p;hsa-miR-183-5p;hsa-miR-21-
ENSG0000	ENSG0000	0.004931	hsa-miR-15a-5p;hsa-miR-15b-5p;hsa-miR-16-5p;hsa-miR-195-
ENSG0000	ENSG0000	0.004931	hsa-miR-15a-5p;hsa-miR-15b-5p;hsa-miR-16-5p;hsa-miR-195-
ENSG0000	ENSG0000	0.000231	hsa-miR-15a-5p;hsa-miR-15b-5p;hsa-miR-16-5p;hsa-miR-195-
ENSG0000	ENSG0000	0.004931	hsa-miR-1260a;hsa-miR-1260b;hsa-miR-143-3p;hsa-miR-29b-
ENSG0000	ENSG0000	0.000425	hsa-miR-103a-3p;hsa-miR-107;hsa-miR-15a-5p;hsa-miR-15b-
ENSG0000	ENSG0000	0.006532	hsa-miR-107;hsa-miR-15a-5p;hsa-miR-15b-5p;hsa-miR-195-5p
ENSG0000	ENSG0000	0.004931	hsa-miR-17-3p;hsa-miR-17-5p;hsa-miR-3065-5p;hsa-miR-31-
ENSG0000	ENSG0000	0.000391	hsa-miR-107;hsa-miR-15a-5p;hsa-miR-15b-5p;hsa-miR-195-5p
ENSG0000	ENSG0000	4.25E-07	hsa-miR-30a-3p;hsa-miR-30b-3p;hsa-miR-30c-1-3p;hsa-miR-
ENSG0000	ENSG0000	0.000346	hsa-miR-103a-3p;hsa-miR-107;hsa-miR-15a-5p;hsa-miR-15b-
ENSG0000	ENSG0000	2.34E-07	hsa-miR-15a-5p;hsa-miR-15b-5p;hsa-miR-16-5p;hsa-miR-195-
ENSG0000	ENSG0000	0.008255	hsa-miR-204-5p;hsa-miR-30a-3p;hsa-miR-30d-3p;hsa-miR-30
ENSG0000	ENSG0000	0.00863	hsa-miR-124-3p;hsa-miR-129-1-3p;hsa-miR-129-2-3p;hsa-mi
ENSG0000	ENSG0000	0.003378	hsa-miR-15a-5p;hsa-miR-15b-5p;hsa-miR-195-5p;hsa-miR-19
ENSG0000	ENSG0000	0.006414	hsa-miR-15a-5p;hsa-miR-15b-5p;hsa-miR-16-5p;hsa-miR-195-
ENSG0000	ENSG0000	4.29E-05	hsa-miR-15a-5p;hsa-miR-15b-5p;hsa-miR-16-5p;hsa-miR-195-
ENSG0000	ENSG0000	0.001191	hsa-miR-222-5p;hsa-miR-30a-3p;hsa-miR-30d-3p;hsa-miR-30
ENSG0000	ENSG0000	0.009334	hsa-miR-125b-2-3p;hsa-miR-3065-3p;hsa-miR-30b-3p;hsa-mi
ENSG0000	ENSG0000	1.25E-05	hsa-miR-124-3p;hsa-miR-142-3p;hsa-miR-15a-5p;hsa-miR-15
ENSG0000	ENSG0000	1.82E-05	hsa-miR-101-3p;hsa-miR-103a-3p;hsa-miR-107;hsa-miR-15a-
ENSG0000	ENSG0000	0.000756	hsa-miR-107;hsa-miR-15a-5p;hsa-miR-15b-5p;hsa-miR-195-5p
ENSG0000	ENSG0000	0.005818	hsa-miR-132-3p;hsa-miR-17-5p;hsa-miR-1976;hsa-miR-20b-5p
ENSG0000	ENSG0000	0.000764	hsa-miR-17-5p;hsa-miR-204-5p;hsa-miR-20b-5p;hsa-miR-93-
ENSG0000	ENSG0000	0.00863	hsa-miR-1226-3p;hsa-miR-138-5p;hsa-miR-143-3p;hsa-miR-1
ENSG0000	ENSG0000	8.61E-06	hsa-miR-103a-3p;hsa-miR-107;hsa-miR-15a-5p;hsa-miR-15b-
ENSG0000	ENSG0000	0.007579	hsa-miR-197-3p;hsa-miR-224-5p;hsa-miR-3065-3p;hsa-miR-3
ENSG0000	ENSG0000	0.000409	hsa-miR-15a-5p;hsa-miR-15b-5p;hsa-miR-16-5p;hsa-miR-195-
ENSG0000	ENSG0000	0.005267	hsa-miR-124-3p;hsa-miR-193a-3p;hsa-miR-193b-3p;hsa-miR-
ENSG0000	ENSG0000	0.002532	hsa-miR-1207-5p;hsa-miR-140-3p;hsa-miR-15a-5p;hsa-miR-1
ENSG0000	ENSG0000	0.004042	hsa-miR-15a-5p;hsa-miR-15b-5p;hsa-miR-16-5p;hsa-miR-195-
ENSG0000	ENSG0000	0.000637	hsa-miR-1293;hsa-miR-15a-5p;hsa-miR-15b-5p;hsa-miR-16-5p
ENSG0000	ENSG0000	0.005654	hsa-miR-126-5p;hsa-miR-15a-5p;hsa-miR-15b-5p;hsa-miR-16-
ENSG0000	ENSG0000	0.005267	hsa-miR-1254;hsa-miR-214-3p;hsa-miR-361-3p;hsa-miR-3619-
ENSG0000	ENSG0000	0.009064	hsa-miR-143-3p;hsa-miR-15a-5p;hsa-miR-15b-5p;hsa-miR-16-
ENSG0000	ENSG0000	0.008453	hsa-miR-103a-3p;hsa-miR-107;hsa-miR-30b-3p;hsa-miR-30c-1
ENSG0000	ENSG0000	0.008373	hsa-miR-15a-5p;hsa-miR-15b-5p;hsa-miR-16-5p;hsa-miR-17-
ENSG0000	ENSG0000	8.27E-05	hsa-miR-15a-5p;hsa-miR-15b-5p;hsa-miR-16-5p;hsa-miR-195-
ENSG0000	ENSG0000	0.000231	hsa-miR-17-5p;hsa-miR-20b-5p;hsa-miR-7-5p;hsa-miR-93-5p
ENSG0000	ENSG0000	3.49E-07	hsa-miR-107;hsa-miR-126-5p;hsa-miR-1293;hsa-miR-15a-5p;h
ENSG0000	ENSG0000	6.40E-05	hsa-miR-125b-2-3p;hsa-miR-1267;hsa-miR-140-3p;hsa-miR-1
ENSG0000	ENSG0000	0.000933	hsa-miR-142-3p;hsa-miR-186-3p;hsa-miR-30a-3p;hsa-miR-30
ENSG0000	ENSG0000	0.002799	hsa-miR-15a-5p;hsa-miR-15b-5p;hsa-miR-16-5p;hsa-miR-195-
ENSG0000	ENSG0000	5.23E-05	hsa-miR-15a-5p;hsa-miR-15b-5p;hsa-miR-16-5p;hsa-miR-186-
ENSG0000	ENSG0000	0.001726	hsa-miR-1207-5p;hsa-miR-1260a;hsa-miR-1260b;hsa-miR-140-
ENSG0000	ENSG0000	0.001221	hsa-miR-204-5p;hsa-miR-224-5p;hsa-miR-335-5p;hsa-miR-9-
ENSG0000	ENSG0000	0.006271	hsa-miR-107;hsa-miR-15a-5p;hsa-miR-16-5p;hsa-miR-17-3p;h
ENSG0000	ENSG0000	7.47E-06	hsa-miR-15a-5p;hsa-miR-15b-5p;hsa-miR-16-5p;hsa-miR-195-

ENSG0000	ENSG0000	8.45E-06	hsa-miR-107;hsa-miR-15a-5p;hsa-miR-15b-5p;hsa-miR-16-5p;
ENSG0000	ENSG0000	0.000779	hsa-miR-15a-5p;hsa-miR-15b-5p;hsa-miR-16-5p;hsa-miR-17-5
ENSG0000	ENSG0000	0.004042	hsa-miR-1293;hsa-miR-15a-5p;hsa-miR-15b-5p;hsa-miR-195-5
ENSG0000	ENSG0000	0.006452	hsa-miR-101-3p;hsa-miR-17-5p;hsa-miR-20b-5p;hsa-miR-93-5
ENSG0000	ENSG0000	0.000625	hsa-miR-142-3p;hsa-miR-15a-5p;hsa-miR-15b-5p;hsa-miR-16-
ENSG0000	ENSG0000	0.008373	hsa-miR-101-3p;hsa-miR-30b-3p;hsa-miR-30c-1-3p;hsa-miR-3
ENSG0000	ENSG0000	1.39E-05	hsa-miR-1267;hsa-miR-15a-5p;hsa-miR-15b-5p;hsa-miR-16-5p
ENSG0000	ENSG0000	0.00827	hsa-miR-1243;hsa-miR-23a-3p;hsa-miR-23b-3p;hsa-miR-567;h
ENSG0000	ENSG0000	0.00827	hsa-miR-1243;hsa-miR-23a-3p;hsa-miR-23b-3p;hsa-miR-567;h
ENSG0000	ENSG0000	0.000521	hsa-miR-15a-5p;hsa-miR-15b-5p;hsa-miR-195-5p;hsa-miR-424
ENSG0000	ENSG0000	1.80E-07	hsa-miR-1267;hsa-miR-129-1-3p;hsa-miR-129-2-3p;hsa-miR-1
ENSG0000	ENSG0000	8.28E-05	hsa-miR-1207-5p;hsa-miR-214-3p;hsa-miR-29b-2-5p;hsa-miR-
ENSG0000	ENSG0000	0.000349	hsa-miR-30a-3p;hsa-miR-30b-3p;hsa-miR-34a-5p;hsa-miR-935
ENSG0000	ENSG0000	0.00159	hsa-miR-103a-3p;hsa-miR-107;hsa-miR-124-3p;hsa-miR-17-3p
ENSG0000	ENSG0000	0.005655	hsa-miR-15a-5p;hsa-miR-15b-5p;hsa-miR-195-5p;hsa-miR-23a
ENSG0000	ENSG0000	0.000764	hsa-miR-15a-5p;hsa-miR-15b-5p;hsa-miR-16-5p;hsa-miR-195-
ENSG0000	ENSG0000	3.99E-06	hsa-miR-17-5p;hsa-miR-197-3p;hsa-miR-20b-5p;hsa-miR-27a-
ENSG0000	ENSG0000	3.99E-06	hsa-miR-17-5p;hsa-miR-197-3p;hsa-miR-20b-5p;hsa-miR-27a-
ENSG0000	ENSG0000	3.99E-06	hsa-miR-17-5p;hsa-miR-197-3p;hsa-miR-20b-5p;hsa-miR-27a-
ENSG0000	ENSG0000	3.99E-06	hsa-miR-17-5p;hsa-miR-197-3p;hsa-miR-20b-5p;hsa-miR-27a-
ENSG0000	ENSG0000	3.99E-06	hsa-miR-17-5p;hsa-miR-197-3p;hsa-miR-20b-5p;hsa-miR-27a-
ENSG0000	ENSG0000	3.99E-06	hsa-miR-17-5p;hsa-miR-197-3p;hsa-miR-20b-5p;hsa-miR-27a-
ENSG0000	ENSG0000	0.000888	hsa-miR-101-3p;hsa-miR-143-3p;hsa-miR-17-5p;hsa-miR-204-
ENSG0000	ENSG0000	7.47E-06	hsa-miR-15a-5p;hsa-miR-15b-5p;hsa-miR-16-5p;hsa-miR-195-
ENSG0000	ENSG0000	8.61E-06	hsa-miR-1207-5p;hsa-miR-1226-3p;hsa-miR-15a-5p;hsa-miR-1
ENSG0000	ENSG0000	0.000764	hsa-miR-17-5p;hsa-miR-20b-5p;hsa-miR-34a-5p;hsa-miR-93-5
ENSG0000	ENSG0000	0.000764	hsa-miR-17-5p;hsa-miR-20b-5p;hsa-miR-34a-5p;hsa-miR-93-5
ENSG0000	ENSG0000	0.001376	hsa-miR-15a-5p;hsa-miR-15b-5p;hsa-miR-17-5p;hsa-miR-186-
ENSG0000	ENSG0000	1.69E-05	hsa-miR-103a-3p;hsa-miR-107;hsa-miR-15a-5p;hsa-miR-15b-5
ENSG0000	ENSG0000	8.65E-05	hsa-miR-144-5p;hsa-miR-15a-5p;hsa-miR-15b-5p;hsa-miR-195
ENSG0000	ENSG0000	1.30E-06	hsa-miR-15a-5p;hsa-miR-15b-5p;hsa-miR-16-5p;hsa-miR-195-
ENSG0000	ENSG0000	0.002342	hsa-miR-17-5p;hsa-miR-182-5p;hsa-miR-183-5p;hsa-miR-20b-
ENSG0000	ENSG0000	0.001042	hsa-miR-142-3p;hsa-miR-183-5p;hsa-miR-23a-3p;hsa-miR-27a
ENSG0000	ENSG0000	3.23E-05	hsa-miR-103a-3p;hsa-miR-107;hsa-miR-15a-5p;hsa-miR-15b-5
ENSG0000	ENSG0000	0.000135	hsa-miR-103a-3p;hsa-miR-107;hsa-miR-15a-5p;hsa-miR-15b-5
ENSG0000	ENSG0000	0.000425	hsa-miR-103a-3p;hsa-miR-107;hsa-miR-15a-5p;hsa-miR-15b-5
ENSG0000	ENSG0000	0.000442	hsa-miR-15a-5p;hsa-miR-15b-5p;hsa-miR-16-5p;hsa-miR-195-
ENSG0000	ENSG0000	8.11E-05	hsa-miR-103a-3p;hsa-miR-107;hsa-miR-148b-5p;hsa-miR-15a-
ENSG0000	ENSG0000	0.000278	hsa-miR-124-3p;hsa-miR-15a-5p;hsa-miR-15b-5p;hsa-miR-16-
ENSG0000	ENSG0000	0.000949	hsa-miR-17-5p;hsa-miR-186-3p;hsa-miR-1976;hsa-miR-20b-5p
ENSG0000	ENSG0000	0.007204	hsa-miR-378a-3p;hsa-miR-378c;hsa-miR-378d;hsa-miR-378i;hs
ENSG0000	ENSG0000	0.000719	hsa-miR-101-3p;hsa-miR-107;hsa-miR-151a-5p;hsa-miR-15a-5
ENSG0000	ENSG0000	1.64E-06	hsa-miR-15a-5p;hsa-miR-15b-5p;hsa-miR-16-5p;hsa-miR-195-
ENSG0000	ENSG0000	2.48E-06	hsa-miR-132-3p;hsa-miR-15a-5p;hsa-miR-15b-5p;hsa-miR-16-
ENSG0000	ENSG0000	0.000721	hsa-miR-107;hsa-miR-15a-5p;hsa-miR-15b-5p;hsa-miR-195-5p
ENSG0000	ENSG0000	0.004237	hsa-miR-15a-5p;hsa-miR-15b-5p;hsa-miR-16-5p;hsa-miR-195-
ENSG0000	ENSG0000	0.009076	hsa-miR-132-3p;hsa-miR-212-3p;hsa-miR-27a-3p;hsa-miR-27b
ENSG0000	ENSG0000	0.000408	hsa-miR-101-3p;hsa-miR-103a-3p;hsa-miR-107;hsa-miR-1260a
ENSG0000	ENSG0000	0.001237	hsa-miR-103a-3p;hsa-miR-107;hsa-miR-1976;hsa-miR-204-5p;
ENSG0000	ENSG0000	0.001237	hsa-miR-103a-3p;hsa-miR-107;hsa-miR-1976;hsa-miR-204-5p;
ENSG0000	ENSG0000	0.001221	hsa-miR-1226-3p;hsa-miR-21-3p;hsa-miR-27a-3p;hsa-miR-27b
ENSG0000	ENSG0000	0.000538	hsa-miR-142-3p;hsa-miR-15a-5p;hsa-miR-15b-5p;hsa-miR-16-
ENSG0000	ENSG0000	0.000578	hsa-miR-15a-5p;hsa-miR-15b-5p;hsa-miR-195-5p;hsa-miR-374
ENSG0000	ENSG0000	0.000628	hsa-miR-137;hsa-miR-140-3p;hsa-miR-15a-5p;hsa-miR-15b-5p

ENSG0000	ENSG0000	0.001221	hsa-miR-15a-5p;hsa-miR-15b-5p;hsa-miR-16-5p;hsa-miR-195-
ENSG0000	ENSG0000	6.27E-07	hsa-miR-15a-5p;hsa-miR-15b-5p;hsa-miR-16-5p;hsa-miR-195-
ENSG0000	ENSG0000	5.38E-05	hsa-miR-126-5p;hsa-miR-137;hsa-miR-15a-5p;hsa-miR-15b-5
ENSG0000	ENSG0000	0.000118	hsa-miR-15a-5p;hsa-miR-15b-5p;hsa-miR-16-5p;hsa-miR-195-
ENSG0000	ENSG0000	0.000118	hsa-miR-15a-5p;hsa-miR-15b-5p;hsa-miR-16-5p;hsa-miR-195-
ENSG0000	ENSG0000	5.08E-05	hsa-miR-101-3p;hsa-miR-1207-5p;hsa-miR-148b-5p;hsa-miR-
ENSG0000	ENSG0000	0.004394	hsa-miR-1260a;hsa-miR-1260b;hsa-miR-1293;hsa-miR-30b-3p;
ENSG0000	ENSG0000	0.00392	hsa-miR-126-5p;hsa-miR-16-5p;hsa-miR-193a-3p;hsa-miR-19
ENSG0000	ENSG0000	0.00083	hsa-miR-197-3p;hsa-miR-20b-5p;hsa-miR-224-5p;hsa-miR-34
ENSG0000	ENSG0000	0.006003	hsa-miR-138-5p;hsa-miR-17-5p;hsa-miR-204-5p;hsa-miR-20b-
ENSG0000	ENSG0000	0.000748	hsa-miR-15a-5p;hsa-miR-15b-5p;hsa-miR-195-5p;hsa-miR-53
ENSG0000	ENSG0000	0.004931	hsa-miR-132-3p;hsa-miR-212-3p;hsa-miR-2277-3p;hsa-miR-7
ENSG0000	ENSG0000	0.000204	hsa-miR-137;hsa-miR-15a-5p;hsa-miR-15b-5p;hsa-miR-16-5p;
ENSG0000	ENSG0000	0.006617	hsa-miR-15a-5p;hsa-miR-15b-5p;hsa-miR-16-5p;hsa-miR-195-
ENSG0000	ENSG0000	4.13E-05	hsa-miR-15a-5p;hsa-miR-15b-5p;hsa-miR-16-5p;hsa-miR-195-
ENSG0000	ENSG0000	0.009294	hsa-miR-17-3p;hsa-miR-30b-3p;hsa-miR-30c-1-3p;hsa-miR-3
ENSG0000	ENSG0000	0.000108	hsa-miR-15a-5p;hsa-miR-15b-5p;hsa-miR-16-5p;hsa-miR-17-
ENSG0000	ENSG0000	0.005655	hsa-miR-124-3p;hsa-miR-30b-3p;hsa-miR-30c-1-3p;hsa-miR-
ENSG0000	ENSG0000	0.001091	hsa-miR-15b-5p;hsa-miR-16-5p;hsa-miR-195-5p;hsa-miR-424-
ENSG0000	ENSG0000	0.00392	hsa-miR-15a-5p;hsa-miR-15b-5p;hsa-miR-16-5p;hsa-miR-193
ENSG0000	ENSG0000	0.00392	hsa-miR-15a-5p;hsa-miR-15b-5p;hsa-miR-16-5p;hsa-miR-193
ENSG0000	ENSG0000	0.004931	hsa-miR-15a-5p;hsa-miR-15b-5p;hsa-miR-16-5p;hsa-miR-195-
ENSG0000	ENSG0000	0.000578	hsa-miR-15a-5p;hsa-miR-15b-5p;hsa-miR-195-5p;hsa-miR-36
ENSG0000	ENSG0000	0.000602	hsa-miR-15a-5p;hsa-miR-15b-5p;hsa-miR-16-5p;hsa-miR-181
ENSG0000	ENSG0000	0.00873	hsa-miR-101-3p;hsa-miR-143-3p;hsa-miR-15a-5p;hsa-miR-15
ENSG0000	ENSG0000	0.003117	hsa-miR-103a-3p;hsa-miR-107;hsa-miR-23a-3p;hsa-miR-23b-
ENSG0000	ENSG0000	0.00827	hsa-miR-23a-3p;hsa-miR-23b-3p;hsa-miR-30a-3p;hsa-miR-30
ENSG0000	ENSG0000	2.52E-05	hsa-miR-15a-5p;hsa-miR-15b-5p;hsa-miR-16-5p;hsa-miR-195-
ENSG0000	ENSG0000	0.007311	hsa-miR-1293;hsa-miR-15a-5p;hsa-miR-15b-5p;hsa-miR-16-5
ENSG0000	ENSG0000	0.003653	hsa-miR-1226-3p;hsa-miR-17-5p;hsa-miR-182-5p;hsa-miR-18
ENSG0000	ENSG0000	0.001907	hsa-miR-15a-5p;hsa-miR-15b-5p;hsa-miR-16-5p;hsa-miR-195-
ENSG0000	ENSG0000	0.001907	hsa-miR-15a-5p;hsa-miR-15b-5p;hsa-miR-16-5p;hsa-miR-195-
ENSG0000	ENSG0000	0.00863	hsa-miR-15a-5p;hsa-miR-15b-5p;hsa-miR-16-5p;hsa-miR-195-
ENSG0000	ENSG0000	0.000299	hsa-miR-15a-5p;hsa-miR-15b-5p;hsa-miR-16-5p;hsa-miR-195-
ENSG0000	ENSG0000	0.001877	hsa-miR-1207-5p;hsa-miR-129-1-3p;hsa-miR-129-2-3p;hsa-m
ENSG0000	ENSG0000	0.001877	hsa-miR-1207-5p;hsa-miR-129-1-3p;hsa-miR-129-2-3p;hsa-m
ENSG0000	ENSG0000	4.23E-07	hsa-miR-1260a;hsa-miR-1260b;hsa-miR-15a-5p;hsa-miR-15b-
ENSG0000	ENSG0000	1.39E-07	hsa-miR-1260a;hsa-miR-1260b;hsa-miR-15a-5p;hsa-miR-15b-
ENSG0000	ENSG0000	9.89E-06	hsa-miR-103a-3p;hsa-miR-107;hsa-miR-15a-5p;hsa-miR-15b-
ENSG0000	ENSG0000	0.000903	hsa-miR-1254;hsa-miR-15a-5p;hsa-miR-15b-5p;hsa-miR-195-
ENSG0000	ENSG0000	0.000891	hsa-miR-15a-5p;hsa-miR-15b-5p;hsa-miR-16-5p;hsa-miR-186-
ENSG0000	ENSG0000	2.03E-05	hsa-miR-103a-3p;hsa-miR-107;hsa-miR-148a-5p;hsa-miR-15a-
ENSG0000	ENSG0000	2.03E-05	hsa-miR-103a-3p;hsa-miR-107;hsa-miR-148a-5p;hsa-miR-15a-
ENSG0000	ENSG0000	1.46E-06	hsa-miR-107;hsa-miR-1260a;hsa-miR-1260b;hsa-miR-15a-5p;h
ENSG0000	ENSG0000	0.003133	hsa-miR-15a-5p;hsa-miR-15b-5p;hsa-miR-16-5p;hsa-miR-195-
ENSG0000	ENSG0000	0.000764	hsa-miR-15a-5p;hsa-miR-15b-5p;hsa-miR-16-5p;hsa-miR-195-
ENSG0000	ENSG0000	1.10E-06	hsa-miR-103a-3p;hsa-miR-107;hsa-miR-15a-5p;hsa-miR-15b-
ENSG0000	ENSG0000	5.38E-05	hsa-miR-107;hsa-miR-15a-5p;hsa-miR-15b-5p;hsa-miR-17-5p;
ENSG0000	ENSG0000	0.000442	hsa-miR-142-3p;hsa-miR-17-5p;hsa-miR-20b-5p;hsa-miR-93-
ENSG0000	ENSG0000	0.003504	hsa-miR-1207-5p;hsa-miR-140-3p;hsa-miR-15a-5p;hsa-miR-1
ENSG0000	ENSG0000	0.003504	hsa-miR-1207-5p;hsa-miR-140-3p;hsa-miR-15a-5p;hsa-miR-1
ENSG0000	ENSG0000	1.30E-06	hsa-miR-15a-5p;hsa-miR-15b-5p;hsa-miR-16-5p;hsa-miR-195-
ENSG0000	ENSG0000	0.002649	hsa-miR-31-5p;hsa-miR-335-5p;hsa-miR-501-5p;hsa-miR-9-5
ENSG0000	ENSG0000	2.25E-07	hsa-miR-135a-5p;hsa-miR-15a-5p;hsa-miR-15b-5p;hsa-miR-1

ENSG0000	ENSG0000	0.004931	hsa-miR-124-3p;hsa-miR-186-3p;hsa-miR-23a-3p;hsa-miR-23
ENSG0000	ENSG0000	0.002865	hsa-miR-103a-3p;hsa-miR-107;hsa-miR-15a-5p;hsa-miR-15b-
ENSG0000	ENSG0000	8.45E-06	hsa-miR-103a-3p;hsa-miR-107;hsa-miR-15a-5p;hsa-miR-15b-
ENSG0000	ENSG0000	3.35E-05	hsa-miR-124-3p;hsa-miR-15a-5p;hsa-miR-15b-5p;hsa-miR-16
ENSG0000	ENSG0000	0.004799	hsa-miR-15a-5p;hsa-miR-15b-5p;hsa-miR-16-5p;hsa-miR-195
ENSG0000	ENSG0000	7.57E-06	hsa-miR-15a-5p;hsa-miR-15b-5p;hsa-miR-16-5p;hsa-miR-195
ENSG0000	ENSG0000	0.000721	hsa-miR-15a-5p;hsa-miR-15b-5p;hsa-miR-16-5p;hsa-miR-195
ENSG0000	ENSG0000	0.004931	hsa-miR-15a-5p;hsa-miR-15b-5p;hsa-miR-195-5p;hsa-miR-34
ENSG0000	ENSG0000	0.00159	hsa-miR-15a-5p;hsa-miR-15b-5p;hsa-miR-16-5p;hsa-miR-186
ENSG0000	ENSG0000	0.001907	hsa-miR-15a-5p;hsa-miR-15b-5p;hsa-miR-16-5p;hsa-miR-195
ENSG0000	ENSG0000	0.000548	hsa-miR-132-3p;hsa-miR-138-5p;hsa-miR-23b-3p;hsa-miR-34
ENSG0000	ENSG0000	0.002614	hsa-miR-17-5p;hsa-miR-186-3p;hsa-miR-20b-5p;hsa-miR-30b
ENSG0000	ENSG0000	8.63E-05	hsa-miR-125b-2-3p;hsa-miR-138-5p;hsa-miR-15a-5p;hsa-miR
ENSG0000	ENSG0000	0.000299	hsa-miR-103a-3p;hsa-miR-107;hsa-miR-1260a;hsa-miR-1260b;
ENSG0000	ENSG0000	0.000442	hsa-miR-30a-3p;hsa-miR-30d-3p;hsa-miR-30e-3p;hsa-miR-54
ENSG0000	ENSG0000	0.003097	hsa-miR-140-3p;hsa-miR-182-5p;hsa-miR-183-5p;hsa-miR-19
ENSG0000	ENSG0000	4.45E-06	hsa-miR-107;hsa-miR-124-3p;hsa-miR-1267;hsa-miR-15a-5p;h
ENSG0000	ENSG0000	8.45E-06	hsa-miR-15a-5p;hsa-miR-15b-5p;hsa-miR-16-5p;hsa-miR-195
ENSG0000	ENSG0000	0.003133	hsa-miR-101-3p;hsa-miR-140-3p;hsa-miR-15a-5p;hsa-miR-16
ENSG0000	ENSG0000	5.38E-05	hsa-miR-15a-5p;hsa-miR-15b-5p;hsa-miR-195-5p;hsa-miR-30
ENSG0000	ENSG0000	0.000299	hsa-miR-142-3p;hsa-miR-15a-5p;hsa-miR-15b-5p;hsa-miR-17
ENSG0000	ENSG0000	0.0015	hsa-miR-30a-3p;hsa-miR-30d-3p;hsa-miR-30e-3p;hsa-miR-45
ENSG0000	ENSG0000	0.000748	hsa-miR-15a-5p;hsa-miR-15b-5p;hsa-miR-195-5p;hsa-miR-42
ENSG0000	ENSG0000	0.003671	hsa-miR-15a-5p;hsa-miR-15b-5p;hsa-miR-16-5p;hsa-miR-195
ENSG0000	ENSG0000	0.002845	hsa-miR-17-5p;hsa-miR-20b-5p;hsa-miR-30b-3p;hsa-miR-30c
ENSG0000	ENSG0000	0.003634	hsa-miR-103a-3p;hsa-miR-107;hsa-miR-15a-5p;hsa-miR-15b-
ENSG0000	ENSG0000	1.39E-06	hsa-miR-15a-5p;hsa-miR-15b-5p;hsa-miR-195-5p;hsa-miR-21
ENSG0000	ENSG0000	0.001292	hsa-miR-101-3p;hsa-miR-15a-5p;hsa-miR-15b-5p;hsa-miR-19
ENSG0000	ENSG0000	0.0015	hsa-miR-15a-5p;hsa-miR-15b-5p;hsa-miR-16-5p;hsa-miR-195
ENSG0000	ENSG0000	7.47E-06	hsa-miR-15a-5p;hsa-miR-15b-5p;hsa-miR-16-5p;hsa-miR-195
ENSG0000	ENSG0000	0.000299	hsa-miR-183-5p;hsa-miR-27a-3p;hsa-miR-27b-3p;hsa-miR-34
ENSG0000	ENSG0000	6.75E-05	hsa-miR-1207-5p;hsa-miR-135a-5p;hsa-miR-15a-5p;hsa-miR-
ENSG0000	ENSG0000	0.002545	hsa-miR-16-5p;hsa-miR-186-3p;hsa-miR-204-5p;hsa-miR-30b
ENSG0000	ENSG0000	0.002545	hsa-miR-16-5p;hsa-miR-186-3p;hsa-miR-204-5p;hsa-miR-30b
ENSG0000	ENSG0000	1.78E-05	hsa-miR-15a-5p;hsa-miR-15b-5p;hsa-miR-16-5p;hsa-miR-195
ENSG0000	ENSG0000	0.000359	hsa-miR-129-1-3p;hsa-miR-129-2-3p;hsa-miR-214-3p;hsa-mi
ENSG0000	ENSG0000	0.003133	hsa-miR-142-3p;hsa-miR-15a-5p;hsa-miR-15b-5p;hsa-miR-16
ENSG0000	ENSG0000	0.00889	hsa-miR-124-3p;hsa-miR-1267;hsa-miR-204-5p;hsa-miR-27a-
ENSG0000	ENSG0000	0.004075	hsa-miR-125b-2-3p;hsa-miR-17-5p;hsa-miR-20b-5p;hsa-miR-
ENSG0000	ENSG0000	0.000429	hsa-miR-129-1-3p;hsa-miR-129-2-3p;hsa-miR-15a-5p;hsa-mi
ENSG0000	ENSG0000	0.000748	hsa-miR-103a-3p;hsa-miR-107;hsa-miR-132-3p;hsa-miR-212-
ENSG0000	ENSG0000	0.008373	hsa-miR-103a-3p;hsa-miR-107;hsa-miR-15a-5p;hsa-miR-15b-
ENSG0000	ENSG0000	3.96E-06	hsa-miR-15a-5p;hsa-miR-15b-5p;hsa-miR-16-5p;hsa-miR-195
ENSG0000	ENSG0000	0.003378	hsa-miR-138-5p;hsa-miR-15a-5p;hsa-miR-15b-5p;hsa-miR-19
ENSG0000	ENSG0000	0.000432	hsa-miR-103a-3p;hsa-miR-107;hsa-miR-140-3p;hsa-miR-15a-
ENSG0000	ENSG0000	3.80E-05	hsa-miR-103a-3p;hsa-miR-107;hsa-miR-15a-5p;hsa-miR-15b-
ENSG0000	ENSG0000	0.000203	hsa-miR-15a-5p;hsa-miR-15b-5p;hsa-miR-195-5p;hsa-miR-30
ENSG0000	ENSG0000	0.004075	hsa-miR-1207-5p;hsa-miR-135b-3p;hsa-miR-17-5p;hsa-miR-2
ENSG0000	ENSG0000	0.004127	hsa-miR-143-3p;hsa-miR-17-5p;hsa-miR-183-5p;hsa-miR-20b
ENSG0000	ENSG0000	0.004042	hsa-miR-30a-3p;hsa-miR-30d-3p;hsa-miR-30e-3p;hsa-miR-31
ENSG0000	ENSG0000	0.004042	hsa-miR-30a-3p;hsa-miR-30d-3p;hsa-miR-30e-3p;hsa-miR-31
ENSG0000	ENSG0000	0.003133	hsa-miR-15a-5p;hsa-miR-16-5p;hsa-miR-29b-2-5p;hsa-miR-5
ENSG0000	ENSG0000	1.00E-05	hsa-miR-15a-5p;hsa-miR-15b-5p;hsa-miR-16-5p;hsa-miR-195
ENSG0000	ENSG0000	0.007298	hsa-miR-142-3p;hsa-miR-183-5p;hsa-miR-23a-3p;hsa-miR-23

ENSG0000	ENSG0000	2.92E-05	hsa-miR-15a-5p;hsa-miR-15b-5p;hsa-miR-16-5p;hsa-miR-195-
ENSG0000	ENSG0000	0.006452	hsa-miR-17-3p;hsa-miR-186-3p;hsa-miR-589-3p;hsa-miR-766-
ENSG0000	ENSG0000	2.46E-05	hsa-miR-15a-5p;hsa-miR-15b-5p;hsa-miR-16-5p;hsa-miR-195-
ENSG0000	ENSG0000	0.000103	hsa-miR-15a-5p;hsa-miR-15b-5p;hsa-miR-16-5p;hsa-miR-195-
ENSG0000	ENSG0000	5.61E-05	hsa-miR-124-3p;hsa-miR-186-3p;hsa-miR-30b-3p;hsa-miR-30
ENSG0000	ENSG0000	5.38E-05	hsa-miR-132-3p;hsa-miR-140-3p;hsa-miR-197-3p;hsa-miR-21
ENSG0000	ENSG0000	0.004042	hsa-miR-17-5p;hsa-miR-20b-5p;hsa-miR-222-5p;hsa-miR-30a-
ENSG0000	ENSG0000	0.007298	hsa-miR-148b-5p;hsa-miR-183-5p;hsa-miR-20b-5p;hsa-miR-3
ENSG0000	ENSG0000	0.007298	hsa-miR-148b-5p;hsa-miR-183-5p;hsa-miR-20b-5p;hsa-miR-3
ENSG0000	ENSG0000	0.000521	hsa-miR-15a-5p;hsa-miR-15b-5p;hsa-miR-16-5p;hsa-miR-195-
ENSG0000	ENSG0000	0.006617	hsa-miR-1260a;hsa-miR-1260b;hsa-miR-186-3p;hsa-miR-324-
ENSG0000	ENSG0000	0.001085	hsa-miR-1254;hsa-miR-15a-5p;hsa-miR-15b-5p;hsa-miR-16-5
ENSG0000	ENSG0000	0.002755	hsa-miR-126-5p;hsa-miR-151a-5p;hsa-miR-17-5p;hsa-miR-20
ENSG0000	ENSG0000	0.003231	hsa-miR-1207-5p;hsa-miR-1293;hsa-miR-1976;hsa-miR-214-3
ENSG0000	ENSG0000	0.000442	hsa-miR-17-5p;hsa-miR-20b-5p;hsa-miR-27a-3p;hsa-miR-27b-
ENSG0000	ENSG0000	0.000391	hsa-miR-107;hsa-miR-1293;hsa-miR-17-5p;hsa-miR-186-3p;hs
ENSG0000	ENSG0000	0.002508	hsa-miR-17-5p;hsa-miR-186-3p;hsa-miR-1976;hsa-miR-20b-5
ENSG0000	ENSG0000	0.001073	hsa-miR-15a-5p;hsa-miR-15b-5p;hsa-miR-16-5p;hsa-miR-195-
ENSG0000	ENSG0000	0.005655	hsa-miR-15a-5p;hsa-miR-15b-5p;hsa-miR-195-5p;hsa-miR-42
ENSG0000	ENSG0000	0.006617	hsa-miR-195-5p;hsa-miR-378a-3p;hsa-miR-378c;hsa-miR-378
ENSG0000	ENSG0000	6.15E-05	hsa-miR-15a-5p;hsa-miR-15b-5p;hsa-miR-16-5p;hsa-miR-195-
ENSG0000	ENSG0000	0.000519	hsa-miR-103a-3p;hsa-miR-107;hsa-miR-15a-5p;hsa-miR-15b-
ENSG0000	ENSG0000	0.001907	hsa-miR-15a-5p;hsa-miR-15b-5p;hsa-miR-16-5p;hsa-miR-195-
ENSG0000	ENSG0000	0.001907	hsa-miR-124-3p;hsa-miR-129-1-3p;hsa-miR-129-2-3p;hsa-mi
ENSG0000	ENSG0000	0.002503	hsa-miR-103a-3p;hsa-miR-107;hsa-miR-15a-5p;hsa-miR-15b-
ENSG0000	ENSG0000	4.13E-05	hsa-miR-15a-5p;hsa-miR-15b-5p;hsa-miR-195-5p;hsa-miR-42
ENSG0000	ENSG0000	0.004691	hsa-miR-1243;hsa-miR-124-3p;hsa-miR-16-5p;hsa-miR-1976;h
ENSG0000	ENSG0000	1.23E-05	hsa-miR-15a-5p;hsa-miR-15b-5p;hsa-miR-16-5p;hsa-miR-195-
ENSG0000	ENSG0000	0.003653	hsa-miR-135a-5p;hsa-miR-15a-5p;hsa-miR-15b-5p;hsa-miR-1
ENSG0000	ENSG0000	0.005818	hsa-miR-1976;hsa-miR-214-3p;hsa-miR-224-5p;hsa-miR-3065-
ENSG0000	ENSG0000	0.004557	hsa-miR-17-5p;hsa-miR-20b-5p;hsa-miR-30b-3p;hsa-miR-30c-
ENSG0000	ENSG0000	5.02E-07	hsa-miR-15a-5p;hsa-miR-15b-5p;hsa-miR-16-5p;hsa-miR-195-
ENSG0000	ENSG0000	0.004861	hsa-miR-15a-5p;hsa-miR-15b-5p;hsa-miR-16-5p;hsa-miR-195-
ENSG0000	ENSG0000	0.00873	hsa-miR-140-3p;hsa-miR-148a-5p;hsa-miR-15a-5p;hsa-miR-1
ENSG0000	ENSG0000	0.000719	hsa-miR-15a-5p;hsa-miR-15b-5p;hsa-miR-16-5p;hsa-miR-183-
ENSG0000	ENSG0000	0.001404	hsa-miR-15a-5p;hsa-miR-15b-5p;hsa-miR-16-5p;hsa-miR-195-
ENSG0000	ENSG0000	0.000883	hsa-miR-15a-5p;hsa-miR-15b-5p;hsa-miR-195-5p;hsa-miR-30
ENSG0000	ENSG0000	2.78E-05	hsa-miR-1243;hsa-miR-214-3p;hsa-miR-29b-2-5p;hsa-miR-30
ENSG0000	ENSG0000	0.003671	hsa-miR-30a-3p;hsa-miR-30d-3p;hsa-miR-30e-3p;hsa-miR-44
ENSG0000	ENSG0000	0.000247	hsa-miR-126-5p;hsa-miR-15a-5p;hsa-miR-15b-5p;hsa-miR-17-
ENSG0000	ENSG0000	1.39E-07	hsa-miR-107;hsa-miR-15a-5p;hsa-miR-15b-5p;hsa-miR-16-5p;
ENSG0000	ENSG0000	0.001236	hsa-miR-15a-5p;hsa-miR-15b-5p;hsa-miR-17-5p;hsa-miR-186-
ENSG0000	ENSG0000	0.004799	hsa-miR-183-5p;hsa-miR-324-3p;hsa-miR-378a-3p;hsa-miR-3
ENSG0000	ENSG0000	0.001489	hsa-miR-17-5p;hsa-miR-20b-5p;hsa-miR-30b-3p;hsa-miR-30c-
ENSG0000	ENSG0000	0.000103	hsa-miR-15a-5p;hsa-miR-15b-5p;hsa-miR-16-5p;hsa-miR-195-
ENSG0000	ENSG0000	0.007421	hsa-miR-1207-5p;hsa-miR-1260a;hsa-miR-1260b;hsa-miR-17-
ENSG0000	ENSG0000	0.005952	hsa-miR-15a-5p;hsa-miR-15b-5p;hsa-miR-195-5p;hsa-miR-42
ENSG0000	ENSG0000	7.76E-05	hsa-miR-103a-3p;hsa-miR-107;hsa-miR-1254;hsa-miR-15a-5p;
ENSG0000	ENSG0000	0.001191	hsa-miR-15a-5p;hsa-miR-16-5p;hsa-miR-182-5p;hsa-miR-31-
ENSG0000	ENSG0000	0.003634	hsa-miR-107;hsa-miR-15a-5p;hsa-miR-15b-5p;hsa-miR-183-5
ENSG0000	ENSG0000	6.76E-05	hsa-miR-103a-3p;hsa-miR-107;hsa-miR-15a-5p;hsa-miR-15b-
ENSG0000	ENSG0000	6.15E-05	hsa-miR-15a-5p;hsa-miR-15b-5p;hsa-miR-16-5p;hsa-miR-195-
ENSG0000	ENSG0000	6.15E-05	hsa-miR-15a-5p;hsa-miR-15b-5p;hsa-miR-16-5p;hsa-miR-195-
ENSG0000	ENSG0000	0.000299	hsa-miR-15a-5p;hsa-miR-15b-5p;hsa-miR-195-5p;hsa-miR-42

ENSG0000	ENSG0000	3.58E-05	hsa-miR-15a-5p;hsa-miR-15b-5p;hsa-miR-16-5p;hsa-miR-195
ENSG0000	ENSG0000	0.004931	hsa-miR-378a-3p;hsa-miR-378c;hsa-miR-378d;hsa-miR-378i
ENSG0000	ENSG0000	7.47E-06	hsa-miR-15a-5p;hsa-miR-15b-5p;hsa-miR-16-5p;hsa-miR-195
ENSG0000	ENSG0000	1.46E-07	hsa-miR-103a-3p;hsa-miR-107;hsa-miR-15a-5p;hsa-miR-15b-5
ENSG0000	ENSG0000	0.000139	hsa-miR-142-3p;hsa-miR-15a-5p;hsa-miR-15b-5p;hsa-miR-16
ENSG0000	ENSG0000	0.000933	hsa-miR-15a-5p;hsa-miR-15b-5p;hsa-miR-16-5p;hsa-miR-195
ENSG0000	ENSG0000	0.000721	hsa-miR-15a-5p;hsa-miR-15b-5p;hsa-miR-16-5p;hsa-miR-195
ENSG0000	ENSG0000	3.58E-05	hsa-miR-15a-5p;hsa-miR-16-5p;hsa-miR-17-5p;hsa-miR-195-5
ENSG0000	ENSG0000	3.58E-05	hsa-miR-15a-5p;hsa-miR-16-5p;hsa-miR-17-5p;hsa-miR-195-5
ENSG0000	ENSG0000	0.006617	hsa-miR-15a-5p;hsa-miR-15b-5p;hsa-miR-16-5p;hsa-miR-195
ENSG0000	ENSG0000	0.004843	hsa-miR-1207-5p;hsa-miR-1254;hsa-miR-132-3p;hsa-miR-197
ENSG0000	ENSG0000	3.58E-05	hsa-miR-15a-5p;hsa-miR-15b-5p;hsa-miR-16-5p;hsa-miR-195
ENSG0000	ENSG0000	0.000519	hsa-miR-103a-3p;hsa-miR-107;hsa-miR-15a-5p;hsa-miR-15b-5
ENSG0000	ENSG0000	0.000463	hsa-miR-1207-5p;hsa-miR-1260a;hsa-miR-1260b;hsa-miR-135
ENSG0000	ENSG0000	0.002649	hsa-miR-15a-5p;hsa-miR-15b-5p;hsa-miR-16-5p;hsa-miR-195
ENSG0000	ENSG0000	0.000213	hsa-miR-16-5p;hsa-miR-17-5p;hsa-miR-20b-5p;hsa-miR-21-3
ENSG0000	ENSG0000	0.001796	hsa-miR-101-3p;hsa-miR-15a-5p;hsa-miR-15b-5p;hsa-miR-16
ENSG0000	ENSG0000	0.001877	hsa-miR-193a-3p;hsa-miR-193b-3p;hsa-miR-1976;hsa-miR-42
ENSG0000	ENSG0000	0.001877	hsa-miR-193a-3p;hsa-miR-193b-3p;hsa-miR-1976;hsa-miR-42
ENSG0000	ENSG0000	0.001877	hsa-miR-193a-3p;hsa-miR-193b-3p;hsa-miR-1976;hsa-miR-42
ENSG0000	ENSG0000	0.001877	hsa-miR-193a-3p;hsa-miR-193b-3p;hsa-miR-1976;hsa-miR-42
ENSG0000	ENSG0000	0.001877	hsa-miR-193a-3p;hsa-miR-193b-3p;hsa-miR-1976;hsa-miR-42
ENSG0000	ENSG0000	0.001877	hsa-miR-193a-3p;hsa-miR-193b-3p;hsa-miR-1976;hsa-miR-42
ENSG0000	ENSG0000	0.001877	hsa-miR-193a-3p;hsa-miR-193b-3p;hsa-miR-1976;hsa-miR-42
ENSG0000	ENSG0000	0.000721	hsa-miR-15a-5p;hsa-miR-15b-5p;hsa-miR-16-5p;hsa-miR-195
ENSG0000	ENSG0000	1.44E-06	hsa-miR-15a-5p;hsa-miR-15b-5p;hsa-miR-195-5p;hsa-miR-42
ENSG0000	ENSG0000	0.001125	hsa-miR-17-5p;hsa-miR-186-3p;hsa-miR-20b-5p;hsa-miR-30b
ENSG0000	ENSG0000	0.001125	hsa-miR-17-5p;hsa-miR-186-3p;hsa-miR-20b-5p;hsa-miR-30b
ENSG0000	ENSG0000	0.001125	hsa-miR-17-5p;hsa-miR-186-3p;hsa-miR-20b-5p;hsa-miR-30b
ENSG0000	ENSG0000	0.001125	hsa-miR-17-5p;hsa-miR-186-3p;hsa-miR-20b-5p;hsa-miR-30b
ENSG0000	ENSG0000	0.001125	hsa-miR-17-5p;hsa-miR-186-3p;hsa-miR-20b-5p;hsa-miR-30b
ENSG0000	ENSG0000	0.001125	hsa-miR-17-5p;hsa-miR-186-3p;hsa-miR-20b-5p;hsa-miR-30b
ENSG0000	ENSG0000	0.001125	hsa-miR-17-5p;hsa-miR-186-3p;hsa-miR-20b-5p;hsa-miR-30b
ENSG0000	ENSG0000	0.001297	hsa-miR-125b-2-3p;hsa-miR-15a-5p;hsa-miR-15b-5p;hsa-miR
ENSG0000	ENSG0000	0.007311	hsa-miR-142-3p;hsa-miR-30b-3p;hsa-miR-30c-1-3p;hsa-miR-
ENSG0000	ENSG0000	0.004861	hsa-miR-1260a;hsa-miR-1260b;hsa-miR-204-5p;hsa-miR-27a-
ENSG0000	ENSG0000	0.008247	hsa-miR-1226-3p;hsa-miR-197-3p;hsa-miR-204-5p;hsa-miR-3
ENSG0000	ENSG0000	0.001404	hsa-miR-15a-5p;hsa-miR-15b-5p;hsa-miR-16-5p;hsa-miR-195
ENSG0000	ENSG0000	0.004931	hsa-miR-15a-5p;hsa-miR-15b-5p;hsa-miR-424-5p;hsa-miR-49
ENSG0000	ENSG0000	0.008255	hsa-miR-30a-3p;hsa-miR-30d-3p;hsa-miR-30e-3p;hsa-miR-33
ENSG0000	ENSG0000	0.000279	hsa-miR-15a-5p;hsa-miR-15b-5p;hsa-miR-16-5p;hsa-miR-195
ENSG0000	ENSG0000	7.47E-06	hsa-miR-15a-5p;hsa-miR-15b-5p;hsa-miR-16-5p;hsa-miR-195
ENSG0000	ENSG0000	0.004337	hsa-miR-1207-5p;hsa-miR-15a-5p;hsa-miR-15b-5p;hsa-miR-1
ENSG0000	ENSG0000	1.37E-06	hsa-miR-103a-3p;hsa-miR-107;hsa-miR-125b-2-3p;hsa-miR-1
ENSG0000	ENSG0000	0.000628	hsa-miR-140-3p;hsa-miR-15a-5p;hsa-miR-15b-5p;hsa-miR-19
ENSG0000	ENSG0000	0.000429	hsa-miR-132-3p;hsa-miR-15a-5p;hsa-miR-15b-5p;hsa-miR-16
ENSG0000	ENSG0000	0.001404	hsa-miR-143-3p;hsa-miR-16-5p;hsa-miR-30b-3p;hsa-miR-30c
ENSG0000	ENSG0000	0.000213	hsa-miR-129-1-3p;hsa-miR-129-2-3p;hsa-miR-17-5p;hsa-miR
ENSG0000	ENSG0000	0.007298	hsa-miR-1226-3p;hsa-miR-1260a;hsa-miR-1260b;hsa-miR-186
ENSG0000	ENSG0000	7.47E-06	hsa-miR-15a-5p;hsa-miR-15b-5p;hsa-miR-16-5p;hsa-miR-195
ENSG0000	ENSG0000	0.002023	hsa-miR-15a-5p;hsa-miR-15b-5p;hsa-miR-16-5p;hsa-miR-195
ENSG0000	ENSG0000	0.000221	hsa-miR-15a-5p;hsa-miR-15b-5p;hsa-miR-16-5p;hsa-miR-17-
ENSG0000	ENSG0000	4.59E-05	hsa-miR-103a-3p;hsa-miR-107;hsa-miR-15a-5p;hsa-miR-15b-5
ENSG0000	ENSG0000	0.008255	hsa-miR-17-5p;hsa-miR-20b-5p;hsa-miR-577;hsa-miR-93-5p

ENSG0000	ENSG0000	0.005816	hsa-miR-17-3p;hsa-miR-29b-2-5p;hsa-miR-30b-3p;hsa-miR-3
ENSG0000	ENSG0000	3.95E-05	hsa-miR-15a-5p;hsa-miR-15b-5p;hsa-miR-195-5p;hsa-miR-30
ENSG0000	ENSG0000	0.00392	hsa-miR-107;hsa-miR-1260a;hsa-miR-1260b;hsa-miR-17-3p;hs
ENSG0000	ENSG0000	0.008137	hsa-miR-1293;hsa-miR-17-5p;hsa-miR-204-5p;hsa-miR-20b-5
ENSG0000	ENSG0000	0.009076	hsa-miR-2277-3p;hsa-miR-30c-1-3p;hsa-miR-30c-2-3p;hsa-mi
ENSG0000	ENSG0000	0.007311	hsa-miR-103a-3p;hsa-miR-107;hsa-miR-15a-5p;hsa-miR-15b-5
ENSG0000	ENSG0000	0.000359	hsa-miR-15a-5p;hsa-miR-15b-5p;hsa-miR-16-5p;hsa-miR-195-
ENSG0000	ENSG0000	0.002023	hsa-miR-1293;hsa-miR-15a-5p;hsa-miR-15b-5p;hsa-miR-195-5
ENSG0000	ENSG0000	0.000602	hsa-miR-15a-5p;hsa-miR-15b-5p;hsa-miR-195-5p;hsa-miR-20
ENSG0000	ENSG0000	0.00015	hsa-miR-101-3p;hsa-miR-103a-3p;hsa-miR-107;hsa-miR-1260a
ENSG0000	ENSG0000	0.001297	hsa-miR-129-1-3p;hsa-miR-129-2-3p;hsa-miR-30b-3p;hsa-mi
ENSG0000	ENSG0000	0.000721	hsa-miR-15a-5p;hsa-miR-15b-5p;hsa-miR-195-5p;hsa-miR-19
ENSG0000	ENSG0000	0.000247	hsa-miR-126-5p;hsa-miR-132-3p;hsa-miR-15a-5p;hsa-miR-15
ENSG0000	ENSG0000	0.006274	hsa-miR-1226-3p;hsa-miR-15a-5p;hsa-miR-15b-5p;hsa-miR-1
ENSG0000	ENSG0000	0.000508	hsa-miR-137;hsa-miR-142-3p;hsa-miR-15a-5p;hsa-miR-15b-5
ENSG0000	ENSG0000	0.00016	hsa-miR-143-3p;hsa-miR-15a-5p;hsa-miR-16-5p;hsa-miR-195-
ENSG0000	ENSG0000	0.000875	hsa-miR-1293;hsa-miR-193a-3p;hsa-miR-193b-3p;hsa-miR-19
ENSG0000	ENSG0000	0.003653	hsa-miR-26b-3p;hsa-miR-30b-3p;hsa-miR-30c-1-3p;hsa-miR-
ENSG0000	ENSG0000	7.23E-06	hsa-miR-103a-3p;hsa-miR-107;hsa-miR-15a-5p;hsa-miR-15b-5
ENSG0000	ENSG0000	0.002699	hsa-miR-1207-5p;hsa-miR-15a-5p;hsa-miR-15b-5p;hsa-miR-1
ENSG0000	ENSG0000	0.005654	hsa-miR-1207-5p;hsa-miR-17-3p;hsa-miR-17-5p;hsa-miR-182-
ENSG0000	ENSG0000	0.005654	hsa-miR-1207-5p;hsa-miR-17-3p;hsa-miR-17-5p;hsa-miR-182-
ENSG0000	ENSG0000	0.000721	hsa-miR-17-5p;hsa-miR-20b-5p;hsa-miR-27a-3p;hsa-miR-27b-
ENSG0000	ENSG0000	7.25E-07	hsa-miR-124-3p;hsa-miR-15a-5p;hsa-miR-15b-5p;hsa-miR-16-
ENSG0000	ENSG0000	3.65E-06	hsa-miR-15a-5p;hsa-miR-15b-5p;hsa-miR-16-5p;hsa-miR-195-
ENSG0000	ENSG0000	0.007204	hsa-miR-204-5p;hsa-miR-29b-2-5p;hsa-miR-30a-3p;hsa-miR-
ENSG0000	ENSG0000	0.001895	hsa-miR-1254;hsa-miR-196a-5p;hsa-miR-196b-5p;hsa-miR-20
ENSG0000	ENSG0000	0.001297	hsa-miR-17-5p;hsa-miR-186-3p;hsa-miR-193a-3p;hsa-miR-19
ENSG0000	ENSG0000	0.006274	hsa-miR-1207-5p;hsa-miR-148a-5p;hsa-miR-17-5p;hsa-miR-1
ENSG0000	ENSG0000	1.93E-05	hsa-miR-15a-5p;hsa-miR-16-5p;hsa-miR-195-5p;hsa-miR-214-
ENSG0000	ENSG0000	0.002649	hsa-miR-15a-5p;hsa-miR-15b-5p;hsa-miR-16-5p;hsa-miR-195-
ENSG0000	ENSG0000	3.58E-05	hsa-miR-30a-3p;hsa-miR-30d-3p;hsa-miR-30e-3p;hsa-miR-31
ENSG0000	ENSG0000	0.001872	hsa-miR-1260a;hsa-miR-1260b;hsa-miR-15a-5p;hsa-miR-15b-5
ENSG0000	ENSG0000	6.27E-05	hsa-miR-15a-5p;hsa-miR-15b-5p;hsa-miR-16-5p;hsa-miR-195-
ENSG0000	ENSG0000	0.000101	hsa-miR-15a-5p;hsa-miR-15b-5p;hsa-miR-16-5p;hsa-miR-195-
ENSG0000	ENSG0000	0.000161	hsa-miR-126-5p;hsa-miR-148a-5p;hsa-miR-15a-5p;hsa-miR-1
ENSG0000	ENSG0000	0.006946	hsa-miR-27a-3p;hsa-miR-27b-3p;hsa-miR-30b-3p;hsa-miR-30
ENSG0000	ENSG0000	2.29E-06	hsa-miR-15a-5p;hsa-miR-15b-5p;hsa-miR-16-5p;hsa-miR-195-
ENSG0000	ENSG0000	2.29E-06	hsa-miR-15a-5p;hsa-miR-15b-5p;hsa-miR-16-5p;hsa-miR-195-
ENSG0000	ENSG0000	2.29E-06	hsa-miR-15a-5p;hsa-miR-15b-5p;hsa-miR-16-5p;hsa-miR-195-
ENSG0000	ENSG0000	2.29E-06	hsa-miR-15a-5p;hsa-miR-15b-5p;hsa-miR-16-5p;hsa-miR-195-
ENSG0000	ENSG0000	0.00021	hsa-miR-107;hsa-miR-15a-5p;hsa-miR-15b-5p;hsa-miR-17-5p;
ENSG0000	ENSG0000	0.00021	hsa-miR-107;hsa-miR-15a-5p;hsa-miR-15b-5p;hsa-miR-17-5p;
ENSG0000	ENSG0000	8.95E-07	hsa-miR-103a-3p;hsa-miR-107;hsa-miR-15a-5p;hsa-miR-15b-5
ENSG0000	ENSG0000	0.000225	hsa-miR-124-3p;hsa-miR-17-5p;hsa-miR-20b-5p;hsa-miR-224-
ENSG0000	ENSG0000	0.003133	hsa-miR-140-3p;hsa-miR-17-5p;hsa-miR-197-3p;hsa-miR-20b-
ENSG0000	ENSG0000	1.05E-05	hsa-miR-15a-5p;hsa-miR-15b-5p;hsa-miR-16-5p;hsa-miR-17-5
ENSG0000	ENSG0000	1.30E-06	hsa-miR-15a-5p;hsa-miR-15b-5p;hsa-miR-195-5p;hsa-miR-21
ENSG0000	ENSG0000	0.000103	hsa-miR-15a-5p;hsa-miR-15b-5p;hsa-miR-16-5p;hsa-miR-195-
ENSG0000	ENSG0000	0.000214	hsa-miR-1260a;hsa-miR-1260b;hsa-miR-21-3p;hsa-miR-27a-3
ENSG0000	ENSG0000	0.009076	hsa-miR-1207-5p;hsa-miR-30b-3p;hsa-miR-30c-1-3p;hsa-miR-
ENSG0000	ENSG0000	0.00994	hsa-miR-1293;hsa-miR-214-3p;hsa-miR-30b-3p;hsa-miR-30c-1
ENSG0000	ENSG0000	7.47E-06	hsa-miR-15a-5p;hsa-miR-15b-5p;hsa-miR-16-5p;hsa-miR-195-
ENSG0000	ENSG0000	0.000764	hsa-miR-548n;hsa-miR-561-3p;hsa-miR-7-5p;hsa-miR-7850-5

ENSG0000	ENSG0000	0.000519	hsa-miR-15a-5p;hsa-miR-15b-5p;hsa-miR-16-5p;hsa-miR-186-
ENSG0000	ENSG0000	0.000519	hsa-miR-15a-5p;hsa-miR-15b-5p;hsa-miR-16-5p;hsa-miR-186-
ENSG0000	ENSG0000	0.000519	hsa-miR-15a-5p;hsa-miR-15b-5p;hsa-miR-16-5p;hsa-miR-186-
ENSG0000	ENSG0000	0.000519	hsa-miR-15a-5p;hsa-miR-15b-5p;hsa-miR-16-5p;hsa-miR-186-
ENSG0000	ENSG0000	0.000519	hsa-miR-15a-5p;hsa-miR-15b-5p;hsa-miR-16-5p;hsa-miR-186-
ENSG0000	ENSG0000	0.000519	hsa-miR-15a-5p;hsa-miR-15b-5p;hsa-miR-16-5p;hsa-miR-186-
ENSG0000	ENSG0000	0.000519	hsa-miR-15a-5p;hsa-miR-15b-5p;hsa-miR-16-5p;hsa-miR-186-
ENSG0000	ENSG0000	0.000519	hsa-miR-15a-5p;hsa-miR-15b-5p;hsa-miR-16-5p;hsa-miR-186-
ENSG0000	ENSG0000	0.000519	hsa-miR-15a-5p;hsa-miR-15b-5p;hsa-miR-16-5p;hsa-miR-186-
ENSG0000	ENSG0000	0.000519	hsa-miR-15a-5p;hsa-miR-15b-5p;hsa-miR-16-5p;hsa-miR-186-
ENSG0000	ENSG0000	0.001221	hsa-miR-129-1-3p;hsa-miR-129-2-3p;hsa-miR-330-3p;hsa-mi
ENSG0000	ENSG0000	1.89E-05	hsa-miR-15a-5p;hsa-miR-15b-5p;hsa-miR-16-5p;hsa-miR-195-
ENSG0000	ENSG0000	0.004931	hsa-miR-17-5p;hsa-miR-20b-5p;hsa-miR-589-3p;hsa-miR-93-
ENSG0000	ENSG0000	0.00827	hsa-miR-137;hsa-miR-30b-3p;hsa-miR-30c-1-3p;hsa-miR-30c-
ENSG0000	ENSG0000	0.009076	hsa-miR-183-5p;hsa-miR-1976;hsa-miR-34b-3p;hsa-miR-4487;
ENSG0000	ENSG0000	8.45E-06	hsa-miR-15a-5p;hsa-miR-15b-5p;hsa-miR-16-5p;hsa-miR-195-
ENSG0000	ENSG0000	0.0015	hsa-miR-214-3p;hsa-miR-361-3p;hsa-miR-378a-3p;hsa-miR-3
ENSG0000	ENSG0000	2.50E-05	hsa-miR-1254;hsa-miR-15a-5p;hsa-miR-15b-5p;hsa-miR-16-5
ENSG0000	ENSG0000	0.00392	hsa-miR-30a-3p;hsa-miR-30d-3p;hsa-miR-30e-3p;hsa-miR-36
ENSG0000	ENSG0000	0.000203	hsa-miR-103a-3p;hsa-miR-107;hsa-miR-15a-5p;hsa-miR-15b-
ENSG0000	ENSG0000	0.000883	hsa-miR-15a-5p;hsa-miR-15b-5p;hsa-miR-16-5p;hsa-miR-181
ENSG0000	ENSG0000	0.004042	hsa-miR-129-2-3p;hsa-miR-335-5p;hsa-miR-455-3p;hsa-miR-
ENSG0000	ENSG0000	0.000108	hsa-miR-103a-3p;hsa-miR-107;hsa-miR-15a-5p;hsa-miR-15b-
ENSG0000	ENSG0000	1.04E-06	hsa-miR-15a-5p;hsa-miR-15b-5p;hsa-miR-195-5p;hsa-miR-19
ENSG0000	ENSG0000	0.007693	hsa-miR-1207-5p;hsa-miR-124-3p;hsa-miR-148a-5p;hsa-miR-
ENSG0000	ENSG0000	0.00863	hsa-miR-124-3p;hsa-miR-30a-3p;hsa-miR-30d-3p;hsa-miR-30
ENSG0000	ENSG0000	0.001595	hsa-miR-1226-3p;hsa-miR-15a-5p;hsa-miR-15b-5p;hsa-miR-1
ENSG0000	ENSG0000	0.005083	hsa-miR-142-3p;hsa-miR-15a-5p;hsa-miR-15b-5p;hsa-miR-16-
ENSG0000	ENSG0000	0.000116	hsa-miR-1207-5p;hsa-miR-138-5p;hsa-miR-182-5p;hsa-miR-1
ENSG0000	ENSG0000	0.000299	hsa-miR-15a-5p;hsa-miR-15b-5p;hsa-miR-16-5p;hsa-miR-195-
ENSG0000	ENSG0000	0.001983	hsa-miR-1267;hsa-miR-135b-3p;hsa-miR-15a-5p;hsa-miR-15b-
ENSG0000	ENSG0000	4.29E-05	hsa-miR-103a-3p;hsa-miR-107;hsa-miR-15a-5p;hsa-miR-15b-
ENSG0000	ENSG0000	0.000139	hsa-miR-15a-5p;hsa-miR-15b-5p;hsa-miR-195-5p;hsa-miR-30
ENSG0000	ENSG0000	6.58E-05	hsa-miR-15a-5p;hsa-miR-15b-5p;hsa-miR-16-5p;hsa-miR-195-
ENSG0000	ENSG0000	0.000748	hsa-miR-15a-5p;hsa-miR-15b-5p;hsa-miR-195-5p;hsa-miR-42
ENSG0000	ENSG0000	0.001123	hsa-miR-125b-2-3p;hsa-miR-30a-3p;hsa-miR-30d-3p;hsa-miR-
ENSG0000	ENSG0000	4.68E-05	hsa-miR-103a-3p;hsa-miR-107;hsa-miR-15a-5p;hsa-miR-15b-
ENSG0000	ENSG0000	0.001907	hsa-miR-15a-5p;hsa-miR-15b-5p;hsa-miR-16-5p;hsa-miR-195-
ENSG0000	ENSG0000	0.002265	hsa-miR-193a-3p;hsa-miR-193b-3p;hsa-miR-197-3p;hsa-miR-
ENSG0000	ENSG0000	0.001867	hsa-miR-1207-5p;hsa-miR-15a-5p;hsa-miR-15b-5p;hsa-miR-1
ENSG0000	ENSG0000	3.84E-07	hsa-miR-124-3p;hsa-miR-15a-5p;hsa-miR-15b-5p;hsa-miR-16-
ENSG0000	ENSG0000	6.85E-05	hsa-miR-1260a;hsa-miR-1260b;hsa-miR-15a-5p;hsa-miR-15b-
ENSG0000	ENSG0000	0.00889	hsa-miR-30b-3p;hsa-miR-30c-1-3p;hsa-miR-30c-2-3p;hsa-mi
ENSG0000	ENSG0000	0.000135	hsa-miR-15a-5p;hsa-miR-15b-5p;hsa-miR-16-5p;hsa-miR-195-
ENSG0000	ENSG0000	0.000967	hsa-miR-103a-3p;hsa-miR-107;hsa-miR-15a-5p;hsa-miR-15b-
ENSG0000	ENSG0000	0.005655	hsa-miR-1207-5p;hsa-miR-193a-3p;hsa-miR-193b-3p;hsa-miR-
ENSG0000	ENSG0000	0.000548	hsa-miR-15a-5p;hsa-miR-15b-5p;hsa-miR-195-5p;hsa-miR-19
ENSG0000	ENSG0000	6.84E-07	hsa-miR-103a-3p;hsa-miR-107;hsa-miR-15a-5p;hsa-miR-15b-
ENSG0000	ENSG0000	0.008255	hsa-miR-15a-5p;hsa-miR-16-5p;hsa-miR-548n;hsa-miR-561-3
ENSG0000	ENSG0000	6.58E-05	hsa-miR-15a-5p;hsa-miR-15b-5p;hsa-miR-16-5p;hsa-miR-195-
ENSG0000	ENSG0000	0.000764	hsa-miR-204-5p;hsa-miR-30a-3p;hsa-miR-30d-3p;hsa-miR-30
ENSG0000	ENSG0000	0.0065	hsa-miR-15a-5p;hsa-miR-15b-5p;hsa-miR-16-5p;hsa-miR-195-
ENSG0000	ENSG0000	0.00827	hsa-miR-142-3p;hsa-miR-17-5p;hsa-miR-186-3p;hsa-miR-20b-
ENSG0000	ENSG0000	1.00E-05	hsa-miR-15a-5p;hsa-miR-15b-5p;hsa-miR-16-5p;hsa-miR-195-

ENSG0000	ENSG0000	0.006452	hsa-miR-183-5p;hsa-miR-23a-3p;hsa-miR-23b-3p;hsa-miR-31-
ENSG0000	ENSG0000	0.005655	hsa-miR-324-3p;hsa-miR-361-3p;hsa-miR-377-3p;hsa-miR-545
ENSG0000	ENSG0000	0.005816	hsa-miR-15a-5p;hsa-miR-183-5p;hsa-miR-195-5p;hsa-miR-197
ENSG0000	ENSG0000	7.57E-06	hsa-miR-15a-5p;hsa-miR-15b-5p;hsa-miR-16-5p;hsa-miR-195-
ENSG0000	ENSG0000	1.39E-05	hsa-miR-15a-5p;hsa-miR-15b-5p;hsa-miR-16-5p;hsa-miR-195-
ENSG0000	ENSG0000	0.000601	hsa-miR-103a-3p;hsa-miR-107;hsa-miR-15a-5p;hsa-miR-15b-5
ENSG0000	ENSG0000	6.58E-05	hsa-miR-15a-5p;hsa-miR-15b-5p;hsa-miR-16-5p;hsa-miR-195-
ENSG0000	ENSG0000	0.000108	hsa-miR-140-3p;hsa-miR-17-5p;hsa-miR-182-5p;hsa-miR-20b-
ENSG0000	ENSG0000	5.08E-05	hsa-miR-101-3p;hsa-miR-103a-3p;hsa-miR-107;hsa-miR-15a-5
ENSG0000	ENSG0000	0.000967	hsa-miR-137;hsa-miR-15a-5p;hsa-miR-15b-5p;hsa-miR-16-5p;
ENSG0000	ENSG0000	0.003805	hsa-miR-1254;hsa-miR-193a-3p;hsa-miR-193b-3p;hsa-miR-30b
ENSG0000	ENSG0000	0.000891	hsa-miR-15a-5p;hsa-miR-15b-5p;hsa-miR-16-5p;hsa-miR-195-
ENSG0000	ENSG0000	0.00457	hsa-miR-15a-5p;hsa-miR-15b-5p;hsa-miR-16-5p;hsa-miR-182-
ENSG0000	ENSG0000	0.000299	hsa-miR-1267;hsa-miR-15a-5p;hsa-miR-15b-5p;hsa-miR-16-5p
ENSG0000	ENSG0000	0.002755	hsa-miR-1207-5p;hsa-miR-15a-5p;hsa-miR-15b-5p;hsa-miR-16
ENSG0000	ENSG0000	0.001841	hsa-miR-101-3p;hsa-miR-15a-5p;hsa-miR-16-5p;hsa-miR-497-
ENSG0000	ENSG0000	7.47E-06	hsa-miR-15a-5p;hsa-miR-15b-5p;hsa-miR-16-5p;hsa-miR-195-
ENSG0000	ENSG0000	0.000247	hsa-miR-124-3p;hsa-miR-30a-3p;hsa-miR-30b-3p;hsa-miR-30d
ENSG0000	ENSG0000	0.000278	hsa-miR-15a-5p;hsa-miR-15b-5p;hsa-miR-16-5p;hsa-miR-195-
ENSG0000	ENSG0000	0.009294	hsa-miR-129-1-3p;hsa-miR-129-2-3p;hsa-miR-197-3p;hsa-miR
ENSG0000	ENSG0000	0.004337	hsa-miR-103a-3p;hsa-miR-107;hsa-miR-15a-5p;hsa-miR-15b-5
ENSG0000	ENSG0000	0.00994	hsa-miR-17-3p;hsa-miR-183-5p;hsa-miR-193a-3p;hsa-miR-193
ENSG0000	ENSG0000	0.0015	hsa-miR-144-5p;hsa-miR-17-5p;hsa-miR-20b-5p;hsa-miR-497-
ENSG0000	ENSG0000	0.000359	hsa-miR-1267;hsa-miR-186-3p;hsa-miR-30a-3p;hsa-miR-30d-3
ENSG0000	ENSG0000	0.000161	hsa-miR-137;hsa-miR-15a-5p;hsa-miR-15b-5p;hsa-miR-16-5p;
ENSG0000	ENSG0000	0.000458	hsa-miR-17-5p;hsa-miR-20b-5p;hsa-miR-30a-3p;hsa-miR-30d-
ENSG0000	ENSG0000	0.000623	hsa-miR-15a-5p;hsa-miR-15b-5p;hsa-miR-16-5p;hsa-miR-195-
ENSG0000	ENSG0000	0.003671	hsa-miR-197-3p;hsa-miR-30a-3p;hsa-miR-30d-3p;hsa-miR-30e
ENSG0000	ENSG0000	6.75E-06	hsa-miR-30a-3p;hsa-miR-30d-3p;hsa-miR-30e-3p;hsa-miR-532
ENSG0000	ENSG0000	0.000111	hsa-miR-130b-5p;hsa-miR-30a-3p;hsa-miR-30d-3p;hsa-miR-30
ENSG0000	ENSG0000	0.002709	hsa-miR-30a-3p;hsa-miR-30d-3p;hsa-miR-30e-3p;hsa-miR-520
ENSG0000	ENSG0000	0.000537	hsa-miR-30a-3p;hsa-miR-30d-3p;hsa-miR-30e-3p;hsa-miR-520
ENSG0000	ENSG0000	4.94E-05	hsa-miR-137;hsa-miR-30a-3p;hsa-miR-30d-3p;hsa-miR-30e-3p
ENSG0000	ENSG0000	6.18E-05	hsa-miR-130b-5p;hsa-miR-30a-3p;hsa-miR-30d-3p;hsa-miR-30
ENSG0000	ENSG0000	0.000952	hsa-miR-24-3p;hsa-miR-30a-3p;hsa-miR-30d-3p;hsa-miR-30e-
ENSG0000	ENSG0000	0.001427	hsa-miR-24-3p;hsa-miR-30a-3p;hsa-miR-30d-3p;hsa-miR-30e-
ENSG0000	ENSG0000	2.85E-05	hsa-miR-24-3p;hsa-miR-30a-3p;hsa-miR-30d-3p;hsa-miR-30e-
ENSG0000	ENSG0000	1.79E-06	hsa-miR-30a-3p;hsa-miR-30d-3p;hsa-miR-30e-3p;hsa-miR-9-5
ENSG0000	ENSG0000	2.85E-05	hsa-miR-130b-5p;hsa-miR-30a-3p;hsa-miR-30d-3p;hsa-miR-30
ENSG0000	ENSG0000	0.001016	hsa-miR-24-3p;hsa-miR-30a-3p;hsa-miR-30d-3p;hsa-miR-30e-
ENSG0000	ENSG0000	7.51E-05	hsa-miR-24-3p;hsa-miR-30a-3p;hsa-miR-30d-3p;hsa-miR-30e-
ENSG0000	ENSG0000	0.00578	hsa-miR-24-3p;hsa-miR-30a-3p;hsa-miR-30d-3p;hsa-miR-30e-
ENSG0000	ENSG0000	7.58E-05	hsa-miR-30a-3p;hsa-miR-30d-3p;hsa-miR-30e-3p;hsa-miR-520
ENSG0000	ENSG0000	0.000388	hsa-miR-130b-5p;hsa-miR-30a-3p;hsa-miR-30d-3p;hsa-miR-30
ENSG0000	ENSG0000	1.23E-07	hsa-miR-130b-5p;hsa-miR-30a-3p;hsa-miR-30d-3p;hsa-miR-30
ENSG0000	ENSG0000	1.23E-07	hsa-miR-130b-5p;hsa-miR-30a-3p;hsa-miR-30d-3p;hsa-miR-30
ENSG0000	ENSG0000	0.001154	hsa-miR-30a-3p;hsa-miR-30d-3p;hsa-miR-30e-3p;hsa-miR-520
ENSG0000	ENSG0000	4.55E-06	hsa-miR-137;hsa-miR-30a-3p;hsa-miR-30d-3p;hsa-miR-30e-3p
ENSG0000	ENSG0000	0.00335	hsa-miR-130b-5p;hsa-miR-30a-3p;hsa-miR-30d-3p;hsa-miR-30
ENSG0000	ENSG0000	8.60E-06	hsa-miR-30a-3p;hsa-miR-30d-3p;hsa-miR-30e-3p;hsa-miR-556
ENSG0000	ENSG0000	5.06E-06	hsa-miR-130b-5p;hsa-miR-30a-3p;hsa-miR-30d-3p;hsa-miR-30
ENSG0000	ENSG0000	0.001266	hsa-miR-30a-3p;hsa-miR-30d-3p;hsa-miR-30e-3p;hsa-miR-520
ENSG0000	ENSG0000	2.12E-06	hsa-miR-24-3p;hsa-miR-30a-3p;hsa-miR-30d-3p;hsa-miR-30e-
ENSG0000	ENSG0000	8.60E-06	hsa-miR-24-3p;hsa-miR-30a-3p;hsa-miR-30d-3p;hsa-miR-30e-

ENSG0000	ENSG0000	8.60E-06	hsa-miR-24-3p;hsa-miR-30a-3p;hsa-miR-30d-3p;hsa-miR-30e-
ENSG0000	ENSG0000	0.000104	hsa-miR-130b-5p;hsa-miR-30a-3p;hsa-miR-30d-3p;hsa-miR-3(
ENSG0000	ENSG0000	3.64E-08	hsa-miR-130b-5p;hsa-miR-30a-3p;hsa-miR-30d-3p;hsa-miR-3(
ENSG0000	ENSG0000	1.81E-05	hsa-miR-30a-3p;hsa-miR-30d-3p;hsa-miR-30e-3p;hsa-miR-55(
ENSG0000	ENSG0000	4.55E-06	hsa-miR-24-3p;hsa-miR-30a-3p;hsa-miR-30d-3p;hsa-miR-30e-
ENSG0000	ENSG0000	4.55E-06	hsa-miR-24-3p;hsa-miR-30a-3p;hsa-miR-30d-3p;hsa-miR-30e-
ENSG0000	ENSG0000	0.001266	hsa-miR-24-3p;hsa-miR-30a-3p;hsa-miR-30d-3p;hsa-miR-30e-
ENSG0000	ENSG0000	0.000132	hsa-miR-24-3p;hsa-miR-30a-3p;hsa-miR-30d-3p;hsa-miR-30e-
ENSG0000	ENSG0000	0.000272	hsa-miR-30a-3p;hsa-miR-30d-3p;hsa-miR-30e-3p;hsa-miR-53
ENSG0000	ENSG0000	3.66E-05	hsa-miR-30a-3p;hsa-miR-30d-3p;hsa-miR-30e-3p;hsa-miR-53
ENSG0000	ENSG0000	3.66E-05	hsa-miR-30a-3p;hsa-miR-30d-3p;hsa-miR-30e-3p;hsa-miR-53
ENSG0000	ENSG0000	0.000832	hsa-miR-130b-5p;hsa-miR-30a-3p;hsa-miR-30d-3p;hsa-miR-3(
ENSG0000	ENSG0000	6.65E-05	hsa-miR-30a-3p;hsa-miR-30d-3p;hsa-miR-30e-3p;hsa-miR-44
ENSG0000	ENSG0000	0.000213	hsa-miR-130b-5p;hsa-miR-30a-3p;hsa-miR-30d-3p;hsa-miR-3(
ENSG0000	ENSG0000	0.000235	hsa-miR-24-3p;hsa-miR-30a-3p;hsa-miR-30d-3p;hsa-miR-30e-
ENSG0000	ENSG0000	0.000235	hsa-miR-24-3p;hsa-miR-30a-3p;hsa-miR-30d-3p;hsa-miR-30e-
ENSG0000	ENSG0000	0.000723	hsa-miR-30a-3p;hsa-miR-30d-3p;hsa-miR-30e-3p;hsa-miR-52(
ENSG0000	ENSG0000	4.98E-05	hsa-miR-130b-5p;hsa-miR-30a-3p;hsa-miR-30d-3p;hsa-miR-3(
ENSG0000	ENSG0000	3.96E-05	hsa-miR-30a-3p;hsa-miR-30d-3p;hsa-miR-30e-3p;hsa-miR-44
ENSG0000	ENSG0000	7.09E-05	hsa-miR-137;hsa-miR-30a-3p;hsa-miR-30d-3p;hsa-miR-30e-3
ENSG0000	ENSG0000	2.93E-08	hsa-miR-320a;hsa-miR-320b;hsa-miR-320d;hsa-miR-320e;hsa-
ENSG0000	ENSG0000	1.95E-06	hsa-miR-320a;hsa-miR-320b;hsa-miR-320d;hsa-miR-320e;hsa-
ENSG0000	ENSG0000	0.00682	hsa-miR-320a;hsa-miR-320b;hsa-miR-320d;hsa-miR-4429
ENSG0000	ENSG0000	5.67E-06	hsa-miR-103a-2-5p;hsa-miR-320a;hsa-miR-320b;hsa-miR-320
ENSG0000	ENSG0000	0.000132	hsa-miR-320a;hsa-miR-320b;hsa-miR-320d;hsa-miR-4429;hsa-
ENSG0000	ENSG0000	0.000726	hsa-miR-142-3p;hsa-miR-320a;hsa-miR-375;hsa-miR-5010-3p
ENSG0000	ENSG0000	0.000838	hsa-miR-21-3p;hsa-miR-421;hsa-miR-449b-5p;hsa-miR-500a-
ENSG0000	ENSG0000	0.000476	hsa-miR-125b-5p;hsa-miR-320a;hsa-miR-320b;hsa-miR-320d;h
ENSG0000	ENSG0000	0.007329	hsa-miR-320a;hsa-miR-320b;hsa-miR-320d;hsa-miR-4429
ENSG0000	ENSG0000	0.002967	hsa-miR-320a;hsa-miR-320b;hsa-miR-320d;hsa-miR-4429
ENSG0000	ENSG0000	1.65E-05	hsa-miR-125a-5p;hsa-miR-125b-5p;hsa-miR-320a;hsa-miR-32(
ENSG0000	ENSG0000	0.002565	hsa-miR-2115-5p;hsa-miR-3194-3p;hsa-miR-320b;hsa-miR-32(
ENSG0000	ENSG0000	0.002754	hsa-miR-320a;hsa-miR-320b;hsa-miR-320d;hsa-miR-4429;hsa-
ENSG0000	ENSG0000	1.30E-05	hsa-miR-125b-5p;hsa-miR-320a;hsa-miR-320b;hsa-miR-320d;h
ENSG0000	ENSG0000	1.30E-05	hsa-miR-125b-5p;hsa-miR-320a;hsa-miR-320b;hsa-miR-320d;h
ENSG0000	ENSG0000	0.009616	hsa-miR-2115-5p;hsa-miR-21-3p;hsa-miR-520a-5p;hsa-miR-5(
ENSG0000	ENSG0000	0.002386	hsa-miR-125a-5p;hsa-miR-125b-5p;hsa-miR-146a-3p;hsa-miR-
ENSG0000	ENSG0000	1.30E-05	hsa-miR-320a;hsa-miR-320b;hsa-miR-320d;hsa-miR-340-3p;hs
ENSG0000	ENSG0000	0.005874	hsa-miR-320a;hsa-miR-320b;hsa-miR-320d;hsa-miR-4429
ENSG0000	ENSG0000	4.81E-05	hsa-miR-320a;hsa-miR-320b;hsa-miR-320d;hsa-miR-4429
ENSG0000	ENSG0000	0.002694	hsa-miR-320a;hsa-miR-320b;hsa-miR-320d;hsa-miR-4429
ENSG0000	ENSG0000	0.002438	hsa-miR-320a;hsa-miR-320b;hsa-miR-320d;hsa-miR-4429
ENSG0000	ENSG0000	0.000236	hsa-miR-320a;hsa-miR-320b;hsa-miR-320d;hsa-miR-320e;hsa-
ENSG0000	ENSG0000	0.000236	hsa-miR-320a;hsa-miR-320b;hsa-miR-320d;hsa-miR-320e;hsa-
ENSG0000	ENSG0000	0.004626	hsa-miR-320a;hsa-miR-320b;hsa-miR-320d;hsa-miR-4429
ENSG0000	ENSG0000	0.00014	hsa-miR-199a-3p;hsa-miR-199b-3p;hsa-miR-488-3p;hsa-miR-
ENSG0000	ENSG0000	0.000176	hsa-miR-320a;hsa-miR-320b;hsa-miR-320d;hsa-miR-4429
ENSG0000	ENSG0000	1.06E-05	hsa-miR-320a;hsa-miR-320b;hsa-miR-320d;hsa-miR-4429
ENSG0000	ENSG0000	0.001058	hsa-miR-143-5p;hsa-miR-320a;hsa-miR-320b;hsa-miR-320d;hs
ENSG0000	ENSG0000	0.000102	hsa-miR-21-3p;hsa-miR-320a;hsa-miR-320b;hsa-miR-320d;hsa
ENSG0000	ENSG0000	0.000483	hsa-miR-320a;hsa-miR-320b;hsa-miR-320d;hsa-miR-320e;hsa-
ENSG0000	ENSG0000	0.006335	hsa-miR-320b;hsa-miR-320d;hsa-miR-375;hsa-miR-4429
ENSG0000	ENSG0000	3.47E-05	hsa-miR-320a;hsa-miR-320b;hsa-miR-320d;hsa-miR-4429
ENSG0000	ENSG0000	0.002216	hsa-miR-142-3p;hsa-miR-143-5p;hsa-miR-199a-3p;hsa-miR-1

ENSG0000	ENSG0000	3.47E-05	hsa-miR-320a;hsa-miR-320b;hsa-miR-320d;hsa-miR-4429
ENSG0000	ENSG0000	1.06E-05	hsa-miR-320a;hsa-miR-320b;hsa-miR-320d;hsa-miR-4429
ENSG0000	ENSG0000	0.000358	hsa-miR-125a-5p;hsa-miR-125b-5p;hsa-miR-142-3p;hsa-miR-2
ENSG0000	ENSG0000	4.81E-05	hsa-miR-320a;hsa-miR-320b;hsa-miR-320d;hsa-miR-4429
ENSG0000	ENSG0000	0.000298	hsa-miR-320a;hsa-miR-320b;hsa-miR-320d;hsa-miR-4429;hsa-r
ENSG0000	ENSG0000	0.001759	hsa-miR-125a-5p;hsa-miR-125b-5p;hsa-miR-143-5p;hsa-miR-2
ENSG0000	ENSG0000	0.001096	hsa-miR-125a-5p;hsa-miR-125b-5p;hsa-miR-421;hsa-miR-488-
ENSG0000	ENSG0000	0.00011	hsa-miR-320a;hsa-miR-320b;hsa-miR-320d;hsa-miR-4429
ENSG0000	ENSG0000	0.000531	hsa-miR-320a;hsa-miR-320b;hsa-miR-320d;hsa-miR-4429;hsa-r
ENSG0000	ENSG0000	0.001096	hsa-miR-320a;hsa-miR-320b;hsa-miR-320d;hsa-miR-4429
ENSG0000	ENSG0000	0.000384	hsa-miR-320a;hsa-miR-320b;hsa-miR-320d;hsa-miR-4429
ENSG0000	ENSG0000	0.000337	hsa-miR-142-3p;hsa-miR-199a-3p;hsa-miR-199b-3p;hsa-miR-2
ENSG0000	ENSG0000	0.00326	hsa-miR-146a-3p;hsa-miR-2355-5p;hsa-miR-301a-5p;hsa-miR-
ENSG0000	ENSG0000	0.007863	hsa-miR-421;hsa-miR-5010-3p;hsa-miR-502-5p;hsa-miR-539-5
ENSG0000	ENSG0000	8.05E-05	hsa-miR-143-5p;hsa-miR-2115-5p;hsa-miR-320a;hsa-miR-320b
ENSG0000	ENSG0000	0.001096	hsa-miR-320a;hsa-miR-320b;hsa-miR-320d;hsa-miR-4429
ENSG0000	ENSG0000	0.002539	hsa-miR-2355-5p;hsa-miR-320a;hsa-miR-320b;hsa-miR-320d;h
ENSG0000	ENSG0000	0.001624	hsa-miR-185-5p;hsa-miR-320a;hsa-miR-320b;hsa-miR-320d;hs
ENSG0000	ENSG0000	0.000626	hsa-miR-320a;hsa-miR-320b;hsa-miR-320d;hsa-miR-4429
ENSG0000	ENSG0000	0.000455	hsa-miR-320a;hsa-miR-320b;hsa-miR-320d;hsa-miR-4429
ENSG0000	ENSG0000	0.005874	hsa-miR-143-5p;hsa-miR-199a-3p;hsa-miR-199b-3p;hsa-miR-3
ENSG0000	ENSG0000	0.003115	hsa-miR-143-5p;hsa-miR-2355-5p;hsa-miR-320a;hsa-miR-320b
ENSG0000	ENSG0000	3.60E-06	hsa-miR-320a;hsa-miR-320b;hsa-miR-320d;hsa-miR-4429
ENSG0000	ENSG0000	0.000879	hsa-miR-320a;hsa-miR-320b;hsa-miR-320d;hsa-miR-340-3p;hs
ENSG0000	ENSG0000	0.00502	hsa-miR-146a-3p;hsa-miR-2355-5p;hsa-miR-301a-5p;hsa-miR-
ENSG0000	ENSG0000	0.00502	hsa-miR-146a-3p;hsa-miR-2355-5p;hsa-miR-301a-5p;hsa-miR-
ENSG0000	ENSG0000	0.00682	hsa-miR-320a;hsa-miR-320b;hsa-miR-320d;hsa-miR-4429
ENSG0000	ENSG0000	0.000342	hsa-miR-2115-5p;hsa-miR-320a;hsa-miR-320b;hsa-miR-320d;h
ENSG0000	ENSG0000	0.00012	hsa-miR-146a-3p;hsa-miR-301a-5p;hsa-miR-3194-3p;hsa-miR-
ENSG0000	ENSG0000	0.001376	hsa-miR-21-3p;hsa-miR-320a;hsa-miR-320b;hsa-miR-320d;hsa
ENSG0000	ENSG0000	2.43E-05	hsa-miR-320a;hsa-miR-320b;hsa-miR-320d;hsa-miR-4429
ENSG0000	ENSG0000	0.001244	hsa-miR-320a;hsa-miR-320b;hsa-miR-320d;hsa-miR-4429
ENSG0000	ENSG0000	0.001244	hsa-miR-320a;hsa-miR-320b;hsa-miR-320d;hsa-miR-4429
ENSG0000	ENSG0000	0.001772	hsa-miR-320a;hsa-miR-320b;hsa-miR-320d;hsa-miR-4429
ENSG0000	ENSG0000	8.77E-15	hsa-let-7a-5p;hsa-let-7b-5p;hsa-let-7d-5p;hsa-let-7e-5p;hsa-l
ENSG0000	ENSG0000	4.39E-11	hsa-let-7a-5p;hsa-let-7b-5p;hsa-let-7d-5p;hsa-let-7e-5p;hsa-l
ENSG0000	ENSG0000	3.14E-11	hsa-let-7a-5p;hsa-let-7b-5p;hsa-let-7d-5p;hsa-let-7e-5p;hsa-l
ENSG0000	ENSG0000	1.37E-12	hsa-let-7a-5p;hsa-let-7b-5p;hsa-let-7d-5p;hsa-let-7e-5p;hsa-l
ENSG0000	ENSG0000	4.68E-09	hsa-let-7a-5p;hsa-let-7b-5p;hsa-let-7d-5p;hsa-let-7e-5p;hsa-l
ENSG0000	ENSG0000	1.33E-10	hsa-let-7a-5p;hsa-let-7b-5p;hsa-let-7d-5p;hsa-let-7e-5p;hsa-l
ENSG0000	ENSG0000	2.57E-06	hsa-let-7a-5p;hsa-let-7b-5p;hsa-let-7d-5p;hsa-let-7e-5p;hsa-l
ENSG0000	ENSG0000	7.85E-13	hsa-let-7a-5p;hsa-let-7b-5p;hsa-let-7d-5p;hsa-let-7e-5p;hsa-l
ENSG0000	ENSG0000	7.83E-12	hsa-let-7a-5p;hsa-let-7b-5p;hsa-let-7d-5p;hsa-let-7e-5p;hsa-l
ENSG0000	ENSG0000	6.79E-12	hsa-let-7a-5p;hsa-let-7b-5p;hsa-let-7d-5p;hsa-let-7e-5p;hsa-l
ENSG0000	ENSG0000	7.85E-13	hsa-let-7a-5p;hsa-let-7b-5p;hsa-let-7d-5p;hsa-let-7e-5p;hsa-l
ENSG0000	ENSG0000	3.09E-08	hsa-let-7a-5p;hsa-let-7b-5p;hsa-let-7d-5p;hsa-let-7e-5p;hsa-l
ENSG0000	ENSG0000	3.38E-14	hsa-let-7a-5p;hsa-let-7b-5p;hsa-let-7d-5p;hsa-let-7e-5p;hsa-l
ENSG0000	ENSG0000	6.66E-12	hsa-let-7a-5p;hsa-let-7d-5p;hsa-let-7e-5p;hsa-let-7f-5p;hsa-le
ENSG0000	ENSG0000	3.20E-12	hsa-let-7a-5p;hsa-let-7b-5p;hsa-let-7d-5p;hsa-let-7e-5p;hsa-l
ENSG0000	ENSG0000	2.49E-11	hsa-let-7a-5p;hsa-let-7b-5p;hsa-let-7d-5p;hsa-let-7e-5p;hsa-l
ENSG0000	ENSG0000	1.56E-08	hsa-let-7a-5p;hsa-let-7e-5p;hsa-let-7f-5p;hsa-let-7g-5p;hsa-le
ENSG0000	ENSG0000	2.46E-14	hsa-let-7a-5p;hsa-let-7b-5p;hsa-let-7d-5p;hsa-let-7e-5p;hsa-l
ENSG0000	ENSG0000	6.79E-12	hsa-let-7a-5p;hsa-let-7b-5p;hsa-let-7d-5p;hsa-let-7e-5p;hsa-l
ENSG0000	ENSG0000	1.51E-08	hsa-let-7a-5p;hsa-let-7d-5p;hsa-let-7e-5p;hsa-let-7f-5p;hsa-le

ENSG0000	ENSG0000	8.99E-12	hsa-let-7a-5p;hsa-let-7b-5p;hsa-let-7d-5p;hsa-let-7e-5p;hsa-l
ENSG0000	ENSG0000	3.14E-11	hsa-let-7a-5p;hsa-let-7b-5p;hsa-let-7d-5p;hsa-let-7e-5p;hsa-l
ENSG0000	ENSG0000	6.06E-14	hsa-let-7a-5p;hsa-let-7b-5p;hsa-let-7d-5p;hsa-let-7e-5p;hsa-l
ENSG0000	ENSG0000	4.74E-10	hsa-let-7a-5p;hsa-let-7b-5p;hsa-let-7d-5p;hsa-let-7e-5p;hsa-l
ENSG0000	ENSG0000	4.39E-10	hsa-let-7a-5p;hsa-let-7b-5p;hsa-let-7d-5p;hsa-let-7e-5p;hsa-l
ENSG0000	ENSG0000	2.28E-08	hsa-let-7a-5p;hsa-let-7b-5p;hsa-let-7d-5p;hsa-let-7e-5p;hsa-l
ENSG0000	ENSG0000	2.72E-12	hsa-let-7a-5p;hsa-let-7b-5p;hsa-let-7d-5p;hsa-let-7e-5p;hsa-l
ENSG0000	ENSG0000	6.05E-11	hsa-let-7a-5p;hsa-let-7b-5p;hsa-let-7d-5p;hsa-let-7e-5p;hsa-l
ENSG0000	ENSG0000	1.41E-07	hsa-let-7a-5p;hsa-let-7d-5p;hsa-let-7e-5p;hsa-let-7f-5p;hsa-le
ENSG0000	ENSG0000	1.52E-10	hsa-let-7a-5p;hsa-let-7b-5p;hsa-let-7d-5p;hsa-let-7f-5p;hsa-le
ENSG0000	ENSG0000	1.11E-16	hsa-let-7a-5p;hsa-let-7b-5p;hsa-let-7d-5p;hsa-let-7e-5p;hsa-l
ENSG0000	ENSG0000	1.06E-09	hsa-let-7a-5p;hsa-let-7b-5p;hsa-let-7d-5p;hsa-let-7e-5p;hsa-l
ENSG0000	ENSG0000	3.45E-10	hsa-let-7a-5p;hsa-let-7b-5p;hsa-let-7d-5p;hsa-let-7e-5p;hsa-l
ENSG0000	ENSG0000	1.04E-13	hsa-let-7a-5p;hsa-let-7b-5p;hsa-let-7d-5p;hsa-let-7e-5p;hsa-l
ENSG0000	ENSG0000	1.86E-10	hsa-let-7d-5p;hsa-let-7f-5p;hsa-let-7g-5p;hsa-let-7i-5p
ENSG0000	ENSG0000	6.92E-10	hsa-let-7a-5p;hsa-let-7b-5p;hsa-let-7d-5p;hsa-let-7e-5p;hsa-l
ENSG0000	ENSG0000	4.59E-10	hsa-let-7a-5p;hsa-let-7b-5p;hsa-let-7e-5p;hsa-let-7f-5p;hsa-le
ENSG0000	ENSG0000	3.20E-12	hsa-let-7a-5p;hsa-let-7b-5p;hsa-let-7d-5p;hsa-let-7e-5p;hsa-l
ENSG0000	ENSG0000	4.00E-15	hsa-let-7a-5p;hsa-let-7b-5p;hsa-let-7d-5p;hsa-let-7e-5p;hsa-l
ENSG0000	ENSG0000	2.66E-15	hsa-let-7a-5p;hsa-let-7b-5p;hsa-let-7d-5p;hsa-let-7e-5p;hsa-l
ENSG0000	ENSG0000	2.46E-14	hsa-let-7a-5p;hsa-let-7b-5p;hsa-let-7d-5p;hsa-let-7e-5p;hsa-l
ENSG0000	ENSG0000	3.33E-07	hsa-let-7a-5p;hsa-let-7b-5p;hsa-let-7d-5p;hsa-let-7e-5p;hsa-l
ENSG0000	ENSG0000	5.42E-07	hsa-let-7a-5p;hsa-let-7d-5p;hsa-let-7e-5p;hsa-let-7f-5p
ENSG0000	ENSG0000	8.10E-06	hsa-let-7a-5p;hsa-let-7d-5p;hsa-let-7e-5p;hsa-let-7g-5p
ENSG0000	ENSG0000	7.85E-13	hsa-let-7a-5p;hsa-let-7b-5p;hsa-let-7d-5p;hsa-let-7e-5p;hsa-l
ENSG0000	ENSG0000	5.12E-10	hsa-let-7a-5p;hsa-let-7b-5p;hsa-let-7d-5p;hsa-let-7e-5p;hsa-l
ENSG0000	ENSG0000	3.38E-14	hsa-let-7a-5p;hsa-let-7b-5p;hsa-let-7d-5p;hsa-let-7e-5p;hsa-l
ENSG0000	ENSG0000	3.38E-14	hsa-let-7a-5p;hsa-let-7b-5p;hsa-let-7d-5p;hsa-let-7e-5p;hsa-l
ENSG0000	ENSG0000	3.38E-14	hsa-let-7a-5p;hsa-let-7b-5p;hsa-let-7d-5p;hsa-let-7e-5p;hsa-l
ENSG0000	ENSG0000	1.35E-13	hsa-let-7a-5p;hsa-let-7b-5p;hsa-let-7d-5p;hsa-let-7e-5p;hsa-l
ENSG0000	ENSG0000	8.77E-15	hsa-let-7a-5p;hsa-let-7b-5p;hsa-let-7d-5p;hsa-let-7e-5p;hsa-l
ENSG0000	ENSG0000	8.77E-15	hsa-let-7a-5p;hsa-let-7b-5p;hsa-let-7d-5p;hsa-let-7e-5p;hsa-l
ENSG0000	ENSG0000	8.77E-15	hsa-let-7a-5p;hsa-let-7b-5p;hsa-let-7d-5p;hsa-let-7e-5p;hsa-l
ENSG0000	ENSG0000	8.77E-15	hsa-let-7a-5p;hsa-let-7b-5p;hsa-let-7d-5p;hsa-let-7e-5p;hsa-l
ENSG0000	ENSG0000	8.77E-15	hsa-let-7a-5p;hsa-let-7b-5p;hsa-let-7d-5p;hsa-let-7e-5p;hsa-l
ENSG0000	ENSG0000	8.77E-15	hsa-let-7a-5p;hsa-let-7b-5p;hsa-let-7d-5p;hsa-let-7e-5p;hsa-l
ENSG0000	ENSG0000	8.77E-15	hsa-let-7a-5p;hsa-let-7b-5p;hsa-let-7d-5p;hsa-let-7e-5p;hsa-l
ENSG0000	ENSG0000	5.82E-09	hsa-let-7a-5p;hsa-let-7b-5p;hsa-let-7d-5p;hsa-let-7e-5p;hsa-l
ENSG0000	ENSG0000	1.96E-06	hsa-let-7a-5p;hsa-let-7d-5p;hsa-let-7e-5p;hsa-let-7f-5p;hsa-le
ENSG0000	ENSG0000	3.52E-11	hsa-let-7a-5p;hsa-let-7b-5p;hsa-let-7d-5p;hsa-let-7e-5p;hsa-l
ENSG0000	ENSG0000	2.80E-11	hsa-let-7a-5p;hsa-let-7b-5p;hsa-let-7d-5p;hsa-let-7e-5p;hsa-l
ENSG0000	ENSG0000	1.39E-08	hsa-let-7a-5p;hsa-let-7b-5p;hsa-let-7e-5p;hsa-let-7f-5p;hsa-le
ENSG0000	ENSG0000	3.20E-12	hsa-let-7a-5p;hsa-let-7b-5p;hsa-let-7d-5p;hsa-let-7e-5p;hsa-l
ENSG0000	ENSG0000	3.18E-10	hsa-let-7a-5p;hsa-let-7b-5p;hsa-let-7d-5p;hsa-let-7e-5p;hsa-l
ENSG0000	ENSG0000	2.49E-11	hsa-let-7a-5p;hsa-let-7b-5p;hsa-let-7d-5p;hsa-let-7e-5p;hsa-l
ENSG0000	ENSG0000	2.80E-08	hsa-let-7a-5p;hsa-let-7d-5p;hsa-let-7f-5p;hsa-let-7g-5p;hsa-le
ENSG0000	ENSG0000	1.91E-10	hsa-let-7a-5p;hsa-let-7b-5p;hsa-let-7d-5p;hsa-let-7e-5p;hsa-l
ENSG0000	ENSG0000	5.26E-13	hsa-let-7a-5p;hsa-let-7b-5p;hsa-let-7d-5p;hsa-let-7e-5p;hsa-l
ENSG0000	ENSG0000	6.42E-10	hsa-let-7a-5p;hsa-let-7b-5p;hsa-let-7d-5p;hsa-let-7e-5p;hsa-l
ENSG0000	ENSG0000	2.21E-11	hsa-let-7a-5p;hsa-let-7b-5p;hsa-let-7d-5p;hsa-let-7e-5p;hsa-l
ENSG0000	ENSG0000	8.87E-12	hsa-let-7a-5p;hsa-let-7b-5p;hsa-let-7d-5p;hsa-let-7e-5p;hsa-l
ENSG0000	ENSG0000	1.87E-09	hsa-let-7a-5p;hsa-let-7d-5p;hsa-let-7e-5p;hsa-let-7f-5p;hsa-le
ENSG0000	ENSG0000	5.57E-07	hsa-let-7a-5p;hsa-let-7b-5p;hsa-let-7d-5p;hsa-let-7e-5p;hsa-l
ENSG0000	ENSG0000	5.57E-07	hsa-let-7a-5p;hsa-let-7b-5p;hsa-let-7d-5p;hsa-let-7e-5p;hsa-l

ENSG0000	ENSG0000	2.21E-11	hsa-let-7a-5p;hsa-let-7b-5p;hsa-let-7d-5p;hsa-let-7e-5p;hsa-l
ENSG0000	ENSG0000	3.52E-11	hsa-let-7a-5p;hsa-let-7b-5p;hsa-let-7d-5p;hsa-let-7e-5p;hsa-l
ENSG0000	ENSG0000	2.72E-12	hsa-let-7a-5p;hsa-let-7b-5p;hsa-let-7d-5p;hsa-let-7e-5p;hsa-l
ENSG0000	ENSG0000	1.10E-10	hsa-let-7a-5p;hsa-let-7b-5p;hsa-let-7d-5p;hsa-let-7e-5p;hsa-l
ENSG0000	ENSG0000	1.10E-10	hsa-let-7a-5p;hsa-let-7b-5p;hsa-let-7d-5p;hsa-let-7e-5p;hsa-l
ENSG0000	ENSG0000	4.00E-15	hsa-let-7a-5p;hsa-let-7b-5p;hsa-let-7d-5p;hsa-let-7e-5p;hsa-l
ENSG0000	ENSG0000	1.96E-11	hsa-let-7a-5p;hsa-let-7b-5p;hsa-let-7d-5p;hsa-let-7e-5p;hsa-l
ENSG0000	ENSG0000	6.82E-05	hsa-let-7b-5p;hsa-let-7e-5p;hsa-let-7f-5p;hsa-let-7i-5p
ENSG0000	ENSG0000	2.49E-11	hsa-let-7a-5p;hsa-let-7b-5p;hsa-let-7d-5p;hsa-let-7e-5p;hsa-l
ENSG0000	ENSG0000	7.39E-08	hsa-let-7a-5p;hsa-let-7b-5p;hsa-let-7d-5p;hsa-let-7e-5p;hsa-l
ENSG0000	ENSG0000	3.13E-09	hsa-let-7a-5p;hsa-let-7b-5p;hsa-let-7d-5p;hsa-let-7e-5p;hsa-l
ENSG0000	ENSG0000	3.44E-13	hsa-let-7a-5p;hsa-let-7b-5p;hsa-let-7d-5p;hsa-let-7e-5p;hsa-l
ENSG0000	ENSG0000	3.23E-06	hsa-let-7a-5p;hsa-let-7d-5p;hsa-let-7e-5p;hsa-let-7f-5p;hsa-le
ENSG0000	ENSG0000	7.43E-11	hsa-let-7a-5p;hsa-let-7b-5p;hsa-let-7d-5p;hsa-let-7e-5p;hsa-l
ENSG0000	ENSG0000	5.88E-12	hsa-let-7a-5p;hsa-let-7b-5p;hsa-let-7d-5p;hsa-let-7e-5p;hsa-l
ENSG0000	ENSG0000	5.94E-11	hsa-let-7a-5p;hsa-let-7b-5p;hsa-let-7e-5p;hsa-let-7f-5p;hsa-le
ENSG0000	ENSG0000	1.64E-12	hsa-let-7a-5p;hsa-let-7b-5p;hsa-let-7d-5p;hsa-let-7e-5p;hsa-l
ENSG0000	ENSG0000	1.13E-08	hsa-let-7a-5p;hsa-let-7b-5p;hsa-let-7d-5p;hsa-let-7e-5p;hsa-l
ENSG0000	ENSG0000	2.20E-06	hsa-let-7a-5p;hsa-let-7b-5p;hsa-let-7d-5p;hsa-let-7e-5p;hsa-l
ENSG0000	ENSG0000	2.72E-12	hsa-let-7a-5p;hsa-let-7b-5p;hsa-let-7d-5p;hsa-let-7e-5p;hsa-l
ENSG0000	ENSG0000	1.33E-07	hsa-let-7a-5p;hsa-let-7d-5p;hsa-let-7e-5p;hsa-let-7f-5p;hsa-le
ENSG0000	ENSG0000	5.45E-11	hsa-let-7a-5p;hsa-let-7b-5p;hsa-let-7d-5p;hsa-let-7e-5p;hsa-l
ENSG0000	ENSG0000	1.33E-10	hsa-let-7a-5p;hsa-let-7b-5p;hsa-let-7d-5p;hsa-let-7e-5p;hsa-l
ENSG0000	ENSG0000	6.44E-13	hsa-let-7a-5p;hsa-let-7b-5p;hsa-let-7d-5p;hsa-let-7e-5p;hsa-l
ENSG0000	ENSG0000	9.51E-13	hsa-let-7a-5p;hsa-let-7b-5p;hsa-let-7d-5p;hsa-let-7e-5p;hsa-l
ENSG0000	ENSG0000	1.78E-14	hsa-let-7a-5p;hsa-let-7b-5p;hsa-let-7d-5p;hsa-let-7e-5p;hsa-l
ENSG0000	ENSG0000	1.49E-07	hsa-let-7a-5p;hsa-let-7d-5p;hsa-let-7e-5p;hsa-let-7f-5p;hsa-le
ENSG0000	ENSG0000	1.96E-11	hsa-let-7a-5p;hsa-let-7b-5p;hsa-let-7d-5p;hsa-let-7e-5p;hsa-l
ENSG0000	ENSG0000	1.18E-11	hsa-let-7a-5p;hsa-let-7b-5p;hsa-let-7d-5p;hsa-let-7e-5p;hsa-l
ENSG0000	ENSG0000	6.71E-11	hsa-let-7a-5p;hsa-let-7b-5p;hsa-let-7d-5p;hsa-let-7e-5p;hsa-l
ENSG0000	ENSG0000	5.53E-09	hsa-let-7a-5p;hsa-let-7e-5p;hsa-let-7f-5p;hsa-let-7g-5p;hsa-le
ENSG0000	ENSG0000	6.66E-16	hsa-let-7a-5p;hsa-let-7b-5p;hsa-let-7d-5p;hsa-let-7e-5p;hsa-l
ENSG0000	ENSG0000	1.96E-06	hsa-let-7a-5p;hsa-let-7d-5p;hsa-let-7f-5p;hsa-let-7g-5p;hsa-le
ENSG0000	ENSG0000	2.49E-11	hsa-let-7a-5p;hsa-let-7b-5p;hsa-let-7d-5p;hsa-let-7e-5p;hsa-l
ENSG0000	ENSG0000	1.73E-11	hsa-let-7a-5p;hsa-let-7b-5p;hsa-let-7d-5p;hsa-let-7e-5p;hsa-l
ENSG0000	ENSG0000	1.73E-11	hsa-let-7a-5p;hsa-let-7b-5p;hsa-let-7d-5p;hsa-let-7e-5p;hsa-l
ENSG0000	ENSG0000	2.72E-12	hsa-let-7a-5p;hsa-let-7b-5p;hsa-let-7d-5p;hsa-let-7e-5p;hsa-l
ENSG0000	ENSG0000	8.99E-12	hsa-let-7a-5p;hsa-let-7b-5p;hsa-let-7d-5p;hsa-let-7e-5p;hsa-l
ENSG0000	ENSG0000	3.38E-14	hsa-let-7a-5p;hsa-let-7b-5p;hsa-let-7d-5p;hsa-let-7e-5p;hsa-l
ENSG0000	ENSG0000	1.08E-08	hsa-let-7a-5p;hsa-let-7b-5p;hsa-let-7d-5p;hsa-let-7e-5p;hsa-l
ENSG0000	ENSG0000	5.88E-12	hsa-let-7a-5p;hsa-let-7b-5p;hsa-let-7d-5p;hsa-let-7e-5p;hsa-l
ENSG0000	ENSG0000	1.18E-11	hsa-let-7a-5p;hsa-let-7b-5p;hsa-let-7d-5p;hsa-let-7e-5p;hsa-l
ENSG0000	ENSG0000	1.27E-14	hsa-let-7a-5p;hsa-let-7b-5p;hsa-let-7d-5p;hsa-let-7e-5p;hsa-l
ENSG0000	ENSG0000	3.90E-11	hsa-let-7a-5p;hsa-let-7b-5p;hsa-let-7e-5p;hsa-let-7f-5p;hsa-le
ENSG0000	ENSG0000	7.85E-13	hsa-let-7a-5p;hsa-let-7b-5p;hsa-let-7d-5p;hsa-let-7e-5p;hsa-l
ENSG0000	ENSG0000	1.34E-11	hsa-let-7a-5p;hsa-let-7b-5p;hsa-let-7d-5p;hsa-let-7e-5p;hsa-l
ENSG0000	ENSG0000	3.75E-10	hsa-let-7a-5p;hsa-let-7b-5p;hsa-let-7d-5p;hsa-let-7e-5p;hsa-l
ENSG0000	ENSG0000	2.21E-11	hsa-let-7a-5p;hsa-let-7b-5p;hsa-let-7d-5p;hsa-let-7e-5p;hsa-l
ENSG0000	ENSG0000	4.81E-06	hsa-let-7a-5p;hsa-let-7f-5p;hsa-let-7g-5p;hsa-let-7i-5p
ENSG0000	ENSG0000	7.12E-08	hsa-let-7a-5p;hsa-let-7b-5p;hsa-let-7d-5p;hsa-let-7e-5p;hsa-l
ENSG0000	ENSG0000	8.59E-10	hsa-let-7a-5p;hsa-let-7b-5p;hsa-let-7d-5p;hsa-let-7e-5p;hsa-l
ENSG0000	ENSG0000	8.59E-10	hsa-let-7a-5p;hsa-let-7b-5p;hsa-let-7d-5p;hsa-let-7e-5p;hsa-l
ENSG0000	ENSG0000	1.56E-06	hsa-let-7a-5p;hsa-let-7b-5p;hsa-let-7d-5p;hsa-let-7e-5p;hsa-l
ENSG0000	ENSG0000	3.38E-14	hsa-let-7a-5p;hsa-let-7b-5p;hsa-let-7d-5p;hsa-let-7e-5p;hsa-l

ENSG0000	ENSG0000	6.06E-14	hsa-let-7a-5p;hsa-let-7b-5p;hsa-let-7d-5p;hsa-let-7e-5p;hsa-l
ENSG0000	ENSG0000	6.06E-14	hsa-let-7a-5p;hsa-let-7b-5p;hsa-let-7d-5p;hsa-let-7e-5p;hsa-l
ENSG0000	ENSG0000	6.14E-09	hsa-let-7a-5p;hsa-let-7b-5p;hsa-let-7d-5p;hsa-let-7e-5p;hsa-l
ENSG0000	ENSG0000	5.08E-12	hsa-let-7a-5p;hsa-let-7b-5p;hsa-let-7d-5p;hsa-let-7e-5p;hsa-l
ENSG0000	ENSG0000	1.39E-08	hsa-let-7b-5p;hsa-let-7d-5p;hsa-let-7e-5p;hsa-let-7f-5p;hsa-le
ENSG0000	ENSG0000	1.96E-11	hsa-let-7a-5p;hsa-let-7b-5p;hsa-let-7d-5p;hsa-let-7e-5p;hsa-l
ENSG0000	ENSG0000	1.91E-10	hsa-let-7a-5p;hsa-let-7b-5p;hsa-let-7d-5p;hsa-let-7e-5p;hsa-l
ENSG0000	ENSG0000	5.12E-10	hsa-let-7a-5p;hsa-let-7b-5p;hsa-let-7d-5p;hsa-let-7e-5p;hsa-l
ENSG0000	ENSG0000	1.35E-13	hsa-let-7a-5p;hsa-let-7b-5p;hsa-let-7d-5p;hsa-let-7e-5p;hsa-l
ENSG0000	ENSG0000	1.09E-08	hsa-let-7a-5p;hsa-let-7b-5p;hsa-let-7d-5p;hsa-let-7f-5p;hsa-le
ENSG0000	ENSG0000	2.62E-09	hsa-let-7a-5p;hsa-let-7b-5p;hsa-let-7d-5p;hsa-let-7e-5p;hsa-l
ENSG0000	ENSG0000	2.21E-11	hsa-let-7a-5p;hsa-let-7b-5p;hsa-let-7d-5p;hsa-let-7e-5p;hsa-l
ENSG0000	ENSG0000	1.91E-10	hsa-let-7a-5p;hsa-let-7b-5p;hsa-let-7d-5p;hsa-let-7e-5p;hsa-l
ENSG0000	ENSG0000	1.35E-05	hsa-let-7a-5p;hsa-let-7d-5p;hsa-let-7f-5p;hsa-let-7g-5p;hsa-le
ENSG0000	ENSG0000	4.14E-07	hsa-let-7d-5p;hsa-let-7e-5p;hsa-let-7f-5p;hsa-let-7g-5p;hsa-le
ENSG0000	ENSG0000	4.14E-07	hsa-let-7d-5p;hsa-let-7e-5p;hsa-let-7f-5p;hsa-let-7g-5p;hsa-le
ENSG0000	ENSG0000	3.13E-09	hsa-let-7a-5p;hsa-let-7b-5p;hsa-let-7d-5p;hsa-let-7e-5p;hsa-l
ENSG0000	ENSG0000	1.96E-11	hsa-let-7a-5p;hsa-let-7b-5p;hsa-let-7d-5p;hsa-let-7e-5p;hsa-l
ENSG0000	ENSG0000	4.37E-12	hsa-let-7a-5p;hsa-let-7b-5p;hsa-let-7d-5p;hsa-let-7e-5p;hsa-l
ENSG0000	ENSG0000	8.38E-09	hsa-let-7a-5p;hsa-let-7d-5p;hsa-let-7e-5p;hsa-let-7f-5p;hsa-le
ENSG0000	ENSG0000	8.67E-07	hsa-let-7d-5p;hsa-let-7e-5p;hsa-let-7f-5p;hsa-let-7g-5p;hsa-le
ENSG0000	ENSG0000	1.95E-12	hsa-let-7a-5p;hsa-let-7b-5p;hsa-let-7d-5p;hsa-let-7e-5p;hsa-l
ENSG0000	ENSG0000	1.95E-12	hsa-let-7a-5p;hsa-let-7b-5p;hsa-let-7d-5p;hsa-let-7e-5p;hsa-l
ENSG0000	ENSG0000	1.95E-12	hsa-let-7a-5p;hsa-let-7b-5p;hsa-let-7d-5p;hsa-let-7e-5p;hsa-l
ENSG0000	ENSG0000	5.22E-15	hsa-let-7a-5p;hsa-let-7b-5p;hsa-let-7d-5p;hsa-let-7e-5p;hsa-l
ENSG0000	ENSG0000	1.91E-10	hsa-let-7a-5p;hsa-let-7b-5p;hsa-let-7d-5p;hsa-let-7e-5p;hsa-l
ENSG0000	ENSG0000	2.75E-13	hsa-let-7a-5p;hsa-let-7b-5p;hsa-let-7d-5p;hsa-let-7e-5p;hsa-l
ENSG0000	ENSG0000	2.18E-13	hsa-let-7a-5p;hsa-let-7b-5p;hsa-let-7d-5p;hsa-let-7e-5p;hsa-l
ENSG0000	ENSG0000	5.29E-10	hsa-let-7a-5p;hsa-let-7d-5p;hsa-let-7e-5p;hsa-let-7f-5p;hsa-le
ENSG0000	ENSG0000	5.22E-09	hsa-let-7a-5p;hsa-let-7b-5p;hsa-let-7d-5p;hsa-let-7e-5p;hsa-l
ENSG0000	ENSG0000	5.22E-09	hsa-let-7a-5p;hsa-let-7b-5p;hsa-let-7d-5p;hsa-let-7e-5p;hsa-l
ENSG0000	ENSG0000	7.96E-10	hsa-let-7a-5p;hsa-let-7d-5p;hsa-let-7e-5p;hsa-let-7f-5p;hsa-le
ENSG0000	ENSG0000	6.68E-09	hsa-let-7a-5p;hsa-let-7e-5p;hsa-let-7f-5p;hsa-let-7g-5p;hsa-le
ENSG0000	ENSG0000	4.00E-15	hsa-let-7a-5p;hsa-let-7b-5p;hsa-let-7d-5p;hsa-let-7e-5p;hsa-l
ENSG0000	ENSG0000	1.72E-13	hsa-let-7a-5p;hsa-let-7b-5p;hsa-let-7d-5p;hsa-let-7e-5p;hsa-l
ENSG0000	ENSG0000	3.74E-12	hsa-let-7a-5p;hsa-let-7b-5p;hsa-let-7d-5p;hsa-let-7e-5p;hsa-l
ENSG0000	ENSG0000	1.34E-11	hsa-let-7a-5p;hsa-let-7b-5p;hsa-let-7d-5p;hsa-let-7e-5p;hsa-l
ENSG0000	ENSG0000	1.34E-11	hsa-let-7a-5p;hsa-let-7b-5p;hsa-let-7d-5p;hsa-let-7e-5p;hsa-l
ENSG0000	ENSG0000	2.75E-13	hsa-let-7a-5p;hsa-let-7b-5p;hsa-let-7d-5p;hsa-let-7e-5p;hsa-l
ENSG0000	ENSG0000	4.26E-13	hsa-let-7a-5p;hsa-let-7b-5p;hsa-let-7d-5p;hsa-let-7e-5p;hsa-l
ENSG0000	ENSG0000	1.77E-08	hsa-let-7a-5p;hsa-let-7b-5p;hsa-let-7d-5p;hsa-let-7f-5p;hsa-le
ENSG0000	ENSG0000	1.46E-10	hsa-let-7a-5p;hsa-let-7b-5p;hsa-let-7d-5p;hsa-let-7e-5p;hsa-l
ENSG0000	ENSG0000	1.03E-09	hsa-let-7a-5p;hsa-let-7b-5p;hsa-let-7e-5p;hsa-let-7f-5p;hsa-le
ENSG0000	ENSG0000	3.20E-12	hsa-let-7a-5p;hsa-let-7b-5p;hsa-let-7d-5p;hsa-let-7e-5p;hsa-l
ENSG0000	ENSG0000	9.97E-09	hsa-let-7a-5p;hsa-let-7b-5p;hsa-let-7d-5p;hsa-let-7f-5p;hsa-le
ENSG0000	ENSG0000	5.08E-12	hsa-let-7a-5p;hsa-let-7b-5p;hsa-let-7d-5p;hsa-let-7e-5p;hsa-l
ENSG0000	ENSG0000	2.05E-09	hsa-let-7a-5p;hsa-let-7b-5p;hsa-let-7d-5p;hsa-let-7e-5p;hsa-l
ENSG0000	ENSG0000	2.05E-09	hsa-let-7a-5p;hsa-let-7b-5p;hsa-let-7d-5p;hsa-let-7e-5p;hsa-l
ENSG0000	ENSG0000	6.00E-15	hsa-let-7a-5p;hsa-let-7b-5p;hsa-let-7d-5p;hsa-let-7e-5p;hsa-l
ENSG0000	ENSG0000	6.66E-16	hsa-let-7a-5p;hsa-let-7b-5p;hsa-let-7d-5p;hsa-let-7e-5p;hsa-l
ENSG0000	ENSG0000	2.49E-11	hsa-let-7a-5p;hsa-let-7b-5p;hsa-let-7d-5p;hsa-let-7e-5p;hsa-l
ENSG0000	ENSG0000	1.10E-10	hsa-let-7a-5p;hsa-let-7b-5p;hsa-let-7d-5p;hsa-let-7e-5p;hsa-l
ENSG0000	ENSG0000	1.52E-11	hsa-let-7a-5p;hsa-let-7b-5p;hsa-let-7d-5p;hsa-let-7e-5p;hsa-l
ENSG0000	ENSG0000	1.80E-09	hsa-let-7a-5p;hsa-let-7b-5p;hsa-let-7d-5p;hsa-let-7e-5p;hsa-l

ENSG0000	ENSG0000	4.39E-10	hsa-let-7a-5p;hsa-let-7b-5p;hsa-let-7d-5p;hsa-let-7e-5p;hsa-l
ENSG0000	ENSG0000	2.18E-08	hsa-let-7a-5p;hsa-let-7b-5p;hsa-let-7d-5p;hsa-let-7e-5p;hsa-l
ENSG0000	ENSG0000	3.80E-08	hsa-let-7a-5p;hsa-let-7b-5p;hsa-let-7d-5p;hsa-let-7e-5p;hsa-l
ENSG0000	ENSG0000	2.75E-13	hsa-let-7a-5p;hsa-let-7b-5p;hsa-let-7d-5p;hsa-let-7e-5p;hsa-l
ENSG0000	ENSG0000	9.99E-16	hsa-let-7a-5p;hsa-let-7b-5p;hsa-let-7d-5p;hsa-let-7e-5p;hsa-l
ENSG0000	ENSG0000	6.05E-11	hsa-let-7a-5p;hsa-let-7b-5p;hsa-let-7d-5p;hsa-let-7e-5p;hsa-l
ENSG0000	ENSG0000	2.18E-13	hsa-let-7a-5p;hsa-let-7b-5p;hsa-let-7d-5p;hsa-let-7e-5p;hsa-l
ENSG0000	ENSG0000	2.31E-12	hsa-let-7a-5p;hsa-let-7b-5p;hsa-let-7d-5p;hsa-let-7e-5p;hsa-l
ENSG0000	ENSG0000	1.78E-14	hsa-let-7a-5p;hsa-let-7b-5p;hsa-let-7d-5p;hsa-let-7e-5p;hsa-l
ENSG0000	ENSG0000	6.06E-14	hsa-let-7a-5p;hsa-let-7b-5p;hsa-let-7d-5p;hsa-let-7e-5p;hsa-l
ENSG0000	ENSG0000	6.06E-14	hsa-let-7a-5p;hsa-let-7b-5p;hsa-let-7d-5p;hsa-let-7e-5p;hsa-l
ENSG0000	ENSG0000	5.22E-09	hsa-let-7a-5p;hsa-let-7b-5p;hsa-let-7d-5p;hsa-let-7e-5p;hsa-l
ENSG0000	ENSG0000	3.44E-13	hsa-let-7a-5p;hsa-let-7b-5p;hsa-let-7d-5p;hsa-let-7e-5p;hsa-l
ENSG0000	ENSG0000	1.00E-10	hsa-let-7a-5p;hsa-let-7b-5p;hsa-let-7d-5p;hsa-let-7e-5p;hsa-l
ENSG0000	ENSG0000	3.45E-10	hsa-let-7a-5p;hsa-let-7b-5p;hsa-let-7d-5p;hsa-let-7e-5p;hsa-l
ENSG0000	ENSG0000	8.87E-12	hsa-let-7a-5p;hsa-let-7b-5p;hsa-let-7e-5p;hsa-let-7f-5p;hsa-le
ENSG0000	ENSG0000	6.71E-11	hsa-let-7a-5p;hsa-let-7b-5p;hsa-let-7d-5p;hsa-let-7e-5p;hsa-l
ENSG0000	ENSG0000	6.71E-11	hsa-let-7a-5p;hsa-let-7b-5p;hsa-let-7d-5p;hsa-let-7e-5p;hsa-l
ENSG0000	ENSG0000	2.43E-08	hsa-let-7d-5p;hsa-let-7e-5p;hsa-let-7f-5p;hsa-let-7g-5p;hsa-le
ENSG0000	ENSG0000	7.43E-11	hsa-let-7a-5p;hsa-let-7b-5p;hsa-let-7d-5p;hsa-let-7e-5p;hsa-l
ENSG0000	ENSG0000	1.27E-10	hsa-let-7a-5p;hsa-let-7b-5p;hsa-let-7e-5p;hsa-let-7f-5p;hsa-le
ENSG0000	ENSG0000	8.81E-11	hsa-let-7b-5p;hsa-let-7d-5p;hsa-let-7e-5p;hsa-let-7f-5p;hsa-le
ENSG0000	ENSG0000	6.66E-16	hsa-let-7a-5p;hsa-let-7b-5p;hsa-let-7d-5p;hsa-let-7e-5p;hsa-l
ENSG0000	ENSG0000	8.77E-15	hsa-let-7a-5p;hsa-let-7b-5p;hsa-let-7d-5p;hsa-let-7e-5p;hsa-l
ENSG0000	ENSG0000	8.77E-15	hsa-let-7a-5p;hsa-let-7b-5p;hsa-let-7d-5p;hsa-let-7e-5p;hsa-l
ENSG0000	ENSG0000	1.96E-11	hsa-let-7a-5p;hsa-let-7b-5p;hsa-let-7d-5p;hsa-let-7e-5p;hsa-l
ENSG0000	ENSG0000	5.53E-10	hsa-let-7a-5p;hsa-let-7b-5p;hsa-let-7d-5p;hsa-let-7e-5p;hsa-l
ENSG0000	ENSG0000	1.52E-11	hsa-let-7a-5p;hsa-let-7b-5p;hsa-let-7d-5p;hsa-let-7e-5p;hsa-l
ENSG0000	ENSG0000	4.39E-10	hsa-let-7a-5p;hsa-let-7b-5p;hsa-let-7d-5p;hsa-let-7e-5p;hsa-l
ENSG0000	ENSG0000	5.45E-11	hsa-let-7a-5p;hsa-let-7b-5p;hsa-let-7d-5p;hsa-let-7e-5p;hsa-l
ENSG0000	ENSG0000	3.67E-08	hsa-let-7a-5p;hsa-let-7d-5p;hsa-let-7e-5p;hsa-let-7f-5p;hsa-le
ENSG0000	ENSG0000	3.52E-11	hsa-let-7a-5p;hsa-let-7b-5p;hsa-let-7d-5p;hsa-let-7e-5p;hsa-l
ENSG0000	ENSG0000	4.26E-13	hsa-let-7a-5p;hsa-let-7b-5p;hsa-let-7d-5p;hsa-let-7e-5p;hsa-l
ENSG0000	ENSG0000	1.80E-09	hsa-let-7a-5p;hsa-let-7b-5p;hsa-let-7d-5p;hsa-let-7e-5p;hsa-l
ENSG0000	ENSG0000	4.06E-10	hsa-let-7a-5p;hsa-let-7b-5p;hsa-let-7d-5p;hsa-let-7e-5p;hsa-l
ENSG0000	ENSG0000	9.97E-09	hsa-let-7a-5p;hsa-let-7d-5p;hsa-let-7e-5p;hsa-let-7f-5p;hsa-le
ENSG0000	ENSG0000	2.28E-10	hsa-let-7a-5p;hsa-let-7b-5p;hsa-let-7d-5p;hsa-let-7e-5p;hsa-l
ENSG0000	ENSG0000	2.18E-13	hsa-let-7a-5p;hsa-let-7b-5p;hsa-let-7d-5p;hsa-let-7e-5p;hsa-l
ENSG0000	ENSG0000	3.44E-13	hsa-let-7a-5p;hsa-let-7b-5p;hsa-let-7d-5p;hsa-let-7e-5p;hsa-l
ENSG0000	ENSG0000	2.72E-12	hsa-let-7a-5p;hsa-let-7b-5p;hsa-let-7d-5p;hsa-let-7e-5p;hsa-l
ENSG0000	ENSG0000	2.96E-08	hsa-let-7a-5p;hsa-let-7b-5p;hsa-let-7d-5p;hsa-let-7e-5p;hsa-l
ENSG0000	ENSG0000	1.96E-11	hsa-let-7a-5p;hsa-let-7b-5p;hsa-let-7d-5p;hsa-let-7e-5p;hsa-l
ENSG0000	ENSG0000	1.35E-13	hsa-let-7a-5p;hsa-let-7b-5p;hsa-let-7d-5p;hsa-let-7e-5p;hsa-l
ENSG0000	ENSG0000	3.57E-09	hsa-let-7a-5p;hsa-let-7b-5p;hsa-let-7e-5p;hsa-let-7f-5p;hsa-le
ENSG0000	ENSG0000	1.15E-12	hsa-let-7a-5p;hsa-let-7b-5p;hsa-let-7d-5p;hsa-let-7e-5p;hsa-l
ENSG0000	ENSG0000	1.91E-07	hsa-let-7b-5p;hsa-let-7e-5p;hsa-let-7f-5p;hsa-let-7g-5p;hsa-le
ENSG0000	ENSG0000	4.74E-10	hsa-let-7a-5p;hsa-let-7b-5p;hsa-let-7d-5p;hsa-let-7e-5p;hsa-l
ENSG0000	ENSG0000	8.00E-10	hsa-let-7a-5p;hsa-let-7b-5p;hsa-let-7d-5p;hsa-let-7e-5p;hsa-l
ENSG0000	ENSG0000	1.13E-08	hsa-let-7a-5p;hsa-let-7b-5p;hsa-let-7d-5p;hsa-let-7e-5p;hsa-l
ENSG0000	ENSG0000	6.00E-15	hsa-let-7a-5p;hsa-let-7b-5p;hsa-let-7d-5p;hsa-let-7e-5p;hsa-l
ENSG0000	ENSG0000	3.52E-11	hsa-let-7a-5p;hsa-let-7b-5p;hsa-let-7d-5p;hsa-let-7e-5p;hsa-l
ENSG0000	ENSG0000	1.75E-10	hsa-let-7a-5p;hsa-let-7b-5p;hsa-let-7d-5p;hsa-let-7e-5p;hsa-l
ENSG0000	ENSG0000	4.42E-09	hsa-let-7a-5p;hsa-let-7b-5p;hsa-let-7d-5p;hsa-let-7e-5p;hsa-l
ENSG0000	ENSG0000	1.03E-09	hsa-let-7a-5p;hsa-let-7b-5p;hsa-let-7e-5p;hsa-let-7f-5p;hsa-le

ENSG0000	ENSG0000	2.95E-09	hsa-let-7a-5p;hsa-let-7b-5p;hsa-let-7d-5p;hsa-let-7e-5p;hsa-l
ENSG0000	ENSG0000	3.14E-11	hsa-let-7a-5p;hsa-let-7b-5p;hsa-let-7d-5p;hsa-let-7e-5p;hsa-l
ENSG0000	ENSG0000	9.51E-13	hsa-let-7a-5p;hsa-let-7b-5p;hsa-let-7d-5p;hsa-let-7e-5p;hsa-l
ENSG0000	ENSG0000	2.14E-07	hsa-let-7a-5p;hsa-let-7d-5p;hsa-let-7e-5p;hsa-let-7f-5p;hsa-le
ENSG0000	ENSG0000	5.45E-11	hsa-let-7a-5p;hsa-let-7b-5p;hsa-let-7d-5p;hsa-let-7e-5p;hsa-l
ENSG0000	ENSG0000	8.22E-11	hsa-let-7a-5p;hsa-let-7b-5p;hsa-let-7d-5p;hsa-let-7e-5p;hsa-l
ENSG0000	ENSG0000	1.69E-09	hsa-let-7a-5p;hsa-let-7b-5p;hsa-let-7d-5p;hsa-let-7e-5p;hsa-l
ENSG0000	ENSG0000	2.46E-14	hsa-let-7a-5p;hsa-let-7b-5p;hsa-let-7d-5p;hsa-let-7e-5p;hsa-l
ENSG0000	ENSG0000	2.66E-15	hsa-let-7a-5p;hsa-let-7b-5p;hsa-let-7d-5p;hsa-let-7e-5p;hsa-l
ENSG0000	ENSG0000	3.75E-10	hsa-let-7a-5p;hsa-let-7b-5p;hsa-let-7d-5p;hsa-let-7e-5p;hsa-l
ENSG0000	ENSG0000	1.30E-09	hsa-let-7a-5p;hsa-let-7b-5p;hsa-let-7d-5p;hsa-let-7e-5p;hsa-l
ENSG0000	ENSG0000	8.81E-11	hsa-let-7a-5p;hsa-let-7b-5p;hsa-let-7d-5p;hsa-let-7e-5p;hsa-l
ENSG0000	ENSG0000	1.73E-11	hsa-let-7a-5p;hsa-let-7b-5p;hsa-let-7d-5p;hsa-let-7e-5p;hsa-l
ENSG0000	ENSG0000	5.53E-10	hsa-let-7a-5p;hsa-let-7b-5p;hsa-let-7d-5p;hsa-let-7e-5p;hsa-l
ENSG0000	ENSG0000	8.59E-10	hsa-let-7a-5p;hsa-let-7b-5p;hsa-let-7d-5p;hsa-let-7e-5p;hsa-l
ENSG0000	ENSG0000	1.27E-14	hsa-let-7a-5p;hsa-let-7b-5p;hsa-let-7d-5p;hsa-let-7e-5p;hsa-l
ENSG0000	ENSG0000	1.15E-12	hsa-let-7a-5p;hsa-let-7b-5p;hsa-let-7d-5p;hsa-let-7e-5p;hsa-l
ENSG0000	ENSG0000	5.96E-10	hsa-let-7a-5p;hsa-let-7b-5p;hsa-let-7d-5p;hsa-let-7e-5p;hsa-l
ENSG0000	ENSG0000	4.74E-10	hsa-let-7a-5p;hsa-let-7b-5p;hsa-let-7d-5p;hsa-let-7e-5p;hsa-l
ENSG0000	ENSG0000	3.13E-09	hsa-let-7a-5p;hsa-let-7b-5p;hsa-let-7d-5p;hsa-let-7e-5p;hsa-l
ENSG0000	ENSG0000	1.60E-10	hsa-let-7a-5p;hsa-let-7b-5p;hsa-let-7d-5p;hsa-let-7e-5p;hsa-l
ENSG0000	ENSG0000	1.91E-08	hsa-let-7a-5p;hsa-let-7b-5p;hsa-let-7d-5p;hsa-let-7e-5p;hsa-l
ENSG0000	ENSG0000	2.18E-13	hsa-let-7a-5p;hsa-let-7b-5p;hsa-let-7d-5p;hsa-let-7e-5p;hsa-l
ENSG0000	ENSG0000	1.73E-11	hsa-let-7a-5p;hsa-let-7b-5p;hsa-let-7d-5p;hsa-let-7e-5p;hsa-l
ENSG0000	ENSG0000	5.08E-12	hsa-let-7a-5p;hsa-let-7b-5p;hsa-let-7d-5p;hsa-let-7e-5p;hsa-l
ENSG0000	ENSG0000	3.95E-09	hsa-let-7a-5p;hsa-let-7b-5p;hsa-let-7e-5p;hsa-let-7f-5p;hsa-le
ENSG0000	ENSG0000	1.35E-13	hsa-let-7a-5p;hsa-let-7b-5p;hsa-let-7d-5p;hsa-let-7e-5p;hsa-l
ENSG0000	ENSG0000	1.38E-08	hsa-let-7a-5p;hsa-let-7b-5p;hsa-let-7d-5p;hsa-let-7e-5p;hsa-l
ENSG0000	ENSG0000	4.55E-14	hsa-let-7a-5p;hsa-let-7b-5p;hsa-let-7d-5p;hsa-let-7e-5p;hsa-l
ENSG0000	ENSG0000	1.92E-09	hsa-let-7a-5p;hsa-let-7b-5p;hsa-let-7d-5p;hsa-let-7e-5p;hsa-l
ENSG0000	ENSG0000	3.14E-11	hsa-let-7a-5p;hsa-let-7b-5p;hsa-let-7d-5p;hsa-let-7e-5p;hsa-l
ENSG0000	ENSG0000	2.04E-07	hsa-let-7a-5p;hsa-let-7b-5p;hsa-let-7e-5p;hsa-let-7f-5p;hsa-le
ENSG0000	ENSG0000	6.44E-13	hsa-let-7a-5p;hsa-let-7b-5p;hsa-let-7d-5p;hsa-let-7e-5p;hsa-l
ENSG0000	ENSG0000	6.79E-12	hsa-let-7a-5p;hsa-let-7b-5p;hsa-let-7d-5p;hsa-let-7e-5p;hsa-l
ENSG0000	ENSG0000	2.18E-09	hsa-let-7a-5p;hsa-let-7b-5p;hsa-let-7d-5p;hsa-let-7e-5p;hsa-l
ENSG0000	ENSG0000	8.99E-12	hsa-let-7a-5p;hsa-let-7b-5p;hsa-let-7d-5p;hsa-let-7e-5p;hsa-l
ENSG0000	ENSG0000	6.06E-14	hsa-let-7a-5p;hsa-let-7b-5p;hsa-let-7d-5p;hsa-let-7e-5p;hsa-l
ENSG0000	ENSG0000	3.20E-12	hsa-let-7a-5p;hsa-let-7b-5p;hsa-let-7d-5p;hsa-let-7e-5p;hsa-l
ENSG0000	ENSG0000	5.12E-10	hsa-let-7a-5p;hsa-let-7b-5p;hsa-let-7d-5p;hsa-let-7e-5p;hsa-l
ENSG0000	ENSG0000	1.46E-10	hsa-let-7a-5p;hsa-let-7b-5p;hsa-let-7d-5p;hsa-let-7e-5p;hsa-l
ENSG0000	ENSG0000	2.72E-12	hsa-let-7a-5p;hsa-let-7b-5p;hsa-let-7d-5p;hsa-let-7e-5p;hsa-l
ENSG0000	ENSG0000	7.93E-08	hsa-let-7a-5p;hsa-let-7d-5p;hsa-let-7e-5p;hsa-let-7f-5p;hsa-le
ENSG0000	ENSG0000	7.85E-13	hsa-let-7a-5p;hsa-let-7b-5p;hsa-let-7d-5p;hsa-let-7e-5p;hsa-l
ENSG0000	ENSG0000	2.26E-07	hsa-let-7a-5p;hsa-let-7d-5p;hsa-let-7e-5p;hsa-let-7f-5p;hsa-le
ENSG0000	ENSG0000	9.99E-16	hsa-let-7a-5p;hsa-let-7b-5p;hsa-let-7d-5p;hsa-let-7e-5p;hsa-l
ENSG0000	ENSG0000	2.49E-11	hsa-let-7a-5p;hsa-let-7b-5p;hsa-let-7d-5p;hsa-let-7e-5p;hsa-l
ENSG0000	ENSG0000	7.85E-13	hsa-let-7a-5p;hsa-let-7b-5p;hsa-let-7d-5p;hsa-let-7e-5p;hsa-l
ENSG0000	ENSG0000	3.73E-09	hsa-let-7a-5p;hsa-let-7b-5p;hsa-let-7d-5p;hsa-let-7e-5p;hsa-l
ENSG0000	ENSG0000	1.18E-11	hsa-let-7a-5p;hsa-let-7b-5p;hsa-let-7d-5p;hsa-let-7e-5p;hsa-l
ENSG0000	ENSG0000	2.72E-08	hsa-let-7a-5p;hsa-let-7b-5p;hsa-let-7d-5p;hsa-let-7e-5p;hsa-l
ENSG0000	ENSG0000	0.002434	hsa-miR-15a-5p;hsa-miR-15b-5p;hsa-miR-424-5p;hsa-miR-49
ENSG0000	ENSG0000	1.54E-05	hsa-miR-15a-5p;hsa-miR-15b-5p;hsa-miR-16-5p;hsa-miR-424-
ENSG0000	ENSG0000	2.74E-07	hsa-miR-15a-5p;hsa-miR-15b-5p;hsa-miR-16-5p;hsa-miR-424-
ENSG0000	ENSG0000	1.79E-05	hsa-miR-15a-5p;hsa-miR-15b-5p;hsa-miR-16-5p;hsa-miR-424-

ENSG0000	ENSG0000	6.19E-05	hsa-miR-15a-5p;hsa-miR-15b-5p;hsa-miR-424-5p;hsa-miR-49
ENSG0000	ENSG0000	9.47E-07	hsa-miR-15a-5p;hsa-miR-15b-5p;hsa-miR-16-5p;hsa-miR-424-
ENSG0000	ENSG0000	0.000561	hsa-miR-15a-5p;hsa-miR-15b-5p;hsa-miR-424-5p;hsa-miR-49
ENSG0000	ENSG0000	0.000128	hsa-miR-15a-5p;hsa-miR-15b-5p;hsa-miR-16-5p;hsa-miR-424-
ENSG0000	ENSG0000	0.000128	hsa-miR-15a-5p;hsa-miR-15b-5p;hsa-miR-16-5p;hsa-miR-424-
ENSG0000	ENSG0000	0.000266	hsa-miR-15a-5p;hsa-miR-15b-5p;hsa-miR-16-5p;hsa-miR-200
ENSG0000	ENSG0000	5.09E-06	hsa-miR-15a-5p;hsa-miR-15b-5p;hsa-miR-16-5p;hsa-miR-32-
ENSG0000	ENSG0000	0.001466	hsa-miR-15a-5p;hsa-miR-15b-5p;hsa-miR-16-5p;hsa-miR-193
ENSG0000	ENSG0000	2.61E-06	hsa-miR-15a-5p;hsa-miR-15b-5p;hsa-miR-16-5p;hsa-miR-200
ENSG0000	ENSG0000	6.19E-05	hsa-miR-15a-5p;hsa-miR-16-5p;hsa-miR-200b-3p;hsa-miR-20
ENSG0000	ENSG0000	3.46E-05	hsa-miR-15a-5p;hsa-miR-15b-5p;hsa-miR-16-5p;hsa-miR-652-
ENSG0000	ENSG0000	0.000138	hsa-miR-145-5p;hsa-miR-17-3p;hsa-miR-378a-3p;hsa-miR-42
ENSG0000	ENSG0000	0.000128	hsa-miR-15a-5p;hsa-miR-15b-5p;hsa-miR-16-5p;hsa-miR-424-
ENSG0000	ENSG0000	0.004186	hsa-miR-218-5p;hsa-miR-32-5p;hsa-miR-652-3p;hsa-miR-7-5
ENSG0000	ENSG0000	0.001442	hsa-miR-15a-5p;hsa-miR-15b-5p;hsa-miR-424-5p;hsa-miR-49
ENSG0000	ENSG0000	3.46E-05	hsa-miR-15b-5p;hsa-miR-16-5p;hsa-miR-218-5p;hsa-miR-424-
ENSG0000	ENSG0000	1.73E-05	hsa-miR-15a-5p;hsa-miR-15b-5p;hsa-miR-16-5p;hsa-miR-424-
ENSG0000	ENSG0000	0.001218	hsa-miR-15a-5p;hsa-miR-15b-5p;hsa-miR-16-5p;hsa-miR-424-
ENSG0000	ENSG0000	3.09E-05	hsa-miR-16-5p;hsa-miR-200b-3p;hsa-miR-200c-3p;hsa-miR-5
ENSG0000	ENSG0000	3.42E-07	hsa-miR-15a-5p;hsa-miR-15b-5p;hsa-miR-16-5p;hsa-miR-205-
ENSG0000	ENSG0000	0.001797	hsa-miR-15a-5p;hsa-miR-15b-5p;hsa-miR-16-5p;hsa-miR-205-
ENSG0000	ENSG0000	0.000371	hsa-miR-15a-5p;hsa-miR-15b-5p;hsa-miR-16-5p;hsa-miR-424-
ENSG0000	ENSG0000	0.000234	hsa-miR-15a-5p;hsa-miR-15b-5p;hsa-miR-16-5p;hsa-miR-424-
ENSG0000	ENSG0000	0.00817	hsa-miR-15a-5p;hsa-miR-17-3p;hsa-miR-200b-3p;hsa-miR-20
ENSG0000	ENSG0000	0.000254	hsa-miR-15a-5p;hsa-miR-15b-5p;hsa-miR-16-5p;hsa-miR-424-
ENSG0000	ENSG0000	0.000478	hsa-miR-15a-5p;hsa-miR-15b-5p;hsa-miR-32-5p;hsa-miR-424-
ENSG0000	ENSG0000	1.59E-06	hsa-miR-15a-5p;hsa-miR-15b-5p;hsa-miR-16-5p;hsa-miR-424-
ENSG0000	ENSG0000	0.00014	hsa-miR-15a-5p;hsa-miR-15b-5p;hsa-miR-16-5p;hsa-miR-424-
ENSG0000	ENSG0000	7.15E-07	hsa-miR-15a-5p;hsa-miR-15b-5p;hsa-miR-16-5p;hsa-miR-424-
ENSG0000	ENSG0000	0.000119	hsa-miR-15a-5p;hsa-miR-15b-5p;hsa-miR-424-5p;hsa-miR-49
ENSG0000	ENSG0000	3.03E-05	hsa-miR-15a-5p;hsa-miR-15b-5p;hsa-miR-16-5p;hsa-miR-17-
ENSG0000	ENSG0000	0.001428	hsa-miR-15a-5p;hsa-miR-15b-5p;hsa-miR-16-5p;hsa-miR-424-
ENSG0000	ENSG0000	0.003755	hsa-miR-15a-5p;hsa-miR-15b-5p;hsa-miR-424-5p;hsa-miR-49
ENSG0000	ENSG0000	1.68E-06	hsa-miR-15a-5p;hsa-miR-15b-5p;hsa-miR-16-5p;hsa-miR-218-
ENSG0000	ENSG0000	5.68E-06	hsa-miR-15a-5p;hsa-miR-15b-5p;hsa-miR-16-5p;hsa-miR-424-
ENSG0000	ENSG0000	1.82E-06	hsa-miR-15a-5p;hsa-miR-15b-5p;hsa-miR-16-5p;hsa-miR-32-
ENSG0000	ENSG0000	2.06E-05	hsa-miR-15a-5p;hsa-miR-15b-5p;hsa-miR-16-5p;hsa-miR-424-
ENSG0000	ENSG0000	3.38E-06	hsa-miR-15a-5p;hsa-miR-15b-5p;hsa-miR-16-5p;hsa-miR-17-
ENSG0000	ENSG0000	7.37E-05	hsa-miR-15a-5p;hsa-miR-15b-5p;hsa-miR-424-5p;hsa-miR-49
ENSG0000	ENSG0000	3.42E-07	hsa-miR-15a-5p;hsa-miR-15b-5p;hsa-miR-16-5p;hsa-miR-424-
ENSG0000	ENSG0000	3.09E-05	hsa-miR-15a-5p;hsa-miR-15b-5p;hsa-miR-218-5p;hsa-miR-42
ENSG0000	ENSG0000	0.000102	hsa-miR-200b-3p;hsa-miR-200c-3p;hsa-miR-205-5p;hsa-miR-
ENSG0000	ENSG0000	7.79E-05	hsa-miR-15a-5p;hsa-miR-15b-5p;hsa-miR-16-5p;hsa-miR-424-
ENSG0000	ENSG0000	0.009266	hsa-miR-15a-5p;hsa-miR-15b-5p;hsa-miR-424-5p;hsa-miR-49
ENSG0000	ENSG0000	0.001017	hsa-miR-15a-5p;hsa-miR-15b-5p;hsa-miR-16-5p;hsa-miR-218-
ENSG0000	ENSG0000	3.97E-05	hsa-miR-15a-5p;hsa-miR-15b-5p;hsa-miR-424-5p;hsa-miR-49
ENSG0000	ENSG0000	0.000687	hsa-miR-15a-5p;hsa-miR-15b-5p;hsa-miR-424-5p;hsa-miR-49
ENSG0000	ENSG0000	0.000561	hsa-miR-15a-5p;hsa-miR-15b-5p;hsa-miR-16-5p;hsa-miR-424-
ENSG0000	ENSG0000	6.82E-06	hsa-miR-15a-5p;hsa-miR-15b-5p;hsa-miR-205-5p;hsa-miR-42
ENSG0000	ENSG0000	0.005145	hsa-miR-15a-5p;hsa-miR-205-5p;hsa-miR-424-5p;hsa-miR-49
ENSG0000	ENSG0000	0.006522	hsa-miR-145-5p;hsa-miR-193a-3p;hsa-miR-193b-3p;hsa-miR-
ENSG0000	ENSG0000	1.18E-07	hsa-miR-200b-3p;hsa-miR-200c-3p;hsa-miR-218-5p;hsa-miR-
ENSG0000	ENSG0000	0.000466	hsa-miR-145-5p;hsa-miR-200b-3p;hsa-miR-200c-3p;hsa-miR-
ENSG0000	ENSG0000	0.000459	hsa-miR-15a-5p;hsa-miR-15b-5p;hsa-miR-218-5p;hsa-miR-42

ENSG0000	ENSG0000	7.37E-05	hsa-miR-15a-5p;hsa-miR-16-5p;hsa-miR-200b-3p;hsa-miR-20
ENSG0000	ENSG0000	9.35E-05	hsa-miR-15a-5p;hsa-miR-15b-5p;hsa-miR-32-5p;hsa-miR-424-
ENSG0000	ENSG0000	0.002189	hsa-miR-15a-5p;hsa-miR-15b-5p;hsa-miR-424-5p;hsa-miR-49
ENSG0000	ENSG0000	6.82E-06	hsa-miR-15a-5p;hsa-miR-15b-5p;hsa-miR-16-5p;hsa-miR-424-
ENSG0000	ENSG0000	8.24E-08	hsa-miR-15a-5p;hsa-miR-15b-5p;hsa-miR-16-5p;hsa-miR-424-
ENSG0000	ENSG0000	8.24E-08	hsa-miR-15a-5p;hsa-miR-15b-5p;hsa-miR-16-5p;hsa-miR-424-
ENSG0000	ENSG0000	7.16E-07	hsa-miR-15a-5p;hsa-miR-15b-5p;hsa-miR-16-5p;hsa-miR-193
ENSG0000	ENSG0000	1.13E-05	hsa-miR-15a-5p;hsa-miR-15b-5p;hsa-miR-32-5p;hsa-miR-424-
ENSG0000	ENSG0000	8.09E-05	hsa-miR-15a-5p;hsa-miR-15b-5p;hsa-miR-16-5p;hsa-miR-32-
ENSG0000	ENSG0000	3.97E-05	hsa-miR-15a-5p;hsa-miR-15b-5p;hsa-miR-16-5p;hsa-miR-218-
ENSG0000	ENSG0000	0.000337	hsa-miR-15a-5p;hsa-miR-32-5p;hsa-miR-424-5p;hsa-miR-497-
ENSG0000	ENSG0000	0.00077	hsa-miR-15a-5p;hsa-miR-16-5p;hsa-miR-193a-3p;hsa-miR-19
ENSG0000	ENSG0000	0.000377	hsa-miR-15a-5p;hsa-miR-15b-5p;hsa-miR-424-5p;hsa-miR-49
ENSG0000	ENSG0000	0.003552	hsa-miR-15a-5p;hsa-miR-15b-5p;hsa-miR-424-5p;hsa-miR-49
ENSG0000	ENSG0000	5.07E-07	hsa-miR-15a-5p;hsa-miR-15b-5p;hsa-miR-16-5p;hsa-miR-205-
ENSG0000	ENSG0000	0.000172	hsa-miR-145-5p;hsa-miR-15a-5p;hsa-miR-15b-5p;hsa-miR-16-
ENSG0000	ENSG0000	0.003755	hsa-miR-15a-5p;hsa-miR-15b-5p;hsa-miR-424-5p;hsa-miR-49
ENSG0000	ENSG0000	3.46E-05	hsa-miR-15a-5p;hsa-miR-15b-5p;hsa-miR-424-5p;hsa-miR-49
ENSG0000	ENSG0000	8.24E-08	hsa-miR-15a-5p;hsa-miR-15b-5p;hsa-miR-16-5p;hsa-miR-424-
ENSG0000	ENSG0000	8.24E-08	hsa-miR-15a-5p;hsa-miR-15b-5p;hsa-miR-16-5p;hsa-miR-424-
ENSG0000	ENSG0000	0.000975	hsa-miR-15a-5p;hsa-miR-15b-5p;hsa-miR-424-5p;hsa-miR-49
ENSG0000	ENSG0000	0.000301	hsa-miR-15a-5p;hsa-miR-15b-5p;hsa-miR-424-5p;hsa-miR-49
ENSG0000	ENSG0000	0.002813	hsa-miR-15a-5p;hsa-miR-15b-5p;hsa-miR-424-5p;hsa-miR-49
ENSG0000	ENSG0000	0.000569	hsa-miR-15a-5p;hsa-miR-15b-5p;hsa-miR-424-5p;hsa-miR-49
ENSG0000	ENSG0000	0.00077	hsa-miR-15a-5p;hsa-miR-15b-5p;hsa-miR-16-5p;hsa-miR-424-
ENSG0000	ENSG0000	8.64E-05	hsa-miR-15a-5p;hsa-miR-15b-5p;hsa-miR-16-5p;hsa-miR-424-
ENSG0000	ENSG0000	0.002661	hsa-miR-15a-5p;hsa-miR-15b-5p;hsa-miR-17-3p;hsa-miR-424-
ENSG0000	ENSG0000	3.00E-06	hsa-miR-15a-5p;hsa-miR-15b-5p;hsa-miR-16-5p;hsa-miR-218-
ENSG0000	ENSG0000	0.004649	hsa-miR-15a-5p;hsa-miR-15b-5p;hsa-miR-16-5p;hsa-miR-218-
ENSG0000	ENSG0000	1.59E-06	hsa-miR-15a-5p;hsa-miR-15b-5p;hsa-miR-16-5p;hsa-miR-424-
ENSG0000	ENSG0000	1.59E-06	hsa-miR-15a-5p;hsa-miR-15b-5p;hsa-miR-16-5p;hsa-miR-424-
ENSG0000	ENSG0000	6.29E-05	hsa-miR-15a-5p;hsa-miR-15b-5p;hsa-miR-16-5p;hsa-miR-424-
ENSG0000	ENSG0000	7.34E-05	hsa-miR-145-5p;hsa-miR-15a-5p;hsa-miR-15b-5p;hsa-miR-16-
ENSG0000	ENSG0000	0.000626	hsa-miR-145-5p;hsa-miR-15a-5p;hsa-miR-15b-5p;hsa-miR-21
ENSG0000	ENSG0000	2.06E-05	hsa-miR-15a-5p;hsa-miR-15b-5p;hsa-miR-16-5p;hsa-miR-424-
ENSG0000	ENSG0000	0.001788	hsa-miR-15a-5p;hsa-miR-15b-5p;hsa-miR-424-5p;hsa-miR-49
ENSG0000	ENSG0000	0.001285	hsa-miR-15a-5p;hsa-miR-15b-5p;hsa-miR-424-5p;hsa-miR-49
ENSG0000	ENSG0000	1.79E-05	hsa-miR-15a-5p;hsa-miR-15b-5p;hsa-miR-16-5p;hsa-miR-424-
ENSG0000	ENSG0000	0.000102	hsa-miR-15a-5p;hsa-miR-15b-5p;hsa-miR-16-5p;hsa-miR-32-
ENSG0000	ENSG0000	1.90E-07	hsa-miR-15a-5p;hsa-miR-15b-5p;hsa-miR-16-5p;hsa-miR-424-
ENSG0000	ENSG0000	2.61E-06	hsa-miR-15a-5p;hsa-miR-15b-5p;hsa-miR-16-5p;hsa-miR-32-
ENSG0000	ENSG0000	9.47E-07	hsa-miR-15a-5p;hsa-miR-15b-5p;hsa-miR-16-5p;hsa-miR-424-
ENSG0000	ENSG0000	0.000419	hsa-miR-15a-5p;hsa-miR-15b-5p;hsa-miR-424-5p;hsa-miR-49
ENSG0000	ENSG0000	0.000345	hsa-miR-15a-5p;hsa-miR-15b-5p;hsa-miR-16-5p;hsa-miR-424-
ENSG0000	ENSG0000	0.000515	hsa-miR-15a-5p;hsa-miR-15b-5p;hsa-miR-424-5p;hsa-miR-49
ENSG0000	ENSG0000	0.005676	hsa-miR-193a-3p;hsa-miR-193b-3p;hsa-miR-200b-3p;hsa-miR
ENSG0000	ENSG0000	0.000183	hsa-miR-200b-3p;hsa-miR-200c-3p;hsa-miR-205-5p;hsa-miR-
ENSG0000	ENSG0000	0.002049	hsa-miR-15a-5p;hsa-miR-15b-5p;hsa-miR-424-5p;hsa-miR-49
ENSG0000	ENSG0000	0.001338	hsa-miR-17-3p;hsa-miR-218-5p;hsa-miR-7-5p;hsa-miR-875-3
ENSG0000	ENSG0000	0.000208	hsa-miR-145-5p;hsa-miR-193a-3p;hsa-miR-378a-3p;hsa-miR-
ENSG0000	ENSG0000	1.91E-05	hsa-miR-15a-5p;hsa-miR-15b-5p;hsa-miR-16-5p;hsa-miR-218-
ENSG0000	ENSG0000	9.47E-07	hsa-miR-15a-5p;hsa-miR-15b-5p;hsa-miR-16-5p;hsa-miR-424-
ENSG0000	ENSG0000	8.13E-06	hsa-miR-15a-5p;hsa-miR-15b-5p;hsa-miR-16-5p;hsa-miR-424-
ENSG0000	ENSG0000	0.004378	hsa-miR-15a-5p;hsa-miR-15b-5p;hsa-miR-16-5p;hsa-miR-424-

ENSG0000	ENSG0000	0.000342	hsa-miR-15a-5p;hsa-miR-15b-5p;hsa-miR-16-5p;hsa-miR-200
ENSG0000	ENSG0000	8.38E-06	hsa-miR-15a-5p;hsa-miR-15b-5p;hsa-miR-16-5p;hsa-miR-17-
ENSG0000	ENSG0000	0.002328	hsa-miR-15a-5p;hsa-miR-15b-5p;hsa-miR-16-5p;hsa-miR-218-
ENSG0000	ENSG0000	0.000275	hsa-miR-15a-5p;hsa-miR-15b-5p;hsa-miR-16-5p;hsa-miR-424-
ENSG0000	ENSG0000	2.24E-07	hsa-miR-15a-5p;hsa-miR-15b-5p;hsa-miR-16-5p;hsa-miR-218-
ENSG0000	ENSG0000	1.21E-06	hsa-miR-145-5p;hsa-miR-15a-5p;hsa-miR-15b-5p;hsa-miR-16-
ENSG0000	ENSG0000	5.03E-05	hsa-miR-15a-5p;hsa-miR-15b-5p;hsa-miR-16-5p;hsa-miR-424-
ENSG0000	ENSG0000	3.68E-08	hsa-miR-15a-5p;hsa-miR-15b-5p;hsa-miR-16-5p;hsa-miR-424-
ENSG0000	ENSG0000	0.000491	hsa-miR-15a-5p;hsa-miR-15b-5p;hsa-miR-16-5p;hsa-miR-424-
ENSG0000	ENSG0000	0.000183	hsa-miR-15a-5p;hsa-miR-15b-5p;hsa-miR-424-5p;hsa-miR-49
ENSG0000	ENSG0000	0.000868	hsa-miR-15a-5p;hsa-miR-15b-5p;hsa-miR-16-5p;hsa-miR-424-
ENSG0000	ENSG0000	1.91E-05	hsa-miR-15a-5p;hsa-miR-15b-5p;hsa-miR-16-5p;hsa-miR-218-
ENSG0000	ENSG0000	3.73E-06	hsa-miR-15a-5p;hsa-miR-15b-5p;hsa-miR-16-5p;hsa-miR-424-
ENSG0000	ENSG0000	0.000216	hsa-miR-15a-5p;hsa-miR-15b-5p;hsa-miR-16-5p;hsa-miR-424-
ENSG0000	ENSG0000	7.44E-06	hsa-miR-15a-5p;hsa-miR-15b-5p;hsa-miR-424-5p;hsa-miR-49
ENSG0000	ENSG0000	5.63E-05	hsa-miR-15a-5p;hsa-miR-15b-5p;hsa-miR-16-5p;hsa-miR-424-
ENSG0000	ENSG0000	0.00032	hsa-miR-15a-5p;hsa-miR-15b-5p;hsa-miR-16-5p;hsa-miR-424-
ENSG0000	ENSG0000	0.001442	hsa-miR-15a-5p;hsa-miR-15b-5p;hsa-miR-424-5p;hsa-miR-49
ENSG0000	ENSG0000	0.000254	hsa-miR-15a-5p;hsa-miR-15b-5p;hsa-miR-16-5p;hsa-miR-424-
ENSG0000	ENSG0000	0.009266	hsa-miR-15a-5p;hsa-miR-15b-5p;hsa-miR-424-5p;hsa-miR-49
ENSG0000	ENSG0000	0.003031	hsa-miR-15a-5p;hsa-miR-15b-5p;hsa-miR-16-5p;hsa-miR-424-
ENSG0000	ENSG0000	5.15E-05	hsa-miR-15a-5p;hsa-miR-15b-5p;hsa-miR-16-5p;hsa-miR-218-
ENSG0000	ENSG0000	1.04E-06	hsa-miR-15a-5p;hsa-miR-15b-5p;hsa-miR-200b-3p;hsa-miR-2
ENSG0000	ENSG0000	8.24E-08	hsa-miR-15a-5p;hsa-miR-15b-5p;hsa-miR-16-5p;hsa-miR-424-
ENSG0000	ENSG0000	0.001428	hsa-miR-15a-5p;hsa-miR-15b-5p;hsa-miR-16-5p;hsa-miR-424-
ENSG0000	ENSG0000	1.44E-06	hsa-miR-15a-5p;hsa-miR-15b-5p;hsa-miR-16-5p;hsa-miR-218-
ENSG0000	ENSG0000	2.06E-05	hsa-miR-15a-5p;hsa-miR-15b-5p;hsa-miR-16-5p;hsa-miR-424-
ENSG0000	ENSG0000	5.42E-07	hsa-miR-15a-5p;hsa-miR-15b-5p;hsa-miR-16-5p;hsa-miR-205-
ENSG0000	ENSG0000	0.001788	hsa-miR-15a-5p;hsa-miR-15b-5p;hsa-miR-424-5p;hsa-miR-49
ENSG0000	ENSG0000	0.002335	hsa-miR-15a-5p;hsa-miR-15b-5p;hsa-miR-424-5p;hsa-miR-49
ENSG0000	ENSG0000	2.53E-06	hsa-miR-15a-5p;hsa-miR-15b-5p;hsa-miR-16-5p;hsa-miR-424-
ENSG0000	ENSG0000	1.27E-07	hsa-miR-15a-5p;hsa-miR-15b-5p;hsa-miR-16-5p;hsa-miR-424-
ENSG0000	ENSG0000	0.000183	hsa-miR-15a-5p;hsa-miR-15b-5p;hsa-miR-424-5p;hsa-miR-49
ENSG0000	ENSG0000	3.51E-05	hsa-miR-15a-5p;hsa-miR-15b-5p;hsa-miR-16-5p;hsa-miR-424-
ENSG0000	ENSG0000	0.00038	hsa-miR-15a-5p;hsa-miR-15b-5p;hsa-miR-16-5p;hsa-miR-424-
ENSG0000	ENSG0000	7.16E-07	hsa-miR-15a-5p;hsa-miR-15b-5p;hsa-miR-16-5p;hsa-miR-32-
ENSG0000	ENSG0000	0.000675	hsa-miR-15a-5p;hsa-miR-15b-5p;hsa-miR-16-5p;hsa-miR-423-
ENSG0000	ENSG0000	0.000159	hsa-miR-15a-5p;hsa-miR-15b-5p;hsa-miR-424-5p;hsa-miR-49
ENSG0000	ENSG0000	1.22E-06	hsa-miR-15a-5p;hsa-miR-15b-5p;hsa-miR-16-5p;hsa-miR-32-
ENSG0000	ENSG0000	5.11E-08	hsa-miR-15a-5p;hsa-miR-15b-5p;hsa-miR-16-5p;hsa-miR-424-
ENSG0000	ENSG0000	0.000337	hsa-miR-15a-5p;hsa-miR-15b-5p;hsa-miR-424-5p;hsa-miR-49
ENSG0000	ENSG0000	4.48E-06	hsa-miR-15a-5p;hsa-miR-15b-5p;hsa-miR-16-5p;hsa-miR-424-
ENSG0000	ENSG0000	4.48E-06	hsa-miR-15a-5p;hsa-miR-15b-5p;hsa-miR-16-5p;hsa-miR-424-
ENSG0000	ENSG0000	0.007489	hsa-miR-200b-3p;hsa-miR-200c-3p;hsa-miR-32-5p;hsa-miR-4
ENSG0000	ENSG0000	2.06E-05	hsa-miR-15a-5p;hsa-miR-15b-5p;hsa-miR-218-5p;hsa-miR-42
ENSG0000	ENSG0000	6.50E-06	hsa-miR-15a-5p;hsa-miR-15b-5p;hsa-miR-16-5p;hsa-miR-32-
ENSG0000	ENSG0000	1.56E-09	hsa-miR-15a-5p;hsa-miR-15b-5p;hsa-miR-16-5p;hsa-miR-218-
ENSG0000	ENSG0000	2.71E-05	hsa-miR-15a-5p;hsa-miR-15b-5p;hsa-miR-16-5p;hsa-miR-424-
ENSG0000	ENSG0000	0.000236	hsa-miR-15b-5p;hsa-miR-16-5p;hsa-miR-424-5p;hsa-miR-497-
ENSG0000	ENSG0000	4.24E-05	hsa-miR-15a-5p;hsa-miR-15b-5p;hsa-miR-16-5p;hsa-miR-193
ENSG0000	ENSG0000	4.24E-05	hsa-miR-15a-5p;hsa-miR-15b-5p;hsa-miR-16-5p;hsa-miR-193
ENSG0000	ENSG0000	7.44E-06	hsa-miR-145-5p;hsa-miR-15a-5p;hsa-miR-15b-5p;hsa-miR-16-
ENSG0000	ENSG0000	6.82E-06	hsa-miR-145-5p;hsa-miR-15a-5p;hsa-miR-15b-5p;hsa-miR-42
ENSG0000	ENSG0000	0.004367	hsa-miR-15a-5p;hsa-miR-15b-5p;hsa-miR-16-5p;hsa-miR-424-

ENSG0000	ENSG0000	7.79E-05	hsa-miR-15a-5p;hsa-miR-15b-5p;hsa-miR-16-5p;hsa-miR-424-
ENSG0000	ENSG0000	1.71E-05	hsa-miR-15a-5p;hsa-miR-15b-5p;hsa-miR-16-5p;hsa-miR-32-5
ENSG0000	ENSG0000	0.000233	hsa-miR-15a-5p;hsa-miR-15b-5p;hsa-miR-16-5p;hsa-miR-20a-
ENSG0000	ENSG0000	9.16E-09	hsa-miR-15a-5p;hsa-miR-15b-5p;hsa-miR-16-5p;hsa-miR-200b
ENSG0000	ENSG0000	6.30E-07	hsa-miR-15a-5p;hsa-miR-15b-5p;hsa-miR-16-5p;hsa-miR-218-
ENSG0000	ENSG0000	1.79E-05	hsa-miR-15a-5p;hsa-miR-15b-5p;hsa-miR-16-5p;hsa-miR-424-
ENSG0000	ENSG0000	1.79E-05	hsa-miR-15a-5p;hsa-miR-15b-5p;hsa-miR-16-5p;hsa-miR-424-
ENSG0000	ENSG0000	5.03E-05	hsa-miR-15a-5p;hsa-miR-15b-5p;hsa-miR-16-5p;hsa-miR-20a-
ENSG0000	ENSG0000	0.001836	hsa-miR-15a-5p;hsa-miR-15b-5p;hsa-miR-218-5p;hsa-miR-424
ENSG0000	ENSG0000	4.24E-05	hsa-miR-15a-5p;hsa-miR-15b-5p;hsa-miR-424-5p;hsa-miR-497
ENSG0000	ENSG0000	8.13E-06	hsa-miR-15a-5p;hsa-miR-15b-5p;hsa-miR-16-5p;hsa-miR-424-
ENSG0000	ENSG0000	1.71E-05	hsa-miR-15a-5p;hsa-miR-15b-5p;hsa-miR-16-5p;hsa-miR-193a
ENSG0000	ENSG0000	9.63E-06	hsa-miR-15a-5p;hsa-miR-15b-5p;hsa-miR-16-5p;hsa-miR-424-
ENSG0000	ENSG0000	2.06E-07	hsa-miR-15a-5p;hsa-miR-15b-5p;hsa-miR-16-5p;hsa-miR-193a
ENSG0000	ENSG0000	2.06E-07	hsa-miR-15a-5p;hsa-miR-15b-5p;hsa-miR-16-5p;hsa-miR-193a
ENSG0000	ENSG0000	0.002647	hsa-miR-15a-5p;hsa-miR-15b-5p;hsa-miR-424-5p;hsa-miR-497
ENSG0000	ENSG0000	2.61E-06	hsa-miR-15a-5p;hsa-miR-15b-5p;hsa-miR-16-5p;hsa-miR-32-5
ENSG0000	ENSG0000	0.000337	hsa-miR-15a-5p;hsa-miR-15b-5p;hsa-miR-424-5p;hsa-miR-497
ENSG0000	ENSG0000	8.24E-08	hsa-miR-15a-5p;hsa-miR-15b-5p;hsa-miR-16-5p;hsa-miR-424-
ENSG0000	ENSG0000	0.000254	hsa-miR-15a-5p;hsa-miR-15b-5p;hsa-miR-16-5p;hsa-miR-424-
ENSG0000	ENSG0000	5.76E-05	hsa-miR-15a-5p;hsa-miR-15b-5p;hsa-miR-218-5p;hsa-miR-424
ENSG0000	ENSG0000	0.000216	hsa-miR-15a-5p;hsa-miR-15b-5p;hsa-miR-16-5p;hsa-miR-424-
ENSG0000	ENSG0000	1.59E-06	hsa-miR-15a-5p;hsa-miR-15b-5p;hsa-miR-16-5p;hsa-miR-424-
ENSG0000	ENSG0000	7.65E-08	hsa-miR-15a-5p;hsa-miR-15b-5p;hsa-miR-16-5p;hsa-miR-200b
ENSG0000	ENSG0000	2.74E-07	hsa-miR-15a-5p;hsa-miR-15b-5p;hsa-miR-16-5p;hsa-miR-424-
ENSG0000	ENSG0000	2.79E-05	hsa-miR-15a-5p;hsa-miR-15b-5p;hsa-miR-16-5p;hsa-miR-424-
ENSG0000	ENSG0000	0.000466	hsa-miR-15a-5p;hsa-miR-15b-5p;hsa-miR-424-5p;hsa-miR-497
ENSG0000	ENSG0000	1.79E-05	hsa-miR-15a-5p;hsa-miR-15b-5p;hsa-miR-16-5p;hsa-miR-424-
ENSG0000	ENSG0000	0.000297	hsa-miR-15a-5p;hsa-miR-15b-5p;hsa-miR-16-5p;hsa-miR-424-
ENSG0000	ENSG0000	1.59E-06	hsa-miR-15a-5p;hsa-miR-15b-5p;hsa-miR-16-5p;hsa-miR-424-
ENSG0000	ENSG0000	0.000337	hsa-miR-15a-5p;hsa-miR-15b-5p;hsa-miR-424-5p;hsa-miR-497
ENSG0000	ENSG0000	0.000639	hsa-miR-15a-5p;hsa-miR-15b-5p;hsa-miR-16-5p;hsa-miR-424-
ENSG0000	ENSG0000	1.01E-05	hsa-miR-15a-5p;hsa-miR-15b-5p;hsa-miR-424-5p;hsa-miR-497
ENSG0000	ENSG0000	0.002647	hsa-miR-15a-5p;hsa-miR-15b-5p;hsa-miR-16-5p;hsa-miR-497-
ENSG0000	ENSG0000	2.21E-05	hsa-miR-15a-5p;hsa-miR-15b-5p;hsa-miR-424-5p;hsa-miR-497
ENSG0000	ENSG0000	0.000138	hsa-miR-15a-5p;hsa-miR-15b-5p;hsa-miR-424-5p;hsa-miR-497
ENSG0000	ENSG0000	0.000278	hsa-miR-15a-5p;hsa-miR-15b-5p;hsa-miR-218-5p;hsa-miR-32-
ENSG0000	ENSG0000	3.14E-06	hsa-miR-15a-5p;hsa-miR-15b-5p;hsa-miR-16-5p;hsa-miR-424-
ENSG0000	ENSG0000	0.001664	hsa-miR-15a-5p;hsa-miR-15b-5p;hsa-miR-218-5p;hsa-miR-424
ENSG0000	ENSG0000	0.001091	hsa-miR-15a-5p;hsa-miR-15b-5p;hsa-miR-16-5p;hsa-miR-424-
ENSG0000	ENSG0000	0.000466	hsa-miR-15a-5p;hsa-miR-15b-5p;hsa-miR-16-5p;hsa-miR-205-
ENSG0000	ENSG0000	0.007489	hsa-miR-15a-5p;hsa-miR-15b-5p;hsa-miR-424-5p;hsa-miR-497
ENSG0000	ENSG0000	2.39E-07	hsa-miR-15a-5p;hsa-miR-15b-5p;hsa-miR-16-5p;hsa-miR-200b
ENSG0000	ENSG0000	2.24E-07	hsa-miR-15a-5p;hsa-miR-15b-5p;hsa-miR-200b-3p;hsa-miR-42
ENSG0000	ENSG0000	0.000138	hsa-miR-15a-5p;hsa-miR-15b-5p;hsa-miR-424-5p;hsa-miR-497
ENSG0000	ENSG0000	0.000105	hsa-miR-15a-5p;hsa-miR-15b-5p;hsa-miR-16-5p;hsa-miR-424-
ENSG0000	ENSG0000	0.000626	hsa-miR-15a-5p;hsa-miR-15b-5p;hsa-miR-424-5p;hsa-miR-497
ENSG0000	ENSG0000	6.82E-06	hsa-miR-15a-5p;hsa-miR-15b-5p;hsa-miR-16-5p;hsa-miR-424-
ENSG0000	ENSG0000	7.15E-07	hsa-miR-15a-5p;hsa-miR-15b-5p;hsa-miR-16-5p;hsa-miR-424-
ENSG0000	ENSG0000	8.24E-08	hsa-miR-15a-5p;hsa-miR-15b-5p;hsa-miR-16-5p;hsa-miR-424-
ENSG0000	ENSG0000	0.000275	hsa-miR-15a-5p;hsa-miR-15b-5p;hsa-miR-16-5p;hsa-miR-424-
ENSG0000	ENSG0000	0.000236	hsa-miR-15a-5p;hsa-miR-15b-5p;hsa-miR-424-5p;hsa-miR-497
ENSG0000	ENSG0000	1.23E-06	hsa-miR-15a-5p;hsa-miR-15b-5p;hsa-miR-16-5p;hsa-miR-424-
ENSG0000	ENSG0000	2.37E-05	hsa-miR-15a-5p;hsa-miR-15b-5p;hsa-miR-16-5p;hsa-miR-424-

ENSG0000	ENSG0000	0.000254	hsa-miR-145-5p;hsa-miR-15a-5p;hsa-miR-15b-5p;hsa-miR-16-
ENSG0000	ENSG0000	7.34E-07	hsa-miR-15a-5p;hsa-miR-15b-5p;hsa-miR-16-5p;hsa-miR-32-5
ENSG0000	ENSG0000	7.44E-06	hsa-miR-15a-5p;hsa-miR-15b-5p;hsa-miR-424-5p;hsa-miR-497
ENSG0000	ENSG0000	3.86E-07	hsa-miR-15a-5p;hsa-miR-15b-5p;hsa-miR-16-5p;hsa-miR-424-
ENSG0000	ENSG0000	0.000116	hsa-miR-193a-3p;hsa-miR-193b-3p;hsa-miR-200b-3p;hsa-miR
ENSG0000	ENSG0000	0.000116	hsa-miR-193a-3p;hsa-miR-193b-3p;hsa-miR-200b-3p;hsa-miR
ENSG0000	ENSG0000	1.52E-11	hsa-miR-15a-5p;hsa-miR-15b-5p;hsa-miR-16-5p;hsa-miR-218-
ENSG0000	ENSG0000	2.29E-05	hsa-miR-15a-5p;hsa-miR-15b-5p;hsa-miR-200b-3p;hsa-miR-20
ENSG0000	ENSG0000	0.000216	hsa-miR-15a-5p;hsa-miR-15b-5p;hsa-miR-16-5p;hsa-miR-424-
ENSG0000	ENSG0000	0.004649	hsa-miR-15a-5p;hsa-miR-15b-5p;hsa-miR-424-5p;hsa-miR-497
ENSG0000	ENSG0000	1.73E-05	hsa-miR-15a-5p;hsa-miR-16-5p;hsa-miR-218-5p;hsa-miR-7-5p
ENSG0000	ENSG0000	0.000291	hsa-miR-15a-5p;hsa-miR-15b-5p;hsa-miR-16-5p;hsa-miR-17-3
ENSG0000	ENSG0000	0.000687	hsa-miR-15a-5p;hsa-miR-15b-5p;hsa-miR-424-5p;hsa-miR-497
ENSG0000	ENSG0000	8.13E-06	hsa-miR-15a-5p;hsa-miR-15b-5p;hsa-miR-16-5p;hsa-miR-424-
ENSG0000	ENSG0000	0.002987	hsa-miR-15a-5p;hsa-miR-15b-5p;hsa-miR-424-5p;hsa-miR-497
ENSG0000	ENSG0000	0.001667	hsa-miR-15a-5p;hsa-miR-15b-5p;hsa-miR-424-5p;hsa-miR-497
ENSG0000	ENSG0000	6.19E-05	hsa-miR-15a-5p;hsa-miR-15b-5p;hsa-miR-424-5p;hsa-miR-497
ENSG0000	ENSG0000	0.002487	hsa-miR-15a-5p;hsa-miR-15b-5p;hsa-miR-424-5p;hsa-miR-497
ENSG0000	ENSG0000	0.002647	hsa-miR-15a-5p;hsa-miR-15b-5p;hsa-miR-424-5p;hsa-miR-497
ENSG0000	ENSG0000	0.00014	hsa-miR-15a-5p;hsa-miR-15b-5p;hsa-miR-16-5p;hsa-miR-424-
ENSG0000	ENSG0000	1.23E-06	hsa-miR-15a-5p;hsa-miR-15b-5p;hsa-miR-16-5p;hsa-miR-424-
ENSG0000	ENSG0000	1.23E-06	hsa-miR-15a-5p;hsa-miR-15b-5p;hsa-miR-16-5p;hsa-miR-424-
ENSG0000	ENSG0000	2.78E-05	hsa-miR-15a-5p;hsa-miR-15b-5p;hsa-miR-424-5p;hsa-miR-497
ENSG0000	ENSG0000	7.15E-07	hsa-miR-15a-5p;hsa-miR-15b-5p;hsa-miR-16-5p;hsa-miR-424-
ENSG0000	ENSG0000	9.63E-06	hsa-miR-15a-5p;hsa-miR-15b-5p;hsa-miR-16-5p;hsa-miR-424-
ENSG0000	ENSG0000	2.01E-06	hsa-miR-15a-5p;hsa-miR-15b-5p;hsa-miR-16-5p;hsa-miR-424-
ENSG0000	ENSG0000	1.59E-06	hsa-miR-15a-5p;hsa-miR-15b-5p;hsa-miR-16-5p;hsa-miR-424-
ENSG0000	ENSG0000	1.33E-05	hsa-miR-15a-5p;hsa-miR-15b-5p;hsa-miR-16-5p;hsa-miR-424-
ENSG0000	ENSG0000	0.002325	hsa-miR-15a-5p;hsa-miR-15b-5p;hsa-miR-16-5p;hsa-miR-32-5
ENSG0000	ENSG0000	2.37E-05	hsa-miR-15a-5p;hsa-miR-15b-5p;hsa-miR-16-5p;hsa-miR-424-
ENSG0000	ENSG0000	1.57E-05	hsa-miR-15a-5p;hsa-miR-15b-5p;hsa-miR-16-5p;hsa-miR-32-5
ENSG0000	ENSG0000	0.009636	hsa-miR-15a-5p;hsa-miR-15b-5p;hsa-miR-16-5p;hsa-miR-424-
ENSG0000	ENSG0000	0.009636	hsa-miR-15a-5p;hsa-miR-15b-5p;hsa-miR-16-5p;hsa-miR-424-
ENSG0000	ENSG0000	0.000371	hsa-miR-15a-5p;hsa-miR-15b-5p;hsa-miR-16-5p;hsa-miR-200b
ENSG0000	ENSG0000	2.21E-05	hsa-miR-193a-3p;hsa-miR-193b-3p;hsa-miR-200b-3p;hsa-miR
ENSG0000	ENSG0000	2.21E-05	hsa-miR-193a-3p;hsa-miR-193b-3p;hsa-miR-200b-3p;hsa-miR
ENSG0000	ENSG0000	2.21E-05	hsa-miR-193a-3p;hsa-miR-193b-3p;hsa-miR-200b-3p;hsa-miR
ENSG0000	ENSG0000	2.21E-05	hsa-miR-193a-3p;hsa-miR-193b-3p;hsa-miR-200b-3p;hsa-miR
ENSG0000	ENSG0000	2.21E-05	hsa-miR-193a-3p;hsa-miR-193b-3p;hsa-miR-200b-3p;hsa-miR
ENSG0000	ENSG0000	2.21E-05	hsa-miR-193a-3p;hsa-miR-193b-3p;hsa-miR-200b-3p;hsa-miR
ENSG0000	ENSG0000	2.21E-05	hsa-miR-193a-3p;hsa-miR-193b-3p;hsa-miR-200b-3p;hsa-miR
ENSG0000	ENSG0000	1.59E-06	hsa-miR-15a-5p;hsa-miR-15b-5p;hsa-miR-16-5p;hsa-miR-424-
ENSG0000	ENSG0000	9.69E-07	hsa-miR-15a-5p;hsa-miR-15b-5p;hsa-miR-424-5p;hsa-miR-497
ENSG0000	ENSG0000	0.000128	hsa-miR-15a-5p;hsa-miR-15b-5p;hsa-miR-16-5p;hsa-miR-424-
ENSG0000	ENSG0000	0.00032	hsa-miR-15a-5p;hsa-miR-15b-5p;hsa-miR-16-5p;hsa-miR-424-
ENSG0000	ENSG0000	7.44E-06	hsa-miR-15a-5p;hsa-miR-15b-5p;hsa-miR-424-5p;hsa-miR-497
ENSG0000	ENSG0000	1.54E-05	hsa-miR-15a-5p;hsa-miR-15b-5p;hsa-miR-16-5p;hsa-miR-424-
ENSG0000	ENSG0000	0.00021	hsa-miR-15a-5p;hsa-miR-15b-5p;hsa-miR-16-5p;hsa-miR-193a
ENSG0000	ENSG0000	0.000115	hsa-miR-15a-5p;hsa-miR-15b-5p;hsa-miR-16-5p;hsa-miR-32-5
ENSG0000	ENSG0000	0.000639	hsa-miR-15a-5p;hsa-miR-15b-5p;hsa-miR-16-5p;hsa-miR-424-
ENSG0000	ENSG0000	0.000777	hsa-miR-15a-5p;hsa-miR-15b-5p;hsa-miR-16-5p;hsa-miR-17-3
ENSG0000	ENSG0000	0.000466	hsa-miR-15a-5p;hsa-miR-15b-5p;hsa-miR-424-5p;hsa-miR-497
ENSG0000	ENSG0000	6.29E-05	hsa-miR-15a-5p;hsa-miR-15b-5p;hsa-miR-16-5p;hsa-miR-193a
ENSG0000	ENSG0000	3.44E-06	hsa-miR-15a-5p;hsa-miR-15b-5p;hsa-miR-16-5p;hsa-miR-424-

ENSG0000	ENSG0000	0.000525	hsa-miR-15a-5p;hsa-miR-15b-5p;hsa-miR-16-5p;hsa-miR-424-
ENSG0000	ENSG0000	0.000254	hsa-miR-15a-5p;hsa-miR-15b-5p;hsa-miR-16-5p;hsa-miR-424-
ENSG0000	ENSG0000	0.000183	hsa-miR-15a-5p;hsa-miR-15b-5p;hsa-miR-16-5p;hsa-miR-424-
ENSG0000	ENSG0000	0.000221	hsa-miR-15a-5p;hsa-miR-15b-5p;hsa-miR-20a-3p;hsa-miR-37
ENSG0000	ENSG0000	0.000167	hsa-miR-15a-5p;hsa-miR-15b-5p;hsa-miR-16-5p;hsa-miR-424-
ENSG0000	ENSG0000	4.70E-06	hsa-miR-15a-5p;hsa-miR-15b-5p;hsa-miR-16-5p;hsa-miR-424-
ENSG0000	ENSG0000	0.000896	hsa-miR-15a-5p;hsa-miR-15b-5p;hsa-miR-424-5p;hsa-miR-49
ENSG0000	ENSG0000	0.001058	hsa-miR-15a-5p;hsa-miR-15b-5p;hsa-miR-424-5p;hsa-miR-49
ENSG0000	ENSG0000	0.001504	hsa-miR-15a-5p;hsa-miR-15b-5p;hsa-miR-16-5p;hsa-miR-424-
ENSG0000	ENSG0000	0.000183	hsa-miR-15a-5p;hsa-miR-15b-5p;hsa-miR-424-5p;hsa-miR-49
ENSG0000	ENSG0000	0.000183	hsa-miR-15a-5p;hsa-miR-15b-5p;hsa-miR-424-5p;hsa-miR-49
ENSG0000	ENSG0000	4.47E-05	hsa-miR-15a-5p;hsa-miR-15b-5p;hsa-miR-16-5p;hsa-miR-424-
ENSG0000	ENSG0000	0.000371	hsa-miR-15a-5p;hsa-miR-15b-5p;hsa-miR-16-5p;hsa-miR-424-
ENSG0000	ENSG0000	0.001119	hsa-miR-15a-5p;hsa-miR-15b-5p;hsa-miR-16-5p;hsa-miR-32-
ENSG0000	ENSG0000	2.53E-06	hsa-miR-15a-5p;hsa-miR-16-5p;hsa-miR-218-5p;hsa-miR-424-
ENSG0000	ENSG0000	5.68E-06	hsa-miR-15a-5p;hsa-miR-15b-5p;hsa-miR-16-5p;hsa-miR-424-
ENSG0000	ENSG0000	4.48E-06	hsa-miR-15a-5p;hsa-miR-15b-5p;hsa-miR-16-5p;hsa-miR-424-
ENSG0000	ENSG0000	9.56E-05	hsa-miR-15a-5p;hsa-miR-15b-5p;hsa-miR-16-5p;hsa-miR-424-
ENSG0000	ENSG0000	9.47E-07	hsa-miR-15a-5p;hsa-miR-15b-5p;hsa-miR-16-5p;hsa-miR-424-
ENSG0000	ENSG0000	0.009636	hsa-miR-15a-5p;hsa-miR-15b-5p;hsa-miR-16-5p;hsa-miR-424-
ENSG0000	ENSG0000	2.21E-05	hsa-miR-15a-5p;hsa-miR-16-5p;hsa-miR-424-5p;hsa-miR-497-
ENSG0000	ENSG0000	2.79E-05	hsa-miR-15a-5p;hsa-miR-15b-5p;hsa-miR-16-5p;hsa-miR-17-
ENSG0000	ENSG0000	0.001504	hsa-miR-15a-5p;hsa-miR-15b-5p;hsa-miR-16-5p;hsa-miR-424-
ENSG0000	ENSG0000	5.68E-06	hsa-miR-15a-5p;hsa-miR-15b-5p;hsa-miR-16-5p;hsa-miR-424-
ENSG0000	ENSG0000	2.74E-07	hsa-miR-15a-5p;hsa-miR-15b-5p;hsa-miR-16-5p;hsa-miR-424-
ENSG0000	ENSG0000	0.006847	hsa-miR-15a-5p;hsa-miR-15b-5p;hsa-miR-16-5p;hsa-miR-205-
ENSG0000	ENSG0000	0.000116	hsa-miR-15a-5p;hsa-miR-15b-5p;hsa-miR-16-5p;hsa-miR-424-
ENSG0000	ENSG0000	7.15E-07	hsa-miR-15a-5p;hsa-miR-15b-5p;hsa-miR-16-5p;hsa-miR-424-
ENSG0000	ENSG0000	7.15E-07	hsa-miR-15a-5p;hsa-miR-15b-5p;hsa-miR-16-5p;hsa-miR-424-
ENSG0000	ENSG0000	7.15E-07	hsa-miR-15a-5p;hsa-miR-15b-5p;hsa-miR-16-5p;hsa-miR-424-
ENSG0000	ENSG0000	7.15E-07	hsa-miR-15a-5p;hsa-miR-15b-5p;hsa-miR-16-5p;hsa-miR-424-
ENSG0000	ENSG0000	0.007489	hsa-miR-15a-5p;hsa-miR-15b-5p;hsa-miR-424-5p;hsa-miR-49
ENSG0000	ENSG0000	0.007489	hsa-miR-15a-5p;hsa-miR-15b-5p;hsa-miR-424-5p;hsa-miR-49
ENSG0000	ENSG0000	0.001218	hsa-miR-15a-5p;hsa-miR-15b-5p;hsa-miR-16-5p;hsa-miR-424-
ENSG0000	ENSG0000	0.000275	hsa-miR-15a-5p;hsa-miR-15b-5p;hsa-miR-16-5p;hsa-miR-424-
ENSG0000	ENSG0000	5.35E-06	hsa-miR-15a-5p;hsa-miR-15b-5p;hsa-miR-424-5p;hsa-miR-49
ENSG0000	ENSG0000	2.37E-05	hsa-miR-15a-5p;hsa-miR-15b-5p;hsa-miR-16-5p;hsa-miR-424-
ENSG0000	ENSG0000	2.37E-05	hsa-miR-15a-5p;hsa-miR-15b-5p;hsa-miR-16-5p;hsa-miR-424-
ENSG0000	ENSG0000	2.37E-05	hsa-miR-15a-5p;hsa-miR-15b-5p;hsa-miR-16-5p;hsa-miR-424-
ENSG0000	ENSG0000	2.37E-05	hsa-miR-15a-5p;hsa-miR-15b-5p;hsa-miR-16-5p;hsa-miR-424-
ENSG0000	ENSG0000	2.37E-05	hsa-miR-15a-5p;hsa-miR-15b-5p;hsa-miR-16-5p;hsa-miR-424-
ENSG0000	ENSG0000	2.37E-05	hsa-miR-15a-5p;hsa-miR-15b-5p;hsa-miR-16-5p;hsa-miR-424-
ENSG0000	ENSG0000	2.37E-05	hsa-miR-15a-5p;hsa-miR-15b-5p;hsa-miR-16-5p;hsa-miR-424-
ENSG0000	ENSG0000	2.37E-05	hsa-miR-15a-5p;hsa-miR-15b-5p;hsa-miR-16-5p;hsa-miR-424-
ENSG0000	ENSG0000	2.37E-05	hsa-miR-15a-5p;hsa-miR-15b-5p;hsa-miR-16-5p;hsa-miR-424-
ENSG0000	ENSG0000	2.37E-05	hsa-miR-15a-5p;hsa-miR-15b-5p;hsa-miR-16-5p;hsa-miR-424-
ENSG0000	ENSG0000	0.000377	hsa-miR-145-5p;hsa-miR-205-5p;hsa-miR-218-5p;hsa-miR-32-
ENSG0000	ENSG0000	1.59E-06	hsa-miR-15a-5p;hsa-miR-15b-5p;hsa-miR-16-5p;hsa-miR-424-
ENSG0000	ENSG0000	3.14E-06	hsa-miR-15a-5p;hsa-miR-15b-5p;hsa-miR-16-5p;hsa-miR-424-
ENSG0000	ENSG0000	1.95E-06	hsa-miR-15a-5p;hsa-miR-15b-5p;hsa-miR-16-5p;hsa-miR-424-
ENSG0000	ENSG0000	3.38E-06	hsa-miR-15a-5p;hsa-miR-15b-5p;hsa-miR-16-5p;hsa-miR-424-
ENSG0000	ENSG0000	2.71E-05	hsa-miR-15a-5p;hsa-miR-15b-5p;hsa-miR-16-5p;hsa-miR-424-
ENSG0000	ENSG0000	9.20E-06	hsa-miR-15a-5p;hsa-miR-15b-5p;hsa-miR-16-5p;hsa-miR-218-
ENSG0000	ENSG0000	0.000159	hsa-miR-15a-5p;hsa-miR-15b-5p;hsa-miR-424-5p;hsa-miR-49

ENSG0000	ENSG0000	0.0001	hsa-miR-15a-5p;hsa-miR-15b-5p;hsa-miR-16-5p;hsa-miR-218
ENSG0000	ENSG0000	6.50E-06	hsa-miR-15a-5p;hsa-miR-15b-5p;hsa-miR-16-5p;hsa-miR-20a
ENSG0000	ENSG0000	0.000301	hsa-miR-15a-5p;hsa-miR-15b-5p;hsa-miR-16-5p;hsa-miR-570
ENSG0000	ENSG0000	0.00032	hsa-miR-15a-5p;hsa-miR-15b-5p;hsa-miR-16-5p;hsa-miR-424
ENSG0000	ENSG0000	0.000254	hsa-miR-145-5p;hsa-miR-15a-5p;hsa-miR-15b-5p;hsa-miR-16
ENSG0000	ENSG0000	7.15E-07	hsa-miR-15a-5p;hsa-miR-15b-5p;hsa-miR-16-5p;hsa-miR-424
ENSG0000	ENSG0000	9.47E-07	hsa-miR-15a-5p;hsa-miR-15b-5p;hsa-miR-205-5p;hsa-miR-42
ENSG0000	ENSG0000	0.000208	hsa-miR-15a-5p;hsa-miR-15b-5p;hsa-miR-424-5p;hsa-miR-49
ENSG0000	ENSG0000	2.59E-09	hsa-miR-15a-5p;hsa-miR-15b-5p;hsa-miR-16-5p;hsa-miR-218
ENSG0000	ENSG0000	1.01E-05	hsa-miR-15a-5p;hsa-miR-15b-5p;hsa-miR-424-5p;hsa-miR-49
ENSG0000	ENSG0000	4.70E-06	hsa-miR-15a-5p;hsa-miR-15b-5p;hsa-miR-16-5p;hsa-miR-424
ENSG0000	ENSG0000	1.79E-05	hsa-miR-15a-5p;hsa-miR-15b-5p;hsa-miR-16-5p;hsa-miR-424
ENSG0000	ENSG0000	0.000102	hsa-miR-15a-5p;hsa-miR-15b-5p;hsa-miR-16-5p;hsa-miR-7-5
ENSG0000	ENSG0000	2.06E-05	hsa-miR-15a-5p;hsa-miR-15b-5p;hsa-miR-16-5p;hsa-miR-424
ENSG0000	ENSG0000	0.00068	hsa-miR-15a-5p;hsa-miR-15b-5p;hsa-miR-32-5p;hsa-miR-424
ENSG0000	ENSG0000	1.44E-06	hsa-miR-15a-5p;hsa-miR-15b-5p;hsa-miR-16-5p;hsa-miR-218
ENSG0000	ENSG0000	0.004413	hsa-miR-15a-5p;hsa-miR-15b-5p;hsa-miR-200b-3p;hsa-miR-2
ENSG0000	ENSG0000	0.004413	hsa-miR-15a-5p;hsa-miR-15b-5p;hsa-miR-200b-3p;hsa-miR-2
ENSG0000	ENSG0000	0.004413	hsa-miR-15a-5p;hsa-miR-15b-5p;hsa-miR-200b-3p;hsa-miR-2
ENSG0000	ENSG0000	0.004413	hsa-miR-15a-5p;hsa-miR-15b-5p;hsa-miR-200b-3p;hsa-miR-2
ENSG0000	ENSG0000	0.004413	hsa-miR-15a-5p;hsa-miR-15b-5p;hsa-miR-200b-3p;hsa-miR-2
ENSG0000	ENSG0000	0.004413	hsa-miR-15a-5p;hsa-miR-15b-5p;hsa-miR-200b-3p;hsa-miR-2
ENSG0000	ENSG0000	0.004413	hsa-miR-15a-5p;hsa-miR-15b-5p;hsa-miR-200b-3p;hsa-miR-2
ENSG0000	ENSG0000	0.004413	hsa-miR-15a-5p;hsa-miR-15b-5p;hsa-miR-200b-3p;hsa-miR-2
ENSG0000	ENSG0000	0.000105	hsa-miR-15a-5p;hsa-miR-15b-5p;hsa-miR-16-5p;hsa-miR-424
ENSG0000	ENSG0000	1.59E-06	hsa-miR-15a-5p;hsa-miR-15b-5p;hsa-miR-16-5p;hsa-miR-424
ENSG0000	ENSG0000	1.90E-07	hsa-miR-15a-5p;hsa-miR-15b-5p;hsa-miR-16-5p;hsa-miR-424
ENSG0000	ENSG0000	0.000182	hsa-miR-15a-5p;hsa-miR-15b-5p;hsa-miR-16-5p;hsa-miR-424
ENSG0000	ENSG0000	0.002904	hsa-miR-15a-5p;hsa-miR-15b-5p;hsa-miR-16-5p;hsa-miR-424
ENSG0000	ENSG0000	6.82E-06	hsa-miR-15a-5p;hsa-miR-15b-5p;hsa-miR-16-5p;hsa-miR-424
ENSG0000	ENSG0000	5.11E-08	hsa-miR-15a-5p;hsa-miR-15b-5p;hsa-miR-16-5p;hsa-miR-424
ENSG0000	ENSG0000	0.000428	hsa-miR-15a-5p;hsa-miR-15b-5p;hsa-miR-16-5p;hsa-miR-424
ENSG0000	ENSG0000	2.59E-09	hsa-miR-15a-5p;hsa-miR-15b-5p;hsa-miR-16-5p;hsa-miR-218
ENSG0000	ENSG0000	0.000975	hsa-miR-15a-5p;hsa-miR-15b-5p;hsa-miR-16-5p;hsa-miR-424
ENSG0000	ENSG0000	6.50E-06	hsa-miR-15a-5p;hsa-miR-15b-5p;hsa-miR-16-5p;hsa-miR-32-
ENSG0000	ENSG0000	9.63E-06	hsa-miR-15a-5p;hsa-miR-15b-5p;hsa-miR-16-5p;hsa-miR-424
ENSG0000	ENSG0000	2.32E-05	hsa-miR-15a-5p;hsa-miR-15b-5p;hsa-miR-16-5p;hsa-miR-424
ENSG0000	ENSG0000	0.001836	hsa-miR-15a-5p;hsa-miR-15b-5p;hsa-miR-16-5p;hsa-miR-424
ENSG0000	ENSG0000	0.002328	hsa-miR-15a-5p;hsa-miR-15b-5p;hsa-miR-16-5p;hsa-miR-218
ENSG0000	ENSG0000	1.11E-07	hsa-miR-15a-5p;hsa-miR-15b-5p;hsa-miR-16-5p;hsa-miR-218
ENSG0000	ENSG0000	0.002987	hsa-miR-15a-5p;hsa-miR-15b-5p;hsa-miR-16-5p;hsa-miR-424
ENSG0000	ENSG0000	0.000639	hsa-miR-15a-5p;hsa-miR-15b-5p;hsa-miR-16-5p;hsa-miR-424
ENSG0000	ENSG0000	0.001218	hsa-miR-15a-5p;hsa-miR-15b-5p;hsa-miR-16-5p;hsa-miR-424
ENSG0000	ENSG0000	1.57E-05	hsa-miR-15a-5p;hsa-miR-15b-5p;hsa-miR-16-5p;hsa-miR-218
ENSG0000	ENSG0000	0.000199	hsa-miR-15a-5p;hsa-miR-15b-5p;hsa-miR-16-5p;hsa-miR-424
ENSG0000	ENSG0000	0.002106	hsa-miR-10b-5p;hsa-miR-125a-5p;hsa-miR-125b-5p;hsa-miR-
ENSG0000	ENSG0000	0.008196	hsa-miR-125b-2-3p;hsa-miR-4525;hsa-miR-625-5p;hsa-miR-6
ENSG0000	ENSG0000	0.007828	hsa-miR-125a-5p;hsa-miR-125b-5p;hsa-miR-139-3p;hsa-miR-
ENSG0000	ENSG0000	0.001192	hsa-miR-181b-3p;hsa-miR-31-3p;hsa-miR-34b-5p;hsa-miR-44
ENSG0000	ENSG0000	0.002617	hsa-miR-10a-5p;hsa-miR-10b-5p;hsa-miR-125a-5p;hsa-miR-1
ENSG0000	ENSG0000	3.54E-05	hsa-miR-10b-5p;hsa-miR-125a-5p;hsa-miR-125b-5p;hsa-miR-
ENSG0000	ENSG0000	0.004587	hsa-miR-1226-3p;hsa-miR-140-5p;hsa-miR-193a-3p;hsa-miR-
ENSG0000	ENSG0000	0.005291	hsa-miR-125a-5p;hsa-miR-125b-5p;hsa-miR-185-3p;hsa-miR-
ENSG0000	ENSG0000	0.004798	hsa-miR-139-3p;hsa-miR-1914-3p;hsa-miR-296-5p;hsa-miR-4

ENSG0000	ENSG0000	0.00137	hsa-miR-1226-3p;hsa-miR-197-3p;hsa-miR-3179;hsa-miR-4525
ENSG0000	ENSG0000	0.001626	hsa-miR-140-5p;hsa-miR-30c-1-3p;hsa-miR-4525;hsa-miR-625
ENSG0000	ENSG0000	0.004045	hsa-miR-185-3p;hsa-miR-3179;hsa-miR-3677-5p;hsa-miR-4525
ENSG0000	ENSG0000	0.00311	hsa-miR-193a-3p;hsa-miR-193b-3p;hsa-miR-4690-5p;hsa-miR-
ENSG0000	ENSG0000	0.002287	hsa-miR-193a-3p;hsa-miR-193b-3p;hsa-miR-34b-5p;hsa-miR-
ENSG0000	ENSG0000	0.007828	hsa-miR-125b-2-3p;hsa-miR-185-3p;hsa-miR-3180-3p;hsa-mi
ENSG0000	ENSG0000	0.004181	hsa-miR-10a-5p;hsa-miR-34b-5p;hsa-miR-449c-5p;hsa-miR-94
ENSG0000	ENSG0000	0.003412	hsa-miR-193a-3p;hsa-miR-193b-3p;hsa-miR-30c-1-3p;hsa-mi
ENSG0000	ENSG0000	0.002371	hsa-miR-140-3p;hsa-miR-3179;hsa-miR-342-5p;hsa-miR-4525;
ENSG0000	ENSG0000	0.001441	hsa-miR-10a-5p;hsa-miR-10b-5p;hsa-miR-197-3p;hsa-miR-30
ENSG0000	ENSG0000	0.008829	hsa-miR-342-5p;hsa-miR-34b-5p;hsa-miR-449c-5p;hsa-miR-94
ENSG0000	ENSG0000	0.001997	hsa-miR-4525;hsa-miR-4786-3p;hsa-miR-625-5p;hsa-miR-940
ENSG0000	ENSG0000	0.000465	hsa-miR-10a-5p;hsa-miR-34b-5p;hsa-miR-449c-5p;hsa-miR-50
ENSG0000	ENSG0000	0.003614	hsa-miR-1226-3p;hsa-miR-185-3p;hsa-miR-296-5p;hsa-miR-3
ENSG0000	ENSG0000	0.000317	hsa-miR-185-3p;hsa-miR-34b-5p;hsa-miR-449c-5p;hsa-miR-58
ENSG0000	ENSG0000	2.08E-05	hsa-miR-125a-5p;hsa-miR-125b-5p;hsa-miR-34b-5p;hsa-miR-
ENSG0000	ENSG0000	0.000107	hsa-miR-193a-3p;hsa-miR-193b-3p;hsa-miR-3157-5p;hsa-miR
ENSG0000	ENSG0000	0.001039	hsa-miR-1914-3p;hsa-miR-4525;hsa-miR-615-5p;hsa-miR-625-
ENSG0000	ENSG0000	0.007828	hsa-miR-1226-3p;hsa-miR-197-3p;hsa-miR-30c-1-3p;hsa-miR-
ENSG0000	ENSG0000	0.005964	hsa-miR-3677-5p;hsa-miR-4690-5p;hsa-miR-7-5p;hsa-miR-940
ENSG0000	ENSG0000	0.000217	hsa-miR-3179;hsa-miR-34b-5p;hsa-miR-449c-5p;hsa-miR-4525
ENSG0000	ENSG0000	0.009263	hsa-miR-1914-3p;hsa-miR-30c-1-3p;hsa-miR-34b-5p;hsa-miR-
ENSG0000	ENSG0000	0.009263	hsa-miR-1914-3p;hsa-miR-30c-1-3p;hsa-miR-34b-5p;hsa-miR-
ENSG0000	ENSG0000	0.007386	hsa-miR-10a-5p;hsa-miR-10b-5p;hsa-miR-34b-5p;hsa-miR-449
ENSG0000	ENSG0000	0.006556	hsa-miR-125b-5p;hsa-miR-296-5p;hsa-miR-4726-3p;hsa-miR-
ENSG0000	ENSG0000	0.00137	hsa-miR-34b-5p;hsa-miR-449c-5p;hsa-miR-5008-5p;hsa-miR-
ENSG0000	ENSG0000	0.007594	hsa-miR-1254;hsa-miR-197-3p;hsa-miR-3157-5p;hsa-miR-505-
ENSG0000	ENSG0000	0.005478	hsa-miR-30c-1-3p;hsa-miR-4525;hsa-miR-625-5p;hsa-miR-744
ENSG0000	ENSG0000	0.005794	hsa-miR-185-3p;hsa-miR-3179;hsa-miR-4525;hsa-miR-625-5p;
ENSG0000	ENSG0000	0.005097	hsa-miR-139-3p;hsa-miR-30c-1-3p;hsa-miR-3179;hsa-miR-452
ENSG0000	ENSG0000	1.60E-05	hsa-miR-193a-3p;hsa-miR-193b-3p;hsa-miR-34b-5p;hsa-miR-
ENSG0000	ENSG0000	0.00557	hsa-miR-185-3p;hsa-miR-3157-5p;hsa-miR-3179;hsa-miR-342-
ENSG0000	ENSG0000	0.000743	hsa-miR-140-3p;hsa-miR-34b-5p;hsa-miR-449c-5p;hsa-miR-45
ENSG0000	ENSG0000	0.005718	hsa-miR-193a-3p;hsa-miR-193b-3p;hsa-miR-30c-1-3p;hsa-mi
ENSG0000	ENSG0000	0.008196	hsa-miR-3180-3p;hsa-miR-4786-3p;hsa-miR-625-5p;hsa-miR-
ENSG0000	ENSG0000	0.000768	hsa-miR-1254;hsa-miR-34b-5p;hsa-miR-449c-5p;hsa-miR-940
ENSG0000	ENSG0000	0.001396	hsa-miR-1254;hsa-miR-3157-5p;hsa-miR-34b-5p;hsa-miR-449
ENSG0000	ENSG0000	0.0038	hsa-miR-140-3p;hsa-miR-197-3p;hsa-miR-4525;hsa-miR-625-
ENSG0000	ENSG0000	0.002244	hsa-miR-1254;hsa-miR-296-5p;hsa-miR-5008-5p;hsa-miR-873-
ENSG0000	ENSG0000	0.009494	hsa-miR-30c-1-3p;hsa-miR-3677-5p;hsa-miR-5008-5p;hsa-mi
ENSG0000	ENSG0000	0.000768	hsa-miR-3179;hsa-miR-34b-5p;hsa-miR-449c-5p;hsa-miR-940
ENSG0000	ENSG0000	0.001354	hsa-miR-185-3p;hsa-miR-193a-3p;hsa-miR-193b-3p;hsa-miR-
ENSG0000	ENSG0000	0.008196	hsa-miR-1254;hsa-miR-139-3p;hsa-miR-576-3p;hsa-miR-873-
ENSG0000	ENSG0000	0.008196	hsa-miR-34b-5p;hsa-miR-449c-5p;hsa-miR-873-3p;hsa-miR-94
ENSG0000	ENSG0000	0.000411	hsa-miR-125a-5p;hsa-miR-125b-5p;hsa-miR-140-3p;hsa-miR-
ENSG0000	ENSG0000	0.004514	hsa-miR-10a-5p;hsa-miR-10b-5p;hsa-miR-224-5p;hsa-miR-45
ENSG0000	ENSG0000	0.003619	hsa-miR-10a-5p;hsa-miR-10b-5p;hsa-miR-125a-5p;hsa-miR-1
ENSG0000	ENSG0000	4.45E-06	hsa-miR-29a-3p;hsa-miR-29b-3p;hsa-miR-29c-3p;hsa-miR-62
ENSG0000	ENSG0000	0.000224	hsa-miR-29a-3p;hsa-miR-29b-3p;hsa-miR-29c-3p;hsa-miR-62
ENSG0000	ENSG0000	2.88E-05	hsa-miR-29a-3p;hsa-miR-29b-3p;hsa-miR-29c-3p;hsa-miR-62
ENSG0000	ENSG0000	7.42E-07	hsa-miR-29a-3p;hsa-miR-29b-3p;hsa-miR-29c-3p;hsa-miR-30
ENSG0000	ENSG0000	2.23E-05	hsa-miR-29a-3p;hsa-miR-29b-3p;hsa-miR-29c-3p;hsa-miR-33
ENSG0000	ENSG0000	9.15E-09	hsa-miR-29a-3p;hsa-miR-29b-3p;hsa-miR-29c-3p;hsa-miR-30
ENSG0000	ENSG0000	0.000346	hsa-miR-29a-3p;hsa-miR-29b-3p;hsa-miR-29c-3p;hsa-miR-30

ENSG0000	ENSG0000	1.85E-08	hsa-miR-29a-3p;hsa-miR-29b-3p;hsa-miR-29c-3p;hsa-miR-30
ENSG0000	ENSG0000	1.85E-08	hsa-miR-29a-3p;hsa-miR-29b-3p;hsa-miR-29c-3p;hsa-miR-30
ENSG0000	ENSG0000	1.48E-06	hsa-miR-29a-3p;hsa-miR-29b-3p;hsa-miR-29c-3p;hsa-miR-30
ENSG0000	ENSG0000	6.52E-06	hsa-miR-29a-3p;hsa-miR-29b-3p;hsa-miR-29c-3p;hsa-miR-30
ENSG0000	ENSG0000	3.89E-09	hsa-miR-29a-3p;hsa-miR-29b-3p;hsa-miR-29c-3p;hsa-miR-30
ENSG0000	ENSG0000	1.33E-06	hsa-miR-29a-3p;hsa-miR-29b-3p;hsa-miR-29c-3p;hsa-miR-30
ENSG0000	ENSG0000	3.20E-07	hsa-miR-29a-3p;hsa-miR-29b-3p;hsa-miR-29c-3p;hsa-miR-30
ENSG0000	ENSG0000	0.000116	hsa-miR-29a-3p;hsa-miR-29b-3p;hsa-miR-29c-3p;hsa-miR-58
ENSG0000	ENSG0000	1.94E-07	hsa-miR-29a-3p;hsa-miR-29b-3p;hsa-miR-29c-3p;hsa-miR-30
ENSG0000	ENSG0000	1.58E-08	hsa-miR-29a-3p;hsa-miR-29b-3p;hsa-miR-29c-3p;hsa-miR-30
ENSG0000	ENSG0000	8.40E-07	hsa-miR-29a-3p;hsa-miR-29b-3p;hsa-miR-29c-3p;hsa-miR-58
ENSG0000	ENSG0000	5.67E-05	hsa-miR-29a-3p;hsa-miR-29b-3p;hsa-miR-29c-3p;hsa-miR-30
ENSG0000	ENSG0000	9.15E-09	hsa-miR-29a-3p;hsa-miR-29b-3p;hsa-miR-29c-3p;hsa-miR-30
ENSG0000	ENSG0000	1.19E-06	hsa-miR-29a-3p;hsa-miR-29b-3p;hsa-miR-29c-3p;hsa-miR-30
ENSG0000	ENSG0000	2.88E-05	hsa-miR-29a-3p;hsa-miR-29b-3p;hsa-miR-29c-3p;hsa-miR-30
ENSG0000	ENSG0000	1.10E-07	hsa-miR-29a-3p;hsa-miR-29b-3p;hsa-miR-29c-3p;hsa-miR-30
ENSG0000	ENSG0000	7.42E-07	hsa-miR-29a-3p;hsa-miR-29b-3p;hsa-miR-29c-3p;hsa-miR-30
ENSG0000	ENSG0000	1.06E-06	hsa-miR-29a-3p;hsa-miR-29b-3p;hsa-miR-29c-3p;hsa-miR-30
ENSG0000	ENSG0000	1.90E-05	hsa-miR-29a-3p;hsa-miR-29b-3p;hsa-miR-29c-3p;hsa-miR-30
ENSG0000	ENSG0000	3.20E-07	hsa-miR-29a-3p;hsa-miR-29b-3p;hsa-miR-29c-3p;hsa-miR-30
ENSG0000	ENSG0000	1.48E-06	hsa-miR-29a-3p;hsa-miR-29b-3p;hsa-miR-29c-3p;hsa-miR-30
ENSG0000	ENSG0000	0.00301	hsa-miR-301a-5p;hsa-miR-4328;hsa-miR-486-5p;hsa-miR-652-
ENSG0000	ENSG0000	0.00301	hsa-miR-301a-5p;hsa-miR-4328;hsa-miR-486-5p;hsa-miR-652-
ENSG0000	ENSG0000	0.001768	hsa-miR-199a-5p;hsa-miR-199b-5p;hsa-miR-24-3p;hsa-miR-4
ENSG0000	ENSG0000	3.13E-05	hsa-miR-199a-5p;hsa-miR-199b-5p;hsa-miR-24-3p;hsa-miR-4
ENSG0000	ENSG0000	3.13E-05	hsa-miR-199a-5p;hsa-miR-199b-5p;hsa-miR-24-3p;hsa-miR-4
ENSG0000	ENSG0000	0.006306	hsa-miR-4328;hsa-miR-4664-5p;hsa-miR-616-5p;hsa-miR-629-
ENSG0000	ENSG0000	3.66E-06	hsa-miR-192-3p;hsa-miR-199a-5p;hsa-miR-199b-5p;hsa-miR-
ENSG0000	ENSG0000	0.003748	hsa-miR-23b-5p;hsa-miR-24-3p;hsa-miR-4691-3p;hsa-miR-57
ENSG0000	ENSG0000	0.00959	hsa-miR-199a-5p;hsa-miR-199b-5p;hsa-miR-4691-3p;hsa-miR
ENSG0000	ENSG0000	0.008165	hsa-miR-192-3p;hsa-miR-23b-5p;hsa-miR-301a-5p;hsa-miR-3
ENSG0000	ENSG0000	0.000224	hsa-miR-199a-5p;hsa-miR-199b-5p;hsa-miR-24-3p;hsa-miR-4
ENSG0000	ENSG0000	2.21E-05	hsa-miR-107;hsa-miR-1304-3p;hsa-miR-15a-5p;hsa-miR-15b-
ENSG0000	ENSG0000	0.000494	hsa-miR-15a-5p;hsa-miR-15b-5p;hsa-miR-16-5p;hsa-miR-195
ENSG0000	ENSG0000	1.58E-06	hsa-miR-15a-5p;hsa-miR-15b-5p;hsa-miR-16-5p;hsa-miR-195
ENSG0000	ENSG0000	1.39E-12	hsa-miR-103a-3p;hsa-miR-107;hsa-miR-15a-5p;hsa-miR-15b-
ENSG0000	ENSG0000	1.84E-06	hsa-miR-15a-5p;hsa-miR-15b-5p;hsa-miR-16-5p;hsa-miR-195
ENSG0000	ENSG0000	1.04E-05	hsa-miR-15a-5p;hsa-miR-15b-5p;hsa-miR-195-5p;hsa-miR-42
ENSG0000	ENSG0000	9.30E-12	hsa-miR-103a-3p;hsa-miR-107;hsa-miR-15a-5p;hsa-miR-15b-
ENSG0000	ENSG0000	0.000938	hsa-miR-15a-5p;hsa-miR-15b-5p;hsa-miR-195-5p;hsa-miR-42
ENSG0000	ENSG0000	1.38E-05	hsa-miR-15a-5p;hsa-miR-15b-5p;hsa-miR-16-5p;hsa-miR-195
ENSG0000	ENSG0000	1.38E-05	hsa-miR-15a-5p;hsa-miR-15b-5p;hsa-miR-16-5p;hsa-miR-195
ENSG0000	ENSG0000	0.001368	hsa-miR-15a-5p;hsa-miR-15b-5p;hsa-miR-16-5p;hsa-miR-195
ENSG0000	ENSG0000	9.21E-06	hsa-miR-15a-5p;hsa-miR-15b-5p;hsa-miR-16-5p;hsa-miR-195
ENSG0000	ENSG0000	0.000669	hsa-miR-15a-5p;hsa-miR-15b-5p;hsa-miR-16-1-3p;hsa-miR-1
ENSG0000	ENSG0000	5.28E-06	hsa-miR-15a-5p;hsa-miR-15b-5p;hsa-miR-16-1-3p;hsa-miR-1
ENSG0000	ENSG0000	5.75E-06	hsa-miR-15a-5p;hsa-miR-15b-5p;hsa-miR-16-5p;hsa-miR-195
ENSG0000	ENSG0000	1.38E-05	hsa-miR-15a-5p;hsa-miR-15b-5p;hsa-miR-16-5p;hsa-miR-195
ENSG0000	ENSG0000	1.25E-05	hsa-miR-107;hsa-miR-15a-5p;hsa-miR-15b-5p;hsa-miR-195-5p
ENSG0000	ENSG0000	8.73E-07	hsa-miR-15a-5p;hsa-miR-15b-5p;hsa-miR-16-5p;hsa-miR-195
ENSG0000	ENSG0000	5.75E-06	hsa-miR-15b-5p;hsa-miR-16-5p;hsa-miR-195-5p;hsa-miR-424
ENSG0000	ENSG0000	3.76E-08	hsa-miR-15a-5p;hsa-miR-15b-5p;hsa-miR-16-1-3p;hsa-miR-1
ENSG0000	ENSG0000	4.36E-07	hsa-miR-103a-3p;hsa-miR-107;hsa-miR-15a-5p;hsa-miR-15b-
ENSG0000	ENSG0000	3.04E-10	hsa-miR-103a-3p;hsa-miR-107;hsa-miR-15a-5p;hsa-miR-15b-

ENSG0000	ENSG0000	5.20E-05	hsa-miR-15a-5p;hsa-miR-15b-5p;hsa-miR-21-5p;hsa-miR-424-
ENSG0000	ENSG0000	1.17E-05	hsa-miR-107;hsa-miR-15a-5p;hsa-miR-15b-5p;hsa-miR-16-5p;
ENSG0000	ENSG0000	4.16E-05	hsa-miR-15a-5p;hsa-miR-15b-5p;hsa-miR-16-5p;hsa-miR-195-
ENSG0000	ENSG0000	2.58E-05	hsa-miR-15a-5p;hsa-miR-15b-5p;hsa-miR-16-5p;hsa-miR-195-
ENSG0000	ENSG0000	0.002257	hsa-miR-1304-3p;hsa-miR-15a-5p;hsa-miR-16-5p;hsa-miR-19
ENSG0000	ENSG0000	2.80E-05	hsa-miR-15a-5p;hsa-miR-15b-5p;hsa-miR-16-5p;hsa-miR-195-
ENSG0000	ENSG0000	0.000492	hsa-miR-15a-5p;hsa-miR-15b-5p;hsa-miR-16-5p;hsa-miR-195-
ENSG0000	ENSG0000	0.00416	hsa-miR-15a-5p;hsa-miR-15b-5p;hsa-miR-195-5p;hsa-miR-42
ENSG0000	ENSG0000	2.03E-11	hsa-miR-103a-3p;hsa-miR-107;hsa-miR-15a-5p;hsa-miR-15b-
ENSG0000	ENSG0000	1.51E-05	hsa-miR-15a-5p;hsa-miR-15b-5p;hsa-miR-16-5p;hsa-miR-195-
ENSG0000	ENSG0000	6.07E-12	hsa-miR-103a-3p;hsa-miR-107;hsa-miR-15a-5p;hsa-miR-15b-
ENSG0000	ENSG0000	2.03E-05	hsa-miR-15a-5p;hsa-miR-15b-5p;hsa-miR-195-5p;hsa-miR-42
ENSG0000	ENSG0000	5.30E-08	hsa-miR-103a-3p;hsa-miR-107;hsa-miR-15a-5p;hsa-miR-15b-
ENSG0000	ENSG0000	5.57E-07	hsa-miR-103a-3p;hsa-miR-107;hsa-miR-15a-5p;hsa-miR-15b-
ENSG0000	ENSG0000	0.00071	hsa-miR-15a-5p;hsa-miR-15b-5p;hsa-miR-195-5p;hsa-miR-42
ENSG0000	ENSG0000	3.65E-06	hsa-miR-15a-5p;hsa-miR-15b-5p;hsa-miR-16-5p;hsa-miR-195-
ENSG0000	ENSG0000	5.72E-07	hsa-miR-15a-5p;hsa-miR-15b-5p;hsa-miR-16-5p;hsa-miR-195-
ENSG0000	ENSG0000	3.85E-05	hsa-miR-15a-5p;hsa-miR-15b-5p;hsa-miR-16-5p;hsa-miR-195-
ENSG0000	ENSG0000	2.13E-06	hsa-miR-15a-5p;hsa-miR-15b-5p;hsa-miR-16-5p;hsa-miR-195-
ENSG0000	ENSG0000	5.97E-05	hsa-miR-15a-5p;hsa-miR-15b-5p;hsa-miR-16-5p;hsa-miR-195-
ENSG0000	ENSG0000	1.24E-05	hsa-miR-15a-5p;hsa-miR-15b-5p;hsa-miR-195-5p;hsa-miR-42
ENSG0000	ENSG0000	3.04E-10	hsa-miR-103a-3p;hsa-miR-107;hsa-miR-15a-5p;hsa-miR-15b-
ENSG0000	ENSG0000	9.04E-05	hsa-miR-15a-5p;hsa-miR-15b-5p;hsa-miR-195-5p;hsa-miR-42
ENSG0000	ENSG0000	8.28E-06	hsa-miR-15a-5p;hsa-miR-15b-5p;hsa-miR-16-5p;hsa-miR-195-
ENSG0000	ENSG0000	0.001847	hsa-miR-15a-5p;hsa-miR-15b-5p;hsa-miR-195-5p;hsa-miR-42
ENSG0000	ENSG0000	0.000206	hsa-miR-15a-5p;hsa-miR-15b-5p;hsa-miR-16-5p;hsa-miR-195-
ENSG0000	ENSG0000	7.75E-05	hsa-miR-15a-5p;hsa-miR-15b-5p;hsa-miR-16-5p;hsa-miR-195-
ENSG0000	ENSG0000	4.14E-06	hsa-miR-107;hsa-miR-15a-5p;hsa-miR-15b-5p;hsa-miR-195-5
ENSG0000	ENSG0000	0.000122	hsa-miR-15a-5p;hsa-miR-15b-5p;hsa-miR-195-5p;hsa-miR-42
ENSG0000	ENSG0000	6.40E-05	hsa-miR-15a-5p;hsa-miR-15b-5p;hsa-miR-16-5p;hsa-miR-195-
ENSG0000	ENSG0000	1.29E-08	hsa-miR-103a-3p;hsa-miR-107;hsa-miR-15a-5p;hsa-miR-15b-
ENSG0000	ENSG0000	0.000796	hsa-miR-15a-5p;hsa-miR-15b-5p;hsa-miR-195-5p;hsa-miR-42
ENSG0000	ENSG0000	0.001405	hsa-miR-15a-5p;hsa-miR-15b-5p;hsa-miR-195-5p;hsa-miR-42
ENSG0000	ENSG0000	0.000404	hsa-miR-15a-5p;hsa-miR-15b-5p;hsa-miR-195-5p;hsa-miR-42
ENSG0000	ENSG0000	6.88E-07	hsa-miR-15a-5p;hsa-miR-15b-5p;hsa-miR-16-5p;hsa-miR-195-
ENSG0000	ENSG0000	7.93E-09	hsa-miR-15a-5p;hsa-miR-15b-5p;hsa-miR-16-5p;hsa-miR-195-
ENSG0000	ENSG0000	7.93E-09	hsa-miR-15a-5p;hsa-miR-15b-5p;hsa-miR-16-5p;hsa-miR-195-
ENSG0000	ENSG0000	1.99E-05	hsa-miR-15a-5p;hsa-miR-15b-5p;hsa-miR-16-5p;hsa-miR-195-
ENSG0000	ENSG0000	4.07E-05	hsa-miR-15a-5p;hsa-miR-15b-5p;hsa-miR-195-5p;hsa-miR-42
ENSG0000	ENSG0000	2.37E-07	hsa-miR-103a-3p;hsa-miR-107;hsa-miR-15a-5p;hsa-miR-15b-
ENSG0000	ENSG0000	9.87E-08	hsa-miR-103a-3p;hsa-miR-107;hsa-miR-15a-5p;hsa-miR-15b-
ENSG0000	ENSG0000	6.56E-05	hsa-miR-15a-5p;hsa-miR-15b-5p;hsa-miR-195-5p;hsa-miR-42
ENSG0000	ENSG0000	4.06E-07	hsa-miR-107;hsa-miR-15a-5p;hsa-miR-16-5p;hsa-miR-424-5p
ENSG0000	ENSG0000	0.00067	hsa-miR-15a-5p;hsa-miR-15b-5p;hsa-miR-195-5p;hsa-miR-42
ENSG0000	ENSG0000	1.35E-06	hsa-miR-15a-5p;hsa-miR-15b-5p;hsa-miR-16-5p;hsa-miR-195-
ENSG0000	ENSG0000	2.42E-08	hsa-miR-103a-3p;hsa-miR-107;hsa-miR-15a-5p;hsa-miR-15b-
ENSG0000	ENSG0000	7.98E-08	hsa-miR-107;hsa-miR-15a-5p;hsa-miR-15b-5p;hsa-miR-195-5
ENSG0000	ENSG0000	5.75E-06	hsa-miR-15a-5p;hsa-miR-15b-5p;hsa-miR-195-5p;hsa-miR-42
ENSG0000	ENSG0000	7.93E-09	hsa-miR-15a-5p;hsa-miR-15b-5p;hsa-miR-16-5p;hsa-miR-195-
ENSG0000	ENSG0000	7.93E-09	hsa-miR-15a-5p;hsa-miR-15b-5p;hsa-miR-16-5p;hsa-miR-195-
ENSG0000	ENSG0000	0.000174	hsa-miR-15a-5p;hsa-miR-15b-5p;hsa-miR-195-5p;hsa-miR-42
ENSG0000	ENSG0000	1.27E-09	hsa-miR-15a-5p;hsa-miR-15b-5p;hsa-miR-16-5p;hsa-miR-195-
ENSG0000	ENSG0000	1.58E-06	hsa-miR-107;hsa-miR-15a-5p;hsa-miR-15b-5p;hsa-miR-195-5
ENSG0000	ENSG0000	3.04E-05	hsa-miR-107;hsa-miR-15a-5p;hsa-miR-15b-5p;hsa-miR-195-5

ENSG0000	ENSG0000	0.0001	hsa-miR-15a-5p;hsa-miR-15b-5p;hsa-miR-195-5p;hsa-miR-42
ENSG0000	ENSG0000	8.91E-05	hsa-miR-15a-5p;hsa-miR-15b-5p;hsa-miR-16-5p;hsa-miR-195-
ENSG0000	ENSG0000	6.31E-09	hsa-miR-15a-5p;hsa-miR-15b-5p;hsa-miR-16-5p;hsa-miR-195-
ENSG0000	ENSG0000	8.22E-07	hsa-miR-107;hsa-miR-15a-5p;hsa-miR-15b-5p;hsa-miR-16-1-
ENSG0000	ENSG0000	9.21E-06	hsa-miR-15a-5p;hsa-miR-15b-5p;hsa-miR-16-5p;hsa-miR-195-
ENSG0000	ENSG0000	2.48E-05	hsa-miR-1304-3p;hsa-miR-15a-5p;hsa-miR-15b-5p;hsa-miR-1
ENSG0000	ENSG0000	5.93E-06	hsa-miR-15a-5p;hsa-miR-15b-5p;hsa-miR-16-5p;hsa-miR-195-
ENSG0000	ENSG0000	0.000889	hsa-miR-15a-5p;hsa-miR-15b-5p;hsa-miR-16-5p;hsa-miR-195-
ENSG0000	ENSG0000	1.57E-07	hsa-miR-15a-5p;hsa-miR-15b-5p;hsa-miR-16-5p;hsa-miR-195-
ENSG0000	ENSG0000	1.57E-07	hsa-miR-15a-5p;hsa-miR-15b-5p;hsa-miR-16-5p;hsa-miR-195-
ENSG0000	ENSG0000	0.00099	hsa-miR-107;hsa-miR-15a-5p;hsa-miR-15b-5p;hsa-miR-16-5p
ENSG0000	ENSG0000	6.65E-06	hsa-miR-15a-5p;hsa-miR-15b-5p;hsa-miR-16-5p;hsa-miR-195-
ENSG0000	ENSG0000	1.05E-07	hsa-miR-103a-3p;hsa-miR-107;hsa-miR-1304-3p;hsa-miR-146
ENSG0000	ENSG0000	1.22E-06	hsa-miR-15a-5p;hsa-miR-15b-5p;hsa-miR-16-5p;hsa-miR-195-
ENSG0000	ENSG0000	1.22E-06	hsa-miR-15a-5p;hsa-miR-15b-5p;hsa-miR-16-5p;hsa-miR-195-
ENSG0000	ENSG0000	4.38E-08	hsa-miR-15a-5p;hsa-miR-15b-5p;hsa-miR-16-5p;hsa-miR-195-
ENSG0000	ENSG0000	9.48E-10	hsa-miR-103a-3p;hsa-miR-107;hsa-miR-15a-5p;hsa-miR-15b-
ENSG0000	ENSG0000	1.66E-05	hsa-miR-107;hsa-miR-15a-5p;hsa-miR-15b-5p;hsa-miR-195-5
ENSG0000	ENSG0000	0.000153	hsa-miR-107;hsa-miR-15a-5p;hsa-miR-15b-5p;hsa-miR-195-5
ENSG0000	ENSG0000	7.66E-10	hsa-miR-103a-3p;hsa-miR-107;hsa-miR-15a-5p;hsa-miR-15b-
ENSG0000	ENSG0000	1.73E-05	hsa-miR-15a-5p;hsa-miR-15b-5p;hsa-miR-16-5p;hsa-miR-195-
ENSG0000	ENSG0000	1.84E-08	hsa-miR-15a-5p;hsa-miR-15b-5p;hsa-miR-16-5p;hsa-miR-195-
ENSG0000	ENSG0000	5.28E-06	hsa-miR-15a-5p;hsa-miR-15b-5p;hsa-miR-16-5p;hsa-miR-195-
ENSG0000	ENSG0000	9.30E-08	hsa-miR-15a-5p;hsa-miR-15b-5p;hsa-miR-16-5p;hsa-miR-195-
ENSG0000	ENSG0000	7.32E-05	hsa-miR-15a-5p;hsa-miR-15b-5p;hsa-miR-195-5p;hsa-miR-42
ENSG0000	ENSG0000	6.41E-08	hsa-miR-103a-3p;hsa-miR-107;hsa-miR-15a-5p;hsa-miR-15b-
ENSG0000	ENSG0000	3.21E-06	hsa-miR-107;hsa-miR-15a-5p;hsa-miR-15b-5p;hsa-miR-195-5
ENSG0000	ENSG0000	0.0001	hsa-miR-15a-5p;hsa-miR-15b-5p;hsa-miR-16-5p;hsa-miR-195-
ENSG0000	ENSG0000	0.000377	hsa-miR-15a-5p;hsa-miR-15b-5p;hsa-miR-195-5p;hsa-miR-42
ENSG0000	ENSG0000	4.04E-08	hsa-miR-103a-3p;hsa-miR-107;hsa-miR-15a-5p;hsa-miR-15b-
ENSG0000	ENSG0000	9.30E-08	hsa-miR-15a-5p;hsa-miR-15b-5p;hsa-miR-16-5p;hsa-miR-195-
ENSG0000	ENSG0000	8.22E-07	hsa-miR-15a-5p;hsa-miR-15b-5p;hsa-miR-16-5p;hsa-miR-195-
ENSG0000	ENSG0000	0.000377	hsa-miR-146b-3p;hsa-miR-15a-5p;hsa-miR-15b-5p;hsa-miR-1
ENSG0000	ENSG0000	0.000631	hsa-miR-15a-5p;hsa-miR-15b-5p;hsa-miR-16-5p;hsa-miR-195-
ENSG0000	ENSG0000	0.00586	hsa-miR-15a-5p;hsa-miR-15b-5p;hsa-miR-16-5p;hsa-miR-195-
ENSG0000	ENSG0000	0.000114	hsa-miR-15a-5p;hsa-miR-15b-5p;hsa-miR-16-5p;hsa-miR-195-
ENSG0000	ENSG0000	0.001717	hsa-miR-15a-5p;hsa-miR-15b-5p;hsa-miR-16-5p;hsa-miR-195-
ENSG0000	ENSG0000	1.34E-06	hsa-miR-1304-3p;hsa-miR-15a-5p;hsa-miR-15b-5p;hsa-miR-1
ENSG0000	ENSG0000	6.88E-07	hsa-miR-15a-5p;hsa-miR-15b-5p;hsa-miR-16-5p;hsa-miR-195-
ENSG0000	ENSG0000	1.18E-05	hsa-miR-107;hsa-miR-15a-5p;hsa-miR-15b-5p;hsa-miR-16-1-
ENSG0000	ENSG0000	0.002478	hsa-miR-15a-5p;hsa-miR-15b-5p;hsa-miR-195-5p;hsa-miR-42
ENSG0000	ENSG0000	2.35E-05	hsa-miR-15a-5p;hsa-miR-15b-5p;hsa-miR-16-5p;hsa-miR-195-
ENSG0000	ENSG0000	1.57E-07	hsa-miR-15a-5p;hsa-miR-15b-5p;hsa-miR-16-5p;hsa-miR-195-
ENSG0000	ENSG0000	0.002192	hsa-miR-107;hsa-miR-15a-5p;hsa-miR-16-1-3p;hsa-miR-16-5
ENSG0000	ENSG0000	1.27E-09	hsa-miR-15a-5p;hsa-miR-15b-5p;hsa-miR-16-5p;hsa-miR-195-
ENSG0000	ENSG0000	2.07E-09	hsa-miR-107;hsa-miR-15a-5p;hsa-miR-15b-5p;hsa-miR-16-5p;
ENSG0000	ENSG0000	5.57E-05	hsa-miR-15a-5p;hsa-miR-15b-5p;hsa-miR-16-5p;hsa-miR-195-
ENSG0000	ENSG0000	3.13E-05	hsa-miR-15a-5p;hsa-miR-15b-5p;hsa-miR-195-5p;hsa-miR-42
ENSG0000	ENSG0000	0.000101	hsa-miR-15a-5p;hsa-miR-15b-5p;hsa-miR-16-5p;hsa-miR-195-
ENSG0000	ENSG0000	2.80E-05	hsa-miR-15a-5p;hsa-miR-15b-5p;hsa-miR-16-5p;hsa-miR-195-
ENSG0000	ENSG0000	4.90E-09	hsa-miR-15a-5p;hsa-miR-15b-5p;hsa-miR-16-5p;hsa-miR-195-
ENSG0000	ENSG0000	9.86E-07	hsa-miR-15a-5p;hsa-miR-15b-5p;hsa-miR-16-5p;hsa-miR-195-
ENSG0000	ENSG0000	1.22E-06	hsa-miR-15a-5p;hsa-miR-15b-5p;hsa-miR-195-5p;hsa-miR-42
ENSG0000	ENSG0000	5.93E-06	hsa-miR-15a-5p;hsa-miR-15b-5p;hsa-miR-16-5p;hsa-miR-195-

ENSG0000	ENSG0000	3.57E-05	hsa-miR-15a-5p;hsa-miR-15b-5p;hsa-miR-16-5p;hsa-miR-195-
ENSG0000	ENSG0000	1.56E-07	hsa-miR-15a-5p;hsa-miR-15b-5p;hsa-miR-16-5p;hsa-miR-195-
ENSG0000	ENSG0000	0.000262	hsa-miR-15a-5p;hsa-miR-15b-5p;hsa-miR-195-5p;hsa-miR-42
ENSG0000	ENSG0000	1.27E-09	hsa-miR-15a-5p;hsa-miR-15b-5p;hsa-miR-16-5p;hsa-miR-195-
ENSG0000	ENSG0000	2.80E-05	hsa-miR-15a-5p;hsa-miR-15b-5p;hsa-miR-16-5p;hsa-miR-195-
ENSG0000	ENSG0000	0.001847	hsa-miR-15a-5p;hsa-miR-15b-5p;hsa-miR-195-5p;hsa-miR-42
ENSG0000	ENSG0000	8.55E-08	hsa-miR-103a-3p;hsa-miR-107;hsa-miR-15a-5p;hsa-miR-15b-
ENSG0000	ENSG0000	8.62E-06	hsa-miR-15a-5p;hsa-miR-15b-5p;hsa-miR-16-5p;hsa-miR-195-
ENSG0000	ENSG0000	7.32E-05	hsa-miR-15a-5p;hsa-miR-15b-5p;hsa-miR-195-5p;hsa-miR-42
ENSG0000	ENSG0000	7.93E-09	hsa-miR-15a-5p;hsa-miR-15b-5p;hsa-miR-16-5p;hsa-miR-195-
ENSG0000	ENSG0000	2.21E-08	hsa-miR-103a-3p;hsa-miR-107;hsa-miR-15a-5p;hsa-miR-15b-
ENSG0000	ENSG0000	1.73E-09	hsa-miR-103a-3p;hsa-miR-107;hsa-miR-15a-5p;hsa-miR-15b-
ENSG0000	ENSG0000	9.48E-10	hsa-miR-103a-3p;hsa-miR-107;hsa-miR-15a-5p;hsa-miR-15b-
ENSG0000	ENSG0000	8.72E-08	hsa-miR-15a-5p;hsa-miR-15b-5p;hsa-miR-16-5p;hsa-miR-195-
ENSG0000	ENSG0000	6.38E-07	hsa-miR-103a-3p;hsa-miR-107;hsa-miR-15a-5p;hsa-miR-15b-
ENSG0000	ENSG0000	1.73E-05	hsa-miR-15a-5p;hsa-miR-15b-5p;hsa-miR-16-5p;hsa-miR-195-
ENSG0000	ENSG0000	1.82E-05	hsa-miR-15a-5p;hsa-miR-15b-5p;hsa-miR-16-5p;hsa-miR-195-
ENSG0000	ENSG0000	2.37E-05	hsa-miR-107;hsa-miR-15a-5p;hsa-miR-15b-5p;hsa-miR-195-5
ENSG0000	ENSG0000	2.51E-07	hsa-miR-15a-5p;hsa-miR-15b-5p;hsa-miR-16-5p;hsa-miR-195-
ENSG0000	ENSG0000	1.23E-08	hsa-miR-15a-5p;hsa-miR-15b-5p;hsa-miR-16-5p;hsa-miR-195-
ENSG0000	ENSG0000	8.22E-07	hsa-miR-107;hsa-miR-15a-5p;hsa-miR-15b-5p;hsa-miR-195-5
ENSG0000	ENSG0000	3.65E-06	hsa-miR-15a-5p;hsa-miR-15b-5p;hsa-miR-16-5p;hsa-miR-195-
ENSG0000	ENSG0000	2.86E-09	hsa-miR-103a-3p;hsa-miR-107;hsa-miR-146b-3p;hsa-miR-15a-
ENSG0000	ENSG0000	7.97E-07	hsa-miR-15a-5p;hsa-miR-15b-5p;hsa-miR-16-5p;hsa-miR-195-
ENSG0000	ENSG0000	1.78E-07	hsa-miR-1304-3p;hsa-miR-15a-5p;hsa-miR-15b-5p;hsa-miR-1
ENSG0000	ENSG0000	2.72E-05	hsa-miR-15a-5p;hsa-miR-15b-5p;hsa-miR-195-5p;hsa-miR-42
ENSG0000	ENSG0000	0.00071	hsa-miR-15a-5p;hsa-miR-15b-5p;hsa-miR-16-5p;hsa-miR-195-
ENSG0000	ENSG0000	2.81E-06	hsa-miR-15a-5p;hsa-miR-15b-5p;hsa-miR-16-5p;hsa-miR-195-
ENSG0000	ENSG0000	0.000377	hsa-miR-15a-5p;hsa-miR-15b-5p;hsa-miR-16-5p;hsa-miR-195-
ENSG0000	ENSG0000	2.60E-07	hsa-miR-15a-5p;hsa-miR-15b-5p;hsa-miR-16-5p;hsa-miR-195-
ENSG0000	ENSG0000	4.90E-09	hsa-miR-15a-5p;hsa-miR-15b-5p;hsa-miR-16-5p;hsa-miR-195-
ENSG0000	ENSG0000	5.85E-05	hsa-miR-15a-5p;hsa-miR-15b-5p;hsa-miR-195-5p;hsa-miR-42
ENSG0000	ENSG0000	8.28E-06	hsa-miR-15a-5p;hsa-miR-15b-5p;hsa-miR-16-5p;hsa-miR-195-
ENSG0000	ENSG0000	8.28E-06	hsa-miR-15a-5p;hsa-miR-15b-5p;hsa-miR-16-5p;hsa-miR-195-
ENSG0000	ENSG0000	1.13E-05	hsa-miR-15a-5p;hsa-miR-15b-5p;hsa-miR-16-5p;hsa-miR-195-
ENSG0000	ENSG0000	4.61E-05	hsa-miR-15a-5p;hsa-miR-15b-5p;hsa-miR-16-5p;hsa-miR-195-
ENSG0000	ENSG0000	1.23E-08	hsa-miR-15a-5p;hsa-miR-15b-5p;hsa-miR-16-5p;hsa-miR-195-
ENSG0000	ENSG0000	7.24E-08	hsa-miR-15a-5p;hsa-miR-15b-5p;hsa-miR-16-5p;hsa-miR-195-
ENSG0000	ENSG0000	4.07E-05	hsa-miR-15b-5p;hsa-miR-16-5p;hsa-miR-195-5p;hsa-miR-424-
ENSG0000	ENSG0000	7.08E-06	hsa-miR-15a-5p;hsa-miR-15b-5p;hsa-miR-16-5p;hsa-miR-195-
ENSG0000	ENSG0000	7.08E-06	hsa-miR-15a-5p;hsa-miR-15b-5p;hsa-miR-16-5p;hsa-miR-195-
ENSG0000	ENSG0000	1.22E-06	hsa-miR-15a-5p;hsa-miR-15b-5p;hsa-miR-16-5p;hsa-miR-195-
ENSG0000	ENSG0000	2.72E-05	hsa-miR-15a-5p;hsa-miR-15b-5p;hsa-miR-195-5p;hsa-miR-42
ENSG0000	ENSG0000	0.000561	hsa-miR-15a-5p;hsa-miR-15b-5p;hsa-miR-16-5p;hsa-miR-195-
ENSG0000	ENSG0000	8.28E-06	hsa-miR-15a-5p;hsa-miR-15b-5p;hsa-miR-16-5p;hsa-miR-195-
ENSG0000	ENSG0000	0.00019	hsa-miR-15a-5p;hsa-miR-15b-5p;hsa-miR-16-5p;hsa-miR-195-
ENSG0000	ENSG0000	1.63E-05	hsa-miR-1304-3p;hsa-miR-15a-5p;hsa-miR-15b-5p;hsa-miR-1
ENSG0000	ENSG0000	0.00153	hsa-miR-15a-5p;hsa-miR-15b-5p;hsa-miR-16-5p;hsa-miR-195-
ENSG0000	ENSG0000	9.21E-06	hsa-miR-15a-5p;hsa-miR-15b-5p;hsa-miR-16-5p;hsa-miR-195-
ENSG0000	ENSG0000	1.82E-05	hsa-miR-15a-5p;hsa-miR-15b-5p;hsa-miR-16-5p;hsa-miR-195-
ENSG0000	ENSG0000	1.84E-06	hsa-miR-15a-5p;hsa-miR-15b-5p;hsa-miR-16-5p;hsa-miR-195-
ENSG0000	ENSG0000	1.84E-06	hsa-miR-15a-5p;hsa-miR-15b-5p;hsa-miR-16-5p;hsa-miR-195-
ENSG0000	ENSG0000	3.13E-07	hsa-miR-1304-3p;hsa-miR-15a-5p;hsa-miR-15b-5p;hsa-miR-1
ENSG0000	ENSG0000	0.000134	hsa-miR-15a-5p;hsa-miR-15b-5p;hsa-miR-16-5p;hsa-miR-195-

ENSG0000	ENSG0000	0.002478	hsa-miR-15a-5p;hsa-miR-15b-5p;hsa-miR-195-5p;hsa-miR-42
ENSG0000	ENSG0000	7.08E-06	hsa-miR-15a-5p;hsa-miR-15b-5p;hsa-miR-195-5p;hsa-miR-42
ENSG0000	ENSG0000	8.22E-07	hsa-miR-15a-5p;hsa-miR-15b-5p;hsa-miR-16-5p;hsa-miR-195-
ENSG0000	ENSG0000	6.52E-07	hsa-miR-103a-3p;hsa-miR-107;hsa-miR-15a-5p;hsa-miR-15b-
ENSG0000	ENSG0000	9.77E-07	hsa-miR-15a-5p;hsa-miR-15b-5p;hsa-miR-16-5p;hsa-miR-195-
ENSG0000	ENSG0000	1.35E-10	hsa-miR-103a-3p;hsa-miR-107;hsa-miR-15a-5p;hsa-miR-15b-
ENSG0000	ENSG0000	1.35E-10	hsa-miR-103a-3p;hsa-miR-107;hsa-miR-15a-5p;hsa-miR-15b-
ENSG0000	ENSG0000	1.21E-06	hsa-miR-107;hsa-miR-15a-5p;hsa-miR-15b-5p;hsa-miR-195-5
ENSG0000	ENSG0000	8.15E-05	hsa-miR-15a-5p;hsa-miR-15b-5p;hsa-miR-16-5p;hsa-miR-195-
ENSG0000	ENSG0000	1.56E-07	hsa-miR-15a-5p;hsa-miR-15b-5p;hsa-miR-16-5p;hsa-miR-195-
ENSG0000	ENSG0000	3.57E-09	hsa-miR-103a-3p;hsa-miR-107;hsa-miR-15a-5p;hsa-miR-15b-
ENSG0000	ENSG0000	1.84E-06	hsa-miR-107;hsa-miR-1304-3p;hsa-miR-15a-5p;hsa-miR-15b-
ENSG0000	ENSG0000	0.000453	hsa-miR-1304-3p;hsa-miR-15a-5p;hsa-miR-15b-5p;hsa-miR-1
ENSG0000	ENSG0000	0.000453	hsa-miR-1304-3p;hsa-miR-15a-5p;hsa-miR-15b-5p;hsa-miR-1
ENSG0000	ENSG0000	7.93E-09	hsa-miR-15a-5p;hsa-miR-15b-5p;hsa-miR-16-5p;hsa-miR-195-
ENSG0000	ENSG0000	0.000594	hsa-miR-15a-5p;hsa-miR-16-5p;hsa-miR-30b-3p;hsa-miR-769-
ENSG0000	ENSG0000	4.04E-08	hsa-miR-146b-3p;hsa-miR-15a-5p;hsa-miR-15b-5p;hsa-miR-1
ENSG0000	ENSG0000	0.000453	hsa-miR-15a-5p;hsa-miR-15b-5p;hsa-miR-195-5p;hsa-miR-24-
ENSG0000	ENSG0000	3.17E-08	hsa-miR-103a-3p;hsa-miR-107;hsa-miR-15a-5p;hsa-miR-15b-
ENSG0000	ENSG0000	2.03E-11	hsa-miR-103a-3p;hsa-miR-107;hsa-miR-15a-5p;hsa-miR-15b-
ENSG0000	ENSG0000	4.14E-06	hsa-miR-15a-5p;hsa-miR-15b-5p;hsa-miR-16-5p;hsa-miR-195-
ENSG0000	ENSG0000	3.57E-05	hsa-miR-15a-5p;hsa-miR-15b-5p;hsa-miR-16-5p;hsa-miR-195-
ENSG0000	ENSG0000	2.66E-08	hsa-miR-15a-5p;hsa-miR-15b-5p;hsa-miR-16-5p;hsa-miR-195-
ENSG0000	ENSG0000	1.57E-07	hsa-miR-146b-3p;hsa-miR-15a-5p;hsa-miR-15b-5p;hsa-miR-1
ENSG0000	ENSG0000	3.85E-05	hsa-miR-15a-5p;hsa-miR-15b-5p;hsa-miR-16-5p;hsa-miR-195-
ENSG0000	ENSG0000	8.15E-05	hsa-miR-15a-5p;hsa-miR-15b-5p;hsa-miR-195-5p;hsa-miR-42
ENSG0000	ENSG0000	1.84E-06	hsa-miR-15a-5p;hsa-miR-15b-5p;hsa-miR-16-5p;hsa-miR-195-
ENSG0000	ENSG0000	0.002257	hsa-miR-15a-5p;hsa-miR-15b-5p;hsa-miR-16-5p;hsa-miR-195-
ENSG0000	ENSG0000	8.62E-06	hsa-miR-15a-5p;hsa-miR-15b-5p;hsa-miR-16-5p;hsa-miR-195-
ENSG0000	ENSG0000	5.11E-08	hsa-miR-103a-3p;hsa-miR-107;hsa-miR-15a-5p;hsa-miR-15b-
ENSG0000	ENSG0000	1.57E-07	hsa-miR-15a-5p;hsa-miR-15b-5p;hsa-miR-16-5p;hsa-miR-195-
ENSG0000	ENSG0000	5.85E-05	hsa-miR-15a-5p;hsa-miR-15b-5p;hsa-miR-195-5p;hsa-miR-42
ENSG0000	ENSG0000	0.000415	hsa-miR-15a-5p;hsa-miR-15b-5p;hsa-miR-195-5p;hsa-miR-42
ENSG0000	ENSG0000	7.33E-05	hsa-miR-15a-5p;hsa-miR-15b-5p;hsa-miR-16-5p;hsa-miR-195-
ENSG0000	ENSG0000	1.65E-06	hsa-miR-15a-5p;hsa-miR-15b-5p;hsa-miR-195-5p;hsa-miR-42
ENSG0000	ENSG0000	8.73E-07	hsa-miR-15a-5p;hsa-miR-15b-5p;hsa-miR-16-5p;hsa-miR-195-
ENSG0000	ENSG0000	1.21E-06	hsa-miR-103a-3p;hsa-miR-107;hsa-miR-15a-5p;hsa-miR-15b-
ENSG0000	ENSG0000	3.66E-06	hsa-miR-15a-5p;hsa-miR-15b-5p;hsa-miR-195-5p;hsa-miR-42
ENSG0000	ENSG0000	2.35E-05	hsa-miR-15a-5p;hsa-miR-15b-5p;hsa-miR-195-5p;hsa-miR-42
ENSG0000	ENSG0000	0.002898	hsa-miR-15a-5p;hsa-miR-15b-5p;hsa-miR-195-5p;hsa-miR-42
ENSG0000	ENSG0000	3.13E-07	hsa-miR-15a-5p;hsa-miR-15b-5p;hsa-miR-16-5p;hsa-miR-195-
ENSG0000	ENSG0000	0.002284	hsa-miR-15a-5p;hsa-miR-15b-5p;hsa-miR-195-5p;hsa-miR-42
ENSG0000	ENSG0000	1.27E-09	hsa-miR-15a-5p;hsa-miR-15b-5p;hsa-miR-16-5p;hsa-miR-195-
ENSG0000	ENSG0000	3.69E-07	hsa-miR-1304-3p;hsa-miR-15a-5p;hsa-miR-15b-5p;hsa-miR-1
ENSG0000	ENSG0000	1.24E-05	hsa-miR-15a-5p;hsa-miR-15b-5p;hsa-miR-16-5p;hsa-miR-195-
ENSG0000	ENSG0000	8.15E-05	hsa-miR-15a-5p;hsa-miR-15b-5p;hsa-miR-16-5p;hsa-miR-195-
ENSG0000	ENSG0000	6.75E-06	hsa-miR-103a-3p;hsa-miR-107;hsa-miR-15a-5p;hsa-miR-15b-
ENSG0000	ENSG0000	9.21E-06	hsa-miR-15a-5p;hsa-miR-15b-5p;hsa-miR-16-5p;hsa-miR-195-
ENSG0000	ENSG0000	2.72E-05	hsa-miR-15a-5p;hsa-miR-15b-5p;hsa-miR-195-5p;hsa-miR-42
ENSG0000	ENSG0000	1.18E-06	hsa-miR-103a-3p;hsa-miR-107;hsa-miR-1304-3p;hsa-miR-15a-
ENSG0000	ENSG0000	2.35E-05	hsa-miR-15a-5p;hsa-miR-15b-5p;hsa-miR-195-5p;hsa-miR-42
ENSG0000	ENSG0000	1.08E-08	hsa-miR-103a-3p;hsa-miR-107;hsa-miR-15a-5p;hsa-miR-15b-
ENSG0000	ENSG0000	0.00011	hsa-miR-15a-5p;hsa-miR-15b-5p;hsa-miR-195-5p;hsa-miR-42
ENSG0000	ENSG0000	6.88E-07	hsa-miR-15a-5p;hsa-miR-15b-5p;hsa-miR-16-5p;hsa-miR-195-

ENSG0000	ENSG0000	7.00E-08	hsa-miR-15a-5p;hsa-miR-15b-5p;hsa-miR-16-5p;hsa-miR-195-
ENSG0000	ENSG0000	7.93E-09	hsa-miR-15a-5p;hsa-miR-15b-5p;hsa-miR-16-5p;hsa-miR-195-
ENSG0000	ENSG0000	1.88E-08	hsa-miR-15a-5p;hsa-miR-15b-5p;hsa-miR-16-5p;hsa-miR-195-
ENSG0000	ENSG0000	1.22E-06	hsa-miR-15a-5p;hsa-miR-15b-5p;hsa-miR-16-5p;hsa-miR-195-
ENSG0000	ENSG0000	3.04E-05	hsa-miR-15a-5p;hsa-miR-15b-5p;hsa-miR-16-5p;hsa-miR-195-
ENSG0000	ENSG0000	0.00011	hsa-miR-15a-5p;hsa-miR-15b-5p;hsa-miR-16-5p;hsa-miR-195-
ENSG0000	ENSG0000	4.07E-05	hsa-miR-15a-5p;hsa-miR-15b-5p;hsa-miR-195-5p;hsa-miR-424
ENSG0000	ENSG0000	1.22E-07	hsa-miR-15a-5p;hsa-miR-15b-5p;hsa-miR-16-5p;hsa-miR-195-
ENSG0000	ENSG0000	1.17E-09	hsa-miR-103a-3p;hsa-miR-107;hsa-miR-15a-5p;hsa-miR-15b-5
ENSG0000	ENSG0000	0.000492	hsa-miR-15a-5p;hsa-miR-15b-5p;hsa-miR-16-5p;hsa-miR-195-
ENSG0000	ENSG0000	1.84E-06	hsa-miR-15a-5p;hsa-miR-15b-5p;hsa-miR-16-5p;hsa-miR-195-
ENSG0000	ENSG0000	9.21E-07	hsa-miR-103a-3p;hsa-miR-107;hsa-miR-15a-5p;hsa-miR-15b-5
ENSG0000	ENSG0000	1.22E-06	hsa-miR-15a-5p;hsa-miR-15b-5p;hsa-miR-195-5p;hsa-miR-424
ENSG0000	ENSG0000	0.002102	hsa-miR-15a-5p;hsa-miR-15b-5p;hsa-miR-195-5p;hsa-miR-424
ENSG0000	ENSG0000	3.76E-08	hsa-miR-15a-5p;hsa-miR-15b-5p;hsa-miR-16-5p;hsa-miR-195-
ENSG0000	ENSG0000	9.04E-05	hsa-miR-15a-5p;hsa-miR-15b-5p;hsa-miR-16-5p;hsa-miR-195-
ENSG0000	ENSG0000	3.52E-10	hsa-miR-15a-5p;hsa-miR-15b-5p;hsa-miR-16-5p;hsa-miR-195-
ENSG0000	ENSG0000	8.62E-06	hsa-miR-15a-5p;hsa-miR-15b-5p;hsa-miR-16-5p;hsa-miR-195-
ENSG0000	ENSG0000	0.002578	hsa-miR-15a-5p;hsa-miR-15b-5p;hsa-miR-195-5p;hsa-miR-424
ENSG0000	ENSG0000	2.37E-05	hsa-miR-15a-5p;hsa-miR-15b-5p;hsa-miR-16-5p;hsa-miR-195-
ENSG0000	ENSG0000	3.57E-05	hsa-miR-15a-5p;hsa-miR-15b-5p;hsa-miR-16-5p;hsa-miR-195-
ENSG0000	ENSG0000	5.97E-05	hsa-miR-15a-5p;hsa-miR-15b-5p;hsa-miR-195-5p;hsa-miR-30b
ENSG0000	ENSG0000	0.001462	hsa-miR-15a-5p;hsa-miR-15b-5p;hsa-miR-16-5p;hsa-miR-195-
ENSG0000	ENSG0000	0.000122	hsa-miR-15a-5p;hsa-miR-15b-5p;hsa-miR-195-5p;hsa-miR-424
ENSG0000	ENSG0000	1.60E-08	hsa-miR-107;hsa-miR-15a-5p;hsa-miR-15b-5p;hsa-miR-16-5p;
ENSG0000	ENSG0000	0.000559	hsa-miR-15a-5p;hsa-miR-15b-5p;hsa-miR-195-5p;hsa-miR-424
ENSG0000	ENSG0000	1.51E-05	hsa-miR-107;hsa-miR-15a-5p;hsa-miR-15b-5p;hsa-miR-195-5p
ENSG0000	ENSG0000	0.000122	hsa-miR-15a-5p;hsa-miR-15b-5p;hsa-miR-16-5p;hsa-miR-195-
ENSG0000	ENSG0000	1.88E-08	hsa-miR-15a-5p;hsa-miR-15b-5p;hsa-miR-16-5p;hsa-miR-195-
ENSG0000	ENSG0000	1.04E-05	hsa-miR-15a-5p;hsa-miR-15b-5p;hsa-miR-195-5p;hsa-miR-424
ENSG0000	ENSG0000	4.01E-07	hsa-miR-103a-3p;hsa-miR-107;hsa-miR-1304-3p;hsa-miR-15a-
ENSG0000	ENSG0000	2.58E-05	hsa-miR-107;hsa-miR-15a-5p;hsa-miR-15b-5p;hsa-miR-195-5p
ENSG0000	ENSG0000	2.80E-05	hsa-miR-107;hsa-miR-15a-5p;hsa-miR-15b-5p;hsa-miR-195-5p
ENSG0000	ENSG0000	3.73E-10	hsa-miR-103a-3p;hsa-miR-107;hsa-miR-15a-5p;hsa-miR-15b-5
ENSG0000	ENSG0000	1.22E-07	hsa-miR-15a-5p;hsa-miR-15b-5p;hsa-miR-16-5p;hsa-miR-195-
ENSG0000	ENSG0000	1.22E-07	hsa-miR-15a-5p;hsa-miR-15b-5p;hsa-miR-16-5p;hsa-miR-195-
ENSG0000	ENSG0000	4.62E-06	hsa-miR-15a-5p;hsa-miR-15b-5p;hsa-miR-195-5p;hsa-miR-424
ENSG0000	ENSG0000	6.31E-09	hsa-miR-15a-5p;hsa-miR-15b-5p;hsa-miR-16-5p;hsa-miR-195-
ENSG0000	ENSG0000	1.27E-09	hsa-miR-15a-5p;hsa-miR-15b-5p;hsa-miR-16-5p;hsa-miR-195-
ENSG0000	ENSG0000	6.07E-12	hsa-miR-103a-3p;hsa-miR-107;hsa-miR-15a-5p;hsa-miR-15b-5
ENSG0000	ENSG0000	1.98E-08	hsa-miR-15a-5p;hsa-miR-15b-5p;hsa-miR-16-5p;hsa-miR-195-
ENSG0000	ENSG0000	0.000282	hsa-miR-15a-5p;hsa-miR-15b-5p;hsa-miR-16-5p;hsa-miR-195-
ENSG0000	ENSG0000	2.80E-09	hsa-miR-15a-5p;hsa-miR-15b-5p;hsa-miR-16-5p;hsa-miR-195-
ENSG0000	ENSG0000	1.57E-07	hsa-miR-15a-5p;hsa-miR-15b-5p;hsa-miR-16-5p;hsa-miR-195-
ENSG0000	ENSG0000	1.35E-06	hsa-miR-15a-5p;hsa-miR-15b-5p;hsa-miR-16-5p;hsa-miR-195-
ENSG0000	ENSG0000	0.00301	hsa-miR-15a-5p;hsa-miR-15b-5p;hsa-miR-16-5p;hsa-miR-195-
ENSG0000	ENSG0000	6.31E-09	hsa-miR-15a-5p;hsa-miR-15b-5p;hsa-miR-16-5p;hsa-miR-195-
ENSG0000	ENSG0000	1.17E-09	hsa-miR-103a-3p;hsa-miR-107;hsa-miR-15a-5p;hsa-miR-15b-5
ENSG0000	ENSG0000	2.37E-05	hsa-miR-15a-5p;hsa-miR-15b-5p;hsa-miR-16-5p;hsa-miR-195-
ENSG0000	ENSG0000	0.006996	hsa-miR-146b-3p;hsa-miR-15a-5p;hsa-miR-15b-5p;hsa-miR-19
ENSG0000	ENSG0000	4.90E-09	hsa-miR-15a-5p;hsa-miR-15b-5p;hsa-miR-16-5p;hsa-miR-195-
ENSG0000	ENSG0000	1.12E-05	hsa-miR-15a-5p;hsa-miR-15b-5p;hsa-miR-16-5p;hsa-miR-195-
ENSG0000	ENSG0000	1.12E-05	hsa-miR-15a-5p;hsa-miR-15b-5p;hsa-miR-16-5p;hsa-miR-195-
ENSG0000	ENSG0000	0.00067	hsa-miR-15a-5p;hsa-miR-15b-5p;hsa-miR-16-5p;hsa-miR-195-

ENSG0000	ENSG0000	1.57E-07	hsa-miR-15a-5p;hsa-miR-15b-5p;hsa-miR-16-5p;hsa-miR-195-
ENSG0000	ENSG0000	1.56E-07	hsa-miR-15a-5p;hsa-miR-15b-5p;hsa-miR-195-5p;hsa-miR-42
ENSG0000	ENSG0000	1.38E-05	hsa-miR-15a-5p;hsa-miR-15b-5p;hsa-miR-16-5p;hsa-miR-195-
ENSG0000	ENSG0000	5.73E-08	hsa-miR-15a-5p;hsa-miR-15b-5p;hsa-miR-16-5p;hsa-miR-195-
ENSG0000	ENSG0000	3.58E-08	hsa-miR-15a-5p;hsa-miR-15b-5p;hsa-miR-16-1-3p;hsa-miR-1
ENSG0000	ENSG0000	1.27E-09	hsa-miR-15a-5p;hsa-miR-15b-5p;hsa-miR-16-5p;hsa-miR-195-
ENSG0000	ENSG0000	0.000116	hsa-miR-15a-5p;hsa-miR-15b-5p;hsa-miR-16-5p;hsa-miR-18b-
ENSG0000	ENSG0000	0.000128	hsa-miR-15a-5p;hsa-miR-15b-5p;hsa-miR-16-5p;hsa-miR-195-
ENSG0000	ENSG0000	7.33E-05	hsa-miR-15a-5p;hsa-miR-15b-5p;hsa-miR-16-5p;hsa-miR-195-
ENSG0000	ENSG0000	4.02E-06	hsa-miR-103a-3p;hsa-miR-107;hsa-miR-15a-5p;hsa-miR-15b-
ENSG0000	ENSG0000	8.15E-05	hsa-miR-15a-5p;hsa-miR-15b-5p;hsa-miR-195-5p;hsa-miR-42
ENSG0000	ENSG0000	0.00016	hsa-miR-15a-5p;hsa-miR-15b-5p;hsa-miR-16-5p;hsa-miR-195-
ENSG0000	ENSG0000	1.58E-06	hsa-miR-15a-5p;hsa-miR-15b-5p;hsa-miR-16-5p;hsa-miR-195-
ENSG0000	ENSG0000	1.27E-09	hsa-miR-15a-5p;hsa-miR-15b-5p;hsa-miR-16-5p;hsa-miR-195-
ENSG0000	ENSG0000	6.65E-06	hsa-miR-15a-5p;hsa-miR-15b-5p;hsa-miR-16-5p;hsa-miR-195-
ENSG0000	ENSG0000	1.47E-05	hsa-miR-15a-5p;hsa-miR-15b-5p;hsa-miR-16-5p;hsa-miR-195-
ENSG0000	ENSG0000	5.97E-05	hsa-miR-15a-5p;hsa-miR-15b-5p;hsa-miR-16-5p;hsa-miR-195-
ENSG0000	ENSG0000	4.04E-08	hsa-miR-103a-3p;hsa-miR-107;hsa-miR-15a-5p;hsa-miR-15b-
ENSG0000	ENSG0000	0.00019	hsa-miR-15a-5p;hsa-miR-15b-5p;hsa-miR-16-5p;hsa-miR-195-
ENSG0000	ENSG0000	0.000121	hsa-miR-1304-3p;hsa-miR-15a-5p;hsa-miR-15b-5p;hsa-miR-1
ENSG0000	ENSG0000	3.57E-05	hsa-miR-107;hsa-miR-15a-5p;hsa-miR-15b-5p;hsa-miR-195-5
ENSG0000	ENSG0000	5.10E-10	hsa-miR-103a-3p;hsa-miR-107;hsa-miR-15a-5p;hsa-miR-15b-
ENSG0000	ENSG0000	0.002898	hsa-miR-15a-5p;hsa-miR-15b-5p;hsa-miR-16-5p;hsa-miR-195-
ENSG0000	ENSG0000	4.71E-07	hsa-miR-15a-5p;hsa-miR-15b-5p;hsa-miR-16-5p;hsa-miR-195-
ENSG0000	ENSG0000	0.00016	hsa-miR-15a-5p;hsa-miR-15b-5p;hsa-miR-16-5p;hsa-miR-195-
ENSG0000	ENSG0000	0.00016	hsa-miR-15a-5p;hsa-miR-15b-5p;hsa-miR-195-5p;hsa-miR-42
ENSG0000	ENSG0000	0.00019	hsa-miR-15a-5p;hsa-miR-15b-5p;hsa-miR-195-5p;hsa-miR-42
ENSG0000	ENSG0000	6.03E-07	hsa-miR-103a-3p;hsa-miR-107;hsa-miR-15a-5p;hsa-miR-15b-
ENSG0000	ENSG0000	3.13E-05	hsa-miR-15a-5p;hsa-miR-15b-5p;hsa-miR-195-5p;hsa-miR-42
ENSG0000	ENSG0000	3.13E-05	hsa-miR-15a-5p;hsa-miR-15b-5p;hsa-miR-195-5p;hsa-miR-42
ENSG0000	ENSG0000	0.000932	hsa-miR-15a-5p;hsa-miR-15b-5p;hsa-miR-195-5p;hsa-miR-30
ENSG0000	ENSG0000	4.68E-06	hsa-miR-15a-5p;hsa-miR-15b-5p;hsa-miR-16-5p;hsa-miR-195-
ENSG0000	ENSG0000	4.16E-05	hsa-miR-15a-5p;hsa-miR-15b-5p;hsa-miR-16-5p;hsa-miR-195-
ENSG0000	ENSG0000	5.52E-05	hsa-miR-15a-5p;hsa-miR-15b-5p;hsa-miR-16-1-3p;hsa-miR-1
ENSG0000	ENSG0000	1.24E-05	hsa-miR-15a-5p;hsa-miR-16-5p;hsa-miR-195-5p;hsa-miR-424-
ENSG0000	ENSG0000	1.38E-10	hsa-miR-103a-3p;hsa-miR-107;hsa-miR-15a-5p;hsa-miR-15b-
ENSG0000	ENSG0000	8.28E-06	hsa-miR-15a-5p;hsa-miR-15b-5p;hsa-miR-16-5p;hsa-miR-195-
ENSG0000	ENSG0000	1.02E-05	hsa-miR-15a-5p;hsa-miR-15b-5p;hsa-miR-16-5p;hsa-miR-195-
ENSG0000	ENSG0000	9.30E-08	hsa-miR-15a-5p;hsa-miR-15b-5p;hsa-miR-16-5p;hsa-miR-195-
ENSG0000	ENSG0000	1.12E-05	hsa-miR-1304-3p;hsa-miR-15a-5p;hsa-miR-15b-5p;hsa-miR-1
ENSG0000	ENSG0000	3.66E-06	hsa-miR-15a-5p;hsa-miR-16-5p;hsa-miR-195-5p;hsa-miR-424-
ENSG0000	ENSG0000	6.07E-07	hsa-miR-15a-5p;hsa-miR-15b-5p;hsa-miR-16-5p;hsa-miR-195-
ENSG0000	ENSG0000	3.85E-05	hsa-miR-15a-5p;hsa-miR-15b-5p;hsa-miR-16-5p;hsa-miR-195-
ENSG0000	ENSG0000	0.00018	hsa-miR-15a-5p;hsa-miR-15b-5p;hsa-miR-16-5p;hsa-miR-195-
ENSG0000	ENSG0000	5.72E-07	hsa-miR-15a-5p;hsa-miR-15b-5p;hsa-miR-16-5p;hsa-miR-195-
ENSG0000	ENSG0000	2.66E-08	hsa-miR-15a-5p;hsa-miR-15b-5p;hsa-miR-16-5p;hsa-miR-195-
ENSG0000	ENSG0000	0.000101	hsa-miR-15a-5p;hsa-miR-15b-5p;hsa-miR-16-5p;hsa-miR-195-
ENSG0000	ENSG0000	1.25E-05	hsa-miR-15a-5p;hsa-miR-15b-5p;hsa-miR-16-5p;hsa-miR-195-
ENSG0000	ENSG0000	7.00E-08	hsa-miR-15a-5p;hsa-miR-15b-5p;hsa-miR-16-5p;hsa-miR-195-
ENSG0000	ENSG0000	7.00E-08	hsa-miR-15a-5p;hsa-miR-15b-5p;hsa-miR-16-5p;hsa-miR-195-
ENSG0000	ENSG0000	7.00E-08	hsa-miR-15a-5p;hsa-miR-15b-5p;hsa-miR-16-5p;hsa-miR-195-
ENSG0000	ENSG0000	7.00E-08	hsa-miR-15a-5p;hsa-miR-15b-5p;hsa-miR-16-5p;hsa-miR-195-
ENSG0000	ENSG0000	6.75E-06	hsa-miR-107;hsa-miR-15a-5p;hsa-miR-15b-5p;hsa-miR-195-5
ENSG0000	ENSG0000	6.75E-06	hsa-miR-107;hsa-miR-15a-5p;hsa-miR-15b-5p;hsa-miR-195-5

ENSG0000	ENSG0000	4.36E-07	hsa-miR-103a-3p;hsa-miR-107;hsa-miR-15a-5p;hsa-miR-15b-5
ENSG0000	ENSG0000	3.04E-05	hsa-miR-15a-5p;hsa-miR-15b-5p;hsa-miR-16-5p;hsa-miR-195-
ENSG0000	ENSG0000	8.73E-07	hsa-miR-15a-5p;hsa-miR-15b-5p;hsa-miR-195-5p;hsa-miR-42
ENSG0000	ENSG0000	1.88E-08	hsa-miR-15a-5p;hsa-miR-15b-5p;hsa-miR-16-5p;hsa-miR-195-
ENSG0000	ENSG0000	1.27E-09	hsa-miR-15a-5p;hsa-miR-15b-5p;hsa-miR-16-5p;hsa-miR-195-
ENSG0000	ENSG0000	2.45E-06	hsa-miR-15a-5p;hsa-miR-15b-5p;hsa-miR-16-5p;hsa-miR-195-
ENSG0000	ENSG0000	2.45E-06	hsa-miR-15a-5p;hsa-miR-15b-5p;hsa-miR-16-5p;hsa-miR-195-
ENSG0000	ENSG0000	2.45E-06	hsa-miR-15a-5p;hsa-miR-15b-5p;hsa-miR-16-5p;hsa-miR-195-
ENSG0000	ENSG0000	2.45E-06	hsa-miR-15a-5p;hsa-miR-15b-5p;hsa-miR-16-5p;hsa-miR-195-
ENSG0000	ENSG0000	2.45E-06	hsa-miR-15a-5p;hsa-miR-15b-5p;hsa-miR-16-5p;hsa-miR-195-
ENSG0000	ENSG0000	2.45E-06	hsa-miR-15a-5p;hsa-miR-15b-5p;hsa-miR-16-5p;hsa-miR-195-
ENSG0000	ENSG0000	2.45E-06	hsa-miR-15a-5p;hsa-miR-15b-5p;hsa-miR-16-5p;hsa-miR-195-
ENSG0000	ENSG0000	2.45E-06	hsa-miR-15a-5p;hsa-miR-15b-5p;hsa-miR-16-5p;hsa-miR-195-
ENSG0000	ENSG0000	2.45E-06	hsa-miR-15a-5p;hsa-miR-15b-5p;hsa-miR-16-5p;hsa-miR-195-
ENSG0000	ENSG0000	2.45E-06	hsa-miR-15a-5p;hsa-miR-15b-5p;hsa-miR-16-5p;hsa-miR-195-
ENSG0000	ENSG0000	8.72E-08	hsa-miR-15a-5p;hsa-miR-15b-5p;hsa-miR-16-5p;hsa-miR-195-
ENSG0000	ENSG0000	1.57E-07	hsa-miR-15a-5p;hsa-miR-15b-5p;hsa-miR-16-5p;hsa-miR-195-
ENSG0000	ENSG0000	1.47E-05	hsa-miR-15a-5p;hsa-miR-15b-5p;hsa-miR-16-5p;hsa-miR-424-
ENSG0000	ENSG0000	7.32E-05	hsa-miR-15a-5p;hsa-miR-15b-5p;hsa-miR-16-5p;hsa-miR-195-
ENSG0000	ENSG0000	2.51E-09	hsa-miR-103a-3p;hsa-miR-107;hsa-miR-15a-5p;hsa-miR-15b-5
ENSG0000	ENSG0000	3.05E-06	hsa-miR-15a-5p;hsa-miR-15b-5p;hsa-miR-16-5p;hsa-miR-195-
ENSG0000	ENSG0000	1.42E-09	hsa-miR-103a-3p;hsa-miR-107;hsa-miR-15a-5p;hsa-miR-15b-5
ENSG0000	ENSG0000	1.51E-05	hsa-miR-15a-5p;hsa-miR-15b-5p;hsa-miR-16-5p;hsa-miR-195-
ENSG0000	ENSG0000	2.72E-05	hsa-miR-15a-5p;hsa-miR-15b-5p;hsa-miR-195-5p;hsa-miR-42
ENSG0000	ENSG0000	0.000114	hsa-miR-15a-5p;hsa-miR-15b-5p;hsa-miR-16-5p;hsa-miR-195-
ENSG0000	ENSG0000	1.13E-05	hsa-miR-15a-5p;hsa-miR-15b-5p;hsa-miR-16-5p;hsa-miR-195-
ENSG0000	ENSG0000	5.20E-05	hsa-miR-15a-5p;hsa-miR-15b-5p;hsa-miR-16-5p;hsa-miR-195-
ENSG0000	ENSG0000	3.57E-05	hsa-miR-15a-5p;hsa-miR-15b-5p;hsa-miR-16-5p;hsa-miR-195-
ENSG0000	ENSG0000	0.001323	hsa-miR-15a-5p;hsa-miR-15b-5p;hsa-miR-16-5p;hsa-miR-195-
ENSG0000	ENSG0000	7.00E-08	hsa-miR-15a-5p;hsa-miR-15b-5p;hsa-miR-16-5p;hsa-miR-195-
ENSG0000	ENSG0000	0.000155	hsa-miR-1304-3p;hsa-miR-15a-5p;hsa-miR-15b-5p;hsa-miR-1
ENSG0000	ENSG0000	1.09E-09	hsa-miR-103a-3p;hsa-miR-107;hsa-miR-15a-5p;hsa-miR-15b-5
ENSG0000	ENSG0000	3.57E-05	hsa-miR-15a-5p;hsa-miR-15b-5p;hsa-miR-195-5p;hsa-miR-42
ENSG0000	ENSG0000	1.84E-08	hsa-miR-15a-5p;hsa-miR-15b-5p;hsa-miR-16-5p;hsa-miR-195-
ENSG0000	ENSG0000	1.65E-06	hsa-miR-15a-5p;hsa-miR-15b-5p;hsa-miR-195-5p;hsa-miR-42
ENSG0000	ENSG0000	1.04E-10	hsa-miR-103a-3p;hsa-miR-107;hsa-miR-15a-5p;hsa-miR-15b-5
ENSG0000	ENSG0000	1.84E-06	hsa-miR-15a-5p;hsa-miR-15b-5p;hsa-miR-16-5p;hsa-miR-195-
ENSG0000	ENSG0000	1.73E-05	hsa-miR-15a-5p;hsa-miR-15b-5p;hsa-miR-16-5p;hsa-miR-195-
ENSG0000	ENSG0000	6.31E-09	hsa-miR-15a-5p;hsa-miR-15b-5p;hsa-miR-16-5p;hsa-miR-195-
ENSG0000	ENSG0000	2.13E-06	hsa-miR-15a-5p;hsa-miR-15b-5p;hsa-miR-16-5p;hsa-miR-195-
ENSG0000	ENSG0000	7.83E-05	hsa-miR-15a-5p;hsa-miR-15b-5p;hsa-miR-195-5p;hsa-miR-24-
ENSG0000	ENSG0000	3.21E-06	hsa-miR-15a-5p;hsa-miR-15b-5p;hsa-miR-16-5p;hsa-miR-195-
ENSG0000	ENSG0000	1.08E-08	hsa-miR-103a-3p;hsa-miR-107;hsa-miR-15a-5p;hsa-miR-15b-5
ENSG0000	ENSG0000	2.03E-11	hsa-miR-103a-3p;hsa-miR-107;hsa-miR-15a-5p;hsa-miR-15b-5
ENSG0000	ENSG0000	1.84E-08	hsa-miR-15a-5p;hsa-miR-15b-5p;hsa-miR-16-5p;hsa-miR-195-
ENSG0000	ENSG0000	1.99E-05	hsa-miR-15a-5p;hsa-miR-15b-5p;hsa-miR-16-5p;hsa-miR-195-
ENSG0000	ENSG0000	5.75E-06	hsa-miR-15a-5p;hsa-miR-15b-5p;hsa-miR-16-5p;hsa-miR-195-
ENSG0000	ENSG0000	5.75E-06	hsa-miR-15a-5p;hsa-miR-15b-5p;hsa-miR-16-5p;hsa-miR-195-
ENSG0000	ENSG0000	2.78E-05	hsa-miR-15a-5p;hsa-miR-15b-5p;hsa-miR-16-5p;hsa-miR-195-
ENSG0000	ENSG0000	6.88E-07	hsa-miR-15a-5p;hsa-miR-15b-5p;hsa-miR-16-5p;hsa-miR-195-
ENSG0000	ENSG0000	2.20E-06	hsa-miR-15a-5p;hsa-miR-15b-5p;hsa-miR-16-5p;hsa-miR-195-
ENSG0000	ENSG0000	4.90E-09	hsa-miR-15a-5p;hsa-miR-15b-5p;hsa-miR-16-5p;hsa-miR-195-
ENSG0000	ENSG0000	8.88E-08	hsa-miR-103a-3p;hsa-miR-107;hsa-miR-15a-5p;hsa-miR-15b-5
ENSG0000	ENSG0000	1.84E-08	hsa-miR-15a-5p;hsa-miR-15b-5p;hsa-miR-16-5p;hsa-miR-195-

ENSG0000	ENSG0000	3.20E-10	hsa-miR-103a-3p;hsa-miR-107;hsa-miR-15a-5p;hsa-miR-15b-5
ENSG0000	ENSG0000	3.99E-07	hsa-miR-146b-3p;hsa-miR-15a-5p;hsa-miR-15b-5p;hsa-miR-1(
ENSG0000	ENSG0000	9.77E-07	hsa-miR-15a-5p;hsa-miR-15b-5p;hsa-miR-16-5p;hsa-miR-195-
ENSG0000	ENSG0000	0.000262	hsa-miR-15a-5p;hsa-miR-15b-5p;hsa-miR-16-5p;hsa-miR-195-
ENSG0000	ENSG0000	3.30E-05	hsa-miR-15a-5p;hsa-miR-15b-5p;hsa-miR-16-5p;hsa-miR-195-
ENSG0000	ENSG0000	0.000223	hsa-miR-15a-5p;hsa-miR-15b-5p;hsa-miR-16-5p;hsa-miR-195-
ENSG0000	ENSG0000	0.001717	hsa-miR-15a-5p;hsa-miR-15b-5p;hsa-miR-16-5p;hsa-miR-195-
ENSG0000	ENSG0000	1.27E-09	hsa-miR-15a-5p;hsa-miR-15b-5p;hsa-miR-16-5p;hsa-miR-195-
ENSG0000	ENSG0000	3.86E-07	hsa-miR-15a-5p;hsa-miR-15b-5p;hsa-miR-16-5p;hsa-miR-195-
ENSG0000	ENSG0000	3.30E-05	hsa-miR-15a-5p;hsa-miR-15b-5p;hsa-miR-16-5p;hsa-miR-195-
ENSG0000	ENSG0000	5.32E-09	hsa-miR-103a-3p;hsa-miR-107;hsa-miR-1304-3p;hsa-miR-15a-
ENSG0000	ENSG0000	0.000144	hsa-miR-15a-5p;hsa-miR-15b-5p;hsa-miR-16-5p;hsa-miR-195-
ENSG0000	ENSG0000	9.86E-07	hsa-miR-15a-5p;hsa-miR-15b-5p;hsa-miR-16-5p;hsa-miR-195-
ENSG0000	ENSG0000	2.17E-05	hsa-miR-15a-5p;hsa-miR-15b-5p;hsa-miR-16-5p;hsa-miR-195-
ENSG0000	ENSG0000	0.000723	hsa-miR-145-5p;hsa-miR-194-3p;hsa-miR-199a-5p;hsa-miR-1!
ENSG0000	ENSG0000	4.11E-06	hsa-miR-1226-5p;hsa-miR-1304-5p;hsa-miR-423-5p;hsa-miR-4
ENSG0000	ENSG0000	0.000389	hsa-miR-200a-5p;hsa-miR-200b-5p;hsa-miR-374c-3p;hsa-miR-
ENSG0000	ENSG0000	0.000193	hsa-miR-16-1-3p;hsa-miR-200a-5p;hsa-miR-200b-5p;hsa-miR-
ENSG0000	ENSG0000	8.80E-06	hsa-miR-106b-5p;hsa-miR-17-5p;hsa-miR-20a-5p;hsa-miR-20I
ENSG0000	ENSG0000	0.001406	hsa-miR-107;hsa-miR-139-5p;hsa-miR-15a-5p;hsa-miR-15b-5
ENSG0000	ENSG0000	3.53E-06	hsa-miR-106a-5p;hsa-miR-106b-5p;hsa-miR-15a-5p;hsa-miR-1
ENSG0000	ENSG0000	0.000862	hsa-miR-15a-5p;hsa-miR-15b-5p;hsa-miR-16-5p;hsa-miR-195-
ENSG0000	ENSG0000	0.000627	hsa-miR-106a-5p;hsa-miR-106b-5p;hsa-miR-20a-5p;hsa-miR-2
ENSG0000	ENSG0000	2.14E-08	hsa-miR-103a-3p;hsa-miR-107;hsa-miR-15a-5p;hsa-miR-15b-5
ENSG0000	ENSG0000	0.000988	hsa-miR-15a-5p;hsa-miR-15b-5p;hsa-miR-16-5p;hsa-miR-195-
ENSG0000	ENSG0000	0.00155	hsa-miR-15a-5p;hsa-miR-15b-5p;hsa-miR-195-5p;hsa-miR-424
ENSG0000	ENSG0000	0.000144	hsa-miR-17-5p;hsa-miR-20a-5p;hsa-miR-20b-5p;hsa-miR-27a-
ENSG0000	ENSG0000	0.00692	hsa-miR-185-3p;hsa-miR-192-5p;hsa-miR-365a-3p;hsa-miR-3(
ENSG0000	ENSG0000	1.35E-07	hsa-miR-103a-3p;hsa-miR-107;hsa-miR-15a-5p;hsa-miR-15b-5
ENSG0000	ENSG0000	0.00045	hsa-miR-106b-5p;hsa-miR-17-5p;hsa-miR-185-5p;hsa-miR-20a
ENSG0000	ENSG0000	0.00026	hsa-miR-106a-5p;hsa-miR-106b-5p;hsa-miR-17-5p;hsa-miR-2(
ENSG0000	ENSG0000	0.000154	hsa-miR-106a-5p;hsa-miR-106b-5p;hsa-miR-15a-5p;hsa-miR-1
ENSG0000	ENSG0000	6.80E-05	hsa-miR-548b-5p;hsa-miR-548c-5p;hsa-miR-548d-5p;hsa-miR-
ENSG0000	ENSG0000	0.005887	hsa-miR-15a-5p;hsa-miR-15b-5p;hsa-miR-16-5p;hsa-miR-195-
ENSG0000	ENSG0000	0.005887	hsa-miR-15a-5p;hsa-miR-15b-5p;hsa-miR-16-5p;hsa-miR-195-
ENSG0000	ENSG0000	0.002254	hsa-miR-106a-5p;hsa-miR-106b-5p;hsa-miR-17-5p;hsa-miR-2(
ENSG0000	ENSG0000	3.51E-06	hsa-miR-106b-5p;hsa-miR-17-5p;hsa-miR-20a-5p;hsa-miR-20I
ENSG0000	ENSG0000	0.000627	hsa-miR-15a-5p;hsa-miR-15b-5p;hsa-miR-16-5p;hsa-miR-195-
ENSG0000	ENSG0000	0.009263	hsa-miR-106a-5p;hsa-miR-106b-5p;hsa-miR-17-5p;hsa-miR-2(
ENSG0000	ENSG0000	0.000748	hsa-miR-106b-5p;hsa-miR-17-5p;hsa-miR-20a-5p;hsa-miR-20I
ENSG0000	ENSG0000	4.24E-05	hsa-miR-27a-3p;hsa-miR-27b-3p;hsa-miR-548b-5p;hsa-miR-5
ENSG0000	ENSG0000	0.001961	hsa-miR-106b-5p;hsa-miR-15a-5p;hsa-miR-15b-5p;hsa-miR-1(
ENSG0000	ENSG0000	3.45E-06	hsa-miR-139-5p;hsa-miR-15a-5p;hsa-miR-15b-5p;hsa-miR-16-
ENSG0000	ENSG0000	0.000748	hsa-miR-106b-5p;hsa-miR-20a-5p;hsa-miR-20b-5p;hsa-miR-5(
ENSG0000	ENSG0000	7.37E-05	hsa-miR-106a-5p;hsa-miR-106b-5p;hsa-miR-17-5p;hsa-miR-2(
ENSG0000	ENSG0000	0.002549	hsa-miR-106a-5p;hsa-miR-106b-5p;hsa-miR-17-5p;hsa-miR-2(
ENSG0000	ENSG0000	0.002487	hsa-miR-103a-3p;hsa-miR-107;hsa-miR-127-5p;hsa-miR-500a-
ENSG0000	ENSG0000	0.000893	hsa-miR-15a-5p;hsa-miR-15b-5p;hsa-miR-16-5p;hsa-miR-195-
ENSG0000	ENSG0000	0.000969	hsa-miR-15a-5p;hsa-miR-15b-5p;hsa-miR-16-5p;hsa-miR-195-
ENSG0000	ENSG0000	0.005089	hsa-miR-106a-5p;hsa-miR-106b-5p;hsa-miR-17-5p;hsa-miR-2(
ENSG0000	ENSG0000	0.002662	hsa-miR-106a-5p;hsa-miR-17-5p;hsa-miR-20a-5p;hsa-miR-27a
ENSG0000	ENSG0000	0.005414	hsa-miR-107;hsa-miR-15a-5p;hsa-miR-15b-5p;hsa-miR-195-5
ENSG0000	ENSG0000	0.000148	hsa-miR-15a-5p;hsa-miR-15b-5p;hsa-miR-16-5p;hsa-miR-195-
ENSG0000	ENSG0000	0.000558	hsa-miR-106b-5p;hsa-miR-15a-5p;hsa-miR-16-5p;hsa-miR-17-

ENSG0000	ENSG0000	0.000893	hsa-miR-15b-5p;hsa-miR-16-5p;hsa-miR-195-5p;hsa-miR-424-
ENSG0000	ENSG0000	0.000462	hsa-miR-15a-5p;hsa-miR-15b-5p;hsa-miR-16-5p;hsa-miR-424-
ENSG0000	ENSG0000	0.0005	hsa-miR-103a-3p;hsa-miR-107;hsa-miR-15a-5p;hsa-miR-15b-5
ENSG0000	ENSG0000	0.000387	hsa-miR-106a-5p;hsa-miR-106b-5p;hsa-miR-17-5p;hsa-miR-2
ENSG0000	ENSG0000	1.19E-12	hsa-miR-106b-5p;hsa-miR-17-5p;hsa-miR-192-5p;hsa-miR-20a
ENSG0000	ENSG0000	3.78E-06	hsa-miR-103a-3p;hsa-miR-107;hsa-miR-15a-5p;hsa-miR-15b-5
ENSG0000	ENSG0000	0.006738	hsa-miR-140-3p;hsa-miR-15a-5p;hsa-miR-15b-5p;hsa-miR-424
ENSG0000	ENSG0000	0.000783	hsa-miR-15a-5p;hsa-miR-15b-5p;hsa-miR-195-5p;hsa-miR-27a
ENSG0000	ENSG0000	0.000462	hsa-miR-107;hsa-miR-185-5p;hsa-miR-195-5p;hsa-miR-224-5p
ENSG0000	ENSG0000	0.000587	hsa-miR-195-5p;hsa-miR-548b-5p;hsa-miR-548c-5p;hsa-miR-5
ENSG0000	ENSG0000	0.003073	hsa-miR-107;hsa-miR-15a-5p;hsa-miR-15b-5p;hsa-miR-16-5p;
ENSG0000	ENSG0000	0.000496	hsa-miR-106a-5p;hsa-miR-106b-5p;hsa-miR-16-5p;hsa-miR-1
ENSG0000	ENSG0000	0.005414	hsa-miR-106a-5p;hsa-miR-106b-5p;hsa-miR-17-5p;hsa-miR-2
ENSG0000	ENSG0000	4.68E-05	hsa-miR-106b-5p;hsa-miR-17-5p;hsa-miR-20a-5p;hsa-miR-20b
ENSG0000	ENSG0000	0.00015	hsa-miR-106a-5p;hsa-miR-106b-5p;hsa-miR-18a-3p;hsa-miR-2
ENSG0000	ENSG0000	0.008729	hsa-miR-106a-5p;hsa-miR-106b-5p;hsa-miR-17-5p;hsa-miR-2
ENSG0000	ENSG0000	2.23E-05	hsa-miR-106a-5p;hsa-miR-106b-5p;hsa-miR-127-5p;hsa-miR-1
ENSG0000	ENSG0000	8.12E-05	hsa-miR-505-3p;hsa-miR-548b-5p;hsa-miR-548c-5p;hsa-miR-5
ENSG0000	ENSG0000	0.0006	hsa-miR-106a-5p;hsa-miR-106b-5p;hsa-miR-17-5p;hsa-miR-2
ENSG0000	ENSG0000	0.000331	hsa-miR-15a-5p;hsa-miR-15b-5p;hsa-miR-16-5p;hsa-miR-185-
ENSG0000	ENSG0000	0.000101	hsa-miR-106b-5p;hsa-miR-17-5p;hsa-miR-20a-5p;hsa-miR-20b
ENSG0000	ENSG0000	0.000144	hsa-miR-106a-5p;hsa-miR-106b-5p;hsa-miR-17-5p;hsa-miR-2
ENSG0000	ENSG0000	2.70E-05	hsa-miR-548b-5p;hsa-miR-548c-5p;hsa-miR-548d-5p;hsa-miR-
ENSG0000	ENSG0000	0.009233	hsa-miR-15a-5p;hsa-miR-15b-5p;hsa-miR-192-5p;hsa-miR-195
ENSG0000	ENSG0000	1.10E-08	hsa-miR-103a-3p;hsa-miR-107;hsa-miR-15a-5p;hsa-miR-15b-5
ENSG0000	ENSG0000	0.001073	hsa-miR-127-5p;hsa-miR-15a-5p;hsa-miR-15b-5p;hsa-miR-16-
ENSG0000	ENSG0000	2.75E-09	hsa-miR-103a-3p;hsa-miR-107;hsa-miR-15a-5p;hsa-miR-15b-5
ENSG0000	ENSG0000	0.002536	hsa-miR-106a-5p;hsa-miR-106b-5p;hsa-miR-17-5p;hsa-miR-2
ENSG0000	ENSG0000	3.25E-06	hsa-miR-106a-5p;hsa-miR-106b-5p;hsa-miR-17-5p;hsa-miR-2
ENSG0000	ENSG0000	0.003927	hsa-miR-106a-5p;hsa-miR-106b-5p;hsa-miR-17-5p;hsa-miR-2
ENSG0000	ENSG0000	0.000298	hsa-miR-106a-5p;hsa-miR-106b-5p;hsa-miR-17-5p;hsa-miR-2
ENSG0000	ENSG0000	0.001109	hsa-miR-106b-5p;hsa-miR-17-5p;hsa-miR-195-5p;hsa-miR-20a
ENSG0000	ENSG0000	8.02E-06	hsa-miR-106a-5p;hsa-miR-106b-5p;hsa-miR-17-5p;hsa-miR-2
ENSG0000	ENSG0000	0.000239	hsa-miR-548b-5p;hsa-miR-548c-5p;hsa-miR-548d-5p;hsa-miR-
ENSG0000	ENSG0000	2.19E-05	hsa-miR-15a-5p;hsa-miR-15b-5p;hsa-miR-195-5p;hsa-miR-20b
ENSG0000	ENSG0000	0.0006	hsa-miR-548b-5p;hsa-miR-548c-5p;hsa-miR-548d-5p;hsa-miR-
ENSG0000	ENSG0000	0.005709	hsa-miR-103a-3p;hsa-miR-107;hsa-miR-15a-5p;hsa-miR-15b-5
ENSG0000	ENSG0000	4.47E-06	hsa-miR-106a-5p;hsa-miR-106b-5p;hsa-miR-17-5p;hsa-miR-2
ENSG0000	ENSG0000	6.95E-06	hsa-miR-106a-5p;hsa-miR-106b-5p;hsa-miR-17-5p;hsa-miR-2
ENSG0000	ENSG0000	2.74E-10	hsa-miR-103a-3p;hsa-miR-106a-5p;hsa-miR-106b-5p;hsa-miR-
ENSG0000	ENSG0000	1.33E-05	hsa-miR-548b-5p;hsa-miR-548c-5p;hsa-miR-548d-5p;hsa-miR-
ENSG0000	ENSG0000	0.000298	hsa-miR-106a-5p;hsa-miR-106b-5p;hsa-miR-140-3p;hsa-miR-1
ENSG0000	ENSG0000	0.001128	hsa-miR-106b-5p;hsa-miR-17-5p;hsa-miR-20a-5p;hsa-miR-20b
ENSG0000	ENSG0000	0.000226	hsa-miR-15a-5p;hsa-miR-15b-5p;hsa-miR-16-5p;hsa-miR-195-
ENSG0000	ENSG0000	3.51E-06	hsa-miR-106b-5p;hsa-miR-17-5p;hsa-miR-20a-5p;hsa-miR-20b
ENSG0000	ENSG0000	0.007728	hsa-miR-106a-5p;hsa-miR-106b-5p;hsa-miR-17-5p;hsa-miR-2
ENSG0000	ENSG0000	2.98E-06	hsa-miR-106a-5p;hsa-miR-106b-5p;hsa-miR-17-5p;hsa-miR-1
ENSG0000	ENSG0000	0.003102	hsa-miR-548b-5p;hsa-miR-548c-5p;hsa-miR-548d-5p;hsa-miR-
ENSG0000	ENSG0000	0.00455	hsa-miR-16-5p;hsa-miR-185-5p;hsa-miR-424-5p;hsa-miR-573;
ENSG0000	ENSG0000	0.000338	hsa-miR-15a-5p;hsa-miR-15b-5p;hsa-miR-16-5p;hsa-miR-195-
ENSG0000	ENSG0000	4.50E-05	hsa-miR-103a-3p;hsa-miR-106a-5p;hsa-miR-106b-5p;hsa-miR-
ENSG0000	ENSG0000	0.001128	hsa-miR-15a-5p;hsa-miR-15b-5p;hsa-miR-16-5p;hsa-miR-195-
ENSG0000	ENSG0000	0.000721	hsa-miR-548b-5p;hsa-miR-548c-5p;hsa-miR-548d-5p;hsa-miR-
ENSG0000	ENSG0000	3.03E-08	hsa-miR-106b-5p;hsa-miR-17-5p;hsa-miR-20a-5p;hsa-miR-20b

ENSG0000	ENSG0000	3.03E-08	hsa-miR-106b-5p;hsa-miR-17-5p;hsa-miR-20a-5p;hsa-miR-20
ENSG0000	ENSG0000	3.03E-08	hsa-miR-106b-5p;hsa-miR-17-5p;hsa-miR-20a-5p;hsa-miR-20
ENSG0000	ENSG0000	3.03E-08	hsa-miR-106b-5p;hsa-miR-17-5p;hsa-miR-20a-5p;hsa-miR-20
ENSG0000	ENSG0000	3.03E-08	hsa-miR-106b-5p;hsa-miR-17-5p;hsa-miR-20a-5p;hsa-miR-20
ENSG0000	ENSG0000	3.03E-08	hsa-miR-106b-5p;hsa-miR-17-5p;hsa-miR-20a-5p;hsa-miR-20
ENSG0000	ENSG0000	3.03E-08	hsa-miR-106b-5p;hsa-miR-17-5p;hsa-miR-20a-5p;hsa-miR-20
ENSG0000	ENSG0000	0.002725	hsa-miR-106a-5p;hsa-miR-106b-5p;hsa-miR-1231;hsa-miR-17
ENSG0000	ENSG0000	6.04E-07	hsa-miR-106b-5p;hsa-miR-17-5p;hsa-miR-20a-5p;hsa-miR-20
ENSG0000	ENSG0000	1.04E-07	hsa-miR-106b-5p;hsa-miR-17-5p;hsa-miR-20a-5p;hsa-miR-20
ENSG0000	ENSG0000	0.000861	hsa-miR-15a-5p;hsa-miR-15b-5p;hsa-miR-16-5p;hsa-miR-195
ENSG0000	ENSG0000	0.001829	hsa-miR-15a-5p;hsa-miR-15b-5p;hsa-miR-195-5p;hsa-miR-42
ENSG0000	ENSG0000	2.32E-07	hsa-miR-103a-3p;hsa-miR-107;hsa-miR-15a-5p;hsa-miR-15b-
ENSG0000	ENSG0000	0.000283	hsa-miR-106b-5p;hsa-miR-17-5p;hsa-miR-20a-5p;hsa-miR-20
ENSG0000	ENSG0000	0.001439	hsa-miR-106a-5p;hsa-miR-106b-5p;hsa-miR-17-5p;hsa-miR-2
ENSG0000	ENSG0000	1.71E-10	hsa-miR-15a-5p;hsa-miR-15b-5p;hsa-miR-16-5p;hsa-miR-195
ENSG0000	ENSG0000	0.000542	hsa-miR-15a-5p;hsa-miR-15b-5p;hsa-miR-18a-3p;hsa-miR-19
ENSG0000	ENSG0000	0.001774	hsa-miR-106a-5p;hsa-miR-106b-5p;hsa-miR-127-5p;hsa-miR-
ENSG0000	ENSG0000	1.75E-07	hsa-miR-548b-5p;hsa-miR-548c-5p;hsa-miR-548d-5p;hsa-miR
ENSG0000	ENSG0000	8.21E-06	hsa-miR-1246;hsa-miR-15a-5p;hsa-miR-15b-5p;hsa-miR-16-5
ENSG0000	ENSG0000	1.31E-05	hsa-miR-106a-5p;hsa-miR-106b-5p;hsa-miR-17-5p;hsa-miR-1
ENSG0000	ENSG0000	1.31E-05	hsa-miR-106a-5p;hsa-miR-106b-5p;hsa-miR-17-5p;hsa-miR-1
ENSG0000	ENSG0000	1.31E-05	hsa-miR-106a-5p;hsa-miR-106b-5p;hsa-miR-17-5p;hsa-miR-1
ENSG0000	ENSG0000	0.000627	hsa-miR-106a-5p;hsa-miR-106b-5p;hsa-miR-17-5p;hsa-miR-2
ENSG0000	ENSG0000	3.44E-08	hsa-miR-106a-5p;hsa-miR-106b-5p;hsa-miR-17-5p;hsa-miR-2
ENSG0000	ENSG0000	0.004541	hsa-miR-106a-5p;hsa-miR-106b-5p;hsa-miR-17-5p;hsa-miR-2
ENSG0000	ENSG0000	0.000872	hsa-miR-107;hsa-miR-17-5p;hsa-miR-20a-5p;hsa-miR-20b-5p;
ENSG0000	ENSG0000	0.001109	hsa-miR-106a-5p;hsa-miR-106b-5p;hsa-miR-17-5p;hsa-miR-2
ENSG0000	ENSG0000	4.24E-05	hsa-miR-106a-5p;hsa-miR-106b-5p;hsa-miR-127-5p;hsa-miR-
ENSG0000	ENSG0000	6.03E-06	hsa-miR-106a-5p;hsa-miR-17-5p;hsa-miR-20a-5p;hsa-miR-20
ENSG0000	ENSG0000	0.003566	hsa-miR-140-3p;hsa-miR-20a-3p;hsa-miR-548b-5p;hsa-miR-5
ENSG0000	ENSG0000	2.70E-05	hsa-miR-107;hsa-miR-140-3p;hsa-miR-15a-5p;hsa-miR-15b-5
ENSG0000	ENSG0000	0.004874	hsa-miR-15a-5p;hsa-miR-15b-5p;hsa-miR-16-5p;hsa-miR-195
ENSG0000	ENSG0000	0.008339	hsa-miR-548b-5p;hsa-miR-548c-5p;hsa-miR-548d-5p;hsa-miR
ENSG0000	ENSG0000	5.07E-07	hsa-miR-106a-5p;hsa-miR-106b-5p;hsa-miR-17-5p;hsa-miR-2
ENSG0000	ENSG0000	1.19E-08	hsa-miR-103a-3p;hsa-miR-107;hsa-miR-548b-5p;hsa-miR-548
ENSG0000	ENSG0000	3.39E-05	hsa-miR-103a-3p;hsa-miR-107;hsa-miR-15a-5p;hsa-miR-15b-
ENSG0000	ENSG0000	3.37E-07	hsa-miR-106a-5p;hsa-miR-106b-5p;hsa-miR-15a-5p;hsa-miR-
ENSG0000	ENSG0000	2.34E-06	hsa-miR-139-5p;hsa-miR-224-5p;hsa-miR-361-5p;hsa-miR-54
ENSG0000	ENSG0000	7.82E-05	hsa-miR-548b-5p;hsa-miR-548c-5p;hsa-miR-548d-5p;hsa-miR
ENSG0000	ENSG0000	0.001645	hsa-miR-106a-5p;hsa-miR-106b-5p;hsa-miR-16-5p;hsa-miR-1
ENSG0000	ENSG0000	3.74E-05	hsa-miR-106a-5p;hsa-miR-106b-5p;hsa-miR-143-3p;hsa-miR-
ENSG0000	ENSG0000	0.001836	hsa-miR-106b-5p;hsa-miR-17-5p;hsa-miR-20a-5p;hsa-miR-20
ENSG0000	ENSG0000	0.001073	hsa-miR-106a-5p;hsa-miR-106b-5p;hsa-miR-17-5p;hsa-miR-2
ENSG0000	ENSG0000	0.00598	hsa-miR-106a-5p;hsa-miR-106b-5p;hsa-miR-17-5p;hsa-miR-2
ENSG0000	ENSG0000	6.96E-06	hsa-miR-106a-5p;hsa-miR-106b-5p;hsa-miR-139-5p;hsa-miR-
ENSG0000	ENSG0000	8.02E-06	hsa-miR-106a-5p;hsa-miR-106b-5p;hsa-miR-17-5p;hsa-miR-2
ENSG0000	ENSG0000	0.000799	hsa-miR-106a-5p;hsa-miR-106b-5p;hsa-miR-140-3p;hsa-miR-
ENSG0000	ENSG0000	1.60E-05	hsa-miR-548b-5p;hsa-miR-548c-5p;hsa-miR-548d-5p;hsa-miR
ENSG0000	ENSG0000	4.50E-05	hsa-miR-106a-5p;hsa-miR-106b-5p;hsa-miR-17-5p;hsa-miR-2
ENSG0000	ENSG0000	1.27E-10	hsa-miR-15a-5p;hsa-miR-15b-5p;hsa-miR-195-5p;hsa-miR-36
ENSG0000	ENSG0000	0.000401	hsa-miR-15a-5p;hsa-miR-15b-5p;hsa-miR-16-5p;hsa-miR-195
ENSG0000	ENSG0000	5.75E-06	hsa-miR-15a-5p;hsa-miR-15b-5p;hsa-miR-16-5p;hsa-miR-195
ENSG0000	ENSG0000	5.75E-06	hsa-miR-15a-5p;hsa-miR-15b-5p;hsa-miR-16-5p;hsa-miR-195
ENSG0000	ENSG0000	2.69E-07	hsa-miR-106b-5p;hsa-miR-15a-5p;hsa-miR-15b-5p;hsa-miR-1

ENSG0000	ENSG0000	6.14E-05	hsa-miR-140-3p;hsa-miR-15a-5p;hsa-miR-15b-5p;hsa-miR-19
ENSG0000	ENSG0000	0.000546	hsa-miR-548b-5p;hsa-miR-548c-5p;hsa-miR-548d-5p;hsa-miR
ENSG0000	ENSG0000	0.000861	hsa-miR-106a-5p;hsa-miR-106b-5p;hsa-miR-140-3p;hsa-miR-
ENSG0000	ENSG0000	0.000273	hsa-miR-103a-3p;hsa-miR-107;hsa-miR-15a-5p;hsa-miR-15b-
ENSG0000	ENSG0000	1.52E-05	hsa-miR-103a-3p;hsa-miR-107;hsa-miR-15a-5p;hsa-miR-15b-
ENSG0000	ENSG0000	0.000144	hsa-miR-106a-5p;hsa-miR-106b-5p;hsa-miR-17-5p;hsa-miR-2
ENSG0000	ENSG0000	0.002827	hsa-miR-17-5p;hsa-miR-20a-5p;hsa-miR-224-5p;hsa-miR-27a-
ENSG0000	ENSG0000	0.000104	hsa-miR-15a-5p;hsa-miR-185-5p;hsa-miR-195-5p;hsa-miR-37
ENSG0000	ENSG0000	7.43E-10	hsa-miR-106a-5p;hsa-miR-106b-5p;hsa-miR-17-5p;hsa-miR-2
ENSG0000	ENSG0000	0.006813	hsa-miR-106b-5p;hsa-miR-15a-5p;hsa-miR-16-5p;hsa-miR-17-
ENSG0000	ENSG0000	0.001109	hsa-miR-106a-5p;hsa-miR-106b-5p;hsa-miR-17-5p;hsa-miR-1
ENSG0000	ENSG0000	9.07E-05	hsa-miR-548b-5p;hsa-miR-548c-5p;hsa-miR-548d-5p;hsa-miR
ENSG0000	ENSG0000	0.001021	hsa-miR-548b-5p;hsa-miR-548c-5p;hsa-miR-548d-5p;hsa-miR
ENSG0000	ENSG0000	0.000438	hsa-miR-106b-5p;hsa-miR-17-5p;hsa-miR-20a-5p;hsa-miR-20
ENSG0000	ENSG0000	2.89E-06	hsa-miR-106a-5p;hsa-miR-106b-5p;hsa-miR-1246;hsa-miR-17-
ENSG0000	ENSG0000	0.005709	hsa-miR-548b-5p;hsa-miR-548c-5p;hsa-miR-548d-5p;hsa-miR
ENSG0000	ENSG0000	0.008274	hsa-miR-15a-5p;hsa-miR-15b-5p;hsa-miR-195-5p;hsa-miR-42
ENSG0000	ENSG0000	2.05E-05	hsa-miR-106a-5p;hsa-miR-106b-5p;hsa-miR-17-5p;hsa-miR-1
ENSG0000	ENSG0000	1.11E-09	hsa-miR-106a-5p;hsa-miR-106b-5p;hsa-miR-17-5p;hsa-miR-2
ENSG0000	ENSG0000	0.006047	hsa-miR-17-5p;hsa-miR-20a-5p;hsa-miR-20b-5p;hsa-miR-93-
ENSG0000	ENSG0000	2.14E-06	hsa-miR-106a-5p;hsa-miR-107;hsa-miR-15a-5p;hsa-miR-16-5
ENSG0000	ENSG0000	6.14E-05	hsa-miR-106a-5p;hsa-miR-106b-5p;hsa-miR-17-5p;hsa-miR-2
ENSG0000	ENSG0000	0.004226	hsa-miR-106a-5p;hsa-miR-106b-5p;hsa-miR-17-5p;hsa-miR-2
ENSG0000	ENSG0000	0.00726	hsa-miR-106a-5p;hsa-miR-106b-5p;hsa-miR-17-5p;hsa-miR-2
ENSG0000	ENSG0000	0.000686	hsa-miR-106a-5p;hsa-miR-106b-5p;hsa-miR-1246;hsa-miR-17-
ENSG0000	ENSG0000	0.001411	hsa-miR-548b-5p;hsa-miR-548c-5p;hsa-miR-548d-5p;hsa-miR
ENSG0000	ENSG0000	0.005887	hsa-miR-106a-5p;hsa-miR-106b-5p;hsa-miR-17-5p;hsa-miR-2
ENSG0000	ENSG0000	7.37E-05	hsa-miR-15a-5p;hsa-miR-15b-5p;hsa-miR-16-5p;hsa-miR-195-
ENSG0000	ENSG0000	1.36E-05	hsa-miR-106a-5p;hsa-miR-106b-5p;hsa-miR-17-5p;hsa-miR-2
ENSG0000	ENSG0000	0.009354	hsa-miR-106b-5p;hsa-miR-17-5p;hsa-miR-20a-5p;hsa-miR-20
ENSG0000	ENSG0000	0.000686	hsa-miR-103a-3p;hsa-miR-107;hsa-miR-15a-5p;hsa-miR-15b-
ENSG0000	ENSG0000	0.003377	hsa-miR-107;hsa-miR-140-3p;hsa-miR-15a-5p;hsa-miR-15b-5
ENSG0000	ENSG0000	0.000893	hsa-miR-15a-5p;hsa-miR-15b-5p;hsa-miR-195-5p;hsa-miR-42
ENSG0000	ENSG0000	5.75E-06	hsa-miR-15a-5p;hsa-miR-15b-5p;hsa-miR-16-5p;hsa-miR-195-
ENSG0000	ENSG0000	5.75E-06	hsa-miR-15a-5p;hsa-miR-15b-5p;hsa-miR-16-5p;hsa-miR-195-
ENSG0000	ENSG0000	0.003446	hsa-miR-139-5p;hsa-miR-15a-5p;hsa-miR-15b-5p;hsa-miR-19
ENSG0000	ENSG0000	0.0006	hsa-miR-106a-5p;hsa-miR-106b-5p;hsa-miR-143-3p;hsa-miR-
ENSG0000	ENSG0000	0.00034	hsa-miR-106a-5p;hsa-miR-106b-5p;hsa-miR-17-5p;hsa-miR-2
ENSG0000	ENSG0000	0.000659	hsa-miR-106a-5p;hsa-miR-106b-5p;hsa-miR-20a-5p;hsa-miR-
ENSG0000	ENSG0000	1.03E-06	hsa-miR-548b-5p;hsa-miR-548c-5p;hsa-miR-548d-5p;hsa-miR
ENSG0000	ENSG0000	2.42E-07	hsa-miR-15a-5p;hsa-miR-15b-5p;hsa-miR-16-5p;hsa-miR-195-
ENSG0000	ENSG0000	1.79E-06	hsa-miR-106a-5p;hsa-miR-106b-5p;hsa-miR-17-5p;hsa-miR-2
ENSG0000	ENSG0000	4.17E-05	hsa-miR-106a-5p;hsa-miR-106b-5p;hsa-miR-17-5p;hsa-miR-2
ENSG0000	ENSG0000	0.000862	hsa-miR-107;hsa-miR-15a-5p;hsa-miR-15b-5p;hsa-miR-195-5
ENSG0000	ENSG0000	0.000555	hsa-miR-106b-5p;hsa-miR-17-5p;hsa-miR-20a-5p;hsa-miR-20
ENSG0000	ENSG0000	0.000387	hsa-miR-106a-5p;hsa-miR-106b-5p;hsa-miR-17-5p;hsa-miR-2
ENSG0000	ENSG0000	8.53E-05	hsa-miR-127-5p;hsa-miR-185-5p;hsa-miR-548b-5p;hsa-miR-5
ENSG0000	ENSG0000	9.10E-06	hsa-miR-106a-5p;hsa-miR-106b-5p;hsa-miR-17-5p;hsa-miR-2
ENSG0000	ENSG0000	1.53E-08	hsa-miR-106b-5p;hsa-miR-1246;hsa-miR-17-5p;hsa-miR-185-
ENSG0000	ENSG0000	4.49E-10	hsa-miR-106a-5p;hsa-miR-106b-5p;hsa-miR-127-5p;hsa-miR-
ENSG0000	ENSG0000	0.000105	hsa-miR-106a-5p;hsa-miR-20a-5p;hsa-miR-20b-5p;hsa-miR-9
ENSG0000	ENSG0000	0.002662	hsa-miR-106a-5p;hsa-miR-106b-5p;hsa-miR-17-5p;hsa-miR-2
ENSG0000	ENSG0000	1.19E-06	hsa-miR-15a-5p;hsa-miR-15b-5p;hsa-miR-16-5p;hsa-miR-195-
ENSG0000	ENSG0000	0.000195	hsa-miR-106b-5p;hsa-miR-17-5p;hsa-miR-20a-5p;hsa-miR-20

ENSG0000	ENSG0000	8.63E-09	hsa-miR-106b-5p;hsa-miR-107;hsa-miR-15a-5p;hsa-miR-15b-5
ENSG0000	ENSG0000	0.002068	hsa-miR-106a-5p;hsa-miR-106b-5p;hsa-miR-15a-5p;hsa-miR-1
ENSG0000	ENSG0000	0.000627	hsa-miR-15a-5p;hsa-miR-15b-5p;hsa-miR-16-5p;hsa-miR-195-
ENSG0000	ENSG0000	1.79E-06	hsa-miR-106a-5p;hsa-miR-106b-5p;hsa-miR-1246;hsa-miR-17-
ENSG0000	ENSG0000	0.00034	hsa-miR-106a-5p;hsa-miR-106b-5p;hsa-miR-17-5p;hsa-miR-20
ENSG0000	ENSG0000	3.78E-06	hsa-miR-548b-5p;hsa-miR-548c-5p;hsa-miR-548d-5p;hsa-miR-
ENSG0000	ENSG0000	4.59E-05	hsa-miR-106b-5p;hsa-miR-17-5p;hsa-miR-20a-5p;hsa-miR-93-
ENSG0000	ENSG0000	0.000387	hsa-miR-15a-5p;hsa-miR-15b-5p;hsa-miR-16-5p;hsa-miR-195-
ENSG0000	ENSG0000	0.005709	hsa-miR-103a-3p;hsa-miR-106b-5p;hsa-miR-107;hsa-miR-17-5
ENSG0000	ENSG0000	0.005709	hsa-miR-103a-3p;hsa-miR-106b-5p;hsa-miR-107;hsa-miR-17-5
ENSG0000	ENSG0000	3.03E-08	hsa-miR-106b-5p;hsa-miR-17-5p;hsa-miR-20a-5p;hsa-miR-20b
ENSG0000	ENSG0000	5.89E-05	hsa-miR-106a-5p;hsa-miR-106b-5p;hsa-miR-139-5p;hsa-miR-1
ENSG0000	ENSG0000	0.000407	hsa-miR-103a-3p;hsa-miR-106b-5p;hsa-miR-107;hsa-miR-17-5
ENSG0000	ENSG0000	2.70E-05	hsa-miR-548b-5p;hsa-miR-548c-5p;hsa-miR-548d-5p;hsa-miR-
ENSG0000	ENSG0000	0.000298	hsa-miR-106a-5p;hsa-miR-106b-5p;hsa-miR-17-5p;hsa-miR-18
ENSG0000	ENSG0000	6.03E-06	hsa-miR-1246;hsa-miR-15a-5p;hsa-miR-15b-5p;hsa-miR-16-5p
ENSG0000	ENSG0000	6.03E-06	hsa-miR-1246;hsa-miR-15a-5p;hsa-miR-15b-5p;hsa-miR-16-5p
ENSG0000	ENSG0000	0.001021	hsa-miR-106b-5p;hsa-miR-107;hsa-miR-15a-5p;hsa-miR-15b-5
ENSG0000	ENSG0000	0.003125	hsa-miR-15a-5p;hsa-miR-15b-5p;hsa-miR-16-5p;hsa-miR-195-
ENSG0000	ENSG0000	0.003124	hsa-miR-106a-5p;hsa-miR-106b-5p;hsa-miR-17-5p;hsa-miR-20
ENSG0000	ENSG0000	0.002068	hsa-miR-106a-5p;hsa-miR-106b-5p;hsa-miR-17-5p;hsa-miR-18
ENSG0000	ENSG0000	0.000101	hsa-miR-106a-5p;hsa-miR-106b-5p;hsa-miR-17-5p;hsa-miR-19
ENSG0000	ENSG0000	0.000249	hsa-miR-136-3p;hsa-miR-548b-5p;hsa-miR-548c-5p;hsa-miR-5
ENSG0000	ENSG0000	2.80E-05	hsa-miR-106b-5p;hsa-miR-17-5p;hsa-miR-20a-5p;hsa-miR-20b
ENSG0000	ENSG0000	2.42E-07	hsa-miR-106b-5p;hsa-miR-17-5p;hsa-miR-192-5p;hsa-miR-20a
ENSG0000	ENSG0000	0.007483	hsa-miR-548b-5p;hsa-miR-548c-5p;hsa-miR-548d-5p;hsa-miR-
ENSG0000	ENSG0000	0.001853	hsa-miR-103a-3p;hsa-miR-107;hsa-miR-136-3p;hsa-miR-15a-5
ENSG0000	ENSG0000	6.03E-06	hsa-miR-106a-5p;hsa-miR-106b-5p;hsa-miR-17-5p;hsa-miR-20
ENSG0000	ENSG0000	0.000204	hsa-miR-15a-5p;hsa-miR-15b-5p;hsa-miR-16-5p;hsa-miR-195-
ENSG0000	ENSG0000	0.000204	hsa-miR-15a-5p;hsa-miR-15b-5p;hsa-miR-16-5p;hsa-miR-195-
ENSG0000	ENSG0000	0.002366	hsa-miR-106a-5p;hsa-miR-1246;hsa-miR-127-5p;hsa-miR-17-5
ENSG0000	ENSG0000	3.03E-08	hsa-miR-106b-5p;hsa-miR-17-5p;hsa-miR-20a-5p;hsa-miR-20b
ENSG0000	ENSG0000	8.05E-06	hsa-miR-15a-5p;hsa-miR-15b-5p;hsa-miR-16-5p;hsa-miR-195-
ENSG0000	ENSG0000	0.002055	hsa-miR-106a-5p;hsa-miR-106b-5p;hsa-miR-20a-5p;hsa-miR-2
ENSG0000	ENSG0000	8.15E-07	hsa-miR-103a-3p;hsa-miR-107;hsa-miR-15a-5p;hsa-miR-15b-5
ENSG0000	ENSG0000	1.04E-05	hsa-miR-107;hsa-miR-140-3p;hsa-miR-548b-5p;hsa-miR-548c-
ENSG0000	ENSG0000	0.001186	hsa-miR-107;hsa-miR-15a-5p;hsa-miR-15b-5p;hsa-miR-195-5p
ENSG0000	ENSG0000	0.00712	hsa-miR-106a-5p;hsa-miR-106b-5p;hsa-miR-17-5p;hsa-miR-20
ENSG0000	ENSG0000	0.002336	hsa-miR-106a-5p;hsa-miR-106b-5p;hsa-miR-17-5p;hsa-miR-20
ENSG0000	ENSG0000	1.04E-07	hsa-miR-106b-5p;hsa-miR-17-5p;hsa-miR-20a-5p;hsa-miR-20b
ENSG0000	ENSG0000	0.000558	hsa-miR-106a-5p;hsa-miR-106b-5p;hsa-miR-17-5p;hsa-miR-20
ENSG0000	ENSG0000	0.000558	hsa-miR-106a-5p;hsa-miR-106b-5p;hsa-miR-17-5p;hsa-miR-20
ENSG0000	ENSG0000	0.000102	hsa-miR-548b-5p;hsa-miR-548c-5p;hsa-miR-548d-5p;hsa-miR-
ENSG0000	ENSG0000	0.000542	hsa-miR-107;hsa-miR-15a-5p;hsa-miR-15b-5p;hsa-miR-195-5p
ENSG0000	ENSG0000	0.000721	hsa-miR-548b-5p;hsa-miR-548c-5p;hsa-miR-548d-5p;hsa-miR-
ENSG0000	ENSG0000	1.92E-05	hsa-miR-548b-5p;hsa-miR-548c-5p;hsa-miR-548d-5p;hsa-miR-
ENSG0000	ENSG0000	8.99E-06	hsa-miR-103a-3p;hsa-miR-107;hsa-miR-15a-5p;hsa-miR-15b-5
ENSG0000	ENSG0000	0.00598	hsa-miR-106a-5p;hsa-miR-106b-5p;hsa-miR-17-5p;hsa-miR-20
ENSG0000	ENSG0000	0.000234	hsa-miR-15a-5p;hsa-miR-15b-5p;hsa-miR-16-5p;hsa-miR-185-
ENSG0000	ENSG0000	1.23E-08	hsa-miR-15a-5p;hsa-miR-15b-5p;hsa-miR-16-5p;hsa-miR-195-
ENSG0000	ENSG0000	0.009519	hsa-miR-106a-5p;hsa-miR-106b-5p;hsa-miR-17-5p;hsa-miR-18
ENSG0000	ENSG0000	0.009519	hsa-miR-106a-5p;hsa-miR-106b-5p;hsa-miR-17-5p;hsa-miR-18
ENSG0000	ENSG0000	0.007728	hsa-miR-106a-5p;hsa-miR-106b-5p;hsa-miR-17-5p;hsa-miR-20
ENSG0000	ENSG0000	0.000995	hsa-miR-106a-5p;hsa-miR-106b-5p;hsa-miR-17-5p;hsa-miR-20

ENSG0000	ENSG0000	0.003756	hsa-miR-139-5p;hsa-miR-15a-5p;hsa-miR-15b-5p;hsa-miR-19
ENSG0000	ENSG0000	0.002549	hsa-miR-15a-5p;hsa-miR-15b-5p;hsa-miR-16-5p;hsa-miR-195
ENSG0000	ENSG0000	6.13E-05	hsa-miR-15a-5p;hsa-miR-15b-5p;hsa-miR-16-5p;hsa-miR-195
ENSG0000	ENSG0000	0.009121	hsa-miR-15a-5p;hsa-miR-15b-5p;hsa-miR-195-5p;hsa-miR-42
ENSG0000	ENSG0000	7.25E-05	hsa-miR-103a-3p;hsa-miR-107;hsa-miR-139-5p;hsa-miR-15a-
ENSG0000	ENSG0000	0.001635	hsa-miR-107;hsa-miR-15a-5p;hsa-miR-15b-5p;hsa-miR-195-5
ENSG0000	ENSG0000	0.001073	hsa-miR-106a-5p;hsa-miR-106b-5p;hsa-miR-17-5p;hsa-miR-2
ENSG0000	ENSG0000	6.04E-07	hsa-miR-106b-5p;hsa-miR-17-5p;hsa-miR-20a-5p;hsa-miR-20
ENSG0000	ENSG0000	0.001203	hsa-miR-106a-5p;hsa-miR-106b-5p;hsa-miR-17-5p;hsa-miR-2
ENSG0000	ENSG0000	0.001073	hsa-miR-106a-5p;hsa-miR-106b-5p;hsa-miR-17-5p;hsa-miR-2
ENSG0000	ENSG0000	5.31E-07	hsa-miR-103a-3p;hsa-miR-107;hsa-miR-136-3p;hsa-miR-15a-
ENSG0000	ENSG0000	0.002536	hsa-miR-106a-5p;hsa-miR-106b-5p;hsa-miR-139-5p;hsa-miR-
ENSG0000	ENSG0000	6.13E-05	hsa-miR-15a-5p;hsa-miR-15b-5p;hsa-miR-16-5p;hsa-miR-195
ENSG0000	ENSG0000	0.006386	hsa-miR-106a-5p;hsa-miR-106b-5p;hsa-miR-17-5p;hsa-miR-2
ENSG0000	ENSG0000	0.002451	hsa-miR-106b-5p;hsa-miR-17-5p;hsa-miR-20a-5p;hsa-miR-20
ENSG0000	ENSG0000	0.000473	hsa-miR-15a-5p;hsa-miR-15b-5p;hsa-miR-16-5p;hsa-miR-195
ENSG0000	ENSG0000	9.95E-07	hsa-miR-106a-5p;hsa-miR-106b-5p;hsa-miR-15a-5p;hsa-miR-
ENSG0000	ENSG0000	0.005224	hsa-miR-106a-5p;hsa-miR-106b-5p;hsa-miR-17-5p;hsa-miR-2
ENSG0000	ENSG0000	0.00026	hsa-miR-106a-5p;hsa-miR-106b-5p;hsa-miR-17-5p;hsa-miR-2
ENSG0000	ENSG0000	1.12E-07	hsa-miR-15a-5p;hsa-miR-15b-5p;hsa-miR-16-5p;hsa-miR-195
ENSG0000	ENSG0000	2.74E-05	hsa-miR-106a-5p;hsa-miR-106b-5p;hsa-miR-17-5p;hsa-miR-2
ENSG0000	ENSG0000	0.000401	hsa-miR-15a-5p;hsa-miR-15b-5p;hsa-miR-16-5p;hsa-miR-195
ENSG0000	ENSG0000	1.04E-07	hsa-miR-106b-5p;hsa-miR-17-5p;hsa-miR-20a-5p;hsa-miR-20
ENSG0000	ENSG0000	9.64E-06	hsa-miR-324-3p;hsa-miR-548b-5p;hsa-miR-548c-5p;hsa-miR-
ENSG0000	ENSG0000	3.33E-07	hsa-miR-106a-5p;hsa-miR-106b-5p;hsa-miR-107;hsa-miR-15a
ENSG0000	ENSG0000	5.09E-05	hsa-miR-17-5p;hsa-miR-548b-5p;hsa-miR-548c-5p;hsa-miR-5
ENSG0000	ENSG0000	5.49E-07	hsa-miR-106b-5p;hsa-miR-125b-2-3p;hsa-miR-140-3p;hsa-mi
ENSG0000	ENSG0000	0.002068	hsa-miR-548b-5p;hsa-miR-548c-5p;hsa-miR-548d-5p;hsa-miR
ENSG0000	ENSG0000	0.002068	hsa-miR-548b-5p;hsa-miR-548c-5p;hsa-miR-548d-5p;hsa-miR
ENSG0000	ENSG0000	0.000168	hsa-miR-106a-5p;hsa-miR-106b-5p;hsa-miR-17-5p;hsa-miR-2
ENSG0000	ENSG0000	0.005593	hsa-miR-106a-5p;hsa-miR-106b-5p;hsa-miR-17-5p;hsa-miR-2
ENSG0000	ENSG0000	0.000783	hsa-miR-106a-5p;hsa-miR-106b-5p;hsa-miR-17-5p;hsa-miR-2
ENSG0000	ENSG0000	0.000783	hsa-miR-106a-5p;hsa-miR-106b-5p;hsa-miR-17-5p;hsa-miR-2
ENSG0000	ENSG0000	0.001439	hsa-miR-106a-5p;hsa-miR-106b-5p;hsa-miR-17-5p;hsa-miR-2
ENSG0000	ENSG0000	0.003293	hsa-miR-15a-5p;hsa-miR-15b-5p;hsa-miR-16-5p;hsa-miR-195
ENSG0000	ENSG0000	0.002549	hsa-miR-15a-5p;hsa-miR-15b-5p;hsa-miR-16-5p;hsa-miR-195
ENSG0000	ENSG0000	0.000127	hsa-miR-361-5p;hsa-miR-548b-5p;hsa-miR-548c-5p;hsa-miR-
ENSG0000	ENSG0000	7.37E-05	hsa-miR-106a-5p;hsa-miR-106b-5p;hsa-miR-17-5p;hsa-miR-2
ENSG0000	ENSG0000	8.02E-06	hsa-miR-106a-5p;hsa-miR-106b-5p;hsa-miR-17-5p;hsa-miR-2
ENSG0000	ENSG0000	4.68E-05	hsa-miR-106a-5p;hsa-miR-106b-5p;hsa-miR-17-5p;hsa-miR-2
ENSG0000	ENSG0000	0.001825	hsa-miR-140-3p;hsa-miR-548b-5p;hsa-miR-548c-5p;hsa-miR-
ENSG0000	ENSG0000	0.000298	hsa-miR-106a-5p;hsa-miR-106b-5p;hsa-miR-17-5p;hsa-miR-2
ENSG0000	ENSG0000	4.51E-08	hsa-miR-106a-5p;hsa-miR-106b-5p;hsa-miR-1231;hsa-miR-17
ENSG0000	ENSG0000	6.09E-07	hsa-miR-106a-5p;hsa-miR-106b-5p;hsa-miR-15a-5p;hsa-miR-
ENSG0000	ENSG0000	5.52E-05	hsa-miR-106a-5p;hsa-miR-106b-5p;hsa-miR-139-5p;hsa-miR-
ENSG0000	ENSG0000	1.10E-05	hsa-miR-548b-5p;hsa-miR-548c-5p;hsa-miR-548d-5p;hsa-miR
ENSG0000	ENSG0000	0.002536	hsa-miR-106a-5p;hsa-miR-106b-5p;hsa-miR-17-5p;hsa-miR-2
ENSG0000	ENSG0000	0.000495	hsa-miR-106a-5p;hsa-miR-106b-5p;hsa-miR-17-5p;hsa-miR-2
ENSG0000	ENSG0000	1.19E-06	hsa-miR-106b-5p;hsa-miR-17-5p;hsa-miR-20a-5p;hsa-miR-20
ENSG0000	ENSG0000	2.42E-07	hsa-miR-15a-5p;hsa-miR-15b-5p;hsa-miR-16-5p;hsa-miR-195
ENSG0000	ENSG0000	1.10E-08	hsa-miR-107;hsa-miR-1246;hsa-miR-15a-5p;hsa-miR-15b-5p;h
ENSG0000	ENSG0000	1.63E-09	hsa-miR-106a-5p;hsa-miR-106b-5p;hsa-miR-15a-5p;hsa-miR-
ENSG0000	ENSG0000	0.000331	hsa-miR-106a-5p;hsa-miR-106b-5p;hsa-miR-143-3p;hsa-miR-
ENSG0000	ENSG0000	0.004261	hsa-miR-15a-5p;hsa-miR-15b-5p;hsa-miR-195-5p;hsa-miR-42

ENSG0000	ENSG0000	1.30E-05	hsa-miR-106b-5p;hsa-miR-17-5p;hsa-miR-20a-5p;hsa-miR-20
ENSG0000	ENSG0000	0.001645	hsa-miR-15a-5p;hsa-miR-15b-5p;hsa-miR-16-5p;hsa-miR-195-
ENSG0000	ENSG0000	0.002068	hsa-miR-15a-5p;hsa-miR-15b-5p;hsa-miR-16-5p;hsa-miR-192-
ENSG0000	ENSG0000	3.60E-06	hsa-miR-15a-5p;hsa-miR-15b-5p;hsa-miR-16-5p;hsa-miR-195-
ENSG0000	ENSG0000	0.001732	hsa-miR-106a-5p;hsa-miR-106b-5p;hsa-miR-17-5p;hsa-miR-2
ENSG0000	ENSG0000	0.001732	hsa-miR-106a-5p;hsa-miR-106b-5p;hsa-miR-17-5p;hsa-miR-2
ENSG0000	ENSG0000	0.009354	hsa-miR-15a-5p;hsa-miR-15b-5p;hsa-miR-16-5p;hsa-miR-195-
ENSG0000	ENSG0000	0.001439	hsa-miR-106a-5p;hsa-miR-106b-5p;hsa-miR-17-5p;hsa-miR-2
ENSG0000	ENSG0000	0.003736	hsa-miR-185-5p;hsa-miR-27a-3p;hsa-miR-27b-3p;hsa-miR-54
ENSG0000	ENSG0000	0.000204	hsa-miR-15a-5p;hsa-miR-15b-5p;hsa-miR-195-5p;hsa-miR-42
ENSG0000	ENSG0000	0.000387	hsa-miR-15a-5p;hsa-miR-15b-5p;hsa-miR-16-5p;hsa-miR-195-
ENSG0000	ENSG0000	0.001645	hsa-miR-548b-5p;hsa-miR-548c-5p;hsa-miR-548d-5p;hsa-miR-
ENSG0000	ENSG0000	0.002662	hsa-miR-15a-5p;hsa-miR-15b-5p;hsa-miR-16-5p;hsa-miR-185-
ENSG0000	ENSG0000	0.00748	hsa-miR-140-3p;hsa-miR-500a-3p;hsa-miR-501-3p;hsa-miR-5
ENSG0000	ENSG0000	3.03E-08	hsa-miR-106b-5p;hsa-miR-17-5p;hsa-miR-20a-5p;hsa-miR-20
ENSG0000	ENSG0000	9.10E-06	hsa-miR-103a-3p;hsa-miR-106a-5p;hsa-miR-106b-5p;hsa-miR-
ENSG0000	ENSG0000	2.80E-05	hsa-miR-15a-5p;hsa-miR-15b-5p;hsa-miR-16-5p;hsa-miR-195-
ENSG0000	ENSG0000	5.92E-06	hsa-miR-106b-5p;hsa-miR-17-5p;hsa-miR-20a-5p;hsa-miR-20
ENSG0000	ENSG0000	5.92E-06	hsa-miR-106b-5p;hsa-miR-17-5p;hsa-miR-20a-5p;hsa-miR-20
ENSG0000	ENSG0000	5.92E-06	hsa-miR-106b-5p;hsa-miR-17-5p;hsa-miR-20a-5p;hsa-miR-20
ENSG0000	ENSG0000	5.92E-06	hsa-miR-106b-5p;hsa-miR-17-5p;hsa-miR-20a-5p;hsa-miR-20
ENSG0000	ENSG0000	5.92E-06	hsa-miR-106b-5p;hsa-miR-17-5p;hsa-miR-20a-5p;hsa-miR-20
ENSG0000	ENSG0000	5.92E-06	hsa-miR-106b-5p;hsa-miR-17-5p;hsa-miR-20a-5p;hsa-miR-20
ENSG0000	ENSG0000	1.15E-06	hsa-miR-106a-5p;hsa-miR-106b-5p;hsa-miR-139-5p;hsa-miR-
ENSG0000	ENSG0000	0.000872	hsa-miR-15a-5p;hsa-miR-15b-5p;hsa-miR-18a-3p;hsa-miR-19
ENSG0000	ENSG0000	2.42E-07	hsa-miR-15a-5p;hsa-miR-15b-5p;hsa-miR-16-5p;hsa-miR-195-
ENSG0000	ENSG0000	0.002068	hsa-miR-15a-5p;hsa-miR-15b-5p;hsa-miR-16-5p;hsa-miR-195-
ENSG0000	ENSG0000	0.002451	hsa-miR-106a-5p;hsa-miR-106b-5p;hsa-miR-17-5p;hsa-miR-2
ENSG0000	ENSG0000	3.97E-05	hsa-miR-548b-5p;hsa-miR-548c-5p;hsa-miR-548d-5p;hsa-miR-
ENSG0000	ENSG0000	0.000861	hsa-miR-106a-5p;hsa-miR-106b-5p;hsa-miR-17-5p;hsa-miR-2
ENSG0000	ENSG0000	6.04E-07	hsa-miR-106b-5p;hsa-miR-17-5p;hsa-miR-20a-5p;hsa-miR-20
ENSG0000	ENSG0000	6.04E-07	hsa-miR-106b-5p;hsa-miR-17-5p;hsa-miR-20a-5p;hsa-miR-20
ENSG0000	ENSG0000	0.002662	hsa-miR-106a-5p;hsa-miR-106b-5p;hsa-miR-17-5p;hsa-miR-2
ENSG0000	ENSG0000	1.89E-06	hsa-miR-106a-5p;hsa-miR-106b-5p;hsa-miR-15a-5p;hsa-miR-
ENSG0000	ENSG0000	0.007917	hsa-miR-103a-3p;hsa-miR-107;hsa-miR-15a-5p;hsa-miR-15b-
ENSG0000	ENSG0000	0.000783	hsa-miR-106a-5p;hsa-miR-106b-5p;hsa-miR-17-5p;hsa-miR-2
ENSG0000	ENSG0000	1.10E-05	hsa-miR-106a-5p;hsa-miR-106b-5p;hsa-miR-17-5p;hsa-miR-1
ENSG0000	ENSG0000	8.96E-05	hsa-miR-548b-5p;hsa-miR-548c-5p;hsa-miR-548d-5p;hsa-miR-
ENSG0000	ENSG0000	0.001303	hsa-miR-15a-5p;hsa-miR-15b-5p;hsa-miR-16-5p;hsa-miR-195-
ENSG0000	ENSG0000	0.009121	hsa-miR-15a-5p;hsa-miR-15b-5p;hsa-miR-195-5p;hsa-miR-42
ENSG0000	ENSG0000	5.75E-06	hsa-miR-106b-5p;hsa-miR-17-5p;hsa-miR-20a-5p;hsa-miR-20
ENSG0000	ENSG0000	0.000587	hsa-miR-103a-3p;hsa-miR-106a-5p;hsa-miR-106b-5p;hsa-miR-
ENSG0000	ENSG0000	0.001308	hsa-miR-106a-5p;hsa-miR-106b-5p;hsa-miR-17-5p;hsa-miR-2
ENSG0000	ENSG0000	0.000495	hsa-miR-106a-5p;hsa-miR-106b-5p;hsa-miR-17-5p;hsa-miR-2
ENSG0000	ENSG0000	5.75E-06	hsa-miR-15a-5p;hsa-miR-15b-5p;hsa-miR-16-5p;hsa-miR-195-
ENSG0000	ENSG0000	0.000938	hsa-miR-548b-5p;hsa-miR-548c-5p;hsa-miR-548d-5p;hsa-miR-
ENSG0000	ENSG0000	0.003446	hsa-miR-106b-5p;hsa-miR-17-5p;hsa-miR-20a-5p;hsa-miR-20
ENSG0000	ENSG0000	0.000144	hsa-miR-106a-5p;hsa-miR-106b-5p;hsa-miR-17-5p;hsa-miR-1
ENSG0000	ENSG0000	0.000635	hsa-miR-103a-3p;hsa-miR-107;hsa-miR-139-5p;hsa-miR-15a-
ENSG0000	ENSG0000	1.92E-05	hsa-miR-103a-3p;hsa-miR-107;hsa-miR-15a-5p;hsa-miR-15b-
ENSG0000	ENSG0000	8.15E-07	hsa-miR-103a-3p;hsa-miR-107;hsa-miR-15a-5p;hsa-miR-15b-
ENSG0000	ENSG0000	1.58E-05	hsa-miR-15a-5p;hsa-miR-15b-5p;hsa-miR-16-5p;hsa-miR-195-
ENSG0000	ENSG0000	0.004342	hsa-miR-106a-5p;hsa-miR-106b-5p;hsa-miR-17-5p;hsa-miR-2
ENSG0000	ENSG0000	0.000872	hsa-miR-106a-5p;hsa-miR-106b-5p;hsa-miR-17-5p;hsa-miR-2

ENSG0000	ENSG0000	1.46E-08	hsa-miR-103a-3p;hsa-miR-106a-5p;hsa-miR-107;hsa-miR-15a-
ENSG0000	ENSG0000	1.19E-06	hsa-miR-106b-5p;hsa-miR-17-5p;hsa-miR-20a-5p;hsa-miR-20
ENSG0000	ENSG0000	0.000234	hsa-miR-127-5p;hsa-miR-15a-5p;hsa-miR-15b-5p;hsa-miR-16-
ENSG0000	ENSG0000	0.005089	hsa-miR-106a-5p;hsa-miR-106b-5p;hsa-miR-143-3p;hsa-miR-
ENSG0000	ENSG0000	4.50E-05	hsa-miR-106a-5p;hsa-miR-106b-5p;hsa-miR-17-5p;hsa-miR-2
ENSG0000	ENSG0000	0.000327	hsa-miR-106a-5p;hsa-miR-106b-5p;hsa-miR-17-5p;hsa-miR-2
ENSG0000	ENSG0000	3.92E-06	hsa-miR-106b-5p;hsa-miR-107;hsa-miR-15a-5p;hsa-miR-15b-
ENSG0000	ENSG0000	0.000401	hsa-miR-106b-5p;hsa-miR-17-5p;hsa-miR-20a-5p;hsa-miR-20
ENSG0000	ENSG0000	1.05E-05	hsa-miR-15a-5p;hsa-miR-15b-5p;hsa-miR-16-5p;hsa-miR-195-
ENSG0000	ENSG0000	0.000283	hsa-miR-106a-5p;hsa-miR-106b-5p;hsa-miR-20a-5p;hsa-miR-
ENSG0000	ENSG0000	2.80E-07	hsa-miR-139-5p;hsa-miR-15a-5p;hsa-miR-15b-5p;hsa-miR-16-
ENSG0000	ENSG0000	3.03E-08	hsa-miR-106b-5p;hsa-miR-17-5p;hsa-miR-20a-5p;hsa-miR-20
ENSG0000	ENSG0000	0.000627	hsa-miR-106a-5p;hsa-miR-106b-5p;hsa-miR-17-5p;hsa-miR-2
ENSG0000	ENSG0000	0.001439	hsa-miR-106a-5p;hsa-miR-106b-5p;hsa-miR-17-5p;hsa-miR-2
ENSG0000	ENSG0000	0.000473	hsa-miR-107;hsa-miR-15a-5p;hsa-miR-15b-5p;hsa-miR-195-5
ENSG0000	ENSG0000	0.001836	hsa-miR-15a-5p;hsa-miR-15b-5p;hsa-miR-16-5p;hsa-miR-195-
ENSG0000	ENSG0000	0.005593	hsa-miR-106a-5p;hsa-miR-106b-5p;hsa-miR-17-5p;hsa-miR-2
ENSG0000	ENSG0000	0.008073	hsa-miR-15a-5p;hsa-miR-15b-5p;hsa-miR-16-5p;hsa-miR-195-
ENSG0000	ENSG0000	0.000104	hsa-miR-106a-5p;hsa-miR-106b-5p;hsa-miR-17-5p;hsa-miR-2
ENSG0000	ENSG0000	0.000104	hsa-miR-106a-5p;hsa-miR-106b-5p;hsa-miR-17-5p;hsa-miR-2
ENSG0000	ENSG0000	0.000401	hsa-miR-15a-5p;hsa-miR-15b-5p;hsa-miR-195-5p;hsa-miR-42
ENSG0000	ENSG0000	0.000168	hsa-miR-140-3p;hsa-miR-15a-5p;hsa-miR-15b-5p;hsa-miR-16-
ENSG0000	ENSG0000	0.003102	hsa-miR-106a-5p;hsa-miR-106b-5p;hsa-miR-17-5p;hsa-miR-2
ENSG0000	ENSG0000	0.000473	hsa-miR-106b-5p;hsa-miR-17-5p;hsa-miR-20a-5p;hsa-miR-20
ENSG0000	ENSG0000	0.000234	hsa-miR-106b-5p;hsa-miR-17-5p;hsa-miR-20a-5p;hsa-miR-20
ENSG0000	ENSG0000	2.89E-06	hsa-miR-106a-5p;hsa-miR-106b-5p;hsa-miR-15a-5p;hsa-miR-
ENSG0000	ENSG0000	7.91E-05	hsa-miR-106b-5p;hsa-miR-17-5p;hsa-miR-20a-5p;hsa-miR-20
ENSG0000	ENSG0000	0.000148	hsa-miR-106b-5p;hsa-miR-17-5p;hsa-miR-20a-5p;hsa-miR-20
ENSG0000	ENSG0000	0.005335	hsa-miR-106a-5p;hsa-miR-106b-5p;hsa-miR-17-5p;hsa-miR-1
ENSG0000	ENSG0000	4.59E-05	hsa-miR-15a-5p;hsa-miR-15b-5p;hsa-miR-16-5p;hsa-miR-195-
ENSG0000	ENSG0000	3.60E-06	hsa-miR-15a-5p;hsa-miR-15b-5p;hsa-miR-16-5p;hsa-miR-195-
ENSG0000	ENSG0000	0.000988	hsa-miR-15a-5p;hsa-miR-15b-5p;hsa-miR-195-5p;hsa-miR-42
ENSG0000	ENSG0000	0.000168	hsa-miR-106a-5p;hsa-miR-106b-5p;hsa-miR-17-5p;hsa-miR-2
ENSG0000	ENSG0000	6.80E-05	hsa-miR-15a-5p;hsa-miR-15b-5p;hsa-miR-16-5p;hsa-miR-195-
ENSG0000	ENSG0000	6.80E-05	hsa-miR-15a-5p;hsa-miR-15b-5p;hsa-miR-16-5p;hsa-miR-195-
ENSG0000	ENSG0000	0.001909	hsa-miR-106a-5p;hsa-miR-106b-5p;hsa-miR-17-5p;hsa-miR-1
ENSG0000	ENSG0000	0.000988	hsa-miR-106a-5p;hsa-miR-17-5p;hsa-miR-20a-5p;hsa-miR-20
ENSG0000	ENSG0000	0.0006	hsa-miR-106a-5p;hsa-miR-106b-5p;hsa-miR-17-5p;hsa-miR-1
ENSG0000	ENSG0000	0.000358	hsa-miR-106a-5p;hsa-miR-106b-5p;hsa-miR-17-5p;hsa-miR-2
ENSG0000	ENSG0000	1.33E-05	hsa-miR-548b-5p;hsa-miR-548c-5p;hsa-miR-548d-5p;hsa-miR-
ENSG0000	ENSG0000	0.00034	hsa-miR-106a-5p;hsa-miR-106b-5p;hsa-miR-17-5p;hsa-miR-2
ENSG0000	ENSG0000	0.000169	hsa-miR-106a-5p;hsa-miR-106b-5p;hsa-miR-17-5p;hsa-miR-2
ENSG0000	ENSG0000	3.03E-08	hsa-miR-106b-5p;hsa-miR-17-5p;hsa-miR-20a-5p;hsa-miR-20
ENSG0000	ENSG0000	5.05E-05	hsa-miR-103a-3p;hsa-miR-106b-5p;hsa-miR-107;hsa-miR-17-
ENSG0000	ENSG0000	0.000298	hsa-miR-106a-5p;hsa-miR-106b-5p;hsa-miR-17-5p;hsa-miR-2
ENSG0000	ENSG0000	0.000298	hsa-miR-106a-5p;hsa-miR-106b-5p;hsa-miR-17-5p;hsa-miR-2
ENSG0000	ENSG0000	7.32E-06	hsa-miR-548b-5p;hsa-miR-548c-5p;hsa-miR-548d-5p;hsa-miR-
ENSG0000	ENSG0000	0.004969	hsa-miR-15a-5p;hsa-miR-15b-5p;hsa-miR-16-5p;hsa-miR-195-
ENSG0000	ENSG0000	0.006047	hsa-miR-15a-5p;hsa-miR-15b-5p;hsa-miR-16-5p;hsa-miR-195-
ENSG0000	ENSG0000	8.80E-06	hsa-miR-15a-5p;hsa-miR-15b-5p;hsa-miR-16-5p;hsa-miR-195-
ENSG0000	ENSG0000	0.000462	hsa-miR-140-3p;hsa-miR-500a-3p;hsa-miR-632;hsa-miR-654-
ENSG0000	ENSG0000	1.18E-05	hsa-miR-365a-3p;hsa-miR-365b-3p;hsa-miR-548b-5p;hsa-miR
ENSG0000	ENSG0000	8.53E-08	hsa-miR-15a-5p;hsa-miR-15b-5p;hsa-miR-16-5p;hsa-miR-17-
ENSG0000	ENSG0000	0.000168	hsa-miR-106a-5p;hsa-miR-106b-5p;hsa-miR-17-5p;hsa-miR-2

ENSG0000	ENSG0000	0.00026	hsa-miR-106a-5p;hsa-miR-106b-5p;hsa-miR-17-5p;hsa-miR-2
ENSG0000	ENSG0000	0.000368	hsa-miR-548b-5p;hsa-miR-548c-5p;hsa-miR-548d-5p;hsa-miR-
ENSG0000	ENSG0000	0.000726	hsa-miR-106b-5p;hsa-miR-17-5p;hsa-miR-20a-5p;hsa-miR-20
ENSG0000	ENSG0000	0.000721	hsa-miR-548b-5p;hsa-miR-548c-5p;hsa-miR-548d-5p;hsa-miR-
ENSG0000	ENSG0000	0.005404	hsa-miR-15b-5p;hsa-miR-16-5p;hsa-miR-195-5p;hsa-miR-424-
ENSG0000	ENSG0000	7.91E-05	hsa-miR-15a-5p;hsa-miR-15b-5p;hsa-miR-16-5p;hsa-miR-195-
ENSG0000	ENSG0000	7.91E-05	hsa-miR-15a-5p;hsa-miR-15b-5p;hsa-miR-16-5p;hsa-miR-195-
ENSG0000	ENSG0000	1.71E-05	hsa-miR-548b-5p;hsa-miR-548c-5p;hsa-miR-548d-5p;hsa-miR-
ENSG0000	ENSG0000	0.000204	hsa-miR-15a-5p;hsa-miR-15b-5p;hsa-miR-16-5p;hsa-miR-195-
ENSG0000	ENSG0000	3.03E-08	hsa-miR-106b-5p;hsa-miR-17-5p;hsa-miR-20a-5p;hsa-miR-20
ENSG0000	ENSG0000	0.00376	hsa-miR-106b-5p;hsa-miR-17-5p;hsa-miR-20a-5p;hsa-miR-20
ENSG0000	ENSG0000	0.000132	hsa-miR-106a-5p;hsa-miR-106b-5p;hsa-miR-17-5p;hsa-miR-2
ENSG0000	ENSG0000	0.003756	hsa-miR-15a-5p;hsa-miR-15b-5p;hsa-miR-195-5p;hsa-miR-42
ENSG0000	ENSG0000	6.80E-05	hsa-miR-15a-5p;hsa-miR-15b-5p;hsa-miR-16-5p;hsa-miR-195-
ENSG0000	ENSG0000	0.000387	hsa-miR-106a-5p;hsa-miR-106b-5p;hsa-miR-17-5p;hsa-miR-2
ENSG0000	ENSG0000	6.80E-05	hsa-miR-548b-5p;hsa-miR-548c-5p;hsa-miR-548d-5p;hsa-miR-
ENSG0000	ENSG0000	0.001645	hsa-miR-106b-5p;hsa-miR-17-5p;hsa-miR-20a-5p;hsa-miR-20
ENSG0000	ENSG0000	6.04E-07	hsa-miR-106b-5p;hsa-miR-17-5p;hsa-miR-20a-5p;hsa-miR-20
ENSG0000	ENSG0000	3.51E-05	hsa-miR-106b-5p;hsa-miR-17-5p;hsa-miR-20a-5p;hsa-miR-20
ENSG0000	ENSG0000	0.00452	hsa-miR-143-3p;hsa-miR-15a-5p;hsa-miR-15b-5p;hsa-miR-16-
ENSG0000	ENSG0000	7.82E-05	hsa-miR-15a-5p;hsa-miR-15b-5p;hsa-miR-16-5p;hsa-miR-195-
ENSG0000	ENSG0000	0.001109	hsa-miR-106a-5p;hsa-miR-106b-5p;hsa-miR-17-5p;hsa-miR-2
ENSG0000	ENSG0000	0.000303	hsa-miR-106a-5p;hsa-miR-106b-5p;hsa-miR-17-5p;hsa-miR-1
ENSG0000	ENSG0000	3.03E-08	hsa-miR-106b-5p;hsa-miR-17-5p;hsa-miR-20a-5p;hsa-miR-20
ENSG0000	ENSG0000	3.03E-08	hsa-miR-106b-5p;hsa-miR-17-5p;hsa-miR-20a-5p;hsa-miR-20
ENSG0000	ENSG0000	3.03E-08	hsa-miR-106b-5p;hsa-miR-17-5p;hsa-miR-20a-5p;hsa-miR-20
ENSG0000	ENSG0000	3.03E-08	hsa-miR-106b-5p;hsa-miR-17-5p;hsa-miR-20a-5p;hsa-miR-20
ENSG0000	ENSG0000	0.007481	hsa-miR-15a-5p;hsa-miR-15b-5p;hsa-miR-16-5p;hsa-miR-195-
ENSG0000	ENSG0000	1.92E-05	hsa-miR-106a-5p;hsa-miR-106b-5p;hsa-miR-17-5p;hsa-miR-2
ENSG0000	ENSG0000	0.000104	hsa-miR-15a-5p;hsa-miR-15b-5p;hsa-miR-16-5p;hsa-miR-195-
ENSG0000	ENSG0000	0.000104	hsa-miR-15a-5p;hsa-miR-15b-5p;hsa-miR-16-5p;hsa-miR-195-
ENSG0000	ENSG0000	4.17E-05	hsa-miR-106a-5p;hsa-miR-106b-5p;hsa-miR-17-5p;hsa-miR-2
ENSG0000	ENSG0000	0.008729	hsa-miR-106a-5p;hsa-miR-106b-5p;hsa-miR-17-5p;hsa-miR-2
ENSG0000	ENSG0000	0.002141	hsa-miR-15a-5p;hsa-miR-15b-5p;hsa-miR-16-5p;hsa-miR-195-
ENSG0000	ENSG0000	0.00452	hsa-miR-548b-5p;hsa-miR-548c-5p;hsa-miR-548d-5p;hsa-miR-
ENSG0000	ENSG0000	0.00034	hsa-miR-15a-5p;hsa-miR-15b-5p;hsa-miR-16-5p;hsa-miR-195-
ENSG0000	ENSG0000	0.000939	hsa-miR-185-5p;hsa-miR-548b-5p;hsa-miR-548c-5p;hsa-miR-
ENSG0000	ENSG0000	7.32E-06	hsa-miR-548b-5p;hsa-miR-548c-5p;hsa-miR-548d-5p;hsa-miR-
ENSG0000	ENSG0000	0.001186	hsa-miR-106a-5p;hsa-miR-106b-5p;hsa-miR-17-5p;hsa-miR-2
ENSG0000	ENSG0000	0.004226	hsa-miR-106a-5p;hsa-miR-106b-5p;hsa-miR-17-5p;hsa-miR-2
ENSG0000	ENSG0000	1.94E-05	hsa-miR-106a-5p;hsa-miR-106b-5p;hsa-miR-16-5p;hsa-miR-1
ENSG0000	ENSG0000	0.001084	hsa-miR-15a-5p;hsa-miR-15b-5p;hsa-miR-195-5p;hsa-miR-42
ENSG0000	ENSG0000	4.68E-05	hsa-miR-106b-5p;hsa-miR-17-5p;hsa-miR-20a-5p;hsa-miR-20
ENSG0000	ENSG0000	0.000473	hsa-miR-15a-5p;hsa-miR-15b-5p;hsa-miR-16-5p;hsa-miR-195-
ENSG0000	ENSG0000	0.004002	hsa-miR-106a-5p;hsa-miR-106b-5p;hsa-miR-143-3p;hsa-miR-
ENSG0000	ENSG0000	2.15E-05	hsa-miR-224-5p;hsa-miR-365a-3p;hsa-miR-365b-3p;hsa-miR-
ENSG0000	ENSG0000	0.000331	hsa-miR-106a-5p;hsa-miR-106b-5p;hsa-miR-17-5p;hsa-miR-2
ENSG0000	ENSG0000	0.00353	hsa-miR-103a-3p;hsa-miR-107;hsa-miR-15a-5p;hsa-miR-15b-
ENSG0000	ENSG0000	3.51E-06	hsa-miR-106b-5p;hsa-miR-17-5p;hsa-miR-20a-5p;hsa-miR-93-
ENSG0000	ENSG0000	3.81E-05	hsa-miR-27a-3p;hsa-miR-27b-3p;hsa-miR-548b-5p;hsa-miR-5
ENSG0000	ENSG0000	0.000555	hsa-miR-15a-5p;hsa-miR-15b-5p;hsa-miR-16-5p;hsa-miR-195-
ENSG0000	ENSG0000	1.56E-07	hsa-miR-106a-5p;hsa-miR-17-5p;hsa-miR-20a-5p;hsa-miR-20
ENSG0000	ENSG0000	6.80E-05	hsa-miR-103a-3p;hsa-miR-107;hsa-miR-15a-5p;hsa-miR-15b-
ENSG0000	ENSG0000	6.80E-05	hsa-miR-103a-3p;hsa-miR-107;hsa-miR-15a-5p;hsa-miR-15b-

ENSG0000	ENSG0000	0.002068	hsa-miR-107;hsa-miR-15a-5p;hsa-miR-15b-5p;hsa-miR-185-5
ENSG0000	ENSG0000	0.007483	hsa-miR-548b-5p;hsa-miR-548c-5p;hsa-miR-548d-5p;hsa-miR
ENSG0000	ENSG0000	0.00145	hsa-miR-139-5p;hsa-miR-15a-5p;hsa-miR-15b-5p;hsa-miR-16
ENSG0000	ENSG0000	0.000555	hsa-miR-106a-5p;hsa-miR-106b-5p;hsa-miR-17-5p;hsa-miR-2
ENSG0000	ENSG0000	0.000481	hsa-miR-106a-5p;hsa-miR-106b-5p;hsa-miR-17-5p;hsa-miR-1
ENSG0000	ENSG0000	0.000247	hsa-miR-106a-5p;hsa-miR-106b-5p;hsa-miR-125b-2-3p;hsa-m
ENSG0000	ENSG0000	2.80E-05	hsa-miR-15a-5p;hsa-miR-15b-5p;hsa-miR-16-5p;hsa-miR-195
ENSG0000	ENSG0000	0.004798	hsa-miR-106b-5p;hsa-miR-17-5p;hsa-miR-18a-3p;hsa-miR-20
ENSG0000	ENSG0000	3.74E-05	hsa-miR-103a-3p;hsa-miR-107;hsa-miR-15a-5p;hsa-miR-15b-
ENSG0000	ENSG0000	0.000387	hsa-miR-106a-5p;hsa-miR-106b-5p;hsa-miR-17-5p;hsa-miR-2
ENSG0000	ENSG0000	3.90E-08	hsa-miR-106b-5p;hsa-miR-107;hsa-miR-15a-5p;hsa-miR-15b-
ENSG0000	ENSG0000	2.73E-07	hsa-miR-106b-5p;hsa-miR-17-5p;hsa-miR-20a-5p;hsa-miR-20
ENSG0000	ENSG0000	0.000552	hsa-miR-106b-5p;hsa-miR-140-3p;hsa-miR-15a-5p;hsa-miR-1
ENSG0000	ENSG0000	0.000552	hsa-miR-106b-5p;hsa-miR-140-3p;hsa-miR-15a-5p;hsa-miR-1
ENSG0000	ENSG0000	0.000144	hsa-miR-106a-5p;hsa-miR-106b-5p;hsa-miR-17-5p;hsa-miR-2
ENSG0000	ENSG0000	5.75E-06	hsa-miR-15a-5p;hsa-miR-15b-5p;hsa-miR-16-5p;hsa-miR-195
ENSG0000	ENSG0000	1.04E-07	hsa-miR-106b-5p;hsa-miR-17-5p;hsa-miR-20a-5p;hsa-miR-20
ENSG0000	ENSG0000	0.000646	hsa-miR-106b-5p;hsa-miR-17-5p;hsa-miR-20a-5p;hsa-miR-20
ENSG0000	ENSG0000	3.67E-09	hsa-miR-106b-5p;hsa-miR-1231;hsa-miR-15a-5p;hsa-miR-15b
ENSG0000	ENSG0000	8.12E-05	hsa-miR-106a-5p;hsa-miR-106b-5p;hsa-miR-17-5p;hsa-miR-2
ENSG0000	ENSG0000	0.000267	hsa-miR-103a-3p;hsa-miR-107;hsa-miR-15a-5p;hsa-miR-15b-
ENSG0000	ENSG0000	2.87E-07	hsa-miR-103a-3p;hsa-miR-107;hsa-miR-15a-5p;hsa-miR-15b-
ENSG0000	ENSG0000	8.54E-07	hsa-miR-500a-3p;hsa-miR-501-3p;hsa-miR-502-3p;hsa-miR-5
ENSG0000	ENSG0000	0.000368	hsa-miR-106b-5p;hsa-miR-17-5p;hsa-miR-192-5p;hsa-miR-20
ENSG0000	ENSG0000	0.002055	hsa-miR-15a-5p;hsa-miR-15b-5p;hsa-miR-16-5p;hsa-miR-195
ENSG0000	ENSG0000	0.00481	hsa-miR-15a-5p;hsa-miR-15b-5p;hsa-miR-16-5p;hsa-miR-195
ENSG0000	ENSG0000	1.85E-05	hsa-miR-15a-5p;hsa-miR-15b-5p;hsa-miR-16-5p;hsa-miR-195
ENSG0000	ENSG0000	0.001303	hsa-miR-15a-5p;hsa-miR-15b-5p;hsa-miR-16-5p;hsa-miR-195
ENSG0000	ENSG0000	5.08E-05	hsa-miR-106a-5p;hsa-miR-106b-5p;hsa-miR-17-5p;hsa-miR-2
ENSG0000	ENSG0000	3.25E-06	hsa-miR-106a-5p;hsa-miR-106b-5p;hsa-miR-17-5p;hsa-miR-2
ENSG0000	ENSG0000	0.002886	hsa-miR-136-3p;hsa-miR-15a-5p;hsa-miR-15b-5p;hsa-miR-16
ENSG0000	ENSG0000	0.00145	hsa-miR-15a-5p;hsa-miR-15b-5p;hsa-miR-195-5p;hsa-miR-42
ENSG0000	ENSG0000	5.09E-05	hsa-miR-106a-5p;hsa-miR-106b-5p;hsa-miR-17-5p;hsa-miR-1
ENSG0000	ENSG0000	0.001109	hsa-miR-106a-5p;hsa-miR-106b-5p;hsa-miR-17-5p;hsa-miR-2
ENSG0000	ENSG0000	8.96E-05	hsa-miR-106a-5p;hsa-miR-106b-5p;hsa-miR-17-5p;hsa-miR-1
ENSG0000	ENSG0000	0.000214	hsa-miR-548b-5p;hsa-miR-548c-5p;hsa-miR-548d-5p;hsa-miR
ENSG0000	ENSG0000	0.000104	hsa-miR-1246;hsa-miR-15a-5p;hsa-miR-15b-5p;hsa-miR-16-5
ENSG0000	ENSG0000	8.86E-07	hsa-miR-27a-3p;hsa-miR-27b-3p;hsa-miR-548b-5p;hsa-miR-5
ENSG0000	ENSG0000	8.86E-07	hsa-miR-27a-3p;hsa-miR-27b-3p;hsa-miR-548b-5p;hsa-miR-5
ENSG0000	ENSG0000	0.008073	hsa-miR-106a-5p;hsa-miR-106b-5p;hsa-miR-17-5p;hsa-miR-2
ENSG0000	ENSG0000	6.03E-06	hsa-miR-125b-2-3p;hsa-miR-15a-5p;hsa-miR-15b-5p;hsa-miR
ENSG0000	ENSG0000	0.006389	hsa-miR-106a-5p;hsa-miR-106b-5p;hsa-miR-20a-5p;hsa-miR-
ENSG0000	ENSG0000	1.73E-05	hsa-miR-106a-5p;hsa-miR-106b-5p;hsa-miR-17-5p;hsa-miR-2
ENSG0000	ENSG0000	0.005593	hsa-miR-106b-5p;hsa-miR-17-5p;hsa-miR-20a-5p;hsa-miR-20
ENSG0000	ENSG0000	0.000407	hsa-miR-103a-3p;hsa-miR-107;hsa-miR-15a-5p;hsa-miR-15b-
ENSG0000	ENSG0000	0.005335	hsa-miR-106a-5p;hsa-miR-106b-5p;hsa-miR-143-3p;hsa-miR-
ENSG0000	ENSG0000	1.04E-07	hsa-miR-106b-5p;hsa-miR-17-5p;hsa-miR-20a-5p;hsa-miR-20
ENSG0000	ENSG0000	1.30E-05	hsa-miR-107;hsa-miR-15a-5p;hsa-miR-16-5p;hsa-miR-505-3p;
ENSG0000	ENSG0000	0.002053	hsa-miR-106a-5p;hsa-miR-106b-5p;hsa-miR-17-5p;hsa-miR-2
ENSG0000	ENSG0000	0.001525	hsa-miR-106a-5p;hsa-miR-106b-5p;hsa-miR-17-5p;hsa-miR-2
ENSG0000	ENSG0000	0.000101	hsa-miR-15a-5p;hsa-miR-15b-5p;hsa-miR-16-5p;hsa-miR-195
ENSG0000	ENSG0000	0.00145	hsa-miR-140-3p;hsa-miR-15a-5p;hsa-miR-16-5p;hsa-miR-185
ENSG0000	ENSG0000	0.00748	hsa-miR-15a-5p;hsa-miR-15b-5p;hsa-miR-195-5p;hsa-miR-42
ENSG0000	ENSG0000	9.19E-05	hsa-miR-15a-5p;hsa-miR-15b-5p;hsa-miR-17-5p;hsa-miR-195

ENSG0000	ENSG0000	0.003644	hsa-miR-106a-5p;hsa-miR-106b-5p;hsa-miR-17-5p;hsa-miR-2
ENSG0000	ENSG0000	0.000187	hsa-miR-15a-5p;hsa-miR-15b-5p;hsa-miR-16-5p;hsa-miR-195
ENSG0000	ENSG0000	6.15E-09	hsa-miR-16-5p;hsa-miR-192-5p;hsa-miR-548b-5p;hsa-miR-54
ENSG0000	ENSG0000	0.000861	hsa-miR-106a-5p;hsa-miR-106b-5p;hsa-miR-143-3p;hsa-miR-
ENSG0000	ENSG0000	0.000274	hsa-miR-15a-5p;hsa-miR-15b-5p;hsa-miR-195-5p;hsa-miR-42
ENSG0000	ENSG0000	0.000148	hsa-miR-15a-5p;hsa-miR-15b-5p;hsa-miR-16-5p;hsa-miR-195
ENSG0000	ENSG0000	0.001308	hsa-miR-106a-5p;hsa-miR-106b-5p;hsa-miR-17-5p;hsa-miR-2
ENSG0000	ENSG0000	1.03E-06	hsa-miR-548b-5p;hsa-miR-548c-5p;hsa-miR-548d-5p;hsa-miR
ENSG0000	ENSG0000	0.002068	hsa-miR-103a-3p;hsa-miR-107;hsa-miR-15a-5p;hsa-miR-15b-
ENSG0000	ENSG0000	0.000583	hsa-miR-15a-5p;hsa-miR-15b-5p;hsa-miR-195-5p;hsa-miR-42
ENSG0000	ENSG0000	0.003293	hsa-miR-15a-5p;hsa-miR-15b-5p;hsa-miR-195-5p;hsa-miR-42
ENSG0000	ENSG0000	0.001228	hsa-miR-15a-5p;hsa-miR-15b-5p;hsa-miR-18a-3p;hsa-miR-19
ENSG0000	ENSG0000	1.36E-05	hsa-miR-15a-5p;hsa-miR-15b-5p;hsa-miR-16-5p;hsa-miR-195
ENSG0000	ENSG0000	0.001146	hsa-miR-106a-5p;hsa-miR-106b-5p;hsa-miR-17-5p;hsa-miR-2
ENSG0000	ENSG0000	0.000826	hsa-miR-27a-3p;hsa-miR-27b-3p;hsa-miR-548b-5p;hsa-miR-5
ENSG0000	ENSG0000	0.000234	hsa-miR-106a-5p;hsa-miR-106b-5p;hsa-miR-17-5p;hsa-miR-2
ENSG0000	ENSG0000	2.73E-07	hsa-miR-106b-5p;hsa-miR-17-5p;hsa-miR-20a-5p;hsa-miR-20
ENSG0000	ENSG0000	2.73E-07	hsa-miR-106b-5p;hsa-miR-17-5p;hsa-miR-20a-5p;hsa-miR-20
ENSG0000	ENSG0000	2.42E-07	hsa-miR-15a-5p;hsa-miR-15b-5p;hsa-miR-16-5p;hsa-miR-195
ENSG0000	ENSG0000	7.91E-05	hsa-miR-106b-5p;hsa-miR-17-5p;hsa-miR-20a-5p;hsa-miR-20
ENSG0000	ENSG0000	6.96E-06	hsa-miR-106a-5p;hsa-miR-106b-5p;hsa-miR-17-5p;hsa-miR-1
ENSG0000	ENSG0000	5.75E-06	hsa-miR-106b-5p;hsa-miR-17-5p;hsa-miR-20a-5p;hsa-miR-20
ENSG0000	ENSG0000	0.001829	hsa-miR-15a-5p;hsa-miR-15b-5p;hsa-miR-16-5p;hsa-miR-195
ENSG0000	ENSG0000	2.80E-07	hsa-miR-106a-5p;hsa-miR-106b-5p;hsa-miR-17-5p;hsa-miR-2
ENSG0000	ENSG0000	2.80E-07	hsa-miR-106a-5p;hsa-miR-106b-5p;hsa-miR-17-5p;hsa-miR-2
ENSG0000	ENSG0000	0.00145	hsa-miR-15a-5p;hsa-miR-15b-5p;hsa-miR-16-5p;hsa-miR-195
ENSG0000	ENSG0000	2.19E-05	hsa-miR-106a-5p;hsa-miR-106b-5p;hsa-miR-17-5p;hsa-miR-2
ENSG0000	ENSG0000	0.003452	hsa-miR-103a-3p;hsa-miR-15a-5p;hsa-miR-15b-5p;hsa-miR-1
ENSG0000	ENSG0000	0.000387	hsa-miR-106a-5p;hsa-miR-106b-5p;hsa-miR-17-5p;hsa-miR-2
ENSG0000	ENSG0000	0.000387	hsa-miR-106a-5p;hsa-miR-106b-5p;hsa-miR-17-5p;hsa-miR-2
ENSG0000	ENSG0000	0.000387	hsa-miR-106a-5p;hsa-miR-106b-5p;hsa-miR-17-5p;hsa-miR-2
ENSG0000	ENSG0000	1.60E-05	hsa-miR-548b-5p;hsa-miR-548c-5p;hsa-miR-548d-5p;hsa-miR
ENSG0000	ENSG0000	0.002487	hsa-miR-1231;hsa-miR-18a-3p;hsa-miR-324-3p;hsa-miR-632
ENSG0000	ENSG0000	0.000387	hsa-miR-106a-5p;hsa-miR-106b-5p;hsa-miR-17-5p;hsa-miR-2
ENSG0000	ENSG0000	6.05E-06	hsa-miR-106a-5p;hsa-miR-106b-5p;hsa-miR-125b-2-3p;hsa-m
ENSG0000	ENSG0000	9.64E-06	hsa-miR-106a-5p;hsa-miR-106b-5p;hsa-miR-1231;hsa-miR-17
ENSG0000	ENSG0000	0.000969	hsa-miR-106b-5p;hsa-miR-16-5p;hsa-miR-17-5p;hsa-miR-20a
ENSG0000	ENSG0000	0.000861	hsa-miR-548b-5p;hsa-miR-548c-5p;hsa-miR-548d-5p;hsa-miR
ENSG0000	ENSG0000	0.002886	hsa-miR-106a-5p;hsa-miR-106b-5p;hsa-miR-17-5p;hsa-miR-2
ENSG0000	ENSG0000	1.61E-06	hsa-miR-106a-5p;hsa-miR-106b-5p;hsa-miR-17-5p;hsa-miR-2
ENSG0000	ENSG0000	0.002366	hsa-miR-106a-5p;hsa-miR-17-5p;hsa-miR-20a-5p;hsa-miR-20
ENSG0000	ENSG0000	0.000195	hsa-miR-106a-5p;hsa-miR-106b-5p;hsa-miR-17-5p;hsa-miR-2
ENSG0000	ENSG0000	4.54E-06	hsa-miR-365a-3p;hsa-miR-365b-3p;hsa-miR-548b-5p;hsa-miR
ENSG0000	ENSG0000	0.000338	hsa-miR-106a-5p;hsa-miR-17-5p;hsa-miR-20a-5p;hsa-miR-20
ENSG0000	ENSG0000	0.008729	hsa-miR-103a-3p;hsa-miR-107;hsa-miR-15a-5p;hsa-miR-15b-
ENSG0000	ENSG0000	0.004226	hsa-miR-106a-5p;hsa-miR-106b-5p;hsa-miR-17-5p;hsa-miR-2
ENSG0000	ENSG0000	0.004158	hsa-miR-15a-5p;hsa-miR-15b-5p;hsa-miR-16-5p;hsa-miR-195
ENSG0000	ENSG0000	0.003756	hsa-miR-15a-5p;hsa-miR-15b-5p;hsa-miR-195-5p;hsa-miR-42
ENSG0000	ENSG0000	1.22E-07	hsa-miR-103a-3p;hsa-miR-106a-5p;hsa-miR-106b-5p;hsa-miR
ENSG0000	ENSG0000	0.004255	hsa-miR-106a-5p;hsa-miR-106b-5p;hsa-miR-17-5p;hsa-miR-1
ENSG0000	ENSG0000	0.003293	hsa-miR-15a-5p;hsa-miR-15b-5p;hsa-miR-195-5p;hsa-miR-42
ENSG0000	ENSG0000	0.002055	hsa-miR-106b-5p;hsa-miR-17-5p;hsa-miR-20a-5p;hsa-miR-20
ENSG0000	ENSG0000	1.19E-06	hsa-miR-17-5p;hsa-miR-20a-5p;hsa-miR-20b-5p;hsa-miR-93-
ENSG0000	ENSG0000	0.000102	hsa-miR-103a-3p;hsa-miR-107;hsa-miR-15a-5p;hsa-miR-15b-

ENSG0000	ENSG0000	0.000583	hsa-miR-103a-3p;hsa-miR-107;hsa-miR-27a-3p;hsa-miR-27b-3
ENSG0000	ENSG0000	0.000368	hsa-miR-106a-5p;hsa-miR-106b-5p;hsa-miR-17-5p;hsa-miR-20
ENSG0000	ENSG0000	4.47E-06	hsa-miR-106a-5p;hsa-miR-106b-5p;hsa-miR-17-5p;hsa-miR-20
ENSG0000	ENSG0000	0.004226	hsa-miR-106a-5p;hsa-miR-106b-5p;hsa-miR-17-5p;hsa-miR-20
ENSG0000	ENSG0000	0.004969	hsa-miR-106a-5p;hsa-miR-106b-5p;hsa-miR-17-5p;hsa-miR-20
ENSG0000	ENSG0000	3.39E-05	hsa-miR-15a-5p;hsa-miR-15b-5p;hsa-miR-16-5p;hsa-miR-195-
ENSG0000	ENSG0000	2.80E-07	hsa-miR-106a-5p;hsa-miR-106b-5p;hsa-miR-17-5p;hsa-miR-20
ENSG0000	ENSG0000	4.68E-05	hsa-miR-15a-5p;hsa-miR-15b-5p;hsa-miR-16-5p;hsa-miR-195-
ENSG0000	ENSG0000	5.75E-06	hsa-miR-15a-5p;hsa-miR-15b-5p;hsa-miR-16-5p;hsa-miR-195-
ENSG0000	ENSG0000	3.51E-06	hsa-miR-15a-5p;hsa-miR-15b-5p;hsa-miR-16-5p;hsa-miR-195-
ENSG0000	ENSG0000	0.002068	hsa-miR-361-5p;hsa-miR-548b-5p;hsa-miR-548c-5p;hsa-miR-5
ENSG0000	ENSG0000	1.30E-05	hsa-miR-106a-5p;hsa-miR-106b-5p;hsa-miR-17-5p;hsa-miR-20
ENSG0000	ENSG0000	0.00598	hsa-miR-106a-5p;hsa-miR-106b-5p;hsa-miR-17-5p;hsa-miR-20
ENSG0000	ENSG0000	0.000226	hsa-miR-106a-5p;hsa-miR-106b-5p;hsa-miR-17-5p;hsa-miR-20
ENSG0000	ENSG0000	0.000473	hsa-miR-106a-5p;hsa-miR-106b-5p;hsa-miR-17-5p;hsa-miR-20
ENSG0000	ENSG0000	0.000893	hsa-miR-106b-5p;hsa-miR-17-5p;hsa-miR-20a-5p;hsa-miR-20b
ENSG0000	ENSG0000	0.000204	hsa-miR-15a-5p;hsa-miR-15b-5p;hsa-miR-16-5p;hsa-miR-195-
ENSG0000	ENSG0000	8.80E-06	hsa-miR-106b-5p;hsa-miR-17-5p;hsa-miR-20a-5p;hsa-miR-20b
ENSG0000	ENSG0000	0.000168	hsa-miR-106a-5p;hsa-miR-106b-5p;hsa-miR-17-5p;hsa-miR-20
ENSG0000	ENSG0000	0.000926	hsa-miR-106a-5p;hsa-miR-106b-5p;hsa-miR-17-5p;hsa-miR-20
ENSG0000	ENSG0000	0.000267	hsa-miR-548b-5p;hsa-miR-548c-5p;hsa-miR-548d-5p;hsa-miR-
ENSG0000	ENSG0000	2.90E-09	hsa-miR-106b-5p;hsa-miR-17-5p;hsa-miR-20a-5p;hsa-miR-20b
ENSG0000	ENSG0000	0.000239	hsa-miR-106a-5p;hsa-miR-106b-5p;hsa-miR-17-5p;hsa-miR-20
ENSG0000	ENSG0000	0.002055	hsa-miR-15a-5p;hsa-miR-15b-5p;hsa-miR-16-5p;hsa-miR-195-
ENSG0000	ENSG0000	0.005404	hsa-miR-15a-5p;hsa-miR-15b-5p;hsa-miR-195-5p;hsa-miR-424
ENSG0000	ENSG0000	0.000338	hsa-miR-106a-5p;hsa-miR-106b-5p;hsa-miR-17-5p;hsa-miR-20
ENSG0000	ENSG0000	7.91E-05	hsa-miR-15a-5p;hsa-miR-15b-5p;hsa-miR-16-5p;hsa-miR-195-
ENSG0000	ENSG0000	3.03E-08	hsa-miR-106b-5p;hsa-miR-17-5p;hsa-miR-20a-5p;hsa-miR-20b
ENSG0000	ENSG0000	1.33E-05	hsa-miR-103a-3p;hsa-miR-107;hsa-miR-15a-5p;hsa-miR-15b-5
ENSG0000	ENSG0000	0.000939	hsa-miR-365a-3p;hsa-miR-365b-3p;hsa-miR-548b-5p;hsa-miR
ENSG0000	ENSG0000	7.32E-06	hsa-miR-548b-5p;hsa-miR-548c-5p;hsa-miR-548d-5p;hsa-miR-
ENSG0000	ENSG0000	0.000195	hsa-miR-106a-5p;hsa-miR-106b-5p;hsa-miR-17-5p;hsa-miR-20
ENSG0000	ENSG0000	0.000104	hsa-miR-15a-5p;hsa-miR-15b-5p;hsa-miR-16-5p;hsa-miR-195-
ENSG0000	ENSG0000	3.47E-06	hsa-miR-103a-3p;hsa-miR-106a-5p;hsa-miR-106b-5p;hsa-miR-
ENSG0000	ENSG0000	8.96E-05	hsa-miR-106b-5p;hsa-miR-17-5p;hsa-miR-20a-5p;hsa-miR-20b
ENSG0000	ENSG0000	0.000274	hsa-miR-106b-5p;hsa-miR-17-5p;hsa-miR-20a-5p;hsa-miR-20b
ENSG0000	ENSG0000	0.000204	hsa-miR-15a-5p;hsa-miR-15b-5p;hsa-miR-195-5p;hsa-miR-424
ENSG0000	ENSG0000	0.001203	hsa-miR-548b-5p;hsa-miR-548c-5p;hsa-miR-548d-5p;hsa-miR-
ENSG0000	ENSG0000	0.002199	hsa-miR-185-5p;hsa-miR-548b-5p;hsa-miR-548c-5p;hsa-miR-5
ENSG0000	ENSG0000	0.000169	hsa-miR-548b-5p;hsa-miR-548c-5p;hsa-miR-548d-5p;hsa-miR-
ENSG0000	ENSG0000	5.21E-05	hsa-miR-501-3p;hsa-miR-502-3p;hsa-miR-548b-5p;hsa-miR-54
ENSG0000	ENSG0000	0.000401	hsa-miR-106a-5p;hsa-miR-106b-5p;hsa-miR-17-5p;hsa-miR-20
ENSG0000	ENSG0000	0.000401	hsa-miR-106a-5p;hsa-miR-106b-5p;hsa-miR-17-5p;hsa-miR-20
ENSG0000	ENSG0000	0.002053	hsa-miR-106a-5p;hsa-miR-106b-5p;hsa-miR-17-5p;hsa-miR-20
ENSG0000	ENSG0000	2.32E-06	hsa-miR-106a-5p;hsa-miR-106b-5p;hsa-miR-17-5p;hsa-miR-20
ENSG0000	ENSG0000	0.002662	hsa-miR-106a-5p;hsa-miR-106b-5p;hsa-miR-17-5p;hsa-miR-20
ENSG0000	ENSG0000	0.002662	hsa-miR-106a-5p;hsa-miR-106b-5p;hsa-miR-17-5p;hsa-miR-20
ENSG0000	ENSG0000	0.00279	hsa-miR-548b-5p;hsa-miR-548c-5p;hsa-miR-548d-5p;hsa-miR-
ENSG0000	ENSG0000	7.66E-07	hsa-miR-106a-5p;hsa-miR-106b-5p;hsa-miR-136-3p;hsa-miR-1
ENSG0000	ENSG0000	3.03E-08	hsa-miR-106b-5p;hsa-miR-17-5p;hsa-miR-20a-5p;hsa-miR-20b
ENSG0000	ENSG0000	2.58E-05	hsa-miR-15a-5p;hsa-miR-15b-5p;hsa-miR-16-5p;hsa-miR-195-
ENSG0000	ENSG0000	0.000226	hsa-miR-106a-5p;hsa-miR-106b-5p;hsa-miR-17-5p;hsa-miR-20
ENSG0000	ENSG0000	0.001439	hsa-miR-106a-5p;hsa-miR-106b-5p;hsa-miR-17-5p;hsa-miR-20
ENSG0000	ENSG0000	3.39E-05	hsa-miR-106a-5p;hsa-miR-106b-5p;hsa-miR-17-5p;hsa-miR-20

ENSG0000	ENSG0000	2.73E-07	hsa-miR-15a-5p;hsa-miR-15b-5p;hsa-miR-16-5p;hsa-miR-195-
ENSG0000	ENSG0000	0.000748	hsa-miR-106a-5p;hsa-miR-106b-5p;hsa-miR-17-5p;hsa-miR-20
ENSG0000	ENSG0000	6.14E-05	hsa-miR-106a-5p;hsa-miR-106b-5p;hsa-miR-17-5p;hsa-miR-20
ENSG0000	ENSG0000	0.000101	hsa-miR-15a-5p;hsa-miR-15b-5p;hsa-miR-16-5p;hsa-miR-195-
ENSG0000	ENSG0000	0.000298	hsa-miR-106a-5p;hsa-miR-106b-5p;hsa-miR-17-5p;hsa-miR-20
ENSG0000	ENSG0000	0.008777	hsa-miR-106a-5p;hsa-miR-106b-5p;hsa-miR-17-5p;hsa-miR-20
ENSG0000	ENSG0000	3.49E-05	hsa-miR-15a-5p;hsa-miR-15b-5p;hsa-miR-16-5p;hsa-miR-195-
ENSG0000	ENSG0000	8.79E-05	hsa-miR-106a-5p;hsa-miR-17-5p;hsa-miR-20a-5p;hsa-miR-20b
ENSG0000	ENSG0000	4.47E-06	hsa-miR-106b-5p;hsa-miR-17-5p;hsa-miR-20a-5p;hsa-miR-20b
ENSG0000	ENSG0000	4.75E-06	hsa-miR-106a-5p;hsa-miR-106b-5p;hsa-miR-17-5p;hsa-miR-20
ENSG0000	ENSG0000	5.92E-06	hsa-miR-548b-5p;hsa-miR-548c-5p;hsa-miR-548d-5p;hsa-miR-
ENSG0000	ENSG0000	1.68E-06	hsa-miR-127-5p;hsa-miR-18a-3p;hsa-miR-324-3p;hsa-miR-50
ENSG0000	ENSG0000	6.80E-05	hsa-miR-106a-5p;hsa-miR-106b-5p;hsa-miR-140-3p;hsa-miR-1
ENSG0000	ENSG0000	2.14E-06	hsa-miR-106b-5p;hsa-miR-17-5p;hsa-miR-20a-5p;hsa-miR-20b
ENSG0000	ENSG0000	2.18E-07	hsa-miR-106a-5p;hsa-miR-106b-5p;hsa-miR-15a-5p;hsa-miR-1
ENSG0000	ENSG0000	4.17E-05	hsa-miR-107;hsa-miR-15a-5p;hsa-miR-15b-5p;hsa-miR-16-5p;
ENSG0000	ENSG0000	0.000195	hsa-miR-106a-5p;hsa-miR-106b-5p;hsa-miR-17-5p;hsa-miR-20
ENSG0000	ENSG0000	7.32E-06	hsa-miR-548b-5p;hsa-miR-548c-5p;hsa-miR-548d-5p;hsa-miR-
ENSG0000	ENSG0000	7.31E-07	hsa-miR-106a-5p;hsa-miR-106b-5p;hsa-miR-15a-5p;hsa-miR-1
ENSG0000	ENSG0000	1.80E-05	hsa-miR-106a-5p;hsa-miR-106b-5p;hsa-miR-127-5p;hsa-miR-1
ENSG0000	ENSG0000	0.009705	hsa-miR-548b-5p;hsa-miR-548c-5p;hsa-miR-548d-5p;hsa-miR-
ENSG0000	ENSG0000	0.006389	hsa-miR-107;hsa-miR-15a-5p;hsa-miR-15b-5p;hsa-miR-195-5p
ENSG0000	ENSG0000	0.002549	hsa-miR-106b-5p;hsa-miR-17-5p;hsa-miR-20a-5p;hsa-miR-20b
ENSG0000	ENSG0000	1.79E-06	hsa-miR-106a-5p;hsa-miR-106b-5p;hsa-miR-127-5p;hsa-miR-1
ENSG0000	ENSG0000	3.51E-06	hsa-miR-15a-5p;hsa-miR-15b-5p;hsa-miR-16-5p;hsa-miR-195-
ENSG0000	ENSG0000	0.004002	hsa-miR-106a-5p;hsa-miR-106b-5p;hsa-miR-17-5p;hsa-miR-20
ENSG0000	ENSG0000	0.004226	hsa-miR-106b-5p;hsa-miR-17-5p;hsa-miR-20a-5p;hsa-miR-20b
ENSG0000	ENSG0000	0.002068	hsa-miR-106a-5p;hsa-miR-106b-5p;hsa-miR-17-5p;hsa-miR-20
ENSG0000	ENSG0000	0.00155	hsa-miR-15a-5p;hsa-miR-15b-5p;hsa-miR-195-5p;hsa-miR-424
ENSG0000	ENSG0000	8.79E-05	hsa-miR-106a-5p;hsa-miR-106b-5p;hsa-miR-17-5p;hsa-miR-20
ENSG0000	ENSG0000	9.64E-06	hsa-miR-106a-5p;hsa-miR-106b-5p;hsa-miR-1231;hsa-miR-127
ENSG0000	ENSG0000	0.000154	hsa-miR-106a-5p;hsa-miR-106b-5p;hsa-miR-17-5p;hsa-miR-20
ENSG0000	ENSG0000	0.000226	hsa-miR-106a-5p;hsa-miR-106b-5p;hsa-miR-17-5p;hsa-miR-20
ENSG0000	ENSG0000	0.000226	hsa-miR-106a-5p;hsa-miR-106b-5p;hsa-miR-17-5p;hsa-miR-20
ENSG0000	ENSG0000	0.000101	hsa-miR-106b-5p;hsa-miR-17-5p;hsa-miR-20a-5p;hsa-miR-20b
ENSG0000	ENSG0000	0.000101	hsa-miR-106b-5p;hsa-miR-17-5p;hsa-miR-20a-5p;hsa-miR-20b
ENSG0000	ENSG0000	3.03E-08	hsa-miR-106b-5p;hsa-miR-17-5p;hsa-miR-20a-5p;hsa-miR-20b
ENSG0000	ENSG0000	3.03E-08	hsa-miR-106b-5p;hsa-miR-17-5p;hsa-miR-20a-5p;hsa-miR-20b
ENSG0000	ENSG0000	1.76E-08	hsa-miR-103a-3p;hsa-miR-106a-5p;hsa-miR-106b-5p;hsa-miR-
ENSG0000	ENSG0000	1.37E-06	hsa-miR-548b-5p;hsa-miR-548c-5p;hsa-miR-548d-5p;hsa-miR-
ENSG0000	ENSG0000	1.78E-06	hsa-miR-103a-3p;hsa-miR-107;hsa-miR-15a-5p;hsa-miR-15b-5
ENSG0000	ENSG0000	7.91E-05	hsa-miR-15a-5p;hsa-miR-15b-5p;hsa-miR-16-5p;hsa-miR-195-
ENSG0000	ENSG0000	7.91E-05	hsa-miR-15a-5p;hsa-miR-15b-5p;hsa-miR-16-5p;hsa-miR-195-
ENSG0000	ENSG0000	0.003377	hsa-miR-106a-5p;hsa-miR-106b-5p;hsa-miR-17-5p;hsa-miR-20
ENSG0000	ENSG0000	0.000726	hsa-miR-15a-5p;hsa-miR-15b-5p;hsa-miR-195-5p;hsa-miR-424
ENSG0000	ENSG0000	0.000125	hsa-miR-17-5p;hsa-miR-20a-5p;hsa-miR-20b-5p;hsa-miR-27a-
ENSG0000	ENSG0000	1.19E-06	hsa-miR-15a-5p;hsa-miR-15b-5p;hsa-miR-16-5p;hsa-miR-195-
ENSG0000	ENSG0000	4.47E-06	hsa-miR-106a-5p;hsa-miR-106b-5p;hsa-miR-17-5p;hsa-miR-20
ENSG0000	ENSG0000	0.000938	hsa-miR-106a-5p;hsa-miR-106b-5p;hsa-miR-17-5p;hsa-miR-20
ENSG0000	ENSG0000	0.000938	hsa-miR-548b-5p;hsa-miR-548c-5p;hsa-miR-548d-5p;hsa-miR-
ENSG0000	ENSG0000	2.42E-07	hsa-miR-15a-5p;hsa-miR-15b-5p;hsa-miR-16-5p;hsa-miR-195-
ENSG0000	ENSG0000	3.39E-05	hsa-miR-106a-5p;hsa-miR-106b-5p;hsa-miR-17-5p;hsa-miR-20
ENSG0000	ENSG0000	3.25E-06	hsa-miR-106a-5p;hsa-miR-106b-5p;hsa-miR-17-5p;hsa-miR-20
ENSG0000	ENSG0000	0.001894	hsa-miR-106a-5p;hsa-miR-106b-5p;hsa-miR-17-5p;hsa-miR-20

ENSG0000	ENSG0000	8.95E-08	hsa-miR-103a-3p;hsa-miR-107;hsa-miR-15a-5p;hsa-miR-15b-5
ENSG0000	ENSG0000	0.000555	hsa-miR-15a-5p;hsa-miR-15b-5p;hsa-miR-16-5p;hsa-miR-195-
ENSG0000	ENSG0000	1.19E-06	hsa-miR-106b-5p;hsa-miR-17-5p;hsa-miR-20a-5p;hsa-miR-20b
ENSG0000	ENSG0000	9.53E-09	hsa-miR-106b-5p;hsa-miR-127-5p;hsa-miR-15a-5p;hsa-miR-15
ENSG0000	ENSG0000	0.000126	hsa-miR-15a-5p;hsa-miR-15b-5p;hsa-miR-16-5p;hsa-miR-195-
ENSG0000	ENSG0000	0.000101	hsa-miR-15a-5p;hsa-miR-15b-5p;hsa-miR-16-5p;hsa-miR-195-
ENSG0000	ENSG0000	1.19E-06	hsa-miR-15a-5p;hsa-miR-16-5p;hsa-miR-17-5p;hsa-miR-195-5
ENSG0000	ENSG0000	1.19E-06	hsa-miR-15a-5p;hsa-miR-16-5p;hsa-miR-17-5p;hsa-miR-195-5
ENSG0000	ENSG0000	0.000748	hsa-miR-15a-5p;hsa-miR-15b-5p;hsa-miR-16-5p;hsa-miR-195-
ENSG0000	ENSG0000	0.002068	hsa-miR-106a-5p;hsa-miR-106b-5p;hsa-miR-17-5p;hsa-miR-20
ENSG0000	ENSG0000	7.20E-07	hsa-miR-106a-5p;hsa-miR-106b-5p;hsa-miR-17-5p;hsa-miR-20
ENSG0000	ENSG0000	7.20E-07	hsa-miR-106a-5p;hsa-miR-106b-5p;hsa-miR-17-5p;hsa-miR-20
ENSG0000	ENSG0000	4.50E-05	hsa-miR-140-3p;hsa-miR-548b-5p;hsa-miR-548c-5p;hsa-miR-5
ENSG0000	ENSG0000	8.12E-05	hsa-miR-106a-5p;hsa-miR-106b-5p;hsa-miR-140-3p;hsa-miR-1
ENSG0000	ENSG0000	0.000368	hsa-miR-548b-5p;hsa-miR-548c-5p;hsa-miR-548d-5p;hsa-miR-
ENSG0000	ENSG0000	0.000368	hsa-miR-548b-5p;hsa-miR-548c-5p;hsa-miR-548d-5p;hsa-miR-
ENSG0000	ENSG0000	0.006386	hsa-miR-106a-5p;hsa-miR-106b-5p;hsa-miR-17-5p;hsa-miR-20
ENSG0000	ENSG0000	1.19E-06	hsa-miR-15a-5p;hsa-miR-15b-5p;hsa-miR-16-5p;hsa-miR-195-
ENSG0000	ENSG0000	1.33E-05	hsa-miR-103a-3p;hsa-miR-107;hsa-miR-15a-5p;hsa-miR-15b-5
ENSG0000	ENSG0000	0.002068	hsa-miR-106a-5p;hsa-miR-106b-5p;hsa-miR-17-5p;hsa-miR-20
ENSG0000	ENSG0000	0.001732	hsa-miR-15a-5p;hsa-miR-15b-5p;hsa-miR-16-5p;hsa-miR-185-
ENSG0000	ENSG0000	0.004018	hsa-miR-106a-5p;hsa-miR-106b-5p;hsa-miR-15a-5p;hsa-miR-1
ENSG0000	ENSG0000	0.000105	hsa-miR-15a-5p;hsa-miR-15b-5p;hsa-miR-16-5p;hsa-miR-195-
ENSG0000	ENSG0000	1.61E-06	hsa-miR-106b-5p;hsa-miR-17-5p;hsa-miR-20a-3p;hsa-miR-20a
ENSG0000	ENSG0000	7.68E-07	hsa-miR-106b-5p;hsa-miR-16-5p;hsa-miR-17-5p;hsa-miR-20a-
ENSG0000	ENSG0000	9.07E-05	hsa-miR-500a-3p;hsa-miR-548b-5p;hsa-miR-548c-5p;hsa-miR-
ENSG0000	ENSG0000	1.52E-05	hsa-miR-106a-5p;hsa-miR-106b-5p;hsa-miR-17-5p;hsa-miR-20
ENSG0000	ENSG0000	0.00026	hsa-miR-106a-5p;hsa-miR-106b-5p;hsa-miR-17-5p;hsa-miR-20
ENSG0000	ENSG0000	1.13E-07	hsa-miR-106a-5p;hsa-miR-106b-5p;hsa-miR-139-5p;hsa-miR-1
ENSG0000	ENSG0000	0.000299	hsa-miR-548b-5p;hsa-miR-548c-5p;hsa-miR-548d-5p;hsa-miR-
ENSG0000	ENSG0000	0.000101	hsa-miR-15a-5p;hsa-miR-15b-5p;hsa-miR-16-5p;hsa-miR-195-
ENSG0000	ENSG0000	2.80E-05	hsa-miR-15a-5p;hsa-miR-15b-5p;hsa-miR-195-5p;hsa-miR-424
ENSG0000	ENSG0000	0.005224	hsa-miR-106a-5p;hsa-miR-106b-5p;hsa-miR-17-5p;hsa-miR-20
ENSG0000	ENSG0000	0.005224	hsa-miR-106a-5p;hsa-miR-106b-5p;hsa-miR-17-5p;hsa-miR-20
ENSG0000	ENSG0000	0.005224	hsa-miR-106a-5p;hsa-miR-106b-5p;hsa-miR-17-5p;hsa-miR-20
ENSG0000	ENSG0000	0.005224	hsa-miR-106a-5p;hsa-miR-106b-5p;hsa-miR-17-5p;hsa-miR-20
ENSG0000	ENSG0000	0.005224	hsa-miR-106a-5p;hsa-miR-106b-5p;hsa-miR-17-5p;hsa-miR-20
ENSG0000	ENSG0000	0.005224	hsa-miR-106a-5p;hsa-miR-106b-5p;hsa-miR-17-5p;hsa-miR-20
ENSG0000	ENSG0000	0.005224	hsa-miR-106a-5p;hsa-miR-106b-5p;hsa-miR-17-5p;hsa-miR-20
ENSG0000	ENSG0000	0.000969	hsa-miR-125b-2-3p;hsa-miR-15a-5p;hsa-miR-15b-5p;hsa-miR-
ENSG0000	ENSG0000	1.92E-05	hsa-miR-106a-5p;hsa-miR-106b-5p;hsa-miR-17-5p;hsa-miR-18
ENSG0000	ENSG0000	0.001186	hsa-miR-106a-5p;hsa-miR-106b-5p;hsa-miR-17-5p;hsa-miR-20
ENSG0000	ENSG0000	6.20E-07	hsa-miR-106a-5p;hsa-miR-106b-5p;hsa-miR-185-5p;hsa-miR-2
ENSG0000	ENSG0000	0.000298	hsa-miR-106a-5p;hsa-miR-106b-5p;hsa-miR-17-5p;hsa-miR-20
ENSG0000	ENSG0000	3.06E-05	hsa-miR-500a-3p;hsa-miR-548b-5p;hsa-miR-548c-5p;hsa-miR-
ENSG0000	ENSG0000	0.000862	hsa-miR-15a-5p;hsa-miR-15b-5p;hsa-miR-16-5p;hsa-miR-195-
ENSG0000	ENSG0000	0.007917	hsa-miR-106a-5p;hsa-miR-106b-5p;hsa-miR-17-5p;hsa-miR-20
ENSG0000	ENSG0000	0.002662	hsa-miR-106a-5p;hsa-miR-106b-5p;hsa-miR-17-5p;hsa-miR-20
ENSG0000	ENSG0000	2.42E-07	hsa-miR-15a-5p;hsa-miR-15b-5p;hsa-miR-16-5p;hsa-miR-195-
ENSG0000	ENSG0000	6.80E-05	hsa-miR-548b-5p;hsa-miR-548c-5p;hsa-miR-548d-5p;hsa-miR-
ENSG0000	ENSG0000	4.26E-06	hsa-miR-106a-5p;hsa-miR-106b-5p;hsa-miR-15a-5p;hsa-miR-1
ENSG0000	ENSG0000	1.04E-07	hsa-miR-106b-5p;hsa-miR-17-5p;hsa-miR-20a-5p;hsa-miR-20b
ENSG0000	ENSG0000	1.04E-07	hsa-miR-106b-5p;hsa-miR-17-5p;hsa-miR-20a-5p;hsa-miR-20b
ENSG0000	ENSG0000	0.000424	hsa-miR-15a-5p;hsa-miR-15b-5p;hsa-miR-16-5p;hsa-miR-195-

ENSG0000	ENSG0000	0.004118	hsa-miR-103a-3p;hsa-miR-107;hsa-miR-125b-2-3p;hsa-miR-1
ENSG0000	ENSG0000	1.27E-06	hsa-miR-139-5p;hsa-miR-140-3p;hsa-miR-15a-5p;hsa-miR-15
ENSG0000	ENSG0000	0.003125	hsa-miR-15a-5p;hsa-miR-15b-5p;hsa-miR-16-5p;hsa-miR-185
ENSG0000	ENSG0000	0.000193	hsa-miR-106b-5p;hsa-miR-17-5p;hsa-miR-20a-5p;hsa-miR-20
ENSG0000	ENSG0000	0.001439	hsa-miR-106a-5p;hsa-miR-106b-5p;hsa-miR-17-5p;hsa-miR-2
ENSG0000	ENSG0000	0.00481	hsa-miR-365a-3p;hsa-miR-365b-3p;hsa-miR-624-3p;hsa-miR-
ENSG0000	ENSG0000	0.006738	hsa-miR-15a-5p;hsa-miR-15b-5p;hsa-miR-16-5p;hsa-miR-195
ENSG0000	ENSG0000	2.98E-06	hsa-miR-548b-5p;hsa-miR-548c-5p;hsa-miR-548d-5p;hsa-miR
ENSG0000	ENSG0000	2.42E-07	hsa-miR-15a-5p;hsa-miR-15b-5p;hsa-miR-16-5p;hsa-miR-195
ENSG0000	ENSG0000	0.003125	hsa-miR-15a-5p;hsa-miR-15b-5p;hsa-miR-16-5p;hsa-miR-195
ENSG0000	ENSG0000	1.36E-05	hsa-miR-15a-5p;hsa-miR-15b-5p;hsa-miR-16-5p;hsa-miR-195
ENSG0000	ENSG0000	0.00982	hsa-miR-106a-5p;hsa-miR-106b-5p;hsa-miR-17-5p;hsa-miR-2
ENSG0000	ENSG0000	2.50E-07	hsa-miR-106a-5p;hsa-miR-106b-5p;hsa-miR-15a-5p;hsa-miR-
ENSG0000	ENSG0000	0.009354	hsa-miR-106a-5p;hsa-miR-106b-5p;hsa-miR-17-5p;hsa-miR-2
ENSG0000	ENSG0000	5.25E-06	hsa-miR-103a-3p;hsa-miR-107;hsa-miR-15a-5p;hsa-miR-15b-
ENSG0000	ENSG0000	0.00019	hsa-miR-106a-5p;hsa-miR-106b-5p;hsa-miR-17-5p;hsa-miR-1
ENSG0000	ENSG0000	0.00019	hsa-miR-106a-5p;hsa-miR-106b-5p;hsa-miR-17-5p;hsa-miR-1
ENSG0000	ENSG0000	7.20E-07	hsa-miR-106a-5p;hsa-miR-106b-5p;hsa-miR-17-5p;hsa-miR-2
ENSG0000	ENSG0000	8.02E-06	hsa-miR-106a-5p;hsa-miR-106b-5p;hsa-miR-17-5p;hsa-miR-2
ENSG0000	ENSG0000	0.001228	hsa-miR-106a-5p;hsa-miR-106b-5p;hsa-miR-143-3p;hsa-miR-
ENSG0000	ENSG0000	0.001228	hsa-miR-106a-5p;hsa-miR-106b-5p;hsa-miR-143-3p;hsa-miR-
ENSG0000	ENSG0000	0.007837	hsa-miR-106a-5p;hsa-miR-106b-5p;hsa-miR-143-3p;hsa-miR-
ENSG0000	ENSG0000	1.60E-05	hsa-miR-548b-5p;hsa-miR-548c-5p;hsa-miR-548d-5p;hsa-miR
ENSG0000	ENSG0000	0.005593	hsa-miR-106a-5p;hsa-miR-106b-5p;hsa-miR-17-5p;hsa-miR-2
ENSG0000	ENSG0000	0.006738	hsa-miR-106b-5p;hsa-miR-17-5p;hsa-miR-20a-5p;hsa-miR-20
ENSG0000	ENSG0000	0.00481	hsa-miR-107;hsa-miR-15a-5p;hsa-miR-15b-5p;hsa-miR-195-5
ENSG0000	ENSG0000	3.03E-08	hsa-miR-106b-5p;hsa-miR-17-5p;hsa-miR-20a-5p;hsa-miR-20
ENSG0000	ENSG0000	7.82E-05	hsa-miR-548b-5p;hsa-miR-548c-5p;hsa-miR-548d-5p;hsa-miR
ENSG0000	ENSG0000	0.001186	hsa-miR-106a-5p;hsa-miR-106b-5p;hsa-miR-17-5p;hsa-miR-2
ENSG0000	ENSG0000	0.000988	hsa-miR-106a-5p;hsa-miR-106b-5p;hsa-miR-17-5p;hsa-miR-2
ENSG0000	ENSG0000	0.003125	hsa-miR-106b-5p;hsa-miR-17-5p;hsa-miR-20a-5p;hsa-miR-20
ENSG0000	ENSG0000	0.003125	hsa-miR-106b-5p;hsa-miR-17-5p;hsa-miR-20a-5p;hsa-miR-20
ENSG0000	ENSG0000	2.47E-06	hsa-miR-103a-3p;hsa-miR-107;hsa-miR-15a-5p;hsa-miR-15b-
ENSG0000	ENSG0000	8.82E-07	hsa-miR-106a-5p;hsa-miR-106b-5p;hsa-miR-15a-5p;hsa-miR-
ENSG0000	ENSG0000	4.37E-05	hsa-miR-548b-5p;hsa-miR-548c-5p;hsa-miR-548d-5p;hsa-miR
ENSG0000	ENSG0000	0.000283	hsa-miR-15a-5p;hsa-miR-15b-5p;hsa-miR-16-5p;hsa-miR-195
ENSG0000	ENSG0000	0.002053	hsa-miR-106a-5p;hsa-miR-106b-5p;hsa-miR-139-5p;hsa-miR-
ENSG0000	ENSG0000	9.97E-05	hsa-miR-27a-3p;hsa-miR-27b-3p;hsa-miR-548b-5p;hsa-miR-5
ENSG0000	ENSG0000	0.000154	hsa-miR-106a-5p;hsa-miR-106b-5p;hsa-miR-17-5p;hsa-miR-1
ENSG0000	ENSG0000	4.31E-09	hsa-miR-106a-5p;hsa-miR-106b-5p;hsa-miR-17-5p;hsa-miR-2
ENSG0000	ENSG0000	0.007481	hsa-miR-106b-5p;hsa-miR-17-5p;hsa-miR-20a-5p;hsa-miR-20
ENSG0000	ENSG0000	0.000274	hsa-miR-106b-5p;hsa-miR-17-5p;hsa-miR-20a-5p;hsa-miR-20
ENSG0000	ENSG0000	0.000438	hsa-miR-15a-5p;hsa-miR-15b-5p;hsa-miR-195-5p;hsa-miR-42
ENSG0000	ENSG0000	2.89E-06	hsa-miR-106a-5p;hsa-miR-106b-5p;hsa-miR-17-5p;hsa-miR-2
ENSG0000	ENSG0000	0.008217	hsa-miR-106a-5p;hsa-miR-106b-5p;hsa-miR-17-5p;hsa-miR-2
ENSG0000	ENSG0000	0.00331	hsa-miR-103a-3p;hsa-miR-107;hsa-miR-15a-5p;hsa-miR-15b-
ENSG0000	ENSG0000	0.000144	hsa-miR-106a-5p;hsa-miR-106b-5p;hsa-miR-17-5p;hsa-miR-2
ENSG0000	ENSG0000	0.000144	hsa-miR-106a-5p;hsa-miR-106b-5p;hsa-miR-17-5p;hsa-miR-2
ENSG0000	ENSG0000	0.000144	hsa-miR-106a-5p;hsa-miR-106b-5p;hsa-miR-17-5p;hsa-miR-2
ENSG0000	ENSG0000	0.000144	hsa-miR-106a-5p;hsa-miR-106b-5p;hsa-miR-17-5p;hsa-miR-2
ENSG0000	ENSG0000	0.000144	hsa-miR-106a-5p;hsa-miR-106b-5p;hsa-miR-17-5p;hsa-miR-2
ENSG0000	ENSG0000	0.000144	hsa-miR-106a-5p;hsa-miR-106b-5p;hsa-miR-17-5p;hsa-miR-2
ENSG0000	ENSG0000	0.000144	hsa-miR-106a-5p;hsa-miR-106b-5p;hsa-miR-17-5p;hsa-miR-2
ENSG0000	ENSG0000	0.004541	hsa-miR-106a-5p;hsa-miR-106b-5p;hsa-miR-17-5p;hsa-miR-2

ENSG0000	ENSG0000	0.004261	hsa-miR-15a-5p;hsa-miR-15b-5p;hsa-miR-195-5p;hsa-miR-42
ENSG0000	ENSG0000	0.004261	hsa-miR-15a-5p;hsa-miR-15b-5p;hsa-miR-195-5p;hsa-miR-42
ENSG0000	ENSG0000	0.002254	hsa-miR-106a-5p;hsa-miR-106b-5p;hsa-miR-17-5p;hsa-miR-2
ENSG0000	ENSG0000	0.000298	hsa-miR-15a-5p;hsa-miR-15b-5p;hsa-miR-16-5p;hsa-miR-195
ENSG0000	ENSG0000	0.003124	hsa-miR-15a-5p;hsa-miR-15b-5p;hsa-miR-16-5p;hsa-miR-195
ENSG0000	ENSG0000	3.03E-08	hsa-miR-106b-5p;hsa-miR-17-5p;hsa-miR-20a-5p;hsa-miR-20
ENSG0000	ENSG0000	0.000438	hsa-miR-106a-5p;hsa-miR-106b-5p;hsa-miR-17-5p;hsa-miR-2
ENSG0000	ENSG0000	0.000401	hsa-miR-106a-5p;hsa-miR-106b-5p;hsa-miR-17-5p;hsa-miR-2
ENSG0000	ENSG0000	0.005609	hsa-miR-106b-5p;hsa-miR-17-5p;hsa-miR-20a-5p;hsa-miR-20
ENSG0000	ENSG0000	0.001774	hsa-miR-106a-5p;hsa-miR-106b-5p;hsa-miR-17-5p;hsa-miR-2
ENSG0000	ENSG0000	0.000401	hsa-miR-106a-5p;hsa-miR-106b-5p;hsa-miR-17-5p;hsa-miR-2
ENSG0000	ENSG0000	0.001351	hsa-miR-106b-5p;hsa-miR-15a-5p;hsa-miR-15b-5p;hsa-miR-1
ENSG0000	ENSG0000	0.000401	hsa-miR-106b-5p;hsa-miR-17-5p;hsa-miR-20a-5p;hsa-miR-20
ENSG0000	ENSG0000	0.001774	hsa-miR-548b-5p;hsa-miR-548c-5p;hsa-miR-548d-5p;hsa-miR
ENSG0000	ENSG0000	0.000157	hsa-miR-143-3p;hsa-miR-15a-5p;hsa-miR-16-5p;hsa-miR-195
ENSG0000	ENSG0000	0.000169	hsa-miR-106a-5p;hsa-miR-106b-5p;hsa-miR-17-5p;hsa-miR-1
ENSG0000	ENSG0000	2.73E-07	hsa-miR-106b-5p;hsa-miR-17-5p;hsa-miR-20a-5p;hsa-miR-20
ENSG0000	ENSG0000	0.000988	hsa-miR-106b-5p;hsa-miR-17-5p;hsa-miR-20a-5p;hsa-miR-20
ENSG0000	ENSG0000	0.000123	hsa-miR-106a-5p;hsa-miR-106b-5p;hsa-miR-17-5p;hsa-miR-2
ENSG0000	ENSG0000	0.001836	hsa-miR-106a-5p;hsa-miR-106b-5p;hsa-miR-17-5p;hsa-miR-2
ENSG0000	ENSG0000	6.03E-06	hsa-miR-106a-5p;hsa-miR-106b-5p;hsa-miR-17-5p;hsa-miR-2
ENSG0000	ENSG0000	1.79E-06	hsa-miR-103a-3p;hsa-miR-107;hsa-miR-15a-5p;hsa-miR-15b-
ENSG0000	ENSG0000	0.00379	hsa-miR-15a-5p;hsa-miR-15b-5p;hsa-miR-16-5p;hsa-miR-195
ENSG0000	ENSG0000	1.10E-08	hsa-miR-106b-5p;hsa-miR-127-5p;hsa-miR-17-5p;hsa-miR-20
ENSG0000	ENSG0000	0.002205	hsa-miR-548b-5p;hsa-miR-548c-5p;hsa-miR-548d-5p;hsa-miR
ENSG0000	ENSG0000	5.89E-05	hsa-miR-106a-5p;hsa-miR-106b-5p;hsa-miR-17-5p;hsa-miR-2
ENSG0000	ENSG0000	3.67E-08	hsa-miR-106a-5p;hsa-miR-15a-5p;hsa-miR-15b-5p;hsa-miR-1
ENSG0000	ENSG0000	0.005089	hsa-miR-106a-5p;hsa-miR-106b-5p;hsa-miR-17-5p;hsa-miR-2
ENSG0000	ENSG0000	0.00026	hsa-miR-27a-3p;hsa-miR-27b-3p;hsa-miR-484;hsa-miR-501-3
ENSG0000	ENSG0000	0.002366	hsa-miR-106a-5p;hsa-miR-106b-5p;hsa-miR-17-5p;hsa-miR-2
ENSG0000	ENSG0000	4.14E-06	hsa-miR-106a-5p;hsa-miR-106b-5p;hsa-miR-17-5p;hsa-miR-2
ENSG0000	ENSG0000	4.76E-10	hsa-miR-106a-5p;hsa-miR-106b-5p;hsa-miR-17-5p;hsa-miR-2
ENSG0000	ENSG0000	2.93E-10	hsa-miR-106a-5p;hsa-miR-106b-5p;hsa-miR-1246;hsa-miR-15
ENSG0000	ENSG0000	6.13E-05	hsa-miR-15a-5p;hsa-miR-15b-5p;hsa-miR-16-5p;hsa-miR-195
ENSG0000	ENSG0000	0.004824	hsa-miR-106a-5p;hsa-miR-106b-5p;hsa-miR-17-5p;hsa-miR-2
ENSG0000	ENSG0000	0.007917	hsa-miR-106a-5p;hsa-miR-106b-5p;hsa-miR-143-3p;hsa-miR-
ENSG0000	ENSG0000	0.002068	hsa-miR-106a-5p;hsa-miR-106b-5p;hsa-miR-17-5p;hsa-miR-2
ENSG0000	ENSG0000	0.000969	hsa-miR-106a-5p;hsa-miR-106b-5p;hsa-miR-17-5p;hsa-miR-2
ENSG0000	ENSG0000	0.000263	hsa-miR-106a-5p;hsa-miR-106b-5p;hsa-miR-140-3p;hsa-miR-
ENSG0000	ENSG0000	7.32E-06	hsa-miR-548b-5p;hsa-miR-548c-5p;hsa-miR-548d-5p;hsa-miR
ENSG0000	ENSG0000	5.98E-06	hsa-miR-15a-5p;hsa-miR-15b-5p;hsa-miR-16-5p;hsa-miR-195
ENSG0000	ENSG0000	0.000101	hsa-miR-106a-5p;hsa-miR-106b-5p;hsa-miR-20a-5p;hsa-miR-2
ENSG0000	ENSG0000	1.52E-05	hsa-miR-106a-5p;hsa-miR-106b-5p;hsa-miR-17-5p;hsa-miR-1
ENSG0000	ENSG0000	0.002481	hsa-miR-106a-5p;hsa-miR-106b-5p;hsa-miR-143-3p;hsa-miR-
ENSG0000	ENSG0000	6.80E-05	hsa-miR-125b-2-3p;hsa-miR-143-3p;hsa-miR-27a-3p;hsa-miR
ENSG0000	ENSG0000	7.37E-05	hsa-miR-106a-5p;hsa-miR-106b-5p;hsa-miR-17-5p;hsa-miR-2
ENSG0000	ENSG0000	0.001406	hsa-miR-106b-5p;hsa-miR-17-5p;hsa-miR-20a-5p;hsa-miR-20
ENSG0000	ENSG0000	0.000546	hsa-miR-106a-5p;hsa-miR-106b-5p;hsa-miR-17-5p;hsa-miR-2
ENSG0000	ENSG0000	0.000331	hsa-miR-548b-5p;hsa-miR-548c-5p;hsa-miR-548d-5p;hsa-miR
ENSG0000	ENSG0000	0.001732	hsa-miR-106a-5p;hsa-miR-106b-5p;hsa-miR-17-5p;hsa-miR-2
ENSG0000	ENSG0000	8.21E-06	hsa-miR-106a-5p;hsa-miR-106b-5p;hsa-miR-17-5p;hsa-miR-1
ENSG0000	ENSG0000	0.000583	hsa-miR-15a-5p;hsa-miR-16-5p;hsa-miR-195-5p;hsa-miR-424
ENSG0000	ENSG0000	0.002662	hsa-miR-106a-5p;hsa-miR-106b-5p;hsa-miR-17-5p;hsa-miR-2
ENSG0000	ENSG0000	0.000105	hsa-miR-15a-5p;hsa-miR-15b-5p;hsa-miR-16-5p;hsa-miR-195

ENSG0000	ENSG0000	0.000169	hsa-miR-548b-5p;hsa-miR-548c-5p;hsa-miR-548d-5p;hsa-miR
ENSG0000	ENSG0000	0.000298	hsa-miR-106a-5p;hsa-miR-106b-5p;hsa-miR-17-5p;hsa-miR-2
ENSG0000	ENSG0000	1.31E-05	hsa-miR-106a-5p;hsa-miR-106b-5p;hsa-miR-17-5p;hsa-miR-2
ENSG0000	ENSG0000	0.001836	hsa-miR-106b-5p;hsa-miR-17-5p;hsa-miR-20a-5p;hsa-miR-20
ENSG0000	ENSG0000	2.74E-05	hsa-miR-106a-5p;hsa-miR-106b-5p;hsa-miR-17-5p;hsa-miR-2
ENSG0000	ENSG0000	0.000338	hsa-miR-15a-5p;hsa-miR-15b-5p;hsa-miR-16-5p;hsa-miR-195
ENSG0000	ENSG0000	0.001525	hsa-miR-106a-5p;hsa-miR-106b-5p;hsa-miR-17-5p;hsa-miR-2
ENSG0000	ENSG0000	1.85E-05	hsa-miR-15a-5p;hsa-miR-15b-5p;hsa-miR-16-5p;hsa-miR-195
ENSG0000	ENSG0000	0.000701	hsa-miR-106a-5p;hsa-miR-17-5p;hsa-miR-20a-5p;hsa-miR-20
ENSG0000	ENSG0000	0.008729	hsa-miR-106a-5p;hsa-miR-106b-5p;hsa-miR-17-5p;hsa-miR-2
ENSG0000	ENSG0000	0.000226	hsa-miR-106a-5p;hsa-miR-106b-5p;hsa-miR-17-5p;hsa-miR-2
ENSG0000	ENSG0000	0.000558	hsa-miR-106b-5p;hsa-miR-17-5p;hsa-miR-20a-3p;hsa-miR-20
ENSG0000	ENSG0000	6.46E-06	hsa-miR-106a-5p;hsa-miR-106b-5p;hsa-miR-15a-5p;hsa-miR-
ENSG0000	ENSG0000	0.005414	hsa-miR-15a-5p;hsa-miR-15b-5p;hsa-miR-16-5p;hsa-miR-195
ENSG0000	ENSG0000	2.34E-05	hsa-miR-106a-5p;hsa-miR-106b-5p;hsa-miR-17-5p;hsa-miR-2
ENSG0000	ENSG0000	0.001439	hsa-miR-106a-5p;hsa-miR-106b-5p;hsa-miR-17-5p;hsa-miR-2
ENSG0000	ENSG0000	5.92E-06	hsa-miR-106a-5p;hsa-miR-106b-5p;hsa-miR-17-5p;hsa-miR-2
ENSG0000	ENSG0000	3.03E-08	hsa-miR-106b-5p;hsa-miR-17-5p;hsa-miR-20a-5p;hsa-miR-20
ENSG0000	ENSG0000	4.68E-05	hsa-miR-15a-5p;hsa-miR-15b-5p;hsa-miR-16-5p;hsa-miR-195
ENSG0000	ENSG0000	4.68E-05	hsa-miR-15a-5p;hsa-miR-15b-5p;hsa-miR-16-5p;hsa-miR-195
ENSG0000	ENSG0000	4.68E-05	hsa-miR-15a-5p;hsa-miR-15b-5p;hsa-miR-16-5p;hsa-miR-195
ENSG0000	ENSG0000	4.68E-05	hsa-miR-15a-5p;hsa-miR-15b-5p;hsa-miR-16-5p;hsa-miR-195
ENSG0000	ENSG0000	1.11E-08	hsa-miR-106a-5p;hsa-miR-106b-5p;hsa-miR-107;hsa-miR-15a
ENSG0000	ENSG0000	1.11E-08	hsa-miR-106a-5p;hsa-miR-106b-5p;hsa-miR-107;hsa-miR-15a
ENSG0000	ENSG0000	0.008339	hsa-miR-106a-5p;hsa-miR-106b-5p;hsa-miR-17-5p;hsa-miR-2
ENSG0000	ENSG0000	0.0005	hsa-miR-103a-3p;hsa-miR-107;hsa-miR-15a-5p;hsa-miR-15b-
ENSG0000	ENSG0000	0.005414	hsa-miR-18a-3p;hsa-miR-324-3p;hsa-miR-376a-3p;hsa-miR-9
ENSG0000	ENSG0000	0.000555	hsa-miR-106a-5p;hsa-miR-106b-5p;hsa-miR-17-5p;hsa-miR-2
ENSG0000	ENSG0000	8.92E-08	hsa-miR-106b-5p;hsa-miR-17-5p;hsa-miR-20a-5p;hsa-miR-20
ENSG0000	ENSG0000	2.14E-06	hsa-miR-106b-5p;hsa-miR-17-5p;hsa-miR-20a-5p;hsa-miR-20
ENSG0000	ENSG0000	0.00145	hsa-miR-106b-5p;hsa-miR-140-3p;hsa-miR-17-5p;hsa-miR-20
ENSG0000	ENSG0000	6.05E-06	hsa-miR-106a-5p;hsa-miR-106b-5p;hsa-miR-15a-5p;hsa-miR-
ENSG0000	ENSG0000	0.000195	hsa-miR-106a-5p;hsa-miR-106b-5p;hsa-miR-17-5p;hsa-miR-2
ENSG0000	ENSG0000	0.000473	hsa-miR-106a-5p;hsa-miR-106b-5p;hsa-miR-17-5p;hsa-miR-2
ENSG0000	ENSG0000	3.03E-08	hsa-miR-106b-5p;hsa-miR-17-5p;hsa-miR-20a-5p;hsa-miR-20
ENSG0000	ENSG0000	0.000148	hsa-miR-15a-5p;hsa-miR-15b-5p;hsa-miR-195-5p;hsa-miR-42
ENSG0000	ENSG0000	3.51E-06	hsa-miR-15a-5p;hsa-miR-15b-5p;hsa-miR-16-5p;hsa-miR-195
ENSG0000	ENSG0000	0.000401	hsa-miR-106a-5p;hsa-miR-106b-5p;hsa-miR-17-5p;hsa-miR-2
ENSG0000	ENSG0000	2.73E-07	hsa-miR-106b-5p;hsa-miR-17-5p;hsa-miR-20a-5p;hsa-miR-20
ENSG0000	ENSG0000	0.000783	hsa-miR-106a-5p;hsa-miR-106b-5p;hsa-miR-17-5p;hsa-miR-2
ENSG0000	ENSG0000	1.98E-08	hsa-miR-106b-5p;hsa-miR-17-5p;hsa-miR-20a-5p;hsa-miR-20
ENSG0000	ENSG0000	0.001186	hsa-miR-106a-5p;hsa-miR-106b-5p;hsa-miR-17-5p;hsa-miR-2
ENSG0000	ENSG0000	0.004253	hsa-miR-106a-5p;hsa-miR-106b-5p;hsa-miR-17-5p;hsa-miR-2
ENSG0000	ENSG0000	0.002055	hsa-miR-106a-5p;hsa-miR-106b-5p;hsa-miR-17-5p;hsa-miR-2
ENSG0000	ENSG0000	0.003377	hsa-miR-106a-5p;hsa-miR-106b-5p;hsa-miR-17-5p;hsa-miR-2
ENSG0000	ENSG0000	0.002827	hsa-miR-106b-5p;hsa-miR-17-5p;hsa-miR-20a-5p;hsa-miR-20
ENSG0000	ENSG0000	0.000558	hsa-miR-106a-5p;hsa-miR-106b-5p;hsa-miR-17-5p;hsa-miR-2
ENSG0000	ENSG0000	8.80E-06	hsa-miR-106b-5p;hsa-miR-17-5p;hsa-miR-20a-5p;hsa-miR-20
ENSG0000	ENSG0000	3.03E-08	hsa-miR-106b-5p;hsa-miR-17-5p;hsa-miR-20a-5p;hsa-miR-20
ENSG0000	ENSG0000	0.000496	hsa-miR-106a-5p;hsa-miR-106b-5p;hsa-miR-17-5p;hsa-miR-2
ENSG0000	ENSG0000	2.42E-07	hsa-miR-15a-5p;hsa-miR-15b-5p;hsa-miR-16-5p;hsa-miR-195
ENSG0000	ENSG0000	0.001308	hsa-miR-106a-5p;hsa-miR-106b-5p;hsa-miR-17-5p;hsa-miR-2
ENSG0000	ENSG0000	0.002068	hsa-miR-106a-5p;hsa-miR-106b-5p;hsa-miR-17-5p;hsa-miR-1
ENSG0000	ENSG0000	0.001282	hsa-miR-15a-5p;hsa-miR-15b-5p;hsa-miR-16-5p;hsa-miR-195

ENSG0000	ENSG0000	0.001282	hsa-miR-15a-5p;hsa-miR-15b-5p;hsa-miR-16-5p;hsa-miR-195-
ENSG0000	ENSG0000	0.001282	hsa-miR-15a-5p;hsa-miR-15b-5p;hsa-miR-16-5p;hsa-miR-195-
ENSG0000	ENSG0000	0.001282	hsa-miR-15a-5p;hsa-miR-15b-5p;hsa-miR-16-5p;hsa-miR-195-
ENSG0000	ENSG0000	0.001282	hsa-miR-15a-5p;hsa-miR-15b-5p;hsa-miR-16-5p;hsa-miR-195-
ENSG0000	ENSG0000	0.001282	hsa-miR-15a-5p;hsa-miR-15b-5p;hsa-miR-16-5p;hsa-miR-195-
ENSG0000	ENSG0000	0.001282	hsa-miR-15a-5p;hsa-miR-15b-5p;hsa-miR-16-5p;hsa-miR-195-
ENSG0000	ENSG0000	0.001282	hsa-miR-15a-5p;hsa-miR-15b-5p;hsa-miR-16-5p;hsa-miR-195-
ENSG0000	ENSG0000	0.001282	hsa-miR-15a-5p;hsa-miR-15b-5p;hsa-miR-16-5p;hsa-miR-195-
ENSG0000	ENSG0000	0.001282	hsa-miR-15a-5p;hsa-miR-15b-5p;hsa-miR-16-5p;hsa-miR-195-
ENSG0000	ENSG0000	0.006746	hsa-miR-106a-5p;hsa-miR-106b-5p;hsa-miR-17-5p;hsa-miR-2(
ENSG0000	ENSG0000	0.00604	hsa-miR-106a-5p;hsa-miR-106b-5p;hsa-miR-17-5p;hsa-miR-2(
ENSG0000	ENSG0000	5.77E-05	hsa-miR-136-3p;hsa-miR-139-5p;hsa-miR-548b-5p;hsa-miR-5
ENSG0000	ENSG0000	1.58E-05	hsa-miR-15a-5p;hsa-miR-15b-5p;hsa-miR-16-5p;hsa-miR-195-
ENSG0000	ENSG0000	0.000473	hsa-miR-106b-5p;hsa-miR-17-5p;hsa-miR-20a-5p;hsa-miR-20l
ENSG0000	ENSG0000	2.80E-07	hsa-miR-106a-5p;hsa-miR-106b-5p;hsa-miR-17-5p;hsa-miR-2(
ENSG0000	ENSG0000	0.000101	hsa-miR-15a-5p;hsa-miR-15b-5p;hsa-miR-16-5p;hsa-miR-195-
ENSG0000	ENSG0000	0.008697	hsa-miR-106b-5p;hsa-miR-139-5p;hsa-miR-20a-5p;hsa-miR-2(
ENSG0000	ENSG0000	7.32E-06	hsa-miR-548b-5p;hsa-miR-548c-5p;hsa-miR-548d-5p;hsa-miR-
ENSG0000	ENSG0000	2.74E-05	hsa-miR-106a-5p;hsa-miR-106b-5p;hsa-miR-17-5p;hsa-miR-2(
ENSG0000	ENSG0000	0.000193	hsa-miR-15a-5p;hsa-miR-15b-5p;hsa-miR-16-5p;hsa-miR-424-
ENSG0000	ENSG0000	0.000627	hsa-miR-106a-5p;hsa-miR-106b-5p;hsa-miR-17-5p;hsa-miR-2(
ENSG0000	ENSG0000	0.009121	hsa-miR-15a-5p;hsa-miR-15b-5p;hsa-miR-16-5p;hsa-miR-195-
ENSG0000	ENSG0000	0.008217	hsa-miR-106a-5p;hsa-miR-106b-5p;hsa-miR-17-5p;hsa-miR-2(
ENSG0000	ENSG0000	1.11E-05	hsa-miR-103a-3p;hsa-miR-106a-5p;hsa-miR-106b-5p;hsa-miR-
ENSG0000	ENSG0000	0.000368	hsa-miR-548b-5p;hsa-miR-548c-5p;hsa-miR-548d-5p;hsa-miR-
ENSG0000	ENSG0000	2.70E-05	hsa-miR-103a-3p;hsa-miR-107;hsa-miR-15a-5p;hsa-miR-15b-5
ENSG0000	ENSG0000	5.08E-05	hsa-miR-548b-5p;hsa-miR-548c-5p;hsa-miR-548d-5p;hsa-miR-
ENSG0000	ENSG0000	0.004541	hsa-miR-15a-5p;hsa-miR-15b-5p;hsa-miR-16-5p;hsa-miR-195-
ENSG0000	ENSG0000	1.60E-05	hsa-miR-103a-3p;hsa-miR-107;hsa-miR-15a-5p;hsa-miR-15b-5
ENSG0000	ENSG0000	2.58E-05	hsa-miR-106b-5p;hsa-miR-17-5p;hsa-miR-20a-5p;hsa-miR-20l
ENSG0000	ENSG0000	0.006389	hsa-miR-15a-5p;hsa-miR-15b-5p;hsa-miR-16-5p;hsa-miR-195-
ENSG0000	ENSG0000	3.39E-05	hsa-miR-15a-5p;hsa-miR-15b-5p;hsa-miR-195-5p;hsa-miR-27a
ENSG0000	ENSG0000	0.008729	hsa-miR-15a-5p;hsa-miR-15b-5p;hsa-miR-16-5p;hsa-miR-195-
ENSG0000	ENSG0000	0.000783	hsa-miR-15a-5p;hsa-miR-15b-5p;hsa-miR-16-5p;hsa-miR-195-
ENSG0000	ENSG0000	5.75E-06	hsa-miR-106b-5p;hsa-miR-17-5p;hsa-miR-20a-5p;hsa-miR-20l
ENSG0000	ENSG0000	0.001894	hsa-miR-106a-5p;hsa-miR-106b-5p;hsa-miR-17-5p;hsa-miR-2(
ENSG0000	ENSG0000	0.001909	hsa-miR-106a-5p;hsa-miR-106b-5p;hsa-miR-17-5p;hsa-miR-2(
ENSG0000	ENSG0000	0.003927	hsa-miR-106a-5p;hsa-miR-106b-5p;hsa-miR-17-5p;hsa-miR-2(
ENSG0000	ENSG0000	0.000862	hsa-miR-15a-5p;hsa-miR-15b-5p;hsa-miR-16-5p;hsa-miR-195-
ENSG0000	ENSG0000	6.66E-09	hsa-miR-15a-5p;hsa-miR-15b-5p;hsa-miR-16-5p;hsa-miR-195-
ENSG0000	ENSG0000	0.001774	hsa-miR-106b-5p;hsa-miR-17-5p;hsa-miR-192-5p;hsa-miR-20a
ENSG0000	ENSG0000	0.004798	hsa-miR-106a-5p;hsa-miR-106b-5p;hsa-miR-17-5p;hsa-miR-2(
ENSG0000	ENSG0000	3.31E-05	hsa-miR-15a-5p;hsa-miR-15b-5p;hsa-miR-16-5p;hsa-miR-195-
ENSG0000	ENSG0000	0.000407	hsa-miR-548b-5p;hsa-miR-548c-5p;hsa-miR-548d-5p;hsa-miR-
ENSG0000	ENSG0000	0.008777	hsa-miR-106b-5p;hsa-miR-17-5p;hsa-miR-20a-5p;hsa-miR-20l
ENSG0000	ENSG0000	4.68E-05	hsa-miR-15a-5p;hsa-miR-15b-5p;hsa-miR-16-5p;hsa-miR-195-
ENSG0000	ENSG0000	4.14E-05	hsa-miR-106a-5p;hsa-miR-106b-5p;hsa-miR-15a-5p;hsa-miR-1
ENSG0000	ENSG0000	0.000283	hsa-miR-106b-5p;hsa-miR-17-5p;hsa-miR-20a-5p;hsa-miR-20l
ENSG0000	ENSG0000	1.35E-07	hsa-miR-103a-3p;hsa-miR-107;hsa-miR-127-5p;hsa-miR-15a-5
ENSG0000	ENSG0000	0.001635	hsa-miR-125b-2-3p;hsa-miR-139-5p;hsa-miR-224-5p;hsa-miR-
ENSG0000	ENSG0000	0.00481	hsa-miR-15a-5p;hsa-miR-15b-5p;hsa-miR-195-5p;hsa-miR-424
ENSG0000	ENSG0000	1.30E-05	hsa-miR-15a-5p;hsa-miR-15b-5p;hsa-miR-16-5p;hsa-miR-195-
ENSG0000	ENSG0000	0.002254	hsa-miR-106a-5p;hsa-miR-106b-5p;hsa-miR-17-5p;hsa-miR-2(
ENSG0000	ENSG0000	0.000274	hsa-miR-15a-5p;hsa-miR-15b-5p;hsa-miR-195-5p;hsa-miR-424

ENSG0000	ENSG0000	0.000407	hsa-miR-548b-5p;hsa-miR-548c-5p;hsa-miR-548d-5p;hsa-miR
ENSG0000	ENSG0000	0.001774	hsa-miR-548b-5p;hsa-miR-548c-5p;hsa-miR-548d-5p;hsa-miR
ENSG0000	ENSG0000	0.007917	hsa-miR-548b-5p;hsa-miR-548c-5p;hsa-miR-548d-5p;hsa-miR
ENSG0000	ENSG0000	1.37E-06	hsa-miR-103a-3p;hsa-miR-107;hsa-miR-15a-5p;hsa-miR-15b-
ENSG0000	ENSG0000	0.000104	hsa-miR-15a-5p;hsa-miR-15b-5p;hsa-miR-16-5p;hsa-miR-195
ENSG0000	ENSG0000	0.000327	hsa-miR-106a-5p;hsa-miR-106b-5p;hsa-miR-17-5p;hsa-miR-1
ENSG0000	ENSG0000	0.000495	hsa-miR-106b-5p;hsa-miR-17-5p;hsa-miR-20a-5p;hsa-miR-20
ENSG0000	ENSG0000	0.002451	hsa-miR-106a-5p;hsa-miR-106b-5p;hsa-miR-17-5p;hsa-miR-2
ENSG0000	ENSG0000	2.70E-05	hsa-miR-548b-5p;hsa-miR-548c-5p;hsa-miR-548d-5p;hsa-miR
ENSG0000	ENSG0000	2.70E-05	hsa-miR-548b-5p;hsa-miR-548c-5p;hsa-miR-548d-5p;hsa-miR
ENSG0000	ENSG0000	2.70E-05	hsa-miR-548b-5p;hsa-miR-548c-5p;hsa-miR-548d-5p;hsa-miR
ENSG0000	ENSG0000	2.70E-05	hsa-miR-548b-5p;hsa-miR-548c-5p;hsa-miR-548d-5p;hsa-miR
ENSG0000	ENSG0000	2.70E-05	hsa-miR-548b-5p;hsa-miR-548c-5p;hsa-miR-548d-5p;hsa-miR
ENSG0000	ENSG0000	2.70E-05	hsa-miR-548b-5p;hsa-miR-548c-5p;hsa-miR-548d-5p;hsa-miR
ENSG0000	ENSG0000	2.70E-05	hsa-miR-548b-5p;hsa-miR-548c-5p;hsa-miR-548d-5p;hsa-miR
ENSG0000	ENSG0000	2.70E-05	hsa-miR-548b-5p;hsa-miR-548c-5p;hsa-miR-548d-5p;hsa-miR
ENSG0000	ENSG0000	0.002487	hsa-miR-15a-5p;hsa-miR-15b-5p;hsa-miR-16-5p;hsa-miR-195
ENSG0000	ENSG0000	1.19E-06	hsa-miR-15a-5p;hsa-miR-15b-5p;hsa-miR-16-5p;hsa-miR-195
ENSG0000	ENSG0000	0.000123	hsa-miR-139-5p;hsa-miR-15a-5p;hsa-miR-15b-5p;hsa-miR-16
ENSG0000	ENSG0000	1.60E-05	hsa-miR-548b-5p;hsa-miR-548c-5p;hsa-miR-548d-5p;hsa-miR
ENSG0000	ENSG0000	0.001635	hsa-miR-15a-5p;hsa-miR-15b-5p;hsa-miR-16-5p;hsa-miR-195
ENSG0000	ENSG0000	0.001308	hsa-miR-106a-5p;hsa-miR-106b-5p;hsa-miR-17-5p;hsa-miR-2
ENSG0000	ENSG0000	0.001308	hsa-miR-106a-5p;hsa-miR-106b-5p;hsa-miR-17-5p;hsa-miR-2
ENSG0000	ENSG0000	0.000102	hsa-miR-103a-3p;hsa-miR-107;hsa-miR-15a-5p;hsa-miR-15b-
ENSG0000	ENSG0000	0.000862	hsa-miR-106b-5p;hsa-miR-17-5p;hsa-miR-20a-5p;hsa-miR-20
ENSG0000	ENSG0000	1.10E-08	hsa-miR-103a-3p;hsa-miR-107;hsa-miR-15a-5p;hsa-miR-15b-
ENSG0000	ENSG0000	0.000496	hsa-miR-106b-5p;hsa-miR-17-5p;hsa-miR-18a-3p;hsa-miR-20
ENSG0000	ENSG0000	0.002205	hsa-miR-106a-5p;hsa-miR-106b-5p;hsa-miR-17-5p;hsa-miR-1
ENSG0000	ENSG0000	1.30E-05	hsa-miR-15a-5p;hsa-miR-15b-5p;hsa-miR-16-5p;hsa-miR-195
ENSG0000	ENSG0000	1.36E-05	hsa-miR-106a-5p;hsa-miR-106b-5p;hsa-miR-17-5p;hsa-miR-2
ENSG0000	ENSG0000	2.86E-06	hsa-miR-484;hsa-miR-548b-5p;hsa-miR-548c-5p;hsa-miR-548
ENSG0000	ENSG0000	0.000331	hsa-miR-548b-5p;hsa-miR-548c-5p;hsa-miR-548d-5p;hsa-miR
ENSG0000	ENSG0000	0.007728	hsa-miR-106a-5p;hsa-miR-17-5p;hsa-miR-185-5p;hsa-miR-20
ENSG0000	ENSG0000	0.000123	hsa-miR-106a-5p;hsa-miR-106b-5p;hsa-miR-17-5p;hsa-miR-2
ENSG0000	ENSG0000	0.000799	hsa-miR-103a-3p;hsa-miR-106b-5p;hsa-miR-107;hsa-miR-17-
ENSG0000	ENSG0000	0.000169	hsa-miR-548b-5p;hsa-miR-548c-5p;hsa-miR-548d-5p;hsa-miR
ENSG0000	ENSG0000	0.003124	hsa-miR-106a-5p;hsa-miR-106b-5p;hsa-miR-17-5p;hsa-miR-2
ENSG0000	ENSG0000	0.003124	hsa-miR-106a-5p;hsa-miR-106b-5p;hsa-miR-17-5p;hsa-miR-2
ENSG0000	ENSG0000	0.008073	hsa-miR-15a-5p;hsa-miR-15b-5p;hsa-miR-16-5p;hsa-miR-195
ENSG0000	ENSG0000	0.006386	hsa-miR-106a-5p;hsa-miR-106b-5p;hsa-miR-17-5p;hsa-miR-2
ENSG0000	ENSG0000	0.000893	hsa-miR-15a-5p;hsa-miR-15b-5p;hsa-miR-16-5p;hsa-miR-195
ENSG0000	ENSG0000	0.000893	hsa-miR-15a-5p;hsa-miR-15b-5p;hsa-miR-16-5p;hsa-miR-195
ENSG0000	ENSG0000	1.09E-06	hsa-miR-106a-5p;hsa-miR-106b-5p;hsa-miR-17-5p;hsa-miR-2
ENSG0000	ENSG0000	7.37E-05	hsa-miR-106a-5p;hsa-miR-106b-5p;hsa-miR-17-5p;hsa-miR-2
ENSG0000	ENSG0000	0.000969	hsa-miR-106a-5p;hsa-miR-106b-5p;hsa-miR-17-5p;hsa-miR-2
ENSG0000	ENSG0000	0.001109	hsa-miR-106a-5p;hsa-miR-106b-5p;hsa-miR-143-3p;hsa-miR-
ENSG0000	ENSG0000	0.001109	hsa-miR-106a-5p;hsa-miR-106b-5p;hsa-miR-143-3p;hsa-miR-
ENSG0000	ENSG0000	4.95E-06	hsa-miR-106a-5p;hsa-miR-106b-5p;hsa-miR-17-5p;hsa-miR-2
ENSG0000	ENSG0000	0.00045	hsa-miR-106a-5p;hsa-miR-106b-5p;hsa-miR-17-5p;hsa-miR-2
ENSG0000	ENSG0000	3.39E-05	hsa-miR-15a-5p;hsa-miR-15b-5p;hsa-miR-16-5p;hsa-miR-195
ENSG0000	ENSG0000	0.002451	hsa-miR-106a-5p;hsa-miR-106b-5p;hsa-miR-17-5p;hsa-miR-2
ENSG0000	ENSG0000	0.001525	hsa-miR-106a-5p;hsa-miR-106b-5p;hsa-miR-136-3p;hsa-miR-
ENSG0000	ENSG0000	1.92E-05	hsa-miR-106a-5p;hsa-miR-106b-5p;hsa-miR-125b-2-3p;hsa-m
ENSG0000	ENSG0000	1.92E-05	hsa-miR-106a-5p;hsa-miR-106b-5p;hsa-miR-125b-2-3p;hsa-m

ENSG0000	ENSG0000	0.007728	hsa-miR-106a-5p;hsa-miR-106b-5p;hsa-miR-17-5p;hsa-miR-2
ENSG0000	ENSG0000	2.14E-08	hsa-miR-15a-5p;hsa-miR-15b-5p;hsa-miR-16-5p;hsa-miR-195
ENSG0000	ENSG0000	0.000387	hsa-miR-106a-5p;hsa-miR-106b-5p;hsa-miR-17-5p;hsa-miR-2
ENSG0000	ENSG0000	0.000168	hsa-miR-106a-5p;hsa-miR-106b-5p;hsa-miR-17-5p;hsa-miR-2
ENSG0000	ENSG0000	3.60E-06	hsa-miR-15a-5p;hsa-miR-15b-5p;hsa-miR-16-5p;hsa-miR-195
ENSG0000	ENSG0000	0.000101	hsa-miR-103a-3p;hsa-miR-107;hsa-miR-15a-5p;hsa-miR-15b-
ENSG0000	ENSG0000	0.000168	hsa-miR-106a-5p;hsa-miR-106b-5p;hsa-miR-17-5p;hsa-miR-2
ENSG0000	ENSG0000	1.30E-05	hsa-miR-15a-5p;hsa-miR-15b-5p;hsa-miR-16-5p;hsa-miR-195
ENSG0000	ENSG0000	0.000139	hsa-miR-548b-5p;hsa-miR-548c-5p;hsa-miR-548d-5p;hsa-miR
ENSG0000	ENSG0000	4.90E-09	hsa-miR-106a-5p;hsa-miR-106b-5p;hsa-miR-140-3p;hsa-miR-
ENSG0000	ENSG0000	4.95E-06	hsa-miR-106a-5p;hsa-miR-106b-5p;hsa-miR-17-5p;hsa-miR-1
ENSG0000	ENSG0000	0.001909	hsa-miR-103a-3p;hsa-miR-107;hsa-miR-15a-5p;hsa-miR-15b-
ENSG0000	ENSG0000	0.004969	hsa-miR-15a-5p;hsa-miR-15b-5p;hsa-miR-16-5p;hsa-miR-195
ENSG0000	ENSG0000	0.000555	hsa-miR-15a-5p;hsa-miR-15b-5p;hsa-miR-16-5p;hsa-miR-195
ENSG0000	ENSG0000	2.70E-05	hsa-miR-548b-5p;hsa-miR-548c-5p;hsa-miR-548d-5p;hsa-miR
ENSG0000	ENSG0000	2.35E-06	hsa-miR-106a-5p;hsa-miR-106b-5p;hsa-miR-17-5p;hsa-miR-1
ENSG0000	ENSG0000	3.78E-06	hsa-miR-548b-5p;hsa-miR-548c-5p;hsa-miR-548d-5p;hsa-miR
ENSG0000	ENSG0000	0.002254	hsa-miR-106a-5p;hsa-miR-106b-5p;hsa-miR-17-5p;hsa-miR-2
ENSG0000	ENSG0000	0.001525	hsa-miR-106a-5p;hsa-miR-106b-5p;hsa-miR-140-3p;hsa-miR-
ENSG0000	ENSG0000	0.000117	hsa-miR-106a-5p;hsa-miR-106b-5p;hsa-miR-17-5p;hsa-miR-2
ENSG0000	ENSG0000	0.002451	hsa-miR-15a-5p;hsa-miR-15b-5p;hsa-miR-16-5p;hsa-miR-195
ENSG0000	ENSG0000	0.004255	hsa-miR-15a-5p;hsa-miR-15b-5p;hsa-miR-16-5p;hsa-miR-195
ENSG0000	ENSG0000	2.74E-05	hsa-miR-106a-5p;hsa-miR-106b-5p;hsa-miR-17-5p;hsa-miR-2
ENSG0000	ENSG0000	4.75E-06	hsa-miR-548b-5p;hsa-miR-548c-5p;hsa-miR-548d-5p;hsa-miR
ENSG0000	ENSG0000	8.79E-05	hsa-miR-106a-5p;hsa-miR-106b-5p;hsa-miR-17-5p;hsa-miR-2
ENSG0000	ENSG0000	0.000298	hsa-miR-106a-5p;hsa-miR-106b-5p;hsa-miR-17-5p;hsa-miR-2
ENSG0000	ENSG0000	3.74E-05	hsa-miR-548b-5p;hsa-miR-548c-5p;hsa-miR-548d-5p;hsa-miR
ENSG0000	ENSG0000	1.03E-06	hsa-miR-106b-5p;hsa-miR-143-3p;hsa-miR-17-5p;hsa-miR-20
ENSG0000	ENSG0000	2.42E-07	hsa-miR-15a-5p;hsa-miR-15b-5p;hsa-miR-16-5p;hsa-miR-195
ENSG0000	ENSG0000	7.25E-05	hsa-miR-106a-5p;hsa-miR-106b-5p;hsa-miR-17-5p;hsa-miR-1
ENSG0000	ENSG0000	0.00046	hsa-miR-143-3p;hsa-miR-548b-5p;hsa-miR-548c-5p;hsa-miR-
ENSG0000	ENSG0000	0.005089	hsa-miR-548b-5p;hsa-miR-548c-5p;hsa-miR-548d-5p;hsa-miR
ENSG0000	ENSG0000	2.28E-05	hsa-miR-548b-5p;hsa-miR-548c-5p;hsa-miR-548d-5p;hsa-miR
ENSG0000	ENSG0000	0.000234	hsa-miR-15a-5p;hsa-miR-15b-5p;hsa-miR-16-5p;hsa-miR-195
ENSG0000	ENSG0000	0.000154	hsa-miR-548b-5p;hsa-miR-548c-5p;hsa-miR-548d-5p;hsa-miR
ENSG0000	ENSG0000	0.000154	hsa-miR-548b-5p;hsa-miR-548c-5p;hsa-miR-548d-5p;hsa-miR
ENSG0000	ENSG0000	0.000407	hsa-miR-15a-5p;hsa-miR-15b-5p;hsa-miR-16-5p;hsa-miR-195
ENSG0000	ENSG0000	0.000368	hsa-miR-106a-5p;hsa-miR-106b-5p;hsa-miR-17-5p;hsa-miR-1
ENSG0000	ENSG0000	0.002068	hsa-miR-106b-5p;hsa-miR-17-5p;hsa-miR-20a-5p;hsa-miR-20
ENSG0000	ENSG0000	0.001109	hsa-miR-103a-3p;hsa-miR-107;hsa-miR-15a-5p;hsa-miR-15b-
ENSG0000	ENSG0000	0.002536	hsa-miR-15a-5p;hsa-miR-15b-5p;hsa-miR-16-5p;hsa-miR-195
ENSG0000	ENSG0000	1.52E-05	hsa-miR-548b-5p;hsa-miR-548c-5p;hsa-miR-548d-5p;hsa-miR
ENSG0000	ENSG0000	1.36E-05	hsa-miR-106a-5p;hsa-miR-106b-5p;hsa-miR-17-5p;hsa-miR-2
ENSG0000	ENSG0000	0.002254	hsa-miR-106a-5p;hsa-miR-106b-5p;hsa-miR-17-5p;hsa-miR-2
ENSG0000	ENSG0000	1.31E-05	hsa-miR-106a-5p;hsa-miR-106b-5p;hsa-miR-17-5p;hsa-miR-1
ENSG0000	ENSG0000	0.000627	hsa-miR-106a-5p;hsa-miR-106b-5p;hsa-miR-17-5p;hsa-miR-2
ENSG0000	ENSG0000	0.009354	hsa-miR-15a-5p;hsa-miR-15b-5p;hsa-miR-16-5p;hsa-miR-195
ENSG0000	ENSG0000	1.79E-06	hsa-miR-106a-5p;hsa-miR-106b-5p;hsa-miR-17-5p;hsa-miR-2
ENSG0000	ENSG0000	0.00279	hsa-miR-185-5p;hsa-miR-548b-5p;hsa-miR-548c-5p;hsa-miR-
ENSG0000	ENSG0000	0.008697	hsa-miR-15a-5p;hsa-miR-15b-5p;hsa-miR-16-5p;hsa-miR-195
ENSG0000	ENSG0000	2.19E-05	hsa-miR-106a-5p;hsa-miR-106b-5p;hsa-miR-17-5p;hsa-miR-2
ENSG0000	ENSG0000	7.17E-05	hsa-let-7g-3p;hsa-miR-24-3p;hsa-miR-378a-5p;hsa-miR-493-
ENSG0000	ENSG0000	0.000547	hsa-miR-214-5p;hsa-miR-221-5p;hsa-miR-24-3p;hsa-miR-378
ENSG0000	ENSG0000	0.001155	hsa-miR-214-5p;hsa-miR-221-5p;hsa-miR-24-3p;hsa-miR-378

ENSG0000	ENSG0000	8.49E-13	hsa-let-7a-5p;hsa-let-7b-5p;hsa-let-7d-5p;hsa-let-7e-5p;hsa-l
ENSG0000	ENSG0000	4.01E-09	hsa-let-7a-5p;hsa-let-7b-5p;hsa-let-7d-5p;hsa-let-7e-5p;hsa-l
ENSG0000	ENSG0000	4.04E-11	hsa-let-7a-5p;hsa-let-7b-5p;hsa-let-7d-5p;hsa-let-7e-5p;hsa-l
ENSG0000	ENSG0000	1.11E-12	hsa-let-7a-5p;hsa-let-7b-5p;hsa-let-7d-5p;hsa-let-7e-5p;hsa-l
ENSG0000	ENSG0000	2.56E-10	hsa-let-7a-5p;hsa-let-7b-5p;hsa-let-7d-5p;hsa-let-7e-5p;hsa-l
ENSG0000	ENSG0000	2.59E-12	hsa-let-7a-5p;hsa-let-7b-5p;hsa-let-7d-5p;hsa-let-7e-5p;hsa-l
ENSG0000	ENSG0000	4.08E-06	hsa-let-7a-5p;hsa-let-7b-5p;hsa-let-7d-5p;hsa-let-7e-5p;hsa-l
ENSG0000	ENSG0000	1.84E-07	hsa-let-7a-5p;hsa-let-7b-5p;hsa-let-7f-5p;hsa-miR-222-3p
ENSG0000	ENSG0000	7.44E-11	hsa-let-7a-5p;hsa-let-7b-5p;hsa-let-7d-5p;hsa-let-7e-5p;hsa-l
ENSG0000	ENSG0000	7.28E-10	hsa-let-7a-5p;hsa-let-7b-5p;hsa-let-7d-5p;hsa-let-7e-5p;hsa-l
ENSG0000	ENSG0000	0.002578	hsa-let-7a-5p;hsa-let-7b-5p;hsa-miR-221-3p;hsa-miR-222-3p
ENSG0000	ENSG0000	6.33E-10	hsa-let-7a-5p;hsa-let-7b-5p;hsa-let-7d-5p;hsa-let-7e-5p;hsa-l
ENSG0000	ENSG0000	7.44E-11	hsa-let-7a-5p;hsa-let-7b-5p;hsa-let-7d-5p;hsa-let-7e-5p;hsa-l
ENSG0000	ENSG0000	9.98E-08	hsa-let-7a-5p;hsa-let-7b-5p;hsa-let-7d-5p;hsa-let-7e-5p;hsa-l
ENSG0000	ENSG0000	3.25E-12	hsa-let-7a-5p;hsa-let-7b-5p;hsa-let-7d-5p;hsa-let-7e-5p;hsa-l
ENSG0000	ENSG0000	2.15E-10	hsa-let-7a-5p;hsa-let-7d-5p;hsa-let-7e-5p;hsa-let-7f-5p;hsa-le
ENSG0000	ENSG0000	2.94E-12	hsa-let-7a-5p;hsa-let-7b-5p;hsa-let-7d-5p;hsa-let-7e-5p;hsa-l
ENSG0000	ENSG0000	3.10E-11	hsa-let-7a-5p;hsa-let-7b-5p;hsa-let-7d-5p;hsa-let-7e-5p;hsa-l
ENSG0000	ENSG0000	2.12E-07	hsa-let-7a-5p;hsa-let-7e-5p;hsa-let-7f-5p;hsa-let-7g-5p;hsa-le
ENSG0000	ENSG0000	2.38E-12	hsa-let-7a-5p;hsa-let-7b-5p;hsa-let-7d-5p;hsa-let-7e-5p;hsa-l
ENSG0000	ENSG0000	6.33E-10	hsa-let-7a-5p;hsa-let-7b-5p;hsa-let-7d-5p;hsa-let-7e-5p;hsa-l
ENSG0000	ENSG0000	4.53E-07	hsa-let-7a-5p;hsa-let-7d-5p;hsa-let-7e-5p;hsa-let-7f-5p;hsa-le
ENSG0000	ENSG0000	8.35E-10	hsa-let-7a-5p;hsa-let-7b-5p;hsa-let-7d-5p;hsa-let-7e-5p;hsa-l
ENSG0000	ENSG0000	2.88E-09	hsa-let-7a-5p;hsa-let-7b-5p;hsa-let-7d-5p;hsa-let-7e-5p;hsa-l
ENSG0000	ENSG0000	5.83E-12	hsa-let-7a-5p;hsa-let-7b-5p;hsa-let-7d-5p;hsa-let-7e-5p;hsa-l
ENSG0000	ENSG0000	4.17E-08	hsa-let-7a-5p;hsa-let-7b-5p;hsa-let-7d-5p;hsa-let-7e-5p;hsa-l
ENSG0000	ENSG0000	3.87E-08	hsa-let-7a-5p;hsa-let-7b-5p;hsa-let-7d-5p;hsa-let-7e-5p;hsa-l
ENSG0000	ENSG0000	1.96E-09	hsa-let-7a-5p;hsa-let-7b-5p;hsa-let-7d-5p;hsa-let-7e-5p;hsa-l
ENSG0000	ENSG0000	2.56E-10	hsa-let-7a-5p;hsa-let-7b-5p;hsa-let-7d-5p;hsa-let-7e-5p;hsa-l
ENSG0000	ENSG0000	5.50E-09	hsa-let-7a-5p;hsa-let-7b-5p;hsa-let-7d-5p;hsa-let-7e-5p;hsa-l
ENSG0000	ENSG0000	4.03E-06	hsa-let-7a-5p;hsa-let-7d-5p;hsa-let-7e-5p;hsa-let-7f-5p;hsa-le
ENSG0000	ENSG0000	4.81E-09	hsa-let-7a-5p;hsa-let-7b-5p;hsa-let-7d-5p;hsa-let-7f-5p;hsa-le
ENSG0000	ENSG0000	8.66E-15	hsa-let-7a-5p;hsa-let-7b-5p;hsa-let-7d-5p;hsa-let-7e-5p;hsa-l
ENSG0000	ENSG0000	9.18E-08	hsa-let-7a-5p;hsa-let-7b-5p;hsa-let-7d-5p;hsa-let-7e-5p;hsa-l
ENSG0000	ENSG0000	3.06E-08	hsa-let-7a-5p;hsa-let-7b-5p;hsa-let-7d-5p;hsa-let-7e-5p;hsa-l
ENSG0000	ENSG0000	1.00E-11	hsa-let-7a-5p;hsa-let-7b-5p;hsa-let-7d-5p;hsa-let-7e-5p;hsa-l
ENSG0000	ENSG0000	1.31E-09	hsa-let-7d-5p;hsa-let-7f-5p;hsa-let-7g-5p;hsa-let-7i-5p
ENSG0000	ENSG0000	1.37E-09	hsa-let-7a-5p;hsa-let-7b-5p;hsa-let-7d-5p;hsa-let-7e-5p;hsa-l
ENSG0000	ENSG0000	1.44E-08	hsa-let-7a-5p;hsa-let-7b-5p;hsa-let-7e-5p;hsa-let-7f-5p;hsa-le
ENSG0000	ENSG0000	2.94E-12	hsa-let-7a-5p;hsa-let-7b-5p;hsa-let-7d-5p;hsa-let-7e-5p;hsa-l
ENSG0000	ENSG0000	3.87E-13	hsa-let-7a-5p;hsa-let-7b-5p;hsa-let-7d-5p;hsa-let-7e-5p;hsa-l
ENSG0000	ENSG0000	2.52E-13	hsa-let-7a-5p;hsa-let-7b-5p;hsa-let-7d-5p;hsa-let-7e-5p;hsa-l
ENSG0000	ENSG0000	2.38E-12	hsa-let-7a-5p;hsa-let-7b-5p;hsa-let-7d-5p;hsa-let-7e-5p;hsa-l
ENSG0000	ENSG0000	9.32E-06	hsa-let-7a-5p;hsa-let-7b-5p;hsa-let-7d-5p;hsa-let-7e-5p;hsa-l
ENSG0000	ENSG0000	3.73E-06	hsa-let-7a-5p;hsa-let-7d-5p;hsa-let-7e-5p;hsa-let-7f-5p
ENSG0000	ENSG0000	5.41E-05	hsa-let-7a-5p;hsa-let-7d-5p;hsa-let-7e-5p;hsa-let-7g-5p
ENSG0000	ENSG0000	7.44E-11	hsa-let-7a-5p;hsa-let-7b-5p;hsa-let-7d-5p;hsa-let-7e-5p;hsa-l
ENSG0000	ENSG0000	9.75E-10	hsa-let-7a-5p;hsa-let-7b-5p;hsa-let-7d-5p;hsa-let-7e-5p;hsa-l
ENSG0000	ENSG0000	3.25E-12	hsa-let-7a-5p;hsa-let-7b-5p;hsa-let-7d-5p;hsa-let-7e-5p;hsa-l
ENSG0000	ENSG0000	3.25E-12	hsa-let-7a-5p;hsa-let-7b-5p;hsa-let-7d-5p;hsa-let-7e-5p;hsa-l
ENSG0000	ENSG0000	3.25E-12	hsa-let-7a-5p;hsa-let-7b-5p;hsa-let-7d-5p;hsa-let-7e-5p;hsa-l
ENSG0000	ENSG0000	1.29E-11	hsa-let-7a-5p;hsa-let-7b-5p;hsa-let-7d-5p;hsa-let-7e-5p;hsa-l
ENSG0000	ENSG0000	8.49E-13	hsa-let-7a-5p;hsa-let-7b-5p;hsa-let-7d-5p;hsa-let-7e-5p;hsa-l
ENSG0000	ENSG0000	8.49E-13	hsa-let-7a-5p;hsa-let-7b-5p;hsa-let-7d-5p;hsa-let-7e-5p;hsa-l

ENSG0000	ENSG0000	8.49E-13	hsa-let-7a-5p;hsa-let-7b-5p;hsa-let-7d-5p;hsa-let-7e-5p;hsa-l
ENSG0000	ENSG0000	8.49E-13	hsa-let-7a-5p;hsa-let-7b-5p;hsa-let-7d-5p;hsa-let-7e-5p;hsa-l
ENSG0000	ENSG0000	8.49E-13	hsa-let-7a-5p;hsa-let-7b-5p;hsa-let-7d-5p;hsa-let-7e-5p;hsa-l
ENSG0000	ENSG0000	8.49E-13	hsa-let-7a-5p;hsa-let-7b-5p;hsa-let-7d-5p;hsa-let-7e-5p;hsa-l
ENSG0000	ENSG0000	8.49E-13	hsa-let-7a-5p;hsa-let-7b-5p;hsa-let-7d-5p;hsa-let-7e-5p;hsa-l
ENSG0000	ENSG0000	1.77E-07	hsa-let-7a-5p;hsa-let-7b-5p;hsa-let-7d-5p;hsa-let-7e-5p;hsa-l
ENSG0000	ENSG0000	5.17E-05	hsa-let-7a-5p;hsa-let-7d-5p;hsa-let-7e-5p;hsa-let-7f-5p;hsa-le
ENSG0000	ENSG0000	3.22E-09	hsa-let-7a-5p;hsa-let-7b-5p;hsa-let-7d-5p;hsa-let-7e-5p;hsa-l
ENSG0000	ENSG0000	2.57E-09	hsa-let-7a-5p;hsa-let-7b-5p;hsa-let-7d-5p;hsa-let-7e-5p;hsa-l
ENSG0000	ENSG0000	4.18E-07	hsa-let-7a-5p;hsa-let-7b-5p;hsa-let-7e-5p;hsa-let-7f-5p;hsa-le
ENSG0000	ENSG0000	2.94E-12	hsa-let-7a-5p;hsa-let-7b-5p;hsa-let-7d-5p;hsa-let-7e-5p;hsa-l
ENSG0000	ENSG0000	2.82E-08	hsa-let-7a-5p;hsa-let-7b-5p;hsa-let-7d-5p;hsa-let-7e-5p;hsa-l
ENSG0000	ENSG0000	3.10E-11	hsa-let-7a-5p;hsa-let-7b-5p;hsa-let-7d-5p;hsa-let-7e-5p;hsa-l
ENSG0000	ENSG0000	3.79E-07	hsa-let-7a-5p;hsa-let-7d-5p;hsa-let-7f-5p;hsa-let-7g-5p;hsa-le
ENSG0000	ENSG0000	3.17E-10	hsa-let-7a-5p;hsa-let-7b-5p;hsa-let-7d-5p;hsa-let-7e-5p;hsa-l
ENSG0000	ENSG0000	3.66E-13	hsa-let-7a-5p;hsa-let-7b-5p;hsa-let-7d-5p;hsa-let-7e-5p;hsa-l
ENSG0000	ENSG0000	1.26E-09	hsa-let-7a-5p;hsa-let-7b-5p;hsa-let-7d-5p;hsa-let-7e-5p;hsa-l
ENSG0000	ENSG0000	2.03E-09	hsa-let-7a-5p;hsa-let-7b-5p;hsa-let-7d-5p;hsa-let-7e-5p;hsa-l
ENSG0000	ENSG0000	1.72E-12	hsa-let-7a-5p;hsa-let-7b-5p;hsa-let-7d-5p;hsa-let-7e-5p;hsa-l
ENSG0000	ENSG0000	5.78E-08	hsa-let-7a-5p;hsa-let-7d-5p;hsa-let-7e-5p;hsa-let-7f-5p;hsa-le
ENSG0000	ENSG0000	2.51E-06	hsa-let-7a-5p;hsa-let-7b-5p;hsa-let-7d-5p;hsa-let-7e-5p;hsa-l
ENSG0000	ENSG0000	2.51E-06	hsa-let-7a-5p;hsa-let-7b-5p;hsa-let-7d-5p;hsa-let-7e-5p;hsa-l
ENSG0000	ENSG0000	2.70E-11	hsa-let-7a-5p;hsa-let-7b-5p;hsa-let-7d-5p;hsa-let-7e-5p;hsa-l
ENSG0000	ENSG0000	3.22E-09	hsa-let-7a-5p;hsa-let-7b-5p;hsa-let-7d-5p;hsa-let-7e-5p;hsa-l
ENSG0000	ENSG0000	2.56E-10	hsa-let-7a-5p;hsa-let-7b-5p;hsa-let-7d-5p;hsa-let-7e-5p;hsa-l
ENSG0000	ENSG0000	1.69E-10	hsa-let-7a-5p;hsa-let-7b-5p;hsa-let-7d-5p;hsa-let-7e-5p;hsa-l
ENSG0000	ENSG0000	1.69E-10	hsa-let-7a-5p;hsa-let-7b-5p;hsa-let-7d-5p;hsa-let-7e-5p;hsa-l
ENSG0000	ENSG0000	3.87E-13	hsa-let-7a-5p;hsa-let-7b-5p;hsa-let-7d-5p;hsa-let-7e-5p;hsa-l
ENSG0000	ENSG0000	1.80E-09	hsa-let-7a-5p;hsa-let-7b-5p;hsa-let-7d-5p;hsa-let-7e-5p;hsa-l
ENSG0000	ENSG0000	0.000436	hsa-let-7b-5p;hsa-let-7e-5p;hsa-let-7f-5p;hsa-let-7i-5p
ENSG0000	ENSG0000	2.29E-09	hsa-let-7a-5p;hsa-let-7b-5p;hsa-let-7d-5p;hsa-let-7e-5p;hsa-l
ENSG0000	ENSG0000	5.68E-06	hsa-let-7a-5p;hsa-let-7b-5p;hsa-let-7d-5p;hsa-let-7e-5p;hsa-l
ENSG0000	ENSG0000	2.65E-07	hsa-let-7a-5p;hsa-let-7b-5p;hsa-let-7d-5p;hsa-let-7e-5p;hsa-l
ENSG0000	ENSG0000	3.27E-11	hsa-let-7a-5p;hsa-let-7b-5p;hsa-let-7d-5p;hsa-let-7e-5p;hsa-l
ENSG0000	ENSG0000	5.28E-06	hsa-let-7a-5p;hsa-let-7d-5p;hsa-let-7e-5p;hsa-let-7f-5p;hsa-le
ENSG0000	ENSG0000	6.73E-09	hsa-let-7a-5p;hsa-let-7b-5p;hsa-let-7d-5p;hsa-let-7e-5p;hsa-l
ENSG0000	ENSG0000	5.48E-10	hsa-let-7a-5p;hsa-let-7b-5p;hsa-let-7d-5p;hsa-let-7e-5p;hsa-l
ENSG0000	ENSG0000	1.89E-09	hsa-let-7a-5p;hsa-let-7b-5p;hsa-let-7e-5p;hsa-let-7f-5p;hsa-le
ENSG0000	ENSG0000	1.55E-10	hsa-let-7a-5p;hsa-let-7b-5p;hsa-let-7d-5p;hsa-let-7e-5p;hsa-l
ENSG0000	ENSG0000	9.28E-07	hsa-let-7a-5p;hsa-let-7b-5p;hsa-let-7d-5p;hsa-let-7e-5p;hsa-l
ENSG0000	ENSG0000	2.79E-05	hsa-let-7a-5p;hsa-let-7b-5p;hsa-let-7d-5p;hsa-let-7e-5p;hsa-l
ENSG0000	ENSG0000	2.56E-10	hsa-let-7a-5p;hsa-let-7b-5p;hsa-let-7d-5p;hsa-let-7e-5p;hsa-l
ENSG0000	ENSG0000	1.36E-07	hsa-let-7a-5p;hsa-let-7d-5p;hsa-let-7e-5p;hsa-let-7f-5p;hsa-le
ENSG0000	ENSG0000	4.95E-09	hsa-let-7a-5p;hsa-let-7b-5p;hsa-let-7d-5p;hsa-let-7e-5p;hsa-l
ENSG0000	ENSG0000	2.59E-12	hsa-let-7a-5p;hsa-let-7b-5p;hsa-let-7d-5p;hsa-let-7e-5p;hsa-l
ENSG0000	ENSG0000	6.11E-11	hsa-let-7a-5p;hsa-let-7b-5p;hsa-let-7d-5p;hsa-let-7e-5p;hsa-l
ENSG0000	ENSG0000	9.00E-11	hsa-let-7a-5p;hsa-let-7b-5p;hsa-let-7d-5p;hsa-let-7e-5p;hsa-l
ENSG0000	ENSG0000	7.11E-15	hsa-let-7a-5p;hsa-let-7b-5p;hsa-let-7d-5p;hsa-let-7e-5p;hsa-l
ENSG0000	ENSG0000	4.25E-06	hsa-let-7a-5p;hsa-let-7d-5p;hsa-let-7e-5p;hsa-let-7f-5p;hsa-le
ENSG0000	ENSG0000	1.80E-09	hsa-let-7a-5p;hsa-let-7b-5p;hsa-let-7d-5p;hsa-let-7e-5p;hsa-l
ENSG0000	ENSG0000	1.09E-09	hsa-let-7a-5p;hsa-let-7b-5p;hsa-let-7d-5p;hsa-let-7e-5p;hsa-l
ENSG0000	ENSG0000	6.09E-09	hsa-let-7a-5p;hsa-let-7b-5p;hsa-let-7d-5p;hsa-let-7e-5p;hsa-l
ENSG0000	ENSG0000	7.59E-08	hsa-let-7a-5p;hsa-let-7e-5p;hsa-let-7f-5p;hsa-let-7g-5p;hsa-le
ENSG0000	ENSG0000	5.75E-14	hsa-let-7a-5p;hsa-let-7b-5p;hsa-let-7d-5p;hsa-let-7e-5p;hsa-l

ENSG0000	ENSG0000	2.49E-05	hsa-let-7a-5p;hsa-let-7d-5p;hsa-let-7f-5p;hsa-let-7g-5p;hsa-le
ENSG0000	ENSG0000	2.29E-09	hsa-let-7a-5p;hsa-let-7b-5p;hsa-let-7d-5p;hsa-let-7e-5p;hsa-l
ENSG0000	ENSG0000	2.04E-11	hsa-let-7a-5p;hsa-let-7b-5p;hsa-let-7d-5p;hsa-let-7e-5p;hsa-l
ENSG0000	ENSG0000	2.04E-11	hsa-let-7a-5p;hsa-let-7b-5p;hsa-let-7d-5p;hsa-let-7e-5p;hsa-l
ENSG0000	ENSG0000	2.56E-10	hsa-let-7a-5p;hsa-let-7b-5p;hsa-let-7d-5p;hsa-let-7e-5p;hsa-l
ENSG0000	ENSG0000	9.64E-12	hsa-let-7a-5p;hsa-let-7b-5p;hsa-let-7d-5p;hsa-let-7e-5p;hsa-l
ENSG0000	ENSG0000	3.25E-12	hsa-let-7a-5p;hsa-let-7b-5p;hsa-let-7d-5p;hsa-let-7e-5p;hsa-l
ENSG0000	ENSG0000	3.07E-08	hsa-let-7a-5p;hsa-let-7b-5p;hsa-let-7d-5p;hsa-let-7e-5p;hsa-l
ENSG0000	ENSG0000	5.48E-10	hsa-let-7a-5p;hsa-let-7b-5p;hsa-let-7d-5p;hsa-let-7e-5p;hsa-l
ENSG0000	ENSG0000	1.10E-13	hsa-let-7a-5p;hsa-let-7b-5p;hsa-let-7d-5p;hsa-let-7e-5p;hsa-l
ENSG0000	ENSG0000	1.22E-12	hsa-let-7a-5p;hsa-let-7b-5p;hsa-let-7d-5p;hsa-let-7e-5p;hsa-l
ENSG0000	ENSG0000	1.25E-09	hsa-let-7a-5p;hsa-let-7b-5p;hsa-let-7e-5p;hsa-let-7f-5p;hsa-le
ENSG0000	ENSG0000	7.44E-11	hsa-let-7a-5p;hsa-let-7b-5p;hsa-let-7d-5p;hsa-let-7e-5p;hsa-l
ENSG0000	ENSG0000	1.24E-09	hsa-let-7a-5p;hsa-let-7b-5p;hsa-let-7d-5p;hsa-let-7e-5p;hsa-l
ENSG0000	ENSG0000	3.31E-08	hsa-let-7a-5p;hsa-let-7b-5p;hsa-let-7d-5p;hsa-let-7e-5p;hsa-l
ENSG0000	ENSG0000	2.03E-09	hsa-let-7a-5p;hsa-let-7b-5p;hsa-let-7d-5p;hsa-let-7e-5p;hsa-l
ENSG0000	ENSG0000	3.24E-05	hsa-let-7a-5p;hsa-let-7f-5p;hsa-let-7g-5p;hsa-let-7i-5p
ENSG0000	ENSG0000	5.48E-06	hsa-let-7a-5p;hsa-let-7b-5p;hsa-let-7d-5p;hsa-let-7e-5p;hsa-l
ENSG0000	ENSG0000	7.47E-08	hsa-let-7a-5p;hsa-let-7b-5p;hsa-let-7d-5p;hsa-let-7e-5p;hsa-l
ENSG0000	ENSG0000	7.47E-08	hsa-let-7a-5p;hsa-let-7b-5p;hsa-let-7d-5p;hsa-let-7e-5p;hsa-l
ENSG0000	ENSG0000	7.79E-06	hsa-let-7a-5p;hsa-let-7b-5p;hsa-let-7d-5p;hsa-let-7e-5p;hsa-l
ENSG0000	ENSG0000	3.25E-12	hsa-let-7a-5p;hsa-let-7b-5p;hsa-let-7d-5p;hsa-let-7e-5p;hsa-l
ENSG0000	ENSG0000	5.83E-12	hsa-let-7a-5p;hsa-let-7b-5p;hsa-let-7d-5p;hsa-let-7e-5p;hsa-l
ENSG0000	ENSG0000	5.83E-12	hsa-let-7a-5p;hsa-let-7b-5p;hsa-let-7d-5p;hsa-let-7e-5p;hsa-l
ENSG0000	ENSG0000	1.62E-08	hsa-let-7a-5p;hsa-let-7b-5p;hsa-let-7d-5p;hsa-let-7e-5p;hsa-l
ENSG0000	ENSG0000	4.74E-10	hsa-let-7a-5p;hsa-let-7b-5p;hsa-let-7d-5p;hsa-let-7e-5p;hsa-l
ENSG0000	ENSG0000	4.18E-07	hsa-let-7b-5p;hsa-let-7d-5p;hsa-let-7e-5p;hsa-let-7f-5p;hsa-le
ENSG0000	ENSG0000	1.80E-09	hsa-let-7a-5p;hsa-let-7b-5p;hsa-let-7d-5p;hsa-let-7e-5p;hsa-l
ENSG0000	ENSG0000	3.17E-10	hsa-let-7a-5p;hsa-let-7b-5p;hsa-let-7d-5p;hsa-let-7e-5p;hsa-l
ENSG0000	ENSG0000	4.50E-08	hsa-let-7a-5p;hsa-let-7b-5p;hsa-let-7d-5p;hsa-let-7e-5p;hsa-l
ENSG0000	ENSG0000	1.29E-11	hsa-let-7a-5p;hsa-let-7b-5p;hsa-let-7d-5p;hsa-let-7e-5p;hsa-l
ENSG0000	ENSG0000	7.43E-09	hsa-let-7a-5p;hsa-let-7b-5p;hsa-let-7d-5p;hsa-let-7f-5p;hsa-le
ENSG0000	ENSG0000	6.19E-09	hsa-let-7a-5p;hsa-let-7b-5p;hsa-let-7d-5p;hsa-let-7e-5p;hsa-l
ENSG0000	ENSG0000	2.03E-09	hsa-let-7a-5p;hsa-let-7b-5p;hsa-let-7d-5p;hsa-let-7e-5p;hsa-l
ENSG0000	ENSG0000	3.17E-10	hsa-let-7a-5p;hsa-let-7b-5p;hsa-let-7d-5p;hsa-let-7e-5p;hsa-l
ENSG0000	ENSG0000	5.41E-05	hsa-let-7a-5p;hsa-let-7d-5p;hsa-miR-221-3p;hsa-miR-222-3p
ENSG0000	ENSG0000	0.000162	hsa-let-7a-5p;hsa-let-7d-5p;hsa-let-7f-5p;hsa-let-7g-5p;hsa-le
ENSG0000	ENSG0000	5.43E-06	hsa-let-7d-5p;hsa-let-7e-5p;hsa-let-7f-5p;hsa-let-7g-5p;hsa-le
ENSG0000	ENSG0000	5.43E-06	hsa-let-7d-5p;hsa-let-7e-5p;hsa-let-7f-5p;hsa-let-7g-5p;hsa-le
ENSG0000	ENSG0000	2.65E-07	hsa-let-7a-5p;hsa-let-7b-5p;hsa-let-7d-5p;hsa-let-7e-5p;hsa-l
ENSG0000	ENSG0000	1.80E-09	hsa-let-7a-5p;hsa-let-7b-5p;hsa-let-7d-5p;hsa-let-7e-5p;hsa-l
ENSG0000	ENSG0000	4.08E-10	hsa-let-7a-5p;hsa-let-7b-5p;hsa-let-7d-5p;hsa-let-7e-5p;hsa-l
ENSG0000	ENSG0000	2.54E-07	hsa-let-7a-5p;hsa-let-7d-5p;hsa-let-7e-5p;hsa-let-7f-5p;hsa-le
ENSG0000	ENSG0000	3.56E-07	hsa-let-7d-5p;hsa-let-7e-5p;hsa-let-7f-5p;hsa-let-7g-5p;hsa-le
ENSG0000	ENSG0000	0.000183	hsa-miR-221-3p;hsa-miR-222-3p;hsa-miR-574-5p;hsa-miR-665
ENSG0000	ENSG0000	0.000183	hsa-miR-221-3p;hsa-miR-222-3p;hsa-miR-574-5p;hsa-miR-665
ENSG0000	ENSG0000	1.84E-10	hsa-let-7a-5p;hsa-let-7b-5p;hsa-let-7d-5p;hsa-let-7e-5p;hsa-l
ENSG0000	ENSG0000	1.84E-10	hsa-let-7a-5p;hsa-let-7b-5p;hsa-let-7d-5p;hsa-let-7e-5p;hsa-l
ENSG0000	ENSG0000	1.84E-10	hsa-let-7a-5p;hsa-let-7b-5p;hsa-let-7d-5p;hsa-let-7e-5p;hsa-l
ENSG0000	ENSG0000	2.56E-10	hsa-let-7a-5p;hsa-let-7b-5p;hsa-let-7d-5p;hsa-let-7e-5p;hsa-l
ENSG0000	ENSG0000	1.71E-08	hsa-let-7a-5p;hsa-let-7b-5p;hsa-let-7d-5p;hsa-let-7e-5p;hsa-l
ENSG0000	ENSG0000	2.62E-11	hsa-let-7a-5p;hsa-let-7b-5p;hsa-let-7d-5p;hsa-let-7e-5p;hsa-l
ENSG0000	ENSG0000	2.09E-11	hsa-let-7a-5p;hsa-let-7b-5p;hsa-let-7d-5p;hsa-let-7e-5p;hsa-l
ENSG0000	ENSG0000	1.66E-08	hsa-let-7a-5p;hsa-let-7d-5p;hsa-let-7e-5p;hsa-let-7f-5p;hsa-le

ENSG0000	ENSG0000	4.36E-07	hsa-let-7a-5p;hsa-let-7b-5p;hsa-let-7d-5p;hsa-let-7e-5p;hsa-l
ENSG0000	ENSG0000	4.36E-07	hsa-let-7a-5p;hsa-let-7b-5p;hsa-let-7d-5p;hsa-let-7e-5p;hsa-l
ENSG0000	ENSG0000	2.48E-08	hsa-let-7a-5p;hsa-let-7d-5p;hsa-let-7e-5p;hsa-let-7f-5p;hsa-le
ENSG0000	ENSG0000	9.15E-08	hsa-let-7a-5p;hsa-let-7e-5p;hsa-let-7f-5p;hsa-let-7g-5p;hsa-le
ENSG0000	ENSG0000	3.87E-13	hsa-let-7a-5p;hsa-let-7b-5p;hsa-let-7d-5p;hsa-let-7e-5p;hsa-l
ENSG0000	ENSG0000	1.65E-11	hsa-let-7a-5p;hsa-let-7b-5p;hsa-let-7d-5p;hsa-let-7e-5p;hsa-l
ENSG0000	ENSG0000	3.52E-12	hsa-let-7a-5p;hsa-let-7b-5p;hsa-let-7d-5p;hsa-let-7e-5p;hsa-l
ENSG0000	ENSG0000	1.24E-09	hsa-let-7a-5p;hsa-let-7b-5p;hsa-let-7d-5p;hsa-let-7e-5p;hsa-l
ENSG0000	ENSG0000	1.24E-09	hsa-let-7a-5p;hsa-let-7b-5p;hsa-let-7d-5p;hsa-let-7e-5p;hsa-l
ENSG0000	ENSG0000	2.62E-11	hsa-let-7a-5p;hsa-let-7b-5p;hsa-let-7d-5p;hsa-let-7e-5p;hsa-l
ENSG0000	ENSG0000	4.06E-11	hsa-let-7a-5p;hsa-let-7b-5p;hsa-let-7d-5p;hsa-let-7e-5p;hsa-l
ENSG0000	ENSG0000	5.28E-07	hsa-let-7a-5p;hsa-let-7b-5p;hsa-let-7d-5p;hsa-let-7f-5p;hsa-le
ENSG0000	ENSG0000	1.31E-08	hsa-let-7a-5p;hsa-let-7b-5p;hsa-let-7d-5p;hsa-let-7e-5p;hsa-l
ENSG0000	ENSG0000	3.20E-08	hsa-let-7a-5p;hsa-let-7b-5p;hsa-let-7e-5p;hsa-let-7f-5p;hsa-le
ENSG0000	ENSG0000	3.00E-10	hsa-let-7a-5p;hsa-let-7b-5p;hsa-let-7d-5p;hsa-let-7e-5p;hsa-l
ENSG0000	ENSG0000	1.08E-10	hsa-let-7a-5p;hsa-let-7b-5p;hsa-let-7d-5p;hsa-let-7f-5p;hsa-le
ENSG0000	ENSG0000	4.74E-10	hsa-let-7a-5p;hsa-let-7b-5p;hsa-let-7d-5p;hsa-let-7e-5p;hsa-l
ENSG0000	ENSG0000	4.68E-09	hsa-let-7a-5p;hsa-let-7b-5p;hsa-let-7d-5p;hsa-let-7e-5p;hsa-l
ENSG0000	ENSG0000	4.68E-09	hsa-let-7a-5p;hsa-let-7b-5p;hsa-let-7d-5p;hsa-let-7e-5p;hsa-l
ENSG0000	ENSG0000	5.80E-13	hsa-let-7a-5p;hsa-let-7b-5p;hsa-let-7d-5p;hsa-let-7e-5p;hsa-l
ENSG0000	ENSG0000	5.75E-14	hsa-let-7a-5p;hsa-let-7b-5p;hsa-let-7d-5p;hsa-let-7e-5p;hsa-l
ENSG0000	ENSG0000	2.29E-09	hsa-let-7a-5p;hsa-let-7b-5p;hsa-let-7d-5p;hsa-let-7e-5p;hsa-l
ENSG0000	ENSG0000	1.69E-10	hsa-let-7a-5p;hsa-let-7b-5p;hsa-let-7d-5p;hsa-let-7e-5p;hsa-l
ENSG0000	ENSG0000	1.41E-09	hsa-let-7a-5p;hsa-let-7b-5p;hsa-let-7d-5p;hsa-let-7e-5p;hsa-l
ENSG0000	ENSG0000	4.06E-09	hsa-let-7a-5p;hsa-let-7b-5p;hsa-let-7d-5p;hsa-let-7e-5p;hsa-l
ENSG0000	ENSG0000	8.17E-10	hsa-let-7a-5p;hsa-let-7b-5p;hsa-let-7d-5p;hsa-let-7e-5p;hsa-l
ENSG0000	ENSG0000	6.76E-08	hsa-let-7a-5p;hsa-let-7b-5p;hsa-let-7d-5p;hsa-let-7e-5p;hsa-l
ENSG0000	ENSG0000	2.99E-06	hsa-let-7a-5p;hsa-let-7b-5p;hsa-let-7d-5p;hsa-let-7e-5p;hsa-l
ENSG0000	ENSG0000	2.62E-11	hsa-let-7a-5p;hsa-let-7b-5p;hsa-let-7d-5p;hsa-let-7e-5p;hsa-l
ENSG0000	ENSG0000	9.77E-14	hsa-let-7a-5p;hsa-let-7b-5p;hsa-let-7d-5p;hsa-let-7e-5p;hsa-l
ENSG0000	ENSG0000	5.50E-09	hsa-let-7a-5p;hsa-let-7b-5p;hsa-let-7d-5p;hsa-let-7e-5p;hsa-l
ENSG0000	ENSG0000	2.09E-11	hsa-let-7a-5p;hsa-let-7b-5p;hsa-let-7d-5p;hsa-let-7e-5p;hsa-l
ENSG0000	ENSG0000	2.17E-10	hsa-let-7a-5p;hsa-let-7b-5p;hsa-let-7d-5p;hsa-let-7e-5p;hsa-l
ENSG0000	ENSG0000	1.72E-12	hsa-let-7a-5p;hsa-let-7b-5p;hsa-let-7d-5p;hsa-let-7e-5p;hsa-l
ENSG0000	ENSG0000	5.83E-12	hsa-let-7a-5p;hsa-let-7b-5p;hsa-let-7d-5p;hsa-let-7e-5p;hsa-l
ENSG0000	ENSG0000	5.83E-12	hsa-let-7a-5p;hsa-let-7b-5p;hsa-let-7d-5p;hsa-let-7e-5p;hsa-l
ENSG0000	ENSG0000	1.35E-08	hsa-let-7a-5p;hsa-let-7b-5p;hsa-let-7d-5p;hsa-let-7e-5p;hsa-l
ENSG0000	ENSG0000	3.27E-11	hsa-let-7a-5p;hsa-let-7b-5p;hsa-let-7d-5p;hsa-let-7e-5p;hsa-l
ENSG0000	ENSG0000	9.02E-09	hsa-let-7a-5p;hsa-let-7b-5p;hsa-let-7d-5p;hsa-let-7e-5p;hsa-l
ENSG0000	ENSG0000	3.06E-08	hsa-let-7a-5p;hsa-let-7b-5p;hsa-let-7d-5p;hsa-let-7e-5p;hsa-l
ENSG0000	ENSG0000	2.86E-10	hsa-let-7a-5p;hsa-let-7b-5p;hsa-let-7e-5p;hsa-let-7f-5p;hsa-le
ENSG0000	ENSG0000	6.09E-09	hsa-let-7a-5p;hsa-let-7b-5p;hsa-let-7d-5p;hsa-let-7e-5p;hsa-l
ENSG0000	ENSG0000	6.09E-09	hsa-let-7a-5p;hsa-let-7b-5p;hsa-let-7d-5p;hsa-let-7e-5p;hsa-l
ENSG0000	ENSG0000	3.30E-07	hsa-let-7d-5p;hsa-let-7e-5p;hsa-let-7f-5p;hsa-let-7g-5p;hsa-le
ENSG0000	ENSG0000	1.22E-12	hsa-let-7a-5p;hsa-let-7b-5p;hsa-let-7d-5p;hsa-let-7e-5p;hsa-l
ENSG0000	ENSG0000	4.04E-09	hsa-let-7a-5p;hsa-let-7b-5p;hsa-let-7e-5p;hsa-let-7f-5p;hsa-le
ENSG0000	ENSG0000	2.80E-09	hsa-let-7b-5p;hsa-let-7d-5p;hsa-let-7e-5p;hsa-let-7f-5p;hsa-le
ENSG0000	ENSG0000	5.75E-14	hsa-let-7a-5p;hsa-let-7b-5p;hsa-let-7d-5p;hsa-let-7e-5p;hsa-l
ENSG0000	ENSG0000	8.49E-13	hsa-let-7a-5p;hsa-let-7b-5p;hsa-let-7d-5p;hsa-let-7e-5p;hsa-l
ENSG0000	ENSG0000	8.49E-13	hsa-let-7a-5p;hsa-let-7b-5p;hsa-let-7d-5p;hsa-let-7e-5p;hsa-l
ENSG0000	ENSG0000	1.80E-09	hsa-let-7a-5p;hsa-let-7b-5p;hsa-let-7d-5p;hsa-let-7e-5p;hsa-l
ENSG0000	ENSG0000	4.85E-08	hsa-let-7a-5p;hsa-let-7b-5p;hsa-let-7d-5p;hsa-let-7e-5p;hsa-l
ENSG0000	ENSG0000	1.41E-09	hsa-let-7a-5p;hsa-let-7b-5p;hsa-let-7d-5p;hsa-let-7e-5p;hsa-l
ENSG0000	ENSG0000	3.87E-08	hsa-let-7a-5p;hsa-let-7b-5p;hsa-let-7d-5p;hsa-let-7e-5p;hsa-l

ENSG0000	ENSG0000	7.58E-11	hsa-let-7a-5p;hsa-let-7b-5p;hsa-let-7d-5p;hsa-let-7e-5p;hsa-l
ENSG0000	ENSG0000	1.08E-06	hsa-let-7a-5p;hsa-let-7d-5p;hsa-let-7e-5p;hsa-let-7f-5p;hsa-le
ENSG0000	ENSG0000	3.22E-09	hsa-let-7a-5p;hsa-let-7b-5p;hsa-let-7d-5p;hsa-let-7e-5p;hsa-l
ENSG0000	ENSG0000	4.06E-11	hsa-let-7a-5p;hsa-let-7b-5p;hsa-let-7d-5p;hsa-let-7e-5p;hsa-l
ENSG0000	ENSG0000	4.06E-09	hsa-let-7a-5p;hsa-let-7b-5p;hsa-let-7d-5p;hsa-let-7e-5p;hsa-l
ENSG0000	ENSG0000	3.58E-08	hsa-let-7a-5p;hsa-let-7b-5p;hsa-let-7d-5p;hsa-let-7e-5p;hsa-l
ENSG0000	ENSG0000	3.01E-07	hsa-let-7a-5p;hsa-let-7d-5p;hsa-let-7e-5p;hsa-let-7f-5p;hsa-le
ENSG0000	ENSG0000	4.64E-14	hsa-let-7a-5p;hsa-let-7b-5p;hsa-let-7d-5p;hsa-let-7e-5p;hsa-l
ENSG0000	ENSG0000	2.09E-11	hsa-let-7a-5p;hsa-let-7b-5p;hsa-let-7d-5p;hsa-let-7e-5p;hsa-l
ENSG0000	ENSG0000	3.27E-11	hsa-let-7a-5p;hsa-let-7b-5p;hsa-let-7d-5p;hsa-let-7e-5p;hsa-l
ENSG0000	ENSG0000	2.56E-10	hsa-let-7a-5p;hsa-let-7b-5p;hsa-let-7d-5p;hsa-let-7e-5p;hsa-l
ENSG0000	ENSG0000	2.35E-06	hsa-let-7a-5p;hsa-let-7b-5p;hsa-let-7d-5p;hsa-let-7e-5p;hsa-l
ENSG0000	ENSG0000	2.35E-11	hsa-let-7a-5p;hsa-let-7b-5p;hsa-let-7d-5p;hsa-let-7e-5p;hsa-l
ENSG0000	ENSG0000	7.55E-14	hsa-let-7a-5p;hsa-let-7b-5p;hsa-let-7d-5p;hsa-let-7e-5p;hsa-l
ENSG0000	ENSG0000	2.70E-11	hsa-let-7a-5p;hsa-let-7b-5p;hsa-let-7e-5p;hsa-let-7f-5p;hsa-le
ENSG0000	ENSG0000	1.08E-10	hsa-let-7a-5p;hsa-let-7b-5p;hsa-let-7d-5p;hsa-let-7e-5p;hsa-l
ENSG0000	ENSG0000	2.53E-06	hsa-let-7b-5p;hsa-let-7e-5p;hsa-let-7f-5p;hsa-let-7g-5p;hsa-le
ENSG0000	ENSG0000	4.17E-08	hsa-let-7a-5p;hsa-let-7b-5p;hsa-let-7d-5p;hsa-let-7e-5p;hsa-l
ENSG0000	ENSG0000	6.97E-08	hsa-let-7a-5p;hsa-let-7b-5p;hsa-let-7d-5p;hsa-let-7e-5p;hsa-l
ENSG0000	ENSG0000	8.00E-10	hsa-let-7a-5p;hsa-let-7b-5p;hsa-let-7d-5p;hsa-let-7e-5p;hsa-l
ENSG0000	ENSG0000	5.80E-13	hsa-let-7a-5p;hsa-let-7b-5p;hsa-let-7d-5p;hsa-let-7e-5p;hsa-l
ENSG0000	ENSG0000	3.22E-09	hsa-let-7a-5p;hsa-let-7b-5p;hsa-let-7d-5p;hsa-let-7e-5p;hsa-l
ENSG0000	ENSG0000	2.87E-10	hsa-let-7a-5p;hsa-let-7b-5p;hsa-let-7d-5p;hsa-let-7e-5p;hsa-l
ENSG0000	ENSG0000	2.39E-10	hsa-let-7a-5p;hsa-let-7b-5p;hsa-let-7d-5p;hsa-let-7e-5p;hsa-l
ENSG0000	ENSG0000	3.20E-08	hsa-let-7a-5p;hsa-let-7b-5p;hsa-let-7e-5p;hsa-let-7f-5p;hsa-le
ENSG0000	ENSG0000	1.42E-10	hsa-let-7a-5p;hsa-let-7b-5p;hsa-let-7d-5p;hsa-let-7e-5p;hsa-l
ENSG0000	ENSG0000	2.88E-09	hsa-let-7a-5p;hsa-let-7b-5p;hsa-let-7d-5p;hsa-let-7e-5p;hsa-l
ENSG0000	ENSG0000	9.00E-11	hsa-let-7a-5p;hsa-let-7b-5p;hsa-let-7d-5p;hsa-let-7e-5p;hsa-l
ENSG0000	ENSG0000	6.08E-06	hsa-let-7a-5p;hsa-let-7d-5p;hsa-let-7e-5p;hsa-let-7f-5p;hsa-le
ENSG0000	ENSG0000	4.95E-09	hsa-let-7a-5p;hsa-let-7b-5p;hsa-let-7d-5p;hsa-let-7e-5p;hsa-l
ENSG0000	ENSG0000	7.43E-09	hsa-let-7a-5p;hsa-let-7b-5p;hsa-let-7d-5p;hsa-let-7e-5p;hsa-l
ENSG0000	ENSG0000	1.45E-07	hsa-let-7a-5p;hsa-let-7b-5p;hsa-let-7d-5p;hsa-let-7e-5p;hsa-l
ENSG0000	ENSG0000	2.38E-12	hsa-let-7a-5p;hsa-let-7b-5p;hsa-let-7d-5p;hsa-let-7e-5p;hsa-l
ENSG0000	ENSG0000	2.52E-13	hsa-let-7a-5p;hsa-let-7b-5p;hsa-let-7d-5p;hsa-let-7e-5p;hsa-l
ENSG0000	ENSG0000	6.83E-10	hsa-let-7a-5p;hsa-let-7b-5p;hsa-let-7d-5p;hsa-let-7e-5p;hsa-l
ENSG0000	ENSG0000	1.12E-07	hsa-let-7a-5p;hsa-let-7b-5p;hsa-let-7d-5p;hsa-let-7e-5p;hsa-l
ENSG0000	ENSG0000	2.80E-09	hsa-let-7a-5p;hsa-let-7b-5p;hsa-let-7d-5p;hsa-let-7e-5p;hsa-l
ENSG0000	ENSG0000	1.59E-09	hsa-let-7a-5p;hsa-let-7b-5p;hsa-let-7d-5p;hsa-let-7e-5p;hsa-l
ENSG0000	ENSG0000	4.85E-08	hsa-let-7a-5p;hsa-let-7b-5p;hsa-let-7d-5p;hsa-let-7e-5p;hsa-l
ENSG0000	ENSG0000	7.47E-08	hsa-let-7a-5p;hsa-let-7b-5p;hsa-let-7d-5p;hsa-let-7e-5p;hsa-l
ENSG0000	ENSG0000	1.22E-12	hsa-let-7a-5p;hsa-let-7b-5p;hsa-let-7d-5p;hsa-let-7e-5p;hsa-l
ENSG0000	ENSG0000	1.08E-10	hsa-let-7a-5p;hsa-let-7b-5p;hsa-let-7d-5p;hsa-let-7e-5p;hsa-l
ENSG0000	ENSG0000	5.22E-08	hsa-let-7a-5p;hsa-let-7b-5p;hsa-let-7d-5p;hsa-let-7e-5p;hsa-l
ENSG0000	ENSG0000	4.17E-08	hsa-let-7a-5p;hsa-let-7b-5p;hsa-let-7d-5p;hsa-let-7e-5p;hsa-l
ENSG0000	ENSG0000	1.53E-10	hsa-let-7a-5p;hsa-let-7b-5p;hsa-let-7d-5p;hsa-let-7e-5p;hsa-l
ENSG0000	ENSG0000	1.43E-08	hsa-let-7a-5p;hsa-let-7b-5p;hsa-let-7d-5p;hsa-let-7e-5p;hsa-l
ENSG0000	ENSG0000	1.53E-06	hsa-let-7a-5p;hsa-let-7b-5p;hsa-let-7d-5p;hsa-let-7e-5p;hsa-l
ENSG0000	ENSG0000	5.55E-16	hsa-let-7a-5p;hsa-let-7b-5p;hsa-let-7d-5p;hsa-let-7e-5p;hsa-l
ENSG0000	ENSG0000	2.04E-11	hsa-let-7a-5p;hsa-let-7b-5p;hsa-let-7d-5p;hsa-let-7e-5p;hsa-l
ENSG0000	ENSG0000	5.00E-12	hsa-let-7a-5p;hsa-let-7b-5p;hsa-let-7d-5p;hsa-let-7e-5p;hsa-l
ENSG0000	ENSG0000	1.21E-07	hsa-let-7a-5p;hsa-let-7b-5p;hsa-let-7e-5p;hsa-let-7f-5p;hsa-le
ENSG0000	ENSG0000	1.29E-11	hsa-let-7a-5p;hsa-let-7b-5p;hsa-let-7d-5p;hsa-let-7e-5p;hsa-l
ENSG0000	ENSG0000	4.03E-08	hsa-let-7a-5p;hsa-let-7b-5p;hsa-let-7d-5p;hsa-let-7e-5p;hsa-l
ENSG0000	ENSG0000	4.38E-12	hsa-let-7a-5p;hsa-let-7b-5p;hsa-let-7d-5p;hsa-let-7e-5p;hsa-l

ENSG0000	ENSG0000	4.36E-09	hsa-let-7a-5p;hsa-let-7b-5p;hsa-let-7d-5p;hsa-let-7e-5p;hsa-l
ENSG0000	ENSG0000	2.88E-09	hsa-let-7a-5p;hsa-let-7b-5p;hsa-let-7d-5p;hsa-let-7e-5p;hsa-l
ENSG0000	ENSG0000	2.22E-07	hsa-let-7a-5p;hsa-let-7b-5p;hsa-let-7e-5p;hsa-let-7f-5p;hsa-le
ENSG0000	ENSG0000	4.63E-13	hsa-let-7a-5p;hsa-let-7b-5p;hsa-let-7d-5p;hsa-let-7e-5p;hsa-l
ENSG0000	ENSG0000	6.33E-10	hsa-let-7a-5p;hsa-let-7b-5p;hsa-let-7d-5p;hsa-let-7e-5p;hsa-l
ENSG0000	ENSG0000	1.86E-07	hsa-let-7a-5p;hsa-let-7b-5p;hsa-let-7d-5p;hsa-let-7e-5p;hsa-l
ENSG0000	ENSG0000	9.64E-12	hsa-let-7a-5p;hsa-let-7b-5p;hsa-let-7d-5p;hsa-let-7e-5p;hsa-l
ENSG0000	ENSG0000	2.99E-14	hsa-let-7a-5p;hsa-let-7b-5p;hsa-let-7d-5p;hsa-let-7e-5p;hsa-l
ENSG0000	ENSG0000	2.94E-12	hsa-let-7a-5p;hsa-let-7b-5p;hsa-let-7d-5p;hsa-let-7e-5p;hsa-l
ENSG0000	ENSG0000	9.75E-10	hsa-let-7a-5p;hsa-let-7b-5p;hsa-let-7d-5p;hsa-let-7e-5p;hsa-l
ENSG0000	ENSG0000	1.31E-08	hsa-let-7a-5p;hsa-let-7b-5p;hsa-let-7d-5p;hsa-let-7e-5p;hsa-l
ENSG0000	ENSG0000	2.56E-10	hsa-let-7a-5p;hsa-let-7b-5p;hsa-let-7d-5p;hsa-let-7e-5p;hsa-l
ENSG0000	ENSG0000	7.47E-08	hsa-let-7a-5p;hsa-let-7d-5p;hsa-let-7e-5p;hsa-let-7f-5p;hsa-le
ENSG0000	ENSG0000	5.82E-13	hsa-let-7a-5p;hsa-let-7b-5p;hsa-let-7d-5p;hsa-let-7e-5p;hsa-l
ENSG0000	ENSG0000	6.38E-06	hsa-let-7a-5p;hsa-let-7d-5p;hsa-let-7e-5p;hsa-let-7f-5p;hsa-le
ENSG0000	ENSG0000	9.77E-14	hsa-let-7a-5p;hsa-let-7b-5p;hsa-let-7d-5p;hsa-let-7e-5p;hsa-l
ENSG0000	ENSG0000	2.29E-09	hsa-let-7a-5p;hsa-let-7b-5p;hsa-let-7d-5p;hsa-let-7e-5p;hsa-l
ENSG0000	ENSG0000	7.44E-11	hsa-let-7a-5p;hsa-let-7b-5p;hsa-let-7d-5p;hsa-let-7e-5p;hsa-l
ENSG0000	ENSG0000	3.14E-07	hsa-let-7a-5p;hsa-let-7b-5p;hsa-let-7d-5p;hsa-let-7e-5p;hsa-l
ENSG0000	ENSG0000	1.09E-09	hsa-let-7a-5p;hsa-let-7b-5p;hsa-let-7d-5p;hsa-let-7e-5p;hsa-l
ENSG0000	ENSG0000	8.64E-08	hsa-let-7a-5p;hsa-let-7b-5p;hsa-let-7d-5p;hsa-let-7e-5p;hsa-l
ENSG0000	ENSG0000	0.000594	hsa-miR-107;hsa-miR-125a-5p;hsa-miR-15a-5p;hsa-miR-15b-5
ENSG0000	ENSG0000	1.17E-07	hsa-let-7a-5p;hsa-let-7b-5p;hsa-let-7d-5p;hsa-let-7e-5p;hsa-l
ENSG0000	ENSG0000	3.06E-06	hsa-miR-15a-5p;hsa-miR-15b-5p;hsa-miR-16-5p;hsa-miR-195-
ENSG0000	ENSG0000	3.97E-05	hsa-let-7a-5p;hsa-let-7b-5p;hsa-let-7d-5p;hsa-let-7e-5p;hsa-l
ENSG0000	ENSG0000	2.63E-12	hsa-miR-103a-3p;hsa-miR-107;hsa-miR-15a-5p;hsa-miR-15b-5
ENSG0000	ENSG0000	5.04E-05	hsa-miR-15a-5p;hsa-miR-15b-5p;hsa-miR-16-5p;hsa-miR-195-
ENSG0000	ENSG0000	6.83E-05	hsa-miR-15a-5p;hsa-miR-15b-5p;hsa-miR-195-5p;hsa-miR-424
ENSG0000	ENSG0000	0.000223	hsa-let-7a-5p;hsa-let-7b-5p;hsa-let-7d-5p;hsa-let-7e-5p;hsa-l
ENSG0000	ENSG0000	1.36E-05	hsa-let-7a-5p;hsa-let-7b-5p;hsa-let-7d-5p;hsa-let-7e-5p;hsa-l
ENSG0000	ENSG0000	0.004011	hsa-miR-125a-5p;hsa-miR-125b-5p;hsa-miR-185-3p;hsa-miR-3
ENSG0000	ENSG0000	1.57E-09	hsa-miR-103a-3p;hsa-miR-107;hsa-miR-15a-5p;hsa-miR-15b-5
ENSG0000	ENSG0000	0.000429	hsa-let-7a-5p;hsa-let-7d-5p;hsa-miR-15a-5p;hsa-miR-15b-5p;
ENSG0000	ENSG0000	0.000764	hsa-let-7a-5p;hsa-let-7b-5p;hsa-let-7d-5p;hsa-let-7e-5p;hsa-l
ENSG0000	ENSG0000	5.78E-05	hsa-miR-15a-5p;hsa-miR-15b-5p;hsa-miR-16-5p;hsa-miR-195-
ENSG0000	ENSG0000	5.78E-05	hsa-miR-15a-5p;hsa-miR-15b-5p;hsa-miR-16-5p;hsa-miR-195-
ENSG0000	ENSG0000	0.001231	hsa-miR-15a-5p;hsa-miR-15b-5p;hsa-miR-16-5p;hsa-miR-195-
ENSG0000	ENSG0000	0.000312	hsa-miR-15a-5p;hsa-miR-15b-5p;hsa-miR-16-5p;hsa-miR-195-
ENSG0000	ENSG0000	3.25E-05	hsa-let-7a-5p;hsa-let-7b-5p;hsa-let-7d-5p;hsa-let-7e-5p;hsa-l
ENSG0000	ENSG0000	0.000168	hsa-let-7a-5p;hsa-miR-125b-5p;hsa-miR-15a-5p;hsa-miR-15b-
ENSG0000	ENSG0000	9.01E-05	hsa-let-7a-5p;hsa-let-7b-5p;hsa-let-7f-5p;hsa-miR-125b-5p
ENSG0000	ENSG0000	0.00055	hsa-miR-15a-5p;hsa-miR-15b-5p;hsa-miR-16-5p;hsa-miR-195-
ENSG0000	ENSG0000	0.000487	hsa-miR-15a-5p;hsa-miR-15b-5p;hsa-miR-16-5p;hsa-miR-195-
ENSG0000	ENSG0000	0.000167	hsa-miR-103a-3p;hsa-miR-195-5p;hsa-miR-204-5p;hsa-miR-49
ENSG0000	ENSG0000	0.000438	hsa-miR-107;hsa-miR-15a-5p;hsa-miR-15b-5p;hsa-miR-195-5p
ENSG0000	ENSG0000	9.01E-05	hsa-miR-15a-5p;hsa-miR-15b-5p;hsa-miR-16-5p;hsa-miR-195-
ENSG0000	ENSG0000	0.00055	hsa-miR-15b-5p;hsa-miR-16-5p;hsa-miR-195-5p;hsa-miR-424-
ENSG0000	ENSG0000	0.000282	hsa-miR-15a-5p;hsa-miR-15b-5p;hsa-miR-16-5p;hsa-miR-424-
ENSG0000	ENSG0000	3.86E-06	hsa-miR-103a-3p;hsa-miR-107;hsa-miR-15a-5p;hsa-miR-15b-5
ENSG0000	ENSG0000	5.75E-07	hsa-let-7a-5p;hsa-let-7b-5p;hsa-let-7d-5p;hsa-let-7e-5p;hsa-l
ENSG0000	ENSG0000	0.001795	hsa-miR-125a-5p;hsa-miR-125b-5p;hsa-miR-99a-3p;hsa-miR-9
ENSG0000	ENSG0000	6.55E-05	hsa-let-7a-5p;hsa-let-7b-5p;hsa-let-7d-5p;hsa-let-7e-5p;hsa-l
ENSG0000	ENSG0000	8.33E-08	hsa-miR-103a-3p;hsa-miR-107;hsa-miR-15a-5p;hsa-miR-15b-5
ENSG0000	ENSG0000	0.000478	hsa-let-7b-5p;hsa-miR-15a-5p;hsa-miR-15b-5p;hsa-miR-424-5

ENSG0000	ENSG0000	5.23E-05	hsa-let-7a-5p;hsa-let-7b-5p;hsa-miR-107;hsa-miR-15a-5p;hsa-
ENSG0000	ENSG0000	5.76E-06	hsa-let-7a-5p;hsa-let-7b-5p;hsa-let-7d-5p;hsa-let-7e-5p;hsa-l
ENSG0000	ENSG0000	3.17E-05	hsa-miR-15a-5p;hsa-miR-15b-5p;hsa-miR-16-5p;hsa-miR-195-
ENSG0000	ENSG0000	5.75E-07	hsa-let-7a-5p;hsa-let-7b-5p;hsa-let-7d-5p;hsa-let-7e-5p;hsa-l
ENSG0000	ENSG0000	0.000132	hsa-miR-15a-5p;hsa-miR-15b-5p;hsa-miR-16-5p;hsa-miR-195-
ENSG0000	ENSG0000	1.78E-08	hsa-let-7a-5p;hsa-let-7b-5p;hsa-let-7d-5p;hsa-let-7e-5p;hsa-l
ENSG0000	ENSG0000	0.00106	hsa-miR-15a-5p;hsa-miR-15b-5p;hsa-miR-16-5p;hsa-miR-195-
ENSG0000	ENSG0000	3.77E-06	hsa-let-7a-5p;hsa-let-7d-5p;hsa-let-7e-5p;hsa-let-7f-5p;hsa-le
ENSG0000	ENSG0000	1.04E-10	hsa-miR-103a-3p;hsa-miR-107;hsa-miR-15a-5p;hsa-miR-15b-5
ENSG0000	ENSG0000	5.81E-07	hsa-miR-15a-5p;hsa-miR-15b-5p;hsa-miR-16-5p;hsa-miR-195-
ENSG0000	ENSG0000	9.59E-10	hsa-miR-103a-3p;hsa-miR-107;hsa-miR-15a-5p;hsa-miR-15b-5
ENSG0000	ENSG0000	2.60E-06	hsa-let-7a-5p;hsa-let-7b-5p;hsa-let-7d-5p;hsa-let-7e-5p;hsa-l
ENSG0000	ENSG0000	0.000182	hsa-let-7a-5p;hsa-let-7b-5p;hsa-let-7d-5p;hsa-let-7e-5p;hsa-l
ENSG0000	ENSG0000	1.83E-07	hsa-let-7a-5p;hsa-let-7e-5p;hsa-let-7f-5p;hsa-let-7g-5p;hsa-le
ENSG0000	ENSG0000	3.14E-07	hsa-let-7a-5p;hsa-let-7b-5p;hsa-let-7d-5p;hsa-let-7e-5p;hsa-l
ENSG0000	ENSG0000	4.90E-07	hsa-let-7a-5p;hsa-let-7b-5p;hsa-let-7d-5p;hsa-let-7e-5p;hsa-l
ENSG0000	ENSG0000	0.003867	hsa-let-7a-5p;hsa-let-7d-5p;hsa-let-7e-5p;hsa-let-7f-5p;hsa-le
ENSG0000	ENSG0000	6.87E-07	hsa-let-7a-5p;hsa-let-7b-5p;hsa-let-7d-5p;hsa-let-7e-5p;hsa-l
ENSG0000	ENSG0000	0.000154	hsa-miR-15a-5p;hsa-miR-15b-5p;hsa-miR-195-5p;hsa-miR-42
ENSG0000	ENSG0000	1.45E-05	hsa-miR-103a-3p;hsa-miR-107;hsa-miR-1224-5p;hsa-miR-15a-
ENSG0000	ENSG0000	5.35E-06	hsa-miR-103a-3p;hsa-miR-107;hsa-miR-1270;hsa-miR-15a-5p;
ENSG0000	ENSG0000	0.009455	hsa-miR-15a-5p;hsa-miR-15b-5p;hsa-miR-195-5p;hsa-miR-42
ENSG0000	ENSG0000	2.82E-05	hsa-let-7a-5p;hsa-let-7b-5p;hsa-let-7d-5p;hsa-let-7e-5p;hsa-l
ENSG0000	ENSG0000	0.004979	hsa-miR-103a-2-5p;hsa-miR-24-3p;hsa-miR-374b-3p;hsa-miR-
ENSG0000	ENSG0000	9.68E-06	hsa-miR-15a-5p;hsa-miR-15b-5p;hsa-miR-16-5p;hsa-miR-195-
ENSG0000	ENSG0000	1.30E-05	hsa-miR-15a-5p;hsa-miR-15b-5p;hsa-miR-16-5p;hsa-miR-195-
ENSG0000	ENSG0000	0.001493	hsa-miR-15a-5p;hsa-miR-15b-5p;hsa-miR-16-5p;hsa-miR-195-
ENSG0000	ENSG0000	5.96E-05	hsa-miR-15a-5p;hsa-miR-15b-5p;hsa-miR-16-5p;hsa-miR-195-
ENSG0000	ENSG0000	7.40E-07	hsa-let-7a-5p;hsa-let-7b-5p;hsa-let-7d-5p;hsa-let-7e-5p;hsa-l
ENSG0000	ENSG0000	9.87E-06	hsa-let-7a-5p;hsa-let-7b-5p;hsa-let-7d-5p;hsa-let-7e-5p;hsa-l
ENSG0000	ENSG0000	0.002043	hsa-let-7a-5p;hsa-let-7b-5p;hsa-let-7d-5p;hsa-let-7e-5p;hsa-l
ENSG0000	ENSG0000	0.000124	hsa-miR-125a-5p;hsa-miR-497-5p;hsa-miR-99a-5p;hsa-miR-9
ENSG0000	ENSG0000	2.54E-05	hsa-let-7a-5p;hsa-let-7b-5p;hsa-let-7d-5p;hsa-let-7e-5p;hsa-l
ENSG0000	ENSG0000	3.58E-10	hsa-let-7a-5p;hsa-let-7b-5p;hsa-let-7d-5p;hsa-let-7e-5p;hsa-l
ENSG0000	ENSG0000	0.003398	hsa-miR-103a-2-5p;hsa-miR-4685-3p;hsa-miR-502-5p;hsa-mi
ENSG0000	ENSG0000	0.001364	hsa-let-7a-5p;hsa-let-7d-5p;hsa-let-7e-5p;hsa-let-7f-5p;hsa-le
ENSG0000	ENSG0000	8.50E-05	hsa-miR-15a-5p;hsa-miR-15b-5p;hsa-miR-195-5p;hsa-miR-42
ENSG0000	ENSG0000	8.33E-08	hsa-miR-103a-3p;hsa-miR-107;hsa-miR-15a-5p;hsa-miR-15b-5
ENSG0000	ENSG0000	9.53E-05	hsa-miR-15a-5p;hsa-miR-15b-5p;hsa-miR-195-5p;hsa-miR-22
ENSG0000	ENSG0000	7.00E-05	hsa-let-7a-5p;hsa-let-7b-5p;hsa-let-7d-5p;hsa-let-7f-5p;hsa-le
ENSG0000	ENSG0000	1.35E-09	hsa-let-7a-5p;hsa-let-7b-5p;hsa-let-7d-5p;hsa-let-7e-5p;hsa-l
ENSG0000	ENSG0000	2.60E-06	hsa-miR-15a-5p;hsa-miR-15b-5p;hsa-miR-16-5p;hsa-miR-195-
ENSG0000	ENSG0000	0.006311	hsa-miR-15a-5p;hsa-miR-15b-5p;hsa-miR-195-5p;hsa-miR-42
ENSG0000	ENSG0000	0.002378	hsa-miR-15a-5p;hsa-miR-15b-5p;hsa-miR-16-5p;hsa-miR-195-
ENSG0000	ENSG0000	0.004116	hsa-let-7a-5p;hsa-let-7b-5p;hsa-let-7d-5p;hsa-let-7e-5p;hsa-l
ENSG0000	ENSG0000	0.000557	hsa-miR-149-5p;hsa-miR-15a-5p;hsa-miR-15b-5p;hsa-miR-16-
ENSG0000	ENSG0000	8.66E-07	hsa-miR-107;hsa-miR-15a-5p;hsa-miR-15b-5p;hsa-miR-195-5
ENSG0000	ENSG0000	0.000146	hsa-miR-15a-5p;hsa-miR-15b-5p;hsa-miR-195-5p;hsa-miR-42
ENSG0000	ENSG0000	7.42E-06	hsa-miR-15a-5p;hsa-miR-15b-5p;hsa-miR-16-5p;hsa-miR-195-
ENSG0000	ENSG0000	9.60E-07	hsa-miR-103a-3p;hsa-miR-107;hsa-miR-15a-5p;hsa-miR-15b-5
ENSG0000	ENSG0000	0.000306	hsa-let-7a-5p;hsa-let-7b-5p;hsa-let-7d-5p;hsa-let-7e-5p;hsa-l
ENSG0000	ENSG0000	6.28E-08	hsa-let-7a-5p;hsa-let-7b-5p;hsa-let-7d-5p;hsa-let-7e-5p;hsa-l
ENSG0000	ENSG0000	7.10E-07	hsa-let-7d-5p;hsa-let-7f-5p;hsa-let-7g-5p;hsa-let-7i-5p
ENSG0000	ENSG0000	0.002941	hsa-let-7a-5p;hsa-let-7b-5p;hsa-let-7d-5p;hsa-let-7e-5p;hsa-l

ENSG0000	ENSG0000	4.43E-05	hsa-miR-15a-5p;hsa-miR-15b-5p;hsa-miR-195-5p;hsa-miR-20
ENSG0000	ENSG0000	0.006326	hsa-miR-129-2-3p;hsa-miR-452-3p;hsa-miR-454-3p;hsa-miR-
ENSG0000	ENSG0000	0.005075	hsa-miR-15a-5p;hsa-miR-15b-5p;hsa-miR-195-5p;hsa-miR-42
ENSG0000	ENSG0000	1.62E-05	hsa-miR-15a-5p;hsa-miR-15b-5p;hsa-miR-16-5p;hsa-miR-195
ENSG0000	ENSG0000	1.87E-05	hsa-let-7a-5p;hsa-let-7b-5p;hsa-let-7e-5p;hsa-let-7f-5p;hsa-le
ENSG0000	ENSG0000	0.000164	hsa-let-7a-5p;hsa-let-7g-5p;hsa-miR-217;hsa-miR-330-5p;hsa
ENSG0000	ENSG0000	7.47E-08	hsa-miR-15a-5p;hsa-miR-15b-5p;hsa-miR-16-5p;hsa-miR-195
ENSG0000	ENSG0000	7.47E-08	hsa-miR-15a-5p;hsa-miR-15b-5p;hsa-miR-16-5p;hsa-miR-195
ENSG0000	ENSG0000	0.000731	hsa-miR-15a-5p;hsa-miR-15b-5p;hsa-miR-16-5p;hsa-miR-195
ENSG0000	ENSG0000	2.95E-05	hsa-miR-15a-5p;hsa-miR-15b-5p;hsa-miR-195-5p;hsa-miR-42
ENSG0000	ENSG0000	0.000191	hsa-miR-125a-5p;hsa-miR-125b-5p;hsa-miR-432-5p;hsa-miR-
ENSG0000	ENSG0000	0.00011	hsa-miR-103a-3p;hsa-miR-107;hsa-miR-15a-5p;hsa-miR-15b-
ENSG0000	ENSG0000	2.93E-05	hsa-let-7a-5p;hsa-let-7b-5p;hsa-let-7d-5p;hsa-let-7e-5p;hsa-l
ENSG0000	ENSG0000	4.43E-05	hsa-miR-103a-3p;hsa-miR-107;hsa-miR-15a-5p;hsa-miR-15b-
ENSG0000	ENSG0000	0.000549	hsa-miR-15a-5p;hsa-miR-195-5p;hsa-miR-374b-3p;hsa-miR-4
ENSG0000	ENSG0000	5.48E-08	hsa-let-7a-5p;hsa-let-7b-5p;hsa-let-7d-5p;hsa-let-7e-5p;hsa-l
ENSG0000	ENSG0000	3.61E-08	hsa-let-7a-5p;hsa-let-7b-5p;hsa-let-7d-5p;hsa-let-7e-5p;hsa-l
ENSG0000	ENSG0000	3.14E-07	hsa-let-7a-5p;hsa-let-7b-5p;hsa-let-7d-5p;hsa-let-7e-5p;hsa-l
ENSG0000	ENSG0000	0.003621	hsa-miR-125a-5p;hsa-miR-125b-5p;hsa-miR-15a-5p;hsa-miR-
ENSG0000	ENSG0000	0.003396	hsa-let-7a-5p;hsa-let-7b-5p;hsa-let-7d-5p;hsa-let-7e-5p;hsa-l
ENSG0000	ENSG0000	0.001552	hsa-let-7a-5p;hsa-let-7d-5p;hsa-let-7e-5p;hsa-let-7f-5p
ENSG0000	ENSG0000	5.96E-05	hsa-miR-149-5p;hsa-miR-15a-5p;hsa-miR-15b-5p;hsa-miR-19
ENSG0000	ENSG0000	4.28E-05	hsa-miR-107;hsa-miR-15a-5p;hsa-miR-16-5p;hsa-miR-424-5p
ENSG0000	ENSG0000	0.000392	hsa-let-7a-5p;hsa-let-7d-5p;hsa-let-7e-5p;hsa-let-7g-5p;hsa-
ENSG0000	ENSG0000	0.001619	hsa-miR-15a-5p;hsa-miR-15b-5p;hsa-miR-195-5p;hsa-miR-42
ENSG0000	ENSG0000	8.11E-06	hsa-let-7a-5p;hsa-let-7b-5p;hsa-let-7d-5p;hsa-let-7e-5p;hsa-l
ENSG0000	ENSG0000	0.00246	hsa-let-7a-5p;hsa-miR-125a-5p;hsa-miR-125b-5p;hsa-miR-18
ENSG0000	ENSG0000	0.004642	hsa-miR-129-2-3p;hsa-miR-15a-5p;hsa-miR-15b-5p;hsa-miR-
ENSG0000	ENSG0000	3.55E-05	hsa-miR-15a-5p;hsa-miR-15b-5p;hsa-miR-16-5p;hsa-miR-195
ENSG0000	ENSG0000	0.002314	hsa-let-7a-5p;hsa-let-7b-5p;hsa-let-7d-5p;hsa-let-7e-5p;hsa-l
ENSG0000	ENSG0000	1.78E-08	hsa-let-7a-5p;hsa-let-7b-5p;hsa-let-7d-5p;hsa-let-7e-5p;hsa-l
ENSG0000	ENSG0000	6.17E-10	hsa-let-7a-5p;hsa-let-7b-5p;hsa-let-7d-5p;hsa-let-7e-5p;hsa-l
ENSG0000	ENSG0000	6.17E-10	hsa-let-7a-5p;hsa-let-7b-5p;hsa-let-7d-5p;hsa-let-7e-5p;hsa-l
ENSG0000	ENSG0000	0.000283	hsa-miR-103a-3p;hsa-miR-107;hsa-miR-15a-5p;hsa-miR-15b-
ENSG0000	ENSG0000	0.000271	hsa-miR-107;hsa-miR-15a-5p;hsa-miR-15b-5p;hsa-miR-195-5
ENSG0000	ENSG0000	8.33E-08	hsa-let-7a-5p;hsa-let-7b-5p;hsa-let-7d-5p;hsa-let-7e-5p;hsa-l
ENSG0000	ENSG0000	1.17E-07	hsa-let-7a-5p;hsa-let-7b-5p;hsa-let-7d-5p;hsa-let-7e-5p;hsa-l
ENSG0000	ENSG0000	1.17E-07	hsa-let-7a-5p;hsa-let-7b-5p;hsa-let-7d-5p;hsa-let-7e-5p;hsa-l
ENSG0000	ENSG0000	1.17E-07	hsa-let-7a-5p;hsa-let-7b-5p;hsa-let-7d-5p;hsa-let-7e-5p;hsa-l
ENSG0000	ENSG0000	1.17E-07	hsa-let-7a-5p;hsa-let-7b-5p;hsa-let-7d-5p;hsa-let-7e-5p;hsa-l
ENSG0000	ENSG0000	1.17E-07	hsa-let-7a-5p;hsa-let-7b-5p;hsa-let-7d-5p;hsa-let-7e-5p;hsa-l
ENSG0000	ENSG0000	1.17E-07	hsa-let-7a-5p;hsa-let-7b-5p;hsa-let-7d-5p;hsa-let-7e-5p;hsa-l
ENSG0000	ENSG0000	1.17E-07	hsa-let-7a-5p;hsa-let-7b-5p;hsa-let-7d-5p;hsa-let-7e-5p;hsa-l
ENSG0000	ENSG0000	3.29E-05	hsa-miR-15a-5p;hsa-miR-15b-5p;hsa-miR-195-5p;hsa-miR-42
ENSG0000	ENSG0000	7.47E-08	hsa-miR-15a-5p;hsa-miR-15b-5p;hsa-miR-16-5p;hsa-miR-195
ENSG0000	ENSG0000	7.47E-08	hsa-miR-15a-5p;hsa-miR-15b-5p;hsa-miR-16-5p;hsa-miR-195
ENSG0000	ENSG0000	0.001962	hsa-miR-15a-5p;hsa-miR-15b-5p;hsa-miR-195-5p;hsa-miR-42
ENSG0000	ENSG0000	0.001754	hsa-let-7a-5p;hsa-let-7b-5p;hsa-let-7d-5p;hsa-let-7e-5p;hsa-l
ENSG0000	ENSG0000	1.44E-07	hsa-miR-15a-5p;hsa-miR-15b-5p;hsa-miR-16-5p;hsa-miR-195
ENSG0000	ENSG0000	4.24E-05	hsa-miR-107;hsa-miR-15a-5p;hsa-miR-15b-5p;hsa-miR-195-5
ENSG0000	ENSG0000	1.96E-05	hsa-miR-107;hsa-miR-15a-5p;hsa-miR-15b-5p;hsa-miR-195-5
ENSG0000	ENSG0000	0.006192	hsa-let-7a-5p;hsa-let-7d-5p;hsa-let-7e-5p;hsa-let-7f-5p;hsa-le
ENSG0000	ENSG0000	0.000246	hsa-let-7a-5p;hsa-let-7b-5p;hsa-let-7d-5p;hsa-let-7e-5p;hsa-l
ENSG0000	ENSG0000	0.001032	hsa-miR-15a-5p;hsa-miR-15b-5p;hsa-miR-195-5p;hsa-miR-42

ENSG0000	ENSG0000	0.000202	hsa-let-7a-5p;hsa-let-7b-5p;hsa-let-7d-5p;hsa-let-7e-5p;hsa-l
ENSG0000	ENSG0000	0.000652	hsa-let-7a-5p;hsa-let-7b-5p;hsa-let-7e-5p;hsa-let-7f-5p;hsa-le
ENSG0000	ENSG0000	0.003621	hsa-miR-15a-5p;hsa-miR-15b-5p;hsa-miR-16-5p;hsa-miR-195-
ENSG0000	ENSG0000	7.10E-07	hsa-miR-15a-5p;hsa-miR-15b-5p;hsa-miR-16-5p;hsa-miR-195-
ENSG0000	ENSG0000	0.00221	hsa-miR-103a-3p;hsa-miR-107;hsa-miR-125a-5p;hsa-miR-125b
ENSG0000	ENSG0000	0.002681	hsa-miR-107;hsa-miR-15a-5p;hsa-miR-15b-5p;hsa-miR-195-5p
ENSG0000	ENSG0000	2.93E-05	hsa-let-7a-5p;hsa-let-7b-5p;hsa-let-7d-5p;hsa-let-7e-5p;hsa-l
ENSG0000	ENSG0000	3.38E-05	hsa-miR-15a-5p;hsa-miR-15b-5p;hsa-miR-16-5p;hsa-miR-195-
ENSG0000	ENSG0000	0.000681	hsa-miR-129-2-3p;hsa-miR-15a-5p;hsa-miR-15b-5p;hsa-miR-1
ENSG0000	ENSG0000	0.000191	hsa-miR-15a-5p;hsa-miR-15b-5p;hsa-miR-16-5p;hsa-miR-195-
ENSG0000	ENSG0000	4.39E-05	hsa-let-7a-5p;hsa-let-7b-5p;hsa-let-7d-5p;hsa-let-7e-5p;hsa-l
ENSG0000	ENSG0000	0.000392	hsa-miR-103a-2-5p;hsa-miR-15a-5p;hsa-miR-15b-5p;hsa-miR-
ENSG0000	ENSG0000	0.003416	hsa-miR-149-5p;hsa-miR-24-3p;hsa-miR-532-3p;hsa-miR-660-
ENSG0000	ENSG0000	0.001962	hsa-miR-125a-5p;hsa-miR-125b-5p;hsa-miR-4786-3p;hsa-miR-
ENSG0000	ENSG0000	1.17E-07	hsa-miR-15a-5p;hsa-miR-15b-5p;hsa-miR-16-5p;hsa-miR-195-
ENSG0000	ENSG0000	1.17E-07	hsa-miR-15a-5p;hsa-miR-15b-5p;hsa-miR-16-5p;hsa-miR-195-
ENSG0000	ENSG0000	0.000182	hsa-let-7a-5p;hsa-let-7b-5p;hsa-let-7d-5p;hsa-let-7e-5p;hsa-l
ENSG0000	ENSG0000	0.000217	hsa-miR-15a-5p;hsa-miR-15b-5p;hsa-miR-16-5p;hsa-miR-195-
ENSG0000	ENSG0000	0.00383	hsa-miR-125a-5p;hsa-miR-125b-5p;hsa-miR-195-5p;hsa-miR-4
ENSG0000	ENSG0000	0.000811	hsa-let-7a-5p;hsa-let-7d-5p;hsa-let-7f-5p;hsa-let-7g-5p;hsa-le
ENSG0000	ENSG0000	0.000666	hsa-miR-103a-3p;hsa-miR-107;hsa-miR-15a-5p;hsa-miR-15b-5
ENSG0000	ENSG0000	2.47E-05	hsa-let-7a-5p;hsa-let-7b-5p;hsa-let-7d-5p;hsa-let-7e-5p;hsa-l
ENSG0000	ENSG0000	5.60E-06	hsa-let-7a-5p;hsa-let-7b-5p;hsa-let-7d-5p;hsa-let-7e-5p;hsa-l
ENSG0000	ENSG0000	4.66E-06	hsa-miR-15a-5p;hsa-miR-15b-5p;hsa-miR-16-5p;hsa-miR-195-
ENSG0000	ENSG0000	4.66E-06	hsa-miR-15a-5p;hsa-miR-15b-5p;hsa-miR-16-5p;hsa-miR-195-
ENSG0000	ENSG0000	0.002773	hsa-let-7a-5p;hsa-let-7b-5p;hsa-let-7d-5p;hsa-let-7e-5p;hsa-l
ENSG0000	ENSG0000	4.82E-06	hsa-miR-15a-5p;hsa-miR-15b-5p;hsa-miR-16-5p;hsa-miR-195-
ENSG0000	ENSG0000	1.96E-05	hsa-let-7a-5p;hsa-let-7b-5p;hsa-let-7d-5p;hsa-let-7e-5p;hsa-l
ENSG0000	ENSG0000	4.95E-06	hsa-let-7a-5p;hsa-let-7b-5p;hsa-let-7d-5p;hsa-let-7e-5p;hsa-l
ENSG0000	ENSG0000	2.96E-07	hsa-miR-103a-3p;hsa-miR-107;hsa-miR-15a-5p;hsa-miR-15b-5
ENSG0000	ENSG0000	7.73E-06	hsa-miR-107;hsa-miR-15a-5p;hsa-miR-15b-5p;hsa-miR-195-5p
ENSG0000	ENSG0000	8.37E-05	hsa-let-7a-5p;hsa-let-7d-5p;hsa-let-7e-5p;hsa-let-7f-5p;hsa-le
ENSG0000	ENSG0000	0.002681	hsa-miR-204-5p;hsa-miR-452-3p;hsa-miR-4685-3p;hsa-miR-50
ENSG0000	ENSG0000	0.002681	hsa-miR-204-5p;hsa-miR-452-3p;hsa-miR-4685-3p;hsa-miR-50
ENSG0000	ENSG0000	0.006311	hsa-miR-107;hsa-miR-15a-5p;hsa-miR-15b-5p;hsa-miR-195-5p
ENSG0000	ENSG0000	2.34E-07	hsa-miR-103a-3p;hsa-miR-107;hsa-miR-15a-5p;hsa-miR-15b-5
ENSG0000	ENSG0000	0.001552	hsa-miR-15a-5p;hsa-miR-15b-5p;hsa-miR-16-5p;hsa-miR-195-
ENSG0000	ENSG0000	2.11E-07	hsa-miR-15a-5p;hsa-miR-15b-5p;hsa-miR-16-5p;hsa-miR-195-
ENSG0000	ENSG0000	0.00022	hsa-miR-15a-5p;hsa-miR-15b-5p;hsa-miR-195-5p;hsa-miR-99a
ENSG0000	ENSG0000	1.60E-05	hsa-miR-15a-5p;hsa-miR-15b-5p;hsa-miR-16-5p;hsa-miR-195-
ENSG0000	ENSG0000	1.52E-06	hsa-miR-15a-5p;hsa-miR-15b-5p;hsa-miR-16-5p;hsa-miR-195-
ENSG0000	ENSG0000	0.000164	hsa-let-7a-5p;hsa-let-7b-5p;hsa-let-7d-5p;hsa-let-7e-5p;hsa-l
ENSG0000	ENSG0000	0.000246	hsa-let-7a-5p;hsa-let-7b-5p;hsa-let-7d-5p;hsa-let-7e-5p;hsa-l
ENSG0000	ENSG0000	2.54E-05	hsa-let-7a-5p;hsa-let-7b-5p;hsa-let-7d-5p;hsa-let-7e-5p;hsa-l
ENSG0000	ENSG0000	9.99E-05	hsa-let-7a-5p;hsa-let-7b-5p;hsa-let-7d-5p;hsa-let-7e-5p;hsa-l
ENSG0000	ENSG0000	9.99E-05	hsa-let-7a-5p;hsa-let-7b-5p;hsa-let-7d-5p;hsa-let-7e-5p;hsa-l
ENSG0000	ENSG0000	0.000715	hsa-miR-15a-5p;hsa-miR-15b-5p;hsa-miR-195-5p;hsa-miR-424
ENSG0000	ENSG0000	5.48E-08	hsa-let-7a-5p;hsa-let-7b-5p;hsa-let-7d-5p;hsa-let-7e-5p;hsa-l
ENSG0000	ENSG0000	2.82E-05	hsa-miR-103a-3p;hsa-miR-107;hsa-miR-15a-5p;hsa-miR-15b-5
ENSG0000	ENSG0000	9.53E-05	hsa-miR-107;hsa-miR-15a-5p;hsa-miR-15b-5p;hsa-miR-195-5p
ENSG0000	ENSG0000	0.007713	hsa-miR-15a-5p;hsa-miR-15b-5p;hsa-miR-16-5p;hsa-miR-195-
ENSG0000	ENSG0000	9.42E-05	hsa-miR-15a-5p;hsa-miR-15b-5p;hsa-miR-195-5p;hsa-miR-424
ENSG0000	ENSG0000	1.09E-13	hsa-let-7a-5p;hsa-let-7b-5p;hsa-let-7d-5p;hsa-let-7e-5p;hsa-l
ENSG0000	ENSG0000	1.52E-06	hsa-miR-15a-5p;hsa-miR-15b-5p;hsa-miR-16-5p;hsa-miR-195-

ENSG0000	ENSG0000	2.22E-05	hsa-let-7a-5p;hsa-let-7b-5p;hsa-let-7d-5p;hsa-let-7e-5p;hsa-l
ENSG0000	ENSG0000	1.99E-05	hsa-miR-15a-5p;hsa-miR-15b-5p;hsa-miR-16-5p;hsa-miR-195-
ENSG0000	ENSG0000	2.04E-06	hsa-let-7a-5p;hsa-let-7b-5p;hsa-let-7d-5p;hsa-let-7e-5p;hsa-l
ENSG0000	ENSG0000	0.000891	hsa-miR-15a-5p;hsa-miR-15b-5p;hsa-miR-16-5p;hsa-miR-195-
ENSG0000	ENSG0000	0.000523	hsa-let-7a-5p;hsa-let-7b-5p;hsa-let-7d-5p;hsa-let-7e-5p;hsa-l
ENSG0000	ENSG0000	0.001157	hsa-miR-15a-5p;hsa-miR-15b-5p;hsa-miR-16-5p;hsa-miR-195-
ENSG0000	ENSG0000	9.60E-07	hsa-miR-15a-5p;hsa-miR-15b-5p;hsa-miR-16-5p;hsa-miR-195-
ENSG0000	ENSG0000	5.37E-10	hsa-let-7a-5p;hsa-let-7b-5p;hsa-let-7d-5p;hsa-let-7e-5p;hsa-l
ENSG0000	ENSG0000	0.001574	hsa-miR-107;hsa-miR-125a-5p;hsa-miR-15a-5p;hsa-miR-15b-5
ENSG0000	ENSG0000	4.82E-06	hsa-miR-15a-5p;hsa-miR-16-5p;hsa-miR-99a-3p;hsa-miR-99b-
ENSG0000	ENSG0000	0.009245	hsa-miR-15a-5p;hsa-miR-15b-5p;hsa-miR-195-5p;hsa-miR-31
ENSG0000	ENSG0000	8.36E-06	hsa-let-7a-5p;hsa-let-7b-5p;hsa-let-7d-5p;hsa-let-7e-5p;hsa-l
ENSG0000	ENSG0000	5.08E-05	hsa-let-7a-5p;hsa-let-7b-5p;hsa-let-7d-5p;hsa-let-7e-5p;hsa-l
ENSG0000	ENSG0000	0.002063	hsa-miR-15a-5p;hsa-miR-15b-5p;hsa-miR-16-5p;hsa-miR-195-
ENSG0000	ENSG0000	8.61E-08	hsa-miR-15a-5p;hsa-miR-15b-5p;hsa-miR-16-5p;hsa-miR-195-
ENSG0000	ENSG0000	1.98E-06	hsa-let-7a-5p;hsa-let-7b-5p;hsa-let-7e-5p;hsa-let-7f-5p;hsa-le
ENSG0000	ENSG0000	0.000806	hsa-let-7b-5p;hsa-miR-4685-3p;hsa-miR-532-3p;hsa-miR-660-
ENSG0000	ENSG0000	1.60E-05	hsa-let-7a-5p;hsa-let-7b-5p;hsa-let-7d-5p;hsa-let-7e-5p;hsa-l
ENSG0000	ENSG0000	4.41E-07	hsa-let-7a-5p;hsa-let-7b-5p;hsa-let-7d-5p;hsa-let-7e-5p;hsa-l
ENSG0000	ENSG0000	6.60E-06	hsa-let-7a-5p;hsa-let-7b-5p;hsa-let-7d-5p;hsa-let-7e-5p;hsa-l
ENSG0000	ENSG0000	2.54E-05	hsa-let-7a-5p;hsa-let-7b-5p;hsa-let-7d-5p;hsa-let-7e-5p;hsa-l
ENSG0000	ENSG0000	0.001285	hsa-let-7a-5p;hsa-let-7d-5p;hsa-let-7e-5p;hsa-let-7f-5p;hsa-le
ENSG0000	ENSG0000	1.44E-07	hsa-miR-15a-5p;hsa-miR-15b-5p;hsa-miR-16-5p;hsa-miR-195-
ENSG0000	ENSG0000	3.88E-09	hsa-miR-107;hsa-miR-15a-5p;hsa-miR-15b-5p;hsa-miR-16-5p;
ENSG0000	ENSG0000	1.03E-12	hsa-let-7a-5p;hsa-let-7b-5p;hsa-let-7d-5p;hsa-let-7e-5p;hsa-l
ENSG0000	ENSG0000	0.00026	hsa-miR-15a-5p;hsa-miR-15b-5p;hsa-miR-195-5p;hsa-miR-42
ENSG0000	ENSG0000	1.08E-10	hsa-let-7a-5p;hsa-let-7b-5p;hsa-let-7d-5p;hsa-let-7e-5p;hsa-l
ENSG0000	ENSG0000	0.000147	hsa-miR-15a-5p;hsa-miR-15b-5p;hsa-miR-16-5p;hsa-miR-195-
ENSG0000	ENSG0000	1.90E-06	hsa-miR-15a-5p;hsa-miR-15b-5p;hsa-miR-16-5p;hsa-miR-195-
ENSG0000	ENSG0000	0.000118	hsa-miR-15a-5p;hsa-miR-15b-5p;hsa-miR-16-5p;hsa-miR-195-
ENSG0000	ENSG0000	4.66E-06	hsa-miR-15a-5p;hsa-miR-15b-5p;hsa-miR-195-5p;hsa-miR-42
ENSG0000	ENSG0000	1.87E-05	hsa-miR-129-2-3p;hsa-miR-15a-5p;hsa-miR-15b-5p;hsa-miR-
ENSG0000	ENSG0000	2.50E-05	hsa-miR-1270;hsa-miR-15a-5p;hsa-miR-15b-5p;hsa-miR-16-5
ENSG0000	ENSG0000	6.76E-06	hsa-let-7a-5p;hsa-let-7b-5p;hsa-let-7d-5p;hsa-let-7e-5p;hsa-l
ENSG0000	ENSG0000	1.68E-05	hsa-miR-15a-5p;hsa-miR-15b-5p;hsa-miR-16-5p;hsa-miR-195-
ENSG0000	ENSG0000	9.68E-06	hsa-let-7a-5p;hsa-let-7b-5p;hsa-let-7d-5p;hsa-let-7e-5p;hsa-l
ENSG0000	ENSG0000	0.003118	hsa-miR-15a-5p;hsa-miR-15b-5p;hsa-miR-195-5p;hsa-miR-42
ENSG0000	ENSG0000	1.44E-07	hsa-miR-15a-5p;hsa-miR-15b-5p;hsa-miR-16-5p;hsa-miR-195-
ENSG0000	ENSG0000	0.00106	hsa-miR-15a-5p;hsa-miR-15b-5p;hsa-miR-16-5p;hsa-miR-195-
ENSG0000	ENSG0000	2.30E-07	hsa-let-7a-5p;hsa-let-7b-5p;hsa-let-7d-5p;hsa-let-7e-5p;hsa-l
ENSG0000	ENSG0000	0.000292	hsa-let-7a-5p;hsa-let-7d-5p;hsa-let-7e-5p;hsa-let-7f-5p;hsa-le
ENSG0000	ENSG0000	0.006311	hsa-miR-15a-5p;hsa-miR-15b-5p;hsa-miR-195-5p;hsa-miR-42
ENSG0000	ENSG0000	0.000831	hsa-miR-103a-3p;hsa-miR-107;hsa-miR-15a-5p;hsa-miR-15b-5
ENSG0000	ENSG0000	1.73E-05	hsa-let-7a-5p;hsa-let-7b-5p;hsa-let-7d-5p;hsa-let-7e-5p;hsa-l
ENSG0000	ENSG0000	0.000806	hsa-miR-15a-5p;hsa-miR-15b-5p;hsa-miR-16-5p;hsa-miR-195-
ENSG0000	ENSG0000	0.000715	hsa-miR-15a-5p;hsa-miR-15b-5p;hsa-miR-195-5p;hsa-miR-42
ENSG0000	ENSG0000	9.49E-07	hsa-let-7a-5p;hsa-let-7b-5p;hsa-let-7d-5p;hsa-let-7e-5p;hsa-l
ENSG0000	ENSG0000	7.47E-08	hsa-miR-15a-5p;hsa-miR-15b-5p;hsa-miR-16-5p;hsa-miR-195-
ENSG0000	ENSG0000	6.08E-05	hsa-let-7a-5p;hsa-let-7b-5p;hsa-let-7d-5p;hsa-let-7e-5p;hsa-l
ENSG0000	ENSG0000	0.000261	hsa-miR-103a-3p;hsa-miR-107;hsa-miR-15a-5p;hsa-miR-15b-5
ENSG0000	ENSG0000	3.44E-08	hsa-miR-103a-3p;hsa-miR-107;hsa-miR-15a-5p;hsa-miR-15b-5
ENSG0000	ENSG0000	1.60E-08	hsa-miR-103a-3p;hsa-miR-107;hsa-miR-15a-5p;hsa-miR-15b-5
ENSG0000	ENSG0000	9.50E-06	hsa-miR-15a-5p;hsa-miR-15b-5p;hsa-miR-16-5p;hsa-miR-195-
ENSG0000	ENSG0000	0.000185	hsa-let-7a-5p;hsa-let-7e-5p;hsa-let-7f-5p;hsa-let-7g-5p;hsa-le

ENSG0000	ENSG0000	7.42E-05	hsa-miR-103a-3p;hsa-miR-107;hsa-miR-15a-5p;hsa-miR-15b-5
ENSG0000	ENSG0000	0.001552	hsa-miR-15a-5p;hsa-miR-15b-5p;hsa-miR-16-5p;hsa-miR-195-
ENSG0000	ENSG0000	8.59E-09	hsa-let-7a-5p;hsa-let-7b-5p;hsa-let-7d-5p;hsa-let-7e-5p;hsa-l
ENSG0000	ENSG0000	0.007067	hsa-let-7a-5p;hsa-let-7d-5p;hsa-let-7f-5p;hsa-let-7g-5p;hsa-le
ENSG0000	ENSG0000	0.002681	hsa-miR-1270;hsa-miR-432-5p;hsa-miR-452-3p;hsa-miR-660-3
ENSG0000	ENSG0000	0.000182	hsa-let-7a-5p;hsa-let-7b-5p;hsa-let-7d-5p;hsa-let-7e-5p;hsa-l
ENSG0000	ENSG0000	0.000118	hsa-miR-107;hsa-miR-1224-5p;hsa-miR-15a-5p;hsa-miR-15b-5
ENSG0000	ENSG0000	0.000132	hsa-let-7a-5p;hsa-let-7b-5p;hsa-let-7d-5p;hsa-let-7e-5p;hsa-l
ENSG0000	ENSG0000	0.000132	hsa-let-7a-5p;hsa-let-7b-5p;hsa-let-7d-5p;hsa-let-7e-5p;hsa-l
ENSG0000	ENSG0000	2.54E-05	hsa-let-7a-5p;hsa-let-7b-5p;hsa-let-7d-5p;hsa-let-7e-5p;hsa-l
ENSG0000	ENSG0000	4.95E-06	hsa-miR-15a-5p;hsa-miR-15b-5p;hsa-miR-16-5p;hsa-miR-195-
ENSG0000	ENSG0000	2.66E-09	hsa-miR-15a-5p;hsa-miR-15b-5p;hsa-miR-16-5p;hsa-miR-195-
ENSG0000	ENSG0000	7.42E-05	hsa-let-7a-5p;hsa-let-7b-5p;hsa-let-7d-5p;hsa-let-7e-5p;hsa-l
ENSG0000	ENSG0000	1.99E-05	hsa-miR-107;hsa-miR-15a-5p;hsa-miR-15b-5p;hsa-miR-195-5p
ENSG0000	ENSG0000	4.24E-07	hsa-let-7a-5p;hsa-let-7b-5p;hsa-let-7d-5p;hsa-let-7e-5p;hsa-l
ENSG0000	ENSG0000	0.00011	hsa-miR-15a-5p;hsa-miR-15b-5p;hsa-miR-16-5p;hsa-miR-195-
ENSG0000	ENSG0000	7.37E-10	hsa-let-7a-5p;hsa-let-7b-5p;hsa-let-7d-5p;hsa-let-7e-5p;hsa-l
ENSG0000	ENSG0000	0.000731	hsa-miR-15a-5p;hsa-miR-15b-5p;hsa-miR-16-5p;hsa-miR-195-
ENSG0000	ENSG0000	0.006514	hsa-miR-15a-5p;hsa-miR-15b-5p;hsa-miR-16-5p;hsa-miR-195-
ENSG0000	ENSG0000	5.08E-05	hsa-let-7a-5p;hsa-let-7b-5p;hsa-let-7d-5p;hsa-let-7e-5p;hsa-l
ENSG0000	ENSG0000	1.62E-05	hsa-miR-15a-5p;hsa-miR-15b-5p;hsa-miR-195-5p;hsa-miR-37
ENSG0000	ENSG0000	0.001754	hsa-miR-15a-5p;hsa-miR-15b-5p;hsa-miR-16-5p;hsa-miR-195-
ENSG0000	ENSG0000	8.19E-05	hsa-miR-15a-5p;hsa-miR-15b-5p;hsa-miR-16-5p;hsa-miR-195-
ENSG0000	ENSG0000	5.30E-09	hsa-let-7a-5p;hsa-let-7b-5p;hsa-let-7d-5p;hsa-let-7e-5p;hsa-l
ENSG0000	ENSG0000	2.77E-05	hsa-miR-15a-5p;hsa-miR-15b-5p;hsa-miR-16-5p;hsa-miR-195-
ENSG0000	ENSG0000	4.09E-08	hsa-miR-15a-5p;hsa-miR-15b-5p;hsa-miR-16-5p;hsa-miR-195-
ENSG0000	ENSG0000	0.000549	hsa-miR-15a-5p;hsa-miR-15b-5p;hsa-miR-195-5p;hsa-miR-42
ENSG0000	ENSG0000	2.93E-05	hsa-miR-15a-5p;hsa-miR-15b-5p;hsa-miR-16-5p;hsa-miR-195-
ENSG0000	ENSG0000	2.93E-05	hsa-miR-15a-5p;hsa-miR-15b-5p;hsa-miR-16-5p;hsa-miR-195-
ENSG0000	ENSG0000	6.89E-06	hsa-miR-1270;hsa-miR-15a-5p;hsa-miR-15b-5p;hsa-miR-195-5
ENSG0000	ENSG0000	0.005274	hsa-miR-15a-5p;hsa-miR-15b-5p;hsa-miR-424-5p;hsa-miR-49
ENSG0000	ENSG0000	1.65E-07	hsa-let-7a-5p;hsa-let-7b-5p;hsa-let-7d-5p;hsa-let-7e-5p;hsa-l
ENSG0000	ENSG0000	1.99E-05	hsa-let-7a-5p;hsa-let-7b-5p;hsa-let-7e-5p;hsa-let-7f-5p;hsa-le
ENSG0000	ENSG0000	4.45E-05	hsa-miR-15a-5p;hsa-miR-15b-5p;hsa-miR-16-5p;hsa-miR-195-
ENSG0000	ENSG0000	0.00383	hsa-miR-15a-5p;hsa-miR-15b-5p;hsa-miR-16-5p;hsa-miR-195-
ENSG0000	ENSG0000	1.29E-07	hsa-miR-15a-5p;hsa-miR-15b-5p;hsa-miR-16-5p;hsa-miR-195-
ENSG0000	ENSG0000	5.75E-07	hsa-let-7a-5p;hsa-let-7b-5p;hsa-let-7d-5p;hsa-let-7e-5p;hsa-l
ENSG0000	ENSG0000	8.19E-05	hsa-miR-15a-5p;hsa-miR-15b-5p;hsa-miR-16-5p;hsa-miR-195-
ENSG0000	ENSG0000	0.000357	hsa-miR-15b-5p;hsa-miR-16-5p;hsa-miR-195-5p;hsa-miR-424-
ENSG0000	ENSG0000	0.000669	hsa-miR-15a-5p;hsa-miR-15b-5p;hsa-miR-16-5p;hsa-miR-195-
ENSG0000	ENSG0000	0.000669	hsa-miR-15a-5p;hsa-miR-15b-5p;hsa-miR-16-5p;hsa-miR-195-
ENSG0000	ENSG0000	4.66E-06	hsa-miR-15a-5p;hsa-miR-15b-5p;hsa-miR-16-5p;hsa-miR-195-
ENSG0000	ENSG0000	0.002358	hsa-miR-125a-5p;hsa-miR-125b-5p;hsa-miR-4685-3p;hsa-miR-
ENSG0000	ENSG0000	1.17E-05	hsa-let-7a-5p;hsa-let-7b-5p;hsa-let-7d-5p;hsa-let-7e-5p;hsa-l
ENSG0000	ENSG0000	0.001797	hsa-let-7a-5p;hsa-let-7b-5p;hsa-let-7d-5p;hsa-let-7e-5p;hsa-l
ENSG0000	ENSG0000	0.00022	hsa-miR-15a-5p;hsa-miR-15b-5p;hsa-miR-195-5p;hsa-miR-42
ENSG0000	ENSG0000	1.96E-05	hsa-let-7a-5p;hsa-let-7b-5p;hsa-let-7d-5p;hsa-let-7e-5p;hsa-l
ENSG0000	ENSG0000	2.93E-05	hsa-miR-15a-5p;hsa-miR-15b-5p;hsa-miR-16-5p;hsa-miR-195-
ENSG0000	ENSG0000	0.000728	hsa-let-7a-5p;hsa-let-7b-5p;hsa-let-7d-5p;hsa-let-7e-5p;hsa-l
ENSG0000	ENSG0000	0.000728	hsa-let-7a-5p;hsa-let-7b-5p;hsa-let-7d-5p;hsa-let-7e-5p;hsa-l
ENSG0000	ENSG0000	0.007888	hsa-miR-15a-5p;hsa-miR-15b-5p;hsa-miR-16-5p;hsa-miR-195-
ENSG0000	ENSG0000	0.000415	hsa-miR-15a-5p;hsa-miR-15b-5p;hsa-miR-16-5p;hsa-miR-195-
ENSG0000	ENSG0000	0.003876	hsa-let-7a-5p;hsa-let-7b-5p;hsa-let-7d-5p;hsa-let-7e-5p;hsa-l
ENSG0000	ENSG0000	0.000312	hsa-miR-15a-5p;hsa-miR-15b-5p;hsa-miR-16-5p;hsa-miR-195-

ENSG0000	ENSG0000	1.78E-08	hsa-let-7a-5p;hsa-let-7b-5p;hsa-let-7d-5p;hsa-let-7e-5p;hsa-l
ENSG0000	ENSG0000	0.000663	hsa-miR-15a-5p;hsa-miR-15b-5p;hsa-miR-16-5p;hsa-miR-195-
ENSG0000	ENSG0000	3.77E-06	hsa-miR-15a-5p;hsa-miR-15b-5p;hsa-miR-16-5p;hsa-miR-195-
ENSG0000	ENSG0000	3.77E-06	hsa-miR-15a-5p;hsa-miR-15b-5p;hsa-miR-16-5p;hsa-miR-195-
ENSG0000	ENSG0000	0.000105	hsa-miR-15a-5p;hsa-miR-15b-5p;hsa-miR-16-5p;hsa-miR-195-
ENSG0000	ENSG0000	1.60E-05	hsa-miR-1270;hsa-miR-15a-5p;hsa-miR-15b-5p;hsa-miR-16-5
ENSG0000	ENSG0000	0.009245	hsa-miR-15a-5p;hsa-miR-15b-5p;hsa-miR-195-5p;hsa-miR-42
ENSG0000	ENSG0000	2.09E-06	hsa-miR-15a-5p;hsa-miR-15b-5p;hsa-miR-195-5p;hsa-miR-37
ENSG0000	ENSG0000	1.23E-06	hsa-miR-15a-5p;hsa-miR-15b-5p;hsa-miR-16-5p;hsa-miR-195-
ENSG0000	ENSG0000	3.44E-08	hsa-let-7a-5p;hsa-let-7b-5p;hsa-let-7d-5p;hsa-let-7e-5p;hsa-l
ENSG0000	ENSG0000	3.44E-08	hsa-let-7a-5p;hsa-let-7b-5p;hsa-let-7d-5p;hsa-let-7e-5p;hsa-l
ENSG0000	ENSG0000	0.000306	hsa-miR-103a-3p;hsa-miR-107;hsa-miR-15a-5p;hsa-miR-15b-5
ENSG0000	ENSG0000	0.007713	hsa-miR-15a-5p;hsa-miR-15b-5p;hsa-miR-195-5p;hsa-miR-31
ENSG0000	ENSG0000	8.33E-08	hsa-miR-15a-5p;hsa-miR-15b-5p;hsa-miR-16-5p;hsa-miR-195-
ENSG0000	ENSG0000	1.27E-08	hsa-miR-103a-3p;hsa-miR-107;hsa-miR-15a-5p;hsa-miR-15b-5
ENSG0000	ENSG0000	1.27E-08	hsa-miR-103a-3p;hsa-miR-107;hsa-miR-15a-5p;hsa-miR-15b-5
ENSG0000	ENSG0000	0.00106	hsa-miR-107;hsa-miR-15a-5p;hsa-miR-15b-5p;hsa-miR-195-5
ENSG0000	ENSG0000	0.006416	hsa-miR-15a-5p;hsa-miR-15b-5p;hsa-miR-16-5p;hsa-miR-195-
ENSG0000	ENSG0000	0.004308	hsa-let-7a-5p;hsa-let-7b-5p;hsa-let-7d-5p;hsa-let-7e-5p;hsa-l
ENSG0000	ENSG0000	4.45E-05	hsa-let-7a-5p;hsa-let-7b-5p;hsa-let-7d-5p;hsa-let-7e-5p;hsa-l
ENSG0000	ENSG0000	1.68E-05	hsa-miR-15a-5p;hsa-miR-15b-5p;hsa-miR-16-5p;hsa-miR-195-
ENSG0000	ENSG0000	1.27E-06	hsa-miR-103a-3p;hsa-miR-107;hsa-miR-15a-5p;hsa-miR-15b-5
ENSG0000	ENSG0000	5.04E-05	hsa-miR-107;hsa-miR-15a-5p;hsa-miR-15b-5p;hsa-miR-2278;h
ENSG0000	ENSG0000	1.35E-09	hsa-miR-15a-5p;hsa-miR-15b-5p;hsa-miR-16-5p;hsa-miR-195-
ENSG0000	ENSG0000	0.000147	hsa-miR-15a-5p;hsa-miR-15b-5p;hsa-miR-16-5p;hsa-miR-195-
ENSG0000	ENSG0000	0.003621	hsa-let-7b-5p;hsa-let-7d-5p;hsa-let-7e-5p;hsa-let-7f-5p;hsa-le
ENSG0000	ENSG0000	1.29E-06	hsa-miR-103a-3p;hsa-miR-107;hsa-miR-15a-5p;hsa-miR-15b-5
ENSG0000	ENSG0000	0.000147	hsa-let-7a-5p;hsa-let-7b-5p;hsa-let-7d-5p;hsa-let-7e-5p;hsa-l
ENSG0000	ENSG0000	3.88E-09	hsa-miR-103a-3p;hsa-miR-107;hsa-miR-15a-5p;hsa-miR-15b-5
ENSG0000	ENSG0000	0.001034	hsa-let-7a-5p;hsa-let-7b-5p;hsa-let-7d-5p;hsa-let-7e-5p;hsa-l
ENSG0000	ENSG0000	0.000447	hsa-let-7a-5p;hsa-let-7b-5p;hsa-let-7d-5p;hsa-let-7e-5p;hsa-l
ENSG0000	ENSG0000	0.000127	hsa-miR-15a-5p;hsa-miR-15b-5p;hsa-miR-16-5p;hsa-miR-195-
ENSG0000	ENSG0000	4.36E-12	hsa-let-7a-5p;hsa-let-7b-5p;hsa-let-7d-5p;hsa-let-7e-5p;hsa-l
ENSG0000	ENSG0000	0.000811	hsa-miR-103a-3p;hsa-miR-107;hsa-miR-1224-5p;hsa-miR-20a-
ENSG0000	ENSG0000	1.71E-10	hsa-miR-15a-5p;hsa-miR-15b-5p;hsa-miR-16-5p;hsa-miR-195-
ENSG0000	ENSG0000	0.000806	hsa-miR-15a-5p;hsa-miR-15b-5p;hsa-miR-16-5p;hsa-miR-195-
ENSG0000	ENSG0000	0.000509	hsa-let-7a-5p;hsa-let-7b-5p;hsa-let-7d-5p;hsa-let-7f-5p;hsa-le
ENSG0000	ENSG0000	0.001493	hsa-miR-15a-5p;hsa-miR-15b-5p;hsa-miR-16-5p;hsa-miR-195-
ENSG0000	ENSG0000	0.000811	hsa-miR-15a-5p;hsa-miR-15b-5p;hsa-miR-195-5p;hsa-miR-42
ENSG0000	ENSG0000	0.002028	hsa-let-7a-5p;hsa-let-7b-5p;hsa-let-7d-5p;hsa-let-7e-5p;hsa-l
ENSG0000	ENSG0000	0.000164	hsa-let-7a-5p;hsa-let-7b-5p;hsa-let-7d-5p;hsa-let-7e-5p;hsa-l
ENSG0000	ENSG0000	5.04E-05	hsa-miR-15a-5p;hsa-miR-15b-5p;hsa-miR-16-5p;hsa-miR-195-
ENSG0000	ENSG0000	0.000172	hsa-let-7a-5p;hsa-let-7b-5p;hsa-let-7d-5p;hsa-let-7e-5p;hsa-l
ENSG0000	ENSG0000	0.002855	hsa-let-7a-5p;hsa-let-7d-5p;hsa-miR-15a-5p;hsa-miR-15b-5p;
ENSG0000	ENSG0000	0.002694	hsa-miR-103a-2-5p;hsa-miR-15a-5p;hsa-miR-15b-5p;hsa-miR-
ENSG0000	ENSG0000	0.000806	hsa-miR-15a-5p;hsa-miR-15b-5p;hsa-miR-16-5p;hsa-miR-195-
ENSG0000	ENSG0000	2.22E-05	hsa-miR-103a-3p;hsa-miR-107;hsa-miR-15a-5p;hsa-miR-15b-5
ENSG0000	ENSG0000	0.000223	hsa-let-7d-5p;hsa-let-7e-5p;hsa-let-7f-5p;hsa-let-7g-5p;hsa-le
ENSG0000	ENSG0000	0.000223	hsa-let-7d-5p;hsa-let-7e-5p;hsa-let-7f-5p;hsa-let-7g-5p;hsa-le
ENSG0000	ENSG0000	0.009415	hsa-let-7a-5p;hsa-let-7b-5p;hsa-let-7d-5p;hsa-let-7e-5p;hsa-l
ENSG0000	ENSG0000	1.73E-05	hsa-let-7a-5p;hsa-let-7b-5p;hsa-let-7d-5p;hsa-let-7e-5p;hsa-l
ENSG0000	ENSG0000	1.17E-07	hsa-miR-15a-5p;hsa-miR-15b-5p;hsa-miR-16-5p;hsa-miR-195-
ENSG0000	ENSG0000	5.04E-05	hsa-miR-15a-5p;hsa-miR-15b-5p;hsa-miR-195-5p;hsa-miR-42
ENSG0000	ENSG0000	7.46E-05	hsa-miR-15a-5p;hsa-miR-15b-5p;hsa-miR-16-5p;hsa-miR-195-

ENSG0000	ENSG0000	3.88E-05	hsa-let-7a-5p;hsa-let-7b-5p;hsa-let-7d-5p;hsa-let-7e-5p;hsa-l
ENSG0000	ENSG0000	6.89E-06	hsa-miR-15a-5p;hsa-miR-15b-5p;hsa-miR-195-5p;hsa-miR-42
ENSG0000	ENSG0000	3.04E-06	hsa-miR-15a-5p;hsa-miR-15b-5p;hsa-miR-16-5p;hsa-miR-195-
ENSG0000	ENSG0000	0.002381	hsa-let-7a-5p;hsa-let-7d-5p;hsa-let-7e-5p;hsa-let-7f-5p;hsa-le
ENSG0000	ENSG0000	1.73E-05	hsa-miR-103a-3p;hsa-miR-107;hsa-miR-15a-5p;hsa-miR-15b-5
ENSG0000	ENSG0000	1.88E-05	hsa-miR-15a-5p;hsa-miR-15b-5p;hsa-miR-195-5p;hsa-miR-42
ENSG0000	ENSG0000	1.30E-05	hsa-miR-15a-5p;hsa-miR-15b-5p;hsa-miR-195-5p;hsa-miR-21
ENSG0000	ENSG0000	0.002613	hsa-miR-15a-5p;hsa-miR-15b-5p;hsa-miR-195-5p;hsa-miR-42
ENSG0000	ENSG0000	3.14E-07	hsa-miR-15a-5p;hsa-miR-15b-5p;hsa-miR-16-5p;hsa-miR-195-
ENSG0000	ENSG0000	1.44E-07	hsa-miR-15a-5p;hsa-miR-15b-5p;hsa-miR-16-5p;hsa-miR-195-
ENSG0000	ENSG0000	0.001034	hsa-miR-15a-5p;hsa-miR-15b-5p;hsa-miR-16-5p;hsa-miR-195-
ENSG0000	ENSG0000	0.001136	hsa-miR-15a-5p;hsa-miR-15b-5p;hsa-miR-16-5p;hsa-miR-195-
ENSG0000	ENSG0000	0.000811	hsa-miR-15a-5p;hsa-miR-15b-5p;hsa-miR-16-5p;hsa-miR-195-
ENSG0000	ENSG0000	1.53E-06	hsa-let-7a-5p;hsa-let-7b-5p;hsa-let-7d-5p;hsa-let-7e-5p;hsa-l
ENSG0000	ENSG0000	1.53E-06	hsa-let-7a-5p;hsa-let-7b-5p;hsa-let-7d-5p;hsa-let-7e-5p;hsa-l
ENSG0000	ENSG0000	1.53E-06	hsa-let-7a-5p;hsa-let-7b-5p;hsa-let-7d-5p;hsa-let-7e-5p;hsa-l
ENSG0000	ENSG0000	2.54E-05	hsa-let-7a-5p;hsa-let-7b-5p;hsa-let-7d-5p;hsa-let-7e-5p;hsa-l
ENSG0000	ENSG0000	2.47E-05	hsa-let-7a-5p;hsa-let-7b-5p;hsa-let-7d-5p;hsa-let-7e-5p;hsa-l
ENSG0000	ENSG0000	3.06E-06	hsa-let-7a-5p;hsa-let-7b-5p;hsa-let-7d-5p;hsa-let-7e-5p;hsa-l
ENSG0000	ENSG0000	2.47E-06	hsa-let-7a-5p;hsa-let-7b-5p;hsa-let-7d-5p;hsa-let-7e-5p;hsa-l
ENSG0000	ENSG0000	0.000144	hsa-miR-103a-3p;hsa-miR-107;hsa-miR-15a-5p;hsa-miR-15b-5
ENSG0000	ENSG0000	0.000312	hsa-miR-15a-5p;hsa-miR-15b-5p;hsa-miR-16-5p;hsa-miR-195-
ENSG0000	ENSG0000	1.62E-05	hsa-miR-103a-2-5p;hsa-miR-15a-5p;hsa-miR-15b-5p;hsa-miR-
ENSG0000	ENSG0000	1.45E-05	hsa-miR-103a-3p;hsa-miR-107;hsa-miR-15a-5p;hsa-miR-15b-5
ENSG0000	ENSG0000	1.30E-05	hsa-miR-15a-5p;hsa-miR-15b-5p;hsa-miR-195-5p;hsa-miR-22
ENSG0000	ENSG0000	3.44E-07	hsa-miR-103a-3p;hsa-miR-107;hsa-miR-15a-5p;hsa-miR-15b-5
ENSG0000	ENSG0000	0.000127	hsa-miR-15a-5p;hsa-miR-15b-5p;hsa-miR-195-5p;hsa-miR-22
ENSG0000	ENSG0000	2.18E-05	hsa-let-7a-5p;hsa-let-7d-5p;hsa-let-7e-5p;hsa-let-7f-5p;hsa-le
ENSG0000	ENSG0000	0.000185	hsa-let-7a-5p;hsa-let-7b-5p;hsa-let-7d-5p;hsa-let-7e-5p;hsa-l
ENSG0000	ENSG0000	0.000185	hsa-let-7a-5p;hsa-let-7b-5p;hsa-let-7d-5p;hsa-let-7e-5p;hsa-l
ENSG0000	ENSG0000	0.000312	hsa-let-7a-5p;hsa-let-7d-5p;hsa-let-7e-5p;hsa-let-7f-5p;hsa-le
ENSG0000	ENSG0000	1.62E-05	hsa-miR-15a-5p;hsa-miR-15b-5p;hsa-miR-16-5p;hsa-miR-195-
ENSG0000	ENSG0000	1.08E-06	hsa-miR-15a-5p;hsa-miR-15b-5p;hsa-miR-16-5p;hsa-miR-195-
ENSG0000	ENSG0000	7.47E-08	hsa-miR-15a-5p;hsa-miR-15b-5p;hsa-miR-16-5p;hsa-miR-195-
ENSG0000	ENSG0000	2.10E-06	hsa-miR-15a-5p;hsa-miR-15b-5p;hsa-miR-16-5p;hsa-miR-195-
ENSG0000	ENSG0000	0.00022	hsa-let-7a-5p;hsa-let-7e-5p;hsa-let-7f-5p;hsa-let-7g-5p;hsa-le
ENSG0000	ENSG0000	5.48E-08	hsa-let-7a-5p;hsa-let-7b-5p;hsa-let-7d-5p;hsa-let-7e-5p;hsa-l
ENSG0000	ENSG0000	1.98E-06	hsa-let-7a-5p;hsa-let-7b-5p;hsa-let-7d-5p;hsa-let-7e-5p;hsa-l
ENSG0000	ENSG0000	3.38E-05	hsa-let-7a-5p;hsa-let-7b-5p;hsa-let-7d-5p;hsa-let-7e-5p;hsa-l
ENSG0000	ENSG0000	0.000124	hsa-miR-15a-5p;hsa-miR-15b-5p;hsa-miR-16-5p;hsa-miR-195-
ENSG0000	ENSG0000	0.000106	hsa-let-7a-5p;hsa-let-7b-5p;hsa-let-7d-5p;hsa-let-7e-5p;hsa-l
ENSG0000	ENSG0000	0.000106	hsa-let-7a-5p;hsa-let-7b-5p;hsa-let-7d-5p;hsa-let-7e-5p;hsa-l
ENSG0000	ENSG0000	0.001157	hsa-miR-15a-5p;hsa-miR-15b-5p;hsa-miR-16-5p;hsa-miR-195-
ENSG0000	ENSG0000	1.83E-07	hsa-let-7a-5p;hsa-let-7b-5p;hsa-let-7d-5p;hsa-let-7e-5p;hsa-l
ENSG0000	ENSG0000	4.61E-06	hsa-let-7a-5p;hsa-let-7b-5p;hsa-let-7d-5p;hsa-let-7e-5p;hsa-l
ENSG0000	ENSG0000	0.004397	hsa-let-7a-5p;hsa-let-7b-5p;hsa-let-7d-5p;hsa-let-7f-5p;hsa-le
ENSG0000	ENSG0000	0.000826	hsa-let-7a-5p;hsa-let-7b-5p;hsa-let-7d-5p;hsa-let-7e-5p;hsa-l
ENSG0000	ENSG0000	0.001157	hsa-miR-15a-5p;hsa-miR-15b-5p;hsa-miR-16-5p;hsa-miR-195-
ENSG0000	ENSG0000	0.000357	hsa-miR-15a-5p;hsa-miR-15b-5p;hsa-miR-195-5p;hsa-miR-42
ENSG0000	ENSG0000	2.09E-06	hsa-miR-15a-5p;hsa-miR-15b-5p;hsa-miR-16-5p;hsa-miR-195-
ENSG0000	ENSG0000	3.72E-07	hsa-miR-103a-3p;hsa-miR-107;hsa-miR-15a-5p;hsa-miR-15b-5
ENSG0000	ENSG0000	0.000147	hsa-miR-125a-5p;hsa-miR-125b-5p;hsa-miR-15a-5p;hsa-miR-
ENSG0000	ENSG0000	3.77E-06	hsa-miR-15a-5p;hsa-miR-15b-5p;hsa-miR-16-5p;hsa-miR-195-
ENSG0000	ENSG0000	0.004442	hsa-miR-103a-3p;hsa-miR-107;hsa-miR-15a-5p;hsa-miR-15b-5

ENSG0000	ENSG0000	0.000392	hsa-let-7a-5p;hsa-let-7b-5p;hsa-let-7e-5p;hsa-let-7f-5p;hsa-le
ENSG0000	ENSG0000	2.93E-05	hsa-let-7a-5p;hsa-let-7b-5p;hsa-let-7d-5p;hsa-let-7e-5p;hsa-l
ENSG0000	ENSG0000	4.66E-06	hsa-miR-15a-5p;hsa-miR-15b-5p;hsa-miR-195-5p;hsa-miR-424
ENSG0000	ENSG0000	5.07E-07	hsa-miR-15a-5p;hsa-miR-15b-5p;hsa-miR-16-5p;hsa-miR-195-
ENSG0000	ENSG0000	0.007045	hsa-miR-15a-5p;hsa-miR-15b-5p;hsa-miR-16-5p;hsa-miR-195-
ENSG0000	ENSG0000	0.000467	hsa-let-7a-5p;hsa-let-7b-5p;hsa-let-7d-5p;hsa-let-7f-5p;hsa-le
ENSG0000	ENSG0000	3.44E-07	hsa-let-7a-5p;hsa-let-7b-5p;hsa-let-7d-5p;hsa-let-7e-5p;hsa-l
ENSG0000	ENSG0000	0.001623	hsa-let-7a-5p;hsa-let-7b-5p;hsa-let-7d-5p;hsa-let-7e-5p;hsa-l
ENSG0000	ENSG0000	0.001623	hsa-let-7a-5p;hsa-let-7b-5p;hsa-let-7d-5p;hsa-let-7e-5p;hsa-l
ENSG0000	ENSG0000	8.09E-08	hsa-let-7a-5p;hsa-let-7b-5p;hsa-let-7d-5p;hsa-let-7e-5p;hsa-l
ENSG0000	ENSG0000	1.32E-09	hsa-miR-15a-5p;hsa-miR-15b-5p;hsa-miR-16-5p;hsa-miR-195-
ENSG0000	ENSG0000	0.000806	hsa-miR-15a-5p;hsa-miR-15b-5p;hsa-miR-16-5p;hsa-miR-195-
ENSG0000	ENSG0000	8.59E-09	hsa-let-7a-5p;hsa-let-7b-5p;hsa-let-7d-5p;hsa-let-7e-5p;hsa-l
ENSG0000	ENSG0000	0.009732	hsa-miR-15a-5p;hsa-miR-15b-5p;hsa-miR-195-5p;hsa-miR-424
ENSG0000	ENSG0000	0.000883	hsa-miR-15a-5p;hsa-miR-15b-5p;hsa-miR-16-5p;hsa-miR-195-
ENSG0000	ENSG0000	2.22E-05	hsa-let-7a-5p;hsa-let-7b-5p;hsa-let-7d-5p;hsa-let-7e-5p;hsa-l
ENSG0000	ENSG0000	0.007781	hsa-miR-15a-5p;hsa-miR-15b-5p;hsa-miR-16-5p;hsa-miR-195-
ENSG0000	ENSG0000	0.000652	hsa-let-7a-5p;hsa-let-7b-5p;hsa-let-7d-5p;hsa-let-7e-5p;hsa-l
ENSG0000	ENSG0000	1.29E-06	hsa-let-7a-5p;hsa-let-7b-5p;hsa-let-7d-5p;hsa-let-7e-5p;hsa-l
ENSG0000	ENSG0000	0.001446	hsa-let-7a-5p;hsa-let-7b-5p;hsa-let-7d-5p;hsa-let-7e-5p;hsa-l
ENSG0000	ENSG0000	0.000146	hsa-miR-15a-5p;hsa-miR-15b-5p;hsa-miR-195-5p;hsa-miR-374
ENSG0000	ENSG0000	1.23E-06	hsa-miR-107;hsa-miR-15a-5p;hsa-miR-15b-5p;hsa-miR-16-5p;
ENSG0000	ENSG0000	0.002043	hsa-let-7a-5p;hsa-let-7b-5p;hsa-let-7d-5p;hsa-let-7e-5p;hsa-l
ENSG0000	ENSG0000	0.001262	hsa-miR-15a-5p;hsa-miR-15b-5p;hsa-miR-195-5p;hsa-miR-374
ENSG0000	ENSG0000	0.009172	hsa-miR-103a-3p;hsa-miR-107;hsa-miR-4786-3p;hsa-miR-671-
ENSG0000	ENSG0000	0.003666	hsa-let-7a-5p;hsa-let-7b-5p;hsa-let-7d-5p;hsa-let-7e-5p;hsa-l
ENSG0000	ENSG0000	0.006192	hsa-let-7a-5p;hsa-let-7b-5p;hsa-let-7d-5p;hsa-let-7e-5p;hsa-l
ENSG0000	ENSG0000	1.83E-07	hsa-let-7a-5p;hsa-let-7b-5p;hsa-let-7d-5p;hsa-let-7e-5p;hsa-l
ENSG0000	ENSG0000	1.44E-08	hsa-let-7a-5p;hsa-let-7b-5p;hsa-let-7d-5p;hsa-let-7e-5p;hsa-l
ENSG0000	ENSG0000	6.55E-05	hsa-miR-107;hsa-miR-15a-5p;hsa-miR-15b-5p;hsa-miR-195-5p
ENSG0000	ENSG0000	0.009172	hsa-miR-15a-5p;hsa-miR-15b-5p;hsa-miR-16-5p;hsa-miR-195-
ENSG0000	ENSG0000	2.10E-06	hsa-miR-15a-5p;hsa-miR-15b-5p;hsa-miR-16-5p;hsa-miR-195-
ENSG0000	ENSG0000	6.83E-05	hsa-miR-15a-5p;hsa-miR-15b-5p;hsa-miR-195-5p;hsa-miR-424
ENSG0000	ENSG0000	0.000392	hsa-let-7a-5p;hsa-let-7b-5p;hsa-let-7d-5p;hsa-let-7e-5p;hsa-l
ENSG0000	ENSG0000	2.47E-06	hsa-let-7a-5p;hsa-let-7b-5p;hsa-let-7d-5p;hsa-let-7e-5p;hsa-l
ENSG0000	ENSG0000	2.18E-05	hsa-let-7a-5p;hsa-let-7b-5p;hsa-let-7d-5p;hsa-let-7e-5p;hsa-l
ENSG0000	ENSG0000	0.001112	hsa-miR-103a-3p;hsa-miR-107;hsa-miR-15a-5p;hsa-miR-15b-5
ENSG0000	ENSG0000	0.000969	hsa-miR-107;hsa-miR-15a-5p;hsa-miR-15b-5p;hsa-miR-195-5p
ENSG0000	ENSG0000	2.66E-10	hsa-let-7a-5p;hsa-let-7b-5p;hsa-let-7d-5p;hsa-let-7e-5p;hsa-l
ENSG0000	ENSG0000	0.000147	hsa-miR-107;hsa-miR-15a-5p;hsa-miR-15b-5p;hsa-miR-195-5p
ENSG0000	ENSG0000	6.68E-06	hsa-miR-103a-3p;hsa-miR-107;hsa-miR-15a-5p;hsa-miR-15b-5
ENSG0000	ENSG0000	6.26E-11	hsa-miR-15a-5p;hsa-miR-15b-5p;hsa-miR-16-5p;hsa-miR-195-
ENSG0000	ENSG0000	6.26E-11	hsa-miR-15a-5p;hsa-miR-15b-5p;hsa-miR-16-5p;hsa-miR-195-
ENSG0000	ENSG0000	2.51E-05	hsa-miR-15a-5p;hsa-miR-15b-5p;hsa-miR-195-5p;hsa-miR-424
ENSG0000	ENSG0000	3.44E-08	hsa-let-7a-5p;hsa-let-7b-5p;hsa-let-7d-5p;hsa-let-7e-5p;hsa-l
ENSG0000	ENSG0000	3.44E-08	hsa-let-7a-5p;hsa-let-7b-5p;hsa-let-7d-5p;hsa-let-7e-5p;hsa-l
ENSG0000	ENSG0000	7.10E-07	hsa-miR-15a-5p;hsa-miR-15b-5p;hsa-miR-16-5p;hsa-miR-195-
ENSG0000	ENSG0000	0.000888	hsa-let-7a-5p;hsa-let-7b-5p;hsa-let-7d-5p;hsa-let-7e-5p;hsa-l
ENSG0000	ENSG0000	3.77E-06	hsa-let-7a-5p;hsa-let-7b-5p;hsa-let-7d-5p;hsa-let-7e-5p;hsa-l
ENSG0000	ENSG0000	1.44E-07	hsa-miR-15a-5p;hsa-miR-15b-5p;hsa-miR-16-5p;hsa-miR-195-
ENSG0000	ENSG0000	9.07E-05	hsa-let-7a-5p;hsa-let-7b-5p;hsa-let-7d-5p;hsa-let-7e-5p;hsa-l
ENSG0000	ENSG0000	9.59E-10	hsa-miR-103a-3p;hsa-miR-107;hsa-miR-15a-5p;hsa-miR-15b-5
ENSG0000	ENSG0000	0.000306	hsa-let-7a-5p;hsa-let-7b-5p;hsa-let-7d-5p;hsa-let-7e-5p;hsa-l
ENSG0000	ENSG0000	2.43E-05	hsa-miR-15a-5p;hsa-miR-15b-5p;hsa-miR-16-5p;hsa-miR-195-

ENSG0000	ENSG0000	0.000487	hsa-miR-15a-5p;hsa-miR-15b-5p;hsa-miR-16-5p;hsa-miR-195-
ENSG0000	ENSG0000	2.30E-07	hsa-let-7a-5p;hsa-let-7b-5p;hsa-let-7e-5p;hsa-let-7f-5p;hsa-le
ENSG0000	ENSG0000	3.77E-06	hsa-miR-15a-5p;hsa-miR-15b-5p;hsa-miR-16-5p;hsa-miR-195-
ENSG0000	ENSG0000	2.83E-06	hsa-miR-15a-5p;hsa-miR-15b-5p;hsa-miR-16-5p;hsa-miR-195-
ENSG0000	ENSG0000	3.55E-05	hsa-miR-15a-5p;hsa-miR-15b-5p;hsa-miR-16-5p;hsa-miR-195-
ENSG0000	ENSG0000	0.000429	hsa-let-7a-5p;hsa-let-7b-5p;hsa-let-7d-5p;hsa-let-7e-5p;hsa-l
ENSG0000	ENSG0000	0.000429	hsa-let-7a-5p;hsa-let-7b-5p;hsa-let-7d-5p;hsa-let-7e-5p;hsa-l
ENSG0000	ENSG0000	7.10E-07	hsa-miR-15a-5p;hsa-miR-15b-5p;hsa-miR-16-5p;hsa-miR-195-
ENSG0000	ENSG0000	3.72E-07	hsa-miR-103a-3p;hsa-miR-107;hsa-miR-15a-5p;hsa-miR-15b-5
ENSG0000	ENSG0000	7.00E-05	hsa-let-7d-5p;hsa-let-7e-5p;hsa-let-7f-5p;hsa-let-7g-5p;hsa-le
ENSG0000	ENSG0000	0.001603	hsa-miR-204-5p;hsa-miR-4685-3p;hsa-miR-4786-3p;hsa-miR-9
ENSG0000	ENSG0000	0.000118	hsa-miR-15a-5p;hsa-miR-15b-5p;hsa-miR-16-5p;hsa-miR-195-
ENSG0000	ENSG0000	4.09E-08	hsa-miR-149-5p;hsa-miR-15a-5p;hsa-miR-15b-5p;hsa-miR-16-
ENSG0000	ENSG0000	0.000467	hsa-let-7a-5p;hsa-let-7b-5p;hsa-let-7d-5p;hsa-let-7e-5p;hsa-l
ENSG0000	ENSG0000	5.96E-05	hsa-let-7a-5p;hsa-let-7b-5p;hsa-let-7e-5p;hsa-let-7f-5p;hsa-le
ENSG0000	ENSG0000	3.06E-06	hsa-let-7b-5p;hsa-let-7d-5p;hsa-let-7e-5p;hsa-let-7f-5p;hsa-le
ENSG0000	ENSG0000	2.83E-06	hsa-miR-15a-5p;hsa-miR-15b-5p;hsa-miR-16-5p;hsa-miR-195-
ENSG0000	ENSG0000	8.59E-09	hsa-let-7a-5p;hsa-let-7b-5p;hsa-let-7d-5p;hsa-let-7e-5p;hsa-l
ENSG0000	ENSG0000	3.17E-07	hsa-miR-15a-5p;hsa-miR-15b-5p;hsa-miR-195-5p;hsa-miR-424
ENSG0000	ENSG0000	0.000487	hsa-miR-15a-5p;hsa-miR-15b-5p;hsa-miR-16-5p;hsa-miR-195-
ENSG0000	ENSG0000	0.001374	hsa-miR-15a-5p;hsa-miR-15b-5p;hsa-miR-16-5p;hsa-miR-195-
ENSG0000	ENSG0000	1.17E-07	hsa-let-7a-5p;hsa-let-7b-5p;hsa-let-7d-5p;hsa-let-7e-5p;hsa-l
ENSG0000	ENSG0000	1.17E-07	hsa-let-7a-5p;hsa-let-7b-5p;hsa-let-7d-5p;hsa-let-7e-5p;hsa-l
ENSG0000	ENSG0000	0.000124	hsa-miR-15a-5p;hsa-miR-15b-5p;hsa-miR-424-5p;hsa-miR-497
ENSG0000	ENSG0000	4.24E-05	hsa-miR-15a-5p;hsa-miR-15b-5p;hsa-miR-16-5p;hsa-miR-195-
ENSG0000	ENSG0000	0.000147	hsa-let-7a-5p;hsa-let-7b-5p;hsa-let-7d-5p;hsa-let-7e-5p;hsa-l
ENSG0000	ENSG0000	1.44E-07	hsa-miR-15a-5p;hsa-miR-15b-5p;hsa-miR-16-5p;hsa-miR-195-
ENSG0000	ENSG0000	0.003922	hsa-miR-1224-5p;hsa-miR-15a-5p;hsa-miR-15b-5p;hsa-miR-16
ENSG0000	ENSG0000	8.11E-05	hsa-let-7a-5p;hsa-let-7b-5p;hsa-let-7d-5p;hsa-let-7e-5p;hsa-l
ENSG0000	ENSG0000	0.00529	hsa-miR-15a-5p;hsa-miR-15b-5p;hsa-miR-16-5p;hsa-miR-195-
ENSG0000	ENSG0000	0.000509	hsa-miR-15a-5p;hsa-miR-15b-5p;hsa-miR-16-5p;hsa-miR-195-
ENSG0000	ENSG0000	0.000381	hsa-miR-103a-3p;hsa-miR-107;hsa-miR-15a-5p;hsa-miR-15b-5
ENSG0000	ENSG0000	6.76E-06	hsa-miR-125a-5p;hsa-miR-125b-5p;hsa-miR-15a-5p;hsa-miR-1
ENSG0000	ENSG0000	0.000478	hsa-miR-125b-5p;hsa-miR-15a-5p;hsa-miR-15b-5p;hsa-miR-16
ENSG0000	ENSG0000	1.44E-07	hsa-miR-15a-5p;hsa-miR-15b-5p;hsa-miR-16-5p;hsa-miR-195-
ENSG0000	ENSG0000	2.18E-05	hsa-miR-15a-5p;hsa-miR-15b-5p;hsa-miR-16-5p;hsa-miR-195-
ENSG0000	ENSG0000	0.001333	hsa-miR-15a-5p;hsa-miR-15b-5p;hsa-miR-16-5p;hsa-miR-195-
ENSG0000	ENSG0000	1.34E-05	hsa-let-7a-5p;hsa-let-7b-5p;hsa-let-7d-5p;hsa-let-7e-5p;hsa-l
ENSG0000	ENSG0000	0.000392	hsa-miR-15a-5p;hsa-miR-15b-5p;hsa-miR-16-5p;hsa-miR-195-
ENSG0000	ENSG0000	0.002043	hsa-let-7a-5p;hsa-let-7b-5p;hsa-let-7d-5p;hsa-let-7e-5p;hsa-l
ENSG0000	ENSG0000	5.82E-06	hsa-let-7a-5p;hsa-let-7b-5p;hsa-let-7d-5p;hsa-let-7e-5p;hsa-l
ENSG0000	ENSG0000	1.15E-08	hsa-miR-103a-3p;hsa-miR-107;hsa-miR-1224-5p;hsa-miR-15a-
ENSG0000	ENSG0000	8.49E-08	hsa-let-7a-5p;hsa-let-7d-5p;hsa-let-7e-5p;hsa-let-7f-5p;hsa-le
ENSG0000	ENSG0000	0.000246	hsa-let-7a-5p;hsa-let-7b-5p;hsa-let-7d-5p;hsa-let-7e-5p;hsa-l
ENSG0000	ENSG0000	0.004112	hsa-miR-15a-5p;hsa-miR-15b-5p;hsa-miR-195-5p;hsa-miR-20a
ENSG0000	ENSG0000	0.003033	hsa-miR-107;hsa-miR-15a-5p;hsa-miR-15b-5p;hsa-miR-195-5p
ENSG0000	ENSG0000	4.61E-06	hsa-let-7a-5p;hsa-let-7b-5p;hsa-let-7d-5p;hsa-let-7e-5p;hsa-l
ENSG0000	ENSG0000	8.09E-07	hsa-miR-103a-3p;hsa-miR-107;hsa-miR-15a-5p;hsa-miR-15b-5
ENSG0000	ENSG0000	7.10E-07	hsa-let-7b-5p;hsa-miR-107;hsa-miR-125a-5p;hsa-miR-125b-5p
ENSG0000	ENSG0000	1.04E-05	hsa-miR-15a-5p;hsa-miR-15b-5p;hsa-miR-16-5p;hsa-miR-195-
ENSG0000	ENSG0000	0.001776	hsa-miR-1224-5p;hsa-miR-15a-5p;hsa-miR-15b-5p;hsa-miR-16
ENSG0000	ENSG0000	0.001776	hsa-miR-15a-5p;hsa-miR-15b-5p;hsa-miR-195-5p;hsa-miR-424
ENSG0000	ENSG0000	0.000277	hsa-miR-15a-5p;hsa-miR-15b-5p;hsa-miR-195-5p;hsa-miR-374
ENSG0000	ENSG0000	4.39E-05	hsa-miR-103a-3p;hsa-miR-107;hsa-miR-15a-5p;hsa-miR-15b-5

ENSG0000	ENSG0000	0.00026	hsa-miR-15a-5p;hsa-miR-15b-5p;hsa-miR-195-5p;hsa-miR-42
ENSG0000	ENSG0000	0.00026	hsa-miR-15a-5p;hsa-miR-15b-5p;hsa-miR-195-5p;hsa-miR-42
ENSG0000	ENSG0000	0.002926	hsa-miR-125a-5p;hsa-miR-125b-5p;hsa-miR-15a-5p;hsa-miR-
ENSG0000	ENSG0000	0.000292	hsa-let-7a-5p;hsa-let-7b-5p;hsa-let-7d-5p;hsa-let-7e-5p;hsa-l
ENSG0000	ENSG0000	1.36E-05	hsa-miR-15a-5p;hsa-miR-15b-5p;hsa-miR-16-5p;hsa-miR-195
ENSG0000	ENSG0000	0.000246	hsa-miR-15a-5p;hsa-miR-15b-5p;hsa-miR-16-5p;hsa-miR-195
ENSG0000	ENSG0000	0.001916	hsa-let-7a-5p;hsa-let-7b-5p;hsa-let-7d-5p;hsa-let-7e-5p;hsa-l
ENSG0000	ENSG0000	0.002751	hsa-let-7a-5p;hsa-let-7d-5p;hsa-let-7e-5p;hsa-let-7f-5p;hsa-le
ENSG0000	ENSG0000	0.000204	hsa-let-7a-5p;hsa-let-7b-5p;hsa-let-7d-5p;hsa-let-7e-5p;hsa-l
ENSG0000	ENSG0000	2.47E-06	hsa-let-7a-5p;hsa-let-7b-5p;hsa-let-7d-5p;hsa-let-7e-5p;hsa-l
ENSG0000	ENSG0000	2.34E-07	hsa-let-7a-5p;hsa-let-7b-5p;hsa-let-7d-5p;hsa-let-7e-5p;hsa-l
ENSG0000	ENSG0000	4.95E-06	hsa-miR-15a-5p;hsa-miR-16-5p;hsa-miR-195-5p;hsa-miR-374
ENSG0000	ENSG0000	2.54E-05	hsa-let-7a-5p;hsa-let-7b-5p;hsa-let-7d-5p;hsa-let-7e-5p;hsa-l
ENSG0000	ENSG0000	3.44E-08	hsa-miR-103a-3p;hsa-miR-107;hsa-miR-15a-5p;hsa-miR-15b-
ENSG0000	ENSG0000	2.93E-05	hsa-miR-125a-5p;hsa-miR-15a-5p;hsa-miR-15b-5p;hsa-miR-1
ENSG0000	ENSG0000	0.000147	hsa-let-7a-5p;hsa-let-7b-5p;hsa-let-7d-5p;hsa-let-7e-5p;hsa-l
ENSG0000	ENSG0000	1.57E-06	hsa-let-7a-5p;hsa-let-7b-5p;hsa-let-7d-5p;hsa-let-7e-5p;hsa-l
ENSG0000	ENSG0000	0.001157	hsa-let-7a-5p;hsa-let-7b-5p;hsa-let-7e-5p;hsa-let-7f-5p;hsa-le
ENSG0000	ENSG0000	1.15E-05	hsa-let-7a-5p;hsa-let-7b-5p;hsa-let-7d-5p;hsa-let-7e-5p;hsa-l
ENSG0000	ENSG0000	0.000663	hsa-let-7b-5p;hsa-let-7e-5p;hsa-let-7f-5p;hsa-let-7g-5p;hsa-le
ENSG0000	ENSG0000	3.88E-05	hsa-miR-15a-5p;hsa-miR-15b-5p;hsa-miR-16-5p;hsa-miR-195
ENSG0000	ENSG0000	1.52E-06	hsa-miR-15a-5p;hsa-miR-15b-5p;hsa-miR-16-5p;hsa-miR-195
ENSG0000	ENSG0000	0.002175	hsa-let-7a-5p;hsa-let-7b-5p;hsa-let-7d-5p;hsa-let-7e-5p;hsa-l
ENSG0000	ENSG0000	0.004721	hsa-miR-15a-5p;hsa-miR-15b-5p;hsa-miR-16-5p;hsa-miR-195
ENSG0000	ENSG0000	1.90E-05	hsa-let-7a-5p;hsa-let-7b-5p;hsa-let-7d-5p;hsa-let-7e-5p;hsa-l
ENSG0000	ENSG0000	8.09E-08	hsa-let-7a-5p;hsa-let-7b-5p;hsa-let-7d-5p;hsa-let-7e-5p;hsa-l
ENSG0000	ENSG0000	0.000246	hsa-let-7a-5p;hsa-let-7b-5p;hsa-let-7d-5p;hsa-let-7e-5p;hsa-l
ENSG0000	ENSG0000	0.000961	hsa-let-7a-5p;hsa-let-7b-5p;hsa-let-7d-5p;hsa-let-7e-5p;hsa-l
ENSG0000	ENSG0000	0.003233	hsa-let-7a-5p;hsa-let-7b-5p;hsa-let-7d-5p;hsa-let-7e-5p;hsa-l
ENSG0000	ENSG0000	0.000392	hsa-let-7a-5p;hsa-let-7b-5p;hsa-let-7e-5p;hsa-let-7f-5p;hsa-le
ENSG0000	ENSG0000	1.59E-05	hsa-let-7a-5p;hsa-let-7b-5p;hsa-let-7d-5p;hsa-let-7e-5p;hsa-l
ENSG0000	ENSG0000	7.49E-07	hsa-miR-15a-5p;hsa-miR-16-5p;hsa-miR-195-5p;hsa-miR-424
ENSG0000	ENSG0000	6.33E-05	hsa-miR-15a-5p;hsa-miR-15b-5p;hsa-miR-16-5p;hsa-miR-195
ENSG0000	ENSG0000	6.13E-12	hsa-let-7a-5p;hsa-let-7b-5p;hsa-let-7d-5p;hsa-let-7e-5p;hsa-l
ENSG0000	ENSG0000	7.08E-07	hsa-let-7a-5p;hsa-let-7b-5p;hsa-let-7d-5p;hsa-let-7e-5p;hsa-l
ENSG0000	ENSG0000	0.007469	hsa-miR-15a-5p;hsa-miR-15b-5p;hsa-miR-16-5p;hsa-miR-195
ENSG0000	ENSG0000	1.30E-05	hsa-miR-15a-5p;hsa-miR-15b-5p;hsa-miR-16-5p;hsa-miR-195
ENSG0000	ENSG0000	0.000359	hsa-let-7a-5p;hsa-let-7b-5p;hsa-let-7d-5p;hsa-let-7e-5p;hsa-l
ENSG0000	ENSG0000	3.33E-07	hsa-miR-15a-5p;hsa-miR-15b-5p;hsa-miR-16-5p;hsa-miR-195
ENSG0000	ENSG0000	9.39E-06	hsa-let-7a-5p;hsa-let-7b-5p;hsa-let-7d-5p;hsa-let-7e-5p;hsa-l
ENSG0000	ENSG0000	0.000438	hsa-miR-15a-5p;hsa-miR-15b-5p;hsa-miR-16-5p;hsa-miR-195
ENSG0000	ENSG0000	1.08E-06	hsa-miR-15a-5p;hsa-miR-15b-5p;hsa-miR-16-5p;hsa-miR-195
ENSG0000	ENSG0000	1.08E-06	hsa-miR-15a-5p;hsa-miR-15b-5p;hsa-miR-16-5p;hsa-miR-195
ENSG0000	ENSG0000	1.08E-06	hsa-miR-15a-5p;hsa-miR-15b-5p;hsa-miR-16-5p;hsa-miR-195
ENSG0000	ENSG0000	1.08E-06	hsa-miR-15a-5p;hsa-miR-15b-5p;hsa-miR-16-5p;hsa-miR-195
ENSG0000	ENSG0000	0.000891	hsa-miR-107;hsa-miR-15a-5p;hsa-miR-15b-5p;hsa-miR-195-5
ENSG0000	ENSG0000	0.000891	hsa-miR-107;hsa-miR-15a-5p;hsa-miR-15b-5p;hsa-miR-195-5
ENSG0000	ENSG0000	0.000273	hsa-let-7a-5p;hsa-let-7b-5p;hsa-let-7d-5p;hsa-let-7e-5p;hsa-l
ENSG0000	ENSG0000	3.86E-06	hsa-miR-103a-2-5p;hsa-miR-103a-3p;hsa-miR-107;hsa-miR-1
ENSG0000	ENSG0000	3.14E-07	hsa-let-7a-5p;hsa-let-7b-5p;hsa-let-7d-5p;hsa-let-7e-5p;hsa-l
ENSG0000	ENSG0000	3.61E-08	hsa-let-7a-5p;hsa-let-7b-5p;hsa-let-7d-5p;hsa-let-7e-5p;hsa-l
ENSG0000	ENSG0000	0.001797	hsa-let-7a-5p;hsa-let-7b-5p;hsa-let-7d-5p;hsa-let-7e-5p;hsa-l
ENSG0000	ENSG0000	0.000164	hsa-miR-15a-5p;hsa-miR-15b-5p;hsa-miR-16-5p;hsa-miR-195
ENSG0000	ENSG0000	7.47E-08	hsa-miR-15a-5p;hsa-miR-15b-5p;hsa-miR-195-5p;hsa-miR-42

ENSG0000	ENSG0000	2.10E-06	hsa-miR-15a-5p;hsa-miR-15b-5p;hsa-miR-16-5p;hsa-miR-195-
ENSG0000	ENSG0000	3.47E-05	hsa-let-7a-5p;hsa-let-7b-5p;hsa-let-7d-5p;hsa-let-7e-5p;hsa-l
ENSG0000	ENSG0000	4.24E-05	hsa-let-7a-5p;hsa-let-7b-5p;hsa-let-7d-5p;hsa-let-7e-5p;hsa-l
ENSG0000	ENSG0000	1.44E-07	hsa-miR-15a-5p;hsa-miR-15b-5p;hsa-miR-16-5p;hsa-miR-195-
ENSG0000	ENSG0000	0.000132	hsa-let-7a-5p;hsa-let-7b-5p;hsa-let-7d-5p;hsa-let-7e-5p;hsa-l
ENSG0000	ENSG0000	3.72E-07	hsa-miR-15a-5p;hsa-miR-15b-5p;hsa-miR-16-5p;hsa-miR-195-
ENSG0000	ENSG0000	3.72E-07	hsa-miR-15a-5p;hsa-miR-15b-5p;hsa-miR-16-5p;hsa-miR-195-
ENSG0000	ENSG0000	3.72E-07	hsa-miR-15a-5p;hsa-miR-15b-5p;hsa-miR-16-5p;hsa-miR-195-
ENSG0000	ENSG0000	3.72E-07	hsa-miR-15a-5p;hsa-miR-15b-5p;hsa-miR-16-5p;hsa-miR-195-
ENSG0000	ENSG0000	3.72E-07	hsa-miR-15a-5p;hsa-miR-15b-5p;hsa-miR-16-5p;hsa-miR-195-
ENSG0000	ENSG0000	3.72E-07	hsa-miR-15a-5p;hsa-miR-15b-5p;hsa-miR-16-5p;hsa-miR-195-
ENSG0000	ENSG0000	3.72E-07	hsa-miR-15a-5p;hsa-miR-15b-5p;hsa-miR-16-5p;hsa-miR-195-
ENSG0000	ENSG0000	3.72E-07	hsa-miR-15a-5p;hsa-miR-15b-5p;hsa-miR-16-5p;hsa-miR-195-
ENSG0000	ENSG0000	3.72E-07	hsa-miR-15a-5p;hsa-miR-15b-5p;hsa-miR-16-5p;hsa-miR-195-
ENSG0000	ENSG0000	3.72E-07	hsa-miR-15a-5p;hsa-miR-15b-5p;hsa-miR-16-5p;hsa-miR-195-
ENSG0000	ENSG0000	0.00246	hsa-let-7a-5p;hsa-let-7b-5p;hsa-let-7d-5p;hsa-let-7e-5p;hsa-l
ENSG0000	ENSG0000	9.50E-06	hsa-miR-15a-5p;hsa-miR-15b-5p;hsa-miR-16-5p;hsa-miR-195-
ENSG0000	ENSG0000	3.88E-09	hsa-miR-15a-5p;hsa-miR-15b-5p;hsa-miR-16-5p;hsa-miR-195-
ENSG0000	ENSG0000	3.57E-07	hsa-let-7a-5p;hsa-let-7b-5p;hsa-let-7d-5p;hsa-let-7e-5p;hsa-l
ENSG0000	ENSG0000	1.65E-07	hsa-let-7a-5p;hsa-let-7b-5p;hsa-let-7d-5p;hsa-let-7e-5p;hsa-l
ENSG0000	ENSG0000	0.000105	hsa-miR-15a-5p;hsa-miR-15b-5p;hsa-miR-16-5p;hsa-miR-424-
ENSG0000	ENSG0000	0.005826	hsa-miR-15a-5p;hsa-miR-15b-5p;hsa-miR-16-5p;hsa-miR-195-
ENSG0000	ENSG0000	1.15E-05	hsa-let-7a-5p;hsa-let-7b-5p;hsa-let-7d-5p;hsa-let-7e-5p;hsa-l
ENSG0000	ENSG0000	0.002613	hsa-let-7a-5p;hsa-let-7b-5p;hsa-let-7d-5p;hsa-let-7e-5p;hsa-l
ENSG0000	ENSG0000	1.41E-07	hsa-let-7a-5p;hsa-let-7b-5p;hsa-let-7d-5p;hsa-let-7e-5p;hsa-l
ENSG0000	ENSG0000	8.66E-07	hsa-miR-103a-3p;hsa-miR-107;hsa-miR-15a-5p;hsa-miR-15b-5
ENSG0000	ENSG0000	0.009415	hsa-let-7a-5p;hsa-let-7b-5p;hsa-let-7d-5p;hsa-let-7e-5p;hsa-l
ENSG0000	ENSG0000	5.48E-05	hsa-miR-15a-5p;hsa-miR-15b-5p;hsa-miR-16-5p;hsa-miR-195-
ENSG0000	ENSG0000	4.64E-07	hsa-miR-103a-3p;hsa-miR-107;hsa-miR-15a-5p;hsa-miR-15b-5
ENSG0000	ENSG0000	0.000541	hsa-miR-15a-5p;hsa-miR-15b-5p;hsa-miR-16-5p;hsa-miR-195-
ENSG0000	ENSG0000	0.00022	hsa-miR-15a-5p;hsa-miR-15b-5p;hsa-miR-195-5p;hsa-miR-42
ENSG0000	ENSG0000	0.000144	hsa-miR-149-5p;hsa-miR-15a-5p;hsa-miR-15b-5p;hsa-miR-16-
ENSG0000	ENSG0000	3.44E-07	hsa-miR-15a-5p;hsa-miR-15b-5p;hsa-miR-16-5p;hsa-miR-195-
ENSG0000	ENSG0000	0.002681	hsa-miR-204-5p;hsa-miR-454-3p;hsa-miR-4685-3p;hsa-miR-5
ENSG0000	ENSG0000	0.000891	hsa-let-7a-5p;hsa-let-7b-5p;hsa-let-7d-5p;hsa-let-7e-5p;hsa-l
ENSG0000	ENSG0000	2.47E-06	hsa-let-7a-5p;hsa-let-7b-5p;hsa-let-7d-5p;hsa-let-7e-5p;hsa-l
ENSG0000	ENSG0000	0.004276	hsa-miR-15a-5p;hsa-miR-15b-5p;hsa-miR-16-5p;hsa-miR-195-
ENSG0000	ENSG0000	1.52E-05	hsa-let-7a-5p;hsa-let-7b-5p;hsa-let-7d-5p;hsa-let-7e-5p;hsa-l
ENSG0000	ENSG0000	2.50E-05	hsa-miR-15a-5p;hsa-miR-15b-5p;hsa-miR-16-5p;hsa-miR-195-
ENSG0000	ENSG0000	4.45E-05	hsa-let-7a-5p;hsa-let-7b-5p;hsa-let-7d-5p;hsa-let-7e-5p;hsa-l
ENSG0000	ENSG0000	0.001262	hsa-let-7a-5p;hsa-let-7b-5p;hsa-let-7e-5p;hsa-let-7f-5p;hsa-le
ENSG0000	ENSG0000	1.08E-06	hsa-miR-15a-5p;hsa-miR-15b-5p;hsa-miR-16-5p;hsa-miR-195-
ENSG0000	ENSG0000	0.005399	hsa-miR-15a-5p;hsa-miR-15b-5p;hsa-miR-16-5p;hsa-miR-195-
ENSG0000	ENSG0000	5.48E-08	hsa-miR-103a-3p;hsa-miR-107;hsa-miR-15a-5p;hsa-miR-15b-5
ENSG0000	ENSG0000	1.57E-06	hsa-let-7a-5p;hsa-let-7b-5p;hsa-let-7d-5p;hsa-let-7e-5p;hsa-l
ENSG0000	ENSG0000	0.000305	hsa-miR-15a-5p;hsa-miR-15b-5p;hsa-miR-195-5p;hsa-miR-42
ENSG0000	ENSG0000	2.11E-07	hsa-miR-15a-5p;hsa-miR-15b-5p;hsa-miR-16-5p;hsa-miR-195-
ENSG0000	ENSG0000	6.89E-06	hsa-miR-15a-5p;hsa-miR-15b-5p;hsa-miR-195-5p;hsa-miR-42
ENSG0000	ENSG0000	0.008544	hsa-let-7a-5p;hsa-let-7b-5p;hsa-let-7d-5p;hsa-let-7e-5p;hsa-l
ENSG0000	ENSG0000	2.49E-08	hsa-miR-103a-3p;hsa-miR-107;hsa-miR-15a-5p;hsa-miR-15b-5
ENSG0000	ENSG0000	5.04E-05	hsa-miR-15a-5p;hsa-miR-15b-5p;hsa-miR-16-5p;hsa-miR-195-
ENSG0000	ENSG0000	0.001552	hsa-miR-15a-5p;hsa-miR-15b-5p;hsa-miR-16-5p;hsa-miR-195-
ENSG0000	ENSG0000	7.10E-07	hsa-miR-15a-5p;hsa-miR-15b-5p;hsa-miR-16-5p;hsa-miR-195-
ENSG0000	ENSG0000	5.96E-05	hsa-miR-15a-5p;hsa-miR-15b-5p;hsa-miR-16-5p;hsa-miR-195-

ENSG0000	ENSG0000	5.63E-07	hsa-let-7a-5p;hsa-let-7b-5p;hsa-let-7d-5p;hsa-let-7e-5p;hsa-l
ENSG0000	ENSG0000	0.003164	hsa-miR-15a-5p;hsa-miR-15b-5p;hsa-miR-195-5p;hsa-miR-24-
ENSG0000	ENSG0000	0.006514	hsa-let-7a-5p;hsa-let-7b-5p;hsa-let-7d-5p;hsa-let-7e-5p;hsa-l
ENSG0000	ENSG0000	8.11E-06	hsa-miR-15a-5p;hsa-miR-15b-5p;hsa-miR-16-5p;hsa-miR-195-
ENSG0000	ENSG0000	2.40E-08	hsa-miR-103a-3p;hsa-miR-107;hsa-miR-15a-5p;hsa-miR-15b-5
ENSG0000	ENSG0000	1.04E-10	hsa-miR-103a-3p;hsa-miR-107;hsa-miR-15a-5p;hsa-miR-15b-5
ENSG0000	ENSG0000	2.82E-05	hsa-let-7a-5p;hsa-let-7b-5p;hsa-let-7d-5p;hsa-let-7e-5p;hsa-l
ENSG0000	ENSG0000	2.11E-07	hsa-miR-15a-5p;hsa-miR-15b-5p;hsa-miR-16-5p;hsa-miR-195-
ENSG0000	ENSG0000	0.008233	hsa-let-7a-5p;hsa-let-7b-5p;hsa-let-7e-5p;hsa-let-7f-5p;hsa-le
ENSG0000	ENSG0000	9.42E-05	hsa-miR-15a-5p;hsa-miR-15b-5p;hsa-miR-16-5p;hsa-miR-195-
ENSG0000	ENSG0000	0.00055	hsa-miR-15a-5p;hsa-miR-15b-5p;hsa-miR-16-5p;hsa-miR-195-
ENSG0000	ENSG0000	0.00055	hsa-miR-15a-5p;hsa-miR-15b-5p;hsa-miR-16-5p;hsa-miR-195-
ENSG0000	ENSG0000	4.64E-07	hsa-let-7a-5p;hsa-let-7b-5p;hsa-let-7d-5p;hsa-let-7e-5p;hsa-l
ENSG0000	ENSG0000	5.78E-05	hsa-let-7a-5p;hsa-let-7b-5p;hsa-let-7d-5p;hsa-let-7e-5p;hsa-l
ENSG0000	ENSG0000	0.000142	hsa-miR-125a-5p;hsa-miR-125b-5p;hsa-miR-15a-5p;hsa-miR-1
ENSG0000	ENSG0000	1.62E-05	hsa-miR-15a-5p;hsa-miR-15b-5p;hsa-miR-16-5p;hsa-miR-195-
ENSG0000	ENSG0000	0.001717	hsa-let-7a-5p;hsa-let-7b-5p;hsa-let-7d-5p;hsa-let-7e-5p;hsa-l
ENSG0000	ENSG0000	7.42E-05	hsa-let-7a-5p;hsa-let-7b-5p;hsa-let-7d-5p;hsa-let-7e-5p;hsa-l
ENSG0000	ENSG0000	0.000652	hsa-miR-15a-5p;hsa-miR-195-5p;hsa-miR-21-3p;hsa-miR-424-
ENSG0000	ENSG0000	0.000219	hsa-miR-15a-5p;hsa-miR-15b-5p;hsa-miR-16-5p;hsa-miR-195-
ENSG0000	ENSG0000	4.09E-08	hsa-miR-15a-5p;hsa-miR-15b-5p;hsa-miR-16-5p;hsa-miR-195-
ENSG0000	ENSG0000	4.53E-06	hsa-miR-103a-3p;hsa-miR-107;hsa-miR-15a-5p;hsa-miR-15b-5
ENSG0000	ENSG0000	4.91E-09	hsa-miR-15a-5p;hsa-miR-15b-5p;hsa-miR-16-5p;hsa-miR-195-
ENSG0000	ENSG0000	7.40E-07	hsa-let-7a-5p;hsa-let-7b-5p;hsa-let-7d-5p;hsa-let-7e-5p;hsa-l
ENSG0000	ENSG0000	2.93E-05	hsa-let-7a-5p;hsa-let-7b-5p;hsa-let-7d-5p;hsa-let-7e-5p;hsa-l
ENSG0000	ENSG0000	2.01E-05	hsa-miR-103a-3p;hsa-miR-107;hsa-miR-15a-5p;hsa-miR-15b-5
ENSG0000	ENSG0000	4.24E-06	hsa-miR-15a-5p;hsa-miR-15b-5p;hsa-miR-16-5p;hsa-miR-195-
ENSG0000	ENSG0000	0.000447	hsa-let-7a-5p;hsa-let-7b-5p;hsa-let-7d-5p;hsa-let-7e-5p;hsa-l
ENSG0000	ENSG0000	8.33E-08	hsa-miR-15a-5p;hsa-miR-15b-5p;hsa-miR-16-5p;hsa-miR-195-
ENSG0000	ENSG0000	0.000132	hsa-let-7a-5p;hsa-let-7b-5p;hsa-let-7d-5p;hsa-let-7e-5p;hsa-l
ENSG0000	ENSG0000	0.000182	hsa-miR-15a-5p;hsa-miR-15b-5p;hsa-miR-16-5p;hsa-miR-195-
ENSG0000	ENSG0000	0.002043	hsa-miR-15a-5p;hsa-miR-15b-5p;hsa-miR-16-5p;hsa-miR-195-
ENSG0000	ENSG0000	2.54E-05	hsa-let-7a-5p;hsa-let-7b-5p;hsa-let-7d-5p;hsa-let-7e-5p;hsa-l
ENSG0000	ENSG0000	1.44E-07	hsa-miR-15a-5p;hsa-miR-15b-5p;hsa-miR-16-5p;hsa-miR-195-
ENSG0000	ENSG0000	8.11E-06	hsa-let-7a-5p;hsa-let-7b-5p;hsa-let-7d-5p;hsa-let-7e-5p;hsa-l
ENSG0000	ENSG0000	0.009008	hsa-let-7a-5p;hsa-let-7d-5p;hsa-let-7e-5p;hsa-let-7f-5p;hsa-le
ENSG0000	ENSG0000	1.44E-08	hsa-let-7a-5p;hsa-let-7b-5p;hsa-let-7d-5p;hsa-let-7e-5p;hsa-l
ENSG0000	ENSG0000	8.21E-06	hsa-miR-15a-5p;hsa-miR-15b-5p;hsa-miR-16-5p;hsa-miR-195-
ENSG0000	ENSG0000	1.15E-09	hsa-let-7a-5p;hsa-let-7b-5p;hsa-let-7d-5p;hsa-let-7e-5p;hsa-l
ENSG0000	ENSG0000	8.11E-06	hsa-let-7a-5p;hsa-let-7b-5p;hsa-let-7d-5p;hsa-let-7e-5p;hsa-l
ENSG0000	ENSG0000	9.39E-06	hsa-miR-103a-3p;hsa-miR-107;hsa-miR-15a-5p;hsa-miR-15b-5
ENSG0000	ENSG0000	0.005956	hsa-miR-15a-5p;hsa-miR-15b-5p;hsa-miR-16-5p;hsa-miR-195-
ENSG0000	ENSG0000	0.002781	hsa-let-7a-5p;hsa-let-7b-5p;hsa-let-7d-5p;hsa-let-7e-5p;hsa-l
ENSG0000	ENSG0000	1.02E-05	hsa-let-7a-5p;hsa-let-7b-5p;hsa-let-7d-5p;hsa-let-7e-5p;hsa-l
ENSG0000	ENSG0000	1.34E-05	hsa-miR-15a-5p;hsa-miR-15b-5p;hsa-miR-16-5p;hsa-miR-195-
ENSG0000	ENSG0000	0.004516	hsa-let-7a-5p;hsa-let-7b-5p;hsa-let-7d-5p;hsa-let-7e-5p;hsa-l
ENSG0000	ENSG0000	0.000804	hsa-miR-15a-5p;hsa-miR-15b-5p;hsa-miR-16-5p;hsa-miR-195-
ENSG0000	ENSG0000	1.99E-05	hsa-miR-139-5p;hsa-miR-15a-5p;hsa-miR-15b-5p;hsa-miR-19
ENSG0000	ENSG0000	0.00033	hsa-miR-15a-5p;hsa-miR-15b-5p;hsa-miR-16-5p;hsa-miR-195-
ENSG0000	ENSG0000	0.008993	hsa-miR-139-5p;hsa-miR-30b-5p;hsa-miR-30c-5p;hsa-miR-36
ENSG0000	ENSG0000	2.76E-06	hsa-miR-15a-5p;hsa-miR-15b-5p;hsa-miR-16-5p;hsa-miR-195-
ENSG0000	ENSG0000	1.41E-06	hsa-miR-27a-3p;hsa-miR-27b-3p;hsa-miR-3605-5p;hsa-miR-5
ENSG0000	ENSG0000	1.93E-08	hsa-miR-15a-5p;hsa-miR-15b-5p;hsa-miR-16-5p;hsa-miR-195-
ENSG0000	ENSG0000	0.000941	hsa-miR-519a-3p;hsa-miR-519b-3p;hsa-miR-519c-3p;hsa-miR-

ENSG0000	ENSG0000	1.52E-10	hsa-miR-15a-5p;hsa-miR-15b-5p;hsa-miR-16-5p;hsa-miR-195-
ENSG0000	ENSG0000	5.86E-09	hsa-miR-15a-5p;hsa-miR-15b-5p;hsa-miR-195-5p;hsa-miR-21
ENSG0000	ENSG0000	1.90E-09	hsa-miR-15a-5p;hsa-miR-15b-5p;hsa-miR-16-5p;hsa-miR-195-
ENSG0000	ENSG0000	0.00021	hsa-miR-15a-5p;hsa-miR-15b-5p;hsa-miR-195-5p;hsa-miR-42
ENSG0000	ENSG0000	1.42E-07	hsa-miR-15a-5p;hsa-miR-15b-5p;hsa-miR-16-5p;hsa-miR-195-
ENSG0000	ENSG0000	1.42E-07	hsa-miR-15a-5p;hsa-miR-15b-5p;hsa-miR-16-5p;hsa-miR-195-
ENSG0000	ENSG0000	1.99E-05	hsa-miR-27a-3p;hsa-miR-27b-3p;hsa-miR-30b-5p;hsa-miR-30
ENSG0000	ENSG0000	0.001268	hsa-miR-15a-5p;hsa-miR-15b-5p;hsa-miR-16-5p;hsa-miR-195-
ENSG0000	ENSG0000	2.22E-05	hsa-miR-15a-5p;hsa-miR-15b-5p;hsa-miR-16-5p;hsa-miR-195-
ENSG0000	ENSG0000	0.001892	hsa-miR-139-5p;hsa-miR-15a-5p;hsa-miR-15b-5p;hsa-miR-16-
ENSG0000	ENSG0000	0.002063	hsa-miR-361-3p;hsa-miR-4691-3p;hsa-miR-520a-3p;hsa-miR-
ENSG0000	ENSG0000	0.001892	hsa-miR-519a-3p;hsa-miR-519b-3p;hsa-miR-519c-3p;hsa-miR-
ENSG0000	ENSG0000	9.14E-05	hsa-miR-15a-5p;hsa-miR-15b-5p;hsa-miR-16-5p;hsa-miR-195-
ENSG0000	ENSG0000	0.002655	hsa-miR-519a-3p;hsa-miR-519b-3p;hsa-miR-519c-3p;hsa-miR-
ENSG0000	ENSG0000	2.47E-06	hsa-miR-15a-5p;hsa-miR-15b-5p;hsa-miR-16-5p;hsa-miR-195-
ENSG0000	ENSG0000	5.87E-05	hsa-miR-1185-5p;hsa-miR-214-3p;hsa-miR-3605-5p;hsa-miR-
ENSG0000	ENSG0000	0.000378	hsa-miR-15a-5p;hsa-miR-15b-5p;hsa-miR-195-5p;hsa-miR-42
ENSG0000	ENSG0000	1.44E-05	hsa-miR-15a-5p;hsa-miR-15b-5p;hsa-miR-16-5p;hsa-miR-195-
ENSG0000	ENSG0000	0.000748	hsa-miR-218-5p;hsa-miR-519a-3p;hsa-miR-519b-3p;hsa-miR-
ENSG0000	ENSG0000	3.30E-06	hsa-miR-15b-5p;hsa-miR-16-5p;hsa-miR-195-5p;hsa-miR-218-
ENSG0000	ENSG0000	4.61E-05	hsa-miR-15a-5p;hsa-miR-15b-5p;hsa-miR-16-5p;hsa-miR-424-
ENSG0000	ENSG0000	6.28E-05	hsa-miR-15a-5p;hsa-miR-15b-5p;hsa-miR-16-5p;hsa-miR-195-
ENSG0000	ENSG0000	0.003567	hsa-miR-214-3p;hsa-miR-30b-5p;hsa-miR-30c-5p;hsa-miR-36
ENSG0000	ENSG0000	1.55E-06	hsa-miR-15a-5p;hsa-miR-15b-5p;hsa-miR-16-5p;hsa-miR-195-
ENSG0000	ENSG0000	0.003317	hsa-miR-15a-5p;hsa-miR-15b-5p;hsa-miR-195-5p;hsa-miR-27
ENSG0000	ENSG0000	0.006385	hsa-miR-15a-5p;hsa-miR-15b-5p;hsa-miR-16-5p;hsa-miR-195-
ENSG0000	ENSG0000	0.001089	hsa-miR-16-5p;hsa-miR-519a-3p;hsa-miR-519b-3p;hsa-miR-5
ENSG0000	ENSG0000	8.66E-06	hsa-miR-15a-5p;hsa-miR-519a-3p;hsa-miR-519b-3p;hsa-miR-
ENSG0000	ENSG0000	0.000128	hsa-miR-15a-5p;hsa-miR-15b-5p;hsa-miR-16-5p;hsa-miR-195-
ENSG0000	ENSG0000	3.27E-05	hsa-miR-519a-3p;hsa-miR-519b-3p;hsa-miR-519c-3p;hsa-miR-
ENSG0000	ENSG0000	7.37E-05	hsa-miR-15a-5p;hsa-miR-15b-5p;hsa-miR-16-5p;hsa-miR-195-
ENSG0000	ENSG0000	1.03E-05	hsa-miR-126-5p;hsa-miR-15a-5p;hsa-miR-16-5p;hsa-miR-195-
ENSG0000	ENSG0000	0.00014	hsa-miR-519a-3p;hsa-miR-519b-3p;hsa-miR-519c-3p;hsa-miR-
ENSG0000	ENSG0000	8.12E-05	hsa-miR-15a-5p;hsa-miR-15b-5p;hsa-miR-16-5p;hsa-miR-195-
ENSG0000	ENSG0000	0.000809	hsa-miR-126-5p;hsa-miR-15a-5p;hsa-miR-15b-5p;hsa-miR-16-
ENSG0000	ENSG0000	0.000166	hsa-miR-519a-3p;hsa-miR-519b-3p;hsa-miR-519c-3p;hsa-miR-
ENSG0000	ENSG0000	3.55E-05	hsa-miR-1185-5p;hsa-miR-15a-5p;hsa-miR-15b-5p;hsa-miR-1
ENSG0000	ENSG0000	1.70E-07	hsa-miR-15a-5p;hsa-miR-15b-5p;hsa-miR-16-5p;hsa-miR-195-
ENSG0000	ENSG0000	3.97E-05	hsa-miR-15a-5p;hsa-miR-15b-5p;hsa-miR-16-5p;hsa-miR-195-
ENSG0000	ENSG0000	1.25E-09	hsa-miR-15a-5p;hsa-miR-15b-5p;hsa-miR-16-5p;hsa-miR-195-
ENSG0000	ENSG0000	0.009982	hsa-miR-361-3p;hsa-miR-520a-3p;hsa-miR-520c-3p;hsa-miR-
ENSG0000	ENSG0000	1.61E-05	hsa-miR-15a-5p;hsa-miR-15b-5p;hsa-miR-195-5p;hsa-miR-42
ENSG0000	ENSG0000	0.000159	hsa-miR-15a-5p;hsa-miR-15b-5p;hsa-miR-16-5p;hsa-miR-195-
ENSG0000	ENSG0000	9.79E-05	hsa-miR-519a-3p;hsa-miR-519b-3p;hsa-miR-519c-3p;hsa-miR-
ENSG0000	ENSG0000	5.87E-05	hsa-miR-218-5p;hsa-miR-519a-3p;hsa-miR-519b-3p;hsa-miR-
ENSG0000	ENSG0000	7.09E-07	hsa-miR-15a-5p;hsa-miR-15b-5p;hsa-miR-16-5p;hsa-miR-195-
ENSG0000	ENSG0000	0.000809	hsa-miR-519a-3p;hsa-miR-519b-3p;hsa-miR-519c-3p;hsa-miR-
ENSG0000	ENSG0000	0.00014	hsa-miR-15a-5p;hsa-miR-15b-5p;hsa-miR-195-5p;hsa-miR-42
ENSG0000	ENSG0000	0.008072	hsa-miR-519a-3p;hsa-miR-519b-3p;hsa-miR-519c-3p;hsa-miR-
ENSG0000	ENSG0000	0.003135	hsa-miR-519a-3p;hsa-miR-519b-3p;hsa-miR-519c-3p;hsa-miR-
ENSG0000	ENSG0000	0.001534	hsa-miR-519a-3p;hsa-miR-519b-3p;hsa-miR-519c-3p;hsa-miR-
ENSG0000	ENSG0000	0.006047	hsa-miR-4691-3p;hsa-miR-520a-3p;hsa-miR-520c-3p;hsa-miR-
ENSG0000	ENSG0000	3.83E-07	hsa-miR-15a-5p;hsa-miR-15b-5p;hsa-miR-16-5p;hsa-miR-195-
ENSG0000	ENSG0000	0.000472	hsa-miR-519a-3p;hsa-miR-519b-3p;hsa-miR-519c-3p;hsa-miR-

ENSG0000	ENSG0000	4.61E-05	hsa-miR-15b-5p;hsa-miR-16-5p;hsa-miR-30c-5p;hsa-miR-497-
ENSG0000	ENSG0000	0.003317	hsa-miR-2115-5p;hsa-miR-519a-3p;hsa-miR-519b-3p;hsa-miR-
ENSG0000	ENSG0000	7.24E-10	hsa-miR-15a-5p;hsa-miR-15b-5p;hsa-miR-16-5p;hsa-miR-195-
ENSG0000	ENSG0000	1.03E-05	hsa-miR-15a-5p;hsa-miR-15b-5p;hsa-miR-16-5p;hsa-miR-195-
ENSG0000	ENSG0000	6.57E-09	hsa-miR-15a-5p;hsa-miR-15b-5p;hsa-miR-16-5p;hsa-miR-195-
ENSG0000	ENSG0000	0.000166	hsa-miR-519a-3p;hsa-miR-519b-3p;hsa-miR-519c-3p;hsa-miR-
ENSG0000	ENSG0000	1.89E-05	hsa-miR-15a-5p;hsa-miR-15b-5p;hsa-miR-16-5p;hsa-miR-195-
ENSG0000	ENSG0000	0.000414	hsa-miR-15a-5p;hsa-miR-195-5p;hsa-miR-30b-5p;hsa-miR-30
ENSG0000	ENSG0000	8.20E-09	hsa-miR-15a-5p;hsa-miR-15b-5p;hsa-miR-195-5p;hsa-miR-21
ENSG0000	ENSG0000	1.55E-06	hsa-miR-15a-5p;hsa-miR-15b-5p;hsa-miR-16-5p;hsa-miR-195-
ENSG0000	ENSG0000	6.40E-06	hsa-miR-15a-5p;hsa-miR-15b-5p;hsa-miR-195-5p;hsa-miR-21
ENSG0000	ENSG0000	0.005013	hsa-miR-4726-3p;hsa-miR-520a-3p;hsa-miR-520c-3p;hsa-miR-
ENSG0000	ENSG0000	0.000231	hsa-miR-27a-3p;hsa-miR-27b-3p;hsa-miR-519a-3p;hsa-miR-5
ENSG0000	ENSG0000	1.96E-05	hsa-miR-15a-5p;hsa-miR-15b-5p;hsa-miR-16-5p;hsa-miR-195-
ENSG0000	ENSG0000	0.000565	hsa-miR-15a-5p;hsa-miR-15b-5p;hsa-miR-195-5p;hsa-miR-42
ENSG0000	ENSG0000	0.000207	hsa-miR-519a-3p;hsa-miR-519b-3p;hsa-miR-519c-3p;hsa-miR-
ENSG0000	ENSG0000	2.22E-05	hsa-miR-15a-5p;hsa-miR-15b-5p;hsa-miR-16-5p;hsa-miR-195-
ENSG0000	ENSG0000	0.008082	hsa-miR-15a-5p;hsa-miR-16-5p;hsa-miR-27a-3p;hsa-miR-27b-
ENSG0000	ENSG0000	3.85E-05	hsa-miR-519a-3p;hsa-miR-519b-3p;hsa-miR-519c-3p;hsa-miR-
ENSG0000	ENSG0000	1.61E-05	hsa-miR-519a-3p;hsa-miR-519b-3p;hsa-miR-519c-3p;hsa-miR-
ENSG0000	ENSG0000	0.001641	hsa-miR-15a-5p;hsa-miR-15b-5p;hsa-miR-195-5p;hsa-miR-21
ENSG0000	ENSG0000	5.51E-06	hsa-miR-519a-3p;hsa-miR-519b-3p;hsa-miR-519c-3p;hsa-miR-
ENSG0000	ENSG0000	1.70E-07	hsa-miR-27b-3p;hsa-miR-519a-3p;hsa-miR-519b-3p;hsa-miR-
ENSG0000	ENSG0000	0.00066	hsa-miR-15a-5p;hsa-miR-15b-5p;hsa-miR-16-5p;hsa-miR-195-
ENSG0000	ENSG0000	8.66E-06	hsa-miR-15a-5p;hsa-miR-15b-5p;hsa-miR-195-5p;hsa-miR-36
ENSG0000	ENSG0000	0.000148	hsa-miR-15a-5p;hsa-miR-15b-5p;hsa-miR-195-5p;hsa-miR-42
ENSG0000	ENSG0000	2.07E-05	hsa-miR-15a-5p;hsa-miR-15b-5p;hsa-miR-16-5p;hsa-miR-195-
ENSG0000	ENSG0000	8.52E-07	hsa-miR-218-5p;hsa-miR-27a-3p;hsa-miR-27b-3p;hsa-miR-51
ENSG0000	ENSG0000	2.33E-05	hsa-miR-15a-5p;hsa-miR-15b-5p;hsa-miR-195-5p;hsa-miR-42
ENSG0000	ENSG0000	1.90E-06	hsa-miR-15a-5p;hsa-miR-195-5p;hsa-miR-424-5p;hsa-miR-49
ENSG0000	ENSG0000	0.00814	hsa-miR-139-5p;hsa-miR-218-5p;hsa-miR-497-5p;hsa-miR-52
ENSG0000	ENSG0000	0.000873	hsa-miR-16-5p;hsa-miR-218-5p;hsa-miR-519a-3p;hsa-miR-51
ENSG0000	ENSG0000	0.008772	hsa-miR-214-3p;hsa-miR-3619-5p;hsa-miR-520a-3p;hsa-miR-
ENSG0000	ENSG0000	2.33E-05	hsa-miR-519a-3p;hsa-miR-519b-3p;hsa-miR-519c-3p;hsa-miR-
ENSG0000	ENSG0000	0.000165	hsa-miR-15a-5p;hsa-miR-15b-5p;hsa-miR-195-5p;hsa-miR-21
ENSG0000	ENSG0000	0.000256	hsa-miR-139-5p;hsa-miR-3605-5p;hsa-miR-520a-3p;hsa-miR-
ENSG0000	ENSG0000	0.002809	hsa-miR-15a-5p;hsa-miR-15b-5p;hsa-miR-195-5p;hsa-miR-42
ENSG0000	ENSG0000	0.006421	hsa-miR-519a-3p;hsa-miR-519b-3p;hsa-miR-519c-3p;hsa-miR-
ENSG0000	ENSG0000	0.000637	hsa-miR-15a-5p;hsa-miR-15b-5p;hsa-miR-195-5p;hsa-miR-42
ENSG0000	ENSG0000	1.02E-06	hsa-miR-15a-5p;hsa-miR-15b-5p;hsa-miR-16-5p;hsa-miR-195-
ENSG0000	ENSG0000	4.25E-09	hsa-miR-15a-5p;hsa-miR-15b-5p;hsa-miR-16-5p;hsa-miR-195-
ENSG0000	ENSG0000	4.25E-09	hsa-miR-15a-5p;hsa-miR-15b-5p;hsa-miR-16-5p;hsa-miR-195-
ENSG0000	ENSG0000	5.46E-05	hsa-miR-15a-5p;hsa-miR-15b-5p;hsa-miR-16-5p;hsa-miR-195-
ENSG0000	ENSG0000	1.89E-06	hsa-miR-15a-5p;hsa-miR-15b-5p;hsa-miR-195-5p;hsa-miR-27
ENSG0000	ENSG0000	0.002244	hsa-miR-519a-3p;hsa-miR-519b-3p;hsa-miR-519c-3p;hsa-miR-
ENSG0000	ENSG0000	3.20E-06	hsa-miR-15a-5p;hsa-miR-15b-5p;hsa-miR-16-5p;hsa-miR-195-
ENSG0000	ENSG0000	1.21E-06	hsa-miR-15a-5p;hsa-miR-15b-5p;hsa-miR-16-5p;hsa-miR-195-
ENSG0000	ENSG0000	0.001089	hsa-miR-15b-5p;hsa-miR-27a-3p;hsa-miR-27b-3p;hsa-miR-36
ENSG0000	ENSG0000	6.03E-05	hsa-miR-15a-5p;hsa-miR-195-5p;hsa-miR-424-5p;hsa-miR-49
ENSG0000	ENSG0000	5.45E-07	hsa-miR-27a-3p;hsa-miR-27b-3p;hsa-miR-519a-3p;hsa-miR-5
ENSG0000	ENSG0000	0.008524	hsa-miR-519a-3p;hsa-miR-519b-3p;hsa-miR-519c-3p;hsa-miR-
ENSG0000	ENSG0000	9.08E-05	hsa-miR-519a-3p;hsa-miR-519b-3p;hsa-miR-519c-3p;hsa-miR-
ENSG0000	ENSG0000	0.001977	hsa-miR-195-5p;hsa-miR-519a-3p;hsa-miR-519b-3p;hsa-miR-
ENSG0000	ENSG0000	0.000267	hsa-miR-139-5p;hsa-miR-218-5p;hsa-miR-27b-3p;hsa-miR-42

ENSG0000	ENSG0000	8.73E-06	hsa-miR-519a-3p;hsa-miR-519b-3p;hsa-miR-519c-3p;hsa-miR-
ENSG0000	ENSG0000	6.94E-05	hsa-miR-15a-5p;hsa-miR-15b-5p;hsa-miR-195-5p;hsa-miR-42
ENSG0000	ENSG0000	0.000312	hsa-miR-27a-3p;hsa-miR-27b-3p;hsa-miR-4726-3p;hsa-miR-5
ENSG0000	ENSG0000	0.001342	hsa-miR-519a-3p;hsa-miR-519b-3p;hsa-miR-519c-3p;hsa-miR-
ENSG0000	ENSG0000	0.000609	hsa-miR-218-5p;hsa-miR-520a-3p;hsa-miR-520c-3p;hsa-miR-
ENSG0000	ENSG0000	0.001169	hsa-miR-15a-5p;hsa-miR-15b-5p;hsa-miR-195-5p;hsa-miR-42
ENSG0000	ENSG0000	0.000682	hsa-miR-519a-3p;hsa-miR-519b-3p;hsa-miR-519c-3p;hsa-miR-
ENSG0000	ENSG0000	0.00383	hsa-miR-519a-3p;hsa-miR-519b-3p;hsa-miR-519c-3p;hsa-miR-
ENSG0000	ENSG0000	2.29E-06	hsa-miR-15a-5p;hsa-miR-15b-5p;hsa-miR-16-5p;hsa-miR-195-
ENSG0000	ENSG0000	0.004696	hsa-miR-218-5p;hsa-miR-27a-3p;hsa-miR-27b-3p;hsa-miR-42
ENSG0000	ENSG0000	0.001342	hsa-miR-519a-3p;hsa-miR-519b-3p;hsa-miR-519c-3p;hsa-miR-
ENSG0000	ENSG0000	0.000682	hsa-miR-15a-5p;hsa-miR-15b-5p;hsa-miR-16-5p;hsa-miR-195-
ENSG0000	ENSG0000	0.001253	hsa-miR-15a-5p;hsa-miR-15b-5p;hsa-miR-195-5p;hsa-miR-42
ENSG0000	ENSG0000	8.01E-08	hsa-miR-218-5p;hsa-miR-27a-3p;hsa-miR-27b-3p;hsa-miR-36
ENSG0000	ENSG0000	3.30E-06	hsa-miR-15a-5p;hsa-miR-15b-5p;hsa-miR-195-5p;hsa-miR-42
ENSG0000	ENSG0000	4.25E-09	hsa-miR-15a-5p;hsa-miR-15b-5p;hsa-miR-16-5p;hsa-miR-195-
ENSG0000	ENSG0000	4.25E-09	hsa-miR-15a-5p;hsa-miR-15b-5p;hsa-miR-16-5p;hsa-miR-195-
ENSG0000	ENSG0000	1.72E-05	hsa-miR-139-5p;hsa-miR-15a-5p;hsa-miR-15b-5p;hsa-miR-19
ENSG0000	ENSG0000	0.008993	hsa-miR-519a-3p;hsa-miR-519b-3p;hsa-miR-519c-3p;hsa-miR-
ENSG0000	ENSG0000	0.005969	hsa-miR-519a-3p;hsa-miR-519b-3p;hsa-miR-519c-3p;hsa-miR-
ENSG0000	ENSG0000	2.18E-08	hsa-miR-15a-5p;hsa-miR-15b-5p;hsa-miR-16-5p;hsa-miR-195-
ENSG0000	ENSG0000	5.22E-05	hsa-miR-15a-5p;hsa-miR-15b-5p;hsa-miR-195-5p;hsa-miR-42
ENSG0000	ENSG0000	0.000117	hsa-miR-519a-3p;hsa-miR-519b-3p;hsa-miR-519c-3p;hsa-miR-
ENSG0000	ENSG0000	0.000117	hsa-miR-27a-3p;hsa-miR-27b-3p;hsa-miR-519a-3p;hsa-miR-5
ENSG0000	ENSG0000	7.44E-06	hsa-miR-126-5p;hsa-miR-15a-5p;hsa-miR-15b-5p;hsa-miR-19
ENSG0000	ENSG0000	7.46E-06	hsa-miR-15a-5p;hsa-miR-15b-5p;hsa-miR-195-5p;hsa-miR-42
ENSG0000	ENSG0000	0.000307	hsa-miR-15a-5p;hsa-miR-15b-5p;hsa-miR-16-5p;hsa-miR-195-
ENSG0000	ENSG0000	1.08E-07	hsa-miR-15a-5p;hsa-miR-15b-5p;hsa-miR-16-5p;hsa-miR-195-
ENSG0000	ENSG0000	0.000472	hsa-miR-15a-5p;hsa-miR-15b-5p;hsa-miR-195-5p;hsa-miR-21
ENSG0000	ENSG0000	0.009131	hsa-miR-15a-5p;hsa-miR-16-5p;hsa-miR-520a-3p;hsa-miR-52
ENSG0000	ENSG0000	3.23E-09	hsa-miR-15a-5p;hsa-miR-15b-5p;hsa-miR-16-5p;hsa-miR-195-
ENSG0000	ENSG0000	0.001344	hsa-miR-15a-5p;hsa-miR-15b-5p;hsa-miR-195-5p;hsa-miR-42
ENSG0000	ENSG0000	3.59E-08	hsa-miR-15a-5p;hsa-miR-15b-5p;hsa-miR-16-5p;hsa-miR-195-
ENSG0000	ENSG0000	0.000194	hsa-miR-15a-5p;hsa-miR-15b-5p;hsa-miR-16-5p;hsa-miR-195-
ENSG0000	ENSG0000	0.006047	hsa-miR-519a-3p;hsa-miR-519b-3p;hsa-miR-519c-3p;hsa-miR-
ENSG0000	ENSG0000	1.70E-07	hsa-miR-15a-5p;hsa-miR-15b-5p;hsa-miR-16-5p;hsa-miR-195-
ENSG0000	ENSG0000	1.70E-07	hsa-miR-15a-5p;hsa-miR-15b-5p;hsa-miR-16-5p;hsa-miR-195-
ENSG0000	ENSG0000	1.51E-05	hsa-miR-15a-5p;hsa-miR-15b-5p;hsa-miR-16-5p;hsa-miR-195-
ENSG0000	ENSG0000	0.008524	hsa-miR-519a-3p;hsa-miR-519b-3p;hsa-miR-519c-3p;hsa-miR-
ENSG0000	ENSG0000	5.51E-06	hsa-miR-218-5p;hsa-miR-519a-3p;hsa-miR-519b-3p;hsa-miR-
ENSG0000	ENSG0000	1.99E-05	hsa-miR-15a-5p;hsa-miR-15b-5p;hsa-miR-16-5p;hsa-miR-195-
ENSG0000	ENSG0000	1.99E-05	hsa-miR-15a-5p;hsa-miR-15b-5p;hsa-miR-16-5p;hsa-miR-195-
ENSG0000	ENSG0000	7.42E-07	hsa-miR-15a-5p;hsa-miR-15b-5p;hsa-miR-16-5p;hsa-miR-195-
ENSG0000	ENSG0000	0.001581	hsa-miR-15a-5p;hsa-miR-15b-5p;hsa-miR-195-5p;hsa-miR-21
ENSG0000	ENSG0000	0.001581	hsa-miR-519a-3p;hsa-miR-519b-3p;hsa-miR-519c-3p;hsa-miR-
ENSG0000	ENSG0000	3.92E-06	hsa-miR-15a-5p;hsa-miR-15b-5p;hsa-miR-16-5p;hsa-miR-195-
ENSG0000	ENSG0000	0.000495	hsa-miR-15a-5p;hsa-miR-15b-5p;hsa-miR-195-5p;hsa-miR-42
ENSG0000	ENSG0000	6.79E-05	hsa-miR-15a-5p;hsa-miR-15b-5p;hsa-miR-195-5p;hsa-miR-27
ENSG0000	ENSG0000	3.30E-06	hsa-miR-15a-5p;hsa-miR-15b-5p;hsa-miR-16-5p;hsa-miR-195-
ENSG0000	ENSG0000	0.000267	hsa-miR-15a-5p;hsa-miR-15b-5p;hsa-miR-16-5p;hsa-miR-195-
ENSG0000	ENSG0000	1.68E-12	hsa-miR-15a-5p;hsa-miR-15b-5p;hsa-miR-16-5p;hsa-miR-195-
ENSG0000	ENSG0000	0.000916	hsa-miR-27a-3p;hsa-miR-27b-3p;hsa-miR-4726-3p;hsa-miR-5
ENSG0000	ENSG0000	0.000413	hsa-miR-139-5p;hsa-miR-15a-5p;hsa-miR-15b-5p;hsa-miR-19
ENSG0000	ENSG0000	6.45E-07	hsa-miR-15a-5p;hsa-miR-15b-5p;hsa-miR-16-5p;hsa-miR-195-

ENSG0000	ENSG0000	9.02E-08	hsa-miR-15a-5p;hsa-miR-15b-5p;hsa-miR-16-5p;hsa-miR-195-
ENSG0000	ENSG0000	4.92E-05	hsa-miR-369-3p;hsa-miR-519a-3p;hsa-miR-519b-3p;hsa-miR-
ENSG0000	ENSG0000	4.64E-06	hsa-miR-15a-5p;hsa-miR-15b-5p;hsa-miR-195-5p;hsa-miR-42
ENSG0000	ENSG0000	1.03E-05	hsa-miR-139-5p;hsa-miR-15a-5p;hsa-miR-15b-5p;hsa-miR-16-
ENSG0000	ENSG0000	6.40E-06	hsa-miR-15a-5p;hsa-miR-15b-5p;hsa-miR-195-5p;hsa-miR-42
ENSG0000	ENSG0000	0.00144	hsa-miR-15a-5p;hsa-miR-15b-5p;hsa-miR-16-5p;hsa-miR-195-
ENSG0000	ENSG0000	0.004105	hsa-miR-519a-3p;hsa-miR-519b-3p;hsa-miR-519c-3p;hsa-miR-
ENSG0000	ENSG0000	2.59E-07	hsa-miR-15a-5p;hsa-miR-15b-5p;hsa-miR-195-5p;hsa-miR-21
ENSG0000	ENSG0000	0.001858	hsa-miR-519a-3p;hsa-miR-519b-3p;hsa-miR-519c-3p;hsa-miR-
ENSG0000	ENSG0000	5.45E-07	hsa-miR-27a-3p;hsa-miR-27b-3p;hsa-miR-519a-3p;hsa-miR-5
ENSG0000	ENSG0000	2.57E-08	hsa-miR-15a-5p;hsa-miR-15b-5p;hsa-miR-16-5p;hsa-miR-195-
ENSG0000	ENSG0000	6.28E-05	hsa-miR-139-5p;hsa-miR-218-5p;hsa-miR-30c-5p;hsa-miR-51
ENSG0000	ENSG0000	9.02E-08	hsa-miR-15a-5p;hsa-miR-15b-5p;hsa-miR-16-5p;hsa-miR-195-
ENSG0000	ENSG0000	1.26E-06	hsa-miR-15a-5p;hsa-miR-15b-5p;hsa-miR-16-5p;hsa-miR-195-
ENSG0000	ENSG0000	0.000122	hsa-miR-15a-5p;hsa-miR-15b-5p;hsa-miR-16-5p;hsa-miR-195-
ENSG0000	ENSG0000	1.08E-10	hsa-miR-126-5p;hsa-miR-15a-5p;hsa-miR-15b-5p;hsa-miR-16-
ENSG0000	ENSG0000	2.50E-06	hsa-miR-1185-5p;hsa-miR-519a-3p;hsa-miR-519b-3p;hsa-miR-
ENSG0000	ENSG0000	0.001952	hsa-miR-15a-5p;hsa-miR-15b-5p;hsa-miR-16-5p;hsa-miR-195-
ENSG0000	ENSG0000	0.000132	hsa-miR-1185-5p;hsa-miR-214-3p;hsa-miR-3605-5p;hsa-miR-
ENSG0000	ENSG0000	0.001581	hsa-miR-519a-3p;hsa-miR-519b-3p;hsa-miR-519c-3p;hsa-miR-
ENSG0000	ENSG0000	4.29E-06	hsa-miR-15a-5p;hsa-miR-15b-5p;hsa-miR-16-5p;hsa-miR-195-
ENSG0000	ENSG0000	4.54E-05	hsa-miR-15a-5p;hsa-miR-15b-5p;hsa-miR-16-5p;hsa-miR-195-
ENSG0000	ENSG0000	8.92E-05	hsa-miR-15a-5p;hsa-miR-15b-5p;hsa-miR-16-5p;hsa-miR-195-
ENSG0000	ENSG0000	2.27E-11	hsa-miR-15a-5p;hsa-miR-15b-5p;hsa-miR-16-5p;hsa-miR-195-
ENSG0000	ENSG0000	8.50E-05	hsa-miR-126-5p;hsa-miR-15a-5p;hsa-miR-15b-5p;hsa-miR-16-
ENSG0000	ENSG0000	0.000865	hsa-miR-15a-5p;hsa-miR-15b-5p;hsa-miR-195-5p;hsa-miR-27
ENSG0000	ENSG0000	0.003317	hsa-miR-519a-3p;hsa-miR-519b-3p;hsa-miR-519c-3p;hsa-miR-
ENSG0000	ENSG0000	0.003317	hsa-miR-519a-3p;hsa-miR-519b-3p;hsa-miR-519c-3p;hsa-miR-
ENSG0000	ENSG0000	0.00814	hsa-miR-519a-3p;hsa-miR-519b-3p;hsa-miR-519c-3p;hsa-miR-
ENSG0000	ENSG0000	0.000359	hsa-miR-15a-5p;hsa-miR-15b-5p;hsa-miR-16-5p;hsa-miR-195-
ENSG0000	ENSG0000	6.45E-07	hsa-miR-15a-5p;hsa-miR-15b-5p;hsa-miR-16-5p;hsa-miR-195-
ENSG0000	ENSG0000	2.50E-06	hsa-miR-519a-3p;hsa-miR-519b-3p;hsa-miR-519c-3p;hsa-miR-
ENSG0000	ENSG0000	0.001732	hsa-miR-519a-3p;hsa-miR-519b-3p;hsa-miR-519c-3p;hsa-miR-
ENSG0000	ENSG0000	1.21E-06	hsa-miR-15a-5p;hsa-miR-15b-5p;hsa-miR-195-5p;hsa-miR-51
ENSG0000	ENSG0000	0.001848	hsa-miR-15a-5p;hsa-miR-16-5p;hsa-miR-195-5p;hsa-miR-218-
ENSG0000	ENSG0000	2.18E-08	hsa-miR-15a-5p;hsa-miR-15b-5p;hsa-miR-16-5p;hsa-miR-195-
ENSG0000	ENSG0000	1.70E-07	hsa-miR-15a-5p;hsa-miR-15b-5p;hsa-miR-16-5p;hsa-miR-195-
ENSG0000	ENSG0000	2.55E-10	hsa-miR-15a-5p;hsa-miR-15b-5p;hsa-miR-16-5p;hsa-miR-195-
ENSG0000	ENSG0000	2.77E-05	hsa-miR-15a-5p;hsa-miR-15b-5p;hsa-miR-195-5p;hsa-miR-42
ENSG0000	ENSG0000	2.72E-07	hsa-miR-15a-5p;hsa-miR-15b-5p;hsa-miR-16-5p;hsa-miR-195-
ENSG0000	ENSG0000	1.83E-12	hsa-miR-15a-5p;hsa-miR-15b-5p;hsa-miR-16-5p;hsa-miR-195-
ENSG0000	ENSG0000	1.81E-07	hsa-miR-15a-5p;hsa-miR-15b-5p;hsa-miR-16-5p;hsa-miR-195-
ENSG0000	ENSG0000	0.000691	hsa-miR-126-5p;hsa-miR-519a-3p;hsa-miR-519b-3p;hsa-miR-
ENSG0000	ENSG0000	0.000691	hsa-miR-126-5p;hsa-miR-519a-3p;hsa-miR-519b-3p;hsa-miR-
ENSG0000	ENSG0000	6.68E-05	hsa-miR-15a-5p;hsa-miR-15b-5p;hsa-miR-16-5p;hsa-miR-195-
ENSG0000	ENSG0000	4.50E-07	hsa-miR-15a-5p;hsa-miR-15b-5p;hsa-miR-195-5p;hsa-miR-42
ENSG0000	ENSG0000	7.60E-07	hsa-miR-15a-5p;hsa-miR-15b-5p;hsa-miR-16-5p;hsa-miR-195-
ENSG0000	ENSG0000	0.000107	hsa-miR-15a-5p;hsa-miR-15b-5p;hsa-miR-16-5p;hsa-miR-195-
ENSG0000	ENSG0000	0.000359	hsa-miR-218-5p;hsa-miR-519a-3p;hsa-miR-519b-3p;hsa-miR-
ENSG0000	ENSG0000	0.000609	hsa-miR-15a-5p;hsa-miR-15b-5p;hsa-miR-195-5p;hsa-miR-21
ENSG0000	ENSG0000	1.99E-05	hsa-miR-16-5p;hsa-miR-30b-5p;hsa-miR-30c-5p;hsa-miR-361-
ENSG0000	ENSG0000	1.13E-07	hsa-miR-30b-5p;hsa-miR-30c-5p;hsa-miR-3605-5p;hsa-miR-4
ENSG0000	ENSG0000	2.16E-10	hsa-miR-15a-5p;hsa-miR-15b-5p;hsa-miR-16-5p;hsa-miR-195-
ENSG0000	ENSG0000	0.003135	hsa-miR-139-5p;hsa-miR-218-5p;hsa-miR-27a-3p;hsa-miR-27

ENSG0000	ENSG0000	2.16E-06	hsa-miR-15a-5p;hsa-miR-15b-5p;hsa-miR-195-5p;hsa-miR-42
ENSG0000	ENSG0000	2.18E-08	hsa-miR-15a-5p;hsa-miR-15b-5p;hsa-miR-16-5p;hsa-miR-195-
ENSG0000	ENSG0000	8.12E-05	hsa-miR-15a-5p;hsa-miR-15b-5p;hsa-miR-16-5p;hsa-miR-195-
ENSG0000	ENSG0000	0.007215	hsa-miR-4726-3p;hsa-miR-520a-3p;hsa-miR-520c-3p;hsa-miR-
ENSG0000	ENSG0000	0.002419	hsa-miR-27a-3p;hsa-miR-27b-3p;hsa-miR-519a-3p;hsa-miR-5
ENSG0000	ENSG0000	0.000267	hsa-miR-126-5p;hsa-miR-15b-5p;hsa-miR-16-5p;hsa-miR-30b
ENSG0000	ENSG0000	6.81E-06	hsa-miR-15a-5p;hsa-miR-15b-5p;hsa-miR-195-5p;hsa-miR-42
ENSG0000	ENSG0000	0.001569	hsa-miR-15a-5p;hsa-miR-15b-5p;hsa-miR-16-5p;hsa-miR-195-
ENSG0000	ENSG0000	0.003317	hsa-miR-519a-3p;hsa-miR-519b-3p;hsa-miR-519c-3p;hsa-miR-
ENSG0000	ENSG0000	0.000962	hsa-miR-519a-3p;hsa-miR-519b-3p;hsa-miR-519c-3p;hsa-miR-
ENSG0000	ENSG0000	5.51E-06	hsa-miR-15a-5p;hsa-miR-15b-5p;hsa-miR-16-5p;hsa-miR-195-
ENSG0000	ENSG0000	4.64E-06	hsa-miR-15a-5p;hsa-miR-15b-5p;hsa-miR-195-5p;hsa-miR-30
ENSG0000	ENSG0000	4.25E-09	hsa-miR-15a-5p;hsa-miR-15b-5p;hsa-miR-16-5p;hsa-miR-195-
ENSG0000	ENSG0000	0.001069	hsa-miR-519a-3p;hsa-miR-519b-3p;hsa-miR-519c-3p;hsa-miR-
ENSG0000	ENSG0000	7.89E-05	hsa-miR-139-5p;hsa-miR-15a-5p;hsa-miR-15b-5p;hsa-miR-16-
ENSG0000	ENSG0000	0.000231	hsa-miR-519a-3p;hsa-miR-519b-3p;hsa-miR-519c-3p;hsa-miR-
ENSG0000	ENSG0000	3.19E-07	hsa-miR-15a-5p;hsa-miR-15b-5p;hsa-miR-16-5p;hsa-miR-195-
ENSG0000	ENSG0000	2.01E-10	hsa-miR-15a-5p;hsa-miR-15b-5p;hsa-miR-16-5p;hsa-miR-195-
ENSG0000	ENSG0000	1.47E-06	hsa-miR-15a-5p;hsa-miR-15b-5p;hsa-miR-16-5p;hsa-miR-195-
ENSG0000	ENSG0000	0.000472	hsa-miR-361-3p;hsa-miR-519a-3p;hsa-miR-519b-3p;hsa-miR-
ENSG0000	ENSG0000	9.74E-09	hsa-miR-15a-5p;hsa-miR-15b-5p;hsa-miR-195-5p;hsa-miR-42
ENSG0000	ENSG0000	0.000267	hsa-miR-15a-5p;hsa-miR-15b-5p;hsa-miR-16-5p;hsa-miR-195-
ENSG0000	ENSG0000	4.64E-06	hsa-miR-214-3p;hsa-miR-3605-5p;hsa-miR-3619-5p;hsa-miR-
ENSG0000	ENSG0000	0.004696	hsa-miR-15a-5p;hsa-miR-15b-5p;hsa-miR-16-5p;hsa-miR-195-
ENSG0000	ENSG0000	0.000691	hsa-miR-15a-5p;hsa-miR-15b-5p;hsa-miR-195-5p;hsa-miR-42
ENSG0000	ENSG0000	1.77E-10	hsa-miR-15a-5p;hsa-miR-15b-5p;hsa-miR-16-5p;hsa-miR-195-
ENSG0000	ENSG0000	0.000311	hsa-miR-519a-3p;hsa-miR-519b-3p;hsa-miR-519c-3p;hsa-miR-
ENSG0000	ENSG0000	8.88E-11	hsa-miR-139-5p;hsa-miR-15a-5p;hsa-miR-15b-5p;hsa-miR-16-
ENSG0000	ENSG0000	2.22E-05	hsa-miR-214-3p;hsa-miR-3619-5p;hsa-miR-519a-3p;hsa-miR-
ENSG0000	ENSG0000	0.005013	hsa-miR-519a-3p;hsa-miR-519b-3p;hsa-miR-519c-3p;hsa-miR-
ENSG0000	ENSG0000	2.77E-05	hsa-miR-15a-5p;hsa-miR-15b-5p;hsa-miR-195-5p;hsa-miR-42
ENSG0000	ENSG0000	7.46E-06	hsa-miR-15a-5p;hsa-miR-15b-5p;hsa-miR-16-5p;hsa-miR-195-
ENSG0000	ENSG0000	0.005343	hsa-miR-519a-3p;hsa-miR-519b-3p;hsa-miR-519c-3p;hsa-miR-
ENSG0000	ENSG0000	0.001775	hsa-miR-15a-5p;hsa-miR-15b-5p;hsa-miR-16-5p;hsa-miR-195-
ENSG0000	ENSG0000	4.13E-06	hsa-miR-15a-5p;hsa-miR-15b-5p;hsa-miR-16-5p;hsa-miR-195-
ENSG0000	ENSG0000	0.002532	hsa-miR-15a-5p;hsa-miR-15b-5p;hsa-miR-16-5p;hsa-miR-195-
ENSG0000	ENSG0000	2.33E-05	hsa-miR-15a-5p;hsa-miR-15b-5p;hsa-miR-195-5p;hsa-miR-42
ENSG0000	ENSG0000	0.001253	hsa-miR-15a-5p;hsa-miR-15b-5p;hsa-miR-16-5p;hsa-miR-195-
ENSG0000	ENSG0000	0.003292	hsa-miR-218-5p;hsa-miR-519a-3p;hsa-miR-519b-3p;hsa-miR-
ENSG0000	ENSG0000	2.64E-07	hsa-miR-15a-5p;hsa-miR-15b-5p;hsa-miR-16-5p;hsa-miR-195-
ENSG0000	ENSG0000	0.005013	hsa-miR-218-5p;hsa-miR-519a-3p;hsa-miR-519b-3p;hsa-miR-
ENSG0000	ENSG0000	0.000587	hsa-miR-15a-5p;hsa-miR-15b-5p;hsa-miR-16-5p;hsa-miR-195-
ENSG0000	ENSG0000	4.33E-06	hsa-miR-15a-5p;hsa-miR-15b-5p;hsa-miR-16-5p;hsa-miR-195-
ENSG0000	ENSG0000	2.31E-09	hsa-miR-15a-5p;hsa-miR-15b-5p;hsa-miR-16-5p;hsa-miR-195-
ENSG0000	ENSG0000	1.33E-05	hsa-miR-519a-3p;hsa-miR-519b-3p;hsa-miR-519c-3p;hsa-miR-
ENSG0000	ENSG0000	1.45E-07	hsa-miR-126-5p;hsa-miR-15a-5p;hsa-miR-15b-5p;hsa-miR-19
ENSG0000	ENSG0000	6.21E-08	hsa-miR-15a-5p;hsa-miR-15b-5p;hsa-miR-16-5p;hsa-miR-195-
ENSG0000	ENSG0000	6.21E-08	hsa-miR-15a-5p;hsa-miR-15b-5p;hsa-miR-16-5p;hsa-miR-195-
ENSG0000	ENSG0000	0.000863	hsa-miR-519a-3p;hsa-miR-519b-3p;hsa-miR-519c-3p;hsa-miR-
ENSG0000	ENSG0000	0.008993	hsa-miR-519a-3p;hsa-miR-519b-3p;hsa-miR-519c-3p;hsa-miR-
ENSG0000	ENSG0000	0.001416	hsa-miR-27a-3p;hsa-miR-27b-3p;hsa-miR-519a-3p;hsa-miR-5
ENSG0000	ENSG0000	6.94E-05	hsa-miR-15a-5p;hsa-miR-15b-5p;hsa-miR-218-5p;hsa-miR-42
ENSG0000	ENSG0000	0.007121	hsa-miR-214-3p;hsa-miR-3619-5p;hsa-miR-519a-3p;hsa-miR-
ENSG0000	ENSG0000	2.82E-05	hsa-miR-15a-5p;hsa-miR-15b-5p;hsa-miR-16-5p;hsa-miR-195-

ENSG0000	ENSG0000	0.000686	hsa-miR-15a-5p;hsa-miR-15b-5p;hsa-miR-16-5p;hsa-miR-195-
ENSG0000	ENSG0000	8.88E-11	hsa-miR-15a-5p;hsa-miR-15b-5p;hsa-miR-16-5p;hsa-miR-195-
ENSG0000	ENSG0000	5.46E-06	hsa-miR-15a-5p;hsa-miR-15b-5p;hsa-miR-16-5p;hsa-miR-195-
ENSG0000	ENSG0000	1.47E-06	hsa-miR-27a-3p;hsa-miR-27b-3p;hsa-miR-30b-5p;hsa-miR-30
ENSG0000	ENSG0000	0.001436	hsa-miR-27a-3p;hsa-miR-27b-3p;hsa-miR-361-5p;hsa-miR-52
ENSG0000	ENSG0000	7.95E-05	hsa-miR-519a-3p;hsa-miR-519b-3p;hsa-miR-519c-3p;hsa-miR-
ENSG0000	ENSG0000	3.85E-05	hsa-miR-15b-5p;hsa-miR-16-5p;hsa-miR-195-5p;hsa-miR-424-
ENSG0000	ENSG0000	0.000112	hsa-miR-15a-5p;hsa-miR-15b-5p;hsa-miR-16-5p;hsa-miR-195-
ENSG0000	ENSG0000	0.000112	hsa-miR-15a-5p;hsa-miR-15b-5p;hsa-miR-16-5p;hsa-miR-195-
ENSG0000	ENSG0000	1.99E-05	hsa-miR-15a-5p;hsa-miR-15b-5p;hsa-miR-16-5p;hsa-miR-195-
ENSG0000	ENSG0000	2.33E-05	hsa-miR-15a-5p;hsa-miR-15b-5p;hsa-miR-195-5p;hsa-miR-42
ENSG0000	ENSG0000	0.002419	hsa-miR-15a-5p;hsa-miR-15b-5p;hsa-miR-16-5p;hsa-miR-195-
ENSG0000	ENSG0000	0.000873	hsa-miR-519a-3p;hsa-miR-519b-3p;hsa-miR-519c-3p;hsa-miR-
ENSG0000	ENSG0000	1.96E-05	hsa-miR-15a-5p;hsa-miR-15b-5p;hsa-miR-16-5p;hsa-miR-195-
ENSG0000	ENSG0000	0.007969	hsa-miR-27a-3p;hsa-miR-27b-3p;hsa-miR-30b-5p;hsa-miR-30
ENSG0000	ENSG0000	0.003489	hsa-miR-519a-3p;hsa-miR-519b-3p;hsa-miR-519c-3p;hsa-miR-
ENSG0000	ENSG0000	0.000724	hsa-miR-15a-5p;hsa-miR-15b-5p;hsa-miR-16-5p;hsa-miR-195-
ENSG0000	ENSG0000	0.000121	hsa-miR-15a-5p;hsa-miR-15b-5p;hsa-miR-16-5p;hsa-miR-195-
ENSG0000	ENSG0000	0.000606	hsa-miR-1185-5p;hsa-miR-126-5p;hsa-miR-15a-5p;hsa-miR-1
ENSG0000	ENSG0000	3.23E-09	hsa-miR-15a-5p;hsa-miR-15b-5p;hsa-miR-16-5p;hsa-miR-195-
ENSG0000	ENSG0000	0.000245	hsa-miR-30b-5p;hsa-miR-30c-5p;hsa-miR-519a-3p;hsa-miR-5
ENSG0000	ENSG0000	0.000117	hsa-miR-4726-3p;hsa-miR-519a-3p;hsa-miR-519b-3p;hsa-miR-
ENSG0000	ENSG0000	3.65E-06	hsa-miR-15a-5p;hsa-miR-15b-5p;hsa-miR-16-5p;hsa-miR-195-
ENSG0000	ENSG0000	3.30E-06	hsa-miR-15a-5p;hsa-miR-15b-5p;hsa-miR-16-5p;hsa-miR-195-
ENSG0000	ENSG0000	3.30E-06	hsa-miR-15a-5p;hsa-miR-15b-5p;hsa-miR-16-5p;hsa-miR-195-
ENSG0000	ENSG0000	0.00144	hsa-miR-519a-3p;hsa-miR-519b-3p;hsa-miR-519c-3p;hsa-miR-
ENSG0000	ENSG0000	0.00144	hsa-miR-519a-3p;hsa-miR-519b-3p;hsa-miR-519c-3p;hsa-miR-
ENSG0000	ENSG0000	0.000228	hsa-miR-15a-5p;hsa-miR-15b-5p;hsa-miR-16-5p;hsa-miR-195-
ENSG0000	ENSG0000	1.15E-05	hsa-miR-15a-5p;hsa-miR-15b-5p;hsa-miR-16-5p;hsa-miR-195-
ENSG0000	ENSG0000	0.004394	hsa-miR-4726-3p;hsa-miR-520a-3p;hsa-miR-520c-3p;hsa-miR-
ENSG0000	ENSG0000	0.000113	hsa-miR-15a-5p;hsa-miR-15b-5p;hsa-miR-195-5p;hsa-miR-21
ENSG0000	ENSG0000	0.000539	hsa-miR-519a-3p;hsa-miR-519b-3p;hsa-miR-519c-3p;hsa-miR-
ENSG0000	ENSG0000	1.25E-07	hsa-miR-15a-5p;hsa-miR-15b-5p;hsa-miR-195-5p;hsa-miR-42
ENSG0000	ENSG0000	1.26E-06	hsa-miR-15a-5p;hsa-miR-15b-5p;hsa-miR-16-5p;hsa-miR-195-
ENSG0000	ENSG0000	0.006421	hsa-miR-519a-3p;hsa-miR-519b-3p;hsa-miR-519c-3p;hsa-miR-
ENSG0000	ENSG0000	9.14E-05	hsa-miR-15a-5p;hsa-miR-15b-5p;hsa-miR-16-5p;hsa-miR-195-
ENSG0000	ENSG0000	0.00144	hsa-miR-15a-5p;hsa-miR-15b-5p;hsa-miR-195-5p;hsa-miR-21
ENSG0000	ENSG0000	0.002366	hsa-miR-27a-3p;hsa-miR-27b-3p;hsa-miR-519a-3p;hsa-miR-5
ENSG0000	ENSG0000	1.55E-06	hsa-miR-15a-5p;hsa-miR-15b-5p;hsa-miR-16-5p;hsa-miR-195-
ENSG0000	ENSG0000	1.96E-05	hsa-miR-15a-5p;hsa-miR-15b-5p;hsa-miR-16-5p;hsa-miR-195-
ENSG0000	ENSG0000	1.96E-05	hsa-miR-15a-5p;hsa-miR-15b-5p;hsa-miR-16-5p;hsa-miR-195-
ENSG0000	ENSG0000	0.000809	hsa-miR-15a-5p;hsa-miR-15b-5p;hsa-miR-195-5p;hsa-miR-42
ENSG0000	ENSG0000	2.64E-07	hsa-miR-139-5p;hsa-miR-15a-5p;hsa-miR-15b-5p;hsa-miR-16-
ENSG0000	ENSG0000	2.62E-06	hsa-miR-15a-5p;hsa-miR-15b-5p;hsa-miR-16-5p;hsa-miR-195-
ENSG0000	ENSG0000	1.15E-05	hsa-miR-15a-5p;hsa-miR-15b-5p;hsa-miR-16-5p;hsa-miR-195-
ENSG0000	ENSG0000	6.03E-05	hsa-miR-15a-5p;hsa-miR-15b-5p;hsa-miR-424-5p;hsa-miR-49
ENSG0000	ENSG0000	0.001912	hsa-miR-15a-5p;hsa-miR-15b-5p;hsa-miR-16-5p;hsa-miR-195-
ENSG0000	ENSG0000	0.001912	hsa-miR-15a-5p;hsa-miR-15b-5p;hsa-miR-16-5p;hsa-miR-195-
ENSG0000	ENSG0000	4.25E-09	hsa-miR-15a-5p;hsa-miR-15b-5p;hsa-miR-16-5p;hsa-miR-195-
ENSG0000	ENSG0000	9.14E-05	hsa-miR-30b-5p;hsa-miR-30c-5p;hsa-miR-520a-3p;hsa-miR-52
ENSG0000	ENSG0000	0.001084	hsa-miR-3605-5p;hsa-miR-519a-3p;hsa-miR-519b-3p;hsa-miR-
ENSG0000	ENSG0000	1.25E-09	hsa-miR-15a-5p;hsa-miR-15b-5p;hsa-miR-16-5p;hsa-miR-195-
ENSG0000	ENSG0000	3.93E-07	hsa-miR-15a-5p;hsa-miR-15b-5p;hsa-miR-195-5p;hsa-miR-21
ENSG0000	ENSG0000	0.006421	hsa-miR-214-3p;hsa-miR-27a-3p;hsa-miR-27b-3p;hsa-miR-36

ENSG0000	ENSG0000	0.006421	hsa-miR-214-3p;hsa-miR-27a-3p;hsa-miR-27b-3p;hsa-miR-36
ENSG0000	ENSG0000	5.26E-06	hsa-miR-15a-5p;hsa-miR-15b-5p;hsa-miR-16-5p;hsa-miR-195-
ENSG0000	ENSG0000	1.70E-07	hsa-miR-15a-5p;hsa-miR-15b-5p;hsa-miR-16-5p;hsa-miR-195-
ENSG0000	ENSG0000	0.007215	hsa-miR-218-5p;hsa-miR-27a-3p;hsa-miR-27b-3p;hsa-miR-62
ENSG0000	ENSG0000	4.58E-07	hsa-miR-15a-5p;hsa-miR-15b-5p;hsa-miR-16-5p;hsa-miR-195-
ENSG0000	ENSG0000	0.000537	hsa-miR-15a-5p;hsa-miR-15b-5p;hsa-miR-16-5p;hsa-miR-195-
ENSG0000	ENSG0000	1.93E-08	hsa-miR-15a-5p;hsa-miR-15b-5p;hsa-miR-16-5p;hsa-miR-195-
ENSG0000	ENSG0000	5.51E-06	hsa-miR-15a-5p;hsa-miR-15b-5p;hsa-miR-16-5p;hsa-miR-195-
ENSG0000	ENSG0000	1.03E-05	hsa-miR-15a-5p;hsa-miR-15b-5p;hsa-miR-16-5p;hsa-miR-195-
ENSG0000	ENSG0000	0.00681	hsa-miR-369-3p;hsa-miR-519a-3p;hsa-miR-519b-3p;hsa-miR-
ENSG0000	ENSG0000	9.08E-05	hsa-miR-15a-5p;hsa-miR-15b-5p;hsa-miR-195-5p;hsa-miR-42
ENSG0000	ENSG0000	0.000179	hsa-miR-218-5p;hsa-miR-519a-3p;hsa-miR-519b-3p;hsa-miR-
ENSG0000	ENSG0000	2.22E-05	hsa-miR-218-5p;hsa-miR-519a-3p;hsa-miR-519b-3p;hsa-miR-
ENSG0000	ENSG0000	3.30E-06	hsa-miR-15a-5p;hsa-miR-15b-5p;hsa-miR-16-5p;hsa-miR-195-
ENSG0000	ENSG0000	2.82E-05	hsa-miR-15a-5p;hsa-miR-15b-5p;hsa-miR-218-5p;hsa-miR-51
ENSG0000	ENSG0000	4.13E-06	hsa-miR-218-5p;hsa-miR-519a-3p;hsa-miR-519b-3p;hsa-miR-
ENSG0000	ENSG0000	0.000458	hsa-miR-15a-5p;hsa-miR-15b-5p;hsa-miR-16-5p;hsa-miR-195-
ENSG0000	ENSG0000	0.000135	hsa-miR-15a-5p;hsa-miR-15b-5p;hsa-miR-16-5p;hsa-miR-195-
ENSG0000	ENSG0000	0.004105	hsa-miR-218-5p;hsa-miR-520a-3p;hsa-miR-520c-3p;hsa-miR-
ENSG0000	ENSG0000	9.79E-05	hsa-miR-15a-5p;hsa-miR-15b-5p;hsa-miR-16-5p;hsa-miR-195-
ENSG0000	ENSG0000	7.84E-11	hsa-miR-15a-5p;hsa-miR-15b-5p;hsa-miR-16-5p;hsa-miR-195-
ENSG0000	ENSG0000	1.45E-07	hsa-miR-15a-5p;hsa-miR-15b-5p;hsa-miR-195-5p;hsa-miR-42
ENSG0000	ENSG0000	0.000262	hsa-miR-15a-5p;hsa-miR-15b-5p;hsa-miR-195-5p;hsa-miR-36
ENSG0000	ENSG0000	0.009479	hsa-miR-4726-3p;hsa-miR-520a-3p;hsa-miR-520c-3p;hsa-miR-
ENSG0000	ENSG0000	2.11E-06	hsa-miR-15a-5p;hsa-miR-15b-5p;hsa-miR-16-5p;hsa-miR-195-
ENSG0000	ENSG0000	6.69E-07	hsa-miR-15a-5p;hsa-miR-15b-5p;hsa-miR-195-5p;hsa-miR-42
ENSG0000	ENSG0000	1.44E-05	hsa-miR-15a-5p;hsa-miR-15b-5p;hsa-miR-16-5p;hsa-miR-195-
ENSG0000	ENSG0000	8.92E-07	hsa-miR-214-3p;hsa-miR-3619-5p;hsa-miR-519a-3p;hsa-miR-
ENSG0000	ENSG0000	2.29E-07	hsa-miR-218-5p;hsa-miR-519a-3p;hsa-miR-519b-3p;hsa-miR-
ENSG0000	ENSG0000	0.000809	hsa-miR-15a-5p;hsa-miR-15b-5p;hsa-miR-16-5p;hsa-miR-195-
ENSG0000	ENSG0000	1.11E-11	hsa-miR-15a-5p;hsa-miR-15b-5p;hsa-miR-195-5p;hsa-miR-21
ENSG0000	ENSG0000	1.95E-05	hsa-miR-15a-5p;hsa-miR-15b-5p;hsa-miR-195-5p;hsa-miR-42
ENSG0000	ENSG0000	1.70E-05	hsa-miR-15a-5p;hsa-miR-15b-5p;hsa-miR-195-5p;hsa-miR-21
ENSG0000	ENSG0000	3.94E-07	hsa-miR-15a-5p;hsa-miR-15b-5p;hsa-miR-16-5p;hsa-miR-195-
ENSG0000	ENSG0000	0.000769	hsa-miR-15a-5p;hsa-miR-15b-5p;hsa-miR-195-5p;hsa-miR-21
ENSG0000	ENSG0000	1.33E-05	hsa-miR-218-5p;hsa-miR-519a-3p;hsa-miR-519b-3p;hsa-miR-
ENSG0000	ENSG0000	2.18E-08	hsa-miR-15a-5p;hsa-miR-15b-5p;hsa-miR-16-5p;hsa-miR-195-
ENSG0000	ENSG0000	4.23E-07	hsa-miR-15a-5p;hsa-miR-15b-5p;hsa-miR-16-5p;hsa-miR-195-
ENSG0000	ENSG0000	3.02E-07	hsa-miR-15a-5p;hsa-miR-15b-5p;hsa-miR-16-5p;hsa-miR-195-
ENSG0000	ENSG0000	2.64E-07	hsa-miR-15a-5p;hsa-miR-15b-5p;hsa-miR-16-5p;hsa-miR-195-
ENSG0000	ENSG0000	0.000312	hsa-miR-519a-3p;hsa-miR-519b-3p;hsa-miR-519c-3p;hsa-miR-
ENSG0000	ENSG0000	0.001916	hsa-miR-126-5p;hsa-miR-15a-5p;hsa-miR-15b-5p;hsa-miR-19
ENSG0000	ENSG0000	0.000186	hsa-miR-218-5p;hsa-miR-519a-3p;hsa-miR-519b-3p;hsa-miR-
ENSG0000	ENSG0000	0.000186	hsa-miR-218-5p;hsa-miR-519a-3p;hsa-miR-519b-3p;hsa-miR-
ENSG0000	ENSG0000	0.000186	hsa-miR-218-5p;hsa-miR-519a-3p;hsa-miR-519b-3p;hsa-miR-
ENSG0000	ENSG0000	0.002063	hsa-miR-519a-3p;hsa-miR-519b-3p;hsa-miR-519c-3p;hsa-miR-
ENSG0000	ENSG0000	0.008072	hsa-miR-218-5p;hsa-miR-520a-3p;hsa-miR-520c-3p;hsa-miR-
ENSG0000	ENSG0000	5.87E-05	hsa-miR-519a-3p;hsa-miR-519b-3p;hsa-miR-519c-3p;hsa-miR-
ENSG0000	ENSG0000	0.002969	hsa-miR-15a-5p;hsa-miR-15b-5p;hsa-miR-195-5p;hsa-miR-42
ENSG0000	ENSG0000	0.001534	hsa-miR-2115-5p;hsa-miR-30b-5p;hsa-miR-30c-5p;hsa-miR-3
ENSG0000	ENSG0000	7.39E-08	hsa-miR-15a-5p;hsa-miR-15b-5p;hsa-miR-16-5p;hsa-miR-195-
ENSG0000	ENSG0000	2.33E-05	hsa-miR-15a-5p;hsa-miR-15b-5p;hsa-miR-195-5p;hsa-miR-42
ENSG0000	ENSG0000	1.53E-07	hsa-miR-15a-5p;hsa-miR-15b-5p;hsa-miR-195-5p;hsa-miR-42
ENSG0000	ENSG0000	1.95E-05	hsa-miR-15a-5p;hsa-miR-15b-5p;hsa-miR-195-5p;hsa-miR-42

ENSG0000	ENSG0000	2.82E-05	hsa-miR-15a-5p;hsa-miR-15b-5p;hsa-miR-16-5p;hsa-miR-195-
ENSG0000	ENSG0000	0.000132	hsa-miR-15a-5p;hsa-miR-15b-5p;hsa-miR-195-5p;hsa-miR-42
ENSG0000	ENSG0000	0.003317	hsa-miR-214-3p;hsa-miR-3619-5p;hsa-miR-424-5p;hsa-miR-4
ENSG0000	ENSG0000	0.000816	hsa-miR-218-5p;hsa-miR-519a-3p;hsa-miR-519b-3p;hsa-miR-
ENSG0000	ENSG0000	1.02E-06	hsa-miR-15a-5p;hsa-miR-15b-5p;hsa-miR-16-5p;hsa-miR-195-
ENSG0000	ENSG0000	1.25E-09	hsa-miR-15a-5p;hsa-miR-15b-5p;hsa-miR-16-5p;hsa-miR-195-
ENSG0000	ENSG0000	4.25E-09	hsa-miR-15a-5p;hsa-miR-15b-5p;hsa-miR-16-5p;hsa-miR-195-
ENSG0000	ENSG0000	3.21E-07	hsa-miR-15a-5p;hsa-miR-15b-5p;hsa-miR-16-5p;hsa-miR-195-
ENSG0000	ENSG0000	0.000724	hsa-miR-214-3p;hsa-miR-3619-5p;hsa-miR-519a-3p;hsa-miR-
ENSG0000	ENSG0000	0.00144	hsa-miR-519a-3p;hsa-miR-519b-3p;hsa-miR-519c-3p;hsa-miR-
ENSG0000	ENSG0000	0.000472	hsa-miR-519a-3p;hsa-miR-519b-3p;hsa-miR-519c-3p;hsa-miR-
ENSG0000	ENSG0000	1.99E-05	hsa-miR-15a-5p;hsa-miR-15b-5p;hsa-miR-16-5p;hsa-miR-195-
ENSG0000	ENSG0000	5.15E-07	hsa-miR-15a-5p;hsa-miR-15b-5p;hsa-miR-16-5p;hsa-miR-195-
ENSG0000	ENSG0000	0.000865	hsa-miR-126-5p;hsa-miR-214-3p;hsa-miR-3619-5p;hsa-miR-5
ENSG0000	ENSG0000	0.000637	hsa-miR-27a-3p;hsa-miR-27b-3p;hsa-miR-520a-3p;hsa-miR-5
ENSG0000	ENSG0000	8.12E-05	hsa-miR-519a-3p;hsa-miR-519b-3p;hsa-miR-519c-3p;hsa-miR-
ENSG0000	ENSG0000	0.001581	hsa-miR-15a-5p;hsa-miR-15b-5p;hsa-miR-16-5p;hsa-miR-195-
ENSG0000	ENSG0000	1.89E-06	hsa-miR-15a-5p;hsa-miR-15b-5p;hsa-miR-195-5p;hsa-miR-42
ENSG0000	ENSG0000	2.83E-09	hsa-miR-15a-5p;hsa-miR-15b-5p;hsa-miR-16-5p;hsa-miR-195-
ENSG0000	ENSG0000	4.64E-06	hsa-miR-15a-5p;hsa-miR-15b-5p;hsa-miR-16-5p;hsa-miR-195-
ENSG0000	ENSG0000	0.000809	hsa-miR-15a-5p;hsa-miR-15b-5p;hsa-miR-16-5p;hsa-miR-195-
ENSG0000	ENSG0000	3.30E-06	hsa-miR-15a-5p;hsa-miR-15b-5p;hsa-miR-16-5p;hsa-miR-195-
ENSG0000	ENSG0000	9.20E-05	hsa-miR-15a-5p;hsa-miR-15b-5p;hsa-miR-16-5p;hsa-miR-195-
ENSG0000	ENSG0000	4.50E-07	hsa-miR-15a-5p;hsa-miR-15b-5p;hsa-miR-195-5p;hsa-miR-42
ENSG0000	ENSG0000	0.004499	hsa-miR-15a-5p;hsa-miR-15b-5p;hsa-miR-195-5p;hsa-miR-42
ENSG0000	ENSG0000	2.96E-08	hsa-miR-15a-5p;hsa-miR-15b-5p;hsa-miR-16-5p;hsa-miR-195-
ENSG0000	ENSG0000	0.003079	hsa-miR-519a-3p;hsa-miR-519b-3p;hsa-miR-519c-3p;hsa-miR-
ENSG0000	ENSG0000	4.41E-10	hsa-miR-15a-5p;hsa-miR-15b-5p;hsa-miR-16-5p;hsa-miR-195-
ENSG0000	ENSG0000	3.17E-05	hsa-miR-369-3p;hsa-miR-519a-3p;hsa-miR-519b-3p;hsa-miR-
ENSG0000	ENSG0000	3.17E-05	hsa-miR-369-3p;hsa-miR-519a-3p;hsa-miR-519b-3p;hsa-miR-
ENSG0000	ENSG0000	0.00144	hsa-miR-519a-3p;hsa-miR-519b-3p;hsa-miR-519c-3p;hsa-miR-
ENSG0000	ENSG0000	2.18E-13	hsa-miR-15a-5p;hsa-miR-15b-5p;hsa-miR-16-5p;hsa-miR-195-
ENSG0000	ENSG0000	0.000686	hsa-miR-519a-3p;hsa-miR-519b-3p;hsa-miR-519c-3p;hsa-miR-
ENSG0000	ENSG0000	0.000135	hsa-miR-15a-5p;hsa-miR-15b-5p;hsa-miR-16-5p;hsa-miR-195-
ENSG0000	ENSG0000	1.26E-06	hsa-miR-15a-5p;hsa-miR-15b-5p;hsa-miR-195-5p;hsa-miR-42
ENSG0000	ENSG0000	8.76E-10	hsa-miR-15a-5p;hsa-miR-15b-5p;hsa-miR-16-5p;hsa-miR-195-
ENSG0000	ENSG0000	0.007635	hsa-miR-15a-5p;hsa-miR-15b-5p;hsa-miR-16-5p;hsa-miR-195-
ENSG0000	ENSG0000	1.89E-05	hsa-miR-15a-5p;hsa-miR-15b-5p;hsa-miR-195-5p;hsa-miR-30
ENSG0000	ENSG0000	0.002639	hsa-miR-520a-3p;hsa-miR-520c-3p;hsa-miR-526b-3p;hsa-miR-
ENSG0000	ENSG0000	0.000235	hsa-miR-15a-5p;hsa-miR-15b-5p;hsa-miR-16-5p;hsa-miR-195-
ENSG0000	ENSG0000	9.04E-10	hsa-miR-126-5p;hsa-miR-15a-5p;hsa-miR-15b-5p;hsa-miR-19
ENSG0000	ENSG0000	1.34E-09	hsa-miR-15a-5p;hsa-miR-15b-5p;hsa-miR-16-5p;hsa-miR-195-
ENSG0000	ENSG0000	5.86E-07	hsa-miR-15a-5p;hsa-miR-15b-5p;hsa-miR-195-5p;hsa-miR-42
ENSG0000	ENSG0000	0.001732	hsa-miR-214-3p;hsa-miR-30b-5p;hsa-miR-30c-5p;hsa-miR-36
ENSG0000	ENSG0000	0.000163	hsa-miR-218-5p;hsa-miR-369-3p;hsa-miR-520a-3p;hsa-miR-5
ENSG0000	ENSG0000	0.000453	hsa-miR-15a-5p;hsa-miR-15b-5p;hsa-miR-195-5p;hsa-miR-42
ENSG0000	ENSG0000	0.001732	hsa-miR-15a-5p;hsa-miR-15b-5p;hsa-miR-16-5p;hsa-miR-195-
ENSG0000	ENSG0000	3.21E-07	hsa-miR-15a-5p;hsa-miR-15b-5p;hsa-miR-16-5p;hsa-miR-195-
ENSG0000	ENSG0000	0.005197	hsa-miR-218-5p;hsa-miR-4726-3p;hsa-miR-520a-3p;hsa-miR-
ENSG0000	ENSG0000	0.001822	hsa-miR-519a-3p;hsa-miR-519b-3p;hsa-miR-519c-3p;hsa-miR-
ENSG0000	ENSG0000	5.86E-09	hsa-miR-15a-5p;hsa-miR-15b-5p;hsa-miR-195-5p;hsa-miR-30
ENSG0000	ENSG0000	0.001745	hsa-miR-3605-5p;hsa-miR-361-5p;hsa-miR-520a-3p;hsa-miR-
ENSG0000	ENSG0000	5.81E-05	hsa-miR-15a-5p;hsa-miR-15b-5p;hsa-miR-16-5p;hsa-miR-195-
ENSG0000	ENSG0000	0.000107	hsa-miR-30b-5p;hsa-miR-519a-3p;hsa-miR-519b-3p;hsa-miR-

ENSG0000	ENSG0000	0.000748	hsa-miR-15a-5p;hsa-miR-15b-5p;hsa-miR-195-5p;hsa-miR-424
ENSG0000	ENSG0000	0.000809	hsa-miR-15a-5p;hsa-miR-15b-5p;hsa-miR-195-5p;hsa-miR-424
ENSG0000	ENSG0000	1.66E-07	hsa-miR-15a-5p;hsa-miR-15b-5p;hsa-miR-16-5p;hsa-miR-195-
ENSG0000	ENSG0000	1.25E-07	hsa-miR-15a-5p;hsa-miR-15b-5p;hsa-miR-16-5p;hsa-miR-195-
ENSG0000	ENSG0000	1.25E-07	hsa-miR-15a-5p;hsa-miR-15b-5p;hsa-miR-16-5p;hsa-miR-195-
ENSG0000	ENSG0000	0.008993	hsa-miR-218-5p;hsa-miR-520a-3p;hsa-miR-520c-3p;hsa-miR-5
ENSG0000	ENSG0000	2.50E-06	hsa-miR-15a-5p;hsa-miR-15b-5p;hsa-miR-195-5p;hsa-miR-424
ENSG0000	ENSG0000	1.08E-07	hsa-miR-15a-5p;hsa-miR-15b-5p;hsa-miR-16-5p;hsa-miR-195-
ENSG0000	ENSG0000	2.18E-08	hsa-miR-15a-5p;hsa-miR-15b-5p;hsa-miR-16-5p;hsa-miR-195-
ENSG0000	ENSG0000	9.14E-05	hsa-miR-519a-3p;hsa-miR-519b-3p;hsa-miR-519c-3p;hsa-miR-
ENSG0000	ENSG0000	0.006047	hsa-miR-519a-3p;hsa-miR-519b-3p;hsa-miR-519c-3p;hsa-miR-
ENSG0000	ENSG0000	1.25E-09	hsa-miR-15a-5p;hsa-miR-15b-5p;hsa-miR-16-5p;hsa-miR-195-
ENSG0000	ENSG0000	1.55E-06	hsa-miR-15a-5p;hsa-miR-15b-5p;hsa-miR-16-5p;hsa-miR-195-
ENSG0000	ENSG0000	0.00383	hsa-miR-15a-5p;hsa-miR-15b-5p;hsa-miR-16-5p;hsa-miR-195-
ENSG0000	ENSG0000	2.29E-07	hsa-miR-15a-5p;hsa-miR-15b-5p;hsa-miR-16-5p;hsa-miR-195-
ENSG0000	ENSG0000	1.70E-07	hsa-miR-15a-5p;hsa-miR-15b-5p;hsa-miR-16-5p;hsa-miR-195-
ENSG0000	ENSG0000	5.46E-05	hsa-miR-519a-3p;hsa-miR-519b-3p;hsa-miR-519c-3p;hsa-miR-
ENSG0000	ENSG0000	0.008072	hsa-miR-519a-3p;hsa-miR-519b-3p;hsa-miR-519c-3p;hsa-miR-
ENSG0000	ENSG0000	2.29E-06	hsa-miR-15a-5p;hsa-miR-15b-5p;hsa-miR-16-5p;hsa-miR-195-
ENSG0000	ENSG0000	8.12E-05	hsa-miR-1185-5p;hsa-miR-3605-5p;hsa-miR-519a-3p;hsa-miR-
ENSG0000	ENSG0000	0.001169	hsa-miR-218-5p;hsa-miR-519a-3p;hsa-miR-519b-3p;hsa-miR-5
ENSG0000	ENSG0000	1.08E-07	hsa-miR-15a-5p;hsa-miR-15b-5p;hsa-miR-16-5p;hsa-miR-195-
ENSG0000	ENSG0000	4.64E-06	hsa-miR-15a-5p;hsa-miR-15b-5p;hsa-miR-16-5p;hsa-miR-195-
ENSG0000	ENSG0000	3.44E-07	hsa-miR-15a-5p;hsa-miR-15b-5p;hsa-miR-16-5p;hsa-miR-195-
ENSG0000	ENSG0000	3.07E-08	hsa-miR-15a-5p;hsa-miR-15b-5p;hsa-miR-195-5p;hsa-miR-214
ENSG0000	ENSG0000	1.00E-05	hsa-miR-15a-5p;hsa-miR-15b-5p;hsa-miR-16-5p;hsa-miR-195-
ENSG0000	ENSG0000	1.61E-05	hsa-miR-15a-5p;hsa-miR-15b-5p;hsa-miR-195-5p;hsa-miR-30b
ENSG0000	ENSG0000	1.33E-05	hsa-miR-519a-3p;hsa-miR-519b-3p;hsa-miR-519c-3p;hsa-miR-
ENSG0000	ENSG0000	0.006159	hsa-miR-15a-5p;hsa-miR-15b-5p;hsa-miR-16-5p;hsa-miR-195-
ENSG0000	ENSG0000	0.006159	hsa-miR-15a-5p;hsa-miR-15b-5p;hsa-miR-16-5p;hsa-miR-195-
ENSG0000	ENSG0000	0.001581	hsa-miR-519a-3p;hsa-miR-519b-3p;hsa-miR-519c-3p;hsa-miR-
ENSG0000	ENSG0000	0.001581	hsa-miR-519a-3p;hsa-miR-519b-3p;hsa-miR-519c-3p;hsa-miR-
ENSG0000	ENSG0000	5.33E-08	hsa-miR-139-5p;hsa-miR-15a-5p;hsa-miR-15b-5p;hsa-miR-16-
ENSG0000	ENSG0000	1.70E-07	hsa-miR-15a-5p;hsa-miR-15b-5p;hsa-miR-16-5p;hsa-miR-195-
ENSG0000	ENSG0000	2.16E-10	hsa-miR-15a-5p;hsa-miR-15b-5p;hsa-miR-195-5p;hsa-miR-424
ENSG0000	ENSG0000	3.55E-05	hsa-miR-15a-5p;hsa-miR-15b-5p;hsa-miR-16-5p;hsa-miR-195-
ENSG0000	ENSG0000	0.001307	hsa-miR-519a-3p;hsa-miR-519b-3p;hsa-miR-519c-3p;hsa-miR-
ENSG0000	ENSG0000	0.004394	hsa-miR-519a-3p;hsa-miR-519b-3p;hsa-miR-519c-3p;hsa-miR-
ENSG0000	ENSG0000	0.000344	hsa-miR-519a-3p;hsa-miR-519b-3p;hsa-miR-519c-3p;hsa-miR-
ENSG0000	ENSG0000	9.26E-06	hsa-miR-15a-5p;hsa-miR-15b-5p;hsa-miR-16-5p;hsa-miR-195-
ENSG0000	ENSG0000	0.000495	hsa-miR-519a-3p;hsa-miR-519b-3p;hsa-miR-519c-3p;hsa-miR-
ENSG0000	ENSG0000	1.99E-05	hsa-miR-15a-5p;hsa-miR-15b-5p;hsa-miR-424-5p;hsa-miR-497
ENSG0000	ENSG0000	2.76E-06	hsa-miR-15a-5p;hsa-miR-15b-5p;hsa-miR-16-5p;hsa-miR-195-
ENSG0000	ENSG0000	0.006421	hsa-miR-2115-5p;hsa-miR-218-5p;hsa-miR-27a-3p;hsa-miR-27
ENSG0000	ENSG0000	2.18E-08	hsa-miR-15a-5p;hsa-miR-15b-5p;hsa-miR-16-5p;hsa-miR-195-
ENSG0000	ENSG0000	2.40E-08	hsa-miR-15a-5p;hsa-miR-15b-5p;hsa-miR-16-5p;hsa-miR-195-
ENSG0000	ENSG0000	0.000465	hsa-miR-15a-5p;hsa-miR-15b-5p;hsa-miR-16-5p;hsa-miR-195-
ENSG0000	ENSG0000	0.000245	hsa-miR-15a-5p;hsa-miR-15b-5p;hsa-miR-16-5p;hsa-miR-195-
ENSG0000	ENSG0000	6.84E-05	hsa-miR-15a-5p;hsa-miR-15b-5p;hsa-miR-16-5p;hsa-miR-195-
ENSG0000	ENSG0000	5.46E-06	hsa-miR-139-5p;hsa-miR-15a-5p;hsa-miR-15b-5p;hsa-miR-195
ENSG0000	ENSG0000	0.000207	hsa-miR-15a-5p;hsa-miR-15b-5p;hsa-miR-16-5p;hsa-miR-195-
ENSG0000	ENSG0000	0.000771	hsa-miR-15a-5p;hsa-miR-15b-5p;hsa-miR-16-5p;hsa-miR-195-
ENSG0000	ENSG0000	2.18E-08	hsa-miR-15a-5p;hsa-miR-15b-5p;hsa-miR-16-5p;hsa-miR-195-
ENSG0000	ENSG0000	8.92E-07	hsa-miR-15a-5p;hsa-miR-15b-5p;hsa-miR-16-5p;hsa-miR-195-

ENSG0000	ENSG0000	0.000228	hsa-miR-15a-5p;hsa-miR-15b-5p;hsa-miR-16-5p;hsa-miR-195-
ENSG0000	ENSG0000	0.002853	hsa-miR-1185-5p;hsa-miR-126-5p;hsa-miR-3605-5p;hsa-miR-3
ENSG0000	ENSG0000	6.03E-09	hsa-miR-15a-5p;hsa-miR-15b-5p;hsa-miR-16-5p;hsa-miR-195-
ENSG0000	ENSG0000	6.68E-05	hsa-miR-519a-3p;hsa-miR-519b-3p;hsa-miR-519c-3p;hsa-miR-
ENSG0000	ENSG0000	1.25E-09	hsa-miR-15a-5p;hsa-miR-15b-5p;hsa-miR-16-5p;hsa-miR-195-
ENSG0000	ENSG0000	0.005939	hsa-miR-519a-3p;hsa-miR-519b-3p;hsa-miR-519c-3p;hsa-miR-
ENSG0000	ENSG0000	0.000724	hsa-miR-15a-5p;hsa-miR-15b-5p;hsa-miR-16-5p;hsa-miR-195-
ENSG0000	ENSG0000	0.007215	hsa-miR-16-5p;hsa-miR-519a-3p;hsa-miR-519b-3p;hsa-miR-51
ENSG0000	ENSG0000	0.004926	hsa-miR-1185-5p;hsa-miR-3605-5p;hsa-miR-520a-3p;hsa-miR-
ENSG0000	ENSG0000	0.000537	hsa-miR-15a-5p;hsa-miR-15b-5p;hsa-miR-195-5p;hsa-miR-54
ENSG0000	ENSG0000	4.92E-05	hsa-miR-15a-5p;hsa-miR-15b-5p;hsa-miR-16-5p;hsa-miR-195-
ENSG0000	ENSG0000	0.006527	hsa-miR-15a-5p;hsa-miR-15b-5p;hsa-miR-16-5p;hsa-miR-195-
ENSG0000	ENSG0000	6.47E-07	hsa-miR-15a-5p;hsa-miR-15b-5p;hsa-miR-16-5p;hsa-miR-195-
ENSG0000	ENSG0000	0.000207	hsa-miR-15a-5p;hsa-miR-15b-5p;hsa-miR-16-5p;hsa-miR-195-
ENSG0000	ENSG0000	4.96E-05	hsa-miR-1185-5p;hsa-miR-139-5p;hsa-miR-3605-5p;hsa-miR-5
ENSG0000	ENSG0000	0.000262	hsa-miR-126-5p;hsa-miR-218-5p;hsa-miR-27a-3p;hsa-miR-27b
ENSG0000	ENSG0000	0.000207	hsa-miR-15a-5p;hsa-miR-15b-5p;hsa-miR-195-5p;hsa-miR-42
ENSG0000	ENSG0000	0.000163	hsa-miR-27a-3p;hsa-miR-27b-3p;hsa-miR-30b-5p;hsa-miR-30
ENSG0000	ENSG0000	0.000163	hsa-miR-27a-3p;hsa-miR-27b-3p;hsa-miR-30b-5p;hsa-miR-30
ENSG0000	ENSG0000	0.000256	hsa-miR-15a-5p;hsa-miR-15b-5p;hsa-miR-195-5p;hsa-miR-42
ENSG0000	ENSG0000	0.000682	hsa-miR-15a-5p;hsa-miR-15b-5p;hsa-miR-16-5p;hsa-miR-195-
ENSG0000	ENSG0000	0.001069	hsa-miR-519a-3p;hsa-miR-519b-3p;hsa-miR-519c-3p;hsa-miR-
ENSG0000	ENSG0000	0.001069	hsa-miR-519a-3p;hsa-miR-519b-3p;hsa-miR-519c-3p;hsa-miR-
ENSG0000	ENSG0000	0.001069	hsa-miR-519a-3p;hsa-miR-519b-3p;hsa-miR-519c-3p;hsa-miR-
ENSG0000	ENSG0000	0.001069	hsa-miR-519a-3p;hsa-miR-519b-3p;hsa-miR-519c-3p;hsa-miR-
ENSG0000	ENSG0000	0.001069	hsa-miR-519a-3p;hsa-miR-519b-3p;hsa-miR-519c-3p;hsa-miR-
ENSG0000	ENSG0000	0.001069	hsa-miR-519a-3p;hsa-miR-519b-3p;hsa-miR-519c-3p;hsa-miR-
ENSG0000	ENSG0000	0.001069	hsa-miR-519a-3p;hsa-miR-519b-3p;hsa-miR-519c-3p;hsa-miR-
ENSG0000	ENSG0000	0.000231	hsa-miR-519a-3p;hsa-miR-519b-3p;hsa-miR-519c-3p;hsa-miR-
ENSG0000	ENSG0000	2.77E-05	hsa-miR-15a-5p;hsa-miR-15b-5p;hsa-miR-195-5p;hsa-miR-42
ENSG0000	ENSG0000	2.77E-05	hsa-miR-15a-5p;hsa-miR-15b-5p;hsa-miR-195-5p;hsa-miR-42
ENSG0000	ENSG0000	0.004213	hsa-miR-139-5p;hsa-miR-15a-5p;hsa-miR-15b-5p;hsa-miR-19
ENSG0000	ENSG0000	7.44E-06	hsa-miR-218-5p;hsa-miR-519a-3p;hsa-miR-519b-3p;hsa-miR-5
ENSG0000	ENSG0000	5.45E-07	hsa-miR-126-5p;hsa-miR-15a-5p;hsa-miR-15b-5p;hsa-miR-16-
ENSG0000	ENSG0000	0.000128	hsa-miR-15a-5p;hsa-miR-15b-5p;hsa-miR-16-5p;hsa-miR-195-
ENSG0000	ENSG0000	0.007215	hsa-miR-2115-5p;hsa-miR-218-5p;hsa-miR-30b-5p;hsa-miR-30
ENSG0000	ENSG0000	6.69E-07	hsa-miR-15a-5p;hsa-miR-15b-5p;hsa-miR-195-5p;hsa-miR-30b
ENSG0000	ENSG0000	0.002244	hsa-miR-4691-3p;hsa-miR-520a-3p;hsa-miR-520c-3p;hsa-miR-
ENSG0000	ENSG0000	0.002809	hsa-miR-218-5p;hsa-miR-369-3p;hsa-miR-520a-3p;hsa-miR-52
ENSG0000	ENSG0000	0.000194	hsa-miR-15a-5p;hsa-miR-15b-5p;hsa-miR-16-5p;hsa-miR-195-
ENSG0000	ENSG0000	0.000539	hsa-miR-27a-3p;hsa-miR-27b-3p;hsa-miR-30b-5p;hsa-miR-30
ENSG0000	ENSG0000	3.02E-07	hsa-miR-15a-5p;hsa-miR-16-5p;hsa-miR-195-5p;hsa-miR-218-
ENSG0000	ENSG0000	5.87E-05	hsa-miR-27a-3p;hsa-miR-27b-3p;hsa-miR-30b-5p;hsa-miR-30
ENSG0000	ENSG0000	0.004394	hsa-miR-519a-3p;hsa-miR-519b-3p;hsa-miR-519c-3p;hsa-miR-
ENSG0000	ENSG0000	0.00681	hsa-miR-2115-5p;hsa-miR-4726-3p;hsa-miR-520a-3p;hsa-miR-
ENSG0000	ENSG0000	0.000962	hsa-miR-519a-3p;hsa-miR-519b-3p;hsa-miR-519c-3p;hsa-miR-
ENSG0000	ENSG0000	0.00144	hsa-miR-519a-3p;hsa-miR-519b-3p;hsa-miR-519c-3p;hsa-miR-
ENSG0000	ENSG0000	8.16E-07	hsa-miR-15a-5p;hsa-miR-15b-5p;hsa-miR-16-5p;hsa-miR-195-
ENSG0000	ENSG0000	1.96E-05	hsa-miR-15a-5p;hsa-miR-15b-5p;hsa-miR-16-5p;hsa-miR-195-
ENSG0000	ENSG0000	0.003871	hsa-miR-369-3p;hsa-miR-4691-3p;hsa-miR-519a-3p;hsa-miR-5
ENSG0000	ENSG0000	0.002206	hsa-miR-30b-5p;hsa-miR-519a-3p;hsa-miR-519b-3p;hsa-miR-5
ENSG0000	ENSG0000	8.50E-05	hsa-miR-15a-5p;hsa-miR-15b-5p;hsa-miR-195-5p;hsa-miR-51
ENSG0000	ENSG0000	0.006243	hsa-miR-30b-5p;hsa-miR-30c-5p;hsa-miR-520a-3p;hsa-miR-52
ENSG0000	ENSG0000	0.000186	hsa-miR-27a-3p;hsa-miR-27b-3p;hsa-miR-520a-3p;hsa-miR-52

ENSG0000	ENSG0000	8.75E-08	hsa-miR-15a-5p;hsa-miR-15b-5p;hsa-miR-16-5p;hsa-miR-195-
ENSG0000	ENSG0000	1.90E-09	hsa-miR-15a-5p;hsa-miR-15b-5p;hsa-miR-16-5p;hsa-miR-195-
ENSG0000	ENSG0000	0.006819	hsa-miR-27a-3p;hsa-miR-27b-3p;hsa-miR-520a-3p;hsa-miR-52
ENSG0000	ENSG0000	3.68E-07	hsa-miR-15a-5p;hsa-miR-15b-5p;hsa-miR-16-5p;hsa-miR-195-
ENSG0000	ENSG0000	0.0087	hsa-miR-27a-3p;hsa-miR-27b-3p;hsa-miR-3605-5p;hsa-miR-51
ENSG0000	ENSG0000	0.000686	hsa-miR-519a-3p;hsa-miR-519b-3p;hsa-miR-519c-3p;hsa-miR-
ENSG0000	ENSG0000	3.55E-05	hsa-miR-3615;hsa-miR-519a-3p;hsa-miR-519b-3p;hsa-miR-519
ENSG0000	ENSG0000	0.002853	hsa-miR-4691-3p;hsa-miR-520a-3p;hsa-miR-520c-3p;hsa-miR-
ENSG0000	ENSG0000	0.002244	hsa-miR-27a-3p;hsa-miR-27b-3p;hsa-miR-520a-3p;hsa-miR-52
ENSG0000	ENSG0000	1.11E-11	hsa-miR-15a-5p;hsa-miR-16-5p;hsa-miR-195-5p;hsa-miR-214-
ENSG0000	ENSG0000	1.00E-05	hsa-miR-15a-5p;hsa-miR-15b-5p;hsa-miR-16-5p;hsa-miR-195-
ENSG0000	ENSG0000	0.000117	hsa-miR-15a-5p;hsa-miR-15b-5p;hsa-miR-16-5p;hsa-miR-195-
ENSG0000	ENSG0000	0.000152	hsa-miR-30c-5p;hsa-miR-519a-3p;hsa-miR-519b-3p;hsa-miR-5
ENSG0000	ENSG0000	4.29E-06	hsa-miR-519a-3p;hsa-miR-519b-3p;hsa-miR-519c-3p;hsa-miR-
ENSG0000	ENSG0000	8.50E-05	hsa-miR-15a-5p;hsa-miR-15b-5p;hsa-miR-16-5p;hsa-miR-195-
ENSG0000	ENSG0000	2.71E-08	hsa-miR-15a-5p;hsa-miR-15b-5p;hsa-miR-16-5p;hsa-miR-195-
ENSG0000	ENSG0000	1.93E-08	hsa-miR-15a-5p;hsa-miR-15b-5p;hsa-miR-16-5p;hsa-miR-195-
ENSG0000	ENSG0000	3.53E-06	hsa-miR-126-5p;hsa-miR-15a-5p;hsa-miR-15b-5p;hsa-miR-16-
ENSG0000	ENSG0000	3.17E-05	hsa-miR-15a-5p;hsa-miR-15b-5p;hsa-miR-16-5p;hsa-miR-195-
ENSG0000	ENSG0000	1.90E-11	hsa-miR-15a-5p;hsa-miR-15b-5p;hsa-miR-16-5p;hsa-miR-195-
ENSG0000	ENSG0000	1.90E-11	hsa-miR-15a-5p;hsa-miR-15b-5p;hsa-miR-16-5p;hsa-miR-195-
ENSG0000	ENSG0000	1.90E-11	hsa-miR-15a-5p;hsa-miR-15b-5p;hsa-miR-16-5p;hsa-miR-195-
ENSG0000	ENSG0000	1.90E-11	hsa-miR-15a-5p;hsa-miR-15b-5p;hsa-miR-16-5p;hsa-miR-195-
ENSG0000	ENSG0000	0.000406	hsa-miR-15a-5p;hsa-miR-15b-5p;hsa-miR-195-5p;hsa-miR-424
ENSG0000	ENSG0000	0.000406	hsa-miR-15a-5p;hsa-miR-15b-5p;hsa-miR-195-5p;hsa-miR-424
ENSG0000	ENSG0000	0.000378	hsa-miR-519a-3p;hsa-miR-519b-3p;hsa-miR-519c-3p;hsa-miR-
ENSG0000	ENSG0000	0.00053	hsa-miR-15a-5p;hsa-miR-15b-5p;hsa-miR-16-5p;hsa-miR-195-
ENSG0000	ENSG0000	1.26E-06	hsa-miR-425-5p;hsa-miR-519a-3p;hsa-miR-519b-3p;hsa-miR-5
ENSG0000	ENSG0000	6.24E-11	hsa-miR-15a-5p;hsa-miR-15b-5p;hsa-miR-16-5p;hsa-miR-195-
ENSG0000	ENSG0000	0.001307	hsa-miR-519a-3p;hsa-miR-519b-3p;hsa-miR-519c-3p;hsa-miR-
ENSG0000	ENSG0000	0.000472	hsa-miR-519a-3p;hsa-miR-519b-3p;hsa-miR-519c-3p;hsa-miR-
ENSG0000	ENSG0000	0.000413	hsa-miR-519a-3p;hsa-miR-519b-3p;hsa-miR-519c-3p;hsa-miR-
ENSG0000	ENSG0000	4.48E-11	hsa-miR-15a-5p;hsa-miR-15b-5p;hsa-miR-195-5p;hsa-miR-214
ENSG0000	ENSG0000	3.21E-07	hsa-miR-15a-5p;hsa-miR-15b-5p;hsa-miR-16-5p;hsa-miR-195-
ENSG0000	ENSG0000	0.001581	hsa-miR-519a-3p;hsa-miR-519b-3p;hsa-miR-519c-3p;hsa-miR-
ENSG0000	ENSG0000	2.18E-08	hsa-miR-15a-5p;hsa-miR-15b-5p;hsa-miR-16-5p;hsa-miR-195-
ENSG0000	ENSG0000	0.004696	hsa-miR-519a-3p;hsa-miR-519b-3p;hsa-miR-519c-3p;hsa-miR-
ENSG0000	ENSG0000	0.006421	hsa-miR-519a-3p;hsa-miR-519b-3p;hsa-miR-519c-3p;hsa-miR-
ENSG0000	ENSG0000	4.64E-06	hsa-miR-15a-5p;hsa-miR-15b-5p;hsa-miR-16-5p;hsa-miR-195-
ENSG0000	ENSG0000	4.64E-06	hsa-miR-15a-5p;hsa-miR-15b-5p;hsa-miR-16-5p;hsa-miR-195-
ENSG0000	ENSG0000	4.64E-06	hsa-miR-15a-5p;hsa-miR-15b-5p;hsa-miR-16-5p;hsa-miR-195-
ENSG0000	ENSG0000	4.64E-06	hsa-miR-15a-5p;hsa-miR-15b-5p;hsa-miR-16-5p;hsa-miR-195-
ENSG0000	ENSG0000	4.64E-06	hsa-miR-15a-5p;hsa-miR-15b-5p;hsa-miR-16-5p;hsa-miR-195-
ENSG0000	ENSG0000	4.64E-06	hsa-miR-15a-5p;hsa-miR-15b-5p;hsa-miR-16-5p;hsa-miR-195-
ENSG0000	ENSG0000	4.64E-06	hsa-miR-15a-5p;hsa-miR-15b-5p;hsa-miR-16-5p;hsa-miR-195-
ENSG0000	ENSG0000	4.64E-06	hsa-miR-15a-5p;hsa-miR-15b-5p;hsa-miR-16-5p;hsa-miR-195-
ENSG0000	ENSG0000	4.64E-06	hsa-miR-15a-5p;hsa-miR-15b-5p;hsa-miR-16-5p;hsa-miR-195-
ENSG0000	ENSG0000	4.64E-06	hsa-miR-15a-5p;hsa-miR-15b-5p;hsa-miR-16-5p;hsa-miR-195-
ENSG0000	ENSG0000	0.000132	hsa-miR-519a-3p;hsa-miR-519b-3p;hsa-miR-519c-3p;hsa-miR-
ENSG0000	ENSG0000	1.47E-06	hsa-miR-15a-5p;hsa-miR-15b-5p;hsa-miR-16-5p;hsa-miR-195-
ENSG0000	ENSG0000	1.70E-07	hsa-miR-15a-5p;hsa-miR-15b-5p;hsa-miR-16-5p;hsa-miR-195-
ENSG0000	ENSG0000	6.05E-05	hsa-miR-139-5p;hsa-miR-218-5p;hsa-miR-519a-3p;hsa-miR-51
ENSG0000	ENSG0000	3.94E-07	hsa-miR-15a-5p;hsa-miR-15b-5p;hsa-miR-16-5p;hsa-miR-424-
ENSG0000	ENSG0000	0.001069	hsa-miR-15a-5p;hsa-miR-15b-5p;hsa-miR-16-5p;hsa-miR-195-

ENSG0000	ENSG0000	0.00053	hsa-miR-15a-5p;hsa-miR-15b-5p;hsa-miR-195-5p;hsa-miR-21
ENSG0000	ENSG0000	8.66E-06	hsa-miR-15a-5p;hsa-miR-15b-5p;hsa-miR-16-5p;hsa-miR-195
ENSG0000	ENSG0000	0.00358	hsa-miR-519a-3p;hsa-miR-519b-3p;hsa-miR-519c-3p;hsa-miR
ENSG0000	ENSG0000	0.000194	hsa-miR-15a-5p;hsa-miR-15b-5p;hsa-miR-16-5p;hsa-miR-195
ENSG0000	ENSG0000	5.46E-06	hsa-miR-15a-5p;hsa-miR-15b-5p;hsa-miR-16-5p;hsa-miR-195
ENSG0000	ENSG0000	2.82E-06	hsa-miR-15a-5p;hsa-miR-15b-5p;hsa-miR-16-5p;hsa-miR-195
ENSG0000	ENSG0000	0.004105	hsa-miR-126-5p;hsa-miR-519a-3p;hsa-miR-519b-3p;hsa-miR-
ENSG0000	ENSG0000	3.54E-08	hsa-miR-15a-5p;hsa-miR-15b-5p;hsa-miR-195-5p;hsa-miR-27
ENSG0000	ENSG0000	4.57E-05	hsa-miR-15a-5p;hsa-miR-15b-5p;hsa-miR-16-5p;hsa-miR-195
ENSG0000	ENSG0000	2.82E-05	hsa-miR-15a-5p;hsa-miR-15b-5p;hsa-miR-16-5p;hsa-miR-195
ENSG0000	ENSG0000	0.00231	hsa-miR-218-5p;hsa-miR-361-5p;hsa-miR-519a-3p;hsa-miR-5
ENSG0000	ENSG0000	0.001436	hsa-miR-369-3p;hsa-miR-519a-3p;hsa-miR-519b-3p;hsa-miR-
ENSG0000	ENSG0000	0.009479	hsa-miR-2115-5p;hsa-miR-520a-3p;hsa-miR-520c-3p;hsa-miR
ENSG0000	ENSG0000	0.000771	hsa-miR-15a-5p;hsa-miR-15b-5p;hsa-miR-16-5p;hsa-miR-195
ENSG0000	ENSG0000	6.65E-07	hsa-miR-15a-5p;hsa-miR-15b-5p;hsa-miR-16-5p;hsa-miR-195
ENSG0000	ENSG0000	2.86E-05	hsa-miR-15a-5p;hsa-miR-15b-5p;hsa-miR-16-5p;hsa-miR-195
ENSG0000	ENSG0000	6.37E-08	hsa-miR-15a-5p;hsa-miR-15b-5p;hsa-miR-16-5p;hsa-miR-195
ENSG0000	ENSG0000	3.41E-05	hsa-miR-15a-5p;hsa-miR-15b-5p;hsa-miR-16-5p;hsa-miR-195
ENSG0000	ENSG0000	1.61E-05	hsa-miR-30b-5p;hsa-miR-30c-5p;hsa-miR-519a-3p;hsa-miR-5
ENSG0000	ENSG0000	3.30E-06	hsa-miR-15a-5p;hsa-miR-15b-5p;hsa-miR-195-5p;hsa-miR-42
ENSG0000	ENSG0000	3.27E-05	hsa-miR-15a-5p;hsa-miR-15b-5p;hsa-miR-195-5p;hsa-miR-42
ENSG0000	ENSG0000	1.65E-10	hsa-miR-15a-5p;hsa-miR-15b-5p;hsa-miR-16-5p;hsa-miR-195
ENSG0000	ENSG0000	6.69E-07	hsa-miR-15a-5p;hsa-miR-15b-5p;hsa-miR-195-5p;hsa-miR-42
ENSG0000	ENSG0000	2.05E-08	hsa-miR-15a-5p;hsa-miR-15b-5p;hsa-miR-16-5p;hsa-miR-195
ENSG0000	ENSG0000	3.30E-06	hsa-miR-15a-5p;hsa-miR-15b-5p;hsa-miR-16-5p;hsa-miR-195
ENSG0000	ENSG0000	0.000379	hsa-miR-30b-5p;hsa-miR-30c-5p;hsa-miR-519a-3p;hsa-miR-5
ENSG0000	ENSG0000	0.000267	hsa-miR-15a-5p;hsa-miR-15b-5p;hsa-miR-16-5p;hsa-miR-195
ENSG0000	ENSG0000	1.08E-07	hsa-miR-15a-5p;hsa-miR-15b-5p;hsa-miR-16-5p;hsa-miR-195
ENSG0000	ENSG0000	0.000117	hsa-miR-519a-3p;hsa-miR-519b-3p;hsa-miR-519c-3p;hsa-miR
ENSG0000	ENSG0000	6.57E-09	hsa-miR-139-5p;hsa-miR-15a-5p;hsa-miR-15b-5p;hsa-miR-16
ENSG0000	ENSG0000	0.002101	hsa-miR-15a-5p;hsa-miR-15b-5p;hsa-miR-195-5p;hsa-miR-42
ENSG0000	ENSG0000	3.19E-07	hsa-miR-15a-5p;hsa-miR-15b-5p;hsa-miR-16-5p;hsa-miR-195
ENSG0000	ENSG0000	0.001534	hsa-miR-15a-5p;hsa-miR-15b-5p;hsa-miR-195-5p;hsa-miR-36
ENSG0000	ENSG0000	0.001534	hsa-miR-15a-5p;hsa-miR-15b-5p;hsa-miR-195-5p;hsa-miR-36
ENSG0000	ENSG0000	0.001534	hsa-miR-15a-5p;hsa-miR-15b-5p;hsa-miR-195-5p;hsa-miR-36
ENSG0000	ENSG0000	0.001534	hsa-miR-15a-5p;hsa-miR-15b-5p;hsa-miR-195-5p;hsa-miR-36
ENSG0000	ENSG0000	0.001534	hsa-miR-15a-5p;hsa-miR-15b-5p;hsa-miR-195-5p;hsa-miR-36
ENSG0000	ENSG0000	0.001534	hsa-miR-15a-5p;hsa-miR-15b-5p;hsa-miR-195-5p;hsa-miR-36
ENSG0000	ENSG0000	0.001534	hsa-miR-15a-5p;hsa-miR-15b-5p;hsa-miR-195-5p;hsa-miR-36
ENSG0000	ENSG0000	0.001534	hsa-miR-15a-5p;hsa-miR-15b-5p;hsa-miR-195-5p;hsa-miR-36
ENSG0000	ENSG0000	2.82E-05	hsa-miR-15a-5p;hsa-miR-15b-5p;hsa-miR-16-5p;hsa-miR-195
ENSG0000	ENSG0000	0.000112	hsa-miR-15a-5p;hsa-miR-15b-5p;hsa-miR-195-5p;hsa-miR-47
ENSG0000	ENSG0000	1.70E-07	hsa-miR-15a-5p;hsa-miR-15b-5p;hsa-miR-16-5p;hsa-miR-195
ENSG0000	ENSG0000	6.97E-06	hsa-miR-519a-3p;hsa-miR-519b-3p;hsa-miR-519c-3p;hsa-miR
ENSG0000	ENSG0000	1.22E-08	hsa-miR-15a-5p;hsa-miR-15b-5p;hsa-miR-16-5p;hsa-miR-195
ENSG0000	ENSG0000	0.001253	hsa-miR-519a-3p;hsa-miR-519b-3p;hsa-miR-519c-3p;hsa-miR
ENSG0000	ENSG0000	4.13E-06	hsa-miR-15a-5p;hsa-miR-15b-5p;hsa-miR-16-5p;hsa-miR-195
ENSG0000	ENSG0000	0.000285	hsa-miR-30b-5p;hsa-miR-30c-5p;hsa-miR-519a-3p;hsa-miR-5
ENSG0000	ENSG0000	9.14E-05	hsa-miR-15a-5p;hsa-miR-15b-5p;hsa-miR-16-5p;hsa-miR-195
ENSG0000	ENSG0000	9.14E-05	hsa-miR-15a-5p;hsa-miR-15b-5p;hsa-miR-16-5p;hsa-miR-195
ENSG0000	ENSG0000	0.000686	hsa-miR-519a-3p;hsa-miR-519b-3p;hsa-miR-519c-3p;hsa-miR
ENSG0000	ENSG0000	1.47E-06	hsa-miR-27a-3p;hsa-miR-27b-3p;hsa-miR-30b-5p;hsa-miR-30
ENSG0000	ENSG0000	1.47E-06	hsa-miR-27a-3p;hsa-miR-27b-3p;hsa-miR-30b-5p;hsa-miR-30
ENSG0000	ENSG0000	2.67E-05	hsa-miR-15a-5p;hsa-miR-15b-5p;hsa-miR-16-5p;hsa-miR-195

ENSG0000	ENSG0000	1.51E-05	hsa-miR-27a-3p;hsa-miR-27b-3p;hsa-miR-519a-3p;hsa-miR-5
ENSG0000	ENSG0000	0.007635	hsa-miR-519a-3p;hsa-miR-519b-3p;hsa-miR-519c-3p;hsa-miR-
ENSG0000	ENSG0000	3.54E-08	hsa-miR-15a-5p;hsa-miR-15b-5p;hsa-miR-16-5p;hsa-miR-195
ENSG0000	ENSG0000	9.14E-05	hsa-miR-214-3p;hsa-miR-27a-3p;hsa-miR-27b-3p;hsa-miR-36
ENSG0000	ENSG0000	1.93E-08	hsa-miR-15a-5p;hsa-miR-15b-5p;hsa-miR-16-5p;hsa-miR-195
ENSG0000	ENSG0000	2.31E-09	hsa-miR-15a-5p;hsa-miR-15b-5p;hsa-miR-16-5p;hsa-miR-195
ENSG0000	ENSG0000	1.41E-05	hsa-miR-15a-5p;hsa-miR-15b-5p;hsa-miR-16-5p;hsa-miR-195
ENSG0000	ENSG0000	1.65E-10	hsa-miR-15a-5p;hsa-miR-15b-5p;hsa-miR-16-5p;hsa-miR-195
ENSG0000	ENSG0000	9.08E-05	hsa-miR-519a-3p;hsa-miR-519b-3p;hsa-miR-519c-3p;hsa-miR-
ENSG0000	ENSG0000	0.000406	hsa-miR-15a-5p;hsa-miR-15b-5p;hsa-miR-16-5p;hsa-miR-195
ENSG0000	ENSG0000	2.82E-05	hsa-miR-15a-5p;hsa-miR-15b-5p;hsa-miR-16-5p;hsa-miR-195
ENSG0000	ENSG0000	1.55E-06	hsa-miR-15a-5p;hsa-miR-15b-5p;hsa-miR-16-5p;hsa-miR-195
ENSG0000	ENSG0000	0.00681	hsa-miR-4726-3p;hsa-miR-520a-3p;hsa-miR-520c-3p;hsa-miR-
ENSG0000	ENSG0000	0.003567	hsa-miR-15a-5p;hsa-miR-15b-5p;hsa-miR-16-5p;hsa-miR-195
ENSG0000	ENSG0000	9.79E-05	hsa-miR-15a-5p;hsa-miR-15b-5p;hsa-miR-16-5p;hsa-miR-195
ENSG0000	ENSG0000	0.000865	hsa-miR-15a-5p;hsa-miR-15b-5p;hsa-miR-16-5p;hsa-miR-195
ENSG0000	ENSG0000	4.54E-05	hsa-miR-15a-5p;hsa-miR-15b-5p;hsa-miR-16-5p;hsa-miR-195
ENSG0000	ENSG0000	0.000287	hsa-miR-15a-5p;hsa-miR-15b-5p;hsa-miR-195-5p;hsa-miR-21
ENSG0000	ENSG0000	0.000359	hsa-miR-519a-3p;hsa-miR-519b-3p;hsa-miR-519c-3p;hsa-miR-
ENSG0000	ENSG0000	2.18E-08	hsa-miR-15a-5p;hsa-miR-15b-5p;hsa-miR-16-5p;hsa-miR-195
ENSG0000	ENSG0000	0.002206	hsa-miR-425-5p;hsa-miR-519a-3p;hsa-miR-519b-3p;hsa-miR-
ENSG0000	ENSG0000	1.53E-08	hsa-miR-15a-5p;hsa-miR-15b-5p;hsa-miR-16-5p;hsa-miR-195
ENSG0000	ENSG0000	0.004956	hsa-miR-4726-3p;hsa-miR-520a-3p;hsa-miR-520c-3p;hsa-miR-
ENSG0000	ENSG0000	0.000941	hsa-miR-15a-5p;hsa-miR-15b-5p;hsa-miR-16-5p;hsa-miR-195
ENSG0000	ENSG0000	2.50E-05	hsa-miR-15a-5p;hsa-miR-15b-5p;hsa-miR-16-5p;hsa-miR-195
ENSG0000	ENSG0000	6.28E-05	hsa-miR-15a-5p;hsa-miR-15b-5p;hsa-miR-16-5p;hsa-miR-195
ENSG0000	ENSG0000	0.000228	hsa-miR-519a-3p;hsa-miR-519b-3p;hsa-miR-519c-3p;hsa-miR-
ENSG0000	ENSG0000	0.00681	hsa-miR-519a-3p;hsa-miR-519b-3p;hsa-miR-519c-3p;hsa-miR-
ENSG0000	ENSG0000	5.26E-06	hsa-miR-15a-5p;hsa-miR-15b-5p;hsa-miR-16-5p;hsa-miR-195
ENSG0000	ENSG0000	0.000359	hsa-miR-519a-3p;hsa-miR-519b-3p;hsa-miR-519c-3p;hsa-miR-
ENSG0000	ENSG0000	6.05E-05	hsa-miR-15a-5p;hsa-miR-15b-5p;hsa-miR-16-5p;hsa-miR-195
ENSG0000	ENSG0000	0.000311	hsa-miR-218-5p;hsa-miR-519a-3p;hsa-miR-519b-3p;hsa-miR-
ENSG0000	ENSG0000	1.22E-08	hsa-let-7a-5p;hsa-let-7b-5p;hsa-let-7d-5p;hsa-let-7f-5p;hsa-le
ENSG0000	ENSG0000	1.28E-05	hsa-let-7a-5p;hsa-let-7b-5p;hsa-let-7d-5p;hsa-let-7f-5p;hsa-le
ENSG0000	ENSG0000	2.15E-08	hsa-let-7a-5p;hsa-let-7b-5p;hsa-let-7d-5p;hsa-let-7f-5p;hsa-le
ENSG0000	ENSG0000	7.70E-07	hsa-let-7a-5p;hsa-let-7b-5p;hsa-let-7d-5p;hsa-let-7f-5p;hsa-le
ENSG0000	ENSG0000	5.56E-05	hsa-let-7a-5p;hsa-let-7b-5p;hsa-let-7d-5p;hsa-let-7f-5p;hsa-le
ENSG0000	ENSG0000	3.10E-05	hsa-let-7a-5p;hsa-let-7b-5p;hsa-let-7d-5p;hsa-let-7f-5p;hsa-le
ENSG0000	ENSG0000	0.000482	hsa-let-7a-5p;hsa-let-7b-5p;hsa-let-7d-5p;hsa-let-7g-5p;hsa-l
ENSG0000	ENSG0000	4.86E-07	hsa-let-7a-5p;hsa-let-7b-5p;hsa-let-7d-5p;hsa-let-7f-5p;hsa-le
ENSG0000	ENSG0000	3.17E-06	hsa-let-7a-5p;hsa-let-7b-5p;hsa-let-7d-5p;hsa-let-7f-5p;hsa-le
ENSG0000	ENSG0000	2.83E-06	hsa-let-7a-5p;hsa-let-7b-5p;hsa-let-7d-5p;hsa-let-7f-5p;hsa-le
ENSG0000	ENSG0000	4.86E-07	hsa-let-7a-5p;hsa-let-7b-5p;hsa-let-7d-5p;hsa-let-7f-5p;hsa-le
ENSG0000	ENSG0000	0.000303	hsa-let-7a-5p;hsa-let-7b-5p;hsa-let-7d-5p;hsa-let-7f-5p;hsa-le
ENSG0000	ENSG0000	3.68E-08	hsa-let-7a-5p;hsa-let-7b-5p;hsa-let-7d-5p;hsa-let-7f-5p;hsa-le
ENSG0000	ENSG0000	2.48E-10	hsa-let-7a-5p;hsa-let-7d-5p;hsa-let-7f-5p;hsa-let-7g-5p;hsa-le
ENSG0000	ENSG0000	1.53E-06	hsa-let-7a-5p;hsa-let-7b-5p;hsa-let-7d-5p;hsa-let-7f-5p;hsa-le
ENSG0000	ENSG0000	8.10E-06	hsa-let-7a-5p;hsa-let-7b-5p;hsa-let-7d-5p;hsa-let-7f-5p;hsa-le
ENSG0000	ENSG0000	0.000153	hsa-let-7a-5p;hsa-let-7f-5p;hsa-let-7g-5p;hsa-let-7i-5p
ENSG0000	ENSG0000	2.85E-08	hsa-let-7a-5p;hsa-let-7b-5p;hsa-let-7d-5p;hsa-let-7f-5p;hsa-le
ENSG0000	ENSG0000	2.83E-06	hsa-let-7a-5p;hsa-let-7b-5p;hsa-let-7d-5p;hsa-let-7f-5p;hsa-le
ENSG0000	ENSG0000	0.000359	hsa-let-7a-5p;hsa-let-7d-5p;hsa-let-7f-5p;hsa-let-7g-5p;hsa-le
ENSG0000	ENSG0000	1.50E-10	hsa-let-7a-5p;hsa-let-7b-5p;hsa-let-7d-5p;hsa-let-7f-5p;hsa-le
ENSG0000	ENSG0000	9.77E-06	hsa-let-7a-5p;hsa-let-7b-5p;hsa-let-7d-5p;hsa-let-7f-5p;hsa-le

ENSG0000	ENSG0000	1.80E-11	hsa-let-7a-5p;hsa-let-7b-5p;hsa-let-7d-5p;hsa-let-7f-5p;hsa-le
ENSG0000	ENSG0000	0.000748	hsa-miR-196b-5p;hsa-miR-19a-5p;hsa-miR-19b-1-5p;hsa-miR-
ENSG0000	ENSG0000	8.50E-05	hsa-let-7a-5p;hsa-let-7b-5p;hsa-let-7d-5p;hsa-let-7f-5p;hsa-le
ENSG0000	ENSG0000	7.99E-05	hsa-let-7a-5p;hsa-let-7b-5p;hsa-let-7d-5p;hsa-let-7f-5p;hsa-le
ENSG0000	ENSG0000	0.001639	hsa-let-7a-5p;hsa-let-7b-5p;hsa-let-7d-5p;hsa-let-7f-5p;hsa-le
ENSG0000	ENSG0000	1.34E-06	hsa-let-7a-5p;hsa-let-7b-5p;hsa-let-7d-5p;hsa-let-7f-5p;hsa-le
ENSG0000	ENSG0000	4.45E-08	hsa-let-7a-5p;hsa-let-7b-5p;hsa-let-7d-5p;hsa-let-7f-5p;hsa-le
ENSG0000	ENSG0000	0.001929	hsa-let-7a-5p;hsa-let-7d-5p;hsa-let-7f-5p;hsa-let-7g-5p;hsa-le
ENSG0000	ENSG0000	3.50E-07	hsa-let-7a-5p;hsa-let-7b-5p;hsa-let-7d-5p;hsa-let-7f-5p;hsa-le
ENSG0000	ENSG0000	2.94E-10	hsa-let-7a-5p;hsa-let-7b-5p;hsa-let-7d-5p;hsa-let-7f-5p;hsa-le
ENSG0000	ENSG0000	1.03E-06	hsa-let-7a-5p;hsa-let-7b-5p;hsa-let-7d-5p;hsa-let-7f-5p;hsa-le
ENSG0000	ENSG0000	6.62E-05	hsa-let-7a-5p;hsa-let-7b-5p;hsa-let-7d-5p;hsa-let-7f-5p;hsa-le
ENSG0000	ENSG0000	9.29E-08	hsa-let-7a-5p;hsa-let-7b-5p;hsa-let-7d-5p;hsa-let-7f-5p;hsa-le
ENSG0000	ENSG0000	1.94E-08	hsa-let-7d-5p;hsa-let-7f-5p;hsa-let-7g-5p;hsa-let-7i-5p
ENSG0000	ENSG0000	0.000114	hsa-let-7a-5p;hsa-let-7b-5p;hsa-let-7d-5p;hsa-let-7f-5p;hsa-le
ENSG0000	ENSG0000	2.37E-05	hsa-let-7a-5p;hsa-let-7b-5p;hsa-let-7f-5p;hsa-let-7g-5p;hsa-le
ENSG0000	ENSG0000	1.53E-06	hsa-let-7a-5p;hsa-let-7b-5p;hsa-let-7d-5p;hsa-let-7f-5p;hsa-le
ENSG0000	ENSG0000	6.40E-09	hsa-let-7a-5p;hsa-let-7b-5p;hsa-let-7d-5p;hsa-let-7f-5p;hsa-le
ENSG0000	ENSG0000	4.50E-09	hsa-let-7a-5p;hsa-let-7b-5p;hsa-let-7d-5p;hsa-let-7f-5p;hsa-le
ENSG0000	ENSG0000	2.85E-08	hsa-let-7a-5p;hsa-let-7b-5p;hsa-let-7d-5p;hsa-let-7f-5p;hsa-le
ENSG0000	ENSG0000	0.0005	hsa-let-7a-5p;hsa-let-7b-5p;hsa-let-7d-5p;hsa-let-7g-5p;hsa-l
ENSG0000	ENSG0000	6.38E-12	hsa-let-7a-5p;hsa-let-7b-5p;hsa-let-7d-5p;hsa-let-7f-5p;hsa-le
ENSG0000	ENSG0000	9.03E-05	hsa-let-7a-5p;hsa-let-7b-5p;hsa-let-7d-5p;hsa-let-7f-5p;hsa-le
ENSG0000	ENSG0000	6.56E-10	hsa-let-7a-5p;hsa-let-7b-5p;hsa-let-7d-5p;hsa-let-7f-5p;hsa-le
ENSG0000	ENSG0000	3.68E-08	hsa-let-7a-5p;hsa-let-7b-5p;hsa-let-7d-5p;hsa-let-7f-5p;hsa-le
ENSG0000	ENSG0000	3.68E-08	hsa-let-7a-5p;hsa-let-7b-5p;hsa-let-7d-5p;hsa-let-7f-5p;hsa-le
ENSG0000	ENSG0000	1.15E-07	hsa-let-7a-5p;hsa-let-7b-5p;hsa-let-7d-5p;hsa-let-7f-5p;hsa-le
ENSG0000	ENSG0000	1.22E-08	hsa-let-7a-5p;hsa-let-7b-5p;hsa-let-7d-5p;hsa-let-7f-5p;hsa-le
ENSG0000	ENSG0000	1.22E-08	hsa-let-7a-5p;hsa-let-7b-5p;hsa-let-7d-5p;hsa-let-7f-5p;hsa-le
ENSG0000	ENSG0000	1.22E-08	hsa-let-7a-5p;hsa-let-7b-5p;hsa-let-7d-5p;hsa-let-7f-5p;hsa-le
ENSG0000	ENSG0000	1.22E-08	hsa-let-7a-5p;hsa-let-7b-5p;hsa-let-7d-5p;hsa-let-7f-5p;hsa-le
ENSG0000	ENSG0000	1.22E-08	hsa-let-7a-5p;hsa-let-7b-5p;hsa-let-7d-5p;hsa-let-7f-5p;hsa-le
ENSG0000	ENSG0000	1.22E-08	hsa-let-7a-5p;hsa-let-7b-5p;hsa-let-7d-5p;hsa-let-7f-5p;hsa-le
ENSG0000	ENSG0000	1.22E-08	hsa-let-7a-5p;hsa-let-7b-5p;hsa-let-7d-5p;hsa-let-7f-5p;hsa-le
ENSG0000	ENSG0000	0.000173	hsa-let-7a-5p;hsa-let-7b-5p;hsa-let-7d-5p;hsa-let-7g-5p;hsa-l
ENSG0000	ENSG0000	0.000364	hsa-let-7a-5p;hsa-let-7d-5p;hsa-let-7f-5p;hsa-let-7g-5p;hsa-le
ENSG0000	ENSG0000	1.07E-05	hsa-let-7a-5p;hsa-let-7b-5p;hsa-let-7d-5p;hsa-let-7f-5p;hsa-le
ENSG0000	ENSG0000	8.90E-06	hsa-let-7a-5p;hsa-let-7b-5p;hsa-let-7d-5p;hsa-let-7f-5p;hsa-le
ENSG0000	ENSG0000	2.67E-05	hsa-let-7a-5p;hsa-let-7b-5p;hsa-let-7f-5p;hsa-let-7g-5p;hsa-le
ENSG0000	ENSG0000	1.53E-06	hsa-let-7a-5p;hsa-let-7b-5p;hsa-let-7d-5p;hsa-let-7f-5p;hsa-le
ENSG0000	ENSG0000	6.21E-05	hsa-let-7a-5p;hsa-let-7b-5p;hsa-let-7d-5p;hsa-let-7f-5p;hsa-le
ENSG0000	ENSG0000	8.10E-06	hsa-let-7a-5p;hsa-let-7b-5p;hsa-let-7d-5p;hsa-let-7f-5p;hsa-le
ENSG0000	ENSG0000	5.15E-05	hsa-miR-196a-5p;hsa-miR-196b-5p;hsa-miR-19a-5p;hsa-miR-1
ENSG0000	ENSG0000	5.15E-05	hsa-miR-196a-5p;hsa-miR-196b-5p;hsa-miR-19a-5p;hsa-miR-1
ENSG0000	ENSG0000	5.15E-05	hsa-miR-196a-5p;hsa-miR-196b-5p;hsa-miR-19a-5p;hsa-miR-1
ENSG0000	ENSG0000	5.15E-05	hsa-miR-196a-5p;hsa-miR-196b-5p;hsa-miR-19a-5p;hsa-miR-1
ENSG0000	ENSG0000	5.15E-05	hsa-miR-196a-5p;hsa-miR-196b-5p;hsa-miR-19a-5p;hsa-miR-1
ENSG0000	ENSG0000	4.14E-07	hsa-let-7a-5p;hsa-let-7d-5p;hsa-let-7f-5p;hsa-let-7g-5p;hsa-le
ENSG0000	ENSG0000	4.14E-05	hsa-let-7a-5p;hsa-let-7b-5p;hsa-let-7d-5p;hsa-let-7f-5p;hsa-le
ENSG0000	ENSG0000	3.50E-07	hsa-let-7a-5p;hsa-let-7b-5p;hsa-let-7d-5p;hsa-let-7f-5p;hsa-le
ENSG0000	ENSG0000	8.89E-06	hsa-let-7a-5p;hsa-let-7b-5p;hsa-let-7d-5p;hsa-let-7f-5p;hsa-le
ENSG0000	ENSG0000	7.36E-06	hsa-let-7a-5p;hsa-let-7b-5p;hsa-let-7d-5p;hsa-let-7f-5p;hsa-le
ENSG0000	ENSG0000	1.04E-06	hsa-let-7a-5p;hsa-let-7b-5p;hsa-let-7d-5p;hsa-let-7f-5p;hsa-le
ENSG0000	ENSG0000	7.15E-05	hsa-let-7a-5p;hsa-let-7d-5p;hsa-let-7f-5p;hsa-let-7g-5p;hsa-le

ENSG0000	ENSG0000	0.000107	hsa-let-7a-5p;hsa-let-7b-5p;hsa-let-7d-5p;hsa-let-7f-5p;hsa-le
ENSG0000	ENSG0000	0.000107	hsa-let-7a-5p;hsa-let-7b-5p;hsa-let-7d-5p;hsa-let-7f-5p;hsa-le
ENSG0000	ENSG0000	7.36E-06	hsa-let-7a-5p;hsa-let-7b-5p;hsa-let-7d-5p;hsa-let-7f-5p;hsa-le
ENSG0000	ENSG0000	1.07E-05	hsa-let-7a-5p;hsa-let-7b-5p;hsa-let-7d-5p;hsa-let-7f-5p;hsa-le
ENSG0000	ENSG0000	1.34E-06	hsa-let-7a-5p;hsa-let-7b-5p;hsa-let-7d-5p;hsa-let-7f-5p;hsa-le
ENSG0000	ENSG0000	2.67E-05	hsa-let-7a-5p;hsa-let-7b-5p;hsa-let-7d-5p;hsa-let-7f-5p;hsa-le
ENSG0000	ENSG0000	2.67E-05	hsa-let-7a-5p;hsa-let-7b-5p;hsa-let-7d-5p;hsa-let-7f-5p;hsa-le
ENSG0000	ENSG0000	6.40E-09	hsa-let-7a-5p;hsa-let-7b-5p;hsa-let-7d-5p;hsa-let-7f-5p;hsa-le
ENSG0000	ENSG0000	0.001303	hsa-miR-196a-5p;hsa-miR-196b-5p;hsa-miR-33a-3p;hsa-miR-4
ENSG0000	ENSG0000	1.27E-08	hsa-let-7a-5p;hsa-let-7b-5p;hsa-let-7d-5p;hsa-let-7f-5p;hsa-le
ENSG0000	ENSG0000	0.00501	hsa-let-7b-5p;hsa-let-7f-5p;hsa-let-7i-5p;hsa-miR-1-3p
ENSG0000	ENSG0000	8.10E-06	hsa-let-7a-5p;hsa-let-7b-5p;hsa-let-7d-5p;hsa-let-7f-5p;hsa-le
ENSG0000	ENSG0000	0.003851	hsa-let-7a-5p;hsa-let-7b-5p;hsa-let-7d-5p;hsa-let-7f-5p;hsa-le
ENSG0000	ENSG0000	0.000368	hsa-let-7a-5p;hsa-let-7b-5p;hsa-let-7d-5p;hsa-let-7f-5p;hsa-le
ENSG0000	ENSG0000	2.47E-07	hsa-let-7a-5p;hsa-let-7b-5p;hsa-let-7d-5p;hsa-let-7f-5p;hsa-le
ENSG0000	ENSG0000	0.00365	hsa-let-7a-5p;hsa-let-7d-5p;hsa-let-7f-5p;hsa-let-7g-5p;hsa-le
ENSG0000	ENSG0000	1.95E-05	hsa-let-7a-5p;hsa-let-7b-5p;hsa-let-7d-5p;hsa-let-7f-5p;hsa-le
ENSG0000	ENSG0000	3.30E-09	hsa-let-7a-5p;hsa-let-7b-5p;hsa-let-7d-5p;hsa-let-7f-5p;hsa-le
ENSG0000	ENSG0000	0.001	hsa-miR-16-1-3p;hsa-miR-19a-5p;hsa-miR-19b-1-5p;hsa-miR-
ENSG0000	ENSG0000	1.40E-07	hsa-let-7a-5p;hsa-let-7b-5p;hsa-let-7f-5p;hsa-let-7g-5p;hsa-le
ENSG0000	ENSG0000	8.90E-07	hsa-let-7a-5p;hsa-let-7b-5p;hsa-let-7d-5p;hsa-let-7f-5p;hsa-le
ENSG0000	ENSG0000	0.000124	hsa-let-7a-5p;hsa-let-7b-5p;hsa-let-7d-5p;hsa-let-7f-5p;hsa-le
ENSG0000	ENSG0000	3.03E-07	hsa-let-7a-5p;hsa-let-7b-5p;hsa-let-7d-5p;hsa-let-7g-5p;hsa-r
ENSG0000	ENSG0000	1.38E-09	hsa-let-7a-5p;hsa-let-7b-5p;hsa-let-7d-5p;hsa-let-7f-5p;hsa-le
ENSG0000	ENSG0000	0.001852	hsa-let-7a-5p;hsa-let-7d-5p;hsa-let-7f-5p;hsa-let-7g-5p;hsa-le
ENSG0000	ENSG0000	1.52E-05	hsa-let-7a-5p;hsa-let-7b-5p;hsa-let-7d-5p;hsa-let-7f-5p;hsa-le
ENSG0000	ENSG0000	2.02E-06	hsa-let-7a-5p;hsa-let-7b-5p;hsa-let-7d-5p;hsa-let-7f-5p;hsa-le
ENSG0000	ENSG0000	4.14E-07	hsa-let-7a-5p;hsa-let-7b-5p;hsa-let-7d-5p;hsa-let-7f-5p;hsa-le
ENSG0000	ENSG0000	5.69E-07	hsa-let-7a-5p;hsa-let-7b-5p;hsa-let-7d-5p;hsa-let-7f-5p;hsa-le
ENSG0000	ENSG0000	2.17E-08	hsa-let-7a-5p;hsa-let-7b-5p;hsa-let-7d-5p;hsa-let-7f-5p;hsa-le
ENSG0000	ENSG0000	0.002008	hsa-let-7a-5p;hsa-let-7d-5p;hsa-let-7f-5p;hsa-let-7g-5p;hsa-le
ENSG0000	ENSG0000	6.67E-06	hsa-let-7a-5p;hsa-let-7b-5p;hsa-let-7d-5p;hsa-let-7f-5p;hsa-le
ENSG0000	ENSG0000	4.42E-06	hsa-let-7a-5p;hsa-let-7b-5p;hsa-let-7d-5p;hsa-let-7f-5p;hsa-le
ENSG0000	ENSG0000	1.80E-05	hsa-let-7a-5p;hsa-let-7b-5p;hsa-let-7d-5p;hsa-let-7f-5p;hsa-le
ENSG0000	ENSG0000	6.97E-05	hsa-let-7a-5p;hsa-let-7f-5p;hsa-let-7g-5p;hsa-let-7i-5p
ENSG0000	ENSG0000	1.35E-09	hsa-let-7a-5p;hsa-let-7b-5p;hsa-let-7d-5p;hsa-let-7f-5p;hsa-le
ENSG0000	ENSG0000	4.26E-06	hsa-let-7a-5p;hsa-let-7d-5p;hsa-let-7f-5p;hsa-let-7g-5p;hsa-le
ENSG0000	ENSG0000	8.10E-06	hsa-let-7a-5p;hsa-let-7b-5p;hsa-let-7d-5p;hsa-let-7f-5p;hsa-le
ENSG0000	ENSG0000	6.03E-06	hsa-let-7a-5p;hsa-let-7b-5p;hsa-let-7d-5p;hsa-let-7f-5p;hsa-le
ENSG0000	ENSG0000	6.03E-06	hsa-let-7a-5p;hsa-let-7b-5p;hsa-let-7d-5p;hsa-let-7f-5p;hsa-le
ENSG0000	ENSG0000	1.34E-06	hsa-let-7a-5p;hsa-let-7b-5p;hsa-let-7d-5p;hsa-let-7f-5p;hsa-le
ENSG0000	ENSG0000	3.55E-06	hsa-let-7a-5p;hsa-let-7b-5p;hsa-let-7d-5p;hsa-let-7f-5p;hsa-le
ENSG0000	ENSG0000	3.68E-08	hsa-let-7a-5p;hsa-let-7b-5p;hsa-let-7d-5p;hsa-let-7f-5p;hsa-le
ENSG0000	ENSG0000	1.23E-05	hsa-let-7a-5p;hsa-let-7b-5p;hsa-let-7d-5p;hsa-let-7f-5p;hsa-le
ENSG0000	ENSG0000	2.52E-06	hsa-let-7a-5p;hsa-let-7b-5p;hsa-let-7d-5p;hsa-let-7f-5p;hsa-le
ENSG0000	ENSG0000	4.42E-06	hsa-let-7a-5p;hsa-let-7b-5p;hsa-let-7d-5p;hsa-let-7f-5p;hsa-le
ENSG0000	ENSG0000	1.64E-08	hsa-let-7a-5p;hsa-let-7b-5p;hsa-let-7d-5p;hsa-let-7f-5p;hsa-le
ENSG0000	ENSG0000	0.000352	hsa-miR-19a-5p;hsa-miR-19b-1-5p;hsa-miR-33a-3p;hsa-miR-4
ENSG0000	ENSG0000	0.000352	hsa-miR-19a-5p;hsa-miR-19b-1-5p;hsa-miR-33a-3p;hsa-miR-4
ENSG0000	ENSG0000	3.36E-06	hsa-let-7a-5p;hsa-let-7b-5p;hsa-let-7f-5p;hsa-let-7g-5p;hsa-le
ENSG0000	ENSG0000	4.86E-07	hsa-let-7a-5p;hsa-let-7b-5p;hsa-let-7d-5p;hsa-let-7f-5p;hsa-le
ENSG0000	ENSG0000	4.91E-06	hsa-let-7a-5p;hsa-let-7b-5p;hsa-let-7d-5p;hsa-let-7f-5p;hsa-le
ENSG0000	ENSG0000	7.05E-05	hsa-let-7a-5p;hsa-let-7b-5p;hsa-let-7d-5p;hsa-let-7f-5p;hsa-le
ENSG0000	ENSG0000	7.36E-06	hsa-let-7a-5p;hsa-let-7b-5p;hsa-let-7d-5p;hsa-let-7f-5p;hsa-le

ENSG0000	ENSG0000	0.000931	hsa-let-7a-5p;hsa-let-7b-5p;hsa-let-7g-5p;hsa-miR-196b-5p
ENSG0000	ENSG0000	2.37E-05	hsa-let-7a-5p;hsa-let-7f-5p;hsa-let-7g-5p;hsa-let-7i-5p;hsa-m
ENSG0000	ENSG0000	0.003749	hsa-let-7a-5p;hsa-let-7b-5p;hsa-let-7d-5p;hsa-let-7f-5p;hsa-le
ENSG0000	ENSG0000	0.000135	hsa-let-7a-5p;hsa-let-7b-5p;hsa-let-7d-5p;hsa-let-7f-5p;hsa-le
ENSG0000	ENSG0000	0.000135	hsa-let-7a-5p;hsa-let-7b-5p;hsa-let-7d-5p;hsa-let-7f-5p;hsa-le
ENSG0000	ENSG0000	3.68E-08	hsa-let-7a-5p;hsa-let-7b-5p;hsa-let-7d-5p;hsa-let-7f-5p;hsa-le
ENSG0000	ENSG0000	1.17E-09	hsa-let-7a-5p;hsa-let-7b-5p;hsa-let-7d-5p;hsa-let-7f-5p;hsa-le
ENSG0000	ENSG0000	1.17E-09	hsa-let-7a-5p;hsa-let-7b-5p;hsa-let-7d-5p;hsa-let-7f-5p;hsa-le
ENSG0000	ENSG0000	0.000615	hsa-let-7a-5p;hsa-let-7b-5p;hsa-let-7d-5p;hsa-let-7f-5p;hsa-le
ENSG0000	ENSG0000	2.23E-06	hsa-let-7a-5p;hsa-let-7b-5p;hsa-let-7d-5p;hsa-let-7f-5p;hsa-le
ENSG0000	ENSG0000	0.000338	hsa-let-7b-5p;hsa-let-7d-5p;hsa-let-7f-5p;hsa-let-7g-5p;hsa-le
ENSG0000	ENSG0000	6.67E-06	hsa-let-7a-5p;hsa-let-7b-5p;hsa-let-7d-5p;hsa-let-7f-5p;hsa-le
ENSG0000	ENSG0000	4.14E-05	hsa-let-7a-5p;hsa-let-7b-5p;hsa-let-7d-5p;hsa-let-7f-5p;hsa-le
ENSG0000	ENSG0000	9.03E-05	hsa-let-7a-5p;hsa-let-7b-5p;hsa-let-7d-5p;hsa-let-7f-5p;hsa-le
ENSG0000	ENSG0000	2.56E-09	hsa-let-7a-5p;hsa-let-7b-5p;hsa-let-7d-5p;hsa-let-7f-5p;hsa-le
ENSG0000	ENSG0000	2.11E-05	hsa-let-7a-5p;hsa-let-7b-5p;hsa-let-7d-5p;hsa-let-7f-5p;hsa-le
ENSG0000	ENSG0000	3.27E-05	hsa-let-7a-5p;hsa-let-7b-5p;hsa-let-7d-5p;hsa-let-7f-5p;hsa-le
ENSG0000	ENSG0000	7.36E-06	hsa-let-7a-5p;hsa-let-7b-5p;hsa-let-7d-5p;hsa-let-7f-5p;hsa-le
ENSG0000	ENSG0000	4.14E-05	hsa-let-7a-5p;hsa-let-7b-5p;hsa-let-7d-5p;hsa-let-7f-5p;hsa-le
ENSG0000	ENSG0000	0.0005	hsa-let-7a-5p;hsa-let-7d-5p;hsa-let-7f-5p;hsa-let-7g-5p;hsa-le
ENSG0000	ENSG0000	0.000149	hsa-let-7d-5p;hsa-let-7f-5p;hsa-let-7g-5p;hsa-let-7i-5p;hsa-m
ENSG0000	ENSG0000	0.000149	hsa-let-7d-5p;hsa-let-7f-5p;hsa-let-7g-5p;hsa-let-7i-5p;hsa-m
ENSG0000	ENSG0000	0.000368	hsa-let-7a-5p;hsa-let-7b-5p;hsa-let-7d-5p;hsa-let-7f-5p;hsa-le
ENSG0000	ENSG0000	2.58E-05	hsa-miR-1-3p;hsa-miR-146a-3p;hsa-miR-19a-5p;hsa-miR-19b-
ENSG0000	ENSG0000	6.67E-06	hsa-let-7a-5p;hsa-let-7b-5p;hsa-let-7d-5p;hsa-let-7f-5p;hsa-le
ENSG0000	ENSG0000	1.98E-06	hsa-let-7a-5p;hsa-let-7b-5p;hsa-let-7d-5p;hsa-let-7f-5p;hsa-le
ENSG0000	ENSG0000	0.000229	hsa-let-7a-5p;hsa-let-7d-5p;hsa-let-7f-5p;hsa-let-7g-5p;hsa-le
ENSG0000	ENSG0000	0.003028	hsa-let-7d-5p;hsa-let-7f-5p;hsa-let-7g-5p;hsa-let-7i-5p
ENSG0000	ENSG0000	9.49E-10	hsa-let-7a-5p;hsa-let-7b-5p;hsa-let-7d-5p;hsa-let-7f-5p;hsa-le
ENSG0000	ENSG0000	9.49E-10	hsa-let-7a-5p;hsa-let-7b-5p;hsa-let-7d-5p;hsa-let-7f-5p;hsa-le
ENSG0000	ENSG0000	9.49E-10	hsa-let-7a-5p;hsa-let-7b-5p;hsa-let-7d-5p;hsa-let-7f-5p;hsa-le
ENSG0000	ENSG0000	1.34E-06	hsa-let-7a-5p;hsa-let-7b-5p;hsa-let-7d-5p;hsa-let-7f-5p;hsa-le
ENSG0000	ENSG0000	4.14E-05	hsa-let-7a-5p;hsa-let-7b-5p;hsa-let-7d-5p;hsa-let-7f-5p;hsa-le
ENSG0000	ENSG0000	2.06E-07	hsa-let-7a-5p;hsa-let-7b-5p;hsa-let-7d-5p;hsa-let-7f-5p;hsa-le
ENSG0000	ENSG0000	1.70E-07	hsa-let-7a-5p;hsa-let-7b-5p;hsa-let-7d-5p;hsa-let-7f-5p;hsa-le
ENSG0000	ENSG0000	2.65E-05	hsa-let-7a-5p;hsa-let-7d-5p;hsa-let-7f-5p;hsa-let-7g-5p;hsa-le
ENSG0000	ENSG0000	6.15E-05	hsa-let-7a-5p;hsa-let-7b-5p;hsa-let-7d-5p;hsa-let-7f-5p;hsa-le
ENSG0000	ENSG0000	6.15E-05	hsa-let-7a-5p;hsa-let-7b-5p;hsa-let-7d-5p;hsa-let-7f-5p;hsa-le
ENSG0000	ENSG0000	3.66E-05	hsa-let-7a-5p;hsa-let-7d-5p;hsa-let-7f-5p;hsa-let-7g-5p;hsa-le
ENSG0000	ENSG0000	8.04E-05	hsa-let-7a-5p;hsa-let-7f-5p;hsa-let-7g-5p;hsa-let-7i-5p
ENSG0000	ENSG0000	6.40E-09	hsa-let-7a-5p;hsa-let-7b-5p;hsa-let-7d-5p;hsa-let-7f-5p;hsa-le
ENSG0000	ENSG0000	1.40E-07	hsa-let-7a-5p;hsa-let-7b-5p;hsa-let-7d-5p;hsa-let-7f-5p;hsa-le
ENSG0000	ENSG0000	1.74E-06	hsa-let-7a-5p;hsa-let-7b-5p;hsa-let-7d-5p;hsa-let-7f-5p;hsa-le
ENSG0000	ENSG0000	4.91E-06	hsa-let-7a-5p;hsa-let-7b-5p;hsa-let-7d-5p;hsa-let-7f-5p;hsa-le
ENSG0000	ENSG0000	4.91E-06	hsa-let-7a-5p;hsa-let-7b-5p;hsa-let-7d-5p;hsa-let-7f-5p;hsa-le
ENSG0000	ENSG0000	2.06E-07	hsa-let-7a-5p;hsa-let-7b-5p;hsa-let-7d-5p;hsa-let-7f-5p;hsa-le
ENSG0000	ENSG0000	2.95E-07	hsa-let-7a-5p;hsa-let-7b-5p;hsa-let-7d-5p;hsa-let-7f-5p;hsa-le
ENSG0000	ENSG0000	3.34E-05	hsa-let-7a-5p;hsa-let-7b-5p;hsa-let-7d-5p;hsa-let-7f-5p;hsa-le
ENSG0000	ENSG0000	2.20E-06	hsa-let-7a-5p;hsa-let-7b-5p;hsa-let-7d-5p;hsa-let-7f-5p;hsa-le
ENSG0000	ENSG0000	2.23E-06	hsa-let-7a-5p;hsa-let-7b-5p;hsa-let-7f-5p;hsa-let-7g-5p;hsa-le
ENSG0000	ENSG0000	1.53E-06	hsa-let-7a-5p;hsa-let-7b-5p;hsa-let-7d-5p;hsa-let-7f-5p;hsa-le
ENSG0000	ENSG0000	1.95E-05	hsa-let-7a-5p;hsa-let-7b-5p;hsa-let-7d-5p;hsa-let-7f-5p;hsa-le
ENSG0000	ENSG0000	2.23E-06	hsa-let-7a-5p;hsa-let-7b-5p;hsa-let-7d-5p;hsa-let-7f-5p;hsa-le
ENSG0000	ENSG0000	2.60E-05	hsa-let-7a-5p;hsa-let-7b-5p;hsa-let-7d-5p;hsa-let-7f-5p;hsa-le

ENSG0000	ENSG0000	2.60E-05	hsa-let-7a-5p;hsa-let-7b-5p;hsa-let-7d-5p;hsa-let-7f-5p;hsa-le
ENSG0000	ENSG0000	8.91E-09	hsa-let-7a-5p;hsa-let-7b-5p;hsa-let-7d-5p;hsa-let-7f-5p;hsa-le
ENSG0000	ENSG0000	1.35E-09	hsa-let-7a-5p;hsa-let-7b-5p;hsa-let-7d-5p;hsa-let-7f-5p;hsa-le
ENSG0000	ENSG0000	4.08E-07	hsa-let-7a-5p;hsa-let-7b-5p;hsa-let-7d-5p;hsa-let-7f-5p;hsa-le
ENSG0000	ENSG0000	2.67E-05	hsa-let-7a-5p;hsa-let-7b-5p;hsa-let-7d-5p;hsa-let-7f-5p;hsa-le
ENSG0000	ENSG0000	2.55E-07	hsa-let-7a-5p;hsa-let-7b-5p;hsa-let-7d-5p;hsa-let-7f-5p;hsa-le
ENSG0000	ENSG0000	1.82E-06	hsa-let-7a-5p;hsa-let-7b-5p;hsa-let-7d-5p;hsa-let-7f-5p;hsa-le
ENSG0000	ENSG0000	7.99E-05	hsa-let-7a-5p;hsa-let-7b-5p;hsa-let-7d-5p;hsa-let-7f-5p;hsa-le
ENSG0000	ENSG0000	0.001586	hsa-let-7a-5p;hsa-let-7b-5p;hsa-let-7d-5p;hsa-let-7f-5p;hsa-le
ENSG0000	ENSG0000	4.57E-05	hsa-let-7a-5p;hsa-let-7b-5p;hsa-let-7d-5p;hsa-let-7f-5p;hsa-le
ENSG0000	ENSG0000	1.02E-10	hsa-let-7a-5p;hsa-let-7b-5p;hsa-let-7d-5p;hsa-let-7f-5p;hsa-le
ENSG0000	ENSG0000	1.48E-13	hsa-let-7a-5p;hsa-let-7b-5p;hsa-let-7d-5p;hsa-let-7f-5p;hsa-le
ENSG0000	ENSG0000	1.65E-05	hsa-let-7a-5p;hsa-let-7b-5p;hsa-let-7d-5p;hsa-let-7f-5p;hsa-le
ENSG0000	ENSG0000	1.70E-07	hsa-let-7a-5p;hsa-let-7b-5p;hsa-let-7d-5p;hsa-let-7f-5p;hsa-le
ENSG0000	ENSG0000	1.18E-06	hsa-let-7a-5p;hsa-let-7b-5p;hsa-let-7d-5p;hsa-let-7f-5p;hsa-le
ENSG0000	ENSG0000	2.17E-08	hsa-let-7a-5p;hsa-let-7b-5p;hsa-let-7d-5p;hsa-let-7f-5p;hsa-le
ENSG0000	ENSG0000	5.96E-08	hsa-let-7a-5p;hsa-let-7b-5p;hsa-let-7d-5p;hsa-let-7f-5p;hsa-le
ENSG0000	ENSG0000	5.96E-08	hsa-let-7a-5p;hsa-let-7b-5p;hsa-let-7d-5p;hsa-let-7f-5p;hsa-le
ENSG0000	ENSG0000	0.000543	hsa-let-7a-5p;hsa-let-7b-5p;hsa-let-7d-5p;hsa-let-7f-5p;hsa-le
ENSG0000	ENSG0000	2.47E-07	hsa-let-7a-5p;hsa-let-7b-5p;hsa-let-7d-5p;hsa-let-7f-5p;hsa-le
ENSG0000	ENSG0000	2.47E-05	hsa-let-7a-5p;hsa-let-7b-5p;hsa-let-7d-5p;hsa-let-7f-5p;hsa-le
ENSG0000	ENSG0000	6.62E-05	hsa-let-7a-5p;hsa-let-7b-5p;hsa-let-7d-5p;hsa-let-7f-5p;hsa-le
ENSG0000	ENSG0000	1.04E-06	hsa-let-7a-5p;hsa-let-7b-5p;hsa-let-7f-5p;hsa-let-7g-5p;hsa-le
ENSG0000	ENSG0000	1.80E-05	hsa-let-7a-5p;hsa-let-7b-5p;hsa-let-7d-5p;hsa-let-7f-5p;hsa-le
ENSG0000	ENSG0000	1.80E-05	hsa-let-7a-5p;hsa-let-7b-5p;hsa-let-7d-5p;hsa-let-7f-5p;hsa-le
ENSG0000	ENSG0000	3.50E-07	hsa-let-7d-5p;hsa-let-7f-5p;hsa-let-7g-5p;hsa-let-7i-5p;hsa-m
ENSG0000	ENSG0000	1.95E-05	hsa-let-7a-5p;hsa-let-7b-5p;hsa-let-7d-5p;hsa-let-7f-5p;hsa-le
ENSG0000	ENSG0000	2.95E-07	hsa-let-7a-5p;hsa-let-7b-5p;hsa-let-7f-5p;hsa-let-7g-5p;hsa-le
ENSG0000	ENSG0000	2.06E-07	hsa-let-7b-5p;hsa-let-7d-5p;hsa-let-7f-5p;hsa-let-7g-5p;hsa-le
ENSG0000	ENSG0000	1.35E-09	hsa-let-7a-5p;hsa-let-7b-5p;hsa-let-7d-5p;hsa-let-7f-5p;hsa-le
ENSG0000	ENSG0000	1.22E-08	hsa-let-7a-5p;hsa-let-7b-5p;hsa-let-7d-5p;hsa-let-7f-5p;hsa-le
ENSG0000	ENSG0000	1.22E-08	hsa-let-7a-5p;hsa-let-7b-5p;hsa-let-7d-5p;hsa-let-7f-5p;hsa-le
ENSG0000	ENSG0000	6.67E-06	hsa-let-7a-5p;hsa-let-7b-5p;hsa-let-7d-5p;hsa-let-7f-5p;hsa-le
ENSG0000	ENSG0000	5.07E-07	hsa-let-7a-5p;hsa-let-7b-5p;hsa-let-7d-5p;hsa-let-7f-5p;hsa-le
ENSG0000	ENSG0000	5.45E-06	hsa-let-7a-5p;hsa-let-7b-5p;hsa-let-7d-5p;hsa-let-7f-5p;hsa-le
ENSG0000	ENSG0000	8.81E-10	hsa-let-7a-5p;hsa-let-7b-5p;hsa-let-7d-5p;hsa-let-7f-5p;hsa-le
ENSG0000	ENSG0000	1.52E-05	hsa-let-7a-5p;hsa-let-7b-5p;hsa-let-7d-5p;hsa-let-7f-5p;hsa-le
ENSG0000	ENSG0000	0.000706	hsa-let-7a-5p;hsa-let-7d-5p;hsa-let-7f-5p;hsa-let-7g-5p;hsa-le
ENSG0000	ENSG0000	1.07E-05	hsa-let-7a-5p;hsa-let-7b-5p;hsa-let-7d-5p;hsa-let-7f-5p;hsa-le
ENSG0000	ENSG0000	2.95E-07	hsa-let-7a-5p;hsa-let-7b-5p;hsa-let-7d-5p;hsa-let-7f-5p;hsa-le
ENSG0000	ENSG0000	6.63E-06	hsa-miR-19a-5p;hsa-miR-19b-1-5p;hsa-miR-33a-3p;hsa-miR-4
ENSG0000	ENSG0000	0.00024	hsa-let-7a-5p;hsa-let-7b-5p;hsa-let-7d-5p;hsa-let-7f-5p;hsa-le
ENSG0000	ENSG0000	7.51E-05	hsa-let-7a-5p;hsa-let-7b-5p;hsa-let-7d-5p;hsa-let-7f-5p;hsa-le
ENSG0000	ENSG0000	0.000262	hsa-let-7a-5p;hsa-let-7d-5p;hsa-let-7f-5p;hsa-let-7g-5p;hsa-le
ENSG0000	ENSG0000	4.76E-05	hsa-let-7a-5p;hsa-let-7b-5p;hsa-let-7d-5p;hsa-let-7f-5p;hsa-le
ENSG0000	ENSG0000	1.70E-07	hsa-let-7a-5p;hsa-let-7b-5p;hsa-let-7d-5p;hsa-let-7f-5p;hsa-le
ENSG0000	ENSG0000	2.47E-07	hsa-let-7a-5p;hsa-let-7b-5p;hsa-let-7d-5p;hsa-let-7f-5p;hsa-le
ENSG0000	ENSG0000	4.82E-08	hsa-let-7a-5p;hsa-let-7b-5p;hsa-let-7d-5p;hsa-let-7f-5p;hsa-le
ENSG0000	ENSG0000	0.001984	hsa-let-7a-5p;hsa-let-7b-5p;hsa-let-7d-5p;hsa-let-7f-5p;hsa-le
ENSG0000	ENSG0000	6.67E-06	hsa-let-7a-5p;hsa-let-7b-5p;hsa-let-7d-5p;hsa-let-7f-5p;hsa-le
ENSG0000	ENSG0000	1.15E-07	hsa-let-7a-5p;hsa-let-7b-5p;hsa-let-7d-5p;hsa-let-7f-5p;hsa-le
ENSG0000	ENSG0000	0.000118	hsa-let-7a-5p;hsa-let-7b-5p;hsa-let-7f-5p;hsa-let-7g-5p;hsa-le
ENSG0000	ENSG0000	6.63E-07	hsa-let-7a-5p;hsa-let-7b-5p;hsa-let-7d-5p;hsa-let-7f-5p;hsa-le
ENSG0000	ENSG0000	0.000998	hsa-let-7b-5p;hsa-let-7f-5p;hsa-let-7g-5p;hsa-let-7i-5p

ENSG0000	ENSG0000	8.50E-05	hsa-let-7a-5p;hsa-let-7b-5p;hsa-let-7d-5p;hsa-let-7f-5p;hsa-le
ENSG0000	ENSG0000	7.57E-07	hsa-let-7a-5p;hsa-let-7b-5p;hsa-let-7d-5p;hsa-let-7f-5p;hsa-le
ENSG0000	ENSG0000	0.000124	hsa-let-7a-5p;hsa-let-7b-5p;hsa-let-7d-5p;hsa-let-7f-5p;hsa-le
ENSG0000	ENSG0000	8.91E-09	hsa-let-7a-5p;hsa-let-7b-5p;hsa-let-7d-5p;hsa-let-7f-5p;hsa-le
ENSG0000	ENSG0000	1.07E-05	hsa-let-7a-5p;hsa-let-7b-5p;hsa-let-7d-5p;hsa-let-7f-5p;hsa-le
ENSG0000	ENSG0000	3.86E-05	hsa-let-7a-5p;hsa-let-7b-5p;hsa-let-7d-5p;hsa-let-7f-5p;hsa-le
ENSG0000	ENSG0000	4.77E-06	hsa-let-7a-5p;hsa-let-7b-5p;hsa-let-7d-5p;hsa-let-7f-5p;hsa-le
ENSG0000	ENSG0000	2.23E-06	hsa-let-7a-5p;hsa-let-7b-5p;hsa-let-7f-5p;hsa-let-7g-5p;hsa-le
ENSG0000	ENSG0000	3.65E-05	hsa-let-7a-5p;hsa-let-7b-5p;hsa-let-7d-5p;hsa-let-7f-5p;hsa-le
ENSG0000	ENSG0000	9.77E-06	hsa-let-7a-5p;hsa-let-7b-5p;hsa-let-7d-5p;hsa-let-7f-5p;hsa-le
ENSG0000	ENSG0000	5.69E-07	hsa-let-7a-5p;hsa-let-7b-5p;hsa-let-7d-5p;hsa-let-7f-5p;hsa-le
ENSG0000	ENSG0000	0.002628	hsa-let-7a-5p;hsa-let-7d-5p;hsa-let-7f-5p;hsa-let-7g-5p;hsa-le
ENSG0000	ENSG0000	3.96E-08	hsa-let-7a-5p;hsa-let-7b-5p;hsa-let-7d-5p;hsa-let-7f-5p;hsa-le
ENSG0000	ENSG0000	6.25E-08	hsa-let-7a-5p;hsa-let-7b-5p;hsa-let-7d-5p;hsa-let-7f-5p;hsa-le
ENSG0000	ENSG0000	0.000229	hsa-let-7a-5p;hsa-let-7b-5p;hsa-let-7d-5p;hsa-let-7f-5p;hsa-le
ENSG0000	ENSG0000	6.35E-12	hsa-let-7a-5p;hsa-let-7b-5p;hsa-let-7d-5p;hsa-let-7f-5p;hsa-le
ENSG0000	ENSG0000	4.50E-09	hsa-let-7a-5p;hsa-let-7b-5p;hsa-let-7d-5p;hsa-let-7f-5p;hsa-le
ENSG0000	ENSG0000	7.05E-05	hsa-let-7a-5p;hsa-let-7b-5p;hsa-let-7d-5p;hsa-let-7f-5p;hsa-le
ENSG0000	ENSG0000	0.000187	hsa-let-7a-5p;hsa-let-7b-5p;hsa-let-7d-5p;hsa-let-7f-5p;hsa-le
ENSG0000	ENSG0000	5.15E-09	hsa-let-7a-5p;hsa-let-7b-5p;hsa-let-7d-5p;hsa-let-7g-5p;hsa-l
ENSG0000	ENSG0000	6.03E-06	hsa-let-7a-5p;hsa-let-7b-5p;hsa-let-7d-5p;hsa-let-7f-5p;hsa-le
ENSG0000	ENSG0000	9.58E-05	hsa-let-7a-5p;hsa-let-7b-5p;hsa-let-7d-5p;hsa-let-7f-5p;hsa-le
ENSG0000	ENSG0000	1.17E-05	hsa-let-7a-5p;hsa-let-7b-5p;hsa-let-7d-5p;hsa-let-7f-5p;hsa-le
ENSG0000	ENSG0000	1.64E-08	hsa-let-7a-5p;hsa-let-7b-5p;hsa-let-7d-5p;hsa-let-7f-5p;hsa-le
ENSG0000	ENSG0000	6.63E-07	hsa-let-7a-5p;hsa-let-7b-5p;hsa-let-7d-5p;hsa-let-7f-5p;hsa-le
ENSG0000	ENSG0000	8.29E-06	hsa-let-7a-5p;hsa-let-7b-5p;hsa-let-7d-5p;hsa-let-7f-5p;hsa-le
ENSG0000	ENSG0000	8.50E-05	hsa-let-7a-5p;hsa-let-7b-5p;hsa-let-7d-5p;hsa-let-7f-5p;hsa-le
ENSG0000	ENSG0000	3.85E-05	hsa-let-7a-5p;hsa-let-7b-5p;hsa-let-7d-5p;hsa-let-7f-5p;hsa-le
ENSG0000	ENSG0000	3.59E-05	hsa-let-7a-5p;hsa-let-7b-5p;hsa-let-7d-5p;hsa-let-7f-5p;hsa-le
ENSG0000	ENSG0000	0.001435	hsa-let-7a-5p;hsa-let-7b-5p;hsa-let-7d-5p;hsa-let-7f-5p;hsa-le
ENSG0000	ENSG0000	1.70E-07	hsa-let-7a-5p;hsa-let-7b-5p;hsa-let-7d-5p;hsa-let-7f-5p;hsa-le
ENSG0000	ENSG0000	6.03E-06	hsa-let-7a-5p;hsa-let-7b-5p;hsa-let-7d-5p;hsa-let-7f-5p;hsa-le
ENSG0000	ENSG0000	2.23E-06	hsa-let-7a-5p;hsa-let-7b-5p;hsa-let-7d-5p;hsa-let-7f-5p;hsa-le
ENSG0000	ENSG0000	0.000128	hsa-let-7a-5p;hsa-let-7b-5p;hsa-let-7f-5p;hsa-let-7g-5p;hsa-le
ENSG0000	ENSG0000	1.15E-07	hsa-let-7a-5p;hsa-let-7b-5p;hsa-let-7d-5p;hsa-let-7f-5p;hsa-le
ENSG0000	ENSG0000	0.001127	hsa-let-7a-5p;hsa-let-7b-5p;hsa-let-7d-5p;hsa-let-7f-5p;hsa-le
ENSG0000	ENSG0000	1.29E-11	hsa-let-7a-5p;hsa-let-7b-5p;hsa-let-7d-5p;hsa-let-7f-5p;hsa-le
ENSG0000	ENSG0000	0.000253	hsa-let-7a-5p;hsa-let-7b-5p;hsa-let-7d-5p;hsa-let-7f-5p;hsa-le
ENSG0000	ENSG0000	5.10E-07	hsa-let-7a-5p;hsa-let-7b-5p;hsa-let-7d-5p;hsa-let-7f-5p;hsa-le
ENSG0000	ENSG0000	0.002532	hsa-let-7a-5p;hsa-let-7b-5p;hsa-let-7f-5p;hsa-let-7g-5p;hsa-le
ENSG0000	ENSG0000	0.006147	hsa-miR-1-3p;hsa-miR-19a-5p;hsa-miR-19b-1-5p;hsa-miR-54
ENSG0000	ENSG0000	4.14E-07	hsa-let-7a-5p;hsa-let-7b-5p;hsa-let-7d-5p;hsa-let-7f-5p;hsa-le
ENSG0000	ENSG0000	2.83E-06	hsa-let-7a-5p;hsa-let-7b-5p;hsa-let-7d-5p;hsa-let-7f-5p;hsa-le
ENSG0000	ENSG0000	0.000278	hsa-let-7a-5p;hsa-let-7b-5p;hsa-let-7d-5p;hsa-let-7f-5p;hsa-le
ENSG0000	ENSG0000	5.31E-09	hsa-let-7a-5p;hsa-let-7b-5p;hsa-let-7d-5p;hsa-let-7f-5p;hsa-le
ENSG0000	ENSG0000	0.002098	hsa-miR-196a-5p;hsa-miR-196b-5p;hsa-miR-19a-5p;hsa-miR-
ENSG0000	ENSG0000	5.96E-08	hsa-let-7a-5p;hsa-let-7b-5p;hsa-let-7d-5p;hsa-let-7f-5p;hsa-le
ENSG0000	ENSG0000	1.53E-06	hsa-let-7a-5p;hsa-let-7b-5p;hsa-let-7d-5p;hsa-let-7f-5p;hsa-le
ENSG0000	ENSG0000	7.19E-06	hsa-let-7a-5p;hsa-let-7b-5p;hsa-let-7d-5p;hsa-let-7f-5p;hsa-le
ENSG0000	ENSG0000	3.34E-05	hsa-let-7a-5p;hsa-let-7b-5p;hsa-let-7d-5p;hsa-let-7f-5p;hsa-le
ENSG0000	ENSG0000	1.34E-06	hsa-let-7a-5p;hsa-let-7b-5p;hsa-let-7d-5p;hsa-let-7f-5p;hsa-le
ENSG0000	ENSG0000	0.00126	hsa-let-7a-5p;hsa-let-7d-5p;hsa-let-7f-5p;hsa-let-7g-5p;hsa-le
ENSG0000	ENSG0000	4.86E-07	hsa-let-7a-5p;hsa-let-7b-5p;hsa-let-7d-5p;hsa-let-7f-5p;hsa-le
ENSG0000	ENSG0000	3.65E-05	hsa-let-7a-5p;hsa-let-7d-5p;hsa-let-7f-5p;hsa-let-7g-5p;hsa-le

ENSG0000	ENSG0000	2.07E-09	hsa-let-7a-5p;hsa-let-7b-5p;hsa-let-7d-5p;hsa-let-7f-5p;hsa-le
ENSG0000	ENSG0000	8.10E-06	hsa-let-7a-5p;hsa-let-7b-5p;hsa-let-7d-5p;hsa-let-7f-5p;hsa-le
ENSG0000	ENSG0000	4.86E-07	hsa-let-7a-5p;hsa-let-7b-5p;hsa-let-7d-5p;hsa-let-7f-5p;hsa-le
ENSG0000	ENSG0000	2.88E-07	hsa-let-7a-5p;hsa-let-7b-5p;hsa-let-7d-5p;hsa-let-7f-5p;hsa-le
ENSG0000	ENSG0000	7.17E-09	hsa-let-7a-5p;hsa-let-7b-5p;hsa-let-7d-5p;hsa-let-7f-5p;hsa-le
ENSG0000	ENSG0000	0.001863	hsa-let-7a-5p;hsa-let-7b-5p;hsa-let-7d-5p;hsa-let-7f-5p;hsa-le
ENSG0000	ENSG0000	9.20E-05	hsa-miR-101-3p;hsa-miR-27a-3p;hsa-miR-27b-3p;hsa-miR-9-5
ENSG0000	ENSG0000	3.41E-06	hsa-miR-132-3p;hsa-miR-1-3p;hsa-miR-27b-3p;hsa-miR-7-5p
ENSG0000	ENSG0000	9.79E-05	hsa-miR-132-3p;hsa-miR-27a-3p;hsa-miR-27b-3p;hsa-miR-361
ENSG0000	ENSG0000	0.000404	hsa-miR-132-3p;hsa-miR-1-3p;hsa-miR-27a-3p;hsa-miR-27b-3
ENSG0000	ENSG0000	0.000298	hsa-miR-27a-3p;hsa-miR-27b-3p;hsa-miR-7-5p;hsa-miR-9-5p
ENSG0000	ENSG0000	9.65E-06	hsa-miR-27a-3p;hsa-miR-27b-3p;hsa-miR-361-5p;hsa-miR-7-5
ENSG0000	ENSG0000	0.000317	hsa-miR-25-3p;hsa-miR-30a-5p;hsa-miR-30d-5p;hsa-miR-30e-
ENSG0000	ENSG0000	1.76E-05	hsa-miR-148a-3p;hsa-miR-30a-5p;hsa-miR-30d-5p;hsa-miR-30
ENSG0000	ENSG0000	1.32E-05	hsa-miR-101-3p;hsa-miR-181a-5p;hsa-miR-30a-5p;hsa-miR-30
ENSG0000	ENSG0000	6.39E-05	hsa-miR-24-3p;hsa-miR-30a-5p;hsa-miR-30d-5p;hsa-miR-30e-
ENSG0000	ENSG0000	4.35E-05	hsa-miR-24-3p;hsa-miR-30a-5p;hsa-miR-30d-5p;hsa-miR-30e-
ENSG0000	ENSG0000	0.000124	hsa-miR-15a-5p;hsa-miR-30a-5p;hsa-miR-30d-5p;hsa-miR-30e
ENSG0000	ENSG0000	1.85E-08	hsa-miR-101-3p;hsa-miR-30a-5p;hsa-miR-30d-5p;hsa-miR-30e
ENSG0000	ENSG0000	8.70E-06	hsa-miR-181a-5p;hsa-miR-30a-5p;hsa-miR-30d-5p;hsa-miR-30
ENSG0000	ENSG0000	1.72E-07	hsa-miR-181a-5p;hsa-miR-30a-5p;hsa-miR-30d-5p;hsa-miR-30
ENSG0000	ENSG0000	0.000546	hsa-miR-101-3p;hsa-miR-141-3p;hsa-miR-15a-5p;hsa-miR-181
ENSG0000	ENSG0000	1.22E-06	hsa-miR-30a-5p;hsa-miR-30d-5p;hsa-miR-30e-5p;hsa-miR-99a
ENSG0000	ENSG0000	9.81E-05	hsa-miR-25-3p;hsa-miR-30a-5p;hsa-miR-30d-5p;hsa-miR-30e-
ENSG0000	ENSG0000	8.31E-05	hsa-miR-101-3p;hsa-miR-30a-5p;hsa-miR-30d-5p;hsa-miR-30e
ENSG0000	ENSG0000	4.81E-05	hsa-miR-30a-5p;hsa-miR-30d-5p;hsa-miR-30e-5p;hsa-miR-576
ENSG0000	ENSG0000	5.82E-05	hsa-miR-15a-5p;hsa-miR-30a-5p;hsa-miR-30d-5p;hsa-miR-30e
ENSG0000	ENSG0000	0.001667	hsa-miR-148a-3p;hsa-miR-30a-5p;hsa-miR-30d-5p;hsa-miR-30
ENSG0000	ENSG0000	8.27E-08	hsa-miR-101-3p;hsa-miR-181a-5p;hsa-miR-30a-5p;hsa-miR-30
ENSG0000	ENSG0000	6.61E-08	hsa-miR-15a-5p;hsa-miR-30a-5p;hsa-miR-30d-5p;hsa-miR-30e
ENSG0000	ENSG0000	3.73E-05	hsa-miR-181a-5p;hsa-miR-25-3p;hsa-miR-30a-5p;hsa-miR-30d
ENSG0000	ENSG0000	3.54E-05	hsa-miR-25-3p;hsa-miR-30a-5p;hsa-miR-30d-5p;hsa-miR-30e-
ENSG0000	ENSG0000	0.000106	hsa-miR-25-3p;hsa-miR-30a-5p;hsa-miR-30d-5p;hsa-miR-30e-
ENSG0000	ENSG0000	0.001178	hsa-miR-141-3p;hsa-miR-30a-5p;hsa-miR-30d-5p;hsa-miR-30e
ENSG0000	ENSG0000	8.70E-06	hsa-miR-30a-5p;hsa-miR-30d-5p;hsa-miR-30e-5p;hsa-miR-375
ENSG0000	ENSG0000	0.001992	hsa-miR-15a-5p;hsa-miR-30a-5p;hsa-miR-30d-5p;hsa-miR-30e
ENSG0000	ENSG0000	0.001992	hsa-miR-15a-5p;hsa-miR-30a-5p;hsa-miR-30d-5p;hsa-miR-30e
ENSG0000	ENSG0000	0.000162	hsa-miR-15a-5p;hsa-miR-24-3p;hsa-miR-30a-5p;hsa-miR-30d-
ENSG0000	ENSG0000	0.000519	hsa-miR-181a-5p;hsa-miR-30a-5p;hsa-miR-30d-5p;hsa-miR-30
ENSG0000	ENSG0000	9.38E-07	hsa-miR-15a-5p;hsa-miR-181a-5p;hsa-miR-30a-5p;hsa-miR-30
ENSG0000	ENSG0000	0.002602	hsa-miR-101-3p;hsa-miR-30a-5p;hsa-miR-30d-5p;hsa-miR-30e
ENSG0000	ENSG0000	0.000697	hsa-miR-148a-3p;hsa-miR-30a-5p;hsa-miR-30d-5p;hsa-miR-30
ENSG0000	ENSG0000	0.005196	hsa-miR-25-3p;hsa-miR-30a-5p;hsa-miR-30d-5p;hsa-miR-30e-
ENSG0000	ENSG0000	1.35E-05	hsa-miR-25-3p;hsa-miR-30a-5p;hsa-miR-30d-5p;hsa-miR-30e-
ENSG0000	ENSG0000	0.000398	hsa-miR-15a-5p;hsa-miR-30a-5p;hsa-miR-30d-5p;hsa-miR-30e
ENSG0000	ENSG0000	4.45E-06	hsa-miR-15a-5p;hsa-miR-30a-5p;hsa-miR-30d-5p;hsa-miR-30e
ENSG0000	ENSG0000	8.70E-06	hsa-miR-15a-5p;hsa-miR-30a-5p;hsa-miR-30d-5p;hsa-miR-30e
ENSG0000	ENSG0000	0.004676	hsa-miR-101-3p;hsa-miR-148a-3p;hsa-miR-15a-5p;hsa-miR-24
ENSG0000	ENSG0000	0.004676	hsa-miR-101-3p;hsa-miR-148a-3p;hsa-miR-15a-5p;hsa-miR-24
ENSG0000	ENSG0000	1.17E-05	hsa-miR-24-3p;hsa-miR-30a-5p;hsa-miR-30d-5p;hsa-miR-30e-
ENSG0000	ENSG0000	3.93E-05	hsa-miR-24-3p;hsa-miR-30a-5p;hsa-miR-30d-5p;hsa-miR-30e-
ENSG0000	ENSG0000	4.45E-06	hsa-miR-181a-5p;hsa-miR-30a-5p;hsa-miR-30d-5p;hsa-miR-30
ENSG0000	ENSG0000	4.45E-06	hsa-miR-181a-5p;hsa-miR-30a-5p;hsa-miR-30d-5p;hsa-miR-30
ENSG0000	ENSG0000	7.03E-07	hsa-miR-15a-5p;hsa-miR-30a-5p;hsa-miR-30d-5p;hsa-miR-30e

ENSG0000	ENSG0000	6.18E-06	hsa-miR-181a-5p;hsa-miR-25-3p;hsa-miR-30a-5p;hsa-miR-30
ENSG0000	ENSG0000	0.000193	hsa-miR-24-3p;hsa-miR-25-3p;hsa-miR-30a-5p;hsa-miR-30d-
ENSG0000	ENSG0000	0.000486	hsa-miR-15a-5p;hsa-miR-24-3p;hsa-miR-30a-5p;hsa-miR-30d-
ENSG0000	ENSG0000	0.003746	hsa-miR-101-3p;hsa-miR-30a-5p;hsa-miR-30d-5p;hsa-miR-30
ENSG0000	ENSG0000	0.000106	hsa-miR-148a-3p;hsa-miR-30a-5p;hsa-miR-30d-5p;hsa-miR-3
ENSG0000	ENSG0000	0.000281	hsa-miR-24-3p;hsa-miR-30a-5p;hsa-miR-30d-5p;hsa-miR-30e-
ENSG0000	ENSG0000	0.000493	hsa-miR-24-3p;hsa-miR-30a-5p;hsa-miR-30d-5p;hsa-miR-30e-
ENSG0000	ENSG0000	0.000633	hsa-miR-141-3p;hsa-miR-30a-5p;hsa-miR-30d-5p;hsa-miR-30
ENSG0000	ENSG0000	0.000519	hsa-miR-24-3p;hsa-miR-30a-5p;hsa-miR-30d-5p;hsa-miR-30e-
ENSG0000	ENSG0000	8.31E-05	hsa-miR-25-3p;hsa-miR-30a-5p;hsa-miR-30d-5p;hsa-miR-30e-
ENSG0000	ENSG0000	0.000144	hsa-miR-101-3p;hsa-miR-30a-5p;hsa-miR-30d-5p;hsa-miR-30
ENSG0000	ENSG0000	4.23E-06	hsa-miR-181a-5p;hsa-miR-25-3p;hsa-miR-30a-5p;hsa-miR-30
ENSG0000	ENSG0000	0.000209	hsa-miR-15a-5p;hsa-miR-15b-5p;hsa-miR-195-5p;hsa-miR-66
ENSG0000	ENSG0000	0.000316	hsa-miR-15a-5p;hsa-miR-15b-5p;hsa-miR-16-5p;hsa-miR-195-
ENSG0000	ENSG0000	2.97E-06	hsa-miR-15a-5p;hsa-miR-15b-5p;hsa-miR-16-5p;hsa-miR-195-
ENSG0000	ENSG0000	1.20E-07	hsa-miR-15a-5p;hsa-miR-15b-5p;hsa-miR-16-5p;hsa-miR-195-
ENSG0000	ENSG0000	3.35E-06	hsa-miR-15a-5p;hsa-miR-15b-5p;hsa-miR-16-5p;hsa-miR-195-
ENSG0000	ENSG0000	3.17E-07	hsa-miR-15a-5p;hsa-miR-15b-5p;hsa-miR-16-5p;hsa-miR-195-
ENSG0000	ENSG0000	1.68E-05	hsa-miR-15a-5p;hsa-miR-15b-5p;hsa-miR-16-5p;hsa-miR-195-
ENSG0000	ENSG0000	1.68E-05	hsa-miR-15a-5p;hsa-miR-15b-5p;hsa-miR-16-5p;hsa-miR-195-
ENSG0000	ENSG0000	3.92E-05	hsa-miR-15a-5p;hsa-miR-15b-5p;hsa-miR-16-5p;hsa-miR-195-
ENSG0000	ENSG0000	1.22E-05	hsa-miR-15a-5p;hsa-miR-15b-5p;hsa-miR-16-5p;hsa-miR-195-
ENSG0000	ENSG0000	0.002147	hsa-miR-15a-5p;hsa-miR-15b-5p;hsa-miR-16-5p;hsa-miR-195-
ENSG0000	ENSG0000	3.17E-07	hsa-miR-15a-5p;hsa-miR-15b-5p;hsa-miR-16-5p;hsa-miR-195-
ENSG0000	ENSG0000	1.68E-05	hsa-miR-15a-5p;hsa-miR-15b-5p;hsa-miR-16-5p;hsa-miR-195-
ENSG0000	ENSG0000	4.73E-08	hsa-miR-15a-5p;hsa-miR-15b-5p;hsa-miR-16-5p;hsa-miR-195-
ENSG0000	ENSG0000	3.61E-06	hsa-miR-15a-5p;hsa-miR-15b-5p;hsa-miR-16-5p;hsa-miR-195-
ENSG0000	ENSG0000	2.02E-06	hsa-miR-15a-5p;hsa-miR-15b-5p;hsa-miR-16-5p;hsa-miR-195-
ENSG0000	ENSG0000	0.000743	hsa-miR-15a-5p;hsa-miR-15b-5p;hsa-miR-16-5p;hsa-miR-195-
ENSG0000	ENSG0000	4.13E-05	hsa-miR-15a-5p;hsa-miR-15b-5p;hsa-miR-16-5p;hsa-miR-195-
ENSG0000	ENSG0000	2.80E-05	hsa-miR-15a-5p;hsa-miR-15b-5p;hsa-miR-16-5p;hsa-miR-195-
ENSG0000	ENSG0000	3.00E-05	hsa-miR-15a-5p;hsa-miR-15b-5p;hsa-miR-16-5p;hsa-miR-195-
ENSG0000	ENSG0000	3.00E-05	hsa-miR-15a-5p;hsa-miR-15b-5p;hsa-miR-16-5p;hsa-miR-195-
ENSG0000	ENSG0000	4.77E-07	hsa-miR-15a-5p;hsa-miR-15b-5p;hsa-miR-16-5p;hsa-miR-195-
ENSG0000	ENSG0000	1.82E-05	hsa-miR-15a-5p;hsa-miR-15b-5p;hsa-miR-16-5p;hsa-miR-195-
ENSG0000	ENSG0000	2.54E-07	hsa-miR-15a-5p;hsa-miR-15b-5p;hsa-miR-16-5p;hsa-miR-195-
ENSG0000	ENSG0000	0.000201	hsa-miR-15a-5p;hsa-miR-15b-5p;hsa-miR-16-5p;hsa-miR-195-
ENSG0000	ENSG0000	0.000131	hsa-miR-15a-5p;hsa-miR-15b-5p;hsa-miR-16-5p;hsa-miR-195-
ENSG0000	ENSG0000	5.79E-06	hsa-miR-15a-5p;hsa-miR-15b-5p;hsa-miR-16-5p;hsa-miR-195-
ENSG0000	ENSG0000	1.32E-06	hsa-miR-15a-5p;hsa-miR-15b-5p;hsa-miR-16-5p;hsa-miR-195-
ENSG0000	ENSG0000	3.88E-05	hsa-miR-15a-5p;hsa-miR-15b-5p;hsa-miR-16-5p;hsa-miR-195-
ENSG0000	ENSG0000	3.76E-06	hsa-miR-15a-5p;hsa-miR-15b-5p;hsa-miR-16-5p;hsa-miR-195-
ENSG0000	ENSG0000	5.55E-05	hsa-miR-15a-5p;hsa-miR-15b-5p;hsa-miR-16-5p;hsa-miR-195-
ENSG0000	ENSG0000	2.02E-06	hsa-miR-15a-5p;hsa-miR-15b-5p;hsa-miR-16-5p;hsa-miR-195-
ENSG0000	ENSG0000	1.12E-05	hsa-miR-15a-5p;hsa-miR-15b-5p;hsa-miR-16-5p;hsa-miR-195-
ENSG0000	ENSG0000	3.83E-06	hsa-miR-15a-5p;hsa-miR-15b-5p;hsa-miR-195-5p;hsa-miR-22
ENSG0000	ENSG0000	1.22E-05	hsa-miR-15a-5p;hsa-miR-15b-5p;hsa-miR-16-5p;hsa-miR-195-
ENSG0000	ENSG0000	2.30E-05	hsa-miR-15a-5p;hsa-miR-15b-5p;hsa-miR-16-5p;hsa-miR-195-
ENSG0000	ENSG0000	5.87E-05	hsa-miR-15a-5p;hsa-miR-15b-5p;hsa-miR-16-5p;hsa-miR-195-
ENSG0000	ENSG0000	4.94E-05	hsa-miR-15a-5p;hsa-miR-15b-5p;hsa-miR-195-5p;hsa-miR-22
ENSG0000	ENSG0000	8.96E-05	hsa-miR-142-3p;hsa-miR-15a-5p;hsa-miR-15b-5p;hsa-miR-19
ENSG0000	ENSG0000	1.53E-06	hsa-miR-15a-5p;hsa-miR-15b-5p;hsa-miR-16-5p;hsa-miR-195-
ENSG0000	ENSG0000	4.73E-08	hsa-miR-15a-5p;hsa-miR-15b-5p;hsa-miR-16-5p;hsa-miR-195-
ENSG0000	ENSG0000	4.73E-08	hsa-miR-15a-5p;hsa-miR-15b-5p;hsa-miR-16-5p;hsa-miR-195-

ENSG0000	ENSG0000	4.63E-07	hsa-miR-142-3p;hsa-miR-15a-5p;hsa-miR-15b-5p;hsa-miR-16-
ENSG0000	ENSG0000	8.10E-05	hsa-miR-15a-5p;hsa-miR-15b-5p;hsa-miR-16-5p;hsa-miR-195-
ENSG0000	ENSG0000	4.94E-05	hsa-miR-15a-5p;hsa-miR-15b-5p;hsa-miR-16-5p;hsa-miR-195-
ENSG0000	ENSG0000	0.008633	hsa-miR-15a-5p;hsa-miR-15b-5p;hsa-miR-16-5p;hsa-miR-195-
ENSG0000	ENSG0000	2.62E-06	hsa-miR-15a-5p;hsa-miR-15b-5p;hsa-miR-16-5p;hsa-miR-195-
ENSG0000	ENSG0000	0.000137	hsa-miR-15a-5p;hsa-miR-15b-5p;hsa-miR-16-5p;hsa-miR-195-
ENSG0000	ENSG0000	4.73E-08	hsa-miR-15a-5p;hsa-miR-15b-5p;hsa-miR-16-5p;hsa-miR-195-
ENSG0000	ENSG0000	4.73E-08	hsa-miR-15a-5p;hsa-miR-15b-5p;hsa-miR-16-5p;hsa-miR-195-
ENSG0000	ENSG0000	6.70E-11	hsa-miR-15a-5p;hsa-miR-15b-5p;hsa-miR-16-5p;hsa-miR-195-
ENSG0000	ENSG0000	3.20E-05	hsa-miR-15a-5p;hsa-miR-15b-5p;hsa-miR-195-5p;hsa-miR-31
ENSG0000	ENSG0000	7.69E-05	hsa-miR-15a-5p;hsa-miR-15b-5p;hsa-miR-16-5p;hsa-miR-195-
ENSG0000	ENSG0000	3.35E-10	hsa-miR-15a-5p;hsa-miR-15b-5p;hsa-miR-16-5p;hsa-miR-195-
ENSG0000	ENSG0000	1.22E-05	hsa-miR-15a-5p;hsa-miR-15b-5p;hsa-miR-16-5p;hsa-miR-195-
ENSG0000	ENSG0000	8.55E-06	hsa-miR-15a-5p;hsa-miR-15b-5p;hsa-miR-16-5p;hsa-miR-195-
ENSG0000	ENSG0000	5.55E-05	hsa-miR-15a-5p;hsa-miR-15b-5p;hsa-miR-16-5p;hsa-miR-195-
ENSG0000	ENSG0000	4.77E-07	hsa-miR-15a-5p;hsa-miR-15b-5p;hsa-miR-16-5p;hsa-miR-195-
ENSG0000	ENSG0000	4.77E-07	hsa-miR-15a-5p;hsa-miR-15b-5p;hsa-miR-16-5p;hsa-miR-195-
ENSG0000	ENSG0000	9.37E-06	hsa-miR-15a-5p;hsa-miR-15b-5p;hsa-miR-16-5p;hsa-miR-195-
ENSG0000	ENSG0000	0.001135	hsa-miR-15a-5p;hsa-miR-15b-5p;hsa-miR-16-5p;hsa-miR-195-
ENSG0000	ENSG0000	6.61E-08	hsa-miR-15a-5p;hsa-miR-15b-5p;hsa-miR-16-5p;hsa-miR-195-
ENSG0000	ENSG0000	6.61E-08	hsa-miR-15a-5p;hsa-miR-15b-5p;hsa-miR-16-5p;hsa-miR-195-
ENSG0000	ENSG0000	2.34E-09	hsa-miR-15a-5p;hsa-miR-15b-5p;hsa-miR-16-5p;hsa-miR-195-
ENSG0000	ENSG0000	3.76E-06	hsa-miR-15a-5p;hsa-miR-15b-5p;hsa-miR-16-5p;hsa-miR-195-
ENSG0000	ENSG0000	3.35E-06	hsa-miR-15a-5p;hsa-miR-15b-5p;hsa-miR-16-5p;hsa-miR-195-
ENSG0000	ENSG0000	9.69E-07	hsa-miR-15a-5p;hsa-miR-15b-5p;hsa-miR-16-5p;hsa-miR-195-
ENSG0000	ENSG0000	9.00E-08	hsa-miR-15a-5p;hsa-miR-15b-5p;hsa-miR-16-5p;hsa-miR-195-
ENSG0000	ENSG0000	7.79E-06	hsa-miR-15a-5p;hsa-miR-15b-5p;hsa-miR-16-5p;hsa-miR-195-
ENSG0000	ENSG0000	3.17E-07	hsa-miR-15a-5p;hsa-miR-15b-5p;hsa-miR-16-5p;hsa-miR-195-
ENSG0000	ENSG0000	4.21E-06	hsa-miR-142-3p;hsa-miR-15a-5p;hsa-miR-15b-5p;hsa-miR-19
ENSG0000	ENSG0000	3.88E-05	hsa-miR-15a-5p;hsa-miR-15b-5p;hsa-miR-16-5p;hsa-miR-195-
ENSG0000	ENSG0000	5.79E-06	hsa-miR-15a-5p;hsa-miR-15b-5p;hsa-miR-16-5p;hsa-miR-195-
ENSG0000	ENSG0000	3.00E-05	hsa-miR-15a-5p;hsa-miR-15b-5p;hsa-miR-16-5p;hsa-miR-195-
ENSG0000	ENSG0000	3.17E-07	hsa-miR-15a-5p;hsa-miR-15b-5p;hsa-miR-16-5p;hsa-miR-195-
ENSG0000	ENSG0000	1.76E-06	hsa-miR-15a-5p;hsa-miR-15b-5p;hsa-miR-16-5p;hsa-miR-195-
ENSG0000	ENSG0000	0.001445	hsa-miR-15a-5p;hsa-miR-15b-5p;hsa-miR-16-5p;hsa-miR-195-
ENSG0000	ENSG0000	3.88E-05	hsa-miR-15a-5p;hsa-miR-15b-5p;hsa-miR-16-5p;hsa-miR-195-
ENSG0000	ENSG0000	0.002576	hsa-miR-15a-5p;hsa-miR-15b-5p;hsa-miR-16-5p;hsa-miR-195-
ENSG0000	ENSG0000	9.42E-05	hsa-miR-15a-5p;hsa-miR-15b-5p;hsa-miR-16-5p;hsa-miR-195-
ENSG0000	ENSG0000	0.0009	hsa-miR-15a-5p;hsa-miR-15b-5p;hsa-miR-16-5p;hsa-miR-195-
ENSG0000	ENSG0000	3.20E-05	hsa-miR-15a-5p;hsa-miR-15b-5p;hsa-miR-16-5p;hsa-miR-195-
ENSG0000	ENSG0000	1.53E-06	hsa-miR-15a-5p;hsa-miR-15b-5p;hsa-miR-16-5p;hsa-miR-195-
ENSG0000	ENSG0000	4.55E-06	hsa-miR-15a-5p;hsa-miR-15b-5p;hsa-miR-16-5p;hsa-miR-195-
ENSG0000	ENSG0000	1.32E-06	hsa-miR-15a-5p;hsa-miR-15b-5p;hsa-miR-16-5p;hsa-miR-195-
ENSG0000	ENSG0000	7.79E-06	hsa-miR-15a-5p;hsa-miR-15b-5p;hsa-miR-16-5p;hsa-miR-195-
ENSG0000	ENSG0000	6.70E-11	hsa-miR-15a-5p;hsa-miR-15b-5p;hsa-miR-16-5p;hsa-miR-195-
ENSG0000	ENSG0000	4.77E-07	hsa-miR-15a-5p;hsa-miR-15b-5p;hsa-miR-16-5p;hsa-miR-195-
ENSG0000	ENSG0000	5.24E-05	hsa-miR-15a-5p;hsa-miR-15b-5p;hsa-miR-16-5p;hsa-miR-195-
ENSG0000	ENSG0000	2.49E-06	hsa-miR-142-3p;hsa-miR-15a-5p;hsa-miR-15b-5p;hsa-miR-16-
ENSG0000	ENSG0000	3.00E-05	hsa-miR-15a-5p;hsa-miR-15b-5p;hsa-miR-16-5p;hsa-miR-195-
ENSG0000	ENSG0000	3.28E-08	hsa-miR-15a-5p;hsa-miR-15b-5p;hsa-miR-16-5p;hsa-miR-195-
ENSG0000	ENSG0000	2.62E-05	hsa-miR-15a-5p;hsa-miR-15b-5p;hsa-miR-16-5p;hsa-miR-195-
ENSG0000	ENSG0000	8.55E-06	hsa-miR-15a-5p;hsa-miR-15b-5p;hsa-miR-16-5p;hsa-miR-195-
ENSG0000	ENSG0000	3.64E-05	hsa-miR-15a-5p;hsa-miR-15b-5p;hsa-miR-16-5p;hsa-miR-195-
ENSG0000	ENSG0000	8.38E-09	hsa-miR-15a-5p;hsa-miR-15b-5p;hsa-miR-16-5p;hsa-miR-195-

ENSG0000	ENSG0000	6.70E-11	hsa-miR-15a-5p;hsa-miR-15b-5p;hsa-miR-16-5p;hsa-miR-195-
ENSG0000	ENSG0000	6.58E-07	hsa-miR-1226-3p;hsa-miR-15a-5p;hsa-miR-15b-5p;hsa-miR-16
ENSG0000	ENSG0000	0.000254	hsa-miR-15a-5p;hsa-miR-15b-5p;hsa-miR-16-5p;hsa-miR-195-
ENSG0000	ENSG0000	4.77E-07	hsa-miR-15a-5p;hsa-miR-15b-5p;hsa-miR-16-5p;hsa-miR-195-
ENSG0000	ENSG0000	4.73E-08	hsa-miR-15a-5p;hsa-miR-15b-5p;hsa-miR-16-5p;hsa-miR-195-
ENSG0000	ENSG0000	0.000131	hsa-miR-15a-5p;hsa-miR-15b-5p;hsa-miR-16-5p;hsa-miR-195-
ENSG0000	ENSG0000	5.22E-06	hsa-miR-15a-5p;hsa-miR-15b-5p;hsa-miR-16-5p;hsa-miR-195-
ENSG0000	ENSG0000	3.76E-06	hsa-miR-15a-5p;hsa-miR-15b-5p;hsa-miR-16-5p;hsa-miR-195-
ENSG0000	ENSG0000	4.66E-09	hsa-miR-15a-5p;hsa-miR-15b-5p;hsa-miR-16-5p;hsa-miR-195-
ENSG0000	ENSG0000	9.69E-07	hsa-miR-15a-5p;hsa-miR-15b-5p;hsa-miR-16-5p;hsa-miR-195-
ENSG0000	ENSG0000	2.11E-05	hsa-miR-15a-5p;hsa-miR-15b-5p;hsa-miR-16-5p;hsa-miR-195-
ENSG0000	ENSG0000	6.91E-07	hsa-miR-15a-5p;hsa-miR-15b-5p;hsa-miR-16-5p;hsa-miR-195-
ENSG0000	ENSG0000	6.61E-08	hsa-miR-15a-5p;hsa-miR-15b-5p;hsa-miR-16-5p;hsa-miR-195-
ENSG0000	ENSG0000	5.79E-06	hsa-miR-15a-5p;hsa-miR-15b-5p;hsa-miR-16-5p;hsa-miR-195-
ENSG0000	ENSG0000	0.000924	hsa-miR-15a-5p;hsa-miR-15b-5p;hsa-miR-16-5p;hsa-miR-195-
ENSG0000	ENSG0000	4.63E-07	hsa-miR-142-3p;hsa-miR-15a-5p;hsa-miR-15b-5p;hsa-miR-16-
ENSG0000	ENSG0000	0.000364	hsa-miR-15a-5p;hsa-miR-15b-5p;hsa-miR-16-5p;hsa-miR-195-
ENSG0000	ENSG0000	4.39E-05	hsa-miR-15a-5p;hsa-miR-15b-5p;hsa-miR-16-5p;hsa-miR-195-
ENSG0000	ENSG0000	4.69E-06	hsa-miR-15a-5p;hsa-miR-15b-5p;hsa-miR-16-5p;hsa-miR-195-
ENSG0000	ENSG0000	2.27E-05	hsa-miR-15a-5p;hsa-miR-15b-5p;hsa-miR-16-5p;hsa-miR-195-
ENSG0000	ENSG0000	1.39E-08	hsa-miR-15a-5p;hsa-miR-15b-5p;hsa-miR-16-5p;hsa-miR-195-
ENSG0000	ENSG0000	3.28E-08	hsa-miR-15a-5p;hsa-miR-15b-5p;hsa-miR-16-5p;hsa-miR-195-
ENSG0000	ENSG0000	1.12E-05	hsa-miR-15a-5p;hsa-miR-15b-5p;hsa-miR-16-5p;hsa-miR-195-
ENSG0000	ENSG0000	1.12E-05	hsa-miR-15a-5p;hsa-miR-15b-5p;hsa-miR-16-5p;hsa-miR-195-
ENSG0000	ENSG0000	1.44E-05	hsa-miR-15a-5p;hsa-miR-15b-5p;hsa-miR-16-5p;hsa-miR-195-
ENSG0000	ENSG0000	2.62E-06	hsa-miR-15a-5p;hsa-miR-15b-5p;hsa-miR-16-5p;hsa-miR-195-
ENSG0000	ENSG0000	6.61E-08	hsa-miR-15a-5p;hsa-miR-15b-5p;hsa-miR-16-5p;hsa-miR-195-
ENSG0000	ENSG0000	4.69E-06	hsa-miR-15a-5p;hsa-miR-15b-5p;hsa-miR-16-5p;hsa-miR-195-
ENSG0000	ENSG0000	3.91E-07	hsa-miR-15a-5p;hsa-miR-15b-5p;hsa-miR-16-5p;hsa-miR-195-
ENSG0000	ENSG0000	3.91E-07	hsa-miR-15a-5p;hsa-miR-15b-5p;hsa-miR-16-5p;hsa-miR-195-
ENSG0000	ENSG0000	6.61E-08	hsa-miR-15a-5p;hsa-miR-15b-5p;hsa-miR-16-5p;hsa-miR-195-
ENSG0000	ENSG0000	1.51E-05	hsa-miR-15a-5p;hsa-miR-15b-5p;hsa-miR-16-5p;hsa-miR-195-
ENSG0000	ENSG0000	1.12E-05	hsa-miR-15a-5p;hsa-miR-15b-5p;hsa-miR-16-5p;hsa-miR-195-
ENSG0000	ENSG0000	0.000143	hsa-miR-15a-5p;hsa-miR-15b-5p;hsa-miR-16-5p;hsa-miR-195-
ENSG0000	ENSG0000	6.00E-06	hsa-miR-15a-5p;hsa-miR-15b-5p;hsa-miR-16-5p;hsa-miR-195-
ENSG0000	ENSG0000	1.22E-05	hsa-miR-15a-5p;hsa-miR-15b-5p;hsa-miR-16-5p;hsa-miR-195-
ENSG0000	ENSG0000	2.11E-05	hsa-miR-15a-5p;hsa-miR-15b-5p;hsa-miR-16-5p;hsa-miR-195-
ENSG0000	ENSG0000	3.35E-06	hsa-miR-15a-5p;hsa-miR-15b-5p;hsa-miR-16-5p;hsa-miR-195-
ENSG0000	ENSG0000	3.35E-06	hsa-miR-15a-5p;hsa-miR-15b-5p;hsa-miR-16-5p;hsa-miR-195-
ENSG0000	ENSG0000	8.21E-07	hsa-miR-15a-5p;hsa-miR-15b-5p;hsa-miR-16-5p;hsa-miR-195-
ENSG0000	ENSG0000	7.79E-06	hsa-miR-15a-5p;hsa-miR-15b-5p;hsa-miR-16-5p;hsa-miR-195-
ENSG0000	ENSG0000	1.76E-06	hsa-miR-15a-5p;hsa-miR-15b-5p;hsa-miR-16-5p;hsa-miR-195-
ENSG0000	ENSG0000	0.000143	hsa-miR-15a-5p;hsa-miR-15b-5p;hsa-miR-16-5p;hsa-miR-195-
ENSG0000	ENSG0000	2.02E-06	hsa-miR-15a-5p;hsa-miR-15b-5p;hsa-miR-16-5p;hsa-miR-195-
ENSG0000	ENSG0000	1.12E-05	hsa-miR-15a-5p;hsa-miR-15b-5p;hsa-miR-16-5p;hsa-miR-195-
ENSG0000	ENSG0000	1.12E-05	hsa-miR-15a-5p;hsa-miR-15b-5p;hsa-miR-16-5p;hsa-miR-195-
ENSG0000	ENSG0000	4.69E-06	hsa-miR-15a-5p;hsa-miR-15b-5p;hsa-miR-16-5p;hsa-miR-195-
ENSG0000	ENSG0000	8.38E-09	hsa-miR-15a-5p;hsa-miR-15b-5p;hsa-miR-16-5p;hsa-miR-195-
ENSG0000	ENSG0000	7.79E-06	hsa-miR-15a-5p;hsa-miR-15b-5p;hsa-miR-16-5p;hsa-miR-195-
ENSG0000	ENSG0000	0.000294	hsa-miR-15a-5p;hsa-miR-15b-5p;hsa-miR-16-5p;hsa-miR-195-
ENSG0000	ENSG0000	0.000294	hsa-miR-15a-5p;hsa-miR-15b-5p;hsa-miR-16-5p;hsa-miR-195-
ENSG0000	ENSG0000	4.73E-08	hsa-miR-15a-5p;hsa-miR-15b-5p;hsa-miR-16-5p;hsa-miR-195-
ENSG0000	ENSG0000	3.00E-05	hsa-miR-15a-5p;hsa-miR-15b-5p;hsa-miR-16-5p;hsa-miR-195-
ENSG0000	ENSG0000	2.62E-05	hsa-miR-15a-5p;hsa-miR-15b-5p;hsa-miR-16-5p;hsa-miR-195-

ENSG0000	ENSG0000	4.77E-07	hsa-miR-15a-5p;hsa-miR-15b-5p;hsa-miR-16-5p;hsa-miR-195-
ENSG0000	ENSG0000	6.41E-06	hsa-miR-15a-5p;hsa-miR-15b-5p;hsa-miR-16-5p;hsa-miR-195-
ENSG0000	ENSG0000	2.02E-06	hsa-miR-15a-5p;hsa-miR-15b-5p;hsa-miR-16-5p;hsa-miR-195-
ENSG0000	ENSG0000	1.20E-07	hsa-miR-15a-5p;hsa-miR-15b-5p;hsa-miR-16-5p;hsa-miR-195-
ENSG0000	ENSG0000	4.77E-07	hsa-miR-15a-5p;hsa-miR-15b-5p;hsa-miR-16-5p;hsa-miR-195-
ENSG0000	ENSG0000	3.88E-05	hsa-miR-15a-5p;hsa-miR-15b-5p;hsa-miR-16-5p;hsa-miR-195-
ENSG0000	ENSG0000	3.35E-06	hsa-miR-15a-5p;hsa-miR-15b-5p;hsa-miR-16-5p;hsa-miR-195-
ENSG0000	ENSG0000	0.001135	hsa-miR-15a-5p;hsa-miR-15b-5p;hsa-miR-16-5p;hsa-miR-195-
ENSG0000	ENSG0000	4.77E-07	hsa-miR-15a-5p;hsa-miR-15b-5p;hsa-miR-16-5p;hsa-miR-195-
ENSG0000	ENSG0000	3.42E-05	hsa-miR-15a-5p;hsa-miR-15b-5p;hsa-miR-16-5p;hsa-miR-195-
ENSG0000	ENSG0000	4.77E-07	hsa-miR-15a-5p;hsa-miR-15b-5p;hsa-miR-16-5p;hsa-miR-195-
ENSG0000	ENSG0000	0.000274	hsa-miR-142-3p;hsa-miR-15a-5p;hsa-miR-15b-5p;hsa-miR-19
ENSG0000	ENSG0000	6.55E-05	hsa-miR-15a-5p;hsa-miR-15b-5p;hsa-miR-16-5p;hsa-miR-195-
ENSG0000	ENSG0000	4.73E-08	hsa-miR-15a-5p;hsa-miR-15b-5p;hsa-miR-16-5p;hsa-miR-195-
ENSG0000	ENSG0000	3.00E-05	hsa-miR-15a-5p;hsa-miR-15b-5p;hsa-miR-16-5p;hsa-miR-195-
ENSG0000	ENSG0000	8.21E-07	hsa-miR-15a-5p;hsa-miR-15b-5p;hsa-miR-16-5p;hsa-miR-195-
ENSG0000	ENSG0000	6.70E-11	hsa-miR-15a-5p;hsa-miR-15b-5p;hsa-miR-16-5p;hsa-miR-195-
ENSG0000	ENSG0000	0.000104	hsa-miR-15a-5p;hsa-miR-15b-5p;hsa-miR-16-5p;hsa-miR-195-
ENSG0000	ENSG0000	6.91E-07	hsa-miR-15a-5p;hsa-miR-15b-5p;hsa-miR-16-5p;hsa-miR-195-
ENSG0000	ENSG0000	6.23E-08	hsa-miR-142-3p;hsa-miR-15a-5p;hsa-miR-15b-5p;hsa-miR-16-
ENSG0000	ENSG0000	1.22E-05	hsa-miR-15a-5p;hsa-miR-15b-5p;hsa-miR-16-5p;hsa-miR-195-
ENSG0000	ENSG0000	1.44E-05	hsa-miR-15a-5p;hsa-miR-15b-5p;hsa-miR-16-5p;hsa-miR-195-
ENSG0000	ENSG0000	1.53E-06	hsa-miR-15a-5p;hsa-miR-15b-5p;hsa-miR-16-5p;hsa-miR-195-
ENSG0000	ENSG0000	2.54E-07	hsa-miR-15a-5p;hsa-miR-15b-5p;hsa-miR-16-5p;hsa-miR-195-
ENSG0000	ENSG0000	4.73E-08	hsa-miR-15a-5p;hsa-miR-15b-5p;hsa-miR-16-5p;hsa-miR-195-
ENSG0000	ENSG0000	1.00E-09	hsa-miR-15a-5p;hsa-miR-15b-5p;hsa-miR-16-5p;hsa-miR-195-
ENSG0000	ENSG0000	6.61E-08	hsa-miR-15a-5p;hsa-miR-15b-5p;hsa-miR-16-5p;hsa-miR-195-
ENSG0000	ENSG0000	3.20E-05	hsa-miR-15a-5p;hsa-miR-15b-5p;hsa-miR-16-5p;hsa-miR-195-
ENSG0000	ENSG0000	6.41E-06	hsa-miR-15a-5p;hsa-miR-15b-5p;hsa-miR-16-5p;hsa-miR-195-
ENSG0000	ENSG0000	3.91E-07	hsa-miR-15a-5p;hsa-miR-15b-5p;hsa-miR-16-5p;hsa-miR-195-
ENSG0000	ENSG0000	4.21E-06	hsa-miR-15a-5p;hsa-miR-15b-5p;hsa-miR-16-5p;hsa-miR-195-
ENSG0000	ENSG0000	3.00E-05	hsa-miR-15a-5p;hsa-miR-15b-5p;hsa-miR-16-5p;hsa-miR-195-
ENSG0000	ENSG0000	3.35E-06	hsa-miR-15a-5p;hsa-miR-15b-5p;hsa-miR-16-5p;hsa-miR-195-
ENSG0000	ENSG0000	0.000225	hsa-miR-15a-5p;hsa-miR-15b-5p;hsa-miR-16-5p;hsa-miR-195-
ENSG0000	ENSG0000	1.56E-07	hsa-miR-15a-5p;hsa-miR-15b-5p;hsa-miR-16-5p;hsa-miR-195-
ENSG0000	ENSG0000	5.22E-06	hsa-miR-15a-5p;hsa-miR-15b-5p;hsa-miR-16-5p;hsa-miR-195-
ENSG0000	ENSG0000	4.66E-09	hsa-miR-15a-5p;hsa-miR-15b-5p;hsa-miR-16-5p;hsa-miR-195-
ENSG0000	ENSG0000	4.77E-07	hsa-miR-15a-5p;hsa-miR-15b-5p;hsa-miR-16-5p;hsa-miR-195-
ENSG0000	ENSG0000	2.62E-05	hsa-miR-15a-5p;hsa-miR-15b-5p;hsa-miR-16-5p;hsa-miR-195-
ENSG0000	ENSG0000	3.64E-05	hsa-miR-15a-5p;hsa-miR-15b-5p;hsa-miR-16-5p;hsa-miR-195-
ENSG0000	ENSG0000	4.21E-05	hsa-miR-15a-5p;hsa-miR-15b-5p;hsa-miR-16-5p;hsa-miR-195-
ENSG0000	ENSG0000	1.76E-06	hsa-miR-15a-5p;hsa-miR-15b-5p;hsa-miR-16-5p;hsa-miR-195-
ENSG0000	ENSG0000	7.07E-06	hsa-miR-15a-5p;hsa-miR-15b-5p;hsa-miR-16-5p;hsa-miR-195-
ENSG0000	ENSG0000	1.00E-09	hsa-miR-15a-5p;hsa-miR-15b-5p;hsa-miR-16-5p;hsa-miR-195-
ENSG0000	ENSG0000	3.40E-06	hsa-miR-15a-5p;hsa-miR-15b-5p;hsa-miR-16-5p;hsa-miR-195-
ENSG0000	ENSG0000	1.82E-05	hsa-miR-15a-5p;hsa-miR-15b-5p;hsa-miR-16-5p;hsa-miR-195-
ENSG0000	ENSG0000	3.91E-07	hsa-miR-15a-5p;hsa-miR-15b-5p;hsa-miR-16-5p;hsa-miR-195-
ENSG0000	ENSG0000	3.91E-07	hsa-miR-15a-5p;hsa-miR-15b-5p;hsa-miR-16-5p;hsa-miR-195-
ENSG0000	ENSG0000	3.35E-10	hsa-miR-15a-5p;hsa-miR-15b-5p;hsa-miR-16-5p;hsa-miR-195-
ENSG0000	ENSG0000	6.70E-11	hsa-miR-15a-5p;hsa-miR-15b-5p;hsa-miR-16-5p;hsa-miR-195-
ENSG0000	ENSG0000	2.54E-07	hsa-miR-15a-5p;hsa-miR-15b-5p;hsa-miR-16-5p;hsa-miR-195-
ENSG0000	ENSG0000	2.13E-08	hsa-miR-142-3p;hsa-miR-15a-5p;hsa-miR-15b-5p;hsa-miR-16-
ENSG0000	ENSG0000	1.68E-05	hsa-miR-15a-5p;hsa-miR-15b-5p;hsa-miR-16-5p;hsa-miR-195-
ENSG0000	ENSG0000	5.76E-07	hsa-miR-15a-5p;hsa-miR-15b-5p;hsa-miR-16-5p;hsa-miR-195-

ENSG0000	ENSG0000	4.77E-07	hsa-miR-15a-5p;hsa-miR-15b-5p;hsa-miR-16-5p;hsa-miR-195-
ENSG0000	ENSG0000	2.62E-06	hsa-miR-15a-5p;hsa-miR-15b-5p;hsa-miR-16-5p;hsa-miR-195-
ENSG0000	ENSG0000	0.000201	hsa-miR-15a-5p;hsa-miR-15b-5p;hsa-miR-16-5p;hsa-miR-195-
ENSG0000	ENSG0000	3.35E-10	hsa-miR-15a-5p;hsa-miR-15b-5p;hsa-miR-16-5p;hsa-miR-195-
ENSG0000	ENSG0000	4.21E-06	hsa-miR-15a-5p;hsa-miR-15b-5p;hsa-miR-16-5p;hsa-miR-195-
ENSG0000	ENSG0000	2.62E-05	hsa-miR-15a-5p;hsa-miR-15b-5p;hsa-miR-16-5p;hsa-miR-195-
ENSG0000	ENSG0000	3.28E-08	hsa-miR-15a-5p;hsa-miR-15b-5p;hsa-miR-16-5p;hsa-miR-195-
ENSG0000	ENSG0000	0.000722	hsa-miR-15a-5p;hsa-miR-15b-5p;hsa-miR-16-5p;hsa-miR-195-
ENSG0000	ENSG0000	0.000722	hsa-miR-15a-5p;hsa-miR-15b-5p;hsa-miR-16-5p;hsa-miR-195-
ENSG0000	ENSG0000	4.13E-05	hsa-miR-15a-5p;hsa-miR-15b-5p;hsa-miR-16-5p;hsa-miR-195-
ENSG0000	ENSG0000	4.77E-07	hsa-miR-15a-5p;hsa-miR-15b-5p;hsa-miR-16-5p;hsa-miR-195-
ENSG0000	ENSG0000	1.68E-05	hsa-miR-15a-5p;hsa-miR-15b-5p;hsa-miR-16-5p;hsa-miR-195-
ENSG0000	ENSG0000	3.64E-05	hsa-miR-15a-5p;hsa-miR-15b-5p;hsa-miR-16-5p;hsa-miR-195-
ENSG0000	ENSG0000	2.97E-06	hsa-miR-15a-5p;hsa-miR-15b-5p;hsa-miR-16-5p;hsa-miR-195-
ENSG0000	ENSG0000	6.70E-11	hsa-miR-15a-5p;hsa-miR-15b-5p;hsa-miR-16-5p;hsa-miR-195-
ENSG0000	ENSG0000	0.000643	hsa-miR-15a-5p;hsa-miR-15b-5p;hsa-miR-16-5p;hsa-miR-195-
ENSG0000	ENSG0000	0.000104	hsa-miR-15a-5p;hsa-miR-15b-5p;hsa-miR-16-5p;hsa-miR-195-
ENSG0000	ENSG0000	6.55E-05	hsa-miR-15a-5p;hsa-miR-15b-5p;hsa-miR-16-5p;hsa-miR-195-
ENSG0000	ENSG0000	0.000403	hsa-miR-15a-5p;hsa-miR-15b-5p;hsa-miR-16-5p;hsa-miR-195-
ENSG0000	ENSG0000	9.37E-06	hsa-miR-15a-5p;hsa-miR-15b-5p;hsa-miR-16-5p;hsa-miR-195-
ENSG0000	ENSG0000	2.97E-06	hsa-miR-15a-5p;hsa-miR-15b-5p;hsa-miR-16-5p;hsa-miR-195-
ENSG0000	ENSG0000	6.70E-11	hsa-miR-15a-5p;hsa-miR-15b-5p;hsa-miR-16-5p;hsa-miR-195-
ENSG0000	ENSG0000	9.37E-06	hsa-miR-15a-5p;hsa-miR-15b-5p;hsa-miR-16-5p;hsa-miR-195-
ENSG0000	ENSG0000	8.21E-07	hsa-miR-15a-5p;hsa-miR-15b-5p;hsa-miR-16-5p;hsa-miR-195-
ENSG0000	ENSG0000	5.55E-05	hsa-miR-15a-5p;hsa-miR-15b-5p;hsa-miR-16-5p;hsa-miR-195-
ENSG0000	ENSG0000	3.00E-05	hsa-miR-15a-5p;hsa-miR-15b-5p;hsa-miR-16-5p;hsa-miR-195-
ENSG0000	ENSG0000	4.82E-06	hsa-miR-142-3p;hsa-miR-15a-5p;hsa-miR-15b-5p;hsa-miR-16-
ENSG0000	ENSG0000	0.000662	hsa-miR-15a-5p;hsa-miR-15b-5p;hsa-miR-195-5p;hsa-miR-66
ENSG0000	ENSG0000	2.11E-05	hsa-miR-15a-5p;hsa-miR-15b-5p;hsa-miR-16-5p;hsa-miR-195-
ENSG0000	ENSG0000	0.000193	hsa-miR-15a-5p;hsa-miR-15b-5p;hsa-miR-16-5p;hsa-miR-195-
ENSG0000	ENSG0000	1.14E-06	hsa-miR-15a-5p;hsa-miR-15b-5p;hsa-miR-16-5p;hsa-miR-195-
ENSG0000	ENSG0000	9.37E-06	hsa-miR-15a-5p;hsa-miR-15b-5p;hsa-miR-16-5p;hsa-miR-195-
ENSG0000	ENSG0000	0.000137	hsa-miR-15a-5p;hsa-miR-15b-5p;hsa-miR-16-5p;hsa-miR-195-
ENSG0000	ENSG0000	7.07E-06	hsa-miR-15a-5p;hsa-miR-15b-5p;hsa-miR-16-5p;hsa-miR-195-
ENSG0000	ENSG0000	9.89E-07	hsa-miR-1226-3p;hsa-miR-15a-5p;hsa-miR-15b-5p;hsa-miR-1
ENSG0000	ENSG0000	5.56E-06	hsa-miR-142-3p;hsa-miR-15a-5p;hsa-miR-15b-5p;hsa-miR-16-
ENSG0000	ENSG0000	1.32E-06	hsa-miR-15a-5p;hsa-miR-15b-5p;hsa-miR-16-5p;hsa-miR-195-
ENSG0000	ENSG0000	1.12E-05	hsa-miR-15a-5p;hsa-miR-15b-5p;hsa-miR-16-5p;hsa-miR-195-
ENSG0000	ENSG0000	1.32E-05	hsa-miR-15a-5p;hsa-miR-15b-5p;hsa-miR-16-5p;hsa-miR-195-
ENSG0000	ENSG0000	3.17E-07	hsa-miR-15a-5p;hsa-miR-15b-5p;hsa-miR-16-5p;hsa-miR-195-
ENSG0000	ENSG0000	3.78E-05	hsa-miR-142-3p;hsa-miR-15a-5p;hsa-miR-15b-5p;hsa-miR-16-
ENSG0000	ENSG0000	3.28E-08	hsa-miR-15a-5p;hsa-miR-15b-5p;hsa-miR-16-5p;hsa-miR-195-
ENSG0000	ENSG0000	3.88E-05	hsa-miR-15a-5p;hsa-miR-15b-5p;hsa-miR-16-5p;hsa-miR-195-
ENSG0000	ENSG0000	0.000137	hsa-miR-15a-5p;hsa-miR-15b-5p;hsa-miR-16-5p;hsa-miR-195-
ENSG0000	ENSG0000	1.32E-06	hsa-miR-15a-5p;hsa-miR-15b-5p;hsa-miR-16-5p;hsa-miR-195-
ENSG0000	ENSG0000	1.20E-07	hsa-miR-15a-5p;hsa-miR-15b-5p;hsa-miR-16-5p;hsa-miR-195-
ENSG0000	ENSG0000	2.49E-06	hsa-miR-15a-5p;hsa-miR-15b-5p;hsa-miR-16-5p;hsa-miR-195-
ENSG0000	ENSG0000	1.56E-05	hsa-miR-15a-5p;hsa-miR-15b-5p;hsa-miR-16-5p;hsa-miR-195-
ENSG0000	ENSG0000	2.54E-07	hsa-miR-15a-5p;hsa-miR-15b-5p;hsa-miR-16-5p;hsa-miR-195-
ENSG0000	ENSG0000	2.54E-07	hsa-miR-15a-5p;hsa-miR-15b-5p;hsa-miR-16-5p;hsa-miR-195-
ENSG0000	ENSG0000	2.54E-07	hsa-miR-15a-5p;hsa-miR-15b-5p;hsa-miR-16-5p;hsa-miR-195-
ENSG0000	ENSG0000	2.54E-07	hsa-miR-15a-5p;hsa-miR-15b-5p;hsa-miR-16-5p;hsa-miR-195-
ENSG0000	ENSG0000	0.000114	hsa-miR-15a-5p;hsa-miR-15b-5p;hsa-miR-16-5p;hsa-miR-195-
ENSG0000	ENSG0000	3.20E-05	hsa-miR-15a-5p;hsa-miR-15b-5p;hsa-miR-16-5p;hsa-miR-195-

ENSG0000	ENSG0000	1.00E-09	hsa-miR-15a-5p;hsa-miR-15b-5p;hsa-miR-16-5p;hsa-miR-195-
ENSG0000	ENSG0000	6.70E-11	hsa-miR-15a-5p;hsa-miR-15b-5p;hsa-miR-16-5p;hsa-miR-195-
ENSG0000	ENSG0000	4.21E-06	hsa-miR-15a-5p;hsa-miR-15b-5p;hsa-miR-16-5p;hsa-miR-195-
ENSG0000	ENSG0000	4.21E-06	hsa-miR-15a-5p;hsa-miR-15b-5p;hsa-miR-16-5p;hsa-miR-195-
ENSG0000	ENSG0000	4.21E-06	hsa-miR-15a-5p;hsa-miR-15b-5p;hsa-miR-16-5p;hsa-miR-195-
ENSG0000	ENSG0000	4.21E-06	hsa-miR-15a-5p;hsa-miR-15b-5p;hsa-miR-16-5p;hsa-miR-195-
ENSG0000	ENSG0000	4.21E-06	hsa-miR-15a-5p;hsa-miR-15b-5p;hsa-miR-16-5p;hsa-miR-195-
ENSG0000	ENSG0000	4.21E-06	hsa-miR-15a-5p;hsa-miR-15b-5p;hsa-miR-16-5p;hsa-miR-195-
ENSG0000	ENSG0000	4.21E-06	hsa-miR-15a-5p;hsa-miR-15b-5p;hsa-miR-16-5p;hsa-miR-195-
ENSG0000	ENSG0000	4.21E-06	hsa-miR-15a-5p;hsa-miR-15b-5p;hsa-miR-16-5p;hsa-miR-195-
ENSG0000	ENSG0000	4.21E-06	hsa-miR-15a-5p;hsa-miR-15b-5p;hsa-miR-16-5p;hsa-miR-195-
ENSG0000	ENSG0000	4.21E-06	hsa-miR-15a-5p;hsa-miR-15b-5p;hsa-miR-16-5p;hsa-miR-195-
ENSG0000	ENSG0000	4.66E-09	hsa-miR-15a-5p;hsa-miR-15b-5p;hsa-miR-16-5p;hsa-miR-195-
ENSG0000	ENSG0000	4.77E-07	hsa-miR-15a-5p;hsa-miR-15b-5p;hsa-miR-16-5p;hsa-miR-195-
ENSG0000	ENSG0000	4.21E-06	hsa-miR-15a-5p;hsa-miR-15b-5p;hsa-miR-16-5p;hsa-miR-195-
ENSG0000	ENSG0000	6.41E-06	hsa-miR-15a-5p;hsa-miR-15b-5p;hsa-miR-16-5p;hsa-miR-195-
ENSG0000	ENSG0000	5.55E-05	hsa-miR-15a-5p;hsa-miR-15b-5p;hsa-miR-16-5p;hsa-miR-195-
ENSG0000	ENSG0000	4.69E-06	hsa-miR-15a-5p;hsa-miR-15b-5p;hsa-miR-16-5p;hsa-miR-195-
ENSG0000	ENSG0000	1.82E-05	hsa-miR-15a-5p;hsa-miR-15b-5p;hsa-miR-16-5p;hsa-miR-195-
ENSG0000	ENSG0000	9.42E-05	hsa-miR-15a-5p;hsa-miR-15b-5p;hsa-miR-16-5p;hsa-miR-195-
ENSG0000	ENSG0000	1.44E-05	hsa-miR-15a-5p;hsa-miR-15b-5p;hsa-miR-16-5p;hsa-miR-195-
ENSG0000	ENSG0000	3.47E-08	hsa-miR-1226-3p;hsa-miR-15a-5p;hsa-miR-15b-5p;hsa-miR-1
ENSG0000	ENSG0000	8.44E-07	hsa-miR-142-3p;hsa-miR-15a-5p;hsa-miR-15b-5p;hsa-miR-16-
ENSG0000	ENSG0000	0.000722	hsa-miR-15a-5p;hsa-miR-15b-5p;hsa-miR-16-5p;hsa-miR-195-
ENSG0000	ENSG0000	2.54E-07	hsa-miR-15a-5p;hsa-miR-15b-5p;hsa-miR-16-5p;hsa-miR-195-
ENSG0000	ENSG0000	4.21E-05	hsa-miR-15a-5p;hsa-miR-15b-5p;hsa-miR-16-5p;hsa-miR-195-
ENSG0000	ENSG0000	9.00E-08	hsa-miR-15a-5p;hsa-miR-15b-5p;hsa-miR-16-5p;hsa-miR-195-
ENSG0000	ENSG0000	1.14E-06	hsa-miR-15a-5p;hsa-miR-15b-5p;hsa-miR-16-5p;hsa-miR-195-
ENSG0000	ENSG0000	3.35E-06	hsa-miR-15a-5p;hsa-miR-15b-5p;hsa-miR-16-5p;hsa-miR-195-
ENSG0000	ENSG0000	9.69E-07	hsa-miR-15a-5p;hsa-miR-15b-5p;hsa-miR-16-5p;hsa-miR-195-
ENSG0000	ENSG0000	3.35E-10	hsa-miR-15a-5p;hsa-miR-15b-5p;hsa-miR-16-5p;hsa-miR-195-
ENSG0000	ENSG0000	3.76E-06	hsa-miR-15a-5p;hsa-miR-15b-5p;hsa-miR-16-5p;hsa-miR-195-
ENSG0000	ENSG0000	5.22E-06	hsa-miR-15a-5p;hsa-miR-15b-5p;hsa-miR-16-5p;hsa-miR-195-
ENSG0000	ENSG0000	1.44E-05	hsa-miR-15a-5p;hsa-miR-15b-5p;hsa-miR-16-5p;hsa-miR-195-
ENSG0000	ENSG0000	4.77E-07	hsa-miR-15a-5p;hsa-miR-15b-5p;hsa-miR-16-5p;hsa-miR-195-
ENSG0000	ENSG0000	9.00E-08	hsa-miR-15a-5p;hsa-miR-15b-5p;hsa-miR-16-5p;hsa-miR-195-
ENSG0000	ENSG0000	2.27E-05	hsa-miR-15a-5p;hsa-miR-15b-5p;hsa-miR-16-5p;hsa-miR-195-
ENSG0000	ENSG0000	3.17E-07	hsa-miR-15a-5p;hsa-miR-15b-5p;hsa-miR-16-5p;hsa-miR-195-
ENSG0000	ENSG0000	3.17E-07	hsa-miR-15a-5p;hsa-miR-15b-5p;hsa-miR-16-5p;hsa-miR-195-
ENSG0000	ENSG0000	0.000244	hsa-miR-15a-5p;hsa-miR-15b-5p;hsa-miR-16-5p;hsa-miR-195-
ENSG0000	ENSG0000	1.53E-06	hsa-miR-15a-5p;hsa-miR-15b-5p;hsa-miR-16-5p;hsa-miR-195-
ENSG0000	ENSG0000	1.20E-07	hsa-miR-15a-5p;hsa-miR-15b-5p;hsa-miR-16-5p;hsa-miR-195-
ENSG0000	ENSG0000	3.28E-08	hsa-miR-15a-5p;hsa-miR-15b-5p;hsa-miR-16-5p;hsa-miR-195-
ENSG0000	ENSG0000	4.66E-05	hsa-miR-15a-5p;hsa-miR-15b-5p;hsa-miR-16-5p;hsa-miR-195-
ENSG0000	ENSG0000	9.00E-08	hsa-miR-15a-5p;hsa-miR-15b-5p;hsa-miR-16-5p;hsa-miR-195-
ENSG0000	ENSG0000	9.42E-05	hsa-miR-15a-5p;hsa-miR-15b-5p;hsa-miR-16-5p;hsa-miR-195-
ENSG0000	ENSG0000	1.44E-05	hsa-miR-15a-5p;hsa-miR-15b-5p;hsa-miR-16-5p;hsa-miR-195-
ENSG0000	ENSG0000	2.02E-06	hsa-miR-15a-5p;hsa-miR-15b-5p;hsa-miR-16-5p;hsa-miR-195-
ENSG0000	ENSG0000	1.56E-05	hsa-miR-15a-5p;hsa-miR-15b-5p;hsa-miR-16-5p;hsa-miR-195-
ENSG0000	ENSG0000	3.42E-05	hsa-miR-15a-5p;hsa-miR-15b-5p;hsa-miR-16-5p;hsa-miR-195-
ENSG0000	ENSG0000	0.000163	hsa-miR-15a-5p;hsa-miR-15b-5p;hsa-miR-16-5p;hsa-miR-195-
ENSG0000	ENSG0000	0.0009	hsa-miR-15a-5p;hsa-miR-15b-5p;hsa-miR-16-5p;hsa-miR-195-
ENSG0000	ENSG0000	6.70E-11	hsa-miR-15a-5p;hsa-miR-15b-5p;hsa-miR-16-5p;hsa-miR-195-
ENSG0000	ENSG0000	9.69E-07	hsa-miR-15a-5p;hsa-miR-15b-5p;hsa-miR-16-5p;hsa-miR-195-

ENSG0000	ENSG0000	7.78E-07	hsa-miR-1226-3p;hsa-miR-15a-5p;hsa-miR-15b-5p;hsa-miR-1
ENSG0000	ENSG0000	1.78E-06	hsa-miR-15a-5p;hsa-miR-15b-5p;hsa-miR-16-5p;hsa-miR-195-
ENSG0000	ENSG0000	3.61E-06	hsa-miR-15a-5p;hsa-miR-15b-5p;hsa-miR-16-5p;hsa-miR-195-
ENSG0000	ENSG0000	2.62E-05	hsa-miR-15a-5p;hsa-miR-15b-5p;hsa-miR-16-5p;hsa-miR-195-
ENSG0000	ENSG0000	2.44E-05	hsa-miR-15a-5p;hsa-miR-15b-5p;hsa-miR-16-5p;hsa-miR-195-
ENSG0000	ENSG0000	1.44E-05	hsa-miR-107;hsa-miR-125a-5p;hsa-miR-1304-3p;hsa-miR-143-
ENSG0000	ENSG0000	0.008165	hsa-miR-106b-5p;hsa-miR-15a-5p;hsa-miR-15b-5p;hsa-miR-1
ENSG0000	ENSG0000	1.09E-05	hsa-let-7a-5p;hsa-let-7b-5p;hsa-let-7d-5p;hsa-let-7e-5p;hsa-l
ENSG0000	ENSG0000	0.001582	hsa-miR-15a-5p;hsa-miR-15b-5p;hsa-miR-16-5p;hsa-miR-195-
ENSG0000	ENSG0000	1.01E-05	hsa-miR-181a-5p;hsa-miR-181b-5p;hsa-miR-181c-5p;hsa-miR-
ENSG0000	ENSG0000	0.009332	hsa-miR-106b-5p;hsa-miR-1-3p;hsa-miR-20a-5p;hsa-miR-27a-
ENSG0000	ENSG0000	0.000873	hsa-let-7a-5p;hsa-let-7b-5p;hsa-let-7d-5p;hsa-let-7e-5p;hsa-l
ENSG0000	ENSG0000	3.07E-11	hsa-miR-103a-3p;hsa-miR-107;hsa-miR-15a-5p;hsa-miR-15b-5
ENSG0000	ENSG0000	3.67E-05	hsa-miR-15a-5p;hsa-miR-15b-5p;hsa-miR-16-5p;hsa-miR-195-
ENSG0000	ENSG0000	1.52E-05	hsa-miR-15a-5p;hsa-miR-15b-5p;hsa-miR-195-5p;hsa-miR-21
ENSG0000	ENSG0000	0.000619	hsa-let-7a-5p;hsa-let-7b-5p;hsa-let-7d-5p;hsa-let-7e-5p;hsa-l
ENSG0000	ENSG0000	2.03E-05	hsa-let-7a-5p;hsa-let-7b-5p;hsa-let-7d-5p;hsa-let-7e-5p;hsa-l
ENSG0000	ENSG0000	3.18E-07	hsa-miR-103a-3p;hsa-miR-107;hsa-miR-15a-5p;hsa-miR-15b-5
ENSG0000	ENSG0000	0.009645	hsa-miR-106b-5p;hsa-miR-181a-5p;hsa-miR-181b-5p;hsa-miR
ENSG0000	ENSG0000	6.01E-05	hsa-let-7a-5p;hsa-let-7d-5p;hsa-miR-106b-5p;hsa-miR-130a-3
ENSG0000	ENSG0000	0.000654	hsa-let-7a-5p;hsa-let-7b-5p;hsa-let-7d-5p;hsa-let-7e-5p;hsa-l
ENSG0000	ENSG0000	2.62E-06	hsa-miR-15a-5p;hsa-miR-15b-5p;hsa-miR-16-5p;hsa-miR-195-
ENSG0000	ENSG0000	2.62E-06	hsa-miR-15a-5p;hsa-miR-15b-5p;hsa-miR-16-5p;hsa-miR-195-
ENSG0000	ENSG0000	0.000551	hsa-miR-15a-5p;hsa-miR-15b-5p;hsa-miR-16-5p;hsa-miR-195-
ENSG0000	ENSG0000	0.003365	hsa-miR-107;hsa-miR-1304-3p;hsa-miR-362-3p;hsa-miR-603
ENSG0000	ENSG0000	0.009332	hsa-miR-15a-5p;hsa-miR-15b-5p;hsa-miR-16-5p;hsa-miR-195-
ENSG0000	ENSG0000	0.001344	hsa-miR-106b-5p;hsa-miR-181a-5p;hsa-miR-181b-5p;hsa-miR
ENSG0000	ENSG0000	0.001129	hsa-let-7a-5p;hsa-let-7b-5p;hsa-let-7d-5p;hsa-let-7e-5p;hsa-l
ENSG0000	ENSG0000	2.68E-09	hsa-let-7a-5p;hsa-miR-125b-5p;hsa-miR-15a-5p;hsa-miR-15b-
ENSG0000	ENSG0000	3.91E-06	hsa-let-7a-5p;hsa-let-7b-5p;hsa-let-7f-5p;hsa-miR-125b-5p;hs
ENSG0000	ENSG0000	0.000348	hsa-miR-103a-3p;hsa-miR-107;hsa-miR-127-5p;hsa-miR-140-5
ENSG0000	ENSG0000	0.006267	hsa-miR-15a-5p;hsa-miR-15b-5p;hsa-miR-16-5p;hsa-miR-195-
ENSG0000	ENSG0000	1.90E-05	hsa-miR-15a-5p;hsa-miR-15b-5p;hsa-miR-16-5p;hsa-miR-195-
ENSG0000	ENSG0000	0.00033	hsa-miR-125a-5p;hsa-miR-200b-3p;hsa-miR-200c-3p;hsa-miR-
ENSG0000	ENSG0000	0.002048	hsa-miR-103a-3p;hsa-miR-195-5p;hsa-miR-222-3p;hsa-miR-4
ENSG0000	ENSG0000	0.002947	hsa-miR-107;hsa-miR-15a-5p;hsa-miR-15b-5p;hsa-miR-195-5
ENSG0000	ENSG0000	0.001139	hsa-miR-15a-5p;hsa-miR-15b-5p;hsa-miR-16-5p;hsa-miR-195-
ENSG0000	ENSG0000	0.003683	hsa-miR-199a-5p;hsa-miR-199b-5p;hsa-miR-200b-3p;hsa-miR
ENSG0000	ENSG0000	0.008423	hsa-let-7a-5p;hsa-let-7b-5p;hsa-miR-106b-5p;hsa-miR-15a-5
ENSG0000	ENSG0000	0.006267	hsa-miR-15b-5p;hsa-miR-16-5p;hsa-miR-195-5p;hsa-miR-424-
ENSG0000	ENSG0000	0.003365	hsa-miR-15a-5p;hsa-miR-15b-5p;hsa-miR-16-5p;hsa-miR-424-
ENSG0000	ENSG0000	0.001182	hsa-miR-103a-3p;hsa-miR-107;hsa-miR-15a-5p;hsa-miR-15b-5
ENSG0000	ENSG0000	1.10E-07	hsa-let-7a-5p;hsa-let-7b-5p;hsa-let-7d-5p;hsa-let-7e-5p;hsa-l
ENSG0000	ENSG0000	0.003683	hsa-let-7a-5p;hsa-let-7b-5p;hsa-let-7d-5p;hsa-let-7e-5p;hsa-l
ENSG0000	ENSG0000	1.40E-05	hsa-miR-103a-3p;hsa-miR-107;hsa-miR-15a-5p;hsa-miR-15b-5
ENSG0000	ENSG0000	0.000235	hsa-let-7b-5p;hsa-miR-140-3p;hsa-miR-15a-5p;hsa-miR-15b-5
ENSG0000	ENSG0000	1.01E-05	hsa-let-7a-5p;hsa-let-7b-5p;hsa-miR-107;hsa-miR-15a-5p;hsa-
ENSG0000	ENSG0000	0.000619	hsa-miR-106b-5p;hsa-miR-130a-3p;hsa-miR-130b-3p;hsa-miR
ENSG0000	ENSG0000	0.000366	hsa-miR-103a-3p;hsa-miR-107;hsa-miR-199a-5p;hsa-miR-199
ENSG0000	ENSG0000	2.95E-05	hsa-miR-15a-5p;hsa-miR-16-5p;hsa-miR-23a-3p;hsa-miR-23b-
ENSG0000	ENSG0000	1.90E-05	hsa-let-7a-5p;hsa-let-7b-5p;hsa-let-7d-5p;hsa-let-7e-5p;hsa-l
ENSG0000	ENSG0000	0.003016	hsa-miR-15a-5p;hsa-miR-15b-5p;hsa-miR-16-5p;hsa-miR-195-
ENSG0000	ENSG0000	0.000951	hsa-miR-130a-3p;hsa-miR-130b-3p;hsa-miR-301a-3p;hsa-miR-
ENSG0000	ENSG0000	8.44E-05	hsa-let-7a-5p;hsa-let-7b-5p;hsa-let-7d-5p;hsa-let-7e-5p;hsa-l

ENSG0000	ENSG0000	0.002947	hsa-miR-106b-5p;hsa-miR-130a-3p;hsa-miR-130b-3p;hsa-miR
ENSG0000	ENSG0000	0.001471	hsa-let-7a-5p;hsa-let-7b-5p;hsa-let-7d-5p;hsa-let-7e-5p;hsa-l
ENSG0000	ENSG0000	5.11E-05	hsa-miR-106b-5p;hsa-miR-1-3p;hsa-miR-181a-5p;hsa-miR-18
ENSG0000	ENSG0000	0.001681	hsa-miR-106b-5p;hsa-miR-127-5p;hsa-miR-1304-3p;hsa-miR-
ENSG0000	ENSG0000	0.003698	hsa-miR-103a-3p;hsa-miR-143-5p;hsa-miR-15a-5p;hsa-miR-1
ENSG0000	ENSG0000	3.25E-06	hsa-let-7a-5p;hsa-let-7b-5p;hsa-let-7d-5p;hsa-let-7e-5p;hsa-l
ENSG0000	ENSG0000	0.0074	hsa-miR-15a-5p;hsa-miR-15b-5p;hsa-miR-16-5p;hsa-miR-194-
ENSG0000	ENSG0000	0.001198	hsa-miR-103a-3p;hsa-miR-107;hsa-miR-125a-5p;hsa-miR-130a
ENSG0000	ENSG0000	0.000168	hsa-let-7a-5p;hsa-let-7d-5p;hsa-let-7e-5p;hsa-let-7f-5p;hsa-le
ENSG0000	ENSG0000	1.15E-05	hsa-miR-130b-3p;hsa-miR-15a-5p;hsa-miR-15b-5p;hsa-miR-1
ENSG0000	ENSG0000	3.61E-05	hsa-miR-103a-3p;hsa-miR-107;hsa-miR-130a-3p;hsa-miR-130b
ENSG0000	ENSG0000	0.000154	hsa-miR-1224-5p;hsa-miR-15a-5p;hsa-miR-15b-5p;hsa-miR-1
ENSG0000	ENSG0000	7.57E-07	hsa-miR-103a-3p;hsa-miR-107;hsa-miR-15a-5p;hsa-miR-15b-
ENSG0000	ENSG0000	3.20E-06	hsa-miR-127-5p;hsa-miR-15a-5p;hsa-miR-15b-5p;hsa-miR-16-
ENSG0000	ENSG0000	1.97E-07	hsa-miR-103a-3p;hsa-miR-107;hsa-miR-15a-5p;hsa-miR-15b-
ENSG0000	ENSG0000	7.31E-06	hsa-let-7a-5p;hsa-let-7b-5p;hsa-let-7d-5p;hsa-let-7e-5p;hsa-l
ENSG0000	ENSG0000	0.000486	hsa-let-7a-5p;hsa-let-7b-5p;hsa-let-7d-5p;hsa-let-7e-5p;hsa-l
ENSG0000	ENSG0000	0.001182	hsa-miR-106b-5p;hsa-miR-130a-3p;hsa-miR-130b-3p;hsa-miR
ENSG0000	ENSG0000	0.000235	hsa-let-7a-5p;hsa-let-7e-5p;hsa-let-7f-5p;hsa-let-7g-5p;hsa-le
ENSG0000	ENSG0000	2.79E-05	hsa-let-7a-5p;hsa-let-7b-5p;hsa-let-7d-5p;hsa-let-7e-5p;hsa-l
ENSG0000	ENSG0000	0.000681	hsa-let-7a-5p;hsa-let-7b-5p;hsa-let-7d-5p;hsa-let-7e-5p;hsa-l
ENSG0000	ENSG0000	0.00239	hsa-let-7a-5p;hsa-let-7b-5p;hsa-miR-106b-5p;hsa-miR-125b-
ENSG0000	ENSG0000	0.00239	hsa-let-7a-5p;hsa-let-7b-5p;hsa-miR-106b-5p;hsa-miR-125b-
ENSG0000	ENSG0000	0.001635	hsa-miR-106b-5p;hsa-miR-125a-5p;hsa-miR-125b-5p;hsa-miR
ENSG0000	ENSG0000	5.70E-08	hsa-let-7a-5p;hsa-let-7b-5p;hsa-let-7d-5p;hsa-let-7e-5p;hsa-l
ENSG0000	ENSG0000	0.000434	hsa-miR-15a-5p;hsa-miR-15b-5p;hsa-miR-195-5p;hsa-miR-33
ENSG0000	ENSG0000	0.000535	hsa-miR-125b-5p;hsa-miR-1-3p;hsa-miR-16-5p;hsa-miR-200a-
ENSG0000	ENSG0000	0.003365	hsa-miR-342-3p;hsa-miR-362-3p;hsa-miR-603;hsa-miR-873-3
ENSG0000	ENSG0000	0.000259	hsa-miR-103a-3p;hsa-miR-107;hsa-miR-1224-5p;hsa-miR-1-3
ENSG0000	ENSG0000	0.000392	hsa-miR-103a-3p;hsa-miR-106b-5p;hsa-miR-107;hsa-miR-15a-
ENSG0000	ENSG0000	0.002223	hsa-miR-107;hsa-miR-181a-5p;hsa-miR-181b-5p;hsa-miR-181
ENSG0000	ENSG0000	0.000124	hsa-let-7a-5p;hsa-let-7b-5p;hsa-let-7d-5p;hsa-let-7e-5p;hsa-l
ENSG0000	ENSG0000	0.002651	hsa-miR-181a-5p;hsa-miR-181b-5p;hsa-miR-181c-5p;hsa-miR-
ENSG0000	ENSG0000	6.67E-05	hsa-miR-181a-5p;hsa-miR-181b-5p;hsa-miR-181c-5p;hsa-miR-
ENSG0000	ENSG0000	0.000206	hsa-miR-106b-5p;hsa-miR-130a-3p;hsa-miR-130b-3p;hsa-miR
ENSG0000	ENSG0000	4.42E-05	hsa-miR-125b-5p;hsa-miR-181a-5p;hsa-miR-181b-5p;hsa-miR
ENSG0000	ENSG0000	0.003744	hsa-miR-15a-5p;hsa-miR-15b-5p;hsa-miR-16-5p;hsa-miR-195-
ENSG0000	ENSG0000	0.004383	hsa-let-7b-5p;hsa-miR-199a-5p;hsa-miR-214-3p;hsa-miR-27a-
ENSG0000	ENSG0000	0.0081	hsa-let-7b-5p;hsa-miR-1-3p;hsa-miR-143-5p;hsa-miR-16-5p;h
ENSG0000	ENSG0000	0.002616	hsa-miR-106b-5p;hsa-miR-1304-3p;hsa-miR-130a-3p;hsa-miR-
ENSG0000	ENSG0000	6.07E-06	hsa-miR-15a-5p;hsa-miR-15b-5p;hsa-miR-16-5p;hsa-miR-195-
ENSG0000	ENSG0000	2.19E-05	hsa-miR-15a-5p;hsa-miR-15b-5p;hsa-miR-16-5p;hsa-miR-181
ENSG0000	ENSG0000	0.009977	hsa-miR-181a-5p;hsa-miR-181b-5p;hsa-miR-181c-5p;hsa-miR-
ENSG0000	ENSG0000	4.57E-05	hsa-miR-15a-5p;hsa-miR-15b-5p;hsa-miR-16-5p;hsa-miR-195-
ENSG0000	ENSG0000	6.25E-05	hsa-let-7a-5p;hsa-let-7b-5p;hsa-let-7d-5p;hsa-let-7e-5p;hsa-l
ENSG0000	ENSG0000	4.50E-07	hsa-let-7g-5p;hsa-miR-1-3p;hsa-miR-140-3p;hsa-miR-200b-3
ENSG0000	ENSG0000	0.002589	hsa-let-7a-5p;hsa-let-7b-5p;hsa-let-7d-5p;hsa-let-7e-5p;hsa-l
ENSG0000	ENSG0000	0.00064	hsa-let-7a-5p;hsa-let-7b-5p;hsa-let-7d-5p;hsa-let-7e-5p;hsa-l
ENSG0000	ENSG0000	0.003092	hsa-miR-106b-5p;hsa-miR-1304-3p;hsa-miR-1-3p;hsa-miR-14
ENSG0000	ENSG0000	0.000286	hsa-let-7a-5p;hsa-let-7b-5p;hsa-let-7d-5p;hsa-let-7e-5p;hsa-l
ENSG0000	ENSG0000	3.22E-09	hsa-let-7a-5p;hsa-let-7b-5p;hsa-let-7d-5p;hsa-let-7e-5p;hsa-l
ENSG0000	ENSG0000	0.007752	hsa-miR-125b-2-3p;hsa-miR-130a-3p;hsa-miR-130b-3p;hsa-m
ENSG0000	ENSG0000	0.007752	hsa-miR-222-3p;hsa-miR-3180-3p;hsa-miR-455-3p;hsa-miR-6
ENSG0000	ENSG0000	0.003299	hsa-miR-103a-3p;hsa-miR-107;hsa-miR-222-3p;hsa-miR-23a-

ENSG0000	ENSG0000	2.08E-05	hsa-miR-15a-5p;hsa-miR-15b-5p;hsa-miR-195-5p;hsa-miR-21
ENSG0000	ENSG0000	1.40E-05	hsa-miR-103a-3p;hsa-miR-107;hsa-miR-15a-5p;hsa-miR-15b-
ENSG0000	ENSG0000	0.000572	hsa-miR-15a-5p;hsa-miR-15b-5p;hsa-miR-195-5p;hsa-miR-32
ENSG0000	ENSG0000	0.00249	hsa-let-7a-5p;hsa-let-7b-5p;hsa-let-7d-5p;hsa-let-7f-5p;hsa-le
ENSG0000	ENSG0000	4.84E-05	hsa-miR-106b-5p;hsa-miR-125a-5p;hsa-miR-125b-5p;hsa-miR
ENSG0000	ENSG0000	1.46E-07	hsa-let-7a-5p;hsa-let-7b-5p;hsa-let-7d-5p;hsa-let-7e-5p;hsa-l
ENSG0000	ENSG0000	0.000348	hsa-miR-181a-5p;hsa-miR-181b-5p;hsa-miR-181c-5p;hsa-miR-
ENSG0000	ENSG0000	0.001817	hsa-miR-15a-5p;hsa-miR-15b-5p;hsa-miR-16-5p;hsa-miR-195-
ENSG0000	ENSG0000	0.004719	hsa-miR-15a-5p;hsa-miR-15b-5p;hsa-miR-18a-3p;hsa-miR-19
ENSG0000	ENSG0000	6.31E-05	hsa-miR-15a-5p;hsa-miR-15b-5p;hsa-miR-16-5p;hsa-miR-195-
ENSG0000	ENSG0000	0.002947	hsa-miR-107;hsa-miR-199a-5p;hsa-miR-199b-5p;hsa-miR-20a-
ENSG0000	ENSG0000	1.01E-05	hsa-miR-125a-5p;hsa-miR-125b-5p;hsa-miR-181b-5p;hsa-miR
ENSG0000	ENSG0000	0.006781	hsa-miR-1224-5p;hsa-miR-23a-3p;hsa-miR-23b-3p;hsa-miR-6
ENSG0000	ENSG0000	0.000785	hsa-miR-107;hsa-miR-140-3p;hsa-miR-15a-5p;hsa-miR-15b-5
ENSG0000	ENSG0000	0.000913	hsa-miR-15a-5p;hsa-miR-15b-5p;hsa-miR-195-5p;hsa-miR-36
ENSG0000	ENSG0000	0.005251	hsa-miR-15a-5p;hsa-miR-15b-5p;hsa-miR-16-5p;hsa-miR-195-
ENSG0000	ENSG0000	0.003016	hsa-miR-130a-3p;hsa-miR-130b-3p;hsa-miR-27a-3p;hsa-miR-
ENSG0000	ENSG0000	3.10E-06	hsa-miR-106b-5p;hsa-miR-130a-3p;hsa-miR-130b-3p;hsa-miR
ENSG0000	ENSG0000	7.98E-05	hsa-miR-103a-3p;hsa-miR-107;hsa-miR-15a-5p;hsa-miR-15b-
ENSG0000	ENSG0000	0.000338	hsa-miR-106b-5p;hsa-miR-130a-3p;hsa-miR-130b-3p;hsa-miR
ENSG0000	ENSG0000	0.002661	hsa-miR-17-3p;hsa-miR-222-3p;hsa-miR-2467-5p;hsa-miR-36
ENSG0000	ENSG0000	0.0081	hsa-miR-130a-3p;hsa-miR-130b-3p;hsa-miR-16-5p;hsa-miR-3
ENSG0000	ENSG0000	2.95E-05	hsa-miR-199a-5p;hsa-miR-200b-3p;hsa-miR-200c-3p;hsa-miR-
ENSG0000	ENSG0000	0.000479	hsa-let-7a-5p;hsa-let-7b-5p;hsa-let-7d-5p;hsa-let-7e-5p;hsa-l
ENSG0000	ENSG0000	0.000101	hsa-let-7a-5p;hsa-let-7b-5p;hsa-let-7d-5p;hsa-let-7e-5p;hsa-l
ENSG0000	ENSG0000	1.01E-05	hsa-let-7d-5p;hsa-let-7f-5p;hsa-let-7g-5p;hsa-let-7i-5p
ENSG0000	ENSG0000	0.000654	hsa-miR-130a-3p;hsa-miR-130b-3p;hsa-miR-143-5p;hsa-miR-
ENSG0000	ENSG0000	0.0011	hsa-let-7a-5p;hsa-let-7b-5p;hsa-let-7d-5p;hsa-let-7e-5p;hsa-l
ENSG0000	ENSG0000	0.004014	hsa-miR-130a-3p;hsa-miR-15a-5p;hsa-miR-15b-5p;hsa-miR-1
ENSG0000	ENSG0000	0.001828	hsa-miR-15a-5p;hsa-miR-16-5p;hsa-miR-200b-3p;hsa-miR-20
ENSG0000	ENSG0000	0.006762	hsa-miR-106b-5p;hsa-miR-130a-3p;hsa-miR-130b-3p;hsa-miR
ENSG0000	ENSG0000	7.77E-05	hsa-miR-16-5p;hsa-miR-195-5p;hsa-miR-199a-3p;hsa-miR-20
ENSG0000	ENSG0000	0.0074	hsa-miR-106b-5p;hsa-miR-130a-3p;hsa-miR-130b-3p;hsa-miR
ENSG0000	ENSG0000	0.000253	hsa-miR-15a-5p;hsa-miR-15b-5p;hsa-miR-181a-5p;hsa-miR-1
ENSG0000	ENSG0000	0.000654	hsa-miR-15a-5p;hsa-miR-15b-5p;hsa-miR-16-5p;hsa-miR-195-
ENSG0000	ENSG0000	0.006079	hsa-let-7a-5p;hsa-let-7b-5p;hsa-let-7e-5p;hsa-let-7f-5p;hsa-le
ENSG0000	ENSG0000	8.11E-05	hsa-let-7a-5p;hsa-let-7g-5p;hsa-miR-1-3p;hsa-miR-181c-5p;h
ENSG0000	ENSG0000	3.91E-06	hsa-miR-15a-5p;hsa-miR-15b-5p;hsa-miR-16-5p;hsa-miR-195-
ENSG0000	ENSG0000	3.91E-06	hsa-miR-15a-5p;hsa-miR-15b-5p;hsa-miR-16-5p;hsa-miR-195-
ENSG0000	ENSG0000	0.001131	hsa-miR-106b-5p;hsa-miR-15a-5p;hsa-miR-15b-5p;hsa-miR-1
ENSG0000	ENSG0000	1.81E-05	hsa-miR-140-3p;hsa-miR-15a-5p;hsa-miR-15b-5p;hsa-miR-19
ENSG0000	ENSG0000	0.005416	hsa-miR-103a-3p;hsa-miR-107;hsa-miR-181b-5p;hsa-miR-181
ENSG0000	ENSG0000	0.00374	hsa-miR-27a-3p;hsa-miR-27b-3p;hsa-miR-362-3p;hsa-miR-54
ENSG0000	ENSG0000	0.007516	hsa-miR-127-5p;hsa-miR-378a-5p;hsa-miR-455-3p;hsa-miR-8
ENSG0000	ENSG0000	3.03E-05	hsa-miR-125a-5p;hsa-miR-125b-5p;hsa-miR-199a-5p;hsa-miR-
ENSG0000	ENSG0000	0.006079	hsa-miR-1224-5p;hsa-miR-200b-3p;hsa-miR-200c-3p;hsa-miR-
ENSG0000	ENSG0000	2.54E-05	hsa-miR-103a-3p;hsa-miR-107;hsa-miR-15a-5p;hsa-miR-15b-
ENSG0000	ENSG0000	7.31E-06	hsa-let-7a-5p;hsa-let-7b-5p;hsa-let-7d-5p;hsa-let-7e-5p;hsa-l
ENSG0000	ENSG0000	0.000974	hsa-miR-103a-3p;hsa-miR-107;hsa-miR-1-3p;hsa-miR-15a-5p;
ENSG0000	ENSG0000	2.97E-05	hsa-miR-18a-3p;hsa-miR-199a-5p;hsa-miR-23a-3p;hsa-miR-3
ENSG0000	ENSG0000	0.006079	hsa-miR-125a-5p;hsa-miR-125b-5p;hsa-miR-199a-5p;hsa-miR-
ENSG0000	ENSG0000	0.001851	hsa-miR-15a-5p;hsa-miR-195-5p;hsa-miR-424-5p;hsa-miR-49
ENSG0000	ENSG0000	5.28E-06	hsa-let-7a-5p;hsa-let-7b-5p;hsa-let-7d-5p;hsa-let-7e-5p;hsa-l
ENSG0000	ENSG0000	3.54E-06	hsa-let-7a-5p;hsa-let-7b-5p;hsa-let-7d-5p;hsa-let-7e-5p;hsa-l

ENSG0000	ENSG0000	2.79E-05	hsa-let-7a-5p;hsa-let-7b-5p;hsa-let-7d-5p;hsa-let-7e-5p;hsa-l
ENSG0000	ENSG0000	0.000529	hsa-miR-106b-5p;hsa-miR-125a-5p;hsa-miR-125b-5p;hsa-miR
ENSG0000	ENSG0000	7.79E-05	hsa-let-7a-5p;hsa-let-7b-5p;hsa-let-7d-5p;hsa-let-7e-5p;hsa-l
ENSG0000	ENSG0000	2.95E-05	hsa-miR-125b-5p;hsa-miR-140-5p;hsa-miR-27a-3p;hsa-miR-2
ENSG0000	ENSG0000	6.67E-05	hsa-miR-140-3p;hsa-miR-362-3p;hsa-miR-455-3p;hsa-miR-60
ENSG0000	ENSG0000	0.002661	hsa-let-7a-5p;hsa-let-7d-5p;hsa-let-7e-5p;hsa-let-7f-5p;hsa-m
ENSG0000	ENSG0000	0.002651	hsa-miR-103a-3p;hsa-miR-107;hsa-miR-143-5p;hsa-miR-545-
ENSG0000	ENSG0000	0.000342	hsa-miR-15a-5p;hsa-miR-15b-5p;hsa-miR-195-5p;hsa-miR-42
ENSG0000	ENSG0000	0.000558	hsa-miR-107;hsa-miR-15a-5p;hsa-miR-16-5p;hsa-miR-424-5p
ENSG0000	ENSG0000	0.000572	hsa-let-7a-5p;hsa-let-7b-5p;hsa-let-7d-5p;hsa-let-7e-5p;hsa-l
ENSG0000	ENSG0000	0.000558	hsa-miR-199a-5p;hsa-miR-199b-5p;hsa-miR-362-3p;hsa-miR-
ENSG0000	ENSG0000	6.67E-05	hsa-miR-107;hsa-miR-27a-3p;hsa-miR-27b-3p;hsa-miR-374a-
ENSG0000	ENSG0000	0.003036	hsa-let-7a-5p;hsa-miR-125a-5p;hsa-miR-125b-5p;hsa-miR-13
ENSG0000	ENSG0000	2.31E-05	hsa-miR-1-3p;hsa-miR-15a-5p;hsa-miR-15b-5p;hsa-miR-16-5
ENSG0000	ENSG0000	0.009141	hsa-let-7a-5p;hsa-let-7b-5p;hsa-let-7d-5p;hsa-let-7e-5p;hsa-l
ENSG0000	ENSG0000	1.48E-08	hsa-let-7a-5p;hsa-let-7b-5p;hsa-let-7d-5p;hsa-let-7e-5p;hsa-l
ENSG0000	ENSG0000	1.48E-08	hsa-let-7a-5p;hsa-let-7b-5p;hsa-let-7d-5p;hsa-let-7e-5p;hsa-l
ENSG0000	ENSG0000	1.48E-08	hsa-let-7a-5p;hsa-let-7b-5p;hsa-let-7d-5p;hsa-let-7e-5p;hsa-l
ENSG0000	ENSG0000	0.005949	hsa-miR-103a-3p;hsa-miR-107;hsa-miR-143-5p;hsa-miR-15a-
ENSG0000	ENSG0000	1.40E-05	hsa-let-7a-5p;hsa-let-7b-5p;hsa-let-7d-5p;hsa-let-7e-5p;hsa-l
ENSG0000	ENSG0000	0.000443	hsa-miR-1-3p;hsa-miR-27a-3p;hsa-miR-27b-3p;hsa-miR-362-
ENSG0000	ENSG0000	7.57E-07	hsa-let-7a-5p;hsa-let-7b-5p;hsa-let-7d-5p;hsa-let-7e-5p;hsa-l
ENSG0000	ENSG0000	7.57E-07	hsa-let-7a-5p;hsa-let-7b-5p;hsa-let-7d-5p;hsa-let-7e-5p;hsa-l
ENSG0000	ENSG0000	7.57E-07	hsa-let-7a-5p;hsa-let-7b-5p;hsa-let-7d-5p;hsa-let-7e-5p;hsa-l
ENSG0000	ENSG0000	7.57E-07	hsa-let-7a-5p;hsa-let-7b-5p;hsa-let-7d-5p;hsa-let-7e-5p;hsa-l
ENSG0000	ENSG0000	7.57E-07	hsa-let-7a-5p;hsa-let-7b-5p;hsa-let-7d-5p;hsa-let-7e-5p;hsa-l
ENSG0000	ENSG0000	7.57E-07	hsa-let-7a-5p;hsa-let-7b-5p;hsa-let-7d-5p;hsa-let-7e-5p;hsa-l
ENSG0000	ENSG0000	7.57E-07	hsa-let-7a-5p;hsa-let-7b-5p;hsa-let-7d-5p;hsa-let-7e-5p;hsa-l
ENSG0000	ENSG0000	7.08E-05	hsa-miR-15a-5p;hsa-miR-15b-5p;hsa-miR-195-5p;hsa-miR-19
ENSG0000	ENSG0000	3.91E-06	hsa-miR-15a-5p;hsa-miR-15b-5p;hsa-miR-16-5p;hsa-miR-195-
ENSG0000	ENSG0000	3.91E-06	hsa-miR-15a-5p;hsa-miR-15b-5p;hsa-miR-16-5p;hsa-miR-195-
ENSG0000	ENSG0000	0.007579	hsa-miR-15a-5p;hsa-miR-15b-5p;hsa-miR-195-5p;hsa-miR-19
ENSG0000	ENSG0000	0.000781	hsa-let-7a-5p;hsa-let-7b-5p;hsa-let-7d-5p;hsa-let-7e-5p;hsa-l
ENSG0000	ENSG0000	0.000812	hsa-miR-103a-3p;hsa-miR-107;hsa-miR-17-3p;hsa-miR-3065-
ENSG0000	ENSG0000	2.09E-06	hsa-miR-15a-5p;hsa-miR-15b-5p;hsa-miR-16-5p;hsa-miR-195-
ENSG0000	ENSG0000	0.007752	hsa-miR-130a-3p;hsa-miR-130b-3p;hsa-miR-301a-3p;hsa-miR-
ENSG0000	ENSG0000	0.008919	hsa-miR-140-3p;hsa-miR-181a-5p;hsa-miR-181b-5p;hsa-miR-
ENSG0000	ENSG0000	2.92E-05	hsa-miR-107;hsa-miR-15a-5p;hsa-miR-15b-5p;hsa-miR-195-5
ENSG0000	ENSG0000	0.00273	hsa-miR-106b-5p;hsa-miR-143-5p;hsa-miR-20a-5p;hsa-miR-2
ENSG0000	ENSG0000	2.02E-06	hsa-miR-107;hsa-miR-15a-5p;hsa-miR-15b-5p;hsa-miR-181a-
ENSG0000	ENSG0000	1.50E-06	hsa-miR-106b-5p;hsa-miR-127-5p;hsa-miR-15a-5p;hsa-miR-1
ENSG0000	ENSG0000	5.06E-08	hsa-let-7a-5p;hsa-let-7b-5p;hsa-let-7d-5p;hsa-let-7e-5p;hsa-l
ENSG0000	ENSG0000	2.22E-05	hsa-let-7a-5p;hsa-let-7b-5p;hsa-let-7e-5p;hsa-let-7f-5p;hsa-le
ENSG0000	ENSG0000	1.01E-05	hsa-miR-15a-5p;hsa-miR-15b-5p;hsa-miR-16-5p;hsa-miR-195-
ENSG0000	ENSG0000	0.000792	hsa-miR-106b-5p;hsa-miR-107;hsa-miR-15a-5p;hsa-miR-15b-
ENSG0000	ENSG0000	0.000334	hsa-let-7a-5p;hsa-let-7b-5p;hsa-let-7d-5p;hsa-let-7e-5p;hsa-l
ENSG0000	ENSG0000	6.31E-05	hsa-miR-130a-3p;hsa-miR-15a-5p;hsa-miR-15b-5p;hsa-miR-1
ENSG0000	ENSG0000	0.00374	hsa-miR-106b-5p;hsa-miR-20a-5p;hsa-miR-24-1-5p;hsa-miR-
ENSG0000	ENSG0000	0.004428	hsa-miR-1304-3p;hsa-miR-15a-5p;hsa-miR-15b-5p;hsa-miR-1
ENSG0000	ENSG0000	0.001548	hsa-miR-200b-3p;hsa-miR-200c-3p;hsa-miR-374a-5p;hsa-miR-
ENSG0000	ENSG0000	0.000207	hsa-miR-15a-5p;hsa-miR-15b-5p;hsa-miR-16-5p;hsa-miR-195-
ENSG0000	ENSG0000	0.00481	hsa-miR-130a-3p;hsa-miR-130b-3p;hsa-miR-15a-5p;hsa-miR-
ENSG0000	ENSG0000	7.15E-08	hsa-miR-103a-3p;hsa-miR-106b-5p;hsa-miR-107;hsa-miR-130
ENSG0000	ENSG0000	7.15E-08	hsa-miR-103a-3p;hsa-miR-106b-5p;hsa-miR-107;hsa-miR-130

ENSG0000	ENSG0000	0.002274	hsa-miR-106b-5p;hsa-miR-130a-3p;hsa-miR-130b-3p;hsa-miR
ENSG0000	ENSG0000	9.67E-06	hsa-miR-106b-5p;hsa-miR-130a-3p;hsa-miR-18a-3p;hsa-miR-1
ENSG0000	ENSG0000	0.000128	hsa-miR-15a-5p;hsa-miR-15b-5p;hsa-miR-16-5p;hsa-miR-195-
ENSG0000	ENSG0000	0.000128	hsa-miR-15a-5p;hsa-miR-15b-5p;hsa-miR-16-5p;hsa-miR-195-
ENSG0000	ENSG0000	0.002223	hsa-let-7a-5p;hsa-let-7b-5p;hsa-let-7d-5p;hsa-let-7e-5p;hsa-l
ENSG0000	ENSG0000	0.006799	hsa-miR-15a-5p;hsa-miR-15b-5p;hsa-miR-16-5p;hsa-miR-195-
ENSG0000	ENSG0000	0.008919	hsa-miR-130a-3p;hsa-miR-130b-3p;hsa-miR-301a-3p;hsa-miR-
ENSG0000	ENSG0000	0.000141	hsa-miR-106b-5p;hsa-miR-130a-3p;hsa-miR-130b-3p;hsa-miR
ENSG0000	ENSG0000	0.008919	hsa-miR-106b-5p;hsa-miR-141-3p;hsa-miR-20a-5p;hsa-miR-2
ENSG0000	ENSG0000	0.000485	hsa-let-7a-5p;hsa-let-7d-5p;hsa-let-7f-5p;hsa-let-7g-5p;hsa-le
ENSG0000	ENSG0000	0.004779	hsa-miR-103a-3p;hsa-miR-107;hsa-miR-1304-3p;hsa-miR-15a-
ENSG0000	ENSG0000	1.62E-06	hsa-let-7a-5p;hsa-let-7b-5p;hsa-let-7d-5p;hsa-let-7e-5p;hsa-l
ENSG0000	ENSG0000	0.000128	hsa-miR-106b-5p;hsa-miR-130a-3p;hsa-miR-130b-3p;hsa-miR
ENSG0000	ENSG0000	0.007516	hsa-miR-181a-5p;hsa-miR-181b-5p;hsa-miR-181c-5p;hsa-miR-
ENSG0000	ENSG0000	5.65E-05	hsa-let-7a-5p;hsa-let-7b-5p;hsa-let-7d-5p;hsa-let-7e-5p;hsa-l
ENSG0000	ENSG0000	0.001548	hsa-miR-15a-5p;hsa-miR-15b-5p;hsa-miR-16-5p;hsa-miR-195-
ENSG0000	ENSG0000	0.001548	hsa-miR-15a-5p;hsa-miR-15b-5p;hsa-miR-16-5p;hsa-miR-195-
ENSG0000	ENSG0000	6.67E-05	hsa-miR-15a-5p;hsa-miR-15b-5p;hsa-miR-16-5p;hsa-miR-195-
ENSG0000	ENSG0000	0.004251	hsa-miR-15a-5p;hsa-miR-15b-5p;hsa-miR-181a-5p;hsa-miR-18
ENSG0000	ENSG0000	0.004251	hsa-miR-106b-5p;hsa-miR-130a-3p;hsa-miR-130b-3p;hsa-miR
ENSG0000	ENSG0000	0.001999	hsa-let-7a-5p;hsa-let-7b-5p;hsa-let-7d-5p;hsa-let-7e-5p;hsa-l
ENSG0000	ENSG0000	0.000217	hsa-let-7a-5p;hsa-let-7b-5p;hsa-let-7d-5p;hsa-let-7e-5p;hsa-l
ENSG0000	ENSG0000	4.57E-05	hsa-miR-103a-3p;hsa-miR-107;hsa-miR-15a-5p;hsa-miR-15b-5
ENSG0000	ENSG0000	0.001139	hsa-miR-181a-5p;hsa-miR-181b-5p;hsa-miR-181c-5p;hsa-miR-
ENSG0000	ENSG0000	0.004719	hsa-miR-107;hsa-miR-15a-5p;hsa-miR-15b-5p;hsa-miR-195-5p
ENSG0000	ENSG0000	3.67E-05	hsa-miR-103a-3p;hsa-miR-107;hsa-miR-15a-5p;hsa-miR-15b-5
ENSG0000	ENSG0000	1.91E-08	hsa-miR-15a-5p;hsa-miR-15b-5p;hsa-miR-16-5p;hsa-miR-195-
ENSG0000	ENSG0000	2.48E-05	hsa-miR-130a-3p;hsa-miR-130b-3p;hsa-miR-199a-5p;hsa-miR-
ENSG0000	ENSG0000	0.001396	hsa-miR-1304-3p;hsa-miR-130a-3p;hsa-miR-130b-3p;hsa-miR-
ENSG0000	ENSG0000	0.001396	hsa-miR-1304-3p;hsa-miR-130a-3p;hsa-miR-130b-3p;hsa-miR-
ENSG0000	ENSG0000	0.00111	hsa-let-7a-5p;hsa-let-7b-5p;hsa-let-7d-5p;hsa-let-7e-5p;hsa-l
ENSG0000	ENSG0000	0.00111	hsa-let-7a-5p;hsa-let-7b-5p;hsa-let-7d-5p;hsa-let-7e-5p;hsa-l
ENSG0000	ENSG0000	0.005416	hsa-miR-15a-5p;hsa-miR-15b-5p;hsa-miR-16-5p;hsa-miR-195-
ENSG0000	ENSG0000	7.08E-05	hsa-miR-15a-5p;hsa-miR-15b-5p;hsa-miR-16-5p;hsa-miR-195-
ENSG0000	ENSG0000	0.000429	hsa-let-7a-5p;hsa-let-7b-5p;hsa-let-7d-5p;hsa-let-7e-5p;hsa-l
ENSG0000	ENSG0000	0.003016	hsa-let-7a-5p;hsa-let-7b-5p;hsa-let-7d-5p;hsa-let-7e-5p;hsa-l
ENSG0000	ENSG0000	5.94E-06	hsa-let-7a-5p;hsa-let-7b-5p;hsa-let-7d-5p;hsa-let-7e-5p;hsa-l
ENSG0000	ENSG0000	0.007579	hsa-miR-1304-3p;hsa-miR-141-3p;hsa-miR-143-5p;hsa-miR-2
ENSG0000	ENSG0000	0.002179	hsa-let-7a-5p;hsa-let-7b-5p;hsa-let-7d-5p;hsa-let-7e-5p;hsa-l
ENSG0000	ENSG0000	0.002179	hsa-let-7a-5p;hsa-let-7b-5p;hsa-let-7d-5p;hsa-let-7e-5p;hsa-l
ENSG0000	ENSG0000	0.00249	hsa-miR-15a-5p;hsa-miR-15b-5p;hsa-miR-195-5p;hsa-miR-42
ENSG0000	ENSG0000	5.28E-06	hsa-let-7a-5p;hsa-let-7b-5p;hsa-let-7d-5p;hsa-let-7e-5p;hsa-l
ENSG0000	ENSG0000	0.00273	hsa-miR-103a-3p;hsa-miR-107;hsa-miR-15a-5p;hsa-miR-15b-5
ENSG0000	ENSG0000	0.000572	hsa-miR-107;hsa-miR-15a-5p;hsa-miR-15b-5p;hsa-miR-195-5p
ENSG0000	ENSG0000	0.001803	hsa-miR-106b-5p;hsa-miR-130a-3p;hsa-miR-130b-3p;hsa-miR
ENSG0000	ENSG0000	0.000101	hsa-miR-130b-3p;hsa-miR-141-3p;hsa-miR-200a-3p;hsa-miR-2
ENSG0000	ENSG0000	3.76E-05	hsa-miR-15a-5p;hsa-miR-15b-5p;hsa-miR-195-5p;hsa-miR-21
ENSG0000	ENSG0000	2.34E-11	hsa-let-7a-5p;hsa-let-7b-5p;hsa-let-7d-5p;hsa-let-7e-5p;hsa-l
ENSG0000	ENSG0000	2.95E-05	hsa-miR-103a-3p;hsa-miR-1-3p;hsa-miR-181b-5p;hsa-miR-22
ENSG0000	ENSG0000	7.08E-05	hsa-miR-15a-5p;hsa-miR-15b-5p;hsa-miR-16-5p;hsa-miR-195-
ENSG0000	ENSG0000	9.36E-05	hsa-let-7a-5p;hsa-let-7b-5p;hsa-let-7d-5p;hsa-let-7e-5p;hsa-l
ENSG0000	ENSG0000	0.000101	hsa-miR-1-3p;hsa-miR-15a-5p;hsa-miR-15b-5p;hsa-miR-16-5p
ENSG0000	ENSG0000	0.000345	hsa-miR-1-3p;hsa-miR-15a-5p;hsa-miR-15b-5p;hsa-miR-16-5p
ENSG0000	ENSG0000	0.000619	hsa-miR-106b-5p;hsa-miR-15a-5p;hsa-miR-15b-5p;hsa-miR-1

ENSG0000	ENSG0000	0.005165	hsa-miR-106b-5p;hsa-miR-181a-5p;hsa-miR-181b-5p;hsa-miR
ENSG0000	ENSG0000	1.57E-08	hsa-let-7a-5p;hsa-let-7b-5p;hsa-let-7d-5p;hsa-let-7e-5p;hsa-l
ENSG0000	ENSG0000	0.004251	hsa-miR-1-3p;hsa-miR-151a-3p;hsa-miR-214-3p;hsa-miR-362-
ENSG0000	ENSG0000	0.004251	hsa-miR-106b-5p;hsa-miR-130a-3p;hsa-miR-130b-3p;hsa-miR
ENSG0000	ENSG0000	3.61E-05	hsa-miR-200b-3p;hsa-miR-200c-3p;hsa-miR-27a-3p;hsa-miR-2
ENSG0000	ENSG0000	0.000802	hsa-miR-15a-5p;hsa-miR-15b-5p;hsa-miR-16-5p;hsa-miR-17-
ENSG0000	ENSG0000	0.000278	hsa-miR-1224-5p;hsa-miR-140-3p;hsa-miR-15a-5p;hsa-miR-1
ENSG0000	ENSG0000	0.005964	hsa-let-7a-5p;hsa-let-7b-5p;hsa-let-7d-5p;hsa-let-7e-5p;hsa-l
ENSG0000	ENSG0000	0.000429	hsa-miR-1304-3p;hsa-miR-15a-5p;hsa-miR-15b-5p;hsa-miR-1
ENSG0000	ENSG0000	0.004383	hsa-let-7b-5p;hsa-miR-324-5p;hsa-miR-362-3p;hsa-miR-378a-
ENSG0000	ENSG0000	6.95E-07	hsa-miR-15a-5p;hsa-miR-15b-5p;hsa-miR-16-5p;hsa-miR-195-
ENSG0000	ENSG0000	3.82E-07	hsa-let-7a-5p;hsa-let-7b-5p;hsa-let-7d-5p;hsa-let-7e-5p;hsa-l
ENSG0000	ENSG0000	2.90E-11	hsa-miR-106b-5p;hsa-miR-107;hsa-miR-125a-5p;hsa-miR-1-3
ENSG0000	ENSG0000	3.39E-05	hsa-miR-106b-5p;hsa-miR-125b-2-3p;hsa-miR-140-3p;hsa-mi
ENSG0000	ENSG0000	0.00148	hsa-let-7a-5p;hsa-let-7b-5p;hsa-let-7d-5p;hsa-let-7e-5p;hsa-l
ENSG0000	ENSG0000	0.002947	hsa-let-7a-5p;hsa-let-7b-5p;hsa-let-7d-5p;hsa-let-7e-5p;hsa-l
ENSG0000	ENSG0000	0.001057	hsa-miR-15a-5p;hsa-miR-15b-5p;hsa-miR-16-5p;hsa-miR-195-
ENSG0000	ENSG0000	0.001135	hsa-let-7a-5p;hsa-let-7b-5p;hsa-let-7e-5p;hsa-let-7f-5p;hsa-le
ENSG0000	ENSG0000	0.001817	hsa-miR-106b-5p;hsa-miR-15a-5p;hsa-miR-15b-5p;hsa-miR-1
ENSG0000	ENSG0000	0.001205	hsa-let-7b-5p;hsa-miR-188-3p;hsa-miR-342-3p;hsa-miR-532-
ENSG0000	ENSG0000	3.08E-06	hsa-let-7a-5p;hsa-let-7b-5p;hsa-let-7d-5p;hsa-let-7e-5p;hsa-l
ENSG0000	ENSG0000	2.42E-05	hsa-let-7a-5p;hsa-let-7b-5p;hsa-let-7d-5p;hsa-let-7e-5p;hsa-l
ENSG0000	ENSG0000	0.006079	hsa-miR-130a-3p;hsa-miR-130b-3p;hsa-miR-151a-3p;hsa-miR-
ENSG0000	ENSG0000	0.000226	hsa-miR-200b-3p;hsa-miR-200c-3p;hsa-miR-374a-5p;hsa-miR-
ENSG0000	ENSG0000	4.49E-06	hsa-let-7a-5p;hsa-let-7b-5p;hsa-let-7d-5p;hsa-let-7e-5p;hsa-l
ENSG0000	ENSG0000	0.003957	hsa-miR-140-3p;hsa-miR-15a-5p;hsa-miR-15b-5p;hsa-miR-18
ENSG0000	ENSG0000	5.94E-06	hsa-let-7a-5p;hsa-let-7b-5p;hsa-let-7d-5p;hsa-let-7e-5p;hsa-l
ENSG0000	ENSG0000	2.09E-06	hsa-miR-15a-5p;hsa-miR-15b-5p;hsa-miR-16-5p;hsa-miR-195-
ENSG0000	ENSG0000	1.09E-05	hsa-miR-107;hsa-miR-15a-5p;hsa-miR-15b-5p;hsa-miR-16-5p;
ENSG0000	ENSG0000	0.004383	hsa-miR-141-3p;hsa-miR-185-3p;hsa-miR-200a-3p;hsa-miR-2
ENSG0000	ENSG0000	2.72E-10	hsa-let-7a-5p;hsa-let-7b-5p;hsa-let-7d-5p;hsa-let-7e-5p;hsa-l
ENSG0000	ENSG0000	0.005101	hsa-miR-15a-5p;hsa-miR-15b-5p;hsa-miR-195-5p;hsa-miR-42
ENSG0000	ENSG0000	1.73E-08	hsa-let-7a-5p;hsa-let-7b-5p;hsa-let-7d-5p;hsa-let-7e-5p;hsa-l
ENSG0000	ENSG0000	1.15E-05	hsa-miR-130a-3p;hsa-miR-130b-3p;hsa-miR-15a-5p;hsa-miR-
ENSG0000	ENSG0000	4.93E-05	hsa-miR-15a-5p;hsa-miR-15b-5p;hsa-miR-16-5p;hsa-miR-195-
ENSG0000	ENSG0000	5.17E-05	hsa-miR-15a-5p;hsa-miR-15b-5p;hsa-miR-16-5p;hsa-miR-181
ENSG0000	ENSG0000	0.000117	hsa-miR-15a-5p;hsa-miR-15b-5p;hsa-miR-195-5p;hsa-miR-42
ENSG0000	ENSG0000	0.001217	hsa-miR-15a-5p;hsa-miR-15b-5p;hsa-miR-16-5p;hsa-miR-195-
ENSG0000	ENSG0000	0.00055	hsa-miR-15a-5p;hsa-miR-15b-5p;hsa-miR-16-5p;hsa-miR-181
ENSG0000	ENSG0000	0.0042	hsa-miR-199a-3p;hsa-miR-222-5p;hsa-miR-323a-3p;hsa-miR-
ENSG0000	ENSG0000	0.000485	hsa-let-7a-5p;hsa-let-7b-5p;hsa-let-7d-5p;hsa-let-7e-5p;hsa-l
ENSG0000	ENSG0000	0.00273	hsa-miR-103a-3p;hsa-miR-106b-5p;hsa-miR-107;hsa-miR-17-
ENSG0000	ENSG0000	0.00374	hsa-miR-130a-3p;hsa-miR-130b-3p;hsa-miR-301a-3p;hsa-miR-
ENSG0000	ENSG0000	1.48E-07	hsa-miR-15a-5p;hsa-miR-15b-5p;hsa-miR-181a-5p;hsa-miR-1
ENSG0000	ENSG0000	0.000191	hsa-miR-106b-5p;hsa-miR-125a-5p;hsa-miR-125b-5p;hsa-miR
ENSG0000	ENSG0000	0.000226	hsa-miR-15a-5p;hsa-miR-15b-5p;hsa-miR-16-5p;hsa-miR-195-
ENSG0000	ENSG0000	0.000558	hsa-miR-106b-5p;hsa-miR-127-5p;hsa-miR-181a-5p;hsa-miR-
ENSG0000	ENSG0000	0.001344	hsa-miR-106b-5p;hsa-miR-20a-5p;hsa-miR-27a-3p;hsa-miR-2
ENSG0000	ENSG0000	0.001344	hsa-miR-106b-5p;hsa-miR-20a-5p;hsa-miR-27a-3p;hsa-miR-2
ENSG0000	ENSG0000	0.001344	hsa-miR-106b-5p;hsa-miR-20a-5p;hsa-miR-27a-3p;hsa-miR-2
ENSG0000	ENSG0000	0.001344	hsa-miR-106b-5p;hsa-miR-20a-5p;hsa-miR-27a-3p;hsa-miR-2
ENSG0000	ENSG0000	0.001344	hsa-miR-106b-5p;hsa-miR-20a-5p;hsa-miR-27a-3p;hsa-miR-2
ENSG0000	ENSG0000	0.001344	hsa-miR-106b-5p;hsa-miR-20a-5p;hsa-miR-27a-3p;hsa-miR-2
ENSG0000	ENSG0000	0.000102	hsa-let-7a-5p;hsa-let-7b-5p;hsa-let-7d-5p;hsa-let-7e-5p;hsa-l

ENSG0000	ENSG0000	0.000104	hsa-miR-15a-5p;hsa-miR-15b-5p;hsa-miR-18a-3p;hsa-miR-19
ENSG0000	ENSG0000	2.09E-06	hsa-miR-15a-5p;hsa-miR-15b-5p;hsa-miR-16-5p;hsa-miR-195-
ENSG0000	ENSG0000	2.08E-05	hsa-let-7a-5p;hsa-let-7b-5p;hsa-let-7d-5p;hsa-let-7e-5p;hsa-l
ENSG0000	ENSG0000	0.007752	hsa-miR-1304-3p;hsa-miR-15a-5p;hsa-miR-16-5p;hsa-miR-24-
ENSG0000	ENSG0000	1.90E-08	hsa-let-7a-5p;hsa-let-7d-5p;hsa-let-7e-5p;hsa-let-7f-5p;hsa-le
ENSG0000	ENSG0000	0.001666	hsa-miR-103a-3p;hsa-miR-107;hsa-miR-15a-5p;hsa-miR-15b-5
ENSG0000	ENSG0000	1.15E-05	hsa-let-7a-5p;hsa-let-7b-5p;hsa-let-7d-5p;hsa-let-7e-5p;hsa-l
ENSG0000	ENSG0000	0.001205	hsa-miR-15a-5p;hsa-miR-15b-5p;hsa-miR-16-5p;hsa-miR-195-
ENSG0000	ENSG0000	0.000226	hsa-miR-130a-3p;hsa-miR-130b-3p;hsa-miR-301a-3p;hsa-miR-
ENSG0000	ENSG0000	5.65E-05	hsa-miR-15a-5p;hsa-miR-15b-5p;hsa-miR-195-5p;hsa-miR-20
ENSG0000	ENSG0000	7.60E-07	hsa-let-7a-5p;hsa-let-7b-5p;hsa-let-7d-5p;hsa-let-7e-5p;hsa-l
ENSG0000	ENSG0000	3.91E-06	hsa-miR-15a-5p;hsa-miR-15b-5p;hsa-miR-16-5p;hsa-miR-195-
ENSG0000	ENSG0000	0.001337	hsa-let-7a-5p;hsa-let-7b-5p;hsa-let-7d-5p;hsa-let-7e-5p;hsa-l
ENSG0000	ENSG0000	0.001555	hsa-miR-103a-3p;hsa-miR-107;hsa-miR-15a-5p;hsa-miR-15b-5
ENSG0000	ENSG0000	8.44E-05	hsa-miR-103a-3p;hsa-miR-107;hsa-miR-15a-5p;hsa-miR-15b-5
ENSG0000	ENSG0000	3.24E-09	hsa-miR-103a-3p;hsa-miR-107;hsa-miR-130a-3p;hsa-miR-130b
ENSG0000	ENSG0000	0.00013	hsa-miR-15a-5p;hsa-miR-15b-5p;hsa-miR-16-5p;hsa-miR-195-
ENSG0000	ENSG0000	0.000535	hsa-let-7a-5p;hsa-let-7e-5p;hsa-let-7f-5p;hsa-let-7g-5p;hsa-le
ENSG0000	ENSG0000	0.000883	hsa-miR-103a-3p;hsa-miR-107;hsa-miR-15a-5p;hsa-miR-15b-5
ENSG0000	ENSG0000	0.002661	hsa-miR-127-5p;hsa-miR-15a-5p;hsa-miR-15b-5p;hsa-miR-16-
ENSG0000	ENSG0000	0.009977	hsa-miR-103a-3p;hsa-miR-107;hsa-miR-143-5p;hsa-miR-362-3
ENSG0000	ENSG0000	0.000408	hsa-miR-103a-3p;hsa-miR-107;hsa-miR-1304-3p;hsa-miR-214-
ENSG0000	ENSG0000	8.86E-07	hsa-let-7a-5p;hsa-let-7b-5p;hsa-let-7d-5p;hsa-let-7e-5p;hsa-l
ENSG0000	ENSG0000	0.000486	hsa-let-7a-5p;hsa-let-7b-5p;hsa-let-7d-5p;hsa-let-7e-5p;hsa-l
ENSG0000	ENSG0000	0.00029	hsa-miR-106b-5p;hsa-miR-107;hsa-miR-1224-5p;hsa-miR-15a-
ENSG0000	ENSG0000	0.001604	hsa-let-7a-5p;hsa-let-7b-5p;hsa-let-7d-5p;hsa-let-7e-5p;hsa-l
ENSG0000	ENSG0000	0.001604	hsa-let-7a-5p;hsa-let-7b-5p;hsa-let-7d-5p;hsa-let-7e-5p;hsa-l
ENSG0000	ENSG0000	0.000286	hsa-let-7a-5p;hsa-let-7b-5p;hsa-let-7d-5p;hsa-let-7e-5p;hsa-l
ENSG0000	ENSG0000	1.63E-06	hsa-miR-15a-5p;hsa-miR-15b-5p;hsa-miR-16-5p;hsa-miR-195-
ENSG0000	ENSG0000	6.62E-06	hsa-miR-15a-5p;hsa-miR-15b-5p;hsa-miR-16-5p;hsa-miR-195-
ENSG0000	ENSG0000	0.000883	hsa-let-7a-5p;hsa-let-7b-5p;hsa-let-7d-5p;hsa-let-7e-5p;hsa-l
ENSG0000	ENSG0000	9.61E-07	hsa-miR-107;hsa-miR-15a-5p;hsa-miR-15b-5p;hsa-miR-195-5p
ENSG0000	ENSG0000	3.70E-05	hsa-let-7a-5p;hsa-let-7b-5p;hsa-let-7d-5p;hsa-let-7e-5p;hsa-l
ENSG0000	ENSG0000	0.003744	hsa-miR-15a-5p;hsa-miR-15b-5p;hsa-miR-16-5p;hsa-miR-195-
ENSG0000	ENSG0000	2.53E-07	hsa-let-7a-5p;hsa-let-7b-5p;hsa-let-7d-5p;hsa-let-7e-5p;hsa-l
ENSG0000	ENSG0000	3.91E-06	hsa-let-7d-5p;hsa-miR-141-3p;hsa-miR-200a-3p;hsa-miR-200b
ENSG0000	ENSG0000	0.001851	hsa-miR-106b-5p;hsa-miR-130a-3p;hsa-miR-130b-3p;hsa-miR
ENSG0000	ENSG0000	0.001851	hsa-miR-106b-5p;hsa-miR-130a-3p;hsa-miR-130b-3p;hsa-miR
ENSG0000	ENSG0000	0.000595	hsa-let-7a-5p;hsa-let-7b-5p;hsa-let-7d-5p;hsa-let-7e-5p;hsa-l
ENSG0000	ENSG0000	0.004383	hsa-miR-15a-5p;hsa-miR-15b-5p;hsa-miR-195-5p;hsa-miR-42
ENSG0000	ENSG0000	0.0041	hsa-miR-130a-3p;hsa-miR-130b-3p;hsa-miR-301a-3p;hsa-miR-
ENSG0000	ENSG0000	6.93E-05	hsa-miR-140-3p;hsa-miR-15a-5p;hsa-miR-15b-5p;hsa-miR-16-
ENSG0000	ENSG0000	0.000429	hsa-miR-107;hsa-miR-143-5p;hsa-miR-181a-5p;hsa-miR-181b-
ENSG0000	ENSG0000	0.000792	hsa-miR-106b-5p;hsa-miR-130a-3p;hsa-miR-130b-3p;hsa-miR
ENSG0000	ENSG0000	8.40E-11	hsa-let-7a-5p;hsa-let-7b-5p;hsa-let-7d-5p;hsa-let-7e-5p;hsa-l
ENSG0000	ENSG0000	0.000366	hsa-miR-15a-5p;hsa-miR-15b-5p;hsa-miR-16-5p;hsa-miR-195-
ENSG0000	ENSG0000	2.17E-06	hsa-miR-15a-5p;hsa-miR-15b-5p;hsa-miR-16-5p;hsa-miR-195-
ENSG0000	ENSG0000	0.001851	hsa-miR-15a-5p;hsa-miR-15b-5p;hsa-miR-195-5p;hsa-miR-42
ENSG0000	ENSG0000	5.29E-05	hsa-miR-15a-5p;hsa-miR-15b-5p;hsa-miR-16-5p;hsa-miR-195-
ENSG0000	ENSG0000	5.29E-05	hsa-miR-15a-5p;hsa-miR-15b-5p;hsa-miR-16-5p;hsa-miR-195-
ENSG0000	ENSG0000	0.000184	hsa-miR-106b-5p;hsa-miR-140-5p;hsa-miR-181a-5p;hsa-miR-1
ENSG0000	ENSG0000	0.005165	hsa-miR-140-3p;hsa-miR-200b-3p;hsa-miR-200c-3p;hsa-miR-4
ENSG0000	ENSG0000	0.003324	hsa-miR-106b-5p;hsa-miR-181a-5p;hsa-miR-181b-5p;hsa-miR
ENSG0000	ENSG0000	0.006079	hsa-miR-130a-3p;hsa-miR-130b-3p;hsa-miR-199a-3p;hsa-miR-

ENSG0000	ENSG0000	0.00017	hsa-miR-15a-5p;hsa-miR-15b-5p;hsa-miR-195-5p;hsa-miR-43
ENSG0000	ENSG0000	0.000342	hsa-miR-15a-5p;hsa-miR-15b-5p;hsa-miR-23a-3p;hsa-miR-23
ENSG0000	ENSG0000	0.007516	hsa-miR-181a-5p;hsa-miR-181b-5p;hsa-miR-181c-5p;hsa-miR
ENSG0000	ENSG0000	1.12E-06	hsa-let-7a-5p;hsa-let-7b-5p;hsa-let-7d-5p;hsa-let-7e-5p;hsa-l
ENSG0000	ENSG0000	0.0011	hsa-miR-103a-3p;hsa-miR-106b-5p;hsa-miR-107;hsa-miR-130
ENSG0000	ENSG0000	0.002651	hsa-miR-130a-3p;hsa-miR-130b-3p;hsa-miR-301a-3p;hsa-miR
ENSG0000	ENSG0000	0.000792	hsa-let-7a-5p;hsa-let-7b-5p;hsa-let-7e-5p;hsa-let-7f-5p;hsa-le
ENSG0000	ENSG0000	0.000518	hsa-miR-15a-5p;hsa-miR-15b-5p;hsa-miR-16-5p;hsa-miR-195
ENSG0000	ENSG0000	0.007752	hsa-miR-15a-5p;hsa-miR-15b-5p;hsa-miR-16-5p;hsa-miR-195
ENSG0000	ENSG0000	6.62E-06	hsa-miR-15a-5p;hsa-miR-15b-5p;hsa-miR-16-5p;hsa-miR-195
ENSG0000	ENSG0000	8.44E-05	hsa-let-7a-5p;hsa-let-7b-5p;hsa-let-7d-5p;hsa-let-7e-5p;hsa-l
ENSG0000	ENSG0000	0.000485	hsa-miR-15a-5p;hsa-miR-15b-5p;hsa-miR-16-5p;hsa-miR-195
ENSG0000	ENSG0000	0.001135	hsa-miR-15b-5p;hsa-miR-16-5p;hsa-miR-194-3p;hsa-miR-195
ENSG0000	ENSG0000	0.007516	hsa-miR-15a-5p;hsa-miR-15b-5p;hsa-miR-16-5p;hsa-miR-195
ENSG0000	ENSG0000	0.007516	hsa-miR-15a-5p;hsa-miR-15b-5p;hsa-miR-16-5p;hsa-miR-195
ENSG0000	ENSG0000	0.001548	hsa-miR-15a-5p;hsa-miR-15b-5p;hsa-miR-16-5p;hsa-miR-195
ENSG0000	ENSG0000	0.005568	hsa-let-7a-5p;hsa-let-7b-5p;hsa-let-7d-5p;hsa-let-7e-5p;hsa-l
ENSG0000	ENSG0000	0.000125	hsa-let-7a-5p;hsa-let-7b-5p;hsa-let-7d-5p;hsa-let-7e-5p;hsa-l
ENSG0000	ENSG0000	0.004383	hsa-miR-15a-5p;hsa-miR-15b-5p;hsa-miR-195-5p;hsa-miR-42
ENSG0000	ENSG0000	0.003278	hsa-miR-15a-5p;hsa-miR-15b-5p;hsa-miR-16-5p;hsa-miR-18a
ENSG0000	ENSG0000	2.70E-07	hsa-let-7a-5p;hsa-let-7b-5p;hsa-let-7d-5p;hsa-let-7e-5p;hsa-l
ENSG0000	ENSG0000	0.008423	hsa-miR-15a-5p;hsa-miR-15b-5p;hsa-miR-16-5p;hsa-miR-195
ENSG0000	ENSG0000	6.42E-05	hsa-let-7a-5p;hsa-let-7b-5p;hsa-let-7d-5p;hsa-let-7e-5p;hsa-l
ENSG0000	ENSG0000	9.26E-05	hsa-let-7a-5p;hsa-let-7b-5p;hsa-let-7d-5p;hsa-let-7e-5p;hsa-l
ENSG0000	ENSG0000	9.26E-05	hsa-let-7a-5p;hsa-let-7b-5p;hsa-let-7d-5p;hsa-let-7e-5p;hsa-l
ENSG0000	ENSG0000	0.001139	hsa-miR-181a-5p;hsa-miR-181b-5p;hsa-miR-181c-5p;hsa-miR
ENSG0000	ENSG0000	0.006267	hsa-miR-103a-3p;hsa-miR-107;hsa-miR-23a-3p;hsa-miR-23b-
ENSG0000	ENSG0000	1.11E-06	hsa-miR-15a-5p;hsa-miR-15b-5p;hsa-miR-16-5p;hsa-miR-195
ENSG0000	ENSG0000	3.25E-06	hsa-let-7a-5p;hsa-let-7b-5p;hsa-let-7d-5p;hsa-let-7e-5p;hsa-l
ENSG0000	ENSG0000	0.004552	hsa-miR-15a-5p;hsa-miR-15b-5p;hsa-miR-16-5p;hsa-miR-195
ENSG0000	ENSG0000	0.000284	hsa-miR-1-3p;hsa-miR-15a-5p;hsa-miR-15b-5p;hsa-miR-16-5
ENSG0000	ENSG0000	0.000284	hsa-miR-1-3p;hsa-miR-15a-5p;hsa-miR-15b-5p;hsa-miR-16-5
ENSG0000	ENSG0000	0.009842	hsa-miR-103a-3p;hsa-miR-107;hsa-miR-130a-3p;hsa-miR-130
ENSG0000	ENSG0000	0.004807	hsa-miR-130a-3p;hsa-miR-140-3p;hsa-miR-23a-3p;hsa-miR-2
ENSG0000	ENSG0000	0.002216	hsa-miR-1304-3p;hsa-miR-15a-5p;hsa-miR-15b-5p;hsa-miR-1
ENSG0000	ENSG0000	2.49E-05	hsa-miR-1-3p;hsa-miR-151a-3p;hsa-miR-15a-5p;hsa-miR-15b
ENSG0000	ENSG0000	5.28E-06	hsa-miR-181a-5p;hsa-miR-181b-5p;hsa-miR-181c-5p;hsa-miR
ENSG0000	ENSG0000	4.97E-07	hsa-miR-15a-5p;hsa-miR-15b-5p;hsa-miR-188-3p;hsa-miR-19
ENSG0000	ENSG0000	0.000101	hsa-miR-15a-5p;hsa-miR-15b-5p;hsa-miR-16-5p;hsa-miR-195
ENSG0000	ENSG0000	0.0074	hsa-miR-106b-5p;hsa-miR-143-5p;hsa-miR-199a-3p;hsa-miR-
ENSG0000	ENSG0000	4.95E-07	hsa-let-7a-5p;hsa-let-7b-5p;hsa-let-7d-5p;hsa-let-7e-5p;hsa-l
ENSG0000	ENSG0000	4.95E-07	hsa-let-7a-5p;hsa-let-7b-5p;hsa-let-7d-5p;hsa-let-7e-5p;hsa-l
ENSG0000	ENSG0000	4.49E-06	hsa-miR-103a-3p;hsa-miR-107;hsa-miR-15a-5p;hsa-miR-15b-
ENSG0000	ENSG0000	0.000126	hsa-miR-15a-5p;hsa-miR-15b-5p;hsa-miR-16-5p;hsa-miR-195
ENSG0000	ENSG0000	0.003168	hsa-miR-18a-3p;hsa-miR-1913;hsa-miR-3157-3p;hsa-miR-339
ENSG0000	ENSG0000	7.31E-06	hsa-miR-103a-3p;hsa-miR-107;hsa-miR-15a-5p;hsa-miR-15b-
ENSG0000	ENSG0000	7.31E-06	hsa-miR-103a-3p;hsa-miR-107;hsa-miR-15a-5p;hsa-miR-15b-
ENSG0000	ENSG0000	0.001793	hsa-miR-107;hsa-miR-15a-5p;hsa-miR-15b-5p;hsa-miR-188-3
ENSG0000	ENSG0000	0.002866	hsa-miR-15a-5p;hsa-miR-15b-5p;hsa-miR-16-5p;hsa-miR-195
ENSG0000	ENSG0000	0.000443	hsa-miR-27a-3p;hsa-miR-27b-3p;hsa-miR-3065-3p;hsa-miR-3
ENSG0000	ENSG0000	0.000518	hsa-let-7a-5p;hsa-let-7b-5p;hsa-let-7d-5p;hsa-let-7e-5p;hsa-l
ENSG0000	ENSG0000	8.61E-06	hsa-miR-1-3p;hsa-miR-15a-5p;hsa-miR-15b-5p;hsa-miR-16-5
ENSG0000	ENSG0000	2.49E-05	hsa-miR-103a-3p;hsa-miR-107;hsa-miR-15a-5p;hsa-miR-15b-
ENSG0000	ENSG0000	0.008919	hsa-let-7b-5p;hsa-miR-27a-3p;hsa-miR-27b-3p;hsa-miR-323a

ENSG0000	ENSG0000	4.04E-06	hsa-miR-106b-5p;hsa-miR-107;hsa-miR-1304-3p;hsa-miR-15a-
ENSG0000	ENSG0000	0.000169	hsa-miR-106b-5p;hsa-miR-1304-3p;hsa-miR-140-3p;hsa-miR-
ENSG0000	ENSG0000	0.000169	hsa-miR-106b-5p;hsa-miR-1304-3p;hsa-miR-140-3p;hsa-miR-
ENSG0000	ENSG0000	3.91E-06	hsa-miR-15a-5p;hsa-miR-15b-5p;hsa-miR-16-5p;hsa-miR-195-
ENSG0000	ENSG0000	1.68E-06	hsa-miR-106b-5p;hsa-miR-15a-5p;hsa-miR-15b-5p;hsa-miR-1
ENSG0000	ENSG0000	0.003683	hsa-miR-2467-5p;hsa-miR-27a-3p;hsa-miR-27b-3p;hsa-miR-3
ENSG0000	ENSG0000	1.14E-06	hsa-miR-103a-3p;hsa-miR-107;hsa-miR-15a-5p;hsa-miR-15b-
ENSG0000	ENSG0000	0.0074	hsa-let-7a-5p;hsa-let-7b-5p;hsa-let-7d-5p;hsa-let-7e-5p;hsa-l
ENSG0000	ENSG0000	4.32E-08	hsa-miR-103a-3p;hsa-miR-107;hsa-miR-143-5p;hsa-miR-15a-
ENSG0000	ENSG0000	0.000771	hsa-let-7a-5p;hsa-let-7b-5p;hsa-let-7d-5p;hsa-let-7e-5p;hsa-l
ENSG0000	ENSG0000	1.92E-06	hsa-miR-15a-5p;hsa-miR-15b-5p;hsa-miR-16-5p;hsa-miR-195-
ENSG0000	ENSG0000	2.21E-11	hsa-let-7a-5p;hsa-let-7b-5p;hsa-let-7d-5p;hsa-let-7e-5p;hsa-l
ENSG0000	ENSG0000	3.69E-08	hsa-miR-15a-5p;hsa-miR-15b-5p;hsa-miR-16-5p;hsa-miR-195-
ENSG0000	ENSG0000	0.008919	hsa-miR-15a-5p;hsa-miR-15b-5p;hsa-miR-16-5p;hsa-miR-195-
ENSG0000	ENSG0000	0.007516	hsa-miR-130a-3p;hsa-miR-130b-3p;hsa-miR-301a-3p;hsa-miR-
ENSG0000	ENSG0000	0.001548	hsa-miR-15a-5p;hsa-miR-15b-5p;hsa-miR-195-5p;hsa-miR-34
ENSG0000	ENSG0000	0.000429	hsa-miR-130a-3p;hsa-miR-130b-3p;hsa-miR-200b-3p;hsa-miR
ENSG0000	ENSG0000	0.000429	hsa-let-7a-5p;hsa-let-7b-5p;hsa-let-7d-5p;hsa-let-7e-5p;hsa-l
ENSG0000	ENSG0000	0.001851	hsa-miR-15a-5p;hsa-miR-15b-5p;hsa-miR-16-5p;hsa-miR-195-
ENSG0000	ENSG0000	1.32E-05	hsa-let-7a-5p;hsa-let-7d-5p;hsa-miR-130a-3p;hsa-miR-15a-5p
ENSG0000	ENSG0000	9.60E-05	hsa-miR-181a-5p;hsa-miR-181b-5p;hsa-miR-181c-5p;hsa-miR-
ENSG0000	ENSG0000	4.50E-07	hsa-miR-141-3p;hsa-miR-200a-3p;hsa-miR-200b-3p;hsa-miR-
ENSG0000	ENSG0000	0.007516	hsa-miR-130a-3p;hsa-miR-130b-3p;hsa-miR-301a-3p;hsa-miR-
ENSG0000	ENSG0000	1.09E-05	hsa-miR-125b-2-3p;hsa-miR-141-3p;hsa-miR-15a-5p;hsa-miR-
ENSG0000	ENSG0000	9.36E-05	hsa-miR-103a-3p;hsa-miR-107;hsa-miR-15a-5p;hsa-miR-15b-
ENSG0000	ENSG0000	0.00273	hsa-let-7d-5p;hsa-let-7e-5p;hsa-let-7f-5p;hsa-let-7g-5p;hsa-le
ENSG0000	ENSG0000	0.00273	hsa-let-7d-5p;hsa-let-7e-5p;hsa-let-7f-5p;hsa-let-7g-5p;hsa-le
ENSG0000	ENSG0000	0.001955	hsa-let-7a-5p;hsa-let-7b-5p;hsa-let-7d-5p;hsa-let-7e-5p;hsa-l
ENSG0000	ENSG0000	0.0074	hsa-let-7a-5p;hsa-let-7b-5p;hsa-let-7d-5p;hsa-let-7e-5p;hsa-l
ENSG0000	ENSG0000	0.007959	hsa-let-7b-5p;hsa-miR-130a-3p;hsa-miR-130b-3p;hsa-miR-22
ENSG0000	ENSG0000	7.57E-07	hsa-miR-15a-5p;hsa-miR-15b-5p;hsa-miR-16-5p;hsa-miR-195-
ENSG0000	ENSG0000	0.001851	hsa-miR-15a-5p;hsa-miR-15b-5p;hsa-miR-195-5p;hsa-miR-42
ENSG0000	ENSG0000	7.83E-05	hsa-miR-15a-5p;hsa-miR-15b-5p;hsa-miR-16-5p;hsa-miR-191
ENSG0000	ENSG0000	0.002329	hsa-let-7a-5p;hsa-let-7b-5p;hsa-let-7d-5p;hsa-let-7e-5p;hsa-l
ENSG0000	ENSG0000	0.00017	hsa-miR-15a-5p;hsa-miR-15b-5p;hsa-miR-195-5p;hsa-miR-42
ENSG0000	ENSG0000	0.001139	hsa-miR-15a-5p;hsa-miR-15b-5p;hsa-miR-16-5p;hsa-miR-195-
ENSG0000	ENSG0000	0.006781	hsa-miR-181a-5p;hsa-miR-181b-5p;hsa-miR-181c-5p;hsa-miR-
ENSG0000	ENSG0000	0.000378	hsa-miR-103a-3p;hsa-miR-107;hsa-miR-15a-5p;hsa-miR-15b-
ENSG0000	ENSG0000	1.18E-07	hsa-miR-15a-5p;hsa-miR-15b-5p;hsa-miR-195-5p;hsa-miR-21
ENSG0000	ENSG0000	6.07E-06	hsa-miR-15a-5p;hsa-miR-15b-5p;hsa-miR-181a-5p;hsa-miR-1
ENSG0000	ENSG0000	0.003282	hsa-miR-15a-5p;hsa-miR-15b-5p;hsa-miR-18a-3p;hsa-miR-19
ENSG0000	ENSG0000	0.000276	hsa-miR-15a-5p;hsa-miR-15b-5p;hsa-miR-16-5p;hsa-miR-195-
ENSG0000	ENSG0000	2.09E-06	hsa-miR-15a-5p;hsa-miR-15b-5p;hsa-miR-16-5p;hsa-miR-195-
ENSG0000	ENSG0000	4.88E-05	hsa-miR-1304-3p;hsa-miR-143-5p;hsa-miR-15a-5p;hsa-miR-1
ENSG0000	ENSG0000	2.08E-05	hsa-miR-15a-5p;hsa-miR-15b-5p;hsa-miR-16-5p;hsa-miR-195-
ENSG0000	ENSG0000	6.93E-05	hsa-miR-15a-5p;hsa-miR-15b-5p;hsa-miR-16-5p;hsa-miR-181
ENSG0000	ENSG0000	3.68E-09	hsa-let-7a-5p;hsa-let-7b-5p;hsa-let-7d-5p;hsa-let-7e-5p;hsa-l
ENSG0000	ENSG0000	3.68E-09	hsa-let-7a-5p;hsa-let-7b-5p;hsa-let-7d-5p;hsa-let-7e-5p;hsa-l
ENSG0000	ENSG0000	3.68E-09	hsa-let-7a-5p;hsa-let-7b-5p;hsa-let-7d-5p;hsa-let-7e-5p;hsa-l
ENSG0000	ENSG0000	5.94E-06	hsa-let-7a-5p;hsa-let-7b-5p;hsa-let-7d-5p;hsa-let-7e-5p;hsa-l
ENSG0000	ENSG0000	0.007752	hsa-miR-1304-3p;hsa-miR-130a-3p;hsa-miR-130b-3p;hsa-miR-
ENSG0000	ENSG0000	0.009977	hsa-miR-127-5p;hsa-miR-1-3p;hsa-miR-27a-3p;hsa-miR-27b-
ENSG0000	ENSG0000	0.0081	hsa-miR-106b-5p;hsa-miR-125b-2-3p;hsa-miR-200b-3p;hsa-m
ENSG0000	ENSG0000	0.003698	hsa-let-7a-5p;hsa-let-7b-5p;hsa-let-7d-5p;hsa-let-7e-5p;hsa-l

ENSG0000	ENSG0000	0.002048	hsa-miR-103a-3p;hsa-miR-107;hsa-miR-1-3p;hsa-miR-362-3p
ENSG0000	ENSG0000	0.000235	hsa-let-7a-5p;hsa-let-7b-5p;hsa-let-7d-5p;hsa-let-7e-5p;hsa-l
ENSG0000	ENSG0000	0.000192	hsa-let-7a-5p;hsa-let-7b-5p;hsa-let-7d-5p;hsa-let-7e-5p;hsa-l
ENSG0000	ENSG0000	1.11E-06	hsa-miR-15a-5p;hsa-miR-15b-5p;hsa-miR-16-5p;hsa-miR-195-
ENSG0000	ENSG0000	7.98E-05	hsa-miR-15a-5p;hsa-miR-15b-5p;hsa-miR-195-5p;hsa-miR-20
ENSG0000	ENSG0000	1.50E-09	hsa-miR-103a-3p;hsa-miR-106b-5p;hsa-miR-107;hsa-miR-130
ENSG0000	ENSG0000	0.000535	hsa-miR-15a-5p;hsa-miR-15b-5p;hsa-miR-181b-5p;hsa-miR-1
ENSG0000	ENSG0000	0.007516	hsa-miR-200b-3p;hsa-miR-200c-3p;hsa-miR-3677-5p;hsa-miR-
ENSG0000	ENSG0000	0.000583	hsa-miR-1304-3p;hsa-miR-1913;hsa-miR-296-5p;hsa-miR-392
ENSG0000	ENSG0000	0.005899	hsa-miR-143-5p;hsa-miR-17-3p;hsa-miR-3065-3p;hsa-miR-32
ENSG0000	ENSG0000	0.000518	hsa-miR-103a-3p;hsa-miR-107;hsa-miR-15a-5p;hsa-miR-15b-
ENSG0000	ENSG0000	0.004251	hsa-miR-15a-5p;hsa-miR-15b-5p;hsa-miR-194-3p;hsa-miR-19
ENSG0000	ENSG0000	0.0042	hsa-miR-103a-3p;hsa-miR-107;hsa-miR-27a-3p;hsa-miR-27b-
ENSG0000	ENSG0000	0.000244	hsa-let-7a-5p;hsa-let-7d-5p;hsa-let-7e-5p;hsa-let-7f-5p;hsa-l
ENSG0000	ENSG0000	9.35E-05	hsa-let-7a-5p;hsa-let-7b-5p;hsa-let-7d-5p;hsa-let-7e-5p;hsa-l
ENSG0000	ENSG0000	9.35E-05	hsa-let-7a-5p;hsa-let-7b-5p;hsa-let-7d-5p;hsa-let-7e-5p;hsa-l
ENSG0000	ENSG0000	6.31E-05	hsa-let-7a-5p;hsa-let-7d-5p;hsa-let-7e-5p;hsa-let-7f-5p;hsa-l
ENSG0000	ENSG0000	8.11E-06	hsa-miR-15a-5p;hsa-miR-15b-5p;hsa-miR-16-5p;hsa-miR-195-
ENSG0000	ENSG0000	3.54E-06	hsa-miR-15a-5p;hsa-miR-15b-5p;hsa-miR-16-5p;hsa-miR-195-
ENSG0000	ENSG0000	3.91E-06	hsa-miR-15a-5p;hsa-miR-15b-5p;hsa-miR-16-5p;hsa-miR-195-
ENSG0000	ENSG0000	2.95E-05	hsa-miR-15a-5p;hsa-miR-15b-5p;hsa-miR-16-5p;hsa-miR-195-
ENSG0000	ENSG0000	0.004383	hsa-let-7a-5p;hsa-let-7e-5p;hsa-let-7f-5p;hsa-let-7g-5p;hsa-l
ENSG0000	ENSG0000	0.000112	hsa-miR-106b-5p;hsa-miR-130a-3p;hsa-miR-130b-3p;hsa-miR
ENSG0000	ENSG0000	5.28E-06	hsa-let-7a-5p;hsa-let-7b-5p;hsa-let-7d-5p;hsa-let-7e-5p;hsa-l
ENSG0000	ENSG0000	0.003744	hsa-miR-106b-5p;hsa-miR-130a-3p;hsa-miR-130b-3p;hsa-miR
ENSG0000	ENSG0000	0.003365	hsa-miR-130a-3p;hsa-miR-130b-3p;hsa-miR-301a-3p;hsa-miR-
ENSG0000	ENSG0000	1.81E-05	hsa-let-7a-5p;hsa-let-7b-5p;hsa-let-7d-5p;hsa-let-7e-5p;hsa-l
ENSG0000	ENSG0000	0.000792	hsa-miR-106b-5p;hsa-miR-181a-5p;hsa-miR-181b-5p;hsa-miR
ENSG0000	ENSG0000	0.002061	hsa-let-7a-5p;hsa-let-7b-5p;hsa-let-7d-5p;hsa-let-7e-5p;hsa-l
ENSG0000	ENSG0000	0.008919	hsa-miR-130a-3p;hsa-miR-130b-3p;hsa-miR-301a-3p;hsa-miR-
ENSG0000	ENSG0000	0.000754	hsa-miR-106b-5p;hsa-miR-130a-3p;hsa-miR-20a-5p;hsa-miR-
ENSG0000	ENSG0000	0.001548	hsa-miR-15a-5p;hsa-miR-15b-5p;hsa-miR-16-5p;hsa-miR-195-
ENSG0000	ENSG0000	0.005568	hsa-let-7a-5p;hsa-let-7b-5p;hsa-let-7d-5p;hsa-let-7e-5p;hsa-l
ENSG0000	ENSG0000	0.005568	hsa-let-7a-5p;hsa-let-7b-5p;hsa-let-7d-5p;hsa-let-7e-5p;hsa-l
ENSG0000	ENSG0000	0.001999	hsa-miR-15a-5p;hsa-miR-15b-5p;hsa-miR-16-5p;hsa-miR-195-
ENSG0000	ENSG0000	2.82E-07	hsa-let-7a-5p;hsa-let-7b-5p;hsa-let-7d-5p;hsa-let-7e-5p;hsa-l
ENSG0000	ENSG0000	0.000496	hsa-miR-106b-5p;hsa-miR-125a-5p;hsa-miR-130a-3p;hsa-miR-
ENSG0000	ENSG0000	0.00013	hsa-miR-106b-5p;hsa-miR-20a-5p;hsa-miR-27a-3p;hsa-miR-2
ENSG0000	ENSG0000	4.57E-05	hsa-let-7a-5p;hsa-let-7b-5p;hsa-let-7d-5p;hsa-let-7e-5p;hsa-l
ENSG0000	ENSG0000	0.000572	hsa-miR-141-3p;hsa-miR-200a-3p;hsa-miR-296-3p;hsa-miR-3
ENSG0000	ENSG0000	0.000725	hsa-let-7a-5p;hsa-let-7b-5p;hsa-let-7d-5p;hsa-let-7e-5p;hsa-l
ENSG0000	ENSG0000	0.002048	hsa-miR-130a-3p;hsa-miR-130b-3p;hsa-miR-301a-3p;hsa-miR-
ENSG0000	ENSG0000	0.006079	hsa-miR-130a-3p;hsa-miR-130b-3p;hsa-miR-27a-3p;hsa-miR-
ENSG0000	ENSG0000	0.004251	hsa-miR-15a-5p;hsa-miR-15b-5p;hsa-miR-16-5p;hsa-miR-195-
ENSG0000	ENSG0000	0.000785	hsa-miR-1304-3p;hsa-miR-181a-5p;hsa-miR-181b-5p;hsa-miR-
ENSG0000	ENSG0000	0.001135	hsa-miR-15a-5p;hsa-miR-15b-5p;hsa-miR-195-5p;hsa-miR-42
ENSG0000	ENSG0000	9.60E-05	hsa-miR-15a-5p;hsa-miR-15b-5p;hsa-miR-16-5p;hsa-miR-195-
ENSG0000	ENSG0000	6.67E-06	hsa-miR-103a-3p;hsa-miR-107;hsa-miR-140-5p;hsa-miR-15a-
ENSG0000	ENSG0000	0.001851	hsa-miR-15a-5p;hsa-miR-15b-5p;hsa-miR-16-5p;hsa-miR-195-
ENSG0000	ENSG0000	1.15E-05	hsa-miR-130a-3p;hsa-miR-130b-3p;hsa-miR-181a-5p;hsa-miR-
ENSG0000	ENSG0000	0.002624	hsa-let-7a-5p;hsa-let-7b-5p;hsa-let-7e-5p;hsa-let-7f-5p;hsa-l
ENSG0000	ENSG0000	5.29E-05	hsa-let-7a-5p;hsa-let-7b-5p;hsa-let-7d-5p;hsa-let-7e-5p;hsa-l
ENSG0000	ENSG0000	2.83E-07	hsa-miR-141-3p;hsa-miR-15a-5p;hsa-miR-15b-5p;hsa-miR-19
ENSG0000	ENSG0000	0.001803	hsa-miR-181a-5p;hsa-miR-181b-5p;hsa-miR-181c-5p;hsa-miR-

ENSG0000	ENSG0000	0.00096	hsa-miR-214-3p;hsa-miR-342-3p;hsa-miR-3619-5p;hsa-miR-3
ENSG0000	ENSG0000	0.009645	hsa-miR-106b-5p;hsa-miR-130a-3p;hsa-miR-130b-3p;hsa-miR
ENSG0000	ENSG0000	0.009645	hsa-miR-106b-5p;hsa-miR-130a-3p;hsa-miR-130b-3p;hsa-miR
ENSG0000	ENSG0000	0.005949	hsa-miR-15a-5p;hsa-miR-15b-5p;hsa-miR-195-5p;hsa-miR-31
ENSG0000	ENSG0000	2.48E-05	hsa-miR-15a-5p;hsa-miR-15b-5p;hsa-miR-16-5p;hsa-miR-195
ENSG0000	ENSG0000	7.48E-05	hsa-miR-1304-3p;hsa-miR-130a-3p;hsa-miR-130b-3p;hsa-miR
ENSG0000	ENSG0000	0.002947	hsa-miR-181a-5p;hsa-miR-181b-5p;hsa-miR-181c-5p;hsa-miR
ENSG0000	ENSG0000	0.002947	hsa-miR-181a-5p;hsa-miR-181b-5p;hsa-miR-181c-5p;hsa-miR
ENSG0000	ENSG0000	0.003744	hsa-miR-106b-5p;hsa-miR-130a-3p;hsa-miR-130b-3p;hsa-miR
ENSG0000	ENSG0000	1.73E-06	hsa-let-7a-5p;hsa-let-7b-5p;hsa-let-7d-5p;hsa-let-7e-5p;hsa-l
ENSG0000	ENSG0000	8.24E-05	hsa-let-7a-5p;hsa-let-7b-5p;hsa-let-7d-5p;hsa-let-7e-5p;hsa-l
ENSG0000	ENSG0000	8.24E-05	hsa-let-7a-5p;hsa-let-7b-5p;hsa-let-7d-5p;hsa-let-7e-5p;hsa-l
ENSG0000	ENSG0000	7.67E-06	hsa-let-7a-5p;hsa-let-7b-5p;hsa-let-7d-5p;hsa-let-7e-5p;hsa-l
ENSG0000	ENSG0000	7.46E-08	hsa-miR-15a-5p;hsa-miR-15b-5p;hsa-miR-16-5p;hsa-miR-195
ENSG0000	ENSG0000	0.001205	hsa-miR-15a-5p;hsa-miR-15b-5p;hsa-miR-16-5p;hsa-miR-191
ENSG0000	ENSG0000	8.86E-07	hsa-let-7a-5p;hsa-let-7b-5p;hsa-let-7d-5p;hsa-let-7e-5p;hsa-l
ENSG0000	ENSG0000	2.39E-07	hsa-miR-130a-3p;hsa-miR-130b-3p;hsa-miR-140-3p;hsa-miR-
ENSG0000	ENSG0000	5.17E-05	hsa-miR-1-3p;hsa-miR-143-5p;hsa-miR-15a-5p;hsa-miR-15b-
ENSG0000	ENSG0000	9.60E-05	hsa-miR-106b-5p;hsa-miR-130a-3p;hsa-miR-181a-5p;hsa-miR
ENSG0000	ENSG0000	0.001548	hsa-miR-130a-3p;hsa-miR-130b-3p;hsa-miR-301a-3p;hsa-miR
ENSG0000	ENSG0000	0.002223	hsa-let-7a-5p;hsa-let-7b-5p;hsa-let-7d-5p;hsa-let-7e-5p;hsa-l
ENSG0000	ENSG0000	0.000114	hsa-let-7a-5p;hsa-let-7b-5p;hsa-let-7d-5p;hsa-let-7e-5p;hsa-l
ENSG0000	ENSG0000	5.17E-05	hsa-let-7a-5p;hsa-let-7b-5p;hsa-let-7d-5p;hsa-let-7e-5p;hsa-l
ENSG0000	ENSG0000	0.000913	hsa-miR-106b-5p;hsa-miR-15a-5p;hsa-miR-15b-5p;hsa-miR-1
ENSG0000	ENSG0000	7.34E-08	hsa-miR-107;hsa-miR-15a-5p;hsa-miR-15b-5p;hsa-miR-16-5p;
ENSG0000	ENSG0000	0.002386	hsa-let-7a-5p;hsa-let-7b-5p;hsa-let-7d-5p;hsa-let-7e-5p;hsa-l
ENSG0000	ENSG0000	0.008848	hsa-miR-106b-5p;hsa-miR-15a-5p;hsa-miR-15b-5p;hsa-miR-1
ENSG0000	ENSG0000	0.000913	hsa-miR-103a-3p;hsa-miR-107;hsa-miR-127-5p;hsa-miR-1-3p;
ENSG0000	ENSG0000	0.00013	hsa-miR-125a-5p;hsa-miR-140-5p;hsa-miR-181a-5p;hsa-miR-
ENSG0000	ENSG0000	0.005346	hsa-let-7a-5p;hsa-let-7b-5p;hsa-let-7d-5p;hsa-let-7e-5p;hsa-l
ENSG0000	ENSG0000	0.001803	hsa-miR-130a-3p;hsa-miR-130b-3p;hsa-miR-188-3p;hsa-miR-
ENSG0000	ENSG0000	2.21E-08	hsa-let-7a-5p;hsa-let-7b-5p;hsa-let-7d-5p;hsa-let-7e-5p;hsa-l
ENSG0000	ENSG0000	1.46E-06	hsa-let-7a-5p;hsa-let-7b-5p;hsa-let-7d-5p;hsa-let-7e-5p;hsa-l
ENSG0000	ENSG0000	0.004807	hsa-miR-143-5p;hsa-miR-15a-5p;hsa-miR-15b-5p;hsa-miR-16
ENSG0000	ENSG0000	2.95E-05	hsa-miR-15a-5p;hsa-miR-15b-5p;hsa-miR-16-5p;hsa-miR-195
ENSG0000	ENSG0000	0.001205	hsa-miR-181a-5p;hsa-miR-181b-5p;hsa-miR-181c-5p;hsa-miR
ENSG0000	ENSG0000	0.009977	hsa-miR-130a-3p;hsa-miR-130b-3p;hsa-miR-301a-3p;hsa-miR
ENSG0000	ENSG0000	0.001493	hsa-miR-15a-5p;hsa-miR-15b-5p;hsa-miR-195-5p;hsa-miR-42
ENSG0000	ENSG0000	2.05E-07	hsa-let-7a-5p;hsa-let-7b-5p;hsa-let-7d-5p;hsa-let-7e-5p;hsa-l
ENSG0000	ENSG0000	0.001001	hsa-miR-130a-3p;hsa-miR-130b-3p;hsa-miR-181a-5p;hsa-miR
ENSG0000	ENSG0000	0.003744	hsa-miR-181a-5p;hsa-miR-181b-5p;hsa-miR-181c-5p;hsa-miR
ENSG0000	ENSG0000	0.001396	hsa-let-7a-5p;hsa-let-7b-5p;hsa-let-7d-5p;hsa-let-7e-5p;hsa-l
ENSG0000	ENSG0000	0.001076	hsa-miR-103a-3p;hsa-miR-106b-5p;hsa-miR-107;hsa-miR-130
ENSG0000	ENSG0000	0.000332	hsa-miR-107;hsa-miR-15a-5p;hsa-miR-15b-5p;hsa-miR-195-5
ENSG0000	ENSG0000	1.07E-07	hsa-let-7a-5p;hsa-let-7b-5p;hsa-let-7d-5p;hsa-let-7e-5p;hsa-l
ENSG0000	ENSG0000	0.001793	hsa-miR-107;hsa-miR-15a-5p;hsa-miR-15b-5p;hsa-miR-195-5
ENSG0000	ENSG0000	0.008919	hsa-miR-362-3p;hsa-miR-545-3p;hsa-miR-603;hsa-miR-873-5
ENSG0000	ENSG0000	2.27E-05	hsa-miR-103a-3p;hsa-miR-107;hsa-miR-15a-5p;hsa-miR-15b-
ENSG0000	ENSG0000	0.001493	hsa-miR-181a-5p;hsa-miR-181b-5p;hsa-miR-181c-5p;hsa-miR
ENSG0000	ENSG0000	7.67E-06	hsa-miR-15a-5p;hsa-miR-15b-5p;hsa-miR-16-5p;hsa-miR-188
ENSG0000	ENSG0000	7.67E-06	hsa-miR-15a-5p;hsa-miR-15b-5p;hsa-miR-16-5p;hsa-miR-188
ENSG0000	ENSG0000	0.000583	hsa-miR-15a-5p;hsa-miR-15b-5p;hsa-miR-195-5p;hsa-miR-42
ENSG0000	ENSG0000	6.25E-05	hsa-let-7a-5p;hsa-let-7b-5p;hsa-let-7d-5p;hsa-let-7e-5p;hsa-l
ENSG0000	ENSG0000	6.25E-05	hsa-let-7a-5p;hsa-let-7b-5p;hsa-let-7d-5p;hsa-let-7e-5p;hsa-l

ENSG0000	ENSG0000	1.01E-05	hsa-miR-15a-5p;hsa-miR-15b-5p;hsa-miR-16-5p;hsa-miR-195-
ENSG0000	ENSG0000	0.0036	hsa-let-7a-5p;hsa-let-7b-5p;hsa-let-7d-5p;hsa-let-7e-5p;hsa-l
ENSG0000	ENSG0000	0.000284	hsa-let-7a-5p;hsa-let-7b-5p;hsa-let-7d-5p;hsa-let-7e-5p;hsa-l
ENSG0000	ENSG0000	2.09E-06	hsa-miR-15a-5p;hsa-miR-15b-5p;hsa-miR-16-5p;hsa-miR-195-
ENSG0000	ENSG0000	0.000754	hsa-miR-106b-5p;hsa-miR-181a-5p;hsa-miR-181b-5p;hsa-miR
ENSG0000	ENSG0000	0.007332	hsa-let-7a-5p;hsa-let-7b-5p;hsa-let-7d-5p;hsa-let-7e-5p;hsa-l
ENSG0000	ENSG0000	8.84E-09	hsa-miR-103a-3p;hsa-miR-107;hsa-miR-15a-5p;hsa-miR-15b-5
ENSG0000	ENSG0000	0.00641	hsa-let-7a-5p;hsa-let-7b-5p;hsa-let-7d-5p;hsa-let-7e-5p;hsa-l
ENSG0000	ENSG0000	0.000951	hsa-miR-15a-5p;hsa-miR-15b-5p;hsa-miR-16-5p;hsa-miR-195-
ENSG0000	ENSG0000	0.000681	hsa-miR-106b-5p;hsa-miR-127-5p;hsa-miR-15a-5p;hsa-miR-1
ENSG0000	ENSG0000	2.08E-05	hsa-let-7a-5p;hsa-let-7b-5p;hsa-let-7e-5p;hsa-let-7f-5p;hsa-le
ENSG0000	ENSG0000	0.000168	hsa-miR-15a-5p;hsa-miR-15b-5p;hsa-miR-16-5p;hsa-miR-195-
ENSG0000	ENSG0000	0.000128	hsa-miR-15a-5p;hsa-miR-15b-5p;hsa-miR-16-5p;hsa-miR-195-
ENSG0000	ENSG0000	1.01E-05	hsa-miR-140-5p;hsa-miR-15a-5p;hsa-miR-16-5p;hsa-miR-195-
ENSG0000	ENSG0000	1.01E-05	hsa-miR-140-5p;hsa-miR-15a-5p;hsa-miR-16-5p;hsa-miR-195-
ENSG0000	ENSG0000	0.001344	hsa-miR-15a-5p;hsa-miR-15b-5p;hsa-miR-16-5p;hsa-miR-195-
ENSG0000	ENSG0000	0.002651	hsa-miR-106b-5p;hsa-miR-141-3p;hsa-miR-200a-3p;hsa-miR-
ENSG0000	ENSG0000	0.002651	hsa-miR-106b-5p;hsa-miR-141-3p;hsa-miR-200a-3p;hsa-miR-
ENSG0000	ENSG0000	0.000301	hsa-let-7a-5p;hsa-let-7b-5p;hsa-let-7d-5p;hsa-let-7e-5p;hsa-l
ENSG0000	ENSG0000	0.000301	hsa-let-7a-5p;hsa-let-7b-5p;hsa-let-7d-5p;hsa-let-7e-5p;hsa-l
ENSG0000	ENSG0000	0.001139	hsa-miR-17-3p;hsa-miR-185-3p;hsa-miR-374a-5p;hsa-miR-74
ENSG0000	ENSG0000	7.74E-08	hsa-miR-15a-5p;hsa-miR-15b-5p;hsa-miR-16-5p;hsa-miR-195-
ENSG0000	ENSG0000	5.65E-05	hsa-miR-103a-3p;hsa-miR-107;hsa-miR-15a-5p;hsa-miR-15b-5
ENSG0000	ENSG0000	6.67E-06	hsa-let-7d-5p;hsa-let-7e-5p;hsa-let-7f-5p;hsa-let-7g-5p;hsa-le
ENSG0000	ENSG0000	0.00029	hsa-miR-15a-5p;hsa-miR-15b-5p;hsa-miR-16-5p;hsa-miR-195-
ENSG0000	ENSG0000	0.000378	hsa-miR-106b-5p;hsa-miR-15a-5p;hsa-miR-15b-5p;hsa-miR-1
ENSG0000	ENSG0000	0.000812	hsa-miR-15a-5p;hsa-miR-15b-5p;hsa-miR-16-5p;hsa-miR-195-
ENSG0000	ENSG0000	0.005723	hsa-let-7a-5p;hsa-let-7b-5p;hsa-let-7d-5p;hsa-let-7e-5p;hsa-l
ENSG0000	ENSG0000	0.000117	hsa-miR-181a-5p;hsa-miR-181b-5p;hsa-miR-181c-5p;hsa-miR-
ENSG0000	ENSG0000	0.000785	hsa-miR-130a-3p;hsa-miR-181a-5p;hsa-miR-181c-5p;hsa-miR-
ENSG0000	ENSG0000	0.000785	hsa-miR-130a-3p;hsa-miR-181a-5p;hsa-miR-181c-5p;hsa-miR-
ENSG0000	ENSG0000	0.001344	hsa-miR-181a-5p;hsa-miR-181b-5p;hsa-miR-181c-5p;hsa-miR-
ENSG0000	ENSG0000	0.007579	hsa-miR-125b-2-3p;hsa-miR-130a-3p;hsa-miR-130b-3p;hsa-m
ENSG0000	ENSG0000	0.002153	hsa-let-7a-5p;hsa-let-7b-5p;hsa-let-7e-5p;hsa-let-7f-5p;hsa-le
ENSG0000	ENSG0000	0.000235	hsa-let-7b-5p;hsa-let-7d-5p;hsa-let-7e-5p;hsa-let-7f-5p;hsa-le
ENSG0000	ENSG0000	4.10E-06	hsa-miR-106b-5p;hsa-miR-141-3p;hsa-miR-15a-5p;hsa-miR-1
ENSG0000	ENSG0000	0.0042	hsa-miR-200b-3p;hsa-miR-200c-3p;hsa-miR-429;hsa-miR-660-
ENSG0000	ENSG0000	0.0042	hsa-miR-200b-3p;hsa-miR-200c-3p;hsa-miR-429;hsa-miR-660-
ENSG0000	ENSG0000	0.0042	hsa-miR-200b-3p;hsa-miR-200c-3p;hsa-miR-429;hsa-miR-660-
ENSG0000	ENSG0000	0.0042	hsa-miR-200b-3p;hsa-miR-200c-3p;hsa-miR-429;hsa-miR-660-
ENSG0000	ENSG0000	0.0042	hsa-miR-200b-3p;hsa-miR-200c-3p;hsa-miR-429;hsa-miR-660-
ENSG0000	ENSG0000	0.0042	hsa-miR-200b-3p;hsa-miR-200c-3p;hsa-miR-429;hsa-miR-660-
ENSG0000	ENSG0000	0.0042	hsa-miR-200b-3p;hsa-miR-200c-3p;hsa-miR-429;hsa-miR-660-
ENSG0000	ENSG0000	0.007516	hsa-miR-27a-3p;hsa-miR-27b-3p;hsa-miR-342-3p;hsa-miR-87
ENSG0000	ENSG0000	0.000128	hsa-miR-15a-5p;hsa-miR-15b-5p;hsa-miR-16-5p;hsa-miR-195-
ENSG0000	ENSG0000	8.86E-07	hsa-let-7a-5p;hsa-let-7b-5p;hsa-let-7d-5p;hsa-let-7e-5p;hsa-l
ENSG0000	ENSG0000	2.17E-07	hsa-miR-15a-5p;hsa-miR-15b-5p;hsa-miR-195-5p;hsa-miR-42
ENSG0000	ENSG0000	0.000681	hsa-miR-125b-2-3p;hsa-miR-15a-5p;hsa-miR-15b-5p;hsa-miR-
ENSG0000	ENSG0000	0.0041	hsa-miR-106b-5p;hsa-miR-130a-3p;hsa-miR-130b-3p;hsa-miR
ENSG0000	ENSG0000	0.0081	hsa-miR-1-3p;hsa-miR-143-5p;hsa-miR-181a-5p;hsa-miR-181
ENSG0000	ENSG0000	0.002466	hsa-miR-15a-5p;hsa-miR-15b-5p;hsa-miR-16-5p;hsa-miR-195-
ENSG0000	ENSG0000	2.70E-05	hsa-miR-130a-3p;hsa-miR-130b-3p;hsa-miR-200b-3p;hsa-miR
ENSG0000	ENSG0000	7.57E-07	hsa-let-7a-5p;hsa-let-7b-5p;hsa-let-7d-5p;hsa-let-7e-5p;hsa-l
ENSG0000	ENSG0000	7.57E-07	hsa-let-7a-5p;hsa-let-7b-5p;hsa-let-7d-5p;hsa-let-7e-5p;hsa-l

ENSG0000	ENSG0000	0.001548	hsa-miR-15a-5p;hsa-miR-15b-5p;hsa-miR-424-5p;hsa-miR-49
ENSG0000	ENSG0000	0.000235	hsa-miR-15a-5p;hsa-miR-15b-5p;hsa-miR-16-5p;hsa-miR-195-
ENSG0000	ENSG0000	7.00E-05	hsa-let-7a-5p;hsa-let-7b-5p;hsa-let-7d-5p;hsa-let-7e-5p;hsa-l
ENSG0000	ENSG0000	2.09E-06	hsa-miR-15a-5p;hsa-miR-15b-5p;hsa-miR-16-5p;hsa-miR-195-
ENSG0000	ENSG0000	6.04E-06	hsa-miR-106b-5p;hsa-miR-1224-5p;hsa-miR-130a-3p;hsa-miR-
ENSG0000	ENSG0000	0.000812	hsa-miR-181a-5p;hsa-miR-181b-5p;hsa-miR-181c-5p;hsa-miR-
ENSG0000	ENSG0000	1.87E-06	hsa-let-7a-5p;hsa-let-7b-5p;hsa-let-7d-5p;hsa-let-7e-5p;hsa-l
ENSG0000	ENSG0000	0.001635	hsa-miR-15a-5p;hsa-miR-15b-5p;hsa-miR-16-5p;hsa-miR-195-
ENSG0000	ENSG0000	1.99E-07	hsa-miR-103a-3p;hsa-miR-107;hsa-miR-125b-2-3p;hsa-miR-14
ENSG0000	ENSG0000	8.46E-06	hsa-miR-125a-5p;hsa-miR-125b-5p;hsa-miR-140-3p;hsa-miR-1
ENSG0000	ENSG0000	0.006799	hsa-miR-15a-5p;hsa-miR-15b-5p;hsa-miR-16-5p;hsa-miR-195-
ENSG0000	ENSG0000	0.008816	hsa-miR-125b-5p;hsa-miR-15a-5p;hsa-miR-15b-5p;hsa-miR-1
ENSG0000	ENSG0000	0.001548	hsa-miR-130a-3p;hsa-miR-130b-3p;hsa-miR-301a-3p;hsa-miR-
ENSG0000	ENSG0000	2.09E-06	hsa-miR-15a-5p;hsa-miR-15b-5p;hsa-miR-16-5p;hsa-miR-195-
ENSG0000	ENSG0000	4.80E-06	hsa-miR-15a-5p;hsa-miR-15b-5p;hsa-miR-16-5p;hsa-miR-195-
ENSG0000	ENSG0000	0.001432	hsa-let-7a-5p;hsa-let-7b-5p;hsa-let-7d-5p;hsa-let-7e-5p;hsa-l
ENSG0000	ENSG0000	2.72E-08	hsa-miR-106b-5p;hsa-miR-130a-3p;hsa-miR-130b-3p;hsa-miR
ENSG0000	ENSG0000	0.007959	hsa-let-7a-5p;hsa-let-7b-5p;hsa-let-7d-5p;hsa-let-7e-5p;hsa-l
ENSG0000	ENSG0000	4.57E-05	hsa-let-7a-5p;hsa-let-7b-5p;hsa-let-7d-5p;hsa-let-7e-5p;hsa-l
ENSG0000	ENSG0000	0.00029	hsa-miR-106b-5p;hsa-miR-130a-3p;hsa-miR-130b-3p;hsa-miR
ENSG0000	ENSG0000	2.19E-07	hsa-miR-103a-3p;hsa-miR-107;hsa-miR-1224-5p;hsa-miR-15a-
ENSG0000	ENSG0000	0.000112	hsa-let-7a-5p;hsa-let-7d-5p;hsa-let-7e-5p;hsa-let-7f-5p;hsa-le
ENSG0000	ENSG0000	2.55E-05	hsa-let-7a-5p;hsa-let-7b-5p;hsa-let-7d-5p;hsa-let-7e-5p;hsa-l
ENSG0000	ENSG0000	0.007516	hsa-miR-107;hsa-miR-17-3p;hsa-miR-188-3p;hsa-miR-545-3p
ENSG0000	ENSG0000	0.005899	hsa-miR-107;hsa-miR-15a-5p;hsa-miR-15b-5p;hsa-miR-195-5p
ENSG0000	ENSG0000	0.009977	hsa-miR-106b-5p;hsa-miR-181a-5p;hsa-miR-181b-5p;hsa-miR
ENSG0000	ENSG0000	0.000342	hsa-let-7a-5p;hsa-let-7b-5p;hsa-let-7d-5p;hsa-let-7e-5p;hsa-l
ENSG0000	ENSG0000	4.71E-06	hsa-miR-103a-3p;hsa-miR-107;hsa-miR-141-3p;hsa-miR-15a-5
ENSG0000	ENSG0000	0.003282	hsa-miR-106b-5p;hsa-miR-15a-5p;hsa-miR-15b-5p;hsa-miR-1
ENSG0000	ENSG0000	0.0041	hsa-miR-130a-3p;hsa-miR-130b-3p;hsa-miR-194-3p;hsa-miR-2
ENSG0000	ENSG0000	1.01E-05	hsa-let-7b-5p;hsa-miR-107;hsa-miR-125a-5p;hsa-miR-125b-5p
ENSG0000	ENSG0000	0.000434	hsa-miR-15a-5p;hsa-miR-15b-5p;hsa-miR-16-5p;hsa-miR-195-
ENSG0000	ENSG0000	0.001396	hsa-miR-1224-5p;hsa-miR-1-3p;hsa-miR-15a-5p;hsa-miR-15b-
ENSG0000	ENSG0000	0.002048	hsa-miR-106b-5p;hsa-miR-1-3p;hsa-miR-20a-5p;hsa-miR-362-
ENSG0000	ENSG0000	0.004115	hsa-miR-181a-5p;hsa-miR-181b-5p;hsa-miR-181c-5p;hsa-miR-
ENSG0000	ENSG0000	0.006799	hsa-miR-15a-5p;hsa-miR-15b-5p;hsa-miR-195-5p;hsa-miR-424
ENSG0000	ENSG0000	0.001493	hsa-miR-181a-5p;hsa-miR-181b-5p;hsa-miR-181c-5p;hsa-miR-
ENSG0000	ENSG0000	0.001493	hsa-miR-181a-5p;hsa-miR-181b-5p;hsa-miR-181c-5p;hsa-miR-
ENSG0000	ENSG0000	0.008423	hsa-miR-15a-5p;hsa-miR-15b-5p;hsa-miR-194-3p;hsa-miR-19
ENSG0000	ENSG0000	0.002329	hsa-miR-1304-3p;hsa-miR-130a-3p;hsa-miR-130b-3p;hsa-miR-
ENSG0000	ENSG0000	0.001699	hsa-miR-103a-3p;hsa-miR-107;hsa-miR-15a-5p;hsa-miR-15b-5
ENSG0000	ENSG0000	0.001582	hsa-miR-1304-3p;hsa-miR-130a-3p;hsa-miR-130b-3p;hsa-miR-
ENSG0000	ENSG0000	7.34E-08	hsa-miR-15a-5p;hsa-miR-15b-5p;hsa-miR-181a-5p;hsa-miR-18
ENSG0000	ENSG0000	0.005101	hsa-miR-15a-5p;hsa-miR-15b-5p;hsa-miR-195-5p;hsa-miR-424
ENSG0000	ENSG0000	1.55E-05	hsa-let-7a-5p;hsa-let-7b-5p;hsa-let-7d-5p;hsa-let-7e-5p;hsa-l
ENSG0000	ENSG0000	0.004807	hsa-miR-15a-5p;hsa-miR-15b-5p;hsa-miR-16-5p;hsa-miR-195-
ENSG0000	ENSG0000	2.55E-05	hsa-miR-15a-5p;hsa-miR-15b-5p;hsa-miR-16-5p;hsa-miR-195-
ENSG0000	ENSG0000	0.007414	hsa-let-7a-5p;hsa-let-7b-5p;hsa-let-7d-5p;hsa-let-7e-5p;hsa-l
ENSG0000	ENSG0000	4.40E-05	hsa-miR-106b-5p;hsa-miR-130a-3p;hsa-miR-130b-3p;hsa-miR
ENSG0000	ENSG0000	0.004552	hsa-miR-181a-5p;hsa-miR-181b-5p;hsa-miR-181c-5p;hsa-miR-
ENSG0000	ENSG0000	0.004357	hsa-let-7a-5p;hsa-let-7b-5p;hsa-let-7d-5p;hsa-let-7e-5p;hsa-l
ENSG0000	ENSG0000	0.000192	hsa-let-7a-5p;hsa-let-7b-5p;hsa-let-7d-5p;hsa-let-7e-5p;hsa-l
ENSG0000	ENSG0000	0.000654	hsa-miR-106b-5p;hsa-miR-199a-5p;hsa-miR-199b-5p;hsa-miR
ENSG0000	ENSG0000	3.67E-05	hsa-let-7a-5p;hsa-let-7b-5p;hsa-let-7d-5p;hsa-let-7e-5p;hsa-l

ENSG0000	ENSG0000	0.001828	hsa-miR-15a-5p;hsa-miR-16-5p;hsa-miR-195-5p;hsa-miR-424-
ENSG0000	ENSG0000	0.0041	hsa-miR-106b-5p;hsa-miR-130a-3p;hsa-miR-18a-3p;hsa-miR-1
ENSG0000	ENSG0000	0.0081	hsa-miR-1304-3p;hsa-miR-130a-3p;hsa-miR-130b-3p;hsa-miR-
ENSG0000	ENSG0000	5.94E-06	hsa-let-7a-5p;hsa-let-7b-5p;hsa-let-7d-5p;hsa-let-7e-5p;hsa-l
ENSG0000	ENSG0000	6.07E-06	hsa-miR-103a-3p;hsa-miR-107;hsa-miR-15a-5p;hsa-miR-15b-5
ENSG0000	ENSG0000	0.001817	hsa-miR-125a-5p;hsa-miR-15a-5p;hsa-miR-15b-5p;hsa-miR-16
ENSG0000	ENSG0000	0.001205	hsa-miR-106b-5p;hsa-miR-127-5p;hsa-miR-20a-5p;hsa-miR-27
ENSG0000	ENSG0000	0.000754	hsa-miR-181a-5p;hsa-miR-181b-5p;hsa-miR-181c-5p;hsa-miR-
ENSG0000	ENSG0000	0.000378	hsa-let-7a-5p;hsa-let-7b-5p;hsa-let-7d-5p;hsa-let-7e-5p;hsa-l
ENSG0000	ENSG0000	1.40E-05	hsa-let-7a-5p;hsa-let-7b-5p;hsa-let-7d-5p;hsa-let-7e-5p;hsa-l
ENSG0000	ENSG0000	1.35E-05	hsa-let-7a-5p;hsa-let-7b-5p;hsa-let-7e-5p;hsa-let-7f-5p;hsa-le
ENSG0000	ENSG0000	1.65E-05	hsa-let-7a-5p;hsa-let-7b-5p;hsa-let-7d-5p;hsa-let-7e-5p;hsa-l
ENSG0000	ENSG0000	0.002179	hsa-miR-106b-5p;hsa-miR-130a-3p;hsa-miR-130b-3p;hsa-miR
ENSG0000	ENSG0000	0.008919	hsa-let-7b-5p;hsa-miR-181a-5p;hsa-miR-181b-5p;hsa-miR-18
ENSG0000	ENSG0000	7.48E-05	hsa-miR-106b-5p;hsa-miR-15a-5p;hsa-miR-15b-5p;hsa-miR-1
ENSG0000	ENSG0000	5.28E-06	hsa-miR-15a-5p;hsa-miR-15b-5p;hsa-miR-16-5p;hsa-miR-195-
ENSG0000	ENSG0000	0.008534	hsa-let-7a-5p;hsa-let-7b-5p;hsa-let-7d-5p;hsa-let-7e-5p;hsa-l
ENSG0000	ENSG0000	0.001655	hsa-miR-1304-3p;hsa-miR-130a-3p;hsa-miR-143-5p;hsa-miR-1
ENSG0000	ENSG0000	0.001303	hsa-let-7a-5p;hsa-let-7b-5p;hsa-let-7d-5p;hsa-let-7e-5p;hsa-l
ENSG0000	ENSG0000	0.003163	hsa-let-7a-5p;hsa-let-7b-5p;hsa-let-7d-5p;hsa-let-7e-5p;hsa-l
ENSG0000	ENSG0000	7.67E-06	hsa-let-7a-5p;hsa-let-7b-5p;hsa-let-7d-5p;hsa-let-7e-5p;hsa-l
ENSG0000	ENSG0000	0.001344	hsa-miR-106b-5p;hsa-miR-130a-3p;hsa-miR-130b-3p;hsa-miR
ENSG0000	ENSG0000	0.002661	hsa-let-7b-5p;hsa-miR-181a-5p;hsa-miR-181b-5p;hsa-miR-18
ENSG0000	ENSG0000	0.003299	hsa-miR-188-3p;hsa-miR-200b-3p;hsa-miR-200c-3p;hsa-miR-3
ENSG0000	ENSG0000	0.000696	hsa-let-7a-5p;hsa-let-7b-5p;hsa-let-7d-5p;hsa-let-7e-5p;hsa-l
ENSG0000	ENSG0000	0.000886	hsa-let-7a-5p;hsa-let-7b-5p;hsa-let-7d-5p;hsa-let-7e-5p;hsa-l
ENSG0000	ENSG0000	0.000145	hsa-let-7a-5p;hsa-let-7b-5p;hsa-let-7d-5p;hsa-let-7e-5p;hsa-l
ENSG0000	ENSG0000	1.18E-07	hsa-miR-15a-5p;hsa-miR-16-5p;hsa-miR-195-5p;hsa-miR-214-
ENSG0000	ENSG0000	0.000812	hsa-miR-15a-5p;hsa-miR-15b-5p;hsa-miR-16-5p;hsa-miR-195-
ENSG0000	ENSG0000	7.27E-09	hsa-let-7a-5p;hsa-let-7b-5p;hsa-let-7d-5p;hsa-let-7e-5p;hsa-l
ENSG0000	ENSG0000	0.000102	hsa-let-7a-5p;hsa-let-7b-5p;hsa-let-7d-5p;hsa-let-7e-5p;hsa-l
ENSG0000	ENSG0000	0.005949	hsa-miR-15a-5p;hsa-miR-15b-5p;hsa-miR-16-5p;hsa-miR-188-
ENSG0000	ENSG0000	6.25E-05	hsa-miR-15a-5p;hsa-miR-15b-5p;hsa-miR-16-5p;hsa-miR-195-
ENSG0000	ENSG0000	0.001085	hsa-let-7a-5p;hsa-let-7b-5p;hsa-let-7d-5p;hsa-let-7e-5p;hsa-l
ENSG0000	ENSG0000	0.008848	hsa-miR-1304-3p;hsa-miR-181a-5p;hsa-miR-181b-5p;hsa-miR-
ENSG0000	ENSG0000	0.001205	hsa-miR-125b-5p;hsa-miR-130a-3p;hsa-miR-130b-3p;hsa-miR
ENSG0000	ENSG0000	0.002651	hsa-miR-15a-5p;hsa-miR-15b-5p;hsa-miR-188-3p;hsa-miR-19
ENSG0000	ENSG0000	8.86E-07	hsa-miR-15a-5p;hsa-miR-15b-5p;hsa-miR-16-5p;hsa-miR-188-
ENSG0000	ENSG0000	0.001635	hsa-let-7a-5p;hsa-let-7b-5p;hsa-let-7d-5p;hsa-let-7e-5p;hsa-l
ENSG0000	ENSG0000	0.002616	hsa-miR-106b-5p;hsa-miR-15a-5p;hsa-miR-15b-5p;hsa-miR-1
ENSG0000	ENSG0000	0.000595	hsa-miR-15a-5p;hsa-miR-15b-5p;hsa-miR-16-5p;hsa-miR-195-
ENSG0000	ENSG0000	0.001999	hsa-miR-106b-5p;hsa-miR-130a-3p;hsa-miR-130b-3p;hsa-miR
ENSG0000	ENSG0000	1.97E-07	hsa-miR-15a-5p;hsa-miR-15b-5p;hsa-miR-16-5p;hsa-miR-195-
ENSG0000	ENSG0000	1.97E-07	hsa-miR-15a-5p;hsa-miR-15b-5p;hsa-miR-16-5p;hsa-miR-195-
ENSG0000	ENSG0000	1.97E-07	hsa-miR-15a-5p;hsa-miR-15b-5p;hsa-miR-16-5p;hsa-miR-195-
ENSG0000	ENSG0000	1.97E-07	hsa-miR-15a-5p;hsa-miR-15b-5p;hsa-miR-16-5p;hsa-miR-195-
ENSG0000	ENSG0000	0.006781	hsa-miR-181a-5p;hsa-miR-181b-5p;hsa-miR-181c-5p;hsa-miR-
ENSG0000	ENSG0000	0.006781	hsa-miR-181a-5p;hsa-miR-181b-5p;hsa-miR-181c-5p;hsa-miR-
ENSG0000	ENSG0000	0.000184	hsa-miR-106b-5p;hsa-miR-107;hsa-miR-143-5p;hsa-miR-15a-5
ENSG0000	ENSG0000	0.000184	hsa-miR-106b-5p;hsa-miR-107;hsa-miR-143-5p;hsa-miR-15a-5
ENSG0000	ENSG0000	0.006762	hsa-miR-106b-5p;hsa-miR-181a-5p;hsa-miR-181b-5p;hsa-miR
ENSG0000	ENSG0000	0.004014	hsa-miR-130a-3p;hsa-miR-130b-3p;hsa-miR-1-3p;hsa-miR-16-
ENSG0000	ENSG0000	0.00024	hsa-miR-181a-5p;hsa-miR-181b-5p;hsa-miR-181c-5p;hsa-miR-
ENSG0000	ENSG0000	0.00091	hsa-let-7a-5p;hsa-let-7b-5p;hsa-let-7d-5p;hsa-let-7e-5p;hsa-l

ENSG0000	ENSG0000	6.24E-05	hsa-miR-103a-3p;hsa-miR-107;hsa-miR-15a-5p;hsa-miR-15b-5
ENSG0000	ENSG0000	2.79E-05	hsa-let-7a-5p;hsa-let-7b-5p;hsa-let-7d-5p;hsa-let-7e-5p;hsa-l
ENSG0000	ENSG0000	0.000792	hsa-miR-130a-3p;hsa-miR-130b-3p;hsa-miR-23a-3p;hsa-miR-2
ENSG0000	ENSG0000	3.54E-06	hsa-let-7a-5p;hsa-let-7b-5p;hsa-let-7d-5p;hsa-let-7e-5p;hsa-l
ENSG0000	ENSG0000	1.35E-05	hsa-miR-106b-5p;hsa-miR-130a-3p;hsa-miR-15a-5p;hsa-miR-1
ENSG0000	ENSG0000	0.003283	hsa-miR-106b-5p;hsa-miR-130a-3p;hsa-miR-130b-3p;hsa-miR
ENSG0000	ENSG0000	0.000792	hsa-miR-106b-5p;hsa-miR-130a-3p;hsa-miR-20a-5p;hsa-miR-3
ENSG0000	ENSG0000	0.004383	hsa-miR-130a-3p;hsa-miR-130b-3p;hsa-miR-301a-3p;hsa-miR-
ENSG0000	ENSG0000	4.07E-09	hsa-miR-15a-5p;hsa-miR-15b-5p;hsa-miR-195-5p;hsa-miR-214
ENSG0000	ENSG0000	2.95E-05	hsa-miR-15a-5p;hsa-miR-15b-5p;hsa-miR-16-5p;hsa-miR-195-
ENSG0000	ENSG0000	0.000284	hsa-miR-181a-5p;hsa-miR-181b-5p;hsa-miR-181c-5p;hsa-miR-
ENSG0000	ENSG0000	0.004251	hsa-miR-130a-3p;hsa-miR-130b-3p;hsa-miR-301a-3p;hsa-miR-
ENSG0000	ENSG0000	0.002216	hsa-miR-1304-3p;hsa-miR-130a-3p;hsa-miR-130b-3p;hsa-miR-
ENSG0000	ENSG0000	0.000117	hsa-miR-106b-5p;hsa-miR-181a-5p;hsa-miR-181b-5p;hsa-miR
ENSG0000	ENSG0000	0.000169	hsa-let-7a-5p;hsa-let-7b-5p;hsa-let-7d-5p;hsa-let-7e-5p;hsa-l
ENSG0000	ENSG0000	0.001582	hsa-let-7a-5p;hsa-let-7b-5p;hsa-let-7d-5p;hsa-let-7e-5p;hsa-l
ENSG0000	ENSG0000	2.09E-06	hsa-miR-15a-5p;hsa-miR-15b-5p;hsa-miR-16-5p;hsa-miR-195-
ENSG0000	ENSG0000	0.004552	hsa-miR-106b-5p;hsa-miR-130a-3p;hsa-miR-130b-3p;hsa-miR
ENSG0000	ENSG0000	0.001604	hsa-let-7a-5p;hsa-let-7b-5p;hsa-let-7d-5p;hsa-let-7e-5p;hsa-l
ENSG0000	ENSG0000	0.000408	hsa-miR-15a-5p;hsa-miR-15b-5p;hsa-miR-16-5p;hsa-miR-195-
ENSG0000	ENSG0000	0.000408	hsa-miR-15a-5p;hsa-miR-15b-5p;hsa-miR-16-5p;hsa-miR-195-
ENSG0000	ENSG0000	0.000408	hsa-miR-15a-5p;hsa-miR-15b-5p;hsa-miR-16-5p;hsa-miR-195-
ENSG0000	ENSG0000	0.000408	hsa-miR-15a-5p;hsa-miR-15b-5p;hsa-miR-16-5p;hsa-miR-195-
ENSG0000	ENSG0000	0.000408	hsa-miR-15a-5p;hsa-miR-15b-5p;hsa-miR-16-5p;hsa-miR-195-
ENSG0000	ENSG0000	0.000408	hsa-miR-15a-5p;hsa-miR-15b-5p;hsa-miR-16-5p;hsa-miR-195-
ENSG0000	ENSG0000	0.000408	hsa-miR-15a-5p;hsa-miR-15b-5p;hsa-miR-16-5p;hsa-miR-195-
ENSG0000	ENSG0000	0.000408	hsa-miR-15a-5p;hsa-miR-15b-5p;hsa-miR-16-5p;hsa-miR-195-
ENSG0000	ENSG0000	0.000408	hsa-miR-15a-5p;hsa-miR-15b-5p;hsa-miR-16-5p;hsa-miR-195-
ENSG0000	ENSG0000	0.000408	hsa-miR-15a-5p;hsa-miR-15b-5p;hsa-miR-16-5p;hsa-miR-195-
ENSG0000	ENSG0000	0.00978	hsa-let-7a-5p;hsa-let-7b-5p;hsa-let-7d-5p;hsa-let-7e-5p;hsa-l
ENSG0000	ENSG0000	0.00013	hsa-miR-15a-5p;hsa-miR-15b-5p;hsa-miR-16-5p;hsa-miR-195-
ENSG0000	ENSG0000	0.000128	hsa-miR-15a-5p;hsa-miR-15b-5p;hsa-miR-16-5p;hsa-miR-195-
ENSG0000	ENSG0000	9.26E-05	hsa-let-7a-5p;hsa-let-7b-5p;hsa-let-7d-5p;hsa-let-7e-5p;hsa-l
ENSG0000	ENSG0000	1.14E-07	hsa-miR-106b-5p;hsa-miR-130a-3p;hsa-miR-130b-3p;hsa-miR
ENSG0000	ENSG0000	1.12E-06	hsa-let-7a-5p;hsa-let-7b-5p;hsa-let-7d-5p;hsa-let-7e-5p;hsa-l
ENSG0000	ENSG0000	0.004383	hsa-miR-130a-3p;hsa-miR-130b-3p;hsa-miR-301a-3p;hsa-miR-
ENSG0000	ENSG0000	0.004383	hsa-miR-130a-3p;hsa-miR-130b-3p;hsa-miR-301a-3p;hsa-miR-
ENSG0000	ENSG0000	0.000276	hsa-miR-15a-5p;hsa-miR-15b-5p;hsa-miR-16-5p;hsa-miR-424-
ENSG0000	ENSG0000	5.65E-05	hsa-miR-15a-5p;hsa-miR-15b-5p;hsa-miR-16-5p;hsa-miR-181a
ENSG0000	ENSG0000	0.000785	hsa-let-7a-5p;hsa-let-7b-5p;hsa-let-7d-5p;hsa-let-7e-5p;hsa-l
ENSG0000	ENSG0000	3.81E-05	hsa-let-7a-5p;hsa-let-7b-5p;hsa-let-7d-5p;hsa-let-7e-5p;hsa-l
ENSG0000	ENSG0000	0.004251	hsa-miR-188-5p;hsa-miR-222-5p;hsa-miR-324-5p;hsa-miR-342
ENSG0000	ENSG0000	1.92E-06	hsa-miR-103a-3p;hsa-miR-107;hsa-miR-143-5p;hsa-miR-15a-5
ENSG0000	ENSG0000	0.000584	hsa-let-7a-5p;hsa-let-7b-5p;hsa-let-7d-5p;hsa-let-7e-5p;hsa-l
ENSG0000	ENSG0000	0.001206	hsa-miR-15a-5p;hsa-miR-15b-5p;hsa-miR-16-5p;hsa-miR-194-
ENSG0000	ENSG0000	0.000583	hsa-miR-1304-3p;hsa-miR-130a-3p;hsa-miR-130b-3p;hsa-miR-
ENSG0000	ENSG0000	8.46E-06	hsa-miR-103a-3p;hsa-miR-107;hsa-miR-15a-5p;hsa-miR-15b-5
ENSG0000	ENSG0000	0.000777	hsa-miR-15a-5p;hsa-miR-15b-5p;hsa-miR-16-5p;hsa-miR-195-
ENSG0000	ENSG0000	8.11E-06	hsa-miR-15a-5p;hsa-miR-15b-5p;hsa-miR-195-5p;hsa-miR-27a
ENSG0000	ENSG0000	0.000434	hsa-miR-1-3p;hsa-miR-200b-3p;hsa-miR-200c-3p;hsa-miR-362
ENSG0000	ENSG0000	0.002624	hsa-miR-15a-5p;hsa-miR-15b-5p;hsa-miR-16-5p;hsa-miR-195-
ENSG0000	ENSG0000	7.77E-05	hsa-miR-106b-5p;hsa-miR-181a-5p;hsa-miR-181b-5p;hsa-miR
ENSG0000	ENSG0000	0.00017	hsa-miR-181a-5p;hsa-miR-181b-5p;hsa-miR-181c-5p;hsa-miR-
ENSG0000	ENSG0000	3.79E-05	hsa-let-7a-5p;hsa-let-7b-5p;hsa-let-7d-5p;hsa-let-7e-5p;hsa-l

ENSG0000	ENSG0000	1.69E-05	hsa-let-7a-5p;hsa-let-7b-5p;hsa-let-7d-5p;hsa-let-7e-5p;hsa-l
ENSG0000	ENSG0000	2.31E-05	hsa-let-7a-5p;hsa-let-7b-5p;hsa-let-7d-5p;hsa-let-7e-5p;hsa-l
ENSG0000	ENSG0000	0.001493	hsa-miR-130a-3p;hsa-miR-130b-3p;hsa-miR-301a-3p;hsa-miR-
ENSG0000	ENSG0000	0.008816	hsa-miR-15a-5p;hsa-miR-15b-5p;hsa-miR-16-5p;hsa-miR-195-
ENSG0000	ENSG0000	0.000332	hsa-let-7a-5p;hsa-let-7b-5p;hsa-let-7d-5p;hsa-let-7e-5p;hsa-l
ENSG0000	ENSG0000	4.03E-07	hsa-miR-130a-3p;hsa-miR-130b-3p;hsa-miR-15a-5p;hsa-miR-1
ENSG0000	ENSG0000	0.009985	hsa-miR-106b-5p;hsa-miR-130a-3p;hsa-miR-130b-3p;hsa-miR
ENSG0000	ENSG0000	0.001548	hsa-miR-24-1-5p;hsa-miR-24-2-5p;hsa-miR-362-3p;hsa-miR-6
ENSG0000	ENSG0000	0.000518	hsa-let-7a-5p;hsa-let-7b-5p;hsa-let-7d-5p;hsa-let-7e-5p;hsa-l
ENSG0000	ENSG0000	0.009977	hsa-miR-23a-3p;hsa-miR-23b-3p;hsa-miR-27a-3p;hsa-miR-27
ENSG0000	ENSG0000	0.002223	hsa-let-7a-5p;hsa-let-7b-5p;hsa-let-7e-5p;hsa-let-7f-5p;hsa-le
ENSG0000	ENSG0000	3.54E-06	hsa-miR-15a-5p;hsa-miR-15b-5p;hsa-miR-16-5p;hsa-miR-195-
ENSG0000	ENSG0000	3.18E-07	hsa-miR-103a-3p;hsa-miR-107;hsa-miR-127-5p;hsa-miR-15a-5
ENSG0000	ENSG0000	0.000126	hsa-let-7a-5p;hsa-let-7b-5p;hsa-let-7d-5p;hsa-let-7e-5p;hsa-l
ENSG0000	ENSG0000	0.005899	hsa-miR-15a-5p;hsa-miR-15b-5p;hsa-miR-195-5p;hsa-miR-42
ENSG0000	ENSG0000	1.07E-05	hsa-miR-15a-5p;hsa-miR-15b-5p;hsa-miR-16-5p;hsa-miR-195-
ENSG0000	ENSG0000	0.00017	hsa-miR-15a-5p;hsa-miR-15b-5p;hsa-miR-195-5p;hsa-miR-42
ENSG0000	ENSG0000	0.000244	hsa-miR-199a-5p;hsa-miR-199b-5p;hsa-miR-222-3p;hsa-miR-
ENSG0000	ENSG0000	0.000244	hsa-miR-199a-5p;hsa-miR-199b-5p;hsa-miR-222-3p;hsa-miR-
ENSG0000	ENSG0000	0.002661	hsa-miR-181a-5p;hsa-miR-181b-5p;hsa-miR-181c-5p;hsa-miR-
ENSG0000	ENSG0000	0.002061	hsa-miR-181a-5p;hsa-miR-181b-5p;hsa-miR-181c-5p;hsa-miR-
ENSG0000	ENSG0000	0.003365	hsa-miR-181a-5p;hsa-miR-181b-5p;hsa-miR-181c-5p;hsa-miR-
ENSG0000	ENSG0000	0.003365	hsa-miR-181a-5p;hsa-miR-181b-5p;hsa-miR-181c-5p;hsa-miR-
ENSG0000	ENSG0000	0.008816	hsa-miR-127-5p;hsa-miR-143-5p;hsa-miR-362-3p;hsa-miR-45
ENSG0000	ENSG0000	0.000438	hsa-let-7a-5p;hsa-let-7b-5p;hsa-let-7d-5p;hsa-let-7e-5p;hsa-l
ENSG0000	ENSG0000	4.48E-06	hsa-miR-103a-3p;hsa-miR-107;hsa-miR-15a-5p;hsa-miR-15b-5
ENSG0000	ENSG0000	0.001851	hsa-miR-15a-5p;hsa-miR-15b-5p;hsa-miR-16-5p;hsa-miR-195-
ENSG0000	ENSG0000	0.000286	hsa-miR-106b-5p;hsa-miR-130a-3p;hsa-miR-130b-3p;hsa-miR
ENSG0000	ENSG0000	0.002153	hsa-miR-1304-3p;hsa-miR-199a-3p;hsa-miR-222-5p;hsa-miR-
ENSG0000	ENSG0000	1.01E-05	hsa-miR-15a-5p;hsa-miR-15b-5p;hsa-miR-16-5p;hsa-miR-195-
ENSG0000	ENSG0000	0.001129	hsa-miR-103a-3p;hsa-miR-106b-5p;hsa-miR-1304-3p;hsa-miR-
ENSG0000	ENSG0000	5.12E-07	hsa-miR-15a-5p;hsa-miR-15b-5p;hsa-miR-16-5p;hsa-miR-188-
ENSG0000	ENSG0000	0.005101	hsa-miR-127-5p;hsa-miR-1-3p;hsa-miR-222-5p;hsa-miR-374a-
ENSG0000	ENSG0000	4.84E-05	hsa-let-7a-5p;hsa-let-7b-5p;hsa-let-7d-5p;hsa-let-7e-5p;hsa-l
ENSG0000	ENSG0000	0.001069	hsa-let-7a-5p;hsa-let-7b-5p;hsa-let-7d-5p;hsa-let-7e-5p;hsa-l
ENSG0000	ENSG0000	8.44E-05	hsa-miR-15a-5p;hsa-miR-15b-5p;hsa-miR-16-5p;hsa-miR-195-
ENSG0000	ENSG0000	4.57E-05	hsa-miR-15a-5p;hsa-miR-15b-5p;hsa-miR-181a-5p;hsa-miR-1
ENSG0000	ENSG0000	4.57E-05	hsa-miR-15a-5p;hsa-miR-15b-5p;hsa-miR-181a-5p;hsa-miR-1
ENSG0000	ENSG0000	4.57E-05	hsa-miR-15a-5p;hsa-miR-15b-5p;hsa-miR-181a-5p;hsa-miR-1
ENSG0000	ENSG0000	4.57E-05	hsa-miR-15a-5p;hsa-miR-15b-5p;hsa-miR-181a-5p;hsa-miR-1
ENSG0000	ENSG0000	4.57E-05	hsa-miR-15a-5p;hsa-miR-15b-5p;hsa-miR-181a-5p;hsa-miR-1
ENSG0000	ENSG0000	4.57E-05	hsa-miR-15a-5p;hsa-miR-15b-5p;hsa-miR-181a-5p;hsa-miR-1
ENSG0000	ENSG0000	4.57E-05	hsa-miR-15a-5p;hsa-miR-15b-5p;hsa-miR-181a-5p;hsa-miR-1
ENSG0000	ENSG0000	4.57E-05	hsa-miR-15a-5p;hsa-miR-15b-5p;hsa-miR-181a-5p;hsa-miR-1
ENSG0000	ENSG0000	1.73E-06	hsa-miR-103a-3p;hsa-miR-107;hsa-miR-15a-5p;hsa-miR-15b-5
ENSG0000	ENSG0000	7.57E-07	hsa-miR-103a-3p;hsa-miR-107;hsa-miR-15a-5p;hsa-miR-15b-5
ENSG0000	ENSG0000	6.27E-08	hsa-let-7a-5p;hsa-let-7b-5p;hsa-let-7d-5p;hsa-let-7e-5p;hsa-l
ENSG0000	ENSG0000	0.008816	hsa-miR-140-3p;hsa-miR-194-3p;hsa-miR-27b-3p;hsa-miR-36
ENSG0000	ENSG0000	0.008816	hsa-miR-140-3p;hsa-miR-194-3p;hsa-miR-27b-3p;hsa-miR-36
ENSG0000	ENSG0000	0.008816	hsa-miR-140-3p;hsa-miR-194-3p;hsa-miR-27b-3p;hsa-miR-36
ENSG0000	ENSG0000	1.07E-05	hsa-miR-15a-5p;hsa-miR-15b-5p;hsa-miR-16-5p;hsa-miR-195-
ENSG0000	ENSG0000	0.004924	hsa-let-7a-5p;hsa-let-7b-5p;hsa-let-7e-5p;hsa-let-7f-5p;hsa-le
ENSG0000	ENSG0000	7.60E-07	hsa-miR-130a-3p;hsa-miR-130b-3p;hsa-miR-143-5p;hsa-miR-1
ENSG0000	ENSG0000	0.006267	hsa-miR-15a-5p;hsa-miR-15b-5p;hsa-miR-16-5p;hsa-miR-195-

ENSG0000	ENSG0000	0.006267	hsa-miR-15a-5p;hsa-miR-15b-5p;hsa-miR-16-5p;hsa-miR-195-
ENSG0000	ENSG0000	6.93E-05	hsa-let-7a-5p;hsa-let-7b-5p;hsa-let-7d-5p;hsa-let-7e-5p;hsa-l
ENSG0000	ENSG0000	0.000122	hsa-let-7a-5p;hsa-let-7b-5p;hsa-let-7d-5p;hsa-let-7e-5p;hsa-l
ENSG0000	ENSG0000	0.001493	hsa-miR-181a-5p;hsa-miR-181b-5p;hsa-miR-181c-5p;hsa-miR-
ENSG0000	ENSG0000	0.005109	hsa-miR-125a-5p;hsa-miR-125b-5p;hsa-miR-15a-5p;hsa-miR-1
ENSG0000	ENSG0000	7.98E-05	hsa-miR-15a-5p;hsa-miR-15b-5p;hsa-miR-16-5p;hsa-miR-195-
ENSG0000	ENSG0000	0.000353	hsa-let-7a-5p;hsa-let-7b-5p;hsa-let-7d-5p;hsa-let-7e-5p;hsa-l
ENSG0000	ENSG0000	0.000883	hsa-let-7a-5p;hsa-let-7b-5p;hsa-let-7d-5p;hsa-let-7e-5p;hsa-l
ENSG0000	ENSG0000	0.006267	hsa-miR-214-3p;hsa-miR-27a-3p;hsa-miR-27b-3p;hsa-miR-36
ENSG0000	ENSG0000	0.003283	hsa-miR-106b-5p;hsa-miR-125b-2-3p;hsa-miR-20a-5p;hsa-mi
ENSG0000	ENSG0000	0.003283	hsa-miR-106b-5p;hsa-miR-125b-2-3p;hsa-miR-20a-5p;hsa-mi
ENSG0000	ENSG0000	0.000529	hsa-miR-130a-3p;hsa-miR-130b-3p;hsa-miR-140-5p;hsa-miR-1
ENSG0000	ENSG0000	1.66E-05	hsa-miR-15a-5p;hsa-miR-15b-5p;hsa-miR-16-5p;hsa-miR-195-
ENSG0000	ENSG0000	2.17E-06	hsa-miR-15a-5p;hsa-miR-15b-5p;hsa-miR-16-5p;hsa-miR-195-
ENSG0000	ENSG0000	0.000184	hsa-miR-103a-3p;hsa-miR-107;hsa-miR-15a-5p;hsa-miR-15b-5
ENSG0000	ENSG0000	1.07E-05	hsa-miR-15a-5p;hsa-miR-15b-5p;hsa-miR-16-5p;hsa-miR-195-
ENSG0000	ENSG0000	6.07E-06	hsa-let-7a-5p;hsa-let-7b-5p;hsa-let-7d-5p;hsa-let-7e-5p;hsa-l
ENSG0000	ENSG0000	6.93E-05	hsa-miR-106b-5p;hsa-miR-140-3p;hsa-miR-199a-3p;hsa-miR-2
ENSG0000	ENSG0000	0.000334	hsa-let-7a-5p;hsa-let-7b-5p;hsa-let-7d-5p;hsa-let-7e-5p;hsa-l
ENSG0000	ENSG0000	0.002624	hsa-miR-15a-5p;hsa-miR-15b-5p;hsa-miR-16-5p;hsa-miR-195-
ENSG0000	ENSG0000	0.002805	hsa-let-7a-5p;hsa-let-7b-5p;hsa-let-7d-5p;hsa-let-7e-5p;hsa-l
ENSG0000	ENSG0000	0.000951	hsa-miR-15a-5p;hsa-miR-15b-5p;hsa-miR-16-5p;hsa-miR-195-
ENSG0000	ENSG0000	0.009985	hsa-let-7a-5p;hsa-let-7b-5p;hsa-let-7d-5p;hsa-let-7e-5p;hsa-l
ENSG0000	ENSG0000	0.003744	hsa-miR-1304-3p;hsa-miR-181a-5p;hsa-miR-181b-5p;hsa-miR-
ENSG0000	ENSG0000	0.002223	hsa-miR-143-5p;hsa-miR-15a-5p;hsa-miR-15b-5p;hsa-miR-16-
ENSG0000	ENSG0000	0.006898	hsa-miR-125b-2-3p;hsa-miR-130a-3p;hsa-miR-130b-3p;hsa-m
ENSG0000	ENSG0000	0.000485	hsa-miR-125a-5p;hsa-miR-125b-5p;hsa-miR-140-3p;hsa-miR-1
ENSG0000	ENSG0000	0.002386	hsa-miR-15a-5p;hsa-miR-15b-5p;hsa-miR-16-5p;hsa-miR-194-
ENSG0000	ENSG0000	4.93E-05	hsa-miR-141-3p;hsa-miR-200a-3p;hsa-miR-200b-3p;hsa-miR-2
ENSG0000	ENSG0000	0.000278	hsa-miR-1224-5p;hsa-miR-140-3p;hsa-miR-15a-5p;hsa-miR-15
ENSG0000	ENSG0000	0.000856	hsa-miR-1224-5p;hsa-miR-140-3p;hsa-miR-15a-5p;hsa-miR-15
ENSG0000	ENSG0000	0.000535	hsa-miR-106b-5p;hsa-miR-130a-3p;hsa-miR-130b-3p;hsa-miR
ENSG0000	ENSG0000	0.001596	hsa-let-7a-5p;hsa-let-7b-5p;hsa-let-7d-5p;hsa-let-7e-5p;hsa-l
ENSG0000	ENSG0000	2.09E-06	hsa-miR-15a-5p;hsa-miR-15b-5p;hsa-miR-16-5p;hsa-miR-195-
ENSG0000	ENSG0000	8.44E-05	hsa-let-7a-5p;hsa-let-7b-5p;hsa-let-7d-5p;hsa-let-7e-5p;hsa-l
ENSG0000	ENSG0000	1.46E-06	hsa-let-7a-5p;hsa-let-7b-5p;hsa-let-7d-5p;hsa-let-7e-5p;hsa-l
ENSG0000	ENSG0000	2.39E-07	hsa-miR-15a-5p;hsa-miR-15b-5p;hsa-miR-16-5p;hsa-miR-181a
ENSG0000	ENSG0000	0.002947	hsa-miR-103a-3p;hsa-miR-107;hsa-miR-125a-5p;hsa-miR-125b
ENSG0000	ENSG0000	4.06E-08	hsa-let-7a-5p;hsa-let-7b-5p;hsa-let-7d-5p;hsa-let-7e-5p;hsa-l
ENSG0000	ENSG0000	0.000572	hsa-let-7a-5p;hsa-let-7b-5p;hsa-let-7d-5p;hsa-let-7e-5p;hsa-l
ENSG0000	ENSG0000	1.45E-05	hsa-miR-103a-3p;hsa-miR-107;hsa-miR-1304-3p;hsa-miR-143-
ENSG0000	ENSG0000	0.004357	hsa-miR-15a-5p;hsa-miR-15b-5p;hsa-miR-16-5p;hsa-miR-195-
ENSG0000	ENSG0000	0.0074	hsa-miR-1304-3p;hsa-miR-181a-5p;hsa-miR-181b-5p;hsa-miR-
ENSG0000	ENSG0000	0.001131	hsa-let-7a-5p;hsa-let-7b-5p;hsa-let-7d-5p;hsa-let-7e-5p;hsa-l
ENSG0000	ENSG0000	0.0081	hsa-miR-106b-5p;hsa-miR-125a-5p;hsa-miR-125b-5p;hsa-miR
ENSG0000	ENSG0000	0.005165	hsa-miR-130a-3p;hsa-miR-130b-3p;hsa-miR-301a-3p;hsa-miR-
ENSG0000	ENSG0000	5.17E-05	hsa-miR-130a-3p;hsa-miR-130b-3p;hsa-miR-15a-5p;hsa-miR-1
ENSG0000	ENSG0000	0.001274	hsa-miR-15a-5p;hsa-miR-15b-5p;hsa-miR-16-5p;hsa-miR-195-
ENSG0000	ENSG0000	0.003168	hsa-miR-130a-3p;hsa-miR-130b-3p;hsa-miR-301a-3p;hsa-miR-
ENSG0000	ENSG0000	0.008919	hsa-miR-27a-3p;hsa-miR-27b-3p;hsa-miR-376a-5p;hsa-miR-4
ENSG0000	ENSG0000	0.000874	hsa-miR-24-3p;hsa-miR-518b;hsa-miR-518d-3p;hsa-miR-518f-
ENSG0000	ENSG0000	0.005044	hsa-miR-186-3p;hsa-miR-18a-5p;hsa-miR-421;hsa-miR-432-5
ENSG0000	ENSG0000	0.002531	hsa-miR-18a-5p;hsa-miR-18b-5p;hsa-miR-3200-5p;hsa-miR-8
ENSG0000	ENSG0000	0.002767	hsa-miR-18b-5p;hsa-miR-24-3p;hsa-miR-28-5p;hsa-miR-3065-

ENSG0000	ENSG0000	0.000792	hsa-miR-3065-5p;hsa-miR-338-5p;hsa-miR-362-3p;hsa-miR-8
ENSG0000	ENSG0000	0.004196	hsa-miR-149-5p;hsa-miR-518b;hsa-miR-518d-3p;hsa-miR-518
ENSG0000	ENSG0000	0.002903	hsa-miR-186-3p;hsa-miR-28-5p;hsa-miR-708-5p;hsa-miR-873-
ENSG0000	ENSG0000	0.006096	hsa-miR-28-5p;hsa-miR-3194-3p;hsa-miR-362-3p;hsa-miR-70
ENSG0000	ENSG0000	0.000792	hsa-miR-183-5p;hsa-miR-28-5p;hsa-miR-338-3p;hsa-miR-708-
ENSG0000	ENSG0000	0.00193	hsa-miR-24-3p;hsa-miR-3194-3p;hsa-miR-518b;hsa-miR-518d
ENSG0000	ENSG0000	0.003048	hsa-miR-18a-5p;hsa-miR-18b-5p;hsa-miR-338-5p;hsa-miR-55
ENSG0000	ENSG0000	0.000792	hsa-miR-186-3p;hsa-miR-18a-5p;hsa-miR-18b-5p;hsa-miR-36
ENSG0000	ENSG0000	0.001615	hsa-miR-24-3p;hsa-miR-3194-3p;hsa-miR-323a-5p;hsa-miR-3
ENSG0000	ENSG0000	0.001546	hsa-miR-192-5p;hsa-miR-338-5p;hsa-miR-362-3p;hsa-miR-99
ENSG0000	ENSG0000	0.001373	hsa-miR-183-5p;hsa-miR-186-3p;hsa-miR-518b;hsa-miR-518d
ENSG0000	ENSG0000	0.009282	hsa-miR-183-5p;hsa-miR-24-3p;hsa-miR-99a-3p;hsa-miR-99b-
ENSG0000	ENSG0000	0.009715	hsa-miR-186-3p;hsa-miR-24-3p;hsa-miR-28-5p;hsa-miR-708-
ENSG0000	ENSG0000	6.85E-06	hsa-miR-107;hsa-miR-122-5p;hsa-miR-129-5p;hsa-miR-1304-
ENSG0000	ENSG0000	9.03E-05	hsa-let-7a-5p;hsa-let-7b-5p;hsa-let-7d-5p;hsa-let-7f-5p;hsa-l
ENSG0000	ENSG0000	0.002245	hsa-miR-139-5p;hsa-miR-30b-5p;hsa-miR-30c-5p;hsa-miR-32-
ENSG0000	ENSG0000	0.000159	hsa-miR-15a-5p;hsa-miR-15b-5p;hsa-miR-16-5p;hsa-miR-195
ENSG0000	ENSG0000	0.000101	hsa-miR-103a-3p;hsa-miR-32-5p;hsa-miR-92a-3p;hsa-miR-92
ENSG0000	ENSG0000	0.006932	hsa-miR-20b-5p;hsa-miR-27a-3p;hsa-miR-27b-3p;hsa-miR-51
ENSG0000	ENSG0000	0.000109	hsa-let-7a-5p;hsa-let-7b-5p;hsa-let-7d-5p;hsa-let-7f-5p;hsa-l
ENSG0000	ENSG0000	6.85E-10	hsa-miR-103a-3p;hsa-miR-107;hsa-miR-15a-5p;hsa-miR-15b-
ENSG0000	ENSG0000	2.32E-05	hsa-miR-15a-5p;hsa-miR-15b-5p;hsa-miR-16-5p;hsa-miR-195
ENSG0000	ENSG0000	0.004798	hsa-miR-122-5p;hsa-miR-1304-3p;hsa-miR-3194-3p;hsa-miR-
ENSG0000	ENSG0000	0.000119	hsa-miR-15a-5p;hsa-miR-15b-5p;hsa-miR-195-5p;hsa-miR-21
ENSG0000	ENSG0000	0.001811	hsa-miR-17-5p;hsa-miR-20b-5p;hsa-miR-27a-3p;hsa-miR-27b
ENSG0000	ENSG0000	0.007539	hsa-let-7a-5p;hsa-let-7b-5p;hsa-let-7d-5p;hsa-let-7f-5p;hsa-l
ENSG0000	ENSG0000	0.000628	hsa-let-7a-5p;hsa-let-7b-5p;hsa-let-7d-5p;hsa-let-7f-5p;hsa-l
ENSG0000	ENSG0000	1.95E-07	hsa-miR-103a-3p;hsa-miR-107;hsa-miR-15a-5p;hsa-miR-15b-
ENSG0000	ENSG0000	0.000149	hsa-let-7a-5p;hsa-let-7b-5p;hsa-let-7d-5p;hsa-let-7f-5p;hsa-l
ENSG0000	ENSG0000	0.007173	hsa-miR-326;hsa-miR-4487;hsa-miR-484;hsa-miR-518c-5p
ENSG0000	ENSG0000	0.000853	hsa-let-7a-5p;hsa-let-7d-5p;hsa-miR-101-3p;hsa-miR-15a-5p;
ENSG0000	ENSG0000	0.007173	hsa-miR-32-5p;hsa-miR-34a-5p;hsa-miR-92a-3p;hsa-miR-92b
ENSG0000	ENSG0000	7.46E-05	hsa-miR-15a-5p;hsa-miR-15b-5p;hsa-miR-16-5p;hsa-miR-195
ENSG0000	ENSG0000	7.46E-05	hsa-miR-15a-5p;hsa-miR-15b-5p;hsa-miR-16-5p;hsa-miR-195
ENSG0000	ENSG0000	0.001227	hsa-miR-27a-3p;hsa-miR-27b-3p;hsa-miR-30b-5p;hsa-miR-30
ENSG0000	ENSG0000	3.83E-05	hsa-miR-15a-5p;hsa-miR-15b-5p;hsa-miR-16-5p;hsa-miR-195
ENSG0000	ENSG0000	0.005969	hsa-miR-30b-5p;hsa-miR-30c-5p;hsa-miR-32-5p;hsa-miR-92a-
ENSG0000	ENSG0000	0.005021	hsa-miR-140-3p;hsa-miR-146a-3p;hsa-miR-301a-5p;hsa-miR-
ENSG0000	ENSG0000	0.000559	hsa-let-7a-5p;hsa-let-7b-5p;hsa-let-7d-5p;hsa-let-7g-5p;hsa-
ENSG0000	ENSG0000	1.21E-09	hsa-let-7a-5p;hsa-miR-139-5p;hsa-miR-15a-3p;hsa-miR-15a-
ENSG0000	ENSG0000	0.000901	hsa-let-7a-5p;hsa-let-7b-5p;hsa-let-7f-5p;hsa-miR-101-3p
ENSG0000	ENSG0000	0.008446	hsa-miR-15a-3p;hsa-miR-15a-5p;hsa-miR-16-5p;hsa-miR-192-
ENSG0000	ENSG0000	0.003354	hsa-miR-1304-3p;hsa-miR-24-3p;hsa-miR-326;hsa-miR-342-3
ENSG0000	ENSG0000	0.003984	hsa-miR-17-5p;hsa-miR-20b-5p;hsa-miR-34a-5p;hsa-miR-519
ENSG0000	ENSG0000	0.005022	hsa-miR-15a-5p;hsa-miR-15b-5p;hsa-miR-16-5p;hsa-miR-195
ENSG0000	ENSG0000	0.005969	hsa-miR-30a-3p;hsa-miR-30d-3p;hsa-miR-30e-3p;hsa-miR-57
ENSG0000	ENSG0000	0.006034	hsa-miR-224-5p;hsa-miR-30b-5p;hsa-miR-34a-5p;hsa-miR-38
ENSG0000	ENSG0000	7.44E-06	hsa-miR-103a-3p;hsa-miR-195-5p;hsa-miR-204-5p;hsa-miR-3
ENSG0000	ENSG0000	0.002063	hsa-miR-107;hsa-miR-15a-5p;hsa-miR-15b-5p;hsa-miR-195-5
ENSG0000	ENSG0000	0.000901	hsa-miR-15a-5p;hsa-miR-15b-5p;hsa-miR-16-5p;hsa-miR-195
ENSG0000	ENSG0000	0.004798	hsa-miR-17-5p;hsa-miR-20b-5p;hsa-miR-218-5p;hsa-miR-448
ENSG0000	ENSG0000	0.000468	hsa-let-7b-5p;hsa-miR-148b-3p;hsa-miR-24-3p;hsa-miR-29a-
ENSG0000	ENSG0000	4.97E-05	hsa-miR-15b-5p;hsa-miR-16-5p;hsa-miR-195-5p;hsa-miR-218
ENSG0000	ENSG0000	0.002682	hsa-miR-15a-5p;hsa-miR-15b-5p;hsa-miR-16-5p;hsa-miR-424

ENSG0000	ENSG0000	0.002856	hsa-miR-103a-3p;hsa-miR-107;hsa-miR-15a-5p;hsa-miR-15b-5
ENSG0000	ENSG0000	5.39E-05	hsa-let-7a-5p;hsa-let-7b-5p;hsa-let-7d-5p;hsa-let-7f-5p;hsa-le
ENSG0000	ENSG0000	1.26E-07	hsa-miR-103a-3p;hsa-miR-107;hsa-miR-122-5p;hsa-miR-214-3
ENSG0000	ENSG0000	8.78E-06	hsa-miR-103a-3p;hsa-miR-107;hsa-miR-15a-5p;hsa-miR-15b-5
ENSG0000	ENSG0000	0.001144	hsa-let-7b-5p;hsa-miR-140-3p;hsa-miR-15a-5p;hsa-miR-15b-5
ENSG0000	ENSG0000	0.000338	hsa-miR-15a-5p;hsa-miR-15b-5p;hsa-miR-195-5p;hsa-miR-27a
ENSG0000	ENSG0000	4.12E-08	hsa-miR-107;hsa-miR-195-5p;hsa-miR-224-5p;hsa-miR-29a-3p
ENSG0000	ENSG0000	3.13E-08	hsa-let-7a-5p;hsa-let-7b-5p;hsa-miR-107;hsa-miR-15a-5p;hsa-
ENSG0000	ENSG0000	0.007539	hsa-miR-16-5p;hsa-miR-17-5p;hsa-miR-20b-5p;hsa-miR-519a-
ENSG0000	ENSG0000	1.09E-05	hsa-let-7a-5p;hsa-let-7b-5p;hsa-let-7d-5p;hsa-let-7f-5p;hsa-le
ENSG0000	ENSG0000	0.000439	hsa-miR-15a-5p;hsa-miR-15b-5p;hsa-miR-16-5p;hsa-miR-195-
ENSG0000	ENSG0000	7.89E-06	hsa-let-7d-5p;hsa-miR-29a-5p;hsa-miR-29b-3p;hsa-miR-29c-3
ENSG0000	ENSG0000	0.003905	hsa-miR-140-3p;hsa-miR-29a-3p;hsa-miR-29b-3p;hsa-miR-29c
ENSG0000	ENSG0000	0.000391	hsa-let-7a-5p;hsa-let-7b-5p;hsa-let-7d-5p;hsa-let-7f-5p;hsa-le
ENSG0000	ENSG0000	0.006245	hsa-let-7a-5p;hsa-let-7b-5p;hsa-let-7d-5p;hsa-let-7f-5p;hsa-le
ENSG0000	ENSG0000	0.004525	hsa-miR-20a-3p;hsa-miR-519a-3p;hsa-miR-519b-3p;hsa-miR-5
ENSG0000	ENSG0000	0.004131	hsa-miR-17-5p;hsa-miR-20b-5p;hsa-miR-218-5p;hsa-miR-93-5
ENSG0000	ENSG0000	4.66E-06	hsa-miR-126-5p;hsa-miR-1304-3p;hsa-miR-15a-5p;hsa-miR-16
ENSG0000	ENSG0000	2.46E-05	hsa-let-7a-5p;hsa-let-7b-5p;hsa-let-7d-5p;hsa-let-7f-5p;hsa-le
ENSG0000	ENSG0000	0.009859	hsa-miR-103a-3p;hsa-miR-107;hsa-miR-126-5p;hsa-miR-20a-3
ENSG0000	ENSG0000	0.001127	hsa-let-7a-5p;hsa-let-7d-5p;hsa-let-7f-5p;hsa-let-7g-5p;hsa-le
ENSG0000	ENSG0000	0.001196	hsa-miR-126-5p;hsa-miR-15a-5p;hsa-miR-15b-5p;hsa-miR-16-
ENSG0000	ENSG0000	0.006801	hsa-miR-145-5p;hsa-miR-29a-3p;hsa-miR-32-5p;hsa-miR-92a-
ENSG0000	ENSG0000	0.003354	hsa-miR-103a-3p;hsa-miR-107;hsa-miR-218-5p;hsa-miR-34a-5
ENSG0000	ENSG0000	0.001694	hsa-miR-122-5p;hsa-miR-1304-3p;hsa-miR-143-3p;hsa-miR-31
ENSG0000	ENSG0000	0.000334	hsa-miR-129-5p;hsa-miR-146a-3p;hsa-miR-15a-5p;hsa-miR-15
ENSG0000	ENSG0000	4.66E-07	hsa-miR-103a-3p;hsa-miR-107;hsa-miR-15a-5p;hsa-miR-15b-5
ENSG0000	ENSG0000	8.72E-05	hsa-miR-15a-5p;hsa-miR-15b-5p;hsa-miR-16-5p;hsa-miR-195-
ENSG0000	ENSG0000	1.21E-07	hsa-miR-103a-3p;hsa-miR-107;hsa-miR-15a-5p;hsa-miR-15b-5
ENSG0000	ENSG0000	0.001261	hsa-let-7a-5p;hsa-let-7b-5p;hsa-let-7d-5p;hsa-let-7f-5p;hsa-le
ENSG0000	ENSG0000	0.006331	hsa-let-7a-5p;hsa-let-7b-5p;hsa-let-7d-5p;hsa-let-7f-5p;hsa-le
ENSG0000	ENSG0000	3.32E-05	hsa-miR-101-3p;hsa-miR-17-5p;hsa-miR-20b-5p;hsa-miR-32-5
ENSG0000	ENSG0000	0.003354	hsa-miR-218-5p;hsa-miR-29b-3p;hsa-miR-30c-5p;hsa-miR-342
ENSG0000	ENSG0000	0.003349	hsa-miR-143-3p;hsa-miR-29a-3p;hsa-miR-29b-3p;hsa-miR-29c
ENSG0000	ENSG0000	0.006801	hsa-let-7a-5p;hsa-let-7f-5p;hsa-let-7g-5p;hsa-let-7i-5p;hsa-m
ENSG0000	ENSG0000	0.005022	hsa-miR-126-5p;hsa-miR-17-5p;hsa-miR-20b-5p;hsa-miR-93-5
ENSG0000	ENSG0000	0.001384	hsa-miR-132-3p;hsa-miR-145-5p;hsa-miR-30a-3p;hsa-miR-30c
ENSG0000	ENSG0000	1.85E-05	hsa-let-7a-5p;hsa-let-7b-5p;hsa-let-7d-5p;hsa-let-7f-5p;hsa-le
ENSG0000	ENSG0000	7.46E-05	hsa-let-7a-5p;hsa-let-7b-5p;hsa-let-7d-5p;hsa-let-7f-5p;hsa-le
ENSG0000	ENSG0000	0.000628	hsa-let-7a-5p;hsa-miR-17-5p;hsa-miR-20b-5p;hsa-miR-330-3p
ENSG0000	ENSG0000	0.005926	hsa-let-7a-5p;hsa-let-7b-5p;hsa-miR-17-5p;hsa-miR-20b-5p;h
ENSG0000	ENSG0000	0.005926	hsa-let-7a-5p;hsa-let-7b-5p;hsa-miR-17-5p;hsa-miR-20b-5p;h
ENSG0000	ENSG0000	0.000309	hsa-miR-15a-5p;hsa-miR-15b-5p;hsa-miR-195-5p;hsa-miR-20b
ENSG0000	ENSG0000	0.000382	hsa-miR-122-5p;hsa-miR-132-3p;hsa-miR-145-5p;hsa-miR-16-
ENSG0000	ENSG0000	0.007512	hsa-miR-103a-3p;hsa-miR-107;hsa-miR-15a-5p;hsa-miR-15b-5
ENSG0000	ENSG0000	0.006034	hsa-miR-17-5p;hsa-miR-20b-5p;hsa-miR-218-5p;hsa-miR-93-5
ENSG0000	ENSG0000	5.47E-05	hsa-miR-17-5p;hsa-miR-20b-5p;hsa-miR-3194-3p;hsa-miR-501
ENSG0000	ENSG0000	0.000332	hsa-miR-218-5p;hsa-miR-29b-3p;hsa-miR-519a-3p;hsa-miR-51
ENSG0000	ENSG0000	4.77E-05	hsa-miR-103a-3p;hsa-miR-107;hsa-miR-15a-5p;hsa-miR-15b-5
ENSG0000	ENSG0000	1.76E-06	hsa-let-7a-5p;hsa-let-7b-5p;hsa-let-7d-5p;hsa-let-7f-5p;hsa-le
ENSG0000	ENSG0000	0.002214	hsa-miR-17-5p;hsa-miR-20b-5p;hsa-miR-24-3p;hsa-miR-29a-3
ENSG0000	ENSG0000	0.00069	hsa-miR-129-5p;hsa-miR-145-5p;hsa-miR-192-5p;hsa-miR-20b
ENSG0000	ENSG0000	0.003349	hsa-miR-129-5p;hsa-miR-30a-3p;hsa-miR-30d-3p;hsa-miR-30e
ENSG0000	ENSG0000	6.54E-05	hsa-miR-15a-5p;hsa-miR-15b-5p;hsa-miR-16-5p;hsa-miR-195-

ENSG0000	ENSG0000	0.003349	hsa-let-7b-5p;hsa-miR-101-3p;hsa-miR-1260b;hsa-miR-214-3
ENSG0000	ENSG0000	0.000568	hsa-miR-17-5p;hsa-miR-20b-5p;hsa-miR-224-5p;hsa-miR-519
ENSG0000	ENSG0000	0.002682	hsa-miR-15b-5p;hsa-miR-16-5p;hsa-miR-30c-5p;hsa-miR-497
ENSG0000	ENSG0000	0.003354	hsa-miR-29a-3p;hsa-miR-29b-3p;hsa-miR-29c-3p;hsa-miR-48
ENSG0000	ENSG0000	4.18E-05	hsa-miR-15a-5p;hsa-miR-15b-5p;hsa-miR-16-5p;hsa-miR-195
ENSG0000	ENSG0000	0.001836	hsa-miR-15a-5p;hsa-miR-15b-5p;hsa-miR-16-5p;hsa-miR-195
ENSG0000	ENSG0000	2.90E-05	hsa-miR-15a-5p;hsa-miR-15b-5p;hsa-miR-16-5p;hsa-miR-195
ENSG0000	ENSG0000	0.000382	hsa-let-7a-5p;hsa-let-7b-5p;hsa-let-7d-5p;hsa-let-7f-5p;hsa-l
ENSG0000	ENSG0000	0.009324	hsa-miR-101-3p;hsa-miR-139-5p;hsa-miR-29a-3p;hsa-miR-29
ENSG0000	ENSG0000	1.93E-05	hsa-let-7a-5p;hsa-let-7b-5p;hsa-let-7d-5p;hsa-let-7f-5p;hsa-l
ENSG0000	ENSG0000	1.71E-05	hsa-let-7a-5p;hsa-let-7b-5p;hsa-let-7d-5p;hsa-let-7f-5p;hsa-l
ENSG0000	ENSG0000	2.30E-05	hsa-miR-222-5p;hsa-miR-30a-3p;hsa-miR-30d-3p;hsa-miR-30
ENSG0000	ENSG0000	0.001106	hsa-let-7a-5p;hsa-let-7b-5p;hsa-let-7d-5p;hsa-let-7f-5p;hsa-l
ENSG0000	ENSG0000	9.35E-08	hsa-let-7a-5p;hsa-let-7b-5p;hsa-let-7d-5p;hsa-let-7f-5p;hsa-l
ENSG0000	ENSG0000	0.001544	hsa-miR-122-5p;hsa-miR-129-5p;hsa-miR-1304-3p;hsa-miR-1
ENSG0000	ENSG0000	0.001849	hsa-let-7a-5p;hsa-let-7d-5p;hsa-let-7f-5p;hsa-let-7g-5p;hsa-l
ENSG0000	ENSG0000	3.13E-09	hsa-miR-129-5p;hsa-miR-15a-5p;hsa-miR-15b-5p;hsa-miR-19
ENSG0000	ENSG0000	0.001127	hsa-miR-129-5p;hsa-miR-32-5p;hsa-miR-548b-3p;hsa-miR-92
ENSG0000	ENSG0000	8.78E-06	hsa-miR-103a-3p;hsa-miR-107;hsa-miR-15a-5p;hsa-miR-15b-
ENSG0000	ENSG0000	6.35E-06	hsa-miR-132-3p;hsa-miR-15a-5p;hsa-miR-15b-5p;hsa-miR-19
ENSG0000	ENSG0000	0.001811	hsa-let-7a-5p;hsa-let-7b-5p;hsa-let-7d-5p;hsa-let-7f-5p;hsa-l
ENSG0000	ENSG0000	0.002411	hsa-miR-17-5p;hsa-miR-20b-5p;hsa-miR-30b-5p;hsa-miR-30c
ENSG0000	ENSG0000	2.71E-06	hsa-let-7a-5p;hsa-let-7b-5p;hsa-let-7d-5p;hsa-let-7f-5p;hsa-l
ENSG0000	ENSG0000	0.000185	hsa-miR-192-5p;hsa-miR-224-5p;hsa-miR-27a-3p;hsa-miR-27
ENSG0000	ENSG0000	0.001261	hsa-miR-15a-5p;hsa-miR-15b-5p;hsa-miR-16-5p;hsa-miR-195
ENSG0000	ENSG0000	5.07E-06	hsa-miR-15a-5p;hsa-miR-15b-5p;hsa-miR-16-5p;hsa-miR-195
ENSG0000	ENSG0000	0.002682	hsa-miR-32-5p;hsa-miR-550a-3p;hsa-miR-92a-3p;hsa-miR-92
ENSG0000	ENSG0000	0.007173	hsa-miR-129-5p;hsa-miR-15a-5p;hsa-miR-16-5p;hsa-miR-574
ENSG0000	ENSG0000	0.007173	hsa-miR-129-5p;hsa-miR-15a-5p;hsa-miR-16-5p;hsa-miR-574
ENSG0000	ENSG0000	1.00E-05	hsa-let-7b-5p;hsa-miR-122-5p;hsa-miR-20a-3p;hsa-miR-27a-
ENSG0000	ENSG0000	0.00375	hsa-let-7a-5p;hsa-let-7b-5p;hsa-let-7d-5p;hsa-let-7f-5p;hsa-l
ENSG0000	ENSG0000	0.00039	hsa-miR-107;hsa-miR-145-5p;hsa-miR-17-5p;hsa-miR-20b-5p;
ENSG0000	ENSG0000	7.19E-06	hsa-miR-17-5p;hsa-miR-20b-5p;hsa-miR-27b-3p;hsa-miR-519
ENSG0000	ENSG0000	0.003936	hsa-miR-129-5p;hsa-miR-15a-5p;hsa-miR-15b-5p;hsa-miR-16
ENSG0000	ENSG0000	1.07E-06	hsa-miR-107;hsa-miR-140-3p;hsa-miR-15a-5p;hsa-miR-15b-5
ENSG0000	ENSG0000	0.000853	hsa-miR-15a-5p;hsa-miR-15b-5p;hsa-miR-16-5p;hsa-miR-195
ENSG0000	ENSG0000	8.32E-05	hsa-miR-218-5p;hsa-miR-224-5p;hsa-miR-27a-3p;hsa-miR-27
ENSG0000	ENSG0000	0.000159	hsa-miR-129-5p;hsa-miR-17-5p;hsa-miR-20b-5p;hsa-miR-27a
ENSG0000	ENSG0000	4.05E-07	hsa-miR-101-3p;hsa-miR-103a-3p;hsa-miR-107;hsa-miR-15a-
ENSG0000	ENSG0000	0.000202	hsa-miR-15a-5p;hsa-miR-17-5p;hsa-miR-195-5p;hsa-miR-20b
ENSG0000	ENSG0000	0.003275	hsa-miR-101-3p;hsa-miR-139-5p;hsa-miR-148b-3p;hsa-miR-2
ENSG0000	ENSG0000	0.006034	hsa-miR-103a-3p;hsa-miR-107;hsa-miR-7-5p;hsa-miR-922
ENSG0000	ENSG0000	1.62E-06	hsa-miR-15a-5p;hsa-miR-15b-5p;hsa-miR-16-5p;hsa-miR-29b
ENSG0000	ENSG0000	0.00353	hsa-let-7b-5p;hsa-miR-16-5p;hsa-miR-29a-3p;hsa-miR-29b-3
ENSG0000	ENSG0000	0.005784	hsa-miR-16-5p;hsa-miR-218-5p;hsa-miR-24-3p;hsa-miR-342-
ENSG0000	ENSG0000	0.000338	hsa-miR-218-5p;hsa-miR-320a;hsa-miR-32-5p;hsa-miR-4708-
ENSG0000	ENSG0000	0.004245	hsa-let-7a-5p;hsa-let-7b-5p;hsa-let-7d-5p;hsa-let-7f-5p;hsa-l
ENSG0000	ENSG0000	6.77E-05	hsa-let-7a-5p;hsa-let-7b-5p;hsa-let-7d-5p;hsa-let-7f-5p;hsa-l
ENSG0000	ENSG0000	7.89E-06	hsa-let-7d-5p;hsa-let-7f-5p;hsa-let-7g-5p;hsa-let-7i-5p
ENSG0000	ENSG0000	0.003493	hsa-miR-17-5p;hsa-miR-20b-5p;hsa-miR-214-3p;hsa-miR-32-
ENSG0000	ENSG0000	0.000332	hsa-miR-222-5p;hsa-miR-30a-3p;hsa-miR-30d-3p;hsa-miR-30
ENSG0000	ENSG0000	0.000728	hsa-miR-143-3p;hsa-miR-17-5p;hsa-miR-20b-5p;hsa-miR-218
ENSG0000	ENSG0000	0.002022	hsa-miR-145-5p;hsa-miR-32-5p;hsa-miR-92a-3p;hsa-miR-92b
ENSG0000	ENSG0000	1.23E-05	hsa-miR-17-5p;hsa-miR-20b-5p;hsa-miR-30b-5p;hsa-miR-30c

ENSG0000	ENSG0000	0.000123	hsa-miR-148b-3p;hsa-miR-15a-5p;hsa-miR-15b-5p;hsa-miR-1
ENSG0000	ENSG0000	0.004641	hsa-miR-17-5p;hsa-miR-20b-5p;hsa-miR-519a-3p;hsa-miR-51
ENSG0000	ENSG0000	0.008446	hsa-miR-129-5p;hsa-miR-143-3p;hsa-miR-361-5p;hsa-miR-37
ENSG0000	ENSG0000	0.000439	hsa-miR-15a-5p;hsa-miR-15b-5p;hsa-miR-195-5p;hsa-miR-32
ENSG0000	ENSG0000	0.003945	hsa-miR-132-3p;hsa-miR-15a-5p;hsa-miR-15b-5p;hsa-miR-19
ENSG0000	ENSG0000	5.34E-05	hsa-miR-15a-5p;hsa-miR-15b-5p;hsa-miR-16-5p;hsa-miR-195
ENSG0000	ENSG0000	0.00084	hsa-let-7a-5p;hsa-let-7b-5p;hsa-let-7f-5p;hsa-let-7g-5p;hsa-l
ENSG0000	ENSG0000	0.001336	hsa-let-7a-5p;hsa-let-7g-5p;hsa-miR-143-3p;hsa-miR-30a-3p;
ENSG0000	ENSG0000	9.47E-08	hsa-miR-15a-5p;hsa-miR-15b-5p;hsa-miR-16-5p;hsa-miR-195
ENSG0000	ENSG0000	9.47E-08	hsa-miR-15a-5p;hsa-miR-15b-5p;hsa-miR-16-5p;hsa-miR-195
ENSG0000	ENSG0000	4.25E-08	hsa-miR-15a-5p;hsa-miR-15b-5p;hsa-miR-16-5p;hsa-miR-17-
ENSG0000	ENSG0000	8.09E-08	hsa-miR-140-3p;hsa-miR-15a-5p;hsa-miR-15b-5p;hsa-miR-19
ENSG0000	ENSG0000	0.008469	hsa-miR-101-3p;hsa-miR-30a-3p;hsa-miR-30d-3p;hsa-miR-30
ENSG0000	ENSG0000	0.00084	hsa-miR-122-5p;hsa-miR-1304-3p;hsa-miR-24-3p;hsa-miR-29
ENSG0000	ENSG0000	2.91E-05	hsa-miR-140-3p;hsa-miR-17-5p;hsa-miR-20b-5p;hsa-miR-342
ENSG0000	ENSG0000	4.49E-08	hsa-miR-101-3p;hsa-miR-103a-3p;hsa-miR-107;hsa-miR-15a-
ENSG0000	ENSG0000	0.000217	hsa-let-7a-5p;hsa-let-7b-5p;hsa-let-7d-5p;hsa-let-7f-5p;hsa-l
ENSG0000	ENSG0000	0.000123	hsa-miR-103a-3p;hsa-miR-107;hsa-miR-15a-5p;hsa-miR-15b-
ENSG0000	ENSG0000	0.00978	hsa-miR-17-5p;hsa-miR-204-5p;hsa-miR-20b-5p;hsa-miR-671
ENSG0000	ENSG0000	0.001836	hsa-miR-132-3p;hsa-miR-145-5p;hsa-miR-15b-5p;hsa-miR-27
ENSG0000	ENSG0000	0.00084	hsa-miR-17-5p;hsa-miR-224-5p;hsa-miR-27a-3p;hsa-miR-27b
ENSG0000	ENSG0000	0.00134	hsa-miR-15a-5p;hsa-miR-195-5p;hsa-miR-32-5p;hsa-miR-424
ENSG0000	ENSG0000	3.47E-06	hsa-let-7a-5p;hsa-let-7b-5p;hsa-let-7d-5p;hsa-let-7f-5p;hsa-l
ENSG0000	ENSG0000	2.32E-06	hsa-let-7a-5p;hsa-let-7b-5p;hsa-let-7d-5p;hsa-let-7f-5p;hsa-l
ENSG0000	ENSG0000	1.85E-05	hsa-let-7a-5p;hsa-let-7b-5p;hsa-let-7d-5p;hsa-let-7f-5p;hsa-l
ENSG0000	ENSG0000	0.002682	hsa-miR-188-3p;hsa-miR-32-5p;hsa-miR-92a-3p;hsa-miR-92b
ENSG0000	ENSG0000	5.07E-10	hsa-miR-101-3p;hsa-miR-15a-5p;hsa-miR-16-5p;hsa-miR-17-
ENSG0000	ENSG0000	0.001048	hsa-miR-122-5p;hsa-miR-17-5p;hsa-miR-195-5p;hsa-miR-20b
ENSG0000	ENSG0000	0.005696	hsa-let-7a-5p;hsa-let-7b-5p;hsa-let-7d-5p;hsa-let-7g-5p;hsa-
ENSG0000	ENSG0000	0.002022	hsa-miR-139-5p;hsa-miR-218-5p;hsa-miR-27b-3p;hsa-miR-34
ENSG0000	ENSG0000	5.76E-05	hsa-miR-132-3p;hsa-miR-143-3p;hsa-miR-15b-5p;hsa-miR-32
ENSG0000	ENSG0000	0.001626	hsa-miR-1260b;hsa-miR-15a-5p;hsa-miR-188-3p;hsa-miR-227
ENSG0000	ENSG0000	0.008703	hsa-miR-15a-5p;hsa-miR-15b-5p;hsa-miR-195-5p;hsa-miR-42
ENSG0000	ENSG0000	2.19E-05	hsa-miR-107;hsa-miR-15a-5p;hsa-miR-16-5p;hsa-miR-34a-5p;
ENSG0000	ENSG0000	0.000338	hsa-let-7a-5p;hsa-let-7d-5p;hsa-let-7g-5p;hsa-miR-145-5p;hs
ENSG0000	ENSG0000	0.001811	hsa-miR-101-3p;hsa-miR-103a-3p;hsa-miR-126-5p;hsa-miR-2
ENSG0000	ENSG0000	0.000817	hsa-miR-17-5p;hsa-miR-20b-5p;hsa-miR-218-5p;hsa-miR-32-
ENSG0000	ENSG0000	0.002033	hsa-miR-15a-5p;hsa-miR-15b-5p;hsa-miR-195-5p;hsa-miR-42
ENSG0000	ENSG0000	6.46E-07	hsa-let-7a-5p;hsa-let-7b-5p;hsa-let-7d-5p;hsa-let-7f-5p;hsa-l
ENSG0000	ENSG0000	0.002682	hsa-miR-122-5p;hsa-miR-29a-3p;hsa-miR-29b-3p;hsa-miR-29
ENSG0000	ENSG0000	0.001681	hsa-miR-145-5p;hsa-miR-218-5p;hsa-miR-29a-3p;hsa-miR-29
ENSG0000	ENSG0000	0.002682	hsa-miR-143-3p;hsa-miR-32-5p;hsa-miR-92a-3p;hsa-miR-92b
ENSG0000	ENSG0000	1.09E-05	hsa-miR-148b-3p;hsa-miR-17-5p;hsa-miR-20b-5p;hsa-miR-32
ENSG0000	ENSG0000	0.000193	hsa-miR-101-3p;hsa-miR-204-5p;hsa-miR-24-3p;hsa-miR-27b
ENSG0000	ENSG0000	1.46E-05	hsa-miR-15a-5p;hsa-miR-15b-5p;hsa-miR-16-5p;hsa-miR-195
ENSG0000	ENSG0000	0.006116	hsa-let-7a-5p;hsa-let-7b-5p;hsa-let-7d-5p;hsa-let-7f-5p;hsa-l
ENSG0000	ENSG0000	3.36E-08	hsa-let-7a-5p;hsa-let-7b-5p;hsa-miR-218-5p;hsa-miR-2278;hs
ENSG0000	ENSG0000	2.46E-05	hsa-let-7a-5p;hsa-let-7b-5p;hsa-let-7d-5p;hsa-let-7f-5p;hsa-l
ENSG0000	ENSG0000	0.000248	hsa-let-7a-5p;hsa-let-7b-5p;hsa-let-7d-5p;hsa-let-7f-5p;hsa-l
ENSG0000	ENSG0000	0.000248	hsa-let-7a-5p;hsa-let-7b-5p;hsa-let-7d-5p;hsa-let-7f-5p;hsa-l
ENSG0000	ENSG0000	0.00105	hsa-miR-103a-3p;hsa-miR-107;hsa-miR-145-5p;hsa-miR-15a-
ENSG0000	ENSG0000	0.000493	hsa-miR-107;hsa-miR-140-3p;hsa-miR-148b-3p;hsa-miR-15a-
ENSG0000	ENSG0000	0.000684	hsa-let-7a-5p;hsa-let-7b-5p;hsa-let-7d-5p;hsa-let-7f-5p;hsa-l
ENSG0000	ENSG0000	1.73E-05	hsa-miR-143-3p;hsa-miR-145-5p;hsa-miR-192-5p;hsa-miR-20

ENSG0000	ENSG0000	0.000293	hsa-miR-103a-3p;hsa-miR-107;hsa-miR-218-5p;hsa-miR-30b-5
ENSG0000	ENSG0000	9.03E-05	hsa-let-7a-5p;hsa-let-7b-5p;hsa-let-7d-5p;hsa-let-7f-5p;hsa-le
ENSG0000	ENSG0000	9.03E-05	hsa-let-7a-5p;hsa-let-7b-5p;hsa-let-7d-5p;hsa-let-7f-5p;hsa-le
ENSG0000	ENSG0000	9.03E-05	hsa-let-7a-5p;hsa-let-7b-5p;hsa-let-7d-5p;hsa-let-7f-5p;hsa-le
ENSG0000	ENSG0000	9.03E-05	hsa-let-7a-5p;hsa-let-7b-5p;hsa-let-7d-5p;hsa-let-7f-5p;hsa-le
ENSG0000	ENSG0000	9.03E-05	hsa-let-7a-5p;hsa-let-7b-5p;hsa-let-7d-5p;hsa-let-7f-5p;hsa-le
ENSG0000	ENSG0000	9.03E-05	hsa-let-7a-5p;hsa-let-7b-5p;hsa-let-7d-5p;hsa-let-7f-5p;hsa-le
ENSG0000	ENSG0000	9.03E-05	hsa-let-7a-5p;hsa-let-7b-5p;hsa-let-7d-5p;hsa-let-7f-5p;hsa-le
ENSG0000	ENSG0000	0.000566	hsa-miR-15a-5p;hsa-miR-15b-5p;hsa-miR-195-5p;hsa-miR-424
ENSG0000	ENSG0000	9.47E-08	hsa-miR-15a-5p;hsa-miR-15b-5p;hsa-miR-16-5p;hsa-miR-195-
ENSG0000	ENSG0000	9.47E-08	hsa-miR-15a-5p;hsa-miR-15b-5p;hsa-miR-16-5p;hsa-miR-195-
ENSG0000	ENSG0000	0.005609	hsa-miR-139-5p;hsa-miR-15a-5p;hsa-miR-15b-5p;hsa-miR-195
ENSG0000	ENSG0000	2.49E-06	hsa-let-7a-5p;hsa-let-7b-5p;hsa-let-7d-5p;hsa-let-7g-5p;hsa-l
ENSG0000	ENSG0000	3.65E-05	hsa-miR-103a-3p;hsa-miR-107;hsa-miR-29a-3p;hsa-miR-29b-3
ENSG0000	ENSG0000	1.62E-06	hsa-miR-15a-5p;hsa-miR-15b-5p;hsa-miR-16-5p;hsa-miR-195-
ENSG0000	ENSG0000	0.001144	hsa-miR-107;hsa-miR-15a-5p;hsa-miR-15b-5p;hsa-miR-195-5p
ENSG0000	ENSG0000	0.000309	hsa-miR-101-3p;hsa-miR-32-5p;hsa-miR-582-5p;hsa-miR-7-5p
ENSG0000	ENSG0000	2.77E-08	hsa-miR-122-5p;hsa-miR-17-5p;hsa-miR-20b-5p;hsa-miR-27a-
ENSG0000	ENSG0000	7.37E-06	hsa-miR-107;hsa-miR-126-5p;hsa-miR-15a-5p;hsa-miR-15b-5p
ENSG0000	ENSG0000	2.71E-05	hsa-let-7a-5p;hsa-let-7d-5p;hsa-let-7f-5p;hsa-let-7g-5p;hsa-le
ENSG0000	ENSG0000	0.008204	hsa-let-7a-5p;hsa-let-7b-5p;hsa-let-7d-5p;hsa-let-7f-5p;hsa-le
ENSG0000	ENSG0000	6.54E-05	hsa-miR-15a-5p;hsa-miR-15b-5p;hsa-miR-17-5p;hsa-miR-195-
ENSG0000	ENSG0000	0.000345	hsa-let-7a-5p;hsa-let-7b-5p;hsa-let-7d-5p;hsa-let-7f-5p;hsa-le
ENSG0000	ENSG0000	0.005471	hsa-let-7a-5p;hsa-let-7b-5p;hsa-let-7f-5p;hsa-let-7g-5p;hsa-le
ENSG0000	ENSG0000	7.89E-06	hsa-miR-15a-5p;hsa-miR-15b-5p;hsa-miR-16-5p;hsa-miR-195-
ENSG0000	ENSG0000	5.76E-05	hsa-miR-148b-3p;hsa-miR-218-5p;hsa-miR-30a-3p;hsa-miR-30
ENSG0000	ENSG0000	0.006116	hsa-miR-122-5p;hsa-miR-1304-3p;hsa-miR-27a-3p;hsa-miR-27
ENSG0000	ENSG0000	0.000154	hsa-miR-30a-3p;hsa-miR-30b-5p;hsa-miR-30c-5p;hsa-miR-30c
ENSG0000	ENSG0000	0.001681	hsa-miR-1260b;hsa-miR-188-3p;hsa-miR-32-5p;hsa-miR-92a-3
ENSG0000	ENSG0000	6.72E-06	hsa-miR-107;hsa-miR-15a-5p;hsa-miR-15b-5p;hsa-miR-17-5p;
ENSG0000	ENSG0000	0.000196	hsa-miR-29a-3p;hsa-miR-29b-3p;hsa-miR-29c-3p;hsa-miR-319
ENSG0000	ENSG0000	0.001261	hsa-let-7a-5p;hsa-let-7b-5p;hsa-let-7d-5p;hsa-let-7f-5p;hsa-le
ENSG0000	ENSG0000	5.89E-07	hsa-miR-101-3p;hsa-miR-15a-5p;hsa-miR-15b-5p;hsa-miR-16-
ENSG0000	ENSG0000	0.002852	hsa-miR-17-5p;hsa-miR-20b-5p;hsa-miR-30b-5p;hsa-miR-30c-
ENSG0000	ENSG0000	0.00084	hsa-miR-15a-5p;hsa-miR-15b-5p;hsa-miR-16-5p;hsa-miR-195-
ENSG0000	ENSG0000	0.00105	hsa-let-7a-5p;hsa-let-7b-5p;hsa-let-7d-5p;hsa-let-7f-5p;hsa-le
ENSG0000	ENSG0000	0.000142	hsa-miR-103a-3p;hsa-miR-107;hsa-miR-17-5p;hsa-miR-20a-3p
ENSG0000	ENSG0000	0.000142	hsa-miR-103a-3p;hsa-miR-107;hsa-miR-17-5p;hsa-miR-20a-3p
ENSG0000	ENSG0000	0.001106	hsa-miR-139-5p;hsa-miR-15a-3p;hsa-miR-17-5p;hsa-miR-20b-
ENSG0000	ENSG0000	1.23E-05	hsa-let-7b-5p;hsa-miR-101-3p;hsa-miR-143-3p;hsa-miR-16-5p
ENSG0000	ENSG0000	9.03E-05	hsa-miR-15a-5p;hsa-miR-15b-5p;hsa-miR-16-5p;hsa-miR-195-
ENSG0000	ENSG0000	9.03E-05	hsa-miR-15a-5p;hsa-miR-15b-5p;hsa-miR-16-5p;hsa-miR-195-
ENSG0000	ENSG0000	0.001489	hsa-let-7a-5p;hsa-let-7b-5p;hsa-let-7d-5p;hsa-let-7f-5p;hsa-le
ENSG0000	ENSG0000	0.000966	hsa-miR-122-5p;hsa-miR-15a-5p;hsa-miR-15b-5p;hsa-miR-16-
ENSG0000	ENSG0000	6.94E-07	hsa-miR-103a-3p;hsa-miR-107;hsa-miR-122-5p;hsa-miR-17-5p
ENSG0000	ENSG0000	0.008204	hsa-miR-17-5p;hsa-miR-20b-5p;hsa-miR-29a-3p;hsa-miR-519a
ENSG0000	ENSG0000	0.005969	hsa-miR-15a-3p;hsa-miR-224-5p;hsa-miR-24-3p;hsa-miR-501-
ENSG0000	ENSG0000	4.15E-08	hsa-miR-101-3p;hsa-miR-103a-3p;hsa-miR-107;hsa-miR-122-5
ENSG0000	ENSG0000	0.000722	hsa-miR-218-5p;hsa-miR-30b-5p;hsa-miR-30c-5p;hsa-miR-92a
ENSG0000	ENSG0000	7.19E-06	hsa-miR-17-5p;hsa-miR-20b-5p;hsa-miR-218-5p;hsa-miR-519a
ENSG0000	ENSG0000	0.000278	hsa-let-7a-5p;hsa-let-7b-5p;hsa-let-7d-5p;hsa-let-7f-5p;hsa-le
ENSG0000	ENSG0000	8.70E-05	hsa-miR-15a-5p;hsa-miR-15b-5p;hsa-miR-16-5p;hsa-miR-195-
ENSG0000	ENSG0000	8.70E-05	hsa-miR-15a-5p;hsa-miR-15b-5p;hsa-miR-16-5p;hsa-miR-195-
ENSG0000	ENSG0000	0.000178	hsa-miR-17-5p;hsa-miR-20b-5p;hsa-miR-30b-5p;hsa-miR-30c-

ENSG0000	ENSG0000	0.008703	hsa-miR-15a-3p;hsa-miR-24-3p;hsa-miR-342-3p;hsa-miR-4708
ENSG0000	ENSG0000	5.22E-05	hsa-miR-15a-5p;hsa-miR-15b-5p;hsa-miR-16-5p;hsa-miR-195-
ENSG0000	ENSG0000	0.003115	hsa-miR-145-5p;hsa-miR-15a-5p;hsa-miR-15b-5p;hsa-miR-195
ENSG0000	ENSG0000	0.005784	hsa-let-7a-5p;hsa-let-7b-5p;hsa-let-7d-5p;hsa-let-7f-5p;hsa-le
ENSG0000	ENSG0000	0.004934	hsa-miR-101-3p;hsa-miR-1304-3p;hsa-miR-139-5p;hsa-miR-14
ENSG0000	ENSG0000	0.000154	hsa-let-7a-5p;hsa-let-7b-5p;hsa-let-7d-5p;hsa-let-7f-5p;hsa-le
ENSG0000	ENSG0000	2.90E-05	hsa-miR-103a-3p;hsa-miR-107;hsa-miR-15a-5p;hsa-miR-15b-5
ENSG0000	ENSG0000	0.002887	hsa-miR-107;hsa-miR-15a-5p;hsa-miR-15b-5p;hsa-miR-195-5p
ENSG0000	ENSG0000	0.0031	hsa-miR-107;hsa-miR-15a-5p;hsa-miR-15b-5p;hsa-miR-195-5p
ENSG0000	ENSG0000	0.002062	hsa-miR-1304-3p;hsa-miR-17-5p;hsa-miR-20b-5p;hsa-miR-30a
ENSG0000	ENSG0000	0.001501	hsa-miR-122-5p;hsa-miR-1260b;hsa-miR-1304-3p;hsa-miR-15a
ENSG0000	ENSG0000	2.39E-06	hsa-miR-103a-3p;hsa-miR-107;hsa-miR-122-5p;hsa-miR-15a-5
ENSG0000	ENSG0000	2.02E-06	hsa-miR-15a-5p;hsa-miR-15b-5p;hsa-miR-16-5p;hsa-miR-195-
ENSG0000	ENSG0000	0.006807	hsa-miR-27a-3p;hsa-miR-27b-3p;hsa-miR-29a-3p;hsa-miR-29b
ENSG0000	ENSG0000	0.006807	hsa-miR-27a-3p;hsa-miR-27b-3p;hsa-miR-29a-3p;hsa-miR-29b
ENSG0000	ENSG0000	0.000437	hsa-miR-214-3p;hsa-miR-218-5p;hsa-miR-24-3p;hsa-miR-7-5p
ENSG0000	ENSG0000	1.16E-08	hsa-miR-15a-5p;hsa-miR-15b-5p;hsa-miR-16-5p;hsa-miR-195-
ENSG0000	ENSG0000	0.002062	hsa-miR-143-3p;hsa-miR-145-5p;hsa-miR-214-3p;hsa-miR-24-
ENSG0000	ENSG0000	0.003258	hsa-let-7a-5p;hsa-let-7b-5p;hsa-let-7d-5p;hsa-let-7f-5p;hsa-le
ENSG0000	ENSG0000	0.003258	hsa-let-7a-5p;hsa-let-7b-5p;hsa-let-7d-5p;hsa-let-7f-5p;hsa-le
ENSG0000	ENSG0000	0.000179	hsa-miR-148b-3p;hsa-miR-204-5p;hsa-miR-30a-3p;hsa-miR-30
ENSG0000	ENSG0000	0.007467	hsa-miR-204-5p;hsa-miR-218-5p;hsa-miR-27a-3p;hsa-miR-27b
ENSG0000	ENSG0000	0.000246	hsa-miR-192-5p;hsa-miR-29a-3p;hsa-miR-29a-5p;hsa-miR-29c
ENSG0000	ENSG0000	0.003349	hsa-miR-139-5p;hsa-miR-15a-5p;hsa-miR-15b-5p;hsa-miR-195
ENSG0000	ENSG0000	1.50E-05	hsa-miR-15a-5p;hsa-miR-15b-5p;hsa-miR-16-5p;hsa-miR-195-
ENSG0000	ENSG0000	3.47E-06	hsa-miR-15a-5p;hsa-miR-15b-5p;hsa-miR-16-5p;hsa-miR-195-
ENSG0000	ENSG0000	4.73E-05	hsa-let-7a-5p;hsa-let-7b-5p;hsa-let-7d-5p;hsa-let-7f-5p;hsa-le
ENSG0000	ENSG0000	1.38E-05	hsa-miR-218-5p;hsa-miR-222-5p;hsa-miR-30a-3p;hsa-miR-30b
ENSG0000	ENSG0000	0.002033	hsa-let-7a-5p;hsa-let-7b-5p;hsa-let-7d-5p;hsa-let-7f-5p;hsa-le
ENSG0000	ENSG0000	3.36E-06	hsa-let-7a-5p;hsa-let-7b-5p;hsa-let-7d-5p;hsa-let-7f-5p;hsa-le
ENSG0000	ENSG0000	0.00978	hsa-miR-15a-5p;hsa-miR-15b-5p;hsa-miR-195-5p;hsa-miR-424
ENSG0000	ENSG0000	3.47E-06	hsa-let-7a-5p;hsa-let-7b-5p;hsa-let-7d-5p;hsa-let-7f-5p;hsa-le
ENSG0000	ENSG0000	7.26E-05	hsa-miR-101-3p;hsa-miR-103a-3p;hsa-miR-107;hsa-miR-139-5
ENSG0000	ENSG0000	0.002397	hsa-miR-107;hsa-miR-15a-5p;hsa-miR-15b-5p;hsa-miR-195-5p
ENSG0000	ENSG0000	0.000178	hsa-miR-17-5p;hsa-miR-204-5p;hsa-miR-20b-5p;hsa-miR-93-5
ENSG0000	ENSG0000	0.004641	hsa-miR-17-5p;hsa-miR-204-5p;hsa-miR-20b-5p;hsa-miR-519a
ENSG0000	ENSG0000	2.90E-06	hsa-miR-30b-5p;hsa-miR-30c-5p;hsa-miR-32-5p;hsa-miR-92a-
ENSG0000	ENSG0000	0.00353	hsa-miR-27a-3p;hsa-miR-27b-3p;hsa-miR-519a-3p;hsa-miR-51
ENSG0000	ENSG0000	4.13E-14	hsa-let-7a-5p;hsa-let-7b-5p;hsa-let-7d-5p;hsa-let-7f-5p;hsa-le
ENSG0000	ENSG0000	0.006245	hsa-miR-101-3p;hsa-miR-129-5p;hsa-miR-204-5p;hsa-miR-320
ENSG0000	ENSG0000	0.002856	hsa-miR-139-5p;hsa-miR-17-5p;hsa-miR-20b-5p;hsa-miR-218-
ENSG0000	ENSG0000	4.97E-05	hsa-miR-15a-5p;hsa-miR-15b-5p;hsa-miR-16-5p;hsa-miR-195-
ENSG0000	ENSG0000	0.000305	hsa-let-7a-5p;hsa-let-7b-5p;hsa-let-7d-5p;hsa-let-7f-5p;hsa-le
ENSG0000	ENSG0000	0.000568	hsa-miR-15a-5p;hsa-miR-15b-5p;hsa-miR-16-5p;hsa-miR-195-
ENSG0000	ENSG0000	0.007711	hsa-miR-218-5p;hsa-miR-224-5p;hsa-miR-30a-3p;hsa-miR-30c
ENSG0000	ENSG0000	1.20E-05	hsa-miR-126-5p;hsa-miR-15a-5p;hsa-miR-15b-5p;hsa-miR-16-
ENSG0000	ENSG0000	0.008446	hsa-miR-129-5p;hsa-miR-32-5p;hsa-miR-34a-5p;hsa-miR-574-
ENSG0000	ENSG0000	0.004131	hsa-miR-20b-5p;hsa-miR-519a-3p;hsa-miR-519b-3p;hsa-miR-5
ENSG0000	ENSG0000	9.74E-05	hsa-let-7a-5p;hsa-let-7b-5p;hsa-let-7d-5p;hsa-let-7f-5p;hsa-le
ENSG0000	ENSG0000	0.003115	hsa-miR-129-5p;hsa-miR-15a-3p;hsa-miR-214-3p;hsa-miR-32-
ENSG0000	ENSG0000	0.003115	hsa-miR-17-5p;hsa-miR-20b-5p;hsa-miR-519a-3p;hsa-miR-519
ENSG0000	ENSG0000	0.000487	hsa-miR-15a-5p;hsa-miR-15b-5p;hsa-miR-16-5p;hsa-miR-195-
ENSG0000	ENSG0000	0.00369	hsa-let-7a-5p;hsa-let-7b-5p;hsa-let-7d-5p;hsa-let-7f-5p;hsa-le
ENSG0000	ENSG0000	0.001336	hsa-miR-1304-3p;hsa-miR-15a-5p;hsa-miR-15b-5p;hsa-miR-16

ENSG0000	ENSG0000	3.59E-05	hsa-miR-140-3p;hsa-miR-143-3p;hsa-miR-218-5p;hsa-miR-22
ENSG0000	ENSG0000	5.07E-06	hsa-miR-15a-5p;hsa-miR-15b-5p;hsa-miR-16-5p;hsa-miR-195
ENSG0000	ENSG0000	2.39E-06	hsa-let-7a-5p;hsa-let-7b-5p;hsa-let-7d-5p;hsa-let-7f-5p;hsa-le
ENSG0000	ENSG0000	5.22E-05	hsa-miR-17-5p;hsa-miR-20b-5p;hsa-miR-7-5p;hsa-miR-93-5p
ENSG0000	ENSG0000	2.91E-07	hsa-miR-107;hsa-miR-126-5p;hsa-miR-145-5p;hsa-miR-15a-5p
ENSG0000	ENSG0000	0.001487	hsa-miR-129-5p;hsa-miR-140-3p;hsa-miR-15a-5p;hsa-miR-15b
ENSG0000	ENSG0000	2.90E-06	hsa-let-7a-5p;hsa-miR-122-5p;hsa-miR-139-5p;hsa-miR-148b
ENSG0000	ENSG0000	0.000856	hsa-let-7a-5p;hsa-let-7d-5p;hsa-let-7f-5p;hsa-let-7g-5p;hsa-le
ENSG0000	ENSG0000	0.000202	hsa-let-7a-5p;hsa-let-7b-5p;hsa-let-7d-5p;hsa-let-7f-5p;hsa-le
ENSG0000	ENSG0000	0.009324	hsa-let-7a-5p;hsa-let-7b-5p;hsa-let-7d-5p;hsa-let-7f-5p;hsa-le
ENSG0000	ENSG0000	0.000338	hsa-miR-15a-3p;hsa-miR-17-5p;hsa-miR-20b-5p;hsa-miR-227
ENSG0000	ENSG0000	0.000338	hsa-miR-15a-3p;hsa-miR-17-5p;hsa-miR-20b-5p;hsa-miR-227
ENSG0000	ENSG0000	0.007539	hsa-miR-30a-3p;hsa-miR-30d-3p;hsa-miR-30e-3p;hsa-miR-32
ENSG0000	ENSG0000	0.000113	hsa-miR-15a-5p;hsa-miR-15b-5p;hsa-miR-16-5p;hsa-miR-195
ENSG0000	ENSG0000	0.005211	hsa-miR-32-5p;hsa-miR-542-3p;hsa-miR-616-5p;hsa-miR-92a
ENSG0000	ENSG0000	0.008446	hsa-miR-126-5p;hsa-miR-17-5p;hsa-miR-20b-5p;hsa-miR-470
ENSG0000	ENSG0000	0.000105	hsa-let-7a-5p;hsa-let-7b-5p;hsa-let-7f-5p;hsa-let-7g-5p;hsa-le
ENSG0000	ENSG0000	0.000437	hsa-miR-17-5p;hsa-miR-20b-5p;hsa-miR-519a-3p;hsa-miR-51
ENSG0000	ENSG0000	4.18E-05	hsa-miR-32-5p;hsa-miR-519a-3p;hsa-miR-519b-3p;hsa-miR-5
ENSG0000	ENSG0000	0.00353	hsa-miR-17-5p;hsa-miR-20b-5p;hsa-miR-519a-3p;hsa-miR-51
ENSG0000	ENSG0000	4.68E-07	hsa-miR-15a-5p;hsa-miR-15b-5p;hsa-miR-17-5p;hsa-miR-195
ENSG0000	ENSG0000	0.000707	hsa-miR-1260b;hsa-miR-139-5p;hsa-miR-140-3p;hsa-miR-17-
ENSG0000	ENSG0000	0.000908	hsa-let-7b-5p;hsa-miR-188-3p;hsa-miR-29a-3p;hsa-miR-342-
ENSG0000	ENSG0000	0.000288	hsa-miR-146a-3p;hsa-miR-204-5p;hsa-miR-224-5p;hsa-miR-9
ENSG0000	ENSG0000	0.009324	hsa-miR-1304-3p;hsa-miR-20b-5p;hsa-miR-218-5p;hsa-miR-3
ENSG0000	ENSG0000	0.003984	hsa-let-7a-5p;hsa-let-7b-5p;hsa-let-7d-5p;hsa-let-7f-5p;hsa-le
ENSG0000	ENSG0000	0.003275	hsa-miR-129-5p;hsa-miR-1304-3p;hsa-miR-143-3p;hsa-miR-2
ENSG0000	ENSG0000	9.03E-05	hsa-miR-129-5p;hsa-miR-218-5p;hsa-miR-29a-3p;hsa-miR-29
ENSG0000	ENSG0000	0.005283	hsa-let-7a-5p;hsa-let-7b-5p;hsa-let-7d-5p;hsa-let-7f-5p;hsa-le
ENSG0000	ENSG0000	3.40E-07	hsa-let-7a-5p;hsa-let-7b-5p;hsa-let-7d-5p;hsa-let-7g-5p;hsa-
ENSG0000	ENSG0000	2.68E-05	hsa-let-7a-5p;hsa-let-7b-5p;hsa-let-7d-5p;hsa-let-7f-5p;hsa-le
ENSG0000	ENSG0000	1.62E-06	hsa-miR-15a-5p;hsa-miR-15b-5p;hsa-miR-16-5p;hsa-miR-195
ENSG0000	ENSG0000	1.10E-09	hsa-miR-107;hsa-miR-15a-5p;hsa-miR-15b-5p;hsa-miR-16-5p;
ENSG0000	ENSG0000	7.23E-13	hsa-let-7a-5p;hsa-let-7b-5p;hsa-let-7d-5p;hsa-let-7f-5p;hsa-le
ENSG0000	ENSG0000	0.000568	hsa-miR-146a-3p;hsa-miR-15a-5p;hsa-miR-15b-5p;hsa-miR-1
ENSG0000	ENSG0000	0.000127	hsa-miR-101-3p;hsa-miR-17-5p;hsa-miR-20b-5p;hsa-miR-574
ENSG0000	ENSG0000	6.80E-09	hsa-let-7a-5p;hsa-let-7b-5p;hsa-let-7d-5p;hsa-let-7f-5p;hsa-le
ENSG0000	ENSG0000	6.22E-06	hsa-miR-15a-5p;hsa-miR-15b-5p;hsa-miR-16-5p;hsa-miR-192
ENSG0000	ENSG0000	3.65E-05	hsa-miR-15a-5p;hsa-miR-15b-5p;hsa-miR-16-5p;hsa-miR-195
ENSG0000	ENSG0000	0.000951	hsa-miR-126-5p;hsa-miR-17-5p;hsa-miR-20b-5p;hsa-miR-320
ENSG0000	ENSG0000	0.000951	hsa-miR-126-5p;hsa-miR-17-5p;hsa-miR-20b-5p;hsa-miR-320
ENSG0000	ENSG0000	0.000951	hsa-miR-129-5p;hsa-miR-15a-5p;hsa-miR-15b-5p;hsa-miR-16
ENSG0000	ENSG0000	8.70E-05	hsa-miR-15a-5p;hsa-miR-15b-5p;hsa-miR-195-5p;hsa-miR-42
ENSG0000	ENSG0000	0.009859	hsa-miR-29a-3p;hsa-miR-29b-3p;hsa-miR-29c-3p;hsa-miR-32
ENSG0000	ENSG0000	0.000134	hsa-miR-15a-5p;hsa-miR-15b-5p;hsa-miR-16-5p;hsa-miR-195
ENSG0000	ENSG0000	0.006915	hsa-miR-15a-5p;hsa-miR-15b-5p;hsa-miR-16-5p;hsa-miR-195
ENSG0000	ENSG0000	0.007711	hsa-miR-140-3p;hsa-miR-2278;hsa-miR-501-3p;hsa-miR-502-
ENSG0000	ENSG0000	0.000331	hsa-let-7a-5p;hsa-let-7b-5p;hsa-let-7d-5p;hsa-let-7f-5p;hsa-le
ENSG0000	ENSG0000	0.000566	hsa-miR-145-5p;hsa-miR-146a-3p;hsa-miR-301a-5p;hsa-miR-
ENSG0000	ENSG0000	0.000901	hsa-miR-129-5p;hsa-miR-30a-3p;hsa-miR-30c-5p;hsa-miR-34
ENSG0000	ENSG0000	8.70E-05	hsa-miR-16-5p;hsa-miR-30b-5p;hsa-miR-30c-5p;hsa-miR-361
ENSG0000	ENSG0000	0.006116	hsa-miR-20b-5p;hsa-miR-30b-5p;hsa-miR-30c-5p;hsa-miR-34
ENSG0000	ENSG0000	1.49E-07	hsa-miR-15a-5p;hsa-miR-15b-5p;hsa-miR-16-5p;hsa-miR-195
ENSG0000	ENSG0000	0.00097	hsa-miR-17-5p;hsa-miR-20b-5p;hsa-miR-27a-3p;hsa-miR-27b

ENSG0000	ENSG0000	0.00097	hsa-miR-17-5p;hsa-miR-20b-5p;hsa-miR-27a-3p;hsa-miR-27b
ENSG0000	ENSG0000	0.00097	hsa-miR-17-5p;hsa-miR-20b-5p;hsa-miR-27a-3p;hsa-miR-27b
ENSG0000	ENSG0000	0.00097	hsa-miR-17-5p;hsa-miR-20b-5p;hsa-miR-27a-3p;hsa-miR-27b
ENSG0000	ENSG0000	0.00097	hsa-miR-17-5p;hsa-miR-20b-5p;hsa-miR-27a-3p;hsa-miR-27b
ENSG0000	ENSG0000	0.00097	hsa-miR-17-5p;hsa-miR-20b-5p;hsa-miR-27a-3p;hsa-miR-27b
ENSG0000	ENSG0000	2.28E-05	hsa-miR-101-3p;hsa-miR-139-5p;hsa-miR-143-3p;hsa-miR-17
ENSG0000	ENSG0000	0.002738	hsa-let-7a-5p;hsa-let-7b-5p;hsa-let-7d-5p;hsa-let-7f-5p;hsa-l
ENSG0000	ENSG0000	0.009324	hsa-miR-15a-5p;hsa-miR-15b-5p;hsa-miR-195-5p;hsa-miR-37
ENSG0000	ENSG0000	1.62E-06	hsa-miR-15a-5p;hsa-miR-15b-5p;hsa-miR-16-5p;hsa-miR-195
ENSG0000	ENSG0000	0.005274	hsa-miR-15a-5p;hsa-miR-15b-5p;hsa-miR-16-5p;hsa-miR-195
ENSG0000	ENSG0000	3.13E-09	hsa-let-7a-5p;hsa-let-7b-5p;hsa-let-7d-5p;hsa-let-7f-5p;hsa-l
ENSG0000	ENSG0000	7.06E-06	hsa-let-7a-5p;hsa-let-7d-5p;hsa-let-7f-5p;hsa-let-7g-5p;hsa-l
ENSG0000	ENSG0000	0.009972	hsa-miR-143-3p;hsa-miR-20a-3p;hsa-miR-24-3p;hsa-miR-342
ENSG0000	ENSG0000	0.000178	hsa-miR-17-5p;hsa-miR-20b-5p;hsa-miR-34a-5p;hsa-miR-93-
ENSG0000	ENSG0000	0.000178	hsa-miR-17-5p;hsa-miR-20b-5p;hsa-miR-34a-5p;hsa-miR-93-
ENSG0000	ENSG0000	5.89E-05	hsa-miR-103a-3p;hsa-miR-107;hsa-miR-15a-5p;hsa-miR-15b-
ENSG0000	ENSG0000	0.001833	hsa-miR-1304-3p;hsa-miR-17-5p;hsa-miR-20b-5p;hsa-miR-51
ENSG0000	ENSG0000	0.005274	hsa-let-7a-5p;hsa-let-7b-5p;hsa-let-7d-5p;hsa-let-7f-5p;hsa-l
ENSG0000	ENSG0000	0.000232	hsa-miR-17-5p;hsa-miR-20b-5p;hsa-miR-24-3p;hsa-miR-519a
ENSG0000	ENSG0000	0.000908	hsa-miR-15a-5p;hsa-miR-15b-5p;hsa-miR-16-5p;hsa-miR-195
ENSG0000	ENSG0000	0.000278	hsa-miR-15a-5p;hsa-miR-15b-5p;hsa-miR-195-5p;hsa-miR-30
ENSG0000	ENSG0000	2.17E-05	hsa-let-7a-5p;hsa-let-7b-5p;hsa-let-7d-5p;hsa-let-7f-5p;hsa-l
ENSG0000	ENSG0000	1.23E-05	hsa-miR-218-5p;hsa-miR-29a-3p;hsa-miR-29b-3p;hsa-miR-29
ENSG0000	ENSG0000	9.47E-08	hsa-miR-15a-5p;hsa-miR-15b-5p;hsa-miR-16-5p;hsa-miR-195
ENSG0000	ENSG0000	0.006034	hsa-miR-129-5p;hsa-miR-30b-5p;hsa-miR-30c-5p;hsa-miR-34
ENSG0000	ENSG0000	0.003583	hsa-let-7a-5p;hsa-let-7b-5p;hsa-let-7d-5p;hsa-let-7f-5p;hsa-l
ENSG0000	ENSG0000	2.71E-06	hsa-miR-103a-3p;hsa-miR-107;hsa-miR-145-5p;hsa-miR-29c-
ENSG0000	ENSG0000	0.00978	hsa-miR-17-5p;hsa-miR-20b-5p;hsa-miR-519a-3p;hsa-miR-51
ENSG0000	ENSG0000	0.003637	hsa-miR-103a-3p;hsa-miR-107;hsa-miR-139-5p;hsa-miR-15a-
ENSG0000	ENSG0000	5.72E-08	hsa-miR-103a-3p;hsa-miR-107;hsa-miR-122-5p;hsa-miR-15a-
ENSG0000	ENSG0000	2.24E-08	hsa-miR-103a-3p;hsa-miR-107;hsa-miR-15a-5p;hsa-miR-15b-
ENSG0000	ENSG0000	0.000101	hsa-miR-15a-5p;hsa-miR-15b-5p;hsa-miR-16-5p;hsa-miR-195
ENSG0000	ENSG0000	2.65E-08	hsa-miR-103a-3p;hsa-miR-107;hsa-miR-15a-5p;hsa-miR-15b-
ENSG0000	ENSG0000	1.23E-05	hsa-miR-17-5p;hsa-miR-20b-5p;hsa-miR-30b-5p;hsa-miR-30c
ENSG0000	ENSG0000	0.00134	hsa-miR-103a-3p;hsa-miR-107;hsa-miR-30b-5p;hsa-miR-30c-
ENSG0000	ENSG0000	3.59E-05	hsa-miR-103a-3p;hsa-miR-107;hsa-miR-1304-3p;hsa-miR-214
ENSG0000	ENSG0000	0.009859	hsa-miR-32-5p;hsa-miR-542-3p;hsa-miR-92a-3p;hsa-miR-92b
ENSG0000	ENSG0000	1.16E-05	hsa-let-7a-5p;hsa-let-7b-5p;hsa-let-7d-5p;hsa-let-7f-5p;hsa-l
ENSG0000	ENSG0000	0.001196	hsa-miR-122-5p;hsa-miR-17-5p;hsa-miR-20b-5p;hsa-miR-222
ENSG0000	ENSG0000	0.000338	hsa-miR-204-5p;hsa-miR-30a-3p;hsa-miR-30b-5p;hsa-miR-30
ENSG0000	ENSG0000	0.00018	hsa-miR-101-3p;hsa-miR-107;hsa-miR-15a-5p;hsa-miR-15b-5
ENSG0000	ENSG0000	0.002397	hsa-miR-29a-3p;hsa-miR-29b-3p;hsa-miR-29c-3p;hsa-miR-32
ENSG0000	ENSG0000	0.003349	hsa-miR-146a-3p;hsa-miR-17-5p;hsa-miR-20b-5p;hsa-miR-30
ENSG0000	ENSG0000	0.005609	hsa-let-7a-5p;hsa-let-7b-5p;hsa-let-7d-5p;hsa-let-7f-5p;hsa-l
ENSG0000	ENSG0000	1.01E-06	hsa-miR-15a-5p;hsa-miR-15b-5p;hsa-miR-16-5p;hsa-miR-195
ENSG0000	ENSG0000	0.002411	hsa-miR-20b-5p;hsa-miR-519a-3p;hsa-miR-519b-3p;hsa-miR-
ENSG0000	ENSG0000	1.27E-10	hsa-miR-132-3p;hsa-miR-139-5p;hsa-miR-15a-5p;hsa-miR-15
ENSG0000	ENSG0000	0.001434	hsa-miR-17-5p;hsa-miR-20b-5p;hsa-miR-214-3p;hsa-miR-519
ENSG0000	ENSG0000	0.000748	hsa-miR-17-5p;hsa-miR-20b-5p;hsa-miR-24-3p;hsa-miR-519a
ENSG0000	ENSG0000	0.000568	hsa-miR-107;hsa-miR-15a-5p;hsa-miR-15b-5p;hsa-miR-195-5
ENSG0000	ENSG0000	0.000248	hsa-let-7a-5p;hsa-let-7b-5p;hsa-let-7d-5p;hsa-let-7f-5p;hsa-l
ENSG0000	ENSG0000	0.002738	hsa-miR-15a-5p;hsa-miR-15b-5p;hsa-miR-16-5p;hsa-miR-195
ENSG0000	ENSG0000	0.007173	hsa-miR-32-5p;hsa-miR-452-5p;hsa-miR-92a-3p;hsa-miR-92b
ENSG0000	ENSG0000	7.89E-06	hsa-miR-218-5p;hsa-miR-32-5p;hsa-miR-92a-3p;hsa-miR-92b

ENSG0000	ENSG0000	1.54E-08	hsa-let-7a-5p;hsa-let-7b-5p;hsa-let-7d-5p;hsa-let-7f-5p;hsa-le
ENSG0000	ENSG0000	0.008703	hsa-miR-29a-3p;hsa-miR-29b-3p;hsa-miR-29c-3p;hsa-miR-342
ENSG0000	ENSG0000	3.05E-06	hsa-miR-15a-5p;hsa-miR-15b-5p;hsa-miR-16-5p;hsa-miR-195-
ENSG0000	ENSG0000	0.002063	hsa-let-7a-5p;hsa-let-7b-5p;hsa-let-7d-5p;hsa-let-7f-5p;hsa-le
ENSG0000	ENSG0000	0.003349	hsa-miR-15a-5p;hsa-miR-15b-5p;hsa-miR-195-5p;hsa-miR-424
ENSG0000	ENSG0000	4.95E-07	hsa-miR-140-3p;hsa-miR-15a-5p;hsa-miR-15b-5p;hsa-miR-16-
ENSG0000	ENSG0000	0.003564	hsa-miR-17-5p;hsa-miR-20b-5p;hsa-miR-218-5p;hsa-miR-312
ENSG0000	ENSG0000	3.14E-11	hsa-let-7a-5p;hsa-let-7b-5p;hsa-let-7d-5p;hsa-let-7f-5p;hsa-le
ENSG0000	ENSG0000	0.000105	hsa-miR-103a-3p;hsa-miR-107;hsa-miR-30b-5p;hsa-miR-30c-5
ENSG0000	ENSG0000	0.000288	hsa-miR-15a-5p;hsa-miR-15b-5p;hsa-miR-16-5p;hsa-miR-195-
ENSG0000	ENSG0000	1.50E-06	hsa-miR-15a-5p;hsa-miR-15b-5p;hsa-miR-16-5p;hsa-miR-195-
ENSG0000	ENSG0000	0.00134	hsa-miR-126-5p;hsa-miR-15a-5p;hsa-miR-15b-5p;hsa-miR-195
ENSG0000	ENSG0000	4.14E-09	hsa-miR-122-5p;hsa-miR-15a-5p;hsa-miR-15b-5p;hsa-miR-16-
ENSG0000	ENSG0000	4.14E-09	hsa-miR-122-5p;hsa-miR-15a-5p;hsa-miR-15b-5p;hsa-miR-16-
ENSG0000	ENSG0000	0.000104	hsa-miR-101-3p;hsa-miR-17-5p;hsa-miR-195-5p;hsa-miR-20b-
ENSG0000	ENSG0000	0.00134	hsa-miR-17-5p;hsa-miR-20b-5p;hsa-miR-519a-3p;hsa-miR-519
ENSG0000	ENSG0000	0.001434	hsa-miR-107;hsa-miR-122-5p;hsa-miR-16-5p;hsa-miR-218-5p;
ENSG0000	ENSG0000	0.001434	hsa-miR-107;hsa-miR-122-5p;hsa-miR-16-5p;hsa-miR-218-5p;
ENSG0000	ENSG0000	0.002245	hsa-miR-17-5p;hsa-miR-20b-5p;hsa-miR-24-3p;hsa-miR-378a-
ENSG0000	ENSG0000	3.21E-07	hsa-miR-17-5p;hsa-miR-204-5p;hsa-miR-20b-5p;hsa-miR-224-
ENSG0000	ENSG0000	0.001626	hsa-miR-15a-5p;hsa-miR-15b-5p;hsa-miR-195-5p;hsa-miR-432
ENSG0000	ENSG0000	0.008703	hsa-miR-15a-5p;hsa-miR-15b-5p;hsa-miR-218-5p;hsa-miR-424
ENSG0000	ENSG0000	0.008469	hsa-miR-1260b;hsa-miR-224-5p;hsa-miR-30b-5p;hsa-miR-30c-
ENSG0000	ENSG0000	0.000119	hsa-let-7a-5p;hsa-let-7b-5p;hsa-let-7d-5p;hsa-let-7f-5p;hsa-le
ENSG0000	ENSG0000	0.000648	hsa-miR-103a-3p;hsa-miR-107;hsa-miR-148b-3p;hsa-miR-17-5
ENSG0000	ENSG0000	0.003905	hsa-let-7a-5p;hsa-let-7b-5p;hsa-let-7f-5p;hsa-let-7g-5p;hsa-le
ENSG0000	ENSG0000	7.50E-06	hsa-miR-15a-5p;hsa-miR-15b-5p;hsa-miR-16-5p;hsa-miR-195-
ENSG0000	ENSG0000	1.84E-07	hsa-miR-15a-5p;hsa-miR-15b-5p;hsa-miR-16-5p;hsa-miR-195-
ENSG0000	ENSG0000	0.002397	hsa-let-7a-5p;hsa-let-7b-5p;hsa-let-7d-5p;hsa-let-7f-5p;hsa-le
ENSG0000	ENSG0000	5.03E-06	hsa-miR-15a-5p;hsa-miR-15b-5p;hsa-miR-16-5p;hsa-miR-17-5
ENSG0000	ENSG0000	0.000101	hsa-miR-27a-3p;hsa-miR-27b-3p;hsa-miR-30b-5p;hsa-miR-30
ENSG0000	ENSG0000	0.00978	hsa-miR-122-5p;hsa-miR-1304-3p;hsa-miR-519a-3p;hsa-miR-5
ENSG0000	ENSG0000	0.005211	hsa-miR-15b-5p;hsa-miR-16-5p;hsa-miR-195-5p;hsa-miR-424-
ENSG0000	ENSG0000	0.006034	hsa-miR-15a-5p;hsa-miR-15b-5p;hsa-miR-16-5p;hsa-miR-195-
ENSG0000	ENSG0000	0.006034	hsa-miR-15a-5p;hsa-miR-15b-5p;hsa-miR-16-5p;hsa-miR-195-
ENSG0000	ENSG0000	1.84E-07	hsa-miR-145-5p;hsa-miR-15a-5p;hsa-miR-15b-5p;hsa-miR-16-
ENSG0000	ENSG0000	0.003945	hsa-let-7a-5p;hsa-let-7b-5p;hsa-let-7d-5p;hsa-let-7f-5p;hsa-le
ENSG0000	ENSG0000	6.73E-05	hsa-let-7a-5p;hsa-let-7b-5p;hsa-let-7d-5p;hsa-let-7f-5p;hsa-le
ENSG0000	ENSG0000	0.000468	hsa-miR-145-5p;hsa-miR-15a-5p;hsa-miR-15b-5p;hsa-miR-195
ENSG0000	ENSG0000	1.02E-06	hsa-let-7a-5p;hsa-let-7b-5p;hsa-let-7d-5p;hsa-let-7f-5p;hsa-le
ENSG0000	ENSG0000	0.001261	hsa-miR-15a-5p;hsa-miR-15b-5p;hsa-miR-16-5p;hsa-miR-195-
ENSG0000	ENSG0000	0.006737	hsa-let-7a-5p;hsa-let-7b-5p;hsa-let-7d-5p;hsa-let-7f-5p;hsa-le
ENSG0000	ENSG0000	5.22E-05	hsa-miR-129-5p;hsa-miR-30a-3p;hsa-miR-30d-3p;hsa-miR-30
ENSG0000	ENSG0000	0.001694	hsa-miR-129-5p;hsa-miR-17-5p;hsa-miR-20b-5p;hsa-miR-214-
ENSG0000	ENSG0000	0.009739	hsa-let-7a-5p;hsa-let-7b-5p;hsa-let-7d-5p;hsa-let-7f-5p;hsa-le
ENSG0000	ENSG0000	0.009739	hsa-let-7a-5p;hsa-let-7b-5p;hsa-let-7d-5p;hsa-let-7f-5p;hsa-le
ENSG0000	ENSG0000	0.002628	hsa-miR-17-5p;hsa-miR-20b-5p;hsa-miR-330-3p;hsa-miR-519
ENSG0000	ENSG0000	0.001147	hsa-miR-15a-5p;hsa-miR-15b-5p;hsa-miR-16-5p;hsa-miR-195-
ENSG0000	ENSG0000	9.35E-05	hsa-miR-101-3p;hsa-miR-1304-3p;hsa-miR-143-3p;hsa-miR-1
ENSG0000	ENSG0000	0.000183	hsa-let-7a-5p;hsa-let-7b-5p;hsa-let-7d-5p;hsa-let-7f-5p;hsa-le
ENSG0000	ENSG0000	5.07E-06	hsa-miR-15a-5p;hsa-miR-15b-5p;hsa-miR-16-5p;hsa-miR-195-
ENSG0000	ENSG0000	0.000227	hsa-miR-17-5p;hsa-miR-20b-5p;hsa-miR-30b-5p;hsa-miR-30c-
ENSG0000	ENSG0000	2.46E-05	hsa-let-7a-5p;hsa-let-7b-5p;hsa-let-7d-5p;hsa-let-7f-5p;hsa-le
ENSG0000	ENSG0000	0.000118	hsa-miR-15a-5p;hsa-miR-15b-5p;hsa-miR-16-5p;hsa-miR-195-

ENSG0000	ENSG0000	0.00134	hsa-miR-15a-5p;hsa-miR-15b-5p;hsa-miR-16-5p;hsa-miR-195-
ENSG0000	ENSG0000	0.00134	hsa-miR-15a-5p;hsa-miR-15b-5p;hsa-miR-16-5p;hsa-miR-195-
ENSG0000	ENSG0000	0.002738	hsa-miR-17-5p;hsa-miR-20b-5p;hsa-miR-519a-3p;hsa-miR-51
ENSG0000	ENSG0000	0.002738	hsa-miR-17-5p;hsa-miR-20b-5p;hsa-miR-519a-3p;hsa-miR-51
ENSG0000	ENSG0000	0.000196	hsa-miR-1304-3p;hsa-miR-15a-5p;hsa-miR-15b-5p;hsa-miR-1
ENSG0000	ENSG0000	0.000728	hsa-miR-15a-5p;hsa-miR-15b-5p;hsa-miR-16-5p;hsa-miR-195-
ENSG0000	ENSG0000	5.05E-06	hsa-miR-1260b;hsa-miR-15a-5p;hsa-miR-15b-5p;hsa-miR-188-
ENSG0000	ENSG0000	6.77E-05	hsa-miR-1260b;hsa-miR-15a-5p;hsa-miR-15b-5p;hsa-miR-16-
ENSG0000	ENSG0000	0.001196	hsa-miR-17-5p;hsa-miR-20b-5p;hsa-miR-34a-5p;hsa-miR-519
ENSG0000	ENSG0000	0.000382	hsa-let-7a-5p;hsa-let-7b-5p;hsa-let-7d-5p;hsa-let-7f-5p;hsa-l
ENSG0000	ENSG0000	0.000382	hsa-let-7a-5p;hsa-let-7b-5p;hsa-let-7d-5p;hsa-let-7f-5p;hsa-l
ENSG0000	ENSG0000	0.009213	hsa-miR-122-5p;hsa-miR-129-5p;hsa-miR-143-3p;hsa-miR-17-
ENSG0000	ENSG0000	0.000277	hsa-miR-103a-3p;hsa-miR-107;hsa-miR-146a-3p;hsa-miR-15a-
ENSG0000	ENSG0000	7.95E-06	hsa-miR-15a-5p;hsa-miR-15b-5p;hsa-miR-195-5p;hsa-miR-21
ENSG0000	ENSG0000	8.48E-05	hsa-miR-15a-5p;hsa-miR-15b-5p;hsa-miR-16-5p;hsa-miR-195-
ENSG0000	ENSG0000	0.008446	hsa-miR-129-5p;hsa-miR-32-5p;hsa-miR-92a-3p;hsa-miR-92b-
ENSG0000	ENSG0000	0.00978	hsa-miR-129-5p;hsa-miR-204-5p;hsa-miR-32-5p;hsa-miR-450
ENSG0000	ENSG0000	4.68E-07	hsa-miR-103a-3p;hsa-miR-107;hsa-miR-15a-5p;hsa-miR-15b-
ENSG0000	ENSG0000	4.68E-07	hsa-miR-103a-3p;hsa-miR-107;hsa-miR-15a-5p;hsa-miR-15b-
ENSG0000	ENSG0000	0.001196	hsa-miR-107;hsa-miR-1260b;hsa-miR-15a-5p;hsa-miR-15b-5p;
ENSG0000	ENSG0000	0.000331	hsa-miR-139-5p;hsa-miR-15a-5p;hsa-miR-15b-5p;hsa-miR-16-
ENSG0000	ENSG0000	0.002577	hsa-let-7a-5p;hsa-let-7b-5p;hsa-let-7d-5p;hsa-let-7f-5p;hsa-l
ENSG0000	ENSG0000	0.000391	hsa-miR-188-3p;hsa-miR-204-5p;hsa-miR-30b-5p;hsa-miR-30
ENSG0000	ENSG0000	9.16E-07	hsa-let-7a-5p;hsa-let-7b-5p;hsa-let-7d-5p;hsa-let-7f-5p;hsa-l
ENSG0000	ENSG0000	0.000178	hsa-miR-15a-5p;hsa-miR-15b-5p;hsa-miR-16-5p;hsa-miR-195-
ENSG0000	ENSG0000	5.25E-12	hsa-miR-103a-3p;hsa-miR-107;hsa-miR-15a-5p;hsa-miR-15b-
ENSG0000	ENSG0000	0.007173	hsa-let-7b-5p;hsa-miR-129-5p;hsa-miR-27a-3p;hsa-miR-27b-
ENSG0000	ENSG0000	2.10E-07	hsa-miR-107;hsa-miR-1304-3p;hsa-miR-15a-5p;hsa-miR-15b-
ENSG0000	ENSG0000	0.000641	hsa-miR-218-5p;hsa-miR-29a-3p;hsa-miR-29b-3p;hsa-miR-29
ENSG0000	ENSG0000	1.95E-05	hsa-miR-122-5p;hsa-miR-129-5p;hsa-miR-1304-3p;hsa-miR-1
ENSG0000	ENSG0000	1.95E-05	hsa-miR-122-5p;hsa-miR-129-5p;hsa-miR-1304-3p;hsa-miR-1
ENSG0000	ENSG0000	2.71E-06	hsa-miR-15a-5p;hsa-miR-15b-5p;hsa-miR-16-5p;hsa-miR-195-
ENSG0000	ENSG0000	3.23E-05	hsa-miR-29a-3p;hsa-miR-29b-3p;hsa-miR-29c-3p;hsa-miR-34
ENSG0000	ENSG0000	1.00E-10	hsa-miR-15a-5p;hsa-miR-15b-5p;hsa-miR-16-5p;hsa-miR-17-
ENSG0000	ENSG0000	0.001366	hsa-miR-15a-5p;hsa-miR-15b-5p;hsa-miR-195-5p;hsa-miR-21
ENSG0000	ENSG0000	0.00018	hsa-miR-103a-3p;hsa-miR-107;hsa-miR-15a-5p;hsa-miR-15b-
ENSG0000	ENSG0000	0.001196	hsa-let-7a-5p;hsa-let-7b-5p;hsa-let-7d-5p;hsa-let-7f-5p;hsa-l
ENSG0000	ENSG0000	4.66E-07	hsa-miR-103a-3p;hsa-miR-107;hsa-miR-15a-5p;hsa-miR-15b-
ENSG0000	ENSG0000	0.000596	hsa-let-7a-5p;hsa-let-7b-5p;hsa-let-7d-5p;hsa-let-7f-5p;hsa-l
ENSG0000	ENSG0000	0.003115	hsa-miR-15a-5p;hsa-miR-15b-5p;hsa-miR-16-5p;hsa-miR-195-
ENSG0000	ENSG0000	3.68E-09	hsa-let-7a-5p;hsa-let-7b-5p;hsa-let-7d-5p;hsa-let-7f-5p;hsa-l
ENSG0000	ENSG0000	0.000331	hsa-miR-103a-3p;hsa-miR-107;hsa-miR-20a-3p;hsa-miR-218-
ENSG0000	ENSG0000	1.16E-05	hsa-miR-15a-5p;hsa-miR-15b-5p;hsa-miR-16-5p;hsa-miR-195-
ENSG0000	ENSG0000	0.000908	hsa-miR-15a-5p;hsa-miR-15b-5p;hsa-miR-16-5p;hsa-miR-195-
ENSG0000	ENSG0000	0.001227	hsa-miR-15a-5p;hsa-miR-15b-5p;hsa-miR-195-5p;hsa-miR-34
ENSG0000	ENSG0000	0.005211	hsa-miR-132-3p;hsa-miR-20a-3p;hsa-miR-32-5p;hsa-miR-92a-
ENSG0000	ENSG0000	0.001836	hsa-miR-15a-5p;hsa-miR-15b-5p;hsa-miR-16-5p;hsa-miR-195-
ENSG0000	ENSG0000	0.002088	hsa-miR-15a-5p;hsa-miR-15b-5p;hsa-miR-195-5p;hsa-miR-33
ENSG0000	ENSG0000	0.000901	hsa-let-7a-5p;hsa-let-7b-5p;hsa-let-7d-5p;hsa-let-7f-5p;hsa-l
ENSG0000	ENSG0000	0.000268	hsa-let-7a-5p;hsa-let-7b-5p;hsa-let-7d-5p;hsa-let-7f-5p;hsa-l
ENSG0000	ENSG0000	0.00134	hsa-miR-15a-5p;hsa-miR-15b-5p;hsa-miR-16-5p;hsa-miR-195-
ENSG0000	ENSG0000	9.54E-09	hsa-let-7a-5p;hsa-let-7d-5p;hsa-miR-101-3p;hsa-miR-148b-3
ENSG0000	ENSG0000	0.007699	hsa-miR-129-5p;hsa-miR-140-3p;hsa-miR-15a-5p;hsa-miR-15
ENSG0000	ENSG0000	0.007173	hsa-miR-15a-5p;hsa-miR-15b-5p;hsa-miR-16-5p;hsa-miR-195-

ENSG0000	ENSG0000	0.002022	hsa-miR-17-5p;hsa-miR-20b-5p;hsa-miR-3194-3p;hsa-miR-93-
ENSG0000	ENSG0000	5.47E-05	hsa-miR-103a-3p;hsa-miR-107;hsa-miR-1260b;hsa-miR-15a-5p
ENSG0000	ENSG0000	0.007539	hsa-let-7d-5p;hsa-let-7f-5p;hsa-let-7g-5p;hsa-let-7i-5p;hsa-m
ENSG0000	ENSG0000	0.007539	hsa-let-7d-5p;hsa-let-7f-5p;hsa-let-7g-5p;hsa-let-7i-5p;hsa-m
ENSG0000	ENSG0000	0.000314	hsa-let-7a-5p;hsa-let-7b-5p;hsa-let-7d-5p;hsa-let-7f-5p;hsa-le
ENSG0000	ENSG0000	0.000127	hsa-miR-107;hsa-miR-15a-5p;hsa-miR-16-5p;hsa-miR-29a-3p;
ENSG0000	ENSG0000	0.005274	hsa-let-7a-5p;hsa-let-7b-5p;hsa-let-7d-5p;hsa-let-7f-5p;hsa-le
ENSG0000	ENSG0000	4.66E-07	hsa-miR-15a-5p;hsa-miR-15b-5p;hsa-miR-16-5p;hsa-miR-195-
ENSG0000	ENSG0000	0.007711	hsa-miR-15a-5p;hsa-miR-15b-5p;hsa-miR-195-5p;hsa-miR-424
ENSG0000	ENSG0000	0.001167	hsa-miR-129-5p;hsa-miR-15a-5p;hsa-miR-15b-5p;hsa-miR-17-
ENSG0000	ENSG0000	0.001048	hsa-miR-15a-5p;hsa-miR-15b-5p;hsa-miR-16-5p;hsa-miR-195-
ENSG0000	ENSG0000	0.000768	hsa-miR-129-5p;hsa-miR-1304-3p;hsa-miR-143-3p;hsa-miR-17
ENSG0000	ENSG0000	0.000401	hsa-miR-122-5p;hsa-miR-1304-3p;hsa-miR-17-5p;hsa-miR-20b
ENSG0000	ENSG0000	0.001681	hsa-miR-24-3p;hsa-miR-30a-3p;hsa-miR-30d-3p;hsa-miR-30e-
ENSG0000	ENSG0000	0.001227	hsa-miR-101-3p;hsa-miR-139-5p;hsa-miR-92a-3p;hsa-miR-92b
ENSG0000	ENSG0000	0.007673	hsa-let-7a-5p;hsa-let-7b-5p;hsa-let-7d-5p;hsa-let-7f-5p;hsa-le
ENSG0000	ENSG0000	0.002682	hsa-miR-30a-3p;hsa-miR-30d-3p;hsa-miR-30e-3p;hsa-miR-342
ENSG0000	ENSG0000	0.000127	hsa-miR-15a-5p;hsa-miR-15b-5p;hsa-miR-195-5p;hsa-miR-424
ENSG0000	ENSG0000	5.76E-05	hsa-miR-15a-5p;hsa-miR-15b-5p;hsa-miR-16-5p;hsa-miR-195-
ENSG0000	ENSG0000	0.003275	hsa-let-7a-5p;hsa-let-7d-5p;hsa-let-7f-5p;hsa-let-7g-5p;hsa-le
ENSG0000	ENSG0000	0.005021	hsa-miR-214-3p;hsa-miR-519a-3p;hsa-miR-519b-3p;hsa-miR-5
ENSG0000	ENSG0000	0.002988	hsa-miR-17-5p;hsa-miR-20b-5p;hsa-miR-24-3p;hsa-miR-330-3
ENSG0000	ENSG0000	0.005609	hsa-miR-24-3p;hsa-miR-29a-3p;hsa-miR-29b-3p;hsa-miR-29c-
ENSG0000	ENSG0000	0.008446	hsa-miR-218-5p;hsa-miR-519a-3p;hsa-miR-519b-3p;hsa-miR-5
ENSG0000	ENSG0000	0.001196	hsa-miR-103a-3p;hsa-miR-107;hsa-miR-15a-5p;hsa-miR-15b-5
ENSG0000	ENSG0000	1.51E-06	hsa-miR-15a-5p;hsa-miR-15b-5p;hsa-miR-195-5p;hsa-miR-214
ENSG0000	ENSG0000	0.000288	hsa-miR-140-3p;hsa-miR-32-5p;hsa-miR-92a-3p;hsa-miR-92b-
ENSG0000	ENSG0000	0.000288	hsa-miR-140-3p;hsa-miR-32-5p;hsa-miR-92a-3p;hsa-miR-92b-
ENSG0000	ENSG0000	0.002852	hsa-miR-15a-5p;hsa-miR-15b-5p;hsa-miR-195-5p;hsa-miR-424
ENSG0000	ENSG0000	0.007711	hsa-miR-24-3p;hsa-miR-30a-3p;hsa-miR-30d-3p;hsa-miR-30e-
ENSG0000	ENSG0000	0.007711	hsa-miR-24-3p;hsa-miR-30a-3p;hsa-miR-30d-3p;hsa-miR-30e-
ENSG0000	ENSG0000	2.74E-05	hsa-miR-101-3p;hsa-miR-15a-5p;hsa-miR-15b-5p;hsa-miR-195
ENSG0000	ENSG0000	0.000196	hsa-miR-15a-5p;hsa-miR-15b-5p;hsa-miR-16-5p;hsa-miR-195-
ENSG0000	ENSG0000	0.008204	hsa-miR-107;hsa-miR-1260b;hsa-miR-218-5p;hsa-miR-30a-3p;
ENSG0000	ENSG0000	0.001157	hsa-let-7d-5p;hsa-let-7f-5p;hsa-let-7g-5p;hsa-let-7i-5p;hsa-m
ENSG0000	ENSG0000	0.000248	hsa-miR-17-5p;hsa-miR-20b-5p;hsa-miR-218-5p;hsa-miR-519a
ENSG0000	ENSG0000	1.62E-06	hsa-miR-15a-5p;hsa-miR-15b-5p;hsa-miR-16-5p;hsa-miR-195-
ENSG0000	ENSG0000	6.76E-05	hsa-miR-17-5p;hsa-miR-20b-5p;hsa-miR-218-5p;hsa-miR-30b
ENSG0000	ENSG0000	0.000131	hsa-miR-1304-3p;hsa-miR-15a-5p;hsa-miR-15b-5p;hsa-miR-16
ENSG0000	ENSG0000	0.000154	hsa-miR-15a-5p;hsa-miR-15b-5p;hsa-miR-16-5p;hsa-miR-195-
ENSG0000	ENSG0000	2.01E-07	hsa-miR-214-3p;hsa-miR-218-5p;hsa-miR-30a-3p;hsa-miR-30
ENSG0000	ENSG0000	0.001833	hsa-miR-122-5p;hsa-miR-148b-3p;hsa-miR-27b-5p;hsa-miR-3
ENSG0000	ENSG0000	4.41E-05	hsa-miR-15a-5p;hsa-miR-15b-5p;hsa-miR-16-5p;hsa-miR-195-
ENSG0000	ENSG0000	1.67E-09	hsa-let-7a-5p;hsa-let-7b-5p;hsa-let-7d-5p;hsa-let-7f-5p;hsa-le
ENSG0000	ENSG0000	1.67E-09	hsa-let-7a-5p;hsa-let-7b-5p;hsa-let-7d-5p;hsa-let-7f-5p;hsa-le
ENSG0000	ENSG0000	1.67E-09	hsa-let-7a-5p;hsa-let-7b-5p;hsa-let-7d-5p;hsa-let-7f-5p;hsa-le
ENSG0000	ENSG0000	0.005609	hsa-let-7a-5p;hsa-let-7b-5p;hsa-let-7d-5p;hsa-let-7f-5p;hsa-le
ENSG0000	ENSG0000	0.00084	hsa-miR-17-5p;hsa-miR-20a-3p;hsa-miR-20b-5p;hsa-miR-34a-
ENSG0000	ENSG0000	0.000596	hsa-let-7a-5p;hsa-let-7b-5p;hsa-let-7d-5p;hsa-let-7f-5p;hsa-le
ENSG0000	ENSG0000	2.53E-05	hsa-miR-17-5p;hsa-miR-20b-5p;hsa-miR-519a-3p;hsa-miR-51
ENSG0000	ENSG0000	0.001144	hsa-let-7a-5p;hsa-let-7b-5p;hsa-let-7d-5p;hsa-let-7f-5p;hsa-le
ENSG0000	ENSG0000	0.00097	hsa-let-7a-5p;hsa-let-7b-5p;hsa-let-7d-5p;hsa-let-7f-5p;hsa-le
ENSG0000	ENSG0000	0.003905	hsa-miR-1260b;hsa-miR-188-3p;hsa-miR-320a;hsa-miR-425-5p
ENSG0000	ENSG0000	0.000253	hsa-miR-15a-5p;hsa-miR-15b-5p;hsa-miR-16-5p;hsa-miR-195-

ENSG0000	ENSG0000	0.003349	hsa-miR-15a-5p;hsa-miR-15b-5p;hsa-miR-195-5p;hsa-miR-42
ENSG0000	ENSG0000	3.80E-09	hsa-miR-103a-3p;hsa-miR-107;hsa-miR-122-5p;hsa-miR-1304-
ENSG0000	ENSG0000	0.000382	hsa-miR-15a-5p;hsa-miR-15b-5p;hsa-miR-195-5p;hsa-miR-22
ENSG0000	ENSG0000	7.50E-06	hsa-miR-103a-3p;hsa-miR-107;hsa-miR-15a-5p;hsa-miR-15b-5
ENSG0000	ENSG0000	7.88E-05	hsa-miR-15a-5p;hsa-miR-15b-5p;hsa-miR-195-5p;hsa-miR-22
ENSG0000	ENSG0000	0.003354	hsa-miR-103a-3p;hsa-miR-107;hsa-miR-27a-3p;hsa-miR-27b-3
ENSG0000	ENSG0000	0.00978	hsa-miR-148b-3p;hsa-miR-24-3p;hsa-miR-32-5p;hsa-miR-92a-
ENSG0000	ENSG0000	0.00978	hsa-miR-148b-3p;hsa-miR-24-3p;hsa-miR-32-5p;hsa-miR-92a-
ENSG0000	ENSG0000	0.000966	hsa-let-7a-5p;hsa-let-7d-5p;hsa-let-7f-5p;hsa-let-7g-5p;hsa-le
ENSG0000	ENSG0000	0.00978	hsa-miR-218-5p;hsa-miR-32-5p;hsa-miR-4487;hsa-miR-92a-3p
ENSG0000	ENSG0000	0.009859	hsa-miR-32-5p;hsa-miR-342-3p;hsa-miR-92a-3p;hsa-miR-92b-
ENSG0000	ENSG0000	0.001434	hsa-let-7a-5p;hsa-let-7d-5p;hsa-let-7f-5p;hsa-let-7g-5p;hsa-le
ENSG0000	ENSG0000	5.34E-05	hsa-miR-15a-5p;hsa-miR-15b-5p;hsa-miR-16-5p;hsa-miR-195-
ENSG0000	ENSG0000	0.001681	hsa-miR-103a-3p;hsa-miR-107;hsa-miR-122-5p;hsa-miR-24-3p
ENSG0000	ENSG0000	3.59E-05	hsa-miR-15a-5p;hsa-miR-15b-5p;hsa-miR-16-5p;hsa-miR-195-
ENSG0000	ENSG0000	2.71E-06	hsa-miR-15a-5p;hsa-miR-15b-5p;hsa-miR-16-5p;hsa-miR-195-
ENSG0000	ENSG0000	2.30E-05	hsa-miR-15a-5p;hsa-miR-15b-5p;hsa-miR-16-5p;hsa-miR-195-
ENSG0000	ENSG0000	0.000127	hsa-miR-17-5p;hsa-miR-20b-5p;hsa-miR-502-5p;hsa-miR-92a-
ENSG0000	ENSG0000	0.00134	hsa-miR-132-3p;hsa-miR-140-3p;hsa-miR-218-5p;hsa-miR-30a
ENSG0000	ENSG0000	0.003349	hsa-let-7a-5p;hsa-let-7f-5p;hsa-let-7g-5p;hsa-let-7i-5p;hsa-m
ENSG0000	ENSG0000	0.000277	hsa-miR-17-5p;hsa-miR-20b-5p;hsa-miR-214-3p;hsa-miR-32-5
ENSG0000	ENSG0000	4.97E-05	hsa-let-7a-5p;hsa-let-7b-5p;hsa-let-7d-5p;hsa-let-7f-5p;hsa-le
ENSG0000	ENSG0000	0.002738	hsa-miR-17-5p;hsa-miR-20b-5p;hsa-miR-519a-3p;hsa-miR-51
ENSG0000	ENSG0000	1.14E-05	hsa-let-7a-5p;hsa-let-7b-5p;hsa-let-7d-5p;hsa-let-7f-5p;hsa-le
ENSG0000	ENSG0000	4.00E-08	hsa-miR-17-5p;hsa-miR-20b-5p;hsa-miR-222-5p;hsa-miR-30a-
ENSG0000	ENSG0000	0.006932	hsa-let-7a-5p;hsa-let-7b-5p;hsa-let-7d-5p;hsa-let-7f-5p;hsa-le
ENSG0000	ENSG0000	0.001227	hsa-miR-15a-5p;hsa-miR-15b-5p;hsa-miR-16-5p;hsa-miR-195-
ENSG0000	ENSG0000	4.60E-06	hsa-miR-17-5p;hsa-miR-20b-5p;hsa-miR-32-5p;hsa-miR-92a-3
ENSG0000	ENSG0000	0.000159	hsa-let-7a-5p;hsa-let-7b-5p;hsa-let-7d-5p;hsa-let-7f-5p;hsa-le
ENSG0000	ENSG0000	0.000101	hsa-miR-17-5p;hsa-miR-20b-5p;hsa-miR-27a-3p;hsa-miR-27b-
ENSG0000	ENSG0000	0.001562	hsa-let-7a-5p;hsa-let-7b-5p;hsa-let-7d-5p;hsa-let-7f-5p;hsa-le
ENSG0000	ENSG0000	0.005274	hsa-miR-17-5p;hsa-miR-20b-5p;hsa-miR-3194-3p;hsa-miR-51
ENSG0000	ENSG0000	0.004481	hsa-miR-148b-3p;hsa-miR-204-5p;hsa-miR-27a-3p;hsa-miR-2
ENSG0000	ENSG0000	0.000539	hsa-miR-129-5p;hsa-miR-15a-5p;hsa-miR-15b-5p;hsa-miR-16-
ENSG0000	ENSG0000	0.000817	hsa-miR-15a-5p;hsa-miR-15b-5p;hsa-miR-195-5p;hsa-miR-42
ENSG0000	ENSG0000	5.05E-06	hsa-miR-15a-5p;hsa-miR-15b-5p;hsa-miR-16-5p;hsa-miR-195-
ENSG0000	ENSG0000	3.59E-05	hsa-miR-103a-3p;hsa-miR-107;hsa-miR-15a-5p;hsa-miR-15b-5
ENSG0000	ENSG0000	1.61E-08	hsa-miR-15a-5p;hsa-miR-15b-5p;hsa-miR-16-5p;hsa-miR-195-
ENSG0000	ENSG0000	0.000539	hsa-miR-224-5p;hsa-miR-29a-3p;hsa-miR-29b-3p;hsa-miR-29
ENSG0000	ENSG0000	0.00134	hsa-miR-129-5p;hsa-miR-29c-3p;hsa-miR-30a-3p;hsa-miR-30
ENSG0000	ENSG0000	0.009263	hsa-miR-103a-3p;hsa-miR-107;hsa-miR-15a-5p;hsa-miR-15b-5
ENSG0000	ENSG0000	0.008469	hsa-let-7a-5p;hsa-let-7b-5p;hsa-let-7f-5p;hsa-let-7g-5p;hsa-le
ENSG0000	ENSG0000	0.001261	hsa-let-7a-5p;hsa-let-7b-5p;hsa-let-7d-5p;hsa-let-7f-5p;hsa-le
ENSG0000	ENSG0000	8.70E-05	hsa-miR-15a-5p;hsa-miR-15b-5p;hsa-miR-195-5p;hsa-miR-42
ENSG0000	ENSG0000	0.003349	hsa-miR-17-5p;hsa-miR-20b-5p;hsa-miR-32-5p;hsa-miR-92a-3
ENSG0000	ENSG0000	0.003349	hsa-miR-17-5p;hsa-miR-20b-5p;hsa-miR-32-5p;hsa-miR-92a-3
ENSG0000	ENSG0000	0.002214	hsa-miR-148b-3p;hsa-miR-17-5p;hsa-miR-20b-5p;hsa-miR-27
ENSG0000	ENSG0000	9.52E-07	hsa-miR-15a-5p;hsa-miR-15b-5p;hsa-miR-16-5p;hsa-miR-195-
ENSG0000	ENSG0000	0.000391	hsa-miR-15a-5p;hsa-miR-15b-5p;hsa-miR-16-5p;hsa-miR-195-
ENSG0000	ENSG0000	0.003905	hsa-miR-30b-5p;hsa-miR-30c-5p;hsa-miR-32-5p;hsa-miR-92a-
ENSG0000	ENSG0000	0.002738	hsa-miR-17-5p;hsa-miR-20b-5p;hsa-miR-519a-3p;hsa-miR-51
ENSG0000	ENSG0000	0.008469	hsa-let-7a-5p;hsa-let-7b-5p;hsa-let-7d-5p;hsa-let-7f-5p;hsa-le
ENSG0000	ENSG0000	8.84E-06	hsa-let-7a-5p;hsa-let-7b-5p;hsa-let-7d-5p;hsa-let-7f-5p;hsa-le
ENSG0000	ENSG0000	8.84E-06	hsa-let-7a-5p;hsa-let-7b-5p;hsa-let-7d-5p;hsa-let-7f-5p;hsa-le

ENSG0000	ENSG0000	5.05E-06	hsa-let-7a-5p;hsa-let-7b-5p;hsa-let-7d-5p;hsa-let-7f-5p;hsa-le
ENSG0000	ENSG0000	5.12E-10	hsa-miR-15a-5p;hsa-miR-15b-5p;hsa-miR-16-5p;hsa-miR-195-
ENSG0000	ENSG0000	0.00097	hsa-miR-17-5p;hsa-miR-20b-5p;hsa-miR-519a-3p;hsa-miR-51
ENSG0000	ENSG0000	0.007173	hsa-miR-15a-5p;hsa-miR-15b-5p;hsa-miR-16-5p;hsa-miR-195-
ENSG0000	ENSG0000	5.77E-07	hsa-let-7a-5p;hsa-let-7b-5p;hsa-let-7d-5p;hsa-let-7f-5p;hsa-le
ENSG0000	ENSG0000	0.005699	hsa-miR-140-3p;hsa-miR-15a-5p;hsa-miR-15b-5p;hsa-miR-19
ENSG0000	ENSG0000	5.73E-07	hsa-miR-15a-5p;hsa-miR-15b-5p;hsa-miR-16-5p;hsa-miR-195-
ENSG0000	ENSG0000	0.006331	hsa-let-7a-5p;hsa-let-7b-5p;hsa-let-7d-5p;hsa-let-7f-5p;hsa-le
ENSG0000	ENSG0000	0.000768	hsa-miR-129-5p;hsa-miR-15a-5p;hsa-miR-15b-5p;hsa-miR-19
ENSG0000	ENSG0000	0.002682	hsa-miR-15a-5p;hsa-miR-16-5p;hsa-miR-218-5p;hsa-miR-7-5p
ENSG0000	ENSG0000	0.000951	hsa-let-7a-5p;hsa-let-7b-5p;hsa-let-7d-5p;hsa-let-7f-5p;hsa-le
ENSG0000	ENSG0000	0.006394	hsa-let-7a-5p;hsa-let-7b-5p;hsa-let-7d-5p;hsa-let-7f-5p;hsa-le
ENSG0000	ENSG0000	0.000901	hsa-miR-30a-3p;hsa-miR-30d-3p;hsa-miR-30e-3p;hsa-miR-44
ENSG0000	ENSG0000	0.000628	hsa-miR-15a-5p;hsa-miR-16-5p;hsa-miR-20a-3p;hsa-miR-26b-
ENSG0000	ENSG0000	2.19E-05	hsa-miR-17-5p;hsa-miR-20b-5p;hsa-miR-30b-5p;hsa-miR-30c-
ENSG0000	ENSG0000	9.45E-05	hsa-miR-126-5p;hsa-miR-15a-5p;hsa-miR-15b-5p;hsa-miR-17-
ENSG0000	ENSG0000	4.00E-08	hsa-miR-107;hsa-miR-15a-5p;hsa-miR-15b-5p;hsa-miR-16-5p;
ENSG0000	ENSG0000	0.001489	hsa-miR-15a-5p;hsa-miR-15b-5p;hsa-miR-17-5p;hsa-miR-195-
ENSG0000	ENSG0000	9.45E-05	hsa-miR-103a-3p;hsa-miR-107;hsa-miR-214-3p;hsa-miR-30b-5
ENSG0000	ENSG0000	0.004098	hsa-let-7a-5p;hsa-let-7b-5p;hsa-let-7d-5p;hsa-let-7f-5p;hsa-le
ENSG0000	ENSG0000	0.007992	hsa-let-7a-5p;hsa-let-7b-5p;hsa-let-7d-5p;hsa-let-7f-5p;hsa-le
ENSG0000	ENSG0000	0.007539	hsa-miR-122-5p;hsa-miR-204-5p;hsa-miR-30a-3p;hsa-miR-30
ENSG0000	ENSG0000	0.000159	hsa-let-7a-5p;hsa-let-7b-5p;hsa-let-7d-5p;hsa-let-7f-5p;hsa-le
ENSG0000	ENSG0000	9.52E-07	hsa-let-7a-5p;hsa-let-7b-5p;hsa-let-7d-5p;hsa-let-7f-5p;hsa-le
ENSG0000	ENSG0000	1.71E-06	hsa-miR-107;hsa-miR-15a-5p;hsa-miR-15b-5p;hsa-miR-195-5p
ENSG0000	ENSG0000	2.30E-05	hsa-miR-15a-5p;hsa-miR-15b-5p;hsa-miR-16-5p;hsa-miR-195-
ENSG0000	ENSG0000	1.00E-05	hsa-miR-15a-5p;hsa-miR-15b-5p;hsa-miR-195-5p;hsa-miR-30b
ENSG0000	ENSG0000	0.000908	hsa-miR-17-5p;hsa-miR-20b-5p;hsa-miR-30b-5p;hsa-miR-30c-
ENSG0000	ENSG0000	0.000908	hsa-miR-17-5p;hsa-miR-20b-5p;hsa-miR-30b-5p;hsa-miR-30c-
ENSG0000	ENSG0000	0.00097	hsa-let-7a-5p;hsa-let-7b-5p;hsa-let-7d-5p;hsa-let-7f-5p;hsa-le
ENSG0000	ENSG0000	0.000966	hsa-let-7a-5p;hsa-let-7b-5p;hsa-let-7d-5p;hsa-let-7f-5p;hsa-le
ENSG0000	ENSG0000	0.000657	hsa-miR-103a-3p;hsa-miR-107;hsa-miR-1304-3p;hsa-miR-15a-
ENSG0000	ENSG0000	0.001068	hsa-miR-107;hsa-miR-15a-5p;hsa-miR-15b-5p;hsa-miR-195-5p
ENSG0000	ENSG0000	0.00641	hsa-miR-139-5p;hsa-miR-192-5p;hsa-miR-29a-3p;hsa-miR-29b
ENSG0000	ENSG0000	1.01E-06	hsa-let-7a-5p;hsa-let-7b-5p;hsa-let-7d-5p;hsa-let-7f-5p;hsa-le
ENSG0000	ENSG0000	0.005274	hsa-miR-107;hsa-miR-15a-5p;hsa-miR-15b-5p;hsa-miR-195-5p
ENSG0000	ENSG0000	1.30E-05	hsa-miR-103a-3p;hsa-miR-107;hsa-miR-15a-5p;hsa-miR-15b-5
ENSG0000	ENSG0000	6.16E-10	hsa-miR-129-5p;hsa-miR-15a-5p;hsa-miR-15b-5p;hsa-miR-16-
ENSG0000	ENSG0000	6.16E-10	hsa-miR-129-5p;hsa-miR-15a-5p;hsa-miR-15b-5p;hsa-miR-16-
ENSG0000	ENSG0000	3.59E-05	hsa-miR-15a-5p;hsa-miR-15b-5p;hsa-miR-195-5p;hsa-miR-42
ENSG0000	ENSG0000	2.88E-07	hsa-let-7a-5p;hsa-let-7b-5p;hsa-let-7d-5p;hsa-let-7f-5p;hsa-le
ENSG0000	ENSG0000	2.88E-07	hsa-let-7a-5p;hsa-let-7b-5p;hsa-let-7d-5p;hsa-let-7f-5p;hsa-le
ENSG0000	ENSG0000	0.00014	hsa-miR-17-5p;hsa-miR-20b-5p;hsa-miR-26b-3p;hsa-miR-27a-
ENSG0000	ENSG0000	7.89E-06	hsa-miR-15a-5p;hsa-miR-15b-5p;hsa-miR-16-5p;hsa-miR-195-
ENSG0000	ENSG0000	0.00134	hsa-let-7a-5p;hsa-let-7b-5p;hsa-let-7d-5p;hsa-let-7f-5p;hsa-le
ENSG0000	ENSG0000	1.62E-06	hsa-miR-15a-5p;hsa-miR-15b-5p;hsa-miR-16-5p;hsa-miR-195-
ENSG0000	ENSG0000	4.97E-05	hsa-miR-17-5p;hsa-miR-20b-5p;hsa-miR-519a-3p;hsa-miR-51
ENSG0000	ENSG0000	0.001068	hsa-miR-17-5p;hsa-miR-20b-5p;hsa-miR-330-3p;hsa-miR-519
ENSG0000	ENSG0000	1.21E-07	hsa-miR-103a-3p;hsa-miR-107;hsa-miR-15a-5p;hsa-miR-15b-5
ENSG0000	ENSG0000	0.000332	hsa-miR-145-5p;hsa-miR-29a-3p;hsa-miR-29b-3p;hsa-miR-29
ENSG0000	ENSG0000	0.000277	hsa-let-7a-5p;hsa-let-7b-5p;hsa-let-7d-5p;hsa-let-7f-5p;hsa-le
ENSG0000	ENSG0000	8.78E-06	hsa-miR-15a-5p;hsa-miR-15b-5p;hsa-miR-16-5p;hsa-miR-195-
ENSG0000	ENSG0000	0.000447	hsa-miR-15a-5p;hsa-miR-15b-5p;hsa-miR-16-5p;hsa-miR-17-5
ENSG0000	ENSG0000	0.001384	hsa-let-7a-5p;hsa-let-7b-5p;hsa-let-7f-5p;hsa-let-7g-5p;hsa-le

ENSG0000	ENSG0000	0.000119	hsa-miR-15a-5p;hsa-miR-15b-5p;hsa-miR-16-5p;hsa-miR-195-
ENSG0000	ENSG0000	4.66E-07	hsa-miR-15a-5p;hsa-miR-15b-5p;hsa-miR-16-5p;hsa-miR-195-
ENSG0000	ENSG0000	7.89E-06	hsa-miR-15a-5p;hsa-miR-16-5p;hsa-miR-17-5p;hsa-miR-195-5
ENSG0000	ENSG0000	7.89E-06	hsa-miR-15a-5p;hsa-miR-16-5p;hsa-miR-17-5p;hsa-miR-195-5
ENSG0000	ENSG0000	0.007539	hsa-miR-1304-3p;hsa-miR-17-5p;hsa-miR-20b-5p;hsa-miR-31
ENSG0000	ENSG0000	0.00097	hsa-miR-15a-5p;hsa-miR-15b-5p;hsa-miR-16-5p;hsa-miR-195-
ENSG0000	ENSG0000	0.005274	hsa-miR-17-5p;hsa-miR-20b-5p;hsa-miR-374a-3p;hsa-miR-51
ENSG0000	ENSG0000	0.002109	hsa-miR-17-5p;hsa-miR-20b-5p;hsa-miR-218-5p;hsa-miR-93-5
ENSG0000	ENSG0000	0.002109	hsa-miR-17-5p;hsa-miR-20b-5p;hsa-miR-218-5p;hsa-miR-93-5
ENSG0000	ENSG0000	0.000439	hsa-miR-140-3p;hsa-miR-17-5p;hsa-miR-20b-5p;hsa-miR-218-
ENSG0000	ENSG0000	0.005022	hsa-miR-26b-3p;hsa-miR-32-5p;hsa-miR-92a-3p;hsa-miR-92b-
ENSG0000	ENSG0000	0.007512	hsa-miR-15a-5p;hsa-miR-15b-5p;hsa-miR-16-5p;hsa-miR-195-
ENSG0000	ENSG0000	0.000179	hsa-let-7a-5p;hsa-let-7b-5p;hsa-let-7d-5p;hsa-let-7f-5p;hsa-le
ENSG0000	ENSG0000	0.000179	hsa-let-7a-5p;hsa-let-7b-5p;hsa-let-7d-5p;hsa-let-7f-5p;hsa-le
ENSG0000	ENSG0000	0.008469	hsa-miR-29a-5p;hsa-miR-30a-3p;hsa-miR-30d-3p;hsa-miR-30e
ENSG0000	ENSG0000	0.008469	hsa-miR-29a-5p;hsa-miR-30a-3p;hsa-miR-30d-3p;hsa-miR-30e
ENSG0000	ENSG0000	7.89E-06	hsa-miR-15a-5p;hsa-miR-15b-5p;hsa-miR-16-5p;hsa-miR-195-
ENSG0000	ENSG0000	3.59E-05	hsa-miR-103a-3p;hsa-miR-107;hsa-miR-15a-5p;hsa-miR-15b-5
ENSG0000	ENSG0000	4.39E-06	hsa-miR-15a-5p;hsa-miR-15b-5p;hsa-miR-16-5p;hsa-miR-195-
ENSG0000	ENSG0000	0.000168	hsa-miR-1260b;hsa-miR-15a-5p;hsa-miR-15b-5p;hsa-miR-188-
ENSG0000	ENSG0000	3.65E-05	hsa-miR-15a-5p;hsa-miR-15b-5p;hsa-miR-16-5p;hsa-miR-195-
ENSG0000	ENSG0000	0.002411	hsa-miR-15a-5p;hsa-miR-15b-5p;hsa-miR-195-5p;hsa-miR-30
ENSG0000	ENSG0000	0.005022	hsa-miR-103a-3p;hsa-miR-107;hsa-miR-129-5p;hsa-miR-30b-5
ENSG0000	ENSG0000	0.000919	hsa-miR-101-3p;hsa-miR-148b-3p;hsa-miR-15a-5p;hsa-miR-1
ENSG0000	ENSG0000	0.000919	hsa-miR-101-3p;hsa-miR-148b-3p;hsa-miR-15a-5p;hsa-miR-1
ENSG0000	ENSG0000	0.003349	hsa-miR-204-5p;hsa-miR-30a-3p;hsa-miR-30d-3p;hsa-miR-30e
ENSG0000	ENSG0000	0.000332	hsa-miR-129-5p;hsa-miR-17-5p;hsa-miR-20a-3p;hsa-miR-20b-
ENSG0000	ENSG0000	0.001681	hsa-miR-16-5p;hsa-miR-17-5p;hsa-miR-20b-5p;hsa-miR-27b-3
ENSG0000	ENSG0000	0.008703	hsa-let-7a-5p;hsa-let-7b-5p;hsa-let-7f-5p;hsa-let-7g-5p;hsa-le
ENSG0000	ENSG0000	1.85E-05	hsa-let-7b-5p;hsa-let-7d-5p;hsa-let-7f-5p;hsa-let-7g-5p;hsa-le
ENSG0000	ENSG0000	3.42E-08	hsa-miR-101-3p;hsa-miR-139-5p;hsa-miR-15a-5p;hsa-miR-15
ENSG0000	ENSG0000	9.03E-05	hsa-miR-15a-5p;hsa-miR-15b-5p;hsa-miR-16-5p;hsa-miR-195-
ENSG0000	ENSG0000	5.77E-07	hsa-let-7a-5p;hsa-let-7b-5p;hsa-let-7d-5p;hsa-let-7f-5p;hsa-le
ENSG0000	ENSG0000	6.33E-06	hsa-miR-15a-5p;hsa-miR-15b-5p;hsa-miR-195-5p;hsa-miR-42
ENSG0000	ENSG0000	0.002397	hsa-miR-17-5p;hsa-miR-20b-5p;hsa-miR-519a-3p;hsa-miR-51
ENSG0000	ENSG0000	0.000582	hsa-miR-17-5p;hsa-miR-20b-5p;hsa-miR-342-3p;hsa-miR-34a-
ENSG0000	ENSG0000	0.001849	hsa-miR-1260b;hsa-miR-188-3p;hsa-miR-20b-5p;hsa-miR-30a-
ENSG0000	ENSG0000	0.007173	hsa-miR-1260b;hsa-miR-204-5p;hsa-miR-27a-3p;hsa-miR-27b-
ENSG0000	ENSG0000	0.001655	hsa-miR-15a-5p;hsa-miR-15b-5p;hsa-miR-16-5p;hsa-miR-195-
ENSG0000	ENSG0000	9.03E-05	hsa-let-7a-5p;hsa-let-7b-5p;hsa-let-7d-5p;hsa-let-7f-5p;hsa-le
ENSG0000	ENSG0000	9.03E-05	hsa-let-7a-5p;hsa-let-7b-5p;hsa-let-7d-5p;hsa-let-7f-5p;hsa-le
ENSG0000	ENSG0000	0.001227	hsa-miR-15a-5p;hsa-miR-15b-5p;hsa-miR-424-5p;hsa-miR-49
ENSG0000	ENSG0000	0.000179	hsa-miR-30a-3p;hsa-miR-30d-3p;hsa-miR-30e-3p;hsa-miR-33
ENSG0000	ENSG0000	0.000159	hsa-miR-15a-5p;hsa-miR-15b-5p;hsa-miR-16-5p;hsa-miR-195-
ENSG0000	ENSG0000	0.000332	hsa-miR-2278;hsa-miR-29a-3p;hsa-miR-29b-3p;hsa-miR-29c-3
ENSG0000	ENSG0000	6.22E-06	hsa-let-7a-5p;hsa-let-7b-5p;hsa-let-7d-5p;hsa-let-7f-5p;hsa-le
ENSG0000	ENSG0000	1.62E-06	hsa-miR-15a-5p;hsa-miR-15b-5p;hsa-miR-16-5p;hsa-miR-195-
ENSG0000	ENSG0000	0.000688	hsa-miR-15a-5p;hsa-miR-15b-5p;hsa-miR-16-5p;hsa-miR-17-5
ENSG0000	ENSG0000	0.001903	hsa-let-7a-5p;hsa-let-7b-5p;hsa-let-7d-5p;hsa-let-7f-5p;hsa-le
ENSG0000	ENSG0000	0.000596	hsa-miR-15a-5p;hsa-miR-15b-5p;hsa-miR-16-5p;hsa-miR-195-
ENSG0000	ENSG0000	0.000205	hsa-miR-103a-3p;hsa-miR-107;hsa-miR-1260b;hsa-miR-15a-5p
ENSG0000	ENSG0000	0.000331	hsa-miR-139-5p;hsa-miR-140-3p;hsa-miR-15a-5p;hsa-miR-15
ENSG0000	ENSG0000	1.62E-06	hsa-miR-15a-5p;hsa-miR-15b-5p;hsa-miR-16-5p;hsa-miR-195-
ENSG0000	ENSG0000	0.000158	hsa-miR-15a-5p;hsa-miR-15b-5p;hsa-miR-16-5p;hsa-miR-195-

ENSG0000	ENSG0000	0.000196	hsa-miR-15a-5p;hsa-miR-15b-5p;hsa-miR-16-5p;hsa-miR-195-
ENSG0000	ENSG0000	0.00018	hsa-let-7a-5p;hsa-let-7b-5p;hsa-let-7d-5p;hsa-let-7f-5p;hsa-le
ENSG0000	ENSG0000	7.03E-07	hsa-miR-15a-5p;hsa-miR-15b-5p;hsa-miR-16-5p;hsa-miR-17-5
ENSG0000	ENSG0000	0.002988	hsa-let-7a-5p;hsa-let-7b-5p;hsa-let-7d-5p;hsa-let-7f-5p;hsa-le
ENSG0000	ENSG0000	1.13E-08	hsa-miR-103a-3p;hsa-miR-107;hsa-miR-15a-5p;hsa-miR-15b-5
ENSG0000	ENSG0000	1.18E-05	hsa-let-7a-5p;hsa-let-7d-5p;hsa-let-7f-5p;hsa-let-7g-5p;hsa-le
ENSG0000	ENSG0000	0.004265	hsa-miR-17-5p;hsa-miR-20b-5p;hsa-miR-4487;hsa-miR-519a-3
ENSG0000	ENSG0000	0.008204	hsa-let-7a-5p;hsa-let-7b-5p;hsa-let-7d-5p;hsa-let-7f-5p;hsa-le
ENSG0000	ENSG0000	0.000159	hsa-miR-17-5p;hsa-miR-20b-5p;hsa-miR-218-5p;hsa-miR-30b
ENSG0000	ENSG0000	0.001562	hsa-let-7a-5p;hsa-let-7b-5p;hsa-let-7d-5p;hsa-let-7f-5p;hsa-le
ENSG0000	ENSG0000	0.000118	hsa-miR-103a-3p;hsa-miR-107;hsa-miR-15a-5p;hsa-miR-15b-5
ENSG0000	ENSG0000	0.000128	hsa-miR-15a-5p;hsa-miR-15b-5p;hsa-miR-16-5p;hsa-miR-17-5
ENSG0000	ENSG0000	3.23E-05	hsa-miR-15a-5p;hsa-miR-15b-5p;hsa-miR-16-5p;hsa-miR-195-
ENSG0000	ENSG0000	0.008043	hsa-miR-139-5p;hsa-miR-17-5p;hsa-miR-20b-5p;hsa-miR-519a
ENSG0000	ENSG0000	0.004131	hsa-miR-204-5p;hsa-miR-30b-5p;hsa-miR-30c-5p;hsa-miR-502
ENSG0000	ENSG0000	0.000966	hsa-miR-15a-5p;hsa-miR-15b-5p;hsa-miR-195-5p;hsa-miR-319
ENSG0000	ENSG0000	0.001127	hsa-miR-27a-3p;hsa-miR-27b-3p;hsa-miR-30b-5p;hsa-miR-30c
ENSG0000	ENSG0000	0.001127	hsa-miR-27a-3p;hsa-miR-27b-3p;hsa-miR-30b-5p;hsa-miR-30c
ENSG0000	ENSG0000	0.003564	hsa-miR-122-5p;hsa-miR-129-5p;hsa-miR-17-5p;hsa-miR-20b-
ENSG0000	ENSG0000	0.000641	hsa-miR-129-5p;hsa-miR-218-5p;hsa-miR-3194-3p;hsa-miR-43
ENSG0000	ENSG0000	0.00025	hsa-miR-101-3p;hsa-miR-103a-3p;hsa-miR-107;hsa-miR-1260b
ENSG0000	ENSG0000	0.001811	hsa-miR-17-5p;hsa-miR-20b-5p;hsa-miR-519a-3p;hsa-miR-519
ENSG0000	ENSG0000	0.001811	hsa-miR-17-5p;hsa-miR-20b-5p;hsa-miR-519a-3p;hsa-miR-519
ENSG0000	ENSG0000	0.001811	hsa-miR-17-5p;hsa-miR-20b-5p;hsa-miR-519a-3p;hsa-miR-519
ENSG0000	ENSG0000	0.001811	hsa-miR-17-5p;hsa-miR-20b-5p;hsa-miR-519a-3p;hsa-miR-519
ENSG0000	ENSG0000	0.001811	hsa-miR-17-5p;hsa-miR-20b-5p;hsa-miR-519a-3p;hsa-miR-519
ENSG0000	ENSG0000	0.001811	hsa-miR-17-5p;hsa-miR-20b-5p;hsa-miR-519a-3p;hsa-miR-519
ENSG0000	ENSG0000	0.001811	hsa-miR-17-5p;hsa-miR-20b-5p;hsa-miR-519a-3p;hsa-miR-519
ENSG0000	ENSG0000	6.77E-05	hsa-miR-15a-5p;hsa-miR-15b-5p;hsa-miR-195-5p;hsa-miR-330
ENSG0000	ENSG0000	0.003905	hsa-miR-15a-5p;hsa-miR-15b-5p;hsa-miR-195-5p;hsa-miR-424
ENSG0000	ENSG0000	0.000232	hsa-miR-27a-3p;hsa-miR-27b-3p;hsa-miR-361-5p;hsa-miR-374
ENSG0000	ENSG0000	0.001336	hsa-miR-17-5p;hsa-miR-20b-5p;hsa-miR-218-5p;hsa-miR-4487
ENSG0000	ENSG0000	1.37E-06	hsa-miR-126-5p;hsa-miR-132-3p;hsa-miR-15a-5p;hsa-miR-15b
ENSG0000	ENSG0000	0.002033	hsa-miR-129-5p;hsa-miR-15a-5p;hsa-miR-15b-5p;hsa-miR-16-
ENSG0000	ENSG0000	1.16E-08	hsa-miR-15a-5p;hsa-miR-15b-5p;hsa-miR-195-5p;hsa-miR-29a
ENSG0000	ENSG0000	7.52E-05	hsa-let-7a-5p;hsa-let-7b-5p;hsa-let-7d-5p;hsa-let-7f-5p;hsa-le
ENSG0000	ENSG0000	0.002214	hsa-miR-122-5p;hsa-miR-1304-3p;hsa-miR-17-5p;hsa-miR-20b
ENSG0000	ENSG0000	0.006923	hsa-miR-101-3p;hsa-miR-1304-3p;hsa-miR-17-5p;hsa-miR-20b
ENSG0000	ENSG0000	0.003912	hsa-let-7a-5p;hsa-let-7d-5p;hsa-let-7f-5p;hsa-let-7g-5p;hsa-le
ENSG0000	ENSG0000	9.84E-05	hsa-miR-15a-5p;hsa-miR-15b-5p;hsa-miR-16-5p;hsa-miR-17-5
ENSG0000	ENSG0000	0.000118	hsa-miR-145-5p;hsa-miR-27a-3p;hsa-miR-27b-3p;hsa-miR-29a
ENSG0000	ENSG0000	0.002852	hsa-miR-126-5p;hsa-miR-15a-3p;hsa-miR-224-5p;hsa-miR-27a
ENSG0000	ENSG0000	0.002856	hsa-let-7a-5p;hsa-let-7b-5p;hsa-let-7d-5p;hsa-let-7f-5p;hsa-le
ENSG0000	ENSG0000	0.00097	hsa-let-7a-5p;hsa-let-7b-5p;hsa-let-7d-5p;hsa-let-7f-5p;hsa-le
ENSG0000	ENSG0000	2.10E-07	hsa-let-7a-5p;hsa-let-7b-5p;hsa-let-7d-5p;hsa-let-7f-5p;hsa-le
ENSG0000	ENSG0000	1.38E-05	hsa-miR-143-3p;hsa-miR-15a-5p;hsa-miR-16-5p;hsa-miR-195-
ENSG0000	ENSG0000	0.002852	hsa-miR-103a-3p;hsa-miR-107;hsa-miR-32-5p;hsa-miR-92a-3p
ENSG0000	ENSG0000	0.000641	hsa-miR-143-3p;hsa-miR-29a-3p;hsa-miR-29b-3p;hsa-miR-29c
ENSG0000	ENSG0000	0.000332	hsa-miR-27a-3p;hsa-miR-27b-3p;hsa-miR-30b-5p;hsa-miR-30c
ENSG0000	ENSG0000	0.002887	hsa-miR-17-5p;hsa-miR-20b-5p;hsa-miR-4487;hsa-miR-502-5p
ENSG0000	ENSG0000	0.007711	hsa-miR-129-5p;hsa-miR-1304-3p;hsa-miR-17-5p;hsa-miR-20b
ENSG0000	ENSG0000	0.001227	hsa-miR-218-5p;hsa-miR-222-5p;hsa-miR-27a-3p;hsa-miR-27b
ENSG0000	ENSG0000	0.000232	hsa-miR-17-5p;hsa-miR-20b-5p;hsa-miR-519a-3p;hsa-miR-519
ENSG0000	ENSG0000	6.54E-05	hsa-miR-17-5p;hsa-miR-20b-5p;hsa-miR-342-3p;hsa-miR-382-

ENSG0000	ENSG0000	0.005609	hsa-let-7a-5p;hsa-let-7b-5p;hsa-let-7d-5p;hsa-let-7f-5p;hsa-le
ENSG0000	ENSG0000	3.79E-06	hsa-miR-103a-3p;hsa-miR-107;hsa-miR-15a-5p;hsa-miR-15b-5
ENSG0000	ENSG0000	0.001261	hsa-miR-15a-5p;hsa-miR-15b-5p;hsa-miR-16-5p;hsa-miR-195-
ENSG0000	ENSG0000	0.000908	hsa-miR-17-5p;hsa-miR-20b-5p;hsa-miR-27a-3p;hsa-miR-27b-
ENSG0000	ENSG0000	0.005274	hsa-let-7a-5p;hsa-let-7b-5p;hsa-let-7d-5p;hsa-let-7f-5p;hsa-le
ENSG0000	ENSG0000	0.000684	hsa-let-7a-5p;hsa-let-7b-5p;hsa-let-7d-5p;hsa-let-7f-5p;hsa-le
ENSG0000	ENSG0000	0.003932	hsa-miR-15a-5p;hsa-miR-15b-5p;hsa-miR-17-5p;hsa-miR-195-
ENSG0000	ENSG0000	7.37E-06	hsa-let-7a-5p;hsa-let-7b-5p;hsa-let-7f-5p;hsa-let-7g-5p;hsa-le
ENSG0000	ENSG0000	8.44E-09	hsa-let-7a-5p;hsa-let-7b-5p;hsa-let-7d-5p;hsa-let-7f-5p;hsa-le
ENSG0000	ENSG0000	0.009294	hsa-miR-17-5p;hsa-miR-20b-5p;hsa-miR-29a-5p;hsa-miR-519
ENSG0000	ENSG0000	0.005471	hsa-miR-17-5p;hsa-miR-20b-5p;hsa-miR-24-3p;hsa-miR-342-
ENSG0000	ENSG0000	4.56E-05	hsa-miR-15a-5p;hsa-miR-15b-5p;hsa-miR-16-5p;hsa-miR-17-
ENSG0000	ENSG0000	4.97E-05	hsa-miR-15a-5p;hsa-miR-15b-5p;hsa-miR-16-5p;hsa-miR-195-
ENSG0000	ENSG0000	0.003493	hsa-miR-101-3p;hsa-miR-122-5p;hsa-miR-129-5p;hsa-miR-13
ENSG0000	ENSG0000	0.000154	hsa-miR-204-5p;hsa-miR-30a-3p;hsa-miR-30d-3p;hsa-miR-30
ENSG0000	ENSG0000	0.000593	hsa-miR-140-3p;hsa-miR-145-5p;hsa-miR-15a-3p;hsa-miR-17-
ENSG0000	ENSG0000	0.002925	hsa-miR-1304-3p;hsa-miR-15a-5p;hsa-miR-15b-5p;hsa-miR-1
ENSG0000	ENSG0000	0.000771	hsa-let-7a-5p;hsa-let-7b-5p;hsa-let-7d-5p;hsa-let-7f-5p;hsa-le
ENSG0000	ENSG0000	0.001795	hsa-let-7a-5p;hsa-let-7b-5p;hsa-let-7d-5p;hsa-let-7f-5p;hsa-le
ENSG0000	ENSG0000	0.006034	hsa-miR-218-5p;hsa-miR-29a-3p;hsa-miR-29b-3p;hsa-miR-29
ENSG0000	ENSG0000	6.76E-05	hsa-let-7a-5p;hsa-let-7b-5p;hsa-let-7d-5p;hsa-let-7f-5p;hsa-le
ENSG0000	ENSG0000	0.00097	hsa-miR-17-5p;hsa-miR-20b-5p;hsa-miR-519a-3p;hsa-miR-51
ENSG0000	ENSG0000	2.82E-07	hsa-let-7a-5p;hsa-let-7b-5p;hsa-let-7d-5p;hsa-let-7f-5p;hsa-le
ENSG0000	ENSG0000	0.001731	hsa-let-7a-5p;hsa-let-7b-5p;hsa-let-7d-5p;hsa-let-7f-5p;hsa-le
ENSG0000	ENSG0000	9.91E-08	hsa-let-7a-5p;hsa-let-7b-5p;hsa-let-7f-5p;hsa-let-7g-5p;hsa-le
ENSG0000	ENSG0000	0.001049	hsa-let-7a-5p;hsa-let-7b-5p;hsa-let-7d-5p;hsa-let-7f-5p;hsa-le
ENSG0000	ENSG0000	2.53E-05	hsa-miR-15a-5p;hsa-miR-16-5p;hsa-miR-195-5p;hsa-miR-214-
ENSG0000	ENSG0000	0.000641	hsa-miR-15a-5p;hsa-miR-15b-5p;hsa-miR-16-5p;hsa-miR-195-
ENSG0000	ENSG0000	2.77E-08	hsa-let-7a-5p;hsa-let-7b-5p;hsa-let-7d-5p;hsa-let-7f-5p;hsa-le
ENSG0000	ENSG0000	0.00046	hsa-let-7a-5p;hsa-let-7b-5p;hsa-let-7d-5p;hsa-let-7f-5p;hsa-le
ENSG0000	ENSG0000	0.009324	hsa-miR-1260b;hsa-miR-17-5p;hsa-miR-20b-5p;hsa-miR-501-
ENSG0000	ENSG0000	0.00044	hsa-miR-148b-3p;hsa-miR-16-5p;hsa-miR-29c-3p;hsa-miR-43
ENSG0000	ENSG0000	0.00105	hsa-miR-1260b;hsa-miR-148b-3p;hsa-miR-15a-5p;hsa-miR-15
ENSG0000	ENSG0000	4.18E-05	hsa-miR-15a-5p;hsa-miR-15b-5p;hsa-miR-16-5p;hsa-miR-195-
ENSG0000	ENSG0000	0.00069	hsa-let-7a-5p;hsa-let-7b-5p;hsa-let-7d-5p;hsa-let-7f-5p;hsa-le
ENSG0000	ENSG0000	0.002109	hsa-miR-15a-5p;hsa-miR-15b-5p;hsa-miR-188-3p;hsa-miR-19
ENSG0000	ENSG0000	5.77E-07	hsa-miR-15a-5p;hsa-miR-15b-5p;hsa-miR-16-5p;hsa-miR-188-
ENSG0000	ENSG0000	1.64E-07	hsa-let-7a-5p;hsa-let-7b-5p;hsa-let-7d-5p;hsa-let-7f-5p;hsa-le
ENSG0000	ENSG0000	0.00641	hsa-miR-126-5p;hsa-miR-15a-5p;hsa-miR-15b-5p;hsa-miR-16-
ENSG0000	ENSG0000	0.009324	hsa-miR-15a-5p;hsa-miR-15b-5p;hsa-miR-16-5p;hsa-miR-195-
ENSG0000	ENSG0000	2.32E-06	hsa-miR-15a-5p;hsa-miR-15b-5p;hsa-miR-16-5p;hsa-miR-195-
ENSG0000	ENSG0000	2.32E-06	hsa-miR-15a-5p;hsa-miR-15b-5p;hsa-miR-16-5p;hsa-miR-195-
ENSG0000	ENSG0000	2.32E-06	hsa-miR-15a-5p;hsa-miR-15b-5p;hsa-miR-16-5p;hsa-miR-195-
ENSG0000	ENSG0000	2.32E-06	hsa-miR-15a-5p;hsa-miR-15b-5p;hsa-miR-16-5p;hsa-miR-195-
ENSG0000	ENSG0000	2.00E-05	hsa-miR-107;hsa-miR-15a-5p;hsa-miR-15b-5p;hsa-miR-17-5p;
ENSG0000	ENSG0000	2.00E-05	hsa-miR-107;hsa-miR-15a-5p;hsa-miR-15b-5p;hsa-miR-17-5p;
ENSG0000	ENSG0000	0.001849	hsa-let-7a-5p;hsa-let-7b-5p;hsa-let-7d-5p;hsa-let-7f-5p;hsa-le
ENSG0000	ENSG0000	0.000164	hsa-miR-103a-3p;hsa-miR-107;hsa-miR-15a-5p;hsa-miR-15b-5
ENSG0000	ENSG0000	0.003905	hsa-miR-425-5p;hsa-miR-519a-3p;hsa-miR-519b-3p;hsa-miR-5
ENSG0000	ENSG0000	0.000196	hsa-let-7a-5p;hsa-let-7b-5p;hsa-let-7d-5p;hsa-let-7f-5p;hsa-le
ENSG0000	ENSG0000	3.59E-05	hsa-let-7a-5p;hsa-let-7b-5p;hsa-let-7d-5p;hsa-let-7f-5p;hsa-le
ENSG0000	ENSG0000	0.000641	hsa-miR-17-5p;hsa-miR-20b-5p;hsa-miR-224-5p;hsa-miR-93-5
ENSG0000	ENSG0000	1.27E-07	hsa-miR-15a-5p;hsa-miR-15b-5p;hsa-miR-16-5p;hsa-miR-17-5
ENSG0000	ENSG0000	0.000391	hsa-miR-17-5p;hsa-miR-20b-5p;hsa-miR-518c-5p;hsa-miR-51

ENSG0000	ENSG0000	0.000568	hsa-miR-122-5p;hsa-miR-17-5p;hsa-miR-20b-5p;hsa-miR-519
ENSG0000	ENSG0000	2.71E-06	hsa-miR-15a-5p;hsa-miR-15b-5p;hsa-miR-195-5p;hsa-miR-21
ENSG0000	ENSG0000	2.30E-05	hsa-miR-15a-5p;hsa-miR-15b-5p;hsa-miR-16-5p;hsa-miR-195
ENSG0000	ENSG0000	0.008703	hsa-miR-24-3p;hsa-miR-29a-3p;hsa-miR-29b-3p;hsa-miR-29c
ENSG0000	ENSG0000	0.003354	hsa-miR-1260b;hsa-miR-27a-3p;hsa-miR-27b-3p;hsa-miR-574
ENSG0000	ENSG0000	0.009859	hsa-miR-27b-3p;hsa-miR-374a-3p;hsa-miR-501-3p;hsa-miR-5
ENSG0000	ENSG0000	0.004798	hsa-miR-122-5p;hsa-miR-30b-5p;hsa-miR-30c-5p;hsa-miR-31
ENSG0000	ENSG0000	7.88E-05	hsa-miR-17-5p;hsa-miR-20b-5p;hsa-miR-32-5p;hsa-miR-519a
ENSG0000	ENSG0000	8.84E-05	hsa-let-7a-5p;hsa-let-7b-5p;hsa-let-7d-5p;hsa-let-7f-5p;hsa-l
ENSG0000	ENSG0000	0.006801	hsa-let-7a-5p;hsa-let-7b-5p;hsa-let-7d-5p;hsa-let-7g-5p;hsa-
ENSG0000	ENSG0000	1.62E-06	hsa-miR-15a-5p;hsa-miR-15b-5p;hsa-miR-16-5p;hsa-miR-195
ENSG0000	ENSG0000	0.003212	hsa-miR-17-5p;hsa-miR-20b-5p;hsa-miR-24-3p;hsa-miR-519a
ENSG0000	ENSG0000	0.000278	hsa-miR-15a-5p;hsa-miR-15b-5p;hsa-miR-16-5p;hsa-miR-195
ENSG0000	ENSG0000	0.000278	hsa-miR-15a-5p;hsa-miR-15b-5p;hsa-miR-16-5p;hsa-miR-195
ENSG0000	ENSG0000	0.000278	hsa-miR-15a-5p;hsa-miR-15b-5p;hsa-miR-16-5p;hsa-miR-195
ENSG0000	ENSG0000	0.000278	hsa-miR-15a-5p;hsa-miR-15b-5p;hsa-miR-16-5p;hsa-miR-195
ENSG0000	ENSG0000	0.000278	hsa-miR-15a-5p;hsa-miR-15b-5p;hsa-miR-16-5p;hsa-miR-195
ENSG0000	ENSG0000	0.000278	hsa-miR-15a-5p;hsa-miR-15b-5p;hsa-miR-16-5p;hsa-miR-195
ENSG0000	ENSG0000	0.000278	hsa-miR-15a-5p;hsa-miR-15b-5p;hsa-miR-16-5p;hsa-miR-195
ENSG0000	ENSG0000	0.000278	hsa-miR-15a-5p;hsa-miR-15b-5p;hsa-miR-16-5p;hsa-miR-195
ENSG0000	ENSG0000	0.000278	hsa-miR-15a-5p;hsa-miR-15b-5p;hsa-miR-16-5p;hsa-miR-195
ENSG0000	ENSG0000	0.000278	hsa-miR-15a-5p;hsa-miR-15b-5p;hsa-miR-16-5p;hsa-miR-195
ENSG0000	ENSG0000	0.001562	hsa-miR-145-5p;hsa-miR-204-5p;hsa-miR-218-5p;hsa-miR-32
ENSG0000	ENSG0000	2.90E-06	hsa-miR-15a-5p;hsa-miR-15b-5p;hsa-miR-16-5p;hsa-miR-195
ENSG0000	ENSG0000	0.001227	hsa-miR-17-5p;hsa-miR-20b-5p;hsa-miR-589-3p;hsa-miR-93-
ENSG0000	ENSG0000	4.66E-07	hsa-miR-15a-5p;hsa-miR-15b-5p;hsa-miR-16-5p;hsa-miR-195
ENSG0000	ENSG0000	0.000845	hsa-miR-101-3p;hsa-miR-139-5p;hsa-miR-20b-5p;hsa-miR-21
ENSG0000	ENSG0000	0.000119	hsa-let-7a-5p;hsa-let-7b-5p;hsa-let-7d-5p;hsa-let-7f-5p;hsa-l
ENSG0000	ENSG0000	0.001681	hsa-miR-15a-5p;hsa-miR-15b-5p;hsa-miR-16-5p;hsa-miR-424
ENSG0000	ENSG0000	0.003115	hsa-let-7a-5p;hsa-let-7b-5p;hsa-let-7d-5p;hsa-let-7f-5p;hsa-l
ENSG0000	ENSG0000	0.006034	hsa-miR-30a-3p;hsa-miR-30d-3p;hsa-miR-30e-3p;hsa-miR-61
ENSG0000	ENSG0000	0.002062	hsa-let-7a-5p;hsa-let-7b-5p;hsa-let-7d-5p;hsa-let-7f-5p;hsa-l
ENSG0000	ENSG0000	0.003115	hsa-miR-129-5p;hsa-miR-222-5p;hsa-miR-30a-3p;hsa-miR-30
ENSG0000	ENSG0000	9.89E-06	hsa-miR-103a-3p;hsa-miR-107;hsa-miR-122-5p;hsa-miR-15a-
ENSG0000	ENSG0000	7.98E-05	hsa-let-7a-5p;hsa-let-7b-5p;hsa-let-7d-5p;hsa-let-7f-5p;hsa-l
ENSG0000	ENSG0000	4.41E-05	hsa-miR-103a-3p;hsa-miR-107;hsa-miR-15a-5p;hsa-miR-15b-
ENSG0000	ENSG0000	0.002588	hsa-miR-15a-5p;hsa-miR-15b-5p;hsa-miR-16-5p;hsa-miR-195
ENSG0000	ENSG0000	0.004465	hsa-miR-129-5p;hsa-miR-148b-3p;hsa-miR-17-5p;hsa-miR-20
ENSG0000	ENSG0000	5.34E-05	hsa-miR-15a-5p;hsa-miR-15b-5p;hsa-miR-195-5p;hsa-miR-27
ENSG0000	ENSG0000	0.000487	hsa-miR-129-5p;hsa-miR-15a-5p;hsa-miR-15b-5p;hsa-miR-16
ENSG0000	ENSG0000	5.39E-05	hsa-miR-129-5p;hsa-miR-15a-5p;hsa-miR-15b-5p;hsa-miR-16
ENSG0000	ENSG0000	0.000515	hsa-miR-129-5p;hsa-miR-17-5p;hsa-miR-20b-5p;hsa-miR-218
ENSG0000	ENSG0000	0.000487	hsa-let-7a-5p;hsa-let-7b-5p;hsa-let-7d-5p;hsa-let-7f-5p;hsa-l
ENSG0000	ENSG0000	6.80E-06	hsa-let-7a-5p;hsa-let-7b-5p;hsa-let-7d-5p;hsa-let-7f-5p;hsa-l
ENSG0000	ENSG0000	0.00013	hsa-let-7a-5p;hsa-let-7b-5p;hsa-let-7d-5p;hsa-let-7f-5p;hsa-l
ENSG0000	ENSG0000	0.006801	hsa-miR-148b-3p;hsa-miR-15a-5p;hsa-miR-15b-5p;hsa-miR-1
ENSG0000	ENSG0000	0.001068	hsa-let-7a-5p;hsa-let-7b-5p;hsa-let-7d-5p;hsa-let-7f-5p;hsa-l
ENSG0000	ENSG0000	0.000345	hsa-miR-15a-5p;hsa-miR-15b-5p;hsa-miR-16-5p;hsa-miR-195
ENSG0000	ENSG0000	0.008911	hsa-miR-2278;hsa-miR-32-5p;hsa-miR-501-3p;hsa-miR-502-3
ENSG0000	ENSG0000	0.006116	hsa-miR-122-5p;hsa-miR-129-5p;hsa-miR-1304-3p;hsa-miR-1
ENSG0000	ENSG0000	0.001833	hsa-let-7a-5p;hsa-let-7b-5p;hsa-let-7d-5p;hsa-let-7f-5p;hsa-l
ENSG0000	ENSG0000	3.59E-05	hsa-miR-15a-5p;hsa-miR-15b-5p;hsa-miR-16-5p;hsa-miR-195
ENSG0000	ENSG0000	0.009961	hsa-miR-1304-3p;hsa-miR-15a-5p;hsa-miR-15b-5p;hsa-miR-1
ENSG0000	ENSG0000	6.38E-10	hsa-miR-17-5p;hsa-miR-20b-5p;hsa-miR-29a-3p;hsa-miR-29b

ENSG0000	ENSG0000	1.95E-07	hsa-miR-103a-3p;hsa-miR-107;hsa-miR-15a-5p;hsa-miR-15b-5
ENSG0000	ENSG0000	0.000684	hsa-let-7a-5p;hsa-let-7b-5p;hsa-let-7d-5p;hsa-let-7f-5p;hsa-le
ENSG0000	ENSG0000	8.78E-06	hsa-miR-15a-5p;hsa-miR-15b-5p;hsa-miR-195-5p;hsa-miR-30a
ENSG0000	ENSG0000	3.34E-07	hsa-miR-15a-5p;hsa-miR-15b-5p;hsa-miR-16-5p;hsa-miR-195-
ENSG0000	ENSG0000	0.000127	hsa-miR-15a-5p;hsa-miR-15b-5p;hsa-miR-195-5p;hsa-miR-424
ENSG0000	ENSG0000	2.01E-07	hsa-miR-103a-3p;hsa-miR-107;hsa-miR-15a-5p;hsa-miR-15b-5
ENSG0000	ENSG0000	0.00134	hsa-miR-15a-5p;hsa-miR-15b-5p;hsa-miR-16-5p;hsa-miR-195-
ENSG0000	ENSG0000	0.001855	hsa-miR-17-5p;hsa-miR-20b-5p;hsa-miR-30b-5p;hsa-miR-30c-
ENSG0000	ENSG0000	0.008703	hsa-miR-129-5p;hsa-miR-1304-3p;hsa-miR-222-5p;hsa-miR-27
ENSG0000	ENSG0000	0.002022	hsa-miR-15a-5p;hsa-miR-15b-5p;hsa-miR-16-5p;hsa-miR-195-
ENSG0000	ENSG0000	7.89E-06	hsa-miR-15a-5p;hsa-miR-15b-5p;hsa-miR-16-5p;hsa-miR-195-
ENSG0000	ENSG0000	0.006801	hsa-let-7a-5p;hsa-miR-101-3p;hsa-miR-129-5p;hsa-miR-582-5
ENSG0000	ENSG0000	3.09E-06	hsa-miR-1260b;hsa-miR-139-5p;hsa-miR-15a-5p;hsa-miR-15b-
ENSG0000	ENSG0000	0.005022	hsa-miR-32-5p;hsa-miR-542-3p;hsa-miR-92a-3p;hsa-miR-92b-
ENSG0000	ENSG0000	0.000309	hsa-let-7a-5p;hsa-let-7b-5p;hsa-let-7d-5p;hsa-let-7f-5p;hsa-le
ENSG0000	ENSG0000	0.001157	hsa-miR-15a-5p;hsa-miR-15b-5p;hsa-miR-195-5p;hsa-miR-24-
ENSG0000	ENSG0000	4.44E-09	hsa-miR-15a-5p;hsa-miR-15b-5p;hsa-miR-16-5p;hsa-miR-195-
ENSG0000	ENSG0000	5.39E-05	hsa-miR-103a-3p;hsa-miR-107;hsa-miR-15a-5p;hsa-miR-15b-5
ENSG0000	ENSG0000	0.006034	hsa-miR-15a-5p;hsa-miR-15b-5p;hsa-miR-195-5p;hsa-miR-448
ENSG0000	ENSG0000	4.66E-07	hsa-miR-103a-3p;hsa-miR-107;hsa-miR-15a-5p;hsa-miR-15b-5
ENSG0000	ENSG0000	0.000389	hsa-let-7a-5p;hsa-let-7b-5p;hsa-let-7d-5p;hsa-let-7f-5p;hsa-le
ENSG0000	ENSG0000	7.44E-06	hsa-miR-15a-5p;hsa-miR-15b-5p;hsa-miR-16-5p;hsa-miR-195-
ENSG0000	ENSG0000	0.000178	hsa-miR-204-5p;hsa-miR-30a-3p;hsa-miR-30d-3p;hsa-miR-30e
ENSG0000	ENSG0000	1.01E-05	hsa-miR-17-5p;hsa-miR-204-5p;hsa-miR-20b-5p;hsa-miR-24-3
ENSG0000	ENSG0000	0.005022	hsa-miR-15a-5p;hsa-miR-15b-5p;hsa-miR-16-5p;hsa-miR-195-
ENSG0000	ENSG0000	0.005022	hsa-miR-15a-5p;hsa-miR-15b-5p;hsa-miR-16-5p;hsa-miR-195-
ENSG0000	ENSG0000	0.000468	hsa-miR-139-5p;hsa-miR-30a-3p;hsa-miR-30d-3p;hsa-miR-30e
ENSG0000	ENSG0000	0.002088	hsa-let-7a-5p;hsa-let-7b-5p;hsa-let-7d-5p;hsa-let-7f-5p;hsa-le
ENSG0000	ENSG0000	0.00097	hsa-miR-17-5p;hsa-miR-20b-5p;hsa-miR-519a-3p;hsa-miR-519
ENSG0000	ENSG0000	1.09E-05	hsa-let-7a-5p;hsa-let-7b-5p;hsa-let-7d-5p;hsa-let-7f-5p;hsa-le
ENSG0000	ENSG0000	0.00978	hsa-miR-126-5p;hsa-miR-145-5p;hsa-miR-378a-5p;hsa-miR-50
ENSG0000	ENSG0000	0.000101	hsa-miR-27a-3p;hsa-miR-27b-3p;hsa-miR-30b-5p;hsa-miR-30c
ENSG0000	ENSG0000	0.000101	hsa-miR-27a-3p;hsa-miR-27b-3p;hsa-miR-30b-5p;hsa-miR-30c
ENSG0000	ENSG0000	0.000966	hsa-miR-17-5p;hsa-miR-20b-5p;hsa-miR-27a-3p;hsa-miR-27b-
ENSG0000	ENSG0000	0.000345	hsa-miR-17-5p;hsa-miR-20b-5p;hsa-miR-29a-5p;hsa-miR-326;
ENSG0000	ENSG0000	5.34E-05	hsa-miR-15a-5p;hsa-miR-15b-5p;hsa-miR-16-5p;hsa-miR-195-
ENSG0000	ENSG0000	0.007695	hsa-let-7a-5p;hsa-let-7b-5p;hsa-let-7d-5p;hsa-let-7f-5p;hsa-le
ENSG0000	ENSG0000	0.001489	hsa-miR-143-3p;hsa-miR-30b-5p;hsa-miR-30c-5p;hsa-miR-320
ENSG0000	ENSG0000	5.77E-07	hsa-miR-15a-5p;hsa-miR-15b-5p;hsa-miR-16-5p;hsa-miR-195-
ENSG0000	ENSG0000	1.50E-06	hsa-miR-15a-5p;hsa-miR-15b-5p;hsa-miR-16-5p;hsa-miR-195-
ENSG0000	ENSG0000	0.009859	hsa-miR-1304-3p;hsa-miR-24-3p;hsa-miR-501-3p;hsa-miR-502
ENSG0000	ENSG0000	0.000552	hsa-miR-103a-3p;hsa-miR-107;hsa-miR-15a-5p;hsa-miR-15b-5
ENSG0000	ENSG0000	1.16E-08	hsa-miR-15a-5p;hsa-miR-15b-5p;hsa-miR-16-5p;hsa-miR-195-
ENSG0000	ENSG0000	0.000382	hsa-let-7a-5p;hsa-let-7b-5p;hsa-let-7d-5p;hsa-let-7f-5p;hsa-le
ENSG0000	ENSG0000	0.000331	hsa-miR-140-3p;hsa-miR-17-5p;hsa-miR-20b-5p;hsa-miR-222-
ENSG0000	ENSG0000	0.001626	hsa-miR-218-5p;hsa-miR-29a-3p;hsa-miR-29b-3p;hsa-miR-29c
ENSG0000	ENSG0000	0.001261	hsa-let-7a-5p;hsa-let-7b-5p;hsa-let-7d-5p;hsa-let-7f-5p;hsa-le
ENSG0000	ENSG0000	0.005211	hsa-miR-122-5p;hsa-miR-32-5p;hsa-miR-432-5p;hsa-miR-92a-
ENSG0000	ENSG0000	0.005211	hsa-miR-122-5p;hsa-miR-32-5p;hsa-miR-432-5p;hsa-miR-92a-
ENSG0000	ENSG0000	0.000487	hsa-miR-101-3p;hsa-miR-103a-3p;hsa-miR-107;hsa-miR-15a-5
ENSG0000	ENSG0000	7.50E-06	hsa-miR-15a-5p;hsa-miR-15b-5p;hsa-miR-16-5p;hsa-miR-195-
ENSG0000	ENSG0000	8.78E-06	hsa-miR-146a-3p;hsa-miR-15a-5p;hsa-miR-15b-5p;hsa-miR-16
ENSG0000	ENSG0000	0.004525	hsa-miR-30b-5p;hsa-miR-30c-5p;hsa-miR-32-5p;hsa-miR-92a-
ENSG0000	ENSG0000	8.69E-06	hsa-miR-122-5p;hsa-miR-15a-5p;hsa-miR-15b-5p;hsa-miR-16-

ENSG0000	ENSG0000	4.18E-05	hsa-miR-17-5p;hsa-miR-20b-5p;hsa-miR-4487;hsa-miR-519a-[illegible]
ENSG0000	ENSG0000	0.001106	hsa-let-7a-5p;hsa-let-7b-5p;hsa-let-7d-5p;hsa-let-7f-5p;hsa-le
ENSG0000	ENSG0000	0.003021	hsa-let-7a-5p;hsa-let-7d-5p;hsa-let-7f-5p;hsa-let-7g-5p;hsa-le
ENSG0000	ENSG0000	1.62E-06	hsa-miR-15a-5p;hsa-miR-15b-5p;hsa-miR-16-5p;hsa-miR-195-
ENSG0000	ENSG0000	0.000391	hsa-let-7a-5p;hsa-let-7b-5p;hsa-let-7d-5p;hsa-let-7f-5p;hsa-le
ENSG0000	ENSG0000	0.000178	hsa-miR-1304-3p;hsa-miR-30a-3p;hsa-miR-30d-3p;hsa-miR-3[illegible]
ENSG0000	ENSG0000	0.000473	hsa-miR-29a-3p;hsa-miR-29b-3p;hsa-miR-29c-3p;hsa-miR-32-
ENSG0000	ENSG0000	1.73E-05	hsa-let-7a-5p;hsa-let-7b-5p;hsa-let-7d-5p;hsa-let-7f-5p;hsa-le
ENSG0000	ENSG0000	1.38E-07	hsa-miR-15a-5p;hsa-miR-15b-5p;hsa-miR-16-5p;hsa-miR-195-
ENSG0000	ENSG0000	0.009324	hsa-miR-103a-3p;hsa-miR-107;hsa-miR-30b-5p;hsa-miR-30c-[illegible]
ENSG0000	ENSG0000	1.23E-06	hsa-let-7a-5p;hsa-let-7b-5p;hsa-let-7d-5p;hsa-let-7f-5p;hsa-le
ENSG0000	ENSG0000	6.35E-06	hsa-let-7a-5p;hsa-let-7b-5p;hsa-let-7d-5p;hsa-let-7f-5p;hsa-le
ENSG0000	ENSG0000	0.001048	hsa-miR-103a-3p;hsa-miR-107;hsa-miR-1304-3p;hsa-miR-15a-
ENSG0000	ENSG0000	0.00978	hsa-miR-126-5p;hsa-miR-32-5p;hsa-miR-518c-5p;hsa-miR-92a
ENSG0000	ENSG0000	0.000196	hsa-miR-17-5p;hsa-miR-20b-5p;hsa-miR-519a-3p;hsa-miR-51[illegible]
ENSG0000	ENSG0000	0.006801	hsa-miR-140-3p;hsa-miR-204-5p;hsa-miR-24-3p;hsa-miR-342-
ENSG0000	ENSG0000	4.39E-06	hsa-miR-122-5p;hsa-miR-129-5p;hsa-miR-15a-5p;hsa-miR-15[illegible]
ENSG0000	ENSG0000	2.88E-07	hsa-miR-17-5p;hsa-miR-20b-5p;hsa-miR-30a-3p;hsa-miR-30d-
ENSG0000	ENSG0000	7.84E-05	hsa-miR-320a;hsa-miR-320b;hsa-miR-320c;hsa-miR-320d;hsa-[illegible]
ENSG0000	ENSG0000	0.00698	hsa-miR-107;hsa-miR-15a-5p;hsa-miR-15b-5p;hsa-miR-195-5[illegible]
ENSG0000	ENSG0000	0.001836	hsa-miR-320a;hsa-miR-320b;hsa-miR-320c;hsa-miR-320d;hsa-[illegible]
ENSG0000	ENSG0000	0.001022	hsa-miR-148a-3p;hsa-miR-301a-3p;hsa-miR-301b-3p;hsa-miR-
ENSG0000	ENSG0000	1.57E-05	hsa-miR-15a-5p;hsa-miR-15b-5p;hsa-miR-16-5p;hsa-miR-195-
ENSG0000	ENSG0000	0.003556	hsa-miR-130a-3p;hsa-miR-130b-3p;hsa-miR-301a-3p;hsa-miR-
ENSG0000	ENSG0000	1.89E-08	hsa-miR-103a-3p;hsa-miR-107;hsa-miR-15a-5p;hsa-miR-15b-[illegible]
ENSG0000	ENSG0000	0.005625	hsa-miR-130a-3p;hsa-miR-130b-3p;hsa-miR-221-5p;hsa-miR-[illegible]
ENSG0000	ENSG0000	0.000168	hsa-miR-15a-5p;hsa-miR-15b-5p;hsa-miR-16-5p;hsa-miR-195-
ENSG0000	ENSG0000	0.000105	hsa-miR-15a-5p;hsa-miR-15b-5p;hsa-miR-195-5p;hsa-miR-31-
ENSG0000	ENSG0000	1.65E-07	hsa-miR-103a-3p;hsa-miR-107;hsa-miR-15a-5p;hsa-miR-15b-[illegible]
ENSG0000	ENSG0000	0.006209	hsa-miR-130a-3p;hsa-miR-130b-3p;hsa-miR-301a-3p;hsa-miR-
ENSG0000	ENSG0000	0.002043	hsa-miR-15a-5p;hsa-miR-15b-5p;hsa-miR-16-5p;hsa-miR-195-
ENSG0000	ENSG0000	0.002043	hsa-miR-15a-5p;hsa-miR-15b-5p;hsa-miR-16-5p;hsa-miR-195-
ENSG0000	ENSG0000	0.0003	hsa-miR-320a;hsa-miR-320b;hsa-miR-320c;hsa-miR-320d;hsa-[illegible]
ENSG0000	ENSG0000	0.006244	hsa-miR-15a-5p;hsa-miR-15b-5p;hsa-miR-16-5p;hsa-miR-195-
ENSG0000	ENSG0000	0.004648	hsa-miR-15a-5p;hsa-miR-15b-5p;hsa-miR-16-5p;hsa-miR-195-
ENSG0000	ENSG0000	0.002043	hsa-miR-15a-5p;hsa-miR-15b-5p;hsa-miR-16-5p;hsa-miR-195-
ENSG0000	ENSG0000	0.002841	hsa-let-7g-3p;hsa-miR-130a-3p;hsa-miR-130b-3p;hsa-miR-30[illegible]
ENSG0000	ENSG0000	0.000114	hsa-miR-103a-3p;hsa-miR-195-5p;hsa-miR-221-5p;hsa-miR-3[illegible]
ENSG0000	ENSG0000	0.000336	hsa-miR-107;hsa-miR-15a-5p;hsa-miR-15b-5p;hsa-miR-195-5[illegible]
ENSG0000	ENSG0000	0.00083	hsa-miR-15a-5p;hsa-miR-15b-5p;hsa-miR-16-5p;hsa-miR-195-
ENSG0000	ENSG0000	0.000416	hsa-miR-130a-3p;hsa-miR-148a-3p;hsa-miR-24-3p;hsa-miR-3[illegible]
ENSG0000	ENSG0000	0.000512	hsa-miR-138-5p;hsa-miR-15b-5p;hsa-miR-16-5p;hsa-miR-195-
ENSG0000	ENSG0000	0.002478	hsa-miR-15a-5p;hsa-miR-15b-5p;hsa-miR-16-5p;hsa-miR-424-
ENSG0000	ENSG0000	0.000609	hsa-miR-103a-3p;hsa-miR-107;hsa-miR-15a-5p;hsa-miR-15b-[illegible]
ENSG0000	ENSG0000	7.46E-06	hsa-miR-103a-3p;hsa-miR-107;hsa-miR-15a-5p;hsa-miR-15b-[illegible]
ENSG0000	ENSG0000	0.007638	hsa-miR-15a-5p;hsa-miR-15b-5p;hsa-miR-195-5p;hsa-miR-50[illegible]
ENSG0000	ENSG0000	0.0008	hsa-miR-107;hsa-miR-15a-5p;hsa-miR-15b-5p;hsa-miR-16-5p;
ENSG0000	ENSG0000	0.000265	hsa-miR-103a-3p;hsa-miR-107;hsa-miR-199a-5p;hsa-miR-199[illegible]
ENSG0000	ENSG0000	0.005446	hsa-miR-320a;hsa-miR-320b;hsa-miR-320c;hsa-miR-320d;hsa-[illegible]
ENSG0000	ENSG0000	0.001768	hsa-miR-1254;hsa-miR-15a-5p;hsa-miR-15b-5p;hsa-miR-16-5[illegible]
ENSG0000	ENSG0000	0.004123	hsa-miR-130a-3p;hsa-miR-130b-3p;hsa-miR-301a-3p;hsa-miR-
ENSG0000	ENSG0000	0.001819	hsa-miR-130a-3p;hsa-miR-130b-3p;hsa-miR-148a-3p;hsa-miR-
ENSG0000	ENSG0000	0.005621	hsa-miR-130a-3p;hsa-miR-130b-3p;hsa-miR-301a-3p;hsa-miR-
ENSG0000	ENSG0000	0.000926	hsa-miR-15a-5p;hsa-miR-15b-5p;hsa-miR-16-5p;hsa-miR-195-

ENSG0000	ENSG0000	0.000213	hsa-miR-1306-5p;hsa-miR-15a-5p;hsa-miR-16-5p;hsa-miR-19
ENSG0000	ENSG0000	0.001037	hsa-miR-15a-5p;hsa-miR-15b-5p;hsa-miR-16-5p;hsa-miR-195-
ENSG0000	ENSG0000	0.002212	hsa-miR-103a-3p;hsa-miR-107;hsa-miR-130a-3p;hsa-miR-130
ENSG0000	ENSG0000	0.003577	hsa-miR-130a-3p;hsa-miR-130b-3p;hsa-miR-301a-3p;hsa-miR-
ENSG0000	ENSG0000	1.30E-06	hsa-miR-103a-3p;hsa-miR-107;hsa-miR-130a-3p;hsa-miR-130
ENSG0000	ENSG0000	2.05E-08	hsa-miR-103a-3p;hsa-miR-107;hsa-miR-15a-5p;hsa-miR-15b-
ENSG0000	ENSG0000	1.37E-06	hsa-miR-1306-5p;hsa-miR-130b-5p;hsa-miR-15a-5p;hsa-miR-
ENSG0000	ENSG0000	4.16E-09	hsa-miR-103a-3p;hsa-miR-107;hsa-miR-15a-5p;hsa-miR-15b-
ENSG0000	ENSG0000	0.002191	hsa-miR-15a-5p;hsa-miR-15b-5p;hsa-miR-195-5p;hsa-miR-42
ENSG0000	ENSG0000	0.001131	hsa-miR-1254;hsa-miR-1271-3p;hsa-miR-550a-3-5p;hsa-miR-
ENSG0000	ENSG0000	0.001893	hsa-miR-103a-3p;hsa-miR-107;hsa-miR-15a-5p;hsa-miR-15b-
ENSG0000	ENSG0000	0.003139	hsa-miR-103a-3p;hsa-miR-107;hsa-miR-15a-5p;hsa-miR-15b-
ENSG0000	ENSG0000	0.001903	hsa-miR-130a-3p;hsa-miR-130b-3p;hsa-miR-24-3p;hsa-miR-3
ENSG0000	ENSG0000	0.000403	hsa-miR-15a-5p;hsa-miR-15b-5p;hsa-miR-16-5p;hsa-miR-195-
ENSG0000	ENSG0000	0.008945	hsa-miR-130a-3p;hsa-miR-130b-3p;hsa-miR-301a-3p;hsa-miR-
ENSG0000	ENSG0000	0.007638	hsa-miR-130a-3p;hsa-miR-130b-3p;hsa-miR-301a-3p;hsa-miR-
ENSG0000	ENSG0000	0.00034	hsa-miR-15a-5p;hsa-miR-15b-5p;hsa-miR-16-5p;hsa-miR-195-
ENSG0000	ENSG0000	0.006708	hsa-miR-15a-5p;hsa-miR-15b-5p;hsa-miR-16-5p;hsa-miR-195-
ENSG0000	ENSG0000	0.001396	hsa-miR-15a-5p;hsa-miR-15b-5p;hsa-miR-16-5p;hsa-miR-195-
ENSG0000	ENSG0000	0.009245	hsa-miR-130a-3p;hsa-miR-130b-3p;hsa-miR-301a-3p;hsa-miR-
ENSG0000	ENSG0000	0.002859	hsa-miR-15a-3p;hsa-miR-15a-5p;hsa-miR-15b-5p;hsa-miR-16-
ENSG0000	ENSG0000	0.000865	hsa-miR-125b-2-3p;hsa-miR-130a-3p;hsa-miR-130b-3p;hsa-m
ENSG0000	ENSG0000	0.001255	hsa-miR-15a-5p;hsa-miR-15b-5p;hsa-miR-195-5p;hsa-miR-42
ENSG0000	ENSG0000	4.64E-08	hsa-let-7g-3p;hsa-miR-103a-3p;hsa-miR-107;hsa-miR-15a-5p;
ENSG0000	ENSG0000	0.000342	hsa-miR-15a-5p;hsa-miR-15b-5p;hsa-miR-195-5p;hsa-miR-22
ENSG0000	ENSG0000	0.009245	hsa-miR-130a-3p;hsa-miR-130b-3p;hsa-miR-301a-3p;hsa-miR-
ENSG0000	ENSG0000	0.000186	hsa-miR-15a-5p;hsa-miR-15b-5p;hsa-miR-16-5p;hsa-miR-195-
ENSG0000	ENSG0000	0.00015	hsa-miR-15a-5p;hsa-miR-15b-5p;hsa-miR-18a-3p;hsa-miR-19
ENSG0000	ENSG0000	0.004752	hsa-miR-130a-3p;hsa-miR-130b-3p;hsa-miR-301a-3p;hsa-miR-
ENSG0000	ENSG0000	0.001819	hsa-miR-107;hsa-miR-138-5p;hsa-miR-199a-5p;hsa-miR-199b-
ENSG0000	ENSG0000	0.001001	hsa-miR-125b-2-3p;hsa-miR-149-5p;hsa-miR-15a-5p;hsa-miR-
ENSG0000	ENSG0000	6.75E-05	hsa-miR-107;hsa-miR-140-3p;hsa-miR-15a-5p;hsa-miR-15b-5
ENSG0000	ENSG0000	0.003167	hsa-miR-15a-5p;hsa-miR-15b-5p;hsa-miR-195-5p;hsa-miR-42
ENSG0000	ENSG0000	0.00313	hsa-miR-15a-5p;hsa-miR-15b-5p;hsa-miR-16-5p;hsa-miR-195-
ENSG0000	ENSG0000	0.006209	hsa-miR-130a-3p;hsa-miR-130b-3p;hsa-miR-301a-3p;hsa-miR-
ENSG0000	ENSG0000	4.64E-05	hsa-miR-103a-3p;hsa-miR-107;hsa-miR-15a-5p;hsa-miR-15b-
ENSG0000	ENSG0000	0.000808	hsa-miR-130a-3p;hsa-miR-130b-3p;hsa-miR-15a-5p;hsa-miR-
ENSG0000	ENSG0000	0.000728	hsa-miR-1306-5p;hsa-miR-3127-3p;hsa-miR-378a-3p;hsa-miR-
ENSG0000	ENSG0000	0.003577	hsa-miR-320a;hsa-miR-320b;hsa-miR-320c;hsa-miR-320d;hsa-
ENSG0000	ENSG0000	0.000227	hsa-miR-130a-3p;hsa-miR-130b-3p;hsa-miR-16-5p;hsa-miR-2
ENSG0000	ENSG0000	0.003048	hsa-miR-130a-3p;hsa-miR-130b-3p;hsa-miR-301a-3p;hsa-miR-
ENSG0000	ENSG0000	4.76E-08	hsa-miR-130a-3p;hsa-miR-148a-3p;hsa-miR-15a-5p;hsa-miR-
ENSG0000	ENSG0000	0.003395	hsa-miR-1254;hsa-miR-130b-5p;hsa-miR-378a-3p;hsa-miR-37
ENSG0000	ENSG0000	0.001947	hsa-miR-338-3p;hsa-miR-4422;hsa-miR-532-3p;hsa-miR-766-
ENSG0000	ENSG0000	0.001947	hsa-miR-338-3p;hsa-miR-4422;hsa-miR-532-3p;hsa-miR-766-
ENSG0000	ENSG0000	0.001947	hsa-miR-338-3p;hsa-miR-4422;hsa-miR-532-3p;hsa-miR-766-
ENSG0000	ENSG0000	0.001947	hsa-miR-338-3p;hsa-miR-4422;hsa-miR-532-3p;hsa-miR-766-
ENSG0000	ENSG0000	0.000416	hsa-miR-15a-5p;hsa-miR-15b-5p;hsa-miR-16-5p;hsa-miR-195-
ENSG0000	ENSG0000	2.38E-06	hsa-miR-15a-5p;hsa-miR-15b-5p;hsa-miR-16-5p;hsa-miR-195-
ENSG0000	ENSG0000	2.38E-06	hsa-miR-15a-5p;hsa-miR-15b-5p;hsa-miR-16-5p;hsa-miR-195-
ENSG0000	ENSG0000	0.003154	hsa-miR-15a-5p;hsa-miR-15b-5p;hsa-miR-16-5p;hsa-miR-195-
ENSG0000	ENSG0000	9.18E-05	hsa-miR-140-3p;hsa-miR-15a-5p;hsa-miR-15b-5p;hsa-miR-19
ENSG0000	ENSG0000	0.005199	hsa-miR-103a-3p;hsa-miR-107;hsa-miR-15a-5p;hsa-miR-15b-
ENSG0000	ENSG0000	0.000103	hsa-miR-103a-3p;hsa-miR-107;hsa-miR-15a-5p;hsa-miR-15b-

ENSG0000	ENSG0000	0.001197	hsa-miR-15a-5p;hsa-miR-195-5p;hsa-miR-374b-3p;hsa-miR-4
ENSG0000	ENSG0000	0.000342	hsa-miR-140-3p;hsa-miR-320a;hsa-miR-320b;hsa-miR-320c;hsa
ENSG0000	ENSG0000	0.007304	hsa-miR-1306-5p;hsa-miR-130a-3p;hsa-miR-130b-3p;hsa-miR
ENSG0000	ENSG0000	0.000799	hsa-miR-125b-2-3p;hsa-miR-130a-3p;hsa-miR-130b-3p;hsa-m
ENSG0000	ENSG0000	0.001525	hsa-miR-130a-3p;hsa-miR-130b-3p;hsa-miR-301a-3p;hsa-miR
ENSG0000	ENSG0000	0.000162	hsa-miR-199a-5p;hsa-miR-199b-5p;hsa-miR-345-5p;hsa-miR-
ENSG0000	ENSG0000	0.000162	hsa-miR-199a-5p;hsa-miR-199b-5p;hsa-miR-345-5p;hsa-miR-
ENSG0000	ENSG0000	2.47E-05	hsa-miR-149-5p;hsa-miR-15a-5p;hsa-miR-15b-5p;hsa-miR-19
ENSG0000	ENSG0000	0.000405	hsa-miR-107;hsa-miR-15a-5p;hsa-miR-16-5p;hsa-miR-424-5p
ENSG0000	ENSG0000	0.005045	hsa-miR-130a-3p;hsa-miR-130b-3p;hsa-miR-301a-3p;hsa-miR
ENSG0000	ENSG0000	0.005045	hsa-miR-130a-3p;hsa-miR-130b-3p;hsa-miR-301a-3p;hsa-miR
ENSG0000	ENSG0000	0.00569	hsa-miR-130a-3p;hsa-miR-130b-3p;hsa-miR-185-3p;hsa-miR-
ENSG0000	ENSG0000	0.000113	hsa-miR-15a-5p;hsa-miR-15b-5p;hsa-miR-16-5p;hsa-miR-195
ENSG0000	ENSG0000	0.000609	hsa-miR-130a-3p;hsa-miR-130b-3p;hsa-miR-301a-3p;hsa-miR
ENSG0000	ENSG0000	0.006244	hsa-miR-130a-3p;hsa-miR-130b-3p;hsa-miR-301a-3p;hsa-miR
ENSG0000	ENSG0000	0.003395	hsa-miR-103a-3p;hsa-miR-107;hsa-miR-15a-5p;hsa-miR-15b-
ENSG0000	ENSG0000	0.001021	hsa-miR-130a-3p;hsa-miR-130b-3p;hsa-miR-301a-3p;hsa-miR
ENSG0000	ENSG0000	0.000419	hsa-miR-107;hsa-miR-140-3p;hsa-miR-148a-3p;hsa-miR-15a-
ENSG0000	ENSG0000	0.000512	hsa-miR-15a-5p;hsa-miR-15b-5p;hsa-miR-195-5p;hsa-miR-42
ENSG0000	ENSG0000	2.38E-06	hsa-miR-15a-5p;hsa-miR-15b-5p;hsa-miR-16-5p;hsa-miR-195
ENSG0000	ENSG0000	2.38E-06	hsa-miR-15a-5p;hsa-miR-15b-5p;hsa-miR-16-5p;hsa-miR-195
ENSG0000	ENSG0000	0.000159	hsa-miR-15a-5p;hsa-miR-15b-5p;hsa-miR-195-5p;hsa-miR-19
ENSG0000	ENSG0000	1.49E-06	hsa-miR-15a-5p;hsa-miR-15b-5p;hsa-miR-16-5p;hsa-miR-195
ENSG0000	ENSG0000	0.000865	hsa-miR-130a-3p;hsa-miR-130b-3p;hsa-miR-301a-3p;hsa-miR
ENSG0000	ENSG0000	0.001438	hsa-miR-320a;hsa-miR-320b;hsa-miR-320c;hsa-miR-320d;hsa-
ENSG0000	ENSG0000	0.001021	hsa-miR-107;hsa-miR-15a-5p;hsa-miR-15b-5p;hsa-miR-195-5
ENSG0000	ENSG0000	0.002453	hsa-miR-130a-3p;hsa-miR-130b-3p;hsa-miR-2355-5p;hsa-miR
ENSG0000	ENSG0000	0.005136	hsa-miR-107;hsa-miR-15a-5p;hsa-miR-15b-5p;hsa-miR-195-5
ENSG0000	ENSG0000	0.004796	hsa-miR-1254;hsa-miR-130a-3p;hsa-miR-130b-3p;hsa-miR-30
ENSG0000	ENSG0000	0.004796	hsa-miR-138-5p;hsa-miR-15a-5p;hsa-miR-15b-5p;hsa-miR-16
ENSG0000	ENSG0000	7.23E-06	hsa-miR-15a-5p;hsa-miR-15b-5p;hsa-miR-16-5p;hsa-miR-195
ENSG0000	ENSG0000	0.003556	hsa-miR-107;hsa-miR-15a-5p;hsa-miR-15b-5p;hsa-miR-195-5
ENSG0000	ENSG0000	4.16E-06	hsa-miR-130a-3p;hsa-miR-15a-5p;hsa-miR-15b-5p;hsa-miR-1
ENSG0000	ENSG0000	0.000738	hsa-miR-15a-5p;hsa-miR-15b-5p;hsa-miR-16-5p;hsa-miR-195
ENSG0000	ENSG0000	2.36E-05	hsa-miR-130a-3p;hsa-miR-130b-3p;hsa-miR-130b-5p;hsa-miR
ENSG0000	ENSG0000	0.004752	hsa-miR-125b-2-3p;hsa-miR-149-5p;hsa-miR-221-5p;hsa-miR
ENSG0000	ENSG0000	0.003943	hsa-miR-130a-3p;hsa-miR-130b-3p;hsa-miR-199a-5p;hsa-miR
ENSG0000	ENSG0000	0.004255	hsa-miR-130a-3p;hsa-miR-18a-3p;hsa-miR-1913;hsa-miR-301
ENSG0000	ENSG0000	6.22E-06	hsa-miR-15a-5p;hsa-miR-15b-5p;hsa-miR-16-5p;hsa-miR-195
ENSG0000	ENSG0000	6.22E-06	hsa-miR-15a-5p;hsa-miR-15b-5p;hsa-miR-16-5p;hsa-miR-195
ENSG0000	ENSG0000	0.000135	hsa-miR-15a-5p;hsa-miR-15b-5p;hsa-miR-16-5p;hsa-miR-195
ENSG0000	ENSG0000	0.000823	hsa-miR-130a-3p;hsa-miR-130b-3p;hsa-miR-301a-3p;hsa-miR
ENSG0000	ENSG0000	0.000823	hsa-miR-130a-3p;hsa-miR-130b-3p;hsa-miR-301a-3p;hsa-miR
ENSG0000	ENSG0000	7.84E-05	hsa-miR-15a-5p;hsa-miR-15b-5p;hsa-miR-16-5p;hsa-miR-195
ENSG0000	ENSG0000	7.84E-05	hsa-miR-15a-5p;hsa-miR-15b-5p;hsa-miR-16-5p;hsa-miR-195
ENSG0000	ENSG0000	4.79E-05	hsa-miR-15a-5p;hsa-miR-15b-5p;hsa-miR-16-5p;hsa-miR-195
ENSG0000	ENSG0000	0.000654	hsa-miR-320a;hsa-miR-320b;hsa-miR-320c;hsa-miR-320d;hsa-
ENSG0000	ENSG0000	0.000654	hsa-miR-320a;hsa-miR-320b;hsa-miR-320c;hsa-miR-320d;hsa-
ENSG0000	ENSG0000	2.47E-05	hsa-miR-103a-3p;hsa-miR-107;hsa-miR-15a-5p;hsa-miR-15b-
ENSG0000	ENSG0000	8.56E-05	hsa-miR-107;hsa-miR-15a-5p;hsa-miR-15b-5p;hsa-miR-195-5
ENSG0000	ENSG0000	0.006209	hsa-miR-221-5p;hsa-miR-31-5p;hsa-miR-548d-3p;hsa-miR-76
ENSG0000	ENSG0000	1.98E-05	hsa-miR-103a-3p;hsa-miR-107;hsa-miR-15a-5p;hsa-miR-15b-
ENSG0000	ENSG0000	2.88E-07	hsa-miR-15a-5p;hsa-miR-15b-5p;hsa-miR-16-5p;hsa-miR-195
ENSG0000	ENSG0000	8.21E-07	hsa-miR-130a-3p;hsa-miR-130b-3p;hsa-miR-199a-5p;hsa-miR

ENSG0000	ENSG0000	0.004514	hsa-miR-130a-3p;hsa-miR-130b-3p;hsa-miR-140-3p;hsa-miR-
ENSG0000	ENSG0000	0.004514	hsa-miR-130a-3p;hsa-miR-130b-3p;hsa-miR-140-3p;hsa-miR-
ENSG0000	ENSG0000	0.000638	hsa-miR-15a-5p;hsa-miR-15b-5p;hsa-miR-16-5p;hsa-miR-195-
ENSG0000	ENSG0000	4.40E-05	hsa-miR-15a-5p;hsa-miR-15b-5p;hsa-miR-16-5p;hsa-miR-195-
ENSG0000	ENSG0000	0.000731	hsa-miR-130a-3p;hsa-miR-130b-3p;hsa-miR-301a-3p;hsa-miR-
ENSG0000	ENSG0000	0.008945	hsa-miR-15a-5p;hsa-miR-15b-5p;hsa-miR-195-5p;hsa-miR-42
ENSG0000	ENSG0000	0.000331	hsa-miR-103a-3p;hsa-miR-107;hsa-miR-15a-5p;hsa-miR-15b-
ENSG0000	ENSG0000	0.002146	hsa-miR-107;hsa-miR-15a-5p;hsa-miR-15b-5p;hsa-miR-195-5
ENSG0000	ENSG0000	4.37E-06	hsa-miR-320a;hsa-miR-320b;hsa-miR-320c;hsa-miR-320d;hsa-
ENSG0000	ENSG0000	0.000647	hsa-miR-15a-5p;hsa-miR-15b-5p;hsa-miR-195-5p;hsa-miR-29
ENSG0000	ENSG0000	0.000609	hsa-miR-148a-3p;hsa-miR-199a-5p;hsa-miR-378a-3p;hsa-miR-
ENSG0000	ENSG0000	0.0002	hsa-miR-103a-3p;hsa-miR-107;hsa-miR-130b-5p;hsa-miR-15a-
ENSG0000	ENSG0000	7.90E-05	hsa-miR-130a-3p;hsa-miR-130b-3p;hsa-miR-140-3p;hsa-miR-
ENSG0000	ENSG0000	4.40E-05	hsa-miR-15a-5p;hsa-miR-15b-5p;hsa-miR-16-5p;hsa-miR-195-
ENSG0000	ENSG0000	7.84E-05	hsa-miR-320a;hsa-miR-320b;hsa-miR-320c;hsa-miR-320d;hsa-
ENSG0000	ENSG0000	0.001868	hsa-miR-1226-5p;hsa-miR-320a;hsa-miR-320b;hsa-miR-320c;h
ENSG0000	ENSG0000	0.000505	hsa-miR-15a-5p;hsa-miR-15b-5p;hsa-miR-16-5p;hsa-miR-195-
ENSG0000	ENSG0000	0.002594	hsa-miR-130a-3p;hsa-miR-301a-3p;hsa-miR-301b-3p;hsa-miR-
ENSG0000	ENSG0000	0.004752	hsa-miR-130a-3p;hsa-miR-130b-3p;hsa-miR-301a-3p;hsa-miR-
ENSG0000	ENSG0000	0.002793	hsa-miR-130a-3p;hsa-miR-130b-3p;hsa-miR-301a-3p;hsa-miR-
ENSG0000	ENSG0000	0.006564	hsa-miR-15a-5p;hsa-miR-15b-5p;hsa-miR-16-5p;hsa-miR-195-
ENSG0000	ENSG0000	0.002552	hsa-miR-149-5p;hsa-miR-15a-5p;hsa-miR-16-5p;hsa-miR-221-
ENSG0000	ENSG0000	0.000503	hsa-miR-130a-3p;hsa-miR-130b-3p;hsa-miR-130b-5p;hsa-miR
ENSG0000	ENSG0000	4.64E-05	hsa-miR-15a-5p;hsa-miR-15b-5p;hsa-miR-16-5p;hsa-miR-195-
ENSG0000	ENSG0000	0.005826	hsa-miR-130a-3p;hsa-miR-130b-3p;hsa-miR-301a-3p;hsa-miR-
ENSG0000	ENSG0000	1.41E-06	hsa-miR-1306-5p;hsa-miR-15a-5p;hsa-miR-15b-5p;hsa-miR-1
ENSG0000	ENSG0000	0.001619	hsa-miR-320a;hsa-miR-320b;hsa-miR-320c;hsa-miR-320d;hsa-
ENSG0000	ENSG0000	0.00034	hsa-miR-130a-3p;hsa-miR-130b-3p;hsa-miR-301a-3p;hsa-miR-
ENSG0000	ENSG0000	0.004423	hsa-miR-1306-5p;hsa-miR-15a-5p;hsa-miR-16-5p;hsa-miR-19
ENSG0000	ENSG0000	0.004025	hsa-miR-130a-3p;hsa-miR-130b-3p;hsa-miR-148a-3p;hsa-miR-
ENSG0000	ENSG0000	0.008414	hsa-miR-1295a;hsa-miR-140-3p;hsa-miR-15a-5p;hsa-miR-15b-
ENSG0000	ENSG0000	1.49E-06	hsa-miR-15a-5p;hsa-miR-15b-5p;hsa-miR-16-5p;hsa-miR-195-
ENSG0000	ENSG0000	3.94E-07	hsa-miR-107;hsa-miR-15a-5p;hsa-miR-15b-5p;hsa-miR-16-5p;
ENSG0000	ENSG0000	0.003048	hsa-miR-15a-3p;hsa-miR-185-3p;hsa-miR-31-5p;hsa-miR-318
ENSG0000	ENSG0000	0.003556	hsa-miR-15a-5p;hsa-miR-15b-5p;hsa-miR-195-5p;hsa-miR-42
ENSG0000	ENSG0000	6.66E-07	hsa-miR-130a-3p;hsa-miR-130b-3p;hsa-miR-15a-5p;hsa-miR-
ENSG0000	ENSG0000	3.28E-05	hsa-miR-15a-5p;hsa-miR-15b-5p;hsa-miR-16-5p;hsa-miR-195-
ENSG0000	ENSG0000	0.000416	hsa-miR-103a-3p;hsa-miR-107;hsa-miR-4487;hsa-miR-493-5p;
ENSG0000	ENSG0000	0.007955	hsa-miR-130a-3p;hsa-miR-130b-3p;hsa-miR-301a-3p;hsa-miR-
ENSG0000	ENSG0000	7.84E-05	hsa-miR-15a-5p;hsa-miR-15b-5p;hsa-miR-195-5p;hsa-miR-42
ENSG0000	ENSG0000	1.60E-08	hsa-miR-15a-5p;hsa-miR-15b-5p;hsa-miR-16-5p;hsa-miR-195-
ENSG0000	ENSG0000	0.001438	hsa-miR-15a-5p;hsa-miR-15b-5p;hsa-miR-16-5p;hsa-miR-195-
ENSG0000	ENSG0000	0.001197	hsa-miR-140-3p;hsa-miR-2278;hsa-miR-500a-3p;hsa-miR-501-
ENSG0000	ENSG0000	3.28E-05	hsa-miR-1254;hsa-miR-1271-3p;hsa-miR-149-5p;hsa-miR-550
ENSG0000	ENSG0000	3.28E-05	hsa-miR-1254;hsa-miR-1271-3p;hsa-miR-149-5p;hsa-miR-550
ENSG0000	ENSG0000	0.002594	hsa-miR-130a-3p;hsa-miR-130b-3p;hsa-miR-301a-3p;hsa-miR-
ENSG0000	ENSG0000	0.000163	hsa-miR-15a-5p;hsa-miR-15b-5p;hsa-miR-16-5p;hsa-miR-195-
ENSG0000	ENSG0000	0.001255	hsa-miR-18a-3p;hsa-miR-1913;hsa-miR-3187-3p;hsa-miR-324-
ENSG0000	ENSG0000	3.63E-05	hsa-miR-1271-3p;hsa-miR-148a-3p;hsa-miR-3127-3p;hsa-miR-
ENSG0000	ENSG0000	0.000336	hsa-miR-15a-5p;hsa-miR-15b-5p;hsa-miR-18a-3p;hsa-miR-19
ENSG0000	ENSG0000	2.01E-06	hsa-miR-320a;hsa-miR-320b;hsa-miR-320c;hsa-miR-320d;hsa-
ENSG0000	ENSG0000	1.49E-06	hsa-miR-15a-5p;hsa-miR-15b-5p;hsa-miR-16-5p;hsa-miR-195-
ENSG0000	ENSG0000	0.000395	hsa-miR-1271-3p;hsa-miR-361-3p;hsa-miR-550a-3-5p;hsa-mi
ENSG0000	ENSG0000	0.008788	hsa-miR-130a-3p;hsa-miR-130b-3p;hsa-miR-24-3p;hsa-miR-3

ENSG0000	ENSG0000	0.009424	hsa-miR-15a-5p;hsa-miR-15b-5p;hsa-miR-195-5p;hsa-miR-31
ENSG0000	ENSG0000	0.002978	hsa-miR-103a-3p;hsa-miR-107;hsa-miR-15a-5p;hsa-miR-15b-
ENSG0000	ENSG0000	0.007638	hsa-let-7g-3p;hsa-miR-130a-3p;hsa-miR-301a-3p;hsa-miR-30
ENSG0000	ENSG0000	0.006647	hsa-miR-15a-5p;hsa-miR-15b-5p;hsa-miR-16-5p;hsa-miR-195
ENSG0000	ENSG0000	5.68E-06	hsa-miR-130a-3p;hsa-miR-130b-3p;hsa-miR-301a-3p;hsa-miR
ENSG0000	ENSG0000	0.008414	hsa-miR-130a-3p;hsa-miR-130b-3p;hsa-miR-301a-3p;hsa-miR
ENSG0000	ENSG0000	0.008945	hsa-miR-15a-5p;hsa-miR-15b-5p;hsa-miR-195-5p;hsa-miR-42
ENSG0000	ENSG0000	2.38E-06	hsa-miR-15a-5p;hsa-miR-15b-5p;hsa-miR-16-5p;hsa-miR-195
ENSG0000	ENSG0000	0.006708	hsa-miR-130a-3p;hsa-miR-130b-3p;hsa-miR-185-3p;hsa-miR-
ENSG0000	ENSG0000	0.006708	hsa-miR-130a-3p;hsa-miR-130b-3p;hsa-miR-185-3p;hsa-miR-
ENSG0000	ENSG0000	1.11E-05	hsa-miR-320a;hsa-miR-320b;hsa-miR-320c;hsa-miR-320d;hsa-
ENSG0000	ENSG0000	0.008414	hsa-miR-1226-5p;hsa-miR-320a;hsa-miR-320b;hsa-miR-320c;h
ENSG0000	ENSG0000	4.61E-05	hsa-miR-103a-3p;hsa-miR-107;hsa-miR-15a-5p;hsa-miR-15b-
ENSG0000	ENSG0000	0.005627	hsa-miR-130b-5p;hsa-miR-3187-3p;hsa-miR-378a-3p;hsa-miR
ENSG0000	ENSG0000	7.11E-11	hsa-miR-103a-3p;hsa-miR-107;hsa-miR-130a-3p;hsa-miR-130
ENSG0000	ENSG0000	9.32E-05	hsa-miR-15a-5p;hsa-miR-15b-5p;hsa-miR-16-5p;hsa-miR-195
ENSG0000	ENSG0000	0.002552	hsa-miR-103a-3p;hsa-miR-107;hsa-miR-15a-5p;hsa-miR-15b-
ENSG0000	ENSG0000	0.000405	hsa-miR-130b-5p;hsa-miR-3187-3p;hsa-miR-588;hsa-miR-766
ENSG0000	ENSG0000	0.005446	hsa-miR-378a-3p;hsa-miR-378c;hsa-miR-378d;hsa-miR-378i;hs
ENSG0000	ENSG0000	0.009146	hsa-miR-378a-3p;hsa-miR-378c;hsa-miR-378d;hsa-miR-378i
ENSG0000	ENSG0000	0.000824	hsa-miR-107;hsa-miR-15a-5p;hsa-miR-15b-5p;hsa-miR-195-5
ENSG0000	ENSG0000	1.19E-05	hsa-miR-15a-5p;hsa-miR-15b-5p;hsa-miR-16-5p;hsa-miR-195
ENSG0000	ENSG0000	1.58E-07	hsa-miR-15a-5p;hsa-miR-15b-5p;hsa-miR-16-5p;hsa-miR-195
ENSG0000	ENSG0000	5.70E-06	hsa-miR-107;hsa-miR-130b-5p;hsa-miR-15a-5p;hsa-miR-15b-
ENSG0000	ENSG0000	0.000403	hsa-miR-1226-5p;hsa-miR-15a-5p;hsa-miR-15b-5p;hsa-miR-1
ENSG0000	ENSG0000	0.001375	hsa-let-7g-3p;hsa-miR-103a-3p;hsa-miR-107;hsa-miR-15a-5p;
ENSG0000	ENSG0000	0.003154	hsa-miR-15a-5p;hsa-miR-15b-5p;hsa-miR-16-5p;hsa-miR-195
ENSG0000	ENSG0000	0.007044	hsa-miR-130a-3p;hsa-miR-130b-3p;hsa-miR-301a-3p;hsa-miR
ENSG0000	ENSG0000	0.007044	hsa-miR-130a-3p;hsa-miR-130b-3p;hsa-miR-301a-3p;hsa-miR
ENSG0000	ENSG0000	0.000416	hsa-miR-15a-5p;hsa-miR-15b-5p;hsa-miR-195-5p;hsa-miR-37
ENSG0000	ENSG0000	0.007939	hsa-miR-15a-5p;hsa-miR-15b-5p;hsa-miR-16-5p;hsa-miR-195
ENSG0000	ENSG0000	0.000289	hsa-miR-140-3p;hsa-miR-15a-5p;hsa-miR-15b-5p;hsa-miR-16
ENSG0000	ENSG0000	0.003556	hsa-miR-130a-3p;hsa-miR-130b-3p;hsa-miR-301a-3p;hsa-miR
ENSG0000	ENSG0000	0.000265	hsa-miR-15a-5p;hsa-miR-15b-5p;hsa-miR-16-5p;hsa-miR-195
ENSG0000	ENSG0000	1.32E-06	hsa-miR-15a-5p;hsa-miR-15b-5p;hsa-miR-16-5p;hsa-miR-195
ENSG0000	ENSG0000	1.98E-05	hsa-miR-1271-3p;hsa-miR-15a-5p;hsa-miR-15b-5p;hsa-miR-1
ENSG0000	ENSG0000	0.00111	hsa-miR-15a-5p;hsa-miR-15b-5p;hsa-miR-16-5p;hsa-miR-195
ENSG0000	ENSG0000	0.00111	hsa-miR-15a-5p;hsa-miR-15b-5p;hsa-miR-16-5p;hsa-miR-195
ENSG0000	ENSG0000	0.004025	hsa-miR-130a-3p;hsa-miR-130b-3p;hsa-miR-301a-3p;hsa-miR
ENSG0000	ENSG0000	0.006915	hsa-miR-342-3p;hsa-miR-378a-3p;hsa-miR-378c;hsa-miR-378
ENSG0000	ENSG0000	0.00143	hsa-miR-1271-3p;hsa-miR-138-5p;hsa-miR-15a-5p;hsa-miR-1
ENSG0000	ENSG0000	0.007955	hsa-miR-15a-5p;hsa-miR-15b-5p;hsa-miR-424-5p;hsa-miR-49
ENSG0000	ENSG0000	0.00698	hsa-miR-103a-3p;hsa-miR-107;hsa-miR-130a-3p;hsa-miR-130
ENSG0000	ENSG0000	0.000162	hsa-miR-130a-3p;hsa-miR-130b-3p;hsa-miR-301a-3p;hsa-miR
ENSG0000	ENSG0000	7.34E-07	hsa-miR-15a-5p;hsa-miR-15b-5p;hsa-miR-16-5p;hsa-miR-195
ENSG0000	ENSG0000	4.05E-06	hsa-miR-15a-5p;hsa-miR-15b-5p;hsa-miR-16-5p;hsa-miR-195
ENSG0000	ENSG0000	0.001868	hsa-miR-15a-5p;hsa-miR-15b-5p;hsa-miR-16-5p;hsa-miR-195
ENSG0000	ENSG0000	0.001287	hsa-miR-1226-5p;hsa-miR-125b-2-3p;hsa-miR-130b-5p;hsa-m
ENSG0000	ENSG0000	0.004752	hsa-miR-15b-5p;hsa-miR-16-5p;hsa-miR-195-5p;hsa-miR-424
ENSG0000	ENSG0000	0.001903	hsa-miR-130a-3p;hsa-miR-130b-3p;hsa-miR-28-5p;hsa-miR-3
ENSG0000	ENSG0000	0.005588	hsa-miR-15a-5p;hsa-miR-15b-5p;hsa-miR-16-5p;hsa-miR-195
ENSG0000	ENSG0000	0.005588	hsa-miR-15a-5p;hsa-miR-15b-5p;hsa-miR-16-5p;hsa-miR-195
ENSG0000	ENSG0000	0.001131	hsa-miR-15a-5p;hsa-miR-15b-5p;hsa-miR-16-5p;hsa-miR-195
ENSG0000	ENSG0000	0.003048	hsa-miR-15a-5p;hsa-miR-15b-5p;hsa-miR-195-5p;hsa-miR-42

ENSG0000	ENSG0000	2.69E-05	hsa-miR-15a-5p;hsa-miR-15b-5p;hsa-miR-16-5p;hsa-miR-195-
ENSG0000	ENSG0000	0.008486	hsa-miR-130a-3p;hsa-miR-130b-3p;hsa-miR-148a-3p;hsa-miR-
ENSG0000	ENSG0000	0.008486	hsa-miR-130a-3p;hsa-miR-130b-3p;hsa-miR-148a-3p;hsa-miR-
ENSG0000	ENSG0000	4.16E-06	hsa-miR-15a-5p;hsa-miR-15b-5p;hsa-miR-16-5p;hsa-miR-195-
ENSG0000	ENSG0000	0.006708	hsa-miR-130a-3p;hsa-miR-138-5p;hsa-miR-301a-3p;hsa-miR-3
ENSG0000	ENSG0000	0.002841	hsa-miR-15a-5p;hsa-miR-15b-5p;hsa-miR-16-5p;hsa-miR-195-
ENSG0000	ENSG0000	1.98E-05	hsa-miR-130b-5p;hsa-miR-15a-5p;hsa-miR-15b-5p;hsa-miR-1
ENSG0000	ENSG0000	1.98E-05	hsa-miR-130b-5p;hsa-miR-15a-5p;hsa-miR-15b-5p;hsa-miR-1
ENSG0000	ENSG0000	5.18E-05	hsa-miR-320a;hsa-miR-320b;hsa-miR-320c;hsa-miR-320d;hsa-r
ENSG0000	ENSG0000	0.003167	hsa-miR-130a-3p;hsa-miR-140-3p;hsa-miR-301a-3p;hsa-miR-3
ENSG0000	ENSG0000	0.003577	hsa-miR-15a-5p;hsa-miR-15b-5p;hsa-miR-16-5p;hsa-miR-195-
ENSG0000	ENSG0000	0.004593	hsa-miR-15a-5p;hsa-miR-15b-5p;hsa-miR-195-5p;hsa-miR-42
ENSG0000	ENSG0000	5.98E-05	hsa-miR-15a-5p;hsa-miR-15b-5p;hsa-miR-195-5p;hsa-miR-37
ENSG0000	ENSG0000	5.88E-05	hsa-miR-15a-5p;hsa-miR-15b-5p;hsa-miR-16-5p;hsa-miR-195-
ENSG0000	ENSG0000	0.003668	hsa-miR-103a-3p;hsa-miR-107;hsa-miR-15a-5p;hsa-miR-15b-5
ENSG0000	ENSG0000	7.46E-06	hsa-miR-15a-5p;hsa-miR-15b-5p;hsa-miR-16-5p;hsa-miR-195-
ENSG0000	ENSG0000	2.69E-05	hsa-miR-103a-3p;hsa-miR-107;hsa-miR-15a-5p;hsa-miR-15b-5
ENSG0000	ENSG0000	2.69E-05	hsa-miR-103a-3p;hsa-miR-107;hsa-miR-15a-5p;hsa-miR-15b-5
ENSG0000	ENSG0000	8.14E-08	hsa-miR-15a-5p;hsa-miR-16-5p;hsa-miR-320a;hsa-miR-320b;h
ENSG0000	ENSG0000	0.001037	hsa-miR-107;hsa-miR-15a-5p;hsa-miR-15b-5p;hsa-miR-195-5p
ENSG0000	ENSG0000	3.18E-07	hsa-miR-320a;hsa-miR-320b;hsa-miR-320c;hsa-miR-320d;hsa-r
ENSG0000	ENSG0000	0.000163	hsa-miR-15a-5p;hsa-miR-15b-5p;hsa-miR-16-5p;hsa-miR-195-
ENSG0000	ENSG0000	1.25E-05	hsa-miR-103a-3p;hsa-miR-107;hsa-miR-15a-5p;hsa-miR-15b-5
ENSG0000	ENSG0000	0.001197	hsa-miR-107;hsa-miR-15a-5p;hsa-miR-15b-5p;hsa-miR-2278;h
ENSG0000	ENSG0000	8.14E-08	hsa-miR-15a-5p;hsa-miR-15b-5p;hsa-miR-16-5p;hsa-miR-195-
ENSG0000	ENSG0000	7.84E-05	hsa-miR-320a;hsa-miR-320b;hsa-miR-320c;hsa-miR-320d;hsa-r
ENSG0000	ENSG0000	0.000152	hsa-miR-103a-3p;hsa-miR-107;hsa-miR-15a-5p;hsa-miR-15b-5
ENSG0000	ENSG0000	3.94E-07	hsa-miR-103a-3p;hsa-miR-107;hsa-miR-15a-5p;hsa-miR-15b-5
ENSG0000	ENSG0000	0.003577	hsa-miR-125b-2-3p;hsa-miR-130a-3p;hsa-miR-24-3p;hsa-miR-
ENSG0000	ENSG0000	0.002793	hsa-miR-15a-5p;hsa-miR-15b-5p;hsa-miR-16-5p;hsa-miR-195-
ENSG0000	ENSG0000	1.89E-08	hsa-miR-15a-5p;hsa-miR-15b-5p;hsa-miR-16-5p;hsa-miR-195-
ENSG0000	ENSG0000	0.000823	hsa-miR-15a-5p;hsa-miR-15b-5p;hsa-miR-16-5p;hsa-miR-195-
ENSG0000	ENSG0000	0.006209	hsa-let-7g-3p;hsa-miR-15a-5p;hsa-miR-15b-5p;hsa-miR-195-5
ENSG0000	ENSG0000	5.98E-05	hsa-miR-130a-3p;hsa-miR-130b-3p;hsa-miR-148a-3p;hsa-miR-
ENSG0000	ENSG0000	0.001131	hsa-miR-15a-5p;hsa-miR-15b-5p;hsa-miR-195-5p;hsa-miR-34
ENSG0000	ENSG0000	0.001868	hsa-let-7g-3p;hsa-miR-15a-5p;hsa-miR-15b-5p;hsa-miR-195-5
ENSG0000	ENSG0000	0.002146	hsa-miR-320a;hsa-miR-320b;hsa-miR-320c;hsa-miR-320d;hsa-r
ENSG0000	ENSG0000	0.000168	hsa-miR-15a-5p;hsa-miR-15b-5p;hsa-miR-16-5p;hsa-miR-195-
ENSG0000	ENSG0000	0.001616	hsa-miR-130a-3p;hsa-miR-148a-3p;hsa-miR-15a-5p;hsa-miR-1
ENSG0000	ENSG0000	0.000654	hsa-miR-130a-3p;hsa-miR-130b-3p;hsa-miR-301a-3p;hsa-miR-
ENSG0000	ENSG0000	0.000723	hsa-miR-1269b;hsa-miR-140-3p;hsa-miR-15a-5p;hsa-miR-15b-
ENSG0000	ENSG0000	7.99E-05	hsa-miR-125b-2-3p;hsa-miR-138-5p;hsa-miR-15a-5p;hsa-miR-
ENSG0000	ENSG0000	0.001293	hsa-miR-103a-3p;hsa-miR-107;hsa-miR-15a-5p;hsa-miR-15b-5
ENSG0000	ENSG0000	0.002146	hsa-miR-130a-3p;hsa-miR-130b-3p;hsa-miR-130b-5p;hsa-miR
ENSG0000	ENSG0000	6.22E-06	hsa-miR-15a-5p;hsa-miR-15b-5p;hsa-miR-16-5p;hsa-miR-195-
ENSG0000	ENSG0000	0.001197	hsa-miR-15a-5p;hsa-miR-15b-5p;hsa-miR-195-5p;hsa-miR-42
ENSG0000	ENSG0000	0.000189	hsa-miR-15a-5p;hsa-miR-15b-5p;hsa-miR-16-5p;hsa-miR-195-
ENSG0000	ENSG0000	0.002594	hsa-miR-107;hsa-miR-130a-3p;hsa-miR-301a-3p;hsa-miR-301b
ENSG0000	ENSG0000	6.56E-06	hsa-miR-15a-5p;hsa-miR-15b-5p;hsa-miR-195-5p;hsa-miR-33
ENSG0000	ENSG0000	0.00083	hsa-miR-15a-5p;hsa-miR-15b-5p;hsa-miR-16-5p;hsa-miR-195-
ENSG0000	ENSG0000	0.00468	hsa-miR-103a-3p;hsa-miR-107;hsa-miR-15a-5p;hsa-miR-15b-5
ENSG0000	ENSG0000	0.000223	hsa-miR-320a;hsa-miR-320b;hsa-miR-320c;hsa-miR-320d;hsa-r
ENSG0000	ENSG0000	0.0003	hsa-miR-15a-5p;hsa-miR-15b-5p;hsa-miR-195-5p;hsa-miR-42
ENSG0000	ENSG0000	0.002594	hsa-miR-15a-5p;hsa-miR-15b-5p;hsa-miR-195-5p;hsa-miR-42

ENSG0000	ENSG0000	0.000115	hsa-let-7g-3p;hsa-miR-3177-5p;hsa-miR-320a;hsa-miR-320b;h
ENSG0000	ENSG0000	0.006097	hsa-miR-15a-5p;hsa-miR-15b-5p;hsa-miR-18a-3p;hsa-miR-19
ENSG0000	ENSG0000	0.000174	hsa-miR-15a-5p;hsa-miR-15b-5p;hsa-miR-16-5p;hsa-miR-195-
ENSG0000	ENSG0000	0.005446	hsa-miR-3127-3p;hsa-miR-3187-3p;hsa-miR-4726-3p;hsa-miR-
ENSG0000	ENSG0000	1.49E-06	hsa-miR-15a-5p;hsa-miR-15b-5p;hsa-miR-16-5p;hsa-miR-195-
ENSG0000	ENSG0000	0.007615	hsa-miR-15a-5p;hsa-miR-15b-5p;hsa-miR-16-5p;hsa-miR-195-
ENSG0000	ENSG0000	0.009146	hsa-miR-15a-5p;hsa-miR-15b-5p;hsa-miR-16-5p;hsa-miR-195-
ENSG0000	ENSG0000	0.002859	hsa-miR-24-3p;hsa-miR-296-5p;hsa-miR-3943;hsa-miR-4523;h
ENSG0000	ENSG0000	0.000865	hsa-miR-130a-3p;hsa-miR-130b-3p;hsa-miR-24-3p;hsa-miR-3
ENSG0000	ENSG0000	0.003556	hsa-miR-320a;hsa-miR-320b;hsa-miR-320c;hsa-miR-320d;hsa-
ENSG0000	ENSG0000	0.001742	hsa-miR-103a-3p;hsa-miR-107;hsa-miR-15a-5p;hsa-miR-15b-
ENSG0000	ENSG0000	0.006244	hsa-miR-15a-5p;hsa-miR-15b-5p;hsa-miR-16-5p;hsa-miR-195-
ENSG0000	ENSG0000	0.000416	hsa-miR-138-5p;hsa-miR-15a-5p;hsa-miR-15b-5p;hsa-miR-19
ENSG0000	ENSG0000	8.16E-07	hsa-miR-103a-3p;hsa-miR-107;hsa-miR-130a-3p;hsa-miR-130
ENSG0000	ENSG0000	0.00034	hsa-miR-15a-5p;hsa-miR-15b-5p;hsa-miR-195-5p;hsa-miR-22
ENSG0000	ENSG0000	4.54E-05	hsa-miR-103a-3p;hsa-miR-107;hsa-miR-130b-5p;hsa-miR-15a-
ENSG0000	ENSG0000	0.002793	hsa-miR-15a-5p;hsa-miR-15b-5p;hsa-miR-195-5p;hsa-miR-22
ENSG0000	ENSG0000	0.008945	hsa-miR-130a-3p;hsa-miR-130b-3p;hsa-miR-301a-3p;hsa-miR-
ENSG0000	ENSG0000	9.18E-05	hsa-miR-125b-2-3p;hsa-miR-2355-5p;hsa-miR-28-5p;hsa-miR-
ENSG0000	ENSG0000	0.000416	hsa-miR-15a-5p;hsa-miR-15b-5p;hsa-miR-16-5p;hsa-miR-195-
ENSG0000	ENSG0000	0.000895	hsa-miR-130a-3p;hsa-miR-130b-3p;hsa-miR-18a-3p;hsa-miR-
ENSG0000	ENSG0000	3.17E-05	hsa-miR-15a-5p;hsa-miR-15b-5p;hsa-miR-16-5p;hsa-miR-195-
ENSG0000	ENSG0000	0.0031	hsa-miR-1271-3p;hsa-miR-550a-3-5p;hsa-miR-550a-5p;hsa-m
ENSG0000	ENSG0000	2.06E-09	hsa-miR-15a-5p;hsa-miR-15b-5p;hsa-miR-16-5p;hsa-miR-195-
ENSG0000	ENSG0000	2.11E-05	hsa-miR-15a-5p;hsa-miR-15b-5p;hsa-miR-16-5p;hsa-miR-195-
ENSG0000	ENSG0000	0.000896	hsa-miR-130a-3p;hsa-miR-301a-3p;hsa-miR-301b-3p;hsa-miR-
ENSG0000	ENSG0000	0.005588	hsa-miR-199a-5p;hsa-miR-199b-5p;hsa-miR-361-3p;hsa-miR-
ENSG0000	ENSG0000	0.001197	hsa-miR-130a-3p;hsa-miR-140-3p;hsa-miR-301a-3p;hsa-miR-
ENSG0000	ENSG0000	0.000223	hsa-miR-130a-3p;hsa-miR-130b-3p;hsa-miR-301a-3p;hsa-miR-
ENSG0000	ENSG0000	7.99E-05	hsa-miR-130a-3p;hsa-miR-130b-3p;hsa-miR-24-3p;hsa-miR-3
ENSG0000	ENSG0000	0.004648	hsa-miR-130a-3p;hsa-miR-301a-3p;hsa-miR-301b-3p;hsa-miR-
ENSG0000	ENSG0000	0.001131	hsa-miR-15a-5p;hsa-miR-15b-5p;hsa-miR-16-5p;hsa-miR-195-
ENSG0000	ENSG0000	0.005136	hsa-miR-1254;hsa-miR-15a-5p;hsa-miR-15b-5p;hsa-miR-16-5
ENSG0000	ENSG0000	0.00055	hsa-miR-130a-3p;hsa-miR-130b-3p;hsa-miR-301a-3p;hsa-miR-
ENSG0000	ENSG0000	0.002219	hsa-let-7g-3p;hsa-miR-130a-3p;hsa-miR-130b-3p;hsa-miR-24-
ENSG0000	ENSG0000	6.56E-06	hsa-miR-130a-3p;hsa-miR-130b-3p;hsa-miR-24-3p;hsa-miR-3
ENSG0000	ENSG0000	0.000738	hsa-miR-130a-3p;hsa-miR-130b-3p;hsa-miR-148a-3p;hsa-miR-
ENSG0000	ENSG0000	0.004752	hsa-miR-15a-5p;hsa-miR-15b-5p;hsa-miR-195-5p;hsa-miR-42
ENSG0000	ENSG0000	0.000865	hsa-miR-1269b;hsa-miR-195-5p;hsa-miR-378a-3p;hsa-miR-37
ENSG0000	ENSG0000	0.002478	hsa-miR-130b-5p;hsa-miR-532-3p;hsa-miR-629-3p;hsa-miR-7
ENSG0000	ENSG0000	4.37E-06	hsa-miR-15a-5p;hsa-miR-15b-5p;hsa-miR-16-5p;hsa-miR-195-
ENSG0000	ENSG0000	3.06E-05	hsa-miR-103a-3p;hsa-miR-107;hsa-miR-15a-5p;hsa-miR-15b-
ENSG0000	ENSG0000	0.00468	hsa-miR-15a-5p;hsa-miR-15b-5p;hsa-miR-16-5p;hsa-miR-195-
ENSG0000	ENSG0000	0.000168	hsa-miR-15a-5p;hsa-miR-15b-5p;hsa-miR-16-5p;hsa-miR-195-
ENSG0000	ENSG0000	0.000654	hsa-miR-1271-3p;hsa-miR-138-5p;hsa-miR-148a-3p;hsa-miR-
ENSG0000	ENSG0000	8.45E-09	hsa-miR-1269b;hsa-miR-130a-3p;hsa-miR-130b-3p;hsa-miR-1
ENSG0000	ENSG0000	0.002453	hsa-miR-130a-3p;hsa-miR-130b-3p;hsa-miR-130b-5p;hsa-miR
ENSG0000	ENSG0000	0.002453	hsa-miR-130a-3p;hsa-miR-130b-3p;hsa-miR-130b-5p;hsa-miR
ENSG0000	ENSG0000	0.004648	hsa-miR-3127-3p;hsa-miR-361-3p;hsa-miR-3943;hsa-miR-766-
ENSG0000	ENSG0000	7.84E-05	hsa-miR-15a-5p;hsa-miR-15b-5p;hsa-miR-195-5p;hsa-miR-42
ENSG0000	ENSG0000	0.006081	hsa-miR-3199;hsa-miR-320a;hsa-miR-320b;hsa-miR-320c;hsa-
ENSG0000	ENSG0000	1.53E-05	hsa-miR-15a-5p;hsa-miR-15b-5p;hsa-miR-16-5p;hsa-miR-195-
ENSG0000	ENSG0000	3.83E-05	hsa-miR-130a-3p;hsa-miR-130b-3p;hsa-miR-130b-5p;hsa-miR
ENSG0000	ENSG0000	4.49E-08	hsa-miR-15a-5p;hsa-miR-15b-5p;hsa-miR-16-5p;hsa-miR-195-

ENSG0000	ENSG0000	7.99E-05	hsa-miR-15a-5p;hsa-miR-15b-5p;hsa-miR-16-5p;hsa-miR-191
ENSG0000	ENSG0000	7.54E-05	hsa-miR-130a-3p;hsa-miR-130b-3p;hsa-miR-140-3p;hsa-miR-
ENSG0000	ENSG0000	0.000824	hsa-miR-15a-5p;hsa-miR-15b-5p;hsa-miR-16-5p;hsa-miR-195
ENSG0000	ENSG0000	7.84E-05	hsa-miR-130a-3p;hsa-miR-130b-3p;hsa-miR-301a-3p;hsa-miR
ENSG0000	ENSG0000	0.003556	hsa-miR-18a-3p;hsa-miR-1913;hsa-miR-3187-3p;hsa-miR-324
ENSG0000	ENSG0000	0.001022	hsa-miR-320a;hsa-miR-320b;hsa-miR-320c;hsa-miR-320d;hsa-r
ENSG0000	ENSG0000	0.003167	hsa-miR-15a-5p;hsa-miR-15b-5p;hsa-miR-195-5p;hsa-miR-37
ENSG0000	ENSG0000	5.88E-05	hsa-miR-107;hsa-miR-15a-5p;hsa-miR-15b-5p;hsa-miR-16-5p;
ENSG0000	ENSG0000	0.000609	hsa-miR-1913;hsa-miR-324-3p;hsa-miR-378a-3p;hsa-miR-378
ENSG0000	ENSG0000	0.000441	hsa-miR-107;hsa-miR-15a-5p;hsa-miR-15b-5p;hsa-miR-195-5
ENSG0000	ENSG0000	0.003167	hsa-miR-15a-5p;hsa-miR-15b-5p;hsa-miR-16-5p;hsa-miR-195
ENSG0000	ENSG0000	2.11E-05	hsa-miR-15a-5p;hsa-miR-15b-5p;hsa-miR-16-5p;hsa-miR-195
ENSG0000	ENSG0000	0.000168	hsa-miR-130a-3p;hsa-miR-130b-3p;hsa-miR-301a-3p;hsa-miR
ENSG0000	ENSG0000	0.000105	hsa-miR-15a-5p;hsa-miR-15b-5p;hsa-miR-195-5p;hsa-miR-42
ENSG0000	ENSG0000	0.005588	hsa-miR-130a-3p;hsa-miR-301a-3p;hsa-miR-301b-3p;hsa-miR
ENSG0000	ENSG0000	0.006149	hsa-miR-130a-3p;hsa-miR-130b-3p;hsa-miR-301a-3p;hsa-miR
ENSG0000	ENSG0000	0.004255	hsa-miR-107;hsa-miR-15a-5p;hsa-miR-15b-5p;hsa-miR-195-5
ENSG0000	ENSG0000	0.00468	hsa-miR-107;hsa-miR-15a-5p;hsa-miR-15b-5p;hsa-miR-195-5
ENSG0000	ENSG0000	7.35E-05	hsa-miR-103a-3p;hsa-miR-107;hsa-miR-15a-5p;hsa-miR-15b-
ENSG0000	ENSG0000	2.58E-07	hsa-miR-1306-5p;hsa-miR-15a-5p;hsa-miR-15b-5p;hsa-miR-1
ENSG0000	ENSG0000	2.58E-07	hsa-miR-1306-5p;hsa-miR-15a-5p;hsa-miR-15b-5p;hsa-miR-1
ENSG0000	ENSG0000	0.000395	hsa-miR-15a-5p;hsa-miR-15b-5p;hsa-miR-195-5p;hsa-miR-42
ENSG0000	ENSG0000	7.23E-06	hsa-miR-15a-5p;hsa-miR-15b-5p;hsa-miR-16-5p;hsa-miR-195
ENSG0000	ENSG0000	0.001131	hsa-miR-378a-3p;hsa-miR-378c;hsa-miR-378d;hsa-miR-378i
ENSG0000	ENSG0000	1.49E-06	hsa-miR-15a-5p;hsa-miR-15b-5p;hsa-miR-16-5p;hsa-miR-195
ENSG0000	ENSG0000	0.002793	hsa-miR-130a-3p;hsa-miR-130b-3p;hsa-miR-301a-3p;hsa-miR
ENSG0000	ENSG0000	1.02E-07	hsa-miR-103a-3p;hsa-miR-107;hsa-miR-15a-5p;hsa-miR-15b-
ENSG0000	ENSG0000	0.000609	hsa-miR-15a-5p;hsa-miR-15b-5p;hsa-miR-16-5p;hsa-miR-195
ENSG0000	ENSG0000	0.000591	hsa-miR-106b-3p;hsa-miR-149-5p;hsa-miR-24-3p;hsa-miR-31
ENSG0000	ENSG0000	0.000105	hsa-miR-15a-5p;hsa-miR-15b-5p;hsa-miR-16-5p;hsa-miR-195
ENSG0000	ENSG0000	7.99E-05	hsa-miR-15a-5p;hsa-miR-15b-5p;hsa-miR-16-5p;hsa-miR-195
ENSG0000	ENSG0000	0.000865	hsa-miR-15a-5p;hsa-miR-15b-5p;hsa-miR-16-5p;hsa-miR-195
ENSG0000	ENSG0000	0.002594	hsa-miR-1254;hsa-miR-1271-3p;hsa-miR-149-5p;hsa-miR-550
ENSG0000	ENSG0000	7.23E-06	hsa-miR-15a-5p;hsa-miR-15b-5p;hsa-miR-16-5p;hsa-miR-195
ENSG0000	ENSG0000	0.005625	hsa-miR-1269b;hsa-miR-24-3p;hsa-miR-3199;hsa-miR-378a-3
ENSG0000	ENSG0000	3.06E-05	hsa-miR-103a-3p;hsa-miR-107;hsa-miR-15a-5p;hsa-miR-15b-
ENSG0000	ENSG0000	1.32E-06	hsa-miR-149-5p;hsa-miR-15a-5p;hsa-miR-15b-5p;hsa-miR-16
ENSG0000	ENSG0000	0.000972	hsa-miR-125b-2-3p;hsa-miR-130a-3p;hsa-miR-130b-3p;hsa-m
ENSG0000	ENSG0000	7.99E-05	hsa-miR-15a-5p;hsa-miR-15b-5p;hsa-miR-16-5p;hsa-miR-195
ENSG0000	ENSG0000	5.68E-06	hsa-miR-15a-5p;hsa-miR-15b-5p;hsa-miR-195-5p;hsa-miR-42
ENSG0000	ENSG0000	0.000385	hsa-miR-125b-2-3p;hsa-miR-15a-5p;hsa-miR-15b-5p;hsa-miR
ENSG0000	ENSG0000	0.002552	hsa-miR-130a-3p;hsa-miR-130b-3p;hsa-miR-221-5p;hsa-miR-
ENSG0000	ENSG0000	0.00099	hsa-miR-130a-3p;hsa-miR-130b-3p;hsa-miR-2355-5p;hsa-miR
ENSG0000	ENSG0000	0.002552	hsa-miR-130a-3p;hsa-miR-130b-3p;hsa-miR-301a-3p;hsa-miR
ENSG0000	ENSG0000	7.84E-05	hsa-miR-15a-5p;hsa-miR-15b-5p;hsa-miR-3685;hsa-miR-424-
ENSG0000	ENSG0000	0.000138	hsa-miR-15a-5p;hsa-miR-15b-5p;hsa-miR-16-5p;hsa-miR-195
ENSG0000	ENSG0000	0.006097	hsa-miR-1269b;hsa-miR-185-3p;hsa-miR-18a-3p;hsa-miR-191
ENSG0000	ENSG0000	1.49E-06	hsa-miR-15a-5p;hsa-miR-15b-5p;hsa-miR-16-5p;hsa-miR-195
ENSG0000	ENSG0000	0.001877	hsa-miR-130a-3p;hsa-miR-130b-3p;hsa-miR-15a-5p;hsa-miR-
ENSG0000	ENSG0000	0.00569	hsa-miR-1254;hsa-miR-1306-5p;hsa-miR-130a-3p;hsa-miR-13
ENSG0000	ENSG0000	0.000895	hsa-miR-15a-5p;hsa-miR-15b-5p;hsa-miR-16-5p;hsa-miR-195
ENSG0000	ENSG0000	8.36E-06	hsa-miR-103a-3p;hsa-miR-107;hsa-miR-125b-2-3p;hsa-miR-1
ENSG0000	ENSG0000	0.000289	hsa-miR-1306-5p;hsa-miR-140-3p;hsa-miR-15a-5p;hsa-miR-1
ENSG0000	ENSG0000	7.84E-05	hsa-miR-130a-3p;hsa-miR-130b-3p;hsa-miR-301a-3p;hsa-miR

ENSG0000	ENSG0000	1.49E-06	hsa-miR-15a-5p;hsa-miR-15b-5p;hsa-miR-16-5p;hsa-miR-195-
ENSG0000	ENSG0000	0.000135	hsa-let-7g-3p;hsa-miR-15a-5p;hsa-miR-15b-5p;hsa-miR-16-5
ENSG0000	ENSG0000	0.000174	hsa-miR-15a-5p;hsa-miR-15b-5p;hsa-miR-16-5p;hsa-miR-195-
ENSG0000	ENSG0000	3.78E-06	hsa-miR-130a-3p;hsa-miR-130b-3p;hsa-miR-15a-5p;hsa-miR-
ENSG0000	ENSG0000	3.37E-05	hsa-miR-103a-3p;hsa-miR-107;hsa-miR-15a-5p;hsa-miR-15b-
ENSG0000	ENSG0000	0.003668	hsa-miR-1226-5p;hsa-miR-15a-5p;hsa-miR-15b-5p;hsa-miR-1
ENSG0000	ENSG0000	0.001836	hsa-miR-149-5p;hsa-miR-378a-3p;hsa-miR-378c;hsa-miR-378
ENSG0000	ENSG0000	0.001262	hsa-miR-16-5p;hsa-miR-320a;hsa-miR-320b;hsa-miR-320c;hsa-
ENSG0000	ENSG0000	9.94E-05	hsa-miR-103a-3p;hsa-miR-107;hsa-miR-15a-5p;hsa-miR-15b-
ENSG0000	ENSG0000	1.95E-07	hsa-miR-130a-3p;hsa-miR-130b-3p;hsa-miR-148a-3p;hsa-miR-
ENSG0000	ENSG0000	0.000275	hsa-miR-15a-5p;hsa-miR-15b-5p;hsa-miR-16-5p;hsa-miR-195-
ENSG0000	ENSG0000	0.007457	hsa-miR-1306-5p;hsa-miR-199a-5p;hsa-miR-199b-5p;hsa-miR-
ENSG0000	ENSG0000	0.004514	hsa-miR-15a-5p;hsa-miR-15b-5p;hsa-miR-195-5p;hsa-miR-42
ENSG0000	ENSG0000	0.005621	hsa-miR-15a-5p;hsa-miR-15b-5p;hsa-miR-195-5p;hsa-miR-37
ENSG0000	ENSG0000	0.00143	hsa-miR-130a-3p;hsa-miR-130b-3p;hsa-miR-301a-3p;hsa-miR-
ENSG0000	ENSG0000	0.003395	hsa-miR-103a-3p;hsa-miR-107;hsa-miR-1306-5p;hsa-miR-15a-
ENSG0000	ENSG0000	0.001021	hsa-miR-130a-3p;hsa-miR-130b-3p;hsa-miR-24-3p;hsa-miR-3
ENSG0000	ENSG0000	5.88E-05	hsa-miR-15a-5p;hsa-miR-15b-5p;hsa-miR-195-5p;hsa-miR-29
ENSG0000	ENSG0000	0.003556	hsa-miR-15a-5p;hsa-miR-15b-5p;hsa-miR-195-5p;hsa-miR-42
ENSG0000	ENSG0000	0.00055	hsa-miR-15a-5p;hsa-miR-15b-5p;hsa-miR-16-5p;hsa-miR-195-
ENSG0000	ENSG0000	6.90E-05	hsa-miR-15a-5p;hsa-miR-15b-5p;hsa-miR-16-5p;hsa-miR-195-
ENSG0000	ENSG0000	0.002191	hsa-miR-130a-3p;hsa-miR-130b-3p;hsa-miR-301a-3p;hsa-miR-
ENSG0000	ENSG0000	0.002439	hsa-let-7g-3p;hsa-miR-1271-3p;hsa-miR-15a-5p;hsa-miR-15b-
ENSG0000	ENSG0000	0.003493	hsa-miR-1254;hsa-miR-149-5p;hsa-miR-15a-3p;hsa-miR-18a-
ENSG0000	ENSG0000	0.000136	hsa-miR-15a-5p;hsa-miR-16-5p;hsa-miR-195-5p;hsa-miR-374
ENSG0000	ENSG0000	0.000503	hsa-miR-130a-3p;hsa-miR-18a-3p;hsa-miR-1913;hsa-miR-301
ENSG0000	ENSG0000	0.005136	hsa-miR-130a-3p;hsa-miR-130b-3p;hsa-miR-148a-3p;hsa-miR-
ENSG0000	ENSG0000	3.21E-06	hsa-miR-103a-3p;hsa-miR-107;hsa-miR-15a-5p;hsa-miR-15b-
ENSG0000	ENSG0000	0.005621	hsa-miR-15a-5p;hsa-miR-15b-5p;hsa-miR-16-5p;hsa-miR-195-
ENSG0000	ENSG0000	0.001836	hsa-miR-130a-3p;hsa-miR-301a-3p;hsa-miR-301b-3p;hsa-miR-
ENSG0000	ENSG0000	0.003556	hsa-miR-320a;hsa-miR-320b;hsa-miR-320c;hsa-miR-320d;hsa-
ENSG0000	ENSG0000	0.004819	hsa-miR-130a-3p;hsa-miR-130b-3p;hsa-miR-1913;hsa-miR-30
ENSG0000	ENSG0000	6.86E-05	hsa-miR-1271-3p;hsa-miR-199a-5p;hsa-miR-199b-5p;hsa-miR-
ENSG0000	ENSG0000	0.001947	hsa-miR-1254;hsa-miR-1271-3p;hsa-miR-550a-3-5p;hsa-miR-
ENSG0000	ENSG0000	0.004796	hsa-miR-130a-3p;hsa-miR-130b-3p;hsa-miR-24-3p;hsa-miR-3
ENSG0000	ENSG0000	0.00143	hsa-miR-15a-5p;hsa-miR-15b-5p;hsa-miR-16-5p;hsa-miR-195-
ENSG0000	ENSG0000	3.00E-06	hsa-miR-15a-5p;hsa-miR-15b-5p;hsa-miR-16-5p;hsa-miR-195-
ENSG0000	ENSG0000	0.002431	hsa-miR-130a-3p;hsa-miR-15a-5p;hsa-miR-15b-5p;hsa-miR-1
ENSG0000	ENSG0000	0.001836	hsa-miR-320a;hsa-miR-320b;hsa-miR-320c;hsa-miR-320d;hsa-
ENSG0000	ENSG0000	0.000865	hsa-miR-130a-3p;hsa-miR-130b-3p;hsa-miR-130b-5p;hsa-miR
ENSG0000	ENSG0000	0.0003	hsa-miR-15a-5p;hsa-miR-16-5p;hsa-miR-195-5p;hsa-miR-424-
ENSG0000	ENSG0000	3.28E-05	hsa-miR-15a-5p;hsa-miR-15b-5p;hsa-miR-16-5p;hsa-miR-195-
ENSG0000	ENSG0000	0.006708	hsa-miR-15a-5p;hsa-miR-15b-5p;hsa-miR-16-5p;hsa-miR-195-
ENSG0000	ENSG0000	0.008613	hsa-miR-130a-3p;hsa-miR-130b-3p;hsa-miR-301a-3p;hsa-miR-
ENSG0000	ENSG0000	0.001836	hsa-miR-130a-3p;hsa-miR-130b-3p;hsa-miR-301a-3p;hsa-miR-
ENSG0000	ENSG0000	0.001836	hsa-miR-130a-3p;hsa-miR-130b-3p;hsa-miR-301a-3p;hsa-miR-
ENSG0000	ENSG0000	0.00034	hsa-miR-15a-5p;hsa-miR-15b-5p;hsa-miR-16-5p;hsa-miR-195-
ENSG0000	ENSG0000	0.000405	hsa-miR-199a-5p;hsa-miR-199b-5p;hsa-miR-2355-5p;hsa-miR-
ENSG0000	ENSG0000	7.99E-05	hsa-miR-130a-3p;hsa-miR-130b-3p;hsa-miR-148a-3p;hsa-miR-
ENSG0000	ENSG0000	1.02E-05	hsa-miR-15a-5p;hsa-miR-15b-5p;hsa-miR-16-5p;hsa-miR-195-
ENSG0000	ENSG0000	5.35E-05	hsa-miR-15a-5p;hsa-miR-15b-5p;hsa-miR-16-5p;hsa-miR-195-
ENSG0000	ENSG0000	3.17E-05	hsa-miR-15a-5p;hsa-miR-15b-5p;hsa-miR-16-5p;hsa-miR-195-
ENSG0000	ENSG0000	3.17E-05	hsa-miR-15a-5p;hsa-miR-15b-5p;hsa-miR-16-5p;hsa-miR-195-
ENSG0000	ENSG0000	3.17E-05	hsa-miR-15a-5p;hsa-miR-15b-5p;hsa-miR-16-5p;hsa-miR-195-

ENSG0000	ENSG0000	3.17E-05	hsa-miR-15a-5p;hsa-miR-15b-5p;hsa-miR-16-5p;hsa-miR-195-
ENSG0000	ENSG0000	2.69E-06	hsa-miR-107;hsa-miR-1271-3p;hsa-miR-15a-5p;hsa-miR-15b-5
ENSG0000	ENSG0000	2.69E-06	hsa-miR-107;hsa-miR-1271-3p;hsa-miR-15a-5p;hsa-miR-15b-5
ENSG0000	ENSG0000	0.002373	hsa-miR-130a-3p;hsa-miR-130b-3p;hsa-miR-130b-5p;hsa-miR
ENSG0000	ENSG0000	0.00246	hsa-miR-103a-3p;hsa-miR-107;hsa-miR-15a-5p;hsa-miR-15b-5
ENSG0000	ENSG0000	0.003556	hsa-miR-130a-3p;hsa-miR-130b-3p;hsa-miR-301a-3p;hsa-miR-
ENSG0000	ENSG0000	0.003556	hsa-miR-130a-3p;hsa-miR-130b-3p;hsa-miR-301a-3p;hsa-miR-
ENSG0000	ENSG0000	5.95E-06	hsa-miR-1226-5p;hsa-miR-130a-3p;hsa-miR-15a-5p;hsa-miR-1
ENSG0000	ENSG0000	0.003556	hsa-miR-130a-3p;hsa-miR-301a-3p;hsa-miR-301b-3p;hsa-miR-
ENSG0000	ENSG0000	0.000416	hsa-miR-130a-3p;hsa-miR-130b-3p;hsa-miR-301a-3p;hsa-miR-
ENSG0000	ENSG0000	2.38E-06	hsa-miR-15a-5p;hsa-miR-15b-5p;hsa-miR-195-5p;hsa-miR-42
ENSG0000	ENSG0000	0.000505	hsa-miR-130a-3p;hsa-miR-130b-3p;hsa-miR-301a-3p;hsa-miR-
ENSG0000	ENSG0000	2.11E-05	hsa-miR-15a-5p;hsa-miR-15b-5p;hsa-miR-16-5p;hsa-miR-195-
ENSG0000	ENSG0000	0.000405	hsa-miR-15a-5p;hsa-miR-16-5p;hsa-miR-493-5p;hsa-miR-532-
ENSG0000	ENSG0000	0.000647	hsa-miR-130a-3p;hsa-miR-130b-3p;hsa-miR-301a-3p;hsa-miR-
ENSG0000	ENSG0000	0.000136	hsa-miR-140-3p;hsa-miR-320a;hsa-miR-320b;hsa-miR-320c;hs
ENSG0000	ENSG0000	0.009146	hsa-miR-3685;hsa-miR-4422;hsa-miR-501-3p;hsa-miR-502-3p
ENSG0000	ENSG0000	0.001947	hsa-miR-1913;hsa-miR-3199;hsa-miR-324-3p;hsa-miR-615-5p
ENSG0000	ENSG0000	1.61E-05	hsa-miR-130a-3p;hsa-miR-130b-3p;hsa-miR-24-3p;hsa-miR-3
ENSG0000	ENSG0000	0.002453	hsa-miR-130a-3p;hsa-miR-130b-3p;hsa-miR-24-3p;hsa-miR-3
ENSG0000	ENSG0000	0.000275	hsa-miR-130a-3p;hsa-miR-130b-3p;hsa-miR-301a-3p;hsa-miR-
ENSG0000	ENSG0000	1.49E-06	hsa-miR-15a-5p;hsa-miR-15b-5p;hsa-miR-16-5p;hsa-miR-195-
ENSG0000	ENSG0000	0.002841	hsa-miR-130a-3p;hsa-miR-130b-3p;hsa-miR-149-5p;hsa-miR-
ENSG0000	ENSG0000	0.00386	hsa-miR-130a-3p;hsa-miR-130b-3p;hsa-miR-148a-3p;hsa-miR-
ENSG0000	ENSG0000	7.84E-05	hsa-miR-1254;hsa-miR-1271-3p;hsa-miR-505-5p;hsa-miR-550a
ENSG0000	ENSG0000	0.007955	hsa-miR-130a-3p;hsa-miR-130b-3p;hsa-miR-301a-3p;hsa-miR-
ENSG0000	ENSG0000	3.06E-07	hsa-miR-15a-5p;hsa-miR-15b-5p;hsa-miR-16-5p;hsa-miR-195-
ENSG0000	ENSG0000	3.06E-07	hsa-miR-15a-5p;hsa-miR-15b-5p;hsa-miR-16-5p;hsa-miR-195-
ENSG0000	ENSG0000	3.06E-07	hsa-miR-15a-5p;hsa-miR-15b-5p;hsa-miR-16-5p;hsa-miR-195-
ENSG0000	ENSG0000	3.06E-07	hsa-miR-15a-5p;hsa-miR-15b-5p;hsa-miR-16-5p;hsa-miR-195-
ENSG0000	ENSG0000	3.06E-07	hsa-miR-15a-5p;hsa-miR-15b-5p;hsa-miR-16-5p;hsa-miR-195-
ENSG0000	ENSG0000	3.06E-07	hsa-miR-15a-5p;hsa-miR-15b-5p;hsa-miR-16-5p;hsa-miR-195-
ENSG0000	ENSG0000	3.06E-07	hsa-miR-15a-5p;hsa-miR-15b-5p;hsa-miR-16-5p;hsa-miR-195-
ENSG0000	ENSG0000	3.06E-07	hsa-miR-15a-5p;hsa-miR-15b-5p;hsa-miR-16-5p;hsa-miR-195-
ENSG0000	ENSG0000	3.06E-07	hsa-miR-15a-5p;hsa-miR-15b-5p;hsa-miR-16-5p;hsa-miR-195-
ENSG0000	ENSG0000	3.06E-07	hsa-miR-15a-5p;hsa-miR-15b-5p;hsa-miR-16-5p;hsa-miR-195-
ENSG0000	ENSG0000	9.32E-05	hsa-miR-15a-5p;hsa-miR-15b-5p;hsa-miR-16-5p;hsa-miR-195-
ENSG0000	ENSG0000	7.99E-05	hsa-miR-15a-5p;hsa-miR-15b-5p;hsa-miR-16-5p;hsa-miR-195-
ENSG0000	ENSG0000	0.003048	hsa-miR-130a-3p;hsa-miR-130b-3p;hsa-miR-301a-3p;hsa-miR-
ENSG0000	ENSG0000	0.003048	hsa-miR-130a-3p;hsa-miR-130b-3p;hsa-miR-301a-3p;hsa-miR-
ENSG0000	ENSG0000	0.000174	hsa-miR-361-3p;hsa-miR-378a-3p;hsa-miR-378c;hsa-miR-378
ENSG0000	ENSG0000	7.71E-08	hsa-miR-1254;hsa-miR-1271-3p;hsa-miR-15a-5p;hsa-miR-15b-
ENSG0000	ENSG0000	0.008945	hsa-miR-15a-5p;hsa-miR-15b-5p;hsa-miR-16-5p;hsa-miR-195-
ENSG0000	ENSG0000	0.00246	hsa-miR-15a-5p;hsa-miR-15b-5p;hsa-miR-195-5p;hsa-miR-31
ENSG0000	ENSG0000	6.75E-05	hsa-miR-103a-3p;hsa-miR-107;hsa-miR-15a-5p;hsa-miR-15b-5
ENSG0000	ENSG0000	0.009146	hsa-miR-130b-5p;hsa-miR-221-5p;hsa-miR-4664-5p;hsa-miR-
ENSG0000	ENSG0000	0.001487	hsa-miR-148a-3p;hsa-miR-185-3p;hsa-miR-2355-5p;hsa-miR-
ENSG0000	ENSG0000	0.002859	hsa-miR-15a-5p;hsa-miR-15b-5p;hsa-miR-16-5p;hsa-miR-195-
ENSG0000	ENSG0000	0.000395	hsa-miR-130a-3p;hsa-miR-130b-3p;hsa-miR-301a-3p;hsa-miR-
ENSG0000	ENSG0000	4.03E-07	hsa-miR-103a-3p;hsa-miR-107;hsa-miR-130b-5p;hsa-miR-15a-
ENSG0000	ENSG0000	7.35E-05	hsa-miR-15a-5p;hsa-miR-15b-5p;hsa-miR-16-5p;hsa-miR-195-
ENSG0000	ENSG0000	0.003048	hsa-miR-15a-5p;hsa-miR-15b-5p;hsa-miR-195-5p;hsa-miR-42
ENSG0000	ENSG0000	0.001768	hsa-miR-130a-3p;hsa-miR-130b-3p;hsa-miR-2355-5p;hsa-miR-
ENSG0000	ENSG0000	0.001616	hsa-miR-15a-5p;hsa-miR-15b-5p;hsa-miR-16-5p;hsa-miR-195-

ENSG0000	ENSG0000	0.003556	hsa-miR-130a-3p;hsa-miR-130b-3p;hsa-miR-301a-3p;hsa-miR-
ENSG0000	ENSG0000	0.000105	hsa-miR-130a-3p;hsa-miR-130b-3p;hsa-miR-148a-3p;hsa-miR-
ENSG0000	ENSG0000	0.001021	hsa-miR-148a-3p;hsa-miR-15a-5p;hsa-miR-15b-5p;hsa-miR-16
ENSG0000	ENSG0000	2.60E-06	hsa-miR-320a;hsa-miR-320b;hsa-miR-320c;hsa-miR-320d;hsa-r
ENSG0000	ENSG0000	1.16E-06	hsa-miR-130a-3p;hsa-miR-130b-3p;hsa-miR-15a-5p;hsa-miR-1
ENSG0000	ENSG0000	0.006915	hsa-miR-320a;hsa-miR-320b;hsa-miR-320c;hsa-miR-320d;hsa-r
ENSG0000	ENSG0000	0.003493	hsa-miR-130a-3p;hsa-miR-130b-3p;hsa-miR-301a-3p;hsa-miR-
ENSG0000	ENSG0000	3.17E-05	hsa-miR-15a-5p;hsa-miR-15b-5p;hsa-miR-16-5p;hsa-miR-195-
ENSG0000	ENSG0000	0.000591	hsa-miR-1254;hsa-miR-1271-3p;hsa-miR-550a-3-5p;hsa-miR-5
ENSG0000	ENSG0000	3.00E-06	hsa-miR-103a-3p;hsa-miR-107;hsa-miR-15a-5p;hsa-miR-15b-5
ENSG0000	ENSG0000	0.000609	hsa-miR-15a-5p;hsa-miR-15b-5p;hsa-miR-195-5p;hsa-miR-424
ENSG0000	ENSG0000	2.88E-07	hsa-miR-15a-5p;hsa-miR-15b-5p;hsa-miR-16-5p;hsa-miR-195-
ENSG0000	ENSG0000	0.005045	hsa-miR-1306-5p;hsa-miR-130a-3p;hsa-miR-130b-3p;hsa-miR-
ENSG0000	ENSG0000	0.009146	hsa-miR-130a-3p;hsa-miR-301a-3p;hsa-miR-301b-3p;hsa-miR-
ENSG0000	ENSG0000	0.000114	hsa-miR-15a-5p;hsa-miR-15b-5p;hsa-miR-195-5p;hsa-miR-424
ENSG0000	ENSG0000	0.006244	hsa-miR-199a-5p;hsa-miR-199b-5p;hsa-miR-2355-5p;hsa-miR-
ENSG0000	ENSG0000	2.36E-06	hsa-miR-103a-3p;hsa-miR-107;hsa-miR-15a-5p;hsa-miR-15b-5
ENSG0000	ENSG0000	0.001197	hsa-miR-15a-5p;hsa-miR-15b-5p;hsa-miR-16-5p;hsa-miR-195-
ENSG0000	ENSG0000	0.007959	hsa-miR-130a-3p;hsa-miR-130b-3p;hsa-miR-130b-5p;hsa-miR
ENSG0000	ENSG0000	7.23E-06	hsa-miR-15a-5p;hsa-miR-15b-5p;hsa-miR-16-5p;hsa-miR-195-
ENSG0000	ENSG0000	0.001396	hsa-miR-15a-5p;hsa-miR-15b-5p;hsa-miR-16-5p;hsa-miR-195-
ENSG0000	ENSG0000	5.30E-06	hsa-miR-1269b;hsa-miR-15a-5p;hsa-miR-15b-5p;hsa-miR-16-5
ENSG0000	ENSG0000	4.54E-05	hsa-miR-103a-3p;hsa-miR-107;hsa-miR-15a-5p;hsa-miR-15b-5
ENSG0000	ENSG0000	0.000654	hsa-miR-15a-5p;hsa-miR-15b-5p;hsa-miR-195-5p;hsa-miR-448
ENSG0000	ENSG0000	2.05E-08	hsa-miR-103a-3p;hsa-miR-107;hsa-miR-15a-5p;hsa-miR-15b-5
ENSG0000	ENSG0000	6.56E-06	hsa-miR-15a-5p;hsa-miR-15b-5p;hsa-miR-16-5p;hsa-miR-195-
ENSG0000	ENSG0000	0.000252	hsa-miR-3187-3p;hsa-miR-320a;hsa-miR-320b;hsa-miR-320c;h
ENSG0000	ENSG0000	2.99E-07	hsa-miR-130a-3p;hsa-miR-130b-3p;hsa-miR-15a-5p;hsa-miR-1
ENSG0000	ENSG0000	0.004648	hsa-miR-15a-5p;hsa-miR-15b-5p;hsa-miR-16-5p;hsa-miR-195-
ENSG0000	ENSG0000	0.004648	hsa-miR-15a-5p;hsa-miR-15b-5p;hsa-miR-16-5p;hsa-miR-195-
ENSG0000	ENSG0000	0.000759	hsa-miR-15a-5p;hsa-miR-15b-5p;hsa-miR-16-5p;hsa-miR-195-
ENSG0000	ENSG0000	4.64E-05	hsa-miR-15a-5p;hsa-miR-15b-5p;hsa-miR-16-5p;hsa-miR-195-
ENSG0000	ENSG0000	0.001255	hsa-miR-1913;hsa-miR-324-3p;hsa-miR-361-3p;hsa-miR-378g;
ENSG0000	ENSG0000	0.000266	hsa-miR-130a-3p;hsa-miR-130b-3p;hsa-miR-15a-5p;hsa-miR-1
ENSG0000	ENSG0000	0.001947	hsa-miR-15a-5p;hsa-miR-15b-5p;hsa-miR-16-5p;hsa-miR-195-
ENSG0000	ENSG0000	1.32E-06	hsa-miR-15a-5p;hsa-miR-15b-5p;hsa-miR-16-5p;hsa-miR-195-
ENSG0000	ENSG0000	2.63E-13	hsa-miR-103a-3p;hsa-miR-107;hsa-miR-1226-5p;hsa-miR-1271
ENSG0000	ENSG0000	2.88E-07	hsa-miR-15a-5p;hsa-miR-15b-5p;hsa-miR-16-5p;hsa-miR-195-
ENSG0000	ENSG0000	2.89E-05	hsa-miR-130a-3p;hsa-miR-130b-3p;hsa-miR-301a-3p;hsa-miR-
ENSG0000	ENSG0000	0.008613	hsa-miR-130a-3p;hsa-miR-130b-3p;hsa-miR-24-3p;hsa-miR-30
ENSG0000	ENSG0000	0.000409	hsa-miR-103a-3p;hsa-miR-107;hsa-miR-1306-5p;hsa-miR-15a-
ENSG0000	ENSG0000	0.000291	hsa-miR-15a-5p;hsa-miR-15b-5p;hsa-miR-16-5p;hsa-miR-195-
ENSG0000	ENSG0000	3.28E-05	hsa-miR-320a;hsa-miR-320b;hsa-miR-320c;hsa-miR-320d;hsa-r
ENSG0000	ENSG0000	0.000609	hsa-miR-15a-5p;hsa-miR-15b-5p;hsa-miR-16-5p;hsa-miR-195-
ENSG0000	ENSG0000	0.000258	hsa-miR-15a-5p;hsa-miR-15b-5p;hsa-miR-16-5p;hsa-miR-195-
ENSG0000	ENSG0000	0.00034	hsa-miR-130a-3p;hsa-miR-130b-3p;hsa-miR-301a-3p;hsa-miR-
ENSG0000	ENSG0000	1.49E-06	hsa-miR-15a-5p;hsa-miR-15b-5p;hsa-miR-16-5p;hsa-miR-195-
ENSG0000	ENSG0000	0.00022	hsa-miR-15a-5p;hsa-miR-15b-5p;hsa-miR-16-5p;hsa-miR-195-
ENSG0000	ENSG0000	0.000895	hsa-miR-103a-3p;hsa-miR-107;hsa-miR-15a-5p;hsa-miR-15b-5
ENSG0000	ENSG0000	0.000134	hsa-let-7g-3p;hsa-miR-130b-5p;hsa-miR-15a-5p;hsa-miR-15b-
ENSG0000	ENSG0000	0.008945	hsa-miR-1254;hsa-miR-1271-3p;hsa-miR-550a-3-5p;hsa-miR-5
ENSG0000	ENSG0000	0.000395	hsa-miR-130a-3p;hsa-miR-130b-3p;hsa-miR-301a-3p;hsa-miR-
ENSG0000	ENSG0000	4.50E-07	hsa-miR-130a-3p;hsa-miR-130b-3p;hsa-miR-15a-5p;hsa-miR-1
ENSG0000	ENSG0000	0.00143	hsa-let-7g-3p;hsa-miR-199a-5p;hsa-miR-199b-5p;hsa-miR-235

ENSG0000	ENSG0000	0.000654	hsa-miR-1271-3p;hsa-miR-199a-5p;hsa-miR-199b-5p;hsa-miR-
ENSG0000	ENSG0000	0.003493	hsa-miR-15a-5p;hsa-miR-15b-5p;hsa-miR-16-5p;hsa-miR-195-
ENSG0000	ENSG0000	0.004123	hsa-miR-320a;hsa-miR-320b;hsa-miR-320c;hsa-miR-320d;hsa-r
ENSG0000	ENSG0000	0.004123	hsa-miR-320a;hsa-miR-320b;hsa-miR-320c;hsa-miR-320d;hsa-r
ENSG0000	ENSG0000	0.006209	hsa-miR-320a;hsa-miR-320b;hsa-miR-320c;hsa-miR-320d;hsa-r
ENSG0000	ENSG0000	0.00034	hsa-miR-130a-3p;hsa-miR-130b-3p;hsa-miR-24-3p;hsa-miR-30
ENSG0000	ENSG0000	0.00034	hsa-miR-130a-3p;hsa-miR-130b-3p;hsa-miR-24-3p;hsa-miR-30
ENSG0000	ENSG0000	0.00034	hsa-miR-130a-3p;hsa-miR-130b-3p;hsa-miR-24-3p;hsa-miR-30
ENSG0000	ENSG0000	0.00034	hsa-miR-130a-3p;hsa-miR-130b-3p;hsa-miR-24-3p;hsa-miR-30
ENSG0000	ENSG0000	0.002191	hsa-miR-130a-3p;hsa-miR-130b-3p;hsa-miR-301a-3p;hsa-miR-
ENSG0000	ENSG0000	3.24E-07	hsa-miR-218-1-3p;hsa-miR-320a;hsa-miR-320b;hsa-miR-320d;
ENSG0000	ENSG0000	9.63E-06	hsa-miR-320a;hsa-miR-320b;hsa-miR-320d;hsa-miR-4429;hsa-r
ENSG0000	ENSG0000	0.002434	hsa-miR-320a;hsa-miR-320b;hsa-miR-320d;hsa-miR-4429
ENSG0000	ENSG0000	0.004635	hsa-miR-200a-5p;hsa-miR-200b-5p;hsa-miR-4691-3p;hsa-miR-
ENSG0000	ENSG0000	1.34E-06	hsa-miR-320a;hsa-miR-320b;hsa-miR-320d;hsa-miR-4429;hsa-r
ENSG0000	ENSG0000	0.003253	hsa-miR-182-5p;hsa-miR-4726-3p;hsa-miR-9-5p;hsa-miR-96-5
ENSG0000	ENSG0000	3.27E-05	hsa-miR-2467-5p;hsa-miR-320a;hsa-miR-320b;hsa-miR-320d;h
ENSG0000	ENSG0000	0.000242	hsa-miR-200a-5p;hsa-miR-214-3p;hsa-miR-320a;hsa-miR-5010
ENSG0000	ENSG0000	0.001491	hsa-miR-130b-5p;hsa-miR-22-5p;hsa-miR-26b-3p;hsa-miR-87
ENSG0000	ENSG0000	0.00398	hsa-miR-130b-5p;hsa-miR-182-5p;hsa-miR-9-5p;hsa-miR-96-5
ENSG0000	ENSG0000	0.002624	hsa-miR-22-5p;hsa-miR-450b-5p;hsa-miR-9-5p;hsa-miR-96-5p
ENSG0000	ENSG0000	0.003033	hsa-miR-130b-5p;hsa-miR-4482-5p;hsa-miR-5010-3p;hsa-miR-
ENSG0000	ENSG0000	0.001491	hsa-miR-320a;hsa-miR-320b;hsa-miR-320d;hsa-miR-4429
ENSG0000	ENSG0000	1.93E-05	hsa-miR-320a;hsa-miR-320b;hsa-miR-320d;hsa-miR-4429;hsa-r
ENSG0000	ENSG0000	0.001027	hsa-miR-320a;hsa-miR-320b;hsa-miR-320d;hsa-miR-4429
ENSG0000	ENSG0000	0.00481	hsa-miR-320a;hsa-miR-320b;hsa-miR-320d;hsa-miR-4429
ENSG0000	ENSG0000	0.00996	hsa-miR-124-3p;hsa-miR-182-5p;hsa-miR-214-3p;hsa-miR-24
ENSG0000	ENSG0000	7.40E-05	hsa-miR-320a;hsa-miR-320b;hsa-miR-320d;hsa-miR-4429;hsa-r
ENSG0000	ENSG0000	8.69E-05	hsa-miR-320a;hsa-miR-320b;hsa-miR-320d;hsa-miR-4429
ENSG0000	ENSG0000	8.69E-05	hsa-miR-320a;hsa-miR-320b;hsa-miR-320d;hsa-miR-4429
ENSG0000	ENSG0000	8.69E-05	hsa-miR-320a;hsa-miR-320b;hsa-miR-320d;hsa-miR-4429
ENSG0000	ENSG0000	0.002084	hsa-miR-320a;hsa-miR-320b;hsa-miR-320d;hsa-miR-4429
ENSG0000	ENSG0000	1.54E-05	hsa-miR-320a;hsa-miR-320b;hsa-miR-320d;hsa-miR-4429
ENSG0000	ENSG0000	0.00093	hsa-miR-320a;hsa-miR-320b;hsa-miR-320d;hsa-miR-4429
ENSG0000	ENSG0000	0.004245	hsa-miR-22-5p;hsa-miR-26b-3p;hsa-miR-5008-5p;hsa-miR-87
ENSG0000	ENSG0000	0.000839	hsa-miR-320a;hsa-miR-320b;hsa-miR-320d;hsa-miR-4429
ENSG0000	ENSG0000	0.004521	hsa-miR-320a;hsa-miR-320b;hsa-miR-320d;hsa-miR-4429
ENSG0000	ENSG0000	0.004521	hsa-miR-320a;hsa-miR-320b;hsa-miR-320d;hsa-miR-4429
ENSG0000	ENSG0000	0.001626	hsa-miR-320a;hsa-miR-320b;hsa-miR-320d;hsa-miR-4429
ENSG0000	ENSG0000	1.80E-06	hsa-miR-320a;hsa-miR-320b;hsa-miR-320d;hsa-miR-4429;hsa-r
ENSG0000	ENSG0000	3.34E-06	hsa-miR-320a;hsa-miR-320b;hsa-miR-320d;hsa-miR-4429
ENSG0000	ENSG0000	0.002823	hsa-miR-320a;hsa-miR-320b;hsa-miR-320d;hsa-miR-4429
ENSG0000	ENSG0000	0.002624	hsa-miR-320a;hsa-miR-320b;hsa-miR-320d;hsa-miR-4429
ENSG0000	ENSG0000	0.007184	hsa-miR-320a;hsa-miR-320b;hsa-miR-320d;hsa-miR-4429
ENSG0000	ENSG0000	0.00107	hsa-miR-1224-5p;hsa-miR-320a;hsa-miR-320b;hsa-miR-320d;h
ENSG0000	ENSG0000	0.003485	hsa-miR-200a-5p;hsa-miR-200b-5p;hsa-miR-5010-3p;hsa-miR-
ENSG0000	ENSG0000	0.002254	hsa-miR-22-5p;hsa-miR-320b;hsa-miR-320d;hsa-miR-4429
ENSG0000	ENSG0000	1.11E-05	hsa-miR-320a;hsa-miR-320b;hsa-miR-320d;hsa-miR-4429
ENSG0000	ENSG0000	1.11E-05	hsa-miR-320a;hsa-miR-320b;hsa-miR-320d;hsa-miR-4429
ENSG0000	ENSG0000	3.34E-06	hsa-miR-320a;hsa-miR-320b;hsa-miR-320d;hsa-miR-4429
ENSG0000	ENSG0000	0.000839	hsa-miR-1224-5p;hsa-miR-124-3p;hsa-miR-5010-3p;hsa-miR-8
ENSG0000	ENSG0000	3.24E-07	hsa-miR-124-3p;hsa-miR-320a;hsa-miR-320b;hsa-miR-320d;hs
ENSG0000	ENSG0000	4.35E-06	hsa-miR-182-5p;hsa-miR-320a;hsa-miR-320b;hsa-miR-320d;hs
ENSG0000	ENSG0000	3.57E-05	hsa-miR-320a;hsa-miR-320b;hsa-miR-320d;hsa-miR-4429

ENSG0000	ENSG0000	0.001626	hsa-miR-320a;hsa-miR-320b;hsa-miR-320d;hsa-miR-4429
ENSG0000	ENSG0000	0.000369	hsa-miR-320a;hsa-miR-320b;hsa-miR-320d;hsa-miR-4429
ENSG0000	ENSG0000	0.00511	hsa-miR-320a;hsa-miR-320b;hsa-miR-320d;hsa-miR-4429
ENSG0000	ENSG0000	0.000126	hsa-miR-320a;hsa-miR-320b;hsa-miR-320d;hsa-miR-4429
ENSG0000	ENSG0000	0.006154	hsa-miR-124-3p;hsa-miR-450b-5p;hsa-miR-452-5p;hsa-miR-4
ENSG0000	ENSG0000	0.002823	hsa-miR-22-5p;hsa-miR-5010-3p;hsa-miR-539-5p;hsa-miR-87
ENSG0000	ENSG0000	0.006088	hsa-miR-130b-5p;hsa-miR-141-5p;hsa-miR-2467-5p;hsa-miR-
ENSG0000	ENSG0000	0.002254	hsa-miR-320a;hsa-miR-320b;hsa-miR-320d;hsa-miR-4429
ENSG0000	ENSG0000	0.000369	hsa-miR-320a;hsa-miR-320b;hsa-miR-320d;hsa-miR-4429
ENSG0000	ENSG0000	0.00398	hsa-miR-320a;hsa-miR-320b;hsa-miR-320d;hsa-miR-4429
ENSG0000	ENSG0000	0.000208	hsa-miR-320a;hsa-miR-320b;hsa-miR-320d;hsa-miR-4429
ENSG0000	ENSG0000	0.001144	hsa-miR-320a;hsa-miR-320b;hsa-miR-320d;hsa-miR-4429;hsa-
ENSG0000	ENSG0000	0.00015	hsa-miR-320a;hsa-miR-320b;hsa-miR-320d;hsa-miR-4429
ENSG0000	ENSG0000	0.009754	hsa-miR-182-5p;hsa-miR-2467-5p;hsa-miR-873-5p;hsa-miR-9
ENSG0000	ENSG0000	1.13E-06	hsa-miR-320a;hsa-miR-320b;hsa-miR-320d;hsa-miR-4429
ENSG0000	ENSG0000	0.002434	hsa-miR-320a;hsa-miR-320b;hsa-miR-320d;hsa-miR-4429
ENSG0000	ENSG0000	0.009289	hsa-miR-320a;hsa-miR-320b;hsa-miR-320d;hsa-miR-4429
ENSG0000	ENSG0000	0.002434	hsa-miR-320a;hsa-miR-320b;hsa-miR-320d;hsa-miR-4429
ENSG0000	ENSG0000	0.005749	hsa-miR-320a;hsa-miR-320b;hsa-miR-320d;hsa-miR-4429
ENSG0000	ENSG0000	0.003485	hsa-miR-320a;hsa-miR-320b;hsa-miR-320d;hsa-miR-4429
ENSG0000	ENSG0000	0.000105	hsa-miR-320a;hsa-miR-320b;hsa-miR-320d;hsa-miR-4429;hsa-
ENSG0000	ENSG0000	7.75E-06	hsa-miR-320a;hsa-miR-320b;hsa-miR-320d;hsa-miR-4429
ENSG0000	ENSG0000	0.00042	hsa-miR-320a;hsa-miR-320b;hsa-miR-320d;hsa-miR-4429
ENSG0000	ENSG0000	0.00042	hsa-miR-320a;hsa-miR-320b;hsa-miR-320d;hsa-miR-4429
ENSG0000	ENSG0000	0.000604	hsa-miR-320a;hsa-miR-320b;hsa-miR-320d;hsa-miR-4429
ENSG0000	ENSG0000	0.005086	hsa-miR-30a-5p;hsa-miR-30b-5p;hsa-miR-30c-5p;hsa-miR-30
ENSG0000	ENSG0000	0.000284	hsa-miR-125a-5p;hsa-miR-125b-5p;hsa-miR-185-3p;hsa-miR-
ENSG0000	ENSG0000	0.008357	hsa-miR-1305;hsa-miR-186-5p;hsa-miR-1976;hsa-miR-19a-3p;
ENSG0000	ENSG0000	0.000942	hsa-miR-30a-5p;hsa-miR-30b-5p;hsa-miR-30c-5p;hsa-miR-30
ENSG0000	ENSG0000	1.32E-05	hsa-miR-30a-5p;hsa-miR-30b-5p;hsa-miR-30c-5p;hsa-miR-30
ENSG0000	ENSG0000	9.78E-05	hsa-miR-204-5p;hsa-miR-30a-5p;hsa-miR-30b-5p;hsa-miR-30
ENSG0000	ENSG0000	1.81E-05	hsa-miR-101-3p;hsa-miR-18a-5p;hsa-miR-30a-5p;hsa-miR-30
ENSG0000	ENSG0000	0.001089	hsa-miR-186-5p;hsa-miR-30b-5p;hsa-miR-30c-5p;hsa-miR-30
ENSG0000	ENSG0000	0.000797	hsa-miR-141-5p;hsa-miR-204-5p;hsa-miR-26a-5p;hsa-miR-26
ENSG0000	ENSG0000	0.004171	hsa-miR-1304-3p;hsa-miR-1976;hsa-miR-204-5p;hsa-miR-26a-
ENSG0000	ENSG0000	0.000797	hsa-miR-30a-3p;hsa-miR-30d-3p;hsa-miR-30e-3p;hsa-miR-66
ENSG0000	ENSG0000	4.39E-05	hsa-miR-17-3p;hsa-miR-204-5p;hsa-miR-378a-3p;hsa-miR-37
ENSG0000	ENSG0000	0.001474	hsa-miR-30a-5p;hsa-miR-30b-5p;hsa-miR-30d-5p;hsa-miR-30
ENSG0000	ENSG0000	0.000197	hsa-miR-30a-5p;hsa-miR-30b-5p;hsa-miR-30c-5p;hsa-miR-30
ENSG0000	ENSG0000	1.79E-06	hsa-miR-1976;hsa-miR-30a-5p;hsa-miR-30b-5p;hsa-miR-30c-5
ENSG0000	ENSG0000	0.003356	hsa-miR-18a-5p;hsa-miR-26a-5p;hsa-miR-26b-5p;hsa-miR-30
ENSG0000	ENSG0000	0.007658	hsa-miR-10a-3p;hsa-miR-1304-3p;hsa-miR-19a-3p;hsa-miR-1
ENSG0000	ENSG0000	0.000797	hsa-miR-140-5p;hsa-miR-30a-5p;hsa-miR-30c-5p;hsa-miR-34
ENSG0000	ENSG0000	0.005086	hsa-miR-30a-5p;hsa-miR-30b-5p;hsa-miR-30d-5p;hsa-miR-30
ENSG0000	ENSG0000	0.007282	hsa-miR-141-5p;hsa-miR-3065-3p;hsa-miR-338-3p;hsa-miR-5
ENSG0000	ENSG0000	0.000604	hsa-miR-30a-5p;hsa-miR-30b-5p;hsa-miR-30c-5p;hsa-miR-30
ENSG0000	ENSG0000	0.009951	hsa-miR-101-3p;hsa-miR-30a-3p;hsa-miR-30d-3p;hsa-miR-30
ENSG0000	ENSG0000	0.001853	hsa-miR-144-3p;hsa-miR-16-1-3p;hsa-miR-186-5p;hsa-miR-1
ENSG0000	ENSG0000	0.002643	hsa-miR-30a-5p;hsa-miR-30b-5p;hsa-miR-30c-5p;hsa-miR-30
ENSG0000	ENSG0000	0.006708	hsa-miR-1304-3p;hsa-miR-222-5p;hsa-miR-30a-3p;hsa-miR-3
ENSG0000	ENSG0000	7.84E-09	hsa-miR-30a-5p;hsa-miR-30b-5p;hsa-miR-30c-5p;hsa-miR-30
ENSG0000	ENSG0000	4.61E-08	hsa-miR-222-5p;hsa-miR-30a-3p;hsa-miR-30d-3p;hsa-miR-30
ENSG0000	ENSG0000	2.35E-08	hsa-miR-19a-3p;hsa-miR-19b-3p;hsa-miR-296-5p;hsa-miR-30
ENSG0000	ENSG0000	1.23E-09	hsa-miR-101-3p;hsa-miR-30a-5p;hsa-miR-30b-5p;hsa-miR-30

ENSG0000	ENSG0000	0.00433	hsa-miR-30a-3p;hsa-miR-30d-3p;hsa-miR-30e-3p;hsa-miR-36
ENSG0000	ENSG0000	0.002475	hsa-miR-30a-3p;hsa-miR-30d-3p;hsa-miR-30e-3p;hsa-miR-36
ENSG0000	ENSG0000	2.41E-06	hsa-miR-125a-5p;hsa-miR-125b-5p;hsa-miR-30a-5p;hsa-miR-
ENSG0000	ENSG0000	6.97E-05	hsa-miR-30a-5p;hsa-miR-30b-5p;hsa-miR-30c-5p;hsa-miR-30
ENSG0000	ENSG0000	0.001216	hsa-miR-101-3p;hsa-miR-1976;hsa-miR-378a-5p;hsa-miR-582
ENSG0000	ENSG0000	0.000692	hsa-miR-30a-5p;hsa-miR-30b-5p;hsa-miR-30c-5p;hsa-miR-30
ENSG0000	ENSG0000	0.00371	hsa-miR-101-3p;hsa-miR-144-3p;hsa-miR-16-1-3p;hsa-miR-1
ENSG0000	ENSG0000	0.007225	hsa-miR-378a-3p;hsa-miR-378c;hsa-miR-378d;hsa-miR-378i
ENSG0000	ENSG0000	3.23E-06	hsa-miR-30a-5p;hsa-miR-30b-5p;hsa-miR-30c-5p;hsa-miR-30
ENSG0000	ENSG0000	1.03E-07	hsa-miR-17-3p;hsa-miR-222-5p;hsa-miR-26a-5p;hsa-miR-306
ENSG0000	ENSG0000	1.79E-06	hsa-miR-30a-5p;hsa-miR-30b-5p;hsa-miR-30c-5p;hsa-miR-30
ENSG0000	ENSG0000	2.77E-05	hsa-miR-125a-5p;hsa-miR-125b-5p;hsa-miR-30a-3p;hsa-miR-
ENSG0000	ENSG0000	7.84E-09	hsa-miR-30a-5p;hsa-miR-30b-5p;hsa-miR-30c-5p;hsa-miR-30
ENSG0000	ENSG0000	0.004042	hsa-miR-125a-5p;hsa-miR-125b-5p;hsa-miR-26a-5p;hsa-miR-
ENSG0000	ENSG0000	7.76E-06	hsa-miR-125b-5p;hsa-miR-144-3p;hsa-miR-30a-5p;hsa-miR-3
ENSG0000	ENSG0000	4.04E-11	hsa-miR-186-5p;hsa-miR-21-3p;hsa-miR-26a-5p;hsa-miR-26b
ENSG0000	ENSG0000	0.004042	hsa-miR-125a-5p;hsa-miR-125b-5p;hsa-miR-144-3p;hsa-miR-
ENSG0000	ENSG0000	1.09E-05	hsa-miR-101-3p;hsa-miR-125a-5p;hsa-miR-125b-5p;hsa-miR-
ENSG0000	ENSG0000	0.000631	hsa-miR-16-1-3p;hsa-miR-19a-3p;hsa-miR-19b-3p;hsa-miR-2
ENSG0000	ENSG0000	0.009008	hsa-miR-1304-3p;hsa-miR-204-5p;hsa-miR-26a-5p;hsa-miR-2
ENSG0000	ENSG0000	0.000355	hsa-miR-185-3p;hsa-miR-30a-5p;hsa-miR-30b-5p;hsa-miR-30
ENSG0000	ENSG0000	3.30E-05	hsa-miR-101-3p;hsa-miR-30b-5p;hsa-miR-30c-5p;hsa-miR-30
ENSG0000	ENSG0000	0.000165	hsa-miR-101-3p;hsa-miR-144-3p;hsa-miR-204-5p;hsa-miR-29
ENSG0000	ENSG0000	9.26E-06	hsa-miR-193a-3p;hsa-miR-193b-3p;hsa-miR-30a-5p;hsa-miR-
ENSG0000	ENSG0000	3.85E-05	hsa-miR-204-5p;hsa-miR-30a-5p;hsa-miR-30c-5p;hsa-miR-30
ENSG0000	ENSG0000	0.006456	hsa-miR-30a-5p;hsa-miR-30b-5p;hsa-miR-30c-5p;hsa-miR-30
ENSG0000	ENSG0000	1.30E-05	hsa-miR-1276;hsa-miR-30a-5p;hsa-miR-30b-5p;hsa-miR-30c-
ENSG0000	ENSG0000	0.001211	hsa-miR-193a-3p;hsa-miR-193b-3p;hsa-miR-30a-5p;hsa-miR-
ENSG0000	ENSG0000	0.000867	hsa-miR-101-3p;hsa-miR-30a-5p;hsa-miR-30b-5p;hsa-miR-30
ENSG0000	ENSG0000	1.05E-05	hsa-miR-1276;hsa-miR-1914-3p;hsa-miR-30a-5p;hsa-miR-30b
ENSG0000	ENSG0000	0.007282	hsa-miR-30a-5p;hsa-miR-30b-5p;hsa-miR-30c-5p;hsa-miR-30
ENSG0000	ENSG0000	6.10E-11	hsa-miR-1276;hsa-miR-1305;hsa-miR-30a-3p;hsa-miR-30a-5p;
ENSG0000	ENSG0000	0.002953	hsa-miR-30a-5p;hsa-miR-30b-5p;hsa-miR-30c-5p;hsa-miR-30
ENSG0000	ENSG0000	5.23E-05	hsa-miR-30a-5p;hsa-miR-30b-5p;hsa-miR-30c-5p;hsa-miR-30
ENSG0000	ENSG0000	0.005713	hsa-miR-144-3p;hsa-miR-16-1-3p;hsa-miR-548o-3p;hsa-miR-
ENSG0000	ENSG0000	0.005908	hsa-miR-101-3p;hsa-miR-1305;hsa-miR-19a-3p;hsa-miR-19b-
ENSG0000	ENSG0000	0.000496	hsa-miR-30a-5p;hsa-miR-30b-5p;hsa-miR-30c-5p;hsa-miR-30
ENSG0000	ENSG0000	0.001086	hsa-miR-193a-3p;hsa-miR-30a-5p;hsa-miR-30b-5p;hsa-miR-3
ENSG0000	ENSG0000	0.008927	hsa-miR-17-3p;hsa-miR-18a-5p;hsa-miR-193a-3p;hsa-miR-19
ENSG0000	ENSG0000	0.008927	hsa-miR-17-3p;hsa-miR-18a-5p;hsa-miR-193a-3p;hsa-miR-19
ENSG0000	ENSG0000	1.79E-06	hsa-miR-101-3p;hsa-miR-144-3p;hsa-miR-204-5p;hsa-miR-26
ENSG0000	ENSG0000	0.008072	hsa-miR-125a-5p;hsa-miR-125b-5p;hsa-miR-19a-3p;hsa-miR-
ENSG0000	ENSG0000	0.00644	hsa-miR-378a-3p;hsa-miR-378c;hsa-miR-378d;hsa-miR-378i
ENSG0000	ENSG0000	0.000117	hsa-miR-30a-5p;hsa-miR-30b-5p;hsa-miR-30c-5p;hsa-miR-30
ENSG0000	ENSG0000	9.14E-07	hsa-miR-30a-5p;hsa-miR-30b-5p;hsa-miR-30c-5p;hsa-miR-30
ENSG0000	ENSG0000	0.001805	hsa-miR-185-3p;hsa-miR-30a-5p;hsa-miR-30b-5p;hsa-miR-30
ENSG0000	ENSG0000	4.14E-07	hsa-miR-30a-5p;hsa-miR-30b-5p;hsa-miR-30c-5p;hsa-miR-30
ENSG0000	ENSG0000	0.002561	hsa-miR-1304-3p;hsa-miR-30a-5p;hsa-miR-30b-5p;hsa-miR-3
ENSG0000	ENSG0000	0.005713	hsa-miR-30b-5p;hsa-miR-30c-5p;hsa-miR-30d-5p;hsa-miR-30
ENSG0000	ENSG0000	2.68E-07	hsa-miR-204-5p;hsa-miR-30a-5p;hsa-miR-30b-5p;hsa-miR-30
ENSG0000	ENSG0000	0.000492	hsa-miR-204-5p;hsa-miR-30a-3p;hsa-miR-30d-3p;hsa-miR-30
ENSG0000	ENSG0000	1.94E-06	hsa-miR-18a-5p;hsa-miR-296-5p;hsa-miR-30a-5p;hsa-miR-30
ENSG0000	ENSG0000	1.87E-09	hsa-miR-16-1-3p;hsa-miR-222-5p;hsa-miR-30a-3p;hsa-miR-3
ENSG0000	ENSG0000	0.005394	hsa-miR-1914-3p;hsa-miR-30a-3p;hsa-miR-30d-3p;hsa-miR-3

ENSG0000	ENSG0000	4.61E-08	hsa-miR-30a-5p;hsa-miR-30b-5p;hsa-miR-30c-5p;hsa-miR-30
ENSG0000	ENSG0000	0.004362	hsa-miR-101-3p;hsa-miR-144-3p;hsa-miR-26a-5p;hsa-miR-26
ENSG0000	ENSG0000	4.14E-07	hsa-miR-30a-5p;hsa-miR-30b-5p;hsa-miR-30c-5p;hsa-miR-30
ENSG0000	ENSG0000	1.56E-05	hsa-miR-1976;hsa-miR-222-5p;hsa-miR-30a-5p;hsa-miR-30b-
ENSG0000	ENSG0000	0.00081	hsa-miR-193a-3p;hsa-miR-378a-3p;hsa-miR-378c;hsa-miR-37
ENSG0000	ENSG0000	0.002886	hsa-miR-101-3p;hsa-miR-18a-5p;hsa-miR-19a-3p;hsa-miR-19
ENSG0000	ENSG0000	0.005896	hsa-miR-125a-5p;hsa-miR-125b-5p;hsa-miR-141-5p;hsa-miR-
ENSG0000	ENSG0000	8.71E-05	hsa-miR-144-3p;hsa-miR-16-1-3p;hsa-miR-30a-5p;hsa-miR-3
ENSG0000	ENSG0000	0.002101	hsa-miR-101-3p;hsa-miR-144-3p;hsa-miR-19a-3p;hsa-miR-19
ENSG0000	ENSG0000	0.003094	hsa-miR-19a-3p;hsa-miR-19b-3p;hsa-miR-30a-5p;hsa-miR-30
ENSG0000	ENSG0000	0.000629	hsa-miR-10a-3p;hsa-miR-1305;hsa-miR-17-3p;hsa-miR-26a-5
ENSG0000	ENSG0000	0.005253	hsa-miR-125a-5p;hsa-miR-140-5p;hsa-miR-16-1-3p;hsa-miR-
ENSG0000	ENSG0000	0.000217	hsa-miR-1304-3p;hsa-miR-186-5p;hsa-miR-193a-3p;hsa-miR-
ENSG0000	ENSG0000	0.002893	hsa-miR-30a-3p;hsa-miR-30d-3p;hsa-miR-30e-3p;hsa-miR-76
ENSG0000	ENSG0000	0.002101	hsa-miR-1276;hsa-miR-30a-3p;hsa-miR-30d-3p;hsa-miR-30e-
ENSG0000	ENSG0000	0.00515	hsa-miR-1276;hsa-miR-17-3p;hsa-miR-193a-3p;hsa-miR-193b-
ENSG0000	ENSG0000	0.00082	hsa-miR-19a-3p;hsa-miR-19b-3p;hsa-miR-26a-5p;hsa-miR-26
ENSG0000	ENSG0000	0.000942	hsa-miR-30a-5p;hsa-miR-30b-5p;hsa-miR-30c-5p;hsa-miR-30
ENSG0000	ENSG0000	9.77E-06	hsa-miR-19a-3p;hsa-miR-30a-5p;hsa-miR-30b-5p;hsa-miR-30
ENSG0000	ENSG0000	4.50E-05	hsa-miR-10a-3p;hsa-miR-125b-5p;hsa-miR-16-1-3p;hsa-miR-
ENSG0000	ENSG0000	0.003479	hsa-miR-185-3p;hsa-miR-186-5p;hsa-miR-28-5p;hsa-miR-296-
ENSG0000	ENSG0000	1.55E-06	hsa-miR-17-3p;hsa-miR-18a-5p;hsa-miR-19b-3p;hsa-miR-30a-
ENSG0000	ENSG0000	1.94E-05	hsa-miR-17-3p;hsa-miR-30b-5p;hsa-miR-30c-5p;hsa-miR-30d-
ENSG0000	ENSG0000	0.002094	hsa-miR-19a-3p;hsa-miR-19b-3p;hsa-miR-30a-5p;hsa-miR-30
ENSG0000	ENSG0000	2.43E-05	hsa-miR-101-3p;hsa-miR-144-3p;hsa-miR-17-3p;hsa-miR-306
ENSG0000	ENSG0000	0.000204	hsa-miR-30a-3p;hsa-miR-30a-5p;hsa-miR-30c-5p;hsa-miR-30
ENSG0000	ENSG0000	1.70E-05	hsa-miR-101-3p;hsa-miR-30a-5p;hsa-miR-30b-5p;hsa-miR-30
ENSG0000	ENSG0000	1.30E-05	hsa-miR-140-5p;hsa-miR-30a-5p;hsa-miR-30b-5p;hsa-miR-30
ENSG0000	ENSG0000	1.30E-05	hsa-miR-140-5p;hsa-miR-30a-5p;hsa-miR-30b-5p;hsa-miR-30
ENSG0000	ENSG0000	1.32E-05	hsa-miR-30b-5p;hsa-miR-30c-5p;hsa-miR-30d-5p;hsa-miR-30
ENSG0000	ENSG0000	0.001836	hsa-miR-125a-5p;hsa-miR-125b-5p;hsa-miR-19a-3p;hsa-miR-
ENSG0000	ENSG0000	9.14E-07	hsa-miR-30a-5p;hsa-miR-30b-5p;hsa-miR-30c-5p;hsa-miR-30
ENSG0000	ENSG0000	0.002857	hsa-miR-186-5p;hsa-miR-30a-5p;hsa-miR-30b-5p;hsa-miR-30
ENSG0000	ENSG0000	1.94E-05	hsa-miR-30a-5p;hsa-miR-30b-5p;hsa-miR-30c-5p;hsa-miR-30
ENSG0000	ENSG0000	0.000345	hsa-miR-19b-3p;hsa-miR-30a-5p;hsa-miR-30b-5p;hsa-miR-30
ENSG0000	ENSG0000	3.55E-07	hsa-miR-1276;hsa-miR-30a-5p;hsa-miR-30b-5p;hsa-miR-30c-
ENSG0000	ENSG0000	7.84E-09	hsa-miR-30a-5p;hsa-miR-30b-5p;hsa-miR-30c-5p;hsa-miR-30
ENSG0000	ENSG0000	0.000867	hsa-miR-30a-5p;hsa-miR-30b-5p;hsa-miR-30c-5p;hsa-miR-30
ENSG0000	ENSG0000	0.002655	hsa-miR-1304-3p;hsa-miR-141-5p;hsa-miR-378a-3p;hsa-miR-
ENSG0000	ENSG0000	0.000496	hsa-miR-30a-5p;hsa-miR-30b-5p;hsa-miR-30c-5p;hsa-miR-30
ENSG0000	ENSG0000	6.35E-05	hsa-miR-30b-5p;hsa-miR-30c-5p;hsa-miR-30d-5p;hsa-miR-30
ENSG0000	ENSG0000	0.001434	hsa-miR-30a-5p;hsa-miR-30b-5p;hsa-miR-30c-5p;hsa-miR-30
ENSG0000	ENSG0000	0.008072	hsa-miR-378a-3p;hsa-miR-378c;hsa-miR-378d;hsa-miR-378i
ENSG0000	ENSG0000	4.59E-07	hsa-miR-30a-5p;hsa-miR-30b-5p;hsa-miR-30c-5p;hsa-miR-30
ENSG0000	ENSG0000	0.003146	hsa-miR-1976;hsa-miR-222-5p;hsa-miR-30a-3p;hsa-miR-30d-
ENSG0000	ENSG0000	0.002475	hsa-miR-378a-3p;hsa-miR-378c;hsa-miR-378d;hsa-miR-378i
ENSG0000	ENSG0000	8.70E-06	hsa-miR-204-5p;hsa-miR-30a-3p;hsa-miR-30a-5p;hsa-miR-30
ENSG0000	ENSG0000	3.85E-05	hsa-miR-101-3p;hsa-miR-144-3p;hsa-miR-545-5p;hsa-miR-58
ENSG0000	ENSG0000	7.84E-09	hsa-miR-30a-5p;hsa-miR-30b-5p;hsa-miR-30c-5p;hsa-miR-30
ENSG0000	ENSG0000	0.005713	hsa-miR-26a-5p;hsa-miR-26b-5p;hsa-miR-582-5p;hsa-miR-66
ENSG0000	ENSG0000	0.000376	hsa-miR-30a-5p;hsa-miR-30b-5p;hsa-miR-30d-5p;hsa-miR-30
ENSG0000	ENSG0000	0.000492	hsa-miR-125a-5p;hsa-miR-140-5p;hsa-miR-19a-3p;hsa-miR-1
ENSG0000	ENSG0000	0.000615	hsa-miR-101-3p;hsa-miR-10a-3p;hsa-miR-140-5p;hsa-miR-14
ENSG0000	ENSG0000	0.003356	hsa-miR-1304-3p;hsa-miR-26a-5p;hsa-miR-26b-5p;hsa-miR-6

ENSG0000	ENSG0000	0.002101	hsa-miR-101-3p;hsa-miR-140-5p;hsa-miR-144-3p;hsa-miR-58
ENSG0000	ENSG0000	0.000942	hsa-miR-30a-5p;hsa-miR-30b-5p;hsa-miR-30c-5p;hsa-miR-30
ENSG0000	ENSG0000	0.002094	hsa-miR-30a-5p;hsa-miR-30b-5p;hsa-miR-30c-5p;hsa-miR-30
ENSG0000	ENSG0000	0.000112	hsa-miR-101-3p;hsa-miR-140-5p;hsa-miR-144-3p;hsa-miR-19
ENSG0000	ENSG0000	0.000152	hsa-miR-19a-3p;hsa-miR-19b-3p;hsa-miR-342-3p;hsa-miR-37
ENSG0000	ENSG0000	0.007039	hsa-miR-30a-5p;hsa-miR-30b-5p;hsa-miR-30c-5p;hsa-miR-30
ENSG0000	ENSG0000	0.000692	hsa-miR-1914-3p;hsa-miR-30a-3p;hsa-miR-30d-3p;hsa-miR-3
ENSG0000	ENSG0000	9.66E-07	hsa-miR-1305;hsa-miR-30a-5p;hsa-miR-30b-5p;hsa-miR-30c-
ENSG0000	ENSG0000	0.005086	hsa-miR-30a-5p;hsa-miR-30b-5p;hsa-miR-30c-5p;hsa-miR-30
ENSG0000	ENSG0000	0.007793	hsa-miR-1243;hsa-miR-19a-3p;hsa-miR-19b-3p;hsa-miR-342-
ENSG0000	ENSG0000	4.14E-07	hsa-miR-30a-5p;hsa-miR-30b-5p;hsa-miR-30c-5p;hsa-miR-30
ENSG0000	ENSG0000	6.65E-05	hsa-miR-19a-3p;hsa-miR-19b-3p;hsa-miR-26a-5p;hsa-miR-26
ENSG0000	ENSG0000	7.50E-06	hsa-miR-10a-3p;hsa-miR-1243;hsa-miR-1276;hsa-miR-19a-3p;
ENSG0000	ENSG0000	0.000744	hsa-miR-1304-3p;hsa-miR-186-5p;hsa-miR-1976;hsa-miR-26a-
ENSG0000	ENSG0000	7.25E-06	hsa-miR-125a-5p;hsa-miR-125b-5p;hsa-miR-19a-3p;hsa-miR-
ENSG0000	ENSG0000	0.007658	hsa-miR-1304-3p;hsa-miR-3677-5p;hsa-miR-499a-3p;hsa-miR-
ENSG0000	ENSG0000	0.006475	hsa-miR-193a-3p;hsa-miR-193b-3p;hsa-miR-21-3p;hsa-miR-3
ENSG0000	ENSG0000	0.001125	hsa-miR-1976;hsa-miR-19a-3p;hsa-miR-19b-3p;hsa-miR-26a-
ENSG0000	ENSG0000	0.000116	hsa-miR-1305;hsa-miR-186-5p;hsa-miR-26a-5p;hsa-miR-26b-
ENSG0000	ENSG0000	0.000415	hsa-miR-30a-5p;hsa-miR-30b-5p;hsa-miR-30c-5p;hsa-miR-30
ENSG0000	ENSG0000	0.000307	hsa-miR-204-5p;hsa-miR-30a-5p;hsa-miR-30b-5p;hsa-miR-30
ENSG0000	ENSG0000	0.002101	hsa-miR-19a-3p;hsa-miR-19b-3p;hsa-miR-26a-5p;hsa-miR-26
ENSG0000	ENSG0000	0.000116	hsa-miR-1914-3p;hsa-miR-204-5p;hsa-miR-30a-5p;hsa-miR-3
ENSG0000	ENSG0000	0.009955	hsa-miR-1304-3p;hsa-miR-19a-3p;hsa-miR-19b-3p;hsa-miR-5
ENSG0000	ENSG0000	0.001018	hsa-miR-1304-3p;hsa-miR-3065-3p;hsa-miR-30a-5p;hsa-miR-
ENSG0000	ENSG0000	0.001018	hsa-miR-1304-3p;hsa-miR-3065-3p;hsa-miR-30a-5p;hsa-miR-
ENSG0000	ENSG0000	0.006456	hsa-miR-1305;hsa-miR-28-5p;hsa-miR-342-3p;hsa-miR-708-5
ENSG0000	ENSG0000	0.001089	hsa-miR-30a-5p;hsa-miR-30b-5p;hsa-miR-30c-5p;hsa-miR-30
ENSG0000	ENSG0000	9.12E-05	hsa-miR-30a-5p;hsa-miR-30b-5p;hsa-miR-30c-5p;hsa-miR-30
ENSG0000	ENSG0000	0.002284	hsa-miR-1243;hsa-miR-1914-3p;hsa-miR-378a-3p;hsa-miR-37
ENSG0000	ENSG0000	0.002643	hsa-miR-1276;hsa-miR-1914-3p;hsa-miR-222-5p;hsa-miR-342-
ENSG0000	ENSG0000	0.002643	hsa-miR-1276;hsa-miR-1914-3p;hsa-miR-222-5p;hsa-miR-342-
ENSG0000	ENSG0000	0.009481	hsa-miR-30a-5p;hsa-miR-30b-5p;hsa-miR-30c-5p;hsa-miR-30
ENSG0000	ENSG0000	0.005947	hsa-miR-1304-3p;hsa-miR-1914-3p;hsa-miR-1976;hsa-miR-26
ENSG0000	ENSG0000	1.58E-07	hsa-miR-30a-5p;hsa-miR-30b-5p;hsa-miR-30c-5p;hsa-miR-30
ENSG0000	ENSG0000	0.000223	hsa-miR-30a-5p;hsa-miR-30b-5p;hsa-miR-30c-5p;hsa-miR-30
ENSG0000	ENSG0000	0.000524	hsa-miR-140-5p;hsa-miR-1914-3p;hsa-miR-26a-5p;hsa-miR-2
ENSG0000	ENSG0000	0.000232	hsa-miR-144-3p;hsa-miR-338-3p;hsa-miR-545-5p;hsa-miR-58
ENSG0000	ENSG0000	2.20E-05	hsa-miR-30a-3p;hsa-miR-30d-3p;hsa-miR-30e-3p;hsa-miR-54
ENSG0000	ENSG0000	0.000972	hsa-miR-193a-3p;hsa-miR-193b-3p;hsa-miR-30a-5p;hsa-miR-
ENSG0000	ENSG0000	1.58E-07	hsa-miR-30a-5p;hsa-miR-30b-5p;hsa-miR-30c-5p;hsa-miR-30
ENSG0000	ENSG0000	0.001597	hsa-miR-141-5p;hsa-miR-186-5p;hsa-miR-18a-5p;hsa-miR-19
ENSG0000	ENSG0000	0.000284	hsa-miR-30a-3p;hsa-miR-30d-3p;hsa-miR-30e-3p;hsa-miR-48
ENSG0000	ENSG0000	3.85E-05	hsa-miR-30a-3p;hsa-miR-30d-3p;hsa-miR-30e-3p;hsa-miR-34
ENSG0000	ENSG0000	0.004429	hsa-miR-378a-3p;hsa-miR-378c;hsa-miR-378d;hsa-miR-378i
ENSG0000	ENSG0000	3.85E-05	hsa-miR-30a-5p;hsa-miR-30b-5p;hsa-miR-30c-5p;hsa-miR-30
ENSG0000	ENSG0000	0.000165	hsa-miR-144-3p;hsa-miR-17-3p;hsa-miR-30a-3p;hsa-miR-30d-
ENSG0000	ENSG0000	0.000165	hsa-miR-144-3p;hsa-miR-17-3p;hsa-miR-30a-3p;hsa-miR-30d-
ENSG0000	ENSG0000	7.25E-06	hsa-miR-26a-5p;hsa-miR-30a-5p;hsa-miR-30b-5p;hsa-miR-30
ENSG0000	ENSG0000	0.000358	hsa-miR-10a-3p;hsa-miR-1304-3p;hsa-miR-1976;hsa-miR-222-
ENSG0000	ENSG0000	0.003867	hsa-miR-30a-3p;hsa-miR-30d-3p;hsa-miR-30e-3p;hsa-miR-36
ENSG0000	ENSG0000	1.32E-05	hsa-miR-30a-5p;hsa-miR-30b-5p;hsa-miR-30c-5p;hsa-miR-30
ENSG0000	ENSG0000	0.007039	hsa-miR-19a-3p;hsa-miR-19b-3p;hsa-miR-30a-3p;hsa-miR-30
ENSG0000	ENSG0000	0.000265	hsa-miR-204-5p;hsa-miR-30a-5p;hsa-miR-30b-5p;hsa-miR-30

ENSG0000	ENSG0000	0.002094	hsa-miR-19a-3p;hsa-miR-19b-3p;hsa-miR-28-5p;hsa-miR-423-
ENSG0000	ENSG0000	5.37E-06	hsa-miR-10a-3p;hsa-miR-16-1-3p;hsa-miR-186-5p;hsa-miR-22
ENSG0000	ENSG0000	0.008981	hsa-miR-125b-5p;hsa-miR-1276;hsa-miR-28-5p;hsa-miR-708-5
ENSG0000	ENSG0000	0.006334	hsa-miR-19b-3p;hsa-miR-30a-5p;hsa-miR-30b-5p;hsa-miR-30
ENSG0000	ENSG0000	4.59E-07	hsa-miR-30a-5p;hsa-miR-30b-5p;hsa-miR-30c-5p;hsa-miR-30
ENSG0000	ENSG0000	4.96E-10	hsa-miR-193a-3p;hsa-miR-193b-3p;hsa-miR-30a-5p;hsa-miR-3
ENSG0000	ENSG0000	1.27E-05	hsa-miR-26a-5p;hsa-miR-26b-5p;hsa-miR-30a-5p;hsa-miR-30
ENSG0000	ENSG0000	0.000692	hsa-miR-222-5p;hsa-miR-30a-3p;hsa-miR-30d-3p;hsa-miR-30
ENSG0000	ENSG0000	2.20E-05	hsa-miR-101-3p;hsa-miR-144-3p;hsa-miR-499a-3p;hsa-miR-58
ENSG0000	ENSG0000	2.09E-05	hsa-miR-18a-5p;hsa-miR-1976;hsa-miR-30a-3p;hsa-miR-30d-3
ENSG0000	ENSG0000	9.77E-06	hsa-miR-30a-5p;hsa-miR-30b-5p;hsa-miR-30c-5p;hsa-miR-30
ENSG0000	ENSG0000	0.003652	hsa-miR-101-3p;hsa-miR-1304-3p;hsa-miR-144-3p;hsa-miR-54
ENSG0000	ENSG0000	6.60E-06	hsa-miR-186-5p;hsa-miR-19a-3p;hsa-miR-19b-3p;hsa-miR-30
ENSG0000	ENSG0000	6.40E-05	hsa-miR-1304-3p;hsa-miR-222-5p;hsa-miR-26a-5p;hsa-miR-26
ENSG0000	ENSG0000	0.008072	hsa-miR-378a-3p;hsa-miR-378c;hsa-miR-378d;hsa-miR-378i
ENSG0000	ENSG0000	8.22E-05	hsa-miR-101-3p;hsa-miR-144-3p;hsa-miR-30a-5p;hsa-miR-30
ENSG0000	ENSG0000	9.12E-05	hsa-miR-30a-5p;hsa-miR-30b-5p;hsa-miR-30c-5p;hsa-miR-30
ENSG0000	ENSG0000	0.002953	hsa-miR-378a-3p;hsa-miR-378c;hsa-miR-378d;hsa-miR-378i;hs
ENSG0000	ENSG0000	0.002101	hsa-miR-30b-5p;hsa-miR-30c-5p;hsa-miR-30d-5p;hsa-miR-30
ENSG0000	ENSG0000	2.21E-08	hsa-miR-144-3p;hsa-miR-16-1-3p;hsa-miR-30a-5p;hsa-miR-30
ENSG0000	ENSG0000	5.43E-05	hsa-miR-30a-5p;hsa-miR-30b-5p;hsa-miR-30c-5p;hsa-miR-30
ENSG0000	ENSG0000	0.000152	hsa-miR-1976;hsa-miR-19a-3p;hsa-miR-19b-3p;hsa-miR-26a-5
ENSG0000	ENSG0000	0.001216	hsa-miR-125a-5p;hsa-miR-125b-5p;hsa-miR-1976;hsa-miR-660
ENSG0000	ENSG0000	0.003717	hsa-miR-1976;hsa-miR-222-5p;hsa-miR-342-3p;hsa-miR-423-5
ENSG0000	ENSG0000	0.008891	hsa-miR-1305;hsa-miR-222-5p;hsa-miR-26a-5p;hsa-miR-26b-5
ENSG0000	ENSG0000	0.001634	hsa-miR-19a-3p;hsa-miR-19b-3p;hsa-miR-26a-5p;hsa-miR-26
ENSG0000	ENSG0000	0.009429	hsa-miR-1243;hsa-miR-186-5p;hsa-miR-1976;hsa-miR-342-3p;
ENSG0000	ENSG0000	0.001634	hsa-miR-141-5p;hsa-miR-30a-3p;hsa-miR-30d-3p;hsa-miR-30
ENSG0000	ENSG0000	0.009955	hsa-miR-1304-3p;hsa-miR-1976;hsa-miR-19a-3p;hsa-miR-19b-
ENSG0000	ENSG0000	0.000684	hsa-miR-19a-3p;hsa-miR-26a-5p;hsa-miR-26b-5p;hsa-miR-30
ENSG0000	ENSG0000	0.008072	hsa-miR-1976;hsa-miR-30a-3p;hsa-miR-30d-3p;hsa-miR-30e-3
ENSG0000	ENSG0000	0.007039	hsa-miR-101-3p;hsa-miR-144-3p;hsa-miR-17-3p;hsa-miR-19a-
ENSG0000	ENSG0000	0.009008	hsa-miR-1976;hsa-miR-3065-3p;hsa-miR-3619-5p;hsa-miR-548
ENSG0000	ENSG0000	6.66E-05	hsa-miR-21-3p;hsa-miR-30a-5p;hsa-miR-30b-5p;hsa-miR-30c-
ENSG0000	ENSG0000	0.000197	hsa-miR-1305;hsa-miR-141-5p;hsa-miR-144-3p;hsa-miR-193a-
ENSG0000	ENSG0000	0.000197	hsa-miR-1305;hsa-miR-141-5p;hsa-miR-144-3p;hsa-miR-193a-
ENSG0000	ENSG0000	0.007039	hsa-miR-125a-5p;hsa-miR-125b-5p;hsa-miR-19a-3p;hsa-miR-2
ENSG0000	ENSG0000	0.005394	hsa-miR-101-3p;hsa-miR-144-3p;hsa-miR-378a-5p;hsa-miR-54
ENSG0000	ENSG0000	7.84E-09	hsa-miR-30a-5p;hsa-miR-30b-5p;hsa-miR-30c-5p;hsa-miR-30
ENSG0000	ENSG0000	0.004051	hsa-miR-19a-3p;hsa-miR-19b-3p;hsa-miR-28-5p;hsa-miR-548
ENSG0000	ENSG0000	8.65E-06	hsa-miR-30a-5p;hsa-miR-30b-5p;hsa-miR-30c-5p;hsa-miR-30
ENSG0000	ENSG0000	0.000376	hsa-miR-30b-5p;hsa-miR-30c-5p;hsa-miR-30d-5p;hsa-miR-30
ENSG0000	ENSG0000	0.005519	hsa-miR-141-5p;hsa-miR-204-5p;hsa-miR-30a-5p;hsa-miR-30
ENSG0000	ENSG0000	3.23E-06	hsa-miR-30a-5p;hsa-miR-30b-5p;hsa-miR-30c-5p;hsa-miR-30
ENSG0000	ENSG0000	0.002475	hsa-miR-1304-3p;hsa-miR-1305;hsa-miR-26a-5p;hsa-miR-26b-
ENSG0000	ENSG0000	5.46E-05	hsa-miR-30a-5p;hsa-miR-30b-5p;hsa-miR-30c-5p;hsa-miR-30
ENSG0000	ENSG0000	0.00644	hsa-miR-378a-3p;hsa-miR-378c;hsa-miR-378d;hsa-miR-378i
ENSG0000	ENSG0000	0.001082	hsa-miR-1976;hsa-miR-30a-5p;hsa-miR-30b-5p;hsa-miR-30c-5
ENSG0000	ENSG0000	0.001434	hsa-miR-1305;hsa-miR-19a-3p;hsa-miR-19b-3p;hsa-miR-26a-5
ENSG0000	ENSG0000	0.000187	hsa-miR-30a-5p;hsa-miR-30b-5p;hsa-miR-30c-5p;hsa-miR-30
ENSG0000	ENSG0000	9.79E-05	hsa-miR-30a-3p;hsa-miR-30d-3p;hsa-miR-30e-3p;hsa-miR-64
ENSG0000	ENSG0000	0.009481	hsa-miR-30a-5p;hsa-miR-30b-5p;hsa-miR-30c-5p;hsa-miR-30
ENSG0000	ENSG0000	1.94E-05	hsa-miR-30a-5p;hsa-miR-30b-5p;hsa-miR-30c-5p;hsa-miR-30
ENSG0000	ENSG0000	1.94E-05	hsa-miR-30a-5p;hsa-miR-30b-5p;hsa-miR-30c-5p;hsa-miR-30

ENSG0000	ENSG0000	5.10E-07	hsa-miR-19a-3p;hsa-miR-19b-3p;hsa-miR-30a-5p;hsa-miR-30
ENSG0000	ENSG0000	5.10E-07	hsa-miR-19a-3p;hsa-miR-19b-3p;hsa-miR-30a-5p;hsa-miR-30
ENSG0000	ENSG0000	0.00403	hsa-miR-19a-3p;hsa-miR-19b-3p;hsa-miR-30a-5p;hsa-miR-30
ENSG0000	ENSG0000	0.001089	hsa-miR-1304-3p;hsa-miR-1976;hsa-miR-26a-5p;hsa-miR-26b-
ENSG0000	ENSG0000	4.61E-08	hsa-miR-30a-5p;hsa-miR-30b-5p;hsa-miR-30c-5p;hsa-miR-30
ENSG0000	ENSG0000	0.001853	hsa-miR-30a-5p;hsa-miR-30b-5p;hsa-miR-30c-5p;hsa-miR-30
ENSG0000	ENSG0000	0.000281	hsa-miR-378a-3p;hsa-miR-378c;hsa-miR-378d;hsa-miR-378i
ENSG0000	ENSG0000	0.008314	hsa-miR-1304-3p;hsa-miR-193a-3p;hsa-miR-1976;hsa-miR-20
ENSG0000	ENSG0000	0.009741	hsa-miR-125a-5p;hsa-miR-125b-5p;hsa-miR-28-5p;hsa-miR-4
ENSG0000	ENSG0000	0.000415	hsa-miR-30a-5p;hsa-miR-30b-5p;hsa-miR-30c-5p;hsa-miR-30
ENSG0000	ENSG0000	0.001216	hsa-miR-30a-5p;hsa-miR-30b-5p;hsa-miR-30d-5p;hsa-miR-30
ENSG0000	ENSG0000	0.005394	hsa-miR-30a-5p;hsa-miR-30b-5p;hsa-miR-30c-5p;hsa-miR-30
ENSG0000	ENSG0000	1.03E-07	hsa-miR-19a-3p;hsa-miR-19b-3p;hsa-miR-30a-5p;hsa-miR-30
ENSG0000	ENSG0000	5.43E-05	hsa-miR-144-3p;hsa-miR-204-5p;hsa-miR-30a-3p;hsa-miR-30
ENSG0000	ENSG0000	4.07E-06	hsa-miR-30a-5p;hsa-miR-30b-5p;hsa-miR-30c-5p;hsa-miR-30
ENSG0000	ENSG0000	0.000307	hsa-miR-185-3p;hsa-miR-19a-3p;hsa-miR-30a-5p;hsa-miR-30
ENSG0000	ENSG0000	0.002357	hsa-miR-30a-5p;hsa-miR-30b-5p;hsa-miR-30c-5p;hsa-miR-30
ENSG0000	ENSG0000	7.84E-09	hsa-miR-30a-5p;hsa-miR-30b-5p;hsa-miR-30c-5p;hsa-miR-30
ENSG0000	ENSG0000	0.000797	hsa-miR-193a-3p;hsa-miR-193b-3p;hsa-miR-1976;hsa-miR-66
ENSG0000	ENSG0000	0.000797	hsa-miR-193a-3p;hsa-miR-193b-3p;hsa-miR-1976;hsa-miR-66
ENSG0000	ENSG0000	0.000797	hsa-miR-193a-3p;hsa-miR-193b-3p;hsa-miR-1976;hsa-miR-66
ENSG0000	ENSG0000	0.000797	hsa-miR-193a-3p;hsa-miR-193b-3p;hsa-miR-1976;hsa-miR-66
ENSG0000	ENSG0000	0.000797	hsa-miR-193a-3p;hsa-miR-193b-3p;hsa-miR-1976;hsa-miR-66
ENSG0000	ENSG0000	0.000797	hsa-miR-193a-3p;hsa-miR-193b-3p;hsa-miR-1976;hsa-miR-66
ENSG0000	ENSG0000	0.000797	hsa-miR-193a-3p;hsa-miR-193b-3p;hsa-miR-1976;hsa-miR-66
ENSG0000	ENSG0000	0.000376	hsa-miR-101-3p;hsa-miR-144-3p;hsa-miR-21-3p;hsa-miR-582-
ENSG0000	ENSG0000	0.002953	hsa-miR-19a-3p;hsa-miR-30b-5p;hsa-miR-30c-5p;hsa-miR-30
ENSG0000	ENSG0000	0.004462	hsa-miR-1914-3p;hsa-miR-193a-3p;hsa-miR-193b-3p;hsa-miR
ENSG0000	ENSG0000	3.15E-06	hsa-miR-30a-5p;hsa-miR-30b-5p;hsa-miR-30c-5p;hsa-miR-30
ENSG0000	ENSG0000	0.003356	hsa-miR-378a-3p;hsa-miR-378c;hsa-miR-378d;hsa-miR-378i
ENSG0000	ENSG0000	0.000942	hsa-miR-16-1-3p;hsa-miR-222-5p;hsa-miR-30a-3p;hsa-miR-3
ENSG0000	ENSG0000	0.001253	hsa-miR-30a-5p;hsa-miR-30b-5p;hsa-miR-30c-5p;hsa-miR-30
ENSG0000	ENSG0000	6.39E-05	hsa-miR-144-3p;hsa-miR-16-1-3p;hsa-miR-1976;hsa-miR-30a-
ENSG0000	ENSG0000	0.001089	hsa-miR-1243;hsa-miR-141-5p;hsa-miR-193a-3p;hsa-miR-193
ENSG0000	ENSG0000	3.75E-05	hsa-miR-19a-3p;hsa-miR-19b-3p;hsa-miR-28-5p;hsa-miR-296-
ENSG0000	ENSG0000	0.000951	hsa-miR-30a-5p;hsa-miR-30b-5p;hsa-miR-30c-5p;hsa-miR-30
ENSG0000	ENSG0000	3.77E-06	hsa-miR-204-5p;hsa-miR-30a-5p;hsa-miR-30b-5p;hsa-miR-30
ENSG0000	ENSG0000	1.30E-05	hsa-miR-193a-3p;hsa-miR-30a-5p;hsa-miR-30b-5p;hsa-miR-3
ENSG0000	ENSG0000	1.30E-05	hsa-miR-193a-3p;hsa-miR-30a-5p;hsa-miR-30b-5p;hsa-miR-3
ENSG0000	ENSG0000	0.000675	hsa-miR-30a-5p;hsa-miR-30b-5p;hsa-miR-30c-5p;hsa-miR-30
ENSG0000	ENSG0000	1.94E-05	hsa-miR-30a-5p;hsa-miR-30b-5p;hsa-miR-30c-5p;hsa-miR-30
ENSG0000	ENSG0000	0.000149	hsa-miR-30a-5p;hsa-miR-30b-5p;hsa-miR-30c-5p;hsa-miR-30
ENSG0000	ENSG0000	0.002857	hsa-miR-101-3p;hsa-miR-1304-3p;hsa-miR-144-3p;hsa-miR-1
ENSG0000	ENSG0000	0.001434	hsa-miR-1976;hsa-miR-26a-5p;hsa-miR-26b-5p;hsa-miR-3065-
ENSG0000	ENSG0000	0.000319	hsa-miR-26a-5p;hsa-miR-30a-5p;hsa-miR-30b-5p;hsa-miR-30
ENSG0000	ENSG0000	0.000117	hsa-miR-193a-3p;hsa-miR-193b-3p;hsa-miR-30b-5p;hsa-miR-
ENSG0000	ENSG0000	0.009741	hsa-miR-186-5p;hsa-miR-28-5p;hsa-miR-338-3p;hsa-miR-545-
ENSG0000	ENSG0000	0.009955	hsa-miR-1305;hsa-miR-30a-3p;hsa-miR-30d-3p;hsa-miR-30e-
ENSG0000	ENSG0000	0.003867	hsa-miR-30b-5p;hsa-miR-30c-5p;hsa-miR-30d-5p;hsa-miR-30
ENSG0000	ENSG0000	1.03E-07	hsa-miR-30a-5p;hsa-miR-30b-5p;hsa-miR-30c-5p;hsa-miR-30
ENSG0000	ENSG0000	0.000422	hsa-miR-1976;hsa-miR-30a-5p;hsa-miR-30b-5p;hsa-miR-30c-
ENSG0000	ENSG0000	6.97E-05	hsa-miR-30a-5p;hsa-miR-30b-5p;hsa-miR-30c-5p;hsa-miR-30
ENSG0000	ENSG0000	0.000496	hsa-miR-30a-3p;hsa-miR-30d-3p;hsa-miR-30e-3p;hsa-miR-64
ENSG0000	ENSG0000	0.002475	hsa-miR-204-5p;hsa-miR-30a-3p;hsa-miR-30d-3p;hsa-miR-30

ENSG0000	ENSG0000	0.000351	hsa-miR-1304-3p;hsa-miR-1305;hsa-miR-30a-5p;hsa-miR-30b-
ENSG0000	ENSG0000	0.000117	hsa-miR-30a-5p;hsa-miR-30b-5p;hsa-miR-30c-5p;hsa-miR-30
ENSG0000	ENSG0000	0.002464	hsa-miR-1304-3p;hsa-miR-144-3p;hsa-miR-16-1-3p;hsa-miR-
ENSG0000	ENSG0000	0.003146	hsa-miR-125a-5p;hsa-miR-125b-5p;hsa-miR-1276;hsa-miR-54
ENSG0000	ENSG0000	0.005086	hsa-miR-1305;hsa-miR-1976;hsa-miR-26a-5p;hsa-miR-26b-5p;
ENSG0000	ENSG0000	7.85E-06	hsa-miR-101-3p;hsa-miR-1276;hsa-miR-19a-3p;hsa-miR-26a-
ENSG0000	ENSG0000	2.09E-05	hsa-miR-30a-3p;hsa-miR-30a-5p;hsa-miR-30b-5p;hsa-miR-30
ENSG0000	ENSG0000	7.84E-09	hsa-miR-30a-5p;hsa-miR-30b-5p;hsa-miR-30c-5p;hsa-miR-30
ENSG0000	ENSG0000	9.14E-07	hsa-miR-30a-5p;hsa-miR-30b-5p;hsa-miR-30c-5p;hsa-miR-30
ENSG0000	ENSG0000	0.00548	hsa-miR-1304-3p;hsa-miR-30a-5p;hsa-miR-30c-5p;hsa-miR-3
ENSG0000	ENSG0000	0.000604	hsa-miR-1304-3p;hsa-miR-1976;hsa-miR-26a-5p;hsa-miR-26b-
ENSG0000	ENSG0000	1.94E-05	hsa-miR-30a-5p;hsa-miR-30b-5p;hsa-miR-30c-5p;hsa-miR-30
ENSG0000	ENSG0000	0.003652	hsa-miR-1914-3p;hsa-miR-19a-3p;hsa-miR-19b-3p;hsa-miR-4
ENSG0000	ENSG0000	0.000604	hsa-miR-1305;hsa-miR-144-3p;hsa-miR-19a-3p;hsa-miR-19b-
ENSG0000	ENSG0000	7.84E-09	hsa-miR-30a-5p;hsa-miR-30b-5p;hsa-miR-30c-5p;hsa-miR-30
ENSG0000	ENSG0000	0.009741	hsa-miR-296-5p;hsa-miR-30a-3p;hsa-miR-30d-3p;hsa-miR-30
ENSG0000	ENSG0000	0.000284	hsa-miR-378a-3p;hsa-miR-378c;hsa-miR-378d;hsa-miR-378i;hs
ENSG0000	ENSG0000	4.64E-05	hsa-miR-10a-3p;hsa-miR-16-1-3p;hsa-miR-222-5p;hsa-miR-3
ENSG0000	ENSG0000	0.004362	hsa-miR-30a-5p;hsa-miR-30b-5p;hsa-miR-30c-5p;hsa-miR-30
ENSG0000	ENSG0000	2.87E-07	hsa-miR-30a-5p;hsa-miR-30b-5p;hsa-miR-30c-5p;hsa-miR-30
ENSG0000	ENSG0000	0.000345	hsa-miR-30a-3p;hsa-miR-30d-3p;hsa-miR-30e-3p;hsa-miR-54
ENSG0000	ENSG0000	0.000942	hsa-miR-1305;hsa-miR-30a-5p;hsa-miR-30c-5p;hsa-miR-30d-
ENSG0000	ENSG0000	0.000284	hsa-miR-30a-3p;hsa-miR-30d-3p;hsa-miR-30e-3p;hsa-miR-54
ENSG0000	ENSG0000	1.79E-06	hsa-miR-30a-5p;hsa-miR-30b-5p;hsa-miR-30c-5p;hsa-miR-30
ENSG0000	ENSG0000	0.000415	hsa-miR-30a-5p;hsa-miR-30b-5p;hsa-miR-30c-5p;hsa-miR-30
ENSG0000	ENSG0000	0.00081	hsa-miR-19a-3p;hsa-miR-30a-3p;hsa-miR-30d-3p;hsa-miR-30
ENSG0000	ENSG0000	0.005908	hsa-miR-101-3p;hsa-miR-1976;hsa-miR-296-5p;hsa-miR-582-
ENSG0000	ENSG0000	0.001955	hsa-miR-19a-3p;hsa-miR-19b-3p;hsa-miR-30a-5p;hsa-miR-30
ENSG0000	ENSG0000	2.58E-06	hsa-miR-1304-3p;hsa-miR-1305;hsa-miR-19a-3p;hsa-miR-19b-
ENSG0000	ENSG0000	0.001253	hsa-miR-30a-5p;hsa-miR-30b-5p;hsa-miR-30c-5p;hsa-miR-30
ENSG0000	ENSG0000	0.000577	hsa-miR-19a-3p;hsa-miR-19b-3p;hsa-miR-26a-5p;hsa-miR-26
ENSG0000	ENSG0000	0.006796	hsa-miR-30a-3p;hsa-miR-30d-3p;hsa-miR-30e-3p;hsa-miR-54
ENSG0000	ENSG0000	0.008981	hsa-miR-101-3p;hsa-miR-144-3p;hsa-miR-1976;hsa-miR-582-
ENSG0000	ENSG0000	0.00082	hsa-miR-125a-5p;hsa-miR-125b-5p;hsa-miR-30a-3p;hsa-miR-
ENSG0000	ENSG0000	0.001086	hsa-miR-185-3p;hsa-miR-193a-3p;hsa-miR-193b-3p;hsa-miR-
ENSG0000	ENSG0000	3.88E-05	hsa-miR-204-5p;hsa-miR-30a-3p;hsa-miR-30d-3p;hsa-miR-30
ENSG0000	ENSG0000	0.002115	hsa-miR-204-5p;hsa-miR-30a-5p;hsa-miR-30b-5p;hsa-miR-30
ENSG0000	ENSG0000	4.14E-07	hsa-miR-30a-5p;hsa-miR-30b-5p;hsa-miR-30c-5p;hsa-miR-30
ENSG0000	ENSG0000	4.14E-07	hsa-miR-30a-5p;hsa-miR-30b-5p;hsa-miR-30c-5p;hsa-miR-30
ENSG0000	ENSG0000	1.39E-05	hsa-miR-193a-3p;hsa-miR-193b-3p;hsa-miR-30a-5p;hsa-miR-
ENSG0000	ENSG0000	9.79E-05	hsa-miR-30a-3p;hsa-miR-30d-3p;hsa-miR-30e-3p;hsa-miR-64
ENSG0000	ENSG0000	0.005255	hsa-miR-144-3p;hsa-miR-1914-3p;hsa-miR-3619-5p;hsa-miR-
ENSG0000	ENSG0000	1.94E-05	hsa-miR-30a-5p;hsa-miR-30b-5p;hsa-miR-30c-5p;hsa-miR-30
ENSG0000	ENSG0000	0.005713	hsa-miR-19a-3p;hsa-miR-19b-3p;hsa-miR-26a-5p;hsa-miR-26
ENSG0000	ENSG0000	9.79E-05	hsa-miR-101-3p;hsa-miR-1243;hsa-miR-144-3p;hsa-miR-499a-
ENSG0000	ENSG0000	0.00081	hsa-miR-30a-5p;hsa-miR-30b-5p;hsa-miR-30c-5p;hsa-miR-30
ENSG0000	ENSG0000	0.000345	hsa-miR-30a-5p;hsa-miR-30b-5p;hsa-miR-30c-5p;hsa-miR-30
ENSG0000	ENSG0000	0.000942	hsa-miR-1914-3p;hsa-miR-26a-5p;hsa-miR-26b-5p;hsa-miR-3
ENSG0000	ENSG0000	3.75E-05	hsa-miR-101-3p;hsa-miR-125b-5p;hsa-miR-140-5p;hsa-miR-3
ENSG0000	ENSG0000	0.004429	hsa-miR-30b-5p;hsa-miR-30c-5p;hsa-miR-30d-5p;hsa-miR-30
ENSG0000	ENSG0000	3.88E-05	hsa-miR-1304-3p;hsa-miR-30a-3p;hsa-miR-30d-3p;hsa-miR-3
ENSG0000	ENSG0000	0.000197	hsa-miR-125a-5p;hsa-miR-125b-5p;hsa-miR-30a-5p;hsa-miR-
ENSG0000	ENSG0000	0.003867	hsa-miR-30a-3p;hsa-miR-30d-3p;hsa-miR-30e-3p;hsa-miR-66
ENSG0000	ENSG0000	0.000284	hsa-miR-30a-3p;hsa-miR-30d-3p;hsa-miR-30e-3p;hsa-miR-34

ENSG0000	ENSG0000	0.005908	hsa-miR-30a-5p;hsa-miR-30b-5p;hsa-miR-30c-5p;hsa-miR-30
ENSG0000	ENSG0000	0.000152	hsa-miR-1304-3p;hsa-miR-222-5p;hsa-miR-26a-5p;hsa-miR-2
ENSG0000	ENSG0000	0.002398	hsa-miR-122-5p;hsa-miR-1307-3p;hsa-miR-3605-5p;hsa-miR-
ENSG0000	ENSG0000	0.000243	hsa-miR-122-5p;hsa-miR-1914-3p;hsa-miR-3605-5p;hsa-miR-
ENSG0000	ENSG0000	0.00489	hsa-miR-1307-3p;hsa-miR-1914-3p;hsa-miR-423-5p;hsa-miR-
ENSG0000	ENSG0000	0.000358	hsa-miR-548ak;hsa-miR-548am-5p;hsa-miR-548c-5p;hsa-miR-
ENSG0000	ENSG0000	1.79E-07	hsa-miR-548ak;hsa-miR-548am-5p;hsa-miR-548c-5p;hsa-miR-
ENSG0000	ENSG0000	4.46E-09	hsa-miR-142-5p;hsa-miR-548ak;hsa-miR-548am-5p;hsa-miR-5
ENSG0000	ENSG0000	2.24E-11	hsa-miR-142-5p;hsa-miR-548ak;hsa-miR-548am-5p;hsa-miR-5
ENSG0000	ENSG0000	3.11E-07	hsa-miR-145-5p;hsa-miR-548ak;hsa-miR-548am-5p;hsa-miR-5
ENSG0000	ENSG0000	1.11E-09	hsa-miR-142-5p;hsa-miR-548ak;hsa-miR-548am-5p;hsa-miR-5
ENSG0000	ENSG0000	7.63E-08	hsa-miR-548ak;hsa-miR-548am-5p;hsa-miR-548c-5p;hsa-miR-
ENSG0000	ENSG0000	3.70E-10	hsa-miR-142-5p;hsa-miR-548ak;hsa-miR-548am-5p;hsa-miR-5
ENSG0000	ENSG0000	4.79E-08	hsa-miR-30b-5p;hsa-miR-548ak;hsa-miR-548am-5p;hsa-miR-5
ENSG0000	ENSG0000	9.15E-12	hsa-miR-142-5p;hsa-miR-548ak;hsa-miR-548am-5p;hsa-miR-5
ENSG0000	ENSG0000	7.34E-06	hsa-miR-548ak;hsa-miR-548am-5p;hsa-miR-548c-5p;hsa-miR-
ENSG0000	ENSG0000	5.93E-08	hsa-miR-548ak;hsa-miR-548am-5p;hsa-miR-548c-5p;hsa-miR-
ENSG0000	ENSG0000	1.79E-07	hsa-miR-548ak;hsa-miR-548am-5p;hsa-miR-548c-5p;hsa-miR-
ENSG0000	ENSG0000	1.05E-08	hsa-miR-548ak;hsa-miR-548am-5p;hsa-miR-548c-5p;hsa-miR-
ENSG0000	ENSG0000	1.16E-06	hsa-miR-106a-5p;hsa-miR-548ak;hsa-miR-548am-5p;hsa-miR-
ENSG0000	ENSG0000	0.000109	hsa-miR-548ak;hsa-miR-548am-5p;hsa-miR-548c-5p;hsa-miR-
ENSG0000	ENSG0000	2.08E-05	hsa-miR-548ak;hsa-miR-548am-5p;hsa-miR-548c-5p;hsa-miR-
ENSG0000	ENSG0000	1.29E-08	hsa-miR-548ak;hsa-miR-548am-5p;hsa-miR-548c-5p;hsa-miR-
ENSG0000	ENSG0000	7.28E-08	hsa-miR-548ak;hsa-miR-548am-5p;hsa-miR-548c-5p;hsa-miR-
ENSG0000	ENSG0000	4.79E-09	hsa-miR-548ak;hsa-miR-548am-5p;hsa-miR-548c-5p;hsa-miR-
ENSG0000	ENSG0000	8.46E-10	hsa-miR-548ak;hsa-miR-548am-5p;hsa-miR-548c-5p;hsa-miR-
ENSG0000	ENSG0000	5.84E-07	hsa-miR-548ak;hsa-miR-548am-5p;hsa-miR-548c-5p;hsa-miR-
ENSG0000	ENSG0000	4.30E-08	hsa-miR-548ak;hsa-miR-548am-5p;hsa-miR-548c-5p;hsa-miR-
ENSG0000	ENSG0000	4.30E-08	hsa-miR-106a-5p;hsa-miR-548ak;hsa-miR-548am-5p;hsa-miR-
ENSG0000	ENSG0000	1.42E-06	hsa-miR-548ak;hsa-miR-548am-5p;hsa-miR-548c-5p;hsa-miR-
ENSG0000	ENSG0000	8.89E-08	hsa-miR-548ak;hsa-miR-548am-5p;hsa-miR-548c-5p;hsa-miR-
ENSG0000	ENSG0000	7.17E-07	hsa-miR-548ak;hsa-miR-548am-5p;hsa-miR-548c-5p;hsa-miR-
ENSG0000	ENSG0000	5.33E-08	hsa-miR-106a-5p;hsa-miR-548ak;hsa-miR-548am-5p;hsa-miR-
ENSG0000	ENSG0000	1.30E-07	hsa-miR-382-5p;hsa-miR-548ak;hsa-miR-548am-5p;hsa-miR-5
ENSG0000	ENSG0000	4.11E-09	hsa-miR-548ak;hsa-miR-548am-5p;hsa-miR-548c-5p;hsa-miR-
ENSG0000	ENSG0000	5.97E-06	hsa-miR-548ak;hsa-miR-548am-5p;hsa-miR-548c-5p;hsa-miR-
ENSG0000	ENSG0000	1.55E-07	hsa-miR-224-3p;hsa-miR-30b-5p;hsa-miR-30c-5p;hsa-miR-54
ENSG0000	ENSG0000	1.80E-10	hsa-miR-548ak;hsa-miR-548am-5p;hsa-miR-548c-5p;hsa-miR-
ENSG0000	ENSG0000	7.63E-08	hsa-miR-548ak;hsa-miR-548am-5p;hsa-miR-548c-5p;hsa-miR-
ENSG0000	ENSG0000	3.30E-06	hsa-miR-548ak;hsa-miR-548am-5p;hsa-miR-548c-5p;hsa-miR-
ENSG0000	ENSG0000	6.86E-05	hsa-miR-548ak;hsa-miR-548am-5p;hsa-miR-548c-5p;hsa-miR-
ENSG0000	ENSG0000	1.29E-06	hsa-miR-548ak;hsa-miR-548am-5p;hsa-miR-548c-5p;hsa-miR-
ENSG0000	ENSG0000	2.62E-07	hsa-miR-548ak;hsa-miR-548am-5p;hsa-miR-548c-5p;hsa-miR-
ENSG0000	ENSG0000	1.70E-06	hsa-miR-548ak;hsa-miR-548am-5p;hsa-miR-548c-5p;hsa-miR-
ENSG0000	ENSG0000	1.03E-09	hsa-miR-548ak;hsa-miR-548am-5p;hsa-miR-548c-5p;hsa-miR-
ENSG0000	ENSG0000	6.09E-09	hsa-miR-142-5p;hsa-miR-548ak;hsa-miR-548am-5p;hsa-miR-5
ENSG0000	ENSG0000	2.31E-07	hsa-miR-548ak;hsa-miR-548am-5p;hsa-miR-548c-5p;hsa-miR-
ENSG0000	ENSG0000	8.81E-07	hsa-miR-548ak;hsa-miR-548am-5p;hsa-miR-548c-5p;hsa-miR-
ENSG0000	ENSG0000	1.16E-06	hsa-miR-548ak;hsa-miR-548am-5p;hsa-miR-548c-5p;hsa-miR-
ENSG0000	ENSG0000	1.16E-06	hsa-miR-548ak;hsa-miR-548am-5p;hsa-miR-548c-5p;hsa-miR-
ENSG0000	ENSG0000	2.73E-05	hsa-miR-548ak;hsa-miR-548am-5p;hsa-miR-548c-5p;hsa-miR-
ENSG0000	ENSG0000	1.86E-05	hsa-miR-548ak;hsa-miR-548am-5p;hsa-miR-548c-5p;hsa-miR-
ENSG0000	ENSG0000	6.89E-12	hsa-miR-106a-5p;hsa-miR-142-5p;hsa-miR-30b-5p;hsa-miR-3
ENSG0000	ENSG0000	7.19E-12	hsa-miR-142-5p;hsa-miR-548ak;hsa-miR-548am-5p;hsa-miR-5

ENSG0000	ENSG0000	0.000304	hsa-miR-548ak;hsa-miR-548am-5p;hsa-miR-548c-5p;hsa-miR-
ENSG0000	ENSG0000	1.57E-07	hsa-miR-548ak;hsa-miR-548am-5p;hsa-miR-548c-5p;hsa-miR-
ENSG0000	ENSG0000	4.94E-10	hsa-miR-142-5p;hsa-miR-548ak;hsa-miR-548am-5p;hsa-miR-5
ENSG0000	ENSG0000	2.31E-07	hsa-miR-548ak;hsa-miR-548am-5p;hsa-miR-548c-5p;hsa-miR-
ENSG0000	ENSG0000	2.20E-06	hsa-miR-548ak;hsa-miR-548am-5p;hsa-miR-548c-5p;hsa-miR-
ENSG0000	ENSG0000	4.00E-08	hsa-miR-548ak;hsa-miR-548am-5p;hsa-miR-548c-5p;hsa-miR-
ENSG0000	ENSG0000	4.34E-12	hsa-miR-30b-5p;hsa-miR-30c-5p;hsa-miR-548ak;hsa-miR-548a
ENSG0000	ENSG0000	4.79E-08	hsa-miR-548ak;hsa-miR-548am-5p;hsa-miR-548c-5p;hsa-miR-
ENSG0000	ENSG0000	2.73E-08	hsa-miR-548ak;hsa-miR-548am-5p;hsa-miR-548c-5p;hsa-miR-
ENSG0000	ENSG0000	1.70E-06	hsa-miR-548ak;hsa-miR-548am-5p;hsa-miR-548c-5p;hsa-miR-
ENSG0000	ENSG0000	1.85E-06	hsa-miR-548ak;hsa-miR-548am-5p;hsa-miR-548c-5p;hsa-miR-
ENSG0000	ENSG0000	1.79E-07	hsa-miR-548ak;hsa-miR-548am-5p;hsa-miR-548c-5p;hsa-miR-
ENSG0000	ENSG0000	1.09E-05	hsa-miR-548ak;hsa-miR-548am-5p;hsa-miR-548c-5p;hsa-miR-
ENSG0000	ENSG0000	2.31E-06	hsa-miR-548ak;hsa-miR-548am-5p;hsa-miR-548c-5p;hsa-miR-
ENSG0000	ENSG0000	3.65E-10	hsa-miR-142-5p;hsa-miR-548ak;hsa-miR-548am-5p;hsa-miR-5
ENSG0000	ENSG0000	3.14E-07	hsa-miR-142-5p;hsa-miR-548ak;hsa-miR-548am-5p;hsa-miR-5
ENSG0000	ENSG0000	8.39E-06	hsa-miR-548ak;hsa-miR-548am-5p;hsa-miR-548c-5p;hsa-miR-
ENSG0000	ENSG0000	3.30E-06	hsa-miR-548ak;hsa-miR-548am-5p;hsa-miR-548c-5p;hsa-miR-
ENSG0000	ENSG0000	1.42E-09	hsa-miR-106a-5p;hsa-miR-548ak;hsa-miR-548am-5p;hsa-miR-
ENSG0000	ENSG0000	6.86E-05	hsa-miR-548ak;hsa-miR-548am-5p;hsa-miR-548c-5p;hsa-miR-
ENSG0000	ENSG0000	4.52E-05	hsa-miR-548ak;hsa-miR-548am-5p;hsa-miR-548c-5p;hsa-miR-
ENSG0000	ENSG0000	1.07E-06	hsa-miR-548ak;hsa-miR-548am-5p;hsa-miR-548c-5p;hsa-miR-
ENSG0000	ENSG0000	1.47E-08	hsa-miR-548ak;hsa-miR-548am-5p;hsa-miR-548c-5p;hsa-miR-
ENSG0000	ENSG0000	1.03E-10	hsa-miR-142-5p;hsa-miR-548ak;hsa-miR-548am-5p;hsa-miR-5
ENSG0000	ENSG0000	1.03E-10	hsa-miR-142-5p;hsa-miR-548ak;hsa-miR-548am-5p;hsa-miR-5
ENSG0000	ENSG0000	8.39E-09	hsa-miR-548ak;hsa-miR-548am-5p;hsa-miR-548c-5p;hsa-miR-
ENSG0000	ENSG0000	4.11E-09	hsa-miR-548ak;hsa-miR-548am-5p;hsa-miR-548c-5p;hsa-miR-
ENSG0000	ENSG0000	3.66E-09	hsa-miR-142-5p;hsa-miR-548ak;hsa-miR-548am-5p;hsa-miR-5
ENSG0000	ENSG0000	0.000821	hsa-miR-548ak;hsa-miR-548am-5p;hsa-miR-548c-5p;hsa-miR-
ENSG0000	ENSG0000	1.16E-11	hsa-miR-142-5p;hsa-miR-548ak;hsa-miR-548am-5p;hsa-miR-5
ENSG0000	ENSG0000	7.28E-08	hsa-miR-548ak;hsa-miR-548am-5p;hsa-miR-548c-5p;hsa-miR-
ENSG0000	ENSG0000	2.14E-08	hsa-miR-548ak;hsa-miR-548am-5p;hsa-miR-548c-5p;hsa-miR-
ENSG0000	ENSG0000	2.08E-05	hsa-miR-548ak;hsa-miR-548am-5p;hsa-miR-548c-5p;hsa-miR-
ENSG0000	ENSG0000	6.49E-07	hsa-miR-548ak;hsa-miR-548am-5p;hsa-miR-548c-5p;hsa-miR-
ENSG0000	ENSG0000	1.86E-05	hsa-miR-142-5p;hsa-miR-548ak;hsa-miR-548am-5p;hsa-miR-5
ENSG0000	ENSG0000	1.12E-07	hsa-miR-30b-5p;hsa-miR-30c-5p;hsa-miR-548ak;hsa-miR-548a
ENSG0000	ENSG0000	2.34E-08	hsa-miR-548ak;hsa-miR-548am-5p;hsa-miR-548c-5p;hsa-miR-
ENSG0000	ENSG0000	2.81E-06	hsa-miR-548ak;hsa-miR-548am-5p;hsa-miR-548c-5p;hsa-miR-
ENSG0000	ENSG0000	2.20E-05	hsa-miR-548ak;hsa-miR-548am-5p;hsa-miR-548c-5p;hsa-miR-
ENSG0000	ENSG0000	4.20E-07	hsa-miR-548ak;hsa-miR-548am-5p;hsa-miR-548c-5p;hsa-miR-
ENSG0000	ENSG0000	4.15E-06	hsa-miR-548ak;hsa-miR-548am-5p;hsa-miR-548c-5p;hsa-miR-
ENSG0000	ENSG0000	1.60E-06	hsa-miR-548ak;hsa-miR-548am-5p;hsa-miR-548c-5p;hsa-miR-
ENSG0000	ENSG0000	2.05E-06	hsa-miR-106a-5p;hsa-miR-548ak;hsa-miR-548am-5p;hsa-miR-
ENSG0000	ENSG0000	0.004279	hsa-miR-106a-5p;hsa-miR-142-5p;hsa-miR-145-5p;hsa-miR-2
ENSG0000	ENSG0000	0.004279	hsa-miR-106a-5p;hsa-miR-142-5p;hsa-miR-145-5p;hsa-miR-2
ENSG0000	ENSG0000	3.33E-12	hsa-miR-142-5p;hsa-miR-548ak;hsa-miR-548am-5p;hsa-miR-5
ENSG0000	ENSG0000	5.97E-06	hsa-miR-548ak;hsa-miR-548am-5p;hsa-miR-548c-5p;hsa-miR-
ENSG0000	ENSG0000	3.65E-10	hsa-miR-548ak;hsa-miR-548am-5p;hsa-miR-548c-5p;hsa-miR-
ENSG0000	ENSG0000	2.60E-06	hsa-miR-106a-5p;hsa-miR-548ak;hsa-miR-548am-5p;hsa-miR-
ENSG0000	ENSG0000	2.45E-05	hsa-miR-548ak;hsa-miR-548am-5p;hsa-miR-548c-5p;hsa-miR-
ENSG0000	ENSG0000	5.27E-09	hsa-miR-548ak;hsa-miR-548am-5p;hsa-miR-548c-5p;hsa-miR-
ENSG0000	ENSG0000	2.60E-06	hsa-miR-548ak;hsa-miR-548am-5p;hsa-miR-548c-5p;hsa-miR-
ENSG0000	ENSG0000	2.60E-06	hsa-miR-548ak;hsa-miR-548am-5p;hsa-miR-548c-5p;hsa-miR-
ENSG0000	ENSG0000	2.20E-06	hsa-miR-548ak;hsa-miR-548am-5p;hsa-miR-548c-5p;hsa-miR-

ENSG0000	ENSG0000	2.42E-08	hsa-miR-548ak;hsa-miR-548am-5p;hsa-miR-548c-5p;hsa-miR-
ENSG0000	ENSG0000	4.31E-05	hsa-miR-548ak;hsa-miR-548am-5p;hsa-miR-548c-5p;hsa-miR-
ENSG0000	ENSG0000	2.73E-08	hsa-miR-548ak;hsa-miR-548am-5p;hsa-miR-548c-5p;hsa-miR-
ENSG0000	ENSG0000	2.73E-08	hsa-miR-548ak;hsa-miR-548am-5p;hsa-miR-548c-5p;hsa-miR-
ENSG0000	ENSG0000	5.93E-06	hsa-miR-106a-5p;hsa-miR-30b-5p;hsa-miR-30c-5p;hsa-miR-54
ENSG0000	ENSG0000	4.79E-08	hsa-miR-548ak;hsa-miR-548am-5p;hsa-miR-548c-5p;hsa-miR-
ENSG0000	ENSG0000	3.85E-06	hsa-miR-548ak;hsa-miR-548am-5p;hsa-miR-548c-5p;hsa-miR-
ENSG0000	ENSG0000	3.12E-09	hsa-miR-106a-5p;hsa-miR-142-5p;hsa-miR-548ak;hsa-miR-548
ENSG0000	ENSG0000	5.84E-07	hsa-miR-548ak;hsa-miR-548am-5p;hsa-miR-548c-5p;hsa-miR-
ENSG0000	ENSG0000	1.79E-07	hsa-miR-548ak;hsa-miR-548am-5p;hsa-miR-548c-5p;hsa-miR-
ENSG0000	ENSG0000	1.40E-10	hsa-miR-548ak;hsa-miR-548am-5p;hsa-miR-548c-5p;hsa-miR-
ENSG0000	ENSG0000	1.16E-11	hsa-miR-142-5p;hsa-miR-548ak;hsa-miR-548am-5p;hsa-miR-5
ENSG0000	ENSG0000	2.04E-07	hsa-miR-548ak;hsa-miR-548am-5p;hsa-miR-548c-5p;hsa-miR-
ENSG0000	ENSG0000	4.12E-11	hsa-miR-142-5p;hsa-miR-548ak;hsa-miR-548am-5p;hsa-miR-5
ENSG0000	ENSG0000	1.17E-12	hsa-miR-142-5p;hsa-miR-145-5p;hsa-miR-30b-5p;hsa-miR-30
ENSG0000	ENSG0000	1.24E-06	hsa-miR-106a-5p;hsa-miR-548ak;hsa-miR-548am-5p;hsa-miR-
ENSG0000	ENSG0000	1.71E-07	hsa-miR-548ak;hsa-miR-548am-5p;hsa-miR-548c-5p;hsa-miR-
ENSG0000	ENSG0000	5.18E-06	hsa-miR-548ak;hsa-miR-548am-5p;hsa-miR-548c-5p;hsa-miR-
ENSG0000	ENSG0000	1.32E-08	hsa-miR-106a-5p;hsa-miR-548ak;hsa-miR-548am-5p;hsa-miR-
ENSG0000	ENSG0000	1.30E-07	hsa-miR-106a-5p;hsa-miR-548ak;hsa-miR-548am-5p;hsa-miR-
ENSG0000	ENSG0000	3.65E-10	hsa-miR-548ak;hsa-miR-548am-5p;hsa-miR-548c-5p;hsa-miR-
ENSG0000	ENSG0000	3.98E-07	hsa-miR-30b-5p;hsa-miR-30c-5p;hsa-miR-548ak;hsa-miR-548a
ENSG0000	ENSG0000	1.11E-05	hsa-miR-548ak;hsa-miR-548am-5p;hsa-miR-548c-5p;hsa-miR-
ENSG0000	ENSG0000	5.68E-10	hsa-miR-142-5p;hsa-miR-548ak;hsa-miR-548am-5p;hsa-miR-5
ENSG0000	ENSG0000	9.79E-08	hsa-miR-142-5p;hsa-miR-548ak;hsa-miR-548am-5p;hsa-miR-5
ENSG0000	ENSG0000	1.13E-08	hsa-miR-548ak;hsa-miR-548am-5p;hsa-miR-548c-5p;hsa-miR-
ENSG0000	ENSG0000	6.57E-08	hsa-miR-137;hsa-miR-548ak;hsa-miR-548am-5p;hsa-miR-548c
ENSG0000	ENSG0000	0.003398	hsa-miR-106a-5p;hsa-miR-145-5p;hsa-miR-224-3p;hsa-miR-57
ENSG0000	ENSG0000	6.09E-09	hsa-miR-142-5p;hsa-miR-548ak;hsa-miR-548am-5p;hsa-miR-5
ENSG0000	ENSG0000	4.34E-12	hsa-miR-142-5p;hsa-miR-548ak;hsa-miR-548am-5p;hsa-miR-5
ENSG0000	ENSG0000	1.08E-07	hsa-miR-106a-5p;hsa-miR-30c-5p;hsa-miR-548ak;hsa-miR-548
ENSG0000	ENSG0000	2.73E-08	hsa-miR-548ak;hsa-miR-548am-5p;hsa-miR-548c-5p;hsa-miR-
ENSG0000	ENSG0000	2.98E-09	hsa-miR-548ak;hsa-miR-548am-5p;hsa-miR-548c-5p;hsa-miR-
ENSG0000	ENSG0000	1.07E-06	hsa-miR-548ak;hsa-miR-548am-5p;hsa-miR-548c-5p;hsa-miR-
ENSG0000	ENSG0000	6.86E-06	hsa-miR-145-5p;hsa-miR-548ak;hsa-miR-548am-5p;hsa-miR-5
ENSG0000	ENSG0000	7.47E-10	hsa-miR-142-5p;hsa-miR-548ak;hsa-miR-548am-5p;hsa-miR-5
ENSG0000	ENSG0000	7.47E-10	hsa-miR-142-5p;hsa-miR-548ak;hsa-miR-548am-5p;hsa-miR-5
ENSG0000	ENSG0000	4.15E-06	hsa-miR-548ak;hsa-miR-548am-5p;hsa-miR-548c-5p;hsa-miR-
ENSG0000	ENSG0000	1.09E-06	hsa-miR-548ak;hsa-miR-548am-5p;hsa-miR-548c-5p;hsa-miR-
ENSG0000	ENSG0000	7.63E-08	hsa-miR-548ak;hsa-miR-548am-5p;hsa-miR-548c-5p;hsa-miR-
ENSG0000	ENSG0000	7.63E-08	hsa-miR-548ak;hsa-miR-548am-5p;hsa-miR-548c-5p;hsa-miR-
ENSG0000	ENSG0000	7.63E-08	hsa-miR-548ak;hsa-miR-548am-5p;hsa-miR-548c-5p;hsa-miR-
ENSG0000	ENSG0000	7.63E-08	hsa-miR-548ak;hsa-miR-548am-5p;hsa-miR-548c-5p;hsa-miR-
ENSG0000	ENSG0000	7.63E-08	hsa-miR-548ak;hsa-miR-548am-5p;hsa-miR-548c-5p;hsa-miR-
ENSG0000	ENSG0000	7.63E-08	hsa-miR-548ak;hsa-miR-548am-5p;hsa-miR-548c-5p;hsa-miR-
ENSG0000	ENSG0000	7.63E-08	hsa-miR-548ak;hsa-miR-548am-5p;hsa-miR-548c-5p;hsa-miR-
ENSG0000	ENSG0000	7.63E-08	hsa-miR-548ak;hsa-miR-548am-5p;hsa-miR-548c-5p;hsa-miR-
ENSG0000	ENSG0000	8.46E-10	hsa-miR-548ak;hsa-miR-548am-5p;hsa-miR-548c-5p;hsa-miR-
ENSG0000	ENSG0000	8.88E-08	hsa-miR-548ak;hsa-miR-548am-5p;hsa-miR-548c-5p;hsa-miR-
ENSG0000	ENSG0000	2.42E-08	hsa-miR-548ak;hsa-miR-548am-5p;hsa-miR-548c-5p;hsa-miR-
ENSG0000	ENSG0000	4.20E-07	hsa-miR-548ak;hsa-miR-548am-5p;hsa-miR-548c-5p;hsa-miR-
ENSG0000	ENSG0000	0.000903	hsa-miR-106a-5p;hsa-miR-30b-5p;hsa-miR-30c-5p;hsa-miR-57
ENSG0000	ENSG0000	0.000248	hsa-miR-548ak;hsa-miR-548am-5p;hsa-miR-548c-5p;hsa-miR-
ENSG0000	ENSG0000	7.28E-08	hsa-miR-548ak;hsa-miR-548am-5p;hsa-miR-548c-5p;hsa-miR-

ENSG0000	ENSG0000	2.24E-11	hsa-miR-548ak;hsa-miR-548am-5p;hsa-miR-548c-5p;hsa-miR-
ENSG0000	ENSG0000	1.29E-08	hsa-miR-548ak;hsa-miR-548am-5p;hsa-miR-548c-5p;hsa-miR-
ENSG0000	ENSG0000	2.30E-10	hsa-miR-548ak;hsa-miR-548am-5p;hsa-miR-548c-5p;hsa-miR-
ENSG0000	ENSG0000	2.13E-09	hsa-miR-548ak;hsa-miR-548am-5p;hsa-miR-548c-5p;hsa-miR-
ENSG0000	ENSG0000	2.42E-07	hsa-miR-548ak;hsa-miR-548am-5p;hsa-miR-548c-5p;hsa-miR-
ENSG0000	ENSG0000	6.21E-07	hsa-miR-548ak;hsa-miR-548am-5p;hsa-miR-548c-5p;hsa-miR-
ENSG0000	ENSG0000	1.24E-09	hsa-miR-548ak;hsa-miR-548am-5p;hsa-miR-548c-5p;hsa-miR-
ENSG0000	ENSG0000	8.05E-08	hsa-miR-548ak;hsa-miR-548am-5p;hsa-miR-548c-5p;hsa-miR-
ENSG0000	ENSG0000	8.05E-08	hsa-miR-548ak;hsa-miR-548am-5p;hsa-miR-548c-5p;hsa-miR-
ENSG0000	ENSG0000	8.59E-09	hsa-miR-548ak;hsa-miR-548am-5p;hsa-miR-548c-5p;hsa-miR-
ENSG0000	ENSG0000	1.60E-06	hsa-miR-548ak;hsa-miR-548am-5p;hsa-miR-548c-5p;hsa-miR-
ENSG0000	ENSG0000	0.002245	hsa-miR-1304-3p;hsa-miR-139-5p;hsa-miR-15a-5p;hsa-miR-1
ENSG0000	ENSG0000	0.000692	hsa-miR-15a-5p;hsa-miR-15b-5p;hsa-miR-16-5p;hsa-miR-17-
ENSG0000	ENSG0000	0.004362	hsa-miR-1-3p;hsa-miR-20b-5p;hsa-miR-27a-3p;hsa-miR-27b-
ENSG0000	ENSG0000	0.001656	hsa-miR-17-5p;hsa-miR-20b-5p;hsa-miR-27a-3p;hsa-miR-27b
ENSG0000	ENSG0000	0.000209	hsa-miR-124-3p;hsa-miR-17-5p;hsa-miR-181a-5p;hsa-miR-18
ENSG0000	ENSG0000	4.64E-06	hsa-miR-17-5p;hsa-miR-181a-5p;hsa-miR-181b-5p;hsa-miR-2
ENSG0000	ENSG0000	1.74E-07	hsa-miR-15b-5p;hsa-miR-181a-5p;hsa-miR-34a-5p;hsa-miR-7
ENSG0000	ENSG0000	1.14E-13	hsa-miR-139-5p;hsa-miR-15a-5p;hsa-miR-15b-5p;hsa-miR-16
ENSG0000	ENSG0000	6.94E-06	hsa-miR-124-3p;hsa-miR-1-3p;hsa-miR-15a-5p;hsa-miR-96-5
ENSG0000	ENSG0000	0.002911	hsa-miR-17-5p;hsa-miR-20b-5p;hsa-miR-34a-5p;hsa-miR-885
ENSG0000	ENSG0000	4.29E-05	hsa-miR-15a-5p;hsa-miR-16-5p;hsa-miR-34a-5p;hsa-miR-483
ENSG0000	ENSG0000	0.004439	hsa-miR-124-3p;hsa-miR-182-5p;hsa-miR-27a-3p;hsa-miR-27
ENSG0000	ENSG0000	0.005826	hsa-miR-15a-5p;hsa-miR-15b-5p;hsa-miR-16-5p;hsa-miR-9-5
ENSG0000	ENSG0000	0.001726	hsa-miR-17-5p;hsa-miR-181a-5p;hsa-miR-27a-3p;hsa-miR-27
ENSG0000	ENSG0000	0.006238	hsa-miR-149-5p;hsa-miR-182-5p;hsa-miR-9-5p;hsa-miR-96-5
ENSG0000	ENSG0000	0.004039	hsa-miR-15a-5p;hsa-miR-16-5p;hsa-miR-17-5p;hsa-miR-182-
ENSG0000	ENSG0000	0.000145	hsa-miR-15b-5p;hsa-miR-16-5p;hsa-miR-218-5p;hsa-miR-34a
ENSG0000	ENSG0000	0.004701	hsa-miR-23b-3p;hsa-miR-27b-3p;hsa-miR-483-3p;hsa-miR-9-
ENSG0000	ENSG0000	0.001199	hsa-miR-15a-5p;hsa-miR-15b-5p;hsa-miR-34a-5p;hsa-miR-44
ENSG0000	ENSG0000	8.17E-05	hsa-miR-124-3p;hsa-miR-15a-5p;hsa-miR-15b-5p;hsa-miR-16
ENSG0000	ENSG0000	5.16E-07	hsa-miR-15a-5p;hsa-miR-16-5p;hsa-miR-23a-3p;hsa-miR-23b
ENSG0000	ENSG0000	2.96E-09	hsa-miR-1-3p;hsa-miR-17-5p;hsa-miR-181a-5p;hsa-miR-181b
ENSG0000	ENSG0000	0.000195	hsa-miR-126-5p;hsa-miR-1304-3p;hsa-miR-1-3p;hsa-miR-15a
ENSG0000	ENSG0000	0.00027	hsa-miR-17-5p;hsa-miR-181a-5p;hsa-miR-181b-5p;hsa-miR-2
ENSG0000	ENSG0000	0.009674	hsa-miR-15a-5p;hsa-miR-15b-5p;hsa-miR-16-5p;hsa-miR-197
ENSG0000	ENSG0000	0.004039	hsa-miR-124-3p;hsa-miR-219a-1-3p;hsa-miR-27a-3p;hsa-miR-
ENSG0000	ENSG0000	1.17E-06	hsa-miR-126-5p;hsa-miR-15a-5p;hsa-miR-15b-5p;hsa-miR-16
ENSG0000	ENSG0000	0.0061	hsa-miR-17-5p;hsa-miR-181a-5p;hsa-miR-181b-5p;hsa-miR-2
ENSG0000	ENSG0000	0.000145	hsa-miR-126-5p;hsa-miR-17-5p;hsa-miR-20b-5p;hsa-miR-93-
ENSG0000	ENSG0000	0.002667	hsa-miR-17-5p;hsa-miR-20b-5p;hsa-miR-23b-3p;hsa-miR-93-
ENSG0000	ENSG0000	0.004197	hsa-miR-17-5p;hsa-miR-20b-5p;hsa-miR-218-5p;hsa-miR-24-
ENSG0000	ENSG0000	0.004197	hsa-miR-17-5p;hsa-miR-20b-5p;hsa-miR-218-5p;hsa-miR-24-
ENSG0000	ENSG0000	0.000563	hsa-miR-1-3p;hsa-miR-16-5p;hsa-miR-27b-3p;hsa-miR-7-5p
ENSG0000	ENSG0000	0.000177	hsa-miR-17-5p;hsa-miR-20b-5p;hsa-miR-218-5p;hsa-miR-93-
ENSG0000	ENSG0000	0.000949	hsa-miR-149-5p;hsa-miR-24-3p;hsa-miR-27a-3p;hsa-miR-27b
ENSG0000	ENSG0000	0.001208	hsa-miR-15a-5p;hsa-miR-15b-5p;hsa-miR-16-5p;hsa-miR-17-
ENSG0000	ENSG0000	0.000191	hsa-miR-181a-5p;hsa-miR-181b-5p;hsa-miR-23a-3p;hsa-miR-
ENSG0000	ENSG0000	0.002223	hsa-miR-15a-5p;hsa-miR-15b-5p;hsa-miR-16-5p;hsa-miR-218
ENSG0000	ENSG0000	0.000675	hsa-miR-1304-3p;hsa-miR-17-5p;hsa-miR-20b-5p;hsa-miR-48
ENSG0000	ENSG0000	0.001853	hsa-miR-15a-5p;hsa-miR-15b-5p;hsa-miR-16-5p;hsa-miR-181
ENSG0000	ENSG0000	0.001009	hsa-miR-182-5p;hsa-miR-27a-3p;hsa-miR-942-5p;hsa-miR-9-
ENSG0000	ENSG0000	0.000181	hsa-miR-182-5p;hsa-miR-197-3p;hsa-miR-218-5p;hsa-miR-23
ENSG0000	ENSG0000	0.000122	hsa-miR-15a-5p;hsa-miR-15b-5p;hsa-miR-16-5p;hsa-miR-34a

ENSG0000	ENSG0000	6.26E-05	hsa-miR-139-5p;hsa-miR-181a-5p;hsa-miR-338-3p;hsa-miR-3
ENSG0000	ENSG0000	4.21E-06	hsa-miR-1-3p;hsa-miR-17-5p;hsa-miR-20b-5p;hsa-miR-93-5p
ENSG0000	ENSG0000	0.000301	hsa-miR-1304-3p;hsa-miR-1-3p;hsa-miR-17-5p;hsa-miR-181a
ENSG0000	ENSG0000	0.002765	hsa-miR-15a-5p;hsa-miR-15b-5p;hsa-miR-16-5p;hsa-miR-181
ENSG0000	ENSG0000	0.001832	hsa-miR-20b-5p;hsa-miR-218-5p;hsa-miR-432-5p;hsa-miR-93
ENSG0000	ENSG0000	2.88E-05	hsa-miR-17-5p;hsa-miR-181a-5p;hsa-miR-181b-5p;hsa-miR-2
ENSG0000	ENSG0000	0.00469	hsa-miR-124-3p;hsa-miR-17-5p;hsa-miR-182-5p;hsa-miR-20b
ENSG0000	ENSG0000	4.40E-05	hsa-miR-15a-5p;hsa-miR-15b-5p;hsa-miR-16-5p;hsa-miR-27a
ENSG0000	ENSG0000	0.004362	hsa-miR-17-5p;hsa-miR-197-3p;hsa-miR-20b-5p;hsa-miR-93-
ENSG0000	ENSG0000	0.005433	hsa-miR-17-5p;hsa-miR-20b-5p;hsa-miR-27a-3p;hsa-miR-338
ENSG0000	ENSG0000	0.001137	hsa-miR-1304-3p;hsa-miR-17-5p;hsa-miR-181b-5p;hsa-miR-2
ENSG0000	ENSG0000	0.004197	hsa-miR-139-5p;hsa-miR-17-5p;hsa-miR-20b-5p;hsa-miR-218
ENSG0000	ENSG0000	0.000214	hsa-miR-17-5p;hsa-miR-20b-5p;hsa-miR-27b-3p;hsa-miR-93-
ENSG0000	ENSG0000	0.000117	hsa-miR-197-3p;hsa-miR-24-3p;hsa-miR-34a-5p;hsa-miR-449
ENSG0000	ENSG0000	0.00136	hsa-miR-149-5p;hsa-miR-15a-5p;hsa-miR-15b-5p;hsa-miR-16
ENSG0000	ENSG0000	0.000248	hsa-miR-124-3p;hsa-miR-218-5p;hsa-miR-27a-3p;hsa-miR-27
ENSG0000	ENSG0000	9.23E-05	hsa-miR-17-5p;hsa-miR-20b-5p;hsa-miR-27a-3p;hsa-miR-27b
ENSG0000	ENSG0000	0.002438	hsa-miR-181a-5p;hsa-miR-181b-5p;hsa-miR-27b-3p;hsa-miR-
ENSG0000	ENSG0000	0.007788	hsa-miR-124-3p;hsa-miR-126-5p;hsa-miR-1304-3p;hsa-miR-2
ENSG0000	ENSG0000	0.000291	hsa-miR-124-3p;hsa-miR-17-5p;hsa-miR-20b-5p;hsa-miR-218
ENSG0000	ENSG0000	0.001832	hsa-miR-24-3p;hsa-miR-338-3p;hsa-miR-34a-5p;hsa-miR-449
ENSG0000	ENSG0000	0.000319	hsa-miR-15a-5p;hsa-miR-15b-5p;hsa-miR-197-3p;hsa-miR-21
ENSG0000	ENSG0000	0.004039	hsa-miR-139-5p;hsa-miR-17-5p;hsa-miR-20b-5p;hsa-miR-93-
ENSG0000	ENSG0000	0.001094	hsa-miR-15a-5p;hsa-miR-15b-5p;hsa-miR-181a-5p;hsa-miR-1
ENSG0000	ENSG0000	9.32E-06	hsa-miR-124-3p;hsa-miR-15a-5p;hsa-miR-15b-5p;hsa-miR-16
ENSG0000	ENSG0000	0.003169	hsa-miR-124-3p;hsa-miR-218-5p;hsa-miR-23a-3p;hsa-miR-23
ENSG0000	ENSG0000	0.000319	hsa-miR-1-3p;hsa-miR-15a-5p;hsa-miR-15b-5p;hsa-miR-16-5
ENSG0000	ENSG0000	0.003169	hsa-miR-17-5p;hsa-miR-27a-3p;hsa-miR-27b-3p;hsa-miR-483
ENSG0000	ENSG0000	0.002667	hsa-miR-23a-3p;hsa-miR-23b-3p;hsa-miR-27a-3p;hsa-miR-27
ENSG0000	ENSG0000	6.35E-07	hsa-miR-15a-5p;hsa-miR-16-5p;hsa-miR-17-5p;hsa-miR-181a
ENSG0000	ENSG0000	3.60E-08	hsa-miR-139-5p;hsa-miR-1-3p;hsa-miR-218-5p;hsa-miR-23b-
ENSG0000	ENSG0000	0.007785	hsa-miR-124-3p;hsa-miR-16-5p;hsa-miR-17-5p;hsa-miR-20b-
ENSG0000	ENSG0000	0.007589	hsa-miR-17-5p;hsa-miR-20b-5p;hsa-miR-7-5p;hsa-miR-93-5p
ENSG0000	ENSG0000	0.003678	hsa-miR-1304-3p;hsa-miR-17-5p;hsa-miR-20b-5p;hsa-miR-27
ENSG0000	ENSG0000	0.001656	hsa-miR-124-3p;hsa-miR-126-5p;hsa-miR-34a-5p;hsa-miR-44
ENSG0000	ENSG0000	0.000584	hsa-miR-124-3p;hsa-miR-17-5p;hsa-miR-197-3p;hsa-miR-20b
ENSG0000	ENSG0000	0.000949	hsa-miR-17-5p;hsa-miR-20b-5p;hsa-miR-218-5p;hsa-miR-93-
ENSG0000	ENSG0000	0.005826	hsa-miR-17-5p;hsa-miR-20b-5p;hsa-miR-34a-5p;hsa-miR-449
ENSG0000	ENSG0000	6.66E-06	hsa-miR-23b-3p;hsa-miR-24-3p;hsa-miR-27b-3p;hsa-miR-34a
ENSG0000	ENSG0000	4.64E-06	hsa-miR-1-3p;hsa-miR-15a-5p;hsa-miR-15b-5p;hsa-miR-16-5
ENSG0000	ENSG0000	0.000928	hsa-miR-181a-5p;hsa-miR-218-5p;hsa-miR-27a-3p;hsa-miR-2
ENSG0000	ENSG0000	5.81E-05	hsa-miR-17-5p;hsa-miR-20b-5p;hsa-miR-218-5p;hsa-miR-219
ENSG0000	ENSG0000	0.001853	hsa-miR-17-5p;hsa-miR-20b-5p;hsa-miR-27a-3p;hsa-miR-27b
ENSG0000	ENSG0000	0.001492	hsa-miR-126-5p;hsa-miR-15a-5p;hsa-miR-15b-5p;hsa-miR-18
ENSG0000	ENSG0000	0.000497	hsa-miR-1-3p;hsa-miR-17-5p;hsa-miR-182-5p;hsa-miR-20b-5
ENSG0000	ENSG0000	0.000205	hsa-miR-15a-5p;hsa-miR-15b-5p;hsa-miR-17-5p;hsa-miR-20b
ENSG0000	ENSG0000	0.001726	hsa-miR-17-5p;hsa-miR-181a-5p;hsa-miR-181b-5p;hsa-miR-2
ENSG0000	ENSG0000	0.001987	hsa-miR-124-3p;hsa-miR-182-5p;hsa-miR-34a-5p;hsa-miR-44
ENSG0000	ENSG0000	0.00978	hsa-miR-1304-3p;hsa-miR-23a-3p;hsa-miR-23b-3p;hsa-miR-2
ENSG0000	ENSG0000	2.57E-06	hsa-miR-15a-5p;hsa-miR-15b-5p;hsa-miR-17-5p;hsa-miR-20b
ENSG0000	ENSG0000	0.004362	hsa-miR-15a-5p;hsa-miR-15b-5p;hsa-miR-16-5p;hsa-miR-9-5
ENSG0000	ENSG0000	0.003169	hsa-miR-15a-5p;hsa-miR-15b-5p;hsa-miR-16-5p;hsa-miR-218
ENSG0000	ENSG0000	0.002765	hsa-miR-124-3p;hsa-miR-15a-5p;hsa-miR-15b-5p;hsa-miR-16
ENSG0000	ENSG0000	2.38E-06	hsa-miR-139-5p;hsa-miR-17-5p;hsa-miR-20b-5p;hsa-miR-218

ENSG0000	ENSG0000	0.000655	hsa-miR-124-3p;hsa-miR-1304-3p;hsa-miR-197-3p;hsa-miR-2
ENSG0000	ENSG0000	0.00313	hsa-miR-15a-5p;hsa-miR-15b-5p;hsa-miR-16-5p;hsa-miR-483
ENSG0000	ENSG0000	0.000519	hsa-miR-17-5p;hsa-miR-181a-5p;hsa-miR-181b-5p;hsa-miR-1
ENSG0000	ENSG0000	0.003443	hsa-miR-15a-5p;hsa-miR-15b-5p;hsa-miR-16-5p;hsa-miR-197
ENSG0000	ENSG0000	2.19E-06	hsa-miR-17-5p;hsa-miR-181a-5p;hsa-miR-181b-5p;hsa-miR-1
ENSG0000	ENSG0000	0.00304	hsa-miR-17-5p;hsa-miR-197-3p;hsa-miR-20b-5p;hsa-miR-483
ENSG0000	ENSG0000	0.003966	hsa-miR-1304-3p;hsa-miR-181a-5p;hsa-miR-181b-5p;hsa-miR
ENSG0000	ENSG0000	0.000214	hsa-miR-17-5p;hsa-miR-20b-5p;hsa-miR-218-5p;hsa-miR-93-
ENSG0000	ENSG0000	0.000941	hsa-miR-17-5p;hsa-miR-20b-5p;hsa-miR-218-5p;hsa-miR-483
ENSG0000	ENSG0000	0.000231	hsa-miR-15a-5p;hsa-miR-15b-5p;hsa-miR-181a-5p;hsa-miR-1
ENSG0000	ENSG0000	0.000655	hsa-miR-1304-3p;hsa-miR-139-5p;hsa-miR-218-5p;hsa-miR-2
ENSG0000	ENSG0000	0.001492	hsa-miR-15a-5p;hsa-miR-15b-5p;hsa-miR-16-5p;hsa-miR-664
ENSG0000	ENSG0000	0.00262	hsa-miR-17-5p;hsa-miR-20b-5p;hsa-miR-218-5p;hsa-miR-7-5
ENSG0000	ENSG0000	0.000715	hsa-miR-181a-5p;hsa-miR-181b-5p;hsa-miR-182-5p;hsa-miR-
ENSG0000	ENSG0000	0.001068	hsa-miR-15a-5p;hsa-miR-15b-5p;hsa-miR-27a-3p;hsa-miR-27
ENSG0000	ENSG0000	1.21E-06	hsa-miR-15a-5p;hsa-miR-15b-5p;hsa-miR-16-5p;hsa-miR-27a
ENSG0000	ENSG0000	0.002045	hsa-miR-17-5p;hsa-miR-197-3p;hsa-miR-20b-5p;hsa-miR-23a
ENSG0000	ENSG0000	0.002045	hsa-miR-17-5p;hsa-miR-197-3p;hsa-miR-20b-5p;hsa-miR-23a
ENSG0000	ENSG0000	0.004951	hsa-miR-124-3p;hsa-miR-149-5p;hsa-miR-218-5p;hsa-miR-27
ENSG0000	ENSG0000	0.001713	hsa-miR-17-5p;hsa-miR-20b-5p;hsa-miR-27a-3p;hsa-miR-27b
ENSG0000	ENSG0000	0.000646	hsa-miR-139-5p;hsa-miR-15a-5p;hsa-miR-15b-5p;hsa-miR-7-
ENSG0000	ENSG0000	1.51E-06	hsa-miR-149-5p;hsa-miR-181a-5p;hsa-miR-181b-5p;hsa-miR-
ENSG0000	ENSG0000	4.21E-06	hsa-miR-17-5p;hsa-miR-181a-5p;hsa-miR-20b-5p;hsa-miR-93
ENSG0000	ENSG0000	0.000882	hsa-miR-182-5p;hsa-miR-34a-5p;hsa-miR-449a;hsa-miR-483-
ENSG0000	ENSG0000	0.000882	hsa-miR-182-5p;hsa-miR-34a-5p;hsa-miR-449a;hsa-miR-483-
ENSG0000	ENSG0000	0.000882	hsa-miR-182-5p;hsa-miR-34a-5p;hsa-miR-449a;hsa-miR-483-
ENSG0000	ENSG0000	0.000882	hsa-miR-182-5p;hsa-miR-34a-5p;hsa-miR-449a;hsa-miR-483-
ENSG0000	ENSG0000	0.000882	hsa-miR-182-5p;hsa-miR-34a-5p;hsa-miR-449a;hsa-miR-483-
ENSG0000	ENSG0000	0.000882	hsa-miR-182-5p;hsa-miR-34a-5p;hsa-miR-449a;hsa-miR-483-
ENSG0000	ENSG0000	0.000882	hsa-miR-182-5p;hsa-miR-34a-5p;hsa-miR-449a;hsa-miR-483-
ENSG0000	ENSG0000	0.000882	hsa-miR-182-5p;hsa-miR-34a-5p;hsa-miR-449a;hsa-miR-483-
ENSG0000	ENSG0000	0.006238	hsa-miR-1-3p;hsa-miR-17-5p;hsa-miR-20b-5p;hsa-miR-93-5p
ENSG0000	ENSG0000	0.000595	hsa-miR-181a-5p;hsa-miR-181b-5p;hsa-miR-218-5p;hsa-miR-
ENSG0000	ENSG0000	0.000159	hsa-miR-15a-5p;hsa-miR-15b-5p;hsa-miR-16-5p;hsa-miR-218
ENSG0000	ENSG0000	1.56E-05	hsa-miR-139-5p;hsa-miR-17-5p;hsa-miR-197-3p;hsa-miR-20b
ENSG0000	ENSG0000	0.000738	hsa-miR-1-3p;hsa-miR-15a-5p;hsa-miR-15b-5p;hsa-miR-16-5
ENSG0000	ENSG0000	2.30E-05	hsa-miR-126-5p;hsa-miR-15a-5p;hsa-miR-15b-5p;hsa-miR-16
ENSG0000	ENSG0000	0.00313	hsa-miR-15a-5p;hsa-miR-16-5p;hsa-miR-181a-5p;hsa-miR-18
ENSG0000	ENSG0000	0.000471	hsa-miR-17-5p;hsa-miR-20b-5p;hsa-miR-24-3p;hsa-miR-27a-
ENSG0000	ENSG0000	0.006669	hsa-miR-182-5p;hsa-miR-23a-3p;hsa-miR-23b-3p;hsa-miR-66
ENSG0000	ENSG0000	0.000646	hsa-miR-15a-5p;hsa-miR-15b-5p;hsa-miR-16-5p;hsa-miR-218
ENSG0000	ENSG0000	1.19E-06	hsa-miR-17-5p;hsa-miR-20b-5p;hsa-miR-7-5p;hsa-miR-93-5p
ENSG0000	ENSG0000	1.52E-08	hsa-miR-126-5p;hsa-miR-1-3p;hsa-miR-15a-5p;hsa-miR-15b-
ENSG0000	ENSG0000	0.000243	hsa-miR-15a-5p;hsa-miR-15b-5p;hsa-miR-17-5p;hsa-miR-23a
ENSG0000	ENSG0000	0.007589	hsa-miR-1304-3p;hsa-miR-17-5p;hsa-miR-20b-5p;hsa-miR-93
ENSG0000	ENSG0000	0.002911	hsa-miR-15a-5p;hsa-miR-15b-5p;hsa-miR-16-5p;hsa-miR-24-
ENSG0000	ENSG0000	0.003169	hsa-miR-149-5p;hsa-miR-197-3p;hsa-miR-27a-3p;hsa-miR-27
ENSG0000	ENSG0000	0.000402	hsa-miR-197-3p;hsa-miR-23a-3p;hsa-miR-23b-3p;hsa-miR-33
ENSG0000	ENSG0000	0.00859	hsa-miR-124-3p;hsa-miR-126-5p;hsa-miR-24-3p;hsa-miR-7-5
ENSG0000	ENSG0000	3.89E-05	hsa-miR-15a-5p;hsa-miR-15b-5p;hsa-miR-17-5p;hsa-miR-20b
ENSG0000	ENSG0000	0.002365	hsa-miR-139-5p;hsa-miR-17-5p;hsa-miR-20b-5p;hsa-miR-27a
ENSG0000	ENSG0000	0.005433	hsa-miR-1304-3p;hsa-miR-20b-5p;hsa-miR-218-5p;hsa-miR-9
ENSG0000	ENSG0000	0.004032	hsa-miR-16-5p;hsa-miR-181a-5p;hsa-miR-181b-5p;hsa-miR-2
ENSG0000	ENSG0000	0.000117	hsa-miR-182-5p;hsa-miR-23a-3p;hsa-miR-23b-3p;hsa-miR-96

ENSG0000	ENSG0000	0.007788	hsa-miR-15a-5p;hsa-miR-16-5p;hsa-miR-181a-5p;hsa-miR-18
ENSG0000	ENSG0000	0.003733	hsa-miR-1-3p;hsa-miR-17-5p;hsa-miR-20b-5p;hsa-miR-93-5p
ENSG0000	ENSG0000	0.000214	hsa-miR-15a-5p;hsa-miR-15b-5p;hsa-miR-16-5p;hsa-miR-34a-
ENSG0000	ENSG0000	3.48E-06	hsa-miR-15a-5p;hsa-miR-15b-5p;hsa-miR-16-5p;hsa-miR-17-
ENSG0000	ENSG0000	8.46E-05	hsa-miR-15a-5p;hsa-miR-15b-5p;hsa-miR-16-5p;hsa-miR-27a-
ENSG0000	ENSG0000	0.009674	hsa-miR-15a-5p;hsa-miR-15b-5p;hsa-miR-16-5p;hsa-miR-218-
ENSG0000	ENSG0000	0.001185	hsa-miR-126-5p;hsa-miR-17-5p;hsa-miR-20b-5p;hsa-miR-320
ENSG0000	ENSG0000	0.001185	hsa-miR-126-5p;hsa-miR-17-5p;hsa-miR-20b-5p;hsa-miR-320
ENSG0000	ENSG0000	0.003443	hsa-miR-23a-3p;hsa-miR-23b-3p;hsa-miR-7-5p;hsa-miR-876-
ENSG0000	ENSG0000	0.003443	hsa-miR-23a-3p;hsa-miR-23b-3p;hsa-miR-7-5p;hsa-miR-876-
ENSG0000	ENSG0000	0.001185	hsa-miR-15a-5p;hsa-miR-15b-5p;hsa-miR-16-5p;hsa-miR-181
ENSG0000	ENSG0000	0.003169	hsa-miR-15a-5p;hsa-miR-15b-5p;hsa-miR-16-5p;hsa-miR-320
ENSG0000	ENSG0000	0.001726	hsa-miR-15a-5p;hsa-miR-15b-5p;hsa-miR-16-5p;hsa-miR-181
ENSG0000	ENSG0000	0.001853	hsa-miR-124-3p;hsa-miR-17-5p;hsa-miR-20b-5p;hsa-miR-34a-
ENSG0000	ENSG0000	0.000304	hsa-miR-181a-5p;hsa-miR-181b-5p;hsa-miR-483-3p;hsa-miR-
ENSG0000	ENSG0000	6.36E-09	hsa-miR-15a-5p;hsa-miR-15b-5p;hsa-miR-181a-5p;hsa-miR-1
ENSG0000	ENSG0000	0.003733	hsa-miR-182-5p;hsa-miR-34a-5p;hsa-miR-483-3p;hsa-miR-96-
ENSG0000	ENSG0000	4.64E-06	hsa-miR-17-5p;hsa-miR-197-3p;hsa-miR-20b-5p;hsa-miR-27a-
ENSG0000	ENSG0000	4.64E-06	hsa-miR-17-5p;hsa-miR-197-3p;hsa-miR-20b-5p;hsa-miR-27a-
ENSG0000	ENSG0000	4.64E-06	hsa-miR-17-5p;hsa-miR-197-3p;hsa-miR-20b-5p;hsa-miR-27a-
ENSG0000	ENSG0000	4.64E-06	hsa-miR-17-5p;hsa-miR-197-3p;hsa-miR-20b-5p;hsa-miR-27a-
ENSG0000	ENSG0000	4.64E-06	hsa-miR-17-5p;hsa-miR-197-3p;hsa-miR-20b-5p;hsa-miR-27a-
ENSG0000	ENSG0000	4.64E-06	hsa-miR-17-5p;hsa-miR-197-3p;hsa-miR-20b-5p;hsa-miR-27a-
ENSG0000	ENSG0000	1.18E-05	hsa-miR-139-5p;hsa-miR-17-5p;hsa-miR-20b-5p;hsa-miR-218-
ENSG0000	ENSG0000	0.001384	hsa-miR-15a-5p;hsa-miR-15b-5p;hsa-miR-16-5p;hsa-miR-182-
ENSG0000	ENSG0000	0.001606	hsa-miR-17-5p;hsa-miR-20b-5p;hsa-miR-24-3p;hsa-miR-338-
ENSG0000	ENSG0000	4.21E-06	hsa-miR-17-5p;hsa-miR-20b-5p;hsa-miR-34a-5p;hsa-miR-93-
ENSG0000	ENSG0000	4.21E-06	hsa-miR-17-5p;hsa-miR-20b-5p;hsa-miR-34a-5p;hsa-miR-93-
ENSG0000	ENSG0000	0.001068	hsa-miR-15a-5p;hsa-miR-15b-5p;hsa-miR-17-5p;hsa-miR-197-
ENSG0000	ENSG0000	0.005058	hsa-miR-1304-3p;hsa-miR-17-5p;hsa-miR-20b-5p;hsa-miR-9-
ENSG0000	ENSG0000	0.001492	hsa-miR-17-5p;hsa-miR-20b-5p;hsa-miR-24-3p;hsa-miR-93-5
ENSG0000	ENSG0000	0.000214	hsa-miR-15a-5p;hsa-miR-15b-5p;hsa-miR-16-5p;hsa-miR-218-
ENSG0000	ENSG0000	0.001656	hsa-miR-15a-5p;hsa-miR-15b-5p;hsa-miR-34a-5p;hsa-miR-44
ENSG0000	ENSG0000	0.003169	hsa-miR-124-3p;hsa-miR-182-5p;hsa-miR-34a-5p;hsa-miR-44
ENSG0000	ENSG0000	0.003733	hsa-miR-1304-3p;hsa-miR-17-5p;hsa-miR-20b-5p;hsa-miR-93-
ENSG0000	ENSG0000	0.000402	hsa-miR-17-5p;hsa-miR-182-5p;hsa-miR-20b-5p;hsa-miR-93-
ENSG0000	ENSG0000	0.007078	hsa-miR-139-5p;hsa-miR-15a-5p;hsa-miR-15b-5p;hsa-miR-16-
ENSG0000	ENSG0000	0.002021	hsa-miR-15a-5p;hsa-miR-15b-5p;hsa-miR-16-5p;hsa-miR-218-
ENSG0000	ENSG0000	0.000853	hsa-miR-15a-5p;hsa-miR-15b-5p;hsa-miR-17-5p;hsa-miR-20b-
ENSG0000	ENSG0000	0.000419	hsa-miR-124-3p;hsa-miR-15a-5p;hsa-miR-15b-5p;hsa-miR-16-
ENSG0000	ENSG0000	0.00859	hsa-miR-15a-5p;hsa-miR-15b-5p;hsa-miR-17-5p;hsa-miR-20b-
ENSG0000	ENSG0000	0.008079	hsa-miR-1304-3p;hsa-miR-218-5p;hsa-miR-27a-3p;hsa-miR-2
ENSG0000	ENSG0000	3.18E-05	hsa-miR-139-5p;hsa-miR-15a-5p;hsa-miR-15b-5p;hsa-miR-16-
ENSG0000	ENSG0000	0.001009	hsa-miR-149-5p;hsa-miR-17-5p;hsa-miR-20b-5p;hsa-miR-24-
ENSG0000	ENSG0000	0.001094	hsa-miR-124-3p;hsa-miR-17-5p;hsa-miR-181a-5p;hsa-miR-18
ENSG0000	ENSG0000	0.00134	hsa-miR-17-5p;hsa-miR-20b-5p;hsa-miR-885-5p;hsa-miR-93-
ENSG0000	ENSG0000	0.00134	hsa-miR-17-5p;hsa-miR-20b-5p;hsa-miR-885-5p;hsa-miR-93-
ENSG0000	ENSG0000	0.004609	hsa-miR-1304-3p;hsa-miR-15a-5p;hsa-miR-15b-5p;hsa-miR-1
ENSG0000	ENSG0000	0.001009	hsa-miR-15a-5p;hsa-miR-15b-5p;hsa-miR-16-5p;hsa-miR-20b-
ENSG0000	ENSG0000	0.000445	hsa-miR-15a-5p;hsa-miR-15b-5p;hsa-miR-16-5p;hsa-miR-20b-
ENSG0000	ENSG0000	0.000445	hsa-miR-15a-5p;hsa-miR-15b-5p;hsa-miR-16-5p;hsa-miR-20b-
ENSG0000	ENSG0000	0.000773	hsa-miR-17-5p;hsa-miR-181a-5p;hsa-miR-181b-5p;hsa-miR-2
ENSG0000	ENSG0000	0.00027	hsa-miR-17-5p;hsa-miR-181a-5p;hsa-miR-181b-5p;hsa-miR-2
ENSG0000	ENSG0000	0.003169	hsa-miR-1-3p;hsa-miR-149-5p;hsa-miR-34a-5p;hsa-miR-449a

ENSG0000	ENSG0000	0.000122	hsa-miR-15a-5p;hsa-miR-15b-5p;hsa-miR-218-5p;hsa-miR-23
ENSG0000	ENSG0000	0.002911	hsa-miR-1304-3p;hsa-miR-17-5p;hsa-miR-20b-5p;hsa-miR-93
ENSG0000	ENSG0000	0.006669	hsa-miR-17-5p;hsa-miR-20b-5p;hsa-miR-484;hsa-miR-93-5p
ENSG0000	ENSG0000	0.002667	hsa-miR-17-5p;hsa-miR-197-3p;hsa-miR-20b-5p;hsa-miR-93-
ENSG0000	ENSG0000	0.002667	hsa-miR-17-5p;hsa-miR-197-3p;hsa-miR-20b-5p;hsa-miR-93-
ENSG0000	ENSG0000	5.58E-05	hsa-miR-15a-5p;hsa-miR-15b-5p;hsa-miR-16-5p;hsa-miR-23a
ENSG0000	ENSG0000	3.18E-05	hsa-miR-15a-5p;hsa-miR-15b-5p;hsa-miR-16-5p;hsa-miR-218
ENSG0000	ENSG0000	1.10E-05	hsa-miR-15a-5p;hsa-miR-15b-5p;hsa-miR-16-5p;hsa-miR-17-
ENSG0000	ENSG0000	0.008161	hsa-miR-17-5p;hsa-miR-20b-5p;hsa-miR-27a-3p;hsa-miR-27b
ENSG0000	ENSG0000	0.009356	hsa-miR-1304-3p;hsa-miR-15a-5p;hsa-miR-15b-5p;hsa-miR-1
ENSG0000	ENSG0000	3.21E-06	hsa-miR-15a-5p;hsa-miR-15b-5p;hsa-miR-16-5p;hsa-miR-197
ENSG0000	ENSG0000	0.007119	hsa-miR-15a-5p;hsa-miR-15b-5p;hsa-miR-16-5p;hsa-miR-218
ENSG0000	ENSG0000	1.28E-05	hsa-miR-17-5p;hsa-miR-182-5p;hsa-miR-20b-5p;hsa-miR-338
ENSG0000	ENSG0000	0.00134	hsa-miR-1-3p;hsa-miR-15a-5p;hsa-miR-15b-5p;hsa-miR-16-5
ENSG0000	ENSG0000	0.00134	hsa-miR-1-3p;hsa-miR-15a-5p;hsa-miR-15b-5p;hsa-miR-16-5
ENSG0000	ENSG0000	0.002223	hsa-miR-124-3p;hsa-miR-17-5p;hsa-miR-20b-5p;hsa-miR-219
ENSG0000	ENSG0000	0.002223	hsa-miR-124-3p;hsa-miR-17-5p;hsa-miR-20b-5p;hsa-miR-219
ENSG0000	ENSG0000	4.93E-05	hsa-miR-17-5p;hsa-miR-20b-5p;hsa-miR-218-5p;hsa-miR-876
ENSG0000	ENSG0000	0.000359	hsa-miR-1304-3p;hsa-miR-15a-5p;hsa-miR-15b-5p;hsa-miR-1
ENSG0000	ENSG0000	0.002911	hsa-miR-1-3p;hsa-miR-15a-5p;hsa-miR-15b-5p;hsa-miR-16-5
ENSG0000	ENSG0000	0.000853	hsa-miR-1304-3p;hsa-miR-17-5p;hsa-miR-20b-5p;hsa-miR-24
ENSG0000	ENSG0000	4.51E-09	hsa-miR-181a-5p;hsa-miR-181b-5p;hsa-miR-23a-3p;hsa-miR-
ENSG0000	ENSG0000	1.49E-05	hsa-miR-17-5p;hsa-miR-20b-5p;hsa-miR-219a-1-3p;hsa-miR-
ENSG0000	ENSG0000	0.000197	hsa-miR-15a-5p;hsa-miR-15b-5p;hsa-miR-16-5p;hsa-miR-181
ENSG0000	ENSG0000	0.004039	hsa-miR-15a-5p;hsa-miR-15b-5p;hsa-miR-16-5p;hsa-miR-24-
ENSG0000	ENSG0000	0.004039	hsa-miR-15a-5p;hsa-miR-15b-5p;hsa-miR-16-5p;hsa-miR-24-
ENSG0000	ENSG0000	0.001832	hsa-miR-139-5p;hsa-miR-15a-5p;hsa-miR-15b-5p;hsa-miR-16
ENSG0000	ENSG0000	0.001069	hsa-miR-27a-3p;hsa-miR-27b-3p;hsa-miR-34a-5p;hsa-miR-44
ENSG0000	ENSG0000	0.005707	hsa-miR-1304-3p;hsa-miR-17-5p;hsa-miR-20b-5p;hsa-miR-21
ENSG0000	ENSG0000	4.21E-06	hsa-miR-1-3p;hsa-miR-15a-5p;hsa-miR-15b-5p;hsa-miR-16-5
ENSG0000	ENSG0000	0.000291	hsa-miR-15a-5p;hsa-miR-15b-5p;hsa-miR-16-5p;hsa-miR-34a
ENSG0000	ENSG0000	2.64E-05	hsa-miR-124-3p;hsa-miR-1-3p;hsa-miR-17-5p;hsa-miR-197-3
ENSG0000	ENSG0000	6.66E-06	hsa-miR-1304-3p;hsa-miR-15a-5p;hsa-miR-15b-5p;hsa-miR-1
ENSG0000	ENSG0000	2.36E-06	hsa-miR-1-3p;hsa-miR-17-5p;hsa-miR-20b-5p;hsa-miR-93-5p
ENSG0000	ENSG0000	0.000619	hsa-miR-1304-3p;hsa-miR-15a-5p;hsa-miR-15b-5p;hsa-miR-1
ENSG0000	ENSG0000	0.000619	hsa-miR-1304-3p;hsa-miR-15a-5p;hsa-miR-15b-5p;hsa-miR-1
ENSG0000	ENSG0000	1.17E-06	hsa-miR-15a-5p;hsa-miR-15b-5p;hsa-miR-16-5p;hsa-miR-17-
ENSG0000	ENSG0000	3.18E-05	hsa-miR-124-3p;hsa-miR-23a-3p;hsa-miR-23b-3p;hsa-miR-32
ENSG0000	ENSG0000	0.002438	hsa-miR-124-3p;hsa-miR-15a-5p;hsa-miR-15b-5p;hsa-miR-16
ENSG0000	ENSG0000	0.000839	hsa-miR-17-5p;hsa-miR-20b-5p;hsa-miR-219a-1-3p;hsa-miR-
ENSG0000	ENSG0000	0.001853	hsa-miR-15a-5p;hsa-miR-15b-5p;hsa-miR-16-5p;hsa-miR-7-5
ENSG0000	ENSG0000	0.004701	hsa-miR-124-3p;hsa-miR-17-5p;hsa-miR-20b-5p;hsa-miR-93-
ENSG0000	ENSG0000	0.002021	hsa-miR-182-5p;hsa-miR-320a;hsa-miR-885-5p;hsa-miR-96-5
ENSG0000	ENSG0000	0.000107	hsa-miR-15a-5p;hsa-miR-15b-5p;hsa-miR-16-5p;hsa-miR-338
ENSG0000	ENSG0000	2.57E-07	hsa-miR-181a-5p;hsa-miR-181b-5p;hsa-miR-23b-3p;hsa-miR-
ENSG0000	ENSG0000	6.82E-05	hsa-miR-149-5p;hsa-miR-218-5p;hsa-miR-24-3p;hsa-miR-483
ENSG0000	ENSG0000	4.29E-05	hsa-miR-124-3p;hsa-miR-15a-5p;hsa-miR-16-5p;hsa-miR-9-5
ENSG0000	ENSG0000	0.003191	hsa-miR-15a-5p;hsa-miR-15b-5p;hsa-miR-17-5p;hsa-miR-20b
ENSG0000	ENSG0000	0.003326	hsa-miR-15a-5p;hsa-miR-15b-5p;hsa-miR-16-5p;hsa-miR-27a
ENSG0000	ENSG0000	0.007589	hsa-miR-1304-3p;hsa-miR-197-3p;hsa-miR-432-5p;hsa-miR-4
ENSG0000	ENSG0000	0.001384	hsa-miR-15a-5p;hsa-miR-15b-5p;hsa-miR-16-5p;hsa-miR-182
ENSG0000	ENSG0000	0.000563	hsa-miR-15a-5p;hsa-miR-15b-5p;hsa-miR-181a-5p;hsa-miR-1
ENSG0000	ENSG0000	0.008161	hsa-miR-124-3p;hsa-miR-15a-5p;hsa-miR-15b-5p;hsa-miR-21
ENSG0000	ENSG0000	0.000177	hsa-miR-17-5p;hsa-miR-20b-5p;hsa-miR-218-5p;hsa-miR-93-

ENSG0000	ENSG0000	0.000117	hsa-miR-27a-3p;hsa-miR-27b-3p;hsa-miR-34a-5p;hsa-miR-44
ENSG0000	ENSG0000	0.005504	hsa-miR-1304-3p;hsa-miR-15a-5p;hsa-miR-15b-5p;hsa-miR-1
ENSG0000	ENSG0000	0.004039	hsa-miR-1-3p;hsa-miR-17-5p;hsa-miR-20b-5p;hsa-miR-93-5p
ENSG0000	ENSG0000	1.10E-05	hsa-miR-15a-5p;hsa-miR-15b-5p;hsa-miR-16-5p;hsa-miR-181
ENSG0000	ENSG0000	0.003169	hsa-miR-17-5p;hsa-miR-20b-5p;hsa-miR-218-5p;hsa-miR-93-
ENSG0000	ENSG0000	0.003169	hsa-miR-17-5p;hsa-miR-20b-5p;hsa-miR-218-5p;hsa-miR-93-
ENSG0000	ENSG0000	0.003169	hsa-miR-17-5p;hsa-miR-20b-5p;hsa-miR-218-5p;hsa-miR-93-
ENSG0000	ENSG0000	0.00134	hsa-miR-124-3p;hsa-miR-1-3p;hsa-miR-27a-3p;hsa-miR-27b-
ENSG0000	ENSG0000	0.003169	hsa-miR-124-3p;hsa-miR-17-5p;hsa-miR-20b-5p;hsa-miR-34a
ENSG0000	ENSG0000	3.01E-05	hsa-miR-1-3p;hsa-miR-15a-5p;hsa-miR-16-5p;hsa-miR-181b-
ENSG0000	ENSG0000	0.004701	hsa-miR-17-5p;hsa-miR-20b-5p;hsa-miR-7-5p;hsa-miR-93-5p
ENSG0000	ENSG0000	0.000715	hsa-miR-16-5p;hsa-miR-17-5p;hsa-miR-20b-5p;hsa-miR-218-
ENSG0000	ENSG0000	0.005797	hsa-miR-149-5p;hsa-miR-17-5p;hsa-miR-20b-5p;hsa-miR-93-
ENSG0000	ENSG0000	0.00213	hsa-miR-1304-3p;hsa-miR-15a-5p;hsa-miR-15b-5p;hsa-miR-1
ENSG0000	ENSG0000	0.002438	hsa-miR-15a-5p;hsa-miR-15b-5p;hsa-miR-34a-5p;hsa-miR-44
ENSG0000	ENSG0000	0.003852	hsa-miR-17-5p;hsa-miR-20b-5p;hsa-miR-218-5p;hsa-miR-432
ENSG0000	ENSG0000	0.004362	hsa-miR-124-3p;hsa-miR-27a-3p;hsa-miR-27b-3p;hsa-miR-66
ENSG0000	ENSG0000	0.003169	hsa-miR-139-5p;hsa-miR-181b-5p;hsa-miR-23a-3p;hsa-miR-2
ENSG0000	ENSG0000	0.001361	hsa-miR-17-5p;hsa-miR-181a-5p;hsa-miR-181b-5p;hsa-miR-2
ENSG0000	ENSG0000	0.000738	hsa-miR-17-5p;hsa-miR-181a-5p;hsa-miR-181b-5p;hsa-miR-2
ENSG0000	ENSG0000	2.36E-06	hsa-miR-17-5p;hsa-miR-20b-5p;hsa-miR-27a-3p;hsa-miR-27b
ENSG0000	ENSG0000	0.005247	hsa-miR-17-5p;hsa-miR-181a-5p;hsa-miR-181b-5p;hsa-miR-2
ENSG0000	ENSG0000	0.008079	hsa-miR-17-5p;hsa-miR-20b-5p;hsa-miR-27a-3p;hsa-miR-27b
ENSG0000	ENSG0000	0.000231	hsa-miR-15a-5p;hsa-miR-15b-5p;hsa-miR-16-5p;hsa-miR-432
ENSG0000	ENSG0000	0.000231	hsa-miR-1304-3p;hsa-miR-181a-5p;hsa-miR-181b-5p;hsa-miR
ENSG0000	ENSG0000	0.008784	hsa-miR-1304-3p;hsa-miR-17-5p;hsa-miR-197-3p;hsa-miR-20
ENSG0000	ENSG0000	0.005111	hsa-miR-15a-5p;hsa-miR-15b-5p;hsa-miR-16-5p;hsa-miR-17-
ENSG0000	ENSG0000	0.004701	hsa-miR-17-5p;hsa-miR-20b-5p;hsa-miR-484;hsa-miR-93-5p
ENSG0000	ENSG0000	0.005222	hsa-miR-17-5p;hsa-miR-20b-5p;hsa-miR-219a-1-3p;hsa-miR-
ENSG0000	ENSG0000	0.002021	hsa-miR-15a-5p;hsa-miR-15b-5p;hsa-miR-16-5p;hsa-miR-197
ENSG0000	ENSG0000	2.36E-06	hsa-miR-15a-5p;hsa-miR-15b-5p;hsa-miR-16-5p;hsa-miR-218
ENSG0000	ENSG0000	0.003191	hsa-miR-17-5p;hsa-miR-181a-5p;hsa-miR-181b-5p;hsa-miR-2
ENSG0000	ENSG0000	0.00859	hsa-miR-1-3p;hsa-miR-15a-5p;hsa-miR-15b-5p;hsa-miR-16-5
ENSG0000	ENSG0000	7.72E-06	hsa-miR-17-5p;hsa-miR-181a-5p;hsa-miR-181b-5p;hsa-miR-2
ENSG0000	ENSG0000	0.006595	hsa-miR-17-5p;hsa-miR-181a-5p;hsa-miR-181b-5p;hsa-miR-2
ENSG0000	ENSG0000	7.32E-05	hsa-miR-15a-5p;hsa-miR-16-5p;hsa-miR-218-5p;hsa-miR-7-5
ENSG0000	ENSG0000	0.00026	hsa-miR-126-5p;hsa-miR-15a-5p;hsa-miR-15b-5p;hsa-miR-17
ENSG0000	ENSG0000	0.003169	hsa-miR-1-3p;hsa-miR-181a-5p;hsa-miR-181b-5p;hsa-miR-24
ENSG0000	ENSG0000	0.001606	hsa-miR-15a-5p;hsa-miR-15b-5p;hsa-miR-17-5p;hsa-miR-20b
ENSG0000	ENSG0000	0.008547	hsa-miR-17-5p;hsa-miR-20b-5p;hsa-miR-218-5p;hsa-miR-484;
ENSG0000	ENSG0000	0.003169	hsa-miR-1304-3p;hsa-miR-218-5p;hsa-miR-7-5p;hsa-miR-9-5
ENSG0000	ENSG0000	0.000126	hsa-miR-1304-3p;hsa-miR-15a-5p;hsa-miR-15b-5p;hsa-miR-1
ENSG0000	ENSG0000	0.000782	hsa-miR-15a-5p;hsa-miR-15b-5p;hsa-miR-16-5p;hsa-miR-27a
ENSG0000	ENSG0000	0.000256	hsa-miR-181a-5p;hsa-miR-181b-5p;hsa-miR-23a-3p;hsa-miR-
ENSG0000	ENSG0000	0.002128	hsa-miR-17-5p;hsa-miR-20b-5p;hsa-miR-218-5p;hsa-miR-483
ENSG0000	ENSG0000	0.000346	hsa-miR-17-5p;hsa-miR-20b-5p;hsa-miR-27a-3p;hsa-miR-27b
ENSG0000	ENSG0000	5.95E-06	hsa-miR-17-5p;hsa-miR-181a-5p;hsa-miR-181b-5p;hsa-miR-2
ENSG0000	ENSG0000	5.60E-06	hsa-miR-15a-5p;hsa-miR-15b-5p;hsa-miR-16-5p;hsa-miR-17-
ENSG0000	ENSG0000	5.66E-05	hsa-miR-17-5p;hsa-miR-20b-5p;hsa-miR-218-5p;hsa-miR-93-
ENSG0000	ENSG0000	5.66E-05	hsa-miR-17-5p;hsa-miR-20b-5p;hsa-miR-218-5p;hsa-miR-93-
ENSG0000	ENSG0000	0.005433	hsa-miR-23a-3p;hsa-miR-23b-3p;hsa-miR-24-3p;hsa-miR-338
ENSG0000	ENSG0000	0.00859	hsa-miR-15a-5p;hsa-miR-15b-5p;hsa-miR-16-5p;hsa-miR-432
ENSG0000	ENSG0000	3.30E-07	hsa-miR-149-5p;hsa-miR-15a-5p;hsa-miR-15b-5p;hsa-miR-16
ENSG0000	ENSG0000	1.93E-05	hsa-miR-16-5p;hsa-miR-17-5p;hsa-miR-20b-5p;hsa-miR-27b-

ENSG0000	ENSG0000	2.55E-05	hsa-miR-139-5p;hsa-miR-15a-5p;hsa-miR-15b-5p;hsa-miR-16-
ENSG0000	ENSG0000	0.000853	hsa-miR-17-5p;hsa-miR-20b-5p;hsa-miR-34a-5p;hsa-miR-449
ENSG0000	ENSG0000	0.009674	hsa-miR-218-5p;hsa-miR-27a-3p;hsa-miR-27b-3p;hsa-miR-7-
ENSG0000	ENSG0000	0.009451	hsa-miR-15a-5p;hsa-miR-15b-5p;hsa-miR-16-5p;hsa-miR-17-
ENSG0000	ENSG0000	0.00313	hsa-miR-1304-3p;hsa-miR-24-3p;hsa-miR-27a-3p;hsa-miR-27
ENSG0000	ENSG0000	0.001832	hsa-miR-139-5p;hsa-miR-15a-5p;hsa-miR-15b-5p;hsa-miR-9-
ENSG0000	ENSG0000	0.003443	hsa-miR-15a-5p;hsa-miR-15b-5p;hsa-miR-16-5p;hsa-miR-483-
ENSG0000	ENSG0000	0.001009	hsa-miR-1304-3p;hsa-miR-17-5p;hsa-miR-20b-5p;hsa-miR-66
ENSG0000	ENSG0000	0.003169	hsa-miR-1304-3p;hsa-miR-1-3p;hsa-miR-27a-3p;hsa-miR-27b-
ENSG0000	ENSG0000	0.003443	hsa-miR-15a-5p;hsa-miR-15b-5p;hsa-miR-16-5p;hsa-miR-432-
ENSG0000	ENSG0000	0.000374	hsa-miR-15a-5p;hsa-miR-15b-5p;hsa-miR-16-5p;hsa-miR-17-
ENSG0000	ENSG0000	0.001185	hsa-miR-124-3p;hsa-miR-17-5p;hsa-miR-181a-5p;hsa-miR-18
ENSG0000	ENSG0000	0.009674	hsa-miR-15a-5p;hsa-miR-15b-5p;hsa-miR-16-5p;hsa-miR-27a-
ENSG0000	ENSG0000	0.001887	hsa-miR-1304-3p;hsa-miR-17-5p;hsa-miR-20b-5p;hsa-miR-23
ENSG0000	ENSG0000	0.001199	hsa-miR-17-5p;hsa-miR-20b-5p;hsa-miR-218-5p;hsa-miR-7-5
ENSG0000	ENSG0000	0.000107	hsa-miR-17-5p;hsa-miR-181a-5p;hsa-miR-181b-5p;hsa-miR-2
ENSG0000	ENSG0000	0.00674	hsa-miR-1304-3p;hsa-miR-17-5p;hsa-miR-20b-5p;hsa-miR-21
ENSG0000	ENSG0000	0.007119	hsa-miR-15a-5p;hsa-miR-15b-5p;hsa-miR-16-5p;hsa-miR-24-
ENSG0000	ENSG0000	4.73E-06	hsa-miR-15a-5p;hsa-miR-15b-5p;hsa-miR-16-5p;hsa-miR-17-
ENSG0000	ENSG0000	0.003443	hsa-miR-1-3p;hsa-miR-15a-5p;hsa-miR-15b-5p;hsa-miR-16-5
ENSG0000	ENSG0000	0.000551	hsa-miR-126-5p;hsa-miR-17-5p;hsa-miR-20b-5p;hsa-miR-218-
ENSG0000	ENSG0000	0.000256	hsa-miR-181a-5p;hsa-miR-181b-5p;hsa-miR-27a-3p;hsa-miR-
ENSG0000	ENSG0000	0.000256	hsa-miR-181a-5p;hsa-miR-181b-5p;hsa-miR-27a-3p;hsa-miR-
ENSG0000	ENSG0000	0.00014	hsa-miR-17-5p;hsa-miR-20b-5p;hsa-miR-23a-3p;hsa-miR-338-
ENSG0000	ENSG0000	0.00014	hsa-miR-17-5p;hsa-miR-20b-5p;hsa-miR-23a-3p;hsa-miR-338-
ENSG0000	ENSG0000	0.00014	hsa-miR-17-5p;hsa-miR-20b-5p;hsa-miR-23a-3p;hsa-miR-338-
ENSG0000	ENSG0000	0.00014	hsa-miR-17-5p;hsa-miR-20b-5p;hsa-miR-23a-3p;hsa-miR-338-
ENSG0000	ENSG0000	0.00014	hsa-miR-17-5p;hsa-miR-20b-5p;hsa-miR-23a-3p;hsa-miR-338-
ENSG0000	ENSG0000	0.00014	hsa-miR-17-5p;hsa-miR-20b-5p;hsa-miR-23a-3p;hsa-miR-338-
ENSG0000	ENSG0000	0.00014	hsa-miR-17-5p;hsa-miR-20b-5p;hsa-miR-23a-3p;hsa-miR-338-
ENSG0000	ENSG0000	4.93E-05	hsa-miR-15a-5p;hsa-miR-15b-5p;hsa-miR-181a-5p;hsa-miR-1
ENSG0000	ENSG0000	0.00026	hsa-miR-126-5p;hsa-miR-15a-5p;hsa-miR-15b-5p;hsa-miR-16-
ENSG0000	ENSG0000	3.43E-05	hsa-miR-126-5p;hsa-miR-17-5p;hsa-miR-181a-5p;hsa-miR-18
ENSG0000	ENSG0000	0.005222	hsa-miR-1304-3p;hsa-miR-149-5p;hsa-miR-17-5p;hsa-miR-20
ENSG0000	ENSG0000	0.000722	hsa-miR-1304-3p;hsa-miR-17-5p;hsa-miR-20b-5p;hsa-miR-21
ENSG0000	ENSG0000	0.00313	hsa-miR-1304-3p;hsa-miR-218-5p;hsa-miR-34a-5p;hsa-miR-4
ENSG0000	ENSG0000	0.009418	hsa-miR-15a-5p;hsa-miR-15b-5p;hsa-miR-16-5p;hsa-miR-17-
ENSG0000	ENSG0000	0.007119	hsa-miR-181a-5p;hsa-miR-181b-5p;hsa-miR-27a-3p;hsa-miR-
ENSG0000	ENSG0000	0.00134	hsa-miR-1304-3p;hsa-miR-17-5p;hsa-miR-20b-5p;hsa-miR-93-
ENSG0000	ENSG0000	0.002223	hsa-miR-1-3p;hsa-miR-17-5p;hsa-miR-20b-5p;hsa-miR-93-5p
ENSG0000	ENSG0000	0.000145	hsa-miR-1-3p;hsa-miR-149-5p;hsa-miR-182-5p;hsa-miR-96-5
ENSG0000	ENSG0000	0.004039	hsa-miR-15a-5p;hsa-miR-15b-5p;hsa-miR-16-5p;hsa-miR-7-5
ENSG0000	ENSG0000	2.30E-05	hsa-miR-17-5p;hsa-miR-182-5p;hsa-miR-20b-5p;hsa-miR-34a-
ENSG0000	ENSG0000	2.30E-05	hsa-miR-17-5p;hsa-miR-182-5p;hsa-miR-20b-5p;hsa-miR-34a-
ENSG0000	ENSG0000	9.89E-06	hsa-miR-17-5p;hsa-miR-20b-5p;hsa-miR-27a-3p;hsa-miR-27b-
ENSG0000	ENSG0000	0.000145	hsa-miR-181a-5p;hsa-miR-181b-5p;hsa-miR-23a-3p;hsa-miR-
ENSG0000	ENSG0000	0.000325	hsa-miR-17-5p;hsa-miR-20b-5p;hsa-miR-27a-3p;hsa-miR-27b-
ENSG0000	ENSG0000	2.30E-07	hsa-miR-124-3p;hsa-miR-15a-5p;hsa-miR-15b-5p;hsa-miR-16-
ENSG0000	ENSG0000	0.009713	hsa-miR-1304-3p;hsa-miR-17-5p;hsa-miR-197-3p;hsa-miR-20
ENSG0000	ENSG0000	0.005826	hsa-miR-17-5p;hsa-miR-20b-5p;hsa-miR-7-5p;hsa-miR-93-5p
ENSG0000	ENSG0000	0.00213	hsa-miR-124-3p;hsa-miR-17-5p;hsa-miR-20b-5p;hsa-miR-27a-
ENSG0000	ENSG0000	0.000362	hsa-miR-181a-5p;hsa-miR-181b-5p;hsa-miR-27a-3p;hsa-miR-
ENSG0000	ENSG0000	0.006195	hsa-miR-126-5p;hsa-miR-1304-3p;hsa-miR-218-5p;hsa-miR-2
ENSG0000	ENSG0000	0.001283	hsa-miR-15a-5p;hsa-miR-15b-5p;hsa-miR-16-5p;hsa-miR-23a-

ENSG0000	ENSG0000	0.000773	hsa-miR-1304-3p;hsa-miR-17-5p;hsa-miR-20b-5p;hsa-miR-23
ENSG0000	ENSG0000	0.004039	hsa-miR-1304-3p;hsa-miR-17-5p;hsa-miR-20b-5p;hsa-miR-93
ENSG0000	ENSG0000	8.46E-05	hsa-miR-126-5p;hsa-miR-15a-5p;hsa-miR-15b-5p;hsa-miR-16
ENSG0000	ENSG0000	0.004951	hsa-miR-15a-5p;hsa-miR-15b-5p;hsa-miR-17-5p;hsa-miR-20b
ENSG0000	ENSG0000	0.004951	hsa-miR-15a-5p;hsa-miR-15b-5p;hsa-miR-17-5p;hsa-miR-20b
ENSG0000	ENSG0000	0.00353	hsa-miR-17-5p;hsa-miR-181a-5p;hsa-miR-181b-5p;hsa-miR-2
ENSG0000	ENSG0000	1.60E-05	hsa-miR-124-3p;hsa-miR-17-5p;hsa-miR-20b-5p;hsa-miR-93-
ENSG0000	ENSG0000	0.001832	hsa-miR-17-5p;hsa-miR-197-3p;hsa-miR-20b-5p;hsa-miR-93-
ENSG0000	ENSG0000	0.001492	hsa-miR-15a-5p;hsa-miR-15b-5p;hsa-miR-16-5p;hsa-miR-17-
ENSG0000	ENSG0000	0.000107	hsa-miR-181a-5p;hsa-miR-181b-5p;hsa-miR-27a-3p;hsa-miR-
ENSG0000	ENSG0000	0.006669	hsa-miR-1304-3p;hsa-miR-17-5p;hsa-miR-20b-5p;hsa-miR-93
ENSG0000	ENSG0000	0.000445	hsa-miR-1304-3p;hsa-miR-17-5p;hsa-miR-20b-5p;hsa-miR-24
ENSG0000	ENSG0000	8.17E-07	hsa-miR-17-5p;hsa-miR-181a-5p;hsa-miR-181b-5p;hsa-miR-2
ENSG0000	ENSG0000	0.000928	hsa-miR-149-5p;hsa-miR-17-5p;hsa-miR-20b-5p;hsa-miR-24-
ENSG0000	ENSG0000	0.001003	hsa-miR-1304-3p;hsa-miR-17-5p;hsa-miR-197-3p;hsa-miR-20
ENSG0000	ENSG0000	1.05E-05	hsa-miR-139-5p;hsa-miR-181a-5p;hsa-miR-181b-5p;hsa-miR-
ENSG0000	ENSG0000	0.00014	hsa-miR-15a-5p;hsa-miR-15b-5p;hsa-miR-16-5p;hsa-miR-181
ENSG0000	ENSG0000	0.002438	hsa-miR-181a-5p;hsa-miR-181b-5p;hsa-miR-23a-3p;hsa-miR-
ENSG0000	ENSG0000	4.17E-05	hsa-miR-15a-5p;hsa-miR-15b-5p;hsa-miR-16-5p;hsa-miR-23a
ENSG0000	ENSG0000	0.006238	hsa-miR-15a-5p;hsa-miR-15b-5p;hsa-miR-16-5p;hsa-miR-218
ENSG0000	ENSG0000	0.000646	hsa-miR-15a-5p;hsa-miR-15b-5p;hsa-miR-27a-3p;hsa-miR-27
ENSG0000	ENSG0000	0.000773	hsa-miR-149-5p;hsa-miR-15a-5p;hsa-miR-15b-5p;hsa-miR-16
ENSG0000	ENSG0000	5.31E-07	hsa-miR-17-5p;hsa-miR-181a-5p;hsa-miR-181b-5p;hsa-miR-2
ENSG0000	ENSG0000	4.29E-05	hsa-miR-181a-5p;hsa-miR-181b-5p;hsa-miR-23a-3p;hsa-miR-
ENSG0000	ENSG0000	0.004951	hsa-miR-17-5p;hsa-miR-20b-5p;hsa-miR-23a-3p;hsa-miR-23b
ENSG0000	ENSG0000	0.001199	hsa-miR-15a-5p;hsa-miR-15b-5p;hsa-miR-16-5p;hsa-miR-197
ENSG0000	ENSG0000	0.00978	hsa-miR-1304-3p;hsa-miR-17-5p;hsa-miR-20b-5p;hsa-miR-48
ENSG0000	ENSG0000	0.00134	hsa-miR-23a-3p;hsa-miR-23b-3p;hsa-miR-27a-3p;hsa-miR-27
ENSG0000	ENSG0000	0.000551	hsa-miR-17-5p;hsa-miR-20b-5p;hsa-miR-27a-3p;hsa-miR-27b
ENSG0000	ENSG0000	0.002786	hsa-miR-1304-3p;hsa-miR-15a-5p;hsa-miR-15b-5p;hsa-miR-1
ENSG0000	ENSG0000	4.29E-05	hsa-miR-15a-5p;hsa-miR-15b-5p;hsa-miR-16-5p;hsa-miR-218
ENSG0000	ENSG0000	0.000181	hsa-miR-182-5p;hsa-miR-197-3p;hsa-miR-34a-5p;hsa-miR-44
ENSG0000	ENSG0000	0.003733	hsa-miR-17-5p;hsa-miR-20b-5p;hsa-miR-27b-3p;hsa-miR-93-
ENSG0000	ENSG0000	0.000191	hsa-miR-17-5p;hsa-miR-20b-5p;hsa-miR-218-5p;hsa-miR-23a
ENSG0000	ENSG0000	0.001492	hsa-miR-1304-3p;hsa-miR-197-3p;hsa-miR-27a-3p;hsa-miR-2
ENSG0000	ENSG0000	0.000419	hsa-miR-15a-5p;hsa-miR-15b-5p;hsa-miR-16-5p;hsa-miR-7-5
ENSG0000	ENSG0000	1.74E-07	hsa-miR-124-3p;hsa-miR-15a-5p;hsa-miR-15b-5p;hsa-miR-16
ENSG0000	ENSG0000	0.001492	hsa-miR-139-5p;hsa-miR-15a-5p;hsa-miR-15b-5p;hsa-miR-16
ENSG0000	ENSG0000	1.28E-05	hsa-miR-15a-5p;hsa-miR-15b-5p;hsa-miR-16-5p;hsa-miR-197
ENSG0000	ENSG0000	0.002594	hsa-miR-15a-5p;hsa-miR-15b-5p;hsa-miR-181a-5p;hsa-miR-1
ENSG0000	ENSG0000	0.002594	hsa-miR-15a-5p;hsa-miR-15b-5p;hsa-miR-181a-5p;hsa-miR-1
ENSG0000	ENSG0000	0.002594	hsa-miR-15a-5p;hsa-miR-15b-5p;hsa-miR-181a-5p;hsa-miR-1
ENSG0000	ENSG0000	0.002594	hsa-miR-15a-5p;hsa-miR-15b-5p;hsa-miR-181a-5p;hsa-miR-1
ENSG0000	ENSG0000	0.002594	hsa-miR-15a-5p;hsa-miR-15b-5p;hsa-miR-181a-5p;hsa-miR-1
ENSG0000	ENSG0000	0.002594	hsa-miR-15a-5p;hsa-miR-15b-5p;hsa-miR-181a-5p;hsa-miR-1
ENSG0000	ENSG0000	0.002594	hsa-miR-15a-5p;hsa-miR-15b-5p;hsa-miR-181a-5p;hsa-miR-1
ENSG0000	ENSG0000	0.002594	hsa-miR-15a-5p;hsa-miR-15b-5p;hsa-miR-181a-5p;hsa-miR-1
ENSG0000	ENSG0000	0.000595	hsa-miR-15a-5p;hsa-miR-15b-5p;hsa-miR-16-5p;hsa-miR-181
ENSG0000	ENSG0000	0.003743	hsa-miR-181a-5p;hsa-miR-181b-5p;hsa-miR-218-5p;hsa-miR-
ENSG0000	ENSG0000	0.008161	hsa-miR-124-3p;hsa-miR-1-3p;hsa-miR-17-5p;hsa-miR-20b-5
ENSG0000	ENSG0000	0.000715	hsa-miR-17-5p;hsa-miR-20b-5p;hsa-miR-34a-5p;hsa-miR-449
ENSG0000	ENSG0000	0.003443	hsa-miR-17-5p;hsa-miR-20b-5p;hsa-miR-27a-3p;hsa-miR-27b
ENSG0000	ENSG0000	0.000646	hsa-miR-15a-5p;hsa-miR-15b-5p;hsa-miR-16-5p;hsa-miR-9-5
ENSG0000	ENSG0000	1.75E-06	hsa-miR-15a-5p;hsa-miR-15b-5p;hsa-miR-16-5p;hsa-miR-27a

ENSG0000	ENSG0000	4.29E-05	hsa-miR-15a-5p;hsa-miR-15b-5p;hsa-miR-16-5p;hsa-miR-218-
ENSG0000	ENSG0000	6.10E-07	hsa-miR-17-5p;hsa-miR-182-5p;hsa-miR-20b-5p;hsa-miR-27a-
ENSG0000	ENSG0000	0.005433	hsa-miR-182-5p;hsa-miR-24-3p;hsa-miR-338-3p;hsa-miR-96-5
ENSG0000	ENSG0000	0.001492	hsa-miR-1304-3p;hsa-miR-17-5p;hsa-miR-20b-5p;hsa-miR-24-
ENSG0000	ENSG0000	0.004197	hsa-miR-1304-3p;hsa-miR-17-5p;hsa-miR-20b-5p;hsa-miR-24-
ENSG0000	ENSG0000	4.90E-06	hsa-miR-15a-5p;hsa-miR-15b-5p;hsa-miR-16-5p;hsa-miR-182-
ENSG0000	ENSG0000	1.86E-05	hsa-miR-15a-5p;hsa-miR-15b-5p;hsa-miR-16-5p;hsa-miR-182-
ENSG0000	ENSG0000	1.04E-06	hsa-miR-15a-5p;hsa-miR-15b-5p;hsa-miR-16-5p;hsa-miR-181a
ENSG0000	ENSG0000	0.001832	hsa-miR-23a-3p;hsa-miR-23b-3p;hsa-miR-24-3p;hsa-miR-876-
ENSG0000	ENSG0000	0.003326	hsa-miR-1304-3p;hsa-miR-15a-5p;hsa-miR-15b-5p;hsa-miR-16
ENSG0000	ENSG0000	1.56E-05	hsa-miR-15a-5p;hsa-miR-15b-5p;hsa-miR-16-5p;hsa-miR-23a-
ENSG0000	ENSG0000	0.009674	hsa-miR-1304-3p;hsa-miR-181a-5p;hsa-miR-181b-5p;hsa-miR-
ENSG0000	ENSG0000	0.001185	hsa-miR-15a-5p;hsa-miR-15b-5p;hsa-miR-16-5p;hsa-miR-218-
ENSG0000	ENSG0000	0.001094	hsa-miR-15a-5p;hsa-miR-15b-5p;hsa-miR-16-5p;hsa-miR-23a-
ENSG0000	ENSG0000	0.000218	hsa-miR-188-3p;hsa-miR-199a-5p;hsa-miR-199b-5p;hsa-miR-4
ENSG0000	ENSG0000	1.96E-05	hsa-miR-199a-5p;hsa-miR-199b-5p;hsa-miR-27a-3p;hsa-miR-2
ENSG0000	ENSG0000	1.26E-05	hsa-miR-27a-3p;hsa-miR-27b-3p;hsa-miR-4685-3p;hsa-miR-53
ENSG0000	ENSG0000	2.97E-06	hsa-miR-27a-3p;hsa-miR-27b-3p;hsa-miR-532-3p;hsa-miR-629
ENSG0000	ENSG0000	0.001411	hsa-miR-15a-3p;hsa-miR-199a-5p;hsa-miR-199b-5p;hsa-miR-4
ENSG0000	ENSG0000	1.16E-08	hsa-miR-188-3p;hsa-miR-27a-3p;hsa-miR-27b-3p;hsa-miR-468
ENSG0000	ENSG0000	0.000178	hsa-miR-199a-5p;hsa-miR-199b-5p;hsa-miR-27a-3p;hsa-miR-2
ENSG0000	ENSG0000	0.00043	hsa-miR-27a-3p;hsa-miR-27b-3p;hsa-miR-532-3p;hsa-miR-629
ENSG0000	ENSG0000	7.07E-06	hsa-miR-27a-3p;hsa-miR-27b-3p;hsa-miR-4685-3p;hsa-miR-62
ENSG0000	ENSG0000	7.40E-06	hsa-miR-15a-3p;hsa-miR-199a-5p;hsa-miR-199b-5p;hsa-miR-2
ENSG0000	ENSG0000	0.000949	hsa-miR-199a-5p;hsa-miR-199b-5p;hsa-miR-27a-3p;hsa-miR-2
ENSG0000	ENSG0000	1.50E-07	hsa-miR-199a-5p;hsa-miR-199b-5p;hsa-miR-27a-3p;hsa-miR-2
ENSG0000	ENSG0000	1.50E-07	hsa-miR-199a-5p;hsa-miR-199b-5p;hsa-miR-27a-3p;hsa-miR-2
ENSG0000	ENSG0000	1.22E-05	hsa-miR-188-3p;hsa-miR-199a-5p;hsa-miR-199b-5p;hsa-miR-6
ENSG0000	ENSG0000	1.23E-11	hsa-miR-320a;hsa-miR-320b;hsa-miR-320c;hsa-miR-320d;hsa-m
ENSG0000	ENSG0000	4.03E-10	hsa-miR-320a;hsa-miR-320b;hsa-miR-320c;hsa-miR-320d;hsa-m
ENSG0000	ENSG0000	1.16E-08	hsa-miR-320a;hsa-miR-320b;hsa-miR-320c;hsa-miR-320d;hsa-m
ENSG0000	ENSG0000	5.26E-11	hsa-miR-320a;hsa-miR-320b;hsa-miR-320c;hsa-miR-320d;hsa-m
ENSG0000	ENSG0000	1.45E-09	hsa-miR-320a;hsa-miR-320b;hsa-miR-320c;hsa-miR-320d;hsa-m
ENSG0000	ENSG0000	3.72E-06	hsa-miR-320a;hsa-miR-320b;hsa-miR-320c;hsa-miR-320d;hsa-m
ENSG0000	ENSG0000	5.86E-09	hsa-miR-320a;hsa-miR-320b;hsa-miR-320c;hsa-miR-320d;hsa-m
ENSG0000	ENSG0000	1.29E-08	hsa-miR-320a;hsa-miR-320b;hsa-miR-320c;hsa-miR-320d;hsa-m
ENSG0000	ENSG0000	3.51E-09	hsa-miR-320a;hsa-miR-320b;hsa-miR-320c;hsa-miR-320d;hsa-m
ENSG0000	ENSG0000	1.11E-07	hsa-miR-320a;hsa-miR-320b;hsa-miR-320c;hsa-miR-320d;hsa-m
ENSG0000	ENSG0000	3.03E-08	hsa-miR-320a;hsa-miR-320b;hsa-miR-320c;hsa-miR-320d;hsa-m
ENSG0000	ENSG0000	4.29E-06	hsa-miR-320b;hsa-miR-320c;hsa-miR-320d;hsa-miR-4429
ENSG0000	ENSG0000	4.25E-08	hsa-miR-320a;hsa-miR-320b;hsa-miR-320c;hsa-miR-320d;hsa-m
ENSG0000	ENSG0000	1.25E-10	hsa-miR-320a;hsa-miR-320b;hsa-miR-320c;hsa-miR-320d;hsa-m
ENSG0000	ENSG0000	1.25E-10	hsa-miR-320a;hsa-miR-320b;hsa-miR-320c;hsa-miR-320d;hsa-m
ENSG0000	ENSG0000	4.28E-07	hsa-miR-320a;hsa-miR-320b;hsa-miR-320c;hsa-miR-320d;hsa-m
ENSG0000	ENSG0000	1.25E-10	hsa-miR-320a;hsa-miR-320b;hsa-miR-320c;hsa-miR-320d;hsa-m
ENSG0000	ENSG0000	9.33E-09	hsa-miR-320a;hsa-miR-320b;hsa-miR-320c;hsa-miR-320d;hsa-m
ENSG0000	ENSG0000	1.23E-11	hsa-miR-320a;hsa-miR-320b;hsa-miR-320c;hsa-miR-320d;hsa-m
ENSG0000	ENSG0000	3.06E-09	hsa-miR-320a;hsa-miR-320b;hsa-miR-320c;hsa-miR-320d;hsa-m
ENSG0000	ENSG0000	2.66E-09	hsa-miR-320a;hsa-miR-320b;hsa-miR-320c;hsa-miR-320d;hsa-m
ENSG0000	ENSG0000	2.78E-08	hsa-miR-320a;hsa-miR-320b;hsa-miR-320c;hsa-miR-320d;hsa-m
ENSG0000	ENSG0000	2.78E-08	hsa-miR-320a;hsa-miR-320b;hsa-miR-320c;hsa-miR-320d;hsa-m
ENSG0000	ENSG0000	6.61E-09	hsa-miR-320a;hsa-miR-320b;hsa-miR-320c;hsa-miR-320d;hsa-m
ENSG0000	ENSG0000	7.14E-11	hsa-miR-320a;hsa-miR-320b;hsa-miR-320c;hsa-miR-320d;hsa-m
ENSG0000	ENSG0000	1.55E-12	hsa-miR-320a;hsa-miR-320b;hsa-miR-320c;hsa-miR-320d;hsa-m

ENSG0000	ENSG0000	1.43E-08	hsa-miR-320a;hsa-miR-320b;hsa-miR-320c;hsa-miR-320d;hsa-r
ENSG0000	ENSG0000	1.29E-08	hsa-miR-320a;hsa-miR-320b;hsa-miR-320c;hsa-miR-320d;hsa-r
ENSG0000	ENSG0000	5.40E-08	hsa-miR-320a;hsa-miR-320b;hsa-miR-320c;hsa-miR-320d;hsa-r
ENSG0000	ENSG0000	6.30E-08	hsa-miR-320a;hsa-miR-320b;hsa-miR-320c;hsa-miR-320d;hsa-r
ENSG0000	ENSG0000	1.51E-06	hsa-miR-320b;hsa-miR-320c;hsa-miR-320d;hsa-miR-4429
ENSG0000	ENSG0000	6.30E-05	hsa-miR-320b;hsa-miR-320c;hsa-miR-320d;hsa-miR-4429
ENSG0000	ENSG0000	7.92E-12	hsa-miR-320a;hsa-miR-320b;hsa-miR-320c;hsa-miR-320d;hsa-r
ENSG0000	ENSG0000	7.92E-12	hsa-miR-320a;hsa-miR-320b;hsa-miR-320c;hsa-miR-320d;hsa-r
ENSG0000	ENSG0000	1.55E-12	hsa-miR-320a;hsa-miR-320b;hsa-miR-320c;hsa-miR-320d;hsa-r
ENSG0000	ENSG0000	1.57E-06	hsa-miR-320a;hsa-miR-320b;hsa-miR-320c;hsa-miR-320d;hsa-r
ENSG0000	ENSG0000	1.23E-11	hsa-miR-320a;hsa-miR-320b;hsa-miR-320c;hsa-miR-320d;hsa-r
ENSG0000	ENSG0000	3.51E-09	hsa-miR-320a;hsa-miR-320b;hsa-miR-320c;hsa-miR-320d;hsa-r
ENSG0000	ENSG0000	3.80E-11	hsa-miR-320a;hsa-miR-320b;hsa-miR-320c;hsa-miR-320d;hsa-r
ENSG0000	ENSG0000	2.55E-06	hsa-miR-320a;hsa-miR-320b;hsa-miR-320c;hsa-miR-320d;hsa-r
ENSG0000	ENSG0000	6.61E-09	hsa-miR-320a;hsa-miR-320b;hsa-miR-320c;hsa-miR-320d;hsa-r
ENSG0000	ENSG0000	8.72E-10	hsa-miR-320a;hsa-miR-320b;hsa-miR-320c;hsa-miR-320d;hsa-r
ENSG0000	ENSG0000	3.31E-08	hsa-miR-320a;hsa-miR-320b;hsa-miR-320c;hsa-miR-320d;hsa-r
ENSG0000	ENSG0000	1.36E-07	hsa-miR-320a;hsa-miR-320b;hsa-miR-320c;hsa-miR-320d;hsa-r
ENSG0000	ENSG0000	2.06E-10	hsa-miR-320a;hsa-miR-320b;hsa-miR-320c;hsa-miR-320d;hsa-r
ENSG0000	ENSG0000	6.90E-06	hsa-miR-320a;hsa-miR-320b;hsa-miR-320c;hsa-miR-320d;hsa-r
ENSG0000	ENSG0000	1.04E-08	hsa-miR-320a;hsa-miR-320b;hsa-miR-320c;hsa-miR-320d;hsa-r
ENSG0000	ENSG0000	8.72E-10	hsa-miR-320a;hsa-miR-320b;hsa-miR-320c;hsa-miR-320d;hsa-r
ENSG0000	ENSG0000	1.23E-06	hsa-miR-320a;hsa-miR-320b;hsa-miR-320c;hsa-miR-320d;hsa-r
ENSG0000	ENSG0000	2.32E-08	hsa-miR-320a;hsa-miR-320b;hsa-miR-320c;hsa-miR-320d;hsa-r
ENSG0000	ENSG0000	4.03E-10	hsa-miR-320a;hsa-miR-320b;hsa-miR-320c;hsa-miR-320d;hsa-r
ENSG0000	ENSG0000	6.79E-08	hsa-miR-320a;hsa-miR-320b;hsa-miR-320c;hsa-miR-320d;hsa-r
ENSG0000	ENSG0000	2.60E-10	hsa-miR-320a;hsa-miR-320b;hsa-miR-320c;hsa-miR-320d;hsa-r
ENSG0000	ENSG0000	3.29E-07	hsa-miR-320a;hsa-miR-320b;hsa-miR-320c;hsa-miR-320d;hsa-r
ENSG0000	ENSG0000	3.45E-13	hsa-miR-320a;hsa-miR-320b;hsa-miR-320c;hsa-miR-320d;hsa-r
ENSG0000	ENSG0000	1.16E-08	hsa-miR-320a;hsa-miR-320b;hsa-miR-320c;hsa-miR-320d;hsa-r
ENSG0000	ENSG0000	7.86E-08	hsa-miR-320a;hsa-miR-320b;hsa-miR-320c;hsa-miR-320d;hsa-r
ENSG0000	ENSG0000	1.16E-08	hsa-miR-320a;hsa-miR-320b;hsa-miR-320c;hsa-miR-320d;hsa-r
ENSG0000	ENSG0000	3.92E-08	hsa-miR-320a;hsa-miR-320b;hsa-miR-320c;hsa-miR-320d;hsa-r
ENSG0000	ENSG0000	1.57E-06	hsa-miR-320a;hsa-miR-320b;hsa-miR-320c;hsa-miR-320d;hsa-r
ENSG0000	ENSG0000	1.92E-08	hsa-miR-320a;hsa-miR-320b;hsa-miR-320c;hsa-miR-320d;hsa-r
ENSG0000	ENSG0000	5.84E-08	hsa-miR-320a;hsa-miR-320b;hsa-miR-320c;hsa-miR-320d;hsa-r
ENSG0000	ENSG0000	4.87E-12	hsa-miR-320a;hsa-miR-320b;hsa-miR-320c;hsa-miR-320d;hsa-r
ENSG0000	ENSG0000	1.04E-09	hsa-miR-320a;hsa-miR-320b;hsa-miR-320c;hsa-miR-320d;hsa-r
ENSG0000	ENSG0000	1.04E-09	hsa-miR-320a;hsa-miR-320b;hsa-miR-320c;hsa-miR-320d;hsa-r
ENSG0000	ENSG0000	1.70E-09	hsa-miR-320a;hsa-miR-320b;hsa-miR-320c;hsa-miR-320d;hsa-r
ENSG0000	ENSG0000	6.23E-06	hsa-miR-1260a;hsa-miR-1260b;hsa-miR-188-5p;hsa-miR-4726-
ENSG0000	ENSG0000	0.003902	hsa-miR-30c-1-3p;hsa-miR-30c-2-3p;hsa-miR-3913-5p;hsa-mi
ENSG0000	ENSG0000	0.000352	hsa-miR-149-5p;hsa-miR-21-3p;hsa-miR-34a-5p;hsa-miR-362-
ENSG0000	ENSG0000	0.003345	hsa-miR-2110;hsa-miR-30c-1-3p;hsa-miR-30c-2-3p;hsa-miR-4
ENSG0000	ENSG0000	0.00505	hsa-miR-24-3p;hsa-miR-30c-1-3p;hsa-miR-30c-2-3p;hsa-miR-
ENSG0000	ENSG0000	0.003764	hsa-miR-221-5p;hsa-miR-30c-1-3p;hsa-miR-30c-2-3p;hsa-miR
ENSG0000	ENSG0000	0.000534	hsa-miR-29a-3p;hsa-miR-29b-3p;hsa-miR-29c-3p;hsa-miR-34a
ENSG0000	ENSG0000	0.00108	hsa-miR-1260a;hsa-miR-1260b;hsa-miR-188-3p;hsa-miR-30c-1
ENSG0000	ENSG0000	0.008566	hsa-miR-30c-1-3p;hsa-miR-30c-2-3p;hsa-miR-331-3p;hsa-miR
ENSG0000	ENSG0000	0.004263	hsa-miR-29a-3p;hsa-miR-29b-3p;hsa-miR-29c-3p;hsa-miR-34a
ENSG0000	ENSG0000	0.001054	hsa-miR-24-3p;hsa-miR-29a-3p;hsa-miR-29b-3p;hsa-miR-29c-
ENSG0000	ENSG0000	0.000269	hsa-miR-2355-5p;hsa-miR-30c-1-3p;hsa-miR-30c-2-3p;hsa-mi
ENSG0000	ENSG0000	0.008566	hsa-miR-29a-3p;hsa-miR-29b-3p;hsa-miR-29c-3p;hsa-miR-624
ENSG0000	ENSG0000	0.00194	hsa-miR-34a-5p;hsa-miR-4525;hsa-miR-625-5p;hsa-miR-873-3

ENSG0000	ENSG0000	0.00194	hsa-miR-140-3p;hsa-miR-34a-5p;hsa-miR-449a;hsa-miR-624-5
ENSG0000	ENSG0000	0.000122	hsa-miR-21-3p;hsa-miR-29a-3p;hsa-miR-29b-3p;hsa-miR-29c-
ENSG0000	ENSG0000	0.001208	hsa-miR-29a-3p;hsa-miR-29b-3p;hsa-miR-29c-3p;hsa-miR-34a
ENSG0000	ENSG0000	0.001202	hsa-miR-29a-3p;hsa-miR-29c-3p;hsa-miR-4525;hsa-miR-625-5
ENSG0000	ENSG0000	9.23E-05	hsa-miR-140-3p;hsa-miR-29a-3p;hsa-miR-29b-3p;hsa-miR-29c
ENSG0000	ENSG0000	0.007327	hsa-miR-21-3p;hsa-miR-2355-5p;hsa-miR-30c-1-3p;hsa-miR-3
ENSG0000	ENSG0000	5.46E-05	hsa-miR-24-3p;hsa-miR-30c-1-3p;hsa-miR-30c-2-3p;hsa-miR-
ENSG0000	ENSG0000	0.006407	hsa-miR-2355-5p;hsa-miR-30c-1-3p;hsa-miR-30c-2-3p;hsa-mi
ENSG0000	ENSG0000	0.005562	hsa-miR-192-5p;hsa-miR-29a-3p;hsa-miR-29b-3p;hsa-miR-29c
ENSG0000	ENSG0000	0.009022	hsa-miR-24-3p;hsa-miR-29a-3p;hsa-miR-29b-3p;hsa-miR-29c-
ENSG0000	ENSG0000	0.003345	hsa-miR-30c-1-3p;hsa-miR-30c-2-3p;hsa-miR-3913-5p;hsa-mi
ENSG0000	ENSG0000	0.009805	hsa-miR-30c-1-3p;hsa-miR-30c-2-3p;hsa-miR-505-5p;hsa-miR
ENSG0000	ENSG0000	0.000155	hsa-miR-29a-3p;hsa-miR-29b-3p;hsa-miR-29c-3p;hsa-miR-484
ENSG0000	ENSG0000	0.000131	hsa-miR-29a-3p;hsa-miR-29c-3p;hsa-miR-3065-3p;hsa-miR-33
ENSG0000	ENSG0000	0.003345	hsa-miR-1260a;hsa-miR-1260b;hsa-miR-188-3p;hsa-miR-24-3p
ENSG0000	ENSG0000	0.000727	hsa-miR-2110;hsa-miR-29a-3p;hsa-miR-29b-3p;hsa-miR-29c-3
ENSG0000	ENSG0000	0.005478	hsa-miR-21-3p;hsa-miR-30c-1-3p;hsa-miR-30c-2-3p;hsa-miR-
ENSG0000	ENSG0000	0.009172	hsa-miR-3065-3p;hsa-miR-30c-1-3p;hsa-miR-30c-2-3p;hsa-mi
ENSG0000	ENSG0000	0.003563	hsa-miR-24-3p;hsa-miR-3065-3p;hsa-miR-4525;hsa-miR-625-5
ENSG0000	ENSG0000	0.001732	hsa-miR-29a-3p;hsa-miR-29b-3p;hsa-miR-29c-3p;hsa-miR-306
ENSG0000	ENSG0000	0.001202	hsa-miR-29a-3p;hsa-miR-29b-3p;hsa-miR-29c-3p;hsa-miR-338
ENSG0000	ENSG0000	0.001202	hsa-miR-29a-3p;hsa-miR-29b-3p;hsa-miR-29c-3p;hsa-miR-338
ENSG0000	ENSG0000	8.08E-08	hsa-miR-192-5p;hsa-miR-24-3p;hsa-miR-30c-1-3p;hsa-miR-30
ENSG0000	ENSG0000	0.005478	hsa-miR-34a-5p;hsa-miR-4525;hsa-miR-625-5p;hsa-miR-873-5
ENSG0000	ENSG0000	0.00505	hsa-miR-24-3p;hsa-miR-29a-3p;hsa-miR-29b-3p;hsa-miR-29c-
ENSG0000	ENSG0000	0.001054	hsa-miR-1260a;hsa-miR-1260b;hsa-miR-188-3p;hsa-miR-873-5
ENSG0000	ENSG0000	9.57E-06	hsa-miR-1226-3p;hsa-miR-29a-3p;hsa-miR-29b-3p;hsa-miR-29
ENSG0000	ENSG0000	0.008712	hsa-miR-30c-1-3p;hsa-miR-30c-2-3p;hsa-miR-3187-3p;hsa-mi
ENSG0000	ENSG0000	0.007987	hsa-miR-24-3p;hsa-miR-29a-3p;hsa-miR-29b-3p;hsa-miR-34a-
ENSG0000	ENSG0000	0.004287	hsa-miR-188-3p;hsa-miR-24-3p;hsa-miR-30c-1-3p;hsa-miR-30
ENSG0000	ENSG0000	0.000588	hsa-miR-29a-3p;hsa-miR-29b-3p;hsa-miR-29c-3p;hsa-miR-642
ENSG0000	ENSG0000	0.000271	hsa-miR-1226-3p;hsa-miR-1260a;hsa-miR-1260b;hsa-miR-188-
ENSG0000	ENSG0000	2.68E-05	hsa-miR-29a-3p;hsa-miR-29b-3p;hsa-miR-29c-3p;hsa-miR-306
ENSG0000	ENSG0000	6.69E-05	hsa-miR-1260a;hsa-miR-1260b;hsa-miR-24-3p;hsa-miR-30c-1-
ENSG0000	ENSG0000	0.000131	hsa-miR-30c-1-3p;hsa-miR-30c-2-3p;hsa-miR-4525;hsa-miR-4
ENSG0000	ENSG0000	0.000588	hsa-miR-29a-3p;hsa-miR-29b-3p;hsa-miR-29c-3p;hsa-miR-542
ENSG0000	ENSG0000	0.008566	hsa-miR-30c-1-3p;hsa-miR-30c-2-3p;hsa-miR-331-3p;hsa-miR
ENSG0000	ENSG0000	0.00154	hsa-miR-149-5p;hsa-miR-188-3p;hsa-miR-221-5p;hsa-miR-24-
ENSG0000	ENSG0000	0.001444	hsa-miR-24-3p;hsa-miR-30c-1-3p;hsa-miR-30c-2-3p;hsa-miR-
ENSG0000	ENSG0000	0.00984	hsa-miR-1260a;hsa-miR-1260b;hsa-miR-29a-3p;hsa-miR-29b-3
ENSG0000	ENSG0000	7.79E-05	hsa-miR-140-3p;hsa-miR-29a-3p;hsa-miR-29b-3p;hsa-miR-29c
ENSG0000	ENSG0000	0.001537	hsa-miR-21-3p;hsa-miR-29a-3p;hsa-miR-29b-3p;hsa-miR-29c-
ENSG0000	ENSG0000	0.006407	hsa-miR-24-3p;hsa-miR-30c-1-3p;hsa-miR-30c-2-3p;hsa-miR-
ENSG0000	ENSG0000	0.005478	hsa-miR-34a-5p;hsa-miR-449a;hsa-miR-4525;hsa-miR-625-5p
ENSG0000	ENSG0000	0.004263	hsa-miR-24-3p;hsa-miR-30c-1-3p;hsa-miR-30c-2-3p;hsa-miR-
ENSG0000	ENSG0000	0.001542	hsa-miR-149-5p;hsa-miR-2110;hsa-miR-2355-5p;hsa-miR-29b-
ENSG0000	ENSG0000	0.001671	hsa-miR-24-3p;hsa-miR-30c-1-3p;hsa-miR-30c-2-3p;hsa-miR-
ENSG0000	ENSG0000	1.57E-06	hsa-miR-1226-3p;hsa-miR-29a-3p;hsa-miR-29b-3p;hsa-miR-29
ENSG0000	ENSG0000	1.57E-06	hsa-miR-1226-3p;hsa-miR-29a-3p;hsa-miR-29b-3p;hsa-miR-29
ENSG0000	ENSG0000	0.001542	hsa-miR-30c-1-3p;hsa-miR-30c-2-3p;hsa-miR-340-3p;hsa-miR
ENSG0000	ENSG0000	0.002304	hsa-miR-1260a;hsa-miR-1260b;hsa-miR-188-3p;hsa-miR-24-3p
ENSG0000	ENSG0000	0.00039	hsa-miR-24-3p;hsa-miR-30c-1-3p;hsa-miR-30c-2-3p;hsa-miR-
ENSG0000	ENSG0000	0.000859	hsa-miR-149-5p;hsa-miR-3065-3p;hsa-miR-30c-1-3p;hsa-miR-
ENSG0000	ENSG0000	0.007093	hsa-miR-140-3p;hsa-miR-188-3p;hsa-miR-2110;hsa-miR-3065-

ENSG0000	ENSG0000	0.009022	hsa-miR-140-3p;hsa-miR-362-3p;hsa-miR-4525;hsa-miR-491-5
ENSG0000	ENSG0000	0.002151	hsa-miR-1226-3p;hsa-miR-29a-3p;hsa-miR-29b-3p;hsa-miR-2
ENSG0000	ENSG0000	0.007987	hsa-miR-24-3p;hsa-miR-30c-1-3p;hsa-miR-30c-2-3p;hsa-miR-
ENSG0000	ENSG0000	0.007093	hsa-miR-221-5p;hsa-miR-24-3p;hsa-miR-30c-1-3p;hsa-miR-3
ENSG0000	ENSG0000	0.000859	hsa-miR-1260a;hsa-miR-1260b;hsa-miR-140-3p;hsa-miR-188-
ENSG0000	ENSG0000	0.000565	hsa-miR-1260a;hsa-miR-1260b;hsa-miR-140-3p;hsa-miR-3065
ENSG0000	ENSG0000	0.008567	hsa-miR-30c-1-3p;hsa-miR-30c-2-3p;hsa-miR-362-3p;hsa-mi
ENSG0000	ENSG0000	0.000477	hsa-miR-24-3p;hsa-miR-29a-3p;hsa-miR-30c-1-3p;hsa-miR-3
ENSG0000	ENSG0000	0.000378	hsa-miR-221-5p;hsa-miR-3187-3p;hsa-miR-34a-5p;hsa-miR-4
ENSG0000	ENSG0000	0.007705	hsa-miR-1260a;hsa-miR-1260b;hsa-miR-188-3p;hsa-miR-30c-1
ENSG0000	ENSG0000	0.008566	hsa-miR-221-5p;hsa-miR-30c-1-3p;hsa-miR-30c-2-3p;hsa-mi
ENSG0000	ENSG0000	0.000984	hsa-miR-30c-1-3p;hsa-miR-30c-2-3p;hsa-miR-34a-5p;hsa-mi
ENSG0000	ENSG0000	0.000449	hsa-miR-24-3p;hsa-miR-30c-1-3p;hsa-miR-30c-2-3p;hsa-miR-
ENSG0000	ENSG0000	0.002667	hsa-miR-140-3p;hsa-miR-30c-1-3p;hsa-miR-30c-2-3p;hsa-mi
ENSG0000	ENSG0000	0.002667	hsa-miR-140-3p;hsa-miR-30c-1-3p;hsa-miR-30c-2-3p;hsa-mi
ENSG0000	ENSG0000	0.002667	hsa-miR-140-3p;hsa-miR-30c-1-3p;hsa-miR-30c-2-3p;hsa-mi
ENSG0000	ENSG0000	0.002667	hsa-miR-140-3p;hsa-miR-30c-1-3p;hsa-miR-30c-2-3p;hsa-mi
ENSG0000	ENSG0000	0.002667	hsa-miR-140-3p;hsa-miR-30c-1-3p;hsa-miR-30c-2-3p;hsa-mi
ENSG0000	ENSG0000	0.006407	hsa-miR-139-3p;hsa-miR-362-3p;hsa-miR-491-5p;hsa-miR-87
ENSG0000	ENSG0000	0.00194	hsa-miR-24-3p;hsa-miR-30c-1-3p;hsa-miR-30c-2-3p;hsa-miR-
ENSG0000	ENSG0000	2.74E-05	hsa-miR-29a-3p;hsa-miR-29b-3p;hsa-miR-29c-3p;hsa-miR-30
ENSG0000	ENSG0000	0.001754	hsa-miR-1260a;hsa-miR-1260b;hsa-miR-188-3p;hsa-miR-2355
ENSG0000	ENSG0000	0.001444	hsa-miR-2110;hsa-miR-34a-5p;hsa-miR-4286;hsa-miR-449a;hs
ENSG0000	ENSG0000	0.001988	hsa-miR-24-3p;hsa-miR-30c-1-3p;hsa-miR-30c-2-3p;hsa-miR-
ENSG0000	ENSG0000	0.001988	hsa-miR-24-3p;hsa-miR-30c-1-3p;hsa-miR-30c-2-3p;hsa-miR-
ENSG0000	ENSG0000	0.005478	hsa-miR-24-3p;hsa-miR-30c-1-3p;hsa-miR-30c-2-3p;hsa-miR-
ENSG0000	ENSG0000	0.006407	hsa-miR-24-3p;hsa-miR-30c-1-3p;hsa-miR-30c-2-3p;hsa-miR-
ENSG0000	ENSG0000	0.006407	hsa-miR-24-3p;hsa-miR-30c-1-3p;hsa-miR-30c-2-3p;hsa-miR-
ENSG0000	ENSG0000	0.002667	hsa-miR-140-3p;hsa-miR-30c-1-3p;hsa-miR-30c-2-3p;hsa-mi
ENSG0000	ENSG0000	0.00154	hsa-miR-24-3p;hsa-miR-30c-1-3p;hsa-miR-30c-2-3p;hsa-miR-
ENSG0000	ENSG0000	0.002123	hsa-miR-221-5p;hsa-miR-24-3p;hsa-miR-3187-3p;hsa-miR-47
ENSG0000	ENSG0000	2.50E-09	hsa-miR-1226-3p;hsa-miR-29a-3p;hsa-miR-29b-3p;hsa-miR-2
ENSG0000	ENSG0000	0.000296	hsa-miR-29a-3p;hsa-miR-29b-3p;hsa-miR-29c-3p;hsa-miR-30
ENSG0000	ENSG0000	0.000129	hsa-miR-192-5p;hsa-miR-24-3p;hsa-miR-30c-1-3p;hsa-miR-3
ENSG0000	ENSG0000	0.003245	hsa-miR-1226-3p;hsa-miR-29a-3p;hsa-miR-29b-3p;hsa-miR-3
ENSG0000	ENSG0000	0.000428	hsa-miR-1226-3p;hsa-miR-24-3p;hsa-miR-30c-1-3p;hsa-miR-
ENSG0000	ENSG0000	0.00505	hsa-miR-24-3p;hsa-miR-30c-1-3p;hsa-miR-30c-2-3p;hsa-miR-
ENSG0000	ENSG0000	3.98E-05	hsa-miR-221-5p;hsa-miR-24-3p;hsa-miR-30c-1-3p;hsa-miR-3
ENSG0000	ENSG0000	3.56E-05	hsa-miR-1260a;hsa-miR-1260b;hsa-miR-188-3p;hsa-miR-30c-1
ENSG0000	ENSG0000	0.009172	hsa-miR-29b-3p;hsa-miR-30c-1-3p;hsa-miR-30c-2-3p;hsa-mi
ENSG0000	ENSG0000	0.000797	hsa-miR-29a-3p;hsa-miR-29b-3p;hsa-miR-29c-3p;hsa-miR-30
ENSG0000	ENSG0000	0.009494	hsa-miR-30c-1-3p;hsa-miR-30c-2-3p;hsa-miR-3913-5p;hsa-mi
ENSG0000	ENSG0000	0.008712	hsa-miR-2110;hsa-miR-331-3p;hsa-miR-362-3p;hsa-miR-491-
ENSG0000	ENSG0000	0.009471	hsa-miR-29a-3p;hsa-miR-29b-3p;hsa-miR-29c-3p;hsa-miR-33
ENSG0000	ENSG0000	0.005819	hsa-miR-1226-3p;hsa-miR-24-3p;hsa-miR-30c-1-3p;hsa-miR-
ENSG0000	ENSG0000	0.002193	hsa-miR-30c-1-3p;hsa-miR-30c-2-3p;hsa-miR-34a-5p;hsa-mi
ENSG0000	ENSG0000	0.00984	hsa-miR-140-3p;hsa-miR-2355-5p;hsa-miR-29a-3p;hsa-miR-2
ENSG0000	ENSG0000	0.005485	hsa-miR-2110;hsa-miR-34a-5p;hsa-miR-449a;hsa-miR-4525;hs
ENSG0000	ENSG0000	2.96E-05	hsa-miR-29a-3p;hsa-miR-29b-3p;hsa-miR-29c-3p;hsa-miR-30
ENSG0000	ENSG0000	3.92E-07	hsa-miR-24-3p;hsa-miR-29a-3p;hsa-miR-29b-3p;hsa-miR-29c-
ENSG0000	ENSG0000	0.000859	hsa-miR-1260a;hsa-miR-1260b;hsa-miR-140-3p;hsa-miR-30c-1
ENSG0000	ENSG0000	0.00194	hsa-miR-192-5p;hsa-miR-3187-3p;hsa-miR-588;hsa-miR-873-
ENSG0000	ENSG0000	9.81E-05	hsa-miR-24-3p;hsa-miR-29a-3p;hsa-miR-29b-3p;hsa-miR-29c-
ENSG0000	ENSG0000	0.003902	hsa-miR-30c-1-3p;hsa-miR-30c-2-3p;hsa-miR-34a-5p;hsa-mi

ENSG0000	ENSG0000	0.002471	hsa-miR-24-3p;hsa-miR-30c-1-3p;hsa-miR-30c-2-3p;hsa-miR-
ENSG0000	ENSG0000	0.009172	hsa-miR-24-3p;hsa-miR-30c-1-3p;hsa-miR-30c-2-3p;hsa-miR-
ENSG0000	ENSG0000	0.004263	hsa-miR-1226-3p;hsa-miR-29a-3p;hsa-miR-29b-3p;hsa-miR-29
ENSG0000	ENSG0000	0.007705	hsa-miR-30c-1-3p;hsa-miR-30c-2-3p;hsa-miR-3913-5p;hsa-mi
ENSG0000	ENSG0000	0.000378	hsa-miR-24-3p;hsa-miR-30c-1-3p;hsa-miR-30c-2-3p;hsa-miR-
ENSG0000	ENSG0000	0.000426	hsa-miR-29a-3p;hsa-miR-29b-3p;hsa-miR-29c-3p;hsa-miR-30
ENSG0000	ENSG0000	6.69E-05	hsa-miR-149-5p;hsa-miR-221-5p;hsa-miR-24-3p;hsa-miR-30c-
ENSG0000	ENSG0000	0.006407	hsa-miR-188-3p;hsa-miR-30c-1-3p;hsa-miR-30c-2-3p;hsa-miR
ENSG0000	ENSG0000	0.001697	hsa-miR-24-3p;hsa-miR-30c-1-3p;hsa-miR-30c-2-3p;hsa-miR-
ENSG0000	ENSG0000	0.000181	hsa-miR-24-3p;hsa-miR-3065-3p;hsa-miR-30c-1-3p;hsa-miR-3
ENSG0000	ENSG0000	0.000127	hsa-miR-24-3p;hsa-miR-30c-1-3p;hsa-miR-30c-2-3p;hsa-miR-
ENSG0000	ENSG0000	4.25E-05	hsa-miR-29a-3p;hsa-miR-29b-3p;hsa-miR-29c-3p;hsa-miR-30
ENSG0000	ENSG0000	0.002667	hsa-miR-1260a;hsa-miR-1260b;hsa-miR-24-3p;hsa-miR-3913-5
ENSG0000	ENSG0000	0.009805	hsa-miR-29a-3p;hsa-miR-29b-3p;hsa-miR-29c-3p;hsa-miR-338
ENSG0000	ENSG0000	0.000239	hsa-miR-1260a;hsa-miR-1260b;hsa-miR-188-3p;hsa-miR-221-5
ENSG0000	ENSG0000	0.000894	hsa-miR-24-3p;hsa-miR-30c-1-3p;hsa-miR-30c-2-3p;hsa-miR-
ENSG0000	ENSG0000	0.002946	hsa-miR-24-3p;hsa-miR-30c-1-3p;hsa-miR-30c-2-3p;hsa-miR-
ENSG0000	ENSG0000	0.004195	hsa-miR-221-5p;hsa-miR-2355-5p;hsa-miR-30c-1-3p;hsa-miR-
ENSG0000	ENSG0000	0.002471	hsa-miR-1260a;hsa-miR-1260b;hsa-miR-2355-5p;hsa-miR-24-3
ENSG0000	ENSG0000	0.003902	hsa-miR-29a-3p;hsa-miR-29b-3p;hsa-miR-338-3p;hsa-miR-873
ENSG0000	ENSG0000	0.002676	hsa-miR-139-3p;hsa-miR-29a-3p;hsa-miR-29b-3p;hsa-miR-29
ENSG0000	ENSG0000	0.009983	hsa-miR-221-5p;hsa-miR-30c-1-3p;hsa-miR-30c-2-3p;hsa-miR
ENSG0000	ENSG0000	0.009022	hsa-miR-24-3p;hsa-miR-30c-1-3p;hsa-miR-30c-2-3p;hsa-miR-
ENSG0000	ENSG0000	0.002382	hsa-miR-1260a;hsa-miR-1260b;hsa-miR-149-5p;hsa-miR-24-3p
ENSG0000	ENSG0000	0.001732	hsa-miR-24-3p;hsa-miR-30c-1-3p;hsa-miR-30c-2-3p;hsa-miR-
ENSG0000	ENSG0000	0.001184	hsa-miR-24-3p;hsa-miR-30c-1-3p;hsa-miR-30c-2-3p;hsa-miR-
ENSG0000	ENSG0000	4.25E-05	hsa-miR-30c-1-3p;hsa-miR-30c-2-3p;hsa-miR-4525;hsa-miR-4
ENSG0000	ENSG0000	0.003764	hsa-miR-139-3p;hsa-miR-30c-1-3p;hsa-miR-30c-2-3p;hsa-miR
ENSG0000	ENSG0000	0.000713	hsa-miR-29a-3p;hsa-miR-29b-3p;hsa-miR-29c-3p;hsa-miR-30
ENSG0000	ENSG0000	4.55E-05	hsa-miR-139-3p;hsa-miR-2355-5p;hsa-miR-30c-1-3p;hsa-miR-
ENSG0000	ENSG0000	2.68E-05	hsa-miR-29a-3p;hsa-miR-29b-3p;hsa-miR-29c-3p;hsa-miR-30
ENSG0000	ENSG0000	0.000481	hsa-miR-140-3p;hsa-miR-188-3p;hsa-miR-34a-5p;hsa-miR-449
ENSG0000	ENSG0000	0.000239	hsa-miR-1260a;hsa-miR-1260b;hsa-miR-188-3p;hsa-miR-362-3
ENSG0000	ENSG0000	0.004019	hsa-miR-1226-3p;hsa-miR-24-3p;hsa-miR-3913-5p;hsa-miR-54
ENSG0000	ENSG0000	0.00593	hsa-miR-24-3p;hsa-miR-30c-1-3p;hsa-miR-30c-2-3p;hsa-miR-
ENSG0000	ENSG0000	0.003068	hsa-miR-2355-5p;hsa-miR-29a-3p;hsa-miR-29b-3p;hsa-miR-29
ENSG0000	ENSG0000	0.002667	hsa-miR-29a-3p;hsa-miR-29b-3p;hsa-miR-29c-3p;hsa-miR-30
ENSG0000	ENSG0000	0.009172	hsa-miR-24-3p;hsa-miR-30c-1-3p;hsa-miR-30c-2-3p;hsa-miR-
ENSG0000	ENSG0000	0.001646	hsa-miR-149-5p;hsa-miR-24-3p;hsa-miR-29a-3p;hsa-miR-29b-
ENSG0000	ENSG0000	0.000198	hsa-miR-24-3p;hsa-miR-30c-1-3p;hsa-miR-30c-2-3p;hsa-miR-
ENSG0000	ENSG0000	0.003846	hsa-miR-3065-3p;hsa-miR-30c-1-3p;hsa-miR-30c-2-3p;hsa-mi
ENSG0000	ENSG0000	0.009172	hsa-miR-29a-3p;hsa-miR-29b-3p;hsa-miR-29c-3p;hsa-miR-34
ENSG0000	ENSG0000	0.001697	hsa-miR-2110;hsa-miR-24-3p;hsa-miR-30c-1-3p;hsa-miR-30c-
ENSG0000	ENSG0000	0.00505	hsa-miR-24-3p;hsa-miR-30c-1-3p;hsa-miR-30c-2-3p;hsa-miR-
ENSG0000	ENSG0000	0.00154	hsa-miR-2110;hsa-miR-29a-3p;hsa-miR-29b-3p;hsa-miR-29c-3
ENSG0000	ENSG0000	0.000292	hsa-miR-29a-3p;hsa-miR-29b-3p;hsa-miR-29c-3p;hsa-miR-30
ENSG0000	ENSG0000	0.001294	hsa-miR-1260a;hsa-miR-1260b;hsa-miR-188-3p;hsa-miR-3065-
ENSG0000	ENSG0000	0.001537	hsa-miR-3065-3p;hsa-miR-30c-1-3p;hsa-miR-30c-2-3p;hsa-mi
ENSG0000	ENSG0000	1.00E-05	hsa-miR-1260a;hsa-miR-1260b;hsa-miR-139-3p;hsa-miR-188-3
ENSG0000	ENSG0000	1.64E-06	hsa-miR-2355-5p;hsa-miR-29a-3p;hsa-miR-29b-3p;hsa-miR-4
ENSG0000	ENSG0000	0.006699	hsa-miR-30c-1-3p;hsa-miR-30c-2-3p;hsa-miR-3187-3p;hsa-mi
ENSG0000	ENSG0000	0.001732	hsa-miR-29a-3p;hsa-miR-29b-3p;hsa-miR-29c-3p;hsa-miR-30
ENSG0000	ENSG0000	1.70E-05	hsa-miR-24-3p;hsa-miR-29a-3p;hsa-miR-29b-3p;hsa-miR-29c-
ENSG0000	ENSG0000	0.004645	hsa-miR-29a-3p;hsa-miR-29b-3p;hsa-miR-29c-3p;hsa-miR-42

ENSG0000	ENSG0000	0.001697	hsa-miR-221-5p;hsa-miR-24-3p;hsa-miR-30c-1-3p;hsa-miR-30
ENSG0000	ENSG0000	0.00095	hsa-miR-21-3p;hsa-miR-29a-3p;hsa-miR-29b-3p;hsa-miR-29c-
ENSG0000	ENSG0000	0.000669	hsa-miR-30c-1-3p;hsa-miR-30c-2-3p;hsa-miR-3187-3p;hsa-mi
ENSG0000	ENSG0000	0.009494	hsa-miR-30c-1-3p;hsa-miR-30c-2-3p;hsa-miR-340-3p;hsa-miR
ENSG0000	ENSG0000	0.000661	hsa-miR-24-3p;hsa-miR-30c-1-3p;hsa-miR-30c-2-3p;hsa-miR-
ENSG0000	ENSG0000	0.000883	hsa-miR-2355-5p;hsa-miR-24-3p;hsa-miR-30c-1-3p;hsa-miR-3
ENSG0000	ENSG0000	0.000687	hsa-miR-29a-3p;hsa-miR-29b-3p;hsa-miR-29c-3p;hsa-miR-873
ENSG0000	ENSG0000	0.000467	hsa-miR-24-3p;hsa-miR-30c-1-3p;hsa-miR-30c-2-3p;hsa-miR-
ENSG0000	ENSG0000	4.92E-05	hsa-miR-139-3p;hsa-miR-24-3p;hsa-miR-29a-3p;hsa-miR-29b-
ENSG0000	ENSG0000	0.000122	hsa-miR-1260a;hsa-miR-1260b;hsa-miR-188-3p;hsa-miR-873-5
ENSG0000	ENSG0000	0.000687	hsa-miR-1260a;hsa-miR-1260b;hsa-miR-188-3p;hsa-miR-362-3
ENSG0000	ENSG0000	0.000797	hsa-miR-29a-3p;hsa-miR-29b-3p;hsa-miR-29c-3p;hsa-miR-306
ENSG0000	ENSG0000	1.88E-05	hsa-miR-34a-5p;hsa-miR-449a;hsa-miR-4525;hsa-miR-505-5p;
ENSG0000	ENSG0000	0.001022	hsa-miR-24-3p;hsa-miR-30c-1-3p;hsa-miR-30c-2-3p;hsa-miR-
ENSG0000	ENSG0000	0.009471	hsa-miR-24-3p;hsa-miR-3065-3p;hsa-miR-30c-1-3p;hsa-miR-3
ENSG0000	ENSG0000	0.003245	hsa-miR-3187-3p;hsa-miR-34a-5p;hsa-miR-449a;hsa-miR-588
ENSG0000	ENSG0000	0.000819	hsa-miR-24-3p;hsa-miR-30c-1-3p;hsa-miR-30c-2-3p;hsa-miR-
ENSG0000	ENSG0000	0.007434	hsa-miR-30c-1-3p;hsa-miR-30c-2-3p;hsa-miR-362-3p;hsa-miR
ENSG0000	ENSG0000	2.74E-05	hsa-miR-29a-3p;hsa-miR-29b-3p;hsa-miR-29c-3p;hsa-miR-306
ENSG0000	ENSG0000	0.000118	hsa-miR-139-3p;hsa-miR-30c-1-3p;hsa-miR-30c-2-3p;hsa-miR
ENSG0000	ENSG0000	0.000271	hsa-miR-24-3p;hsa-miR-30c-1-3p;hsa-miR-30c-2-3p;hsa-miR-
ENSG0000	ENSG0000	0.009983	hsa-miR-222-5p;hsa-miR-24-3p;hsa-miR-30c-1-3p;hsa-miR-30
ENSG0000	ENSG0000	0.00154	hsa-miR-188-3p;hsa-miR-362-3p;hsa-miR-4738-3p;hsa-miR-64
ENSG0000	ENSG0000	0.007297	hsa-miR-30c-1-3p;hsa-miR-30c-2-3p;hsa-miR-3913-5p;hsa-mi
ENSG0000	ENSG0000	0.004019	hsa-miR-24-3p;hsa-miR-30c-1-3p;hsa-miR-30c-2-3p;hsa-miR-
ENSG0000	ENSG0000	0.000361	hsa-miR-149-5p;hsa-miR-2355-5p;hsa-miR-29a-3p;hsa-miR-29
ENSG0000	ENSG0000	0.001184	hsa-miR-24-3p;hsa-miR-30c-1-3p;hsa-miR-30c-2-3p;hsa-miR-
ENSG0000	ENSG0000	0.001363	hsa-miR-1260a;hsa-miR-1260b;hsa-miR-140-3p;hsa-miR-188-3
ENSG0000	ENSG0000	0.003902	hsa-miR-1260a;hsa-miR-1260b;hsa-miR-188-3p;hsa-miR-873-5
ENSG0000	ENSG0000	0.007093	hsa-miR-140-3p;hsa-miR-188-3p;hsa-miR-2110;hsa-miR-3065-
ENSG0000	ENSG0000	0.006828	hsa-miR-140-3p;hsa-miR-188-3p;hsa-miR-2110;hsa-miR-3065-
ENSG0000	ENSG0000	0.009172	hsa-miR-30c-1-3p;hsa-miR-30c-2-3p;hsa-miR-338-3p;hsa-miR
ENSG0000	ENSG0000	0.009172	hsa-miR-30c-1-3p;hsa-miR-30c-2-3p;hsa-miR-338-3p;hsa-miR
ENSG0000	ENSG0000	0.005819	hsa-miR-24-3p;hsa-miR-30c-1-3p;hsa-miR-30c-2-3p;hsa-miR-
ENSG0000	ENSG0000	0.005819	hsa-miR-24-3p;hsa-miR-30c-1-3p;hsa-miR-30c-2-3p;hsa-miR-
ENSG0000	ENSG0000	0.009172	hsa-miR-30c-1-3p;hsa-miR-30c-2-3p;hsa-miR-338-3p;hsa-miR
ENSG0000	ENSG0000	0.003764	hsa-miR-24-3p;hsa-miR-30c-1-3p;hsa-miR-30c-2-3p;hsa-miR-
ENSG0000	ENSG0000	0.003764	hsa-miR-24-3p;hsa-miR-30c-1-3p;hsa-miR-30c-2-3p;hsa-miR-
ENSG0000	ENSG0000	5.81E-05	hsa-miR-27a-3p;hsa-miR-27b-3p;hsa-miR-30a-3p;hsa-miR-30
ENSG0000	ENSG0000	5.04E-07	hsa-miR-30a-3p;hsa-miR-30d-3p;hsa-miR-30e-3p;hsa-miR-33
ENSG0000	ENSG0000	0.000354	hsa-miR-141-5p;hsa-miR-27a-3p;hsa-miR-27b-3p;hsa-miR-33
ENSG0000	ENSG0000	0.000423	hsa-miR-30a-3p;hsa-miR-30d-3p;hsa-miR-30e-3p;hsa-miR-33
ENSG0000	ENSG0000	0.005961	hsa-miR-30a-3p;hsa-miR-30d-3p;hsa-miR-30e-3p;hsa-miR-33
ENSG0000	ENSG0000	7.21E-06	hsa-miR-30a-3p;hsa-miR-30d-3p;hsa-miR-30e-3p;hsa-miR-33
ENSG0000	ENSG0000	6.03E-06	hsa-miR-30a-3p;hsa-miR-30d-3p;hsa-miR-30e-3p;hsa-miR-33
ENSG0000	ENSG0000	0.001973	hsa-miR-30a-3p;hsa-miR-30d-3p;hsa-miR-30e-3p;hsa-miR-33
ENSG0000	ENSG0000	1.58E-05	hsa-miR-30a-3p;hsa-miR-30d-3p;hsa-miR-30e-3p;hsa-miR-33
ENSG0000	ENSG0000	7.21E-06	hsa-miR-30a-3p;hsa-miR-30d-3p;hsa-miR-30e-3p;hsa-miR-33
ENSG0000	ENSG0000	0.000239	hsa-miR-30a-3p;hsa-miR-30d-3p;hsa-miR-30e-3p;hsa-miR-33
ENSG0000	ENSG0000	0.000928	hsa-miR-30a-3p;hsa-miR-30d-3p;hsa-miR-30e-3p;hsa-miR-33
ENSG0000	ENSG0000	0.003429	hsa-miR-200b-5p;hsa-miR-30a-3p;hsa-miR-30d-3p;hsa-miR-30
ENSG0000	ENSG0000	0.001114	hsa-miR-30a-3p;hsa-miR-30d-3p;hsa-miR-30e-3p;hsa-miR-33
ENSG0000	ENSG0000	8.55E-06	hsa-miR-30a-3p;hsa-miR-30d-3p;hsa-miR-30e-3p;hsa-miR-33
ENSG0000	ENSG0000	0.000304	hsa-miR-27a-3p;hsa-miR-27b-3p;hsa-miR-30a-3p;hsa-miR-30

ENSG0000	ENSG0000	0.000304	hsa-miR-27a-3p;hsa-miR-27b-3p;hsa-miR-30a-3p;hsa-miR-30
ENSG0000	ENSG0000	0.001563	hsa-miR-141-5p;hsa-miR-27a-3p;hsa-miR-27b-3p;hsa-miR-33
ENSG0000	ENSG0000	0.000112	hsa-miR-30a-3p;hsa-miR-30d-3p;hsa-miR-30e-3p;hsa-miR-33
ENSG0000	ENSG0000	3.82E-05	hsa-miR-16-2-3p;hsa-miR-195-3p;hsa-miR-28-5p;hsa-miR-30
ENSG0000	ENSG0000	0.003059	hsa-miR-30a-3p;hsa-miR-30d-3p;hsa-miR-30e-3p;hsa-miR-33
ENSG0000	ENSG0000	4.82E-07	hsa-miR-16-2-3p;hsa-miR-195-3p;hsa-miR-27a-3p;hsa-miR-27
ENSG0000	ENSG0000	0.000376	hsa-miR-30a-3p;hsa-miR-30d-3p;hsa-miR-30e-3p;hsa-miR-33
ENSG0000	ENSG0000	0.001325	hsa-miR-30a-3p;hsa-miR-30d-3p;hsa-miR-30e-3p;hsa-miR-33
ENSG0000	ENSG0000	0.000292	hsa-miR-30a-3p;hsa-miR-30d-3p;hsa-miR-30e-3p;hsa-miR-33
ENSG0000	ENSG0000	0.004126	hsa-miR-30a-3p;hsa-miR-30d-3p;hsa-miR-30e-3p;hsa-miR-33
ENSG0000	ENSG0000	2.34E-07	hsa-miR-30a-3p;hsa-miR-30d-3p;hsa-miR-30e-3p;hsa-miR-33
ENSG0000	ENSG0000	0.001973	hsa-miR-27a-3p;hsa-miR-27b-3p;hsa-miR-335-3p;hsa-miR-48
ENSG0000	ENSG0000	9.41E-05	hsa-miR-30a-3p;hsa-miR-30d-3p;hsa-miR-30e-3p;hsa-miR-33
ENSG0000	ENSG0000	0.001501	hsa-miR-30a-3p;hsa-miR-30d-3p;hsa-miR-30e-3p;hsa-miR-33
ENSG0000	ENSG0000	5.51E-07	hsa-miR-16-2-3p;hsa-miR-195-3p;hsa-miR-27b-3p;hsa-miR-3
ENSG0000	ENSG0000	0.004509	hsa-miR-30a-3p;hsa-miR-30d-3p;hsa-miR-30e-3p;hsa-miR-43
ENSG0000	ENSG0000	1.54E-07	hsa-miR-30a-3p;hsa-miR-30d-3p;hsa-miR-30e-3p;hsa-miR-33
ENSG0000	ENSG0000	0.000399	hsa-miR-30a-3p;hsa-miR-30d-3p;hsa-miR-30e-3p;hsa-miR-33
ENSG0000	ENSG0000	0.000143	hsa-miR-30a-3p;hsa-miR-30d-3p;hsa-miR-30e-3p;hsa-miR-33
ENSG0000	ENSG0000	1.49E-07	hsa-miR-30a-3p;hsa-miR-30d-3p;hsa-miR-30e-3p;hsa-miR-33
ENSG0000	ENSG0000	1.82E-05	hsa-miR-30a-3p;hsa-miR-30d-3p;hsa-miR-30e-3p;hsa-miR-33
ENSG0000	ENSG0000	1.82E-05	hsa-miR-30a-3p;hsa-miR-30d-3p;hsa-miR-30e-3p;hsa-miR-33
ENSG0000	ENSG0000	0.000143	hsa-miR-30a-3p;hsa-miR-30d-3p;hsa-miR-30e-3p;hsa-miR-46
ENSG0000	ENSG0000	7.85E-05	hsa-miR-30a-3p;hsa-miR-30d-3p;hsa-miR-30e-3p;hsa-miR-33
ENSG0000	ENSG0000	3.84E-05	hsa-miR-141-5p;hsa-miR-30a-3p;hsa-miR-30d-3p;hsa-miR-30
ENSG0000	ENSG0000	0.000503	hsa-miR-30a-3p;hsa-miR-30d-3p;hsa-miR-30e-3p;hsa-miR-33
ENSG0000	ENSG0000	1.58E-05	hsa-miR-30a-3p;hsa-miR-30d-3p;hsa-miR-30e-3p;hsa-miR-33
ENSG0000	ENSG0000	8.60E-05	hsa-miR-16-2-3p;hsa-miR-195-3p;hsa-miR-27a-3p;hsa-miR-27
ENSG0000	ENSG0000	0.003766	hsa-miR-30a-3p;hsa-miR-30d-3p;hsa-miR-30e-3p;hsa-miR-33
ENSG0000	ENSG0000	3.42E-05	hsa-miR-30a-3p;hsa-miR-30d-3p;hsa-miR-30e-3p;hsa-miR-33
ENSG0000	ENSG0000	1.27E-06	hsa-miR-30a-3p;hsa-miR-30d-3p;hsa-miR-30e-3p;hsa-miR-33
ENSG0000	ENSG0000	0.002631	hsa-miR-30a-3p;hsa-miR-30d-3p;hsa-miR-30e-3p;hsa-miR-33
ENSG0000	ENSG0000	7.21E-06	hsa-miR-16-2-3p;hsa-miR-195-3p;hsa-miR-27a-3p;hsa-miR-27
ENSG0000	ENSG0000	2.08E-05	hsa-miR-30a-3p;hsa-miR-30d-3p;hsa-miR-30e-3p;hsa-miR-33
ENSG0000	ENSG0000	1.58E-05	hsa-miR-30a-3p;hsa-miR-30d-3p;hsa-miR-30e-3p;hsa-miR-33
ENSG0000	ENSG0000	0.004004	hsa-miR-30a-3p;hsa-miR-30d-3p;hsa-miR-30e-3p;hsa-miR-33
ENSG0000	ENSG0000	0.000132	hsa-miR-30a-3p;hsa-miR-30d-3p;hsa-miR-30e-3p;hsa-miR-33
ENSG0000	ENSG0000	1.20E-06	hsa-miR-320a;hsa-miR-320b;hsa-miR-320c;hsa-miR-320d;hsa-r
ENSG0000	ENSG0000	0.006496	hsa-miR-129-5p;hsa-miR-15a-5p;hsa-miR-15b-5p;hsa-miR-19
ENSG0000	ENSG0000	6.96E-05	hsa-miR-320a;hsa-miR-320b;hsa-miR-320c;hsa-miR-320d;hsa-r
ENSG0000	ENSG0000	0.000338	hsa-miR-15a-5p;hsa-miR-15b-5p;hsa-miR-16-5p;hsa-miR-195-
ENSG0000	ENSG0000	3.05E-06	hsa-miR-15a-5p;hsa-miR-15b-5p;hsa-miR-16-5p;hsa-miR-195-
ENSG0000	ENSG0000	3.36E-07	hsa-miR-15a-5p;hsa-miR-15b-5p;hsa-miR-16-5p;hsa-miR-195-
ENSG0000	ENSG0000	3.27E-05	hsa-miR-15a-5p;hsa-miR-15b-5p;hsa-miR-195-5p;hsa-miR-21
ENSG0000	ENSG0000	0.004104	hsa-miR-192-5p;hsa-miR-22-3p;hsa-miR-326;hsa-miR-34a-5p;
ENSG0000	ENSG0000	1.35E-05	hsa-miR-15a-5p;hsa-miR-15b-5p;hsa-miR-16-5p;hsa-miR-195-
ENSG0000	ENSG0000	0.00879	hsa-miR-181a-5p;hsa-miR-181b-5p;hsa-miR-181c-5p;hsa-miR-
ENSG0000	ENSG0000	0.003181	hsa-miR-21-3p;hsa-miR-34a-5p;hsa-miR-92a-3p;hsa-miR-92b-
ENSG0000	ENSG0000	1.18E-05	hsa-miR-15a-5p;hsa-miR-15b-5p;hsa-miR-16-5p;hsa-miR-195-
ENSG0000	ENSG0000	1.18E-05	hsa-miR-15a-5p;hsa-miR-15b-5p;hsa-miR-16-5p;hsa-miR-195-
ENSG0000	ENSG0000	0.005882	hsa-miR-181a-5p;hsa-miR-181b-5p;hsa-miR-181c-5p;hsa-miR-
ENSG0000	ENSG0000	0.000113	hsa-miR-320a;hsa-miR-320b;hsa-miR-320c;hsa-miR-320d;hsa-r
ENSG0000	ENSG0000	0.00223	hsa-miR-181a-5p;hsa-miR-181b-5p;hsa-miR-181c-5p;hsa-miR-
ENSG0000	ENSG0000	6.03E-07	hsa-miR-137;hsa-miR-15a-5p;hsa-miR-15b-5p;hsa-miR-16-5p;

ENSG0000	ENSG0000	0.007808	hsa-miR-34a-5p;hsa-miR-34c-5p;hsa-miR-449a;hsa-miR-449b-
ENSG0000	ENSG0000	3.16E-06	hsa-miR-15b-5p;hsa-miR-181a-5p;hsa-miR-34a-5p;hsa-miR-7-
ENSG0000	ENSG0000	0.00024	hsa-miR-137;hsa-miR-140-3p;hsa-miR-146a-3p;hsa-miR-301a-
ENSG0000	ENSG0000	1.54E-08	hsa-miR-15a-5p;hsa-miR-15b-5p;hsa-miR-16-5p;hsa-miR-181
ENSG0000	ENSG0000	0.002201	hsa-miR-15a-5p;hsa-miR-15b-5p;hsa-miR-16-5p;hsa-miR-195-
ENSG0000	ENSG0000	0.003387	hsa-miR-15a-5p;hsa-miR-15b-5p;hsa-miR-16-5p;hsa-miR-195-
ENSG0000	ENSG0000	4.24E-05	hsa-miR-135a-5p;hsa-miR-135b-5p;hsa-miR-195-5p;hsa-miR-
ENSG0000	ENSG0000	0.000379	hsa-miR-15a-5p;hsa-miR-15b-5p;hsa-miR-16-5p;hsa-miR-195-
ENSG0000	ENSG0000	0.000196	hsa-miR-15b-5p;hsa-miR-16-5p;hsa-miR-195-5p;hsa-miR-34a-
ENSG0000	ENSG0000	0.001155	hsa-miR-15a-5p;hsa-miR-15b-5p;hsa-miR-16-5p;hsa-miR-424-
ENSG0000	ENSG0000	0.002493	hsa-miR-15a-5p;hsa-miR-15b-5p;hsa-miR-16-5p;hsa-miR-195-
ENSG0000	ENSG0000	0.000198	hsa-miR-15a-5p;hsa-miR-15b-5p;hsa-miR-16-5p;hsa-miR-195-
ENSG0000	ENSG0000	3.67E-05	hsa-miR-140-3p;hsa-miR-15a-5p;hsa-miR-15b-5p;hsa-miR-19
ENSG0000	ENSG0000	0.001155	hsa-miR-137;hsa-miR-185-5p;hsa-miR-195-5p;hsa-miR-21-3p
ENSG0000	ENSG0000	2.18E-05	hsa-miR-137;hsa-miR-15a-5p;hsa-miR-15b-5p;hsa-miR-16-5p;
ENSG0000	ENSG0000	0.00223	hsa-miR-320a;hsa-miR-320b;hsa-miR-320c;hsa-miR-320d;hsa-
ENSG0000	ENSG0000	1.11E-05	hsa-miR-15a-5p;hsa-miR-15b-5p;hsa-miR-16-5p;hsa-miR-195-
ENSG0000	ENSG0000	0.000183	hsa-miR-34a-5p;hsa-miR-34c-5p;hsa-miR-449a;hsa-miR-449b-
ENSG0000	ENSG0000	0.006386	hsa-miR-15a-5p;hsa-miR-15b-5p;hsa-miR-16-5p;hsa-miR-195-
ENSG0000	ENSG0000	0.001112	hsa-miR-127-5p;hsa-miR-15a-5p;hsa-miR-16-5p;hsa-miR-195-
ENSG0000	ENSG0000	0.000256	hsa-miR-15a-5p;hsa-miR-15b-5p;hsa-miR-16-5p;hsa-miR-185-
ENSG0000	ENSG0000	0.006936	hsa-miR-15a-5p;hsa-miR-15b-5p;hsa-miR-16-5p;hsa-miR-181
ENSG0000	ENSG0000	6.47E-06	hsa-miR-129-5p;hsa-miR-146a-3p;hsa-miR-15a-5p;hsa-miR-1
ENSG0000	ENSG0000	2.47E-05	hsa-miR-15a-5p;hsa-miR-15b-5p;hsa-miR-16-5p;hsa-miR-195-
ENSG0000	ENSG0000	0.000104	hsa-miR-127-5p;hsa-miR-130b-5p;hsa-miR-15a-5p;hsa-miR-1
ENSG0000	ENSG0000	9.64E-06	hsa-miR-15a-5p;hsa-miR-15b-5p;hsa-miR-16-5p;hsa-miR-195-
ENSG0000	ENSG0000	0.000993	hsa-miR-1260a;hsa-miR-1260b;hsa-miR-195-5p;hsa-miR-199a-
ENSG0000	ENSG0000	0.000869	hsa-miR-15a-5p;hsa-miR-15b-5p;hsa-miR-195-5p;hsa-miR-42
ENSG0000	ENSG0000	0.007808	hsa-miR-135a-5p;hsa-miR-137;hsa-miR-16-5p;hsa-miR-7-5p
ENSG0000	ENSG0000	0.002531	hsa-miR-15a-5p;hsa-miR-15b-5p;hsa-miR-195-5p;hsa-miR-42
ENSG0000	ENSG0000	0.00015	hsa-miR-129-5p;hsa-miR-181a-5p;hsa-miR-181b-5p;hsa-miR-
ENSG0000	ENSG0000	0.009617	hsa-miR-181a-5p;hsa-miR-181b-5p;hsa-miR-181c-5p;hsa-miR-
ENSG0000	ENSG0000	0.000841	hsa-miR-15a-5p;hsa-miR-15b-5p;hsa-miR-16-5p;hsa-miR-195-
ENSG0000	ENSG0000	0.000134	hsa-miR-1260a;hsa-miR-1260b;hsa-miR-137;hsa-miR-199a-5p;
ENSG0000	ENSG0000	9.29E-06	hsa-miR-15a-5p;hsa-miR-15b-5p;hsa-miR-16-5p;hsa-miR-195-
ENSG0000	ENSG0000	1.39E-07	hsa-miR-137;hsa-miR-15a-5p;hsa-miR-15b-5p;hsa-miR-16-5p;
ENSG0000	ENSG0000	4.38E-07	hsa-miR-15a-5p;hsa-miR-15b-5p;hsa-miR-16-5p;hsa-miR-195-
ENSG0000	ENSG0000	0.00385	hsa-miR-1260a;hsa-miR-1260b;hsa-miR-129-5p;hsa-miR-31-3
ENSG0000	ENSG0000	0.000109	hsa-miR-148a-5p;hsa-miR-21-3p;hsa-miR-34c-5p;hsa-miR-44
ENSG0000	ENSG0000	0.000137	hsa-miR-15a-5p;hsa-miR-15b-5p;hsa-miR-16-5p;hsa-miR-181
ENSG0000	ENSG0000	1.70E-07	hsa-miR-129-5p;hsa-miR-15a-5p;hsa-miR-15b-5p;hsa-miR-19
ENSG0000	ENSG0000	0.003767	hsa-miR-129-5p;hsa-miR-500a-5p;hsa-miR-92a-3p;hsa-miR-9
ENSG0000	ENSG0000	0.000198	hsa-miR-15a-5p;hsa-miR-15b-5p;hsa-miR-16-5p;hsa-miR-195-
ENSG0000	ENSG0000	0.004727	hsa-miR-15a-5p;hsa-miR-15b-5p;hsa-miR-195-5p;hsa-miR-42
ENSG0000	ENSG0000	4.26E-05	hsa-miR-147b;hsa-miR-15a-5p;hsa-miR-15b-5p;hsa-miR-16-5
ENSG0000	ENSG0000	0.00392	hsa-miR-1260a;hsa-miR-1260b;hsa-miR-140-3p;hsa-miR-194-
ENSG0000	ENSG0000	0.008861	hsa-miR-130b-5p;hsa-miR-146a-3p;hsa-miR-301a-5p;hsa-miR-
ENSG0000	ENSG0000	0.008861	hsa-miR-130b-5p;hsa-miR-146a-3p;hsa-miR-301a-5p;hsa-miR-
ENSG0000	ENSG0000	0.008861	hsa-miR-130b-5p;hsa-miR-146a-3p;hsa-miR-301a-5p;hsa-miR-
ENSG0000	ENSG0000	0.008861	hsa-miR-130b-5p;hsa-miR-146a-3p;hsa-miR-301a-5p;hsa-miR-
ENSG0000	ENSG0000	0.008861	hsa-miR-130b-5p;hsa-miR-146a-3p;hsa-miR-301a-5p;hsa-miR-
ENSG0000	ENSG0000	0.008861	hsa-miR-130b-5p;hsa-miR-146a-3p;hsa-miR-301a-5p;hsa-miR-
ENSG0000	ENSG0000	0.008861	hsa-miR-130b-5p;hsa-miR-146a-3p;hsa-miR-301a-5p;hsa-miR-
ENSG0000	ENSG0000	0.0018	hsa-miR-34a-5p;hsa-miR-34b-5p;hsa-miR-34c-5p;hsa-miR-44

ENSG0000	ENSG0000	0.002257	hsa-miR-129-5p;hsa-miR-15a-5p;hsa-miR-15b-5p;hsa-miR-16-
ENSG0000	ENSG0000	0.000963	hsa-miR-140-3p;hsa-miR-15a-5p;hsa-miR-15b-5p;hsa-miR-19
ENSG0000	ENSG0000	0.006528	hsa-miR-15a-5p;hsa-miR-15b-5p;hsa-miR-195-5p;hsa-miR-42
ENSG0000	ENSG0000	0.00385	hsa-miR-135a-5p;hsa-miR-15a-5p;hsa-miR-15b-5p;hsa-miR-1
ENSG0000	ENSG0000	0.001221	hsa-miR-15a-5p;hsa-miR-15b-5p;hsa-miR-195-5p;hsa-miR-42
ENSG0000	ENSG0000	0.007223	hsa-miR-320a;hsa-miR-320b;hsa-miR-320c;hsa-miR-320d;hsa-
ENSG0000	ENSG0000	6.78E-05	hsa-miR-320a;hsa-miR-320b;hsa-miR-320c;hsa-miR-320d;hsa-
ENSG0000	ENSG0000	0.000183	hsa-miR-181a-5p;hsa-miR-181b-5p;hsa-miR-181c-5p;hsa-miR-
ENSG0000	ENSG0000	0.004215	hsa-miR-192-5p;hsa-miR-34a-5p;hsa-miR-34c-5p;hsa-miR-44
ENSG0000	ENSG0000	0.000109	hsa-miR-15a-5p;hsa-miR-15b-5p;hsa-miR-195-5p;hsa-miR-34
ENSG0000	ENSG0000	0.000351	hsa-miR-15a-5p;hsa-miR-15b-5p;hsa-miR-195-5p;hsa-miR-42
ENSG0000	ENSG0000	0.000177	hsa-miR-15a-5p;hsa-miR-15b-5p;hsa-miR-181a-5p;hsa-miR-1
ENSG0000	ENSG0000	0.000134	hsa-miR-15a-5p;hsa-miR-15b-5p;hsa-miR-16-5p;hsa-miR-195-
ENSG0000	ENSG0000	1.91E-08	hsa-miR-15a-5p;hsa-miR-15b-5p;hsa-miR-16-5p;hsa-miR-195-
ENSG0000	ENSG0000	1.91E-08	hsa-miR-15a-5p;hsa-miR-15b-5p;hsa-miR-16-5p;hsa-miR-195-
ENSG0000	ENSG0000	0.00095	hsa-miR-15a-5p;hsa-miR-15b-5p;hsa-miR-16-5p;hsa-miR-195-
ENSG0000	ENSG0000	2.02E-06	hsa-miR-140-3p;hsa-miR-15a-5p;hsa-miR-15b-5p;hsa-miR-19
ENSG0000	ENSG0000	8.14E-06	hsa-miR-15a-5p;hsa-miR-15b-5p;hsa-miR-16-5p;hsa-miR-185-
ENSG0000	ENSG0000	0.003008	hsa-miR-15a-5p;hsa-miR-15b-5p;hsa-miR-16-5p;hsa-miR-195-
ENSG0000	ENSG0000	0.000399	hsa-miR-15a-5p;hsa-miR-185-5p;hsa-miR-195-5p;hsa-miR-42
ENSG0000	ENSG0000	0.000731	hsa-miR-140-3p;hsa-miR-320a;hsa-miR-320b;hsa-miR-320c;hs
ENSG0000	ENSG0000	9.93E-07	hsa-miR-15a-5p;hsa-miR-16-5p;hsa-miR-181a-5p;hsa-miR-18
ENSG0000	ENSG0000	0.006386	hsa-miR-320a;hsa-miR-320b;hsa-miR-320c;hsa-miR-320d;hsa-
ENSG0000	ENSG0000	0.000724	hsa-miR-34a-5p;hsa-miR-34b-5p;hsa-miR-34c-5p;hsa-miR-44
ENSG0000	ENSG0000	0.000468	hsa-miR-15a-5p;hsa-miR-15b-5p;hsa-miR-195-5p;hsa-miR-22-
ENSG0000	ENSG0000	1.97E-07	hsa-miR-15a-5p;hsa-miR-16-5p;hsa-miR-34a-5p;hsa-miR-34b-
ENSG0000	ENSG0000	0.003743	hsa-miR-34a-5p;hsa-miR-34b-5p;hsa-miR-34c-5p;hsa-miR-44
ENSG0000	ENSG0000	7.55E-05	hsa-miR-15a-5p;hsa-miR-15b-5p;hsa-miR-195-5p;hsa-miR-42
ENSG0000	ENSG0000	9.07E-05	hsa-miR-34a-5p;hsa-miR-34b-5p;hsa-miR-34c-5p;hsa-miR-44
ENSG0000	ENSG0000	2.61E-06	hsa-miR-15a-5p;hsa-miR-15b-5p;hsa-miR-16-5p;hsa-miR-181
ENSG0000	ENSG0000	0.000789	hsa-miR-15a-5p;hsa-miR-15b-5p;hsa-miR-16-5p;hsa-miR-192-
ENSG0000	ENSG0000	0.002531	hsa-miR-140-3p;hsa-miR-15a-5p;hsa-miR-15b-5p;hsa-miR-19
ENSG0000	ENSG0000	0.000196	hsa-miR-15a-5p;hsa-miR-15b-5p;hsa-miR-195-5p;hsa-miR-42
ENSG0000	ENSG0000	1.91E-08	hsa-miR-15a-5p;hsa-miR-15b-5p;hsa-miR-16-5p;hsa-miR-195-
ENSG0000	ENSG0000	1.91E-08	hsa-miR-15a-5p;hsa-miR-15b-5p;hsa-miR-16-5p;hsa-miR-195-
ENSG0000	ENSG0000	0.000277	hsa-miR-15a-5p;hsa-miR-15b-5p;hsa-miR-195-5p;hsa-miR-19
ENSG0000	ENSG0000	6.47E-07	hsa-miR-15a-5p;hsa-miR-15b-5p;hsa-miR-16-5p;hsa-miR-195-
ENSG0000	ENSG0000	0.003181	hsa-miR-140-3p;hsa-miR-181a-5p;hsa-miR-181b-5p;hsa-miR-
ENSG0000	ENSG0000	0.00879	hsa-miR-320a;hsa-miR-320b;hsa-miR-320c;hsa-miR-320d;hsa-
ENSG0000	ENSG0000	0.002557	hsa-miR-15a-5p;hsa-miR-15b-5p;hsa-miR-195-5p;hsa-miR-42
ENSG0000	ENSG0000	0.00684	hsa-miR-199a-5p;hsa-miR-7-5p;hsa-miR-92a-3p;hsa-miR-92b-
ENSG0000	ENSG0000	0.001592	hsa-miR-15a-5p;hsa-miR-15b-5p;hsa-miR-181a-5p;hsa-miR-1
ENSG0000	ENSG0000	0.000841	hsa-miR-127-5p;hsa-miR-15a-5p;hsa-miR-15b-5p;hsa-miR-19
ENSG0000	ENSG0000	3.16E-06	hsa-miR-15a-5p;hsa-miR-15b-5p;hsa-miR-16-5p;hsa-miR-195-
ENSG0000	ENSG0000	0.005151	hsa-miR-1260a;hsa-miR-1260b;hsa-miR-92a-3p;hsa-miR-92b-
ENSG0000	ENSG0000	0.000363	hsa-miR-137;hsa-miR-15a-5p;hsa-miR-15b-5p;hsa-miR-16-5p;
ENSG0000	ENSG0000	0.001411	hsa-miR-15a-5p;hsa-miR-15b-5p;hsa-miR-16-5p;hsa-miR-195-
ENSG0000	ENSG0000	1.54E-06	hsa-miR-135a-5p;hsa-miR-15a-5p;hsa-miR-15b-5p;hsa-miR-1
ENSG0000	ENSG0000	1.54E-06	hsa-miR-135a-5p;hsa-miR-15a-5p;hsa-miR-15b-5p;hsa-miR-1
ENSG0000	ENSG0000	0.00024	hsa-miR-15a-5p;hsa-miR-15b-5p;hsa-miR-16-5p;hsa-miR-195-
ENSG0000	ENSG0000	7.72E-05	hsa-miR-1260a;hsa-miR-1260b;hsa-miR-15a-5p;hsa-miR-15b-
ENSG0000	ENSG0000	0.00266	hsa-miR-129-5p;hsa-miR-181a-5p;hsa-miR-181b-5p;hsa-miR-
ENSG0000	ENSG0000	0.000519	hsa-miR-15a-5p;hsa-miR-15b-5p;hsa-miR-16-5p;hsa-miR-195-
ENSG0000	ENSG0000	0.000519	hsa-miR-15a-5p;hsa-miR-15b-5p;hsa-miR-16-5p;hsa-miR-195-

ENSG0000	ENSG0000	9.28E-07	hsa-miR-181a-5p;hsa-miR-181b-5p;hsa-miR-181c-5p;hsa-miR-
ENSG0000	ENSG0000	2.12E-05	hsa-miR-15a-5p;hsa-miR-15b-5p;hsa-miR-16-5p;hsa-miR-195-
ENSG0000	ENSG0000	0.000251	hsa-miR-320a;hsa-miR-320b;hsa-miR-320c;hsa-miR-320d;hsa-r
ENSG0000	ENSG0000	0.000251	hsa-miR-320a;hsa-miR-320b;hsa-miR-320c;hsa-miR-320d;hsa-r
ENSG0000	ENSG0000	0.00013	hsa-miR-15a-5p;hsa-miR-15b-5p;hsa-miR-181a-5p;hsa-miR-18
ENSG0000	ENSG0000	0.000468	hsa-miR-15a-5p;hsa-miR-15b-5p;hsa-miR-16-5p;hsa-miR-195-
ENSG0000	ENSG0000	0.004104	hsa-miR-15a-5p;hsa-miR-15b-5p;hsa-miR-195-5p;hsa-miR-21-
ENSG0000	ENSG0000	0.001433	hsa-miR-130b-5p;hsa-miR-181a-5p;hsa-miR-181b-5p;hsa-miR
ENSG0000	ENSG0000	0.001433	hsa-miR-130b-5p;hsa-miR-181a-5p;hsa-miR-181b-5p;hsa-miR
ENSG0000	ENSG0000	0.002688	hsa-miR-15a-5p;hsa-miR-15b-5p;hsa-miR-195-5p;hsa-miR-33a
ENSG0000	ENSG0000	0.00538	hsa-miR-20b-3p;hsa-miR-34a-5p;hsa-miR-34c-5p;hsa-miR-423
ENSG0000	ENSG0000	0.00538	hsa-miR-20b-3p;hsa-miR-34a-5p;hsa-miR-34c-5p;hsa-miR-423
ENSG0000	ENSG0000	4.22E-06	hsa-miR-15a-5p;hsa-miR-15b-5p;hsa-miR-16-5p;hsa-miR-195-
ENSG0000	ENSG0000	0.008766	hsa-miR-194-3p;hsa-miR-34a-5p;hsa-miR-34c-5p;hsa-miR-449
ENSG0000	ENSG0000	5.41E-06	hsa-miR-15a-5p;hsa-miR-15b-5p;hsa-miR-16-5p;hsa-miR-185-
ENSG0000	ENSG0000	1.95E-06	hsa-miR-15a-5p;hsa-miR-15b-5p;hsa-miR-16-5p;hsa-miR-195-
ENSG0000	ENSG0000	0.007223	hsa-miR-21-3p;hsa-miR-320e;hsa-miR-92a-3p;hsa-miR-92b-3p
ENSG0000	ENSG0000	0.000317	hsa-miR-34a-5p;hsa-miR-34b-5p;hsa-miR-34c-5p;hsa-miR-449
ENSG0000	ENSG0000	0.001221	hsa-miR-15a-5p;hsa-miR-15b-5p;hsa-miR-195-5p;hsa-miR-22-
ENSG0000	ENSG0000	0.000179	hsa-miR-15a-5p;hsa-miR-15b-5p;hsa-miR-16-5p;hsa-miR-195-
ENSG0000	ENSG0000	7.33E-07	hsa-miR-15a-5p;hsa-miR-15b-5p;hsa-miR-16-5p;hsa-miR-195-
ENSG0000	ENSG0000	0.00266	hsa-miR-34a-5p;hsa-miR-34c-5p;hsa-miR-449a;hsa-miR-449b-
ENSG0000	ENSG0000	0.004502	hsa-miR-33a-3p;hsa-miR-34a-5p;hsa-miR-34c-5p;hsa-miR-449
ENSG0000	ENSG0000	0.003743	hsa-miR-15a-5p;hsa-miR-15b-5p;hsa-miR-195-5p;hsa-miR-424
ENSG0000	ENSG0000	0.002115	hsa-miR-15a-5p;hsa-miR-15b-5p;hsa-miR-16-5p;hsa-miR-195-
ENSG0000	ENSG0000	9.35E-05	hsa-miR-15a-5p;hsa-miR-15b-5p;hsa-miR-195-5p;hsa-miR-34b
ENSG0000	ENSG0000	0.000251	hsa-miR-320a;hsa-miR-320b;hsa-miR-320c;hsa-miR-320d;hsa-r
ENSG0000	ENSG0000	0.008536	hsa-miR-34a-5p;hsa-miR-34b-5p;hsa-miR-34c-5p;hsa-miR-423
ENSG0000	ENSG0000	0.008536	hsa-miR-34a-5p;hsa-miR-34b-5p;hsa-miR-34c-5p;hsa-miR-423
ENSG0000	ENSG0000	0.008536	hsa-miR-34a-5p;hsa-miR-34b-5p;hsa-miR-34c-5p;hsa-miR-423
ENSG0000	ENSG0000	0.008536	hsa-miR-34a-5p;hsa-miR-34b-5p;hsa-miR-34c-5p;hsa-miR-423
ENSG0000	ENSG0000	0.008536	hsa-miR-34a-5p;hsa-miR-34b-5p;hsa-miR-34c-5p;hsa-miR-423
ENSG0000	ENSG0000	0.008536	hsa-miR-34a-5p;hsa-miR-34b-5p;hsa-miR-34c-5p;hsa-miR-423
ENSG0000	ENSG0000	0.008536	hsa-miR-34a-5p;hsa-miR-34b-5p;hsa-miR-34c-5p;hsa-miR-423
ENSG0000	ENSG0000	0.008536	hsa-miR-34a-5p;hsa-miR-34b-5p;hsa-miR-34c-5p;hsa-miR-423
ENSG0000	ENSG0000	0.000164	hsa-miR-1260a;hsa-miR-1260b;hsa-miR-137;hsa-miR-181a-5p;
ENSG0000	ENSG0000	0.00095	hsa-miR-15a-5p;hsa-miR-15b-5p;hsa-miR-195-5p;hsa-miR-214
ENSG0000	ENSG0000	0.002767	hsa-miR-181a-5p;hsa-miR-181b-5p;hsa-miR-181c-5p;hsa-miR-
ENSG0000	ENSG0000	0.001442	hsa-miR-130b-5p;hsa-miR-15a-5p;hsa-miR-15b-5p;hsa-miR-1
ENSG0000	ENSG0000	0.002003	hsa-miR-129-5p;hsa-miR-320a;hsa-miR-320b;hsa-miR-320c;hsa
ENSG0000	ENSG0000	1.35E-05	hsa-miR-15a-5p;hsa-miR-15b-5p;hsa-miR-16-5p;hsa-miR-195-
ENSG0000	ENSG0000	0.005955	hsa-miR-181a-5p;hsa-miR-181b-5p;hsa-miR-181c-5p;hsa-miR-
ENSG0000	ENSG0000	1.20E-06	hsa-miR-320a;hsa-miR-320b;hsa-miR-320c;hsa-miR-320d;hsa-r
ENSG0000	ENSG0000	0.004215	hsa-miR-320a;hsa-miR-320b;hsa-miR-320c;hsa-miR-320d;hsa-r
ENSG0000	ENSG0000	0.000164	hsa-miR-15a-5p;hsa-miR-15b-5p;hsa-miR-16-5p;hsa-miR-195-
ENSG0000	ENSG0000	0.00747	hsa-miR-15a-5p;hsa-miR-15b-5p;hsa-miR-16-5p;hsa-miR-195-
ENSG0000	ENSG0000	0.007152	hsa-miR-140-3p;hsa-miR-15a-5p;hsa-miR-15b-5p;hsa-miR-16-
ENSG0000	ENSG0000	0.007519	hsa-miR-15a-5p;hsa-miR-15b-5p;hsa-miR-16-5p;hsa-miR-195-
ENSG0000	ENSG0000	1.20E-05	hsa-miR-15a-5p;hsa-miR-15b-5p;hsa-miR-16-5p;hsa-miR-195-
ENSG0000	ENSG0000	0.007808	hsa-miR-15a-5p;hsa-miR-15b-5p;hsa-miR-16-5p;hsa-miR-195-
ENSG0000	ENSG0000	0.001247	hsa-miR-15a-5p;hsa-miR-15b-5p;hsa-miR-16-5p;hsa-miR-195-
ENSG0000	ENSG0000	0.003743	hsa-miR-320a;hsa-miR-320b;hsa-miR-320c;hsa-miR-320d;hsa-r
ENSG0000	ENSG0000	0.007808	hsa-miR-33a-3p;hsa-miR-671-5p;hsa-miR-92a-3p;hsa-miR-92b
ENSG0000	ENSG0000	5.12E-05	hsa-miR-127-5p;hsa-miR-16-5p;hsa-miR-181a-5p;hsa-miR-181

ENSG0000	ENSG0000	0.007877	hsa-miR-15a-5p;hsa-miR-16-5p;hsa-miR-195-5p;hsa-miR-346;
ENSG0000	ENSG0000	0.00018	hsa-miR-15a-5p;hsa-miR-16-5p;hsa-miR-181a-5p;hsa-miR-18
ENSG0000	ENSG0000	0.005882	hsa-miR-132-5p;hsa-miR-34a-5p;hsa-miR-34c-5p;hsa-miR-44
ENSG0000	ENSG0000	6.47E-07	hsa-miR-15a-5p;hsa-miR-15b-5p;hsa-miR-16-5p;hsa-miR-195
ENSG0000	ENSG0000	1.54E-06	hsa-miR-15a-5p;hsa-miR-15b-5p;hsa-miR-16-5p;hsa-miR-195
ENSG0000	ENSG0000	0.000678	hsa-miR-1270;hsa-miR-15a-5p;hsa-miR-15b-5p;hsa-miR-16-5
ENSG0000	ENSG0000	0.000164	hsa-miR-146a-3p;hsa-miR-15a-5p;hsa-miR-15b-5p;hsa-miR-1
ENSG0000	ENSG0000	0.000196	hsa-miR-146a-3p;hsa-miR-181a-5p;hsa-miR-181b-5p;hsa-miR
ENSG0000	ENSG0000	9.26E-06	hsa-miR-15a-5p;hsa-miR-15b-5p;hsa-miR-16-5p;hsa-miR-195
ENSG0000	ENSG0000	0.001442	hsa-miR-15a-5p;hsa-miR-15b-5p;hsa-miR-16-5p;hsa-miR-192
ENSG0000	ENSG0000	1.20E-05	hsa-miR-15a-5p;hsa-miR-15b-5p;hsa-miR-16-5p;hsa-miR-195
ENSG0000	ENSG0000	3.27E-05	hsa-miR-34a-5p;hsa-miR-34b-5p;hsa-miR-34c-5p;hsa-miR-44
ENSG0000	ENSG0000	0.00587	hsa-miR-320a;hsa-miR-320b;hsa-miR-320c;hsa-miR-320d;hsa-
ENSG0000	ENSG0000	0.00587	hsa-miR-320a;hsa-miR-320b;hsa-miR-320c;hsa-miR-320d;hsa-
ENSG0000	ENSG0000	4.31E-07	hsa-miR-129-5p;hsa-miR-15a-5p;hsa-miR-15b-5p;hsa-miR-16
ENSG0000	ENSG0000	0.000841	hsa-miR-214-3p;hsa-miR-34a-5p;hsa-miR-34c-5p;hsa-miR-44
ENSG0000	ENSG0000	2.89E-05	hsa-miR-15a-5p;hsa-miR-15b-5p;hsa-miR-195-5p;hsa-miR-42
ENSG0000	ENSG0000	2.01E-08	hsa-miR-15a-5p;hsa-miR-15b-5p;hsa-miR-16-5p;hsa-miR-195
ENSG0000	ENSG0000	1.04E-06	hsa-miR-1270;hsa-miR-15a-5p;hsa-miR-15b-5p;hsa-miR-16-5
ENSG0000	ENSG0000	0.000239	hsa-miR-15a-5p;hsa-miR-15b-5p;hsa-miR-181a-5p;hsa-miR-1
ENSG0000	ENSG0000	7.29E-05	hsa-miR-15a-5p;hsa-miR-15b-5p;hsa-miR-16-5p;hsa-miR-195
ENSG0000	ENSG0000	0.000183	hsa-miR-127-5p;hsa-miR-181a-5p;hsa-miR-181b-5p;hsa-miR-
ENSG0000	ENSG0000	0.003065	hsa-miR-15a-5p;hsa-miR-15b-5p;hsa-miR-195-5p;hsa-miR-42
ENSG0000	ENSG0000	0.00015	hsa-miR-320a;hsa-miR-320b;hsa-miR-320c;hsa-miR-320d;hsa-
ENSG0000	ENSG0000	6.47E-07	hsa-miR-15a-5p;hsa-miR-15b-5p;hsa-miR-16-5p;hsa-miR-195
ENSG0000	ENSG0000	3.93E-05	hsa-miR-1226-3p;hsa-miR-15a-5p;hsa-miR-15b-5p;hsa-miR-1
ENSG0000	ENSG0000	0.00244	hsa-miR-15a-5p;hsa-miR-15b-5p;hsa-miR-16-5p;hsa-miR-194
ENSG0000	ENSG0000	0.003181	hsa-miR-15a-5p;hsa-miR-15b-5p;hsa-miR-16-5p;hsa-miR-195
ENSG0000	ENSG0000	4.15E-08	hsa-miR-15a-5p;hsa-miR-15b-5p;hsa-miR-195-5p;hsa-miR-34
ENSG0000	ENSG0000	0.007969	hsa-miR-34a-5p;hsa-miR-34c-5p;hsa-miR-423-5p;hsa-miR-44
ENSG0000	ENSG0000	0.00266	hsa-miR-34a-5p;hsa-miR-34c-5p;hsa-miR-449a;hsa-miR-449b-
ENSG0000	ENSG0000	1.91E-08	hsa-miR-15a-5p;hsa-miR-15b-5p;hsa-miR-16-5p;hsa-miR-195
ENSG0000	ENSG0000	3.99E-06	hsa-miR-320a;hsa-miR-320b;hsa-miR-320c;hsa-miR-320d;hsa-
ENSG0000	ENSG0000	7.29E-05	hsa-miR-34a-5p;hsa-miR-34c-5p;hsa-miR-449a;hsa-miR-449b-
ENSG0000	ENSG0000	0.000731	hsa-miR-15a-5p;hsa-miR-15b-5p;hsa-miR-16-5p;hsa-miR-195
ENSG0000	ENSG0000	0.000468	hsa-miR-15a-5p;hsa-miR-15b-5p;hsa-miR-16-5p;hsa-miR-195
ENSG0000	ENSG0000	4.14E-05	hsa-miR-15a-5p;hsa-miR-15b-5p;hsa-miR-16-5p;hsa-miR-195
ENSG0000	ENSG0000	5.41E-06	hsa-miR-127-5p;hsa-miR-15a-5p;hsa-miR-15b-5p;hsa-miR-16
ENSG0000	ENSG0000	1.70E-07	hsa-miR-15a-5p;hsa-miR-15b-5p;hsa-miR-16-5p;hsa-miR-195
ENSG0000	ENSG0000	1.20E-06	hsa-miR-15a-5p;hsa-miR-15b-5p;hsa-miR-16-5p;hsa-miR-195
ENSG0000	ENSG0000	1.53E-05	hsa-miR-130b-5p;hsa-miR-15a-5p;hsa-miR-15b-5p;hsa-miR-1
ENSG0000	ENSG0000	0.000111	hsa-miR-15a-5p;hsa-miR-15b-5p;hsa-miR-16-5p;hsa-miR-195
ENSG0000	ENSG0000	0.005955	hsa-miR-34a-5p;hsa-miR-34c-5p;hsa-miR-449a;hsa-miR-449b-
ENSG0000	ENSG0000	0.005955	hsa-miR-34a-5p;hsa-miR-34c-5p;hsa-miR-449a;hsa-miR-449b-
ENSG0000	ENSG0000	0.007508	hsa-miR-1260a;hsa-miR-1260b;hsa-miR-135a-5p;hsa-miR-15a-
ENSG0000	ENSG0000	2.73E-06	hsa-miR-15a-5p;hsa-miR-15b-5p;hsa-miR-16-5p;hsa-miR-195
ENSG0000	ENSG0000	0.001221	hsa-miR-15a-5p;hsa-miR-15b-5p;hsa-miR-195-5p;hsa-miR-42
ENSG0000	ENSG0000	5.50E-08	hsa-miR-137;hsa-miR-140-3p;hsa-miR-15a-5p;hsa-miR-15b-5
ENSG0000	ENSG0000	0.007519	hsa-miR-181a-5p;hsa-miR-181b-5p;hsa-miR-181c-5p;hsa-miR
ENSG0000	ENSG0000	0.000119	hsa-miR-15a-5p;hsa-miR-15b-5p;hsa-miR-16-5p;hsa-miR-195
ENSG0000	ENSG0000	3.86E-07	hsa-miR-15a-5p;hsa-miR-15b-5p;hsa-miR-16-5p;hsa-miR-195
ENSG0000	ENSG0000	0.000399	hsa-miR-137;hsa-miR-15a-5p;hsa-miR-15b-5p;hsa-miR-195-5
ENSG0000	ENSG0000	0.000317	hsa-miR-15a-5p;hsa-miR-15b-5p;hsa-miR-16-5p;hsa-miR-195
ENSG0000	ENSG0000	0.000317	hsa-miR-15a-5p;hsa-miR-15b-5p;hsa-miR-16-5p;hsa-miR-195

ENSG0000	ENSG0000	0.002916	hsa-miR-132-5p;hsa-miR-320e;hsa-miR-885-3p;hsa-miR-922;h
ENSG0000	ENSG0000	0.002916	hsa-miR-132-5p;hsa-miR-320e;hsa-miR-885-3p;hsa-miR-922;h
ENSG0000	ENSG0000	0.001411	hsa-miR-194-3p;hsa-miR-34a-5p;hsa-miR-34c-5p;hsa-miR-423
ENSG0000	ENSG0000	0.000692	hsa-miR-1270;hsa-miR-15a-5p;hsa-miR-15b-5p;hsa-miR-195-5
ENSG0000	ENSG0000	0.003311	hsa-miR-15a-5p;hsa-miR-15b-5p;hsa-miR-33a-3p;hsa-miR-424
ENSG0000	ENSG0000	0.000519	hsa-miR-181a-5p;hsa-miR-181b-5p;hsa-miR-181c-5p;hsa-miR-
ENSG0000	ENSG0000	2.00E-12	hsa-miR-137;hsa-miR-15a-5p;hsa-miR-15b-5p;hsa-miR-16-5p;
ENSG0000	ENSG0000	0.00223	hsa-miR-15a-5p;hsa-miR-15b-5p;hsa-miR-16-5p;hsa-miR-195-
ENSG0000	ENSG0000	1.20E-06	hsa-miR-15a-5p;hsa-miR-15b-5p;hsa-miR-16-5p;hsa-miR-195-
ENSG0000	ENSG0000	8.55E-05	hsa-miR-181a-5p;hsa-miR-181b-5p;hsa-miR-181c-5p;hsa-miR-
ENSG0000	ENSG0000	7.85E-05	hsa-miR-15a-5p;hsa-miR-15b-5p;hsa-miR-16-5p;hsa-miR-195-
ENSG0000	ENSG0000	2.41E-05	hsa-miR-15b-5p;hsa-miR-16-5p;hsa-miR-194-3p;hsa-miR-195-
ENSG0000	ENSG0000	0.00266	hsa-miR-15a-5p;hsa-miR-15b-5p;hsa-miR-16-5p;hsa-miR-195-
ENSG0000	ENSG0000	0.00266	hsa-miR-15a-5p;hsa-miR-15b-5p;hsa-miR-16-5p;hsa-miR-195-
ENSG0000	ENSG0000	1.20E-06	hsa-miR-15a-5p;hsa-miR-15b-5p;hsa-miR-16-5p;hsa-miR-195-
ENSG0000	ENSG0000	0.001221	hsa-miR-15a-5p;hsa-miR-15b-5p;hsa-miR-195-5p;hsa-miR-424
ENSG0000	ENSG0000	0.000317	hsa-miR-15a-5p;hsa-miR-15b-5p;hsa-miR-16-5p;hsa-miR-181a
ENSG0000	ENSG0000	0.003594	hsa-miR-15a-5p;hsa-miR-15b-5p;hsa-miR-16-5p;hsa-miR-195-
ENSG0000	ENSG0000	6.03E-07	hsa-miR-15a-5p;hsa-miR-15b-5p;hsa-miR-16-5p;hsa-miR-195-
ENSG0000	ENSG0000	0.004502	hsa-miR-15a-5p;hsa-miR-15b-5p;hsa-miR-16-5p;hsa-miR-195-
ENSG0000	ENSG0000	4.49E-05	hsa-miR-130b-5p;hsa-miR-15a-5p;hsa-miR-15b-5p;hsa-miR-1(
ENSG0000	ENSG0000	4.49E-05	hsa-miR-130b-5p;hsa-miR-15a-5p;hsa-miR-15b-5p;hsa-miR-1(
ENSG0000	ENSG0000	7.00E-07	hsa-miR-129-5p;hsa-miR-320a;hsa-miR-320b;hsa-miR-320c;hsa
ENSG0000	ENSG0000	0.005151	hsa-miR-15a-5p;hsa-miR-15b-5p;hsa-miR-16-5p;hsa-miR-195-
ENSG0000	ENSG0000	0.001247	hsa-miR-1270;hsa-miR-15a-5p;hsa-miR-15b-5p;hsa-miR-16-5p
ENSG0000	ENSG0000	0.000238	hsa-miR-1260a;hsa-miR-1260b;hsa-miR-127-5p;hsa-miR-15a-5
ENSG0000	ENSG0000	1.07E-06	hsa-miR-1260a;hsa-miR-1260b;hsa-miR-15a-5p;hsa-miR-15b-5
ENSG0000	ENSG0000	1.18E-06	hsa-miR-1260a;hsa-miR-1260b;hsa-miR-15a-5p;hsa-miR-15b-5
ENSG0000	ENSG0000	1.63E-10	hsa-miR-146a-3p;hsa-miR-15a-5p;hsa-miR-15b-5p;hsa-miR-1(
ENSG0000	ENSG0000	0.000198	hsa-miR-15a-5p;hsa-miR-15b-5p;hsa-miR-16-5p;hsa-miR-195-
ENSG0000	ENSG0000	4.90E-06	hsa-miR-148a-5p;hsa-miR-15a-5p;hsa-miR-15b-5p;hsa-miR-1(
ENSG0000	ENSG0000	4.90E-06	hsa-miR-148a-5p;hsa-miR-15a-5p;hsa-miR-15b-5p;hsa-miR-1(
ENSG0000	ENSG0000	1.91E-08	hsa-miR-15a-5p;hsa-miR-16-5p;hsa-miR-320a;hsa-miR-320b;h
ENSG0000	ENSG0000	0.000256	hsa-miR-1260a;hsa-miR-1260b;hsa-miR-15a-5p;hsa-miR-15b-5
ENSG0000	ENSG0000	3.99E-06	hsa-miR-320a;hsa-miR-320b;hsa-miR-320c;hsa-miR-320d;hsa-r
ENSG0000	ENSG0000	0.004215	hsa-miR-15a-5p;hsa-miR-15b-5p;hsa-miR-16-5p;hsa-miR-195-
ENSG0000	ENSG0000	0.000198	hsa-miR-34a-5p;hsa-miR-34b-5p;hsa-miR-34c-5p;hsa-miR-449
ENSG0000	ENSG0000	0.00223	hsa-miR-34a-5p;hsa-miR-34c-5p;hsa-miR-449a;hsa-miR-449b-
ENSG0000	ENSG0000	7.29E-05	hsa-miR-15a-5p;hsa-miR-15b-5p;hsa-miR-16-5p;hsa-miR-195-
ENSG0000	ENSG0000	6.29E-11	hsa-miR-15a-5p;hsa-miR-15b-5p;hsa-miR-16-5p;hsa-miR-195-
ENSG0000	ENSG0000	7.00E-07	hsa-miR-15a-5p;hsa-miR-15b-5p;hsa-miR-16-5p;hsa-miR-195-
ENSG0000	ENSG0000	0.000156	hsa-miR-34a-5p;hsa-miR-34b-5p;hsa-miR-34c-5p;hsa-miR-449
ENSG0000	ENSG0000	6.48E-08	hsa-miR-135a-5p;hsa-miR-15a-5p;hsa-miR-15b-5p;hsa-miR-1(
ENSG0000	ENSG0000	2.89E-05	hsa-miR-320a;hsa-miR-320b;hsa-miR-320c;hsa-miR-320d;hsa-r
ENSG0000	ENSG0000	0.00587	hsa-miR-15a-5p;hsa-miR-15b-5p;hsa-miR-16-5p;hsa-miR-195-
ENSG0000	ENSG0000	2.47E-05	hsa-miR-15a-5p;hsa-miR-15b-5p;hsa-miR-16-5p;hsa-miR-195-
ENSG0000	ENSG0000	0.00013	hsa-miR-15a-5p;hsa-miR-15b-5p;hsa-miR-16-5p;hsa-miR-195-
ENSG0000	ENSG0000	0.000379	hsa-miR-137;hsa-miR-140-3p;hsa-miR-92a-3p;hsa-miR-92b-3p
ENSG0000	ENSG0000	3.05E-06	hsa-miR-15a-5p;hsa-miR-15b-5p;hsa-miR-16-5p;hsa-miR-195-
ENSG0000	ENSG0000	0.003181	hsa-miR-15a-5p;hsa-miR-15b-5p;hsa-miR-16-5p;hsa-miR-195-
ENSG0000	ENSG0000	0.002115	hsa-miR-15a-5p;hsa-miR-15b-5p;hsa-miR-16-5p;hsa-miR-195-
ENSG0000	ENSG0000	0.000633	hsa-miR-15a-5p;hsa-miR-15b-5p;hsa-miR-195-5p;hsa-miR-424
ENSG0000	ENSG0000	0.000731	hsa-miR-192-5p;hsa-miR-320a;hsa-miR-320b;hsa-miR-320c;hsa
ENSG0000	ENSG0000	0.000399	hsa-miR-15a-5p;hsa-miR-15b-5p;hsa-miR-16-5p;hsa-miR-195-

ENSG0000	ENSG0000	1.07E-06	hsa-miR-181a-5p;hsa-miR-181b-5p;hsa-miR-181c-5p;hsa-miR-
ENSG0000	ENSG0000	0.003181	hsa-miR-15a-5p;hsa-miR-15b-5p;hsa-miR-16-5p;hsa-miR-195
ENSG0000	ENSG0000	5.14E-05	hsa-miR-1260a;hsa-miR-1260b;hsa-miR-15a-5p;hsa-miR-15b-
ENSG0000	ENSG0000	1.54E-06	hsa-miR-15a-5p;hsa-miR-15b-5p;hsa-miR-16-5p;hsa-miR-195
ENSG0000	ENSG0000	0.004215	hsa-miR-140-3p;hsa-miR-15a-5p;hsa-miR-16-5p;hsa-miR-185
ENSG0000	ENSG0000	0.002916	hsa-miR-15a-5p;hsa-miR-15b-5p;hsa-miR-195-5p;hsa-miR-42
ENSG0000	ENSG0000	0.000519	hsa-miR-135a-5p;hsa-miR-135b-5p;hsa-miR-92a-3p;hsa-miR-
ENSG0000	ENSG0000	4.24E-05	hsa-miR-15a-5p;hsa-miR-15b-5p;hsa-miR-195-5p;hsa-miR-42
ENSG0000	ENSG0000	0.000379	hsa-miR-15a-5p;hsa-miR-15b-5p;hsa-miR-16-5p;hsa-miR-195
ENSG0000	ENSG0000	8.35E-05	hsa-miR-320a;hsa-miR-320b;hsa-miR-320c;hsa-miR-320d;hsa-
ENSG0000	ENSG0000	3.15E-07	hsa-miR-15a-5p;hsa-miR-15b-5p;hsa-miR-195-5p;hsa-miR-21
ENSG0000	ENSG0000	6.56E-07	hsa-miR-15a-5p;hsa-miR-15b-5p;hsa-miR-181a-5p;hsa-miR-1
ENSG0000	ENSG0000	0.007969	hsa-miR-320a;hsa-miR-320b;hsa-miR-320c;hsa-miR-320d;hsa-
ENSG0000	ENSG0000	5.48E-05	hsa-miR-15a-5p;hsa-miR-15b-5p;hsa-miR-16-5p;hsa-miR-195
ENSG0000	ENSG0000	0.003854	hsa-miR-15a-5p;hsa-miR-15b-5p;hsa-miR-195-5p;hsa-miR-34
ENSG0000	ENSG0000	6.47E-07	hsa-miR-15a-5p;hsa-miR-15b-5p;hsa-miR-16-5p;hsa-miR-195
ENSG0000	ENSG0000	0.0018	hsa-miR-34a-5p;hsa-miR-34c-5p;hsa-miR-449a;hsa-miR-449b-
ENSG0000	ENSG0000	0.002531	hsa-miR-1260a;hsa-miR-1260b;hsa-miR-194-3p;hsa-miR-326;h
ENSG0000	ENSG0000	1.52E-05	hsa-miR-135a-5p;hsa-miR-135b-5p;hsa-miR-15a-5p;hsa-miR-
ENSG0000	ENSG0000	4.26E-05	hsa-miR-15a-5p;hsa-miR-15b-5p;hsa-miR-16-5p;hsa-miR-195
ENSG0000	ENSG0000	0.00684	hsa-miR-214-3p;hsa-miR-922;hsa-miR-92a-3p;hsa-miR-92b-3
ENSG0000	ENSG0000	7.85E-05	hsa-miR-15a-5p;hsa-miR-15b-5p;hsa-miR-16-5p;hsa-miR-181
ENSG0000	ENSG0000	0.008536	hsa-miR-129-5p;hsa-miR-34a-5p;hsa-miR-34c-5p;hsa-miR-423
ENSG0000	ENSG0000	1.53E-05	hsa-miR-1260a;hsa-miR-1260b;hsa-miR-320a;hsa-miR-320b;hs
ENSG0000	ENSG0000	5.89E-06	hsa-miR-15a-5p;hsa-miR-15b-5p;hsa-miR-16-5p;hsa-miR-195
ENSG0000	ENSG0000	0.001221	hsa-miR-15a-5p;hsa-miR-15b-5p;hsa-miR-195-5p;hsa-miR-42
ENSG0000	ENSG0000	0.000109	hsa-miR-15a-5p;hsa-miR-15b-5p;hsa-miR-181b-5p;hsa-miR-1
ENSG0000	ENSG0000	0.00047	hsa-miR-130b-5p;hsa-miR-15a-5p;hsa-miR-15b-5p;hsa-miR-1
ENSG0000	ENSG0000	5.66E-10	hsa-miR-15a-5p;hsa-miR-15b-5p;hsa-miR-194-3p;hsa-miR-19
ENSG0000	ENSG0000	0.00047	hsa-miR-194-3p;hsa-miR-214-3p;hsa-miR-423-5p;hsa-miR-42
ENSG0000	ENSG0000	0.000134	hsa-miR-15a-5p;hsa-miR-15b-5p;hsa-miR-16-5p;hsa-miR-195
ENSG0000	ENSG0000	9.64E-06	hsa-miR-15a-5p;hsa-miR-15b-5p;hsa-miR-16-5p;hsa-miR-195
ENSG0000	ENSG0000	7.00E-07	hsa-miR-15a-5p;hsa-miR-15b-5p;hsa-miR-16-5p;hsa-miR-195
ENSG0000	ENSG0000	9.28E-06	hsa-miR-15a-5p;hsa-miR-15b-5p;hsa-miR-16-5p;hsa-miR-195
ENSG0000	ENSG0000	0.000519	hsa-miR-15a-5p;hsa-miR-15b-5p;hsa-miR-16-5p;hsa-miR-195
ENSG0000	ENSG0000	0.001592	hsa-miR-15a-5p;hsa-miR-15b-5p;hsa-miR-16-5p;hsa-miR-195
ENSG0000	ENSG0000	0.006386	hsa-miR-214-3p;hsa-miR-34a-5p;hsa-miR-34c-5p;hsa-miR-44
ENSG0000	ENSG0000	0.007223	hsa-miR-181a-5p;hsa-miR-181b-5p;hsa-miR-181c-5p;hsa-miR-
ENSG0000	ENSG0000	0.000268	hsa-miR-127-5p;hsa-miR-181a-2-3p;hsa-miR-3943;hsa-miR-4
ENSG0000	ENSG0000	0.005882	hsa-miR-129-5p;hsa-miR-15a-5p;hsa-miR-15b-5p;hsa-miR-16
ENSG0000	ENSG0000	0.000239	hsa-miR-15a-5p;hsa-miR-15b-5p;hsa-miR-195-5p;hsa-miR-42
ENSG0000	ENSG0000	1.84E-05	hsa-miR-15a-5p;hsa-miR-15b-5p;hsa-miR-16-5p;hsa-miR-195
ENSG0000	ENSG0000	0.000546	hsa-miR-15a-5p;hsa-miR-15b-5p;hsa-miR-16-5p;hsa-miR-195
ENSG0000	ENSG0000	3.36E-07	hsa-miR-15a-5p;hsa-miR-15b-5p;hsa-miR-16-5p;hsa-miR-195
ENSG0000	ENSG0000	5.26E-06	hsa-miR-181a-5p;hsa-miR-181b-5p;hsa-miR-181c-5p;hsa-miR-
ENSG0000	ENSG0000	2.89E-05	hsa-miR-15a-5p;hsa-miR-15b-5p;hsa-miR-195-5p;hsa-miR-42
ENSG0000	ENSG0000	0.000268	hsa-miR-1260a;hsa-miR-1260b;hsa-miR-92a-3p;hsa-miR-92b-
ENSG0000	ENSG0000	4.60E-06	hsa-miR-15a-5p;hsa-miR-15b-5p;hsa-miR-16-5p;hsa-miR-195
ENSG0000	ENSG0000	0.000731	hsa-miR-135a-5p;hsa-miR-135b-5p;hsa-miR-15a-5p;hsa-miR-
ENSG0000	ENSG0000	1.28E-08	hsa-miR-15a-5p;hsa-miR-15b-5p;hsa-miR-16-5p;hsa-miR-195
ENSG0000	ENSG0000	0.003181	hsa-miR-15a-5p;hsa-miR-15b-5p;hsa-miR-16-5p;hsa-miR-195
ENSG0000	ENSG0000	0.004722	hsa-miR-140-3p;hsa-miR-148a-5p;hsa-miR-15a-5p;hsa-miR-1
ENSG0000	ENSG0000	0.001176	hsa-miR-15a-5p;hsa-miR-15b-5p;hsa-miR-16-5p;hsa-miR-195
ENSG0000	ENSG0000	0.001927	hsa-miR-15a-5p;hsa-miR-15b-5p;hsa-miR-16-5p;hsa-miR-195

ENSG0000	ENSG0000	0.000748	hsa-miR-129-5p;hsa-miR-15a-5p;hsa-miR-15b-5p;hsa-miR-19
ENSG0000	ENSG0000	0.000396	hsa-miR-320a;hsa-miR-320b;hsa-miR-320c;hsa-miR-320d;hsa-
ENSG0000	ENSG0000	0.006528	hsa-miR-15a-5p;hsa-miR-15b-5p;hsa-miR-195-5p;hsa-miR-42
ENSG0000	ENSG0000	1.18E-06	hsa-miR-15a-5p;hsa-miR-15b-5p;hsa-miR-16-5p;hsa-miR-195
ENSG0000	ENSG0000	0.002237	hsa-miR-1270;hsa-miR-127-5p;hsa-miR-194-3p;hsa-miR-320e;
ENSG0000	ENSG0000	9.28E-06	hsa-miR-15a-5p;hsa-miR-15b-5p;hsa-miR-16-5p;hsa-miR-195
ENSG0000	ENSG0000	0.000317	hsa-miR-1260a;hsa-miR-1260b;hsa-miR-181a-5p;hsa-miR-181
ENSG0000	ENSG0000	0.000396	hsa-miR-15a-5p;hsa-miR-15b-5p;hsa-miR-195-5p;hsa-miR-42
ENSG0000	ENSG0000	0.000841	hsa-miR-146a-3p;hsa-miR-181a-5p;hsa-miR-181b-5p;hsa-miR
ENSG0000	ENSG0000	0.004861	hsa-miR-1270;hsa-miR-129-5p;hsa-miR-135a-5p;hsa-miR-135
ENSG0000	ENSG0000	0.003733	hsa-miR-15a-5p;hsa-miR-15b-5p;hsa-miR-16-5p;hsa-miR-195
ENSG0000	ENSG0000	1.07E-06	hsa-miR-129-5p;hsa-miR-15a-5p;hsa-miR-15b-5p;hsa-miR-16
ENSG0000	ENSG0000	1.07E-06	hsa-miR-129-5p;hsa-miR-15a-5p;hsa-miR-15b-5p;hsa-miR-16
ENSG0000	ENSG0000	9.64E-06	hsa-miR-15a-5p;hsa-miR-15b-5p;hsa-miR-195-5p;hsa-miR-42
ENSG0000	ENSG0000	3.16E-06	hsa-miR-15a-5p;hsa-miR-15b-5p;hsa-miR-16-5p;hsa-miR-195
ENSG0000	ENSG0000	6.47E-07	hsa-miR-15a-5p;hsa-miR-15b-5p;hsa-miR-16-5p;hsa-miR-195
ENSG0000	ENSG0000	4.88E-07	hsa-miR-15a-5p;hsa-miR-15b-5p;hsa-miR-16-5p;hsa-miR-195
ENSG0000	ENSG0000	1.93E-05	hsa-miR-15a-5p;hsa-miR-15b-5p;hsa-miR-16-5p;hsa-miR-195
ENSG0000	ENSG0000	0.003387	hsa-miR-127-5p;hsa-miR-15a-5p;hsa-miR-15b-5p;hsa-miR-16
ENSG0000	ENSG0000	3.27E-05	hsa-miR-15a-5p;hsa-miR-15b-5p;hsa-miR-16-5p;hsa-miR-195
ENSG0000	ENSG0000	2.47E-05	hsa-miR-15a-5p;hsa-miR-15b-5p;hsa-miR-16-5p;hsa-miR-195
ENSG0000	ENSG0000	0.000285	hsa-miR-15a-5p;hsa-miR-15b-5p;hsa-miR-16-5p;hsa-miR-195
ENSG0000	ENSG0000	0.006106	hsa-miR-15a-5p;hsa-miR-15b-5p;hsa-miR-16-5p;hsa-miR-195
ENSG0000	ENSG0000	3.16E-06	hsa-miR-15a-5p;hsa-miR-15b-5p;hsa-miR-16-5p;hsa-miR-195
ENSG0000	ENSG0000	0.000546	hsa-miR-15a-5p;hsa-miR-15b-5p;hsa-miR-16-5p;hsa-miR-195
ENSG0000	ENSG0000	3.81E-06	hsa-miR-15a-5p;hsa-miR-15b-5p;hsa-miR-16-5p;hsa-miR-185
ENSG0000	ENSG0000	0.000365	hsa-miR-1260a;hsa-miR-1260b;hsa-miR-135a-5p;hsa-miR-135
ENSG0000	ENSG0000	0.000268	hsa-miR-15a-5p;hsa-miR-15b-5p;hsa-miR-16-5p;hsa-miR-195
ENSG0000	ENSG0000	2.47E-05	hsa-miR-15a-5p;hsa-miR-15b-5p;hsa-miR-16-5p;hsa-miR-195
ENSG0000	ENSG0000	2.04E-06	hsa-miR-15a-5p;hsa-miR-15b-5p;hsa-miR-195-5p;hsa-miR-42
ENSG0000	ENSG0000	0.003387	hsa-miR-15a-5p;hsa-miR-15b-5p;hsa-miR-16-5p;hsa-miR-195
ENSG0000	ENSG0000	0.000841	hsa-miR-148a-5p;hsa-miR-194-3p;hsa-miR-34a-5p;hsa-miR-3
ENSG0000	ENSG0000	0.000361	hsa-miR-15a-5p;hsa-miR-15b-5p;hsa-miR-16-5p;hsa-miR-195
ENSG0000	ENSG0000	0.000519	hsa-miR-15a-5p;hsa-miR-15b-5p;hsa-miR-424-5p;hsa-miR-49
ENSG0000	ENSG0000	1.53E-05	hsa-miR-34a-5p;hsa-miR-34c-5p;hsa-miR-449a;hsa-miR-449b-
ENSG0000	ENSG0000	1.53E-05	hsa-miR-34a-5p;hsa-miR-34c-5p;hsa-miR-449a;hsa-miR-449b-
ENSG0000	ENSG0000	3.67E-05	hsa-miR-15a-5p;hsa-miR-15b-5p;hsa-miR-16-5p;hsa-miR-195
ENSG0000	ENSG0000	6.47E-07	hsa-miR-15a-5p;hsa-miR-15b-5p;hsa-miR-16-5p;hsa-miR-195
ENSG0000	ENSG0000	0.000465	hsa-miR-15a-5p;hsa-miR-15b-5p;hsa-miR-16-5p;hsa-miR-195
ENSG0000	ENSG0000	0.004504	hsa-miR-15a-5p;hsa-miR-15b-5p;hsa-miR-16-5p;hsa-miR-195
ENSG0000	ENSG0000	8.71E-05	hsa-miR-1260a;hsa-miR-1260b;hsa-miR-15a-5p;hsa-miR-15b-
ENSG0000	ENSG0000	0.000633	hsa-miR-140-3p;hsa-miR-15a-5p;hsa-miR-15b-5p;hsa-miR-19
ENSG0000	ENSG0000	0.001591	hsa-miR-15a-5p;hsa-miR-15b-5p;hsa-miR-16-5p;hsa-miR-185
ENSG0000	ENSG0000	6.47E-07	hsa-miR-15a-5p;hsa-miR-15b-5p;hsa-miR-16-5p;hsa-miR-195
ENSG0000	ENSG0000	0.00024	hsa-miR-15a-5p;hsa-miR-15b-5p;hsa-miR-16-5p;hsa-miR-195
ENSG0000	ENSG0000	0.005151	hsa-miR-15a-5p;hsa-miR-15b-5p;hsa-miR-16-5p;hsa-miR-195
ENSG0000	ENSG0000	0.000256	hsa-miR-15a-5p;hsa-miR-15b-5p;hsa-miR-16-5p;hsa-miR-195
ENSG0000	ENSG0000	0.003854	hsa-miR-140-3p;hsa-miR-194-3p;hsa-miR-34a-5p;hsa-miR-34
ENSG0000	ENSG0000	0.000857	hsa-miR-15a-5p;hsa-miR-15b-5p;hsa-miR-16-5p;hsa-miR-195
ENSG0000	ENSG0000	0.008136	hsa-miR-130b-5p;hsa-miR-140-3p;hsa-miR-16-5p;hsa-miR-18
ENSG0000	ENSG0000	0.002237	hsa-miR-16-5p;hsa-miR-320a;hsa-miR-320b;hsa-miR-320c;hsa-
ENSG0000	ENSG0000	0.004502	hsa-miR-15a-5p;hsa-miR-15b-5p;hsa-miR-16-5p;hsa-miR-195
ENSG0000	ENSG0000	6.91E-05	hsa-miR-15a-5p;hsa-miR-15b-5p;hsa-miR-16-5p;hsa-miR-181
ENSG0000	ENSG0000	7.13E-06	hsa-miR-15a-5p;hsa-miR-15b-5p;hsa-miR-16-5p;hsa-miR-195

ENSG0000	ENSG0000	0.005282	hsa-miR-1260a;hsa-miR-1260b;hsa-miR-129-5p;hsa-miR-500a-
ENSG0000	ENSG0000	0.00024	hsa-miR-15a-5p;hsa-miR-15b-5p;hsa-miR-195-5p;hsa-miR-42
ENSG0000	ENSG0000	0.000317	hsa-miR-15a-5p;hsa-miR-15b-5p;hsa-miR-194-3p;hsa-miR-19
ENSG0000	ENSG0000	0.000789	hsa-miR-1260a;hsa-miR-1260b;hsa-miR-15a-5p;hsa-miR-15b-
ENSG0000	ENSG0000	7.65E-08	hsa-miR-15a-5p;hsa-miR-15b-5p;hsa-miR-181a-5p;hsa-miR-1
ENSG0000	ENSG0000	0.001433	hsa-miR-15a-5p;hsa-miR-15b-5p;hsa-miR-195-5p;hsa-miR-42
ENSG0000	ENSG0000	0.000153	hsa-miR-15a-5p;hsa-miR-15b-5p;hsa-miR-16-5p;hsa-miR-195
ENSG0000	ENSG0000	0.000113	hsa-miR-31-3p;hsa-miR-34a-5p;hsa-miR-34c-5p;hsa-miR-449
ENSG0000	ENSG0000	1.11E-05	hsa-miR-1226-3p;hsa-miR-129-5p;hsa-miR-15a-5p;hsa-miR-1
ENSG0000	ENSG0000	0.000295	hsa-miR-137;hsa-miR-15a-5p;hsa-miR-15b-5p;hsa-miR-16-5p;
ENSG0000	ENSG0000	0.004502	hsa-miR-181a-5p;hsa-miR-181b-5p;hsa-miR-181c-5p;hsa-miR
ENSG0000	ENSG0000	0.000317	hsa-miR-34a-5p;hsa-miR-34b-5p;hsa-miR-34c-5p;hsa-miR-44
ENSG0000	ENSG0000	7.29E-05	hsa-miR-34a-5p;hsa-miR-34c-5p;hsa-miR-449a;hsa-miR-449b-
ENSG0000	ENSG0000	0.00049	hsa-miR-15a-5p;hsa-miR-16-5p;hsa-miR-195-5p;hsa-miR-424
ENSG0000	ENSG0000	9.29E-06	hsa-miR-15a-5p;hsa-miR-15b-5p;hsa-miR-16-5p;hsa-miR-195
ENSG0000	ENSG0000	0.000317	hsa-miR-15a-5p;hsa-miR-15b-5p;hsa-miR-16-5p;hsa-miR-195
ENSG0000	ENSG0000	0.002201	hsa-miR-181a-5p;hsa-miR-181b-5p;hsa-miR-181c-5p;hsa-miR
ENSG0000	ENSG0000	0.001433	hsa-miR-320a;hsa-miR-320b;hsa-miR-320c;hsa-miR-320d;hsa-
ENSG0000	ENSG0000	0.007519	hsa-miR-181a-5p;hsa-miR-181b-5p;hsa-miR-181c-5p;hsa-miR
ENSG0000	ENSG0000	0.007607	hsa-miR-199a-5p;hsa-miR-320a;hsa-miR-320b;hsa-miR-320c;
ENSG0000	ENSG0000	0.007223	hsa-miR-192-5p;hsa-miR-3661;hsa-miR-650;hsa-miR-92a-3p;h
ENSG0000	ENSG0000	2.73E-06	hsa-miR-1270;hsa-miR-127-5p;hsa-miR-140-3p;hsa-miR-185-
ENSG0000	ENSG0000	0.000414	hsa-let-7i-3p;hsa-miR-15a-5p;hsa-miR-15b-5p;hsa-miR-16-5p
ENSG0000	ENSG0000	7.33E-07	hsa-miR-15a-5p;hsa-miR-15b-5p;hsa-miR-16-5p;hsa-miR-195
ENSG0000	ENSG0000	0.007332	hsa-miR-194-3p;hsa-miR-34a-5p;hsa-miR-34b-5p;hsa-miR-34
ENSG0000	ENSG0000	0.000724	hsa-miR-320a;hsa-miR-320b;hsa-miR-320c;hsa-miR-320d;hsa-
ENSG0000	ENSG0000	0.005955	hsa-miR-181a-2-3p;hsa-miR-181a-5p;hsa-miR-181b-5p;hsa-m
ENSG0000	ENSG0000	0.00799	hsa-miR-1295a;hsa-miR-147b;hsa-miR-320e;hsa-miR-490-3p;h
ENSG0000	ENSG0000	6.75E-06	hsa-miR-15a-5p;hsa-miR-16-5p;hsa-miR-195-5p;hsa-miR-214
ENSG0000	ENSG0000	0.000268	hsa-miR-15a-5p;hsa-miR-15b-5p;hsa-miR-16-5p;hsa-miR-195
ENSG0000	ENSG0000	0.009481	hsa-miR-15a-5p;hsa-miR-15b-5p;hsa-miR-16-5p;hsa-miR-195
ENSG0000	ENSG0000	0.000338	hsa-miR-140-3p;hsa-miR-34a-5p;hsa-miR-34c-5p;hsa-miR-42
ENSG0000	ENSG0000	0.000338	hsa-miR-140-3p;hsa-miR-34a-5p;hsa-miR-34c-5p;hsa-miR-42
ENSG0000	ENSG0000	0.000546	hsa-miR-31-3p;hsa-miR-34a-5p;hsa-miR-34c-5p;hsa-miR-449
ENSG0000	ENSG0000	0.004215	hsa-miR-146a-3p;hsa-miR-181a-5p;hsa-miR-181b-5p;hsa-miR
ENSG0000	ENSG0000	7.38E-07	hsa-miR-1260a;hsa-miR-1260b;hsa-miR-15a-5p;hsa-miR-15b-
ENSG0000	ENSG0000	9.29E-06	hsa-miR-15a-5p;hsa-miR-15b-5p;hsa-miR-16-5p;hsa-miR-195
ENSG0000	ENSG0000	3.05E-06	hsa-miR-15a-5p;hsa-miR-15b-5p;hsa-miR-16-5p;hsa-miR-195
ENSG0000	ENSG0000	0.006506	hsa-miR-148a-5p;hsa-miR-15a-5p;hsa-miR-15b-5p;hsa-miR-1
ENSG0000	ENSG0000	0.003065	hsa-miR-15a-5p;hsa-miR-15b-5p;hsa-miR-16-5p;hsa-miR-195
ENSG0000	ENSG0000	4.88E-07	hsa-miR-15a-5p;hsa-miR-15b-5p;hsa-miR-16-5p;hsa-miR-195
ENSG0000	ENSG0000	4.88E-07	hsa-miR-15a-5p;hsa-miR-15b-5p;hsa-miR-16-5p;hsa-miR-195
ENSG0000	ENSG0000	4.88E-07	hsa-miR-15a-5p;hsa-miR-15b-5p;hsa-miR-16-5p;hsa-miR-195
ENSG0000	ENSG0000	4.88E-07	hsa-miR-15a-5p;hsa-miR-15b-5p;hsa-miR-16-5p;hsa-miR-195
ENSG0000	ENSG0000	0.002493	hsa-miR-15a-5p;hsa-miR-15b-5p;hsa-miR-16-5p;hsa-miR-195
ENSG0000	ENSG0000	0.007519	hsa-miR-15a-5p;hsa-miR-15b-5p;hsa-miR-16-5p;hsa-miR-195
ENSG0000	ENSG0000	0.005151	hsa-miR-34a-5p;hsa-miR-34c-5p;hsa-miR-449a;hsa-miR-449b-
ENSG0000	ENSG0000	7.00E-07	hsa-miR-15a-5p;hsa-miR-15b-5p;hsa-miR-195-5p;hsa-miR-21
ENSG0000	ENSG0000	9.28E-06	hsa-miR-15a-5p;hsa-miR-15b-5p;hsa-miR-16-5p;hsa-miR-195
ENSG0000	ENSG0000	9.64E-06	hsa-miR-22-3p;hsa-miR-34a-5p;hsa-miR-34b-5p;hsa-miR-34c-
ENSG0000	ENSG0000	9.64E-06	hsa-miR-22-3p;hsa-miR-34a-5p;hsa-miR-34b-5p;hsa-miR-34c-
ENSG0000	ENSG0000	4.26E-05	hsa-miR-140-3p;hsa-miR-320a;hsa-miR-320b;hsa-miR-320c;hs
ENSG0000	ENSG0000	0.007223	hsa-miR-34a-5p;hsa-miR-34c-5p;hsa-miR-423-5p;hsa-miR-44
ENSG0000	ENSG0000	6.47E-07	hsa-miR-15a-5p;hsa-miR-15b-5p;hsa-miR-16-5p;hsa-miR-195

ENSG0000	ENSG0000	0.000546	hsa-miR-15a-5p;hsa-miR-15b-5p;hsa-miR-16-5p;hsa-miR-195-
ENSG0000	ENSG0000	0.000546	hsa-miR-15a-5p;hsa-miR-15b-5p;hsa-miR-16-5p;hsa-miR-195-
ENSG0000	ENSG0000	0.000546	hsa-miR-15a-5p;hsa-miR-15b-5p;hsa-miR-16-5p;hsa-miR-195-
ENSG0000	ENSG0000	0.000546	hsa-miR-15a-5p;hsa-miR-15b-5p;hsa-miR-16-5p;hsa-miR-195-
ENSG0000	ENSG0000	0.000546	hsa-miR-15a-5p;hsa-miR-15b-5p;hsa-miR-16-5p;hsa-miR-195-
ENSG0000	ENSG0000	0.000546	hsa-miR-15a-5p;hsa-miR-15b-5p;hsa-miR-16-5p;hsa-miR-195-
ENSG0000	ENSG0000	0.000546	hsa-miR-15a-5p;hsa-miR-15b-5p;hsa-miR-16-5p;hsa-miR-195-
ENSG0000	ENSG0000	0.000546	hsa-miR-15a-5p;hsa-miR-15b-5p;hsa-miR-16-5p;hsa-miR-195-
ENSG0000	ENSG0000	0.000546	hsa-miR-15a-5p;hsa-miR-15b-5p;hsa-miR-16-5p;hsa-miR-195-
ENSG0000	ENSG0000	0.000546	hsa-miR-15a-5p;hsa-miR-15b-5p;hsa-miR-16-5p;hsa-miR-195-
ENSG0000	ENSG0000	4.14E-05	hsa-miR-15a-5p;hsa-miR-15b-5p;hsa-miR-16-5p;hsa-miR-195-
ENSG0000	ENSG0000	2.47E-05	hsa-miR-15a-5p;hsa-miR-15b-5p;hsa-miR-16-5p;hsa-miR-195-
ENSG0000	ENSG0000	0.000598	hsa-miR-15a-5p;hsa-miR-15b-5p;hsa-miR-16-5p;hsa-miR-424-
ENSG0000	ENSG0000	6.56E-05	hsa-miR-15a-5p;hsa-miR-15b-5p;hsa-miR-16-5p;hsa-miR-181a
ENSG0000	ENSG0000	0.005882	hsa-miR-181a-5p;hsa-miR-181b-5p;hsa-miR-181c-5p;hsa-miR-
ENSG0000	ENSG0000	0.000963	hsa-miR-15a-5p;hsa-miR-15b-5p;hsa-miR-16-5p;hsa-miR-195-
ENSG0000	ENSG0000	0.000137	hsa-miR-15a-5p;hsa-miR-15b-5p;hsa-miR-16-5p;hsa-miR-181a
ENSG0000	ENSG0000	7.85E-05	hsa-miR-130b-5p;hsa-miR-15a-5p;hsa-miR-15b-5p;hsa-miR-1
ENSG0000	ENSG0000	0.003733	hsa-miR-15a-5p;hsa-miR-15b-5p;hsa-miR-16-5p;hsa-miR-195-
ENSG0000	ENSG0000	0.001221	hsa-miR-15a-5p;hsa-miR-15b-5p;hsa-miR-195-5p;hsa-miR-42
ENSG0000	ENSG0000	7.20E-05	hsa-miR-129-5p;hsa-miR-15a-5p;hsa-miR-15b-5p;hsa-miR-16-
ENSG0000	ENSG0000	6.78E-05	hsa-miR-129-5p;hsa-miR-15a-5p;hsa-miR-15b-5p;hsa-miR-16-
ENSG0000	ENSG0000	0.002557	hsa-miR-1226-3p;hsa-miR-15a-5p;hsa-miR-15b-5p;hsa-miR-1
ENSG0000	ENSG0000	9.28E-07	hsa-miR-320a;hsa-miR-320b;hsa-miR-320c;hsa-miR-320d;hsa-r
ENSG0000	ENSG0000	5.86E-05	hsa-miR-15a-5p;hsa-miR-15b-5p;hsa-miR-16-5p;hsa-miR-195-
ENSG0000	ENSG0000	0.009781	hsa-miR-146a-3p;hsa-miR-34a-5p;hsa-miR-34b-5p;hsa-miR-3
ENSG0000	ENSG0000	9.64E-06	hsa-miR-15a-5p;hsa-miR-15b-5p;hsa-miR-16-5p;hsa-miR-195-
ENSG0000	ENSG0000	7.33E-07	hsa-miR-127-5p;hsa-miR-15a-5p;hsa-miR-15b-5p;hsa-miR-19
ENSG0000	ENSG0000	0.000317	hsa-miR-194-3p;hsa-miR-34a-5p;hsa-miR-34c-5p;hsa-miR-44
ENSG0000	ENSG0000	0.000198	hsa-miR-15a-5p;hsa-miR-15b-5p;hsa-miR-195-5p;hsa-miR-33a
ENSG0000	ENSG0000	1.95E-06	hsa-miR-15a-5p;hsa-miR-15b-5p;hsa-miR-16-5p;hsa-miR-195-
ENSG0000	ENSG0000	4.24E-05	hsa-miR-15a-5p;hsa-miR-15b-5p;hsa-miR-195-5p;hsa-miR-42
ENSG0000	ENSG0000	6.96E-05	hsa-miR-135a-5p;hsa-miR-135b-5p;hsa-miR-181a-5p;hsa-miR-
ENSG0000	ENSG0000	0.000869	hsa-miR-1226-3p;hsa-miR-181a-5p;hsa-miR-181b-5p;hsa-miR-
ENSG0000	ENSG0000	0.004727	hsa-miR-34a-5p;hsa-miR-34c-5p;hsa-miR-449a;hsa-miR-449b-
ENSG0000	ENSG0000	7.13E-06	hsa-miR-15a-5p;hsa-miR-15b-5p;hsa-miR-16-5p;hsa-miR-195-
ENSG0000	ENSG0000	0.000399	hsa-miR-15a-5p;hsa-miR-15b-5p;hsa-miR-16-5p;hsa-miR-195-
ENSG0000	ENSG0000	0.000724	hsa-miR-15a-5p;hsa-miR-15b-5p;hsa-miR-16-5p;hsa-miR-195-
ENSG0000	ENSG0000	3.16E-06	hsa-miR-15a-5p;hsa-miR-15b-5p;hsa-miR-16-5p;hsa-miR-195-
ENSG0000	ENSG0000	5.30E-06	hsa-miR-1260a;hsa-miR-1260b;hsa-miR-15a-5p;hsa-miR-15b-5
ENSG0000	ENSG0000	0.000212	hsa-miR-15a-5p;hsa-miR-15b-5p;hsa-miR-195-5p;hsa-miR-42
ENSG0000	ENSG0000	0.000731	hsa-miR-15a-5p;hsa-miR-15b-5p;hsa-miR-16-5p;hsa-miR-195-
ENSG0000	ENSG0000	0.003271	hsa-miR-1270;hsa-miR-15a-5p;hsa-miR-15b-5p;hsa-miR-181a-
ENSG0000	ENSG0000	0.003271	hsa-miR-1270;hsa-miR-15a-5p;hsa-miR-15b-5p;hsa-miR-181a-
ENSG0000	ENSG0000	0.003271	hsa-miR-1270;hsa-miR-15a-5p;hsa-miR-15b-5p;hsa-miR-181a-
ENSG0000	ENSG0000	0.003271	hsa-miR-1270;hsa-miR-15a-5p;hsa-miR-15b-5p;hsa-miR-181a-
ENSG0000	ENSG0000	0.003271	hsa-miR-1270;hsa-miR-15a-5p;hsa-miR-15b-5p;hsa-miR-181a-
ENSG0000	ENSG0000	0.003271	hsa-miR-1270;hsa-miR-15a-5p;hsa-miR-15b-5p;hsa-miR-181a-
ENSG0000	ENSG0000	0.003271	hsa-miR-1270;hsa-miR-15a-5p;hsa-miR-15b-5p;hsa-miR-181a-
ENSG0000	ENSG0000	0.003271	hsa-miR-1270;hsa-miR-15a-5p;hsa-miR-15b-5p;hsa-miR-181a-
ENSG0000	ENSG0000	8.52E-08	hsa-miR-15a-5p;hsa-miR-15b-5p;hsa-miR-16-5p;hsa-miR-181a
ENSG0000	ENSG0000	2.47E-05	hsa-miR-15a-5p;hsa-miR-15b-5p;hsa-miR-16-5p;hsa-miR-195-
ENSG0000	ENSG0000	0.008536	hsa-miR-135a-5p;hsa-miR-135b-5p;hsa-miR-185-5p;hsa-miR-2
ENSG0000	ENSG0000	1.95E-06	hsa-miR-15a-5p;hsa-miR-15b-5p;hsa-miR-16-5p;hsa-miR-195-

ENSG0000	ENSG0000	4.14E-05	hsa-miR-34a-5p;hsa-miR-34c-5p;hsa-miR-449a;hsa-miR-449b-
ENSG0000	ENSG0000	0.004929	hsa-miR-15a-5p;hsa-miR-15b-5p;hsa-miR-16-5p;hsa-miR-195-
ENSG0000	ENSG0000	0.002201	hsa-miR-15a-5p;hsa-miR-15b-5p;hsa-miR-16-5p;hsa-miR-195-
ENSG0000	ENSG0000	0.002201	hsa-miR-15a-5p;hsa-miR-15b-5p;hsa-miR-16-5p;hsa-miR-195-
ENSG0000	ENSG0000	0.003387	hsa-miR-34a-5p;hsa-miR-34b-5p;hsa-miR-34c-5p;hsa-miR-44
ENSG0000	ENSG0000	0.000134	hsa-miR-15a-5p;hsa-miR-15b-5p;hsa-miR-16-5p;hsa-miR-195-
ENSG0000	ENSG0000	0.001753	hsa-miR-320a;hsa-miR-320b;hsa-miR-320c;hsa-miR-320d;hsa-
ENSG0000	ENSG0000	0.003065	hsa-miR-181a-5p;hsa-miR-181b-5p;hsa-miR-181c-5p;hsa-miR-
ENSG0000	ENSG0000	0.000903	hsa-miR-15a-5p;hsa-miR-15b-5p;hsa-miR-16-5p;hsa-miR-195-
ENSG0000	ENSG0000	0.004104	hsa-miR-21-3p;hsa-miR-320a;hsa-miR-320b;hsa-miR-320c;hsa-
ENSG0000	ENSG0000	3.86E-07	hsa-miR-15a-5p;hsa-miR-15b-5p;hsa-miR-16-5p;hsa-miR-195-
ENSG0000	ENSG0000	1.98E-06	hsa-miR-15a-5p;hsa-miR-15b-5p;hsa-miR-16-5p;hsa-miR-195-
ENSG0000	ENSG0000	1.95E-06	hsa-miR-15a-5p;hsa-miR-15b-5p;hsa-miR-16-5p;hsa-miR-195-
ENSG0000	ENSG0000	0.00747	hsa-miR-185-5p;hsa-miR-34a-5p;hsa-miR-34b-5p;hsa-miR-34
ENSG0000	ENSG0000	9.04E-07	hsa-miR-137;hsa-miR-15a-5p;hsa-miR-15b-5p;hsa-miR-16-5p;
ENSG0000	ENSG0000	1.20E-05	hsa-miR-320a;hsa-miR-320b;hsa-miR-320c;hsa-miR-320d;hsa-
ENSG0000	ENSG0000	1.55E-06	hsa-miR-146a-3p;hsa-miR-15a-5p;hsa-miR-15b-5p;hsa-miR-1
ENSG0000	ENSG0000	1.90E-05	hsa-miR-34a-5p;hsa-miR-34c-5p;hsa-miR-382-5p;hsa-miR-44
ENSG0000	ENSG0000	0.003743	hsa-miR-3943;hsa-miR-650;hsa-miR-671-5p;hsa-miR-92a-3p;h
ENSG0000	ENSG0000	7.86E-05	hsa-miR-15a-5p;hsa-miR-15b-5p;hsa-miR-16-5p;hsa-miR-195-
ENSG0000	ENSG0000	0.00167	hsa-miR-1260a;hsa-miR-1260b;hsa-miR-140-3p;hsa-miR-326;h
ENSG0000	ENSG0000	0.001753	hsa-miR-15a-5p;hsa-miR-15b-5p;hsa-miR-16-5p;hsa-miR-195-
ENSG0000	ENSG0000	0.004418	hsa-miR-15a-5p;hsa-miR-15b-5p;hsa-miR-16-5p;hsa-miR-194-
ENSG0000	ENSG0000	0.007152	hsa-miR-140-3p;hsa-miR-15a-5p;hsa-miR-15b-5p;hsa-miR-16-
ENSG0000	ENSG0000	6.47E-07	hsa-miR-15a-5p;hsa-miR-15b-5p;hsa-miR-16-5p;hsa-miR-195-
ENSG0000	ENSG0000	0.008536	hsa-miR-137;hsa-miR-199a-5p;hsa-miR-326;hsa-miR-33a-3p;h
ENSG0000	ENSG0000	8.17E-10	hsa-miR-15a-5p;hsa-miR-15b-5p;hsa-miR-16-5p;hsa-miR-181
ENSG0000	ENSG0000	0.008136	hsa-miR-1226-3p;hsa-miR-15a-5p;hsa-miR-15b-5p;hsa-miR-1
ENSG0000	ENSG0000	0.004504	hsa-miR-15a-5p;hsa-miR-15b-5p;hsa-miR-16-5p;hsa-miR-195-
ENSG0000	ENSG0000	0.009711	hsa-miR-130b-5p;hsa-miR-15a-5p;hsa-miR-15b-5p;hsa-miR-1
ENSG0000	ENSG0000	0.003743	hsa-miR-137;hsa-miR-518c-5p;hsa-miR-650;hsa-miR-92a-3p;h
ENSG0000	ENSG0000	0.001409	hsa-miR-1295a;hsa-miR-130b-5p;hsa-miR-192-5p;hsa-miR-19
ENSG0000	ENSG0000	0.000201	hsa-miR-129-5p;hsa-miR-137;hsa-miR-15a-5p;hsa-miR-15b-5
ENSG0000	ENSG0000	0.005384	hsa-miR-15a-5p;hsa-miR-15b-5p;hsa-miR-16-5p;hsa-miR-195-
ENSG0000	ENSG0000	0.000198	hsa-miR-127-5p;hsa-miR-320a;hsa-miR-320b;hsa-miR-320c;hs
ENSG0000	ENSG0000	0.000198	hsa-miR-127-5p;hsa-miR-320a;hsa-miR-320b;hsa-miR-320c;hs
ENSG0000	ENSG0000	3.67E-05	hsa-miR-127-5p;hsa-miR-320a;hsa-miR-320b;hsa-miR-320c;hs
ENSG0000	ENSG0000	3.45E-06	hsa-miR-320a;hsa-miR-320b;hsa-miR-320d;hsa-miR-320e;hsa-
ENSG0000	ENSG0000	7.76E-05	hsa-miR-142-3p;hsa-miR-378a-3p;hsa-miR-378c;hsa-miR-378
ENSG0000	ENSG0000	7.76E-05	hsa-miR-142-3p;hsa-miR-378a-3p;hsa-miR-378c;hsa-miR-378
ENSG0000	ENSG0000	9.56E-05	hsa-miR-320a;hsa-miR-320b;hsa-miR-320d;hsa-miR-320e;hsa-
ENSG0000	ENSG0000	7.62E-08	hsa-let-7a-5p;hsa-let-7b-5p;hsa-let-7d-5p;hsa-let-7e-5p;hsa-l
ENSG0000	ENSG0000	5.56E-07	hsa-miR-181a-5p;hsa-miR-181b-5p;hsa-miR-181c-5p;hsa-miR-
ENSG0000	ENSG0000	2.69E-06	hsa-let-7a-5p;hsa-let-7b-5p;hsa-let-7d-5p;hsa-let-7e-5p;hsa-l
ENSG0000	ENSG0000	0.000152	hsa-let-7a-5p;hsa-let-7b-5p;hsa-let-7d-5p;hsa-let-7e-5p;hsa-l
ENSG0000	ENSG0000	6.54E-07	hsa-let-7a-5p;hsa-let-7b-5p;hsa-let-7d-5p;hsa-let-7e-5p;hsa-l
ENSG0000	ENSG0000	0.000432	hsa-miR-374b-5p;hsa-miR-378a-3p;hsa-miR-378c;hsa-miR-37
ENSG0000	ENSG0000	1.62E-05	hsa-let-7a-5p;hsa-let-7b-5p;hsa-let-7d-5p;hsa-let-7e-5p;hsa-l
ENSG0000	ENSG0000	7.76E-05	hsa-let-7a-5p;hsa-let-7b-5p;hsa-let-7d-5p;hsa-let-7e-5p;hsa-l
ENSG0000	ENSG0000	0.00682	hsa-let-7b-5p;hsa-miR-181a-5p;hsa-miR-181b-5p;hsa-miR-18
ENSG0000	ENSG0000	1.39E-05	hsa-miR-103a-2-5p;hsa-miR-320a;hsa-miR-320b;hsa-miR-320
ENSG0000	ENSG0000	0.003079	hsa-miR-130a-5p;hsa-miR-23a-3p;hsa-miR-23b-3p;hsa-miR-2
ENSG0000	ENSG0000	0.003079	hsa-miR-142-3p;hsa-miR-181a-5p;hsa-miR-181b-5p;hsa-miR-
ENSG0000	ENSG0000	0.005563	hsa-let-7a-5p;hsa-let-7b-5p;hsa-let-7d-5p;hsa-let-7e-5p;hsa-l

ENSG0000	ENSG0000	0.008084	hsa-let-7a-5p;hsa-miR-181a-5p;hsa-miR-181b-5p;hsa-miR-181
ENSG0000	ENSG0000	9.15E-06	hsa-miR-1299;hsa-miR-378a-3p;hsa-miR-378c;hsa-miR-378d;hs
ENSG0000	ENSG0000	0.000251	hsa-miR-142-3p;hsa-miR-23b-3p;hsa-miR-27b-3p;hsa-miR-518
ENSG0000	ENSG0000	5.38E-06	hsa-let-7a-5p;hsa-let-7b-5p;hsa-let-7d-5p;hsa-let-7e-5p;hsa-l
ENSG0000	ENSG0000	4.41E-05	hsa-let-7a-5p;hsa-let-7b-5p;hsa-let-7d-5p;hsa-let-7e-5p;hsa-l
ENSG0000	ENSG0000	0.003079	hsa-miR-320a;hsa-miR-320b;hsa-miR-320d;hsa-miR-4429
ENSG0000	ENSG0000	3.62E-06	hsa-let-7a-5p;hsa-let-7b-5p;hsa-let-7d-5p;hsa-let-7e-5p;hsa-l
ENSG0000	ENSG0000	7.11E-05	hsa-miR-130a-5p;hsa-miR-23a-3p;hsa-miR-23b-3p;hsa-miR-23
ENSG0000	ENSG0000	5.38E-06	hsa-let-7a-5p;hsa-let-7b-5p;hsa-let-7d-5p;hsa-let-7e-5p;hsa-l
ENSG0000	ENSG0000	0.000857	hsa-let-7a-5p;hsa-let-7b-5p;hsa-let-7d-5p;hsa-let-7e-5p;hsa-l
ENSG0000	ENSG0000	7.32E-06	hsa-miR-129-5p;hsa-miR-130a-5p;hsa-miR-23a-3p;hsa-miR-23
ENSG0000	ENSG0000	2.77E-07	hsa-let-7a-5p;hsa-let-7b-5p;hsa-let-7d-5p;hsa-let-7e-5p;hsa-l
ENSG0000	ENSG0000	0.001435	hsa-miR-142-3p;hsa-miR-181a-5p;hsa-miR-320a;hsa-miR-375
ENSG0000	ENSG0000	2.64E-06	hsa-let-7a-5p;hsa-let-7d-5p;hsa-let-7e-5p;hsa-let-7f-5p;hsa-le
ENSG0000	ENSG0000	1.62E-06	hsa-let-7a-5p;hsa-let-7b-5p;hsa-let-7d-5p;hsa-let-7e-5p;hsa-l
ENSG0000	ENSG0000	1.41E-05	hsa-let-7a-5p;hsa-let-7b-5p;hsa-let-7d-5p;hsa-let-7e-5p;hsa-l
ENSG0000	ENSG0000	3.00E-05	hsa-let-7a-5p;hsa-let-7e-5p;hsa-let-7f-5p;hsa-let-7g-5p;hsa-le
ENSG0000	ENSG0000	2.06E-07	hsa-let-7a-5p;hsa-let-7b-5p;hsa-let-7d-5p;hsa-let-7e-5p;hsa-l
ENSG0000	ENSG0000	3.88E-05	hsa-let-7a-5p;hsa-let-7b-5p;hsa-let-7d-5p;hsa-let-7e-5p;hsa-l
ENSG0000	ENSG0000	0.002851	hsa-let-7a-5p;hsa-let-7d-5p;hsa-let-7e-5p;hsa-let-7f-5p;hsa-le
ENSG0000	ENSG0000	5.00E-05	hsa-let-7a-5p;hsa-let-7b-5p;hsa-let-7d-5p;hsa-let-7e-5p;hsa-l
ENSG0000	ENSG0000	0.000124	hsa-miR-130a-5p;hsa-miR-181a-5p;hsa-miR-181b-5p;hsa-miR-
ENSG0000	ENSG0000	0.000152	hsa-let-7a-5p;hsa-let-7b-5p;hsa-let-7d-5p;hsa-let-7e-5p;hsa-l
ENSG0000	ENSG0000	0.000173	hsa-miR-142-3p;hsa-miR-181a-5p;hsa-miR-181b-5p;hsa-miR-1
ENSG0000	ENSG0000	1.87E-05	hsa-miR-129-5p;hsa-miR-181a-5p;hsa-miR-181b-5p;hsa-miR-1
ENSG0000	ENSG0000	3.78E-06	hsa-miR-181a-5p;hsa-miR-181b-5p;hsa-miR-181c-5p;hsa-miR-
ENSG0000	ENSG0000	1.32E-05	hsa-miR-130a-5p;hsa-miR-23a-3p;hsa-miR-23b-3p;hsa-miR-23
ENSG0000	ENSG0000	0.006456	hsa-miR-181a-5p;hsa-miR-181b-5p;hsa-miR-181c-5p;hsa-miR-
ENSG0000	ENSG0000	4.85E-07	hsa-let-7a-5p;hsa-let-7b-5p;hsa-let-7d-5p;hsa-let-7e-5p;hsa-l
ENSG0000	ENSG0000	0.000272	hsa-let-7a-5p;hsa-let-7b-5p;hsa-let-7d-5p;hsa-let-7e-5p;hsa-l
ENSG0000	ENSG0000	0.00143	hsa-let-7a-5p;hsa-let-7b-5p;hsa-let-7d-5p;hsa-let-7e-5p;hsa-l
ENSG0000	ENSG0000	0.009059	hsa-let-7a-5p;hsa-let-7b-5p;hsa-let-7d-5p;hsa-let-7e-5p;hsa-l
ENSG0000	ENSG0000	1.69E-05	hsa-let-7a-5p;hsa-let-7b-5p;hsa-let-7d-5p;hsa-let-7e-5p;hsa-l
ENSG0000	ENSG0000	3.92E-06	hsa-let-7a-5p;hsa-let-7b-5p;hsa-let-7d-5p;hsa-let-7e-5p;hsa-l
ENSG0000	ENSG0000	0.004217	hsa-let-7a-5p;hsa-let-7d-5p;hsa-let-7e-5p;hsa-let-7f-5p;hsa-le
ENSG0000	ENSG0000	4.96E-05	hsa-let-7a-5p;hsa-let-7b-5p;hsa-let-7d-5p;hsa-let-7f-5p;hsa-le
ENSG0000	ENSG0000	0.001435	hsa-miR-142-3p;hsa-miR-181a-5p;hsa-miR-181b-5p;hsa-miR-1
ENSG0000	ENSG0000	8.72E-10	hsa-let-7a-5p;hsa-let-7b-5p;hsa-let-7d-5p;hsa-let-7e-5p;hsa-l
ENSG0000	ENSG0000	0.002918	hsa-let-7a-5p;hsa-let-7b-5p;hsa-let-7d-5p;hsa-let-7e-5p;hsa-l
ENSG0000	ENSG0000	0.002743	hsa-miR-130a-5p;hsa-miR-23a-3p;hsa-miR-23b-3p;hsa-miR-23
ENSG0000	ENSG0000	2.08E-05	hsa-miR-1299;hsa-miR-378a-3p;hsa-miR-378c;hsa-miR-378d;hs
ENSG0000	ENSG0000	0.00682	hsa-miR-181a-5p;hsa-miR-181b-5p;hsa-miR-181c-5p;hsa-miR-
ENSG0000	ENSG0000	0.001098	hsa-let-7a-5p;hsa-miR-320a;hsa-miR-320b;hsa-miR-320d;hsa-m
ENSG0000	ENSG0000	0.0002	hsa-let-7a-5p;hsa-let-7b-5p;hsa-let-7d-5p;hsa-let-7e-5p;hsa-l
ENSG0000	ENSG0000	8.11E-07	hsa-let-7a-5p;hsa-let-7b-5p;hsa-let-7d-5p;hsa-let-7e-5p;hsa-l
ENSG0000	ENSG0000	5.56E-07	hsa-let-7d-5p;hsa-let-7f-5p;hsa-let-7g-5p;hsa-let-7i-5p
ENSG0000	ENSG0000	0.002072	hsa-let-7a-5p;hsa-let-7b-5p;hsa-let-7d-5p;hsa-let-7e-5p;hsa-l
ENSG0000	ENSG0000	0.001098	hsa-miR-129-5p;hsa-miR-378a-3p;hsa-miR-378c;hsa-miR-378c
ENSG0000	ENSG0000	0.000136	hsa-let-7a-5p;hsa-let-7b-5p;hsa-let-7e-5p;hsa-let-7f-5p;hsa-le
ENSG0000	ENSG0000	0.008084	hsa-miR-181b-5p;hsa-miR-181c-5p;hsa-miR-27b-3p;hsa-miR-4
ENSG0000	ENSG0000	0.00877	hsa-miR-130a-5p;hsa-miR-23a-3p;hsa-miR-23b-3p;hsa-miR-23
ENSG0000	ENSG0000	1.96E-05	hsa-let-7a-5p;hsa-let-7b-5p;hsa-let-7d-5p;hsa-let-7e-5p;hsa-l
ENSG0000	ENSG0000	3.56E-08	hsa-let-7a-5p;hsa-let-7b-5p;hsa-let-7d-5p;hsa-let-7e-5p;hsa-l
ENSG0000	ENSG0000	2.35E-08	hsa-let-7a-5p;hsa-let-7b-5p;hsa-let-7d-5p;hsa-let-7e-5p;hsa-l

ENSG0000	ENSG0000	2.06E-07	hsa-let-7a-5p;hsa-let-7b-5p;hsa-let-7d-5p;hsa-let-7e-5p;hsa-l
ENSG0000	ENSG0000	0.000104	hsa-miR-130a-5p;hsa-miR-23a-3p;hsa-miR-23b-3p;hsa-miR-2
ENSG0000	ENSG0000	0.005695	hsa-miR-320a;hsa-miR-320b;hsa-miR-320d;hsa-miR-4429
ENSG0000	ENSG0000	0.000701	hsa-miR-103a-2-5p;hsa-miR-130a-5p;hsa-miR-23a-3p;hsa-miI
ENSG0000	ENSG0000	0.002667	hsa-miR-181a-5p;hsa-miR-181b-5p;hsa-miR-320a;hsa-miR-32
ENSG0000	ENSG0000	0.009186	hsa-let-7a-5p;hsa-let-7b-5p;hsa-let-7d-5p;hsa-let-7e-5p;hsa-l
ENSG0000	ENSG0000	0.001877	hsa-miR-130a-5p;hsa-miR-23a-3p;hsa-miR-23b-3p;hsa-miR-2
ENSG0000	ENSG0000	0.00124	hsa-let-7a-5p;hsa-let-7d-5p;hsa-let-7e-5p;hsa-let-7f-5p
ENSG0000	ENSG0000	0.008084	hsa-miR-130a-5p;hsa-miR-23a-3p;hsa-miR-23b-3p;hsa-miR-2
ENSG0000	ENSG0000	0.002188	hsa-let-7a-5p;hsa-let-7d-5p;hsa-let-7e-5p;hsa-let-7g-5p;hsa-r
ENSG0000	ENSG0000	5.38E-06	hsa-let-7a-5p;hsa-let-7b-5p;hsa-let-7d-5p;hsa-let-7e-5p;hsa-l
ENSG0000	ENSG0000	0.00017	hsa-miR-129-5p;hsa-miR-130a-5p;hsa-miR-23a-3p;hsa-miR-2
ENSG0000	ENSG0000	0.000293	hsa-let-7a-5p;hsa-let-7b-5p;hsa-let-7d-5p;hsa-let-7e-5p;hsa-l
ENSG0000	ENSG0000	0.003346	hsa-let-7a-5p;hsa-let-7b-5p;hsa-miR-181a-5p;hsa-miR-27a-3
ENSG0000	ENSG0000	2.77E-07	hsa-let-7a-5p;hsa-let-7b-5p;hsa-let-7d-5p;hsa-let-7e-5p;hsa-l
ENSG0000	ENSG0000	2.77E-07	hsa-let-7a-5p;hsa-let-7b-5p;hsa-let-7d-5p;hsa-let-7e-5p;hsa-l
ENSG0000	ENSG0000	2.77E-07	hsa-let-7a-5p;hsa-let-7b-5p;hsa-let-7d-5p;hsa-let-7e-5p;hsa-l
ENSG0000	ENSG0000	2.18E-05	hsa-miR-130a-5p;hsa-miR-23a-3p;hsa-miR-23b-3p;hsa-miR-2
ENSG0000	ENSG0000	0.001498	hsa-miR-378a-3p;hsa-miR-378c;hsa-miR-378d;hsa-miR-378i;hs
ENSG0000	ENSG0000	1.03E-06	hsa-let-7a-5p;hsa-let-7b-5p;hsa-let-7d-5p;hsa-let-7e-5p;hsa-l
ENSG0000	ENSG0000	7.62E-08	hsa-let-7a-5p;hsa-let-7b-5p;hsa-let-7d-5p;hsa-let-7e-5p;hsa-l
ENSG0000	ENSG0000	7.62E-08	hsa-let-7a-5p;hsa-let-7b-5p;hsa-let-7d-5p;hsa-let-7e-5p;hsa-l
ENSG0000	ENSG0000	7.62E-08	hsa-let-7a-5p;hsa-let-7b-5p;hsa-let-7d-5p;hsa-let-7e-5p;hsa-l
ENSG0000	ENSG0000	7.62E-08	hsa-let-7a-5p;hsa-let-7b-5p;hsa-let-7d-5p;hsa-let-7e-5p;hsa-l
ENSG0000	ENSG0000	7.62E-08	hsa-let-7a-5p;hsa-let-7b-5p;hsa-let-7d-5p;hsa-let-7e-5p;hsa-l
ENSG0000	ENSG0000	7.62E-08	hsa-let-7a-5p;hsa-let-7b-5p;hsa-let-7d-5p;hsa-let-7e-5p;hsa-l
ENSG0000	ENSG0000	7.62E-08	hsa-let-7a-5p;hsa-let-7b-5p;hsa-let-7d-5p;hsa-let-7e-5p;hsa-l
ENSG0000	ENSG0000	0.000185	hsa-let-7a-5p;hsa-let-7b-5p;hsa-let-7d-5p;hsa-let-7e-5p;hsa-l
ENSG0000	ENSG0000	0.006037	hsa-miR-223-5p;hsa-miR-320a;hsa-miR-320b;hsa-miR-320d;hs
ENSG0000	ENSG0000	0.000252	hsa-let-7a-5p;hsa-let-7d-5p;hsa-let-7e-5p;hsa-let-7f-5p;hsa-le
ENSG0000	ENSG0000	0.000168	hsa-let-7a-5p;hsa-let-7b-5p;hsa-let-7d-5p;hsa-let-7e-5p;hsa-l
ENSG0000	ENSG0000	1.01E-08	hsa-let-7a-5p;hsa-let-7b-5p;hsa-let-7d-5p;hsa-let-7e-5p;hsa-l
ENSG0000	ENSG0000	0.000449	hsa-let-7a-5p;hsa-let-7b-5p;hsa-let-7e-5p;hsa-let-7f-5p;hsa-le
ENSG0000	ENSG0000	0.007673	hsa-miR-130a-5p;hsa-miR-23a-3p;hsa-miR-23b-3p;hsa-miR-2
ENSG0000	ENSG0000	1.96E-05	hsa-let-7a-5p;hsa-let-7b-5p;hsa-let-7d-5p;hsa-let-7e-5p;hsa-l
ENSG0000	ENSG0000	0.000184	hsa-let-7a-5p;hsa-let-7b-5p;hsa-let-7d-5p;hsa-let-7e-5p;hsa-l
ENSG0000	ENSG0000	0.000124	hsa-let-7a-5p;hsa-let-7b-5p;hsa-let-7d-5p;hsa-let-7e-5p;hsa-l
ENSG0000	ENSG0000	1.03E-06	hsa-miR-27a-3p;hsa-miR-27b-3p;hsa-miR-378a-3p;hsa-miR-3
ENSG0000	ENSG0000	0.000615	hsa-let-7a-5p;hsa-let-7d-5p;hsa-let-7f-5p;hsa-let-7g-5p;hsa-le
ENSG0000	ENSG0000	0.000695	hsa-miR-378a-3p;hsa-miR-378c;hsa-miR-378d;hsa-miR-378i;hs
ENSG0000	ENSG0000	0.000717	hsa-let-7a-5p;hsa-let-7b-5p;hsa-let-7d-5p;hsa-let-7e-5p;hsa-l
ENSG0000	ENSG0000	0.000532	hsa-miR-129-5p;hsa-miR-181a-5p;hsa-miR-181b-5p;hsa-miR-
ENSG0000	ENSG0000	3.71E-06	hsa-let-7a-5p;hsa-let-7b-5p;hsa-let-7d-5p;hsa-let-7e-5p;hsa-l
ENSG0000	ENSG0000	0.001952	hsa-let-7a-5p;hsa-let-7b-5p;hsa-let-7d-5p;hsa-let-7e-5p;hsa-l
ENSG0000	ENSG0000	0.000532	hsa-miR-320a;hsa-miR-320b;hsa-miR-320d;hsa-miR-4429
ENSG0000	ENSG0000	0.000532	hsa-miR-320a;hsa-miR-320b;hsa-miR-320d;hsa-miR-4429
ENSG0000	ENSG0000	0.000111	hsa-let-7a-5p;hsa-let-7b-5p;hsa-let-7d-5p;hsa-let-7e-5p;hsa-l
ENSG0000	ENSG0000	3.46E-06	hsa-let-7a-5p;hsa-let-7b-5p;hsa-let-7d-5p;hsa-let-7e-5p;hsa-l
ENSG0000	ENSG0000	0.000478	hsa-let-7a-5p;hsa-let-7d-5p;hsa-let-7e-5p;hsa-let-7f-5p;hsa-le
ENSG0000	ENSG0000	9.15E-06	hsa-miR-130a-5p;hsa-miR-23a-3p;hsa-miR-23b-3p;hsa-miR-2
ENSG0000	ENSG0000	0.00983	hsa-miR-320a;hsa-miR-320b;hsa-miR-320d;hsa-miR-374a-5p;h
ENSG0000	ENSG0000	0.00014	hsa-let-7a-5p;hsa-let-7b-5p;hsa-let-7d-5p;hsa-let-7e-5p;hsa-l
ENSG0000	ENSG0000	0.00014	hsa-let-7a-5p;hsa-let-7b-5p;hsa-let-7d-5p;hsa-let-7e-5p;hsa-l
ENSG0000	ENSG0000	0.000111	hsa-let-7a-5p;hsa-let-7b-5p;hsa-let-7d-5p;hsa-let-7e-5p;hsa-l

ENSG0000	ENSG0000	0.000168	hsa-let-7a-5p;hsa-let-7b-5p;hsa-let-7d-5p;hsa-let-7e-5p;hsa-l
ENSG0000	ENSG0000	1.69E-05	hsa-let-7a-5p;hsa-let-7b-5p;hsa-let-7d-5p;hsa-let-7e-5p;hsa-l
ENSG0000	ENSG0000	6.42E-05	hsa-let-7a-5p;hsa-let-7b-5p;hsa-let-7d-5p;hsa-let-7e-5p;hsa-l
ENSG0000	ENSG0000	6.42E-05	hsa-let-7a-5p;hsa-let-7b-5p;hsa-let-7d-5p;hsa-let-7e-5p;hsa-l
ENSG0000	ENSG0000	3.56E-08	hsa-let-7a-5p;hsa-let-7b-5p;hsa-let-7d-5p;hsa-let-7e-5p;hsa-l
ENSG0000	ENSG0000	1.41E-05	hsa-miR-129-5p;hsa-miR-130a-5p;hsa-miR-181a-5p;hsa-miR-1
ENSG0000	ENSG0000	0.000532	hsa-miR-320a;hsa-miR-320b;hsa-miR-320d;hsa-miR-4429
ENSG0000	ENSG0000	0.000196	hsa-miR-181a-5p;hsa-miR-181b-5p;hsa-miR-181c-5p;hsa-miR-
ENSG0000	ENSG0000	0.000281	hsa-miR-1299;hsa-miR-181a-5p;hsa-miR-181b-5p;hsa-miR-18
ENSG0000	ENSG0000	0.00023	hsa-miR-378a-3p;hsa-miR-378c;hsa-miR-378d;hsa-miR-378i;hs
ENSG0000	ENSG0000	1.03E-06	hsa-let-7a-5p;hsa-let-7b-5p;hsa-let-7d-5p;hsa-let-7e-5p;hsa-l
ENSG0000	ENSG0000	0.001653	hsa-miR-129-5p;hsa-miR-320a;hsa-miR-320b;hsa-miR-320d;hs
ENSG0000	ENSG0000	0.000124	hsa-let-7a-5p;hsa-let-7b-5p;hsa-let-7d-5p;hsa-let-7e-5p;hsa-l
ENSG0000	ENSG0000	3.06E-06	hsa-let-7a-5p;hsa-let-7e-5p;hsa-miR-130a-5p;hsa-miR-23a-3p
ENSG0000	ENSG0000	9.81E-05	hsa-miR-320a;hsa-miR-320b;hsa-miR-320d;hsa-miR-4429
ENSG0000	ENSG0000	0.005182	hsa-miR-320a;hsa-miR-320b;hsa-miR-320d;hsa-miR-4429
ENSG0000	ENSG0000	0.000576	hsa-let-7a-5p;hsa-let-7b-5p;hsa-let-7d-5p;hsa-let-7e-5p;hsa-l
ENSG0000	ENSG0000	0.006787	hsa-let-7a-5p;hsa-let-7b-5p;hsa-let-7d-5p;hsa-let-7e-5p;hsa-l
ENSG0000	ENSG0000	0.000891	hsa-miR-130a-5p;hsa-miR-181a-5p;hsa-miR-181b-5p;hsa-miR-
ENSG0000	ENSG0000	2.49E-06	hsa-let-7a-5p;hsa-let-7b-5p;hsa-let-7d-5p;hsa-let-7e-5p;hsa-l
ENSG0000	ENSG0000	0.00143	hsa-miR-129-5p;hsa-miR-130a-5p;hsa-miR-23a-3p;hsa-miR-23
ENSG0000	ENSG0000	0.001651	hsa-miR-142-3p;hsa-miR-181a-5p;hsa-miR-181b-5p;hsa-miR-1
ENSG0000	ENSG0000	4.32E-05	hsa-let-7a-5p;hsa-let-7b-5p;hsa-let-7d-5p;hsa-let-7e-5p;hsa-l
ENSG0000	ENSG0000	3.41E-05	hsa-let-7a-5p;hsa-let-7b-5p;hsa-let-7d-5p;hsa-let-7e-5p;hsa-l
ENSG0000	ENSG0000	0.001743	hsa-miR-130a-5p;hsa-miR-23a-3p;hsa-miR-23b-3p;hsa-miR-23
ENSG0000	ENSG0000	4.96E-05	hsa-miR-320a;hsa-miR-320b;hsa-miR-320d;hsa-miR-374a-5p;h
ENSG0000	ENSG0000	2.08E-05	hsa-let-7a-5p;hsa-let-7b-5p;hsa-let-7e-5p;hsa-let-7f-5p;hsa-le
ENSG0000	ENSG0000	1.06E-05	hsa-let-7a-5p;hsa-let-7b-5p;hsa-let-7d-5p;hsa-let-7e-5p;hsa-l
ENSG0000	ENSG0000	1.87E-05	hsa-miR-130a-5p;hsa-miR-223-5p;hsa-miR-23a-3p;hsa-miR-23
ENSG0000	ENSG0000	0.005024	hsa-let-7a-5p;hsa-let-7b-5p;hsa-let-7d-5p;hsa-let-7e-5p;hsa-l
ENSG0000	ENSG0000	0.0002	hsa-let-7a-5p;hsa-let-7b-5p;hsa-let-7d-5p;hsa-let-7e-5p;hsa-l
ENSG0000	ENSG0000	1.69E-05	hsa-let-7a-5p;hsa-let-7b-5p;hsa-let-7d-5p;hsa-let-7e-5p;hsa-l
ENSG0000	ENSG0000	3.47E-06	hsa-let-7a-5p;hsa-let-7b-5p;hsa-let-7d-5p;hsa-let-7e-5p;hsa-l
ENSG0000	ENSG0000	0.000437	hsa-miR-181a-5p;hsa-miR-181b-5p;hsa-miR-181c-5p;hsa-miR-
ENSG0000	ENSG0000	1.08E-06	hsa-let-7a-5p;hsa-let-7b-5p;hsa-let-7d-5p;hsa-let-7e-5p;hsa-l
ENSG0000	ENSG0000	0.004224	hsa-miR-320a;hsa-miR-320b;hsa-miR-320d;hsa-miR-320e;hsa-r
ENSG0000	ENSG0000	0.004224	hsa-miR-320a;hsa-miR-320b;hsa-miR-320d;hsa-miR-320e;hsa-r
ENSG0000	ENSG0000	8.01E-05	hsa-miR-129-5p;hsa-miR-181a-5p;hsa-miR-181b-5p;hsa-miR-1
ENSG0000	ENSG0000	0.00877	hsa-miR-320a;hsa-miR-320b;hsa-miR-320d;hsa-miR-4429
ENSG0000	ENSG0000	4.48E-06	hsa-let-7a-5p;hsa-let-7b-5p;hsa-let-7d-5p;hsa-let-7e-5p;hsa-l
ENSG0000	ENSG0000	0.000906	hsa-miR-181a-5p;hsa-miR-181b-5p;hsa-miR-181c-5p;hsa-miR-
ENSG0000	ENSG0000	1.30E-06	hsa-miR-130a-5p;hsa-miR-181a-5p;hsa-miR-181b-5p;hsa-miR-
ENSG0000	ENSG0000	6.43E-06	hsa-let-7a-5p;hsa-let-7b-5p;hsa-let-7d-5p;hsa-let-7e-5p;hsa-l
ENSG0000	ENSG0000	0.000354	hsa-miR-320a;hsa-miR-320b;hsa-miR-320d;hsa-miR-4429
ENSG0000	ENSG0000	1.50E-07	hsa-let-7a-5p;hsa-let-7b-5p;hsa-let-7d-5p;hsa-let-7e-5p;hsa-l
ENSG0000	ENSG0000	0.000966	hsa-let-7a-5p;hsa-let-7d-5p;hsa-let-7e-5p;hsa-let-7f-5p;hsa-le
ENSG0000	ENSG0000	1.10E-05	hsa-let-7a-5p;hsa-let-7b-5p;hsa-let-7d-5p;hsa-let-7e-5p;hsa-l
ENSG0000	ENSG0000	6.36E-05	hsa-let-7a-5p;hsa-let-7b-5p;hsa-let-7d-5p;hsa-let-7e-5p;hsa-l
ENSG0000	ENSG0000	4.43E-06	hsa-let-7a-5p;hsa-let-7b-5p;hsa-let-7d-5p;hsa-let-7e-5p;hsa-l
ENSG0000	ENSG0000	4.60E-07	hsa-miR-223-5p;hsa-miR-320a;hsa-miR-320b;hsa-miR-320d;hs
ENSG0000	ENSG0000	7.32E-06	hsa-miR-142-3p;hsa-miR-23a-3p;hsa-miR-27a-3p;hsa-miR-27b
ENSG0000	ENSG0000	0.000478	hsa-miR-1299;hsa-miR-130a-5p;hsa-miR-23a-3p;hsa-miR-23b-
ENSG0000	ENSG0000	0.003044	hsa-miR-320e;hsa-miR-378a-3p;hsa-miR-378c;hsa-miR-378d;hs
ENSG0000	ENSG0000	9.15E-06	hsa-let-7a-5p;hsa-let-7e-5p;hsa-let-7f-5p;hsa-let-7g-5p;hsa-le

ENSG0000	ENSG0000	0.000312	hsa-miR-378a-3p;hsa-miR-378c;hsa-miR-378d;hsa-miR-378i;hs
ENSG0000	ENSG0000	5.57E-09	hsa-let-7a-5p;hsa-let-7b-5p;hsa-let-7d-5p;hsa-let-7e-5p;hsa-l
ENSG0000	ENSG0000	3.46E-06	hsa-miR-1299;hsa-miR-378a-3p;hsa-miR-378c;hsa-miR-378d;h
ENSG0000	ENSG0000	0.000532	hsa-miR-130a-5p;hsa-miR-23a-3p;hsa-miR-23b-3p;hsa-miR-2
ENSG0000	ENSG0000	0.000124	hsa-let-7a-5p;hsa-let-7b-5p;hsa-let-7d-5p;hsa-let-7e-5p;hsa-l
ENSG0000	ENSG0000	8.96E-05	hsa-let-7a-5p;hsa-let-7b-5p;hsa-let-7d-5p;hsa-let-7e-5p;hsa-l
ENSG0000	ENSG0000	8.96E-05	hsa-let-7a-5p;hsa-let-7b-5p;hsa-let-7d-5p;hsa-let-7e-5p;hsa-l
ENSG0000	ENSG0000	1.37E-06	hsa-let-7a-5p;hsa-let-7b-5p;hsa-let-7d-5p;hsa-let-7e-5p;hsa-l
ENSG0000	ENSG0000	0.000224	hsa-miR-130a-5p;hsa-miR-23a-3p;hsa-miR-23b-3p;hsa-miR-2
ENSG0000	ENSG0000	5.00E-05	hsa-let-7a-5p;hsa-let-7b-5p;hsa-let-7d-5p;hsa-let-7e-5p;hsa-l
ENSG0000	ENSG0000	2.77E-07	hsa-let-7a-5p;hsa-let-7b-5p;hsa-let-7d-5p;hsa-let-7e-5p;hsa-l
ENSG0000	ENSG0000	0.004816	hsa-let-7a-5p;hsa-let-7b-5p;hsa-let-7d-5p;hsa-let-7e-5p;hsa-l
ENSG0000	ENSG0000	3.41E-05	hsa-let-7a-5p;hsa-let-7b-5p;hsa-let-7d-5p;hsa-let-7e-5p;hsa-l
ENSG0000	ENSG0000	6.45E-06	hsa-let-7a-5p;hsa-let-7b-5p;hsa-let-7d-5p;hsa-let-7e-5p;hsa-l
ENSG0000	ENSG0000	0.000251	hsa-miR-181c-3p;hsa-miR-378a-3p;hsa-miR-378c;hsa-miR-37
ENSG0000	ENSG0000	0.004251	hsa-miR-130a-5p;hsa-miR-23a-3p;hsa-miR-23b-3p;hsa-miR-2
ENSG0000	ENSG0000	9.58E-11	hsa-let-7a-5p;hsa-let-7b-5p;hsa-let-7d-5p;hsa-let-7e-5p;hsa-l
ENSG0000	ENSG0000	1.40E-05	hsa-let-7a-5p;hsa-let-7b-5p;hsa-let-7e-5p;hsa-let-7f-5p;hsa-l
ENSG0000	ENSG0000	2.01E-07	hsa-miR-130a-5p;hsa-miR-23a-3p;hsa-miR-23b-3p;hsa-miR-2
ENSG0000	ENSG0000	0.000766	hsa-miR-130a-5p;hsa-miR-23a-3p;hsa-miR-23b-3p;hsa-miR-2
ENSG0000	ENSG0000	1.69E-08	hsa-miR-181a-5p;hsa-miR-181b-5p;hsa-miR-181c-5p;hsa-miR-
ENSG0000	ENSG0000	3.56E-07	hsa-let-7a-5p;hsa-let-7b-5p;hsa-let-7d-5p;hsa-let-7e-5p;hsa-l
ENSG0000	ENSG0000	7.14E-05	hsa-let-7a-5p;hsa-let-7b-5p;hsa-let-7d-5p;hsa-let-7e-5p;hsa-l
ENSG0000	ENSG0000	3.20E-05	hsa-let-7a-5p;hsa-let-7b-5p;hsa-let-7d-5p;hsa-let-7e-5p;hsa-l
ENSG0000	ENSG0000	0.000111	hsa-let-7a-5p;hsa-let-7b-5p;hsa-let-7d-5p;hsa-let-7e-5p;hsa-l
ENSG0000	ENSG0000	0.001221	hsa-let-7a-5p;hsa-let-7f-5p;hsa-let-7g-5p;hsa-let-7i-5p;hsa-m
ENSG0000	ENSG0000	2.61E-05	hsa-let-7a-5p;hsa-let-7b-5p;hsa-let-7d-5p;hsa-let-7e-5p;hsa-l
ENSG0000	ENSG0000	8.10E-05	hsa-let-7a-5p;hsa-let-7b-5p;hsa-let-7d-5p;hsa-let-7e-5p;hsa-l
ENSG0000	ENSG0000	8.10E-05	hsa-let-7a-5p;hsa-let-7b-5p;hsa-let-7d-5p;hsa-let-7e-5p;hsa-l
ENSG0000	ENSG0000	7.11E-05	hsa-miR-181a-5p;hsa-miR-181b-5p;hsa-miR-181c-5p;hsa-miR-
ENSG0000	ENSG0000	0.000437	hsa-miR-130a-5p;hsa-miR-23a-3p;hsa-miR-23b-3p;hsa-miR-2
ENSG0000	ENSG0000	0.006522	hsa-let-7a-5p;hsa-let-7b-5p;hsa-let-7d-5p;hsa-let-7e-5p;hsa-l
ENSG0000	ENSG0000	0.001354	hsa-miR-130a-5p;hsa-miR-223-5p;hsa-miR-23a-3p;hsa-miR-2
ENSG0000	ENSG0000	0.000224	hsa-miR-27a-3p;hsa-miR-27b-3p;hsa-miR-320b;hsa-miR-320d
ENSG0000	ENSG0000	2.77E-07	hsa-let-7a-5p;hsa-let-7b-5p;hsa-let-7d-5p;hsa-let-7e-5p;hsa-l
ENSG0000	ENSG0000	3.37E-05	hsa-miR-130a-5p;hsa-miR-23a-3p;hsa-miR-23b-3p;hsa-miR-2
ENSG0000	ENSG0000	2.25E-06	hsa-miR-129-5p;hsa-miR-320a;hsa-miR-320b;hsa-miR-320d;hs
ENSG0000	ENSG0000	0.007434	hsa-miR-130a-5p;hsa-miR-23a-3p;hsa-miR-23b-3p;hsa-miR-2
ENSG0000	ENSG0000	8.92E-08	hsa-miR-130a-5p;hsa-miR-23a-3p;hsa-miR-23b-3p;hsa-miR-2
ENSG0000	ENSG0000	0.000906	hsa-miR-130a-5p;hsa-miR-23a-3p;hsa-miR-23b-3p;hsa-miR-2
ENSG0000	ENSG0000	0.000581	hsa-miR-181a-5p;hsa-miR-181b-5p;hsa-miR-181c-5p;hsa-miR-
ENSG0000	ENSG0000	0.000581	hsa-miR-181a-5p;hsa-miR-181b-5p;hsa-miR-181c-5p;hsa-miR-
ENSG0000	ENSG0000	3.55E-13	hsa-miR-130a-5p;hsa-miR-181a-5p;hsa-miR-181b-5p;hsa-miR-
ENSG0000	ENSG0000	4.85E-07	hsa-let-7a-5p;hsa-let-7b-5p;hsa-let-7d-5p;hsa-let-7e-5p;hsa-l
ENSG0000	ENSG0000	4.85E-07	hsa-let-7a-5p;hsa-let-7b-5p;hsa-let-7d-5p;hsa-let-7e-5p;hsa-l
ENSG0000	ENSG0000	7.11E-05	hsa-miR-320a;hsa-miR-320b;hsa-miR-320d;hsa-miR-4429
ENSG0000	ENSG0000	2.18E-05	hsa-miR-320a;hsa-miR-320b;hsa-miR-320d;hsa-miR-4429
ENSG0000	ENSG0000	0.000668	hsa-let-7a-5p;hsa-let-7b-5p;hsa-let-7d-5p;hsa-let-7e-5p;hsa-l
ENSG0000	ENSG0000	2.99E-05	hsa-let-7a-5p;hsa-let-7b-5p;hsa-let-7d-5p;hsa-let-7e-5p;hsa-l
ENSG0000	ENSG0000	0.000641	hsa-let-7b-5p;hsa-miR-129-5p;hsa-miR-27a-3p;hsa-miR-27b-
ENSG0000	ENSG0000	7.84E-08	hsa-let-7b-5p;hsa-let-7d-5p;hsa-let-7e-5p;hsa-let-7f-5p;hsa-l
ENSG0000	ENSG0000	2.48E-11	hsa-miR-130a-5p;hsa-miR-23a-3p;hsa-miR-23b-3p;hsa-miR-2
ENSG0000	ENSG0000	1.00E-04	hsa-let-7a-5p;hsa-let-7b-5p;hsa-let-7d-5p;hsa-let-7e-5p;hsa-l
ENSG0000	ENSG0000	1.49E-05	hsa-let-7a-5p;hsa-let-7b-5p;hsa-let-7d-5p;hsa-let-7e-5p;hsa-l

ENSG0000	ENSG0000	4.56E-05	hsa-let-7a-5p;hsa-let-7b-5p;hsa-let-7d-5p;hsa-let-7e-5p;hsa-l
ENSG0000	ENSG0000	1.03E-06	hsa-let-7a-5p;hsa-let-7b-5p;hsa-let-7d-5p;hsa-let-7e-5p;hsa-l
ENSG0000	ENSG0000	0.002168	hsa-let-7a-5p;hsa-let-7b-5p;hsa-let-7d-5p;hsa-let-7f-5p;hsa-le
ENSG0000	ENSG0000	0.000272	hsa-let-7a-5p;hsa-let-7b-5p;hsa-let-7d-5p;hsa-let-7e-5p;hsa-l
ENSG0000	ENSG0000	1.24E-05	hsa-let-7a-5p;hsa-let-7b-5p;hsa-let-7d-5p;hsa-let-7e-5p;hsa-l
ENSG0000	ENSG0000	0.00215	hsa-miR-181c-5p;hsa-miR-223-5p;hsa-miR-374a-5p;hsa-miR-3
ENSG0000	ENSG0000	0.000695	hsa-miR-320a;hsa-miR-320b;hsa-miR-320d;hsa-miR-4429;hsa-r
ENSG0000	ENSG0000	0.000717	hsa-let-7a-5p;hsa-let-7b-5p;hsa-let-7d-5p;hsa-let-7e-5p;hsa-l
ENSG0000	ENSG0000	0.000532	hsa-miR-181a-5p;hsa-miR-181b-5p;hsa-miR-181c-5p;hsa-miR-
ENSG0000	ENSG0000	0.000917	hsa-miR-103a-2-5p;hsa-miR-181a-5p;hsa-miR-181b-5p;hsa-m
ENSG0000	ENSG0000	0.006456	hsa-let-7d-5p;hsa-let-7e-5p;hsa-let-7f-5p;hsa-let-7g-5p;hsa-le
ENSG0000	ENSG0000	0.006456	hsa-let-7d-5p;hsa-let-7e-5p;hsa-let-7f-5p;hsa-let-7g-5p;hsa-le
ENSG0000	ENSG0000	0.001605	hsa-let-7a-5p;hsa-let-7b-5p;hsa-let-7d-5p;hsa-let-7e-5p;hsa-l
ENSG0000	ENSG0000	1.00E-04	hsa-let-7a-5p;hsa-let-7b-5p;hsa-let-7d-5p;hsa-let-7e-5p;hsa-l
ENSG0000	ENSG0000	4.05E-05	hsa-miR-130a-5p;hsa-miR-223-5p;hsa-miR-23a-3p;hsa-miR-23
ENSG0000	ENSG0000	2.60E-05	hsa-let-7a-5p;hsa-let-7b-5p;hsa-let-7d-5p;hsa-let-7e-5p;hsa-l
ENSG0000	ENSG0000	0.000139	hsa-miR-378a-3p;hsa-miR-378c;hsa-miR-378d;hsa-miR-378i;hs
ENSG0000	ENSG0000	3.51E-05	hsa-let-7a-5p;hsa-let-7d-5p;hsa-let-7e-5p;hsa-let-7f-5p;hsa-le
ENSG0000	ENSG0000	0.000766	hsa-miR-130a-5p;hsa-miR-23a-3p;hsa-miR-23b-3p;hsa-miR-23
ENSG0000	ENSG0000	1.32E-05	hsa-miR-130a-5p;hsa-miR-23a-3p;hsa-miR-23b-3p;hsa-miR-23
ENSG0000	ENSG0000	0.000224	hsa-miR-320a;hsa-miR-320b;hsa-miR-320d;hsa-miR-4429
ENSG0000	ENSG0000	0.001221	hsa-miR-320a;hsa-miR-320b;hsa-miR-320d;hsa-miR-4429;hsa-r
ENSG0000	ENSG0000	0.007356	hsa-miR-130a-5p;hsa-miR-23a-3p;hsa-miR-23b-3p;hsa-miR-23
ENSG0000	ENSG0000	0.000284	hsa-miR-1299;hsa-miR-181a-5p;hsa-miR-181b-5p;hsa-miR-181
ENSG0000	ENSG0000	0.002325	hsa-let-7d-5p;hsa-let-7e-5p;hsa-let-7f-5p;hsa-let-7g-5p;hsa-le
ENSG0000	ENSG0000	0.00624	hsa-miR-130a-5p;hsa-miR-23a-3p;hsa-miR-23b-3p;hsa-miR-23
ENSG0000	ENSG0000	0.000615	hsa-miR-142-3p;hsa-miR-181a-5p;hsa-miR-181b-5p;hsa-miR-1
ENSG0000	ENSG0000	1.25E-05	hsa-let-7a-5p;hsa-let-7b-5p;hsa-let-7d-5p;hsa-let-7e-5p;hsa-l
ENSG0000	ENSG0000	1.25E-05	hsa-let-7a-5p;hsa-let-7b-5p;hsa-let-7d-5p;hsa-let-7e-5p;hsa-l
ENSG0000	ENSG0000	1.25E-05	hsa-let-7a-5p;hsa-let-7b-5p;hsa-let-7d-5p;hsa-let-7e-5p;hsa-l
ENSG0000	ENSG0000	1.69E-05	hsa-let-7a-5p;hsa-let-7b-5p;hsa-let-7d-5p;hsa-let-7e-5p;hsa-l
ENSG0000	ENSG0000	0.000111	hsa-let-7a-5p;hsa-let-7b-5p;hsa-let-7d-5p;hsa-let-7e-5p;hsa-l
ENSG0000	ENSG0000	2.02E-06	hsa-let-7a-5p;hsa-let-7b-5p;hsa-let-7d-5p;hsa-let-7e-5p;hsa-l
ENSG0000	ENSG0000	1.63E-06	hsa-let-7a-5p;hsa-let-7b-5p;hsa-let-7d-5p;hsa-let-7e-5p;hsa-l
ENSG0000	ENSG0000	0.00215	hsa-miR-320a;hsa-miR-320b;hsa-miR-320d;hsa-miR-4429
ENSG0000	ENSG0000	0.000155	hsa-let-7a-5p;hsa-let-7d-5p;hsa-let-7e-5p;hsa-let-7f-5p;hsa-le
ENSG0000	ENSG0000	0.002545	hsa-let-7a-5p;hsa-let-7b-5p;hsa-let-7d-5p;hsa-let-7e-5p;hsa-l
ENSG0000	ENSG0000	0.002545	hsa-let-7a-5p;hsa-let-7b-5p;hsa-let-7d-5p;hsa-let-7e-5p;hsa-l
ENSG0000	ENSG0000	1.92E-06	hsa-let-7a-5p;hsa-let-7d-5p;hsa-let-7e-5p;hsa-let-7f-5p;hsa-le
ENSG0000	ENSG0000	0.000136	hsa-miR-130a-5p;hsa-miR-142-3p;hsa-miR-181b-5p;hsa-miR-2
ENSG0000	ENSG0000	0.000165	hsa-let-7a-5p;hsa-let-7e-5p;hsa-let-7f-5p;hsa-let-7g-5p;hsa-le
ENSG0000	ENSG0000	3.56E-08	hsa-let-7a-5p;hsa-let-7b-5p;hsa-let-7d-5p;hsa-let-7e-5p;hsa-l
ENSG0000	ENSG0000	6.72E-08	hsa-let-7a-5p;hsa-let-7b-5p;hsa-let-7d-5p;hsa-let-7e-5p;hsa-l
ENSG0000	ENSG0000	2.26E-05	hsa-let-7a-5p;hsa-let-7b-5p;hsa-let-7d-5p;hsa-let-7e-5p;hsa-l
ENSG0000	ENSG0000	7.14E-05	hsa-let-7a-5p;hsa-let-7b-5p;hsa-let-7d-5p;hsa-let-7e-5p;hsa-l
ENSG0000	ENSG0000	7.14E-05	hsa-let-7a-5p;hsa-let-7b-5p;hsa-let-7d-5p;hsa-let-7e-5p;hsa-l
ENSG0000	ENSG0000	2.02E-06	hsa-let-7a-5p;hsa-let-7b-5p;hsa-let-7d-5p;hsa-let-7e-5p;hsa-l
ENSG0000	ENSG0000	3.05E-06	hsa-let-7a-5p;hsa-let-7b-5p;hsa-let-7d-5p;hsa-let-7e-5p;hsa-l
ENSG0000	ENSG0000	0.003248	hsa-let-7a-5p;hsa-let-7b-5p;hsa-let-7d-5p;hsa-let-7f-5p;hsa-le
ENSG0000	ENSG0000	0.001098	hsa-miR-181a-5p;hsa-miR-181b-5p;hsa-miR-181c-5p;hsa-miR-
ENSG0000	ENSG0000	1.09E-05	hsa-let-7a-5p;hsa-let-7b-5p;hsa-let-7d-5p;hsa-let-7e-5p;hsa-l
ENSG0000	ENSG0000	0.00877	hsa-miR-181c-3p;hsa-miR-27a-3p;hsa-miR-27b-3p;hsa-miR-32
ENSG0000	ENSG0000	4.99E-05	hsa-miR-130a-5p;hsa-miR-23a-3p;hsa-miR-23b-3p;hsa-miR-23
ENSG0000	ENSG0000	0.000766	hsa-miR-130a-5p;hsa-miR-23a-3p;hsa-miR-23b-3p;hsa-miR-23

ENSG0000	ENSG0000	0.000879	hsa-miR-181a-5p;hsa-miR-181b-5p;hsa-miR-181c-5p;hsa-miR-
ENSG0000	ENSG0000	0.000312	hsa-miR-378a-3p;hsa-miR-378c;hsa-miR-378d;hsa-miR-378i;hs
ENSG0000	ENSG0000	0.003079	hsa-miR-181a-5p;hsa-miR-181b-5p;hsa-miR-181c-5p;hsa-miR-
ENSG0000	ENSG0000	0.000879	hsa-miR-378a-3p;hsa-miR-378c;hsa-miR-378d;hsa-miR-378i;hs
ENSG0000	ENSG0000	4.99E-05	hsa-miR-130a-5p;hsa-miR-23a-3p;hsa-miR-23b-3p;hsa-miR-2
ENSG0000	ENSG0000	1.00E-04	hsa-miR-181a-5p;hsa-miR-181b-5p;hsa-miR-181c-5p;hsa-miR-
ENSG0000	ENSG0000	2.99E-05	hsa-let-7a-5p;hsa-let-7b-5p;hsa-let-7e-5p;hsa-let-7f-5p;hsa-le
ENSG0000	ENSG0000	1.96E-05	hsa-let-7a-5p;hsa-let-7b-5p;hsa-let-7d-5p;hsa-let-7e-5p;hsa-l
ENSG0000	ENSG0000	0.001889	hsa-miR-130a-5p;hsa-miR-23a-3p;hsa-miR-23b-3p;hsa-miR-2
ENSG0000	ENSG0000	3.00E-05	hsa-miR-130a-5p;hsa-miR-23a-3p;hsa-miR-23b-3p;hsa-miR-2
ENSG0000	ENSG0000	0.002018	hsa-let-7a-5p;hsa-let-7b-5p;hsa-let-7d-5p;hsa-let-7f-5p;hsa-le
ENSG0000	ENSG0000	2.99E-05	hsa-let-7a-5p;hsa-let-7b-5p;hsa-let-7d-5p;hsa-let-7e-5p;hsa-l
ENSG0000	ENSG0000	0.001086	hsa-let-7a-5p;hsa-let-7b-5p;hsa-let-7d-5p;hsa-let-7e-5p;hsa-l
ENSG0000	ENSG0000	0.001086	hsa-let-7a-5p;hsa-let-7b-5p;hsa-let-7d-5p;hsa-let-7e-5p;hsa-l
ENSG0000	ENSG0000	5.27E-08	hsa-let-7a-5p;hsa-let-7b-5p;hsa-let-7d-5p;hsa-let-7e-5p;hsa-l
ENSG0000	ENSG0000	5.57E-09	hsa-let-7a-5p;hsa-let-7b-5p;hsa-let-7d-5p;hsa-let-7e-5p;hsa-l
ENSG0000	ENSG0000	0.000437	hsa-miR-130a-5p;hsa-miR-23a-3p;hsa-miR-23b-3p;hsa-miR-2
ENSG0000	ENSG0000	1.41E-05	hsa-let-7a-5p;hsa-let-7b-5p;hsa-let-7d-5p;hsa-let-7e-5p;hsa-l
ENSG0000	ENSG0000	0.000766	hsa-miR-320a;hsa-miR-320b;hsa-miR-320d;hsa-miR-4429
ENSG0000	ENSG0000	6.44E-09	hsa-let-7a-5p;hsa-let-7b-5p;hsa-let-7d-5p;hsa-let-7e-5p;hsa-l
ENSG0000	ENSG0000	8.01E-05	hsa-let-7a-5p;hsa-let-7b-5p;hsa-let-7d-5p;hsa-let-7e-5p;hsa-l
ENSG0000	ENSG0000	0.004434	hsa-let-7a-5p;hsa-let-7b-5p;hsa-let-7d-5p;hsa-let-7e-5p;hsa-l
ENSG0000	ENSG0000	0.00143	hsa-let-7a-5p;hsa-let-7b-5p;hsa-let-7d-5p;hsa-let-7e-5p;hsa-l
ENSG0000	ENSG0000	0.002399	hsa-let-7a-5p;hsa-let-7b-5p;hsa-let-7d-5p;hsa-let-7e-5p;hsa-l
ENSG0000	ENSG0000	0.00023	hsa-miR-378a-3p;hsa-miR-378c;hsa-miR-378d;hsa-miR-378i;hs
ENSG0000	ENSG0000	9.80E-06	hsa-let-7a-5p;hsa-let-7b-5p;hsa-let-7d-5p;hsa-let-7e-5p;hsa-l
ENSG0000	ENSG0000	2.02E-06	hsa-let-7a-5p;hsa-let-7b-5p;hsa-let-7d-5p;hsa-let-7e-5p;hsa-l
ENSG0000	ENSG0000	9.33E-09	hsa-let-7a-5p;hsa-let-7b-5p;hsa-let-7d-5p;hsa-let-7e-5p;hsa-l
ENSG0000	ENSG0000	0.000269	hsa-let-7a-5p;hsa-let-7b-5p;hsa-let-7d-5p;hsa-let-7e-5p;hsa-l
ENSG0000	ENSG0000	3.11E-15	hsa-let-7a-5p;hsa-let-7b-5p;hsa-let-7d-5p;hsa-let-7e-5p;hsa-l
ENSG0000	ENSG0000	0.001653	hsa-miR-181a-5p;hsa-miR-181b-5p;hsa-miR-181c-5p;hsa-miR-
ENSG0000	ENSG0000	1.46E-05	hsa-let-7a-5p;hsa-let-7b-5p;hsa-let-7d-5p;hsa-let-7e-5p;hsa-l
ENSG0000	ENSG0000	1.50E-07	hsa-let-7a-5p;hsa-let-7b-5p;hsa-let-7d-5p;hsa-let-7e-5p;hsa-l
ENSG0000	ENSG0000	4.05E-05	hsa-miR-130a-5p;hsa-miR-23a-3p;hsa-miR-23b-3p;hsa-miR-2
ENSG0000	ENSG0000	1.08E-07	hsa-miR-130a-5p;hsa-miR-181a-5p;hsa-miR-181b-5p;hsa-miR-
ENSG0000	ENSG0000	4.85E-07	hsa-let-7a-5p;hsa-let-7b-5p;hsa-let-7d-5p;hsa-let-7e-5p;hsa-l
ENSG0000	ENSG0000	4.85E-07	hsa-let-7a-5p;hsa-let-7b-5p;hsa-let-7d-5p;hsa-let-7e-5p;hsa-l
ENSG0000	ENSG0000	0.002545	hsa-let-7a-5p;hsa-let-7b-5p;hsa-let-7d-5p;hsa-let-7e-5p;hsa-l
ENSG0000	ENSG0000	2.49E-06	hsa-let-7a-5p;hsa-let-7b-5p;hsa-let-7d-5p;hsa-let-7e-5p;hsa-l
ENSG0000	ENSG0000	3.45E-06	hsa-miR-378a-3p;hsa-miR-378c;hsa-miR-378d;hsa-miR-378i;hs
ENSG0000	ENSG0000	0.000541	hsa-miR-130a-5p;hsa-miR-23a-3p;hsa-miR-23b-3p;hsa-miR-2
ENSG0000	ENSG0000	0.000414	hsa-let-7a-5p;hsa-let-7b-5p;hsa-let-7d-5p;hsa-let-7e-5p;hsa-l
ENSG0000	ENSG0000	0.0002	hsa-let-7a-5p;hsa-let-7b-5p;hsa-let-7d-5p;hsa-let-7e-5p;hsa-l
ENSG0000	ENSG0000	3.46E-06	hsa-let-7a-5p;hsa-let-7b-5p;hsa-let-7e-5p;hsa-let-7f-5p;hsa-le
ENSG0000	ENSG0000	0.000294	hsa-let-7a-5p;hsa-let-7b-5p;hsa-let-7d-5p;hsa-let-7e-5p;hsa-l
ENSG0000	ENSG0000	0.000294	hsa-let-7a-5p;hsa-let-7b-5p;hsa-let-7d-5p;hsa-let-7e-5p;hsa-l
ENSG0000	ENSG0000	0.000695	hsa-miR-1299;hsa-miR-130a-5p;hsa-miR-23a-3p;hsa-miR-23b-
ENSG0000	ENSG0000	0.005637	hsa-miR-378a-3p;hsa-miR-378c;hsa-miR-378d;hsa-miR-378i;hs
ENSG0000	ENSG0000	0.00035	hsa-miR-130a-5p;hsa-miR-223-5p;hsa-miR-23a-3p;hsa-miR-2
ENSG0000	ENSG0000	1.20E-08	hsa-let-7d-5p;hsa-let-7e-5p;hsa-let-7f-5p;hsa-let-7g-5p;hsa-le
ENSG0000	ENSG0000	0.00998	hsa-miR-130a-5p;hsa-miR-23a-3p;hsa-miR-23b-3p;hsa-miR-2
ENSG0000	ENSG0000	0.000321	hsa-let-7a-5p;hsa-let-7b-5p;hsa-let-7d-5p;hsa-let-7e-5p;hsa-l
ENSG0000	ENSG0000	2.45E-05	hsa-miR-130a-5p;hsa-miR-23a-3p;hsa-miR-23b-3p;hsa-miR-2
ENSG0000	ENSG0000	0.000224	hsa-miR-130a-5p;hsa-miR-23a-3p;hsa-miR-23b-3p;hsa-miR-2

ENSG0000	ENSG0000	4.22E-05	hsa-let-7a-5p;hsa-let-7b-5p;hsa-let-7e-5p;hsa-let-7f-5p;hsa-le
ENSG0000	ENSG0000	3.00E-05	hsa-let-7b-5p;hsa-let-7d-5p;hsa-let-7e-5p;hsa-let-7f-5p;hsa-le
ENSG0000	ENSG0000	0.004701	hsa-miR-223-5p;hsa-miR-374a-5p;hsa-miR-374b-5p;hsa-miR-!
ENSG0000	ENSG0000	5.57E-09	hsa-let-7a-5p;hsa-let-7b-5p;hsa-let-7d-5p;hsa-let-7e-5p;hsa-l
ENSG0000	ENSG0000	9.56E-05	hsa-miR-181a-5p;hsa-miR-181b-5p;hsa-miR-181c-5p;hsa-miR-
ENSG0000	ENSG0000	7.62E-08	hsa-let-7a-5p;hsa-let-7b-5p;hsa-let-7d-5p;hsa-let-7e-5p;hsa-l
ENSG0000	ENSG0000	7.62E-08	hsa-let-7a-5p;hsa-let-7b-5p;hsa-let-7d-5p;hsa-let-7e-5p;hsa-l
ENSG0000	ENSG0000	1.03E-06	hsa-let-7a-5p;hsa-let-7b-5p;hsa-let-7d-5p;hsa-let-7e-5p;hsa-l
ENSG0000	ENSG0000	1.40E-06	hsa-miR-181a-5p;hsa-miR-181b-5p;hsa-miR-181c-5p;hsa-miR-
ENSG0000	ENSG0000	4.96E-05	hsa-let-7a-5p;hsa-let-7b-5p;hsa-let-7d-5p;hsa-let-7e-5p;hsa-l
ENSG0000	ENSG0000	8.01E-05	hsa-let-7a-5p;hsa-let-7b-5p;hsa-let-7d-5p;hsa-let-7e-5p;hsa-l
ENSG0000	ENSG0000	0.000252	hsa-let-7a-5p;hsa-let-7b-5p;hsa-let-7d-5p;hsa-let-7e-5p;hsa-l
ENSG0000	ENSG0000	0.000245	hsa-let-7a-5p;hsa-let-7b-5p;hsa-let-7d-5p;hsa-let-7e-5p;hsa-l
ENSG0000	ENSG0000	0.0002	hsa-let-7a-5p;hsa-let-7d-5p;hsa-let-7e-5p;hsa-let-7f-5p;hsa-le
ENSG0000	ENSG0000	9.56E-05	hsa-miR-378a-3p;hsa-miR-378c;hsa-miR-378d;hsa-miR-378i;hs
ENSG0000	ENSG0000	0.000168	hsa-let-7a-5p;hsa-let-7b-5p;hsa-let-7d-5p;hsa-let-7e-5p;hsa-l
ENSG0000	ENSG0000	9.81E-05	hsa-miR-130a-5p;hsa-miR-23a-3p;hsa-miR-23b-3p;hsa-miR-2:
ENSG0000	ENSG0000	3.05E-06	hsa-let-7a-5p;hsa-let-7b-5p;hsa-let-7d-5p;hsa-let-7e-5p;hsa-l
ENSG0000	ENSG0000	6.91E-05	hsa-miR-130a-5p;hsa-miR-181a-5p;hsa-miR-181b-5p;hsa-miR-
ENSG0000	ENSG0000	5.10E-05	hsa-miR-181a-5p;hsa-miR-181b-5p;hsa-miR-181c-5p;hsa-miR-
ENSG0000	ENSG0000	5.10E-05	hsa-miR-181a-5p;hsa-miR-181b-5p;hsa-miR-181c-5p;hsa-miR-
ENSG0000	ENSG0000	0.00215	hsa-miR-181a-5p;hsa-miR-181b-5p;hsa-miR-181c-5p;hsa-miR-
ENSG0000	ENSG0000	4.36E-06	hsa-let-7a-5p;hsa-let-7b-5p;hsa-let-7d-5p;hsa-let-7e-5p;hsa-l
ENSG0000	ENSG0000	0.000233	hsa-let-7a-5p;hsa-let-7b-5p;hsa-let-7d-5p;hsa-let-7e-5p;hsa-l
ENSG0000	ENSG0000	0.002018	hsa-let-7a-5p;hsa-let-7d-5p;hsa-let-7e-5p;hsa-let-7f-5p;hsa-le
ENSG0000	ENSG0000	0.000478	hsa-miR-181a-5p;hsa-miR-181b-5p;hsa-miR-181c-5p;hsa-miR-
ENSG0000	ENSG0000	0.00083	hsa-let-7a-5p;hsa-let-7b-5p;hsa-let-7d-5p;hsa-let-7e-5p;hsa-l
ENSG0000	ENSG0000	1.63E-06	hsa-let-7a-5p;hsa-let-7b-5p;hsa-let-7d-5p;hsa-let-7e-5p;hsa-l
ENSG0000	ENSG0000	0.00881	hsa-miR-129-5p;hsa-miR-181a-5p;hsa-miR-181b-5p;hsa-miR-:
ENSG0000	ENSG0000	2.49E-06	hsa-let-7a-5p;hsa-let-7b-5p;hsa-let-7d-5p;hsa-let-7e-5p;hsa-l
ENSG0000	ENSG0000	1.37E-06	hsa-let-7a-5p;hsa-let-7b-5p;hsa-let-7d-5p;hsa-let-7e-5p;hsa-l
ENSG0000	ENSG0000	3.56E-08	hsa-miR-130a-5p;hsa-miR-181a-5p;hsa-miR-181b-5p;hsa-miR-
ENSG0000	ENSG0000	1.00E-04	hsa-let-7a-5p;hsa-let-7b-5p;hsa-let-7d-5p;hsa-let-7e-5p;hsa-l
ENSG0000	ENSG0000	0.00215	hsa-miR-320a;hsa-miR-320b;hsa-miR-320d;hsa-miR-4429
ENSG0000	ENSG0000	1.03E-06	hsa-let-7a-5p;hsa-let-7b-5p;hsa-let-7d-5p;hsa-let-7e-5p;hsa-l
ENSG0000	ENSG0000	1.19E-06	hsa-let-7a-5p;hsa-let-7b-5p;hsa-let-7e-5p;hsa-let-7f-5p;hsa-le
ENSG0000	ENSG0000	0.007098	hsa-miR-296-3p;hsa-miR-320a;hsa-miR-320b;hsa-miR-320d;hs
ENSG0000	ENSG0000	7.00E-11	hsa-let-7a-5p;hsa-let-7b-5p;hsa-let-7d-5p;hsa-let-7e-5p;hsa-l
ENSG0000	ENSG0000	0.003346	hsa-let-7b-5p;hsa-let-7e-5p;hsa-let-7f-5p;hsa-let-7g-5p;hsa-le
ENSG0000	ENSG0000	0.000641	hsa-let-7b-5p;hsa-miR-181a-5p;hsa-miR-181b-5p;hsa-miR-18:
ENSG0000	ENSG0000	0.000272	hsa-let-7a-5p;hsa-let-7b-5p;hsa-let-7d-5p;hsa-let-7e-5p;hsa-l
ENSG0000	ENSG0000	0.002329	hsa-let-7a-5p;hsa-let-7b-5p;hsa-let-7d-5p;hsa-let-7e-5p;hsa-l
ENSG0000	ENSG0000	0.00124	hsa-miR-320a;hsa-miR-320b;hsa-miR-320d;hsa-miR-4429
ENSG0000	ENSG0000	0.000274	hsa-let-7a-5p;hsa-let-7b-5p;hsa-let-7d-5p;hsa-let-7e-5p;hsa-l
ENSG0000	ENSG0000	5.27E-08	hsa-let-7a-5p;hsa-let-7b-5p;hsa-let-7d-5p;hsa-let-7e-5p;hsa-l
ENSG0000	ENSG0000	5.76E-06	hsa-let-7b-5p;hsa-miR-181a-5p;hsa-miR-181b-5p;hsa-miR-18:
ENSG0000	ENSG0000	2.01E-05	hsa-let-7a-5p;hsa-let-7b-5p;hsa-let-7d-5p;hsa-let-7e-5p;hsa-l
ENSG0000	ENSG0000	0.001354	hsa-miR-181a-5p;hsa-miR-181b-5p;hsa-miR-181c-5p;hsa-miR-
ENSG0000	ENSG0000	0.000665	hsa-let-7a-5p;hsa-let-7b-5p;hsa-let-7d-5p;hsa-let-7e-5p;hsa-l
ENSG0000	ENSG0000	0.000474	hsa-let-7a-5p;hsa-let-7b-5p;hsa-let-7d-5p;hsa-let-7e-5p;hsa-l
ENSG0000	ENSG0000	0.000281	hsa-let-7a-5p;hsa-let-7b-5p;hsa-let-7e-5p;hsa-let-7f-5p;hsa-le
ENSG0000	ENSG0000	0.000309	hsa-let-7a-5p;hsa-let-7b-5p;hsa-let-7d-5p;hsa-let-7e-5p;hsa-l
ENSG0000	ENSG0000	0.000152	hsa-let-7a-5p;hsa-let-7b-5p;hsa-let-7d-5p;hsa-let-7e-5p;hsa-l
ENSG0000	ENSG0000	4.39E-07	hsa-let-7a-5p;hsa-let-7b-5p;hsa-let-7d-5p;hsa-let-7e-5p;hsa-l

ENSG0000	ENSG0000	0.006198	hsa-let-7a-5p;hsa-let-7d-5p;hsa-let-7e-5p;hsa-let-7f-5p;hsa-le
ENSG0000	ENSG0000	3.47E-06	hsa-let-7a-5p;hsa-let-7b-5p;hsa-let-7d-5p;hsa-let-7e-5p;hsa-l
ENSG0000	ENSG0000	0.00035	hsa-let-7a-5p;hsa-let-7b-5p;hsa-let-7d-5p;hsa-let-7e-5p;hsa-l
ENSG0000	ENSG0000	0.003461	hsa-miR-130a-5p;hsa-miR-142-3p;hsa-miR-23a-3p;hsa-miR-23
ENSG0000	ENSG0000	2.77E-05	hsa-let-7a-5p;hsa-let-7b-5p;hsa-let-7d-5p;hsa-let-7e-5p;hsa-l
ENSG0000	ENSG0000	2.06E-07	hsa-let-7a-5p;hsa-let-7b-5p;hsa-let-7d-5p;hsa-let-7e-5p;hsa-l
ENSG0000	ENSG0000	0.000196	hsa-miR-130a-5p;hsa-miR-142-3p;hsa-miR-23a-3p;hsa-miR-23
ENSG0000	ENSG0000	2.35E-08	hsa-let-7a-5p;hsa-let-7b-5p;hsa-let-7d-5p;hsa-let-7e-5p;hsa-l
ENSG0000	ENSG0000	0.001256	hsa-let-7a-5p;hsa-let-7b-5p;hsa-let-7d-5p;hsa-let-7e-5p;hsa-l
ENSG0000	ENSG0000	0.001653	hsa-miR-130a-5p;hsa-miR-223-5p;hsa-miR-23a-3p;hsa-miR-23
ENSG0000	ENSG0000	0.004701	hsa-miR-130a-5p;hsa-miR-23a-3p;hsa-miR-23b-3p;hsa-miR-23
ENSG0000	ENSG0000	0.000415	hsa-miR-181a-5p;hsa-miR-181b-5p;hsa-miR-181c-5p;hsa-miR-
ENSG0000	ENSG0000	0.000906	hsa-miR-320a;hsa-miR-320b;hsa-miR-320d;hsa-miR-4429
ENSG0000	ENSG0000	0.00343	hsa-let-7a-5p;hsa-let-7b-5p;hsa-let-7d-5p;hsa-let-7e-5p;hsa-l
ENSG0000	ENSG0000	3.00E-05	hsa-let-7a-5p;hsa-let-7b-5p;hsa-let-7d-5p;hsa-let-7e-5p;hsa-l
ENSG0000	ENSG0000	8.96E-05	hsa-let-7a-5p;hsa-let-7b-5p;hsa-let-7d-5p;hsa-let-7e-5p;hsa-l
ENSG0000	ENSG0000	0.001497	hsa-miR-129-5p;hsa-miR-181a-5p;hsa-miR-181b-5p;hsa-miR-1
ENSG0000	ENSG0000	0.001728	hsa-let-7a-5p;hsa-let-7b-5p;hsa-let-7d-5p;hsa-let-7e-5p;hsa-l
ENSG0000	ENSG0000	0.002467	hsa-let-7a-5p;hsa-let-7b-5p;hsa-let-7d-5p;hsa-let-7e-5p;hsa-l
ENSG0000	ENSG0000	1.08E-07	hsa-let-7a-5p;hsa-let-7b-5p;hsa-let-7d-5p;hsa-let-7e-5p;hsa-l
ENSG0000	ENSG0000	4.49E-06	hsa-miR-378a-3p;hsa-miR-378c;hsa-miR-378d;hsa-miR-378i;hs
ENSG0000	ENSG0000	0.001435	hsa-miR-130a-5p;hsa-miR-23a-3p;hsa-miR-23b-3p;hsa-miR-23
ENSG0000	ENSG0000	0.004701	hsa-miR-181a-5p;hsa-miR-181b-5p;hsa-miR-181c-5p;hsa-miR-
ENSG0000	ENSG0000	7.65E-06	hsa-let-7a-5p;hsa-let-7b-5p;hsa-let-7d-5p;hsa-let-7e-5p;hsa-l
ENSG0000	ENSG0000	0.003079	hsa-let-7b-5p;hsa-miR-375;hsa-miR-500a-3p;hsa-miR-589-3p
ENSG0000	ENSG0000	0.001837	hsa-let-7a-5p;hsa-let-7b-5p;hsa-let-7d-5p;hsa-let-7e-5p;hsa-l
ENSG0000	ENSG0000	0.000272	hsa-let-7a-5p;hsa-let-7b-5p;hsa-let-7d-5p;hsa-let-7e-5p;hsa-l
ENSG0000	ENSG0000	7.65E-06	hsa-miR-130a-5p;hsa-miR-181a-5p;hsa-miR-181b-5p;hsa-miR-
ENSG0000	ENSG0000	0.000284	hsa-miR-130a-5p;hsa-miR-23a-3p;hsa-miR-23b-3p;hsa-miR-23
ENSG0000	ENSG0000	5.91E-05	hsa-let-7a-5p;hsa-let-7b-5p;hsa-let-7d-5p;hsa-let-7e-5p;hsa-l
ENSG0000	ENSG0000	0.000437	hsa-miR-103a-2-5p;hsa-miR-181a-5p;hsa-miR-181b-5p;hsa-m
ENSG0000	ENSG0000	3.18E-09	hsa-miR-130a-5p;hsa-miR-181a-5p;hsa-miR-181b-5p;hsa-miR-
ENSG0000	ENSG0000	1.36E-07	hsa-let-7a-5p;hsa-let-7b-5p;hsa-let-7d-5p;hsa-let-7e-5p;hsa-l
ENSG0000	ENSG0000	0.002101	hsa-let-7a-5p;hsa-let-7b-5p;hsa-let-7d-5p;hsa-let-7e-5p;hsa-l
ENSG0000	ENSG0000	7.85E-05	hsa-miR-1299;hsa-miR-378a-3p;hsa-miR-378c;hsa-miR-378d;h
ENSG0000	ENSG0000	1.63E-06	hsa-let-7a-5p;hsa-let-7b-5p;hsa-let-7d-5p;hsa-let-7e-5p;hsa-l
ENSG0000	ENSG0000	8.96E-05	hsa-let-7a-5p;hsa-let-7b-5p;hsa-let-7d-5p;hsa-let-7e-5p;hsa-l
ENSG0000	ENSG0000	7.46E-06	hsa-miR-320a;hsa-miR-320b;hsa-miR-320d;hsa-miR-4429
ENSG0000	ENSG0000	0.001997	hsa-miR-320a;hsa-miR-320b;hsa-miR-320d;hsa-miR-4429;hsa-r
ENSG0000	ENSG0000	1.30E-08	hsa-let-7a-5p;hsa-let-7b-5p;hsa-let-7d-5p;hsa-let-7e-5p;hsa-l
ENSG0000	ENSG0000	0.009414	hsa-miR-130a-5p;hsa-miR-23a-3p;hsa-miR-23b-3p;hsa-miR-23
ENSG0000	ENSG0000	2.49E-06	hsa-miR-130a-5p;hsa-miR-142-3p;hsa-miR-23a-3p;hsa-miR-23
ENSG0000	ENSG0000	0.001877	hsa-miR-1246;hsa-miR-223-5p;hsa-miR-320a;hsa-miR-320b;hsa
ENSG0000	ENSG0000	0.000917	hsa-let-7a-5p;hsa-let-7b-5p;hsa-let-7e-5p;hsa-let-7f-5p;hsa-le
ENSG0000	ENSG0000	1.03E-06	hsa-let-7a-5p;hsa-let-7b-5p;hsa-let-7d-5p;hsa-let-7e-5p;hsa-l
ENSG0000	ENSG0000	2.18E-05	hsa-miR-130a-5p;hsa-miR-23a-3p;hsa-miR-23b-3p;hsa-miR-23
ENSG0000	ENSG0000	0.001819	hsa-miR-129-5p;hsa-miR-181a-5p;hsa-miR-181b-5p;hsa-miR-1
ENSG0000	ENSG0000	0.000641	hsa-miR-130a-5p;hsa-miR-23a-3p;hsa-miR-23b-3p;hsa-miR-23
ENSG0000	ENSG0000	0.00124	hsa-miR-181a-5p;hsa-miR-181b-5p;hsa-miR-181c-5p;hsa-miR-
ENSG0000	ENSG0000	0.000224	hsa-miR-181a-5p;hsa-miR-181b-5p;hsa-miR-181c-5p;hsa-miR-
ENSG0000	ENSG0000	0.001435	hsa-miR-181a-5p;hsa-miR-181b-5p;hsa-miR-181c-5p;hsa-miR-
ENSG0000	ENSG0000	0.000224	hsa-miR-181a-5p;hsa-miR-181b-5p;hsa-miR-181c-5p;hsa-miR-
ENSG0000	ENSG0000	0.000224	hsa-miR-181a-5p;hsa-miR-181b-5p;hsa-miR-181c-5p;hsa-miR-
ENSG0000	ENSG0000	0.004251	hsa-miR-129-5p;hsa-miR-27a-3p;hsa-miR-27b-3p;hsa-miR-37

ENSG0000	ENSG0000	3.69E-07	hsa-let-7a-5p;hsa-let-7b-5p;hsa-let-7d-5p;hsa-let-7e-5p;hsa-l
ENSG0000	ENSG0000	0.001024	hsa-let-7a-5p;hsa-let-7b-5p;hsa-let-7d-5p;hsa-let-7e-5p;hsa-l
ENSG0000	ENSG0000	0.000152	hsa-let-7a-5p;hsa-let-7b-5p;hsa-let-7d-5p;hsa-let-7e-5p;hsa-l
ENSG0000	ENSG0000	0.009494	hsa-miR-130a-5p;hsa-miR-23a-3p;hsa-miR-23b-3p;hsa-miR-2
ENSG0000	ENSG0000	0.000541	hsa-miR-130a-5p;hsa-miR-23a-3p;hsa-miR-23b-3p;hsa-miR-2
ENSG0000	ENSG0000	0.001363	hsa-let-7a-5p;hsa-let-7b-5p;hsa-let-7e-5p;hsa-let-7f-5p;hsa-le
ENSG0000	ENSG0000	4.48E-06	hsa-let-7a-5p;hsa-let-7b-5p;hsa-let-7d-5p;hsa-let-7e-5p;hsa-l
ENSG0000	ENSG0000	3.62E-06	hsa-let-7a-5p;hsa-let-7b-5p;hsa-let-7d-5p;hsa-let-7e-5p;hsa-l
ENSG0000	ENSG0000	0.000766	hsa-miR-181a-5p;hsa-miR-181b-5p;hsa-miR-181c-5p;hsa-miR-
ENSG0000	ENSG0000	0.009494	hsa-miR-142-3p;hsa-miR-181c-5p;hsa-miR-27a-3p;hsa-miR-27
ENSG0000	ENSG0000	0.000223	hsa-let-7a-5p;hsa-let-7b-5p;hsa-let-7d-5p;hsa-let-7e-5p;hsa-l
ENSG0000	ENSG0000	4.86E-06	hsa-let-7a-5p;hsa-let-7b-5p;hsa-let-7d-5p;hsa-let-7e-5p;hsa-l
ENSG0000	ENSG0000	0.002392	hsa-miR-181a-5p;hsa-miR-181b-5p;hsa-miR-181c-5p;hsa-miR-
ENSG0000	ENSG0000	0.000132	hsa-miR-130a-5p;hsa-miR-23a-3p;hsa-miR-23b-3p;hsa-miR-2
ENSG0000	ENSG0000	5.00E-05	hsa-miR-27a-3p;hsa-miR-27b-3p;hsa-miR-320a;hsa-miR-320b;
ENSG0000	ENSG0000	0.003086	hsa-miR-129-5p;hsa-miR-130a-5p;hsa-miR-23a-3p;hsa-miR-2
ENSG0000	ENSG0000	0.001889	hsa-miR-130a-5p;hsa-miR-23a-3p;hsa-miR-23b-3p;hsa-miR-2
ENSG0000	ENSG0000	9.81E-05	hsa-miR-130a-5p;hsa-miR-23a-3p;hsa-miR-23b-3p;hsa-miR-2
ENSG0000	ENSG0000	4.85E-07	hsa-let-7a-5p;hsa-let-7b-5p;hsa-let-7d-5p;hsa-let-7e-5p;hsa-l
ENSG0000	ENSG0000	5.81E-05	hsa-miR-181a-5p;hsa-miR-181b-5p;hsa-miR-181c-5p;hsa-miR-
ENSG0000	ENSG0000	1.62E-06	hsa-let-7a-5p;hsa-let-7b-5p;hsa-let-7d-5p;hsa-let-7e-5p;hsa-l
ENSG0000	ENSG0000	4.99E-05	hsa-miR-320a;hsa-miR-320b;hsa-miR-320d;hsa-miR-4429
ENSG0000	ENSG0000	0.000293	hsa-let-7a-5p;hsa-let-7b-5p;hsa-let-7d-5p;hsa-let-7e-5p;hsa-l
ENSG0000	ENSG0000	0.000571	hsa-let-7a-5p;hsa-let-7b-5p;hsa-let-7d-5p;hsa-let-7e-5p;hsa-l
ENSG0000	ENSG0000	9.33E-08	hsa-let-7a-5p;hsa-let-7b-5p;hsa-let-7d-5p;hsa-let-7e-5p;hsa-l
ENSG0000	ENSG0000	0.002467	hsa-let-7a-5p;hsa-let-7d-5p;hsa-let-7e-5p;hsa-let-7f-5p;hsa-le
ENSG0000	ENSG0000	3.56E-07	hsa-let-7a-5p;hsa-let-7b-5p;hsa-let-7d-5p;hsa-let-7e-5p;hsa-l
ENSG0000	ENSG0000	9.33E-09	hsa-let-7a-5p;hsa-let-7b-5p;hsa-let-7d-5p;hsa-let-7e-5p;hsa-l
ENSG0000	ENSG0000	0.009414	hsa-miR-142-3p;hsa-miR-27a-3p;hsa-miR-27b-3p;hsa-miR-51
ENSG0000	ENSG0000	0.001617	hsa-miR-129-5p;hsa-miR-378a-3p;hsa-miR-378c;hsa-miR-378
ENSG0000	ENSG0000	0.005182	hsa-miR-130a-5p;hsa-miR-23a-3p;hsa-miR-23b-3p;hsa-miR-2
ENSG0000	ENSG0000	0.000124	hsa-let-7a-5p;hsa-let-7b-5p;hsa-let-7d-5p;hsa-let-7e-5p;hsa-l
ENSG0000	ENSG0000	5.38E-06	hsa-let-7a-5p;hsa-let-7b-5p;hsa-let-7d-5p;hsa-let-7e-5p;hsa-l
ENSG0000	ENSG0000	0.007749	hsa-let-7a-5p;hsa-let-7b-5p;hsa-let-7d-5p;hsa-let-7e-5p;hsa-l
ENSG0000	ENSG0000	6.36E-05	hsa-let-7a-5p;hsa-let-7b-5p;hsa-let-7d-5p;hsa-let-7e-5p;hsa-l
ENSG0000	ENSG0000	0.002971	hsa-let-7a-5p;hsa-let-7b-5p;hsa-let-7d-5p;hsa-let-7e-5p;hsa-l
ENSG0000	ENSG0000	0.002434	hsa-miR-320a;hsa-miR-320b;hsa-miR-320d;hsa-miR-4429
ENSG0000	ENSG0000	0.002434	hsa-miR-320a;hsa-miR-320b;hsa-miR-320d;hsa-miR-4429
ENSG0000	ENSG0000	0.003441	hsa-miR-320a;hsa-miR-320b;hsa-miR-320d;hsa-miR-4429
ENSG0000	ENSG0000	4.48E-05	hsa-miR-106b-5p;hsa-miR-20a-5p;hsa-miR-26a-5p;hsa-miR-2
ENSG0000	ENSG0000	0.000731	hsa-miR-320a;hsa-miR-320b;hsa-miR-320d;hsa-miR-4429
ENSG0000	ENSG0000	0.00817	hsa-miR-320a;hsa-miR-320b;hsa-miR-320d;hsa-miR-4429
ENSG0000	ENSG0000	5.91E-05	hsa-miR-103a-3p;hsa-miR-32-5p;hsa-miR-449a;hsa-miR-92a-
ENSG0000	ENSG0000	0.003527	hsa-miR-106b-5p;hsa-miR-20a-5p;hsa-miR-519a-3p;hsa-miR-
ENSG0000	ENSG0000	2.26E-05	hsa-miR-137;hsa-miR-200c-3p;hsa-miR-26a-5p;hsa-miR-26b-
ENSG0000	ENSG0000	0.008573	hsa-miR-106b-5p;hsa-miR-20a-5p;hsa-miR-378a-5p;hsa-miR-
ENSG0000	ENSG0000	0.004401	hsa-miR-21-3p;hsa-miR-32-5p;hsa-miR-34a-5p;hsa-miR-92a-
ENSG0000	ENSG0000	0.002135	hsa-miR-106b-5p;hsa-miR-124-3p;hsa-miR-20a-5p;hsa-miR-4
ENSG0000	ENSG0000	0.000173	hsa-miR-320a;hsa-miR-320b;hsa-miR-320d;hsa-miR-4429;hsa-
ENSG0000	ENSG0000	1.69E-05	hsa-miR-200b-3p;hsa-miR-200c-3p;hsa-miR-34a-5p;hsa-miR-
ENSG0000	ENSG0000	4.72E-07	hsa-miR-182-5p;hsa-miR-200b-3p;hsa-miR-200c-3p;hsa-miR-
ENSG0000	ENSG0000	0.005201	hsa-miR-192-5p;hsa-miR-194-5p;hsa-miR-200b-3p;hsa-miR-2
ENSG0000	ENSG0000	0.000314	hsa-miR-106b-5p;hsa-miR-194-5p;hsa-miR-20a-5p;hsa-miR-3
ENSG0000	ENSG0000	0.009494	hsa-miR-10b-3p;hsa-miR-30a-3p;hsa-miR-30d-3p;hsa-miR-30

ENSG0000	ENSG0000	0.009494	hsa-miR-29a-3p;hsa-miR-29b-3p;hsa-miR-29c-3p;hsa-miR-34a
ENSG0000	ENSG0000	3.32E-06	hsa-miR-103a-3p;hsa-miR-135a-5p;hsa-miR-135b-5p;hsa-miR-
ENSG0000	ENSG0000	2.90E-05	hsa-miR-1271-5p;hsa-miR-182-5p;hsa-miR-187-5p;hsa-miR-2
ENSG0000	ENSG0000	0.004401	hsa-miR-26a-5p;hsa-miR-32-5p;hsa-miR-548ap-3p;hsa-miR-9
ENSG0000	ENSG0000	0.004343	hsa-miR-10a-3p;hsa-miR-187-5p;hsa-miR-221-3p;hsa-miR-22
ENSG0000	ENSG0000	7.80E-06	hsa-miR-107;hsa-miR-137;hsa-miR-21-3p;hsa-miR-29a-3p;hsa-
ENSG0000	ENSG0000	5.58E-07	hsa-miR-107;hsa-miR-124-3p;hsa-miR-137;hsa-miR-187-5p;hs
ENSG0000	ENSG0000	0.002774	hsa-miR-187-5p;hsa-miR-200c-3p;hsa-miR-221-3p;hsa-miR-22
ENSG0000	ENSG0000	0.001542	hsa-miR-1299;hsa-miR-519a-3p;hsa-miR-519b-3p;hsa-miR-51
ENSG0000	ENSG0000	0.00214	hsa-miR-29a-3p;hsa-miR-29b-3p;hsa-miR-29c-3p;hsa-miR-42
ENSG0000	ENSG0000	0.002489	hsa-miR-519a-3p;hsa-miR-519b-3p;hsa-miR-519c-3p;hsa-miR-
ENSG0000	ENSG0000	0.002465	hsa-miR-106b-5p;hsa-miR-20a-5p;hsa-miR-3065-5p;hsa-miR-
ENSG0000	ENSG0000	2.87E-05	hsa-miR-106b-5p;hsa-miR-20a-5p;hsa-miR-26a-5p;hsa-miR-2
ENSG0000	ENSG0000	0.000221	hsa-miR-194-5p;hsa-miR-29a-3p;hsa-miR-29b-3p;hsa-miR-29
ENSG0000	ENSG0000	5.91E-05	hsa-miR-124-3p;hsa-miR-29a-3p;hsa-miR-29b-3p;hsa-miR-29
ENSG0000	ENSG0000	0.000741	hsa-miR-132-3p;hsa-miR-194-5p;hsa-miR-30a-3p;hsa-miR-30
ENSG0000	ENSG0000	0.005201	hsa-miR-30a-3p;hsa-miR-30d-3p;hsa-miR-30e-3p;hsa-miR-55
ENSG0000	ENSG0000	0.001791	hsa-miR-106b-5p;hsa-miR-200b-3p;hsa-miR-20a-5p;hsa-miR-
ENSG0000	ENSG0000	0.001551	hsa-miR-132-3p;hsa-miR-135a-5p;hsa-miR-137;hsa-miR-200a-
ENSG0000	ENSG0000	2.66E-07	hsa-miR-106b-5p;hsa-miR-141-3p;hsa-miR-200a-3p;hsa-miR-
ENSG0000	ENSG0000	0.002027	hsa-miR-29b-3p;hsa-miR-519a-3p;hsa-miR-519b-3p;hsa-miR-
ENSG0000	ENSG0000	9.72E-05	hsa-miR-182-5p;hsa-miR-194-5p;hsa-miR-200b-3p;hsa-miR-2
ENSG0000	ENSG0000	0.003489	hsa-miR-106b-5p;hsa-miR-20a-5p;hsa-miR-29a-3p;hsa-miR-2
ENSG0000	ENSG0000	0.001235	hsa-miR-192-5p;hsa-miR-26a-5p;hsa-miR-29b-3p;hsa-miR-51
ENSG0000	ENSG0000	0.00982	hsa-miR-106b-5p;hsa-miR-20a-5p;hsa-miR-520a-3p;hsa-miR-
ENSG0000	ENSG0000	0.00214	hsa-miR-106b-5p;hsa-miR-20a-5p;hsa-miR-519a-3p;hsa-miR-
ENSG0000	ENSG0000	4.54E-06	hsa-miR-29a-3p;hsa-miR-29b-3p;hsa-miR-29c-3p;hsa-miR-40
ENSG0000	ENSG0000	0.002027	hsa-miR-29a-3p;hsa-miR-29b-3p;hsa-miR-29c-3p;hsa-miR-40
ENSG0000	ENSG0000	0.003688	hsa-miR-29a-3p;hsa-miR-29b-3p;hsa-miR-29c-3p;hsa-miR-40
ENSG0000	ENSG0000	4.59E-05	hsa-miR-1271-5p;hsa-miR-130b-5p;hsa-miR-135a-5p;hsa-miR-
ENSG0000	ENSG0000	0.000149	hsa-miR-187-5p;hsa-miR-221-3p;hsa-miR-222-3p;hsa-miR-29
ENSG0000	ENSG0000	1.33E-05	hsa-miR-30a-3p;hsa-miR-30d-3p;hsa-miR-30e-3p;hsa-miR-54
ENSG0000	ENSG0000	4.06E-08	hsa-miR-187-5p;hsa-miR-221-3p;hsa-miR-222-3p;hsa-miR-26
ENSG0000	ENSG0000	0.001551	hsa-miR-21-3p;hsa-miR-421;hsa-miR-449a;hsa-miR-449b-5p;h
ENSG0000	ENSG0000	9.75E-05	hsa-miR-106b-5p;hsa-miR-135a-5p;hsa-miR-135b-5p;hsa-miR
ENSG0000	ENSG0000	0.000741	hsa-miR-30a-3p;hsa-miR-30d-3p;hsa-miR-30e-3p;hsa-miR-33
ENSG0000	ENSG0000	0.002525	hsa-miR-221-3p;hsa-miR-519a-3p;hsa-miR-519b-3p;hsa-miR-
ENSG0000	ENSG0000	0.00063	hsa-miR-26b-5p;hsa-miR-29a-3p;hsa-miR-29b-3p;hsa-miR-33
ENSG0000	ENSG0000	0.001615	hsa-miR-124-3p;hsa-miR-137;hsa-miR-32-5p;hsa-miR-92a-3p
ENSG0000	ENSG0000	4.34E-05	hsa-miR-124-3p;hsa-miR-4708-3p;hsa-miR-519a-3p;hsa-miR-
ENSG0000	ENSG0000	0.001359	hsa-miR-106b-5p;hsa-miR-20a-5p;hsa-miR-26b-5p;hsa-miR-3
ENSG0000	ENSG0000	0.001306	hsa-miR-519a-3p;hsa-miR-519b-3p;hsa-miR-519c-3p;hsa-miR-
ENSG0000	ENSG0000	0.000483	hsa-miR-519a-3p;hsa-miR-519b-3p;hsa-miR-519c-3p;hsa-miR-
ENSG0000	ENSG0000	4.14E-05	hsa-miR-20a-5p;hsa-miR-519a-3p;hsa-miR-519b-3p;hsa-miR-
ENSG0000	ENSG0000	2.50E-05	hsa-miR-10a-3p;hsa-miR-124-3p;hsa-miR-3065-5p;hsa-miR-4
ENSG0000	ENSG0000	0.000341	hsa-miR-106b-5p;hsa-miR-20a-5p;hsa-miR-26b-5p;hsa-miR-3
ENSG0000	ENSG0000	0.00817	hsa-miR-221-3p;hsa-miR-222-3p;hsa-miR-32-5p;hsa-miR-92a-
ENSG0000	ENSG0000	0.001988	hsa-miR-200b-3p;hsa-miR-200c-3p;hsa-miR-320a;hsa-miR-320
ENSG0000	ENSG0000	0.000129	hsa-miR-320a;hsa-miR-320b;hsa-miR-320d;hsa-miR-32-5p;hsa
ENSG0000	ENSG0000	0.000221	hsa-miR-20a-5p;hsa-miR-519a-3p;hsa-miR-519b-3p;hsa-miR-
ENSG0000	ENSG0000	0.002747	hsa-miR-106b-5p;hsa-miR-20a-5p;hsa-miR-26a-5p;hsa-miR-2
ENSG0000	ENSG0000	0.000173	hsa-miR-26a-5p;hsa-miR-30a-3p;hsa-miR-30d-3p;hsa-miR-30
ENSG0000	ENSG0000	0.007715	hsa-miR-106b-5p;hsa-miR-20a-5p;hsa-miR-3065-5p;hsa-miR-
ENSG0000	ENSG0000	0.005201	hsa-miR-106b-5p;hsa-miR-20a-5p;hsa-miR-520a-3p;hsa-miR-

ENSG0000	ENSG0000	0.002434	hsa-miR-106b-5p;hsa-miR-20a-5p;hsa-miR-519a-3p;hsa-miR-5
ENSG0000	ENSG0000	0.000534	hsa-miR-200b-3p;hsa-miR-30a-3p;hsa-miR-30d-3p;hsa-miR-30
ENSG0000	ENSG0000	0.004343	hsa-miR-221-3p;hsa-miR-222-3p;hsa-miR-30a-3p;hsa-miR-30
ENSG0000	ENSG0000	0.000918	hsa-miR-135a-5p;hsa-miR-135b-5p;hsa-miR-200b-3p;hsa-miR
ENSG0000	ENSG0000	0.000918	hsa-miR-135a-5p;hsa-miR-135b-5p;hsa-miR-200b-3p;hsa-miR
ENSG0000	ENSG0000	0.007081	hsa-miR-192-5p;hsa-miR-26a-5p;hsa-miR-26b-5p;hsa-miR-29
ENSG0000	ENSG0000	0.000128	hsa-miR-124-3p;hsa-miR-26a-5p;hsa-miR-26b-5p;hsa-miR-32
ENSG0000	ENSG0000	0.000269	hsa-miR-26a-5p;hsa-miR-26b-5p;hsa-miR-519a-3p;hsa-miR-51
ENSG0000	ENSG0000	4.63E-08	hsa-miR-106b-5p;hsa-miR-141-3p;hsa-miR-200a-3p;hsa-miR-
ENSG0000	ENSG0000	1.60E-05	hsa-miR-103a-3p;hsa-miR-107;hsa-miR-423-5p;hsa-miR-519a-
ENSG0000	ENSG0000	0.000195	hsa-miR-103a-3p;hsa-miR-107;hsa-miR-141-3p;hsa-miR-200a-
ENSG0000	ENSG0000	0.001091	hsa-miR-340-5p;hsa-miR-34a-5p;hsa-miR-449a;hsa-miR-449b-
ENSG0000	ENSG0000	0.000741	hsa-miR-519a-3p;hsa-miR-519b-3p;hsa-miR-519c-3p;hsa-miR-
ENSG0000	ENSG0000	0.009364	hsa-miR-137;hsa-miR-182-5p;hsa-miR-340-5p;hsa-miR-96-5p
ENSG0000	ENSG0000	0.007081	hsa-miR-4698;hsa-miR-519a-3p;hsa-miR-519b-3p;hsa-miR-51
ENSG0000	ENSG0000	0.000882	hsa-miR-103a-3p;hsa-miR-124-3p;hsa-miR-29a-3p;hsa-miR-3
ENSG0000	ENSG0000	0.002878	hsa-miR-106b-5p;hsa-miR-20a-5p;hsa-miR-32-5p;hsa-miR-52
ENSG0000	ENSG0000	0.001615	hsa-miR-212-5p;hsa-miR-29a-3p;hsa-miR-29b-3p;hsa-miR-29
ENSG0000	ENSG0000	0.002027	hsa-miR-124-3p;hsa-miR-192-5p;hsa-miR-29b-3p;hsa-miR-42
ENSG0000	ENSG0000	3.09E-06	hsa-miR-106b-5p;hsa-miR-20a-5p;hsa-miR-32-5p;hsa-miR-34
ENSG0000	ENSG0000	0.00112	hsa-miR-137;hsa-miR-200b-3p;hsa-miR-200c-3p;hsa-miR-519
ENSG0000	ENSG0000	1.86E-05	hsa-miR-103a-3p;hsa-miR-107;hsa-miR-29a-3p;hsa-miR-29b-
ENSG0000	ENSG0000	0.004584	hsa-miR-106b-5p;hsa-miR-200b-3p;hsa-miR-200c-3p;hsa-miR-
ENSG0000	ENSG0000	0.002414	hsa-miR-103a-3p;hsa-miR-107;hsa-miR-10b-3p;hsa-miR-34a-
ENSG0000	ENSG0000	0.007715	hsa-miR-519a-3p;hsa-miR-519b-3p;hsa-miR-519c-3p;hsa-miR-
ENSG0000	ENSG0000	0.003535	hsa-miR-106b-5p;hsa-miR-20a-5p;hsa-miR-32-5p;hsa-miR-51
ENSG0000	ENSG0000	2.50E-05	hsa-miR-10a-3p;hsa-miR-124-3p;hsa-miR-1271-5p;hsa-miR-1
ENSG0000	ENSG0000	0.000904	hsa-miR-29a-3p;hsa-miR-29b-3p;hsa-miR-29c-3p;hsa-miR-31
ENSG0000	ENSG0000	1.69E-07	hsa-miR-103a-3p;hsa-miR-106b-5p;hsa-miR-107;hsa-miR-141-
ENSG0000	ENSG0000	1.69E-07	hsa-miR-103a-3p;hsa-miR-106b-5p;hsa-miR-107;hsa-miR-141-
ENSG0000	ENSG0000	0.000424	hsa-miR-106b-5p;hsa-miR-200b-3p;hsa-miR-200c-3p;hsa-miR-
ENSG0000	ENSG0000	0.005201	hsa-miR-103a-3p;hsa-miR-107;hsa-miR-32-5p;hsa-miR-92a-3
ENSG0000	ENSG0000	4.14E-05	hsa-miR-106b-5p;hsa-miR-132-3p;hsa-miR-141-3p;hsa-miR-1
ENSG0000	ENSG0000	0.001265	hsa-miR-103a-3p;hsa-miR-107;hsa-miR-520a-3p;hsa-miR-520
ENSG0000	ENSG0000	0.001918	hsa-miR-194-5p;hsa-miR-221-3p;hsa-miR-222-3p;hsa-miR-30
ENSG0000	ENSG0000	0.000483	hsa-miR-106b-5p;hsa-miR-20a-5p;hsa-miR-519a-3p;hsa-miR-5
ENSG0000	ENSG0000	0.000173	hsa-miR-1271-5p;hsa-miR-182-5p;hsa-miR-21-3p;hsa-miR-42
ENSG0000	ENSG0000	0.003688	hsa-miR-320a;hsa-miR-320b;hsa-miR-320d;hsa-miR-4429
ENSG0000	ENSG0000	0.003688	hsa-miR-320a;hsa-miR-320b;hsa-miR-320d;hsa-miR-4429
ENSG0000	ENSG0000	0.003919	hsa-miR-103a-3p;hsa-miR-107;hsa-miR-10b-3p;hsa-miR-33a-
ENSG0000	ENSG0000	0.008573	hsa-miR-106b-5p;hsa-miR-20a-5p;hsa-miR-519a-3p;hsa-miR-5
ENSG0000	ENSG0000	0.00529	hsa-miR-1271-5p;hsa-miR-182-5p;hsa-miR-34a-5p;hsa-miR-5
ENSG0000	ENSG0000	0.001265	hsa-miR-141-3p;hsa-miR-26a-5p;hsa-miR-26b-5p;hsa-miR-29
ENSG0000	ENSG0000	9.71E-07	hsa-miR-106b-5p;hsa-miR-10b-3p;hsa-miR-187-5p;hsa-miR-2
ENSG0000	ENSG0000	0.002504	hsa-miR-124-3p;hsa-miR-29a-3p;hsa-miR-29b-3p;hsa-miR-29
ENSG0000	ENSG0000	0.002504	hsa-miR-124-3p;hsa-miR-29a-3p;hsa-miR-29b-3p;hsa-miR-29
ENSG0000	ENSG0000	0.003688	hsa-miR-34a-5p;hsa-miR-449a;hsa-miR-449b-5p;hsa-miR-548
ENSG0000	ENSG0000	0.006093	hsa-miR-30a-3p;hsa-miR-30d-3p;hsa-miR-30e-3p;hsa-miR-37
ENSG0000	ENSG0000	4.98E-10	hsa-miR-26a-5p;hsa-miR-26b-5p;hsa-miR-33a-3p;hsa-miR-34
ENSG0000	ENSG0000	0.003165	hsa-miR-130b-5p;hsa-miR-30a-3p;hsa-miR-30d-3p;hsa-miR-30
ENSG0000	ENSG0000	0.003688	hsa-miR-320a;hsa-miR-320b;hsa-miR-320d;hsa-miR-4429
ENSG0000	ENSG0000	0.001161	hsa-miR-106b-5p;hsa-miR-132-3p;hsa-miR-200b-3p;hsa-miR-
ENSG0000	ENSG0000	7.94E-05	hsa-miR-106b-5p;hsa-miR-200b-3p;hsa-miR-200c-3p;hsa-miR-
ENSG0000	ENSG0000	0.00214	hsa-miR-141-3p;hsa-miR-200a-3p;hsa-miR-200b-3p;hsa-miR-

ENSG0000	ENSG0000	3.63E-05	hsa-miR-1271-5p;hsa-miR-1299;hsa-miR-182-5p;hsa-miR-34a-
ENSG0000	ENSG0000	3.63E-05	hsa-miR-1271-5p;hsa-miR-1299;hsa-miR-182-5p;hsa-miR-34a-
ENSG0000	ENSG0000	3.63E-05	hsa-miR-1271-5p;hsa-miR-1299;hsa-miR-182-5p;hsa-miR-34a-
ENSG0000	ENSG0000	3.63E-05	hsa-miR-1271-5p;hsa-miR-1299;hsa-miR-182-5p;hsa-miR-34a-
ENSG0000	ENSG0000	3.63E-05	hsa-miR-1271-5p;hsa-miR-1299;hsa-miR-182-5p;hsa-miR-34a-
ENSG0000	ENSG0000	3.63E-05	hsa-miR-1271-5p;hsa-miR-1299;hsa-miR-182-5p;hsa-miR-34a-
ENSG0000	ENSG0000	3.63E-05	hsa-miR-1271-5p;hsa-miR-1299;hsa-miR-182-5p;hsa-miR-34a-
ENSG0000	ENSG0000	3.63E-05	hsa-miR-1271-5p;hsa-miR-1299;hsa-miR-182-5p;hsa-miR-34a-
ENSG0000	ENSG0000	0.002027	hsa-miR-10b-3p;hsa-miR-29a-3p;hsa-miR-29b-3p;hsa-miR-29
ENSG0000	ENSG0000	0.009494	hsa-miR-519a-3p;hsa-miR-519b-3p;hsa-miR-519c-3p;hsa-miR-
ENSG0000	ENSG0000	4.48E-05	hsa-miR-320a;hsa-miR-320b;hsa-miR-320d;hsa-miR-34a-5p;hs
ENSG0000	ENSG0000	0.004307	hsa-miR-30a-3p;hsa-miR-30d-3p;hsa-miR-30e-3p;hsa-miR-33
ENSG0000	ENSG0000	0.000136	hsa-miR-106b-5p;hsa-miR-10a-3p;hsa-miR-20a-5p;hsa-miR-3
ENSG0000	ENSG0000	0.002507	hsa-miR-106b-5p;hsa-miR-519a-3p;hsa-miR-519b-3p;hsa-miR
ENSG0000	ENSG0000	0.008573	hsa-miR-106b-5p;hsa-miR-20a-5p;hsa-miR-519a-3p;hsa-miR-
ENSG0000	ENSG0000	3.39E-05	hsa-miR-10a-3p;hsa-miR-141-3p;hsa-miR-182-5p;hsa-miR-20
ENSG0000	ENSG0000	0.001514	hsa-miR-106b-5p;hsa-miR-107;hsa-miR-200b-3p;hsa-miR-200
ENSG0000	ENSG0000	0.007081	hsa-miR-141-3p;hsa-miR-30a-3p;hsa-miR-30d-3p;hsa-miR-30
ENSG0000	ENSG0000	0.005201	hsa-miR-30a-3p;hsa-miR-30d-3p;hsa-miR-30e-3p;hsa-miR-32-
ENSG0000	ENSG0000	0.006183	hsa-miR-106b-5p;hsa-miR-20a-5p;hsa-miR-212-5p;hsa-miR-5
ENSG0000	ENSG0000	0.003535	hsa-miR-26a-5p;hsa-miR-26b-5p;hsa-miR-30a-3p;hsa-miR-30
ENSG0000	ENSG0000	0.000882	hsa-miR-320a;hsa-miR-320b;hsa-miR-320d;hsa-miR-374a-5p;h
ENSG0000	ENSG0000	0.005201	hsa-miR-106b-5p;hsa-miR-20a-5p;hsa-miR-376a-3p;hsa-miR-
ENSG0000	ENSG0000	0.002489	hsa-miR-1271-5p;hsa-miR-182-5p;hsa-miR-301a-5p;hsa-miR-
ENSG0000	ENSG0000	0.00023	hsa-miR-106b-5p;hsa-miR-20a-5p;hsa-miR-519a-3p;hsa-miR-
ENSG0000	ENSG0000	1.69E-05	hsa-miR-32-5p;hsa-miR-33a-3p;hsa-miR-409-3p;hsa-miR-519
ENSG0000	ENSG0000	0.000269	hsa-miR-106b-5p;hsa-miR-187-5p;hsa-miR-20a-5p;hsa-miR-5
ENSG0000	ENSG0000	0.000552	hsa-miR-106b-5p;hsa-miR-20a-5p;hsa-miR-409-3p;hsa-miR-5
ENSG0000	ENSG0000	2.84E-06	hsa-miR-135b-5p;hsa-miR-200b-3p;hsa-miR-200c-3p;hsa-miR-
ENSG0000	ENSG0000	0.001641	hsa-miR-107;hsa-miR-10a-3p;hsa-miR-194-5p;hsa-miR-26a-5
ENSG0000	ENSG0000	0.00817	hsa-miR-30a-3p;hsa-miR-30d-3p;hsa-miR-30e-3p;hsa-miR-33
ENSG0000	ENSG0000	0.008573	hsa-miR-3194-3p;hsa-miR-34a-5p;hsa-miR-449a;hsa-miR-449
ENSG0000	ENSG0000	0.003527	hsa-miR-1271-5p;hsa-miR-135b-5p;hsa-miR-222-3p;hsa-miR-
ENSG0000	ENSG0000	0.002747	hsa-miR-187-5p;hsa-miR-221-3p;hsa-miR-222-3p;hsa-miR-34
ENSG0000	ENSG0000	6.12E-05	hsa-miR-106b-5p;hsa-miR-20a-5p;hsa-miR-320a;hsa-miR-320
ENSG0000	ENSG0000	6.12E-05	hsa-miR-106b-5p;hsa-miR-20a-5p;hsa-miR-320a;hsa-miR-320
ENSG0000	ENSG0000	0.00763	hsa-miR-106b-5p;hsa-miR-20a-5p;hsa-miR-212-5p;hsa-miR-3
ENSG0000	ENSG0000	0.000741	hsa-miR-182-5p;hsa-miR-212-5p;hsa-miR-29a-3p;hsa-miR-29
ENSG0000	ENSG0000	0.003535	hsa-miR-103a-3p;hsa-miR-106b-5p;hsa-miR-107;hsa-miR-124-
ENSG0000	ENSG0000	0.002507	hsa-miR-221-3p;hsa-miR-222-3p;hsa-miR-26a-5p;hsa-miR-26
ENSG0000	ENSG0000	0.000596	hsa-miR-106b-5p;hsa-miR-141-3p;hsa-miR-200a-3p;hsa-miR-
ENSG0000	ENSG0000	0.002831	hsa-miR-106b-5p;hsa-miR-1271-5p;hsa-miR-182-5p;hsa-miR-
ENSG0000	ENSG0000	0.002507	hsa-miR-320a;hsa-miR-320b;hsa-miR-320d;hsa-miR-4429
ENSG0000	ENSG0000	0.00488	hsa-miR-106b-5p;hsa-miR-20a-5p;hsa-miR-519a-3p;hsa-miR-
ENSG0000	ENSG0000	1.30E-05	hsa-miR-200a-3p;hsa-miR-200b-3p;hsa-miR-200c-3p;hsa-miR-
ENSG0000	ENSG0000	4.98E-05	hsa-miR-124-3p;hsa-miR-1271-5p;hsa-miR-182-5p;hsa-miR-3
ENSG0000	ENSG0000	0.000287	hsa-miR-103a-3p;hsa-miR-106b-5p;hsa-miR-107;hsa-miR-20a-
ENSG0000	ENSG0000	0.000382	hsa-miR-34a-5p;hsa-miR-449a;hsa-miR-449b-5p;hsa-miR-469
ENSG0000	ENSG0000	0.000169	hsa-miR-320a;hsa-miR-320b;hsa-miR-320d;hsa-miR-4429
ENSG0000	ENSG0000	0.000104	hsa-miR-34a-5p;hsa-miR-449a;hsa-miR-449b-5p;hsa-miR-548
ENSG0000	ENSG0000	0.004799	hsa-miR-26a-5p;hsa-miR-26b-5p;hsa-miR-320a;hsa-miR-320b;
ENSG0000	ENSG0000	1.20E-06	hsa-miR-103a-3p;hsa-miR-107;hsa-miR-135a-5p;hsa-miR-29c-
ENSG0000	ENSG0000	0.000882	hsa-miR-106b-5p;hsa-miR-20a-5p;hsa-miR-4698;hsa-miR-519
ENSG0000	ENSG0000	0.000104	hsa-miR-106b-5p;hsa-miR-20a-5p;hsa-miR-221-3p;hsa-miR-2

ENSG0000	ENSG0000	0.000482	hsa-miR-124-3p;hsa-miR-519a-3p;hsa-miR-519b-3p;hsa-miR-[illegible]
ENSG0000	ENSG0000	0.000758	hsa-miR-103a-3p;hsa-miR-107;hsa-miR-340-5p;hsa-miR-519a-
ENSG0000	ENSG0000	9.11E-05	hsa-miR-192-5p;hsa-miR-222-3p;hsa-miR-320a;hsa-miR-340-[illegible]
ENSG0000	ENSG0000	0.004799	hsa-miR-106b-5p;hsa-miR-20a-5p;hsa-miR-212-5p;hsa-miR-3[illegible]
ENSG0000	ENSG0000	0.000226	hsa-miR-103a-3p;hsa-miR-107;hsa-miR-20a-5p;hsa-miR-340-[illegible]
ENSG0000	ENSG0000	0.000882	hsa-miR-103a-3p;hsa-miR-107;hsa-miR-519a-3p;hsa-miR-519[illegible]
ENSG0000	ENSG0000	0.000476	hsa-miR-106b-5p;hsa-miR-20a-5p;hsa-miR-30a-3p;hsa-miR-3[illegible]
ENSG0000	ENSG0000	0.000166	hsa-miR-137;hsa-miR-29a-3p;hsa-miR-29b-3p;hsa-miR-29c-3[illegible]
ENSG0000	ENSG0000	1.30E-05	hsa-miR-106b-5p;hsa-miR-20a-5p;hsa-miR-340-5p;hsa-miR-4[illegible]
ENSG0000	ENSG0000	0.003527	hsa-miR-106b-5p;hsa-miR-20a-5p;hsa-miR-423-5p;hsa-miR-5[illegible]
ENSG0000	ENSG0000	0.00763	hsa-miR-106b-5p;hsa-miR-20a-5p;hsa-miR-4698;hsa-miR-519[illegible]
ENSG0000	ENSG0000	0.000535	hsa-miR-130b-5p;hsa-miR-30a-3p;hsa-miR-30d-3p;hsa-miR-3[illegible]
ENSG0000	ENSG0000	0.000535	hsa-miR-130b-5p;hsa-miR-30a-3p;hsa-miR-30d-3p;hsa-miR-3[illegible]
ENSG0000	ENSG0000	0.000332	hsa-miR-124-3p;hsa-miR-20a-5p;hsa-miR-26a-5p;hsa-miR-26[illegible]
ENSG0000	ENSG0000	0.000535	hsa-miR-141-3p;hsa-miR-200a-3p;hsa-miR-200b-3p;hsa-miR-[illegible]
ENSG0000	ENSG0000	0.000293	hsa-miR-106b-5p;hsa-miR-187-5p;hsa-miR-20a-5p;hsa-miR-2[illegible]
ENSG0000	ENSG0000	0.001091	hsa-miR-519a-3p;hsa-miR-519b-3p;hsa-miR-519c-3p;hsa-miR-
ENSG0000	ENSG0000	0.000552	hsa-miR-221-3p;hsa-miR-222-3p;hsa-miR-29a-3p;hsa-miR-29[illegible]
ENSG0000	ENSG0000	0.000552	hsa-miR-221-3p;hsa-miR-222-3p;hsa-miR-29a-3p;hsa-miR-29[illegible]
ENSG0000	ENSG0000	4.68E-06	hsa-miR-106b-5p;hsa-miR-200b-3p;hsa-miR-200c-3p;hsa-miR-
ENSG0000	ENSG0000	0.000648	hsa-miR-200b-3p;hsa-miR-200c-3p;hsa-miR-20a-5p;hsa-miR-[illegible]
ENSG0000	ENSG0000	0.004211	hsa-miR-106b-5p;hsa-miR-20a-5p;hsa-miR-378a-5p;hsa-miR-[illegible]
ENSG0000	ENSG0000	0.001057	hsa-miR-106b-5p;hsa-miR-20a-5p;hsa-miR-29a-3p;hsa-miR-2[illegible]
ENSG0000	ENSG0000	0.00214	hsa-miR-137;hsa-miR-30a-3p;hsa-miR-30d-3p;hsa-miR-30e-3[illegible]
ENSG0000	ENSG0000	0.003221	hsa-miR-103a-3p;hsa-miR-106b-5p;hsa-miR-107;hsa-miR-130[illegible]
ENSG0000	ENSG0000	0.000269	hsa-miR-106b-5p;hsa-miR-20a-5p;hsa-miR-32-3p;hsa-miR-33[illegible]
ENSG0000	ENSG0000	0.000269	hsa-miR-106b-5p;hsa-miR-20a-5p;hsa-miR-32-3p;hsa-miR-33[illegible]
ENSG0000	ENSG0000	0.000129	hsa-miR-137;hsa-miR-21-3p;hsa-miR-320a;hsa-miR-320b;hsa-[illegible]
ENSG0000	ENSG0000	0.009364	hsa-miR-135a-5p;hsa-miR-135b-5p;hsa-miR-26a-5p;hsa-miR-[illegible]
ENSG0000	ENSG0000	0.005504	hsa-miR-519a-3p;hsa-miR-519b-3p;hsa-miR-519c-3p;hsa-miR-
ENSG0000	ENSG0000	0.006183	hsa-miR-29a-3p;hsa-miR-29b-3p;hsa-miR-29c-3p;hsa-miR-62[illegible]
ENSG0000	ENSG0000	0.00529	hsa-miR-106b-5p;hsa-miR-141-3p;hsa-miR-200a-3p;hsa-miR-[illegible]
ENSG0000	ENSG0000	0.000379	hsa-miR-106b-5p;hsa-miR-10a-3p;hsa-miR-200b-3p;hsa-miR-[illegible]
ENSG0000	ENSG0000	1.76E-07	hsa-miR-1271-5p;hsa-miR-141-3p;hsa-miR-182-5p;hsa-miR-2[illegible]
ENSG0000	ENSG0000	1.97E-05	hsa-miR-106b-5p;hsa-miR-1271-5p;hsa-miR-182-5p;hsa-miR-[illegible]
ENSG0000	ENSG0000	0.000535	hsa-miR-320a;hsa-miR-320b;hsa-miR-320d;hsa-miR-4429
ENSG0000	ENSG0000	0.00135	hsa-miR-124-3p;hsa-miR-20a-5p;hsa-miR-4698;hsa-miR-519a-
ENSG0000	ENSG0000	0.00135	hsa-miR-124-3p;hsa-miR-20a-5p;hsa-miR-4698;hsa-miR-519a-
ENSG0000	ENSG0000	0.006382	hsa-miR-106b-5p;hsa-miR-20a-5p;hsa-miR-212-5p;hsa-miR-3[illegible]
ENSG0000	ENSG0000	1.88E-08	hsa-miR-106b-5p;hsa-miR-187-5p;hsa-miR-20a-5p;hsa-miR-2[illegible]
ENSG0000	ENSG0000	0.000972	hsa-miR-137;hsa-miR-141-3p;hsa-miR-200a-3p;hsa-miR-34a-[illegible]
ENSG0000	ENSG0000	0.000535	hsa-miR-320a;hsa-miR-320b;hsa-miR-320d;hsa-miR-4429
ENSG0000	ENSG0000	0.000169	hsa-miR-320a;hsa-miR-320b;hsa-miR-320d;hsa-miR-4429
ENSG0000	ENSG0000	0.001918	hsa-miR-106b-5p;hsa-miR-20a-5p;hsa-miR-212-5p;hsa-miR-3[illegible]
ENSG0000	ENSG0000	4.72E-07	hsa-miR-103a-3p;hsa-miR-107;hsa-miR-26a-5p;hsa-miR-29a-[illegible]
ENSG0000	ENSG0000	0.000379	hsa-miR-29a-3p;hsa-miR-29b-3p;hsa-miR-29c-3p;hsa-miR-30[illegible]
ENSG0000	ENSG0000	0.000145	hsa-miR-29a-3p;hsa-miR-29b-3p;hsa-miR-29c-3p;hsa-miR-51[illegible]
ENSG0000	ENSG0000	3.22E-05	hsa-miR-187-5p;hsa-miR-221-3p;hsa-miR-222-3p;hsa-miR-34[illegible]
ENSG0000	ENSG0000	0.009494	hsa-miR-187-5p;hsa-miR-221-3p;hsa-miR-222-3p;hsa-miR-26[illegible]
ENSG0000	ENSG0000	4.48E-05	hsa-miR-124-3p;hsa-miR-320a;hsa-miR-320b;hsa-miR-320d;hs[illegible]
ENSG0000	ENSG0000	0.008573	hsa-miR-124-3p;hsa-miR-200b-3p;hsa-miR-200c-3p;hsa-miR-[illegible]
ENSG0000	ENSG0000	0.00268	hsa-miR-200b-3p;hsa-miR-200c-3p;hsa-miR-374a-5p;hsa-miR-
ENSG0000	ENSG0000	0.004401	hsa-miR-187-5p;hsa-miR-222-3p;hsa-miR-99a-3p;hsa-miR-99[illegible]
ENSG0000	ENSG0000	0.001791	hsa-miR-106b-5p;hsa-miR-141-3p;hsa-miR-194-5p;hsa-miR-2[illegible]

ENSG0000	ENSG0000	0.000245	hsa-miR-449a;hsa-miR-449b-5p;hsa-miR-4708-3p;hsa-miR-51
ENSG0000	ENSG0000	1.99E-06	hsa-miR-1271-5p;hsa-miR-182-5p;hsa-miR-192-5p;hsa-miR-3
ENSG0000	ENSG0000	9.57E-05	hsa-miR-200b-3p;hsa-miR-200c-3p;hsa-miR-519a-3p;hsa-miR-
ENSG0000	ENSG0000	0.000129	hsa-miR-200a-3p;hsa-miR-221-3p;hsa-miR-222-3p;hsa-miR-2
ENSG0000	ENSG0000	1.99E-06	hsa-miR-132-3p;hsa-miR-200c-3p;hsa-miR-34a-5p;hsa-miR-4
ENSG0000	ENSG0000	1.33E-05	hsa-miR-141-3p;hsa-miR-200a-3p;hsa-miR-200b-3p;hsa-miR-
ENSG0000	ENSG0000	0.000293	hsa-miR-106b-5p;hsa-miR-20a-5p;hsa-miR-519a-3p;hsa-miR-
ENSG0000	ENSG0000	0.001091	hsa-miR-106b-5p;hsa-miR-20a-5p;hsa-miR-3194-3p;hsa-miR-
ENSG0000	ENSG0000	5.91E-05	hsa-miR-30a-3p;hsa-miR-30d-3p;hsa-miR-30e-3p;hsa-miR-54
ENSG0000	ENSG0000	6.55E-05	hsa-miR-107;hsa-miR-124-3p;hsa-miR-29a-3p;hsa-miR-29b-3
ENSG0000	ENSG0000	0.000274	hsa-miR-106b-5p;hsa-miR-130b-5p;hsa-miR-20a-5p;hsa-miR-
ENSG0000	ENSG0000	0.00495	hsa-miR-106b-5p;hsa-miR-20a-5p;hsa-miR-29b-2-5p;hsa-miR
ENSG0000	ENSG0000	0.007081	hsa-miR-30a-3p;hsa-miR-30d-3p;hsa-miR-30e-3p;hsa-miR-40
ENSG0000	ENSG0000	0.002525	hsa-miR-519a-3p;hsa-miR-519b-3p;hsa-miR-519c-3p;hsa-miR-
ENSG0000	ENSG0000	0.000601	hsa-miR-519a-3p;hsa-miR-519b-3p;hsa-miR-519c-3p;hsa-miR-
ENSG0000	ENSG0000	0.001615	hsa-miR-320a;hsa-miR-320b;hsa-miR-320d;hsa-miR-4429
ENSG0000	ENSG0000	0.000363	hsa-miR-320a;hsa-miR-320b;hsa-miR-320d;hsa-miR-4429;hsa-
ENSG0000	ENSG0000	0.000115	hsa-miR-106b-5p;hsa-miR-20a-5p;hsa-miR-519a-3p;hsa-miR-
ENSG0000	ENSG0000	0.00023	hsa-miR-34a-5p;hsa-miR-449a;hsa-miR-449b-5p;hsa-miR-545-
ENSG0000	ENSG0000	0.001306	hsa-miR-30a-3p;hsa-miR-30d-3p;hsa-miR-30e-3p;hsa-miR-32-
ENSG0000	ENSG0000	0.003919	hsa-miR-340-5p;hsa-miR-519a-3p;hsa-miR-519b-3p;hsa-miR-
ENSG0000	ENSG0000	0.009364	hsa-miR-106b-5p;hsa-miR-20a-5p;hsa-miR-520a-3p;hsa-miR-
ENSG0000	ENSG0000	0.000169	hsa-miR-130b-5p;hsa-miR-30a-3p;hsa-miR-30d-3p;hsa-miR-3
ENSG0000	ENSG0000	5.85E-06	hsa-miR-106b-5p;hsa-miR-124-3p;hsa-miR-20a-5p;hsa-miR-3
ENSG0000	ENSG0000	0.003919	hsa-miR-106b-5p;hsa-miR-20a-5p;hsa-miR-4698;hsa-miR-520
ENSG0000	ENSG0000	0.009494	hsa-miR-10a-3p;hsa-miR-30a-3p;hsa-miR-30d-3p;hsa-miR-30
ENSG0000	ENSG0000	0.000173	hsa-miR-106b-5p;hsa-miR-20a-5p;hsa-miR-519a-3p;hsa-miR-
ENSG0000	ENSG0000	0.003688	hsa-miR-21-3p;hsa-miR-32-3p;hsa-miR-340-5p;hsa-miR-4698
ENSG0000	ENSG0000	0.000173	hsa-miR-132-3p;hsa-miR-421;hsa-miR-449a;hsa-miR-449b-5p;
ENSG0000	ENSG0000	0.000972	hsa-miR-106b-5p;hsa-miR-20a-5p;hsa-miR-502-5p;hsa-miR-9
ENSG0000	ENSG0000	0.00135	hsa-miR-106b-5p;hsa-miR-20a-5p;hsa-miR-340-5p;hsa-miR-5
ENSG0000	ENSG0000	1.69E-07	hsa-miR-106b-5p;hsa-miR-20a-5p;hsa-miR-30a-3p;hsa-miR-3
ENSG0000	ENSG0000	4.48E-05	hsa-miR-106b-5p;hsa-miR-10b-3p;hsa-miR-20a-5p;hsa-miR-3
ENSG0000	ENSG0000	0.009812	hsa-miR-106b-5p;hsa-miR-135a-5p;hsa-miR-135b-5p;hsa-miR
ENSG0000	ENSG0000	8.07E-05	hsa-miR-106b-5p;hsa-miR-20a-5p;hsa-miR-29b-2-5p;hsa-miR
ENSG0000	ENSG0000	0.00692	hsa-miR-141-3p;hsa-miR-200a-3p;hsa-miR-3065-5p;hsa-miR-
ENSG0000	ENSG0000	0.001988	hsa-miR-26a-5p;hsa-miR-26b-5p;hsa-miR-29b-2-5p;hsa-miR-
ENSG0000	ENSG0000	0.001551	hsa-miR-106b-5p;hsa-miR-20a-5p;hsa-miR-520a-3p;hsa-miR-
ENSG0000	ENSG0000	2.90E-05	hsa-miR-200b-3p;hsa-miR-200c-3p;hsa-miR-29a-3p;hsa-miR-
ENSG0000	ENSG0000	0.004307	hsa-miR-124-3p;hsa-miR-29c-3p;hsa-miR-30a-3p;hsa-miR-30
ENSG0000	ENSG0000	0.000476	hsa-miR-132-3p;hsa-miR-26a-5p;hsa-miR-26b-5p;hsa-miR-32
ENSG0000	ENSG0000	0.007715	hsa-miR-130b-5p;hsa-miR-137;hsa-miR-32-5p;hsa-miR-5690;h
ENSG0000	ENSG0000	0.007715	hsa-miR-130b-5p;hsa-miR-137;hsa-miR-32-5p;hsa-miR-5690;h
ENSG0000	ENSG0000	0.007939	hsa-miR-200b-3p;hsa-miR-200c-3p;hsa-miR-221-3p;hsa-miR-
ENSG0000	ENSG0000	1.97E-05	hsa-miR-135a-5p;hsa-miR-135b-5p;hsa-miR-374a-5p;hsa-miR
ENSG0000	ENSG0000	0.000149	hsa-miR-124-3p;hsa-miR-200b-3p;hsa-miR-200c-3p;hsa-miR-
ENSG0000	ENSG0000	0.000149	hsa-miR-124-3p;hsa-miR-200b-3p;hsa-miR-200c-3p;hsa-miR-
ENSG0000	ENSG0000	0.007715	hsa-miR-106b-5p;hsa-miR-20a-5p;hsa-miR-519a-3p;hsa-miR-
ENSG0000	ENSG0000	0.005201	hsa-miR-320a;hsa-miR-320b;hsa-miR-320d;hsa-miR-4429
ENSG0000	ENSG0000	0.00239	hsa-miR-106b-5p;hsa-miR-1299;hsa-miR-20a-5p;hsa-miR-320
ENSG0000	ENSG0000	0.000551	hsa-miR-107;hsa-miR-141-3p;hsa-miR-200a-3p;hsa-miR-26a-
ENSG0000	ENSG0000	0.000299	hsa-miR-106b-5p;hsa-miR-20a-5p;hsa-miR-519a-3p;hsa-miR-
ENSG0000	ENSG0000	0.001542	hsa-miR-200a-3p;hsa-miR-519a-3p;hsa-miR-519b-3p;hsa-miR
ENSG0000	ENSG0000	7.04E-05	hsa-miR-106b-5p;hsa-miR-130b-5p;hsa-miR-200b-3p;hsa-miR

ENSG0000	ENSG0000	0.001619	hsa-miR-106b-5p;hsa-miR-1299;hsa-miR-519a-3p;hsa-miR-519
ENSG0000	ENSG0000	0.001265	hsa-miR-106b-5p;hsa-miR-141-3p;hsa-miR-200a-3p;hsa-miR-2
ENSG0000	ENSG0000	0.001265	hsa-miR-106b-5p;hsa-miR-141-3p;hsa-miR-200a-3p;hsa-miR-2
ENSG0000	ENSG0000	9.90E-06	hsa-miR-187-5p;hsa-miR-222-3p;hsa-miR-519a-3p;hsa-miR-51
ENSG0000	ENSG0000	0.008573	hsa-miR-106b-5p;hsa-miR-20a-5p;hsa-miR-212-5p;hsa-miR-52
ENSG0000	ENSG0000	2.50E-05	hsa-miR-106b-5p;hsa-miR-141-3p;hsa-miR-200a-3p;hsa-miR-2
ENSG0000	ENSG0000	0.00692	hsa-miR-106b-5p;hsa-miR-20a-5p;hsa-miR-519a-3p;hsa-miR-5
ENSG0000	ENSG0000	3.39E-05	hsa-miR-106b-5p;hsa-miR-20a-5p;hsa-miR-34a-5p;hsa-miR-44
ENSG0000	ENSG0000	0.002189	hsa-miR-106b-5p;hsa-miR-130b-5p;hsa-miR-20a-5p;hsa-miR-3
ENSG0000	ENSG0000	0.000535	hsa-miR-137;hsa-miR-32-3p;hsa-miR-32-5p;hsa-miR-92a-3p
ENSG0000	ENSG0000	0.001301	hsa-miR-200b-3p;hsa-miR-200c-3p;hsa-miR-519a-3p;hsa-miR-
ENSG0000	ENSG0000	0.000269	hsa-miR-32-5p;hsa-miR-34a-5p;hsa-miR-449a;hsa-miR-449b-5
ENSG0000	ENSG0000	0.000269	hsa-miR-32-5p;hsa-miR-34a-5p;hsa-miR-449a;hsa-miR-449b-5
ENSG0000	ENSG0000	0.002314	hsa-miR-106b-5p;hsa-miR-141-3p;hsa-miR-200a-3p;hsa-miR-2
ENSG0000	ENSG0000	0.001619	hsa-miR-106b-5p;hsa-miR-187-5p;hsa-miR-20a-5p;hsa-miR-22
ENSG0000	ENSG0000	0.000379	hsa-miR-29a-3p;hsa-miR-29b-3p;hsa-miR-29c-3p;hsa-miR-548
ENSG0000	ENSG0000	0.000376	hsa-miR-106b-5p;hsa-miR-124-3p;hsa-miR-20a-5p;hsa-miR-51
ENSG0000	ENSG0000	0.001453	hsa-miR-106b-5p;hsa-miR-192-5p;hsa-miR-20a-5p;hsa-miR-21
ENSG0000	ENSG0000	0.001453	hsa-miR-106b-5p;hsa-miR-192-5p;hsa-miR-20a-5p;hsa-miR-21
ENSG0000	ENSG0000	0.003919	hsa-miR-1271-5p;hsa-miR-132-3p;hsa-miR-182-5p;hsa-miR-54
ENSG0000	ENSG0000	0.005201	hsa-miR-106b-5p;hsa-miR-20a-5p;hsa-miR-520a-3p;hsa-miR-5
ENSG0000	ENSG0000	0.000814	hsa-miR-106b-5p;hsa-miR-130b-5p;hsa-miR-200b-3p;hsa-miR
ENSG0000	ENSG0000	0.006555	hsa-miR-106b-5p;hsa-miR-20a-5p;hsa-miR-212-5p;hsa-miR-26
ENSG0000	ENSG0000	0.003784	hsa-miR-106b-5p;hsa-miR-20a-5p;hsa-miR-212-5p;hsa-miR-50
ENSG0000	ENSG0000	0.006332	hsa-miR-106b-5p;hsa-miR-20a-5p;hsa-miR-212-5p;hsa-miR-32
ENSG0000	ENSG0000	0.005504	hsa-miR-106b-5p;hsa-miR-20a-5p;hsa-miR-519a-3p;hsa-miR-5
ENSG0000	ENSG0000	0.005504	hsa-miR-106b-5p;hsa-miR-20a-5p;hsa-miR-519a-3p;hsa-miR-5
ENSG0000	ENSG0000	0.005504	hsa-miR-106b-5p;hsa-miR-20a-5p;hsa-miR-519a-3p;hsa-miR-5
ENSG0000	ENSG0000	0.005504	hsa-miR-106b-5p;hsa-miR-20a-5p;hsa-miR-519a-3p;hsa-miR-5
ENSG0000	ENSG0000	0.005504	hsa-miR-106b-5p;hsa-miR-20a-5p;hsa-miR-519a-3p;hsa-miR-5
ENSG0000	ENSG0000	0.005504	hsa-miR-106b-5p;hsa-miR-20a-5p;hsa-miR-519a-3p;hsa-miR-5
ENSG0000	ENSG0000	0.005504	hsa-miR-106b-5p;hsa-miR-20a-5p;hsa-miR-519a-3p;hsa-miR-5
ENSG0000	ENSG0000	6.96E-05	hsa-miR-187-5p;hsa-miR-221-3p;hsa-miR-222-3p;hsa-miR-519
ENSG0000	ENSG0000	0.00268	hsa-miR-106b-5p;hsa-miR-20a-5p;hsa-miR-519a-3p;hsa-miR-5
ENSG0000	ENSG0000	0.000173	hsa-miR-106b-5p;hsa-miR-10b-3p;hsa-miR-194-5p;hsa-miR-20
ENSG0000	ENSG0000	0.006838	hsa-miR-10a-3p;hsa-miR-26a-5p;hsa-miR-26b-5p;hsa-miR-34a
ENSG0000	ENSG0000	0.000972	hsa-miR-29a-3p;hsa-miR-29b-3p;hsa-miR-3065-5p;hsa-miR-42
ENSG0000	ENSG0000	3.20E-06	hsa-miR-34a-5p;hsa-miR-376a-3p;hsa-miR-449a;hsa-miR-449b
ENSG0000	ENSG0000	0.004307	hsa-miR-29b-2-5p;hsa-miR-30a-3p;hsa-miR-30d-3p;hsa-miR-3
ENSG0000	ENSG0000	0.001551	hsa-miR-103a-3p;hsa-miR-107;hsa-miR-32-5p;hsa-miR-548ap-
ENSG0000	ENSG0000	0.006382	hsa-miR-106b-5p;hsa-miR-20a-5p;hsa-miR-502-5p;hsa-miR-51
ENSG0000	ENSG0000	0.009364	hsa-miR-187-5p;hsa-miR-221-3p;hsa-miR-222-3p;hsa-miR-520
ENSG0000	ENSG0000	0.00488	hsa-miR-106b-5p;hsa-miR-20a-5p;hsa-miR-519a-3p;hsa-miR-5
ENSG0000	ENSG0000	0.000136	hsa-miR-106b-5p;hsa-miR-1271-5p;hsa-miR-182-5p;hsa-miR-2
ENSG0000	ENSG0000	0.000136	hsa-miR-106b-5p;hsa-miR-1271-5p;hsa-miR-182-5p;hsa-miR-2
ENSG0000	ENSG0000	0.00039	hsa-miR-124-3p;hsa-miR-29a-3p;hsa-miR-29b-3p;hsa-miR-29c
ENSG0000	ENSG0000	0.000269	hsa-miR-212-5p;hsa-miR-320a;hsa-miR-320b;hsa-miR-320d;hs
ENSG0000	ENSG0000	0.004065	hsa-miR-106b-5p;hsa-miR-20a-5p;hsa-miR-3065-5p;hsa-miR-3
ENSG0000	ENSG0000	0.009166	hsa-miR-106b-5p;hsa-miR-20a-5p;hsa-miR-374a-5p;hsa-miR-5
ENSG0000	ENSG0000	7.10E-05	hsa-miR-29b-2-5p;hsa-miR-30a-3p;hsa-miR-30d-3p;hsa-miR-3
ENSG0000	ENSG0000	0.002434	hsa-miR-106b-5p;hsa-miR-20a-5p;hsa-miR-212-5p;hsa-miR-52
ENSG0000	ENSG0000	0.00817	hsa-miR-320a;hsa-miR-320b;hsa-miR-320d;hsa-miR-4429
ENSG0000	ENSG0000	0.000465	hsa-miR-106b-5p;hsa-miR-130b-5p;hsa-miR-20a-5p;hsa-miR-5
ENSG0000	ENSG0000	0.003784	hsa-miR-34a-5p;hsa-miR-423-5p;hsa-miR-449a;hsa-miR-449b-

ENSG0000	ENSG0000	0.003784	hsa-miR-34a-5p;hsa-miR-423-5p;hsa-miR-449a;hsa-miR-449b-
ENSG0000	ENSG0000	0.000195	hsa-miR-449a;hsa-miR-449b-5p;hsa-miR-519a-3p;hsa-miR-51
ENSG0000	ENSG0000	0.000382	hsa-miR-519a-3p;hsa-miR-519b-3p;hsa-miR-519c-3p;hsa-miR-
ENSG0000	ENSG0000	0.001619	hsa-miR-106b-5p;hsa-miR-130b-5p;hsa-miR-20a-5p;hsa-miR-
ENSG0000	ENSG0000	0.003919	hsa-miR-124-3p;hsa-miR-200b-3p;hsa-miR-200c-3p;hsa-miR-
ENSG0000	ENSG0000	0.000269	hsa-miR-519a-3p;hsa-miR-519b-3p;hsa-miR-519c-3p;hsa-miR-
ENSG0000	ENSG0000	0.000325	hsa-miR-106b-5p;hsa-miR-20a-5p;hsa-miR-26a-5p;hsa-miR-3
ENSG0000	ENSG0000	0.003309	hsa-miR-1299;hsa-miR-29a-3p;hsa-miR-29b-3p;hsa-miR-29c-
ENSG0000	ENSG0000	0.001177	hsa-miR-106b-5p;hsa-miR-1271-5p;hsa-miR-20a-5p;hsa-miR-
ENSG0000	ENSG0000	0.000269	hsa-miR-106b-5p;hsa-miR-20a-5p;hsa-miR-519a-3p;hsa-miR-
ENSG0000	ENSG0000	0.000904	hsa-miR-34a-5p;hsa-miR-449a;hsa-miR-449b-5p;hsa-miR-548
ENSG0000	ENSG0000	0.000245	hsa-miR-106b-5p;hsa-miR-137;hsa-miR-20a-5p;hsa-miR-3065-
ENSG0000	ENSG0000	0.006093	hsa-miR-320a;hsa-miR-320b;hsa-miR-320d;hsa-miR-4429
ENSG0000	ENSG0000	2.90E-05	hsa-miR-106b-5p;hsa-miR-20a-5p;hsa-miR-212-5p;hsa-miR-3
ENSG0000	ENSG0000	0.002434	hsa-miR-106b-5p;hsa-miR-20a-5p;hsa-miR-29b-2-5p;hsa-miR
ENSG0000	ENSG0000	0.001542	hsa-miR-340-5p;hsa-miR-519a-3p;hsa-miR-519b-3p;hsa-miR-
ENSG0000	ENSG0000	0.001799	hsa-miR-106b-5p;hsa-miR-200b-3p;hsa-miR-200c-3p;hsa-miR
ENSG0000	ENSG0000	0.004885	hsa-miR-103a-3p;hsa-miR-106b-5p;hsa-miR-107;hsa-miR-129
ENSG0000	ENSG0000	0.008573	hsa-miR-10a-3p;hsa-miR-30a-3p;hsa-miR-30d-3p;hsa-miR-30
ENSG0000	ENSG0000	0.00495	hsa-miR-106b-5p;hsa-miR-20a-5p;hsa-miR-32-5p;hsa-miR-33
ENSG0000	ENSG0000	0.005818	hsa-miR-194-5p;hsa-miR-519a-3p;hsa-miR-519b-3p;hsa-miR-
ENSG0000	ENSG0000	0.004982	hsa-miR-106b-5p;hsa-miR-20a-5p;hsa-miR-374a-5p;hsa-miR-
ENSG0000	ENSG0000	0.000904	hsa-miR-124-3p;hsa-miR-30a-3p;hsa-miR-30d-3p;hsa-miR-30
ENSG0000	ENSG0000	0.007373	hsa-miR-187-5p;hsa-miR-221-3p;hsa-miR-222-3p;hsa-miR-46
ENSG0000	ENSG0000	5.91E-05	hsa-miR-320a;hsa-miR-320b;hsa-miR-320d;hsa-miR-4429
ENSG0000	ENSG0000	6.00E-08	hsa-miR-106b-5p;hsa-miR-194-5p;hsa-miR-20a-5p;hsa-miR-2
ENSG0000	ENSG0000	0.002489	hsa-miR-30a-3p;hsa-miR-30d-3p;hsa-miR-30e-3p;hsa-miR-33
ENSG0000	ENSG0000	1.97E-05	hsa-miR-1271-5p;hsa-miR-182-5p;hsa-miR-34a-5p;hsa-miR-3
ENSG0000	ENSG0000	0.000688	hsa-miR-106b-5p;hsa-miR-141-3p;hsa-miR-200a-3p;hsa-miR-
ENSG0000	ENSG0000	0.000136	hsa-miR-103a-3p;hsa-miR-107;hsa-miR-130b-5p;hsa-miR-519
ENSG0000	ENSG0000	0.006093	hsa-miR-130b-5p;hsa-miR-29a-3p;hsa-miR-29b-3p;hsa-miR-2
ENSG0000	ENSG0000	0.003535	hsa-miR-30a-3p;hsa-miR-30d-3p;hsa-miR-30e-3p;hsa-miR-33
ENSG0000	ENSG0000	0.000104	hsa-miR-30a-3p;hsa-miR-30d-3p;hsa-miR-30e-3p;hsa-miR-34
ENSG0000	ENSG0000	0.000601	hsa-miR-519a-3p;hsa-miR-519b-3p;hsa-miR-519c-3p;hsa-miR-
ENSG0000	ENSG0000	0.006838	hsa-miR-106b-5p;hsa-miR-20a-5p;hsa-miR-423-5p;hsa-miR-5
ENSG0000	ENSG0000	0.009364	hsa-miR-135a-5p;hsa-miR-135b-5p;hsa-miR-141-3p;hsa-miR-
ENSG0000	ENSG0000	5.91E-05	hsa-miR-34a-5p;hsa-miR-449a;hsa-miR-449b-5p;hsa-miR-548
ENSG0000	ENSG0000	0.00488	hsa-miR-106b-5p;hsa-miR-20a-5p;hsa-miR-340-5p;hsa-miR-5
ENSG0000	ENSG0000	0.001777	hsa-miR-103a-3p;hsa-miR-106b-5p;hsa-miR-107;hsa-miR-124-
ENSG0000	ENSG0000	0.004211	hsa-miR-21-3p;hsa-miR-378a-5p;hsa-miR-519a-3p;hsa-miR-5
ENSG0000	ENSG0000	0.000465	hsa-miR-106b-5p;hsa-miR-130b-5p;hsa-miR-20a-5p;hsa-miR-
ENSG0000	ENSG0000	0.00529	hsa-miR-106b-5p;hsa-miR-20a-5p;hsa-miR-340-5p;hsa-miR-3
ENSG0000	ENSG0000	0.002525	hsa-miR-106b-5p;hsa-miR-20a-5p;hsa-miR-32-3p;hsa-miR-51
ENSG0000	ENSG0000	0.000105	hsa-miR-320a;hsa-miR-320b;hsa-miR-320d;hsa-miR-32-5p;hsa
ENSG0000	ENSG0000	0.000535	hsa-miR-301a-5p;hsa-miR-3194-3p;hsa-miR-320a;hsa-miR-32
ENSG0000	ENSG0000	4.43E-06	hsa-miR-187-5p;hsa-miR-21-3p;hsa-miR-221-3p;hsa-miR-222-
ENSG0000	ENSG0000	3.28E-05	hsa-miR-103a-3p;hsa-miR-107;hsa-miR-320a;hsa-miR-320b;hs
ENSG0000	ENSG0000	0.006183	hsa-miR-106b-5p;hsa-miR-1271-5p;hsa-miR-182-5p;hsa-miR-
ENSG0000	ENSG0000	0.006183	hsa-miR-519a-3p;hsa-miR-519b-3p;hsa-miR-519c-3p;hsa-miR-
ENSG0000	ENSG0000	0.000379	hsa-miR-320a;hsa-miR-320b;hsa-miR-320d;hsa-miR-4429
ENSG0000	ENSG0000	0.000535	hsa-miR-34a-5p;hsa-miR-449a;hsa-miR-449b-5p;hsa-miR-548
ENSG0000	ENSG0000	0.004799	hsa-miR-1271-5p;hsa-miR-137;hsa-miR-182-5p;hsa-miR-5690;
ENSG0000	ENSG0000	0.000918	hsa-miR-1271-5p;hsa-miR-182-5p;hsa-miR-34a-5p;hsa-miR-4
ENSG0000	ENSG0000	0.000379	hsa-miR-141-3p;hsa-miR-200a-3p;hsa-miR-200b-3p;hsa-miR-

ENSG0000	ENSG0000	0.001551	hsa-miR-106b-5p;hsa-miR-20a-5p;hsa-miR-519a-3p;hsa-miR-5
ENSG0000	ENSG0000	0.00982	hsa-miR-106b-5p;hsa-miR-20a-5p;hsa-miR-32-5p;hsa-miR-52(
ENSG0000	ENSG0000	0.000173	hsa-miR-135a-5p;hsa-miR-135b-5p;hsa-miR-141-3p;hsa-miR-2
ENSG0000	ENSG0000	5.22E-06	hsa-miR-141-3p;hsa-miR-200a-3p;hsa-miR-29a-3p;hsa-miR-29
ENSG0000	ENSG0000	0.000904	hsa-miR-106b-5p;hsa-miR-20a-5p;hsa-miR-519a-3p;hsa-miR-5
ENSG0000	ENSG0000	0.00268	hsa-miR-106b-5p;hsa-miR-200b-3p;hsa-miR-200c-3p;hsa-miR-
ENSG0000	ENSG0000	0.003527	hsa-miR-106b-5p;hsa-miR-20a-5p;hsa-miR-378a-5p;hsa-miR-4
ENSG0000	ENSG0000	8.61E-08	hsa-miR-106b-5p;hsa-miR-194-5p;hsa-miR-20a-5p;hsa-miR-30
ENSG0000	ENSG0000	0.009364	hsa-miR-106b-5p;hsa-miR-20a-5p;hsa-miR-520a-3p;hsa-miR-5
ENSG0000	ENSG0000	0.009364	hsa-miR-124-3p;hsa-miR-519a-3p;hsa-miR-519b-3p;hsa-miR-5
ENSG0000	ENSG0000	0.003919	hsa-miR-26a-5p;hsa-miR-26b-5p;hsa-miR-29b-2-5p;hsa-miR-3
ENSG0000	ENSG0000	1.59E-10	hsa-let-7a-5p;hsa-let-7b-5p;hsa-let-7e-5p;hsa-let-7f-5p;hsa-le
ENSG0000	ENSG0000	1.94E-07	hsa-let-7a-5p;hsa-let-7b-5p;hsa-let-7e-5p;hsa-let-7f-5p;hsa-le
ENSG0000	ENSG0000	1.47E-07	hsa-let-7a-5p;hsa-let-7b-5p;hsa-let-7e-5p;hsa-let-7f-5p;hsa-le
ENSG0000	ENSG0000	1.07E-08	hsa-let-7a-5p;hsa-let-7b-5p;hsa-let-7e-5p;hsa-let-7f-5p;hsa-le
ENSG0000	ENSG0000	1.19E-08	hsa-let-7a-5p;hsa-let-7b-5p;hsa-let-7e-5p;hsa-let-7f-5p;hsa-le
ENSG0000	ENSG0000	4.89E-07	hsa-let-7a-5p;hsa-let-7b-5p;hsa-let-7e-5p;hsa-let-7f-5p;hsa-le
ENSG0000	ENSG0000	0.000809	hsa-let-7a-5p;hsa-let-7b-5p;hsa-let-7e-5p;hsa-let-7g-5p;hsa-l
ENSG0000	ENSG0000	1.84E-07	hsa-let-7a-5p;hsa-let-7b-5p;hsa-let-7f-5p;hsa-miR-125b-5p
ENSG0000	ENSG0000	6.72E-09	hsa-let-7a-5p;hsa-let-7b-5p;hsa-let-7e-5p;hsa-let-7f-5p;hsa-le
ENSG0000	ENSG0000	4.60E-08	hsa-let-7a-5p;hsa-let-7b-5p;hsa-let-7e-5p;hsa-let-7f-5p;hsa-le
ENSG0000	ENSG0000	6.33E-10	hsa-let-7a-5p;hsa-let-7b-5p;hsa-let-7e-5p;hsa-let-7f-5p;hsa-le
ENSG0000	ENSG0000	6.72E-09	hsa-let-7a-5p;hsa-let-7b-5p;hsa-let-7e-5p;hsa-let-7f-5p;hsa-le
ENSG0000	ENSG0000	4.36E-05	hsa-let-7a-5p;hsa-let-7b-5p;hsa-let-7e-5p;hsa-let-7f-5p;hsa-le
ENSG0000	ENSG0000	4.87E-10	hsa-let-7a-5p;hsa-let-7b-5p;hsa-let-7e-5p;hsa-let-7f-5p;hsa-le
ENSG0000	ENSG0000	2.63E-08	hsa-let-7a-5p;hsa-let-7e-5p;hsa-let-7f-5p;hsa-let-7g-5p;hsa-le
ENSG0000	ENSG0000	2.18E-08	hsa-let-7a-5p;hsa-let-7b-5p;hsa-let-7e-5p;hsa-let-7f-5p;hsa-le
ENSG0000	ENSG0000	1.21E-07	hsa-let-7a-5p;hsa-let-7b-5p;hsa-let-7e-5p;hsa-let-7f-5p;hsa-le
ENSG0000	ENSG0000	2.80E-09	hsa-let-7a-5p;hsa-let-7e-5p;hsa-let-7f-5p;hsa-let-7g-5p;hsa-le
ENSG0000	ENSG0000	3.75E-10	hsa-let-7a-5p;hsa-let-7b-5p;hsa-let-7e-5p;hsa-let-7f-5p;hsa-le
ENSG0000	ENSG0000	6.33E-10	hsa-let-7a-5p;hsa-let-7b-5p;hsa-let-7e-5p;hsa-let-7f-5p;hsa-le
ENSG0000	ENSG0000	1.36E-05	hsa-let-7a-5p;hsa-let-7e-5p;hsa-let-7f-5p;hsa-let-7g-5p;hsa-le
ENSG0000	ENSG0000	8.35E-10	hsa-let-7a-5p;hsa-let-7b-5p;hsa-let-7e-5p;hsa-let-7f-5p;hsa-le
ENSG0000	ENSG0000	4.04E-11	hsa-let-7a-5p;hsa-let-7b-5p;hsa-let-7e-5p;hsa-let-7f-5p;hsa-le
ENSG0000	ENSG0000	7.94E-10	hsa-let-7a-5p;hsa-let-7b-5p;hsa-let-7e-5p;hsa-let-7f-5p;hsa-le
ENSG0000	ENSG0000	1.41E-06	hsa-let-7a-5p;hsa-let-7b-5p;hsa-let-7e-5p;hsa-let-7f-5p;hsa-le
ENSG0000	ENSG0000	1.32E-06	hsa-let-7a-5p;hsa-let-7b-5p;hsa-let-7e-5p;hsa-let-7f-5p;hsa-le
ENSG0000	ENSG0000	3.41E-05	hsa-let-7a-5p;hsa-let-7b-5p;hsa-let-7e-5p;hsa-let-7f-5p;hsa-le
ENSG0000	ENSG0000	1.90E-08	hsa-let-7a-5p;hsa-let-7b-5p;hsa-let-7e-5p;hsa-let-7f-5p;hsa-le
ENSG0000	ENSG0000	2.54E-07	hsa-let-7a-5p;hsa-let-7b-5p;hsa-let-7e-5p;hsa-let-7f-5p;hsa-le
ENSG0000	ENSG0000	4.03E-06	hsa-let-7a-5p;hsa-let-7e-5p;hsa-let-7f-5p;hsa-let-7g-5p;hsa-le
ENSG0000	ENSG0000	3.30E-07	hsa-let-7a-5p;hsa-let-7b-5p;hsa-let-7f-5p;hsa-let-7g-5p;hsa-le
ENSG0000	ENSG0000	3.73E-12	hsa-let-7a-5p;hsa-let-7b-5p;hsa-let-7e-5p;hsa-let-7f-5p;hsa-le
ENSG0000	ENSG0000	2.74E-06	hsa-let-7a-5p;hsa-let-7b-5p;hsa-let-7e-5p;hsa-let-7f-5p;hsa-le
ENSG0000	ENSG0000	3.06E-08	hsa-let-7a-5p;hsa-let-7b-5p;hsa-let-7e-5p;hsa-let-7f-5p;hsa-le
ENSG0000	ENSG0000	1.00E-11	hsa-let-7a-5p;hsa-let-7b-5p;hsa-let-7e-5p;hsa-let-7f-5p;hsa-le
ENSG0000	ENSG0000	1.92E-06	hsa-let-7a-5p;hsa-let-7b-5p;hsa-let-7e-5p;hsa-let-7f-5p;hsa-le
ENSG0000	ENSG0000	1.44E-08	hsa-let-7a-5p;hsa-let-7b-5p;hsa-let-7e-5p;hsa-let-7f-5p;hsa-le
ENSG0000	ENSG0000	2.18E-08	hsa-let-7a-5p;hsa-let-7b-5p;hsa-let-7e-5p;hsa-let-7f-5p;hsa-le
ENSG0000	ENSG0000	8.30E-11	hsa-let-7a-5p;hsa-let-7b-5p;hsa-let-7e-5p;hsa-let-7f-5p;hsa-le
ENSG0000	ENSG0000	5.82E-11	hsa-let-7a-5p;hsa-let-7b-5p;hsa-let-7e-5p;hsa-let-7f-5p;hsa-le
ENSG0000	ENSG0000	3.75E-10	hsa-let-7a-5p;hsa-let-7b-5p;hsa-let-7e-5p;hsa-let-7f-5p;hsa-le
ENSG0000	ENSG0000	0.000162	hsa-let-7a-5p;hsa-let-7b-5p;hsa-let-7e-5p;hsa-let-7g-5p;hsa-l
ENSG0000	ENSG0000	6.72E-09	hsa-let-7a-5p;hsa-let-7b-5p;hsa-let-7e-5p;hsa-let-7f-5p;hsa-le

ENSG0000	ENSG0000	4.50E-08	hsa-let-7a-5p;hsa-let-7b-5p;hsa-let-7e-5p;hsa-let-7f-5p;hsa-le
ENSG0000	ENSG0000	4.87E-10	hsa-let-7a-5p;hsa-let-7b-5p;hsa-let-7e-5p;hsa-let-7f-5p;hsa-le
ENSG0000	ENSG0000	1.51E-14	hsa-let-7a-5p;hsa-let-7b-5p;hsa-let-7e-5p;hsa-let-7f-5p;hsa-le
ENSG0000	ENSG0000	1.51E-14	hsa-let-7a-5p;hsa-let-7b-5p;hsa-let-7e-5p;hsa-let-7f-5p;hsa-le
ENSG0000	ENSG0000	1.29E-11	hsa-let-7a-5p;hsa-let-7b-5p;hsa-let-7e-5p;hsa-let-7f-5p;hsa-le
ENSG0000	ENSG0000	1.59E-10	hsa-let-7a-5p;hsa-let-7b-5p;hsa-let-7e-5p;hsa-let-7f-5p;hsa-le
ENSG0000	ENSG0000	1.59E-10	hsa-let-7a-5p;hsa-let-7b-5p;hsa-let-7e-5p;hsa-let-7f-5p;hsa-le
ENSG0000	ENSG0000	1.59E-10	hsa-let-7a-5p;hsa-let-7b-5p;hsa-let-7e-5p;hsa-let-7f-5p;hsa-le
ENSG0000	ENSG0000	1.59E-10	hsa-let-7a-5p;hsa-let-7b-5p;hsa-let-7e-5p;hsa-let-7f-5p;hsa-le
ENSG0000	ENSG0000	1.59E-10	hsa-let-7a-5p;hsa-let-7b-5p;hsa-let-7e-5p;hsa-let-7f-5p;hsa-le
ENSG0000	ENSG0000	1.59E-10	hsa-let-7a-5p;hsa-let-7b-5p;hsa-let-7e-5p;hsa-let-7f-5p;hsa-le
ENSG0000	ENSG0000	1.59E-10	hsa-let-7a-5p;hsa-let-7b-5p;hsa-let-7e-5p;hsa-let-7f-5p;hsa-le
ENSG0000	ENSG0000	6.33E-06	hsa-let-7a-5p;hsa-let-7b-5p;hsa-let-7e-5p;hsa-let-7g-5p;hsa-l
ENSG0000	ENSG0000	0.000655	hsa-let-7a-5p;hsa-let-7e-5p;hsa-let-7f-5p;hsa-let-7g-5p;hsa-le
ENSG0000	ENSG0000	1.62E-07	hsa-let-7a-5p;hsa-let-7b-5p;hsa-let-7e-5p;hsa-let-7f-5p;hsa-le
ENSG0000	ENSG0000	1.33E-07	hsa-let-7a-5p;hsa-let-7b-5p;hsa-let-7e-5p;hsa-let-7f-5p;hsa-le
ENSG0000	ENSG0000	4.18E-07	hsa-let-7a-5p;hsa-let-7b-5p;hsa-let-7e-5p;hsa-let-7f-5p;hsa-le
ENSG0000	ENSG0000	2.18E-08	hsa-let-7a-5p;hsa-let-7b-5p;hsa-let-7e-5p;hsa-let-7f-5p;hsa-le
ENSG0000	ENSG0000	1.01E-06	hsa-let-7a-5p;hsa-let-7b-5p;hsa-let-7e-5p;hsa-let-7f-5p;hsa-le
ENSG0000	ENSG0000	1.21E-07	hsa-let-7a-5p;hsa-let-7b-5p;hsa-let-7e-5p;hsa-let-7f-5p;hsa-le
ENSG0000	ENSG0000	1.79E-05	hsa-let-7a-5p;hsa-let-7f-5p;hsa-let-7g-5p;hsa-let-7i-5p
ENSG0000	ENSG0000	6.62E-07	hsa-let-7a-5p;hsa-let-7b-5p;hsa-let-7e-5p;hsa-let-7f-5p;hsa-le
ENSG0000	ENSG0000	4.81E-09	hsa-let-7a-5p;hsa-let-7b-5p;hsa-let-7e-5p;hsa-let-7f-5p;hsa-le
ENSG0000	ENSG0000	1.81E-06	hsa-let-7a-5p;hsa-let-7b-5p;hsa-let-7e-5p;hsa-let-7f-5p;hsa-le
ENSG0000	ENSG0000	1.09E-07	hsa-let-7a-5p;hsa-let-7b-5p;hsa-let-7e-5p;hsa-let-7f-5p;hsa-le
ENSG0000	ENSG0000	3.31E-08	hsa-let-7a-5p;hsa-let-7b-5p;hsa-let-7e-5p;hsa-let-7f-5p;hsa-le
ENSG0000	ENSG0000	2.53E-06	hsa-let-7a-5p;hsa-let-7e-5p;hsa-let-7f-5p;hsa-let-7g-5p;hsa-le
ENSG0000	ENSG0000	2.51E-06	hsa-let-7a-5p;hsa-let-7b-5p;hsa-let-7e-5p;hsa-let-7f-5p;hsa-le
ENSG0000	ENSG0000	2.51E-06	hsa-let-7a-5p;hsa-let-7b-5p;hsa-let-7e-5p;hsa-let-7f-5p;hsa-le
ENSG0000	ENSG0000	2.03E-09	hsa-let-7a-5p;hsa-let-7b-5p;hsa-let-7e-5p;hsa-let-7f-5p;hsa-le
ENSG0000	ENSG0000	1.62E-07	hsa-let-7a-5p;hsa-let-7b-5p;hsa-let-7e-5p;hsa-let-7f-5p;hsa-le
ENSG0000	ENSG0000	1.90E-08	hsa-let-7a-5p;hsa-let-7b-5p;hsa-let-7e-5p;hsa-let-7f-5p;hsa-le
ENSG0000	ENSG0000	4.18E-07	hsa-let-7a-5p;hsa-let-7b-5p;hsa-let-7e-5p;hsa-let-7f-5p;hsa-le
ENSG0000	ENSG0000	4.18E-07	hsa-let-7a-5p;hsa-let-7b-5p;hsa-let-7e-5p;hsa-let-7f-5p;hsa-le
ENSG0000	ENSG0000	8.30E-11	hsa-let-7a-5p;hsa-let-7b-5p;hsa-let-7e-5p;hsa-let-7f-5p;hsa-le
ENSG0000	ENSG0000	9.89E-08	hsa-let-7a-5p;hsa-let-7b-5p;hsa-let-7e-5p;hsa-let-7f-5p;hsa-le
ENSG0000	ENSG0000	0.000436	hsa-let-7b-5p;hsa-let-7e-5p;hsa-let-7f-5p;hsa-let-7i-5p
ENSG0000	ENSG0000	1.21E-07	hsa-let-7a-5p;hsa-let-7b-5p;hsa-let-7e-5p;hsa-let-7f-5p;hsa-le
ENSG0000	ENSG0000	0.000183	hsa-let-7a-5p;hsa-let-7e-5p;hsa-miR-125a-5p;hsa-miR-125b-5
ENSG0000	ENSG0000	8.85E-05	hsa-let-7a-5p;hsa-let-7b-5p;hsa-let-7e-5p;hsa-let-7f-5p;hsa-le
ENSG0000	ENSG0000	6.70E-06	hsa-let-7a-5p;hsa-let-7b-5p;hsa-let-7e-5p;hsa-let-7f-5p;hsa-le
ENSG0000	ENSG0000	3.37E-09	hsa-let-7a-5p;hsa-let-7b-5p;hsa-let-7e-5p;hsa-let-7f-5p;hsa-le
ENSG0000	ENSG0000	0.000965	hsa-let-7a-5p;hsa-let-7e-5p;hsa-let-7f-5p;hsa-let-7g-5p;hsa-le
ENSG0000	ENSG0000	3.01E-07	hsa-let-7a-5p;hsa-let-7b-5p;hsa-let-7e-5p;hsa-let-7f-5p;hsa-le
ENSG0000	ENSG0000	3.62E-08	hsa-let-7a-5p;hsa-let-7b-5p;hsa-let-7e-5p;hsa-let-7f-5p;hsa-le
ENSG0000	ENSG0000	1.89E-09	hsa-let-7a-5p;hsa-let-7b-5p;hsa-let-7e-5p;hsa-let-7f-5p;hsa-le
ENSG0000	ENSG0000	1.25E-08	hsa-let-7a-5p;hsa-let-7b-5p;hsa-let-7e-5p;hsa-let-7f-5p;hsa-le
ENSG0000	ENSG0000	1.93E-05	hsa-let-7a-5p;hsa-let-7b-5p;hsa-let-7e-5p;hsa-let-7f-5p;hsa-le
ENSG0000	ENSG0000	2.79E-05	hsa-let-7a-5p;hsa-let-7b-5p;hsa-let-7e-5p;hsa-let-7g-5p;hsa-r
ENSG0000	ENSG0000	1.90E-08	hsa-let-7a-5p;hsa-let-7b-5p;hsa-let-7e-5p;hsa-let-7f-5p;hsa-le
ENSG0000	ENSG0000	7.83E-05	hsa-let-7a-5p;hsa-let-7e-5p;hsa-let-7f-5p;hsa-let-7g-5p;hsa-le
ENSG0000	ENSG0000	2.33E-07	hsa-let-7a-5p;hsa-let-7b-5p;hsa-let-7e-5p;hsa-let-7f-5p;hsa-le
ENSG0000	ENSG0000	4.89E-07	hsa-let-7a-5p;hsa-let-7b-5p;hsa-let-7e-5p;hsa-let-7f-5p;hsa-le
ENSG0000	ENSG0000	5.70E-09	hsa-let-7a-5p;hsa-let-7b-5p;hsa-let-7e-5p;hsa-let-7f-5p;hsa-le

ENSG0000	ENSG0000	7.89E-09	hsa-let-7a-5p;hsa-let-7b-5p;hsa-let-7e-5p;hsa-let-7f-5p;hsa-le
ENSG0000	ENSG0000	2.86E-10	hsa-let-7a-5p;hsa-let-7b-5p;hsa-let-7e-5p;hsa-let-7f-5p;hsa-le
ENSG0000	ENSG0000	4.25E-06	hsa-let-7a-5p;hsa-let-7e-5p;hsa-let-7f-5p;hsa-let-7g-5p;hsa-le
ENSG0000	ENSG0000	1.80E-09	hsa-let-7a-5p;hsa-let-7b-5p;hsa-let-7e-5p;hsa-let-7f-5p;hsa-le
ENSG0000	ENSG0000	6.47E-08	hsa-let-7a-5p;hsa-let-7b-5p;hsa-let-7e-5p;hsa-let-7f-5p;hsa-le
ENSG0000	ENSG0000	6.09E-09	hsa-let-7a-5p;hsa-let-7b-5p;hsa-let-7e-5p;hsa-let-7f-5p;hsa-le
ENSG0000	ENSG0000	7.59E-08	hsa-let-7a-5p;hsa-let-7e-5p;hsa-let-7f-5p;hsa-let-7g-5p;hsa-le
ENSG0000	ENSG0000	1.73E-11	hsa-let-7a-5p;hsa-let-7b-5p;hsa-let-7e-5p;hsa-let-7f-5p;hsa-le
ENSG0000	ENSG0000	0.000477	hsa-let-7a-5p;hsa-let-7f-5p;hsa-let-7g-5p;hsa-let-7i-5p
ENSG0000	ENSG0000	1.21E-07	hsa-let-7a-5p;hsa-let-7b-5p;hsa-let-7e-5p;hsa-let-7f-5p;hsa-le
ENSG0000	ENSG0000	8.92E-08	hsa-let-7a-5p;hsa-let-7b-5p;hsa-let-7e-5p;hsa-let-7f-5p;hsa-le
ENSG0000	ENSG0000	8.92E-08	hsa-let-7a-5p;hsa-let-7b-5p;hsa-let-7e-5p;hsa-let-7f-5p;hsa-le
ENSG0000	ENSG0000	1.90E-08	hsa-let-7a-5p;hsa-let-7b-5p;hsa-let-7e-5p;hsa-let-7f-5p;hsa-le
ENSG0000	ENSG0000	5.16E-08	hsa-let-7a-5p;hsa-let-7b-5p;hsa-let-7e-5p;hsa-let-7f-5p;hsa-le
ENSG0000	ENSG0000	4.87E-10	hsa-let-7a-5p;hsa-let-7b-5p;hsa-let-7e-5p;hsa-let-7f-5p;hsa-le
ENSG0000	ENSG0000	8.85E-07	hsa-let-7a-5p;hsa-let-7b-5p;hsa-let-7e-5p;hsa-let-7f-5p;hsa-le
ENSG0000	ENSG0000	3.62E-08	hsa-let-7a-5p;hsa-let-7b-5p;hsa-let-7e-5p;hsa-let-7f-5p;hsa-le
ENSG0000	ENSG0000	6.47E-08	hsa-let-7a-5p;hsa-let-7b-5p;hsa-let-7e-5p;hsa-let-7f-5p;hsa-le
ENSG0000	ENSG0000	2.15E-10	hsa-let-7a-5p;hsa-let-7b-5p;hsa-let-7e-5p;hsa-let-7f-5p;hsa-le
ENSG0000	ENSG0000	1.25E-09	hsa-let-7a-5p;hsa-let-7b-5p;hsa-let-7e-5p;hsa-let-7f-5p;hsa-le
ENSG0000	ENSG0000	6.72E-09	hsa-let-7a-5p;hsa-let-7b-5p;hsa-let-7e-5p;hsa-let-7f-5p;hsa-le
ENSG0000	ENSG0000	7.21E-08	hsa-let-7a-5p;hsa-let-7b-5p;hsa-let-7e-5p;hsa-let-7f-5p;hsa-le
ENSG0000	ENSG0000	1.16E-06	hsa-let-7a-5p;hsa-let-7b-5p;hsa-let-7e-5p;hsa-let-7f-5p;hsa-le
ENSG0000	ENSG0000	1.09E-07	hsa-let-7a-5p;hsa-let-7b-5p;hsa-let-7e-5p;hsa-let-7f-5p;hsa-le
ENSG0000	ENSG0000	3.24E-05	hsa-let-7a-5p;hsa-let-7f-5p;hsa-let-7g-5p;hsa-let-7i-5p
ENSG0000	ENSG0000	8.58E-05	hsa-let-7a-5p;hsa-let-7b-5p;hsa-let-7e-5p;hsa-let-7f-5p;hsa-le
ENSG0000	ENSG0000	7.47E-08	hsa-let-7a-5p;hsa-let-7b-5p;hsa-let-7e-5p;hsa-let-7f-5p;hsa-le
ENSG0000	ENSG0000	7.47E-08	hsa-let-7a-5p;hsa-let-7b-5p;hsa-let-7e-5p;hsa-let-7f-5p;hsa-le
ENSG0000	ENSG0000	7.79E-06	hsa-let-7a-5p;hsa-let-7b-5p;hsa-let-7e-5p;hsa-let-7f-5p;hsa-le
ENSG0000	ENSG0000	4.87E-10	hsa-let-7a-5p;hsa-let-7b-5p;hsa-let-7e-5p;hsa-let-7f-5p;hsa-le
ENSG0000	ENSG0000	7.94E-10	hsa-let-7a-5p;hsa-let-7b-5p;hsa-let-7e-5p;hsa-let-7f-5p;hsa-le
ENSG0000	ENSG0000	7.94E-10	hsa-let-7a-5p;hsa-let-7b-5p;hsa-let-7e-5p;hsa-let-7f-5p;hsa-le
ENSG0000	ENSG0000	1.17E-05	hsa-let-7a-5p;hsa-let-7b-5p;hsa-let-7e-5p;hsa-let-7f-5p;hsa-le
ENSG0000	ENSG0000	4.74E-10	hsa-let-7a-5p;hsa-let-7b-5p;hsa-let-7e-5p;hsa-let-7f-5p;hsa-le
ENSG0000	ENSG0000	1.28E-05	hsa-let-7b-5p;hsa-let-7e-5p;hsa-let-7f-5p;hsa-let-7g-5p;hsa-le
ENSG0000	ENSG0000	9.89E-08	hsa-let-7a-5p;hsa-let-7b-5p;hsa-let-7e-5p;hsa-let-7f-5p;hsa-le
ENSG0000	ENSG0000	1.71E-08	hsa-let-7a-5p;hsa-let-7b-5p;hsa-let-7e-5p;hsa-let-7f-5p;hsa-le
ENSG0000	ENSG0000	1.50E-06	hsa-let-7a-5p;hsa-let-7b-5p;hsa-let-7e-5p;hsa-let-7f-5p;hsa-le
ENSG0000	ENSG0000	1.54E-09	hsa-let-7a-5p;hsa-let-7b-5p;hsa-let-7e-5p;hsa-let-7f-5p;hsa-le
ENSG0000	ENSG0000	1.05E-05	hsa-let-7a-5p;hsa-let-7b-5p;hsa-let-7f-5p;hsa-let-7g-5p;hsa-le
ENSG0000	ENSG0000	2.22E-07	hsa-let-7a-5p;hsa-let-7b-5p;hsa-let-7e-5p;hsa-let-7f-5p;hsa-le
ENSG0000	ENSG0000	1.09E-07	hsa-let-7a-5p;hsa-let-7b-5p;hsa-let-7e-5p;hsa-let-7f-5p;hsa-le
ENSG0000	ENSG0000	6.62E-07	hsa-let-7a-5p;hsa-let-7b-5p;hsa-let-7e-5p;hsa-let-7f-5p;hsa-le
ENSG0000	ENSG0000	0.002059	hsa-let-7a-5p;hsa-let-7f-5p;hsa-let-7g-5p;hsa-let-7i-5p
ENSG0000	ENSG0000	5.43E-06	hsa-let-7e-5p;hsa-let-7f-5p;hsa-let-7g-5p;hsa-let-7i-5p;hsa-m
ENSG0000	ENSG0000	5.43E-06	hsa-let-7e-5p;hsa-let-7f-5p;hsa-let-7g-5p;hsa-let-7i-5p;hsa-m
ENSG0000	ENSG0000	6.70E-06	hsa-let-7a-5p;hsa-let-7b-5p;hsa-let-7e-5p;hsa-let-7f-5p;hsa-le
ENSG0000	ENSG0000	9.89E-08	hsa-let-7a-5p;hsa-let-7b-5p;hsa-let-7e-5p;hsa-let-7f-5p;hsa-le
ENSG0000	ENSG0000	2.82E-08	hsa-let-7a-5p;hsa-let-7b-5p;hsa-let-7e-5p;hsa-let-7f-5p;hsa-le
ENSG0000	ENSG0000	8.50E-06	hsa-let-7a-5p;hsa-let-7e-5p;hsa-let-7f-5p;hsa-let-7g-5p;hsa-le
ENSG0000	ENSG0000	1.12E-05	hsa-let-7e-5p;hsa-let-7f-5p;hsa-let-7g-5p;hsa-let-7i-5p;hsa-m
ENSG0000	ENSG0000	1.44E-08	hsa-let-7a-5p;hsa-let-7b-5p;hsa-let-7e-5p;hsa-let-7f-5p;hsa-le
ENSG0000	ENSG0000	1.44E-08	hsa-let-7a-5p;hsa-let-7b-5p;hsa-let-7e-5p;hsa-let-7f-5p;hsa-le
ENSG0000	ENSG0000	1.44E-08	hsa-let-7a-5p;hsa-let-7b-5p;hsa-let-7e-5p;hsa-let-7f-5p;hsa-le

ENSG0000	ENSG0000	1.90E-08	hsa-let-7a-5p;hsa-let-7b-5p;hsa-let-7e-5p;hsa-let-7f-5p;hsa-le
ENSG0000	ENSG0000	6.62E-07	hsa-let-7a-5p;hsa-let-7b-5p;hsa-let-7e-5p;hsa-let-7f-5p;hsa-le
ENSG0000	ENSG0000	2.80E-09	hsa-let-7a-5p;hsa-let-7b-5p;hsa-let-7e-5p;hsa-let-7f-5p;hsa-le
ENSG0000	ENSG0000	2.31E-09	hsa-let-7a-5p;hsa-let-7b-5p;hsa-let-7e-5p;hsa-let-7f-5p;hsa-le
ENSG0000	ENSG0000	9.08E-07	hsa-let-7a-5p;hsa-let-7e-5p;hsa-let-7f-5p;hsa-let-7g-5p;hsa-le
ENSG0000	ENSG0000	1.02E-05	hsa-let-7a-5p;hsa-let-7b-5p;hsa-let-7e-5p;hsa-let-7f-5p;hsa-le
ENSG0000	ENSG0000	1.02E-05	hsa-let-7a-5p;hsa-let-7b-5p;hsa-let-7e-5p;hsa-let-7f-5p;hsa-le
ENSG0000	ENSG0000	1.26E-06	hsa-let-7a-5p;hsa-let-7e-5p;hsa-let-7f-5p;hsa-let-7g-5p;hsa-le
ENSG0000	ENSG0000	9.15E-08	hsa-let-7a-5p;hsa-let-7e-5p;hsa-let-7f-5p;hsa-let-7g-5p;hsa-le
ENSG0000	ENSG0000	8.30E-11	hsa-let-7a-5p;hsa-let-7b-5p;hsa-let-7e-5p;hsa-let-7f-5p;hsa-le
ENSG0000	ENSG0000	1.89E-09	hsa-let-7a-5p;hsa-let-7b-5p;hsa-let-7e-5p;hsa-let-7f-5p;hsa-le
ENSG0000	ENSG0000	2.48E-08	hsa-let-7a-5p;hsa-let-7b-5p;hsa-let-7e-5p;hsa-let-7f-5p;hsa-le
ENSG0000	ENSG0000	7.21E-08	hsa-let-7a-5p;hsa-let-7b-5p;hsa-let-7e-5p;hsa-let-7f-5p;hsa-le
ENSG0000	ENSG0000	7.21E-08	hsa-let-7a-5p;hsa-let-7b-5p;hsa-let-7e-5p;hsa-let-7f-5p;hsa-le
ENSG0000	ENSG0000	2.80E-09	hsa-let-7a-5p;hsa-let-7b-5p;hsa-let-7e-5p;hsa-let-7f-5p;hsa-le
ENSG0000	ENSG0000	4.04E-09	hsa-let-7a-5p;hsa-let-7b-5p;hsa-let-7e-5p;hsa-let-7f-5p;hsa-le
ENSG0000	ENSG0000	1.55E-05	hsa-let-7a-5p;hsa-let-7b-5p;hsa-let-7f-5p;hsa-let-7g-5p;hsa-le
ENSG0000	ENSG0000	5.28E-07	hsa-let-7a-5p;hsa-let-7b-5p;hsa-let-7e-5p;hsa-let-7f-5p;hsa-le
ENSG0000	ENSG0000	3.20E-08	hsa-let-7a-5p;hsa-let-7b-5p;hsa-let-7e-5p;hsa-let-7f-5p;hsa-le
ENSG0000	ENSG0000	2.18E-08	hsa-let-7a-5p;hsa-let-7b-5p;hsa-let-7e-5p;hsa-let-7f-5p;hsa-le
ENSG0000	ENSG0000	9.78E-06	hsa-let-7a-5p;hsa-let-7b-5p;hsa-let-7f-5p;hsa-let-7g-5p;hsa-le
ENSG0000	ENSG0000	5.00E-12	hsa-let-7a-5p;hsa-let-7b-5p;hsa-let-7e-5p;hsa-let-7f-5p;hsa-le
ENSG0000	ENSG0000	4.72E-06	hsa-let-7a-5p;hsa-let-7b-5p;hsa-let-7e-5p;hsa-let-7f-5p;hsa-le
ENSG0000	ENSG0000	4.72E-06	hsa-let-7a-5p;hsa-let-7b-5p;hsa-let-7e-5p;hsa-let-7f-5p;hsa-le
ENSG0000	ENSG0000	1.16E-10	hsa-let-7a-5p;hsa-let-7b-5p;hsa-let-7e-5p;hsa-let-7f-5p;hsa-le
ENSG0000	ENSG0000	1.73E-11	hsa-let-7a-5p;hsa-let-7b-5p;hsa-let-7e-5p;hsa-let-7f-5p;hsa-le
ENSG0000	ENSG0000	1.21E-07	hsa-let-7a-5p;hsa-let-7b-5p;hsa-let-7e-5p;hsa-let-7f-5p;hsa-le
ENSG0000	ENSG0000	4.18E-07	hsa-let-7a-5p;hsa-let-7b-5p;hsa-let-7e-5p;hsa-let-7f-5p;hsa-le
ENSG0000	ENSG0000	8.03E-08	hsa-let-7a-5p;hsa-let-7b-5p;hsa-let-7e-5p;hsa-let-7f-5p;hsa-le
ENSG0000	ENSG0000	1.54E-07	hsa-let-7a-5p;hsa-let-7b-5p;hsa-let-7e-5p;hsa-let-7f-5p;hsa-le
ENSG0000	ENSG0000	1.32E-06	hsa-let-7a-5p;hsa-let-7b-5p;hsa-let-7e-5p;hsa-let-7f-5p;hsa-le
ENSG0000	ENSG0000	1.75E-06	hsa-let-7a-5p;hsa-let-7b-5p;hsa-let-7e-5p;hsa-let-7f-5p;hsa-le
ENSG0000	ENSG0000	2.99E-06	hsa-let-7a-5p;hsa-let-7b-5p;hsa-let-7e-5p;hsa-let-7f-5p;hsa-le
ENSG0000	ENSG0000	2.80E-09	hsa-let-7a-5p;hsa-let-7b-5p;hsa-let-7e-5p;hsa-let-7f-5p;hsa-le
ENSG0000	ENSG0000	2.67E-11	hsa-let-7a-5p;hsa-let-7b-5p;hsa-let-7e-5p;hsa-let-7f-5p;hsa-le
ENSG0000	ENSG0000	2.54E-07	hsa-let-7a-5p;hsa-let-7b-5p;hsa-let-7e-5p;hsa-let-7f-5p;hsa-le
ENSG0000	ENSG0000	2.31E-09	hsa-let-7a-5p;hsa-let-7b-5p;hsa-let-7e-5p;hsa-let-7f-5p;hsa-le
ENSG0000	ENSG0000	1.66E-08	hsa-let-7a-5p;hsa-let-7b-5p;hsa-let-7e-5p;hsa-let-7f-5p;hsa-le
ENSG0000	ENSG0000	2.86E-10	hsa-let-7a-5p;hsa-let-7b-5p;hsa-let-7e-5p;hsa-let-7f-5p;hsa-le
ENSG0000	ENSG0000	7.94E-10	hsa-let-7a-5p;hsa-let-7b-5p;hsa-let-7e-5p;hsa-let-7f-5p;hsa-le
ENSG0000	ENSG0000	7.94E-10	hsa-let-7a-5p;hsa-let-7b-5p;hsa-let-7e-5p;hsa-let-7f-5p;hsa-le
ENSG0000	ENSG0000	1.02E-05	hsa-let-7a-5p;hsa-let-7b-5p;hsa-let-7e-5p;hsa-let-7f-5p;hsa-le
ENSG0000	ENSG0000	3.37E-09	hsa-let-7a-5p;hsa-let-7b-5p;hsa-let-7e-5p;hsa-let-7f-5p;hsa-le
ENSG0000	ENSG0000	3.86E-07	hsa-let-7a-5p;hsa-let-7b-5p;hsa-let-7e-5p;hsa-let-7f-5p;hsa-le
ENSG0000	ENSG0000	1.08E-06	hsa-let-7a-5p;hsa-let-7b-5p;hsa-let-7e-5p;hsa-let-7f-5p;hsa-le
ENSG0000	ENSG0000	2.86E-10	hsa-let-7a-5p;hsa-let-7b-5p;hsa-let-7e-5p;hsa-let-7f-5p;hsa-le
ENSG0000	ENSG0000	6.09E-09	hsa-let-7a-5p;hsa-let-7b-5p;hsa-let-7e-5p;hsa-let-7f-5p;hsa-le
ENSG0000	ENSG0000	6.09E-09	hsa-let-7a-5p;hsa-let-7b-5p;hsa-let-7e-5p;hsa-let-7f-5p;hsa-le
ENSG0000	ENSG0000	3.30E-07	hsa-let-7e-5p;hsa-let-7f-5p;hsa-let-7g-5p;hsa-let-7i-5p;hsa-m
ENSG0000	ENSG0000	3.01E-07	hsa-let-7a-5p;hsa-let-7b-5p;hsa-let-7e-5p;hsa-let-7f-5p;hsa-le
ENSG0000	ENSG0000	4.04E-09	hsa-let-7a-5p;hsa-let-7b-5p;hsa-let-7e-5p;hsa-let-7f-5p;hsa-le
ENSG0000	ENSG0000	2.12E-07	hsa-let-7b-5p;hsa-let-7e-5p;hsa-let-7f-5p;hsa-let-7g-5p;hsa-le
ENSG0000	ENSG0000	1.73E-11	hsa-let-7a-5p;hsa-let-7b-5p;hsa-let-7e-5p;hsa-let-7f-5p;hsa-le
ENSG0000	ENSG0000	1.59E-10	hsa-let-7a-5p;hsa-let-7b-5p;hsa-let-7e-5p;hsa-let-7f-5p;hsa-le

ENSG0000	ENSG0000	1.59E-10	hsa-let-7a-5p;hsa-let-7b-5p;hsa-let-7e-5p;hsa-let-7f-5p;hsa-le
ENSG0000	ENSG0000	9.89E-08	hsa-let-7a-5p;hsa-let-7b-5p;hsa-let-7e-5p;hsa-let-7f-5p;hsa-le
ENSG0000	ENSG0000	1.60E-06	hsa-let-7a-5p;hsa-let-7b-5p;hsa-let-7e-5p;hsa-let-7f-5p;hsa-le
ENSG0000	ENSG0000	8.03E-08	hsa-let-7a-5p;hsa-let-7b-5p;hsa-let-7e-5p;hsa-let-7f-5p;hsa-le
ENSG0000	ENSG0000	1.32E-06	hsa-let-7a-5p;hsa-let-7b-5p;hsa-let-7e-5p;hsa-let-7f-5p;hsa-le
ENSG0000	ENSG0000	2.33E-07	hsa-let-7a-5p;hsa-let-7b-5p;hsa-let-7e-5p;hsa-let-7f-5p;hsa-le
ENSG0000	ENSG0000	2.79E-05	hsa-let-7a-5p;hsa-let-7e-5p;hsa-let-7f-5p;hsa-let-7g-5p;hsa-le
ENSG0000	ENSG0000	1.62E-07	hsa-let-7a-5p;hsa-let-7b-5p;hsa-let-7e-5p;hsa-let-7f-5p;hsa-le
ENSG0000	ENSG0000	4.04E-09	hsa-let-7a-5p;hsa-let-7b-5p;hsa-let-7e-5p;hsa-let-7f-5p;hsa-le
ENSG0000	ENSG0000	4.25E-06	hsa-let-7a-5p;hsa-let-7b-5p;hsa-let-7e-5p;hsa-let-7f-5p;hsa-le
ENSG0000	ENSG0000	3.58E-08	hsa-let-7a-5p;hsa-let-7b-5p;hsa-let-7e-5p;hsa-let-7f-5p;hsa-le
ENSG0000	ENSG0000	9.78E-06	hsa-let-7a-5p;hsa-let-7e-5p;hsa-let-7f-5p;hsa-let-7g-5p;hsa-le
ENSG0000	ENSG0000	7.65E-07	hsa-let-7a-5p;hsa-let-7b-5p;hsa-let-7e-5p;hsa-let-7f-5p;hsa-le
ENSG0000	ENSG0000	2.31E-09	hsa-let-7a-5p;hsa-let-7b-5p;hsa-let-7e-5p;hsa-let-7f-5p;hsa-le
ENSG0000	ENSG0000	3.37E-09	hsa-let-7a-5p;hsa-let-7b-5p;hsa-let-7e-5p;hsa-let-7f-5p;hsa-le
ENSG0000	ENSG0000	1.90E-08	hsa-let-7a-5p;hsa-let-7b-5p;hsa-let-7e-5p;hsa-let-7f-5p;hsa-le
ENSG0000	ENSG0000	4.21E-05	hsa-let-7a-5p;hsa-let-7b-5p;hsa-let-7e-5p;hsa-let-7f-5p;hsa-le
ENSG0000	ENSG0000	9.89E-08	hsa-let-7a-5p;hsa-let-7b-5p;hsa-let-7e-5p;hsa-let-7f-5p;hsa-le
ENSG0000	ENSG0000	1.54E-09	hsa-let-7a-5p;hsa-let-7b-5p;hsa-let-7e-5p;hsa-let-7f-5p;hsa-le
ENSG0000	ENSG0000	1.09E-07	hsa-let-7a-5p;hsa-let-7b-5p;hsa-let-7e-5p;hsa-let-7f-5p;hsa-le
ENSG0000	ENSG0000	9.23E-09	hsa-let-7a-5p;hsa-let-7b-5p;hsa-let-7e-5p;hsa-let-7f-5p;hsa-le
ENSG0000	ENSG0000	2.53E-06	hsa-let-7b-5p;hsa-let-7e-5p;hsa-let-7f-5p;hsa-let-7g-5p;hsa-le
ENSG0000	ENSG0000	1.41E-06	hsa-let-7a-5p;hsa-let-7b-5p;hsa-let-7e-5p;hsa-let-7f-5p;hsa-le
ENSG0000	ENSG0000	6.97E-08	hsa-let-7a-5p;hsa-let-7b-5p;hsa-let-7e-5p;hsa-let-7f-5p;hsa-le
ENSG0000	ENSG0000	9.28E-07	hsa-let-7a-5p;hsa-let-7b-5p;hsa-let-7e-5p;hsa-let-7f-5p;hsa-le
ENSG0000	ENSG0000	1.16E-10	hsa-let-7a-5p;hsa-let-7b-5p;hsa-let-7e-5p;hsa-let-7f-5p;hsa-le
ENSG0000	ENSG0000	1.62E-07	hsa-let-7a-5p;hsa-let-7b-5p;hsa-let-7e-5p;hsa-let-7f-5p;hsa-le
ENSG0000	ENSG0000	6.14E-07	hsa-let-7a-5p;hsa-let-7b-5p;hsa-let-7e-5p;hsa-let-7f-5p;hsa-le
ENSG0000	ENSG0000	8.91E-06	hsa-let-7a-5p;hsa-let-7b-5p;hsa-let-7e-5p;hsa-let-7f-5p;hsa-le
ENSG0000	ENSG0000	3.20E-08	hsa-let-7a-5p;hsa-let-7b-5p;hsa-let-7e-5p;hsa-let-7f-5p;hsa-le
ENSG0000	ENSG0000	6.38E-06	hsa-let-7a-5p;hsa-let-7b-5p;hsa-let-7e-5p;hsa-let-7f-5p;hsa-le
ENSG0000	ENSG0000	1.47E-07	hsa-let-7a-5p;hsa-let-7b-5p;hsa-let-7e-5p;hsa-let-7f-5p;hsa-le
ENSG0000	ENSG0000	7.89E-09	hsa-let-7a-5p;hsa-let-7b-5p;hsa-let-7e-5p;hsa-let-7f-5p;hsa-le
ENSG0000	ENSG0000	0.000114	hsa-let-7a-5p;hsa-let-7e-5p;hsa-let-7f-5p;hsa-let-7g-5p;hsa-le
ENSG0000	ENSG0000	2.33E-07	hsa-let-7a-5p;hsa-let-7b-5p;hsa-let-7e-5p;hsa-let-7f-5p;hsa-le
ENSG0000	ENSG0000	3.28E-07	hsa-let-7a-5p;hsa-let-7b-5p;hsa-let-7e-5p;hsa-let-7f-5p;hsa-le
ENSG0000	ENSG0000	1.45E-07	hsa-let-7a-5p;hsa-let-7b-5p;hsa-let-7e-5p;hsa-let-7f-5p;hsa-le
ENSG0000	ENSG0000	3.75E-10	hsa-let-7a-5p;hsa-let-7b-5p;hsa-let-7e-5p;hsa-let-7f-5p;hsa-le
ENSG0000	ENSG0000	5.82E-11	hsa-let-7a-5p;hsa-let-7b-5p;hsa-let-7e-5p;hsa-let-7f-5p;hsa-le
ENSG0000	ENSG0000	3.31E-08	hsa-let-7a-5p;hsa-let-7b-5p;hsa-let-7e-5p;hsa-let-7f-5p;hsa-le
ENSG0000	ENSG0000	1.12E-07	hsa-let-7a-5p;hsa-let-7b-5p;hsa-let-7e-5p;hsa-let-7f-5p;hsa-le
ENSG0000	ENSG0000	2.12E-07	hsa-let-7a-5p;hsa-let-7b-5p;hsa-let-7e-5p;hsa-let-7g-5p;hsa-l
ENSG0000	ENSG0000	8.92E-08	hsa-let-7a-5p;hsa-let-7b-5p;hsa-let-7e-5p;hsa-let-7f-5p;hsa-le
ENSG0000	ENSG0000	1.60E-06	hsa-let-7a-5p;hsa-let-7b-5p;hsa-let-7e-5p;hsa-let-7f-5p;hsa-le
ENSG0000	ENSG0000	7.47E-08	hsa-let-7a-5p;hsa-let-7b-5p;hsa-let-7e-5p;hsa-let-7f-5p;hsa-le
ENSG0000	ENSG0000	2.15E-10	hsa-let-7a-5p;hsa-let-7b-5p;hsa-let-7e-5p;hsa-let-7f-5p;hsa-le
ENSG0000	ENSG0000	1.08E-10	hsa-let-7a-5p;hsa-let-7b-5p;hsa-let-7e-5p;hsa-let-7f-5p;hsa-le
ENSG0000	ENSG0000	1.70E-06	hsa-let-7a-5p;hsa-let-7b-5p;hsa-let-7e-5p;hsa-let-7f-5p;hsa-le
ENSG0000	ENSG0000	8.93E-10	hsa-let-7a-5p;hsa-let-7b-5p;hsa-let-7e-5p;hsa-let-7f-5p;hsa-le
ENSG0000	ENSG0000	6.70E-06	hsa-let-7a-5p;hsa-let-7b-5p;hsa-let-7e-5p;hsa-let-7f-5p;hsa-le
ENSG0000	ENSG0000	5.70E-07	hsa-let-7a-5p;hsa-let-7b-5p;hsa-let-7e-5p;hsa-let-7f-5p;hsa-le
ENSG0000	ENSG0000	1.53E-06	hsa-let-7a-5p;hsa-let-7b-5p;hsa-let-7e-5p;hsa-let-7f-5p;hsa-le
ENSG0000	ENSG0000	2.31E-09	hsa-let-7a-5p;hsa-let-7b-5p;hsa-let-7e-5p;hsa-let-7f-5p;hsa-le
ENSG0000	ENSG0000	8.92E-08	hsa-let-7a-5p;hsa-let-7b-5p;hsa-let-7e-5p;hsa-let-7f-5p;hsa-le

ENSG0000	ENSG0000	3.20E-08	hsa-let-7a-5p;hsa-let-7b-5p;hsa-let-7e-5p;hsa-let-7f-5p;hsa-le
ENSG0000	ENSG0000	1.21E-07	hsa-let-7a-5p;hsa-let-7b-5p;hsa-let-7e-5p;hsa-let-7f-5p;hsa-le
ENSG0000	ENSG0000	1.54E-09	hsa-let-7a-5p;hsa-let-7b-5p;hsa-let-7e-5p;hsa-let-7f-5p;hsa-le
ENSG0000	ENSG0000	2.26E-05	hsa-let-7a-5p;hsa-let-7b-5p;hsa-let-7e-5p;hsa-let-7f-5p;hsa-le
ENSG0000	ENSG0000	6.25E-10	hsa-let-7a-5p;hsa-let-7b-5p;hsa-let-7e-5p;hsa-let-7f-5p;hsa-le
ENSG0000	ENSG0000	4.48E-06	hsa-let-7a-5p;hsa-let-7b-5p;hsa-let-7e-5p;hsa-let-7f-5p;hsa-le
ENSG0000	ENSG0000	1.47E-07	hsa-let-7a-5p;hsa-let-7b-5p;hsa-let-7e-5p;hsa-let-7f-5p;hsa-le
ENSG0000	ENSG0000	2.22E-07	hsa-let-7a-5p;hsa-let-7b-5p;hsa-let-7e-5p;hsa-let-7f-5p;hsa-le
ENSG0000	ENSG0000	5.70E-09	hsa-let-7a-5p;hsa-let-7b-5p;hsa-let-7e-5p;hsa-let-7f-5p;hsa-le
ENSG0000	ENSG0000	6.33E-10	hsa-let-7a-5p;hsa-let-7b-5p;hsa-let-7e-5p;hsa-let-7f-5p;hsa-le
ENSG0000	ENSG0000	4.97E-06	hsa-let-7a-5p;hsa-let-7b-5p;hsa-let-7e-5p;hsa-let-7f-5p;hsa-le
ENSG0000	ENSG0000	5.16E-08	hsa-let-7a-5p;hsa-let-7b-5p;hsa-let-7e-5p;hsa-let-7f-5p;hsa-le
ENSG0000	ENSG0000	7.94E-10	hsa-let-7a-5p;hsa-let-7b-5p;hsa-let-7e-5p;hsa-let-7f-5p;hsa-le
ENSG0000	ENSG0000	2.18E-08	hsa-let-7a-5p;hsa-let-7b-5p;hsa-let-7e-5p;hsa-let-7f-5p;hsa-le
ENSG0000	ENSG0000	4.50E-08	hsa-let-7a-5p;hsa-let-7b-5p;hsa-let-7e-5p;hsa-let-7f-5p;hsa-le
ENSG0000	ENSG0000	1.31E-08	hsa-let-7a-5p;hsa-let-7b-5p;hsa-let-7e-5p;hsa-let-7f-5p;hsa-le
ENSG0000	ENSG0000	1.90E-08	hsa-let-7a-5p;hsa-let-7b-5p;hsa-let-7e-5p;hsa-let-7f-5p;hsa-le
ENSG0000	ENSG0000	5.18E-05	hsa-let-7a-5p;hsa-let-7e-5p;hsa-let-7f-5p;hsa-let-7g-5p;hsa-le
ENSG0000	ENSG0000	6.72E-09	hsa-let-7a-5p;hsa-let-7b-5p;hsa-let-7e-5p;hsa-let-7f-5p;hsa-le
ENSG0000	ENSG0000	0.000119	hsa-let-7a-5p;hsa-let-7e-5p;hsa-let-7f-5p;hsa-let-7g-5p;hsa-le
ENSG0000	ENSG0000	2.67E-11	hsa-let-7a-5p;hsa-let-7b-5p;hsa-let-7e-5p;hsa-let-7f-5p;hsa-le
ENSG0000	ENSG0000	1.21E-07	hsa-let-7a-5p;hsa-let-7b-5p;hsa-let-7e-5p;hsa-let-7f-5p;hsa-le
ENSG0000	ENSG0000	6.72E-09	hsa-let-7a-5p;hsa-let-7b-5p;hsa-let-7e-5p;hsa-let-7f-5p;hsa-le
ENSG0000	ENSG0000	7.74E-06	hsa-let-7a-5p;hsa-let-7b-5p;hsa-let-7e-5p;hsa-let-7f-5p;hsa-le
ENSG0000	ENSG0000	6.47E-08	hsa-let-7a-5p;hsa-let-7b-5p;hsa-let-7e-5p;hsa-let-7f-5p;hsa-le
ENSG0000	ENSG0000	3.93E-05	hsa-let-7a-5p;hsa-let-7b-5p;hsa-let-7e-5p;hsa-let-7f-5p;hsa-le
ENSG0000	ENSG0000	1.01E-05	hsa-miR-30a-5p;hsa-miR-30b-5p;hsa-miR-30c-5p;hsa-miR-30c
ENSG0000	ENSG0000	2.48E-07	hsa-miR-30a-5p;hsa-miR-30b-5p;hsa-miR-30c-5p;hsa-miR-30c
ENSG0000	ENSG0000	2.59E-09	hsa-miR-30a-5p;hsa-miR-30b-5p;hsa-miR-30c-5p;hsa-miR-30c
ENSG0000	ENSG0000	2.48E-07	hsa-miR-30a-5p;hsa-miR-30b-5p;hsa-miR-30c-5p;hsa-miR-30c
ENSG0000	ENSG0000	8.65E-06	hsa-miR-30a-5p;hsa-miR-30b-5p;hsa-miR-30c-5p;hsa-miR-30c
ENSG0000	ENSG0000	1.44E-05	hsa-miR-30b-5p;hsa-miR-30c-5p;hsa-miR-30d-5p;hsa-miR-30e
ENSG0000	ENSG0000	2.31E-05	hsa-miR-30a-5p;hsa-miR-30b-5p;hsa-miR-30c-5p;hsa-miR-30c
ENSG0000	ENSG0000	2.58E-08	hsa-miR-30a-5p;hsa-miR-30b-5p;hsa-miR-30d-5p;hsa-miR-30e
ENSG0000	ENSG0000	6.63E-08	hsa-miR-30a-5p;hsa-miR-30b-5p;hsa-miR-30c-5p;hsa-miR-30c
ENSG0000	ENSG0000	7.93E-09	hsa-miR-30a-5p;hsa-miR-30b-5p;hsa-miR-30c-5p;hsa-miR-30c
ENSG0000	ENSG0000	1.01E-05	hsa-miR-30a-5p;hsa-miR-30b-5p;hsa-miR-30d-5p;hsa-miR-30e
ENSG0000	ENSG0000	3.93E-10	hsa-miR-140-3p;hsa-miR-30a-5p;hsa-miR-30b-5p;hsa-miR-30c
ENSG0000	ENSG0000	7.95E-07	hsa-miR-30a-5p;hsa-miR-30b-5p;hsa-miR-30c-5p;hsa-miR-30c
ENSG0000	ENSG0000	1.32E-12	hsa-miR-30a-5p;hsa-miR-30b-5p;hsa-miR-30c-5p;hsa-miR-30c
ENSG0000	ENSG0000	3.04E-06	hsa-miR-30a-5p;hsa-miR-30b-5p;hsa-miR-30c-5p;hsa-miR-30c
ENSG0000	ENSG0000	2.76E-11	hsa-miR-30a-5p;hsa-miR-30b-5p;hsa-miR-30c-5p;hsa-miR-30c
ENSG0000	ENSG0000	1.01E-07	hsa-miR-30a-5p;hsa-miR-30b-5p;hsa-miR-30c-5p;hsa-miR-30c
ENSG0000	ENSG0000	1.48E-08	hsa-miR-30a-5p;hsa-miR-30b-5p;hsa-miR-30c-5p;hsa-miR-30c
ENSG0000	ENSG0000	1.77E-07	hsa-miR-30a-5p;hsa-miR-30b-5p;hsa-miR-30c-5p;hsa-miR-30c
ENSG0000	ENSG0000	6.02E-10	hsa-miR-30a-5p;hsa-miR-30b-5p;hsa-miR-30c-5p;hsa-miR-30c
ENSG0000	ENSG0000	3.29E-10	hsa-miR-30a-5p;hsa-miR-30b-5p;hsa-miR-30c-5p;hsa-miR-30c
ENSG0000	ENSG0000	1.32E-12	hsa-miR-30a-5p;hsa-miR-30b-5p;hsa-miR-30c-5p;hsa-miR-30c
ENSG0000	ENSG0000	1.30E-06	hsa-miR-30a-5p;hsa-miR-30b-5p;hsa-miR-30c-5p;hsa-miR-30c
ENSG0000	ENSG0000	1.03E-09	hsa-miR-21-3p;hsa-miR-30a-5p;hsa-miR-30b-5p;hsa-miR-30c-
ENSG0000	ENSG0000	7.95E-07	hsa-miR-30a-5p;hsa-miR-30b-5p;hsa-miR-30c-5p;hsa-miR-30c
ENSG0000	ENSG0000	2.84E-05	hsa-miR-30b-5p;hsa-miR-30c-5p;hsa-miR-30d-5p;hsa-miR-30e
ENSG0000	ENSG0000	1.46E-06	hsa-miR-30a-5p;hsa-miR-30b-5p;hsa-miR-30c-5p;hsa-miR-30c
ENSG0000	ENSG0000	8.73E-07	hsa-miR-30a-5p;hsa-miR-30c-5p;hsa-miR-30d-5p;hsa-miR-30e

ENSG0000	ENSG0000	2.25E-06	hsa-miR-30a-5p;hsa-miR-30b-5p;hsa-miR-30c-5p;hsa-miR-30c
ENSG0000	ENSG0000	4.25E-08	hsa-miR-30a-5p;hsa-miR-30b-5p;hsa-miR-30c-5p;hsa-miR-30c
ENSG0000	ENSG0000	5.86E-08	hsa-miR-30a-5p;hsa-miR-30c-5p;hsa-miR-30d-5p;hsa-miR-30e
ENSG0000	ENSG0000	1.82E-06	hsa-miR-30a-5p;hsa-miR-30b-5p;hsa-miR-30c-5p;hsa-miR-30c
ENSG0000	ENSG0000	9.07E-05	hsa-miR-30a-5p;hsa-miR-30b-5p;hsa-miR-30c-5p;hsa-miR-30c
ENSG0000	ENSG0000	1.45E-05	hsa-miR-30a-5p;hsa-miR-30b-5p;hsa-miR-30c-5p;hsa-miR-30c
ENSG0000	ENSG0000	5.35E-08	hsa-miR-30a-5p;hsa-miR-30b-5p;hsa-miR-30c-5p;hsa-miR-30c
ENSG0000	ENSG0000	9.03E-07	hsa-miR-30a-5p;hsa-miR-30b-5p;hsa-miR-30c-5p;hsa-miR-30c
ENSG0000	ENSG0000	1.10E-08	hsa-miR-30a-5p;hsa-miR-30b-5p;hsa-miR-30c-5p;hsa-miR-30c
ENSG0000	ENSG0000	1.22E-07	hsa-miR-30a-5p;hsa-miR-30b-5p;hsa-miR-30c-5p;hsa-miR-30c
ENSG0000	ENSG0000	2.25E-06	hsa-miR-30a-5p;hsa-miR-30b-5p;hsa-miR-30c-5p;hsa-miR-30c
ENSG0000	ENSG0000	2.58E-08	hsa-miR-30a-5p;hsa-miR-30b-5p;hsa-miR-30c-5p;hsa-miR-30c
ENSG0000	ENSG0000	1.65E-10	hsa-miR-30a-5p;hsa-miR-30b-5p;hsa-miR-30c-5p;hsa-miR-30c
ENSG0000	ENSG0000	1.66E-05	hsa-miR-30a-5p;hsa-miR-30b-5p;hsa-miR-30c-5p;hsa-miR-30c
ENSG0000	ENSG0000	7.35E-11	hsa-miR-30a-5p;hsa-miR-30b-5p;hsa-miR-30c-5p;hsa-miR-30c
ENSG0000	ENSG0000	3.68E-06	hsa-miR-30a-5p;hsa-miR-30b-5p;hsa-miR-30c-5p;hsa-miR-30c
ENSG0000	ENSG0000	9.70E-06	hsa-miR-30b-5p;hsa-miR-30c-5p;hsa-miR-30d-5p;hsa-miR-30e
ENSG0000	ENSG0000	1.67E-09	hsa-miR-30a-5p;hsa-miR-30b-5p;hsa-miR-30c-5p;hsa-miR-30c
ENSG0000	ENSG0000	5.31E-07	hsa-miR-30a-5p;hsa-miR-30b-5p;hsa-miR-30c-5p;hsa-miR-30c
ENSG0000	ENSG0000	4.21E-05	hsa-miR-30a-5p;hsa-miR-30c-5p;hsa-miR-30d-5p;hsa-miR-30e
ENSG0000	ENSG0000	3.82E-06	hsa-miR-30a-5p;hsa-miR-30b-5p;hsa-miR-30c-5p;hsa-miR-30c
ENSG0000	ENSG0000	0.000217	hsa-miR-30b-5p;hsa-miR-30c-5p;hsa-miR-30d-5p;hsa-miR-30e
ENSG0000	ENSG0000	7.92E-12	hsa-miR-30a-5p;hsa-miR-30b-5p;hsa-miR-30c-5p;hsa-miR-30c
ENSG0000	ENSG0000	7.35E-11	hsa-miR-30a-5p;hsa-miR-30b-5p;hsa-miR-30c-5p;hsa-miR-30c
ENSG0000	ENSG0000	3.97E-07	hsa-miR-30a-5p;hsa-miR-30b-5p;hsa-miR-30c-5p;hsa-miR-30c
ENSG0000	ENSG0000	3.32E-05	hsa-miR-30a-5p;hsa-miR-30c-5p;hsa-miR-30d-5p;hsa-miR-30e
ENSG0000	ENSG0000	6.38E-05	hsa-miR-30a-5p;hsa-miR-30b-5p;hsa-miR-30c-5p;hsa-miR-30c
ENSG0000	ENSG0000	8.55E-05	hsa-miR-30a-5p;hsa-miR-30b-5p;hsa-miR-30c-5p;hsa-miR-30c
ENSG0000	ENSG0000	2.48E-07	hsa-miR-30a-5p;hsa-miR-30b-5p;hsa-miR-30c-5p;hsa-miR-30c
ENSG0000	ENSG0000	3.33E-08	hsa-miR-30a-5p;hsa-miR-30b-5p;hsa-miR-30c-5p;hsa-miR-30c
ENSG0000	ENSG0000	0.002545	hsa-miR-30b-5p;hsa-miR-30c-5p;hsa-miR-30d-5p;hsa-miR-30e
ENSG0000	ENSG0000	0.000897	hsa-miR-140-3p;hsa-miR-30a-5p;hsa-miR-30b-5p;hsa-miR-30c
ENSG0000	ENSG0000	4.60E-07	hsa-miR-30a-5p;hsa-miR-30b-5p;hsa-miR-30c-5p;hsa-miR-30c
ENSG0000	ENSG0000	5.04E-07	hsa-miR-30b-5p;hsa-miR-30c-5p;hsa-miR-30d-5p;hsa-miR-30e
ENSG0000	ENSG0000	8.34E-05	hsa-miR-30b-5p;hsa-miR-30c-5p;hsa-miR-30d-5p;hsa-miR-30e
ENSG0000	ENSG0000	2.65E-07	hsa-miR-30a-5p;hsa-miR-30c-5p;hsa-miR-30e-5p;hsa-miR-34a
ENSG0000	ENSG0000	3.82E-06	hsa-miR-30a-5p;hsa-miR-30b-5p;hsa-miR-30d-5p;hsa-miR-30e
ENSG0000	ENSG0000	4.25E-08	hsa-miR-30a-5p;hsa-miR-30b-5p;hsa-miR-30c-5p;hsa-miR-30c
ENSG0000	ENSG0000	4.25E-08	hsa-miR-30a-5p;hsa-miR-30b-5p;hsa-miR-30c-5p;hsa-miR-30c
ENSG0000	ENSG0000	3.70E-07	hsa-miR-30b-5p;hsa-miR-30c-5p;hsa-miR-30d-5p;hsa-miR-30e
ENSG0000	ENSG0000	5.76E-05	hsa-miR-30b-5p;hsa-miR-30c-5p;hsa-miR-30d-5p;hsa-miR-30e
ENSG0000	ENSG0000	1.65E-10	hsa-miR-30a-5p;hsa-miR-30b-5p;hsa-miR-30c-5p;hsa-miR-30c
ENSG0000	ENSG0000	2.46E-05	hsa-miR-30a-5p;hsa-miR-30b-5p;hsa-miR-30c-5p;hsa-miR-30c
ENSG0000	ENSG0000	3.87E-09	hsa-miR-30a-5p;hsa-miR-30b-5p;hsa-miR-30c-5p;hsa-miR-30c
ENSG0000	ENSG0000	5.35E-06	hsa-miR-30a-5p;hsa-miR-30b-5p;hsa-miR-30d-5p;hsa-miR-30e
ENSG0000	ENSG0000	2.58E-08	hsa-miR-30a-5p;hsa-miR-30b-5p;hsa-miR-30c-5p;hsa-miR-30c
ENSG0000	ENSG0000	1.32E-12	hsa-miR-30a-5p;hsa-miR-30b-5p;hsa-miR-30c-5p;hsa-miR-30c
ENSG0000	ENSG0000	1.82E-06	hsa-miR-30a-5p;hsa-miR-30b-5p;hsa-miR-30c-5p;hsa-miR-30c
ENSG0000	ENSG0000	1.22E-07	hsa-miR-30a-5p;hsa-miR-30b-5p;hsa-miR-30c-5p;hsa-miR-30c
ENSG0000	ENSG0000	7.86E-08	hsa-miR-30b-5p;hsa-miR-30c-5p;hsa-miR-30d-5p;hsa-miR-30e
ENSG0000	ENSG0000	3.97E-07	hsa-miR-30a-5p;hsa-miR-30b-5p;hsa-miR-30c-5p;hsa-miR-30c
ENSG0000	ENSG0000	2.59E-09	hsa-miR-30a-5p;hsa-miR-30b-5p;hsa-miR-30c-5p;hsa-miR-30c
ENSG0000	ENSG0000	4.91E-05	hsa-miR-30a-5p;hsa-miR-30b-5p;hsa-miR-30c-5p;hsa-miR-30c
ENSG0000	ENSG0000	1.32E-12	hsa-miR-30a-5p;hsa-miR-30b-5p;hsa-miR-30c-5p;hsa-miR-30c

ENSG0000	ENSG0000	5.04E-07	hsa-miR-30a-5p;hsa-miR-30b-5p;hsa-miR-30d-5p;hsa-miR-30
ENSG0000	ENSG0000	2.48E-07	hsa-miR-30a-5p;hsa-miR-30b-5p;hsa-miR-30c-5p;hsa-miR-30
ENSG0000	ENSG0000	6.09E-07	hsa-miR-30a-5p;hsa-miR-30b-5p;hsa-miR-30c-5p;hsa-miR-30
ENSG0000	ENSG0000	2.49E-06	hsa-miR-30a-5p;hsa-miR-30b-5p;hsa-miR-30c-5p;hsa-miR-30
ENSG0000	ENSG0000	3.41E-07	hsa-miR-30a-5p;hsa-miR-30b-5p;hsa-miR-30c-5p;hsa-miR-30
ENSG0000	ENSG0000	2.54E-06	hsa-miR-30a-5p;hsa-miR-30b-5p;hsa-miR-30c-5p;hsa-miR-30
ENSG0000	ENSG0000	1.01E-05	hsa-miR-30a-5p;hsa-miR-30b-5p;hsa-miR-30c-5p;hsa-miR-30
ENSG0000	ENSG0000	7.35E-11	hsa-miR-30a-5p;hsa-miR-30b-5p;hsa-miR-30c-5p;hsa-miR-30
ENSG0000	ENSG0000	6.21E-07	hsa-miR-192-3p;hsa-miR-21-3p;hsa-miR-30a-5p;hsa-miR-30b-
ENSG0000	ENSG0000	0.001149	hsa-miR-30a-5p;hsa-miR-30b-5p;hsa-miR-30c-5p;hsa-miR-30
ENSG0000	ENSG0000	1.66E-05	hsa-miR-30a-5p;hsa-miR-30b-5p;hsa-miR-30c-5p;hsa-miR-30
ENSG0000	ENSG0000	1.01E-07	hsa-miR-30a-5p;hsa-miR-30b-5p;hsa-miR-30c-5p;hsa-miR-30
ENSG0000	ENSG0000	6.97E-07	hsa-miR-30a-5p;hsa-miR-30b-5p;hsa-miR-30c-5p;hsa-miR-30
ENSG0000	ENSG0000	3.99E-08	hsa-miR-140-3p;hsa-miR-30a-5p;hsa-miR-30b-5p;hsa-miR-30
ENSG0000	ENSG0000	5.82E-06	hsa-miR-140-3p;hsa-miR-30a-5p;hsa-miR-30b-5p;hsa-miR-30
ENSG0000	ENSG0000	5.82E-06	hsa-miR-140-3p;hsa-miR-30a-5p;hsa-miR-30b-5p;hsa-miR-30
ENSG0000	ENSG0000	2.92E-07	hsa-miR-30a-5p;hsa-miR-30b-5p;hsa-miR-30c-5p;hsa-miR-30
ENSG0000	ENSG0000	1.97E-08	hsa-miR-30a-5p;hsa-miR-30b-5p;hsa-miR-30c-5p;hsa-miR-30
ENSG0000	ENSG0000	5.82E-06	hsa-miR-30a-5p;hsa-miR-30b-5p;hsa-miR-30c-5p;hsa-miR-30
ENSG0000	ENSG0000	1.90E-05	hsa-miR-30a-5p;hsa-miR-30b-5p;hsa-miR-30c-5p;hsa-miR-30
ENSG0000	ENSG0000	2.76E-11	hsa-miR-30a-5p;hsa-miR-30b-5p;hsa-miR-30c-5p;hsa-miR-30
ENSG0000	ENSG0000	3.04E-06	hsa-miR-30a-5p;hsa-miR-30b-5p;hsa-miR-30c-5p;hsa-miR-30
ENSG0000	ENSG0000	0.000179	hsa-miR-30a-5p;hsa-miR-30b-5p;hsa-miR-30c-5p;hsa-miR-30
ENSG0000	ENSG0000	2.03E-06	hsa-miR-140-3p;hsa-miR-30a-5p;hsa-miR-30c-5p;hsa-miR-30
ENSG0000	ENSG0000	2.76E-11	hsa-miR-30a-5p;hsa-miR-30b-5p;hsa-miR-30c-5p;hsa-miR-30
ENSG0000	ENSG0000	7.93E-09	hsa-miR-30a-5p;hsa-miR-30b-5p;hsa-miR-30c-5p;hsa-miR-30
ENSG0000	ENSG0000	2.58E-08	hsa-miR-30a-5p;hsa-miR-30b-5p;hsa-miR-30c-5p;hsa-miR-30
ENSG0000	ENSG0000	2.59E-09	hsa-miR-30a-5p;hsa-miR-30b-5p;hsa-miR-30c-5p;hsa-miR-30
ENSG0000	ENSG0000	6.09E-07	hsa-miR-30a-5p;hsa-miR-30b-5p;hsa-miR-30c-5p;hsa-miR-30
ENSG0000	ENSG0000	1.26E-05	hsa-miR-30a-5p;hsa-miR-30b-5p;hsa-miR-30c-5p;hsa-miR-30
ENSG0000	ENSG0000	1.21E-11	hsa-miR-30a-5p;hsa-miR-30b-5p;hsa-miR-30c-5p;hsa-miR-30
ENSG0000	ENSG0000	3.87E-09	hsa-miR-30a-5p;hsa-miR-30b-5p;hsa-miR-30c-5p;hsa-miR-30
ENSG0000	ENSG0000	3.41E-07	hsa-miR-30a-5p;hsa-miR-30b-5p;hsa-miR-30c-5p;hsa-miR-30
ENSG0000	ENSG0000	3.33E-08	hsa-miR-30a-5p;hsa-miR-30b-5p;hsa-miR-30c-5p;hsa-miR-30
ENSG0000	ENSG0000	2.10E-07	hsa-miR-192-3p;hsa-miR-30a-5p;hsa-miR-30c-5p;hsa-miR-30
ENSG0000	ENSG0000	8.90E-06	hsa-miR-30a-5p;hsa-miR-30b-5p;hsa-miR-30c-5p;hsa-miR-30
ENSG0000	ENSG0000	1.97E-08	hsa-miR-30a-5p;hsa-miR-30b-5p;hsa-miR-30c-5p;hsa-miR-30
ENSG0000	ENSG0000	3.20E-06	hsa-miR-30b-5p;hsa-miR-30c-5p;hsa-miR-30d-5p;hsa-miR-30
ENSG0000	ENSG0000	3.87E-09	hsa-miR-30a-5p;hsa-miR-30b-5p;hsa-miR-30c-5p;hsa-miR-30
ENSG0000	ENSG0000	1.48E-07	hsa-miR-30a-5p;hsa-miR-30b-5p;hsa-miR-30c-5p;hsa-miR-30
ENSG0000	ENSG0000	0.00051	hsa-miR-30a-5p;hsa-miR-30c-5p;hsa-miR-30d-5p;hsa-miR-30
ENSG0000	ENSG0000	0.000374	hsa-miR-30a-5p;hsa-miR-30b-5p;hsa-miR-30c-5p;hsa-miR-30
ENSG0000	ENSG0000	2.30E-09	hsa-miR-21-3p;hsa-miR-30a-5p;hsa-miR-30b-5p;hsa-miR-30c-
ENSG0000	ENSG0000	1.32E-12	hsa-miR-30a-5p;hsa-miR-30b-5p;hsa-miR-30c-5p;hsa-miR-30
ENSG0000	ENSG0000	1.67E-09	hsa-miR-30a-5p;hsa-miR-30b-5p;hsa-miR-30c-5p;hsa-miR-30
ENSG0000	ENSG0000	1.35E-05	hsa-miR-30a-5p;hsa-miR-30b-5p;hsa-miR-30c-5p;hsa-miR-30
ENSG0000	ENSG0000	5.04E-07	hsa-miR-30b-5p;hsa-miR-30c-5p;hsa-miR-30d-5p;hsa-miR-30
ENSG0000	ENSG0000	0.000134	hsa-miR-30a-5p;hsa-miR-30b-5p;hsa-miR-30c-5p;hsa-miR-30
ENSG0000	ENSG0000	6.02E-10	hsa-miR-30a-5p;hsa-miR-30b-5p;hsa-miR-30c-5p;hsa-miR-30
ENSG0000	ENSG0000	1.02E-06	hsa-miR-30a-5p;hsa-miR-30b-5p;hsa-miR-30c-5p;hsa-miR-30
ENSG0000	ENSG0000	3.54E-07	hsa-miR-30a-5p;hsa-miR-30b-5p;hsa-miR-30c-5p;hsa-miR-30
ENSG0000	ENSG0000	3.97E-10	hsa-miR-30a-5p;hsa-miR-30b-5p;hsa-miR-30c-5p;hsa-miR-30
ENSG0000	ENSG0000	1.90E-05	hsa-miR-30a-5p;hsa-miR-30b-5p;hsa-miR-30c-5p;hsa-miR-30
ENSG0000	ENSG0000	3.87E-09	hsa-miR-30a-5p;hsa-miR-30b-5p;hsa-miR-30c-5p;hsa-miR-30

ENSG0000	ENSG0000	3.87E-09	hsa-miR-30a-5p;hsa-miR-30b-5p;hsa-miR-30c-5p;hsa-miR-30
ENSG0000	ENSG0000	3.33E-08	hsa-miR-30a-5p;hsa-miR-30b-5p;hsa-miR-30c-5p;hsa-miR-30
ENSG0000	ENSG0000	3.33E-08	hsa-miR-30a-5p;hsa-miR-30b-5p;hsa-miR-30c-5p;hsa-miR-30
ENSG0000	ENSG0000	0.000192	hsa-miR-30a-5p;hsa-miR-30b-5p;hsa-miR-30e-5p;hsa-miR-54
ENSG0000	ENSG0000	7.92E-12	hsa-miR-30a-5p;hsa-miR-30b-5p;hsa-miR-30c-5p;hsa-miR-30
ENSG0000	ENSG0000	2.31E-05	hsa-miR-30a-5p;hsa-miR-30b-5p;hsa-miR-30c-5p;hsa-miR-30
ENSG0000	ENSG0000	5.31E-07	hsa-miR-30a-5p;hsa-miR-30b-5p;hsa-miR-30c-5p;hsa-miR-30
ENSG0000	ENSG0000	1.01E-07	hsa-miR-30a-5p;hsa-miR-30b-5p;hsa-miR-30c-5p;hsa-miR-30
ENSG0000	ENSG0000	0.003973	hsa-miR-30a-5p;hsa-miR-30c-5p;hsa-miR-30d-5p;hsa-miR-30
ENSG0000	ENSG0000	1.76E-06	hsa-miR-30a-5p;hsa-miR-30b-5p;hsa-miR-30d-5p;hsa-miR-30
ENSG0000	ENSG0000	1.82E-06	hsa-miR-30a-5p;hsa-miR-30b-5p;hsa-miR-30c-5p;hsa-miR-30
ENSG0000	ENSG0000	1.10E-08	hsa-miR-30a-5p;hsa-miR-30b-5p;hsa-miR-30c-5p;hsa-miR-30
ENSG0000	ENSG0000	1.48E-07	hsa-miR-30a-5p;hsa-miR-30b-5p;hsa-miR-30c-5p;hsa-miR-30
ENSG0000	ENSG0000	2.84E-05	hsa-miR-30a-5p;hsa-miR-30b-5p;hsa-miR-30d-5p;hsa-miR-30
ENSG0000	ENSG0000	6.97E-07	hsa-miR-30a-5p;hsa-miR-30b-5p;hsa-miR-30c-5p;hsa-miR-30
ENSG0000	ENSG0000	1.32E-12	hsa-miR-30a-5p;hsa-miR-30b-5p;hsa-miR-30c-5p;hsa-miR-30
ENSG0000	ENSG0000	3.47E-05	hsa-miR-30b-5p;hsa-miR-30c-5p;hsa-miR-30d-5p;hsa-miR-30
ENSG0000	ENSG0000	0.003007	hsa-miR-30a-5p;hsa-miR-30b-5p;hsa-miR-30c-5p;hsa-miR-30
ENSG0000	ENSG0000	1.22E-07	hsa-miR-30a-5p;hsa-miR-30b-5p;hsa-miR-30c-5p;hsa-miR-30
ENSG0000	ENSG0000	1.47E-07	hsa-miR-140-3p;hsa-miR-30a-5p;hsa-miR-30b-5p;hsa-miR-30
ENSG0000	ENSG0000	3.41E-07	hsa-miR-30a-5p;hsa-miR-30b-5p;hsa-miR-30c-5p;hsa-miR-30
ENSG0000	ENSG0000	5.28E-06	hsa-miR-30a-5p;hsa-miR-30b-5p;hsa-miR-30c-5p;hsa-miR-30
ENSG0000	ENSG0000	5.51E-06	hsa-miR-30a-5p;hsa-miR-30b-5p;hsa-miR-30c-5p;hsa-miR-30
ENSG0000	ENSG0000	1.48E-08	hsa-miR-30a-5p;hsa-miR-30b-5p;hsa-miR-30c-5p;hsa-miR-30
ENSG0000	ENSG0000	4.25E-08	hsa-miR-30a-5p;hsa-miR-30b-5p;hsa-miR-30c-5p;hsa-miR-30
ENSG0000	ENSG0000	4.25E-08	hsa-miR-30a-5p;hsa-miR-30b-5p;hsa-miR-30c-5p;hsa-miR-30
ENSG0000	ENSG0000	7.38E-06	hsa-miR-30a-5p;hsa-miR-30b-5p;hsa-miR-30c-5p;hsa-miR-30
ENSG0000	ENSG0000	3.87E-09	hsa-miR-30a-5p;hsa-miR-30b-5p;hsa-miR-30c-5p;hsa-miR-30
ENSG0000	ENSG0000	3.33E-08	hsa-miR-30a-5p;hsa-miR-30b-5p;hsa-miR-30c-5p;hsa-miR-30
ENSG0000	ENSG0000	4.04E-06	hsa-miR-30a-5p;hsa-miR-30b-5p;hsa-miR-30c-5p;hsa-miR-30
ENSG0000	ENSG0000	1.44E-05	hsa-miR-30b-5p;hsa-miR-30c-5p;hsa-miR-30d-5p;hsa-miR-30
ENSG0000	ENSG0000	6.26E-06	hsa-miR-30b-5p;hsa-miR-30c-5p;hsa-miR-30d-5p;hsa-miR-30
ENSG0000	ENSG0000	1.10E-08	hsa-miR-30a-5p;hsa-miR-30b-5p;hsa-miR-30c-5p;hsa-miR-30
ENSG0000	ENSG0000	7.23E-06	hsa-miR-30a-5p;hsa-miR-30b-5p;hsa-miR-30c-5p;hsa-miR-30
ENSG0000	ENSG0000	0.00163	hsa-miR-30a-5p;hsa-miR-30b-5p;hsa-miR-30d-5p;hsa-miR-30
ENSG0000	ENSG0000	6.06E-05	hsa-miR-30a-5p;hsa-miR-30b-5p;hsa-miR-30c-5p;hsa-miR-30
ENSG0000	ENSG0000	1.48E-08	hsa-miR-30a-5p;hsa-miR-30b-5p;hsa-miR-30c-5p;hsa-miR-30
ENSG0000	ENSG0000	2.26E-05	hsa-miR-30a-5p;hsa-miR-30b-5p;hsa-miR-30c-5p;hsa-miR-30
ENSG0000	ENSG0000	2.92E-07	hsa-miR-30a-5p;hsa-miR-30b-5p;hsa-miR-30c-5p;hsa-miR-30
ENSG0000	ENSG0000	0.000244	hsa-miR-30a-5p;hsa-miR-30b-5p;hsa-miR-30c-5p;hsa-miR-30
ENSG0000	ENSG0000	2.49E-06	hsa-miR-30a-5p;hsa-miR-30b-5p;hsa-miR-30c-5p;hsa-miR-30
ENSG0000	ENSG0000	1.32E-12	hsa-miR-30a-5p;hsa-miR-30b-5p;hsa-miR-30c-5p;hsa-miR-30
ENSG0000	ENSG0000	1.65E-10	hsa-miR-30a-5p;hsa-miR-30b-5p;hsa-miR-30c-5p;hsa-miR-30
ENSG0000	ENSG0000	0.000244	hsa-miR-30a-5p;hsa-miR-30c-5p;hsa-miR-30d-5p;hsa-miR-30
ENSG0000	ENSG0000	3.87E-09	hsa-miR-30a-5p;hsa-miR-30b-5p;hsa-miR-30c-5p;hsa-miR-30
ENSG0000	ENSG0000	5.75E-06	hsa-miR-30a-5p;hsa-miR-30b-5p;hsa-miR-30c-5p;hsa-miR-30
ENSG0000	ENSG0000	1.32E-12	hsa-miR-30a-5p;hsa-miR-30b-5p;hsa-miR-30c-5p;hsa-miR-30
ENSG0000	ENSG0000	0.007182	hsa-miR-30a-5p;hsa-miR-30c-5p;hsa-miR-30d-5p;hsa-miR-30
ENSG0000	ENSG0000	8.65E-06	hsa-miR-30a-5p;hsa-miR-30b-5p;hsa-miR-30c-5p;hsa-miR-30
ENSG0000	ENSG0000	7.95E-07	hsa-miR-30a-5p;hsa-miR-30b-5p;hsa-miR-30c-5p;hsa-miR-30
ENSG0000	ENSG0000	1.27E-05	hsa-miR-30a-5p;hsa-miR-30c-5p;hsa-miR-30d-5p;hsa-miR-30
ENSG0000	ENSG0000	3.29E-10	hsa-miR-30a-5p;hsa-miR-30b-5p;hsa-miR-30c-5p;hsa-miR-30
ENSG0000	ENSG0000	1.01E-07	hsa-miR-30a-5p;hsa-miR-30b-5p;hsa-miR-30c-5p;hsa-miR-30
ENSG0000	ENSG0000	1.78E-05	hsa-miR-30a-5p;hsa-miR-30b-5p;hsa-miR-30c-5p;hsa-miR-30

ENSG0000	ENSG0000	3.41E-07	hsa-miR-30a-5p;hsa-miR-30b-5p;hsa-miR-30c-5p;hsa-miR-30
ENSG0000	ENSG0000	2.46E-05	hsa-miR-30a-5p;hsa-miR-30b-5p;hsa-miR-30c-5p;hsa-miR-30
ENSG0000	ENSG0000	1.90E-05	hsa-miR-30a-5p;hsa-miR-30b-5p;hsa-miR-30c-5p;hsa-miR-30
ENSG0000	ENSG0000	7.35E-11	hsa-miR-30a-5p;hsa-miR-30b-5p;hsa-miR-30c-5p;hsa-miR-30
ENSG0000	ENSG0000	7.35E-11	hsa-miR-30a-5p;hsa-miR-30b-5p;hsa-miR-30c-5p;hsa-miR-30
ENSG0000	ENSG0000	7.38E-06	hsa-miR-30a-5p;hsa-miR-30b-5p;hsa-miR-30c-5p;hsa-miR-30
ENSG0000	ENSG0000	3.87E-09	hsa-miR-30a-5p;hsa-miR-30b-5p;hsa-miR-30c-5p;hsa-miR-30
ENSG0000	ENSG0000	2.10E-07	hsa-miR-30a-5p;hsa-miR-30b-5p;hsa-miR-30c-5p;hsa-miR-30
ENSG0000	ENSG0000	6.02E-05	hsa-miR-30a-5p;hsa-miR-30b-5p;hsa-miR-30d-5p;hsa-miR-30
ENSG0000	ENSG0000	8.24E-08	hsa-miR-30a-5p;hsa-miR-30b-5p;hsa-miR-30c-5p;hsa-miR-30
ENSG0000	ENSG0000	3.68E-06	hsa-miR-30a-5p;hsa-miR-30b-5p;hsa-miR-30c-5p;hsa-miR-30
ENSG0000	ENSG0000	7.29E-06	hsa-miR-30b-5p;hsa-miR-30c-5p;hsa-miR-30d-5p;hsa-miR-30
ENSG0000	ENSG0000	2.76E-06	hsa-miR-30a-5p;hsa-miR-30b-5p;hsa-miR-30c-5p;hsa-miR-30
ENSG0000	ENSG0000	2.03E-06	hsa-miR-30a-5p;hsa-miR-30b-5p;hsa-miR-30c-5p;hsa-miR-30
ENSG0000	ENSG0000	9.33E-06	hsa-miR-15a-5p;hsa-miR-15b-5p;hsa-miR-195-5p;hsa-miR-49
ENSG0000	ENSG0000	1.43E-05	hsa-miR-15a-5p;hsa-miR-15b-5p;hsa-miR-16-5p;hsa-miR-195
ENSG0000	ENSG0000	2.86E-10	hsa-miR-15a-5p;hsa-miR-15b-5p;hsa-miR-16-5p;hsa-miR-195
ENSG0000	ENSG0000	4.49E-12	hsa-miR-15a-5p;hsa-miR-15b-5p;hsa-miR-16-5p;hsa-miR-195
ENSG0000	ENSG0000	3.34E-10	hsa-miR-15a-5p;hsa-miR-15b-5p;hsa-miR-16-5p;hsa-miR-195
ENSG0000	ENSG0000	2.34E-08	hsa-miR-15a-5p;hsa-miR-15b-5p;hsa-miR-195-5p;hsa-miR-49
ENSG0000	ENSG0000	1.59E-11	hsa-miR-15a-5p;hsa-miR-15b-5p;hsa-miR-16-5p;hsa-miR-195
ENSG0000	ENSG0000	2.53E-06	hsa-miR-15a-5p;hsa-miR-15b-5p;hsa-miR-195-5p;hsa-miR-49
ENSG0000	ENSG0000	2.67E-09	hsa-miR-15a-5p;hsa-miR-15b-5p;hsa-miR-16-5p;hsa-miR-195
ENSG0000	ENSG0000	2.67E-09	hsa-miR-15a-5p;hsa-miR-15b-5p;hsa-miR-16-5p;hsa-miR-195
ENSG0000	ENSG0000	3.70E-07	hsa-miR-15a-5p;hsa-miR-15b-5p;hsa-miR-16-5p;hsa-miR-195
ENSG0000	ENSG0000	1.76E-09	hsa-miR-15a-5p;hsa-miR-15b-5p;hsa-miR-16-5p;hsa-miR-195
ENSG0000	ENSG0000	0.000106	hsa-miR-15a-5p;hsa-miR-15b-5p;hsa-miR-16-5p;hsa-miR-195
ENSG0000	ENSG0000	1.28E-08	hsa-miR-15a-5p;hsa-miR-15b-5p;hsa-miR-16-5p;hsa-miR-195
ENSG0000	ENSG0000	2.67E-09	hsa-miR-15a-5p;hsa-miR-15b-5p;hsa-miR-16-5p;hsa-miR-195
ENSG0000	ENSG0000	6.55E-07	hsa-miR-15a-5p;hsa-miR-15b-5p;hsa-miR-195-5p;hsa-miR-49
ENSG0000	ENSG0000	1.90E-09	hsa-miR-15a-5p;hsa-miR-15b-5p;hsa-miR-16-5p;hsa-miR-195
ENSG0000	ENSG0000	3.17E-08	hsa-miR-15a-5p;hsa-miR-15b-5p;hsa-miR-16-5p;hsa-miR-195
ENSG0000	ENSG0000	1.75E-10	hsa-miR-15a-5p;hsa-miR-15b-5p;hsa-miR-16-5p;hsa-miR-195
ENSG0000	ENSG0000	3.70E-07	hsa-miR-15a-5p;hsa-miR-15b-5p;hsa-miR-16-5p;hsa-miR-195
ENSG0000	ENSG0000	8.49E-09	hsa-miR-15a-5p;hsa-miR-15b-5p;hsa-miR-16-5p;hsa-miR-195
ENSG0000	ENSG0000	5.14E-09	hsa-miR-15a-5p;hsa-miR-15b-5p;hsa-miR-16-5p;hsa-miR-195
ENSG0000	ENSG0000	5.43E-05	hsa-miR-15a-5p;hsa-miR-16-5p;hsa-miR-195-5p;hsa-miR-497
ENSG0000	ENSG0000	5.61E-09	hsa-miR-15a-5p;hsa-miR-15b-5p;hsa-miR-16-5p;hsa-miR-195
ENSG0000	ENSG0000	1.27E-06	hsa-miR-15a-5p;hsa-miR-15b-5p;hsa-miR-16-5p;hsa-miR-195
ENSG0000	ENSG0000	1.28E-05	hsa-miR-15a-5p;hsa-miR-15b-5p;hsa-miR-195-5p;hsa-miR-49
ENSG0000	ENSG0000	2.71E-11	hsa-miR-15a-5p;hsa-miR-15b-5p;hsa-miR-16-5p;hsa-miR-195
ENSG0000	ENSG0000	2.95E-09	hsa-miR-15a-5p;hsa-miR-15b-5p;hsa-miR-16-5p;hsa-miR-195
ENSG0000	ENSG0000	1.19E-11	hsa-miR-15a-5p;hsa-miR-15b-5p;hsa-miR-16-5p;hsa-miR-195
ENSG0000	ENSG0000	4.64E-08	hsa-miR-15a-5p;hsa-miR-15b-5p;hsa-miR-195-5p;hsa-miR-49
ENSG0000	ENSG0000	6.62E-08	hsa-miR-15a-5p;hsa-miR-15b-5p;hsa-miR-16-5p;hsa-miR-195
ENSG0000	ENSG0000	3.80E-08	hsa-miR-15a-5p;hsa-miR-15b-5p;hsa-miR-16-5p;hsa-miR-195
ENSG0000	ENSG0000	1.88E-06	hsa-miR-15a-5p;hsa-miR-15b-5p;hsa-miR-195-5p;hsa-miR-49
ENSG0000	ENSG0000	6.76E-10	hsa-miR-15a-5p;hsa-miR-15b-5p;hsa-miR-16-5p;hsa-miR-195
ENSG0000	ENSG0000	1.01E-10	hsa-miR-15a-5p;hsa-miR-15b-5p;hsa-miR-16-5p;hsa-miR-195
ENSG0000	ENSG0000	7.84E-09	hsa-miR-15a-5p;hsa-miR-15b-5p;hsa-miR-16-5p;hsa-miR-195
ENSG0000	ENSG0000	3.87E-10	hsa-miR-15a-5p;hsa-miR-15b-5p;hsa-miR-16-5p;hsa-miR-195
ENSG0000	ENSG0000	1.24E-08	hsa-miR-15a-5p;hsa-miR-15b-5p;hsa-miR-16-5p;hsa-miR-195
ENSG0000	ENSG0000	2.81E-08	hsa-miR-15a-5p;hsa-miR-15b-5p;hsa-miR-195-5p;hsa-miR-49
ENSG0000	ENSG0000	1.75E-10	hsa-miR-15a-5p;hsa-miR-15b-5p;hsa-miR-16-5p;hsa-miR-195

ENSG0000	ENSG0000	2.16E-07	hsa-miR-15a-5p;hsa-miR-15b-5p;hsa-miR-195-5p;hsa-miR-49
ENSG0000	ENSG0000	1.58E-09	hsa-miR-15a-5p;hsa-miR-15b-5p;hsa-miR-16-5p;hsa-miR-195-
ENSG0000	ENSG0000	5.25E-06	hsa-miR-15a-5p;hsa-miR-15b-5p;hsa-miR-195-5p;hsa-miR-49
ENSG0000	ENSG0000	5.11E-07	hsa-miR-15a-5p;hsa-miR-15b-5p;hsa-miR-16-5p;hsa-miR-195-
ENSG0000	ENSG0000	2.13E-07	hsa-miR-15a-5p;hsa-miR-15b-5p;hsa-miR-16-5p;hsa-miR-195-
ENSG0000	ENSG0000	2.66E-07	hsa-miR-15a-5p;hsa-miR-15b-5p;hsa-miR-195-5p;hsa-miR-49
ENSG0000	ENSG0000	2.94E-07	hsa-miR-15a-5p;hsa-miR-15b-5p;hsa-miR-195-5p;hsa-miR-49
ENSG0000	ENSG0000	1.34E-08	hsa-miR-15a-5p;hsa-miR-15b-5p;hsa-miR-16-5p;hsa-miR-195-
ENSG0000	ENSG0000	6.27E-08	hsa-miR-15a-5p;hsa-miR-15b-5p;hsa-miR-195-5p;hsa-miR-49
ENSG0000	ENSG0000	2.12E-06	hsa-miR-15a-5p;hsa-miR-15b-5p;hsa-miR-195-5p;hsa-miR-49
ENSG0000	ENSG0000	3.90E-06	hsa-miR-15a-5p;hsa-miR-15b-5p;hsa-miR-195-5p;hsa-miR-49
ENSG0000	ENSG0000	1.03E-06	hsa-miR-15a-5p;hsa-miR-15b-5p;hsa-miR-195-5p;hsa-miR-49
ENSG0000	ENSG0000	1.22E-10	hsa-miR-15a-5p;hsa-miR-15b-5p;hsa-miR-16-5p;hsa-miR-195-
ENSG0000	ENSG0000	1.32E-12	hsa-miR-15a-5p;hsa-miR-15b-5p;hsa-miR-16-5p;hsa-miR-195-
ENSG0000	ENSG0000	1.32E-12	hsa-miR-15a-5p;hsa-miR-15b-5p;hsa-miR-16-5p;hsa-miR-195-
ENSG0000	ENSG0000	3.93E-09	hsa-miR-15a-5p;hsa-miR-15b-5p;hsa-miR-16-5p;hsa-miR-195-
ENSG0000	ENSG0000	9.48E-08	hsa-miR-15a-5p;hsa-miR-15b-5p;hsa-miR-195-5p;hsa-miR-49
ENSG0000	ENSG0000	2.03E-08	hsa-miR-15a-5p;hsa-miR-15b-5p;hsa-miR-16-5p;hsa-miR-195-
ENSG0000	ENSG0000	1.07E-08	hsa-miR-15a-5p;hsa-miR-15b-5p;hsa-miR-16-5p;hsa-miR-195-
ENSG0000	ENSG0000	1.55E-07	hsa-miR-15a-5p;hsa-miR-15b-5p;hsa-miR-195-5p;hsa-miR-49
ENSG0000	ENSG0000	1.77E-06	hsa-miR-15a-5p;hsa-miR-15b-5p;hsa-miR-195-5p;hsa-miR-49
ENSG0000	ENSG0000	1.04E-05	hsa-miR-15a-5p;hsa-miR-15b-5p;hsa-miR-16-5p;hsa-miR-195-
ENSG0000	ENSG0000	2.44E-10	hsa-miR-15a-5p;hsa-miR-15b-5p;hsa-miR-16-5p;hsa-miR-195-
ENSG0000	ENSG0000	4.03E-08	hsa-miR-15a-5p;hsa-miR-15b-5p;hsa-miR-16-5p;hsa-miR-195-
ENSG0000	ENSG0000	1.88E-06	hsa-miR-15a-5p;hsa-miR-15b-5p;hsa-miR-195-5p;hsa-miR-49
ENSG0000	ENSG0000	1.28E-08	hsa-miR-15a-5p;hsa-miR-15b-5p;hsa-miR-195-5p;hsa-miR-49
ENSG0000	ENSG0000	1.32E-12	hsa-miR-15a-5p;hsa-miR-15b-5p;hsa-miR-16-5p;hsa-miR-195-
ENSG0000	ENSG0000	1.32E-12	hsa-miR-15a-5p;hsa-miR-15b-5p;hsa-miR-16-5p;hsa-miR-195-
ENSG0000	ENSG0000	4.29E-07	hsa-miR-15a-5p;hsa-miR-15b-5p;hsa-miR-195-5p;hsa-miR-49
ENSG0000	ENSG0000	2.66E-12	hsa-miR-15a-5p;hsa-miR-15b-5p;hsa-miR-16-5p;hsa-miR-195-
ENSG0000	ENSG0000	1.22E-07	hsa-miR-15a-5p;hsa-miR-15b-5p;hsa-miR-195-5p;hsa-miR-49
ENSG0000	ENSG0000	1.36E-06	hsa-miR-15a-5p;hsa-miR-15b-5p;hsa-miR-195-5p;hsa-miR-49
ENSG0000	ENSG0000	2.40E-07	hsa-miR-15a-5p;hsa-miR-15b-5p;hsa-miR-195-5p;hsa-miR-49
ENSG0000	ENSG0000	1.90E-08	hsa-miR-15a-5p;hsa-miR-15b-5p;hsa-miR-16-5p;hsa-miR-195-
ENSG0000	ENSG0000	1.33E-11	hsa-miR-15a-5p;hsa-miR-15b-5p;hsa-miR-16-5p;hsa-miR-195-
ENSG0000	ENSG0000	1.76E-09	hsa-miR-15a-5p;hsa-miR-15b-5p;hsa-miR-16-5p;hsa-miR-195-
ENSG0000	ENSG0000	1.01E-05	hsa-miR-15a-5p;hsa-miR-15b-5p;hsa-miR-195-5p;hsa-miR-49
ENSG0000	ENSG0000	1.12E-09	hsa-miR-15a-5p;hsa-miR-15b-5p;hsa-miR-16-5p;hsa-miR-195-
ENSG0000	ENSG0000	2.39E-06	hsa-miR-15a-5p;hsa-miR-15b-5p;hsa-miR-16-5p;hsa-miR-195-
ENSG0000	ENSG0000	2.71E-11	hsa-miR-15a-5p;hsa-miR-15b-5p;hsa-miR-16-5p;hsa-miR-195-
ENSG0000	ENSG0000	2.71E-11	hsa-miR-15a-5p;hsa-miR-15b-5p;hsa-miR-16-5p;hsa-miR-195-
ENSG0000	ENSG0000	1.26E-09	hsa-miR-15a-5p;hsa-miR-15b-5p;hsa-miR-16-5p;hsa-miR-195-
ENSG0000	ENSG0000	6.50E-07	hsa-miR-15a-5p;hsa-miR-15b-5p;hsa-miR-16-5p;hsa-miR-195-
ENSG0000	ENSG0000	2.66E-09	hsa-miR-15a-5p;hsa-miR-15b-5p;hsa-miR-16-5p;hsa-miR-195-
ENSG0000	ENSG0000	2.66E-09	hsa-miR-15a-5p;hsa-miR-15b-5p;hsa-miR-16-5p;hsa-miR-195-
ENSG0000	ENSG0000	9.30E-11	hsa-miR-15a-5p;hsa-miR-15b-5p;hsa-miR-16-5p;hsa-miR-195-
ENSG0000	ENSG0000	3.87E-10	hsa-miR-15a-5p;hsa-miR-15b-5p;hsa-miR-16-5p;hsa-miR-195-
ENSG0000	ENSG0000	8.28E-07	hsa-miR-15a-5p;hsa-miR-15b-5p;hsa-miR-195-5p;hsa-miR-49
ENSG0000	ENSG0000	5.25E-06	hsa-miR-15a-5p;hsa-miR-15b-5p;hsa-miR-195-5p;hsa-miR-49
ENSG0000	ENSG0000	3.34E-10	hsa-miR-15a-5p;hsa-miR-15b-5p;hsa-miR-16-5p;hsa-miR-195-
ENSG0000	ENSG0000	3.95E-08	hsa-miR-15a-5p;hsa-miR-15b-5p;hsa-miR-16-5p;hsa-miR-195-
ENSG0000	ENSG0000	3.09E-12	hsa-miR-15a-5p;hsa-miR-15b-5p;hsa-miR-16-5p;hsa-miR-195-
ENSG0000	ENSG0000	9.89E-10	hsa-miR-15a-5p;hsa-miR-15b-5p;hsa-miR-16-5p;hsa-miR-195-
ENSG0000	ENSG0000	1.59E-11	hsa-miR-15a-5p;hsa-miR-15b-5p;hsa-miR-16-5p;hsa-miR-195-

ENSG0000	ENSG0000	1.74E-07	hsa-miR-15a-5p;hsa-miR-15b-5p;hsa-miR-195-5p;hsa-miR-49
ENSG0000	ENSG0000	7.84E-09	hsa-miR-15a-5p;hsa-miR-15b-5p;hsa-miR-16-5p;hsa-miR-195-
ENSG0000	ENSG0000	2.16E-07	hsa-miR-15a-5p;hsa-miR-15b-5p;hsa-miR-195-5p;hsa-miR-49
ENSG0000	ENSG0000	2.40E-07	hsa-miR-15a-5p;hsa-miR-15b-5p;hsa-miR-16-5p;hsa-miR-195-
ENSG0000	ENSG0000	9.62E-07	hsa-miR-15a-5p;hsa-miR-15b-5p;hsa-miR-195-5p;hsa-miR-49
ENSG0000	ENSG0000	5.61E-09	hsa-miR-15a-5p;hsa-miR-15b-5p;hsa-miR-16-5p;hsa-miR-195-
ENSG0000	ENSG0000	1.59E-11	hsa-miR-15a-5p;hsa-miR-15b-5p;hsa-miR-16-5p;hsa-miR-195-
ENSG0000	ENSG0000	1.46E-10	hsa-miR-15a-5p;hsa-miR-15b-5p;hsa-miR-16-5p;hsa-miR-195-
ENSG0000	ENSG0000	8.98E-07	hsa-miR-15a-5p;hsa-miR-15b-5p;hsa-miR-16-5p;hsa-miR-195-
ENSG0000	ENSG0000	1.66E-06	hsa-miR-15a-5p;hsa-miR-15b-5p;hsa-miR-16-5p;hsa-miR-195-
ENSG0000	ENSG0000	1.96E-06	hsa-miR-15a-5p;hsa-miR-15b-5p;hsa-miR-16-5p;hsa-miR-195-
ENSG0000	ENSG0000	2.47E-08	hsa-miR-15a-5p;hsa-miR-15b-5p;hsa-miR-16-5p;hsa-miR-195-
ENSG0000	ENSG0000	4.77E-07	hsa-miR-15a-5p;hsa-miR-15b-5p;hsa-miR-16-5p;hsa-miR-195-
ENSG0000	ENSG0000	6.11E-09	hsa-miR-15a-5p;hsa-miR-15b-5p;hsa-miR-16-5p;hsa-miR-195-
ENSG0000	ENSG0000	1.22E-10	hsa-miR-15a-5p;hsa-miR-15b-5p;hsa-miR-16-5p;hsa-miR-195-
ENSG0000	ENSG0000	6.04E-06	hsa-miR-15a-5p;hsa-miR-15b-5p;hsa-miR-16-5p;hsa-miR-195-
ENSG0000	ENSG0000	5.41E-08	hsa-miR-15a-5p;hsa-miR-15b-5p;hsa-miR-16-5p;hsa-miR-195-
ENSG0000	ENSG0000	9.89E-10	hsa-miR-15a-5p;hsa-miR-15b-5p;hsa-miR-16-5p;hsa-miR-195-
ENSG0000	ENSG0000	4.50E-05	hsa-miR-15a-5p;hsa-miR-16-5p;hsa-miR-195-5p;hsa-miR-497-
ENSG0000	ENSG0000	2.66E-12	hsa-miR-15a-5p;hsa-miR-15b-5p;hsa-miR-16-5p;hsa-miR-195-
ENSG0000	ENSG0000	2.71E-11	hsa-miR-15a-5p;hsa-miR-15b-5p;hsa-miR-16-5p;hsa-miR-195-
ENSG0000	ENSG0000	1.15E-08	hsa-miR-15a-5p;hsa-miR-15b-5p;hsa-miR-16-5p;hsa-miR-195-
ENSG0000	ENSG0000	7.23E-08	hsa-miR-15a-5p;hsa-miR-15b-5p;hsa-miR-195-5p;hsa-miR-49
ENSG0000	ENSG0000	2.17E-08	hsa-miR-15a-5p;hsa-miR-15b-5p;hsa-miR-16-5p;hsa-miR-195-
ENSG0000	ENSG0000	5.61E-09	hsa-miR-15a-5p;hsa-miR-15b-5p;hsa-miR-16-5p;hsa-miR-195-
ENSG0000	ENSG0000	1.31E-09	hsa-miR-15a-5p;hsa-miR-15b-5p;hsa-miR-16-5p;hsa-miR-195-
ENSG0000	ENSG0000	4.71E-09	hsa-miR-15a-5p;hsa-miR-15b-5p;hsa-miR-16-5p;hsa-miR-195-
ENSG0000	ENSG0000	2.66E-09	hsa-miR-15a-5p;hsa-miR-15b-5p;hsa-miR-195-5p;hsa-miR-49
ENSG0000	ENSG0000	1.12E-09	hsa-miR-15a-5p;hsa-miR-15b-5p;hsa-miR-16-5p;hsa-miR-195-
ENSG0000	ENSG0000	7.22E-09	hsa-miR-15a-5p;hsa-miR-15b-5p;hsa-miR-16-5p;hsa-miR-195-
ENSG0000	ENSG0000	3.35E-10	hsa-miR-15a-5p;hsa-miR-15b-5p;hsa-miR-16-5p;hsa-miR-195-
ENSG0000	ENSG0000	6.55E-07	hsa-miR-15a-5p;hsa-miR-15b-5p;hsa-miR-195-5p;hsa-miR-49
ENSG0000	ENSG0000	2.66E-12	hsa-miR-15a-5p;hsa-miR-15b-5p;hsa-miR-16-5p;hsa-miR-195-
ENSG0000	ENSG0000	5.61E-09	hsa-miR-15a-5p;hsa-miR-15b-5p;hsa-miR-16-5p;hsa-miR-195-
ENSG0000	ENSG0000	5.25E-06	hsa-miR-15a-5p;hsa-miR-15b-5p;hsa-miR-195-5p;hsa-miR-49
ENSG0000	ENSG0000	9.00E-08	hsa-miR-15a-5p;hsa-miR-15b-5p;hsa-miR-16-5p;hsa-miR-195-
ENSG0000	ENSG0000	1.94E-08	hsa-miR-15a-5p;hsa-miR-15b-5p;hsa-miR-16-5p;hsa-miR-195-
ENSG0000	ENSG0000	1.74E-07	hsa-miR-15a-5p;hsa-miR-15b-5p;hsa-miR-195-5p;hsa-miR-49
ENSG0000	ENSG0000	1.32E-12	hsa-miR-15a-5p;hsa-miR-15b-5p;hsa-miR-16-5p;hsa-miR-195-
ENSG0000	ENSG0000	3.80E-08	hsa-miR-15a-5p;hsa-miR-15b-5p;hsa-miR-16-5p;hsa-miR-195-
ENSG0000	ENSG0000	5.92E-10	hsa-miR-15a-5p;hsa-miR-15b-5p;hsa-miR-16-5p;hsa-miR-195-
ENSG0000	ENSG0000	3.87E-10	hsa-miR-15a-5p;hsa-miR-15b-5p;hsa-miR-16-5p;hsa-miR-195-
ENSG0000	ENSG0000	1.86E-10	hsa-miR-15a-5p;hsa-miR-15b-5p;hsa-miR-16-5p;hsa-miR-195-
ENSG0000	ENSG0000	8.28E-07	hsa-miR-15a-5p;hsa-miR-15b-5p;hsa-miR-195-5p;hsa-miR-49
ENSG0000	ENSG0000	3.95E-08	hsa-miR-15a-5p;hsa-miR-15b-5p;hsa-miR-16-5p;hsa-miR-195-
ENSG0000	ENSG0000	8.93E-07	hsa-miR-15a-5p;hsa-miR-15b-5p;hsa-miR-16-5p;hsa-miR-195-
ENSG0000	ENSG0000	1.11E-06	hsa-miR-15a-5p;hsa-miR-15b-5p;hsa-miR-195-5p;hsa-miR-49
ENSG0000	ENSG0000	4.37E-11	hsa-miR-15a-5p;hsa-miR-15b-5p;hsa-miR-16-5p;hsa-miR-195-
ENSG0000	ENSG0000	2.06E-12	hsa-miR-15a-5p;hsa-miR-15b-5p;hsa-miR-16-5p;hsa-miR-195-
ENSG0000	ENSG0000	7.23E-08	hsa-miR-15a-5p;hsa-miR-15b-5p;hsa-miR-195-5p;hsa-miR-49
ENSG0000	ENSG0000	6.76E-10	hsa-miR-15a-5p;hsa-miR-15b-5p;hsa-miR-16-5p;hsa-miR-195-
ENSG0000	ENSG0000	4.94E-07	hsa-miR-15a-5p;hsa-miR-15b-5p;hsa-miR-16-5p;hsa-miR-195-
ENSG0000	ENSG0000	3.93E-09	hsa-miR-15a-5p;hsa-miR-15b-5p;hsa-miR-16-5p;hsa-miR-195-
ENSG0000	ENSG0000	1.44E-07	hsa-miR-15a-5p;hsa-miR-15b-5p;hsa-miR-16-5p;hsa-miR-195-

ENSG0000	ENSG0000	6.27E-08	hsa-miR-15a-5p;hsa-miR-15b-5p;hsa-miR-195-5p;hsa-miR-49
ENSG0000	ENSG0000	1.88E-06	hsa-miR-15a-5p;hsa-miR-15b-5p;hsa-miR-16-5p;hsa-miR-195-
ENSG0000	ENSG0000	5.16E-10	hsa-miR-15a-5p;hsa-miR-15b-5p;hsa-miR-16-5p;hsa-miR-195-
ENSG0000	ENSG0000	9.62E-07	hsa-miR-15a-5p;hsa-miR-15b-5p;hsa-miR-16-5p;hsa-miR-195-
ENSG0000	ENSG0000	5.58E-10	hsa-miR-15a-5p;hsa-miR-15b-5p;hsa-miR-16-5p;hsa-miR-195-
ENSG0000	ENSG0000	8.14E-13	hsa-miR-15a-5p;hsa-miR-15b-5p;hsa-miR-16-5p;hsa-miR-195-
ENSG0000	ENSG0000	1.38E-07	hsa-miR-15a-5p;hsa-miR-15b-5p;hsa-miR-195-5p;hsa-miR-49
ENSG0000	ENSG0000	1.58E-09	hsa-miR-15a-5p;hsa-miR-15b-5p;hsa-miR-16-5p;hsa-miR-195-
ENSG0000	ENSG0000	1.58E-09	hsa-miR-15a-5p;hsa-miR-15b-5p;hsa-miR-16-5p;hsa-miR-195-
ENSG0000	ENSG0000	2.18E-09	hsa-miR-15a-5p;hsa-miR-15b-5p;hsa-miR-16-5p;hsa-miR-195-
ENSG0000	ENSG0000	1.08E-07	hsa-miR-15a-5p;hsa-miR-15b-5p;hsa-miR-16-5p;hsa-miR-195-
ENSG0000	ENSG0000	2.06E-12	hsa-miR-15a-5p;hsa-miR-15b-5p;hsa-miR-16-5p;hsa-miR-195-
ENSG0000	ENSG0000	5.16E-10	hsa-miR-15a-5p;hsa-miR-15b-5p;hsa-miR-16-5p;hsa-miR-195-
ENSG0000	ENSG0000	9.48E-08	hsa-miR-15b-5p;hsa-miR-16-5p;hsa-miR-195-5p;hsa-miR-497-
ENSG0000	ENSG0000	1.58E-08	hsa-miR-15a-5p;hsa-miR-15b-5p;hsa-miR-16-5p;hsa-miR-195-
ENSG0000	ENSG0000	1.58E-08	hsa-miR-15a-5p;hsa-miR-15b-5p;hsa-miR-16-5p;hsa-miR-195-
ENSG0000	ENSG0000	2.66E-09	hsa-miR-15a-5p;hsa-miR-15b-5p;hsa-miR-16-5p;hsa-miR-195-
ENSG0000	ENSG0000	6.27E-08	hsa-miR-15a-5p;hsa-miR-15b-5p;hsa-miR-195-5p;hsa-miR-49
ENSG0000	ENSG0000	1.38E-07	hsa-miR-15a-5p;hsa-miR-15b-5p;hsa-miR-16-5p;hsa-miR-195-
ENSG0000	ENSG0000	1.58E-09	hsa-miR-15a-5p;hsa-miR-15b-5p;hsa-miR-16-5p;hsa-miR-195-
ENSG0000	ENSG0000	4.27E-08	hsa-miR-15a-5p;hsa-miR-15b-5p;hsa-miR-16-5p;hsa-miR-195-
ENSG0000	ENSG0000	5.34E-08	hsa-miR-15a-5p;hsa-miR-15b-5p;hsa-miR-16-5p;hsa-miR-195-
ENSG0000	ENSG0000	1.76E-05	hsa-miR-15a-5p;hsa-miR-15b-5p;hsa-miR-16-5p;hsa-miR-195-
ENSG0000	ENSG0000	1.76E-09	hsa-miR-15a-5p;hsa-miR-15b-5p;hsa-miR-16-5p;hsa-miR-195-
ENSG0000	ENSG0000	3.57E-09	hsa-miR-15a-5p;hsa-miR-15b-5p;hsa-miR-16-5p;hsa-miR-195-
ENSG0000	ENSG0000	3.34E-10	hsa-miR-15a-5p;hsa-miR-15b-5p;hsa-miR-16-5p;hsa-miR-195-
ENSG0000	ENSG0000	3.34E-10	hsa-miR-15a-5p;hsa-miR-15b-5p;hsa-miR-16-5p;hsa-miR-195-
ENSG0000	ENSG0000	3.34E-08	hsa-miR-15a-5p;hsa-miR-15b-5p;hsa-miR-16-5p;hsa-miR-195-
ENSG0000	ENSG0000	9.89E-10	hsa-miR-15a-5p;hsa-miR-15b-5p;hsa-miR-16-5p;hsa-miR-195-
ENSG0000	ENSG0000	7.23E-06	hsa-miR-15a-5p;hsa-miR-15b-5p;hsa-miR-195-5p;hsa-miR-49
ENSG0000	ENSG0000	1.58E-08	hsa-miR-15a-5p;hsa-miR-15b-5p;hsa-miR-195-5p;hsa-miR-49
ENSG0000	ENSG0000	1.46E-10	hsa-miR-15a-5p;hsa-miR-15b-5p;hsa-miR-16-5p;hsa-miR-195-
ENSG0000	ENSG0000	4.27E-08	hsa-miR-15a-5p;hsa-miR-15b-5p;hsa-miR-16-5p;hsa-miR-195-
ENSG0000	ENSG0000	1.75E-10	hsa-miR-15a-5p;hsa-miR-15b-5p;hsa-miR-16-5p;hsa-miR-195-
ENSG0000	ENSG0000	1.58E-09	hsa-miR-15a-5p;hsa-miR-15b-5p;hsa-miR-16-5p;hsa-miR-195-
ENSG0000	ENSG0000	1.58E-09	hsa-miR-15a-5p;hsa-miR-15b-5p;hsa-miR-16-5p;hsa-miR-195-
ENSG0000	ENSG0000	1.27E-06	hsa-miR-15a-5p;hsa-miR-15b-5p;hsa-miR-195-5p;hsa-miR-49
ENSG0000	ENSG0000	1.94E-07	hsa-miR-15a-5p;hsa-miR-15b-5p;hsa-miR-16-5p;hsa-miR-195-
ENSG0000	ENSG0000	3.35E-10	hsa-miR-15a-5p;hsa-miR-15b-5p;hsa-miR-16-5p;hsa-miR-195-
ENSG0000	ENSG0000	9.89E-10	hsa-miR-15a-5p;hsa-miR-15b-5p;hsa-miR-16-5p;hsa-miR-195-
ENSG0000	ENSG0000	1.33E-05	hsa-miR-15a-5p;hsa-miR-15b-5p;hsa-miR-16-5p;hsa-miR-195-
ENSG0000	ENSG0000	1.33E-05	hsa-miR-15a-5p;hsa-miR-15b-5p;hsa-miR-16-5p;hsa-miR-195-
ENSG0000	ENSG0000	1.32E-12	hsa-miR-15a-5p;hsa-miR-15b-5p;hsa-miR-16-5p;hsa-miR-195-
ENSG0000	ENSG0000	5.61E-09	hsa-miR-15a-5p;hsa-miR-15b-5p;hsa-miR-16-5p;hsa-miR-195-
ENSG0000	ENSG0000	1.33E-05	hsa-miR-15a-5p;hsa-miR-15b-5p;hsa-miR-195-5p;hsa-miR-49
ENSG0000	ENSG0000	4.71E-09	hsa-miR-15a-5p;hsa-miR-15b-5p;hsa-miR-16-5p;hsa-miR-195-
ENSG0000	ENSG0000	2.71E-11	hsa-miR-15a-5p;hsa-miR-15b-5p;hsa-miR-16-5p;hsa-miR-195-
ENSG0000	ENSG0000	7.70E-10	hsa-miR-15a-5p;hsa-miR-15b-5p;hsa-miR-16-5p;hsa-miR-195-
ENSG0000	ENSG0000	8.30E-08	hsa-miR-15a-5p;hsa-miR-15b-5p;hsa-miR-16-5p;hsa-miR-195-
ENSG0000	ENSG0000	4.49E-12	hsa-miR-15a-5p;hsa-miR-15b-5p;hsa-miR-16-5p;hsa-miR-195-
ENSG0000	ENSG0000	1.94E-08	hsa-miR-15a-5p;hsa-miR-15b-5p;hsa-miR-16-5p;hsa-miR-195-
ENSG0000	ENSG0000	7.84E-09	hsa-miR-15a-5p;hsa-miR-15b-5p;hsa-miR-16-5p;hsa-miR-195-
ENSG0000	ENSG0000	1.94E-07	hsa-miR-15a-5p;hsa-miR-15b-5p;hsa-miR-195-5p;hsa-miR-49
ENSG0000	ENSG0000	3.34E-10	hsa-miR-15a-5p;hsa-miR-15b-5p;hsa-miR-16-5p;hsa-miR-195-

ENSG0000	ENSG0000	6.50E-07	hsa-miR-15a-5p;hsa-miR-15b-5p;hsa-miR-16-5p;hsa-miR-195-
ENSG0000	ENSG0000	1.94E-08	hsa-miR-15a-5p;hsa-miR-15b-5p;hsa-miR-16-5p;hsa-miR-195-
ENSG0000	ENSG0000	6.65E-09	hsa-miR-15a-5p;hsa-miR-15b-5p;hsa-miR-16-5p;hsa-miR-195-
ENSG0000	ENSG0000	2.71E-11	hsa-miR-15a-5p;hsa-miR-15b-5p;hsa-miR-16-5p;hsa-miR-195-
ENSG0000	ENSG0000	1.38E-07	hsa-miR-15a-5p;hsa-miR-15b-5p;hsa-miR-195-5p;hsa-miR-49
ENSG0000	ENSG0000	1.23E-05	hsa-miR-15a-5p;hsa-miR-15b-5p;hsa-miR-195-5p;hsa-miR-49
ENSG0000	ENSG0000	1.54E-08	hsa-miR-15a-5p;hsa-miR-15b-5p;hsa-miR-16-5p;hsa-miR-195-
ENSG0000	ENSG0000	3.62E-09	hsa-miR-15a-5p;hsa-miR-15b-5p;hsa-miR-195-5p;hsa-miR-49
ENSG0000	ENSG0000	1.90E-09	hsa-miR-15a-5p;hsa-miR-15b-5p;hsa-miR-16-5p;hsa-miR-195-
ENSG0000	ENSG0000	5.61E-09	hsa-miR-15a-5p;hsa-miR-15b-5p;hsa-miR-16-5p;hsa-miR-195-
ENSG0000	ENSG0000	8.11E-09	hsa-miR-15a-5p;hsa-miR-15b-5p;hsa-miR-195-5p;hsa-miR-49
ENSG0000	ENSG0000	5.41E-08	hsa-miR-15a-5p;hsa-miR-15b-5p;hsa-miR-195-5p;hsa-miR-49
ENSG0000	ENSG0000	8.59E-06	hsa-miR-15a-5p;hsa-miR-15b-5p;hsa-miR-195-5p;hsa-miR-49
ENSG0000	ENSG0000	5.46E-11	hsa-miR-15a-5p;hsa-miR-15b-5p;hsa-miR-16-5p;hsa-miR-195-
ENSG0000	ENSG0000	6.62E-06	hsa-miR-15a-5p;hsa-miR-15b-5p;hsa-miR-195-5p;hsa-miR-49
ENSG0000	ENSG0000	2.66E-12	hsa-miR-15a-5p;hsa-miR-15b-5p;hsa-miR-16-5p;hsa-miR-195-
ENSG0000	ENSG0000	2.80E-08	hsa-miR-15a-5p;hsa-miR-15b-5p;hsa-miR-16-5p;hsa-miR-195-
ENSG0000	ENSG0000	2.81E-08	hsa-miR-15a-5p;hsa-miR-15b-5p;hsa-miR-16-5p;hsa-miR-195-
ENSG0000	ENSG0000	1.94E-07	hsa-miR-15a-5p;hsa-miR-15b-5p;hsa-miR-16-5p;hsa-miR-195-
ENSG0000	ENSG0000	0.000636	hsa-miR-15a-5p;hsa-miR-15b-5p;hsa-miR-195-5p;hsa-miR-49
ENSG0000	ENSG0000	4.11E-06	hsa-miR-15a-5p;hsa-miR-15b-5p;hsa-miR-195-5p;hsa-miR-49
ENSG0000	ENSG0000	1.76E-09	hsa-miR-15a-5p;hsa-miR-15b-5p;hsa-miR-16-5p;hsa-miR-195-
ENSG0000	ENSG0000	6.27E-08	hsa-miR-15a-5p;hsa-miR-15b-5p;hsa-miR-195-5p;hsa-miR-49
ENSG0000	ENSG0000	8.95E-06	hsa-miR-15a-5p;hsa-miR-15b-5p;hsa-miR-195-5p;hsa-miR-49
ENSG0000	ENSG0000	5.41E-08	hsa-miR-15a-5p;hsa-miR-15b-5p;hsa-miR-195-5p;hsa-miR-49
ENSG0000	ENSG0000	2.18E-09	hsa-miR-15a-5p;hsa-miR-15b-5p;hsa-miR-16-5p;hsa-miR-195-
ENSG0000	ENSG0000	2.66E-07	hsa-miR-15a-5p;hsa-miR-15b-5p;hsa-miR-195-5p;hsa-miR-49
ENSG0000	ENSG0000	1.22E-10	hsa-miR-15a-5p;hsa-miR-15b-5p;hsa-miR-16-5p;hsa-miR-195-
ENSG0000	ENSG0000	1.19E-11	hsa-miR-15a-5p;hsa-miR-15b-5p;hsa-miR-16-5p;hsa-miR-195-
ENSG0000	ENSG0000	1.32E-12	hsa-miR-15a-5p;hsa-miR-15b-5p;hsa-miR-16-5p;hsa-miR-195-
ENSG0000	ENSG0000	3.99E-11	hsa-miR-15a-5p;hsa-miR-15b-5p;hsa-miR-16-5p;hsa-miR-195-
ENSG0000	ENSG0000	2.66E-09	hsa-miR-15a-5p;hsa-miR-15b-5p;hsa-miR-16-5p;hsa-miR-195-
ENSG0000	ENSG0000	6.11E-09	hsa-miR-15a-5p;hsa-miR-15b-5p;hsa-miR-16-5p;hsa-miR-195-
ENSG0000	ENSG0000	2.66E-07	hsa-miR-15a-5p;hsa-miR-15b-5p;hsa-miR-16-5p;hsa-miR-195-
ENSG0000	ENSG0000	9.48E-08	hsa-miR-15a-5p;hsa-miR-15b-5p;hsa-miR-195-5p;hsa-miR-49
ENSG0000	ENSG0000	2.09E-11	hsa-miR-15a-5p;hsa-miR-15b-5p;hsa-miR-16-5p;hsa-miR-195-
ENSG0000	ENSG0000	4.48E-10	hsa-miR-15a-5p;hsa-miR-15b-5p;hsa-miR-16-5p;hsa-miR-195-
ENSG0000	ENSG0000	1.27E-06	hsa-miR-15a-5p;hsa-miR-15b-5p;hsa-miR-16-5p;hsa-miR-195-
ENSG0000	ENSG0000	3.34E-10	hsa-miR-15a-5p;hsa-miR-15b-5p;hsa-miR-16-5p;hsa-miR-195-
ENSG0000	ENSG0000	2.29E-06	hsa-miR-15a-5p;hsa-miR-15b-5p;hsa-miR-16-5p;hsa-miR-195-
ENSG0000	ENSG0000	2.66E-09	hsa-miR-15a-5p;hsa-miR-15b-5p;hsa-miR-195-5p;hsa-miR-49
ENSG0000	ENSG0000	6.04E-06	hsa-miR-15a-5p;hsa-miR-15b-5p;hsa-miR-195-5p;hsa-miR-49
ENSG0000	ENSG0000	6.36E-12	hsa-miR-15a-5p;hsa-miR-15b-5p;hsa-miR-16-5p;hsa-miR-195-
ENSG0000	ENSG0000	2.16E-07	hsa-miR-15a-5p;hsa-miR-15b-5p;hsa-miR-16-5p;hsa-miR-195-
ENSG0000	ENSG0000	5.75E-14	hsa-miR-15a-5p;hsa-miR-15b-5p;hsa-miR-16-5p;hsa-miR-195-
ENSG0000	ENSG0000	1.94E-08	hsa-miR-15a-5p;hsa-miR-15b-5p;hsa-miR-16-5p;hsa-miR-195-
ENSG0000	ENSG0000	7.55E-06	hsa-miR-15a-5p;hsa-miR-15b-5p;hsa-miR-195-5p;hsa-miR-49
ENSG0000	ENSG0000	4.71E-09	hsa-miR-15a-5p;hsa-miR-15b-5p;hsa-miR-16-5p;hsa-miR-195-
ENSG0000	ENSG0000	1.56E-06	hsa-miR-15a-5p;hsa-miR-15b-5p;hsa-miR-16-5p;hsa-miR-195-
ENSG0000	ENSG0000	2.39E-06	hsa-miR-15a-5p;hsa-miR-15b-5p;hsa-miR-195-5p;hsa-miR-49
ENSG0000	ENSG0000	3.98E-07	hsa-miR-15a-5p;hsa-miR-15b-5p;hsa-miR-16-5p;hsa-miR-195-
ENSG0000	ENSG0000	2.94E-07	hsa-miR-15a-5p;hsa-miR-15b-5p;hsa-miR-195-5p;hsa-miR-49
ENSG0000	ENSG0000	1.46E-10	hsa-miR-15a-5p;hsa-miR-15b-5p;hsa-miR-16-5p;hsa-miR-195-
ENSG0000	ENSG0000	1.46E-06	hsa-miR-15a-5p;hsa-miR-15b-5p;hsa-miR-195-5p;hsa-miR-49

ENSG0000	ENSG0000	7.67E-07	hsa-miR-15a-5p;hsa-miR-15b-5p;hsa-miR-195-5p;hsa-miR-49
ENSG0000	ENSG0000	2.94E-07	hsa-miR-15a-5p;hsa-miR-15b-5p;hsa-miR-16-5p;hsa-miR-195
ENSG0000	ENSG0000	3.99E-11	hsa-miR-15a-5p;hsa-miR-15b-5p;hsa-miR-16-5p;hsa-miR-195
ENSG0000	ENSG0000	2.34E-08	hsa-miR-15a-5p;hsa-miR-15b-5p;hsa-miR-195-5p;hsa-miR-49
ENSG0000	ENSG0000	4.77E-06	hsa-miR-15a-5p;hsa-miR-15b-5p;hsa-miR-16-5p;hsa-miR-195
ENSG0000	ENSG0000	1.19E-06	hsa-miR-15a-5p;hsa-miR-15b-5p;hsa-miR-195-5p;hsa-miR-49
ENSG0000	ENSG0000	1.27E-06	hsa-miR-15a-5p;hsa-miR-15b-5p;hsa-miR-195-5p;hsa-miR-49
ENSG0000	ENSG0000	2.95E-09	hsa-miR-15a-5p;hsa-miR-15b-5p;hsa-miR-16-5p;hsa-miR-195
ENSG0000	ENSG0000	2.09E-11	hsa-miR-15a-5p;hsa-miR-15b-5p;hsa-miR-16-5p;hsa-miR-195
ENSG0000	ENSG0000	2.09E-11	hsa-miR-15a-5p;hsa-miR-15b-5p;hsa-miR-16-5p;hsa-miR-195
ENSG0000	ENSG0000	1.03E-08	hsa-miR-15a-5p;hsa-miR-15b-5p;hsa-miR-195-5p;hsa-miR-49
ENSG0000	ENSG0000	1.33E-11	hsa-miR-15a-5p;hsa-miR-15b-5p;hsa-miR-16-5p;hsa-miR-195
ENSG0000	ENSG0000	2.66E-12	hsa-miR-15a-5p;hsa-miR-15b-5p;hsa-miR-16-5p;hsa-miR-195
ENSG0000	ENSG0000	1.19E-11	hsa-miR-15a-5p;hsa-miR-15b-5p;hsa-miR-16-5p;hsa-miR-195
ENSG0000	ENSG0000	1.75E-10	hsa-miR-15a-5p;hsa-miR-15b-5p;hsa-miR-16-5p;hsa-miR-195
ENSG0000	ENSG0000	7.10E-07	hsa-miR-15a-5p;hsa-miR-15b-5p;hsa-miR-16-5p;hsa-miR-195
ENSG0000	ENSG0000	3.46E-11	hsa-miR-15a-5p;hsa-miR-15b-5p;hsa-miR-16-5p;hsa-miR-195
ENSG0000	ENSG0000	2.71E-11	hsa-miR-15a-5p;hsa-miR-15b-5p;hsa-miR-16-5p;hsa-miR-195
ENSG0000	ENSG0000	2.44E-10	hsa-miR-15a-5p;hsa-miR-15b-5p;hsa-miR-16-5p;hsa-miR-195
ENSG0000	ENSG0000	8.95E-06	hsa-miR-15a-5p;hsa-miR-15b-5p;hsa-miR-16-5p;hsa-miR-195
ENSG0000	ENSG0000	1.33E-11	hsa-miR-15a-5p;hsa-miR-15b-5p;hsa-miR-16-5p;hsa-miR-195
ENSG0000	ENSG0000	4.48E-10	hsa-miR-15a-5p;hsa-miR-15b-5p;hsa-miR-16-5p;hsa-miR-195
ENSG0000	ENSG0000	4.71E-09	hsa-miR-15a-5p;hsa-miR-15b-5p;hsa-miR-16-5p;hsa-miR-195
ENSG0000	ENSG0000	0.000152	hsa-miR-15a-5p;hsa-miR-15b-5p;hsa-miR-195-5p;hsa-miR-49
ENSG0000	ENSG0000	1.31E-09	hsa-miR-15a-5p;hsa-miR-15b-5p;hsa-miR-16-5p;hsa-miR-195
ENSG0000	ENSG0000	3.56E-07	hsa-miR-15a-5p;hsa-miR-15b-5p;hsa-miR-16-5p;hsa-miR-195
ENSG0000	ENSG0000	3.56E-07	hsa-miR-15a-5p;hsa-miR-15b-5p;hsa-miR-16-5p;hsa-miR-195
ENSG0000	ENSG0000	1.77E-06	hsa-miR-15a-5p;hsa-miR-15b-5p;hsa-miR-16-5p;hsa-miR-195
ENSG0000	ENSG0000	2.71E-11	hsa-miR-15a-5p;hsa-miR-15b-5p;hsa-miR-16-5p;hsa-miR-195
ENSG0000	ENSG0000	3.35E-10	hsa-miR-15a-5p;hsa-miR-15b-5p;hsa-miR-195-5p;hsa-miR-49
ENSG0000	ENSG0000	2.67E-09	hsa-miR-15a-5p;hsa-miR-15b-5p;hsa-miR-16-5p;hsa-miR-195
ENSG0000	ENSG0000	7.22E-09	hsa-miR-15a-5p;hsa-miR-15b-5p;hsa-miR-16-5p;hsa-miR-195
ENSG0000	ENSG0000	2.86E-10	hsa-miR-15a-5p;hsa-miR-15b-5p;hsa-miR-16-5p;hsa-miR-195
ENSG0000	ENSG0000	2.66E-12	hsa-miR-15a-5p;hsa-miR-15b-5p;hsa-miR-16-5p;hsa-miR-195
ENSG0000	ENSG0000	3.06E-07	hsa-miR-15a-5p;hsa-miR-15b-5p;hsa-miR-16-5p;hsa-miR-195
ENSG0000	ENSG0000	2.80E-08	hsa-miR-15a-5p;hsa-miR-15b-5p;hsa-miR-16-5p;hsa-miR-195
ENSG0000	ENSG0000	1.54E-08	hsa-miR-15a-5p;hsa-miR-15b-5p;hsa-miR-16-5p;hsa-miR-195
ENSG0000	ENSG0000	1.65E-07	hsa-miR-15a-5p;hsa-miR-15b-5p;hsa-miR-16-5p;hsa-miR-195
ENSG0000	ENSG0000	1.94E-07	hsa-miR-15a-5p;hsa-miR-15b-5p;hsa-miR-195-5p;hsa-miR-49
ENSG0000	ENSG0000	3.91E-07	hsa-miR-15a-5p;hsa-miR-15b-5p;hsa-miR-16-5p;hsa-miR-195
ENSG0000	ENSG0000	1.22E-07	hsa-miR-15a-5p;hsa-miR-15b-5p;hsa-miR-16-5p;hsa-miR-195
ENSG0000	ENSG0000	2.66E-12	hsa-miR-15a-5p;hsa-miR-15b-5p;hsa-miR-16-5p;hsa-miR-195
ENSG0000	ENSG0000	1.26E-09	hsa-miR-15a-5p;hsa-miR-15b-5p;hsa-miR-16-5p;hsa-miR-195
ENSG0000	ENSG0000	3.34E-08	hsa-miR-15a-5p;hsa-miR-15b-5p;hsa-miR-16-5p;hsa-miR-195
ENSG0000	ENSG0000	1.24E-08	hsa-miR-15a-5p;hsa-miR-15b-5p;hsa-miR-16-5p;hsa-miR-195
ENSG0000	ENSG0000	5.61E-09	hsa-miR-15a-5p;hsa-miR-15b-5p;hsa-miR-16-5p;hsa-miR-195
ENSG0000	ENSG0000	4.27E-08	hsa-miR-15a-5p;hsa-miR-15b-5p;hsa-miR-16-5p;hsa-miR-195
ENSG0000	ENSG0000	3.09E-05	hsa-miR-15a-5p;hsa-miR-15b-5p;hsa-miR-195-5p;hsa-miR-49
ENSG0000	ENSG0000	3.57E-09	hsa-miR-15a-5p;hsa-miR-15b-5p;hsa-miR-16-5p;hsa-miR-195
ENSG0000	ENSG0000	8.59E-06	hsa-miR-15a-5p;hsa-miR-15b-5p;hsa-miR-16-5p;hsa-miR-195
ENSG0000	ENSG0000	8.30E-11	hsa-miR-15a-5p;hsa-miR-15b-5p;hsa-miR-16-5p;hsa-miR-195
ENSG0000	ENSG0000	3.91E-07	hsa-miR-15a-5p;hsa-miR-15b-5p;hsa-miR-16-5p;hsa-miR-195
ENSG0000	ENSG0000	3.91E-07	hsa-miR-15a-5p;hsa-miR-15b-5p;hsa-miR-195-5p;hsa-miR-49
ENSG0000	ENSG0000	4.68E-07	hsa-miR-15a-5p;hsa-miR-15b-5p;hsa-miR-195-5p;hsa-miR-49

ENSG0000	ENSG0000	4.03E-08	hsa-miR-15a-5p;hsa-miR-15b-5p;hsa-miR-16-5p;hsa-miR-195-
ENSG0000	ENSG0000	7.23E-08	hsa-miR-15a-5p;hsa-miR-15b-5p;hsa-miR-195-5p;hsa-miR-49
ENSG0000	ENSG0000	7.23E-08	hsa-miR-15a-5p;hsa-miR-15b-5p;hsa-miR-195-5p;hsa-miR-49
ENSG0000	ENSG0000	2.48E-05	hsa-miR-15a-5p;hsa-miR-15b-5p;hsa-miR-195-5p;hsa-miR-49
ENSG0000	ENSG0000	8.74E-10	hsa-miR-15a-5p;hsa-miR-15b-5p;hsa-miR-16-5p;hsa-miR-195-
ENSG0000	ENSG0000	8.49E-09	hsa-miR-15a-5p;hsa-miR-15b-5p;hsa-miR-16-5p;hsa-miR-195-
ENSG0000	ENSG0000	1.22E-06	hsa-miR-15a-5p;hsa-miR-15b-5p;hsa-miR-16-5p;hsa-miR-195-
ENSG0000	ENSG0000	2.81E-08	hsa-miR-15a-5p;hsa-miR-16-5p;hsa-miR-195-5p;hsa-miR-497-
ENSG0000	ENSG0000	1.01E-10	hsa-miR-15a-5p;hsa-miR-15b-5p;hsa-miR-16-5p;hsa-miR-195-
ENSG0000	ENSG0000	1.58E-09	hsa-miR-15a-5p;hsa-miR-15b-5p;hsa-miR-16-5p;hsa-miR-195-
ENSG0000	ENSG0000	1.96E-09	hsa-miR-15a-5p;hsa-miR-15b-5p;hsa-miR-16-5p;hsa-miR-195-
ENSG0000	ENSG0000	1.59E-11	hsa-miR-15a-5p;hsa-miR-15b-5p;hsa-miR-16-5p;hsa-miR-195-
ENSG0000	ENSG0000	3.56E-07	hsa-miR-15a-5p;hsa-miR-15b-5p;hsa-miR-16-5p;hsa-miR-195-
ENSG0000	ENSG0000	8.11E-09	hsa-miR-15a-5p;hsa-miR-16-5p;hsa-miR-195-5p;hsa-miR-497-
ENSG0000	ENSG0000	1.31E-09	hsa-miR-15a-5p;hsa-miR-15b-5p;hsa-miR-16-5p;hsa-miR-195-
ENSG0000	ENSG0000	7.84E-09	hsa-miR-15a-5p;hsa-miR-15b-5p;hsa-miR-16-5p;hsa-miR-195-
ENSG0000	ENSG0000	4.03E-08	hsa-miR-15a-5p;hsa-miR-15b-5p;hsa-miR-16-5p;hsa-miR-195-
ENSG0000	ENSG0000	1.01E-10	hsa-miR-15a-5p;hsa-miR-15b-5p;hsa-miR-16-5p;hsa-miR-195-
ENSG0000	ENSG0000	4.49E-12	hsa-miR-15a-5p;hsa-miR-15b-5p;hsa-miR-16-5p;hsa-miR-195-
ENSG0000	ENSG0000	3.71E-06	hsa-miR-15a-5p;hsa-miR-15b-5p;hsa-miR-16-5p;hsa-miR-195-
ENSG0000	ENSG0000	2.41E-09	hsa-miR-15a-5p;hsa-miR-15b-5p;hsa-miR-16-5p;hsa-miR-195-
ENSG0000	ENSG0000	1.19E-11	hsa-miR-15a-5p;hsa-miR-15b-5p;hsa-miR-16-5p;hsa-miR-195-
ENSG0000	ENSG0000	1.19E-11	hsa-miR-15a-5p;hsa-miR-15b-5p;hsa-miR-16-5p;hsa-miR-195-
ENSG0000	ENSG0000	1.19E-11	hsa-miR-15a-5p;hsa-miR-15b-5p;hsa-miR-16-5p;hsa-miR-195-
ENSG0000	ENSG0000	1.19E-11	hsa-miR-15a-5p;hsa-miR-15b-5p;hsa-miR-16-5p;hsa-miR-195-
ENSG0000	ENSG0000	4.11E-06	hsa-miR-15a-5p;hsa-miR-15b-5p;hsa-miR-195-5p;hsa-miR-49
ENSG0000	ENSG0000	4.11E-06	hsa-miR-15a-5p;hsa-miR-15b-5p;hsa-miR-195-5p;hsa-miR-49
ENSG0000	ENSG0000	3.17E-08	hsa-miR-15a-5p;hsa-miR-15b-5p;hsa-miR-16-5p;hsa-miR-195-
ENSG0000	ENSG0000	6.11E-09	hsa-miR-15a-5p;hsa-miR-15b-5p;hsa-miR-16-5p;hsa-miR-195-
ENSG0000	ENSG0000	1.90E-09	hsa-miR-15a-5p;hsa-miR-15b-5p;hsa-miR-195-5p;hsa-miR-49
ENSG0000	ENSG0000	3.99E-11	hsa-miR-15a-5p;hsa-miR-15b-5p;hsa-miR-16-5p;hsa-miR-195-
ENSG0000	ENSG0000	2.66E-12	hsa-miR-15a-5p;hsa-miR-15b-5p;hsa-miR-16-5p;hsa-miR-195-
ENSG0000	ENSG0000	4.48E-10	hsa-miR-15a-5p;hsa-miR-15b-5p;hsa-miR-16-5p;hsa-miR-195-
ENSG0000	ENSG0000	4.48E-10	hsa-miR-15a-5p;hsa-miR-15b-5p;hsa-miR-16-5p;hsa-miR-195-
ENSG0000	ENSG0000	4.48E-10	hsa-miR-15a-5p;hsa-miR-15b-5p;hsa-miR-16-5p;hsa-miR-195-
ENSG0000	ENSG0000	4.48E-10	hsa-miR-15a-5p;hsa-miR-15b-5p;hsa-miR-16-5p;hsa-miR-195-
ENSG0000	ENSG0000	4.48E-10	hsa-miR-15a-5p;hsa-miR-15b-5p;hsa-miR-16-5p;hsa-miR-195-
ENSG0000	ENSG0000	4.48E-10	hsa-miR-15a-5p;hsa-miR-15b-5p;hsa-miR-16-5p;hsa-miR-195-
ENSG0000	ENSG0000	4.48E-10	hsa-miR-15a-5p;hsa-miR-15b-5p;hsa-miR-16-5p;hsa-miR-195-
ENSG0000	ENSG0000	4.48E-10	hsa-miR-15a-5p;hsa-miR-15b-5p;hsa-miR-16-5p;hsa-miR-195-
ENSG0000	ENSG0000	4.48E-10	hsa-miR-15a-5p;hsa-miR-15b-5p;hsa-miR-16-5p;hsa-miR-195-
ENSG0000	ENSG0000	4.48E-10	hsa-miR-15a-5p;hsa-miR-15b-5p;hsa-miR-16-5p;hsa-miR-195-
ENSG0000	ENSG0000	1.86E-10	hsa-miR-15a-5p;hsa-miR-15b-5p;hsa-miR-16-5p;hsa-miR-195-
ENSG0000	ENSG0000	2.71E-11	hsa-miR-15a-5p;hsa-miR-15b-5p;hsa-miR-16-5p;hsa-miR-195-
ENSG0000	ENSG0000	3.34E-08	hsa-miR-15a-5p;hsa-miR-15b-5p;hsa-miR-16-5p;hsa-miR-497-
ENSG0000	ENSG0000	1.74E-07	hsa-miR-15a-5p;hsa-miR-15b-5p;hsa-miR-16-5p;hsa-miR-195-
ENSG0000	ENSG0000	7.70E-10	hsa-miR-15a-5p;hsa-miR-15b-5p;hsa-miR-16-5p;hsa-miR-195-
ENSG0000	ENSG0000	1.24E-08	hsa-miR-15a-5p;hsa-miR-15b-5p;hsa-miR-16-5p;hsa-miR-195-
ENSG0000	ENSG0000	5.16E-10	hsa-miR-15a-5p;hsa-miR-15b-5p;hsa-miR-16-5p;hsa-miR-195-
ENSG0000	ENSG0000	2.95E-09	hsa-miR-15a-5p;hsa-miR-15b-5p;hsa-miR-16-5p;hsa-miR-195-
ENSG0000	ENSG0000	6.27E-08	hsa-miR-15a-5p;hsa-miR-15b-5p;hsa-miR-195-5p;hsa-miR-49
ENSG0000	ENSG0000	2.47E-08	hsa-miR-15a-5p;hsa-miR-15b-5p;hsa-miR-16-5p;hsa-miR-195-
ENSG0000	ENSG0000	2.18E-09	hsa-miR-15a-5p;hsa-miR-15b-5p;hsa-miR-16-5p;hsa-miR-195-
ENSG0000	ENSG0000	1.22E-07	hsa-miR-15a-5p;hsa-miR-15b-5p;hsa-miR-16-5p;hsa-miR-195-

ENSG0000	ENSG0000	7.22E-09	hsa-miR-15a-5p;hsa-miR-15b-5p;hsa-miR-16-5p;hsa-miR-195-
ENSG0000	ENSG0000	3.56E-07	hsa-miR-15a-5p;hsa-miR-15b-5p;hsa-miR-16-5p;hsa-miR-195-
ENSG0000	ENSG0000	1.19E-11	hsa-miR-15a-5p;hsa-miR-15b-5p;hsa-miR-16-5p;hsa-miR-195-
ENSG0000	ENSG0000	3.98E-07	hsa-miR-15a-5p;hsa-miR-15b-5p;hsa-miR-16-5p;hsa-miR-195-
ENSG0000	ENSG0000	1.28E-08	hsa-miR-15a-5p;hsa-miR-15b-5p;hsa-miR-195-5p;hsa-miR-49
ENSG0000	ENSG0000	8.30E-08	hsa-miR-15a-5p;hsa-miR-15b-5p;hsa-miR-195-5p;hsa-miR-49
ENSG0000	ENSG0000	3.09E-12	hsa-miR-15a-5p;hsa-miR-15b-5p;hsa-miR-16-5p;hsa-miR-195-
ENSG0000	ENSG0000	3.62E-09	hsa-miR-15a-5p;hsa-miR-15b-5p;hsa-miR-195-5p;hsa-miR-49
ENSG0000	ENSG0000	8.30E-11	hsa-miR-15a-5p;hsa-miR-15b-5p;hsa-miR-16-5p;hsa-miR-195-
ENSG0000	ENSG0000	3.34E-10	hsa-miR-15a-5p;hsa-miR-15b-5p;hsa-miR-16-5p;hsa-miR-195-
ENSG0000	ENSG0000	3.95E-08	hsa-miR-15a-5p;hsa-miR-15b-5p;hsa-miR-16-5p;hsa-miR-195-
ENSG0000	ENSG0000	1.33E-11	hsa-miR-15a-5p;hsa-miR-15b-5p;hsa-miR-16-5p;hsa-miR-195-
ENSG0000	ENSG0000	3.87E-10	hsa-miR-15a-5p;hsa-miR-15b-5p;hsa-miR-16-5p;hsa-miR-195-
ENSG0000	ENSG0000	3.00E-06	hsa-miR-15a-5p;hsa-miR-15b-5p;hsa-miR-195-5p;hsa-miR-49
ENSG0000	ENSG0000	5.92E-10	hsa-miR-15a-5p;hsa-miR-15b-5p;hsa-miR-16-5p;hsa-miR-195-
ENSG0000	ENSG0000	2.18E-09	hsa-miR-15a-5p;hsa-miR-15b-5p;hsa-miR-16-5p;hsa-miR-195-
ENSG0000	ENSG0000	2.71E-11	hsa-miR-15a-5p;hsa-miR-15b-5p;hsa-miR-16-5p;hsa-miR-195-
ENSG0000	ENSG0000	3.09E-12	hsa-miR-15a-5p;hsa-miR-15b-5p;hsa-miR-16-5p;hsa-miR-195-
ENSG0000	ENSG0000	3.93E-09	hsa-miR-15a-5p;hsa-miR-15b-5p;hsa-miR-16-5p;hsa-miR-195-
ENSG0000	ENSG0000	1.28E-08	hsa-miR-15a-5p;hsa-miR-15b-5p;hsa-miR-16-5p;hsa-miR-195-
ENSG0000	ENSG0000	1.28E-08	hsa-miR-15a-5p;hsa-miR-15b-5p;hsa-miR-16-5p;hsa-miR-195-
ENSG0000	ENSG0000	8.56E-08	hsa-miR-15a-5p;hsa-miR-15b-5p;hsa-miR-16-5p;hsa-miR-195-
ENSG0000	ENSG0000	1.22E-10	hsa-miR-15a-5p;hsa-miR-15b-5p;hsa-miR-16-5p;hsa-miR-195-
ENSG0000	ENSG0000	4.82E-09	hsa-miR-15a-5p;hsa-miR-15b-5p;hsa-miR-16-5p;hsa-miR-195-
ENSG0000	ENSG0000	8.14E-13	hsa-miR-15a-5p;hsa-miR-15b-5p;hsa-miR-16-5p;hsa-miR-195-
ENSG0000	ENSG0000	9.92E-09	hsa-miR-15a-5p;hsa-miR-15b-5p;hsa-miR-16-5p;hsa-miR-195-
ENSG0000	ENSG0000	3.09E-12	hsa-miR-15a-5p;hsa-miR-15b-5p;hsa-miR-16-5p;hsa-miR-195-
ENSG0000	ENSG0000	2.47E-08	hsa-miR-15a-5p;hsa-miR-15b-5p;hsa-miR-16-5p;hsa-miR-195-
ENSG0000	ENSG0000	2.18E-09	hsa-miR-15a-5p;hsa-miR-15b-5p;hsa-miR-16-5p;hsa-miR-195-
ENSG0000	ENSG0000	1.75E-10	hsa-miR-15a-5p;hsa-miR-15b-5p;hsa-miR-16-5p;hsa-miR-195-
ENSG0000	ENSG0000	6.55E-07	hsa-miR-15a-5p;hsa-miR-15b-5p;hsa-miR-16-5p;hsa-miR-195-
ENSG0000	ENSG0000	6.65E-09	hsa-miR-15a-5p;hsa-miR-15b-5p;hsa-miR-16-5p;hsa-miR-195-
ENSG0000	ENSG0000	5.05E-08	hsa-miR-15a-5p;hsa-miR-15b-5p;hsa-miR-16-5p;hsa-miR-195-
ENSG0000	ENSG0000	4.77E-07	hsa-miR-15a-5p;hsa-miR-15b-5p;hsa-miR-16-5p;hsa-miR-195-
ENSG0000	ENSG0000	4.14E-05	hsa-miR-15a-5p;hsa-miR-15b-5p;hsa-miR-195-5p;hsa-miR-49
ENSG0000	ENSG0000	2.66E-12	hsa-miR-15a-5p;hsa-miR-15b-5p;hsa-miR-16-5p;hsa-miR-195-
ENSG0000	ENSG0000	6.76E-11	hsa-miR-15a-5p;hsa-miR-15b-5p;hsa-miR-16-5p;hsa-miR-195-
ENSG0000	ENSG0000	1.46E-06	hsa-miR-15a-5p;hsa-miR-15b-5p;hsa-miR-16-5p;hsa-miR-195-
ENSG0000	ENSG0000	1.54E-08	hsa-miR-15a-5p;hsa-miR-15b-5p;hsa-miR-16-5p;hsa-miR-195-
ENSG0000	ENSG0000	3.17E-08	hsa-miR-15a-5p;hsa-miR-15b-5p;hsa-miR-16-5p;hsa-miR-195-
ENSG0000	ENSG0000	4.71E-09	hsa-miR-15a-5p;hsa-miR-15b-5p;hsa-miR-16-5p;hsa-miR-195-
ENSG0000	ENSG0000	4.30E-09	hsa-miR-15a-5p;hsa-miR-15b-5p;hsa-miR-16-5p;hsa-miR-195-
ENSG0000	ENSG0000	0.002495	hsa-miR-130a-3p;hsa-miR-130b-3p;hsa-miR-301a-3p;hsa-miR-
ENSG0000	ENSG0000	0.006694	hsa-miR-130a-3p;hsa-miR-130b-3p;hsa-miR-301a-3p;hsa-miR-
ENSG0000	ENSG0000	0.003984	hsa-miR-130a-3p;hsa-miR-130b-3p;hsa-miR-301a-3p;hsa-miR-
ENSG0000	ENSG0000	2.56E-05	hsa-miR-125b-5p;hsa-miR-135a-5p;hsa-miR-135b-5p;hsa-miR
ENSG0000	ENSG0000	0.005422	hsa-miR-122-5p;hsa-miR-130b-3p;hsa-miR-200b-3p;hsa-miR-
ENSG0000	ENSG0000	0.00318	hsa-miR-1270;hsa-miR-148a-3p;hsa-miR-148b-3p;hsa-miR-45
ENSG0000	ENSG0000	0.000838	hsa-miR-122-5p;hsa-miR-130a-3p;hsa-miR-130b-3p;hsa-miR-
ENSG0000	ENSG0000	0.007199	hsa-miR-126-5p;hsa-miR-200b-3p;hsa-miR-200c-3p;hsa-miR-
ENSG0000	ENSG0000	1.09E-06	hsa-miR-125b-5p;hsa-miR-148a-3p;hsa-miR-16-1-3p;hsa-miR-
ENSG0000	ENSG0000	0.001997	hsa-miR-130a-3p;hsa-miR-130b-3p;hsa-miR-148a-3p;hsa-miR-
ENSG0000	ENSG0000	0.003992	hsa-miR-130a-3p;hsa-miR-130b-3p;hsa-miR-301a-3p;hsa-miR-
ENSG0000	ENSG0000	0.000262	hsa-miR-125a-5p;hsa-miR-200b-3p;hsa-miR-200c-3p;hsa-miR-

ENSG0000	ENSG0000	0.000486	hsa-miR-199a-5p;hsa-miR-200b-3p;hsa-miR-200c-3p;hsa-miR-
ENSG0000	ENSG0000	1.44E-05	hsa-miR-130a-3p;hsa-miR-148a-3p;hsa-miR-148b-3p;hsa-miR-
ENSG0000	ENSG0000	0.006573	hsa-miR-200b-3p;hsa-miR-200c-3p;hsa-miR-429;hsa-miR-7-5
ENSG0000	ENSG0000	0.002823	hsa-miR-130a-3p;hsa-miR-130b-3p;hsa-miR-301a-3p;hsa-miR-
ENSG0000	ENSG0000	0.002392	hsa-miR-122-5p;hsa-miR-130a-3p;hsa-miR-130b-3p;hsa-miR-
ENSG0000	ENSG0000	0.00286	hsa-miR-130a-3p;hsa-miR-130b-3p;hsa-miR-148a-3p;hsa-miR-
ENSG0000	ENSG0000	0.001982	hsa-miR-122-5p;hsa-miR-130a-3p;hsa-miR-130b-3p;hsa-miR-
ENSG0000	ENSG0000	0.004799	hsa-miR-200b-3p;hsa-miR-200c-3p;hsa-miR-30c-1-3p;hsa-mi
ENSG0000	ENSG0000	9.60E-05	hsa-miR-125a-5p;hsa-miR-130a-3p;hsa-miR-130b-3p;hsa-miR-
ENSG0000	ENSG0000	0.009309	hsa-miR-130a-3p;hsa-miR-130b-3p;hsa-miR-301a-3p;hsa-miR-
ENSG0000	ENSG0000	1.70E-05	hsa-miR-130a-3p;hsa-miR-130b-3p;hsa-miR-218-5p;hsa-miR-
ENSG0000	ENSG0000	0.000332	hsa-miR-140-5p;hsa-miR-148a-3p;hsa-miR-218-5p;hsa-miR-30
ENSG0000	ENSG0000	0.003683	hsa-miR-125a-5p;hsa-miR-130a-3p;hsa-miR-130b-3p;hsa-miR-
ENSG0000	ENSG0000	0.000168	hsa-miR-122-5p;hsa-miR-125b-5p;hsa-miR-135a-5p;hsa-miR-
ENSG0000	ENSG0000	0.004515	hsa-miR-130a-3p;hsa-miR-130b-3p;hsa-miR-24-3p;hsa-miR-30
ENSG0000	ENSG0000	0.001997	hsa-miR-130a-3p;hsa-miR-130b-3p;hsa-miR-26b-3p;hsa-miR-
ENSG0000	ENSG0000	0.000653	hsa-miR-130a-3p;hsa-miR-130b-3p;hsa-miR-16-1-3p;hsa-miR-
ENSG0000	ENSG0000	0.002618	hsa-miR-130a-3p;hsa-miR-130b-3p;hsa-miR-301a-3p;hsa-miR-
ENSG0000	ENSG0000	0.000438	hsa-miR-122-5p;hsa-miR-130a-3p;hsa-miR-130b-3p;hsa-miR-
ENSG0000	ENSG0000	0.003567	hsa-miR-130a-3p;hsa-miR-130b-3p;hsa-miR-301a-3p;hsa-miR-
ENSG0000	ENSG0000	0.003118	hsa-miR-122-5p;hsa-miR-130a-3p;hsa-miR-130b-3p;hsa-miR-
ENSG0000	ENSG0000	0.00318	hsa-miR-130a-3p;hsa-miR-130b-3p;hsa-miR-301a-3p;hsa-miR-
ENSG0000	ENSG0000	0.001461	hsa-miR-130a-3p;hsa-miR-130b-3p;hsa-miR-218-5p;hsa-miR-
ENSG0000	ENSG0000	0.003984	hsa-miR-130a-3p;hsa-miR-130b-3p;hsa-miR-301a-3p;hsa-miR-
ENSG0000	ENSG0000	0.002488	hsa-miR-130a-3p;hsa-miR-130b-3p;hsa-miR-301a-3p;hsa-miR-
ENSG0000	ENSG0000	0.009309	hsa-miR-125b-5p;hsa-miR-200b-3p;hsa-miR-200c-3p;hsa-miR-
ENSG0000	ENSG0000	0.001043	hsa-miR-130a-3p;hsa-miR-130b-3p;hsa-miR-218-5p;hsa-miR-
ENSG0000	ENSG0000	9.76E-05	hsa-miR-1270;hsa-miR-140-5p;hsa-miR-148a-3p;hsa-miR-148
ENSG0000	ENSG0000	1.43E-08	hsa-miR-148a-3p;hsa-miR-199a-5p;hsa-miR-200b-3p;hsa-miR-
ENSG0000	ENSG0000	0.002194	hsa-miR-130a-3p;hsa-miR-130b-3p;hsa-miR-301a-3p;hsa-miR-
ENSG0000	ENSG0000	0.000741	hsa-miR-148a-3p;hsa-miR-200b-3p;hsa-miR-200c-3p;hsa-miR-
ENSG0000	ENSG0000	0.001851	hsa-miR-130a-3p;hsa-miR-148a-3p;hsa-miR-148b-3p;hsa-miR-
ENSG0000	ENSG0000	1.49E-05	hsa-miR-130a-3p;hsa-miR-130b-3p;hsa-miR-151a-3p;hsa-miR-
ENSG0000	ENSG0000	0.000171	hsa-miR-200b-3p;hsa-miR-200c-3p;hsa-miR-218-5p;hsa-miR-
ENSG0000	ENSG0000	7.35E-05	hsa-miR-130a-3p;hsa-miR-130b-3p;hsa-miR-148a-3p;hsa-miR-
ENSG0000	ENSG0000	0.001238	hsa-miR-130a-3p;hsa-miR-130b-3p;hsa-miR-301a-3p;hsa-miR-
ENSG0000	ENSG0000	2.22E-05	hsa-miR-122-5p;hsa-miR-130a-3p;hsa-miR-130b-3p;hsa-miR-
ENSG0000	ENSG0000	2.22E-05	hsa-miR-122-5p;hsa-miR-130a-3p;hsa-miR-130b-3p;hsa-miR-
ENSG0000	ENSG0000	0.000317	hsa-miR-122-5p;hsa-miR-130a-3p;hsa-miR-130b-3p;hsa-miR-
ENSG0000	ENSG0000	0.002193	hsa-miR-125a-5p;hsa-miR-125b-5p;hsa-miR-130a-3p;hsa-miR-
ENSG0000	ENSG0000	0.002331	hsa-miR-122-5p;hsa-miR-130a-3p;hsa-miR-130b-3p;hsa-miR-
ENSG0000	ENSG0000	0.003118	hsa-miR-130a-3p;hsa-miR-148a-3p;hsa-miR-148b-3p;hsa-miR-
ENSG0000	ENSG0000	0.000155	hsa-miR-125a-5p;hsa-miR-125b-5p;hsa-miR-151a-3p;hsa-miR-
ENSG0000	ENSG0000	0.000278	hsa-miR-122-5p;hsa-miR-130a-3p;hsa-miR-130b-3p;hsa-miR-
ENSG0000	ENSG0000	0.00218	hsa-miR-130a-3p;hsa-miR-130b-3p;hsa-miR-301a-3p;hsa-miR-
ENSG0000	ENSG0000	0.003984	hsa-miR-130a-3p;hsa-miR-130b-3p;hsa-miR-301a-3p;hsa-miR-
ENSG0000	ENSG0000	0.009869	hsa-miR-200b-3p;hsa-miR-200c-3p;hsa-miR-218-5p;hsa-miR-
ENSG0000	ENSG0000	0.003567	hsa-miR-130a-3p;hsa-miR-130b-3p;hsa-miR-301a-3p;hsa-miR-
ENSG0000	ENSG0000	0.000943	hsa-miR-130a-3p;hsa-miR-130b-3p;hsa-miR-16-1-3p;hsa-miR-
ENSG0000	ENSG0000	0.00286	hsa-miR-140-5p;hsa-miR-199a-5p;hsa-miR-30c-1-3p;hsa-miR-
ENSG0000	ENSG0000	0.003278	hsa-miR-122-5p;hsa-miR-130a-3p;hsa-miR-130b-3p;hsa-miR-
ENSG0000	ENSG0000	0.009309	hsa-miR-130b-3p;hsa-miR-27a-5p;hsa-miR-30c-1-3p;hsa-miR-
ENSG0000	ENSG0000	0.000115	hsa-miR-200b-3p;hsa-miR-200c-3p;hsa-miR-24-3p;hsa-miR-42
ENSG0000	ENSG0000	0.000347	hsa-miR-122-5p;hsa-miR-130a-3p;hsa-miR-130b-3p;hsa-miR-

ENSG0000	ENSG0000	0.004319	hsa-miR-122-5p;hsa-miR-130a-3p;hsa-miR-130b-3p;hsa-miR-1
ENSG0000	ENSG0000	0.000872	hsa-miR-130a-3p;hsa-miR-200b-3p;hsa-miR-200c-3p;hsa-miR-
ENSG0000	ENSG0000	0.000748	hsa-miR-130a-3p;hsa-miR-130b-3p;hsa-miR-301a-3p;hsa-miR-
ENSG0000	ENSG0000	4.93E-05	hsa-miR-130a-3p;hsa-miR-130b-3p;hsa-miR-218-5p;hsa-miR-3
ENSG0000	ENSG0000	2.96E-07	hsa-miR-200c-3p;hsa-miR-218-5p;hsa-miR-362-5p;hsa-miR-45
ENSG0000	ENSG0000	0.000239	hsa-miR-122-5p;hsa-miR-130a-3p;hsa-miR-130b-3p;hsa-miR-3
ENSG0000	ENSG0000	0.001058	hsa-miR-130a-3p;hsa-miR-130b-3p;hsa-miR-301a-3p;hsa-miR-
ENSG0000	ENSG0000	0.000249	hsa-miR-200c-3p;hsa-miR-24-3p;hsa-miR-30c-1-3p;hsa-miR-3
ENSG0000	ENSG0000	4.50E-07	hsa-miR-130a-3p;hsa-miR-130b-3p;hsa-miR-199a-5p;hsa-miR-
ENSG0000	ENSG0000	0.001626	hsa-miR-122-5p;hsa-miR-130a-3p;hsa-miR-130b-3p;hsa-miR-3
ENSG0000	ENSG0000	0.001626	hsa-miR-122-5p;hsa-miR-130a-3p;hsa-miR-130b-3p;hsa-miR-3
ENSG0000	ENSG0000	0.004664	hsa-miR-122-5p;hsa-miR-130a-3p;hsa-miR-130b-3p;hsa-miR-3
ENSG0000	ENSG0000	0.006694	hsa-miR-122-5p;hsa-miR-218-5p;hsa-miR-23a-3p;hsa-miR-23b
ENSG0000	ENSG0000	0.00049	hsa-miR-130a-3p;hsa-miR-130b-3p;hsa-miR-200b-3p;hsa-miR
ENSG0000	ENSG0000	0.002495	hsa-miR-130b-3p;hsa-miR-200b-3p;hsa-miR-200c-3p;hsa-miR-
ENSG0000	ENSG0000	0.000918	hsa-miR-122-5p;hsa-miR-130a-3p;hsa-miR-130b-3p;hsa-miR-2
ENSG0000	ENSG0000	0.001583	hsa-miR-130a-3p;hsa-miR-130b-3p;hsa-miR-135b-3p;hsa-miR
ENSG0000	ENSG0000	0.00318	hsa-miR-130a-3p;hsa-miR-130b-3p;hsa-miR-301a-3p;hsa-miR-
ENSG0000	ENSG0000	0.001058	hsa-miR-130a-3p;hsa-miR-130b-3p;hsa-miR-301a-3p;hsa-miR-
ENSG0000	ENSG0000	0.00068	hsa-miR-130a-3p;hsa-miR-130b-3p;hsa-miR-148a-3p;hsa-miR-
ENSG0000	ENSG0000	0.003683	hsa-miR-130a-3p;hsa-miR-130b-3p;hsa-miR-16-1-3p;hsa-miR-
ENSG0000	ENSG0000	0.0014	hsa-miR-125a-5p;hsa-miR-126-5p;hsa-miR-140-5p;hsa-miR-16
ENSG0000	ENSG0000	2.16E-05	hsa-miR-122-5p;hsa-miR-1270;hsa-miR-148a-3p;hsa-miR-148b
ENSG0000	ENSG0000	0.006694	hsa-miR-130a-3p;hsa-miR-130b-3p;hsa-miR-193b-5p;hsa-miR
ENSG0000	ENSG0000	0.002151	hsa-miR-135b-3p;hsa-miR-183-3p;hsa-miR-193b-5p;hsa-miR-1
ENSG0000	ENSG0000	0.000168	hsa-miR-130a-3p;hsa-miR-130b-3p;hsa-miR-301a-3p;hsa-miR-
ENSG0000	ENSG0000	0.000748	hsa-miR-135b-5p;hsa-miR-200b-3p;hsa-miR-200c-3p;hsa-miR-
ENSG0000	ENSG0000	1.64E-05	hsa-miR-130a-3p;hsa-miR-130b-3p;hsa-miR-148a-3p;hsa-miR-
ENSG0000	ENSG0000	5.08E-05	hsa-miR-122-5p;hsa-miR-130a-3p;hsa-miR-185-3p;hsa-miR-30
ENSG0000	ENSG0000	0.002194	hsa-miR-185-3p;hsa-miR-200b-3p;hsa-miR-200c-3p;hsa-miR-2
ENSG0000	ENSG0000	0.000954	hsa-miR-130a-3p;hsa-miR-130b-3p;hsa-miR-218-5p;hsa-miR-3
ENSG0000	ENSG0000	0.000573	hsa-miR-122-5p;hsa-miR-130a-3p;hsa-miR-130b-3p;hsa-miR-3
ENSG0000	ENSG0000	0.001944	hsa-miR-122-5p;hsa-miR-130a-3p;hsa-miR-130b-3p;hsa-miR-2
ENSG0000	ENSG0000	1.16E-05	hsa-miR-130a-3p;hsa-miR-130b-3p;hsa-miR-218-5p;hsa-miR-3
ENSG0000	ENSG0000	0.00039	hsa-miR-125a-5p;hsa-miR-125b-5p;hsa-miR-130a-3p;hsa-miR-
ENSG0000	ENSG0000	0.001919	hsa-miR-125b-5p;hsa-miR-148a-3p;hsa-miR-148b-3p;hsa-miR
ENSG0000	ENSG0000	1.56E-05	hsa-miR-130a-3p;hsa-miR-130b-3p;hsa-miR-301a-3p;hsa-miR-
ENSG0000	ENSG0000	0.007658	hsa-miR-130a-3p;hsa-miR-130b-3p;hsa-miR-185-3p;hsa-miR-3
ENSG0000	ENSG0000	0.007658	hsa-miR-130a-3p;hsa-miR-130b-3p;hsa-miR-185-3p;hsa-miR-3
ENSG0000	ENSG0000	0.001497	hsa-miR-122-5p;hsa-miR-130a-3p;hsa-miR-130b-3p;hsa-miR-1
ENSG0000	ENSG0000	1.26E-07	hsa-miR-130a-3p;hsa-miR-130b-3p;hsa-miR-148a-3p;hsa-miR-
ENSG0000	ENSG0000	0.004916	hsa-miR-130a-3p;hsa-miR-130b-3p;hsa-miR-301a-3p;hsa-miR-
ENSG0000	ENSG0000	0.000237	hsa-miR-200b-3p;hsa-miR-200c-3p;hsa-miR-429;hsa-miR-488-
ENSG0000	ENSG0000	0.003984	hsa-miR-24-3p;hsa-miR-30c-1-3p;hsa-miR-30c-2-3p;hsa-miR-
ENSG0000	ENSG0000	0.000137	hsa-miR-130a-3p;hsa-miR-148b-3p;hsa-miR-200b-3p;hsa-miR
ENSG0000	ENSG0000	0.004434	hsa-miR-130a-3p;hsa-miR-130b-3p;hsa-miR-301a-3p;hsa-miR-
ENSG0000	ENSG0000	0.004434	hsa-miR-130a-3p;hsa-miR-130b-3p;hsa-miR-301a-3p;hsa-miR-
ENSG0000	ENSG0000	0.003683	hsa-miR-122-5p;hsa-miR-130a-3p;hsa-miR-130b-3p;hsa-miR-3
ENSG0000	ENSG0000	0.007743	hsa-miR-130a-3p;hsa-miR-218-5p;hsa-miR-301a-3p;hsa-miR-3
ENSG0000	ENSG0000	0.000237	hsa-miR-122-5p;hsa-miR-130a-3p;hsa-miR-130b-3p;hsa-miR-3
ENSG0000	ENSG0000	0.004319	hsa-miR-130a-3p;hsa-miR-130b-3p;hsa-miR-218-5p;hsa-miR-3
ENSG0000	ENSG0000	0.000791	hsa-miR-140-5p;hsa-miR-200b-3p;hsa-miR-200c-3p;hsa-miR-3
ENSG0000	ENSG0000	0.002625	hsa-miR-130a-3p;hsa-miR-130b-3p;hsa-miR-148a-3p;hsa-miR-
ENSG0000	ENSG0000	0.000203	hsa-miR-130a-3p;hsa-miR-130b-3p;hsa-miR-301a-3p;hsa-miR-

ENSG0000	ENSG0000	0.005985	hsa-miR-500a-5p;hsa-miR-501-5p;hsa-miR-584-5p;hsa-miR-9-
ENSG0000	ENSG0000	0.009279	hsa-miR-130a-3p;hsa-miR-130b-3p;hsa-miR-301a-3p;hsa-miR-
ENSG0000	ENSG0000	0.009279	hsa-miR-130a-3p;hsa-miR-130b-3p;hsa-miR-301a-3p;hsa-miR-
ENSG0000	ENSG0000	0.00318	hsa-miR-24-3p;hsa-miR-30c-1-3p;hsa-miR-30c-2-3p;hsa-miR-
ENSG0000	ENSG0000	0.003119	hsa-miR-130a-3p;hsa-miR-130b-3p;hsa-miR-148a-3p;hsa-miR-
ENSG0000	ENSG0000	0.003119	hsa-miR-130a-3p;hsa-miR-130b-3p;hsa-miR-148a-3p;hsa-miR-
ENSG0000	ENSG0000	0.003682	hsa-miR-122-5p;hsa-miR-24-3p;hsa-miR-30c-1-3p;hsa-miR-3
ENSG0000	ENSG0000	0.000748	hsa-miR-23b-3p;hsa-miR-362-5p;hsa-miR-501-5p;hsa-miR-87
ENSG0000	ENSG0000	0.000131	hsa-miR-130a-3p;hsa-miR-23a-3p;hsa-miR-23b-3p;hsa-miR-3
ENSG0000	ENSG0000	0.00582	hsa-miR-30c-1-3p;hsa-miR-30c-2-3p;hsa-miR-501-5p;hsa-mi
ENSG0000	ENSG0000	0.00886	hsa-miR-130a-3p;hsa-miR-130b-3p;hsa-miR-148a-3p;hsa-miR-
ENSG0000	ENSG0000	0.000393	hsa-miR-130a-3p;hsa-miR-130b-3p;hsa-miR-218-5p;hsa-miR-
ENSG0000	ENSG0000	0.00286	hsa-miR-122-5p;hsa-miR-130a-3p;hsa-miR-148b-3p;hsa-miR-
ENSG0000	ENSG0000	0.009309	hsa-miR-130a-3p;hsa-miR-24-3p;hsa-miR-301a-3p;hsa-miR-3
ENSG0000	ENSG0000	0.000636	hsa-miR-130a-3p;hsa-miR-148a-3p;hsa-miR-148b-3p;hsa-miR-
ENSG0000	ENSG0000	1.86E-06	hsa-miR-130a-3p;hsa-miR-130b-3p;hsa-miR-148a-3p;hsa-miR-
ENSG0000	ENSG0000	0.000145	hsa-miR-130a-3p;hsa-miR-130b-3p;hsa-miR-200b-3p;hsa-miR
ENSG0000	ENSG0000	0.000352	hsa-miR-130a-3p;hsa-miR-148a-3p;hsa-miR-148b-3p;hsa-miR-
ENSG0000	ENSG0000	3.87E-05	hsa-miR-122-5p;hsa-miR-130a-3p;hsa-miR-130b-3p;hsa-miR-
ENSG0000	ENSG0000	5.76E-05	hsa-miR-130a-3p;hsa-miR-130b-3p;hsa-miR-148a-3p;hsa-miR-
ENSG0000	ENSG0000	0.005096	hsa-miR-122-5p;hsa-miR-183-3p;hsa-miR-23a-3p;hsa-miR-23
ENSG0000	ENSG0000	0.00318	hsa-miR-122-5p;hsa-miR-140-5p;hsa-miR-218-5p;hsa-miR-24-
ENSG0000	ENSG0000	0.00014	hsa-miR-130a-3p;hsa-miR-148b-3p;hsa-miR-218-5p;hsa-miR-
ENSG0000	ENSG0000	0.006573	hsa-miR-130a-3p;hsa-miR-130b-3p;hsa-miR-301a-3p;hsa-miR-
ENSG0000	ENSG0000	0.001669	hsa-miR-183-3p;hsa-miR-218-5p;hsa-miR-30c-1-3p;hsa-miR-
ENSG0000	ENSG0000	0.002495	hsa-miR-125a-5p;hsa-miR-125b-5p;hsa-miR-488-3p;hsa-miR-
ENSG0000	ENSG0000	0.007863	hsa-miR-148a-3p;hsa-miR-148b-3p;hsa-miR-30c-1-3p;hsa-mi
ENSG0000	ENSG0000	0.000748	hsa-miR-193b-5p;hsa-miR-23a-3p;hsa-miR-23b-3p;hsa-miR-9-
ENSG0000	ENSG0000	0.001467	hsa-miR-130a-3p;hsa-miR-130b-3p;hsa-miR-218-5p;hsa-miR-
ENSG0000	ENSG0000	0.001467	hsa-miR-130a-3p;hsa-miR-130b-3p;hsa-miR-218-5p;hsa-miR-
ENSG0000	ENSG0000	0.001467	hsa-miR-130a-3p;hsa-miR-130b-3p;hsa-miR-218-5p;hsa-miR-
ENSG0000	ENSG0000	0.000378	hsa-miR-130a-3p;hsa-miR-130b-3p;hsa-miR-24-3p;hsa-miR-3
ENSG0000	ENSG0000	0.005433	hsa-miR-130a-3p;hsa-miR-130b-3p;hsa-miR-301a-3p;hsa-miR-
ENSG0000	ENSG0000	0.000438	hsa-miR-24-3p;hsa-miR-30c-1-3p;hsa-miR-30c-2-3p;hsa-miR-
ENSG0000	ENSG0000	0.004434	hsa-miR-130a-3p;hsa-miR-218-5p;hsa-miR-301a-3p;hsa-miR-
ENSG0000	ENSG0000	0.007199	hsa-miR-130a-3p;hsa-miR-130b-3p;hsa-miR-301a-3p;hsa-miR-
ENSG0000	ENSG0000	0.000262	hsa-miR-130a-3p;hsa-miR-130b-3p;hsa-miR-301a-3p;hsa-miR-
ENSG0000	ENSG0000	2.52E-06	hsa-miR-122-5p;hsa-miR-130a-3p;hsa-miR-130b-3p;hsa-miR-
ENSG0000	ENSG0000	0.007743	hsa-miR-125a-5p;hsa-miR-126-5p;hsa-miR-130a-3p;hsa-miR-
ENSG0000	ENSG0000	0.003486	hsa-miR-135b-3p;hsa-miR-183-3p;hsa-miR-199a-5p;hsa-miR-
ENSG0000	ENSG0000	0.001184	hsa-miR-122-5p;hsa-miR-130a-3p;hsa-miR-130b-3p;hsa-miR-
ENSG0000	ENSG0000	0.001595	hsa-miR-130a-3p;hsa-miR-130b-3p;hsa-miR-135a-5p;hsa-miR-
ENSG0000	ENSG0000	1.88E-07	hsa-miR-122-5p;hsa-miR-130a-3p;hsa-miR-130b-3p;hsa-miR-
ENSG0000	ENSG0000	0.000171	hsa-miR-130a-3p;hsa-miR-130b-3p;hsa-miR-148a-3p;hsa-miR-
ENSG0000	ENSG0000	0.00318	hsa-miR-135a-5p;hsa-miR-135b-5p;hsa-miR-185-3p;hsa-miR-
ENSG0000	ENSG0000	0.001982	hsa-miR-130a-3p;hsa-miR-16-1-3p;hsa-miR-301a-3p;hsa-miR-
ENSG0000	ENSG0000	0.00013	hsa-miR-130a-3p;hsa-miR-130b-3p;hsa-miR-148a-3p;hsa-miR-
ENSG0000	ENSG0000	0.007199	hsa-miR-130a-3p;hsa-miR-130b-3p;hsa-miR-301a-3p;hsa-miR-
ENSG0000	ENSG0000	0.007199	hsa-miR-130a-3p;hsa-miR-130b-3p;hsa-miR-301a-3p;hsa-miR-
ENSG0000	ENSG0000	0.007165	hsa-miR-122-5p;hsa-miR-130a-3p;hsa-miR-130b-3p;hsa-miR-
ENSG0000	ENSG0000	0.007165	hsa-miR-122-5p;hsa-miR-130a-3p;hsa-miR-130b-3p;hsa-miR-
ENSG0000	ENSG0000	0.000314	hsa-miR-122-5p;hsa-miR-130a-3p;hsa-miR-130b-3p;hsa-miR-
ENSG0000	ENSG0000	8.48E-06	hsa-miR-130a-3p;hsa-miR-130b-3p;hsa-miR-301a-3p;hsa-miR-
ENSG0000	ENSG0000	0.000892	hsa-miR-200b-3p;hsa-miR-200c-3p;hsa-miR-24-3p;hsa-miR-4

ENSG0000	ENSG0000	0.001938	hsa-miR-130a-3p;hsa-miR-130b-3p;hsa-miR-200b-3p;hsa-miR
ENSG0000	ENSG0000	0.000115	hsa-miR-130a-3p;hsa-miR-130b-3p;hsa-miR-301a-3p;hsa-miR-
ENSG0000	ENSG0000	0.002039	hsa-miR-122-5p;hsa-miR-130a-3p;hsa-miR-130b-3p;hsa-miR-
ENSG0000	ENSG0000	0.004434	hsa-miR-130a-3p;hsa-miR-130b-3p;hsa-miR-301a-3p;hsa-miR-
ENSG0000	ENSG0000	0.000621	hsa-miR-122-5p;hsa-miR-130a-3p;hsa-miR-301a-3p;hsa-miR-
ENSG0000	ENSG0000	0.003119	hsa-miR-130a-3p;hsa-miR-130b-3p;hsa-miR-16-1-3p;hsa-miR-
ENSG0000	ENSG0000	0.007863	hsa-miR-130a-3p;hsa-miR-130b-3p;hsa-miR-301a-3p;hsa-miR-
ENSG0000	ENSG0000	0.007658	hsa-miR-130a-3p;hsa-miR-130b-3p;hsa-miR-301a-3p;hsa-miR-
ENSG0000	ENSG0000	0.00286	hsa-miR-23a-3p;hsa-miR-23b-3p;hsa-miR-24-3p;hsa-miR-30c-
ENSG0000	ENSG0000	0.003118	hsa-miR-200b-3p;hsa-miR-200c-3p;hsa-miR-23a-3p;hsa-miR-
ENSG0000	ENSG0000	0.001797	hsa-miR-130a-3p;hsa-miR-130b-3p;hsa-miR-301a-3p;hsa-miR-
ENSG0000	ENSG0000	0.00049	hsa-miR-122-5p;hsa-miR-130a-3p;hsa-miR-130b-3p;hsa-miR-
ENSG0000	ENSG0000	6.52E-05	hsa-miR-130a-3p;hsa-miR-130b-3p;hsa-miR-200b-3p;hsa-miR
ENSG0000	ENSG0000	0.009804	hsa-miR-122-5p;hsa-miR-130a-3p;hsa-miR-130b-3p;hsa-miR-
ENSG0000	ENSG0000	0.000115	hsa-miR-130a-3p;hsa-miR-130b-3p;hsa-miR-301a-3p;hsa-miR-
ENSG0000	ENSG0000	0.002151	hsa-miR-130a-3p;hsa-miR-130b-3p;hsa-miR-151a-3p;hsa-miR-
ENSG0000	ENSG0000	0.000201	hsa-miR-130a-3p;hsa-miR-130b-3p;hsa-miR-200b-3p;hsa-miR
ENSG0000	ENSG0000	0.000775	hsa-miR-122-5p;hsa-miR-130a-3p;hsa-miR-130b-3p;hsa-miR-
ENSG0000	ENSG0000	0.000539	hsa-miR-130a-3p;hsa-miR-130b-3p;hsa-miR-148a-3p;hsa-miR-
ENSG0000	ENSG0000	0.002392	hsa-miR-122-5p;hsa-miR-130a-3p;hsa-miR-130b-3p;hsa-miR-
ENSG0000	ENSG0000	3.79E-05	hsa-miR-122-5p;hsa-miR-130a-3p;hsa-miR-130b-3p;hsa-miR-
ENSG0000	ENSG0000	3.96E-05	hsa-miR-200b-3p;hsa-miR-200c-3p;hsa-miR-218-5p;hsa-miR-
ENSG0000	ENSG0000	0.00014	hsa-miR-122-5p;hsa-miR-130a-3p;hsa-miR-130b-3p;hsa-miR-
ENSG0000	ENSG0000	0.005407	hsa-miR-135a-5p;hsa-miR-135b-5p;hsa-miR-24-3p;hsa-miR-3
ENSG0000	ENSG0000	0.001043	hsa-miR-130a-3p;hsa-miR-130b-3p;hsa-miR-148a-3p;hsa-miR-
ENSG0000	ENSG0000	0.00051	hsa-miR-23a-3p;hsa-miR-23b-3p;hsa-miR-500a-5p;hsa-miR-5
ENSG0000	ENSG0000	0.005731	hsa-miR-130a-3p;hsa-miR-130b-3p;hsa-miR-301a-3p;hsa-miR-
ENSG0000	ENSG0000	0.000116	hsa-miR-130a-3p;hsa-miR-148b-3p;hsa-miR-301a-3p;hsa-miR-
ENSG0000	ENSG0000	0.003278	hsa-miR-130a-3p;hsa-miR-130b-3p;hsa-miR-24-3p;hsa-miR-3
ENSG0000	ENSG0000	0.003567	hsa-miR-130a-3p;hsa-miR-130b-3p;hsa-miR-301a-3p;hsa-miR-
ENSG0000	ENSG0000	0.002618	hsa-miR-148a-3p;hsa-miR-148b-3p;hsa-miR-218-5p;hsa-miR-
ENSG0000	ENSG0000	0.004319	hsa-miR-122-5p;hsa-miR-130a-3p;hsa-miR-130b-3p;hsa-miR-
ENSG0000	ENSG0000	2.58E-06	hsa-miR-130a-3p;hsa-miR-130b-3p;hsa-miR-148a-3p;hsa-miR-
ENSG0000	ENSG0000	1.15E-05	hsa-miR-130a-3p;hsa-miR-130b-3p;hsa-miR-135a-5p;hsa-miR-
ENSG0000	ENSG0000	0.009279	hsa-miR-130a-3p;hsa-miR-130b-3p;hsa-miR-301a-3p;hsa-miR-
ENSG0000	ENSG0000	0.000116	hsa-miR-122-5p;hsa-miR-130a-3p;hsa-miR-130b-3p;hsa-miR-
ENSG0000	ENSG0000	0.000116	hsa-miR-122-5p;hsa-miR-130a-3p;hsa-miR-130b-3p;hsa-miR-
ENSG0000	ENSG0000	8.67E-11	hsa-miR-125b-5p;hsa-miR-130a-3p;hsa-miR-130b-3p;hsa-miR
ENSG0000	ENSG0000	0.000289	hsa-miR-130a-3p;hsa-miR-130b-3p;hsa-miR-148a-3p;hsa-miR-
ENSG0000	ENSG0000	0.000246	hsa-miR-122-5p;hsa-miR-130a-3p;hsa-miR-130b-3p;hsa-miR-
ENSG0000	ENSG0000	0.000237	hsa-miR-130a-3p;hsa-miR-130b-3p;hsa-miR-301a-3p;hsa-miR-
ENSG0000	ENSG0000	0.00286	hsa-miR-16-1-3p;hsa-miR-23a-3p;hsa-miR-23b-3p;hsa-miR-3
ENSG0000	ENSG0000	1.78E-05	hsa-miR-130a-3p;hsa-miR-130b-3p;hsa-miR-23a-3p;hsa-miR-
ENSG0000	ENSG0000	0.000943	hsa-miR-200b-3p;hsa-miR-200c-3p;hsa-miR-429;hsa-miR-488-
ENSG0000	ENSG0000	0.006573	hsa-miR-130a-3p;hsa-miR-130b-3p;hsa-miR-301a-3p;hsa-miR-
ENSG0000	ENSG0000	0.003738	hsa-miR-125a-5p;hsa-miR-130b-3p;hsa-miR-16-1-3p;hsa-miR-
ENSG0000	ENSG0000	0.002495	hsa-miR-122-5p;hsa-miR-130a-3p;hsa-miR-301a-3p;hsa-miR-
ENSG0000	ENSG0000	0.002194	hsa-miR-130a-3p;hsa-miR-130b-3p;hsa-miR-301a-3p;hsa-miR-
ENSG0000	ENSG0000	0.002495	hsa-miR-130a-3p;hsa-miR-130b-3p;hsa-miR-301a-3p;hsa-miR-
ENSG0000	ENSG0000	0.000657	hsa-miR-122-5p;hsa-miR-130a-3p;hsa-miR-130b-3p;hsa-miR-
ENSG0000	ENSG0000	0.001058	hsa-miR-122-5p;hsa-miR-130a-3p;hsa-miR-130b-3p;hsa-miR-
ENSG0000	ENSG0000	9.52E-05	hsa-miR-130a-3p;hsa-miR-130b-3p;hsa-miR-24-3p;hsa-miR-3
ENSG0000	ENSG0000	0.000943	hsa-miR-130a-3p;hsa-miR-130b-3p;hsa-miR-24-3p;hsa-miR-3
ENSG0000	ENSG0000	0.00014	hsa-miR-122-5p;hsa-miR-130a-3p;hsa-miR-130b-3p;hsa-miR-

ENSG0000	ENSG0000	0.003992	hsa-miR-130a-3p;hsa-miR-130b-3p;hsa-miR-24-3p;hsa-miR-30
ENSG0000	ENSG0000	0.000104	hsa-miR-1270;hsa-miR-130a-3p;hsa-miR-130b-3p;hsa-miR-14
ENSG0000	ENSG0000	0.000573	hsa-miR-122-5p;hsa-miR-130a-3p;hsa-miR-130b-3p;hsa-miR-
ENSG0000	ENSG0000	1.01E-05	hsa-miR-130a-3p;hsa-miR-130b-3p;hsa-miR-200b-3p;hsa-miR
ENSG0000	ENSG0000	0.0002	hsa-miR-130a-3p;hsa-miR-130b-3p;hsa-miR-135b-3p;hsa-miR
ENSG0000	ENSG0000	0.0002	hsa-miR-130a-3p;hsa-miR-130b-3p;hsa-miR-135b-3p;hsa-miR
ENSG0000	ENSG0000	2.28E-05	hsa-miR-122-5p;hsa-miR-130a-3p;hsa-miR-130b-3p;hsa-miR-
ENSG0000	ENSG0000	0.000217	hsa-miR-122-5p;hsa-miR-130a-3p;hsa-miR-130b-3p;hsa-miR-
ENSG0000	ENSG0000	0.002495	hsa-miR-130a-3p;hsa-miR-130b-3p;hsa-miR-301a-3p;hsa-miR
ENSG0000	ENSG0000	0.000314	hsa-miR-122-5p;hsa-miR-130a-3p;hsa-miR-130b-3p;hsa-miR-
ENSG0000	ENSG0000	3.36E-06	hsa-miR-130a-3p;hsa-miR-130b-3p;hsa-miR-148a-3p;hsa-miR
ENSG0000	ENSG0000	0.003985	hsa-miR-122-5p;hsa-miR-130a-3p;hsa-miR-130b-3p;hsa-miR-
ENSG0000	ENSG0000	0.004916	hsa-miR-1270;hsa-miR-185-3p;hsa-miR-500a-5p;hsa-miR-501
ENSG0000	ENSG0000	0.004244	hsa-miR-122-5p;hsa-miR-193b-5p;hsa-miR-24-3p;hsa-miR-30
ENSG0000	ENSG0000	0.001238	hsa-miR-130a-3p;hsa-miR-218-5p;hsa-miR-301a-3p;hsa-miR-
ENSG0000	ENSG0000	0.004664	hsa-miR-122-5p;hsa-miR-130a-3p;hsa-miR-130b-3p;hsa-miR-
ENSG0000	ENSG0000	0.00049	hsa-miR-122-5p;hsa-miR-130a-3p;hsa-miR-130b-3p;hsa-miR-
ENSG0000	ENSG0000	0.001797	hsa-miR-122-5p;hsa-miR-130a-3p;hsa-miR-130b-3p;hsa-miR-
ENSG0000	ENSG0000	0.000352	hsa-miR-125a-5p;hsa-miR-125b-5p;hsa-miR-331-5p;hsa-miR-
ENSG0000	ENSG0000	0.001442	hsa-miR-135a-5p;hsa-miR-135b-5p;hsa-miR-199a-5p;hsa-miR
ENSG0000	ENSG0000	0.006246	hsa-miR-125a-5p;hsa-miR-125b-5p;hsa-miR-24-3p;hsa-miR-3
ENSG0000	ENSG0000	0.007303	hsa-miR-122-5p;hsa-miR-126-5p;hsa-miR-130a-3p;hsa-miR-1
ENSG0000	ENSG0000	0.000872	hsa-miR-130a-3p;hsa-miR-130b-3p;hsa-miR-148a-3p;hsa-miR
ENSG0000	ENSG0000	3.43E-05	hsa-miR-122-5p;hsa-miR-130a-3p;hsa-miR-130b-3p;hsa-miR-
ENSG0000	ENSG0000	0.000288	hsa-miR-122-5p;hsa-miR-130a-3p;hsa-miR-130b-3p;hsa-miR-
ENSG0000	ENSG0000	0.001919	hsa-miR-130a-3p;hsa-miR-130b-3p;hsa-miR-301a-3p;hsa-miR
ENSG0000	ENSG0000	0.001055	hsa-miR-135a-5p;hsa-miR-200b-3p;hsa-miR-200c-3p;hsa-miR
ENSG0000	ENSG0000	0.005985	hsa-miR-23a-3p;hsa-miR-23b-3p;hsa-miR-24-3p;hsa-miR-876
ENSG0000	ENSG0000	0.001043	hsa-miR-125a-5p;hsa-miR-125b-5p;hsa-miR-193b-5p;hsa-miR
ENSG0000	ENSG0000	0.000414	hsa-miR-130a-3p;hsa-miR-130b-3p;hsa-miR-301a-3p;hsa-miR
ENSG0000	ENSG0000	0.000104	hsa-miR-122-5p;hsa-miR-130a-3p;hsa-miR-130b-3p;hsa-miR-
ENSG0000	ENSG0000	0.00218	hsa-miR-122-5p;hsa-miR-130a-3p;hsa-miR-130b-3p;hsa-miR-
ENSG0000	ENSG0000	1.16E-05	hsa-miR-122-5p;hsa-miR-130a-3p;hsa-miR-130b-3p;hsa-miR-
ENSG0000	ENSG0000	1.16E-05	hsa-miR-122-5p;hsa-miR-130a-3p;hsa-miR-130b-3p;hsa-miR-
ENSG0000	ENSG0000	1.16E-05	hsa-miR-122-5p;hsa-miR-130a-3p;hsa-miR-130b-3p;hsa-miR-
ENSG0000	ENSG0000	1.16E-05	hsa-miR-122-5p;hsa-miR-130a-3p;hsa-miR-130b-3p;hsa-miR-
ENSG0000	ENSG0000	0.00014	hsa-miR-130a-3p;hsa-miR-130b-3p;hsa-miR-218-5p;hsa-miR-
ENSG0000	ENSG0000	3.77E-06	hsa-miR-30a-5p;hsa-miR-30b-5p;hsa-miR-30c-5p;hsa-miR-30
ENSG0000	ENSG0000	9.07E-08	hsa-miR-30a-5p;hsa-miR-30b-5p;hsa-miR-30c-5p;hsa-miR-30
ENSG0000	ENSG0000	9.34E-10	hsa-miR-30a-5p;hsa-miR-30b-5p;hsa-miR-30c-5p;hsa-miR-30
ENSG0000	ENSG0000	9.07E-08	hsa-miR-30a-5p;hsa-miR-30b-5p;hsa-miR-30c-5p;hsa-miR-30
ENSG0000	ENSG0000	7.49E-08	hsa-miR-30a-5p;hsa-miR-30b-5p;hsa-miR-30c-5p;hsa-miR-30
ENSG0000	ENSG0000	6.75E-06	hsa-miR-30b-5p;hsa-miR-30c-5p;hsa-miR-30d-5p;hsa-miR-30
ENSG0000	ENSG0000	8.69E-06	hsa-miR-30a-5p;hsa-miR-30b-5p;hsa-miR-30c-5p;hsa-miR-30
ENSG0000	ENSG0000	9.37E-09	hsa-miR-30a-5p;hsa-miR-30b-5p;hsa-miR-30d-5p;hsa-miR-30
ENSG0000	ENSG0000	1.02E-06	hsa-miR-30a-5p;hsa-miR-30b-5p;hsa-miR-30c-5p;hsa-miR-30
ENSG0000	ENSG0000	2.87E-09	hsa-miR-30a-5p;hsa-miR-30b-5p;hsa-miR-30c-5p;hsa-miR-30
ENSG0000	ENSG0000	0.000111	hsa-miR-30a-5p;hsa-miR-30b-5p;hsa-miR-30d-5p;hsa-miR-30
ENSG0000	ENSG0000	4.78E-07	hsa-miR-30a-5p;hsa-miR-30b-5p;hsa-miR-30c-5p;hsa-miR-30
ENSG0000	ENSG0000	2.92E-07	hsa-miR-30a-5p;hsa-miR-30b-5p;hsa-miR-30c-5p;hsa-miR-30
ENSG0000	ENSG0000	4.75E-13	hsa-miR-30a-5p;hsa-miR-30b-5p;hsa-miR-30c-5p;hsa-miR-30
ENSG0000	ENSG0000	1.13E-06	hsa-miR-30a-5p;hsa-miR-30b-5p;hsa-miR-30c-5p;hsa-miR-30
ENSG0000	ENSG0000	9.93E-12	hsa-miR-30a-5p;hsa-miR-30b-5p;hsa-miR-30c-5p;hsa-miR-30
ENSG0000	ENSG0000	3.67E-08	hsa-miR-30a-5p;hsa-miR-30b-5p;hsa-miR-30c-5p;hsa-miR-30

ENSG0000	ENSG0000	5.37E-09	hsa-miR-30a-5p;hsa-miR-30b-5p;hsa-miR-30c-5p;hsa-miR-30d
ENSG0000	ENSG0000	6.45E-08	hsa-miR-30a-5p;hsa-miR-30b-5p;hsa-miR-30c-5p;hsa-miR-30d
ENSG0000	ENSG0000	2.17E-10	hsa-miR-30a-5p;hsa-miR-30b-5p;hsa-miR-30c-5p;hsa-miR-30d
ENSG0000	ENSG0000	1.18E-10	hsa-miR-30a-5p;hsa-miR-30b-5p;hsa-miR-30c-5p;hsa-miR-30d
ENSG0000	ENSG0000	4.75E-13	hsa-miR-30a-5p;hsa-miR-30b-5p;hsa-miR-30c-5p;hsa-miR-30d
ENSG0000	ENSG0000	4.78E-07	hsa-miR-30a-5p;hsa-miR-30b-5p;hsa-miR-30c-5p;hsa-miR-30d
ENSG0000	ENSG0000	8.54E-08	hsa-miR-30a-5p;hsa-miR-30b-5p;hsa-miR-30c-5p;hsa-miR-30d
ENSG0000	ENSG0000	2.92E-07	hsa-miR-30a-5p;hsa-miR-30b-5p;hsa-miR-30c-5p;hsa-miR-30d
ENSG0000	ENSG0000	1.34E-05	hsa-miR-30b-5p;hsa-miR-30c-5p;hsa-miR-30d-5p;hsa-miR-30e
ENSG0000	ENSG0000	5.37E-07	hsa-miR-30a-5p;hsa-miR-30b-5p;hsa-miR-30c-5p;hsa-miR-30d
ENSG0000	ENSG0000	2.87E-09	hsa-miR-143-3p;hsa-miR-30a-5p;hsa-miR-30c-5p;hsa-miR-30d
ENSG0000	ENSG0000	8.31E-07	hsa-miR-30a-5p;hsa-miR-30b-5p;hsa-miR-30c-5p;hsa-miR-30d
ENSG0000	ENSG0000	1.54E-08	hsa-miR-30a-5p;hsa-miR-30b-5p;hsa-miR-30c-5p;hsa-miR-30d
ENSG0000	ENSG0000	3.66E-05	hsa-miR-30a-5p;hsa-miR-30c-5p;hsa-miR-30d-5p;hsa-miR-30e
ENSG0000	ENSG0000	6.71E-07	hsa-miR-30a-5p;hsa-miR-30b-5p;hsa-miR-30c-5p;hsa-miR-30d
ENSG0000	ENSG0000	0.000404	hsa-miR-30a-5p;hsa-miR-30b-5p;hsa-miR-30c-5p;hsa-miR-30d
ENSG0000	ENSG0000	5.43E-06	hsa-miR-30a-5p;hsa-miR-30b-5p;hsa-miR-30c-5p;hsa-miR-30d
ENSG0000	ENSG0000	1.94E-08	hsa-miR-30a-5p;hsa-miR-30b-5p;hsa-miR-30c-5p;hsa-miR-30d
ENSG0000	ENSG0000	3.32E-07	hsa-miR-30a-5p;hsa-miR-30b-5p;hsa-miR-30c-5p;hsa-miR-30d
ENSG0000	ENSG0000	3.97E-09	hsa-miR-30a-5p;hsa-miR-30b-5p;hsa-miR-30c-5p;hsa-miR-30d
ENSG0000	ENSG0000	4.46E-08	hsa-miR-30a-5p;hsa-miR-30b-5p;hsa-miR-30c-5p;hsa-miR-30d
ENSG0000	ENSG0000	8.31E-07	hsa-miR-30a-5p;hsa-miR-30b-5p;hsa-miR-30c-5p;hsa-miR-30d
ENSG0000	ENSG0000	8.63E-05	hsa-miR-30b-5p;hsa-miR-30d-5p;hsa-miR-30e-5p;hsa-miR-51
ENSG0000	ENSG0000	9.37E-09	hsa-miR-30a-5p;hsa-miR-30b-5p;hsa-miR-30c-5p;hsa-miR-30d
ENSG0000	ENSG0000	5.94E-11	hsa-miR-30a-5p;hsa-miR-30b-5p;hsa-miR-30c-5p;hsa-miR-30d
ENSG0000	ENSG0000	6.24E-06	hsa-miR-30a-5p;hsa-miR-30b-5p;hsa-miR-30c-5p;hsa-miR-30d
ENSG0000	ENSG0000	2.64E-11	hsa-miR-30a-5p;hsa-miR-30b-5p;hsa-miR-30c-5p;hsa-miR-30d
ENSG0000	ENSG0000	1.05E-06	hsa-miR-30a-5p;hsa-miR-30b-5p;hsa-miR-30c-5p;hsa-miR-30d
ENSG0000	ENSG0000	4.55E-06	hsa-miR-30b-5p;hsa-miR-30c-5p;hsa-miR-30d-5p;hsa-miR-30e
ENSG0000	ENSG0000	6.02E-10	hsa-miR-30a-5p;hsa-miR-30b-5p;hsa-miR-30c-5p;hsa-miR-30d
ENSG0000	ENSG0000	1.94E-07	hsa-miR-30a-5p;hsa-miR-30b-5p;hsa-miR-30c-5p;hsa-miR-30d
ENSG0000	ENSG0000	1.99E-05	hsa-miR-30a-5p;hsa-miR-30c-5p;hsa-miR-30d-5p;hsa-miR-30e
ENSG0000	ENSG0000	1.44E-10	hsa-miR-30a-5p;hsa-miR-30b-5p;hsa-miR-30c-5p;hsa-miR-30d
ENSG0000	ENSG0000	0.000104	hsa-miR-30b-5p;hsa-miR-30c-5p;hsa-miR-30d-5p;hsa-miR-30e
ENSG0000	ENSG0000	2.84E-12	hsa-miR-30a-5p;hsa-miR-30b-5p;hsa-miR-30c-5p;hsa-miR-30d
ENSG0000	ENSG0000	2.64E-11	hsa-miR-30a-5p;hsa-miR-30b-5p;hsa-miR-30c-5p;hsa-miR-30d
ENSG0000	ENSG0000	1.45E-07	hsa-miR-30a-5p;hsa-miR-30b-5p;hsa-miR-30c-5p;hsa-miR-30d
ENSG0000	ENSG0000	0.000284	hsa-miR-30a-5p;hsa-miR-30c-5p;hsa-miR-30d-5p;hsa-miR-30e
ENSG0000	ENSG0000	2.43E-05	hsa-miR-30a-5p;hsa-miR-30b-5p;hsa-miR-30c-5p;hsa-miR-30d
ENSG0000	ENSG0000	3.27E-05	hsa-miR-30a-5p;hsa-miR-30b-5p;hsa-miR-30c-5p;hsa-miR-30d
ENSG0000	ENSG0000	9.07E-08	hsa-miR-30a-5p;hsa-miR-30b-5p;hsa-miR-30c-5p;hsa-miR-30d
ENSG0000	ENSG0000	1.21E-08	hsa-miR-30a-5p;hsa-miR-30b-5p;hsa-miR-30c-5p;hsa-miR-30d
ENSG0000	ENSG0000	8.46E-05	hsa-miR-30b-5p;hsa-miR-30c-5p;hsa-miR-30d-5p;hsa-miR-30e
ENSG0000	ENSG0000	0.002713	hsa-miR-30a-5p;hsa-miR-30b-5p;hsa-miR-30c-5p;hsa-miR-30d
ENSG0000	ENSG0000	1.69E-07	hsa-miR-30a-5p;hsa-miR-30b-5p;hsa-miR-30c-5p;hsa-miR-30d
ENSG0000	ENSG0000	2.34E-07	hsa-miR-30b-5p;hsa-miR-30c-5p;hsa-miR-30d-5p;hsa-miR-30e
ENSG0000	ENSG0000	1.84E-08	hsa-miR-30b-5p;hsa-miR-30c-5p;hsa-miR-30d-5p;hsa-miR-30e
ENSG0000	ENSG0000	1.79E-06	hsa-miR-30a-5p;hsa-miR-30b-5p;hsa-miR-30d-5p;hsa-miR-30
ENSG0000	ENSG0000	1.54E-08	hsa-miR-30a-5p;hsa-miR-30b-5p;hsa-miR-30c-5p;hsa-miR-30d
ENSG0000	ENSG0000	1.54E-08	hsa-miR-30a-5p;hsa-miR-30b-5p;hsa-miR-30c-5p;hsa-miR-30d
ENSG0000	ENSG0000	1.72E-07	hsa-miR-30b-5p;hsa-miR-30c-5p;hsa-miR-30d-5p;hsa-miR-30e
ENSG0000	ENSG0000	0.00044	hsa-miR-30b-5p;hsa-miR-30c-5p;hsa-miR-30d-5p;hsa-miR-30e
ENSG0000	ENSG0000	5.94E-11	hsa-miR-30a-5p;hsa-miR-30b-5p;hsa-miR-30c-5p;hsa-miR-30d
ENSG0000	ENSG0000	9.26E-06	hsa-miR-30a-5p;hsa-miR-30b-5p;hsa-miR-30c-5p;hsa-miR-30d

ENSG0000	ENSG0000	5.43E-12	hsa-miR-30a-5p;hsa-miR-30b-5p;hsa-miR-30c-5p;hsa-miR-30
ENSG0000	ENSG0000	2.50E-06	hsa-miR-30a-5p;hsa-miR-30b-5p;hsa-miR-30d-5p;hsa-miR-30
ENSG0000	ENSG0000	9.37E-09	hsa-miR-30a-5p;hsa-miR-30b-5p;hsa-miR-30c-5p;hsa-miR-30
ENSG0000	ENSG0000	4.75E-13	hsa-miR-30a-5p;hsa-miR-30b-5p;hsa-miR-30c-5p;hsa-miR-30
ENSG0000	ENSG0000	6.71E-07	hsa-miR-30a-5p;hsa-miR-30b-5p;hsa-miR-30c-5p;hsa-miR-30
ENSG0000	ENSG0000	4.46E-08	hsa-miR-30a-5p;hsa-miR-30b-5p;hsa-miR-30c-5p;hsa-miR-30
ENSG0000	ENSG0000	3.64E-08	hsa-miR-30b-5p;hsa-miR-30c-5p;hsa-miR-30d-5p;hsa-miR-30
ENSG0000	ENSG0000	1.45E-07	hsa-miR-30a-5p;hsa-miR-30b-5p;hsa-miR-30c-5p;hsa-miR-30
ENSG0000	ENSG0000	9.34E-10	hsa-miR-30a-5p;hsa-miR-30b-5p;hsa-miR-30c-5p;hsa-miR-30
ENSG0000	ENSG0000	1.87E-05	hsa-miR-30a-5p;hsa-miR-30b-5p;hsa-miR-30c-5p;hsa-miR-30
ENSG0000	ENSG0000	4.75E-13	hsa-miR-30a-5p;hsa-miR-30b-5p;hsa-miR-30c-5p;hsa-miR-30
ENSG0000	ENSG0000	2.34E-07	hsa-miR-30a-5p;hsa-miR-30b-5p;hsa-miR-30d-5p;hsa-miR-30
ENSG0000	ENSG0000	9.07E-08	hsa-miR-30a-5p;hsa-miR-30b-5p;hsa-miR-30c-5p;hsa-miR-30
ENSG0000	ENSG0000	2.24E-07	hsa-miR-30a-5p;hsa-miR-30b-5p;hsa-miR-30c-5p;hsa-miR-30
ENSG0000	ENSG0000	2.00E-10	hsa-miR-30a-5p;hsa-miR-30b-5p;hsa-miR-30c-5p;hsa-miR-30
ENSG0000	ENSG0000	1.25E-07	hsa-miR-30a-5p;hsa-miR-30b-5p;hsa-miR-30c-5p;hsa-miR-30
ENSG0000	ENSG0000	2.08E-05	hsa-miR-30a-5p;hsa-miR-30b-5p;hsa-miR-30c-5p;hsa-miR-30
ENSG0000	ENSG0000	3.77E-06	hsa-miR-30a-5p;hsa-miR-30b-5p;hsa-miR-30c-5p;hsa-miR-30
ENSG0000	ENSG0000	2.64E-11	hsa-miR-30a-5p;hsa-miR-30b-5p;hsa-miR-30c-5p;hsa-miR-30
ENSG0000	ENSG0000	0.000613	hsa-miR-30a-5p;hsa-miR-30b-5p;hsa-miR-30c-5p;hsa-miR-30
ENSG0000	ENSG0000	0.000462	hsa-miR-30a-5p;hsa-miR-30b-5p;hsa-miR-30c-5p;hsa-miR-30
ENSG0000	ENSG0000	6.24E-06	hsa-miR-30a-5p;hsa-miR-30b-5p;hsa-miR-30c-5p;hsa-miR-30
ENSG0000	ENSG0000	3.67E-08	hsa-miR-30a-5p;hsa-miR-30b-5p;hsa-miR-30c-5p;hsa-miR-30
ENSG0000	ENSG0000	2.56E-07	hsa-miR-30a-5p;hsa-miR-30b-5p;hsa-miR-30c-5p;hsa-miR-30
ENSG0000	ENSG0000	6.71E-07	hsa-miR-30a-5p;hsa-miR-30b-5p;hsa-miR-30c-5p;hsa-miR-30
ENSG0000	ENSG0000	4.13E-05	hsa-miR-30a-5p;hsa-miR-30b-5p;hsa-miR-30c-5p;hsa-miR-30
ENSG0000	ENSG0000	4.13E-05	hsa-miR-30a-5p;hsa-miR-30b-5p;hsa-miR-30c-5p;hsa-miR-30
ENSG0000	ENSG0000	1.07E-07	hsa-miR-30a-5p;hsa-miR-30b-5p;hsa-miR-30c-5p;hsa-miR-30
ENSG0000	ENSG0000	7.15E-09	hsa-miR-30a-5p;hsa-miR-30b-5p;hsa-miR-30c-5p;hsa-miR-30
ENSG0000	ENSG0000	4.13E-05	hsa-miR-30a-5p;hsa-miR-30b-5p;hsa-miR-30c-5p;hsa-miR-30
ENSG0000	ENSG0000	7.15E-06	hsa-miR-30a-5p;hsa-miR-30b-5p;hsa-miR-30c-5p;hsa-miR-30
ENSG0000	ENSG0000	9.93E-12	hsa-miR-30a-5p;hsa-miR-30b-5p;hsa-miR-30c-5p;hsa-miR-30
ENSG0000	ENSG0000	1.13E-06	hsa-miR-30a-5p;hsa-miR-30b-5p;hsa-miR-30c-5p;hsa-miR-30
ENSG0000	ENSG0000	3.16E-06	hsa-miR-30a-5p;hsa-miR-30b-5p;hsa-miR-30c-5p;hsa-miR-30
ENSG0000	ENSG0000	3.10E-05	hsa-miR-30a-5p;hsa-miR-30c-5p;hsa-miR-30d-5p;hsa-miR-30
ENSG0000	ENSG0000	9.93E-12	hsa-miR-30a-5p;hsa-miR-30b-5p;hsa-miR-30c-5p;hsa-miR-30
ENSG0000	ENSG0000	2.87E-09	hsa-miR-30a-5p;hsa-miR-30b-5p;hsa-miR-30c-5p;hsa-miR-30
ENSG0000	ENSG0000	9.37E-09	hsa-miR-30a-5p;hsa-miR-30b-5p;hsa-miR-30c-5p;hsa-miR-30
ENSG0000	ENSG0000	9.34E-10	hsa-miR-30a-5p;hsa-miR-30b-5p;hsa-miR-30c-5p;hsa-miR-30
ENSG0000	ENSG0000	2.24E-07	hsa-miR-30a-5p;hsa-miR-30b-5p;hsa-miR-30c-5p;hsa-miR-30
ENSG0000	ENSG0000	4.71E-06	hsa-miR-30a-5p;hsa-miR-30b-5p;hsa-miR-30c-5p;hsa-miR-30
ENSG0000	ENSG0000	9.34E-10	hsa-miR-30a-5p;hsa-miR-30b-5p;hsa-miR-30c-5p;hsa-miR-30
ENSG0000	ENSG0000	1.40E-09	hsa-miR-30a-5p;hsa-miR-30b-5p;hsa-miR-30c-5p;hsa-miR-30
ENSG0000	ENSG0000	1.25E-07	hsa-miR-30a-5p;hsa-miR-30b-5p;hsa-miR-30c-5p;hsa-miR-30
ENSG0000	ENSG0000	1.21E-08	hsa-miR-30a-5p;hsa-miR-30b-5p;hsa-miR-30c-5p;hsa-miR-30
ENSG0000	ENSG0000	5.21E-06	hsa-miR-30a-5p;hsa-miR-30c-5p;hsa-miR-30d-5p;hsa-miR-30
ENSG0000	ENSG0000	5.87E-05	hsa-miR-30a-5p;hsa-miR-30b-5p;hsa-miR-30c-5p;hsa-miR-30
ENSG0000	ENSG0000	7.15E-09	hsa-miR-30a-5p;hsa-miR-30b-5p;hsa-miR-30c-5p;hsa-miR-30
ENSG0000	ENSG0000	1.49E-06	hsa-miR-30b-5p;hsa-miR-30c-5p;hsa-miR-30d-5p;hsa-miR-30
ENSG0000	ENSG0000	1.40E-09	hsa-miR-30a-5p;hsa-miR-30b-5p;hsa-miR-30c-5p;hsa-miR-30
ENSG0000	ENSG0000	5.38E-08	hsa-miR-30a-5p;hsa-miR-30b-5p;hsa-miR-30c-5p;hsa-miR-30
ENSG0000	ENSG0000	0.000247	hsa-miR-30a-5p;hsa-miR-30c-5p;hsa-miR-30d-5p;hsa-miR-30
ENSG0000	ENSG0000	0.000147	hsa-miR-30a-5p;hsa-miR-30b-5p;hsa-miR-30c-5p;hsa-miR-30
ENSG0000	ENSG0000	6.45E-08	hsa-miR-30a-5p;hsa-miR-30b-5p;hsa-miR-30c-5p;hsa-miR-30

ENSG0000	ENSG0000	4.75E-13	hsa-miR-30a-5p;hsa-miR-30b-5p;hsa-miR-30c-5p;hsa-miR-30
ENSG0000	ENSG0000	6.02E-10	hsa-miR-30a-5p;hsa-miR-30b-5p;hsa-miR-30c-5p;hsa-miR-30
ENSG0000	ENSG0000	5.06E-06	hsa-miR-30a-5p;hsa-miR-30b-5p;hsa-miR-30c-5p;hsa-miR-30
ENSG0000	ENSG0000	2.34E-07	hsa-miR-30b-5p;hsa-miR-30c-5p;hsa-miR-30d-5p;hsa-miR-30
ENSG0000	ENSG0000	5.16E-05	hsa-miR-30a-5p;hsa-miR-30b-5p;hsa-miR-30c-5p;hsa-miR-30
ENSG0000	ENSG0000	2.17E-10	hsa-miR-30a-5p;hsa-miR-30b-5p;hsa-miR-30c-5p;hsa-miR-30
ENSG0000	ENSG0000	3.76E-07	hsa-miR-30a-5p;hsa-miR-30b-5p;hsa-miR-30c-5p;hsa-miR-30
ENSG0000	ENSG0000	4.06E-06	hsa-miR-30a-5p;hsa-miR-30b-5p;hsa-miR-30c-5p;hsa-miR-30
ENSG0000	ENSG0000	1.54E-08	hsa-miR-30a-5p;hsa-miR-30b-5p;hsa-miR-30c-5p;hsa-miR-30
ENSG0000	ENSG0000	7.15E-06	hsa-miR-30a-5p;hsa-miR-30b-5p;hsa-miR-30c-5p;hsa-miR-30
ENSG0000	ENSG0000	1.40E-09	hsa-miR-30a-5p;hsa-miR-30b-5p;hsa-miR-30c-5p;hsa-miR-30
ENSG0000	ENSG0000	1.40E-09	hsa-miR-30a-5p;hsa-miR-30b-5p;hsa-miR-30c-5p;hsa-miR-30
ENSG0000	ENSG0000	8.00E-11	hsa-miR-30a-5p;hsa-miR-30b-5p;hsa-miR-30c-5p;hsa-miR-30
ENSG0000	ENSG0000	8.00E-11	hsa-miR-30a-5p;hsa-miR-30b-5p;hsa-miR-30c-5p;hsa-miR-30
ENSG0000	ENSG0000	2.84E-12	hsa-miR-30a-5p;hsa-miR-30b-5p;hsa-miR-30c-5p;hsa-miR-30
ENSG0000	ENSG0000	8.69E-06	hsa-miR-30a-5p;hsa-miR-30b-5p;hsa-miR-30c-5p;hsa-miR-30
ENSG0000	ENSG0000	1.94E-07	hsa-miR-30a-5p;hsa-miR-30b-5p;hsa-miR-30c-5p;hsa-miR-30
ENSG0000	ENSG0000	3.15E-10	hsa-miR-30a-5p;hsa-miR-30b-5p;hsa-miR-30c-5p;hsa-miR-30
ENSG0000	ENSG0000	0.001996	hsa-miR-30a-5p;hsa-miR-30c-5p;hsa-miR-30d-5p;hsa-miR-30
ENSG0000	ENSG0000	8.22E-07	hsa-miR-30a-5p;hsa-miR-30b-5p;hsa-miR-30d-5p;hsa-miR-30
ENSG0000	ENSG0000	6.71E-07	hsa-miR-30a-5p;hsa-miR-30b-5p;hsa-miR-30c-5p;hsa-miR-30
ENSG0000	ENSG0000	3.97E-09	hsa-miR-30a-5p;hsa-miR-30b-5p;hsa-miR-30c-5p;hsa-miR-30
ENSG0000	ENSG0000	5.38E-08	hsa-miR-30a-5p;hsa-miR-30b-5p;hsa-miR-30c-5p;hsa-miR-30
ENSG0000	ENSG0000	1.34E-05	hsa-miR-30a-5p;hsa-miR-30b-5p;hsa-miR-30d-5p;hsa-miR-30
ENSG0000	ENSG0000	2.56E-07	hsa-miR-30a-5p;hsa-miR-30b-5p;hsa-miR-30c-5p;hsa-miR-30
ENSG0000	ENSG0000	4.75E-13	hsa-miR-30a-5p;hsa-miR-30b-5p;hsa-miR-30c-5p;hsa-miR-30
ENSG0000	ENSG0000	1.64E-05	hsa-miR-30b-5p;hsa-miR-30c-5p;hsa-miR-30d-5p;hsa-miR-30
ENSG0000	ENSG0000	0.001243	hsa-miR-30a-5p;hsa-miR-30b-5p;hsa-miR-30c-5p;hsa-miR-30
ENSG0000	ENSG0000	4.46E-08	hsa-miR-30a-5p;hsa-miR-30b-5p;hsa-miR-30c-5p;hsa-miR-30
ENSG0000	ENSG0000	1.96E-06	hsa-miR-30a-5p;hsa-miR-30b-5p;hsa-miR-30c-5p;hsa-miR-30
ENSG0000	ENSG0000	1.25E-07	hsa-miR-30a-5p;hsa-miR-30b-5p;hsa-miR-30c-5p;hsa-miR-30
ENSG0000	ENSG0000	1.96E-06	hsa-miR-30a-5p;hsa-miR-30b-5p;hsa-miR-30c-5p;hsa-miR-30
ENSG0000	ENSG0000	1.59E-06	hsa-miR-30a-5p;hsa-miR-30b-5p;hsa-miR-30c-5p;hsa-miR-30
ENSG0000	ENSG0000	5.37E-09	hsa-miR-30a-5p;hsa-miR-30b-5p;hsa-miR-30c-5p;hsa-miR-30
ENSG0000	ENSG0000	1.54E-08	hsa-miR-30a-5p;hsa-miR-30b-5p;hsa-miR-30c-5p;hsa-miR-30
ENSG0000	ENSG0000	1.54E-08	hsa-miR-30a-5p;hsa-miR-30b-5p;hsa-miR-30c-5p;hsa-miR-30
ENSG0000	ENSG0000	2.75E-06	hsa-miR-30a-5p;hsa-miR-30b-5p;hsa-miR-30c-5p;hsa-miR-30
ENSG0000	ENSG0000	1.40E-09	hsa-miR-30a-5p;hsa-miR-30b-5p;hsa-miR-30c-5p;hsa-miR-30
ENSG0000	ENSG0000	1.21E-08	hsa-miR-30a-5p;hsa-miR-30b-5p;hsa-miR-30c-5p;hsa-miR-30
ENSG0000	ENSG0000	2.94E-08	hsa-miR-30a-5p;hsa-miR-30b-5p;hsa-miR-30c-5p;hsa-miR-30
ENSG0000	ENSG0000	6.75E-06	hsa-miR-30b-5p;hsa-miR-30c-5p;hsa-miR-30d-5p;hsa-miR-30
ENSG0000	ENSG0000	2.93E-06	hsa-miR-30b-5p;hsa-miR-30c-5p;hsa-miR-30d-5p;hsa-miR-30
ENSG0000	ENSG0000	3.97E-09	hsa-miR-30a-5p;hsa-miR-30b-5p;hsa-miR-30c-5p;hsa-miR-30
ENSG0000	ENSG0000	4.94E-05	hsa-miR-30a-5p;hsa-miR-30b-5p;hsa-miR-30c-5p;hsa-miR-30
ENSG0000	ENSG0000	0.000804	hsa-miR-30a-5p;hsa-miR-30b-5p;hsa-miR-30d-5p;hsa-miR-30
ENSG0000	ENSG0000	2.31E-05	hsa-miR-30a-5p;hsa-miR-30b-5p;hsa-miR-30c-5p;hsa-miR-30
ENSG0000	ENSG0000	5.37E-09	hsa-miR-30a-5p;hsa-miR-30b-5p;hsa-miR-30c-5p;hsa-miR-30
ENSG0000	ENSG0000	0.000127	hsa-miR-30a-5p;hsa-miR-30b-5p;hsa-miR-30c-5p;hsa-miR-30
ENSG0000	ENSG0000	1.07E-07	hsa-miR-30a-5p;hsa-miR-30b-5p;hsa-miR-30c-5p;hsa-miR-30
ENSG0000	ENSG0000	9.50E-05	hsa-miR-30a-5p;hsa-miR-30b-5p;hsa-miR-30c-5p;hsa-miR-30
ENSG0000	ENSG0000	1.63E-08	hsa-miR-30a-5p;hsa-miR-30b-5p;hsa-miR-30c-5p;hsa-miR-30
ENSG0000	ENSG0000	4.75E-13	hsa-miR-30a-5p;hsa-miR-30b-5p;hsa-miR-30c-5p;hsa-miR-30
ENSG0000	ENSG0000	5.94E-11	hsa-miR-30a-5p;hsa-miR-30b-5p;hsa-miR-30c-5p;hsa-miR-30
ENSG0000	ENSG0000	0.000117	hsa-miR-30a-5p;hsa-miR-30c-5p;hsa-miR-30d-5p;hsa-miR-30

ENSG0000	ENSG0000	1.40E-09	hsa-miR-30a-5p;hsa-miR-30b-5p;hsa-miR-30c-5p;hsa-miR-30
ENSG0000	ENSG0000	2.14E-06	hsa-miR-30a-5p;hsa-miR-30b-5p;hsa-miR-30c-5p;hsa-miR-30
ENSG0000	ENSG0000	4.75E-13	hsa-miR-30a-5p;hsa-miR-30b-5p;hsa-miR-30c-5p;hsa-miR-30
ENSG0000	ENSG0000	0.003665	hsa-miR-30a-5p;hsa-miR-30c-5p;hsa-miR-30d-5p;hsa-miR-30
ENSG0000	ENSG0000	3.23E-06	hsa-miR-30a-5p;hsa-miR-30b-5p;hsa-miR-30c-5p;hsa-miR-30
ENSG0000	ENSG0000	2.92E-07	hsa-miR-30a-5p;hsa-miR-30b-5p;hsa-miR-30c-5p;hsa-miR-30
ENSG0000	ENSG0000	5.94E-06	hsa-miR-30a-5p;hsa-miR-30c-5p;hsa-miR-30d-5p;hsa-miR-30
ENSG0000	ENSG0000	1.18E-10	hsa-miR-30a-5p;hsa-miR-30b-5p;hsa-miR-30c-5p;hsa-miR-30
ENSG0000	ENSG0000	3.67E-08	hsa-miR-30a-5p;hsa-miR-30b-5p;hsa-miR-30c-5p;hsa-miR-30
ENSG0000	ENSG0000	6.68E-06	hsa-miR-30a-5p;hsa-miR-30b-5p;hsa-miR-30c-5p;hsa-miR-30
ENSG0000	ENSG0000	1.25E-07	hsa-miR-30a-5p;hsa-miR-30b-5p;hsa-miR-30c-5p;hsa-miR-30
ENSG0000	ENSG0000	2.71E-07	hsa-miR-30a-5p;hsa-miR-30b-5p;hsa-miR-30c-5p;hsa-miR-30
ENSG0000	ENSG0000	1.97E-07	hsa-miR-30a-5p;hsa-miR-30b-5p;hsa-miR-30c-5p;hsa-miR-30
ENSG0000	ENSG0000	2.64E-11	hsa-miR-30a-5p;hsa-miR-30b-5p;hsa-miR-30c-5p;hsa-miR-30
ENSG0000	ENSG0000	2.64E-11	hsa-miR-30a-5p;hsa-miR-30b-5p;hsa-miR-30c-5p;hsa-miR-30
ENSG0000	ENSG0000	6.16E-08	hsa-miR-143-3p;hsa-miR-30a-5p;hsa-miR-30b-5p;hsa-miR-30
ENSG0000	ENSG0000	1.40E-09	hsa-miR-30a-5p;hsa-miR-30b-5p;hsa-miR-30c-5p;hsa-miR-30
ENSG0000	ENSG0000	7.67E-08	hsa-miR-30a-5p;hsa-miR-30b-5p;hsa-miR-30c-5p;hsa-miR-30
ENSG0000	ENSG0000	6.71E-07	hsa-miR-30a-5p;hsa-miR-30b-5p;hsa-miR-30d-5p;hsa-miR-30
ENSG0000	ENSG0000	3.00E-08	hsa-miR-30a-5p;hsa-miR-30b-5p;hsa-miR-30c-5p;hsa-miR-30
ENSG0000	ENSG0000	3.53E-10	hsa-miR-30a-5p;hsa-miR-30b-5p;hsa-miR-30c-5p;hsa-miR-30
ENSG0000	ENSG0000	3.41E-06	hsa-miR-30b-5p;hsa-miR-30c-5p;hsa-miR-30d-5p;hsa-miR-30
ENSG0000	ENSG0000	1.84E-08	hsa-miR-30a-5p;hsa-miR-30b-5p;hsa-miR-30c-5p;hsa-miR-30
ENSG0000	ENSG0000	7.48E-07	hsa-miR-30a-5p;hsa-miR-30b-5p;hsa-miR-30c-5p;hsa-miR-30
ENSG0000	ENSG0000	0.001832	hsa-miR-107;hsa-miR-15a-5p;hsa-miR-15b-5p;hsa-miR-188-3
ENSG0000	ENSG0000	0.006736	hsa-miR-30b-5p;hsa-miR-30c-5p;hsa-miR-30d-5p;hsa-miR-30
ENSG0000	ENSG0000	2.66E-05	hsa-miR-15a-5p;hsa-miR-15b-5p;hsa-miR-16-5p;hsa-miR-195
ENSG0000	ENSG0000	8.67E-11	hsa-miR-103a-3p;hsa-miR-107;hsa-miR-15a-5p;hsa-miR-15b-
ENSG0000	ENSG0000	1.22E-07	hsa-miR-15a-5p;hsa-miR-15b-5p;hsa-miR-16-5p;hsa-miR-195
ENSG0000	ENSG0000	9.30E-08	hsa-miR-15a-5p;hsa-miR-15b-5p;hsa-miR-195-5p;hsa-miR-21
ENSG0000	ENSG0000	8.03E-10	hsa-miR-103a-3p;hsa-miR-107;hsa-miR-15a-5p;hsa-miR-15b-
ENSG0000	ENSG0000	0.002541	hsa-miR-30b-5p;hsa-miR-30c-5p;hsa-miR-30d-5p;hsa-miR-30
ENSG0000	ENSG0000	0.009158	hsa-miR-15a-5p;hsa-miR-15b-5p;hsa-miR-195-5p;hsa-miR-42
ENSG0000	ENSG0000	3.07E-06	hsa-miR-15a-5p;hsa-miR-15b-5p;hsa-miR-16-5p;hsa-miR-195
ENSG0000	ENSG0000	3.07E-06	hsa-miR-15a-5p;hsa-miR-15b-5p;hsa-miR-16-5p;hsa-miR-195
ENSG0000	ENSG0000	9.04E-05	hsa-miR-30b-5p;hsa-miR-30c-5p;hsa-miR-30d-5p;hsa-miR-30
ENSG0000	ENSG0000	0.000199	hsa-miR-15a-5p;hsa-miR-15b-5p;hsa-miR-16-5p;hsa-miR-195
ENSG0000	ENSG0000	0.002541	hsa-miR-30b-5p;hsa-miR-30c-5p;hsa-miR-30d-5p;hsa-miR-30
ENSG0000	ENSG0000	0.005908	hsa-miR-181a-5p;hsa-miR-30b-5p;hsa-miR-30c-5p;hsa-miR-3
ENSG0000	ENSG0000	0.002853	hsa-miR-30b-5p;hsa-miR-30c-5p;hsa-miR-30d-5p;hsa-miR-30
ENSG0000	ENSG0000	0.000106	hsa-miR-15a-5p;hsa-miR-15b-5p;hsa-miR-16-5p;hsa-miR-181
ENSG0000	ENSG0000	0.000403	hsa-miR-15a-5p;hsa-miR-15b-5p;hsa-miR-16-5p;hsa-miR-195
ENSG0000	ENSG0000	0.000312	hsa-miR-15a-5p;hsa-miR-15b-5p;hsa-miR-16-5p;hsa-miR-195
ENSG0000	ENSG0000	0.000491	hsa-miR-224-5p;hsa-miR-30b-5p;hsa-miR-30d-5p;hsa-miR-30
ENSG0000	ENSG0000	2.97E-05	hsa-miR-107;hsa-miR-15a-5p;hsa-miR-15b-5p;hsa-miR-195-5
ENSG0000	ENSG0000	6.55E-05	hsa-miR-15a-5p;hsa-miR-15b-5p;hsa-miR-16-5p;hsa-miR-195
ENSG0000	ENSG0000	0.000403	hsa-miR-15b-5p;hsa-miR-16-5p;hsa-miR-195-5p;hsa-miR-424
ENSG0000	ENSG0000	0.000206	hsa-miR-15a-5p;hsa-miR-15b-5p;hsa-miR-16-5p;hsa-miR-424
ENSG0000	ENSG0000	0.000114	hsa-miR-103a-3p;hsa-miR-107;hsa-miR-15a-5p;hsa-miR-15b-
ENSG0000	ENSG0000	2.64E-06	hsa-miR-103a-3p;hsa-miR-107;hsa-miR-214-3p;hsa-miR-30b-
ENSG0000	ENSG0000	4.31E-08	hsa-miR-103a-3p;hsa-miR-107;hsa-miR-15a-5p;hsa-miR-15b-
ENSG0000	ENSG0000	0.000206	hsa-miR-107;hsa-miR-195-5p;hsa-miR-224-5p;hsa-miR-30c-5
ENSG0000	ENSG0000	0.000134	hsa-miR-107;hsa-miR-15a-5p;hsa-miR-15b-5p;hsa-miR-16-5p;
ENSG0000	ENSG0000	0.000262	hsa-miR-30d-5p;hsa-miR-30e-5p;hsa-miR-3194-5p;hsa-miR-4

ENSG0000	ENSG0000	1.51E-06	hsa-miR-15a-5p;hsa-miR-16-5p;hsa-miR-23a-3p;hsa-miR-23b-
ENSG0000	ENSG0000	0.000147	hsa-miR-15a-5p;hsa-miR-15b-5p;hsa-miR-16-5p;hsa-miR-195-
ENSG0000	ENSG0000	0.000837	hsa-miR-1224-3p;hsa-miR-1260a;hsa-miR-1260b;hsa-miR-197-
ENSG0000	ENSG0000	0.000837	hsa-miR-1224-3p;hsa-miR-1260a;hsa-miR-1260b;hsa-miR-197-
ENSG0000	ENSG0000	0.000837	hsa-miR-1224-3p;hsa-miR-1260a;hsa-miR-1260b;hsa-miR-197-
ENSG0000	ENSG0000	0.000837	hsa-miR-1224-3p;hsa-miR-1260a;hsa-miR-1260b;hsa-miR-197-
ENSG0000	ENSG0000	0.000627	hsa-miR-15a-5p;hsa-miR-15b-5p;hsa-miR-16-5p;hsa-miR-195-
ENSG0000	ENSG0000	0.000206	hsa-miR-30b-5p;hsa-miR-30c-5p;hsa-miR-30d-5p;hsa-miR-30
ENSG0000	ENSG0000	8.72E-05	hsa-miR-15a-5p;hsa-miR-15b-5p;hsa-miR-16-5p;hsa-miR-195-
ENSG0000	ENSG0000	0.008895	hsa-miR-103a-3p;hsa-miR-107;hsa-miR-1260a;hsa-miR-1260b;
ENSG0000	ENSG0000	9.34E-06	hsa-miR-15a-5p;hsa-miR-15b-5p;hsa-miR-16-5p;hsa-miR-182-
ENSG0000	ENSG0000	0.002883	hsa-miR-15a-5p;hsa-miR-15b-5p;hsa-miR-195-5p;hsa-miR-30
ENSG0000	ENSG0000	4.85E-11	hsa-miR-103a-3p;hsa-miR-107;hsa-miR-15a-5p;hsa-miR-15b-
ENSG0000	ENSG0000	0.00319	hsa-miR-1224-3p;hsa-miR-1260a;hsa-miR-1260b;hsa-miR-191-
ENSG0000	ENSG0000	3.84E-05	hsa-miR-15a-5p;hsa-miR-15b-5p;hsa-miR-16-5p;hsa-miR-195-
ENSG0000	ENSG0000	4.88E-10	hsa-miR-103a-3p;hsa-miR-107;hsa-miR-15a-5p;hsa-miR-15b-
ENSG0000	ENSG0000	0.000105	hsa-miR-15a-5p;hsa-miR-15b-5p;hsa-miR-195-5p;hsa-miR-42
ENSG0000	ENSG0000	0.000314	hsa-miR-103a-3p;hsa-miR-107;hsa-miR-15a-5p;hsa-miR-15b-
ENSG0000	ENSG0000	2.04E-05	hsa-miR-103a-3p;hsa-miR-107;hsa-miR-15a-5p;hsa-miR-15b-
ENSG0000	ENSG0000	0.006736	hsa-miR-15a-5p;hsa-miR-15b-5p;hsa-miR-195-5p;hsa-miR-42
ENSG0000	ENSG0000	0.00111	hsa-miR-24-3p;hsa-miR-30b-5p;hsa-miR-30c-5p;hsa-miR-30d-
ENSG0000	ENSG0000	5.58E-06	hsa-miR-15a-5p;hsa-miR-15b-5p;hsa-miR-16-5p;hsa-miR-195-
ENSG0000	ENSG0000	0.00015	hsa-miR-1224-3p;hsa-miR-1260a;hsa-miR-1260b;hsa-miR-214-
ENSG0000	ENSG0000	0.000711	hsa-miR-24-3p;hsa-miR-30b-5p;hsa-miR-30c-5p;hsa-miR-30d-
ENSG0000	ENSG0000	0.000206	hsa-miR-15b-5p;hsa-miR-16-5p;hsa-miR-30c-5p;hsa-miR-497-
ENSG0000	ENSG0000	1.77E-08	hsa-miR-15a-5p;hsa-miR-15b-5p;hsa-miR-16-5p;hsa-miR-195-
ENSG0000	ENSG0000	0.000133	hsa-miR-15a-5p;hsa-miR-15b-5p;hsa-miR-16-5p;hsa-miR-181
ENSG0000	ENSG0000	0.005286	hsa-miR-182-5p;hsa-miR-197-3p;hsa-miR-23a-3p;hsa-miR-30
ENSG0000	ENSG0000	1.54E-07	hsa-miR-15a-5p;hsa-miR-15b-5p;hsa-miR-16-5p;hsa-miR-195-
ENSG0000	ENSG0000	0.001346	hsa-miR-1224-3p;hsa-miR-1260a;hsa-miR-1260b;hsa-miR-188-
ENSG0000	ENSG0000	5.11E-07	hsa-miR-30b-5p;hsa-miR-30c-5p;hsa-miR-30d-5p;hsa-miR-30
ENSG0000	ENSG0000	0.000257	hsa-miR-103a-3p;hsa-miR-107;hsa-miR-1224-3p;hsa-miR-126
ENSG0000	ENSG0000	0.000235	hsa-miR-15a-5p;hsa-miR-15b-5p;hsa-miR-16-5p;hsa-miR-195-
ENSG0000	ENSG0000	0.000312	hsa-miR-15a-5p;hsa-miR-195-5p;hsa-miR-30b-5p;hsa-miR-30
ENSG0000	ENSG0000	3.48E-06	hsa-miR-30b-5p;hsa-miR-30c-5p;hsa-miR-30d-5p;hsa-miR-30
ENSG0000	ENSG0000	1.30E-07	hsa-miR-15a-5p;hsa-miR-15b-5p;hsa-miR-195-5p;hsa-miR-21
ENSG0000	ENSG0000	4.31E-08	hsa-miR-103a-3p;hsa-miR-107;hsa-miR-15a-5p;hsa-miR-15b-
ENSG0000	ENSG0000	6.01E-05	hsa-miR-15a-5p;hsa-miR-15b-5p;hsa-miR-195-5p;hsa-miR-21
ENSG0000	ENSG0000	0.000105	hsa-miR-181a-5p;hsa-miR-30b-5p;hsa-miR-30c-5p;hsa-miR-30
ENSG0000	ENSG0000	0.000177	hsa-miR-15a-5p;hsa-miR-15b-5p;hsa-miR-16-5p;hsa-miR-195-
ENSG0000	ENSG0000	1.68E-05	hsa-miR-15a-5p;hsa-miR-15b-5p;hsa-miR-1913;hsa-miR-195-
ENSG0000	ENSG0000	0.000199	hsa-miR-1246;hsa-miR-15a-5p;hsa-miR-15b-5p;hsa-miR-16-5
ENSG0000	ENSG0000	0.000327	hsa-miR-30b-5p;hsa-miR-30c-5p;hsa-miR-30d-5p;hsa-miR-30
ENSG0000	ENSG0000	0.000177	hsa-miR-23a-3p;hsa-miR-30b-5p;hsa-miR-30c-5p;hsa-miR-30
ENSG0000	ENSG0000	0.006325	hsa-miR-15a-5p;hsa-miR-15b-5p;hsa-miR-16-5p;hsa-miR-195-
ENSG0000	ENSG0000	6.63E-06	hsa-miR-107;hsa-miR-1224-3p;hsa-miR-15a-5p;hsa-miR-15b-
ENSG0000	ENSG0000	0.000894	hsa-miR-15a-5p;hsa-miR-15b-5p;hsa-miR-195-5p;hsa-miR-42
ENSG0000	ENSG0000	0.001683	hsa-miR-15a-5p;hsa-miR-15b-5p;hsa-miR-16-5p;hsa-miR-195-
ENSG0000	ENSG0000	5.45E-07	hsa-miR-103a-3p;hsa-miR-107;hsa-miR-15a-5p;hsa-miR-15b-
ENSG0000	ENSG0000	0.009706	hsa-miR-15a-5p;hsa-miR-195-5p;hsa-miR-331-3p;hsa-miR-42
ENSG0000	ENSG0000	7.48E-07	hsa-miR-181a-5p;hsa-miR-30b-5p;hsa-miR-30c-5p;hsa-miR-30
ENSG0000	ENSG0000	0.000224	hsa-miR-1913;hsa-miR-214-3p;hsa-miR-22-3p;hsa-miR-324-3
ENSG0000	ENSG0000	2.00E-05	hsa-miR-30b-5p;hsa-miR-30c-5p;hsa-miR-30d-5p;hsa-miR-30
ENSG0000	ENSG0000	0.001341	hsa-miR-15a-5p;hsa-miR-15b-5p;hsa-miR-195-5p;hsa-miR-19

ENSG0000	ENSG0000	0.00292	hsa-miR-15a-5p;hsa-miR-15b-5p;hsa-miR-195-5p;hsa-miR-424
ENSG0000	ENSG0000	6.23E-05	hsa-miR-15a-5p;hsa-miR-15b-5p;hsa-miR-181a-5p;hsa-miR-19
ENSG0000	ENSG0000	1.01E-05	hsa-miR-15a-5p;hsa-miR-15b-5p;hsa-miR-16-5p;hsa-miR-195-
ENSG0000	ENSG0000	4.55E-08	hsa-miR-15a-5p;hsa-miR-15b-5p;hsa-miR-16-5p;hsa-miR-195-
ENSG0000	ENSG0000	4.55E-08	hsa-miR-15a-5p;hsa-miR-15b-5p;hsa-miR-16-5p;hsa-miR-195-
ENSG0000	ENSG0000	0.000471	hsa-miR-15a-5p;hsa-miR-15b-5p;hsa-miR-16-5p;hsa-miR-195-
ENSG0000	ENSG0000	0.000243	hsa-miR-15a-5p;hsa-miR-15b-5p;hsa-miR-195-5p;hsa-miR-424
ENSG0000	ENSG0000	5.11E-07	hsa-miR-30b-5p;hsa-miR-30c-5p;hsa-miR-30d-5p;hsa-miR-30e
ENSG0000	ENSG0000	0.00111	hsa-miR-212-3p;hsa-miR-30b-5p;hsa-miR-30c-5p;hsa-miR-30d
ENSG0000	ENSG0000	3.10E-05	hsa-miR-1224-3p;hsa-miR-1260a;hsa-miR-1260b;hsa-miR-188-
ENSG0000	ENSG0000	7.77E-07	hsa-miR-103a-3p;hsa-miR-107;hsa-miR-15a-5p;hsa-miR-15b-5
ENSG0000	ENSG0000	2.41E-05	hsa-miR-103a-3p;hsa-miR-107;hsa-miR-15a-5p;hsa-miR-15b-5
ENSG0000	ENSG0000	0.003553	hsa-miR-15a-5p;hsa-miR-195-5p;hsa-miR-424-5p;hsa-miR-497
ENSG0000	ENSG0000	0.000428	hsa-miR-103a-3p;hsa-miR-1207-5p;hsa-miR-1913;hsa-miR-191
ENSG0000	ENSG0000	0.009158	hsa-miR-1913;hsa-miR-23a-3p;hsa-miR-23b-3p;hsa-miR-324-3
ENSG0000	ENSG0000	0.000122	hsa-miR-1260a;hsa-miR-1260b;hsa-miR-15a-5p;hsa-miR-188-3
ENSG0000	ENSG0000	0.003307	hsa-miR-1224-3p;hsa-miR-1246;hsa-miR-1913;hsa-miR-324-3p
ENSG0000	ENSG0000	3.74E-05	hsa-miR-15a-5p;hsa-miR-15b-5p;hsa-miR-195-5p;hsa-miR-22-
ENSG0000	ENSG0000	3.10E-05	hsa-miR-107;hsa-miR-15a-5p;hsa-miR-16-5p;hsa-miR-424-5p
ENSG0000	ENSG0000	0.005795	hsa-miR-30b-5p;hsa-miR-30c-5p;hsa-miR-30d-5p;hsa-miR-30e
ENSG0000	ENSG0000	0.006313	hsa-miR-15a-5p;hsa-miR-15b-5p;hsa-miR-195-5p;hsa-miR-424
ENSG0000	ENSG0000	0.000126	hsa-miR-1207-5p;hsa-miR-182-5p;hsa-miR-30b-5p;hsa-miR-30
ENSG0000	ENSG0000	0.005286	hsa-miR-30b-5p;hsa-miR-30c-5p;hsa-miR-30d-5p;hsa-miR-30e
ENSG0000	ENSG0000	0.004742	hsa-miR-1224-3p;hsa-miR-1913;hsa-miR-23a-3p;hsa-miR-23b-
ENSG0000	ENSG0000	0.000661	hsa-miR-1207-5p;hsa-miR-15a-5p;hsa-miR-15b-5p;hsa-miR-16
ENSG0000	ENSG0000	1.41E-06	hsa-miR-15a-5p;hsa-miR-15b-5p;hsa-miR-16-5p;hsa-miR-191-
ENSG0000	ENSG0000	0.008829	hsa-miR-30b-5p;hsa-miR-30c-5p;hsa-miR-30d-5p;hsa-miR-30e
ENSG0000	ENSG0000	2.24E-05	hsa-miR-103a-3p;hsa-miR-107;hsa-miR-1225-5p;hsa-miR-15a-
ENSG0000	ENSG0000	1.93E-05	hsa-miR-107;hsa-miR-15a-5p;hsa-miR-15b-5p;hsa-miR-195-5p
ENSG0000	ENSG0000	0.000206	hsa-miR-224-5p;hsa-miR-30c-5p;hsa-miR-30d-5p;hsa-miR-30e
ENSG0000	ENSG0000	0.000224	hsa-miR-103a-3p;hsa-miR-107;hsa-miR-30b-5p;hsa-miR-30c-5
ENSG0000	ENSG0000	2.21E-05	hsa-miR-15a-5p;hsa-miR-15b-5p;hsa-miR-195-5p;hsa-miR-424
ENSG0000	ENSG0000	4.55E-08	hsa-miR-15a-5p;hsa-miR-15b-5p;hsa-miR-16-5p;hsa-miR-195-
ENSG0000	ENSG0000	4.55E-08	hsa-miR-15a-5p;hsa-miR-15b-5p;hsa-miR-16-5p;hsa-miR-195-
ENSG0000	ENSG0000	0.000156	hsa-miR-15a-5p;hsa-miR-15b-5p;hsa-miR-195-5p;hsa-miR-424
ENSG0000	ENSG0000	1.04E-07	hsa-miR-15a-5p;hsa-miR-15b-5p;hsa-miR-16-5p;hsa-miR-195-
ENSG0000	ENSG0000	0.000707	hsa-miR-30b-5p;hsa-miR-30c-5p;hsa-miR-30d-5p;hsa-miR-30e
ENSG0000	ENSG0000	2.66E-05	hsa-miR-107;hsa-miR-15a-5p;hsa-miR-15b-5p;hsa-miR-195-5p
ENSG0000	ENSG0000	0.006736	hsa-miR-1224-3p;hsa-miR-1260a;hsa-miR-1260b;hsa-miR-24-3
ENSG0000	ENSG0000	0.001505	hsa-miR-30b-5p;hsa-miR-30c-5p;hsa-miR-30d-5p;hsa-miR-30e
ENSG0000	ENSG0000	0.003943	hsa-miR-1207-5p;hsa-miR-24-3p;hsa-miR-342-3p;hsa-miR-469
ENSG0000	ENSG0000	9.72E-05	hsa-miR-107;hsa-miR-15a-5p;hsa-miR-15b-5p;hsa-miR-181a-5
ENSG0000	ENSG0000	0.000711	hsa-miR-15a-5p;hsa-miR-15b-5p;hsa-miR-195-5p;hsa-miR-424
ENSG0000	ENSG0000	0.002395	hsa-miR-15a-5p;hsa-miR-15b-5p;hsa-miR-16-5p;hsa-miR-195-
ENSG0000	ENSG0000	5.11E-07	hsa-miR-15a-5p;hsa-miR-15b-5p;hsa-miR-16-5p;hsa-miR-195-
ENSG0000	ENSG0000	0.000837	hsa-miR-30b-5p;hsa-miR-30c-5p;hsa-miR-30d-5p;hsa-miR-30e
ENSG0000	ENSG0000	0.006335	hsa-miR-30b-5p;hsa-miR-30c-5p;hsa-miR-30d-5p;hsa-miR-30e
ENSG0000	ENSG0000	0.000262	hsa-miR-30b-5p;hsa-miR-30c-5p;hsa-miR-30d-5p;hsa-miR-30e
ENSG0000	ENSG0000	0.00199	hsa-miR-107;hsa-miR-15a-5p;hsa-miR-15b-5p;hsa-miR-195-5p
ENSG0000	ENSG0000	0.000199	hsa-miR-15a-5p;hsa-miR-15b-5p;hsa-miR-16-5p;hsa-miR-195-
ENSG0000	ENSG0000	0.000126	hsa-miR-1246;hsa-miR-30b-5p;hsa-miR-30c-5p;hsa-miR-30d-5
ENSG0000	ENSG0000	0.002061	hsa-miR-15a-5p;hsa-miR-15b-5p;hsa-miR-1915-3p;hsa-miR-19
ENSG0000	ENSG0000	1.08E-05	hsa-miR-15a-5p;hsa-miR-15b-5p;hsa-miR-16-5p;hsa-miR-195-
ENSG0000	ENSG0000	0.001725	hsa-miR-103a-3p;hsa-miR-107;hsa-miR-181a-5p;hsa-miR-214-

ENSG0000	ENSG0000	0.001725	hsa-miR-103a-3p;hsa-miR-107;hsa-miR-181a-5p;hsa-miR-214-
ENSG0000	ENSG0000	0.005155	hsa-miR-103a-3p;hsa-miR-107;hsa-miR-30b-5p;hsa-miR-30d-
ENSG0000	ENSG0000	6.56E-08	hsa-miR-1246;hsa-miR-15a-5p;hsa-miR-15b-5p;hsa-miR-16-5
ENSG0000	ENSG0000	6.56E-08	hsa-miR-1246;hsa-miR-15a-5p;hsa-miR-15b-5p;hsa-miR-16-5
ENSG0000	ENSG0000	0.000281	hsa-miR-107;hsa-miR-1260a;hsa-miR-1260b;hsa-miR-15a-5p;h
ENSG0000	ENSG0000	1.27E-05	hsa-miR-15a-5p;hsa-miR-15b-5p;hsa-miR-16-5p;hsa-miR-195-
ENSG0000	ENSG0000	1.87E-06	hsa-miR-103a-3p;hsa-miR-107;hsa-miR-1224-3p;hsa-miR-122
ENSG0000	ENSG0000	0.000491	hsa-miR-30b-5p;hsa-miR-30c-5p;hsa-miR-30d-5p;hsa-miR-30
ENSG0000	ENSG0000	9.04E-05	hsa-miR-15a-5p;hsa-miR-15b-5p;hsa-miR-16-5p;hsa-miR-195-
ENSG0000	ENSG0000	9.04E-05	hsa-miR-15a-5p;hsa-miR-15b-5p;hsa-miR-16-5p;hsa-miR-195-
ENSG0000	ENSG0000	1.22E-05	hsa-miR-30b-5p;hsa-miR-30c-5p;hsa-miR-30d-5p;hsa-miR-30
ENSG0000	ENSG0000	3.48E-06	hsa-miR-15a-5p;hsa-miR-15b-5p;hsa-miR-16-5p;hsa-miR-195-
ENSG0000	ENSG0000	0.000799	hsa-miR-15a-5p;hsa-miR-15b-5p;hsa-miR-181a-5p;hsa-miR-1
ENSG0000	ENSG0000	1.54E-07	hsa-miR-103a-3p;hsa-miR-107;hsa-miR-15a-5p;hsa-miR-15b-
ENSG0000	ENSG0000	0.000385	hsa-miR-107;hsa-miR-15a-5p;hsa-miR-15b-5p;hsa-miR-195-5
ENSG0000	ENSG0000	6.86E-06	hsa-miR-30b-5p;hsa-miR-30c-5p;hsa-miR-30d-5p;hsa-miR-30
ENSG0000	ENSG0000	0.000785	hsa-miR-107;hsa-miR-15a-5p;hsa-miR-15b-5p;hsa-miR-195-5
ENSG0000	ENSG0000	0.000177	hsa-miR-30b-5p;hsa-miR-30c-5p;hsa-miR-30d-5p;hsa-miR-30
ENSG0000	ENSG0000	1.22E-07	hsa-miR-103a-3p;hsa-miR-107;hsa-miR-15a-5p;hsa-miR-15b-
ENSG0000	ENSG0000	0.001146	hsa-miR-15a-5p;hsa-miR-15b-5p;hsa-miR-16-5p;hsa-miR-195-
ENSG0000	ENSG0000	0.000327	hsa-miR-214-3p;hsa-miR-24-3p;hsa-miR-3619-5p;hsa-miR-7-
ENSG0000	ENSG0000	2.03E-06	hsa-miR-22-3p;hsa-miR-30b-5p;hsa-miR-30c-5p;hsa-miR-30d-
ENSG0000	ENSG0000	1.29E-07	hsa-miR-15a-5p;hsa-miR-15b-5p;hsa-miR-16-5p;hsa-miR-195-
ENSG0000	ENSG0000	0.006908	hsa-miR-197-3p;hsa-miR-24-3p;hsa-miR-342-3p;hsa-miR-571
ENSG0000	ENSG0000	0.004362	hsa-miR-30b-5p;hsa-miR-30c-5p;hsa-miR-30d-5p;hsa-miR-30
ENSG0000	ENSG0000	0.006335	hsa-miR-1913;hsa-miR-3194-3p;hsa-miR-324-3p;hsa-miR-877-
ENSG0000	ENSG0000	0.00015	hsa-miR-15a-5p;hsa-miR-15b-5p;hsa-miR-195-5p;hsa-miR-22-
ENSG0000	ENSG0000	4.12E-08	hsa-miR-15a-5p;hsa-miR-15b-5p;hsa-miR-16-5p;hsa-miR-195-
ENSG0000	ENSG0000	9.31E-07	hsa-miR-15a-5p;hsa-miR-15b-5p;hsa-miR-16-5p;hsa-miR-195-
ENSG0000	ENSG0000	0.000491	hsa-miR-15a-5p;hsa-miR-15b-5p;hsa-miR-195-5p;hsa-miR-42
ENSG0000	ENSG0000	1.04E-08	hsa-miR-197-3p;hsa-miR-30b-5p;hsa-miR-30c-5p;hsa-miR-30
ENSG0000	ENSG0000	1.53E-05	hsa-miR-103a-3p;hsa-miR-107;hsa-miR-15a-5p;hsa-miR-15b-
ENSG0000	ENSG0000	6.01E-05	hsa-miR-107;hsa-miR-15a-5p;hsa-miR-15b-5p;hsa-miR-195-5
ENSG0000	ENSG0000	0.005795	hsa-miR-15a-5p;hsa-miR-15b-5p;hsa-miR-16-5p;hsa-miR-195-
ENSG0000	ENSG0000	6.86E-06	hsa-miR-30b-5p;hsa-miR-30c-5p;hsa-miR-30d-5p;hsa-miR-30
ENSG0000	ENSG0000	0.00199	hsa-miR-1224-3p;hsa-miR-1260a;hsa-miR-1260b;hsa-miR-181
ENSG0000	ENSG0000	5.54E-05	hsa-miR-15a-5p;hsa-miR-15b-5p;hsa-miR-195-5p;hsa-miR-21
ENSG0000	ENSG0000	0.00112	hsa-miR-1224-3p;hsa-miR-1260a;hsa-miR-1260b;hsa-miR-188-
ENSG0000	ENSG0000	3.16E-05	hsa-miR-1913;hsa-miR-30b-5p;hsa-miR-30c-5p;hsa-miR-30d-
ENSG0000	ENSG0000	9.34E-06	hsa-miR-103a-3p;hsa-miR-107;hsa-miR-15a-5p;hsa-miR-15b-
ENSG0000	ENSG0000	0.00073	hsa-miR-1825;hsa-miR-197-3p;hsa-miR-23a-3p;hsa-miR-23b-
ENSG0000	ENSG0000	9.31E-07	hsa-miR-15a-5p;hsa-miR-15b-5p;hsa-miR-16-5p;hsa-miR-195-
ENSG0000	ENSG0000	1.24E-05	hsa-miR-15a-5p;hsa-miR-15b-5p;hsa-miR-16-5p;hsa-miR-195-
ENSG0000	ENSG0000	0.000566	hsa-miR-15a-5p;hsa-miR-15b-5p;hsa-miR-16-5p;hsa-miR-195-
ENSG0000	ENSG0000	8.00E-05	hsa-miR-15a-5p;hsa-miR-15b-5p;hsa-miR-16-5p;hsa-miR-195-
ENSG0000	ENSG0000	0.000969	hsa-miR-15a-5p;hsa-miR-15b-5p;hsa-miR-16-5p;hsa-miR-188-
ENSG0000	ENSG0000	0.000751	hsa-miR-15a-5p;hsa-miR-15b-5p;hsa-miR-16-5p;hsa-miR-195-
ENSG0000	ENSG0000	8.87E-10	hsa-miR-15a-5p;hsa-miR-15b-5p;hsa-miR-16-5p;hsa-miR-195-
ENSG0000	ENSG0000	0.005019	hsa-miR-107;hsa-miR-15a-5p;hsa-miR-15b-5p;hsa-miR-16-5p;
ENSG0000	ENSG0000	0.001262	hsa-miR-15a-5p;hsa-miR-15b-5p;hsa-miR-195-5p;hsa-miR-23
ENSG0000	ENSG0000	0.000126	hsa-miR-15a-5p;hsa-miR-15b-5p;hsa-miR-16-5p;hsa-miR-192-
ENSG0000	ENSG0000	9.24E-06	hsa-miR-15a-5p;hsa-miR-15b-5p;hsa-miR-16-5p;hsa-miR-195-
ENSG0000	ENSG0000	0.002541	hsa-miR-30b-5p;hsa-miR-30c-5p;hsa-miR-30d-5p;hsa-miR-30
ENSG0000	ENSG0000	0.000592	hsa-miR-30b-5p;hsa-miR-30c-5p;hsa-miR-30d-5p;hsa-miR-30

ENSG0000	ENSG0000	0.004408	hsa-miR-15a-5p;hsa-miR-16-5p;hsa-miR-1913;hsa-miR-1915-3
ENSG0000	ENSG0000	0.009284	hsa-miR-1207-5p;hsa-miR-1260a;hsa-miR-1260b;hsa-miR-188-
ENSG0000	ENSG0000	3.63E-05	hsa-miR-1224-3p;hsa-miR-1260a;hsa-miR-1260b;hsa-miR-15a-
ENSG0000	ENSG0000	0.000431	hsa-miR-224-5p;hsa-miR-30b-5p;hsa-miR-30c-5p;hsa-miR-30c
ENSG0000	ENSG0000	1.04E-07	hsa-miR-15a-5p;hsa-miR-15b-5p;hsa-miR-16-5p;hsa-miR-195-
ENSG0000	ENSG0000	0.000122	hsa-miR-30b-5p;hsa-miR-30c-5p;hsa-miR-30d-5p;hsa-miR-30e
ENSG0000	ENSG0000	1.98E-09	hsa-miR-107;hsa-miR-1246;hsa-miR-15a-5p;hsa-miR-15b-5p;h
ENSG0000	ENSG0000	0.001449	hsa-miR-15a-5p;hsa-miR-15b-5p;hsa-miR-16-5p;hsa-miR-195-
ENSG0000	ENSG0000	0.000177	hsa-miR-15a-5p;hsa-miR-15b-5p;hsa-miR-195-5p;hsa-miR-424
ENSG0000	ENSG0000	0.002736	hsa-miR-15a-5p;hsa-miR-15b-5p;hsa-miR-16-5p;hsa-miR-195-
ENSG0000	ENSG0000	0.000686	hsa-miR-15a-5p;hsa-miR-15b-5p;hsa-miR-16-5p;hsa-miR-195-
ENSG0000	ENSG0000	1.26E-06	hsa-miR-15a-5p;hsa-miR-15b-5p;hsa-miR-16-5p;hsa-miR-195-
ENSG0000	ENSG0000	6.99E-05	hsa-miR-15a-5p;hsa-miR-15b-5p;hsa-miR-16-5p;hsa-miR-181a
ENSG0000	ENSG0000	3.11E-06	hsa-miR-15a-5p;hsa-miR-15b-5p;hsa-miR-195-5p;hsa-miR-424
ENSG0000	ENSG0000	0.000121	hsa-miR-15a-5p;hsa-miR-15b-5p;hsa-miR-16-5p;hsa-miR-195-
ENSG0000	ENSG0000	0.00012	hsa-miR-15a-5p;hsa-miR-15b-5p;hsa-miR-16-5p;hsa-miR-181a
ENSG0000	ENSG0000	0.000837	hsa-miR-181a-5p;hsa-miR-30b-5p;hsa-miR-30d-5p;hsa-miR-30
ENSG0000	ENSG0000	1.84E-05	hsa-miR-15a-5p;hsa-miR-15b-5p;hsa-miR-181a-5p;hsa-miR-19
ENSG0000	ENSG0000	0.001363	hsa-miR-1224-3p;hsa-miR-1260a;hsa-miR-1260b;hsa-miR-188-
ENSG0000	ENSG0000	0.000707	hsa-miR-30b-5p;hsa-miR-30c-5p;hsa-miR-30d-5p;hsa-miR-30e
ENSG0000	ENSG0000	0.000707	hsa-miR-30b-5p;hsa-miR-30c-5p;hsa-miR-30d-5p;hsa-miR-30e
ENSG0000	ENSG0000	3.11E-06	hsa-miR-16-5p;hsa-miR-30b-5p;hsa-miR-30c-5p;hsa-miR-30d-
ENSG0000	ENSG0000	6.68E-14	hsa-miR-15a-5p;hsa-miR-15b-5p;hsa-miR-16-5p;hsa-miR-195-
ENSG0000	ENSG0000	0.00292	hsa-miR-181a-5p;hsa-miR-30b-5p;hsa-miR-30c-5p;hsa-miR-30
ENSG0000	ENSG0000	0.000122	hsa-miR-30b-5p;hsa-miR-30c-5p;hsa-miR-30d-5p;hsa-miR-30e
ENSG0000	ENSG0000	2.64E-06	hsa-miR-15a-5p;hsa-miR-15b-5p;hsa-miR-1913;hsa-miR-195-5
ENSG0000	ENSG0000	1.04E-07	hsa-miR-15a-5p;hsa-miR-15b-5p;hsa-miR-16-5p;hsa-miR-195-
ENSG0000	ENSG0000	9.34E-06	hsa-miR-1207-5p;hsa-miR-15a-5p;hsa-miR-15b-5p;hsa-miR-16
ENSG0000	ENSG0000	8.64E-05	hsa-miR-15b-5p;hsa-miR-16-5p;hsa-miR-30b-5p;hsa-miR-30d
ENSG0000	ENSG0000	0.004224	hsa-miR-15a-5p;hsa-miR-15b-5p;hsa-miR-195-5p;hsa-miR-19
ENSG0000	ENSG0000	0.000475	hsa-miR-103a-3p;hsa-miR-107;hsa-miR-15a-5p;hsa-miR-15b-5
ENSG0000	ENSG0000	0.000592	hsa-miR-15a-5p;hsa-miR-15b-5p;hsa-miR-16-5p;hsa-miR-195-
ENSG0000	ENSG0000	4.40E-05	hsa-miR-15a-5p;hsa-miR-15b-5p;hsa-miR-195-5p;hsa-miR-30b
ENSG0000	ENSG0000	4.55E-08	hsa-miR-15a-5p;hsa-miR-15b-5p;hsa-miR-16-5p;hsa-miR-195-
ENSG0000	ENSG0000	1.22E-05	hsa-miR-1224-3p;hsa-miR-1260a;hsa-miR-1260b;hsa-miR-188-
ENSG0000	ENSG0000	2.87E-05	hsa-miR-30b-5p;hsa-miR-30c-5p;hsa-miR-30d-5p;hsa-miR-30e
ENSG0000	ENSG0000	5.11E-07	hsa-miR-30b-5p;hsa-miR-30c-5p;hsa-miR-30d-5p;hsa-miR-30e
ENSG0000	ENSG0000	2.04E-05	hsa-miR-103a-3p;hsa-miR-107;hsa-miR-15a-5p;hsa-miR-15b-5
ENSG0000	ENSG0000	3.01E-07	hsa-miR-103a-3p;hsa-miR-107;hsa-miR-15a-5p;hsa-miR-15b-5
ENSG0000	ENSG0000	1.54E-07	hsa-miR-103a-3p;hsa-miR-107;hsa-miR-15a-5p;hsa-miR-15b-5
ENSG0000	ENSG0000	6.86E-06	hsa-miR-15a-5p;hsa-miR-15b-5p;hsa-miR-16-5p;hsa-miR-195-
ENSG0000	ENSG0000	0.001528	hsa-miR-30b-5p;hsa-miR-30c-5p;hsa-miR-30d-5p;hsa-miR-30e
ENSG0000	ENSG0000	4.35E-05	hsa-miR-103a-3p;hsa-miR-107;hsa-miR-15a-5p;hsa-miR-15b-5
ENSG0000	ENSG0000	2.00E-05	hsa-miR-30b-5p;hsa-miR-30c-5p;hsa-miR-30d-5p;hsa-miR-30e
ENSG0000	ENSG0000	0.001146	hsa-miR-15a-5p;hsa-miR-15b-5p;hsa-miR-16-5p;hsa-miR-195-
ENSG0000	ENSG0000	2.16E-06	hsa-miR-103a-3p;hsa-miR-107;hsa-miR-30b-5p;hsa-miR-30c-5
ENSG0000	ENSG0000	0.000491	hsa-miR-103a-3p;hsa-miR-107;hsa-miR-214-3p;hsa-miR-224-5
ENSG0000	ENSG0000	9.04E-05	hsa-miR-30b-5p;hsa-miR-30c-5p;hsa-miR-30d-5p;hsa-miR-30e
ENSG0000	ENSG0000	0.005919	hsa-miR-30b-5p;hsa-miR-30c-5p;hsa-miR-30d-5p;hsa-miR-30e
ENSG0000	ENSG0000	5.11E-07	hsa-miR-30b-5p;hsa-miR-30c-5p;hsa-miR-30d-5p;hsa-miR-30e
ENSG0000	ENSG0000	0.000571	hsa-miR-107;hsa-miR-15a-5p;hsa-miR-15b-5p;hsa-miR-195-5p
ENSG0000	ENSG0000	4.43E-09	hsa-miR-15a-5p;hsa-miR-15b-5p;hsa-miR-16-5p;hsa-miR-195-
ENSG0000	ENSG0000	7.85E-08	hsa-miR-15a-5p;hsa-miR-15b-5p;hsa-miR-16-5p;hsa-miR-195-
ENSG0000	ENSG0000	1.24E-05	hsa-miR-107;hsa-miR-15a-5p;hsa-miR-15b-5p;hsa-miR-195-5p

ENSG0000	ENSG0000	6.97E-05	hsa-miR-15a-5p;hsa-miR-15b-5p;hsa-miR-16-5p;hsa-miR-195-
ENSG0000	ENSG0000	0.004805	hsa-miR-1207-5p;hsa-miR-1224-3p;hsa-miR-1260a;hsa-miR-12
ENSG0000	ENSG0000	1.00E-06	hsa-miR-103a-3p;hsa-miR-107;hsa-miR-1224-3p;hsa-miR-126
ENSG0000	ENSG0000	5.54E-05	hsa-miR-15a-5p;hsa-miR-15b-5p;hsa-miR-16-5p;hsa-miR-195-
ENSG0000	ENSG0000	0.00414	hsa-miR-15a-5p;hsa-miR-15b-5p;hsa-miR-16-5p;hsa-miR-195-
ENSG0000	ENSG0000	0.00015	hsa-miR-15a-5p;hsa-miR-15b-5p;hsa-miR-195-5p;hsa-miR-42
ENSG0000	ENSG0000	3.88E-06	hsa-miR-15a-5p;hsa-miR-15b-5p;hsa-miR-16-5p;hsa-miR-195-
ENSG0000	ENSG0000	1.84E-05	hsa-miR-103a-3p;hsa-miR-107;hsa-miR-30b-5p;hsa-miR-30c-5
ENSG0000	ENSG0000	2.00E-05	hsa-miR-15a-5p;hsa-miR-15b-5p;hsa-miR-16-5p;hsa-miR-195-
ENSG0000	ENSG0000	2.48E-08	hsa-miR-15a-5p;hsa-miR-15b-5p;hsa-miR-16-5p;hsa-miR-195-
ENSG0000	ENSG0000	0.000376	hsa-miR-15a-5p;hsa-miR-15b-5p;hsa-miR-195-5p;hsa-miR-42
ENSG0000	ENSG0000	1.71E-05	hsa-miR-15a-5p;hsa-miR-15b-5p;hsa-miR-16-5p;hsa-miR-195-
ENSG0000	ENSG0000	1.71E-05	hsa-miR-15a-5p;hsa-miR-15b-5p;hsa-miR-16-5p;hsa-miR-195-
ENSG0000	ENSG0000	0.004809	hsa-miR-30b-5p;hsa-miR-30c-5p;hsa-miR-30d-5p;hsa-miR-30
ENSG0000	ENSG0000	3.74E-05	hsa-miR-15a-5p;hsa-miR-15b-5p;hsa-miR-23a-3p;hsa-miR-23
ENSG0000	ENSG0000	2.25E-06	hsa-miR-1224-3p;hsa-miR-1260a;hsa-miR-1260b;hsa-miR-224-
ENSG0000	ENSG0000	3.10E-05	hsa-miR-1224-3p;hsa-miR-1260a;hsa-miR-1260b;hsa-miR-24-3
ENSG0000	ENSG0000	0.000491	hsa-miR-1224-3p;hsa-miR-1260a;hsa-miR-1260b;hsa-miR-188-
ENSG0000	ENSG0000	0.00319	hsa-miR-30b-5p;hsa-miR-30c-5p;hsa-miR-30d-5p;hsa-miR-30
ENSG0000	ENSG0000	2.25E-06	hsa-miR-15a-5p;hsa-miR-15b-5p;hsa-miR-16-5p;hsa-miR-195-
ENSG0000	ENSG0000	0.002853	hsa-miR-15a-5p;hsa-miR-15b-5p;hsa-miR-16-5p;hsa-miR-195-
ENSG0000	ENSG0000	7.85E-08	hsa-miR-15a-5p;hsa-miR-15b-5p;hsa-miR-16-5p;hsa-miR-195-
ENSG0000	ENSG0000	3.88E-06	hsa-miR-15a-5p;hsa-miR-15b-5p;hsa-miR-16-5p;hsa-miR-195-
ENSG0000	ENSG0000	0.006736	hsa-miR-30b-5p;hsa-miR-30c-5p;hsa-miR-30d-5p;hsa-miR-30
ENSG0000	ENSG0000	6.86E-06	hsa-miR-30b-5p;hsa-miR-30c-5p;hsa-miR-30d-5p;hsa-miR-30
ENSG0000	ENSG0000	0.000243	hsa-miR-15b-5p;hsa-miR-16-5p;hsa-miR-195-5p;hsa-miR-424-
ENSG0000	ENSG0000	0.000491	hsa-miR-15a-5p;hsa-miR-15b-5p;hsa-miR-16-5p;hsa-miR-195-
ENSG0000	ENSG0000	0.000491	hsa-miR-15a-5p;hsa-miR-15b-5p;hsa-miR-16-5p;hsa-miR-195-
ENSG0000	ENSG0000	9.04E-05	hsa-miR-15a-5p;hsa-miR-15b-5p;hsa-miR-16-5p;hsa-miR-195-
ENSG0000	ENSG0000	0.003553	hsa-miR-1224-3p;hsa-miR-1260a;hsa-miR-1260b;hsa-miR-877-
ENSG0000	ENSG0000	0.00015	hsa-miR-15a-5p;hsa-miR-15b-5p;hsa-miR-195-5p;hsa-miR-42
ENSG0000	ENSG0000	2.48E-05	hsa-miR-15a-5p;hsa-miR-15b-5p;hsa-miR-16-5p;hsa-miR-195-
ENSG0000	ENSG0000	0.000177	hsa-miR-15a-5p;hsa-miR-15b-5p;hsa-miR-16-5p;hsa-miR-195-
ENSG0000	ENSG0000	0.001816	hsa-miR-1207-5p;hsa-miR-1915-3p;hsa-miR-192-3p;hsa-miR-2
ENSG0000	ENSG0000	0.001035	hsa-miR-1207-5p;hsa-miR-15a-5p;hsa-miR-15b-5p;hsa-miR-1
ENSG0000	ENSG0000	0.006581	hsa-miR-15a-5p;hsa-miR-15b-5p;hsa-miR-16-5p;hsa-miR-195-
ENSG0000	ENSG0000	0.000403	hsa-miR-103a-3p;hsa-miR-107;hsa-miR-23a-3p;hsa-miR-23b-3
ENSG0000	ENSG0000	1.97E-05	hsa-miR-15a-5p;hsa-miR-15b-5p;hsa-miR-16-5p;hsa-miR-195-
ENSG0000	ENSG0000	0.000426	hsa-miR-15a-5p;hsa-miR-15b-5p;hsa-miR-16-5p;hsa-miR-195-
ENSG0000	ENSG0000	2.16E-06	hsa-miR-15a-5p;hsa-miR-15b-5p;hsa-miR-16-5p;hsa-miR-195-
ENSG0000	ENSG0000	2.16E-06	hsa-miR-15a-5p;hsa-miR-15b-5p;hsa-miR-16-5p;hsa-miR-195-
ENSG0000	ENSG0000	0.000983	hsa-miR-15a-5p;hsa-miR-15b-5p;hsa-miR-16-5p;hsa-miR-195-
ENSG0000	ENSG0000	0.000106	hsa-miR-15a-5p;hsa-miR-15b-5p;hsa-miR-16-5p;hsa-miR-195-
ENSG0000	ENSG0000	0.000217	hsa-miR-1224-3p;hsa-miR-1260a;hsa-miR-1260b;hsa-miR-15a-
ENSG0000	ENSG0000	2.90E-11	hsa-miR-1224-3p;hsa-miR-1260a;hsa-miR-1260b;hsa-miR-15a-
ENSG0000	ENSG0000	1.25E-09	hsa-miR-1224-3p;hsa-miR-1260a;hsa-miR-1260b;hsa-miR-15a-
ENSG0000	ENSG0000	0.009284	hsa-miR-1224-3p;hsa-miR-1260a;hsa-miR-1260b;hsa-miR-188-
ENSG0000	ENSG0000	3.21E-08	hsa-miR-103a-3p;hsa-miR-107;hsa-miR-15a-5p;hsa-miR-15b-5
ENSG0000	ENSG0000	1.52E-05	hsa-miR-15a-5p;hsa-miR-15b-5p;hsa-miR-16-5p;hsa-miR-195-
ENSG0000	ENSG0000	9.42E-08	hsa-miR-103a-3p;hsa-miR-107;hsa-miR-15a-5p;hsa-miR-15b-5
ENSG0000	ENSG0000	9.42E-08	hsa-miR-103a-3p;hsa-miR-107;hsa-miR-15a-5p;hsa-miR-15b-5
ENSG0000	ENSG0000	8.54E-07	hsa-miR-107;hsa-miR-1260a;hsa-miR-1260b;hsa-miR-15a-5p;h
ENSG0000	ENSG0000	0.000105	hsa-miR-1246;hsa-miR-30b-5p;hsa-miR-30c-5p;hsa-miR-30d-5
ENSG0000	ENSG0000	5.15E-05	hsa-miR-15a-5p;hsa-miR-15b-5p;hsa-miR-16-5p;hsa-miR-195-

ENSG0000	ENSG0000	6.01E-05	hsa-miR-188-3p;hsa-miR-30b-5p;hsa-miR-30c-5p;hsa-miR-30
ENSG0000	ENSG0000	1.22E-05	hsa-miR-15a-5p;hsa-miR-15b-5p;hsa-miR-16-5p;hsa-miR-195-
ENSG0000	ENSG0000	0.001505	hsa-miR-30b-5p;hsa-miR-30c-5p;hsa-miR-30d-5p;hsa-miR-30
ENSG0000	ENSG0000	4.12E-08	hsa-miR-103a-3p;hsa-miR-107;hsa-miR-15a-5p;hsa-miR-15b-
ENSG0000	ENSG0000	0.000376	hsa-miR-107;hsa-miR-15a-5p;hsa-miR-15b-5p;hsa-miR-424-5
ENSG0000	ENSG0000	1.65E-05	hsa-miR-1207-5p;hsa-miR-15a-5p;hsa-miR-15b-5p;hsa-miR-1
ENSG0000	ENSG0000	1.65E-05	hsa-miR-1207-5p;hsa-miR-15a-5p;hsa-miR-15b-5p;hsa-miR-1
ENSG0000	ENSG0000	0.002853	hsa-miR-30b-5p;hsa-miR-30c-5p;hsa-miR-30d-5p;hsa-miR-30
ENSG0000	ENSG0000	4.55E-08	hsa-miR-15a-5p;hsa-miR-15b-5p;hsa-miR-16-5p;hsa-miR-195-
ENSG0000	ENSG0000	0.000403	hsa-miR-30b-5p;hsa-miR-30c-5p;hsa-miR-30d-5p;hsa-miR-30
ENSG0000	ENSG0000	6.73E-08	hsa-miR-15a-5p;hsa-miR-15b-5p;hsa-miR-16-5p;hsa-miR-181
ENSG0000	ENSG0000	1.65E-05	hsa-miR-15a-5p;hsa-miR-15b-5p;hsa-miR-195-5p;hsa-miR-24-
ENSG0000	ENSG0000	4.79E-08	hsa-miR-103a-3p;hsa-miR-107;hsa-miR-15a-5p;hsa-miR-15b-
ENSG0000	ENSG0000	1.98E-09	hsa-miR-103a-3p;hsa-miR-107;hsa-miR-15a-5p;hsa-miR-15b-
ENSG0000	ENSG0000	8.05E-05	hsa-miR-15a-5p;hsa-miR-15b-5p;hsa-miR-16-5p;hsa-miR-195-
ENSG0000	ENSG0000	0.002254	hsa-miR-15a-5p;hsa-miR-15b-5p;hsa-miR-16-5p;hsa-miR-195-
ENSG0000	ENSG0000	2.04E-07	hsa-miR-15a-5p;hsa-miR-15b-5p;hsa-miR-16-5p;hsa-miR-195-
ENSG0000	ENSG0000	3.48E-06	hsa-miR-30b-5p;hsa-miR-30c-5p;hsa-miR-30d-5p;hsa-miR-30
ENSG0000	ENSG0000	0.000592	hsa-miR-15a-5p;hsa-miR-15b-5p;hsa-miR-16-5p;hsa-miR-195-
ENSG0000	ENSG0000	9.04E-05	hsa-miR-15a-5p;hsa-miR-15b-5p;hsa-miR-195-5p;hsa-miR-34
ENSG0000	ENSG0000	0.000133	hsa-miR-15a-5p;hsa-miR-15b-5p;hsa-miR-16-5p;hsa-miR-195-
ENSG0000	ENSG0000	0.000558	hsa-miR-15a-5p;hsa-miR-15b-5p;hsa-miR-195-5p;hsa-miR-42
ENSG0000	ENSG0000	0.002378	hsa-miR-1207-5p;hsa-miR-30b-5p;hsa-miR-30c-5p;hsa-miR-3
ENSG0000	ENSG0000	2.16E-06	hsa-miR-1246;hsa-miR-15a-5p;hsa-miR-15b-5p;hsa-miR-16-5
ENSG0000	ENSG0000	0.000338	hsa-miR-1207-5p;hsa-miR-15a-5p;hsa-miR-15b-5p;hsa-miR-1
ENSG0000	ENSG0000	3.66E-05	hsa-miR-15a-5p;hsa-miR-15b-5p;hsa-miR-16-5p;hsa-miR-195-
ENSG0000	ENSG0000	2.30E-11	hsa-miR-103a-3p;hsa-miR-107;hsa-miR-1224-3p;hsa-miR-122
ENSG0000	ENSG0000	3.48E-06	hsa-miR-30b-5p;hsa-miR-30c-5p;hsa-miR-30d-5p;hsa-miR-30
ENSG0000	ENSG0000	1.98E-09	hsa-miR-15a-5p;hsa-miR-15b-5p;hsa-miR-16-5p;hsa-miR-195-
ENSG0000	ENSG0000	0.000376	hsa-miR-15a-5p;hsa-miR-15b-5p;hsa-miR-195-5p;hsa-miR-42
ENSG0000	ENSG0000	4.09E-05	hsa-miR-15a-5p;hsa-miR-15b-5p;hsa-miR-16-5p;hsa-miR-191
ENSG0000	ENSG0000	4.60E-06	hsa-miR-15a-5p;hsa-miR-15b-5p;hsa-miR-195-5p;hsa-miR-42
ENSG0000	ENSG0000	6.55E-05	hsa-miR-15a-5p;hsa-miR-15b-5p;hsa-miR-16-5p;hsa-miR-195-
ENSG0000	ENSG0000	9.34E-06	hsa-miR-103a-3p;hsa-miR-107;hsa-miR-15a-5p;hsa-miR-15b-
ENSG0000	ENSG0000	1.30E-08	hsa-miR-15a-5p;hsa-miR-15b-5p;hsa-miR-195-5p;hsa-miR-21
ENSG0000	ENSG0000	0.000206	hsa-miR-30b-5p;hsa-miR-30c-5p;hsa-miR-30d-5p;hsa-miR-30
ENSG0000	ENSG0000	8.09E-06	hsa-miR-15a-5p;hsa-miR-15b-5p;hsa-miR-181a-5p;hsa-miR-1
ENSG0000	ENSG0000	0.001623	hsa-miR-15a-5p;hsa-miR-15b-5p;hsa-miR-1913;hsa-miR-195-
ENSG0000	ENSG0000	3.97E-06	hsa-miR-15a-5p;hsa-miR-15b-5p;hsa-miR-16-5p;hsa-miR-195-
ENSG0000	ENSG0000	0.006313	hsa-miR-107;hsa-miR-1224-3p;hsa-miR-1260a;hsa-miR-1260b;
ENSG0000	ENSG0000	1.04E-07	hsa-miR-15a-5p;hsa-miR-15b-5p;hsa-miR-16-5p;hsa-miR-195-
ENSG0000	ENSG0000	0.000491	hsa-miR-30b-5p;hsa-miR-30c-5p;hsa-miR-30d-5p;hsa-miR-30
ENSG0000	ENSG0000	0.006736	hsa-miR-1224-3p;hsa-miR-1260a;hsa-miR-1260b;hsa-miR-7-5
ENSG0000	ENSG0000	1.40E-07	hsa-miR-1207-5p;hsa-miR-15a-5p;hsa-miR-15b-5p;hsa-miR-1
ENSG0000	ENSG0000	3.06E-06	hsa-miR-15a-5p;hsa-miR-15b-5p;hsa-miR-16-5p;hsa-miR-195-
ENSG0000	ENSG0000	3.11E-06	hsa-miR-197-3p;hsa-miR-30b-5p;hsa-miR-30c-5p;hsa-miR-30
ENSG0000	ENSG0000	5.69E-10	hsa-miR-15a-5p;hsa-miR-15b-5p;hsa-miR-16-5p;hsa-miR-181
ENSG0000	ENSG0000	1.24E-05	hsa-miR-1224-3p;hsa-miR-1260a;hsa-miR-1260b;hsa-miR-188-
ENSG0000	ENSG0000	0.000542	hsa-miR-103a-3p;hsa-miR-107;hsa-miR-15a-5p;hsa-miR-15b-
ENSG0000	ENSG0000	1.63E-06	hsa-miR-15a-5p;hsa-miR-15b-5p;hsa-miR-16-5p;hsa-miR-195-
ENSG0000	ENSG0000	0.00015	hsa-miR-15a-5p;hsa-miR-15b-5p;hsa-miR-195-5p;hsa-miR-42
ENSG0000	ENSG0000	0.000314	hsa-miR-103a-3p;hsa-miR-107;hsa-miR-15a-5p;hsa-miR-15b-
ENSG0000	ENSG0000	0.000126	hsa-miR-15a-5p;hsa-miR-15b-5p;hsa-miR-195-5p;hsa-miR-42
ENSG0000	ENSG0000	9.04E-05	hsa-miR-30b-5p;hsa-miR-30c-5p;hsa-miR-30d-5p;hsa-miR-30

ENSG0000	ENSG0000	2.25E-06	hsa-miR-103a-3p;hsa-miR-107;hsa-miR-15a-5p;hsa-miR-15b-5
ENSG0000	ENSG0000	0.000799	hsa-miR-15a-5p;hsa-miR-15b-5p;hsa-miR-195-5p;hsa-miR-424
ENSG0000	ENSG0000	5.45E-07	hsa-miR-15a-5p;hsa-miR-15b-5p;hsa-miR-16-5p;hsa-miR-195-
ENSG0000	ENSG0000	6.61E-07	hsa-miR-15a-5p;hsa-miR-15b-5p;hsa-miR-16-5p;hsa-miR-195-
ENSG0000	ENSG0000	4.55E-08	hsa-miR-15a-5p;hsa-miR-15b-5p;hsa-miR-16-5p;hsa-miR-195-
ENSG0000	ENSG0000	1.51E-06	hsa-miR-15a-5p;hsa-miR-15b-5p;hsa-miR-16-5p;hsa-miR-195-
ENSG0000	ENSG0000	4.60E-06	hsa-miR-30b-5p;hsa-miR-30c-5p;hsa-miR-30d-5p;hsa-miR-30e
ENSG0000	ENSG0000	0.00319	hsa-miR-30b-5p;hsa-miR-30c-5p;hsa-miR-30d-5p;hsa-miR-30e
ENSG0000	ENSG0000	9.04E-05	hsa-miR-15a-5p;hsa-miR-15b-5p;hsa-miR-16-5p;hsa-miR-195-
ENSG0000	ENSG0000	2.22E-05	hsa-miR-1224-3p;hsa-miR-1260a;hsa-miR-1260b;hsa-miR-188-
ENSG0000	ENSG0000	1.06E-05	hsa-miR-15a-5p;hsa-miR-15b-5p;hsa-miR-16-5p;hsa-miR-195-
ENSG0000	ENSG0000	0.000592	hsa-miR-30b-5p;hsa-miR-30c-5p;hsa-miR-30d-5p;hsa-miR-30e
ENSG0000	ENSG0000	0.002254	hsa-miR-192-3p;hsa-miR-30c-5p;hsa-miR-30d-5p;hsa-miR-30e
ENSG0000	ENSG0000	0.006335	hsa-miR-15a-5p;hsa-miR-15b-5p;hsa-miR-16-5p;hsa-miR-195-
ENSG0000	ENSG0000	0.000243	hsa-miR-15a-5p;hsa-miR-15b-5p;hsa-miR-195-5p;hsa-miR-424
ENSG0000	ENSG0000	1.29E-06	hsa-miR-15a-5p;hsa-miR-15b-5p;hsa-miR-16-5p;hsa-miR-195-
ENSG0000	ENSG0000	1.94E-07	hsa-miR-103a-3p;hsa-miR-107;hsa-miR-15a-5p;hsa-miR-15b-5
ENSG0000	ENSG0000	0.000403	hsa-miR-30b-5p;hsa-miR-30c-5p;hsa-miR-30d-5p;hsa-miR-30e
ENSG0000	ENSG0000	3.16E-05	hsa-miR-15a-5p;hsa-miR-15b-5p;hsa-miR-16-5p;hsa-miR-195-
ENSG0000	ENSG0000	0.000707	hsa-miR-30b-5p;hsa-miR-30c-5p;hsa-miR-30d-5p;hsa-miR-30e
ENSG0000	ENSG0000	0.000122	hsa-miR-30b-5p;hsa-miR-30c-5p;hsa-miR-30d-5p;hsa-miR-30e
ENSG0000	ENSG0000	0.002394	hsa-miR-103a-3p;hsa-miR-107;hsa-miR-15a-5p;hsa-miR-15b-5
ENSG0000	ENSG0000	0.00015	hsa-miR-30b-5p;hsa-miR-30c-5p;hsa-miR-30d-5p;hsa-miR-30e
ENSG0000	ENSG0000	3.11E-06	hsa-miR-15a-5p;hsa-miR-15b-5p;hsa-miR-195-5p;hsa-miR-424
ENSG0000	ENSG0000	0.001993	hsa-miR-1260a;hsa-miR-1260b;hsa-miR-188-3p;hsa-miR-214-3
ENSG0000	ENSG0000	3.10E-07	hsa-miR-15a-5p;hsa-miR-15b-5p;hsa-miR-16-5p;hsa-miR-195-
ENSG0000	ENSG0000	0.000631	hsa-miR-15a-5p;hsa-miR-15b-5p;hsa-miR-16-5p;hsa-miR-195-
ENSG0000	ENSG0000	0.00199	hsa-miR-30b-5p;hsa-miR-30c-5p;hsa-miR-30d-5p;hsa-miR-30e
ENSG0000	ENSG0000	7.96E-10	hsa-miR-15a-5p;hsa-miR-15b-5p;hsa-miR-16-5p;hsa-miR-195-
ENSG0000	ENSG0000	1.74E-06	hsa-miR-15a-5p;hsa-miR-15b-5p;hsa-miR-16-5p;hsa-miR-1913
ENSG0000	ENSG0000	0.001346	hsa-miR-1224-3p;hsa-miR-15a-5p;hsa-miR-15b-5p;hsa-miR-19
ENSG0000	ENSG0000	0.000571	hsa-miR-15a-5p;hsa-miR-15b-5p;hsa-miR-16-5p;hsa-miR-195-
ENSG0000	ENSG0000	5.11E-07	hsa-miR-30b-5p;hsa-miR-30c-5p;hsa-miR-30d-5p;hsa-miR-30e
ENSG0000	ENSG0000	0.005523	hsa-miR-15a-5p;hsa-miR-15b-5p;hsa-miR-16-5p;hsa-miR-195-
ENSG0000	ENSG0000	6.55E-05	hsa-miR-30b-5p;hsa-miR-30c-5p;hsa-miR-30d-5p;hsa-miR-30e
ENSG0000	ENSG0000	3.27E-06	hsa-miR-15a-5p;hsa-miR-15b-5p;hsa-miR-195-5p;hsa-miR-30b
ENSG0000	ENSG0000	4.60E-06	hsa-miR-181a-5p;hsa-miR-30b-5p;hsa-miR-30c-5p;hsa-miR-30
ENSG0000	ENSG0000	3.10E-05	hsa-miR-30b-5p;hsa-miR-30c-5p;hsa-miR-30d-5p;hsa-miR-30e
ENSG0000	ENSG0000	0.000894	hsa-miR-15a-5p;hsa-miR-15b-5p;hsa-miR-195-5p;hsa-miR-424
ENSG0000	ENSG0000	1.25E-09	hsa-miR-107;hsa-miR-15a-5p;hsa-miR-15b-5p;hsa-miR-16-5p;
ENSG0000	ENSG0000	4.40E-05	hsa-miR-1224-3p;hsa-miR-1260a;hsa-miR-1260b;hsa-miR-1913
ENSG0000	ENSG0000	0.005155	hsa-miR-15a-5p;hsa-miR-15b-5p;hsa-miR-195-5p;hsa-miR-424
ENSG0000	ENSG0000	5.54E-07	hsa-miR-103a-3p;hsa-miR-107;hsa-miR-214-3p;hsa-miR-30b-5
ENSG0000	ENSG0000	0.000347	hsa-miR-107;hsa-miR-15a-5p;hsa-miR-15b-5p;hsa-miR-195-5p
ENSG0000	ENSG0000	9.04E-05	hsa-miR-1224-3p;hsa-miR-1260a;hsa-miR-1260b;hsa-miR-188-
ENSG0000	ENSG0000	0.006908	hsa-miR-15a-5p;hsa-miR-15b-5p;hsa-miR-16-5p;hsa-miR-195-
ENSG0000	ENSG0000	1.51E-06	hsa-miR-15a-5p;hsa-miR-15b-5p;hsa-miR-16-5p;hsa-miR-195-
ENSG0000	ENSG0000	0.000592	hsa-miR-1260a;hsa-miR-1260b;hsa-miR-181a-5p;hsa-miR-188-
ENSG0000	ENSG0000	7.86E-11	hsa-miR-15a-5p;hsa-miR-15b-5p;hsa-miR-195-5p;hsa-miR-30b
ENSG0000	ENSG0000	0.000122	hsa-miR-30b-5p;hsa-miR-30c-5p;hsa-miR-30d-5p;hsa-miR-30e
ENSG0000	ENSG0000	0.000122	hsa-miR-30b-5p;hsa-miR-30c-5p;hsa-miR-30d-5p;hsa-miR-30e
ENSG0000	ENSG0000	0.000592	hsa-miR-30b-5p;hsa-miR-30c-5p;hsa-miR-30d-5p;hsa-miR-30e
ENSG0000	ENSG0000	0.000592	hsa-miR-30b-5p;hsa-miR-30c-5p;hsa-miR-30d-5p;hsa-miR-30e
ENSG0000	ENSG0000	0.003749	hsa-miR-103a-3p;hsa-miR-107;hsa-miR-15a-5p;hsa-miR-15b-5

ENSG0000	ENSG0000	1.51E-06	hsa-miR-30b-5p;hsa-miR-30c-5p;hsa-miR-30d-5p;hsa-miR-30
ENSG0000	ENSG0000	0.000627	hsa-miR-107;hsa-miR-15a-5p;hsa-miR-15b-5p;hsa-miR-195-5
ENSG0000	ENSG0000	0.000686	hsa-miR-107;hsa-miR-15a-5p;hsa-miR-15b-5p;hsa-miR-195-5
ENSG0000	ENSG0000	0.004362	hsa-miR-30b-5p;hsa-miR-30c-5p;hsa-miR-30d-5p;hsa-miR-30
ENSG0000	ENSG0000	3.57E-06	hsa-miR-103a-3p;hsa-miR-107;hsa-miR-15a-5p;hsa-miR-15b-
ENSG0000	ENSG0000	1.28E-09	hsa-miR-1224-3p;hsa-miR-15a-5p;hsa-miR-15b-5p;hsa-miR-1
ENSG0000	ENSG0000	1.28E-09	hsa-miR-1224-3p;hsa-miR-15a-5p;hsa-miR-15b-5p;hsa-miR-1
ENSG0000	ENSG0000	1.68E-05	hsa-miR-15a-5p;hsa-miR-15b-5p;hsa-miR-195-5p;hsa-miR-42
ENSG0000	ENSG0000	0.00016	hsa-miR-1224-3p;hsa-miR-1260a;hsa-miR-1260b;hsa-miR-188-
ENSG0000	ENSG0000	5.11E-07	hsa-miR-15a-5p;hsa-miR-15b-5p;hsa-miR-16-5p;hsa-miR-195-
ENSG0000	ENSG0000	0.000686	hsa-miR-1224-3p;hsa-miR-1260a;hsa-miR-1260b;hsa-miR-24-
ENSG0000	ENSG0000	1.04E-07	hsa-miR-15a-5p;hsa-miR-15b-5p;hsa-miR-16-5p;hsa-miR-195-
ENSG0000	ENSG0000	4.88E-10	hsa-miR-103a-3p;hsa-miR-107;hsa-miR-15a-5p;hsa-miR-15b-
ENSG0000	ENSG0000	0.000206	hsa-miR-103a-3p;hsa-miR-107;hsa-miR-197-3p;hsa-miR-877-
ENSG0000	ENSG0000	1.52E-05	hsa-miR-15a-5p;hsa-miR-15b-5p;hsa-miR-16-5p;hsa-miR-195-
ENSG0000	ENSG0000	2.33E-06	hsa-miR-15a-5p;hsa-miR-15b-5p;hsa-miR-16-5p;hsa-miR-195-
ENSG0000	ENSG0000	1.74E-06	hsa-miR-15a-5p;hsa-miR-15b-5p;hsa-miR-16-5p;hsa-miR-195-
ENSG0000	ENSG0000	2.22E-05	hsa-miR-15a-5p;hsa-miR-15b-5p;hsa-miR-16-5p;hsa-miR-195-
ENSG0000	ENSG0000	5.11E-07	hsa-miR-15a-5p;hsa-miR-15b-5p;hsa-miR-16-5p;hsa-miR-195-
ENSG0000	ENSG0000	9.87E-09	hsa-miR-103a-3p;hsa-miR-107;hsa-miR-1225-5p;hsa-miR-15a-
ENSG0000	ENSG0000	7.20E-06	hsa-miR-15a-5p;hsa-miR-15b-5p;hsa-miR-16-5p;hsa-miR-195-
ENSG0000	ENSG0000	1.11E-06	hsa-miR-1207-5p;hsa-miR-1225-5p;hsa-miR-1260a;hsa-miR-1
ENSG0000	ENSG0000	1.26E-06	hsa-miR-15a-5p;hsa-miR-15b-5p;hsa-miR-16-5p;hsa-miR-195-
ENSG0000	ENSG0000	3.19E-07	hsa-miR-15a-5p;hsa-miR-15b-5p;hsa-miR-195-5p;hsa-miR-30
ENSG0000	ENSG0000	2.21E-05	hsa-miR-103a-3p;hsa-miR-107;hsa-miR-30b-5p;hsa-miR-30d-
ENSG0000	ENSG0000	0.000177	hsa-miR-1915-3p;hsa-miR-30b-5p;hsa-miR-30c-5p;hsa-miR-3
ENSG0000	ENSG0000	0.000262	hsa-miR-30b-5p;hsa-miR-30c-5p;hsa-miR-30d-5p;hsa-miR-30
ENSG0000	ENSG0000	0.000636	hsa-miR-15a-5p;hsa-miR-15b-5p;hsa-miR-16-5p;hsa-miR-182
ENSG0000	ENSG0000	0.000636	hsa-miR-15a-5p;hsa-miR-15b-5p;hsa-miR-16-5p;hsa-miR-182
ENSG0000	ENSG0000	0.00015	hsa-miR-24-3p;hsa-miR-30b-5p;hsa-miR-30c-5p;hsa-miR-30d-
ENSG0000	ENSG0000	0.005286	hsa-miR-191-5p;hsa-miR-30b-5p;hsa-miR-30d-5p;hsa-miR-30
ENSG0000	ENSG0000	0.000631	hsa-miR-24-3p;hsa-miR-30b-5p;hsa-miR-30c-5p;hsa-miR-30d-
ENSG0000	ENSG0000	5.11E-07	hsa-miR-30b-5p;hsa-miR-30c-5p;hsa-miR-30d-5p;hsa-miR-30
ENSG0000	ENSG0000	1.74E-06	hsa-miR-15a-5p;hsa-miR-15b-5p;hsa-miR-16-5p;hsa-miR-195-
ENSG0000	ENSG0000	2.10E-07	hsa-miR-15a-5p;hsa-miR-15b-5p;hsa-miR-195-5p;hsa-miR-42
ENSG0000	ENSG0000	0.000312	hsa-miR-15a-5p;hsa-miR-15b-5p;hsa-miR-16-5p;hsa-miR-195-
ENSG0000	ENSG0000	0.006335	hsa-miR-30b-5p;hsa-miR-30c-5p;hsa-miR-30d-5p;hsa-miR-30
ENSG0000	ENSG0000	0.00012	hsa-miR-15a-5p;hsa-miR-15b-5p;hsa-miR-16-5p;hsa-miR-195-
ENSG0000	ENSG0000	9.04E-05	hsa-miR-15a-5p;hsa-miR-15b-5p;hsa-miR-424-5p;hsa-miR-49
ENSG0000	ENSG0000	2.66E-05	hsa-miR-15a-5p;hsa-miR-15b-5p;hsa-miR-16-5p;hsa-miR-195-
ENSG0000	ENSG0000	1.04E-07	hsa-miR-15a-5p;hsa-miR-15b-5p;hsa-miR-16-5p;hsa-miR-195-
ENSG0000	ENSG0000	0.009284	hsa-miR-15a-5p;hsa-miR-15b-5p;hsa-miR-16-5p;hsa-miR-195-
ENSG0000	ENSG0000	0.00063	hsa-miR-15a-5p;hsa-miR-15b-5p;hsa-miR-16-5p;hsa-miR-195-
ENSG0000	ENSG0000	0.000307	hsa-miR-1207-5p;hsa-miR-15a-5p;hsa-miR-15b-5p;hsa-miR-1
ENSG0000	ENSG0000	7.55E-08	hsa-miR-103a-3p;hsa-miR-107;hsa-miR-1224-3p;hsa-miR-126
ENSG0000	ENSG0000	0.000558	hsa-miR-15a-5p;hsa-miR-15b-5p;hsa-miR-195-5p;hsa-miR-42
ENSG0000	ENSG0000	0.001232	hsa-miR-15a-5p;hsa-miR-15b-5p;hsa-miR-16-5p;hsa-miR-195-
ENSG0000	ENSG0000	0.00319	hsa-miR-15a-5p;hsa-miR-15b-5p;hsa-miR-16-5p;hsa-miR-195-
ENSG0000	ENSG0000	1.04E-07	hsa-miR-15a-5p;hsa-miR-15b-5p;hsa-miR-16-5p;hsa-miR-195-
ENSG0000	ENSG0000	0.000138	hsa-miR-15a-5p;hsa-miR-15b-5p;hsa-miR-16-5p;hsa-miR-195-
ENSG0000	ENSG0000	0.001528	hsa-miR-30b-5p;hsa-miR-30c-5p;hsa-miR-30d-5p;hsa-miR-30
ENSG0000	ENSG0000	0.000983	hsa-miR-15a-5p;hsa-miR-15b-5p;hsa-miR-16-5p;hsa-miR-195-
ENSG0000	ENSG0000	0.001563	hsa-miR-15a-5p;hsa-miR-15b-5p;hsa-miR-16-5p;hsa-miR-195-
ENSG0000	ENSG0000	6.73E-08	hsa-miR-103a-3p;hsa-miR-107;hsa-miR-15a-5p;hsa-miR-15b-

ENSG0000	ENSG0000	0.001035	hsa-miR-15a-5p;hsa-miR-15b-5p;hsa-miR-16-5p;hsa-miR-195-
ENSG0000	ENSG0000	0.000327	hsa-miR-30b-5p;hsa-miR-30c-5p;hsa-miR-30d-5p;hsa-miR-30
ENSG0000	ENSG0000	0.000491	hsa-miR-107;hsa-miR-1260a;hsa-miR-1260b;hsa-miR-188-3p
ENSG0000	ENSG0000	0.002254	hsa-miR-107;hsa-miR-15a-5p;hsa-miR-15b-5p;hsa-miR-195-5
ENSG0000	ENSG0000	3.94E-07	hsa-miR-103a-3p;hsa-miR-107;hsa-miR-15a-5p;hsa-miR-15b-
ENSG0000	ENSG0000	3.10E-05	hsa-miR-1224-3p;hsa-miR-1260a;hsa-miR-1260b;hsa-miR-188-
ENSG0000	ENSG0000	0.007675	hsa-miR-15a-5p;hsa-miR-15b-5p;hsa-miR-16-5p;hsa-miR-181
ENSG0000	ENSG0000	6.45E-06	hsa-miR-15a-5p;hsa-miR-15b-5p;hsa-miR-16-5p;hsa-miR-195-
ENSG0000	ENSG0000	0.000138	hsa-miR-1207-5p;hsa-miR-15a-5p;hsa-miR-15b-5p;hsa-miR-1
ENSG0000	ENSG0000	0.000327	hsa-miR-30b-5p;hsa-miR-30c-5p;hsa-miR-30d-5p;hsa-miR-30
ENSG0000	ENSG0000	0.000138	hsa-miR-15a-5p;hsa-miR-15b-5p;hsa-miR-195-5p;hsa-miR-31
ENSG0000	ENSG0000	4.61E-05	hsa-miR-181a-5p;hsa-miR-30b-5p;hsa-miR-30c-5p;hsa-miR-3
ENSG0000	ENSG0000	4.61E-05	hsa-miR-181a-5p;hsa-miR-30b-5p;hsa-miR-30c-5p;hsa-miR-3
ENSG0000	ENSG0000	0.000177	hsa-miR-15a-5p;hsa-miR-15b-5p;hsa-miR-195-5p;hsa-miR-31
ENSG0000	ENSG0000	2.81E-08	hsa-miR-103a-3p;hsa-miR-107;hsa-miR-1224-3p;hsa-miR-122
ENSG0000	ENSG0000	7.00E-07	hsa-miR-15a-5p;hsa-miR-15b-5p;hsa-miR-181a-5p;hsa-miR-1
ENSG0000	ENSG0000	0.000177	hsa-miR-15a-5p;hsa-miR-15b-5p;hsa-miR-195-5p;hsa-miR-42
ENSG0000	ENSG0000	7.85E-06	hsa-miR-15a-5p;hsa-miR-15b-5p;hsa-miR-16-5p;hsa-miR-195-
ENSG0000	ENSG0000	0.001056	hsa-miR-15a-5p;hsa-miR-15b-5p;hsa-miR-16-5p;hsa-miR-195-
ENSG0000	ENSG0000	2.73E-09	hsa-miR-15a-5p;hsa-miR-15b-5p;hsa-miR-195-5p;hsa-miR-30
ENSG0000	ENSG0000	3.66E-05	hsa-miR-1915-3p;hsa-miR-30b-5p;hsa-miR-30c-5p;hsa-miR-3
ENSG0000	ENSG0000	0.000912	hsa-miR-15a-5p;hsa-miR-15b-5p;hsa-miR-16-5p;hsa-miR-195-
ENSG0000	ENSG0000	0.000426	hsa-miR-181a-5p;hsa-miR-30b-5p;hsa-miR-30c-5p;hsa-miR-3
ENSG0000	ENSG0000	0.000283	hsa-miR-1207-5p;hsa-miR-30b-5p;hsa-miR-30c-5p;hsa-miR-3
ENSG0000	ENSG0000	5.74E-05	hsa-miR-15a-5p;hsa-miR-16-5p;hsa-miR-195-5p;hsa-miR-424-
ENSG0000	ENSG0000	0.001328	hsa-miR-30b-5p;hsa-miR-30c-5p;hsa-miR-30d-5p;hsa-miR-30
ENSG0000	ENSG0000	1.26E-05	hsa-miR-30b-5p;hsa-miR-30c-5p;hsa-miR-30d-5p;hsa-miR-30
ENSG0000	ENSG0000	1.77E-08	hsa-miR-103a-3p;hsa-miR-107;hsa-miR-15a-5p;hsa-miR-15b-
ENSG0000	ENSG0000	1.38E-06	hsa-miR-1207-5p;hsa-miR-15a-5p;hsa-miR-15b-5p;hsa-miR-1
ENSG0000	ENSG0000	0.000742	hsa-miR-188-3p;hsa-miR-1913;hsa-miR-197-3p;hsa-miR-30b-
ENSG0000	ENSG0000	1.68E-05	hsa-miR-30b-5p;hsa-miR-30c-5p;hsa-miR-30d-5p;hsa-miR-30
ENSG0000	ENSG0000	2.26E-05	hsa-miR-1246;hsa-miR-15a-5p;hsa-miR-15b-5p;hsa-miR-16-5
ENSG0000	ENSG0000	9.31E-07	hsa-miR-15a-5p;hsa-miR-15b-5p;hsa-miR-16-5p;hsa-miR-195-
ENSG0000	ENSG0000	5.81E-08	hsa-miR-15a-5p;hsa-miR-15b-5p;hsa-miR-16-5p;hsa-miR-182
ENSG0000	ENSG0000	0.000283	hsa-miR-30b-5p;hsa-miR-30c-5p;hsa-miR-30d-5p;hsa-miR-30
ENSG0000	ENSG0000	0.000631	hsa-miR-1224-3p;hsa-miR-1260a;hsa-miR-1260b;hsa-miR-188-
ENSG0000	ENSG0000	5.11E-07	hsa-miR-30b-5p;hsa-miR-30c-5p;hsa-miR-30d-5p;hsa-miR-30
ENSG0000	ENSG0000	5.01E-12	hsa-miR-15a-5p;hsa-miR-16-5p;hsa-miR-195-5p;hsa-miR-214-
ENSG0000	ENSG0000	4.59E-05	hsa-miR-15a-5p;hsa-miR-15b-5p;hsa-miR-16-5p;hsa-miR-195-
ENSG0000	ENSG0000	0.000133	hsa-miR-15a-5p;hsa-miR-15b-5p;hsa-miR-16-5p;hsa-miR-195-
ENSG0000	ENSG0000	1.22E-05	hsa-miR-30b-5p;hsa-miR-30c-5p;hsa-miR-30d-5p;hsa-miR-30
ENSG0000	ENSG0000	2.75E-06	hsa-miR-1260a;hsa-miR-1260b;hsa-miR-15a-5p;hsa-miR-15b-
ENSG0000	ENSG0000	8.09E-06	hsa-miR-15a-5p;hsa-miR-15b-5p;hsa-miR-16-5p;hsa-miR-195-
ENSG0000	ENSG0000	0.00016	hsa-miR-15a-5p;hsa-miR-15b-5p;hsa-miR-188-3p;hsa-miR-19
ENSG0000	ENSG0000	4.79E-09	hsa-miR-15a-5p;hsa-miR-15b-5p;hsa-miR-16-5p;hsa-miR-188-
ENSG0000	ENSG0000	0.00028	hsa-miR-15a-5p;hsa-miR-15b-5p;hsa-miR-16-5p;hsa-miR-195-
ENSG0000	ENSG0000	4.88E-10	hsa-miR-15a-5p;hsa-miR-15b-5p;hsa-miR-16-5p;hsa-miR-195-
ENSG0000	ENSG0000	4.88E-10	hsa-miR-15a-5p;hsa-miR-15b-5p;hsa-miR-16-5p;hsa-miR-195-
ENSG0000	ENSG0000	4.88E-10	hsa-miR-15a-5p;hsa-miR-15b-5p;hsa-miR-16-5p;hsa-miR-195-
ENSG0000	ENSG0000	4.88E-10	hsa-miR-15a-5p;hsa-miR-15b-5p;hsa-miR-16-5p;hsa-miR-195-
ENSG0000	ENSG0000	1.02E-05	hsa-miR-107;hsa-miR-15a-5p;hsa-miR-15b-5p;hsa-miR-1913;h
ENSG0000	ENSG0000	1.02E-05	hsa-miR-107;hsa-miR-15a-5p;hsa-miR-15b-5p;hsa-miR-1913;h
ENSG0000	ENSG0000	1.53E-05	hsa-miR-103a-3p;hsa-miR-107;hsa-miR-15a-5p;hsa-miR-15b-
ENSG0000	ENSG0000	0.00218	hsa-miR-1913;hsa-miR-23a-3p;hsa-miR-23b-3p;hsa-miR-3194-

ENSG0000	ENSG0000	0.000751	hsa-miR-15a-5p;hsa-miR-15b-5p;hsa-miR-16-5p;hsa-miR-195-
ENSG0000	ENSG0000	7.49E-10	hsa-miR-15a-5p;hsa-miR-15b-5p;hsa-miR-195-5p;hsa-miR-21
ENSG0000	ENSG0000	0.000122	hsa-miR-30b-5p;hsa-miR-30c-5p;hsa-miR-30d-5p;hsa-miR-30
ENSG0000	ENSG0000	1.51E-06	hsa-miR-15a-5p;hsa-miR-15b-5p;hsa-miR-16-5p;hsa-miR-195-
ENSG0000	ENSG0000	0.000327	hsa-miR-1224-3p;hsa-miR-1260a;hsa-miR-1260b;hsa-miR-24-
ENSG0000	ENSG0000	0.004158	hsa-miR-30b-5p;hsa-miR-30c-5p;hsa-miR-30d-5p;hsa-miR-30
ENSG0000	ENSG0000	5.11E-07	hsa-miR-30b-5p;hsa-miR-30c-5p;hsa-miR-30d-5p;hsa-miR-30
ENSG0000	ENSG0000	1.04E-07	hsa-miR-15a-5p;hsa-miR-15b-5p;hsa-miR-16-5p;hsa-miR-195-
ENSG0000	ENSG0000	4.40E-05	hsa-miR-15a-5p;hsa-miR-15b-5p;hsa-miR-16-5p;hsa-miR-195-
ENSG0000	ENSG0000	4.40E-05	hsa-miR-15a-5p;hsa-miR-15b-5p;hsa-miR-16-5p;hsa-miR-195-
ENSG0000	ENSG0000	4.40E-05	hsa-miR-15a-5p;hsa-miR-15b-5p;hsa-miR-16-5p;hsa-miR-195-
ENSG0000	ENSG0000	4.40E-05	hsa-miR-15a-5p;hsa-miR-15b-5p;hsa-miR-16-5p;hsa-miR-195-
ENSG0000	ENSG0000	4.40E-05	hsa-miR-15a-5p;hsa-miR-15b-5p;hsa-miR-16-5p;hsa-miR-195-
ENSG0000	ENSG0000	4.40E-05	hsa-miR-15a-5p;hsa-miR-15b-5p;hsa-miR-16-5p;hsa-miR-195-
ENSG0000	ENSG0000	4.40E-05	hsa-miR-15a-5p;hsa-miR-15b-5p;hsa-miR-16-5p;hsa-miR-195-
ENSG0000	ENSG0000	4.40E-05	hsa-miR-15a-5p;hsa-miR-15b-5p;hsa-miR-16-5p;hsa-miR-195-
ENSG0000	ENSG0000	4.40E-05	hsa-miR-15a-5p;hsa-miR-15b-5p;hsa-miR-16-5p;hsa-miR-195-
ENSG0000	ENSG0000	4.40E-05	hsa-miR-15a-5p;hsa-miR-15b-5p;hsa-miR-16-5p;hsa-miR-195-
ENSG0000	ENSG0000	6.86E-06	hsa-miR-15a-5p;hsa-miR-15b-5p;hsa-miR-16-5p;hsa-miR-195-
ENSG0000	ENSG0000	0.007675	hsa-miR-1224-3p;hsa-miR-1260a;hsa-miR-1260b;hsa-miR-24-
ENSG0000	ENSG0000	1.74E-06	hsa-miR-15a-5p;hsa-miR-15b-5p;hsa-miR-16-5p;hsa-miR-195-
ENSG0000	ENSG0000	7.08E-05	hsa-miR-15a-5p;hsa-miR-15b-5p;hsa-miR-16-5p;hsa-miR-424-
ENSG0000	ENSG0000	0.000491	hsa-miR-15a-5p;hsa-miR-15b-5p;hsa-miR-16-5p;hsa-miR-181
ENSG0000	ENSG0000	4.55E-07	hsa-miR-103a-3p;hsa-miR-107;hsa-miR-15a-5p;hsa-miR-15b-
ENSG0000	ENSG0000	3.09E-07	hsa-miR-15a-5p;hsa-miR-15b-5p;hsa-miR-16-5p;hsa-miR-192-
ENSG0000	ENSG0000	2.43E-07	hsa-miR-103a-3p;hsa-miR-107;hsa-miR-15a-5p;hsa-miR-15b-
ENSG0000	ENSG0000	0.000347	hsa-miR-15a-5p;hsa-miR-15b-5p;hsa-miR-16-5p;hsa-miR-195-
ENSG0000	ENSG0000	0.00015	hsa-miR-15a-5p;hsa-miR-15b-5p;hsa-miR-195-5p;hsa-miR-42
ENSG0000	ENSG0000	0.003113	hsa-miR-15a-5p;hsa-miR-15b-5p;hsa-miR-16-5p;hsa-miR-195-
ENSG0000	ENSG0000	0.005908	hsa-miR-24-3p;hsa-miR-30b-5p;hsa-miR-30c-5p;hsa-miR-30d-
ENSG0000	ENSG0000	0.000251	hsa-miR-15a-5p;hsa-miR-15b-5p;hsa-miR-16-5p;hsa-miR-195-
ENSG0000	ENSG0000	0.005795	hsa-miR-30b-5p;hsa-miR-30c-5p;hsa-miR-30d-5p;hsa-miR-30
ENSG0000	ENSG0000	0.000327	hsa-miR-15a-5p;hsa-miR-15b-5p;hsa-miR-16-5p;hsa-miR-195-
ENSG0000	ENSG0000	1.36E-05	hsa-miR-15a-5p;hsa-miR-15b-5p;hsa-miR-16-5p;hsa-miR-195-
ENSG0000	ENSG0000	0.001563	hsa-miR-103a-3p;hsa-miR-107;hsa-miR-1224-3p;hsa-miR-23a-
ENSG0000	ENSG0000	6.61E-07	hsa-miR-15a-5p;hsa-miR-15b-5p;hsa-miR-16-5p;hsa-miR-195-
ENSG0000	ENSG0000	2.00E-05	hsa-miR-30b-5p;hsa-miR-30c-5p;hsa-miR-30d-5p;hsa-miR-30
ENSG0000	ENSG0000	0.003242	hsa-miR-1224-3p;hsa-miR-15a-5p;hsa-miR-15b-5p;hsa-miR-1
ENSG0000	ENSG0000	0.001328	hsa-miR-30b-5p;hsa-miR-30c-5p;hsa-miR-30d-5p;hsa-miR-30
ENSG0000	ENSG0000	3.07E-08	hsa-miR-103a-3p;hsa-miR-107;hsa-miR-15a-5p;hsa-miR-15b-
ENSG0000	ENSG0000	0.000208	hsa-miR-15a-5p;hsa-miR-15b-5p;hsa-miR-195-5p;hsa-miR-42
ENSG0000	ENSG0000	1.29E-07	hsa-miR-15a-5p;hsa-miR-15b-5p;hsa-miR-16-5p;hsa-miR-195-
ENSG0000	ENSG0000	4.60E-06	hsa-miR-15a-5p;hsa-miR-15b-5p;hsa-miR-195-5p;hsa-miR-42
ENSG0000	ENSG0000	0.000327	hsa-miR-301a-5p;hsa-miR-30b-5p;hsa-miR-30c-5p;hsa-miR-3
ENSG0000	ENSG0000	4.26E-10	hsa-miR-103a-3p;hsa-miR-107;hsa-miR-15a-5p;hsa-miR-15b-
ENSG0000	ENSG0000	3.16E-05	hsa-miR-15a-5p;hsa-miR-15b-5p;hsa-miR-16-5p;hsa-miR-195-
ENSG0000	ENSG0000	0.000501	hsa-miR-1913;hsa-miR-30b-5p;hsa-miR-30c-5p;hsa-miR-30d-
ENSG0000	ENSG0000	5.09E-06	hsa-miR-1207-5p;hsa-miR-15a-5p;hsa-miR-15b-5p;hsa-miR-1
ENSG0000	ENSG0000	5.11E-07	hsa-miR-15a-5p;hsa-miR-15b-5p;hsa-miR-16-5p;hsa-miR-195-
ENSG0000	ENSG0000	7.57E-09	hsa-miR-1260a;hsa-miR-1260b;hsa-miR-15a-5p;hsa-miR-15b-
ENSG0000	ENSG0000	0.002087	hsa-miR-15a-5p;hsa-miR-15b-5p;hsa-miR-195-5p;hsa-miR-24-
ENSG0000	ENSG0000	4.67E-06	hsa-miR-15a-5p;hsa-miR-15b-5p;hsa-miR-16-5p;hsa-miR-195-
ENSG0000	ENSG0000	0.00259	hsa-miR-1207-5p;hsa-miR-1913;hsa-miR-1915-3p;hsa-miR-32
ENSG0000	ENSG0000	1.66E-07	hsa-miR-103a-3p;hsa-miR-107;hsa-miR-15a-5p;hsa-miR-15b-

ENSG0000	ENSG0000	9.04E-05	hsa-miR-1224-3p;hsa-miR-1260a;hsa-miR-1260b;hsa-miR-188-
ENSG0000	ENSG0000	0.007514	hsa-miR-1224-3p;hsa-miR-1260a;hsa-miR-1260b;hsa-miR-188-
ENSG0000	ENSG0000	0.000491	hsa-miR-15a-5p;hsa-miR-15b-5p;hsa-miR-195-5p;hsa-miR-19
ENSG0000	ENSG0000	4.85E-11	hsa-miR-103a-3p;hsa-miR-107;hsa-miR-15a-5p;hsa-miR-15b-5
ENSG0000	ENSG0000	1.29E-07	hsa-miR-15a-5p;hsa-miR-15b-5p;hsa-miR-16-5p;hsa-miR-195-
ENSG0000	ENSG0000	5.54E-05	hsa-miR-15a-5p;hsa-miR-15b-5p;hsa-miR-16-5p;hsa-miR-191
ENSG0000	ENSG0000	0.002237	hsa-miR-24-3p;hsa-miR-30b-5p;hsa-miR-30c-5p;hsa-miR-30d-
ENSG0000	ENSG0000	0.000403	hsa-miR-15a-5p;hsa-miR-15b-5p;hsa-miR-16-5p;hsa-miR-195-
ENSG0000	ENSG0000	0.000403	hsa-miR-15a-5p;hsa-miR-15b-5p;hsa-miR-16-5p;hsa-miR-195-
ENSG0000	ENSG0000	6.86E-06	hsa-miR-30b-5p;hsa-miR-30c-5p;hsa-miR-30d-5p;hsa-miR-30
ENSG0000	ENSG0000	6.86E-06	hsa-miR-30b-5p;hsa-miR-30c-5p;hsa-miR-30d-5p;hsa-miR-30
ENSG0000	ENSG0000	0.002311	hsa-miR-15a-5p;hsa-miR-15b-5p;hsa-miR-16-5p;hsa-miR-195-
ENSG0000	ENSG0000	1.01E-05	hsa-miR-15a-5p;hsa-miR-15b-5p;hsa-miR-16-5p;hsa-miR-195-
ENSG0000	ENSG0000	6.59E-06	hsa-miR-15a-5p;hsa-miR-15b-5p;hsa-miR-16-5p;hsa-miR-195-
ENSG0000	ENSG0000	2.48E-08	hsa-miR-15a-5p;hsa-miR-15b-5p;hsa-miR-16-5p;hsa-miR-195-
ENSG0000	ENSG0000	2.24E-06	hsa-miR-103a-3p;hsa-miR-107;hsa-miR-15a-5p;hsa-miR-15b-5
ENSG0000	ENSG0000	0.000122	hsa-miR-30b-5p;hsa-miR-30c-5p;hsa-miR-30d-5p;hsa-miR-30
ENSG0000	ENSG0000	1.29E-07	hsa-miR-15a-5p;hsa-miR-15b-5p;hsa-miR-16-5p;hsa-miR-195-
ENSG0000	ENSG0000	1.02E-05	hsa-miR-103a-3p;hsa-miR-107;hsa-miR-15a-5p;hsa-miR-15b-5
ENSG0000	ENSG0000	0.000251	hsa-miR-15a-5p;hsa-miR-15b-5p;hsa-miR-16-5p;hsa-miR-195-
ENSG0000	ENSG0000	1.52E-05	hsa-miR-15a-5p;hsa-miR-15b-5p;hsa-miR-16-5p;hsa-miR-195-
ENSG0000	ENSG0000	0.00218	hsa-miR-15a-5p;hsa-miR-15b-5p;hsa-miR-16-5p;hsa-miR-182-
ENSG0000	ENSG0000	0.002254	hsa-miR-30b-5p;hsa-miR-30c-5p;hsa-miR-30d-5p;hsa-miR-30
ENSG0000	ENSG0000	1.20E-05	hsa-miR-15a-5p;hsa-miR-15b-5p;hsa-miR-16-5p;hsa-miR-182-
ENSG0000	ENSG0000	0.000217	hsa-miR-1207-5p;hsa-miR-15a-5p;hsa-miR-15b-5p;hsa-miR-1
ENSG0000	ENSG0000	8.05E-05	hsa-miR-1207-5p;hsa-miR-1224-3p;hsa-miR-1260a;hsa-miR-1
ENSG0000	ENSG0000	0.000969	hsa-miR-15a-5p;hsa-miR-15b-5p;hsa-miR-16-5p;hsa-miR-188-
ENSG0000	ENSG0000	0.003874	hsa-miR-15a-5p;hsa-miR-15b-5p;hsa-miR-188-3p;hsa-miR-19
ENSG0000	ENSG0000	2.39E-07	hsa-miR-1224-3p;hsa-miR-1260a;hsa-miR-1260b;hsa-miR-30b-
ENSG0000	ENSG0000	1.04E-07	hsa-miR-15a-5p;hsa-miR-15b-5p;hsa-miR-16-5p;hsa-miR-195-
ENSG0000	ENSG0000	0.001528	hsa-miR-30b-5p;hsa-miR-30c-5p;hsa-miR-30d-5p;hsa-miR-30
ENSG0000	ENSG0000	9.16E-09	hsa-miR-15a-5p;hsa-miR-15b-5p;hsa-miR-16-5p;hsa-miR-181
ENSG0000	ENSG0000	2.97E-05	hsa-miR-103a-3p;hsa-miR-107;hsa-miR-181a-5p;hsa-miR-30b-
ENSG0000	ENSG0000	0.004809	hsa-miR-1915-3p;hsa-miR-23a-3p;hsa-miR-23b-3p;hsa-miR-2
ENSG0000	ENSG0000	0.005155	hsa-miR-15a-5p;hsa-miR-15b-5p;hsa-miR-16-5p;hsa-miR-195-
ENSG0000	ENSG0000	4.68E-06	hsa-miR-103a-3p;hsa-miR-107;hsa-miR-1225-5p;hsa-miR-15a-
ENSG0000	ENSG0000	0.000114	hsa-miR-15a-5p;hsa-miR-15b-5p;hsa-miR-16-5p;hsa-miR-195-
ENSG0000	ENSG0000	7.20E-06	hsa-miR-15a-5p;hsa-miR-15b-5p;hsa-miR-16-5p;hsa-miR-195-
ENSG0000	ENSG0000	6.29E-06	hsa-miR-15a-5p;hsa-miR-15b-5p;hsa-miR-16-5p;hsa-miR-195-
ENSG0000	ENSG0000	0.001656	hsa-miR-1225-5p;hsa-miR-30b-5p;hsa-miR-30c-5p;hsa-miR-3
ENSG0000	ENSG0000	1.60E-05	hsa-miR-130a-3p;hsa-miR-148a-3p;hsa-miR-27a-3p;hsa-miR-
ENSG0000	ENSG0000	3.57E-07	hsa-miR-130a-3p;hsa-miR-148a-3p;hsa-miR-27a-3p;hsa-miR-
ENSG0000	ENSG0000	1.64E-05	hsa-miR-124-3p;hsa-miR-34b-5p;hsa-miR-449c-5p;hsa-miR-9
ENSG0000	ENSG0000	1.97E-07	hsa-miR-124-3p;hsa-miR-34b-5p;hsa-miR-34c-5p;hsa-miR-44
ENSG0000	ENSG0000	0.001791	hsa-miR-124-3p;hsa-miR-34b-5p;hsa-miR-34c-5p;hsa-miR-44
ENSG0000	ENSG0000	6.68E-06	hsa-miR-124-3p;hsa-miR-34b-5p;hsa-miR-34c-5p;hsa-miR-44
ENSG0000	ENSG0000	1.08E-05	hsa-miR-34b-5p;hsa-miR-34c-5p;hsa-miR-449b-5p;hsa-miR-4
ENSG0000	ENSG0000	2.22E-05	hsa-miR-124-3p;hsa-miR-34b-5p;hsa-miR-34c-5p;hsa-miR-44
ENSG0000	ENSG0000	1.08E-05	hsa-miR-124-3p;hsa-miR-34b-5p;hsa-miR-34c-5p;hsa-miR-44
ENSG0000	ENSG0000	0.000477	hsa-miR-34b-5p;hsa-miR-34c-5p;hsa-miR-449b-5p;hsa-miR-4
ENSG0000	ENSG0000	1.13E-06	hsa-miR-34b-5p;hsa-miR-34c-5p;hsa-miR-449b-5p;hsa-miR-4
ENSG0000	ENSG0000	1.24E-06	hsa-miR-34b-5p;hsa-miR-34c-5p;hsa-miR-449b-5p;hsa-miR-4
ENSG0000	ENSG0000	0.000259	hsa-miR-34b-5p;hsa-miR-34c-5p;hsa-miR-449b-5p;hsa-miR-4
ENSG0000	ENSG0000	0.000259	hsa-miR-34b-5p;hsa-miR-34c-5p;hsa-miR-449b-5p;hsa-miR-4

ENSG0000	ENSG0000	0.000259	hsa-miR-34b-5p;hsa-miR-34c-5p;hsa-miR-449b-5p;hsa-miR-44
ENSG0000	ENSG0000	0.000259	hsa-miR-34b-5p;hsa-miR-34c-5p;hsa-miR-449b-5p;hsa-miR-44
ENSG0000	ENSG0000	0.000259	hsa-miR-34b-5p;hsa-miR-34c-5p;hsa-miR-449b-5p;hsa-miR-44
ENSG0000	ENSG0000	0.000259	hsa-miR-34b-5p;hsa-miR-34c-5p;hsa-miR-449b-5p;hsa-miR-44
ENSG0000	ENSG0000	0.000259	hsa-miR-34b-5p;hsa-miR-34c-5p;hsa-miR-449b-5p;hsa-miR-44
ENSG0000	ENSG0000	0.000259	hsa-miR-34b-5p;hsa-miR-34c-5p;hsa-miR-449b-5p;hsa-miR-44
ENSG0000	ENSG0000	0.000213	hsa-miR-34b-5p;hsa-miR-34c-5p;hsa-miR-449b-5p;hsa-miR-44
ENSG0000	ENSG0000	1.49E-06	hsa-miR-34b-5p;hsa-miR-34c-5p;hsa-miR-449b-5p;hsa-miR-44
ENSG0000	ENSG0000	1.67E-05	hsa-miR-34b-5p;hsa-miR-34c-5p;hsa-miR-449b-5p;hsa-miR-44
ENSG0000	ENSG0000	5.21E-06	hsa-miR-34b-5p;hsa-miR-34c-5p;hsa-miR-449b-5p;hsa-miR-44
ENSG0000	ENSG0000	1.64E-06	hsa-miR-34b-5p;hsa-miR-34c-5p;hsa-miR-449b-5p;hsa-miR-44
ENSG0000	ENSG0000	1.77E-05	hsa-miR-34b-5p;hsa-miR-34c-5p;hsa-miR-449b-5p;hsa-miR-44
ENSG0000	ENSG0000	1.24E-06	hsa-miR-34b-5p;hsa-miR-34c-5p;hsa-miR-449b-5p;hsa-miR-44
ENSG0000	ENSG0000	8.09E-05	hsa-miR-34b-5p;hsa-miR-34c-5p;hsa-miR-449b-5p;hsa-miR-44
ENSG0000	ENSG0000	0.000558	hsa-miR-34b-5p;hsa-miR-34c-5p;hsa-miR-449b-5p;hsa-miR-44
ENSG0000	ENSG0000	6.59E-07	hsa-miR-34b-5p;hsa-miR-34c-5p;hsa-miR-449b-5p;hsa-miR-44
ENSG0000	ENSG0000	6.59E-07	hsa-miR-34b-5p;hsa-miR-34c-5p;hsa-miR-449b-5p;hsa-miR-44
ENSG0000	ENSG0000	0.000673	hsa-miR-34b-5p;hsa-miR-34c-5p;hsa-miR-449b-5p;hsa-miR-44
ENSG0000	ENSG0000	0.002516	hsa-miR-34b-5p;hsa-miR-34c-5p;hsa-miR-449b-5p;hsa-miR-44
ENSG0000	ENSG0000	1.13E-06	hsa-miR-34b-5p;hsa-miR-34c-5p;hsa-miR-449b-5p;hsa-miR-44
ENSG0000	ENSG0000	9.86E-06	hsa-miR-34b-5p;hsa-miR-34c-5p;hsa-miR-449b-5p;hsa-miR-44
ENSG0000	ENSG0000	0.000372	hsa-miR-34b-5p;hsa-miR-34c-5p;hsa-miR-449b-5p;hsa-miR-44
ENSG0000	ENSG0000	7.51E-05	hsa-miR-34b-5p;hsa-miR-34c-5p;hsa-miR-449b-5p;hsa-miR-44
ENSG0000	ENSG0000	3.26E-05	hsa-miR-148a-5p;hsa-miR-17-5p;hsa-miR-20b-5p;hsa-miR-26a
ENSG0000	ENSG0000	0.002987	hsa-miR-320a;hsa-miR-320b;hsa-miR-320d;hsa-miR-4429;hsa-
ENSG0000	ENSG0000	0.008521	hsa-miR-106a-5p;hsa-miR-1293;hsa-miR-15a-5p;hsa-miR-15b-
ENSG0000	ENSG0000	0.000945	hsa-let-7a-5p;hsa-let-7b-5p;hsa-let-7e-5p;hsa-let-7f-5p;hsa-le
ENSG0000	ENSG0000	0.003537	hsa-miR-15a-5p;hsa-miR-15b-5p;hsa-miR-16-5p;hsa-miR-195-
ENSG0000	ENSG0000	6.02E-07	hsa-miR-103a-3p;hsa-miR-107;hsa-miR-15a-5p;hsa-miR-15b-5
ENSG0000	ENSG0000	0.001008	hsa-miR-1247-5p;hsa-miR-15a-5p;hsa-miR-15b-5p;hsa-miR-16
ENSG0000	ENSG0000	0.001271	hsa-miR-15a-5p;hsa-miR-15b-5p;hsa-miR-195-5p;hsa-miR-31-
ENSG0000	ENSG0000	0.003102	hsa-miR-1305;hsa-miR-137;hsa-miR-145-5p;hsa-miR-26a-5p;h
ENSG0000	ENSG0000	0.003027	hsa-let-7a-5p;hsa-let-7b-5p;hsa-let-7e-5p;hsa-let-7f-5p;hsa-le
ENSG0000	ENSG0000	0.000232	hsa-let-7a-5p;hsa-let-7b-5p;hsa-let-7e-5p;hsa-let-7f-5p;hsa-le
ENSG0000	ENSG0000	0.002265	hsa-miR-125a-5p;hsa-miR-125b-5p;hsa-miR-138-5p;hsa-miR-1
ENSG0000	ENSG0000	5.85E-07	hsa-miR-103a-3p;hsa-miR-107;hsa-miR-15a-5p;hsa-miR-15b-5
ENSG0000	ENSG0000	0.007276	hsa-let-7a-5p;hsa-miR-101-3p;hsa-miR-106a-5p;hsa-miR-15a-
ENSG0000	ENSG0000	0.00214	hsa-let-7a-5p;hsa-let-7b-5p;hsa-let-7e-5p;hsa-let-7f-5p;hsa-le
ENSG0000	ENSG0000	0.005522	hsa-miR-15a-5p;hsa-miR-15b-5p;hsa-miR-16-5p;hsa-miR-195-
ENSG0000	ENSG0000	0.005522	hsa-miR-15a-5p;hsa-miR-15b-5p;hsa-miR-16-5p;hsa-miR-195-
ENSG0000	ENSG0000	0.002894	hsa-miR-142-3p;hsa-miR-17-5p;hsa-miR-204-5p;hsa-miR-20b-
ENSG0000	ENSG0000	0.002987	hsa-miR-139-3p;hsa-miR-186-5p;hsa-miR-5010-3p;hsa-miR-50
ENSG0000	ENSG0000	0.002987	hsa-miR-139-3p;hsa-miR-186-5p;hsa-miR-5010-3p;hsa-miR-50
ENSG0000	ENSG0000	7.36E-05	hsa-let-7a-5p;hsa-let-7b-5p;hsa-let-7e-5p;hsa-let-7g-5p;hsa-l
ENSG0000	ENSG0000	3.86E-08	hsa-let-7a-5p;hsa-miR-125b-5p;hsa-miR-136-5p;hsa-miR-15a-
ENSG0000	ENSG0000	0.005071	hsa-miR-124-3p;hsa-miR-15a-5p;hsa-miR-22-3p;hsa-miR-96-5
ENSG0000	ENSG0000	0.009899	hsa-miR-129-5p;hsa-miR-204-5p;hsa-miR-26a-5p;hsa-miR-26b
ENSG0000	ENSG0000	0.000218	hsa-let-7a-5p;hsa-let-7b-5p;hsa-let-7f-5p;hsa-miR-101-3p;hsa
ENSG0000	ENSG0000	2.82E-07	hsa-miR-125a-3p;hsa-miR-146a-5p;hsa-miR-15a-5p;hsa-miR-1
ENSG0000	ENSG0000	0.003102	hsa-miR-1299;hsa-miR-15a-5p;hsa-miR-15b-5p;hsa-miR-16-5p
ENSG0000	ENSG0000	0.000159	hsa-miR-1299;hsa-miR-145-5p;hsa-miR-17-3p;hsa-miR-204-5p
ENSG0000	ENSG0000	0.000557	hsa-miR-103a-3p;hsa-miR-195-5p;hsa-miR-204-5p;hsa-miR-22
ENSG0000	ENSG0000	0.003102	hsa-miR-138-5p;hsa-miR-15b-5p;hsa-miR-16-5p;hsa-miR-195-
ENSG0000	ENSG0000	0.000151	hsa-miR-15a-5p;hsa-miR-15b-5p;hsa-miR-16-5p;hsa-miR-26a-

ENSG0000	ENSG0000	0.000541	hsa-let-7a-5p;hsa-let-7b-5p;hsa-let-7e-5p;hsa-let-7f-5p;hsa-le
ENSG0000	ENSG0000	0.005485	hsa-miR-106a-5p;hsa-miR-17-5p;hsa-miR-204-5p;hsa-miR-20b
ENSG0000	ENSG0000	0.001882	hsa-miR-103a-3p;hsa-miR-107;hsa-miR-15a-5p;hsa-miR-15b-5
ENSG0000	ENSG0000	0.00762	hsa-miR-107;hsa-miR-137;hsa-miR-185-5p;hsa-miR-195-5p;hsa
ENSG0000	ENSG0000	1.33E-08	hsa-let-7a-5p;hsa-let-7b-5p;hsa-miR-107;hsa-miR-124-3p;hsa-
ENSG0000	ENSG0000	0.005071	hsa-miR-103a-3p;hsa-miR-107;hsa-miR-199a-5p;hsa-miR-199b
ENSG0000	ENSG0000	0.008563	hsa-let-7a-5p;hsa-let-7b-5p;hsa-let-7e-5p;hsa-let-7f-5p;hsa-le
ENSG0000	ENSG0000	0.001782	hsa-let-7a-5p;hsa-let-7b-5p;hsa-let-7e-5p;hsa-let-7f-5p;hsa-le
ENSG0000	ENSG0000	0.007492	hsa-miR-34a-5p;hsa-miR-34c-5p;hsa-miR-449a;hsa-miR-449b-
ENSG0000	ENSG0000	0.004694	hsa-miR-15a-5p;hsa-miR-15b-5p;hsa-miR-16-5p;hsa-miR-186-
ENSG0000	ENSG0000	7.51E-06	hsa-miR-106a-5p;hsa-miR-126-5p;hsa-miR-1304-3p;hsa-miR-1
ENSG0000	ENSG0000	0.003479	hsa-miR-103a-3p;hsa-miR-125a-3p;hsa-miR-15a-5p;hsa-miR-1
ENSG0000	ENSG0000	0.002791	hsa-let-7a-5p;hsa-let-7b-5p;hsa-let-7e-5p;hsa-let-7f-5p;hsa-le
ENSG0000	ENSG0000	0.008292	hsa-miR-103a-3p;hsa-miR-107;hsa-miR-126-5p;hsa-miR-142-3
ENSG0000	ENSG0000	0.008039	hsa-miR-103a-3p;hsa-miR-107;hsa-miR-125a-5p;hsa-miR-136-
ENSG0000	ENSG0000	0.008602	hsa-miR-124-3p;hsa-miR-2355-5p;hsa-miR-27a-3p;hsa-miR-27
ENSG0000	ENSG0000	7.92E-07	hsa-miR-1224-5p;hsa-miR-129-5p;hsa-miR-146a-3p;hsa-miR-1
ENSG0000	ENSG0000	1.11E-08	hsa-miR-103a-3p;hsa-miR-107;hsa-miR-146a-5p;hsa-miR-146b
ENSG0000	ENSG0000	0.000527	hsa-miR-1293;hsa-miR-15a-5p;hsa-miR-15b-5p;hsa-miR-16-5p
ENSG0000	ENSG0000	3.96E-06	hsa-miR-103a-3p;hsa-miR-107;hsa-miR-15a-5p;hsa-miR-15b-5
ENSG0000	ENSG0000	0.008602	hsa-let-7a-5p;hsa-let-7b-5p;hsa-let-7e-5p;hsa-let-7f-5p;hsa-le
ENSG0000	ENSG0000	0.006937	hsa-let-7a-5p;hsa-let-7b-5p;hsa-let-7e-5p;hsa-let-7f-5p;hsa-le
ENSG0000	ENSG0000	0.000387	hsa-let-7a-5p;hsa-let-7b-5p;hsa-let-7e-5p;hsa-let-7f-5p;hsa-le
ENSG0000	ENSG0000	0.000105	hsa-let-7a-5p;hsa-let-7b-5p;hsa-let-7e-5p;hsa-let-7f-5p;hsa-le
ENSG0000	ENSG0000	0.001096	hsa-miR-125a-5p;hsa-miR-125b-5p;hsa-miR-139-3p;hsa-miR-1
ENSG0000	ENSG0000	4.56E-06	hsa-let-7b-5p;hsa-miR-124-3p;hsa-miR-129-1-3p;hsa-miR-129
ENSG0000	ENSG0000	0.000694	hsa-miR-15a-5p;hsa-miR-15b-5p;hsa-miR-195-5p;hsa-miR-20b
ENSG0000	ENSG0000	2.39E-05	hsa-miR-125b-5p;hsa-miR-132-3p;hsa-miR-137;hsa-miR-145-5
ENSG0000	ENSG0000	0.002287	hsa-miR-106a-5p;hsa-miR-17-5p;hsa-miR-20b-5p;hsa-miR-26a
ENSG0000	ENSG0000	0.000788	hsa-miR-103a-3p;hsa-miR-106a-5p;hsa-miR-107;hsa-miR-1270
ENSG0000	ENSG0000	0.00094	hsa-let-7a-5p;hsa-let-7b-5p;hsa-let-7e-5p;hsa-let-7f-5p;hsa-le
ENSG0000	ENSG0000	0.000172	hsa-miR-129-5p;hsa-miR-142-3p;hsa-miR-145-5p;hsa-miR-192
ENSG0000	ENSG0000	0.000266	hsa-miR-17-5p;hsa-miR-204-5p;hsa-miR-20b-5p;hsa-miR-26a-
ENSG0000	ENSG0000	3.40E-05	hsa-let-7b-5p;hsa-miR-101-3p;hsa-miR-1224-3p;hsa-miR-124-
ENSG0000	ENSG0000	0.000908	hsa-miR-15a-5p;hsa-miR-15b-5p;hsa-miR-16-5p;hsa-miR-195-
ENSG0000	ENSG0000	0.003027	hsa-miR-137;hsa-miR-15a-5p;hsa-miR-15b-5p;hsa-miR-16-5p;
ENSG0000	ENSG0000	0.001256	hsa-miR-15a-5p;hsa-miR-15b-5p;hsa-miR-16-5p;hsa-miR-195-
ENSG0000	ENSG0000	0.002219	hsa-let-7a-5p;hsa-let-7b-5p;hsa-let-7e-5p;hsa-let-7f-5p;hsa-le
ENSG0000	ENSG0000	0.00026	hsa-miR-17-5p;hsa-miR-19a-3p;hsa-miR-19b-3p;hsa-miR-20b-
ENSG0000	ENSG0000	0.000706	hsa-let-7a-5p;hsa-let-7b-5p;hsa-let-7e-5p;hsa-let-7f-5p;hsa-le
ENSG0000	ENSG0000	0.001883	hsa-miR-129-5p;hsa-miR-1304-3p;hsa-miR-140-3p;hsa-miR-16
ENSG0000	ENSG0000	0.000437	hsa-miR-124-3p;hsa-miR-125a-3p;hsa-miR-15a-5p;hsa-miR-19
ENSG0000	ENSG0000	0.008602	hsa-miR-1224-3p;hsa-miR-129-5p;hsa-miR-199a-5p;hsa-miR-1
ENSG0000	ENSG0000	0.001678	hsa-miR-129-5p;hsa-miR-15a-5p;hsa-miR-15b-5p;hsa-miR-195
ENSG0000	ENSG0000	0.000378	hsa-miR-103a-3p;hsa-miR-107;hsa-miR-15a-5p;hsa-miR-15b-5
ENSG0000	ENSG0000	0.008563	hsa-miR-132-3p;hsa-miR-15a-5p;hsa-miR-15b-5p;hsa-miR-195
ENSG0000	ENSG0000	0.003525	hsa-miR-125a-5p;hsa-miR-125b-5p;hsa-miR-142-3p;hsa-miR-1
ENSG0000	ENSG0000	0.000218	hsa-let-7a-5p;hsa-let-7b-5p;hsa-let-7e-5p;hsa-let-7f-5p;hsa-le
ENSG0000	ENSG0000	0.005522	hsa-miR-125a-3p;hsa-miR-125b-5p;hsa-miR-136-5p;hsa-miR-1
ENSG0000	ENSG0000	0.000195	hsa-miR-129-1-3p;hsa-miR-15a-5p;hsa-miR-15b-5p;hsa-miR-1
ENSG0000	ENSG0000	0.00762	hsa-miR-16-5p;hsa-miR-204-5p;hsa-miR-4685-3p;hsa-miR-469
ENSG0000	ENSG0000	0.006478	hsa-miR-106a-5p;hsa-miR-1224-3p;hsa-miR-17-5p;hsa-miR-20
ENSG0000	ENSG0000	1.78E-05	hsa-miR-107;hsa-miR-138-5p;hsa-miR-145-5p;hsa-miR-17-5p;
ENSG0000	ENSG0000	0.005239	hsa-miR-106a-5p;hsa-miR-17-5p;hsa-miR-20b-5p;hsa-miR-27b

ENSG0000	ENSG0000	0.008292	hsa-miR-140-3p;hsa-miR-27a-3p;hsa-miR-27b-3p;hsa-miR-54
ENSG0000	ENSG0000	0.000342	hsa-miR-24-1-5p;hsa-miR-34a-5p;hsa-miR-34b-5p;hsa-miR-3
ENSG0000	ENSG0000	0.000184	hsa-miR-107;hsa-miR-1224-3p;hsa-miR-140-3p;hsa-miR-15a-
ENSG0000	ENSG0000	0.007276	hsa-miR-15a-5p;hsa-miR-15b-5p;hsa-miR-16-5p;hsa-miR-195
ENSG0000	ENSG0000	0.009943	hsa-miR-124-3p;hsa-miR-224-5p;hsa-miR-27a-3p;hsa-miR-27
ENSG0000	ENSG0000	0.000216	hsa-miR-101-3p;hsa-miR-103a-3p;hsa-miR-107;hsa-miR-15a-
ENSG0000	ENSG0000	0.005924	hsa-miR-125b-5p;hsa-miR-138-5p;hsa-miR-214-5p;hsa-miR-2
ENSG0000	ENSG0000	4.49E-08	hsa-let-7f-5p;hsa-miR-106a-5p;hsa-miR-125a-3p;hsa-miR-136
ENSG0000	ENSG0000	0.007424	hsa-let-7a-5p;hsa-let-7b-5p;hsa-let-7e-5p;hsa-let-7f-5p;hsa-l
ENSG0000	ENSG0000	0.000288	hsa-let-7a-5p;hsa-let-7b-5p;hsa-let-7e-5p;hsa-let-7f-5p;hsa-l
ENSG0000	ENSG0000	0.007313	hsa-let-7a-5p;hsa-let-7b-5p;hsa-let-7e-5p;hsa-let-7f-5p;hsa-l
ENSG0000	ENSG0000	0.004669	hsa-miR-106a-5p;hsa-miR-124-3p;hsa-miR-17-5p;hsa-miR-20
ENSG0000	ENSG0000	1.52E-05	hsa-let-7a-5p;hsa-miR-17-3p;hsa-miR-192-5p;hsa-miR-204-5
ENSG0000	ENSG0000	0.000144	hsa-miR-15a-5p;hsa-miR-15b-5p;hsa-miR-195-5p;hsa-miR-19
ENSG0000	ENSG0000	0.006639	hsa-miR-106a-5p;hsa-miR-17-5p;hsa-miR-20b-5p;hsa-miR-30
ENSG0000	ENSG0000	0.001172	hsa-miR-15a-5p;hsa-miR-15b-5p;hsa-miR-16-5p;hsa-miR-195
ENSG0000	ENSG0000	0.005485	hsa-let-7a-5p;hsa-let-7b-5p;hsa-let-7e-5p;hsa-let-7f-5p;hsa-l
ENSG0000	ENSG0000	0.000218	hsa-miR-15a-5p;hsa-miR-15b-5p;hsa-miR-16-5p;hsa-miR-195
ENSG0000	ENSG0000	0.000218	hsa-miR-15a-5p;hsa-miR-15b-5p;hsa-miR-16-5p;hsa-miR-195
ENSG0000	ENSG0000	0.000896	hsa-miR-124-3p;hsa-miR-142-3p;hsa-miR-15a-5p;hsa-miR-15
ENSG0000	ENSG0000	0.002345	hsa-miR-140-3p;hsa-miR-15a-5p;hsa-miR-15b-5p;hsa-miR-19
ENSG0000	ENSG0000	0.004397	hsa-miR-125a-3p;hsa-miR-146a-5p;hsa-miR-196a-3p;hsa-miR
ENSG0000	ENSG0000	0.000612	hsa-miR-101-3p;hsa-miR-103a-3p;hsa-miR-107;hsa-miR-15a-
ENSG0000	ENSG0000	0.008602	hsa-let-7a-5p;hsa-let-7b-5p;hsa-let-7e-5p;hsa-let-7f-5p;hsa-l
ENSG0000	ENSG0000	0.007492	hsa-miR-199a-5p;hsa-miR-29b-1-5p;hsa-miR-424-5p;hsa-miR
ENSG0000	ENSG0000	2.59E-06	hsa-miR-125a-5p;hsa-miR-125b-5p;hsa-miR-146a-5p;hsa-miR
ENSG0000	ENSG0000	0.009585	hsa-miR-124-3p;hsa-miR-27a-3p;hsa-miR-34a-3p;hsa-miR-42
ENSG0000	ENSG0000	0.001008	hsa-miR-15a-5p;hsa-miR-185-5p;hsa-miR-195-5p;hsa-miR-37
ENSG0000	ENSG0000	6.34E-05	hsa-let-7a-5p;hsa-let-7b-5p;hsa-let-7e-5p;hsa-let-7f-5p;hsa-l
ENSG0000	ENSG0000	4.07E-05	hsa-let-7a-5p;hsa-let-7b-5p;hsa-let-7e-5p;hsa-let-7f-5p;hsa-l
ENSG0000	ENSG0000	7.32E-06	hsa-let-7a-5p;hsa-let-7b-5p;hsa-let-7e-5p;hsa-let-7f-5p;hsa-l
ENSG0000	ENSG0000	0.000528	hsa-miR-101-3p;hsa-miR-125a-5p;hsa-miR-125b-5p;hsa-miR-
ENSG0000	ENSG0000	0.007279	hsa-miR-103a-3p;hsa-miR-107;hsa-miR-302a-3p;hsa-miR-30c-
ENSG0000	ENSG0000	0.000523	hsa-miR-27b-3p;hsa-miR-34a-3p;hsa-miR-34a-5p;hsa-miR-34
ENSG0000	ENSG0000	0.00762	hsa-miR-19a-3p;hsa-miR-19b-3p;hsa-miR-29b-1-5p;hsa-miR-
ENSG0000	ENSG0000	0.00758	hsa-let-7b-5p;hsa-miR-106a-5p;hsa-miR-124-3p;hsa-miR-16-
ENSG0000	ENSG0000	0.000465	hsa-miR-125b-5p;hsa-miR-27a-3p;hsa-miR-27b-3p;hsa-miR-9
ENSG0000	ENSG0000	9.49E-05	hsa-miR-145-5p;hsa-miR-199a-5p;hsa-miR-199b-5p;hsa-miR-
ENSG0000	ENSG0000	9.49E-05	hsa-miR-145-5p;hsa-miR-199a-5p;hsa-miR-199b-5p;hsa-miR-
ENSG0000	ENSG0000	0.009459	hsa-miR-106a-5p;hsa-miR-1224-3p;hsa-miR-145-5p;hsa-miR-
ENSG0000	ENSG0000	4.94E-05	hsa-miR-125a-3p;hsa-miR-149-5p;hsa-miR-15a-5p;hsa-miR-1
ENSG0000	ENSG0000	1.54E-07	hsa-miR-106a-5p;hsa-miR-107;hsa-miR-15a-5p;hsa-miR-16-5
ENSG0000	ENSG0000	2.59E-06	hsa-let-7a-5p;hsa-let-7e-5p;hsa-let-7g-5p;hsa-miR-129-2-3p;
ENSG0000	ENSG0000	0.00034	hsa-miR-101-3p;hsa-miR-103a-3p;hsa-miR-124-3p;hsa-miR-1
ENSG0000	ENSG0000	0.006494	hsa-miR-106a-5p;hsa-miR-124-3p;hsa-miR-1305;hsa-miR-17-
ENSG0000	ENSG0000	0.005423	hsa-miR-136-3p;hsa-miR-146a-5p;hsa-miR-146b-5p;hsa-miR-
ENSG0000	ENSG0000	0.008563	hsa-let-7a-5p;hsa-let-7b-5p;hsa-let-7e-5p;hsa-let-7f-5p;hsa-l
ENSG0000	ENSG0000	0.007492	hsa-miR-145-5p;hsa-miR-199a-5p;hsa-miR-199b-5p;hsa-miR-
ENSG0000	ENSG0000	0.007279	hsa-miR-2114-5p;hsa-miR-30b-3p;hsa-miR-30c-1-3p;hsa-miR
ENSG0000	ENSG0000	1.39E-05	hsa-miR-138-5p;hsa-miR-146a-5p;hsa-miR-146b-5p;hsa-miR-
ENSG0000	ENSG0000	0.000437	hsa-miR-106a-5p;hsa-miR-17-5p;hsa-miR-19a-3p;hsa-miR-20
ENSG0000	ENSG0000	0.001008	hsa-miR-101-3p;hsa-miR-146a-5p;hsa-miR-204-5p;hsa-miR-2
ENSG0000	ENSG0000	0.000119	hsa-miR-146a-5p;hsa-miR-15a-5p;hsa-miR-15b-5p;hsa-miR-1
ENSG0000	ENSG0000	0.004916	hsa-let-7a-5p;hsa-let-7b-5p;hsa-let-7e-5p;hsa-let-7f-5p;hsa-l

ENSG0000	ENSG0000	0.008318	hsa-let-7a-5p;hsa-let-7b-5p;hsa-miR-2278;hsa-miR-25-3p;hsa-
ENSG0000	ENSG0000	8.26E-05	hsa-let-7a-5p;hsa-let-7b-5p;hsa-let-7e-5p;hsa-let-7f-5p;hsa-le
ENSG0000	ENSG0000	0.000523	hsa-let-7a-5p;hsa-let-7b-5p;hsa-let-7e-5p;hsa-let-7f-5p;hsa-le
ENSG0000	ENSG0000	0.000523	hsa-let-7a-5p;hsa-let-7b-5p;hsa-let-7e-5p;hsa-let-7f-5p;hsa-le
ENSG0000	ENSG0000	0.002551	hsa-miR-103a-3p;hsa-miR-107;hsa-miR-145-5p;hsa-miR-15a-5
ENSG0000	ENSG0000	0.000378	hsa-let-7a-5p;hsa-let-7b-5p;hsa-let-7e-5p;hsa-let-7f-5p;hsa-le
ENSG0000	ENSG0000	0.000945	hsa-let-7a-5p;hsa-let-7b-5p;hsa-let-7e-5p;hsa-let-7f-5p;hsa-le
ENSG0000	ENSG0000	0.000945	hsa-let-7a-5p;hsa-let-7b-5p;hsa-let-7e-5p;hsa-let-7f-5p;hsa-le
ENSG0000	ENSG0000	0.000945	hsa-let-7a-5p;hsa-let-7b-5p;hsa-let-7e-5p;hsa-let-7f-5p;hsa-le
ENSG0000	ENSG0000	0.000945	hsa-let-7a-5p;hsa-let-7b-5p;hsa-let-7e-5p;hsa-let-7f-5p;hsa-le
ENSG0000	ENSG0000	0.000945	hsa-let-7a-5p;hsa-let-7b-5p;hsa-let-7e-5p;hsa-let-7f-5p;hsa-le
ENSG0000	ENSG0000	0.000945	hsa-let-7a-5p;hsa-let-7b-5p;hsa-let-7e-5p;hsa-let-7f-5p;hsa-le
ENSG0000	ENSG0000	0.000945	hsa-let-7a-5p;hsa-let-7b-5p;hsa-let-7e-5p;hsa-let-7f-5p;hsa-le
ENSG0000	ENSG0000	0.000218	hsa-miR-15a-5p;hsa-miR-15b-5p;hsa-miR-16-5p;hsa-miR-195-
ENSG0000	ENSG0000	0.000218	hsa-miR-15a-5p;hsa-miR-15b-5p;hsa-miR-16-5p;hsa-miR-195-
ENSG0000	ENSG0000	0.003951	hsa-let-7a-5p;hsa-let-7b-5p;hsa-let-7e-5p;hsa-let-7g-5p;hsa-l
ENSG0000	ENSG0000	3.51E-05	hsa-miR-15a-5p;hsa-miR-15b-5p;hsa-miR-16-5p;hsa-miR-195-
ENSG0000	ENSG0000	0.004397	hsa-miR-106a-5p;hsa-miR-125a-3p;hsa-miR-17-5p;hsa-miR-20
ENSG0000	ENSG0000	0.002636	hsa-miR-19a-3p;hsa-miR-19b-3p;hsa-miR-3179;hsa-miR-320a;
ENSG0000	ENSG0000	0.005734	hsa-miR-138-5p;hsa-miR-19a-3p;hsa-miR-19b-3p;hsa-miR-320
ENSG0000	ENSG0000	0.00218	hsa-miR-142-3p;hsa-miR-146a-5p;hsa-miR-146b-5p;hsa-miR-4
ENSG0000	ENSG0000	0.005071	hsa-miR-103a-3p;hsa-miR-107;hsa-miR-2115-5p;hsa-miR-7-5p
ENSG0000	ENSG0000	0.004776	hsa-miR-199a-5p;hsa-miR-199b-5p;hsa-miR-30c-1-3p;hsa-miR
ENSG0000	ENSG0000	0.00094	hsa-miR-106a-5p;hsa-miR-17-5p;hsa-miR-19a-3p;hsa-miR-19b
ENSG0000	ENSG0000	0.001976	hsa-miR-107;hsa-miR-126-5p;hsa-miR-15a-5p;hsa-miR-15b-5p
ENSG0000	ENSG0000	0.002684	hsa-let-7a-5p;hsa-let-7e-5p;hsa-let-7f-5p;hsa-let-7g-5p;hsa-le
ENSG0000	ENSG0000	0.000674	hsa-miR-106a-5p;hsa-miR-15a-5p;hsa-miR-15b-5p;hsa-miR-17
ENSG0000	ENSG0000	0.007847	hsa-let-7a-5p;hsa-let-7b-5p;hsa-let-7e-5p;hsa-let-7f-5p;hsa-le
ENSG0000	ENSG0000	0.003464	hsa-miR-124-3p;hsa-miR-125b-5p;hsa-miR-186-5p;hsa-miR-19
ENSG0000	ENSG0000	0.000165	hsa-miR-15a-5p;hsa-miR-15b-5p;hsa-miR-16-5p;hsa-miR-195-
ENSG0000	ENSG0000	0.006619	hsa-miR-107;hsa-miR-15a-5p;hsa-miR-15b-5p;hsa-miR-17-5p;
ENSG0000	ENSG0000	0.005374	hsa-let-7b-5p;hsa-miR-106a-5p;hsa-miR-125a-3p;hsa-miR-15a
ENSG0000	ENSG0000	0.002545	hsa-let-7a-5p;hsa-let-7b-5p;hsa-let-7e-5p;hsa-let-7f-5p;hsa-le
ENSG0000	ENSG0000	4.21E-05	hsa-miR-101-3p;hsa-miR-1305;hsa-miR-137;hsa-miR-15a-5p;h
ENSG0000	ENSG0000	0.008822	hsa-miR-129-1-3p;hsa-miR-129-2-3p;hsa-miR-1304-3p;hsa-m
ENSG0000	ENSG0000	0.005485	hsa-miR-15a-5p;hsa-miR-15b-5p;hsa-miR-16-5p;hsa-miR-195-
ENSG0000	ENSG0000	1.49E-06	hsa-let-7b-5p;hsa-miR-101-3p;hsa-miR-137;hsa-miR-16-5p;hsa
ENSG0000	ENSG0000	0.000945	hsa-miR-15a-5p;hsa-miR-15b-5p;hsa-miR-16-5p;hsa-miR-195-
ENSG0000	ENSG0000	0.000945	hsa-miR-15a-5p;hsa-miR-15b-5p;hsa-miR-16-5p;hsa-miR-195-
ENSG0000	ENSG0000	0.006937	hsa-let-7a-5p;hsa-let-7b-5p;hsa-let-7e-5p;hsa-let-7f-5p;hsa-le
ENSG0000	ENSG0000	0.000541	hsa-miR-15b-5p;hsa-miR-17-3p;hsa-miR-19a-3p;hsa-miR-3065
ENSG0000	ENSG0000	0.000631	hsa-miR-125a-5p;hsa-miR-125b-5p;hsa-miR-132-3p;hsa-miR-1
ENSG0000	ENSG0000	0.005239	hsa-miR-106a-5p;hsa-miR-132-3p;hsa-miR-17-5p;hsa-miR-192
ENSG0000	ENSG0000	0.002987	hsa-miR-15a-5p;hsa-miR-15b-5p;hsa-miR-16-5p;hsa-miR-195-
ENSG0000	ENSG0000	0.002987	hsa-miR-15a-5p;hsa-miR-15b-5p;hsa-miR-16-5p;hsa-miR-195-
ENSG0000	ENSG0000	0.000836	hsa-miR-138-5p;hsa-miR-139-3p;hsa-miR-145-5p;hsa-miR-29b
ENSG0000	ENSG0000	0.002619	hsa-let-7a-5p;hsa-let-7b-5p;hsa-let-7e-5p;hsa-let-7f-5p;hsa-le
ENSG0000	ENSG0000	0.001019	hsa-miR-15a-5p;hsa-miR-15b-5p;hsa-miR-16-5p;hsa-miR-195-
ENSG0000	ENSG0000	0.004068	hsa-miR-125b-5p;hsa-miR-320a;hsa-miR-320b;hsa-miR-320d;h
ENSG0000	ENSG0000	0.004068	hsa-miR-125b-5p;hsa-miR-320a;hsa-miR-320b;hsa-miR-320d;h
ENSG0000	ENSG0000	0.000154	hsa-let-7a-5p;hsa-let-7b-5p;hsa-let-7e-5p;hsa-let-7f-5p;hsa-le
ENSG0000	ENSG0000	0.008292	hsa-let-7a-5p;hsa-let-7b-5p;hsa-let-7e-5p;hsa-let-7f-5p;hsa-le
ENSG0000	ENSG0000	0.005152	hsa-miR-103a-3p;hsa-miR-107;hsa-miR-15a-5p;hsa-miR-15b-5
ENSG0000	ENSG0000	0.000185	hsa-miR-101-3p;hsa-miR-106a-5p;hsa-miR-1247-5p;hsa-miR-1

ENSG0000	ENSG0000	0.006619	hsa-miR-192-5p;hsa-miR-19a-3p;hsa-miR-19b-3p;hsa-miR-20
ENSG0000	ENSG0000	0.006619	hsa-miR-192-5p;hsa-miR-19a-3p;hsa-miR-19b-3p;hsa-miR-20
ENSG0000	ENSG0000	0.004286	hsa-miR-103a-3p;hsa-miR-107;hsa-miR-15a-5p;hsa-miR-15b-
ENSG0000	ENSG0000	0.008292	hsa-miR-136-5p;hsa-miR-199a-5p;hsa-miR-199b-5p;hsa-miR-
ENSG0000	ENSG0000	4.56E-06	hsa-miR-15a-5p;hsa-miR-15b-5p;hsa-miR-16-5p;hsa-miR-195
ENSG0000	ENSG0000	0.005002	hsa-let-7a-5p;hsa-let-7b-5p;hsa-let-7e-5p;hsa-let-7f-5p;hsa-le
ENSG0000	ENSG0000	0.005002	hsa-let-7a-5p;hsa-let-7b-5p;hsa-let-7e-5p;hsa-let-7f-5p;hsa-le
ENSG0000	ENSG0000	0.005071	hsa-miR-145-5p;hsa-miR-146a-5p;hsa-miR-34a-5p;hsa-miR-34
ENSG0000	ENSG0000	0.002743	hsa-miR-124-3p;hsa-miR-137;hsa-miR-149-5p;hsa-miR-1914-
ENSG0000	ENSG0000	0.005239	hsa-miR-34a-5p;hsa-miR-34b-5p;hsa-miR-34c-5p;hsa-miR-44
ENSG0000	ENSG0000	0.003102	hsa-miR-15a-5p;hsa-miR-15b-5p;hsa-miR-16-5p;hsa-miR-195
ENSG0000	ENSG0000	0.006112	hsa-let-7a-5p;hsa-let-7b-5p;hsa-let-7e-5p;hsa-let-7f-5p;hsa-le
ENSG0000	ENSG0000	0.000549	hsa-let-7a-5p;hsa-let-7b-5p;hsa-let-7e-5p;hsa-let-7f-5p;hsa-le
ENSG0000	ENSG0000	0.001652	hsa-let-7a-5p;hsa-let-7b-5p;hsa-let-7e-5p;hsa-let-7f-5p;hsa-le
ENSG0000	ENSG0000	0.001652	hsa-let-7a-5p;hsa-let-7b-5p;hsa-let-7e-5p;hsa-let-7f-5p;hsa-le
ENSG0000	ENSG0000	6.56E-05	hsa-miR-124-3p;hsa-miR-142-3p;hsa-miR-15a-5p;hsa-miR-15
ENSG0000	ENSG0000	0.000491	hsa-let-7a-5p;hsa-let-7b-5p;hsa-let-7e-5p;hsa-let-7f-5p;hsa-le
ENSG0000	ENSG0000	1.63E-05	hsa-miR-101-3p;hsa-miR-103a-3p;hsa-miR-107;hsa-miR-15a-
ENSG0000	ENSG0000	0.000112	hsa-miR-107;hsa-miR-1305;hsa-miR-15a-5p;hsa-miR-15b-5p;h
ENSG0000	ENSG0000	0.003238	hsa-miR-17-5p;hsa-miR-204-5p;hsa-miR-20b-5p;hsa-miR-93-
ENSG0000	ENSG0000	0.003479	hsa-miR-1299;hsa-miR-146a-5p;hsa-miR-146b-5p;hsa-miR-34
ENSG0000	ENSG0000	0.003479	hsa-miR-1299;hsa-miR-146a-5p;hsa-miR-146b-5p;hsa-miR-34
ENSG0000	ENSG0000	0.003479	hsa-miR-1299;hsa-miR-146a-5p;hsa-miR-146b-5p;hsa-miR-34
ENSG0000	ENSG0000	0.003479	hsa-miR-1299;hsa-miR-146a-5p;hsa-miR-146b-5p;hsa-miR-34
ENSG0000	ENSG0000	0.003479	hsa-miR-1299;hsa-miR-146a-5p;hsa-miR-146b-5p;hsa-miR-34
ENSG0000	ENSG0000	0.003479	hsa-miR-1299;hsa-miR-146a-5p;hsa-miR-146b-5p;hsa-miR-34
ENSG0000	ENSG0000	0.003479	hsa-miR-1299;hsa-miR-146a-5p;hsa-miR-146b-5p;hsa-miR-34
ENSG0000	ENSG0000	0.003479	hsa-miR-1299;hsa-miR-146a-5p;hsa-miR-146b-5p;hsa-miR-34
ENSG0000	ENSG0000	0.009459	hsa-miR-15a-5p;hsa-miR-15b-5p;hsa-miR-195-5p;hsa-miR-36
ENSG0000	ENSG0000	0.002987	hsa-miR-106a-5p;hsa-miR-146a-5p;hsa-miR-15b-5p;hsa-miR-
ENSG0000	ENSG0000	0.009899	hsa-miR-145-5p;hsa-miR-5690;hsa-miR-629-3p;hsa-miR-766-
ENSG0000	ENSG0000	6.43E-09	hsa-let-7a-5p;hsa-let-7b-5p;hsa-let-7e-5p;hsa-let-7f-5p;hsa-le
ENSG0000	ENSG0000	6.71E-06	hsa-miR-146a-5p;hsa-miR-146b-5p;hsa-miR-15a-5p;hsa-miR-
ENSG0000	ENSG0000	0.000465	hsa-miR-125b-5p;hsa-miR-17-5p;hsa-miR-19a-3p;hsa-miR-34
ENSG0000	ENSG0000	0.000465	hsa-miR-125b-5p;hsa-miR-17-5p;hsa-miR-19a-3p;hsa-miR-34
ENSG0000	ENSG0000	0.000465	hsa-miR-125b-5p;hsa-miR-17-5p;hsa-miR-19a-3p;hsa-miR-34
ENSG0000	ENSG0000	0.000465	hsa-miR-125b-5p;hsa-miR-17-5p;hsa-miR-19a-3p;hsa-miR-34
ENSG0000	ENSG0000	0.000465	hsa-miR-125b-5p;hsa-miR-17-5p;hsa-miR-19a-3p;hsa-miR-34
ENSG0000	ENSG0000	0.000465	hsa-miR-125b-5p;hsa-miR-17-5p;hsa-miR-19a-3p;hsa-miR-34
ENSG0000	ENSG0000	0.000465	hsa-miR-125b-5p;hsa-miR-17-5p;hsa-miR-19a-3p;hsa-miR-34
ENSG0000	ENSG0000	0.000465	hsa-miR-125b-5p;hsa-miR-17-5p;hsa-miR-19a-3p;hsa-miR-34
ENSG0000	ENSG0000	0.000357	hsa-miR-320a;hsa-miR-320b;hsa-miR-320d;hsa-miR-34a-5p;hs
ENSG0000	ENSG0000	0.006619	hsa-miR-125a-3p;hsa-miR-15a-5p;hsa-miR-15b-5p;hsa-miR-1
ENSG0000	ENSG0000	0.003027	hsa-miR-106a-5p;hsa-miR-126-5p;hsa-miR-15a-5p;hsa-miR-1
ENSG0000	ENSG0000	0.003323	hsa-miR-129-5p;hsa-miR-15a-3p;hsa-miR-186-5p;hsa-miR-30
ENSG0000	ENSG0000	0.006639	hsa-miR-101-3p;hsa-miR-19a-3p;hsa-miR-19b-3p;hsa-miR-47
ENSG0000	ENSG0000	0.007423	hsa-miR-1305;hsa-miR-15a-5p;hsa-miR-15b-5p;hsa-miR-16-5
ENSG0000	ENSG0000	0.00265	hsa-miR-1224-5p;hsa-miR-138-5p;hsa-miR-140-3p;hsa-miR-1
ENSG0000	ENSG0000	0.002265	hsa-miR-149-5p;hsa-miR-15a-5p;hsa-miR-16-5p;hsa-miR-196
ENSG0000	ENSG0000	0.006112	hsa-miR-1304-3p;hsa-miR-15a-5p;hsa-miR-15b-5p;hsa-miR-1
ENSG0000	ENSG0000	0.003238	hsa-miR-500a-3p;hsa-miR-501-3p;hsa-miR-502-3p;hsa-miR-5
ENSG0000	ENSG0000	0.003238	hsa-miR-500a-3p;hsa-miR-501-3p;hsa-miR-502-3p;hsa-miR-5
ENSG0000	ENSG0000	0.002545	hsa-miR-1224-3p;hsa-miR-1270;hsa-miR-146a-5p;hsa-miR-14
ENSG0000	ENSG0000	0.008563	hsa-miR-199a-5p;hsa-miR-199b-5p;hsa-miR-204-5p;hsa-miR-

ENSG0000	ENSG0000	0.001172	hsa-miR-15a-5p;hsa-miR-15b-5p;hsa-miR-16-5p;hsa-miR-195-
ENSG0000	ENSG0000	0.001008	hsa-let-7a-5p;hsa-let-7b-5p;hsa-let-7e-5p;hsa-let-7f-5p;hsa-le
ENSG0000	ENSG0000	0.001019	hsa-miR-17-5p;hsa-miR-20b-5p;hsa-miR-7-5p;hsa-miR-93-5p
ENSG0000	ENSG0000	6.64E-06	hsa-miR-106a-5p;hsa-miR-107;hsa-miR-125a-3p;hsa-miR-125a
ENSG0000	ENSG0000	0.002133	hsa-miR-129-5p;hsa-miR-1299;hsa-miR-1304-3p;hsa-miR-139-
ENSG0000	ENSG0000	0.006146	hsa-miR-136-5p;hsa-miR-320a;hsa-miR-320b;hsa-miR-320d;hs
ENSG0000	ENSG0000	0.006639	hsa-miR-106a-5p;hsa-miR-126-5p;hsa-miR-17-5p;hsa-miR-20b
ENSG0000	ENSG0000	0.002345	hsa-let-7a-5p;hsa-let-7b-5p;hsa-let-7e-5p;hsa-let-7f-5p;hsa-le
ENSG0000	ENSG0000	0.008602	hsa-miR-106a-5p;hsa-miR-15a-5p;hsa-miR-15b-5p;hsa-miR-17
ENSG0000	ENSG0000	0.005071	hsa-miR-146b-5p;hsa-miR-199a-5p;hsa-miR-199b-5p;hsa-miR
ENSG0000	ENSG0000	0.005071	hsa-miR-146a-3p;hsa-miR-146a-5p;hsa-miR-204-5p;hsa-miR-2
ENSG0000	ENSG0000	0.003152	hsa-let-7a-5p;hsa-let-7b-5p;hsa-let-7e-5p;hsa-let-7f-5p;hsa-le
ENSG0000	ENSG0000	0.00102	hsa-let-7a-5p;hsa-let-7b-5p;hsa-let-7e-5p;hsa-let-7g-5p;hsa-r
ENSG0000	ENSG0000	0.000549	hsa-let-7a-5p;hsa-let-7b-5p;hsa-let-7e-5p;hsa-let-7f-5p;hsa-le
ENSG0000	ENSG0000	3.51E-05	hsa-miR-15a-5p;hsa-miR-15b-5p;hsa-miR-16-5p;hsa-miR-195-
ENSG0000	ENSG0000	1.78E-06	hsa-miR-107;hsa-miR-15a-5p;hsa-miR-15b-5p;hsa-miR-16-5p;
ENSG0000	ENSG0000	4.76E-05	hsa-miR-125b-5p;hsa-miR-1271-3p;hsa-miR-132-3p;hsa-miR-1
ENSG0000	ENSG0000	4.27E-10	hsa-let-7a-5p;hsa-let-7b-5p;hsa-let-7e-5p;hsa-let-7f-5p;hsa-le
ENSG0000	ENSG0000	0.006619	hsa-miR-1293;hsa-miR-146a-3p;hsa-miR-15a-5p;hsa-miR-15b-
ENSG0000	ENSG0000	0.004203	hsa-miR-101-3p;hsa-miR-17-5p;hsa-miR-20b-5p;hsa-miR-568
ENSG0000	ENSG0000	1.13E-09	hsa-let-7a-5p;hsa-let-7b-5p;hsa-let-7e-5p;hsa-let-7f-5p;hsa-le
ENSG0000	ENSG0000	0.001344	hsa-miR-15a-5p;hsa-miR-15b-5p;hsa-miR-16-5p;hsa-miR-195-
ENSG0000	ENSG0000	0.006639	hsa-miR-34a-5p;hsa-miR-34b-5p;hsa-miR-34c-5p;hsa-miR-449
ENSG0000	ENSG0000	0.002987	hsa-miR-15a-5p;hsa-miR-15b-5p;hsa-miR-195-5p;hsa-miR-424
ENSG0000	ENSG0000	4.03E-07	hsa-miR-129-1-3p;hsa-miR-129-2-3p;hsa-miR-15a-5p;hsa-miR
ENSG0000	ENSG0000	0.002636	hsa-miR-1270;hsa-miR-15a-5p;hsa-miR-15b-5p;hsa-miR-16-5p
ENSG0000	ENSG0000	0.004286	hsa-miR-140-3p;hsa-miR-2278;hsa-miR-483-3p;hsa-miR-500a-
ENSG0000	ENSG0000	0.001897	hsa-let-7a-5p;hsa-let-7b-5p;hsa-let-7e-5p;hsa-let-7f-5p;hsa-le
ENSG0000	ENSG0000	0.003027	hsa-miR-103a-3p;hsa-miR-106a-5p;hsa-miR-107;hsa-miR-1224
ENSG0000	ENSG0000	0.00187	hsa-miR-106a-5p;hsa-miR-125a-5p;hsa-miR-125b-5p;hsa-miR-
ENSG0000	ENSG0000	0.003238	hsa-miR-15a-5p;hsa-miR-15b-5p;hsa-miR-16-5p;hsa-miR-195-
ENSG0000	ENSG0000	0.007492	hsa-miR-19a-3p;hsa-miR-19b-3p;hsa-miR-25-3p;hsa-miR-93-5
ENSG0000	ENSG0000	1.96E-05	hsa-miR-17-5p;hsa-miR-20b-5p;hsa-miR-27a-3p;hsa-miR-27b-
ENSG0000	ENSG0000	1.96E-05	hsa-miR-17-5p;hsa-miR-20b-5p;hsa-miR-27a-3p;hsa-miR-27b-
ENSG0000	ENSG0000	1.96E-05	hsa-miR-17-5p;hsa-miR-20b-5p;hsa-miR-27a-3p;hsa-miR-27b-
ENSG0000	ENSG0000	1.96E-05	hsa-miR-17-5p;hsa-miR-20b-5p;hsa-miR-27a-3p;hsa-miR-27b-
ENSG0000	ENSG0000	1.96E-05	hsa-miR-17-5p;hsa-miR-20b-5p;hsa-miR-27a-3p;hsa-miR-27b-
ENSG0000	ENSG0000	1.96E-05	hsa-miR-17-5p;hsa-miR-20b-5p;hsa-miR-27a-3p;hsa-miR-27b-
ENSG0000	ENSG0000	0.000342	hsa-miR-1247-5p;hsa-miR-320a;hsa-miR-320b;hsa-miR-320d;h
ENSG0000	ENSG0000	3.51E-05	hsa-miR-15a-5p;hsa-miR-15b-5p;hsa-miR-16-5p;hsa-miR-195-
ENSG0000	ENSG0000	0.001701	hsa-miR-1226-3p;hsa-miR-146a-5p;hsa-miR-146b-5p;hsa-miR-
ENSG0000	ENSG0000	0.000282	hsa-let-7a-5p;hsa-let-7b-5p;hsa-let-7e-5p;hsa-let-7f-5p;hsa-le
ENSG0000	ENSG0000	0.008563	hsa-miR-101-3p;hsa-miR-15b-5p;hsa-miR-34b-5p;hsa-miR-449
ENSG0000	ENSG0000	0.001935	hsa-let-7a-5p;hsa-let-7e-5p;hsa-let-7f-5p;hsa-let-7g-5p;hsa-le
ENSG0000	ENSG0000	1.39E-05	hsa-miR-17-5p;hsa-miR-19a-3p;hsa-miR-19b-3p;hsa-miR-20b-
ENSG0000	ENSG0000	1.39E-05	hsa-miR-17-5p;hsa-miR-19a-3p;hsa-miR-19b-3p;hsa-miR-20b-
ENSG0000	ENSG0000	0.001701	hsa-let-7a-5p;hsa-let-7b-5p;hsa-let-7e-5p;hsa-let-7f-5p;hsa-le
ENSG0000	ENSG0000	6.56E-05	hsa-miR-15a-5p;hsa-miR-15b-5p;hsa-miR-195-5p;hsa-miR-26a
ENSG0000	ENSG0000	0.000896	hsa-let-7a-5p;hsa-let-7b-5p;hsa-let-7e-5p;hsa-let-7f-5p;hsa-le
ENSG0000	ENSG0000	0.000218	hsa-miR-15a-5p;hsa-miR-15b-5p;hsa-miR-16-5p;hsa-miR-195-
ENSG0000	ENSG0000	0.000487	hsa-miR-186-5p;hsa-miR-320a;hsa-miR-320b;hsa-miR-320d;hs
ENSG0000	ENSG0000	0.003238	hsa-miR-34a-5p;hsa-miR-34c-5p;hsa-miR-449a;hsa-miR-449b-
ENSG0000	ENSG0000	0.001386	hsa-miR-146a-5p;hsa-miR-146b-5p;hsa-miR-19a-3p;hsa-miR-1
ENSG0000	ENSG0000	0.005734	hsa-miR-142-3p;hsa-miR-26b-5p;hsa-miR-27a-3p;hsa-miR-27b

ENSG0000	ENSG0000	0.006045	hsa-miR-103a-3p;hsa-miR-107;hsa-miR-15a-5p;hsa-miR-15b-5
ENSG0000	ENSG0000	0.002303	hsa-miR-103a-3p;hsa-miR-107;hsa-miR-15a-5p;hsa-miR-15b-5
ENSG0000	ENSG0000	0.001256	hsa-miR-103a-3p;hsa-miR-107;hsa-miR-15a-5p;hsa-miR-15b-5
ENSG0000	ENSG0000	0.001914	hsa-miR-15a-5p;hsa-miR-15b-5p;hsa-miR-16-5p;hsa-miR-195-
ENSG0000	ENSG0000	0.001019	hsa-miR-129-5p;hsa-miR-19a-3p;hsa-miR-19b-3p;hsa-miR-50
ENSG0000	ENSG0000	0.001019	hsa-miR-19a-3p;hsa-miR-19b-3p;hsa-miR-34a-5p;hsa-miR-9-5
ENSG0000	ENSG0000	0.002265	hsa-miR-103a-3p;hsa-miR-106a-5p;hsa-miR-107;hsa-miR-15a-
ENSG0000	ENSG0000	0.000523	hsa-miR-124-3p;hsa-miR-15a-5p;hsa-miR-15b-5p;hsa-miR-16-
ENSG0000	ENSG0000	0.006146	hsa-miR-103a-3p;hsa-miR-107;hsa-miR-1304-3p;hsa-miR-224-
ENSG0000	ENSG0000	0.003525	hsa-miR-103a-3p;hsa-miR-107;hsa-miR-129-5p;hsa-miR-3065-
ENSG0000	ENSG0000	0.000834	hsa-let-7a-5p;hsa-let-7b-5p;hsa-let-7e-5p;hsa-let-7f-5p;hsa-le
ENSG0000	ENSG0000	0.004694	hsa-miR-107;hsa-miR-129-5p;hsa-miR-17-5p;hsa-miR-19a-3p;
ENSG0000	ENSG0000	0.008292	hsa-miR-1299;hsa-miR-378a-3p;hsa-miR-378c;hsa-miR-378d;h
ENSG0000	ENSG0000	0.000343	hsa-miR-101-3p;hsa-miR-107;hsa-miR-1224-5p;hsa-miR-15a-5
ENSG0000	ENSG0000	0.007443	hsa-let-7a-5p;hsa-let-7b-5p;hsa-let-7e-5p;hsa-let-7f-5p;hsa-le
ENSG0000	ENSG0000	0.001678	hsa-miR-15a-5p;hsa-miR-15b-5p;hsa-miR-16-5p;hsa-miR-195-
ENSG0000	ENSG0000	2.28E-06	hsa-miR-132-3p;hsa-miR-15a-5p;hsa-miR-15b-5p;hsa-miR-16-
ENSG0000	ENSG0000	0.009895	hsa-miR-106a-5p;hsa-miR-1224-3p;hsa-miR-17-5p;hsa-miR-1
ENSG0000	ENSG0000	4.76E-05	hsa-miR-107;hsa-miR-1247-5p;hsa-miR-15a-5p;hsa-miR-15b-5
ENSG0000	ENSG0000	1.27E-06	hsa-let-7a-5p;hsa-let-7b-5p;hsa-let-7e-5p;hsa-let-7f-5p;hsa-le
ENSG0000	ENSG0000	6.93E-05	hsa-let-7a-5p;hsa-let-7b-5p;hsa-let-7e-5p;hsa-let-7f-5p;hsa-le
ENSG0000	ENSG0000	0.00437	hsa-miR-103a-3p;hsa-miR-107;hsa-miR-204-5p;hsa-miR-30c-1
ENSG0000	ENSG0000	0.00437	hsa-miR-103a-3p;hsa-miR-107;hsa-miR-204-5p;hsa-miR-30c-1
ENSG0000	ENSG0000	5.64E-05	hsa-miR-142-3p;hsa-miR-15a-5p;hsa-miR-15b-5p;hsa-miR-16-
ENSG0000	ENSG0000	0.005423	hsa-miR-1305;hsa-miR-15a-5p;hsa-miR-15b-5p;hsa-miR-195-5
ENSG0000	ENSG0000	0.001897	hsa-miR-137;hsa-miR-140-3p;hsa-miR-15a-5p;hsa-miR-15b-5p
ENSG0000	ENSG0000	1.23E-05	hsa-let-7a-5p;hsa-let-7b-5p;hsa-let-7e-5p;hsa-let-7f-5p;hsa-le
ENSG0000	ENSG0000	0.005071	hsa-miR-15a-5p;hsa-miR-15b-5p;hsa-miR-16-5p;hsa-miR-195-
ENSG0000	ENSG0000	0.000125	hsa-miR-15a-5p;hsa-miR-15b-5p;hsa-miR-16-5p;hsa-miR-195-
ENSG0000	ENSG0000	0.000206	hsa-miR-126-5p;hsa-miR-1271-3p;hsa-miR-137;hsa-miR-15a-5
ENSG0000	ENSG0000	0.000157	hsa-miR-15a-5p;hsa-miR-15b-5p;hsa-miR-16-5p;hsa-miR-195-
ENSG0000	ENSG0000	0.000157	hsa-miR-15a-5p;hsa-miR-15b-5p;hsa-miR-16-5p;hsa-miR-195-
ENSG0000	ENSG0000	0.003522	hsa-miR-106a-5p;hsa-miR-19a-3p;hsa-miR-19b-3p;hsa-miR-2
ENSG0000	ENSG0000	0.000557	hsa-miR-1270;hsa-miR-139-3p;hsa-miR-15a-5p;hsa-miR-15b-5
ENSG0000	ENSG0000	0.002987	hsa-miR-124-3p;hsa-miR-1305;hsa-miR-26a-5p;hsa-miR-26b-5
ENSG0000	ENSG0000	0.006639	hsa-let-7a-5p;hsa-let-7b-5p;hsa-let-7e-5p;hsa-let-7f-5p;hsa-le
ENSG0000	ENSG0000	0.001494	hsa-let-7a-5p;hsa-let-7b-5p;hsa-let-7e-5p;hsa-let-7f-5p;hsa-le
ENSG0000	ENSG0000	6.74E-05	hsa-miR-137;hsa-miR-154-5p;hsa-miR-15a-5p;hsa-miR-15b-5p
ENSG0000	ENSG0000	0.002894	hsa-miR-15a-5p;hsa-miR-15b-5p;hsa-miR-16-5p;hsa-miR-195-
ENSG0000	ENSG0000	0.000357	hsa-miR-15a-5p;hsa-miR-15b-5p;hsa-miR-16-5p;hsa-miR-195-
ENSG0000	ENSG0000	0.008563	hsa-let-7a-5p;hsa-let-7b-5p;hsa-let-7e-5p;hsa-let-7f-5p;hsa-le
ENSG0000	ENSG0000	8.62E-05	hsa-miR-15a-5p;hsa-miR-15b-5p;hsa-miR-16-5p;hsa-miR-17-5
ENSG0000	ENSG0000	0.004068	hsa-miR-125a-5p;hsa-miR-125b-5p;hsa-miR-34b-5p;hsa-miR-
ENSG0000	ENSG0000	0.002413	hsa-miR-1226-3p;hsa-miR-148a-5p;hsa-miR-15a-3p;hsa-miR-
ENSG0000	ENSG0000	0.002987	hsa-miR-145-5p;hsa-miR-15a-5p;hsa-miR-15b-5p;hsa-miR-16-
ENSG0000	ENSG0000	0.008201	hsa-let-7a-5p;hsa-let-7b-5p;hsa-let-7e-5p;hsa-let-7f-5p;hsa-le
ENSG0000	ENSG0000	0.002545	hsa-miR-15a-5p;hsa-miR-15b-5p;hsa-miR-16-5p;hsa-miR-181
ENSG0000	ENSG0000	0.003238	hsa-miR-17-5p;hsa-miR-186-5p;hsa-miR-20b-5p;hsa-miR-93-5
ENSG0000	ENSG0000	4.21E-05	hsa-miR-15a-5p;hsa-miR-15b-5p;hsa-miR-16-5p;hsa-miR-186-
ENSG0000	ENSG0000	0.009102	hsa-miR-106a-5p;hsa-miR-1243;hsa-miR-142-3p;hsa-miR-17-5
ENSG0000	ENSG0000	0.001926	hsa-miR-106a-5p;hsa-miR-1224-3p;hsa-miR-129-5p;hsa-miR-
ENSG0000	ENSG0000	0.002791	hsa-let-7a-5p;hsa-let-7b-5p;hsa-let-7e-5p;hsa-let-7f-5p;hsa-le
ENSG0000	ENSG0000	0.002303	hsa-miR-106a-5p;hsa-miR-1226-3p;hsa-miR-17-5p;hsa-miR-2
ENSG0000	ENSG0000	9.57E-05	hsa-miR-1226-3p;hsa-miR-125a-5p;hsa-miR-125b-5p;hsa-miR-

ENSG0000	ENSG0000	0.004286	hsa-miR-15a-5p;hsa-miR-15b-5p;hsa-miR-16-5p;hsa-miR-195
ENSG0000	ENSG0000	0.004286	hsa-miR-15a-5p;hsa-miR-15b-5p;hsa-miR-16-5p;hsa-miR-195
ENSG0000	ENSG0000	0.002048	hsa-miR-129-5p;hsa-miR-320a;hsa-miR-320b;hsa-miR-320d;hs
ENSG0000	ENSG0000	0.00124	hsa-miR-1270;hsa-miR-15a-5p;hsa-miR-15b-5p;hsa-miR-16-5
ENSG0000	ENSG0000	9.57E-05	hsa-miR-1224-3p;hsa-miR-15a-5p;hsa-miR-15b-5p;hsa-miR-1
ENSG0000	ENSG0000	0.002319	hsa-miR-154-5p;hsa-miR-17-5p;hsa-miR-19a-3p;hsa-miR-19b
ENSG0000	ENSG0000	0.001494	hsa-miR-1224-3p;hsa-miR-15a-5p;hsa-miR-15b-5p;hsa-miR-1
ENSG0000	ENSG0000	0.000159	hsa-let-7a-5p;hsa-let-7b-5p;hsa-let-7e-5p;hsa-let-7f-5p;hsa-l
ENSG0000	ENSG0000	0.000159	hsa-let-7a-5p;hsa-let-7b-5p;hsa-let-7e-5p;hsa-let-7f-5p;hsa-l
ENSG0000	ENSG0000	4.70E-07	hsa-miR-103a-3p;hsa-miR-107;hsa-miR-146a-3p;hsa-miR-15a
ENSG0000	ENSG0000	0.008001	hsa-miR-15a-5p;hsa-miR-15b-5p;hsa-miR-16-5p;hsa-miR-195
ENSG0000	ENSG0000	6.24E-06	hsa-miR-103a-3p;hsa-miR-107;hsa-miR-148a-5p;hsa-miR-15a
ENSG0000	ENSG0000	6.24E-06	hsa-miR-103a-3p;hsa-miR-107;hsa-miR-148a-5p;hsa-miR-15a
ENSG0000	ENSG0000	0.000218	hsa-miR-15a-5p;hsa-miR-16-5p;hsa-miR-320a;hsa-miR-320b;h
ENSG0000	ENSG0000	6.61E-06	hsa-miR-107;hsa-miR-15a-5p;hsa-miR-15b-5p;hsa-miR-185-5
ENSG0000	ENSG0000	0.000487	hsa-miR-320a;hsa-miR-320b;hsa-miR-320d;hsa-miR-4429;hsa-
ENSG0000	ENSG0000	0.008001	hsa-miR-2278;hsa-miR-34a-5p;hsa-miR-34b-5p;hsa-miR-34c-
ENSG0000	ENSG0000	0.000631	hsa-miR-125a-5p;hsa-miR-125b-5p;hsa-miR-2110;hsa-miR-27
ENSG0000	ENSG0000	0.00026	hsa-miR-15a-5p;hsa-miR-15b-5p;hsa-miR-16-5p;hsa-miR-195
ENSG0000	ENSG0000	3.86E-08	hsa-miR-103a-3p;hsa-miR-107;hsa-miR-15a-5p;hsa-miR-15b-
ENSG0000	ENSG0000	0.005239	hsa-let-7b-5p;hsa-miR-129-5p;hsa-miR-1305;hsa-miR-27a-3p;
ENSG0000	ENSG0000	9.81E-08	hsa-miR-107;hsa-miR-1304-3p;hsa-miR-15a-5p;hsa-miR-15b-
ENSG0000	ENSG0000	0.001914	hsa-miR-142-3p;hsa-miR-17-5p;hsa-miR-20b-5p;hsa-miR-93-
ENSG0000	ENSG0000	0.00712	hsa-miR-129-5p;hsa-miR-1299;hsa-miR-1304-3p;hsa-miR-140
ENSG0000	ENSG0000	0.00712	hsa-miR-129-5p;hsa-miR-1299;hsa-miR-1304-3p;hsa-miR-140
ENSG0000	ENSG0000	1.74E-05	hsa-miR-15a-5p;hsa-miR-15b-5p;hsa-miR-16-5p;hsa-miR-195
ENSG0000	ENSG0000	0.000127	hsa-miR-146b-3p;hsa-miR-15a-5p;hsa-miR-15b-5p;hsa-miR-1
ENSG0000	ENSG0000	0.000357	hsa-miR-124-3p;hsa-miR-145-5p;hsa-miR-320a;hsa-miR-320b;
ENSG0000	ENSG0000	0.002928	hsa-miR-1299;hsa-miR-146a-5p;hsa-miR-146b-5p;hsa-miR-15
ENSG0000	ENSG0000	0.001019	hsa-miR-140-3p;hsa-miR-19a-3p;hsa-miR-19b-3p;hsa-miR-87
ENSG0000	ENSG0000	0.00014	hsa-miR-103a-3p;hsa-miR-107;hsa-miR-15a-5p;hsa-miR-15b-
ENSG0000	ENSG0000	0.00915	hsa-let-7a-5p;hsa-let-7b-5p;hsa-let-7e-5p;hsa-let-7f-5p;hsa-l
ENSG0000	ENSG0000	0.003323	hsa-miR-124-3p;hsa-miR-15a-5p;hsa-miR-15b-5p;hsa-miR-16
ENSG0000	ENSG0000	9.86E-06	hsa-let-7a-5p;hsa-let-7b-5p;hsa-let-7e-5p;hsa-let-7f-5p;hsa-l
ENSG0000	ENSG0000	8.51E-06	hsa-miR-15a-5p;hsa-miR-15b-5p;hsa-miR-16-5p;hsa-miR-195
ENSG0000	ENSG0000	1.73E-05	hsa-miR-136-5p;hsa-miR-146b-3p;hsa-miR-15a-5p;hsa-miR-1
ENSG0000	ENSG0000	0.007492	hsa-miR-146a-5p;hsa-miR-146b-5p;hsa-miR-362-3p;hsa-miR-
ENSG0000	ENSG0000	0.007492	hsa-miR-146a-5p;hsa-miR-146b-5p;hsa-miR-362-3p;hsa-miR-
ENSG0000	ENSG0000	0.009102	hsa-let-7a-5p;hsa-let-7b-5p;hsa-let-7f-5p;hsa-let-7g-5p;hsa-l
ENSG0000	ENSG0000	0.008847	hsa-miR-136-3p;hsa-miR-15a-5p;hsa-miR-15b-5p;hsa-miR-16
ENSG0000	ENSG0000	0.001417	hsa-let-7a-5p;hsa-let-7b-5p;hsa-let-7e-5p;hsa-let-7f-5p;hsa-l
ENSG0000	ENSG0000	0.004286	hsa-miR-15a-5p;hsa-miR-15b-5p;hsa-miR-16-5p;hsa-miR-186
ENSG0000	ENSG0000	9.57E-05	hsa-miR-132-3p;hsa-miR-138-5p;hsa-miR-199b-5p;hsa-miR-3
ENSG0000	ENSG0000	0.002898	hsa-miR-106a-5p;hsa-miR-17-5p;hsa-miR-1914-3p;hsa-miR-2
ENSG0000	ENSG0000	0.005239	hsa-miR-138-5p;hsa-miR-15a-5p;hsa-miR-15b-5p;hsa-miR-16
ENSG0000	ENSG0000	0.002791	hsa-miR-106a-5p;hsa-miR-17-5p;hsa-miR-20b-5p;hsa-miR-30
ENSG0000	ENSG0000	0.006937	hsa-miR-103a-3p;hsa-miR-107;hsa-miR-1224-3p;hsa-miR-130
ENSG0000	ENSG0000	0.000557	hsa-miR-107;hsa-miR-124-3p;hsa-miR-15a-5p;hsa-miR-16-5p;
ENSG0000	ENSG0000	0.005366	hsa-let-7a-5p;hsa-let-7b-5p;hsa-let-7e-5p;hsa-let-7f-5p;hsa-l
ENSG0000	ENSG0000	0.00014	hsa-miR-15a-5p;hsa-miR-15b-5p;hsa-miR-16-5p;hsa-miR-195
ENSG0000	ENSG0000	0.007279	hsa-miR-101-3p;hsa-miR-140-3p;hsa-miR-15a-5p;hsa-miR-16
ENSG0000	ENSG0000	0.000206	hsa-miR-15a-5p;hsa-miR-15b-5p;hsa-miR-195-5p;hsa-miR-30
ENSG0000	ENSG0000	0.005912	hsa-miR-129-5p;hsa-miR-142-3p;hsa-miR-15a-5p;hsa-miR-15
ENSG0000	ENSG0000	0.009102	hsa-miR-125a-3p;hsa-miR-15a-5p;hsa-miR-15b-5p;hsa-miR-1

ENSG0000	ENSG0000	0.000557	hsa-miR-15a-5p;hsa-miR-15b-5p;hsa-miR-195-5p;hsa-miR-33
ENSG0000	ENSG0000	0.008318	hsa-miR-101-3p;hsa-miR-106a-5p;hsa-miR-1224-5p;hsa-miR-
ENSG0000	ENSG0000	0.001207	hsa-miR-136-5p;hsa-miR-320a;hsa-miR-320b;hsa-miR-320d;hs
ENSG0000	ENSG0000	0.000231	hsa-miR-15a-5p;hsa-miR-15b-5p;hsa-miR-195-5p;hsa-miR-36
ENSG0000	ENSG0000	0.005038	hsa-miR-145-5p;hsa-miR-196a-3p;hsa-miR-2114-5p;hsa-miR-
ENSG0000	ENSG0000	0.002346	hsa-miR-101-3p;hsa-miR-15a-5p;hsa-miR-15b-5p;hsa-miR-19
ENSG0000	ENSG0000	0.002097	hsa-miR-106a-5p;hsa-miR-125a-3p;hsa-miR-145-5p;hsa-miR-
ENSG0000	ENSG0000	0.000123	hsa-miR-17-5p;hsa-miR-19a-3p;hsa-miR-19b-3p;hsa-miR-20b
ENSG0000	ENSG0000	0.000123	hsa-miR-17-5p;hsa-miR-19a-3p;hsa-miR-19b-3p;hsa-miR-20b
ENSG0000	ENSG0000	3.51E-05	hsa-miR-15a-5p;hsa-miR-15b-5p;hsa-miR-16-5p;hsa-miR-195
ENSG0000	ENSG0000	0.000342	hsa-miR-27a-3p;hsa-miR-27b-3p;hsa-miR-34a-5p;hsa-miR-34
ENSG0000	ENSG0000	0.008292	hsa-miR-15a-5p;hsa-miR-15b-5p;hsa-miR-16-5p;hsa-miR-195
ENSG0000	ENSG0000	0.007279	hsa-miR-142-3p;hsa-miR-15a-5p;hsa-miR-15b-5p;hsa-miR-16
ENSG0000	ENSG0000	0.003525	hsa-miR-106a-5p;hsa-miR-1305;hsa-miR-17-5p;hsa-miR-20b-
ENSG0000	ENSG0000	0.001498	hsa-let-7a-5p;hsa-let-7b-5p;hsa-let-7e-5p;hsa-let-7f-5p;hsa-le
ENSG0000	ENSG0000	0.001498	hsa-let-7a-5p;hsa-let-7b-5p;hsa-let-7e-5p;hsa-let-7f-5p;hsa-le
ENSG0000	ENSG0000	0.001498	hsa-let-7a-5p;hsa-let-7b-5p;hsa-let-7e-5p;hsa-let-7f-5p;hsa-le
ENSG0000	ENSG0000	0.000549	hsa-let-7a-5p;hsa-let-7b-5p;hsa-let-7e-5p;hsa-let-7f-5p;hsa-le
ENSG0000	ENSG0000	0.006112	hsa-miR-106a-5p;hsa-miR-154-5p;hsa-miR-17-5p;hsa-miR-20
ENSG0000	ENSG0000	0.009899	hsa-miR-199a-5p;hsa-miR-199b-5p;hsa-miR-19a-3p;hsa-miR-
ENSG0000	ENSG0000	0.000118	hsa-let-7a-5p;hsa-let-7b-5p;hsa-let-7e-5p;hsa-let-7f-5p;hsa-le
ENSG0000	ENSG0000	0.008563	hsa-miR-106a-5p;hsa-miR-129-5p;hsa-miR-146a-3p;hsa-miR-
ENSG0000	ENSG0000	0.002894	hsa-let-7a-5p;hsa-let-7b-5p;hsa-let-7e-5p;hsa-let-7f-5p;hsa-le
ENSG0000	ENSG0000	0.001172	hsa-miR-1299;hsa-miR-138-5p;hsa-miR-15a-5p;hsa-miR-15b-
ENSG0000	ENSG0000	2.30E-06	hsa-miR-103a-3p;hsa-miR-106a-5p;hsa-miR-107;hsa-miR-130
ENSG0000	ENSG0000	0.000908	hsa-miR-15a-5p;hsa-miR-15b-5p;hsa-miR-195-5p;hsa-miR-22
ENSG0000	ENSG0000	0.004111	hsa-miR-103a-3p;hsa-miR-107;hsa-miR-15a-5p;hsa-miR-15b-
ENSG0000	ENSG0000	6.28E-06	hsa-miR-15a-5p;hsa-miR-15b-5p;hsa-miR-195-5p;hsa-miR-22
ENSG0000	ENSG0000	0.006112	hsa-miR-106a-5p;hsa-miR-129-5p;hsa-miR-17-5p;hsa-miR-20
ENSG0000	ENSG0000	0.009895	hsa-let-7a-5p;hsa-let-7e-5p;hsa-let-7f-5p;hsa-let-7g-5p;hsa-le
ENSG0000	ENSG0000	0.001172	hsa-miR-15a-5p;hsa-miR-15b-5p;hsa-miR-16-5p;hsa-miR-195
ENSG0000	ENSG0000	0.002987	hsa-miR-106a-5p;hsa-miR-17-5p;hsa-miR-20b-5p;hsa-miR-55
ENSG0000	ENSG0000	0.001084	hsa-miR-1270;hsa-miR-196a-3p;hsa-miR-2114-5p;hsa-miR-22
ENSG0000	ENSG0000	0.000342	hsa-miR-15a-5p;hsa-miR-15b-5p;hsa-miR-16-5p;hsa-miR-195
ENSG0000	ENSG0000	0.000218	hsa-miR-15a-5p;hsa-miR-15b-5p;hsa-miR-16-5p;hsa-miR-195
ENSG0000	ENSG0000	2.02E-07	hsa-miR-15a-5p;hsa-miR-15b-5p;hsa-miR-16-5p;hsa-miR-195
ENSG0000	ENSG0000	0.000491	hsa-let-7a-5p;hsa-let-7b-5p;hsa-let-7e-5p;hsa-let-7f-5p;hsa-le
ENSG0000	ENSG0000	0.002345	hsa-let-7a-5p;hsa-let-7b-5p;hsa-let-7e-5p;hsa-let-7f-5p;hsa-le
ENSG0000	ENSG0000	0.002987	hsa-miR-15a-5p;hsa-miR-15b-5p;hsa-miR-16-5p;hsa-miR-195
ENSG0000	ENSG0000	0.003537	hsa-let-7a-5p;hsa-let-7b-5p;hsa-let-7e-5p;hsa-let-7f-5p;hsa-le
ENSG0000	ENSG0000	0.000123	hsa-miR-17-5p;hsa-miR-20b-5p;hsa-miR-22-3p;hsa-miR-27a-
ENSG0000	ENSG0000	0.003537	hsa-miR-129-1-3p;hsa-miR-129-2-3p;hsa-miR-136-5p;hsa-mi
ENSG0000	ENSG0000	0.00488	hsa-miR-107;hsa-miR-1224-3p;hsa-miR-1224-5p;hsa-miR-129
ENSG0000	ENSG0000	0.009043	hsa-miR-106a-5p;hsa-miR-136-5p;hsa-miR-17-5p;hsa-miR-20
ENSG0000	ENSG0000	9.57E-05	hsa-miR-15a-5p;hsa-miR-15b-5p;hsa-miR-16-5p;hsa-miR-195
ENSG0000	ENSG0000	0.002048	hsa-miR-129-5p;hsa-miR-196a-3p;hsa-miR-2278;hsa-miR-29b
ENSG0000	ENSG0000	0.006146	hsa-miR-103a-3p;hsa-miR-107;hsa-miR-15a-5p;hsa-miR-15b-
ENSG0000	ENSG0000	3.68E-05	hsa-miR-15a-5p;hsa-miR-15b-5p;hsa-miR-16-5p;hsa-miR-186
ENSG0000	ENSG0000	0.000127	hsa-miR-132-3p;hsa-miR-199a-5p;hsa-miR-199b-5p;hsa-miR-
ENSG0000	ENSG0000	0.005423	hsa-miR-3194-3p;hsa-miR-34a-3p;hsa-miR-34b-5p;hsa-miR-4
ENSG0000	ENSG0000	0.001165	hsa-let-7a-5p;hsa-let-7b-5p;hsa-let-7e-5p;hsa-let-7f-5p;hsa-le
ENSG0000	ENSG0000	0.002545	hsa-let-7a-5p;hsa-let-7b-5p;hsa-let-7e-5p;hsa-let-7f-5p;hsa-le
ENSG0000	ENSG0000	0.002987	hsa-miR-15a-5p;hsa-miR-15b-5p;hsa-miR-195-5p;hsa-miR-42
ENSG0000	ENSG0000	0.003093	hsa-miR-106a-5p;hsa-miR-142-3p;hsa-miR-17-5p;hsa-miR-19

ENSG0000	ENSG0000	0.004776	hsa-miR-1224-3p;hsa-miR-125a-3p;hsa-miR-136-5p;hsa-miR-1
ENSG0000	ENSG0000	0.000151	hsa-miR-15a-5p;hsa-miR-15b-5p;hsa-miR-16-5p;hsa-miR-195-
ENSG0000	ENSG0000	6.77E-08	hsa-let-7a-5p;hsa-let-7b-5p;hsa-let-7e-5p;hsa-let-7f-5p;hsa-le
ENSG0000	ENSG0000	0.002376	hsa-let-7a-5p;hsa-let-7b-5p;hsa-let-7e-5p;hsa-let-7f-5p;hsa-le
ENSG0000	ENSG0000	0.002376	hsa-let-7a-5p;hsa-let-7b-5p;hsa-let-7e-5p;hsa-let-7f-5p;hsa-le
ENSG0000	ENSG0000	9.57E-05	hsa-let-7a-5p;hsa-let-7b-5p;hsa-let-7e-5p;hsa-let-7f-5p;hsa-le
ENSG0000	ENSG0000	2.67E-06	hsa-miR-15a-5p;hsa-miR-15b-5p;hsa-miR-195-5p;hsa-miR-19a
ENSG0000	ENSG0000	4.96E-06	hsa-miR-15a-5p;hsa-miR-15b-5p;hsa-miR-16-5p;hsa-miR-195-
ENSG0000	ENSG0000	0.000834	hsa-let-7a-5p;hsa-let-7b-5p;hsa-let-7e-5p;hsa-let-7f-5p;hsa-le
ENSG0000	ENSG0000	0.004068	hsa-miR-154-5p;hsa-miR-17-5p;hsa-miR-19a-3p;hsa-miR-19b-
ENSG0000	ENSG0000	0.006478	hsa-miR-129-5p;hsa-miR-15a-5p;hsa-miR-15b-5p;hsa-miR-19
ENSG0000	ENSG0000	2.51E-06	hsa-let-7a-5p;hsa-let-7b-5p;hsa-let-7e-5p;hsa-let-7f-5p;hsa-le
ENSG0000	ENSG0000	0.004091	hsa-let-7a-5p;hsa-let-7b-5p;hsa-let-7e-5p;hsa-let-7f-5p;hsa-le
ENSG0000	ENSG0000	0.004777	hsa-let-7a-5p;hsa-let-7b-5p;hsa-let-7e-5p;hsa-let-7f-5p;hsa-le
ENSG0000	ENSG0000	0.001678	hsa-miR-1304-3p;hsa-miR-1305;hsa-miR-2467-5p;hsa-miR-26a
ENSG0000	ENSG0000	4.70E-05	hsa-miR-106a-5p;hsa-miR-126-5p;hsa-miR-15a-5p;hsa-miR-15
ENSG0000	ENSG0000	0.001494	hsa-miR-107;hsa-miR-15a-5p;hsa-miR-15b-5p;hsa-miR-16-5p;
ENSG0000	ENSG0000	0.002287	hsa-miR-106a-5p;hsa-miR-15a-5p;hsa-miR-15b-5p;hsa-miR-1
ENSG0000	ENSG0000	0.008001	hsa-miR-146a-3p;hsa-miR-1913;hsa-miR-378a-3p;hsa-miR-37
ENSG0000	ENSG0000	0.005684	hsa-let-7a-5p;hsa-let-7b-5p;hsa-let-7e-5p;hsa-let-7f-5p;hsa-le
ENSG0000	ENSG0000	0.009585	hsa-miR-129-5p;hsa-miR-132-3p;hsa-miR-19a-3p;hsa-miR-19b
ENSG0000	ENSG0000	2.70E-05	hsa-let-7a-5p;hsa-let-7b-5p;hsa-let-7e-5p;hsa-let-7f-5p;hsa-le
ENSG0000	ENSG0000	0.001207	hsa-let-7a-5p;hsa-let-7b-5p;hsa-let-7e-5p;hsa-let-7f-5p;hsa-le
ENSG0000	ENSG0000	0.006357	hsa-miR-107;hsa-miR-15a-5p;hsa-miR-15b-5p;hsa-miR-195-5p
ENSG0000	ENSG0000	0.000465	hsa-miR-15a-5p;hsa-miR-15b-5p;hsa-miR-16-5p;hsa-miR-195-
ENSG0000	ENSG0000	0.001008	hsa-miR-124-3p;hsa-miR-1305;hsa-miR-142-3p;hsa-miR-19a-
ENSG0000	ENSG0000	0.005239	hsa-miR-142-3p;hsa-miR-17-5p;hsa-miR-19a-3p;hsa-miR-19b-
ENSG0000	ENSG0000	0.005239	hsa-miR-142-3p;hsa-miR-17-5p;hsa-miR-19a-3p;hsa-miR-19b-
ENSG0000	ENSG0000	0.002894	hsa-let-7a-5p;hsa-let-7b-5p;hsa-let-7e-5p;hsa-let-7f-5p;hsa-le
ENSG0000	ENSG0000	0.000447	hsa-let-7a-5p;hsa-let-7b-5p;hsa-let-7e-5p;hsa-let-7f-5p;hsa-le
ENSG0000	ENSG0000	0.001459	hsa-miR-107;hsa-miR-125a-3p;hsa-miR-15a-5p;hsa-miR-15b-
ENSG0000	ENSG0000	3.97E-05	hsa-let-7a-5p;hsa-let-7b-5p;hsa-let-7e-5p;hsa-let-7f-5p;hsa-le
ENSG0000	ENSG0000	0.005366	hsa-miR-107;hsa-miR-154-5p;hsa-miR-15a-5p;hsa-miR-15b-5p
ENSG0000	ENSG0000	0.005239	hsa-miR-1305;hsa-miR-362-3p;hsa-miR-4511;hsa-miR-545-3p;
ENSG0000	ENSG0000	0.000527	hsa-miR-103a-3p;hsa-miR-107;hsa-miR-15a-5p;hsa-miR-15b-
ENSG0000	ENSG0000	8.24E-08	hsa-miR-1224-3p;hsa-miR-129-5p;hsa-miR-15a-5p;hsa-miR-15
ENSG0000	ENSG0000	8.24E-08	hsa-miR-1224-3p;hsa-miR-129-5p;hsa-miR-15a-5p;hsa-miR-15
ENSG0000	ENSG0000	0.002319	hsa-miR-15a-5p;hsa-miR-15b-5p;hsa-miR-195-5p;hsa-miR-42
ENSG0000	ENSG0000	0.004397	hsa-let-7a-5p;hsa-let-7b-5p;hsa-let-7e-5p;hsa-let-7f-5p;hsa-le
ENSG0000	ENSG0000	0.004397	hsa-let-7a-5p;hsa-let-7b-5p;hsa-let-7e-5p;hsa-let-7f-5p;hsa-le
ENSG0000	ENSG0000	0.000165	hsa-miR-15a-5p;hsa-miR-15b-5p;hsa-miR-16-5p;hsa-miR-195-
ENSG0000	ENSG0000	0.001008	hsa-let-7a-5p;hsa-let-7b-5p;hsa-let-7e-5p;hsa-let-7f-5p;hsa-le
ENSG0000	ENSG0000	3.51E-05	hsa-miR-15a-5p;hsa-miR-15b-5p;hsa-miR-16-5p;hsa-miR-195-
ENSG0000	ENSG0000	3.96E-06	hsa-miR-103a-3p;hsa-miR-107;hsa-miR-15a-5p;hsa-miR-15b-
ENSG0000	ENSG0000	0.000378	hsa-miR-142-3p;hsa-miR-15a-5p;hsa-miR-15b-5p;hsa-miR-16-
ENSG0000	ENSG0000	0.005522	hsa-miR-15a-5p;hsa-miR-15b-5p;hsa-miR-16-5p;hsa-miR-17-
ENSG0000	ENSG0000	0.008292	hsa-let-7a-5p;hsa-let-7b-5p;hsa-let-7e-5p;hsa-let-7f-5p;hsa-le
ENSG0000	ENSG0000	0.006639	hsa-miR-15a-5p;hsa-miR-15b-5p;hsa-miR-16-5p;hsa-miR-195-
ENSG0000	ENSG0000	0.000945	hsa-miR-15a-5p;hsa-miR-15b-5p;hsa-miR-16-5p;hsa-miR-195-
ENSG0000	ENSG0000	0.000165	hsa-miR-15a-5p;hsa-miR-16-5p;hsa-miR-17-5p;hsa-miR-195-
ENSG0000	ENSG0000	0.000165	hsa-miR-15a-5p;hsa-miR-16-5p;hsa-miR-17-5p;hsa-miR-195-
ENSG0000	ENSG0000	0.005734	hsa-miR-106a-5p;hsa-miR-17-5p;hsa-miR-20b-5p;hsa-miR-7-
ENSG0000	ENSG0000	0.005734	hsa-miR-106a-5p;hsa-miR-17-5p;hsa-miR-20b-5p;hsa-miR-7-
ENSG0000	ENSG0000	0.000165	hsa-miR-15a-5p;hsa-miR-15b-5p;hsa-miR-16-5p;hsa-miR-195-

ENSG0000	ENSG0000	0.00034	hsa-miR-103a-3p;hsa-miR-107;hsa-miR-15a-5p;hsa-miR-15b-5
ENSG0000	ENSG0000	0.006146	hsa-let-7e-5p;hsa-let-7f-5p;hsa-let-7g-5p;hsa-let-7i-5p;hsa-m
ENSG0000	ENSG0000	0.001344	hsa-miR-149-5p;hsa-miR-15a-5p;hsa-miR-15b-5p;hsa-miR-16-
ENSG0000	ENSG0000	0.000284	hsa-let-7a-5p;hsa-let-7b-5p;hsa-let-7e-5p;hsa-let-7f-5p;hsa-le
ENSG0000	ENSG0000	0.009899	hsa-miR-129-5p;hsa-miR-137;hsa-miR-17-5p;hsa-miR-20b-5p;
ENSG0000	ENSG0000	0.00218	hsa-miR-16-5p;hsa-miR-17-5p;hsa-miR-19a-3p;hsa-miR-19b-3
ENSG0000	ENSG0000	0.007423	hsa-miR-1224-5p;hsa-miR-1305;hsa-miR-25-3p;hsa-miR-302a-
ENSG0000	ENSG0000	0.001641	hsa-miR-106a-5p;hsa-miR-17-5p;hsa-miR-20b-5p;hsa-miR-302
ENSG0000	ENSG0000	0.003537	hsa-let-7b-5p;hsa-let-7e-5p;hsa-let-7f-5p;hsa-let-7g-5p;hsa-le
ENSG0000	ENSG0000	0.009943	hsa-miR-101-3p;hsa-miR-106a-5p;hsa-miR-15a-5p;hsa-miR-15
ENSG0000	ENSG0000	0.00014	hsa-miR-146a-5p;hsa-miR-146b-5p;hsa-miR-15a-5p;hsa-miR-1
ENSG0000	ENSG0000	0.000834	hsa-let-7a-5p;hsa-let-7b-5p;hsa-let-7e-5p;hsa-let-7f-5p;hsa-le
ENSG0000	ENSG0000	1.39E-05	hsa-miR-15a-5p;hsa-miR-15b-5p;hsa-miR-195-5p;hsa-miR-424
ENSG0000	ENSG0000	0.000949	hsa-miR-106a-5p;hsa-miR-17-5p;hsa-miR-20b-5p;hsa-miR-21
ENSG0000	ENSG0000	0.000949	hsa-miR-106a-5p;hsa-miR-17-5p;hsa-miR-20b-5p;hsa-miR-21
ENSG0000	ENSG0000	0.000949	hsa-miR-106a-5p;hsa-miR-17-5p;hsa-miR-20b-5p;hsa-miR-21
ENSG0000	ENSG0000	0.000949	hsa-miR-106a-5p;hsa-miR-17-5p;hsa-miR-20b-5p;hsa-miR-21
ENSG0000	ENSG0000	0.000949	hsa-miR-106a-5p;hsa-miR-17-5p;hsa-miR-20b-5p;hsa-miR-21
ENSG0000	ENSG0000	0.000949	hsa-miR-106a-5p;hsa-miR-17-5p;hsa-miR-20b-5p;hsa-miR-21
ENSG0000	ENSG0000	0.000949	hsa-miR-106a-5p;hsa-miR-17-5p;hsa-miR-20b-5p;hsa-miR-21
ENSG0000	ENSG0000	0.000632	hsa-miR-106a-5p;hsa-miR-17-5p;hsa-miR-19a-3p;hsa-miR-19b
ENSG0000	ENSG0000	0.009899	hsa-miR-149-5p;hsa-miR-199a-5p;hsa-miR-199b-5p;hsa-miR-3
ENSG0000	ENSG0000	0.001207	hsa-miR-124-3p;hsa-miR-3157-5p;hsa-miR-501-3p;hsa-miR-50
ENSG0000	ENSG0000	0.007847	hsa-miR-15a-5p;hsa-miR-15b-5p;hsa-miR-16-5p;hsa-miR-195-
ENSG0000	ENSG0000	0.000945	hsa-let-7a-5p;hsa-let-7b-5p;hsa-let-7e-5p;hsa-let-7f-5p;hsa-le
ENSG0000	ENSG0000	0.000945	hsa-let-7a-5p;hsa-let-7b-5p;hsa-let-7e-5p;hsa-let-7f-5p;hsa-le
ENSG0000	ENSG0000	0.003537	hsa-miR-15a-5p;hsa-miR-15b-5p;hsa-miR-16-5p;hsa-miR-195-
ENSG0000	ENSG0000	0.000127	hsa-let-7a-5p;hsa-let-7b-5p;hsa-let-7e-5p;hsa-let-7f-5p;hsa-le
ENSG0000	ENSG0000	3.51E-05	hsa-miR-15a-5p;hsa-miR-15b-5p;hsa-miR-16-5p;hsa-miR-195-
ENSG0000	ENSG0000	0.001019	hsa-miR-17-5p;hsa-miR-20b-5p;hsa-miR-541-5p;hsa-miR-93-5
ENSG0000	ENSG0000	0.001096	hsa-miR-139-3p;hsa-miR-15a-5p;hsa-miR-15b-5p;hsa-miR-16-
ENSG0000	ENSG0000	0.006869	hsa-miR-103a-3p;hsa-miR-107;hsa-miR-1224-3p;hsa-miR-15a-
ENSG0000	ENSG0000	1.52E-05	hsa-miR-125a-5p;hsa-miR-125b-5p;hsa-miR-140-3p;hsa-miR-1
ENSG0000	ENSG0000	1.97E-05	hsa-miR-132-3p;hsa-miR-15a-5p;hsa-miR-15b-5p;hsa-miR-16-
ENSG0000	ENSG0000	5.79E-05	hsa-miR-129-1-3p;hsa-miR-129-2-3p;hsa-miR-17-5p;hsa-miR-
ENSG0000	ENSG0000	3.51E-05	hsa-miR-15a-5p;hsa-miR-15b-5p;hsa-miR-16-5p;hsa-miR-195-
ENSG0000	ENSG0000	0.001799	hsa-miR-15a-5p;hsa-miR-15b-5p;hsa-miR-16-5p;hsa-miR-195-
ENSG0000	ENSG0000	0.000387	hsa-miR-15a-5p;hsa-miR-15b-5p;hsa-miR-16-5p;hsa-miR-186-
ENSG0000	ENSG0000	5.43E-05	hsa-miR-106a-5p;hsa-miR-15a-5p;hsa-miR-15b-5p;hsa-miR-16
ENSG0000	ENSG0000	4.49E-05	hsa-let-7a-5p;hsa-let-7b-5p;hsa-let-7e-5p;hsa-let-7f-5p;hsa-le
ENSG0000	ENSG0000	3.03E-05	hsa-miR-103a-3p;hsa-miR-107;hsa-miR-1224-5p;hsa-miR-15a-
ENSG0000	ENSG0000	0.002643	hsa-miR-106a-5p;hsa-miR-142-3p;hsa-miR-17-5p;hsa-miR-192
ENSG0000	ENSG0000	0.002643	hsa-miR-106a-5p;hsa-miR-142-3p;hsa-miR-17-5p;hsa-miR-192
ENSG0000	ENSG0000	0.006639	hsa-miR-106a-5p;hsa-miR-17-5p;hsa-miR-20b-5p;hsa-miR-302
ENSG0000	ENSG0000	7.99E-06	hsa-let-7a-5p;hsa-let-7e-5p;hsa-let-7f-5p;hsa-let-7g-5p;hsa-le
ENSG0000	ENSG0000	0.00172	hsa-miR-106a-5p;hsa-miR-1224-3p;hsa-miR-1224-5p;hsa-miR-
ENSG0000	ENSG0000	0.009943	hsa-let-7a-5p;hsa-let-7b-5p;hsa-let-7e-5p;hsa-let-7f-5p;hsa-le
ENSG0000	ENSG0000	0.005297	hsa-miR-1304-3p;hsa-miR-15a-5p;hsa-miR-15b-5p;hsa-miR-19
ENSG0000	ENSG0000	6.56E-05	hsa-miR-107;hsa-miR-146a-5p;hsa-miR-146b-5p;hsa-miR-15a-
ENSG0000	ENSG0000	0.00085	hsa-miR-106a-5p;hsa-miR-15a-5p;hsa-miR-15b-5p;hsa-miR-16
ENSG0000	ENSG0000	0.002265	hsa-miR-19a-3p;hsa-miR-19b-3p;hsa-miR-28-5p;hsa-miR-34c-
ENSG0000	ENSG0000	0.000165	hsa-let-7b-5p;hsa-miR-107;hsa-miR-125a-5p;hsa-miR-125b-5p
ENSG0000	ENSG0000	0.003525	hsa-miR-15a-5p;hsa-miR-15b-5p;hsa-miR-16-5p;hsa-miR-195-
ENSG0000	ENSG0000	0.006641	hsa-miR-106a-5p;hsa-miR-125a-3p;hsa-miR-1304-3p;hsa-miR-

ENSG0000	ENSG0000	0.007279	hsa-miR-17-5p;hsa-miR-204-5p;hsa-miR-20b-5p;hsa-miR-221-
ENSG0000	ENSG0000	0.005734	hsa-miR-146a-5p;hsa-miR-146b-5p;hsa-miR-19a-3p;hsa-miR-3
ENSG0000	ENSG0000	0.000447	hsa-miR-1293;hsa-miR-15a-5p;hsa-miR-15b-5p;hsa-miR-195-5
ENSG0000	ENSG0000	0.008602	hsa-miR-15a-5p;hsa-miR-15b-5p;hsa-miR-195-5p;hsa-miR-30
ENSG0000	ENSG0000	0.006619	hsa-miR-15a-5p;hsa-miR-15b-5p;hsa-miR-186-5p;hsa-miR-19
ENSG0000	ENSG0000	0.003952	hsa-miR-126-5p;hsa-miR-132-3p;hsa-miR-15a-5p;hsa-miR-15
ENSG0000	ENSG0000	0.000231	hsa-miR-125a-3p;hsa-miR-136-5p;hsa-miR-34a-3p;hsa-miR-3
ENSG0000	ENSG0000	0.000316	hsa-miR-1226-3p;hsa-miR-129-5p;hsa-miR-15a-5p;hsa-miR-1
ENSG0000	ENSG0000	0.000165	hsa-miR-15a-5p;hsa-miR-16-5p;hsa-miR-374b-3p;hsa-miR-56
ENSG0000	ENSG0000	0.000284	hsa-let-7a-5p;hsa-let-7e-5p;hsa-let-7f-5p;hsa-let-7g-5p;hsa-l
ENSG0000	ENSG0000	0.001206	hsa-miR-1224-5p;hsa-miR-1271-3p;hsa-miR-1299;hsa-miR-13
ENSG0000	ENSG0000	0.008318	hsa-miR-145-5p;hsa-miR-146b-3p;hsa-miR-25-3p;hsa-miR-26
ENSG0000	ENSG0000	0.005239	hsa-miR-34a-5p;hsa-miR-34b-5p;hsa-miR-34c-5p;hsa-miR-44
ENSG0000	ENSG0000	0.004397	hsa-miR-126-5p;hsa-miR-15a-3p;hsa-miR-224-5p;hsa-miR-27
ENSG0000	ENSG0000	0.003238	hsa-miR-34a-5p;hsa-miR-34c-5p;hsa-miR-449a;hsa-miR-449b-
ENSG0000	ENSG0000	0.000631	hsa-let-7a-5p;hsa-let-7b-5p;hsa-let-7e-5p;hsa-let-7f-5p;hsa-l
ENSG0000	ENSG0000	0.005423	hsa-miR-136-5p;hsa-miR-17-5p;hsa-miR-199a-5p;hsa-miR-19
ENSG0000	ENSG0000	0.004286	hsa-let-7a-5p;hsa-let-7b-5p;hsa-let-7e-5p;hsa-let-7f-5p;hsa-l
ENSG0000	ENSG0000	0.008292	hsa-miR-15a-5p;hsa-miR-16-5p;hsa-miR-195-5p;hsa-miR-374
ENSG0000	ENSG0000	0.002146	hsa-let-7a-5p;hsa-let-7b-5p;hsa-let-7e-5p;hsa-let-7f-5p;hsa-l
ENSG0000	ENSG0000	0.000159	hsa-miR-103a-3p;hsa-miR-107;hsa-miR-15a-5p;hsa-miR-15b-5
ENSG0000	ENSG0000	0.003614	hsa-let-7a-5p;hsa-let-7b-5p;hsa-let-7e-5p;hsa-let-7f-5p;hsa-l
ENSG0000	ENSG0000	0.000266	hsa-miR-145-5p;hsa-miR-17-3p;hsa-miR-17-5p;hsa-miR-199a-
ENSG0000	ENSG0000	0.000266	hsa-miR-145-5p;hsa-miR-17-3p;hsa-miR-17-5p;hsa-miR-199a-
ENSG0000	ENSG0000	0.00014	hsa-miR-17-5p;hsa-miR-20b-5p;hsa-miR-214-5p;hsa-miR-27a-
ENSG0000	ENSG0000	0.007443	hsa-miR-106a-5p;hsa-miR-129-5p;hsa-miR-17-5p;hsa-miR-20
ENSG0000	ENSG0000	0.008001	hsa-let-7a-5p;hsa-let-7b-5p;hsa-let-7e-5p;hsa-let-7f-5p;hsa-l
ENSG0000	ENSG0000	0.007492	hsa-miR-15a-5p;hsa-miR-16-5p;hsa-miR-339-5p;hsa-miR-361-
ENSG0000	ENSG0000	0.000184	hsa-let-7a-5p;hsa-let-7b-5p;hsa-let-7e-5p;hsa-let-7f-5p;hsa-l
ENSG0000	ENSG0000	0.002643	hsa-let-7b-5p;hsa-let-7e-5p;hsa-let-7f-5p;hsa-let-7g-5p;hsa-l
ENSG0000	ENSG0000	0.005485	hsa-miR-106a-5p;hsa-miR-17-5p;hsa-miR-20b-5p;hsa-miR-27
ENSG0000	ENSG0000	8.18E-10	hsa-miR-106a-5p;hsa-miR-124-3p;hsa-miR-1305;hsa-miR-146
ENSG0000	ENSG0000	6.34E-05	hsa-miR-15a-5p;hsa-miR-15b-5p;hsa-miR-16-5p;hsa-miR-195-
ENSG0000	ENSG0000	0.000961	hsa-miR-106a-5p;hsa-miR-124-3p;hsa-miR-137;hsa-miR-140-3
ENSG0000	ENSG0000	0.003537	hsa-miR-1304-3p;hsa-miR-2115-5p;hsa-miR-34a-5p;hsa-miR-3
ENSG0000	ENSG0000	0.000161	hsa-let-7a-5p;hsa-let-7b-5p;hsa-let-7e-5p;hsa-let-7f-5p;hsa-l
ENSG0000	ENSG0000	0.005522	hsa-miR-106a-5p;hsa-miR-17-5p;hsa-miR-1913;hsa-miR-193b-
ENSG0000	ENSG0000	0.004068	hsa-let-7a-5p;hsa-let-7b-5p;hsa-let-7e-5p;hsa-let-7f-5p;hsa-l
ENSG0000	ENSG0000	0.000316	hsa-let-7a-5p;hsa-let-7b-5p;hsa-let-7e-5p;hsa-let-7f-5p;hsa-l
ENSG0000	ENSG0000	0.004232	hsa-let-7a-5p;hsa-let-7b-5p;hsa-let-7e-5p;hsa-let-7f-5p;hsa-l
ENSG0000	ENSG0000	0.000231	hsa-miR-15a-5p;hsa-miR-16-5p;hsa-miR-195-5p;hsa-miR-361
ENSG0000	ENSG0000	0.007847	hsa-miR-106a-5p;hsa-miR-146a-3p;hsa-miR-17-5p;hsa-miR-2
ENSG0000	ENSG0000	0.001344	hsa-miR-15a-5p;hsa-miR-15b-5p;hsa-miR-16-5p;hsa-miR-195-
ENSG0000	ENSG0000	0.000266	hsa-let-7a-5p;hsa-let-7b-5p;hsa-let-7e-5p;hsa-let-7f-5p;hsa-l
ENSG0000	ENSG0000	0.002776	hsa-let-7a-5p;hsa-let-7b-5p;hsa-let-7e-5p;hsa-let-7f-5p;hsa-l
ENSG0000	ENSG0000	0.000802	hsa-miR-125a-3p;hsa-miR-140-3p;hsa-miR-1914-3p;hsa-miR-3
ENSG0000	ENSG0000	0.000802	hsa-miR-125a-3p;hsa-miR-140-3p;hsa-miR-1914-3p;hsa-miR-3
ENSG0000	ENSG0000	0.003238	hsa-miR-136-5p;hsa-miR-382-3p;hsa-miR-485-5p;hsa-miR-54
ENSG0000	ENSG0000	0.000159	hsa-miR-15a-5p;hsa-miR-15b-5p;hsa-miR-16-5p;hsa-miR-195-
ENSG0000	ENSG0000	0.000836	hsa-miR-199a-5p;hsa-miR-199b-5p;hsa-miR-2355-5p;hsa-miR-
ENSG0000	ENSG0000	0.000834	hsa-miR-15a-5p;hsa-miR-15b-5p;hsa-miR-16-5p;hsa-miR-195-
ENSG0000	ENSG0000	0.000706	hsa-miR-106a-5p;hsa-miR-126-5p;hsa-miR-148a-5p;hsa-miR-1
ENSG0000	ENSG0000	0.006112	hsa-miR-142-3p;hsa-miR-17-5p;hsa-miR-19a-3p;hsa-miR-19b-
ENSG0000	ENSG0000	4.07E-05	hsa-miR-125a-3p;hsa-miR-15a-5p;hsa-miR-15b-5p;hsa-miR-1

ENSG0000	ENSG0000	4.07E-05	hsa-miR-125a-3p;hsa-miR-15a-5p;hsa-miR-15b-5p;hsa-miR-16
ENSG0000	ENSG0000	4.07E-05	hsa-miR-125a-3p;hsa-miR-15a-5p;hsa-miR-15b-5p;hsa-miR-16
ENSG0000	ENSG0000	4.07E-05	hsa-miR-125a-3p;hsa-miR-15a-5p;hsa-miR-15b-5p;hsa-miR-16
ENSG0000	ENSG0000	0.002743	hsa-miR-106a-5p;hsa-miR-107;hsa-miR-1271-3p;hsa-miR-15a-
ENSG0000	ENSG0000	0.002743	hsa-miR-106a-5p;hsa-miR-107;hsa-miR-1271-3p;hsa-miR-15a-
ENSG0000	ENSG0000	0.001574	hsa-miR-129-5p;hsa-miR-1305;hsa-miR-186-5p;hsa-miR-196a-
ENSG0000	ENSG0000	0.001574	hsa-miR-129-5p;hsa-miR-1305;hsa-miR-186-5p;hsa-miR-196a-
ENSG0000	ENSG0000	0.000162	hsa-miR-103a-3p;hsa-miR-107;hsa-miR-139-3p;hsa-miR-15a-5
ENSG0000	ENSG0000	0.000342	hsa-let-7a-5p;hsa-let-7b-5p;hsa-let-7e-5p;hsa-let-7f-5p;hsa-le
ENSG0000	ENSG0000	0.000125	hsa-miR-124-3p;hsa-miR-17-5p;hsa-miR-20b-5p;hsa-miR-224-
ENSG0000	ENSG0000	0.001976	hsa-miR-106a-5p;hsa-miR-15a-5p;hsa-miR-15b-5p;hsa-miR-16
ENSG0000	ENSG0000	1.74E-05	hsa-miR-15a-5p;hsa-miR-15b-5p;hsa-miR-195-5p;hsa-miR-36
ENSG0000	ENSG0000	2.02E-07	hsa-miR-15a-5p;hsa-miR-15b-5p;hsa-miR-16-5p;hsa-miR-195-
ENSG0000	ENSG0000	3.96E-06	hsa-miR-22-3p;hsa-miR-34a-5p;hsa-miR-34b-5p;hsa-miR-34c-
ENSG0000	ENSG0000	3.96E-06	hsa-miR-22-3p;hsa-miR-34a-5p;hsa-miR-34b-5p;hsa-miR-34c-
ENSG0000	ENSG0000	0.009899	hsa-miR-1224-3p;hsa-miR-19a-3p;hsa-miR-19b-3p;hsa-miR-2
ENSG0000	ENSG0000	0.008292	hsa-miR-140-3p;hsa-miR-320a;hsa-miR-320b;hsa-miR-320d;hs
ENSG0000	ENSG0000	0.004669	hsa-miR-1914-3p;hsa-miR-19a-3p;hsa-miR-19b-3p;hsa-miR-3
ENSG0000	ENSG0000	0.005485	hsa-miR-1305;hsa-miR-17-5p;hsa-miR-19a-3p;hsa-miR-19b-3
ENSG0000	ENSG0000	0.003027	hsa-miR-106a-5p;hsa-miR-1271-3p;hsa-miR-129-5p;hsa-miR-
ENSG0000	ENSG0000	3.51E-05	hsa-miR-15a-5p;hsa-miR-15b-5p;hsa-miR-16-5p;hsa-miR-195-
ENSG0000	ENSG0000	0.006146	hsa-miR-15a-5p;hsa-miR-15b-5p;hsa-miR-16-5p;hsa-miR-195-
ENSG0000	ENSG0000	0.006146	hsa-miR-15a-5p;hsa-miR-15b-5p;hsa-miR-16-5p;hsa-miR-195-
ENSG0000	ENSG0000	0.006146	hsa-miR-15a-5p;hsa-miR-15b-5p;hsa-miR-16-5p;hsa-miR-195-
ENSG0000	ENSG0000	0.006146	hsa-miR-15a-5p;hsa-miR-15b-5p;hsa-miR-16-5p;hsa-miR-195-
ENSG0000	ENSG0000	0.006146	hsa-miR-15a-5p;hsa-miR-15b-5p;hsa-miR-16-5p;hsa-miR-195-
ENSG0000	ENSG0000	0.006146	hsa-miR-15a-5p;hsa-miR-15b-5p;hsa-miR-16-5p;hsa-miR-195-
ENSG0000	ENSG0000	0.006146	hsa-miR-15a-5p;hsa-miR-15b-5p;hsa-miR-16-5p;hsa-miR-195-
ENSG0000	ENSG0000	0.006146	hsa-miR-15a-5p;hsa-miR-15b-5p;hsa-miR-16-5p;hsa-miR-195-
ENSG0000	ENSG0000	0.006146	hsa-miR-15a-5p;hsa-miR-15b-5p;hsa-miR-16-5p;hsa-miR-195-
ENSG0000	ENSG0000	0.006146	hsa-miR-15a-5p;hsa-miR-15b-5p;hsa-miR-16-5p;hsa-miR-195-
ENSG0000	ENSG0000	0.000123	hsa-miR-15a-5p;hsa-miR-15b-5p;hsa-miR-16-5p;hsa-miR-195-
ENSG0000	ENSG0000	0.002987	hsa-miR-106a-5p;hsa-miR-17-5p;hsa-miR-20b-5p;hsa-miR-58
ENSG0000	ENSG0000	1.73E-05	hsa-miR-15a-5p;hsa-miR-15b-5p;hsa-miR-16-5p;hsa-miR-195-
ENSG0000	ENSG0000	0.006639	hsa-let-7a-5p;hsa-let-7b-5p;hsa-let-7e-5p;hsa-let-7f-5p;hsa-le
ENSG0000	ENSG0000	0.00218	hsa-miR-361-3p;hsa-miR-362-3p;hsa-miR-378a-3p;hsa-miR-37
ENSG0000	ENSG0000	5.79E-05	hsa-miR-1271-3p;hsa-miR-15a-5p;hsa-miR-15b-5p;hsa-miR-16
ENSG0000	ENSG0000	0.009895	hsa-miR-106a-5p;hsa-miR-17-5p;hsa-miR-204-5p;hsa-miR-20
ENSG0000	ENSG0000	0.001019	hsa-miR-24-1-5p;hsa-miR-24-2-5p;hsa-miR-5582-3p;hsa-miR-
ENSG0000	ENSG0000	0.001019	hsa-miR-24-1-5p;hsa-miR-24-2-5p;hsa-miR-5582-3p;hsa-miR-
ENSG0000	ENSG0000	0.001019	hsa-miR-24-1-5p;hsa-miR-24-2-5p;hsa-miR-5582-3p;hsa-miR-
ENSG0000	ENSG0000	0.001019	hsa-miR-24-1-5p;hsa-miR-24-2-5p;hsa-miR-5582-3p;hsa-miR-
ENSG0000	ENSG0000	0.001019	hsa-miR-24-1-5p;hsa-miR-24-2-5p;hsa-miR-5582-3p;hsa-miR-
ENSG0000	ENSG0000	0.002894	hsa-let-7b-5p;hsa-miR-124-3p;hsa-miR-136-5p;hsa-miR-29a-5
ENSG0000	ENSG0000	0.00443	hsa-let-7a-5p;hsa-let-7b-5p;hsa-let-7e-5p;hsa-let-7f-5p;hsa-le
ENSG0000	ENSG0000	0.003323	hsa-miR-103a-3p;hsa-miR-107;hsa-miR-15a-5p;hsa-miR-15b-5
ENSG0000	ENSG0000	0.004635	hsa-let-7a-5p;hsa-let-7b-5p;hsa-let-7e-5p;hsa-let-7f-5p;hsa-le
ENSG0000	ENSG0000	0.001897	hsa-miR-103a-3p;hsa-miR-107;hsa-miR-15a-5p;hsa-miR-15b-5
ENSG0000	ENSG0000	0.001172	hsa-miR-15a-5p;hsa-miR-15b-5p;hsa-miR-195-5p;hsa-miR-26
ENSG0000	ENSG0000	0.007423	hsa-let-7a-5p;hsa-let-7b-5p;hsa-let-7e-5p;hsa-let-7f-5p;hsa-le
ENSG0000	ENSG0000	0.003538	hsa-let-7a-5p;hsa-let-7b-5p;hsa-let-7e-5p;hsa-let-7f-5p;hsa-le
ENSG0000	ENSG0000	0.000119	hsa-let-7a-5p;hsa-let-7b-5p;hsa-let-7e-5p;hsa-let-7f-5p;hsa-le
ENSG0000	ENSG0000	0.009899	hsa-miR-139-3p;hsa-miR-30c-1-3p;hsa-miR-30c-2-3p;hsa-miR
ENSG0000	ENSG0000	0.001914	hsa-miR-320a;hsa-miR-320b;hsa-miR-320d;hsa-miR-4429

ENSG0000	ENSG0000	0.004111	hsa-let-7a-5p;hsa-let-7b-5p;hsa-let-7e-5p;hsa-let-7f-5p;hsa-le
ENSG0000	ENSG0000	3.20E-05	hsa-miR-146a-3p;hsa-miR-146a-5p;hsa-miR-146b-5p;hsa-miR-
ENSG0000	ENSG0000	0.002319	hsa-miR-15a-5p;hsa-miR-15b-5p;hsa-miR-16-5p;hsa-miR-195-
ENSG0000	ENSG0000	0.000491	hsa-miR-103a-3p;hsa-miR-107;hsa-miR-15a-5p;hsa-miR-15b-5
ENSG0000	ENSG0000	0.000945	hsa-miR-34a-5p;hsa-miR-34c-5p;hsa-miR-449a;hsa-miR-449b-
ENSG0000	ENSG0000	0.008001	hsa-miR-15a-5p;hsa-miR-15b-5p;hsa-miR-195-5p;hsa-miR-19
ENSG0000	ENSG0000	5.71E-05	hsa-miR-15a-5p;hsa-miR-15b-5p;hsa-miR-16-5p;hsa-miR-195-
ENSG0000	ENSG0000	0.004203	hsa-miR-15a-5p;hsa-miR-15b-5p;hsa-miR-195-5p;hsa-miR-42
ENSG0000	ENSG0000	0.006406	hsa-miR-146a-3p;hsa-miR-199a-5p;hsa-miR-199b-5p;hsa-miR-
ENSG0000	ENSG0000	0.006406	hsa-miR-146a-3p;hsa-miR-199a-5p;hsa-miR-199b-5p;hsa-miR-
ENSG0000	ENSG0000	0.000116	hsa-miR-103a-3p;hsa-miR-107;hsa-miR-15a-5p;hsa-miR-15b-5
ENSG0000	ENSG0000	0.004286	hsa-miR-15a-5p;hsa-miR-15b-5p;hsa-miR-16-5p;hsa-miR-195-
ENSG0000	ENSG0000	0.004492	hsa-miR-181a-2-3p;hsa-miR-186-5p;hsa-miR-204-5p;hsa-miR-
ENSG0000	ENSG0000	2.67E-06	hsa-miR-124-3p;hsa-miR-15a-5p;hsa-miR-15b-5p;hsa-miR-16-
ENSG0000	ENSG0000	4.94E-05	hsa-miR-15a-5p;hsa-miR-15b-5p;hsa-miR-16-5p;hsa-miR-195-
ENSG0000	ENSG0000	0.003525	hsa-let-7a-5p;hsa-let-7b-5p;hsa-let-7e-5p;hsa-let-7f-5p;hsa-le
ENSG0000	ENSG0000	0.002303	hsa-miR-15a-5p;hsa-miR-15b-5p;hsa-miR-16-5p;hsa-miR-195-
ENSG0000	ENSG0000	1.11E-08	hsa-miR-103a-3p;hsa-miR-107;hsa-miR-146a-5p;hsa-miR-146
ENSG0000	ENSG0000	1.63E-05	hsa-let-7a-5p;hsa-let-7b-5p;hsa-let-7e-5p;hsa-let-7f-5p;hsa-le
ENSG0000	ENSG0000	0.008149	hsa-miR-106a-5p;hsa-miR-139-3p;hsa-miR-17-5p;hsa-miR-19
ENSG0000	ENSG0000	0.000834	hsa-miR-15a-5p;hsa-miR-16-5p;hsa-miR-2355-5p;hsa-miR-48
ENSG0000	ENSG0000	0.000557	hsa-miR-15a-5p;hsa-miR-15b-5p;hsa-miR-16-5p;hsa-miR-195-
ENSG0000	ENSG0000	0.008164	hsa-let-7a-5p;hsa-let-7b-5p;hsa-let-7e-5p;hsa-let-7f-5p;hsa-le
ENSG0000	ENSG0000	0.001914	hsa-miR-34a-5p;hsa-miR-34c-5p;hsa-miR-449a;hsa-miR-449b-
ENSG0000	ENSG0000	2.69E-09	hsa-let-7a-5p;hsa-let-7b-5p;hsa-let-7e-5p;hsa-let-7f-5p;hsa-le
ENSG0000	ENSG0000	0.000216	hsa-miR-15a-5p;hsa-miR-15b-5p;hsa-miR-16-5p;hsa-miR-195-
ENSG0000	ENSG0000	0.007287	hsa-let-7a-5p;hsa-let-7b-5p;hsa-let-7e-5p;hsa-let-7f-5p;hsa-le
ENSG0000	ENSG0000	9.49E-05	hsa-miR-15a-5p;hsa-miR-15b-5p;hsa-miR-16-5p;hsa-miR-195-
ENSG0000	ENSG0000	0.001559	hsa-miR-1304-3p;hsa-miR-1305;hsa-miR-146a-3p;hsa-miR-30
ENSG0000	ENSG0000	0.005485	hsa-miR-106a-5p;hsa-miR-1293;hsa-miR-1305;hsa-miR-137;hs
ENSG0000	ENSG0000	0.007279	hsa-miR-106a-5p;hsa-miR-1224-3p;hsa-miR-17-5p;hsa-miR-2
ENSG0000	ENSG0000	0.000125	hsa-miR-15a-5p;hsa-miR-15b-5p;hsa-miR-16-5p;hsa-miR-195-
ENSG0000	ENSG0000	2.97E-07	hsa-miR-103a-3p;hsa-miR-107;hsa-miR-1271-3p;hsa-miR-15a-
ENSG0000	ENSG0000	5.71E-05	hsa-miR-15a-5p;hsa-miR-15b-5p;hsa-miR-16-5p;hsa-miR-195-
ENSG0000	ENSG0000	0.004397	hsa-let-7a-5p;hsa-let-7b-5p;hsa-let-7e-5p;hsa-let-7f-5p;hsa-le
ENSG0000	ENSG0000	0.007279	hsa-miR-106a-5p;hsa-miR-140-3p;hsa-miR-17-5p;hsa-miR-20
ENSG0000	ENSG0000	0.000157	hsa-let-7a-5p;hsa-let-7b-5p;hsa-let-7e-5p;hsa-let-7f-5p;hsa-le
ENSG0000	ENSG0000	0.002743	hsa-miR-101-3p;hsa-miR-185-5p;hsa-miR-2110;hsa-miR-2467-
ENSG0000	ENSG0000	0.006406	hsa-miR-106a-5p;hsa-miR-1224-3p;hsa-miR-125a-3p;hsa-miR-
ENSG0000	ENSG0000	0.007423	hsa-miR-101-3p;hsa-miR-103a-3p;hsa-miR-107;hsa-miR-15a-5
ENSG0000	ENSG0000	0.001165	hsa-miR-137;hsa-miR-146b-3p;hsa-miR-15a-5p;hsa-miR-15b-5
ENSG0000	ENSG0000	0.000125	hsa-miR-146a-5p;hsa-miR-146b-5p;hsa-miR-320a;hsa-miR-32
ENSG0000	ENSG0000	0.00187	hsa-let-7a-5p;hsa-let-7b-5p;hsa-let-7e-5p;hsa-let-7f-5p;hsa-le
ENSG0000	ENSG0000	0.001882	hsa-miR-146a-3p;hsa-miR-15a-5p;hsa-miR-15b-5p;hsa-miR-1
ENSG0000	ENSG0000	0.004776	hsa-miR-137;hsa-miR-145-5p;hsa-miR-196a-3p;hsa-miR-25-3
ENSG0000	ENSG0000	0.00036	hsa-miR-15a-5p;hsa-miR-15b-5p;hsa-miR-16-5p;hsa-miR-195-
ENSG0000	ENSG0000	0.00265	hsa-miR-1224-5p;hsa-miR-138-5p;hsa-miR-140-3p;hsa-miR-1
ENSG0000	ENSG0000	0.005466	hsa-miR-1224-5p;hsa-miR-138-5p;hsa-miR-140-3p;hsa-miR-1
ENSG0000	ENSG0000	0.00762	hsa-miR-1243;hsa-miR-125a-5p;hsa-miR-125b-5p;hsa-miR-50
ENSG0000	ENSG0000	3.51E-05	hsa-miR-15a-5p;hsa-miR-15b-5p;hsa-miR-16-5p;hsa-miR-195-
ENSG0000	ENSG0000	0.00915	hsa-miR-124-3p;hsa-miR-137;hsa-miR-145-5p;hsa-miR-199a-5
ENSG0000	ENSG0000	0.002303	hsa-let-7a-5p;hsa-let-7b-5p;hsa-let-7e-5p;hsa-let-7f-5p;hsa-le
ENSG0000	ENSG0000	0.001207	hsa-let-7a-5p;hsa-let-7b-5p;hsa-let-7e-5p;hsa-let-7f-5p;hsa-le
ENSG0000	ENSG0000	0.000388	hsa-miR-106a-5p;hsa-miR-17-5p;hsa-miR-196a-3p;hsa-miR-2

ENSG0000	ENSG0000	0.000186	hsa-let-7a-5p;hsa-let-7b-5p;hsa-let-7e-5p;hsa-let-7f-5p;hsa-le
ENSG0000	ENSG0000	0.003302	hsa-miR-103a-3p;hsa-miR-107;hsa-miR-1304-3p;hsa-miR-136-
ENSG0000	ENSG0000	0.003012	hsa-miR-125a-3p;hsa-miR-1271-3p;hsa-miR-192-5p;hsa-miR-
ENSG0000	ENSG0000	0.004068	hsa-miR-1271-3p;hsa-miR-199a-5p;hsa-miR-199b-5p;hsa-miR
ENSG0000	ENSG0000	0.003525	hsa-miR-106a-5p;hsa-miR-17-5p;hsa-miR-20b-5p;hsa-miR-29
ENSG0000	ENSG0000	4.99E-05	hsa-miR-101-3p;hsa-miR-144-3p;hsa-miR-199a-3p;hsa-miR-1
ENSG0000	ENSG0000	0.002578	hsa-miR-185-5p;hsa-miR-199a-3p;hsa-miR-199b-3p;hsa-miR-
ENSG0000	ENSG0000	0.000655	hsa-miR-132-3p;hsa-miR-185-5p;hsa-miR-212-3p;hsa-miR-22
ENSG0000	ENSG0000	0.000362	hsa-miR-101-3p;hsa-miR-144-3p;hsa-miR-199a-3p;hsa-miR-1
ENSG0000	ENSG0000	0.000978	hsa-miR-101-3p;hsa-miR-144-3p;hsa-miR-199a-3p;hsa-miR-1
ENSG0000	ENSG0000	0.000648	hsa-miR-101-3p;hsa-miR-144-3p;hsa-miR-199a-3p;hsa-miR-1
ENSG0000	ENSG0000	0.001379	hsa-miR-101-3p;hsa-miR-144-3p;hsa-miR-199a-3p;hsa-miR-1
ENSG0000	ENSG0000	0.000345	hsa-miR-101-3p;hsa-miR-144-3p;hsa-miR-185-5p;hsa-miR-49
ENSG0000	ENSG0000	2.30E-08	hsa-miR-106b-5p;hsa-miR-17-5p;hsa-miR-20a-5p;hsa-miR-20
ENSG0000	ENSG0000	6.35E-06	hsa-miR-106b-5p;hsa-miR-17-5p;hsa-miR-20a-5p;hsa-miR-20
ENSG0000	ENSG0000	1.43E-07	hsa-miR-106b-5p;hsa-miR-17-5p;hsa-miR-20a-5p;hsa-miR-20
ENSG0000	ENSG0000	2.50E-07	hsa-miR-103a-2-5p;hsa-miR-106b-5p;hsa-miR-17-5p;hsa-miR
ENSG0000	ENSG0000	0.001466	hsa-miR-101-3p;hsa-miR-106b-5p;hsa-miR-17-5p;hsa-miR-20
ENSG0000	ENSG0000	2.83E-06	hsa-miR-106b-5p;hsa-miR-17-5p;hsa-miR-20a-5p;hsa-miR-20
ENSG0000	ENSG0000	0.00015	hsa-miR-106b-5p;hsa-miR-17-5p;hsa-miR-19a-3p;hsa-miR-20
ENSG0000	ENSG0000	3.07E-08	hsa-miR-106b-5p;hsa-miR-17-5p;hsa-miR-20a-5p;hsa-miR-20
ENSG0000	ENSG0000	0.000217	hsa-miR-106b-5p;hsa-miR-17-5p;hsa-miR-20a-5p;hsa-miR-20
ENSG0000	ENSG0000	2.50E-06	hsa-miR-106b-5p;hsa-miR-17-5p;hsa-miR-20a-5p;hsa-miR-20
ENSG0000	ENSG0000	0.007962	hsa-miR-101-3p;hsa-miR-106b-5p;hsa-miR-19a-3p;hsa-miR-2
ENSG0000	ENSG0000	7.37E-05	hsa-miR-106b-5p;hsa-miR-20a-5p;hsa-miR-20b-5p;hsa-miR-9
ENSG0000	ENSG0000	2.50E-06	hsa-miR-106b-5p;hsa-miR-17-5p;hsa-miR-20a-5p;hsa-miR-20
ENSG0000	ENSG0000	0.000212	hsa-miR-106b-5p;hsa-miR-17-5p;hsa-miR-20a-5p;hsa-miR-20
ENSG0000	ENSG0000	0.000459	hsa-miR-106b-5p;hsa-miR-17-5p;hsa-miR-20a-5p;hsa-miR-20
ENSG0000	ENSG0000	1.09E-05	hsa-miR-106b-5p;hsa-miR-17-5p;hsa-miR-20a-5p;hsa-miR-20
ENSG0000	ENSG0000	7.61E-06	hsa-miR-106b-5p;hsa-miR-17-5p;hsa-miR-20a-5p;hsa-miR-20
ENSG0000	ENSG0000	0.009611	hsa-miR-106b-5p;hsa-miR-17-5p;hsa-miR-20a-5p;hsa-miR-20
ENSG0000	ENSG0000	1.60E-07	hsa-miR-106b-5p;hsa-miR-17-5p;hsa-miR-19a-3p;hsa-miR-19
ENSG0000	ENSG0000	0.000414	hsa-miR-106b-5p;hsa-miR-17-5p;hsa-miR-20a-5p;hsa-miR-93
ENSG0000	ENSG0000	0.000875	hsa-miR-106b-5p;hsa-miR-17-5p;hsa-miR-20a-5p;hsa-miR-20
ENSG0000	ENSG0000	1.30E-07	hsa-miR-106b-5p;hsa-miR-17-5p;hsa-miR-20a-5p;hsa-miR-20
ENSG0000	ENSG0000	2.76E-05	hsa-miR-101-3p;hsa-miR-106b-5p;hsa-miR-20a-5p;hsa-miR-2
ENSG0000	ENSG0000	1.41E-05	hsa-miR-106b-5p;hsa-miR-17-5p;hsa-miR-20a-5p;hsa-miR-20
ENSG0000	ENSG0000	5.06E-06	hsa-miR-106b-5p;hsa-miR-17-5p;hsa-miR-20a-5p;hsa-miR-20
ENSG0000	ENSG0000	8.09E-05	hsa-miR-106b-5p;hsa-miR-17-5p;hsa-miR-20a-5p;hsa-miR-20
ENSG0000	ENSG0000	4.83E-05	hsa-miR-103a-2-5p;hsa-miR-199a-5p;hsa-miR-199b-5p;hsa-m
ENSG0000	ENSG0000	2.91E-07	hsa-miR-106b-5p;hsa-miR-17-5p;hsa-miR-20a-5p;hsa-miR-20
ENSG0000	ENSG0000	1.31E-07	hsa-miR-106b-5p;hsa-miR-17-5p;hsa-miR-20a-5p;hsa-miR-20
ENSG0000	ENSG0000	0.006361	hsa-miR-199a-5p;hsa-miR-199b-5p;hsa-miR-19a-3p;hsa-miR-
ENSG0000	ENSG0000	0.00111	hsa-miR-106b-5p;hsa-miR-17-5p;hsa-miR-20a-5p;hsa-miR-20
ENSG0000	ENSG0000	4.91E-08	hsa-miR-101-3p;hsa-miR-106b-5p;hsa-miR-17-5p;hsa-miR-19
ENSG0000	ENSG0000	1.73E-07	hsa-miR-106b-5p;hsa-miR-17-5p;hsa-miR-20a-5p;hsa-miR-20
ENSG0000	ENSG0000	0.001246	hsa-miR-106b-5p;hsa-miR-17-5p;hsa-miR-20b-5p;hsa-miR-93
ENSG0000	ENSG0000	8.59E-06	hsa-miR-106b-5p;hsa-miR-17-5p;hsa-miR-20a-5p;hsa-miR-20
ENSG0000	ENSG0000	5.86E-07	hsa-miR-106b-5p;hsa-miR-17-5p;hsa-miR-20a-5p;hsa-miR-20
ENSG0000	ENSG0000	5.86E-07	hsa-miR-106b-5p;hsa-miR-17-5p;hsa-miR-20a-5p;hsa-miR-20
ENSG0000	ENSG0000	4.04E-07	hsa-miR-106b-5p;hsa-miR-17-5p;hsa-miR-199a-5p;hsa-miR-1
ENSG0000	ENSG0000	6.06E-09	hsa-miR-106b-5p;hsa-miR-17-5p;hsa-miR-20a-5p;hsa-miR-20
ENSG0000	ENSG0000	2.26E-07	hsa-miR-106b-5p;hsa-miR-17-5p;hsa-miR-20a-5p;hsa-miR-20
ENSG0000	ENSG0000	0.000176	hsa-miR-101-3p;hsa-miR-106b-5p;hsa-miR-20b-5p;hsa-miR-2

ENSG0000	ENSG0000	5.97E-05	hsa-miR-106b-5p;hsa-miR-17-5p;hsa-miR-20a-5p;hsa-miR-20
ENSG0000	ENSG0000	0.000304	hsa-miR-106b-5p;hsa-miR-17-5p;hsa-miR-20a-5p;hsa-miR-20
ENSG0000	ENSG0000	0.000991	hsa-miR-106b-5p;hsa-miR-17-5p;hsa-miR-20a-5p;hsa-miR-20
ENSG0000	ENSG0000	1.82E-07	hsa-miR-101-3p;hsa-miR-106b-5p;hsa-miR-17-5p;hsa-miR-20
ENSG0000	ENSG0000	3.67E-08	hsa-miR-103a-2-5p;hsa-miR-106b-5p;hsa-miR-17-5p;hsa-miR
ENSG0000	ENSG0000	4.68E-05	hsa-miR-106b-5p;hsa-miR-17-5p;hsa-miR-20a-5p;hsa-miR-20
ENSG0000	ENSG0000	3.91E-06	hsa-miR-106b-5p;hsa-miR-17-5p;hsa-miR-20a-5p;hsa-miR-20
ENSG0000	ENSG0000	3.07E-08	hsa-miR-106b-5p;hsa-miR-17-5p;hsa-miR-20a-5p;hsa-miR-20
ENSG0000	ENSG0000	0.000181	hsa-miR-106b-5p;hsa-miR-17-5p;hsa-miR-20a-5p;hsa-miR-20
ENSG0000	ENSG0000	5.01E-05	hsa-miR-106b-5p;hsa-miR-17-5p;hsa-miR-20a-5p;hsa-miR-20
ENSG0000	ENSG0000	5.08E-05	hsa-miR-106b-5p;hsa-miR-17-5p;hsa-miR-20a-5p;hsa-miR-20
ENSG0000	ENSG0000	7.14E-11	hsa-miR-106b-5p;hsa-miR-17-5p;hsa-miR-20a-5p;hsa-miR-20
ENSG0000	ENSG0000	7.14E-11	hsa-miR-106b-5p;hsa-miR-17-5p;hsa-miR-20a-5p;hsa-miR-20
ENSG0000	ENSG0000	7.14E-11	hsa-miR-106b-5p;hsa-miR-17-5p;hsa-miR-20a-5p;hsa-miR-20
ENSG0000	ENSG0000	7.14E-11	hsa-miR-106b-5p;hsa-miR-17-5p;hsa-miR-20a-5p;hsa-miR-20
ENSG0000	ENSG0000	7.14E-11	hsa-miR-106b-5p;hsa-miR-17-5p;hsa-miR-20a-5p;hsa-miR-20
ENSG0000	ENSG0000	7.14E-11	hsa-miR-106b-5p;hsa-miR-17-5p;hsa-miR-20a-5p;hsa-miR-20
ENSG0000	ENSG0000	7.14E-11	hsa-miR-106b-5p;hsa-miR-17-5p;hsa-miR-20a-5p;hsa-miR-20
ENSG0000	ENSG0000	0.004816	hsa-miR-106b-5p;hsa-miR-17-5p;hsa-miR-20a-5p;hsa-miR-20
ENSG0000	ENSG0000	1.15E-14	hsa-miR-106b-5p;hsa-miR-17-5p;hsa-miR-19a-3p;hsa-miR-19
ENSG0000	ENSG0000	0.000311	hsa-miR-106b-5p;hsa-miR-17-5p;hsa-miR-199a-5p;hsa-miR-1
ENSG0000	ENSG0000	2.49E-10	hsa-miR-106b-5p;hsa-miR-17-5p;hsa-miR-20a-5p;hsa-miR-20
ENSG0000	ENSG0000	7.18E-06	hsa-miR-103a-2-5p;hsa-miR-106b-5p;hsa-miR-20a-5p;hsa-mi
ENSG0000	ENSG0000	0.00013	hsa-miR-106b-5p;hsa-miR-20a-5p;hsa-miR-20b-5p;hsa-miR-9
ENSG0000	ENSG0000	8.73E-07	hsa-miR-106b-5p;hsa-miR-17-5p;hsa-miR-20a-5p;hsa-miR-20
ENSG0000	ENSG0000	3.62E-05	hsa-miR-106b-5p;hsa-miR-17-5p;hsa-miR-20a-5p;hsa-miR-20
ENSG0000	ENSG0000	0.007241	hsa-miR-106b-5p;hsa-miR-17-5p;hsa-miR-20a-5p;hsa-miR-20
ENSG0000	ENSG0000	0.000192	hsa-miR-106b-5p;hsa-miR-17-5p;hsa-miR-20a-5p;hsa-miR-20
ENSG0000	ENSG0000	2.28E-05	hsa-miR-106b-5p;hsa-miR-17-5p;hsa-miR-20a-5p;hsa-miR-20
ENSG0000	ENSG0000	2.28E-05	hsa-miR-106b-5p;hsa-miR-17-5p;hsa-miR-20a-5p;hsa-miR-20
ENSG0000	ENSG0000	2.28E-05	hsa-miR-106b-5p;hsa-miR-17-5p;hsa-miR-20a-5p;hsa-miR-20
ENSG0000	ENSG0000	1.68E-05	hsa-miR-106b-5p;hsa-miR-17-5p;hsa-miR-20a-5p;hsa-miR-20
ENSG0000	ENSG0000	0.0007	hsa-miR-106b-5p;hsa-miR-17-5p;hsa-miR-20a-5p;hsa-miR-20
ENSG0000	ENSG0000	0.000108	hsa-miR-106b-5p;hsa-miR-17-5p;hsa-miR-20a-5p;hsa-miR-20
ENSG0000	ENSG0000	2.28E-05	hsa-miR-17-5p;hsa-miR-199a-5p;hsa-miR-199b-5p;hsa-miR-2
ENSG0000	ENSG0000	0.000132	hsa-miR-106b-5p;hsa-miR-17-5p;hsa-miR-20a-5p;hsa-miR-20
ENSG0000	ENSG0000	0.00408	hsa-miR-106b-5p;hsa-miR-17-5p;hsa-miR-20a-5p;hsa-miR-20
ENSG0000	ENSG0000	0.00017	hsa-miR-106b-5p;hsa-miR-17-5p;hsa-miR-20a-5p;hsa-miR-20
ENSG0000	ENSG0000	1.39E-05	hsa-miR-17-5p;hsa-miR-20a-5p;hsa-miR-20b-5p;hsa-miR-93-
ENSG0000	ENSG0000	0.000731	hsa-miR-101-3p;hsa-miR-19a-3p;hsa-miR-19b-3p;hsa-miR-30
ENSG0000	ENSG0000	2.92E-06	hsa-miR-106b-5p;hsa-miR-17-5p;hsa-miR-20a-5p;hsa-miR-20
ENSG0000	ENSG0000	0.000123	hsa-miR-106b-5p;hsa-miR-17-5p;hsa-miR-20a-5p;hsa-miR-20
ENSG0000	ENSG0000	0.000669	hsa-miR-106b-5p;hsa-miR-17-5p;hsa-miR-20a-5p;hsa-miR-20
ENSG0000	ENSG0000	0.000181	hsa-miR-106b-5p;hsa-miR-17-5p;hsa-miR-20a-5p;hsa-miR-20
ENSG0000	ENSG0000	9.68E-06	hsa-miR-106b-5p;hsa-miR-17-5p;hsa-miR-20a-5p;hsa-miR-20
ENSG0000	ENSG0000	6.71E-06	hsa-miR-106b-5p;hsa-miR-17-5p;hsa-miR-20a-5p;hsa-miR-20
ENSG0000	ENSG0000	2.76E-05	hsa-miR-106b-5p;hsa-miR-17-5p;hsa-miR-20a-5p;hsa-miR-20
ENSG0000	ENSG0000	0.000141	hsa-miR-106b-5p;hsa-miR-17-5p;hsa-miR-20a-5p;hsa-miR-20
ENSG0000	ENSG0000	1.51E-05	hsa-miR-106b-5p;hsa-miR-17-5p;hsa-miR-20a-5p;hsa-miR-20
ENSG0000	ENSG0000	3.71E-07	hsa-miR-106b-5p;hsa-miR-17-5p;hsa-miR-20a-5p;hsa-miR-20
ENSG0000	ENSG0000	0.000357	hsa-miR-106b-5p;hsa-miR-17-5p;hsa-miR-20a-5p;hsa-miR-20
ENSG0000	ENSG0000	5.08E-05	hsa-miR-106b-5p;hsa-miR-17-5p;hsa-miR-20a-5p;hsa-miR-20
ENSG0000	ENSG0000	0.000825	hsa-miR-199a-5p;hsa-miR-30a-3p;hsa-miR-30e-3p;hsa-miR-9
ENSG0000	ENSG0000	3.62E-05	hsa-miR-106b-5p;hsa-miR-17-5p;hsa-miR-20a-5p;hsa-miR-20

ENSG0000	ENSG0000	0.000108	hsa-miR-106b-5p;hsa-miR-17-5p;hsa-miR-20a-5p;hsa-miR-20l
ENSG0000	ENSG0000	4.51E-06	hsa-miR-106b-5p;hsa-miR-17-5p;hsa-miR-20a-5p;hsa-miR-20l
ENSG0000	ENSG0000	0.000232	hsa-miR-17-5p;hsa-miR-199a-5p;hsa-miR-19a-3p;hsa-miR-20a
ENSG0000	ENSG0000	1.78E-07	hsa-miR-106b-5p;hsa-miR-17-5p;hsa-miR-19a-3p;hsa-miR-19l
ENSG0000	ENSG0000	0.00016	hsa-miR-101-3p;hsa-miR-106b-5p;hsa-miR-17-5p;hsa-miR-20l
ENSG0000	ENSG0000	1.58E-08	hsa-miR-106b-5p;hsa-miR-17-5p;hsa-miR-199a-5p;hsa-miR-1
ENSG0000	ENSG0000	0.009565	hsa-miR-199a-5p;hsa-miR-199b-5p;hsa-miR-19a-3p;hsa-miR-1
ENSG0000	ENSG0000	0.005643	hsa-miR-106b-5p;hsa-miR-17-5p;hsa-miR-20a-5p;hsa-miR-20l
ENSG0000	ENSG0000	1.22E-05	hsa-miR-106b-5p;hsa-miR-17-5p;hsa-miR-20a-5p;hsa-miR-20l
ENSG0000	ENSG0000	3.62E-05	hsa-miR-106b-5p;hsa-miR-17-5p;hsa-miR-20a-5p;hsa-miR-20l
ENSG0000	ENSG0000	0.000159	hsa-miR-106b-5p;hsa-miR-20a-5p;hsa-miR-20b-5p;hsa-miR-9
ENSG0000	ENSG0000	0.000832	hsa-miR-106b-5p;hsa-miR-17-5p;hsa-miR-20a-5p;hsa-miR-20l
ENSG0000	ENSG0000	2.21E-07	hsa-miR-106b-5p;hsa-miR-17-5p;hsa-miR-19a-3p;hsa-miR-19l
ENSG0000	ENSG0000	7.37E-05	hsa-miR-17-5p;hsa-miR-20a-5p;hsa-miR-20b-5p;hsa-miR-93-
ENSG0000	ENSG0000	7.12E-05	hsa-miR-106b-5p;hsa-miR-17-5p;hsa-miR-20a-5p;hsa-miR-20l
ENSG0000	ENSG0000	2.13E-06	hsa-miR-106b-5p;hsa-miR-17-5p;hsa-miR-20a-5p;hsa-miR-20l
ENSG0000	ENSG0000	5.89E-06	hsa-miR-106b-5p;hsa-miR-17-5p;hsa-miR-20a-5p;hsa-miR-20l
ENSG0000	ENSG0000	1.13E-05	hsa-miR-106b-5p;hsa-miR-17-5p;hsa-miR-20a-5p;hsa-miR-20l
ENSG0000	ENSG0000	2.45E-05	hsa-miR-106b-5p;hsa-miR-17-5p;hsa-miR-19a-3p;hsa-miR-20a
ENSG0000	ENSG0000	2.51E-05	hsa-miR-106b-5p;hsa-miR-17-5p;hsa-miR-19a-3p;hsa-miR-20a
ENSG0000	ENSG0000	5.80E-07	hsa-miR-106b-5p;hsa-miR-17-5p;hsa-miR-20a-5p;hsa-miR-20l
ENSG0000	ENSG0000	4.30E-05	hsa-miR-106b-5p;hsa-miR-17-5p;hsa-miR-20a-5p;hsa-miR-20l
ENSG0000	ENSG0000	0.000798	hsa-miR-106b-5p;hsa-miR-17-5p;hsa-miR-20a-5p;hsa-miR-20l
ENSG0000	ENSG0000	8.09E-05	hsa-miR-106b-5p;hsa-miR-17-5p;hsa-miR-20a-5p;hsa-miR-20l
ENSG0000	ENSG0000	9.68E-06	hsa-miR-106b-5p;hsa-miR-17-5p;hsa-miR-20a-5p;hsa-miR-20l
ENSG0000	ENSG0000	0.001178	hsa-miR-106b-5p;hsa-miR-20a-5p;hsa-miR-20b-5p;hsa-miR-9
ENSG0000	ENSG0000	3.53E-10	hsa-miR-106b-5p;hsa-miR-17-5p;hsa-miR-20a-5p;hsa-miR-20l
ENSG0000	ENSG0000	1.52E-06	hsa-miR-106b-5p;hsa-miR-17-5p;hsa-miR-20a-5p;hsa-miR-20l
ENSG0000	ENSG0000	0.000933	hsa-miR-19a-3p;hsa-miR-19b-3p;hsa-miR-491-5p;hsa-miR-96-
ENSG0000	ENSG0000	1.80E-06	hsa-miR-106b-5p;hsa-miR-17-5p;hsa-miR-20a-5p;hsa-miR-20l
ENSG0000	ENSG0000	1.09E-05	hsa-miR-106b-5p;hsa-miR-17-5p;hsa-miR-20a-5p;hsa-miR-20l
ENSG0000	ENSG0000	1.60E-07	hsa-miR-106b-5p;hsa-miR-17-5p;hsa-miR-19a-3p;hsa-miR-19l
ENSG0000	ENSG0000	0.006567	hsa-miR-106b-5p;hsa-miR-17-5p;hsa-miR-20a-5p;hsa-miR-20l
ENSG0000	ENSG0000	6.71E-06	hsa-miR-106b-5p;hsa-miR-17-5p;hsa-miR-20a-5p;hsa-miR-20l
ENSG0000	ENSG0000	6.45E-05	hsa-miR-106b-5p;hsa-miR-17-5p;hsa-miR-20a-5p;hsa-miR-20l
ENSG0000	ENSG0000	5.90E-06	hsa-miR-106b-5p;hsa-miR-17-5p;hsa-miR-20a-5p;hsa-miR-20l
ENSG0000	ENSG0000	1.52E-06	hsa-miR-106b-5p;hsa-miR-17-5p;hsa-miR-20a-5p;hsa-miR-20l
ENSG0000	ENSG0000	0.0007	hsa-miR-106b-5p;hsa-miR-17-5p;hsa-miR-20a-5p;hsa-miR-20l
ENSG0000	ENSG0000	1.06E-06	hsa-miR-106b-5p;hsa-miR-17-5p;hsa-miR-20a-5p;hsa-miR-20l
ENSG0000	ENSG0000	9.68E-06	hsa-miR-106b-5p;hsa-miR-17-5p;hsa-miR-20a-5p;hsa-miR-20l
ENSG0000	ENSG0000	8.98E-06	hsa-miR-106b-5p;hsa-miR-17-5p;hsa-miR-19a-3p;hsa-miR-19l
ENSG0000	ENSG0000	4.21E-07	hsa-miR-106b-5p;hsa-miR-17-5p;hsa-miR-20a-5p;hsa-miR-93-
ENSG0000	ENSG0000	4.29E-05	hsa-miR-106b-5p;hsa-miR-17-5p;hsa-miR-19a-3p;hsa-miR-19l
ENSG0000	ENSG0000	4.29E-05	hsa-miR-106b-5p;hsa-miR-17-5p;hsa-miR-19a-3p;hsa-miR-19l
ENSG0000	ENSG0000	7.14E-11	hsa-miR-106b-5p;hsa-miR-17-5p;hsa-miR-20a-5p;hsa-miR-20l
ENSG0000	ENSG0000	1.36E-05	hsa-miR-106b-5p;hsa-miR-17-5p;hsa-miR-20a-5p;hsa-miR-20l
ENSG0000	ENSG0000	1.07E-06	hsa-miR-106b-5p;hsa-miR-17-5p;hsa-miR-20a-5p;hsa-miR-20l
ENSG0000	ENSG0000	5.97E-05	hsa-miR-106b-5p;hsa-miR-17-5p;hsa-miR-20a-5p;hsa-miR-20l
ENSG0000	ENSG0000	5.61E-06	hsa-miR-106b-5p;hsa-miR-17-5p;hsa-miR-199a-5p;hsa-miR-1
ENSG0000	ENSG0000	0.000726	hsa-miR-106b-5p;hsa-miR-17-5p;hsa-miR-20a-5p;hsa-miR-20l
ENSG0000	ENSG0000	0.000299	hsa-miR-101-3p;hsa-miR-106b-5p;hsa-miR-17-5p;hsa-miR-20a
ENSG0000	ENSG0000	1.78E-07	hsa-miR-106b-5p;hsa-miR-17-5p;hsa-miR-19a-3p;hsa-miR-19l
ENSG0000	ENSG0000	0.0007	hsa-miR-106b-5p;hsa-miR-17-5p;hsa-miR-20a-5p;hsa-miR-20l
ENSG0000	ENSG0000	2.54E-07	hsa-miR-106b-5p;hsa-miR-17-5p;hsa-miR-20a-5p;hsa-miR-20l

ENSG0000	ENSG0000	0.000555	hsa-miR-106b-5p;hsa-miR-17-5p;hsa-miR-20a-5p;hsa-miR-24-
ENSG0000	ENSG0000	2.91E-07	hsa-miR-106b-5p;hsa-miR-17-5p;hsa-miR-20a-5p;hsa-miR-20l
ENSG0000	ENSG0000	0.000243	hsa-miR-101-3p;hsa-miR-17-5p;hsa-miR-20a-5p;hsa-miR-20b-
ENSG0000	ENSG0000	7.14E-11	hsa-miR-106b-5p;hsa-miR-17-5p;hsa-miR-20a-5p;hsa-miR-20l
ENSG0000	ENSG0000	0.000176	hsa-miR-106b-5p;hsa-miR-20a-5p;hsa-miR-20b-5p;hsa-miR-93
ENSG0000	ENSG0000	0.000878	hsa-miR-106b-5p;hsa-miR-17-5p;hsa-miR-20b-5p;hsa-miR-93
ENSG0000	ENSG0000	3.79E-06	hsa-miR-101-3p;hsa-miR-106b-5p;hsa-miR-17-5p;hsa-miR-19a
ENSG0000	ENSG0000	0.000764	hsa-miR-106b-5p;hsa-miR-17-5p;hsa-miR-20a-5p;hsa-miR-20l
ENSG0000	ENSG0000	2.49E-10	hsa-miR-106b-5p;hsa-miR-17-5p;hsa-miR-20a-5p;hsa-miR-20l
ENSG0000	ENSG0000	1.51E-05	hsa-miR-106b-5p;hsa-miR-17-5p;hsa-miR-20a-5p;hsa-miR-20l
ENSG0000	ENSG0000	1.51E-05	hsa-miR-106b-5p;hsa-miR-17-5p;hsa-miR-20a-5p;hsa-miR-20l
ENSG0000	ENSG0000	0.000984	hsa-miR-106b-5p;hsa-miR-17-5p;hsa-miR-20a-5p;hsa-miR-20l
ENSG0000	ENSG0000	4.05E-05	hsa-miR-106b-5p;hsa-miR-17-5p;hsa-miR-20a-5p;hsa-miR-20l
ENSG0000	ENSG0000	0.000696	hsa-miR-106b-5p;hsa-miR-17-5p;hsa-miR-20a-5p;hsa-miR-20l
ENSG0000	ENSG0000	4.44E-07	hsa-miR-106b-5p;hsa-miR-17-5p;hsa-miR-20a-5p;hsa-miR-20l
ENSG0000	ENSG0000	2.00E-05	hsa-miR-103a-2-5p;hsa-miR-199a-5p;hsa-miR-199b-5p;hsa-m
ENSG0000	ENSG0000	0.005943	hsa-miR-106b-5p;hsa-miR-20a-5p;hsa-miR-20b-5p;hsa-miR-93
ENSG0000	ENSG0000	0.000181	hsa-miR-106b-5p;hsa-miR-17-5p;hsa-miR-20a-5p;hsa-miR-20l
ENSG0000	ENSG0000	0.000416	hsa-miR-106b-5p;hsa-miR-17-5p;hsa-miR-20a-5p;hsa-miR-20l
ENSG0000	ENSG0000	2.45E-05	hsa-miR-106b-5p;hsa-miR-17-5p;hsa-miR-20a-5p;hsa-miR-20l
ENSG0000	ENSG0000	2.76E-05	hsa-miR-106b-5p;hsa-miR-17-5p;hsa-miR-20a-5p;hsa-miR-20l
ENSG0000	ENSG0000	1.48E-09	hsa-miR-106b-5p;hsa-miR-17-5p;hsa-miR-20a-5p;hsa-miR-20l
ENSG0000	ENSG0000	0.000141	hsa-miR-106b-5p;hsa-miR-17-5p;hsa-miR-20a-5p;hsa-miR-20l
ENSG0000	ENSG0000	2.76E-05	hsa-miR-106b-5p;hsa-miR-17-5p;hsa-miR-20a-5p;hsa-miR-20l
ENSG0000	ENSG0000	4.68E-05	hsa-miR-101-3p;hsa-miR-17-5p;hsa-miR-19a-3p;hsa-miR-19b-
ENSG0000	ENSG0000	0.000272	hsa-miR-17-5p;hsa-miR-19b-3p;hsa-miR-20a-5p;hsa-miR-30a-
ENSG0000	ENSG0000	0.000257	hsa-miR-106b-5p;hsa-miR-17-5p;hsa-miR-20a-5p;hsa-miR-20l
ENSG0000	ENSG0000	0.009015	hsa-miR-24-3p;hsa-miR-28-3p;hsa-miR-30a-3p;hsa-miR-30e-3
ENSG0000	ENSG0000	0.00015	hsa-miR-106b-5p;hsa-miR-17-5p;hsa-miR-20a-5p;hsa-miR-20l
ENSG0000	ENSG0000	5.97E-05	hsa-miR-106b-5p;hsa-miR-17-5p;hsa-miR-20a-5p;hsa-miR-20l
ENSG0000	ENSG0000	0.005943	hsa-miR-19a-3p;hsa-miR-2110;hsa-miR-24-3p;hsa-miR-28-3p
ENSG0000	ENSG0000	6.97E-05	hsa-miR-106b-5p;hsa-miR-17-5p;hsa-miR-20a-5p;hsa-miR-20l
ENSG0000	ENSG0000	0.000123	hsa-miR-106b-5p;hsa-miR-17-5p;hsa-miR-20a-5p;hsa-miR-20l
ENSG0000	ENSG0000	7.61E-06	hsa-miR-106b-5p;hsa-miR-17-5p;hsa-miR-20a-5p;hsa-miR-20l
ENSG0000	ENSG0000	8.22E-06	hsa-miR-106b-5p;hsa-miR-17-5p;hsa-miR-20a-5p;hsa-miR-20l
ENSG0000	ENSG0000	2.49E-10	hsa-miR-106b-5p;hsa-miR-17-5p;hsa-miR-20a-5p;hsa-miR-20l
ENSG0000	ENSG0000	0.000321	hsa-miR-106b-5p;hsa-miR-17-5p;hsa-miR-20a-5p;hsa-miR-20l
ENSG0000	ENSG0000	2.15E-06	hsa-miR-106b-5p;hsa-miR-17-5p;hsa-miR-20a-5p;hsa-miR-20l
ENSG0000	ENSG0000	5.16E-06	hsa-miR-106b-5p;hsa-miR-17-5p;hsa-miR-20a-5p;hsa-miR-20l
ENSG0000	ENSG0000	0.000132	hsa-miR-106b-5p;hsa-miR-17-5p;hsa-miR-20a-5p;hsa-miR-20l
ENSG0000	ENSG0000	2.06E-05	hsa-miR-106b-5p;hsa-miR-17-5p;hsa-miR-20a-5p;hsa-miR-20l
ENSG0000	ENSG0000	2.06E-05	hsa-miR-106b-5p;hsa-miR-17-5p;hsa-miR-20a-5p;hsa-miR-20l
ENSG0000	ENSG0000	3.62E-05	hsa-miR-106b-5p;hsa-miR-17-5p;hsa-miR-20a-5p;hsa-miR-20l
ENSG0000	ENSG0000	0.000378	hsa-miR-106b-5p;hsa-miR-17-5p;hsa-miR-20a-5p;hsa-miR-20l
ENSG0000	ENSG0000	0.000991	hsa-miR-19a-3p;hsa-miR-19b-3p;hsa-miR-30a-3p;hsa-miR-30e
ENSG0000	ENSG0000	2.50E-06	hsa-miR-106b-5p;hsa-miR-17-5p;hsa-miR-20a-5p;hsa-miR-20l
ENSG0000	ENSG0000	1.67E-05	hsa-miR-106b-5p;hsa-miR-17-5p;hsa-miR-20a-5p;hsa-miR-20l
ENSG0000	ENSG0000	7.46E-06	hsa-miR-106b-5p;hsa-miR-17-5p;hsa-miR-20a-5p;hsa-miR-20l
ENSG0000	ENSG0000	8.59E-06	hsa-miR-106b-5p;hsa-miR-17-5p;hsa-miR-20a-5p;hsa-miR-20l
ENSG0000	ENSG0000	0.001562	hsa-miR-106b-5p;hsa-miR-17-5p;hsa-miR-20a-5p;hsa-miR-20l
ENSG0000	ENSG0000	1.51E-05	hsa-miR-106b-5p;hsa-miR-17-5p;hsa-miR-20a-5p;hsa-miR-20l
ENSG0000	ENSG0000	0.000557	hsa-miR-106b-5p;hsa-miR-17-5p;hsa-miR-20a-5p;hsa-miR-20l
ENSG0000	ENSG0000	0.000414	hsa-miR-106b-5p;hsa-miR-20a-5p;hsa-miR-20b-5p;hsa-miR-93
ENSG0000	ENSG0000	1.87E-05	hsa-miR-106b-5p;hsa-miR-17-5p;hsa-miR-20a-5p;hsa-miR-20l

ENSG0000	ENSG0000	1.36E-05	hsa-miR-106b-5p;hsa-miR-17-5p;hsa-miR-20a-5p;hsa-miR-20[illegible]
ENSG0000	ENSG0000	2.78E-05	hsa-miR-199a-5p;hsa-miR-199b-5p;hsa-miR-30a-3p;hsa-miR-[illegible]
ENSG0000	ENSG0000	2.94E-09	hsa-miR-106b-5p;hsa-miR-17-5p;hsa-miR-20a-5p;hsa-miR-20[illegible]
ENSG0000	ENSG0000	0.0001	hsa-miR-106b-5p;hsa-miR-17-5p;hsa-miR-20a-5p;hsa-miR-20[illegible]
ENSG0000	ENSG0000	5.08E-05	hsa-miR-106b-5p;hsa-miR-17-5p;hsa-miR-20a-5p;hsa-miR-20[illegible]
ENSG0000	ENSG0000	3.11E-10	hsa-miR-101-3p;hsa-miR-106b-5p;hsa-miR-17-5p;hsa-miR-20[illegible]
ENSG0000	ENSG0000	4.30E-05	hsa-miR-106b-5p;hsa-miR-17-5p;hsa-miR-20a-5p;hsa-miR-20[illegible]
ENSG0000	ENSG0000	4.30E-05	hsa-miR-106b-5p;hsa-miR-17-5p;hsa-miR-20a-5p;hsa-miR-20[illegible]
ENSG0000	ENSG0000	0.001229	hsa-miR-106b-5p;hsa-miR-17-5p;hsa-miR-20a-5p;hsa-miR-20[illegible]
ENSG0000	ENSG0000	3.62E-05	hsa-miR-106b-5p;hsa-miR-17-5p;hsa-miR-20a-5p;hsa-miR-20[illegible]
ENSG0000	ENSG0000	7.14E-11	hsa-miR-106b-5p;hsa-miR-17-5p;hsa-miR-20a-5p;hsa-miR-20[illegible]
ENSG0000	ENSG0000	6.97E-05	hsa-miR-106b-5p;hsa-miR-17-5p;hsa-miR-20a-5p;hsa-miR-20[illegible]
ENSG0000	ENSG0000	3.59E-05	hsa-miR-106b-5p;hsa-miR-19a-3p;hsa-miR-19b-3p;hsa-miR-2[illegible]
ENSG0000	ENSG0000	6.59E-07	hsa-miR-106b-5p;hsa-miR-19a-3p;hsa-miR-19b-3p;hsa-miR-9[illegible]
ENSG0000	ENSG0000	2.50E-06	hsa-miR-106b-5p;hsa-miR-17-5p;hsa-miR-20a-5p;hsa-miR-20[illegible]
ENSG0000	ENSG0000	2.50E-06	hsa-miR-106b-5p;hsa-miR-17-5p;hsa-miR-20a-5p;hsa-miR-20[illegible]
ENSG0000	ENSG0000	2.50E-06	hsa-miR-106b-5p;hsa-miR-17-5p;hsa-miR-20a-5p;hsa-miR-20[illegible]
ENSG0000	ENSG0000	2.50E-06	hsa-miR-106b-5p;hsa-miR-17-5p;hsa-miR-20a-5p;hsa-miR-20[illegible]
ENSG0000	ENSG0000	2.50E-06	hsa-miR-106b-5p;hsa-miR-17-5p;hsa-miR-20a-5p;hsa-miR-20[illegible]
ENSG0000	ENSG0000	2.50E-06	hsa-miR-106b-5p;hsa-miR-17-5p;hsa-miR-20a-5p;hsa-miR-20[illegible]
ENSG0000	ENSG0000	8.33E-07	hsa-miR-101-3p;hsa-miR-106b-5p;hsa-miR-17-5p;hsa-miR-20[illegible]
ENSG0000	ENSG0000	3.12E-06	hsa-miR-106b-5p;hsa-miR-17-5p;hsa-miR-20a-5p;hsa-miR-20[illegible]
ENSG0000	ENSG0000	9.73E-05	hsa-miR-106b-5p;hsa-miR-19a-3p;hsa-miR-19b-3p;hsa-miR-2[illegible]
ENSG0000	ENSG0000	0.000108	hsa-miR-106b-5p;hsa-miR-17-5p;hsa-miR-20a-5p;hsa-miR-20[illegible]
ENSG0000	ENSG0000	1.15E-14	hsa-miR-106b-5p;hsa-miR-17-5p;hsa-miR-19a-3p;hsa-miR-19[illegible]
ENSG0000	ENSG0000	1.15E-14	hsa-miR-106b-5p;hsa-miR-17-5p;hsa-miR-19a-3p;hsa-miR-19[illegible]
ENSG0000	ENSG0000	6.45E-05	hsa-miR-106b-5p;hsa-miR-17-5p;hsa-miR-20a-5p;hsa-miR-20[illegible]
ENSG0000	ENSG0000	0.000272	hsa-miR-106b-5p;hsa-miR-17-5p;hsa-miR-20a-5p;hsa-miR-20[illegible]
ENSG0000	ENSG0000	0.000383	hsa-miR-106b-5p;hsa-miR-17-5p;hsa-miR-20a-5p;hsa-miR-20[illegible]
ENSG0000	ENSG0000	1.11E-07	hsa-miR-106b-5p;hsa-miR-17-5p;hsa-miR-20a-5p;hsa-miR-20[illegible]
ENSG0000	ENSG0000	1.48E-08	hsa-miR-106b-5p;hsa-miR-17-5p;hsa-miR-20a-5p;hsa-miR-20[illegible]
ENSG0000	ENSG0000	0.000288	hsa-miR-106b-5p;hsa-miR-17-5p;hsa-miR-20a-5p;hsa-miR-20[illegible]
ENSG0000	ENSG0000	3.31E-05	hsa-miR-106b-5p;hsa-miR-17-5p;hsa-miR-20a-5p;hsa-miR-20[illegible]
ENSG0000	ENSG0000	1.36E-05	hsa-miR-106b-5p;hsa-miR-17-5p;hsa-miR-20a-5p;hsa-miR-20[illegible]
ENSG0000	ENSG0000	0.000338	hsa-miR-106b-5p;hsa-miR-17-5p;hsa-miR-20a-5p;hsa-miR-20[illegible]
ENSG0000	ENSG0000	5.09E-07	hsa-miR-106b-5p;hsa-miR-17-5p;hsa-miR-20a-5p;hsa-miR-20[illegible]
ENSG0000	ENSG0000	0.000117	hsa-miR-106b-5p;hsa-miR-17-5p;hsa-miR-20a-5p;hsa-miR-20[illegible]
ENSG0000	ENSG0000	0.0012	hsa-miR-106b-5p;hsa-miR-17-5p;hsa-miR-20a-5p;hsa-miR-20[illegible]
ENSG0000	ENSG0000	2.28E-05	hsa-miR-106b-5p;hsa-miR-17-5p;hsa-miR-20a-5p;hsa-miR-20[illegible]
ENSG0000	ENSG0000	0.000517	hsa-miR-17-5p;hsa-miR-20a-5p;hsa-miR-20b-5p;hsa-miR-93-[illegible]
ENSG0000	ENSG0000	2.94E-09	hsa-miR-106b-5p;hsa-miR-17-5p;hsa-miR-20a-5p;hsa-miR-20[illegible]
ENSG0000	ENSG0000	0.000459	hsa-miR-106b-5p;hsa-miR-17-5p;hsa-miR-20a-5p;hsa-miR-20[illegible]
ENSG0000	ENSG0000	2.31E-06	hsa-miR-17-5p;hsa-miR-19a-3p;hsa-miR-19b-3p;hsa-miR-20a-[illegible]
ENSG0000	ENSG0000	1.01E-07	hsa-miR-106b-5p;hsa-miR-17-5p;hsa-miR-20a-5p;hsa-miR-20[illegible]
ENSG0000	ENSG0000	0.003672	hsa-miR-2110;hsa-miR-30a-3p;hsa-miR-30e-3p;hsa-miR-491-[illegible]
ENSG0000	ENSG0000	0.000192	hsa-miR-106b-5p;hsa-miR-17-5p;hsa-miR-20a-5p;hsa-miR-20[illegible]
ENSG0000	ENSG0000	4.30E-05	hsa-miR-101-3p;hsa-miR-106b-5p;hsa-miR-17-5p;hsa-miR-20[illegible]
ENSG0000	ENSG0000	4.05E-05	hsa-miR-101-3p;hsa-miR-106b-5p;hsa-miR-17-5p;hsa-miR-20[illegible]
ENSG0000	ENSG0000	1.27E-06	hsa-miR-106b-5p;hsa-miR-17-5p;hsa-miR-20a-5p;hsa-miR-20[illegible]
ENSG0000	ENSG0000	0.000984	hsa-miR-106b-5p;hsa-miR-17-5p;hsa-miR-20a-5p;hsa-miR-20[illegible]
ENSG0000	ENSG0000	3.25E-05	hsa-miR-106b-5p;hsa-miR-20a-5p;hsa-miR-20b-5p;hsa-miR-9[illegible]
ENSG0000	ENSG0000	7.14E-11	hsa-miR-106b-5p;hsa-miR-17-5p;hsa-miR-20a-5p;hsa-miR-20[illegible]
ENSG0000	ENSG0000	1.68E-05	hsa-miR-106b-5p;hsa-miR-17-5p;hsa-miR-20a-5p;hsa-miR-20[illegible]
ENSG0000	ENSG0000	1.69E-06	hsa-miR-106b-5p;hsa-miR-17-5p;hsa-miR-20a-5p;hsa-miR-20[illegible]

ENSG0000	ENSG0000	0.000132	hsa-miR-106b-5p;hsa-miR-17-5p;hsa-miR-20a-5p;hsa-miR-20b
ENSG0000	ENSG0000	0.004981	hsa-miR-20a-5p;hsa-miR-20b-5p;hsa-miR-491-5p;hsa-miR-93-
ENSG0000	ENSG0000	0.004981	hsa-miR-20a-5p;hsa-miR-20b-5p;hsa-miR-491-5p;hsa-miR-93-
ENSG0000	ENSG0000	0.004981	hsa-miR-20a-5p;hsa-miR-20b-5p;hsa-miR-491-5p;hsa-miR-93-
ENSG0000	ENSG0000	1.95E-09	hsa-miR-106b-5p;hsa-miR-17-5p;hsa-miR-19a-3p;hsa-miR-19b
ENSG0000	ENSG0000	1.95E-09	hsa-miR-106b-5p;hsa-miR-17-5p;hsa-miR-19a-3p;hsa-miR-19b
ENSG0000	ENSG0000	0.000304	hsa-miR-106b-5p;hsa-miR-17-5p;hsa-miR-20a-5p;hsa-miR-20b
ENSG0000	ENSG0000	1.52E-06	hsa-miR-106b-5p;hsa-miR-17-5p;hsa-miR-20a-5p;hsa-miR-20b
ENSG0000	ENSG0000	0.000595	hsa-miR-106b-5p;hsa-miR-17-5p;hsa-miR-20a-5p;hsa-miR-20b
ENSG0000	ENSG0000	7.15E-07	hsa-miR-106b-5p;hsa-miR-17-5p;hsa-miR-20a-5p;hsa-miR-20b
ENSG0000	ENSG0000	0.000595	hsa-miR-106b-5p;hsa-miR-20a-5p;hsa-miR-20b-5p;hsa-miR-93
ENSG0000	ENSG0000	2.26E-07	hsa-miR-106b-5p;hsa-miR-17-5p;hsa-miR-20a-5p;hsa-miR-20b
ENSG0000	ENSG0000	1.08E-10	hsa-miR-106b-5p;hsa-miR-17-5p;hsa-miR-19a-3p;hsa-miR-19b
ENSG0000	ENSG0000	1.28E-05	hsa-miR-106b-5p;hsa-miR-17-5p;hsa-miR-20a-5p;hsa-miR-20b
ENSG0000	ENSG0000	5.16E-06	hsa-miR-106b-5p;hsa-miR-17-5p;hsa-miR-20a-5p;hsa-miR-20b
ENSG0000	ENSG0000	3.26E-11	hsa-miR-101-3p;hsa-miR-106b-5p;hsa-miR-17-5p;hsa-miR-19a
ENSG0000	ENSG0000	0.000639	hsa-miR-106b-5p;hsa-miR-17-5p;hsa-miR-20a-5p;hsa-miR-20b
ENSG0000	ENSG0000	0.000639	hsa-miR-106b-5p;hsa-miR-17-5p;hsa-miR-20a-5p;hsa-miR-20b
ENSG0000	ENSG0000	9.35E-05	hsa-miR-17-5p;hsa-miR-20a-5p;hsa-miR-20b-5p;hsa-miR-93-5
ENSG0000	ENSG0000	4.52E-06	hsa-miR-106b-5p;hsa-miR-17-5p;hsa-miR-20a-5p;hsa-miR-20b
ENSG0000	ENSG0000	0.001317	hsa-miR-106b-5p;hsa-miR-17-5p;hsa-miR-20a-5p;hsa-miR-20b
ENSG0000	ENSG0000	0.000611	hsa-miR-106b-5p;hsa-miR-17-5p;hsa-miR-20a-5p;hsa-miR-20b
ENSG0000	ENSG0000	9.68E-06	hsa-miR-106b-5p;hsa-miR-17-5p;hsa-miR-20a-5p;hsa-miR-20b
ENSG0000	ENSG0000	3.03E-05	hsa-miR-106b-5p;hsa-miR-17-5p;hsa-miR-20a-5p;hsa-miR-20b
ENSG0000	ENSG0000	7.14E-11	hsa-miR-106b-5p;hsa-miR-17-5p;hsa-miR-20a-5p;hsa-miR-20b
ENSG0000	ENSG0000	3.09E-06	hsa-miR-106b-5p;hsa-miR-17-5p;hsa-miR-19a-3p;hsa-miR-19b
ENSG0000	ENSG0000	8.59E-06	hsa-miR-106b-5p;hsa-miR-17-5p;hsa-miR-20a-5p;hsa-miR-20b
ENSG0000	ENSG0000	8.59E-06	hsa-miR-106b-5p;hsa-miR-17-5p;hsa-miR-20a-5p;hsa-miR-20b
ENSG0000	ENSG0000	3.37E-05	hsa-miR-106b-5p;hsa-miR-17-5p;hsa-miR-20a-5p;hsa-miR-20b
ENSG0000	ENSG0000	0.00013	hsa-miR-17-5p;hsa-miR-20a-5p;hsa-miR-20b-5p;hsa-miR-93-5
ENSG0000	ENSG0000	5.16E-06	hsa-miR-106b-5p;hsa-miR-17-5p;hsa-miR-20a-5p;hsa-miR-20b
ENSG0000	ENSG0000	7.61E-06	hsa-miR-106b-5p;hsa-miR-17-5p;hsa-miR-20a-5p;hsa-miR-20b
ENSG0000	ENSG0000	0.00072	hsa-miR-199a-5p;hsa-miR-199b-5p;hsa-miR-27a-5p;hsa-miR-2
ENSG0000	ENSG0000	7.46E-06	hsa-miR-106b-5p;hsa-miR-17-5p;hsa-miR-20a-5p;hsa-miR-20b
ENSG0000	ENSG0000	7.14E-11	hsa-miR-106b-5p;hsa-miR-17-5p;hsa-miR-20a-5p;hsa-miR-20b
ENSG0000	ENSG0000	8.53E-08	hsa-miR-101-3p;hsa-miR-106b-5p;hsa-miR-17-5p;hsa-miR-19a
ENSG0000	ENSG0000	2.51E-05	hsa-miR-106b-5p;hsa-miR-17-5p;hsa-miR-20a-5p;hsa-miR-20b
ENSG0000	ENSG0000	0.007732	hsa-miR-106b-5p;hsa-miR-17-5p;hsa-miR-20a-5p;hsa-miR-20b
ENSG0000	ENSG0000	1.09E-05	hsa-miR-106b-5p;hsa-miR-17-5p;hsa-miR-20a-5p;hsa-miR-20b
ENSG0000	ENSG0000	0.000181	hsa-miR-106b-5p;hsa-miR-17-5p;hsa-miR-20a-5p;hsa-miR-20b
ENSG0000	ENSG0000	1.48E-09	hsa-miR-106b-5p;hsa-miR-17-5p;hsa-miR-20a-5p;hsa-miR-20b
ENSG0000	ENSG0000	0.000243	hsa-miR-106b-5p;hsa-miR-17-5p;hsa-miR-20a-5p;hsa-miR-20b
ENSG0000	ENSG0000	3.37E-05	hsa-miR-106b-5p;hsa-miR-17-5p;hsa-miR-20a-5p;hsa-miR-20b
ENSG0000	ENSG0000	9.65E-08	hsa-miR-106b-5p;hsa-miR-17-5p;hsa-miR-20a-5p;hsa-miR-20b
ENSG0000	ENSG0000	0.007416	hsa-miR-19a-3p;hsa-miR-19b-3p;hsa-miR-28-3p;hsa-miR-30a-
ENSG0000	ENSG0000	8.22E-06	hsa-miR-106b-5p;hsa-miR-17-5p;hsa-miR-19a-3p;hsa-miR-19b
ENSG0000	ENSG0000	0.00015	hsa-miR-106b-5p;hsa-miR-17-5p;hsa-miR-20a-5p;hsa-miR-20b
ENSG0000	ENSG0000	7.14E-11	hsa-miR-106b-5p;hsa-miR-17-5p;hsa-miR-20a-5p;hsa-miR-20b
ENSG0000	ENSG0000	7.14E-11	hsa-miR-106b-5p;hsa-miR-17-5p;hsa-miR-20a-5p;hsa-miR-20b
ENSG0000	ENSG0000	7.14E-11	hsa-miR-106b-5p;hsa-miR-17-5p;hsa-miR-20a-5p;hsa-miR-20b
ENSG0000	ENSG0000	7.14E-11	hsa-miR-106b-5p;hsa-miR-17-5p;hsa-miR-20a-5p;hsa-miR-20b
ENSG0000	ENSG0000	1.83E-07	hsa-miR-106b-5p;hsa-miR-17-5p;hsa-miR-20a-5p;hsa-miR-20b
ENSG0000	ENSG0000	1.52E-06	hsa-miR-106b-5p;hsa-miR-17-5p;hsa-miR-20a-5p;hsa-miR-20b
ENSG0000	ENSG0000	0.000204	hsa-miR-106b-5p;hsa-miR-17-5p;hsa-miR-20a-5p;hsa-miR-20b

ENSG0000	ENSG0000	1.36E-06	hsa-miR-106b-5p;hsa-miR-17-5p;hsa-miR-20a-5p;hsa-miR-20l
ENSG0000	ENSG0000	0.0001	hsa-miR-106b-5p;hsa-miR-17-5p;hsa-miR-20a-5p;hsa-miR-20l
ENSG0000	ENSG0000	0.000321	hsa-miR-106b-5p;hsa-miR-17-5p;hsa-miR-20a-5p;hsa-miR-20l
ENSG0000	ENSG0000	1.56E-11	hsa-miR-106b-5p;hsa-miR-17-5p;hsa-miR-19a-3p;hsa-miR-19l
ENSG0000	ENSG0000	1.51E-05	hsa-miR-106b-5p;hsa-miR-17-5p;hsa-miR-19a-3p;hsa-miR-19l
ENSG0000	ENSG0000	0.000376	hsa-miR-106b-5p;hsa-miR-17-5p;hsa-miR-20a-5p;hsa-miR-20l
ENSG0000	ENSG0000	5.08E-05	hsa-miR-106b-5p;hsa-miR-17-5p;hsa-miR-20a-5p;hsa-miR-20l
ENSG0000	ENSG0000	0.007068	hsa-miR-106b-5p;hsa-miR-17-5p;hsa-miR-20a-5p;hsa-miR-20l
ENSG0000	ENSG0000	6.46E-14	hsa-miR-106b-5p;hsa-miR-17-5p;hsa-miR-19b-3p;hsa-miR-20a
ENSG0000	ENSG0000	1.80E-06	hsa-miR-106b-5p;hsa-miR-17-5p;hsa-miR-19a-3p;hsa-miR-20a
ENSG0000	ENSG0000	0.001802	hsa-miR-106b-5p;hsa-miR-17-5p;hsa-miR-20a-5p;hsa-miR-20l
ENSG0000	ENSG0000	0.001248	hsa-miR-106b-5p;hsa-miR-17-5p;hsa-miR-20a-5p;hsa-miR-20l
ENSG0000	ENSG0000	3.59E-05	hsa-miR-106b-5p;hsa-miR-17-5p;hsa-miR-20a-5p;hsa-miR-20l
ENSG0000	ENSG0000	1.09E-05	hsa-miR-106b-5p;hsa-miR-17-5p;hsa-miR-20a-5p;hsa-miR-20l
ENSG0000	ENSG0000	1.95E-09	hsa-miR-106b-5p;hsa-miR-17-5p;hsa-miR-19a-3p;hsa-miR-19l
ENSG0000	ENSG0000	6.60E-10	hsa-miR-106b-5p;hsa-miR-17-5p;hsa-miR-20a-5p;hsa-miR-20l
ENSG0000	ENSG0000	0.006577	hsa-miR-106b-5p;hsa-miR-17-5p;hsa-miR-20b-5p;hsa-miR-93
ENSG0000	ENSG0000	0.006577	hsa-miR-106b-5p;hsa-miR-17-5p;hsa-miR-20b-5p;hsa-miR-93
ENSG0000	ENSG0000	4.51E-06	hsa-miR-106b-5p;hsa-miR-17-5p;hsa-miR-20a-5p;hsa-miR-20l
ENSG0000	ENSG0000	2.49E-10	hsa-miR-106b-5p;hsa-miR-17-5p;hsa-miR-20a-5p;hsa-miR-20l
ENSG0000	ENSG0000	2.13E-06	hsa-miR-106b-5p;hsa-miR-17-5p;hsa-miR-20a-5p;hsa-miR-20l
ENSG0000	ENSG0000	7.51E-05	hsa-miR-106b-5p;hsa-miR-17-5p;hsa-miR-20a-5p;hsa-miR-20l
ENSG0000	ENSG0000	5.51E-05	hsa-miR-106b-5p;hsa-miR-17-5p;hsa-miR-20a-5p;hsa-miR-20l
ENSG0000	ENSG0000	1.80E-06	hsa-miR-106b-5p;hsa-miR-17-5p;hsa-miR-20a-5p;hsa-miR-20l
ENSG0000	ENSG0000	1.73E-07	hsa-miR-106b-5p;hsa-miR-17-5p;hsa-miR-20a-5p;hsa-miR-20l
ENSG0000	ENSG0000	0.000288	hsa-miR-106b-5p;hsa-miR-17-5p;hsa-miR-20a-5p;hsa-miR-20l
ENSG0000	ENSG0000	5.51E-05	hsa-miR-106b-5p;hsa-miR-17-5p;hsa-miR-20a-5p;hsa-miR-20l
ENSG0000	ENSG0000	8.22E-06	hsa-miR-101-3p;hsa-miR-106b-5p;hsa-miR-17-5p;hsa-miR-20a
ENSG0000	ENSG0000	1.87E-05	hsa-miR-106b-5p;hsa-miR-17-5p;hsa-miR-20a-5p;hsa-miR-20l
ENSG0000	ENSG0000	0.001154	hsa-miR-106b-5p;hsa-miR-17-5p;hsa-miR-20a-5p;hsa-miR-20l
ENSG0000	ENSG0000	0.000595	hsa-miR-106b-5p;hsa-miR-17-5p;hsa-miR-20a-5p;hsa-miR-20l
ENSG0000	ENSG0000	1.36E-06	hsa-miR-106b-5p;hsa-miR-17-5p;hsa-miR-19a-3p;hsa-miR-19l
ENSG0000	ENSG0000	1.21E-06	hsa-miR-106b-5p;hsa-miR-19a-3p;hsa-miR-19b-3p;hsa-miR-2(
ENSG0000	ENSG0000	7.15E-07	hsa-miR-106b-5p;hsa-miR-17-5p;hsa-miR-20a-5p;hsa-miR-20l
ENSG0000	ENSG0000	8.22E-06	hsa-miR-103a-2-5p;hsa-miR-106b-5p;hsa-miR-17-5p;hsa-miR-
ENSG0000	ENSG0000	0.001398	hsa-miR-106b-5p;hsa-miR-17-5p;hsa-miR-20a-5p;hsa-miR-20l
ENSG0000	ENSG0000	2.49E-10	hsa-miR-106b-5p;hsa-miR-17-5p;hsa-miR-20a-5p;hsa-miR-20l
ENSG0000	ENSG0000	0.000217	hsa-miR-106b-5p;hsa-miR-17-5p;hsa-miR-20a-5p;hsa-miR-20l
ENSG0000	ENSG0000	0.00017	hsa-miR-106b-5p;hsa-miR-17-5p;hsa-miR-20a-5p;hsa-miR-20l
ENSG0000	ENSG0000	0.00615	hsa-miR-17-5p;hsa-miR-20a-5p;hsa-miR-20b-5p;hsa-miR-93-!
ENSG0000	ENSG0000	8.71E-05	hsa-miR-106b-5p;hsa-miR-17-5p;hsa-miR-20a-5p;hsa-miR-20l
ENSG0000	ENSG0000	3.01E-07	hsa-miR-106b-5p;hsa-miR-17-5p;hsa-miR-20a-5p;hsa-miR-20l
ENSG0000	ENSG0000	0.000172	hsa-miR-106b-5p;hsa-miR-17-5p;hsa-miR-20a-5p;hsa-miR-20l
ENSG0000	ENSG0000	3.77E-06	hsa-miR-19a-3p;hsa-miR-19b-3p;hsa-miR-20a-5p;hsa-miR-20l
ENSG0000	ENSG0000	0.0001	hsa-miR-106b-5p;hsa-miR-17-5p;hsa-miR-20b-5p;hsa-miR-24
ENSG0000	ENSG0000	1.51E-06	hsa-miR-101-3p;hsa-miR-106b-5p;hsa-miR-17-5p;hsa-miR-20a
ENSG0000	ENSG0000	0.001678	hsa-miR-106b-5p;hsa-miR-17-5p;hsa-miR-20a-5p;hsa-miR-20l
ENSG0000	ENSG0000	5.72E-05	hsa-miR-106b-5p;hsa-miR-17-5p;hsa-miR-20a-5p;hsa-miR-20l
ENSG0000	ENSG0000	0.000459	hsa-miR-106b-5p;hsa-miR-17-5p;hsa-miR-20a-5p;hsa-miR-20l
ENSG0000	ENSG0000	2.78E-05	hsa-miR-106b-5p;hsa-miR-17-5p;hsa-miR-20a-5p;hsa-miR-20l
ENSG0000	ENSG0000	2.66E-15	hsa-miR-106b-5p;hsa-miR-17-5p;hsa-miR-19a-3p;hsa-miR-19l
ENSG0000	ENSG0000	2.66E-15	hsa-miR-106b-5p;hsa-miR-17-5p;hsa-miR-19a-3p;hsa-miR-19l
ENSG0000	ENSG0000	2.26E-07	hsa-miR-106b-5p;hsa-miR-17-5p;hsa-miR-20a-5p;hsa-miR-20l
ENSG0000	ENSG0000	1.51E-05	hsa-miR-106b-5p;hsa-miR-17-5p;hsa-miR-20a-5p;hsa-miR-20l

ENSG0000	ENSG0000	1.48E-08	hsa-miR-106b-5p;hsa-miR-17-5p;hsa-miR-20a-5p;hsa-miR-20
ENSG0000	ENSG0000	2.30E-08	hsa-miR-106b-5p;hsa-miR-17-5p;hsa-miR-20a-5p;hsa-miR-20
ENSG0000	ENSG0000	2.30E-08	hsa-miR-106b-5p;hsa-miR-17-5p;hsa-miR-20a-5p;hsa-miR-20
ENSG0000	ENSG0000	0.000383	hsa-miR-19a-3p;hsa-miR-19b-3p;hsa-miR-30a-3p;hsa-miR-30
ENSG0000	ENSG0000	8.73E-07	hsa-miR-106b-5p;hsa-miR-17-5p;hsa-miR-20a-5p;hsa-miR-20
ENSG0000	ENSG0000	1.09E-05	hsa-miR-106b-5p;hsa-miR-17-5p;hsa-miR-20a-5p;hsa-miR-20
ENSG0000	ENSG0000	1.09E-05	hsa-miR-106b-5p;hsa-miR-17-5p;hsa-miR-20a-5p;hsa-miR-20
ENSG0000	ENSG0000	1.09E-05	hsa-miR-106b-5p;hsa-miR-17-5p;hsa-miR-20a-5p;hsa-miR-20
ENSG0000	ENSG0000	0.000232	hsa-miR-106b-5p;hsa-miR-17-5p;hsa-miR-20a-5p;hsa-miR-20
ENSG0000	ENSG0000	2.83E-06	hsa-miR-106b-5p;hsa-miR-17-5p;hsa-miR-20a-5p;hsa-miR-20
ENSG0000	ENSG0000	9.11E-06	hsa-miR-106b-5p;hsa-miR-17-5p;hsa-miR-199a-5p;hsa-miR-1
ENSG0000	ENSG0000	1.87E-05	hsa-miR-106b-5p;hsa-miR-17-5p;hsa-miR-20a-5p;hsa-miR-20
ENSG0000	ENSG0000	0.001879	hsa-miR-106b-5p;hsa-miR-17-5p;hsa-miR-20a-5p;hsa-miR-20
ENSG0000	ENSG0000	5.91E-06	hsa-miR-199a-5p;hsa-miR-199b-5p;hsa-miR-19a-3p;hsa-miR-
ENSG0000	ENSG0000	2.51E-05	hsa-miR-106b-5p;hsa-miR-17-5p;hsa-miR-20a-5p;hsa-miR-20
ENSG0000	ENSG0000	6.97E-05	hsa-miR-106b-5p;hsa-miR-17-5p;hsa-miR-20a-5p;hsa-miR-20
ENSG0000	ENSG0000	9.65E-08	hsa-miR-106b-5p;hsa-miR-17-5p;hsa-miR-20a-5p;hsa-miR-20
ENSG0000	ENSG0000	0.002621	hsa-miR-17-5p;hsa-miR-20a-5p;hsa-miR-20b-5p;hsa-miR-93-
ENSG0000	ENSG0000	0.001451	hsa-miR-106b-5p;hsa-miR-17-5p;hsa-miR-20a-5p;hsa-miR-20
ENSG0000	ENSG0000	5.90E-06	hsa-miR-106b-5p;hsa-miR-17-5p;hsa-miR-20a-5p;hsa-miR-20
ENSG0000	ENSG0000	3.78E-05	hsa-miR-17-5p;hsa-miR-20a-5p;hsa-miR-20b-5p;hsa-miR-93-
ENSG0000	ENSG0000	0.0001	hsa-miR-106b-5p;hsa-miR-17-5p;hsa-miR-19b-3p;hsa-miR-20
ENSG0000	ENSG0000	2.88E-06	hsa-miR-106b-5p;hsa-miR-17-5p;hsa-miR-19a-3p;hsa-miR-19
ENSG0000	ENSG0000	0.000395	hsa-miR-106b-5p;hsa-miR-17-5p;hsa-miR-20a-5p;hsa-miR-20
ENSG0000	ENSG0000	7.61E-06	hsa-miR-106b-5p;hsa-miR-17-5p;hsa-miR-20a-5p;hsa-miR-20
ENSG0000	ENSG0000	1.03E-08	hsa-miR-17-5p;hsa-miR-20a-5p;hsa-miR-20b-5p;hsa-miR-93-
ENSG0000	ENSG0000	5.51E-05	hsa-miR-106b-5p;hsa-miR-17-5p;hsa-miR-20a-5p;hsa-miR-20
ENSG0000	ENSG0000	2.26E-07	hsa-miR-106b-5p;hsa-miR-17-5p;hsa-miR-20a-5p;hsa-miR-20
ENSG0000	ENSG0000	0.0001	hsa-miR-106b-5p;hsa-miR-17-5p;hsa-miR-20a-5p;hsa-miR-20
ENSG0000	ENSG0000	0.000868	hsa-miR-106b-5p;hsa-miR-17-5p;hsa-miR-20a-5p;hsa-miR-20
ENSG0000	ENSG0000	0.000383	hsa-miR-106b-5p;hsa-miR-17-5p;hsa-miR-20a-5p;hsa-miR-20
ENSG0000	ENSG0000	1.52E-06	hsa-miR-199a-5p;hsa-miR-199b-5p;hsa-miR-24-3p;hsa-miR-3
ENSG0000	ENSG0000	1.52E-06	hsa-miR-199a-5p;hsa-miR-199b-5p;hsa-miR-24-3p;hsa-miR-3
ENSG0000	ENSG0000	2.30E-08	hsa-miR-106b-5p;hsa-miR-17-5p;hsa-miR-20a-5p;hsa-miR-20
ENSG0000	ENSG0000	2.67E-06	hsa-miR-106b-5p;hsa-miR-17-5p;hsa-miR-20a-5p;hsa-miR-20
ENSG0000	ENSG0000	1.60E-06	hsa-miR-106b-5p;hsa-miR-17-5p;hsa-miR-19a-3p;hsa-miR-19
ENSG0000	ENSG0000	0.000141	hsa-miR-106b-5p;hsa-miR-17-5p;hsa-miR-20a-5p;hsa-miR-20
ENSG0000	ENSG0000	5.29E-09	hsa-miR-106b-5p;hsa-miR-17-5p;hsa-miR-19a-3p;hsa-miR-19
ENSG0000	ENSG0000	3.46E-08	hsa-miR-106b-5p;hsa-miR-17-5p;hsa-miR-20a-5p;hsa-miR-20
ENSG0000	ENSG0000	9.28E-06	hsa-miR-106b-5p;hsa-miR-17-5p;hsa-miR-20a-5p;hsa-miR-20
ENSG0000	ENSG0000	2.30E-08	hsa-miR-106b-5p;hsa-miR-17-5p;hsa-miR-20a-5p;hsa-miR-20
ENSG0000	ENSG0000	1.55E-07	hsa-miR-106b-5p;hsa-miR-17-5p;hsa-miR-19a-3p;hsa-miR-20
ENSG0000	ENSG0000	0.000395	hsa-miR-106b-5p;hsa-miR-17-5p;hsa-miR-20a-5p;hsa-miR-20
ENSG0000	ENSG0000	0.001296	hsa-miR-101-3p;hsa-miR-106b-5p;hsa-miR-17-5p;hsa-miR-20
ENSG0000	ENSG0000	1.42E-07	hsa-miR-106b-5p;hsa-miR-17-5p;hsa-miR-20a-5p;hsa-miR-20
ENSG0000	ENSG0000	6.32E-07	hsa-miR-106b-5p;hsa-miR-17-5p;hsa-miR-19a-3p;hsa-miR-19
ENSG0000	ENSG0000	0.000636	hsa-miR-106b-5p;hsa-miR-17-5p;hsa-miR-20a-5p;hsa-miR-20
ENSG0000	ENSG0000	0.000775	hsa-miR-106b-5p;hsa-miR-17-5p;hsa-miR-20a-5p;hsa-miR-20
ENSG0000	ENSG0000	0.000376	hsa-miR-106b-5p;hsa-miR-17-5p;hsa-miR-20a-5p;hsa-miR-20
ENSG0000	ENSG0000	1.06E-06	hsa-miR-106b-5p;hsa-miR-17-5p;hsa-miR-19b-3p;hsa-miR-20
ENSG0000	ENSG0000	7.14E-11	hsa-miR-106b-5p;hsa-miR-17-5p;hsa-miR-20a-5p;hsa-miR-20
ENSG0000	ENSG0000	5.90E-06	hsa-miR-106b-5p;hsa-miR-17-5p;hsa-miR-20a-5p;hsa-miR-20
ENSG0000	ENSG0000	0.001866	hsa-miR-106b-5p;hsa-miR-17-5p;hsa-miR-20a-5p;hsa-miR-20
ENSG0000	ENSG0000	5.08E-05	hsa-miR-199a-5p;hsa-miR-199b-5p;hsa-miR-19a-3p;hsa-miR-

ENSG0000	ENSG0000	0.001982	hsa-miR-106b-5p;hsa-miR-17-5p;hsa-miR-20a-5p;hsa-miR-20
ENSG0000	ENSG0000	1.07E-06	hsa-miR-106b-5p;hsa-miR-17-5p;hsa-miR-19a-3p;hsa-miR-19
ENSG0000	ENSG0000	2.35E-08	hsa-miR-106b-5p;hsa-miR-17-5p;hsa-miR-19a-3p;hsa-miR-19
ENSG0000	ENSG0000	2.67E-06	hsa-miR-106b-5p;hsa-miR-17-5p;hsa-miR-20a-5p;hsa-miR-20
ENSG0000	ENSG0000	0.000944	hsa-miR-106b-5p;hsa-miR-17-5p;hsa-miR-20a-5p;hsa-miR-20
ENSG0000	ENSG0000	4.36E-05	hsa-miR-106b-5p;hsa-miR-17-5p;hsa-miR-20a-5p;hsa-miR-20
ENSG0000	ENSG0000	4.36E-05	hsa-miR-106b-5p;hsa-miR-17-5p;hsa-miR-20a-5p;hsa-miR-20
ENSG0000	ENSG0000	0.000217	hsa-miR-106b-5p;hsa-miR-17-5p;hsa-miR-19a-3p;hsa-miR-20
ENSG0000	ENSG0000	1.27E-06	hsa-miR-106b-5p;hsa-miR-17-5p;hsa-miR-20a-5p;hsa-miR-20
ENSG0000	ENSG0000	6.45E-05	hsa-miR-106b-5p;hsa-miR-17-5p;hsa-miR-20a-5p;hsa-miR-20
ENSG0000	ENSG0000	6.45E-05	hsa-miR-106b-5p;hsa-miR-17-5p;hsa-miR-20a-5p;hsa-miR-20
ENSG0000	ENSG0000	0.007978	hsa-miR-106b-5p;hsa-miR-17-5p;hsa-miR-20a-5p;hsa-miR-20
ENSG0000	ENSG0000	7.14E-11	hsa-miR-106b-5p;hsa-miR-17-5p;hsa-miR-20a-5p;hsa-miR-20
ENSG0000	ENSG0000	6.71E-06	hsa-miR-106b-5p;hsa-miR-17-5p;hsa-miR-20a-5p;hsa-miR-20
ENSG0000	ENSG0000	3.62E-05	hsa-miR-106b-5p;hsa-miR-17-5p;hsa-miR-20a-5p;hsa-miR-20
ENSG0000	ENSG0000	0.000107	hsa-miR-101-3p;hsa-miR-106b-5p;hsa-miR-17-5p;hsa-miR-20
ENSG0000	ENSG0000	0.000107	hsa-miR-101-3p;hsa-miR-106b-5p;hsa-miR-17-5p;hsa-miR-20
ENSG0000	ENSG0000	1.27E-06	hsa-miR-106b-5p;hsa-miR-17-5p;hsa-miR-20a-5p;hsa-miR-20
ENSG0000	ENSG0000	0.00333	hsa-miR-106b-5p;hsa-miR-17-5p;hsa-miR-20a-5p;hsa-miR-20
ENSG0000	ENSG0000	5.97E-05	hsa-miR-106b-5p;hsa-miR-17-5p;hsa-miR-20a-5p;hsa-miR-20
ENSG0000	ENSG0000	7.37E-05	hsa-miR-106b-5p;hsa-miR-17-5p;hsa-miR-20b-5p;hsa-miR-93
ENSG0000	ENSG0000	2.13E-06	hsa-miR-106b-5p;hsa-miR-17-5p;hsa-miR-20a-5p;hsa-miR-20
ENSG0000	ENSG0000	8.59E-06	hsa-miR-106b-5p;hsa-miR-17-5p;hsa-miR-20a-5p;hsa-miR-20
ENSG0000	ENSG0000	6.75E-05	hsa-miR-101-3p;hsa-miR-106b-5p;hsa-miR-17-5p;hsa-miR-20
ENSG0000	ENSG0000	8.32E-05	hsa-miR-17-5p;hsa-miR-20a-5p;hsa-miR-20b-5p;hsa-miR-93-
ENSG0000	ENSG0000	3.57E-11	hsa-miR-106b-5p;hsa-miR-17-5p;hsa-miR-19a-3p;hsa-miR-19
ENSG0000	ENSG0000	2.13E-06	hsa-miR-106b-5p;hsa-miR-17-5p;hsa-miR-20a-5p;hsa-miR-20
ENSG0000	ENSG0000	0.002621	hsa-miR-106b-5p;hsa-miR-17-5p;hsa-miR-20a-5p;hsa-miR-20
ENSG0000	ENSG0000	0.002772	hsa-miR-106b-5p;hsa-miR-17-5p;hsa-miR-20a-5p;hsa-miR-20
ENSG0000	ENSG0000	1.51E-05	hsa-miR-106b-5p;hsa-miR-17-5p;hsa-miR-20a-5p;hsa-miR-20
ENSG0000	ENSG0000	0.004947	hsa-miR-106b-5p;hsa-miR-17-5p;hsa-miR-20a-5p;hsa-miR-20
ENSG0000	ENSG0000	5.37E-09	hsa-miR-106b-5p;hsa-miR-17-5p;hsa-miR-20a-5p;hsa-miR-20
ENSG0000	ENSG0000	0.000193	hsa-miR-106b-5p;hsa-miR-17-5p;hsa-miR-20a-5p;hsa-miR-20
ENSG0000	ENSG0000	4.05E-06	hsa-miR-106b-5p;hsa-miR-17-5p;hsa-miR-199a-5p;hsa-miR-1
ENSG0000	ENSG0000	4.05E-06	hsa-miR-106b-5p;hsa-miR-17-5p;hsa-miR-199a-5p;hsa-miR-1
ENSG0000	ENSG0000	0.000288	hsa-miR-106b-5p;hsa-miR-17-5p;hsa-miR-20a-5p;hsa-miR-20
ENSG0000	ENSG0000	5.90E-06	hsa-miR-106b-5p;hsa-miR-17-5p;hsa-miR-20a-5p;hsa-miR-20
ENSG0000	ENSG0000	5.97E-05	hsa-miR-106b-5p;hsa-miR-17-5p;hsa-miR-20a-5p;hsa-miR-20
ENSG0000	ENSG0000	0.004687	hsa-miR-106b-5p;hsa-miR-17-5p;hsa-miR-20a-5p;hsa-miR-20
ENSG0000	ENSG0000	0.000476	hsa-miR-106b-5p;hsa-miR-17-5p;hsa-miR-20a-5p;hsa-miR-20
ENSG0000	ENSG0000	9.68E-06	hsa-miR-106b-5p;hsa-miR-17-5p;hsa-miR-20a-5p;hsa-miR-20
ENSG0000	ENSG0000	1.06E-06	hsa-miR-106b-5p;hsa-miR-17-5p;hsa-miR-20a-5p;hsa-miR-20
ENSG0000	ENSG0000	0.000376	hsa-miR-106b-5p;hsa-miR-17-5p;hsa-miR-20a-5p;hsa-miR-20
ENSG0000	ENSG0000	0.006361	hsa-miR-106b-5p;hsa-miR-17-5p;hsa-miR-20a-5p;hsa-miR-20
ENSG0000	ENSG0000	0.0001	hsa-miR-106b-5p;hsa-miR-17-5p;hsa-miR-20a-5p;hsa-miR-20
ENSG0000	ENSG0000	5.08E-05	hsa-miR-106b-5p;hsa-miR-17-5p;hsa-miR-20a-5p;hsa-miR-20
ENSG0000	ENSG0000	2.92E-06	hsa-miR-106b-5p;hsa-miR-17-5p;hsa-miR-20a-5p;hsa-miR-20
ENSG0000	ENSG0000	0.000354	hsa-miR-106b-5p;hsa-miR-17-5p;hsa-miR-20b-5p;hsa-miR-93
ENSG0000	ENSG0000	0.000115	hsa-miR-106b-5p;hsa-miR-17-5p;hsa-miR-20a-5p;hsa-miR-20
ENSG0000	ENSG0000	6.71E-06	hsa-miR-106b-5p;hsa-miR-17-5p;hsa-miR-20a-5p;hsa-miR-20
ENSG0000	ENSG0000	6.71E-06	hsa-miR-106b-5p;hsa-miR-17-5p;hsa-miR-20a-5p;hsa-miR-20
ENSG0000	ENSG0000	5.21E-11	hsa-miR-106b-5p;hsa-miR-17-5p;hsa-miR-19a-3p;hsa-miR-19
ENSG0000	ENSG0000	5.21E-11	hsa-miR-106b-5p;hsa-miR-17-5p;hsa-miR-19a-3p;hsa-miR-19
ENSG0000	ENSG0000	7.14E-11	hsa-miR-106b-5p;hsa-miR-17-5p;hsa-miR-20a-5p;hsa-miR-20

ENSG0000	ENSG0000	7.14E-11	hsa-miR-106b-5p;hsa-miR-17-5p;hsa-miR-20a-5p;hsa-miR-20
ENSG0000	ENSG0000	0.000243	hsa-miR-106b-5p;hsa-miR-17-5p;hsa-miR-20a-5p;hsa-miR-20
ENSG0000	ENSG0000	8.09E-05	hsa-miR-106b-5p;hsa-miR-17-5p;hsa-miR-20a-5p;hsa-miR-20
ENSG0000	ENSG0000	0.001317	hsa-miR-17-5p;hsa-miR-20a-5p;hsa-miR-20b-5p;hsa-miR-93-
ENSG0000	ENSG0000	3.29E-09	hsa-miR-106b-5p;hsa-miR-17-5p;hsa-miR-19a-3p;hsa-miR-20
ENSG0000	ENSG0000	0.000115	hsa-miR-106b-5p;hsa-miR-17-5p;hsa-miR-20a-5p;hsa-miR-20
ENSG0000	ENSG0000	1.27E-06	hsa-miR-106b-5p;hsa-miR-17-5p;hsa-miR-20a-5p;hsa-miR-20
ENSG0000	ENSG0000	0.001246	hsa-miR-106b-5p;hsa-miR-17-5p;hsa-miR-20a-5p;hsa-miR-20
ENSG0000	ENSG0000	1.73E-07	hsa-miR-106b-5p;hsa-miR-17-5p;hsa-miR-20a-5p;hsa-miR-20
ENSG0000	ENSG0000	4.68E-05	hsa-miR-106b-5p;hsa-miR-17-5p;hsa-miR-20a-5p;hsa-miR-20
ENSG0000	ENSG0000	1.03E-08	hsa-miR-106b-5p;hsa-miR-17-5p;hsa-miR-20a-5p;hsa-miR-20
ENSG0000	ENSG0000	0.002621	hsa-miR-199a-5p;hsa-miR-199b-5p;hsa-miR-30a-3p;hsa-miR-
ENSG0000	ENSG0000	2.51E-05	hsa-miR-106b-5p;hsa-miR-17-5p;hsa-miR-20a-5p;hsa-miR-20
ENSG0000	ENSG0000	1.01E-07	hsa-miR-106b-5p;hsa-miR-17-5p;hsa-miR-19a-3p;hsa-miR-19
ENSG0000	ENSG0000	4.97E-08	hsa-miR-106b-5p;hsa-miR-17-5p;hsa-miR-20a-5p;hsa-miR-20
ENSG0000	ENSG0000	4.97E-08	hsa-miR-106b-5p;hsa-miR-17-5p;hsa-miR-20a-5p;hsa-miR-20
ENSG0000	ENSG0000	4.13E-06	hsa-miR-106b-5p;hsa-miR-17-5p;hsa-miR-20a-5p;hsa-miR-20
ENSG0000	ENSG0000	0.00015	hsa-miR-106b-5p;hsa-miR-17-5p;hsa-miR-20a-5p;hsa-miR-20
ENSG0000	ENSG0000	2.56E-06	hsa-miR-106b-5p;hsa-miR-17-5p;hsa-miR-20a-5p;hsa-miR-20
ENSG0000	ENSG0000	0.000288	hsa-miR-106b-5p;hsa-miR-17-5p;hsa-miR-20a-5p;hsa-miR-20
ENSG0000	ENSG0000	9.65E-08	hsa-miR-106b-5p;hsa-miR-17-5p;hsa-miR-20a-5p;hsa-miR-20
ENSG0000	ENSG0000	1.45E-10	hsa-miR-106b-5p;hsa-miR-17-5p;hsa-miR-19a-3p;hsa-miR-19
ENSG0000	ENSG0000	2.51E-05	hsa-miR-106b-5p;hsa-miR-17-5p;hsa-miR-20a-5p;hsa-miR-20
ENSG0000	ENSG0000	7.61E-06	hsa-miR-106b-5p;hsa-miR-17-5p;hsa-miR-20a-5p;hsa-miR-20
ENSG0000	ENSG0000	0.000376	hsa-miR-106b-5p;hsa-miR-17-5p;hsa-miR-20a-5p;hsa-miR-20
ENSG0000	ENSG0000	1.78E-07	hsa-miR-101-3p;hsa-miR-106b-5p;hsa-miR-17-5p;hsa-miR-19
ENSG0000	ENSG0000	7.58E-06	hsa-miR-106b-5p;hsa-miR-17-5p;hsa-miR-20a-5p;hsa-miR-20
ENSG0000	ENSG0000	7.58E-06	hsa-miR-106b-5p;hsa-miR-17-5p;hsa-miR-20a-5p;hsa-miR-20
ENSG0000	ENSG0000	7.58E-06	hsa-miR-106b-5p;hsa-miR-17-5p;hsa-miR-20a-5p;hsa-miR-20
ENSG0000	ENSG0000	7.58E-06	hsa-miR-106b-5p;hsa-miR-17-5p;hsa-miR-20a-5p;hsa-miR-20
ENSG0000	ENSG0000	7.58E-06	hsa-miR-106b-5p;hsa-miR-17-5p;hsa-miR-20a-5p;hsa-miR-20
ENSG0000	ENSG0000	7.58E-06	hsa-miR-106b-5p;hsa-miR-17-5p;hsa-miR-20a-5p;hsa-miR-20
ENSG0000	ENSG0000	7.58E-06	hsa-miR-106b-5p;hsa-miR-17-5p;hsa-miR-20a-5p;hsa-miR-20
ENSG0000	ENSG0000	5.90E-06	hsa-miR-106b-5p;hsa-miR-17-5p;hsa-miR-20a-5p;hsa-miR-20
ENSG0000	ENSG0000	4.75E-08	hsa-miR-106b-5p;hsa-miR-17-5p;hsa-miR-19a-3p;hsa-miR-19
ENSG0000	ENSG0000	7.18E-06	hsa-miR-106b-5p;hsa-miR-19a-3p;hsa-miR-20a-5p;hsa-miR-2
ENSG0000	ENSG0000	2.90E-07	hsa-miR-103a-2-5p;hsa-miR-106b-5p;hsa-miR-17-5p;hsa-miR
ENSG0000	ENSG0000	6.05E-05	hsa-miR-106b-5p;hsa-miR-17-5p;hsa-miR-20a-5p;hsa-miR-20
ENSG0000	ENSG0000	0.002211	hsa-miR-106b-5p;hsa-miR-17-5p;hsa-miR-20a-5p;hsa-miR-20
ENSG0000	ENSG0000	6.45E-05	hsa-miR-106b-5p;hsa-miR-17-5p;hsa-miR-20a-5p;hsa-miR-20
ENSG0000	ENSG0000	2.17E-05	hsa-miR-106b-5p;hsa-miR-17-5p;hsa-miR-19a-3p;hsa-miR-19
ENSG0000	ENSG0000	2.49E-10	hsa-miR-106b-5p;hsa-miR-17-5p;hsa-miR-20a-5p;hsa-miR-20
ENSG0000	ENSG0000	2.49E-10	hsa-miR-106b-5p;hsa-miR-17-5p;hsa-miR-20a-5p;hsa-miR-20
ENSG0000	ENSG0000	7.61E-05	hsa-miR-106b-5p;hsa-miR-17-5p;hsa-miR-19a-3p;hsa-miR-19
ENSG0000	ENSG0000	5.80E-07	hsa-miR-106b-5p;hsa-miR-17-5p;hsa-miR-20a-5p;hsa-miR-20
ENSG0000	ENSG0000	3.62E-05	hsa-miR-106b-5p;hsa-miR-17-5p;hsa-miR-20a-5p;hsa-miR-20
ENSG0000	ENSG0000	0.00023	hsa-miR-106b-5p;hsa-miR-17-5p;hsa-miR-20a-5p;hsa-miR-20
ENSG0000	ENSG0000	3.01E-07	hsa-miR-106b-5p;hsa-miR-17-5p;hsa-miR-19a-3p;hsa-miR-19
ENSG0000	ENSG0000	2.09E-06	hsa-miR-106b-5p;hsa-miR-17-5p;hsa-miR-19a-3p;hsa-miR-19
ENSG0000	ENSG0000	3.31E-05	hsa-miR-106b-5p;hsa-miR-17-5p;hsa-miR-20a-5p;hsa-miR-20
ENSG0000	ENSG0000	3.31E-05	hsa-miR-106b-5p;hsa-miR-17-5p;hsa-miR-20a-5p;hsa-miR-20
ENSG0000	ENSG0000	4.97E-08	hsa-miR-106b-5p;hsa-miR-17-5p;hsa-miR-20a-5p;hsa-miR-20
ENSG0000	ENSG0000	3.71E-07	hsa-miR-106b-5p;hsa-miR-17-5p;hsa-miR-20a-5p;hsa-miR-20
ENSG0000	ENSG0000	1.93E-06	hsa-miR-106b-5p;hsa-miR-17-5p;hsa-miR-19a-3p;hsa-miR-19

ENSG0000	ENSG0000	0.000482	hsa-miR-106b-5p;hsa-miR-17-5p;hsa-miR-20a-5p;hsa-miR-20
ENSG0000	ENSG0000	0.000482	hsa-miR-106b-5p;hsa-miR-17-5p;hsa-miR-20a-5p;hsa-miR-20
ENSG0000	ENSG0000	0.001866	hsa-miR-106b-5p;hsa-miR-17-5p;hsa-miR-20a-5p;hsa-miR-20
ENSG0000	ENSG0000	4.04E-07	hsa-miR-106b-5p;hsa-miR-17-5p;hsa-miR-19a-3p;hsa-miR-19
ENSG0000	ENSG0000	8.32E-05	hsa-miR-106b-5p;hsa-miR-17-5p;hsa-miR-20a-5p;hsa-miR-20
ENSG0000	ENSG0000	0.001248	hsa-miR-106b-5p;hsa-miR-17-5p;hsa-miR-20a-5p;hsa-miR-20
ENSG0000	ENSG0000	7.14E-11	hsa-miR-106b-5p;hsa-miR-17-5p;hsa-miR-20a-5p;hsa-miR-20
ENSG0000	ENSG0000	3.03E-05	hsa-miR-106b-5p;hsa-miR-17-5p;hsa-miR-20a-5p;hsa-miR-20
ENSG0000	ENSG0000	9.35E-05	hsa-miR-106b-5p;hsa-miR-17-5p;hsa-miR-20a-5p;hsa-miR-20
ENSG0000	ENSG0000	0.002987	hsa-miR-17-5p;hsa-miR-20a-5p;hsa-miR-20b-5p;hsa-miR-93-
ENSG0000	ENSG0000	1.22E-05	hsa-miR-106b-5p;hsa-miR-17-5p;hsa-miR-20a-5p;hsa-miR-20
ENSG0000	ENSG0000	1.22E-05	hsa-miR-106b-5p;hsa-miR-17-5p;hsa-miR-20a-5p;hsa-miR-20
ENSG0000	ENSG0000	0.000482	hsa-miR-106b-5p;hsa-miR-17-5p;hsa-miR-20a-5p;hsa-miR-20
ENSG0000	ENSG0000	0.000217	hsa-miR-106b-5p;hsa-miR-17-5p;hsa-miR-20a-5p;hsa-miR-20
ENSG0000	ENSG0000	0.000506	hsa-miR-106b-5p;hsa-miR-17-5p;hsa-miR-20a-5p;hsa-miR-20
ENSG0000	ENSG0000	0.00013	hsa-miR-106b-5p;hsa-miR-17-5p;hsa-miR-20b-5p;hsa-miR-93
ENSG0000	ENSG0000	0.000212	hsa-miR-199a-5p;hsa-miR-199b-5p;hsa-miR-19a-3p;hsa-miR-
ENSG0000	ENSG0000	0.000115	hsa-miR-106b-5p;hsa-miR-17-5p;hsa-miR-20a-5p;hsa-miR-20
ENSG0000	ENSG0000	0.000731	hsa-miR-106b-5p;hsa-miR-17-5p;hsa-miR-20a-5p;hsa-miR-20
ENSG0000	ENSG0000	4.05E-05	hsa-miR-106b-5p;hsa-miR-17-5p;hsa-miR-20a-5p;hsa-miR-20
ENSG0000	ENSG0000	3.31E-05	hsa-miR-106b-5p;hsa-miR-17-5p;hsa-miR-20a-5p;hsa-miR-20
ENSG0000	ENSG0000	2.67E-06	hsa-miR-106b-5p;hsa-miR-17-5p;hsa-miR-20a-5p;hsa-miR-20
ENSG0000	ENSG0000	3.62E-05	hsa-miR-106b-5p;hsa-miR-17-5p;hsa-miR-20a-5p;hsa-miR-20
ENSG0000	ENSG0000	0.000192	hsa-miR-106b-5p;hsa-miR-17-5p;hsa-miR-20a-5p;hsa-miR-20
ENSG0000	ENSG0000	4.51E-06	hsa-miR-106b-5p;hsa-miR-17-5p;hsa-miR-20a-5p;hsa-miR-20
ENSG0000	ENSG0000	4.51E-06	hsa-miR-106b-5p;hsa-miR-17-5p;hsa-miR-20a-5p;hsa-miR-20
ENSG0000	ENSG0000	4.51E-06	hsa-miR-106b-5p;hsa-miR-17-5p;hsa-miR-20a-5p;hsa-miR-20
ENSG0000	ENSG0000	4.51E-06	hsa-miR-106b-5p;hsa-miR-17-5p;hsa-miR-20a-5p;hsa-miR-20
ENSG0000	ENSG0000	4.51E-06	hsa-miR-106b-5p;hsa-miR-17-5p;hsa-miR-20a-5p;hsa-miR-20
ENSG0000	ENSG0000	4.51E-06	hsa-miR-106b-5p;hsa-miR-17-5p;hsa-miR-20a-5p;hsa-miR-20
ENSG0000	ENSG0000	4.51E-06	hsa-miR-106b-5p;hsa-miR-17-5p;hsa-miR-20a-5p;hsa-miR-20
ENSG0000	ENSG0000	0.000108	hsa-miR-106b-5p;hsa-miR-17-5p;hsa-miR-20a-5p;hsa-miR-20
ENSG0000	ENSG0000	0.001317	hsa-miR-106b-5p;hsa-miR-17-5p;hsa-miR-20a-5p;hsa-miR-20
ENSG0000	ENSG0000	5.51E-05	hsa-miR-106b-5p;hsa-miR-17-5p;hsa-miR-20a-5p;hsa-miR-20
ENSG0000	ENSG0000	3.12E-06	hsa-miR-103a-2-5p;hsa-miR-106b-5p;hsa-miR-17-5p;hsa-miR-
ENSG0000	ENSG0000	7.14E-11	hsa-miR-106b-5p;hsa-miR-17-5p;hsa-miR-20a-5p;hsa-miR-20
ENSG0000	ENSG0000	0.000272	hsa-miR-106b-5p;hsa-miR-17-5p;hsa-miR-20a-5p;hsa-miR-20
ENSG0000	ENSG0000	1.22E-05	hsa-miR-106b-5p;hsa-miR-17-5p;hsa-miR-20a-5p;hsa-miR-20
ENSG0000	ENSG0000	4.36E-05	hsa-miR-106b-5p;hsa-miR-17-5p;hsa-miR-20a-5p;hsa-miR-20
ENSG0000	ENSG0000	0.001451	hsa-miR-106b-5p;hsa-miR-17-5p;hsa-miR-20a-5p;hsa-miR-20
ENSG0000	ENSG0000	0.000217	hsa-miR-106b-5p;hsa-miR-17-5p;hsa-miR-20a-5p;hsa-miR-20
ENSG0000	ENSG0000	0.002379	hsa-miR-101-3p;hsa-miR-106b-5p;hsa-miR-17-5p;hsa-miR-20
ENSG0000	ENSG0000	1.31E-05	hsa-miR-101-3p;hsa-miR-106b-5p;hsa-miR-17-5p;hsa-miR-20
ENSG0000	ENSG0000	4.36E-05	hsa-miR-106b-5p;hsa-miR-17-5p;hsa-miR-20a-5p;hsa-miR-20
ENSG0000	ENSG0000	0.001025	hsa-miR-106b-5p;hsa-miR-17-5p;hsa-miR-20b-5p;hsa-miR-24
ENSG0000	ENSG0000	4.63E-10	hsa-miR-106b-5p;hsa-miR-17-5p;hsa-miR-199a-5p;hsa-miR-1
ENSG0000	ENSG0000	3.03E-05	hsa-miR-106b-5p;hsa-miR-17-5p;hsa-miR-19a-3p;hsa-miR-20
ENSG0000	ENSG0000	6.60E-10	hsa-miR-106b-5p;hsa-miR-17-5p;hsa-miR-20a-5p;hsa-miR-20
ENSG0000	ENSG0000	3.39E-06	hsa-miR-106b-5p;hsa-miR-17-5p;hsa-miR-20a-5p;hsa-miR-20
ENSG0000	ENSG0000	3.91E-06	hsa-miR-106b-5p;hsa-miR-17-5p;hsa-miR-20a-5p;hsa-miR-20
ENSG0000	ENSG0000	0.000159	hsa-miR-106b-5p;hsa-miR-17-5p;hsa-miR-20b-5p;hsa-miR-93
ENSG0000	ENSG0000	0.000984	hsa-miR-106b-5p;hsa-miR-17-5p;hsa-miR-20a-5p;hsa-miR-20
ENSG0000	ENSG0000	2.91E-07	hsa-miR-106b-5p;hsa-miR-17-5p;hsa-miR-20a-5p;hsa-miR-20
ENSG0000	ENSG0000	0.005643	hsa-miR-106b-5p;hsa-miR-17-5p;hsa-miR-20a-5p;hsa-miR-20

ENSG0000	ENSG0000	5.39E-09	hsa-miR-106b-5p;hsa-miR-17-5p;hsa-miR-199a-5p;hsa-miR-1
ENSG0000	ENSG0000	5.39E-09	hsa-miR-106b-5p;hsa-miR-17-5p;hsa-miR-199a-5p;hsa-miR-1
ENSG0000	ENSG0000	0.000821	hsa-miR-106b-5p;hsa-miR-17-5p;hsa-miR-20a-5p;hsa-miR-20
ENSG0000	ENSG0000	2.91E-07	hsa-miR-106b-5p;hsa-miR-17-5p;hsa-miR-20a-5p;hsa-miR-20
ENSG0000	ENSG0000	1.36E-05	hsa-miR-106b-5p;hsa-miR-17-5p;hsa-miR-20a-5p;hsa-miR-20
ENSG0000	ENSG0000	0.003249	hsa-miR-17-5p;hsa-miR-19a-3p;hsa-miR-20a-5p;hsa-miR-20b-
ENSG0000	ENSG0000	0.000459	hsa-miR-106b-5p;hsa-miR-17-5p;hsa-miR-20a-5p;hsa-miR-20
ENSG0000	ENSG0000	9.87E-07	hsa-miR-106b-5p;hsa-miR-17-5p;hsa-miR-19a-3p;hsa-miR-19
ENSG0000	ENSG0000	1.09E-05	hsa-miR-106b-5p;hsa-miR-17-5p;hsa-miR-20a-5p;hsa-miR-20
ENSG0000	ENSG0000	1.04E-05	hsa-miR-106b-5p;hsa-miR-17-5p;hsa-miR-20a-5p;hsa-miR-20
ENSG0000	ENSG0000	1.87E-05	hsa-miR-106b-5p;hsa-miR-17-5p;hsa-miR-20a-5p;hsa-miR-20
ENSG0000	ENSG0000	0.001057	hsa-miR-106b-5p;hsa-miR-17-5p;hsa-miR-20a-5p;hsa-miR-20
ENSG0000	ENSG0000	6.05E-05	hsa-miR-101-3p;hsa-miR-106b-5p;hsa-miR-17-5p;hsa-miR-20
ENSG0000	ENSG0000	5.08E-05	hsa-miR-106b-5p;hsa-miR-17-5p;hsa-miR-20a-5p;hsa-miR-20
ENSG0000	ENSG0000	2.51E-05	hsa-miR-106b-5p;hsa-miR-17-5p;hsa-miR-20a-5p;hsa-miR-20
ENSG0000	ENSG0000	2.88E-06	hsa-miR-106b-5p;hsa-miR-17-5p;hsa-miR-199a-5p;hsa-miR-1
ENSG0000	ENSG0000	2.17E-05	hsa-miR-106b-5p;hsa-miR-17-5p;hsa-miR-19a-3p;hsa-miR-19
ENSG0000	ENSG0000	0.001266	hsa-miR-106b-5p;hsa-miR-17-5p;hsa-miR-20a-5p;hsa-miR-20
ENSG0000	ENSG0000	0.000611	hsa-miR-106b-5p;hsa-miR-17-5p;hsa-miR-20a-5p;hsa-miR-20
ENSG0000	ENSG0000	1.39E-05	hsa-miR-106b-5p;hsa-miR-20a-5p;hsa-miR-20b-5p;hsa-miR-9
ENSG0000	ENSG0000	2.51E-05	hsa-miR-106b-5p;hsa-miR-17-5p;hsa-miR-20a-5p;hsa-miR-20
ENSG0000	ENSG0000	0.000798	hsa-miR-106b-5p;hsa-miR-17-5p;hsa-miR-20a-5p;hsa-miR-20
ENSG0000	ENSG0000	0.000393	hsa-miR-106b-5p;hsa-miR-17-5p;hsa-miR-20a-5p;hsa-miR-20
ENSG0000	ENSG0000	2.50E-06	hsa-miR-106b-5p;hsa-miR-17-5p;hsa-miR-20a-5p;hsa-miR-20
ENSG0000	ENSG0000	0.001866	hsa-miR-106b-5p;hsa-miR-17-5p;hsa-miR-20a-5p;hsa-miR-20
ENSG0000	ENSG0000	0.000531	hsa-miR-106b-5p;hsa-miR-17-5p;hsa-miR-20a-5p;hsa-miR-20
ENSG0000	ENSG0000	7.51E-05	hsa-miR-106b-5p;hsa-miR-17-5p;hsa-miR-20a-5p;hsa-miR-20
ENSG0000	ENSG0000	4.30E-05	hsa-miR-106b-5p;hsa-miR-17-5p;hsa-miR-20a-5p;hsa-miR-20
ENSG0000	ENSG0000	1.68E-05	hsa-miR-106b-5p;hsa-miR-17-5p;hsa-miR-20a-5p;hsa-miR-20
ENSG0000	ENSG0000	0.000555	hsa-miR-106b-5p;hsa-miR-17-5p;hsa-miR-20a-5p;hsa-miR-93-
ENSG0000	ENSG0000	6.45E-05	hsa-miR-106b-5p;hsa-miR-17-5p;hsa-miR-20a-5p;hsa-miR-20
ENSG0000	ENSG0000	8.59E-06	hsa-miR-106b-5p;hsa-miR-17-5p;hsa-miR-20a-5p;hsa-miR-20
ENSG0000	ENSG0000	0.000414	hsa-miR-106b-5p;hsa-miR-17-5p;hsa-miR-20a-5p;hsa-miR-20
ENSG0000	ENSG0000	6.71E-06	hsa-miR-106b-5p;hsa-miR-17-5p;hsa-miR-20a-5p;hsa-miR-20
ENSG0000	ENSG0000	0.000132	hsa-miR-106b-5p;hsa-miR-17-5p;hsa-miR-20a-5p;hsa-miR-20
ENSG0000	ENSG0000	0.00017	hsa-miR-106b-5p;hsa-miR-17-5p;hsa-miR-20a-5p;hsa-miR-20
ENSG0000	ENSG0000	0.000132	hsa-miR-106b-5p;hsa-miR-17-5p;hsa-miR-20a-5p;hsa-miR-20
ENSG0000	ENSG0000	0.000354	hsa-miR-17-5p;hsa-miR-20a-5p;hsa-miR-20b-5p;hsa-miR-93-
ENSG0000	ENSG0000	0.000204	hsa-miR-106b-5p;hsa-miR-17-5p;hsa-miR-20a-5p;hsa-miR-20
ENSG0000	ENSG0000	6.71E-06	hsa-miR-106b-5p;hsa-miR-17-5p;hsa-miR-20a-5p;hsa-miR-20
ENSG0000	ENSG0000	5.81E-07	hsa-miR-103a-2-5p;hsa-miR-106b-5p;hsa-miR-17-5p;hsa-miR-
ENSG0000	ENSG0000	0.000181	hsa-miR-106b-5p;hsa-miR-17-5p;hsa-miR-20a-5p;hsa-miR-20
ENSG0000	ENSG0000	3.31E-05	hsa-miR-106b-5p;hsa-miR-17-5p;hsa-miR-20a-5p;hsa-miR-20
ENSG0000	ENSG0000	3.62E-05	hsa-miR-106b-5p;hsa-miR-17-5p;hsa-miR-20a-5p;hsa-miR-20
ENSG0000	ENSG0000	1.14E-07	hsa-miR-106b-5p;hsa-miR-17-5p;hsa-miR-19a-3p;hsa-miR-19
ENSG0000	ENSG0000	2.50E-06	hsa-miR-106b-5p;hsa-miR-17-5p;hsa-miR-20a-5p;hsa-miR-20
ENSG0000	ENSG0000	7.14E-11	hsa-miR-106b-5p;hsa-miR-17-5p;hsa-miR-20a-5p;hsa-miR-20
ENSG0000	ENSG0000	7.64E-07	hsa-miR-106b-5p;hsa-miR-17-5p;hsa-miR-20a-5p;hsa-miR-20
ENSG0000	ENSG0000	7.64E-07	hsa-miR-106b-5p;hsa-miR-17-5p;hsa-miR-20a-5p;hsa-miR-20
ENSG0000	ENSG0000	0.000141	hsa-miR-106b-5p;hsa-miR-17-5p;hsa-miR-20a-5p;hsa-miR-20
ENSG0000	ENSG0000	0.0007	hsa-miR-106b-5p;hsa-miR-17-5p;hsa-miR-20a-5p;hsa-miR-20
ENSG0000	ENSG0000	0.007962	hsa-miR-106b-5p;hsa-miR-17-5p;hsa-miR-20a-5p;hsa-miR-20
ENSG0000	ENSG0000	5.72E-05	hsa-miR-106b-5p;hsa-miR-17-5p;hsa-miR-20a-5p;hsa-miR-20
ENSG0000	ENSG0000	9.17E-09	hsa-miR-106b-5p;hsa-miR-17-5p;hsa-miR-20a-5p;hsa-miR-20

ENSG0000	ENSG0000	5.37E-09	hsa-miR-106b-5p;hsa-miR-17-5p;hsa-miR-20a-5p;hsa-miR-20
ENSG0000	ENSG0000	0.00013	hsa-miR-106b-5p;hsa-miR-17-5p;hsa-miR-20b-5p;hsa-miR-93
ENSG0000	ENSG0000	0.000825	hsa-miR-106b-5p;hsa-miR-17-5p;hsa-miR-19a-3p;hsa-miR-20
ENSG0000	ENSG0000	4.38E-09	hsa-miR-106b-5p;hsa-miR-17-5p;hsa-miR-19a-3p;hsa-miR-19
ENSG0000	ENSG0000	1.21E-05	hsa-miR-106b-5p;hsa-miR-19a-3p;hsa-miR-19b-3p;hsa-miR-2
ENSG0000	ENSG0000	5.01E-05	hsa-miR-106b-5p;hsa-miR-17-5p;hsa-miR-20a-5p;hsa-miR-20
ENSG0000	ENSG0000	7.14E-11	hsa-miR-106b-5p;hsa-miR-17-5p;hsa-miR-20a-5p;hsa-miR-20
ENSG0000	ENSG0000	4.36E-05	hsa-miR-106b-5p;hsa-miR-17-5p;hsa-miR-20a-5p;hsa-miR-20
ENSG0000	ENSG0000	6.60E-10	hsa-miR-106b-5p;hsa-miR-17-5p;hsa-miR-20a-5p;hsa-miR-20
ENSG0000	ENSG0000	2.06E-05	hsa-miR-106b-5p;hsa-miR-17-5p;hsa-miR-20a-5p;hsa-miR-20
ENSG0000	ENSG0000	0.0001	hsa-miR-106b-5p;hsa-miR-17-5p;hsa-miR-20a-5p;hsa-miR-20
ENSG0000	ENSG0000	3.03E-05	hsa-miR-106b-5p;hsa-miR-17-5p;hsa-miR-20a-5p;hsa-miR-20
ENSG0000	ENSG0000	0.006248	hsa-miR-106b-5p;hsa-miR-17-5p;hsa-miR-20a-5p;hsa-miR-20
ENSG0000	ENSG0000	0.000304	hsa-miR-106b-5p;hsa-miR-17-5p;hsa-miR-20a-5p;hsa-miR-20
ENSG0000	ENSG0000	0.0007	hsa-miR-106b-5p;hsa-miR-17-5p;hsa-miR-20a-5p;hsa-miR-20
ENSG0000	ENSG0000	0.000176	hsa-miR-106b-5p;hsa-miR-17-5p;hsa-miR-20a-5p;hsa-miR-20
ENSG0000	ENSG0000	8.09E-05	hsa-miR-106b-5p;hsa-miR-17-5p;hsa-miR-20a-5p;hsa-miR-20
ENSG0000	ENSG0000	1.07E-08	hsa-miR-106b-5p;hsa-miR-17-5p;hsa-miR-19a-3p;hsa-miR-19
ENSG0000	ENSG0000	5.81E-07	hsa-miR-106b-5p;hsa-miR-17-5p;hsa-miR-20a-5p;hsa-miR-20
ENSG0000	ENSG0000	5.61E-06	hsa-miR-106b-5p;hsa-miR-17-5p;hsa-miR-20a-5p;hsa-miR-20
ENSG0000	ENSG0000	2.30E-08	hsa-miR-106b-5p;hsa-miR-17-5p;hsa-miR-20a-5p;hsa-miR-20
ENSG0000	ENSG0000	0.000395	hsa-miR-106b-5p;hsa-miR-17-5p;hsa-miR-20a-5p;hsa-miR-20
ENSG0000	ENSG0000	7.14E-11	hsa-miR-106b-5p;hsa-miR-17-5p;hsa-miR-20a-5p;hsa-miR-20
ENSG0000	ENSG0000	6.97E-05	hsa-miR-106b-5p;hsa-miR-17-5p;hsa-miR-20a-5p;hsa-miR-20
ENSG0000	ENSG0000	0.000459	hsa-miR-106b-5p;hsa-miR-17-5p;hsa-miR-20a-5p;hsa-miR-20
ENSG0000	ENSG0000	1.51E-06	hsa-miR-106b-5p;hsa-miR-17-5p;hsa-miR-20a-5p;hsa-miR-20
ENSG0000	ENSG0000	0.000775	hsa-miR-106b-5p;hsa-miR-17-5p;hsa-miR-20a-5p;hsa-miR-20
ENSG0000	ENSG0000	0.000583	hsa-miR-106b-5p;hsa-miR-17-5p;hsa-miR-20a-5p;hsa-miR-20
ENSG0000	ENSG0000	0.000531	hsa-miR-106b-5p;hsa-miR-17-5p;hsa-miR-20a-5p;hsa-miR-20
ENSG0000	ENSG0000	1.52E-06	hsa-miR-106b-5p;hsa-miR-17-5p;hsa-miR-20a-5p;hsa-miR-20
ENSG0000	ENSG0000	2.30E-08	hsa-miR-106b-5p;hsa-miR-17-5p;hsa-miR-20a-5p;hsa-miR-20
ENSG0000	ENSG0000	0.000257	hsa-miR-106b-5p;hsa-miR-17-5p;hsa-miR-20a-5p;hsa-miR-20
ENSG0000	ENSG0000	7.00E-08	hsa-miR-101-3p;hsa-miR-106b-5p;hsa-miR-19a-3p;hsa-miR-1
ENSG0000	ENSG0000	1.06E-06	hsa-miR-106b-5p;hsa-miR-17-5p;hsa-miR-20a-5p;hsa-miR-20
ENSG0000	ENSG0000	0.000904	hsa-miR-106b-5p;hsa-miR-17-5p;hsa-miR-20a-5p;hsa-miR-20
ENSG0000	ENSG0000	1.68E-05	hsa-miR-106b-5p;hsa-miR-17-5p;hsa-miR-20a-5p;hsa-miR-20
ENSG0000	ENSG0000	1.31E-05	hsa-miR-106b-5p;hsa-miR-17-5p;hsa-miR-20a-5p;hsa-miR-20
ENSG0000	ENSG0000	0.000821	hsa-miR-106b-5p;hsa-miR-17-5p;hsa-miR-19a-3p;hsa-miR-20
ENSG0000	ENSG0000	0.001398	hsa-miR-106b-5p;hsa-miR-17-5p;hsa-miR-20a-5p;hsa-miR-20
ENSG0000	ENSG0000	5.08E-05	hsa-miR-106b-5p;hsa-miR-17-5p;hsa-miR-20a-5p;hsa-miR-20
ENSG0000	ENSG0000	5.08E-05	hsa-miR-106b-5p;hsa-miR-17-5p;hsa-miR-20a-5p;hsa-miR-20
ENSG0000	ENSG0000	7.00E-08	hsa-miR-106b-5p;hsa-miR-17-5p;hsa-miR-20a-5p;hsa-miR-20
ENSG0000	ENSG0000	1.51E-05	hsa-miR-106b-5p;hsa-miR-17-5p;hsa-miR-19a-3p;hsa-miR-19
ENSG0000	ENSG0000	1.48E-08	hsa-miR-106b-5p;hsa-miR-17-5p;hsa-miR-20a-5p;hsa-miR-20
ENSG0000	ENSG0000	4.68E-05	hsa-miR-106b-5p;hsa-miR-17-5p;hsa-miR-20a-5p;hsa-miR-20
ENSG0000	ENSG0000	0.000944	hsa-miR-106b-5p;hsa-miR-17-5p;hsa-miR-20a-5p;hsa-miR-20
ENSG0000	ENSG0000	0.000204	hsa-miR-106b-5p;hsa-miR-17-5p;hsa-miR-20a-5p;hsa-miR-20
ENSG0000	ENSG0000	9.35E-05	hsa-miR-106b-5p;hsa-miR-17-5p;hsa-miR-20a-5p;hsa-miR-20
ENSG0000	ENSG0000	0.001178	hsa-miR-106b-5p;hsa-miR-17-5p;hsa-miR-20b-5p;hsa-miR-93
ENSG0000	ENSG0000	0.000192	hsa-miR-106b-5p;hsa-miR-17-5p;hsa-miR-20a-5p;hsa-miR-20
ENSG0000	ENSG0000	0.000437	hsa-miR-106b-5p;hsa-miR-17-5p;hsa-miR-20a-5p;hsa-miR-20
ENSG0000	ENSG0000	0.000731	hsa-miR-106b-5p;hsa-miR-17-5p;hsa-miR-20a-5p;hsa-miR-20
ENSG0000	ENSG0000	0.000311	hsa-miR-106b-5p;hsa-miR-17-5p;hsa-miR-20a-5p;hsa-miR-20
ENSG0000	ENSG0000	8.73E-07	hsa-miR-106b-5p;hsa-miR-17-5p;hsa-miR-20a-5p;hsa-miR-20

ENSG0000	ENSG0000	5.51E-05	hsa-miR-106b-5p;hsa-miR-17-5p;hsa-miR-20a-5p;hsa-miR-20
ENSG0000	ENSG0000	2.45E-05	hsa-miR-101-3p;hsa-miR-106b-5p;hsa-miR-17-5p;hsa-miR-20
ENSG0000	ENSG0000	0.000414	hsa-miR-19a-3p;hsa-miR-19b-3p;hsa-miR-30a-3p;hsa-miR-30
ENSG0000	ENSG0000	0.000192	hsa-miR-106b-5p;hsa-miR-17-5p;hsa-miR-20a-5p;hsa-miR-20
ENSG0000	ENSG0000	1.48E-08	hsa-miR-106b-5p;hsa-miR-17-5p;hsa-miR-19a-3p;hsa-miR-19
ENSG0000	ENSG0000	1.28E-07	hsa-miR-106b-5p;hsa-miR-17-5p;hsa-miR-19a-3p;hsa-miR-19
ENSG0000	ENSG0000	1.80E-05	hsa-miR-106b-5p;hsa-miR-17-5p;hsa-miR-19a-3p;hsa-miR-19
ENSG0000	ENSG0000	0.000376	hsa-miR-106b-5p;hsa-miR-17-5p;hsa-miR-20a-5p;hsa-miR-20
ENSG0000	ENSG0000	0.006798	hsa-miR-106b-5p;hsa-miR-17-5p;hsa-miR-20a-5p;hsa-miR-20
ENSG0000	ENSG0000	3.31E-05	hsa-miR-106b-5p;hsa-miR-17-5p;hsa-miR-20a-5p;hsa-miR-20
ENSG0000	ENSG0000	3.31E-05	hsa-miR-106b-5p;hsa-miR-17-5p;hsa-miR-20a-5p;hsa-miR-20
ENSG0000	ENSG0000	7.70E-08	hsa-miR-106b-5p;hsa-miR-17-5p;hsa-miR-20a-5p;hsa-miR-20
ENSG0000	ENSG0000	0.000991	hsa-miR-106b-5p;hsa-miR-17-5p;hsa-miR-20a-5p;hsa-miR-20
ENSG0000	ENSG0000	0.001545	hsa-miR-106b-5p;hsa-miR-17-5p;hsa-miR-20a-5p;hsa-miR-20
ENSG0000	ENSG0000	0.00023	hsa-miR-106b-5p;hsa-miR-17-5p;hsa-miR-20a-5p;hsa-miR-20
ENSG0000	ENSG0000	5.80E-07	hsa-miR-106b-5p;hsa-miR-17-5p;hsa-miR-20a-5p;hsa-miR-20
ENSG0000	ENSG0000	0.002083	hsa-miR-17-5p;hsa-miR-20a-5p;hsa-miR-20b-5p;hsa-miR-93-
ENSG0000	ENSG0000	3.91E-06	hsa-miR-106b-5p;hsa-miR-17-5p;hsa-miR-20a-5p;hsa-miR-20
ENSG0000	ENSG0000	1.72E-06	hsa-miR-106b-5p;hsa-miR-17-5p;hsa-miR-19a-3p;hsa-miR-19
ENSG0000	ENSG0000	7.51E-05	hsa-miR-106b-5p;hsa-miR-17-5p;hsa-miR-20a-5p;hsa-miR-20
ENSG0000	ENSG0000	7.51E-05	hsa-miR-106b-5p;hsa-miR-17-5p;hsa-miR-20a-5p;hsa-miR-20
ENSG0000	ENSG0000	9.64E-06	hsa-miR-106b-5p;hsa-miR-17-5p;hsa-miR-20a-5p;hsa-miR-20
ENSG0000	ENSG0000	7.00E-08	hsa-miR-106b-5p;hsa-miR-17-5p;hsa-miR-20a-5p;hsa-miR-20
ENSG0000	ENSG0000	2.50E-06	hsa-miR-106b-5p;hsa-miR-17-5p;hsa-miR-20a-5p;hsa-miR-20
ENSG0000	ENSG0000	2.51E-05	hsa-miR-106b-5p;hsa-miR-17-5p;hsa-miR-20a-5p;hsa-miR-20
ENSG0000	ENSG0000	0.000132	hsa-miR-106b-5p;hsa-miR-17-5p;hsa-miR-20a-5p;hsa-miR-20
ENSG0000	ENSG0000	0.000132	hsa-miR-106b-5p;hsa-miR-17-5p;hsa-miR-20a-5p;hsa-miR-20
ENSG0000	ENSG0000	0.000254	hsa-miR-106b-5p;hsa-miR-17-5p;hsa-miR-20a-5p;hsa-miR-20
ENSG0000	ENSG0000	1.43E-07	hsa-miR-106b-5p;hsa-miR-17-5p;hsa-miR-19a-3p;hsa-miR-19
ENSG0000	ENSG0000	0.000113	hsa-miR-106b-5p;hsa-miR-17-5p;hsa-miR-19a-3p;hsa-miR-19
ENSG0000	ENSG0000	0.007024	hsa-miR-106b-5p;hsa-miR-17-5p;hsa-miR-20a-5p;hsa-miR-20
ENSG0000	ENSG0000	5.97E-05	hsa-miR-106b-5p;hsa-miR-17-5p;hsa-miR-20a-5p;hsa-miR-20
ENSG0000	ENSG0000	0.00017	hsa-miR-106b-5p;hsa-miR-17-5p;hsa-miR-20a-5p;hsa-miR-20
ENSG0000	ENSG0000	5.90E-06	hsa-miR-106b-5p;hsa-miR-17-5p;hsa-miR-20a-5p;hsa-miR-20
ENSG0000	ENSG0000	5.90E-06	hsa-miR-106b-5p;hsa-miR-17-5p;hsa-miR-20a-5p;hsa-miR-20
ENSG0000	ENSG0000	1.21E-05	hsa-miR-106b-5p;hsa-miR-17-5p;hsa-miR-20a-5p;hsa-miR-20
ENSG0000	ENSG0000	0.000726	hsa-miR-19a-3p;hsa-miR-19b-3p;hsa-miR-30a-3p;hsa-miR-30
ENSG0000	ENSG0000	0.000181	hsa-miR-106b-5p;hsa-miR-17-5p;hsa-miR-20a-5p;hsa-miR-20
ENSG0000	ENSG0000	1.09E-05	hsa-miR-106b-5p;hsa-miR-17-5p;hsa-miR-20a-5p;hsa-miR-20
ENSG0000	ENSG0000	5.16E-06	hsa-miR-106b-5p;hsa-miR-17-5p;hsa-miR-20a-5p;hsa-miR-20
ENSG0000	ENSG0000	5.16E-06	hsa-miR-106b-5p;hsa-miR-17-5p;hsa-miR-20a-5p;hsa-miR-20
ENSG0000	ENSG0000	3.61E-09	hsa-miR-106b-5p;hsa-miR-17-5p;hsa-miR-20a-5p;hsa-miR-20
ENSG0000	ENSG0000	0.002288	hsa-miR-101-3p;hsa-miR-2110;hsa-miR-491-5p;hsa-miR-96-5
ENSG0000	ENSG0000	1.22E-05	hsa-miR-106b-5p;hsa-miR-17-5p;hsa-miR-20a-5p;hsa-miR-20
ENSG0000	ENSG0000	7.61E-06	hsa-miR-106b-5p;hsa-miR-17-5p;hsa-miR-20a-5p;hsa-miR-20
ENSG0000	ENSG0000	2.83E-06	hsa-miR-106b-5p;hsa-miR-17-5p;hsa-miR-20a-5p;hsa-miR-20
ENSG0000	ENSG0000	2.44E-08	hsa-miR-106b-5p;hsa-miR-17-5p;hsa-miR-20a-5p;hsa-miR-20
ENSG0000	ENSG0000	2.28E-05	hsa-miR-106b-5p;hsa-miR-17-5p;hsa-miR-20a-5p;hsa-miR-20
ENSG0000	ENSG0000	1.06E-06	hsa-miR-106b-5p;hsa-miR-17-5p;hsa-miR-20a-5p;hsa-miR-20
ENSG0000	ENSG0000	2.92E-06	hsa-miR-106b-5p;hsa-miR-17-5p;hsa-miR-20a-5p;hsa-miR-20
ENSG0000	ENSG0000	4.68E-05	hsa-miR-106b-5p;hsa-miR-17-5p;hsa-miR-20a-5p;hsa-miR-20
ENSG0000	ENSG0000	1.98E-10	hsa-miR-106b-5p;hsa-miR-17-5p;hsa-miR-19a-3p;hsa-miR-19
ENSG0000	ENSG0000	6.97E-05	hsa-miR-106b-5p;hsa-miR-17-5p;hsa-miR-20a-5p;hsa-miR-20
ENSG0000	ENSG0000	0.001317	hsa-miR-106b-5p;hsa-miR-17-5p;hsa-miR-20a-5p;hsa-miR-20

ENSG0000	ENSG0000	0.000669	hsa-miR-106b-5p;hsa-miR-17-5p;hsa-miR-20a-5p;hsa-miR-20
ENSG0000	ENSG0000	0.003527	hsa-miR-106b-5p;hsa-miR-17-5p;hsa-miR-20a-5p;hsa-miR-20
ENSG0000	ENSG0000	5.51E-05	hsa-miR-106b-5p;hsa-miR-17-5p;hsa-miR-20a-5p;hsa-miR-20
ENSG0000	ENSG0000	1.01E-07	hsa-miR-106b-5p;hsa-miR-17-5p;hsa-miR-19a-3p;hsa-miR-19
ENSG0000	ENSG0000	5.80E-07	hsa-miR-106b-5p;hsa-miR-17-5p;hsa-miR-20a-5p;hsa-miR-20
ENSG0000	ENSG0000	5.51E-05	hsa-miR-106b-5p;hsa-miR-17-5p;hsa-miR-20a-5p;hsa-miR-20
ENSG0000	ENSG0000	2.28E-05	hsa-miR-106b-5p;hsa-miR-17-5p;hsa-miR-20a-5p;hsa-miR-20
ENSG0000	ENSG0000	1.68E-05	hsa-miR-106b-5p;hsa-miR-17-5p;hsa-miR-20a-5p;hsa-miR-20
ENSG0000	ENSG0000	3.53E-10	hsa-miR-106b-5p;hsa-miR-17-5p;hsa-miR-20a-5p;hsa-miR-20
ENSG0000	ENSG0000	8.73E-07	hsa-miR-106b-5p;hsa-miR-17-5p;hsa-miR-20a-5p;hsa-miR-20
ENSG0000	ENSG0000	0.000135	hsa-miR-320a;hsa-miR-320b;hsa-miR-320d;hsa-miR-320e;hsa-
ENSG0000	ENSG0000	0.003031	hsa-miR-320a;hsa-miR-320b;hsa-miR-320d;hsa-miR-320e;hsa-
ENSG0000	ENSG0000	0.002682	hsa-miR-103a-2-5p;hsa-miR-1305;hsa-miR-15b-3p;hsa-miR-1
ENSG0000	ENSG0000	0.001377	hsa-miR-149-5p;hsa-miR-25-3p;hsa-miR-32-5p;hsa-miR-363-
ENSG0000	ENSG0000	0.000509	hsa-miR-103a-2-5p;hsa-miR-320a;hsa-miR-320b;hsa-miR-320
ENSG0000	ENSG0000	0.008772	hsa-miR-181a-5p;hsa-miR-181b-5p;hsa-miR-181c-5p;hsa-miR
ENSG0000	ENSG0000	0.000169	hsa-miR-148a-5p;hsa-miR-148b-5p;hsa-miR-181c-3p;hsa-miR
ENSG0000	ENSG0000	0.004674	hsa-miR-30a-3p;hsa-miR-30d-3p;hsa-miR-30e-3p;hsa-miR-32
ENSG0000	ENSG0000	0.002975	hsa-miR-106a-5p;hsa-miR-141-5p;hsa-miR-181a-5p;hsa-miR-
ENSG0000	ENSG0000	0.002416	hsa-miR-25-3p;hsa-miR-32-5p;hsa-miR-363-3p;hsa-miR-500a
ENSG0000	ENSG0000	0.000145	hsa-miR-25-3p;hsa-miR-421;hsa-miR-92a-3p;hsa-miR-9-3p
ENSG0000	ENSG0000	0.000909	hsa-miR-106a-5p;hsa-miR-181a-5p;hsa-miR-181b-5p;hsa-miR
ENSG0000	ENSG0000	0.008772	hsa-miR-320a;hsa-miR-320b;hsa-miR-320d;hsa-miR-4429;hsa-
ENSG0000	ENSG0000	5.69E-05	hsa-miR-181b-3p;hsa-miR-25-3p;hsa-miR-32-5p;hsa-miR-363
ENSG0000	ENSG0000	0.005742	hsa-miR-181a-5p;hsa-miR-181b-5p;hsa-miR-181c-5p;hsa-miR
ENSG0000	ENSG0000	0.006169	hsa-miR-106a-5p;hsa-miR-126-5p;hsa-miR-30a-3p;hsa-miR-3
ENSG0000	ENSG0000	0.000182	hsa-miR-129-1-3p;hsa-miR-129-2-3p;hsa-miR-421;hsa-miR-4
ENSG0000	ENSG0000	0.009966	hsa-miR-10b-3p;hsa-miR-25-3p;hsa-miR-32-5p;hsa-miR-363-
ENSG0000	ENSG0000	5.06E-05	hsa-miR-181b-5p;hsa-miR-181c-5p;hsa-miR-19a-3p;hsa-miR-
ENSG0000	ENSG0000	0.001488	hsa-miR-106a-5p;hsa-miR-181a-5p;hsa-miR-181b-5p;hsa-miR
ENSG0000	ENSG0000	0.008341	hsa-miR-181a-5p;hsa-miR-342-3p;hsa-miR-377-3p;hsa-miR-4
ENSG0000	ENSG0000	0.006283	hsa-miR-141-5p;hsa-miR-25-3p;hsa-miR-3065-3p;hsa-miR-32
ENSG0000	ENSG0000	0.005742	hsa-miR-181a-5p;hsa-miR-181b-5p;hsa-miR-181c-5p;hsa-miR
ENSG0000	ENSG0000	1.77E-06	hsa-miR-181a-5p;hsa-miR-181b-5p;hsa-miR-181c-5p;hsa-miR
ENSG0000	ENSG0000	5.60E-05	hsa-miR-181a-5p;hsa-miR-181b-5p;hsa-miR-181c-5p;hsa-miR
ENSG0000	ENSG0000	0.00329	hsa-miR-181a-5p;hsa-miR-181b-5p;hsa-miR-181c-5p;hsa-miR
ENSG0000	ENSG0000	3.32E-05	hsa-miR-222-5p;hsa-miR-30a-3p;hsa-miR-30d-3p;hsa-miR-30
ENSG0000	ENSG0000	0.001705	hsa-miR-25-3p;hsa-miR-32-5p;hsa-miR-363-3p;hsa-miR-500a
ENSG0000	ENSG0000	0.005788	hsa-miR-548a-5p;hsa-miR-548ab;hsa-miR-548b-5p;hsa-miR-5
ENSG0000	ENSG0000	0.000475	hsa-miR-129-1-3p;hsa-miR-27a-3p;hsa-miR-3065-3p;hsa-miR
ENSG0000	ENSG0000	0.000379	hsa-miR-25-3p;hsa-miR-32-5p;hsa-miR-363-3p;hsa-miR-550a
ENSG0000	ENSG0000	0.001808	hsa-miR-129-1-3p;hsa-miR-129-2-3p;hsa-miR-141-5p;hsa-mi
ENSG0000	ENSG0000	0.005562	hsa-miR-10b-3p;hsa-miR-222-5p;hsa-miR-30a-3p;hsa-miR-30
ENSG0000	ENSG0000	0.005562	hsa-miR-10b-3p;hsa-miR-222-5p;hsa-miR-30a-3p;hsa-miR-30
ENSG0000	ENSG0000	0.005562	hsa-miR-10b-3p;hsa-miR-222-5p;hsa-miR-30a-3p;hsa-miR-30
ENSG0000	ENSG0000	0.005562	hsa-miR-10b-3p;hsa-miR-222-5p;hsa-miR-30a-3p;hsa-miR-30
ENSG0000	ENSG0000	0.005562	hsa-miR-10b-3p;hsa-miR-222-5p;hsa-miR-30a-3p;hsa-miR-30
ENSG0000	ENSG0000	0.005562	hsa-miR-10b-3p;hsa-miR-222-5p;hsa-miR-30a-3p;hsa-miR-30
ENSG0000	ENSG0000	0.002087	hsa-miR-27a-3p;hsa-miR-27b-3p;hsa-miR-513a-5p;hsa-miR-5
ENSG0000	ENSG0000	0.009966	hsa-miR-106a-5p;hsa-miR-27a-3p;hsa-miR-27b-3p;hsa-miR-4
ENSG0000	ENSG0000	0.000341	hsa-miR-141-5p;hsa-miR-23a-3p;hsa-miR-361-5p;hsa-miR-37
ENSG0000	ENSG0000	1.72E-05	hsa-miR-25-3p;hsa-miR-320a;hsa-miR-320b;hsa-miR-320d;hsa
ENSG0000	ENSG0000	2.83E-06	hsa-miR-222-5p;hsa-miR-26a-5p;hsa-miR-3065-3p;hsa-miR-3
ENSG0000	ENSG0000	0.003031	hsa-miR-19b-1-5p;hsa-miR-25-3p;hsa-miR-32-5p;hsa-miR-36

ENSG0000	ENSG0000	0.000581	hsa-miR-369-3p;hsa-miR-548a-5p;hsa-miR-548ab;hsa-miR-54
ENSG0000	ENSG0000	9.11E-06	hsa-miR-132-3p;hsa-miR-181a-5p;hsa-miR-181b-5p;hsa-miR-
ENSG0000	ENSG0000	0.009487	hsa-miR-181c-5p;hsa-miR-30a-3p;hsa-miR-30d-3p;hsa-miR-3
ENSG0000	ENSG0000	0.007019	hsa-miR-103a-2-5p;hsa-miR-132-3p;hsa-miR-212-3p;hsa-miR-
ENSG0000	ENSG0000	0.00329	hsa-miR-132-3p;hsa-miR-212-3p;hsa-miR-27a-3p;hsa-miR-27
ENSG0000	ENSG0000	3.85E-05	hsa-miR-19a-3p;hsa-miR-26a-5p;hsa-miR-27a-3p;hsa-miR-27
ENSG0000	ENSG0000	0.00109	hsa-miR-130a-5p;hsa-miR-23a-3p;hsa-miR-23b-3p;hsa-miR-2
ENSG0000	ENSG0000	0.002678	hsa-miR-181a-5p;hsa-miR-181b-5p;hsa-miR-193b-3p;hsa-miR
ENSG0000	ENSG0000	0.003748	hsa-miR-19a-3p;hsa-miR-19b-3p;hsa-miR-222-5p;hsa-miR-32
ENSG0000	ENSG0000	0.001083	hsa-miR-106a-5p;hsa-miR-19a-3p;hsa-miR-19b-3p;hsa-miR-5
ENSG0000	ENSG0000	0.003748	hsa-miR-25-3p;hsa-miR-32-5p;hsa-miR-363-3p;hsa-miR-92a-
ENSG0000	ENSG0000	0.00945	hsa-miR-369-3p;hsa-miR-382-5p;hsa-miR-548a-5p;hsa-miR-5
ENSG0000	ENSG0000	0.002684	hsa-miR-1307-3p;hsa-miR-193b-3p;hsa-miR-2115-5p;hsa-miR
ENSG0000	ENSG0000	0.001729	hsa-miR-25-3p;hsa-miR-32-5p;hsa-miR-363-3p;hsa-miR-92a-
ENSG0000	ENSG0000	0.00024	hsa-miR-181a-5p;hsa-miR-25-3p;hsa-miR-26a-5p;hsa-miR-27
ENSG0000	ENSG0000	0.00041	hsa-miR-130a-5p;hsa-miR-23a-3p;hsa-miR-23b-3p;hsa-miR-3
ENSG0000	ENSG0000	0.00041	hsa-miR-25-3p;hsa-miR-32-5p;hsa-miR-363-3p;hsa-miR-92a-
ENSG0000	ENSG0000	0.003031	hsa-miR-548a-5p;hsa-miR-548ab;hsa-miR-548b-5p;hsa-miR-5
ENSG0000	ENSG0000	4.75E-06	hsa-miR-106a-5p;hsa-miR-19a-3p;hsa-miR-19b-3p;hsa-miR-2
ENSG0000	ENSG0000	0.002895	hsa-miR-19a-5p;hsa-miR-19b-1-5p;hsa-miR-342-3p;hsa-miR-
ENSG0000	ENSG0000	0.002416	hsa-miR-126-5p;hsa-miR-181a-5p;hsa-miR-181b-5p;hsa-miR-
ENSG0000	ENSG0000	0.003493	hsa-miR-130a-5p;hsa-miR-23a-3p;hsa-miR-23b-3p;hsa-miR-2
ENSG0000	ENSG0000	0.002087	hsa-miR-1305;hsa-miR-30a-3p;hsa-miR-30d-3p;hsa-miR-30e-
ENSG0000	ENSG0000	0.006688	hsa-miR-548a-5p;hsa-miR-548ab;hsa-miR-548b-5p;hsa-miR-5
ENSG0000	ENSG0000	0.007019	hsa-miR-25-3p;hsa-miR-32-5p;hsa-miR-363-3p;hsa-miR-4786-
ENSG0000	ENSG0000	0.000343	hsa-miR-130a-5p;hsa-miR-181a-5p;hsa-miR-181c-5p;hsa-miR-
ENSG0000	ENSG0000	0.000343	hsa-miR-130a-5p;hsa-miR-181a-5p;hsa-miR-181c-5p;hsa-miR-
ENSG0000	ENSG0000	0.003031	hsa-miR-19a-5p;hsa-miR-19b-1-5p;hsa-miR-200a-5p;hsa-miR-
ENSG0000	ENSG0000	0.003031	hsa-miR-19a-5p;hsa-miR-19b-1-5p;hsa-miR-200a-5p;hsa-miR-
ENSG0000	ENSG0000	0.003031	hsa-miR-19a-5p;hsa-miR-19b-1-5p;hsa-miR-200a-5p;hsa-miR-
ENSG0000	ENSG0000	0.003031	hsa-miR-19a-5p;hsa-miR-19b-1-5p;hsa-miR-200a-5p;hsa-miR-
ENSG0000	ENSG0000	0.003031	hsa-miR-19a-5p;hsa-miR-19b-1-5p;hsa-miR-200a-5p;hsa-miR-
ENSG0000	ENSG0000	0.001377	hsa-miR-106a-5p;hsa-miR-132-3p;hsa-miR-212-3p;hsa-miR-2
ENSG0000	ENSG0000	0.000671	hsa-miR-1307-3p;hsa-miR-181a-5p;hsa-miR-181b-5p;hsa-miR
ENSG0000	ENSG0000	0.004674	hsa-miR-1305;hsa-miR-22-3p;hsa-miR-421;hsa-miR-4328
ENSG0000	ENSG0000	0.00768	hsa-miR-181a-5p;hsa-miR-181b-5p;hsa-miR-181c-5p;hsa-miR-
ENSG0000	ENSG0000	4.58E-06	hsa-miR-181a-5p;hsa-miR-181b-5p;hsa-miR-181c-5p;hsa-miR-
ENSG0000	ENSG0000	0.002286	hsa-miR-25-3p;hsa-miR-32-5p;hsa-miR-363-3p;hsa-miR-92a-
ENSG0000	ENSG0000	0.001705	hsa-miR-19a-5p;hsa-miR-19b-1-5p;hsa-miR-382-5p;hsa-miR-
ENSG0000	ENSG0000	0.001099	hsa-miR-320a;hsa-miR-320b;hsa-miR-320d;hsa-miR-4429;hsa-
ENSG0000	ENSG0000	0.001099	hsa-miR-320a;hsa-miR-320b;hsa-miR-320d;hsa-miR-4429;hsa-
ENSG0000	ENSG0000	0.00363	hsa-miR-19a-3p;hsa-miR-200a-5p;hsa-miR-200b-5p;hsa-miR-
ENSG0000	ENSG0000	0.000927	hsa-miR-130b-5p;hsa-miR-181a-5p;hsa-miR-181b-5p;hsa-miR
ENSG0000	ENSG0000	0.000927	hsa-miR-130b-5p;hsa-miR-181a-5p;hsa-miR-181b-5p;hsa-miR
ENSG0000	ENSG0000	0.001273	hsa-miR-181a-5p;hsa-miR-181b-5p;hsa-miR-181c-5p;hsa-miR-
ENSG0000	ENSG0000	0.003107	hsa-miR-32-5p;hsa-miR-363-3p;hsa-miR-542-3p;hsa-miR-548
ENSG0000	ENSG0000	0.004078	hsa-miR-106a-5p;hsa-miR-10b-3p;hsa-miR-148a-5p;hsa-miR-
ENSG0000	ENSG0000	0.006259	hsa-miR-212-3p;hsa-miR-25-3p;hsa-miR-320e;hsa-miR-32-5p;
ENSG0000	ENSG0000	0.000113	hsa-miR-320a;hsa-miR-320b;hsa-miR-320d;hsa-miR-342-3p;hs
ENSG0000	ENSG0000	0.005788	hsa-miR-181a-5p;hsa-miR-181b-5p;hsa-miR-181c-5p;hsa-miR-
ENSG0000	ENSG0000	0.000633	hsa-miR-181a-5p;hsa-miR-181b-5p;hsa-miR-181c-5p;hsa-miR-
ENSG0000	ENSG0000	0.009697	hsa-miR-15b-3p;hsa-miR-320a;hsa-miR-320b;hsa-miR-320d;hs
ENSG0000	ENSG0000	0.00531	hsa-miR-106a-5p;hsa-miR-1305;hsa-miR-130a-5p;hsa-miR-20
ENSG0000	ENSG0000	0.001273	hsa-miR-25-3p;hsa-miR-32-5p;hsa-miR-363-3p;hsa-miR-92a-

ENSG0000	ENSG0000	4.49E-05	hsa-miR-181a-5p;hsa-miR-181b-5p;hsa-miR-181c-5p;hsa-miR-
ENSG0000	ENSG0000	0.004817	hsa-miR-130a-5p;hsa-miR-141-5p;hsa-miR-19a-3p;hsa-miR-1
ENSG0000	ENSG0000	0.000135	hsa-miR-320a;hsa-miR-320b;hsa-miR-320d;hsa-miR-4429;hsa-
ENSG0000	ENSG0000	0.005742	hsa-miR-106a-5p;hsa-miR-181a-5p;hsa-miR-181b-5p;hsa-miR-
ENSG0000	ENSG0000	0.003818	hsa-miR-1305;hsa-miR-26a-5p;hsa-miR-342-3p;hsa-miR-377-
ENSG0000	ENSG0000	0.000207	hsa-miR-130a-5p;hsa-miR-23a-3p;hsa-miR-23b-3p;hsa-miR-2
ENSG0000	ENSG0000	0.009487	hsa-miR-1305;hsa-miR-409-5p;hsa-miR-548a-5p;hsa-miR-548
ENSG0000	ENSG0000	2.91E-05	hsa-miR-19a-3p;hsa-miR-19b-3p;hsa-miR-25-3p;hsa-miR-26a-
ENSG0000	ENSG0000	6.92E-05	hsa-miR-130a-5p;hsa-miR-19a-3p;hsa-miR-19a-5p;hsa-miR-1
ENSG0000	ENSG0000	0.002895	hsa-miR-320a;hsa-miR-320b;hsa-miR-320d;hsa-miR-4429;hsa-
ENSG0000	ENSG0000	0.00101	hsa-miR-19a-3p;hsa-miR-19b-3p;hsa-miR-3194-3p;hsa-miR-3
ENSG0000	ENSG0000	0.001326	hsa-miR-25-3p;hsa-miR-32-5p;hsa-miR-363-3p;hsa-miR-542-
ENSG0000	ENSG0000	0.007019	hsa-miR-148b-5p;hsa-miR-149-5p;hsa-miR-27a-3p;hsa-miR-2
ENSG0000	ENSG0000	0.00062	hsa-miR-106a-5p;hsa-miR-181a-5p;hsa-miR-181b-5p;hsa-miR-
ENSG0000	ENSG0000	0.001736	hsa-miR-106a-5p;hsa-miR-126-5p;hsa-miR-320a;hsa-miR-320
ENSG0000	ENSG0000	0.001736	hsa-miR-106a-5p;hsa-miR-126-5p;hsa-miR-320a;hsa-miR-320
ENSG0000	ENSG0000	0.002505	hsa-miR-19a-3p;hsa-miR-19b-3p;hsa-miR-25-3p;hsa-miR-32-
ENSG0000	ENSG0000	5.07E-06	hsa-miR-129-1-3p;hsa-miR-129-2-3p;hsa-miR-19a-3p;hsa-mi
ENSG0000	ENSG0000	0.003208	hsa-miR-19a-5p;hsa-miR-19b-1-5p;hsa-miR-4328;hsa-miR-45
ENSG0000	ENSG0000	2.25E-05	hsa-miR-130a-5p;hsa-miR-181a-5p;hsa-miR-181b-5p;hsa-miR-
ENSG0000	ENSG0000	3.69E-08	hsa-miR-181a-5p;hsa-miR-181b-5p;hsa-miR-19a-3p;hsa-miR-
ENSG0000	ENSG0000	0.006961	hsa-miR-25-3p;hsa-miR-32-5p;hsa-miR-363-3p;hsa-miR-92a-
ENSG0000	ENSG0000	0.000917	hsa-miR-103a-2-5p;hsa-miR-181a-5p;hsa-miR-181b-5p;hsa-m
ENSG0000	ENSG0000	0.000669	hsa-miR-320a;hsa-miR-320b;hsa-miR-320d;hsa-miR-4429;hsa-
ENSG0000	ENSG0000	1.93E-05	hsa-miR-181a-5p;hsa-miR-181b-5p;hsa-miR-181c-5p;hsa-miR-
ENSG0000	ENSG0000	0.004136	hsa-miR-106a-5p;hsa-miR-19a-3p;hsa-miR-19b-3p;hsa-miR-2
ENSG0000	ENSG0000	0.001273	hsa-miR-132-3p;hsa-miR-19a-3p;hsa-miR-19b-3p;hsa-miR-21
ENSG0000	ENSG0000	0.00055	hsa-miR-19a-3p;hsa-miR-19b-3p;hsa-miR-548a-5p;hsa-miR-5
ENSG0000	ENSG0000	0.000831	hsa-miR-129-1-3p;hsa-miR-129-2-3p;hsa-miR-181b-3p;hsa-m
ENSG0000	ENSG0000	0.00041	hsa-miR-320a;hsa-miR-320b;hsa-miR-320d;hsa-miR-4429
ENSG0000	ENSG0000	0.003486	hsa-miR-19a-3p;hsa-miR-19b-3p;hsa-miR-26a-5p;hsa-miR-32
ENSG0000	ENSG0000	0.001273	hsa-miR-25-3p;hsa-miR-32-5p;hsa-miR-375;hsa-miR-92a-3p
ENSG0000	ENSG0000	0.003809	hsa-miR-193b-3p;hsa-miR-19a-5p;hsa-miR-19b-1-5p;hsa-miR-
ENSG0000	ENSG0000	0.002527	hsa-miR-130b-5p;hsa-miR-27a-3p;hsa-miR-27b-3p;hsa-miR-4
ENSG0000	ENSG0000	0.000255	hsa-miR-25-3p;hsa-miR-32-5p;hsa-miR-363-3p;hsa-miR-4793-
ENSG0000	ENSG0000	0.008676	hsa-miR-106a-5p;hsa-miR-222-5p;hsa-miR-30a-3p;hsa-miR-3
ENSG0000	ENSG0000	0.008066	hsa-miR-129-1-3p;hsa-miR-129-2-3p;hsa-miR-130b-5p;hsa-m
ENSG0000	ENSG0000	0.008341	hsa-miR-130a-5p;hsa-miR-23a-3p;hsa-miR-23b-3p;hsa-miR-4
ENSG0000	ENSG0000	0.003809	hsa-miR-25-3p;hsa-miR-3065-3p;hsa-miR-32-5p;hsa-miR-363-
ENSG0000	ENSG0000	0.001273	hsa-miR-130b-5p;hsa-miR-30a-3p;hsa-miR-30d-3p;hsa-miR-3
ENSG0000	ENSG0000	0.001273	hsa-miR-130b-5p;hsa-miR-30a-3p;hsa-miR-30d-3p;hsa-miR-3
ENSG0000	ENSG0000	0.006548	hsa-miR-132-3p;hsa-miR-212-3p;hsa-miR-27a-3p;hsa-miR-27
ENSG0000	ENSG0000	9.83E-06	hsa-miR-106a-5p;hsa-miR-148b-5p;hsa-miR-181a-5p;hsa-miR-
ENSG0000	ENSG0000	0.00768	hsa-miR-19a-5p;hsa-miR-19b-1-5p;hsa-miR-27a-3p;hsa-miR-
ENSG0000	ENSG0000	0.001099	hsa-miR-181a-5p;hsa-miR-181b-5p;hsa-miR-181c-5p;hsa-miR-
ENSG0000	ENSG0000	0.000864	hsa-miR-181a-5p;hsa-miR-25-3p;hsa-miR-32-5p;hsa-miR-363-
ENSG0000	ENSG0000	0.009966	hsa-miR-1305;hsa-miR-548a-5p;hsa-miR-548ab;hsa-miR-548b-
ENSG0000	ENSG0000	0.000135	hsa-miR-132-3p;hsa-miR-181a-5p;hsa-miR-181b-5p;hsa-miR-
ENSG0000	ENSG0000	2.80E-07	hsa-miR-130a-5p;hsa-miR-154-5p;hsa-miR-23a-3p;hsa-miR-2
ENSG0000	ENSG0000	1.40E-07	hsa-miR-181a-5p;hsa-miR-181b-5p;hsa-miR-181c-5p;hsa-miR-
ENSG0000	ENSG0000	0.002416	hsa-miR-181a-5p;hsa-miR-181b-5p;hsa-miR-181c-5p;hsa-miR-
ENSG0000	ENSG0000	0.000267	hsa-miR-320a;hsa-miR-320b;hsa-miR-320d;hsa-miR-4429;hsa-
ENSG0000	ENSG0000	0.000379	hsa-miR-25-3p;hsa-miR-32-5p;hsa-miR-363-3p;hsa-miR-5582-
ENSG0000	ENSG0000	0.008772	hsa-miR-130b-5p;hsa-miR-25-3p;hsa-miR-363-3p;hsa-miR-64

ENSG0000	ENSG0000	0.000254	hsa-miR-19a-5p;hsa-miR-19b-1-5p;hsa-miR-361-5p;hsa-miR-4
ENSG0000	ENSG0000	0.001662	hsa-miR-130a-5p;hsa-miR-23a-3p;hsa-miR-23b-3p;hsa-miR-30
ENSG0000	ENSG0000	0.002445	hsa-miR-27a-3p;hsa-miR-27b-3p;hsa-miR-320b;hsa-miR-320d;
ENSG0000	ENSG0000	0.001273	hsa-miR-320a;hsa-miR-320b;hsa-miR-320d;hsa-miR-4429
ENSG0000	ENSG0000	0.006259	hsa-miR-342-3p;hsa-miR-545-3p;hsa-miR-548at-5p;hsa-miR-5
ENSG0000	ENSG0000	4.01E-07	hsa-miR-130a-5p;hsa-miR-181a-5p;hsa-miR-181b-5p;hsa-miR-
ENSG0000	ENSG0000	0.004674	hsa-miR-25-3p;hsa-miR-32-5p;hsa-miR-363-3p;hsa-miR-92a-3
ENSG0000	ENSG0000	0.00015	hsa-miR-19a-3p;hsa-miR-19b-3p;hsa-miR-26a-5p;hsa-miR-27a
ENSG0000	ENSG0000	0.001705	hsa-miR-25-3p;hsa-miR-32-5p;hsa-miR-363-3p;hsa-miR-450b-
ENSG0000	ENSG0000	0.001273	hsa-miR-320a;hsa-miR-320b;hsa-miR-320d;hsa-miR-4429
ENSG0000	ENSG0000	1.93E-05	hsa-miR-320a;hsa-miR-320b;hsa-miR-320d;hsa-miR-4429;hsa-
ENSG0000	ENSG0000	0.004674	hsa-miR-27a-3p;hsa-miR-27b-3p;hsa-miR-3065-3p;hsa-miR-51
ENSG0000	ENSG0000	0.000215	hsa-miR-25-3p;hsa-miR-26a-5p;hsa-miR-32-5p;hsa-miR-342-3
ENSG0000	ENSG0000	0.001377	hsa-miR-1305;hsa-miR-27a-3p;hsa-miR-27b-3p;hsa-miR-323a-
ENSG0000	ENSG0000	0.005562	hsa-miR-181a-5p;hsa-miR-181b-5p;hsa-miR-181c-5p;hsa-miR-
ENSG0000	ENSG0000	0.008676	hsa-miR-181a-5p;hsa-miR-25-3p;hsa-miR-27a-3p;hsa-miR-27b
ENSG0000	ENSG0000	3.48E-07	hsa-miR-130a-5p;hsa-miR-23a-3p;hsa-miR-23b-3p;hsa-miR-32
ENSG0000	ENSG0000	0.009888	hsa-miR-141-5p;hsa-miR-187-5p;hsa-miR-99a-3p;hsa-miR-99b
ENSG0000	ENSG0000	0.001374	hsa-miR-30a-3p;hsa-miR-30d-3p;hsa-miR-30e-3p;hsa-miR-548
ENSG0000	ENSG0000	0.000113	hsa-miR-132-3p;hsa-miR-181a-5p;hsa-miR-181b-5p;hsa-miR-1
ENSG0000	ENSG0000	0.00055	hsa-miR-27a-3p;hsa-miR-27b-3p;hsa-miR-450b-5p;hsa-miR-54
ENSG0000	ENSG0000	0.00055	hsa-miR-27a-3p;hsa-miR-27b-3p;hsa-miR-450b-5p;hsa-miR-54
ENSG0000	ENSG0000	0.009966	hsa-miR-27a-3p;hsa-miR-27b-3p;hsa-miR-513a-5p;hsa-miR-54
ENSG0000	ENSG0000	0.000669	hsa-miR-181b-5p;hsa-miR-19a-3p;hsa-miR-19b-3p;hsa-miR-42
ENSG0000	ENSG0000	0.001729	hsa-miR-25-3p;hsa-miR-32-5p;hsa-miR-363-3p;hsa-miR-92a-3
ENSG0000	ENSG0000	4.58E-06	hsa-miR-30a-3p;hsa-miR-30d-3p;hsa-miR-30e-3p;hsa-miR-369
ENSG0000	ENSG0000	0.004978	hsa-miR-548a-5p;hsa-miR-548ab;hsa-miR-548b-5p;hsa-miR-54
ENSG0000	ENSG0000	0.003748	hsa-miR-30a-3p;hsa-miR-30d-3p;hsa-miR-30e-3p;hsa-miR-342
ENSG0000	ENSG0000	0.003031	hsa-miR-548a-5p;hsa-miR-548ab;hsa-miR-548b-5p;hsa-miR-54
ENSG0000	ENSG0000	0.000379	hsa-miR-320a;hsa-miR-320b;hsa-miR-320d;hsa-miR-4429;hsa-
ENSG0000	ENSG0000	0.000669	hsa-miR-27a-3p;hsa-miR-27b-3p;hsa-miR-4328;hsa-miR-513a-
ENSG0000	ENSG0000	3.04E-08	hsa-miR-129-1-3p;hsa-miR-129-2-3p;hsa-miR-30a-3p;hsa-miR
ENSG0000	ENSG0000	0.00041	hsa-miR-130b-5p;hsa-miR-30a-3p;hsa-miR-30d-3p;hsa-miR-30
ENSG0000	ENSG0000	0.005788	hsa-miR-320a;hsa-miR-320b;hsa-miR-320d;hsa-miR-4429;hsa-
ENSG0000	ENSG0000	0.002895	hsa-miR-19a-3p;hsa-miR-19b-3p;hsa-miR-25-3p;hsa-miR-32-5
ENSG0000	ENSG0000	0.000625	hsa-miR-126-5p;hsa-miR-27a-3p;hsa-miR-27b-3p;hsa-miR-513
ENSG0000	ENSG0000	0.000255	hsa-miR-25-3p;hsa-miR-32-5p;hsa-miR-342-3p;hsa-miR-363-3
ENSG0000	ENSG0000	0.002156	hsa-miR-132-3p;hsa-miR-138-1-3p;hsa-miR-212-3p;hsa-miR-3
ENSG0000	ENSG0000	6.43E-06	hsa-miR-106a-5p;hsa-miR-181a-5p;hsa-miR-181b-5p;hsa-miR-
ENSG0000	ENSG0000	1.34E-05	hsa-miR-106a-5p;hsa-miR-181a-5p;hsa-miR-181b-5p;hsa-miR-
ENSG0000	ENSG0000	0.006548	hsa-miR-106a-5p;hsa-miR-10b-3p;hsa-miR-27a-3p;hsa-miR-27
ENSG0000	ENSG0000	0.003748	hsa-miR-25-3p;hsa-miR-32-5p;hsa-miR-363-3p;hsa-miR-92a-3
ENSG0000	ENSG0000	0.006259	hsa-miR-181a-5p;hsa-miR-181b-5p;hsa-miR-181c-5p;hsa-miR-
ENSG0000	ENSG0000	0.000255	hsa-miR-181c-3p;hsa-miR-27a-3p;hsa-miR-27b-3p;hsa-miR-32
ENSG0000	ENSG0000	0.006259	hsa-miR-1307-3p;hsa-miR-138-1-3p;hsa-miR-545-3p;hsa-miR-
ENSG0000	ENSG0000	0.006259	hsa-miR-1307-3p;hsa-miR-222-5p;hsa-miR-26a-5p;hsa-miR-30
ENSG0000	ENSG0000	0.000939	hsa-miR-181a-5p;hsa-miR-181b-5p;hsa-miR-181c-5p;hsa-miR-
ENSG0000	ENSG0000	0.008772	hsa-miR-181a-5p;hsa-miR-181b-5p;hsa-miR-181c-5p;hsa-miR-
ENSG0000	ENSG0000	0.009966	hsa-miR-548a-5p;hsa-miR-548ab;hsa-miR-548b-5p;hsa-miR-54
ENSG0000	ENSG0000	0.000342	hsa-miR-129-1-3p;hsa-miR-129-2-3p;hsa-miR-30a-3p;hsa-miR
ENSG0000	ENSG0000	3.13E-07	hsa-miR-132-3p;hsa-miR-181a-5p;hsa-miR-181b-5p;hsa-miR-1
ENSG0000	ENSG0000	1.20E-08	hsa-miR-1305;hsa-miR-181a-5p;hsa-miR-181b-5p;hsa-miR-18
ENSG0000	ENSG0000	0.007735	hsa-miR-15b-3p;hsa-miR-181a-5p;hsa-miR-181b-5p;hsa-miR-1
ENSG0000	ENSG0000	0.000207	hsa-miR-138-1-3p;hsa-miR-25-3p;hsa-miR-32-5p;hsa-miR-363

ENSG0000	ENSG0000	0.003502	hsa-miR-320a;hsa-miR-320b;hsa-miR-320d;hsa-miR-4429;hsa-
ENSG0000	ENSG0000	0.004978	hsa-miR-106a-5p;hsa-miR-25-3p;hsa-miR-32-5p;hsa-miR-363-
ENSG0000	ENSG0000	0.004978	hsa-miR-106a-5p;hsa-miR-25-3p;hsa-miR-32-5p;hsa-miR-363-
ENSG0000	ENSG0000	0.001729	hsa-miR-1305;hsa-miR-181a-5p;hsa-miR-181b-5p;hsa-miR-18
ENSG0000	ENSG0000	0.000135	hsa-miR-25-3p;hsa-miR-32-5p;hsa-miR-363-3p;hsa-miR-377-
ENSG0000	ENSG0000	0.000726	hsa-miR-1305;hsa-miR-141-5p;hsa-miR-181a-5p;hsa-miR-181
ENSG0000	ENSG0000	0.000726	hsa-miR-1305;hsa-miR-141-5p;hsa-miR-181a-5p;hsa-miR-181
ENSG0000	ENSG0000	0.000113	hsa-miR-154-5p;hsa-miR-181a-5p;hsa-miR-181b-5p;hsa-miR-
ENSG0000	ENSG0000	0.006961	hsa-miR-1305;hsa-miR-130a-5p;hsa-miR-23a-3p;hsa-miR-23b-
ENSG0000	ENSG0000	0.008772	hsa-miR-548a-5p;hsa-miR-548ab;hsa-miR-548b-5p;hsa-miR-5
ENSG0000	ENSG0000	0.001705	hsa-miR-320a;hsa-miR-320b;hsa-miR-320d;hsa-miR-4429;hsa-
ENSG0000	ENSG0000	0.001845	hsa-miR-369-3p;hsa-miR-548a-5p;hsa-miR-548ab;hsa-miR-54
ENSG0000	ENSG0000	0.009888	hsa-miR-181a-5p;hsa-miR-181b-5p;hsa-miR-181c-5p;hsa-miR-
ENSG0000	ENSG0000	0.003605	hsa-miR-548a-5p;hsa-miR-548ab;hsa-miR-548b-5p;hsa-miR-5
ENSG0000	ENSG0000	0.000198	hsa-miR-130a-5p;hsa-miR-181a-5p;hsa-miR-181b-5p;hsa-miR-
ENSG0000	ENSG0000	0.005273	hsa-miR-27a-3p;hsa-miR-27b-3p;hsa-miR-32-5p;hsa-miR-363-
ENSG0000	ENSG0000	0.002956	hsa-miR-181a-5p;hsa-miR-181b-5p;hsa-miR-181c-5p;hsa-miR-
ENSG0000	ENSG0000	4.88E-05	hsa-miR-19a-3p;hsa-miR-200a-5p;hsa-miR-200b-5p;hsa-miR-
ENSG0000	ENSG0000	0.006961	hsa-miR-106a-5p;hsa-miR-181a-5p;hsa-miR-181b-5p;hsa-miR-
ENSG0000	ENSG0000	0.008261	hsa-miR-154-5p;hsa-miR-19a-3p;hsa-miR-19b-3p;hsa-miR-25-
ENSG0000	ENSG0000	0.00217	hsa-miR-193b-3p;hsa-miR-548a-5p;hsa-miR-548ab;hsa-miR-5
ENSG0000	ENSG0000	0.009404	hsa-miR-132-3p;hsa-miR-212-3p;hsa-miR-25-3p;hsa-miR-32-
ENSG0000	ENSG0000	0.009487	hsa-miR-369-3p;hsa-miR-548a-5p;hsa-miR-548ab;hsa-miR-54
ENSG0000	ENSG0000	0.009487	hsa-miR-369-3p;hsa-miR-548a-5p;hsa-miR-548ab;hsa-miR-54
ENSG0000	ENSG0000	0.002895	hsa-miR-181a-5p;hsa-miR-181b-5p;hsa-miR-181c-5p;hsa-miR-
ENSG0000	ENSG0000	0.005788	hsa-miR-181a-5p;hsa-miR-181b-5p;hsa-miR-181c-5p;hsa-miR-
ENSG0000	ENSG0000	0.004978	hsa-miR-181c-3p;hsa-miR-30a-3p;hsa-miR-30d-3p;hsa-miR-3
ENSG0000	ENSG0000	0.002527	hsa-miR-19a-3p;hsa-miR-19b-3p;hsa-miR-27b-3p;hsa-miR-43
ENSG0000	ENSG0000	0.000969	hsa-miR-25-3p;hsa-miR-32-5p;hsa-miR-363-3p;hsa-miR-548a-
ENSG0000	ENSG0000	0.008772	hsa-miR-181a-5p;hsa-miR-181b-5p;hsa-miR-181c-5p;hsa-miR-
ENSG0000	ENSG0000	0.000333	hsa-miR-106a-5p;hsa-miR-130b-5p;hsa-miR-191-3p;hsa-miR-
ENSG0000	ENSG0000	0.00126	hsa-miR-25-3p;hsa-miR-363-3p;hsa-miR-409-5p;hsa-miR-548
ENSG0000	ENSG0000	0.001273	hsa-miR-25-3p;hsa-miR-32-5p;hsa-miR-363-3p;hsa-miR-92a-
ENSG0000	ENSG0000	0.001547	hsa-miR-154-5p;hsa-miR-450b-5p;hsa-miR-548a-5p;hsa-miR-
ENSG0000	ENSG0000	0.00768	hsa-miR-19a-5p;hsa-miR-19b-1-5p;hsa-miR-450b-5p;hsa-miR-
ENSG0000	ENSG0000	0.005788	hsa-miR-548a-5p;hsa-miR-548ab;hsa-miR-548b-5p;hsa-miR-5
ENSG0000	ENSG0000	0.007208	hsa-miR-106a-5p;hsa-miR-181a-5p;hsa-miR-181b-5p;hsa-miR-
ENSG0000	ENSG0000	0.008676	hsa-miR-10b-3p;hsa-miR-27a-3p;hsa-miR-361-5p;hsa-miR-36
ENSG0000	ENSG0000	0.008341	hsa-miR-25-3p;hsa-miR-32-5p;hsa-miR-363-3p;hsa-miR-92a-
ENSG0000	ENSG0000	0.001729	hsa-miR-130a-5p;hsa-miR-23a-3p;hsa-miR-23b-3p;hsa-miR-4
ENSG0000	ENSG0000	0.002445	hsa-miR-342-3p;hsa-miR-548a-5p;hsa-miR-548ab;hsa-miR-54
ENSG0000	ENSG0000	0.001729	hsa-miR-27a-3p;hsa-miR-27b-3p;hsa-miR-513a-5p;hsa-miR-5
ENSG0000	ENSG0000	0.000625	hsa-miR-181a-5p;hsa-miR-181b-5p;hsa-miR-181c-5p;hsa-miR-
ENSG0000	ENSG0000	0.007019	hsa-miR-375;hsa-miR-548a-5p;hsa-miR-548ab;hsa-miR-548b-
ENSG0000	ENSG0000	0.004674	hsa-miR-25-3p;hsa-miR-32-5p;hsa-miR-363-3p;hsa-miR-92a-
ENSG0000	ENSG0000	0.008261	hsa-miR-130b-5p;hsa-miR-27a-3p;hsa-miR-27b-3p;hsa-miR-4
ENSG0000	ENSG0000	0.00248	hsa-miR-106a-5p;hsa-miR-126-5p;hsa-miR-27a-3p;hsa-miR-2
ENSG0000	ENSG0000	0.003329	hsa-miR-19a-3p;hsa-miR-19b-3p;hsa-miR-342-3p;hsa-miR-37
ENSG0000	ENSG0000	0.000198	hsa-miR-181a-5p;hsa-miR-181b-5p;hsa-miR-181c-5p;hsa-miR-
ENSG0000	ENSG0000	0.000198	hsa-miR-181a-5p;hsa-miR-181b-5p;hsa-miR-181c-5p;hsa-miR-
ENSG0000	ENSG0000	0.00041	hsa-miR-10b-3p;hsa-miR-27a-3p;hsa-miR-27b-3p;hsa-miR-36
ENSG0000	ENSG0000	8.56E-07	hsa-miR-181a-5p;hsa-miR-181b-5p;hsa-miR-181c-5p;hsa-miR-
ENSG0000	ENSG0000	0.004252	hsa-miR-126-5p;hsa-miR-27a-3p;hsa-miR-27b-3p;hsa-miR-54
ENSG0000	ENSG0000	0.007917	hsa-miR-132-3p;hsa-miR-141-5p;hsa-miR-212-3p;hsa-miR-25-

ENSG0000	ENSG0000	0.004674	hsa-miR-27a-3p;hsa-miR-27b-3p;hsa-miR-342-3p;hsa-miR-51
ENSG0000	ENSG0000	0.001118	hsa-miR-148b-5p;hsa-miR-25-3p;hsa-miR-32-5p;hsa-miR-363
ENSG0000	ENSG0000	0.001729	hsa-miR-222-5p;hsa-miR-27a-3p;hsa-miR-27b-3p;hsa-miR-55
ENSG0000	ENSG0000	2.03E-08	hsa-miR-1305;hsa-miR-130a-5p;hsa-miR-181a-5p;hsa-miR-18
ENSG0000	ENSG0000	0.005788	hsa-miR-320a;hsa-miR-320b;hsa-miR-320d;hsa-miR-4429;hsa-
ENSG0000	ENSG0000	0.000536	hsa-miR-132-3p;hsa-miR-181a-5p;hsa-miR-181b-5p;hsa-miR-
ENSG0000	ENSG0000	0.00945	hsa-miR-106a-5p;hsa-miR-342-3p;hsa-miR-500a-5p;hsa-miR-5
ENSG0000	ENSG0000	0.009888	hsa-miR-1305;hsa-miR-181a-5p;hsa-miR-181b-5p;hsa-miR-18
ENSG0000	ENSG0000	0.002087	hsa-miR-30a-3p;hsa-miR-30d-3p;hsa-miR-30e-3p;hsa-miR-32
ENSG0000	ENSG0000	0.000282	hsa-miR-154-5p;hsa-miR-548a-5p;hsa-miR-548ab;hsa-miR-54
ENSG0000	ENSG0000	0.001662	hsa-miR-181a-5p;hsa-miR-181b-5p;hsa-miR-181c-5p;hsa-miR
ENSG0000	ENSG0000	0.007843	hsa-miR-181a-5p;hsa-miR-181b-5p;hsa-miR-181c-5p;hsa-miR
ENSG0000	ENSG0000	0.006961	hsa-miR-25-3p;hsa-miR-32-5p;hsa-miR-363-3p;hsa-miR-92a-
ENSG0000	ENSG0000	0.001326	hsa-miR-181a-5p;hsa-miR-181b-5p;hsa-miR-181c-5p;hsa-miR
ENSG0000	ENSG0000	0.001326	hsa-miR-181a-5p;hsa-miR-181b-5p;hsa-miR-181c-5p;hsa-miR
ENSG0000	ENSG0000	0.005788	hsa-miR-130a-5p;hsa-miR-19a-3p;hsa-miR-19b-3p;hsa-miR-2
ENSG0000	ENSG0000	0.001547	hsa-miR-141-5p;hsa-miR-15b-3p;hsa-miR-19a-5p;hsa-miR-19
ENSG0000	ENSG0000	0.002158	hsa-miR-130a-5p;hsa-miR-23a-3p;hsa-miR-23b-3p;hsa-miR-2
ENSG0000	ENSG0000	0.002895	hsa-miR-130a-5p;hsa-miR-23a-3p;hsa-miR-23b-3p;hsa-miR-3
ENSG0000	ENSG0000	0.002156	hsa-miR-181a-5p;hsa-miR-181b-5p;hsa-miR-181c-5p;hsa-miR
ENSG0000	ENSG0000	0.000509	hsa-miR-19a-3p;hsa-miR-19b-3p;hsa-miR-27a-3p;hsa-miR-27
ENSG0000	ENSG0000	2.53E-07	hsa-miR-181b-3p;hsa-miR-25-3p;hsa-miR-320a;hsa-miR-320b;
ENSG0000	ENSG0000	0.004923	hsa-miR-106a-5p;hsa-miR-25-3p;hsa-miR-32-5p;hsa-miR-363
ENSG0000	ENSG0000	1.14E-05	hsa-miR-181a-5p;hsa-miR-181c-5p;hsa-miR-193b-3p;hsa-miR
ENSG0000	ENSG0000	0.006961	hsa-miR-126-5p;hsa-miR-369-3p;hsa-miR-548at-5p;hsa-miR-5
ENSG0000	ENSG0000	0.001014	hsa-miR-200a-5p;hsa-miR-200b-5p;hsa-miR-377-3p;hsa-miR-
ENSG0000	ENSG0000	0.009966	hsa-miR-548a-5p;hsa-miR-548ab;hsa-miR-548b-5p;hsa-miR-5
ENSG0000	ENSG0000	0.000509	hsa-miR-130a-5p;hsa-miR-23a-3p;hsa-miR-23b-3p;hsa-miR-4
ENSG0000	ENSG0000	0.001099	hsa-miR-30a-3p;hsa-miR-30d-3p;hsa-miR-30e-3p;hsa-miR-61
ENSG0000	ENSG0000	0.004923	hsa-miR-130a-5p;hsa-miR-181a-5p;hsa-miR-181b-5p;hsa-miR
ENSG0000	ENSG0000	0.000939	hsa-miR-103a-2-5p;hsa-miR-222-5p;hsa-miR-30a-3p;hsa-miR
ENSG0000	ENSG0000	0.000864	hsa-miR-103a-2-5p;hsa-miR-181a-5p;hsa-miR-181b-5p;hsa-m
ENSG0000	ENSG0000	6.31E-07	hsa-miR-130a-5p;hsa-miR-181a-5p;hsa-miR-181b-3p;hsa-miR
ENSG0000	ENSG0000	4.58E-06	hsa-miR-320a;hsa-miR-320b;hsa-miR-320d;hsa-miR-4429;hsa-
ENSG0000	ENSG0000	0.006252	hsa-miR-148a-5p;hsa-miR-19a-3p;hsa-miR-19b-3p;hsa-miR-3
ENSG0000	ENSG0000	0.002156	hsa-miR-130a-5p;hsa-miR-23a-3p;hsa-miR-23b-3p;hsa-miR-2
ENSG0000	ENSG0000	0.003486	hsa-miR-1305;hsa-miR-19a-3p;hsa-miR-19b-3p;hsa-miR-30a-
ENSG0000	ENSG0000	0.009888	hsa-miR-130a-5p;hsa-miR-23a-3p;hsa-miR-23b-3p;hsa-miR-6
ENSG0000	ENSG0000	0.003031	hsa-miR-181a-5p;hsa-miR-181b-5p;hsa-miR-181c-5p;hsa-miR
ENSG0000	ENSG0000	0.009966	hsa-miR-25-3p;hsa-miR-32-5p;hsa-miR-363-3p;hsa-miR-4786
ENSG0000	ENSG0000	0.006759	hsa-miR-193b-3p;hsa-miR-369-3p;hsa-miR-450b-5p;hsa-miR-
ENSG0000	ENSG0000	0.00041	hsa-miR-193b-3p;hsa-miR-19a-5p;hsa-miR-19b-1-5p;hsa-miR
ENSG0000	ENSG0000	0.000864	hsa-miR-25-3p;hsa-miR-32-5p;hsa-miR-363-3p;hsa-miR-542-
ENSG0000	ENSG0000	0.003329	hsa-miR-548a-5p;hsa-miR-548ab;hsa-miR-548ax;hsa-miR-548
ENSG0000	ENSG0000	0.000409	hsa-miR-130b-5p;hsa-miR-25-3p;hsa-miR-32-5p;hsa-miR-363
ENSG0000	ENSG0000	0.002956	hsa-miR-19a-3p;hsa-miR-19b-3p;hsa-miR-377-3p;hsa-miR-54
ENSG0000	ENSG0000	0.008676	hsa-miR-450b-5p;hsa-miR-548a-5p;hsa-miR-548ab;hsa-miR-5
ENSG0000	ENSG0000	0.004978	hsa-miR-126-5p;hsa-miR-19a-3p;hsa-miR-19b-3p;hsa-miR-22
ENSG0000	ENSG0000	0.004978	hsa-miR-30a-3p;hsa-miR-30d-3p;hsa-miR-30e-3p;hsa-miR-36
ENSG0000	ENSG0000	0.007843	hsa-miR-106a-5p;hsa-miR-181c-5p;hsa-miR-27a-3p;hsa-miR-2
ENSG0000	ENSG0000	0.009888	hsa-miR-181a-5p;hsa-miR-181b-5p;hsa-miR-181c-5p;hsa-miR
ENSG0000	ENSG0000	0.000607	hsa-miR-193b-3p;hsa-miR-2115-5p;hsa-miR-320a;hsa-miR-32
ENSG0000	ENSG0000	0.000909	hsa-miR-27a-3p;hsa-miR-27b-3p;hsa-miR-99a-3p;hsa-miR-99
ENSG0000	ENSG0000	0.000113	hsa-miR-103a-2-5p;hsa-miR-27a-3p;hsa-miR-27b-3p;hsa-miR

ENSG0000	ENSG0000	0.001273	hsa-miR-25-3p;hsa-miR-32-5p;hsa-miR-363-3p;hsa-miR-92a-
ENSG0000	ENSG0000	0.000131	hsa-miR-181a-5p;hsa-miR-181b-5p;hsa-miR-181c-5p;hsa-miR-
ENSG0000	ENSG0000	0.001943	hsa-miR-19a-3p;hsa-miR-19a-5p;hsa-miR-19b-1-5p;hsa-miR-
ENSG0000	ENSG0000	0.000207	hsa-miR-187-5p;hsa-miR-27a-3p;hsa-miR-27b-3p;hsa-miR-32
ENSG0000	ENSG0000	0.004393	hsa-miR-19a-5p;hsa-miR-19b-1-5p;hsa-miR-320a;hsa-miR-32
ENSG0000	ENSG0000	0.004738	hsa-miR-19a-5p;hsa-miR-19b-1-5p;hsa-miR-30a-3p;hsa-miR-
ENSG0000	ENSG0000	0.001729	hsa-miR-130a-5p;hsa-miR-130b-5p;hsa-miR-23a-3p;hsa-miR-
ENSG0000	ENSG0000	0.008341	hsa-miR-19a-5p;hsa-miR-19b-1-5p;hsa-miR-421;hsa-miR-450
ENSG0000	ENSG0000	0.001491	hsa-miR-25-3p;hsa-miR-32-5p;hsa-miR-363-3p;hsa-miR-548a-
ENSG0000	ENSG0000	0.00768	hsa-miR-1307-3p;hsa-miR-25-3p;hsa-miR-32-5p;hsa-miR-363-
ENSG0000	ENSG0000	0.00768	hsa-miR-1307-3p;hsa-miR-25-3p;hsa-miR-32-5p;hsa-miR-363-
ENSG0000	ENSG0000	0.001729	hsa-miR-10b-3p;hsa-miR-181a-5p;hsa-miR-181b-5p;hsa-miR-
ENSG0000	ENSG0000	0.001729	hsa-miR-10b-3p;hsa-miR-181a-5p;hsa-miR-181b-5p;hsa-miR-
ENSG0000	ENSG0000	0.001729	hsa-miR-10b-3p;hsa-miR-181a-5p;hsa-miR-181b-5p;hsa-miR-
ENSG0000	ENSG0000	0.001729	hsa-miR-10b-3p;hsa-miR-181a-5p;hsa-miR-181b-5p;hsa-miR-
ENSG0000	ENSG0000	5.69E-05	hsa-miR-320a;hsa-miR-320b;hsa-miR-320d;hsa-miR-4429;hsa-
ENSG0000	ENSG0000	0.004923	hsa-miR-369-3p;hsa-miR-548a-5p;hsa-miR-548ab;hsa-miR-54
ENSG0000	ENSG0000	0.006688	hsa-miR-3200-5p;hsa-miR-548a-5p;hsa-miR-548ab;hsa-miR-5
ENSG0000	ENSG0000	0.001326	hsa-miR-369-3p;hsa-miR-548a-5p;hsa-miR-548ab;hsa-miR-54
ENSG0000	ENSG0000	0.006259	hsa-miR-369-3p;hsa-miR-548a-5p;hsa-miR-548ab;hsa-miR-54
ENSG0000	ENSG0000	0.003748	hsa-miR-25-3p;hsa-miR-32-5p;hsa-miR-363-3p;hsa-miR-92a-
ENSG0000	ENSG0000	0.003748	hsa-miR-25-3p;hsa-miR-32-5p;hsa-miR-363-3p;hsa-miR-92a-
ENSG0000	ENSG0000	0.003748	hsa-miR-25-3p;hsa-miR-32-5p;hsa-miR-363-3p;hsa-miR-92a-
ENSG0000	ENSG0000	0.003748	hsa-miR-25-3p;hsa-miR-32-5p;hsa-miR-363-3p;hsa-miR-92a-
ENSG0000	ENSG0000	0.003748	hsa-miR-25-3p;hsa-miR-32-5p;hsa-miR-363-3p;hsa-miR-92a-
ENSG0000	ENSG0000	0.003748	hsa-miR-25-3p;hsa-miR-32-5p;hsa-miR-363-3p;hsa-miR-92a-
ENSG0000	ENSG0000	0.003748	hsa-miR-25-3p;hsa-miR-32-5p;hsa-miR-363-3p;hsa-miR-92a-
ENSG0000	ENSG0000	0.001729	hsa-miR-10b-3p;hsa-miR-181a-5p;hsa-miR-181b-5p;hsa-miR-
ENSG0000	ENSG0000	0.001729	hsa-miR-10b-3p;hsa-miR-181a-5p;hsa-miR-181b-5p;hsa-miR-
ENSG0000	ENSG0000	0.001491	hsa-miR-129-1-3p;hsa-miR-129-2-3p;hsa-miR-25-3p;hsa-miR-
ENSG0000	ENSG0000	0.000804	hsa-miR-200a-5p;hsa-miR-200b-5p;hsa-miR-548a-5p;hsa-miR-
ENSG0000	ENSG0000	0.003486	hsa-miR-181a-5p;hsa-miR-181b-5p;hsa-miR-181c-5p;hsa-miR-
ENSG0000	ENSG0000	0.006283	hsa-miR-132-3p;hsa-miR-212-3p;hsa-miR-548a-5p;hsa-miR-5
ENSG0000	ENSG0000	0.006283	hsa-miR-132-3p;hsa-miR-212-3p;hsa-miR-548a-5p;hsa-miR-5
ENSG0000	ENSG0000	0.001729	hsa-miR-10b-3p;hsa-miR-181a-5p;hsa-miR-181b-5p;hsa-miR-
ENSG0000	ENSG0000	0.001729	hsa-miR-10b-3p;hsa-miR-181a-5p;hsa-miR-181b-5p;hsa-miR-
ENSG0000	ENSG0000	0.002895	hsa-miR-126-5p;hsa-miR-25-3p;hsa-miR-32-5p;hsa-miR-363-
ENSG0000	ENSG0000	0.001729	hsa-miR-10b-3p;hsa-miR-181a-5p;hsa-miR-181b-5p;hsa-miR-
ENSG0000	ENSG0000	0.001729	hsa-miR-10b-3p;hsa-miR-181a-5p;hsa-miR-181b-5p;hsa-miR-
ENSG0000	ENSG0000	0.001729	hsa-miR-10b-3p;hsa-miR-181a-5p;hsa-miR-181b-5p;hsa-miR-
ENSG0000	ENSG0000	0.001729	hsa-miR-10b-3p;hsa-miR-181a-5p;hsa-miR-181b-5p;hsa-miR-
ENSG0000	ENSG0000	0.001729	hsa-miR-10b-3p;hsa-miR-181a-5p;hsa-miR-181b-5p;hsa-miR-
ENSG0000	ENSG0000	0.001729	hsa-miR-10b-3p;hsa-miR-181a-5p;hsa-miR-181b-5p;hsa-miR-
ENSG0000	ENSG0000	0.001729	hsa-miR-10b-3p;hsa-miR-181a-5p;hsa-miR-181b-5p;hsa-miR-
ENSG0000	ENSG0000	0.001729	hsa-miR-10b-3p;hsa-miR-181a-5p;hsa-miR-181b-5p;hsa-miR-
ENSG0000	ENSG0000	0.004252	hsa-miR-106a-5p;hsa-miR-30a-3p;hsa-miR-30d-3p;hsa-miR-3
ENSG0000	ENSG0000	8.96E-05	hsa-miR-19a-5p;hsa-miR-19b-1-5p;hsa-miR-30a-3p;hsa-miR-
ENSG0000	ENSG0000	0.001845	hsa-miR-320a;hsa-miR-320b;hsa-miR-320d;hsa-miR-382-5p;hs
ENSG0000	ENSG0000	0.009888	hsa-miR-25-3p;hsa-miR-32-5p;hsa-miR-363-3p;hsa-miR-92a-
ENSG0000	ENSG0000	7.25E-06	hsa-miR-29a-3p;hsa-miR-29b-3p;hsa-miR-29c-3p;hsa-miR-76
ENSG0000	ENSG0000	8.85E-07	hsa-miR-29a-3p;hsa-miR-29b-3p;hsa-miR-29c-3p;hsa-miR-76
ENSG0000	ENSG0000	8.73E-06	hsa-miR-181a-5p;hsa-miR-181b-5p;hsa-miR-181c-5p;hsa-miR-
ENSG0000	ENSG0000	0.000548	hsa-miR-181a-5p;hsa-miR-181b-5p;hsa-miR-181c-5p;hsa-miR-
ENSG0000	ENSG0000	1.59E-05	hsa-miR-129-5p;hsa-miR-20a-3p;hsa-miR-26a-5p;hsa-miR-26

ENSG0000	ENSG0000	0.0022	hsa-miR-127-5p;hsa-miR-26a-5p;hsa-miR-26b-5p;hsa-miR-87
ENSG0000	ENSG0000	0.000268	hsa-miR-181a-5p;hsa-miR-181b-5p;hsa-miR-181c-5p;hsa-miR-
ENSG0000	ENSG0000	0.002371	hsa-miR-129-5p;hsa-miR-181b-5p;hsa-miR-181c-5p;hsa-miR-
ENSG0000	ENSG0000	0.000828	hsa-miR-181a-5p;hsa-miR-181b-5p;hsa-miR-181c-5p;hsa-miR-
ENSG0000	ENSG0000	0.0022	hsa-miR-181a-5p;hsa-miR-181b-5p;hsa-miR-181c-5p;hsa-miR-
ENSG0000	ENSG0000	4.66E-07	hsa-miR-129-5p;hsa-miR-181a-5p;hsa-miR-181b-5p;hsa-miR-
ENSG0000	ENSG0000	4.65E-05	hsa-miR-181a-5p;hsa-miR-181b-5p;hsa-miR-181c-5p;hsa-miR-
ENSG0000	ENSG0000	0.000232	hsa-miR-181a-5p;hsa-miR-181b-5p;hsa-miR-181c-5p;hsa-miR-
ENSG0000	ENSG0000	0.000244	hsa-miR-181a-5p;hsa-miR-181b-5p;hsa-miR-181c-5p;hsa-miR-
ENSG0000	ENSG0000	0.009498	hsa-miR-26a-5p;hsa-miR-26b-5p;hsa-miR-382-3p;hsa-miR-48
ENSG0000	ENSG0000	0.000455	hsa-miR-144-5p;hsa-miR-181a-5p;hsa-miR-181b-5p;hsa-miR-
ENSG0000	ENSG0000	0.002071	hsa-miR-181c-5p;hsa-miR-217;hsa-miR-4328;hsa-miR-543
ENSG0000	ENSG0000	0.002931	hsa-miR-181a-5p;hsa-miR-181b-5p;hsa-miR-181c-5p;hsa-miR-
ENSG0000	ENSG0000	0.002773	hsa-miR-129-5p;hsa-miR-26a-5p;hsa-miR-26b-5p;hsa-miR-37
ENSG0000	ENSG0000	8.13E-08	hsa-miR-17-3p;hsa-miR-181a-5p;hsa-miR-181c-5p;hsa-miR-2
ENSG0000	ENSG0000	8.13E-08	hsa-miR-17-3p;hsa-miR-181a-5p;hsa-miR-181c-5p;hsa-miR-2
ENSG0000	ENSG0000	8.06E-07	hsa-miR-129-5p;hsa-miR-181a-5p;hsa-miR-181b-5p;hsa-miR-
ENSG0000	ENSG0000	2.49E-07	hsa-miR-181a-5p;hsa-miR-181b-5p;hsa-miR-181c-5p;hsa-miR-
ENSG0000	ENSG0000	0.000232	hsa-miR-181a-5p;hsa-miR-181b-5p;hsa-miR-181c-5p;hsa-miR-
ENSG0000	ENSG0000	0.000199	hsa-miR-129-5p;hsa-miR-181a-5p;hsa-miR-181b-5p;hsa-miR-
ENSG0000	ENSG0000	0.000979	hsa-miR-17-3p;hsa-miR-181a-5p;hsa-miR-181b-5p;hsa-miR-1
ENSG0000	ENSG0000	0.006923	hsa-miR-181a-5p;hsa-miR-181b-5p;hsa-miR-181c-5p;hsa-miR-
ENSG0000	ENSG0000	0.000113	hsa-miR-17-3p;hsa-miR-181a-5p;hsa-miR-181b-5p;hsa-miR-1
ENSG0000	ENSG0000	0.001716	hsa-miR-129-5p;hsa-miR-181a-5p;hsa-miR-181b-5p;hsa-miR-
ENSG0000	ENSG0000	1.14E-06	hsa-miR-181a-5p;hsa-miR-181b-5p;hsa-miR-181c-5p;hsa-miR-
ENSG0000	ENSG0000	0.003624	hsa-miR-129-5p;hsa-miR-26a-5p;hsa-miR-26b-5p;hsa-miR-62
ENSG0000	ENSG0000	0.000133	hsa-miR-181a-5p;hsa-miR-181b-5p;hsa-miR-181c-5p;hsa-miR-
ENSG0000	ENSG0000	0.002071	hsa-miR-181a-5p;hsa-miR-181b-5p;hsa-miR-181c-5p;hsa-miR-
ENSG0000	ENSG0000	3.05E-05	hsa-miR-181a-5p;hsa-miR-181b-5p;hsa-miR-181c-5p;hsa-miR-
ENSG0000	ENSG0000	0.002773	hsa-miR-181a-5p;hsa-miR-181b-5p;hsa-miR-181c-5p;hsa-miR-
ENSG0000	ENSG0000	0.002071	hsa-miR-181a-5p;hsa-miR-181b-5p;hsa-miR-181c-5p;hsa-miR-
ENSG0000	ENSG0000	0.001607	hsa-miR-181a-5p;hsa-miR-181b-5p;hsa-miR-181c-5p;hsa-miR-
ENSG0000	ENSG0000	0.001607	hsa-miR-181a-5p;hsa-miR-181b-5p;hsa-miR-181c-5p;hsa-miR-
ENSG0000	ENSG0000	0.008143	hsa-miR-181a-5p;hsa-miR-181b-5p;hsa-miR-181c-5p;hsa-miR-
ENSG0000	ENSG0000	5.35E-08	hsa-miR-144-5p;hsa-miR-181a-5p;hsa-miR-181b-5p;hsa-miR-
ENSG0000	ENSG0000	2.96E-05	hsa-miR-181a-5p;hsa-miR-181b-5p;hsa-miR-181c-5p;hsa-miR-
ENSG0000	ENSG0000	0.004422	hsa-miR-181a-5p;hsa-miR-181b-5p;hsa-miR-181c-5p;hsa-miR-
ENSG0000	ENSG0000	0.008469	hsa-miR-129-5p;hsa-miR-26a-5p;hsa-miR-26b-5p;hsa-miR-87
ENSG0000	ENSG0000	3.05E-05	hsa-miR-181a-5p;hsa-miR-181b-5p;hsa-miR-181c-5p;hsa-miR-
ENSG0000	ENSG0000	9.98E-05	hsa-miR-181a-5p;hsa-miR-181b-5p;hsa-miR-181c-5p;hsa-miR-
ENSG0000	ENSG0000	0.000548	hsa-miR-181a-5p;hsa-miR-181b-5p;hsa-miR-181c-5p;hsa-miR-
ENSG0000	ENSG0000	0.00017	hsa-miR-181a-5p;hsa-miR-181b-5p;hsa-miR-181c-5p;hsa-miR-
ENSG0000	ENSG0000	0.00046	hsa-miR-181a-5p;hsa-miR-181b-5p;hsa-miR-181c-5p;hsa-miR-
ENSG0000	ENSG0000	0.000112	hsa-miR-129-5p;hsa-miR-181a-5p;hsa-miR-181b-5p;hsa-miR-
ENSG0000	ENSG0000	0.000772	hsa-miR-181a-5p;hsa-miR-181b-5p;hsa-miR-181c-5p;hsa-miR-
ENSG0000	ENSG0000	0.000413	hsa-miR-181a-5p;hsa-miR-181b-5p;hsa-miR-181c-5p;hsa-miR-
ENSG0000	ENSG0000	5.32E-06	hsa-miR-181a-5p;hsa-miR-181b-5p;hsa-miR-181c-5p;hsa-miR-
ENSG0000	ENSG0000	2.66E-06	hsa-miR-181a-5p;hsa-miR-181b-5p;hsa-miR-181c-5p;hsa-miR-
ENSG0000	ENSG0000	0.008143	hsa-miR-20a-3p;hsa-miR-217;hsa-miR-3157-5p;hsa-miR-382-
ENSG0000	ENSG0000	0.000273	hsa-miR-181a-5p;hsa-miR-181b-5p;hsa-miR-181c-5p;hsa-miR-
ENSG0000	ENSG0000	0.000244	hsa-miR-181a-5p;hsa-miR-181b-5p;hsa-miR-181c-5p;hsa-miR-
ENSG0000	ENSG0000	0.000205	hsa-miR-181a-5p;hsa-miR-181b-5p;hsa-miR-181c-5p;hsa-miR-
ENSG0000	ENSG0000	3.86E-07	hsa-miR-181b-5p;hsa-miR-181c-5p;hsa-miR-26a-5p;hsa-miR-
ENSG0000	ENSG0000	0.000112	hsa-miR-181a-5p;hsa-miR-181b-5p;hsa-miR-181c-5p;hsa-miR-

ENSG0000	ENSG0000	0.000797	hsa-miR-129-5p;hsa-miR-181a-5p;hsa-miR-181b-5p;hsa-miR-
ENSG0000	ENSG0000	1.79E-05	hsa-miR-181a-5p;hsa-miR-181b-5p;hsa-miR-181c-5p;hsa-miR-
ENSG0000	ENSG0000	0.000244	hsa-miR-181a-5p;hsa-miR-181b-5p;hsa-miR-181c-5p;hsa-miR-
ENSG0000	ENSG0000	0.003095	hsa-miR-129-5p;hsa-miR-181a-5p;hsa-miR-181b-5p;hsa-miR-
ENSG0000	ENSG0000	0.000132	hsa-miR-181a-5p;hsa-miR-181b-5p;hsa-miR-181c-5p;hsa-miR-
ENSG0000	ENSG0000	0.000455	hsa-miR-181a-5p;hsa-miR-181b-5p;hsa-miR-181c-5p;hsa-miR-
ENSG0000	ENSG0000	6.18E-07	hsa-miR-181a-5p;hsa-miR-181b-5p;hsa-miR-181c-5p;hsa-miR-
ENSG0000	ENSG0000	0.000837	hsa-miR-129-5p;hsa-miR-181a-5p;hsa-miR-181b-5p;hsa-miR-
ENSG0000	ENSG0000	7.37E-05	hsa-miR-181a-5p;hsa-miR-181b-5p;hsa-miR-181c-5p;hsa-miR-
ENSG0000	ENSG0000	1.04E-06	hsa-miR-181a-5p;hsa-miR-181b-5p;hsa-miR-181c-5p;hsa-miR-
ENSG0000	ENSG0000	0.000404	hsa-miR-129-5p;hsa-miR-181a-5p;hsa-miR-181b-5p;hsa-miR-
ENSG0000	ENSG0000	0.001948	hsa-miR-181a-5p;hsa-miR-181b-5p;hsa-miR-181c-5p;hsa-miR-
ENSG0000	ENSG0000	0.000603	hsa-miR-124-3p;hsa-miR-30b-5p;hsa-miR-30c-5p;hsa-miR-38
ENSG0000	ENSG0000	1.17E-05	hsa-miR-148b-3p;hsa-miR-29a-3p;hsa-miR-29b-3p;hsa-miR-2
ENSG0000	ENSG0000	4.23E-06	hsa-miR-29a-3p;hsa-miR-29b-3p;hsa-miR-29c-3p;hsa-miR-30
ENSG0000	ENSG0000	1.23E-08	hsa-miR-185-5p;hsa-miR-29a-3p;hsa-miR-29b-3p;hsa-miR-29
ENSG0000	ENSG0000	0.000503	hsa-miR-124-3p;hsa-miR-221-3p;hsa-miR-29a-3p;hsa-miR-29
ENSG0000	ENSG0000	0.000135	hsa-miR-148b-3p;hsa-miR-221-3p;hsa-miR-29a-3p;hsa-miR-2
ENSG0000	ENSG0000	3.68E-08	hsa-miR-124-3p;hsa-miR-29a-3p;hsa-miR-29b-3p;hsa-miR-29
ENSG0000	ENSG0000	1.99E-05	hsa-miR-185-5p;hsa-miR-29a-3p;hsa-miR-29b-3p;hsa-miR-29
ENSG0000	ENSG0000	0.000546	hsa-miR-29a-3p;hsa-miR-29b-3p;hsa-miR-29c-3p;hsa-miR-30
ENSG0000	ENSG0000	4.81E-05	hsa-miR-185-5p;hsa-miR-30b-5p;hsa-miR-30c-5p;hsa-miR-57
ENSG0000	ENSG0000	6.31E-06	hsa-miR-29a-3p;hsa-miR-29b-3p;hsa-miR-29c-3p;hsa-miR-57
ENSG0000	ENSG0000	0.000144	hsa-miR-29a-3p;hsa-miR-29b-3p;hsa-miR-29c-3p;hsa-miR-36
ENSG0000	ENSG0000	0.002439	hsa-miR-124-3p;hsa-miR-29a-3p;hsa-miR-29b-3p;hsa-miR-29
ENSG0000	ENSG0000	0.002439	hsa-miR-124-3p;hsa-miR-29a-3p;hsa-miR-29b-3p;hsa-miR-29
ENSG0000	ENSG0000	0.000326	hsa-miR-29a-3p;hsa-miR-29b-3p;hsa-miR-29c-3p;hsa-miR-30
ENSG0000	ENSG0000	8.31E-05	hsa-miR-148b-3p;hsa-miR-29c-3p;hsa-miR-369-3p;hsa-miR-5
ENSG0000	ENSG0000	1.01E-05	hsa-miR-124-3p;hsa-miR-30b-5p;hsa-miR-30c-5p;hsa-miR-57
ENSG0000	ENSG0000	0.000166	hsa-miR-221-3p;hsa-miR-29a-3p;hsa-miR-29b-3p;hsa-miR-29
ENSG0000	ENSG0000	8.31E-05	hsa-miR-221-3p;hsa-miR-29a-3p;hsa-miR-29b-3p;hsa-miR-36
ENSG0000	ENSG0000	8.31E-05	hsa-miR-221-3p;hsa-miR-29a-3p;hsa-miR-29b-3p;hsa-miR-36
ENSG0000	ENSG0000	0.002602	hsa-miR-148b-3p;hsa-miR-29a-3p;hsa-miR-29b-3p;hsa-miR-2
ENSG0000	ENSG0000	0.000106	hsa-miR-148b-3p;hsa-miR-221-3p;hsa-miR-29a-3p;hsa-miR-3
ENSG0000	ENSG0000	0.000843	hsa-miR-151a-5p;hsa-miR-185-5p;hsa-miR-29a-3p;hsa-miR-2
ENSG0000	ENSG0000	2.50E-06	hsa-miR-29a-3p;hsa-miR-29b-3p;hsa-miR-29c-3p;hsa-miR-36
ENSG0000	ENSG0000	0.000398	hsa-miR-221-3p;hsa-miR-29a-3p;hsa-miR-29b-3p;hsa-miR-29
ENSG0000	ENSG0000	0.000376	hsa-miR-124-3p;hsa-miR-30b-5p;hsa-miR-30c-5p;hsa-miR-36
ENSG0000	ENSG0000	5.30E-05	hsa-miR-185-5p;hsa-miR-29a-3p;hsa-miR-29b-3p;hsa-miR-29
ENSG0000	ENSG0000	4.22E-05	hsa-miR-148b-3p;hsa-miR-29a-3p;hsa-miR-29b-3p;hsa-miR-2
ENSG0000	ENSG0000	3.51E-05	hsa-miR-29a-3p;hsa-miR-29b-3p;hsa-miR-29c-3p;hsa-miR-30
ENSG0000	ENSG0000	2.25E-05	hsa-miR-185-5p;hsa-miR-29a-3p;hsa-miR-29b-3p;hsa-miR-29
ENSG0000	ENSG0000	6.00E-09	hsa-miR-29a-3p;hsa-miR-29b-3p;hsa-miR-29c-3p;hsa-miR-30
ENSG0000	ENSG0000	1.76E-05	hsa-miR-124-3p;hsa-miR-29a-3p;hsa-miR-29b-3p;hsa-miR-29
ENSG0000	ENSG0000	1.56E-07	hsa-miR-29a-3p;hsa-miR-29b-3p;hsa-miR-29c-3p;hsa-miR-30
ENSG0000	ENSG0000	0.000801	hsa-miR-29a-3p;hsa-miR-29b-3p;hsa-miR-29c-3p;hsa-miR-57
ENSG0000	ENSG0000	0.005899	hsa-miR-151a-5p;hsa-miR-29a-3p;hsa-miR-29b-3p;hsa-miR-2
ENSG0000	ENSG0000	0.003522	hsa-miR-17-3p;hsa-miR-185-3p;hsa-miR-423-5p;hsa-miR-744
ENSG0000	ENSG0000	0.000311	hsa-miR-185-3p;hsa-miR-326;hsa-miR-330-5p;hsa-miR-34a-5
ENSG0000	ENSG0000	6.09E-05	hsa-miR-17-5p;hsa-miR-20b-5p;hsa-miR-30a-3p;hsa-miR-30d
ENSG0000	ENSG0000	0.001839	hsa-miR-1226-5p;hsa-miR-1914-3p;hsa-miR-423-5p;hsa-miR-
ENSG0000	ENSG0000	9.70E-06	hsa-miR-106b-3p;hsa-miR-30a-3p;hsa-miR-30d-3p;hsa-miR-3
ENSG0000	ENSG0000	0.003618	hsa-miR-2355-5p;hsa-miR-30a-3p;hsa-miR-30d-3p;hsa-miR-3
ENSG0000	ENSG0000	0.000225	hsa-miR-1306-5p;hsa-miR-17-5p;hsa-miR-20b-5p;hsa-miR-30

ENSG0000	ENSG0000	0.000141	hsa-miR-17-5p;hsa-miR-1914-3p;hsa-miR-20b-5p;hsa-miR-34
ENSG0000	ENSG0000	9.46E-05	hsa-miR-17-5p;hsa-miR-20b-5p;hsa-miR-30a-3p;hsa-miR-30d
ENSG0000	ENSG0000	0.004261	hsa-miR-17-5p;hsa-miR-20b-5p;hsa-miR-3176;hsa-miR-93-5p
ENSG0000	ENSG0000	0.000664	hsa-miR-1306-5p;hsa-miR-17-5p;hsa-miR-20b-5p;hsa-miR-93
ENSG0000	ENSG0000	0.00794	hsa-miR-20b-5p;hsa-miR-30a-3p;hsa-miR-30d-3p;hsa-miR-30
ENSG0000	ENSG0000	0.00037	hsa-miR-1306-5p;hsa-miR-17-5p;hsa-miR-20b-5p;hsa-miR-47
ENSG0000	ENSG0000	0.007856	hsa-miR-17-5p;hsa-miR-20b-5p;hsa-miR-3176;hsa-miR-93-5p
ENSG0000	ENSG0000	0.001268	hsa-miR-17-5p;hsa-miR-20b-5p;hsa-miR-301a-5p;hsa-miR-76
ENSG0000	ENSG0000	0.001202	hsa-miR-1306-5p;hsa-miR-30a-3p;hsa-miR-30d-3p;hsa-miR-3
ENSG0000	ENSG0000	0.001202	hsa-miR-1306-5p;hsa-miR-30a-3p;hsa-miR-30d-3p;hsa-miR-3
ENSG0000	ENSG0000	0.001202	hsa-miR-1306-5p;hsa-miR-30a-3p;hsa-miR-30d-3p;hsa-miR-3
ENSG0000	ENSG0000	0.001202	hsa-miR-1306-5p;hsa-miR-30a-3p;hsa-miR-30d-3p;hsa-miR-3
ENSG0000	ENSG0000	0.001202	hsa-miR-1306-5p;hsa-miR-30a-3p;hsa-miR-30d-3p;hsa-miR-3
ENSG0000	ENSG0000	0.001202	hsa-miR-1306-5p;hsa-miR-30a-3p;hsa-miR-30d-3p;hsa-miR-3
ENSG0000	ENSG0000	0.003758	hsa-miR-2355-5p;hsa-miR-30a-3p;hsa-miR-30d-3p;hsa-miR-3
ENSG0000	ENSG0000	0.005788	hsa-miR-1306-5p;hsa-miR-17-5p;hsa-miR-20b-5p;hsa-miR-34
ENSG0000	ENSG0000	1.13E-06	hsa-miR-17-3p;hsa-miR-30a-3p;hsa-miR-30d-3p;hsa-miR-30e
ENSG0000	ENSG0000	0.002877	hsa-miR-17-5p;hsa-miR-20b-5p;hsa-miR-744-3p;hsa-miR-93-
ENSG0000	ENSG0000	0.001839	hsa-miR-1306-5p;hsa-miR-17-5p;hsa-miR-20b-5p;hsa-miR-93
ENSG0000	ENSG0000	0.000553	hsa-miR-30a-3p;hsa-miR-30d-3p;hsa-miR-30e-3p;hsa-miR-32
ENSG0000	ENSG0000	0.002318	hsa-miR-30a-3p;hsa-miR-30d-3p;hsa-miR-30e-3p;hsa-miR-34
ENSG0000	ENSG0000	0.000744	hsa-miR-1306-5p;hsa-miR-17-5p;hsa-miR-20b-5p;hsa-miR-23
ENSG0000	ENSG0000	0.001655	hsa-miR-17-3p;hsa-miR-30a-3p;hsa-miR-30d-3p;hsa-miR-30e
ENSG0000	ENSG0000	0.009104	hsa-miR-17-5p;hsa-miR-20b-5p;hsa-miR-2278;hsa-miR-93-5p
ENSG0000	ENSG0000	0.008675	hsa-miR-34a-5p;hsa-miR-374b-3p;hsa-miR-449a;hsa-miR-744
ENSG0000	ENSG0000	0.007077	hsa-miR-1306-5p;hsa-miR-17-5p;hsa-miR-20b-5p;hsa-miR-47
ENSG0000	ENSG0000	0.002681	hsa-miR-17-5p;hsa-miR-20b-5p;hsa-miR-34a-5p;hsa-miR-449
ENSG0000	ENSG0000	0.000595	hsa-miR-30a-3p;hsa-miR-326;hsa-miR-34a-5p;hsa-miR-449a
ENSG0000	ENSG0000	0.002511	hsa-miR-30a-3p;hsa-miR-30d-3p;hsa-miR-30e-3p;hsa-miR-45
ENSG0000	ENSG0000	3.77E-05	hsa-miR-17-3p;hsa-miR-17-5p;hsa-miR-20b-5p;hsa-miR-326;h
ENSG0000	ENSG0000	0.000672	hsa-miR-106b-3p;hsa-miR-17-3p;hsa-miR-17-5p;hsa-miR-301
ENSG0000	ENSG0000	0.000672	hsa-miR-106b-3p;hsa-miR-17-3p;hsa-miR-17-5p;hsa-miR-301
ENSG0000	ENSG0000	0.005098	hsa-miR-1306-5p;hsa-miR-17-5p;hsa-miR-20b-5p;hsa-miR-93
ENSG0000	ENSG0000	0.000636	hsa-miR-17-5p;hsa-miR-20b-5p;hsa-miR-2355-5p;hsa-miR-30
ENSG0000	ENSG0000	0.001839	hsa-miR-30a-3p;hsa-miR-30d-3p;hsa-miR-30e-3p;hsa-miR-31
ENSG0000	ENSG0000	0.009547	hsa-miR-17-3p;hsa-miR-17-5p;hsa-miR-20b-5p;hsa-miR-93-5
ENSG0000	ENSG0000	0.008556	hsa-miR-1914-3p;hsa-miR-30a-3p;hsa-miR-30d-3p;hsa-miR-3
ENSG0000	ENSG0000	0.008556	hsa-miR-1914-3p;hsa-miR-30a-3p;hsa-miR-30d-3p;hsa-miR-3
ENSG0000	ENSG0000	0.006377	hsa-miR-185-3p;hsa-miR-3176;hsa-miR-423-5p;hsa-miR-744-
ENSG0000	ENSG0000	0.006377	hsa-miR-185-3p;hsa-miR-3176;hsa-miR-423-5p;hsa-miR-744-
ENSG0000	ENSG0000	3.99E-05	hsa-miR-106b-3p;hsa-miR-1226-5p;hsa-miR-1914-3p;hsa-miR
ENSG0000	ENSG0000	0.000246	hsa-miR-1226-5p;hsa-miR-185-3p;hsa-miR-1914-3p;hsa-miR-
ENSG0000	ENSG0000	0.00016	hsa-miR-1914-3p;hsa-miR-30a-3p;hsa-miR-30d-3p;hsa-miR-3
ENSG0000	ENSG0000	0.00214	hsa-miR-1226-5p;hsa-miR-185-3p;hsa-miR-34a-5p;hsa-miR-4
ENSG0000	ENSG0000	0.00214	hsa-miR-1226-5p;hsa-miR-185-3p;hsa-miR-34a-5p;hsa-miR-4
ENSG0000	ENSG0000	0.00214	hsa-miR-1226-5p;hsa-miR-185-3p;hsa-miR-34a-5p;hsa-miR-4
ENSG0000	ENSG0000	0.00214	hsa-miR-1226-5p;hsa-miR-185-3p;hsa-miR-34a-5p;hsa-miR-4
ENSG0000	ENSG0000	0.00214	hsa-miR-1226-5p;hsa-miR-185-3p;hsa-miR-34a-5p;hsa-miR-4
ENSG0000	ENSG0000	0.00214	hsa-miR-1226-5p;hsa-miR-185-3p;hsa-miR-34a-5p;hsa-miR-4
ENSG0000	ENSG0000	0.00214	hsa-miR-1226-5p;hsa-miR-185-3p;hsa-miR-34a-5p;hsa-miR-4
ENSG0000	ENSG0000	0.00214	hsa-miR-1226-5p;hsa-miR-185-3p;hsa-miR-34a-5p;hsa-miR-4
ENSG0000	ENSG0000	0.005098	hsa-miR-17-5p;hsa-miR-20b-5p;hsa-miR-760;hsa-miR-93-5p
ENSG0000	ENSG0000	0.009104	hsa-miR-17-5p;hsa-miR-20b-5p;hsa-miR-744-3p;hsa-miR-93-
ENSG0000	ENSG0000	0.004437	hsa-miR-17-3p;hsa-miR-17-5p;hsa-miR-20b-5p;hsa-miR-30a-

ENSG0000	ENSG0000	0.002318	hsa-miR-17-5p;hsa-miR-20b-5p;hsa-miR-2278;hsa-miR-93-5p
ENSG0000	ENSG0000	0.002318	hsa-miR-17-5p;hsa-miR-20b-5p;hsa-miR-2278;hsa-miR-93-5p
ENSG0000	ENSG0000	0.003522	hsa-miR-17-5p;hsa-miR-20b-5p;hsa-miR-4726-3p;hsa-miR-93-
ENSG0000	ENSG0000	4.74E-05	hsa-miR-17-3p;hsa-miR-30a-3p;hsa-miR-30d-3p;hsa-miR-30e-
ENSG0000	ENSG0000	0.007467	hsa-miR-17-5p;hsa-miR-20b-5p;hsa-miR-2278;hsa-miR-93-5p
ENSG0000	ENSG0000	5.21E-06	hsa-miR-1306-5p;hsa-miR-17-3p;hsa-miR-17-5p;hsa-miR-20b-
ENSG0000	ENSG0000	0.000472	hsa-miR-1306-5p;hsa-miR-17-5p;hsa-miR-20b-5p;hsa-miR-93-
ENSG0000	ENSG0000	0.000472	hsa-miR-1306-5p;hsa-miR-17-5p;hsa-miR-20b-5p;hsa-miR-93-
ENSG0000	ENSG0000	0.000472	hsa-miR-1306-5p;hsa-miR-17-5p;hsa-miR-20b-5p;hsa-miR-93-
ENSG0000	ENSG0000	0.000472	hsa-miR-1306-5p;hsa-miR-17-5p;hsa-miR-20b-5p;hsa-miR-93-
ENSG0000	ENSG0000	0.000472	hsa-miR-1306-5p;hsa-miR-17-5p;hsa-miR-20b-5p;hsa-miR-93-
ENSG0000	ENSG0000	0.000472	hsa-miR-1306-5p;hsa-miR-17-5p;hsa-miR-20b-5p;hsa-miR-93-
ENSG0000	ENSG0000	0.005098	hsa-miR-17-5p;hsa-miR-20b-5p;hsa-miR-4726-3p;hsa-miR-93-
ENSG0000	ENSG0000	1.76E-06	hsa-miR-17-5p;hsa-miR-20b-5p;hsa-miR-34a-5p;hsa-miR-93-
ENSG0000	ENSG0000	1.76E-06	hsa-miR-17-5p;hsa-miR-20b-5p;hsa-miR-34a-5p;hsa-miR-93-
ENSG0000	ENSG0000	0.0054	hsa-miR-17-5p;hsa-miR-20b-5p;hsa-miR-2355-5p;hsa-miR-93-
ENSG0000	ENSG0000	0.000664	hsa-miR-17-5p;hsa-miR-185-3p;hsa-miR-20b-5p;hsa-miR-93-
ENSG0000	ENSG0000	0.000818	hsa-miR-1226-5p;hsa-miR-17-3p;hsa-miR-1914-3p;hsa-miR-4
ENSG0000	ENSG0000	0.001434	hsa-miR-185-3p;hsa-miR-34a-5p;hsa-miR-423-5p;hsa-miR-44
ENSG0000	ENSG0000	0.009886	hsa-miR-1306-5p;hsa-miR-17-5p;hsa-miR-20b-5p;hsa-miR-31
ENSG0000	ENSG0000	4.59E-05	hsa-miR-17-5p;hsa-miR-20b-5p;hsa-miR-30a-3p;hsa-miR-30d-
ENSG0000	ENSG0000	0.000283	hsa-miR-17-5p;hsa-miR-20b-5p;hsa-miR-301a-5p;hsa-miR-93-
ENSG0000	ENSG0000	0.008244	hsa-miR-17-5p;hsa-miR-20b-5p;hsa-miR-3176;hsa-miR-340-3
ENSG0000	ENSG0000	0.000177	hsa-miR-17-5p;hsa-miR-1914-3p;hsa-miR-20b-5p;hsa-miR-42
ENSG0000	ENSG0000	0.000336	hsa-miR-17-5p;hsa-miR-20b-5p;hsa-miR-30a-3p;hsa-miR-30d-
ENSG0000	ENSG0000	2.35E-05	hsa-miR-17-5p;hsa-miR-20b-5p;hsa-miR-30a-3p;hsa-miR-30d-
ENSG0000	ENSG0000	0.000798	hsa-miR-17-5p;hsa-miR-1914-3p;hsa-miR-20b-5p;hsa-miR-42
ENSG0000	ENSG0000	1.70E-05	hsa-miR-1914-3p;hsa-miR-30a-3p;hsa-miR-30d-3p;hsa-miR-3
ENSG0000	ENSG0000	0.000335	hsa-miR-1226-5p;hsa-miR-185-3p;hsa-miR-30a-3p;hsa-miR-3
ENSG0000	ENSG0000	0.003082	hsa-miR-17-5p;hsa-miR-20b-5p;hsa-miR-4726-3p;hsa-miR-93-
ENSG0000	ENSG0000	4.59E-05	hsa-miR-17-5p;hsa-miR-20b-5p;hsa-miR-3176;hsa-miR-34a-5
ENSG0000	ENSG0000	0.003049	hsa-miR-17-5p;hsa-miR-20b-5p;hsa-miR-3176;hsa-miR-34a-5
ENSG0000	ENSG0000	0.000595	hsa-miR-17-5p;hsa-miR-20b-5p;hsa-miR-2278;hsa-miR-93-5p
ENSG0000	ENSG0000	0.00215	hsa-miR-17-5p;hsa-miR-185-3p;hsa-miR-20b-5p;hsa-miR-93-
ENSG0000	ENSG0000	3.08E-07	hsa-miR-17-5p;hsa-miR-1914-3p;hsa-miR-20b-5p;hsa-miR-31
ENSG0000	ENSG0000	0.008675	hsa-miR-1306-5p;hsa-miR-326;hsa-miR-330-5p;hsa-miR-4726-
ENSG0000	ENSG0000	0.001268	hsa-miR-17-5p;hsa-miR-20b-5p;hsa-miR-326;hsa-miR-330-5p;
ENSG0000	ENSG0000	0.006728	hsa-miR-17-5p;hsa-miR-20b-5p;hsa-miR-4726-3p;hsa-miR-93-
ENSG0000	ENSG0000	0.000111	hsa-miR-17-5p;hsa-miR-20b-5p;hsa-miR-30a-3p;hsa-miR-30d-
ENSG0000	ENSG0000	0.002441	hsa-miR-17-5p;hsa-miR-20b-5p;hsa-miR-30a-3p;hsa-miR-30d-
ENSG0000	ENSG0000	0.000692	hsa-miR-20b-5p;hsa-miR-30a-3p;hsa-miR-30d-3p;hsa-miR-30
ENSG0000	ENSG0000	0.000595	hsa-miR-17-3p;hsa-miR-30a-3p;hsa-miR-30d-3p;hsa-miR-30e-
ENSG0000	ENSG0000	0.000595	hsa-miR-17-3p;hsa-miR-30a-3p;hsa-miR-30d-3p;hsa-miR-30e-
ENSG0000	ENSG0000	0.001917	hsa-miR-2278;hsa-miR-326;hsa-miR-330-5p;hsa-miR-34a-5p;h
ENSG0000	ENSG0000	0.006039	hsa-miR-30a-3p;hsa-miR-30d-3p;hsa-miR-30e-3p;hsa-miR-47
ENSG0000	ENSG0000	0.006377	hsa-miR-1306-5p;hsa-miR-326;hsa-miR-330-5p;hsa-miR-760
ENSG0000	ENSG0000	0.002318	hsa-miR-30a-3p;hsa-miR-30d-3p;hsa-miR-30e-3p;hsa-miR-34
ENSG0000	ENSG0000	0.00215	hsa-miR-17-5p;hsa-miR-20b-5p;hsa-miR-3176;hsa-miR-93-5p
ENSG0000	ENSG0000	0.002681	hsa-miR-17-5p;hsa-miR-20b-5p;hsa-miR-3176;hsa-miR-93-5p
ENSG0000	ENSG0000	0.004474	hsa-miR-17-5p;hsa-miR-20b-5p;hsa-miR-2278;hsa-miR-4726-
ENSG0000	ENSG0000	0.007467	hsa-miR-17-5p;hsa-miR-20b-5p;hsa-miR-301a-5p;hsa-miR-93-
ENSG0000	ENSG0000	0.002441	hsa-miR-30a-3p;hsa-miR-30d-3p;hsa-miR-30e-3p;hsa-miR-34
ENSG0000	ENSG0000	1.70E-05	hsa-miR-17-5p;hsa-miR-20b-5p;hsa-miR-30a-3p;hsa-miR-30d-
ENSG0000	ENSG0000	0.002986	hsa-miR-17-5p;hsa-miR-20b-5p;hsa-miR-3176;hsa-miR-34a-5

ENSG0000	ENSG0000	0.00215	hsa-miR-17-5p;hsa-miR-20b-5p;hsa-miR-744-3p;hsa-miR-93-5
ENSG0000	ENSG0000	0.000643	hsa-miR-17-5p;hsa-miR-20b-5p;hsa-miR-326;hsa-miR-330-5p;
ENSG0000	ENSG0000	0.000643	hsa-miR-17-5p;hsa-miR-20b-5p;hsa-miR-326;hsa-miR-330-5p;
ENSG0000	ENSG0000	0.008876	hsa-miR-1306-5p;hsa-miR-17-5p;hsa-miR-20b-5p;hsa-miR-235
ENSG0000	ENSG0000	0.008876	hsa-miR-1306-5p;hsa-miR-17-5p;hsa-miR-20b-5p;hsa-miR-235
ENSG0000	ENSG0000	0.000597	hsa-miR-17-5p;hsa-miR-20b-5p;hsa-miR-374b-3p;hsa-miR-74
ENSG0000	ENSG0000	0.000476	hsa-miR-17-5p;hsa-miR-20b-5p;hsa-miR-2278;hsa-miR-340-3p
ENSG0000	ENSG0000	0.006539	hsa-miR-1226-5p;hsa-miR-17-5p;hsa-miR-185-3p;hsa-miR-20b
ENSG0000	ENSG0000	0.000512	hsa-miR-1306-5p;hsa-miR-17-5p;hsa-miR-20b-5p;hsa-miR-74
ENSG0000	ENSG0000	0.007467	hsa-miR-30a-3p;hsa-miR-30d-3p;hsa-miR-30e-3p;hsa-miR-74
ENSG0000	ENSG0000	0.008259	hsa-miR-1306-5p;hsa-miR-17-5p;hsa-miR-20b-5p;hsa-miR-93-
ENSG0000	ENSG0000	0.002681	hsa-miR-17-5p;hsa-miR-20b-5p;hsa-miR-2278;hsa-miR-93-5p
ENSG0000	ENSG0000	0.006039	hsa-miR-17-5p;hsa-miR-20b-5p;hsa-miR-423-5p;hsa-miR-93-5
ENSG0000	ENSG0000	0.00035	hsa-miR-1306-5p;hsa-miR-17-5p;hsa-miR-20b-5p;hsa-miR-32
ENSG0000	ENSG0000	0.000156	hsa-miR-17-5p;hsa-miR-20b-5p;hsa-miR-3176;hsa-miR-93-5p
ENSG0000	ENSG0000	2.49E-05	hsa-miR-17-5p;hsa-miR-20b-5p;hsa-miR-34a-5p;hsa-miR-449a
ENSG0000	ENSG0000	0.00794	hsa-miR-20b-5p;hsa-miR-30a-3p;hsa-miR-30d-3p;hsa-miR-30
ENSG0000	ENSG0000	0.004279	hsa-miR-17-5p;hsa-miR-20b-5p;hsa-miR-326;hsa-miR-330-5p;
ENSG0000	ENSG0000	0.008259	hsa-miR-1306-5p;hsa-miR-17-5p;hsa-miR-20b-5p;hsa-miR-93-
ENSG0000	ENSG0000	0.004807	hsa-miR-17-5p;hsa-miR-20b-5p;hsa-miR-340-3p;hsa-miR-93-5
ENSG0000	ENSG0000	6.66E-05	hsa-miR-17-3p;hsa-miR-17-5p;hsa-miR-20b-5p;hsa-miR-34a-5
ENSG0000	ENSG0000	6.66E-05	hsa-miR-17-3p;hsa-miR-17-5p;hsa-miR-20b-5p;hsa-miR-34a-5
ENSG0000	ENSG0000	0.008259	hsa-miR-1306-5p;hsa-miR-326;hsa-miR-330-5p;hsa-miR-340-3
ENSG0000	ENSG0000	0.00215	hsa-miR-17-5p;hsa-miR-20b-5p;hsa-miR-374b-3p;hsa-miR-93
ENSG0000	ENSG0000	0.003297	hsa-miR-17-5p;hsa-miR-374b-3p;hsa-miR-744-3p;hsa-miR-93-
ENSG0000	ENSG0000	0.000418	hsa-miR-1306-5p;hsa-miR-326;hsa-miR-330-5p;hsa-miR-744-3
ENSG0000	ENSG0000	0.000531	hsa-miR-1914-3p;hsa-miR-34a-5p;hsa-miR-423-5p;hsa-miR-44
ENSG0000	ENSG0000	0.000531	hsa-miR-1914-3p;hsa-miR-34a-5p;hsa-miR-423-5p;hsa-miR-44
ENSG0000	ENSG0000	0.009104	hsa-miR-17-5p;hsa-miR-20b-5p;hsa-miR-4726-3p;hsa-miR-93-
ENSG0000	ENSG0000	0.009104	hsa-miR-17-5p;hsa-miR-20b-5p;hsa-miR-2278;hsa-miR-93-5p
ENSG0000	ENSG0000	0.000997	hsa-miR-17-5p;hsa-miR-20b-5p;hsa-miR-2355-5p;hsa-miR-93-
ENSG0000	ENSG0000	0.002681	hsa-miR-1226-5p;hsa-miR-1306-5p;hsa-miR-1914-3p;hsa-miR-
ENSG0000	ENSG0000	0.001917	hsa-miR-17-5p;hsa-miR-20b-5p;hsa-miR-30a-3p;hsa-miR-30d-
ENSG0000	ENSG0000	0.001917	hsa-miR-17-5p;hsa-miR-20b-5p;hsa-miR-30a-3p;hsa-miR-30d-
ENSG0000	ENSG0000	0.001314	hsa-miR-1914-3p;hsa-miR-34a-5p;hsa-miR-423-5p;hsa-miR-44
ENSG0000	ENSG0000	0.000155	hsa-miR-17-5p;hsa-miR-20b-5p;hsa-miR-30a-3p;hsa-miR-30d-
ENSG0000	ENSG0000	0.00794	hsa-miR-17-5p;hsa-miR-20b-5p;hsa-miR-2278;hsa-miR-423-5p
ENSG0000	ENSG0000	0.000138	hsa-miR-1306-5p;hsa-miR-17-5p;hsa-miR-20b-5p;hsa-miR-227
ENSG0000	ENSG0000	0.004192	hsa-miR-1306-5p;hsa-miR-17-5p;hsa-miR-20b-5p;hsa-miR-374
ENSG0000	ENSG0000	0.000553	hsa-miR-1306-5p;hsa-miR-17-5p;hsa-miR-20b-5p;hsa-miR-235
ENSG0000	ENSG0000	0.003297	hsa-miR-17-5p;hsa-miR-20b-5p;hsa-miR-4523;hsa-miR-93-5p
ENSG0000	ENSG0000	0.003297	hsa-miR-17-5p;hsa-miR-20b-5p;hsa-miR-4523;hsa-miR-93-5p
ENSG0000	ENSG0000	0.008675	hsa-miR-17-5p;hsa-miR-1914-3p;hsa-miR-20b-5p;hsa-miR-423
ENSG0000	ENSG0000	0.001399	hsa-miR-17-3p;hsa-miR-17-5p;hsa-miR-1914-3p;hsa-miR-20b-
ENSG0000	ENSG0000	0.00026	hsa-miR-17-5p;hsa-miR-20b-5p;hsa-miR-34a-5p;hsa-miR-449a
ENSG0000	ENSG0000	0.000643	hsa-miR-17-5p;hsa-miR-20b-5p;hsa-miR-326;hsa-miR-330-5p;
ENSG0000	ENSG0000	0.000597	hsa-miR-17-5p;hsa-miR-20b-5p;hsa-miR-2355-5p;hsa-miR-317
ENSG0000	ENSG0000	0.00032	hsa-miR-17-5p;hsa-miR-20b-5p;hsa-miR-30a-3p;hsa-miR-30d-
ENSG0000	ENSG0000	0.000818	hsa-miR-17-5p;hsa-miR-20b-5p;hsa-miR-340-3p;hsa-miR-93-5
ENSG0000	ENSG0000	0.004807	hsa-miR-17-5p;hsa-miR-20b-5p;hsa-miR-4726-3p;hsa-miR-93-
ENSG0000	ENSG0000	0.005713	hsa-miR-17-5p;hsa-miR-20b-5p;hsa-miR-340-3p;hsa-miR-93-5
ENSG0000	ENSG0000	0.000448	hsa-miR-17-5p;hsa-miR-20b-5p;hsa-miR-340-3p;hsa-miR-34a-
ENSG0000	ENSG0000	4.47E-07	hsa-miR-17-5p;hsa-miR-20b-5p;hsa-miR-30a-3p;hsa-miR-30d-
ENSG0000	ENSG0000	6.86E-06	hsa-miR-218-5p;hsa-miR-30b-3p;hsa-miR-30c-1-3p;hsa-miR-3

ENSG0000	ENSG0000	2.80E-05	hsa-miR-218-5p;hsa-miR-30b-3p;hsa-miR-30c-1-3p;hsa-miR-3
ENSG0000	ENSG0000	5.91E-05	hsa-miR-218-5p;hsa-miR-30b-3p;hsa-miR-30c-1-3p;hsa-miR-3
ENSG0000	ENSG0000	9.50E-06	hsa-miR-23a-3p;hsa-miR-30b-3p;hsa-miR-30c-1-3p;hsa-miR-3
ENSG0000	ENSG0000	0.000202	hsa-miR-218-5p;hsa-miR-30b-3p;hsa-miR-30c-1-3p;hsa-miR-3
ENSG0000	ENSG0000	0.000804	hsa-miR-218-5p;hsa-miR-30b-3p;hsa-miR-30c-1-3p;hsa-miR-3
ENSG0000	ENSG0000	0.00013	hsa-miR-218-5p;hsa-miR-30b-3p;hsa-miR-30c-1-3p;hsa-miR-3
ENSG0000	ENSG0000	4.37E-05	hsa-miR-23a-3p;hsa-miR-30b-3p;hsa-miR-30c-1-3p;hsa-miR-3
ENSG0000	ENSG0000	7.47E-05	hsa-miR-218-5p;hsa-miR-30b-3p;hsa-miR-30c-1-3p;hsa-miR-3
ENSG0000	ENSG0000	1.66E-06	hsa-miR-218-5p;hsa-miR-30b-3p;hsa-miR-30c-1-3p;hsa-miR-3
ENSG0000	ENSG0000	2.06E-05	hsa-miR-218-5p;hsa-miR-30b-3p;hsa-miR-30c-1-3p;hsa-miR-3
ENSG0000	ENSG0000	5.10E-05	hsa-miR-218-5p;hsa-miR-30b-3p;hsa-miR-30c-1-3p;hsa-miR-3
ENSG0000	ENSG0000	0.000256	hsa-miR-218-5p;hsa-miR-30b-3p;hsa-miR-30c-1-3p;hsa-miR-3
ENSG0000	ENSG0000	1.11E-05	hsa-miR-218-5p;hsa-miR-30b-3p;hsa-miR-30c-1-3p;hsa-miR-3
ENSG0000	ENSG0000	8.28E-06	hsa-miR-30b-3p;hsa-miR-30c-1-3p;hsa-miR-30c-2-3p;hsa-miR
ENSG0000	ENSG0000	6.35E-07	hsa-miR-218-5p;hsa-miR-30b-3p;hsa-miR-30c-1-3p;hsa-miR-3
ENSG0000	ENSG0000	8.10E-06	hsa-miR-218-5p;hsa-miR-30b-3p;hsa-miR-30c-1-3p;hsa-miR-3
ENSG0000	ENSG0000	3.93E-05	hsa-miR-218-5p;hsa-miR-30b-3p;hsa-miR-30c-1-3p;hsa-miR-3
ENSG0000	ENSG0000	0.000182	hsa-miR-218-5p;hsa-miR-30b-3p;hsa-miR-30c-1-3p;hsa-miR-3
ENSG0000	ENSG0000	6.82E-05	hsa-miR-218-5p;hsa-miR-30b-3p;hsa-miR-30c-1-3p;hsa-miR-3
ENSG0000	ENSG0000	0.00013	hsa-miR-218-5p;hsa-miR-30b-3p;hsa-miR-30c-1-3p;hsa-miR-3
ENSG0000	ENSG0000	0.00013	hsa-miR-218-5p;hsa-miR-30b-3p;hsa-miR-30c-1-3p;hsa-miR-3
ENSG0000	ENSG0000	7.14E-05	hsa-miR-23a-3p;hsa-miR-30b-3p;hsa-miR-30c-1-3p;hsa-miR-3
ENSG0000	ENSG0000	8.78E-06	hsa-miR-23a-3p;hsa-miR-30b-3p;hsa-miR-30c-1-3p;hsa-miR-3
ENSG0000	ENSG0000	5.36E-05	hsa-miR-218-5p;hsa-miR-30b-3p;hsa-miR-30c-1-3p;hsa-miR-3
ENSG0000	ENSG0000	2.06E-05	hsa-miR-218-5p;hsa-miR-30b-3p;hsa-miR-30c-1-3p;hsa-miR-3
ENSG0000	ENSG0000	0.000163	hsa-miR-218-5p;hsa-miR-30b-3p;hsa-miR-30c-1-3p;hsa-miR-3
ENSG0000	ENSG0000	0.000508	hsa-miR-218-5p;hsa-miR-23a-3p;hsa-miR-30b-3p;hsa-miR-30
ENSG0000	ENSG0000	6.86E-06	hsa-miR-218-5p;hsa-miR-30b-3p;hsa-miR-30c-1-3p;hsa-miR-3
ENSG0000	ENSG0000	0.000273	hsa-miR-30b-3p;hsa-miR-30c-1-3p;hsa-miR-30c-2-3p;hsa-miR
ENSG0000	ENSG0000	3.15E-05	hsa-miR-30c-1-3p;hsa-miR-30c-2-3p;hsa-miR-330-5p;hsa-miR
ENSG0000	ENSG0000	0.000824	hsa-miR-23a-3p;hsa-miR-30b-3p;hsa-miR-30c-1-3p;hsa-miR-3
ENSG0000	ENSG0000	0.002024	hsa-miR-218-5p;hsa-miR-30b-3p;hsa-miR-30c-1-3p;hsa-miR-3
ENSG0000	ENSG0000	0.000731	hsa-miR-17-5p;hsa-miR-20b-5p;hsa-miR-33b-3p;hsa-miR-93-
ENSG0000	ENSG0000	2.84E-06	hsa-let-7a-5p;hsa-let-7b-5p;hsa-let-7d-5p;hsa-let-7e-5p;hsa-l
ENSG0000	ENSG0000	0.001014	hsa-let-7a-5p;hsa-let-7b-5p;hsa-let-7d-5p;hsa-let-7e-5p;hsa-l
ENSG0000	ENSG0000	0.00982	hsa-miR-185-3p;hsa-miR-193b-5p;hsa-miR-19a-5p;hsa-miR-1
ENSG0000	ENSG0000	0.005504	hsa-miR-17-5p;hsa-miR-20b-5p;hsa-miR-2681-5p;hsa-miR-42
ENSG0000	ENSG0000	2.17E-05	hsa-let-7a-5p;hsa-let-7b-5p;hsa-let-7d-5p;hsa-let-7e-5p;hsa-l
ENSG0000	ENSG0000	3.49E-05	hsa-let-7a-5p;hsa-let-7b-5p;hsa-let-7d-5p;hsa-let-7e-5p;hsa-l
ENSG0000	ENSG0000	4.43E-06	hsa-miR-125a-5p;hsa-miR-125b-5p;hsa-miR-185-3p;hsa-miR-
ENSG0000	ENSG0000	0.001542	hsa-miR-17-5p;hsa-miR-20b-5p;hsa-miR-378a-5p;hsa-miR-52
ENSG0000	ENSG0000	2.23E-05	hsa-let-7a-5p;hsa-let-7b-5p;hsa-let-7d-5p;hsa-let-7e-5p;hsa-l
ENSG0000	ENSG0000	0.008536	hsa-miR-130a-3p;hsa-miR-17-5p;hsa-miR-19a-5p;hsa-miR-19
ENSG0000	ENSG0000	0.004211	hsa-miR-125b-5p;hsa-miR-221-5p;hsa-miR-30b-3p;hsa-miR-3
ENSG0000	ENSG0000	0.000314	hsa-let-7a-5p;hsa-miR-125b-5p;hsa-miR-148a-3p;hsa-miR-17-
ENSG0000	ENSG0000	2.95E-05	hsa-let-7a-5p;hsa-let-7b-5p;hsa-let-7f-5p;hsa-miR-125b-5p;hs
ENSG0000	ENSG0000	0.000465	hsa-miR-17-5p;hsa-miR-193b-5p;hsa-miR-20b-5p;hsa-miR-52
ENSG0000	ENSG0000	0.001551	hsa-miR-378a-3p;hsa-miR-378c;hsa-miR-378d;hsa-miR-378i;hs
ENSG0000	ENSG0000	0.001828	hsa-let-7b-5p;hsa-miR-130a-3p;hsa-miR-148a-3p;hsa-miR-14
ENSG0000	ENSG0000	1.97E-05	hsa-let-7a-5p;hsa-let-7b-5p;hsa-let-7d-5p;hsa-let-7e-5p;hsa-l
ENSG0000	ENSG0000	0.002244	hsa-miR-17-5p;hsa-miR-20b-5p;hsa-miR-520a-3p;hsa-miR-52
ENSG0000	ENSG0000	0.001161	hsa-let-7a-5p;hsa-let-7b-5p;hsa-let-7d-5p;hsa-let-7e-5p;hsa-l
ENSG0000	ENSG0000	0.001034	hsa-let-7a-5p;hsa-let-7b-5p;hsa-let-7d-5p;hsa-let-7e-5p;hsa-l
ENSG0000	ENSG0000	1.97E-05	hsa-let-7a-5p;hsa-let-7b-5p;hsa-let-7d-5p;hsa-let-7e-5p;hsa-l

ENSG0000	ENSG0000	0.004799	hsa-miR-130a-3p;hsa-miR-148a-3p;hsa-miR-17-5p;hsa-miR-18
ENSG0000	ENSG0000	4.83E-07	hsa-let-7a-5p;hsa-let-7b-5p;hsa-let-7d-5p;hsa-let-7e-5p;hsa-l
ENSG0000	ENSG0000	0.002507	hsa-miR-17-5p;hsa-miR-181a-5p;hsa-miR-20b-5p;hsa-miR-93-
ENSG0000	ENSG0000	9.90E-06	hsa-let-7a-5p;hsa-let-7b-5p;hsa-let-7d-5p;hsa-let-7e-5p;hsa-l
ENSG0000	ENSG0000	0.004211	hsa-miR-17-5p;hsa-miR-181a-5p;hsa-miR-20b-5p;hsa-miR-42
ENSG0000	ENSG0000	2.37E-07	hsa-let-7a-5p;hsa-let-7d-5p;hsa-let-7e-5p;hsa-let-7f-5p;hsa-le
ENSG0000	ENSG0000	0.005504	hsa-miR-17-5p;hsa-miR-20b-5p;hsa-miR-520a-3p;hsa-miR-52
ENSG0000	ENSG0000	4.37E-05	hsa-miR-1271-3p;hsa-miR-17-5p;hsa-miR-20b-5p;hsa-miR-30
ENSG0000	ENSG0000	1.04E-05	hsa-let-7a-5p;hsa-let-7b-5p;hsa-let-7d-5p;hsa-let-7e-5p;hsa-l
ENSG0000	ENSG0000	1.62E-05	hsa-let-7a-5p;hsa-let-7b-5p;hsa-let-7d-5p;hsa-let-7e-5p;hsa-l
ENSG0000	ENSG0000	0.004379	hsa-miR-130a-3p;hsa-miR-17-5p;hsa-miR-181a-5p;hsa-miR-2
ENSG0000	ENSG0000	0.000551	hsa-let-7a-5p;hsa-let-7e-5p;hsa-let-7f-5p;hsa-let-7g-5p;hsa-le
ENSG0000	ENSG0000	4.96E-07	hsa-let-7a-5p;hsa-let-7b-5p;hsa-let-7d-5p;hsa-let-7e-5p;hsa-l
ENSG0000	ENSG0000	2.47E-05	hsa-let-7a-5p;hsa-let-7b-5p;hsa-let-7d-5p;hsa-let-7e-5p;hsa-l
ENSG0000	ENSG0000	0.009494	hsa-let-7a-5p;hsa-miR-17-5p;hsa-miR-20b-5p;hsa-miR-331-3
ENSG0000	ENSG0000	0.00983	hsa-let-7a-5p;hsa-let-7b-5p;hsa-miR-125b-5p;hsa-miR-17-5p;
ENSG0000	ENSG0000	0.00983	hsa-let-7a-5p;hsa-let-7b-5p;hsa-miR-125b-5p;hsa-miR-17-5p;
ENSG0000	ENSG0000	0.00983	hsa-let-7a-5p;hsa-let-7d-5p;hsa-let-7e-5p;hsa-let-7f-5p;hsa-le
ENSG0000	ENSG0000	4.70E-09	hsa-let-7a-5p;hsa-let-7b-5p;hsa-let-7d-5p;hsa-let-7e-5p;hsa-l
ENSG0000	ENSG0000	0.000731	hsa-miR-1254;hsa-miR-1271-3p;hsa-miR-550a-3-5p;hsa-miR-
ENSG0000	ENSG0000	0.008573	hsa-let-7a-5p;hsa-let-7e-5p;hsa-let-7f-5p;hsa-miR-20b-5p;hsa
ENSG0000	ENSG0000	0.000598	hsa-miR-17-5p;hsa-miR-20b-5p;hsa-miR-500a-3p;hsa-miR-50
ENSG0000	ENSG0000	2.17E-05	hsa-let-7a-5p;hsa-let-7b-5p;hsa-let-7d-5p;hsa-let-7e-5p;hsa-l
ENSG0000	ENSG0000	0.001235	hsa-miR-130a-3p;hsa-miR-192-5p;hsa-miR-193b-3p;hsa-miR-
ENSG0000	ENSG0000	0.002205	hsa-miR-17-5p;hsa-miR-19a-5p;hsa-miR-19b-1-5p;hsa-miR-2
ENSG0000	ENSG0000	8.63E-06	hsa-miR-125b-5p;hsa-miR-181a-5p;hsa-miR-193b-3p;hsa-miR
ENSG0000	ENSG0000	0.009364	hsa-miR-218-1-3p;hsa-miR-450b-5p;hsa-miR-507;hsa-miR-93-
ENSG0000	ENSG0000	8.61E-08	hsa-let-7a-5p;hsa-let-7b-5p;hsa-let-7d-5p;hsa-let-7e-5p;hsa-l
ENSG0000	ENSG0000	0.000501	hsa-let-7a-5p;hsa-let-7b-5p;hsa-let-7d-5p;hsa-let-7e-5p;hsa-l
ENSG0000	ENSG0000	1.22E-06	hsa-let-7a-5p;hsa-let-7b-5p;hsa-let-7d-5p;hsa-let-7e-5p;hsa-l
ENSG0000	ENSG0000	5.48E-06	hsa-miR-1271-3p;hsa-miR-214-5p;hsa-miR-3200-3p;hsa-miR-
ENSG0000	ENSG0000	0.000482	hsa-let-7a-5p;hsa-let-7b-5p;hsa-let-7d-5p;hsa-let-7e-5p;hsa-l
ENSG0000	ENSG0000	0.000274	hsa-let-7a-5p;hsa-let-7b-5p;hsa-let-7d-5p;hsa-let-7e-5p;hsa-l
ENSG0000	ENSG0000	0.002189	hsa-let-7a-5p;hsa-let-7d-5p;hsa-let-7e-5p;hsa-let-7f-5p;hsa-le
ENSG0000	ENSG0000	0.000882	hsa-let-7a-5p;hsa-let-7b-5p;hsa-let-7d-5p;hsa-let-7f-5p;hsa-le
ENSG0000	ENSG0000	0.000145	hsa-miR-125a-5p;hsa-miR-125b-5p;hsa-miR-17-5p;hsa-miR-18
ENSG0000	ENSG0000	8.04E-10	hsa-let-7a-5p;hsa-let-7b-5p;hsa-let-7d-5p;hsa-let-7e-5p;hsa-l
ENSG0000	ENSG0000	0.000918	hsa-miR-17-5p;hsa-miR-18a-3p;hsa-miR-1913;hsa-miR-20b-5
ENSG0000	ENSG0000	0.000918	hsa-miR-17-5p;hsa-miR-18a-3p;hsa-miR-1913;hsa-miR-20b-5
ENSG0000	ENSG0000	0.000918	hsa-miR-17-5p;hsa-miR-18a-3p;hsa-miR-1913;hsa-miR-20b-5
ENSG0000	ENSG0000	1.45E-05	hsa-let-7a-5p;hsa-let-7b-5p;hsa-let-7d-5p;hsa-let-7e-5p;hsa-l
ENSG0000	ENSG0000	0.005855	hsa-miR-17-5p;hsa-miR-20b-5p;hsa-miR-221-5p;hsa-miR-378
ENSG0000	ENSG0000	0.007373	hsa-miR-17-5p;hsa-miR-20b-5p;hsa-miR-520a-3p;hsa-miR-52
ENSG0000	ENSG0000	0.000483	hsa-miR-17-5p;hsa-miR-20b-5p;hsa-miR-545-3p;hsa-miR-558
ENSG0000	ENSG0000	0.003784	hsa-miR-130a-3p;hsa-miR-17-5p;hsa-miR-20b-5p;hsa-miR-45
ENSG0000	ENSG0000	0.002027	hsa-let-7a-5p;hsa-let-7b-5p;hsa-miR-143-3p;hsa-miR-181a-5
ENSG0000	ENSG0000	0.002027	hsa-let-7a-5p;hsa-let-7b-5p;hsa-miR-143-3p;hsa-miR-181a-5
ENSG0000	ENSG0000	0.000381	hsa-let-7a-5p;hsa-let-7b-5p;hsa-let-7d-5p;hsa-let-7e-5p;hsa-l
ENSG0000	ENSG0000	2.76E-05	hsa-let-7a-5p;hsa-let-7b-5p;hsa-let-7d-5p;hsa-let-7e-5p;hsa-l
ENSG0000	ENSG0000	4.54E-06	hsa-let-7d-5p;hsa-let-7f-5p;hsa-let-7g-5p;hsa-let-7i-5p
ENSG0000	ENSG0000	3.97E-05	hsa-let-7a-5p;hsa-let-7b-5p;hsa-let-7d-5p;hsa-let-7e-5p;hsa-l
ENSG0000	ENSG0000	0.006183	hsa-let-7a-5p;hsa-miR-192-5p;hsa-miR-30b-3p;hsa-miR-30c-1
ENSG0000	ENSG0000	0.005818	hsa-miR-17-5p;hsa-miR-193b-5p;hsa-miR-20b-5p;hsa-miR-52
ENSG0000	ENSG0000	0.000347	hsa-miR-1254;hsa-miR-378a-3p;hsa-miR-378c;hsa-miR-378d;h

ENSG0000	ENSG0000	0.000601	hsa-miR-17-5p;hsa-miR-20b-5p;hsa-miR-520a-3p;hsa-miR-52[illegible]
ENSG0000	ENSG0000	0.00214	hsa-miR-1271-3p;hsa-miR-221-5p;hsa-miR-339-5p;hsa-miR-5[illegible]
ENSG0000	ENSG0000	0.000363	hsa-let-7a-5p;hsa-let-7b-5p;hsa-let-7e-5p;hsa-let-7f-5p;hsa-le
ENSG0000	ENSG0000	0.000552	hsa-let-7a-5p;hsa-let-7b-5p;hsa-let-7d-5p;hsa-let-7e-5p;hsa-l
ENSG0000	ENSG0000	0.005504	hsa-miR-17-5p;hsa-miR-20b-5p;hsa-miR-214-5p;hsa-miR-671-
ENSG0000	ENSG0000	1.36E-06	hsa-let-7a-5p;hsa-let-7b-5p;hsa-let-7d-5p;hsa-let-7e-5p;hsa-l
ENSG0000	ENSG0000	9.07E-07	hsa-let-7a-5p;hsa-let-7b-5p;hsa-let-7d-5p;hsa-let-7e-5p;hsa-l
ENSG0000	ENSG0000	4.96E-07	hsa-let-7a-5p;hsa-let-7b-5p;hsa-let-7d-5p;hsa-let-7e-5p;hsa-l
ENSG0000	ENSG0000	0.002326	hsa-miR-125a-5p;hsa-miR-125b-5p;hsa-miR-17-5p;hsa-miR-1[illegible]
ENSG0000	ENSG0000	0.000535	hsa-let-7a-5p;hsa-let-7b-5p;hsa-let-7d-5p;hsa-let-7e-5p;hsa-l
ENSG0000	ENSG0000	0.00817	hsa-let-7a-5p;hsa-let-7d-5p;hsa-let-7e-5p;hsa-let-7f-5p
ENSG0000	ENSG0000	0.001619	hsa-miR-17-5p;hsa-miR-18a-3p;hsa-miR-1913;hsa-miR-20b-5[illegible]
ENSG0000	ENSG0000	0.004343	hsa-let-7a-5p;hsa-let-7d-5p;hsa-let-7e-5p;hsa-let-7g-5p;hsa-r
ENSG0000	ENSG0000	0.005469	hsa-miR-17-5p;hsa-miR-20b-5p;hsa-miR-30b-3p;hsa-miR-30c-
ENSG0000	ENSG0000	0.002878	hsa-miR-17-5p;hsa-miR-20b-5p;hsa-miR-520a-3p;hsa-miR-52[illegible]
ENSG0000	ENSG0000	1.31E-08	hsa-let-7a-5p;hsa-let-7b-5p;hsa-let-7d-5p;hsa-let-7e-5p;hsa-l
ENSG0000	ENSG0000	0.007081	hsa-miR-17-5p;hsa-miR-20b-5p;hsa-miR-5586-3p;hsa-miR-93-
ENSG0000	ENSG0000	4.61E-06	hsa-let-7a-5p;hsa-let-7b-5p;hsa-let-7d-5p;hsa-let-7e-5p;hsa-l
ENSG0000	ENSG0000	6.98E-07	hsa-let-7a-5p;hsa-let-7b-5p;hsa-let-7d-5p;hsa-let-7e-5p;hsa-l
ENSG0000	ENSG0000	8.44E-11	hsa-let-7a-5p;hsa-let-7b-5p;hsa-let-7d-5p;hsa-let-7e-5p;hsa-l
ENSG0000	ENSG0000	8.44E-11	hsa-let-7a-5p;hsa-let-7b-5p;hsa-let-7d-5p;hsa-let-7e-5p;hsa-l
ENSG0000	ENSG0000	0.009364	hsa-miR-19a-5p;hsa-miR-19b-1-5p;hsa-miR-450b-5p;hsa-miR-
ENSG0000	ENSG0000	3.10E-06	hsa-let-7a-5p;hsa-let-7b-5p;hsa-let-7d-5p;hsa-let-7e-5p;hsa-l
ENSG0000	ENSG0000	2.84E-06	hsa-let-7a-5p;hsa-let-7b-5p;hsa-let-7d-5p;hsa-let-7e-5p;hsa-l
ENSG0000	ENSG0000	2.84E-06	hsa-let-7a-5p;hsa-let-7b-5p;hsa-let-7d-5p;hsa-let-7e-5p;hsa-l
ENSG0000	ENSG0000	2.84E-06	hsa-let-7a-5p;hsa-let-7b-5p;hsa-let-7d-5p;hsa-let-7e-5p;hsa-l
ENSG0000	ENSG0000	2.84E-06	hsa-let-7a-5p;hsa-let-7b-5p;hsa-let-7d-5p;hsa-let-7e-5p;hsa-l
ENSG0000	ENSG0000	2.84E-06	hsa-let-7a-5p;hsa-let-7b-5p;hsa-let-7d-5p;hsa-let-7e-5p;hsa-l
ENSG0000	ENSG0000	2.84E-06	hsa-let-7a-5p;hsa-let-7b-5p;hsa-let-7d-5p;hsa-let-7e-5p;hsa-l
ENSG0000	ENSG0000	2.84E-06	hsa-let-7a-5p;hsa-let-7b-5p;hsa-let-7d-5p;hsa-let-7e-5p;hsa-l
ENSG0000	ENSG0000	2.87E-05	hsa-let-7a-5p;hsa-let-7b-5p;hsa-let-7d-5p;hsa-let-7e-5p;hsa-l
ENSG0000	ENSG0000	0.006823	hsa-let-7a-5p;hsa-let-7d-5p;hsa-let-7e-5p;hsa-let-7f-5p;hsa-le
ENSG0000	ENSG0000	0.003862	hsa-let-7a-5p;hsa-let-7b-5p;hsa-let-7d-5p;hsa-let-7e-5p;hsa-l
ENSG0000	ENSG0000	3.21E-07	hsa-let-7a-5p;hsa-let-7b-5p;hsa-let-7d-5p;hsa-let-7e-5p;hsa-l
ENSG0000	ENSG0000	0.000515	hsa-let-7a-5p;hsa-let-7b-5p;hsa-let-7e-5p;hsa-let-7f-5p;hsa-le
ENSG0000	ENSG0000	0.004818	hsa-let-7b-5p;hsa-miR-17-5p;hsa-miR-20b-5p;hsa-miR-221-5[illegible]
ENSG0000	ENSG0000	1.11E-07	hsa-let-7a-5p;hsa-let-7b-5p;hsa-let-7d-5p;hsa-let-7e-5p;hsa-l
ENSG0000	ENSG0000	0.003306	hsa-miR-17-5p;hsa-miR-181a-5p;hsa-miR-193b-5p;hsa-miR-2[illegible]
ENSG0000	ENSG0000	1.62E-05	hsa-let-7a-5p;hsa-let-7b-5p;hsa-let-7d-5p;hsa-let-7e-5p;hsa-l
ENSG0000	ENSG0000	0.00817	hsa-miR-196a-5p;hsa-miR-196b-5p;hsa-miR-19a-5p;hsa-miR-[illegible]
ENSG0000	ENSG0000	0.00817	hsa-miR-196a-5p;hsa-miR-196b-5p;hsa-miR-19a-5p;hsa-miR-[illegible]
ENSG0000	ENSG0000	0.00817	hsa-miR-196a-5p;hsa-miR-196b-5p;hsa-miR-19a-5p;hsa-miR-[illegible]
ENSG0000	ENSG0000	0.00817	hsa-miR-196a-5p;hsa-miR-196b-5p;hsa-miR-19a-5p;hsa-miR-[illegible]
ENSG0000	ENSG0000	0.00817	hsa-miR-196a-5p;hsa-miR-196b-5p;hsa-miR-19a-5p;hsa-miR-[illegible]
ENSG0000	ENSG0000	0.006183	hsa-let-7a-5p;hsa-let-7d-5p;hsa-let-7f-5p;hsa-let-7g-5p;hsa-le
ENSG0000	ENSG0000	0.001177	hsa-miR-378a-3p;hsa-miR-378c;hsa-miR-378d;hsa-miR-378i;hs
ENSG0000	ENSG0000	0.000907	hsa-let-7a-5p;hsa-let-7b-5p;hsa-let-7d-5p;hsa-let-7e-5p;hsa-l
ENSG0000	ENSG0000	0.004401	hsa-miR-130a-3p;hsa-miR-17-5p;hsa-miR-20b-5p;hsa-miR-93-
ENSG0000	ENSG0000	0.000117	hsa-let-7a-5p;hsa-let-7b-5p;hsa-let-7d-5p;hsa-let-7e-5p;hsa-l
ENSG0000	ENSG0000	0.005201	hsa-miR-19a-5p;hsa-miR-19b-1-5p;hsa-miR-450b-5p;hsa-miR-
ENSG0000	ENSG0000	0.000175	hsa-let-7a-5p;hsa-let-7b-5p;hsa-let-7d-5p;hsa-let-7e-5p;hsa-l
ENSG0000	ENSG0000	2.20E-07	hsa-let-7a-5p;hsa-let-7b-5p;hsa-let-7d-5p;hsa-let-7e-5p;hsa-l
ENSG0000	ENSG0000	5.48E-06	hsa-let-7a-5p;hsa-let-7b-5p;hsa-let-7d-5p;hsa-let-7e-5p;hsa-l
ENSG0000	ENSG0000	0.006986	hsa-let-7a-5p;hsa-let-7d-5p;hsa-let-7e-5p;hsa-let-7f-5p;hsa-le

ENSG0000	ENSG0000	0.003165	hsa-miR-125a-5p;hsa-miR-17-5p;hsa-miR-20b-5p;hsa-miR-52
ENSG0000	ENSG0000	0.003165	hsa-miR-125a-5p;hsa-miR-17-5p;hsa-miR-20b-5p;hsa-miR-52
ENSG0000	ENSG0000	3.27E-07	hsa-let-7a-5p;hsa-let-7b-5p;hsa-let-7d-5p;hsa-let-7e-5p;hsa-l
ENSG0000	ENSG0000	3.27E-07	hsa-let-7a-5p;hsa-let-7b-5p;hsa-let-7d-5p;hsa-let-7e-5p;hsa-l
ENSG0000	ENSG0000	9.23E-05	hsa-let-7a-5p;hsa-let-7b-5p;hsa-let-7d-5p;hsa-let-7e-5p;hsa-l
ENSG0000	ENSG0000	2.50E-05	hsa-let-7a-5p;hsa-let-7b-5p;hsa-let-7d-5p;hsa-let-7e-5p;hsa-l
ENSG0000	ENSG0000	8.72E-08	hsa-let-7a-5p;hsa-let-7b-5p;hsa-let-7d-5p;hsa-let-7e-5p;hsa-l
ENSG0000	ENSG0000	0.001514	hsa-miR-1254;hsa-miR-17-5p;hsa-miR-20b-5p;hsa-miR-214-5
ENSG0000	ENSG0000	0.002326	hsa-let-7a-5p;hsa-let-7b-5p;hsa-let-7d-5p;hsa-let-7e-5p;hsa-l
ENSG0000	ENSG0000	0.002326	hsa-let-7a-5p;hsa-let-7b-5p;hsa-let-7d-5p;hsa-let-7e-5p;hsa-l
ENSG0000	ENSG0000	1.36E-06	hsa-let-7a-5p;hsa-let-7b-5p;hsa-let-7d-5p;hsa-let-7e-5p;hsa-l
ENSG0000	ENSG0000	0.005818	hsa-miR-17-5p;hsa-miR-20b-5p;hsa-miR-520a-3p;hsa-miR-52
ENSG0000	ENSG0000	0.000104	hsa-miR-17-5p;hsa-miR-181a-5p;hsa-miR-20b-5p;hsa-miR-93
ENSG0000	ENSG0000	0.007939	hsa-miR-130a-3p;hsa-miR-17-5p;hsa-miR-193b-3p;hsa-miR-2
ENSG0000	ENSG0000	0.005818	hsa-miR-17-5p;hsa-miR-20b-5p;hsa-miR-30c-1-3p;hsa-miR-3
ENSG0000	ENSG0000	0.002489	hsa-miR-148a-3p;hsa-miR-378a-3p;hsa-miR-378c;hsa-miR-37
ENSG0000	ENSG0000	0.00982	hsa-miR-17-5p;hsa-miR-196a-5p;hsa-miR-196b-5p;hsa-miR-5
ENSG0000	ENSG0000	1.20E-05	hsa-let-7a-5p;hsa-let-7b-5p;hsa-let-7d-5p;hsa-let-7e-5p;hsa-l
ENSG0000	ENSG0000	2.20E-06	hsa-let-7a-5p;hsa-let-7b-5p;hsa-let-7d-5p;hsa-let-7e-5p;hsa-l
ENSG0000	ENSG0000	8.03E-05	hsa-let-7a-5p;hsa-let-7b-5p;hsa-let-7d-5p;hsa-let-7e-5p;hsa-l
ENSG0000	ENSG0000	5.91E-05	hsa-let-7a-5p;hsa-let-7e-5p;hsa-miR-148a-3p;hsa-miR-148b-
ENSG0000	ENSG0000	0.004993	hsa-let-7a-5p;hsa-let-7d-5p;hsa-let-7e-5p;hsa-let-7f-5p;hsa-le
ENSG0000	ENSG0000	0.006183	hsa-miR-17-5p;hsa-miR-20b-5p;hsa-miR-520a-3p;hsa-miR-52
ENSG0000	ENSG0000	0.000341	hsa-let-7a-5p;hsa-let-7b-5p;hsa-let-7d-5p;hsa-let-7e-5p;hsa-l
ENSG0000	ENSG0000	2.10E-05	hsa-let-7a-5p;hsa-let-7b-5p;hsa-let-7d-5p;hsa-let-7e-5p;hsa-l
ENSG0000	ENSG0000	0.000416	hsa-miR-1271-3p;hsa-miR-17-5p;hsa-miR-20b-5p;hsa-miR-22
ENSG0000	ENSG0000	0.003309	hsa-miR-17-5p;hsa-miR-20b-5p;hsa-miR-520a-3p;hsa-miR-52
ENSG0000	ENSG0000	0.00039	hsa-let-7a-5p;hsa-let-7b-5p;hsa-let-7e-5p;hsa-let-7f-5p;hsa-le
ENSG0000	ENSG0000	0.000314	hsa-let-7a-5p;hsa-let-7b-5p;hsa-let-7d-5p;hsa-let-7e-5p;hsa-l
ENSG0000	ENSG0000	1.46E-05	hsa-let-7a-5p;hsa-let-7b-5p;hsa-let-7d-5p;hsa-let-7e-5p;hsa-l
ENSG0000	ENSG0000	9.28E-07	hsa-let-7a-5p;hsa-let-7b-5p;hsa-let-7d-5p;hsa-let-7e-5p;hsa-l
ENSG0000	ENSG0000	2.69E-05	hsa-let-7a-5p;hsa-let-7d-5p;hsa-let-7e-5p;hsa-let-7f-5p;hsa-le
ENSG0000	ENSG0000	0.000269	hsa-miR-125b-5p;hsa-miR-1271-3p;hsa-miR-20b-5p;hsa-miR-
ENSG0000	ENSG0000	9.08E-07	hsa-let-7a-5p;hsa-let-7b-5p;hsa-let-7d-5p;hsa-let-7e-5p;hsa-l
ENSG0000	ENSG0000	0.001619	hsa-miR-17-5p;hsa-miR-193b-5p;hsa-miR-20b-5p;hsa-miR-37
ENSG0000	ENSG0000	0.002747	hsa-miR-19a-5p;hsa-miR-19b-1-5p;hsa-miR-2681-5p;hsa-miR
ENSG0000	ENSG0000	1.60E-05	hsa-let-7a-5p;hsa-let-7b-5p;hsa-let-7d-5p;hsa-let-7e-5p;hsa-l
ENSG0000	ENSG0000	0.000379	hsa-miR-1254;hsa-miR-1271-3p;hsa-miR-550a-3-5p;hsa-miR-5
ENSG0000	ENSG0000	0.000379	hsa-miR-1254;hsa-miR-1271-3p;hsa-miR-550a-3-5p;hsa-miR-5
ENSG0000	ENSG0000	0.004799	hsa-miR-1271-3p;hsa-miR-214-5p;hsa-miR-3065-3p;hsa-miR-
ENSG0000	ENSG0000	0.000131	hsa-miR-125a-5p;hsa-miR-125b-5p;hsa-miR-130a-3p;hsa-miR
ENSG0000	ENSG0000	1.32E-06	hsa-miR-125b-5p;hsa-miR-1271-3p;hsa-miR-148a-3p;hsa-miR
ENSG0000	ENSG0000	0.004307	hsa-miR-214-5p;hsa-miR-30b-3p;hsa-miR-30c-1-3p;hsa-miR-
ENSG0000	ENSG0000	0.000196	hsa-let-7a-5p;hsa-let-7b-5p;hsa-let-7d-5p;hsa-let-7e-5p;hsa-l
ENSG0000	ENSG0000	0.00023	hsa-miR-1271-3p;hsa-miR-361-3p;hsa-miR-550a-3-5p;hsa-mi
ENSG0000	ENSG0000	3.46E-07	hsa-let-7a-5p;hsa-let-7b-5p;hsa-let-7d-5p;hsa-let-7e-5p;hsa-l
ENSG0000	ENSG0000	0.000626	hsa-let-7a-5p;hsa-let-7d-5p;hsa-let-7e-5p;hsa-let-7f-5p;hsa-le
ENSG0000	ENSG0000	0.005855	hsa-miR-17-5p;hsa-miR-20b-5p;hsa-miR-221-5p;hsa-miR-500
ENSG0000	ENSG0000	1.56E-06	hsa-let-7a-5p;hsa-let-7b-5p;hsa-let-7d-5p;hsa-let-7e-5p;hsa-l
ENSG0000	ENSG0000	0.000293	hsa-let-7a-5p;hsa-let-7b-5p;hsa-let-7d-5p;hsa-let-7e-5p;hsa-l
ENSG0000	ENSG0000	0.006332	hsa-let-7a-5p;hsa-let-7b-5p;hsa-let-7d-5p;hsa-let-7e-5p;hsa-l
ENSG0000	ENSG0000	0.004379	hsa-miR-30b-3p;hsa-miR-30c-1-3p;hsa-miR-30c-2-3p;hsa-mi
ENSG0000	ENSG0000	0.001551	hsa-let-7a-5p;hsa-let-7e-5p;hsa-let-7f-5p;hsa-let-7g-5p;hsa-le
ENSG0000	ENSG0000	0.000909	hsa-miR-1254;hsa-miR-1271-3p;hsa-miR-17-5p;hsa-miR-20b-

ENSG0000	ENSG0000	0.002599	hsa-miR-143-3p;hsa-miR-17-5p;hsa-miR-20b-5p;hsa-miR-30b
ENSG0000	ENSG0000	1.42E-06	hsa-miR-1254;hsa-miR-125a-5p;hsa-miR-125b-5p;hsa-miR-12
ENSG0000	ENSG0000	5.37E-05	hsa-miR-2681-5p;hsa-miR-324-5p;hsa-miR-378a-3p;hsa-miR-3
ENSG0000	ENSG0000	2.22E-07	hsa-let-7a-5p;hsa-let-7b-5p;hsa-let-7d-5p;hsa-let-7e-5p;hsa-l
ENSG0000	ENSG0000	0.002205	hsa-miR-17-5p;hsa-miR-20b-5p;hsa-miR-33b-3p;hsa-miR-450l
ENSG0000	ENSG0000	0.006093	hsa-miR-378a-3p;hsa-miR-378c;hsa-miR-378d;hsa-miR-378i
ENSG0000	ENSG0000	1.15E-05	hsa-let-7a-5p;hsa-let-7d-5p;hsa-let-7f-5p;hsa-let-7g-5p;hsa-le
ENSG0000	ENSG0000	0.002979	hsa-miR-17-5p;hsa-miR-193b-5p;hsa-miR-20b-5p;hsa-miR-52
ENSG0000	ENSG0000	0.000598	hsa-let-7a-5p;hsa-let-7b-5p;hsa-let-7d-5p;hsa-let-7e-5p;hsa-l
ENSG0000	ENSG0000	0.002205	hsa-let-7a-5p;hsa-let-7b-5p;hsa-let-7d-5p;hsa-let-7e-5p;hsa-l
ENSG0000	ENSG0000	0.002205	hsa-let-7a-5p;hsa-let-7b-5p;hsa-let-7d-5p;hsa-let-7e-5p;hsa-l
ENSG0000	ENSG0000	0.00692	hsa-miR-2681-5p;hsa-miR-3065-3p;hsa-miR-30c-1-3p;hsa-miR
ENSG0000	ENSG0000	6.96E-05	hsa-let-7a-5p;hsa-let-7b-5p;hsa-let-7d-5p;hsa-let-7e-5p;hsa-l
ENSG0000	ENSG0000	3.39E-05	hsa-let-7a-5p;hsa-let-7b-5p;hsa-let-7d-5p;hsa-let-7e-5p;hsa-l
ENSG0000	ENSG0000	9.90E-06	hsa-let-7a-5p;hsa-let-7b-5p;hsa-let-7d-5p;hsa-let-7e-5p;hsa-l
ENSG0000	ENSG0000	2.10E-05	hsa-let-7a-5p;hsa-let-7b-5p;hsa-let-7d-5p;hsa-let-7e-5p;hsa-l
ENSG0000	ENSG0000	6.30E-05	hsa-miR-1254;hsa-miR-1271-3p;hsa-miR-130a-3p;hsa-miR-21
ENSG0000	ENSG0000	8.02E-08	hsa-let-7a-5p;hsa-let-7b-5p;hsa-let-7d-5p;hsa-let-7e-5p;hsa-l
ENSG0000	ENSG0000	0.003688	hsa-miR-17-5p;hsa-miR-20b-5p;hsa-miR-5586-3p;hsa-miR-93
ENSG0000	ENSG0000	0.006183	hsa-miR-17-5p;hsa-miR-20b-5p;hsa-miR-520a-3p;hsa-miR-52
ENSG0000	ENSG0000	0.001142	hsa-miR-17-5p;hsa-miR-20b-5p;hsa-miR-214-5p;hsa-miR-30b
ENSG0000	ENSG0000	0.001142	hsa-miR-17-5p;hsa-miR-20b-5p;hsa-miR-214-5p;hsa-miR-30b
ENSG0000	ENSG0000	0.002878	hsa-miR-19a-5p;hsa-miR-19b-1-5p;hsa-miR-450b-5p;hsa-miR
ENSG0000	ENSG0000	3.98E-06	hsa-let-7a-5p;hsa-let-7b-5p;hsa-let-7d-5p;hsa-let-7e-5p;hsa-l
ENSG0000	ENSG0000	0.003221	hsa-miR-130a-3p;hsa-miR-148a-3p;hsa-miR-148b-3p;hsa-miR
ENSG0000	ENSG0000	0.000269	hsa-miR-17-5p;hsa-miR-19a-5p;hsa-miR-19b-1-5p;hsa-miR-2
ENSG0000	ENSG0000	0.000269	hsa-miR-17-5p;hsa-miR-19a-5p;hsa-miR-19b-1-5p;hsa-miR-2
ENSG0000	ENSG0000	0.000269	hsa-let-7a-5p;hsa-let-7b-5p;hsa-let-7e-5p;hsa-let-7f-5p;hsa-le
ENSG0000	ENSG0000	0.005405	hsa-miR-1271-3p;hsa-miR-30b-3p;hsa-miR-30c-1-3p;hsa-miR
ENSG0000	ENSG0000	0.000166	hsa-let-7a-5p;hsa-let-7b-5p;hsa-let-7d-5p;hsa-let-7e-5p;hsa-l
ENSG0000	ENSG0000	0.001021	hsa-let-7b-5p;hsa-miR-17-5p;hsa-miR-20b-5p;hsa-miR-520a-3
ENSG0000	ENSG0000	0.006986	hsa-miR-125a-5p;hsa-miR-125b-5p;hsa-miR-192-5p;hsa-miR-2
ENSG0000	ENSG0000	0.008573	hsa-miR-17-5p;hsa-miR-20b-5p;hsa-miR-520a-3p;hsa-miR-52
ENSG0000	ENSG0000	0.000332	hsa-let-7a-5p;hsa-let-7b-5p;hsa-let-7d-5p;hsa-let-7e-5p;hsa-l
ENSG0000	ENSG0000	0.000418	hsa-let-7a-5p;hsa-let-7b-5p;hsa-let-7d-5p;hsa-let-7e-5p;hsa-l
ENSG0000	ENSG0000	0.00529	hsa-miR-17-5p;hsa-miR-20b-5p;hsa-miR-221-5p;hsa-miR-520a
ENSG0000	ENSG0000	2.20E-07	hsa-let-7a-5p;hsa-let-7b-5p;hsa-let-7d-5p;hsa-let-7e-5p;hsa-l
ENSG0000	ENSG0000	0.002244	hsa-let-7a-5p;hsa-let-7f-5p;hsa-let-7g-5p;hsa-let-7i-5p;hsa-m
ENSG0000	ENSG0000	0.000252	hsa-let-7a-5p;hsa-let-7b-5p;hsa-let-7d-5p;hsa-let-7e-5p;hsa-l
ENSG0000	ENSG0000	0.000252	hsa-let-7a-5p;hsa-let-7b-5p;hsa-let-7d-5p;hsa-let-7e-5p;hsa-l
ENSG0000	ENSG0000	0.004818	hsa-miR-1271-3p;hsa-miR-214-5p;hsa-miR-30b-3p;hsa-miR-3
ENSG0000	ENSG0000	0.000547	hsa-miR-17-5p;hsa-miR-20b-5p;hsa-miR-214-5p;hsa-miR-30b
ENSG0000	ENSG0000	0.003465	hsa-let-7a-5p;hsa-let-7b-5p;hsa-let-7d-5p;hsa-let-7e-5p;hsa-l
ENSG0000	ENSG0000	0.004148	hsa-miR-17-5p;hsa-miR-18a-3p;hsa-miR-1913;hsa-miR-20b-5
ENSG0000	ENSG0000	9.90E-06	hsa-let-7a-5p;hsa-let-7b-5p;hsa-let-7d-5p;hsa-let-7e-5p;hsa-l
ENSG0000	ENSG0000	0.00214	hsa-miR-17-5p;hsa-miR-19a-5p;hsa-miR-19b-1-5p;hsa-miR-2
ENSG0000	ENSG0000	0.006382	hsa-miR-17-5p;hsa-miR-20b-5p;hsa-miR-378a-5p;hsa-miR-52
ENSG0000	ENSG0000	0.005405	hsa-miR-1254;hsa-miR-17-5p;hsa-miR-20b-5p;hsa-miR-3200-3
ENSG0000	ENSG0000	0.001922	hsa-miR-143-3p;hsa-miR-17-5p;hsa-miR-193b-5p;hsa-miR-20l
ENSG0000	ENSG0000	1.69E-05	hsa-let-7a-5p;hsa-let-7b-5p;hsa-let-7d-5p;hsa-let-7e-5p;hsa-l
ENSG0000	ENSG0000	1.69E-05	hsa-let-7a-5p;hsa-let-7b-5p;hsa-let-7d-5p;hsa-let-7e-5p;hsa-l
ENSG0000	ENSG0000	0.000476	hsa-miR-1254;hsa-miR-1271-3p;hsa-miR-143-3p;hsa-miR-30c-
ENSG0000	ENSG0000	0.000201	hsa-let-7a-5p;hsa-let-7b-5p;hsa-let-7d-5p;hsa-let-7e-5p;hsa-l
ENSG0000	ENSG0000	0.006206	hsa-miR-17-5p;hsa-miR-18a-3p;hsa-miR-1913;hsa-miR-196a-5

ENSG0000	ENSG0000	0.000813	hsa-let-7a-5p;hsa-let-7b-5p;hsa-let-7d-5p;hsa-let-7e-5p;hsa-l
ENSG0000	ENSG0000	0.008651	hsa-miR-17-5p;hsa-miR-18a-3p;hsa-miR-1913;hsa-miR-20b-5p
ENSG0000	ENSG0000	0.005504	hsa-miR-17-5p;hsa-miR-20b-5p;hsa-miR-520a-3p;hsa-miR-520
ENSG0000	ENSG0000	0.0001	hsa-let-7b-5p;hsa-let-7d-5p;hsa-let-7e-5p;hsa-let-7f-5p;hsa-le
ENSG0000	ENSG0000	0.000826	hsa-miR-17-5p;hsa-miR-20b-5p;hsa-miR-507;hsa-miR-520a-3p
ENSG0000	ENSG0000	0.002434	hsa-let-7a-5p;hsa-let-7b-5p;hsa-let-7d-5p;hsa-let-7e-5p;hsa-l
ENSG0000	ENSG0000	6.11E-06	hsa-let-7a-5p;hsa-let-7b-5p;hsa-let-7d-5p;hsa-let-7e-5p;hsa-l
ENSG0000	ENSG0000	0.008651	hsa-let-7a-5p;hsa-let-7b-5p;hsa-let-7d-5p;hsa-let-7e-5p;hsa-l
ENSG0000	ENSG0000	0.008573	hsa-miR-19a-5p;hsa-miR-19b-1-5p;hsa-miR-450b-5p;hsa-miR-
ENSG0000	ENSG0000	2.33E-07	hsa-let-7a-5p;hsa-let-7b-5p;hsa-let-7d-5p;hsa-let-7e-5p;hsa-l
ENSG0000	ENSG0000	0.002489	hsa-miR-17-5p;hsa-miR-20b-5p;hsa-miR-520a-3p;hsa-miR-520
ENSG0000	ENSG0000	0.000379	hsa-let-7a-5p;hsa-let-7b-5p;hsa-let-7d-5p;hsa-let-7f-5p;hsa-le
ENSG0000	ENSG0000	0.003059	hsa-miR-17-5p;hsa-miR-20b-5p;hsa-miR-606;hsa-miR-93-5p
ENSG0000	ENSG0000	0.000534	hsa-miR-17-5p;hsa-miR-18a-3p;hsa-miR-1913;hsa-miR-20b-5p
ENSG0000	ENSG0000	0.003453	hsa-let-7a-5p;hsa-let-7b-5p;hsa-let-7d-5p;hsa-let-7e-5p;hsa-l
ENSG0000	ENSG0000	1.39E-05	hsa-let-7a-5p;hsa-let-7b-5p;hsa-let-7d-5p;hsa-let-7e-5p;hsa-l
ENSG0000	ENSG0000	0.003919	hsa-miR-17-5p;hsa-miR-185-3p;hsa-miR-20b-5p;hsa-miR-520a
ENSG0000	ENSG0000	0.00692	hsa-miR-1271-3p;hsa-miR-214-5p;hsa-miR-221-5p;hsa-miR-55
ENSG0000	ENSG0000	0.00495	hsa-miR-1271-3p;hsa-miR-214-5p;hsa-miR-30b-3p;hsa-miR-30
ENSG0000	ENSG0000	0.004343	hsa-let-7a-5p;hsa-let-7d-5p;hsa-miR-130a-3p;hsa-miR-148a-3
ENSG0000	ENSG0000	0.001918	hsa-let-7a-5p;hsa-let-7d-5p;hsa-let-7f-5p;hsa-let-7g-5p;hsa-le
ENSG0000	ENSG0000	0.001161	hsa-miR-130a-3p;hsa-miR-148a-3p;hsa-miR-148b-3p;hsa-miR-
ENSG0000	ENSG0000	0.001091	hsa-miR-17-5p;hsa-miR-20b-5p;hsa-miR-520a-3p;hsa-miR-520
ENSG0000	ENSG0000	7.52E-05	hsa-let-7a-5p;hsa-let-7b-5p;hsa-let-7d-5p;hsa-let-7e-5p;hsa-l
ENSG0000	ENSG0000	8.07E-05	hsa-let-7a-5p;hsa-let-7b-5p;hsa-let-7d-5p;hsa-let-7e-5p;hsa-l
ENSG0000	ENSG0000	0.000356	hsa-miR-17-5p;hsa-miR-193b-5p;hsa-miR-19a-5p;hsa-miR-19b
ENSG0000	ENSG0000	0.005855	hsa-miR-143-3p;hsa-miR-17-5p;hsa-miR-20b-5p;hsa-miR-361-
ENSG0000	ENSG0000	0.008573	hsa-miR-148a-3p;hsa-miR-148b-3p;hsa-miR-30b-3p;hsa-miR-3
ENSG0000	ENSG0000	0.000717	hsa-let-7a-5p;hsa-let-7b-5p;hsa-let-7d-5p;hsa-let-7e-5p;hsa-l
ENSG0000	ENSG0000	0.001551	hsa-miR-378a-3p;hsa-miR-378c;hsa-miR-378d;hsa-miR-378i;hs
ENSG0000	ENSG0000	0.005855	hsa-let-7a-5p;hsa-let-7d-5p;hsa-let-7e-5p;hsa-let-7f-5p;hsa-le
ENSG0000	ENSG0000	0.002525	hsa-miR-221-5p;hsa-miR-3619-5p;hsa-miR-520a-3p;hsa-miR-5
ENSG0000	ENSG0000	0.005405	hsa-miR-17-5p;hsa-miR-20b-5p;hsa-miR-30b-3p;hsa-miR-30c-
ENSG0000	ENSG0000	0.002599	hsa-miR-17-5p;hsa-miR-20b-5p;hsa-miR-30b-3p;hsa-miR-485
ENSG0000	ENSG0000	0.00042	hsa-let-7d-5p;hsa-let-7e-5p;hsa-let-7f-5p;hsa-let-7g-5p;hsa-le
ENSG0000	ENSG0000	0.000552	hsa-miR-17-5p;hsa-miR-18a-3p;hsa-miR-1913;hsa-miR-20b-5p
ENSG0000	ENSG0000	0.001306	hsa-miR-17-5p;hsa-miR-20b-5p;hsa-miR-520a-3p;hsa-miR-520
ENSG0000	ENSG0000	1.74E-11	hsa-let-7a-5p;hsa-let-7b-5p;hsa-let-7d-5p;hsa-let-7e-5p;hsa-l
ENSG0000	ENSG0000	1.74E-11	hsa-let-7a-5p;hsa-let-7b-5p;hsa-let-7d-5p;hsa-let-7e-5p;hsa-l
ENSG0000	ENSG0000	1.74E-11	hsa-let-7a-5p;hsa-let-7b-5p;hsa-let-7d-5p;hsa-let-7e-5p;hsa-l
ENSG0000	ENSG0000	8.63E-06	hsa-let-7a-5p;hsa-let-7b-5p;hsa-let-7d-5p;hsa-let-7e-5p;hsa-l
ENSG0000	ENSG0000	0.003768	hsa-let-7a-5p;hsa-let-7b-5p;hsa-let-7d-5p;hsa-let-7e-5p;hsa-l
ENSG0000	ENSG0000	0.003535	hsa-miR-17-5p;hsa-miR-20b-5p;hsa-miR-324-5p;hsa-miR-378a
ENSG0000	ENSG0000	0.00692	hsa-miR-17-5p;hsa-miR-20b-5p;hsa-miR-520a-3p;hsa-miR-520
ENSG0000	ENSG0000	6.59E-05	hsa-let-7a-5p;hsa-let-7b-5p;hsa-let-7d-5p;hsa-let-7e-5p;hsa-l
ENSG0000	ENSG0000	0.001551	hsa-miR-17-5p;hsa-miR-20b-5p;hsa-miR-520a-3p;hsa-miR-520
ENSG0000	ENSG0000	5.37E-05	hsa-let-7a-5p;hsa-let-7b-5p;hsa-let-7d-5p;hsa-let-7e-5p;hsa-l
ENSG0000	ENSG0000	5.90E-05	hsa-let-7a-5p;hsa-let-7d-5p;hsa-let-7e-5p;hsa-let-7f-5p;hsa-le
ENSG0000	ENSG0000	0.00692	hsa-let-7a-5p;hsa-let-7b-5p;hsa-let-7d-5p;hsa-let-7e-5p;hsa-l
ENSG0000	ENSG0000	0.00692	hsa-let-7a-5p;hsa-let-7b-5p;hsa-let-7d-5p;hsa-let-7e-5p;hsa-l
ENSG0000	ENSG0000	0.003527	hsa-let-7a-5p;hsa-let-7d-5p;hsa-let-7e-5p;hsa-let-7f-5p;hsa-le
ENSG0000	ENSG0000	0.001828	hsa-let-7a-5p;hsa-let-7e-5p;hsa-let-7f-5p;hsa-let-7g-5p;hsa-le
ENSG0000	ENSG0000	0.001641	hsa-miR-130a-3p;hsa-miR-17-5p;hsa-miR-181a-5p;hsa-miR-19
ENSG0000	ENSG0000	0.007939	hsa-miR-17-5p;hsa-miR-20b-5p;hsa-miR-218-1-3p;hsa-miR-48

ENSG0000	ENSG0000	1.36E-06	hsa-let-7a-5p;hsa-let-7b-5p;hsa-let-7d-5p;hsa-let-7e-5p;hsa-l
ENSG0000	ENSG0000	4.34E-05	hsa-let-7a-5p;hsa-let-7b-5p;hsa-let-7d-5p;hsa-let-7e-5p;hsa-l
ENSG0000	ENSG0000	1.25E-05	hsa-let-7a-5p;hsa-let-7b-5p;hsa-let-7d-5p;hsa-let-7e-5p;hsa-l
ENSG0000	ENSG0000	5.31E-05	hsa-let-7a-5p;hsa-let-7b-5p;hsa-let-7d-5p;hsa-let-7e-5p;hsa-l
ENSG0000	ENSG0000	5.31E-05	hsa-let-7a-5p;hsa-let-7b-5p;hsa-let-7d-5p;hsa-let-7e-5p;hsa-l
ENSG0000	ENSG0000	0.006183	hsa-miR-17-5p;hsa-miR-20b-5p;hsa-miR-520a-3p;hsa-miR-52
ENSG0000	ENSG0000	0.007615	hsa-miR-17-5p;hsa-miR-20b-5p;hsa-miR-3619-5p;hsa-miR-52
ENSG0000	ENSG0000	6.59E-05	hsa-let-7a-5p;hsa-let-7b-5p;hsa-let-7d-5p;hsa-let-7e-5p;hsa-l
ENSG0000	ENSG0000	1.05E-05	hsa-let-7a-5p;hsa-let-7b-5p;hsa-let-7d-5p;hsa-let-7e-5p;hsa-l
ENSG0000	ENSG0000	0.00014	hsa-let-7a-5p;hsa-let-7b-5p;hsa-let-7d-5p;hsa-let-7f-5p;hsa-le
ENSG0000	ENSG0000	0.002979	hsa-let-7a-5p;hsa-let-7b-5p;hsa-let-7d-5p;hsa-let-7e-5p;hsa-l
ENSG0000	ENSG0000	0.000458	hsa-miR-17-5p;hsa-miR-20b-5p;hsa-miR-221-5p;hsa-miR-378
ENSG0000	ENSG0000	0.001177	hsa-miR-17-5p;hsa-miR-193b-5p;hsa-miR-20b-5p;hsa-miR-52
ENSG0000	ENSG0000	3.09E-05	hsa-miR-1271-3p;hsa-miR-148a-3p;hsa-miR-148b-3p;hsa-miR
ENSG0000	ENSG0000	0.006555	hsa-miR-17-5p;hsa-miR-192-5p;hsa-miR-193b-5p;hsa-miR-20
ENSG0000	ENSG0000	0.007121	hsa-miR-17-5p;hsa-miR-193b-5p;hsa-miR-20b-5p;hsa-miR-22
ENSG0000	ENSG0000	1.77E-05	hsa-let-7a-5p;hsa-let-7b-5p;hsa-let-7e-5p;hsa-let-7f-5p;hsa-le
ENSG0000	ENSG0000	8.18E-05	hsa-let-7a-5p;hsa-let-7b-5p;hsa-let-7d-5p;hsa-let-7e-5p;hsa-l
ENSG0000	ENSG0000	0.00817	hsa-miR-1271-3p;hsa-miR-214-5p;hsa-miR-550a-3-5p;hsa-mi
ENSG0000	ENSG0000	0.004799	hsa-miR-181a-5p;hsa-miR-193b-3p;hsa-miR-2681-5p;hsa-miR
ENSG0000	ENSG0000	0.004799	hsa-miR-181a-5p;hsa-miR-193b-3p;hsa-miR-2681-5p;hsa-miR
ENSG0000	ENSG0000	6.24E-05	hsa-let-7a-5p;hsa-let-7b-5p;hsa-let-7d-5p;hsa-let-7f-5p;hsa-le
ENSG0000	ENSG0000	1.67E-09	hsa-let-7a-5p;hsa-let-7b-5p;hsa-let-7d-5p;hsa-let-7e-5p;hsa-l
ENSG0000	ENSG0000	0.000179	hsa-let-7a-5p;hsa-let-7b-5p;hsa-let-7d-5p;hsa-let-7e-5p;hsa-l
ENSG0000	ENSG0000	0.000179	hsa-let-7a-5p;hsa-let-7b-5p;hsa-let-7d-5p;hsa-let-7e-5p;hsa-l
ENSG0000	ENSG0000	1.99E-06	hsa-let-7a-5p;hsa-let-7b-5p;hsa-let-7d-5p;hsa-let-7e-5p;hsa-l
ENSG0000	ENSG0000	0.001828	hsa-miR-17-5p;hsa-miR-20b-5p;hsa-miR-520a-3p;hsa-miR-52
ENSG0000	ENSG0000	0.000598	hsa-miR-17-5p;hsa-miR-20b-5p;hsa-miR-214-5p;hsa-miR-30b
ENSG0000	ENSG0000	0.002878	hsa-miR-17-5p;hsa-miR-20b-5p;hsa-miR-520a-3p;hsa-miR-52
ENSG0000	ENSG0000	0.009494	hsa-miR-17-5p;hsa-miR-20b-5p;hsa-miR-520a-3p;hsa-miR-52
ENSG0000	ENSG0000	2.22E-07	hsa-let-7a-5p;hsa-let-7b-5p;hsa-let-7d-5p;hsa-let-7e-5p;hsa-l
ENSG0000	ENSG0000	0.003784	hsa-miR-17-5p;hsa-miR-20b-5p;hsa-miR-520a-3p;hsa-miR-52
ENSG0000	ENSG0000	0.000382	hsa-miR-130a-3p;hsa-miR-17-5p;hsa-miR-181a-5p;hsa-miR-2
ENSG0000	ENSG0000	0.002878	hsa-miR-17-5p;hsa-miR-20b-5p;hsa-miR-33b-3p;hsa-miR-93-
ENSG0000	ENSG0000	0.002945	hsa-let-7a-5p;hsa-let-7b-5p;hsa-let-7d-5p;hsa-let-7e-5p;hsa-l
ENSG0000	ENSG0000	0.003165	hsa-miR-17-5p;hsa-miR-20b-5p;hsa-miR-520a-3p;hsa-miR-52
ENSG0000	ENSG0000	5.10E-11	hsa-let-7a-5p;hsa-let-7b-5p;hsa-let-7d-5p;hsa-let-7e-5p;hsa-l
ENSG0000	ENSG0000	8.72E-06	hsa-let-7a-5p;hsa-let-7b-5p;hsa-let-7d-5p;hsa-let-7e-5p;hsa-l
ENSG0000	ENSG0000	5.36E-10	hsa-let-7a-5p;hsa-let-7b-5p;hsa-let-7d-5p;hsa-let-7e-5p;hsa-l
ENSG0000	ENSG0000	0.000108	hsa-let-7a-5p;hsa-let-7b-5p;hsa-let-7d-5p;hsa-let-7e-5p;hsa-l
ENSG0000	ENSG0000	0.001177	hsa-miR-17-5p;hsa-miR-20b-5p;hsa-miR-331-3p;hsa-miR-520
ENSG0000	ENSG0000	0.000341	hsa-let-7a-5p;hsa-let-7b-5p;hsa-let-7d-5p;hsa-let-7e-5p;hsa-l
ENSG0000	ENSG0000	0.002489	hsa-miR-1913;hsa-miR-378a-3p;hsa-miR-378c;hsa-miR-378d;h
ENSG0000	ENSG0000	1.55E-07	hsa-let-7a-5p;hsa-let-7b-5p;hsa-let-7d-5p;hsa-let-7e-5p;hsa-l
ENSG0000	ENSG0000	4.07E-08	hsa-let-7a-5p;hsa-let-7b-5p;hsa-let-7d-5p;hsa-let-7e-5p;hsa-l
ENSG0000	ENSG0000	4.03E-10	hsa-let-7a-5p;hsa-let-7b-5p;hsa-let-7d-5p;hsa-let-7e-5p;hsa-l
ENSG0000	ENSG0000	0.001988	hsa-miR-17-5p;hsa-miR-20b-5p;hsa-miR-30b-3p;hsa-miR-30c
ENSG0000	ENSG0000	0.003784	hsa-miR-17-5p;hsa-miR-20b-5p;hsa-miR-520a-3p;hsa-miR-52
ENSG0000	ENSG0000	1.07E-06	hsa-let-7a-5p;hsa-let-7b-5p;hsa-let-7d-5p;hsa-let-7e-5p;hsa-l
ENSG0000	ENSG0000	0.007715	hsa-miR-17-5p;hsa-miR-20b-5p;hsa-miR-520a-3p;hsa-miR-52
ENSG0000	ENSG0000	0.007715	hsa-miR-17-5p;hsa-miR-20b-5p;hsa-miR-520a-3p;hsa-miR-52
ENSG0000	ENSG0000	5.37E-05	hsa-let-7a-5p;hsa-let-7b-5p;hsa-let-7d-5p;hsa-let-7e-5p;hsa-l
ENSG0000	ENSG0000	0.00042	hsa-let-7a-5p;hsa-let-7b-5p;hsa-let-7d-5p;hsa-let-7e-5p;hsa-l
ENSG0000	ENSG0000	5.48E-06	hsa-let-7a-5p;hsa-let-7b-5p;hsa-let-7d-5p;hsa-let-7e-5p;hsa-l

ENSG0000	ENSG0000	0.003243	hsa-miR-148a-3p;hsa-miR-148b-3p;hsa-miR-196a-5p;hsa-miR-
ENSG0000	ENSG0000	0.003243	hsa-miR-148a-3p;hsa-miR-148b-3p;hsa-miR-196a-5p;hsa-miR-
ENSG0000	ENSG0000	0.005818	hsa-miR-1271-3p;hsa-miR-30b-3p;hsa-miR-30c-1-3p;hsa-miR-
ENSG0000	ENSG0000	0.004211	hsa-miR-1254;hsa-miR-17-5p;hsa-miR-20b-5p;hsa-miR-520a-3
ENSG0000	ENSG0000	1.69E-05	hsa-let-7a-5p;hsa-let-7b-5p;hsa-let-7d-5p;hsa-let-7e-5p;hsa-l
ENSG0000	ENSG0000	1.69E-05	hsa-let-7a-5p;hsa-let-7b-5p;hsa-let-7d-5p;hsa-let-7e-5p;hsa-l
ENSG0000	ENSG0000	0.006332	hsa-miR-17-5p;hsa-miR-20b-5p;hsa-miR-30b-3p;hsa-miR-30c-
ENSG0000	ENSG0000	0.000159	hsa-let-7a-5p;hsa-let-7b-5p;hsa-let-7d-5p;hsa-let-7e-5p;hsa-l
ENSG0000	ENSG0000	8.34E-06	hsa-let-7a-5p;hsa-let-7b-5p;hsa-let-7d-5p;hsa-let-7e-5p;hsa-l
ENSG0000	ENSG0000	4.48E-05	hsa-miR-2681-5p;hsa-miR-378a-3p;hsa-miR-378c;hsa-miR-378
ENSG0000	ENSG0000	0.003059	hsa-miR-17-5p;hsa-miR-181a-5p;hsa-miR-20b-5p;hsa-miR-93-
ENSG0000	ENSG0000	0.002135	hsa-let-7a-5p;hsa-let-7b-5p;hsa-let-7d-5p;hsa-let-7e-5p;hsa-l
ENSG0000	ENSG0000	0.00624	hsa-let-7a-5p;hsa-let-7b-5p;hsa-let-7d-5p;hsa-let-7e-5p;hsa-l
ENSG0000	ENSG0000	7.10E-05	hsa-let-7a-5p;hsa-let-7b-5p;hsa-let-7e-5p;hsa-let-7f-5p;hsa-le
ENSG0000	ENSG0000	0.001492	hsa-let-7a-5p;hsa-let-7b-5p;hsa-let-7d-5p;hsa-let-7e-5p;hsa-l
ENSG0000	ENSG0000	0.001492	hsa-let-7a-5p;hsa-let-7b-5p;hsa-let-7d-5p;hsa-let-7e-5p;hsa-l
ENSG0000	ENSG0000	0.008536	hsa-miR-17-5p;hsa-miR-193b-5p;hsa-miR-20b-5p;hsa-miR-52
ENSG0000	ENSG0000	0.006332	hsa-miR-1271-3p;hsa-miR-214-5p;hsa-miR-30b-3p;hsa-miR-3
ENSG0000	ENSG0000	1.23E-06	hsa-let-7d-5p;hsa-let-7e-5p;hsa-let-7f-5p;hsa-let-7g-5p;hsa-le
ENSG0000	ENSG0000	2.36E-09	hsa-let-7a-5p;hsa-let-7b-5p;hsa-let-7d-5p;hsa-let-7e-5p;hsa-l
ENSG0000	ENSG0000	9.72E-05	hsa-let-7a-5p;hsa-let-7b-5p;hsa-let-7e-5p;hsa-let-7f-5p;hsa-le
ENSG0000	ENSG0000	0.008573	hsa-miR-17-5p;hsa-miR-20b-5p;hsa-miR-520a-3p;hsa-miR-52
ENSG0000	ENSG0000	0.000551	hsa-let-7b-5p;hsa-let-7d-5p;hsa-let-7e-5p;hsa-let-7f-5p;hsa-le
ENSG0000	ENSG0000	2.22E-07	hsa-let-7a-5p;hsa-let-7b-5p;hsa-let-7d-5p;hsa-let-7e-5p;hsa-l
ENSG0000	ENSG0000	0.000341	hsa-miR-17-5p;hsa-miR-20b-5p;hsa-miR-30b-3p;hsa-miR-30c-
ENSG0000	ENSG0000	0.000341	hsa-miR-17-5p;hsa-miR-20b-5p;hsa-miR-30b-3p;hsa-miR-30c-
ENSG0000	ENSG0000	0.000341	hsa-miR-17-5p;hsa-miR-20b-5p;hsa-miR-30b-3p;hsa-miR-30c-
ENSG0000	ENSG0000	0.000341	hsa-miR-17-5p;hsa-miR-20b-5p;hsa-miR-30b-3p;hsa-miR-30c-
ENSG0000	ENSG0000	0.000341	hsa-miR-17-5p;hsa-miR-20b-5p;hsa-miR-30b-3p;hsa-miR-30c-
ENSG0000	ENSG0000	0.000341	hsa-miR-17-5p;hsa-miR-20b-5p;hsa-miR-30b-3p;hsa-miR-30c-
ENSG0000	ENSG0000	0.000341	hsa-miR-17-5p;hsa-miR-20b-5p;hsa-miR-30b-3p;hsa-miR-30c-
ENSG0000	ENSG0000	0.00692	hsa-miR-17-5p;hsa-miR-20b-5p;hsa-miR-30c-1-3p;hsa-miR-3
ENSG0000	ENSG0000	0.000341	hsa-miR-193b-5p;hsa-miR-30b-3p;hsa-miR-30c-1-3p;hsa-miR-
ENSG0000	ENSG0000	0.009494	hsa-miR-17-5p;hsa-miR-20b-5p;hsa-miR-520a-3p;hsa-miR-52
ENSG0000	ENSG0000	2.84E-06	hsa-let-7a-5p;hsa-let-7b-5p;hsa-let-7d-5p;hsa-let-7e-5p;hsa-l
ENSG0000	ENSG0000	2.84E-06	hsa-let-7a-5p;hsa-let-7b-5p;hsa-let-7d-5p;hsa-let-7e-5p;hsa-l
ENSG0000	ENSG0000	0.008542	hsa-miR-130a-3p;hsa-miR-17-5p;hsa-miR-19a-5p;hsa-miR-19
ENSG0000	ENSG0000	0.000476	hsa-let-7a-5p;hsa-let-7b-5p;hsa-let-7d-5p;hsa-let-7e-5p;hsa-l
ENSG0000	ENSG0000	0.000145	hsa-let-7a-5p;hsa-let-7b-5p;hsa-let-7d-5p;hsa-let-7e-5p;hsa-l
ENSG0000	ENSG0000	0.002878	hsa-miR-19a-5p;hsa-miR-19b-1-5p;hsa-miR-450b-5p;hsa-miR-
ENSG0000	ENSG0000	0.007081	hsa-miR-17-5p;hsa-miR-20b-5p;hsa-miR-642b-3p;hsa-miR-93
ENSG0000	ENSG0000	0.001994	hsa-let-7a-5p;hsa-let-7b-5p;hsa-let-7d-5p;hsa-let-7e-5p;hsa-l
ENSG0000	ENSG0000	0.002076	hsa-let-7a-5p;hsa-let-7b-5p;hsa-let-7d-5p;hsa-let-7e-5p;hsa-l
ENSG0000	ENSG0000	1.24E-08	hsa-let-7a-5p;hsa-let-7b-5p;hsa-let-7d-5p;hsa-let-7e-5p;hsa-l
ENSG0000	ENSG0000	0.001994	hsa-miR-130a-3p;hsa-miR-17-5p;hsa-miR-181a-5p;hsa-miR-2
ENSG0000	ENSG0000	0.006986	hsa-miR-17-5p;hsa-miR-192-5p;hsa-miR-20b-5p;hsa-miR-520
ENSG0000	ENSG0000	0.006986	hsa-miR-17-5p;hsa-miR-192-5p;hsa-miR-20b-5p;hsa-miR-520
ENSG0000	ENSG0000	0.000601	hsa-miR-17-5p;hsa-miR-20b-5p;hsa-miR-520a-3p;hsa-miR-52
ENSG0000	ENSG0000	0.00817	hsa-miR-378a-3p;hsa-miR-378c;hsa-miR-378d;hsa-miR-378i
ENSG0000	ENSG0000	0.003862	hsa-let-7a-5p;hsa-let-7b-5p;hsa-let-7d-5p;hsa-let-7e-5p;hsa-l
ENSG0000	ENSG0000	9.72E-05	hsa-let-7a-5p;hsa-let-7b-5p;hsa-let-7d-5p;hsa-let-7e-5p;hsa-l
ENSG0000	ENSG0000	0.006382	hsa-miR-130a-3p;hsa-miR-148a-3p;hsa-miR-148b-3p;hsa-miR-
ENSG0000	ENSG0000	0.003002	hsa-miR-17-5p;hsa-miR-20b-5p;hsa-miR-30b-3p;hsa-miR-30c-
ENSG0000	ENSG0000	0.006183	hsa-miR-17-5p;hsa-miR-20b-5p;hsa-miR-221-5p;hsa-miR-339-

ENSG0000	ENSG0000	0.006332	hsa-miR-17-5p;hsa-miR-18a-3p;hsa-miR-1913;hsa-miR-20b-5
ENSG0000	ENSG0000	0.002794	hsa-miR-17-5p;hsa-miR-193b-5p;hsa-miR-20b-5p;hsa-miR-30
ENSG0000	ENSG0000	0.001619	hsa-miR-17-5p;hsa-miR-20b-5p;hsa-miR-501-3p;hsa-miR-502
ENSG0000	ENSG0000	1.94E-07	hsa-let-7a-5p;hsa-let-7b-5p;hsa-let-7d-5p;hsa-let-7e-5p;hsa-l
ENSG0000	ENSG0000	0.000534	hsa-miR-17-5p;hsa-miR-20b-5p;hsa-miR-30c-1-3p;hsa-miR-3
ENSG0000	ENSG0000	0.000458	hsa-let-7a-5p;hsa-let-7b-5p;hsa-let-7d-5p;hsa-let-7e-5p;hsa-l
ENSG0000	ENSG0000	0.006838	hsa-let-7a-5p;hsa-let-7d-5p;hsa-let-7e-5p;hsa-let-7f-5p;hsa-le
ENSG0000	ENSG0000	7.92E-06	hsa-let-7a-5p;hsa-let-7b-5p;hsa-let-7d-5p;hsa-let-7e-5p;hsa-l
ENSG0000	ENSG0000	5.37E-05	hsa-let-7a-5p;hsa-let-7b-5p;hsa-let-7d-5p;hsa-let-7e-5p;hsa-l
ENSG0000	ENSG0000	7.35E-07	hsa-let-7a-5p;hsa-let-7b-5p;hsa-let-7d-5p;hsa-let-7e-5p;hsa-l
ENSG0000	ENSG0000	0.000482	hsa-let-7a-5p;hsa-let-7b-5p;hsa-let-7d-5p;hsa-let-7e-5p;hsa-l
ENSG0000	ENSG0000	3.94E-05	hsa-let-7a-5p;hsa-let-7b-5p;hsa-let-7d-5p;hsa-let-7e-5p;hsa-l
ENSG0000	ENSG0000	0.000483	hsa-miR-17-5p;hsa-miR-20b-5p;hsa-miR-214-5p;hsa-miR-485
ENSG0000	ENSG0000	0.002831	hsa-miR-17-5p;hsa-miR-20b-5p;hsa-miR-500a-3p;hsa-miR-52
ENSG0000	ENSG0000	0.000476	hsa-let-7a-5p;hsa-let-7b-5p;hsa-let-7d-5p;hsa-let-7e-5p;hsa-l
ENSG0000	ENSG0000	3.10E-06	hsa-let-7a-5p;hsa-let-7b-5p;hsa-let-7d-5p;hsa-let-7e-5p;hsa-l
ENSG0000	ENSG0000	0.00268	hsa-let-7a-5p;hsa-let-7b-5p;hsa-let-7e-5p;hsa-let-7f-5p;hsa-le
ENSG0000	ENSG0000	0.000649	hsa-miR-1254;hsa-miR-1271-3p;hsa-miR-17-5p;hsa-miR-20b-
ENSG0000	ENSG0000	2.90E-07	hsa-let-7a-5p;hsa-let-7b-5p;hsa-let-7d-5p;hsa-let-7e-5p;hsa-l
ENSG0000	ENSG0000	0.001265	hsa-miR-1254;hsa-miR-1271-3p;hsa-miR-550a-3-5p;hsa-miR-
ENSG0000	ENSG0000	0.007081	hsa-miR-1254;hsa-miR-1271-3p;hsa-miR-550a-3-5p;hsa-miR-
ENSG0000	ENSG0000	0.000547	hsa-let-7a-5p;hsa-let-7b-5p;hsa-let-7d-5p;hsa-let-7e-5p;hsa-l
ENSG0000	ENSG0000	0.000977	hsa-let-7a-5p;hsa-let-7b-5p;hsa-let-7d-5p;hsa-let-7e-5p;hsa-l
ENSG0000	ENSG0000	0.000172	hsa-miR-17-5p;hsa-miR-18a-3p;hsa-miR-1913;hsa-miR-193b-
ENSG0000	ENSG0000	0.005135	hsa-let-7a-5p;hsa-let-7b-5p;hsa-let-7d-5p;hsa-let-7e-5p;hsa-l
ENSG0000	ENSG0000	1.99E-06	hsa-let-7a-5p;hsa-let-7b-5p;hsa-let-7d-5p;hsa-let-7e-5p;hsa-l
ENSG0000	ENSG0000	3.59E-06	hsa-let-7a-5p;hsa-let-7b-5p;hsa-let-7d-5p;hsa-let-7e-5p;hsa-l
ENSG0000	ENSG0000	0.00063	hsa-miR-17-5p;hsa-miR-18a-3p;hsa-miR-1913;hsa-miR-20b-5
ENSG0000	ENSG0000	3.22E-05	hsa-let-7a-5p;hsa-let-7b-5p;hsa-let-7d-5p;hsa-let-7e-5p;hsa-l
ENSG0000	ENSG0000	5.96E-06	hsa-let-7a-5p;hsa-let-7b-5p;hsa-let-7d-5p;hsa-let-7e-5p;hsa-l
ENSG0000	ENSG0000	1.77E-05	hsa-let-7a-5p;hsa-let-7b-5p;hsa-let-7e-5p;hsa-let-7f-5p;hsa-le
ENSG0000	ENSG0000	0.003535	hsa-let-7a-5p;hsa-let-7b-5p;hsa-let-7d-5p;hsa-let-7e-5p;hsa-l
ENSG0000	ENSG0000	2.40E-05	hsa-let-7a-5p;hsa-let-7b-5p;hsa-let-7d-5p;hsa-let-7e-5p;hsa-l
ENSG0000	ENSG0000	0.009494	hsa-miR-17-5p;hsa-miR-20b-5p;hsa-miR-520a-3p;hsa-miR-52
ENSG0000	ENSG0000	0.001791	hsa-miR-17-5p;hsa-miR-20b-5p;hsa-miR-500a-3p;hsa-miR-50
ENSG0000	ENSG0000	0.000245	hsa-let-7a-5p;hsa-let-7b-5p;hsa-let-7d-5p;hsa-let-7e-5p;hsa-l
ENSG0000	ENSG0000	0.000483	hsa-miR-125b-5p;hsa-miR-130a-3p;hsa-miR-148a-3p;hsa-miR
ENSG0000	ENSG0000	0.00983	hsa-miR-17-5p;hsa-miR-20b-5p;hsa-miR-520a-3p;hsa-miR-52
ENSG0000	ENSG0000	2.26E-07	hsa-let-7a-5p;hsa-let-7b-5p;hsa-let-7d-5p;hsa-let-7e-5p;hsa-l
ENSG0000	ENSG0000	0.003919	hsa-miR-17-5p;hsa-miR-20b-5p;hsa-miR-2681-5p;hsa-miR-52
ENSG0000	ENSG0000	0.003309	hsa-miR-17-5p;hsa-miR-20b-5p;hsa-miR-520a-3p;hsa-miR-52
ENSG0000	ENSG0000	0.000156	hsa-miR-1271-3p;hsa-miR-17-5p;hsa-miR-18a-3p;hsa-miR-19
ENSG0000	ENSG0000	0.000156	hsa-miR-1271-3p;hsa-miR-17-5p;hsa-miR-18a-3p;hsa-miR-19
ENSG0000	ENSG0000	0.000579	hsa-let-7a-5p;hsa-let-7b-5p;hsa-let-7d-5p;hsa-let-7e-5p;hsa-l
ENSG0000	ENSG0000	2.75E-08	hsa-let-7a-5p;hsa-let-7b-5p;hsa-let-7d-5p;hsa-let-7e-5p;hsa-l
ENSG0000	ENSG0000	9.07E-07	hsa-let-7a-5p;hsa-let-7b-5p;hsa-let-7d-5p;hsa-let-7e-5p;hsa-l
ENSG0000	ENSG0000	8.76E-05	hsa-let-7a-5p;hsa-let-7b-5p;hsa-let-7d-5p;hsa-let-7e-5p;hsa-l
ENSG0000	ENSG0000	0.001799	hsa-miR-19a-5p;hsa-miR-19b-1-5p;hsa-miR-3619-5p;hsa-miR
ENSG0000	ENSG0000	0.007715	hsa-miR-214-5p;hsa-miR-30b-3p;hsa-miR-30c-1-3p;hsa-miR-
ENSG0000	ENSG0000	0.004343	hsa-miR-1254;hsa-miR-17-5p;hsa-miR-20b-5p;hsa-miR-520a-
ENSG0000	ENSG0000	0.006382	hsa-miR-17-5p;hsa-miR-193b-5p;hsa-miR-20b-5p;hsa-miR-52
ENSG0000	ENSG0000	0.004818	hsa-miR-17-5p;hsa-miR-20b-5p;hsa-miR-378a-5p;hsa-miR-48
ENSG0000	ENSG0000	0.000972	hsa-miR-1271-3p;hsa-miR-33b-3p;hsa-miR-550a-3-5p;hsa-mi
ENSG0000	ENSG0000	0.001655	hsa-miR-17-5p;hsa-miR-20b-5p;hsa-miR-5006-3p;hsa-miR-52

ENSG0000	ENSG0000	0.000731	hsa-miR-17-5p;hsa-miR-181a-5p;hsa-miR-20b-5p;hsa-miR-93-
ENSG0000	ENSG0000	0.005819	hsa-let-7a-5p;hsa-let-7b-5p;hsa-let-7d-5p;hsa-let-7e-5p;hsa-l
ENSG0000	ENSG0000	6.60E-06	hsa-let-7a-5p;hsa-let-7b-5p;hsa-let-7d-5p;hsa-let-7e-5p;hsa-l
ENSG0000	ENSG0000	2.17E-05	hsa-miR-1271-3p;hsa-miR-17-5p;hsa-miR-20b-5p;hsa-miR-21
ENSG0000	ENSG0000	0.009208	hsa-miR-1254;hsa-miR-17-5p;hsa-miR-20b-5p;hsa-miR-22-3p;
ENSG0000	ENSG0000	4.48E-05	hsa-miR-1254;hsa-miR-1271-3p;hsa-miR-4254;hsa-miR-550a-
ENSG0000	ENSG0000	0.002205	hsa-let-7a-5p;hsa-let-7b-5p;hsa-let-7d-5p;hsa-let-7e-5p;hsa-l
ENSG0000	ENSG0000	0.006093	hsa-miR-193b-5p;hsa-miR-450b-5p;hsa-miR-483-5p;hsa-miR-
ENSG0000	ENSG0000	5.21E-06	hsa-let-7a-5p;hsa-let-7b-5p;hsa-let-7d-5p;hsa-let-7e-5p;hsa-l
ENSG0000	ENSG0000	0.001057	hsa-let-7a-5p;hsa-let-7b-5p;hsa-let-7d-5p;hsa-let-7e-5p;hsa-l
ENSG0000	ENSG0000	3.98E-06	hsa-let-7a-5p;hsa-let-7b-5p;hsa-let-7d-5p;hsa-let-7e-5p;hsa-l
ENSG0000	ENSG0000	0.001551	hsa-miR-17-5p;hsa-miR-20b-5p;hsa-miR-520a-3p;hsa-miR-52
ENSG0000	ENSG0000	0.000904	hsa-miR-361-3p;hsa-miR-378a-3p;hsa-miR-378c;hsa-miR-378
ENSG0000	ENSG0000	0.000904	hsa-miR-1254;hsa-miR-1271-3p;hsa-miR-545-3p;hsa-miR-550
ENSG0000	ENSG0000	0.003527	hsa-miR-17-5p;hsa-miR-20b-5p;hsa-miR-5006-3p;hsa-miR-52
ENSG0000	ENSG0000	2.90E-05	hsa-let-7a-5p;hsa-let-7b-5p;hsa-let-7d-5p;hsa-let-7e-5p;hsa-l
ENSG0000	ENSG0000	0.000706	hsa-let-7a-5p;hsa-let-7b-5p;hsa-let-7d-5p;hsa-let-7e-5p;hsa-l
ENSG0000	ENSG0000	0.000119	hsa-let-7a-5p;hsa-let-7b-5p;hsa-let-7d-5p;hsa-let-7e-5p;hsa-l
ENSG0000	ENSG0000	5.82E-07	hsa-let-7a-5p;hsa-let-7b-5p;hsa-let-7d-5p;hsa-let-7e-5p;hsa-l
ENSG0000	ENSG0000	0.000535	hsa-miR-17-5p;hsa-miR-181a-5p;hsa-miR-20b-5p;hsa-miR-93-
ENSG0000	ENSG0000	4.68E-06	hsa-let-7a-5p;hsa-let-7b-5p;hsa-let-7d-5p;hsa-let-7e-5p;hsa-l
ENSG0000	ENSG0000	7.35E-05	hsa-let-7a-5p;hsa-let-7b-5p;hsa-let-7d-5p;hsa-let-7e-5p;hsa-l
ENSG0000	ENSG0000	5.18E-06	hsa-let-7a-5p;hsa-let-7b-5p;hsa-let-7d-5p;hsa-let-7e-5p;hsa-l
ENSG0000	ENSG0000	0.004584	hsa-miR-17-5p;hsa-miR-193b-5p;hsa-miR-20b-5p;hsa-miR-37
ENSG0000	ENSG0000	0.005201	hsa-miR-130a-3p;hsa-miR-148a-3p;hsa-miR-148b-3p;hsa-miR-
ENSG0000	ENSG0000	0.000424	hsa-let-7a-5p;hsa-let-7b-5p;hsa-let-7d-5p;hsa-let-7e-5p;hsa-l
ENSG0000	ENSG0000	0.001988	hsa-miR-125a-5p;hsa-miR-125b-5p;hsa-miR-143-3p;hsa-miR-
ENSG0000	ENSG0000	0.000129	hsa-let-7a-5p;hsa-let-7b-5p;hsa-let-7d-5p;hsa-let-7e-5p;hsa-l
ENSG0000	ENSG0000	0.000598	hsa-let-7a-5p;hsa-let-7b-5p;hsa-let-7e-5p;hsa-let-7f-5p;hsa-le
ENSG0000	ENSG0000	0.000379	hsa-miR-1254;hsa-miR-1271-3p;hsa-miR-550a-3-5p;hsa-miR-
ENSG0000	ENSG0000	3.48E-05	hsa-let-7a-5p;hsa-let-7b-5p;hsa-let-7d-5p;hsa-let-7e-5p;hsa-l
ENSG0000	ENSG0000	0.00817	hsa-miR-181a-5p;hsa-miR-19a-5p;hsa-miR-19b-1-5p;hsa-miR-
ENSG0000	ENSG0000	0.002979	hsa-miR-17-5p;hsa-miR-18a-3p;hsa-miR-1913;hsa-miR-20b-5
ENSG0000	ENSG0000	0.00488	hsa-miR-193b-3p;hsa-miR-193b-5p;hsa-miR-19a-5p;hsa-miR-
ENSG0000	ENSG0000	6.00E-08	hsa-let-7a-5p;hsa-let-7b-5p;hsa-let-7d-5p;hsa-let-7e-5p;hsa-l
ENSG0000	ENSG0000	0.000164	hsa-let-7a-5p;hsa-let-7b-5p;hsa-let-7d-5p;hsa-let-7e-5p;hsa-l
ENSG0000	ENSG0000	3.85E-07	hsa-let-7a-5p;hsa-let-7b-5p;hsa-let-7d-5p;hsa-let-7e-5p;hsa-l
ENSG0000	ENSG0000	0.003768	hsa-miR-1254;hsa-miR-17-5p;hsa-miR-20b-5p;hsa-miR-485-5
ENSG0000	ENSG0000	0.000758	hsa-miR-17-5p;hsa-miR-193b-3p;hsa-miR-20b-5p;hsa-miR-52
ENSG0000	ENSG0000	0.006675	hsa-miR-17-5p;hsa-miR-19a-5p;hsa-miR-19b-1-5p;hsa-miR-2
ENSG0000	ENSG0000	0.00014	hsa-let-7a-5p;hsa-let-7b-5p;hsa-let-7d-5p;hsa-let-7e-5p;hsa-l
ENSG0000	ENSG0000	3.09E-06	hsa-let-7a-5p;hsa-let-7b-5p;hsa-let-7d-5p;hsa-let-7e-5p;hsa-l
ENSG0000	ENSG0000	0.007373	hsa-miR-1254;hsa-miR-143-3p;hsa-miR-17-5p;hsa-miR-20b-5
ENSG0000	ENSG0000	0.007373	hsa-miR-1254;hsa-miR-143-3p;hsa-miR-17-5p;hsa-miR-20b-5
ENSG0000	ENSG0000	0.000195	hsa-let-7a-5p;hsa-let-7b-5p;hsa-let-7d-5p;hsa-let-7e-5p;hsa-l
ENSG0000	ENSG0000	4.43E-06	hsa-let-7a-5p;hsa-let-7b-5p;hsa-let-7d-5p;hsa-let-7e-5p;hsa-l
ENSG0000	ENSG0000	0.000552	hsa-miR-1254;hsa-miR-1271-3p;hsa-miR-33b-3p;hsa-miR-425
ENSG0000	ENSG0000	7.04E-05	hsa-miR-130a-3p;hsa-miR-148a-3p;hsa-miR-148b-3p;hsa-miR-
ENSG0000	ENSG0000	0.006183	hsa-miR-17-5p;hsa-miR-20b-5p;hsa-miR-520a-3p;hsa-miR-52
ENSG0000	ENSG0000	4.90E-06	hsa-miR-1271-3p;hsa-miR-196a-5p;hsa-miR-196b-5p;hsa-miR-
ENSG0000	ENSG0000	1.32E-06	hsa-let-7a-5p;hsa-let-7b-5p;hsa-let-7d-5p;hsa-let-7e-5p;hsa-l
ENSG0000	ENSG0000	0.000382	hsa-miR-19a-5p;hsa-miR-19b-1-5p;hsa-miR-421;hsa-miR-450
ENSG0000	ENSG0000	1.15E-06	hsa-let-7a-5p;hsa-let-7b-5p;hsa-let-7d-5p;hsa-let-7e-5p;hsa-l
ENSG0000	ENSG0000	5.90E-05	hsa-miR-17-5p;hsa-miR-18a-3p;hsa-miR-1913;hsa-miR-20b-5

ENSG0000	ENSG0000	0.005504	hsa-miR-1254;hsa-miR-1271-3p;hsa-miR-378a-5p;hsa-miR-55
ENSG0000	ENSG0000	0.007081	hsa-miR-1271-3p;hsa-miR-214-5p;hsa-miR-550a-3-5p;hsa-mi
ENSG0000	ENSG0000	0.008651	hsa-let-7a-5p;hsa-let-7b-5p;hsa-let-7d-5p;hsa-let-7e-5p;hsa-l
ENSG0000	ENSG0000	0.00023	hsa-miR-17-5p;hsa-miR-18a-3p;hsa-miR-1913;hsa-miR-20b-5
ENSG0000	ENSG0000	0.000688	hsa-let-7a-5p;hsa-let-7b-5p;hsa-let-7d-5p;hsa-let-7e-5p;hsa-l
ENSG0000	ENSG0000	0.004799	hsa-miR-17-5p;hsa-miR-20b-5p;hsa-miR-421;hsa-miR-520a-3
ENSG0000	ENSG0000	0.001021	hsa-miR-125a-5p;hsa-miR-125b-5p;hsa-miR-181a-5p;hsa-miR
ENSG0000	ENSG0000	0.000482	hsa-let-7a-5p;hsa-let-7b-5p;hsa-let-7d-5p;hsa-let-7e-5p;hsa-l
ENSG0000	ENSG0000	0.003784	hsa-miR-17-5p;hsa-miR-20b-5p;hsa-miR-520a-3p;hsa-miR-52
ENSG0000	ENSG0000	0.00982	hsa-miR-17-5p;hsa-miR-20b-5p;hsa-miR-500a-3p;hsa-miR-52
ENSG0000	ENSG0000	0.000252	hsa-let-7a-5p;hsa-let-7d-5p;hsa-let-7e-5p;hsa-let-7f-5p;hsa-le
ENSG0000	ENSG0000	8.18E-05	hsa-miR-1271-3p;hsa-miR-214-5p;hsa-miR-30b-3p;hsa-miR-3
ENSG0000	ENSG0000	0.00817	hsa-miR-143-3p;hsa-miR-17-5p;hsa-miR-20b-5p;hsa-miR-93-
ENSG0000	ENSG0000	0.001615	hsa-miR-125a-5p;hsa-miR-125b-5p;hsa-miR-5006-3p;hsa-miR
ENSG0000	ENSG0000	0.000743	hsa-miR-17-5p;hsa-miR-18a-3p;hsa-miR-1913;hsa-miR-20b-5
ENSG0000	ENSG0000	0.000166	hsa-let-7a-5p;hsa-let-7b-5p;hsa-let-7d-5p;hsa-let-7e-5p;hsa-l
ENSG0000	ENSG0000	3.69E-07	hsa-let-7a-5p;hsa-let-7b-5p;hsa-let-7d-5p;hsa-let-7e-5p;hsa-l
ENSG0000	ENSG0000	0.004516	hsa-miR-17-5p;hsa-miR-20b-5p;hsa-miR-30b-3p;hsa-miR-30c
ENSG0000	ENSG0000	0.005405	hsa-miR-361-3p;hsa-miR-378a-3p;hsa-miR-378c;hsa-miR-378
ENSG0000	ENSG0000	0.000598	hsa-let-7a-5p;hsa-let-7b-5p;hsa-let-7d-5p;hsa-let-7e-5p;hsa-l
ENSG0000	ENSG0000	1.97E-05	hsa-let-7a-5p;hsa-let-7b-5p;hsa-let-7d-5p;hsa-let-7e-5p;hsa-l
ENSG0000	ENSG0000	0.000398	hsa-let-7a-5p;hsa-let-7b-5p;hsa-let-7d-5p;hsa-let-7e-5p;hsa-l
ENSG0000	ENSG0000	4.59E-05	hsa-let-7a-5p;hsa-let-7b-5p;hsa-let-7d-5p;hsa-let-7e-5p;hsa-l
ENSG0000	ENSG0000	0.002943	hsa-miR-1271-3p;hsa-miR-192-5p;hsa-miR-214-5p;hsa-miR-2
ENSG0000	ENSG0000	0.000918	hsa-miR-17-5p;hsa-miR-18a-3p;hsa-miR-1913;hsa-miR-20b-5
ENSG0000	ENSG0000	0.003527	hsa-miR-17-5p;hsa-miR-20b-5p;hsa-miR-378a-5p;hsa-miR-52
ENSG0000	ENSG0000	0.009287	hsa-miR-1254;hsa-miR-1271-3p;hsa-miR-143-3p;hsa-miR-193
ENSG0000	ENSG0000	0.003688	hsa-miR-1271-3p;hsa-miR-214-5p;hsa-miR-550a-3-5p;hsa-mi
ENSG0000	ENSG0000	0.001306	hsa-miR-17-5p;hsa-miR-20b-5p;hsa-miR-520a-3p;hsa-miR-52
ENSG0000	ENSG0000	0.000462	hsa-miR-204-5p;hsa-miR-29a-3p;hsa-miR-29b-3p;hsa-miR-29
ENSG0000	ENSG0000	0.000892	hsa-miR-214-3p;hsa-miR-29a-3p;hsa-miR-29b-3p;hsa-miR-29
ENSG0000	ENSG0000	1.04E-05	hsa-miR-137;hsa-miR-29a-3p;hsa-miR-29b-3p;hsa-miR-29c-3
ENSG0000	ENSG0000	0.005768	hsa-miR-137;hsa-miR-29a-3p;hsa-miR-29b-3p;hsa-miR-29c-3
ENSG0000	ENSG0000	0.001939	hsa-miR-1185-5p;hsa-miR-1224-5p;hsa-miR-1277-5p;hsa-miR
ENSG0000	ENSG0000	0.001705	hsa-miR-1277-5p;hsa-miR-218-5p;hsa-miR-29a-3p;hsa-miR-2
ENSG0000	ENSG0000	0.004547	hsa-miR-137;hsa-miR-204-5p;hsa-miR-2355-3p;hsa-miR-5006
ENSG0000	ENSG0000	0.000707	hsa-miR-1277-5p;hsa-miR-29a-3p;hsa-miR-29b-3p;hsa-miR-3
ENSG0000	ENSG0000	0.000163	hsa-miR-29a-3p;hsa-miR-29b-3p;hsa-miR-29c-3p;hsa-miR-30
ENSG0000	ENSG0000	0.000267	hsa-miR-204-3p;hsa-miR-29a-3p;hsa-miR-29b-3p;hsa-miR-29
ENSG0000	ENSG0000	0.003953	hsa-miR-193b-3p;hsa-miR-29a-3p;hsa-miR-29b-3p;hsa-miR-2
ENSG0000	ENSG0000	5.27E-05	hsa-miR-218-5p;hsa-miR-29a-3p;hsa-miR-29b-3p;hsa-miR-29
ENSG0000	ENSG0000	2.23E-06	hsa-miR-29a-3p;hsa-miR-29b-3p;hsa-miR-29c-3p;hsa-miR-30
ENSG0000	ENSG0000	0.001757	hsa-miR-204-5p;hsa-miR-29a-3p;hsa-miR-29b-3p;hsa-miR-49
ENSG0000	ENSG0000	0.0006	hsa-miR-1277-5p;hsa-miR-204-5p;hsa-miR-218-5p;hsa-miR-5
ENSG0000	ENSG0000	5.27E-05	hsa-miR-29a-3p;hsa-miR-29b-3p;hsa-miR-29c-3p;hsa-miR-50
ENSG0000	ENSG0000	0.009222	hsa-miR-29a-3p;hsa-miR-29b-3p;hsa-miR-29c-3p;hsa-miR-30
ENSG0000	ENSG0000	0.009093	hsa-miR-214-3p;hsa-miR-29a-3p;hsa-miR-29b-3p;hsa-miR-29
ENSG0000	ENSG0000	3.37E-06	hsa-miR-137;hsa-miR-149-5p;hsa-miR-204-5p;hsa-miR-218-5
ENSG0000	ENSG0000	4.45E-06	hsa-miR-29a-3p;hsa-miR-29b-3p;hsa-miR-29c-3p;hsa-miR-30
ENSG0000	ENSG0000	4.45E-06	hsa-miR-29a-3p;hsa-miR-29b-3p;hsa-miR-29c-3p;hsa-miR-30
ENSG0000	ENSG0000	0.000384	hsa-miR-1185-5p;hsa-miR-214-3p;hsa-miR-3605-5p;hsa-miR-
ENSG0000	ENSG0000	0.007139	hsa-miR-1224-5p;hsa-miR-214-3p;hsa-miR-218-5p;hsa-miR-3
ENSG0000	ENSG0000	4.45E-06	hsa-miR-1185-5p;hsa-miR-1224-5p;hsa-miR-218-5p;hsa-miR-
ENSG0000	ENSG0000	0.003874	hsa-miR-1277-5p;hsa-miR-218-5p;hsa-miR-29a-3p;hsa-miR-2

ENSG0000	ENSG0000	2.48E-10	hsa-miR-1185-5p;hsa-miR-1224-5p;hsa-miR-218-5p;hsa-miR-2
ENSG0000	ENSG0000	0.000536	hsa-miR-1185-5p;hsa-miR-1224-5p;hsa-miR-204-5p;hsa-miR-5
ENSG0000	ENSG0000	9.55E-07	hsa-miR-218-5p;hsa-miR-29a-3p;hsa-miR-29b-3p;hsa-miR-29c
ENSG0000	ENSG0000	0.000206	hsa-miR-29a-3p;hsa-miR-29b-3p;hsa-miR-29c-3p;hsa-miR-360
ENSG0000	ENSG0000	1.63E-05	hsa-miR-137;hsa-miR-29a-3p;hsa-miR-29b-3p;hsa-miR-29c-3p
ENSG0000	ENSG0000	0.001274	hsa-miR-29a-3p;hsa-miR-29b-3p;hsa-miR-29c-3p;hsa-miR-306
ENSG0000	ENSG0000	0.001561	hsa-miR-204-5p;hsa-miR-29a-3p;hsa-miR-29b-3p;hsa-miR-29c
ENSG0000	ENSG0000	9.55E-07	hsa-miR-29a-3p;hsa-miR-29b-3p;hsa-miR-29c-3p;hsa-miR-306
ENSG0000	ENSG0000	0.000285	hsa-miR-29a-3p;hsa-miR-29b-3p;hsa-miR-29c-3p;hsa-miR-306
ENSG0000	ENSG0000	2.23E-06	hsa-miR-218-5p;hsa-miR-29a-3p;hsa-miR-29b-3p;hsa-miR-29c
ENSG0000	ENSG0000	7.22E-05	hsa-miR-29a-3p;hsa-miR-29b-3p;hsa-miR-29c-3p;hsa-miR-306
ENSG0000	ENSG0000	4.45E-05	hsa-miR-29a-3p;hsa-miR-29b-3p;hsa-miR-29c-3p;hsa-miR-306
ENSG0000	ENSG0000	0.0006	hsa-miR-29a-3p;hsa-miR-29b-3p;hsa-miR-29c-3p;hsa-miR-488
ENSG0000	ENSG0000	0.000422	hsa-miR-2355-3p;hsa-miR-29a-3p;hsa-miR-29b-3p;hsa-miR-29
ENSG0000	ENSG0000	0.005915	hsa-miR-1224-5p;hsa-miR-193b-3p;hsa-miR-204-5p;hsa-miR-2
ENSG0000	ENSG0000	0.000384	hsa-miR-29a-3p;hsa-miR-29b-3p;hsa-miR-29c-3p;hsa-miR-306
ENSG0000	ENSG0000	0.006426	hsa-miR-204-5p;hsa-miR-29a-3p;hsa-miR-29b-3p;hsa-miR-29c
ENSG0000	ENSG0000	0.001033	hsa-miR-29a-3p;hsa-miR-29b-3p;hsa-miR-29c-3p;hsa-miR-497
ENSG0000	ENSG0000	0.000183	hsa-miR-1224-5p;hsa-miR-29a-3p;hsa-miR-29b-3p;hsa-miR-29
ENSG0000	ENSG0000	0.009093	hsa-miR-29a-3p;hsa-miR-29b-3p;hsa-miR-29c-3p;hsa-miR-306
ENSG0000	ENSG0000	0.000735	hsa-miR-1224-5p;hsa-miR-1277-5p;hsa-miR-193b-3p;hsa-miR-
ENSG0000	ENSG0000	2.23E-06	hsa-miR-29a-3p;hsa-miR-29b-3p;hsa-miR-29c-3p;hsa-miR-306
ENSG0000	ENSG0000	0.001982	hsa-miR-204-5p;hsa-miR-29a-3p;hsa-miR-29b-3p;hsa-miR-29c
ENSG0000	ENSG0000	1.25E-05	hsa-miR-1277-5p;hsa-miR-29a-3p;hsa-miR-29b-3p;hsa-miR-29
ENSG0000	ENSG0000	0.000568	hsa-miR-149-5p;hsa-miR-29a-3p;hsa-miR-29b-3p;hsa-miR-29c
ENSG0000	ENSG0000	5.95E-06	hsa-miR-1277-5p;hsa-miR-193b-3p;hsa-miR-29a-3p;hsa-miR-2
ENSG0000	ENSG0000	1.33E-05	hsa-miR-1277-5p;hsa-miR-29a-3p;hsa-miR-29b-3p;hsa-miR-29
ENSG0000	ENSG0000	0.000505	hsa-miR-29a-3p;hsa-miR-29b-3p;hsa-miR-29c-3p;hsa-miR-501
ENSG0000	ENSG0000	6.41E-07	hsa-miR-218-5p;hsa-miR-29a-3p;hsa-miR-29b-3p;hsa-miR-29c
ENSG0000	ENSG0000	0.000163	hsa-miR-29a-3p;hsa-miR-29b-3p;hsa-miR-29c-3p;hsa-miR-306
ENSG0000	ENSG0000	0.00023	hsa-miR-29a-3p;hsa-miR-29b-3p;hsa-miR-29c-3p;hsa-miR-306
ENSG0000	ENSG0000	0.000462	hsa-miR-29a-3p;hsa-miR-29b-3p;hsa-miR-29c-3p;hsa-miR-488
ENSG0000	ENSG0000	2.80E-05	hsa-miR-204-5p;hsa-miR-29a-3p;hsa-miR-29b-3p;hsa-miR-29c
ENSG0000	ENSG0000	3.10E-05	hsa-miR-204-5p;hsa-miR-497-5p;hsa-miR-5006-3p;hsa-miR-64
ENSG0000	ENSG0000	0.000707	hsa-miR-1277-5p;hsa-miR-204-5p;hsa-miR-5006-3p;hsa-miR-5
ENSG0000	ENSG0000	0.000673	hsa-miR-1277-5p;hsa-miR-214-3p;hsa-miR-29a-3p;hsa-miR-29
ENSG0000	ENSG0000	0.004759	hsa-miR-149-5p;hsa-miR-218-5p;hsa-miR-497-5p;hsa-miR-647
ENSG0000	ENSG0000	7.22E-05	hsa-miR-29a-3p;hsa-miR-29b-3p;hsa-miR-29c-3p;hsa-miR-306
ENSG0000	ENSG0000	0.000766	hsa-miR-204-5p;hsa-miR-214-3p;hsa-miR-378g;hsa-miR-5006-
ENSG0000	ENSG0000	0.000766	hsa-miR-204-5p;hsa-miR-214-3p;hsa-miR-378g;hsa-miR-5006-
ENSG0000	ENSG0000	0.000766	hsa-miR-204-5p;hsa-miR-214-3p;hsa-miR-378g;hsa-miR-5006-
ENSG0000	ENSG0000	6.51E-07	hsa-miR-218-5p;hsa-miR-29a-3p;hsa-miR-29b-3p;hsa-miR-29c
ENSG0000	ENSG0000	6.03E-06	hsa-miR-218-5p;hsa-miR-29a-3p;hsa-miR-29b-3p;hsa-miR-29c
ENSG0000	ENSG0000	0.007352	hsa-miR-149-5p;hsa-miR-204-3p;hsa-miR-29a-3p;hsa-miR-29b
ENSG0000	ENSG0000	4.45E-05	hsa-miR-1185-5p;hsa-miR-1224-5p;hsa-miR-137;hsa-miR-3605
ENSG0000	ENSG0000	0.007139	hsa-miR-1224-5p;hsa-miR-214-3p;hsa-miR-218-5p;hsa-miR-30
ENSG0000	ENSG0000	0.00693	hsa-miR-1224-5p;hsa-miR-214-3p;hsa-miR-218-5p;hsa-miR-30
ENSG0000	ENSG0000	0.004509	hsa-miR-129-5p;hsa-miR-2681-5p;hsa-miR-3194-3p;hsa-miR-3
ENSG0000	ENSG0000	0.001382	hsa-miR-129-5p;hsa-miR-186-5p;hsa-miR-2681-5p;hsa-miR-32
ENSG0000	ENSG0000	0.00144	hsa-miR-129-5p;hsa-miR-185-5p;hsa-miR-186-5p;hsa-miR-217
ENSG0000	ENSG0000	1.17E-05	hsa-miR-137;hsa-miR-150-5p;hsa-miR-26a-5p;hsa-miR-27a-3p
ENSG0000	ENSG0000	0.001606	hsa-miR-137;hsa-miR-1-3p;hsa-miR-27a-3p;hsa-miR-27b-3p
ENSG0000	ENSG0000	0.000838	hsa-miR-155-5p;hsa-miR-27a-3p;hsa-miR-27b-3p;hsa-miR-9-5
ENSG0000	ENSG0000	0.000956	hsa-miR-126-5p;hsa-miR-1-3p;hsa-miR-150-5p;hsa-miR-320a

ENSG0000	ENSG0000	0.000124	hsa-miR-126-5p;hsa-miR-155-5p;hsa-miR-27a-3p;hsa-miR-27l
ENSG0000	ENSG0000	0.002653	hsa-let-7d-5p;hsa-miR-126-5p;hsa-miR-150-5p;hsa-miR-22-3
ENSG0000	ENSG0000	0.000248	hsa-miR-1-3p;hsa-miR-150-5p;hsa-miR-27a-3p;hsa-miR-27b-
ENSG0000	ENSG0000	0.001227	hsa-let-7d-5p;hsa-miR-155-5p;hsa-miR-27a-3p;hsa-miR-27b-
ENSG0000	ENSG0000	0.003854	hsa-let-7d-5p;hsa-miR-126-5p;hsa-miR-150-5p;hsa-miR-92a-
ENSG0000	ENSG0000	2.53E-05	hsa-miR-1-3p;hsa-miR-26a-5p;hsa-miR-27a-3p;hsa-miR-27b-
ENSG0000	ENSG0000	7.03E-07	hsa-miR-1-3p;hsa-miR-155-5p;hsa-miR-27a-3p;hsa-miR-27b-
ENSG0000	ENSG0000	0.001857	hsa-miR-126-5p;hsa-miR-150-5p;hsa-miR-27a-3p;hsa-miR-27l
ENSG0000	ENSG0000	0.000155	hsa-miR-155-5p;hsa-miR-26a-5p;hsa-miR-27a-3p;hsa-miR-27l
ENSG0000	ENSG0000	0.00138	hsa-miR-137;hsa-miR-150-5p;hsa-miR-27a-3p;hsa-miR-27b-3
ENSG0000	ENSG0000	0.002602	hsa-let-7d-5p;hsa-miR-150-5p;hsa-miR-27a-3p;hsa-miR-27b-
ENSG0000	ENSG0000	3.20E-05	hsa-miR-204-5p;hsa-miR-424-5p;hsa-miR-497-5p;hsa-miR-57
ENSG0000	ENSG0000	0.001445	hsa-miR-3199;hsa-miR-424-5p;hsa-miR-497-5p;hsa-miR-5581-
ENSG0000	ENSG0000	5.19E-05	hsa-miR-140-5p;hsa-miR-204-5p;hsa-miR-424-5p;hsa-miR-49
ENSG0000	ENSG0000	7.69E-05	hsa-miR-140-5p;hsa-miR-3199;hsa-miR-424-5p;hsa-miR-497-
ENSG0000	ENSG0000	9.55E-07	hsa-miR-148a-3p;hsa-miR-301a-3p;hsa-miR-301b-3p;hsa-miR-
ENSG0000	ENSG0000	3.54E-08	hsa-miR-130a-3p;hsa-miR-130b-3p;hsa-miR-301a-3p;hsa-miR-
ENSG0000	ENSG0000	1.53E-06	hsa-miR-130a-3p;hsa-miR-130b-3p;hsa-miR-301a-3p;hsa-miR-
ENSG0000	ENSG0000	6.88E-08	hsa-miR-130a-3p;hsa-miR-130b-3p;hsa-miR-301a-3p;hsa-miR-
ENSG0000	ENSG0000	0.002862	hsa-miR-130b-3p;hsa-miR-301a-3p;hsa-miR-301b-3p;hsa-miR
ENSG0000	ENSG0000	3.81E-06	hsa-miR-148a-3p;hsa-miR-148b-3p;hsa-miR-152-3p;hsa-miR-
ENSG0000	ENSG0000	0.000119	hsa-miR-130a-3p;hsa-miR-301a-3p;hsa-miR-301b-3p;hsa-miR-
ENSG0000	ENSG0000	1.41E-07	hsa-miR-130a-3p;hsa-miR-130b-3p;hsa-miR-301a-3p;hsa-miR-
ENSG0000	ENSG0000	7.19E-12	hsa-miR-130a-3p;hsa-miR-130b-3p;hsa-miR-148a-3p;hsa-miR-
ENSG0000	ENSG0000	4.85E-06	hsa-miR-130a-3p;hsa-miR-130b-3p;hsa-miR-301a-3p;hsa-miR-
ENSG0000	ENSG0000	8.31E-07	hsa-miR-130a-3p;hsa-miR-130b-3p;hsa-miR-301a-3p;hsa-miR-
ENSG0000	ENSG0000	1.18E-12	hsa-miR-130a-3p;hsa-miR-148a-3p;hsa-miR-148b-3p;hsa-miR-
ENSG0000	ENSG0000	7.90E-06	hsa-miR-130a-3p;hsa-miR-130b-3p;hsa-miR-301a-3p;hsa-miR-
ENSG0000	ENSG0000	1.79E-06	hsa-miR-130a-3p;hsa-miR-130b-3p;hsa-miR-301a-3p;hsa-miR-
ENSG0000	ENSG0000	4.22E-08	hsa-miR-130a-3p;hsa-miR-130b-3p;hsa-miR-301a-3p;hsa-miR-
ENSG0000	ENSG0000	4.60E-07	hsa-miR-130a-3p;hsa-miR-130b-3p;hsa-miR-301a-3p;hsa-miR-
ENSG0000	ENSG0000	8.56E-11	hsa-miR-130a-3p;hsa-miR-130b-3p;hsa-miR-148a-3p;hsa-miR-
ENSG0000	ENSG0000	3.71E-07	hsa-miR-130a-3p;hsa-miR-130b-3p;hsa-miR-301a-3p;hsa-miR-
ENSG0000	ENSG0000	1.44E-10	hsa-miR-130a-3p;hsa-miR-130b-3p;hsa-miR-148a-3p;hsa-miR-
ENSG0000	ENSG0000	2.34E-07	hsa-miR-130a-3p;hsa-miR-130b-3p;hsa-miR-301a-3p;hsa-miR-
ENSG0000	ENSG0000	2.17E-09	hsa-miR-130a-3p;hsa-miR-130b-3p;hsa-miR-301a-3p;hsa-miR-
ENSG0000	ENSG0000	1.39E-08	hsa-miR-130a-3p;hsa-miR-148a-3p;hsa-miR-148b-3p;hsa-miR-
ENSG0000	ENSG0000	7.03E-06	hsa-miR-130a-3p;hsa-miR-130b-3p;hsa-miR-301a-3p;hsa-miR-
ENSG0000	ENSG0000	7.56E-07	hsa-miR-130a-3p;hsa-miR-130b-3p;hsa-miR-301a-3p;hsa-miR-
ENSG0000	ENSG0000	5.86E-06	hsa-miR-130a-3p;hsa-miR-130b-3p;hsa-miR-301a-3p;hsa-miR-
ENSG0000	ENSG0000	2.62E-06	hsa-miR-130a-3p;hsa-miR-130b-3p;hsa-miR-301a-3p;hsa-miR-
ENSG0000	ENSG0000	4.85E-06	hsa-miR-130a-3p;hsa-miR-130b-3p;hsa-miR-301a-3p;hsa-miR-
ENSG0000	ENSG0000	1.07E-07	hsa-miR-130a-3p;hsa-miR-130b-3p;hsa-miR-301a-3p;hsa-miR-
ENSG0000	ENSG0000	5.10E-07	hsa-miR-130a-3p;hsa-miR-130b-3p;hsa-miR-301a-3p;hsa-miR-
ENSG0000	ENSG0000	6.24E-07	hsa-miR-130a-3p;hsa-miR-130b-3p;hsa-miR-301a-3p;hsa-miR-
ENSG0000	ENSG0000	5.87E-08	hsa-miR-130a-3p;hsa-miR-130b-3p;hsa-miR-301a-3p;hsa-miR-
ENSG0000	ENSG0000	6.24E-07	hsa-miR-130a-3p;hsa-miR-130b-3p;hsa-miR-301a-3p;hsa-miR-
ENSG0000	ENSG0000	1.54E-05	hsa-miR-130a-3p;hsa-miR-301a-3p;hsa-miR-301b-3p;hsa-miR-
ENSG0000	ENSG0000	4.55E-06	hsa-miR-130a-3p;hsa-miR-130b-3p;hsa-miR-301a-3p;hsa-miR-
ENSG0000	ENSG0000	4.99E-08	hsa-miR-130a-3p;hsa-miR-130b-3p;hsa-miR-301a-3p;hsa-miR-
ENSG0000	ENSG0000	1.88E-06	hsa-miR-130a-3p;hsa-miR-301a-3p;hsa-miR-301b-3p;hsa-miR-
ENSG0000	ENSG0000	1.94E-06	hsa-miR-130a-3p;hsa-miR-130b-3p;hsa-miR-301a-3p;hsa-miR-
ENSG0000	ENSG0000	6.88E-08	hsa-miR-130a-3p;hsa-miR-130b-3p;hsa-miR-301a-3p;hsa-miR-
ENSG0000	ENSG0000	3.25E-06	hsa-miR-130a-3p;hsa-miR-130b-3p;hsa-miR-301a-3p;hsa-miR-

ENSG0000	ENSG0000	1.41E-06	hsa-miR-130a-3p;hsa-miR-130b-3p;hsa-miR-301a-3p;hsa-miR-
ENSG0000	ENSG0000	2.96E-08	hsa-miR-130a-3p;hsa-miR-130b-3p;hsa-miR-301a-3p;hsa-miR-
ENSG0000	ENSG0000	7.04E-10	hsa-miR-130a-3p;hsa-miR-148a-3p;hsa-miR-148b-3p;hsa-miR-
ENSG0000	ENSG0000	3.73E-06	hsa-miR-130a-3p;hsa-miR-130b-3p;hsa-miR-301a-3p;hsa-miR-
ENSG0000	ENSG0000	1.29E-06	hsa-miR-130a-3p;hsa-miR-130b-3p;hsa-miR-301a-3p;hsa-miR-
ENSG0000	ENSG0000	1.94E-06	hsa-miR-130a-3p;hsa-miR-130b-3p;hsa-miR-301a-3p;hsa-miR-
ENSG0000	ENSG0000	1.94E-06	hsa-miR-130a-3p;hsa-miR-130b-3p;hsa-miR-301a-3p;hsa-miR-
ENSG0000	ENSG0000	6.46E-12	hsa-miR-130a-3p;hsa-miR-130b-3p;hsa-miR-148a-3p;hsa-miR-
ENSG0000	ENSG0000	1.33E-08	hsa-miR-130a-3p;hsa-miR-130b-3p;hsa-miR-301a-3p;hsa-miR-
ENSG0000	ENSG0000	2.26E-06	hsa-miR-130a-3p;hsa-miR-130b-3p;hsa-miR-301a-3p;hsa-miR-
ENSG0000	ENSG0000	3.32E-07	hsa-miR-130a-3p;hsa-miR-130b-3p;hsa-miR-301a-3p;hsa-miR-
ENSG0000	ENSG0000	3.32E-07	hsa-miR-130a-3p;hsa-miR-130b-3p;hsa-miR-301a-3p;hsa-miR-
ENSG0000	ENSG0000	2.62E-06	hsa-miR-130a-3p;hsa-miR-130b-3p;hsa-miR-301a-3p;hsa-miR-
ENSG0000	ENSG0000	1.30E-05	hsa-miR-130a-3p;hsa-miR-130b-3p;hsa-miR-301a-3p;hsa-miR-
ENSG0000	ENSG0000	1.36E-05	hsa-miR-130a-3p;hsa-miR-130b-3p;hsa-miR-301a-3p;hsa-miR-
ENSG0000	ENSG0000	0.000223	hsa-miR-130a-3p;hsa-miR-301a-3p;hsa-miR-301b-3p;hsa-miR-
ENSG0000	ENSG0000	9.88E-11	hsa-miR-130a-3p;hsa-miR-148a-3p;hsa-miR-148b-3p;hsa-miR-
ENSG0000	ENSG0000	4.22E-08	hsa-miR-130a-3p;hsa-miR-130b-3p;hsa-miR-301a-3p;hsa-miR-
ENSG0000	ENSG0000	4.14E-07	hsa-miR-130a-3p;hsa-miR-130b-3p;hsa-miR-301a-3p;hsa-miR-
ENSG0000	ENSG0000	6.88E-08	hsa-miR-130a-3p;hsa-miR-130b-3p;hsa-miR-301a-3p;hsa-miR-
ENSG0000	ENSG0000	5.87E-08	hsa-miR-130a-3p;hsa-miR-130b-3p;hsa-miR-301a-3p;hsa-miR-
ENSG0000	ENSG0000	1.61E-07	hsa-miR-130a-3p;hsa-miR-130b-3p;hsa-miR-301a-3p;hsa-miR-
ENSG0000	ENSG0000	4.26E-06	hsa-miR-130a-3p;hsa-miR-130b-3p;hsa-miR-301a-3p;hsa-miR-
ENSG0000	ENSG0000	2.00E-05	hsa-miR-130a-3p;hsa-miR-301a-3p;hsa-miR-301b-3p;hsa-miR-
ENSG0000	ENSG0000	0.000185	hsa-miR-130a-3p;hsa-miR-130b-3p;hsa-miR-301a-3p;hsa-miR-
ENSG0000	ENSG0000	2.82E-06	hsa-miR-130a-3p;hsa-miR-130b-3p;hsa-miR-301a-3p;hsa-miR-
ENSG0000	ENSG0000	2.33E-05	hsa-miR-130a-3p;hsa-miR-130b-3p;hsa-miR-301a-3p;hsa-miR-
ENSG0000	ENSG0000	4.59E-05	hsa-miR-130a-3p;hsa-miR-301a-3p;hsa-miR-301b-3p;hsa-miR-
ENSG0000	ENSG0000	6.63E-09	hsa-miR-130a-3p;hsa-miR-130b-3p;hsa-miR-301a-3p;hsa-miR-
ENSG0000	ENSG0000	1.94E-06	hsa-miR-130a-3p;hsa-miR-130b-3p;hsa-miR-301a-3p;hsa-miR-
ENSG0000	ENSG0000	6.23E-06	hsa-miR-130a-3p;hsa-miR-130b-3p;hsa-miR-301a-3p;hsa-miR-
ENSG0000	ENSG0000	6.63E-09	hsa-miR-130a-3p;hsa-miR-130b-3p;hsa-miR-301a-3p;hsa-miR-
ENSG0000	ENSG0000	4.20E-08	hsa-miR-130a-3p;hsa-miR-130b-3p;hsa-miR-301a-3p;hsa-miR-
ENSG0000	ENSG0000	1.83E-07	hsa-miR-130a-3p;hsa-miR-130b-3p;hsa-miR-301a-3p;hsa-miR-
ENSG0000	ENSG0000	6.75E-05	hsa-miR-130a-3p;hsa-miR-301a-3p;hsa-miR-301b-3p;hsa-miR-
ENSG0000	ENSG0000	0.000312	hsa-miR-130a-3p;hsa-miR-301a-3p;hsa-miR-301b-3p;hsa-miR-
ENSG0000	ENSG0000	1.57E-09	hsa-miR-130a-3p;hsa-miR-130b-3p;hsa-miR-301a-3p;hsa-miR-
ENSG0000	ENSG0000	2.97E-07	hsa-miR-130a-3p;hsa-miR-130b-3p;hsa-miR-301a-3p;hsa-miR-
ENSG0000	ENSG0000	2.97E-07	hsa-miR-130a-3p;hsa-miR-130b-3p;hsa-miR-301a-3p;hsa-miR-
ENSG0000	ENSG0000	9.97E-07	hsa-miR-130a-3p;hsa-miR-130b-3p;hsa-miR-301a-3p;hsa-miR-
ENSG0000	ENSG0000	3.73E-06	hsa-miR-130a-3p;hsa-miR-130b-3p;hsa-miR-301a-3p;hsa-miR-
ENSG0000	ENSG0000	6.23E-06	hsa-miR-130a-3p;hsa-miR-130b-3p;hsa-miR-301a-3p;hsa-miR-
ENSG0000	ENSG0000	2.10E-06	hsa-miR-130a-3p;hsa-miR-130b-3p;hsa-miR-301a-3p;hsa-miR-
ENSG0000	ENSG0000	2.19E-06	hsa-miR-130a-3p;hsa-miR-301a-3p;hsa-miR-301b-3p;hsa-miR-
ENSG0000	ENSG0000	4.99E-08	hsa-miR-130a-3p;hsa-miR-130b-3p;hsa-miR-301a-3p;hsa-miR-
ENSG0000	ENSG0000	1.83E-07	hsa-miR-130a-3p;hsa-miR-130b-3p;hsa-miR-301a-3p;hsa-miR-
ENSG0000	ENSG0000	1.84E-10	hsa-miR-130a-3p;hsa-miR-130b-3p;hsa-miR-148a-3p;hsa-miR-
ENSG0000	ENSG0000	7.56E-07	hsa-miR-130a-3p;hsa-miR-130b-3p;hsa-miR-301a-3p;hsa-miR-
ENSG0000	ENSG0000	0.000121	hsa-miR-130a-3p;hsa-miR-130b-3p;hsa-miR-301a-3p;hsa-miR-
ENSG0000	ENSG0000	3.48E-06	hsa-miR-130a-3p;hsa-miR-130b-3p;hsa-miR-301a-3p;hsa-miR-
ENSG0000	ENSG0000	1.53E-06	hsa-miR-130a-3p;hsa-miR-130b-3p;hsa-miR-301a-3p;hsa-miR-
ENSG0000	ENSG0000	2.55E-05	hsa-miR-130a-3p;hsa-miR-130b-3p;hsa-miR-301a-3p;hsa-miR-
ENSG0000	ENSG0000	2.45E-08	hsa-miR-130a-3p;hsa-miR-130b-3p;hsa-miR-301a-3p;hsa-miR-
ENSG0000	ENSG0000	1.55E-13	hsa-miR-130a-3p;hsa-miR-130b-3p;hsa-miR-148a-3p;hsa-miR-

ENSG0000	ENSG0000	2.77E-05	hsa-miR-130a-3p;hsa-miR-301a-3p;hsa-miR-301b-3p;hsa-miR-
ENSG0000	ENSG0000	1.29E-06	hsa-miR-130a-3p;hsa-miR-130b-3p;hsa-miR-301a-3p;hsa-miR-
ENSG0000	ENSG0000	9.29E-08	hsa-miR-130a-3p;hsa-miR-130b-3p;hsa-miR-301a-3p;hsa-miR-
ENSG0000	ENSG0000	0.000274	hsa-miR-130a-3p;hsa-miR-130b-3p;hsa-miR-301a-3p;hsa-miR-
ENSG0000	ENSG0000	2.45E-08	hsa-miR-130a-3p;hsa-miR-130b-3p;hsa-miR-301a-3p;hsa-miR-
ENSG0000	ENSG0000	1.23E-05	hsa-miR-130a-3p;hsa-miR-130b-3p;hsa-miR-301a-3p;hsa-miR-
ENSG0000	ENSG0000	2.19E-06	hsa-miR-148a-3p;hsa-miR-148b-3p;hsa-miR-152-3p;hsa-miR-3
ENSG0000	ENSG0000	3.97E-10	hsa-miR-130a-3p;hsa-miR-130b-3p;hsa-miR-148a-3p;hsa-miR-
ENSG0000	ENSG0000	7.03E-06	hsa-miR-130a-3p;hsa-miR-130b-3p;hsa-miR-301a-3p;hsa-miR-
ENSG0000	ENSG0000	5.10E-07	hsa-miR-130a-3p;hsa-miR-301a-3p;hsa-miR-301b-3p;hsa-miR-
ENSG0000	ENSG0000	3.24E-11	hsa-miR-130a-3p;hsa-miR-130b-3p;hsa-miR-301a-3p;hsa-miR-
ENSG0000	ENSG0000	5.65E-07	hsa-miR-130a-3p;hsa-miR-130b-3p;hsa-miR-301a-3p;hsa-miR-
ENSG0000	ENSG0000	1.79E-06	hsa-miR-130a-3p;hsa-miR-130b-3p;hsa-miR-301a-3p;hsa-miR-
ENSG0000	ENSG0000	1.79E-06	hsa-miR-130a-3p;hsa-miR-130b-3p;hsa-miR-301a-3p;hsa-miR-
ENSG0000	ENSG0000	2.60E-07	hsa-miR-130a-3p;hsa-miR-130b-3p;hsa-miR-148a-3p;hsa-miR-
ENSG0000	ENSG0000	9.41E-11	hsa-miR-130a-3p;hsa-miR-130b-3p;hsa-miR-148a-3p;hsa-miR-
ENSG0000	ENSG0000	9.29E-08	hsa-miR-130a-3p;hsa-miR-130b-3p;hsa-miR-301a-3p;hsa-miR-
ENSG0000	ENSG0000	1.47E-07	hsa-miR-130a-3p;hsa-miR-148b-3p;hsa-miR-152-3p;hsa-miR-3
ENSG0000	ENSG0000	8.01E-08	hsa-miR-130a-3p;hsa-miR-130b-3p;hsa-miR-301a-3p;hsa-miR-
ENSG0000	ENSG0000	8.01E-08	hsa-miR-130a-3p;hsa-miR-130b-3p;hsa-miR-301a-3p;hsa-miR-
ENSG0000	ENSG0000	7.56E-07	hsa-miR-130a-3p;hsa-miR-130b-3p;hsa-miR-301a-3p;hsa-miR-
ENSG0000	ENSG0000	0.000715	hsa-miR-130a-3p;hsa-miR-301a-3p;hsa-miR-301b-3p;hsa-miR-
ENSG0000	ENSG0000	3.54E-08	hsa-miR-130a-3p;hsa-miR-130b-3p;hsa-miR-301a-3p;hsa-miR-
ENSG0000	ENSG0000	9.11E-07	hsa-miR-130a-3p;hsa-miR-130b-3p;hsa-miR-301a-3p;hsa-miR-
ENSG0000	ENSG0000	2.64E-07	hsa-miR-130a-3p;hsa-miR-130b-3p;hsa-miR-301a-3p;hsa-miR-
ENSG0000	ENSG0000	1.33E-10	hsa-miR-130a-3p;hsa-miR-130b-3p;hsa-miR-148a-3p;hsa-miR-
ENSG0000	ENSG0000	1.11E-09	hsa-miR-130a-3p;hsa-miR-130b-3p;hsa-miR-301a-3p;hsa-miR-
ENSG0000	ENSG0000	2.82E-06	hsa-miR-130a-3p;hsa-miR-130b-3p;hsa-miR-301a-3p;hsa-miR-
ENSG0000	ENSG0000	2.26E-06	hsa-miR-130a-3p;hsa-miR-130b-3p;hsa-miR-301a-3p;hsa-miR-
ENSG0000	ENSG0000	2.26E-06	hsa-miR-130a-3p;hsa-miR-130b-3p;hsa-miR-301a-3p;hsa-miR-
ENSG0000	ENSG0000	5.86E-06	hsa-miR-130a-3p;hsa-miR-130b-3p;hsa-miR-301a-3p;hsa-miR-
ENSG0000	ENSG0000	1.41E-06	hsa-miR-130a-3p;hsa-miR-130b-3p;hsa-miR-301a-3p;hsa-miR-
ENSG0000	ENSG0000	1.70E-10	hsa-miR-130a-3p;hsa-miR-130b-3p;hsa-miR-148a-3p;hsa-miR-
ENSG0000	ENSG0000	1.70E-10	hsa-miR-130a-3p;hsa-miR-130b-3p;hsa-miR-148a-3p;hsa-miR-
ENSG0000	ENSG0000	6.35E-05	hsa-miR-130a-3p;hsa-miR-301a-3p;hsa-miR-301b-3p;hsa-miR-
ENSG0000	ENSG0000	2.43E-07	hsa-miR-130a-3p;hsa-miR-130b-3p;hsa-miR-301a-3p;hsa-miR-
ENSG0000	ENSG0000	2.92E-05	hsa-miR-130a-3p;hsa-miR-130b-3p;hsa-miR-301a-3p;hsa-miR-
ENSG0000	ENSG0000	1.17E-05	hsa-miR-130a-3p;hsa-miR-301a-3p;hsa-miR-301b-3p;hsa-miR-
ENSG0000	ENSG0000	6.62E-06	hsa-miR-130a-3p;hsa-miR-130b-3p;hsa-miR-301a-3p;hsa-miR-
ENSG0000	ENSG0000	6.62E-06	hsa-miR-130a-3p;hsa-miR-130b-3p;hsa-miR-301a-3p;hsa-miR-
ENSG0000	ENSG0000	6.62E-06	hsa-miR-130a-3p;hsa-miR-130b-3p;hsa-miR-301a-3p;hsa-miR-
ENSG0000	ENSG0000	6.62E-06	hsa-miR-130a-3p;hsa-miR-130b-3p;hsa-miR-301a-3p;hsa-miR-
ENSG0000	ENSG0000	4.80E-05	hsa-miR-130a-3p;hsa-miR-130b-3p;hsa-miR-301a-3p;hsa-miR-
ENSG0000	ENSG0000	4.26E-06	hsa-miR-130a-3p;hsa-miR-130b-3p;hsa-miR-301a-3p;hsa-miR-
ENSG0000	ENSG0000	8.61E-11	hsa-miR-130a-3p;hsa-miR-130b-3p;hsa-miR-148a-3p;hsa-miR-
ENSG0000	ENSG0000	5.65E-07	hsa-miR-130a-3p;hsa-miR-130b-3p;hsa-miR-301a-3p;hsa-miR-
ENSG0000	ENSG0000	8.49E-09	hsa-miR-130a-3p;hsa-miR-148b-3p;hsa-miR-152-3p;hsa-miR-3
ENSG0000	ENSG0000	4.90E-06	hsa-miR-130a-3p;hsa-miR-301a-3p;hsa-miR-301b-3p;hsa-miR-
ENSG0000	ENSG0000	1.28E-05	hsa-miR-130a-3p;hsa-miR-301a-3p;hsa-miR-301b-3p;hsa-miR-
ENSG0000	ENSG0000	1.21E-08	hsa-miR-130a-3p;hsa-miR-148a-3p;hsa-miR-148b-3p;hsa-miR-
ENSG0000	ENSG0000	2.22E-16	hsa-miR-130a-3p;hsa-miR-130b-3p;hsa-miR-148a-3p;hsa-miR-
ENSG0000	ENSG0000	2.58E-08	hsa-miR-130a-3p;hsa-miR-130b-3p;hsa-miR-301a-3p;hsa-miR-
ENSG0000	ENSG0000	2.62E-06	hsa-miR-130a-3p;hsa-miR-130b-3p;hsa-miR-301a-3p;hsa-miR-
ENSG0000	ENSG0000	7.39E-11	hsa-miR-130a-3p;hsa-miR-148a-3p;hsa-miR-148b-3p;hsa-miR-

ENSG0000	ENSG0000	5.14E-09	hsa-miR-130a-3p;hsa-miR-130b-3p;hsa-miR-301a-3p;hsa-miR-
ENSG0000	ENSG0000	7.71E-13	hsa-miR-130a-3p;hsa-miR-130b-3p;hsa-miR-148a-3p;hsa-miR-
ENSG0000	ENSG0000	1.44E-10	hsa-miR-130a-3p;hsa-miR-148b-3p;hsa-miR-152-3p;hsa-miR-
ENSG0000	ENSG0000	1.41E-07	hsa-miR-130a-3p;hsa-miR-130b-3p;hsa-miR-301a-3p;hsa-miR-
ENSG0000	ENSG0000	1.11E-05	hsa-miR-130a-3p;hsa-miR-130b-3p;hsa-miR-301a-3p;hsa-miR-
ENSG0000	ENSG0000	2.19E-06	hsa-miR-130a-3p;hsa-miR-301a-3p;hsa-miR-301b-3p;hsa-miR-
ENSG0000	ENSG0000	2.55E-05	hsa-miR-130a-3p;hsa-miR-130b-3p;hsa-miR-301a-3p;hsa-miR-
ENSG0000	ENSG0000	4.60E-07	hsa-miR-130a-3p;hsa-miR-130b-3p;hsa-miR-301a-3p;hsa-miR-
ENSG0000	ENSG0000	2.64E-07	hsa-miR-130a-3p;hsa-miR-130b-3p;hsa-miR-301a-3p;hsa-miR-
ENSG0000	ENSG0000	2.64E-07	hsa-miR-130a-3p;hsa-miR-130b-3p;hsa-miR-301a-3p;hsa-miR-
ENSG0000	ENSG0000	2.64E-07	hsa-miR-130a-3p;hsa-miR-130b-3p;hsa-miR-301a-3p;hsa-miR-
ENSG0000	ENSG0000	9.91E-06	hsa-miR-130a-3p;hsa-miR-130b-3p;hsa-miR-301a-3p;hsa-miR-
ENSG0000	ENSG0000	5.87E-08	hsa-miR-130a-3p;hsa-miR-130b-3p;hsa-miR-301a-3p;hsa-miR-
ENSG0000	ENSG0000	2.62E-06	hsa-miR-130a-3p;hsa-miR-130b-3p;hsa-miR-301a-3p;hsa-miR-
ENSG0000	ENSG0000	1.44E-05	hsa-miR-130a-3p;hsa-miR-130b-3p;hsa-miR-301a-3p;hsa-miR-
ENSG0000	ENSG0000	1.07E-07	hsa-miR-130a-3p;hsa-miR-130b-3p;hsa-miR-301a-3p;hsa-miR-
ENSG0000	ENSG0000	2.26E-06	hsa-miR-130a-3p;hsa-miR-130b-3p;hsa-miR-301a-3p;hsa-miR-
ENSG0000	ENSG0000	3.48E-06	hsa-miR-130a-3p;hsa-miR-130b-3p;hsa-miR-301a-3p;hsa-miR-
ENSG0000	ENSG0000	0.000426	hsa-miR-130a-3p;hsa-miR-301a-3p;hsa-miR-301b-3p;hsa-miR-
ENSG0000	ENSG0000	5.52E-06	hsa-miR-130a-3p;hsa-miR-301a-3p;hsa-miR-301b-3p;hsa-miR-
ENSG0000	ENSG0000	9.37E-06	hsa-miR-130a-3p;hsa-miR-130b-3p;hsa-miR-301a-3p;hsa-miR-
ENSG0000	ENSG0000	1.61E-07	hsa-miR-130a-3p;hsa-miR-130b-3p;hsa-miR-301a-3p;hsa-miR-
ENSG0000	ENSG0000	1.57E-09	hsa-miR-130a-3p;hsa-miR-130b-3p;hsa-miR-301a-3p;hsa-miR-
ENSG0000	ENSG0000	6.63E-09	hsa-miR-130a-3p;hsa-miR-130b-3p;hsa-miR-301a-3p;hsa-miR-
ENSG0000	ENSG0000	5.26E-07	hsa-miR-130a-3p;hsa-miR-301a-3p;hsa-miR-301b-3p;hsa-miR-
ENSG0000	ENSG0000	3.92E-05	hsa-miR-130a-3p;hsa-miR-130b-3p;hsa-miR-301a-3p;hsa-miR-
ENSG0000	ENSG0000	2.07E-07	hsa-miR-130a-3p;hsa-miR-130b-3p;hsa-miR-301a-3p;hsa-miR-
ENSG0000	ENSG0000	3.46E-05	hsa-miR-130a-3p;hsa-miR-130b-3p;hsa-miR-301a-3p;hsa-miR-
ENSG0000	ENSG0000	7.65E-10	hsa-miR-130a-3p;hsa-miR-130b-3p;hsa-miR-301a-3p;hsa-miR-
ENSG0000	ENSG0000	1.55E-13	hsa-miR-130a-3p;hsa-miR-130b-3p;hsa-miR-148a-3p;hsa-miR-
ENSG0000	ENSG0000	6.48E-07	hsa-miR-148a-3p;hsa-miR-148b-3p;hsa-miR-152-3p;hsa-miR-
ENSG0000	ENSG0000	1.84E-05	hsa-miR-130a-3p;hsa-miR-301a-3p;hsa-miR-301b-3p;hsa-miR-
ENSG0000	ENSG0000	2.21E-12	hsa-miR-130a-3p;hsa-miR-130b-3p;hsa-miR-148a-3p;hsa-miR-
ENSG0000	ENSG0000	1.61E-07	hsa-miR-130a-3p;hsa-miR-130b-3p;hsa-miR-301a-3p;hsa-miR-
ENSG0000	ENSG0000	1.61E-07	hsa-miR-130a-3p;hsa-miR-130b-3p;hsa-miR-301a-3p;hsa-miR-
ENSG0000	ENSG0000	1.93E-05	hsa-miR-130a-3p;hsa-miR-130b-3p;hsa-miR-301a-3p;hsa-miR-
ENSG0000	ENSG0000	1.66E-06	hsa-miR-130a-3p;hsa-miR-130b-3p;hsa-miR-301a-3p;hsa-miR-
ENSG0000	ENSG0000	1.66E-06	hsa-miR-130a-3p;hsa-miR-130b-3p;hsa-miR-301a-3p;hsa-miR-
ENSG0000	ENSG0000	4.60E-07	hsa-miR-130a-3p;hsa-miR-130b-3p;hsa-miR-301a-3p;hsa-miR-
ENSG0000	ENSG0000	1.61E-07	hsa-miR-130a-3p;hsa-miR-130b-3p;hsa-miR-301a-3p;hsa-miR-
ENSG0000	ENSG0000	9.37E-06	hsa-miR-130a-3p;hsa-miR-130b-3p;hsa-miR-301a-3p;hsa-miR-
ENSG0000	ENSG0000	1.17E-05	hsa-miR-130a-3p;hsa-miR-130b-3p;hsa-miR-301a-3p;hsa-miR-
ENSG0000	ENSG0000	5.11E-10	hsa-miR-130a-3p;hsa-miR-130b-3p;hsa-miR-301a-3p;hsa-miR-
ENSG0000	ENSG0000	4.04E-09	hsa-miR-130a-3p;hsa-miR-130b-3p;hsa-miR-148a-3p;hsa-miR-
ENSG0000	ENSG0000	3.73E-06	hsa-miR-130a-3p;hsa-miR-130b-3p;hsa-miR-301a-3p;hsa-miR-
ENSG0000	ENSG0000	8.01E-08	hsa-miR-130a-3p;hsa-miR-130b-3p;hsa-miR-301a-3p;hsa-miR-
ENSG0000	ENSG0000	6.48E-07	hsa-miR-130a-3p;hsa-miR-301a-3p;hsa-miR-301b-3p;hsa-miR-
ENSG0000	ENSG0000	1.75E-05	hsa-miR-130a-3p;hsa-miR-130b-3p;hsa-miR-301a-3p;hsa-miR-
ENSG0000	ENSG0000	8.31E-07	hsa-miR-130a-3p;hsa-miR-130b-3p;hsa-miR-301a-3p;hsa-miR-
ENSG0000	ENSG0000	1.66E-06	hsa-miR-130a-3p;hsa-miR-130b-3p;hsa-miR-301a-3p;hsa-miR-
ENSG0000	ENSG0000	1.83E-07	hsa-miR-130a-3p;hsa-miR-130b-3p;hsa-miR-301a-3p;hsa-miR-
ENSG0000	ENSG0000	1.79E-06	hsa-miR-130a-3p;hsa-miR-130b-3p;hsa-miR-301a-3p;hsa-miR-
ENSG0000	ENSG0000	1.29E-06	hsa-miR-130a-3p;hsa-miR-130b-3p;hsa-miR-301a-3p;hsa-miR-
ENSG0000	ENSG0000	0.000274	hsa-miR-130a-3p;hsa-miR-130b-3p;hsa-miR-301a-3p;hsa-miR-

ENSG0000	ENSG0000	3.03E-06	hsa-miR-130a-3p;hsa-miR-130b-3p;hsa-miR-301a-3p;hsa-miR-
ENSG0000	ENSG0000	1.11E-05	hsa-miR-130a-3p;hsa-miR-130b-3p;hsa-miR-301a-3p;hsa-miR-
ENSG0000	ENSG0000	0.000586	hsa-miR-130a-3p;hsa-miR-130b-3p;hsa-miR-301a-3p;hsa-miR-
ENSG0000	ENSG0000	3.32E-07	hsa-miR-130a-3p;hsa-miR-130b-3p;hsa-miR-301a-3p;hsa-miR-
ENSG0000	ENSG0000	7.56E-07	hsa-miR-130a-3p;hsa-miR-130b-3p;hsa-miR-301a-3p;hsa-miR-
ENSG0000	ENSG0000	0.000547	hsa-miR-130a-3p;hsa-miR-301a-3p;hsa-miR-301b-3p;hsa-miR-
ENSG0000	ENSG0000	8.46E-08	hsa-miR-130a-3p;hsa-miR-130b-3p;hsa-miR-301a-3p;hsa-miR-
ENSG0000	ENSG0000	7.56E-07	hsa-miR-130a-3p;hsa-miR-130b-3p;hsa-miR-301a-3p;hsa-miR-
ENSG0000	ENSG0000	7.00E-05	hsa-miR-130a-3p;hsa-miR-130b-3p;hsa-miR-301a-3p;hsa-miR-
ENSG0000	ENSG0000	6.27E-05	hsa-miR-130a-3p;hsa-miR-130b-3p;hsa-miR-301a-3p;hsa-miR-
ENSG0000	ENSG0000	1.30E-05	hsa-miR-130a-3p;hsa-miR-130b-3p;hsa-miR-301a-3p;hsa-miR-
ENSG0000	ENSG0000	5.11E-10	hsa-miR-130a-3p;hsa-miR-130b-3p;hsa-miR-301a-3p;hsa-miR-
ENSG0000	ENSG0000	2.82E-06	hsa-miR-130a-3p;hsa-miR-130b-3p;hsa-miR-301a-3p;hsa-miR-
ENSG0000	ENSG0000	1.09E-06	hsa-miR-130a-3p;hsa-miR-130b-3p;hsa-miR-301a-3p;hsa-miR-
ENSG0000	ENSG0000	7.00E-05	hsa-miR-130a-3p;hsa-miR-130b-3p;hsa-miR-301a-3p;hsa-miR-
ENSG0000	ENSG0000	3.48E-06	hsa-miR-130a-3p;hsa-miR-130b-3p;hsa-miR-301a-3p;hsa-miR-
ENSG0000	ENSG0000	2.03E-05	hsa-miR-130a-3p;hsa-miR-130b-3p;hsa-miR-301a-3p;hsa-miR-
ENSG0000	ENSG0000	9.04E-13	hsa-miR-130a-3p;hsa-miR-130b-3p;hsa-miR-148a-3p;hsa-miR-
ENSG0000	ENSG0000	5.86E-06	hsa-miR-130a-3p;hsa-miR-130b-3p;hsa-miR-301a-3p;hsa-miR-
ENSG0000	ENSG0000	4.60E-07	hsa-miR-130a-3p;hsa-miR-130b-3p;hsa-miR-301a-3p;hsa-miR-
ENSG0000	ENSG0000	6.88E-08	hsa-miR-130a-3p;hsa-miR-130b-3p;hsa-miR-301a-3p;hsa-miR-
ENSG0000	ENSG0000	4.43E-05	hsa-miR-130a-3p;hsa-miR-130b-3p;hsa-miR-301a-3p;hsa-miR-
ENSG0000	ENSG0000	2.02E-08	hsa-miR-130a-3p;hsa-miR-130b-3p;hsa-miR-301a-3p;hsa-miR-
ENSG0000	ENSG0000	3.22E-05	hsa-miR-130a-3p;hsa-miR-301a-3p;hsa-miR-301b-3p;hsa-miR-
ENSG0000	ENSG0000	2.55E-12	hsa-miR-130a-3p;hsa-miR-130b-3p;hsa-miR-148a-3p;hsa-miR-
ENSG0000	ENSG0000	7.46E-06	hsa-miR-130a-3p;hsa-miR-130b-3p;hsa-miR-301a-3p;hsa-miR-
ENSG0000	ENSG0000	1.12E-10	hsa-miR-130a-3p;hsa-miR-148b-3p;hsa-miR-152-3p;hsa-miR-3
ENSG0000	ENSG0000	1.84E-05	hsa-miR-130a-3p;hsa-miR-301a-3p;hsa-miR-301b-3p;hsa-miR-
ENSG0000	ENSG0000	2.67E-05	hsa-miR-130a-3p;hsa-miR-130b-3p;hsa-miR-301a-3p;hsa-miR-
ENSG0000	ENSG0000	6.62E-06	hsa-miR-130a-3p;hsa-miR-130b-3p;hsa-miR-301a-3p;hsa-miR-
ENSG0000	ENSG0000	4.26E-06	hsa-miR-130a-3p;hsa-miR-130b-3p;hsa-miR-301a-3p;hsa-miR-
ENSG0000	ENSG0000	6.27E-05	hsa-miR-130a-3p;hsa-miR-130b-3p;hsa-miR-301a-3p;hsa-miR-
ENSG0000	ENSG0000	0.001155	hsa-miR-130a-3p;hsa-miR-301a-3p;hsa-miR-301b-3p;hsa-miR-
ENSG0000	ENSG0000	5.87E-08	hsa-miR-130a-3p;hsa-miR-130b-3p;hsa-miR-301a-3p;hsa-miR-
ENSG0000	ENSG0000	9.11E-07	hsa-miR-130a-3p;hsa-miR-130b-3p;hsa-miR-301a-3p;hsa-miR-
ENSG0000	ENSG0000	3.82E-12	hsa-miR-130a-3p;hsa-miR-130b-3p;hsa-miR-148a-3p;hsa-miR-
ENSG0000	ENSG0000	1.09E-06	hsa-miR-130a-3p;hsa-miR-130b-3p;hsa-miR-301a-3p;hsa-miR-
ENSG0000	ENSG0000	2.26E-06	hsa-miR-130a-3p;hsa-miR-130b-3p;hsa-miR-301a-3p;hsa-miR-
ENSG0000	ENSG0000	1.65E-08	hsa-miR-130a-3p;hsa-miR-130b-3p;hsa-miR-301a-3p;hsa-miR-
ENSG0000	ENSG0000	1.65E-08	hsa-miR-130a-3p;hsa-miR-130b-3p;hsa-miR-301a-3p;hsa-miR-
ENSG0000	ENSG0000	2.22E-16	hsa-miR-130a-3p;hsa-miR-130b-3p;hsa-miR-148a-3p;hsa-miR-
ENSG0000	ENSG0000	1.41E-06	hsa-miR-130a-3p;hsa-miR-130b-3p;hsa-miR-301a-3p;hsa-miR-
ENSG0000	ENSG0000	6.36E-12	hsa-miR-130a-3p;hsa-miR-130b-3p;hsa-miR-148a-3p;hsa-miR-
ENSG0000	ENSG0000	0.000289	hsa-miR-130a-3p;hsa-miR-130b-3p;hsa-miR-301a-3p;hsa-miR-
ENSG0000	ENSG0000	8.86E-06	hsa-miR-130a-3p;hsa-miR-130b-3p;hsa-miR-301a-3p;hsa-miR-
ENSG0000	ENSG0000	3.54E-08	hsa-miR-130a-3p;hsa-miR-130b-3p;hsa-miR-301a-3p;hsa-miR-
ENSG0000	ENSG0000	3.54E-08	hsa-miR-130a-3p;hsa-miR-130b-3p;hsa-miR-301a-3p;hsa-miR-
ENSG0000	ENSG0000	5.24E-05	hsa-miR-130a-3p;hsa-miR-301a-3p;hsa-miR-301b-3p;hsa-miR-
ENSG0000	ENSG0000	1.41E-07	hsa-miR-130a-3p;hsa-miR-130b-3p;hsa-miR-301a-3p;hsa-miR-
ENSG0000	ENSG0000	2.92E-06	hsa-miR-130a-3p;hsa-miR-301a-3p;hsa-miR-301b-3p;hsa-miR-
ENSG0000	ENSG0000	2.96E-08	hsa-miR-130a-3p;hsa-miR-130b-3p;hsa-miR-301a-3p;hsa-miR-
ENSG0000	ENSG0000	3.54E-08	hsa-miR-130a-3p;hsa-miR-130b-3p;hsa-miR-301a-3p;hsa-miR-
ENSG0000	ENSG0000	9.11E-07	hsa-miR-130a-3p;hsa-miR-130b-3p;hsa-miR-301a-3p;hsa-miR-
ENSG0000	ENSG0000	1.83E-07	hsa-miR-130a-3p;hsa-miR-130b-3p;hsa-miR-301a-3p;hsa-miR-

ENSG0000	ENSG0000	1.33E-08	hsa-miR-130a-3p;hsa-miR-130b-3p;hsa-miR-301a-3p;hsa-miR-
ENSG0000	ENSG0000	1.11E-05	hsa-miR-130a-3p;hsa-miR-130b-3p;hsa-miR-301a-3p;hsa-miR-
ENSG0000	ENSG0000	1.61E-07	hsa-miR-130a-3p;hsa-miR-130b-3p;hsa-miR-301a-3p;hsa-miR-
ENSG0000	ENSG0000	2.02E-08	hsa-miR-130a-3p;hsa-miR-130b-3p;hsa-miR-301a-3p;hsa-miR-
ENSG0000	ENSG0000	8.31E-07	hsa-miR-130a-3p;hsa-miR-130b-3p;hsa-miR-301a-3p;hsa-miR-
ENSG0000	ENSG0000	1.66E-12	hsa-miR-130a-3p;hsa-miR-130b-3p;hsa-miR-148a-3p;hsa-miR-
ENSG0000	ENSG0000	9.29E-08	hsa-miR-130a-3p;hsa-miR-130b-3p;hsa-miR-301a-3p;hsa-miR-
ENSG0000	ENSG0000	1.75E-05	hsa-miR-130a-3p;hsa-miR-130b-3p;hsa-miR-301a-3p;hsa-miR-
ENSG0000	ENSG0000	9.97E-07	hsa-miR-130a-3p;hsa-miR-130b-3p;hsa-miR-301a-3p;hsa-miR-
ENSG0000	ENSG0000	2.96E-08	hsa-miR-130a-3p;hsa-miR-130b-3p;hsa-miR-301a-3p;hsa-miR-
ENSG0000	ENSG0000	2.96E-08	hsa-miR-130a-3p;hsa-miR-130b-3p;hsa-miR-301a-3p;hsa-miR-
ENSG0000	ENSG0000	0.002453	hsa-miR-130a-3p;hsa-miR-301a-3p;hsa-miR-301b-3p;hsa-miR-
ENSG0000	ENSG0000	2.94E-09	hsa-miR-130a-3p;hsa-miR-130b-3p;hsa-miR-301a-3p;hsa-miR-
ENSG0000	ENSG0000	1.94E-06	hsa-miR-130a-3p;hsa-miR-130b-3p;hsa-miR-301a-3p;hsa-miR-
ENSG0000	ENSG0000	3.54E-08	hsa-miR-130a-3p;hsa-miR-130b-3p;hsa-miR-301a-3p;hsa-miR-
ENSG0000	ENSG0000	4.60E-07	hsa-miR-130a-3p;hsa-miR-130b-3p;hsa-miR-301a-3p;hsa-miR-
ENSG0000	ENSG0000	2.79E-05	hsa-miR-130a-3p;hsa-miR-130b-3p;hsa-miR-301a-3p;hsa-miR-
ENSG0000	ENSG0000	0.00199	hsa-miR-130a-3p;hsa-miR-301a-3p;hsa-miR-301b-3p;hsa-miR-
ENSG0000	ENSG0000	4.44E-16	hsa-miR-130a-3p;hsa-miR-130b-3p;hsa-miR-148a-3p;hsa-miR-
ENSG0000	ENSG0000	1.66E-06	hsa-miR-130a-3p;hsa-miR-130b-3p;hsa-miR-301a-3p;hsa-miR-
ENSG0000	ENSG0000	5.17E-06	hsa-miR-130a-3p;hsa-miR-130b-3p;hsa-miR-301a-3p;hsa-miR-
ENSG0000	ENSG0000	1.36E-06	hsa-miR-130a-3p;hsa-miR-301a-3p;hsa-miR-301b-3p;hsa-miR-
ENSG0000	ENSG0000	9.97E-07	hsa-miR-130a-3p;hsa-miR-130b-3p;hsa-miR-301a-3p;hsa-miR-
ENSG0000	ENSG0000	3.73E-06	hsa-miR-130a-3p;hsa-miR-130b-3p;hsa-miR-301a-3p;hsa-miR-
ENSG0000	ENSG0000	3.32E-07	hsa-miR-130a-3p;hsa-miR-130b-3p;hsa-miR-301a-3p;hsa-miR-
ENSG0000	ENSG0000	1.14E-06	hsa-miR-130a-3p;hsa-miR-301a-3p;hsa-miR-301b-3p;hsa-miR-
ENSG0000	ENSG0000	0.000117	hsa-miR-130a-3p;hsa-miR-130b-3p;hsa-miR-301a-3p;hsa-miR-
ENSG0000	ENSG0000	3.32E-07	hsa-miR-130a-3p;hsa-miR-130b-3p;hsa-miR-301a-3p;hsa-miR-
ENSG0000	ENSG0000	1.67E-05	hsa-miR-130a-3p;hsa-miR-130b-3p;hsa-miR-301a-3p;hsa-miR-
ENSG0000	ENSG0000	7.25E-05	hsa-miR-130a-3p;hsa-miR-130b-3p;hsa-miR-301a-3p;hsa-miR-
ENSG0000	ENSG0000	1.85E-08	hsa-miR-130a-3p;hsa-miR-130b-3p;hsa-miR-148a-3p;hsa-miR-
ENSG0000	ENSG0000	7.56E-07	hsa-miR-130a-3p;hsa-miR-130b-3p;hsa-miR-301a-3p;hsa-miR-
ENSG0000	ENSG0000	2.10E-06	hsa-miR-130a-3p;hsa-miR-130b-3p;hsa-miR-301a-3p;hsa-miR-
ENSG0000	ENSG0000	9.11E-07	hsa-miR-130a-3p;hsa-miR-130b-3p;hsa-miR-301a-3p;hsa-miR-
ENSG0000	ENSG0000	1.66E-06	hsa-miR-130a-3p;hsa-miR-130b-3p;hsa-miR-301a-3p;hsa-miR-
ENSG0000	ENSG0000	4.26E-06	hsa-miR-130a-3p;hsa-miR-130b-3p;hsa-miR-301a-3p;hsa-miR-
ENSG0000	ENSG0000	1.92E-12	hsa-miR-130a-3p;hsa-miR-130b-3p;hsa-miR-148a-3p;hsa-miR-
ENSG0000	ENSG0000	1.19E-06	hsa-miR-130a-3p;hsa-miR-130b-3p;hsa-miR-301a-3p;hsa-miR-
ENSG0000	ENSG0000	2.26E-06	hsa-miR-130a-3p;hsa-miR-130b-3p;hsa-miR-301a-3p;hsa-miR-
ENSG0000	ENSG0000	9.91E-06	hsa-miR-130a-3p;hsa-miR-130b-3p;hsa-miR-301a-3p;hsa-miR-
ENSG0000	ENSG0000	2.45E-08	hsa-miR-130a-3p;hsa-miR-130b-3p;hsa-miR-301a-3p;hsa-miR-
ENSG0000	ENSG0000	0.000125	hsa-miR-130a-3p;hsa-miR-301a-3p;hsa-miR-301b-3p;hsa-miR-
ENSG0000	ENSG0000	2.94E-09	hsa-miR-130a-3p;hsa-miR-130b-3p;hsa-miR-301a-3p;hsa-miR-
ENSG0000	ENSG0000	1.09E-06	hsa-miR-130a-3p;hsa-miR-130b-3p;hsa-miR-301a-3p;hsa-miR-
ENSG0000	ENSG0000	4.14E-07	hsa-miR-130a-3p;hsa-miR-130b-3p;hsa-miR-301a-3p;hsa-miR-
ENSG0000	ENSG0000	2.45E-08	hsa-miR-130a-3p;hsa-miR-130b-3p;hsa-miR-301a-3p;hsa-miR-
ENSG0000	ENSG0000	2.45E-08	hsa-miR-130a-3p;hsa-miR-130b-3p;hsa-miR-301a-3p;hsa-miR-
ENSG0000	ENSG0000	2.45E-08	hsa-miR-130a-3p;hsa-miR-130b-3p;hsa-miR-301a-3p;hsa-miR-
ENSG0000	ENSG0000	2.45E-08	hsa-miR-130a-3p;hsa-miR-130b-3p;hsa-miR-301a-3p;hsa-miR-
ENSG0000	ENSG0000	2.02E-08	hsa-miR-130a-3p;hsa-miR-130b-3p;hsa-miR-301a-3p;hsa-miR-
ENSG0000	ENSG0000	2.95E-08	hsa-let-7a-5p;hsa-let-7b-5p;hsa-let-7d-5p;hsa-let-7e-5p;hsa-l
ENSG0000	ENSG0000	0.002911	hsa-miR-204-5p;hsa-miR-365a-3p;hsa-miR-365b-3p;hsa-miR-3
ENSG0000	ENSG0000	8.35E-07	hsa-let-7a-5p;hsa-let-7b-5p;hsa-let-7d-5p;hsa-let-7e-5p;hsa-l
ENSG0000	ENSG0000	0.000103	hsa-miR-103a-3p;hsa-miR-107;hsa-miR-188-5p;hsa-miR-204-5

ENSG0000	ENSG0000	0.005062	hsa-miR-194-3p;hsa-miR-23a-3p;hsa-miR-23b-3p;hsa-miR-23
ENSG0000	ENSG0000	6.40E-05	hsa-let-7a-5p;hsa-let-7b-5p;hsa-let-7d-5p;hsa-let-7e-5p;hsa-l
ENSG0000	ENSG0000	2.26E-07	hsa-let-7a-5p;hsa-let-7b-5p;hsa-let-7d-5p;hsa-let-7e-5p;hsa-l
ENSG0000	ENSG0000	0.000178	hsa-let-7a-5p;hsa-let-7b-5p;hsa-let-7d-5p;hsa-let-7e-5p;hsa-l
ENSG0000	ENSG0000	2.89E-05	hsa-let-7a-5p;hsa-let-7b-5p;hsa-let-7d-5p;hsa-let-7e-5p;hsa-l
ENSG0000	ENSG0000	5.82E-05	hsa-miR-27a-3p;hsa-miR-27b-3p;hsa-miR-30b-5p;hsa-miR-30
ENSG0000	ENSG0000	6.40E-05	hsa-miR-181a-5p;hsa-miR-218-5p;hsa-miR-30b-5p;hsa-miR-30
ENSG0000	ENSG0000	0.003885	hsa-miR-126-5p;hsa-miR-200b-3p;hsa-miR-200c-3p;hsa-miR-3
ENSG0000	ENSG0000	0.008158	hsa-let-7a-5p;hsa-let-7b-5p;hsa-let-7d-5p;hsa-let-7e-5p;hsa-l
ENSG0000	ENSG0000	5.61E-05	hsa-let-7a-5p;hsa-miR-181a-5p;hsa-miR-200b-3p;hsa-miR-200
ENSG0000	ENSG0000	0.003408	hsa-miR-1179;hsa-miR-181a-5p;hsa-miR-27a-3p;hsa-miR-27b-
ENSG0000	ENSG0000	5.80E-09	hsa-let-7a-5p;hsa-let-7b-5p;hsa-let-7d-5p;hsa-let-7e-5p;hsa-l
ENSG0000	ENSG0000	0.009309	hsa-miR-103a-3p;hsa-miR-107;hsa-miR-30b-5p;hsa-miR-30c-5
ENSG0000	ENSG0000	1.48E-06	hsa-let-7a-5p;hsa-let-7b-5p;hsa-let-7d-5p;hsa-let-7e-5p;hsa-l
ENSG0000	ENSG0000	8.61E-08	hsa-let-7a-5p;hsa-let-7b-5p;hsa-let-7d-5p;hsa-let-7e-5p;hsa-l
ENSG0000	ENSG0000	2.16E-06	hsa-let-7a-5p;hsa-let-7b-5p;hsa-let-7d-5p;hsa-let-7e-5p;hsa-l
ENSG0000	ENSG0000	0.005198	hsa-let-7a-5p;hsa-let-7b-5p;hsa-let-7d-5p;hsa-let-7e-5p;hsa-l
ENSG0000	ENSG0000	3.63E-09	hsa-let-7a-5p;hsa-let-7b-5p;hsa-let-7d-5p;hsa-let-7e-5p;hsa-l
ENSG0000	ENSG0000	1.18E-09	hsa-let-7a-5p;hsa-let-7d-5p;hsa-let-7e-5p;hsa-let-7f-5p;hsa-le
ENSG0000	ENSG0000	5.66E-07	hsa-let-7a-5p;hsa-let-7b-5p;hsa-let-7d-5p;hsa-let-7e-5p;hsa-l
ENSG0000	ENSG0000	5.19E-05	hsa-let-7a-5p;hsa-let-7b-5p;hsa-let-7d-5p;hsa-let-7e-5p;hsa-l
ENSG0000	ENSG0000	0.00017	hsa-miR-140-5p;hsa-miR-218-5p;hsa-miR-30c-5p;hsa-miR-377
ENSG0000	ENSG0000	0.000193	hsa-let-7a-5p;hsa-let-7e-5p;hsa-let-7f-5p;hsa-let-7g-5p;hsa-le
ENSG0000	ENSG0000	8.01E-08	hsa-let-7a-5p;hsa-let-7b-5p;hsa-let-7d-5p;hsa-let-7e-5p;hsa-l
ENSG0000	ENSG0000	8.61E-08	hsa-let-7a-5p;hsa-let-7b-5p;hsa-let-7d-5p;hsa-let-7e-5p;hsa-l
ENSG0000	ENSG0000	0.000211	hsa-let-7a-5p;hsa-let-7d-5p;hsa-let-7e-5p;hsa-let-7f-5p;hsa-le
ENSG0000	ENSG0000	2.06E-05	hsa-let-7a-5p;hsa-let-7b-5p;hsa-let-7d-5p;hsa-let-7e-5p;hsa-l
ENSG0000	ENSG0000	0.000447	hsa-miR-107;hsa-miR-181a-5p;hsa-miR-23a-3p;hsa-miR-23b-3
ENSG0000	ENSG0000	6.47E-06	hsa-let-7a-5p;hsa-let-7b-5p;hsa-let-7d-5p;hsa-let-7e-5p;hsa-l
ENSG0000	ENSG0000	0.006455	hsa-miR-181a-5p;hsa-miR-200b-3p;hsa-miR-377-3p;hsa-miR-5
ENSG0000	ENSG0000	1.19E-07	hsa-miR-218-5p;hsa-miR-23a-3p;hsa-miR-23b-3p;hsa-miR-23
ENSG0000	ENSG0000	0.000373	hsa-miR-103a-3p;hsa-miR-107;hsa-miR-204-5p;hsa-miR-374a-
ENSG0000	ENSG0000	0.003539	hsa-miR-1179;hsa-miR-218-5p;hsa-miR-23a-3p;hsa-miR-30c-5
ENSG0000	ENSG0000	2.16E-10	hsa-let-7a-5p;hsa-let-7b-5p;hsa-let-7d-5p;hsa-let-7e-5p;hsa-l
ENSG0000	ENSG0000	9.61E-07	hsa-let-7g-5p;hsa-miR-200b-3p;hsa-miR-200c-3p;hsa-miR-218
ENSG0000	ENSG0000	0.000682	hsa-let-7a-5p;hsa-let-7b-5p;hsa-let-7d-5p;hsa-let-7e-5p;hsa-l
ENSG0000	ENSG0000	9.66E-05	hsa-let-7a-5p;hsa-let-7b-5p;hsa-let-7d-5p;hsa-let-7e-5p;hsa-l
ENSG0000	ENSG0000	4.76E-07	hsa-let-7a-5p;hsa-let-7b-5p;hsa-let-7d-5p;hsa-let-7e-5p;hsa-l
ENSG0000	ENSG0000	1.01E-07	hsa-let-7a-5p;hsa-let-7b-5p;hsa-let-7d-5p;hsa-let-7e-5p;hsa-l
ENSG0000	ENSG0000	0.008978	hsa-let-7a-5p;hsa-let-7d-5p;hsa-let-7e-5p;hsa-let-7f-5p;hsa-le
ENSG0000	ENSG0000	0.001442	hsa-miR-103a-3p;hsa-miR-107;hsa-miR-23a-3p;hsa-miR-23b-3
ENSG0000	ENSG0000	2.30E-05	hsa-let-7a-5p;hsa-let-7b-5p;hsa-let-7d-5p;hsa-let-7f-5p;hsa-le
ENSG0000	ENSG0000	3.31E-10	hsa-let-7a-5p;hsa-let-7b-5p;hsa-let-7d-5p;hsa-let-7e-5p;hsa-l
ENSG0000	ENSG0000	0.007514	hsa-miR-27a-3p;hsa-miR-374a-5p;hsa-miR-377-3p;hsa-miR-50
ENSG0000	ENSG0000	3.43E-05	hsa-let-7a-5p;hsa-let-7b-5p;hsa-let-7d-5p;hsa-let-7e-5p;hsa-l
ENSG0000	ENSG0000	0.001314	hsa-let-7a-5p;hsa-miR-23a-3p;hsa-miR-30b-5p;hsa-miR-30c-5
ENSG0000	ENSG0000	9.52E-06	hsa-let-7a-5p;hsa-let-7b-5p;hsa-let-7d-5p;hsa-let-7e-5p;hsa-l
ENSG0000	ENSG0000	1.30E-08	hsa-let-7a-5p;hsa-let-7b-5p;hsa-let-7d-5p;hsa-let-7e-5p;hsa-l
ENSG0000	ENSG0000	3.24E-07	hsa-let-7d-5p;hsa-let-7f-5p;hsa-let-7g-5p;hsa-let-7i-5p
ENSG0000	ENSG0000	0.000936	hsa-let-7a-5p;hsa-let-7b-5p;hsa-let-7d-5p;hsa-let-7e-5p;hsa-l
ENSG0000	ENSG0000	6.41E-05	hsa-let-7a-5p;hsa-let-7b-5p;hsa-let-7e-5p;hsa-let-7f-5p;hsa-le
ENSG0000	ENSG0000	0.0055	hsa-miR-132-3p;hsa-miR-27b-3p;hsa-miR-30b-5p;hsa-miR-30
ENSG0000	ENSG0000	5.05E-06	hsa-miR-200b-3p;hsa-miR-200c-3p;hsa-miR-218-5p;hsa-miR-2
ENSG0000	ENSG0000	7.99E-06	hsa-let-7a-5p;hsa-let-7b-5p;hsa-let-7d-5p;hsa-let-7e-5p;hsa-l

ENSG0000	ENSG0000	0.003652	hsa-miR-132-3p;hsa-miR-27a-3p;hsa-miR-27b-3p;hsa-miR-36
ENSG0000	ENSG0000	1.37E-08	hsa-let-7a-5p;hsa-let-7b-5p;hsa-let-7d-5p;hsa-let-7e-5p;hsa-l
ENSG0000	ENSG0000	9.03E-09	hsa-let-7a-5p;hsa-let-7b-5p;hsa-let-7d-5p;hsa-let-7e-5p;hsa-l
ENSG0000	ENSG0000	8.01E-08	hsa-let-7a-5p;hsa-let-7b-5p;hsa-let-7d-5p;hsa-let-7e-5p;hsa-l
ENSG0000	ENSG0000	0.000534	hsa-miR-23a-3p;hsa-miR-23b-3p;hsa-miR-23c;hsa-miR-27a-3p
ENSG0000	ENSG0000	0.004408	hsa-let-7a-5p;hsa-let-7b-5p;hsa-let-7d-5p;hsa-let-7e-5p;hsa-l
ENSG0000	ENSG0000	5.03E-05	hsa-miR-218-5p;hsa-miR-23b-3p;hsa-miR-27b-3p;hsa-miR-42
ENSG0000	ENSG0000	5.03E-05	hsa-let-7a-5p;hsa-let-7d-5p;hsa-let-7e-5p;hsa-let-7f-5p;hsa-m
ENSG0000	ENSG0000	0.008682	hsa-let-7a-5p;hsa-let-7d-5p;hsa-let-7e-5p;hsa-let-7g-5p
ENSG0000	ENSG0000	8.00E-12	hsa-let-7a-5p;hsa-let-7b-5p;hsa-let-7d-5p;hsa-let-7e-5p;hsa-l
ENSG0000	ENSG0000	2.22E-06	hsa-miR-107;hsa-miR-27a-3p;hsa-miR-27b-3p;hsa-miR-374a-5
ENSG0000	ENSG0000	0.000728	hsa-let-7a-5p;hsa-let-7b-5p;hsa-let-7d-5p;hsa-let-7e-5p;hsa-l
ENSG0000	ENSG0000	1.99E-06	hsa-let-7a-5p;hsa-let-7b-5p;hsa-miR-181a-5p;hsa-miR-218-5p
ENSG0000	ENSG0000	1.08E-07	hsa-let-7a-5p;hsa-let-7b-5p;hsa-let-7d-5p;hsa-let-7e-5p;hsa-l
ENSG0000	ENSG0000	1.08E-07	hsa-let-7a-5p;hsa-let-7b-5p;hsa-let-7d-5p;hsa-let-7e-5p;hsa-l
ENSG0000	ENSG0000	1.08E-07	hsa-let-7a-5p;hsa-let-7b-5p;hsa-let-7d-5p;hsa-let-7e-5p;hsa-l
ENSG0000	ENSG0000	4.07E-07	hsa-let-7a-5p;hsa-let-7b-5p;hsa-let-7d-5p;hsa-let-7e-5p;hsa-l
ENSG0000	ENSG0000	0.004754	hsa-miR-200b-3p;hsa-miR-200c-3p;hsa-miR-218-5p;hsa-miR-2
ENSG0000	ENSG0000	0.001098	hsa-miR-103a-3p;hsa-miR-107;hsa-miR-218-5p;hsa-miR-30b-5
ENSG0000	ENSG0000	2.95E-08	hsa-let-7a-5p;hsa-let-7b-5p;hsa-let-7d-5p;hsa-let-7e-5p;hsa-l
ENSG0000	ENSG0000	2.95E-08	hsa-let-7a-5p;hsa-let-7b-5p;hsa-let-7d-5p;hsa-let-7e-5p;hsa-l
ENSG0000	ENSG0000	2.95E-08	hsa-let-7a-5p;hsa-let-7b-5p;hsa-let-7d-5p;hsa-let-7e-5p;hsa-l
ENSG0000	ENSG0000	2.95E-08	hsa-let-7a-5p;hsa-let-7b-5p;hsa-let-7d-5p;hsa-let-7e-5p;hsa-l
ENSG0000	ENSG0000	2.95E-08	hsa-let-7a-5p;hsa-let-7b-5p;hsa-let-7d-5p;hsa-let-7e-5p;hsa-l
ENSG0000	ENSG0000	2.95E-08	hsa-let-7a-5p;hsa-let-7b-5p;hsa-let-7d-5p;hsa-let-7e-5p;hsa-l
ENSG0000	ENSG0000	2.95E-08	hsa-let-7a-5p;hsa-let-7b-5p;hsa-let-7d-5p;hsa-let-7e-5p;hsa-l
ENSG0000	ENSG0000	0.000629	hsa-let-7a-5p;hsa-let-7b-5p;hsa-let-7d-5p;hsa-let-7e-5p;hsa-l
ENSG0000	ENSG0000	0.006971	hsa-miR-218-5p;hsa-miR-30b-5p;hsa-miR-30c-5p;hsa-miR-36
ENSG0000	ENSG0000	0.002956	hsa-miR-107;hsa-miR-126-5p;hsa-miR-181a-5p;hsa-miR-188-5
ENSG0000	ENSG0000	7.08E-05	hsa-let-7a-5p;hsa-let-7d-5p;hsa-let-7e-5p;hsa-let-7f-5p;hsa-le
ENSG0000	ENSG0000	0.009964	hsa-miR-188-5p;hsa-miR-200b-3p;hsa-miR-200c-3p;hsa-miR-2
ENSG0000	ENSG0000	7.08E-05	hsa-let-7a-5p;hsa-let-7b-5p;hsa-let-7d-5p;hsa-let-7e-5p;hsa-l
ENSG0000	ENSG0000	4.85E-07	hsa-let-7a-5p;hsa-let-7b-5p;hsa-let-7d-5p;hsa-let-7e-5p;hsa-l
ENSG0000	ENSG0000	0.002628	hsa-miR-365a-3p;hsa-miR-365b-3p;hsa-miR-4693-5p;hsa-miR
ENSG0000	ENSG0000	2.50E-06	hsa-let-7a-5p;hsa-let-7b-5p;hsa-let-7e-5p;hsa-let-7f-5p;hsa-le
ENSG0000	ENSG0000	0.003984	hsa-miR-23a-3p;hsa-miR-23b-3p;hsa-miR-23c;hsa-miR-27a-3p
ENSG0000	ENSG0000	7.99E-06	hsa-let-7a-5p;hsa-let-7b-5p;hsa-let-7d-5p;hsa-let-7e-5p;hsa-l
ENSG0000	ENSG0000	0.008084	hsa-miR-126-5p;hsa-miR-30b-5p;hsa-miR-30c-5p;hsa-miR-36
ENSG0000	ENSG0000	7.01E-05	hsa-let-7a-5p;hsa-let-7b-5p;hsa-let-7d-5p;hsa-let-7e-5p;hsa-l
ENSG0000	ENSG0000	0.00088	hsa-miR-103a-3p;hsa-miR-107;hsa-miR-181a-5p;hsa-miR-200c
ENSG0000	ENSG0000	0.00088	hsa-miR-103a-3p;hsa-miR-107;hsa-miR-181a-5p;hsa-miR-200c
ENSG0000	ENSG0000	0.006455	hsa-miR-218-5p;hsa-miR-23a-3p;hsa-miR-23b-3p;hsa-miR-23c
ENSG0000	ENSG0000	5.19E-05	hsa-let-7a-5p;hsa-let-7b-5p;hsa-let-7d-5p;hsa-let-7e-5p;hsa-l
ENSG0000	ENSG0000	0.000331	hsa-let-7a-5p;hsa-let-7d-5p;hsa-let-7f-5p;hsa-let-7g-5p;hsa-le
ENSG0000	ENSG0000	0.000314	hsa-let-7a-5p;hsa-let-7b-5p;hsa-let-7d-5p;hsa-let-7e-5p;hsa-l
ENSG0000	ENSG0000	1.48E-06	hsa-let-7a-5p;hsa-let-7b-5p;hsa-let-7d-5p;hsa-let-7e-5p;hsa-l
ENSG0000	ENSG0000	0.001314	hsa-miR-181a-5p;hsa-miR-200b-3p;hsa-miR-200c-3p;hsa-miR
ENSG0000	ENSG0000	0.00088	hsa-let-7a-5p;hsa-let-7b-5p;hsa-let-7d-5p;hsa-let-7e-5p;hsa-l
ENSG0000	ENSG0000	4.66E-05	hsa-let-7a-5p;hsa-let-7b-5p;hsa-let-7d-5p;hsa-let-7e-5p;hsa-l
ENSG0000	ENSG0000	1.57E-06	hsa-let-7a-5p;hsa-let-7b-5p;hsa-let-7d-5p;hsa-let-7e-5p;hsa-l
ENSG0000	ENSG0000	0.00023	hsa-let-7a-5p;hsa-let-7d-5p;hsa-let-7e-5p;hsa-let-7f-5p;hsa-le
ENSG0000	ENSG0000	0.001006	hsa-miR-23a-3p;hsa-miR-23b-3p;hsa-miR-23c;hsa-miR-374a-5
ENSG0000	ENSG0000	0.002549	hsa-miR-200b-3p;hsa-miR-200c-3p;hsa-miR-204-5p;hsa-miR-3
ENSG0000	ENSG0000	0.002364	hsa-miR-103a-3p;hsa-miR-107;hsa-miR-1179;hsa-miR-500a-5p

ENSG0000	ENSG0000	2.78E-05	hsa-let-7a-5p;hsa-let-7b-5p;hsa-let-7d-5p;hsa-let-7e-5p;hsa-l
ENSG0000	ENSG0000	2.78E-05	hsa-let-7a-5p;hsa-let-7b-5p;hsa-let-7d-5p;hsa-let-7e-5p;hsa-l
ENSG0000	ENSG0000	0.000103	hsa-miR-140-5p;hsa-miR-4693-5p;hsa-miR-500a-5p;hsa-miR-5
ENSG0000	ENSG0000	4.47E-06	hsa-let-7a-5p;hsa-let-7b-5p;hsa-let-7d-5p;hsa-let-7e-5p;hsa-l
ENSG0000	ENSG0000	0.000548	hsa-miR-218-5p;hsa-miR-30b-5p;hsa-miR-30c-5p;hsa-miR-374
ENSG0000	ENSG0000	6.40E-07	hsa-let-7a-5p;hsa-let-7b-5p;hsa-let-7d-5p;hsa-let-7e-5p;hsa-l
ENSG0000	ENSG0000	6.89E-06	hsa-let-7a-5p;hsa-let-7b-5p;hsa-let-7d-5p;hsa-let-7e-5p;hsa-l
ENSG0000	ENSG0000	0.006455	hsa-miR-3613-3p;hsa-miR-365a-3p;hsa-miR-365b-3p;hsa-miR-
ENSG0000	ENSG0000	0.000194	hsa-let-7a-5p;hsa-let-7b-5p;hsa-let-7d-5p;hsa-let-7e-5p;hsa-l
ENSG0000	ENSG0000	0.000194	hsa-let-7a-5p;hsa-let-7b-5p;hsa-let-7d-5p;hsa-let-7e-5p;hsa-l
ENSG0000	ENSG0000	1.37E-08	hsa-let-7a-5p;hsa-let-7b-5p;hsa-let-7d-5p;hsa-let-7e-5p;hsa-l
ENSG0000	ENSG0000	0.003176	hsa-miR-181a-5p;hsa-miR-218-5p;hsa-miR-23a-3p;hsa-miR-23
ENSG0000	ENSG0000	1.21E-13	hsa-let-7a-5p;hsa-let-7b-5p;hsa-let-7d-5p;hsa-let-7e-5p;hsa-l
ENSG0000	ENSG0000	5.19E-05	hsa-let-7a-5p;hsa-let-7b-5p;hsa-let-7d-5p;hsa-let-7e-5p;hsa-l
ENSG0000	ENSG0000	0.000739	hsa-let-7a-5p;hsa-let-7e-5p;hsa-miR-23a-3p;hsa-miR-23b-3p;
ENSG0000	ENSG0000	0.000168	hsa-let-7a-5p;hsa-let-7b-5p;hsa-let-7d-5p;hsa-let-7e-5p;hsa-l
ENSG0000	ENSG0000	0.00017	hsa-miR-200b-3p;hsa-miR-200c-3p;hsa-miR-27a-3p;hsa-miR-2
ENSG0000	ENSG0000	0.000653	hsa-let-7a-5p;hsa-let-7b-5p;hsa-let-7d-5p;hsa-let-7e-5p;hsa-l
ENSG0000	ENSG0000	9.90E-07	hsa-let-7a-5p;hsa-let-7b-5p;hsa-let-7d-5p;hsa-let-7e-5p;hsa-l
ENSG0000	ENSG0000	0.000486	hsa-miR-107;hsa-miR-126-5p;hsa-miR-140-5p;hsa-miR-181a-5
ENSG0000	ENSG0000	0.003577	hsa-miR-23a-3p;hsa-miR-23b-3p;hsa-miR-23c;hsa-miR-27a-3p
ENSG0000	ENSG0000	0.000137	hsa-let-7a-5p;hsa-let-7b-5p;hsa-let-7d-5p;hsa-let-7e-5p;hsa-l
ENSG0000	ENSG0000	1.09E-06	hsa-let-7a-5p;hsa-let-7b-5p;hsa-let-7d-5p;hsa-let-7e-5p;hsa-l
ENSG0000	ENSG0000	2.27E-08	hsa-let-7a-5p;hsa-let-7b-5p;hsa-let-7e-5p;hsa-let-7f-5p;hsa-le
ENSG0000	ENSG0000	1.47E-08	hsa-let-7a-5p;hsa-let-7b-5p;hsa-let-7d-5p;hsa-let-7e-5p;hsa-l
ENSG0000	ENSG0000	0.000458	hsa-let-7a-5p;hsa-let-7b-5p;hsa-let-7d-5p;hsa-let-7e-5p;hsa-l
ENSG0000	ENSG0000	9.52E-06	hsa-let-7a-5p;hsa-let-7b-5p;hsa-let-7d-5p;hsa-let-7e-5p;hsa-l
ENSG0000	ENSG0000	4.76E-07	hsa-let-7a-5p;hsa-let-7b-5p;hsa-let-7d-5p;hsa-let-7e-5p;hsa-l
ENSG0000	ENSG0000	0.008598	hsa-let-7a-5p;hsa-let-7d-5p;hsa-let-7e-5p;hsa-let-7f-5p;hsa-le
ENSG0000	ENSG0000	1.08E-06	hsa-let-7a-5p;hsa-let-7b-5p;hsa-let-7d-5p;hsa-let-7e-5p;hsa-l
ENSG0000	ENSG0000	2.91E-07	hsa-let-7a-5p;hsa-let-7b-5p;hsa-let-7d-5p;hsa-let-7e-5p;hsa-l
ENSG0000	ENSG0000	9.82E-08	hsa-let-7a-5p;hsa-let-7b-5p;hsa-let-7d-5p;hsa-let-7e-5p;hsa-l
ENSG0000	ENSG0000	0.00132	hsa-miR-200b-3p;hsa-miR-200c-3p;hsa-miR-30b-5p;hsa-miR-3
ENSG0000	ENSG0000	9.55E-06	hsa-miR-181a-5p;hsa-miR-218-5p;hsa-miR-23a-3p;hsa-miR-23
ENSG0000	ENSG0000	0.000462	hsa-miR-140-5p;hsa-miR-30b-5p;hsa-miR-30c-5p;hsa-miR-543
ENSG0000	ENSG0000	0.000462	hsa-miR-140-5p;hsa-miR-30b-5p;hsa-miR-30c-5p;hsa-miR-543
ENSG0000	ENSG0000	2.58E-06	hsa-let-7a-5p;hsa-let-7b-5p;hsa-let-7d-5p;hsa-let-7e-5p;hsa-l
ENSG0000	ENSG0000	1.75E-09	hsa-let-7a-5p;hsa-let-7b-5p;hsa-let-7d-5p;hsa-let-7e-5p;hsa-l
ENSG0000	ENSG0000	0.000386	hsa-let-7a-5p;hsa-let-7d-5p;hsa-let-7e-5p;hsa-let-7f-5p;hsa-le
ENSG0000	ENSG0000	3.93E-06	hsa-let-7a-5p;hsa-let-7b-5p;hsa-let-7d-5p;hsa-let-7e-5p;hsa-l
ENSG0000	ENSG0000	0.002911	hsa-miR-200b-3p;hsa-miR-200c-3p;hsa-miR-30b-5p;hsa-miR-4
ENSG0000	ENSG0000	1.07E-08	hsa-let-7a-5p;hsa-let-7b-5p;hsa-let-7d-5p;hsa-let-7e-5p;hsa-l
ENSG0000	ENSG0000	0.003885	hsa-miR-188-5p;hsa-miR-27a-3p;hsa-miR-27b-3p;hsa-miR-361
ENSG0000	ENSG0000	1.43E-05	hsa-let-7a-5p;hsa-let-7b-5p;hsa-let-7d-5p;hsa-let-7e-5p;hsa-l
ENSG0000	ENSG0000	0.003539	hsa-miR-103a-3p;hsa-miR-107;hsa-miR-212-5p;hsa-miR-218-5
ENSG0000	ENSG0000	7.33E-05	hsa-let-7a-5p;hsa-let-7e-5p;hsa-let-7f-5p;hsa-let-7g-5p;hsa-le
ENSG0000	ENSG0000	0.000222	hsa-miR-103a-3p;hsa-miR-107;hsa-miR-30b-5p;hsa-miR-30c-5
ENSG0000	ENSG0000	2.13E-09	hsa-let-7a-5p;hsa-let-7b-5p;hsa-let-7d-5p;hsa-let-7e-5p;hsa-l
ENSG0000	ENSG0000	0.000453	hsa-let-7a-5p;hsa-let-7d-5p;hsa-let-7f-5p;hsa-let-7g-5p;hsa-le
ENSG0000	ENSG0000	5.19E-05	hsa-let-7a-5p;hsa-let-7b-5p;hsa-let-7d-5p;hsa-let-7e-5p;hsa-l
ENSG0000	ENSG0000	3.74E-05	hsa-let-7a-5p;hsa-let-7b-5p;hsa-let-7d-5p;hsa-let-7e-5p;hsa-l
ENSG0000	ENSG0000	3.74E-05	hsa-let-7a-5p;hsa-let-7b-5p;hsa-let-7d-5p;hsa-let-7e-5p;hsa-l
ENSG0000	ENSG0000	6.89E-06	hsa-let-7a-5p;hsa-let-7b-5p;hsa-let-7d-5p;hsa-let-7e-5p;hsa-l
ENSG0000	ENSG0000	2.06E-05	hsa-let-7a-5p;hsa-let-7b-5p;hsa-let-7d-5p;hsa-let-7e-5p;hsa-l

ENSG0000	ENSG0000	1.08E-07	hsa-let-7a-5p;hsa-let-7b-5p;hsa-let-7d-5p;hsa-let-7e-5p;hsa-l
ENSG0000	ENSG0000	1.89E-06	hsa-let-7a-5p;hsa-let-7b-5p;hsa-let-7d-5p;hsa-let-7e-5p;hsa-l
ENSG0000	ENSG0000	1.40E-05	hsa-let-7a-5p;hsa-let-7b-5p;hsa-let-7d-5p;hsa-let-7e-5p;hsa-l
ENSG0000	ENSG0000	0.002956	hsa-miR-107;hsa-miR-181a-5p;hsa-miR-188-5p;hsa-miR-204-5
ENSG0000	ENSG0000	2.64E-05	hsa-let-7a-5p;hsa-let-7b-5p;hsa-let-7d-5p;hsa-let-7e-5p;hsa-l
ENSG0000	ENSG0000	0.001683	hsa-miR-103a-3p;hsa-miR-107;hsa-miR-30b-5p;hsa-miR-30c-5
ENSG0000	ENSG0000	0.002628	hsa-miR-218-5p;hsa-miR-23a-3p;hsa-miR-23b-3p;hsa-miR-23c
ENSG0000	ENSG0000	1.18E-09	hsa-let-7a-5p;hsa-let-7b-5p;hsa-let-7d-5p;hsa-let-7e-5p;hsa-l
ENSG0000	ENSG0000	6.41E-06	hsa-let-7a-5p;hsa-let-7b-5p;hsa-let-7e-5p;hsa-let-7f-5p;hsa-le
ENSG0000	ENSG0000	0.000462	hsa-miR-23a-3p;hsa-miR-23b-3p;hsa-miR-23c;hsa-miR-501-5p
ENSG0000	ENSG0000	0.004177	hsa-miR-181a-5p;hsa-miR-3613-3p;hsa-miR-365a-3p;hsa-miR-
ENSG0000	ENSG0000	2.16E-06	hsa-let-7a-5p;hsa-let-7b-5p;hsa-let-7d-5p;hsa-let-7e-5p;hsa-l
ENSG0000	ENSG0000	4.38E-06	hsa-miR-27a-3p;hsa-miR-27b-3p;hsa-miR-30b-5p;hsa-miR-30c
ENSG0000	ENSG0000	2.97E-05	hsa-let-7a-5p;hsa-let-7b-5p;hsa-let-7d-5p;hsa-let-7e-5p;hsa-l
ENSG0000	ENSG0000	1.04E-05	hsa-let-7a-5p;hsa-let-7b-5p;hsa-let-7d-5p;hsa-let-7e-5p;hsa-l
ENSG0000	ENSG0000	4.66E-05	hsa-let-7a-5p;hsa-let-7b-5p;hsa-let-7d-5p;hsa-let-7e-5p;hsa-l
ENSG0000	ENSG0000	0.000207	hsa-let-7a-5p;hsa-let-7b-5p;hsa-let-7g-5p;hsa-miR-140-5p;hsa
ENSG0000	ENSG0000	0.000665	hsa-let-7a-5p;hsa-let-7f-5p;hsa-let-7g-5p;hsa-let-7i-5p;hsa-m
ENSG0000	ENSG0000	1.45E-08	hsa-let-7a-5p;hsa-let-7b-5p;hsa-let-7d-5p;hsa-let-7e-5p;hsa-l
ENSG0000	ENSG0000	0.001121	hsa-let-7a-5p;hsa-let-7b-5p;hsa-let-7d-5p;hsa-let-7e-5p;hsa-l
ENSG0000	ENSG0000	0.001121	hsa-let-7a-5p;hsa-let-7b-5p;hsa-let-7d-5p;hsa-let-7e-5p;hsa-l
ENSG0000	ENSG0000	1.28E-05	hsa-miR-103a-3p;hsa-miR-107;hsa-miR-23a-3p;hsa-miR-23b-3
ENSG0000	ENSG0000	0.006405	hsa-let-7a-5p;hsa-let-7b-5p;hsa-let-7d-5p;hsa-let-7e-5p;hsa-l
ENSG0000	ENSG0000	0.000998	hsa-miR-200b-3p;hsa-miR-200c-3p;hsa-miR-218-5p;hsa-miR-2
ENSG0000	ENSG0000	0.004645	hsa-miR-188-5p;hsa-miR-200b-3p;hsa-miR-218-5p;hsa-miR-30
ENSG0000	ENSG0000	1.08E-07	hsa-let-7a-5p;hsa-let-7b-5p;hsa-let-7d-5p;hsa-let-7e-5p;hsa-l
ENSG0000	ENSG0000	0.004645	hsa-miR-204-5p;hsa-miR-23a-3p;hsa-miR-23b-3p;hsa-miR-23c
ENSG0000	ENSG0000	5.82E-05	hsa-miR-23a-3p;hsa-miR-23b-3p;hsa-miR-23c;hsa-miR-374a-5
ENSG0000	ENSG0000	0.000548	hsa-miR-23a-3p;hsa-miR-23b-3p;hsa-miR-23c;hsa-miR-4693-5
ENSG0000	ENSG0000	4.74E-07	hsa-miR-181a-5p;hsa-miR-23a-3p;hsa-miR-23b-3p;hsa-miR-23
ENSG0000	ENSG0000	1.90E-07	hsa-let-7a-5p;hsa-let-7b-5p;hsa-let-7d-5p;hsa-let-7e-5p;hsa-l
ENSG0000	ENSG0000	1.90E-07	hsa-let-7a-5p;hsa-let-7b-5p;hsa-let-7d-5p;hsa-let-7e-5p;hsa-l
ENSG0000	ENSG0000	0.005452	hsa-let-7a-5p;hsa-let-7b-5p;hsa-let-7d-5p;hsa-let-7e-5p;hsa-l
ENSG0000	ENSG0000	1.22E-05	hsa-let-7a-5p;hsa-let-7b-5p;hsa-let-7d-5p;hsa-let-7e-5p;hsa-l
ENSG0000	ENSG0000	0.00506	hsa-miR-103a-3p;hsa-miR-107;hsa-miR-377-3p;hsa-miR-449b-
ENSG0000	ENSG0000	0.000386	hsa-let-7b-5p;hsa-miR-27a-3p;hsa-miR-27b-3p;hsa-miR-543
ENSG0000	ENSG0000	0.001337	hsa-let-7b-5p;hsa-let-7d-5p;hsa-let-7e-5p;hsa-let-7f-5p;hsa-le
ENSG0000	ENSG0000	3.93E-06	hsa-let-7a-5p;hsa-let-7b-5p;hsa-let-7d-5p;hsa-let-7e-5p;hsa-l
ENSG0000	ENSG0000	0.000314	hsa-let-7a-5p;hsa-let-7b-5p;hsa-let-7d-5p;hsa-let-7e-5p;hsa-l
ENSG0000	ENSG0000	1.73E-06	hsa-let-7a-5p;hsa-let-7b-5p;hsa-let-7d-5p;hsa-let-7e-5p;hsa-l
ENSG0000	ENSG0000	1.73E-08	hsa-let-7a-5p;hsa-let-7b-5p;hsa-let-7d-5p;hsa-let-7e-5p;hsa-l
ENSG0000	ENSG0000	0.003214	hsa-miR-103a-3p;hsa-miR-107;hsa-miR-218-5p;hsa-miR-377-3
ENSG0000	ENSG0000	0.00108	hsa-let-7a-5p;hsa-let-7b-5p;hsa-let-7d-5p;hsa-let-7f-5p;hsa-le
ENSG0000	ENSG0000	0.005687	hsa-miR-218-5p;hsa-miR-23a-3p;hsa-miR-23b-3p;hsa-miR-23c
ENSG0000	ENSG0000	0.000551	hsa-let-7a-5p;hsa-let-7b-5p;hsa-let-7d-5p;hsa-let-7e-5p;hsa-l
ENSG0000	ENSG0000	4.66E-05	hsa-let-7a-5p;hsa-let-7b-5p;hsa-let-7d-5p;hsa-let-7e-5p;hsa-l
ENSG0000	ENSG0000	0.000314	hsa-let-7a-5p;hsa-let-7b-5p;hsa-let-7d-5p;hsa-let-7e-5p;hsa-l
ENSG0000	ENSG0000	0.001205	hsa-let-7a-5p;hsa-let-7d-5p;hsa-miR-218-5p;hsa-miR-3613-3p
ENSG0000	ENSG0000	1.65E-05	hsa-miR-132-3p;hsa-miR-181a-5p;hsa-miR-200c-3p;hsa-miR-2
ENSG0000	ENSG0000	0.003176	hsa-miR-103a-3p;hsa-miR-107;hsa-miR-374a-5p;hsa-miR-500a
ENSG0000	ENSG0000	0.003652	hsa-let-7d-5p;hsa-let-7e-5p;hsa-let-7f-5p;hsa-let-7g-5p;hsa-le
ENSG0000	ENSG0000	0.003652	hsa-let-7d-5p;hsa-let-7e-5p;hsa-let-7f-5p;hsa-let-7g-5p;hsa-le
ENSG0000	ENSG0000	0.000653	hsa-let-7a-5p;hsa-let-7b-5p;hsa-let-7d-5p;hsa-let-7e-5p;hsa-l
ENSG0000	ENSG0000	4.18E-05	hsa-let-7a-5p;hsa-let-7b-5p;hsa-let-7d-5p;hsa-let-7e-5p;hsa-l

ENSG0000	ENSG0000	0.003539	hsa-miR-194-3p;hsa-miR-365a-3p;hsa-miR-365b-3p;hsa-miR-4
ENSG0000	ENSG0000	7.92E-07	hsa-let-7a-5p;hsa-let-7b-5p;hsa-let-7d-5p;hsa-let-7e-5p;hsa-l
ENSG0000	ENSG0000	0.000115	hsa-let-7a-5p;hsa-let-7d-5p;hsa-let-7e-5p;hsa-let-7f-5p;hsa-le
ENSG0000	ENSG0000	0.001161	hsa-let-7d-5p;hsa-let-7e-5p;hsa-let-7f-5p;hsa-let-7g-5p;hsa-le
ENSG0000	ENSG0000	0.000331	hsa-miR-181a-5p;hsa-miR-204-5p;hsa-miR-27b-3p;hsa-miR-30
ENSG0000	ENSG0000	8.58E-10	hsa-let-7a-5p;hsa-let-7b-5p;hsa-let-7d-5p;hsa-let-7e-5p;hsa-l
ENSG0000	ENSG0000	8.58E-10	hsa-let-7a-5p;hsa-let-7b-5p;hsa-let-7d-5p;hsa-let-7e-5p;hsa-l
ENSG0000	ENSG0000	8.58E-10	hsa-let-7a-5p;hsa-let-7b-5p;hsa-let-7d-5p;hsa-let-7e-5p;hsa-l
ENSG0000	ENSG0000	6.89E-06	hsa-let-7a-5p;hsa-let-7b-5p;hsa-let-7d-5p;hsa-let-7e-5p;hsa-l
ENSG0000	ENSG0000	4.18E-05	hsa-let-7a-5p;hsa-let-7b-5p;hsa-let-7d-5p;hsa-let-7e-5p;hsa-l
ENSG0000	ENSG0000	7.85E-05	hsa-miR-103a-3p;hsa-miR-107;hsa-miR-132-3p;hsa-miR-377-3
ENSG0000	ENSG0000	8.02E-07	hsa-let-7a-5p;hsa-let-7b-5p;hsa-let-7d-5p;hsa-let-7e-5p;hsa-l
ENSG0000	ENSG0000	0.001683	hsa-miR-377-3p;hsa-miR-500a-5p;hsa-miR-501-5p;hsa-miR-54
ENSG0000	ENSG0000	6.45E-07	hsa-let-7a-5p;hsa-let-7b-5p;hsa-let-7d-5p;hsa-let-7e-5p;hsa-l
ENSG0000	ENSG0000	0.008682	hsa-miR-103a-3p;hsa-miR-107;hsa-miR-204-5p;hsa-miR-3613-
ENSG0000	ENSG0000	0.00017	hsa-miR-103a-3p;hsa-miR-107;hsa-miR-27a-3p;hsa-miR-27b-3
ENSG0000	ENSG0000	7.31E-05	hsa-let-7a-5p;hsa-let-7d-5p;hsa-let-7e-5p;hsa-let-7f-5p;hsa-le
ENSG0000	ENSG0000	0.004806	hsa-let-7a-5p;hsa-let-7b-5p;hsa-let-7d-5p;hsa-let-7e-5p;hsa-l
ENSG0000	ENSG0000	0.004806	hsa-let-7a-5p;hsa-let-7b-5p;hsa-let-7d-5p;hsa-let-7e-5p;hsa-l
ENSG0000	ENSG0000	6.71E-07	hsa-let-7a-5p;hsa-let-7d-5p;hsa-let-7e-5p;hsa-let-7f-5p;hsa-le
ENSG0000	ENSG0000	8.74E-05	hsa-let-7a-5p;hsa-let-7e-5p;hsa-let-7f-5p;hsa-let-7g-5p;hsa-le
ENSG0000	ENSG0000	1.37E-08	hsa-let-7a-5p;hsa-let-7b-5p;hsa-let-7d-5p;hsa-let-7e-5p;hsa-l
ENSG0000	ENSG0000	0.002119	hsa-miR-196a-5p;hsa-miR-196b-5p;hsa-miR-30b-5p;hsa-miR-3
ENSG0000	ENSG0000	5.14E-07	hsa-let-7a-5p;hsa-let-7b-5p;hsa-let-7d-5p;hsa-let-7e-5p;hsa-l
ENSG0000	ENSG0000	9.24E-06	hsa-let-7a-5p;hsa-let-7b-5p;hsa-let-7d-5p;hsa-let-7e-5p;hsa-l
ENSG0000	ENSG0000	2.97E-05	hsa-let-7a-5p;hsa-let-7b-5p;hsa-let-7d-5p;hsa-let-7e-5p;hsa-l
ENSG0000	ENSG0000	2.97E-05	hsa-let-7a-5p;hsa-let-7b-5p;hsa-let-7d-5p;hsa-let-7e-5p;hsa-l
ENSG0000	ENSG0000	3.83E-08	hsa-let-7a-5p;hsa-let-7b-5p;hsa-let-7d-5p;hsa-let-7e-5p;hsa-l
ENSG0000	ENSG0000	1.21E-06	hsa-let-7a-5p;hsa-let-7b-5p;hsa-let-7d-5p;hsa-let-7e-5p;hsa-l
ENSG0000	ENSG0000	0.001638	hsa-let-7a-5p;hsa-let-7b-5p;hsa-let-7d-5p;hsa-let-7f-5p;hsa-le
ENSG0000	ENSG0000	0.000248	hsa-let-7a-5p;hsa-let-7b-5p;hsa-let-7d-5p;hsa-let-7e-5p;hsa-l
ENSG0000	ENSG0000	1.22E-05	hsa-let-7a-5p;hsa-let-7b-5p;hsa-let-7e-5p;hsa-let-7f-5p;hsa-le
ENSG0000	ENSG0000	7.99E-06	hsa-let-7a-5p;hsa-let-7b-5p;hsa-let-7d-5p;hsa-let-7e-5p;hsa-l
ENSG0000	ENSG0000	0.000193	hsa-miR-140-5p;hsa-miR-23a-3p;hsa-miR-23b-3p;hsa-miR-23c
ENSG0000	ENSG0000	0.000137	hsa-let-7a-5p;hsa-let-7b-5p;hsa-let-7d-5p;hsa-let-7f-5p;hsa-le
ENSG0000	ENSG0000	1.22E-05	hsa-let-7a-5p;hsa-let-7b-5p;hsa-let-7d-5p;hsa-let-7e-5p;hsa-l
ENSG0000	ENSG0000	0.002283	hsa-let-7a-5p;hsa-let-7b-5p;hsa-let-7d-5p;hsa-let-7e-5p;hsa-l
ENSG0000	ENSG0000	0.002283	hsa-let-7a-5p;hsa-let-7b-5p;hsa-let-7d-5p;hsa-let-7e-5p;hsa-l
ENSG0000	ENSG0000	5.03E-10	hsa-let-7a-5p;hsa-let-7b-5p;hsa-let-7d-5p;hsa-let-7e-5p;hsa-l
ENSG0000	ENSG0000	3.38E-11	hsa-let-7a-5p;hsa-let-7b-5p;hsa-let-7d-5p;hsa-let-7e-5p;hsa-l
ENSG0000	ENSG0000	0.000262	hsa-miR-23a-3p;hsa-miR-23b-3p;hsa-miR-23c;hsa-miR-3613-3
ENSG0000	ENSG0000	5.19E-05	hsa-let-7a-5p;hsa-let-7b-5p;hsa-let-7d-5p;hsa-let-7e-5p;hsa-l
ENSG0000	ENSG0000	0.000194	hsa-let-7a-5p;hsa-let-7b-5p;hsa-let-7d-5p;hsa-let-7e-5p;hsa-l
ENSG0000	ENSG0000	3.34E-05	hsa-let-7a-5p;hsa-let-7b-5p;hsa-let-7d-5p;hsa-let-7e-5p;hsa-l
ENSG0000	ENSG0000	8.72E-06	hsa-let-7a-5p;hsa-let-7b-5p;hsa-let-7d-5p;hsa-let-7e-5p;hsa-l
ENSG0000	ENSG0000	0.000638	hsa-let-7a-5p;hsa-let-7b-5p;hsa-let-7d-5p;hsa-let-7e-5p;hsa-l
ENSG0000	ENSG0000	0.004645	hsa-miR-103a-3p;hsa-miR-107;hsa-miR-30b-5p;hsa-miR-30c-5
ENSG0000	ENSG0000	0.000905	hsa-let-7a-5p;hsa-let-7b-5p;hsa-let-7d-5p;hsa-let-7e-5p;hsa-l
ENSG0000	ENSG0000	0.000357	hsa-let-7a-5p;hsa-let-7b-5p;hsa-let-7d-5p;hsa-let-7e-5p;hsa-l
ENSG0000	ENSG0000	5.10E-11	hsa-let-7a-5p;hsa-let-7b-5p;hsa-let-7d-5p;hsa-let-7e-5p;hsa-l
ENSG0000	ENSG0000	8.65E-13	hsa-let-7a-5p;hsa-let-7b-5p;hsa-let-7d-5p;hsa-let-7e-5p;hsa-l
ENSG0000	ENSG0000	0.000115	hsa-let-7a-5p;hsa-let-7b-5p;hsa-let-7d-5p;hsa-let-7e-5p;hsa-l
ENSG0000	ENSG0000	3.64E-11	hsa-let-7a-5p;hsa-let-7b-5p;hsa-let-7d-5p;hsa-let-7e-5p;hsa-l
ENSG0000	ENSG0000	5.91E-06	hsa-let-7a-5p;hsa-let-7b-5p;hsa-let-7d-5p;hsa-let-7e-5p;hsa-l

ENSG0000	ENSG0000	5.84E-08	hsa-let-7a-5p;hsa-let-7b-5p;hsa-let-7d-5p;hsa-let-7e-5p;hsa-l
ENSG0000	ENSG0000	0.000462	hsa-miR-181a-5p;hsa-miR-23a-3p;hsa-miR-23b-3p;hsa-miR-23
ENSG0000	ENSG0000	7.05E-09	hsa-let-7a-5p;hsa-let-7b-5p;hsa-let-7d-5p;hsa-let-7e-5p;hsa-l
ENSG0000	ENSG0000	7.05E-09	hsa-let-7a-5p;hsa-let-7b-5p;hsa-let-7d-5p;hsa-let-7e-5p;hsa-l
ENSG0000	ENSG0000	0.001055	hsa-let-7a-5p;hsa-let-7b-5p;hsa-let-7d-5p;hsa-let-7e-5p;hsa-l
ENSG0000	ENSG0000	9.90E-07	hsa-let-7a-5p;hsa-let-7b-5p;hsa-let-7d-5p;hsa-let-7e-5p;hsa-l
ENSG0000	ENSG0000	0.002911	hsa-miR-23a-3p;hsa-miR-23b-3p;hsa-miR-23c;hsa-miR-377-3p
ENSG0000	ENSG0000	0.000179	hsa-let-7a-5p;hsa-let-7b-5p;hsa-let-7d-5p;hsa-let-7e-5p;hsa-l
ENSG0000	ENSG0000	0.000521	hsa-let-7a-5p;hsa-let-7b-5p;hsa-let-7d-5p;hsa-let-7e-5p;hsa-l
ENSG0000	ENSG0000	1.57E-06	hsa-let-7a-5p;hsa-let-7b-5p;hsa-let-7e-5p;hsa-let-7f-5p;hsa-le
ENSG0000	ENSG0000	0.000126	hsa-let-7a-5p;hsa-let-7b-5p;hsa-let-7d-5p;hsa-let-7e-5p;hsa-l
ENSG0000	ENSG0000	0.000126	hsa-let-7a-5p;hsa-let-7b-5p;hsa-let-7d-5p;hsa-let-7e-5p;hsa-l
ENSG0000	ENSG0000	0.001442	hsa-miR-200b-3p;hsa-miR-200c-3p;hsa-miR-23a-3p;hsa-miR-2
ENSG0000	ENSG0000	3.49E-09	hsa-let-7d-5p;hsa-let-7e-5p;hsa-let-7f-5p;hsa-let-7g-5p;hsa-le
ENSG0000	ENSG0000	0.000137	hsa-let-7a-5p;hsa-let-7b-5p;hsa-let-7d-5p;hsa-let-7e-5p;hsa-l
ENSG0000	ENSG0000	0.00017	hsa-miR-30b-5p;hsa-miR-30c-5p;hsa-miR-365a-3p;hsa-miR-36
ENSG0000	ENSG0000	1.21E-06	hsa-let-7a-5p;hsa-let-7b-5p;hsa-let-7e-5p;hsa-let-7f-5p;hsa-le
ENSG0000	ENSG0000	1.38E-05	hsa-let-7b-5p;hsa-let-7d-5p;hsa-let-7e-5p;hsa-let-7f-5p;hsa-le
ENSG0000	ENSG0000	2.13E-09	hsa-let-7a-5p;hsa-let-7b-5p;hsa-let-7d-5p;hsa-let-7e-5p;hsa-l
ENSG0000	ENSG0000	2.95E-08	hsa-let-7a-5p;hsa-let-7b-5p;hsa-let-7d-5p;hsa-let-7e-5p;hsa-l
ENSG0000	ENSG0000	2.95E-08	hsa-let-7a-5p;hsa-let-7b-5p;hsa-let-7d-5p;hsa-let-7e-5p;hsa-l
ENSG0000	ENSG0000	2.16E-08	hsa-let-7a-5p;hsa-let-7b-5p;hsa-let-7d-5p;hsa-let-7e-5p;hsa-l
ENSG0000	ENSG0000	0.000122	hsa-let-7a-5p;hsa-let-7b-5p;hsa-let-7d-5p;hsa-let-7e-5p;hsa-l
ENSG0000	ENSG0000	2.32E-07	hsa-let-7a-5p;hsa-let-7b-5p;hsa-let-7d-5p;hsa-let-7e-5p;hsa-l
ENSG0000	ENSG0000	1.39E-07	hsa-let-7a-5p;hsa-let-7b-5p;hsa-let-7d-5p;hsa-let-7e-5p;hsa-l
ENSG0000	ENSG0000	0.000104	hsa-let-7a-5p;hsa-let-7b-5p;hsa-let-7d-5p;hsa-let-7e-5p;hsa-l
ENSG0000	ENSG0000	0.003025	hsa-let-7a-5p;hsa-let-7d-5p;hsa-let-7e-5p;hsa-let-7f-5p;hsa-le
ENSG0000	ENSG0000	7.29E-06	hsa-let-7a-5p;hsa-let-7b-5p;hsa-let-7d-5p;hsa-let-7e-5p;hsa-l
ENSG0000	ENSG0000	0.002119	hsa-miR-212-5p;hsa-miR-218-5p;hsa-miR-30b-5p;hsa-miR-30
ENSG0000	ENSG0000	1.21E-06	hsa-let-7a-5p;hsa-let-7b-5p;hsa-let-7d-5p;hsa-let-7e-5p;hsa-l
ENSG0000	ENSG0000	2.67E-05	hsa-miR-181a-5p;hsa-miR-27a-3p;hsa-miR-27b-3p;hsa-miR-30
ENSG0000	ENSG0000	2.67E-05	hsa-miR-181a-5p;hsa-miR-27a-3p;hsa-miR-27b-3p;hsa-miR-30
ENSG0000	ENSG0000	1.99E-05	hsa-miR-200b-3p;hsa-miR-200c-3p;hsa-miR-218-5p;hsa-miR-4
ENSG0000	ENSG0000	6.22E-05	hsa-let-7a-5p;hsa-let-7b-5p;hsa-let-7d-5p;hsa-let-7e-5p;hsa-l
ENSG0000	ENSG0000	0.000597	hsa-let-7a-5p;hsa-let-7b-5p;hsa-let-7d-5p;hsa-let-7e-5p;hsa-l
ENSG0000	ENSG0000	1.59E-05	hsa-let-7a-5p;hsa-let-7d-5p;hsa-let-7e-5p;hsa-let-7f-5p;hsa-le
ENSG0000	ENSG0000	2.34E-05	hsa-miR-181a-5p;hsa-miR-27a-3p;hsa-miR-27b-3p;hsa-miR-30
ENSG0000	ENSG0000	0.000364	hsa-let-7a-5p;hsa-let-7b-5p;hsa-let-7d-5p;hsa-let-7e-5p;hsa-l
ENSG0000	ENSG0000	6.45E-07	hsa-let-7a-5p;hsa-let-7b-5p;hsa-let-7d-5p;hsa-let-7e-5p;hsa-l
ENSG0000	ENSG0000	9.90E-07	hsa-let-7a-5p;hsa-let-7b-5p;hsa-let-7d-5p;hsa-let-7e-5p;hsa-l
ENSG0000	ENSG0000	0.00017	hsa-miR-27a-3p;hsa-miR-27b-3p;hsa-miR-30b-5p;hsa-miR-30
ENSG0000	ENSG0000	6.89E-06	hsa-let-7a-5p;hsa-let-7b-5p;hsa-let-7d-5p;hsa-let-7e-5p;hsa-l
ENSG0000	ENSG0000	0.001006	hsa-miR-103a-3p;hsa-miR-107;hsa-miR-500a-5p;hsa-miR-501-
ENSG0000	ENSG0000	0.005011	hsa-let-7a-5p;hsa-let-7b-5p;hsa-let-7d-5p;hsa-let-7e-5p;hsa-l
ENSG0000	ENSG0000	4.74E-07	hsa-miR-181a-5p;hsa-miR-23a-3p;hsa-miR-23b-3p;hsa-miR-23
ENSG0000	ENSG0000	3.93E-06	hsa-let-7a-5p;hsa-let-7b-5p;hsa-let-7d-5p;hsa-let-7e-5p;hsa-l
ENSG0000	ENSG0000	4.07E-07	hsa-let-7a-5p;hsa-let-7b-5p;hsa-let-7d-5p;hsa-let-7e-5p;hsa-l
ENSG0000	ENSG0000	3.64E-07	hsa-let-7a-5p;hsa-let-7b-5p;hsa-let-7e-5p;hsa-let-7f-5p;hsa-le
ENSG0000	ENSG0000	9.36E-09	hsa-let-7a-5p;hsa-let-7b-5p;hsa-let-7d-5p;hsa-let-7e-5p;hsa-l
ENSG0000	ENSG0000	1.99E-06	hsa-let-7b-5p;hsa-let-7e-5p;hsa-let-7f-5p;hsa-let-7g-5p;hsa-le
ENSG0000	ENSG0000	0.000104	hsa-let-7a-5p;hsa-let-7b-5p;hsa-let-7d-5p;hsa-let-7e-5p;hsa-l
ENSG0000	ENSG0000	3.17E-08	hsa-let-7a-5p;hsa-let-7b-5p;hsa-let-7d-5p;hsa-let-7e-5p;hsa-l
ENSG0000	ENSG0000	0.000458	hsa-let-7a-5p;hsa-let-7b-5p;hsa-let-7d-5p;hsa-let-7e-5p;hsa-l
ENSG0000	ENSG0000	2.03E-08	hsa-let-7a-5p;hsa-let-7b-5p;hsa-let-7d-5p;hsa-let-7e-5p;hsa-l

ENSG0000	ENSG0000	7.29E-06	hsa-let-7a-5p;hsa-let-7b-5p;hsa-let-7d-5p;hsa-let-7e-5p;hsa-l
ENSG0000	ENSG0000	0.001153	hsa-miR-103a-3p;hsa-miR-107;hsa-miR-196a-5p;hsa-miR-196b
ENSG0000	ENSG0000	3.82E-05	hsa-let-7a-5p;hsa-let-7b-5p;hsa-let-7d-5p;hsa-let-7e-5p;hsa-l
ENSG0000	ENSG0000	4.76E-07	hsa-let-7a-5p;hsa-let-7b-5p;hsa-let-7d-5p;hsa-let-7e-5p;hsa-l
ENSG0000	ENSG0000	6.47E-12	hsa-let-7a-5p;hsa-let-7b-5p;hsa-let-7e-5p;hsa-let-7f-5p;hsa-le
ENSG0000	ENSG0000	0.000107	hsa-let-7a-5p;hsa-let-7b-5p;hsa-let-7d-5p;hsa-let-7e-5p;hsa-l
ENSG0000	ENSG0000	6.40E-05	hsa-let-7a-5p;hsa-let-7b-5p;hsa-let-7d-5p;hsa-let-7e-5p;hsa-l
ENSG0000	ENSG0000	1.51E-07	hsa-let-7a-5p;hsa-let-7b-5p;hsa-let-7d-5p;hsa-let-7e-5p;hsa-l
ENSG0000	ENSG0000	0.000104	hsa-let-7a-5p;hsa-let-7b-5p;hsa-let-7d-5p;hsa-let-7e-5p;hsa-l
ENSG0000	ENSG0000	1.76E-06	hsa-let-7a-5p;hsa-let-7b-5p;hsa-let-7d-5p;hsa-let-7e-5p;hsa-l
ENSG0000	ENSG0000	0.000363	hsa-let-7a-5p;hsa-let-7b-5p;hsa-let-7d-5p;hsa-let-7e-5p;hsa-l
ENSG0000	ENSG0000	6.61E-11	hsa-let-7a-5p;hsa-let-7b-5p;hsa-let-7d-5p;hsa-let-7e-5p;hsa-l
ENSG0000	ENSG0000	0.009309	hsa-miR-212-5p;hsa-miR-23a-3p;hsa-miR-23b-3p;hsa-miR-23
ENSG0000	ENSG0000	9.03E-09	hsa-let-7a-5p;hsa-let-7b-5p;hsa-let-7d-5p;hsa-let-7e-5p;hsa-l
ENSG0000	ENSG0000	0.000558	hsa-let-7a-5p;hsa-let-7b-5p;hsa-let-7d-5p;hsa-let-7e-5p;hsa-l
ENSG0000	ENSG0000	0.006971	hsa-miR-218-5p;hsa-miR-23a-3p;hsa-miR-23b-3p;hsa-miR-23
ENSG0000	ENSG0000	0.002911	hsa-miR-23a-3p;hsa-miR-23b-3p;hsa-miR-23c;hsa-miR-3613-
ENSG0000	ENSG0000	0.002364	hsa-miR-181a-5p;hsa-miR-27a-3p;hsa-miR-27b-3p;hsa-miR-50
ENSG0000	ENSG0000	5.75E-06	hsa-let-7a-5p;hsa-let-7b-5p;hsa-let-7d-5p;hsa-let-7e-5p;hsa-l
ENSG0000	ENSG0000	3.83E-08	hsa-let-7a-5p;hsa-let-7b-5p;hsa-let-7d-5p;hsa-let-7e-5p;hsa-l
ENSG0000	ENSG0000	0.006561	hsa-miR-103a-3p;hsa-miR-107;hsa-miR-23a-3p;hsa-miR-23b-
ENSG0000	ENSG0000	3.74E-05	hsa-let-7a-5p;hsa-let-7b-5p;hsa-let-7d-5p;hsa-let-7e-5p;hsa-l
ENSG0000	ENSG0000	0.000776	hsa-let-7a-5p;hsa-let-7b-5p;hsa-let-7d-5p;hsa-let-7e-5p;hsa-l
ENSG0000	ENSG0000	0.001121	hsa-let-7a-5p;hsa-let-7b-5p;hsa-let-7d-5p;hsa-let-7e-5p;hsa-l
ENSG0000	ENSG0000	4.19E-08	hsa-let-7a-5p;hsa-let-7b-5p;hsa-let-7d-5p;hsa-let-7e-5p;hsa-l
ENSG0000	ENSG0000	0.000873	hsa-miR-23a-3p;hsa-miR-23b-3p;hsa-miR-23c;hsa-miR-500a-
ENSG0000	ENSG0000	3.08E-06	hsa-let-7a-5p;hsa-let-7b-5p;hsa-let-7d-5p;hsa-let-7e-5p;hsa-l
ENSG0000	ENSG0000	0.000827	hsa-let-7a-5p;hsa-let-7b-5p;hsa-let-7d-5p;hsa-let-7e-5p;hsa-l
ENSG0000	ENSG0000	0.000104	hsa-let-7a-5p;hsa-let-7b-5p;hsa-let-7d-5p;hsa-let-7e-5p;hsa-l
ENSG0000	ENSG0000	0.000476	hsa-miR-181a-5p;hsa-miR-23a-3p;hsa-miR-23b-3p;hsa-miR-2
ENSG0000	ENSG0000	0.003212	hsa-let-7a-5p;hsa-let-7b-5p;hsa-let-7d-5p;hsa-let-7e-5p;hsa-l
ENSG0000	ENSG0000	0.001153	hsa-miR-196a-5p;hsa-miR-196b-5p;hsa-miR-27a-3p;hsa-miR-
ENSG0000	ENSG0000	7.85E-05	hsa-miR-181a-5p;hsa-miR-23a-3p;hsa-miR-23b-3p;hsa-miR-2
ENSG0000	ENSG0000	3.11E-08	hsa-let-7a-5p;hsa-let-7b-5p;hsa-let-7d-5p;hsa-let-7e-5p;hsa-l
ENSG0000	ENSG0000	0.003413	hsa-let-7a-5p;hsa-let-7b-5p;hsa-let-7d-5p;hsa-let-7e-5p;hsa-l
ENSG0000	ENSG0000	6.45E-07	hsa-let-7a-5p;hsa-let-7b-5p;hsa-let-7d-5p;hsa-let-7e-5p;hsa-l
ENSG0000	ENSG0000	3.74E-05	hsa-let-7a-5p;hsa-let-7b-5p;hsa-let-7d-5p;hsa-let-7e-5p;hsa-l
ENSG0000	ENSG0000	1.22E-05	hsa-let-7a-5p;hsa-let-7b-5p;hsa-let-7d-5p;hsa-let-7e-5p;hsa-l
ENSG0000	ENSG0000	0.005385	hsa-miR-103a-3p;hsa-miR-107;hsa-miR-23a-3p;hsa-miR-23b-
ENSG0000	ENSG0000	0.000222	hsa-miR-23a-3p;hsa-miR-23b-3p;hsa-miR-23c;hsa-miR-27a-3
ENSG0000	ENSG0000	0.000447	hsa-let-7a-5p;hsa-let-7b-5p;hsa-let-7e-5p;hsa-let-7f-5p;hsa-le
ENSG0000	ENSG0000	4.07E-07	hsa-let-7a-5p;hsa-let-7b-5p;hsa-let-7d-5p;hsa-let-7e-5p;hsa-l
ENSG0000	ENSG0000	0.000386	hsa-miR-23a-3p;hsa-miR-23b-3p;hsa-miR-23c;hsa-miR-543
ENSG0000	ENSG0000	2.72E-06	hsa-let-7a-5p;hsa-let-7b-5p;hsa-let-7d-5p;hsa-let-7e-5p;hsa-l
ENSG0000	ENSG0000	1.48E-10	hsa-let-7a-5p;hsa-let-7b-5p;hsa-let-7d-5p;hsa-let-7e-5p;hsa-l
ENSG0000	ENSG0000	0.00217	hsa-let-7a-5p;hsa-let-7b-5p;hsa-let-7d-5p;hsa-let-7e-5p;hsa-l
ENSG0000	ENSG0000	6.40E-05	hsa-let-7a-5p;hsa-let-7b-5p;hsa-let-7d-5p;hsa-let-7e-5p;hsa-l
ENSG0000	ENSG0000	0.002911	hsa-miR-23a-3p;hsa-miR-23b-3p;hsa-miR-23c;hsa-miR-374a-
ENSG0000	ENSG0000	0.002784	hsa-let-7a-5p;hsa-let-7b-5p;hsa-let-7e-5p;hsa-let-7f-5p;hsa-le
ENSG0000	ENSG0000	1.79E-06	hsa-let-7a-5p;hsa-let-7b-5p;hsa-let-7d-5p;hsa-let-7e-5p;hsa-l
ENSG0000	ENSG0000	1.27E-06	hsa-let-7a-5p;hsa-let-7b-5p;hsa-let-7d-5p;hsa-let-7e-5p;hsa-l
ENSG0000	ENSG0000	4.38E-06	hsa-miR-27a-3p;hsa-miR-27b-3p;hsa-miR-30b-5p;hsa-miR-30
ENSG0000	ENSG0000	4.38E-06	hsa-miR-27a-3p;hsa-miR-27b-3p;hsa-miR-30b-5p;hsa-miR-30
ENSG0000	ENSG0000	7.67E-05	hsa-let-7a-5p;hsa-let-7b-5p;hsa-let-7d-5p;hsa-let-7e-5p;hsa-l

ENSG0000	ENSG0000	1.21E-07	hsa-let-7a-5p;hsa-let-7b-5p;hsa-let-7d-5p;hsa-let-7e-5p;hsa-l
ENSG0000	ENSG0000	1.90E-07	hsa-let-7a-5p;hsa-let-7b-5p;hsa-let-7d-5p;hsa-let-7e-5p;hsa-l
ENSG0000	ENSG0000	7.99E-06	hsa-let-7a-5p;hsa-let-7b-5p;hsa-let-7d-5p;hsa-let-7e-5p;hsa-l
ENSG0000	ENSG0000	0.000728	hsa-let-7a-5p;hsa-let-7b-5p;hsa-let-7d-5p;hsa-let-7e-5p;hsa-l
ENSG0000	ENSG0000	0.000248	hsa-let-7a-5p;hsa-let-7b-5p;hsa-let-7d-5p;hsa-let-7e-5p;hsa-l
ENSG0000	ENSG0000	2.95E-05	hsa-miR-200b-3p;hsa-miR-200c-3p;hsa-miR-204-5p;hsa-miR-2
ENSG0000	ENSG0000	6.89E-06	hsa-let-7a-5p;hsa-let-7b-5p;hsa-let-7d-5p;hsa-let-7e-5p;hsa-l
ENSG0000	ENSG0000	2.06E-05	hsa-let-7a-5p;hsa-let-7b-5p;hsa-miR-140-5p;hsa-miR-30b-5p;
ENSG0000	ENSG0000	0.005687	hsa-let-7a-5p;hsa-let-7d-5p;hsa-let-7e-5p;hsa-let-7f-5p;hsa-le
ENSG0000	ENSG0000	2.16E-06	hsa-let-7a-5p;hsa-let-7b-5p;hsa-let-7d-5p;hsa-let-7e-5p;hsa-l
ENSG0000	ENSG0000	0.003064	hsa-let-7a-5p;hsa-let-7d-5p;hsa-let-7e-5p;hsa-let-7f-5p;hsa-le
ENSG0000	ENSG0000	3.57E-09	hsa-let-7a-5p;hsa-let-7b-5p;hsa-let-7d-5p;hsa-let-7e-5p;hsa-l
ENSG0000	ENSG0000	0.00132	hsa-miR-103a-3p;hsa-miR-107;hsa-miR-181a-5p;hsa-miR-30b-
ENSG0000	ENSG0000	5.19E-05	hsa-let-7a-5p;hsa-let-7b-5p;hsa-let-7d-5p;hsa-let-7e-5p;hsa-l
ENSG0000	ENSG0000	2.16E-06	hsa-let-7a-5p;hsa-let-7b-5p;hsa-let-7d-5p;hsa-let-7e-5p;hsa-l
ENSG0000	ENSG0000	0.006442	hsa-miR-103a-3p;hsa-miR-107;hsa-miR-374a-5p;hsa-miR-500a
ENSG0000	ENSG0000	0.000139	hsa-let-7a-5p;hsa-let-7b-5p;hsa-let-7d-5p;hsa-let-7e-5p;hsa-l
ENSG0000	ENSG0000	2.64E-05	hsa-let-7a-5p;hsa-let-7b-5p;hsa-let-7d-5p;hsa-let-7e-5p;hsa-l
ENSG0000	ENSG0000	0.004652	hsa-let-7a-5p;hsa-let-7b-5p;hsa-let-7d-5p;hsa-let-7e-5p;hsa-l
ENSG0000	ENSG0000	4.66E-06	hsa-miR-17-5p;hsa-miR-20b-5p;hsa-miR-26a-5p;hsa-miR-33a-
ENSG0000	ENSG0000	0.000124	hsa-miR-320a;hsa-miR-320b;hsa-miR-320c;hsa-miR-320d
ENSG0000	ENSG0000	0.000128	hsa-miR-320a;hsa-miR-320b;hsa-miR-320c;hsa-miR-320d;hsa-r
ENSG0000	ENSG0000	0.002609	hsa-miR-320a;hsa-miR-320b;hsa-miR-320c;hsa-miR-320d;hsa-r
ENSG0000	ENSG0000	0.007781	hsa-miR-124-3p;hsa-miR-17-5p;hsa-miR-181a-5p;hsa-miR-20b
ENSG0000	ENSG0000	2.77E-05	hsa-miR-106a-5p;hsa-miR-135b-5p;hsa-miR-218-5p;hsa-miR-5
ENSG0000	ENSG0000	9.85E-05	hsa-miR-129-5p;hsa-miR-181a-5p;hsa-miR-183-5p;hsa-miR-19
ENSG0000	ENSG0000	0.008421	hsa-miR-106a-5p;hsa-miR-17-5p;hsa-miR-20b-5p;hsa-miR-93-
ENSG0000	ENSG0000	0.006787	hsa-miR-106a-5p;hsa-miR-17-5p;hsa-miR-20b-5p;hsa-miR-21
ENSG0000	ENSG0000	1.88E-05	hsa-miR-1976;hsa-miR-320a;hsa-miR-320b;hsa-miR-320c;hsa-r
ENSG0000	ENSG0000	0.000414	hsa-miR-17-5p;hsa-miR-181a-5p;hsa-miR-20b-5p;hsa-miR-26a
ENSG0000	ENSG0000	0.003867	hsa-miR-33a-3p;hsa-miR-409-3p;hsa-miR-548a-5p;hsa-miR-54
ENSG0000	ENSG0000	0.00383	hsa-miR-106a-5p;hsa-miR-17-5p;hsa-miR-20b-5p;hsa-miR-93-
ENSG0000	ENSG0000	0.009964	hsa-miR-106a-5p;hsa-miR-17-5p;hsa-miR-20b-5p;hsa-miR-34a
ENSG0000	ENSG0000	1.15E-05	hsa-miR-17-5p;hsa-miR-20b-5p;hsa-miR-548a-5p;hsa-miR-548
ENSG0000	ENSG0000	0.00494	hsa-miR-124-3p;hsa-miR-137;hsa-miR-20b-5p;hsa-miR-26a-5p
ENSG0000	ENSG0000	3.55E-05	hsa-miR-1976;hsa-miR-320a;hsa-miR-320b;hsa-miR-320c;hsa-r
ENSG0000	ENSG0000	0.003118	hsa-miR-106a-5p;hsa-miR-129-5p;hsa-miR-17-5p;hsa-miR-181
ENSG0000	ENSG0000	1.08E-06	hsa-miR-17-5p;hsa-miR-181a-5p;hsa-miR-20b-5p;hsa-miR-218
ENSG0000	ENSG0000	0.003695	hsa-miR-106a-5p;hsa-miR-20b-5p;hsa-miR-548k;hsa-miR-548l
ENSG0000	ENSG0000	2.86E-05	hsa-miR-106a-5p;hsa-miR-126-5p;hsa-miR-17-5p;hsa-miR-20b
ENSG0000	ENSG0000	0.001754	hsa-miR-106a-5p;hsa-miR-17-5p;hsa-miR-181a-5p;hsa-miR-20
ENSG0000	ENSG0000	0.005274	hsa-miR-129-5p;hsa-miR-1976;hsa-miR-26a-5p;hsa-miR-660-3
ENSG0000	ENSG0000	0.005826	hsa-miR-106a-5p;hsa-miR-17-5p;hsa-miR-20b-5p;hsa-miR-93-
ENSG0000	ENSG0000	0.001006	hsa-miR-129-5p;hsa-miR-26a-5p;hsa-miR-548ar-3p;hsa-miR-5
ENSG0000	ENSG0000	0.007166	hsa-miR-106a-5p;hsa-miR-17-5p;hsa-miR-1976;hsa-miR-20b-5
ENSG0000	ENSG0000	0.005956	hsa-miR-106a-5p;hsa-miR-17-5p;hsa-miR-181a-5p;hsa-miR-20
ENSG0000	ENSG0000	3.29E-05	hsa-miR-106a-5p;hsa-miR-126-5p;hsa-miR-17-5p;hsa-miR-20b
ENSG0000	ENSG0000	0.009172	hsa-miR-106a-5p;hsa-miR-17-5p;hsa-miR-20b-5p;hsa-miR-93-
ENSG0000	ENSG0000	0.000707	hsa-miR-17-5p;hsa-miR-1976;hsa-miR-20b-5p;hsa-miR-218-5p
ENSG0000	ENSG0000	0.000707	hsa-miR-17-5p;hsa-miR-1976;hsa-miR-20b-5p;hsa-miR-218-5p
ENSG0000	ENSG0000	0.000961	hsa-miR-106a-5p;hsa-miR-17-5p;hsa-miR-20b-5p;hsa-miR-93-
ENSG0000	ENSG0000	0.005075	hsa-miR-1179;hsa-miR-548a-5p;hsa-miR-548h-5p;hsa-miR-548
ENSG0000	ENSG0000	2.09E-06	hsa-miR-106a-5p;hsa-miR-17-5p;hsa-miR-183-5p;hsa-miR-20b
ENSG0000	ENSG0000	0.001262	hsa-miR-106a-5p;hsa-miR-17-5p;hsa-miR-20b-5p;hsa-miR-26a

ENSG0000	ENSG0000	0.005826	hsa-miR-548a-5p;hsa-miR-548h-5p;hsa-miR-548k;hsa-miR-54[illegible]
ENSG0000	ENSG0000	0.005274	hsa-miR-17-5p;hsa-miR-20b-5p;hsa-miR-26a-5p;hsa-miR-93-[illegible]
ENSG0000	ENSG0000	0.002358	hsa-miR-124-3p;hsa-miR-137;hsa-miR-26a-5p;hsa-miR-320c
ENSG0000	ENSG0000	0.008314	hsa-miR-137;hsa-miR-181a-5p;hsa-miR-33a-3p;hsa-miR-548ar
ENSG0000	ENSG0000	0.006326	hsa-miR-106a-5p;hsa-miR-17-5p;hsa-miR-20b-5p;hsa-miR-39[illegible]
ENSG0000	ENSG0000	0.007045	hsa-miR-1179;hsa-miR-1976;hsa-miR-218-5p;hsa-miR-93-5p
ENSG0000	ENSG0000	0.005908	hsa-miR-106a-5p;hsa-miR-137;hsa-miR-20b-5p;hsa-miR-3179;
ENSG0000	ENSG0000	0.001795	hsa-miR-17-5p;hsa-miR-181a-5p;hsa-miR-20b-5p;hsa-miR-93[illegible]
ENSG0000	ENSG0000	0.0047	hsa-miR-106a-5p;hsa-miR-17-5p;hsa-miR-1976;hsa-miR-20b-[illegible]
ENSG0000	ENSG0000	0.000132	hsa-miR-106a-5p;hsa-miR-1179;hsa-miR-124-3p;hsa-miR-137;
ENSG0000	ENSG0000	0.003118	hsa-miR-106a-5p;hsa-miR-17-5p;hsa-miR-1976;hsa-miR-20b-[illegible]
ENSG0000	ENSG0000	0.003118	hsa-miR-106a-5p;hsa-miR-17-5p;hsa-miR-1976;hsa-miR-20b-[illegible]
ENSG0000	ENSG0000	0.003118	hsa-miR-106a-5p;hsa-miR-17-5p;hsa-miR-1976;hsa-miR-20b-[illegible]
ENSG0000	ENSG0000	0.004116	hsa-miR-106a-5p;hsa-miR-1306-3p;hsa-miR-17-5p;hsa-miR-2[illegible]
ENSG0000	ENSG0000	3.27E-05	hsa-miR-106a-5p;hsa-miR-1179;hsa-miR-129-5p;hsa-miR-17-[illegible]
ENSG0000	ENSG0000	0.00011	hsa-miR-106a-5p;hsa-miR-135b-5p;hsa-miR-17-5p;hsa-miR-2[illegible]
ENSG0000	ENSG0000	0.000806	hsa-miR-106a-5p;hsa-miR-17-5p;hsa-miR-20b-5p;hsa-miR-93[illegible]
ENSG0000	ENSG0000	0.000478	hsa-miR-106a-5p;hsa-miR-129-5p;hsa-miR-17-5p;hsa-miR-20[illegible]
ENSG0000	ENSG0000	0.009964	hsa-miR-320a;hsa-miR-320b;hsa-miR-320c;hsa-miR-320d
ENSG0000	ENSG0000	0.002855	hsa-miR-218-5p;hsa-miR-320a;hsa-miR-320b;hsa-miR-320c;hs[illegible]
ENSG0000	ENSG0000	0.005075	hsa-miR-1179;hsa-miR-129-5p;hsa-miR-3929;hsa-miR-5582-3[illegible]
ENSG0000	ENSG0000	0.004126	hsa-miR-106a-5p;hsa-miR-17-5p;hsa-miR-1976;hsa-miR-20b-[illegible]
ENSG0000	ENSG0000	8.61E-08	hsa-miR-106a-5p;hsa-miR-124-3p;hsa-miR-17-5p;hsa-miR-18[illegible]
ENSG0000	ENSG0000	0.007713	hsa-miR-17-5p;hsa-miR-20b-5p;hsa-miR-590-3p;hsa-miR-93-[illegible]
ENSG0000	ENSG0000	0.000961	hsa-miR-106a-5p;hsa-miR-17-5p;hsa-miR-20b-5p;hsa-miR-93[illegible]
ENSG0000	ENSG0000	0.008323	hsa-miR-106a-5p;hsa-miR-17-5p;hsa-miR-1976;hsa-miR-20b-[illegible]
ENSG0000	ENSG0000	8.19E-05	hsa-miR-548a-5p;hsa-miR-548ar-3p;hsa-miR-548h-5p;hsa-mi[illegible]
ENSG0000	ENSG0000	0.00106	hsa-miR-106a-5p;hsa-miR-17-5p;hsa-miR-183-5p;hsa-miR-20[illegible]
ENSG0000	ENSG0000	0.008871	hsa-miR-548a-5p;hsa-miR-548at-5p;hsa-miR-548h-5p;hsa-mi[illegible]
ENSG0000	ENSG0000	0.000715	hsa-miR-106a-5p;hsa-miR-17-5p;hsa-miR-20b-5p;hsa-miR-67[illegible]
ENSG0000	ENSG0000	0.000917	hsa-miR-2110;hsa-miR-320a;hsa-miR-320b;hsa-miR-320c;hsa-[illegible]
ENSG0000	ENSG0000	0.000246	hsa-miR-106a-5p;hsa-miR-17-5p;hsa-miR-20b-5p;hsa-miR-23[illegible]
ENSG0000	ENSG0000	1.51E-06	hsa-miR-17-5p;hsa-miR-181a-5p;hsa-miR-20b-5p;hsa-miR-32[illegible]
ENSG0000	ENSG0000	0.001028	hsa-miR-106a-5p;hsa-miR-20b-5p;hsa-miR-218-5p;hsa-miR-2[illegible]
ENSG0000	ENSG0000	0.0047	hsa-miR-106a-5p;hsa-miR-17-5p;hsa-miR-20b-5p;hsa-miR-59[illegible]
ENSG0000	ENSG0000	3.97E-05	hsa-miR-106a-5p;hsa-miR-1179;hsa-miR-17-5p;hsa-miR-20b-[illegible]
ENSG0000	ENSG0000	0.00383	hsa-miR-17-5p;hsa-miR-20b-5p;hsa-miR-590-3p;hsa-miR-93-[illegible]
ENSG0000	ENSG0000	0.001006	hsa-miR-106a-5p;hsa-miR-124-3p;hsa-miR-17-5p;hsa-miR-20[illegible]
ENSG0000	ENSG0000	0.000357	hsa-miR-106a-5p;hsa-miR-17-5p;hsa-miR-20b-5p;hsa-miR-21[illegible]
ENSG0000	ENSG0000	0.001333	hsa-miR-106a-5p;hsa-miR-17-5p;hsa-miR-20b-5p;hsa-miR-93[illegible]
ENSG0000	ENSG0000	0.003416	hsa-miR-129-5p;hsa-miR-26a-5p;hsa-miR-548ar-3p;hsa-miR-5
ENSG0000	ENSG0000	0.001071	hsa-miR-106a-5p;hsa-miR-17-5p;hsa-miR-1976;hsa-miR-20b-[illegible]
ENSG0000	ENSG0000	0.009455	hsa-miR-106a-5p;hsa-miR-17-5p;hsa-miR-20b-5p;hsa-miR-31[illegible]
ENSG0000	ENSG0000	0.009964	hsa-miR-106a-5p;hsa-miR-17-5p;hsa-miR-20b-5p;hsa-miR-93[illegible]
ENSG0000	ENSG0000	1.30E-05	hsa-miR-106a-5p;hsa-miR-17-5p;hsa-miR-20b-5p;hsa-miR-21[illegible]
ENSG0000	ENSG0000	0.002681	hsa-miR-106a-5p;hsa-miR-17-5p;hsa-miR-20b-5p;hsa-miR-93[illegible]
ENSG0000	ENSG0000	0.001374	hsa-miR-3179;hsa-miR-320a;hsa-miR-320b;hsa-miR-320c;hsa-[illegible]
ENSG0000	ENSG0000	0.004276	hsa-miR-26a-5p;hsa-miR-34a-5p;hsa-miR-548ar-3p;hsa-miR-5
ENSG0000	ENSG0000	0.003033	hsa-miR-17-5p;hsa-miR-20b-5p;hsa-miR-218-5p;hsa-miR-93-[illegible]
ENSG0000	ENSG0000	0.007713	hsa-miR-106a-5p;hsa-miR-17-5p;hsa-miR-20b-5p;hsa-miR-93[illegible]
ENSG0000	ENSG0000	0.000202	hsa-miR-106a-5p;hsa-miR-17-5p;hsa-miR-181a-5p;hsa-miR-2[illegible]
ENSG0000	ENSG0000	0.00026	hsa-miR-17-5p;hsa-miR-20b-5p;hsa-miR-218-5p;hsa-miR-641;
ENSG0000	ENSG0000	0.000185	hsa-miR-106a-5p;hsa-miR-17-5p;hsa-miR-20b-5p;hsa-miR-54[illegible]
ENSG0000	ENSG0000	0.009964	hsa-miR-106a-5p;hsa-miR-17-5p;hsa-miR-20b-5p;hsa-miR-93[illegible]

ENSG0000	ENSG0000	0.003033	hsa-miR-548a-5p;hsa-miR-548h-5p;hsa-miR-548k;hsa-miR-548
ENSG0000	ENSG0000	0.004516	hsa-miR-106a-5p;hsa-miR-129-5p;hsa-miR-17-5p;hsa-miR-20b
ENSG0000	ENSG0000	0.001962	hsa-miR-106a-5p;hsa-miR-17-5p;hsa-miR-20b-5p;hsa-miR-218
ENSG0000	ENSG0000	0.000182	hsa-miR-17-5p;hsa-miR-20b-5p;hsa-miR-548at-5p;hsa-miR-54
ENSG0000	ENSG0000	0.001297	hsa-miR-106a-5p;hsa-miR-17-5p;hsa-miR-181a-5p;hsa-miR-19
ENSG0000	ENSG0000	0.001619	hsa-miR-106a-5p;hsa-miR-17-5p;hsa-miR-181a-5p;hsa-miR-20
ENSG0000	ENSG0000	0.000669	hsa-miR-218-5p;hsa-miR-5010-3p;hsa-miR-548k;hsa-miR-548l
ENSG0000	ENSG0000	5.43E-05	hsa-miR-106a-5p;hsa-miR-17-5p;hsa-miR-20b-5p;hsa-miR-218
ENSG0000	ENSG0000	0.005616	hsa-miR-106a-5p;hsa-miR-17-5p;hsa-miR-20b-5p;hsa-miR-218
ENSG0000	ENSG0000	0.000669	hsa-miR-320a;hsa-miR-320b;hsa-miR-320c;hsa-miR-320d
ENSG0000	ENSG0000	0.000669	hsa-miR-320a;hsa-miR-320b;hsa-miR-320c;hsa-miR-320d
ENSG0000	ENSG0000	0.008871	hsa-miR-4286;hsa-miR-5010-3p;hsa-miR-548a-5p;hsa-miR-548
ENSG0000	ENSG0000	0.002163	hsa-miR-106a-5p;hsa-miR-17-5p;hsa-miR-20b-5p;hsa-miR-590
ENSG0000	ENSG0000	0.002163	hsa-miR-106a-5p;hsa-miR-17-5p;hsa-miR-20b-5p;hsa-miR-590
ENSG0000	ENSG0000	0.007045	hsa-miR-548a-5p;hsa-miR-548h-5p;hsa-miR-548k;hsa-miR-548
ENSG0000	ENSG0000	0.004681	hsa-miR-124-3p;hsa-miR-137;hsa-miR-2110;hsa-miR-218-5p;h
ENSG0000	ENSG0000	0.009732	hsa-miR-106a-5p;hsa-miR-17-5p;hsa-miR-1976;hsa-miR-20b-5
ENSG0000	ENSG0000	0.004346	hsa-miR-183-5p;hsa-miR-26a-5p;hsa-miR-33a-3p;hsa-miR-34a
ENSG0000	ENSG0000	0.000669	hsa-miR-320a;hsa-miR-320b;hsa-miR-320c;hsa-miR-320d
ENSG0000	ENSG0000	0.003695	hsa-miR-106a-5p;hsa-miR-17-5p;hsa-miR-1976;hsa-miR-20b-5
ENSG0000	ENSG0000	1.68E-05	hsa-miR-17-5p;hsa-miR-181a-5p;hsa-miR-20b-5p;hsa-miR-93-
ENSG0000	ENSG0000	4.28E-05	hsa-miR-129-5p;hsa-miR-28-5p;hsa-miR-590-3p;hsa-miR-708-
ENSG0000	ENSG0000	0.002163	hsa-miR-129-5p;hsa-miR-320a;hsa-miR-320b;hsa-miR-320c;hsa
ENSG0000	ENSG0000	0.005956	hsa-miR-106a-5p;hsa-miR-17-5p;hsa-miR-20b-5p;hsa-miR-218
ENSG0000	ENSG0000	1.29E-07	hsa-miR-320a;hsa-miR-320b;hsa-miR-320c;hsa-miR-320d;hsa-m
ENSG0000	ENSG0000	0.006416	hsa-miR-320a;hsa-miR-320b;hsa-miR-320c;hsa-miR-320d
ENSG0000	ENSG0000	0.008314	hsa-miR-106a-5p;hsa-miR-126-5p;hsa-miR-17-5p;hsa-miR-20b
ENSG0000	ENSG0000	0.000961	hsa-miR-1976;hsa-miR-2355-5p;hsa-miR-5010-3p;hsa-miR-590
ENSG0000	ENSG0000	0.001157	hsa-miR-106a-5p;hsa-miR-17-5p;hsa-miR-20b-5p;hsa-miR-766
ENSG0000	ENSG0000	0.004681	hsa-miR-218-5p;hsa-miR-26a-5p;hsa-miR-548a-5p;hsa-miR-54
ENSG0000	ENSG0000	4.39E-05	hsa-miR-106a-5p;hsa-miR-126-5p;hsa-miR-17-5p;hsa-miR-181
ENSG0000	ENSG0000	0.005826	hsa-miR-22-3p;hsa-miR-5010-3p;hsa-miR-548k;hsa-miR-548n
ENSG0000	ENSG0000	0.004116	hsa-miR-33a-3p;hsa-miR-409-3p;hsa-miR-548a-5p;hsa-miR-54
ENSG0000	ENSG0000	0.004116	hsa-miR-33a-3p;hsa-miR-409-3p;hsa-miR-548a-5p;hsa-miR-54
ENSG0000	ENSG0000	0.006416	hsa-miR-106a-5p;hsa-miR-17-5p;hsa-miR-20b-5p;hsa-miR-93-
ENSG0000	ENSG0000	0.002855	hsa-miR-106a-5p;hsa-miR-17-5p;hsa-miR-20b-5p;hsa-miR-428
ENSG0000	ENSG0000	0.002855	hsa-miR-106a-5p;hsa-miR-17-5p;hsa-miR-20b-5p;hsa-miR-428
ENSG0000	ENSG0000	0.005826	hsa-miR-320a;hsa-miR-320b;hsa-miR-320c;hsa-miR-320d
ENSG0000	ENSG0000	0.00383	hsa-miR-106a-5p;hsa-miR-17-5p;hsa-miR-20b-5p;hsa-miR-93-
ENSG0000	ENSG0000	0.000961	hsa-miR-106a-5p;hsa-miR-126-5p;hsa-miR-17-5p;hsa-miR-20b
ENSG0000	ENSG0000	0.009172	hsa-miR-106a-5p;hsa-miR-17-5p;hsa-miR-20b-5p;hsa-miR-93-
ENSG0000	ENSG0000	2.18E-05	hsa-miR-106a-5p;hsa-miR-17-5p;hsa-miR-20b-5p;hsa-miR-218
ENSG0000	ENSG0000	0.002163	hsa-miR-106a-5p;hsa-miR-17-5p;hsa-miR-20b-5p;hsa-miR-409
ENSG0000	ENSG0000	0.005274	hsa-miR-548a-5p;hsa-miR-548h-5p;hsa-miR-548k;hsa-miR-548
ENSG0000	ENSG0000	0.001195	hsa-miR-106a-5p;hsa-miR-17-5p;hsa-miR-20b-5p;hsa-miR-218
ENSG0000	ENSG0000	0.00106	hsa-miR-106a-5p;hsa-miR-17-5p;hsa-miR-20b-5p;hsa-miR-548
ENSG0000	ENSG0000	1.29E-06	hsa-miR-106a-5p;hsa-miR-126-5p;hsa-miR-17-5p;hsa-miR-20b
ENSG0000	ENSG0000	1.29E-06	hsa-miR-106a-5p;hsa-miR-126-5p;hsa-miR-17-5p;hsa-miR-20b
ENSG0000	ENSG0000	0.001603	hsa-miR-3177-5p;hsa-miR-320a;hsa-miR-320b;hsa-miR-320c;h
ENSG0000	ENSG0000	0.004126	hsa-miR-28-5p;hsa-miR-3177-5p;hsa-miR-324-5p;hsa-miR-548
ENSG0000	ENSG0000	0.000223	hsa-miR-106a-5p;hsa-miR-124-3p;hsa-miR-17-5p;hsa-miR-20b
ENSG0000	ENSG0000	0.000414	hsa-miR-17-5p;hsa-miR-20b-5p;hsa-miR-28-5p;hsa-miR-708-5
ENSG0000	ENSG0000	0.000414	hsa-miR-17-5p;hsa-miR-20b-5p;hsa-miR-28-5p;hsa-miR-708-5
ENSG0000	ENSG0000	0.000414	hsa-miR-17-5p;hsa-miR-20b-5p;hsa-miR-28-5p;hsa-miR-708-5

ENSG0000	ENSG0000	0.000414	hsa-miR-17-5p;hsa-miR-20b-5p;hsa-miR-28-5p;hsa-miR-708-
ENSG0000	ENSG0000	0.000414	hsa-miR-17-5p;hsa-miR-20b-5p;hsa-miR-28-5p;hsa-miR-708-
ENSG0000	ENSG0000	0.000414	hsa-miR-17-5p;hsa-miR-20b-5p;hsa-miR-28-5p;hsa-miR-708-
[truncated: 576,964 more chars]
